# Role of GuaB, the inosine-5′-monophosphate dehydrogenase of uropathogenic *Escherichia coli* pathogenicity: a key factor for bladder infection

**DOI:** 10.1128/spectrum.00221-25

**Published:** 2025-06-17

**Authors:** Mizuki Shimokawa, Ayako Takita, Kazutomo Suzue, Ayuko Kimura, Himari Tabo, Yumika Sato, Yuya Yanagita, Takuya Sadahira, Haruyoshi Tomita, Hidetada Hirakawa

**Affiliations:** 1 Department of Bacteriology, Graduate School of Medicine, Gunma University 12925, Maebashi, Japan; 2 Department of Infectious Diseases and Host Defense, Graduate School of Medicine, Gunma University 12925, Maebashi, Japan; 3 Graduate School of Health Sciences, Gunma Paz University 74041 , Takasaki, Japan; 4 Department of Urology, Graduate School of Medicine, Dentistry and Pharmaceutical Sciences, Okayama University 199491 , Okayama, Japan; 5 Laboratory of Bacterial Drug Resistance, Graduate School of Medicine, Gunma University 12925, Maebashi, Japan; ICON plc, London, United Kingdom

**Keywords:** urinary tract infection (UTI), uropathogenic *Escherichia coli *(UPEC), bacterial pathogenesis, proteome, antimicrobial resistance (AMR), virulence, inosine monophosphate dehydrogenase (IMPDH), cystitis, pyelonephritis, biofilm

## Abstract

Uropathogenic *Escherichia coli* (UPEC) is the primary causative agent of urinary tract infections (UTIs). This bacterium infects the bladder through the urinary tract, and some bacteria ascend to the kidneys, leading to more severe conditions such as pyelonephritis and sepsis. The infection of the bladder by UPEC is a critical initial step in the development of UTIs. Once inside the bladder, UPEC forms microcolonies both within and outside bladder epithelial cells, allowing it to persist in the bladder by resisting urine flow, innate immunity, and antimicrobials. In this study, to look for novel factors of UPEC that contribute to bladder infection and persistence, we analyzed proteins expressed at significant levels by UPEC in the bladder using a UTI mouse model. Mass spectrometry detected over 30 candidate proteins, including those already reported to play important roles in bladder infection, such as OmpA, components of the iron acquisition system, and the Tol/Pal system. Among them, GuaB (inosine-5′-monophosphate dehydrogenase) emerged as a key factor for bladder infection. The GuaB mutant showed impaired growth in urine-mimicking conditions and reduced infection efficiency, attributed to decreased GMP production. GuaB expression was upregulated in urine-like conditions, influenced by urea. This highlights GuaB’s role in UPEC pathogenicity. These findings suggest GuaB as a potential target for new therapeutic strategies against UPEC-related UTIs, especially amid growing antimicrobial resistance.

**IMPORTANCE:**

Uropathogenic *Escherichia coli* (UPEC) is the most common cause of urinary tract infections (UTIs). UTI caused by UPEC is often recurrent, and repeated use of antimicrobial agents is feared to lead to the spread of drug-resistant strains. In fact, quinolone-resistant and extended spectrum β-lactamase-producing strains have been rapidly increasing since 2000. Therefore, improvement of current treatment methods, including new therapeutic agents against UPEC, is desired. In this study, we analyzed proteins significantly expressed in the bladder of UTI mice by proteomic analysis in order to identify new factors contributing to UPEC infection of the bladder. Among them, we found GuaB (inosine-5′-monophosphate dehydrogenase), which is important for bladder infection. Furthermore, we characterized the role of GuaB in bladder infection and the mechanism by which GuaB induces its expression in urine. Our findings will contribute not only to further understanding of UPEC pathogenesis but also to the development of new antimicrobial strategies.

## INTRODUCTION

Uropathogenic *Escherichia coli* (UPEC) is the leading causative agent of urinary tract infections (UTIs), accounting for more than 80% of all cases (1, 2). When UPEC enters the urinary tract and reaches the bladder, it causes cystitis. One of the major challenges in the treatment of UPEC-mediated UTIs is the high recurrence rate. It has been reported that approximately 25% of patients experience recurrence within 6 months, even after receiving antimicrobial therapy (3, 4). Moreover, the increase and spread of antibiotic-resistant strains, including quinolone-resistant strains and extended spectrum β-lactamase-producing strains, pose serious concerns in clinical settings (5, 6). These issues highlight the urgent need for the development of new therapeutic strategies to improve the management of UPEC infections. One promising approach to developing such strategies is to identify novel molecular targets for antibacterial drug development. In addition to well-characterized targets such as ribosomes, cell wall synthesis enzymes, and nucleic acid synthesis-related proteins, the discovery of new targets could facilitate the development of innovative antimicrobials with enhanced efficacy and effectiveness against drug-resistant strains.

It is well established that type 1 fimbriae, flagella, and iron acquisition systems are key virulence factors that contribute to UPEC’s ability to establish and sustain infection in the bladder. Type 1 fimbriae are the predominant fimbrial structures of UPEC and are essential for bacterial adhesion to and invasion of bladder epithelial cells (7, 8). Flagella enhance bacterial fitness within the bladder environment, promote microcolony formation, and facilitate the ascending movement of bacteria from the bladder to the kidneys (9–12). Within the bladder, UPEC competes with host cells for limited iron availability, making efficient iron acquisition critical for bacterial growth and survival in this niche (13, 14).

Like many other bacterial species, UPEC possesses the capacity to synthesize guanosine monophosphate (GMP) and adenosine monophosphate (AMP), precursors of the purine nucleotides guanine and adenine, from ribose-5-phosphate via inosine-5′-monophosphate (IMP) (15, 16). This pathway is known as *de novo* purine biosynthesis. Previous studies have demonstrated that *de novo* purine biosynthesis contributes to the intracellular survival and proliferation of UPEC within bladder epithelial cells (17, 18).

Some *in vitro* and *in vivo* expression studies have shown that type 1 fimbriae, flagella, and proteins involved in the iron uptake system are highly expressed during bladder infection (19–23). It may be for the purpose that the above factors, which contribute to the establishment and persistence of infection in the bladder, are highly expressed at the site of infection. Based on this background, we hypothesized that, in addition to these known factors, there may be other proteins that are highly expressed during bladder infection and contribute to UPEC pathogenicity. To test this hypothesis, we aimed to identify such proteins by performing a proteomic analysis of UPEC during bladder infection using a murine model of UTI.

Specifically, we infected mice transurethrally with UPEC and harvested bladder-associated bacteria 48 hours post-infection for proteomic profiling. This analysis revealed a set of proteins, including ribosomal proteins and chaperones, as well as at least 31 candidate proteins. To assess their roles in pathogenesis, we constructed gene deletion mutants corresponding to these candidate proteins and evaluated their contribution to UPEC virulence. Among these, we identified GuaB (inosine-5′-monophosphate dehydrogenase, IMPDH), a key enzyme in the *de novo* purine biosynthesis pathway involved in GMP synthesis, as a critical factor for UPEC pathogenicity in the bladder. Furthermore, we characterized the role of GuaB in the urinary environment, highlighting its potential as a novel therapeutic target.

## MATERIALS AND METHODS

### Bacterial strains, host cells, and culture conditions

The bacterial strains and plasmids are listed in Table 1. The UPEC GU2018_CL13 strain was originally isolated from the urine and blood of a patient with pyelonephritis (accession number: AP029000) (24). Unless otherwise indicated, bacteria were grown in Luria-Bertani (LB) medium. When culturing in modified artificial urine medium (mAUM) mimicking urine (25), 0.05% glycine was added for growth promotion. The cell growth was monitored by absorbance at 600 nm. For marker selection and maintenance of plasmids, antibiotics were added to growth media at the following concentrations: 30 µg/mL chloramphenicol and 50 µg/mL kanamycin. HTB-44 cells, the human uroepithelial cells, were cultured in Eagle’s minimal essential medium containing 10% HyClone FetalClone III serum (HyClone Laboratories, Inc., Logan, UT, USA) at 37°C and in an atmosphere of 5% CO_2_.

**TABLE 1:** Strains and plasmids used in this study^*a*^

Strain or plasmid	Relevant genotype/phenotype	Reference
Strains		
GU2018_CL13	Parent strain	(24)
GU2018_CL13ΔartPJ1QMJ2	*artPJ1QMJ2* mutant from GU2018_CL13	This work
GU2018_CL13Δ*gntRKU*	*gntRKU* mutant from GU2018_CL13	This work
GU2018_CL13Δ*mtlRDA*	*mtlRDA* mutant from GU2018_CL13	This work
GU2018_CL13Δ*sitABCD*	*sitABCD* mutant from GU2018_CL13	This work
GU2018_CL13ΔacrRAB	*acrRAB* mutant from GU2018_CL13	This work
GU2018_CL13Δ*azoR*	*azoR* mutant from GU2018_CL13	This work
GU2018_CL13Δblc	*blc* mutant from GU2018_CL13	This work
GU2018_CL13ΔgatD	*gatD* mutant from GU2018_CL13	This work
GU2018_CL13ΔguaB	*guaB* mutant from GU2018_CL13	This work
GU2018_CL13ΔselD	*selD* mutant from GU2018_CL13	This work
Plasmids		
pKO3	Temperature sensitive vector for gene targeting, *sacB*, Cm^R^	(26)
pTH18kr	Low copy plasmid, Km^R^	(27)
pTH18krguaB	*guaB* expression plasmid, Km^R^	This work
pNN387	*S*ingle-copy plasmid with promoterless *lacZ*, Cm^R^	(28)
pNNguaB-P	*guaB* promoter reporter, Cm^R^	This work

^*a*^ Cm^R^, chloramphenicol resistance; Km^R^, kanamycin resistance.

### UTIs in mice

To estimate UPEC virulence within the urinary tract, we used a UTI mouse model (11). Twenty-four hour static cultures of the UPEC were harvested and re-suspended in phosphate-buffered saline (PBS). The bacterial suspensions (1 × 10^8^ colony forming unit [CFU]) were administered to 8-week-old C3H/HeN female mice (CLEA Japan, Inc., Tokyo, Japan) via transurethral catheterization. Mice were euthanized 48 hours post-infection, and the entire bladder of each mouse was aseptically removed and homogenized in PBS. The numbers of bacterial CFU were determined by counting colonies grown on LB agar.

### Protein preparation and mass spectrometry analyses

The homogenate of the bladder prepared from UPEC-infected mice was vortexed for 5 minutes. To remove as much extract as possible from the disrupted bladder tissue, the supernatant was discarded after centrifugation, and the remaining pellet was resuspended in 1 mL of SDS-lysis buffer (20 mM Tris-HCl, pH 6.8, 2% SDS, 6.8% glycerol, 20 mM dithiothreitol) containing a proteinase inhibitor cocktail (Nacalai tesque, Kyoto, Japan). The bacteria and residual bladder tissue were disrupted by sonication, and the supernatant was collected through centrifugation. Protein concentration was determined by using the Protein Assay Coomassie Brilliant Blue (CBB) Cleanup Kit (Nacalai Tesque, Kyoto, Japan) and a Bradford dye reagent protein assay (Bio-Rad, Hercules, CA, USA). Proteins (10 µg) were separated by SDS-polyacrylamide gel electrophoresis (SDS-PAGE) with a 5%–20% gel and stained with CBB. Protein bands observed in the infected samples but not in the uninfected sample were excised and subjected to in-gel digestion using trypsin (Promega Corp., Madison, WI, USA) at 37°C overnight. Peptides were desalted using stage tips filled with C18 Empore disc membranes (3M, St. Paul, MN, USA) and evaporated in a vacuum concentrator. Samples dissolved in 0.1% formic acid were separated on a reversed phase (RP) LC (Dionex Ultimate 3000 nano HPLC system; Thermo Fisher Scientific Inc., Waltham, MA, USA) and analyzed on an LTQ Orbitrap Velos MS (Thermo Fisher Scientific Inc., Waltham, MA, USA) equipped with a nanoscale C18 capillary LC column (NTCC-360/75-3-125; Nikkyo Technos, Tokyo, Japan). RPLC was performed in a 115 minute acetonitrile gradient (2%–33%), and the eluent was electrosprayed with an electrospray ionization source in positive ion mode. MS/MS analyses were performed in data-dependent acquisition mode at an *m*/*z* range of 350–1,200 and subsequent product ion scans for each of the 15 most intense ions in the full MS scan. MS/MS spectra are searched against a protein sequence database as shown in Table S1, using MaxQuant software ver. 1.6.14 (29) at 1% false discovery rate threshold with slight alteration in default setting (main search peptide tolerance: 6 ppm). The mass spectrometry (MS) proteomics data have been deposited to the ProteomeXchange Consortium via the jPOST (30) with data set identifier JPST003552.

### Cloning and mutant constructions

The in-frame gene deletions were performed using the temperature-sensitive vector pKO3, as previously described, through homologous recombination (26). For homologous recombination, the flanking DNA included 450 bp upstream and downstream of the target gene. For the deletion of *artPJ1QMJ2*, *gntRKU*, *mtlRDA*, and *sitABCD*, the flanking DNA was amplified by sequence overlap extension PCR using primer pairs delta1/delta2 and delta3/delta4. These deletion constructs were ligated into the BamHI- and SalI-digested pKO3 vector. For the deletion of *acrRAB*, *azoR*, *blc*, *gatD*, *guaB*, and *selD*, the flanking DNA including upstream and downstream regions of each target gene was amplified using primer pairs FW1/RV1 and FW2/RV2 and cloned into the pKO3 vector using the In-fusion Cloning Kit (Takara Bio, Shiga, Japan). These plasmids were introduced into UPEC, and sucrose-resistant/chloramphenicol-sensitive colonies were selected at 30°C. The primers are listed in Table 2.

**TABLE 2:** Primers used for mutant and plasmid constructions

Primer	DNA sequence (5′–3′)	Use
artPJ1QMJ2-delta1	gcgggatccgtcgaaccaccttgccagac	*artPJ1QMJ2* mutant construction
artPJ1QMJ2-delta2	ggtgcggctttctgaatcttactgactcattgacactcgtatac	*artPJ1QMJ2* mutant construction
artPJ1QMJ2-delta3	gccagtatacgagtgtcaatgagtcagtaagattcagaaagccg	*artPJ1QMJ2* mutant construction
artPJ1QMJ2-delta4	gcggtcgacatatgtgggatctgttctgc	*artPJ1QMJ2* mutant construction
gntRKU-delta1	gcggcggccgcatctgtatcagatctggatc	*gntRKU* mutant construction
gntRKU-delta2	accgggtaaacaaacttaactcaactttttcatcgtcctgaagg	*gntRKU* mutant construction
gntRKU-delta3	tgtaccttcaggacgatgaaaaagttgagttaagtttgtttacccg	*gntRKU* mutant construction
gntRKU-delta4	gcggtcgactggcatttcttagcctgcgg	*gntRKU* mutant construction
mtlRDA-delta1	gcgggatccgctttgttcacaactgatgg	*mtlRDA* mutant construction
mtlRDA-delta2	agaaggggtgtttttatgtcatcctttcaaaagtaagtactttcgc	*mtlRDA* mutant construction
mtlRDA-delta3	aagcgaaagtacttacttttgaaaggatgacataaaaacacccc	*mtlRDA* mutant construction
mtlRDA-delta4	gcggtcgaccgggttcgcgattctcgcc	*mtlRDA* mutant construction
sitABCD-delta1	gcgggatccacaacccgtttgactgcttc	*sitABCD* mutant construction
sitABCD-delta 2	tcctcggatgttgtgctaattgggcgagtgcataatttagtacc	*sitABCD* mutant construction
sitABCD-delta3	ataggtactaaattatgcactcgcccaattagcacaacatccg	*sitABCD* mutant construction
sitABCD-delta4	gcggtcgactgtattctctgattatgtcg	*sitABCD* mutant construction
acrRAB-deltaFW1	gccgcggaccggatcgaattttgcgcgtttcttcgtcg	*acrRAB* mutant construction
acrRAB-deltaRV1	tgtatcaatggaataaccctgaatctgactccagg	*acrRAB* mutant construction
acrRAB-deltaFW2	agggttattccattgatacaacgtgtaatcactaa	*acrRAB* mutant construction
acrRAB-deltaRV2	gccattctccggtcgagatggaaaaaacttactgacctgga	*acrRAB* mutant construction
azoR-deltaFW1	gccgcggaccggatccccggcgacgatcaccac	*azoR* mutant construction
azoR-deltaRV1	gatcgtgtccggttacatggtgtttccttatagata	*azoR* mutant construction
azoR-deltaFW2	taaccggacacgatcttga	*azoR* mutant construction
azoR-deltaRV2	gccattcccggtcgaatcgggatgtggctgtct	*azoR* mutant construction
blc-deltaFW1	gccgcggaccggatcgaccattgaagatatggcc	*blc* mutant construction
blc-deltaRV1	actcagcactcattacataaatgtttccttactggt	*blc* mutant construction
blc-deltaFW2	taatgagtgctgagtttcag	*blc* mutant construction
blc-deltaRV2	gccattcccggtcgacgcagcaataagcaataacg	*blc* mutant construction
gatD-deltaFW1	gccgcggaccggatcgggcatacggcggtggta	*gatD* mutant construction
gatD-deltaRV1	tggcccgcggtttcacataaaaactcctgattattaag	*gatD* mutant construction
gatD-deltaFW2	tgaaaccgcgggccagcg	*gatD* mutant construction
gatD-deltaRV2	gccattcccggtcgagccgccgggagactgttg	*gatD* mutant construction
guaB-deltaFW1	gccgcggaccggatccttcagttgcagcaatcct	*guaB* mutant construction
guaB-deltaRV1	gggcgaagagaatcacatgggcaatatctcgacc	*guaB* mutant construction
guaB-deltaFW2	tgattctcttcgcccgac	*guaB* mutant construction
guaB-deltaRV2	gccattcccggtcgagccgtggctcatccagac	*guaB* mutant construction
selD-deltaFW1	gccgcggaccggatccgtttcagcgctgtactg	*selD mutant construction*
selD-deltaRV1	cgcatcgacttaacgcatggacatctcctgtcaa	*selD* mutant construction
selD-deltaFW2	cgttaagtcgatgcggttg	*selD* mutant construction
selD-deltaRV2	gccattcccggtcgacccgcgccagtgcagaaac	*selD* mutant construction
pTHguaB-F	gcgggatcccatgtgagcgagatcaaattc	pTH18krguaB construction
pTHguaB-R	gcgaagctttcaggagcccagacggtag	pTH18krguaB construction
guaB-PF	gcggcggccgcttcagttgcagcaatccttc	pNNguaB-P construction
guaB-PR	gcgaagcttgggcaatatctcgaccagag	pNNguaB-P construction

To construct the *guaB* complementation plasmid pHT18krguaB, the *guaB* gene region from 164 bp upstream to the stop codon was PCR amplified using primers pTHguaB-F and pTHguaB-R (listed in Table 2), and cloned into the BamHI and HindIII sites of pTH18kr (27) using in-fusion cloning. We also constructed pNNguaB-P, a *lacZ* reporter plasmid to measure the *guaB/A* promoter activity. We PCR amplified the 500 bp upstream region of *guaB* and ligated these products into pNN387 (28) with a promoterless lacZ. All constructs were confirmed by DNA sequencing.

### Gentamicin assay

The number of bacteria to invade human uroepithelial cells was determined by gentamicin protection assay with some modifications (21). HTB-44 cells were cultured to confluence in 24-well plates, and then we inoculated ~5.0 × 10^6^ bacteria into ~5.0 × 10^5^ host cells. After incubation for 2 hours, the wells were washed once with PBS^+^ (PBS containing 0.5 mM Mg^2+^ and 1 mM Ca^2+^) and incubated them in the presence of gentamicin at 100 µg/mL for another 2 hours. The wells were washed twice with PBS^+^, and the cells were lysed by 0.1% Triton X-100 and plated to determine bacterial numbers.

### Swarming motility assay

To evaluate the motility of the bacteria, its swarming was tested. Bacteria were statically grown overnight at 37°C. The bacterial cultures (2 µL) were spotted onto LB medium containing 0.25% agar and incubated for 16 h at 30°C. The bacterial migration on the agar medium was measured.

### Hemolysin assay

To evaluate the activity of calcium ion-dependent hemolysin produced by UPEC, the bacteria from the overnight culture (5 µL) were spotted on LB agar plates containing 5% sheep erythrocytes and 10 mM CaCl_2_. After 18 hours of incubation at 37°C, the diameter of the transparent (hemolyzed) area (hemolytic ring) was measured.

### RNA extraction and quantitative real-time PCR analyses

Bacteria were grown to the logarithmic growth phase (optical density at 600 nm [OD_600_] ~0.5) in LB medium and mAUM. Total RNA extraction and cDNA synthesis were performed by using the Monarch Total RNA Miniprep Kit (New England Biolabs, Ipswich, MA, USA) and ReverTra Ace qPCR RT Master Mix (Toyobo Co. Ltd., Osaka, Japan). The genomic DNA (gDNA) was digested on the column using DNase I supplied in the RNA Miniprep Kit. In addition, the remaining gDNA was removed by gDNA remover provided in the qPCR RT Master Mix. Real-time PCR mixtures included 2 ng of cDNA and 160 nM primers in Thunderbird Next SYBR qPCR Mix (Toyobo Co. Ltd., Osaka, Japan). Constitutively expressed *rrsA* and *rpoD* genes were used as internal controls. The primers are listed in Table 3.

**TABLE 3:** Primers used for quantitative PCR in this study

Primer	DNA sequence (5′–3′)	Primer	DNA sequence (5′–3′)
rrsA-qPCR-F	cggtggagcatgtggtttaa	rrsA-qPCR-R	gaaaacttccgtggatgtcaaga
rpoD-qPCR-F	caagccgtggtcggaaaa	rpoD-qPCR-R	gggcgcgatgcacttct
guaA-qPCR-F	gtgggcgtgggatgtga	guaA-qPCR-R	gccgcttggattgaagtca
guaB-qPCR-F	acgccaggcagaagaagttc	guaB-qPCR-R	agtcaccacgccagattcg
fimH-qPCR-F	gatgcgggcaactcgatt	fimH-qPCR-R	ccctgcgcgggtgaa
papG-qPCR-F	tcggttggtctgggtcatg	papG-qPCR-R	tgtgtccacgccattaatcg
fis-qPCR-F	ccctgcgtgactcggttaa	fis-qPCR-R	cctgaccattcagttgagcaaa
purR-qPCR-F	tgccggaaagctggattg	purR-qPCR-R	ggcgcgataaccggattc
dnaA-qPCR-F	caggcggaactgagcgata	dnaA-qPCR-R	tcgaggacaaaacggtttgg
crp-qPCR-F	ccgtcaggaaatcggtcaga	crp-qPCR-R	tgcgtcccacggtttca
glpT-qPCR-F	tgcccgcaggtttgattc	glpT-qPCR-R	ccatggcacaaagcccata

### cAMP assay

Intracellular cAMP levels of UPEC were determined by using the cAMP-Glo Assay Kit (Promega Corp., Madison, WI, USA). Bacteria were grown in 16 mL of LB medium or mAUM to late logarithmic phase and harvested. The cell pellets were once washed in PBS and suspended in 0.5 mL of enzyme-linked immunosorbent assay buffer (100 mM phosphate solution containing 0.1% bovine serum albumin [BSA], 400 mM sodium chloride, 1 mM EDTA, and 0.01% sodium azide), then lysed by sonication. The lysate was centrifuged, and the amount of cAMP in the resulting supernatant was quantified according to the manufacturer protocol.

### Promoter assay

To measure the promoter activity of *guaB/A*, the parent strain carrying pNNguaB-P was grown at 37°C. β-Galactosidase activities from *lacZ* expression in cell lysates were determined using a Tropix Galacto-Light Plus Kit (Thermo Fisher Scientific Inc., Waltham, MA, USA) as previously described (31). The β-galactosidase activity was determined as the signal value normalized to an OD_600_ of 1 and recalculated by subtracting the endogenous LacZ activity derived from the *lacZ* encoded on the chromosome of the parent strain.

## RESULTS

### Identification of UPEC proteins infecting the bladder of UTI mice

**Fig 1:**
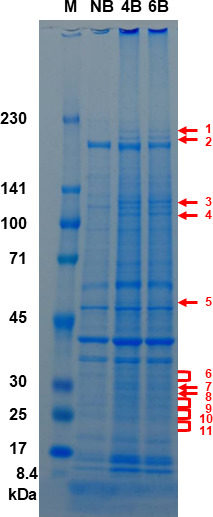
Proteins from the bladders of infected and uninfected mice. Proteins were separated by SDS-PAGE using a 5%–20% acrylamide gel and stained with Coomassie brilliant blue. Lanes labeled NB are proteins isolated from the bladders of uninfected mice, and lanes labeled 4B and 6B are proteins isolated from the bladders of infected mice. Numbers 1–11, pointed to the right in red, indicate protein bands observed in the infected samples but not in the uninfected sample. Locations of molecular mass standards (in kDa) are indicated on the left (lane marked “M”).

To identify UPEC proteins that are highly expressed during bladder infection, UPEC was transurethrally inoculated into six 8-week-old female C3H/HeN mice. At 48 hours post-infection, bladders were excised and homogenized in PBS. A portion of each homogenate was used to determine bacterial counts, while the remainder was subjected to protein extraction via bacterial lysis, as detailed in Materials and Methods. Among the six samples, the two with the highest bacterial loads (4.3 × 10^7^ CFU) were selected for further analysis. Proteins extracted from these samples, along with those from uninfected control bladders, were separated by SDS-PAGE. Eleven gel regions showing in the infected samples (4B and 6B) but not in the uninfected sample were excised for analysis (Fig. 1). To serve as negative controls, gel regions corresponding to the same electrophoretic mobility as the excised bands from infected samples were also collected from the non-infected sample and subjected to MS analysis. Thirty-one proteins consistently detected in both infected samples with scores >5 were listed as candidate infection-related proteins (Table 4).

**TABLE 4:** Candidate proteins expressed at significant levels in the bladder of UTI mice

Protein ID	Protein	MW^*a*^ (kDa)	Score	Coverage (%)
GU2018CL13_04860	Malate dehydrogenase (Mdh)	32.337	213.24	36.5
GU2018CL13_44410	50S ribosomal protein L1 (RplA)	24.729	91.261	18.4
GU2018CL13_04030	50S ribosomal protein L3 (RplC)	22.243	43.691	20.6
GU2018CL13_37760	Chaperone protein (DnaK)	69.114	42.421	2.5
GU2018CL13_04090	30S ribosomal protein S3 (RpsC)	25.983	39.724	18.5
GU2018CL13_37950	2,3-bisphosphoglycerate-dependent phosphoglycerate mutase (GpmB)	28.556	37.856	8.8
GU2018CL13_04040	50S ribosomal protein L4 (RplD)	22.086	35.094	14.9
GU2018CL13_29270	Outer membrane protein A (OmpA)	37.187	34.369	6.9
GU2018CL13_30230	ABC transporter arginine-binding protein 1 (ArtJ_2)	26.842	30.876	6.6
GU2018CL13_26390	Iron/manganese ABC transporter substrate-binding protein (SitA)	33.426	25.654	16.4
GU2018CL13_23830	FMN-dependent NADH-azoreductase (AzoR)	21.641	9.0613	4.5
GU2018CL13_45160	Superoxide dismutase [Mn] (SodA)	23.064	6.4161	4.4
GU2018CL13_31410	Cytochrome bd-II ubiquinol oxidase subunit 1 (CydA)	58.022	6.0057	1.8
GU2018CL13_35130	50S ribosomal protein L31 type B (YkgM)	10.051	5.9128	11.4
GU2018CL13_31320	Cell division coordinator (CpoB)	28.26	5.9004	4.9
GU2018CL13_08880	Phosphoenolpyruvate–protein phosphotransferase (PtsP_1)	83.727	5.8937	1.3
GU2018CL13_41860	Lipocalin (Blc)	19.923	5.8672	5.1
GU2018CL13_33550	Multidrug efflux transporter transcriptional repressor (AcrR)	24.766	5.8565	4.7
GU2018CL13_18210	Colibactin hybrid non-ribosomal peptide synthetase/type I polyketide synthase (ClbB)	352.52	5.855	0.3
GU2018CL13_34090	Protein translocase subunit (SecF)	35.382	5.835	4.0
GU2018CL13_02830	Gluconate operon transcriptional repressor (GntR)	36.434	5.8236	2.4
GU2018CL13_30740	23S rRNA (adenine(1618)-N(6))-methyltransferase (RlmF)	34.179	5.7947	3.6
GU2018CL13_36950	Cell division protein (FtsQ)	31.419	5.7917	3.3
GU2018CL13_20420	Glyceraldehyde-3-phosphate dehydrogenase (GapA)	35.532	5.7759	2.1
GU2018CL13_16850	Galactitol 1-phosphate 5-dehydrogenase (GatD)	37.441	5.7264	3.8
GU2018CL13_01150	L-seryl-tRNA(Sec) selenium transferase (SelA)	50.695	5.7139	2.4
GU2018CL13_01090	Mannitol operon repressor (MtlR)	21.99	5.7041	4.6
GU2018CL13_32640	Enterobactin non-ribosomal peptide synthetase (EntF)	141.88	5.7025	0.6
GU2018CL13_12580	Inosine-5′-monophosphate dehydrogenase (GuaB)	51.995	5.6895	2.3
GU2018CL13_29310	Ribosome modulation factor (Rmf)	6.5074	5.6757	18.2
GU2018CL13_40990	Ribosome biogenesis factor (YjgA)	21.359	5.6728	3.8

^*a*^ MW, molecular weight.

The candidate list included numerous proteins essential for bacterial survival or proliferation, such as ribosomal proteins, ribosomal modification factors, the DnaK chaperone, tricarboxylic acid cycle enzymes, and components of the respiratory chain. Additionally, several well-characterized UPEC virulence factors were identified, including the outer membrane protein OmpA and enzymes involved in siderophore biosynthesis (32, 33). The Tol/Pal system, a protein complex involved in pathogenesis through bladder epithelial invasion and microcolony formation (11), was also noted. Although direct detection of Tol/Pal components was not achieved—likely due to detection limits—peptides corresponding to CpoB, which is encoded by a gene in the same operon, were identified. These findings suggest that multiple proteins critical for UPEC bladder colonization are expressed at significant levels during infection, supporting the utility of this approach for identifying infection-related proteins.

### The *guaB* mutant exhibits reduced colonization ability in the bladder

Ten proteins, excluding housekeeping and previously characterized virulence-related proteins, were selected for functional analysis via gene knockout. For the genes *artJ_2* (GU2018CL13_30230), *sitA* (GU2018CL13_26390), *mtlR* (GU2018CL13_01090), *gntR* (GU2018CL13_02830), and *acrR* (GU2018CL13_33550), adjacent genes with potentially related functions were also deleted.

**Fig 2:**
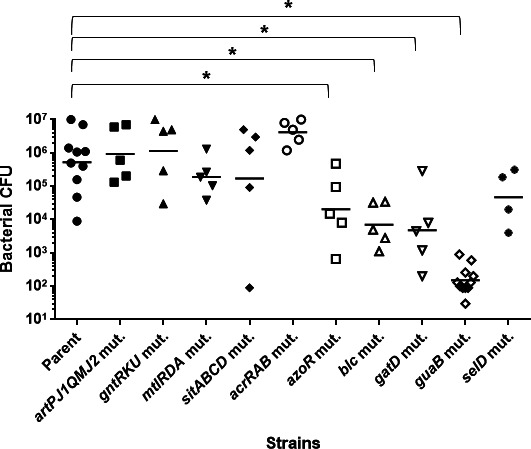
Colonization by the UPEC GU2018_CL13 parent strain and its derivative mutants in the bladders of UTI mice. At 48 hours post-infection, cell numbers of bacteria isolated from the bladder were determined as CFU. Numbers of mice are five except for the parent strain, the *guaB* mutant, and the *selD* mutant. The data for the *selD* mutant are for four mice because one infected mouse died. The parent strain and the *guaB* mutant were also initially tested with five mice, but since the CFU of the *guaB* mutant infected group was very low at this point, therefore another five mice were added to both the parent and *guaB* mutant strains for confirmation. Each data point represents a sample from an individual mouse. Horizontal bars show geometric mean values. *P < 0.05. Asterisks denote significance for values relative to the parent strain. The *P* value was determined by the Mann-Whitney test.

To assess bladder colonization, the parent strain and each mutant were transurethrally inoculated into female C3H/HeN mice, and bacterial loads in the bladder were quantified 48 hours post-infection. Among the mutants, those lacking *azoR*, *blc*, *gatD*, or *guaB* exhibited significantly reduced colonization compared to the parent strain (Fig. 2). Notably, the *guaB* mutant strain (*guaB mut*), which lacks the gene encoding IMP dehydrogenase, showed the most pronounced colonization defect with bacterial counts over 1,000-fold lower than those of the parent strain.

### GuaB is required for bacterial growth in urine and efficient epithelial invasion

**Fig 3:**
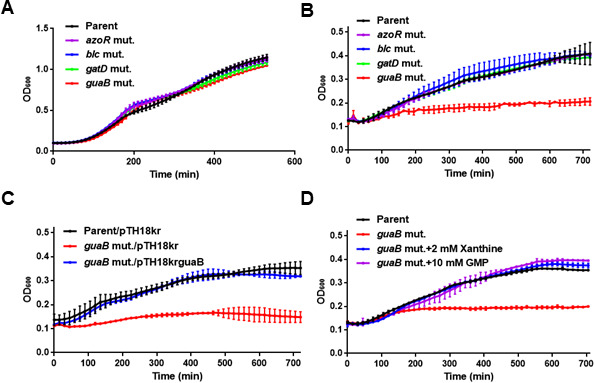
Growth in the parent strain, the *guaB* mutant (*guaB* mut.) or the parent and the *guaB* mutant carrying the pTH18kr empty vector (parent/pTH18kr and *guaB* mut./pTH18kr) or pTH18krguaB, the *guaB* expression plasmid (*guaB* mut./pTH18krguaB). Bacteria were cultured in LB medium (**A**) and mAUM containing glycine with and without 2 mM xanthine or 10 mM guanosine monophosphate (GMP) (**B through D**). Bacterial growth was monitored by measuring OD_600_. Data plotted are the means for two biological replicates; error bars indicate the ranges. We performed this assay twice, then similar results were obtained.

Growth of the parent and *guaB* mutant strains was compared by monitoring culture turbidity. In LB medium, which is commonly used in the laboratory, the *guaB* mutant showed comparable growth to the parent strain (Fig. 3A). However, in mAUM, a medium that mimics the urinary environment (25), the mutant failed to grow (Fig. 3B). This growth defect was rescued by complementation with the *guaB* expression plasmid pTH18kguaB (Fig. 3C). GuaB catalyzes the conversion of inosine-5′-monophosphate (IMP) to xanthosine-5′-phosphate (XMP) (15), which is subsequently converted to GMP by the downstream *guaA* gene product (16). XMP can also be produced from xanthine, bypassing GuaB. Supplementation of mAUM with either xanthine or GMP restored the growth of the *guaB* mutant, indicating that the growth defect arises from insufficient GMP biosynthesis (Fig. 3D).

**Fig 4:**
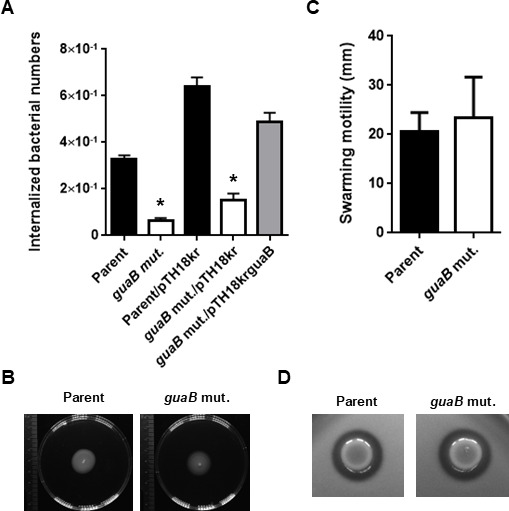
Internalization in uroepithelial cells, swarming motilities, and hemolytic activities of the parent strain, the *guaB* mutant (*guaB* mut.) or the parent and the *guaB* mutant carrying the pTH18kr empty vector (parent/pTH18kr and *guaB* mut./pTH18kr) or pTH18krguaB, the *guaB* expression plasmid (*guaB* mut./pTh18krguaB). (**A**) Numbers of internalized bacteria are represented. Data plotted are the means of three biological replicates; error bars indicate standard deviations, *P < 0.05. Asterisks denote significance for values relative to parent or parent/pTH18kr. The *P* value was determined by the unpaired *t* test. (**B**) Swarming motilities were evaluated as bacterial migrations on LB medium containing 0.25% agar. (**C**) Diameters reflecting bacterial migration on the agar. Data are means from three independent experiments; error bars indicate standard deviations. (**D**) Hemolysis is indicated by areas of transparency around the bacteria on LB agar plates containing 5% sheep erythrocytes and 10 mM CaCl_2_. We performed this experiment twice, then similar results were obtained.

Gentamicin assays were used to quantify bacterial invasion into human urothelial cells. The cell number of intracellular *guaB* mutant was approximately one-third that of the parent strain, while complementation restored invasion to the parental level (Fig. 4A). These results indicate that GuaB is important for UPEC growth in urine and its ability to adhere and invade urinary tract cells.

Flagellar-dependent motility contributes to infection of urothelial cells and fitness in the bladder (9–12). Hemolysin is a molecule that causes injury to urinary tract cells and immune cells (34). We assessed swarming motility and hemolysin activity between the parent and *guaB* mutant strains by measuring the spread of the bacteria on a soft agar medium and the size of the hemolytic ring on sheep blood-containing agar plates. However, no significant differences in flagellar motility or hemolytic activity were observed between the parent and *guaB* mutant strains (Fig. 4B through D).

### GuaB/A expression increases under conditions that mimic urine

**Fig 5:**
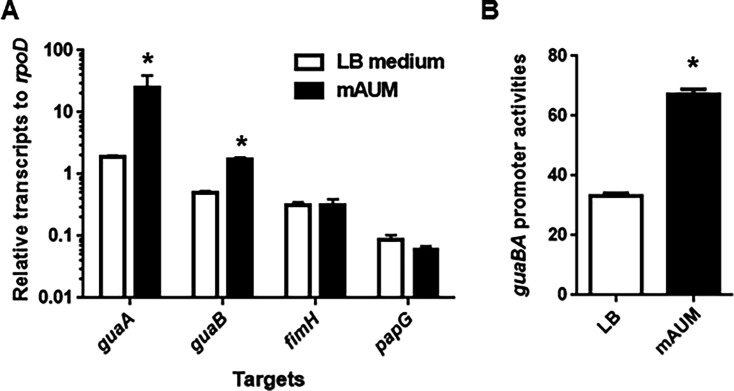
Transcript levels of *guaA*, *guaB*, *fimH*, and *papG* genes and *guaB*/A promoter activities. The parent strain was cultured in LB medium and mAUM containing glycine. (**A**) Transcript levels of target genes were described as relative values to that of *rpoD* (housekeeping gene). Data plotted are the means from three independent experiments; error bars indicate the standard deviations. (**B**) β-Galactosidase activities corresponding to *guaB/A* promoter activities in the parent strain containing pNNguaB-P, the *lacZ* reporter plasmid. Data plotted are the means for three biological replicates; error bars indicate the standard deviations. We performed this experiment twice, then similar results were obtained. *P < 0.05 relative to the value for LB medium. The *P* value was determined by the unpaired *t* test.

Given the essential role of GuaB in urinary tract infection (UTI) and its necessity for growth in mAUM, we investigated the expression levels of the *guaB* and *guaA* genes under urine-mimicking conditions. Real-time PCR analysis showed that both genes were more highly transcribed in mAUM than in the LB medium (Fig. 5A). On the other hand, when comparing the transcription levels of *fimH* and *papG* encoding type 1 and P fimbriae, respectively, no significant differences were observed between the two media. Since *guaB* and *guaA* are co-transcribed from a shared promoter upstream of *guaB*, we constructed a transcriptional fusion plasmid (pNNguaB-P) containing the 500 bp upstream region fused to *lacZ*. Consistent with the real-time PCR results, β-galactosidase assays revealed higher promoter activity in mAUM than in the LB medium (Fig. 5B).

**Fig 6:**
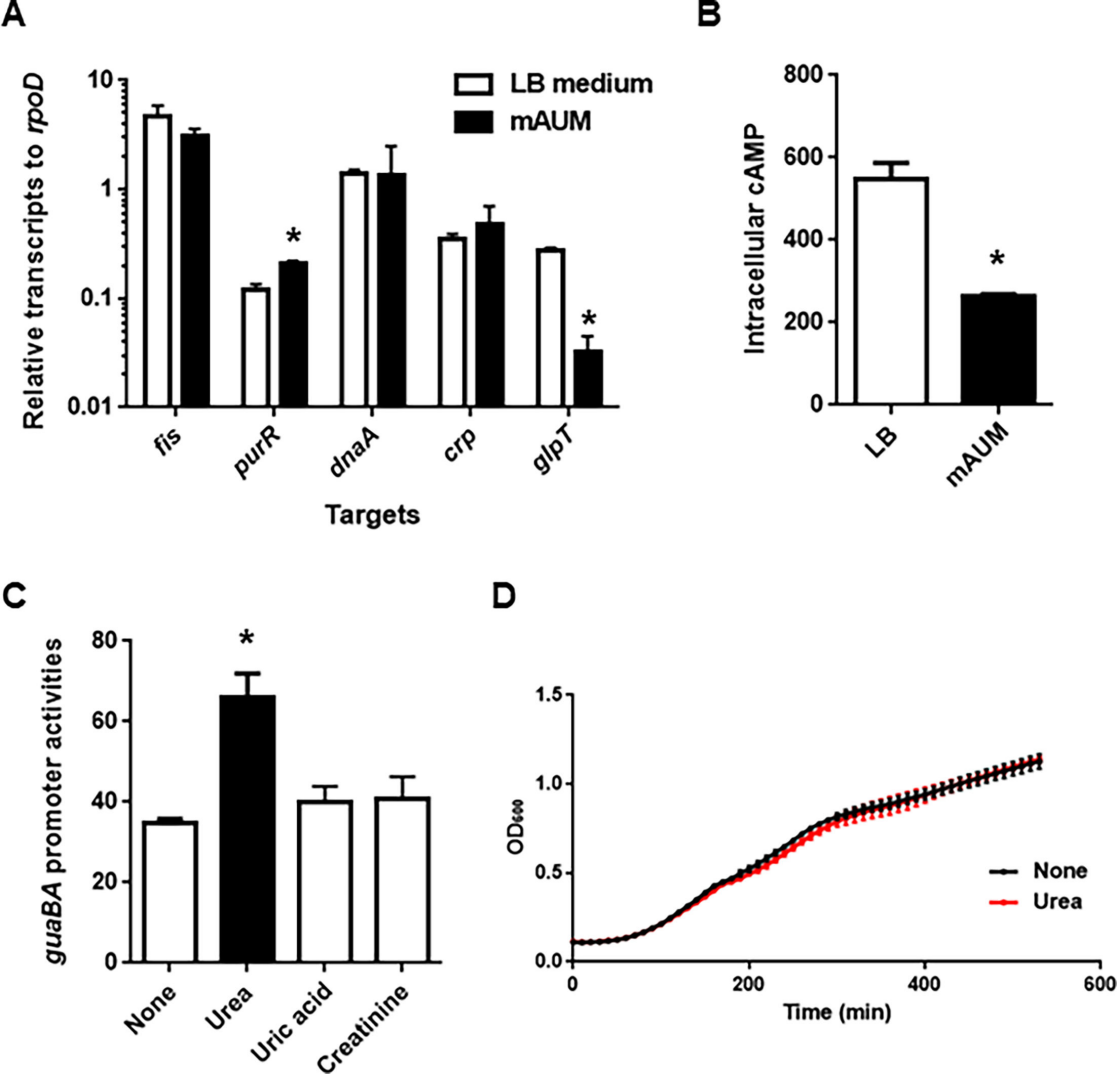
Regulatory factors of the *guaB/A* gene. (**A**) Transcript levels of the *guaB/A*-regulatory genes in the parent strain cultured in LB medium and mAUM containing glycine. Transcript levels of target genes were described as relative values to that of *rpoD* (housekeeping gene). Data plotted are the means from three independent experiments; error bars indicate the standard deviations. (**B**) Intracellular concentration of cAMP in the parent strain cultured in LB medium and mAUM containing glycine. The intracellular concentration of cAMP was the amount of cAMP (represented as pmol) per cells producing 1 mg of protein. Data plotted are the means for three biological replicates; error bars indicate the standard deviations. We performed this experiment twice, then similar results were obtained. (**C**) β-Galactosidase activities corresponding to *guaB/A* promoter activities in the parent strain containing pNNguaB-P, the *lacZ* reporter plasmid. Bacteria were cultured in LB medium with and without urea (170 mM), uric acid (0.4 mM), or creatinine (7 mM). Data plotted are the means for three biological replicates; error bars indicate the standard deviations. We performed this experiment twice, then similar results were obtained. *P < 0.05 relative to the value for plain LB medium. The *P* value was determined by the unpaired *t* test. (**D**) Growth in the parent strain cultured in LB medium with and without 170 mM urea. Bacterial growth was monitored by measuring OD_600_. Data plotted are the means for two biological replicates; error bars indicate the ranges. We performed this assay twice, then similar results were obtained.

The *guaB/A* promoter activity is regulated by Fis, DnaA, PurR, and CRP (35–38). We measured the transcription levels of these regulators in the parent UPEC strain cultured in LB medium and mAUM. Transcriptional analysis revealed no significant changes in *fis*, *dnaA*, or *crp* expression between the two media, while *purR* expression was elevated in mAUM (Fig. 6A). As PurR acts as a repressor of *guaB/A* transcription, its upregulation would be expected to suppress, rather than enhance, *guaB/A* expression. Therefore, PurR is unlikely to mediate the observed induction.

During this quantitative PCR analysis, we found that the transcription level of *glpT*, one of the genes whose transcription is activated by CRP (39), was significantly lower in mAUM culture compared to LB medium (Fig. 6A). In contrast to *glpT*, CRP represses the transcription of *guaB/A* (38). It is known that CRP activity is modulated by intracellular cAMP levels (40). We hypothesized that reduced cAMP levels may underlie the increased *guaB/A* expression. Indeed, we found that intracellular cAMP levels in mAUM were approximately twofold lower than in the LB medium (Fig. 6B), supporting the idea that reduced CRP-mediated repression enhances *guaB/A* expression.

As components of urine, substances such as urea, uric acid, and creatinine are included. To investigate the effects of these components on the expression of *guaB/A*, we cultured UPEC harboring the plasmid pNNguaB-P in an LB medium supplemented with the aforementioned compounds and measured the activity of the *guaB/A* promoter. Among these, only 170 mM urea—consistent with typical urinary concentrations (41)—induced a significant increase in *guaB/A* promoter activity (Fig. 6C). The *guaB/A* promoter is subject to growth rate-dependent control (38, 42). A comparison of bacterial growth with and without urea revealed that the concentration of urea used in this study did not affect bacterial growth (Fig. 6D). These results indicate that GuaB expression is upregulated by physiological concentrations of urea, highlighting its importance in UPEC pathogenesis in the urinary tract.

## DISCUSSION

Most currently used antimicrobials were developed during the 20th century, and since 2000, the pace of novel antimicrobials discovery has markedly declined (43). A major technical challenge lies in the difficulty of identifying effective new drug targets (44, 45). Established antimicrobial targets include ribosomes, enzymes involved in cell wall synthesis, and proteins related to nucleic acid synthesis. Expanding the repertoire of drug targets beyond these could facilitate the development of novel antimicrobial agents.

Ribosomes, cell wall synthesis enzymes, and nucleic acid synthesis-related proteins are universally essential for bacterial growth and survival, both under nutrient-rich laboratory conditions and within host environments during infection. However, certain bacterial factors, while dispensable *in vitro*, are critical for growth, fitness, and survival *in vivo*. Such factors, which have been identified in various bacterial species to differing extents, are increasingly recognized as promising targets for antimicrobials development (46, 47).

In recent years, transposon sequencing (Tn-seq) has been employed to identify candidate factors essential for UPEC survival specifically within the bladder of UTI mouse models (48, 49). The aim of this study was to take a different approach from these prior Tn-seq-based studies by focusing on proteins that are significantly expressed within the bladder. From among these proteins, we sought to identify those that are essential for UPEC survival and growth in the bladder, or that play critical roles in the establishment or persistence of infection. As a result, several candidate proteins meeting these criteria were identified, including some that had not been previously recognized by Tn-seq analyses (Fig. 1; Table 4). Among these, GuaB—a key enzyme involved in *de novo* purine biosynthesis—was found to be required for optimal colonization of the bladder in UTI mouse models (Fig. 2). Previous studies have also suggested that the *de novo* purine biosynthesis pathway contributes to UPEC virulence in the bladder, and GuaB has been identified as one of the fitness factors necessary for bladder colonization (17, 18, 49). Our findings further support the idea that GuaB is a promising target for the development of novel therapeutic strategies against UPEC infections. In addition, we further characterized the specific role of GuaB during bladder infection. GuaB contributes to GMP synthesis, and its deletion leads to impaired bacterial growth in urine (Fig. 3). Although the detailed mechanisms remain unclear, we found that GuaB expression is upregulated in response to urea, a major component of urine (Fig. 5 and 6), indicating that this protein is expressed at significant levels within the urinary environment. These results underscore the importance of GuaB as a critical factor for UPEC pathogenicity in the bladder, where urine accumulates and serves as the primary site of infection. It is known that GuaB expression can be influenced by quantitative changes in certain nutrient sources, such as amino acids or catabolite sugars (38, 50). In this study, we demonstrated that the expression of *guaB* is also affected by urea—despite not serving as a viable nutrient source for *E. coli*. These findings not only highlight the role of GuaB in UPEC pathogenicity but also contribute to a deeper understanding of its physiological functions and regulatory mechanisms of expression.

IMPDH is conserved across both eukaryotes and prokaryotes, and its dysfunction is thought to have a profound impact, particularly in rapidly dividing cells. Accordingly, in eukaryotes, IMPDH has been targeted for immunosuppressive therapy, cancer treatment, and antiviral chemotherapy, leading to the development of various IMPDH inhibitors such as mycophenolic acid, mizoribine, tiazofurin, merimepodib, 5-ethynyl-1-beta-d-ribofuranosylimidazole-4-carboxamide (EICAR), and ribavirin (51). Rapid cell division is also a characteristic of most bacterial cells, and in recent years, IMPDH has gained attention as a potential target for antibacterial chemotherapy. However, many bacteria can synthesize GMP even in the absence of IMPDH activity by salvaging purine compounds such as guanine, xanthine, xanthosine, and guanosine from the environment. This salvage pathway may confer resistance to IMPDH inhibitors. The efficacy of IMPDH inhibitors therefore depends on the availability of these purine sources within specific environmental niches, such as infection sites (52), as well as on the specificity of transporters and enzymes involved in their uptake and metabolism. Although structural differences exist between eukaryotic and bacterial IMPDHs, it remains necessary to identify highly selective compounds that specifically inhibit bacterial IMPDHs. To address these challenges, recent studies have identified promising inhibitory compounds against several bacterial species that are of clinical concern due to the spread of multidrug resistance, including *Mycobacterium tuberculosis*, *Pseudomonas aeruginosa*, and *Acinetobacter* spp. (52–55). In the present study, we demonstrate that GuaB activity is essential for UPEC growth in urine. These findings suggest that IMPDH inhibition could be an effective therapeutic strategy against UPEC-caused UTIs.

Mass spectrometry in this study identified not only GuaB but also proteins previously known to be critical for the establishment and persistence of UPEC infection, such as the outer membrane protein OmpA (32) and proteins involved in iron acquisition systems (33). These findings demonstrate the effectiveness of the study’s approach in identifying novel targets. However, because bacteria colonizing the bladder were collected together with the entire mouse bladder for protein analysis, a substantial proportion of the detected proteins originated from host tissue. This limited the sensitivity for detecting bacterial proteins. Future methodological refinements, such as the selective isolation of bacterial proteins, are expected to enhance sensitivity and enable more comprehensive identification of proteins that meet the defined criteria. Nonetheless, this study contributes to the development of a versatile research platform that could be adapted for the identification of novel therapeutic targets in other pathogens as well.

## Data Availability

The mass spectrometry proteomics data can be obtained from the ProteomeXchange Consortium with data set identifier JPST003552.

## SUPPLEMENTAL MATERIAL

Supplemental legend spectrum.00221-25-s0001.doc Legend for Table S1.

Table S1 spectrum.00221-25-s0002.pdf Protein sequence database used for proteomic peptide identification.

ASM does not own the copyrights to Supplemental Material that may be linked to, or accessed through, an article. The authors have granted ASM a non-exclusive, world-wide license to publish the Supplemental Material files. Please contact the corresponding author directly for reuse.
