# Supplementary material for: Role of GuaB, the inosine-5′-monophosphate dehydrogenase of uropathogenic Escherichia coli pathogenicity: a key factor for bladder infection
Source: Microbiol Spectr. 2025 Jun 17;13(8):e00221-25. doi: 10.1128/spectrum.00221-25 (PMC12323610; doi:10.1128/spectrum.00221-25)
Supplement: Supplemental legend — Legend for Table S1. [file spectrum.00221-25-s0001.doc]

**Table S1. Protein sequence database used for proteomic peptide identification**

This table contains the amino acid sequences of proteins used as a reference database for peptide identification in the proteomic analysis. The sequences represent predicted or annotated proteins derived from a genome of strain UPEC GU2018_CL13.
