# Supplementary material for: Role of GuaB, the inosine-5′-monophosphate dehydrogenase of uropathogenic Escherichia coli pathogenicity: a key factor for bladder infection
Source: Microbiol Spectr. 2025 Jun 17;13(8):e00221-25. doi: 10.1128/spectrum.00221-25 (PMC12323610; doi:10.1128/spectrum.00221-25)
Supplement: Table S1 — Protein sequence database used for proteomic peptide identification. [file spectrum.00221-25-s0002.pdf]

>LFGLNPFC_00001 Tyrosine recombinase XerC
MNNVIPLQNSPERVSLLPIAPGVDFATALSLRRMATSTGATPAYLLAPEVSALLFYMPDQ
RHHMLFATLWNTGMRIGEARMLTPESFDLDGVRPFVRILSEKVRARRGRPPKDEVRLVPL
TDISYVRQMESWMITTRPRRREPLWPVTDETMRNWLKQAVKRAEADGVHFSIPVTPHTFR
HSYIMHMLYHRQPRKVIQALAGHRDPRSMEVYTRVFALDMAATLAVPFTGDGRDAAEILR
TLPPLK
>LFGLNPFC_00002 RepFIB replication protein A
MDKSSGELVTLTPNNNNTVQPVALMRLGVFVPTLKSLKNSKKNTLSRTDATEELTRLSLA
RAEGFDKVEITGPRLDMDNDFKTWVGIIHSFARHNVIGDKVELPFVEFAKLCGIPSSQSS
RRLRERISPSLKRIAGTVISFSRTDEKHTREYITHLVQSAYYDTERDIVQLQADPRLFEL
YQFDRKVLLQLKAINALKRRESAQALYTFIESLPRDPAPISLARLRARLNLKSPVFSQNQ
TVRRAMEQLREIGYLDYTEIQRGRTKFFCIHYRRPRLKAPNDESKENQLPPSPAEKISPE
MAEKLALLEKLGITLDDLEKLFKSR
>LFGLNPFC_00003 hypothetical protein
MTGTAAQTPVMDVMEIPAEPQVFQSGTQSTGLELVDTYLWIFKHFIEGKELTRSLACQVY
IKPYSDRMDSVTFQSVAKRSKVFLNKLQEPTSEMINKRGNTWCWKS
>LFGLNPFC_00004 hypothetical protein
MNADTWTRLQAFRHALELTSCEASLTAGYDHLKDFPAGCSELASQTLTDYLTEDGSNLYS
CIVGMQWDNGPGRYGHVIADPARDYIDLTLDQFPGYHNRIVAEPVESGGQLAADLNREPA
ISTADGIVASPSDKVNYITEQKNRQPMVIITECYKLIRMTARFIPMCQSSQLFQHQVFPR
LFIISDVGSCSLFRNTLDRLATDWKVTLSILSL
>LFGLNPFC_00005 hypothetical protein
MTVRETEENIEQIFPELSNLIDIINKWLSHGNIDPELAHFIIYLLDYLTTTHKSI
>LFGLNPFC_00006 Antitoxin CcdA
MKQRITVTVDSDSYQLLKAYDVNISGLVSTTMQNEARRLRAERWKAENQEGMAEVARFIE
MNGSFADENRDW
>LFGLNPFC_00007 Toxin CcdB
MQFKVYTYKRESRYRLFVDVQSDIIDTPGRRMVIPLASARLLSDKVSRELYPVVHVGDES
WRMMTTDMASVPVSVIGEEVADLSHRENDIKNAINLMFWGI
>LFGLNPFC_00008 Tyrosine recombinase XerC
MSGSVIHSQSAARVPAVYSAGQSPQLPVVIDYPAALALRQMSMVHDELPKYLLAPEVSAL
LHYVPDLRRKMLLATLWNTGARINEALALTRGDFSLAPPYPFVQLATLKQRTEKAARTAG
RTPAGQQTHRLVPLSDSWYVSQLQTMVATLKIPLERRNKRTGRTEKARIWEVTDRTVRTW
IGEAVAAAATDGVTFSVPVTPHTFRHSYAMHMLYAGIPLKVLQSLMGHKSISSTEVYTKV
FALDVAARHRVQFSMPESDAVTMLKNRHA
>LFGLNPFC_00009 hypothetical protein
MVKKPSQQALNRAAVTVEQAEALAQRLADKPYGAPEKPEPEKQCRTTISLGESMLVTIED
LALRNKRNGKDPKNVSAIVRVALEQYLKTLT
>LFGLNPFC_00010 Signal recognition particle 54 kDa protein
MITVVGGNKGGSGKTTIASNLAIALANKGREVCLLNGDLQRTAAKHHAEREAAGLLPAIT
LVEKFDNLTQTLQALNEKFDDVIVDVAGRNSKEFITSGVVAHQIIAPLQCSQPDLDTLTE
LEQQIDAMRNLNPELKVYCLQSMATTNPVLRGNERKEFLEYLEEFPTIQVLDSVICFRKV
YRDCMSNGTGVVETNNTAAKAEIEHLMNEVFGPW
>LFGLNPFC_00011 Plasmid segregation protein ParM
MNVYCDDGSTTIKLAWNDNGKICKSLSQNSFRHGWKVDGLGIRQTFNYELDGKKYTYDEV
SNQSILTTHIEYQYTDVNLLAVHHALLNSGLAPQPVSLTVTLPISEFYTKECQKNELNIQ
RKIENLMRPIRLNKGDVFTIEHVDVMPESLPAVFSRLVVDKVGQFEKSLVVDIGGTTLDV
GVIVGQFDSVSAIHGNSGIGVSSVTKAAMSALRMASSDTSFLVADELIKRRNDPDFVRQV
INDETKTDLVLNTIEGAIASLGEQVVNELGDFHHVNRVYVVGGGAPLIYDSIKTAWHHLG
QKVVMMESPQTALVEAIAAFKEE
>LFGLNPFC_00012 hypothetical protein
MDDERKRKKYTLYLHPEKAADFQTLEAIESVPRSERGELFRNAFISGMALHQLDPRLPVL
LTAILSEEFSADQVVTLLSQTTGWKPSQADIRTVLTELGASQSVEKMPPSATDSVQEAMN
DVRLKMKKLF
>LFGLNPFC_00013 hypothetical protein
MKHRTWITEALRLHFEEHLPRVVAGRRLGVPKSTVCSMFVRFRRAGLSWPLPAGMSEQEL
DACLYGQFSTVPVVRPESTVISEAPVVKKRPRRPNFPYEFKIALVEQSLQPGACVAQIAR
ENGINDNLLFNWRHQYRKGGLLPSGKNMPALLPVTLTPEPDNKIPAPAQEPEQINTPSDS
LCCELVLPAGTLRLKGKLTPALLQTLIREIKGSSH
>LFGLNPFC_00014 hypothetical protein
MISLPAGSRIWLVAGITDMRNGFNGLASKVQNVLKDDPFSGHLFIFRGRRGDQIKVLWAD
SDGLCLFTKRLERGRFVWPVTRDGKVHLTPAQLSMLLEGINWKHPKRTERAGIRI
>LFGLNPFC_00015 IS66 family transposase ISCro1
MDTSLAHENARLRALLQTQQDTIRQMAEYNRLLSQRVAAYASEINRLKALVAKLQRMQFG

KSSEKLRAKTERQIQEAQERISALQEEMAETLGEQYDPVLPSALRQSSARKPLPASLPRE
TRVIRPEEECCPACGGELSSLGCDVSEQLELISSAFKVIETQRPKQACCRCDHIVQAPVP
SKPIARSYAGAGLLAHVVTGKYADHLPLYRQSEIYRRQGVELSRATLGRWTGAVAELLEP
LYDVLRQYVLMPGKVHADDIPVPVQEPGSGKTRTARLWVYVRDDRNAGSQMPPAVWFAYS
PDRKGIHPQNHLAGYSGVLQADAYGGYRALYESGRITEAACMAHARRKIHDVHARAPTYI
TTEALQRIGELYAIEAEVRGCSAEQRLAARKARAAPLMQSLYDWIQQQMKTLSRHSDTAK
AFAYLLKQWDALNVLQ
>LFGLNPFC_00016 IS66 family transposase ISCro1
MFAGSDSGGEHAAVLYSLIGTCRLNNVEPEKWLRYVIEHIQDWPANRVRDLLPWKVDLSS
Q
>LFGLNPFC_00017 hypothetical protein
MLADFTPSGIAQPGLFDEIQPRKNSEKLMKTLDERKRLVRTVLIFTESSDQLSRATDRVP
GLPASPGYVQ
>LFGLNPFC_00018 Protein UmuC
MFALADINSFYASCEKVFRPDLRNEPVIVLSNNDGCVIARSPEAKALGIRMGQPWFQVRQ
MRLEKKIHVFSSNYALYHSMSQRVMAVLESLSPAVEPYSIDEMFIDLRGINHCISPEFFG
HQLREQVKSWTGLTMGVGIAPTKTLAKSAQWATKQWPQFSGVVALTAENRNRILKLLGLQ
PVGEVWGVGRRLTEKLNALGINTALQLAQANTAFIRKNFSVILERTVRELNGESSISLEE
VPPAKQQIVCSRSFGERITDKDAMHQAVVQYAERAAEKLRGERQYCRQVTTFVRTSPFAV
KEPCYSNAAVEKLSLPHRTAGTLLPPHAES
>LFGLNPFC_00019 Protein UmuD
MSTVYHRPADPSGDDSYVRPLFADRCQAGFPSPATDYAEQELDLNSYCISRPAATFFLRT
SGESMNQAGVQNGDLLVVDRAEKPQHGDIVIAEIDGEFTVKRLLLRPRPALEPVSDSPEF
RTLYPENICIFGIVTHVIHRTRELR
>LFGLNPFC_00020 hypothetical protein
MIRIEILFDRQSTKNLKSGTLQALQNEIEQRLKPHYPEIWLRIDQGSAPSVSVTGARNDK
GKERIQSLLEEIWQDDSWLPAA
>LFGLNPFC_00021 hypothetical protein
MSVPQTKAELLLAIDKNFSKLISYLNTIPPEITSDKSMDGHAKGTEMSVRDLVSYLLGWN
ALVVKWIASDAKGLPVDFPETGYKWNQLGLLAQKFYSDYSELSYELLVAELQTVKNEIVN
LINDRTDDILYGRPWYTKWTMGRMISFNTSSPYANANGRLRKWAKNNNISLK
>LFGLNPFC_00022 ISL3 family transposase ISEc53
MDEKSLYAHILNLTAPWQVKSLTLDENAGSVTVTVGIAENTQLTCPTCRKSCSVHDHRHR
KWRHLDTCQFMTLVEADVPRVMCPEHGCQTLPVPWAGSGSRYTLLFESFVLSWLKISTVD
AVRKQLKLSWNAVDGIMTRAVKRGLSRIKKPLSVRHMNVDEVAFKKGHRYITVVSDRDGR
ALALTDDRGTESLASYLRSLTDSQLLAIKTLSMDMNAGYIRAARIHLPNAVEKIAFDRFH
VAKQLGEVVDKTRQNEHPHLPVESRRQAKGTRFLWQYSDKWMTESRQEKLMWLREQMQQT
SQCWTLKELAKNIWDRPWSTERRNDWLQWISRASECDVPMMKNAAKTIKKRLYGILNAMR
HRVSNGNAEALNSKIRLLRIKARGYRNRERFKLGVMFHYGKLNMAY
>LFGLNPFC_00023 hypothetical protein
MPNWCSNRMYFSGEPAKIAEIKRLASGAVTPFYRRATNEGIQLFLAGSAGLLQTTEDVRF
EPCPGLTAAGRGVLSPENITFTRWLKHLQDGVLLDEQNCLMLHELWLQSGTGQRRWEGLP
DDVRETITVHFTAKRGDWCDIWGNEDVSVWWNRLCDNVLSEKTMPFDLLTVLPTRLDIEV
NGFNGGVLNGVPSAYHWYTERYGVKWPCGYDLNISSQGENFIQVDFDTPWCQPESDVVAE
LSRRFGCTLEHWYAEQGCNFCGCVNATMFDRYLPFSCPLRYFRY
>LFGLNPFC_00024 IS21 family transposase IS21
MHELEVLLSRLKMEHLSYHVESLLEQAAKKELNYREFLCMALQQEWNGRHQRGMESRLKQ
ARLPWVKTLEQFDFTFQPGIDRKVVRELAGLAFVERSENVILLGPPGVGKTHLAIALGVK
AVDAGHRVLFMPLDRLIATLMKAKQENRLERQLQQLSYARVLILDEIGYLPMNREEASLF
FRLLNRRYEKASIILTSNKGFADWGEMFGDHVLATAILDRLLHHSTTLNIKGESYRLKEK
RKAGVLTKNTTPISDDEMVKSGQHQ
>LFGLNPFC_00025 hypothetical protein
MLSREDFYMIKQMRQQGAYIVDIATQIGCSERTVRRYLKYPEPPARKTRHKMVKLKPFMD
YIDMRLAENVWNSEVIFAEIKAMGYTGGRSMLRYYIQPKRKMRPSKRTVRFETQPGYQLQ
HDWGEVEVEVAGQRCKVNFAVNTLGFSRRFHVFAAPKQDAEHTYESLVRAFRYFGGCVKT
VLVDNQKAAVLKNNNGKVVFNSGFLLLADHYNFLPRACRPRRARTKGKVERMVKYLKENF
FVRYRRFDSFTHVNQQLEQWIADVADKRELRQFKETPEQRFALEQEHLQPLPDTDFDTSY
FDIRHVSRDSYIEVGGNRYSVPEALCGQPVSIRISLDDELRIYSNEKLVASHRLCSASSG
WQTVPEHHAPLWQQVSQVEHRPLSAYEELL
>LFGLNPFC_00026 hypothetical protein
MLGGADNPNGELPSYITGADSWQLYERGELVDVLWGELEWSSPTDDDELPEVTGPAWIVD
NVAHYGG
>LFGLNPFC_00027 DNA adenine methyltransferase YhdJ
MSRFVLGNCIDVMARIPDNAIDFILTDPPYLVGFRDRSGRTIAGDVNDDWLQPASNEMYR

VLKKDALMVSFYGWNRVDRFMAAWKRAGFSVAGHLVFTKNYTSKSAYVAYRHECAYILAK
GSPARPRNPLPDVLGWKYSGNRHHPTEKPVTSLQPLIESFTHPNAIVLDPFAGSGSTCVA
ALQSGRRYIGIELLEQYHRAGQQRLAAVQRAMQQGAANDNWFEPEAA
>LFGLNPFC_00028 hypothetical protein
MNYAGHEKLRADVAEVANTMCDLRARLNDMEHRCRFDSDVLVERLTRQTLYRANRLFMEA
YTEILELDSCFKD
>LFGLNPFC_00029 hypothetical protein
MYGTCETLCRKLAAKYSGDTPLMLVIWSPEEIQALSDGMDIALTDHEIRTVLARLEDIPE
DQRTESGISSGVAMEIINNVSENRQVTVPAELLASLIQTAEQALWKREWAAWDHGLAVPE
CVTRRQAVVNQVRILLKNNTHEND
>LFGLNPFC_00030 hypothetical protein
MNEMLNALIYRHASNLLLAQGWPEETDVDQRNPKYPGWISIYVRLDAPRLATLLIKRHGG
VLPPLLASAIQKLTGTGAELVLSGSQWQSLPVLPADGTQVSFPYAGEWLAEDEIRAVLGA
VRDAVCSVSYQVAEDARRIRAALTTTGQTLLTRQTRRFRLVVKESDHPCWLDEDDENLPV
VLDAILNRGARFSSVEMYLVSECVEHILSSGLACDMLRIPDEPPRRWFDRGVLREVVREA
RNEIRSMADALAKIRK
>LFGLNPFC_00031 Antirestriction protein KlcA
MQYAKPVTLNVEECDRLSFLPYLFGLDFLYAEASVYTLAKKMMPEYEGGFWHFIRLPDGG
GYMMPDGDRFHLVNGENWFDRTVSADAAGIILTSLVINRQLWLYHDSGDAGLTHLYRMRD
AQLWSHIEFHPECNAIYAALD
>LFGLNPFC_00032 hypothetical protein
MYCTVKEIIRDVLDTDVPDSECVFAVVLTRGDVRHIAQDWSLTDDELETVMQRLDDAFVY
GACDRVVSDIVNELMEEKRASRHVTVPAVMLEKVMALAGSEMKRLYAVGSENGGDGDAFV
REEREAMDVVLQALDGEHMS
>LFGLNPFC_00033 hypothetical protein
MNISTETREILRNYRAVINARRREMGQKPLTTAQIVDEICDFVANQQAVFLGGHYILQGS
RNR
>LFGLNPFC_00034 hypothetical protein
MTISIVSPSAAAVKPRRHPRFRREDIPAPEIDPVLKAFGRHIARSFHRGRGVHIPAMKNT
AFGQVLRTLELKRAFN
>LFGLNPFC_00035 hypothetical protein
MTLAAITMTAPEAASPVQMYRATYSPDDNKLRLYAASRLDPETYKKVHDAGFRWAPKQAL
FVAPAWTPGREDVLLSLAGEIEDEDSTLAERQEARAERFTGYSGKRASESAQALDEVERL
AAMIPPGQPILVGHHSERRARRDAQRIENGMKRAVMLFERAEYWEERARSALLHAKYKER
PDVRWRRIKKIEADLRKAEKTIAQSQKYLTMWRAESLDLNMAKLISSHDHISACFPLDTY
PRPAEKSQYEGSRSLWSALDDDIITTEQAREIAIRCHERQIQHQQRWVNHYQNRLIYERA
MLDESGGVVTRTQDFEPGGQVFSRGEWLTIIRVNKSNGAVSSVTTPNYSFLGYSGTMKVT
PDRITDYKAPSAEEAAVASQAAKRPPVVNYPGEGFREMTKAQWAALPRDCKAVRSVEEAE
DHGAYRYRRTMDNNFRLVNVYITDMKITEIPQK
>LFGLNPFC_00036 Ribosomal RNA small subunit methyltransferase A
MHSQLRERIRLMRARLDNAAPVAEIRAESQLFVSPAPICDRLVTLAEISNRDHILEPSAG
TGAILRAIRDTAPEAMCDAVEINSGLVRYLRENFNGVRVQCGDFMEWQSVQYYSRIIMNP
PFSHGQDIRHILRAFSLLRPGGVLVAVCLNGPRQQEKLLPFSDVREELPRGTFAYTDVPT
MIIRLRA
>LFGLNPFC_00037 hypothetical protein
MTQGWREDKCPSVRAGKKPRVGGAERGRAHAPPLSLRGRSDGGFCLFRPVICLLLMMLLF
LLLLLLLLLLLFFFFLLL
>LFGLNPFC_00038 Plasmid-derived single-stranded DNA-binding protein
MAVRGINKVILVGRLGKDPEVRYIPNGGAVANLQVATSETWRDKQTGEMKEQTEWHRVVL
FGKLAEVAGEYLRKGAQVYIEGQLRTRSWEDNGITRYVTEILVKTTGTMQMLGSAPQQNA
QVQPQPQQNGQPQSADATKKGSAKTKGRGRKAAQPEPQPQPPEGEDYWFSDDIPF
>LFGLNPFC_00039 hypothetical protein
MSEYFRILQGLPDGSFTREQAEAVAAQYRNVFIEDDQGTHFRLVVRNNSGLVWRTWNFED
GAGYWMNHVIRNFGILK
>LFGLNPFC_00040 Nucleoid occlusion protein
MPVTKCEPETTRKASRKYAKTQETVLSALLAQTEEVSVPLASLIKSPLNVRTVPYSAESV
SELAESIKGVGLLQNLVVHTLPGDRYGVAAGGRRLAALNMLAERGIIPADWPVRVKVIPQ
ELATAASMTENGHRRDMHPAEQIAGFRAMAQEGKTPAQIGDLLGYSPRHVQRMLKLADLA
PVILDALAEDRITTEHCQALALENDTARQVQVFEAACQSGWGGKPDVRVIRNLITESEVA
VAGNSKFRFVGADAFSPDELRTDLFSDDEGGYVDCVALDAALLEKLQAVAEFLREAEGWE
WCAGRMEPVGECREDAGTYRCLPEPEAVLTEAEEERLNELMARYDALENQCEESDLLEAE
MKLMRCMAKVRAWTPEMRAGGGVVVSWRYGNVCVQRGVQLRSEDDAADDADRTEQVQEKA
SVEEISLPLLTKMSSERTLAVQAALMQQPDKSLTLLAWTLCLNVFGSGAYSKPAQISLEC
KHYSLTSDAPSGKEGAAFMALMAEKARLAALLPEGWSRDMTTFLSLSQEVLLSLLSFCTA

CSLNGVQTREYGHTSRSPLDTLESAIGFHMRDWWQPTKANFFGHLKKPQIIAALNEAGLS
GAARDAEKMKKGDAAEHAEHHMKDNRWVPGWMCAPRPQTDATERTDNLADAA
>LFGLNPFC_00041 Protein PsiB
MKTELTLNVLHTMNAQEYEDIRAAGSDERRELTHAVMRELDAPDNWTMNGEYGSEFGGFF
PVQVRFTPAHERFHLALCSPGDVSQVWVLVLVNAGGEPFAVVQVQRRFAPEAVSHSLALA
ASLDTQGYSVNDIIHILMAEGGQV
>LFGLNPFC_00042 hypothetical protein
MSARSQALVPLSTEQQAAWRAVAETEKRRHQGNTLAEYPYAGAFFRCLNGSRRISLSDLR
FFMPSLTAEELHGNRLQWLYAIDVLIETQGEVCLLPLPGDAAERLFPSVRFCVRERSRHK
SALVMQKYSRQQAREAEQKARAYQALVAQAEIELAFHSPETVGSWHARWSDRVAEHDLET
LFWQWGERFPSLAGMERWQWQDMPFWQVIAEASLAAKEAGHAVREMERWMVPNKLREVA
>LFGLNPFC_00043 Protein FlmC
MLRQHQDSLLPRFAQGEEGHETTTQLSCLVCVDRVSHTVDIHLSDTKIAVRDSLQRRIQG
GGGFHGLRIR
>LFGLNPFC_00044 hypothetical protein
MSTRNIHVNTASYTILVAGREKNTGEEWDVLEFSSLTELKKYRKSHPEKMAFSYSYALSR
GVDTQFRHINIAEADHFKQFLRQIKRAGLDIRAIC
>LFGLNPFC_00045 hypothetical protein
MRLASRFGRYNSIRRERPLTDDELMQFVPSVFSGDKHESRSERYTYIPTINIINKLRDEG
FQPFFACQSRVRDLGRREYSKHMLRLRREGNINGQEVPEIILLNSHDGSSSYQMIPGIFR
FVCTNGLVCGNNFGEIRVPHKGDIVGQVIEGAYEVLGVFDKVTDNMEAMKEIHLNSDEQH
LFGRAALMVRYEDENKTPVTPEQIITPRRREDKQNDLWTTCQRVQENMIKGGLSGRSASG
KNTRTRAITGIDGDIRINKALWVIAEQFRKWKS
>LFGLNPFC_00046 hypothetical protein
MKKWMLAICLMFINEICQATDCFDLAGRDYKIDPDLLRAISWKESRYRVNAIGINPVTGY
GSGLMQVDSQHFNELDRYGIKPEHLTTDPCMNIYTGAYYLAIAFKKWGVSWEAVGAYNAG
FRKTERQNQRRLAYASEVYRIYTGIKSSKGIRIPVTKKSLPEINSVQNN
>LFGLNPFC_00047 Relaxosome protein TraM
MAKVQAYVSDEIVYKINKIVERRRAEGAKSTDVSFSSISTMLLELGLRVHEAQMERKESA
FNQAEFNKVLLECAVKTQSTVAKILGIESLSPHVSGNPKFEYANMVEDIRDKVSSEMERF
FPENDEE
>LFGLNPFC_00048 Protein TraJ
MCALDRRERPLNSQSVNKYILNVQNIYRNSPVPVCVRNKNRKILYANGAFIELFSREDKP
LSGESYIRLQVEIFLSSLELECQALGHGSAFCRRFNFHGEIYQIRMENVSFYNDESVVLW
QINPFPDYPFFALNQSGSNTNTSDKLTIWNDLSPGTLVVFSFYMLGVGHATIARELGITD
RASEDRIKPVKRKIKEFFEHFDLFRVSCIYKGEIDSLLSIIREFYGVK
>LFGLNPFC_00049 Pilin
MNTVLSVQGASAPVEKKSFFSKFTCLNMLRLVRAVIPVAVLMMLFPELAMAAGKGDLMAK
GQETVKATFGKNSSIVKWIVLAEVLVGAVMYMMTKNIKFLVGFAILSVFIAVGMSVAGF
>LFGLNPFC_00050 hypothetical protein
MSGDENKLKKYRFPETLTNQSRWFGLPLDELIPAAICIGWGIITSKYLFGIGAAVLVYFG
IKKLKKGRGSSWLRDLIYWYMPTALLRGIFHNVPDSCFRQWIK
>LFGLNPFC_00051 hypothetical protein
MEHGARLSTSRVMAIAFIFMSVLIVLSLSVNLIQGVNNYRLQNEQRTAVTPMAFNAPFAV
SQNSADASYLQQMALSFIALRLNVSSETVDASHQALLQYIRPGAQNQMKVILAEEAKRIK
NDNVNSAFFQTSVRVWPQYGRVEIRGVLKTWIGDSKPFTDIKHYILILKRENGVTWLDNF
GETDDEKK
>LFGLNPFC_00052 hypothetical protein
MRKNNTAIIFGSLFFSCSVMAANGTLAPTVVPMVNGGQASIAISNTSPNLFTVPGDRIIA
VNSLDGALTNNEQTASGGVVVATVNKKPFTFILETERGLNLSIQAVPREGAGRTIQLVSD
LRGTGEEAGAWETSTPYESLLVTISQAVRGGKLPAGWYQVPVTKETLQAPAGLSSVADAV
WTGNHLKMVRFAVENKTLSALNIRESDFWQPGTRAVMFSQPASQLLAGARMDVYVIRDGE
GN
>LFGLNPFC_00053 hypothetical protein
MASINTIVKRKQYLWLGIVVVGAASAIGGVLYLSDVDMSGNGEAVAEQEPVPDMTGVVDT
TFDDKVRQHATTEMQVTAAQMQKQYEEIRRELDVLNKQRGDDQRRIEKLGQDNAALAEQV
KALGANPVTATGEPVPQMPASPPGPEGEPQPGNTPVSFPPQGSVAVPPPTAFYPGNGVTP
PPQVTYQSVPVPNRIQRKVFTRNEGKQGTSLPYIPSGSFAKAMLIEGADANASVTGNEST
VPMQLRITGLVEMPNSKTYDATGCFVGLEAWGDVSSERAIVRTRNISCLKDGKTIDMPIK
GHVSFRGKNGIKGEVVMRNGKILGWAWGAGFVDGIGQGMERASQPAVGLGATAAYGAGDV
LKMGIGGGASKAAQTLSDYYIKRAEQYHPVIPIGAGNEVTVVFQDGFQLKTVEEMALERT
QNRAEEDNPESPVPVPPSAESHLNGFNTDQMLKQLGNLNPQQFMSGSQGGGNDGK
>LFGLNPFC_00054 hypothetical protein
MANNMSSRQACHAARYVVARVLRGLFWCLKYTVILPLATMALMALFVLWKDNTTPGKLLV

KEINFVRQTAPAGQFPVSECWFSSSDSSGRSEIQDICHYRAADAADYVRETDRSLMQLVT
ALWATLALMYVSLAAITGKYPVRPGKMKCIRVVTADEHLKEVYTEDASLPGKIRKCPVYL
PDDRTNRNNGDKNEHA
>LFGLNPFC_00055 hypothetical protein
MNMRNINAITALSVPGKTMSDDFMHAVLSNCTTRIVLPAPKEFSSESLPHNFNMAAVGVM
KEGMSMRLFSSPGHIVVAGQFGGGKSASVKELIDRIIQKYKEPFDE
>LFGLNPFC_00056 Protein TraV
MKQISLFIPLLGTLLLSGCAGTSTEFECNATTSDTCMTMEQANEKAKKLEQPADAKPAAA
SLPRLAEGNFRTTTVRAVTAASPSGSGPVVTALPEQKLLAPRPLFTAAREVKTIIPVSSV
ASVTPPRPLRTGEQTAALWIAPYIDNQDVYHQPSSVFFVIKPSAWGKPRIN
>LFGLNPFC_00057 Protein TraR
MSDEADEAYSVTEQLTMTGINRIRQKINVHGIPVYLCEACGNPIPEARRKIFPGVTLCVE
CQAYQEKQRKHYA
>LFGLNPFC_00058 hypothetical protein
MHKSEAEKIDSILDEHQQIYRRHNQINNILAYCACIAWIPCIIAAFGVDGIYLKILFAVL
ACCGAVCFFIFFFTLLPESLMVLSRQSLLQLMRLTEDVPDARQELLNRLLSGKKLTGRDE
QDIRRLWQEKVDAMQESATRQREQDTIRKFTEGNKSE
>LFGLNPFC_00059 hypothetical protein
MPEVVFHVSYFAHFLLPVSPLPFRFFLYTLMQALLPYQDLNIAITPTNIKARTDSKYIDF
ISFSFEAVSTHCPPPFSVSITLFKGLFVLTSIHSPVFPNPLPPCDSIVRRTCTHTSVFGM
GVIFQLPPPLIFFISTRKIKVCILYYLRLYINH
>LFGLNPFC_00060 Protein TraC
MSNNPLEAVTQAVNSLVTALKLPDESAKANEVLGEMSFPQFSRLLPYRDYNQESGLFMND
TTMGFMLEAIPINGANKSIVEALDHMLRTKLPRGIPLCIHLMSSQLVGDRIEYGLREFSW
SGEQAERFNAITRAYYMKAAATQFPLPEGMNLPLTLRHYRVFISYCSPSKKKSRADILEM
ENLVKIIRASLQGASIATQTVDAQAFIDIVGEMINHNPDSLYPKRRQLDPYSDLNYQCVE
DSFDLNVRADYLTLGLRENGRNSTARILNFHLARNPEIAFLWNMADNYSNLLNPELSISC
PFILTLTLVVEDQVKTHSEANLKYMDLEKKSKTSYAKWFPSVEKEAKEWGELRQRLGSGQ
SSVVSYFLNITAFCKDNNETALEVEQDILNSFRKNGFELISPRFNHMRNFLTCLPFMAGK
GLFKQLKEAGVVQRAESFNVANLMPLVADNPLTPAGLLAPTYRNQLAFIDIFFRGMNNTN
YNMAVCGTSGAGKTGLIQPLIRSVLDSGGFAVVFDMGDGYKSLCENMGGVYLDGETLRFN
PFANITDIDQSAERVRDQLSVMASPNGNLDEVHEGLLLQAVRASWLAKENRARIDDVVDF
LKNASDSEQYAGSPTIRSRLDEMIVLLDQYTANGTYGRYFNSDEPSLRDDARMVVLELGG
LEDRPSLLVAVMFSLIIYIENRMYRTPRTLKKLNVIDEGWRLLDFKNRKVGEFIQKGYRT
CRRHTGAYITITQNIVDFDSDKASSAARAAWGNSSYKIILKQSAKEFAKYNQLFPDQFQP
LQRDMIGKFGAAKDQWFSSFLLQVENHSSWHRLFVDPLSRAMYSSDGPDFEFVQQKRREG
MSIHEAVWQLAWKKSGPEMASLEAWLEEHEKYRSVA
>LFGLNPFC_00061 hypothetical protein
MTTTQKTTDVTAPRRSHWWWTVPGCLAMVLLNAAISYGIVRLNAPVTAAFNMKQTVDAFF
DSASQKQLSEAQSKALSARFNTALEASLQAWQQKHHAVILVSPAVVQGAPDITREIQQDI
ARRMRAEP
>LFGLNPFC_00062 hypothetical protein
MRCRGLIALLIWGQSVVAADLGTWGDLWPVKEPDMLTVIMQRLTALEQSGEMGRKMDAFK
ERVIRNSLRPPAVPGIGRTEKYSSRLFDPSVRLAADIRDNEGRVFARQGEVMNPLQYVPF
NQALYFINGDDPAQVAWMKRQTPPTLESKIILVQGSIPEMQKSLDSRVYFDQNGVLCQRL
GIDQVPARVSAVPGDRFLKVEFIPAEEGRK
>LFGLNPFC_00063 hypothetical protein
MKRSLWLLMLFLLAGHVPAASADSACEGRFVNPITDICWSCIFPLSLGSIKVSQGKVPDT
ANPSMPIQICPAPPPLFRRIGLAIGYWEPMALTDVTRSPGCMVNLGFSLPAFGKTAQGTA
KKDEKQVNGAFYHVHWYKYPLTYWLNIITSLGCLEGGDLDIAYLSEIDPTWTDSSLTTIL
NPEAVIFANPIAQGACAADAIASAFNMPLDVLFWCAGSQGSMYPFNGWVSNESSPLQSSL
LVSERMAFKLHRQGMIMETIGKNNAVCNEYPSPILPKERWRYQMVNMYPDSGQCHPFGRS
VMRWETGKNPPNTKKNFGYLMWRKRNCVFL
>LFGLNPFC_00064 hypothetical protein
MATMDSKYIQHLRKISAVLLRLISRTLKLFPFGLVMVPVILFYFAPDSFLAEIIHEWKKA
DISEQVTLVRLYLKVTFTCAFMAVFLNYLFSDEFMPVNSRK
>LFGLNPFC_00065 hypothetical protein
MNRKIFALFALTAGMHLAVHASENVNTPENRQFLKQQENLSRQLREKPDHQLKAWAEKQV
LENPLQRSDNHFLDELVRKQQASQDGKPRQGALYFVSFSIPEEGLKRMLGETRHYGIPAT
LRGMVNNDLKTTAEAVLSLVKDGATDGVQIDPTLFSQYGIRSVPALVVFCSQGYDIIRGN
LRVGQALEKVAATGDCRQVAHDLLAGKGDSGK
>LFGLNPFC_00066 Mating pair stabilization protein TraN
MKRILPLILALVAGMAQADSNSDYRAGSDFARQIQGQGTGSIQGFKPQESIPGYNANPDE
TKYYGGVTAGGDGGLKNDGTTEWATGETGKTITESFMNKPKDILSPDAPFIQTGRDVVNR

ADSIVGNTGQQCSAQQINRSEFTNYTCERDTMVEEYCTRTASITGDWKYTDEYREVTIPH
SQFRFSMNGLKLVFSVTAPVTGTVESASLSVYAAFFFLNSRYTFMNTTFNVGLANGQSDT
YPLSGATGLQVTQGQVLTGSGCTANGNCLPHGNGDRKVYESLVSGASTFTLKLRMKVRDK
EWVPRVEWVESCPFNKADGVLTGTECSEPGGTKTGVMEGKPWNITQACWAYRDKYVTQSA
DNGTCQKYVDNPACTLASRQCAFYSDEGTCLHEYATYSCESKTSGKVMVCGGDVFCLDGE
CDKAQSGQSNDFAEAVSQLAALAAAGKDVAALNGVDVRAFTGQAKFCKKAAAGYSNCCKD
SGWGQDIGLAKCSSDEKALAKAKSNKLTVSVGEFCSKKVLGVCLQKKRSYCQFDSKLAQI
VQQQGRNGQLRIGFGSAKHPDCRGITVDELQKIQFDRLDFTNFYEDLMNNQKIPDSGVLT
QKVKEQIADQLKQAGQ
>LFGLNPFC_00067 hypothetical protein
MKVIFTSNRFIDFLIRLLITAIVISPVIIWSWDTVKETTADGILAAAFVILYSGVLLFIL
YFCFSALTDLQKPDERKSDERNEDE
>LFGLNPFC_00068 hypothetical protein
MNKALLPLLLCCFIFPASGKDAGWQWYNEKINPKEKENKPVPAAPRQEPDIMQKLTALQT
ATKRALYEAILYPGVDNFVKYFRLQNYWTQQAGLFTMSAKKAMLAHPELDYNLQYSHYNG
TVRNQLAADQAQQRQAIAKLAEHYGIMFFYRGQDPIDGQLAQVINGFRDTYGLSVIPVSV
DGVINPLLPDSRTDQGQAQRLGVKYFPAMMLVDPKQGSVRPLSYGFISQDDLAKQFLNVS
EDFKPNF
>LFGLNPFC_00069 hypothetical protein
MHTGDLCFFTGASLFIVGVVGYFFTGPNNKGVYLLSSFIFFVVNVISVCMLPDNFLFLCG
AVMNQCAVPVVSMAVSTIPVITKFDSFRRMFVCIMLSAIWSGVMWFFIISLVK
>LFGLNPFC_00070 hypothetical protein
MKKLSFKEKLEIVRNIIRESLSGNAAFALLVYSLLHSLPIKTFADLFVTILLFIAIIAVV
IWLFLIIYVYFCELFRSHWIAVWFILWSVVINLTIGFGIYDKLAPVISLMK
>LFGLNPFC_00071 Protein TraQ
MISKRRFSLPRLDITGMWVFSLGVWFHIVARLVYSKPWMAFFLAELIAAILVLFGAYQVL
DAWIARVSLEEREALEARQQAMMEGQQEGGHVSH
>LFGLNPFC_00072 hypothetical protein
MSLTKLLPVILLMMATGVQASTRDEIERLWNPQGMAVQPAQPAADTSARTEKPAPRWFRL
SNGRQVNLADWKVVLFMQGHCPYCHQFDPVLKQLAQQYGFSVFPYTLDGQGDTAFPEALP
VPPDVMQTFFPNIPVATPTTFLVNVNTLEALPLLQGATDAASFMARMDTVLQMYGEEKGA
K
>LFGLNPFC_00073 hypothetical protein
MRNKQVVLLIAGISGIATGIIVSLNIPFIRQGLFYPASPVEIVVSLCLTFSVSVVFFVGA
IVGWISVSEIYYSRMNGLNESSEISEETYNERKKK
>LFGLNPFC_00074 hypothetical protein
MKEKRNDAELKNRKTKRDYDYERRVSDIYFDLFFVFVAAGTFLWVIMHSIFDACIDSWKA
DPELNNFRYMWNILMYVIPYTLWAFAGGFLIVYVRNPLNELINGGIRIFRLKRRMRRENK
LREGGNNASH
>LFGLNPFC_00075 hypothetical protein
MLRIKPLLVLCAALLTVTPAASADVNSDMNQFFNKLGFASNTTQPGVWQGQAAGYAYGGS
LYARTQVKNIQLISMTLPDINAGCGGIDAYLGSFSFINGDQLQRFVKQIMSNAAGYFFDL
ALQATVPEIKTAKDFLQKMASDINSMNLSSCQVAQGIVGGLFPQTQVSQQKVCQDIAGES
NIFADWAASRQGCTVGGQSSSVRDKASDKDKERVLKNINIIWDSLSKNRMLDGNKELKEF
IMTLTGTLIFGEDSEITPLPARTTDRDILRAIMEGGTAKVYHCNDSDKCLKVVADANVTI
ARDKALKSQITKLLTSIQNKAVSDTPLDDREKGFISSTTVPVFKYLIDPQMLGVSSSVVY
QLTDYIGYDIMLQYIQELLQQARAMIATGNYPQTVLDSVLENLNQAQQQIAVFQGQVKVQ
QDALLVVDRQMSYMRQQLSARMLSRYQNNYHFGGSTL
>LFGLNPFC_00076 hypothetical protein
MNEVYVIAGGEWLSNNLNAIAAFMGTRTWDSIEKIALTLSVLAVAVMWVQRHNVMDLLGW
VAVFVLISLLVNVRTSVQIIDNSDLVKVHRVDNVPVGLAMPLSLTTRIGHAMVASYEMIF
TQPDSVTYSKTGMLFGAELVSKSTDFLSRNPEIANLFQDYVQNCVMGDIYLNHKYTLEEL
MASADPYTLIFSRPSPLRGVYDSNNNFVTCKDASVSLKDKLNLDTQSGGKTWHYYAQQLF
GGRPDPNLLFSTLIGDSYSYFYGSSKSASQIIRQNVTINALKEGITSYAARNGDSASLVN
LATTSSMEKQRLAHVSIGHVAMRTLPMTQTILTGIAIGIFPLLVLAAVFNKLTLSVLKGY
VFALMWLQSWPMLYAILNSAMTFYAKQNGAPVVLSEISQIQLKYSDLASTAGYLSMMIPP
LSWMMVRGLGAGFSSVYSHFASSAISPTASAAGSVVDGNYSYGNMQTENVNGFSWSTNST
TSFGQMMYQTGSGATATQTRDGNMVMDASGAMSRLPVGINATRQIAAAQQEMAREASNRA
ESALHGFSSSIASAWNSLSQFGSNRGSSDSVTGGADSTMSAQDSMMASRMRSAVESYAKA
HNISNEQATQELASRSTRTSAGMYGDAHAEWGVKPKILGVGGGLGVRGGGRAGIDWEDND
AHTASSNTQSSHNARHDIDARATQDFKEASDYFTSRKVSESGSHTDNNADSRVDQLSAAL
NSAKQSYDQYTTNMTRSHEYAEMASRTESMSGQMSEDLSQQFAQYVMKHAPQDAEAILTN
TSSPEIAERRRAMAWSFVQEQVQPGVDNAWRESRGDIGKGMESVPSGGGSQDIIADHQGH
QAIIEQRTQDSNIRNDVKHQVDNMVTEYKGNIGDTQNSVRGEENIVRGQYSELQNHHKTE

ALSQNNKYNEEKSAQERMPGADSPQELMKRAKEYQDKYKQ
>LFGLNPFC_00077 hypothetical protein
MRCLTHITLVTVIQFIACYLAGWGNAETIFMLFFIVLWQGLFIWLFSQIRKKRNVSDEFK
FSKGVWYITIPVSSLLSPLLSLMVFIIGTLYELRRVSGCVSVREWMQSQVNEQTNEDLHL
DFDNMDFHRTNPATGLPMSGIGVDIGGNSYGYSSKRYDE
>LFGLNPFC_00078 Lipoprotein YlpA
MKMKKLMMVALVSSTLALSGCGAMSTAIKKRNLEVKTQMSETIWLEPASERTVFLQIKNT
SDKDMSGLQGKIADAVKAKGYQVVTSPDKAYYWIQANVLKADKMDLRESQGWLNRGYEGA
AVGAALGAGITGYNSNSAGATLGVGLAAGLVGMAADAMVEDVNYTMITDVQIAERTKATV
TTDNVAALRQGTSGAKIQTSTETGNQHKYQTRVVSNANKVNLKFEEAKPVLEDQLAKSIA
NIL
>LFGLNPFC_00079 hypothetical protein
MTSDNIAEYVGENNQFTEKPVTEYPPVIQDNTVTTKDYLYSNNIDTDLLVVIGGLIVILF
LMMLIMYFINSLRHKELTEILEKISFPEKSGSNLLKPEAVSCNLLSLCVLAPVLCTPRKL
AEVSREIIVASAQEIAWTPGSSRIDGHQGENITWSLSSQSPADKYDLINKTSRVSPDSGR
FYEPDTVLDHRERVRLFIQIQFPHGSDWSEVLRLTDIVTEKVSSGRAAGGYHHRHTGYTW
SVSVN
>LFGLNPFC_00080 Coupling protein TraD
MSFNAKDMTQGGQIASMRIRMFSQIANIMLYCLFIFFWILVGLILWVKISWQTFVNGCIY
WWCTTLEGMRDLIKSQPVYEIQYYGKTFRMNAAQVLHDKYMIWCGEQLWSAFVLATVVAL
VICLITFFVVSWILGRQGKQQSENEVTGGRQLTDNPKDVARMLKKDGKDSDIRIGDLPII
RDSEIQNFCLHGTVGAGKSEVIRRLANYARQRGDMVVIYDRSGEFVKSYYDPSIDKILNP
LDARCAAWDLWKECLTQPDFDNTANTLIPMGTKEDPFWQGSGRTIFAEAAYLMRNDPNRS
YSKLVDTLLSIKIEKLRTFLRNSPAANLVEEKIEKTAISIRAVLTNYVKAIRYLQGIEHN
GDPFTIRDWMRGVREDQKNGWLFISSNADTHASLKPVISMWLSIAIRGLLAMGENRNRRV
WFFCDELPTLHKLPDLVEILPEARKFGGCYVFGIQSYAQLEDIYGEKAAATLFDVMNTRA
FFRSPSHKIAEFAAGEIGEKEHLKASEQYSYGADPVRDGVSTGKDMERQTLVSYSDIQSL
PDLTCYVTLPGPYPAVKLSLKYQARPKVAPEFIPRDINPEMENRLSAVLAAREAEGRQMA
SLFEPEVASGEDVTQAEQPQQPVSSVINDKKSDAGVSVPAGGIEQELKMKPEEEMEQQLP
PGISESGEVVDMAAYEAWQQENHPDIQQHMQRREEVNINVHRERGEDVEPGDDF
>LFGLNPFC_00081 Multifunctional conjugation protein TraI
MLSFSVVKSAGSAGNYYTDKDNYYVLGSMGERWAGQGAEQLGLQGSVDKDVFTRLLEGRL
PDGADLSRMQDGSNKHRPGYDLTFSAPKSVSMMAMLGGDKRLIDAHNQAVDFAVRQVEAL
ASTRVMTDGQSETVLTGNLVMALFNHDTSRDQDPQLHTHVVVANVTQHNGEWKTLSSDKV
GKTGFSENVLANRIAFGKIYQSELRQRVEALGYETEVVGKHGMWEMPGVPVEAFSSRSQA
IREAVGEDASLKSRDVAALDTRKSKQHVDPEIRMAEWMQTLKETGFDIRAYRDAADQRAE
IRTQAPGPASQDGPDVQQAVTQAIAGLSERKVQFTYTDVLARTVGILPPENGVIERARAG
IDEAISREQLIPLDREKGLFTSGIHVLDELSVRALSRDIMKQNRVTVHPEKSVPRTAGYS
DAVSVLAQDRPSLAIVSGQGGAAGQRERVAELVMMAREQGREVQIIAADRRSQMNLKQDE
RLSGELITGRRQLQEGMVFTPGSTVIVDQGEKLSLKETLTLLDGAARHNVQVLITDSGQR
TGTGSALMAMKDAGVNTYRWQGGEQRPATIISEPDRNVRYDRLAGDFAASVKAGEESVAQ
VSGVREQAILTQAIRSELKTQGVLGHPEVTMTALSPVWLDSRSRYLRDMYRPGMVMEQWN
PETRSHDRYVIDRVTAQSHSLTLRDAQGETQVVRISSLDSSWSLFRPEKMPVADGERLRV
TGKIPGLRVSGGDRLQVASVSEDAMTVVVPGRAEPATLPVADSPFTALKLENGWVETPGH
SVSDSATVFASVTQMAMDNATLNGLARSGRDVRLYSSLDETRTAEKLARHPSFTVVSEQI
KARAGETSLETAISLQKTGLHTPAQQAIHLALPVLESKNLAFSMVDLLTEAKSFAAEGTG
FTELGGEINAQIKRGDLLYVDVAKGYGTGLLVSRASYEAEKSILRHILEGKEAVTPLMER
VPGELMETLTSGQRAATRMILETSDRFTVVQGYAGVGKTTQFRAVMSAVNMLPASERPRV
VGLGPTHRAVGEMRSAGVDAQTLASFLHDTQLQQRSGETPDFSNTLFLLDESSMVGNTDM
ARAYALIAAGGGRAVASGDTDQLQAIAPGQPFRLQQTRSAADVVIMKEIVRQTPELREAV
YSLINRDVERALSGLESVKPSQVPRQEGAWAPEHSVTEFSHSQEAKLAEAQQKAMLKGET
FPDVPMTLYEAIVRDYTGRTPEAREQTLIVTHLNEDRRVLNSMIHDAREKAGELGKEQVM
VPVLNTANIRDGELRRLSTWENNPDALALVDSVYHRIAGISKDDGLITLEDAEGNTRLIS
PREAVAEGVTLYTPDKIRVGTGDRMRFTKSDRERGYVANSVWTVTAVSGDSVTLSDGQQT
RVIRPGQERAEQHIDLAYAITAHGAQGASETFAIALEGTEGNRKLMAGFESAYVALSRMK
QHVQVYTDNRQGWTDAINNAVQKGTAHDVLEPKPDREVMNAQRLFSTARELRDVAAGRAV
LRQAGLAGGDSPARFIAPGRKYPQPYVALPAFDRNGKSAGIWLNPLTTDDGNGLRGFSGE
GRVKGSGDAQFVALQGSRNGESLLADNMQDGVRIARDNPDSGVVVRIAGEGRPWNPGAIT
GGRVWGDIPDNSVQPGAGNGEPVTAEVLAQRQAEEAIRRETERRADEIVRKMAENKPDLP
DGKTELAVRDIAGQERDRSAISERETALPESVLRESQREREAVREVARENLLQERLQQME
RDMVRDLQKEKTLGGD
>LFGLNPFC_00082 hypothetical protein
MTTDNTNTTRNDSLAARTDTWLQSFLVWSPGQRDIIKTVALVLMVLDHINLIFQLKQEWM
FLAGRGAFPLFALVWGLNLSRHAHIRQPAINRLWGWGIIAQFAYYLAGFPWYEGNILFAF

AVAAQVLTWCETRSGWRTAAAILLMALWGPLSGTSYGIAGLLMLAVSYRLYRAEDRAERL
ALLACLLAVIPALNLASSDAAAVAGLVMTVLTVGLVSCAGKSLPRFWPGDFFPVFYACHL
AVLGVLAL
>LFGLNPFC_00083 Quorum-quenching protein AidA
MKITDHKLSEGIALTFRVPEGNIKHPLIILCHGFCGIRNVLLPCFANAFTEAGFATITFD
YRGFGESDGERGRLVPAMQTEDIISVINWAEKQECIDNQRIGLWGTSLGGGHVFSAAAQD
QRVKCIVSQLAFADGDVLVTGEMNESERASFLSTLNKMAEKKKNTGKEMFVGVTRVLSDN
ESKVFFEKVKGQYPEMDIKIPFLTVMETLQYKPAESAAKVQCPVLIVIAGQDSVNPPEQG
KALYDAVASGTKELYEEADACHYDIYEGAFFERVAAVQTQWFKKHL
>LFGLNPFC_00084 Fertility inhibition protein
MTEQKRPVLTLKRKTEGETPTRSRKTIINVTTPPKWKVKKQKLAEKAAREAELTAKKAQA
RQALSIYLNLPSLDEAVNTLKPWWPGLFDGDTPRLLACGIRDVLLEDVAQRNIPLSHKKL
RRALKAITRSESYLCAMKAGACRYDTEGYVTEHISQEEEVYAAERLDKIRRQNRIKAELQ
AVLDEQ
>LFGLNPFC_00085 Endonuclease YhcR
MRKYIPLVLFIFSWPVLCADIHGRVVRVLDGDTIEVMDSRKAVRIRLINIDAPEKKQDYG
RWSTDMMKSLVAGKTVTVTYFQRDRYGRILGQVYAPDGMNVNQFMVRAGAAWVYEQYNTD
PVLPVLQNEARQQKRGLWSDADPVPPWIWRHRK
>LFGLNPFC_00086 Hemolysin expression-modulating protein Hha
MAKTKQEWLYQLRRCSSVNTLEKIIHKNRDSLLNSERESFNSAADHRLAELITGKLYDRI
PKEIWKYVR
>LFGLNPFC_00087 hypothetical protein
MKLIIFILIVLIIAALLIRIILRSVNQHSPLLMQLHAAGIRTGDAERILSGGEYWQRQKT
LLTEREVSFMKGLFRIVDMKRWYLCPQVRVADIVQLNGNIRPRSRQWWQLFRMVSQWHVD
VVIVERRSFSIVAAVELDDASHLRPERRRRDILLEEVLRQAGIPLLRSHDARKLLQMTGE
WLNTTGAVQQSPEHRS
>LFGLNPFC_00088 hypothetical protein
MSQIENAVTSSPKRIYRKGNPLTGAEKQRISVSRKKGTHKAINVFIQSELKDDLTQLCKD
SGLTQKEMIEHWILKEKAAMTDANRK
>LFGLNPFC_00089 hypothetical protein
MTDLQQTYYRQVKNPNPVFTPRKGAGTLKFCEKLMEKAVGFTSRFDFAIHVAHARSRGLR
RRMPPVLRRRAIDALLQGLCFHYDPLANRVQCSITTLAIECGLATESGAGKLSITRATRA
LTFLSELGLITYQTEYDPLIGCYIPTDITFTPALFAALDVSEDAVAAARRSRVEWENKQR
KKQGLDTLGMDELIAKAWRFVRERFCSYQTELKSRGIKRARARRDANRERQDIVTLVKRQ
LTREISEGRFSASREAVKREVERRVKERMILSRNRNYSRLATASP
>LFGLNPFC_00090 IS6 family transposase IS26
MNPFKGRHFQRDIILWAVRWYCKYGISYRELQEMLAERGVNVDHSTIYRWVQRYAPEMEK
RLRWYWRNPSDLCPWHMDETYVKVNGRWAYLYRAVDSRGRTVDFYLSSRRNSKAAYLFLG
KILNNVKKWQIPRFINTDKAPAYGRALALLKREGRCPSDVEHRQIKYRNNVIECDHGKLK
RIIGATLGFKSMKTAYATIKGIEVMGALRKGQASAFYYGDPLGEMRLVSRVFEM
>LFGLNPFC_00091 hypothetical protein
METLSTNLQLARLVGVQGTPATIIGDEMIPGAVSWETLEAVVKEKLAVAHAQ
>LFGLNPFC_00092 Thiol-disulfide oxidoreductase ResA
MLSKLRRWLREGAILLVLLAGVIILLDVWRSPQMPAMFDSTPLHTLDGETVTLASISEER
PVLLYFWASWCGICRFTTPDVARLQSEGESVMTIALRSGNDGEVSRWLSRKRVTFPVVND
SGGEISRNWEISVTPTLVVVSKGQVVTTTSGWTSYWGMKLRLWRAAMF
>LFGLNPFC_00093 Na(+)-translocating NADH-quinone reductase subunit C
MRKGKIIVASMMVLCVLIFVAAAAWFMLFQGEMTEPTEEEKQAAILHAAGLMKSETQDKK
SVETLYHRYIIQRHVNLDSGELVAGSSADTARQKCEKLAPERDPAQVRQRCTVADVFFVK
DKNNEIQQVIIPVTGKGAKSMMHAFLALGLDGRTVRNLYYYQQRETPFLGARVEDANWRK
QWPGKRLLDNSGHPALKIVQDKPEHADEYTVDGISGATLTSTGVEKSINYWMGPQGYGQF
LQRLASDRNNLNL
>LFGLNPFC_00094 hypothetical protein
MRNFRNSLLVTLFGLFISMSVWASTNYAPLIEDIEQRLDKTAELYQQQHADEARRTVQMA
YFEVFENLEGPIRINISARKSYEMESAFGEIRRMIGEKKPLADVQARIDWLKAALREVEP
VLDGGHRLVAEEQHNALSRDDIAVHWQESFRTIDDLLAQAVTEYQAGNYSVASQHVQQAH
YQGFKNSEMEMSLRQNRSAKDAASINQQFSSLIALTAQPDHLNDVSYQVTTLLQDIEDIL
PGLPTTRDDQQVAAAQSADNIPAADEGNNRTDWAEVTTGINQSIQDAIARYQGGDAKNAI
LDIQDTYFDRFEASGMENKIGSRDSAFKTTLEAYFTRLVSLMKAGQPVERLNAEASALAQ
DLQKAVTMLGEGEETQWSLLLYSLMIIVREGLEALLIVAAIVAYMVKNNHQDKLPLIRQS
VIVALIASVITAAIFQMLFTNSGASRELLEGITMLIAVVMLFFMSYWLLSKVEARHWKAW
LEGKLSHSLSRGSLVGLWLTSFLAVYREGAETVLFYYALIGDANTVSGHMAIGAGFVIGC
VVLLLAWLVMRYSVVRLPLKPFFMFTGSFMYLMAFVFAGKGVLELVEGKLFQPTLINGFP
EISWLGIYPYVETLLPQVVLLLAALVALWVMRRKSAVPGETIKNNP

>LFGLNPFC_00095 34 kDa membrane antigen
MTMKKTLIASAVMASIFIAPAAFAFKEYPAGEPVTMNEMELAAVYLQPIDMEPRGMGLPA
AKADVHLEADIHAVEGNKNGFGAGEWIPYLTISYTLVNNDTGEKQEGTFMPMVASDGPHY
GANIKMMGVGNYKVTYHIEPPSKAGMHRHTDSETGVGRWWKPFDVSYEFKYVGLN
>LFGLNPFC_00096 hypothetical protein
MSYFFVTTLQVFFCIALLSGVLWSRNDPPSLRPLTWTLLTGLIAGVLAGLFIHGSQPVQL
LLVGAEVMVSLLFVLSFWWASTRIRYLWQGILIFGAARHWALDPNLGGLTSTHVLNTDLL
LNLTAVVLAFAILCLAGVLCAMLLRRIRGLYWPLTLILLVMIWLPLSGNLLLLLMKLQVV
PLGKSLLSFVAKVTNNTALYNWAGAALLLALALCWLPALLRAFRQTRETEEPIAHRLALA
QRRNALRLWLVTIGCAVVVIAGQLWWDKVASQPPQLSEAVPVTLGSDGMVRLPVEQLRDG
KLHRFVWVADDGKAVRFFVINRYPDKLRFGVVFDACLLCGDQGYVMEGNQVICVACGVHI
FIPSIGKAGGCNPVPIENWHNDEKELVIPGKELATGVNYFSTVMTIKVTDPVDGSTLTNT
SADYKYSYGGKTWFFSSEANYERFRETPEQFVPADMREE
>LFGLNPFC_00097 hypothetical protein
MLWRMLRQSWGRNLRRKVLAIITVFLASSLISALLAVSIDIGDKMSRELKSYGANILIEP
AGQAALPALFSESSNPLSGQDFLDEAELPNIKDIFWRNNIVGFAPMLGGEASVEGEPVRI
LGTFFSQPVDIPDEEGYETGQKTVSPYWQVTGDWPQEPAGAEPQTLVGHALARQMGWKPG
DKLTLRTEGEAVQVTVSGILSSGGDEDNQLVMPLSTVQHLLGLPGKVQAIRVSALTVPEN
ELSRRARENLDALNAEEYDLWYCTAYVSSIAHQLEEAISGAEVRPVWQVAASEGVVIDKI
QLLMAVVTVAALVASAMGIASLMTSTIMERAKEIGLMKALGARQWQIMLLFYLEAASSGL
AGGALGCIAGWGLAKAIGVMLFDAPLNFAWIVVPCVLVIAVLIALIGTWFPARRIARLYP
VEVLYGR
>LFGLNPFC_00098 hypothetical protein
MAAKRTMLWLLVWRALRLRFQRVSVVFAALMVGATIVTALFAVWFDINTKMSEELRTFGA
NFYIGPGHGSSMPQQELQSILDQAPQGLVHGASPWLYGMARTELEKVVMVGVWFESLQKL
VPYWQVTGSWIGVSFDDRNAMIGVKLAERLNVQPGDSITLVDHNQRKNLQIKGIVESGDA
TDNMLIVSLDVAQAWLHQPGKISHGLLSVSNDVGQVENYASRLQAQYPDLEIRPVRKVSA
SEGQVLDKIKGLMGLVSIVILALSSLCVNTTLMAIVGERAREFALQKALGSSNGDIVRQI
LLETSIIALAAVACGWVLGYLLAQLLGLTVFNAAISLRLPVLPITLVLSLLVAILAAIVP
VRRAVSVEPAKVLKGE
>LFGLNPFC_00099 Lipoprotein-releasing system ATP-binding protein LolD
MSVTHIPQVAIETRHLYKRFGDVTALDDINLRIAQGEFVAIMGASGSGKTTLMNILTCLD
TATEGQVFLDGTDAAALDEEGRRRFRAEKIGLVFQQFHLIPFLTALENIMLAQHYHSVVD
EAAARKVLEQVGLGHRVTHLPSQLSGGEQQRVCIARALVNEPPVIFADEPTGNLDEENEQ
RVLDLLTDLHRQGRTIVMVTHNPALGQFADRILRLQHGKYLGEEANQHALA
>LFGLNPFC_00100 Thiol-disulfide oxidoreductase ResA
MRWHNVLLLAAAILISGCKEEKLAVGAPAPALAAFDLQGQESGLERWQGKAVYLNFWSAG
CGGCLAEMDTLEALSKKWGDKVVVVAVNTDPETVRINDLLAKHQITYPVVRDQLNITQER
YQVIGTPTSVLIDSEGRVLELHQGMRKPSELEATFARLAAR
>LFGLNPFC_00101 hypothetical protein
MAFIWNDESLAILRENAGILTTEQIAQLLHTNITAVRNMAYRLKLSLRVTAYNHRRIAQV
QALYASETLSLKEIAAKTGLTASTVQYIVYVKSKNKPYATTEYVSFETENAVHYRVQKEF
VDTERSLLDNISDNTRFRELYLTDGTFYCARNIKYEVFISE
>LFGLNPFC_00102 Glucose-1-phosphatase
MKKLILAAAVAGAVLLSSAAQAQTTPEGYQLQQVLMMSRHNLRAPLANNGSVLEQSTPNQ
WPEWDVPGGQLTTKGGVLEIYMGHYMREWLAELGMVTSGECPTPDTVYTYANSLQRTVAT
AQFFITGAFPGCDIPVHHQEKNGHDGPNV
>LFGLNPFC_00103 hypothetical protein
MGTMDPTFNPVITDDSAAFSQKAVQAMEKERSQMQLDDSYQLLAQMTDYKDSPSCKEKQQ
CSLTEAKDAFREGANKQVMSSQADSLIKISRIWADFCPANTSNQPI
>LFGLNPFC_00104 Group II intron-encoded protein LtrA
MLQARLAVELQILRDELLSGHYQPLPARRVYIPKSNGKLRPLGIPALRDRISIRAKVEHP
FRIIKRQFGFVKARYKGLLKNDNQLAMLFTLANLFRVDQMIRQWERSQ
>LFGLNPFC_00105 hypothetical protein
MTFNTSQVSYYMTQRKKGITQHISAMKAGISVRSGRRIEKGEWAKNSVRHWRTRKDPLEA
VWDSMLVPLLKERPALTPTTLLEMLQDKYPGQYPNSLRRTMQRRVCEWKLQYGAEQEVMF
RQRHQPGLRGLSDFTELKGVVVTIAGKLLAHKLYHFRLEWSHWSWMRVVLGGESFSALAE
GLQEALGQLGGVPVEHKTDSLRAAWKQQGEDGRRELTERYAALCQHYGMQGVHNNAGRGH
ENGSVESAHGHLKRRICQALILRGSNDFSTIEEYQAFITQQVMRHNRNNQDLVKEERLHL
KPLPLRRSADYDELTVRVSRSSTINVKHVVYSVPSRLVGQLLRVRLWDDRLSCYVGSSEV
MSCPRVRPEKGKTRARRIDFRHVIDSLAKKPGAFCHATLRNDILPDDEWRRLWRRLCNHL
EPDMAGRLMVHALKLAAGYDDISVVAKGMEQMLNTPGNVDLHRLMRFLGIKEKALPVVNV
KQHNLSSYEQLLRGKGGSQ
>LFGLNPFC_00106 IS21 family transposase ISEc10

MSNIHHLERSLRKLRLTRVGAEWHALEKRALAEGWTPSRYLLTLCNEELLWRESEKLRRY
KKEARLPVAKTLSEYDFSQVPELNGAQFRQLCETTDWVDAGENVLLFGASGLGKSHLAAA
IVDGVVGQGYRARFYSAGELLQELRKARAQLKLNELLLKLDRYRVIVVDDLGYVKRDSAE
TGVLFELIAHRYERGSLVITSNHPFSMWGSIFVDETMAVAAADRLIHHGYMFELKGESYR
KKTAKAVTSAT
>LFGLNPFC_00107 IS3 family transposase IS629
MTKNTRFSPEVRQRAVRMVLESQGEYDSQWATICSIAPKIGCTPETLRVWVRQHERDTGG
GDGGLTTAERQRLKELERENRELRRSNDILRQASAYFAKAEFDRLWKK
>LFGLNPFC_00108 IS91 family transposase ISSbo1
MVRYFGFLANRVCGEKLPQVYRALGMDKPEAVAKVCYAQMVKQFLSRDPFECVLCGGRMV
YRRAIAGLNVSGLKKNARDISLLRYMPA
>LFGLNPFC_00109 putative protein YncE
MNIFTLSKAPLYLLISLFLPTMAMAIDPPERELSRFALKTNYLQSPDEGVYELAFDNASK
KVFAAVTDRVNREANKGYLYSFNSDSLKVENKYTMPYRAFSLAINQDKHQLYIGHTQSAS
LRISMFDTPTGKLVRTSDRLSFKAANAADSRFEHFRHMVYSQDSDTLFVSYSNMLKTAEG
MKPLHKLLMLDGTTLALKGEVKDAYKGTAYGLTMDEKTQKIYVGGRDYINEIDAKNQTLL
RTIPLKDPRPQITSVQNLAVDSASDRAFVVVFDHDDRSGTKDGLYIFDLRDGKQLGYVHT
GAGANAVKYNPKYNELYVTNFTSGTISVVDATKYSITREFNMPVYPNQMVLSDDMDTLYI
GIKEGFNRDWDPDVFVEGAKERILSIDLKKS
>LFGLNPFC_00110 putative TonB-dependent receptor
MNVIKLAIGSGILLLSCGAYSQSISEKTNSDKKGAAEFSPLSVSVGKTTSEQEALEKTGA
TSSRTTDKNLQSLDATVRSMPGTYTQIDPGQGAISVNIRGMSGFGRVNTMVDGITQSFYG
TSTSGTTTHGSTNNMAGVLIDPNLLVAVDVTRGDSSGSEGINALAGSANMRTIGVDDVIF
NGNTYGLRSRFSVGSNGLGRSGMIALGGKSDAFTDTGSIGVMAAVSGSSVYSNFSNGSGI
NSKEFGYDKYMKQNPKSQLYKMDIRPDEFNSFELSARTYENKFTRRDITSDDYYIKYHYT
PFSELIDFNVTASTSRGNQKYRDGSLYTFYKTSAQNRSDALDINNTSRFTVADNDLEFML
GSKLMRTRYDRTIHSAAGDPKANQESIENNPFAPSGQQDISALYTGLKVTRGIWEADFNL
NYTRNRITGYKPACDSRVICVPQGSYDIDDKEGGFNPSVQLSAQVTPWLQPFIGYSKSMR
APNIQEMFFSNSGGASMNPFLKPERAETWQAGFNIDTRDLLVEQDALRFKALAYRSRIQN
YIYSESYLVCSGGRKCSLPEVIGNGWEGISDEYSDNMYIYVNSASDVIAKGFELEMDYDA
GFAFGRLSFSQQQTDQPTSIASTHFGAGDITELPRKYMTLDTGVRFFDNALTLGTIIKYT
GKARRLSPDFEQDEHTGAIIKQDLPQIPTIIDLYGTYEYNRNLTLKLSVQNLMNRDYSEA
LNKLNMMPGLGDETHPANSARGRTWIFGGDIRF
>LFGLNPFC_00111 hypothetical protein
MMNILHFSQSVKWSSWFICSLLLHGLIFLAFIWRFSEVQPAMSPAPAIMLQWAEIIEAPS
SPLSLPVGIAQQESAVTEEKQQTEDRQQRPVTEDSDATIEITRKKKSSDGEKKKTRPPRK
IKAQTSDSNPTAVSSNAAPQALVESSRIAAPFNSDSTKRDNSEASWESRVKGHLNRYKRY
PGDARKRARTGTAVVTFTVNTEGTIVSSFLEISSGTFSLDREAIAVLERAQPLPKPPPEI
LEGGLFKVKMPITFKLKE
>LFGLNPFC_00112 hypothetical protein
MRKFILISMITLASVSLGACRQNVTIKQDAPGQKAFLTDGQIYDLHSGKIISSSELLADL
ATAQHLIIGEKHDNAEHHQIELWLIQNLLIQRPQGSVLLEMLTSEQQPRVNQVKCWLKDN
PVVRDSRVQELLNWQKGWSWEMYGDIVMQLLRGPYPLLNANIGREQILALYKKNEFPKGK
KSTAPVVQEALRETIISMHEGNLESQQLTSMLSIQQQRDRYMARQLLSAPVPSLLIAGGY
HASKSMGVPLHMEDLATGTHPVVLMLAEKGMNITVDHADYVWFVAPDTTKR
>LFGLNPFC_00113 hypothetical protein
MGFHSCAPMSSAPDVHVEENGRRGEYGALPGSLSGGEGQWNENVR
>LFGLNPFC_00114 hypothetical protein
MNGHAHLLYALNIAVRTAPDSSVKALKYAAAIERSLCEKLCADVNYSGLICKNPFHLEWQ
VMEWREEAYTLDELADYLDLSASARRSIDKHYGMGRNCHLFEMTRKWAYRAIRQGWPEFS
QWLDAVIQRVEMYNASLPVPLSPPECRAIGKSIAKYTHRNFTPETFAQYVADTHTLTYVF
VPLALTPR
>LFGLNPFC_00115 hypothetical protein
MNRKYYFNNMWWGWVTGGYMLYMSWDYDFKYRLLFWCISLCGMVLYPVAKWYIEDTALKF
TRPDFWNSGFFTDTPGKMGLLAVYTGTVFILSLPLSMIYILSVIIKRLSVR
>LFGLNPFC_00116 hypothetical protein
MTFEQKKARAIALMDSKKMWRSNYAPPLLRILWRLGIRLPPLPFMPFWQVTVLTGGLWGI
SWGCAMWFIYWGPSGMVAGEAIIISITGGFLSGLLMASFHWWRRKVNRLPPWDDV
>LFGLNPFC_00117 hypothetical protein
MTQSRRPSPLQRRVLIVLAALDEKRPGPVLTRDIERVLEQSGEAPVYGPNLRASCRRLED
AGWLRTLRAPNLQLAVELTDAGRAVAQPLLPAGGTSATDLAVELNGITYQACRGDFVVRL
DGSTCLQLWNKEGRVVRREGDPLEVAQWLQACHDAGMEVRVQINESAAP
>LFGLNPFC_00118 hypothetical protein
MDQEMTFSLSYEQLTRFAEKRIRECNLDSHGVTYLCESAKAGAVLIFWHELAINGYTSMN

AIKRQEIIDADHQRLRKLIWPEDDWK
>LFGLNPFC_00119 IS3 family transposase IS629
MTKNTRFSPEVRQRAIRMVLESQGEYDSQWAAICSIAPKIGCTPETLRVWVRQHERDTGG
GDGGLTTAERQRLKELERENRELRRSNDILRQASAYFAKAEFDRLWKK
>LFGLNPFC_00120 Putative transposase InsK for insertion sequence element IS150
MPLLDKLRKLYGVGPVCSELHIAPSTYYHCQQQRHHPDKRSARAQRDDWLKKEILRVYDG
NHQVYGVRKVWRQLLREGIRVARCTVARLMAVMGLAGVLRGKKVRTTVSRKAVAAGDRVN
RQFVAERPDQLWVADFTYGVPGVQGGHGCLNEPRVCLEY
>LFGLNPFC_00121 hypothetical protein
MIISEMQRKLATWAATDPSLRIQRLLRLITQPEWLAEAARITLSSKGAHTPGVDGVNKTM
LQARLAVELQILRDELLSGHYQPLPARRVYIPKSNGKLRPLGIPALRDRIVQRAMLMAME
PIWESDFHTLSYGFRPERSVHHAIRTVKLQLTDCGETRGRWVIEGDLSSYFDTVHHRLLM
KAVRRRISDARFMTLLWKTIKAGHIDVGLFRAASEGVPQGGVISPLLSNIMLNEFDQYLH
ERYLSGKARKDRWYWNNSIQRGRSTAVRENWQWKPAVAYCRYADDFVLIVKGTKAQAEAI
REECRGVLEGSLKLRLNMDKTKITHVNDGFIFLGHRIIRKRSRYGEMRVVSTIPQEKARN
FAASLTALLSGNYSESKVDMAEQLNRKLKGWAMFYQFVDFKAKVFSYIDRVVFWKLAHWL
ARKYRTGIASLMRWWCKSPKPGQSKTWVLFGKTNHGKLSGEILYWLVGQGKKLFRWRLPE
GNPYLRTETRNTYTSRFTEVAMAFASI
>LFGLNPFC_00122 Putative transposase InsK for insertion sequence element IS150
MAFIIDVFAGYIVGWRVSSSMETTFVLDALEQALWARRPSGTVHHSDKGSQYVSLAYTQR
LKEAGLLASTGSTGDSYDNAMAESINGLYKAEVIHRKSWKNRKRRLSTVWQILKFLRE
>LFGLNPFC_00123 hypothetical protein
MKSLTAVRKKSPNYPVEFKIKMVELSHRPEISVAQLAREHGINDNLLFKWRQYWREGKLR
PPSTTENNVPELLPITLDAEDVVPTTSPRSQPVAAATPESLNISCEVTFRHGSLRLNGAI
SENILNLLIRELKR
>LFGLNPFC_00124 hypothetical protein
MIPLPSGTKIWLVAGITDMRNGFNGLAAKVQTTLKDDPMSGHVFIFRGRNGSQVKLLWST
GDGLCLLTKRLERGRFAWPSARDGKVFLTPAQLAMLLEGIDWRQPKRLLTSLTML
>LFGLNPFC_00125 IS66 family transposase ISEc22
MSSSLPDDINALKRLLAEQEALNRALLEKLNEREREIDHLQAQLDKLRRMNFGSRSEKVS
RRIAQMEADLNRLQKESDRERYADW
>LFGLNPFC_00126 IS66 family transposase ISEc8
MYDPAVQRPLRQTRTRKPFPESLPRDEKRLLPAAPCCPNCGGSLSYLGEDTAEQLELMRS
AFRVIRTVREKHACTQCDAIVQAPAPSRPIERGIAGPGLLARVLTSKYAEHTPLYRQSEI
YGRQGVELSRSLLSGWVDACCRLLSPLEEALHGYVLTDGKLHADDTPVPVLLPGNKKTKT
GRLWTYVRDDRNAGSTLAPAVWFAYSPDRKGIHPQTHLAGFSGVLQADAVTNGAIVLHTQ
RLKSDPGGNLLS
>LFGLNPFC_00127 hypothetical protein
MVTFETVMEIKILHKQGMSSRAIARELGISRNTVKRYLQAKSEPPKYTPRPAVASLLDEY
RDYIRQRIADAHPYKIPATVIAREIRDQGISWRNDHSQGIHSFSLGSSGAGACRSVRN
>LFGLNPFC_00128 hypothetical protein
MQVDWGTMRNGRSPLHVFVAVLGYSRMLYIEFTDNMRYDTLETCHRNAFRFFGGVPREVL
YDNMKTVVLQRDAYQTGQHRFHPSLWQFGKEMGFSPRLCRPFRAQTKGKVERMVQYTRNS
FYIPLMTRLRPMGITVDVETANRHGLRWLHDVANQRKHETIQARPCDRWLEEQQSMLALP
PEKKEYDVHLDENLVNFDKHPLHHPLSIYDSFCRGVA
>LFGLNPFC_00129 IS21 family transposase IS100kyp
MMELQHQRLMVLAGQLQLESLISAAPALSQQAVDQEWSYMDFLEHLLHEEKLARHQRKQA
MYTRMAAFPAVKTFEEYDFTFATGAPQKQLQSLRSLSFIERNENIVLLGPSGVGKTHLAI
AMGYEAVRAGIKVRFTTAADLLLQLSTAQRQGRYKTTLQRGVMAPRLLIIDEIGYLPFSQ
EEAKLFFQVIAKRYEKSAMILTSNLPFGQWDQTFAGDAALTSAMLDRILHHSHVVQIKGE
SYRLRQKRKAGVIAEANPE
>LFGLNPFC_00130 IS66 family transposase ISEc22
MTAEQRLAERQLKTKPLLKSLESWLREKMKTLSRHSELAKAFAYALNQWPALTYYADDGW
AEADNNIAENALRMVSLGRKNYLFFGSDHGGERGALLYSLIGTCKLNGVEPESYLRYVLD
VIADWPINRVGELLPWRVALPTE
>LFGLNPFC_00131 putative protein
MMKNTGYILALCLTASGHVLAHDVWITGKQAENNITAEIGYGHNFPSKGTIPDRRNFFEN
PRIYNGKETITLKPASTDYVYKTESASKDNGYVLSTYMKPGYWSRTSSGWKPVSREGRND
VAYCEFVTKYAKSFIPGEQQMPAQLYQSPTGHELEIIPLSDISRFSEDVKLKVLYKTSPL
AGAIMELDSVSYLKSSRHTHAVEHKHPVHKAELTFVTNEDGIVTVPSLHIGQWLAKVQNK
KSFQDKSLCDETVDVATLSFSRN
>LFGLNPFC_00132 Hemin receptor
MYMNVIRNVICTLIILPIGLQAETSHSSMAKDTITVVATGNQNTVFETPSMVSVVTNDTP
WSQNAVTSAGMLKGVAGLSQTGAGRTNGQTFNLRGYDKSGVLVLVDGIRQLSDMAKSSGT

YLDPALVKRIEVVRGPNSSLYGSGGLGGVVDFRTADAADFLPPGETNGLSLWGNIASGDH
STGSGLTWFGKTGKTDALLSVIMRKRGNIYQSDGERAPNKEKPAALFAKGSVGITDSNKA
GASLRLYRNNTTEPGNPTQTHGDSGLRDRKTVQNDVQFWYQYAPVDNSLINVKSTLYLSD
ITIKTNGHNKTAEWRNNRTSGVNVVNRSHTLIFPGAHQLSYGAEYYRQQQKPEGSATLYP
EGNIDFTSLYFQDEMTMKSYPVNIIVGSRYDRYKSFNPRAGELKAERLSPRAAISVSPTD
WLMMYGSISSAFRAPTMAEMYRDDVHFYRKGKPNYWVPNLNLKPENNITREIGAGIQLDG
LLTGNDRLQLKGGYFGTDARNYIATRVDMKRMRSYSYNVSRARIWGWDMQGNYQSDYFDW
MLSYNRTESMDASSREWLGSGNPDTLISDISIPVGHGGVSAGWRAELSAAATHVKKGDPH
QAGYAVHSFSLSYKPVSVKGFEASVTLDNAFNKLAMNGKGVPLSGRTVSLYTRYQW
>LFGLNPFC_00133 hypothetical protein
MIDVLGPEKRRRRTTQEKIAIVQQSFEPGMTVSLVARQHGVAASQLFLWRKQYQEGSLTA
VAAGEQVVPASELAAAMKQIKELQRLLGKKTMENELLKEAVEYGRAKKWIAHAPLLPGDG
E
>LFGLNPFC_00134 hypothetical protein
MVFRNIETSDKSINFKVIAELAFMSYFIFILTILPGLIYLKMNPQHKLSKRISSAFVASL
MILLVISTQITVLPVIFTHSVIKLSGISDFKIHSYIIKTSEYPEEFFSNAVWGKKNIKPG
EYYSVQAVSMFTTNQFILLCPKDIIRFYRESWKFDLLNVDFDTNTRKKLQEEAAYCVPIS
AISVKRWDMPLQGSKPSN
>LFGLNPFC_00135 Glycolate permease GlcA
MLLIGFSFGALLEGAAGFGAPVAITGALLVGLGFKPLYAAGLCLIANTAPVAFGALGVPI
LVAGQVTGIDPFHIGAMAGRQLPFLSVLVPFWLVAMMDGWKGVKETWPAALVAGGSFAVT
QFFTSNYIGPELPDITSALVSIVSLALFLKVWRPKNTETAISMGQSAGAMVVNKPSSGGP
VPSEYSLGQIIRAWSPFLILTVLVTIWTMKPFKALFAPGGAFYSLVINFQIPHLHQQVLK
AAPIVAQPTPMDAVFKFDPLSAGGTAIFIAAIISIFILGVGIKKGIGVFAETLISLKWPI
LSIGMVLAFAFVTNYSGMSTTLALVLAGTGVMFPFFSPFLGWLGVFLTGSDTSSNALFGS
LQSTTAQQINVSDTLLVAANTSGGVTGKMISPQSIAVACAATGMVGRESELFRYTVKHSL
IFASVIGIITLLQAYVFTGMLVS
>LFGLNPFC_00136 hypothetical protein
MNKKFKYKKSLLAAILSATLLAGCDGGGSGSSSDTPSVDSGSGTLPEVKPDPTPTPEPTP
EPTPDPEPTPDPTPDPEPTPEPEPEPVPTKTGYLTLGGSQRVTGATCNGESSDGFTFTPG
NTVSCVVGSTTIATFNTQSEAARSLRAVDKVSFSLEDAQELANSENKKTNAISLVTSSDS
CPADAEQLCLTFSSVVDRARFEKLYKQIDLATDNFSKLVNEEVENNAATDKAPSTHTSTV
VPVTTEGTKPDLNASFVSANAEQFYQYQPTEIILSEGQLVDSLGNGVAGVDYYTNSGRGV
TDENGKFSFSWGETISFGIDTFELGSVRGNKSTIALTELGDEVRGANIDQLIHRYSTTGQ
NNTRVVPDDVRKVFAEYPNVINEIINLSLSNGATLDEGDQNVVLPNEFIEQFKTGQAKEI
DTAICAKTDGCNEARWFSLTTRNVNDGQIQGVINKLWGVDTNYQSVSKFHVFHDSTNFYG
STGNARGQAVVNISNSAFPILMARNDKNYWLAFGEKRAWDKNELAYITEAPSIVQPENVT
RDTATFNLPFISLGQVGEGKLMVIGNPHYNSILRCPNGYSWGGGVNSKGECTLSGDSDDM
KHFMQNVLRYLSNDIWQPNTKSIMTVGTNLENVYFKKAGQVLGNSAPFAFHEDFTGITVK
QLTSYGDLNPEEIPLLILNGFEYVTQWSGDPYAVPLRADTSKPKLTQQDVTDLIAYLNKG
GSVLIMENVMSNLKEESASSFVRLLDAAGLSMALNKSVVNNDPQGYPDRVRQRRATGIWV
YERYPAADGAQPPYTIDPNTGEVTWKYQQDNKPDDKPKLEVASWQEEVEGKQVTRYAFID
EAEYTTEESLEAAKAKIFEKFPGLQECKDSTYHYEINCLERRPGTDVPVTGGMYVPRYTQ
LNLDADTAKAMVQAADLGTNIQRLYQHELYFRTKGSKGERLNSVDLERLYQNMSVWLWND
TKYRYEEGKEDELGFKTFTEFLNCYANDAYAGGTKCSADLKKSLVDNNMIYGDGSSKAGM
MNPSYPLNYMEKPLTRLMLGRSWWDLNIKVDVEKYPGSVSAKGESVTENISLYSNPTKWF
AGNMQSTGLWAPAQQDVTIKSSASVPVTVTVALADDLTGREKHEVALNRPPRVTKTYTLE
ANGEVTFKVPYGGLIYIKGDSKDDVSANFTFTGVVKAPFYKDGEWKNDLDSPAPLGELES
ASFVYTTPKKNLEASNFTGGVAEFAKDLDTFASSMNDFYGRNDEDGKHRMFTYKNLTGHK
HRFTNDVQISIGDAHSGYPVMNSSFSTNSTTLPTTPLNDWLIWHEVGHNAAETPLNVPGA
TEVANNVLALYMQDRYLGKMNRVADDITVAPEYLDESNGQAWARGGAGDRLLMYAQLKEW
AEENFDIKQWYPDGELPKFYSDRKGMKGWNLFQLMHRKARGDDVGNSTFGGKNYCAESNG
NAADTLMLCASWVAQADLSEFFKKWNPGASAYQLPGATEMSFQGGVSSSAYSTLASLKLP
KPEKGPETINKVTEHKMSAE
>LFGLNPFC_00137 Prepilin peptidase PppA
MLFDVFQQYPAAMPILATVGGLIIGSFLNVVIWRYPIMLRQQMAEFHGETPSTQSKISLA
LPRSHCPHCQQTIRVRDNIPLLSWLMLKGRCRDCQAKISKRYPLVELLTALAFLLASLVW
PESGWGLAVMILSAWLIAASIIDLDNQWLPDVFTQGVLWTGLIAAWAQQSPLTLQDAVTG
VLVGFITFYSLRWIAGIVLRKEALGMGDVLLFAALGGWVGPLSLPNVALIASCCGLIYAV
ITKRGSTTLPFGPCLSLGGIATLYLQALF
>LFGLNPFC_00138 Lipoprotein YghG
MSIKQMPGRVLISLLLSVTGLLSGCASHNENASLLAKKQAQNISQNLPIKSAGYTLVLAQ
SSGTTVKMTIISESGTQTTQTPDAFLTSYQRQMCADPTVKLMITEGINYSITINDTRTGN
QYQRKLDRTTCGIVKA

>LFGLNPFC_00139 Type II secretion system protein C 2
MVRGMFWLMLLIISAKMAYSLWRYFSFSAEYTAVSSSVNKPLRADAKPFDKNDVQLVSQQ
NWFGKYQPVAAPVKQPESAPVAETRLNVVLRGIAFGARPGVVIEEGGKQQVYLQGERLGS
HNAVIEEINRDHVMLRYQGKMERLSLAEEERPPVAVTSKKAASDEAKQAVAEPVVSAPVE
IPAAVRQALAKDPQKIFNYIQLTPVRKEGIVGYAVKPGADRSLFDASGFREGDIAIALNQ
QDFTDPRAMIALMRQLPSMDSIQLTVLRKGARYDISIALR
>LFGLNPFC_00140 Secretin GspD 2
MFWRDMTLSIWRKKTTGLKTKKRLLPLMLAAALCSSPVWAEEATFTANFKDTDLKSFIET
VGANLNKTIIMGPGVQGKVSIRTMTPLNERQYYQLFLNLLEAQGYAVVPMENDVLKVVKS
SAAKVEPLPLVGEGSDNYAGDEMVTKVVPVRNVSVRELAPILRQMIDSAGSGNVVNYDPS
NVIMLTGRASVVERLTEVIQRVDHAGNRTEEVIPLDNASASEIARVLESLTKNSGENQPA
TLKSQIVADERTNSVIVSGDPATRDKMRRLIRRLDSEMERSGNSQVFYLKYSKAEDLVDV
LKQVSGTLTAAKEEAEGTVGSGREVVSIAASKHSNALIVTAPQDIMQSLQSVIEQLDIRR
AQVHVEALIVEVAEGSNINFGVQWGSKDAGLMQFANGTQIPIGTLGAAISAAKPQKGSTV
ISENGATTINPDTNGDLSTLAQLLSGFSGTAVGVVKGDWMALVQAVKNDSSSNVLSTPSI
TTLDNQEAFFMVGQDVPVLTGSTVGSNNSNPFNTVERKKVGIMLKVTPQINEGNAVQMVI
EQEVSKVEGQTSLDVVFGERKLKTTVLANDGELIVLGGLMDDQAGESVAKVPLLGDIPVI
GNLFKSTADKKEKRNLMVFIRPTILRDGMAADGVSQRKYNYMRAEQIYRDEQGLSLMPHT
AQPILPAQNQALPPEVRAFLNAGRTR
>LFGLNPFC_00141 Type II secretion system protein E
MVPVAQETTANTVRLPYSFSRRFSLVAWCEASLEILHVHPLSLSVLQELQRGLNAPFTLR
QIDEAEFEQRLNAVWQRDSSEARQLMEDLGSAEDFFTLAEELPETEDLLESDDDAPIIKL
INAMLAEAIKEGASDIHIETFEKSLVIRFRVDGTLHEMLRPGRKLASLLVSRIKVMARLD
IAEKRVPQDGRIALLLGGRAIDVRVSTMPSAWGERVVLRLLDKNQARLTLERLGLSLELT
AQLRQLLHKPHGIFLVTGPTGSGKSTTLYAGLQELNNHSRNILTVEDPIEYMIEGIGQTQ
VNTRVGMTFARGLRAILRQDPDVVMVGEIRDTETAEIAVQASLTGHLVLSTLHTNTAVGA
ITRLQDMGVEPFLLSSSLTGVMAQRLVRTLCSDCRQAAPATDEEKRLMGISDTHAVTLYH
PQGCPACNHKGFRGRTAIHELIVVDATLRDLIHRQAGELELERYVRQHSAGIRSNGIEKV
LAGETSLDEVLRVTMEA
>LFGLNPFC_00142 Type II secretion system protein F
MALFYYQALERNGRKTKGMIEADSARHARQLLRGKDLIPVHIEARMNASAGGLLQRRRHA
HRRVATADLALFTRQLATLVQAAMPLETCLQAVSEQSEKLHVVKSLGMALRSRIQEGYTLS
DSLREHPRVFDSLFCSMVAAGEKSGHLDVVLNRLADYTEQRQRLKSRLLQAMLYPLVLLV
VATGVVTILLTAVVPKIIEQFDHLGHALPASTRMLIAMSDALQASGVYWLAGLLGLLVLG
QRLLKNPAMRLRWDKTLLRLPVTGRVARGLNTARFSRTLSILTASSVPLLEGIQTAAAVS
ANRYVEQQLLLAADRVREGSSLRAALADLRLFPPMMLYMIASGEQSGELETMLEQAAVNQ
EREFDTQVGLALGLFEPALVVMMAGVVLFIVIAILEPMLQLNNMVGM
>LFGLNPFC_00143 Type II secretion system protein G
MNSLSRTQKPRAGFTLLEVMVVIVILGVLASLVVPNLLGNKEKADRQKAISDIVALENAL
DMYRLDNGRYPTTEQGLEALIQQPANMADARNYRTGGYIKRLPKDPWGNDYQYLSPGEKG
LFDVYTLGADGQENGEGAGADIGNWNLQEFQ
>LFGLNPFC_00144 Type II secretion system protein H
MPERGFTLLEIMLVIFLIGLASAGVVQTFATASEPPAKKAAQDFLTRFAQFKDRAVIEGQ
TLGVLIDPPGYQFMQRRHGQWLPVSATRLSAQVTVPKQVQMLLQPGSDIWQKEYALELQR
RRLTLHDIELELQKEAKKKTPQIRFSPFEPATPFTLRFYSAAQNACWAVKLAHDGALSLN
QCDERMP
>LFGLNPFC_00145 hypothetical protein
MKRGFTLLEVMLALAIFALAAMAVLQIASGTLSNQQILEEKTVAGWVAENQTALLYLMTR
EQRAVRHQGESDMAGSRWYWRTIPLNTGNVLLQAVDIEVSLHDDFSPVIQSRRAWFSAVG
GQQ
>LFGLNPFC_00146 Type II secretion system protein J
MRRARAGFTLLEMLVAIAIFASLALMAQQVTNGVTRVNSAVAGHDQKLNLMQQTMSFLTH
DLTQMMPRPVRGDQGQREPALLAGAGVLVSESGGMRFVRGGVVNPLMRLPRSNLLTVGYR
IHDGYLERLAWPLTDAAGSVKPTTQKLIPADSLRLQFYDGTRWQESWSSVQAIPVAVRIT
LHSPQWGEIERIWLLRGPQLS
>LFGLNPFC_00147 Putative type II secretion system protein K
MITSPPKRGMALVVVLVLLAVMMLVTITLSGRMQQQLGRTRSQQEYQQALWYSASAESLA
LSALSLSLKNEKRVHLAQPWASGPRFFPLPQGQIAVTLRDAQACFNLNALAQPTTASRPL
AVQQLIALISRLDVPAYRAELIAESLWEFIDEDRSVQTRLGREDSEYLARSVPFYAANQP
LADISEMRVVQGMDAGLYQKLKPLVCALPMARQQININTLDVTQSVILEALFDPWLSPVQ
ARALLQQRPAKGWEDVDQFLAQPLLADVDERTKKQLKTILSVDSNYFWLRSDITVNEIEL
TMNSLIVRMGPQHFSVLWHQTGESE
>LFGLNPFC_00148 hypothetical protein
MSSMLEIFFPLCAADPIRWQRHTPDVEHGIWPDVADERLQQWLQTDAIRLYIPGEWISVW

QVELPDVPRKQIPTILPALLEEELNQDIDELHFAPLKIDQQLATVAVIHQQHMRNIAQWL
QANGITRATVAPDWMSIPCGFMAGDAQRVICRIDECRGWSAGRALAPVMFRAQLNEQNLP
ISLTVVGIAPEELSAWAGADAERLTVTALPAITTYGESEGNLLTGPWQPRVSYRKQWARW
RVMILPILLILVALVVERGVTLWSVSEQVAQSRTQAEKKFLTLFPEQKRIVNLRSQVTMA
LKKYRPQADDTRLLAELSAIASTLKSASLSDIEMRGFTFDQKRQTLHLQLRAANFASFDK
LRSALAADYVVQQDALQKEGDAVSGGVTLRRK
>LFGLNPFC_00149 Type II secretion system protein M
MLRDKFIHYFQQWRERQLSRGEHWLTQHLAGRSPREKGMLLAAVVFLFSVGYYVLIWQPL
SERIEQQETMLQQLVAMNTRLKNAAPDIIAARKSATTTPAQVSRVISDSASAHSVAIKRI
AERGENIQVWIEPVVFNDLLKWLNALDEKYALRVTQIDVSAAEKPGMVNVQRLEFGRG
>LFGLNPFC_00150 Polysialic acid transport protein KpsM
MARSGFEVQKVTVEALFLREIRTRFGKFRLGYLWAILEPSAHLLILLGILGYVMHRTMPD
ISFPVFLLNGLIPFFIFSSISKRSIGAIEANQGLFNYRPVKPIDTIIARALLETLIYVAV
YILLMLIVWMTGEYFEITNFLQLVLTWSLLIILSCGVGLIFMVVGKTFPEMQKVLPILLK
PLYFISCIMFPLHSIPKQYWSYLLWNPLVHVVELSREAVMPGYISEGVSLNYLAMFTLVT
LFIGLALYRTREEAMLTS
>LFGLNPFC_00151 Polysialic acid transport ATP-binding protein KpsT
MIKIENLTKSYRTPTGRHYVFKNLNIIFPKGYNIALIGQNGAGKSTLLRIIGGIDRPDSG
NIITEHKISWPVGLAGGFQGSLTGRENVKFVARLYAKRDELNERVDFVEEFSELGKYFDM
PIKTYSSGMRSRLAFGLSMAFKFDYYLIDEITAVGDAKFKKKCSDIFDKIREKSHLIMVS
HSERALKEYCDVAIYLNKEGQGKFYKNVTEAIADYKKDL
>LFGLNPFC_00152 Putative acetyltransferase EpsM
MSKKLIIFGAGGFSKSIIDSLNHKHYELIGFIDKYKSGYHQSYPILGNDIADIENKDNYY
YFIGIGKPSTRKHYLNIIRKHNLRLINIIDKTAILSPNIILGDGIFIGKMCILNRDTRIH
DAVVINTRSLIEHGNEIGCCSNISTNVVLNGDVSVGEETFVGSCTVVNGQLKLGSKSIIG
SGSVVIRNIPSNVVVAGTPTRLIRGNE
>LFGLNPFC_00153 N,N'-diacetyllegionaminic acid synthase
MSNIYIVAEIGCNHNGSVDIAREMILKAKEAGVNAVKFQTFKADKLISAIAPKAEYQIKN
TGELESQLEMTKKLEMKYDDYLHLMEYAVSLNLDVFSTPFDEDSIDFLASLKQKIWKIPS
GELLNLPYLEKIAKLPIPDKKIIISTGMATIDEIKQSVSIFINNKVPVGNITILHCNTEY
PTPFEDVNLNAINDLKKHFPKNNIGFSDHSSGFYAAIAAVPYGITFIEKHFTLDKSMSGP
DHLASIEPDELKHLCIGVRCVEKSLGSNSKVVTASERKNKIVARKSIIAKTEIKKGEVFS
EKNITTKRPGNGISPMEWYNLLGKIAEQDFIPDELIIHSEFKNQGE
>LFGLNPFC_00154 N-acylneuraminate cytidylyltransferase
MRTKIIAIIPARSGSKGLRNKNALMLIDKPLLAYTIEAALQSEMFEKVIVTTDSEQYGAI
AESYGADFLLRPEELATDKASSFEFIKHALSIYTDYESFALLQPTSPFRDSTHIIEAVKL
YQTLEKYQCVVSVTRSNKPSQIIRPLDDYSTLSFFDLDYSKYNRNSIVEYHPNGAIFIAN
KQHYLHTKHFFGRYSLAYIMDKESSLDIDDRMDFELAITIQQKKNRQKILYQNIHNRINE
KRNEFDSVSDITLIGHSLFDYWDVKKINDIEVNNLGIAGINSKEYYEYIIEKELIVNFGE
FVFIFFGTNDIVVSDWKKEDTLWYLKKTCQYIKKKNAASKIYLLSVPPVFGRIDRDNRII
NDLNSYLRENVDFAKFISLDHVLKDSYGNLNKMYTYDGLHFNSNGYTVLENEIAEIVK
>LFGLNPFC_00155 Polysialic acid biosynthesis protein P7
MLRETPEIQLDLAVTGMHCDNAYGNTIHIIEQDNFNIIKVVDININTTSHTHILHSMSVC
LNSFGDFFSNNTYDAVMVLGDRYEIFSVAIAASMHNIPLIHIHGGEKTLANYDEFIRHSI
TKMSKLHLTSTEEYKKRVIQLGEKPGSVFNIGSLGAENALSLHLPNKQELELKYGSLLKR
YFVVVFHPETLSTQSVNDQIDELLSAISFFKNTHDFIFIGSNADTGSDIIQRKVKYFCKE
YKFRYLISIRSEDYLAMIKYSCGLIGNSSSGLIEVPSLKVATINIGDRQKGRVRGASVID
VPVEKNAIVRGINISQDEKFISVVQSSSNPYFKENALINAVRIIKDFIKSKNKDYKDFYD
IPECTTSYD
>LFGLNPFC_00156 hypothetical protein
MTRKKVLCFVFRYDSHFLALKNIFEQIDVDSYDLFFCCLDNSLQEFVKKNLDEKIVVFYP
DDFVCFFTFINIEFIFCSTGGKDLHEIVNTVRTKDTIIISCFPGIVLTSQIEAFISKSNS
HYLLINSPKDIKTYKKICKIIGVPFNGILFGPPWIKNVNINAKSENSCLIVDQVNEPLTP
IKRIEYARFLIRVIQKHPHMNFIFKTRNPLISPDSIVFDIKEYIERFDLKNITFSDDNID
SLISKVEYCITISSSVAIYCLANKIKVYLINGFNHTCNGQCYFSRSGLIVDYNKFNFKHI
PRIKKKWMEENFYYSRDIQHKILNDILKMPSNVNVRTFGIKRSTLIILFLIFFNFFFSLG
PKKIKTLKKIHKVLLRYKKDDI
>LFGLNPFC_00157 hypothetical protein
MIFDASLKKLRKLFVNPIGFFRDSWFFNSKNKAEELLSPLKIKSKNIFIISNLGQLKKAE
SFVQKFSKRSNYLIVLATEKNTEMPKIIVEQINNKLFSSYKVLFIPTFPNVFSLKKVIWF
YNVYNYLVLNSKAKDAYFMSYAQHYAIFVYLFKKNNIRCSLIEEGTGTYKTEKENPVVNI
NFYSEIINSIILFHYPDLKFENVYGTYPILLKKKFNAQKFVEFKGAPSVKSSTRIDNVIH
KYSITRDDIIYANQKYLIEHTLFADSLISILLRIDKPDNARIFIKPHPKEPKKNINAIQK
AIKKAKCRDIILITEPDFLIEPVIKKAKIKHLIGLTSSSLVYAPLVSKRCQSYSIAPLMI

KLCDNDKSQKGINTLRLHFDILKNFDNVKILSDDITSPSLHDKRIFLGE
>LFGLNPFC_00158 hypothetical protein
MQGNALTVLLSGKKYLLLQGPMGPFFNDVAEWLESLGRNAVNVVFNGGDRFYCRHRQYLA
YYQTPKEFPGWLRDLHRQYDFDTILCFGDCRPLHKEAKRWAKSKGIRFLAFEEGYLRPQF
ITVEEDGVNAYSSLPRDPDFYRKLPDMPTPHVENLKPSTMKRIGHAMWYYLMGWHYRHEF
PRYRHHKSFSPWYEARCWVRAYWRKQLYKVTQRKVLPRLMNELDQRYYLAVLQVYNDSQI
RNHSNYNDVRDYINEVMYSFSRKAPKESYLVIKHHPMDRGHRLYRPLIKRLSKEYGLSER
VIYVHDLPMPELLRHAKAVVTINSTAGISALIHNKPLKVMGNALYDIKGLTYQGHLHQFW
QADFKPDMKLFKKFRGYLLMKTQVNWVYYGGNTTNCQHNIY
>LFGLNPFC_00159 hypothetical protein
MIGIYSPGIWRIPHLEKFLAQPCQKLSLLRPVPQEVNAIAVWGHRPSAAKPVAIAKAAGK
PVIRLEDGFVRSLDLGVNGEPPLSLVVDDCGIYYDASKPSALEKLVQDKAGNTALISQAR
EAMHTIVTGDMSKYNLAPAFVADESERTNIVLVVDQTFNDMSVTYGNAGPHEFAAMLEAA
MAENPQAEIWVKVHPDVLEGKKTGYFADLRATQRVRLIAENVSPQSLLRHVSRVYVVTSQ
YGFEALLAGKPVTCFGQPWYASWGLTDDRHPQSALLSARRGSATLEELFAAAYLRYCRYI
DPQTGEVSDLFTVLQWLQLQRRHLQQRNGYLWAPGLTLWKSAILKPFLRTPTNRLSFSRR
CTAASACVVWGVKGEQQWRAEAQRKSLPLWRMEDGFLRSSGLGSDLLPPLSLVLDKRGIY
YDATRPSDLEVLLNHSQLTLAQQMRAEKLRQRLVESKLSKYNLGADFSLPAKAKDKKVIL
VPGQVEDDASIKTGTVSIKSNLELLRTVRERNPHAYIVYKPHPDVLVGNRKGDIPAELTA
ELADYQALDADIIQCIQRADEVHTMTSLSGFEALLHGKHVHCYGLPFYAGWGLTVDEHRC
PRRERKLTLADLIYQALIVYPTYIHPTRLQPITVEEAAEYLIQTPRKPMFITRKKAGRVI
RYYRKLIMFCKVRFG
>LFGLNPFC_00160 3-deoxy-manno-octulosonate cytidylyltransferase
MSKAVIVIPARYGSSRLPGKPLLDIVGKPMIQHVYERALQVAGVAEVWVATDDQRVEKAV
QAFGGKAIMTRNDHESGTDRLVEVMHKVEADIYINLQGDEPMIRPRDVDTLLQGMRDDPA
LPVATLCHAISAEEATEPSTVKVVVNTHQDALYFSRSPIPYPRNAEKARYLKHVGIYAYR
RDVLQNYSQLPESMPEQAESLEQLRLMSAGINIRTFEVAATGPGVDTPACLEKVRALMAQ
ELAENA
>LFGLNPFC_00161 Polysialic acid transport protein KpsD
MKLFKSILLIAACHAAQASAAIDINADPNLTGAAPLTGILNGQQSDTQNMSGFDNTPPPS
PPVVMSRMFGAQLFNGTSADSGATVGFNPDYILNPGDSIQVRLWGAFTFDGALQVDPKGN
IFLPNVGPVKVAGVSNSQLNALVTSKVKEVYQSNVNVYASLLQAQPVKVYVTGFVRNPGL
YGGVTSDSLLNYLIKAGGVDPERGSYVDIVVKRGNRVRSNVNLYDFLLNGKLGLSQFADG
DTIIVGPRQHTFSVQGDVFNSYDFEFRESSIPVTEALSWARPKPGATHITIMRKQGLQKR
SEYYPISSAPGRMLQNGDTLIVSTDRYAGTIQVRVEGAHSGEHAMVLPYGSTMRAVLEKV
RPNSMSQMNAVQLYRPSVAQRQKEMLNLSLQKLEEASLSAQSSTKEEASLRMQEAQLISR
FVAKARTVVPKGEVILNESNIDSVLLEDGDVINIPEKTSLVMVHGEVLFPNAVSWQKGMT
TEDYIEKCGGLTQKSGNARIIVIRQNGAAVNAEDVDSLKPGDEIMVLPKYESKNIEVTRG
ISTILYQLAVGAKVILSL
>LFGLNPFC_00162 hypothetical protein
MLIKVKSAVSWMRARLSAISLADIQKHLAKIIIILAPMAVLLIYLAIFSQPRYMSESKVAI
KRSDDLNSGSLNFGLLLGASNPSSAEDALYLKEYINSPDMLAALDKQLNFREAFSHSGLD
FLNHLSKDETAEGFLKYYKDRINVSYDDKTGLLNIQTQGFSPEFALKFNQTVLKESERFI
NEMSHRIARDQLAFAETEMEKARQRLDASKAELLSYQDNNNVLDPQAQAQAASTLVNTLM
GQKIQMEADLRNLLTYLREDAPQVVSARNAIQSLQAQIDEEQSKITAPQGDKLNRMAVDF
EEIKSKVEFNTELYKLTLTSIEKTRVEAARKLKVLSVISSPQLPQESSFPNIPYLIACWL
LVCCLLFGTLKLLLAVIEDHRD
>LFGLNPFC_00163 Arabinose 5-phosphate isomerase KpsF
MSERHLPDDQSSTIDPYLITSVRQTLAEQGAALQNLSKQLDSGQYQRVLNLIMNCKGHVI
LSGMGKSGHVGRKMSATLASTGTPSFFIHPAEAFHGDLGMITPYDLLILISASGETDEIL
KLVPSLKNFGNRIIAITNNGNSTLAKNADAVLELHMANETCPNNLAPTTSTTLTMAIGDA
LAIAMIRQRKFMPNDFARYHPGGSLGRRLLTRVADVMQHDVPAVQLDASFKTVIQRITSG
CQGMVMVEDAEGGLAGIITDGDLRRFMEKEDSLTSATAAQMMTREPLTLPEDTMIIEAEE
KMQKHRVSTLLVTNKANKVTGLVRIFD
>LFGLNPFC_00164 hypothetical protein
MHLSAAGKTKKMALVDCMRKLLTILNAMLRKNEE
>LFGLNPFC_00166 hypothetical protein
MVIGPFINASAVLLGGVFGALLSQRLPERIRVSMTSIFGLASLGIGILLVVKCANLPAMV
LATLLGALIGEICLLEKGVNTVVAKAHNLFHHSRKKPAHESFIQNFVAIIVLFCASGTGI
FGAMNEGMTGDPSILIAKSFLDFFTAMIFACSLGIAVSVISIPLLIIQLTLAWAAALILP
LTTPSMMADFSAVGGLLLLATGLRICGIKMFPVVNMLPALLLAMPLSAAWTAWFA
>LFGLNPFC_00167 Constitutive ornithine decarboxylase
MKSMNIAASSELVSRLSTHRCVVALGDTDFTDVAAVVITAADSRSGILALLKRTGFHLPV
FLYSEHAVELPAGVTAVINGNEQQWLELESAACQYEENLLPPFYDTLTQYVEMGNSTFAC

PGHQHGAFFKKHPAGRHFYDFFGENIFRADMCNADVKLGDLLIHEGSAKDAQKFAAKVFH
ADKTYFVLNGTSAANKVVTNALLTRGDLVLFDRNNHKSNHHGALIQAGATPVYLEASRNP
FGFIGGIDAHCFNEEYLRQQIRDVAPEKADLPRPFRLAIIQLGTYDGTVYNARQVIDTVG
HLCDYILFDSAWVGYEQFIPMMADSSPLLLELNENDPGIFVTQSVHKQQAGFSQTSQIHK
KDNHIRGQARFCPHKRLNNAFMLHASTSPFYPLFAALDVNAKIHEGESGRRLWAECVELG
IESRKAILARCKLFRPFIPPVVDGKLWQDYPTSVLASDRRFFSFDPGAKWHGFEGYAADQ
YFVDPCKLLLTTQGIDAETGEYSDFGVPATILAHYLRENGIVPEKCDLNSILFLLTPAES
HEKLAQLVAMLAQFEQHIEDDSPLAEVLPSVYNKYPVRYRDYTLRQLCQEMHDLYVSFDV
KDLQKAMFRQQSFPSVVMNPQDAHSAYIRGEVELVRIRDAEGRIAAEGALPYPPGVLCVV
PGEVWGGAVQRYFLALEEGVNLLPGFSPELQGVYSETDANGMKRLYGYVLK
>LFGLNPFC_00168 Nucleoside permease NupG
MNLKLQLKILSFLQFCLWGSWLTTLGSYMFVTLKFDGASIGAVYSSLGIAAVFMPALLGI
VADKWLSAKWVYAICHTIGAITLFMAAEVTTPEAMFLVILINSFAYMPTLGLINTISYYR
LQNAGMDIVTDFPPIRIWGTIGFIMAMWVVSLSGFELSHMQLYIGAALSAILVLFTLTLP
HIPVAKQQANQSWTTLLGLDAFALFKNKRMAIFFIFSMLLGAELQITNMFGNTFLHSFDK
DPMFASSFIVQHASIIMSISQISETLFILTIPFFLSRYGIKNVMMISIVAWILRFALFAY
GDPTPFGTVLLVLSMIVYGCAFDFFNISGSVFVEKEVSPAIRASAQGMFLMMTNGFGCIL
GGIVSGKVVEMYTQNGITDWQTVWLIFAGYSVVLAFAFMAMFKYKHVRVPTGTQTVSH
>LFGLNPFC_00169 Membrane-bound lytic murein transglycosylase C
MKKYLALALIAPLLISCSTTKKGDTYNEAWVKDTNGFDILMGQFAHNIENIWGFKEVVIA
GPKDYVKYTDQYQTRSHINFDDGTITIETIAGTEPAAHLRRAIIKTLLMGDDPSSVDLYS
DVDDITISKEPFLYGQVVDNTGQPIRWEGRASNFADYLLKNRLKSRSNGLRIIYSVTINM
VPNHLDKRAHKYLGMVRQASRKYGVDESLILAIMQTESSFNPYAVSRSDALGLMQVVQHT
AGKDVFRSQGKSGTPSRSFLFDPASNIDTGTAYLAMLNNVYLGGIDNPTSRRYAVITAYN
GGAGSVLRVFSNDKIQAANIINTMTPGDVYQTLTTRHPSAESRRYLYKVNTAQKSYRRR
>LFGLNPFC_00170 putative Fe(2+)-trafficking protein
MSRTIFCTFLQREAEGQDFQLYPGELGKRIYNEISKEAWAQWQHKQTMLINEKKLNMMNA
EHRKLLEQEMVNFLFEGKEVHIEGYTPEDKK
>LFGLNPFC_00171 Adenine DNA glycosylase
MQASQFSAQVLDWYDKYGRKTLPWQIDKTPYKVWLSEVMLQQTQVATVIPYFERFMARFP
TVTDLANAPLDEVLHLWTGLGYYARARNLHKAAQQVATLHGGKFPETFEEVAALPGVGRS
TAGAILSLSLGKHFPILDGNVKRVLARCYAVSGWPGKKEVENKLWSLSEQVTPAVGVERF
NQAMMDLGAMICTRSKPKCSLCPLQNGCIAAANNSWSLYPGKKPKQTLPERTGYFLLLQH
EDEVLLAQRPSSGLWGGLYCFPQFADEESLRQWLAQRQISADNLTQLTAFRHTFSHFHLD
IVPMWLPVSSFTGCMDEDNALWYNLAQPPSVGLAAPVERLLQQLRTGAPV
>LFGLNPFC_00172 tRNA (guanine-N(7)-)-methyltransferase
MKNDVISPEFDENGRPLRRIRSFVRRQGRLTKGQEHALENYWPVMGVEFSEDMLDFPALF
GREAPVTLEIGFGMGASLVAMAKDRPEQDFLGIEVHSPGVGACLSSAHEEGLSNLRVMCH
DAVEVLHKMIPDNSLRMVQLFFPDPWHKARHNKRRIVQVPFAELVKSKLQLGGIFHMATD
WEPYAEHMLEVMSSIDGYKNLSESNDYVPRPASRPVTKFEQRGHRLGHGVWDLMFERVK
>LFGLNPFC_00173 putative protein
MAKNRSRRLRKKMHIDEFQELGFSVAWRFPEGTSEEQIDKTVDDFINEVIEPNKLAFDGS
GYLAWEGLICMQEIGKCTEEHQAIVRKWLEERKLEEVRTSELFDVWWD
>LFGLNPFC_00174 hypothetical protein
MMRKMLLAAALSVTAMTAHADYQCSVTPRDDVIVSPQTVQVKGENGNLVITPDGNVMYNG
KQYSLNAAQREQAKDYQAELRSTLPWIDEGAKSRVEKARIALDKIIVQEMGESSKMRSRL
TKLDAQLKEQMNRIIETRSDGLTFHYKAIDQVRAEGQQLVNQAMGGILQDSINEMGAKAV
LKSGGNPLQNVLGSLGGLQSSIQTEWKKQEKDFQQFGKDVCSRVVTLEDSRKALVGNLK
>LFGLNPFC_00175 L-asparaginase 2
MEFFKKTALAALVMGFSGAALALPNITILATGGTIAGGGDSATKSNYTAGKVGVENLVNA
VPQLKDIANVKGEQVVNIGSQDMNDDVWLTLAKKINTDCDKTDGFVITHGTDTMEETAYF
LDLTVKCDKPVVMVGAMRPSTSMSADGPFNLYNAVVTAADKASANRGVLVVMNDTVLDGR
DVTKTNTTDVATFKSVNYGPLGYIHNGKIDYQRTPARKHTSDTPFDVSKLNELPKVGIVY
NYANASDLPAKALVDAGYDGIVSAGVGNGNLYKTVFDTLATAAKNGTAVVRSSRVPTGAT
TQDAEVDDAKYGFVASGMLNPQKARVLLQLALTQTKDPQQIQQIFNQY
>LFGLNPFC_00176 hypothetical protein
MKKQWIVGTALFMLMTGNVWADGEPPTENILKDQFKKQYHGILKLDVITLKNLDAKGNQA
TWSAEGDVSSSDDLYTWVGQLADYELLEQTWTKDKPVKFSAMLTSKGTPASGWSVNFYSF
QAAASDRGRVVDDIKTNNKYLIVNSEDFNYRFSQLESALNNQNNSIPALKKDVKALDKQM
VAAQKAADAYWGKDANGKQMTREDAFKKIHQQRDDFNKQNDSEAFAVKYDKEVYQPAIAA
CHKQSEECYEVPIQQKRDFDINEQRRQTFLQSQKLSRKLQDDWITLEKGQYPLTMKVSEI
NSKKVAILMKIDDINQANERWKKDTEQLRRNGVIK
>LFGLNPFC_00177 Heme chaperone HemW
MVKLPPLSLYIHIPWCVQKCPYCDFNSHALKGEVPHDDYVQHLLNDLDNDVAYAQGREVQ

TIFIGGGTPSLLSGPAMQTLLDGVRARLPLTADAEITMEANPGTVEADRFVDYQRAGVNR
ISIGVQSFSEEKLKRLGRIHGPQEAKRAAKLASGLGLRSFNLDLMHGLPDQSLEEALGDL
RQAIELNPPHLSWYQLTIEPNTLFGSRPPVLPDDDALWDIFEQGHQLLTAAGYQQYETSA
YSKPGYQCQHNLNYWRFGDYIGIGCGAHGKVTFPDGRILRTTKTRHPRGFMQGRYLESQR
DVEAADKPFEFFMNRFRLLEAAPRVEFSQYTGLSEEVIRPQLEEAIAQGYLTECADYWQI
TEHGKLFLNSLLELFLAE
>LFGLNPFC_00178 dITP/XTP pyrophosphatase
MQKVVLATGNAGKVRELASLLSDFGLDIVAQTDLGVDSAEETGLTFIENAILKARHAAKV
TGLPAIADDSGLAVDALGGAPGIYSARYSGEDATDQKNLQKLLETMKDVPDDQRQARFHC
VLVYLRHAEDPTPLVCHGSWPGVITREPAGTGGFGYDPIFFVPSEGKTAAELTREEKSAI
SHRGQALKLLLDALRNG
>LFGLNPFC_00179 hypothetical protein
MSAVTVNDDGLVLRLYIQPKASRDSIVGLHGDEVKVAITAPPVDGQANSHLVKFLGKQFR
VAKSQVVIEKGELGRHKQIKIINPQQIPPEIAALIN
>LFGLNPFC_00180 hypothetical protein
MNTLTFLLSTVIELYTMVLLLRIWMQWAHCDFYNPFSQFVVKVTQPIIGPLRRVIPAMGP
IDSASLLVAYILSFIKAIVLFKVVTFLPIIWIAGLLILLKTIGLLIFWVLLVMAIMSWVS
QGRSPIEYVLIQLADPLLRPIRRLLPAMGGIDFSPMILVLLLYVINMGVAEVLQATGNML
LPGLWMAL
>LFGLNPFC_00181 Pyridoxal phosphate homeostasis protein
MNDIAHNLAQVRDKISAAATRCGRSPEEITLLAVSKTKPASAIAEAIDAGQRQFGENYVQ
EGVDKIRHFQELSVTGLEWHFIGPLQSNKSRLVAEHFDWCHTIDRLRIATRLNDQRPAEL
PPLNVLIQINISDENSKSGIQLAELDELAAAVAELPRLRLRGLMAIPAPESEYVRQFEVA
RQMAVAFAGLKTRYPHIDTLSLGMSDDMEAAIAAGSTMVRIGTAIFGARDYSKK
>LFGLNPFC_00182 putative protein YggR
MNMEEIVALSVKHNVSDLHLCSAWPARWRIRGRMEAAPFDAPDVEELLREWLDDDQRAIL
LENGQLDFAVSLAENQRLRGSAFAQRQGISLALRLLPSHCPQLEQLGAPPVLPELLKSEN
GLILVTGATGSGKSTTLAAMVGYLNQHADAHILTLEDPVEYLYTSQRCLIQQREIGLHCM
TFASGLRAALREDPDVILLGELRDSETIRLALTAAETGHLVLATLHTRGAAQAVERLVDS
FPAQEKDPVRNQLAGSLRAVLSQKLEVDKQEGRVALFELLINTPAVGNLIREGKTHQLPH
VIQTGQQVGMITFQQSYQQRVKEGRL
>LFGLNPFC_00183 Putative pre-16S rRNA nuclease
MSGTLLAFDFGTKSIGVAVGQRITGTARPLPAIKAQDGTPDWNIIERLLKEWQPDEIIVG
LPLNMDGTEQPLTARARKFANRIHGRFGVEVKLHDERLSTVEARSGLFEQGGYRALNKGK
VDSASAVIILESYFEQGY
>LFGLNPFC_00184 hypothetical protein
MTAPFFPRILGAVAFLNQETEPLTMNLQHHFLIAMPALQDPIFRRSVVYICEHNTNGAMG
IIVNKPLENLKIEGILEKLKITPEPRDESIRLDKPVMLGGPLAEDRGFILHTPPSNFASS
IRISDNTVMTTSRDVLETLGTDKQPSDVLVALGYASWEKGQLEQEILDNAWLTAPADLNI
LFKTPIADRWREAAKLIGVDILTMPGVAGHA
>LFGLNPFC_00185 Glutathione synthetase
MIKLGIVMDPIANINIKKDSSFAMLLEAQRRGYELHYMEMADLYLINGEARARTRTLSVE
QNYDKWYEFTGEQDLPLADLDVILMRKDPPFDTEFIYATYILERAEEKGTLIVNKPQSLR
DCNEKLFTAWFSDLTPETLVTRNKAQLKAFWEKHSDIILKPLDGMGGASIFRVKEGDPNL
GVIAETLTEHGTRYCMAQNYLPAIKDGDKRVLVVDGEPVPYCLARIPQGGETRGNLAAGG
RGEPRPLTESDWKIARQIGPTLKEKGLIFVGLDIIGDRLTEINVTSPTCIREIEAEFPVS
ITGMLMDAIEARLQQQ
>LFGLNPFC_00186 Ribosomal RNA small subunit methyltransferase E
MRIPRIYHPEPLTSHSHIALCEDAANHIGRVLRMGPGQALQLFDGSNQVFDAEITSASKK
SVEVKVLEGQIDDRESPLHIHLGQVMSRGEKMEFTIQKSIELGVSLITPLFSERCGVKLD
SERLNKKLQQWQKIAIAACEQCGRNRVPEIRPAMDLEAWCAEQDEGLKLNLHPRASNSIN
TLPLPVERVRLLIGPEGGLSADEIAMTARYQFTDILLGPRVLRTETTALTAITALQVRFG
DLG
>LFGLNPFC_00187 Endonuclease-1
MYRYLSIAAVVLSAAFSGPALAEGINSFSQAKAAAVKVHADAPGTFYCGCKINWQGKKGV
VDLQSCGYQVRKNENRASRVEWEHVVPAWQFGHQRQCWQDGGRKNCAKDPVYRKMESDMH
NLQPSVGEVNGDRGNFMYSQWNGGEGQYGQCAMKVDFKEKVAEPPARARGAIARTYFYMR
DQYNLTLSRQQTQLFNAWDKMYPVTDWECERDERIAKVQGNHNPYVQRACQARKS
>LFGLNPFC_00188 Protein SprT
MRRLREKLAQANLKLGRNYPEPKLSYTQRGTSAGTAWLESYEIRLNPVLLLENSEAFIEE
VVPHELAHLLVWKHFGRVAPHGKEWKWMMESVLGVPARRTHQFELQSVRRNTFPYRCKCQ
EHQLTVRRHNRVVRGEAVYRCVHCGEQLVAK
>LFGLNPFC_00189 Galactose-proton symporter
MPDAKKQGRSNKAMTFFVCFLAALAGLLFGLDIGVIAGALPFIADEFQITSHTQEWVVSS

MMFGAAVGAVGSGWLSFKLGRKKSLMIGAILFVAGSLFSAAAPNVEVLILSRVLLGLAVG
VASYTAPLYLSEIAPEKIRGSMISMYQLMITIGILGAYLSDTAFSYTGAWRWMLGVIIIP
AILLLIGVFFLPDSPRWFAAKRRFVDAERVLLRLRDTSAEAKRELDEIRESLQVKQSGWA
LFKENSNFRRAVFLGVLLQIMQQFTGMNVIMYYAPKIFELAGYTNTTEQMWGTVIVGLTN
VLATFIAIGLVDRWGRKPTLTLGFLVMAAGMGVLGTMMHIGIHSPSAQYFAIAMLLMFIV
GFAMSAGPLIWVLCSEIQPLKGRDFGITCSTATNWIANMIVGATFLTMLNTLGNANTFWV
YAALNVLFILLTLWLVPETKHVSLEHIERNLMKGRKLREIGAHD
>LFGLNPFC_00190 S-adenosylmethionine synthase
MAKHLFTSESVSEGHPDKIADQISDAVLDAILEQDPKARVACETYVKTGMVLVGGEITTS
AWVDIEEITRNTVREIGYVHSDMGFDANSCAVLSAIGKQSPDINQGVDRADPLEQGAGDQ
GLMFGYATNETDVLMPAPITYAHRLVQRQAEVRKNGTLPWLRPDAKSQVTFQYDDGKIVG
IDAVVLSTQHSEEIDQKSLQEAVMEEIIKPILPAEWLTSATKFFINPTGRFVIGGPMGDC
GLTGRKIIVDTYGGMARHGGGAFSGKDPSKVDRSAAYAARYVAKNIVAAGLADRCEIQVS
YAIGVAEPTSIMVETFGTEKVPSEQLTLLVREFFDLRPYGLIQMLDLLHPIYKETAAYGH
FGREHFPWEKTDKAQLLRDAAGLK
>LFGLNPFC_00191 hypothetical protein
MVADPTTTLQVKNTGSLSVNRYGWINIWMAILGQFFTQFPLFFESCLILLKTWLEIFPDN
AGILRIYLLQFSAIVGYKTRRAA
>LFGLNPFC_00192 Biosynthetic arginine decarboxylase
MSDDMSMGLPSSAGEHGVLRSMQEVAMSSQEASKMLRTYNIAWWGNNYYDVNELGHISVC
PDPDVPEARVDLAQLVKTREAQGQRLPALFCFPQILQHRLRSINAAFKRARESYGYNGDY
FLVYPIKVNQHRRVIESLIHSGEPLGLEAGSKAELMAVLAHAGMTRSVIVCNGYKDREYI
RLALIGEKMGHKVYLVIEKMSEIAIVLDEAERLNVVPRLGVRARLASQGSGKWQSSGGEK
SKFGLAATQVLQLVETLREAGRLDSLQLLHFHLGSQMANIRDIATGVRESARFYVELHKL
GVNIQCFDVGGGLGVDYEGTRSQSDCSVNYGLNEYANNIIWAIGDACEENGLPHPTVITE
SGRAVTAHHTVLVSNIIGVERNEYTVPTAPAEDAPRALQSMWETWQEMHEPGTRRSLREW
LHDSQMDLHDIHIGYSSGTFSLQERAWAEQLYLSMCHEVQKQLDPQNRAHRPIIDELQER
MADKMYVNFSLFQSMPDAWGIDQLFPVLPLEGLDQVPERRAVLLDITCDSDGAIDHYIDG
DGIATTMPMPEYDPENPPMLGFFMVGAYQEILGNMHNLFGDTEAVDVFVFPDGSVEVELS
DEGDTVADMLQYVQLDPKTLLTQFRDQVKKTDLDAELQQQFLEEFEAGLYGYTYLEDE
>LFGLNPFC_00193 hypothetical protein
MKKWKVRSALVALIVLLAGCSSNAQYNSSASGNVGTAWGGDVHSTVHGVSAERAWRDPAE
MIVISYSTNVPSGYDRVYSIRINELEYAIRDGNFKSLPITRVYDSSNNEPRYIVHARVGM
NYQLYVRNYSRNTNYEIVATVDGLDVLNGKQGSLNNNGYIVNAGDSLAIKGFRKDKHTEA
AFQFANVADSYAANSAQGDVRNTGVIGFAAFELQGPAKNALPPCSGQAFPADNNGYAPPP
CRK
>LFGLNPFC_00194 Agmatinase
MSTLGHQYDNSLVSNAFGFLRLPMNFQPYDSDADWVITGVPFDMATSGRAGGRHGPAAIR
QVSTNLAWEHNRFPWNFDMRERLNVVDCGDLVYAFGDAREMSEKLQAHAEKLLAAGKRML
SFGGDHFVTLPLLRAHAKHFGKMALVHFDAHTDTYANGCEFDHGTMFYTAPKEGLIDPNH
SVQIGIRTEFDKDNGFTVLDACQVNDRSVDDIIAQVKQIVGDMPVYLTFDIDCLDPAFAP
GTGTPVIGGLTSDRAIKLVRGLKDLNIVGMDVVEVAPAYDQSEITALAAATLALEMLYIQ
AAKKGE
>LFGLNPFC_00195 Metalloprotease LoiP
MKIRALLVAMSVATVLTGCQNMDSSGLLSSGAEAFQAYSLSDAQVKKLSDQACQEMDSKA
TIAPANSEYARRLTTISRALGDNINGQPVNYKVYMAKDVNAFAMANGCIRVYSGLMDMMT
DNEVEAVLGHEMGHVALGHVKKGMQVALGTNAIRVAAASAGGIVGSLSQSQLGDLGEKLV
NSQFTQRQESEADDYSYDLLRQRGISPAGLATSFEKLAKLEEGRQSSMFDDHPASAERAQ
HIRDRMSADGVK
>LFGLNPFC_00196 Transketolase 1
MSSRKELANAIRALSMDAVQKAKSGHPGAPMGMADIAEVLWRDFLKHNPQNPSWADRDRF
VLSNGHGSMLIYSLLHLTGYDLPMEELKNFRQLHSKTPGHPEVGYTAGVETTTGPLGQGI
ANAVGMAIAEKTLAAQFNRPGHDIVDHYTYAFMGDGCMMEGISHEVCSLAGTLKLGKLIA
FYDDNGISIDGHVEGWFTDDTAMRFEAYGWHVIRDIDGHDAASIKRAIEEARAVTDKPSL
LMCKTIIGFGSPNKAGTHDSHGAPLGDAEIALTREQLGWKYAPFEIPSEIYAQWDAKEAG
QAKESAWNEKFAAYAKAYPQEAAEFTRRMKGEMPSDFDAKAKEFIAKLQANPAKIASRKA
SQNAIEAFGPLLPEFLGGSADLAPSNLTLWSGSKAINEDAAGNYIHYGVREFGMTAIANG
ISLHGGFLPYTSTFLMFVEYARNAVRMAALMKQRQVMVYTHDSIGLGEDGPTHQPVEQIA
SLRVTPNMSTWRPCDQVESAVAWKYGVERQDGPTALILSRQNLAQQERTEEQLANIARGG
YVLKDCAGQPELIFIATGSEVELAVAAYEKLTAEGVKARVVSMPSTDAFDKQDAAYRESV
LPKAVTARVAVEAGIADYWYKYVGLNGAIVGMTTFGESAPAEQLFEEFGFTVDNVVAKAK
ALL
>LFGLNPFC_00197 Modulator of FtsH protease HflC
MKAPVSLTSFRPQKSLAIAIGVLAVVVLPFLSYYTVNEGERGILLRYGKIVKVAEPGLGF

KIPFMESVEKISTRNQAVVYQGLQAYSRDQQPAQMTVSVSFHIKPSEAGAVYTTYNTIEA
LKDRLIVRQLPTQLENVFGQYTAISAVQDRTKLVQDLQNAMRKAVVGPVVIDGVQIENID
FSDAYEKSIEDRMKAEVAIATRKQNLETEKIQAQIAVTQAQAEADSKLAAAKAEAETIRV
RGAAEAETIRLKSAAEAEAIRLRGEALRENPGLVALTTAERWDGKLPDTMIPGSTVPFIS
TK
>LFGLNPFC_00198 Mannitol-specific cryptic phosphotransferase enzyme IIA component
MRLIDYFPELSISVIHSTKDWQEAINFSMASLLAKNYISENYIQAIKDSTISNGPYYILA
PGVAMPHARPECGALKTGMSLTLLKQGVYFPGNDEPIKLLIGLSAADADSHIAAIQALSE
LLCEEEILEQLLTASSEKQLAGIISRG
>LFGLNPFC_00199 PTS system mannitol-specific EIICB component
MENKSARAKVQAFGGFLTAMVIPNIGAFIAWGFITALFIPTGWLPNEHFAKIVGPMITYL
LPVMIGSTGGHLVGGKRGAVMGGIGTIGVIVGAEIPMFLGSMIMGPLGGLVIKYIDKSLE
KRIPAGFEMVINNFSLGIAGMLLCLLGFEVIGPAVLIANTFVKECIEALVHAGYLPLLSV
INEPAKVLFLNNAIDQGVYYPLGMQQASVNGKSIFFMVASNPGPGLGLLLAFTLFGKGMS
KRSAPGAMIIHFLGGIHELYFPYVLMKPLTIIAMIAGGMSGTWMFNLLDGGLVAGPSPGS
IFAYLALTPKGSFLATIAGVTVGTLVSFAITSLILKMEKTVETESDDEFAQSANAVKAMK
QEGAFSLSRVKRIAFVCDAGMGSSAMGATTFRKRLEKAGLAIEVKHYAIENVPADADIVV
THASLEGRVKRVTDKPLILINNYIGDPKLDTLFNQLTAEHKH
>LFGLNPFC_00200 Mannitol-1-phosphate 5-dehydrogenase
MKTKVAAIYGKRDVRLREFELPEITDNELLVNVISDSVCLSTWKAALLGSEHKRVPDDLE
NHPVITGHECAGVIVEVGKNLTGKYKKGQRFVLQPAMGLPGGYSAGYSYEYFGGNATYMI
IPEIAINLGCVLPYHGSYFAAASLAEPMCCIIGAYHANYHTTQYVYEHRMGVKPGGNIAL
LACAGPMGIGAIDYAINGGIQPSRVVVVDIDDKRLAQVQMLLPVELAASKGIELVYMNTK
GVSDPVQTLRALTDDAGFDDVFVYAAVPSVVEMADELLAEDGCLNFFAGPTDKNFKVPFN
FYNVHYNSTHVVGTSGGSTDDMKEAIALSATGGQLQPSFMVTHIGGLDAVPETVLNLPDIP
GGKKLIYNGVTMPLTAIADFAEKGKTDPLFKELARLVEKTHGIWNEQAEKYLLAQFGVDI
GEAAQ
>LFGLNPFC_00201 Fructose-1,6-bisphosphatase 2 class 2
MMSLAWPLFRVTEQAALAAWPQTGCGDKNKIDSLAVTAMRQALNDVAFRGRVVIGEGERY
PL
>LFGLNPFC_00202 Fumarase E
MATLTEDDVLEQLDAQDNLFSFMKTAHSILLQGIRQFLPSLFVDNDEEIVEYAVKPLLAQ
SGPLDDIDVALRLIYALGKMDKWLYADITHFSQFWHYLNEQDETPGFADDMAWDFISNVN
SIIRNASLYDALKAMKFADFAVWSEARFSGMVKTALMLAVTTTLKELTP
>LFGLNPFC_00203 Pantothenate kinase
MKIELTVNGLKVQAQYSDDEIENVHKPLLRMLAALQTVNPQRRTVVFLCAPPGTGKSTLT
TFWEYLAQQDPELPAIQTLPMDGFHHYNSWLDVHQLRPFKGAPETFNVAKLAENLCRVVE
GDCTWPQYDRQKHDPVEDVLHVTAPLVIVEGNWLLLDDEKWCQLAQFCDFSIFIKAPASA
LRERLVGRKLAGGLSLADAEAFYDRTDGPNVRRVLEESLPANLTLMMTATGEYRLMD
>LFGLNPFC_00204 Energy-coupling factor transporter ATP-binding protein EcfA1
MLTLNQISYRWPGAATDCLCDISLQLKQGEWLALTGDNGAGKSTLLRVMAGLLTPTAGTV
MLQQQAMKNLKNRQRAAKIGVLFQEAENQLFHSTVADEIAFGLILQKCPADEITQHTNAA
LQCCQLADTASAHPLDLHSAQRRMVAVACLEALSPPLLLLDEPSRDFDENWLSVFESWLE
KCRQRGTSVVAISHDAAFTRRHFSRVVRLEDGVIRNVNPSDDIHP
>LFGLNPFC_00205 Energy-coupling factor transporter ATP-binding protein EcfA1
MVTLEQFRYCPTHSTHPPFCYDFHYVKPGMVAIFGDNGSGKSTLAQLMAGWYPDFLPGEI
TGTGTLLGTPIGRLPLNEQSATIQLVQQSPYLQLSGCTFSVEEEVAFGPENLCLAEKEIM
ARIDAALALTECQPLRHRHPATLSGGETQRVVIACAIAMQPKLLILDEAFSRLTPQAREM
LLQRLQHWALERGSLIILFERHHTPFLNHCQQAWQLQNGALQPLC
>LFGLNPFC_00206 Energy-coupling factor transporter transmembrane protein EcfT
MHPFTSLTLWALAACTTLILPTQTILPVYSAATFFCLIALKATRRRAKYVVWLMFSLGAG
LWLVHGGWLTEWLSGTPRSPERWAHAITLWLRILAIVSTSQLWMQYVPVQRFIRALFASR
LPPGVAYLFAGPLLVVEQLKRQLAIIHEAQRARGVPLDEGWYQRLRAMPALIIPLTHNAL
NDLAVRGAALDMRAFRINNRRTTLWAPADSTLQRVARYAMILLMLAEFGAWIWLR
>LFGLNPFC_00207 hypothetical protein
MARRHFSSQALVLIVISIAINMIGGQLASMVKLPIFLDSIGTLISAVLLGPVIGMLTGLL
TNLLWGLLTDPIAAAFAPVAMVIGLVAGWLARAGWFRTLPKVVVSGVIITLAVTVVAVPL
RTALFGGVTGSGADLFVAWMHSMGQNLVESVAITVIGANLVDKILTAVIVWLLLRQLPIR
TTRHFPAMAAVR
>LFGLNPFC_00208 hypothetical protein
MMTLPYPYSSSARFYALRLLPGQEVLSQLRAFAQQQQLHAAWIAGCTGSLTDVALRYAGQ
ENTALLSGKFEVIALNGTLEQSGEHLHLCVSDPHGTMLGGHMMPGCTVRTTLELVIGCLE
ELAFSRQLCALSGYDELHISPVK
>LFGLNPFC_00209 D-erythrose-4-phosphate dehydrogenase

MTVRVAINGFGRIGRNVVRALYESGRRAEITVVAINELADAAGMAHLLKYDTSHGRFAWE
VRQERDQLFVGDDAIRVLHERSLQSLPWRELGVDVVLDCTGVYGSREHGEAHIAAGAKKV
LFSHPGSNDLDTTVVYGVNQDQLRAEHRIVSNASCTTNCIIPVIKLLDDAYGIESGTVTT
IHSAMHDQQVIDAYHPDLRRTRAASQSIIPVDTKLAAGITRFFPQFNDRFEAIAVRVPTI
NVTAIDLSVTVKKPVKANEVNLLLQKAAQGAFHGIVDYTELPLVSVDFNHDPHSAIVDGT
QTRVSGAHLIKTLVWCDNEWGFANRMLDTTLAMATVAFR
>LFGLNPFC_00210 Phosphoglycerate kinase
MSVIKMTDLDLAGKRVFIRADLNVPVKDGKVTSDARIRASLPTIELALKQGAKVMVTSHL
GRPTEGEYNEEFSLLPVVNYLKDKLSNPVRLVKDYLDGVDVAEGELVVLENVRFNKGEKK
DDETLSKKYAALCDVFVMDAFGTAHRAQASTHGIGKFADVACAGPLLAAELDALGKALKE
PARPMVAIVGGSKVSTKLTVLDSLSKIADQLIVGGGIANTFIAAQGHDVGKSLYEADLVD
EAKRLLTTCNIPVPSDVRVATEFSETAPATLKSVNDVKADEQILDIGDASAQELAEILKN
AKTILWNGPVGVFEFPNFRKGTEIVANAIADSEAFSIAGGGDTLAAIDLFGIADKISYIS
TGGGAFLEFVEGKVLPAVAMLEERAKK
>LFGLNPFC_00211 Fructose-bisphosphate aldolase class 2
MSKIFDFVKPGVITGDDVQKVFQVAKENNFALPAVNCVGTDSINAVLETAAKVKAPVIVQ
FSNGGASFIAGKGVKSDVPQGAAILGAISGAHHVHQMAEHYGVPVILHTDHCAKKLLPWI
DGLLDAGEKHFAATGKPLFSSHMIDLSEESLQENIEICSKYLERMSKIGMTLEIELGCTG
GEEDGVDNSHMDASALYTQPEDVDYAYTELSKISPRFTIAASFGNVHGVYKPGNVVLTPT
ILRDSQEYVSKKHNLPHNSLNFVFHGGSGSTAQEIKDSVSYGVVKMNIDTDTQWATWEGV
LNYYKENEAYLQGQLGNPKGEDQPNKKYYDPRVWLRAGQTSMIARLEKAFQELNAIDVL
>LFGLNPFC_00212 Small-conductance mechanosensitive channel
MEDLNVVDSINGAGSWLVANQALLLSYAVNIVAALAIIIVGLIIARMISNAVNRLMISRK
IDATVADFLSALVRYGIIAFTLIAALGRVGVQTASVIAVLGAAGLAVGLALQGSLSNLAA
GVLLVMFRPFRAGEYVDLGGVAGTVLSVQIFSTTMRTADGKIIVIPNGKIIAGNIINFSR
EPARRNEFIIGVAYDSDIDQVKQILTDIIQSEDRILKDREMTVRLNELGASSINFVVRVW
SNSGDLQNVYWDVLERIKREFDAAGISFPYPQMDVNFKRVKEDKAA
>LFGLNPFC_00213 Arginine exporter protein ArgO
MFSYYFQGLALGATMILPLGPQNAFVMNQGIRRQYHIMIALLCAISDLVLICAGIFGGSA
LLMQSPWLLALTWGGVAFLLWYGFGAFKTAMSSNIELASAEVLKQGRWKIIATMLAVTWL
NPHVYLDTFVVLGSLGGQLDVEPKRWFALGTISASFLWFFGLAILAAWLAPRLRTAKSQR
IINLVVGCVMWFIALQLARDGIAHAQALFS
>LFGLNPFC_00214 26 kDa periplasmic immunogenic protein
MKFKVIALAALMGISGMAAQANELPDGPHIVTSGTASVDAVPDIATLAIEVNVAAKDAAT
AKKQADERVAQYISFLELNQIAKKDISSANLRTQPDYDYQDGKSILKGYRAVRTVEVTLR
QLDKLNSLLDGALKAGLNEIRSVSLGVAQPDAYKDKARKAAIDNAIHQAQELANGFHRKL
GPVYSVRYHVSNYQPSPMVRMMKADAAPVSAQETYEQAAIQFDDQVDVIFQLEPVDQPPA
KTPAAH
>LFGLNPFC_00215 HTH-type transcriptional regulator CynR
MDIFISKKMRNFILLAQTNNIARAAEKIHMTASRFGKSIAALEEQIGYTLFTRKDNNISL
NKAGQELYQKLFPVYQRLSAIDNEIHNSGRRSRDIVIGIDNTYPTIIFDQLISLGDKYEG
VTAQPVEFSANGVIDNLFDRQLDFIISPQHVSARVQELENLTISELPPLRLGFLVSRRYE
ERQEQELLQELPWLQMRFQNWANFEAMIDANMRPCGINPTIIYRPYSFMAKISDVERGHF
LTVIPHFAWRLVNPATLKYFDAPHRPMYMQEYLYSIRNHRYTATMLQHIAEDRDGTNH
>LFGLNPFC_00216 Methylmalonyl-CoA mutase
MSNVQEWQQLANKELSRREKTVDSLVQQTAEGIAIKPLYTEADLDNLEVTGTLPGLPPYV
RGPRATMYTAQPWTIRQYAGFSTAKESNAFYRRNLAAHRGYDSDNPRVAGDVGKAGVAID
TGEDMKVLFDQIPLDKMSVSMTMNGAVLPVLAFYIVAAEEQGVTSDKLTGTIQNDILKEY
LCRNTYIYPPKPSMRIIADIIAWCSGNMPRFNTISISSYHMGEAGANCVQQVAFTLADRI
EYIKAAISAGLKIDGFVPRLSFFFGIGMDLFMNAAMLRAARYLWSEAVSGFGAQDPYNNV
IRTTIDHCAHPMCRDYLHRYLENASGGHIHHDLSHVFDLHRNLIATGSMLG
>LFGLNPFC_00217 HTH-type transcriptional regulator ArgP
MKRPDYRTLQALDAVIRERGFERAAQKLCITQSAVSQRIKQLENMFGQPLLVRTVPPRPT
EQGQKLLALLRQVELLEEEWLGDEQTGTSTPLLLSLAVNADSLATWLLPALAPVLADSPIR
LNLQVEDETRTQERLRRGEVVGAVSIQHQALPSCLVDKLGALDYLFVSSKPFAEKYFPNG
VTRSALLKAPVVAFDHLDDMHQAFLQQNFDLPPGSVPCHIVNSSEAFVQLARQGTTCCMI
PHLQIEKELASGELIDLTPGLFQRRMLYWHRFAPESRMMRKVTDALLDYGHKVLRQD
>LFGLNPFC_00218 hypothetical protein
MHFAQRVRALVVLNGVALLPQFACKQGLANGELVRLFAPWSGIPRPLYALFAGRKGMPAI
ARYFMDELTTRLANGV
>LFGLNPFC_00219 Ribose-5-phosphate isomerase A
MTQDELKKAVGWAALQYVQPGTIVGVGTGSTAAHFIDALGTMKGQIEGAVSSSDASTEKL
KSLGIHVFDLNEVDSLGIYVDGADEINGHMQMIKGGGAALTREKIIASVAEKFICIADAS
KQVDILGKFPLPVEVIPMARSAVARQLVKLGGRPEYRQGVVTDNGNVILDVHGMEILDPI

AMENAINAIPGVVTVGLFANRGADVALIGTPDGVKTIVK
>LFGLNPFC_00220 D-3-phosphoglycerate dehydrogenase
MAKVSLEKDKIKFLLVEGVHQKALESLRAAGYTNIEFHKGALDDEQLKESIRDAHFIGLR
SRTHLTEDVINAAEKLVAIGCFCIGTNQVDLDAAAKRGIPVFNAPFSNTRSVAELVIGEL
LLLLRGVPEANAKAHRGVWNKLAAGSFEARGKKLGIIGYGHIGTQLGILAESLGMYVYFY
DIENKLPLGNATQVQHLSDLLNMSDVVSLHVPENPSTKNMMGAKEISLMKPGSLLINASR
GTVVDIPALCDALASKHLAGAAIDVFPTEPATNSDPFTSPLCEFDNVLLTPHIGGSTQEA
QENIGLEVAGKLIKYSDNGSTLSAVNFPEVSLPLHGGRRLMHIHENRPGVLTALNKIFAE
QGVNIAAQYLQTSAQMGYVVIDIEADEDVAEKALQAMKAIPGTIRARLLY
>LFGLNPFC_00221 5-formyltetrahydrofolate cyclo-ligase
MTQLPELPLTLSRQEIRKMIRQRRRTLTPEQQQEMGQQAATRMMTYPPVVMAHTVAVFLS
FDGELDTQPLIEQLWRAGKRVYLPVLHPFSAGNLLFLNYHPQSELVMNRLKIHEPKLDVR
DVLPLSRLDVLITPLVAFDEYGQRLGMGGGFYDRTLQNWQHYKMQPVGYAHDCQLVEKLP
VEKWDIPLPAVVTPSKVWEW
>LFGLNPFC_00222 Cell division protein ZapA
MSAQPVDIQIFGRSLRVNCPPDQRDALNQAADDLNQRLQDLKERTRVTNTEQLVFIAALN
ISYELAQEKAKTRDYAASMEQRIRMLQQTIEQALLEQGRITEKTNQNFE
>LFGLNPFC_00223 hypothetical protein
MSIQNEMPGYNEMNQYLNQQGTGLTPAEMHGLISGMICGGNDDSSWLPLLHDLTNEGMAF
GHELAQALRKMHSATSDALQDDGFLFQLYLPDGDDVSVFDRADALAGWVNHFLLGLGVTQ
PKLDKVTGETGEAIDDLRNIAQLGYDEDEDQEELEMSLEEIIEYVRVAALLCHDTFTHPQ
PTAPEVQKPTLH
>LFGLNPFC_00224 Xaa-Pro aminopeptidase
MSDISRQEFQRRRQALVEQMQPGSAALIFAAPEVTRSADSEYPYRQNSDFWYFTGFNEPE
AVLVLIKSDDTHNHSVLFNRVRDLTAEIWFGRRLGQDAAPEKLGVDRALAFSEINQQLYQ
LLNGLDVVYHAQGEHAYADEIVNSALEKLRKGSRQNLTAPATMIDWRPVVHEMRLFKSPE
EIAVLRRAGEITALAHTRAMEKCRPGMFEYHLEGEIHHEFNRHGARYPSYNTIVGSGENG
CILHYTENECELRDGDLVLIDAGCEYKGYAGDITRTFPVNGKFTQAQREIYDIVLESLET
SLRLYRPGTSIQEVTGEVVRIMVSGLVKLGILKGEVDELIAQNAHRPFFMHGLSHWLGLD
VHDVGVYGQDRSRILEPGMVLTVEPGLYIAPDAEVPEQYRGIGIRIEDDIVITETGNENL
TASVVKKPEEIEALMAAARKQ
>LFGLNPFC_00225 2-octaprenyl-6-methoxyphenol hydroxylase
MRVIIVGGGMAGATLALAISRLSHGALPVHLIEATAPESHAHPGFDGRAIALAAGTCQQL
ARIGVWQSLADCATAITTVHVSDRGHAGFVTLAAEDYQLAALGQVVELHNVGQRLFALLR
KAPGVTLHCPDRVANVARTQSHVEVTLESGETLTGRVLVAADGTHSALATVCGVDWQQEP
YEQLAVIANVATSVAHEGRAFERFTQHGPLAMLPMSDGRCSLVWCHPLERREEVLSWSDE
KFCRELQSAFGWRLGKITHAGKRSAYPLALTRAAKPITHRTVLVGNAAQTLHPIAGQGFN
LGMRDVMSLAETLTQAQERGEDMGDYGVLCRYQQRRQSDREATIGVTDSLVHLFANRWTP
LVVGRNIGLMTMELFTPARDVLAQRTLGWVAR
>LFGLNPFC_00226 2-octaprenylphenol hydroxylase
MQSVDVAIVGGGMVGLAVACGLQGSGLRVAVLEQRVPEPLAADAPPQLRVSAINAASEKL
LTRLGVWQDILSRRASCYHGMEVWDKDSFGHISFDDQSMGYSHLGHIVENSVIHYALWNK
ARQSSDITLLAPAELQQVAWGENETFLTLKDGSMLTARLVIGADGANSWLRNKADIPLTF
WDYQHHALVATIRTEEPHDAVARQVFHGEGILAFLPLSDPYLCSIVWSLSPEEALRMQQA
SEDEFNRALNIAFDNRLGLCKVESERLVFPLTGRYARQFAAHRLALVGDAAHTIHPLAGQ
GVNLGFMDAAELIAELKRLHRQGKDIGQYIYLRRYERSRKHSAALMLAGMQGFRDLFSGA
NPAKKLLRDIGLKLADTLPGVKPQLIRQAMGLNDLPEWLR
>LFGLNPFC_00227 Aminomethyltransferase
MAQQTPLYEQHTLCGARMVDFHGWMMPLHYGSQIDEHHAVRTDAGMFDVSHMTIVDLRGS
RTREFLRYLLANDVAKLTKSGKALYSGMLNASGGVIDDLIVYYFTEDFFRLVVNSATREK
DLSWITQHAEPFGIEITVRDDLSMIAVQGPNAQAKAATLFNDAQRQAVEGMKPFFGVQAG
DLFIATTGYTGEAGYEIALPNEKAADFWRALVEAGVKPCGLGARDTLRLEAGMNLYSQEM
DETISPLAANMGWTIAWEPADRDFIGREALEAQREHGTEKLVGLVMTEKGVLRNELPVRF
TDAQGNQHEGIITSGTFSPTLGYSIALARVPEGIGETAIVQIRNREMPVKVTKPVFVRNG
KAVA
>LFGLNPFC_00228 Glycine cleavage system H protein
MSNIPAELKYSKEHEWLRKEADGTYTVGITEHAQELLGDMVFVDLPEVGATVSAGDDCAV
AESVKAASDIYAPVSGEIVAVNDALSDSPELVNSEPYAGGWIFKIKASDESELESLLDAT
AYEALLEDE
>LFGLNPFC_00229 Glycine dehydrogenase (decarboxylating)
MTQTLSQLENSGAFIERHIGPDAAQQQEMLNAVGAQSLNALTGQIVPKDIQLATPPQVGA
PATEYAALAELKAIASRNKRFTSYIGMGYTAVQLPPVILRNMLENPGWYTAYTPYQPEVS
QGRLEALLNFQQVTLDLTGLDMASASLLDEATAAAEAMAMAKRVSKLKNANRFFVASDVH
PQTLDVVRTRAETFGFEVIVDDAQKVLDHQDVFGVLLQQVGTTGEIHDYTALISELKSRK

IVVSVAADIMALVLLTAPGKQGADIVFGSAQRFGVPMGYGGPHAAFFAAKDEYKRSMPGR
IIGVSKDAAGNTALRMAMQTREQHIRREKANSNICTSQVLLANIASLYAVYHGPVGLKRI
ANRIHRLTDILAAGLQQKGLKLRHAHYFDTLCVEVVDKAGVLARAEAAEINLRSDILNAV
GITLDETTTRENVMQLFSVLLGDNHGLDIDTLDKDVAHDSRSIQAAMLRDDEILTHPVFN
RYHSETEMMRYMHSLERKDLALNQAMIPLGSCTMKLNAAAEMIPITWPEFAELHPFCPPE
QAEGYQQMIAQLADWLVKLTGYDAVCMQPNSGAQGEYAGLLAIRHYHESRNEGHRDICLI
PASAHGTNPASAHMAGMQVVVVACDKNGNIDLTDLRAKAEQAGDNLSCIMVTYPSTHGVY
EETIREVCEVVHQFGGQVYLDGANMNAQVGITSPGFIGADVSHLNLHKTFCIPHGGGGPG
MGPIGVKAHLAPFVPGHSVVQIEGMLTRQGAVSAAPFGSASILPISWMYIRMMGAEGLKK
ASQVAILNANYIASRLQDAFPVLYTGRDGRVAHECILDIRPLKEETGISELDIAKRLIDY
GFHAPTMSFPVAGTLMVEPTESESKVELDRFIDAMLAIRAEIDQVKAGVWPLEDNPLVNA
PHIQSELVAEWAHPYSREVAVFPAGVADKYWPTVKRLDDVYGDRNLFCSCVPISEYQ
>LFGLNPFC_00230 6-phospho-beta-glucosidase BglA
MKKLTLPKDFLWGGAVAAHQVEGGWNKGGKGPSICDVLTGGAHGVPREITKEVVPGKYYP
NHEAVDFYGHYKEDIKLFAEMGFKCFRTSIAWTRIFPKGDEAQPNEEGLKFYDDMIDELL
KYNIEPVITLSHFEMPLHLVQQYGSWTNRKVVDFFVRFAEVVFERYKHKVKYWMTFNEIN
NQRNWRAPLFGYCCSGVVYTEHENPEETMYQVLHHQFVASALAVKAAHRINPEMKVGCML
AMVPLYPYSCNPDDVMFAQESMRERYVFTDVQLRGYYPSYVLNEWERRGFNIKMEDGDLD
VLREGTCDYLGFSYYMTNAVKAEGGTGDAISGFEGSVPNPYVKASDWGWQIDPVGLRYAL
CELYERYQKPLFIVENGFGAYDKVEDDGSINDDYRIDYLRAHIEEMKKAVTYDGVDLMGY
TPWGCIDCVSFTTGQYSKRYGFIYVNKHDDGTGDMSRSRKKSFNWYKEVIASNGENL
>LFGLNPFC_00231 hypothetical protein
MQPNDITFFQRFQDDILAGRKTITIRDESESHFKTGDVLRVGRFEDDGYFCTIEVTATST
VTLDTLTEKHAEQENMTLTELKKVIADIYPDQTQFYVIEFKCL
>LFGLNPFC_00232 hypothetical protein
MVQKPLIKQGYSLAEEIANSVSHGIGLVFGIVGLVLLLVQAVDLNASATAITSYSLYGGS
MILLFLASTLYHAIPHQRAKMWLKKFDHCAIYLLIAGTYTPFLLVGLDSPLARGLMIVIW
SLALLGILFKLTIAHRFKILSLVTYLAMGWLSLVVIYEMAVKLAAGSVTLLAVGGVVYSL
GVIFYVCKRIPYNHAIWHGFVLGGSVCHFLAIYLYIGQA
>LFGLNPFC_00233 tRNA-modifying protein YgfZ
MAFTPFPPRQPTASARLPLTLMTLDDWALATITGADSEKYMQGQVTADVSQMTEDQHLLA
AHCDAKGKMWSNLRLFRDGDGFAWIERRSVREPQLTELKKYAVFSKVTIAPDDERVLLGV
AGFQARAALANLFSELPSREKQVVKEGATTLLWFEHPAERFLIVTDEATANMLTDKLRGE
AELNNSQQWLALNIEAGFPVIDAANSGQFIPQATNLQALGGISFKKGCYTGQEMVARAKF
RGANKRALWLLKGSASRLPEAGEDLELKMGENWRRTGTVLAAVKLEDGQVVVQVVMNNDM
EPDSIFRVRDDANTLRIEPLPYSLEE
>LFGLNPFC_00234 FAD assembly factor SdhE
MDINNKARIHWACRRGMRELDISIMPFFEHEYDSLSDDEKRIFIRLLECDDPDLFNWLMN
HGKPADAELEMMVRLIQTRNRERGPVAI
>LFGLNPFC_00235 Inner membrane protein YgfX
MVLWQSDLRVSWRAQWLSLLIHGLVAAVILLMPWPLSYTPLWMVLLSLVVFDCVRSQRRI
NARQGEIRLLMDGRLRWQGQEWSIVKAPWMIKSGMMLRLRSDSGKRQHLWLAADSMDEAE
WRDLRRILLQQETQR
>LFGLNPFC_00236 Flavodoxin 2
MNMGLFYGSSTCYTEMAAEKIRDIIGPELVTLHNLKDDSPKLMEQYDVLILGIPTWDFGE
IQEDWEAVWDQLDDLNLEGKIVALYGLGDQLGYGEWFLDALGMLHDKLSTKGVKFVGYWP
TEGYEFTSPKPVIADGQLFVGLALDETNQYDLSDERIQSWCEQILNEMAEHYA
>LFGLNPFC_00237 Tyrosine recombinase XerD
MKQELARIEQFLDALWLEKNLAENTLNAYRRDLSMMVEWLHHRGLTLATAQSDDLQALLA
ERLEGGYKATSSARLLSAVRRLFQYLYREKFREDDPSAHLASPKLPQRLPKDLSEAQVER
LLQAPLIDQPLELRDKAMLEVLYATGLRVSELVGLTMSDISLRQGVVRVIGKGNKERLVP
LGEEAVYWLETYLEHGRPWLLNGVSIDVLFPSQRAQQMTRQTFWHRIKHYAVLAGIDSEK
LSPHVLRHAFATHLLNHGADLRVVQMLLGHSDLSTTQIYTHVATERLRQLHQQHHPRA
>LFGLNPFC_00238 Thiol:disulfide interchange protein DsbC
MKKGFMLFTLLAAFSGFVQADDAAIQQTLAKMGIKSSDIQPAPVAGMKTVLTNSGVLYIT
DDGKHIIQGPMYDVSGTAPVNVTNKMLLKQLNALEKEMIVYKAPQEKHVITVFTDITCGY
CHKLHEQMADYNALGITVRYLAFPRQGLDSDAEKEMKAIWCAKDKNKAFDDVMAGKSVAP
ASCDVDIADHYALGVQLGVSGTPAVVLSNGTLVPGYQPPKDMKEFLDEHQKMTSGK
>LFGLNPFC_00239 Single-stranded-DNA-specific exonuclease RecJ
MKQQIQLRRREVDETADLPAELPPLLRRLYASRGVRSAQELERSVKGMLPWQQLSGVEKA
VEILYNAFREGTRIIVVGDFDADGATSTALSVLAMRSLGCSNIDYLVPNRFEDGYGLSPE
VVDQAHARGAQLIVTVDNGISSHAGVEHARSLGIPVIVTDHHLPGDTLPAAEAIINPNLR
DCNFPSKSLAGVGVAFYLMLALRTFLRDQGWFDERGIAIPNLAELLDLVALGTVADVVPL
DANNRILTWQGMSRIRAGKCRPGIKALLEVANRDPQKLAASDLGFALGPRLNAAGRLDDM

SVGVALLLCDNIGEARVLANELDALNQTRKEIEQGMQVEALTLCEKLERSRDTLPGGLAM
YHPEWHQGVVGILASRIKERFHRPVIAFAPAGDGTLKGSGRSIQGLHMRDALERLDTLYP
GMMLKFGGHAMAAGLSLEEDKFELFQQRFGELVTEWLDPSLLQGEVVSDGPLSPAEMTME
VAQLLRDAGPWGQMFPEPLFDGHFRLLQQRLVGERHLKVMVEPVGGGPLLDGIAFNVDTA
LWPDNGVREVQLAYKLDINEFRGNRSLQIIIDNIWPI
>LFGLNPFC_00240 Peptide chain release factor RF2
MKQGLEDVSGLLELAVEADDEETFNEAVAELDALEEKLAQLEFRRMFSGEYDSADCYLDI
QAGSGGTEAQDWASMLERMYLRWAESRGFKTEIIEESEGEVAGIKSVTIKISGDYAYGWL
RTETGVHRLVRKSPFDSGGRRHTSFSSAFVYPEVDDDIDIEINPADLRIDVYRASGAGGQ
HVNRTESAVRITHIPTGIVTQCQNDRSQHKNKDQAMKQMKAKLYELEMQKKNAEKQAMED
NKSDIGWGSQIRSYVLDDSRIKDLRTGVETRNTQAVLDGSLDQFIEASLKAGL
>LFGLNPFC_00241 Lysine--tRNA ligase
MSEQHAQGADAVVDLNNELKTRREKLANLREQGIAFPNDFRRDHTSDQLHAEFDGKENEE
LEALNIEVAVAGRMMTRRIMGKASFVTLQDVGGRIQLYVARDDLPEGVYNEQFKKWDLGD
ILGAKGKLFKTKTGELSIHCTELRLLTKALRPLPDKFHGLQDQEARYRQRYLDLISNDES
RNTFKVRSQILSGIRQFMVNRGFMEVETPMMQVIPGGAAARPFITHHNALDLDMYLRIAP
ELYLKRLVVGGFERVFEINRNFRNEGISVRHNPEFTMMELYMAYADYKDLIELTESLFRT
LAQDILGKTEVTYGDVTLDFGKPFEKLTMREAIKKYRPETDMADLDNFDSAKAIAESIGI
HVEKSWGLGRIVTEIFEEVAEAHLIQPTFITEYPAEVSPLARRNDVNPEITDRFEFFIGG
REIGNGFSELNDAEDQAQRFLDQVAAKDAGDDEAMFYDEDYVTALEHGLPPTAGLGIGID
RMVMLFTNSHTIRDVILFPAMRPVK
>LFGLNPFC_00242 Isopentenyl-diphosphate Delta-isomerase
MQTEHVILLNAQGVPTGTLEKYAAHTADTLLHLAFSSWLFNAKGQLLVTRRALSKKAWPG
VWTNSVCGHPQLGESNEEAVIRRCRYELGVEITPPESIYPDFRYRATDPNGIVENEVCPV
FAARTTSALQINDDEVMDYQWCDLAAVLRGIDATPWAFSPWMVMQATNREARKRLSAFTQ
LK
>LFGLNPFC_00243 putative protein YqfG
MNLLMRAIFSLLLLFTLSIPVISDCVAMAIESRFKYMMLLF
>LFGLNPFC_00244 Uric acid transporter UacT
MNAIDSQLPSSSGQDRPTDEVDRILSPGKLIILGLQHVLVMYAGAVAVPLMIGDRLGLSK
EAIAMLISSDLFCCGIVTLLQCIGIGRFMGIRLPVIMSVTFAAVTPMIAIGMNPDIGLLG
IFGATIAAGFITTLLAPLIGRLMPLFPPLVTGVVITSIGLSIIQVGIDWAAGGKGNPQYG
NPVYLGISFAVLIFILLITRYAKGFMSNVAVLLGIVFGFLLSWMMNEVNLSGLHDASWFA
IVTPMSFGMPIFDPVSILTMTAVLIIVFIESMGMFLALGEIVGRKLSSHDIIRGLRVDGV
GTMIGGTFNSFPHTSFSQNVGLVSVTRVHSRWVCIASGIILILFGMVPKMAVLVASIPQF
VLGGAGLVMFGMVLATGIRILSRCNYTTNRYNLYIVAISLGVGMTPTLSHDFFSKLPAVL
QPLLHSGIMLATLSAVVLNVFFNGYQHHADLVKESVSDKDLKVRTVRMWLLMRKLKKNEH
GE
>LFGLNPFC_00245 Ferredoxin--NADP reductase
MNKFIAAEAAECIGCHACEIACAVAHNQENWPLSHSDFRPRIHVVGKGQAANPVACHHCN
NAPCVTACPVNALTFQSDSVQLDEQKCIGCKRCAIACPFGVVEMVDTIAQKCDLCNQRSS
GTQACIDVCPTQALRLMDDKGLQQIKVARQRKTAAGKASSDAQPSRSAALLPVNSRKGAD
KISASERKTHFGEIYCGLDPQQATYESDRCVYCAEKANCNWHCPLHNAIPDYIRLVQEGK
IIEAAELCHQTSSLPEICGRVCPQDRLCEGACTLKDHSGAVSIGNLERYITDTALAMGWR
PDVSKVVPRSEKVAVIGAGPAGLGCADILARAGVQVDVFDRHPEIGGMLTFGIPPFKLDK
TVLSQRREIFTAMGIDFHLNCEIGRDISFNELTAEYDAVFLGVGTYGMMRADLPHEDAPG
VIQALPFLTAHTRQLMGLPESAEYPLTDVEGKRVVVLGGGDTTMDCLRTSIRLNAASVTC
AYRRDEVSMPGSRKEVVNAREEGVEFQFNVQPQYIACDEDGRLTAVGLIRTAMGEPGPDG
RRRPRPVAGSEFELPADVLIMAFGFQAHTMPWLQGSGIKLDKWGLIQTGDVGYLPTQTHL
KKVFAGGDAVHGADLVVTAMAAGRQAARDMLTLFDTKAS
>LFGLNPFC_00246 Hydrogenase-4 component A
MKSLIIVNPADCIGCRTCEVACVVAHPSEQELNADIFLPRLKVQRLDSISAPVMCHQCEN
APCVGACPVGALTMGEQVVQANSARCIGCQSCVSACPFGMITIQSLPGDTRQQIVKCDLC
EQREEGPACVESCPTQALQLLTERELRRVRQQRIVASSENPL
>LFGLNPFC_00247 Guanine/hypoxanthine permease GhxQ
MSGDILQTPDAPKPQGALDNYFKITARGSTVRQEVLAGLTTFLAMVYSVIVVPGMLGKAG
FPPAAVFVATCLVAGFGSLLMGLWANLPMAIGCAISLTAFTAFSLVLGQQISVPVALGAV
FLMGVIFTAISVTGVRTWILRNLPMGIAHGTGIGIGLFLLLIAANGVGMVIKNPIEGLPV
ALGAFTSFPVMMSLLGLAVIFGLEKCRVPGGILLVIIAISIIGLIFDPAVKYHGLVAMPS
LTGEDGNSLIFSLDIMGALQPTVLPSVLALVMTAVFDATGTIRAVAGQANLLDKDNQIIN
GGKALTSDSVSSIFSGLVGAAPAAVYIESAAGTAAGGKTGLTATVVGVLFLMILFLSPLS
FLIPGYATAPALMYVGLLMLSNVSKLDFNDFIDAMAGLVCAVFIVLTCNIVTGIMLGFVT
LVVGRVFAREWQKLNIGTVIITAALVAFYAGGWAI
>LFGLNPFC_00248 Guanine deaminase

MSGEHTLKAVRGSFIDVTRTVDNPEEIASALRFIEDGLLLIKQGKVEWFGEWEDGKHQIP
DTIRVRDYRGKLIVPGFIDTHIHYPQSEMVGAYGEQLLEWLNKHTFPTERRYEDLEYARE
MSAFFIKQLLRNGTTTALVFGTVHPQSVDALFEAASHINMRMIAGKVMMDRNAPDYLLDT
AESSYHQSKELIERWHKNGRLLYAITPRFAPTSSPEQMAMAQRLKEEYPDTWVHTHLCEN
KDEIAWVKSLYPDHDGYLDVYHQYGLTGKNCVFAHCVHLEEKEWDRLSETKSSIAFCPTS
NLYLGSGLFNLKKAWQKKVKVGMGTDIGAGTTFNMLQTLNEAYKVLQLQGYRLSAYEAFY
LATLGGAKSLGLDDLIGNFLPGKEADFVVMEPTATPLQQLRYDNSVSLVDKLFVMMTLGD
DRSIYRTYVDGRLVYERN
>LFGLNPFC_00249 Xanthine permease XanQ
MSDINHAGSDLIFELEDRPPFHQALVGAITHLLAIFVPMVTPALIVGAALQLSAETTAYL
VSMAMIASGIGTWLQVNRYGIVGSGLLSIQSVNFSFVTVMIALGSSMKSDGFHEELIMSS
LLGVSFVGAFLVVGSSFILPYLRRVITPTVSGIVVLMIGLSLIKVGIIDFGGGFAAKSSG
TFGNYEHLGVGLLVLIVVIGFNCCRSPLLRMGGIAIGLCVGYIASLCLGMVDFSSMRNLP
LITIPHPFKYGFSFSFHQFLVVGTIYLLSVLEAVGDITATAMVSRRPIQGEEYQSRLKGG
VLADGLVSVIASAVGSLPLTTFAQNNGVIQMTGVASRYVGRTIAVMLVILGLFPMIGGFF
TTIPSAVLGGAMTLMFSMIAIAGIRIIITNGLKRRETLIVATSLGLGLGVSYDPEIFKIL
PASIYVLVENPICAGGLTAILLNIILPGGYRQENVLPGITSAEEMD
>LFGLNPFC_00250 Putative xanthine dehydrogenase molybdenum-binding subunit XdhA
MIIHFTLNGAPQELTVNPGENVQKLLFNMGMHSVRNSDDGFGFAGSDAIIFNGNIVNASL
LIAAQLEKADIRTAESLGKWNELSLVQQAMVDVGVVQSGYNDPAAALIITDLLDRIDAPT
REEIDDALSGLFSRDAGWQQYYQVIELAVARKNNPQATIDIAPTFRDDLEVIGKHYPKTD
AAKMVQAKPCYVEDRVTADACVIKMLRSPHAHALITHLDVSKAEALPGVVHVITHLNCPD
IYYTPGGQSAPEPSPLDRRMFGKKMRHVGDRVAAVVAESEDIALEALKLIDVEYEVLKPV
MSIDEAMAEDAPVVHDEPVVYVAGAPDTLEDDNSHAAQRGEHMIINFPIGSRPRKNIAAS
IHGHIGDMDKGFADADVIIERTYNSTQAQQCPTETHICFTRMDGDRLVIHASTQVPWHLR
RQVARLVGMKQHKVHVIKERVGGGFGSKQDILLEEVCAWATCVTGRPVLFRYTREEEFIA
NTSRHVAKVTVKLGAKKDGRLTAVKMDFRANTGPYGNHSLTVPCNGPALSLPLYPCDNVD
FQVTTYYSNICPNGAYQGYGAPKGNFAITMALAELAEQLQIDQLEIIERNRVHEGKELKI
LGAIGEGKAPTSVPSAASCALEEILRQGREMIQWSSPKPQNGDWHIGRGVAIIMQKSGIP
DIDQANCMIKLESDGTFIVHSGGADIGTGLDTVVTKLAAEVLHCPPQDVHVISGDTDHAL
FDKGAYASSGTCFSGNAARLAAENLREKILFHGAQMLGEPVADVQLATPGVVRGKKGEVS
FGEIAHKGETGTGFGSLVGTGSYITPDFAFPYGANFAEVAVNTRTGEIRLDKFYALLDCG
TPVNPELALGQIYGATLRAIGHSMSEEIIYDAEGHPLTRDLRSYGAPKIGDIPRDFRAVL
VPSDDKVGPFGAKSISEIGVNGAAPAIATAIHDACGIWLREWHFTPEKILTALEKI
>LFGLNPFC_00251 hypothetical protein
MIEQFFRPDSVEQALELKRRYQDEAVWFAGGSKLNATPTRTDKKIAISLQDLELDWIDWD
NGALRIGAMSRLQPLRDARFIPAALCEALGFVYSRHVRNQSTIGGEIAARQEESVLLPVL
LALDAELVFGNGETLSIEDYLACPCDRLLTEIIIKDPYRTCATRKISRSQAGLTVVTAAV
AITDHDGMRIALDGVASKALRLHDVETQNLEGNALEQAVANAIFPQEDLRGSVAYKRYIT
GVLVADLYADCQQAGEEAV
>LFGLNPFC_00252 Putative aminohydrolase SsnA
MLILKNVTAVQLHPAKVQEGVDIAIENDVIVAIDDALTQRYPDASYKEMHGRIVMPGIVC
SHNHFYSGLSRGIMANIAPCPDFISTLKNLWWRLDRALDEESLYYSGLICSLEAIKSGCT
SVIDHHASPAYIDGSLSTLRNAFLKVGLRAMTCFETTDRNNGIKELQEGVEENIRFARQI
DEAKKAATEPYLVEAHIGAHAPFTVPDAGLEMLREAVKSTGRGLHIHAAEDLYDVSYSHH
WYGKDLLARLAQFDLIDSKTLVAHGLYLSKDDIALLNQRDAFLVHNARSNMNNHVGYNHH
LSDIRNLALGTDGIGSDMFEEMKFAFFKHRDAGGPLWPDSFAKALANGNELMSRNFGAKF
GLLEAGYKADLTICDYNSPTPLLADNIAGHIAFGMGSGSVHSVMVNGVMVYEDRQFNFDC
DSIYAQARKAAASMWRRMDALA
>LFGLNPFC_00253 Putative oxidoreductase YgfK
MGDIMRPIPFEELLTRIFDEYQQQRSIFGIPEQQFYSPVKGKTVSVFGETCATPVGPAAG
PHTQLAQNIVTSWLTGGRFIELKTVQILDRLELEKPCIDAEDECFNTEWSTEFTLLKAWD
EYLKAWFALHLLEAMFQPSDSGKSFIFNMSVGYNLEGIKQPPMQQFIDNMMDASDHPKFA
QYRDALNKLLQDEAFLARHGLQEKRESLQALPARIPTSMVQGVTLSTMHGCPPHEIEAIC
RYMLEEKGLNTFVKLNPTLLGYARVREILDVCGFGYIGLKEESFDHDLKLTQALEMLERL
MALAKEKSLGFGVKLTNTLGTINNKGALPGEEMYMSGRALFPLSINVAAVLSRAFDGKLP
ISYSGGASQLTIRDIFDTGIRPITMATDLLKPGGYLRLSACMRELEGSDAWGLDHVDVER
LNRLAADALTMEYTQKHWKPEERIEVAEDLPLTDCYVAPCVTACAIKQDIPEYIRLLGEH
RYADALELIYQRNALPAITGHICDHQCQYNCTRLDYDSALNIRELKKVALEKGWDEYKQR
WHKPAGSGSRHPVAVIGAGPAGLAAGYFLARAGHPVTLFEREANAGGVVKNIIPQFRIPA
ELIQHDIDFVADHGVKFEYGCSPDLTVEQLKNQGFHYVLIATGTDKNSGVKLAGDNQNVW
KSLPFLREYNKGTALKLGKHVVVVGAGNTAMDCARAALRVPGVEKATVVYRRSLQEMPAW
REEYEEALHDGVEFRFLNNPERFDADGTLTLRVMSLGEPDEKGRRRPVETNETVTLHVDS
LITAIGEQQDTEALNAMGVPLDKNGWPDVDHNGETRLSDVFMIGDVQRGPSSIVAAVGTA

RRATDAILSRENIRSHQNDKYWNNVNPAEIYQRKGDISVTLVNSDDRDAFVAQEAARCLE
CNYVCSKCVDVCPNRANVSIAVPGFQNRFQTLHLDAYCNECGNCAQFCPWNGKPYKDKIT
VFSLSQDFDNSSNPGFLVEDCRVRVRLNNQSWVLNIDSEGQFNNVPPELNDMCRIISHVH
QHHHYLLGRVEV
>LFGLNPFC_00254 Molybdenum cofactor cytidylyltransferase
MSAIDCIITAAGLSSRMGQWKMMLPWQQGTILDTSIKNALQFCSRIILVTGYRGNELHER
YANQSNITIIHNPDYAQGLLTSVKAAVPAVQTEHCFLTHGDMPTLTIDIFRKIWSLRNDG
AILPLHNGIPGHPILVSKPCLMQAIQRPNVTNMRQALLMGEHYSVEIENAEIILDIDTPD
DFITAKKRYTEI
>LFGLNPFC_00255 hypothetical protein
MKSIIDPSALFIDLGAQKRPTVISIVGAGGKTSLLFWLAELFQASGRRVLITTTTHMFMP
TSHWPVVFCRDPAMLPHASFISPISFCFHCWKANQGKVQGFTPEAIDALVQRPECDVILI
EADGSRGMPLKAPDEHEPCIPKSSCCVIAVMGGHILGAKVSTENVHRWSQFADITGLTPD
APLQLSDLVALVRHPQGAFKNVPQGCRRVWFINRFSQCENAIAQSELLQPLQQHNVEAIW
LGDIQEHPAIARRFVN
>LFGLNPFC_00256 hypothetical protein
MNIFTEAAKLEEQNCPFAMAQIVDSRGSTPRHSAQMLVRADGSIVGTIGGGMVERKVIEE
SLQALQERKPRLFHGRMARNGADAVGSDCGGAMSVFISVHGMRPRLVLIGAGHVNRAIAQ
SAALLGFDIAVADIYRESLNPELFPPSTTLLHAESFGAAVEALDIRPDNFVLIATNNQDR
EALDKLIEQPIAWLGLLASRRKVQLFLRQLREKGVAEEHIARLHAPVGYNIGAETPQEIA
ISVLAEILQVKNNAPGGLMMKPSHPSGHQLVVIRGAGDIASGVALRLYHAGFKVIMLEVE
KPTVIRCTVAFAQAVFDGEMTVEGVTARLATSSAEAMKLTERGFIPVMVDPTCSLLDELK
PLCVVDAILAKQNLGTRADMAPVTIALGPGFTAGKDCHAVIETNRGHWLGQVIYSGCAQE
NTGVPGNIMGHTTRRVIRAPAAGIMRSNVKLGDLVKEGDVIAWIGEHEIKAPLTGMVRGL
LNDGLAVVGGFKIGDIDPRGETADFTSVSDKARAIGGGVLEALMMLMHQGVKATKEVLEV
A
>LFGLNPFC_00257 Carbamate kinase 1
MSKKIVLALGGNALGDDLAGQMKAVKITSQAIVDLIAQGHEVIVTHGNGPQVGMINQAFE
AAAKTEAHSPMLPMSVCVALSQGYIGYDLQNALREELLSRGINKPVATLVTQVEVDANDP
AFLNPTKPIGSFFTEQEAELLTKQGYTLKEDAGRGYRRVVASPKPVDIIEKETVKALVDA
GQVVITVGGGGIPVIREGNHLRGASAVIDKDWASARLAEMIDADMLIILTAVEKVAINFG
KENEQWLDRLSLSDAERFIEEGHFAKGSMLPKVEAAASFARSRAGREALITVLSKAKEGI
EGKTGTVICQ
>LFGLNPFC_00258 D-phenylhydantoinase
MRVLIKNGIVVNADGQAKQDLLIESGIVRQLGTDISPQLPCEEIDASGCYVFPGGVDVHT
HFNIDVGIARSCDDFFTGTRAAACGGTTTIIDHMGFGPNGCRLRHQLEVYRGYAAHKAVI
DYSFHGVIQHINHAILDEIPMMVEEGLSSFKLYLTYQYKLNDDEVLQALRRLHESGALTT
VHPENDAAIASKRAEFIAAGLTAPRYHALSRPLECEAEAIARMINLAQIAGNAPLYIVHL
SNGLGLDYLRLARANHQPVWVETCPQYLLLDERSYDTEDGMKFILSPPLRNVREQDKLWC
GISDGAIDVVATDHCTFSMAQRLQISKGDFSRCPNGLPGVENRMQLLFSSGVMTGRISLE
RFVELTSAMPARLFGLWPQKGILAPGSDGDVVIIDPRQSQQIQHRHLHDNADYSPWEGFT
CQGAIVRTLSRGETIFCDGTFTGKAGRGRFLRRKPFVPPVL
>LFGLNPFC_00259 Acetylornithine deacetylase
MAKNIPFKLILEKAKDYQADMTRFLRDMVAIPSESCDEKRVVHRIKEEMEKVGFDKVEID
PMGNVLGYIGHGPRLVAMDAHIDTVGIGNIKNWDFDPYEGMETDELIGGRGTSDQEGGMA
SMVYAGKIIKDLGLEDEYTLLVTGTVQEEDCDGLCWQYIIEQSGIRPEFVVSTEPTDCQV
YRGQRGRMEIRIDVQGVSCHGSAPERGDNAIFKMGPILGELQELSQRLGYDEFLGKGTLT
VSEIFFTSPSRCAVADSCAVSIDRRLTWGETWEGALDEIRALPAVQKANAVVSMYNYDRP
SWTGLVYPTECYFPTWKVEEDHFTVKALVNAYEGLFGKAPVVDKWTFSTNGVSIMGRHGI
PVIGFGPGKEPEAHAPNEKTWKSHLVTCAAMYAAIPLSWLATE
>LFGLNPFC_00260 Diaminopropionate ammonia-lyase
MSVFSLKIDIADNKFFNGETSPLFSQSQAKLARQFHQKIAGYHPTPLCALDDLANLFGVK
KILVKDESKRFGLNAFKMLGGAYAIAQLLCEKYHLDIETLSFEHLKNAIGEKMTFATTTD
GNHGRGVAWAAQQLGQNAVIYMPKGSAQERVDAILNLGAECIVTDMNYDDTVRLTMQHAQ
QHGWEVVQDTAWEGYTKIPTWIMQGYATLADEAVEQMREMGVTPTHVLLQAGVGAMAGGV
LGYLVDVYSPQNLHSIIVEPDKADCIYRSGVKGDIVNVGGDMATIMAGLACGEPNPLGWE
ILRNCATQFISCQDSVAALGMRVLGNPYGNDPRIISGESGAVGLGVLAAVHYHPQRQSLM
EKLALNKDAVVLVISTEGDTDVKHYREVVWEGKHAVAP
>LFGLNPFC_00261 Putative carbamoyltransferase YgeW
MKTVNELIKDINSLTSHLHEKDFLLTWEQTPDELKQVLDVAAALKALRAENISTKVFNSG
LGISVFRDNSTRTRFSYASALNLLGLAQQDLDEGKSQIAHGETVRETANMISFCADAIGI
RDDMYLGAGNAYMREVGAALDDGYKQGVLPQRPALVNLQCDIDHPTQSMADLAWLREHFG
SLENLKGKKIAMTWAYSPSYGKPLSVPQGIIGLMTRFGMDVTLAHPEGYDLIPDVVEVAK
NNAKASGGSFRQVTSMEEAFKDADIVYPKSWAPYKVMEERTELLRANDHEGLKALEKQCL

AQNAQHKDWHCTEEMMELTRDGEALYMHCLPADISGVSCKEGEVTEGVFEKYRIATYKEA
SWKPYIIAAMILSRKYAKPGALLEQLLKEAQERVK
>LFGLNPFC_00262 Anaerobic nitric oxide reductase transcription regulator NorR
MELATTQSVLMQIQPTIQRFARMLASVLQLEVEIVDENLCRVAGTGAYGKFLGRQLSGNS
RLLRHVLETKTEKVVTQSRFDPLCEGCDSKENCREKAFLGTPVILQERCVGVISLIAVTH
EQQEHISDNLREFSDYVRHISTIFVSKLLEDQGPGDNISKIFATMIDNMDQGVLVVDADN
RVQFVNQTALKTLGVVQNNIIGKPVRFRPLTFESNFTHGHMQHIVSWDDKSELIIGQLHN
IQGRQLFLMAFHQSHTSFSVANAPDEPHIEQLVGECRVMRQLKRLISRIAPSPSSVMVVG
ESGTGKEVVARAIHKLSGRRNKPFIAINCAAIPEQLLESELFGYVKGAFTGASANGKTGL
IQAANTGTLFLDEIGDMPLMLQAKLLRAIEAREILPIGASSPIQVDIRIISATNQNLAQF
IAEGKFREDLFYRLNVIPITLPPLRERQEDIELLVHYFLHLHTRRLGSVYPGIAPDVVEI
LRKHRWPGNLRELSNLMEYLVNVVPSGEVIDSTLLPPNLLNNGTTEQSDVTEVSEAHLSL
DDAGGTALEEMEKQMIREALSRHNSKKEVADELGIGIATLYRKIKKYELLNT
>LFGLNPFC_00263 Nicotinate dehydrogenase small FeS subunit
MNHSETITIECTINGMPFQLHAAPGMPLSELLREQGLLSVKQGCCVGECGACTVLVDGTA
IDSCLFLAAWAEGKEIRTLEGEAKGGKLSHVQLAYAKSGAVQCGFCTPGLIMATTAMLAK
PREKPLTITEIRRGLAGNLCRCTGYQMIVNTVLDCEKTK
>LFGLNPFC_00264 Nicotinate dehydrogenase FAD-subunit
MFDFASYHRATTLADAITLLADNPQAKLLAGGTDVLIQLHHHNDRYRHIVDIHNLAELRG
ITQAEDGALRIGSATTFTQLIEDPVIQRNLPALCAAAASIAGPQIRNVATYGGNICNGAT
SADSATPTLIYDAKLELHSPRGVRFVPINGFHTGPGKVSLEHDEILVAFHFPPQPKEHAG
SAHFKYAMRDAMDISTIGCAAHCRLDNGNFSELRLAFGVAAPTPIRCQHAEQTAQNAPLN
LQTLEAISESVLQDVAPRSSWRASKEFRLHLIQTMTQKVISEAVAAAGGKLQ
>LFGLNPFC_00265 Putative xanthine dehydrogenase molybdenum-binding subunit XdhA
MEAREATATGESCMRVDAIAKVTGRARYTDDYVMAGMCYAKYVRSPIAHGYAVSINDEQA
RSLPGVLAIFTWEDVPEIPFATAGHAWTLDENKRDTADRALLTRHVRHHGDAVAIVVARD
ELTAEKAAQLVSIEWQELPVITSPEAALAEDAAPIHNGGNLLKQSTMSTGNVQQTIDAAD
YQVQGHYQTPVIQHCHMESVTSLAWMEDDSRITIVSSTQIPHIVRRVVGQALDIPWSCVR
VIKPFIGGGFGNKQDVLEEPMAAFLTSKLGGIPVKVSLSREECFLATRTRHAFTIDGQMG
VNRDGTLKGYSLDVLSNTGAYASHGHSIASAGGNKVAYLYPRCAYAYSSKTCYTNLPSAG
AMRGYGAPQVVFAVESMLDDAATALGIDPVEIRLRNAAREGDANPLTGKRIYSAGLPECL
EKGRKIFEWEKRRAECQNQQGNLRRGVGVACFSYTSNTWPVGVEIAGARLLMNQDGTINV
QSGATEIGQGADTVFSQMVAETVGVPVSDVRVISTQDTDVTPFDPGAFASRQSYVAAPAL
RSAALLLKEKIIAHAAVMLHQSAMNLTLIKGHIVLVERPEEPLMSLKDLAMDAFYHPERG
GQLSAESSIKTTTNPPAFGCTFVDLTVDIALCKVTINRILNVHDSGHILNPLLAEGQVHG
GMGMGIGWALFEEMIIDAKSGVVRNPNLLDYKMPTMPDLPQLESAFVEINEPQSAYGHKS
LGEPPIIPVAAAIRNAVKMATGVAINTLPLTPKRLYEEFHLAGLI
>LFGLNPFC_00266 Murein hydrolase activator NlpD
MSAGRLNKKSLGIVMLLSVGLLLAGCSGSKSSDTGTYSGSVYTVKRGDTLYRISRTTGTS
VKELARLNGISPPYTIEVGQKLKLGGAKSSSSTRKSTAKSTTKTASVTPSSAVPKSSWPP
VGQRCWLWPTTGKVIMPYSTADGGNKGIDISAPRGTPIYAAGAGKVVYVGNQLRGYGNLI
MIKHSEDYITAYAHNDTMLVNNGQSVKAGQKIATMGSTDAASVRLHFQIRYRATAIDPLR
YLPPQGSKPKC
>LFGLNPFC_00268 Inner membrane transport protein YqeG
MSNIWSKEETLWSFALYGTAVGAGTLFLPIQLGSAGAVVLFITALVAWPLTYWPHKALCQ
FILSSKTSAGEGITGAVTHYYGKKIGNLITTLYFIAFFVVVLIYAVAITNSLTEQLAKHM
VIDLRIRMLVSLGVVLILNLIFLMGRHATIRVMGFLVFPLIAYFLFLSIYLVGSWQPDLL
TTQVEFNQNTLHQIWISIPVMVFAFSHTPIISTFAIDRREKYGEHAMDKCKKIMKVAYLI
ICISVLFFVFSCLLSIPPSYIEAAKEEGVTILSALSMLPNAPAWLSISGIIVAVVAMSKS
FLGTYFGVIEGATEVVKTTLQQVGVKKSRAFNRALSIMLVSLITFIVCCINPNAISMIYA
ISGPLIAMILFIMPTLSTYLIPALKPWRSIGNLITLIVGILCVSVMFFS
>LFGLNPFC_00269 Acetyl-CoA acetyltransferase
MKDVVIVGALRTPIGCFRGALADHSAVELGSLVVKALIERTGVPAYAVDEVILGQVLTAG
AGQNPARQSAIKGGLPNSVSAITINDVCGSGLKALHLATQAIQCGEADIVIAGGQENMSR
APHVLTDSRTGAQLGNSQLVDSLVHDGLWDAFNDYHIGVTAENLAREYGISRQLQDAYAL
SSQQKARAAIDAGRFKDEIVPVITQSNGQTLVVDTDEQPRTDTSAEGLARLNPSFDSLGS
VTAGNASSINDGAAAVMMMSEAKARALNLPVLARIRAFASVGVDPALMGIAPVYATRRCL
ERVGWQLADVDLIEANEAFAAQALSVGKMLEWDERRVNVNGGAIALGHPIGASGCRILVS
LVHEMVKRNARKGLATLCIGGGQGVALTIERDE
>LFGLNPFC_00270 4-deoxy-L-threo-5-hexosulose-uronate ketol-isomerase
MDVRQSIHSAHAKTLDTQGLRNEFLVEKVFVADEYTMVYSHIDRIIVGGIMPVTKTVSVG
GEVGKQLGVSYFLERRELGVINIGGAGTITVDGQCYEIGHRDALYVGKGAKEVVFASIDT
ATPAKFYYNCAPAHTTYPTKKVTPDEVSPVTLGDNLTSNRRTINKYFVPDVLETCQLSMG
LTELAPGNLWNTMPCHTHERRMEVYFYFNMDDDACVFHMMGQPQETRHIVMHNEQAVISP

SWSIHSGVGTKAYTFIWGMVGENQVFDDMDHVAVKDLR
>LFGLNPFC_00271 2-dehydro-3-deoxy-D-gluconate 5-dehydrogenase
MILSAFSLEGKVAVVTGCDTGLGQGMALGLAQAGCDIVGINIVEPTETIKQVTALGRRFL
SLTADLRKIDGIPALLDRAVAEFGHIDILVNNAGLIRREDALEFSEKDWDDVMNLNIKSV
FFMSQAAAKHFIAQGNGGKIINIASMLSFQGGIRVPSYTASKSGVMGVTRLMANEWAKHN
INVNAIAPGYMATNNTQQLRADEQRSAEILDRIPAGRWGLPSDLMGPVVFLASSASDYVN
GYTIAVDGGWLAR
>LFGLNPFC_00272 Arabinose-proton symporter
MVTINTESALTPRPLRDTRRMNMFVSVAAAVAGLLFGLDIGVIAGALPFITDHFVLTSRL
QEWVVSSMMLGAAIGALFNGWLSFRLGRKYSLMAGAILFVLGSIGSAFATSVEMLIAARV
VLGIAVGIASYTAPLYLSEMASENVRGKMISMYQLMVTLGIVLAFLSDTAFSYSGNWRAM
LGVLALPAVLLIILVVFLPNSPRWLAEKGRHIEAEEVLRMLRDTSEKAREELNEIRESLK
LKQGGWALFKINRNVRRAVFLGMLLQAMQQFTGMNIIMYYAPRIFKMAGFTTTEQQMIAT
LVVGLTFMFATFIAVFTVDKAGRKPALKIGFSVMALGTLVLGYCLMQFDNGTASSGLSWL
SVGMTMMCIAGYAMSAAPVVWILCSEIQPLKCRDFGITCSTTTNWVSNMIIGATFLTLLD
SIGAAGTFWLYTALNIAFVGITFWLIPETKNVTLEHIERKLMAGEKLRNIGV
>LFGLNPFC_00273 L-aspartate/glutamate-specific racemase
MKTIGLLGGMSWESTIPYYRLINEGIKQRLGGLHSAQVLLHSVDFHEIEECQRRGEWDKT
GDILAEAALGLQRAGAEGIVLCTNTMHKVADAIESRCSLPFLHIADATGRAITGAGMTRV
ALLGTRYTMEQDFYRGRLTEQFSINCLIPEADERAKINQIIFEELCLGQFTEASRAYYAQ
VIARLAEQGAQGVIFGCTEIGLLVPEERSVLPVFDTAAIHAEDAVAFMLS
>LFGLNPFC_00274 Octopine catabolism/uptake operon regulatory protein OccR
MAAVNLRHIEIFHAVMTAGSLTEAAHLLHTSQPTVSRELARFEKVIGLKLFERIRGRLHP
TVQGLRLFEEVQRSWYGLDRIVSAAESLREFRQGELSIACLPVFSQSFLPQLLQPFLARY
PDVSLNIVPQESPLLEEWLSAQRHDLGLTETLHTPAGTERTELLSLDEVCVLPPGHPLAV
KKVLTPDDFHSENYISLSRTDSYRQLLDQLFTENQVKRRMIVETHSAASVCAMVRAGVGV
SVVNPLTALDYAASGLVVRRFSIAVPFTVSLIRPLHRPSSALVQAFSEHLQAGLPKLVTS
LDAILSSATTA
>LFGLNPFC_00275 Diaminopimelate decarboxylase
MPHSLFSTDTDLTAENLLRLPAEFGCPVWVYDAQIIRRQIAALKQFDVVRFAQKACSNIH
ILRLMREQGVKVDSVSLGEIERALAAGYNPQTHPDDIVFTADVIDQATLERVSELQIPVN
AGSVDMLDQLGQVSPGHRVWLRVNPGFGHGHSQKTNTGGENSKHGIWYTDLPAALDVIQR
HHLQLVGIHMHIGSGVDYAHLEQVCGAMVRQVLEFGQDLQAISAGGGLSIPYQQGEEAVD
TEHYYGLWNAAREQIARHLGHPVKLEIEPGRFLVAQAGVLITQVRSVKQMGSRHFVLVDA
GFNDLMRPAMYGSYHHISALAADGRSLEHAPTVETVVAGPLCESGDVFTQQEGGNVETRA
LPEVKACDYLVLHDTGAYGASMSSNYNSRPLLPEVLFDNGQARLIRRRQTIEELLALELL
>LFGLNPFC_00276 HTH-type transcriptional regulator GalR
MATIKDVARLAGVSVATVSRVINNSPKASEASRLAVHSAMESLSYHPNANARALAQQTTE
TIGLVVGDVSDPFFGAMVKAVEQVAYHTGNFLLIGNGYHNEQKERQAIEQLIRHRCAALV
VHAKMIPDADLASLMKQMPGMVLINRILPGFENRCIALDDRYGAWLATRHLIQQGHTRIG
YLCSNHSISDAEDRLQGYYDALAESGIPANDRLVTFGEPDESGGEQAMTELLGRGRNFTA
VACYNDSMAAGAMGVLNDNGIDVPGEISLIGFDDVLVSRYVRPRLTTVRYPIVTMATQAA
ELALALADNRPLPEITNVFSPTLVRRHSVSTPSLEASHHATSD
>LFGLNPFC_00277 Bifunctional protein Aas
MLFSFFRNLCRVLYRVRVTGDTKALKGERVLITPNHVSFIDGILLALFLPVRPVFAVYTS
ISQQWYMRWLKSFIDFVPLDPTQPMAIKHLVRLVEQGRPVVIFPEGRITTTGSLMKIYDG
AGFVAAKSGATVIPVRIEGAELTHFSRLKGLVKRRLFPQITLHILPPTQVEMPDAPRARD
RRKIAGEMLHQIMMEARMAVRPRETLYESLLSAMYRFGAGKKCVEDVNFTPDSYRKLLTK
TLFVGRILEKYSVEGERIGLMLPNAGISAAVIFGAIARRRIPAMMNYTAGVKGLTSAITA
AEIKTIFTSRQFLDKGKLWHLPEQLTQVRWVYLEDLKADVTTADKVWIFAHLLMPRLAQV
KQQPEEEALILFTSGSEGHPKGVVHSHKSILANVEQIKTIADFTTNDRFMSALPLFHSFG
LTVGLFTPLLTGAEVFLYPSPLHYRIVPELVYDRSCTVLFGTSTFLGHYARFANPYDFYR
LRYVVAGAEKLQESTKQLWQDKFGLRILEGYGVTECAPVVSINVPMAAKPGTVGRILPGM
DARLLSVPGIEEGGRLQLKGPNIMNGYLRVEKPGVLEVPTAENIRGEMERDWYDTGDIVR
FDEQGFVQIQGRAKRFAKIAGEMVSLEMVEQLALGVSPDKVHATAIKSDASKGEALVLFT
TDNELTRDKLQQYAREHGVPELAVPRDIRYLKQMPLLGSGKPDFVTLKSWVDEAEQHDE
>LFGLNPFC_00278 Lysophospholipid transporter LplT
MSESVHTNTSLWSKGMKAVIVAQFLSAFGDNALLFATLALLKAQFYPEWSQPILQMVFVG
AYILFAPFVGQVADSFAKGRVMMFANGLKLLGAASICFGINPFLGYTLVGVGAAAYSPAK
YGILGELTTGSKLVKANGLMEASTIAAILLGSVAGGVLADWHILVALVACALAYGGAVVA
NIYIPKLAAARPGQSWNLISMTRSFLNACTSLWRNGETRFSLVGTSLFWGAGVTLRFLLV
LWVPVALGITDNATPTYLNAMVAIGIVVGAGAAAKLVTLETVSRCMPAGILIGVVVLIFS
LQHELLPAYALLMLIGVLGGFFVVPLNALLQERGKKSVGAGNAIAVQNLGENSAMLLMLG
IYSLAVMVGIPVVPIGIGFGALFALAITALWIWQRRH

>LFGLNPFC_00279 Protein tas
MQYHRIPHSSLEVSTLGLGTMTFGEQNSEADAHAQLDYAVAQGINLIDVAEMYPVPPRPE
TQGLTETYVGNWLAKHGSREKLIIASKVSGPSRNNDKGIRPDQALDRKNIREALHDSLKR
LQTDYLDLYQVHWPQRPTNCFGKLGYSWTDSAPAVSLLDTLDALAEYQRAGKIRYIGVSN
ETAFGVMRYLHLADKHDLPRIVTIQNPYSLLNRSFEVGLAEVSQYEGVELLAYSCLGFGT
LTGKYLNGAKPAGARNTLFSRFTRYSGEQTQKAVAAYVDIARRHGLDPAQMALAFVRRQP
FVASTLLGATTMEQLKTNVESLHLELSEDVLAEIEAVHQVYTYPAP
>LFGLNPFC_00280 putative lipoprotein YgdR
MKKWAVIISAVGLAFAVSGCSSDYVMATKDGRMILTDGKPEIDDDTGLVSYHDQQGNAMQ
INRDDVSQIIER
>LFGLNPFC_00281 hypothetical protein
MLFAWITDPNAWLALGTLTLLEIVLGIDNIIFLSLVVAKLPTAQRAHARRLGLAGAMVMR
LALLASIAWVTRLTNPLFTIFSQEISARDLILLLGGLFLIWKASKEIHESIEGEEEGLKT
RVSSFLGAIVQIMLLDIIFSLDSVITAVGLSDHLFIMMAAVVIAVGVMMFAARSIGDFVE
RHPSVKMLALSFLILVGFTLILESFDIHVPKGYIYFAMFFSIAVESLNLIRNKKNPL
>LFGLNPFC_00282 DNA mismatch repair protein MutH
MSQPRPLLSPPETEEQLLAQAQQLSGYTLGELAALAGLVTPENLKRDKGWIGVLLEIWLG
ASAGSKPEQDFAALGVELKTIPVDSLGRPLETTFVCVAPLTGNSGVTWETSHVRHKLKRV
LWIPVEGERSIPLAKRRVGSPLLWSPNEEEDRQLREDWEELMDMIVLGQIERITARHGEY
LQIRPKAANAKALTEAIGARGERILTLPRGFYLKKNFTSALLARHFLIQ
>LFGLNPFC_00283 hypothetical protein
MLSTESWDNCEKPPLLFPFTALTCDETPVFSGSVLNLVAHSVDKYGIG
>LFGLNPFC_00284 RNA pyrophosphohydrolase
MIDDDGYRPNVGIVICNRQGQVMWARRFGQHSWQFPQGGINPGESAEQAMYRELFEEVGL
SRKDVRILASTRNWLRYKLPKRLVRWDTKPVCIGQKQKWFLLQLVSGDAEINMQTSSTPE
FDGWRWVSYWYPVRQVVSFKRDVYRRVMKEFASVVMSLQENTPKPQNASAYRRKRG
>LFGLNPFC_00285 Phosphoenolpyruvate-dependent phosphotransferase system
MLTRLREIVEKVASAPRLNEALNILVTDICLAMDTEVCSVYLADHDRRCYYLMATRGLKK
PRGRTVTLAFDEGIVGLVGRLAEPINLADAQKHPSFKYIPSVKEERFRAFLGVPIIQRRQ
LLGVLVVQQRELRQYDESEESFLVTLATQMAAILSQSQLTALFGQYRQTRIRALPAAPGV
AIAEGWQDATLPLMEQVYQASTLDPALERERLTGALEEAANEFRRYSKRFAAGAQKETAA
IFDLYSHLLSDTRLRRELFAEVDKGSVAEWAVKTVIEKFAEQFAALSDNYLKERAGDLRA
LGQRLLFHLDDANQGPNAWPERFILVADELSATTLAELPQDRLVGVVVRDGAANSHAAIM
VRALGIPTVMGADIQPSVLHRRTLIVDGYRGELLVDPEPVLLQEYQRLISEEIELSRLAE
DDVNLPAQLKSGERIKVMLNAGLSPEHEEKLGSRIDGIGLYRTEIPFMLQSGFPSEEEQV
AQYQGMLQMFNDKPVTLRTLDVGADKQLPYMPISEENPCLGWRGIRITLDQPEIFLIQVR
AMLRANAATGNLNILLPMVTSLDEVDEARRLIERAGREVEEMIGYEIPKPRIGIMLEVPS
MVFMLPHLAKRVDFISVGTNDLTQYILAVDRNNTRVANIYDSLHPAMLRALAMIAREAEI
HGIDLRLCGEMAGDPMCVAILIGLGYRHLSMNGRSVARVKYLLRRIDFAEAENLAQRSLE
AQLATEVRHQVAAFMERRGMGGLIRGGL
>LFGLNPFC_00286 Phosphatidylglycerol--prolipoprotein diacylglyceryl transferase
MTSSYLHFPEFDPVIFSIGPVALHWYGLMYLVGFIFAMWLATRRANRPGSGWTKNEVENL
LYAGFLGVFLGGRIGYVLFYNFPQFMADPLYLFRVWDGGMSFHGGLIGVIVVMIIFARRT
KRSFFQVSDFIAPLIPFGLGAGRLGNFINGELWGRVDPNFPFAMLFPGSRTEDILLLQTN
PQWQSIFDTYGVLPRHPSQLYELLLEGVVLFIILNLYIRKPRPMGAVSGLFLIGYGAFRI
IVEFFRQPDAQFTGAWVQYISMGQILSIPMIVAGVIMMVWAYRRSPQQHVS
>LFGLNPFC_00287 Thymidylate synthase
MKQYLELMQKVLDEGTQKNDRTGTGTLSIFGHQMRFNLQDGFPLVTTKRCHLRSIIHELL
WFLQGDTNIAYLHENNVTIWDEWADENGDLGPVYGKQWRAWPTPDGRHIDQITTVLNQLK
NDPDSRRIIVSAWNVGELDKMALAPCHAFFQFYVADGKLSCQLYQRSCDVFLGLPFNIAS
YALLVHMMAQQCDLEVGDFVWTGGDTHLYSNHMDQTHLQLSREPRPLPKLIIKRKPESIF
DYRFEDFEIEGYDPHPGIKAPVAI
>LFGLNPFC_00288 hypothetical protein
MKTQRGYTLIETLVAMLILVMLSASGLYGWQYWQQSQRLWQTASQARDYLLYLREDANWH
NRDHSISLIREGTLWCLVSSVAGANTCHGSSPLVFVPRWPEVEMSDLTPSLAFFGLRNTA
WAGHIRFKNSTGEWWLVVSPWGRLRLCQQGETEGCL
>LFGLNPFC_00289 hypothetical protein
MPVKEQGFSLLEVLIAMAISSVLLLGAARFLPALQRESLTNTRKLALEDEIWLRVFTVAK
HLQRAGYCHGSCTGEGLEIVGQGDCVIVQWDANSNGIWDREPVKESDQIGFRLKEHVLET
LRGATSCEGKGWDKVTNPDAIIIDTFQVVRQDVSGFSPVLTVNMHAASKADPQTVVDASY
SVTGSNL
>LFGLNPFC_00290 hypothetical protein
MNREKGVSSLALVLMLLILSSLLLQGMSQQDRSFASRVSMESQSLRRQAIVQSALEWGKM
HSWQTQPAVQCLLYAATGARVCLRLLADNEALLIAGYEGVSLWRTGEVIDGNIVFSPRGW

SDFCPLKERALCQLP
>LFGLNPFC_00291 hypothetical protein
MSASLRNQQGFSLPEVMLAMVLMVMIVTALSGFQRTLMNSLASRNQYQQLWRHGWQQTQL
RAISPPANWQVNRMQTSQAGCVSISVTLVSPGGREGEMTRLHCPNRQ
>LFGLNPFC_00292 RecBCD enzyme subunit RecC
MLRVYHSNRLDVLEALMEFIVERERLDDPFEPEMILVQSTGMAQWLQMTLSQKFGIAANI
DFPLPASFIWDMFVRVLPEIPKESAFNKQSMSWKLMTLLPQLLEREDFTLLRHYLTDDSD
KRKLFQLSSKAADLFDQYLVYRPDWLAQWETGHLVEGLGEAQAWQAPLWKALVEYTDELG
QPRWHRANLYQRFIETLESATTCPPGLPSRVFICGISALPPVYLQALQALGKHIEIHLLF
TNPCRYYWGDIKDPAYLAKLLTRQRRHSFEDRELPLFRDSENAGQLFNSDGEQDVGNPLL
ASWGKLGRDYIYLLSDLESSQELDAFVDVTPDNLLHNIQSDILELENRAVAGVNIEEFSR
SDNKRPLDPLDSSITFHVCHSPQREVEVLHDRLLAMLEEDPTLTPRDIIVMVADIDSYSP
FIQAVFGSAPADRYLPYAISDRRARQSHPVLEAFISLLSLPDSRFVSEDVLALLDVPVLA
ARFDITEEGLRYLRQWVNESGIRWGIDDDNVRELELPATGQHTWRFGLTRMLLGYAMESA
QGEWQSVLPYDESSGLIAELVGHLASLLMQLNIWRRGLAQERPLEEWLPVCRDMLNAFFL
PDAETEAAMTLIEQQWQAIISEGLGAQYGDAVPLSLLRDELALRLDQERISQRFLAGPVN
ICTLMPMRSIPFKVVCLLGMNDGVYPRQLAPLGFDLMSQKPKRGDRSRRDDDRYLFLEAL
ISAQQKLYISYIGRSIQDNSERFPSVLVQELIDYIGQSHYLPGDEALNCDESEARVKAHL
TCHHTRMPFDPQNYQPGNLQSYAREWLPAASQAGKAHSEFVQPLPFTLPETVPLETLQRF
WAHPVRAFFQMRLQVNFRTEDSEIPDTEPFILEGLSRYQINQQLLNVLVEQDDAERLFRR
FRAAGDLPYGAFGEIFWETQCQEMQQLADRVIACRQPGQSMEIDLACNGVQITGWLPQVQ
PDGLLRWRPSLLSVAQGMQLWLEHLVYCASGGNGESRLFLRKDGEWRFPPLAAEQALHYL
SQLIEGYREGMSAPLLVLPESGGAWLKTCYDAQNDAMLDDDSTLQKARTKFLQAYEGNMM
VRGEGDDIWYQRLWRQLTPETMEAIVEQSQRFLLPLFRFNQS
>LFGLNPFC_00293 Protease 3
MPRSIWFKALLLFVALWAPLSQAETGWQPIQETIRKSDKDNRQYQAIRLDNGMVVLLVSD
PQAVKSLSALVVPVGSLEDPEAYQGLAHYLEHMSLMGSKKYPQADSLAEYLKMHGGSHNA
STAPYRTAFYLEVENDALPGAVDRLADAIAEPLLDKKYAERERNAVNAELTMARTRDGMR
MAQVSAETINPAHPGSKFSGGNLETLSDKPGNPVQQALKDFHEKYYSANLMKAVIYSNKP
LPELAKMAADTFGRVPNKESKKPEITVPVVTDAQKGIIIHYVPALPRKVLRVEFRIDNNS
AKFRSKTDELITYLIGNRSPGTLSDWLQKQGLVEGISANSDPIVNGNSGVLAIFASLTDK
GLANRDQVVAAIFSYLNLLREKGIDKQYFDELANVLDIDFRYPSITRDMDYVEWLADTMI
RVPVEHTLDAVNIADRYDAKAVKERLAMMTPQNARIWYISPKEPHNKTAYFVDAPYQVDK
ISEQTFADWQKKAANIALSLPELNPYIPDDFSLIKSEKKYDHPELIVDESNLRVVYAPSR
YFASEPKADVSLILRNPKAMDSARNQVMFALNDYLAGLALDQLSNQASVGGISFSTNANN
GLMVNANGYTQRLPQLFQALLEGYFSYTATEDQLEQAKSWYNQMMDSAEKGKAFEQAIMP
AQMLSQVPYFSRDERRKILPSITLKEVLAYRDTLKSGARPEFMVIGNMTEAQATTLARHV
QKQLGADGSEWCRNKDVVVDKKQSVIFEKAGNSTDSALAAVFVPTGYDEYTSSAYSSLLG
QIVQPWFYNQLRTEEQLGYAVFAFPMSVGRQWGMGFLLQSNDKQPSFLWERYKAFFPTAE
AKLRAMKPEEFAQIQQAVITQMLQAPQTLGEEASKLSKDFDRGNMRFDSRDKIVAQIKLL
TPQKLADFFHQAVVEPQGMAILSQISGSQNGKAEYVHPEGWKVWENVSALQQTMPLMSEK
NE
>LFGLNPFC_00294 RecBCD enzyme subunit RecB
MSDVAETLDPLRLPLQGERLIEASAGTGKTFTIAALYLRLLLGLGGSAAFPRPLTVEELL
VVTFTEAATAELRGRIRSNIHELRIACLRETTDNPLYKRLLEEIDDKAQAAQWLLLAERQ
MDEAAVFTIHGFCQRMLNLNAFESGMLFEQQLIEDESLLRYQACADFWRRHCYPLPREIA
QVVFETWKGPQALLRDINRYLQGEAPVIKAPPPDDETLASRHAQIVARIDAVKQQWRDAV
GELDALIESSGIDRRKFNRSNQAKWIEKISAWAEEETNSYQLPESLEKFSQRFLEDRTKA
GGETPRHPLFEAIDQLLAEPLSIRDLVITRALAEIRETVAREKRRRGELGFDDMLSRLDS
ALRSESGEVLAAAIRTRFPVAMIDEFQDTDPQQYRIFRRIWHHQPETALLLIGDPKQAIY
AFRGADIFTYMKARSEVHAHYTLDTNWRSAPGMVNSVNKLFSQTDDAFMFREIPFIPVKS
AGKNQALRFVFKGETQPAMKMWLMEGESCGVGDYQSTMAQVCAAQICDWLQAGQRGEALL
MNGDDARPVRASDISVLVRSRQEAAQVRDALTLLEIPSVYLSNRDSVFETLEAQEMLWLL
QAVMTPERENTLRSALATSMMGLNALDIETLNNDEHAWDAVVEEFDGYRQIWRKRGVMPM
LRALMSARNIAENLLATAGGERRLTDILHISELLQEAGTQLESEHALVRWLSQHILEPDS
NASSQQMRLESDKHLVQIVTIHKSKGLEYPLVWLPFITNFRVQDQAFYHDRHSFEAVLDL
NAAPESVDLAEAERLAEDLRLLYVALTRSVWHCSLGVAPLVRRRGDKKGDTDVHQSALGR
LLQKGEPQDAAGLRTCIEALCNDDIAWQTAQIGDNQPWQVNDALTAELNARTLQRLPGDN
WRVTSYSGLQQRGHGIAQDLMPRLDVDAAGVVSVVEEPTLTPHQFPRGASPGTFLHSLFE
DLDFTQPIDPNWVQEKLELGGFEPQWELVLTEWITAVLQAPLNETGVSLNQLSDRDKQVE
MEFYLPISEPLIASQLDALIRQFDPLSAGCPPLEFMQVRGMLKGFIDLVFRHEGRYYLLD
YKSNWLGEDSSAYTQQAMAAAMQAHRYDLQYQLYTLALHRYLRHRIADYDYERHFGGVIY
LFLRGVDKEHPQQGIYATRPNAGLIDLMDEMFASMTLEEA
>LFGLNPFC_00295 RecBCD enzyme subunit RecD

MKLQKQLLEAVEHKQLRPLDVQFALTVAGDEHPAVTLAAALLSHDAGEGHVCLPLSRLEN
NEASHPLLATCVSEIGELQNWEECLLASQAVSRGDEPTPMILCGDRLYLNRMWCNERTVA
RFFNEVNHAIEVDEALLAQTLDKLFPVSDEINWQKVAAAVALTRRISVISGGPGTGKTTT
VAKLLAALIQMADGERCRIRLAAPTGKAAARLTESLGKALRQLPLTDEQKKRIPEDASTL
HRLLGAQPGSQRLRHHAGNPLHLDVLVVDEASMIDLPMMSRLIDALPDHARVIFLGDRDQ
LASVEAGAVLGDICAYANAGFTAERAGQLSRLTGSHVPAGTGTEAASLRDSLCLLQKSYR
FGSDSGIGQLAAAINRGDKTAVKTVFQQDFTDIEKRLLQSGEDYIAMLEEALAGYGRYLD
LLQARAEPDLIIQAFNEYQLLCALREGPFGVAGLNERIEQFMQQKRKIHRHPHSRWYEGR
PVMIARNDSALGLFNGDIGIALDRGQGTRVWFAMPDGNIKSVQPSRLPEHETTWAMTVHK
SQGSEFDHAALILPSQRTPVVTRELVYTAVTRARRRLSLYADERILSAAIATRTERRSGL
AALFSSRG
>LFGLNPFC_00296 Amino-acid acetyltransferase
MVKERKTELVEGFRHSVPYINTHRGKTFVIMLGGEAIEHENFSSIVNDIGLLHSLGIRLV
VVYGARPQIDANLAAHHHEPLYHKNIRVTDAKTLELVKQAAGTLQLDITARLSMSLNNTP
LQGAHINVVSGNFIIAQPLGVDDGVDYCHSGRIRRIDEDAIHRQLDSGAIVLMGPVAVSV
TGESFNLTSEEIATQLAIKLKAEKMIGFCSSQGVTNDDGDIVSELFPNEAQARVEAQEEK
GDYNSGTVRFLRGAVKACRSGVRRCHLISYQEDGALLQELFSRDGIGTQIVMESAEQIRR
ATINDIGGILELIRPLEQQGILVRRSREQLEMEIDKFTIIQRDNTTIACAALYPFPEEKI
GEMACVAVHPDYRSSSRGEVLLERIAAQAKQSGLSKLFVLTTRSIHWFQERGFTPVDIDL
LPESKKQLYNYQRKSKVLMADLG
>LFGLNPFC_00297 N-acetylmuramoyl-L-alanine amidase AmiC
MSGSNTAISRRRLLQGAGAMWLLSVSQVSLAAVSQVVAVRVWPASSYTRVTVESNRQLKY
KQFALSNPERVVVDIEDVNLNSVLKGMAAQIRADDPFIKSARVGQFDPQTVRMVFELKQN
VKPQLFALAPVAGFKERLVMDLYPANAQDMQDPLLALLEDYNKGDLEKQVPPAQSGPQPG
KAGRDRPIVIMLDPGHGGEDSGAVGKYKTREKDVVLQIARRLRSLIEKEGNMKVYMTRNE
DIFIPLQVRVAKAQKQRADLFVSIHADAFTSRQPSGSSVFALSTKGATSTAAKYLAQTQN
ASDLIGGVSKSGDRYVDHTMFDMVQSLTIADSLKFGKAVLNKLGKINKLHKNQVEQAGFA
VLKAPDIPSILVETAFISNVEEERKLKTATFQQEVAESILAGIKAYFADGATLARRG
>LFGLNPFC_00298 Transcription antiterminator LicT
MIIEKVMNNNCVQASMNGQEVIISGPGVGYNKKYGMSVPEHPANRIFYVRNEQKNKLYKL
IEHVDIEYVFVAEKIVQYAEKNLNPSLLLILADHISNAISRVVSGIQINNVFLDEIKALY
KAEYAISRDALTIINEQFSVQLPDDEIGFIALHILNNYENSVDYESVRIIELSQIITELI
EVVYNRKVDRSSFNYSRFMMHLKYFSSRVLCNEKIKQKDIGDIYEQFLEKDILLQRAIHE
IERYLYATFKYELILEEKLYLSIRTKVLMD
>LFGLNPFC_00299 PTS system maltose-specific EIICB component
MKQKKAWSFFQSLGKAFMYPIALLSVCGMMLGLGSGLASDDMAKLIPFLAIPIIKTILDF
IVSLGLFAFVNLPVLFAIAIPLGLLKDKEDKAYGAFSGLIGFMAMHLGTNFYLKQHDLLV
VADQMSTHGQTIILGIQSYNTSVLGGIVAGLLVASMYKKIVNLRIPESLGFYSGPRLVPI
ITLIVMSGFGLIIPFIWPPFFNLFMLIGHWISTSGPVGYFFYAVAERVTIPFGLNHLVTS
VFRFTPIGGSAVIGGEEYYGTLNMFMAYVKENAVIPLDLAGKMEQGKLMIQYGLAGAALA
MYRTAHAQNRKAIKALLISGVLTVIIGGVSEPIEFLFLFVSPLLFVFHAFMNGFANMVLP
YMGVKMGFTGDLIQFISFGVLRGTRTGWPIAVCVEVAYFFIYYFVFRWTILKFNLMTVGR
EESNPVTLNVHEDTAIADIPTPDKSELQAAEQMVKALGGKENIKSLDNCVTRLRLTIADM
GLIDEVAIKRAGGIAVVKLDQNTLQVIIGTKVIALRRDMDNYMGIY
>LFGLNPFC_00300 Protein MalY
MDVFNTPVSRKGTYCTQWDFCEDRFGVKDVLPFSISDMDLPIPDAITRALKKRLEHPILG
YSRWQHGEYLNAIVNWYYQQYQTDIKPEWITYSPSVMYSIAKAIELLTSHGDNILVFTPV
YNAFFDVIKHSERNILTASLIIKNEGGYAINWQDFDLKIKSAKMVLLCNPHNPTGTVWSE
EELHKIAASCTRHNVWLCSDEIHSDFVFNRSFTSALKIKKEKVVVFNSISKTFNVPALTG
SYMISTDDNFNHKFRTISRYRDFVNSPSVLNIIATIIAYNECEGWLKSLKAHIASNIQFT
QQYLNENIPELIVRPADGCYFSWIDCSAIGYPFDEFYSRLIHEGKVGIMAGHVYGTEGEG
YLRLNLACGREKLYMGLTRLVSVIKNINQGE
>LFGLNPFC_00301 Arabinose 5-phosphate isomerase KdsD
MNNTDLIYLIKHFMHNELKAVEEVIYSPLSEFANLIKVLQSCQGKVVFIGVGKSGIIARK
LAATFASTGTPSFFVHGTEAVHGDLGMVAKDDVVILISNSGETAEILATLPSLKKMGNYL
ISFTRSHHSSLAISCDLSVEIPVKSEADNLGLAPSCSSTVVLVVGDAVALALSELKKFTR
ADFGLYHPGGALGIKANS
>LFGLNPFC_00302 Hydroxypyruvate reductase
MKNVLVTFPSFSARCVSASKLLRENNFNLIIKNNVEHLLKSESTALRESICAVIAGKDGY
QADTLSLLPGVRIISRFGTGIDNIDLRAAQQSGIVVNNAVGINSNAVAEFIIGLIFASMR
NIPGSYHAMQNGYWGESHVCELQGKRIGLVGYGNIGKTLAKRLSGFDVELLAFDKQPDYQ
VADKAGVQFVSIEDIFMQSHVIIVLLPFSSELENFISHKYLSMMRNGALIINAARGKLLD
EGALLQVIEERNVFAALDVFSSEPLAQFSPLLHAKNIITTPHIAAATVESYQQTGIHVAQ
SIIDYFAGREIKNVL

>LFGLNPFC_00303 hypothetical protein
MASNANFISQFVMGGDPCTYKESGELQAEMSKLTHPARPDVDWRQVEKLSLALFRQNGVE
LQTLVCYVLAITRRQGLAGMADGLGSLDILLQRWADFWPVQVHSRISLLSWVTEKMQQAL
RTLDIQYQDLPQIYRCVQHLSTIETTLQQCELWHMTKLDVLSGQFRNTALRLERLAPQGA
ETTITPPELPRREMNQPKKSEESPQPVFATRSVQQNDKDASPPVPSPEISRQRTWPIFMA
GMVVMAGLGGTGLWGWSQLNQPDALIQRIQLSVMPLPQSLESGELAKLDVKDKALLAQDR
TIAASQMQLEQLNKLPARWPLEQGYRQLRQLDALWPDNPQVRALNAQWRKQRELSALSAE
ALNGYAQAQSQLQRLSAQLDALDERKGRYLTGSELKTAVYGIRQSLKEPPLEELLRQLEE
QKQTGEVSPTLLTQIDTRLNQLLNRYVILLDTKVEQSQ
>LFGLNPFC_00304 hypothetical protein
MSTPSLYEMLTFSFSGELPLEQISERDQLILSVMDNMQRIINCRAGTLAHLPDYGLPDLS
LIHQGMAAGIHGLMRQIEETLLRYEPRLSQIQVELLPQPRPGHLNYLIHAQLPDTGWIRF
DGVFSPEGRIVLRHLKQQERAY
>LFGLNPFC_00305 hypothetical protein
MFPIRFKRPALLCMAMLTVVLSGCGLIQKVVDESKSVASAVFYKQIKILHLDFFSRSALN
TDAEDTPLSTMVHVWQLKTREDFDKADYDTLFMQEEKTLEKDVLAKHTVWVKPEGTASLN
VPLDKETQFVAIIGQFYHPDEKSDSWRLVIKRDELEADKPRSIELMRSDLRLLPLKDK
>LFGLNPFC_00306 hypothetical protein
MDRASQPARTGLTDRLAPDIARINFYRFCQLLEQSQQKAPLGSTDNPAADAVRFRPHPGM
GFPVSELKNVERDVDNPDAPPTVRTTFLGLYGVDSPLPTAYLDYITQRHDGHDAVMAFLD
IFNHRFITQYYRIWRKYNYPASFEAGAVDDISRCLLGLIGLGIPGSENHIATPVSRFLAL
LSVMRLPTRTAEGVTALVGLLAPLTKATVVPHDPQPVILPAPAGLSKNSRISLKTRTLLG
RTGTDVNSQLLLKLYTEDAAEARGWLPGGQLHSDLLVLLRVYLGWRCQARLQLTLPVSLL
PAARLGKQRVQISRTGILRASFAAPATGTVTVSLGRYQGLIPAFSIRNRESMTHVSYSF
>LFGLNPFC_00307 hypothetical protein
MDDLTQRYYEAEMRYLREAGKEFAQAYPDRAAMLNLDKPGARDPYVERLFEGFAFLMGRL
REKLDDDLPELTEGLVSLLWPHYLRTIPSLSVVELSTDHRQMKQSETLSDFQVLSRPVGE
RRTRCVYSATRDITLHPLALPDVSLQYEPDGRSVIRLRFACGPLVGDWSQIDLSRLPLYL
NADSPVACALHRALTLGTQQFWLRLPGQDRRMLGAHFSPMGFEDDDRLWPKGESAFSGYQ
LLLEYFTFREKFMFVALNGLEQVAWPEGITGFEIDVVLNENWPHDLPFDSDNIRLHCVPV
INLFPLEADPLHLSPLENEFLLRPMRIQDGHTEIYSVDNIISSRHTGSQAYVPFSSFRHR
GGMLRHDAPERYYHTRVKRGPSGLHDTWLILGGDAFDSDRMLEDETLSLSLTGTNGQLPR
KALQSTLLDTPVHASQNVLRVRNLCAPTQPCYPPARDRFHWRVLSHLGSNFLSMMDNAEI
LRGTLALYDWTESEMNRRRLEAIVDVQHSLIQRFERGFLLRGVDIQVTLDSNGFAGEGDI
TLFGELLHRFFALYADIHLFTQLTLILQPTGKCLQWTEHHSQRVPG
>LFGLNPFC_00308 hypothetical protein
MNIQAIKEMVNLICSFLFIFFLSSAFVSFGCYAIYELFLWNDIIVYSWGYILIVFLPFTL
YVMSFEILFFAISGRRLSKVTMVRLWLIIKIIIAFSICAVLIFSSIYKKELLSRNYIACS
GIPSGWMPGLATKYVKEKSLCEKNGNN
>LFGLNPFC_00309 hypothetical protein
MYRQEYQMVVTVPTADANDPNWPNKRIQFDTSEWLQQLQYIKIDDHYILNTQYTPIANLD
DFGITLKLQNALNGSDKRLPALYGLAEMDAQKFKDLMRGKIKCEYLRTTFDAETLKPVND
YFLISFTYKDKWYEFETERKISKTSDDGYFLWAFDNTVHEAGYWHNTDPAAYSYRDYQNG
KAVK
>LFGLNPFC_00310 hypothetical protein
MGTGYFLVRGDKTTCGGKIIEGADDHTIMGIPQARDMDRVTCGRYPGMFIIVGGVPETDI
HGRLMAGSLDSQSSCPCKARFIASMMDDTYETDDGGSEPEQHAQSARKNLTSGNPDKKYS
HQIKLQHGENNVSVQDIPYVFILNNNMSLSGKTNQDGETERIYTDTAQKVIALTGKLADS
WLKRGKNFGSLKEIDNRKIELTTEENEPVKYVNWINGRDYIVIVAARTAVTNWIGMEDSK
GNQYRFINCGLEQLQQFPPASKQDSSSQRIMVVFSLGYTQKDIDRINDYTKAHDGRIIYV
KNKDELVSFLNQRKEKGRVIKELVILCHGVIKTASYHYHHEDKDIEKNGMFKHEDIAAVH
ESVFDYDAHVTTYACRAGISDGDKDFSGKDDAGQKDSPAQKMADNWDVMVKAFEMRSDYS
LAYGTGKEIKEAQEYGSVVEKYKKDIDMYNKEKAKGNTEVSPPVKPEGYDEKSKRHADVT
TRDKNEKSGGGPIAPNGAWHMPRTGDSPKGLKSGLQDYQPEEWVQ
>LFGLNPFC_00311 hypothetical protein
MRKSMGKTPMSELLQKLTRSCFADRDALDVARTQAALWQTWLLPVTADTPVGEDPGYHDD
FLRIRDEMNKLSGTDTGLICQLAESLLLTQAKDVRIATYYIWARLHRDGERGLAEGLALL
TGLVERFGTQLLPSRPASRKMALEWLAGEKMLDSLARYPEVAKEDFANIVAALNQLTVSF
TAWPEDQHSPSLMPLINALESRLAQSGGMNAVVPQNSSSVTAPSSPVDAPQVQTITSGRD
LLDQAKVLARYLNEQPQGWLSAHRLMKTLRWDTVHELPPDVDGKTRLAPPRTESRNQLKR
LYAQQNWTELLEQADLMFSTGVSHFWLDIQWYLHQALTKAGVPWDRWTAVIRQDLALLLE
RLPGLENLAWNDGTPFADEVTRNWIAQQVMMREDGAWLAGKAAVPTDDATNDVLALEPEA
LAMADSQGVEAALGWIQTRPGITTARQRLLLRLLMARVAEQYGKNEMALLLLEELDTAAQ
GITLTQWEPELLFEVKARQLKLLRLRAHRYADKALLNRKMEILLGTLVAIDPARAAVLCD

TQHKD
>LFGLNPFC_00312 hypothetical protein
MHTQRYGFWGYLILIIGLAGICALLIIINQDVLIKSGVSARLIWGIWAGLFSLFLLGVVC
SPFWWIRKQRKAQFIYRPKNSAENDQPVSSPSEDILTDLSSNIRLRYGPFWRRKVRLLLV
TGEPEEAEAIAPGLTGQHWLEGDHTVLIYGGRPTAEPDVTLLTALKKLRRSRPLDGIIWP
LTEEQSRQTAQLDKGWRELINGGKRLGFQAPLYLWQVCDDGDYQTGRPLQSVGCLLPERC
TPEQLAVMLEAQTLPLTEQGMSQLLADNRHDFLLRLAHTLAERGIAHWQSVLKPLLAGGA
FSSLRLRGLMFSPPLAAVPEAAPHAWLPSPVWAGVTGDNARGRTVGFPWLRTALMSAVCV
LVIWGAGMTTSFFANRALVQETGIQTARALDTRLPLAEQLVALHTLQGELERLQYRIREG
APWYQRFGLERNQQLLAAAFPGYAQAANRLVRDVAVDHLQQQLNAFVALPPNSPQRTATG
EQRYKQLKALLMTSRPEKADAAFFSTTLMADGLRYENIPEGVRQSVLPSLLTFWTANLPE
HPQWKTSPPPELTGAVRKILLRQIGVRNAENTLYQNVLQQVSRNYADMTLADMTGDTLTE
SLFSTEQTVPGMFTRQAWEGGQVREAIEQVVTARREEIDWVLSDRQQDTSADISPDTLRNR
LTSRYFTDFAGSWLAFLNSIRWKKEDSLSGILDQLTLMADARQSPLIALTDTLAWQAAAG
RENRGLSDSLAKSAQELFNGKEKTPQQSREGNEPVGPLDKTFAPLLRLLGDKAGGGGGDT
QLSLQTYLTRVTRVRLKLQQVTNAPDPQEMTQQLAQTVLQGKTVDLTDTRDYGRLIAASL
GEEWSGFGQALFVRPVEQSWRQVLTPAADSLNRQWQRAIVSHWNQDFAGRYPFKASQNDA
SLPLLAQYLRDDGRINQFIAANLSGVLKREGRYWVADAMNTQGLTVNPDFIRALNRLRDV
ADTAFASGDAGIHFELRAKPARDVMKTHLVIDGQELEYFNQKERWQRFNWPDEQWQPGAS
LSWTSTQAMERILADYRGSWSLIRLLEQAQVTPVDSSTFKVVWKAQDGLPLNYLLRVEQG
KGPLALLELKNFRLPGQVFLTGRSMKDAEEYGEDADE
>LFGLNPFC_00313 hypothetical protein
MGWPVPDIPERKMLPEPVYRRWIILLISMLTVGTLFILSVWNSATYWDIFIYGVLPMLFL
WLCLFGIALNKYEQSVAACISWESERQQVKQLWQHWSQKQLAIVGNVLFTPEEKGMSVLL
GPQEEIPAYPKKARPLFSASRYSLSSIFHDIHQQLTQQFPDYRHYLHTIYVLQPEKWRGE
TVRQAIFHQWDLVPERTNTLNQIQSLYDERFDGLILVVCLQNWPENKPEDTSELVSAQLI
SSSSFVRQHQIPVIAGLGRVMPLEPEELEHNLDVLFEYNQLDNKQLQHVWVSGLDEGTIE
NLMQYAEQHQWSLPKKRPLHMIDHSFGPTGEFIFPVSLAMLSEAAKETEQNHLIIYQSAQ
YAQKKSLCLITRKLYLRT
>LFGLNPFC_00314 hypothetical protein
MPGIVCLGDATTHGGKVITATSTLFINGIQVALVGDLVSCPKHGVNPIQEGDPTATEKGR
NVVVNHCQCACGCRVISSQPENSIES
>LFGLNPFC_00315 hypothetical protein
METEHGITLYKGRKKINGLEISEWWVRKQIMLNSEPSRDYEFNLAIHEDKKNNKQLLKLV
MNYSVDILHADNALTEHELMALWESITGTIKYHPTQW
>LFGLNPFC_00316 hypothetical protein
MVKCHTLINRRNKCLLIVFIVLIGWIIFRPKAYTYSLNDKEKEMLIMLSQHPETRYFGFY
SIELPADYKPTGMVMFIQGSAMIPVETKLQYYPPFLQYMTRYEAELKNTSALDPLDTPYL
KQVHPLSPPMNGVIFERMKAKYTPDFARVLDAWKWENGVTFSVKIEAKDGRATRYDGISK
IAEYSYGYNIPEKKVQLLTILSGLQPRADNQPPSENKLAIQYAQVDASLLGEYELSVDYK
NSNNIKISLQTDNNSYIDSLLDIRYPSNGNRAWYNSI
>LFGLNPFC_00317 hypothetical protein
MTSTMHMDRADKNKVGTVEIEDLRVIPVIFLPGVMGSNLMDKKGKSIWRYDDSMSLMGWS
LPTSGPKERKRLLHPDRVEVDNRGRIPAPPDAQEKLIQLGQQYPEDPSDKEAMDNYTQAV
RDILDNIEPEAKLFGSRKDRGWGEVANASYGSFLDVLQTALYRDKPTKKGETLSATYQQL
LDVPLGLEYGPDSLDEEYLEVIRLYQFPVHVVGYNWLGSNMLSAIRLQEQIKKIVGGYQK
RGMKCHKVILVTHSMGGLVARYFSECLSGNTDVYGIVHGVLPSIGAAATYTRMKRGTENP
ESNPEGYVISHILGRNAAEMVAVFSQSPGPMELLPMNDYGEEWLNIVDRDGSTLTLPKNL
PIKEGIAPEERTYAELYLNRENWWKLVDENLLNPFNTSLNQKQIDTDWNIYENLITESVN
PFHKQIAGKYHINTYSFYGRAKLGDIPEAHLTQENVLWKGSLSMGKKSDISLEPKFIDGR
LDLNEVGNIRTIKDEFSPEEQAWEINTDDGDTYVKIGQRFTLRDSCENGDGTVPLRAGQI
VHKNILERLAVQVSHEAAYRNPVSQAFALRSIIKIAQEVKKDGKMSYSD
>LFGLNPFC_00318 hypothetical protein
MGIQNFDHSHHKLKIRGLKSPVDVLTFTGHEQLSSPFRYDIQFTSSDKAIAPESVLMQDG
AFSLTAPPVQGMPVQTALRTLHGVITGFKHLSSSQDEARYEVRLEPRMALLTRSRQNAIY
QNQTVPQIVEKILRERHQMRGQDFVFNLKSEYPAREQVMQYGEDDLTFVSRLLSEVGIWF
RFATDARLKIEVIEFYDDQSGYERGLTLPLRHPSGLFDGETEAVWGLNTAYSVVEKNVTT
RDYNYRTATAEMMTEQHDATGGDNTTYGEAYHYADNFLQKGDKEAAESGAFYARIRHERY
LNEQAILKGQSTSSLLMPGLEIRVQGDDAPAVFRKGVLITGVTASAARDRSYELTFTAIP
YSERYGYRPALIPRPVMAGTLPARVTSTVKNDIYAHIDKDGRYRVNLDFDRDAWKPGYES
LWVRQSRPYAGDTYGLHLPLLAGTEVSIAFEEGNPDRPYIAGVKHDSAHTDHVTIQNYKR
NVLRTPANNKIRLDDERGKEHIKVSTEYGGKSQLNLGHLVDAGKQQRGEGFELRTDMWGA
VRAKKGIFISADAQDKAQGGQVLDMTDALAQLREAQSLVEALCSATEVAKAELADLQTQKV
MMSEALEELKKSAMLLSAPEGIAQVTPKSLQLSAGENIISTSGKNSDFSVLKKFTVAAGE

TVSLFAQKLGIKIFAGKGKVEIQAQGDEMLLDALKDIRISSSEGRILISAKNEIILTSGG
GYIRIGDGTVECAAPDKIIERGAVWQKFSGQSISQAMQQWDSADFAVTPEILWQQTGKPA
KNQKVQVTRGDGSVVEMTTDEQGRLPIQSGLFVESIKIDLPDSQEN
>LFGLNPFC_00319 Protein ClpV1
MTGNHPAALLRRLNPYCARALDAAASLCQTRAHAEITIEHWLLKLLEQGEGDITVIARRY
EWDIDTLWQSLLAHLDTLPRSVRERPQLSEPLTALIRQAWLIASLEGDDPQIRSQHLLMA
LTEKSMLPACNDLWVLLSLSRVQLERLRPLLDAQSDECPARQPQVTEPLTSALPETATAD
APAKTLTEKQDDALLAVLNRFTEDVTEKARSGRIDPVFGRDTEIRQMVDILSRRRKNNPI
LVGEPGVGKTALVEGLALRITEGNVPDSLKTVHIRTLDLGLLQAGAGVKGEFEQRLKNVI
DAVQKSPEPVLLFIDEAHTIIGAGNQAGGADAANLLKPALARGELRTIAATTWSEYKQYF
ERDAALERRFQMVKVDEPDDDTACLMLRGLKARYAQHHGVHMLDSAIQTAVRLSRRYLTG
RQLPDKAVDLLDTAGARVRMSLDTLPEPLTQLHARLAALDIEREAIEQDSVFYPEASPER
LAELTDLRDELQAEAGHLEAQYQQEKALAQQIMTLRQEGTDSTELQQQLRTHQGFAPLLA
LDVDARAVATVVADWTGIPLSSLLRDEQSDLLSMEQSLENRVVGQRPALCAIAQRLRAAK
TGLTPENGPQGVFLLTGPSGTGKTETALTLADTLFGGEKSLITINLSEYQEPHTVSQLKG
SPPGYVGYGQGGVLTEAVRKRPYSVVLLDEVEKAHRDVMNLFYQVFDRGFMRDGEGREID
FRNTVILMTANLGSDHIMQLLEEKPDATDADLHELLYPLLRDHFQPALMARFQTVIYRPL
GQEAMRTIVEMKLAQVVRRLHQHYGLETEINDSLYDQLTAACLLPDTGARNIDSLLNQQI
LPVLSQQLLAQQAAHRKPAQLRLGWDEEDGIVLEFATEEMQ
>LFGLNPFC_00320 Major exported protein
MAIPAYLWLKDDGGADIKGSVDVQGREGSIEVVALDHDVYIPTDNNTGKLTGTRTHKPFT
FTKEIDASSPYLYKAVTTGQTLKTAEFKFYRINDAGQEVEYFNTTLDNVKLVRVAPLMHD
IKDPGKEKHNHLERIEFRYEKITWTYKDGNIIHSDSWNERPSA
>LFGLNPFC_00321 Outer membrane protein A
MRNTLKQAIVLWGMVLLLVLWSVFISPSGVLRWAGAAAIVLAVAALLIYRRRQAWTEMTG
DAGLSSLPPETYRQPVVLVCGGLSAHLFTDSPVRQVSEGLYLHVPDEEQLVAQVERLLTL
RPAWASQLAVAYTIMPGIHRDVAVLAGRLRRFAHSMATVRRRAGVNVPWLLWSGLSGSPL
PERASSPWFICTGGEVQVATSTENAMPAQWIAQSGVQERSQRLSYLLKAESLMQWLDLNV
LAELNGPEAKCPPLAMAVGLVPSLPAVDNNLWQLWITARTGLTPDIADTGTDDALPFPDA
LLRRLPRQSGFTPLRRACVTMLGVTTVAGIAALCLSATANRQLLRQVGDDLHRFYAVPVE
EFITKARHLSVLKDDATMLDGYYREGEPLRLGLGLYPGERIRQPVLRAIRDWRPPEQKME
VTASLQVQTVRLDSMSLFDVGQARLKDGSTKVLVDALVNIRAKPGWLILVAGYTDATGDE
KSNQQLSLRRAEAVRNWMLQTSDIPATCFAVQGLGESQPAATNDTPQGRAVNRRVEISLV
PRSDACQDVK
>LFGLNPFC_00322 hypothetical protein
MKKDMDINIDALLRDTFLTVVELRQGTTVRHGIELYRHCQRQVELVRERLKDAGFSRENV
EHITYAQCALLDETVLSRSGMDDGQAIWMKNPLQSHFFNTLQAGELLYERMKQVLQEPAP
AQAVLTCFHRVLLLGFRGRYQDPAAPERDQLISTLNGQVAPFGVLPETAVLNVPLSTRQH
PLLHSPFFWLVTLALLLAGVWWGLHHWLNVLVDELLPQSLR
>LFGLNPFC_00323 hypothetical protein
MKIHRPLWAEGTFLSSQQFQQQARWEAFSNDCIAQLCIRHPWGIANVLFDRDALTPGKLK
TQAVRLRFADGTLIDSDVSDLLPLACDLRALKNDSAIVLLALPLAHGNGGNLGQGEQTER
PLRYRQEWQKVQDIYGSDSEDMAVERHALSLRFAHDNNQDYITCPLARLVRDVQGNWTQD
ESYIPPLLAFNAHDGLVQRLDTLLLQLRAKCQRLMAMRRESNQRMADFAVADVSLFWLLN
ALNSAEPVLSDFLRYPAVHPELVWRELARLAGALLTFSLEHNVSAVPPYVHESPSTVFPP
LFSLLSELLEASLPSRVIALDLASLPGNRWKADLHDPRLREEADFYLSVRSSLPAHQVLH
QLPLVCKIGAPDDVTLLINVALNGVQLVPLTSVPAALPLRLENQYFALDMHSDAAKSMLE
SGSCMIYAPGTMGDLKPELFAVLRT
>LFGLNPFC_00324 hypothetical protein
MSVQQEHSTSETATLTTTESGGVYQSLFDKINLTPVSSIQEIDLWQNSETLADASPDERV
TAAIHVLLSCLAKSGEDVVKLDKSLLDFHIDDLDQKISKQLDAVMHHPEFQKVESLWRGT
WFVVQRTDFRKNVRIELLDISKEHLRQDFDDSPEIIQSGLYRHTYIQEYDTPGGEPVASL
ISSYEFDNSPQDIALLRNISRVSAASHMPFIGSVGPKFFLKNSMEEVAAIKDIGNYFDRA
EYIKWKSFRDTDDSRYVGLVMPRVLGRLPYGPDTVPVRSFNYVEEVKGPDHEKYLWTNAS
FAFAANMVKSFVNNGWCVQIRGPQAGGAVADLPIHLYDLGTGNQVKIPSEVMIPETREFE
FANLGFIPLSYYKNRDYACFFSANSAQKPALYDTADATANSRINARLPYIFLLSRIAHYL
KIIQRENIGTTKDRRVLELELNTWIRTLVTEMTDPGDELQASHPLRDGKVIVEDIEDNPG
FFRVRLFAVPHFQIEGMDINLSLVSQMPKAKA
>LFGLNPFC_00325 hypothetical protein
MADSFQNEVPTARVNIKLDLHTGNAKKKVELPLKLLAVGDYSNGKEQRPLSERDKVDINK
NNFNSVMAEFSPAVNLTVEDTLNGNGNEQNIALEFKSLKDFEPEQVAKNIPQLRVLLAMR
NLLRDLKSNLLDNATFRRELENILKDPTLSSELRDELAKIAPQENV
>LFGLNPFC_00330 Membrane-bound lytic murein transglycosylase A
MKGRWVKYLLMGTVVAMLAACSSKPTDRGQQYKDGKFTQPFSLVNQPDAVGAPINAGDFA

EQINHIRNSSPRLYGNQSNVYNAVQEWLRAGGDTRNMRQFGIDAWQMEGVDNYGNVQFTG
YYTPVIQARHTRQGEFQYPIYRMPPKRGRLPSRAEIYAGALSDKYILAYSNSLMDNFIMD
VQGSGYIDFGDGSPLNFFSYAGKNGHAYRSIGKVLIDRGEVKKEDMSMQAIRHWGETHSE
AEVRELLEQNPSFVFFKPQSFAPVKGASAVPLVGRASVASDRSIIPPGTTLLAEVPLLDN
NGKFNGQYELRLMVALDVGGAIKGQHFDIYQGIGPEAGHRAGWYNHYGRVWVLKTAPGAG
NVFSG
>LFGLNPFC_00331 tRNA threonylcarbamoyladenosine dehydratase
MSVVISDAWRQRFGGTARLYGEKALQLFADAHICVVGIGGVGSWAAEALARTGIGAITLI
DMDDVCVTNTNRQIHALRDNVGLAKAEVMAERIRQINPECRVTVVDDFVTPDNVAQYMNA
GYSYVIDAIDSVRPKAALIAYCRRNKIPLVTTGGAGGQIDPTQIQVTDLAKTIQDPLAAK
LRERLKSDFGVVKNSKGKLGVDCVFSTEALVYPQSDGTVCAMKATAEGPKRMDCASGFGA
ATMVTATFGFVAVSHALKKMMAKAARQG
>LFGLNPFC_00332 Sulfur acceptor protein CsdE
MTNPQFAGHPFGTTVTAETLRNTFAPLTQWEDKYRQLIMLGKQLPALPEELKAQAKEIAG
CENRVWLGYTVAENGKMHFFGDSEGRIVRGLLAVLLTAVEGKTAAELQAQSPLALFDELG
LRAQLSASRSQGLNALSEAIIAAAKQV
>LFGLNPFC_00333 Cysteine desulfurase CsdA
MNVFNPAQFRAQFPALQDAGVYLDSAATALKPEAVVEATRQFYSLSAGNVHRSQFAEAQR
LTARYEAAREKVAQLLNASDDKNIVWTRGTTESINMVAQCYARPRLQPGDEIIVSVAEHH
ANLVPWLMVAQQTGAKVVKLPLNAQRLPDVDLLPELITPRSRILALGQMSNVTGGCPDLA
RAITFAHSAGIVVMVDGAQGAVHFPADVQQLDIDFYAFSGHKLYGPTGIGVLYGKPELLE
AMSPWLGGGKMVHEVSFDDFTTQSAPWKLEAGTPNVAGVIGLSAALEWLTDYDINQAESW
SRSLATLAEEALAKRPGFRSFRCLDSSLLAFDFAGVHHSDMVTLLAEYGIALRAGQHCAQ
PLLAELGVTGTLRASFAPYNTKSDVDALVNAVDRALELLVD
>LFGLNPFC_00334 putative lipoprotein YgdR
MKKTAAIISACMLTFALSACSGSNYVMHTNDGRTIVSDGKPQTDNDTGMISYKDANGNKQ
QINRTDVKEMVELDQ
>LFGLNPFC_00335 Glycine cleavage system transcriptional activator
MSKRLPPLNALRVFDAAARHLSFTRAAEELFVTQAAVSHQIKSLEDFLGLKLFRRRNRSL
LLTEEGQSYFLDIKEIFSQLTEATRKLQARSAKGALTVSLLPSFAIHWLVPRLSSFNSAY
PGIDVRIQAVDRQEDKLADDVDVAIFYGRGNWPGLRVEKLYAEYLLPVCSPLLLTGEKPL
KTPEDLAKHTLLHDASRRDWQTYTRQLGLNHINVQQGPIFSHSAMVLQAAIHGQGVALAN
NVMAQSEIEAGRLVCPFNDVLVSKNAFYLVCHDSQAELGKIAAFRQWILAKAAAEQEKFR
FRYEQ
>LFGLNPFC_00336 hypothetical protein
MTSRFMLIFAAISGFIFVALGAFGAHVLSKTMGAVEMGWIQTGLEYQAFHTLAILGLAVA
MQRRISIWFYWSSVFLALGTVLFSGSLYCLALSHLRLWAFVTPVGGVSFLAGWALMLIGA
IRLKRKGVSHE
>LFGLNPFC_00337 Ribosomal RNA large subunit methyltransferase M
MNKVVLLCRPGFEKECAAEITDKAGQREIFGFARVKENAGYVIYECYQPDDGDKLIRELP
FSSLIFARQWFVVGELLQHLPPEDRITPIVGMLQGVVEKGGELRVEVADTNESKELLKFC
RKFTVPLRAALRDAGVLANYETPKRPVVHVFFIAPGCCYTGYSYSSNNSPFYMGIPRLKF
PADAPSRSTLKLEEAFHVFIPADEWDERLANGMWAVDLGACPGGWTYQLVKRNMWVYSVD
NGPMAQSLMDTGQVTWLREDGFKFRPTCSNISWMVCDMVEKPAKVAALMAQWLVNGWCRE
TIFNLKLPMKKRYEEVSHNLAYIQAQLDEHGINAQIQARQLYHDREEVTVHVRRIWAAVG
GRRDER
>LFGLNPFC_00338 putative HTH-type transcriptional regulator YdjF
MKAARQQAIVDLLLNHTSLTTEALSEQLKVSKETIRRDLNELQTQGKILRNHGRAKYIHR
QNQDSGDPFHIRLKSHYAHKADIAREALAWIEEGMVIALDASSTCWYLARQLPDINIQVF
TNSHPICHELGKRERIKLISSGGTLERKYGCYVNPSLISQLKSLEIDLFIFSCEGIDSSG
ALWDSNAINADYKSMLLKRAAQSLLLIDKSKFNRSGEARIGHLDEVTHIISDERQVTTSL
VTA
>LFGLNPFC_00339 L-fucose mutarotase
MLKTISPLISPELLKVLAEMGHGDEIIFSDAHFPAHSMGPQVIRADGLLVSDLLQAIIPL
FELDSYAPPLVMMAAVEGDTLDPEVERRYRNALSLQAPCPDIIRINRFAFYERAQKAFAI
VITGERAKYGNILLKKGVTP
>LFGLNPFC_00340 L-fuculokinase
MKQEVILVLDCGATNVRAIAVNRQGKIVARASTPNASDIAMENNTWHQWSLDAILQRFAD
CCRQINSELTDCHIRGIAVTTFGVDGALVDKQGNLLYPIISWKCPRTAAVMDHIERLISA
QQLQAISGVGAFSFNTLYKLVWLKENHPQLLERAHAWLFISSLINHRLTGEFTTDITMAG
TSQMLDIQQRDFSPQILQATGIPRRLFPRLVEAGEQIGTLQNSAAAMLGLPVGIPVISAG
HDTQFALFGAGAEQNEPVLSSGTWEILMVRSAQVDTSLLSQYAGSTCELDSQAGLYNPGM
QWLASGVLEWVRKLFWTAETPWQILIEEARLIAPGADGVKMQCDLLSCQNAGWQGVTLNT
TRGHFYRAALEGLTTQLQRNLQMLEKIGHFKASELLLVGGGSRNTLWNQIKANMLDIPVK

```
VLDDAETTVAGAALFGWYGVGEFNSPEEARAQIHYQYRYFYPQTEPEFIEEV
>LFGLNPFC_00341 L-fucose isomerase
MKKISLPKIGIRPVIDGRRMGVRESLEEQTMNMAKATAALLTEKLRHACGAAVECVISDT
CIAGMAEAAACEEKFSSQNVGLTITVTPCWCYGSETIDMDPTRPKAIWGFNGTERPGAVY
LAAALAAHSQKGIPAFSIYGHDVQDADDTSIPADVEEKLLRFARAGLAVASMKGKSYLSL
GGVSMGIAGSIVDHNFFESWLGMKVQAVDMTELRRRIDQKIYDEAELEMALAWADKNFRY
GEDENNKQYQRNAEQSRAVLRESLLMAMCIRDMMQGNSKLADIGRVEESLGYNAIAAGFQ
GQRHWTDQYPNGDTAEAILNSSFDWNGVRKPFVVATENDSLNGVAMLMGHQLTGTAQVFA
DVRTYWSPEAIERVTGHKLDGLAEHGIIHLINSGSAALDGSCKQRDSEGKPTMKPHWEIS
QQEADACLAATEWCPAIHEYFRGGGYSSRFLTEGGVPFTMTRVNIIKGLGPVLQIAEGWS
VELPKDVHDILNKRTNSTWPTTWFAPRLTGKGPFTDVYSVMANWGANHGVLTIGHVGADF
ITLASMLRIPVCMHNVEETKVYRPSAWAAHGMDIEGQDYRACQNYGPLYKR
>LFGLNPFC_00342 L-fucose-proton symporter
MGNTSIQTQSYRAVDKDAGQSRSYIIPFALLCSLFFLWAVANNLNDILLPQFQQAFTLTN
FQAGLIQSAFYFGYFIIPIPAGILMKKLSYKAGIITGLFLYAFGAALFWPAAEIMNYTLF
LVGLFIIAAGLGCLETAANPFVTVLGPESSGHFRLNLAQTFNSFGAIIAVVFGQSLILSN
VPHQSQDVLDKMSPEQLSAYKHSLVLSVQTPYMIIVAIVLLVALLIMLTKFPALQSDNHS
DAKQGSFSASLSRLARIRHWRWAVLAQFCYVGAQTACWSYLIRYAVEEIPGMTAGFAANY
LTGTMVCFFIGRFTGTWLISRFAPHKVLAAYALIAMALCLISAFAGGHVGLIALTLCSAF
MSIQYPTIFSLGIKNLGQDTKYGSSFIVMTIIGGGIVTPVMGFVSDAAGNIPTAELLPAL
CFAVIFIFARFRSQTATN
>LFGLNPFC_00343 L-fuculose phosphate aldolase
MERNKLARQIIDTCLEMTRLGLNQGTAGNVSVRYQDGMLITPTGIPYEKLTESHIVFIDG
NGKHEEGKLPSSEWRFHMAAYQSRPDANAVVHNHAVHCTAVSILNRPIPAIHYMIAAAGG
NSIPCAPYATFGTRELSEHVALALKNRKATLLQHHGLIACEANLEKALWLAHEVEVLAQL
YLTTLAITDPVPVLSDEEIAVVLEKFKTYGLRIEE
>LFGLNPFC_00344 Lactaldehyde reductase
MANRMILNETAWFGRGAVGALTDEVKRRGYQKALIVTDKTLVQCGVVAKVIDKMDAAGLA
WAIYDGVVPNPTITVVKEGLDVFQNSGADYLIAIGGGSPQDTCKAIGIISNNPEFADVRS
LEGLSPTNKPSVPILAIPTTAGTAAEVTINYVITDEEKRRKFVCVDPHDIPQVAFIDADM
MDGMPPALKAATGVDALTHAIEGYITRGAWALTDALHIKAIEIIAGALRGTVAGDKDAGE
EMALGQYVAGMGFSNVGLGLVHGMAHPLGAFYNTPHGVANAILLPHVMRYNADFTGEKYR
DIARVMGVKVEGMSLEEARNAAVEAVFALNRDVGIPPHLRDVGVRKEDIPALAQAALDDV
CTGGNPREATLEDIVELYHTAW
>LFGLNPFC_00345 Flap endonuclease Xni
MRGLFPISHPAIACSGIECYPYRLIFKGVIVAVHLLIVDALNLIRRIHAVQGSPCVETCQ
HALDQLIMHSQPTHAVAVFDDENRSSGWRHQRLPDYKAGRPPMPEELHNEMPALRAAFEQ
RGVPCWSASGNEADDLAATLAVKVTQAGHQATIVSTDKGYCQLLSPTLRIRDYFQKRWLD
APFIDKEFGVQPQQLPDYWGLAGISSSKVPGVAGIGPKSATQLLVEFQSLEGIYENLDAV
AEKWRKKLETHKEMAFLCRDIARLQTDLHIDGNLQQLRLVR
>LFGLNPFC_00346 L-serine dehydratase 2
MISVFDIFKIGIGPSSSHTVGPMKAGKQFTDDLIARNLLKDVTRVVVDVYGSLSLTGKGH
HTDIAIIMGLAGNLPDTVDIDSIPGFIQDVNTHGRLMLANGQHEVEFPVDQCMNFHADNL
SLHENGMRITALAGDKVVYSQTYYSIGGGFIVDEEHFGQQDSAPVEVPYPYSSAADLQKH
CQETGLSLSGLMMKNELALHSKEELEQHLANVWEVMRGGIERGISTEGVLPGKLRVPRRA
AALRRMLVSQDKTTTDPMAVVDWINMFALAVNEENAAGGRVVTAPTNGACGIIPAVLAYY
DKFIREVNANSLARYLLVASAIGSLYKMNASISGAEVGCQGEVGVACSMAAAGLAELLGA
SPAQVCIAAEIAMEHNLGLTCDPVAGQVQVPCIERNAIAAVKAVNAARMALRRTSEPRVC
LDKVIETMYETGKDMNAKYRETSRGGLAMKIVACD
>LFGLNPFC_00347 Serine transporter
METTQTSTIASKDSRSAWRKTDTMWMLGLYGTAIGAGVLFLPINAGVGGMIPLIIMAILA
FPMTFFAHRGLTRFVLSGKNPGEDITEVVEEHFGIGAGKLITLLYFFAIYPILLVYSVAI
TNTVESFMSHQLGMTPPPRAILSLILIVGMMTIVRFGEQMIVKAMSILVFPFVGVLMLLA
LYLIPQWNGAALETLSLDTASATGNGLWMTLWLAIPVMVFSFNHSPIISSFAVAKREEYG
DMAEQKCSKILAFAHIMMVLTVMFFVFSCVLSLTPADLAAAKEQNISILSYLANHFNAPI
IAWMAPIIAIIAITKSFLGHYLGAREGFNGMVIKSLRGKGKSIEINKLNRITALFMLVTT
WIVATLNPSILGMIETLGGPIIAMILFLMPMYAIQKVPAMRKYSGHISNVFVVVMGLIAI
SAIFYSLFS
>LFGLNPFC_00348 Pyrimidine/purine nucleotide 5'-monophosphate nucleosidase
MITHISPLGSMDMLSQLEVDMLKRTASSDLYQLFRNCSLAVLNSGSLTDNSKELLSRFEN
FDINVLRRERGVKLELINPPEEAFVDGRIIRALQANLFAVLRDILFVYGQIHNTVRFPNL
NLDNSVHITNLVFSILRNARALHVGEAPNMVVCWGGHSINENEYLYARRVGNQLGLRELN
ICTGCGPGAMEAPMKGAAVGHAQQRYKDSRFIGMTEPSIIAAEPPNPLVNELIIMPDIEK
RLEAFVRIAHGIIIFPGGVGTAEELLYLLGILMNPANKDQVLPLILTGPKESADYFRVLD
```

EFVVHTLGENARRHYRIIIDDAAEVARQMKKSMPLVKENRRDTGDAYSFNWSMRIAPDLQ
MPFEPSHENMANLKLYPDQPVEVLAADLRRAFSGIVAGNVKEVGIRAIEEFGPYKINGDK
EIMRRMDDLLQGFVAQHRMKLPGSAYIPCYEICT
>LFGLNPFC_00349 NADPH-dependent 7-cyano-7-deazaguanine reductase
MSSYANHQALAGLTLGKSTDYRDTYDASLLQGVPRSLNRDPLGLKADNLPFHGTDIWTLY
ELSWLNAKGLPQVAVGHVELDYTSVNLIESKSFKLYLNSFNQTRFNNWDEVRQTLERDLS
TCAQGKVSVALYRLDELEGQPIGHFNGTCIDDQDITIDNYEFTTDYLENATSGEKVVEET
LVSHLLKSNCLITHQPDWGSIQIQYRGRQIDREKLLRYLVSFRHHNEFHEQCVERIFNDL
LRFCQPEKLSVYARYTRRGGLDINPWRSNSDFVPSTTRLVRQ
>LFGLNPFC_00350 Protein Syd
MDDLTAQALKDFTARYCDAWHEEHKSWPLSEELYGVPSPCIISTTEDAVYWQPQPFTGEQ
NVNAVERAFDIVIQPTIHTFYTTQFAGDMHAQFGDIKLTLLQTWSEDDFRRVQENLIGHL
VTQKRLKLPPTLFIATLEEELEVISVCNLSGEVCKETLGTRKRTHLASNLAEFLNQLKPL
L
>LFGLNPFC_00351 putative protein YqcC
MTTHDRVRLQLQALEALLREHQHWRNDEPLPHQFASTQPFFMDTMEPLEWLQWVLIPRMH
DLLDNNQPLPGAFAVAPYYEMALATDHPQRALILAELEKLDALFADDAS
>LFGLNPFC_00352 tRNA pseudouridine synthase C
MLEILYQDEWLVAVNKPSGWLVHRSWLDRDEKVVVMQTVRDLIGQHVFTAHRLDRPTSGV
LLMGLSSEAGRLLAQQFEQHQIQKRYHAIVRGWLMEEAVLDYPLVEELDKIADKFAREDK
GPQPAVTHYRGLATVEMPVATGRYPTTRYGLVELEPKTGRKHQLRRHLAHLRHPIIGDSK
HGDLRQNRSGAEHFGLQRLMLHASQLSLTHPFTGEPLTIHAGLDDTWMQALSQFGWRGLL
PENERVEFSAPSGQDGEISS
>LFGLNPFC_00353 putative protein
MAEIGIFVGTMYGNSLLVAEEAEAILTAQGHKATVFEDPELSDWLPYQDKYVLVVTSTTG
QGDLPDSIVPLFQGIKDSLGFQPNLRYGVIALGDSSYVNFCNGGKQFDALLQEQSAQRVG
EMLLIDASENPEPETESNPWVEHWGTMLS
>LFGLNPFC_00354 putative glucarate transporter
MSSLSQAASSVEKRTNARYWIVVMLFIVTSFNYGDRATLSIAGSEMAKDIGLDPVGMGYV
FSAFSWAYVIGQIPGGWLLDRFGSKRVYFWSIFIWSMFTLLQGFVDIFSGFGIIVALFTL
RFLVGLAEAPSFPGNSRIVAAWFPAQERGTAVSIFNSAQYFATVIFAPIMGWLTHEVGWS
HVFFFMGGLGIVISFIWLKVIHEPNQHPGVNQKELEYIAAGGALINMDQQNTKVKVPFSV
KWGQIKQLLGSRMMIGVYIGQYCINALTYFFITWFPVYLVQARGMSILKAGFVASVPAVC
GFIGGVLGGIISDWLMRRTGSLNIARKTPIVMGMLLSMVMVFCNYVNVEWMIIGFMALAF
FGKGIGALGWAVMADTAPKEISGLSGGLFNMFGNISGIVTPIAIGYIVGTTGSFNGALIY
VGVHALIAVLSYLVLVGDIKRIELKPVAGQ
>LFGLNPFC_00355 Glucarate dehydratase-related protein
MTTQSSPVITDMKVIPVAGHDSMLLNIGGAHNAYFTRNIVVLTDNAGHTGIGEAPGGEVI
YQTLVKAIPMVLGQEVARLNKVVQQVHKGNQAADFDTFGKGAWTFELRVNAVAALEAALL
DLLGQALNVPVCELLGPGKQRDAITVLGYLFYIGDRTKTDLPYLGNTPGNHEWYQLRHQK
AMNSEAVVRLAEASQDRYGFKDFKLKGGVLPGEQEIDTVRALKKRFPDARITVDPNGAWL
LDEAISLCKGLNDVLTYAEDPCGAEQGFSGREVMAEFRRATGLPVATNMIATNWREMGHA
VMLNAVDIPLADPHFWTLSGAVRVAQLCDDWGLTWGCHSNNHFDISLAMFTHVGAAAPGN
PTAIDTHWIWQEGDCRLTKNPLEIKNGKIAVPDAPGLGVELDWEQVQKAHEAYKRLPGGA
RNDAGPMQYLIPGWTFDRKRPVFGRH
>LFGLNPFC_00356 Glucarate dehydratase
MSSQFTTPVVTEMQVIPVAGHDSMLMNLSGAHAPFFTRNIVIIKDNSGHTGVGEIPGGEK
IRKTLEDAIPLVVGKTLGEYKNVLTLVRNTFADRDAGGRGLQTFDLRTTIHVVTGIEAAM
LDLLGQHLGVNVASLLGDGQQRSEVEMLGYLFFVGNRKATPLPYQSQPDDSCDWYRLRHE
EAMTPDAVVRLAEAAYEKYGFNDFKLKGGVLAGEEEAESIVALAQRFPQARITLDPNGAW
SLKEAIKIGTYLKGSLAYAEDPCGAEQGFSGREVMAEFRRATGLPTATNMIATDWRQMGH
TLSLQSVDIPLADPHFWTMQGSVRVAQMCHEFGLTWGSHSNNHFDISLAMFTHVAAAAPG
KITAIDTHWIWQEGNQRLTKEPLEIKGGLVQVPEKPGLGVEIDMDQVMKAHELYQKHGLG
ARDDAMGMQYLIPGWTFDNKRPCMVR
>LFGLNPFC_00357 Signal transduction histidine-protein kinase BarA
MTNYSLRARMMILILAPTVLIGLLLSIFFVVHRYNDLQRQLEDAGASIIEPLAVSTEYGM
SLQNRESIGQLISVLHRRHSDIVRAISVYDENNRLFVTSNFHLDPSSMQLGSNVPFPRQL
TVTRDGDIMILRTPIISESYSPDESPSSDAKNSQNMLGYIALELDLKSVRLQQYKEIFIS
SVMMLFCIGIALIFGWRLMRDVTGPIRNMVNTVDRIRRGQLDSRVEGFMLGELDMLKNGI
NSMAMSLAAYHEEMQHNIDQATSDLRETLEQMEIQNVELDLAKKRAQEAARIKSEFLANM
SHELRTPLNGVIGFTRLTLKTELTPTQRDHLNTIERSANNLLAIINDVLDFSKLEAGKLI
LESIPFPLRSTLDEVVTLLAHSSHDKGLELTLNIKSDVPDNVIGDPLRLQQIITNLVGNA
IKFTENGNIDILVEKRALSNTKVQIEVQIRDTGIGIPERDQSRLFQAFRQADASISRRHG
GTGLGLVITQKLVNEMGGDISFHSLPNRGSTFWFHINLDLNPNIIIEGPSTQCLAGKRLA

YVEPNSAAAQCTLDILSETPLEVVYSPTFSALPPAHYDMMLLGIAVTFREPLTMQHERLA
KAVSMTDFLMLALPCHAQVNAEKLKQDGIGACLLKPLTPTRLLPALTEFCHHKQNTLLPV
TDESKLAMTVMAVDDNPANLKLIGALLEDMVQHVELCDSGHQAVERAKQMPFDLILMDIQ
MPDMDGIRACELIHQLPHQQQTPVIAVTAHAMAGQKEKLLGAGMSDYLAKPIEEERLHNL
LLRYKPGSGISSRVVTPEVNEIVVNPNATLDWQLALRQAAGKTDLARDMLQMLLDFLPEV
RNKVEEQLVGENPEGLVDLIHKLHGSCGYSGVPRMKNLCQLIEQQLRSGTKEEDLEPELL
ELLDEMDNVAREASKILG
>LFGLNPFC_00358 23S rRNA (uracil(1939)-C(5))-methyltransferase RlmD
MAQFYSAKRRTTTRQIITVSVNDLDSFGQGVARHNGKTLFIPGLLPQENVEVTVTEDKKQ
YARAKVVRRLSDSPERETPRCPHFGVCGGCQQQHASVDLQQRSKSAALARLMKHEVSEVI
ADVPWGYRRRARLSLNYLPKTQQLQMGFRKAGSSDIVDVKQCPILVPQLEALLPKVRACL
GSLQAMRHLGHVELVQATSGTLMILRHTAPLSSADREKLECFSHSEGLDLYLAPDSEILE
TVSGEMPWYDSNGLRLTFSPRDFIQVNAGVNQKMVARALEWLDVEPEDCVLDLFCGMGNF
TLPLATQAASVVGVEGVPALVEKGQQNARLNGLQNVTFYHENLEEDVTKQPWAKNGFDKV
LLDPARAGAAGVMQQIIKLEPIRIVYVSCNPATLARDSEALLKAGYTIARLAMLDMFPHT
GHLESMVLFSRVK
>LFGLNPFC_00359 GTP pyrophosphokinase
MVAVRSAHINKAGEFDPEKWIASLGITSQKSCECLAETWAYCLQQTQGHPDASLLLWRGV
EMVEILSTLSMDIDTLRAALLFPLADANVVSEDVLRESVGKSVVNLIHGVRDMAAIRQLK
ATHTDSVSSEQVDNVRRMLLAMVDDFRCVVIKLAERIAHLREVKDAPEDERVLAAKECTN
IYAPLANRLGIGQLKWELEDYCFRYLHPTEYKRIAKLLHERRLDREHYIEEFVGHLRAEM
KAEGVKAEVYGRPKHIYSIWRKMQKKNLAFDELFDVRAVRIVAERLQDCYAALGIVHTHY
RHLPDEFDDYVVANPKPNGYQSIHTVVLGPGGKTVEIQIRTKQMHEDAELGVAAHWKYKEG
AAAGGARSGHEDRIAWLRKLIAWQEEMADSGEMLDEVRSQVFDDRVYVFTPKGDVVDLPA
GSTPLDFAYHIHSDVGHRCIGAKIGGRIVPFTYQLQMGDQIEIITQKQPNPSRDWLNPNL
GYVTTSRGRSKIHAWFRKQDRDKNILAGRQILDDELEHLGISLKEAEKHLLPRYNFNDVD
ELLAAIGGGDIRLNQMVNFLQSQFNKPSAEEQDAAALKQLQQKSYTPQNRSKDNGRVVVE
GVGNLMHHIARCCQPIPGDEIVGFITQGRGISVHRADCEQLAELRSHAPERIVDAVWGES
YSAGYSLVVRVVANDRSGLLRDITTILANEKVNVLGVASRSDTKQQLATIDMTIEIYNLQ
VLGRVLGKLNQVPDVIDARRLHGS
>LFGLNPFC_00360 Antitoxin MazE
MIHSSVKRWGNSPAVRIPATLMQALNLNIDDEVKIDLVDGKLIIEPVRKEPVFTLAELVN
DITPENLHENIDWGEPKDKEVW
>LFGLNPFC_00361 Endoribonuclease toxin MazF
MVSRYVPDTGDLIWVDFDPTKGSEQAGHRPAVVLSPFMYNNKTGMCLCVPCTTQSKGYPF
EVVLSGQERDGVALADQVKSIAWRARGATKKGTVAPEELQLIKAKINVLIG
>LFGLNPFC_00362 Nucleoside triphosphate pyrophosphohydrolase
MNQIDRLLTIMQRLRDPENGCPWDKEQTFATIAPYTLEETYEVLDAIAREDFDDLRGELG
DLLFQVVFYAQMAQEEGRFDFNDICAAISDKLERRHPHVFADSSAENSSEVLARWEQIKT
EERAQKAQHSALDDIPRSLPALMRAQKIQKRCANVGFDWTTLGPVVDKVYEEIDEVMYEA
RQAVVDQAKLEEEMGDLLFATVNLARHLGTKAEIALQKANEKFERRFREVERIVAARGLE
MTGVDLETMEEVWQQVKRQEIDL
>LFGLNPFC_00363 CTP synthase
MTTNYIFVTGGVVSSLGKGIAAASLAAILEARGLNVTIMKLDPYINVDPGTMSPIQHGEV
FVTEDGAETDLDLGHYERFIRTKMSRRNNFTTGRIYSDVLRKERRGDYLGATVQVIPHIT
NAIKERVLEGGEGHDVVLVEIGGTVGDIESLPFLEAIRQMAVEIGREHTLFMHLTLVPYM
AASGEVKTKPTQHSVKELLSIGIQPDILICRSDRAVPANERAKIALFCNVPEKAVISLKD
VDSIYKIPGLLKSQGLDDYICKRFSLNCPEANLSEWEQVIFEEANPVSEVTIGMVGKYIE
LPDAYKSVIEALKHGGLKNRVSVNIKLIDSQDVETRGVEILKGLDAILVPGGFGYRGVEG
MITTARFARENNIPYLGICLGMQVALIDYARHVANMENANSTEFVPDCKYPVVALITEWR
DENGNVEVRSEKSDLGGTMRLGAQQCQLVDDSLVRQLYNAPTIVERHRHRYEVNNMLLKQ
IEDAGLRVAGRSGDDQLVEIIEVPNHPWFVACQFHPEFTSTPRDGHPLFAGFVKAASEFQ
KRQAK
>LFGLNPFC_00364 Enolase
MSKIVKIIGREIIDSRGNPTVEAEVHLEGGFVGMAAAPSGASTGSREALELRDGDKSRFL
GKGVTKAVAAVNGPIAQALIGKDAKDQAGIDKIMIDLDGTENKSKFGANAILAVSLANAK
AAAAAKGMPLYEHIAELNGTPGKYSMPVPMMNIINGGEHADNNVDIQEFMIQPVGAKTVK
EAIRMGSEVFHHLAKVLKAKGMNTAVGDEGGYAPNLGSNAEALAVIAEAVKAAGYELGKD
ITLAMDCAASEFYKDGKYVLAGEGNKAFTSEEFTHFLEELTKQYPIVSIEDGLDESDWDG
FAYQTKVLGDKIQLVGDDLFVTNTKILKEGIEKGIANSILIKFNQIGSLTETLAAIKMAK
DAGYTAVISHRSGETEDATIADLAVGTAAGQIKTGSMSRSDRVAKYNQLIRIEEALGEKA
PYNGRKEIKGQA
>LFGLNPFC_00365 hypothetical protein
MIVMRLIFILFTLWCLPGLAQQIAVPELRQQVTDITGTLSTSEQQSLTQQLQDITHKTRA

QVAVLVVPSTGDDSIEQYATRVFDSWKLGDKQRNDGILLLVAWEDHAVRIEVGYGLEGVV
TDLQAAKIIRDILIPAFKSDDLMGGLTLASENIGALLLNGELPEDRGDYYSINPPIPLSL
AVIILLAVLSYFIVFTDPSNLPWITLTGAIYGMVFLYVAEPGPWTNLIVACGMLTPFAIV
PLVVFWLIVNKKLRAKYKKLSKERASRKGSSSSSSGGGSSGGGFSGGGGSSGGGGASGRW
>LFGLNPFC_00366 hypothetical protein
MRIFLLLLMLCGFQVFAERLPSVTDLTGTLTTEEQSALNQQLQTLEQQNHFQVAVLVTPT
TGGKNIKLAASQRYGVYHWKPVGEKRYGEGILILVVWPEGLASMKIGHGLEQMLPPEQAA
QIVRYHMQPEFEKNNLFAGLTGGIESIAQFTHIKANLSPLDALANHLFANPQLSLPCLAW
TVLMIVAIIVLWRFTSRPGPGIWMISMITPTVWFFCFQDDVIIRRVSVVCFSLFFAAICW
QRLTVVFNMCRVFPMMLAPKNKNAKVKKQKKTPQVRESGRNSLFVVMLIIIVGWCLVFAS
NKDIAFISTIIGLIDHFIGPITIAGIVLLIIVAKFTGNLKLGSGEKSNRKTGNRNAHSRS
SSRNSFRGGGGSSGGGGSSGRW
>LFGLNPFC_00367 hypothetical protein
MRNFIFLMAFFCSSVFATQIPVPESPRYVNDLTGTLTNSEVNTLTNQIKALTQKNHAQLV
VLVVDTTGDETIEQYATRVFDSWKPGDKDRDDGVLLLVAWQDHTVRIEIGYGLEGIITDA
QSGKIIRNSIIPAFKKGDLAGGLQKGINDIESRLTGNNSATITPTDHPLSFSGWWALLVW
AIILTFISARGYIKTLGVICFAAIVLAFVLPIAGFSGSWGVLTTLLCFATPFLAVAIAFT
PFGKKVRDSMRDANQPSRHTRSNSSSHYSSSSSSSFDNDNFSGGGGSSGGGGASGRW
>LFGLNPFC_00368 Protein LemA
MFRIFIAFIFIFNLSGCGYNDIQTYDEQVNASWSEVLNQYQRRTDLIPNLVASIKGYSSH
EQEVLEAVTLARSQANRASSDLQKTPGDEQKLQAWQQAQAQAQVTRTLGQLTIISERYPQ
LKAQELYQNLMVQLEGSENRIAVARGRYIKAIEQYNVTIRKFPAVLTAKVMDYTPKKNYL
PDDVTAVSKAPTIDFSQNANAH
>LFGLNPFC_00369 7-carboxy-7-deazaguanine synthase
MQYPINEMFQTLQGEGYFTGVPAIFIRLQGCPVGCAWCDTKHTWEKLEDREVSLFSILAK
TKESDKWGAASSEDLLAVIGRQGYTARHVVITGGEPCIHDLLPLTDLLEKNGFSCQIETS
GTHEVRCTPNTWVTVSPKLNMRGGYEVLSQALERANEIKHPVGRVRDIEALDELLATLTD
DKPRVIALQPISQKEDATRLCIDTCIARNWRLSMQTHKYLNIA
>LFGLNPFC_00370 Autoinducer-2 kinase
MSKKYIIGIDGGSQSTKVVMYDLEGNVVCEGKGLLQPMHTPDADTAEHPDDDLWASLCFA
GHDLMSQFAGNKEDIVGIGLGSIRCCRALLKADGTPAAPLISWQDARVTRPYEHTNPDVA
YVTSFSGYLTHRLTGEFKDNIANYFGQWPVDYKTWAWSEDAAVMEKFNIPRQMLFDVQMP
GTILGHITPQAALATHFPAGLPVVCTTSDKPVEALGAGLLDDETAVISLGTYIALMMNGK
ALPKDPVAYWPIMSSIPQTLLYEGYGIRKGMWTVSWLRDMLGESLIQDAKAQDLSPEDLL
NKKASCVPPGCNGLMTVLDWLTNPWEPYKRGIMIGFDSSMDYAWIYRSILESVALTLKNN
YDNMCHEMNHFAKHVIITGGGSNSDLFMQIFADVFNLPARRNAINGCASLGAAINTAVGL
GLYPDYATAVDKMVRVKDIFMPVENNAKRYDAMNKGIFKELTKHTDVILKKSYEVMHGEL
GNADSIQSWSNA
>LFGLNPFC_00371 Inner membrane protein YqcE
MQHNSYRRWITLAIISFSGGVSFDLAYLRYIYQIPMAKFMGFSNTEIGLIMSTFGIAAII
FYAPSGVIADKFSHRKMITSAMIITGLLGLIMATYPPLWVMLCIQVAFAITTILMLWSVS
IKAASLLGDHSEQGKIMGWMEGLRGVGVMSLAVFTMWVFSRFAPDDSASLKTVIIIYSVV
YILLGILCWFFVSDNNNLRSANNEEKQSFQLSDILAVLRISTTWYCSMVIFGVFTIYAIL
SYSTNYLTEMYGMSLVAASYMGIVINKIFRALCGPLGGIITTYSKVKSPTRVVQILSIIG
LLALTALLVTNSNPQSVAMGIGLILLLGFTCYASRGLYWACPGEARTPSYIMGTTVGICS
VIGFLPDVFVYPIIGYWQDTLPAAEAYRNMWLMGMAALGMVIIFTFLLFQKIRTADSAPA
MASSK
>LFGLNPFC_00372 2-dehydro-3-deoxy-D-gluconate 5-dehydrogenase
MSIESLNAFSMDFFSLKGKTAIVTGGNSGLGQAFAMALAKAGANVFIPSFVKDNGETKEM
IEKQGVEVDFMQVDITAEGAPQKIIAACCERFGTVDILVNNAGICKLNKVLDFGRADWDP
MIDVNLTAAFELSYEAAKIMIPQKSGKIINICSLFSYLGGQWSPAYSATKHALAGFTKAY
CDELGQYNIQVNGISPGYYATDITLATRSNPETNQRVLDYIPANRWGDTQDLMGAAVFLA
SPASNYVNGHLLVVDGGYLVR
>LFGLNPFC_00373 hypothetical protein
MSLSRAAIVDQLKEIVGADRVITDETVLKKNSIDRFRKFPDIHGIYTLPIPAAVVKLGST
EQVSRVLNFMNAHKINGVPRTGASATEGGLETVVENSVVLDGSAMNQIINIDIENMQATA
QCGVPLEVLENALREKGYTTGHSPQSKPLAQMGGLVATRSIGQFSTLYGAIEDMVVGLEA
VLADGTVTRIKNVPRRAAGPDIRHIIIGNEGALCYITEVTVKIFKFTPENNLFYGYVLED
MKTGFNILREIMVEGYRPSIARLYDAEDGTQHFTHFADGKCVLIFMAEGNPRIAKATGEG
IAEIVARYPQCQRVDSKLIETWFNNLNWGPDKVAAERVQILKTGNMGFTTEVSGCWSCIH
EIYESVINRIRTEFPHADDITMLGGHSSHSYQNGTNMYFVYDYNVVDCKPEEEIDKYHNP
LNKIICEETIRLGGSMVHHHGIGKHRVHWSKLEHGSAWALLEGLKKQFDPNGIMNTGTIY
PIEK
>LFGLNPFC_00374 Inner membrane metabolite transport protein YgcS

MTGRCLFGFSGEKPFLLPDNEGVKMNTSPVRMDDLPLNRFHCRIAALTFGAHLTDGYVLG
VIGYAIIQLTPAMQLTPFMAGMIGGSALLGLFLGSLVLGWISDHIGRQKIFTFSFMLITL
ASFLQFFATTPEHLIGLRILIGIGLGGDYSVGHTLLAEFSPRRHRGVLLGAFSVVWTVGY
VLASIAGHHFISESPEAWRWLLASAALPALLITLLRWGTPESPRWLLRQGRFAEAHAIVH
RYFGPHVLLGDEVATATHKHIKTLFSSRYWRRTAFNSVFFVCLVIPWFVIYTWLPTIAQT
IGLEDALTASLMLNALLIVGALLGLVLTHLLAHRRFLLGSFLLLTATLVVMACLPSGSSL
TLLLFVLFSTTISAVSNLVGILPAESFPTDIRSLGVGFATAMSRLGAAVSTGLLPWVLAQ
WGMQATLLLLAAVLLVGFVVTWLWAPETKALPLVAAGNAGGANEHTVSV
>LFGLNPFC_00375 Protein FixA
MNILLAFKAEPDAGMLAEKEWLAAAQGNIGPDVSLLRSLLGADEQAAAALLLAQRKNGTP
MSLTALSMGDERALHWLRYLMALGFEEAVLLETAADLRFAPEFVALHIAEWQYQNPLDLI
ITGCQSSEGQNGQTPFLLAEMLVWPCFTQVERFTLDAPFITLEQRTEHGVRCCRVRLPAV
IAVRQCGEVALPVPGMRQRMAAGKAEITRETVAAEAPAIQCLQLARPEQRRRAALIDGQT
VAEKAQKLWQDYLRQRMQP
>LFGLNPFC_00376 Protein FixB
MNIAIVTINQEHAAMAGWLAAQDFSGSTLTHWQIEPQPMVAEQVLDALVEQWQRTPAEVV
LFPPGAFGDELSTRLAWRLHGASICQVTSLDIPTVSVRKSHWGNALTATLQTEKRPLCLS
LARQAGADKNATLPSGMQQLNIVPGALPDWLISVENLKNVTRDPLAEARRVLVVGGQGGEA
DNQEIAMLAEKMGAEVGYSRARVMNGGVDAEKVIGISGHLLAPEVCIVVGASGAAALMAG
VRNSKFVVAINHDASAAVFSQADVGVVDDWKVVLEALVTNIHADCQ
>LFGLNPFC_00377 putative protein YgcP
MPLLHLLRQNPVIAAVKDNASLQLAIDSECQFISVLYGNICTISNIVKKIKNAGKYAFIH
VDLLEGASNKEVVIQFLKLVTEADGIISTKASMLKAARAEGFFCIHRLFIVDSISFHNID
KQVAQSNPDCIEILPGCMPKVLGWVTEKIRQPLIAGGLVCDEEDARNAINAGVVALSTTN
TGVWTLAKKLL
>LFGLNPFC_00378 Ferredoxin-like protein FixX
MSVARNLWRAADAPHIVPADSVERQTAQRLINACPAGLFSLTPEGGLRVDYHGCLECGTC
RLLCDESTLQQWRYPPSGFGITYRFG
>LFGLNPFC_00379 Electron transfer flavoprotein-ubiquinone oxidoreductase
MEDDCDIIIIGAGIAGTACALRCARAGLSVLLLERAEIPGSKNLSGGRLYTHALAELLPQ
FHLTAPLERRITHESLSLLTPDGATTFSSLQPGGESWSVLRARFDPWLVAEAEKEGVECI
PGATVDALYEENGRVCGVICGDDILRARYVVLAEGANSVLAERHGLVTRPAGEAMALGIK
EVLTLEPSAIEERFHLENNEGAALLFSGGICDDLPGGAFLYTNQQTLSLGIVCPLSSLTQ
SRVPASELLARFKTHPAVRPLIKNTESLEYGAHLVPEGGLRSMPVQYAGNGWLLVGDALR
SCVNTGISVRGMDMALTGAQAAAQTLISACQHREPQNLFPLYHHNVERSLLWDVLQRYQH
VPALLQRPGWYRVWSGLMQDISRDLWDQGNTPVPPLRQLLWRHLRRHGLWHLTGDVIKSL
RCL
>LFGLNPFC_00380 6-carboxy-5,6,7,8-tetrahydropterin synthase
MMSTTLFKDFTFEAAHRLPHVPQGHKCGRLHGHSFMVRLEITGEVDPHTGWIIDFAELKA
AFKPTYERLDHHYLNDIPGLENPTSEVLAKWIWDQVKPVVPLLSAVMVKETCTAGCIYRG
E
>LFGLNPFC_00381 Sulfite reductase [NADPH] flavoprotein alpha-component
MTTQVPPSALLPLNPEQLARLQAATTDLTPTQLAWVSGYFWGVLNQQPAALAATPAPAAE
MPGITIISASQTGNARRVAEALRDDLLAAKLNVKLVNAGDYKFKQIASEKLLIVVTSTQG
EGEPPEEAVALHKFLFSKKAPKLENTAFAVFSLGDSSYEFFCQSGKDFDSKLAELGGERL
LDRVDADVEYQAAASEWRARVVDALKSRAPVAAPSQSVATGTVNEIHTSPYSKDAPLAAS
LSVNQKITGRNSEKDVRHIEIDLGDSGLRYQPGDALGVWYQNDPALVKELVELLWLKGDE
PVTVEGKTLPLNEALQWHFELTVNTANIVENYATLTRSETLLPLVGDKAKLQHYAATTPI
VDMVRFSPAQLDAEALINLLRPLTPRLYSIASSQAEVENEVHVTVGVVRYDVEGRARAGG
ASSFLADRVEEEGEVRVFIEHNDNFRLPTNPETPVIMIGPGTGIAPFRAFMQQRAADEAP
GKNWLFFGNPHFTEDFLYQVEWQRYVKEGVLTRIDLAWSRDQKEKIYVQDKLREQGAELW
RWINDGAHIYVCGDANRMAKDVEQALLEVIAEFGGMDTEAADEFLSELRVERRYQRDVY
>LFGLNPFC_00382 Sulfite reductase [NADPH] hemoprotein beta-component
MSEKHPGPLVVEGKLTDAERMKLESNYLRGTIAEDLNDGLTGGFKGDNFLLIRFHGMYQQ
DDRDIRAERAEQKLEPRHAMLLRCRLPGGVITTKQWQAIDKFAGENTIYGSIRLTNRQTF
QFHGILKKNVKPVHQMLHSVGLDALATANDMNRNVLCTSNPYESQLHAEAYEWAKKISEH
LLPRTRAYAEIWLDQEKVATTDEEPILGQTYLPRKFKTTVVIPPQNDIDLHANDMNFVAI
AENGKLVGFNLLVGGGLSIEHGNKKTYARTASEFGYLPLEHTLAVAEAVVTTQRDWGNRT
DRKNAKTKYTLERVGVETFKAEVERRAGIKFEPIRPYEFTGRGDRIGWVKGIDDNWHLTL
FIENGRILDYPGRPLKTGLLEIAKIHKGDFRITANQNLIIAGVPESEKAKIEKIAKESGL
MNAVTPQRENSMACVSFPTCPLAMAEAERFLPSFIDNIDNLMAKHGVSDEHIVMRVTGCP
NGCGRAMLAEVGLVGKAPGRYNLHLGGNRIGTRIPRMYKENITEPEILASLDELIGRWAK
EREVGEGFGDFTVRAGIIRPVLDPARDLWD
>LFGLNPFC_00383 Phosphoadenosine phosphosulfate reductase

MSKLDLNALNELPKVDRILALAETNAQLEKLDAEGRVAWALDNLPGEYVLSSSFGIQAAV
SLHLVNQIRPDIPVILTDTGYLFPETYRFIDELTDKLKLNLKVYRATESAAWQEARYGKL
WEQGVEGIEKYNDINKVEPMNRALKELNVQTWFAGLRREQSGSRANLPVLAIQRGVFKVL
PIIDWDNRTIYQYLQKHGLKYHPLWDEGYLSVGDTHTTRKWEPGMAEEETRFFGLKRECG
LHEG
>LFGLNPFC_00384 Toxic protein HokE
MLTKYALVAIIVLCCTVLGFTLMVGDSLCELSIRERGMEFKAVLAYESKK
>LFGLNPFC_00385 hypothetical protein
MFSALRHRTAALALGVCFILPVHASSPKPGDFANTQARHIATFFPGRMTGTPAEMLSADY
IRQQFQQMGYRSDVRTFNSRYIYTARDNRKSWHNVTGSTVIAAHEGKAPQQIIIMAHLDT
YAPLSDADADANLGGLTLQGMDDNAAGLGVMLELAEHLKNTPTEYGIRFVATSGEEEGKL
GAENLLKRMSDTEKKNTLLVINLDNLIVGDKLYFNSGVNTPEAVRKLTRDRALAIARSHG
IAATTNPGLNKNYPKGTGCCNDAEIFDKAGIAVLSVEATNWNLGNKDGYQQRAKTAAFPA
GNSWHDVRLDNQQHIDKALPGRIERRCRDVMRIMLPLVKELAKAS
>LFGLNPFC_00386 Sulfate adenylyltransferase subunit 2
MDQKRLTHLRQLEAESIHIIREVAAEFSNPVMLYSIGKDSSVMLHLARKAFYPGTLPFPL
LHVDTGWKFREMYEFRDRTAKAYGCELLVHKNPEGVAMGINPFVHGSAKHTDIMKTEGLK
QALNKYGFDAAFGGARRDEEKSRAKERIYSFRDRFHRWDPKNQRPELWHNYNGQINKGES
IRVFPLSNWTEQDIWQYIWLENIDIVPLYLAAERPVLERDGMLMMIDDNRINLQSGEVIK
KRMVRFRTLGCWPLTGAVESNAQTLPEIIEEMLVSTTSERQGRVIDRDQAGSMELKKRQG
YF
>LFGLNPFC_00387 Sulfate adenylyltransferase subunit 1
MNTALAQQIANEGGVEAWMIAQQHKSLLRFLTCGSVDDGKSTLIGRLLHDTRQIYEDQLS
SLHNDSKRHGTQGEKLDLALLVDGLQAEREQGITIDVAYRYFSTEKRKFIIADTPGHEQY
TRNMATGASTCELAILLIDARKGVLDQTRRHSFISTLLGIKHLVVAINKMDLVDYSEETF
TRIREDYLTFAGQLPGNLDIRFVPLSALEGDNVASQSESMPWYSGPTLLEVLETVEIQRV
VDAQPMRFPVQYVNRPNLDFRGYAGTLASGRVEVGQRVKVLPSGVESNVARIVTFDGDRE
EAFAGEAITLVLTDEIDISRGDLLLAADEALPAVQSASVDVVWMAEQPLSPGQSYDIKIA
GKKTRARVDGIHYQVDINNLTQREVENLPLNGIGLVDLTFDEPLVLDRYQQNPVTGGLIF
IDRLSNVTVGAGMVHEPVSQATAAPSEFSAFELELNALVRRHFPHWGARDLLGDK
>LFGLNPFC_00388 Adenylyl-sulfate kinase
MALHDENVVWHSHPVTPQQREQHHGHRGVVLWFTGLSGSGKSTVAGALEEALHKLGVSTY
LLDGDNVRHGLCSDLGFSDADRKENIRRVGEVANLMVEAGLVVLTAFISPHRAERQMVRE
RVGEGRFIEVFVDTPLAICEARDPKGLYKKARAGELRNFTGIDSVYEAPESAEIHLNGEQ
LVTNLVQQLLDLLRQNDIIRS
>LFGLNPFC_00389 Inner membrane protein YgbE
MRNSHNITLTNNDSLTEVEDTTWSLPGAVVGFLSWLCALAIPMLIYGSNTLLFFIYTWPF
FLALMPVAVVVGIALHSLMDGKLRYSIVFTLATVGIMFGALFMWLLG
>LFGLNPFC_00390 Cell division protein FtsB
MGKLTLLLLAILVWLQYSLWFGKNGIHDYTRVNNDVAAQQATNAKLKARNDQLFAEIDDL
NGGQEALEERARNELSMTRPGETFYRLVPDASKRAQSAGQNNR
>LFGLNPFC_00391 2-C-methyl-D-erythritol 4-phosphate cytidylyltransferase
MATTHLDVCAVVPAAGFGRRMQTECPKQYLSIGNQTILEHSVHALLAHPRVKRVVIAISP
GDSRFAQLPLANHPRITVVDGGEERADSVLAGLKAAGDAQWVLVHDAARPCLHQDDLARL
LALSETSRTGGILAAPVRDTMKRAEPGKNAIAHTVDRNGLWHALTPQFFPRELLHDCLTR
ALNEGATITDEASALEYCGFHPQLVEGRADNIKVTRPEDLALAEFYLTRTIHQENT
>LFGLNPFC_00392 2-C-methyl-D-erythritol 2,4-cyclodiphosphate synthase
MRIGHGFDVHAFGGEGPIIIGGVRIPYEKGLLAHSDGDVALHALTDALLGAAALGDIGKL
FPDTDPTFKGADSRELLREAWRRIQAKGYALGNVDVTIIAQAPRMLPHIPQMRVFIAEDL
GCHMDDVNVKATTTEKLGFTGRGEGIACEAVALLIKATK
>LFGLNPFC_00393 tRNA pseudouridine synthase D
MIEFDNLTYLHGKPQGTGLLKANPEDFVVVEDLGFEPDGEGEHILVRILKNGCNTRFVAD
ALAKFLKIHAREVSFAGQKDKHAVTEQWLCARVPGKEMPDLSAFQLEGCQVLEYARHKRK
LRLGALKGNAFTLVLREVSNRDDVEQRLIDICVKGVPNYFGAQRFGIGGSNLQGALRWAQ
TNTPVRDRNKRSFWLSAARSALFNQIVAERLKKADVNQVVDGDALQLAGRGSWFVATTEE
LAELQRRVNDKELMITAALPGSGEWGTQREALAFEQAAVAEETELQTLLVREKVEAARRA
MLLYPQQLSWNWWDDVTVEIHFWLPAGSFATSVVRELINTTGDYAHIAE
>LFGLNPFC_00394 5'/3'-nucleotidase SurE
MRILLSNDDGVHAPGIQTLAKALREFADVQVVAPDRNRSGASNSLTLESSLRTFTFENGD
IAVQMGTPTDCVYLGVNALMRPRPDIVVSGINAGPNLGDDVIYSGTVAAAMEGRHLGFPA
LAVSLDGHKHYDTAAAVTCSILRALCKEPLRTGRILNINVPDLPLDQIKGIRVTRCGTRH
PADQVIPQQDPRGNTLYWIGPPGGKCDAGPGTDFAAVDEGYVSITPLHVDLTAHSAQDVV
SDWLNSVGVGTQW
>LFGLNPFC_00395 Protein-L-isoaspartate O-methyltransferase

MVSRRVQALLDQLRAQGIQDELVLNALAAVPREKFVDEAFEQKAWDNIALPIGQGQTISQ
PYMVARMTELLELTPQSRVLEIGTGSGYQTAILAHLVQHVCSVERIKGLQWQARRRLKNL
DLHNVSTRHGDGWQGWQARAPFDAIIVTAAPPEIPTALMTQLDEGGILVLPVGEEHQYLK
RVRRRGGEFIIDTVEAVRFVPLVKGELA
>LFGLNPFC_00396 Murein hydrolase activator NlpD
MSAGSPKFTVRRIAALSLVSLWLAGCSDTSNPPAPVSSVNGNAPANTNSGMLITPPPKMG
TTSTAQPPQIQPVQQPQIQATQQPQIQPVQPVAQQPVQMENGRIVYNRQYGNIPKGSYSG
STYTVKKGDTLFYIAWITGNDFRDLAQRNNIQAPYALNVGQTLQVGNASGTPITGGNAIT
QADAAEQGVVIKPAQNSTVAVASQPTITYSESSGEQSANKMLPNNKPTATTVTAPVTVPT
ASTTEPTVSSTSTSTPISTWRWPTEGKVIETFGASEGGNKGIDIAGSKGQAIIATADGRV
VYAGNALRGYGNLIIIKHNDDYLSAYAHNDTMLVREQQEVKAGQKIATMGSTGTSSTRLH
FEIRYKGKSVNPLRYLPQR
>LFGLNPFC_00397 RNA polymerase sigma factor RpoS
MSQNTLKVHDLNEDAEFDENGVEVFDEKALVEEEPSDNDLAEEELLSQGATQRVLDATQL
YLGEIGYSPLLTAEEEVYFARRALRGDVASRRRMIESNLRLVVKIARRYGNRGLALLDLI
EEGNLGLIRAVEKFDPERGFRFSTYATWWIRQTIERAIMNQTRTIRLPIHIVKELNVYLR
TARELSHKLDHEPSAEEIAEQLDKPVDDVSRMLRLNERITSVDTPLGGDSEKALLDILAD
EKENGPEDTTQDDDMKQSIVKWLFELNAKQREVLARRFGLLGYEAATLEDVGREIGLTRE
RVRQIQVEGLRRLREILQTQGLNIEALFRE
>LFGLNPFC_00398 Flavodoxin FldP
MQNIAVVYYSGYGHTKLVAEKVAQGAHADLISIDNEGNITEQEWEKLDAADAIIFGAPTY
MGGAPWQFKKFADASSKAWFTRKWQDKVFGGFTNSASLNGDKQVTLIYLQTLASQHGGLW
VSLGQAPANVLASTREDVNNLGGSVGLLVQSPADAGADQIPTGDLDTAVKYGERVATITA
RLK
>LFGLNPFC_00399 hypothetical protein
MQGIKIYTILASSLISGSVFAQTEISTANSSTVNASYVEPSAEKIISPSDKLNNLFERNM
SQPYILQKIGERTYYVQRYFYSTTFYVGDKGVLLFDAPEGRGKYLLQAIRDVTPLPVTAL
VYSHYHVDHIGDSPFWNDEAKKEGVNLRIIASKATAEKMQFMNSRLPVATQVLSKKDDQF
KFEKQTIELHRFVKAGHTDDHSVWLLKQEKVAHSPDLLNPDQLPMMGFAVSDTLVYHDSN
LRQVEMLDWKYFIGGHGNIGSHDDFKFQRQFLNDLRDTTIKVRKEESFGKFMNKTANNHA
DFARAQREAIIKKVTEVLRPKYGHMYGYDASMPANIEMAIRLVGSYY
>LFGLNPFC_00400 Inner membrane permease YgbN
MSTITLLCIALAGVIMLLLLVIKAKVQPFVALLLVSLLVALAAGIPAGEVGKVMIAGMGG
VLGSVTIIIGLGAMLGRMIEHSGGAESLANYFSRKLGDKRTIAALTLAAFFLGIPVFFDV
GFIILAPIIYGFAKVAKISPLKFGLPVAGIMLTVHVAVPPHPGPVAAAGLLHADIGWLTI
IGIAISIPVGVVGYFAAKIINKRQYAMSVEVLEQMQLAPASEEGATKLSDKINPPGVALV
TSLIVIPIAIIMAGTVSATLMPPSHPLLGTLQLIGSPMVALMIALVLAFWLLALRRGWSL
QHTSDIMGSALPTAAVVILVTGAGGVFGKVLVESGVGKALANMLQMIDLPLLPAAFIISL
ALRASQGSATVAILTTSGLLSEAVMGLNPIQCVLVTLAACFGGLGASHINDSGFWIVTKY
LGLSVADGLKTWTVLTTILGFTGFLITWCVWLVI
>LFGLNPFC_00401 2-oxo-tetronate isomerase
MPRFAANLSMMFTEVPFIERFAAARKAGFDAVEFLFPYDYSPLQIQKQLEQNHLTLALFN
TAPGDINAGEWGLSALPGREHEARADIDLALEYALALNCEQVHVMAGVVPAGEDAERYRA
VFIDNLRYAADRFAPHGKRILVEALSPGVKPHYLFSSQYQALAIVEEVARDNVFIQLDTF
HAQKVDGNLTHLIRDYAGKYAHVQIAGLPDRHEPDDGEINYPWLFRLFDEVGYQGWIGCE
YKPRGLTEEGLGWFDAWR
>LFGLNPFC_00402 3-oxo-tetronate 4-phosphate decarboxylase
MSDFAKVEQSLREEMTRIASSFFQRGYATGSAGNLSLLLPDGNLLATPTGSCLGNLDPQR
LSKVTADGEWLSGDKPSKEVLFHLALYRNNPRCKAVVHLHSTWSTALSCQEGLDSNNVIR
PFTPYVVMRMGNVPLVPYYRPGDKRIAQDLAELAADNQAFLLANHGPVVCGESLQEAANN
MEELEETAKLIFILGDRPIRYLTAGEIAELRS
>LFGLNPFC_00403 3-oxo-tetronate kinase
MIKIGVIADDFTGATDIASFLVENGLPTVQINGVPTGKMPEAIDALVISLKTRSCPVVEA
TQQSLAALSWLQQQGCKQIYFKYCSTFDSTAKGNIGPVTDALMDALDTPFTVFSPALPVN
GRTVYQGYLFVMNQLLAESGMRHHPVNPMTDSYLPRLVESQSTGRCGVVSAHVFEQGVKA
VRQELARLQQEGYRYAVLDALTEHHLEIQGKALRDAPLVTGGSGLAIGLARQWAQENGNQ
AREAGRPLAGRGVVLSGSCSQMTNRQVAHYRQIAPAREVDVARCLSTETLAAYAHELAEW
VLGQESVLAPLVFATASTDALAAIQQQYGAQKASQAVETLFSQLAARLAAEGVTRFIVAG
GETSGVVTQSLGIKGFHIGPTISPGVPWVNALDKPVSLALKSGNFGDEAFFSRAQREFLS
>LFGLNPFC_00404 L-threonate dehydrogenase
MKTGSEFHVGIVGLGSMGMGAALSCVRAGLSTWGADLNSNACATLKEAGACGVSDNAATF
AEKLDALLVLVVNATQVKQVLFGEKGVAQHLKPGTAVMVSSTIASADAQEIATALAGFGL
EMLDAPVSGGAVKAANGEMTVMASGSDIAFERLAPVLEAVAGKVYRIGAEPGLGSTVKII
HQLLAGVHIAAGAEAMALAARAGIPLDVMYDVVTNAAGNSWMFENRMRHVVDGDYTPHSA

VDIFVKDLGLVADTAKALHFPLPLASTALNMFTSASNAGYGKEDDSAVIKIFSGITLPGA
KS
>LFGLNPFC_00405 HTH-type transcriptional repressor GlcR
MIPVERRQIILEMVAEKGIVSIAELTDRMNVSHMTIRRDLQKLEQQGAVVLVSGGVQSPG
RVAHEPSHQVKTALAMTQKAAIGKLAASLVQPGRCIYLDAGTTTLAIAQHLIHMEPLTVV
TNDFVIADYLLDNSNCTIIHTGGAVCRENRSCVGEAAATMLRSLMIDQAFISASSWSVRG
ISTPAEDKVTVKRAIASASRQRVLVCDATKYGQVATWLALPLSEFDQIITDDGLPESASR
ALAKLDLSLLVAKNE
>LFGLNPFC_00406 Serine/threonine-protein phosphatase 2
MLRLLNQPWFISVKGNHEAMALDAFETGDGNMWLASGGDWFFDLNDSEQQEAIDLLLKFH
HLPHIIEITNDTIKYVIAHADYPGKEYQFGKEIAENELLWPVDRVQKSLNSELQKINGAD
FFIFGHMMFDNIQTFANQIYIDTGSPKSGRLSFYKIK
>LFGLNPFC_00407 IS1 family transposase IS1R
MPGNSPHYGRWPQHDFTSLKKLRPQSVTSRIQPGSDVIVCAEMDEQWGYVGAKSRQRWLF
YAYDRLRKTVVAHVFGERTMATLGRLMSLLSPFDVVIWMTDGWPLYESRLKGKLHVISKR
YTQRIERHNLNLRQHLARLGRKSLSFSKSVELHDKVIGHYLNIKHYQ
>LFGLNPFC_00408 hypothetical protein
MPSTRYQKINAHHYRHIWTVGDIHGDYQLLQSRLHQLSFCPETDLLISGNDSNLLIVFYV
QIMPDDFVMQLHRF
>LFGLNPFC_00409 DNA mismatch repair protein MutS
MSTIENFDAHTPMMQQYLKLKAQHPEILLFYRMGDFYELFYDDAKRASQLLDISLTKRGA
SAGEPIPMAGIPYHAVENYLAKLVNQGESVAICEQIGDPATSKGPVERKVVRIVTPGTIS
DEALLQERQDNLLAAIWQDSKGFGYATLDISSGRFRLSEPADRETMAAELQRTNPAELLY
AEDFAEMSLIEGRRGLRRRPLWEFEIDTARQQLNLQFGTRDLVGFGVENAPRGLCAAGCL
LQYAKDTQRTTLPHIRSITMERQQDSIIMDAATRRNLEITQNLAGGAENTLASVLDCTVT
PMGSRMLKRWLHMPVRDTRVLLERQQTIGALQDFTAELQPVLRQVGDLERILARLALRTA
RPRDLARMRHAFQQLPELRAQLENVDSAPVQALREKMGEFAELRDLLERAIIDTPPVLVR
DGGVIATGYNEELDEWRALADGATDYLERLEVRERERTGLDTLKVGFNAVHGYYIQISRG
QSHLAPINYMRRQTLKNAERYIIPELKEYEDKVLTSKGKALALEKQLYEELFDLLLPHLE
ALQQSASALAELDVLVNLAERAYTLNYTCPTFIDKPGIRITEGRHPVVEQVLNEPFIANP
LNLSPQRRMLIITGPNMGGKSTYMRQTALIALMAYIGSYVPAQKVEIGPIDRIFTRVGAA
DDLASGRSTFMVEMTETANILHNATEYSLVLMDEIGRGTSTYDGLSLAWACAENLANKIK
ALTLFATHYFELTQLPEKMEGVANVHLDALEHGDTIAFMHSVQDGAASKSYGLAVAALAG
VPKEVIKRARQKLRELESISPNAAATQVDGTQMSLLSVPEETSPAVEALENLDPDSLTPR
QALEWIYRLKSLV
>LFGLNPFC_00410 hypothetical protein
MSGKRISREKLTIKKMIDLYQAKCPQASAEPEHYEALFAYAQKRLDKCVFGEEKPACKQC
PVHCYQPAKREEMKQIMRWVGPRMLWRHPILTVRHLIDDKRPVPELPEKYRPKK
>LFGLNPFC_00411 Molybdenum-pterin-binding protein MopA
MAVSARNQLTGTVSAVAMGAVNDEVELTLAGGAKLVAIVTHSSQQALGLAKGKEAIALIK
APWVTLATEDCGLKFSARNQFAGSVSTITEGAVNATVHIKTDAGFEIVAVVTNESQDEMK
LTTGSRVIALIKASAILIATKA
>LFGLNPFC_00412 Formate hydrogenlyase transcriptional activator FhlA
MSYTPMSDLGQQGLFDITRTLLQQPDLASLCEALSQLVKRSALADNAAIVLWQAQTQRAS
YYASREKDTPIKYEDETVLAHGPVRSILSRPDTLHCSYEEFCETWPQLATGGLYPKFGHY
CLMPLAAEGHIFGGCEFIRYDDRPWSEKEFNRLQTFTQIVSVVTEQIQSRVVNNVDYELL
CRERDNFRILVAITNAVLSRLDMDELVSEVAKEIHYYFDIDDISIVLRSHRKNKLNIYST
HYLDKQHPAHEQSEVDEAGTLTERVFKSKEMLLINLHERDDLAPYERMLFNTWGNQIQTL
CLLPLMSGDTMLGVLKLAQCEEKVFTTTNLNLLRQIAERVAIAVDNALAYQEIHRLKERL
VDENLALTEQLNNVDSEFGEIIGRSEAMYSVLKQVEMVAQSDSTVLILGETGTGKELIAR
AIHNLSGRNNRRMVKMNCAAMPAGLLESDLFGHERGAFTGASAQRIGRFELADKSSLFLD
EVGDMPLELQPKLLRVLQEQEFERLGSNKIIQTDVRLIAATNRDLKKMVADREFRSDLYY
RLNVFPIHLPPLRERPEDIPLLAKAFTFKIARRLGRNIDSIPAETLRILSNMEWPGNVRE
LENVIERAVLLTRGNVLQLSLPDIALPEPETPPAATVVAQEGEDEYQLIVRVLKETNGVV
AGPKGAAQRLGLKRTTLLSRMKRLGIDKSALI
>LFGLNPFC_00413 Carbamoyl dehydratase HypE
MNNIQLAHGSGGQAMQQLINSLFMEAFANPWLAEQEDQARLDLAQLVAEGDRLAFSTDSY
VIDPLFFPGGNIGKLAICGTANDVAVSGAIPRYLSCGFILEEGLPMETLKAVVTSMAETA
RAAGIAIVTGDTKVVQRGAADKLFINTAGMGAIPANIHWGAQTLTAGDVLLVSGTLGDHG
ATILNLREQLGLDGELVSDCAVLTPLIQTLRDIPGVKALRDATRGGVNAVVHEFAAACGC
GIELSEAALPVKPAVRGVCELLGLDALNFANEGKLVIAVERNAAEQVLAALHSHPLGKDA
ALIGEVVERKGVRLAGLYGVKRTLDLPHAEPLPRIC
>LFGLNPFC_00414 Hydrogenase maturation factor HypD
MRFVDEYRAPEQVMQLIEHLRERASHLSYTAERPLRIMEVCGGHTHAIFKFGLDQLLPEN

VEFIHGPGCPVCVLPMGRIDTCVEIASHPEVIFCTFGDAMRVPGKQGSLLQAKARGADVR
IVYSPMDALKLAQENPTRKVVFFGLGFETTMPTTAITLQQAKARDVQNFYFFCQHITLIP
TLRSLLEEPDNGIDAFLAPGHVSMVIGTDAYNFIASDFHRPLVVAGFEPLDLLQGVVMLV
EQKIAAHSKVENQYRRVVPDAGNLLAQQAIADVFCVNGDSEWRGLGVIESSGVHLTPDYQ
RFDAEAHFRPAPQQVCDDPRARCGEVLTGKCKPHQCPLFGNTCNPQTAFGALMVSSEGAC
AAWYQYRQQESEA
>LFGLNPFC_00415 Hydrogenase maturation factor HypC
MCIGVPGQIRTIDGNQAKVDVCGIQRDVDLTLVGSCDENGQPRVGQWVLVHVGFAMSVIN
EAEARDTLDALQNMFDVEPDVGALLYGEEK
>LFGLNPFC_00416 Hydrogenase maturation factor HypB
MCTTCGCGEGNLYIEGDEHNPHSAFRSAPFAPAARPKMKITGIKAPEFTPSQTEEGDLHY
GHGEAGTHAPGMSQRRMLEVEIDVLDKNNRLAERNRAHFAARKQLVLNLVSSPGSGKTTL
LTETLMRLKDSVPCAVIEGDQQTVNDAARIRATGTPAIQVNTGKGCHLDAQMIADAAPRL
PLDDNGILFIENVGNLVCPASFDLGEKHKVAVLSVTEGEDKPLKYPHMFAAASLMLLNKV
DLLPYLNFDVEKCIACAREVNPEIEIILISATSGEGMDQWLNWLETQRCA
>LFGLNPFC_00417 Hydrogenase maturation factor HypA
MHEITLCQRALELIEQQAAKHGAKRVTGVWLKIGAFSCVETSSLAFCFDLVCRGSVAEGC
KLHLEEQEAECWCETCQQYVTLLTQRVRRCPQCHGDMLQIVADDGLQIRRIEIDQE
>LFGLNPFC_00418 Formate hydrogenlyase regulatory protein HycA
MTIWEISEKADYIAQRHRRLQDQWHIYCNSLVQGITLSKARLHHAMSCAPDKELCFVLFE
HFRIYVTLADGFNSHTIEYYVETKEGEDKQRIAQAQLSIDGMIDGKVNIRDREQVLEHYL
EKIAGVYDSLYTAIENNVPVNLSQLVKGQSPAA
>LFGLNPFC_00419 Hydrogenase-4 component A
MNRFVIADSTLCIGCHTCEATCSETHRQHGLQSMPRLRVMLNEKESAPQLCHHCEDAPCA
TVCPVNAITRVDGAVQLNESLCVSCKLCGIACPFGAIEFSGSRPLDIPANANTPKAPPAP
PAPARVSTLLDWVPGIRAIAVKCDLCSFDEQGPACVRMCPTKALHLVDNTDIARVSKRKR
ELTFNTDFGDLTLFQQAQSGEAK
>LFGLNPFC_00420 NAD(P)H-quinone oxidoreductase subunit 2, chloroplastic
MSAISLINSGVAWFVAVAVLAFLFSFQKALSGWIAGIGGAVGSLYTAAAGFTVLTGTVGV
SGALSLVSYDVQISPLNAIWLITLGLCGLFVSLYNIDWHRHAQVKCNGLQINMLMAAAVC
AVIASNLGMFVVMAEIMALCAVFLTSNSKEGKLWFALGRLGTLLLAIACWLLWQRYGTLD
LRLLDMRMQQLPLGSDIWLLGVIGFGLLAGIIPLHGWVPQAHANASAPAAALFSTVVMKI
GLLGILTLSLLGGNAPLWWGIALLVLGMITAFVGGLYALMEHNIQRLLAYHTLENIGIIL
LGLGAGVTGIALEQPALIALGLVGGLYHLLNHSLFKSVLFLGAGSVWFRTGHRDIEKLGG
IGKKMPVISIAMLVGLMAMAALPPLNGFAGEWVIYQSFFKLSNSGAFVARLLGPLLAVGL
AITGALAVMCMAKVYGVTFLGAPRTKEAENATCAPLLMSVSVVALAICCVIGGVAAPWLL
PMLSAAVPLPLEPANTTVSQPMITLLLIACPLLPFIIMAICKGDRLPSRSRGAAWVCGYD
HEKSMVITAHGFAMPVKQAFAPVLKLRKWLNPVSLVPGWQCEGSALLFRRMALVELAVLV
VIIVSRGA
>LFGLNPFC_00421 Formate hydrogenlyase subunit 4
MSVLYPLIQALVLFAVAPLLSGITRVARARLHNRRGPGVLQEYRDIIKLLGRQSVGPDAS
GWVFRLTPYVMVGVMLTIATALPVVTVGSPLPPLGDLITLLYLFAIARFFFAISGLDTGS
PFTAIGASREAMLGVLVEPMLLLGLWVAAQAAGSTNISNITDTVYHWPLSQSIPLVLALC
ACAFATFIEMGKLPFDLAEAEQELQEGPLSEYSGSGFGVMKWGISLKQLVVLQMFVGVFI
PWGQMETFTVGGLLLALVIAIVKLVVGVLVIALFENSMARLRLDITPRITWAGFGFAFLA
FVSLLAA
>LFGLNPFC_00422 Formate hydrogenlyase subunit 5
MSEEKLGQHYLAALNEAFPGVVLDHAWQTKDQLTVTVKVNYLPEVVEFLYYKQGGWLSVL
FGNDERKLNGHYAVYYVLSMEKGTKCWVTVRVEVDANKPEYPSVTPRVPAAVWGEREVRD
MYGLIPVGLPDERRLVLPDDWPDELYPLRKDSMDYRQRPAPTTDAETYEFINELGDKKNN
VVPIGPLHVTSDEPGHFRLFVDGENIIDADYRLFYVHRGMEKLAETRMGYNEVTFLSDRV
CGICGFAHSTAYTTSVENAMGIQVPERAQMIRAILLEVERLHSHLLNLGLACHFTGFDSG
FMQFFRVRETSMKMAEILTGARKTYGLNLIGGIRRDLLKDDMIQTRQLAQQMRREVQELV
DVLLSTPNMEQRTVGIGRLDPEIARDFSNVGPMVRASGHARDTRADHPFVGYGLLPMEVH
SEQGCDVISRLKVRINEVYTALNMIDYGLDNLPGGPLMVEGFTYIPHRFALGFAEAPRGD
DIHWSMTGDNQKLYRWRCRAATYANWPTLRYMLRGNTVSDAPLIIGSLDPCYSCTDRMTV
VDVRKKKSKVVPYKELERYSIERKNSPLK
>LFGLNPFC_00423 NAD(P)H-quinone oxidoreductase subunit I, chloroplastic
MFTFIKKVIKTGTATSSYPLEPIAVDKNFRGKPEQNPQQCIGCAACVNACPSNALTVETD
LATGELAWQFDLGRCIFCGRCEEVCPTAAIKLSQEYELAVWKKEDFLQQSRFALCNCRVC
NRPFAVQKEIDYAIALLKHNGDSRAENHRESFETCPECKRQKCLVPSDRIELTRHMKEAI
>LFGLNPFC_00424 Formate hydrogenlyase subunit 7
MSNLLGPRDANGIPVPMTVDESIASMKASLLKKIKRSAYVYRVDCGGCNGCEIEIFATLS
PLFDAERFGIKVVPSPRHADILLFTGAVTRAMRSPALRAWQSAPDPKICISYGACGNSGG

IFHDLYCVWGGTDKIVPVDVYIPGCPPTPAATLYGFAMALGLLEQKIHARGPGEQDEQPA
EILHGDMVQPLRVKVDREARRLAGYRYGRQIADDFLTQLGQGEEQVARWLEAENDPRLNE
IVSHLNHVVEEARIR
>LFGLNPFC_00425 hypothetical protein
MSEKVVFSQLSRKFIDENDATPAEAQQVVYYSLAIGHHLGVIDCLEAALTCPWDDYLAWI
ATLEAGSEARRKMEGVPKYGEIVIDINHVPMLANAFDKARAAQTSQQKEWSTMLLSMLHD
IHQENAIYLMVRRLRD
>LFGLNPFC_00426 Hydrogenase 3 maturation protease
MTDVLLCVGNSMMGDDGAGPLLAEKCAAAPKGNWVVIDGGSAPENDIVAIRELRPTRLLI
VDATDMGLNPGEIRIIDPADIAEMFMMTTHNMPLNYLIDQLKEDIGEVIFLGIQPDIVGF
YYPMTQPIKDAVETVYQRLEGWEGNGGFAQLAVEEE
>LFGLNPFC_00427 Antitoxin HigA
MTANAARAVKATRELVNAVPFLGGSDSEDDYREALELVEYLIEEDDTNPLIDFLASRIAE
YENNNEKFAEFDKAVAAMPVGVALLRTLIDQHNLTYADLKNEIGSKSLVSQILSGQRSLT
ISHIKALSARFGVKPEWFL
>LFGLNPFC_00428 Aryl-phospho-beta-D-glucosidase BglH
MSVFPQGFLWGGALAANQSEGAYREGGKGLTTVDMIPHGEHRMAVKLGLEKRFQLRDDEF
YPSHEATDFYHRYKEDIALMAEMGFKVFRTSIAWSRLFPQGDELTPNQQGIAFYRAVFEE
CKKYGIEPLVTLCHFDVPMHLVTEYGSWRNRKLVELFSRYARTCFEAFDGLVKYWLTFNE
INIMLHSPFSGAGLVFEEGENQDQVKYQAAHHQLVASALATKIAHEVNPQNQVGCMLAGG
NFYPYSCKPEDVWAALEKDRENLFFIDVQARGAYPAYSARVFREKGVTIDKAPSDDEILK
NTVDFVSFSYYASRCASAEMNANNCSAANVVKSLRNPYLQVSDWGWGIDPLGLRITMNMM
YDRYQKPLFLVENGLGAKDEFAANGEINDDYRISYLREHIRAMGEAIADGIPLMGYTTWG
CIDLVSASTGEMSKRYGFVYVDRDDAGNGTLTRTRKKSFWWYKKVIASNGEDLE
>LFGLNPFC_00429 PTS system beta-glucoside-specific EIIBCA component
MAKNYAALASSVITALGGVDNISAVTHCMTRLRFVIKDDQLIDSPTLKTISGVLGVVRSD
NQCQVIIGNTVSQAFQEVVSLLPGDLQPAPPVGKPKLTLRRIGAGVLDALIGTMSPLIPA
IIGGSMVKLLAMILEMSGVLTKGSPTLIILNVIGDGAFFFLPLMVAASAAIKFKTNMSLA
IAIAGVLVHPSFIELMAKAALGEHVEFALIPVTAVKYTYTVIPALVMTWCLSYIERWVDS
ITPAVTKNFLKPMLIVLIAAPLAILLIGPIGIWIGSAISALVYTIHGYLGWLSVAIMGAL
WPLLVMTGMHRVFTPTIIQTIAETGKEGMVMPSEIGANLSLGGSSLAVAWKTKNPELRQT
ALAAAASAIMAGISEPALYGVAIRLKRPLIASLISGFICGAVAGMAGLASHSMAAPGLFT
SVQFFDPANPMSIVWVFAVMALAVVLSFILTLLLGFEDIPVEEATAEARKHQSVQPTVAK
EVSLN
>LFGLNPFC_00430 HTH-type transcriptional regulator AscG
MMTTMLEVAKRAGVSKATVSRVLSGNGYVSQETKDRVFQAVEESGYRPNLLARNLSAKST
QTLGLVVTNTLYHGIYFSELLFHAARMAEEKGRQLLLADGKHSAEEERQAIQYLLDLRCD
AIMIYPRFLSVDEIDDIIDAHSQPIMVLNRRLRKNSSHSVWCDHKQTSFNAVAELITAGH
QEIAFLTGSMDSPTSIERLAGYKDALAQHGIAFNEKLIANGKWTPASGAEGVETLLERGA
KFSALVASNDDMAIGAIKALHERGVAVPEQVSVIGFDDIAIAPYIVPALSSVKIPVTEMI
QEIIGRLIFMLDGGDFSPPKTFSGKLIRRGSLIALSR
>LFGLNPFC_00431 Hydrogenase-4 component A
MNRFIIADASKCIGCRTCEVACVVSHQENQDCASLTPETFLPRIHVIKGVNISTATVCRQ
CEDAPCANVCPNGAISRDKGFVHVMQERCIGCKTCVVACPYGAMEVVVRPVIRNSGAGLN
VRADKAEANKCDLCNHREDGPACMAACPTHALICVDRNKLEQLSAEKRRRTALMF
>LFGLNPFC_00432 Carbamoyltransferase HypF
MAKNTSCGVQLRIRGKVQGVGFRPFVWQLAQQLNLHGDVCNDGDGVEVRLLEDPETFLVQ
LHQHCPPLARIDSVEREPFIWSQLPTEFTIRQSAGGVMNTQIVPDAATCPACLAEMNTPG
ERRWRYPFINCTHCGPRFTIIRAMPYDRPFTVMAAFPLCPACDKEYCDPLDRRFHAQPVA
CPECGPHLEWVSHGEHAEQEALQAAIAQLKMGNIVAIKGIGGFHLACDARNSNAVATLR
ARKHRPAKPLAVMLPVADGLPDAARQLLTTPAAPIVLVDKKYVPELCDDIAPDLNEVGVM
LPANPLQHLLLQELQCPLVMTSGNLSGKPPAISNEQALADLQGIADGFLIHNRDIVQRMD
DSVVRESGEMLRRSRGYVPDALALPPGFKNVPPVLCLGADLKNTFCLVRGEQAVLSQHLG
DLSDDGIQMQWREALRLMQNIYDFTPQYVVHDAHPGYVSSQWAREMNLPTQTVLHHHAHA
AACLAEHLWPLDGGDVIALTLDGIGMGENGALWGGECLRVNYRECQHLGGLPAVALPGGD
LAAKQPWRNLLAQCLRFVPEWQNYSETASVQQQNWSVLARAIERGINAPLASSCGRLFDA
VAAALGCAPATLSYEGEAACALEALAASCHGVTHPVTMPLVDNQLDLATFWQQWLSWQAP
VNQRAWAFHDALAQGFAALMREQATMRGITTLVFSGGVIHNCLLRARLAHYLADFTLLFP
QSLPAGDGGLSLGQGVIVAARWLAGEVQNG
>LFGLNPFC_00433 Nitric oxide reductase FlRd-NAD(+) reductase
MSNGIVIIGSGFAARQLVKNIRKQDASIPLTLIAADSMDEYNKPDLSHVISQGQRADDLT
RQTAGEFAEQFNLRLFPHTWVTDIDAEAHVVKSQNNQWQYDKLVLATGASAFVPPVPGRE
LMLTLNSQQEYRACETQLRDARRVLIVGGGLIGSELAMDFCRAGKAVTLIDNAASILASL
MPPEVSSRLQHRLTEMGVHLLLKSQLQGLEKTDSGILATLDRQRCIEVDAVIAATGLRPE

TALARRAVLTINRGVCVDSYLQTSNADIYALGDCAEINGQVLPFLQPIQLSAMVLAKNLL
GNNTPLKLPAMLVKIKTPELPLHLAGETQRQDLRWQINTERQGMVARGVDDADQLRAFVV
SEDRMKEAFGLLKTLSM
>LFGLNPFC_00434 Anaerobic nitric oxide reductase flavorubredoxin
MSIVVKNNIHWVGQRDWEVRDFHGTEYKTLRGSSYNSYLIREEKNVLIDTVDHKFSREFV
QNLRNEIDLADIDYIVINHAEEDHAGALTELMAQIPDTPIYCTANAIDSINGHHHHPEWN
FNVVKTGDTLDIGNGKQLIFVETPMLHWPDSMMTYLTGDAVLFSNDAFGQHYCDEHLFND
EVDQTELFEQCQRYYANILTPFSRLVTPKITEILGFNLPVDMIATSHGVVWRDNPTQIVE
LYLKWAADYQEDRITIVYDTMSNNTRMMADAIAQGIAETDPRVAVKIFNVARSDKNEILT
NVFRSKGVLVGTSTMNNVMMPKIAGLVEEMTGLRFRNKRASAFGSHGWSGGAVDRLSTRL
QDAGFEMSLSLKAKWRPDQDALELCREHGREIARQWALAPLPQSTVNTVVEEETSAATTA
DLGPRMQCSVCQWIYDPAKGEPMQDVAPGTPWSEVPDNFLCPECSLGKDVFDELASEAK
>LFGLNPFC_00435 Anaerobic nitric oxide reductase transcription regulator NorR
MSFSVDVLANIAIELQRGIGHQDRFQRLITTLRQVLECDASALLRYDSRQFIPLAIDGLA
KDVLGRRFALEGHPRLEAIARAGDVVRFPADSELPDPYDGLIPGQESLKVHACVGLPLFA
GQNLIGALTLDGMQPDQFDVFSDEELRLIAALAAGALSNALLIEQLESQNMLPGDAAPFE
AVKQTQMIGLSPGMTQLKKEIEIVAASDLNVLISGETGTGKELVAKAIHEASPRAVNPLV
YLNCAALPESVAESELFGHVKGAFTGAISNRSGKFEMADNGTLFLDEIGELSLALQAKLL
RVLQYGDIQRVGDDRSLRVDVRVLAATNRDLREEVLAGRFRADLFHRLSVFPLSVPPLRE
RGDDVILLAGYFCEQCRLRLGLSRVVLSAGARNLLQHYNFPGNVRELEHAIHRAVVLSRA
TRSGDEVILEAQHFAFPEVTLPPPEAAAVPVVKQNLREATEAFQRETIRQALAQNHHNWA
ACARMLETDVANLHRLAKRLGLKD
>LFGLNPFC_00436 Arabinose 5-phosphate isomerase GutQ
MSEALLNAGRQTLMLELQEASHLPERLGDDFVRAANIILHCEGKVVVSGIGKSGHIGKKI
AATLASTGTPAFFVHPAEALHGDLGMIESRDVMLFISYSGGAKELDLIIPRLEDKSIALL
AMTGKPTSPLGLAAKAVLDISVEREACPMHLAPTSSTVNTLMMGDALAMAVMQARGFNEE
DFARSHPAGALGARLLNKVHHLMRRDDAIPQVALTASVMDAMLELSRTGLGLVAVCDDQR
LVKGVFTDGDLRRWLVGGGALTTPVNEAMTVGGTTLQSQSRAIDAKEILMKRKITAAPVV
DENGKLTGAINLQDFYQAGII
>LFGLNPFC_00437 Glucitol operon repressor
MKPRQRQAAILEYLQKQGKCSVEELAQYFDTTGTTIRKDLVILEHAGTVIRTYGGVVLNK
EESDPPIDHKTLINTHKKELIAEAAVGFIHDGDSIILDAGSTVLQMVPLLSRFNNITVMT
NSLHIVNALSELDNEQTILMPGGTFRKKSASFHGQLAENAFEHFTFDKLFMGTDGIDLNA
GVTTFNEVYTVSKAMCNAAREVILMADSSKFGRKSPNVVCSLESVDKLITDAGIDPAFRQ
ALEEKGIDVIITGESNE
>LFGLNPFC_00438 hypothetical protein
MVSALITVAVIAWCAQLALGGWQISRFNRAFDTLCQQGRVGVGRSSGRFKPRVVVAIALD
DQQRIVDTLFMKGLTVFARPQKIPAITGMHVGDLQPDVIFPHDPLSQNALSLALKLKRG
>LFGLNPFC_00439 Sorbitol-6-phosphate 2-dehydrogenase
MNQVAVVIGGGQTLGAFLCHGLAAEGYRVAVVDIQSDKAANVAQEINAEYGEGTAYGFGA
DATSEQSVLALSRGVDEIFGRVDLLVYSAGIAKAAFISDFQLGDFDRSLQVNLVGYFLCA
REFSRLMIRDGIQGRIIQINSKSGKVGSKHNSGYSAAKFGGVGLTQSLALDLAEYGITVH
SLMLGNLLKSPMFQSLLPQYATKLGIKPDQVEQYYIDKVPLKRGCDYQDVLNMLLFYASP
KASYCTGQSINVTGGQVMF
>LFGLNPFC_00440 PTS system glucitol/sorbitol-specific EIIA component
MTVIYQTTITRIGASATDALSDQMLITFREGAPADLEEYCFIHCHGELKGALHPGLQFSL
GQHRYPVTAVGSVAEDNLRELGHVTLRFDGLSEAEFPGTVHVAGPVPDDIAPGSVLKFES
VKE
>LFGLNPFC_00441 PTS system glucitol/sorbitol-specific EIIB component
MTRIRIEKGTGGWGGPLELEATPGKKIVYITAGTRPAIVDKLAQLTGWQAIDGFKEGEPA
EAEIGVAVIDCGGTLRCGIYPKRRIPTINIHSTGKSGPLAQYIVEDIYVSGVKEENITVV
GEATPQPSSVGRDYDTSKKITEQSDGLLAKVGMGMGSAVAVLFQSGRDTIDTVLKTILPF
MAFVSALIGIIMASGLGDWIAHGLAPLASHPLGLVMLALICSFPLLSPFLGPGAVIAQVI
GVLIGVQIGLGNIPPHLALPALFAINAQAACDFIPVGLSLAEARQDTVRVGVPSVLVSRF
LTGAPTVLIAWFVSGFIYQ
>LFGLNPFC_00442 PTS system glucitol/sorbitol-specific EIIC component
MIETITHGAEWFIGLFQKGGEVFTGMVTGILPLLISLLVIMNALINFIGGQHRIERFAQRC
AGNPVSRYLLLQCIGTFVFCNPMTLSLGRFMPEKYKPSYYAAASYSCHSMNGLFPHINPG
ELFVVYLGIASGLTTLNLPLGPLAVSYLLVGLVTNFFRGWVTDLTTAIFEKKMGIQLEQKV
RLAGATS
>LFGLNPFC_00443 Membrane-bound lytic murein transglycosylase B
MFKRRYVTLLPLFVLLAACSSKPKPTETETTTGTPSGGFLLEPQHNVMQMGGDFANNPNA
QQFIDKMVNKHGFDRQQLQEILSQAKRLDSVLRLMDNQAPTTSVKPPSGPNGAWLRYRKK
FITPDNVQNGVVFWNQYEDALNRAWQVYGVPPEIIVGIIGVETRWGRVMGKTRILDALAT

LSFNYPRRAEYFSGELETFLLMARDEQDDPLNLKGSFAGAMGYGQFMPSSYKQYAVDFSG
DGHINLWDPVDAIGSVANYFKAHGWVKGDQVAVMANGQAPGLPNGFKTRYSISQLAAAGL
TPQQPLGNHQQASLLRLDVGTGYQYWYGLPNFYTITRYNHSTHYAMAVWQLGQAVALARV
Q
>LFGLNPFC_00444 Nicotinamide-nucleotide amidohydrolase PncC
MTDSELMQLSEQVGQSLKARGATVTTAESCTGGWVAKVITDIAGSSAWFERGFVTYSNEA
KAQMIGVREETLAQHGAVSEPVVVEMAIGALKAARADYAVSISGIAGPDGGSEEKPVGTV
WFAFATARGEGITRRECFSGDRDAVRRQATAYALQTLWQQFLQNT
>LFGLNPFC_00445 Protein RecA
MAIDENKQKALAAALGQIEKQFGKGSIMRLGEDRSMDVETISTGSLSLDIALGAGGLPMG
RIVEIYGPESSGKTTLTLQVIAAAQREGKTCAFIDAEHALDPIYARKLGVDIDNLLCSQP
DTGEQALEICDALARSGAVDVIVVDSVAALTPKAEIEGEIGDSHMGLAARMMSQAMRKLA
GNLKQSNTLLIFINQIRMKIGVMFGNPETTTGGNALKFYASVRLDIRRIGAVKEGENVVG
SETRVKVVKNKIAAPFKQAEFQILYGEGINFYGELVDLGVKEKLIEKAGAWYSYKGEKIG
QGKANATAWLKDNPETAKEIEKKVRELLLSNPNSTPDFSVDDSEGVAETNEDF
>LFGLNPFC_00446 Regulatory protein RecX
MTESTSRRPAYARLLDRAVRILAVRDHSEQELRRKLAAPIMGKNGPEEIDATAEDYERVI
AWCHEHGYLDDSRFVARFIASRSRKGYGPARIRQELNQKGISREATEKAMRECDIDWCAL
ARDQATRKYGEPLPTVFSEKVKIQRFLLYRGYLMEDIQDIWRNFAD
>LFGLNPFC_00447 Alanine--tRNA ligase
MSKSTAEIRQAFLDFFHSKGHQVVASSSLVPHNDPTLLFTNAGMNQFKDVFLGLDKRNYS
RATTSQRCVRAGGKHNDLENVGYTARHHTFFEMLGNFSFGDYFKHDAIQFAWELLTSEKW
FALPKERLWVTVYESDDEAYEIWEKEVGIPRERIIRIGDNKGAPYASDNFWQMGDTGPCG
PCTEIFYDHGDHIWGGPPGSPEEDGDRYIEIWNIVFMQFNRQADGTMEPLPKPSVDTGMG
LERIAAVLQHVNSNYDIDLFRTLIQAVAKVTGATDLSNKSLRVIADHIRSCAFLIADGVM
PSNESRGYVLRRIIRRAVRHGNMLGAKETFFYKLVGPLIDVMGSAGEDLKRQQAQVEQVL
KTEEEQFARTLERGLALLDEELAKLSGDTLDGETAFRLYDTYGFPVDLTADVCRERNIKV
DEAGFDAAMEEQRRRAREASGFGADYNAMIRVDSASEFKGYDHLELNGKVTALFVDGKAV
DAINAGQEAVVVLDQTPFYAESGGQVGDKGELKGANFSFAVEDTQKYGQAIGHIGKLAAG
SLKVGDAVQADVDEARRARIRLNHSATHLMHAALRQVLGTHVSQKGSLVNDKVLRFDFSH
NEAMKPEEIRAVEDLVNAQIRRNLPIETNIMDLEAAKAKGAMALFGEKYDERVRVLSMGD
FSTELCGGTHASRTGDIGLFRIISESGTAAGVRRIEAVTGEGAIATVHADSDRLSEVAHL
LKGDSNNLADKVRSVLERTRQLEKELQQLKEQAAAQESANLSSKAIDVNGVKLLVSELSG
VEPKMLRTMVDDLKNQLGSTIIVLATVAEGKVSLIAGVSKDVTDRVKAGELIGMVAQQVG
GKGGGRPDMAQAGGTDAAALPAALASVKGWVSAKLQ
>LFGLNPFC_00448 Carbon storage regulator
MLILTRRVGETLMIGDEVTVTVLGVKGNQVRIGVNAPKEVSVHREEIYQRIQAEKSQQSS
Y
>LFGLNPFC_00453 Fructose-1-phosphate phosphatase YqaB
MYERYAGLIFDMDGTILDTEPTHRKAWREVLGHYGLQYDVQAMIALNGSPTWRIAQAIIE
LNQADLDPHALAREKTEAVRSMLLDSVEPLPLVEVVKSWHGRRPMAVGTGSESAIAEALL
AHLGLRRYFDAVVAADHVKHHKPAPDTFLLCAQRMGVQPTQCVVFEDADFGIQAARAAGM
DAVDVRLL
>LFGLNPFC_00454 Inner membrane protein YqaA
MSEVLSLFSLFASSFLSATLLPGNSEVVLVAMLLSGVSHPWVLVLTATMGNSLGGLTNVI
LGRFFPLRKTSRWQEKATGWLKRYGAVTLLLSWMPVVGDLLCLLAGWMRISWGPVIFFLC
LGKALRYVAVAAATVQGMMWWH
>LFGLNPFC_00455 Glutamate--cysteine ligase
MIPDVSQALAWLEKHPQALKGIQRGLERETLRVNADGTLATTGHPEALGSALTHKWITTD
FAEALLEFITPVDGDIEHMLTFMRDLHRYTARNMGDERMWPLSMPCYIAEGQDIELAQYG
TSNTGRFKTLYREGLKNRYGALMQTISGVHYNFSLPMAFWQAKCGDISGADAKEKISAGY
FRVIRNYYRFGWVIPYLFGASPAICSSFLQGKPTSLPFEKTECGMYYLPYATSLRLSDLG
YTNKSQSNLGITFNDLYEYVAGLKQAIKTPSEEYAKIGIEKDGKRLQINSNVLQIENELY
APIRPKRVTRSGESPSDALLRGGIEYIEVRSLDINPFSPIGVDEQQVRFLDLFMVWCALA
DAPEMSSSELACTRVNWNRVILEGRKPGLTLGIGCETAQFPLPQVGKDLFRDLKRVAQTL
DSINGGEAYQKVCDELVACFDNPDLTFSARILRSMIDTGIGGTGKAFAEAYRNLLREEPL
EILREEDFVAEREASERRQQEMEAADTEPFAVWLEKHA
>LFGLNPFC_00456 S-ribosylhomocysteine lyase
MPLLDSFTVDHTRMEAPAVRVAKTMNTPHGDAITVFDLRFCVPNKEVMPERGIHTLEHLF
AGFMRNHLNGNGVEIIDISPMGCRTGFYMSLIGTPDEQRVADAWKAAMEDVLKVQDQNQI
PELNVYQCGTYQMHSLQEAQDIARSILERDVRINSNEELALPKEKLQELHI
>LFGLNPFC_00457 Multifunctional CCA protein
MIWQLTDDKRWSALRQRFSWVEEMHHTPQDPEHHGEGDVGVHTEMVLNALITLPEFQQLP
AQQQEVLWAAALLHDVEKRSTTVQENGRIQSPGHARRGELTARQILWRDIPTPFVLREQI

VALVRLHGLPLWLLERPEPERLLLTAAMRIDTRLLALLARADLLGRQSPDQQSMLERIDL
FELFCHEQQCWGKMRPFVSDSARWHYLTHEQSSPDFVPWEAEPFEVILLCGLPGMGKDRY
INEQCQGMDVISLDDMRRRINASPDDKTATGRIVQQAKEEARVFLRQKKPFIWNATNITR
QLRSQLISLFTAYGARVKIVYLEVPWAQWKQQNARREYAVPEAVMMRMASRLEVPQLDEA
HSVEYRMTDR
>LFGLNPFC_00458 hypothetical protein
MNTQRKYGRTWHYPFSPGTTSDDRINADYWQDLQAITQLVHTEKLDGENNCLNRYGVFAR
SHAAPTQSAWTYKIRQRWQLLKNDLGDLELFGENLYAVHSIEYRALEQDFYLFAVRCQDM
WLSWEEVQFYAALFDFPCVPEISGPQPGNDEKSWQRDFLALTNARGTFDPWDTQTCQPCT
LEGIVSRNHDAFSVADFSHNVFKYVRKNHVKTTVHWKRHWQRARMAHEFVYGEQS
>LFGLNPFC_00459 hypothetical protein
MGLKEKCEYHPRKRGRRKKRINNLSECSEPVNGEWKSLGRRKDTPNEWIKQPFTV
>LFGLNPFC_00460 Multidrug export protein EmrB
MQQQKPLEGAQLVIMTIALSLATFMQVLDSTIANVAIPTIAGNLGSSLSQGTWVITSFGV
ANAISIPLTGWLAKRVGEVKLFLWSTIAFAIASWACGVSSSLNMLIFFRVIQGIVAGPLI
PLSQSLLLNNYPPAKRSIALALWSMTVIVAPICGPILGGYISDNYHWGWIFFINVPIGVA
VVLMTLQTLRGRETRTERRRIDAVGLALLVIGIGSLQIMLDRGKELDWFSSQEIIILTVV
AVVAICFLIVWELTDDNPIVDLSLFKSRNFTIGCLCISLAYMLYFGAIVLLPQLLQEVYG
YTATWAGLASAPVGIIPVILSPIIGRFAHKLDMRRLVTFSFIMYAVCFYWRAYTFEPGMD
FGASAWPQFIQGFAVACFFMPLTTITLSGLPPERLAAASSLSNFTRTLAGSIGTSITTTM
WTNRESLHHAQLTESVNPFNPNAQAMYSQLEGLGMTQQQASGWIAQQITNQGLIISANEI
FWMSAGIFLVLLGLVWFAKPPFGAGGGGGGAH
>LFGLNPFC_00461 Multidrug export protein EmrA
MSANAETQTPQQPVKKSGKRKRLLLLLTLLFIIIAVAIGIYWFLVLRHFEETDDAYVAGN
QIQIMSQVSGSVTKVWADNTDFVKEGDVLVTLDPTDARQAFEKAKTALASSVRQTHQLMI
NSKQLQANIEVQKIALAKAQSDYNRRVPLGNANLIGREELQHARDAVTSAQAQLDVAIQQ
YNANQAMILGTKLEDQPAVQQAATEVRNAWLALERTRIVSPMTGYVSRRAVQPGAQISPT
TPLMAVVPATNMWVDANFKETQIANMRIGQPVTITTDIYGDDVKYTGKVVGLDMGTGSAF
SLLPAQNATGNWIKVVQRLPVRIELDQKQLEQYPLRIGLSTLVSVNTTNRDGQVLANKVR
STPVAVSTAREISLAPVNKLIDDIVKANAG
>LFGLNPFC_00462 Transcriptional repressor MprA
MDSSFTPIEQMLKFRASRHEDFPYQEILLTRLCMHMQSKLLENRNKMLKAQGINETLFMA
LITLESQENHSIQPSELSCALGSSRTNATRIADELEKRGWIERRESDNDRRCLHLQLTEK
GHEFLREVLPPQHNCLHQLWSALSTTEKDQLEQITRKLLSRLDQMEQDGVVLEAMS
>LFGLNPFC_00463 hypothetical protein
MSYEVLLLGLLVGTANYCFRYLPLRLRVGNARPTKRGAIGILLDTIGIASICALLVVSTA
PEVMHDTRRFVPTLVGFAVLGASFYKTRSIIIPTLLSALAYGLAWKVMAII
>LFGLNPFC_00464 Inner membrane protein YgaZ
MESPTPQPAPGSATFMEGCKDSLPIVISYIPVAFAFGLNATRLGFSPLESVFFSCIIYAG
ASQFVITAMLAAGSSLWIAALTVMAMDVRHVLYGPSLRSRIIQRLQKSKTALWAFGLTDE
VFAAATAKLVRNNRRWSENWMIGIAFSSWSSWVFGTVIGAFSGSGLLQGYPAVEAALGFM
LPALFMSFLLASFQRKQSLCVTAALVGALAGVTLFSIPVAILAGIVCGCLTALIQAFWQG
APDEL
>LFGLNPFC_00465 putative transporter
MTKPNHELSPALIVLMSIATGLAVASNYYAQPLLDTIARNFSLSASSAGFIVTAAQLGYA
AGLLFLVPLGDMFERRRLIVSMTLLAAGGMLITASSQSLAMMILGTALTGLFSVVAQILV
PLAATLASPDKRGKVVGTIMSGLLLGILLARTVAGLLANLGGWRTVFWVASMLMALMALA
LWRGLPQMKSETHLNYPQLLGSVFSMFISNKILRTRALLGCLTFANFSILWTSMAFLLAA
PPFNYSDGVIGLFGLAGAAGALGARPAGGFADKGKSHHTTTFGLLLLLLSWLAIWFGHTS
VLALIIGILVLDLTVQGVHITNQTVIYRIHPDARNRLTAGYMTSYFIGGAAGSLISASAW
QHGGWAGVCLAGATIALVNLLVWWRGFHRQEAAN
>LFGLNPFC_00466 Glycine betaine/proline betaine-binding periplasmic protein
MRHSVLFATAFATLISTQTFAADLPGKGITVNPVQSTITEETFQTLLVSRALEKLGYTVN
KPSEVDYNVGYTSLASGDATFTAVNWTPLHDNMYEAAGGDKKFYREGVFVNGAAQGYLID
KKTADQYKITNIAQLKDPKIAKLFDTNGDGKADLTGCNPGWGCEGAINHQLAAYGLTNTV
THNQGNYAAMMADTISRYKEGKPVFYYTWTPYWVSNELKPGKDVVWLQVPFSALPGDKNA
DTKLPNGANYGFPVSTMHIVANKAWAEKNPAAAKLFAIMQLPVADINAQNAIMHDGKASE
DDIQGHVDGWIKAHQQQFDGWVNEALAAQK
>LFGLNPFC_00467 Glycine betaine/proline betaine transport system permease protein ProW
MADQNNPWDTTPAADSAAQSADAWGTPATAPTDGGGADWLTSTPAPNVEHFNILDPFHKT
LIPLDSWVTEGIDWVVTHFRPVFQGVRVPVDYILNGFQQLLLGMPAPVAIIVFALIAWQI
SGVGMGVATLVSLIAIGAIGAWSQAMVTLALVLTALLFCIVIGLPLGIWLARSPRAAKII
RPLLDAMQTTPAFVYLVPIVMLFGIGNVPGVVVTIIFALPPIIRLTILGINQVPADLIEA
SRSFGASPSQMLFKVQLPLAMPTIMAGVNQTLMLALSMVVIASMIAVGGLGQMVLRGIGR

LDMGLATVGGVGIVILAIILDRLTQAVGRDSRSRGNRRWYTTGPVGLLTRPFIK
>LFGLNPFC_00468 Glycine betaine/proline betaine transport system ATP-binding protein ProV
MAIKLEIKNLYKIFGEHPQRAFKYIEQGLSKEQILEKTGLSLGVKDASLAIEEGEIFVIM
GLSGSGKSTMVRLLNRLIEPTRGQVLIDGVDIAKISDAELREVRRKKIAMVFQSFALMPH
MTVLDNTAFGMELAGINAEERREKALDALRQVGLENYAHSYPDELSGGMRQRVGLARALA
INPDILLMDEAFSALDPLIRTEMQDELVKLQAKHQRTIVFISHDLDEAMRIGDRIAIMQN
GEVVQVGTPDEILNNPANDYVRTFFRGVDISQVFSAKDIARRTPNGLIRKTPGFGPRSAL
KLLQDEDREYGYVIERGNKFVGAVSIDSLKAALTQQQGLDAALIDAPLAVDAQTPLSELL
SHVGQAPCAVPVVDEDQQYVGIISKGMLLRALDREGVNNG
>LFGLNPFC_00469 Ribonucleoside-diphosphate reductase 2 subunit beta
MKLSRISAINWNKISDDKDLEVWNRLTSNFWLPEKVPLSNDIPAWQTLSVVEQQLTMRVF
TGLTLLDTLQNVIGAPSLMPDALTPHEEAVLSNISFMEAVHARSYSSIFSTLCQTKDVDA
AYAWSEENAPLQRKAQIIQQHYRGDDPLKKKIASVFLESFLFYSGFWLPMYFASRGKLTN
TADLIRLIIRDEAVHGYYIGYKYQKNQEKISQAQREELKSFAFDLLLELYDNELQYTDEL
YAETPWSDDVKAFLCYNANKALMNLGYEPLFPAEMAEVNPAILAALSPNADENHDFFSGS
GSSYVMGKAVETEDEDWNF
>LFGLNPFC_00470 Ribonucleoside-diphosphate reductase 2 subunit alpha
MATTTAERLTQETMDYHALNAMLNLYDSAGRIQFDKDSQAVDAFMTTHVRPNSVAFSSQQ
QRLNWLVNEGYYDESVLNRYSRDFVITLFAHAHASGFRFQTFLGAWKFYTSYTLKTFDGK
RYLEDFADRVTMVALTLAQGDETLATQLTDEMLSGRFQPATPTFLNCGKQQRGELVSCFL
LRIEDNMESIGRAVNSALQLSKRGGGVAFLLSNLREAGAPIKRIENQSSGVIPVMKMLED
AFSYANQLGARQGAGAVYLHAHHPDILRFLDTKRENADEKIRIKTLSLGVVIPDITFHLA
KENAQMALFSPYDVERVYGKPFADVAISEHYDELVADERIRKKYLNARDFFQRLAEIQFE
SGYPYIMYEDTVNRANPIAGRINMSNLCSEILQVNSASEYDENLDYARTGHDISCNLGSL
NIAHTMDSPDFARTVETAVRGLTAVSDMSHIRSVPSIEAGNAASHAIGLGQMNLHGYLAR
EGIAYGSPEALDFTNLYFYTITWHALRTSMLLARERGETFAGFKQSRYASGEYFSQYLQG
NWQPKTAKVGELFARSGITLPTREMWAQLRDDVMRYGIYNQNLQAVPPTGSISYINHATS
SIHPIVAKVEIRKEGKTGRVYYPAPFMTNENLALYQDAYEIGAEKIIDTYAEATRHVDQG
LSLTLFFPDTATTRDINKAQIYAWRKGIKTLYYIRLRQMALEGTEIEGCVSCAL
>LFGLNPFC_00471 Protein NrdI
MSQLVYFSSSSENTQRFIERLGLPAVRIPLNERERIQVDEPYILIVPSYGGGGTAGAVPR
QVIRFLNDEHNRALLRGVIASGNRNFGEAYGRAGDVIARKCSVPWLYRFELMGTQSDIEN
VRKGVTEFWQRQPQNA
>LFGLNPFC_00472 Glutaredoxin-like protein NrdH
MRITIYTRNDCVQCHATKRAMENRGFDFEMINVDRVPEAAEALRAQGFRQLPVVIAGDLS
WSGFRPDMINRLHPAPHAASA
>LFGLNPFC_00473 Alkyl hydroperoxide reductase AhpD
MTTLRQPYYELSPAVYNALVQAKTALENSTLDTTLMELVYLRVSQINGCAFCLEMHSKAL
RKSGVPQHKLDALAGWRVSHHFDERERAALAWAESVTEIARTHAEDEVYQPLLEHFSAAE
ISDLTFAIGLMNCFNRLAVSMRM
>LFGLNPFC_00474 HTH-type transcriptional regulatory protein GabR
MPRYQDIARQLKTAIEQGELKPGARLPSSRTWSQELGVSRSTVENAYAELVAQGWLIRRG
QAGTFVSERIYPQQSTVQVVAFAGESQQPLPFQMGLPALDLFPRELWARVMGRRLRTQTR
FDLALGDVCGEAALREAIVDYLRVSRGIDCQPEQVFITHGYAASIALILHALAKPGNGMW
IEDPGFPLIRPIVTRHDVEILPVPVDDNGLDITSGIQNYPDARFALITPAHQSPLGVALS
LARRHQILEWADRSQAWIIEDDYDSEFRYHGKPLPALKSLDAPQRVIYAGTFSKALFPAL
RCAWLVVPVKQIAQFRHQASLAPCAVPVLWQNTLADFLREGHFWRHLKKMRQHYAQRRQW
IEQALTQQGFQVVPQKGGIQMVIRMIGDDIAHARKANAAGLAVQALSDWRIRSSGEGGLL
LSFTNIVNEGMARQVAQQLRKALS
>LFGLNPFC_00475 putative protein YgaM
MFNRPNRNDVDDGVQDIQNDVNQLADSLESVLKSWGSDAKGEAEAARSKAQALLKETRAR
MHGRTRVQQAARDAVGCADSFVRERPWCSVGTAAAVGIFIGALLSMRKS
>LFGLNPFC_00476 hypothetical protein
MYLRPDEVARVLEKVGFTVDVVTQKAYGYRRGENYVVYVNREARMGRTALVIHPTLKERSS
TLAEPASDIKTCDHYQQFPLYLAGERHEHYGIPHGFSSRVALERYLNGLFGEAS
>LFGLNPFC_00477 L-alanine exporter AlaE
MFSPQSRLRHAVADTFAMVVYCSVVNMCIEVFLSGMSFEQSFYSRLVAIPVNILIAWPYG
MYRDLFMRAARKVSPSGWIKNLADIMAYVTFQSPVYVAILLVVGADWHQIMAAVSSNIVV
SMLMGAVYGYFLDYCRRLFKVSRYQQVKA
>LFGLNPFC_00478 DNA-binding protein StpA
MSVMLQSLNNIRTLRAMAREFSIDVLEEMLEKFRVVTKERREEEEQQQRELAERQEKIST
WLELMKADGINPEELLGDSSAAAPRAGKKRQPRPAKYKFIDVNGETKTWTGQGRTPKPIA
QALAEGKSLDDFLI

>LFGLNPFC_00479 Inner membrane protein YgaP
MALTTISPHDAQELIARGAKLIDIRDADEYLREHIPEADLAPLSVLEQSGLPAKLRREQI
IFHCQAGKRTSNNADKLAAIAAPAEIFLLEDGIDGWKKAGLPVAVNKSQPLPLMRQVQIA
AGGLILIGVILGYTVNSGFFLLSGFVGAGLLFAGISGFCGMARLLDKMPWNQRA
>LFGLNPFC_00480 putative HTH-type transcriptional regulator YgaV
MTELAQLQASAEQAAALLKAMSHPKRLLILCMLSGSPGTSAGELTRITGLSASATSQHLA
RMRDEGLIDSQRDAQRILYSIKNEAVNAIIATLKNVYCP
>LFGLNPFC_00481 hypothetical protein
MGFWRIVITIILPPLGVLLGKGFGWAFIINILLTLLGYIPGLIHAFWVQTRD
>LFGLNPFC_00482 Potassium binding protein Kbp
MGLFNFVKDAGEKLWDAVTGQHDKDDQAKKVQEHLNKTGIPDADKVNIQIADGKATVTGD
GLSQEAKEKILVAVGNISGIASVDDQVKTATPATASQFYTVKSGDTLSAISKQVYGNANL
YNKIFEANKPMLKSPDKIYPGQVLRIPEE
>LFGLNPFC_00483 HTH-type transcriptional repressor GlaR
MTITSLDGYRWLKNDIIRGNFQPDEKLRMSLLTSRYALGVGPLREALSQLVAERLVTVVN
QKGYRVASMSEQELLDIFDARANMEAMLVSLAIARGGDEWEADVLAKAHLLSKLEACDAS
EKMLDEWDLRHQAFHTAIVAGCGSYYLLQMRERLFDLAARYRFIWLRRTVLSVEMLEDKH
DQHQTLTATVLARDTARASELMRQHLLTPIPIIQQAMAGN
>LFGLNPFC_00484 GABA permease
MGQSSQPHELGGGLKSRHVTMLSIAGVIGASLFVGSSVAIAEAGPAVLLAYLFAGLLVVM
IMRMLAEMAVATPDTGSFSTYADKAIGRWAGYTIGWLYWWFWVLVIPLEANIAAMILHSW
VPGIPIWLFSLVITLALTGSNLLSVKNYGEFEFWLALCKVIAILAFIFLGAVAISGFYPY
ADVSGISRLWDSGGFMPNGFGAVLSAMLITMFSFMGAEIVTIAAAESDTPEKHIVRATNS
VIWRISIFYLCSIFVVVALIPWNMPGLKAVGSYRSVLELLNIPHAKLIMDCVILLSVTSC
LNSALYTASRMLYSLSRRGDAPAVMGKINRSKTPYVAVLLSTGAAFLTVVVNYYAPAKVF
KFLIDSSGAIALLVYLVIAVSQLRMRKILRAEGSEIRLRMWLYPWLTWLVIGFITFVLVV
MLFRPAQQLEVLSTGLLAIGIICTVPIMARWKKLIMWQKTPIHNTR
>LFGLNPFC_00485 4-aminobutyrate aminotransferase GabT
MSSNKELMQRRSQAIPRGVGQIHPIFADRAENCRVWDVEGREYLDFAGGIAVLNTGHLHP
KVVAAVEAQLKKLSHTCFQVLAYEPYLELCEIMNQKVPGDFAKKTLLVTTGSEAVENAVK
IARAATKRSGTIAFSGAYHGRTHYTLALTGKVNPYSAGMGLMPGHVYRALYPCPLHGISE
DDAIASIHRIFKNDAAPEDIAAIVIEPVQGEGGFYAATPAFMQRLRALCDEHGIMLIADE
VQSGAGRTGTLFAMEQMGVAPDLTTFAKSIAGGFPLAGVTGRAEVMDAVAPGGLGGTYAG
NPIACVAALEVLKVFEQENLLQKANDLGQKLKDGLLAIAEKHTEIGDVRGLGAMIAIELF
EDGDHSKPDAKLTAEIVARARDKGLILLSCGPYNNVLRILVPLTIEDAQIRQGLEIISQC
FAEAKQ
>LFGLNPFC_00486 Succinate-semialdehyde dehydrogenase [NADP(+)] GabD
MKLNDSNLFRQQALINGEWLDANNGEVIDVTNPANGDKLGSVPKMGADETRAAIDAANRA
LPVWRALTAKERANILRNWFNLMMEHQDDLARLMTLEQGKPLAEAKGEISYAASFIEWFA
EEGKRIYGDTIPGHQADKRLIVIKQPIGVTAAITPWNFPAAMITRKAGPALAAGCTMVLK
PASQTPFSALALAELAIRAGVPAGVFNVVTGSAGAVGNELTSNPLVRKLSFTGSTEIGRQ
LMEQCAKDIKKVSLELGGNAPFIVFDDADLDKAVEGALASKFRNAGQTCVCANRLYVQNG
VYDRFAEKLQQAVSKLHIGNGLEKGVTIGPLIDEKAVAKVEEHIADALEKGARVVCGGKA
HERGGNFFQPTILVDVPANAKVSKEETFGPLAPLFRFKDEADVIAQANDTEFGLAAYFYA
RDLSRVFRVGEALEYGIIGINTGIISNEVAPFGGIKASGLGREGSKYGIEDYLEIKYMCI
GL
>LFGLNPFC_00487 L-2-hydroxyglutarate dehydrogenase
MYDFLIIGGGIIGMSTAMQLIDVYPDARIALLEKEPGPACHQTGHNSGVIHAGVYYTPGS
LKAQFCLAGNRATKAFCDQNGIRYDNCGKMLVATSELEMERMRALWERTAANGIDREWLN
AEELREREPNITGLGGIFVPSSGIVSYREVTAAMAKIFQARGGEIIYNAEVSALSEHKNG
VVICTRQGGEYEASTLISCSGLMADRLVKMLGLEPGFIICPFRGEYFRLAPEHNQIVNHL
IYPIPDPAMPFLGVHLTRMIDGSVTVGPNAVLAFKREGYRKRDFSFSDTLEILGSSGIRR
VLQNHLRSGLGEMKNSLCKSGYLRLVQKYCPRLSLSDLQPWPAGVRAQAVSPDGKLIDDF
LFVTTPRTIHTCNAPSPAATSAIPIGAHIVSKVQTLLASQSNPGRTLRAARSVDALHAAF
NQ
>LFGLNPFC_00488 Glutarate 2-hydroxylase
MNALTAVQNNAVDSGQDYSGFTLIPSAQSPRLLELTFTEQTTNRFLEQVAEWPVQALEYK
SFLRFRVGKILDDLCANQLQPLLLKTLLNRAEGALLINAVGIDDVAQADEMVKLATAVAH
LIGRSNFDAMSGQYYARFVVKNVDNSDSYLRQPHRVMELHNDGTYVEEITDYVLMMKIDE
QNMQGGNSLLLHLDDWEHLDHFFRHPLARRPMRFAAPPSKNVSKDVFHPVFDVDQQGRPV
MRYIDQFVQPKDFEEGVWLSELSDAIETSKGILSVPVPVGKFLLINNLFWLHGRDRFTPH
PDLRRELMRQRGYFAYATHHYQTHQ
>LFGLNPFC_00489 hypothetical protein
MTILVPPSKFCIYSDDSRAGTLNFINSIESIGVKNKGRVIVDLSKVKFASAAASVLFFAI

VNRAQFLTRDPNFIRFKWPKKDDNPSGHRWIVGTGLARALLAGTEEKLNALTREERYFQS
AVEPYEHIVETVLMLQKSALLNNEQLGLLLTAISEALLNVSHHAYEDEGFESDIQLLKGK
RWWQCAWFNRDENKVVFIVCDLGLGIYRSFVPNGDGHSIQNEVSSVERAMLVGESRFVGS
GRGNGSEDIKRPIGAGCEDNETLLILTGRARYSYNSNDSSPRCEKLAEYIPGTLLQWSLV
PRR
>LFGLNPFC_00490 hypothetical protein
MRNIVIAKEFSRTPFGRYTTDSPHSAERFRREFLVPALKGTEQEIVVDFRGIALGVGSSF
LEEAFGGLIRKEGIPKANVKARLIIKSDVPFYKEQIDKFIDLAQPERV
>LFGLNPFC_00491 hypothetical protein
MISTEPMSLVAQIGQYSWCITVVSVCLVFIGWRVAYNNSVKLATRSESKSIIDAISKLVI
EISDISSNYWLSQTTQPKIRASKHRLLRLQKDRTKASVSYLLTILAKAQQVSKLICILES
RGLYIPDEVFSSVLEKATLDCEVAHKLSDADRPVKAQEVIDACMGVIEALHTSFQRYHPP
KKDRTFMQRLKIWFQTVDDWHNDLK
>LFGLNPFC_00492 Putative defective protein IntQ
MGQTKLLKLPRGVTIRKHHQGETINITFTYKGVRCREPLSNLEVTPKNIKYAERTLGEIH
NKIERGTFIYAEYFPRSARLKIFGNAAASKTVKMYLDEYLEICETRKLSPSTIGGYKKCR
SALASLHICPASELTPATLKAWIQSQKTTLKTIRNQLSFLRSALDEAVTDGVLQINPVSL
VTASRYQSDKSEAESSYVVDPLSPAEVDALLAAAGNKQWENLFRFAIHTGLRSSELCALR
WHDIDFVGKTAHVQSASVVGVIKGTKTKAGTRKVELTEEAMLALINQKPFTFMKDATVFE
DPKTNKPWASADAIRKKAWVPTLRKAGIRYRNPYQTRHTFATSHISRGANLFWLAAQMGH
KGPEMLFRHYGQYLKEYDGRASIKDKKALF
>LFGLNPFC_00493 hypothetical protein
MLNLDCVPISTYCKETGETPEAINKRVQRGVWREGVQVLKVEGVKERWIDLSEVAKWARQ
NCSNYRAA
>LFGLNPFC_00494 hypothetical protein
MNQNPFSFYDFLGYLIPGGFFILLMYFCGLTFDLDIVIDLSELLRGQSQIFGILNYASIV
IISYIAGHFISITSAFFIEKYMNKELGYPSKYLFKKLIDTSESICSPSCDERSADKKTKI
KNRIIKCVLCPIILWDFITQKLCYSQSLPFHLANTTWLMIKEGYEKKFITNRQLLQDKNG
LDDDLFRLAYHYVYEFSKQHQTKIQNYVALYGFCRNICLIFIISFWVSVPTFIYRLCTHS
DYLYSLLSIMLSFFFVYVFYVGFVKFYRRYTLEVLMAFAVLQSNDTIR
>LFGLNPFC_00495 hypothetical protein
MSFIKTFSGKHFYYDRINKDDIVINDIAVSLSNICRFAGHLSHFYSVAQHAVLCSQLVPQ
EFAFEALMHDATEAYCQDIPAPLKRLLPDYKRMEEKIDAVIREKYGLPPVMSTPVKYADL
IMLATERRDLGLDDGSFWPVLEGIPATEMFKVIPLSPGHTYGMFMERFKELTES
>LFGLNPFC_00496 hypothetical protein
MSQNLDATAINQIHALISAQGVNEIISKIGADAVALPENFRIHDLEKFNLNRFRFRGALS
TASIDDFTRYSKDLADEGTRCFIDADNMRAVSVLNLGTIDEPGHADNTATLKLKKTAPFS
ALLSVNGERNSQKSLAEWIEDWADYLVGFDANGDAIQATKAAAAVRKITIEANQTADFED
NDFSGKRSLMESVEAKTKDIMPVAFEFKCVPFEGLKERPFKLRLSIITGDRPVLVLRIIQ
LEAVQEEMANEFRDLLVEKFKDSKVETFIGTFTA
>LFGLNPFC_00497 hypothetical protein
MASERSTNVQEFIGELDGGVFETKIGAVLSEVASGVMNTKTKGKVSLNLEIEPFDENRVK
IKHKLSYVRPTNRGKISEEDTTETPMYVNRGGRLTILQEDQGQLLTLAGEPDGKLRAAGH
>LFGLNPFC_00498 hypothetical protein
MGAIYVKRLILSVALIIPIASNASDALNQPSSSLNDGVETFFISCFDMPQETTTDMDACQ
RVQLAQVSWVKNKYSVAALNRLKQDNKDDPQRLQELTASFNAESEAWTELIEKASKSVQV
DYAGGTIAGTAVASRQIGLLELQSHDIWEHWLRFEDSTPPLLPEPKFKSE
>LFGLNPFC_00499 LexA repressor
MKTIHDIRRSNARKLRDGVGGNSSFATMIDREPTQTSRFMGDGATKNIGDSMARHIEKCF
DLPVGWLDQEHQTTNITKKPDVSITNKQITLVPVISWVQAGAWKEVGYSEVDLSTAETYP
CPVPCGEMTYILRVIGDSMIDEYRPGDMIFVDPEVPACHGDDVIALMHDTGETTFKRLIE
DGTQRYLKALNPNWPEPYIKINGNCSIIGTVIFSGKPRRYKIKA
>LFGLNPFC_00500 hypothetical protein
MKAYWDSLTKEQQGELAGKVGSTPGYLRLVFNGYKKASFVLAKKLEQCTSGAITKSDLRP
DIYPKD
>LFGLNPFC_00501 hypothetical protein
MGKHHWKIEKQPEWYVKAVRKTIAALPGGYAEAADWLDVTENALFNRLRADGDQIFPLGW
AMVLQRAGGTHFIADAVAQSANGVFVSLPDVEDVDNADINQRLLEVIEQIGSYSKQIRSA
IEDGVVEPHEKTAINDELYLSISKLQEHAALVYKIFCVSESSDARECAAPGAVACRDCGE
TNA
>LFGLNPFC_00502 hypothetical protein
MNSLTTHYRRSQLIALPVPGGKAKVEYCYAVNVPGDREIVTHSFAEWAVGDFNRQKETVL
CDKLTAGSKITTECPSESFVGNRKHNGLSTSAKAMSMNASVRSNSFVVNSGK
>LFGLNPFC_00503 hypothetical protein

MSTKLTGYVWDGCAASGMKLSSVAIMARLADFSNDEGVCWPSIETIARQIGAGMSTVRTA
IARLEAEGWLTRKARRQGNRNASNVYQLNVAKLQAAAFSQLSDSDPSKSDASKSAPSKFD
ASKSGKKAGFHPSESGGDPSVKSKHDPSDKKTSRPDASQPDTQTAEQEFLTRHPDAVVFS
PKKRQWGTQDDLTCAQWLWKKIIALYEQAAECDGEVVRPKEPNWTAWANEIRLMCVQDGR
THKQICEMYSRVSRDPFWCRNVLSPSKLREKWDELSLRLSPSVSTYTEKREDPYFKASYD
NVDYSQIPAGFRG
>LFGLNPFC_00504 hypothetical protein
MSLLNDVQKFIEAHPGCTSGDIADAFAGYSRQRVLQSASKLRQSGRVAHRCEGDTRRHFP
RLTERAQEPEPQPVRETRPVRNFYVGTNDPRVILCLTRQAEELESRGLYRRAATVWMAAF
RESHSQPERNNFLARRERCLRKSSKRAASGEEWYLSGNYVGA
>LFGLNPFC_00505 hypothetical protein
MSNKYCQALVELRNKPAHELKEVGDQWRTPDNIFWGINTLFGPFVLDLFTDGGNAKCAAY
YTAEDNALAHDWSERLAELKGAAFGNPPYSRASQHEGQYITGMRYIMKHASAMRDKGGRY
VFLIKAATSEVWWPEDADHIAFIRGRIGFELPVWFIPKDEKQVPTGAFFAGAIAVFDKTW
KGPAISYIGRDELEACGEAFLAQVRQQAEKLLREMAA
>LFGLNPFC_00506 LexA repressor
MTTLTQCQQQVLDMLISYQKERGFPPTNQEVATMLGYRSVNAAVEHLRALEKKGVITIKR
GVARGITLHTAVKDDDSEAVGIIRSLLAGKENARLRAAHWLHERGLKV
>LFGLNPFC_00507 Crossover junction endodeoxyribonuclease RusA
MKLILPFPPSVNTYWRHPNKGAFAGKSLISAAGRKFQSAACAAIVEQLRRLPKPTSAPAS
VEIVLFPPDNRIRDLDNYNKALFDALTHAGVWEDDSQVKRMLVEWGPVIPEGKVEITISK
YEKTAGAAA
>LFGLNPFC_00508 hypothetical protein
MNNLMVIDGIEVRRDAYGRYSLNDLHRAAGSLDKHKPAFWLRNEQTERLISELQICNSVN
IEPVNVIRGGNNQGTYVCKELVYAYAMWISPSFHLKVIRTFDMVTSTPEKLSGQAADKMQ
AGVILLDFMRRELNLSNSSVLGACQKLQEAVGLPNLAPRYAIDAPADAPDGSSRPTLSLS
ALLKQYGIRLTANQAYHQMAKLGIVEQRERYSRTAINNIKKFWSLTAKGCMFGKNITSPA
NPRETQPHFFESRFPELLKLLDTVH
>LFGLNPFC_00509 hypothetical protein
MRALLTPEIAPRMGIVLFRPGSELMPLFMQGRVLLEPEPERYSSFASGAVPAASQPLADD
PAIRAVFRNEAVIRRAGGVECLESWLLREKGCQWPHSGWHSENMTTMRHAPGAIRLCWHC
DNLLRDQFTERLESMATDNCARWVLSVVRRDLGFDDSHVVTMPELCWWLVRNDLADALPE
SAARKALRLPKPVVPSVTRESDLVPSVPATSIIQDKAKKVLALKVDPESPESFMLRPKRR
RWVNEKYTRWVKTQSCACCGKPADDPHHLIGHGQGGMGTKAHDLFVLPLCRKHHDELHAD
TVAFEEMYGSQLELIFRFIDRALAIGVLV
>LFGLNPFC_00510 Chaperone protein DnaJ
MNLEALPKYYSPKSPKLSDDASATGSGGLTITDVMAAQGMVQSKAPLGFALFLAKVGVQD
PQFAIEGLLNYAMALDNPTLNKLSEETRLQIIPYLVNFAFADYSRSAASKARCEHCTGTG
FHNVLREVVKHSRSGESVIKEEWVKELCQHCHGKGEVSTACRGCKGKGIVLDEKRTRLHG
TPVYKTCGRCNGNRFSRLPTTLARRHVQKLVPDLTDYQWYKGYADVIDKLVTKCWQEEAY
AETQLRKVTR
>LFGLNPFC_00511 hypothetical protein
MINAVEYRLPLGNISPENLDALSELIVKHSDKFNNYVLSSISDDDIRYSYDYYNFEITEI
DEYGFHFIAPYSYYEGCVDNNFSGEVEGYAEYEIIDNELVFSLEELPWDVK
>LFGLNPFC_00512 hypothetical protein
MPDELECNFRINNKSTYHFTLRFSVGGETAAIHMHEKENLAGAFWLVLLIIAGWGGLVRY
LIDVKQSKATWSWINALAQIVVSGFTGVIGGLISIESGFSIYMILATAGISGAMGSVALT
YFWERLTGVKNAKS
>LFGLNPFC_00513 hypothetical protein
MQNLNPQRKAFLDMVAWSEGTDNGLQPTRNHGYDVIVGGEIFTDYSDHPRRLVTLNPKLK
STAAGRYQLLSRWWDAYRKQLGLKDFSPKSQDAVALQQIKERGALPMIDRGDIRQAIDRC
SNIWASLPGAGYGQFEHKADSLIAKFKEAGGTVKEIQV
>LFGLNPFC_00514 hypothetical protein
MSRVTVIISALVICIIVCLSWAVDNYRDDAIVYKVQRDKATSIIADMQKRQRNVAELDAR
YTKELADANATIESLRADVSAGHKRLQVAATCTKSTTGASGMGDGESPRLTADAELHYYR
LRSGIDKITSQVNYLQEYIRTQCPN
>LFGLNPFC_00515 hypothetical protein
MRIKPQDFKFDVLTVAGYANPKAKTHFAPSASGFNFKWEWEGKIYELHIEQEKLQYPDFI
LERLFDAAIEMAKK
>LFGLNPFC_00516 hypothetical protein
MSIPSLDVHKVVKCAEHKKALDAISLKLKNNIIDSSSEYDFLGDTIIFNKDKKIG
>LFGLNPFC_00517 hypothetical protein
MPPRTPKACRVRGCRQTTTDPSGYCESHKSEGWKQYKPGQSRHQRGYGSKWDVIRARVLK
RDKGLCQLCLHVGVVREAKTVDHIIPKAHGGTDADCNLQSLCWPCHKAKTARERLK

>LFGLNPFC_00518 hypothetical protein
MAGTAGRSGRRPKPTARKALAGNPGKRALNKDEPVFTPIKGVEPPEWFAEEDLPLATIMW
QLTTKELCGQGLLCVTDLAVLERWCVAYEFWRRAVKNIAIQGNTITGAMGGRVKNPELTA
KKEQESEMSSTGAMLGLDPSSRQRLIGLAGQKKATNPFLKIIES
>LFGLNPFC_00519 hypothetical protein
MSRKSYPNVNAANQYARDVVRGKIVACQFVIQACQRHLDDLMAEKSKSFRYRFDKDLAER
AAKFIQLLPHTKGEWAFKRMPITLEPWQLFVICCAFGWVNKGSRLRRFREVYTEIPRKNG
KSAISAGVALYCFACDNEFGAEVYSGATTEKQAWEVFRPARLMCKRTPMLTEAFGIEVNA
SNMNRPEDGARFEPLIGNPGDGSSPHCAVVDEYHEHATDALYTTMLTGMGARRQPLMWAI
TTAGYNIEGPCYDKRREVIEMLNGSVPNDELFGIIYTVDEGDDWTDPQVLEKANPNIGVS
VYREFLLSQQQRAKNNARLANVFKTKHLNIWVSARSAYFNLVSWQSCEDKSLTLEQFEGQ
PCILAFDLARKLDMNSMARLYTREIDGKTHYYSVAPRFWVPYDTVYSVEKNEDRRTAERF
QKWVEMGVLTVTDGAEVDYRYILEEAKAANKISPVSESPIDPFGATGLSHDLADEDLNPI
TIIQNYTNMSDPMKELEAAIESGRFHHDGNPIMTWCIGNVVGKTIPGNDDVVKPVKEQAE
NKIDGAVALIMAVGRAMLYEKEDTLSDHIESYGIRSL
>LFGLNPFC_00520 hypothetical protein
MIMLILAPLVGVLGALLLAYGAWLIYPPAGFVVAGALCLFWSWLVARYLDRTQLSVGGGK
>LFGLNPFC_00521 hypothetical protein
MFFSGLFQRKSDAPVTTPAELAEAIGLSYDTYTGKQISSQRAMRLTAVFSCVRVLAESVG
MLPCNLYHLNGSLKQRATGERLHKLISTHPNGYMTPQEFWELVVTCLCLRGNFYAYKVKA
FGEVAELLPVDPGCVVPKLNSSWEPVYQVTFSDGSTDVLSQEDIWHVRTLTLDGLVGLNP
IAYAREAISLAAATEEHGARLFSNGAVTSGVLRTEQTLSDQAYERLKKDFEERHTGLGNA
HRPMILEMGLDWKSMALNAEDSQFLETRKFQLEEICRLFRVPLHMVQNTDRATFNNIEEL
GLGFINYSLVPYLTRIEQRINTGLVRKSKQGVYYAKFNAGALLRGDMKSRFEAYATGINW
GIYSPNDCRDLEDMNPRPGGDVYLTPMNMTTKPSDGSKAGKQKDNANADETTS
>LFGLNPFC_00522 hypothetical protein
MQTKQRLDVPLSLKSVSDSGEFEGYGSVFGVKDSHDDVVMSGAFAASLRAWSDRKALPAL
LWQHRMDEPIGVYTEMKEDDVGLYVRGRLLIDDDPLAKRAHAHMKAGSLTGLSIGYVLKD
WEYDRSKEAFLLKEIDLWEVSLVTFPSNDEARISDVKNALARGEIPEQKKIERVLRDVGL
SRTQAKAFMAGGYGALSLRDAEDVGSALNALKNLNF
>LFGLNPFC_00523 hypothetical protein
MAVDIKDVEQVAQELQQKFDDFKAKNDKRVDAIEQEKGKLAGQVETLNGKLSELENLKSD
LEKELLELKRPAGGAQNKLATEHKEAFVGFLRKGREDGLRDLERKALQVGTDEDGGYAVP
EALDRNILTLLKDEVVMRQEATVITVGGSDYKKLVNLGGTASGWVGETDARSQTATSKLG
LIEPFMGEIYGNPQATQKMLDDAFFNVEAWINSELATEFAEQEEIAFTTGDGTKKPKGFL
AYESTDETDKVRAFGKLQHIVSGEATAVTADAIIKLIYTLRKAHRTGAKFMMNNNSLFAI
RLLKDSEGNYLWRPGLELGQPSSLAGYGIAENEQMPDIAADAKAIAFGNFKRGYTIVDRI
GTRILRDPYTNKPFVGFYTTKRTGGMLVDSQAIKLLKIAAA
>LFGLNPFC_00524 hypothetical protein
MILKQDLKWSPDGMRVEVIRAGEYDDGALPARVQEIALQAGLAERGISAKSSKAAKEKKA
TTSKEG
>LFGLNPFC_00525 hypothetical protein
MLLTMEEIKAQLRLDEDFDADDRHLQLLACAAQKRTETYLNRKLYAPDETIPDSDPDGLH
LPDDIRLGMLMLISHFYENRSSVTEVEKLDMPQSFGWLVGPYRYFPQ
>LFGLNPFC_00526 hypothetical protein
MKIRQAQTSATYILPDPGELNKRVLIRLRVDMPADNFGVEPQYLITFRTWAKVIQTSATT
WQETAQTGDAITHYITIRYRRGITADYEVVCGDSVYRVKRQRDLNGARRFLLLECTELGE
CRQSHGGNNDDFLFAR
>LFGLNPFC_00527 hypothetical protein
MTTSFLHVDFQQPAEMRFNRARVRRAFVTIGQRHMRDARRLVMRRARSAPGENPGYQTGR
LARSIGYMVPRASKHRPGFMARIAPNQRNGEGNRRITGDFYPAFLFYGVRRGAKRRRSHH
RGASGGSGWRLAPRNNFMVETLEKNRSWTRYFLARELRKSLKPERRRR
>LFGLNPFC_00528 hypothetical protein
MKLTPVIAALRARCPYFENRVAGAAQFKNLPEVGKLRLPAAYVVPGDDSPGENKSQTDYW
QELKEGFSVVVILSNGRDERGQFASYDVVDDVRQMLFKALLGWNPEACGNPITYDGGTLL
DLNRHELIYQFDFSVIRELTEDDTRQQDDLNSLDELRTLAIDVDYLDPGNGSDGDIEHHT
EIPLPS
>LFGLNPFC_00529 hypothetical protein
MFVKPVKGRSVPDPARGDLLPAEGRNVDENNYWLRREAAGDIRRVNKKVNTDDDKL
>LFGLNPFC_00530 hypothetical protein
MTISFNTIPSNTLVPLFYAEMDNQAANTAQDSGASLLIGHANNGAEIVANSLVLMPSADY
ARQICGAGSQLARMVEAYRQTDPFGELYVIAVPESTGAAATVTLTVTGAATETGTVNVYV
GRTRVQVPVTNGDNVTMIASSIQDAINAVPTLPFTASSSAGVVTLTARHKGLCGNEIPVS
LNYYGFGGGEVLPAGVQIAVVTGTAGTGAPVLTGAVAAMADEPFDYIGLPFNDTASVNTL

VTEMNDTSGRWSYARQLYGHVYTAKTGTLSELVTAGDQFNQQHITLAGYEKDTQTPADEL
AASRTARAAVFIRNDPARPTQTGELVGMLPAPKGKRFTMTEQKTLLSHGVATAYVESGVL
RIQRDVTTYRKNAYGVADNSYLDSETLHTSAYVLRKLKSVITSKYGRHKLASDGTRFGPG
QAIVTPAVIKGELLATYRQLERAGIVENYELFKQYLVVERDASDPNRLNTLFPPDYVNQL
RVFAVVNQFRLQYSEESA
>LFGLNPFC_00531 hypothetical protein
MARIGGTCYFKIDGQQLSLTGGIEVPMNRTVNDDIIGLDGSVDRKETHRAPYVKGTFKVP
KNFPVSKITSSDEMTITAELANGQVYVLSSAWLHGEANHNAEEGTVDLEFHGEEGDYQ
>LFGLNPFC_00532 hypothetical protein
MKELELKKPITAHGETLSVLEFDEPTGKDVRELGYPYQMNQDESVRLLAHVVSKYIVRLA
KVPQNSVDQMSPADLNAAAWLVAGFFLQA
>LFGLNPFC_00533 hypothetical protein
MAEFELKALITGVDRLSPALSKMQKKIRGFKRQAEEASQGGLALGGGLAAGLTLSLKSYA
DQENAATGLKVAMMDANGEVGKRFQDINKLAIGLGNQLPGTTADFQNMMQMLVRQGIPAE
NILGGVGKATAYLAVQLKKTPEAAAEFAAKMQDATGTASEDMMGLFDTIQKAFYLGVDDT
NMLSFFTKTSSVLKMVNKDGLQAAQSLAPISVMMDQMGMNGESAGNALRKVIQSGLSVKK
IRDVNKVMARQKLGVQLDFTDGKGSFGGLDNMFRQLAKLRKLTDVKRTGVLKAIFGDDAE
TLQVVNALIDKGKDGYDQIQQKMNKQASLNKRVQAQLGTLSNLWEAMTGTATNGLAAIGG
AFSGDAKNITQWLGELGEKFTKFADENPRVIRGVVGLAAGLAILKLGLMGVGGAISIVSR
IMSMTPIGMIATAIALAAGLIITNWDVVGPYFKKLWETIGPYFEAGRELLKKVFAWSPLG
MVINNWGPVVKWFQDMWDKLKPIIEWFTDSSGDTVDAINSAQWGAGAYDAYGTGIPARGY
TPYQAVDPAQSNNASGATGPNPFMINKASAPKVDGEIKVSFVNSPPGMRVMETRSSGFDV
SHDVGYTRFGR
>LFGLNPFC_00534 hypothetical protein
MTWKDRLQDASFRGVPFKVEEESAGTGRRVETHEYPNRDKPYTEDLGKITFRPSITAYVV
GDDCFDQRDRLIDALNKPGPGTLVHPTYGELKVCVDGEVRVSTSKSEGRIVRFDLKFVEA
GELSYPTSGAATAQTLMSSCSALDDCISDSFSGFSIDGVADFVQNDVVGNASTMLGYVSD
AMKVVDSAVSDAARLLQGDISVLLPPPSSGKNFVEQVQKMWRTGKRLYGNASDLVTMIKT
LSGVSLGSDLQPRGVWKTDSKTTATATQQRNVVASTLRTTAISEAAYAVTRLPAPTTSAV
MQNATVGQSTTPAQSTGWPSVTHPALNNAPAVKNTVDLPTWEELTDIRDTLNTAIDKELS
RTTSDVLFLALRRVKADLNADINTRLEQSARIIQRTPDEVLPALVLAATWFDNAARDADI
IRRNAITHPGFVPVIPLKVPVQ
>LFGLNPFC_00535 hypothetical protein
MNDNVTLRVNGREWNGWTSVRIGAGIERLARDFSVEITRQWPGDEGITTLQPRIKNGSKV
EVLIGDELVITGWVEATPVRYDARSVSTGIAGRSLTADLIDCAAEPTQFNGRSLVQIAQA
LAAPFGIEVVNNGAPSGVIPDVQPDHGETVIEVINKILGQQQALAYDDPHGRLVIGGIGS
TRAHTALVLGENILSCDTEKSIRERFSVYQVAGQRAGNDDDFGEATTSALRARTEDAFIA
RYRPMYIRQTGQATGAGCIARADFEARQRAARTDETTYVVQGWRQGNGTLWQPNQRVIVF
DPVCGFDNTELLVSEVTFTQDQNGTLTEIRVGPPDAYLPEPEAPGARKKKKARVQEDPF
>LFGLNPFC_00536 hypothetical protein
MSTIEAMQRQLLGLIGRAVVKSISAATKCQTVDVSLIAGEPKAGVEHLEPYGFTARANSG
AEAVVLFPDGDRSHAVVVTVSDRRYRLKGLQTGEVAVYDDQGQSVTLTREGIVVDGAGKT
ITFRNAPEARFEMDLEVTGQVKDLCDSSGTTMSAMRLAYNGHRHRENGQGSNTDKPDKAM
EA
>LFGLNPFC_00537 hypothetical protein
MELWLTVNGKRTCASAPLDPLTRAVVISLFTWRRAEPDDNADVPMGWWGDTWPAVQNDRY
GSRLWLLQRSKLTNQLVQTVRGYIRECLQWMIDDGVVSRIDLDILRTGINELGNSITLWR
RDGPVMISLDDLWSAITHGGQ
>LFGLNPFC_00538 hypothetical protein
MADSEFQRPTLAENISMLRNDLFARLDVSDTLRRMDEDVRAKVYAAALHTVYGYIDYLAM
NMLPDLCDESWLARHAAMKRCPRKGATTASGYMRWEGVSDGLKVTAGSVIQRDDLIQYTA
TADATSAGGVLRVPIACSSAGAVGNADDGTSLILVTPVNGLPSSGVADTLTGGFDTEELE
TWRARVIERYYWTPQGGADGDYVVVWAKELPGITRAWAYRHWMGTGTVGVMIASSDLINPI
PEESTETAARQHIGPLAPVAGSDLYVFRPVAHTVDFHIRVTPDTPEIRAAITAELRSFLL
RDGYPQGELKVSRISEAISGANGEYSHQLLAPAENISIAKNELAVLGTISWA
>LFGLNPFC_00539 hypothetical protein
MGVTNDDYIRLLSALLPPGPAWSASDPAIAGAAQSLTRVHQRADALMRELDPRTTTELIN
RWERLCGLPDECIPAGTQTLRQRQQRLDAKVNLAGGINEDFYLAQLAALGRPDATITRYD
KSTFTCSSACTDAVNAPEWRYYWQVNMPAATNSTWMTCGDPCDSALRIWGDTVVECVLNK
LCPSHTYVIFKYPE
>LFGLNPFC_00540 hypothetical protein
MHRIDTKTAQKDKFGAGKNGFTRGNPQTGTPATDLDDDYFDMLQEELCSVVEASGASLEK
ARHDQLLTALRALLLSRKNPFSDIKSDGTVKTALENLGLGEGSALPVGVPVPWPSATPPT
GWLKCNGAAFSAEEYPELAKAYPTNKLPDLRGEFIRGWDDGRGVDSRRAVLSTQEPTVGT

FYVELAIISGTLSGSGAKFTDSVGIGSTSSNITVSNGNDQSVSGTVAVNPVDTRPRNIAF
NYIVRAA
>LFGLNPFC_00541 Prophage tail fiber assembly protein TfaE
MDKAILNSGLVATKAGDITVYNYDGETREYISTSSEYLAVGVGIPAYSCLDAPGIHKAGY
AICRSVNLNSWEYVPDHRGEIVYNTETGESKQITAPGDYPENTTTIAPLTPYDKWDGEKW
VTDTEAQHSAAVDAAEAQRQSLIDAAMASIGLIQLKLQAGRKLMQAETTRLNAVLDYIDA
VTATNTSTAPDVIWPELPEA
>LFGLNPFC_00542 hypothetical protein
MNSVFFSPGSKSFYLQELFPEYEDAGTLPDDVIEITRETYEQFLGLHPEGKEIGADSSGR
PVWINSPPPSKEDEVLTAEMKKISLVSEVNTYINTHQWPGKAAIGRLKGEELAQYNSWLD
YLDALELVDTSCAPDIEWPTPPAVQAR
>LFGLNPFC_00543 hypothetical protein
MPFARYFCIFINVGLGEAAKRNVGNGENQIPDMSFWTVTGGNGNFVIRQPDGLIIQMVTV
SISGPVAMNGMTDNAYAITGSNKSYIATATLPFVFPNKVLGVIPLVSTTAYGGVSSNITG
SYATAVCSFAAVRGNNTIVFKVDKPLNAAFPSDTSVSALIIGR
>LFGLNPFC_00544 DNA-invertase hin
MLIGYVRVSTNDQNTDLQRNALNCAGCELIFEDKISGTKSET
>LFGLNPFC_00545 hypothetical protein
MFFTEAFIKWIKIENHSPQENAVALIEAVLTDLKLVSIFLEAEDDAQIIFETLNGRGAEL
HATDLIRNYIFMCAEHENINAIELYENEWKSFEDKYWSEKQRRGRINKPRMEWLVHATLQ
SERQREIDLSRLYNEYRDYVSKDLSSQRADLQVKRLKQYASQYKELVDGFGTTPISHFGY
RIADYDVTTLYPLALFISIANIADDEKAAMYNDLVSYVVRRAVCGLTPKNYNNVFMNVLR
HLAKTEISSVELRNILNNLNGEASRWPGDSEFLNACINAPLYPGRLDAPKMRSMLTELER
ELCRQVKTEKPDVPNLSNLDIDHLMPQSWYSCWPLENGRMVTNSDATVLNQIVLSGTDLT
PEQLLVRKRQQAISTLGNLTLLNLSVNRSVQNAVFLKKRDALIVHTNLRLNIPLIVKDKW
DEDEILERGKKLGEIALKVWPKHD
>LFGLNPFC_00546 hypothetical protein
MKSETLTVQQLFQDRRQYCVPFYQRAYVWTQQDQWSALLEDILEKVQSRLSGTKPTPHFL
GAVVLEPQSKKGLLGVDSIHIIDGQQRLTTLQYVLASIRLALRATDLSSLEALISPCLKN
SNEDTMRNKEVERFKLWPTFRDQTHFIQSFNVENIDDLRDVFSDSFTQHGTLRKHFNHPP
SLEALCFLLKPL
>LFGLNPFC_00547 hypothetical protein
MDINEFPSGVIEHLGWYVYRLIDPRDGSTFYVGKGKGNRVFAHMRGEVAAVDDDELLNNK
LRQLREIRLAGLDVIHVIHRHGMAEEKTAYEVEAALIDAYPGLTNIMNGAGSNEFGAAHI
KELIATYQPETITFQHKALMISVNRSSKDIDLYDAVRFSWRVSVDRARKAEIILATVRGI
VRGVYIADEWLKSTRENFPEMTSWEADDEFEATQCSRFGFRGRVASPEITQLYIGKKIPD
DLRKKGAMSPVRYSPGF
>LFGLNPFC_00548 hypothetical protein
MTLPKFRNDLQVEANYSINQAREMVGKTVKSVQIGFQKTGVQVHQTEMLIITFTDDTQLA
ISTGSNVVNITSLIGRGGSCELKPADFHVDFDLTWQR
>LFGLNPFC_00549 hypothetical protein
MTNLQLEERFRALEKDYDALISTKYTAQNVLTHTMETYVDSGKYKNWIARVKKLIEDSYG
KESDYYNDFNTVNSRWSSNYNTLIKSYKPLFDAARDDLAHSAVSTNTPQEGSPLSLVLNI
LNRFPTFVRQLKRRYNGRAPLEVNDEYDVQDLIYALLTLHFNDIRAEEYTPSFAGAASRQ
DFLLKKEKIVIEVKKTRESLGAGKVGGELLIDMARYRAHQDCDTLILFVYDPDCYINNPL
GVKTDLESKDAEGKVKVVIAQF
>LFGLNPFC_00550 hypothetical protein
MALDSYASPNIIPELRQPNAAAGKITLGGLDRMAVTPSVKKRRLSMVEFPYAQTLLSDYT
AWKAL
>LFGLNPFC_00552 SsrA-binding protein
MTKKKAHKPGSATIALNKRARHEYFIEEEFEAGLALQGWEVKSLRAGKANISDSYVLLRD
GEAFLFGANITPMAVASTHVVCDPTRTRKLLLNQRELDSLYGRVNREGYTVVALSLYWKN
AWCKVKIGVAKGKKQHDKRSDIKEREWQVDKARIMKNAHR
>LFGLNPFC_00553 Persistence and stress-resistance toxin PasT
MILFVGFLLMEIVMPQISRTALVPYSAEQMYQLVNDVQSYPQFLPGCTGSRILESTPGQM
TAAVDVSKAGISKTFTTRNQLTSNQSILMSLVDGPFKKLIGGWKFTPLSQDACRIEFHLD
FEFTNKLIELAFGRVFKELAANMVQAFTVRAKEVYSAR
>LFGLNPFC_00554 Persistence and stress-resistance antitoxin PasI
MPGKIAVEVAYALPEKQYLQRVTLQEGATVEEAIRASGLLELRTDIDLTKNKVGIYSRPA
KLSDIVHDGDRVEIYRPLIADPKELRRQRAEKSANK
>LFGLNPFC_00555 Outer membrane protein assembly factor BamE
MRCKTLTAAAAVLLMLTAGCSTLERVVYRPDINQGNYLTANDVSKIRVGMTQQQVAYALG
TPLMSDPFGTNTWFYVFRQQPGHEGVTQQTLTLTFNSSGVLTNIDNKPALSGN
>LFGLNPFC_00556 DNA repair protein RecN

MLAQLTISNFAIVRELEIDFHSGMTVITGETGAGKSIAIDALGLCLGGRAEADMVRTGAA
RADLCARFSLKDTPAALRWLEENQLEDGHECLLRRVISSDGRSRGFINGTAVPLSQLREL
GQLLIQIHGQHAHQLLTKPEHQKFLLDGYANETSQLQEMTARYQLWHQSCRDLAHHQQLS
QERAARAELLQYQLKELNEFNPQPGEFEQIDEEYKRLANSGQLLTTSQNALALMADGEDA
NLQSQLYTAKQLVSELIGMDSKLSGVLDMLEEATIQIAEASDELRHYCDRLDLDPNRLFE
LEQRISKQISLARKHHVSPEALPQYYQSLLEEQQQLDDQADSQETLALAVTKHHQQALET
ARALHQQRQHYANELAQLITDSMHALSMPHGQFTIDVKFDEHHLGADGADRIEFRVTTNP
GQPMQPIAKVASGGELSRIALAIQVITARKMETPALIFDEVDVGISGPTAAVVGKLLRQL
GESTQVMCVTHLPQVAGCGHQHYFVSKETDGAMTETHMQSLDKKARLQELARLLGGSEVT
RNTLANAKELLAA
>LFGLNPFC_00557 NAD kinase
MNNHFKCIGIVGHPRHPTALTTHEMLYRWLCTKGYEVIVEQQIAHELQLKNVKTGTLAEI
GQQADLAVVVGGDGNMLGAARTLARYDIKVIGINRGNLGFLTDLDPDNAQQQLADVLEGH
YISEKRFLLEAQVCQQDCQKRISTAINEVVLHPGKVAHMIEFEVYIDEIFAFSQRSDGLI
ISTPTGSTAYSLSAGGPILTPSLDAITLVPMFPHTLSARPLVINSSSTIRLRFSHRRNDL
EISCDSQIALPIQEGEDVLIRRCDYHLNLIHPKDYSYFNTLSTKLGWSKKLF
>LFGLNPFC_00558 Protein GrpE
MSSKEQKTPEGQAPEEIIMDQHEEIEAVEPEASAEQVDPRDEKIANLEAQLAEAQTRERD
GILRVKAEMENLRRRTELDIEKAHKFALEKFINELLPVIDSLDRALEVADKANPDMSAMV
EGIELTLKSMLDVVRKFGVEVIAETNVPLDPNVHQAIAMVESDDVAPGNVLGIMQKGYTL
NGRTIRAAMVTVAKAKD
>LFGLNPFC_00559 hypothetical protein
MVVISAYFSGSETGMMTLNRYRLRHMAKQGNRSAKRVEKLLRKPDRLISLVLIGNNLVNI
LASALGTIVGMRLYGDAGVAIATGVLTFVVLVFAEVLPKTIAALYPEKVAYPSSFLLATL
QILMMPLVWLLNAITRMLMRMMGIKTDIVVSGSLSKEELRTIVHESRSQISRRNQDMLLS
VLDLEKMTVDDIMVPRSEIIGIDINDDWKSILRQLSHSPHGRIVLYRDSLDDAISMLRVR
EAWRLMSEKKEFTKETMLRAADEIYFVPEGTPLSTQLVKFQRNKKKVGLVVNEYGDIQGL
VTVEDILEEIVGDFTTSMSPTLAEEVTPQNDGSVIIDGTANVREINKAFNWHLPEDDART
VNGVILEALEEIPVAGTRVRIGEYDIDILDVQDNMIKQVKVFPVKPLRESVAE
>LFGLNPFC_00560 Inner membrane protein YpjD
MQRLEQRSPDAILLLFLIAQTVDITMPVFALLALVAYSVSLALIVPGLLQKNGGWRRMAI
ISAVIALVCHAIALEARILPDGDSGQNLSLLNVGSLVSLMICTVMTIVASRNRGWLLLPI
VYAFALINLALATFMPNEYITHLEATPGMLVHIGLSLFSYATLIIAALYALQLAWIDYQL
KNKKLAFNQEMPPLMSIERKMFHITQIGVVLLTLTLCTGLFYMHNLFSMENIDKAVLSIV
AWFVYIVLLWGHYHEGWRGRRVVWFNVAGAVILTLAYFGSRIVQQLIS
>LFGLNPFC_00561 Signal recognition particle protein
MFDNLTDRLSRTLRNISGRGRLTEDNVKDTLREVRMALLEADVALPVVREFINRVKEKAV
GHEVNKSLTPGQEFVKIVRNELVAAMGEENQTLNLAAQPPAVVLMAGLQGAGKTTSVGKL
GKFLREKHKKKVLVVSADVYRPAAIKQLETLAEQVGVDFFPSDVGQKPVDIVNAALKEAK
LKFYDVLLVDTAGRLHVDEAMMDEIKQVHASINPVETLFVVDAMTGQDAANTAKAFNEAL
PLTGVVLTKVDGDARGGAALSIRHITGKPIKFLGVGEKTEALEPFHPDRIASRILGMGDV
LSLIEDIESKVDRAQAEKLASKLKKGDGFDLNDFLEQLRQMKNMGGMASLMGKLPGMGQI
PDNVKSQMDDKVLVRMEAIINSMTMKERAKPEIIKGSRKRRIAAGCGMQVQDVNRLLKQF
DDMQRMMKKMKKGGMAKMMRSMKGMMPPGFPGR
>LFGLNPFC_00562 30S ribosomal protein S16
MVTIRLARHGAKKRPFYQVVVADSRNARNGRFIERVGFFNPIASEKEEGTRLDLDRIAHW
VGQGATISDRVAALIKEVNKAA
>LFGLNPFC_00563 Ribosome maturation factor RimM
MSKQLTAQAPVDPIVLGKMGSSYGIRGWLRVFSSTEDAESIFDYQPWFIQKAGQWQQVQL
ESWKHHNQDMIIKLKGVDDRDAANLLTNCEIVVDSSQLPQLEEGDYYWKDLMGCQVVTTE
GYDLGKVVDMMETGSNDVLVIKANLKDAFGIKERLVPFLDGQVIKKVDLTTRSIEVDWDP
GF
>LFGLNPFC_00564 tRNA (guanine-N(1)-)-methyltransferase
MWIGIISLFPEMFRAITDYGVTGRAVKNGLLSIQSWSPRDFTHDRHRTVDDRPYGGGPGM
LMMVQPLRDAIHAAKAAAGEGAKVIYLSPQGRKLDQAGVSELATNQKLILVCGRYEGIDE
RVIQTEIDEEWSIGDYVLSGGELPAMTLIDSVSRFIPGVLGHEASATEDSFAEGLLDCPH
YTRPEVLEGMEVPPVLLSGNHAEIRRWRLKQSLGRTWLRRPELLENLALTEEQARLLAEF
KTEHAQQQHKHDGMA
>LFGLNPFC_00565 50S ribosomal protein L19
MSNIIKQLEQEQMKQDVPSFRPGDTVEVKVWVVEGSKKRLQAFEGVVIAIRNRGLHSAFT
VRKISNGEGVERVFQTHSPVVDSISVKRRGAVRKAKLYYLRERTGKAARIKERLN
>LFGLNPFC_00566 Peptidoglycan-associated lipoprotein
MIKHLLAPLIFTSLILTGCQSPQGKFTPEQVAAMQSYGFTESAGDWSLGLSDAILFAKND
YKLLPESQQQIQTMAAKLASTGLTHARMDGHTDNYGEDSYNEVLSLKRANVVADAWAMGG

QIPRSNLTTQGLGKKYPIASNKTAQGRAENRRVAVVITTP
>LFGLNPFC_00567 Diguanylate cyclase DgcN
MMDNDNSLNKRPTFKRALRNISMTSIFITMMLIWLLLSVTSVLTLKQYAQKNLALTAATM
TYSLEAAVVFADGPAATETLAALGQQGQFSTAEVRDKQQNILASWHYTHKEPGDTFSNFI
SHWLFPAPIIQPIRHNGETIGEVRLTARDSSISHFIWFSLAVLTGCILLASGIAITLTRH
LHNGLVEALKNITDVVHDVRSNRNFSRRVSEERIAEFHRFALDFNSLLDEMEEWQLRLQA
KNAQLLRTALHDPLTGLANRAAFRSGINTLMNNSDARKTSALLFLDGDNFKYINDTWGHA
TGDRVLIEIAKRLAEFGGLRHKAYRLGGDEFAMVLYDVQSESEVQQICSALTQIFNLPFD
LHNGHQTTMTLSIGYAMTIEHASAENLQELADHNMYQAKHQRAEKLVR
>LFGLNPFC_00568 hypothetical protein
MRFSHRLFLLLILLLTGAPILAQEPSDVAKNVRMMVSGIVSYTRWPGLSGPPKLCIFSSS
RFSTALQENAATSLPYLPVIIHTQQEAIISGCNGFYFGNESPTFQMELTEQYPSKALLLI
AEQNTECIIGSAFCLIIHNNDVRFAVNLDALSRSGVKVNPDVLMLARKKNDG
>LFGLNPFC_00569 hypothetical protein
MKKFIAPLLALLVSGCQIDPYTHAPTLTSTDWYDVGMEDAISGSAIKDDDAFSDSQADRG
LYLKGYAEGQKKTCQTDFTYARGLSGKSFPASCNNVENASQLHEVWRKGADENASTIRLN
>LFGLNPFC_00570 Phospho-2-dehydro-3-deoxyheptonate aldolase, Tyr-sensitive
MQKDALNNVHITAEQVLMTPEQLKAAFPLSLQQEAQIADSRKTISDIIAGRDPRLLVVCG
PCSIHDPETALEYARRFKTLAAEVSDSLYLVMRVYFEKPRTTVGWKGLINDPHMDGSFDV
EAGLQIARKLLLELVNMGLPLATEALDPNSPQYLGDLFSWSAIGARTTESQTHREMASGL
SMPVGFKNGTDGSLATAINAMRAAAQPHRFVGINQAGQVALLQTQGNPDGHVILRGGKAP
NYSPADVAQCEKEMEQAGLRPSLMVDCSHGNSNKDYRRQPAVAESVVAQIKDGNRSIIGL
MIESNIHEGNQSSEQPRSEMKYGVSVTDACISWEMTDALLREIHQDLNGQLTARVA
>LFGLNPFC_00571 T-protein
MVAELTALRDQIDEVDKALLNLLAKRLELVAEVGEVKSRFGLPIYVPEREASMLASRRAE
AEALGVPPDLIEDVLRRVMRESYSSENDKGFKTLCPSLRPVVIVGGGGQMGRLFEKMLTL
SGYQVRILEQHDWDRAADIVSDAGMVIVSVPIHVTEQVIGKLPPLPKDCILVDLASVKNG
PLQAMLAAHDGPVLGLHPMFGPDSGSLAKQVVVWCDGRKPEAYQWFLEQIQVWGARLHRI
SAVEHDQNMAFIQALRHFATFAYGLHLAEENVQLEQLLALSSPIYRLELAMVGRLFAQDP
QLYADIIMSSERNLALIKRYYKRFGEAIELLEQGDKQAFIDSFRKVEHWFGDYAQRFQSE
SRVLLRQANDNRQ
>LFGLNPFC_00572 Bifunctional chorismate mutase/prephenate dehydratase
MTSENPLLALREKISALDEKLLALLAERRELAVEVGKAKLLSHRPVRDIDRERDLLERLI
TLGKAHHLDAHYITRLFQLIIEDSVLTQQALLQQHLNKINPHSARIAFLGPKGSYSHLAA
RQYAARHFEQFIESGCAKFADIFNQVETGQADYAVVPIENTSSGAINDVYDLLQHTSLSI
VGEMTLTIDHCLLVSGTTDLSAINTVYSHPQPFQQCSKFLNRYPHWKIEYTESTSAAMEK
VAQAKSPHVAALGSEAGGTLYGLQVLERIEANQRQNFTRFVVLARKAINVSDQVPAKTTL
LMATGQQAGALVEALLVLRNHSLIMTRLESRPIHGNPWEEMFYLDIQANLESAEMQKALK
ELGEITRSMKVLGCYPSENVVPVDPT
>LFGLNPFC_00573 Ribosome-associated inhibitor A
MTMNITSKQMEITPAIRQHVADRLAKLEKWQTHLINPHIILSKEPQGFVADATINTPNGV
LVASGKHEDMYTAINELINKLERQLNKLQHKGEARRAATSVKDANFVEEVEEE
>LFGLNPFC_00574 Outer membrane protein assembly factor BamD
MTRMKYLVAAATLSLFLAGCSGSKEEVPDNPPNEIYATAQQKLQDGNWRQAITQLEALDN
RYPFGPYSQQVQLDLIYAYYKNADLPLAQAAIDRFIRLNPTHPNIDYVMYMRGLTNMALD
DSALQGFFGVDRSDRDPQHARAAFSDFSKLVRGYPNSQYTTDATKRLVFLKDRLAKYEYS
VAEYYTERGAWVAVVNRVEGMLRDYPDTQATRDALPLMENAYRQMQMNAQAEKVAKIIAA
NSSNT
>LFGLNPFC_00575 Ribosomal large subunit pseudouridine synthase D
MAQRVQLTATVSENQLGQRLDQALAEMFPDYSRSRIKEWILDQRVLVNGKVCDKPKEKVL
GGEQVAINAEIEEEARFEPQDIPLDIVYEDEDIIVINKPRDLVVHPGAGNPDGTVLNALL
HYYPPIADVPRAGIVHRLDKDTTGLMVVAKTVPAQTRLVESLQRREITREYEAVAIGHMT
AGGTVDEPISRHPTKRTHMAVHPMGKPAVTHYRIMEHFRVHTRLRLRLETGRTHQIRVHM
AHITHPLVGDPVYGGRPRPPKGASEAFISTLRKFDRQSLHATMLRLYHPISGIEMEWHAP
IPQDMVELIEVMRADFEEHKDEVDWL
>LFGLNPFC_00576 Polyphenol oxidase
MSKLIVPQWPLPKGVAACSSTRIGGVSLPPYDSLNLGAHCGDNPDHVEENRKRLFAAGNL
PSKPVWLEQVHGKDVLKLIGEPYASKRADASYSNTPGTVCAVMTADCLPVLFCNRAGTEV
AAAHAGWRGLCAGVLEETVSCFADNPENILAWLGPAIGPRAFEVGAEVREAFMAVDAEAS
TAFIQHGDKYLADIYQLARQRLASVGVEQIFGGDRCTYTENETFFSYRRDKTTGRMASFI
WLI
>LFGLNPFC_00577 Chaperone protein ClpB
MRLDRLTNKFQLALADAQSLALGHDNQFIEPLHLMSALLNQEGGSVSPLLTSAGINAGQL
RTDINQALNRLPQVEGTGGDVQPSQDLVRVLNLCDKLAQKRGDNFISSELFVLAALESRG

TLADILKAAGATTANITQAIEQMRGGESVNDQGAEDQRQALKKYTIDLTERAEQGKLDPV
IGRDEEIRRTIQVLQRRTKNNPVLIGEPGVGKTAIVEGLAQRIINGEVPEGLKGRRVLAL
DMGALVAGAKYRGEFEERLKGVLNDLAKQEGNVILFIDELHTMVGAGKADGAMDAGNMLK
PALARGELHCVGATTLDEYRQYIEKDAALERRFQKVFVAEPSVEDTIAILRGLKERYELH
HHVQITDPAIVAAATLSHRYIADRQLPDKAIDLIDEAASSIRMQIDSKPEELDRLDRRII
QLKLEQQALMKESDEASKKRLDMLNEELSDKERQYSELEEEWKAEKASLSGTQTIKAELE
QAKIAIEQARRVGDLARMSELQYGKIPELEKQLEAATQLEGKTMRLLRNKVTDAEIAEVL
ARWTGIPVSRMMESEREKLLRMEQELHHRVIGQNEAVDAVSNAIRRSRAGLADPNRPIGS
FLFLGPTGVGKTELCKALANFMFDSDEAMVRIDMSEFMEKHSVSRLVGAPPGYVGYEEGG
YLTEAVRRRPYSVILLDEVEKAHPDVFNILLQVLDDGRLTDGQGRTVDFRNTVVIMTSNL
GSDLIQERFGELDYAHMKELVLGVVSHNFRPEFINRIDEVVVFHPLGEQHIASIAQIQLK
RLYKRLEERGYEIHISDEVLKLLSENGYDPVYGARPLKRAIQQQIENPLAQQILSGELVP
GKVIRLEVNEDRIVAVQ
>LFGLNPFC_00582 hypothetical protein
MKHSFEIKLAAVNHYLAGHAGIISTAKLFQLSHTSLSHWINLFLLHGPRHWIADTSVAIL
LKINFAWFFMLSGILSLYPG
>LFGLNPFC_00583 hypothetical protein
MTRSDDTHENEANMTPEEMKNELRYLRAENAYLKAMQEHLLEKKRQELEKKRKSSRA
>LFGLNPFC_00584 IS3 family transposase IS1397
MQQLGLKSPVRLKKYRSYRGNMGLAAENILQRQFKAEAPCEKWVTDITEFRAGGQKLYLS
PILDLFNGEIVAWETACRPTEELVKRMLNKGLESLAEGEKPLLHSDQGWHYRIKSYQSAL
ADRGLVQSMSRKGNCLDNAVMENFFGHLKEEMYYRRDYRNVEELENAVNEYITYWNQKRI
KLSLGGLSPVEYRTEYQKAG
>LFGLNPFC_00585 hypothetical protein
MEHELHYIGIDTAKEKLDVDVLRPDGRHRTKKFANTTKGHDELVSWLKCHKIDHAHICIE
ATGTYMEPVAECLYDAGYIVSVINPALGKAFAQSEGLRNKTDTVDAACWQSSVVRSALQP
GKRITRLNARCMPW
>LFGLNPFC_00586 IS110 family transposase IS621
MHRQELNRTETAREVQRPSIDAHLLWLEAELKRLEKQIKDLTDDDPDMKHRRKLLESIPG
IGEKTSAVLLAYMV
>LFGLNPFC_00587 IS110 family transposase IS621
MPAMVATSKTEWGRALAANGKKGKVILGSIMRKLAQVAYGVLKSGVPFDASRHNPVAA
>LFGLNPFC_00588 Alpha-ketoglutarate permease
MAESTVTADSKLTSSDTRRRIWAIVGASSGNLIEWFDFYVYSFCSLYFAHIFFPSGNTTT
QLLQTAGVFAAGFLMRPIGGWLFGRIADKHGRKKSMLLSVCMMCFGSLVIACLPGYETIG
TWAPALLLLARLFQGLSVGGEYGTSATYMSEVAVEGRKGFYASFQYVTLIGGQLLALLVV
VVLQHTMEDAVLREWGWRIPFALGAVLAVVALWLRRQLDETSQQETRALKEAGSLKGLWR
NRRAFIMVLGFTAAASLCFYTFTTYMQKYLVNTAGMHANVASGIMTAALFVFMLIQPLIG
ALSDKIGRRTSMLCFGSLATIFTVPILSALQNVSSPYAAFGLVMCALLIVSFYTSISGIL
KAEMFPAQVRALGVGLSYAVANAIFGGSAEYVALSLKSIGMETAFFWYVTLMAVVAFLVS
LTLHRKGKGMRL
>LFGLNPFC_00589 hypothetical protein
MANDSWSGQDKAQHFIASAMLSAAGNEYSQHQGMSRDRSAMFGLMFSVSLGASKELWDSR
PEGSGWSWKDFAWDIAGASTGYTVWQLTRH
>LFGLNPFC_00590 CDP-diacylglycerol--serine O-phosphatidyltransferase
MLSKFKRNKHQQHLAQLPKISQSVDDVDFFYAPADFRETLLEKIASAKQRICIVALYLEQ
DDGGKGILNALYEAKRQRPELDVRVLVDWHRAQRGRIGAAASNTNADWYCRMAQENPGVD
VPVYGVPINTREALGVLHFKGFIIDDSVLYSGASLNDVYLHQHDKYRYDRYHLIRNRKMS
DIMFEWVTQNIMNGRGVNRLDDVNRPKSPEIKNDIRLFRQELRDAAYHFQGDADNDQLSV
TPLVGLGKSSLLNKTIFHLMPCAEQKLTICTPYFNLPAILVRNIIQLLREGKKVEIIVGD
KTANDFYIPEDEPFKIIGALPYLYEINLRRFLSRLQYYVNTDQLVVRLWKDDDNTYHLKG
MWVDDKWMLITGNNLNPRAWRLDLENAILIHDPQLELAPQREKELELIREHTTIVKHYRD
LQSIADYPVKVRKLIRRLRRIRIDRLISRIL
>LFGLNPFC_00591 Peptidyl-lysine N-acetyltransferase PatZ
MSQRGLEALLRPKSIAVIGASMKPNRAGYLMMRNLLAGGFNGPVLPVTPAWKAVLGVLAW
PDIARLPFTPDLAVLCTNASRNLALLEELGEKGCKTCIILSAPASQHEDLRACALRHNMR
LLGPNSLGLLAPWQGLNASFSPVPIKRGKLAFISQSAAVSNTILDWAQQREMGFSYFIAL
GDSLDIDVDELLDYLARDSKTSAILLYLEQLSDARRFVSAARSASRNKPILVIKSGRSPA
AQRLLNTTAGMDPAWDAAIQRAGLLRVQDTHELFSAVETLSHMRPLRGDRLMIISNGAAP
AALALDALWSRNGKLATLSEETCQKLRDALPGHVAISNPLDLRDDASSEHYVKTLDILLH
SQDFDALMVIHSPSAAAPATESAQVLIEAVKHHPRSKYVSLLTNWCGEHSSQEARRLFSE
AGLPTYRTPEGTITAFMHMVEYRRNQKQLRETPALPSNLTSNTAEAHLLLQHAIAEGATS
LDTHEVQPILQAYGMNTLPTWIASNSTEAVHIAKQIGYPVALKLRSPDIPHKSEVQGVML
YLRTANEVQQAANAIFDRVKMAWPQARVHGLLVQSMANRAGAQELRVVVEHDPVFGPLIM

LGEGGVEWRPEDQAVVALPPLNMNLARYLVIQGIKSKKIRARSALRPLDVAGLSQLLVQV
SNLIVDCPEIQRLDIHPLLASGSEFTALDVTLDIAPFEGDNESRLAVRPYPHQLEEWVEL
KNGERCLFRPILPEDEPQLQQFISRVTKEDLYYRYFSEINEFTHEDLANMTQIDYDREMA
FVAVRRIDQTEEILGVIRAISDPDNIDAEFAVLVRSDLKGLGLGRRLMEKLITYTRDHGL
QRLNGITMPNNRGMVALARKLGFNVDIQLEEGIVGLTLNLAQREES
>LFGLNPFC_00592 hypothetical protein
MTENAVLQLRAERIARATRPFLARGNRVRRCQRCLLPEKLCLCSTITPAQAKSRFCLLMF
DTEPMKPSNTGRLIADILPDTVAFQWSRTEPSQDLLDLVQNPDYQPMVVFPASYADEQRE
VIFTPPAGKPPLFIMLDGTWPEARKMFRKSPYLDNLPVISVDLSRLSAYRLREAQAEGQY
CTAEVAIALLDMAGDTRAATGLGEHFTRFKTRYLAGKTQHPGSITAEQLESV
>LFGLNPFC_00593 Thioredoxin 2
MNTVCTHCQAINRIPDDRIEDAAKCGRCGHDLFDGEVINATGETLDKLLKDDLPVVIDFW
APWCGPCRNFAPIFEDVAQERSGKVRFVKVNTEAERELSSRFGIRSIPTIMIFKNGQVVD
MLNGAVPKAPFDSWLNESL
>LFGLNPFC_00594 putative tRNA/rRNA methyltransferase YfiF
MNDEMKGKSGKVKVMYVRSDDDSDKRTHNPRTGKGGGRPGKSRADGGRRPARDDKQSQHR
DRKWEDSPWRTVSRAPGDETPEKADHGGISGKSFIDPEVLRRQRAEETRVYGENACQALF
QSRPEAIVRAWFIQSVTPRFKEALRWMAANRKAYHVVDEAELTKASGTEHHGGVCFLIKK
RNGTTVQQWVSQAGAQDCVLALENESNPHNLGGMMRSCAHFGVKGVVVQDAALLESGAAI
RTAEGGAEYVQPITGDNIVNVLDDFRQAGYTVVTTSSEQGKPLFKTSLPAKMVLVLGQEY
EGLPDAARDPNDLRVKIDGTGNVAGLNISVATGVLLGEWWRQNKA
>LFGLNPFC_00595 Uracil-DNA glycosylase
MANELTWHDVLAEEKQQPYFLNTLQTVASERQSGVTIYPPQKDVFNAFRFTELGDVKVVI
LGQDPYHGPGQAHGLAFSVRPGIATPPSLLNMYKELENTIPGFTRPNHGYLESWARQGVL
LLNTVLTVRAGQAHSHASLGWETFTDKVISLINQHREGVVFLLWGSHAQKKGAIIDKQRH
HVLKAPHPSPLSAHRGFFGCNHFVLANQWLEQRGETPIDWMPVLPAESE
>LFGLNPFC_00596 Autonomous glycyl radical cofactor
MITGIQITKAANDDLLNSFWLLDSEKGEARCIVAKAGFAEDEVVAVSKLGDIEYREVPVE
VKPEVRVEGGQHLNVNVLRRETLEDAVKHPEKYPQLTIRVSGYAVRFNSLTPEQQRDVIA
RTFTESL
>LFGLNPFC_00597 Cysteine/O-acetylserine efflux protein
MTPTLLSAFWTYTLITAMTPGPNNILALSSATSHGFRQSTRVLAGMSLGFLIVMLLCAGI
SFSLAVIDPAAVHLLSWAGAAYIVWLAWKIATSPTKEDGLQTKPISFWASFALQFVNVKI
ILYGVTALSTFVLPQTQALSWVVGVSVLLAMIGTFGNVCWALAGHLFQRLFRQYGRQLNI
VLALLLVYCAVRIFY
>LFGLNPFC_00598 HTH-type transcriptional activator CmpR
MDLRRFITLKTVVEEGSFLRASQKLCCTQSTVTFHIQQLEQEFSVQLFEKIGRRMCLTRE
GKKLLPHIYELTRVMDTLREAAKKESDPDGELRVISGETLLSYRMPQVLQRFRQRAPKVR
LSLQSLNCYVIRDALLNDEADVGVFYRVGNDDALNRRELGEQPLALVASPQIADVDFTEP
GRHNACSFIINEPQCVFRQIFESTLRQRRITVENTIELISIESIKRCVAANIGVSYLPRF
AVVKELKCGELIDLPFGEQSQTITAMCAHHAGKAVSPAMHTFIQCVEECFLPG
>LFGLNPFC_00599 ATP-dependent RNA helicase SrmB
MTVTTFSELELDESLLEALQDKGFTRPTAIQAAAIPPALDGRDVLGSAPTGTGKTAAYLL
PALQHLLDFPRKKSGPPRILILTPTRELAMQVADHARELAKHTHLDIATITGGVAYMNHA
EVFSENQDIVVATTGRLLQYIKEENFDCRAVETLILDEADRMLDMGFAQDIEHIAGETRW
RKQTLLFSATLEGDAIQDFAERLLEDPVEVSANPSTRERKKIHQWYYRADDLEHKTALLV
HLLKQPEATRSIVFVRKRERVHELANWLREAGINNCYLEGEMVQGKRNEAIKRLTEGRVN
VLVATDVAARGIDIPDVSHVFNFDMPRSGDTYLHRIGRTARAGRKGTAISLVEAHDHLLL
GKVGRYIEEPIKARVIDELRPKTRAPSEKQTGKPSKKVLAKRAEKKKAKEKEKPRVKKRH
RDTKNIGKRRKPSGTGVPPQTTEE
>LFGLNPFC_00600 tRNA1(Val) (adenine(37)-N6)-methyltransferase
MSQSTSVFRRNGFTFKQFFVAHDRCAMKVGTDGILLGAWAPVAGVKRCLDIGAGSGLLAL
MLAQRTDDSVMIDAVELESEAAAQAQENINQSPWAERINVHTADILQWITQQTVRFDLII
SNPPYYQQGVECATPQREQARYTTTLDHPSLLTCAAECITEEGFFCVVLPEQIGNGFTEL
ALSMGWHLRLRTDVAENEARLPHRVLLAFSPQAGECFSDRLVIRGPDQNYSEAYTALTQA
FYLFM
>LFGLNPFC_00601 L-aspartate oxidase
MNTLPEHSCDVLIIGSGAAGLSLALRLVDQHQVIVLSKGPVTEGSTFYAQGGIAAVFDET
DSIDSHVEDTLIAGAGICDRHAVEFVASNARSCVQWLIDQGVLFDTHIQPNGEESYHLTR
EGGHSHRRILHAADATGREVETTLVGKAQNHPNIRVLERSNAVDLIVSDKIGLPGTRWVV
GAWVWNRNKETVETCHAKAVVLATGGASKVYQYTTNPDISSGDGIAMAWRAGCRVANLEF
NQFHPTALYHPQARNFLLTEALRGEGAYLKRPDGTRFMPDFDERGELAPRDIVARAIDHE
MKRLGADCMFLDISHKPADFIRQHFPMIYEKLLGLGIDLTKEPVPIVPAAHYTCGGVMVD
DHGRTDVDGLYAIGEVSYTGLHGANRMASNSLLECLVYGWSAAEDISRRIPYAHGVSTLP

PWDESRVENPDERVVIQHNWHELRLFMWDYVGIVRTTKRLARALRRITMLQQEIDEYYAH
FRVSNNLLELRNLVQVAELIVRCAMMRKESRGLHFTLDYPELLTHSGPSILSAGNHYINR
>LFGLNPFC_00602 ECF RNA polymerase sigma-E factor
MSEQLTDQVLVERVQKGDQKAFNLLVVRYQHKVASLVSRYVPSGDVPDVVQEAFIKAYRA
LDSFRGDSAFYTWLYRIAVNTAKNYLVAQGRRPPSSDVDAIEAENFESGGALKEISNPEN
LMLSEELRQIVFRTIESLPEDLRMAITLRELDGLSYEEIAAIMDCPVGTVRSRIFRAREA
IDNKVQPLIRR
>LFGLNPFC_00603 Anti-sigma-E factor RseA
MQKEQLSALMDGETLDSELLNELAHNPEMQKTWESYHLIRDSMRGDTPEVLHFDISSRVM
AAIEEEPVRQPATLIPEAQPAPHQWQKMPFWQKVRPWAAQLTQMGVAACVSLAVIVGVQH
YNGQSETSQQPETPVFNTLPMMGKASPVSLGVPSEATANNGQQQQVQEQRRRINAMLQDY
ELQRRLHSEQLQFEQAQTQQAAVQVPGIQTLGTQSQ
>LFGLNPFC_00604 Sigma-E factor regulatory protein RseB
MKQLWFAMSLVTGSLLFSANASATPASGALLQQMNLASQSLNYELSFISINKQGVESLRY
RHARLDNRPLAQLLQMDGPRREVVQRGNEISYFEPGLEPFTLNGDYIVDSLPSLIYTDFK
RLSPYYDFISVGRTRIADRLCEVIRVVARDGTRYSYIVWMDTESKLPMRVDLLDRDGETL
EQFRVIAFNVNQDISSSMQTLAKANLPPLLSVPVGEKAKFSWTPTWLPQGFSEVSSSRRP
LPTMDNMPIESRLYSDGLFSFSVNVNRATPSSSDQMLRTGRRTVSTSVRDNAEITIVGEL
PPQTAKRIAENIKFGAAQ
>LFGLNPFC_00605 Protein RseC
MIKEWATVVSWQNGQALVSCDVKASCSSCASRAGCGSRVLNKLGPQTTHTIVVPCDEPLV
PGQKVELGIAEGSLLSSALLVYMSPLVGLFLIASLFQLLFASDVAALCGAILGGFGGFLI
ARGYSRKFAARAEWQPIILSVALPPGLVRFETSSEDASQ
>LFGLNPFC_00606 Elongation factor 4
MKNIRNFSIIAHIDHGKSTLSDRIIQICGGLSDREMEAQVLDSMDLERERGITIKAQSVT
LDYKASDGETYQLNFIDTPGHVDFSYEVSRSLAACEGALLVVDAGQGVEAQTLANCYTAM
EMDLEVVPVLNKIDLPAADPERVAEEIEDIVGIDATDAVRCSAKTGVGVQDVLERLVRDI
PPPEGDPEGPLQALIIDSWFDNYLGVVSLIRIKNGTLRKGDKVKVMSTGQTYNADRLGIF
TPKQVDRTELKCGEVGWLVCAIKDIHGAPVGDTLTLARNPAEKALPGFKKVKPQVYAGLF
PVSSDDYEAFRDALGKLSLNDASLFYEPESSSALGFGFRCGFLGLLHMEIIQERLEREYD
LDLITTAPTVVYEVETTSREVIYVDSPSKLPAVNNIYELREPIAECHMLLPQAYLGNVIT
LCVEKRGVQTNMVYHGNQVALTYEIPMAEVVLDFFDRLKSTSRGYASLDYNFKRFQASDM
VRVDVLINGERVDALALITHRGNSQNRGRELVEKMKDLIPRQQFDIAIQAAIGTHIIARS
TVKQLRKNVLAKCYGGDISRKKKLLQKQKEGKKRMKQIGNVELPQEAFLAILHVGKDNK
>LFGLNPFC_00607 Signal peptidase I
MANMFALILVIATLVTGILWCVDKFFFAPKRRERQAAAQAAAGDSLDKATLKKVAPKPGW
LETGASVFFPVLAIVLIVRSFIYEPFQIPSGSMMPTLLIGDFILVEKFAYGIKDPIYQKTL
IETGHPKRGDIVVFKYPEDPKLDYIKRAVGLPGDKVTYDPVSKELTIQPGCSSGQACENA
LPVTYSNVEPSDFVQTFSRRNGGEATSGFFEVPKNETKENGIRLSERKETLGDVTHRILT
VPIAQDQVGMYYQQPGQQLATWIVPPGQYFMMGDNRDNSADSRYWGFVPEANLVGRATAI
WMSFDKQEGEWPTGVRLSRIGGIH
>LFGLNPFC_00608 Ribonuclease 3
MNPIVINRLQRKLGYTFNHQELLQQALTHRSASSKHNERLEFLGDSILSYVIANALYHRF
PRVDEGDMSRMRATLVRGNTLAELAREFELGECLRLGPGELKSGGFRRESILADTVEALI
GGVFLDSDIQTVEKLILNWYQTRLDEISPGDKQKDPKTRLQEYLQGRHLPLPTYLVVQVR
GEAHDQEFTIHCQVSGLSEPVVGTGSSRRKAEQAAAEQALKKLELE
>LFGLNPFC_00609 GTPase Era
MSIDKSYCGFIAIVGRPNVGKSTLLNKLLGQKISITSRKAQTTRHRIVGIHTEGAYQAIY
VDTPGLHMEEKRAINRLMNKAASSSIGDVELVIFVVEGTRWTPDDEMVLNKLRDGKAPVI
LAVNKVDNVQEKADLLPHLQFLASQMNFLDIVPISAETGLNVDTIAAIVRKHLPEATHHF
PEDYITDRSQRFMASEIIREKLMRFLGAELPYSVTVEIERFVSNERGGYDINGLILVERE
GQKKMVIGNKGAKIKTIGIEARKDMQEMFEAPVHLELWVKVKSGWADDERALRSLGYVDD
L
>LFGLNPFC_00610 DNA repair protein RecO
MEGWQRAFVLHSRPWSETSLMLDVFTEESGRVRLVAKGARSKRSTLKGALQPFTPLLLRF
GGRGEVKTLRSAEAVSLALPLSGITLYSGLYINELLSRVLEYETRFSELFFDYLHCIQSL
AGVTGTPEPALRRFELALLGHLGYGVNFTHCAGSGEPVDDTMTYRYREEKGFIASVVIDN
KTFTGRQLKALNAREFPDADTLRAAKRFTRMALKPYLGGKPLKSRELFRQFMPKRTVKTH
YE
>LFGLNPFC_00611 Pyridoxine 5'-phosphate synthase
MAELLLGVNIDHIATLRNARGTAYPDPVQAAFIAEQAGADGITVHLREDRRHITDRDVCI
LRQTLDTRMNLEMAVTEEMLAIAVETKPHFCCLVPEKRQEVTTEGGLDVAGQREKMRDAC
KRLADAGIQVSLFIDADEEQIKAAAEVGAPFIEIHTGCYADAKTDAEQAQELARIAKAAT
FATSLGLKVNAGHGLTYHNVKAIAAIPEMHELNIGHAIIGRAVMTGLKDAVAEMKRLMLE

ARG
>LFGLNPFC_00612 Holo-[acyl-carrier-protein] synthase
MAILGLGTDIVEIARIEAVIARSGERLARRVLSDNEWEIWKTHHQPVRFLAKRFAVKEAA
AKAFGTGIRNGLAFNQFEVFNDELGKPRLRLWGEALKLAEKLGVVNMHVTLADERHYACA
TVIIES
>LFGLNPFC_00613 Ferredoxin YfhL
MALLITKKCINCDMCEPECPNEAISMGEHIYEINSDKCTECVGHYETPTCQKVCPIPNTI
VKDPQHVETEEQLWDKFVLMHHADKI
>LFGLNPFC_00614 HTH-type transcriptional regulator MurR
MNGLLRIRQRYQGLAQSDKKLADYLLLQPDTARHLSSQQLANEAGVSQSSVVKFAQKLGY
KGFPALKLALSEALASQPESPSVPIHNQIRGDDPLRLVGEKLIKENTAAMYATLNVNTEE
KLHECVTMLRSARRIILTGIGASGLVAQNFAWKLMKIGFNAAAVRDMHALLATVQASSPD
DLLLAISYTGVRRELNLAADEMLRVGGKVLAITGFTPNALQQRASHCLYTIAEEQATNSA
SISACHAQGMLTDLLFIALIQQDLELAPERIRHSEALVKKLV
>LFGLNPFC_00615 Phosphatidylglycerophosphatase C
MATHERRVVFFDLDGTLHQQDMFGSFLRYLLRRQPLNALLVLPLLPIIAIALLIKGRAAR
WPMSLLLWGCTFGHNEARLQALQADFVRWFRDNVTAFPLVQERLTTYLLSSDADIWLITG
SPQPLVEAVYFDTPWLPRVNLIASQIQRGYGGWVLTMRCLGHEKVAQLERKIGTPLRLYS
GYSDSNQDNPLLYFCQHRWRVTPRGELQQLE
>LFGLNPFC_00616 tRNA-specific adenosine deaminase
MSEVEFSHEYWMRHAMTLAKRAWDEREVPVGAVLVHNNRVIGEGWNRPIGRHDPTAHAEI
MALRQGGLVMQNYRLIDATLYVTLEPCVMCAGAMIHSRIGRVVFGARDAKTGAAGSLMDV
LHHPGMNHRVEITEGILADECAALLSDFFRMRRQEIKAQKKAQSSTD
>LFGLNPFC_00617 Membrane-bound lytic murein transglycosylase F
MKKLKINYLFIGILALLLAVALWPSIPWFGKADNRIAAIQARGELRVSTIHTPLTYNEIN
GKPFGLDYELAKQFADYLGVKLKVTVRQNISQLFDDLDNGNADLLAAGLVYNSERVKNYQ
PGPTYYSVSQQLVYKVGQYRPRTLGNLTAEQLTVAPGHVVVNDLQTLKDTKFPELSWKVD
DKKGSAELMEDVIEGKLDYTIADSVAISLFQRVHPELAVALDITDEQPVTWFSPLDGDNT
LSAALLDFFNEMNEDGTLARIEEKYLGHGDDFDYVDTRTFLRAVDAVLPQLKPLFEKYAE
EIDWRLLAAIAYQESHWDAQATSPTGVRGMMMLTKNTAQSLGITDRTDAEQSISGGVRYL
QDMMSKVPESVPENERIWFALAAYNMGYAHMLDARALTAKTKGNPDSWADVKQRLPLLSQ
KPYYSKLTYGYARGHEAYAYVENIRKYQISLVGYLQEKEKQATEAAMQLAQDYPAVSPTE
LGKEKFPFLSFLSQSSSNYLTHSPSLLFSRKGSEEKQN
>LFGLNPFC_00618 Phosphoribosylformylglycinamidine synthase
MATYYSHWRHALLIHDGDFISTQTVSSAHQILYNDARFPPLGYTESLEDERLMMEILRGS
PALSAFRINKLLARFQAARLLVHTIYAEYVHFADLNAPLNDDEHAQLERLLKYGPALASH
APQGKLLLVTPRPGTISPWSSKATDIAHNCGLQQVNRLERGVAYYIEAGTLTNEQWQQVT
AELHDRMMETVFFALDDAEQLFAHHQPTPVTSVDLLGQGRQALIDANLRLGLALAEDEID
YLQDAFTRLGRNPNDIELYMFAQANSEHCRHKIFNADWVIDGEQQPKSLFKMIKNTFETT
PDHVLSAYKDNAAVMEGSEVGRYFADHETGRYDFHQEPAHILMKVETHNHPTAISPWPGA
ATGSGGEIRDEGATGRGAKPKAGLVGFSVSNLRIPGFEQPWEEDFGKPERIVTALDIMTE
GPLGGAAFNNEFGRPALNGYFRTYEEKVNSHNGEELRGYHKPIMLAGGIGNIRADHVQKG
EINVGAKLVVLGGPAMNIGLGGGAASSMASGQSDADLDFASVQRDNPEMERRCQEVIDRC
WQLGDANPILFIHDVGAGGLSNAMPELVSDGGRGGKFELRDILSDEPGMSPLEIWCNESQ
ERYVLAVAADQLPLFDELCKRERAPYAVIGEATEELHLSLHDRHFDNQPIDLPLDVLLGK
TPKMTRDVQTLKAKGDALAREGITIADAVKRVLHLPTVAEKTFLVTIGDRSVTGMVARDQ
MVGPWQVPVANCAVTTASLDSYYGEAMAIGERAPVALLDFAASARLAVGEALTNIAATQI
GDIKRIKLSANWMAAAGHPGEDAGLYEAVKAVGEELCPALGLTIPVGKDSMSMKTRWQEG
NEEREMTSPLSLVISAFARVEDVRHTITPQLSTEDNALLLIDLGKGNNALGATALAQVYR
QLGDKPADVRDVAQLKGFYDAIQALVAQRKLLAYHDRSDGGLLVTLAEMAFAGHCGIDAD
IATLGDDRLAALFNEELGAVIQVRAADREAVEAVLAQHGLADCVHYVGQAVSGDRFVITA
NGQTVFSESRTTLRVWWAETTWQMQRLRDNPECADQEHQAKSNDADPGLNVKLSFDINED
VAAPFIATGARPKVAVLREQGVNSHVEMAAAFHRAGFDAIDVHMSDLLAGRTGLEGFHVL
VACGGFSYGDVLGAGEGWAKSILFNDRVRDEFATFFHRPQTLALGVCNGCQMMSNLRELI
PGSELWPRFVRNTSDRFEARFSLVEVTQSPSLLLQGMVGSQMPIAVSHGEGRVEVRDAAH
LAALESKGLVALRYVDNFGKVTETYPANPNGSPNGITAVTTESGRVTIMMPHPERVFRTV
SNSWHPENWGEDGPWMRIFRNARKQLG
>LFGLNPFC_00619 Sensor histidine kinase QseE
MLAFLLILLPLLVLAWQAWQSLNALSDQAALVNRTTLIDARRSEAMTNAALEMERSYRQY
CVLDDPTLAKVYQSQRKRYSEMLDAHAGVLPDDKLYQALRQDLNNLAQLQCNNSGPDAAA
AARLEAFASANTEMVQATRTVVFSRGQQLQREIAERGQYFGWQSLVLFLVSLVMVLLFTR
MIIGPVKNIERMINRLGEGRSLGNSVSFSGPSELRSVGQRILWLSERLSWLESQRHQFLR
HLSHELKTPLASMREGTELLADQVVGPLTPEQKEVVSILDSSSRNLQKLIEQLLDYNRKQ
ADSAVELENVELAPLVETVVSAHSLPARAKMMHTDVDLKATACLAEPMLLMSVLDNLYSN

AVHYGAESGNICLRSSLHGARVYIDVINTGTPIPQEERAMIFEPFFQGSHQRKGAVKGSG
LGLSIARDCIRRMQGELYLVDESGQDVCFRIELPSSKNTK
>LFGLNPFC_00620 Quorum-sensing regulator protein G
MRHIFQRLLPRRLWLAGLPCLALLGCVQSHNKPAIDTPAEEKIPVYQLADYLSTECSDIW
ALQGKSTETNPLYWLRAMDCADRLMPAQSRQQARQYDDGNWQNTFKQGILLADAKITPYE
RRQLVARIDALSTEIPAQVRPLYQLWRDGQALQLQLAEERQRYSKLQQSSDSELDTLRQQ
HHVLQQQLELTTRKLENLTDIERQLSTRKPAGNFSPDTPHESEKPAPSTDEVTPDEP
>LFGLNPFC_00621 Transcriptional regulatory protein QseF
MSHKPAHLLLVDDDPGLLKLLGLRLTSEGYSVVTAESGVEGLRVLNREKVDLVISDLRMD
EMDGMQLFAEIQKVQPGMPVIILTAHGSIPDAVAATQQGVFSFLTKPVDKDALYQAIDDA
LEQSAPATDERWREAIVTRSPLMLRLLEQARLVAQSDVSVLINGQSGTGKEIFAQAIHNA
SPRNSKPFIAINCGALPEQLLESELFGHARGAFTGAVSNREGLFQAAEGGTLFLDEIGDM
PASLQVKLLRVLQERKVRPLGSNRDIDIDVRIISATHRDLPKAMTRGEFREDLYYRLNVV
SLKIPALAERTEDIPLLANHLLRQAAERHKPFVRAFSTDAMKRLMTASWPGNVRQLVNVI
EQCVALTSSPVISDALVEQALEGENTALPTFVEARNQFELNYLRKLLQITKGNVTHAARM
AGRNRTEFYKLLSRHELDANDFKE
>LFGLNPFC_00622 Nitrogen regulatory protein P-II 1
MKKIDAIIKPFKLDDVREALAEVGITGMTVTEVKGFGRQKGHTELYRGAEYMVDFLPKVK
IEIVVPDDIVDTCVDTIIRTAQTGKIGDGKIFVFDVARVIRIRTGEEDDAAI
>LFGLNPFC_00623 Flavohemoprotein
MLDAQTIATVKATIPLLVETGPKLTAHFYDRMFTHNPELKEIFNMSNQRNGDQREALFNA
IAAYASNIENLPALLPAVEKIAQKHTSFQIKPEQYNIVGEHLLATLDEMFSPGQEVLDAW
GKAYGVLANVFINREAEIYNENASKAGGWEGTRDFRIVAKTPRSALITSFELEPVDGGAV
AEYRPGQYLGVWLKPEGFPHQEIRQYSLTRKPDGKGYRIAVKREEGGQVSNWLHNHANVG
DVVKLVAPAGDFFMAVADDTPVTLISAGVGQTPMLAMLDTLAKAGHTAQVNWFHAAENGD
VHAFADEVKELGQSLPRFTAHTWYRQPNEADRAKGQFDSEGLMDLSKLEGAFSDPTMQFY
LCGPVGFMQFAAKQLVDLGVKQENIHYECFGPHKVL
>LFGLNPFC_00624 Serine hydroxymethyltransferase
MLKREMNIADYDAELWQAMEQEKVRQEEHIELIASENYTSPRVMQAQGSQLTNKYAEGYP
GKRYYGGCEYVDIVEQLAIDRAKELFGADYANVQPHSGSQANFAVYTALLEPGDTVLGMN
LAHGGHLTHGSPVNFSGKLYNIVPYGIDATGHIDYADLEKQAKEHKPKMIIGGFSAYSGV
VDWAKMREIADSIGAYLFVDMAHVAGLVAAGVYPNPVPHAHVVTTTTHKTLAGPRGGLIL
AKGGSEELYKKLNSAVFPGGQGGPLMHVIAGKAVALKEAMEPEFKTYQQQVAKNAKAMVE
VFLERGYKVVSGGTDNHLFLVDLVDKNLTGKEADAALGRANITVNKNSVPNDPKSPFVTS
GIRVGTPAITRRGFKEAEAKELAGWMCDVLDSINDEAVIERIKGKVLDICARYPVYA
>LFGLNPFC_00625 Protein mlc
MRACINNQQIRHHNKCVILELLYRQKRANKSTLARLAQISIPAVSNILQELESEKRVVNI
DDESQTRGHSSGTWLIAPEGDWTLCLNVTPTSIECQVANACLSPKGEFEYLQIDAPTPQA
LLSEIEKCWHRHRKLWPDRTINLALAIHGQVDPVTGVSQTMPQAPWATPIEVKYLLEEKL
GIRVMVDNDCVMLALAEKWQNNSQVRDFCVINVDYGIGSSFVINEQIYRGSLYGSGQIGH
TIVNPDGVVCDCGRYGCLETVASLSALKKQARVWLKSQPVNTQLDPEKLTTAQLIAAWQS
GEPWITSWVDHSANAIGLSLYNFLNILNINQIWLYGRSCAFGENWLNTIIRQTGFNPFDR
DEGPSVKATQIGFGQLSRAQQVLGIGYLYVEAQLRQI
>LFGLNPFC_00626 Lipopolysaccharide assembly protein B
MTPVKVWQERVEIPTYETGPQDIHPMFLENRVYQGSSGAVYPYGVTDTLSEQKTLKSWQA
VWLENDYIKVMILPELGGRVHRAWDKVKQRDFVYHNEVIKPALVGLLGPWISGGIEFNWP
QHHRPTTFMPVDFTLEAHDDGAQTVWVGETEPMHGLQVMTGFTLRPDRAALEIASRVYNG
NATPRHFLWWANPAVKGGEGHQSVFPPDVTAVFDHGKRAVSAFPIATGTYYKVDYSAGVD
ISRYKNVPVPTSYMAEKSQYDFVGAWCHDEDGGLLHVANHHIAPGKKQWSWGHSEFGQAW
DKSLTDNNGPYIELMTGIFADNQPDFTWLDAYEEKRFEQYFLPYHSLGMVQNASRDAVIK
LQRSERGIEWGLYAISPLNGYRLAIREIGKCNALLDDAVALTPATAIQGVLHGINPERLT
IELSDADGNIVLSYHEHQSQALPLPDVAKAPLAAQDITSTDEAWFIGQHLEQYHHASRSP
FDYYLRGVALDPLDYRCNLALAMLEYNRADFPQAVAYATQALKRAHALNKNPQCGQASLI
RASAYERQGQYQQAEEDFWRAVWSGNSKAGGYYGLARLAARNGNFDAGLDFCQQSLRACP
TNQEVLCLHNLLLVLSGRQDNARLQREKLLRDYPLNATLWWLNWFDGRSESALVQWRGLC
QGRDVNALMTAGQLINWGMPALAADMLNALDCQRTLPLYLQASLLPKAERGELVVKAIDA
FPQFVRFPNTLEEVAALESIEECWFARHLLACFYYNKRSYGKAIALWQRCVEMSPEFADG
WRGLAIHAWNKQHDYELAARYLDNAYQLAPQDARLLFERDLLDKLSGVTPEKRLARLENN
LEIALKRDDMTAELLNLWHLTGQADKAADILATRKFHPWEGGEGKVTSQFILNQLLRAWQ
HLDAREPQQASELLHAALHYPENLSEGRLPGQTDNDIWFWQAVCANAQGDETEAMRCLRL
AATGDRTINIHSYYNDQPVDYLFWQGMALRLLGEQHTAQQLFSEMKQWAKEMAKTSIEAD
FFAVSQPDLLSLYSDLQQQHKEKCLMVAMLAAAGLGEVAHYESARAELMAINPAWPKAAL
FTTVMPFIFSYVH
>LFGLNPFC_00627 ABC transporter periplasmic-binding protein YphF

MPKKMTRTRNLLLMATLLGSALFARASDKEMTIGAIYLDTQGYYAGVRQGVQDAAKDSSV
QVQLIETNAQGDISKESTFVDTLVERNVDAIILSAVSENGSSRTVRRASEAGIPVICYNT
CINQKGVDKYVSAYLVGDPLEFGKKLGNAAADYFIANKIDQPKIAVINCEAFEVCVQRRK
GFEEVLKTRVPGAQIVANQEGTVLDKAISVGEKLIISTPDLNAIMGESGGATLGAVKAVR
NQNQAGKIAVFGSDMTTEIAQELENNQVLKAVVDISGKKMGNAVFAQTLKVINKQADGEK
VIQVPIDLYTKTEDGKQWLATHVDGLP
>LFGLNPFC_00628 Galactose/methyl galactoside import ATP-binding protein MglA
MFTATEAVPVAKVVAGNKRYPGVVALDNVNFTLNKGEVRALLGKNGAGKSTLIRMLTGSE
RPDSGDIWIGETRLEGDEATLTRRAAELGVRAVYQELSLVEGLTVAENLCLGQWPRRNGM
IDYLQMAQDAQRCLQALGVDVSPEQLVSTLSPAQKQLVEIARVMKGEPRVVILDEPTSSL
ASAEVELVISAVKKMSALGVAVIYVSHRMEEIRRIASCATVMRDGQVAGDVMLENTSTHH
IVSLMLGRDHVDIAPVAPQEIMDQAVLEVRALRHKPKLEDISFTLRCGEVLGIAGLLGAG
RSELLKAIVGLETYEQGEIVINGEKITRPDYGDMLKRGIGYTPENRKEAGIIPWLGVDEN
TVLTNRQKISANGVLQWSTIRRLTEEVMQRMTVKAASSETPIGTLSGGNQQKVVIGRWVY
AASQILLLDEPTRGVDIEAKQQIYRIVRELAAEGKSVVFISSEVEELPLVCDRILLLQHG
TFSQEFHSPVNVDELMSAILSVH
>LFGLNPFC_00629 Ribose import permease protein RbsC
MSASSLPLPQGKSVSLKQFVSRHINEIGLLVVIAILYLVFSLNAPGFISLNNQMNVLRDA
ATIGIAAWAMTLIIISGEIDVSVGPMVAFVSVCLAFLLQFDVPLAIACLLVLLLGALMGT
LAGVLRGVFNVPSFVATLGLWSALRGMGLFMTNALPVPINENEVLDWLGGQFLGVPVSAL
IMMVLFALFVFISRKTAFGRSVFAVGGNATAAQLCGINVRRVRILIFTLSGLLAAVTGIL
LAARLGSGNAGAANGLEFDVIAAVVVGGTALSGGRGSLFGTLLGVLVITLIGNGLVLLGI
NSFFQQVVRGVIIVVAVLANILLTQRSSKAKR
>LFGLNPFC_00630 Sorbitol dehydrogenase
MKTMLAAYLPGNSTVDLREVAVPTPGINQVLIKMKSSGICGSDVHYIYHQHRATAAAPDK
PLYQGFINGHEPCGQIVAMGQGCRHFKEGDRVLVYHISGCGFCPNCRRGFPISCTGEGKA
AYGWQRDGGHAEYLLAEEKDLILLPDALSYEDGAFISCGVGTAYEGILRGEVSGSDNVLV
VGLGPVGMMAMMLAKGRGAKRIIGVDMLPERLAMAKQLGVMDHGYLATTEGLPQIIAELT
HGGADVALDCSGNAAGRLLALQSTADWGRVVYIGETGKVEFEVSADLMHHQRRIIGSWVT
SLFHMEKCAHDLTDWKLWPRNAITHRFSLEQAGDAYALMASGKCGKVVINFPD
>LFGLNPFC_00631 putative protein YphB
MTIYTLSHGPLKLDVSDQGGVIEGFWRDTTPLLRPGKKSGVATDASCFPLVPFANRVSGN
RFVWQGREYQLQPNVEWDAHYLHGDAWLGQWQCVSRSEDSLCLVYEHRSGVYHYRVSQAF
HLTADTLTVTLSVTNKGAETLPFGTGWHPYFPLSPQTRIQAQASCYWREQEQWLAGEFCE
QLPQELDFNQLAPLPRQWVNNGFAGWNGQARIEQPQEGYAIIMETTPPAPCYFIFVSDPA
FDKGYAFDFFCLEPMSHAPDDHHRPEGGDLIALAPGESTISEMSLRVALL
>LFGLNPFC_00632 Inner membrane protein YphA
MNSLRYFDFGTARPVLLLIARIAVVLIFIIFGFPKMMGFGGTVQYMASLGAPMPMLAAII
AVVMEVPAAILIVLGFFTRPLAVLFIFYTLGTAVIGHHYWDMTGDAVGPNMINFWKNVSI
AGAFLLLAITGPGAISLDRR
>LFGLNPFC_00633 putative 3-phenylpropionic acid transporter
MVLQSTRWLALGYFTYFFSYGIFLPFWSVWLKGIGLTPETIGLLLGAGLVARFLGSLLIA
PRVSDPSRLISALRVLALLTLLFAVAFWAGAHVAWLMLVMIGFNLFFSPLVPLTDALANT
WQKQFPLDYGKVRLWGSVAFVIGSALTGKLVSMFDYRVILALLTLGVASMLLGFLIRPTI
QPQGASRQQESTGWSAWLALVRQNWRFLACVCLLQGAHAAYYGFSAIYWQAAGYSASAVG
YLWSLGVVAEVIIFALSNKLFRRCSARDMLLISAICGVVRWGIMGATTALPWLIVVQILH
CGTFTVCHLAAMRYIAARQGSEVIRLQAVYSAVAMGGSIAIMTVFAGFLYQYLGHGVFWV
MALVALPAMFLRPKVVPSC
>LFGLNPFC_00634 hypothetical protein
MMPTLAPPSVLSAPQRRCQILLTLFQPGLTATTATFSELNGVDDDIASLDISETGREILR
YHQLTLTTGYDGSYRVEGTVLNQRLCLFHWLRRGFRLCPLFITSHFTPALKSELKRRGIA
RNFYDDTNLQALVNLCSRRLQKRFETRDIHFLCLYLQYCLLQHHAGITPQFNPLQRRWAE
SCLEFQVAQEIGRHWQRRALQPVPPDEPLFMALLFSMLRVPDPLRDAHQRDRQLRKSIKR
LVNHFRELGNVRFYDEQGLCDQLYTHLAQALNRSLFAIGIDNTLPEEFARLYPRLVRTTR
AALAGFESEYGVHLSDEESGLVAVIFGAWLMQENDLHEKQIILLTGNDSEREAQIEQQLR
ELTLLPLNIKHMSVKVFLQTGAPRGAALIIAPYTMPLPLFSPPLIYTDLTLTTHQQEQIR
KMLESA
>LFGLNPFC_00635 putative protein
MALPVNKRVLKILFILFVVAFCVYLVPRVAINFFYYPDDKIYGPDPWSAESVEFTAKDGT
RLQGWFIPSSTGPADNAIATIIHAHGNAGNMSAHWPLVSWLPERNFNVFMFDYRGFGKSK
GTPSQAGLLDDTQSAINVVRHRSDVNPQRLVLFGQSIGGANILDVIGQGDREGIRAVILD
STFASYATIANQMIPGSGYLLDESYSGENYIASVSPIPLLLIHGKADHVIPWQHSEKLYS
LAKEPKRLILIPDGEHIDAFSDRHGDVYREQMVDFILSALNPQN
>LFGLNPFC_00636 Inositol-1-monophosphatase

MHPMLNIAVRAARKAGNLIAKNYETPDAVEASQKGSNDFVTNVDKAAEAVIIDTIRKSYP
QHTIITEESGELEGTDQDVQWVIDPLDGTTNFIKRLPHFAVSIAVRIKGRTEVAVVYDPM
RNELFTATRGQGAQLNGYRLRGSTARDLDGTILATGFPFKAKQYATTYINIVGKLFNECA
DFRRTGSAALDLAYVAAGRVDGFFEIGLRPWDFAAGELLVREAGGIVSDFTGGHNYMLTG
NIVAGNPRVVKAMLANMRDELSDALKR
>LFGLNPFC_00637 tRNA (cytidine/uridine-2'-O-)-methyltransferase TrmJ
MLQNIRIVLVETSHTGNMGSVARAMKTMGLTNLWLVNPLVKPDSQAIALAAGASDVIGNA
HIVDTLDEALAGCSLVVGTSARSRTLPWPMLDPRECGLKSVAEAANTPVALVFGRERVGL
TNEELQKCHYHVAIAANPEYSSLNLAMAVQVIAYEVRMAWLATQENGEQVEHEETPYPLV
DDLERFYGHLEQTLLATGFIRENHPGQVMNKLRRLFTRARPESQELNILRGILASIEQQN
KGNKAE
>LFGLNPFC_00638 HTH-type transcriptional regulator IscR
MRLTSKGRYAVTAMLDVALNSEAGPVPLADISERQGISLSYLEQLFSRLRKNGLVSSVRG
PGGGYLLGKDASSIAVGEVISAVDESVDATRCQGKGGCQGGDKCLTHALWRDLSDRLTGF
LNNITLGELVNNQEVLDVSGRQHTHDAPRTRTQDAIDVKLRA
>LFGLNPFC_00639 Cysteine desulfurase IscS
MKLPIYLDYSATTPVDPRVAEKMMQFMTMDGTFGNPASRSHRFGWQAEEAVDIARNQIAD
LVGADPREIVFTSGATESDNLAIKGAANFYQKKGKHIITSKTEHKAVLDTCRQLEREGFE
VTYLAPQRNGIIDLKELEAAMRDDTILVSIMHVNNEIGVVQDIAAIGEMCRARGIIYHVD
ATQSVGKLPIDLSQLKVDLMSFSGHKIYGPKGIGALYVRRKPRVRIEAQMHGGGHERGMR
SGTLPVHQIVGMGEAYRIAKEEMATEMERLRGLRNRLWNGIKDIEEVYLNGDLEHGAPNI
LNVSFNYVEGESLIMALKDLAVSSGSACTSASLEPSYVLRALGLNDELAHSSIRFSLGRF
TTEEEIDYTIELVRKSIGRLRDLSPLWEMYKQGVDLNSIEWAHH
>LFGLNPFC_00640 Iron-sulfur cluster assembly scaffold protein IscU
MAYSEKVIDHYENPRNVGSFDNNDENVGSGMVGAPACGDVMKLQIKVNDEGIIEDARFKT
YGCGSAIASSSLVTEWVKGKSLDEAQAIKNTDIAEELELPPVKIHCSILAEDAIKAAIAD
YKSKREAK
>LFGLNPFC_00641 Iron-binding protein IscA
MSITLSDSAAARVNTFLANRGKGFGLRLGVRTSGCSGMAYVLEFVDEPTPEDIVFEDKGV
KVVVDGKSLQFLDGTQLDFVKEGLNEGFKFTNPNVKDECGCGESFHV
>LFGLNPFC_00642 Co-chaperone protein HscB
MDYFTLFGLPARYQLDTQALSLRFQDLQRQYHPDKFASGSQAEQLAAVQQSATINQAWQT
LRHPLMRAEYLLSLHGFDLASEQHTVRDTAFLMEQLELREELDEIEQAKDEARLESFIKR
VKKMFDTRHQLMVEQLDNETWDAAADTVRKLRFLDKLRSSAEQLEEKLLDF
>LFGLNPFC_00643 Chaperone protein HscA
MALLQISEPGLSAAPHQRRLAAGIDLGTTNSLVATVRSGQAETLADHEGRHLLPSVVHYQ
QQGHSVGYDARTNAALDTANTISSVKRLMGRSLADIQQRYPHLPYQFQASENGLPMIETA
AGLLNPVRVSADILKALAARATEALAGELDGVVITVPAYFDDAQRQGTKDAARLAGLHVL
RLLNEPTAAAIAYGLDSGQEGVIAVYDLGGGTFDISILRLSRGVFEVLATGGDSALGGDD
FDHLLADYIREQAGIPDRSDNRVQRELLDAAIAAKIALSDADSVTVNVAGWQGEISREQF
NELIAPLVKRTLLACRRALKDAGVEADEVLEVVMVGGSTRVPLVRERVGEFFGRPPLTSI
DPDKVVAIGAAIQADILVGNKPDSEMLLLDVIPLSLGLETMGGLVEKVIPRNTTIPVARA
QDFTTFKDGQTAMSIHVMQGERELVQDCRSLARFALRGIPALPAGGAHIRVTFQVDADGL
LSVTAMEKSTGVEASIQVKPSYGLTDSEIASMIKDSMSYAEQDVKARMLAEQKVEAARVL
ESLHGALAADAALLSAAERQVIDDAAAHLSEVAQGDDVDAIEQAIKNVDKQTQDFAARRM
DQSVRRALKGHSVDEV
>LFGLNPFC_00644 2Fe-2S ferredoxin
MPKIVILPHQDLCPDGAVLEANSGETILDVALRNGIEIEHACEKSCACTTCHCIVREGFD
SLPESSEQEDDMLDKAWGLEPESRLSCQARVTDEDLVVEIPRYTINHAREH
>LFGLNPFC_00645 Protein IscX
MGLKWTDSREIGEALYDAYPDLDPKTVRFTDMHQWICDLEDFDDDPQASNEKILEAILLV
WLDEAE
>LFGLNPFC_00646 Peptidase B
MTEAMKITLSTQPADARWGEKATYSINNDGITLHLNGADDLGLIQRAARKIDGLGIKHVQ
LSGEGWDADRCWAFWQGYKAPKGIRKVEWPDLDDAQRQELDNRLMIIDWVRDTINAPAEE
LGPSQLAQRAVDLISNVAGDRVTYRITKGEDLREQGYMGLHTVGRGSERSPVLLALDYNP
TGDKEAPVYACLVGKGITFDSGGYSIKQTAFMDSMKSDMGGAATVTGALAFAITRGLNKR
VKLFLCCADNLISGNAFKLGDIITYRNGKKVEVMNTDAEGRLVLADGLIDASAQKPELII
DAATLTGAAKTALGNDYHALFSFDDALAGRLLASAAQENEPFWRLPLAEFHRNQLPSNFA
ELNNTGSAAYPAGASTAAGFLSHFVENYQQGWLHIDCSATYRKAPVEQWSAGATGLGVRT
IANLLTA
>LFGLNPFC_00647 hypothetical protein
MSETKNELEDLLEKAATEPAHRPAFFRTLLESTVWVPGTAAQGEAVVEDSALDLQHWEKE
DGTSVIPFFTSLEALQQAVEDEQAFVVMPVRTLFEMTLGETLFLNAKLPTGKEFMPREIS

LLIGEEGNPLSSQEVLEGGESLILSEVAEPPAQMIDSLTTLFKTIKPVKRAFICSIKENE
EAQPNLLIGIEADGDIEEIIQAAGSVATDTLPGDEPIDICQVKKGEKGISHFITEHIAPF
YERRWGGFLRDFKQNRII
>LFGLNPFC_00648 3-mercaptopyruvate sulfurtransferase
MSTTWFVGADWLAEHIDDPEIQIIDARMASPGQEDRNVAQEYLNGHIPGAVFFDIEALSD
HTSPLPHMLPRPETFAVAMRELGVNQDKHLIVYDEGNLFSAPRAWWMLRTFGVEKVSILG
GGLAGWQRDDLLLEEGAVELPEGEFNAAFNPEAVVKVTDVLLASHENTAQIIDARPATRF
NAEVDEPRPGLRRGHIPGALNVPWTELVREGELKTTDELDAIFFGRGVSYDKPIIVSCGS
GVTAAVVLLALATLDVTNVKLYDGAWSEWGARADLPVEPVK
>LFGLNPFC_00649 Alpha-2-macroglobulin
MKKLRVAACMLMLALAGCDNNDNAPTAVKKDAPSEVTKAASSENVSSAKLSAPERQKLAQ
QSAGKALTLLDLSEVQLDGAATLVLTFSIPLDPDQDFSRVIHVVDKKSGKVDGAWELSHN
LKELRLRHLEPKRDLIVTIGKEVKALNNATFSKDDEKTITTRDIQPSVGFASRGSLLPGK
VVEGLPVMALNVNNVDVNFFRVKPESLPAFISQWEYRNSLANWQSDKLLQMADLVYTGRF
DLNPARNTREKLLLPLGDIKPLQQAGVYLAVMNQAGRYDYSNPATLFTLSDIGVSAHRYH
NRLDIFTQSLENGAAQQGIEVSLLNEKGQTLTQATSDAQGHVQLENDKNAALLLARKNGR
TTLLDLKLPALDLAEFNIAGAPGYSKQFFMFGPRDLYRPGETVILNGLLRDADGKALPDQ
PIKLDVIKPDGQVLRSVVSQPENGLYHFTWPLDSNAATGMWHIRANTGDNQYRMWDFHVE
DFMPERMALNLTGEKTPLTPKDEVKFSVVGYYLYGAPANGNTLQGQLFLRPLREAVSALP
GFEFGDIAAENLSRTLDEVQLTLDDKGRGEVSTESQWKETHSPLQVIFQGSLLESGGRPV
TRRAEQAIWPADALPGIRPQFASKSVYDYRTDSTVKQPIVDEGSNAAFDIVYSDAQGVKK
AVSGLQVRLIRERRDYYWNWSEYEGWQSQFDQKDLIENEQTLDLKADETGKVSFPVEWGA
YRLEVKAPNEAVSSVRFWAGYSWQDNSDGGGAVRPDRVTLKLDKASYCPGDTIKLHIAAP
TAGKGYAMVESSEGPLWWQEIDVPAQGLDLTIPVDKTWNRHDLYLSTLVVRPGDKSRSAT
PKRAVGVLHLPLGDENRRLDLALETPTKMRPNQPLTVKIKASNKNGEMPKQVNVLVSAVD
SGVLNITDYVTPDPWQAFFGQKRYGADIYDIYGQVIEGQGRLAALRFGGDGDELKRGGKP
PVNHVNIVAQQALPVTLNEQGEGSVTLPIGDFNGELRVMAQAWTADDFGSNESKVIVAAP
VIAELNMPRFMASGDTSRLTLDITNLTDKPQKLNVALTASGLLELVSNSPAPVELAPGVR
TTLFIPVRALPGYGDGDIQATISGLALPGETVADQHKQWKIGVRPAFPAQTVNYGTALQP
GETWALPADGLQNFSPVTLEGGQLLLSGKPPLNIARYIKELKAYPYGCLEQTASGLFPSLY
TNAAQLQALGIKGDSDEKRRASVDIGISRLLQMQRDNGGFALWDKNGDEEYWLTAYVMDF
LVRAGEQGYSVPTDAINRGNERLLRYLQDPGMMSIPYADNLKASKFAVQSYAALVLARQQ
KAPLGALREIWEHRADAASGLPLLQLGVALKTMGDAMRGEEAIVLALKTPRNSDERIWLG
DYGSPLRDSALMLSLLEENKLLPDEQYSLLNTLSQQAFGERWLSTQESNALFLAARTLQD
LPGKWQAQTTFSAEPLTGEKAQTSNLNSDQLATLQVTNSGDQPLWLRVDASGYPQSAPLP
ASNVLQIERHILGTDGKSKSLDSLRSGDLVLVWLQVKASNSVPDALVVDLLPAGLELENQ
NLANGSASLEQSGGEVQNLLNQMQQASIKHIEFRDDRFVAAVAVDEYQPVTLVYLARAVT
PGTYQVPQPMVESMYVPQWRATGAADDLLIVRP
>LFGLNPFC_00650 Penicillin-binding protein 1C
MLAAAPFIIILAAWAADKLWPLPLQEVNPARVVVAQDGTPLWRFADADGIWRYPVTIEDV
SPRYLEALINYEDRWFWKHPGVNPFSVARAAWQDLTSGRVISGGSTLTMQVARLLDPHPK
TFGGKIRQLWRALQLEWHLSKREILTLYLNRAPFGGTLQGIGAASWAYLGKSPANLSYSE
AAMLAVLPQAPSRLRPDRWPERAEAARNKVLERMAVQGVWSRERVKESREEPIWLAPRQM
PQLAPLFSRMMLGKSKSDKIVTTLDAGLQRRLEELAQNWKGRLPPRSSLAMIVVDHTDMR
VRGWVGSVDLNDDSRFGHVDMVNAIRSPGSVLKPFVYGLALDEGLIHPASLLQDVPRRTG
DYRPGNFDSGFHGPISMSEALVRSLNLPAVQVLEAYGPKRFAAKLRNVGLPLYLPNGAAP
NLSLILGGAGAKLEDMAAAYTAFARHGKAGKLRLQPDDPLLERPLMSSGAAWIIRRIMAD
EAQPLPDGALPRVAPLAWKTGTSYGYRDAWAIGVNARYVIGIWTGRPDGTPVVGQFGFAS
AVPLLNQVNNILLSRSVNLPEDPRPDSVSRGVICWPGGQSLPEGDGNCRRRLATWLLDGS
QPPTLLLPEQEGINGIRFPIWLDENGKRVAADCPQARQEMINVWPLPLEPWLPASERRAV
RLPPASTICPPYGHDAQLPLQLTGVRDGAIIKRLPGAAEATLPLQSSGGAGERWWFLNGE
PLTERGRNVTLHLTDKGDYQLLVMDDVGQIATVKFVMQ
>LFGLNPFC_00651 Nucleoside diphosphate kinase
MAIERTFSIIKPNAVAKNVIGSIFARFEAAGFKIVGTKMLHLTVEQARGFYAEHDGKPFF
DGLVEFMTSGPIVVSVLEGENAVQRHRDLLGATNPANALAGTLRADYADSLTENGTHGSD
SVESAAREIAYFFGEGEVCPRTR
>LFGLNPFC_00652 Dual-specificity RNA methyltransferase RlmN
MSEQLVTPENVTTKDGKINLLDLNRQQMREFFKDLGEKPFRADQVMKWMYHYCCDNFDEM
TDINKVLRGKLKEVAEIRAPEVVEEQRSSDGTIKWAIAVGDQRVETVYIPEDDRATLCVS
SQVGCALECKFCSTAQQGFNRNLRVSEIIGQVWRAAKIVGAAKVTGQRPITNVVMMGMGE
PLLNLNNVVPAMEIMLDDFGFGLSKRRVTLSTSGVVPALDKLGDMIDVALAISLHAPNDE
IRDEIVPINKKYNIETFLAAVRRYLEKSNANQGRVTIEYVMLDHVNDGTEHAHQLAELLK
DTPCKINLIPWNPFPGAPYGRSSNSRIDRFSKVLMSYGFTTIVRKTRGDDIDAACGQLAG
DVIDRTKRTLRKRMQGEAIDIKAV

>LFGLNPFC_00653 Cytoskeleton protein RodZ
MNTEATHDQNEALTTGARLRNAREQLGLSQQAVAERLCLKVSTVRDIEEDKAPADLASTF
LRGYIRSYARLVHIPEEELLPGLEKQAPLRAAKVAPMQSFSLGKRRKKRDGWLMTFTWLV
LFVVIGLSGAWWWQDHKAQQEEITTMADQSSAELNNNQSQSVPLDTSTTTDQAMATTPTS
PVDTTATNTQTPAVTAPAPAVDPQQNAVVPPSQANVDTAATPAPAATTTPDGAAPLPTDQ
AGVTTPAVDPNALVMNFTADCWLEVTDATGKKLFSGMQRKDGNLNLTGQAPYKLKIGAPA
AVQIQYQGKPVDLSRFIRTNQVARLTLNAEQSPAQ
>LFGLNPFC_00654 4-hydroxy-3-methylbut-2-en-1-yl diphosphate synthase (flavodoxin)
MHNQAPIQRRKSTRIYVGNVPIGDGAPIAVQSMTNTRTTDVEATVNQIKALERVGADIVR
VSVPTMDAAEAFKLIKQQVNVPLVADIHFDYRIALKVAEYGVDCLRINPGNIGNEERIRM
VVDCARDKNIPIRIGVNAGSLEKDLQEKYGEPTPQALLESAMRHVDHLDRLNFDQFKVSV
KASDVFLAVESYRLLAKQIDQPLHLGITEAGGARSGAVKSAIGLGLLLSEGIGDTLRVSL
AADPVEEIKVGFDILKSLRIRSRGINFIACPTCSRQEFDVIGTVNALEQRLEDIITPMDV
SIIGCVVNGPGEALVSTLGVTGGNKKSGLYEDGVRKDRLDNNDMIDQLEARIRAKASQLD
EARRIDVQQVEK
>LFGLNPFC_00655 Histidine--tRNA ligase
MAKNIQAIRGMNDYLPGETAIWQRIEGTLKNVLGSYGYSEIRLPIVEQTPLFKRAIGEVT
DVVEKEMYTFEDRNGDSLTLRPEGTAGCVRAGIEHGLLYNQEQRLWYIGPMFRHERPQKG
RYRQFHQLGCEVFGLQGPDIDAELIMLTARWWRALGISEHVTLELNSIGSLEARANYRDA
LVAFLEQHKEKLDEDCKRRMYTNPLRVLDSKNPEVQALLNDAPALGDYLDEESREHFAGL
CKLLESAGIAYTVNQRLVRGLDYYNRTVFEWVTNSLGSQGTVCAGGRYDGLVEQLGGRAT
PAVGFAMGLERLVLLVQAVNPEFKADPVVDIYLVASGADTQSAAMALAERLRDELPGVKL
MTNHGGGNFKKQFARADKWGARVAVVLGESEVANGTAVVKDLRSGEQTAVAQDSVAAHLR
TLLG
>LFGLNPFC_00656 hypothetical protein
MEIYENENDQVEAVKRFFAENGKALAVGVILGVGALIGWRYWNSHQVDSARSASLAYQNA
VTAVSEGKPDSIPAAEKFAAENKNTYGALASLELAQQFVDKNELEKAAAQLQQGLADTSD
ENLKAVINLRLARVQVQLKQADAALKTLDAIKGEGWAAIVADLRGEALLSKGDKQGARSA
WEAGVKSDVTPALSEMMQMKINNLSI
>LFGLNPFC_00657 Outer membrane protein assembly factor BamB
MQLRKLLLPGLLSVTLLSGCSLFNSEEDVVKMSPLPTVENQFTPTTAWSTSVGSGIGNFY
SNLHPALADNVVYAADRAGLVKALNADDGKEIWSVNLAEKDGWFSKDPALLSGGVTVSGG
HVYIGTEKAQVYALNTSDGTVAWQTKVAGEALSRPVVSDGLVLIHTSNGQLQALNEADGA
VKWTVNLDMPSLSLRGESAPATAFGAAVVGGDNGRVSAVLMEQGQMIWQQRISQATGSTE
IDRLSDVDTTPVVVNGVVFALAYNGNLTALDLRSGQIMWKRELGSVNDFIVDGNRIYLVD
QNDRVMALTIDGGVTLWTQSDLLHRLLTSPVLYNGNLVVGDSEGYLHWINVEDGRFVAQQ
KVDSSGFQTEPVAADGKLLIQAKDGTVYSITR
>LFGLNPFC_00658 GTPase Der
MVPVVALVGRPNVGKSTLFNRLTRTRDALVADFPGLTRDRKYGRAEIEGREFICIDTGGI
DGTEDGVETRMAEQSLLAIEEADVVLFMVDARSGLMPADEAIAKHLRSREKPTFLVANKT
DGLDPDQAVVDFYALGLGEIYPIAASHGRGVLSLLEHVLLPWMEDLAPQEEVDEDAEYWA
QFEAEENGEEEEEDDFDPQSLPIKLAIVGRPNVGKSTLTNRILGEERVVVYDMPGTTRDS
IYIPMERDGREYVLIDTAGVRKRGKITDAVEKFSVIKTLQAIEDANVVMLVIDAREGISD
QDLSLLGFILNSGRSLVIVVNKWDGLSQEVKEQVKETLDFRLGFIDFARVHFISALHGSG
VGNLFESVREAYDSSTRRVGTSMLTRIMTMAVEDHQPPLVRGRRVKLKYAHAGGYNPPIV
VIHGNQVKDLPDSYKRYLMNYFRKSLDVMGSPIRIQFKEGENPYANKRNTLTPTQMRKRK
RLMKHIKKSK
>LFGLNPFC_00659 putative protein YfgJ
MALNCPTCKTPLERNGETAHCATCDKDFTVQALCPECHQALQVLKACGAVDYFCQHGHGL
ISKKRVEFIIA
>LFGLNPFC_00660 hypothetical protein
MLRWKRCIILTFISGAAFAAPEINVKQNESLPDLGSQAAQQDEQTNKGKSLKERGADYVI
NSATQGFENLTPEALKSQARSYLQSQITSTAQSYIEDTLSPYGKVRSNLSIGQGGDLDGS
SIDYFVPWYDNQTTVYFSQFSAQRKEDRTIGNIGLGVRYNFDKYLLGGNIFYDYDFTRGH
RRLGLGAEAWTDYLKFSGNYYHPLSDWKDSEDFDFYEERPARGWDIRAEAWLPAYPQLGG
KIVFEQYYGNEVALFGTDSLEKDPFAVTLGVKYQPVPLIVVGTDFKAGTGDNTDLSVNAT
LNYQFGVPLKDQLDPDKVSAAHSLMGSRHDFVERNNFIVLEYKEKDPLYVTLWLKADVTN
EHPECVIKDTPEEAIGLEKCKWTINALINHHYKIVAASWQAKNNAASWQAKNNAARTLVM
PVIKENTLTEGNNNHWNLVLPAWQYSSDQAEQEKLNTWRVRLALEDEKGNRQNSGVVEIT
VQQDRKIELIVNNIANVPEENNHSHEASAQADGVDGVVMDLDVTDSFGDNTDRNGDALPE
DNLTPQLYDAQDKRVTLTNKPCSTDNPCVFIAKQDKEKGTVTLSSTLPGTYRWKAKAAPY
DDSNYVDVTFLGAEIGGLNAFIYRVGAAKPSNLIGKDKEPLPLNNTYRFVLWRDNNKDGV
FQQVEKLTDEEMVQYDYKWEFTGKSINGEVGAQANTSNEDIVIPATNREAAQTYGAQAGD
GLQGYGLRVLYTKK

>LFGLNPFC_00661 hypothetical protein
MKQDKRRGLTRIALALALAGYCVAPVALAEDSAWVDSGETNIFQGTIPWLYSEGGSATTD
ADRVTLTSDLKGARPQGSETDKRLYSGDKLTVSWEIGDTEGDVDLGGLGDNAKTIDTIRW
MSYKDAQGGDPKELVTKVTSYTLTDADRGRYIGIEITPTTQTGTPNVGTALHLYDVSTAS
GGGSDSDNVAPGPVVNQNLKVAIFVDGTSINLINGSTPIELGKTYVAKLYSDENKNGKFD
AGTDADVTANYDFRWVLSGSSQQLGTSGGIVNSSFDNNNLVIPATNDEARTNLNGPARDG
KEALSIPTNGDGVQGYKLHIIYKHK
>LFGLNPFC_00662 hypothetical protein
MSLTLNKILVLCALLISAMLPGWSWAESAWQDSSDTVGEFNGTVPTADSASIPVYQGSVF
LDPTKTHEVAFTAKPSEFNADVSVSKLLVTNPQDREGDIIATPRWENQTPPAVSLVWADA
ATPDTLLDPQPVADRSFCAQGLAGRSLVAWAQPDPQQTMPLLYLLTSTGYPYESVLMLAD
QKVTLKIAPAQGDLISVSAAGYDESSGAAKMTVGGSITLTVTTKDCVGNVVGNIPFVIKR
KDAENRQGVVNNTAPVKLGTTELTTTATEYRGTTDANGVATVTVTQANGPGVKTPLVASL
AGIAQASETAVIFTVLTSPDVPQATMWGHMPDTLKARDYTFSRPKLAAEVDNEGGTVNDH
NETWSTFTWSGADKHCDILPGMRQFGALATVVPTSVQDVAGWPMQGNFYWSSLAGMSGQH
HAADVSNRSEAQKPDDTTFIVSCVDKEAPDVEPKLVLTPGSYDSTIKAMKVKVGEEASLR
LTITDSKNNDQPLAYYYFSLHLDDGINRKNQTDAAWETHPVQIDGGSNVRKVDAHTYEGI
TDANGEATLTLTQPGGVGVKTHITARMRSDFTASDEKDVIFTVITSPDTDKARMWGHMLG
IIEANNIFKRPRLADETDNEQGSVRENNEDWALFDQNSSMQAECGLGHIPSQSSLHSLFA
AHPANAIGTEYGWPTLQKAYLSAVEETSHASVNLATGNIDTYSGFKQNYLSCSGNEMVAQ
IAATTDRDVSAGSRAQAKVGDTITMTVRTFNALNNAPVPYTAFTITKDMGKNRQGQTTGF
DDPTRGAIEMNGTLYGTSQPSLVYAGTTDAQGFATVEIKQSQGVGLSTPLNIVPVNSYIP
NTVNYNVIFTTLTSPDAVGAQMWGHMDETITVDALTFARPRLAAEVFSPDGTLTENNEVW
SRVSQANASSTSKGGCGANMLPRRSQLSALYDANNGDGVQTVHGWPTQRQPYWSSSPADQ
VPHYYTIALNDGARTVGGSTAVYVSCLTTANNPASSITLEVVDPAQWNAAANAAKLKKGE
TLQVKVTVKDAQGNPLGDIPFTLKRGDGYTRSEEKHVAGSSDALVAPVVVNGGLADETSL
NNTAAAYSAMTGSDGTKILTITRPDTHGTKTSLTARLYSDTTKKATLDTIFTVVTSPDSD
KAKMWGHMPETVTAADGAVFKRPLLLKELSSTSGRTAIAEDNEDWAQFTQAQAISTSSNG
CGSEYVPSQAGLESLYEANRGNAMKTVQGWPVASSYLSSTTGSSSLEQRDFKAVNLSSGT
SSIIPSATKELLTCQTTPIVKASQIVLEAADLTKFDRMNNVVKVKKGEEAVLRVTTKDAQ
GKPVGNTAFTLKRNTSVNRANVSTTTSIASLAVTDAWGNTQNDFLSTTLVIYGVTGADGT
TTFTLKQDQTTGLKTELTAALDSSSSTKSTLPVVFTVLTSPDSPKAKFWGHMAETATGDD
GLIYRRPLLRDENSATTSIGTLVEEGEAWSTFPSGQANDTSINGCGAEYVPTDNELRAIY
AHQGSSALHDAIGWPVSRFYISNTVADTFTQTFTYDVVSLKTGDETQMPSSGGALLSCRT
TPVAVASQIIVEANDTAQFVKVDDTLSALKVKKGEDAVIRVVTKNAQGNSVPNVPFILRR
EGSKNRQNAEMINKSITVINAAGASARMNSSSSLLYGVTGADGTTSFTVKQDDSMGLVTN
MYAQLYQLTIESNKLPVMFTVITSPDTPLASYWGHMPETFTTRSGIAFKRPLLTAEHPAG
QSTMANNESWLSLNTAAKNDVSKSDCGEPYQPLLSEFQELYSEHPNGAIGTDLGLPLTNT
WWAYDKIAYANVWYDQSINLSNGSSSRALSNTVAFVSCLVNPHAVAASIEMTSTALDAEK
TASNDGRPSATAAKGTAIRMTVIVRDSGGNLLPGANFNLIRGTALDRAKNRLDSTYDDLT
IVPVTPAGVNMSLYNNGAQALLTTGSDGKATFDVTQNETYGLATPLTATLMRDTTKSATM
DVIFTVITSPNSPKAKYWGHMPDTFTSRAGVTFKRPLLAAEATLGSSVSNNNESWSYLFY
TNKVTPDCPVEYQPRLNELQGLYNDHPGGTILTDLGLPITAGSGNWWTYEMSTTDALTWY
YGVINLKTGQSTTTINGYALMLCLTQPHSAPASLTLSSTAYDEGRTASNGGTPTSSVKKG
EMLPIVVTIKDANGNPVGGEGVTLKRVQAKSRSGISVSSNTVDDLILDEVTPTSARISFN
QNTSAWSGFTGSDGTITFNVTQNNTVGLVTPFTASLARNPQVTANQDLIFTVVTSPDSAK
ANYWGHMPATLTAVNGAVFERPKLWSELTSTSGVGKINNNNEDWPYFTPTQKSDASVSPC
EVARQPLFNDLSSLSARYPNNTFVTETGWPAYYTWWAEDKSADGKDQSVDLRNGTLYTGS
TKSFQPCLANARSTVSSVTLTSTAFDAATQAAKVKKGEAMSVTVTVKDSAGNTVPNVEFT
LKRGEASPRNAGATLYGNVVAMDDLVVQPLSGSAVTLSESGNTISGMTGADGTASFTLRQ
DNTPGYKMPLTVTLANYASATDTLDAIFTVPTSPNVSSAHFWGHMADTVVVNSKSLHRPL
LTTELPSGANPVSSPIINYENWASAHIIDASKWDIARQCGSIENAPTYNELELLHTVFNS
LGWPSSPSFPYLSSQQCGMDEGTGAQDCSITLINKPGLVTCFQ
>LFGLNPFC_00663 Exodeoxyribonuclease 7 large subunit
MLPSQSPAIFTVSRLNQTVRLLLEHEMGQVWISGEISNFTQPASGHWYFTLKDDTAQVRC
AMFRNSNRRVTFRPQHGQQVLVRANITLYEPRGDYQIIVESMQPAGEGLLQLKYEQLKAK
LQAEGLFDLQYKKSLPSPAHCVGVITSKTGAALHDILHVLKRRDPSLPVIIYPTAVQGDD
APGQIVRAIELANQRNECDVLIVGRGGGSLEDLWSFNDERVARAIFASRIPIVSAVGHET
DVTIADFVADLRAPTPSAAAEVVSRNQQELLRQVQSTHQRLEMAMDYYLANRTRRFTQIH
HRLQQQHPQLRLARQQTMLERLQKRMSFALESQLKRAGQQQQRLTRQLVQQNPQSRIHRA
QTRIQQLEYRLAETLRAQLSATRERFGNAVTHLEAVSPLSTLARGYSVTSAADGAVLKQV
KQVKVGETLTTRLGDGVVISEVSAVTKTRKSRKKTSNP
>LFGLNPFC_00664 Inosine-5'-monophosphate dehydrogenase
MLRIAKEALTFDDVLLVPAHSTVLPNTADLSTQLTKTIRLNIPMLSAAMDTVTEARLAIA

LAQEGGIGFIHKNMSIERQAEEVRRVKKHESGVVTDPQTVLPTTTLREVKELTERNGFAG
YPVVTEENELVGIITGRDVRFVTDLSQPVSVYMTPKERLVTVREGEAREVVLAKMHEKRV
EKALVVDDEFHLIGMITVKDFQKAERKPNACKDEQGRLRVGAAVGAGAGNEERVDALVAA
GVDVLLIDSSHGHSEGVLQRIRETRAKYPDLQIIGGNVATAAGARALAEAGCSAVKVGIG
PGSICTTRIVTGVGVPQITAVADAVEALEGTGIPVIADGGIRFSGDIAKAIAAGASAVMV
GSMLAGTEESPGEIELYQGRSYKSYRGMGSLGAMSKGSSDRYFQSDNAADKLVPEGIEGR
VAYKGRLKEIIHQQMGGLRSCMGLTGCGTIDELRTKAEFVRISGAGIQESHVHDVTITKE
SPNYRLGS
>LFGLNPFC_00665 GMP synthase [glutamine-hydrolyzing]
MTENIHKHRILILDFGSQYTQLVARRVRELGVYCELWAWDVTEAQIRDFNPSGIILSGGP
ESTTEENSPRAPQYVFEAGVPVFGVCYGMQTMAMQLGGHVEASNEREFGYAQVEVVNDSA
LVRGIEDALTADGKPLLDVWMSHGDKVTAIPSDFVTVASTESCPFAIMANEEKRFYGVQF
HPEVTHTRQGMRMLERFVRDICQCEALWTPAKIIDDAVARIREQVGDDKVILGLSGGVDS
SVTAMLLHRAIGKNLTCVFVDNGLLRLNEAEQVLDMFGDHFGLNIVHVPAEDRFLSALAG
ENDPEAKRKIIGRVFVEVFDEEALKLEDVKWLAQGTIYPDVIESAASATGKAHVIKSHHN
VGGLPKEMKMGLVEPLKELFKDEVRKIGLELGLPYDMLYRHPFPGPGLGVRVLGEVKKEY
CDLLRRADAIFIEELRKADLYDKVSQAFTVFLPVRSVGVMGDGRKYDWVVSLRAVETIDF
MTAHWAHLPYDFLGRVSNRIINEVNGISRVVYDISGKPPATIEWE
>LFGLNPFC_00666 hypothetical protein
MKKVFLCAILASLSYPAIASSLQDQLSAVAEAEQQGKNEEQRQHDEWVAERNREIQQEKQ
RRANAQAAANKRAATAAANKKARQDKLDAEATADKKRDQSYEDELRSLEIQKQKLALAKE
EARVKRENEFIDQELKHKAAQTDVVQSEADANRNMTEGGRDLMKSVGKAEENKSDSWFN
>LFGLNPFC_00667 hypothetical protein
MKFKKCLLPVAMLASFTLAGCQSNADDHAADVYQTDQLNTKQETKTVNIISILPAKVAVD
NAQNKRNAQAFGALIGAVAGGVIGHNVGSGSNSGTTAGAVGGGAVGAAAGSMVNDKTLVE
GVSLTYKEGTKVYTSTQVGKECQFTTGLAVVITTTYNETRIQPNTKCPEKS
>LFGLNPFC_00668 Protein YfgG
MRKRHRFNSRMTRIVLLISFIFFFGRFIYSSVGAWQHHQSKKEAQQSTLSVESPVQR
>LFGLNPFC_00669 hypothetical protein
MSHAVDGTYIKAQQDKMVNSSLCKKASVDTYFNVMEVNLIIYNDDVIMMS
>LFGLNPFC_00670 Cyclic di-GMP phosphodiesterase PdeF
MKLNATYIKIRDKWWGLPLFLPSLILPIFAHINTFAHISSGEVFLFYLPLALMISMMMFF
SWAALPGIALGIFVRKYAELGFYETLSLTANFIIIIILCWGGYRVFTPRRNNVSHGDSRL
ISQRLFWQIVFPATLFLILFQFAAFVGLLASRENLVGVMPFNLGTLINYQALLVGNLIGV
PLCYFIIRVVRNPFYLRSYYSQLKQQVDAKVTKKEFAIWLLALGALLLLLCMPLNEKSTI
FSTNYTLSLLLPLMMWGAMRYGYKLISLLWAVVLMISIHSYQNYIPIYPGYTTQLTITSS
SYLVFSFIVNYMAVLATRQRAVVRRIQRLAYVDPVVHLPNVRALNRALRDAPWSALCYLR
IPGMEMLVKNYGIMLRIQYKQKLSHWLSPLLEPGEDVYQLSGNDLALRLNTESHQERITA
LDSHLKQFRFFWDGMPMQPQIGVSYCYVRSPVNHIYLLLGELNTVAELSIVTNAPENMQR
RGAMYLQRELKDKVAMMNRLQRALEHNHFFLMAQPITGMRGDVYHEILLRMKGENDELIS
PDSFLPVAHEFGLSSSIDMWVIEHTLQFMAENRAKMPAHRFAINLSPTSVCQARFPVEVS
QLLAKYQIEAWQLIFEVTESNALTNVKQAQITLQHLQELGCQIAIDDFGTGYASYARLKN
VNADLLKIDGSFIRNIVSNSLDYQIVASICHLARMKKMRVVAEYVENEEIREAVLSLGID
YMQGYLIGKPQPLIDTLNEIEPIRESA
>LFGLNPFC_00671 Exopolyphosphatase
MPIHDKSPRPQEFAAVDLGSNSFHMVIARVVDGAMQIIGRLKQRVHLADGLGPDNMLSEE
AMTRGLNCLSLFAERLQGFSPASVCIVGTHTLRQALNATDFLKRAEKVIPYPIEIISGNE
EARLIFMGVEHTQPEKGRKLVIDIGGGSTELVIGENFEPILVESRRMGCVSFAQLYFPGG
VINKENFQRARMAAAQKLETLTWQFRIQGWNVAMGASGTIKAAHEVLMEMGEKDGIITPE
RLEKLVKEVLRHRNFASLSLPGLSEERKTVFVPGLAILCGVFDALAIRELRLSDGALREG
VLYEMEGRFRHQDVRSRTASSLANQYHIDSEQARRVLDTTMQMYEQWREQQPKLAHPQLE
ALLRWAAMLHEVGLNINHSGLHRHSAYILQNSDLPGFNQEQQLMMATLVRYHRKAIKLDD
LPRFTLFKKKQFLPLIQLLRLGVLLNNQRQATTTPPTLTLITDDSHWTLRFPHDWFSQNA
LVLLDLEKEQEYWEGVAGWRLKIEEESTPEIAA
>LFGLNPFC_00672 Polyphosphate kinase
MGQEKLYIEKELSWLSFNERVLQEAADKSNPLIERMRFLGIYSNNLDEFYKVRFAELKRR
IIISEEQGSNSHSRHLLGKIQSRVLKADQEFDGLYNELLLEMARNQIFLINERQLSVNQQ
NWLRHYFKQYLRQHITPILINPDTDLVQFLKDDYTYLAVEIIRGDTIRYALLEIPSDKVP
RFVNLPPEAPRRRKPMILLDNILRYCLDDIFKGFFDYDALNAYSMKMTRDAEYDLVHEME
ASLMELMSSSLKQRLTAEPVRFVYQRDMPNALVEVLREKLTISRYDSIVPGGRYHNFKDF
INFPNVGKANLVNKPLPRLRHIWFDKAQFRNGFDAIRERDVLLYYPYHTFEHVLELLRQA
SFDPSVLAIKINIYRVAKDSRIIDSMIHAAHNGKKVTVVVELQARFDEEANIHWAKRLTE
AGVHVIFSAPGLKIHAKLFLISRKENGEVVRYAHIGTGNFNEKTARLYTDYSLLTADARI
TNEVRRVFNFIENPYRPVTFDYLMVSPQNSRRLLYEMVDREIANAQQGLPSGITLKLNNL

VDKGLVDRLYAASSSGVPVNLLVRGMCSLIPNLEGISDNIRAISIVDRYLEHDRVYIFEN
GGDKKVYLSSADWMTRNIDYRIEVATPLLDPRLKQRVLDIIDILFSDTVKARYIDKELSN
RYVPRGNRRKVRAQLAIYDYIKSLEQSE
>LFGLNPFC_00673 Phosphoribosylglycinamide formyltransferase
MNIVVLISGNGSNLQAIIDACKTNKIKGTVRAVFSNKADAFGLERARQAGIATHTLITSA
FDSREAYDRELIHEIDMYAPDVVVLAGFMRILSPAFVSHYAGRLLNIHPSLLPKYPGLHT
HRQALENGDEEHGTSVHFVTDELDGGPVILQAKVPVFAGDTEDDITARVQTQEHAIYPLV
ISWFADGRLKMHENAAWLDGQRLPPQGYAADE
>LFGLNPFC_00674 Phosphoribosylformylglycinamidine cyclo-ligase
MTDKTSLSYKDAGVDIDAGNALVGRIKGVVKKTRRPEVMGGLGGFGALCALPQKYREPVL
VSGTDGVGTKLRLAMDLKRHDTIGIDLVAMCVNDLVVQGAEPLFFLDYYATGKLDVNTAS
AVISGIAEGCLQSGCSLVGGETAEMPGMYHGEDYDVAGFCVGVVEKSEIIDGSKVSDGDV
LIALGSSGPHSNGYSLVRKILEVSGCDPQTTELDGKPLADHLLAPTRIYVKSVLELIEKV
DVHAIAHLTGGGFWENIPRVLPDNTQAVIDESSWQWPEVFNWLQTAGNVERHEMYRTFNC
GVGMIIALPAPEVDKALALLNANGENAWKIGIIKTSDSEQRVVIE
>LFGLNPFC_00675 Uracil phosphoribosyltransferase
MKIVEVKHPLVKHKLGLMREQDISTKRFRELASEVGSLLTYEATADLETEKVTIEGWNGP
VEIDQIKGKKITVVPILRAGLGMMDGVLENVPSARISVVGMYRNEETLEPVPYFQKLVSN
IDERMALIVDPMLATGGSVIATIDLLKKAGCSSIKVLVLVAAPEGIAALEKAHPDVELYT
ASIDQGLNEHGYIIPGLGDAGDKIFGTK
>LFGLNPFC_00676 Uracil permease
MTRRAIGVSERPPLLQTIPLSLQHLFAMFGATVLVPVLFHINPATVLLFNGIGTLLYLFI
CKGKIPAYLGSSFAFISPVLLLLPLGYEVALGGFIMCGVLFCLVSFIVKKAGTGWLDVLF
PPAAMGAIVAVIGLELAGVAAGMAGLLPAEGQTPDSKTIIISITTLAVTVLGSVLFRGFL
AIIPILIGVLVGYALSFAMGIVDTTPIINAHWFALPTLYTPRFEWFAILTILPAALVVIA
EHVGHLVVTANIVKKDLLRDPGLHRSMFANGLSTVISGFFGSTPNTTYGENIGVMAITRV
YSTWVIGGAAIFAILLSCVGKLAAAIQMIPLPVMGGVSLLLYGVIGASGIRVLIESKVDY
NKAQNLILTSVILIIGVSGAKVNIGAAELKGMALATIVGIGLSLIFKLISMLRPEEVVLD
AEDADITDK
>LFGLNPFC_00677 DnaA regulatory inactivator Hda
MVNFSRFCEILVEVSLNTPAQLSLPLYLPDDETFASFWPGDNSSLLAALQNVLRQEHSGY
IYLWAREGAGRSHLLHAACAELSQRGDAVGYVPLDKRTWFVPEVLDGMEHLSLVCIDNIE
CIAGDELWEMAIFDLYNRILESGKTRLLITGDRPPRQLNLGLPDLASRLDWGQIYKLQPL
SDEDKLQALQLRARLRGFELPEDVGRFLLKRLDREMRTLFMTLDQLDRASITAQRKLTIP
FVKEILKL
>LFGLNPFC_00678 putative protein YfgD
MTKQVKIYHNPRCSKSRETLNLLKENGVEPEVVLYLETPADAATLRDLLKMLGMNSAREL
MRQKEDLYKELNLADSSLSEEVLIQAMVENPKLMERPIVVANGKARIGRPPEQVLEIVG
>LFGLNPFC_00679 Beta-barrel assembly-enhancing protease
MFRQLKKNLVATLIAAMTIGQVAPAFADSADTLPDMGTSAGSTLSIGQEMQMGDYYVRQL
RGSAPLINDPLLTQYINSLGMRLVSHANSVKTPFHFFLINNDEINAFAFFGGNVVLHSAL
FRYSDNESQLASVMAHEISHVTQRHLARAMEDQQRSAPLTWVGALGSILLAMASPQAGMA
ALTGTLAGTRQGMISFTQQNEQEADRIGIQVLQRSGFDPQAMPTFLEKLLDQARYSSRPP
EILLTHPLPESRLADARNRANQMRPIVVQSSEDFYLAKVRTLGMYNSGRNQLTSDLLDEW
AKGNVRQQRAAQYGRALQAMEANKYDEARKTLQPLLAAEPGNAWYLDLATDIDLGQNKAN
DAINRLKNARDLRTNPVLQLNLANAYLQGGQPQEAANILNRYTFNNKDDSNGWDLLAQAE
AALNNRDQELAARAEGYALAGRLDQAISLLSSASSQVKLGSLQQARYDARIDQLHQLQER
FKPYTKM
>LFGLNPFC_00680 Putative transport protein YhhT
MLEMLMQWYRRRFSDPEAIALLVILVAGFGIIFFFSGLLAPLLVAIVLAYLLEWPTVRLQ
SIGCSRPWATSIVLILFVGILLLMAFVVLPIAWQQGIYLIRDMPGMLNKLSDFAATLPRR
YPALMDAGIIDAMAENMRSRMLTMGDSVVKISLASLVGLLTIAVYLVLVPLMVFFLLKDK
EQMLNAVRRVLPRNRGLAGQVWKEMNQQITNYIRGKVLEMIVVGIATWLGFLLFGLNYSL
LLAVLVGFSVLIPYIGAFVVTIPVVGVALFQFGAGTEFWSCFAVYLIIQALDGNLLVPVL
FSEAVNLHPLVIILSVVIFGGLWGFWGVFFAIPLATLIKAVIHAWPDGQIAQD
>LFGLNPFC_00681 Peroxiredoxin Bcp
MNPLKAGDIAPKFSLPDQDGEQVNLTDFQGQRVLVYFYPKAMTPGCTVQACGLRDNMDEL
KKAGVDVLGISTDKPEKLSRFAEKELLNFTLLSDEDHQVCEQFGVWGEKSFMGKTYDGIH
RISFLIDADGKIEHVFDDFKTSNHHDVVLNWLKEHA
>LFGLNPFC_00682 Glycine cleavage system transcriptional repressor
MTLSSQHYLVITALGADRPGIVNTITRHVSSCGCNIEDSRLAMLGEEFTFIMLLSGSWNA
ITLIESTLPLKGAELDLLIVMKRTTARPRPPMPASVWVQVDVADSPHLIERFTALFDAHH
MNIAELVSRTQPAENERAAQLHIQITAHSPASADAANIEQAFKALCTELNAQGSINVVNY
SQHDEQDGVK

>LFGLNPFC_00683 4-hydroxy-tetrahydrodipicolinate synthase
MFTGSIVAIVTPMDEKGNVCRASLKKLIDYHVASGTSAIVSVGTTGESATLNHDEHADVV
MMTLELADGRIPVIAGTGANATAEAISLTQRFNDSGIVGCLTVTPYYNRPSQEGLYQHFK
AIAEHTDLPQILYNVPSRTGCDLLPETVGRLAKVKNIIGIKEATGNLTRVNQIKELVSDD
FVLLSGDDASALDFMQLGGHGVISVTANVAARDMAQMCKLAAEGHFAEARVINQRLMPLH
NKLFVEPNPIPVKWACKELGLVATDTLRLPMTPITDSGRETVRAALKHAGLL
>LFGLNPFC_00684 Outer membrane protein assembly factor BamC
MAYSVQKSRLAKVAGVSLVLLLAACSSDSRYKRQVSGDEAYLEAAPLAELHAPAGMILPV
TSGDYAIPVTNGSGAVGKALDIRPPAQPLALVSGARTQFTGDTASLLVENGRGNTLWPQV
VSVLQAKNYTITQRDDAGQTLTTDWVQWNRLDEDEQYRGRYQISVKPQGYQQAVTVKLLN
LEQAGKPVADAASMQRYSTEMMNVISAGLDKSATDAANAAQNRASTTMDVQSAADDTGLP
MLVVRGPFNVVWQRLPAALEKVGMKVTDSTRSQGNMAVTYKPLSDSEWHELGASDPGLAS
GDYKLQVGDLDNRSSLQFIDPKGHTLTQSQNDALVAVFQAAFSK
>LFGLNPFC_00685 hypothetical protein
MRRERLIRPTSDKQISCELPLKSRFSADAHVCVSYQKKGPDDSSPVFLLAKRSLEDSYQR
VVLTLSQSMTFRIDEL
>LFGLNPFC_00686 Phosphoribosylaminoimidazole-succinocarboxamide synthase
MQKQAELYRGKAKTVYSTENPDLLVLEFRNDTSAGDGARIEQFDRKGMVNNKFNYFIMSK
LAEAGIPTQMERLLSDTECLVKKLDMVPVECVVRNRAAGSLVKRLGIEEGIELNPPLFDL
FLKNDAMHDPMVNESYCETFGWVSKENLARMKELTYKANDVLKKLFDDAGLILVDFKLEF
GLYKGEVVLGDEFSPDGSRLWDKETLEKMDKDRFRQSLGGLIEAYEAVARRLGVQLD
>LFGLNPFC_00687 putative protein
MRWQGRRESDNVEDRRNSSGGPSMGGPGFRLPSGKGGLILLIVVLVAGYYGVDLTGLMTG
QPVSQQQSTRSISPNEDEAAKFTSVILATTEDTWGQQFEKMGKTYQQPKLVMYRGMTRTG
CGAGQSIMGPFYCPADGTVYIDLSFYDDMKDKLGADGDFAQGYVIAHEVGHHVQKLLGIE
PKVRQLQQNATQAEVNRLSVRMELQADCFAGVWGHSMQQQGVLETGDLEEALNAAQAIGD
DRLQQQSQGRVVPDSFTHGTSQQRYSWFKRGFDSGDPAQCNTFGKSI
>LFGLNPFC_00688 tRNA(Met) cytidine acetyltransferase TmcA
MAELTALHTLTAQMKREGIRRLLVLSGEERWCFDHALKLRDALPGDWLWISPQPDAENHC
SPSALQTLLGREFRHAVFDARQGFDAAAFAALSGTLKAGSWLVLLLPVWDEWENQPDADS
LRWSDCPDPIATPHFVQHFKRVLTANNDAILWRQNQPFSLAHFTPRTDWHPATGAPQPEQ
QQLLQQLLTMPPGVAAVTAARGRGKSALAGQLISRIAGSAIVTAPAKAATDVLAQFAGEK
FRFIAPDALLASDEQADWLVVDEAAAIPAPLLYQLVSRFPRTLLTTTVQGYEGTGRGFLL
KFCARFPHLHRFELQQPIRWAQGCPLEKMVSEALVFDDENFTHTPQGNIVISAFEQTLWR
SEPETPLKVYQLLSGAHYRTSPLDLRRMMDAPGQHFLQAAGENEIAGALWLVDEGGLSQE
LSQAVWAGFRRPRGNLVAQSLAAHGSNPLAATLRGRRVSRIAVHPARQREGTGQQLIAGA
LQYIHDLDYLSVSFGYTEELWRFWQRCGFVLVRMGNHREASSGCYTAMALLPMSDAGKQL
AEREHYRLRRDAQALAQWNGEMLPVDPLNDAVLSDDDWLELAGFAFTHRPLLTSLGCLLR
LLQTSELALPALRGRLQKNASDAQLCTTLKLSGRKLLLVRQREEAAQALFALDDVRTERL
RDRITQWQFFH
>LFGLNPFC_00689 hypothetical protein
MKHDHFVVQSPDKPAQQLLLLFHGVGDNPVAMGEIGSWFAPLFPDALVVSVGGAEPSGNP
AGRQWFSVQGITEDNRQARVNAIMPTFIETVRYWQKQSGVGANATALIGFSQGAIMALES
IKAEPGLASRVIAFNGRYASLPETASTATTIHLIHGGEDPVIDLAHAVAAQEALISAGGD
VTLDIVEDLGHAIDNRSMQLALDHLRYTIPKHYFDEALSGGKPGDDDVIEMM
>LFGLNPFC_00690 hypothetical protein
MDWLAKYWWILVIVFLVGVLLNVIKDLKRVDHKKFLANKPELPPHRDFNDKWDDDDDWPK
KDQPKK
>LFGLNPFC_00691 Succinyl-diaminopimelate desuccinylase
MSCPVIELTQQLIRRPSLSPDDAGCQALLIERLQAIGFTVERMDFADTQNFWAWRGQGET
LAFAGHTDVVPPGDADRWINPPFEPTIRDGMLFGRGAADMKGSLAAMVVAAERFVAQHPN
HTGRLAFLITSDEEASAHNGTVKVVEALMARNERLDYCLVGEPSSIEVVGDVVKNGRRGS
LTCNLTIHGVQGHVAYPHLADNPVHRAAPFINELVAIEWDQGNEFFPATSMQIANIQAGT
GSNNVIPGELFVQFNFRFSTELTDEMIKAQVLALLEKHQLRYTVDWWLSGQPFLTARGKL
VDAVVNAVEHYNEIKPQLLTTGGTSDGRFIARMGAQVVELGPVNATIHKINECVNAADLQ
LLARMYQRIMEQLVA
>LFGLNPFC_00692 Protein YffB
MVTLYGIKNCDTIKKARRWLEANNIDYRFHDYRVDGLDNELLNGFINELGWEALLNTRGT
TWRKLDETTRNKITDAASAATLMTEMPAIIKRPLLCAPGKPMLLGFSESSYQQFFHEV
>LFGLNPFC_00693 Multidrug efflux pump subunit AcrB
MANFFIDRPIFAWVLAILLCLTGTLAIFSLPVEQYPDLAPPNVRVTANYPGASAQTLENT
VTQVIEQNMTGLDNLMYMSSQSSGTGQASVTLSFKAGTDPDEAVQQVQNQLQSAMRKLPQ
AVQNQGVTVRKTGDTNILTIAFVSTDGSMDKQDIADYVASNIQDPLSRVNGVGDIDAYGS
QYSMRIWLDPAKLNSFQMTAKDVTDAIESQNAQIAVGQLGGTPSVDKQALNATINAQSLL

QTPEQFRDITLRVNQDGSEVRLGDVATVEMGAEKYDYLSRFNGKPASGLGVKLASGANEM
ATAELVLNRLDELAQYFPHGLEYKVAYETTSFVKASIEDVVKTLLEAIALVFLVMYLFLQ
NFRATLIPTIAVPVVLMGTFSVLYAFGYSVNTLTMFAMVLAIGLLVDDAIVVVENVERIM
SEEGLTPREATRKSMGQIQGALVGIAMVLSAVFVPMAFFGGTTGAIYRQFSITIVAAMVL
SVLVAMILTPALCATLLKPLKKGEHHGQKGFFAWFNQMFNRNAERYEKGVAKILHRSLRW
IVIYVLLLGGMVFLFLRLPTSFLPLEDRGMFTTSVQLPSGSTQQQTLKVVEQIEKYYFTH
EKDNIMSVFATVGSGPGGNGQNVARMFIRLKDWSERDSKTGTSFAIIERATKAFNKIKEA
RVIASSPPAISGLGSSAGFDMELQDHAGAGHDALMAARNQLLALAAENPELTRVRHNGLD
DSPQLQIDIDQRKAQALGVAIDDINDTLQTAWGSSYVNDFMDRGRVKKVYVQAAAPYRML
PDDINLWYVRNKDGGMVPFSAFATSRWETGSPRLERYNGYSAVEIVGEAAPGVSTGTAMD
IMESLVKQLPNGFGLEWTAMSYQERLSGAQAPALYAISLLVVFLCLAALYESWSVPFSVM
LVVPLGVIGALLATWMRGLENDVYFQVGLLTVIGLSAKNAILIVEFANEMNQKGHDLFEA
TLHACRQRLRPILMTSLAFIFGVLPMATSTGAGSGGQHAVGTGVMGGMISATILAIYFVP
LFFVLVRRRFPLKPRPE
>LFGLNPFC_00694 Nitrate/nitrite sensor protein NarQ
MIVKRPVSASLARAFFYIVLLSILSTGIALLTLASSLRDAEAINIAGSLRMQSYRLGYDL
QSGSPQLNAHRQLFQQALHSPVLTNLNVWYVPEAVKTRYAHLNANWLEMNNRLSKGDLPW
YQANINNYVNQIDLFVLALQHYAERKMLLVVAISLAGGIGIFTLVFFTLRRIRHQVVAPL
NQLVTASQRIEHGQFDSPPLDTSLPNELGLLAKTFNQMSSELHKLYRSLEASVEEKTRDL
HEAKRRLEVLYQCSQALNTSQIDVHCFRHILQIVRDNEAAEYLELNVGDNWRISEGQPNP
ELPMQILPVTMQETVYGELHWQNSHVSSSEPLLNSVSSMLGRGLYFNQAQKHFQQLLLME
ERATIARELHDSLAQVLSYLRIQLTLLKRSIPEDNATAQSIMADFSQALNDAYRQLRELL
TTFRLTLQQADLPSALREMLDTLQNQTSAKLTLDCRLPTLALDAQMQVHLLQIIREAVLN
AMKHANASEIAVSCVTAPDGNHTVYIRDNGIGIGEPKEPEGHYGLNIMRERAERLGGTLT
FSQPSGGGTLVSISFRSAEGEESQLM
>LFGLNPFC_00695 Ferredoxin--NADP reductase
MNRFIMANSQQCLGCHACEIACVMAHNDEQHVLSQHHFHPRITVIKHQQQRSAVTCHHCE
DAPCARSCPNGAISHVDDSIQVNQQKCIGCKSCVVACPFGTMQIVLTPVAAGKVKATAHK
CDLCAGRENGPACVENCPADALQLVTDAALSGMAKSRRLRTARQEQQPWHASTAAQEMPV
MSKVEQMQATPARGEPDKLAIEARKTGFDEIYLPFRADQAQREASRCLKCGEHSVCEWTC
PLHNHIPQWIELVKAGNIDAAVELSHQTNTLPEITGRVCPQDRLCEGACTIRDEHGAVTI
GNIERYISDQALAKGWRPDLSHVTKVDKRVAIIGAGPAGLACADVLTRNGVEVTVYDRHP
EIGGLLTFGIPSFKLDKSLLARRREIFSAMGIHFELNCEVGKDVSLNSLLEQYDAVFVGV
GTYRSMKAGLPNEDAPGVYDALPFLIANTKQVMGLEELPEEPFINTAGLNVVVLGGGDTA
MDCVRTALRHGASNVTCAYRRDEANMPGSKKEVKNAREEGANFEFNVQPVALELNEQGHV
CGIRFLRTRLGEPDAQGRRRPVPVEGSEFVMPADAVIMAFGFNPHGMPWLESHGVTVDKW
GRIIADVESQYRYQTTNPKIFAGGDAVCGADLVVTAMAEGRHAAQGIIDWLGVKSVKSH
>LFGLNPFC_00696 GDP-mannose pyrophosphatase NudK
MTQQITLVKDKILSDNYFTLHNITYDLTRKDGEVIRHKREVYDRGNGATILLYNAKKKTV
VLIRQFRVATWVNGNESGQLIETCAGLLDNDEPEVCIRKEAIEETGYEVGEVRKLFELYM
SPGGVTELIHFFIAEYSDSQRANAGGGVEDEDIEVLELPFSQALEMIKTGEIRDGKTVLL
LNYLQMSHLMD
>LFGLNPFC_00697 hypothetical protein
MSLVWAAPAQRAFSDWQVTCNNQNFCVARNTGDHNGLVMTLSRSAGAHTDAVLRIERGGL
KSPDASEGEIAPRMLLDGEPLALSGDKWRISPWLLVTDDTATITAFLQMIQEGRAITLRD
GNQTISLSGLKAALLFIDAQQKRVGSETAWIKKGDEPPLSVPPAPALKEVAVVNPTPTPL
SLEERNDLLDYGNWRMNGLRCSLDPLRREVNVTALTDDKALMMISCEAGAYNTIDLAWIV
SRKKPLASRPVRLRLPFNNGQETNELELMNATFDEKSRELVTLAKGRGLSDCGIQARWRF
DGQRFRLVRYAAEPTCDNWHGPDAWPTLWITR
>LFGLNPFC_00698 Transketolase 2
MSRKDLANAIRALSMDAVQKANSGHPGAPMGMADIAEVLWNDFLKHNPTDPTWYDRDRFI
LSNGHASMLLYSLLHLTGYDLPLEELKNFRQLHSKTPGHPEIGYTPGVETTTGPLGQGLA
NAVGLAIAERTLAAQFNQPDHEIVDHFTYVFMGDGCLMEGISHEVCSLAGTLGLGKLIGF
YDHNGISIDGETEGWFTDDTAKRFEAYHWHVIHEIDGHDPQAVKEAILEAQSVKDKPSLI
ICRTVIGFGSPNKAGKEEAHGAPLGEEEVALARQKLGWHHPPFEIPKEIYHAWNAREKGE
KAQQRWNEKFAAYKKAHPQLAEEFTRRMSGGLPKDWEKTTQKYINELQANPAKIATRKAS
QNTLNAYGPMLPELLGGSADLAPSNLTIWKGSVSLKEDPAGNYIHYGVREFGMTAIANGI
AHHGGFVPYTATFLMFVEYARNAARMAALMKARQIMVYTHDSIGLGEDGPTHQAVEQLAS
LRLTPNFSTWRPCDQVEAAVGWKLAVERHNGPTALILSRQNLAQVERTPDQVKEIARGGY
VLKDSGGKPDIILIATGSEMEITLQAAEKLAGEGRNVRVVSLPSTDIFDAQDEEYRESVL
PSNVSARVAVEAGIADYWYKYVGLKGAIVGMTGYGESAPADKLFPFFGFTAENIVAKAHK
VLGVKGA
>LFGLNPFC_00699 Transaldolase
MNELDGIKQFTTVVADSGDIESIRHYHPQDATTNPSLLLKAAGLSQYEHLIDDAIAWGKK

NGKTQEQQVVAACDKLAVNFGAEILKIVPGRVSTEVDARLSFDKEKSIEKARHLVDLYQQ
QGVEKSRILIKLASTWEGIRAAEELEKEGINCNLTLLFSFAQARACAEAGVFLISPFVGR
IYDWYQARKPMDPYVVEEDPGVKSVRNIYDYYKQHHYETIVMGASFRRTEQILALTGCDR
LTIAPNLLKELQEKVSPVVRKLIPPSQTFPRPAPVSEAEFRWEHNQDAMAVEKLSEGIRL
FAVDQRKLEDLLAAKL
>LFGLNPFC_00700 NADP-dependent malic enzyme
MDDQLKQSALDFHEFPVPGKIQVSPTKPLATQRDLALAYSPGVAAPCLEIEKDPLKAYKY
TARGNLVAVISNGTAVLGLGNIGALAGKPVMEGKGVLFKKFAGIDVFDIEVDELDPDKFI
EVVAALEPTFGGINLEDIKAPECFYIEQKLRERMNIPVFHDDQHGTAIISTAAILNGLRV
VEKNISDVRMVVSGAGAAAIACMNLLVALGLQKHNIVVCDSKGVIYQGREPNMAETKAAY
AVVDDGKRTLDDVIEGADIFLGCSGPKVLTQEMVKKMARAPMILALANPEPEILPPLAKE
VRPDAIICTGRSDYPNQVNNVLCFPFIFRGALDVGATAINEEMKLAAVRAIAELAHAEQS
EVVASAYGDQDLSFGPEYIIPKPFDPRLIVKIAPAVAKAAMESGVATRPIADFDVYIDKL
TEFVYKTNLFMKPIFSQARKAPKRVVLPEGEEARVLHATQELVTLGLAKPILIGRPNVIE
MRIQKLGLQIKSGVDFEIVNNESDPRFKEYWTEYFQIMKRRGVTQEQAQRALISNPTVIG
AIMVQRGEADAMICGTVGDYHEHFSVVKNVFGYRDGVHTAGAMNALLLPSGNTFIADTYV
NDEPDAEELAEITLMAAETVRRFGIEPRVALLSHSNFGSSDCPSSSKMRQALELVRERAP
ELMIDGEMHGDAALVEAIRNDRMPDSPLKGSANILVMPNMEAARISYNLLRVSSSEGVTV
GPVLMGVAKPVHVLTPIASVRRIVNMVALAVVEAQTQPL
>LFGLNPFC_00701 Ethanolamine utilization protein EutS
MDKERIIQEFVPGKQVTLAHLIAHPGEELAKKIGVPDAGAIGIMTLTPGETAMIAGDLAL
KAAGVHIGFLDRFSGALVIYGSVGAVEEALSQTVSGLGRLLNYTLCEMTKS
>LFGLNPFC_00702 Propanediol utilization protein PduV
MKRIAFVGTVGAGKTTLFNALQGNYTLARKTQAVEFNDNGDIDTPGEYFSHPRWYHALIT
TLQDVDMLIYVHGANDPESRLPAGLLDIGVSKRQIAVISKTDMPDADVAATRKLLLETGF
EEPIFELNSHDPQSVQQLVDYLASLTKQEEAGEKTHHSE
>LFGLNPFC_00703 Ethanolamine utilization protein EutQ
MKKLITANDIREAHARGELAMSVVLRASIITPEAREMADLLGFTITECDESIPVTASVPA
SASADKTESQRIRETIIAQLPEGQFTESLVAQLMEKVMKEKQSLEQGALQPSFKSVTGKG
GIKVIDGSSVKFGRFDGAQPHCVGLTDLVTGDDGSSMAAGFMQWENAFFPWTLNYDEIDM
VLEGELHVRHEGETMIAKAGDVMFIPKGSSIEFGTTSSVKFLYVAWPANWQSL
>LFGLNPFC_00704 hypothetical protein
MKDFITEAWLRANHTLSEGAEIHLPADSRLTPSARELLESRHLRIKFIDEQGRLFVDDEQ
QQPQPVHGLTSSDEHPQACCELCRQPVAKKPDTLTHLSAEKMVAKSDPRLAFRAVLDSTI
ALAVWLQIELAEPWQPWLADIRSRLGNIMRADALGEPLACQAIVGLSDEDLHRLSHQPLR
YLDHDHLVPEASHGRDAALLNLLRTKVRETETVAAQVFITRSFEVLRPDILQALNRLSST
VYVMMILSVTKQPLTVKQIQQRLGETQ
>LFGLNPFC_00705 Ethanolamine utilization protein EutD
MIIKRCRELALRAPARVVFPDALDQRVLKAAQYLHQQGLATPILVANPFELRQFALSHGV
AMDGLQVIDPHGNLAMREEFAHRWLARAGEKTPPDALEKLTDPLMFAAAMVSAGKADVCI
AGNLSSTANVLRAGLRIIGLQPGCKTLSSIFLMLPQYSGPALGFADCSVVPQPTAAQLAD
IALASAETWRAITGEEPRVAMLSFSSNGSARHPCVANVQQATEIVRERAPKLVVDGELQF
DAAFVPEVAAQKAPASPLQGKANVMVFPSLEVGNIGYKIAQRLGGYRAVGPLIQGLAAPM
HDLSRGCSVQEIIELALVAAVPRQTEVNRESSLQTLVE
>LFGLNPFC_00706 Ethanolamine utilization protein EutM
MNGPVLDPDKEESTMEALGMIETRGLVALIEASDAMVKAARVKLVGVKQIGGGLCTAMVR
GDVAACKAATDAGAAAAQRIGELVSVHVIPRPHGDLEEVFPIGLKGDSSNL
>LFGLNPFC_00707 Ethanolamine utilization protein EutN
MKLAVVTGQIVCTVRHHGLAHDKLLMVEMIDPQGNPDGQCAVAIDNIGAGTGEWVLLVSG
SSARQAHKSETSPVDLCVIGIVDEVVSGGQVIFHK
>LFGLNPFC_00708 Gamma-glutamyl phosphate reductase
MNQQDIEQVVKAVLLKMQSSDTPPAAVHEMGVFASLDDAVAAAKVAQQGLKSVAMRQLAI
AAIREAGEKHARDLAELAVSETGMGRVEDKFAKNVAQARGTPGVECLSPQVLTGDNGLTL
IENAPWGVVASVTPSTNPAATVINNAISLIAAGNSVIFAPHPAAKKVSQRAITLLNQAIV
AAGGPENLLVTVANPDIETAQRLFKFPGIGLLVVTGGEAVVEAARKHTNKRLIAAGAGNP
PVVVDETADLARAAQSIVKGASFDNNIICADEKVLIVVDSVADELMRLMEGQHAVKLTAE
QAQQLQPVLLKNIDERGKGTVSRDWVGRDAAKIAAAIGLKVPEQTRLLFVETTAEHPFAV
TELMMPVLPVVRVANVADAIALAVKLEGGCHHTAAMHSRNIENMNQMANAIDTSIFVKNG
PCIAGLGLGGEGWTTMTITTPTGEGVTSARTFVRLRRCVLVDAFRIV
>LFGLNPFC_00709 Cell division protein FtsA
MAHDEQWLTPRLQTAATLCNQTPAATESPLWLGVDLGTCDVVSMVVDRDGQPVAVCLDWA
DVVRDGIVWDFFGAVTIVRRHLDTLEQQFGRRFSHAATSFPPGTDPRISINVLESAGLEV
SHVLDEPTAVADLLQLDNAGVVDIGGGTTGIAIVKKGKVTYSADEATGGHHISLTLAGNR
RISLEEAEQYKRGHGDEIWPAVKPVYEKMADIVARHIEGQGITDLWLAGGSCMQPGVAAL

FCKQFPALQVHLPQHSLFMTPLAIASSGREKAEGIYAK
>LFGLNPFC_00710 NAD-dependent methanol dehydrogenase
MQSELQTALFQAFDTLNLQRVKTFSVPPVTLCGPGAVSSCGQQAQTRGLKHLFVMADSFL
HQAGMTAGLTRSLAVKGIAMTLWPCPVGEPCITDVCAAVAQLRESGCDGVIAFGGGSVLD
AAKAVALLVTNPDSTLAEMSETSVLQPRLPLIAIPTTAGTGSETTNVTVIIDAVSGRKQV
LAHASLMPDVAILDAALTEGVPSHVTAMTGIDALTHAIEAYSALNATPFTDSLAIGAIAM
IGKSLPKAVGYGHDLAARESMLLASCMAGMAFSSAGLGLCHAMAHQPGAALHIPHGLANA
MLLPTVMEFNRMVCRDRFSQIGRALRTKKSDDRDAINAVSELIAEVGIGKRLGDVGATSA
HYGAWAQAALEDICLRSNPRTASLEQIVGLYAAAQ
>LFGLNPFC_00711 hypothetical protein
MGINEIIMYIMMFFMLIAAVDRILSQFGGSARFLGKFGKSIEGSGGQFEEGFMAMGALGL
AMVGMTALAPVLAHVLGPVIIPVYEMLGANPSMFAGTLLACDMGGFFLAKELAGGDVAAW
LYSGLILGSMMGPTIVFSIPVALGIIEPSDRRYLALGVLAGIVTIPIGCIAGGLVAMYSG
VQINGQPVEFTFALILMNMIPVLIVAVLVALGLKFIPEKMINGFQIFAKFLVALITLGLA
AAVVKFLLGWELIPGLDPIFMAPGDKPGEVMRAIEVIGSISCVLLGAYPMVLLLTRWFEK
PLMSVGNVLNMNNIAAAGMVATLANNIPMFGMMKQMDTRGKVINCAFAVSAAFALGDHLG
FAAANMNAMIFPMIVGKLIGGVTAIGVAMMLVPKEDATAAKTEAEAQS
>LFGLNPFC_00712 hypothetical protein
MNTRQLLSVGIDIGTTTTQVIFSHLELVNRAAVSQVPRYEFIKREISWQSPVFFTPVDKQ
GGLKEAELKTLILEQYQAAGIAPESVDSGAIIITGESAKTRNARPAVMALSRSLGDFVVA
SAGPHLESVIAGHGAGAQTLSEQRLCRVLNIDIGGGTANYALFDAGKISGTACLNVGGRL
LETDSQGRVVYAHKPGQMIVDECFGAGTDARSLTGAQLVQVTRRMAELIVEVIDGTLSPL
AQALMQTGLLPAGVTPEIITLSGGVGECYRHQPADPFCFADIGPLLATALHDHPRLREMN
VQFPAQTVRATVIGAGAHTLSLSGSTIWLEGVQLPLRNLPVAIPIDETDLVSAWQQALIQ
LDLCPKTDAYVLALPASLPVRYAAVLTVINALVDFVARFPNPHPLLVVAGQDFGKALGML
LRPQLQQLPLAVIDEVIVRAGDYIDIGTPLFGGSVVPVTVKSLAFPS
>LFGLNPFC_00713 Ethanolamine ammonia-lyase heavy chain
MKLKTTLFGNVYQFKDVKEVLAKANELRSGDVLAGVAAASSQERVAAKQVLSEMTVADIR
NNPVIAYEDDCVTRLIQDDVNETAYNQIKNWSISELREYVLSDETSVDDIAFTRKGLTSE
VVAAVAKICSNADLIYGAKKMPVIKKANTTIGIPGTFSARLQPNDTRDDVQSIAAQIYEG
LSFGVGDAVIGVNPVTDDVENLSRVLDTIYGVIDKFNIPTQGCVLAHVTTQIEAIRRGAP
GGLIFQSICGSEKGLKEFGVELAMLAEARAVGAEFNRIAGENCLYFETGQGSALSAGANF
GADQVTMEARNYGLARHYDPFIVNTVVGFIGPEYLYNDRQIIRAGLEDHFMGKLSGISMG
CDCCYTNHADADQNLNENLMILLATAGCNYIMGMPLGDDIMLNYQTTAFHDTATVRQLLN
LRPSPEFERWLESMGIMANGRLTKRAGDPSLFF
>LFGLNPFC_00714 Ethanolamine ammonia-lyase light chain
MDQKQIEEIVRSVMASMGQAAPAPSEAKCATTNCAAPVTSESCALDLGSAEAKAWIGVEN
PHRADVLTELRRSTVARVCTGRAGPRPRTQALLRFLADHSRSKDTVLKEVPEEWVKAQGL
LEVRSEISDKNLYLTRPDMGRRLCAEAVEALKAQCVANPDVQVVISDGLSTDAITVNYEE
ILPPLMAGLKQAGLKVGTPFFVRYGRVKIEDQIGEILGAKVVILLVGERPGLGQSESLSC
YAVYSPRMATTVEADRTCISNIHQGGTPPVEAAAVIVDLAKRMLEQKASGINMTR
>LFGLNPFC_00715 Ethanolamine utilization protein EutL
MPALDLIRPSVTAMRVIASVNAEFARELKLPPHIRSLGLISADSDDVTYIAADEATKQAM
VEVVYGRSLYAGAAHGPSPTAGEVLIMLGGPNPAEVRAGLDAMVANIENGAAFQWANDAE
NTAFLAHVVSRTGSYLSSTAGITLGDPMAYLVAPPLEATYGIDAALKSADVQLVTYVPPP
SETNYSAAFLTGSQAACKAACNAFTDAVLEIARNPIQRA
>LFGLNPFC_00716 Ethanolamine utilization protein EutK
MINALGLLEVDGMVAAIDAADAMLKAANVRLLSHEVLDPGRLTLVVEGDLAACRAALDAG
CAAAMRTGRVISRKEIGRPDDDTQWLVTGFNRQPKQPVKEPDAPVIVAESADELLALLTS
VRQGMTAGEVAAHFGWPLEKARNALEQLFSAGTLRKRSSRYRLKPH
>LFGLNPFC_00717 hypothetical protein
MKKTRTANLHHLYHEPLPENLKLTPKVEVDNVHQRQTTDVYEHALTITAWQQIYDQLHPG
KFHGEFTEILLDDIQVFREYTGLALRQSCLVWPNSFWFGIPATRGEQGFIGSQCLGSAEI
ATRPGGTEFELSTPDDYTILGVVLSEDVITRQANFLHNPDRVLHMLRSQSALEVKEQHKA
ALWGFVQQALATFCENPENLHQPAVRKVLGDNLLMAMGAMLEEAQPMVTAESISHQSYRR
LLSRAREYVLENMSEPVTVLDLCNQLHVSRRTLQNAFHAILGIGPNAWLKRIRLNAVRRE
LISPWSQSTTVKDAAMQWGFWHLGQFATDYQQLFAEKPSLTLHQRMREWG
>LFGLNPFC_00718 Oxygen-dependent coproporphyrinogen-III oxidase
MMKPDAHQVKQFLLNLQDTICQQLSAVDGAEFVEDSWQREAGGGGRSRVLRNGGVFEQAG
VNFSHVHGEAMPASATAHRPELAGRSFEAMGVSLVVHPHNPYVPTSHANVRFFIAEKPGA
EPVWWFGGGFDLTPFYGFEEDAIHWHRTARDLCLPFGEDVYPRYKKWCDEYFYLKHRNEQ
RGIGGLFFDDLNTPDFDHCFAFMQAVGKGYTDAYLPIVERRKAMAYGERERNFQLYRRGR
YVEFNLVWDRGTLFGLQTGGRTESILMSMPPLVRWEYDYQPKDGSPEAALSEFIKVRDWV
>LFGLNPFC_00719 N-acetylmuramoyl-L-alanine amidase AmiA

MSTFKPLKTLTSRRQVLKAGLAALTLSGMSQAIAKEEPLKTSNGHSKPKAKKSGGRRVVV
LDPGHGGIDTGAIGRNGSKEKHVVLAIAKNVRSILRNHGIDARLTRSGDTFIPLYDRVEI
AHKHGADLFMSIHADGFTNPKAAGASVFALSNRGASSAMAKYLSERENRADEVAGKKATD
KDHLLQQVLFDLVQTDTIKNSLTLGSHILKKIKPVHKLHSRNTEQAAFVVLKSPSVPSVL
VETSFITNPEEERLLGTAAFRQKIATAIAEGVISYFHWFDNQKAHSRKR
>LFGLNPFC_00720 Acetyltransferase YpeA
MEIRVFRQEDFEEVITLWERCDLLRPWNDPEMDIERKMNHDVSLFLVAEVNGEVVGTVMG
GYDGHRGSAYYLGVHPEFRGRGIANALLNRLEKKLIARGCPKIQINVPEDNDMVLGMYER
LGYEHADVLSLGKRLIEDEEY
>LFGLNPFC_00721 Inner membrane protein YfeZ
MKSTEFYPVHYDAHGRLRLPLLFWLVLLLQARTWVLFVIAGASREQGTALLNLFYPDHDN
FWLGLIPGIPAVLAFLLSGRRASFPRIWHVLYFLLLLAQVVLLCWQPWLWLNGESVSGIG
LALVVADIVALIWLLTNRRLRACFNEEKE
>LFGLNPFC_00722 putative protein YfeY
MKSLRLMLCAMPLMLTGCSTMSSVNWSAANPWNWFGSSTKVSEQGVGELTASTPLQEQAI
ADALDGDYRLRSGMKTANGNVVRFFEVMKGDNVAMVINGDQGTISRIDVLDSDIPADTGV
KIGTPFSDLYSKAFGNCQKADSDDNRAVECKAEGSQHISYQFSGEWSGPEGLMPSDDTLK
NWKVSKIIWRR
>LFGLNPFC_00723 Dye-decolorizing peroxidase YfeX
MSQVQSGILPEHCRAAIWIEANVKGEVDALRAASKTFADKLATFEAKFPDAHLGAVVAFG
NNTWRALSGGVGAEELKDFPGYGKGLAPTTQFDVLIHILSLRHDVNFSVAQAAMEAFGDC
IEVKEEIHGFRWVEERDLSGFVDGTENPAGEETRREVAVIKDGVDAGGSYVFVQRWEHNL
KQLNRMSVHDQEMMIGRTKEANEEIDGDERPETSHLTRVDLKEDGKGLKIVRQSLPYGTA
SGTHGLYFCAYCARLHNIEQQLLSMFGDTDGKRDAMLRFTKPVTGGYYFAPSLDKLMAL
>LFGLNPFC_00724 PTS system N-acetylmuramic acid-specific EIIBC component
MAKEISSELLNTILTRVGGPGNIASCGNCMTRLRLGVHDSSLVDPDIKTLEGVKGVILTS
DQVQVVFGPGKAHRAAKAMSELLGEAPVQDAAEIAAQNKRQLKARQTSGVQQFLAKFATI
FTPLIPGFIAAGLLLGIATLIATVMHVTADAQGTLPDALNFMKVFSKGLFTFLVILVGYN
AAQAFGGTGVNGAIIAALFLLGYNPTATTGYYAGFHDFFGLPIDPRGNIIGVLIAAWACA
RIEGMVRRFMPDDLDMLLTSLITLLITATLAYLIIMPLGGWLFEGMSWLFMHLNSNPLGC
AVLAGLFLIAVVFGVHQGFIPVYLALMDSQGFNSLFPILSMAGAGQVGAALALYWRAQPH
SALRSQVRGAIIPGLLGVGEPLIYGVTLPRMKPFVTACLGGAAGGLFIGLIAWWGLPMGL
NSAFGPSGLVALPLMTSAQGILPAMAVYAGGILVAWVSGFIFTTLFGCRNVNLD
>LFGLNPFC_00725 N-acetylmuramic acid 6-phosphate etherase
MQLEKMITEGSNAASAEIDRVSTLEMCRIINDEDKTVPLAVERVLPDIAAAIDVIHTQVS
GGGRLIYLGAGTSGRLGILDASECPPTYGVKPGLVVGLIAGGEYAIQHAVEGAEDSREGG
VNDLKNINLTAQDVVVGIAASGRTPYVIAGLEYARQLGCRTVGISCNPGSAVSSTAEFAI
TPVVGAEVVTGSSRMKAGTAQKLVLNMLSTGLMIKSGKVFGNLMVDVVATNEKLHVRQVN
IVKNATGCNAEQAEAALIACERNCKTAIVMVLKNLDADEAKKCLDQHGGFIRKALEKE
>LFGLNPFC_00726 Oxidoreductase UcpA
MGKLTGKTALITGALQGIGEGIARTFARHGANLILLDISPEIEKLADELCGRGHRCTAVV
ADVRDPASVAAAIKRAKEKEGRIDILVNNAGVCRLGSFLDMSDEDRDFHIDINIKGVWNV
TKAVLPEMIARKDGRIVMMSSVTGDMVADPGETAYALTKAAIVGLTKSLAVEYAQSGIRV
NAICPGYVRTPMAESIARQSNPEDPESVLTEMAKAIPMRRLADPLEVGELAAFLASDESS
YLTGTQNVIDGGSTLPETVSVGI
>LFGLNPFC_00727 Thiosulfate-binding protein
MAVNLLKKNSLVLVASLLLAGHVQATELLNSSYDVSRELFAALNPPFEQQWAKDNGGDKL
TIKQSHAGSSKQALAILQGLKADVVTYNQVTDVQILHDKGKLIPADWQSRLPNNSSPFYS
TMGFLVRKGNPKNIHDWNDLVRSDVKLIFPNPKTSGNARYTYLAAWGAADKADGGDKAKT
EQFMTQFLKNVEVFDTGGRGATTTFAERGLGDVLISFESEVNNIRKQYEAQGFEVVIPKT
NILAEFPVAWVDKNVQANGTEKAAKAYLNWLYSPQAQTIITDYYYRVNNPEVMDKLKDKF
PQTELFRVEDKFGSWTEVMKTHFTSGGELDKLLAAGRK
>LFGLNPFC_00728 Sulfate transport system permease protein CysT
MFAVSSRRVLPGFTLSLGTSLLFVCLILLLPLSALVMQLAQMSWAQYWEVITNPQVVAAY
KVTLLSAFVASIFNGVFGLLMAWILTRYRFPGRTLLDALMDLPFALPTAVAGLTLASLFS
VNGFYGEWLAKFDIKVTYTWLGIAVAMAFTSIPFVVRTVQPVLEELGPEYEEAAETLGAT
RWQSFRKVVLPELSPALVAGVALSFTRSLGEFGAVIFIAGNIAWKTEVTSLMIFVRLQEF
DYPAASAIASVILAASLLLLFSINTLQSRFGRRVVGH
>LFGLNPFC_00729 Sulfate transport system permease protein CysW
MAEVTQLKRYDARPINWGKWFLIGIGMLVSAFILLVPMIYIFVQAFSKGLMPVLQNLADP
DMLHAIWLTVMIALIAVPVNLVFGILLAWLVTRFNFPGRQLLLTLLDIPFAVSPVVAGLV
YLLFYGSNGPLGGWLDEHNLQIMFSWPGMVLVTIFVTCPFVVRELVPVMLSQGSQEDEAA
ILLGASGWQMFRRVTLPNIRWALLYGVVLTNARAIGEFGAVSVVSGSIRGETLSLPLQIE
LLEQDYNTVGSFTAAALLTLMAIITLFLKSMLQWRLENQEKRAQQEEHHEH

>LFGLNPFC_00730 Sulfate/thiosulfate import ATP-binding protein CysA
MSIEIANIKKSFGRTQVLNDISLDIPSGQMVALLGPSGSGKTTLLRIIAGLEHQTSGHIR
FHGTDVSRLHARDRKVGFVFQHYALFRHMTVFDNIAFGLTVLPRRERPNAAAIKAKVTKL
LEMVQLAHLADRYPAQLSGGQKQRVALARALAVEPQILLLDEPFGALDAQVRKELRRWLR
QLHEELKFTSVFVTHDQEEATEVADRVVVMSQGNIEQADAPDQVWREPATRFVLEFMGEV
NRLQGTIRGGQFHVGAHRWPLGYTPAYQGPVDLFLRPWEVDISRRTSLDSPLPVQVLEAS
PKGHYTQLVVQPLGWYNEPLTVVMHGDDAPQRGDRLYVGLQHARLYNGDERIETRDEELA
LAQSA
>LFGLNPFC_00731 Cysteine synthase B
MSTLEQTIGNTPLVKLQRMAPDNGSEVWLKLEGNNPAGSVKDRAALSMIVEAEKRGEIKP
GDVLIEATSGNTGIALAMIAALKGYRMKLLMPDNMSQERRAAMRAYGAELILVTKEQGME
GARDLALEMANRGEGKLLDQFNNPDNPYAHYTTTGPEIWQQTGGRITHFVSSMGTTGTIT
GVSRFMREQSKPVTIVGLQPEEGSSIPGIRRWPAEYLPGIFNASLVDEVLDIHQRDAENT
MRELAVREGIFCGVSSGGAVAGALRVAKANPGAVVVAIICDRGDRYLSTGVFGEEHFSQG
AGI
>LFGLNPFC_00732 hypothetical protein
MKKIVCLVVTLLMTLPAYAKLTAHEEARINAMLEGLAQKKDLIFVRNGDEHTCDEAVSHL
RLKLGNTRNRIDTAEQFIDKVASSSSITGKPYIVKMPGKSDENAQPFLHALIAQTDKTVP
AQ
>LFGLNPFC_00733 Pyridoxine/pyridoxal/pyridoxamine kinase
MSSLLLFNDKSRALQADIVAVQSQVVYGSVGNSIAVPAIKQNGLNVFAVPTVLLSNTPHY
DTFYGGAIPDEWFSGYLRALQERDALRQLRAVTTGYMGTASQIKILAEWLTALRKDHPDL
LIMVDPVIGDIDSGIYVKPDLPEAYRQYLLPLAQGITPNIFELEILTGKDCRDLDSAIAA
AKSLLSDTLKWVVITSASGNEENQEMQVVVVSADSVNVISHSRVKTDLKGTGDLFCAQLI
SGLLKGKALTDAVHRAGLRVLEVMRYTQQHESDELILPPLAEA
>LFGLNPFC_00734 PTS system glucose-specific EIIA component
MGLFDKLKSLVSDDKKDTGTIEIIAPLSGEIVNIEDVPDVVFAEKIVGDGIAIKPTGNKM
VAPVDGTIGKIFETNHAFSIESDSGVELFVHFGIDTVELKGEGFKRIAEEGQRVKVGDTV
IEFDLPLLEEKAKSTLTPVVISNMDEIKELIKLSGSVTVGETPVIRIKK
>LFGLNPFC_00735 Phosphoenolpyruvate-protein phosphotransferase
MISGILASPGIAFGKALLLKEDEIVIDRKKISADQVDQEVERFLSGRAKASAQLETIKTK
AGETFGEEKEAIFEGHIMLLEDEELEQEIIALIKDKHMTADAAAHEVIEGQASALEELDD
EYLKERAADVRDIGKRLLRNILGLKIIDLSAIQDEVILVAADLTPSETAQLNLKKVLGFI
TDAGGRTSHTSIMARSLELPAIVGTGSVTSQVKNDDYLILDAVNNQVYVNPTNEVIDKMR
AVQEQVASEKAELAKLKDLPAITLDGHQVEVCANIGTVRDVEGAERNGAEGVGLYRTEFL
FMDRDTLPTEEEQFAAYKAVAEACGSQAVIVRTMDIGGDKELPYMNFPKEENPFLGWRAI
RIAMDRKEILRDQLRAILRASAFGKLRIMFPMIISVEEVRALRKEIEIYKQELRDEGKAF
DESIEIGVMVETPAAATIARHLAKEVDFFSIGTNDLTQYTLAVDRGNDMISHLYQPMSPS
VLNLIKQVIDASHAEGKWTGMCGELAGDERATLLLLGMGLDEFSMSAISIPRIKKIIRNT
NFEDAKVLAEQALAQPTTDELMTLVNKFIEEKTIC
>LFGLNPFC_00736 Phosphocarrier protein HPr
MFQQEVTITAPNGLHTRPAAQFVKEAKGFTSEITVTSNGKSASAKSLFKLQTLGLTQGTV
VTISAEGEDEQKAVEHLVKLMAELE
>LFGLNPFC_00737 Cysteine synthase A
MSKIFEDNSLTIGHTPLVRLNRIGNGRILAKVESRNPSFSVKCRIGANMIWDAEKRGVLK
PGVELVEPTSGNTGIALAYVAAARGYKLTLTMPETMSIERRKLLKALGANLVLTEGAKGM
KGAIQKAEEIVASNPEKYLLLQQFSNPANPEIHEKTTGPEIWEDTDGQVDVFIAGVGTGG
TLTGVSRYIKGTKGKTDLISVAVEPTDSPVIAQALAGEEIKPGPHKIQGIGAGFIPANLD
LKLVDKVIGITNEEAISTARRLMEEEGILAGISSGAAVAAALKLQEDESFTNKNIVVILP
SSGERYLSTALFADLFTEKELQQ
>LFGLNPFC_00738 Sulfate transporter CysZ
MVSSFTSAPRSGFYYFAQGWKLVSQPGIRRFVILPLLVNILLMGGAFWWLFTQLDVWIPT
LMSYVPDWLQWLSYLLWPLAVISVLLVFGYFFSTIANWIAAPFNGLLAEQLEARLTGATP
PDTGIFGIMKDVPRIMKREWQKFAWYLPRAIVLLILYFIPGIGQTVAPVLWFLFSAWMLA
IQYCDYPFDNHKVPFKEMRTALRTRKITNMQFGALTSLFTMIPLLNLFIMPVAVCGATAM
WVDCYRDKHAMWR
>LFGLNPFC_00739 Cell division protein ZipA
MMQDLRLILIIVGAIAIIALLVHGFWTSRKERSSMFRDRPLKRMKSKRDDDSYDEDVEDD
EGVGEVRVHRVNHAPANAQEHEAARPSPQHQYQPPYASAQPRQPVQQPPEAQVPPQHAPR
PAQPVQQPVQQPAYQPQPEQPLQQPVSPQVASAPQPVHSAPQPAQQAFQPAEPVAAPQPE
PVAEPAPVMDKPKRKEAVIIMNVAAHHGSELNGELLLNSIQQAGFIFGDMNIYHRHLSPD
GSGPALFSLANMVKPGTFDPEMKDFTTPGVTIFMQVPSYGDELQNFKLMLQSAQHIADEV
GGVVLDDQRRMMTPQKLREYQDIIREVKDANA
>LFGLNPFC_00740 DNA ligase

MESIEQQLTELRTTLRHHEYLYHVMDAPEIPDAEYDRLMRELRELETKHPELITPDSPTQ
RVGAAPLAAFSQIRHEVPMLSLDNVFDEESFLAFNKRVQDRLKSNEKVTWCCELKLDGLA
VSILYENGVLVSAATRGDGTTGEDITSNVRTIRAIPLKLHGENIPARLEVRGEVFLPQAG
FEKINEDARRTGGKVFANPRNAAAGSLRQLDPRITAKRPLTFFCYGVGVLEGGELPDTHL
GRLMQFKAWGLPVSDRVTLCESAEEVLAFYHKVEEDRPTLGFDIDGVVIKVNSLAQQEQL
GFVARAPRWAVAFKFPAQEQMTFVRDVEFQVGRTGAITPVARLEPVHVAGVLVSNATLHN
ADEIERLGLRIGDKVVIRRAGDVIPQVVNVVLSERPEDTREVVFPTHCPVCGSDVERVEG
EAVARCTGGLICGAQRKESLKHFVSRRAMDVDGMGDKIIDQLVEKEYVHTPADLFKLTAG
KLTGLERMGPKSAQNVVNALEKAKETTFARFLYALGVREVGEATAAGLAAYFGTLEALEA
ASIEELQKVPDVGIVVASHVHNFFAEESNRNVISELLAEGVHWPEPIVINAEEIDSPFAG
KTVVLTGSLSQMSRDDAKARLVELGAKVAGSVSKKTDLVIAGEAAGSKLAKAQELGIEVI
DETEMLRLLGS
>LFGLNPFC_00741 hypothetical protein
MEKEQLIEIANTIMPFGKYKGRRLIDLPEEYLLWFARKDEFPAGKLGELMQITLLIKTEG
LTQLVQPLKRPL
>LFGLNPFC_00742 hypothetical protein
MKLFRILDPFTLTLITVVLLASFFPARGDFVPFFENLTTAAIALLFFMHGAKLSREAIIA
GGGHWRLHLWVMCSTFVLFPILGVLFAWWKPVNVDPMLYSGFLYLCILPATVQSAIAFTS
MAGGNVAAAVCSASASSLLGIFLSPLLVGLVMNVHGAGGSLEQVGKIMLQLLLPFVLGHL
SRPWIGDWVSRNKKWIAKTDQTSILLVVYTAFSEAVVNGIWHKVGWGSLLFIVVVSCVLL
AIVIVVNVFMARRLGFNKADEITIVFCGSKKSLANGIPMANILFPTSVIGMMVLPLMIFH
QIQLMVCAVLARRYKRQTEQLQAQQESSADKA
>LFGLNPFC_00743 HTH-type transcriptional regulator CynR
MNYSLKQLKVFVTVAQEKSFSRAGERIGLSQSAVSHSVKELENHTGVRLLDRTTREVVLT
DAGQQLALRLERLLDELNSTLRDTGRMGQQLSGKVRVAASQTISAHLIPQCIAESHRCYP
DIQFVLHDRPQQWVMESIRQGDVDFGIVIDPGPVGDLQCEAILSEPFFLLCHRDSALAVE
DYVPWQALQGAKLVLQDYASGSRPLIDAALARNGIQANIVQEIGHPATLFPMVAAGIGIS
ILPALALPLPEGSPLVVKRITPVVERQLMLVRRKNRSLSTAAEALWDVVRDQGNALMAGR
EGDPLYQI
>LFGLNPFC_00744 hypothetical protein
MKKHLFTLTLSSVLAIPAVSHAEFKGGFADIGIHYLDWTSRTTEKSSTKSHKDDFGYLEL
EGGANFSWGEMYGFFDWENFYNDRHDKPGSEQRYTFKNTNRIYLGDTGFNLYLHAYGTYG
SANRVNFHDDMFLYGIGYNFTGSGWWFKPFFAKRYTDQTYYTGDNGYVAGWVAGYSFMLG
SEKFTLTNWNEYEFDRDATYAAGNGGKEGLNGAVALWWNATSHITTGIQYRYADDKLGED
FYQDAIIYSIKFNF
>LFGLNPFC_00745 Purine nucleoside phosphorylase 2
MYQAQFSHNPLYCVDIIKTYKPDFTPRVAFILGSGLGALADQIENAVAISYEKLPGFPVS
TVHGHAGELVLGYLQGVPVACMKGRGHFYEGRGMTIMTDAIRTFKLLGCELLFCTNAAGS
LRPEVGAGSLVALKDHINTMPGTPMVGLNDERFGERFFSLANAYDAEYRALLQKVAKEEG
VPLTEGVFVSYPGPNFETAAEIRMMQIIGGDVVGMSVVPEVISARHCELKVVAVSAITNM
AEGLSDVKLSHAQTLAAAELSKQNFINLICGFLRKIA
>LFGLNPFC_00746 Xanthosine permease
MSIALRLKVMSFLQYFIWGSWLVTLGSYMINTLHFTGANVGMVYSSKGIAAIIMPGIMGI
IADKWLRAERAYMLCHLVCAGVLFYAASVTDPDMMFWVMLVNAMAFMPTIALSNSVSYSC
LAQAGLDPVTAFPPIRVFGTVGFIVAMWAVSLLHLELSSLQLYIASGASLLLSAYALTLP
KIPVAEKKATTSLASKLGLDAFVLFKNPRMAIFFLFAMMLGAVLQITNVFGNPFLHDFAR
NPEFADSFVVKYPSILLSVSQMAEVGFILTIPFFLKRFGIKTVMLMSMVAWTLRFGFFAY
GDPSPTGFILLLLSMIVYGCAFDFFNISGSVFVEQEVDSSIRASAQGLFMTMVNGVGAWV
GSILSGMAVDYFSVDGVKDWHTIWLMFAGYALFLAVIFFFGFKYNHDPEKITHRSVTH
>LFGLNPFC_00747 Hca operon transcriptional activator HcaR
MERVYRTDLKLLRYFLAVAEELHFGRAAARLNMSQPPLSIHIKELENQLGTQLFIRHSRS
VVLTHAGKILMEESRRLLVNANNVLARVEQIGRGEAGRIELGVVGTAMWGRMRPVMRRFL
RENPNVEVLFREKMPAMQMALLERRELDAGIWRMATEPPTGFTSLRLHESAFLVAMPEEH
HLSSFSTVPLEALRDEYFVTMPPVYTDWDFLQRVCQQVGFSPVVIREVNEPQTVLAMVSM
GIGITLIADSYAQMNWPGVIFRPLKERIPADLYIVYETQQVTPALVKLLAALAQ
>LFGLNPFC_00752 Glutamate--tRNA ligase
MKIKTRFAPSPTGYLHVGGARTALYSWLFARNHGGEFVLRIEDTDLERSTPEAIEAIMDG
MNWLSLEWDEGPYYQTKRFDRYNAVIDQMLEEGTAYKCYCSKERLEALREEQMAKGEKPR
YDGRCRHSHEHHADDEPCVVRFANPQEGSVVFDDQIRGPIEFSNQELDDLIIRRTDGSPT
YNFCVVVDDWDMEITHVIRGEDHINNTPRQINILKALKAPVPVYAHVSMINGDDGKKLSK
RHGAVSVMQYRDDGYLPEALLNYLVRLGWSHGDQEIFTREEMIKYFTLNAVSKSASAFNT
DKLLWLNHHYINALPPEYVATHLQWHIEQENIDTRNGPQLADLVKLLGERCKTLKEMAQS
CRYFYEDFAEFDADAAKKHLRPVARQPLEVVRDKLTAITDWTAENVHHAIQATADELEVG
MGKVGMPLRVAVTGAGQSPALDVTVHAIGKTRSIERINKALAFIAERENQH

>LFGLNPFC_00753 hypothetical protein
MTTEELAECLGVAKQTVNRWIREKGWKTEKFPGVKGGRARLILVDTQVCEFIQNTPAFHN
TPMLLEAEEPLAEYAPGIRTPAYRQIISAIDNMTHSEQEKVAQFLSREGIRNFLARLDID
ESA
>LFGLNPFC_00754 hypothetical protein
MFKERMTPDELARLTGYSRQTINKWVRKEGWTTSPKPGVQGGKARLVHVNEQVREYIRNA
ERPEGQGEAPALSGDAPLEVLLVTLAKEMTPVEQKQFTSLLLREGIIGLLQRLGIRDSK
>LFGLNPFC_00757 putative cyclic di-GMP phosphodiesterase PdeA
MFVEHNLIKNIKIFTLAFTLTVVLIQLSRLISPLAVIHSNYIFLAWMPLCVMLSILFIFG
WRGVVPILCGMFCTNLWNLHLSFLQTAVMIGSQAFAVLCACAILRWQLGTRWRYGLTSRY
VWQRLFWLGLVAPIGIKCSMYLVGNFFDFPLKISTFFGDADAIFTVVDLLSLFTAVLIYN
MLFYYLTRMIVSPHFAQILWRRDIAPSLSKEKRAFTLSWLAALSVLLLLMCTPYENDFIA
GYLVPVFFIIFTLGVGKIRYPFLNLTWAVSTLCLLNYNQNFLQGVLTEYSLAFILAVLIS
FSVCLLYMVRIYHRSEWLNRRWHLQALTDPLTLLPNFRALEQAPEQEAGKSFCCLRIDNL
EFMSRHYGLIMRVHCIRSIYRTLLPLMQENEKLYQLPGSELLLVLSGPETEGRLQHMVNI
LNSRQIHWNNTGLDMGYGAAWGRFDGNQETLQPLLGQLSWLAEQSCAHHHVLALDSREEM
VSGQTTKQVLLLNTIRTALDQGDLLLYAQPIRNKEGEGYDEILARLKYDGGIMTPDKFLP
LIAQFNLSARFDLQVLESLLKWLATHPCDKKGPRFSVNLMPLTLLQKNIAGRIIRLFKRY
YISPQAVILEITEEQAFSNAESSMYNIEQLHKFGFRIAIDDFGTGYANYERLKRLQADII
KIDGVFVKDIVTNTLDAMIVRSITDLAKAKSLSVVAEFVETPQQQALLHKLGVQYLQGYL
IGRPQPLAD
>LFGLNPFC_00758 Nucleoside permease NupC
MDRVLHFVLALAVVAILALLVSSDRKKIRIRYVIQLLVIEVLLAWFFLNSDVGLGFVKGF
SEMFEKLLGFANEGTNFVFGSMNDQGLAFFFLKVLCPIVFISALIGILQHIRVLPVIIRA
IGFLLSKVNGMGKLESFNAVSSLILGQSENFIAYKDILGKISRNRMYTMAATAMSTVSMS
IVGAYMTMLEPKYVVAALVLNMFSTFIVLSLINPYRVDASEENIQMSNLHEGQSFFEMLG
EYILAGFKVAIIVAAMLIGFIALIAALNALFATVTGWFGYSISFQGILGYIFYPIAWVMG
VPSSEALQVGSIMATKLVSNEFVAMMDLQKIASTLSPRAEGIISVFLVSFANFSSIGIIA
GAVKGLNEEQGNVVSRFGLKLVYGSTLVSVLSASIAALVL
>LFGLNPFC_00759 Divalent metal cation transporter MntH
MTNYRVESSSGRAARKMRLALMGPAFIAAIGYIDPGNFATNIQAGASFGYQLLWVVVWAN
LMAMLIQILSAKLGIATGKNLAEQIRDHYPRPVVWFYWVQAEIIAMATDLAEFIGAAIGF
KLILGVSLLQGAVLTGIATFLILMLQRRGQKPLEKVIGGLLLFVAAAYIVELIFSQPNLA
QLGKGMVIPSLPTSEAVFLAAGVLGATIMPHVIYLHSSLTQHLHGGSRQQRYSATKWDVA
IAMTIAGFVNLAMMATAAAAFHFSGHTGVADLDEAYLTLQPLLSHAAATVFGLSLVAAGL
SSTVVGTLAGQVVMQGFIRFHIPLWVRRTVTMLPSFIVILMGLDPTRILVMSQVLLSFGI
ALALVPLLIFTSDSKLMGDLVNSKRVKQTGWVIVVLVVALNIWLLVGTALGL
>LFGLNPFC_00760 hypothetical protein
MFRSLFLAAALMAFTPLAANASEITLLPSIKLQIGDRDHYGNYWDGGHWRDRDYWHRNYE
WRKNRWWRHDNGYHRGWDKRKAYERGYREGWRDRDDHRGKGRGHGHRH
>LFGLNPFC_00761 Putative ion-transport protein YfeO
MLHPRARTMLLLSLPAVAIGIASSLILIMVMKIASVLQNLLWQRLPGTLGIAQDSPLWII
GVLTLTGIAVGLVIRFSQGHAGPDPACEPLIGAPVPPSALPGLIVALILGLAGGVSLGPE
HPIMTVNIALAVAIGARLLPRVNRMEWTILASAGTIGALFGTPVAAALIFSQTLNGSNEV
PLWDRLFAPLMAAAAGALTTGLFFHPHFSLPIAHYGQMEMTDILSGAIVAAIAIAAGMVA
VWCLPRLHAMMHQMKNPVFVLGIGGLILGILGVIGGPVSLFKGLDEMQQMVANQAFSTSD
YFLLAVIKLAALVVAAASGFRGGRIFPAVFVGVALGLMLHEHVPAVPAAITVSCAILGIV
LVVTRDGWLSLFMAAVVVPNTTLLPLLCIVMLPAWLLLAGKPMMMVNRQKQQPPHDNV
>LFGLNPFC_00762 Glucokinase
MTKYALVGDVGGTNARLALCDIASGEISQAKTYSGLDYPSLEAVIRVYLEEHKVEVKDGC
IAIACPITGDWVAMTNHTWAFSIAEMKKNLGFSHLEIINDFTAVSMAIPMLKKEHLIQFG
GAEPVEGKPIAVYGAGTGLGVAHLVHVDKRWVSLPGEGGHVDFAPNSEEEGIILEILRAE
IGHVSAERVLSGPGLVNLYRAIVKADNRLPENLKPKDITERALADSCTDCRRALSLFCVI
MGRFGGNLALNLGTFGGVFIAGGIVPRFLEFFKASGFRAAFEDKGRFKEYVHDIPVYLIV
HDNPGLLGSGAHLRQTLGHIL
>LFGLNPFC_00763 PTS system fructose-like EIIB component 1
MSKKLIALCACPMGLAHTFMAAQALEEAAVEAGYEVKIETQGADGIQNRLTAQDIAEATI
IIHSVAVTPEDNERFESRDVYEITLQDAIKNAAGIIKEIEEMIASEQQ
>LFGLNPFC_00764 Fructose-like permease IIC component 1
MAIKKRSATVVHGASGAAAAVKNPQASKSSFWGELPQHVMSGISRMVPTLIMGGVILAFS
QLIAYSWLKIPADIGIMDALNSGKFSGFDLSLLKFAWLSQSFGGVLFGFAIPMFAAFVAN
SIGGKLAFPAGFIGGLMSTQPTQLLNFDPSIMQWATSSPVPSTFIGALIISIVAGYLVKW
MNQKIQLPDFLLAFKTTFLLPILSAIFVMLAMYYVITPFGGWINGGIRTVLTAAGEKGAL
MYAMGIAAATAIDLGGPINKAAGFVAFSFTTDHVLPVTARSIAIVIPPIGLGLATIIDRR

LTGKRLFNAQLYPQGKTAMFLAFMGISEGAIPFALESPITAIPSYMVGAIVGSTAAVWLG
AVQWFPESAIWAWPLVTNLGVYMAGIALGAVITALMVVFLRLMMFRKGKLLIDSL
>LFGLNPFC_00765 Aminopeptidase YpdF
MTLLASLRDWLKAQQLDAVLLSSRQNKQPHLGISTGSGYVVISRESAHILVDSRYYADVE
ARTQGYQLHLLDAMHTLATIVRQIIADEQLQTLGFEGQQVSWETAHRWQSELNAKLVSAT
PDVLRQIKTPEEVEKIRLACGIADRGAEHIRRFIQAGMSECEIAAELEWFMRRQGAEKAS
FDTIVASGWRGALPHGKASDKIVTAGEFVTLDFGALYQGYCSDMTRTLLVNGEGVSAESH
PLFNVYQIVLQAQLAAISAIRPGVRCQQVDDAARRVITEAGYGDYFGHNTGHAIGIEVHE
DPRFSPRDTTTLQPGMLLTVEPGIYLPGQGGVRIEDVVLVTPQGAEVLYAMPKTVLLTGE
A
>LFGLNPFC_00766 Aminopeptidase YpdE
MDLSLLKALSEADAIASSEQEVRQILLEEADRLQKEVRFDGLGSVLIRLNESTGPKVMIC
AHMDEVGFMVRSISREGAIDVLPVGNVRMAARQLQSVRITTREECKIPGLLDGDRQGNDV
SAMRVDIGARSCDEVMQAGIRPGDRVTFDTTFQVLPHQRVMGKAFDDRLGCYLLVTLLRE
LHDAELPAEVWLVASSSEEVGLRGGQTATRAVSPDVAIVLDTACWAKNFDYGAANHRQIG
NGPMLVLSDKSLIAPPKLIAWIETVAAEIGVPLQSDMFSNGGTDGGAVHLTGTGVPTVVM
GPATRHGHCAASIADCRDILQMQQLLSALIQRFTRETVVQLTDFR
>LFGLNPFC_00767 Multiphosphoryl transfer protein 1
MLTIQFLCPLPNGLHARPAWELKEQCSQWQSEITFINHRQNAKADAKSSLALIGTGTLFN
DSCSLNISGSDEEQARRVLEEYIQVRFIDSDSVQPTLAELTAHPLPSSLSRLNPDLLYGN
VLASGVGVGTLTLLQSDSLDSYRVIPASAQDSTLLEHSLATLAEQLNQQLRERDGESKTI
LSAHLSLIQDDEFAGNIRHLMAEQHQGLGAAIISNMEQICAKLSASASDYLRERVSDIRD
ISEQLLHITWPELKPRNNLVLEKPTILVAEDLTPSQFLSLDLKNLAGMILEKTGRTSHTL
ILARASAIPVLSGLPLDAIARYAGQPAVLDAQCGVLAINPNDAVSGYYQVAQTLADKRQK
QQAQAAAQLAYSRDKKRIDIAANIGTALEAPGAFANGAEGVGLFRTEMLYMDRDSAPDEQ
EQFEAYQQVLLAAGDKPIIFRTMDIGGDKSIPYLNIPQEENPFLGYRAVRIYPEFAGLFR
TQLRAILRAASFGNAQLMIPMVHSLDQILWVKGEIQKAIVELKRDGLRHAETITLGIMVE
VPSVCYIIDHFCDEVDFFSIGSNDMTQYLYAVDRNNPRVSPLYNPITPSFLRMLQQIVTA
AHQRGKWVGICGELGGESRYLPLLLGLGLDELSMSSPRIPAVKSQLRQLDSEACRELARQ
ACECRSAQEIEALLTAFAPEEDVRPLLALENIFVDQSFSNKEQAIQFLCGNLGVNGRTEH
PFELEEDVWQREEIVTTGVGFGVAIPHTKSQWIRHSSISIARLVKPVDWQSEMGEVELVI
MLTLGANEGMNHVKVFSQLARKLVNKNFRQSLFAAQDAQSILTLLETELTF
>LFGLNPFC_00768 HTH-type transcriptional activator RhaR
MKAPGLPADQQFFADLFSGLVLNPQLLGRVWFASHPASLPVGSLCIDFPRLDIVLRGEYG
NLLEAKQQRMVEGEMLFIPARAANLPVNNKPVMLLSLVFAPTWLGLSFYDSRTTSLLHPA
RQIQLPSLQRGEGEAMLTALTHLSRSPLEQNIIQPLVLSLLHLCRNVVNMPPGNSQPRGD
FLYHSICNWVQDNYAQPLTRESVAQFFNITPNHLSKLFAQHGTMRFIEYVRWVRMAKARM
ILQKYHLSIHDVAQRCGFPDSDYFCRVFRRQFGLTPGEYSARFQG
>LFGLNPFC_00769 Transcriptional regulatory protein YpdB
MKVIIVEDEFLAQQELSWLIKEHSQMEIVGTFDDGLDVLKFLQHNRVDAIFLDINIPSLD
GVLLAQNISQFAHKPFIVFITAWKEHAVEAFELEAFDYILKPYQESRITGMLQKLEAAWQ
QQQTSSTTPAATVTRENDTINLVKDERIIVTPINDIYYAEAHEKMTFVYTRRESYVMPMN
ITEFCSKLPPSHFFRCHRSFCVNLNKIREIEPWFNNTYILRLKDLDFEVPVSRSKVKEFR
QLMHL
>LFGLNPFC_00770 Sensor histidine kinase YpdA
MHEIFNMLLAVFDRAALMLICLFFLIRIRLFRELLHKSAHSPKELLAVTAIFSLFALFST
WSGVPVEGSLVNVRIIAVMSGGILFGPWVGIITGVIAGIHRYLIDIGGVTAIPCFITSIL
AGCISGWINLKIPKAQRWRVGILGGMLCETLTMILVIVWAPTTALGIDIVSKIGIPMILG
SVCIGFIVLLVQSVEGEKEASAARQAKLALDIANKTLPLFRHVNSESLRKVCEIIRDDIH
ADAVAMTNTDHVLAYVGVGEHNYQNGDDFISPTTRQAMNYGKIIIKNNDEAHRTPEIHSM
LVIPLWEKGVVTGTLKIYYCHAHQITSSLQEMAVGLSQIISTQLEVSRAEQLREMANKAE
LRALQSKINPHFLFNALNAISSSIRLNPDTARQLIFNLSRYLRYNIELKDDEQIDIKKEL
YQIKDYIAIEQARFGDKLTVIYDIDEEVNCCIPSLLIQPLVENAIVHGIQPCKGKGVVTI
SVAECGNRVRIAVRDTGHGIDPKVIERVEANEMPGNKIGLLNVHHRVKLLYGEGLHIRRL
EPGTEIAFYIPNQRTPVASQATLLL
>LFGLNPFC_00771 Glutamate-pyruvate aminotransferase AlaC
MADTRPERRFTRIDRLPPYVFNITAELKMAARRRGEDIIDFSMGNPDGATPPHIVEKLCT
VAQRPDTHGYSTSRGIPRLRRAISRWYQDRYDVEIDPESEAIVTIGSKEGLAHLMLATLD
HGDTVLVPNPSYPIHIYGAVIAGAQVRSVPLVEGVDFFNELERAIRESYPKPKMMILGFP
SNPTAQCVELEFFEKVVALAKRYDVLVVHDLAYADIVYDGWKAPSIMQVPGARDVAVEFF
TLSKSYNMAGWRIGFMVGNKTLVSALARIKSYHDYGTFTPLQVAAIAALEGDQQCVRDIA
EQYKRRRDVLVKGLHEAGWMVEMPKASMYVWAKIPEPYAAMGSLEFAKKLLNEAKVCVSP
GIGFGDYGDTHVRFALIENRDRIRQAIRGIKAMFRADGLLPASSKHIHENAE
>LFGLNPFC_00772 Lipid A biosynthesis palmitoleoyltransferase

MFPKCKFSREFLHPRYWLTWFGLGVLWLWVQLPYPVLCFLGTRIGTMARPFLKRRESIAR
KNLELCFPQHSAKEREKMIAENFRSLGMALVETGMAWFWPDSRVRKWFDVEGLDNLKRAQ
MQNRGVMVVGVHFMSLELGGRVMGLCQPMMATYRPHNNQLMEWVQTRGRMRSNKAMIGRN
NLRGIVSALKKGEAVWFAPDQDYGRKGSSFAPFFAVENVATTNGTYVLSRLSGAAMLTVT
MVRKADYSGYRLYITPEMEGYPTDENQAAAYMNKIIEKEIMRAPEQYLWIHRRFKTRPVG
ESSLYI
>LFGLNPFC_00773 hypothetical protein
MIYLWMFLALCIVCVSGYIGQVLNMISAVSSFFGMVILAALIYYFTMWLTGGNELVTGIF
MFLAPACGLMIRFMVGYGRR
>LFGLNPFC_00774 hypothetical protein
MKVNLMLFSLFLLVSIMACNVFAFSISGGGSEASYKETEKTSAMTTTHSTKIQPSQAILL
KMREDAPPLNLTEEIPPPFPTKANYLIHPVR
>LFGLNPFC_00775 Protein YfdX
MKRLIMATMVTAILASSTVWAADNAPVAAQQQTQQVQQTQKTAAAAERISEQGLYAMRDV
QVARLALFHGDPEKAKELTNEASALLSDDSTEWAKFAKPGKKTNVNDDQYIVINASVGIS
ESYVATPEKEAAIKIANEKMAKGDKKGAMEELRLAGVGVMENQYLMPLKQTRNALADAQK
LLDKKQYYEANLALKGAEDGIIVDSEALFVN
>LFGLNPFC_00776 Formyl-CoA:oxalate CoA-transferase
MSTPLQGIKVLDFTGVQSGPSCTQMLAWFGADVIKIERPGVGDVTRHQLRDIPDIDALYF
TMLNSNKRSIELNTKTAEGKEVMEKLIREADILVENFHPGAIDHMGFTWEHIQEINPRLI
FGSIKGFDECSPYVNVKAYENVAQAAGGAASTTGFWDGPPLVSAAALGDSNTGMHLLIGL
LAALLHREKTGRGQRVTMSMQDAVLNLCRVKLRDQQRLDKLGYLEEYPQYPNGTFGDAVP
RGGNAGGGGQPGWILKCKGWETDPNAYIYFTIQEQNWENTCKAIGKPEWITDPAYSTAHA
RQPHIFDIFAEIEKYTVTIDKHEAVAYLTQFDIPCAPVLSMKEISLDPSLRQSGSVVEVE
QPLRGKYLTVGCPMKFSAFTPDIKAAPLLGEHTAAVLQELGYSDDEIAAMKQNHAI
>LFGLNPFC_00777 Oxalyl-CoA decarboxylase
MSDQLQMTDGMHIIVEALKQNNIDTIYGVVGIPVTDMARHAQAEGIRYIGFRHEQSAGYA
AAASGFLTQKPGICLTVSAPGFLNGLTALANATVNGFPMIMISGSSDRAIVDLQQGDYEE
LDQMNAAKPYAKAAFRVNQPQDLGIALARAIRVSVSGRPGGVYLDLPANVLAATMEKDEA
LTTIVKVENPSPALLPCPKSVTSAISLLAKAERPLIILGKGAAYSQSDEQLREFIESAQI
PFLPMSMAKGILEDTHPLSAAAARSFALANADVVMLVGARLNWLLAHGKKGWAADTQFIQ
LDIEPQEIDSNRPIAVPVVGDIASSMQGMLAELKQNTFTTPLVWRDILNIHKQQNAQKMH
EKLSTDTQPLNYFNALSAVRDVLRENQDIYLVNEGANTLDNARNIIDMYKPRRRLDCGTW
GVMGIGMGYAIGASVTSGSPVVAIEGDSAFGFSGMEIETICRYNLPVTIVIFNNGGIYRG
DGVDLSGAGAPSPTDLLHHARYDKLMDAFRGLGYNVTTTDELRHALTTGIQSRKPTIINV
VIDPAAGTESGHITKLNPKQVAGN
>LFGLNPFC_00778 hypothetical protein
MLTFFIGDLLPIIVIMLLGYFSGRRETFSEDQARAFNKLVLNYALPAALFVSITRANREM
IFADTRLTLVSLVVIVGCFFFSWFGCYKFFKRTHAEAAVCALIAGSPTIGFLGFAVLDPI
YGDSVSTGLVVAIISIIVNAITIPIGLYLLNPSSGADGKKNSNLSALISAAKEPVVWAPV
LATILVLVGVKIPAAWDPTFNLIAKANSGVAVFAAGLTLAAHKFEFSAEIAYNTFLKLIL
MPLALLLVGMACHLNSEHLQMMVLAGALPPAFSGIIIASRFNVYTRTGTASLAVSVLGFV
VTAPLWIYVSRLVS
>LFGLNPFC_00779 Acetyl-CoA:oxalate CoA-transferase
MTNNECKGPFEGLLVIDMTHVLNGPFGTQLLCNMGARVIKVEPPGHGDDTRTFGPYVDGQ
SLYYSFINHGKESVVLDLKNDHDKSIFINMLKQADVLAENFRPGTMEKLGFSWETLQEIN
PRLIYASSSGFGHTGPLKDAPAYDTIIQAMSGIMMETGYPDAPPVRVGTSLADLCGGVYL
FSGIVSALYGREKSQRGAHVDIAMFDATLSFLEHGLMAYIATGKSPQRLGNRHPYMAPFD
VFDTQDKPITICCGNDKLFSVLCQALELTELVNDPRFSSNILRVQNQAILKQYIERTLKT
QAAEVWLARIHEVGVPVAPLLSVAEAINLPQTQARNMLIEAGGIMMPGNPIKISGCADPH
VMPGAATLDQHGEQIRQEFSS
>LFGLNPFC_00780 Sensor protein EvgS
MKFLPYIFLLCCGLWSTISFADGDYIEYRGISSNNRVTLDPLRLSNKELRWLASKKNLVI
AVHKSQTATLLHTDSQQRIRGINADYLNLLKRALNIKLTLREYADHQKAMDALEDGEVDI
VLSHLVASPPLNDDIAATNPLIITFPALVTTLHDSMRPLTSSKPVNIARVANYPPDEVIH
QSFPKATIISFTNLYQALASVSAGQNDYFIGSNIITSSMISRYFTHSLNVVKYYNSPRQY
NFLLTRKDSIVLNEVLNRFVDALTNEVRYEVSQNWLDTGNLAFLNKPLELTEHEKQWIKQ
HPDLKVLENPYSPPYSMTDETGSVRGVMGDILNIITLQTGLNFSPITVSHNIHAGTQLNP
GGWDILPAAIYSEDRENNVSFAEVFITTPYVFVMQKAPDSEQTLKKGMKVAIPYYYELHS
QLKEMYPEVEWIKVDNASAAFHKVKEGELDALVATQLNSRYMIDHYYPNELYHFLIPGVQ
NASLSFAFPRGEPELKDIINKALNAIPPSEVLRLTEKWIKMPNVTIDTWDLYSEQFYIVT
TLSVLLVGSSLLWGFYLLRSVRRRKVIQGDLENQISFRKALSDSLPNPTYVVNWQGNVIS
HNSAFEHYFTDDYYKNAMLPLENSESPFKDVFSNTHEVTAETKENRTIYTQVFEIDNGIE
KRCINHWHTLCNLPASEHAVYICGWQDITETRDLIHALEVERNKAINATVAKSQFLATMS

HEIRTPISSIMGFLELLSGSGLSKEQRVEAISLAYATGQSLLGLIGEILDVDKIESGNYQ
LQPQWVDIPTLVQNTCHSFAAIAASKSIALSCSSTLPERYLVKIDPQAFKQVLSNLLSNA
LKFTTEGAVKITTSLGHIDDNHAVIKMTIMDSGSGLSQEEQQQLFKRYSQTSAGRQQTGS
GLGLMICKELIKNMQGDLSLESHPGIGTIFTITIPVEITQQVAAVEAKAEQPITLPEKLS
ILIADDHPTNRLLLKRQLNLLGYDVDEATDGVQALHKVSMQHYDLLITDVNMPNMDGFEL
TRKLREQNSSLPIWGLTANAQANEREKGLNCGMNLCLFKPLTLDVLKTHLSQLHQVAHIA
PQYRHLDIEALKNNTANDLQLMQEILMTFQHETHKDLPAAFHALEAGDNRTFHQCIHRIH
GAANILNLQKLINISHQLEITPVSDDSKPEILQLLNSVKEHIAELDQEITVFCQQND
>LFGLNPFC_00781 DNA-binding transcriptional activator EvgA
MNAIIIDDHPLAIAAIRNLLIKNDIEILAELTEGGSAVQRVETLKPDIVIIDVDIPGVNG
IQVLETLRKRQYSGIIIIVSAKNDHFYGKHCADAGANGFVSKKEGMNNIIAAIEAAKNGY
CYFPFSLNRFVGSLTSDQQKLDSLSKQEISVMRYILDGKDNNDIAEKMFISNKTVSTYKS
RLMEKLECKSLMDLYTFAQRNKIG
>LFGLNPFC_00782 putative multidrug resistance protein EmrK
MEQVNSNKKHSDRRKYFALLAVVLFIAFSGAYAYWSMELKDMISTDDAYVTGNADPISAQ
VSGSVTVVNHKDTNYVRQGDILVSLDKTDATIALNKAKNNLANIVRQTNKLYLQDKQYSA
EVASARIQYQQSLEDYNRRVPLAKQGVISKEALEHTKDTLISSKAALNAAIQAYKANKAL
VMNTPLNRQPQVIEAADATKEAWLALKRTDIKSPVTGYIAQRSVQVGETVSPGQSLMAVV
PARQMWVNANFKETQLTDVRIGQSVNIISDLYGENVVFHGRVTGINMGTGNAFSLLPAQN
ATGNWIKIVQRVPVEVSLDPKELMEHPLRIGLSMTATIDTKNEDIAEMPDLASTVTSMPA
YTSKALVIDTSPIEKEISNIISHNGQL
>LFGLNPFC_00783 putative multidrug resistance protein EmrY
MAITKSTPAPLTGGTLWCVTIALSLATFMQMLDSTISNVAIPTISGFLGASTDEGTWVIT
SFGVANAIAIPVTGRLAQRIGELRLFLLSVTFFSLSSLMCSLSTNLDVLIFFRVVQGLMA
GPLIPLSQSLLLRNYPPEKRTFALALWSMTVIIAPICGPILGGYICDNFSWGWIFLINVP
MGIIVLTLCLTLLKGRETETSPVKMNLPGLTLLVLGVGGLQIMLDKGRDLDWFNSSTIIL
LTVVSVISLISLVIWESTSENPILDLSLFKSRNFTIGIVSITCAYLFYSGAIVLMPQLLQ
ETMGYNAIWAGLAYAPIGIMPLLISPLIGRYGNKIDMRLLVTFSFLMYAVCYYWRSVTFM
PTIDFTGIILPQFFQGFAVACFFLPLTTISFSGLPDNKFANASSMSNFFRTLSGSVGTSL
TMTLWGRRESLHHSQLTATIDQFNPVFNSSSQIMDKYYGSLSGVLNEINNEITQQSLSIS
ANEIFRMAAIAFILLTVLVWFAKPPFTAKGVG
>LFGLNPFC_00784 D-serine dehydratase
MENAKMNSLIAQYPLVEDLVALKETTWFNPGTTSLAEGLPYVGLTEQDVQDAHARLSRFA
PYLAKAFPETAAAGGIIESELVAIPAMQKRLEKEYHQPIAGQLLLKKDSHLPISGSIKAR
GGIYEVLAHAEKLALEAGLLTLEDDYSKLLSPEFKQFFSQYSIAVGSTGNLGLSIGIMSA
RIGFKVTVHMSADARAWKKKAKLRSHGVTVVEYEQDYGVAVEEGRKAAQSDPNCFFIDDEN
SRTLFLGYSVAGQRLKAQFAQQGRIVNADNPLFVYLPCGVGGGPGGVAFGLKLAFGDHVH
CFFAEPTHSPCMLLGVHTGLHDQISVQDIGIDNLTAADGLAVGRASGFVGRAMERLLDGF
YTLSDQTMYDMLSWLAQEEGIRLEPSALAGMAGPQRVCASVSYQQMHGFSAEQLRNATHL
VWATGGGMVPEEEMNQYLAKGR
>LFGLNPFC_00785 D-serine transporter DsdX
MHSQIWVVSTLLISIVLIVLTIVKFKFHPFLALLLASFFVGTMMGMGPLDMVNAIESGIG
GTLGFLAAVIGLGTILGKMMEVSGAAERIGLTLQRCRWLSADVIMVLVGLICGITLFVEV
GVVLLIPLAFSIAKKTNTSLLKLAIPLCTALMAVHCVVPPHPAALYVANKLGADIGSVIV
YGLLVGLMASLIGGPLFLKFLGQRLPFKPVPTEFADLKVRDEKTLPSLGATLFTILLPIA
LMLVKTIAELNMARESGLYTLLEFIGNPITAMFIAVFVAYYVLGIRQHMSMGTMLTHTEN
GFGSIANILLIIGAGGAFNAILKSSSLADTLAVILSNMHMHPILLAWLVALILHAAVGSA
TVAMMGATAIVAPMLPLYPDISPEIIAIAIGSGAIGCTIVTDSLFWLVKQYCGATLNETF
KYYTTATFIASVIALAGTFLLSFII
>LFGLNPFC_00786 HTH-type transcriptional regulator DsdC
MEPLREIRNRLLNGWQLSKLHTFEVAARHQSFALAAEELSLSPSAVSHRINQLEEELGIQ
LFVRSHRKVELTHEGKRVYWALKSSLDTLNQEILDIKNQELSGTLTLYSRPSIAQCWLVP
ALGDFTRRYPSISLTVLTGNDNVNLQRAGIDLAIYFDDAPSAQLAHHFLMDEEILPVCSP
EYAHRHDLTNTVINLRHCTLLHDRQAWSNDSGTDEWHSWAQHYAVNLPTSSGIGFDRSDL
AVIAAMNHIGVAMGRKRLVQKRLASGELVAPFGDMTVKCHQHYYITTLPGRQWPKIEAFI
TWLREQVKTTS
>LFGLNPFC_00787 Response regulator inhibitor for tor operon
MQHELQPDSLVDLKFIMADTGFGKTFIYDRIKSGDLPKAKVIHGRARWLYRDHCEFKNKL
LSRANG
>LFGLNPFC_00788 hypothetical protein
MHFRVTGEWNGEPFNRVIEAENINDCYDHWMLWAQIAHADVTNIRIEELKEHQAA
>LFGLNPFC_00789 hypothetical protein
MANLQLAVKGEYFDAMIRGEKTEEYRLCNDYWNKRIMFREYDRLIITKGYPKRDDSSRRI
DVPYDGYEIKTITHPHFGDKPVKVFAIKVNIDNE

>LFGLNPFC_00790 hypothetical protein
MVSFAKYTIIDWIAFIQVLLIWFYMAYRSGQWIVSVACSNGWRWWNRKNKKALALASFYE
AFNLNSLQPGSVVVVTTQSGMTIQIHKPKEEGRG
>LFGLNPFC_00791 hypothetical protein
MTKINYQALREAAERAIPAMERLLMLPVDDDLLSEQELKDYGVDIDALNAFKFLTGPETV
LALLDERERNQQYIKRRDQENEEIALTVGKLRVELEAAEKRIAELEAEPVSQTYKLNELS
GNYPVTPDGWISCSERMPAQDDWILIYSKHGEYMAGQVQGEYVELSDGTLSWLGNALYWM
PLPEPPQEVN
>LFGLNPFC_00792 hypothetical protein
MTVCLIDKRRRGQQIPSVEMPNHTWFCVLDIDGMDTLVDTRHYCDTATATPAKAKKMAAL
IENWTPPDGWCNGNDRDWHEKMKGYICDFLRKCNGFRGM
>LFGLNPFC_00793 hypothetical protein
MKQMSLIEMDGFLKGKCIPSDLKVNETNAEYLVRKFGELESKLETALRECSSAGITIDNL
EAKCAKMAAENTSLKQSEKEFNDFCREEFSEWEDDVTETPATDAFLAEVRAQGVDAAIEA
AKNLVAQEYEYKDFKAAQSDCCMHPGSDLVGKVEMTEWLVDFAAQLRKGGNQ
>LFGLNPFC_00794 hypothetical protein
MPAPLYGADDPRRCSGNSVSEVLDKFRKNYDRIMSLPQETKEEKEFRHCIWLAEKEERER
IYQTSIRPFRKATYTHFPEYIDPRLRNYRSRYGAISND
>LFGLNPFC_00795 hypothetical protein
MAHSITVRLNKPAREFQGGENIGFNIRAGVQYYDRQTKKKEWTNYSAVVFAKPGAQADYY
RSVLVEGGIVEITGENIRVDVYQGQNGQSITLELLNAKIGFATSGNSQQQQSSNHQNNPE
YDDSIPF
>LFGLNPFC_00796 hypothetical protein
MSKEFYARLAAIQENLNAPKNQYNSFGKYKYRSCEDILEGVKPLLNGLFLSISDEVVLIG
DRYYVKATATITDGENSHTATALAREEESKKGMDSAQVTGATSSYARKYCLNGLFGIDDA
KDADTDEHKHQQNAAAKQSKPSLTPEQVLKAFTDAAMQKNTVEELKQAFAKAWKMLEGTP
EQHKAQDVYNIRRDELEGATA
>LFGLNPFC_00797 hypothetical protein
MSLATTVKESKLQRRMYTQKALWYRHNGDREGMRVCLNLSRVEVLNQRYFLGPCPF
>LFGLNPFC_00798 hypothetical protein
MRNEIAINHQMLRAAQNKAVIARFIGDSKMWLEANKAMKSAINLPWYRRK
>LFGLNPFC_00799 hypothetical protein
MIYAIAGGARMGAFQLNESLLERITRKLRDGWKRVEVLLCAMK
>LFGLNPFC_00800 hypothetical protein
MRKNQARIKAEEEAKAAAEALAKQIESDHEIAILMDREFDRQREEARLKAEQEKREHEER
LKREAEEKARAEAEAKAKAEIEAAARREAEAKAAAERAERERIEAEQRAQREAKEAAERA
EREKQAAIEAERRKAQEEAERIRREAEAKEQARIAEEKRIKDEEERRAKDKAHRKEVNNK
ILADLIKVGASEDVAKNIITAIVKGEVFATKITY
>LFGLNPFC_00801 hypothetical protein
MSEVTDLVVIEKANAMTVFQSADQIEEILQKVEREVMSFVPDITTAKGRKEIASLAYKVA
QTKTYLDGLGKDLVAELKEIPKLIDANRKTVRDRLDELKAKARQPLTDYEEESGAD
>LFGLNPFC_00802 hypothetical protein
MEEQANKILVELLQKASNGIDAAVSFSQAQIPDVVRQLLTWSFVHSALFQVAGLLLLIAA
MKLPSFARTARNNGERWTSLDGCPNDRYFISSFYYDICTVFAPIFGSIIGVLIIAFNFEW
LKIWLAPKLFLIEYAASLVK
>LFGLNPFC_00803 hypothetical protein
MTTTIDKNQWCGQFKRCNGCKLQSECMVKPEEMFPVMEDGKYVDKWAIRTTAMIARELSK
QNNKAA
>LFGLNPFC_00804 hypothetical protein
MTVVITYLADDNARNRRRARRQAQREQAMQEQRLARKIALKLSGCVRADKAASLGSLRCK
KAEEVESKQNRIYYRKPRSEMGVTCVGRQKMKLGSKPLI
>LFGLNPFC_00805 putative HTH-type transcriptional regulator
MEAVEPYHKRAIDNWYDTTVDCSEDSFWLDVKGDSMTAPAGLSIPEGMIILVDPEVEPRN
GKLVVAKLEGENEATFKKLVIDAGRKFLKPLNPQYPMIEINGNCKIIGVVVDAKLANLP
>LFGLNPFC_00806 hypothetical protein
MYKKDVIDHFGTQRAVAKALGISDAAVSQWKEVIPEKDAYRLEVVTAGALKYQESAYRKA
A
>LFGLNPFC_00807 hypothetical protein
MVRANKRNEALRIESALLNKIAMLGTEKTAEAVGVDKSQISRWKRDWIPKFSMLLAVLEW
GVVDDDMARLARQVASILTNKKRPAATERSDQIQMEF
>LFGLNPFC_00808 hypothetical protein
MRNKGFNPPDTHKEAKRLRFLRSIDERTQISFVKVARTELLKAEARALLPSLPKEEGYTF
IPNAFLEKLLKEDISVSQFNDVLKVFRQGR
>LFGLNPFC_00809 hypothetical protein

MENQKTGYIPLYRSILKQSWAKDVYLRTLWENLLLNAARKPYKANFKGHEWHLQPGQLVV
TAADLGLQLCDRHGKPASRDQVERMLQVFVKEGMISIDGEKQKGRVITITNYHEYAQKMD
NLPAHEAAQTTAHDAAHDEASNSAAFSVHAAHESAHEAAQTTAHHEQEGINKNINNTPLP
PNGGGDGQVKPERRKAERIDYESFLNAYNTEVGDRLPHAVAVNEKRKRRLKKIIPQLKTP
NVDGFRAYVRAFVHQAKPFYFGDNDTGWTADFDYLLREDSLTGVREGKFADRGIA
>LFGLNPFC_00810 Replicative DNA helicase
MRQDIEASVIGGLLIGGLTPTASDVLATLEPEAFSIPLYRKAFEVIRKQARNRNLIDALM
VAEACGEEHFTAILMTSKNCPSAANLKGYAGMVADNYHRRLVLEIMDEMREPIQSGTIDA
SSQAMDELVKRLSAIRKPRDEVKPVRLGEIITDYTDTLDRRLRNGEESDTLKTGIEELDA
ITGGMNAEDLVIIAARPGMGKTELALKIAEGVASRVIPGSDVRRGVLIFSMEMSALQIAE
RSIANAGRMSVSVLRNPASMDDEGWARVANGMSQLADLDVWVVDASRLSVEEIRSIAERH
KQENPNLSLIMADYLGLIEKPKADRNDLAIAHISGSLKAMAKDLKTPVISLSQLSRDVEK
RPNKRPTNADLRDSGSIEQDADSIIMLYREAVYDENSSAAPFAEIIVTKNRFGSLGTVYQ
RFCNGHFVACDQDEARQICTASNAPAARGRRYAQGADV
>LFGLNPFC_00811 hypothetical protein
MVILMDTVNGMCSDAPRAKKCKCGKSPTIFDMENGCQIYCANHAAVVAANYRSAVTEWNN
LKSVREGSHEKTNL
>LFGLNPFC_00812 hypothetical protein
MKKLTFEIRSPAHQQNAIHAVQQILPDPTKPIVVTIQERNRSLDQNRKLWACLGDVSRQV
EWHGRWLDAESWKCVFTAALKQQDVVPNLAGNGFVVIGQSTSRMRVNEFAELLELIQAFG
TERGVKWSDEARLALEWKARWGDRAA
>LFGLNPFC_00813 hypothetical protein
MDYSQLSDFEINKRVFKAIVGAIPLGYPHNADGRSVGNESNGNYRWYDYCNNPEDAEPII
VENRIGIIPAPENGLWKAAHRKVGSDSTPYHMTQDENQLRAAMIVFLMMQDANNA
>LFGLNPFC_00814 hypothetical protein
MLSPSQSLQCQKESVERALTCANCGQKLHVLEVHMCEHCCAELMSDPNSSMYEEEDDE
>LFGLNPFC_00815 hypothetical protein
MNELMNGNAIKMTSIEIAELVGKRHDNVKRTIETLAKNGVIRLPQIEVSERINNLGFNVQ
YEHYVFEGEQGKRDSIVVVAQLSPEFTARLVDRWRELEEAAVNIPKTLPEALRLAADLAE
QKMQLENQLAIVAPKVEFADRVGEASGILIGNFAKVVGIGQNKLFAWMRDHKILIASGSR
RNVPMQEYMDRGYFTVKETAVNTNHGIQISFTTKITGRGQQWLTRKLLDNGMLKVTGEAA
>LFGLNPFC_00816 Putative nuclease YbcO
MANLRKEARGRECQVRIYGVCNGNPETTVLAHYRMAGICGTGMKPDDLIGAWACSACHDE
IDRRTHNLDNKDARLYHLEGVIRTQAILLREGKIKS
>LFGLNPFC_00817 Crossover junction endodeoxyribonuclease RusA
MNEYQFVLPYPPSVNTYWRRRGSQYYISDKGQKYRKDVQQIIRQLKLDIFTKSRLRIKVI
ADVPDSRRRDLDNILKGLLDSLIHAGFAEDDEQFDDIRVIRGVKVPGGRLGIKITELENA
>LFGLNPFC_00818 hypothetical protein
MNATIQTIPELLIQTRGNQTEVARMLSCARGTVLKYNRDSKGERHVIVNGVLMVKQGKRG
RR
>LFGLNPFC_00819 hypothetical protein
MSIRELNLTKEQHEWLNGWLELWGAWVYSGRLEKRMSSVIAKFMESVEPGRIMTRPMCND
DDGMLISQVVDSVMYIDKKAFGILLSYYAHGSSKRAIASYYHATAKPRKMCGRGGEGWRK
PSLATCRNEIDDILKASLFVLYQPMQNAFKMRKRVEKVKHVAVKNLDMQLSI
>LFGLNPFC_00820 hypothetical protein
MKMPEKHDLLAAILAAKEQGIGAILAFAMAYLRGRYNGGAFTKTVIDATMCAIIAWFIRD
LLDFAGLSSNLAYITSVFIGYIGTDSIGSLIKRFAAKKAGVEDGGNQ
>LFGLNPFC_00821 hypothetical protein
MVEINNQRKAFLDMLAWSEGTDNGRQKTRNHGYDVIVGGELFTDYSDHPRKLVTLNPKLK
STAAGRYQLLSRWWDAYRKQLGLKDFSPKSQDAVALQQIKERGALPMIDRGDIRQAIDRC
SNIWASLPGAGYGQFEHKADNLIAKFKEAGGTVREIEV
>LFGLNPFC_00822 hypothetical protein
MSRVTAIISALIICIIVCLSWAVNHYRDNAIAYKEQRDKATSIIADMRKRQRDVAELDAR
YTKELADANATIESLRADVSAGRKRLQVAATCAKSTTGASSMGDGENPRLTADAELNYYR
LRSGIDRITAQVNYLQEYIRTQCLR
>LFGLNPFC_00823 hypothetical protein
MPSMIAIILLIILHIWLCRQGGDHFWSESRLNISLLMLDIEHLARGKGLR
>LFGLNPFC_00824 hypothetical protein
MNYPTVVNDIDFRDLIFVANNDPVTDSFMVAKAFGKLPKNVVRDIERTIEACPPEFDTKL
NFELCYKNNELQNGKPQKFYRLRKDGLMLLVMSYTKKEAMRIKIAYINAFNWMYAMLQVG
HRQFEEERNAVMLEYMKEKDVASMSGRLLNRWGKIKKPQLLARIERLEQHGQTVIPGLTN
>LFGLNPFC_00825 hypothetical protein
MAEIIPMTEEQKFQLEIYKLVMNQNAAAEEAFQFIGTDELKLELFKIHFQSGGANSDITT
RTIEAVRKSKEALDLFTTGA

>LFGLNPFC_00826 hypothetical protein
MARPTKYQEAYAEQARKLCLLGYTDAELADFFEVSESTINKWKLDYPEFSESIKKGKAVA
DAEVSDRLYQRAMGFVAPDIDIRVIENRIVETPLEKYYPPDTTAAIFWLKNRQKDKWRDK
VDHELTGKDGGAIQIETSPMSTLFGK
>LFGLNPFC_00827 putative protein
MTSINPIFEPFIEAHRYKVAKGGRGSGKSWAIARLLVEAARRQPVRILCARELQNSISDS
VIRLLEDTIEREGYTAEFEIQRSMIRHLGTNAEFMFYGIKNNPTKIKSLEGIDICWVEEA
EAVTKESWDILIPTIRKPFSEIWVSFNPKNILDDTYQRFVVNPPDDICLLTVNYTDNPHF
PEVLRLEMEECKRRNPTLYRHIWLGEPVSASDMAIIKREWLEAATDAHKKLGWKAKGAVV
SAHDPSDTGPDAKGYASRHGSVVKRIAEGLLMDINEGADWATSLAIEDGADHYLWDGDGV
GAGLRRQTTEAFSGKKITATMFKGSESPFDEDAPYQAGAWADEVVQGDNVRTIGDVFRNK
RAQFYYALADRLYLTYRAVVHGEYADPDDMLSFDKEAIGEKMLEKLFAELTQIQRKFNNN
GKLELMTKVEMKQKLGIPSPNLADALMMCMHCPESAAQPDYSSYSIPCGVG
>LFGLNPFC_00828 hypothetical protein
MAEKKMTDWHRKVLCNFDNAWSATQDMREQIIEAQRFVRVSGAQWEGSTNAGYSFDEGRF
EHYPRFELNKIARECDRIIGEYRQNRISVKFRPKDDKASEALAKKMNGKFRADYQETSGG
EACDNAFDDAVTGGFGCFRMCADYEDEMDPSNEQRRISLLPVYDPATCVFFDQDSKQYDR
SDAMWAMEMFSMTPKAFEAEYPDSIAAGLSRDETGTQYDWSTPDAIYVGRYYEVRIEKVK
LTAWRNPVSGETAIYDEEQIKDIVDELTDGAFELIGERTVKKRRVYCGLLSGAEWLEEPK
RIPGEHIPLIPVYGRRSFVDNQERIEGHAAKAMDAQRLENLMVSMIADNATQAGGDGIPV
VDVDMIPGPLATHWAERNKKRPAFLPMVSLKNKNGDITAQAQVSSYTPPTQMPPALAGLL
QYTGTAIQQITGASQLENMPSNVATDTVDSIFNRMDTQSYIYMDNMAKSMRRAGVVWLSM
AREVYGSDTPMRIVNEDGSDDVALMTGEVVDRQTGQVIALNDLSQGNYEVTVDVGQSFAT
RRDATVKSLLSMLALIPPGTPKHDLVSSMILDNMDGEGMDDLKEYNRNQLLLSGVIKPRT
PEEQQMVEQAKQQQASQPDPAMVAAQGQLLAGQAELQKAQNEQAAIQVKAFQAQTDAQVA
AANVVKILASADSQQKSDIREALKLLGQFQQQQGDNARADAELVLKSQAQGHAQRMDISS
ILQKSTQQQPQQ
>LFGLNPFC_00829 hypothetical protein
MDQMAENTPEVEIETDASEQIPDDVELAEKVETEDGSESSGNDAEEATETDDDESEQEFY
FGDEKLDSPTSEDGAEHGLVKHLRKTIKEKDRELKELMRQSQKPVEQQPVITQPPRMPKL
DDEDIGFDEEIYQQRMAKWAEDNGKYQQQEMARKQKEQELQAAYQERLSKYQQRVKALKV
PGYQEAEQAVLEEIPIETQNAILFESEKPEIVVLALGRNAELRKQLAEATNPVAIGRLLE
RIESKARIMPKAKTTAATTPTVKGSNGAVINNLDKLKAKALETGDWTPYFAAKKAKK
>LFGLNPFC_00830 hypothetical protein
MANQLAKDLEIMFENYVEGFEAACVVSRNAKKFRPGDTAMQRAGDVLYRPQHYHMNIEEG
LDLSSKTPTALVQRLVPSVFKEPKNILYTLDAREMRDPEHKTEAGRAAGMRLAAQIDSDL
ISMVTQRATNVITMADSTAGTQGRDLWNCAAGIDATMTAIGVPQGINRRSFWNPFNYKDL
AGELGHRAYAQGATLTAYEKAQIPPVASFDSYKTDISGRLPKGSTESLTVSGQPEHKVEA
KDSNGMPVDNRQGTITVSASGLQVGDAFTIAGVNSVHQITKDTTGQPQVFRVLAVSGTTV
TISPKILPVENTDVASRPYANVDAKPAESAAITILNKNAAPANLFWADGSVELMYGKLAF
PTGQGPQVMTATTEQGATLIMSYAFDHIKGVTTARFTTLYGCSVLVPEYTGIVIAGQ
>LFGLNPFC_00831 hypothetical protein
MARTMLYKPGNMITCGQFAVDYVIVDDEEVKSHLKKGWVKTPEETATKQKVAKAEEDGEN
EG
>LFGLNPFC_00832 hypothetical protein
MAKTKGDLVLKALRKAGLYSNATLTDADPQAIEDAINDLEDMMAAWQAKGIELGYQFADT
ENGIMPLPDDDSGIPAWANDGVALKLAVQVCMDNVIQPSDALLTAADSAYQTICIALTKI
PPLERRNDMPRGSGNKSAFTWNRFYIEKDDPST
>LFGLNPFC_00833 hypothetical protein
MPIQQLPLMKGVGKDFRNADYIDYLPVNMLATPKEILNSSGYLRSFPGIAKRSDVNGISR
GVEYNMAQSAVYRVCGGKLYKGESEVGDVAGSGRVSMAHGRTSQAVGVNGQLVEYRYDGT
VKTVSNWPTDSGFTQYELGSVRDITRLRGRYAWSKDGTDSWFITDLEDESHPDRYSAQYR
AESQPDGIIGIGTWRDFIVCFGSSTIEYFSLTGATTVGAALYVAQPSLMVQKGIAGTYCK
TPFADSYAFISNPATGAPSVYIIGSGQVSPIASASIEKILRSYTADELADGVMESLRFDA
HELLIIHLPRHVLVYDASSSANAPQWCVLKTGLYDDVYRAIDFIYEGNQITCGDKLESVT
GKLQFDISSQYDKQQEHLLFTPLFKADNARVFDLEVESSTGVAQYADRLFLSATTDGINY
GREQMIEQNEPFVYDKRVLWKRVGRIRKNVGFKLRVITKSPVTLSGCQIRIE
>LFGLNPFC_00834 hypothetical protein
MADSNLNTPVIVQATRLDTSILPRNIFSQSYLLYVINQGADVGAIAGKANQAGQGAYDAQ
VKNDEQDVELADHDARITANTKAINLLEVRLTTAEGKIVVLRSDVDYLLDEVIDIQAHLV
TVDQRLDGVESDISDIKSDYVSKTVTESQSLASPLDVKTSYSVDGIQVVGARQTGWTAAT
GTPLLGSFNANQSYTVGTTYTQSEVAAIATGLEQARQRILALETALRLHGLID
>LFGLNPFC_00835 hypothetical protein
MITFKPTRNIDLIEAVGNHPDIIAGSNNGDGYDYKPDCRYFEVNVHGQFGGIVYYQEIQP

LTFDCHAMYLPEIRGFSKEIGLAFWRYILTNTTVQCVTSFAARKFRHGQMYCAMIGLKRV
GTIKKYFKGVDDVTFYSATREELIDFLNHGR
>LFGLNPFC_00836 hypothetical protein
MLYAFTLGRKLRGEEPYYPEKGGKGGSSDKSAKYAAEAQKYAADLQNQQFNTIMNNLKLF
TPLAGKYVGSLENLSSLEGQGQALNQYYNSQQYKDLAGQARYQSLAAAEATGGLGSTATS
NQLATIAPTLGQQWLSGQMNNYNNLANIGLGALQGQANAGQTYANNMSQISQQSAALAAA
NANRPSALRQGVSGAASGALLGGGIASALELSTPWGAGIGAGLGLLGSLF
>LFGLNPFC_00837 hypothetical protein
MATWQQGINSGGFLAGIGAQNENAPKASDINATLGLIRENNELAQSGANNVGLTALRGLA
GVADIYKQDQQQKAINAFNKVHADAWASGDPSGLFKFAKENPAFVAQAQQAFSGLNEQQR
NDMGDLAMRANVALSQGPEAYSKFITDNKDRLNRVGANADWMIQTGIQNPEQLSHMLTTM
SLGALGPEKAFAVQDKMVGRQQEQQRINETIRNNDMTNARAIRGQDLSYKAQMARLNHDK
YVFKQSQAALERAGQLQDMDVLSLNSQIAATGIDPLTGKAATSARMSQAKRWLDGNNNYN
NALITGERGIEKIDSLLGKKELEGIGRFEGRNIDGFTSAEGLANRNAIEELKSGAFVQNV
QIMRGMGSLSNAEGQKLENLIAKLDITQPEEVVRKQLSEIRSQYSVFQKVAAREAESMGY
SSSGYDTYVSERKSGSDSNKSGFSSLWGD
>LFGLNPFC_00838 hypothetical protein
MAKAWKDVIASPQYQALTEEQKAQAQAQYFDEVVAPKAGDKWAEARDQFYAAYPPPQQQK
EEPSLMQQAGDWLTGGQSAGQIAEQAGRGLVNIPFDVLQGGASLINAISQGLGGPKVLDD
VYRPAERPTDPYAQAGETIGGYLLPIGTAAKAAGATAKLAGDIGSAGNMIAGSLADAANQ
EGDFAQNAAINGGINIGAQGVLSGVGRVIAPRVSQALGGAALNSANDVSRMAKSGAGRQS
IASQAANVSEDVAKAAESAGIDINALTPGMRSGSRGIAQAEGALASTPGIVQDAHQAAFN
EISSKLSRNLDEFGAASGTASEKSAAIKQRILQNLDQMKDAERAAWDDVRSTMPNQKARM
LNGNAVIQAERSAGIPLTPEMKQFVQANNQGGVTFDGMKAWRAKFADAEQKYKRSGEANA
ARRAGEIRRAITDDMRTMAENGGFLDDWQKANDLSKARLSAQESAESVFGRDLATDALIT
NGVKSLQSSSAKGLNGPAGFHSMIRALPESERVPAISSMLQDAISHGVRGGKADAAGINH
IAEILTPQNVKAISRYSSELGRIADAYGTLARAAVKPQQYIERTGRTANVLRDLDAGLSN
VTSTVLNAIANSTSGAIVGGAGGGIAGAAAGALVGAGLKGAVSKIATTRSGRYAIEKAVQ
EATKAVRAGGSKEALAAAERRFMANKAAVKAIRDAVGNEEFNRLARAGIVASLSGMAQE
>LFGLNPFC_00839 hypothetical protein
MYTTVIAIAIALIVVQYQLASLKQKISDLKTENEALKNSIKDEKNKLSFTISDIEQSIEI
IENNINRLKKEDIHEINDNIKDLKSWLRNVGQIATSTRDKLNTSMDD
>LFGLNPFC_00840 hypothetical protein
MENRISIVVDAAAGPLEQYFAKALANYKNRGTVSAYLGKIGGFERNHHAVLSGIYKSHIR
IPGVDDPWLRTTPIHRRVSDNFLIFAVHNTYPIHIQIIAIIKPDGHEKVKKLLPAIIDIT
EKRFQSLNERELNSLISYK
>LFGLNPFC_00841 hypothetical protein
MAILNQEPGKIENVFSDISTSIERSISDFDRSHSGSLSKKQASEALSKIYCVMSPVEEVC
KKYITFIDILSNGTEEDISSLNIQHDDVDMLNDQISKLDYGIAKLLYTFFIAENSDAWKP
HMSTLTTMKNHSINTFIEYKRLTMGLVTLAMQHIPLSYAEPEEFTEEELASFKKSVEDSH
KRFGMEAPKWKTA
>LFGLNPFC_00842 hypothetical protein
MARDDPHFNFRMPLEVREKLKLRAEANGRSMNSELLQIVQDALSKPSPIAGYRDEAERMA
DQQAEIVKKMVFETLKKMYETR
>LFGLNPFC_00843 hypothetical protein
MSDRKYKNPQVNLRLPVEIKERLIELAEANSRSLNAEMVAALEAWTEKNKHIQALDLATI
ASRLIDLEHDVEKLKCMYGNNKDES
>LFGLNPFC_00844 hypothetical protein
MKVKTLRMPEKLEKILEEKAKEECRSFSAEVIKRVLDSLKREGVMV
>LFGLNPFC_00845 hypothetical protein
MSKECCFCGISESDADQTYIYSKETGRMLCSDCVLDIIRYKHLGCSASISNIGEVYEGKD
ITDRAES
>LFGLNPFC_00846 hypothetical protein
MKSIATAVSTINVPFHGAELYVVNHNGEPYTPMKPIVEGMGLDWKSQHKKISQRFSNGMV
EITIPSAGGVQAMICMALRKLAAWLNSISPNKVRPEIRDKVIQYQEECDDVLYEYWTKGH
VVNPRKAKKALQGKITTEQQEAIKQLVMSRGQSLPKEKQAKAMITMWSSLKSHFGCSYKE
ISEEQFTEALSLAARVPLEGEFIGKQEKKTNELSAKEVNSLVWLWDYANRSQALFRELYQ
ALKQIQSNYSGRCYDYGHEFSYVIGMARDVLINHTRDVDINEPDGPTNLSAWMRLKNKEL
PPSVHNY
>LFGLNPFC_00847 hypothetical protein
MTDITANVIVSMPSQLFTMARSFKAVANGKIYIGKIDTDPVNTENQIQVYVENEDGSHVP
VSQPIIINAAGYPVYNGQIAKFVTVQGHSMAVYDAYGAQQFYFPNVLKYDPDQLRQQLED
TDGANKYPKLQIARWRDSYDVRGWGAIGDGVHDDTSALSELLSVATGGEKIDGRGLTFKV
STLPDVSRFKNARFLFERIPGQPLFYVSEDFIQGELFKITDTPWYNAWTQDKTFVYDNVI

YAPFMAGDRHGVNNLHVAWVRSGDDGKTWTTPEWLTDLHENYPTVNYHCMSMGVVRNRLF
AVIETRTVSGNKLQVAELWDRPMSRSLRVYGGITKAANQQVAYIRITDHGLFAGDFVNFS
NSGVTGVTGNMTVTTVIDKNTFTVTTQNTQDVDQNNEGRYWSFGTSFHSSPWRKTSLGTI
PSFVDGSTPVTEIHSFATISDNSFAVGYHNGDIGPRELGILYFSDAFGSPGSFVRRRIPA
EYEANASEPCVKYYDGILYLTTRGTLSTQPGSSLHRSSDLGTSWNSLRFPNNVHHSNLPF
AKVGDELIIFGSERAFGEWEGGEPDNRYAGNYPRTFMTRVNVNEWSLDNVEWVNVTDQIY
QGGIVNSAVGVGSVCIKDNWLYYIFGGEDFLNPWSIGDNNRKYPYVHDGHPADLYCFRVK
IKQEEFVSRDFVYGATPNRTLPTFMSTSGVRTVPVPVDFTDDVAVQSLTVHAGTSGQVRA
EVKLEGNYAIIAKKVPSDDVTAQRLIVSGGETTSSADGAMITLHGSRSSTPRRAVYNALE
HLFENGDVKPYLDNVNALGGPGNRFSIVYLGSNPVVTSDGTLKTEPVSPDETLLDAWGDV
RYIAYKWLNAVAIKGEEGARIHHGVIAQQLRDVLISHGLMEEESTTCRYAFLCYDDYPAV
YDDVITGQREMPLTDNDGSIIVDEDDNPVMVMEDIIERVEITPAGSRWGVRPDLLFYIEA
AWQRREIERIKARLDLIEGKH
>LFGLNPFC_00848 Prophage integrase IntS
MLTVKQIEAAKPKEKPYRLLDGNGLYLYVPVSGKKVWQLRYKIDGKEKILTVGKYPLMTL
QEARDKAWTARKDISVGIDPVKAKKASSNNNSFSAIYKEWYEHKKQVWSVGYATELAKMF
DDDILPIIGGLEIQDIEPMQLLEVIRRFEDRGAMERANKARRRCGEVFRYAIVTGRAKYN
PAPDLADAMKGYRKKNFPFLPADQIPAFNKALATFSGSIVSLIATKVLRYTALRTKELRS
MLWKNVDFENRIITIDASVMKGRKIHVVPMSDQVVELLTTLSSITKPVSEFVFAGRNDKK
KPICENAVLLVIKQIGYEGLESGHGFRHEFSTIMNEHEWPADAIEVQLAHANGGSVRGIY
NHAQYLDKRREMMQWWADWLDEKVE
>LFGLNPFC_00850 Inner membrane protein YfdC
MDNDKIDQHSDEIEVESEEKERGKKIEIDEDRLPSRAMAIHEHIRQDGEKELERDAMALL
WSAIAAGLSMGASLLAKGIFHVELEGVPGSFLLENLGYTFGFIIVIMARQQLFTENTVTA
VLPVMQKPTMSNVGLLMRLWGIVLLGNILGTGIAAWAFEYMPIFNEETRDAFVKIGMDVM
KNTPSEMFANAIISGWLIATMVWMFPAAGAAKIVVIILMTWLIALGDTTHIVVGSVEILY
LVFNGTLHWSDFIWPFALPTLAGNICGGTFIFALMSHAQIRNDMSNKRKAEARQKAERAE
NIKKNDKNPA
>LFGLNPFC_00851 Intermembrane phospholipid transport system lipoprotein MlaA
MKLRLSALALGTTLLVGCASSGTDQQGRSDPLEGFNRTMYNFNFNVLDPYIVRPVAVAWR
DYVPQPARNGLSNFTGNLEEPAVMVNYFLQGDPYQGMVHFTRFFLNTILGMGGFIDVAGM
ANPKLQRTEPHRFGSTLGHYGVGYGPYVQLPFYGSFTLRDDGGDMADSLYPVLSWLTWPM
SVGKWTLEGIETRAQLLDSDGLLRQSSDPYIMVREAYFQRHDFIANGGELKPQENPNAQA
IQDDLKDIDSE
>LFGLNPFC_00852 Long-chain fatty acid transport protein
MSQKTLFTKSALAVAVALISTQAWSAGFQLNEFSSSGLGRAYSGEGAIADDAGNVSRNPA
LITMFDRPTFSAGAVYIDPDVNISGTSPSGRSLKADNIAPTAWVPNMHFVAPINDQFGWG
ASITSNYGLATEFNDTYAGGSVGGTTDLETMNLNLSGAYRLNNAWSFGLGFNAVYARAKI
ERFAGDLGQLVAGQIRQSPAWLTPQGQALAATANGIDSNTKIAHLNGNQWGFGWNAGILY
ELDKNNRYALTYRSEVKIDFKGNYSSDLNPAFNNYGLPIPTATGGATQSGYLTLNLPEMW
EVSGYNRVAPQWAIHYSLAYTSWSQFQQLKATSTSGDTLFQKHEGFKDAYRIALGTTYYY
DDNWTFRTGIAFDDSPVPAQNRSISIPDQDRFWLSAGTTYAFNKDASVDVGVSYMHGQSV
KINEGPYQFESEGKAWLFGTNFNYAF
>LFGLNPFC_00853 hypothetical protein
MSKCSADETPVCCCMDVGTIMDNSDCTASYSRVFANRAEAEQTLAALTEKARSVESEPCK
ITPTFTEESDGVRLDIDFTFACEAEMLIFQLGLR
>LFGLNPFC_00854 3-ketoacyl-CoA thiolase FadI
MGQVLPLVTRQGDRIAIVSGLRTPFARQATAFHGIPAVDLGKMVVGELLARTEIPAEVIE
QLVFGQVVQMPEAPNIAREIVLGTGMNVHTDAYSVSRACATSFQAVANVAESLMAGTIRA
GIAGGADSSSVLPIGVSKKLARVLVDVNKARTMSQRLKLFSRLRLRDLMPVPPAVAEYST
GLRMGDTAEQMAKTYGITREQQDALAHRSHQRAAQAWSEGKLKEEVMTAFIPPYKQPLVE
DNNIRGNSSLADYAKLRPAFDRKHGTVTAANSTPLTDGAAAVILMTESRAKELGLVPLGY
LRSYAFTAIDVWQDMLLGPAWSTPLALERAGLTMGDLTLIDMHEAFAAQTLANIQLLGSE
RFARDVLGRAHATGEVDESKFNVLGGSIAYGHPFAATGARMITQTLHELRRRGGGFGLVT
ACAAGGLGAAMVLEAE
>LFGLNPFC_00855 Fatty acid oxidation complex subunit alpha
MEMASAFTLNVRLDNIAIITIDVPGEKMNTLKAEFASQVRAIIKQIRENKELRGVVFVSA
KPDNFIAGADINMIGNCKTAQEAEVLARQGQQLMAEIHALPIPVIAAIHGACLGGGLELA
LACHGRVCTDDPKTVLGLPEVQLGLLPGSGGTQRLPRLIGVSTALEMILTGKQLRAKQAV
KLGLVDDVVPHSILLEAAVELAKQDRPSSRPLPVRERILAGPLGRALLFKMVGKKTEHKT
QGNYPATERILEVVETGLAQGTSSGYDAEARAFGELAMTPQSQALRNIFFASTDVKKDPG
SDAPPAPLNSVGILGGGLMGGGIAYVTACKAGLPVRIKDINPRGINHALKYSWDQLEGKV
RRRHLKASERDKQLALISGTTDYCGFAHRDLIIEAVFENLELKQQMVAEVEQNCATHTIF
ASNTSSLPIGDIAAHAARPEQVIGLHFFSPVEKMPLVEIIPHASTSAQTIATTVKLAKKQ

GKTPIVVRDKAGFYVNRILAPYINEAIRMLTEGERIEHIDAALVKFGFPVGPIQLLDEVG
IDTGTKIMPVLEAAYGERFSAPANVVSSILNDDRKGRKNGRGFYLYGQKGRKSKKQVDPA
IYPLIGAQGQGRLSAPQVAERCVMLMLNEAVRCLDEQVIRSVRDGDIGAVFGIGFPPFLG
GPFRYIDSLGAGEVVAIMQRLATQYGSRFTPCDRLVEMSERGESFWKTTATDLQ
>LFGLNPFC_00856 Phosphohistidine phosphatase SixA
MQVFIMRHGDAALDAASDSVRPLTTNGCDESRLMANWLKGQKVEIERVLVSPFLRAEQTL
EEVGDCLNLPSSAEVLPELTPCGDVGLVGAYLQALTNEGVASVLVISHLPLVGYLVAELC
PGETPPMFTTSAIASVTLDESGKGQFNWQMSPCNLKMAKAI
>LFGLNPFC_00857 hypothetical protein
MSKFAKTAIAAAMVMGAVVSSSAFAAGNNGTARFYGTIEDSPCSIVPDDHKLEVDMGDIG
AAKLTGGGTTTPKDFQIRLQDCVFTTETNMETTFTGTNYAGPANTDNYALFNVDSGVAMN
HVSLVIGDTHGKGYKVGEKIVQPIVMDTSTSKGKDKQTLNFKAWLVGETDAPDLGQFETL
TTFQITYLCCGVIQHATDLRLFHKRICVCLITQIFGCGESPAILRWQSLVDQSMHGLMIQ
FNLTPVSLS
>LFGLNPFC_00858 Outer membrane usher protein PapC
MAALGLKEGIAKSLQWTHNDECLKPGQLDGMEVENDLSQSALLLTVPQAYLEYTSSDWDP
PSRWDDGIPGLIADYSLNAQTRHQEQGGEDSHDISGNGTVGANLGAWRFRADWQSDYQHT
RSNDDDDDSSNSTTSKNWDWSRYYAWRALPSLKAKLSLGEDYLNSDIFDGFNYIGSSVST
DDQMLPPNLRGYAPDVSGVAHSSAKVTISQMGRVLYETQVPAGPFRIQDIGDSVSGTLHV
RVEEQNGQVQEYDVTTASMPFLTRQGQVRYKVMMGRPEDWNHKTEGGFFSGGEASWGVAD
GWSLYGGALADKHYQSAAMGVGRDLAQFGALAFDVTHSHVNLDHDSAYGKGKLDGNSFRV
SYAKDFDELNSRVTFAGYRFSEKNFMTMSEYLDANQSDMARTGNDKEMYTITYNQNFAAA
GVSIYLNYSHRTYWDRPEQTNYNLMFSHYFNMGSIRNMSISVTGYRYEYDDNADKGMYLS
MSIPWSDSSTVTYNGSYGSGSDSSQVGYFKRVDDATHYQVNVGTSEQHGSVDGYLSHDGS
LAKVDLSANYHEGEYRSAGIALQGGATLTAHGGALHRTQNMGGTRLLIDADGIANVPVES
NGAPVYTNMFGKAVVADINNYYRNQAYIDLNNLPEDAEATQSVVQATLTEGAIGYRKFKV
ISGQKAMAVLRLRDGSYPPFGAEVKNDEQQQVGIVDDEGNVYLAGVNAGEHMMVFWEGSA
QCEIVLPKPLPADLFSGLLLPCEQKGTAAPDSSAPEIKPVIQDQTRQVTPTEAPTSISAT
Q
>LFGLNPFC_00859 putative fimbrial chaperone YfcS
MSYKLSCPMLVSTALMALLTTASLTAHASVTPDRTRLVFNESDKSISVTLRNNTEKLPYL
AQSWLEDEKGNKITSPLAVLPPVQRIDAMMNGQVKIQALPDIHTLPSDRESLFYYNVREI
PPKSGKANTLQIALQTRIKLFWRPKALEKIDMRRPWQFKVTLTRSGQDYTVNNPTPYHVI
ISDASTQKKGLTAAGFKPLVMPPKTSQPLKAKMASAPVLTYINDYGARMPLIFRCEGNTC
KVDEDQSSKG
>LFGLNPFC_00860 putative fimbrial-like protein YfcR
MKAFFLTGSAALLLACGVTVFPAMAGGKIVNLTLRVLVDGPPPCSVKGSAVEFGNVIIKS
IDGANYRQPVGYTLNCSNSVSDDLQMQLQATTTTINGETVLSTGISGFGIRIQNAADHSL
VAVGNSSWLPFNINTQPDFEAVPVKQSGVELMASEFNATMTMVVDYQ
>LFGLNPFC_00861 putative fimbrial-like protein YfcQ
MKRLAWCLLYGFAGLAQAAINDVTFHGTLVSPPACTISDGKTIEVEFRNVIIDNINGDNF
RQDVPYTITCDPDVRDDAWEMSLTWTGSQTPYDDSAIETDVSGLGIELQQNGQPFKLGTP
LKIDPSTPPTLQAVPVKANDAALSDGTFSAYATLQVDYQ
>LFGLNPFC_00862 putative fimbrial-like protein YfcP
MNLKRISLCAAVLATVLLTPHVQAADNLHFYGNLLSKSCTLVVQGETLAEVHFPTISRKD
LMVTGQSARVPVVFQLKDCKGPAQYEVRVTLTGTEDSEQPGFLALDASSAAQGVGIGMEK
TDGTPVPINNTSGATFVLSNGNNNLNFNAWLQAKSGREVTMGEFTAFLTATFEYI
>LFGLNPFC_00863 putative protein YfcO
MKILRFWACLLVLIFSSTGGLAAKKIATLVTPIGFSAVTTQIHYYLEILTPEGVAYGVYK
QHSRMLQTEKLDLVSWSGNTTAPELTVDDGYEVAKSNCVGLEAYDALSSRTNWICENMVL
SVYHDGPVQGCPWLVSTYTDSVLPYDVPTPYVGPKVHNSSCPVSVATYDISWSEDYVAHS
KVLSLQSTGGTIEKTLPTFLMESGKLCDGRVMDDRGAYCRFVAQMITFSTSGCDSSKVTV
TPNQHPITDKQLHDMVVRVDTSSRQPIDSTCRFQYTLNEL
>LFGLNPFC_00864 Endonuclease MutS2
MKKKTTLSEEDQALFRQLMAGTRKIKQDTIVHRPQRKKISEVPVKRLIQEQADASHYFSD
EFQPLLNTEGPVKYVRPDVSHFEAKKLRRGDYSPELFLDLHGLTQLQAKQELGALIAACR
REHVFCACVMHGHGKHILKQQTPLWLAQHPHVMAFHQAPKEYGGDAALLVLIEVEEWLPP
ELP
>LFGLNPFC_00865 50S ribosomal protein L3 glutamine methyltransferase
MDKIFVDEAVNELQTIQDMLRWSVSRFSAANIWYGHGTDNPWDEAVQLVLPSLYLPLDIP
EDMRTARLTSSEKHRIVERVIRRVNERIPVAYLTNKAWFCGHEFYVDERVLVPRSPIGEL
INNKFAGLISKQPQHILDMCTGSGCIAIACAYAFPEAEVDAVDISPDALAVAEQNIEEHG
LIHNVIPIRSDLFRDLPKVQYDLIVTNPPYVDAEDMSDLPNEYRHEPELGLASGTDGLKL
TRRILGNAADYLADDGVLICEVGNSMVHLMEQYPDVPFTWLEFDNGGDGVFMLTKEQLIA

AREHFAIYKD
>LFGLNPFC_00866 Chorismate synthase
MAGNTIGQLFRVTTFGESHGLALGCIVDGVPPGIPLTEADLQHDLDRRRPGTSRYTTQRR
EPDQVKILSGVFEGVTTGTSIGLLIENTDQRSQDYSAIKDVFRPGHADYTYEQKYGLRDY
RGGGRSSARETAMRVAAGAIAKKYLAEKFGIEIRGCLTQMGDIPLEIKDWSQVEQNPFFC
PDPDKIDALDELMRALKKEGDSIGAKVTVVASGVPAGLGEPVFDRLDADIAHALMSINAV
KGVEIGDGFDVVALRGSQNRDEITKDGFQSNHAGGILGGISSGQKIIAHMALKPTSSITV
PGRTINRFGEEVEMITKGRHDPCVGIRAVPIAEAMLAIVLMDHLLRQRAQNADVKTDIPR
W
>LFGLNPFC_00867 Penicillin-insensitive murein endopeptidase
MNKTAIALLALLASSASLAATPWQKITQPVPGSAQSIGSFSNGCIVGADTLPIQSEHYQV
MRTDQRRYFGHPDLVMFIQRLSRQVSNLGMGTVLIGDMGMPAGGRFNGGHASHQTGLDVD
IFLQLPKTRWTSAQLLRPQALDLVSRDGKHVVPALWKPEIFSLIKLAAQDKDVTRIFVNP
AIKQQLCLDAGTDRDWLRKVRPWFQHRAHMHVRLRCPADSLECEDQPLPPPGDGCGAELQ
SWFEPPKPGTTKPEKKTPPPLPPSCQALLDEHVI
>LFGLNPFC_00868 putative membrane transporter protein YfcA
METFNSLLMVSPLLLGVLFFVAMLAGFIDSIAGGGGLLTIPALMAAGMSPANALATNKLQ
ACGGSISATIYFIRRKVVSLSDQKLNIAMTFVGSMSGALLVQYVQADVLRQILPILVICI
GLYFLLMPKLGEEDRQRRMYGLPFALVAGGCVGFYDGFFGPAAGSFYALAFVTLCGFNLA
KATAHAKLLNATSNIGGLLLFILGGKVIWATGFVMLVGQFLGARMGSRLVLSKGQKLIRP
MIVIVSAVMSAKLLYDSHGQEILHWLGMN
>LFGLNPFC_00869 Elongation factor P hydroxylase
MNSTHHYEQLIEIFNSCFADEFNTRLIKGDDEPIYLPADAEVPYNRIVFAHGFYASAIHE
ISHWCIAGKARRELVDFGYWYCPDGRDAQTQSQFEDVEVKPQALDWLFCVAAGYPFNVSC
DNLEGDFEPDRVVFQRRVHAQVMDYLANGIPERPARFIKALQNYYHTPELTAEQFPWPEA
LS
>LFGLNPFC_00870 hypothetical protein
MIAEFESRILALIDGMVDHASDDELFASGYLRGHLTLAIAELESGDDHSAQAVHTTVSQS
LEKAIGAGELSPRDQALVTDMWEHLFQQASQQ
>LFGLNPFC_00871 tRNA 5-methylaminomethyl-2-thiouridine biosynthesis bifunctional protein MnmC
MKHYSIQPANLEFNAEGTPVSRDFDDVYFSNDNGLEETRYVFLGGNHLEARFPEHPHPLF
VVAESGFGTGLNFLTLWQAFDQFREAHPQAQLQRLHFISFEKFPLTRADLALAHQHWPEL
APWAEQLQAQWPLPLPGCHRLLLDEGRITLDLWFGDINELTSQLDDSLNQKVDAWFLDGF
APAKNPDMWTQNLFNAMARLARPGSTLATFTSAGFVRRGLQEAGFTMQKRKGFGRKREML
CGVMEQTLPLPCSTPWFNRTGSNKQEAAIIGGGIASALLSLALLRRGWQVTLYCADEAPA
LGASGNRQGALYPLLSKHDEALNRFFSNAFTFARRFYDLLPVKFDHDWCGVTQLGWDEKS
QHKIAQMLSMDLPAELAVAVEANAVEQITGVATNCSGITYPQGGWLCPAELTRNVLKLAQ
QQGLQIHYQYQLQDLSRKDDGWLLNFAGDQQATHSVVVLANGHQISRFSQTSSLPVYSVA
GQVSHIPTTPELAELKQVLCYDGYLTPQNTANQHHCIGASYHRGSEETAYSDEDQQQNRQ
RLIDCFPHAQWAKEVDVSGKEARCGVRCATRDHLPMVGNVPDYDATLVEYASLAEKKDEA
VSAPVYDDLFMFAALGSRGLCSAPLCAEILAAQMSEEPIPMDASTLAALNPNRLWVRKLL
KGKAVKAG
>LFGLNPFC_00872 3-oxoacyl-[acyl-carrier-protein] synthase 1
MKRAVITGLGIVSSIGNNQQEVLASLREGRSGITFSQELKDSGMRSHVWGNVKLDTTGLI
DRKVVRFMSDASIYAFLSMEQAIADAGLSPEAYQNNPRVGLIAGSGGGSPRFQVFGADAM
RGPRGLKAVGPYVVTKAMASGVSACLATPFKIHGVNYSISSACATSAHCIGNAVEQIQLG
KQDIVFAGGGEELCWEMACEFDAMGALSTKYNDTPEKASRTYDAHRDGFVIAGGGGMVVV
EELEHALARGAHIYAEIVGYGATSDGADMVAPSGEGAVRCMKMAMHGVDTPIDYLNSHGT
STPVGDVKELAAIREVFGDKSPAISATKAMTGHSLGAAGVQEAIYSLLMLEHGFIAPSIN
IEELDEQAAGLNIVTETTDRELTTVMSNSFGFGGTNATLVMRKLKD
>LFGLNPFC_00873 putative MFS-type transporter YfcJ
MTAVSQTETRSSANFSLFRIAFAVFLTYMTVGLPLPVIPLFVHHELGYGNTMVGIAVGIQ
FLATVLTRGYAGRLADQYGAKRSALQGMLACGLAGGALLLAAILPVPAPFKFALLVVGRL
ILGFGESQLLTGALTWGLGIVGPKHSGKVMSWNGMAIYGALAVGAPLGLLIHSHYGFAAL
ALTTMALPLLAWACNGTVRKVPALAGERPSLWSVVGLIWKPGLGLALQGVGFAVIGTFVS
LYFASKGWAMAGFTLTAFGGAFVVMRVMFGWMPDRFGGVKVAIVSLLVETVGLLLLWQAP
GAWVALAGAALTGAGCSLIFPALGVEVVKRVPSHVRGTALGGYAAFQDIALGVSGPLAGM
LATTFGYSSVFLAGAISAVLGIIVTILSFRRG
>LFGLNPFC_00874 Flagellar regulator flk
MIQPISGPPPGQPPGQGDNLPSGAGNQPLSSQQRTSLESLMTKVTSLTQQQRAELWAGIR
HDIGLSGDSPLLSRHFPAAEHNLAQRLLAAQKSHSARQLLAQLGEYLRLGNNRQAVTDYI
RHNFGQTPLNQLSPEQLKTILTLLQEGKMVIPQPQQREATDRPLLPAEHNALKQLVTKLA
AATGEPSKQIWQSMLELSGVKDGELIPAKLFNHLVTWLQARQTLSQQNTPTLESLQMALK

QPLDASELAALSAYIQQKYGLSAQSSLSSAQAEDILNQLYQRRVKGIEPRDMQPLLNPFP
PMMDTLQNMATRPALWILLVAIILILVWLVR
>LFGLNPFC_00875 Erythronate-4-phosphate dehydrogenase
MKILVDENMPYARDLFSRLGEVTAVPGRPIPVAQLADADALMVRSVTKVNESLLAGKPIK
FVGTATAGTDHVDEAWLKQAGIGFSAAPGCNAIAVVEYVFSSLLMLAERDGFSLHERTVG
IVGVGNVGRRLQARLEALGIKTLLCDPPRADRGDEGDFRSLDELVQHADILTFHTPLFKD
GPYKTLHLADEKLIRSLKPGAILINACRGAVVDNTALLTCLSEGQKLSVVLDVWEGEPEL
NVELLKKVDIGTPHIAGYTLEGKARGTTQVFEAYSKFIGHEQHVALDTLLPAPEFGRITL
HGPLDQPTLKRLVHLVYDVRRDDAPLRKVAGIPGEFDKLRKNYLERREWSSLYVICDDAS
AASLLCKLGFNAVHHPAR
>LFGLNPFC_00876 USG-1 protein
MSEGWNIAVLGATGAVGEALLETLAERQFPVGEIYALARNESAGEQLRFGGKTITVQDAA
EFDWTQAQLAFFVAGKEATATWVEEATNSGCLVIDSSGLFALEPDVPLVVPEVNPFVLTD
YRNRNVIAVPDSLTSQLLAALKPLIDQGGLSRISVTSLISASAQGKKAVDALAGQSAKLL
NGIPIDEEDFFGRQLAFNMLPLLPDSEGSVREERRIVDEVRKILQDEGLMISASVVQAPV
FYGHAQMVNFEALRPLAAEEARDAFAQGEDIVLSEENEFPTQVGDASGSPHLSVGCVRND
YGMPEQVQFWSVADNVRFGGALMAVKIAEKLVQEYLY
>LFGLNPFC_00877 tRNA pseudouridine synthase A
MSDQQQPPVYKIALGIEYDGSRYYGWQRQNEVRSVQEKLEKALSQVANEPITVFCAGRTD
AGVHGTGQVVHFETTAQRKDAAWTLGVNANLPGDIAVRWVKAVPDDFHARFSATARRYRY
IIYNHRLRPAVLSKGVTHFYEPLDAERMHRAAQCLLGENDFTSFRAVQCQSRTPWRNVMH
INVTRHGPYVVVDIKANAFVHHMVRNIVGSLMEVGAHNQPESWIAELLAAKDRTLAAATA
KAEGLYLVAVDYPDRYDLPKPPMGPLFLAD
>LFGLNPFC_00878 Protein DedA
MDLIYFLIDFILHIDVHLAELVAEYGVWVYAILFLILFCETGLVVTPFLPGDSLLFVAGA
LASLETNDLNVHMMVVLMLIAAIVGDAVNYTIGRLFGEKLFSNPNSKIFRRSYLDKTHQF
YEKHGGKTIILARFVPIVRTFAPFVAGMGHMSYRHFAAYNVIGALLWVLLFTYAGYFFGT
LPFIQSNLKLMIVGIIFVSILPGVFEFIRHKRAAARAAK
>LFGLNPFC_00879 Acetyl-coenzyme A carboxylase carboxyl transferase subunit beta
MSWIERIKSNITPTRKASIPEGVWTKCDSCGQVLYRAELERNLEVCPKCDHHMRMTARNR
LHSLLDDEGSLVELGSELEPKDVLKFRDSKKYKDRLASAQKETGEKDALVVMKGTLYGMPV
VAAAFEFAFMGGSMGSVVGARFVRAVEQALEDNCPLICFSASGGARMQEALMSLMQMAKT
SAALAKMQERGLPYISVLTDPTMGGVSASFAMLGDLNIAEPKALIGFAGPRVIEQTVREK
LPPGFQRSEFLIEKGAIDMIVRRPEMRLKLASILAKLMNLPAPNPEAPREGVVVPPVPDQ
EPEA
>LFGLNPFC_00880 Dihydrofolate synthase/folylpolyglutamate synthase
MIIKRTPQAASPLASWLSYLENLHSKTIDLGLERVSQVAARLGVLKPAPFVFTVAGTNGK
GTTCRTLESILMAAGYKVGVYSSPHLVRYTERVRVQGQELPESAHTASFAEIESARGDIS
LTYFEYGTLSALWLFKQAQLDVVILEVGLGGRLDATNIVDADVAVVTSIALDHTDWLGPD
RESIGREKAGIFRSEKPAIVGEPEMPSTIADVAQEKGALLQRRGVEWNYSVTDHDWTFSD
AHGTLENLPLPLVPQPNAATALAALRASGLEVSENAIRDGIASAILPGRFQIVSESPRVI
FDVAHNPHAAEYLTGRMKALPKNGRVLAVIGMLHDKDIAGTLAWLKSVVDDWYCAPLEGP
RGATAEQLLEHLGNGKSFDSVAQAWDAAMADAKAEDTVLVCGSFHTVAHVMEVIDARRSG
GK
>LFGLNPFC_00881 Cell division protein DedD
MASKFQNRLVGTIVLVALGVIVLPGLLDGQKKHYQDEFAAIPLVPKAGDRDEPDMMPAAT
QALPTQPPEGAAEEVRAGDAAAPSLDPATIAANNTEFEPEPAPVVPPKPKPVEPPKPKVE
APPAPKPEPKPVVEEKAAPTGKAYVVQLGALKNADKVNEIVGKLRGAGYRVYTSPSTPVQ
GKITRILVGPDASKDKLKGSLGELKQLSGLSGVVMGYTPN
>LFGLNPFC_00882 Colicin V production protein
MVWIDYAIIAVIAFSSLVSLIRGFVREVLSLVTWGCAFFVASHYYTYLSVWFTGFEDELV
RNGIAIAVLFIATLIVGAIVNFVIGQLVEKTGLSGTDRVLGVCFGALRGVLIVAAILFFL
DSFTGVSKSEDWSKSQLIPQFSFIIRWFFDYLQSSSSFLPRA
>LFGLNPFC_00883 Amidophosphoribosyltransferase
MCGIVGIAGVMPVNQSIYDALTVLQHRGQDAAGIITIDANNCFRLRKANGLVSDVFEARH
MQRLQGNMGIGHVRYPTAGSSSASEAQPFYVNSPYGITLAHNGNLTNAHELRKKLFEEKR
RHINTTSDSEILLNIFASELDNFRHYPLEADNIFAAIAATNRLIRGAYACVAMIIGHGMV
AFRDPNGIRPLVLGKRDIDENRTEYMVASESVALDTLGFDFLRDVAPGEAIYITEEGQLF
TRQCADNPVSNPCLFEYVYFARPDSFIDKISVYSARVNMGTKLGEKIAREWEDLDIDVVI
PIPETSCDIALEIARILGKPYRQGFVKNRYVGRTFIMPGQQLRRKSVRRKLNANRAEFRD
KNVLLVDDSIVRGTTSEQIIEMAREAGAKKVYLASAAPEIRFPNVYGIDMPSATELIAHG
REVDEIRQIIGADGLIFQDLNDLIEAVRAENPDIQQFECSVFNGVYVTKDVDQGYLDFLD
TLRNDDAKAVQRQNEVENLEMHNEG
>LFGLNPFC_00884 Flavin prenyltransferase UbiX

MKRLIVGISGASGAIYGVRLLQVLRDVTDIETHLVMSQAARQTLSLETDFSLREVQALAD
VTHDARDIAASISSGSFQTLGMVILPCSIKTLSGIVHSYTDGLLTRAADVVLKERRPLVL
CVRETPLHLGHLRLMTQAAEIGAVIMPPVPAFYHRPQSLDDVINQTVNRVLDQFAVTLPE
DLFARWQGA
>LFGLNPFC_00885 Lysine/arginine/ornithine-binding periplasmic protein
MKKSILALSLLVGLSAAASSYAALPETVRIGTDTTYAPFSSKDAKGDFVGFDIDLGNEMC
KRMQVKCTWVASDFDALIPSLKAKKIDTIISSLSITDKRQQEIAFSDKLYAADSRLIAAK
GSPIQPTLDSLKGKHVGVLQGSTQEAYANETWRSKGVDVVAYANQDLVYSDLAAGRLDAA
LQDEVAASEGFLKQPAGKDFAFAGPSVKDKKYFGDGTGVGLRKDDAELTAAFNKALNELR
QDGTYDKMAKKYFDFNVYGD
>LFGLNPFC_00886 Histidine-binding periplasmic protein
MKKLVLSLSLVLAFSSATAAFAAIPQNIRIGTDPTYAPFESKNSQGELVGFDIDLAKELC
KRINTQCTFVENPLDALIPSLKAKKIDAIMSSLSITEKRQQEIAFTDKLYAADSRLVVAK
NSDIQPTVESLKGKRIGVLQGTTQETFGNEHWAPKGIEIVSYQGQDNIYSDLTAGRIDAA
FQDEVAASEGFLKQPVGKDYKFGGPSVKDEKLFGVGTGMGLRKEDNELREALNKAFAEMR
ADGTYEKLAKKYFDFDVYGG
>LFGLNPFC_00887 Histidine transport system permease protein HisQ
MLYGFSGVILQGALVTLELAISSVVLAVIIGLIGAGGKLSQNRLSGLIFEGYTTLIRGVP
DLVLMLLIFYGLQIALNTVTEAMGVGQIDIDPMVAGIITLGFIYGAYFTETFRGAFMAVP
KGHIEAATAFGFTRGQVFRRIMFPAMMRYALPGIGNNWQVILKSTALVSLLGLEDVVKAT
QLAGKSTWEPFYFAIVCGVIYLVFTTVSNGVLLFLERRYSVGVKRADL
>LFGLNPFC_00888 Histidine transport system permease protein HisM
MIEILHEYWKPLLWTDGYRFTGVAITLWLLILSVVIGGVLALFLAIGRVSSNKYIQFPIW
LFTYIFRGTPLYVQLLVFYSGMYTLEIVKGTEFLNAFFRSGLNCTVLALTLNTCAYTTEI
FAGAIRSVPHGEIEAARAYGFSTFKMYRCIILPSALRIALPAYSNEVILMLHSTALAFTA
TVPDLLKIARDINAATYQPFTAFGIAAVLYLIISYVLISLFRRAEKRWLQHVKPSSTH
>LFGLNPFC_00889 Histidine transport ATP-binding protein HisP
MSENKLNVIDLHKRYGEHEVLKGVSLQANAGDVISIIGSSGSGKSTFLRCINFLEKPSEG
SIVVNGQTINLVRDKDGQLKVADKNQLRLLRTRLTMVFQHFNLWSHMTVLENVMEAPIQV
LGLSKQEARERAVKYLAKVGIDERAQGKYPVHLSGGQQQRVSIARALAMEPEVLLFDEPT
SALDPELVGEVLRIMQQLAEEGKTMVVVTHEMGFARHVSTHVIFLHQGKIEEEGAPEQLF
GNPQSPRLQQFLKGSLK
>LFGLNPFC_00890 ISNCY family transposase ISRor2
MTISTTSTPHDAVFKSFLRHPDTARDFIDIHLPAPLRKLCDLTTLKLEPNSFIDEDLRQY
YSDLLWSVKTQEGAGYIYVVIEHQSKPEELMAFRMMRYSIAAMQNHLDAGYKELPLVIPM
LFYHGCRSPYPYSLCWLDEFAEPAIARKIYSSAFPLVDITVVPDDEIMQHRKMALLELIQ
KHIRQRDLLGLVDQIVSLLVTGNTNDRQLKALFNYVLQTGDARRFFRAFIGEIAERAPQEK
EKLMTIADRLREEGAMQGKHEEALRIAQEMMEKGFDHEVILTLTRLSPDDLIAPSH
>LFGLNPFC_00891 Epimerase family protein
MNIVITGGTGLIGRYLIPRLLDLGHQITVVTRNPQKASSVLGPRVTLWQGLAGQSNLNGV
DAVINLAGEPIADKRWTHEQKERLCQSRWNITQKLVDLINASDTPPSVLISGSATGYYGD
LGEVVVTEEEPPHNEFTHKLCARWEEIACRAQSDKTRVCLLRTGVVLAPDGGILGKMLPP
FRLGLGGPIGSGRQYLAWIHIDDMVNGILWLLDNELRGPFNMVSPYPVRNEQFAHALGHA
LHRPAILRVPATAIRLLMGESSVLVLGGQRALPKKLEEAGFAFRWYDLEEALADVVR
>LFGLNPFC_00892 Dihydroneopterin triphosphate 2'-epimerase
MAQPAAIIRIKNLRLRTFIGIKEEEINNRQDIVINVTIHYPADKARTSEDINDALNYRTV
TKNIIQHVENNRFSLLEKLTQDVLDIAREHHWVTYAEVEIDKLHALRYADSVSMTLSWQR
>LFGLNPFC_00893 Disulfide-bond oxidoreductase YfcG
MIDLYFAPTPNGHKITLFLEEAGLDYRLIKVDLGKGGQFRPEFLRISPNNKIPAIVDHSP
ADGGEPLSLFESGAILLYLAEKTGLFLSHETRERAVTLQWLFWQVGGLGPMLGQNHHFNH
AAPQTIPYAIERYQVETQRLYHVLNKRLENSPWLGGENYSIADIACWPWVNAWTRQRIDL
AMYPAVKNWHERIRSRPATGLALLKAQHGDERSDS
>LFGLNPFC_00894 Glutathione S-transferase YfcF
MSKPAITLWSDAHFFSPYVLSAWVALQEKGLSFHIKTIDLDSGEHLQPTWQGYGQTRRVP
LLQIDDFELSESSAIAEYLEDRFAPPTWERIYPLDLENRARARQIQAWLRSDLMPIREER
PTDVVFAGAKKAPLTAEGKASAEKLFAMAEHLLALGQPNLFGEWCIADTDLALMINRLVL
HGDEVPERLVDYATFQWQRASVQRFIALSAKQSG
>LFGLNPFC_00895 Phosphodiesterase YfcE
MKLMFASDIHGSLPATERVLELFVQSGAQWLVILGDVLNHGPRNALPEGYAPAKVAERLN
EVAHKVIAVRGNCDSEVDQMLLHFPITAPWQQVLLEKQRLFLTHGHLFGPENLPALNQND
VLVYGHTHLPVAEQRGEIFHFNPGSVSIPKGGNPASYGMLDNDVLSVIALNDQSIIAQVA
INP
>LFGLNPFC_00896 putative Nudix hydrolase YfcD
MEQRRLASTEWVDIVNEENEVIAQASREQMRAQCLRHRATYIVVHDGMGKILVQRRTETK

DFLPGMLDATAGGVVQADEQLLESARREAEEELGIAGVPFAEHGQFYFEDKNCRVWGALF
SCVSHGPFALQEDEVSEVCWLTPEEITARCDEFTPDSLKALALWMKRNAKNEAVETETAE
>LFGLNPFC_00897 hypothetical protein
MPDTLVIIFFVAILTSLATWVVPVGMFDSQEVQYQVDGQTKTRKVVDPHSFRILTNEAGE
PEYHRVQLFTTGDERPGLMNFPFEGLTSGSKYGTAVGIIMFMLVIGGAFGIVMRTGTIDN
GILALIRHTRGNEILFIPALFILFSLGGAIFGMGEEAVAFAIIIAPLMVRLGYDSITTVL
VTYIATQIGFASSWMNPFCVVVAQGIAGVPVLSGSGLRIVVWVIATLIGLIFTMVYASRV
KKNPLLSRVHESDRFFREKQADVEQRPFTFGDWLVLIVLTAVMVWVIWGVIVNAWFIPEI
ASQFFTMGLVIGIIGVVFRLNGMTVNTMASSFTEGARMMIAPALLVGFAKGILLLVGNGE
AGDASVLNTILNSIANAISGLDNAVAAWFMLLFQAVFNFFVTSGSGQAALTMPLLAPLGD
LVGVNRQVTVLAFQFGDGFSHIIYPTSASLMATLGVCRVDFRNWLKVGATLLGLLFIMSS
VVVIGAQLMGYH
>LFGLNPFC_00898 Phosphate acetyltransferase
MSRIIMLIPTGTSVGLTSVSLGVIRAMERKGVRLSVFKPIAQPRTGGDAPDQTTTIVRAN
SSTTTAAEPLKMSYVEGLLSSNQKDVLMEEIIANYHANTKDAEVVLVEGLVPTRKHQFAQ
SLNYEIAKTLNAEIVFVMSQGTDTPEQLKERIELTRNSFGGAKNTNITGVIVNKLNAPVD
EQGRTRPDLSEIFDDSTKAKVNNVDPAKLQESSPLPVLGAVPWSFDLIATRAIDMARHLN
ATIINEGDINTRRVKSVTFCARSIPHMLEHFRAGSLLVTSADRPDVLVAACLAAMNGVEI
GALLLTGGYEMDARISKLCERAFATGLPVFMVNTNTWQTSLSLQSFNLEVPVDDHERIEK
VQEYVANYINADWIDSLTATSERSRRLSPPAFRYQLTELARKAGKRIVLPEGDEPRTVKA
AAICAERGIATCVLLGNPAEINRVAASQGVELGAGIEIVDPEVVRENYVGRLVELRKNKG
MTETVAREQLEDNVVLGTLMLEQDEVDGLVSGAVHTTANTIRPPLQLIKTAPGSSLVSSV
FFMLLPEQVYVVYGDCAINPDPTAEQLAEIAIQSADSAAAFGIEPRVAMLSYSTGTSGAGS
DVEKVREATRLAQEKRPDLMIDGPLQYDAAVMADVAKSKAPNSPVAGRATVFIFPDLNTG
NTTYKAVQRSADLISIGPMLQGMRKPVNDLSRGALVDDIVYTIALTAIQSAQQQ
>LFGLNPFC_00899 Acetate kinase
MSSKLVLVLNCGSSSLKFAIIDAVNGEEYLSGLAECFHLPEARIKWKMDGNKQEAALGAG
AAHSEALNFIVNTILAQKPELSAQLTAIGHRIVHGGEKYTSSVVIDESVIQGIKDAASFA
PLHNPAHLIGIEEALKSFPQLKDKNVAVFDTAFHQTMPEESYLYALPYNLYKEHGIRRYG
AHGTSHFYVTQEAAKMLNKPVEELNIITCHLGNGGSVSAIRNGKCVDTSMGLTPLEGLVM
GTRSGDIDPAIIFHLHDTLGMSVDAINKLLTKESGLLGLTEVTSDCRYVEDNYATKEDAK
RAMDVYCHRLAKYIGAYTALMDGRLDAVVFTGGIGENAAMVRELSLGKLGVLGFEVDHER
NLAARFGKSGFINKEGTRPAVVIPTNEELVIAQDASRLTA
>LFGLNPFC_00900 hypothetical protein
MSTPDNRSVNFFSLFRRGQHYSKMWPLEKRLAPVFVENRVIKMTCYAIRFMPPIAVFTLC
WQIALGGQLGPAVATALFALSLPMQGLWWLGKRSVTPLPPAILNWFYDVRGKLQESGQVL
APVEGKPDYQALADTLKRAFKQLDKTFLDDL
>LFGLNPFC_00901 hypothetical protein
MEMTNAQRLILSNQYKMMTMLDPANAERYRRLQTIIERGYGLQMRELDREFGELKEETCR
TIIDIMEMYHALHVSWSNLQDQQSIDERRVTFLGFDAATEARYLGYVRFMVNVEGRYTHF
DAGTHGFNAQTPMWEKYQRMLNVWHACPRQYHLSANEINQIINA
>LFGLNPFC_00902 Hexitol phosphatase A
MRCKGFLFDLDGTLVDSLPAVERAWSNWARRHGLSPEEVLAFIHGKQAITSLRHFMAGKS
EADIAAEFTRLEHIEATETEGITALPGAIALLNHLNKAGIPWAIVTSGSMPVARARHKIA
GLPAPEVFVTAERVKRGKPEPDAYLLGAQLLGLAPQECVVVEDAPAGVLSGLAAGCHVIA
VNAPADTPRLNEVDLVLHSLEQITVTKQPNGDVIIQ
>LFGLNPFC_00903 hypothetical protein
MNGELIWVLSLLAVAIVLFATGRVRMDAVALFVIVAFALSGTLTVPEVFSGFSDPNVVLI
AALFIIGDGLVRTGVATVMGTWLVKVAGNSEIKMLVLLMLTVAGLGAFMSSTGVVAIFIP
VVLSVAMRMQTSPSRLMMPLSFAGLISGMMTLVATPPNLVVNSELLREGYHGFSFFSVTP
IGLVVLVLGILYMLVMRFMLKGDTQTPQREGWTRRTFRDLIREYRLTGRARRLAIRPGSP
MIGQRLDDLKLRERYGANVIGVERWRRFRRVIVNVNGVSEFRARDVLLIDMSAADVDLRQ
FCSEQLLEPMVLRGEYFSDQALDVGMAEISLIPESELIGKSVREIGFRTRYGLNVVGLKR
NGVALEGSLADEPLLLGDIILVVGNWKLIGMLAKQGRDFVALNLPEEVSEASPAHSQAPH
AIFCLVLMVALMLTDEIPNPVAAIIACLLMGKFRCIDAESAYKSIHWPSIILIVGMMPFA
VALQKTGGVALAVKGLMDIGGGYGPHMMLGCLFVLSAVIGLFISNTATAVLMAPIALAAA
KTMGVSPYPFAMVVAMAASAAFMTPVSSPVNTLVLGPGNYSFSDFVKLGVPFTIIVMAVC
VVMIPMLFPF
>LFGLNPFC_00904 5’-deoxynucleotidase YfbR
MKQSHFFAHLSRLKLINRWPLMRNVRTENVSEHSLQVAMVAHALAAIKNRKFGGNVNAER
IALLAMYHDASEVLTGDLPTPVKYFNSQIAQEYKAIEKIAQQKLVDMVPEELQDIFAPLI
DEHAYSDEEKSLVKQADALCAYLKCLEELAAGNNEFLLAKTRLEATLEARRSQEMDYFME
VFVPSFHLSLDEISQDSPL
>LFGLNPFC_00905 Glutamate-pyruvate aminotransferase AlaA

MSPIEKSSKLENVCYDIRGPVLKEAKRLEEEGNKVLKLNIGNPAPFGFDAPDEILVDVIR
NLPTAQGYCDSKGLYSARKAIMQHYQARGMRDVTVEDIYIGNGVSELIVQAMQALLNSGD
EMLVPAPDYPLWTAAVSLSSGKAVHYLCDESSDWFPDLDDIRAKITPRTRGIVIINPNNP
TGAVYSKELLMEIVEIARQHNLIIFADEIYDKILYDDAEHHSIAPLAPDLLTITFNGLSK
TYRVAGFRQGWMVLNGPKKHAKGYIEGLEMLASMRLCANVPAQHAIQTALGGYQSISEFI
TPGGRLYEQRNRAWELINDIPGVSCVKPRGALYMFPKIDAKRFNIHDDQKMVLDFLLQEK
VLLVQGTAFNWPWPDHFRIVTLPRVDDIELSLSKFARFLSGYHQL
>LFGLNPFC_00906 HTH-type transcriptional regulator HdfR
MISANRPIINLDLDLLRTFVAVADLNTFAAAAAAVCRTQSAVSQQMQRLEQLVGKELFAR
HGRNKLLTEHGIQLLGYARKILRFNDEACSSLMFSNLQGVLTIGASDESADTILPFLLNR
VSSVYPKLALDVRVKRNAYMAEMLESQEVDLMVTTHRPSTFKALNLRTSPTHWYCAAEYV
LQKGEPIPLVLLDDPSPFRDMVLATLNKADIPWRLAYVASTLPAVRAAVKAGLGVTARPV
EMMSPDLRVLSGVDGLPPLPDTEYLLCYDPSSNNELAQVIYQAMESYHNPWQYSPMSAPE
GDDSLLIERDIE
>LFGLNPFC_00907 hypothetical protein
MLLNRVRCEAMEKIRVYFDRNLCRFLPPKESIFLICDTLLSFYNKNAATNSRIFNIIVAG
DFFQVIICAYGA
>LFGLNPFC_00908 NAD(P)H-quinone oxidoreductase subunit 3
MSMSTSTEVIAHHWAFAIFLIVAIGLCCLMLVGGWFLGGRARARSKNVPFESGIDSVGSA
RLRLSAKFYLVAMFFVIFDVEALYLFAWSTSIRESGWVGFVEAAIFIFVLLAGLVYLVRI
GALDWTPARSRRERMNPETNSIANRQR
>LFGLNPFC_00909 NADH-quinone oxidoreductase subunit B
MDYTLTRIDPNGENDRYPLQKQEIVTDPLEQEVNKNVFMGKLNDMVNWGRKNSIWPYNFG
LSCCYVEMVTSFTAVHDVARFGAEVLRASPRQADLMVVAGTCFTKMAPVIQRLYDQMLEP
KWVISMGACANSGGMYDIYSVVQGVDKFIPVDVYIPGCPPRPEAYMQALMLLQESIGKER
RPLSWVVGDQGVYRANMQSERERKRGERIAVTNLRTPDEI
>LFGLNPFC_00910 NADH-quinone oxidoreductase subunit C/D
MVNNMTDLTAQEPAWQTRDHLDDPVIGELRNRFGPDAFTVQATRTGVPVVWIKREQLLEV
GDFLKKLPKPYVMLFDLHGMDERLRTHREGLPAADFSVFYHLISIDRNRDIMLKVALAEN
DLHVPTFTKLFPNANWYERETWDLFGITFDGHPNLRRIMMPQTWKGHPLRKDYPARATEF
SPFELTKAKQDLEMEALTFKPEEWGMKRGTENEDFMFLNLGPNHPSAHGAFRIVLQLDGE
EIVDCVPDIGYHHRGAEKMGERQSWHSYIPYTDRIEYLGGCVNEMPYVLAVEKLAGITVP
DRVNVIRVMLSELFRINSHLLYISTFIQDVGAMTPVFFAFTDRQKIYDLVEAITGFRMHP
AWFRIGGVAHDLPRGWDRLLREFLDWMPKRLASYEKAALQNTILKGRSQGVAAYGAKEAL
EWGTTGAGLRATGIDFDVRKARPYSGYENFDFEIPVGGGVSDCYTRVMLKVEELRQSLRI
LEQCLNNMPEGPFKADHPLTTPPPKERTLQHIETLITHFLQVSWGPVMPANESFQMVEAT
KGINSYYLTSDGSTMSYRTRIRTPSYAHLQQIPAAIRGSLVSDLIVYLGSIDFVMSDVDR
>LFGLNPFC_00911 NADH-quinone oxidoreductase subunit E
MHENQQPQTEAFELSAAEREAIEHEMHHYEDPRAASIEALKIVQKQRGWVPDGAIHAIAD
VLGIPASDVEGVATFYSQIFRQPVGRHVIRYCDSVVCHINGYQGIQAALEKKLNIKPGQT
TFDGRFTLLPTCCLGNCDKGPNMMIDEDTHAHLTPEAIPELLERYK
>LFGLNPFC_00912 NADH-quinone oxidoreductase subunit F
MKNIIRTPETHPLTWRLRDDKQPVWLDEYRSKNGYEGARKALTGLSPDEIVNQVKDSGLK
GRGGAGFSTGLKWSLMPKDESMNIRYLLCNADEMEPGTYKDRLLMEQLPHLLVEGMLISA
FALKAYRGYIFLRGEYIEAAVNLRRAIAEATEAGLLGKNIMGTGFDFELFVHTGAGRYIC
GEETALINSLEGRRANPRSKPPFPATSGAWGKPTCVNNVETLCNVPAILANGVEWYQNIS
KSKDAGTKLMGFSGRVKNPGLWELPFGTTAREILEDYAGGMRDGLKFKAWQPGGAGTDFL
TEAHLDLPMEFESIGKAGSRLGTALAMAVDHEINMVSLVRNLEEFFARESCGWCTPCRDG
LPWSVKILRALERGEGQPGDIETLEQLCRFLGPGKTFCAHAPGAVEPLQSAIKYFREEFE
AGIKQPFSNTHLINGIQPNLLKERW
>LFGLNPFC_00913 NADH-quinone oxidoreductase subunit G
MATIHVDGKEYEVNGADNLLEACLSLGLDIPYFCWHPALGSVGACRQCAVKQYQNAEDTR
GRLVMSCMTPASDGTFISIDDEEAKQFRESVVEWLMTNHPHDCPVCEEGGNCHLQDMTVM
TGHSFRRYRFTKRTHRNQDLGPFISHEMNRCIACYRCVRYYKDYADGTDLGVYGAHDNVY
FGRPEDGTLESEFSGNLVEICPTGVFTDKTHSERYNRKWDMQFAPSICQQCSIGCNISPG
ERYGELRRIENRYNGTVNHYFLCDRGRFGYGYVNLKDRPRQPVQRRGDDFITLNAEQAMQ
GAADILRQSKKVIGIGSPRASVESNFALRELVGEENFYTGIAHGEQERLQLALKVLREGG
IYTPALREIESYDAVLVLGEDVTQTGARVALAVRQAVKGKAREMAAAQKVADWQIAAILN
IGQRAKHPLFVTNVDDTRLDDIAAWTYRAPVEDQARLGFAIAHALDNSAPAVDGIEPELQ
SKIDVIVQALAGAKKPLIISGTNAGSAEVIQAAANVAKALKGRGADVGITMIARSVNSMG
LGIMGGGSLEEALTELETGRADAVVVLENDLHRHASATRVNAALAKAPLVMVVDHQRTAI
MENAHLVLSAASFAESDGTVINNEGRAQRFFQVYDPAYYDSKTVMLESWRWLHSLHSTLL
SREVDWTQLDHVIDAVVAKIPELAGIKDAAPDATFRIRGQKLAREPHRYSGRTAMRANIS
VHEPRQPQDIDTMFTFSMEGNNQPTAHRSQVPFAWAPGWNSPQAWNKFQDEVGGKLRFGD

PGVRLFETSENGLDYFTSVPARFQPQDGKWRIAPYYHLFGSDELSQRAPVFQSRMPQPYI
KLNPADAAKLGVNAGTRVSFSYDGNTVTLPVEIAEGLTAGQVGLPMGMSGIAPVLAGAHL
EDLKEAQQ
>LFGLNPFC_00914 NADH-quinone oxidoreductase subunit H
MSWISPELIEILLTVLKAVVILLVVVTCGAFMSFGERRLLGLFQNRYGPNRVGWGGSLQL
VADMIKMFFKEDWIPKFSDRVIFTLAPMIAFTSLLLAFAIVPVSPGWVVADLNIGILFFL
MMAGLAVYAVLFAGWSSNNKYSLLGAMRASAQTLSYEVFLGLSLMGVVAQAGSFNMTDIV
NSQAHVWNVIPQFFGFITFAIAGVAVCHRHPFDQPEAEQELADGYHIEYSGMKFGLFFVG
EYIGIVTISALMVTLFFGGWQGPLLPPFIWFALKTAFFMMMFILIRASLPRPRYDQVMSF
GWKICLPLTLINLLVTAAVILWQAQ
>LFGLNPFC_00915 NADH-quinone oxidoreductase subunit I
MTLKELLVGFGTQVRSIWMIGLHAFAKRETRMYPEEPVYLPPRYRGRIVLTRDPDGEERC
VACNLCAVACPVGCISLQKAETKDGRWYPEFFRINFSRCIFCGLCEEACPTTAIQLTPDF
EMGEYKRQDLVYEKEDLLISGPGKYPEYNFYRMAGMAIDGKDKGEAENEAKPIDVKSLLP
>LFGLNPFC_00916 NADH-quinone oxidoreductase subunit J
MEFAFYICGLIAILATLRVITHTNPVHALLYLIISLLAISGVFFSLGAYFAGALEIIVYA
GAIMVLFVFVVMMLNLGGSEIEQERQWLKPQVWIGPAILSAIMLVVIVYAILGVNDQGID
GTPISAKAVGITLFGPYVLAVELASMLLLAGLVVAFHVGREERAGEVLSNRKDDSAKRKT
EEHA
>LFGLNPFC_00917 NADH-quinone oxidoreductase subunit K
MIPLQHGLILAAILFVLGLTGLVIRRNLLFMLIGLEIMINASALAFVVAGSYWGQTDGQV
MYILAISLAAAEASIGLALLLQLHRRRQNLNIDSVSEMRG
>LFGLNPFC_00918 NADH-quinone oxidoreductase subunit L
MNMLALTIIILPLIGFVLLAFSRGRWSENVSAIVGVGSVGLAALVTAFIGVDFFANGEQAY
SQPLWTWMSVGDFNIGFNLVLDGLSLTMLSVVTGVGFLIHMYASWYMRGEEGYSRFFAYT
NLFIASMVVLVLADNLLLMYLGWEGVGLCSYLLIGFYYTDPKNGAAAMKAFVVTRVGDVF
LAFALFILYNELGTLNFREMVELAPAHFADGNNMLMWATLMLLGGAVGKSAQLPLQTWLA
DAMAGPTPVSALIHAATMVTAGVYLIARTHGLFLMTPEVLHLVGIVGAVTLLLAGFAALV
QTDIKRVLAYSTMSQIGYMFLALGVQAWDAAIFHLMTHAFFKALLFLASGSVILACHHEQ
NIFKMGGLRKSIPLVYLCFLVGGAALSALPLVTAGFFSKDEILAGAMANGHINLMVAGLV
GAFMTSLYTFRMIFIVFHGKEQIHAHAVKGVTHSLPLIVLLILSTFVGALIVPPLQGVLP
QTTELAHGSMLTLEITSGVVAVVGILLAAWLWLGKRTLVTSIANSAPGRLLGTWWYNAWG
FDWLYDKVFVKPFLGIAWLLKRDPLNSMMNIPAVLSRFAGKGLLLSENGYLRWYVASMSI
GAVVVLALLMVLR
>LFGLNPFC_00919 NADH-quinone oxidoreductase subunit M
MLLPWLILIPFIGGFLCWQTERFGVKVPRWIALVTMGLTLALSLQLWLQGGYSLTQSAGI
PQWQSEFDMPWIPRFGISIHLAIDGLSLLMVVLTGLLGVLAVLCSWKEIEKYQGFFHLNL
MWILGGVIGVFLAIDMFLFFFFWEMMLVPMYFLIALWGHKASDGKTRITAATKFFIYTQA
SGLVMLIAILALVFVHYNATGVWTFNYEELLNTPMSNGVEYLLMLGFFIAFAVKMPVVPL
HGWLPDAHSQAPTAGSVDLAGILLKTAAYGLLRFSLPLFPNASAEFAPIAMWLGVIGIFY
GAWMAFAQTDIKRLIAYTSVSHMGFVLIAIYTGSQLAYQGAVIQMIAHGLSAAGLFILCG
QLYERIHTRDMRMMGGLWSKMKWLPALSLFFAVATLGMPGTGNFVGEFMILFGSFQVVPV
ITVISTFGLVFASVYSLAMLHRAYFGKAKSQIASQELPGMSLRELFMILLLVVLLVLLGF
YPQPILDTSHSAIGNIQQWFVNSVTTTRP
>LFGLNPFC_00920 NADH-quinone oxidoreductase subunit N
MTITPQNLIALLPLLIVGLTVVVVMLSIAWRRNHFLNATLSVIGLNAALVSLWFVGQAGA
MDVTPLMRVDGFAMLYTGLVLLASLATCTFAYPWLEGYNDNKDEFYLLVLIAALGGILLA
NANHLASLFLGIELISLPLFGLVGYAFRQKRSLEASIKYTILSAAASSFLLFGMALVYAQ
SGDLSFVALGKNLGDGMLNEPLLLAGFGLMIVGLGFKLSLVPFHLWTPDVYQGAPAPVST
FLATASKIAIFGVVMRLFLYAPVGDSEAIRVVLAIIAFASIIFGNLMALSQTNIKRLLGY
SSISHLGYLLVALIALQTGEMSMEAVGVYLVGYLFSSLGAFGVVSLMSSPYRGPDADSLF
SYRGLFWHRPILAAVMTVMMLSLAGIPMTLGFIGKFYVLAVGVQAHLWWLVGAVVVGSAI
GLYYYLRVAVSLYLHAPEQPGRDAPSNWQYSAGGIVVLISALLVLVLGVWPQPLISIVRL
AMPLM
>LFGLNPFC_00921 Protein YfbM
MGMIGYFAEIDSEKINQLLESTKKPLMDNIHDTLSGLRRLDIDKRWDFLHFGLTGTSAFD
PAKNDPLSRAVLGEHSLEDGIDGFLGLTWNQELAATIDRLESLDRSELRKQFSIKRLNEM
EIYPGVTFSEELEGQLFASIMLDMEKLISAYRRMLRQGNHALTVIVG
>LFGLNPFC_00922 hypothetical protein
MKKINFAFIILFLFSLPLIIFYQPWVNALPPTPRHASPEQLEKTVRYLTQTVHPRSADNI
DNLNRSAEYIKEVFISNGARVTAQDVPITGGPYKNIVANYGPADGPLIIIGAHYDSVSSY
ENDQLTYTPGADDNASGVAGLLELARLLQQQTPGIGVQLVAYASEEPPFFRSDEMGSAVH
AASLERPVKLMIALEMIGYYDSTPGSQDYPYPAMSWLYPDRGDFIAVVGRMQDINAVRQV
KAALLSSRDLSVYSMNAPGFIPGIDFSDHLNYWQHDIPAVMITDTAFYRNKQYHLPGDIA

DRLNYQKMAQVVDGVTTLLYNSK
>LFGLNPFC_00923 hypothetical protein
MRNKNIIMLLISSLILSGCGPEPEDKKSQQQQSTTPTDQQVLAAQQAATKVAEQSAAKAA
ADAKALAQQEVQQYSDKQALLGRLQAAPKYQHAVREKAASQIANPGTARYQQFDDNPVKQ
VAQNPLATFSLDVDTGSYANVRRFLNQGLLPPPDAVRVEEIVNYFPSDWDIKDKQSIPAS
KPIPFAMRYELAPAPWNEQRTLLKVDILAKDRKSEELPASNLVFLIDTSGSMISDERLPL
IQSSLKLLVKELREQDNIAIVTYAGDSRIALPSISGSHKAEINAAIDSLDAEGSTNGGAG
LEMAYQQAAKGFIKGGINRILLATDGDFNVGIDDPKSIESMVKKQRESGVTLSTLGVGDS
NYNEAMMVRIADVGNGNYSYIDTLSEAQKVLNSEMRQTLITVAKDVKAQIEFNPAWVTEY
RQIGYEKRQLRAEDFNNDNVDAGDIGAGKHITFLFELTLKGQKASIDKLRYAPDNKSAKS
DKTKELAWLKIRWKSPQGKESQLVEFPLAFAIKAPSEDMRFRAAVAAYGQKLRGSEYLNN
TSWQQIKQWAQKAKGEDPQGYRAEFIRLIGLAKDLDNSQN
>LFGLNPFC_00924 Ribonuclease BN
MELIFLGTSAGVPTRTRNVTAILLNLQHPTQSGLWLFDCGEGTQHQLLHTAFNPGKLDKI
FISHLHGDHLFGLPGLLCSRSMSGIIQPLTIYGPHGIREFVETALRISGSWTDYPLEIVE
IGAGEIFDDGLRKVTAYPMEHPLECYGYRIEEHDKPGALNAQALKAAGVPPGPLFQELKA
GKTIMLDDGRQINGADYLAVPVPGKALAIFGDTGPCDAALELAKGVDVMVHEATLDMAME
AKANSRGHSSTRQAAALAREAGVGKLIITHVSSRYDDKGCQHLLRECRSIFPATELANDF
AVFSI
>LFGLNPFC_00925 Protein ElaA
MIDWQDLHHSDLSVSQLYALLQLRCAVFVVEQNCPYQDIDGDDLEGENRHILGWHNGTLV
AYARILKSDDELQPVVIGRVIVSEALRGEKIGQQLMSKALESCTRRWPEKPIYLGAQAHL
QNFYGQFGFIPVTDIYEEDGIPHIGMAREVIQA
>LFGLNPFC_00926 Protein ElaB
MSNQFGDTRIDDDLTLLSETLEEVLRSSGDPADQKYVELKARAEKALDDVKKRVSQASDS
YYYRAKQAVYRADDYVHEKPWQGIGVGAAVGLVLGLLLARR
>LFGLNPFC_00927 Isochorismate synthase MenF
MQSLTTALENLLRHLSQEIPATPGIRVIDIPFPLKDAFDALSWLASQQVYPQFYWQQRNG
DEEAAVLGAITRFTSLDQAQRFLRQHPEHADLRIWGLNAFDPSQGNLLLPRLEWRRCGGK
ATLRLTLFSESSLQHDAIKAKEFIATLVSIKPLPGLHLTTTREQHWPDKTGWTQLIELAT
KTIAEGELDKVVLARATDLHFASPVNAAAMMAASRRLNLNCYHFYMAFDGENAFLGSSPE
RLWRRRDKALRTEALAGTVANHPDDKQAQQLGEWLMADDKNQRENMLVVEDICQRLQADT
QTLDVLPPQVLRLRKVQHLRRCIWTSLNKADDVICLHQLQPTAAVAGLPRDLARQFIARH
EPFTREWYAGSAGYLSLQQSEFCVSLRSAKISGNIVRLYAGAGIVRGSDPEQEWQEIDNK
AAGLRTLLQME
>LFGLNPFC_00928 2-succinyl-5-enolpyruvyl-6-hydroxy-3-cyclohexene-1-carboxylate synthase
MSVSAFNRRWAAVILEALTRHGVRHICIAPGSRSTPLTLAAAENSAFIHHTHFDERGLGH
LALGLAKVSKQPVAVIVTSGTAVANLYPALIEAGLTGEKLILLTADRPPELIDCGANQAI
RQPGMFASHPTHSISLPRPTQDIPARWLVSTIDHALGTLHAGGVHINCPFAEPLYGEMDD
TGISWQQRLGDWWQDDKPWLREAPRRESEKQRDWFFWRQKRGVVVAGRMSAEEGKKVALW
AQTLGWPLIGDVLSQTGGQPLPCADLWLGNAKATSELQQAQIVVQLGSSLTGKRLLQWQAS
CEPEEYWIVDDIEGRLDPAHHRGRRLIANIADWLELHPAEKRQPWCVEIPRLAEQAMQAV
IARRDAFGEAQLAHRISDYLPEQGQLFVGNSLVVRLIDALSQLPAGYPVYSNRGASGIDG
LLSTAAGVQRASGKPTLAIVGDLSALYDLNALALLRQVSAPLVLIVVNNNGGQIFSLLPT
PKSERERFYLMPQNVHFEHAAAMFELKYHRPQNWQELETTLVDAWRTPTTTVIEMVVNDT
DGAQTLQQLLAQVSHL
>LFGLNPFC_00929 2-succinyl-6-hydroxy-2,4-cyclohexadiene-1-carboxylate synthase
MILHAQAKHGKPGLPWLVFLHGFSGDCHEWQEVGEAFADYSRLYVDLPGHGGSATISVDG
FDDVTGLLCKTLVSYNILNFWLVGYSLGGRVAMMAACQEPAGLCGVVVEGGHPGLQNAEQ
RAERQRSDRQWAQRFRTEPLTAVFADWYQQPVFASLNDDQRRELVALRSNNNGATLAAML
EATSLAVQPDLRANLSARTFAFYYLCGERDSKFRALAAELAADCHVIPRAGHNAHRENPA
GVIASLAQILRF
>LFGLNPFC_00930 1,4-dihydroxy-2-naphthoyl-CoA synthase
MIYPDEAMLYAPVEWHDCSEGFEDIRYEKSTDGIAKITINRPQVRNAFRPLTVKEMIQAL
ADARYDDNIGVIILTGAGDKAFCSGGDQKVRGDYGGYKDDSGVHHLNVLDFQRQIRTCPK
PVVAMVAGYSIGGGHVLHMMCDLTIAADNAIFGQTGPKVGSFDGGWGASYMARIVGQKKA
REIWFLCRQYDAKQALDMGLVNTVVPLADLEKETVRWCREMLQNSPMALRCLKAALNADC
DGQAGLQELAGNATMLFYMTEEGQEGRNAFNQKRQPDFSKFKRNP
>LFGLNPFC_00931 o-succinylbenzoate synthase
MRSAQVYRWQIPMDAGVVLRDRRLKTRDGLYVCLREGEREGWGEISPLPGFSQETWEDAQ
SVLLAWVNNWLAGDCEIPQMPSVAFGVSCALAELAETLPQAANYRAAPLCNGDPDDLILK
LADMPGEKVAKVKVGLYEAVRDGMVVNLLLEAIPDLHLRLDANRAWTPLKGQQFAKYVNP
DYRHRIAFLEEPCKTRDDSRAFARETGIAIAWDESLREPDFAFVAEEGVRAVVIKPTLTG
SLDKVREQVQAAHALGLTAVISSSIESSLGLTQLARIAAWLTPDTIPGLDTLDLMQAQQV

RRWPGSPLPLVDVDALERLL
>LFGLNPFC_00932 2-succinylbenzoate--CoA ligase
MIFSDWPWRHWRQVRGEAIALRLNDEQLNWRELCARVDELASSFAVQGVVEGSGVMLRAW
NTPQTLLAWLALLQCGARVLPVNPQLPQPLLEELLPNLTLQFALVPEGENTFPALASLHI
QLVEGAHAAAWQPTRLCSMTLTSGSTGLPKAAVHTYQAHLASAEGVLSLIPFGDHDDWLL
SLPLFHVSGQGIMWRWLYAGARMTVRDKQPLEQMLAGCTHASLVPTQLWRLLVNRSSVSL
KAVLLGGAAIPVELTEQAREQGIRCFCGYGLTEFASTVCAKEADGLADVGSPLPGREVKI
VNDEVWLRAASMAEGYWRNGQRVPLVNDEGWYATRDRGEMHNGKLTIVGRLDNLFFSGGE
GIQPEEVERVIAAHPAVLQVFIVPVADKEFGHRPVAVVEYDQQSVDLDEWVKDKLARFQQ
PVRWLTLPPELKNGGIKISRQALKEWVKRQQ
>LFGLNPFC_00933 Signal transduction protein PmrD
MEWLVKKSCCNKQDNRHVIMLCDAGGAIKMIAEVKSDFAVKVGDLLSPLQNALYCINREK
LHTVKVLSASCYSPDEWERQCKAAGKTQ
>LFGLNPFC_00934 putative 4-amino-4-deoxy-L-arabinose-phosphoundecaprenol flippase subunit ArnF
MGLMWGLFSVIIASAAQLSMGFAASHLPPMTHLWDFIAALLAFGLDARILLLGLLGYLLS
VFCWYKTLHKLALSKAYALLSMSYVLVWIASMVLPGWEGTFSLKALLGVACIMSGLMLIF
LPTTKQRY
>LFGLNPFC_00935 putative 4-amino-4-deoxy-L-arabinose-phosphoundecaprenol flippase subunit ArnE
MIWLTLVFASLLSVAGQLCQKQATCFAAVNKRRKHIVLWLGLALACLGLAMVLWLLVLQN
VPVGIAYPMLSLNFVWVTLAAVKLWHEPVSLRHWCGVAFIIGGIVILGSTV
>LFGLNPFC_00936 Undecaprenyl phosphate-alpha-4-amino-4-deoxy-L-arabinose arabinosyl transferase
MKSVRYLIGLFAFIACYYLLPISTRLLWQPDETRYAEISREMLASGDWIVPHLLGLRYFE
KPIAGYWINSIGQWLFGANNFGVRAGVIFATLLTAALVTWFTLRLWRNKRLALLATVIYL
SLFIVYAIGTYAVLDPFIAFWLVAGMCSFWLAMQAQTWKGKSAGFLLLGITCGMGVMTKG
FLALAVPVLSVLPWVATQKRWKDLFIYGWLAVISCVLTVLPWGLAIAQREPDFWHYFFWV
EHIQRFALDDAQHRAPFWYYLPVIIAGSLPWLGLLPGALYAGWKNRKHSATVYLLSWTIM
PLLFFSVAKGKLPTYILSCFAPLAMLMAHYALLAAKNNPLALRINGWINIAFGVTGIIAT
FVVSPWGPMNTPVWQTFESYKVFCAWSIFSLWAFFGWYTLTNVEKTWPFAALCPLGLALL
VGFSIPDRVMEGKHPQFFVEMTQESLQPSRYILTDSVGVAAGLAWSLQRDDIIMYRQTGE
LKYGLNYPDAKGRFVSGDEFANWLNQHRQEGIITLVLSVDRDEDINSLAIPPADVIDRQE
RLVLIQYRPK
>LFGLNPFC_00937 putative 4-deoxy-4-formamido-L-arabinose-phosphoundecaprenol deformylase ArnD
MTKVGLRIDVDTFRGTREGVPRLLEILSKHNIQASIFFSVGPDNMGRHLWRLVKPQFLWK
MLRSNAASLYGWDILLAGTAWPGKEIGHANADIIREAAKHHEVGLHAWDHHAWQAHSGNW
DRQTMIDDIARGLRTLEEIIGQPVTCSAAAGWRADQQVIEAKEAFHLRYNSDCRGAMPFR
PLLESGTPGTAQIPVTLPTWDEVIGRDVKAEDFNGWLLNRIQRDKGTPVYTIHAEVEGCA
YQHNFVDLLKRAAQEGVTFCPLSELLSGTLPLGQVVRGNIAGREGWLGCQQIAGSH
>LFGLNPFC_00938 Bifunctional polymyxin resistance protein ArnA
MKTVVFAYHDMGCLGIEALLAAGYEISAIFTHTDNPGEKAFYGSVARLAAERGIPVYAPD
NVNHPLWVERIAQLSPEVIFSFYYRHLICDEILQLAPRGAFNLHGSLLPKYRGRAPLNWV
LVNGETETGVTLHRMVKRADAGAIVAQLRVAIAPDDIAITLHHKLCHAARQLLEQTLPAI
KHGNILEIAQRENEATCFGRRTPDDSFLEWHKSASVLHNMVRAVADPWPGAFSYVGNQKF
TVWSSRVHPHASKAQPGSVISVAPLLIACGDGALEIVTGQAGDGITMQGSQLAQTLGLVQ
GSRLNSQPACAARRRTRVLILGVNGFIGNHLTERLLREDHYEVYGLDIGSDAISRFLNHP
HFHFVEGDISIHSEWIEYHVKKCDVVLPLVAIATPIEYTRNPLRVFELDFEENLRIIRYC
VKYRKRIIFPSTSEVYGMCSDKYFDEDHSNLIVGPVNKPRWIYSVSKQLLDRVIWAYGEK
EGLQFTLFRPFNWMGPRLDNLNAARIGSSRAITQLILNLVEGSPIKLIDGGKQKRCFTDI
RDGIEALYRIIENAGNRCDGEIINIGNPENEASIEELGEMLLASFEKHPLRHYFPPFAGF
RVVESSSYYGKGYQDVEHRKPSIRNARRCLNWEPKIDMQETIDETLDFFLRTVDLTDKPS
>LFGLNPFC_00939 Undecaprenyl-phosphate 4-deoxy-4-formamido-L-arabinose transferase
MFEIHPVKKVSVVIPVYNEQESLPELIRRTTAACESLGKEYEILLIDDGSSDNSAHMLVE
ASQAEGSHIVSILLNRNYGQHSAIMAGFSHVTGDLIITLDADLQNPPEEIPRLVAKADEG
YDVVGTVRQNRQDSWFRKTASKMINRLIQRTTGKAMGDYGCMLRAYRRHIVDAMLHCHER
STFIPILANIFARRAIEIPVHHAEREFGESKYSFMRLINLMYDLVTCLTTTPLRMLSLLG
SIIAIGGFSIAVLLVILRLTFGPQWAAEGVFMLFAVLFTFIGAQFIGMGLLGEYIGRIYT
DVRARPRYFVQQVIRPSSKENE
>LFGLNPFC_00940 UDP-4-amino-4-deoxy-L-arabinose--oxoglutarate aminotransferase
MTDGKKMSGFLPFSRPAMGVEELAAVKEVLESGWITTGPKNQALEQAFCQLTGNQHAIAV
SSATAGMHITLMALEIGKGDEVITPSLTWVSTLNMISLLGATPVMVDVDRDTLMVTPEAI
EAAITPRTKAIIPVHYAGAPADIDAIRAIGERYGIAVIEDAAHAVGTYYKGRHIGAKGTA

IFSFHAIKNITCAEGGLIVTDNENLARQLRMLKFHGLGVDAYDRHTWGRAPQAEVLTPGY
KYNLTDINAAIALTQLVKLEHLNTRRREIAQQYQQALAALPFQPLSLPAWPHVHAWHLFI
IRVDEQRCGISRDALMEALKERGIGTGLHFRAAHTQKYYRERFPTLSLPNTEWNSERICS
LPLFPDMTTADADRVITALQQLAGQ
>LFGLNPFC_00941 Lipopolysaccharide core heptose(II)-phosphate phosphatase
MLAFCRSSLKSKKYFIILLALAAIAGLGTHAAWSSNGLPRIDNKTLARLAQQHPVVVLFR
HAERCDRSTNQCLSDKTGITVKGTQDARELGNAFSADIPDFDLYSSNTVRTIQSATWFSA
GKKLTVDKRFLQCGNEIYSAIKDLQRKAPDKNIVIFTHNHCLTYIAKDKRDATFKPDYLD
GLVMHVEKGKVYLDGEFVNH
>LFGLNPFC_00942 Nucleoside triphosphatase NudI
MRQRTIVCPLIQNDGAYLLCKMADDRGVFPGQWALSGGGVEPGERIEEALRREIREELGE
QLLLTEITPWTFSDDIRTKTYADGRKEEIYMIYLIFDCVSANREVKINEEFQDYAWVKPE
DLVHYDLNVATRKTLRLKGLL
>LFGLNPFC_00943 hypothetical protein
MKKIALAGLAGMLLVSASVNAMSISGQAGKEYTNIGVGFGTESTGLALSGNWTHNDDDGD
AAGVGLGLNLPLGPLMATVGGKGVYTNPNDGDEGYAAAVGGGLQWQIGDSFRLFGEYYYS
PDSLSSGIKSYEEANAGARYTIMRPVSIEAGYRYLNLAGKNGNRDNAIADGPYVGVNASF
>LFGLNPFC_00944 Nicotinamide-nucleotide amidohydrolase PncC
MLKVEMLSTGDEVLHGQIVDTNAAWLADFFFHQGLPLSRRNTVGDNLDDLVTILRERSQH
ADVLIVNGGLGPTSDDLSALAAATAKGEGLVLHEAWLKEMERYFHERGRVMAPSNRKQAE
LPASAEFINNPVGTACGFAIQLNRCLMFFTPGVPSEFKVMVEHEILPRLRERFSLPQPPV
CLRLTTFGRSESDLAQSLDTLQLPPGVTMGYRSSMPIIELKLTGPASEEQAMEKLWLDVK
RVAGQSVIFEGTEGLPAQISRELQSRQFSLTLSEQFTGGLLALQLSRADAPLLACEVVPS
QEETLAQTAHWITERRANHFAGLALAVSGLEDEHLNFALATPDGTFALRVHFSATRYSLA
IRQEVCAMMALNMLRRWLNGQDIASEHGWIEVVESMTLSV
>LFGLNPFC_00945 putative HTH-type transcriptional regulator RhmR
MLESSKVPALTRAIDILNLIARIGPCSAATIIDTLGIPKSTAYLLLNELRRQRFLSLDHQ
ENFCLWTRLVELSGHALSKMDLRELARPRLTQLMDTTGLLCHLGIIDNGSAYYILKVESS
ATISVRSHEGKSLSLYRSGIGKCLLAWQPAAVQKSIIEELVWEQMTPTTITHPQRLYEEL
ARIRRQGWSYDNGEDYADVRCVAAPVFNANNELTAAISVVGTRLQINEEYRDYLAGKAIA
CARDISRLLGWKSPFDLQAS
>LFGLNPFC_00946 L-rhamnonate dehydratase
MTLPKIKQVRAWFTGGAIAEKGAGGGDYHDQGANHWIDDHIATPMSKYRDYEQSRQSFGI
NVLGTLVVEVEAENGQTGFAVSTAGEMGCFIVEKHLNRFIEGKCVSDIKLIHDQMLNATL
YYSGSGGLVMNTISCVDLALWDLFGKVVGLPVYKLLGGAVRDEIQFYATGARPDLAKEMG
FIGGKMPTHWGPHDGDAGIRKDAAMVADMREKCGEDFWLMLDCWMSQDVNYATKLAHACA
PYNLKWIEECLPPQQYEGYRELKHNAPAGMMVTSGEHHGTLQSFRTLSETGIDIMQPDVG
WCGGLTTLVEIAAIAKSRGQLVVPHGSSVYSHHAVITFTNTPFSEFLMTSPDCSTMRPQF
DPILLNEPVPVNGRIHKSVLDKPGFGVELNRDCNLKRPYSH
>LFGLNPFC_00947 Inner membrane transport protein RhmT
MSTALLDAVVKKNRARLIPFMLALYVLAFLDRSNIGFAKQTYQIDTGLSNEAYALGAGIF
FVVYAFLGVPANLLMRKLGARTWIGTTTLLWGFLSAAMAWADTEAKFLIVRTLLGAAEAG
FFPGMIYLTSQWFPQRNRASIMGLFYMGAPLALTLGSPLSGALLEMHGFMGHPGWFWMFV
IEGLLAVGAGVFTFFWLDDTPEQARFLSKQEKTLLINQLASEEQQKVTSRLSDALRNGRV
WQLAIIYLTIQVAVYGLIFFLPTQVAALLGTKVGFTASVVTAIPWVAALFGTWLIPRYSD
KTGERRNVAALTLLAAGIGIGLSGLLSPVLAIVALCVAAIGFIAVQPVFWTMPTQLLSGT
ALAAGIGFINLFGAVGGFIAPILRVKAETLFSSDAAGLLTLAAVAVIGSLIIFTLRVNRT
VAQTDVAHH
>LFGLNPFC_00948 2-keto-3-deoxy-L-rhamnonate aldolase
MNALLTNPFKERLRKGEVQIGLWLSSTTAYMAEIAATSGYDWLLIDGEHAPNTIQDLYHQ
LQAVAPYASHPVIRPVEGSKPLIKQVLDIGAQTLLIPMVDTADQARQVVSATRYPPYGER
GVGASVARAARWGRIENYMAQVNDSLCLLVQVESKTALDNLDEILDVEGIDGVFIGPADL
SASLGYPDNAGHPEVQRIIETSIRRIRAAGKAAGFLAVAPDMAQQCLAWGANFVAVGVDT
MLYSDALDQRLAMFKSGKNGPRVKGSY
>LFGLNPFC_00949 hypothetical protein
MTIAERLRQEGHQIGWQEGKLEGMHEQAIKIALRMLEQGIDRDQVLAATQLSEADLAANN
H
>LFGLNPFC_00950 ISNCY family transposase ISRor2
MTESTTSSPHDAVFKTFMFTPETARDFLEIHLPEPLRKLCNLQTLRLEPTSFIEKSLRAY
YSDVLWSVETSDGDGYIYCVIEHQSSAEKNMAFRLMRYATAAMQRHLDKGYDRVPLVVPL
LFYHGETSPYPYSLNWLDEFDDPQLARQLYTEAFPLVDITIVPDDEIMQHRRIALLELIQ
KHIRDRDLIGMVDRITTLLVRGFTNDSQLQTLFNYLLQCGDTSRFTRFIQEIAERSPLQK
ERLMTIAERLRQEGHQIGWQEGKIEGWQEGKLEGLQKGKVEGMHEQAIKIALRMLEQGFE
REIVLATTQLSDADIPNCY

>LFGLNPFC_00951 Anaerobic glycerol-3-phosphate dehydrogenase subunit C
MNDTSFENCIKCTVCTTACPVSRVNPGYPGPKQAGPDGERLRLKDGALYDEALKYCINCK
RCEVACPSDVKIGDIIQRARAKYDTTRPSLRNFVLSHTDLMGSVSTPFAPIVNTATSLKP
VRQLLDAALKIDHRRTLPKYSFGTFRRWYRSVAAQQAQYKDQVAFFHGCFVNYNHPQLGK
DLIKVLNAMGTGVQLLSKEKCCGVPLIANGFTDKARKQAITNVESIREAVGVKGIPVIAT
SSTCTFALRDEYPEVLNVDNKGLRDHIELATRWLWRKLDEGKTLPLKPLPLKVVYHTPCH
MEKMGWTLYTLELLRKIPGLELTVLDSQCCGIAGTYGFKKENYPTSQAIGAPLFRQIEES
GADLVVTDCETCKWQIEMSTSLRCEHPITLLAQALA
>LFGLNPFC_00952 Anaerobic glycerol-3-phosphate dehydrogenase subunit B
MRFDTVIMGGGLAGLLCGLQLQKHGLRCAIVTRGQSALHFSSGSLDLLSHLPDGQPVTDI
HSGLESLRQQAPAHPYTLLGPQRVLDLACQAQALIAESGAQLQGSVELAHQRITPLGTLR
STWLSSPEVPVWPLPAKKICVVGISGLMDFQAHLAAASLRELDLAVETAEIELPELDVLR
NNATEFRAVNIARFLDNEENWPLIIDALIPVANTCEMILMPACFGLADDKLWRWLNEKLP
CSLMLLPTLPPSVLGIRLQNQLQRQFVRQGGVWMPGDEVKKVTCKNGVVNEIWTRNHADI
PLRPRFAVLASGSFFSGGLVAERDGIREPILGLDVLQTATRGEWYKGDFFAPQPWQQFGV
TTDEALRPSQAGQTIENLFAIGSVLGGFDPIAQGCGGGVCAVSALHAAQQIAQRAGGQQ
>LFGLNPFC_00953 Anaerobic glycerol-3-phosphate dehydrogenase subunit A
MKTRDSQSSDVIIIGGGATGAGIARDCALRGLRVILVERHDIATGATGRNHGLLHSGARY
AVTDAESARECISENQILKRIARHCVEPTNGLFITLPEDDLSFQATFIRACEEAGINAEA
IEPQQARIIEPAVNPALIGAVKVPDGTVDPFRLTAANMLDAKEHGAVILTAHEVTGLIRE
GATVCGVRVRNHLTGEIQALHAPVVVNAAGIWGQHIAEYADLRIRMFPAKGSLLIMDHRI
NQHVINRCRKPSDADILVPGDTISLIGTTSLRIDYNEIDDNRVTAEEVDILLREGEKLAP
VMAKTRILRAYSGVRPLVASDDDPSGRNVSRGIVLLDHAERDGLDGFITITGGKLMTYRL
MAEWATDAVCRKLGNTRPCTTADLALPGSQEPAEVTLRNVISLPAPLRGSAVYRHGDRTP
AWLSEGRLHRSLVCECEAVTAGEVQYAVENLNVNSLLDLRRRTRVGMGTCQGELCACRAA
GLLQRFNVTTSAQSIEQLSTFLNERWKGVQPIAWGDALRESEFTRWVYQGLCGLEKEQKD
AL
>LFGLNPFC_00954 Glycerol-3-phosphate transporter
MLSIFKPAPHKARLPTAEIDPTYRRLRWQIFLGIFFGYAAYYLVRKNFALAMPYLVEQGF
SRGDLGFALSGISIAYGFSKFIMGSVSDRSNPRVFLPAGLILAAAVMLFMGFVPWATSSI
AVMFVLLFLCGWFQGMGWPPCGRTMVHWWSQKERGGIVSVWNCAHNVGGGIPPLLFLLGM
AWFNDWHAALYMPAFCAILVALFAFAMMRDTPQSCGLPPIEEYKNDYPDDYNEKAEQELT
AKQIFMQYVLPNKLLWYIAIANVFVYLLRYGILDWSPTYLKEVKHFALDKSSWAYFLYEY
AGIPGTLLCGWMSDKVFRGNRGATGVFFMTLVTIATIVYWMNPAGNPTVDMICMIVIGFL
IYGPVMLIGLHALELAPKKAAGTAAGFTGLFGYLGGSVAASAIVGYTVDFFGWDGGFMVM
IGGSILAVILLIVVMIGEKRRHEQLLQKRNGG
>LFGLNPFC_00955 Glycerophosphodiester phosphodiesterase, periplasmic
MKLTLKNLSMAIMMSTIVMGSSAMAADSNEKIVIAHRGASGYLPEHTLPAKAIAYAQGAD
YLEQDLVMTKDDHLVVLHDHYLDRVTDVADRFPDRARKDGRYYAIDFTLDEIKSLKFTEG
FDIENGKKVQTYPGRFPMGKSDFRVHTFEEEIEFVQGLNHSTGKNIGIYPEIKAPWFHHQ
EGKDIAAKTLEVLKKYGYTGKDDKVYLQCFDADELKRIKNELEPKMGMDLNLVQLIAYTD
WNETQQKQPDGSWVNYNYDWMFKPGAMKQVAEYADGIGPDYHMLIEETSQPGNIKLTGMV
QDAQQNKLVVHPYTVRSDKLPEYTTDVNQLYDALYNKAGVNGLFTDFPDKAVKFLNKE
>LFGLNPFC_00956 Protein InaA
MAVSAKHDEFNHWWATEGDWVEEPNYRRNGMSGVQCVERNGKKLYVKRMTHHLFHSVRYP
FGRPTIVREVAVIKELERAGVIVPKIVFGEAVKVEGEWRALLVTEDMAGFISIADWYARH
AVSPYSDEVRQAMLKAVALAFKKMHSVNRQHGCCYVRHIYVKTEGKAEAGFLDLEKSRRR
LRRDKAINHDFRQLEKYLEPIPKADWEQVKAYYYAM
>LFGLNPFC_00957 Carnitine monooxygenase reductase subunit
MARVTLRITGTQLLCQDEHPSLLAALESHNVAVEYQCREGYCGSCRTRLVAGQVDWIAEP
LAFIQPGEILPCCCRAKGDIEIEM
>LFGLNPFC_00958 Ribonucleoside-diphosphate reductase 1 subunit beta
MAYTTFSQTKNDQLKEPMFFGQPVNVARYDQQKYDIFEKLIEKQLSFFWRPEEVDVSRDR
IDYQALPEHEKHIFISNLKYQTLLDSIQGRSPNVALLPLISIPELETWVETWAFSETIHS
RSYTHIIRNIVNDPSVVFDDIVTNEQIQKRAEGISSYYDELIEMTSYWHLLGEGTHTVNG
KTVTVSLRELKKKLYLCLMSVNALEAIRFYVSFACSFAFAERELMEGNAKIIRLIARDEA
LHLTGTQHMLNLLRSGADDPEMAEIAEECKQECYDLFVQAAQQEKDWADYLFRDGSMIGL
NKDILCQYVEYITNIRMQAVGLDLPFQTRSNPIPWINTWLVSDNVQVAPQEVEVSSYLVG
QIDAEVDTDDLSNFQL
>LFGLNPFC_00959 Ribonucleoside-diphosphate reductase 1 subunit alpha
MNQNLLVTKRDGSTERINLDKIHRVLDWAAEGLHNVSISQVELRSHIQFYDGIKTSDIHE
TIIKAAADLISRDAPDYQYLAARLAIFHLRKKAYGQFEPPALYDHVVKMVEMGKYDNHLL
EDYTEEEFKQMDTFIDHDRDMTFSYAAVKQLEGKYLVQNRVTGEIYESAQFLYILVAACL
FSNYPRETRLQYVKRFYDAVSTFKISLPTPIMSGVRTPTRQFSSCVLIECGDSLDSINAT

SSAIVKYVSQRAGIGINAGRIRALGSPIRGGEAFHTGCIPFYKHFQTAVKSCSQGGVRGG
AATLFYPMWHLEVESLLVLKNNRGVEGNRVRHMDYGVQINKLMYTRLLKGEDITLFSPSD
VPGLYDAFFADQEEFERLYTKYEKDDSIRKQRVKAVELFSLMMQERASTGRIYIQNVDHC
NTHSPFDPAIAPVRQSNLCLEIALPTKPLNDVNDENGEIALCTLSAFNLGAINSLDELEE
LAILAVRALDALLDYQDYPIPAAKRGAMGRRTLGIGVINFAYYLAKHGKRYSDGSANNLT
HKTFEAIQYYLLKASNELAKEQGACPWFNETTYAKGILPIDTYKKDLDAIANEPLHYDWE
ALRESIKTHGLRNSTLSALMPSETSSQISNATNGIEPPRGYVSIKASKDGILRQVVPDYE
HLHDAYELLWEMPGNDGYLQLVGIMQKFIDQSISANTNYDPSRFPSGKVPMQQLLKDLLT
AYKFGVKTLYYQNTRDGAEDAQDDLVPSIQDDGCESGACKI
>LFGLNPFC_00960 putative autotransporter YfaL
MNMRIISLRKEYLSLLPSMIASLFSANGAAAVIDSCQGYDIKANCQASRQSLSGNMQDWS
IADGQWLIFSGMANNASGGAVFLQHSAEFTISPQNETGMTLFADNSVSGEYNNGGAIFAK
ENSTINLANVIFDSNVAGGYGGAIYSAGTNDTGAADLRVTNAVFHNNIANDGKGGAIYTI
NNDVYLSDDAFNNNQAYTSTSYSDGDGGAIDVTDNSTDNTHLSGKTIINNTSFTNNYAEG
YGGAIYTSSTTSPYLIDISVDDNYDQNNGVMIDENNSASGYDHSATAAAGGFMYIGHSVA
EFNIAADKTLVIGNTSNDGAIDSLAGTGVIVKEGAGELVLNADNNAFTGEISIQNGEVTL
GRSDELMNVGDTHCQSDPQDCFGLMVGSTVHSEYQAELNVGNTQQTFVHSLTGFANGILN
IDAGGNVTVNQGGFSGSIQGEGQLTVAQDGSYLLTGAQSMALTGDIVVEDNAVLSLAGNQ
ADLRAMQSDPQSIVLNGGVLDLSDFTTWDGDSSYNDGLQISGSGGTVIGSNDVVDISSGD
DLHIGGSDASQNGVYVVINAGDQRVTLANNNGYLGNTQIASGTLEVSDNSQLGNTSYNRS
VIFTDPQQHSEMDVTTDVDTRSATTGQGRNIEMRADGEIHVEDGVDTQWGGLMADSTGQQ
LDSVSTLTKSGGGTLELTASGTATSAVRVEDGTLKGEAENIIPYVSSLWVGEDGVFETGQ
NQDIRSIDATSGGDIDITDGTVLRLTQQDTNQALDASLFSGDGTLVNATDGVTLAGELNT
NLETDSLTYLSDVTVNGNLTNTSGAVSLQNGVAGNTLTVNGDYTGGGTLLLDSELNGDDS
ASDQLVLNGNTAGNTAVVINPITGIGEPTSTGIKVVDFAADPTQFQNNAQFSLAGSGYVN
MGAYDYTLVEDNNDWYLRSQEVNPTPPPDPDPTPDPDPTPDPDPTPDPEPTPAYQPVLNA
KVGGYFNNLRAANQAFVMERRDHAGGDGQTLNLRVIGGRYHYTAVGQLAQHEDTSTVQLS
GNLFSGHWGDDGEWMLGIVGGYSDNQGDSRSNMTGTRADNQNHGYAVGLTSSWYQHGNQK
QGAWLDSWLQYAWFNNDVSEQDDGVDHYHSSGIIASLEAGYQWLPGHGVVIEPQTQVIYQ
GVQQDDFTAANHARVSQSQGDDIQTRLGLHSEWRTAVGVTPTLDLNYYHDPHATEIEEDG
STISDDAVKQRGEIKVGITGNISQRVSLRGSVAWQKGSDDFAQTAGFLSMTVKW
>LFGLNPFC_00961 Ubiquinone biosynthesis O-methyltransferase
MNAEKSPVNHNVDHEEIAKFEAVASRWWDLEGEFKPLHRINPLRLGYIAERAGGLFGKKV
LDVGCGGGILAESMAREGATVTGLDMGFEPLQVAKLHALESGIQVDYVQETVEEHAAKHA
GQYDVVTCMEMLEHVPDPQSVVRACAQLVKPGGDVFFSTLNRNGKSWLMAVVGAEYILRM
VPKGTHDVKKFIKPAELLGWVDQTSLKERHMTGLHYNPITNSFKLGPGVDVNYMLHTQNK
>LFGLNPFC_00962 DNA gyrase subunit A
MSDLAREITPVNIEEELKSSYLDYAMSVIVGRALPDVRDGLKPVHRRVLYAMNVLGNDWN
KAYKKSARVVGDVIGKYHPHGDSAVYDTIVRMAQPFSLRYMLVDGQGNFGSIDGDSAAAM
RYTEIRLAKIAHELMADLEKETVDFVDNYDGTEKIPDVMPTKIPNLLVNGSSGIAVGMAT
NIPPHNLTEVINGCLAYIDDEDISIEGLMEHIPGPDFPTAAIINGRRGIEEAYRTGRGKV
YIRARAEVEVDAKTGRETIIVHEIPYQVNKARLIEKIAELVKEKRVEGISALRDESDKDG
MRIVIEVKRDAVGEVVLNNLYSQTQLQVSFGINMVALHHGQPKIMNLKDIIAAFVRHRRE
VVTRRTIFELRKARDRAHILEALAVALANIDPIIELIRHAPTPAEAKTALVANPWQLGNV
AAMLERAGDDAARPEWLEPEFGVRDGLYYLTEQQAQAILDLRLQKLTGLEHEKLLDEYKE
LLDQIAELLRILGSADRLMEVIREELELVREQFGDKRRTEITANSADINLEDLITQEDVV
VTLSHQGYVKYQPLSEYEAQRRGGKGKSAARIKEEDFIDRLLVANTHDHILCFSSRGRVY
SMKVYQLPEATRGARGRPIVNLLPLEQDERITAILPVTEFEEGVKVFMATANGTVKKTVL
TEFNRLRTAGKVAIKLVEGDELIGVDLTSGEDEVMLFSAEGKVVRFKESSVRAMGCNTTG
VRGIRLGEGDKVVSLIVPRGDGAILTATQNGYGKRTAVAEYPTKSRATKGVISIKVTERN
GLVVGAVQVDDCDQIMMITDAGTLVRTRVSEISIVGRNTQGVILIRTSEDENVVGLQRVA
EPVDEEDLDTIDGSAAEGDDEIAPEVDVDDEPEEE
>LFGLNPFC_00963 hypothetical protein
MSGEKKAKGWRFYGLVGFGAIALLSAGVWALQYSGSGPEKTLSPLVVHNNLQIDLNEPDL
FLDSDSLSQLPKDLLTIPFLHDVLSEDFVFYYQNHADRLGIEGSIRRIVYEHDLTLKDKL
FSSLLDQPAQAALWHDKQGHLSHYMVLIQRSGLSKLLEPLLFAATSDSQLSKTEISSIKI
NSETIPVYQLRYNGNNVLMFATYQDKILAFSSSDMLFKDDQQDTEATAIASDLLSGKKRW
QASFGLEERTAEKTPVRQRIVVSARLLGFGYQRLVPSFAGMRFEMGNDGWHSFLALNDES
ASVDASFDFTPVWNSMPAGASFCVAVPYSHGIAEEMLSHISQENDKLNGALDGGAGLCWY
EDSKLQTPLFVGQFDGTAEQAQLPGKLFTQNIGAHESKAPEGVLPVSQTQQGEAQIWRRE
VSSRYGQYPKAQAAQPDQLMSDYFFRVSLAMQNKTLLFSLDDTLVNNALQALNKTRPAMV
DVIPTDGIVPLYINPQGMAKLLRNETLTSLPKNLEPVFYNAAQTLLMPKLDALSQQPRYV
MKLAQMEPGAAWQWLPITWQPL
>LFGLNPFC_00964 hypothetical protein

MRHGLLVLICWLYCVVAHSEMLNVEQSGLFRAWFVRIAQEQLRQGPSPRWYQQDCAGLVR
FAANEALKVHDSKWLKSNGLSNQYLPPEMTLTPEQRQLAQNWNQGNGKTGPYVTAINLIQ
YNSQFIGQDINQALPGDMIFFDQGDAQHLMVWMGRYIIYHTGSATKTDNGMRAVSLQQLM
TWKDTRWIPNDSNPNFIGIYRLNFLAR
>LFGLNPFC_00965 hypothetical protein
MRLEAPGRDYRRYQMEEYGGVDVRLYRIPDPMAFLRQQKNLHRIVVQPQYLGDGLNNTLT
WLWDNWYGKSRRVMQRTFSSQSRQNVTQALPELQLGNAIIKPSRYVQNNQFSPLKKYPLV
EQFRYPLWQAKPVEPQQGVKLEGASSNFISPQPGNIYIPLGKQEPGLYLVEAMVGGYRAT
TVVFVSDTVALSKVSGNELLVWTAGKKQGEAKPGSEILWTDGLGVMTRGVTDGSGTLQLQ
HISPERSYILGKDAEGGVFVSENFFYESEIYNTRLYIFTDRPLYRAGDRVDVKVMGREFH
DPLHSSPIVSAPAKLSVLDANGSLLQTVNVTLDARNGGQGSFRLPENAVAGGYELRLAYR
KQVYSSSFRVANYIKPHFEIGLALDKKEFKTGEAVSGKLQLLYPDGEPVKDARVQLSLRA
QQLSMVGNDLRYAGRFPVSLEGSETVSDDNGHVALNLPAADKPSRYLLTVSASDGAAYRV
TTTKEILIERGLAHYSLSTAAQYSNSGESVVFRYAALESSKQVPVTYEWLRLEDRTSHSG
ELQSGGKSFTVNFAKPGNYNLTLRDKDGLILAGLSHAVSGKGSTSHTGTVDIVADKTLYQ
PGETAKMLITFPEPIDEALLTLERDRVEQQSLLSHPANWLTLQRLNDTQYEARVPVSNSF
APNITFSVLYTRNGQYSFQNAGIKVAVPQLDIRVKTDKTHYQPGELVNVELTSSLKGKPV
SAQLTVGVVDEMIYALQPEIAPNIGKFFYPLGRNNVRTSSSLSFISYDQALSSEPVAPGA
TNRSERRVKMLERPRREEVDTAAWMPSLTTDKQGKAYFTFLMPDSLTRWRITARGMNGDG
LVGQGRAYLRSEKSLYMKWSMPTVYRMGDKPAAGLFIFSQQDNEPVALVTKFAGAEMRQT
LTLHKGANYISLTQNIQQSGLLSAELQQNGQVQDSISTKLSFVDNSWPVEQQKNVMLGGG
DNALTLPEQASNIRLQSSETPQEIFRNNLDALVDEPWGGVINTGSRLIPLSLAWRSLADH
QSAAANDIRQMIQDNRLRLMQLAGPGARFTWWGEDGNGDAFLTAWAWYADWQASQALGVT
QQPEYWQHMLDSYAEQADNMPLLHRALVLAWAQEMNLPCKTLLKGLDEAIARRGTKTEDF
SEEDTSDINDSLILDTPESPLADAVANVLTMTLLKKAQLKSTVMPQVQQYAWDKAANSNQ
PLAHTVVLLNSGGDATQAAAILSGLTAEQSTIERALAMNWLAKYMATMPPVVLPAPAGAW
AKHKLTGGGEDWRWVGQGVPDILSFGDELSPQNVQVRWREPAKTAQQSNIPVTVERQLYR
LIPGEEEMSFTLLPVTSNEIDSDALYLDEITLTSEQDAVLRYGQVEVPLPPGADVERTTW
GISVNKPNPAKQQGQLLEKARNEMGELGYMVPVKELTGTVTFRHLLRFSQKGQFVLPPAR
YVRSYAPAQQSVAPGSEWIGMQVK
>LFGLNPFC_00966 hypothetical protein
MNWYRIVWLLALVTLPTLAEETPLQLALRGAQHDQLYQLSSSGVTKVSVLPDTLTTPLGS
LWKLYIYAWLEDTHQPEQPYQCRGNSPEEVYCCQAGESITRDSALVRSCGLYFAPQRLHI
GADMWGQYWQQRQAPAWLASLTTLKPETSVTVKSLLDSLATLPAQNKAQEVLLDVVLDEA
KIGVASMLGSRVRVKTWSWFADDKQEIRQGGFAGWLTDGTPLWVTGSGTSKTVLTRYATA
LNRVLPVPTQVASGQCVLVDLFARYPLKKVTEEKSTTAVKPGVLNGRYRVTFANGNHMTF
VSHGETTLLTVKGKLKLQSHLDREEYVARVLDREAKSTPPEAAKAMTVAIRSYLQQNADR
DGDCLSIPDSSATQRVSASPATVGARTMTAWTQDLIYAGDPVHYHGSRVTEGTLSWRHAT
AQAGQGERYDQILAFAYPDNNLSRWGAPRSTCQLLPKAKAWLAKKMPQWRRVLQDEMGYN
EPDVFAVCRLVSGFPYTDRQQKRLFIRNFFTLQDRLDLTHEYLHLAFDGYPTGLDENYIE
TLTRQLLMD
>LFGLNPFC_00967 hypothetical protein
MRKIFLPLLLVALSPVAHSEGVQEVEIDAPLSGWHPAEGEDASFSQTINYPASSANMADD
QNISAQIRGKIKNYAAAGKVQQGRLVVNGASMPQRIESDGSFARPYIFTEGSNSVQVISP
DGQSRQKMQFYSTPGAGAIRARLRLVLSWDTDNTDLDLHVVTPDGEHAWYGNTVLKNSGA
LDMDVTTGYGPEIFAMPAPVHGRYQVYINYYGGRSETELTTAQLTLITDEGSVNEKQETF
IVPMRNAGELTLVKSFDW
>LFGLNPFC_00968 Acetyl-CoA acetyltransferase
MKNCVIVSAVRTAIGSFNGSLASTSAIDLGATVIKAAIERAKIDSLHVDEVIMGNVLQAG
LGQNPARQALLKSGLAETVCGFTVNKVCGSGLKSVALAAQAIQAGQAQSIVAGGMENMSL
APYLLDAKARSGYRLGDGQVYDVILRDGLMCATHGYHMGITAENVAKEYGITREMQDELA
LHSQRKAAAAIESGAFTAEIVPVNVVTRKKTFVFSQDEFPKADSTAEALGALRPAFDKAG
TVTAGNASGINDGAAALVIMEESSALAAGLTPLARIKSYASGGVPPALMGMGPVPATQKA
LQLAGLQLADIDLIEANEAFAAQFLAVGKTLGFEPEKVNVNGGAIALGHPIGASGARILV
TLLHAMQARDKTLGLATLCIGGGQGIAMVIERLN
>LFGLNPFC_00969 Putative short-chain fatty acid transporter
MIGRISRFMTRFVSRWLPDPLIFAMLLTLLTFVIALWLTPQTPISMVKMWGDGFWNLLAF
GMQMALIIVTGHALASSAPVKSLLRTAASAAKTPVQGVMLVTFFGSVACVINWGFGLVVG
AMFAREVARRVPGSDYPLLIACAYIGFLTWGGGFSGSMPLLAATPGNPVEHIAGLIPVGD
TLFSGFNIFITVALIVVMPFITRMMMPKPSDVVSIDPKLLMEEADFQKQLPKDAPPSERL
EESRILTLIIGALGIAYLAMYFSEHGFNITINTVNLMFMIAGLLLHKTPMAYMRAISAAA
RSTAGILVQFPFYAGIQLMMEHSGLGGLITEFFINVANKDTFPVMTFFSSALINFAVPSG
GGHWVIQGPFVMPAAQALGADLGKSVMAIAYGEQWMNMAQPFWALPALAIAGLGVRDIMG
YCITALLFSGVIFVIGLTLF

>LFGLNPFC_00970 Acetate CoA-transferase subunit beta
MDAKQRIARRVAQELRDGDIVNLGIGLPTMVANYLPEGIHITLQSENGFLGLGPVTTAHP
DLVNAGGQPCGILPGAAMFDSAMSFALIRGGHIDACVLGGLQVDEEANLANWVVPGKMVP
GMGGAMDLVTGSRKVIIAMEHCAKDGSAKILRRCTMPLTAQHAVHMLVTELAVFRFIDGK
MWLTEIADGCDLATVRAKTEARFEVAADLNTQRGDL
>LFGLNPFC_00971 Acetate CoA-transferase subunit alpha
MKTKLMTLQDATGFFRDGMTIMVGGFMGIGTPSRLVEALLESGVRDLTLIANDTAFVDTG
IGPLIVNGRVRKVIASHIGTNPETGRRMISGEMDVVLVPQGTLIEQIRCGGAGLGGFLTP
TGVGSVVEEGKQTLTLDGKTWLLERPLRADLALIRAHRCDTLGNLTYHLSARNFNPLIAL
AADITLVEPDELVETGELQPDHIVTPGAVIDHIIVSQESK
>LFGLNPFC_00972 Regulatory protein AtoC
MTAINRILIVDDEDNVRRMLSTAFALQGFETHCANNGRTALHLFADIHPDVVLMDIRMPE
MDGIKALKEMRSHETRTPVILMTAYAEVETAVEALRCGAFDYVIKPFDLDELNLIVQRAL
QLQSMKKEIRHLHQALSTSWQWGHILTNSPAMMDICKDTAKIALSQASVLISGESGTGKE
LIARAIHYNSRRAKGPFIKVNCAALPESLLESELFGHEKGAFTGAQTLRQGLFERANEGT
LLLDEIGEMPLVLQAKLLRILQEREFERIGGHQTIKVDIRIIAATNRDLQAMVKEGTFRE
DLFYRLNVIHLILPPLRDRREDISLLANHFLQKFSSENQRDIIDIDPMAMSLLTAWSWPG
NIRELSNVIERAVVMNSGPIIFSEDLPPQIRQPVCNAGEAKTAPVGERNLKEEIKRVEKR
IIMEVLEQQEGNRTRTALMLGISRRALMYKLQEYGIDPADV
>LFGLNPFC_00973 Signal transduction histidine-protein kinase AtoS
MHYMKWIYPRRLRNQMILMAILMVIVPTLTIGYIVETEGRSAVLSEKEKKLSAVVNLLNQ
ALGNRYDLYIDLPREERIRALNAELAPITENITHAFPGIGAGYYNKTLDAIITYAPSALY
QNNVGVTIAADHPGREVMRTNTPLVYSGRQVRGDILNSMIPIERNGEILGYIWANELTED
IRRQAWKMDVRIIIVLTAGLLISLLLIVLFSRRLSANIDIITDGLSTLAQNIPTRLPQLP
GEMGQISQSVNNLAQALRETRTLNDLIIENAADGVIAIDRQGDVTTMNPAAEVITGYQRH
ELVGQPYSMLFDNTQFYSPVLDTLEHGTEHVALEISFPGRDRTIELSVTTSRIHNTHGEM
IGALVIFSDLTARKETQRRMAQAERLATLGELMAGVAHEVRNPLTAIRGYVQILRQQTRD
PIHQEYLSVVLKEIDSINKVIQQLLEFSRPRHSQWQQVSLNALVEETLVLVQTAGVQARV
DFISELDNELSPINADRELLKQVLLNILINAVQAISARGKIRIRTWQYSDSQQAISIEDN
GSGIDLSLQKKIFDPFFTTKASGTGLGLALSQRIINAHQGDIRVASLPGYGATFTLILPI
NPQGNQTV
>LFGLNPFC_00974 Sensor histidine kinase RcsC
MKYLASFRTTLKASRYMFRALALVLWLLIAFSSVFYIVNALHQRESEIRQEFNLSSDQAQ
RFIQRTSDVMKELKYIAENRLSAENGVLSPRGRETQTDVPAFEPLFADSDCSAMSNTWRG
SLESLAWFMRYWRDNFSAAYDLNRVFLIGSDNLCMANFGLRDMPVERDTALKALHERINK
YRNAPQDDSGSNLYWISEGPRPGVGYFYALTPVYLANRLQALLGIEQTIRMENFFLPGTL
PMGVTILDENGHTLISLTGPESKIKGDPRWMQERSWFGYTEGFRELVLKKNLPPSSLSIV
YSVPVDKVLERIRMLILNAILLNVLAGAALFTLARMYERRIFIPAESDALRLEEHEQFNR
KIVASAPVGICILRTADGVNILSNELAHTYLNMLTHEDRQRLTQIICGQQVNFVDVLTSN
NTNLQISFVHSRYRNENVAICVLVDVSSRVKMEESLQEMAQAAEQASQSKSMFLATVSHE
LRTPLYGIIGNLDLLQTKELPKGVDRLVTAMNNSSSLLLKIISDILDFSKIESEQLKIEP
REFSPREVMNHITANYLPLVVRKQLGLYCFIEPDVPVALNGDPMRLQQVISNLLSNAIKF
TDTGCIVLHVRADGDYLSIRVRDTGVGIPAKEVVRLFDPFFQVGTGVQRNFQGTGLGLAI
CEKLISMMDGDISVDSEPGMGSQFTVRIPLYGAQYPQKKGVEGLSGKRCWLAVRNASLCQ
FLETSLQRSGIVVTTYEGQEPTPEDVLITDEVVNKKWQGRAVVTFCRRHIGIPLEKAPGE
WVHSVAAPHELPALLARIYLIEMESDDPANALPSTDKAVSDNDDMMILVVDDHPINRSLL
ADQLGSLGYQCKTANDGVDALNVLNKNHIDIVLSDVNMPNMDGYRLTQRIRQLGLTLPVI
GVTANALAEEKQRCLESGMDSCLSKPVTLDVIKQTLTVYAERVRKSRES
>LFGLNPFC_00975 Transcriptional regulatory protein RcsB
MNNMNVIIADDHPIVLFGIRKSLEQIEWVNVVGEFEDSTALINNLPKLDAHVLITDLSMP
GDKYGDGITLIKYIKRHFPSLSIIVLTMNNNPAILSAVLDLDIEGIVLKQGAPTDLPKAL
AALQKGKKFTPESVSRLLEKISAGGYGDKRLSPKESEVLRLFAEGFLVTEIAKKLNRSIK
TISSQKKSAMMKLGVENDIALLNYLSSVTLSPADKD
>LFGLNPFC_00976 Phosphotransferase RcsD
MRQKETTATTRFSLLPGSITRFFLLLIIVLLVTMGVMVQSAVNAWLKDKSYQIVDITHAI
QKRVDTWRYVTWQIYDNIAATTSPSSGEGLQETRLKQDVYYLEKPRRKTEALIFGSHDNS
TLEMTQRMSTYLDTLWGAENVPWSMYYLNGQDNSLVLISTLPLKDLTSGFKESTVSDIVD
SRRAEMLQQANALDERESFSNMRRLAWQNGHYFTLRTTFNQPGHLATVVAFDLPINDLIP
PGMPLDSFRLEPDATATGNNENEKEGTDSVSIHFNSTKIEISSALNSTDMRLVWQVPYGT
LLLDTLQNILLPLLLNIGLLALALFGYTTFRHFSSRSTESVPSTAVNNELRILRAINEEI
VSLLPLGLLVHDQESNRTVISNKIADHLLPHLNLQNITTMAEQHQGIIQATINNELYEIR
MFRSQVAPRTQIFIIRDQDREVLVNKKLKQAQRLYEKNQQGRMTFMKNIGDALKEPAQSL
AESAAKLNAPEGKQLANQADVLVRLVDEIQLANMLADDSWKSETELFSVQDLIDEVVPSV
LPAIKRKGLQLLINNHLKAHDMRRGDRDALRRILLLLMQYAVTSTQLGKITLEVDQDESS

EDRLTFRILDTGEGVSIHEMDNLHFPFINQTQNDRYGKADPLAFWLSDQLARKLGGHLNI
KTRDGLGTRYSVHIKMLATDPEVEEEEERLLDDVCVMVDVTSAEIRNIVTRQLENWGATC
ITPDERLISQDYDIFLTDNPSNLTASGLLLSDDESGVREIGPGQLCVNFNMSNAMQEAVL
QLIEVQLAQEEVTESPLGGDENAQLHASGYYALFVDTVPDDVKRLYTEAATSDFAALAQT
AHRLKGVFAMLNLVPGKQLCETLEHLIREKDVPGIEKYISDIDSYVKSLL
>LFGLNPFC_00977 Outer membrane porin C
MKVKVLSLLVPALLVAGAANAAEVYNKDGNKLDLYGKVDGLHYFSDDKSVDGDQTYMRLG
FKGETQVTDQLTGYGQWEYQIQGNSAENENNSWTRVAFAGLKFQDVGSFDYGRNYGVVYD
VTSWTDVLPEFGGDTYGSDNFMQQRGNGFATYRNTDFFGLVDGLNFAVQYQGKNGSVSGE
GMTNNGRGALRQNGDGVGGSITYDYEGFGIGGAISSSKRTDDQNSPLYIGNGDRAETYTG
GLKYDANNIYLAAQYTQTYNATRVGSLGWANKAQNFEAVAQYQFDFGLRPSLAYLQSKGK
NLGRGYDDEDILKYVDVGATYYFNKNMSTYVDYKINLLDDNQFTRDAGINTDNIVALGLV
YQF
>LFGLNPFC_00978 FAD:protein FMN transferase
MEINFTRVALLAAALFFVGCDQKPQPAKTHATEVTVLEGKTMGTFWRASIPGIDAKRSAE
LKEKIQTQLDADDQLLSTYKKDSALMRFNDSQSLSPWPVSEAMADIVTTSLRIGAKTDGA
MDITVGPLVNLWGFGPEQQPVQIPSQEQIDAMKAKTGLQHLTVINQSHQQYLQKDLPDLY
VDLSTVGEGYAADHLARLMEQEGISRYLVSVGGALNSRGMNGAGQPWRVAIQKPTDKENA
VQAVVDINGHGISTSGSYRNYYELDGKRLSHVIDPQTGRPIEHNLVSVTVIAPTALEADA
WDTGLMVLGPEKAKEVVRREGLAVYMITKEGDSFKTWMSPQFKSFLISEKN
>LFGLNPFC_00979 Bifunctional transcriptional activator/DNA repair enzyme Ada
MKYATCLTDDQRWQSVLARDSNADGEFVFAVRTTGIFCRPSCRARHALRENVSFYANASE
ALAAGFRPCKRCQPDKANPLQHRLDKITHACRLLEQETPVTLEALADQVAMSPFHLHRLF
KATTGMTPKAWQQAWRARRLRELLAKGVSVTTSILNAGFPDSSSYYRKADETLGMTAKQF
RHGGENLAVRYALADCELGRCLVAESERGICAILLGDDDATLISELQQMFPAADSALADL
TFQQHVREVIASLNQRDTPLTLPLDIRGTAFQQQVWQALRTIPCGETVSYQQLANAIGKP
KAVRAVASACAANKLAIVIPCHRVVRGDGTLSGYRWGVSRKAQLLRREAENEER
>LFGLNPFC_00980 Alpha-ketoglutarate-dependent dioxygenase AlkB
MLDLFADAEPWQEPLAAGAVILHRFAFNAAEQLIRDINDVASQSPFRQMVTPGGYTMSVA
MTNCGHLGWTTHRQGYLYSPIDPQTNKPWPAMPQSFHHLCQRAATAAGYPDFQPDACLIN
RYVPGAKLSLHQDKDEPDLRAPIVSVSLGLPAIFQFGGLKRNDPLKRLLLEHGDVVVWGG
ESRLFYHGIQPLKVGGHPLTTDCRYNLTFRQAGKKE
>LFGLNPFC_00981 ABC transporter ATP-binding/permease protein YojI
MELLVLVWRQYRWPFISVMALSLASAALGIGLIAFINQRLIKTADTSLLVLPEFLGLLLL
LMAVTLGSQLALTTLGHHFVYRLRSEFIKRILDTHVERIEQLGSASLLAGLTSDVRNITI
AFVRLPELVQGIILTIGSAAYLWMLSGKMLLVTAIWMAVTIWGGFILVARVYKHMATLRE
TEDKLYTDFQTVLEGRKELTLNRERAEYVFNNLYIPDAQEYRHHIIRADTFHLSAVNWSN
IMMLGAIGLVFWMANSLGWADTNVAATYSLTLLFLRTPLLSAVGALPTLLTAQVAFNKLN
KFALTPFKAEFPRPQAFPNWQTLELRNVTFSYQDNAFSVGPINLTIKRGELLFLIGGNGS
GKSTLAMLLTGLYQPQNGEILLDGKPVSGEQPEDYRKLFSAVFTDVWLFDQLLGPEGQPA
NPQLVEKWLAQLKIAHKLELSNGRIVNLKLSKGQKKRVALLLALAEERDIILLDEWAADQ
DPHFRREFYQVLLPLMQEMGKTIFAISHDDHYFIHADRLLEMRNGQLSELTGEERDAASR
DAVARTA
>LFGLNPFC_00982 Malate:quinone oxidoreductase
MKKVTAMLFSMAVGLNAVSMAAKAKASEEQETDVLLIGGGIMSATLGTYLRELEPEWSMT
MVERLEGVAQESSNGWNNAGTGHSALMELNYTPQNADGSISIEKAVAINEAFQISRQFWA
HQVERGVLRTPRSFINTVPHMSFVWGEDNVNFLRARYAALQQSSLFRGMRYSEDHAQIKE
WAPLVMEGRDPQQKVAATRTEIGTDVNYGEITRQLIASLQKKSNFSLQLSSEVRALKRND
DNTWTVTVADLKNGTAQNIRAKFVFIGAGGAALKLLQESGIPEAKDYAGFPVGGQFLVSE
NPDVVNHHLAKVYGKASVGAPPMSVPHIDTRVLDGKRVVLFGPFATFSTKFLKNGSLWDL
MSSTTTSNVMPMMHVGLDNFDLVKYLVSQVMLSEEDRFEALKEYYPQAKKEDWRLWQAGQ
RVQIIKRDADKGGVLRLGTEVVSDQQGTIAALLGASPGASTAAPIMLNLLEKVFGDRVSS
PQWQATLKAIVPSYGRKLNGDVAATERELQYTSEVLGLKYDKPQAADSTPKPQLKPQPVQ
KEVADIAL
>LFGLNPFC_00983 Ecotin
MKTILPAVLFAAFATTSAWAAESVQPLEKIAPYPQAEKGMKRQVIQLTQQEDESTLKVEL
LIGQTLEVDCNLHRLGGKLESKTLEGWGYDYYVFDKVSSPVSTMMACPDGKKEKKFVTAY
LGDAGMLRYNSKLPIVVYTPDNVDVKYRIWKAEEKIDNAVVR
>LFGLNPFC_00984 hypothetical protein
MHTPIGVKPVAGSKEWREAWQKRAFAHISNGYKHIYIAINSPEIFLLVCFLIRI
>LFGLNPFC_00985 Ferredoxin-type protein NapF
MKIDASRRGILTGRWRKASNGIRPPWSGDESHFLTHCTRCDACINACENNILQRGAGGYP
SVNFKNNECSFCYACAQACPESLFSPRHTRAWDLQFTIGDACLAYQSVECRRCQDSCEPM
AIIFRPTLSGIYQPQLNSQLCNGCGACAASCPVSAITAEYLHAH

>LFGLNPFC_00986 Chaperone NapD
MHTNWQVCSLVVQAKSERISDISTQLNAFTGCEVAVSDAPSGQLIVVVEAEDSETLIQTI
ESVRNVEGVLAVSLVYHQQEEQGEETP
>LFGLNPFC_00987 Periplasmic nitrate reductase
MKLSRRSFMKANAVAAAAAAAGLSVPGVARAVVGQQEAIKWDKAPCRFCGTGCGVLVGTQ
QGRVVACQGDPDAPVNRGLNCIKGYFLPKIMYGKDRLTQPLLRMKNGKYDKEGEFTPITW
DQAFDVMEDKFKTALKEKGPESIGMFGSGQWTIWEGYAASKLFKAGFRSNNIDPNARHCM
ASAVVGFMRTFGMDEPMGCYDDIEQADAFVLWGSNMAEMHPILWSRITNRRLSNQNVTVA
VLSTYQHRSFELADNGIIFTPQSDLVILNYIANYIIQNNAINQDFFSKHVNLRKGATDIG
YGLRPTHPLEKAAKNPGSDASEPMSFEDYKAFVAEYTLEKTAEMTGVPKDQLEQLAQLYA
DPNKKVISYWTMGFNQHTRGVWANNLVYNLHLLTGKISQPGCGPFSLTGQPSACGTAREV
GTFAHRLPADMVVTNEKHRDICEKKWNIPSGTIPAKIGLHAVAQDRALKDGKLNVYWTMC
TNNMQAGPNINEERMPGWRDPRNFIIVSDPYPTVSALAADLILPTAMWVEKEGAYGNAER
RTQFWRQQVQAPGEAKSDLWQLVQFSRRFKTEEVWPEELLAKKPELRGKTLYEVLYATPE
VSKFPVSELAEDQLNDESRELGFYLQKGLFEEYAWFGRGHGHDLAPFDDYHKARGLRWPV
VNGKETQWRYSEGNDPYVKAGEGYKFYGKPDGKAVIFALPFEPAAEAPDEEYDLWLSTGR
VLEHWHTGSMTRRVPELHRAFPEAVLFIHPLDAKARDLRRGDKVKVVSRRGEVISIVETR
GRNRPPQGLVYMPFFDAAQLVNKLTLDATDPLSKETDFKKCAVKLEKV
>LFGLNPFC_00988 Ferredoxin-type protein NapG
MSRSAKPQNGRRRFLRDVVRTAGGLAAVGVALGLQQQTARASGVRLRPPGAINENAFASA
CVRCGQCVQACPYDTLKLATLASGLSAGTPYFVARDIPCEMCEDIPCAKVCPSGALDREI
ESIDDARMGLAVLVDQENCLNFQGLRCDVCYRECPKIDEAITLELERNTRTGKHARFLPT
VHSDACTGCGKCEKVCVLEQPAIKVLPLSLAKGELGHHYRFGWLEGNNGKS
>LFGLNPFC_00989 Ferredoxin-type protein NapH
MANRKRDAGREALEKKGWWRSHRWLVLRRLCQFFVLGMFLSGPWFGVWILHGNYSSSLLF
DTVPLTDPLMTLQSLASGHLPATVALTGAVIITVLYALAGKRLFCSWVCPLNPITDLANW
LRRRFDLNQSATIPRHIRYVLLVVILVGSALTGTLIWEWINPVSLMGRSLVMGFGSGALL
ILALFLFDLLVVEHGWCGHICPVGALYGVLGSKGVITVAATDRQKCNRCMDCFHVCPEPH
VLRAPVLDEQSPVQVTSRDCMTCGRCVDVCSEDVFTITTRWSSGAKS
>LFGLNPFC_00990 Periplasmic nitrate reductase, electron transfer subunit
MKSHDLKKALCQWTAMLALVVSGAVWAANGVDFSQSPEVSGTQEGAIRMPKEQDRMPLNY
VNQPPMIPHSVEGYQVTTNTNRCLQCHGVESYRTTGAPRISPTHFMDSDGKVGAEVAPRR
YFCLQCHVPQADTAPIVGNTFTPSKGYGK
>LFGLNPFC_00991 Cytochrome c-type protein NapC
MGNSDRKPGLIKRLWKWWRTPSRLALGTLLLIGFVGGIVFWGGFNTGMEKANTEEFCISC
HEMRNTVYQEYMDSVHYNNRSGVRATCPDCHVPHEFVPKMIRKLKASKELYGKIFGVIDT
PQKFEAHRLTMAQNEWRRMKDNNSQECRNCHNFEYMDTTAQKSVAAKMHDQAVKDGQTCI
DCHKGIAHKLPDMREVEPGF
>LFGLNPFC_00992 Cytochrome c biogenesis ATP-binding export protein CcmA
MLEARELLCERDERTLFSGLSFTLNAGEWVQITGSNGAGKTTLLRLLTGLSRPDAGEVLW
QGQPLHQVRDSYHQNLLWIGHQPGIKTRLTALENLHFYHRDGDTAQCLEALAQAGLAGFE
DIPVNQLSAGQQRRVALARLWLTRATLWILDEPFTAIDVNGVDRLTQRMAQHTEQGGIVI
LTTHQPLNVAESKIRRISLTQTGAA
>LFGLNPFC_00993 Heme exporter protein B
MMFWRIFRLELRVAFRHSAEIANPLWFFLIVITLFPLSIGPEPQLLARIAPGIIWVAALL
SSLLALERLFRDDLQDGSLEQLMLLPLPLPAAVLAKVMAHWMVTGLPLLILSPLVAMLLG
MDVYGWQVMALTLLLGTPTLGFLGAPGVALTVGLKRGGVLLSILVLPLTIPLLIFATAAM
DAASMHLPVDGYLAILGALLAGTATLSPFATAAALRISIQ
>LFGLNPFC_00994 Heme exporter protein C
MWKTLHQLAIPPRLYQICGWFIPWLAIASVVVLTVGWIWGFGFAPADYQQGNSYRIIYLH
VPAAIWSMGIYASMAVAAFIGLVWQMKMANLAVAAMAPIGAVFTFIALVTGSAWGKPMWG
TWWVWDARLTSELVLLFLYVGVIALWHAFDDRRLAGRAAGILVLIGVVNLPIIHYSVEWW
NTLHQGSTRMQQSIDPAMRSPLRWSIFGFLLLSATLTLMRMRNLILLMEKRRPWVSELIL
KRGRK
>LFGLNPFC_00995 Heme exporter protein D
MTPAFASWNEFFAMGGYAFFVWLAVVMTVIPLVVLVVHSVMQHRAILRGVAQQRAREARL
RAAQQQEAA
>LFGLNPFC_00996 Cytochrome c-type biogenesis protein CcmE
MNIRRKNRLWIACAVLAGLALTIGLVLYALRSNIDLFYTPGEILYGKRETQQMPEVGQRL
RVGGMVMPGSVQRDPNSLKVTFTIYDAEGSVDVSYEGILPDLFREGQGVVVQGELEKGNH
ILAKEVLAKHDENYTPPEVEKAMEANHRRPASVYKDPAS
>LFGLNPFC_00997 Cytochrome c-type biogenesis protein CcmF
MMPEIGNGLLCLALGIALLLSVYPLWGVARGDARMMASSRLFAWLLFMSVAGAFLVLVNA
FVVNDFTVTYYVASNSNTQLPVWYRVAATWGAHEGSLLLWVLLMSGWTFAVAIFSQRIPLD

IVARVLAIMGMVSVGFLLFILFTSNPFSRTLPNFPIEGRDLNPLLQDPGLIFHPPLLYMG
YVGFSVAFAFAIASLLSGRLDSTYARFTRPWTLAAWIFLTLGIVLGSAWAYYELGWGGWW
FWDPVENASFMPWLVGTALMHSLAVTEQRASFKAWTLLLAISAFSLCLLGTFLVRSGVLV
SVHAFASDPARGMFILAFMVLVIGGSLLLFAARGHKVRSRVNNALWSRESLLLANNVLLV
AAMLVVLLGTLLPLVHKQLGLGSISIGEPFFNTMFTWLMVPFALLLGVGPLVRWGRDRPR
KIRNLLIIAFISTLVLSLLLPWLFESKVVAMTVLGLAMACWIAVLAIAEAALRISRGTKT
TFSYWGMVAAHLGLAVTIVGIAFSQNYSVERDVRMKSGDSVDIHEYRFTFRDVKEVTGPN
WRGGVATIGVTRDGKPETVLYAEKRYYNTAGSMMTEAAIDGGITRDLYAALGEELENGAW
AVRLYYKPFVRWIWAGGLMMALGGLLCLFDPRYRKRVNPQKTAPEAA
>LFGLNPFC_00998 Thiol:disulfide interchange protein DsbE
MKRKVLLIPLIIFLAIAAALLWQLARNAEGDDPTNLESALIGKPVPKFRLESLDNPGQFY
QADVLTQGKPVLLNVWATWCPTCRAEHQYLNQLSAQGIRVVGMNYKDDRQKAISWLKELG
NPYALSLFDGDGMLGLDLGVYGAPETFLIDGNGIIRYRHAGDLNPRVWEEEIKPLWEKYS
KEAAQ
>LFGLNPFC_00999 Cytochrome c-type biogenesis protein CcmH
MRFLLGVLMLMISGSALATIDVLQFKDEAQEQQFRQLTEELRCPKCQNNSIADSNSMIAT
DLRQKVYELMQEGKSKKEIVDYMVARYGNFVTYDPPLTPLTVLLWVLPVVAIGIGGWVIY
ARSRRRVRVVLEAFPEQSVPEGKRAGYVVYLPGIVVALIVAGVSYYQTGNYQQVKIWQQA
TAQAPALLDRALDPKADPLNEEEMSRLALGMRTQLQKNPGDIEGWIMLGRVGMALGNASI
ATDAYATAYRLDPKNSDAALGYAEALTRSSDPNDNRLGGELLRQLVRTDHSNIRVLSMYA
FNAFEQQRFGEAVAAWEMMLKLLPANDTRRAVIERSIAQAMQHLSPQESK
>LFGLNPFC_01000 Nitrate/nitrite response regulator protein NarL
MPEATPFQVMIVDDHPLMRRGVRQLLELDSGFEVVAEAGDGASAIDLANRLDIDVILLDL
NMKGMSGLDTLNALRRDGVTAQIIILTVSDASSDVFALIDAGADGYLLKDSDPEVLLEAI
RAGAKGSKVFSERVNQYLREREMFGAEEDPFSVLTERELDVLHELAQGLSNKQIASVLNI
SEQTVKVHIRNLLRKLNVRSRVAATILFLQQRGAQ
>LFGLNPFC_01001 hypothetical protein
MHQSGSVSLCRSAISVLVATALYSPIALASTVEYGETVDGVVLEKDIQLVYGAANNTKIN
PGGEQHI
>LFGLNPFC_01002 hypothetical protein
MGGAVNQTTINNGVLQVYGAANDPTIKGGRGDAAFTLGNAGSVVDISTYEYTLLDNGNHS
WSLAENRVQMPPSTTDVLNMAAAQPLVFDVELDTVRGRLGSVKGVNYDTAMWSSAINSRN
NVNTDAGAGFEQTLTGLTLGIDSRFSREESSTTQAGVVWTF
>LFGLNPFC_01004 Inner membrane protein YejM
MVTHRQRYREKVSQMVSWGHWFALFNILLSLVIGSRYLFIADWPTTLAGRIYSYVSIIGH
FSFLVFATYLLILFPLTFIVGSQRLMRFLSVILATAGMTLLLIDSEVFTRFHLHLNPIVW
QLVINPDENEMARDWQLMFISVPVILLLELVFATWSWQKLRSLTRRRRFARPLAAFLFIA
FIASHVVYIWADANFYRPITMQRANLPLSYPMTARRFLEKHGLLDAQEYQRRLIEQGNPD
AVSVQYPLSELRYRDMGTGQNVLLITVDGLNYSRFEKQMPALAGFAEQNISFTRHMSSGN
TTDNGIFGLFYGISPSYMDGILSTRTPAALITALNQQGYQLGLFSSDGFTSPLYRQALLS
DFSMPSVRTQSDEQTATQWINWLGRYAQEDNRWFSWVSFNGTNIDDSNQQAFARKYSRAA
GNVDDQINRVLNALRDSGKLDNTVVIITAGRGIPLSEEEETFDWSHGHLQVPLVIHWPGT
PAQRINALTDHTDLMTTLMQRLLHVSTPASEYSQGQDLFNPQRRHYWVTAADNDTLAITT
PKKTLVLNNNGKYRTYNLRGERVKDEKPQLSLLLQVLTDEKRFIAN
>LFGLNPFC_01005 hypothetical protein
MPQISRYSDEQVEQLLAELLNILEKHKAPTDLSLMVLGNMVTNLINTSIAPAQRQAIANS
FARALQSSINEDKAH
>LFGLNPFC_01006 Nucleoid-associated protein YejK
MSLDINQIALHQLIKRDEQNLELVLRDSLLEPTETVVEMVAELHRVYSAKNKAYGLFSEE
SELAQTLRLQRQGEEDFLAFSRAATGRLRDELAKYPFADGGFVLFCHYRYLAVEYLLVAV
LSNLSSMRVNENLDINPTHYLDINHADIVARIDLTEWETNPESTRYLTFLKGRVGRKVAD
FFMDFLGASEGLNAKAQNRGLLQAVDDFTAEAQLDKAERQNVRQQVYSYCNEQLQAGEEI
ELESLSKELAGVSEVSFTEFAAEKGYELEESFPADRSTLRQLTKFAGSGGGLTINFDAML
LGERIFWDPATDTLTIKGTPPNLRDQLQRRTSGGN
>LFGLNPFC_01007 50S ribosomal protein L25
MFTINAEVRKEQGKGASRRLRAANKFPAIIYGGKEAPLAVELDHDKVMNMQVKAEFYSEV
LTIVVDGKEIKVKAQDVQRHPYKPKLLHIDFVRA
>LFGLNPFC_01008 Putative DNA repair helicase RadD
MIFTLRPYQQEAVDATLNHFRRHKTPAVIVLPTGAGKSLVIAELARLARGRVLVLAHVKE
LVAQNHEKYQALGLEADIFAAGLKRKESHGKVVFGSVQSVTRNLDAFQGEFSLLIVDECH
RIGDDEESQYQQILTHLTKVNPHLRLLGLTATPFRLGKGWIYQFHYHGMVRGDEKALFRD
CIYELPLRYMIKHGYLTPPERLDMPVVQYDFSRLQAQSNGLFSEADLNRELKKQQRITPH
IISQIMEFAATRKGVMIFAATVEHAKEIVGLLPAEDAALITGDTPGSERDVLIDDFKAQR
FRYLVNVAVLTTGFDAPHVDLIAILRPTESVSLYQQIVGRGLRLAPGKTDCLILDYAGNP

HDLYAPEVGTPKGKSDNVPVQVFCPACGFANTFWGKTTADGTLIEHFGRRCQGWFEDDDG
HREQCDFRFRFKNCPQCNAENDIAARRCRECDTVLVDPDDMLKAALRLKDALVLRCSGMS
LQHGHDEKGEWLKITYYDEDGADVSERFRLQTPAQRTAFEQLFIRPHTRTPGIPLRWITA
ADILAQQALLRHPDFVVARMKGQYWQVREKVFDYEGRFRRAHELRG
>LFGLNPFC_01009 Ribosomal small subunit pseudouridine synthase A
MRLDKFIAQQLGVSRAIAGREIRGNRVTVDGEIVRNAAFKLLPEHDVAYDGNPLAQQHGP
RYFMLNKPQGYVCSTDDPDHPTVLYFLDEPVAWKLHAAGRLDIDTTGLVLMTDDGQWSHR
ITSPRHHCEKTYLVTLESPVADDTAEQFAKGVQLHNEKDLTKPAVLEVITPTQVRLTISE
GRYHQVKRMFAAVGNHVVELHRERIGGITLDADLAPGEYRPLTEEEIASVV
>LFGLNPFC_01010 Bicyclomycin resistance protein
MTTRQHSSFAIVFILGLLAMLMPLSIDMYLPALPVISAQFGVPAGSTQMTLSTYILGFAL
GQLIYGPMADSFGRKPVVLGGTLVFAAAAVACALAQTIDQLIVMRFFHGLAAAAASVVIN
ALMRDIYPKEEFSRMMSFVMLVTTIAPLMAPIVGGWVLVWLSWHYIFWILAVAAILASAM
IFFLIKETLPPERRQPFHIRTTIGNFAALFRHKRVLSYMLASGFSFAGMFSFLSAGPFVY
IEINHVAPENFGYYFALNILFLFVMTIFNSRFVRRIGALNMFRSGLWIQFIMAAWMVISA
LLGLGFWSLVVGVAAFVGCVSMVSSNAMAVILDEFPHMAGTASSLAGTFRFGIGAIVGAL
LSLATFNSAWPMIWSIAFCATSSILFCLYASRPKKR
>LFGLNPFC_01011 putative protein YejG
MTSLQLSIVHRLPQNYRWSAGFAGSKVEPIPQNGPCGDNSLVALKLLSPDGDNAWSVMYK
LSQALSDIEVPCSVLECEGEPCLFVNRQDEFAATCRLKNFGVAIAEPFSNYNPF
>LFGLNPFC_01012 putative ABC transporter ATP-binding protein YejF
MTQTLLAIENLSVGFRHQQTVRTVVNDVSLQIAAGETLALVGESGSGKSVTALSILRLLP
CPPVEYLSGDIRFHGESLLHASDQTLRGIRGNKIAMIFQEPMVSLNPLHTLEKQLYEVLS
LHRGMRREAARGEILNCLDRVGIRQAAKRLTDYPHQLSGGERQRVMIAMALLTRPELLIA
DEPTTALDVSVQAQILQLLRELQGELNMGMLFITHNLSIVRKLAHRVAVMQNGRCVEQNN
AATLFASPTHPYTQKLLNSEPSGDPVPLPEPASTLLDVEQLQVAFPIRKGILKRIVDHNV
VVKNISFTLRAGETLGLVGESGSGKSTTGLALLRLINSQGSIVFDGQPLQNLNRRQLLPI
RHRIQVVFQDPNSSLNPRLNVLQIIEEGLRVHQPTLSAAQREQQVIAVMHEVGLDPETRH
RYPAEFSGGQRQRIAIARALILKPSLIILDEPTSSLDKTVQAQILTLLKSLQQKHQLAYL
FISHDLHVVRALCHQVIVLRQGEVVEQGPCARVFAAPQQEYTRQLLALS
>LFGLNPFC_01013 Inner membrane ABC transporter permease protein YejE
MSRLSPVNQARWARFRHNRRGYWSLWIFLLLFGLSLCSELIANDKPLLVRYDGSWYFPLL
KNYSESDFGGPLASQADYQDPWLKQRLENNGWVLWAPIRFGATSINFATDKPFPSPPSRQ
NWLGTDANGGDVLARILYGTRISVLFGLMLTLCSSVMGVLAGALQGYYGGKVDLWGQRFI
EVWSGMPTLFLIILLSSVVQPNFWWLLAITVLFGWMSLVGVVRAEFLRTRNFDYIRAAQA
LGVSDRSIILRHMLPNAMVATLTFLPFILCSSITTLTSLDFLGFGLPLGSPSLGELLLQG
KNNLQAPWLGITAFLSVAILLSLLIFIGEAVRDAFDPNKAV
>LFGLNPFC_01014 Inner membrane ABC transporter permease protein YejB
MGAYLIRRLLLVIPTLWAIITINFFIVQIAPGGPVDQAIAAIEFGNAGVLPGAGGEGVRA
SHAQTGVGNISDSNYRGGRGLDPEVIAEITHRYGFDKPIHERYFKMLWDYIRFDFGDSLF
RSASVLTLIKDSLPVSITLGLWSTLIIYLVSIPLGIRKAVYNGSRFDVWSSAFIIIGYAI
PAFLFAILLIVFFAGGSYFDLFPLRGLVSANFDSLPWYQKITDYLWHITLPVLATVIGGF
AALTMLTKNSFLDEVRKQYVVTARAKGVSEKNILWKHVFRNAMLLVIAGFPATFISMFFT
GSLLIEVMFSLNGLGLLGYEATVSRDYPVMFGTLYIFTLIGLLLNIVSDISYTLVDPRID
FEGR
>LFGLNPFC_01015 hypothetical protein
MIVRILLLFIALFTFGAQAQAIKESYAFAVLGEPRYAFNFNHFDYVNPAAPKGGQITLSA
LGTFDNFNRYALRGNPGARTEQLYDTLFTTSDDEPGSYYPLIAESARYADDYSWVEVAIN
PRARFHDGSPITARDVEFTFQKFMTEGVPQFRLVYKGTTVKAIAPLTVRIELAKPGKEDM
LSLFSLPVFPEKYWKDHKLSDPLATPPLASGPYRITSWKMGQNIVYSRVKDYWAANLPVN
RGRWNFDTIRYDYYLDDNVAFEAFKAGAFDLRMENDAKNWATRYTGKNFDKKYIIKDEQK
NESAQDTRWLAFNIQRPVFSDRRVREAITLAFDFEWMNKALFYNAWSRTNSYFQNTEYAA
RNYPDAAELVLLAPMKKDLPPEVFTQIYQPPVSKGDGYDRDNLLKADKLLNEAGWVLKGQ
QRVNVTTGQPLSFELLLPASSNSQWVLPFQHSLQRLGINMDIRKVDNSQITNRMRSRDYD
MMPRVWRAMPWPSSDLQISWSSEYINSTYNAPGVQSPVIDSLINQIIAAQGNKEKLLPLG
RALDRVLTWNYYMLPMWYMAEDRLAWWDKFSQPAVRPVYSLGIDTWWYDVNKAAKLPSAR
QQGE
>LFGLNPFC_01016 putative cyclic di-GMP phosphodiesterase PdeN
MFIRAPNSGRKLLLTCIVAGVMIAILVSCLQFLVAWHKHEVTYDTLITDVQKYLDTYFAD
LKSTTDRLQPLTLDTCQQANPELTARAAFSMNVRTFVLVKDKKTFCSSATGEMDIPLNEL
IPALDINKNVDMAILPGTPMVPNKPAIVIWYRNPLLKNSGVFAALNLNLTPSLFYSSRQE
DYDGLALIIGNTALSTFSSRLMNVNELTDMPVRETKIAGIPLTVRLYADDWTWNDVWYAF
LLGGMSGTFVGLLCYYLMSVRMRPGREIMTAIKREQFYVVYQPVVDTQALRVTGLEVLLR
WRHPVAGEIPPDAFINFAEAQKMIVPLTQHLFELIARDAAELEKVLPVGVKFGINIAPAH

LHSESFKADIQKLLTSLPAHHFQIVLEITERDMLKEREATQLFAWLHSVGVEIAIDDFGT
GHSALIYLERFTLDYLKIDRGFINAIGTETITSPVLDAVLTLAKRLNMLTVAEGVETPEQ
ARWLSERGVNFMQGYWISRPLPLDDFVRWLKKPYTPQW
>LFGLNPFC_01017 Murein DD-endopeptidase MepS/Murein LD-carboxypeptidase
MVKSQPILRYILRGIPAIAVAVLLSACSANNTAKNMHPETRAVGSETSSLQASQDEFENL
VRNVDVKSRIMDQYADWKGVRYRLGGSTKKGIDCSGFVQRTFREQFGLELPRSTYEQQEM
GKSVSRSNLRTGDLVLFRAGSTGRHVGIYIGNNQFVHASTSSGVIISSMNEPYWKKRYNE
ARRVLSRS
>LFGLNPFC_01018 Lipid A 1-diphosphate synthase
MIKNLPQIVLLNIVGLALFLSWYIPVNHGFWLPIDADIFYFFNQKLVESKAFLWLVALTN
NRAFDGCSLLAMGMLMLSFWLKENAPGRRRIVIIGLVMLLTAVVLNQLGQALIPVKRASP
TLTFTDINRVSELLSVPTKDASRDSFPGDHGMMLLIFSAFMWRYFGKVAGLIALIIFVVF
AFPRVMIGAHWFTDIIVGSMTVILIGLPWVLLTPLSDRLITFFDKSLPGKNKHFQNK
>LFGLNPFC_01019 Zinc-binding GTPase YeiR
MTRTNLITGFLGSGKTTSILHLLAHKDPNEKWAVLVNEFGEVGIDGALLADSGALLKEIP
GGCMCCVNGLPMQVGLNTLLRQGKPDRLLIEPTGLGHPKQILDLLTAPVYEPWIDLRATL
CILDPRLLLDEKSASNENFRDQLAAADIIVANKSDRATPESEQALERWWQQNGGDRQLIH
SEHGKIDGHLLDLPRRNLAELPASAAHSHQHSVKKGLAALSLPEHQRWRRSLNSGQGHQA
CGWIFDADTVFDTIGILEWARLAPVERVKGVLRIPEGLVRINRQGDDLHIETQNVAPPDS
RIELISSSEADWNALQSALLKLRLATPA
>LFGLNPFC_01020 Mannitol 2-dehydrogenase
MNTIASVTLPHHVHAPRYDRQQLQSRIVHFGFGAFHRAHQALLTDRVLNAQGGDWGICEI
SLFSGDQLMSQLRAQNHLYTVLEKGADGNQAIIVGAVHECLNAKLDSLAAIIEKFCEPQV
AIVSLTITEKGYCIDPAIGALDTSNPRIIHDLQNPEEPHSAPGILVEALKRRRERGLTPF
TVLSCDNIPDNGHVVKNAVLGMAEKRSPELAGWIKEHVSFPGTMVDRIVPAATNESLAEI
SQHLGVNDPCAISCEPFIQWVVEDNFVAGRPAWEVAGVQMVNDVLPWEEMKLRMLNGSHS
FLAYLGYLSGFAHISDCMQDRAFRHAARTLMLDEQAPTLRIKDVDLTQYADKLIARFANP
ALKHKTWQIAMDGSQKLPQRMLAGIRIHLGRETDWSLLALGVAGWMRYVSGVDDAGNAID
VRDPLSDKIRELVAVSSSEQRVTALLSLREIFGDDLPDNPHFVQAIEQAWQQIAQFGAHQ
ALLNTLKI
>LFGLNPFC_01021 Elongation factor P-like protein
MPRANEIKKGMVLNYNGKLLLVKDIDIQSPTARGAATLYKMRFSDVRTGLKVEERFKGDD
IVDTVTLTRRYVDFSYVDGNEYVFMDKEDYTPYTFTKDQIEEELLFMPEGGMPDMQVLTW
DGQLLALELPQTVDLEIVETAPGIKGASASARNKPATLSTGLVIQVPEYLSPGEKIRIHI
EERRYMGRAD
>LFGLNPFC_01022 hypothetical protein
MECRPGCGACCTAPSISSPIPGMPDGKPANTPCIQLDEQQRCKIFTSPLRPKVCAGLQAS
AEMCGNSRQQAMTWLIDLEMLTAP
>LFGLNPFC_01023 Sugar efflux transporter B
MHNSPAVTSAKSFDLTSTAFLIVAFLTGIAGALQTPTLSIFLTDEVHARPAMVGFFFTGS
AVIGILVSQFLAGRSDKRGDRKSLIVFCCLLGVLACTLFAWNRNYFVLLFVGVFLSSFGS
TANPQMFALAREHADKTGREAVMFSSFLRAQVSLAWVIGPPLAYALAMGFSFTVMYLSAA
VAFIVCGVMVWLFLPSMQKELPLATGTVEAPRRNRRDTLLLFVICTLMWGSNSLYIINMP
LFIINELHLPEKLAGVMMGTAAGLEIPTMLIAGYFAKRLGKRFLMRVAAVGGVCFYAGML
MAHSPVILLGLQLLNAIFIGILGGIGMLYFQDLMPGQAGSATTLYTNTSRVGWIIAGSVA
GIVAEIWNYHAVFWFAMVMIIATLFCLLRIKDV
>LFGLNPFC_01024 Multiphosphoryl transfer protein
MFQLSVQDIHPGEKAGDKEEAIRQVAAALVQAGNVAEGYVNGMLAREQQTSTFLGNGIAI
PHGTTDTRDQVLKTGVQVFQFPEGVTWGDGQVAYVAIGIAASSDEHLGLLRQLTHVLSDD
SVAEQLKSATTAEELRALLMGEKQSEQLKLDNEMLTLDIVASDLLTLQALNAARLKEAGA
VDATFVTKAINEQPLNLGQGIWLSDSAEGNLRSAIAVSRAANAFDVDGETAAMLVSVAMN
DDQPIAVLKRLADLLLDNKADRLLKADAATLLTLLTSDDAPTDDVLSAEFVVRNEHGLHA
RPGTMLVNTIKQFNSDITVTNLDGTGKPANGRSLMKVVALGVKKGHRLRFTAQGADAEQA
LKAIGDAIAAGLGEGA
>LFGLNPFC_01025 Tagatose-6-phosphate kinase
MSRRVATITLNPAYDLVGFCPEIERGEVNLVKTTGLHAAGKGINVAKVLKDLGIDVTVGG
FLGKDNQDGFQQLFSELGIANRFQVVQGRTRINVKLTEKDGEVTDFNFSGFEVTPADWER
FVTDSLSWLGQFDMVCVSGSLPSGVSPEAFTDWMTRLRSQCPCIIFDSSREALVAGLKAA
PWLVKPNRRELEIWAGRKLPEMKDVIEAAHALREQGIAHVVISLGAEGALWVNASGEWIA
KPPSVDVVSTVGAGDSMVGGLIYGLLMRESSEHTLRLATAVAALAVSQSNVGITDRPQLA
AMMARVDLQPFN
>LFGLNPFC_01026 PTS system fructose-specific EIIB'BC component
MKTLLIIDANLGQARAYMAKTLLGAAARKAKLEIIDNPNDAEMAIVLGDSIPNDSALNGK
NVWLGDISRAVAHPELFLSEAKGHAKPYTAPVAATAPVAASGPKRVVAVTACPTGVAHTF

MAAEAIETEAKKRGWWVKVETRGSVGAGNAITPEEVAAADLVIVAADIEVDLAKFAGKPM
YRTSTGLALKKTAQELDKAVAEATPYEPAGKTQTATTEGKKESAGAYRHLLTGVSYMLPM
VVAGGLCIALSFAFGIEAFKEPGTLAAALMQIGGGSAFALMVPVLAGYIAFSIADRPGLT
PGLIGGMLAVSTGSGFIGGIIAGFLAGYIAKLISTQLKLPQSMEALKPILIIPLISSLVV
GLAMIYLIGKPVAGILDGLTHWLQTMGTANAVLLGAILGGMMCTDMGGPVNKAAYAFGVG
LLSTQTYGPMAAIMAAGMVPPLAMGLATMVARRKFDKAQQEGGKAALVLGLCFISEGAIP
FAARDPMRVLPCCIVGGALTGAISMAIGAKLMAPHGGLFVLLIPGAITPVLGYLVAIIAG
TLVAGLAYAFLKRPETQIVEKNA
>LFGLNPFC_01027 Pseudouridine kinase
MREKDYIVIIGSANIDVAGYSHHPLNYADSNPGKIKFTPGGVGRNIAHNLALLGKNAWLL
SAVGGDFYGQSLLAQTNQSGVYVDKCLIVPGENTSSYLSLLDNTGEMLVAINDMSISDCI
SAEFLAQHQEFIRGAKVIVADCNLSEEALVWVLENSGETPVFIDPVSAWKCVKIRDHLSK
IHTLKPNRLEAETLSGIALSGREDVAKVAAWFHQHGLNRLVLSMGGDGVYYSDINGESGW
SAPIKTNVINVTGAGDAMMAGLASCWVDGMPFIDSVRFAQGCSSMALACEYTNNPELSIA
NVTSLVENTECLN
>LFGLNPFC_01028 Pseudouridine-5'-phosphate glycosidase
MSELTLSPELLQISAEVQDALKNKKSVVALESTIISHGMPFPQNAQTAIEVEETIRKQGA
VPATIAIIGGVMKVGLSKEEIELLGREGHNVTKVSRRDLPFVVAAGKNGATTVASTMIIA
ALAGIKVFATGGIGGVHRGAEHTFDISADLQELANTNVTVVCAGAKSILDLGLTTEYLET
FGVPLIGYQTKALPAFFCRTSPFDVSIRLDSAREIARAMAVKWQTGLNGGLVVANPIPEQ
FAMPEETINAAIDQAVAEAEEQGVIGKESTPFLLARVAELTGGDSLKSNIQLVFNNAILA
SEIAKEYQRLVG
>LFGLNPFC_01029 Putative pseudouridine transporter
MDIMRSVVGMVVLLAIAFLLSVNKKSISLRTVGAALLLQIAIGGIMLYFPPGKWAVEQAA
LGVHKVMSYSDAGSAFIFGSLVGPKMDVLFDGAGFIFAFRVLPAIIFVTALISLLYYIGV
MGLLIRILGSIFQKALNISKIESFVAVTTIFLGQNEIPAIVKPFIDRMNRNELFTAICSG
MASIAGSMMIGYAGMGVPIDYLLAASLMAIPGGILFARILSPATEPSQVTFENLSFSETP
PKSIIEAAANGAMTGLKIAAGVATVVMAFVAIIALINGIIGGVGGWFGFANVSLESIFGY
VLAPLAWIMGVDWSDANLAGSLIGQKLAINEFVAYLNFSPYLQTSGTLDVKTIAIISFAL
CGFANFGSIGVVVGAFSAISPKRAPEIAQLGLRALAAATLSNLMSATIAGFFIGLA
>LFGLNPFC_01030 Regulatory protein YeiL
MKEIHNNDLKQQLMSESAFKDCFSTDVSADTRLFHFLARDYIVQEGQQPSWLFYLTRGRA
RLYVTLANGRVSLIDFFAAPCFIGEIELIDKDHEPRAVQAIEECWCLALPMKHYRPLLLN
DTLFLRKLCVTLSHKNYRNIVSLTQNQSFPLVNRLAAFILLSQEGDLYHEKHTQAAEYLG
VSYRHLLYVLAQFINDGLLIKSKKGYLIKNRKQLSGLALKMYPENKFSGMMQ
>LFGLNPFC_01031 Pyrimidine-specific ribonucleoside hydrolase RihB
MEKRKIILDCDPGHDDAIAIMMAAKHPAIDLLGITIVAGNQTLDKTLINGLNVCQKLEIN
VPVYAGMPQPIMRQQIVADNIHGETGLDGPVFEPLTRQAENTHAVKYIIDTLMASDGDIT
LVPVGPLSNIAVAMRMQPAILPKIREIVLMGGAYGTGNFTPSAEFNIFADPEAARVVFTS
GVPLVMMGLDLTNQTVCTPDVIARMERAGGPAGELFSDIMNFTLKTQFENYGLAGGPVHD
ATCIGYLINPDGIKTQEMYVEVDVNSGPCYGRTVCDELGVLGKPANTKVGITIDTDWFWG
LVEECVRGYIKTH
>LFGLNPFC_01032 Pseudouridine kinase
MNDREKQILKILRRNPLIQQNEIADILQISRSRVAAHIMDLMRKGLIKGKGYILTEQDYC
VVVGAINMDIRGMADIRYPQAASHPGSVHCSAGGVGRNIAHNLALLGRDVHLISAIGNDF
YGETLLEETRRAGVNVSNCIRLHGHSTATYLAIANKQEETILAINDTHILQQLTPQLLNT
SRDLIRHAGVVLADCNLTPEALEWVFTIADEIPMFVDTVSEFKANKVKNWYSRIHTLKPT
QNELEILWGQPIKDDNNRIRAVNSLHQQGVKRIFVYLKDESVFCSDKDGEQFLLTAPAHT
TVDSFGADDGFMAGLVYSFLEGYSFRDSARFAMACAAISRASGSLNNPTLSADNALSLVP
MV
>LFGLNPFC_01033 Endonuclease 4
MKYIGAHVSAAGGLANAAIRAAEIDATAFALFTKNQRQWRAAPLTTQTIDEFKAACEKYH
YTSAQILPHDSYLINLGHPVAEALEKSRDAFIDEMQRCEQLGLSLLNFHPGSHLMQISEE
DCLARIAESINIALDKTQGVTAVIENTAGQGSNLGFKFEHLAAIIDGVEDKSRVGVCIDT
CHAFAAGYDLRTPAECEKTFADFARIVGFKYLHGMHLNDAKSTFGSRVDRHHSLGEGNIG
HDAFRWIMQDDRFDGIPLILETINPDIWAEEIAWLKAQQTEKAVA
>LFGLNPFC_01034 hypothetical protein
MTNITLQKQHRTLWHFIPGLALSAVITGVALWGGSIPAVAGAGFSALTLAILLGMVLGNT
IYPHIWKSCDGGVLFAKQYLLRLGIILYGFRLTFSQIADVGISGIIIDVLTLSSTFLLAC
FLGQKVFGLDKHTSWLIGAGSSICGAAAVLATEPVVKAEASKVTVAVATVVIFGTVAIFL
YPAIYPLMSQWFSPETFGIYIGSTVHEVAQVVAAGHAISPDAENAAVISKMLRVMMLAPF
LILLAARVKQLSGANSGEKSKITIPWFAILFIVVAIFNSFHLLPQSVVNMLVTLDTFLLA
MAMAALGLTTHVSALKKAGAKPLLMALVLFAWLIVGGGAINYVIQSVIA
>LFGLNPFC_01035 HTH-type transcriptional regulator CysL

MHITLRQLEVFAEVLKSGSTTQASVMLALSQSAVSAALTDLEGQLGVQLFDRVGKRLVVN
EHGRLLYPRALALLEQAVEIEQLFREDNGAIRIYASSTIGNYILPAVIARYRHDYPQLPI
ELSVGNSQDVMQAVLDFRVDIGFIEGPCHSTEIISEPWLEDELVVFAAPTSPLARGPVTL
EQLAAAPWILRERGSGTREIVDYLLLSHLPKFEMAMELGNSEAIKHAVRHGLGISCLSRR
VIEDQLQAGTLSEVAVPLPRLMRTLWRIHHRQKHLSNALRRFLDYCDPANVPR
>LFGLNPFC_01036 Lysine-specific permease
MGSETKTTEAPGLRRELKARHLTMIAIGGSIGTGLFVASGATISQAGPGGALLSYMLIGL
MVYFLMTSLGELAAYMPVSGSFATYGQNYVEEGFGFALGWNYWYNWAVTIAVDLVAAQLV
MSWWFPDTPGWIWSALFLGVIFLLNYISVRGFGEAEYWFSLIKVTTVIVFIIVGVLMIIG
IFKGAQPAGWSNWTIGEAPFAGGFAAMIGVAMIVGFSFQGTELIGIAAGESEDPAKNIPR
AVRQVFWRILLFYVFAILIISLIIPYTDPSLLRNDVKDISVSPFTLVFQHAGLLSAAAVM
NAVILTAVLSAGNSGMYASTRMLYTLACDGKAPRIFAKLSRGGVPRNALYATTVIAGLCF
LTSMFGNQTVYLWLLNTSGMTGFIAWLGIAISHYRFRRGYVLQGHDINDLPYRSGFFPMG
PIFAFILCLIITLGQNYEAFLKDTIDWGGVAATYIGIPLFLIIWFGYKLIKGTHFVRYSE
MKFPQNDKK
>LFGLNPFC_01037 Colicin I receptor
MFRLNPFVRVGLCLSAISCAWPVLAVDDDGETMVVTASSVEQNLKDAPASISVITQEDLQ
RKPVQNLKDVLKEVPGVQLTNEGDNRKGVSIRGLDSSYTLILVDGKRVNSRNAVFRHNDF
DLNWIPVDSIERIEVVRGPMSSLYGSDALGGVVNIITKKIGQKWSGTVTVDTTVQEHRDR
GDTYNGQFFTSGPLIDGVLGMKAYGSLAKREKDDPQNSTTTDTGETPRIEGFSSRDGNVE
FAWTPNQNHDFTAGYGFDRQDRDSDSLDKNRLERQNYSVSHNGRWDYGTSELKYYGEKVE
NKNPGNSSPITSESNTVDGKYTLPLTAINQFLTVGGEWRHDKLSDAVNLTGGTSSKTSAS
QYALFVEDEWRIFEPLALTTGVRMDDHETYGEHWSPRAYLVYNATDTVTVKGGWATAFKA
PSLLQLSPDWTSNSCRGACKIVGSPDLKPETSESWELGLYYMGEEGWLEGVESSVTVFRN
DVKDRISISRTSDVNAAPGYQNFVGFETGANGRRIPVFSYYNVNKARIQGVETELKIPFN
DEWKLSINYTYNDGRDVSNGENKPLSDLPFHTANGTLDWKPLALEDWSFYVSGHYTGQKR
ADSATAKTPGGYTIWNTGAAWQVTKDVKLRAGVLNLGDKDLSRDDYSYNEDGRRYFMAVD
YRF
>LFGLNPFC_01038 S-formylglutathione hydrolase YeiG
MEMLEEHRCFEGWQQRWRHDSSTLNCPMTFSIFLPPPRDHTPPPVLYWLSGLTCNDENFT
TKAGAQRVAAELGIVLVMPDTSPRGEQVANDDGYDLGQGAGFYLNATQPPWATHYRMYDY
LRDELPALIQSQFNVSDRCAISGHSMGGHGALIMALKNPGKYTSVSAFAPIVNPCSVPWG
IKAFSTYLGEDKNAWLEWDSCALMYASNAQDAIPTLIDQGDNDQFLADQLQPAVLAEAAR
QKAWPMTLRIQPGYDHSYYFIASFIEDHLRFHAQYLLK
>LFGLNPFC_01039 GTP cyclohydrolase 1
MPSLSKEAALVHEALVARGLETPLRPPVHEMDNETRKSLIAGHMTEIMQLLNLDLADDSL
METPHRIAKMYVDEIFSGLDYANFPKITLIENKMKVDEMVTVRDITLTSTCEHHFVTIDG
KATVAYIPKDSVIGLSKINRIVQFFAQRPQVQERLTQQILIALQTLLGTNNVAVSIDAVH
YCVKARGIRDATSATTTTSLGGLFKSSQNTRHEFLRAVRHHN
>LFGLNPFC_01040 hypothetical protein
MERNVTLDFVRGVAILGILLLNISAFGLPKAAYLNPAWYGAITPQDAWTWAFLDLIGQVK
FLTLFALLFGAGLQMLLPRGRRWIQSRLTLLVLLGFIHGLLFWDGDILLAYGLVGLICWR
LVRDAPSVKSLFNTGVMLYLVGLGVLLLLGLISDSQTSRAWTPDASAILYEKHWKLHGGV
EAISNRADGVGNSLLALGAQYGWQLAGMMLIGAALMRSGWLKGQFSLRHYRRTGFVLVAI
GVIINLPAIALQWRLDWAYRWCAFLLQMPRELSAPFQAIGYASLFYGFWPQLSRFKLVLA
IACVGRMALTNYLLQTLICTTLFYHLGLFMQFDRLELLAFVIPVWLANIFFSVIWLRYFR
QGPVEWLWRQLTLRAAGPTISKTSR
>LFGLNPFC_01041 HTH-type transcriptional regulator GalS
MITIRDVARQAGVSVATVSRVLNNSTLVSADTREAVMKAVSELDYRPNANAQALATQVSD
TIGVVVMDVSDAFFGALVKAVDLVAQQHQKYVLIGNSYHEAEKERHAIEVLIRQRCNALI
VHSKALSDDELAQFMDNIPGMVLINRVVPGYAHRCVCLDNLSGARMATRMLLNNGHQRIG
YLSSSHGIEDDAMRKAGWMSALKEQDIIPPESWIGTGTPDMPGGEAAMVELLGRNLQLTA
VFAYNDNMAAGALTALKDNGIAIPLHLSIIGFDDIPIARYTDPQLTTVRYPIASMAKLAT
ELALQGAADNIDPRASHCFMPTLVRRHSVATRQNAAAITNSTNQAM
>LFGLNPFC_01042 D-galactose-binding periplasmic protein
MNKKVLTLSAVMASMLFGAAAHAADTRIGVTIYKYDDNFMSVVRKAIEQDAKAAPDVQLL
MNDSQNDQSKQNDQIDVLLAKGVKALAINLVDPAAAGTVIEKARGQNVPVVFFNKEPSRK
ALDSYDKAYYVGTDSKESGIIQGDLIAKHWAANQGWDLNKDGQIQFVLLKGEPGHPDAEA
RTTYVIKELNDKGIKTEQLQLDTAMWDTAQAKDKMDAWLSGPNANKIEVVIANNDAMAMG
AVEALKAHNKSSIPVFGVDALPEALALVKSGALAGTVLNDANNQAKATFDLAKNLADGKG
AADGTNWKIDNKVVRVPYVGVDKDNLAEFSKK
>LFGLNPFC_01043 Galactose/methyl galactoside import ATP-binding protein MglA
MVSSTTPSSGEYLLEMSGINKSFPGVKALDNVNLKVRPHSIHALMGENGAGKSTLLKCLF
GIYQKDSGTILFQGKEIDFHSAKEALENGISMVHQELNLVLQRSVMDNMWLGRYPTKGMF

VDQDKMYRETKAIFDELDIDIDPRAPVGTLSVSQMQMIEIAKAFSYNAKIVIMDEPTSSL
TEKEVNHLFTIIRKLKERGCGIVYISHKMEEIFQLCDEVTVLRDGQWIATEPLAGLTMDK
IIAMMVGRSLNQRFPDKENKPGEVILEVRNLTSLRQPSIRDVSFDLHKGEILGIAGLVGA
KRTDIVETLFGIREKSAGTITLHGKKINNHNANEAINHGFALVTEERRSTGIYAYLDIGF
NSLISNIRNYKNKVGLLDNSRMKSDTQWVIDSMRVKTPGHRTQIGSLSGGNQQKVIIGRW
LLTQPEILMLDEPTRGIDVGAKFEIYQLIAELAKKGKGIIIISSEMPELLGITDRILVMS
NGLVSGIVDTKTTTQSEILRLASLHL
>LFGLNPFC_01044 Galactoside transport system permease protein MglC
MSALNKKSFLTYLKEGGIYVVLLVLLAIIIFQDPTFLSLLNLSNILTQSSVRIIIALGVA
GLIVTQGTDLSAGRQVGLAAVVAATLLQSMDNANKVFPEMATMPIALVILIVCAIGAVIG
LINGLIIAYLNVTPFITTLGTMIIVYGINSLYYDFVGASPISGFDSGFSTFAQGFVALGS
FRLSYITFYALIAVVFVWVLWNKTRFGKNIFAIGGNPEAAKVSGVNVGLNLLMIYALSGV
FYAFGGMLEAGRIGSATNNLGFMYELDAIAACVVGGVSFSGGVGTVIGVVTGVIIFTVIN
YGLTYIGVNPYWQYIIKGAIIIFAVALDSLKYARKK
>LFGLNPFC_01045 NAD-dependent dihydropyrimidine dehydrogenase subunit PreA
MLTKDLSITFCGVKFPNPFCLSSSPVGNCYEMCAKAYDTGWGGVVFKTIGFFIANEVSPR
FDHLVKEDTGFIGFKNMEQIAEHPLEENLAALRRLKEDYPDKVLIASIMGENEQQWEELA
RLVQEAGADMIECNFSCPQMTSHAMGSDVGQSPELVEKYCRAVKRGSTLPMLAKMTPNIG
DMCEVALAAKRGGADGIAAINTVKSITNIDLNQKIGMPIVNGKSSISGYSGKAVKPIALR
FIQQMRTHPELRDFPISGIGGIETWEDAAEFLLLGAATLQVTTGIMQYGYRIVEDMASGL
SHYLADQGFDSLQEMVGLANHNIVPAEDLDRSYIVYPHINLDKCVGCGRCYISCYDGGHQ
AMEWSEKTRTPHCNTEKCVGCLLCGHVCPVGCIDLGEVKFKKGEKEHPVTL
>LFGLNPFC_01046 NAD-dependent dihydropyrimidine dehydrogenase subunit PreT
MPQQNYLDELTPAFTPLLAIKEASRCLLCHDAPCSQACPAQTDPGKFIRSIYFRNFKGAA
ETIRENNALGAVCARVCPTEKLCQSGCTRAGVDAPIDIGRLQRFVTDFEQQTGMEIYQPG
TKTLGKVAIIGAGPAGLQASVTLTNQGYDVTIYEKEAQPGGWLRNGIPQFRLPQSVLDAE
IARIEKMGVTIKCNNEIGKTLTLEQLKAENRAVLVTVGLSSGSGLSLFEHSDVEIAVDFL
QRARQAQGDISIPQSALIIGGGDVAMDVASTLKVLGCQAVTCVAREELDEFPASEKEFTS
ARELGVSIIDGFTPVAVEGNKVTFKHVRLPGELTIAADKIILAVGQHAELAAFSALEPQR
NTIETQHYQTRDPQVFAAGDIVEGDKTVVYAVKTGKEAAGAIHHYLEGACSC
>LFGLNPFC_01047 hypothetical protein
MDVQQFFVVAVFFLIPIFCFREAWKGWRAGAIDKRVKNAPEPVYVWRAKNPGLFFAYMVA
YIGFGILSIGMIVYLIFYR
>LFGLNPFC_01048 hypothetical protein
MLKRVFLSLLVLIGLLLLTVLGLDRWMSWKTAPYIYDELQDLPYRQVGVVLGTAKYYRTG
VINQYYRYRIQGAINAYNSGKVNYLLLSGDNALQSYNEPMTMRKDLIAAGVDPSDIVLDY
AGFRTLDSIVRTRKVFDTNDFIIITQRFHCERALFIALHMGIQAQCYAVPSPKDMLSVRI
REFAARFGALADLYIFKREPRFLGPLVPIPAMHQVPEDAQGYPAVTPEQLLELQKKQGK
>LFGLNPFC_01049 Cytidine deaminase
MHPRFQTAFAQLADNLQSALEPILADKYFPALLTGEQVSSLKSATGLDEDALAFALLPLA
AACARTPLSNFNVGAIARGVSGTWYFGANMEFIGATMQQTVHAEQSAISHAWLSGEKALA
AITVNYTPCGHCRQFMNELNSGLDLRIHLPGREAHALRDYLPDAFGPKDLEIKTLLMDEQ
DHGYALTGDALSQAAIAAANRSHMPYSKSPSGVALECKDGRIFSGSYAENAAFNPTLPPL
QGALILLNLKGYDYPDIQRAVLAEKADAPLIQWDATSATLKALGCHNIDRVLLA
>LFGLNPFC_01050 Inner membrane protein YohK
MMANIWWSLPLTLIVFFAARKLAARYKFPLLNPLLVAMVVIIPFLMLTGISYDSYFKGSE
VLNDLLQPAVVALAYPLYEQLHQIRARWKSIITICFIGSVVAMVTGTSVALLMGASPEIA
ASILPKSVTTPIAMAVGGSIGGIPAISAVCVIFVGILGAVFGHTLLNAMRIRTKAARGLA
MGTASHALGTARCAELDYQEGAFSSLALVLCGIITSLIAPFLFPIILAVMG
>LFGLNPFC_01051 hypothetical protein
MSKTLNIIWQYLRAFVLIYACLYAGIFIASLLPVTIPGSIIGMLILFVLLALQILPAKWV
NPGCYVLIRYMALLFVPIGVGVMQYFDLLRAQFGPVVVSCAVSTLVVFLVVSWSSQLVHG
ERKVVGQKGSEE
>LFGLNPFC_01052 tRNA-dihydrouridine(16) synthase
MRVLLAPMEGVLDSLVRELLTEVNDYDLCITEFVRVVDQLLPVKVFHRICPELQNASRTP
SGTLVRVQLLGQFPQWLAENAARAVELGSWGVDLNCGCPSKTVNGSGGGATLLKDPELIY
QGAKAMREAVPAHLPVSVKVRLGWDSGEKKFEIADAVQQAGATELVVHGRTKEQGYRAEH
IDWQAIGEIRQRLNIPVIANGEIWDWQSAQQCMAISGCDAVMIGRGALNIPNLSRVVKYN
EPRMPWPEVVALLQKYTRLEKQGDTGLYHVARIKQWLSYLRKEYDEATELFQHVRVLNNS
PDIARAIQAIDIDKLR
>LFGLNPFC_01053 Toluene efflux pump outer membrane protein TtgI
MNRDSFYPAIACFPLLLMLAGCAPMHETRQVLSQQTPAAQVDTVLPTALKNGWPDSQWWL
EYHDNQLTSLINNALQSAPDMQVAEQRIQLAEAQAKAIATQDGPQLDFSADMERQKMSAE
GLMGPFALNYPAAGTTGPWYTNGTFGLTAGWHLDIWGKNRAEVTARLGTVKARAAEREQT

RQLLAGSVARLYWEWQTQAALNTVLQQIEKEQNTIIATDRQLYQNGITSSVEGVETDINA
SKTRQQLNDVAGKMKIIEARLNALTNHQTKSLKLKPVALPKVASQLPDELGYSLLARRAD
LQAAHWYVESSLSTIDAAKAAFYPDINLMAFLQQDALHLSDLFRHSAQQMGVTAGLTLPI
FDSGRLNANLDIAKAESNLSIASYNKAVVEAVNDVARAASQVQTLAEKNQHQAQIERDAL
RVVGLAQARFNAGLIAGSRVSEARIPALRERANGLLLQGQWLDASIQLTGALGGGYKR
>LFGLNPFC_01054 putative oxidoreductase YohF
MAQVAIITASDSGIGKECALLLAQQGFDIGITWHSDEEGAKDTARKVVSHGVRAEIVQLD
LGNLPEGAQALEKLIQRLWRIDVLVNNAGAMTKAPFLDMAFDEWRKIFTVDVDGAFLCSQ
IAARQMVKQGQGGRIINITSVHEHTPLPDASAYTAAKHALGGLTKAMALELVRHKILVNA
VAPGAIATPMNGMDGGDVKPDAEPSIPLRRFGTTHEIASLVAWLCSEGANYTTGQSLIVD
GGFMLANPQFKPE
>LFGLNPFC_01055 Inner membrane protein YohD
MDLNTLISQYGYAALVIGSLAEGETVTLLGGVAAHQGLLKFPLVVLSVALGGMIGDQVLY
LCGRRFGGKLLRRFSKHQDKIERAQKLIQRHPYLFVIGTRFMYGFRVIGPTLIGASQLPP
KIFLPLNILGAFAWSLIFTTIGYAGGQVIAPWLHNLDQHLKHWVWLILVVVLVVGVRWWL
KRRGKKKPDHQA
>LFGLNPFC_01056 Inner membrane protein YohC
MSHVWGLFSHPDREMQVINRENETISHHYTHHVLLMAAIPVICAFIGTTQIGWNFGDGTI
LKLSWFTGLALAVLFYGVMLAGVAVMGRVIWWMARNYPQRPSLAHCMVFAGYVATPLFLS
GLVALYPLVWLCALVGTVALFYTGYLLYLGIPSFLNINKEEGLSFSSSTLAIGVLVLEVL
LALTVILWGYGYRLF
>LFGLNPFC_01057 D-alanyl-D-alanine endopeptidase
MPKFRVSLFSLALMLAVPFAPQAVAKTVAATTASQPEIASGSAMIVDLNTNKVIYSNHPD
LVRPIASISKLMTAMVVLDARLPLDEKLKVDISQTPEMKGVYSRVRLNSEISRKDMLLLA
LMSSENRAAASLAHHYPGGYKAFIKAMNAKAKSLGMNNTRFVEPTGLSVHNVSTARDLTK
LLIASKQYPLIGQLSTTREDMATFSNPTYTLPFRNTNHLVYRDNWNIQLTKTGFTNAAGH
CLVMRTVINNKPVALVVMDAFGKYTHFADASRLRTWIETGKVMPVPAAALSYKKQKAAQM
AAAGQTAQND
>LFGLNPFC_01058 Quinone-dependent D-lactate dehydrogenase
MSSMTTTDNKAFLNELARLVGHSHLLTDPAKTARYRKGFRSGQGDALAVVFPGSLLELWR
VLKACVTADKIILMQAANTGLTEGSTPNGNDYDRDIVIISTLRLDKLHVLGKGEQVLAYP
GTTLYSLEKALKPLGREPHSVIGSSCIGASVIGGICNNSGGSLVQRGPAYTEMSLFARIN
EDGKLTLVNHLGIDLGETPEQILSKLDDDRIKDDDVRHDGRHAHDYDYVHRVRDIEADTP
ARYNADPDRLFESSGCAGKLAVFAVRLDTFEAEKNQQVFYIGTNQPEVLTEIRRHILANF
ENLPVAGEYMHRDIYDIAEKYGKDTFLMIDKLGTDKMPFFFNLKGRTDAMLEKVKFFRPH
FTDRAMQKFGHLFPSHLPPRMKNWRDKYEHHLLLKMAGDGVGEAKSWLVDYFKQAEGDFF
VCTPEEGSKAFLHRFAAAGAAIRYQAVHSDEVEDILALDIALRRNDTEWYEHLPPEIDSQ
LVHKLYYGHFMCYVFHQDYIVKKGVDVHVLKEQMLELLQQRGAQYPAEHNVGHLYKAPET
LQKFYRENDPTNSMNPGIGKTSKRKNWQEVK
>LFGLNPFC_01059 Beta-xylosidase
MSLALQPALADDLFGNHPLTPEARDAFVTELLKKMTVDEKIGQLRLISVGPDNPKEAIRE
MIKDGQVGAIFNTVTRQDIRAMQDQVMELSRLKIPLFFAYDVLHGQRTVFPISLGLASSF
NLDAVKTVGRVSAYEAADDGLNMTWAPMVDVSRDPRWGRASEGFGEDTYLTSIMGKTMVE
AMQGKSPADRYSVMTSVKHFAAYGAVEGGKEYNTVDMSPQRLFNDYMPPYKAGLDAGSGA
VMVALNSLNGTPATSDSWLLKDVLRDQWGFKGITVSDHGAIKELIKHGTAADPEDAVRVA
LKSGINMSMSDEYYSKYLPGLIKSGKVTMEELDDAARHVLNVKYDMGLFNDPYSHLGPKE
SDPVDTNAESRLHRKEAREVARESLVLLKNRLETLPLKKSATIAVVGPLADSKRDVMGSW
SAAGVADQSVTVLTGIKNAVGENGKVLYAKGANVTSDKGIIDFLNQYEEAVKVDPRSPQE
MLDEAVQTAKQSDVVVAVVGEAQGMAHEASSRTDITIPQSQRDLIAALKATGKPLVLVLM
NGRPLALVKEDQQADAILETWFAGTEGGNAIADVLFGDYNPSGKLPMSFPRSVGQIPVYY
SHLNTGRPYNADKPNKYTSRYFDEANGALYPFGYGLSYTTFTVSDVKLSAPTMKRDGKVT
ASVQVTNTGKREGATVVQMYLQDVTASMSRPVKQLKGFEKITLKPGETQTVSFPIDIEAL
KFWNQQMKYDAEPGKFNVFIGTDSARVKKGEFELL
>LFGLNPFC_01060 Glycine betaine-binding protein YehZ
MPLSKVWAGSLVLLAAVSLPLQAASPVKVGSKIDTEGALLGNIILQVLESHGVPTVNKVQ
LGTTPVVRGAITSGELDIYPEYTGNGAFFFKDENDTAWKNAQQGYEKVKKLDAEQNKLIW
LTPAPANNTWTIAVRQDVAEKNKLTSLAELSRYLKEGGNFKLAASAEFIERADALPAFEK
AYGFKLGQDQLLSLAGGDTAVTIKAAAQQTSGVNAAMAYGTDGPVAALGLQTLSDPQGVQ
PIYAPAPVVRESVLKEYPQIAQWLQPVFASLDEKTLQQLNASIAVEGLDAKKVAADYLKQ
KGWTK
>LFGLNPFC_01061 Glycine betaine uptake system permease protein YehY
MTYFRINPVLALLLLLTAIAAALPFISYAPNRLVSGEGRHLWQLWPQTLWMLVGVGCAWL
TACFIPAKKGSIFALILAQFVFVLLVWGAGKAATQLAQNGSALARTSLGSGFWLAAALAL
LACSDAIRRISTHPLWRWLLHMQIAIIPLWLLYSGTLNDLSLMKEYANRQDVFDDALAQH

LTLLFGAVLPALVIGVPLGIWCYFSTARQGAIFSLLNVIQTVPSIALFGLLIAPLAALVT
AFPWLGMLGIAGTGMTPALIALVLYALLPLVRGVVVGLNQIPRDVLESARAMGMSGVQRF
LHVQLPLALPVFLRSLRVVMVQTVGMAVIAALIGAGGFGALVFQGLLSSAIDLVLLGVIP
VIVLAVLTDALFDLLIALLKVKRND
>LFGLNPFC_01062 Glycine betaine uptake system ATP-binding protein YehX
MIEFSHVSKLFGAQKAVNDLNLNFQEGSFSVLIGTSGSGKSTTLKMINRLVEHDSGVIRF
AGEEIRSLPVLELRRRMGYAIQSIGLFPHWSVAQNIATVPQLQKWSRARIDDRIDELMAL
LGLEPNLRERYPHQLSGGQQQRVGVARALAADPQVLLMDEPFGALDPVTRGALQQEMTRI
HRLLGRTIVLVTHDIDEALRLAEHLVLMDHGEVVQQGNPLTMLTRPTNDFVRQFFGHSEL
GVRLLSLRSVADYVRREERAEGEALAEEMTLRDALSLFVARGCEVLPVVNTQGEPCGTLH
FQDLLEEA
>LFGLNPFC_01063 Glycine betaine uptake system permease protein YehW
MKMLRDPLFWLIALFVALIFWLPYSQPLFAALFPQLPRPVYQQESFAALALAHFWLVGIS
SLFAVIIGTGAGIAVTRPWGAGFRPLVETIAAVGQTFPPVAVLAIAVPVIGFGLKPAIIA
LILYGVLPVLQATLAGLGAIDASVTEVAKGMGMSRGQRLRKVELPLAAPVILAGVRTSVI
INIGTATIASTVGASTLGTPIIIGLSGFNTAYVIQGALLVALAAIIADRLFERLVQAFSQ
HAK
>LFGLNPFC_01064 hypothetical protein
MRIAKIGVIALFLFMALGGIGGVMLAGYTFILRAG
>LFGLNPFC_01065 HTH-type transcriptional regulator MlrA
MALYTIGEVALLCDINPVTLRAWQRRYGLLKPQRTDGGHRLFNDADIDRIREIKRWIDNG
VQVSKVKMLLSNENVDVQNGWRDQQETLLTYLQSGNLHSLRTWIKERGQDYPAQTLTTHL
FIPLRRRLQCQQATLQALLAILDGVLINYISICLASARKKQGKDALVVGWNIQDTTRLWL
EGWIASQQGWRIDVLAHSLNQLRPELFEGRTLLVWCGDNRTSAQQQQLTSWQEQGHDIFP
LGI
>LFGLNPFC_01066 Sensor histidine kinase BtsS
MYDFNLVLLLLQQMCVFLVIAWLMSKTPLFIPLMQVTVRLPHKFLCYIVFSIFCIMGTWF
GLHIDDSIANTRAIGAVMGGLLGGPVVGGLVGLTGGLHRYSMGGMTALSCMISTIVEGLL
GGLVHSILIRRGRTDKVFNPITAGAVTFVAEMVQMLIILAIARPYEDAVRLVSNIAAPMM
VTNTVGAALFMRILLDKRAMFEKYTSAFSATALKVAASTEGILRQGFNEVNSMKVAQVLY
QELDIGAVAITDREKLLAFTGIGDDHHLPGKPISSTYTLKAIETGEVVYADGNEVPYRCS
LHPQCKLGSTLVIPLRGENQRVMGTIKLYEAKNRLFSSINRTLGEGIAQLLSAQILAGQY
ERQKAMLTQSEIKLLHAQVNPHFLFNALNTIKAVIRRDSEQASQLVQYLSTFFRKNLKRP
SEFVTLADEIEHVNAYLQIEKARFQSRLQVNIAIPQELSQQQLPAFTLQPIVENAIKHGT
SQLLDTGRVAISARREGQHLMLEIEDNAGLYQPVTNASGLGMNLVDKRLRERFGDDYGIS
VACEPDSYTRITLRLPWRDEA
>LFGLNPFC_01067 Transcriptional regulatory protein BtsR
MIKVLIVDDEPLARENLRVFLQEQSDIEIVGECSNAVEGIGAVHKLRPDVLFLDIQMPRI
SGLEMVGMLDPEHRPYIVFLTAFDEYAIKAFEEHAFDYLLKPIDEARLEKTLARLRQERS
KQDVSLLPENQQALKFIPCTGHSRIYLLQMKDVAFVSSRMSGVYVTSHEGKEGFTELTLR
TLESRTPLLRCHRQYLVNLAHLQEIRLEDNGQAELILRNGLTVPVSRRYLKSLKEAIGL
>LFGLNPFC_01068 hypothetical protein
MLSNDILRSVRYILKANNNDLVRILALGNVEATAEQIAVWLRKEDEEGFQRCPDIVLSSF
LNGLIYEKRGKDESAPALEPERRINNNIVLKKLRIAFSLKTDDILAILTEQQFRVSMPEI
TAMMRAPDHKNFRECGDQFLRYFLRGLAARQHVKKS
>LFGLNPFC_01069 putative lipoprotein YehR
MKAFNKLFSLVVASVLVFSLAGCGDKEESKKFSANLNGTEIAITYVYKGDKVLKQSSETK
IQFASIGATTKEDAARTLEPLSAKYKNIAGVEEKLTYTDTYAQENVTIDMEKVDFKALQG
ISGINVSAEDAKKGITMAQMELVMKAAGFKEVK
>LFGLNPFC_01070 hypothetical protein
MNSLRPELLELTPQALTALSNAGFVKRSLKELENGNVPEISHENGALIATFSDGVRTQLA
NGQALKEAQCTCGASGMCRHRVMLVLSYQRLCATAQPTEKKEEEWDPAIWLKELATLPDA
TRKRAQALVAKGITIELFCAPGEIPSARLPMSDVRFYSRSSIRFARCDCIEGTLCEHVVL
AVQAFVEAKTQQAEFTHLIWQMRSEHVTSSDDPFASEEGKTCRQYVQQLSQALWLGGISQ
PPIHYEAAFSRAQQAAERCNWRWVSESLRQLRASVDAFHARASHYHAGECLRQLAALNSR
LNCVQEMARRDSIGEVPPMPWRTVVGAGIAGEAKLDHLRLVSLGMRCWQDIEQYGLRIWF
TDPDTGSILHLSRSWQRSEQENSPAATRRLFSFQAGALAGGQIVSQAAKRSADGELLFAT
RNRLSGVVPLSPDAWLMLSAPLRQPGIVALREYLRQRPPACIRPINQVDNLFILPIAECI
SLGWDSSRQTLDAQVISGEGEDNVLTLSLPASACSPFAVERMAALLQQTDDPVSLVSGFV
SFVEGQLTLEPRVMMTKTRAWALDAETAPVAPLPSASVLPVPSTAHQLLMRCQALLIQLL
HNGWRYQEQSAIGQAELLANDLTAVGFYRLAHVLGQFRNTESEARVEAMNNGVLLCEQLF
PMLQQQG
>LFGLNPFC_01071 hypothetical protein
MSELNDLLTTRELQRWRLILGEAAETTLCGLDDNARQIDHALEWLYGRDPERLQRGERYG

GLGRSNLTTPEWINSIHTLFPQQVIERLESDAVLRYGIEDVVTNLDVLERMQPSESLLRA
VLHTKHLMNPEVLAAARRIVHQVVEEIMARLAKEVRQAFSGVRDRRRRSFISLARNFDFK
STLRANLQHWHPQHGKLYIESPRFNSRIKRHSEQWQLVLLVDQSGSMVDSVIHSAVMAAC
LWQLPGIRTHLVAFDTSVVDLTADVADPVELLMKVQLGGGTNIASAVEYGRQLIEQPAKS
VIILVSDFYEGGSSSLLTHQVKKCVQSGIKVLGLAALDSNATPCYDHDTAQALVNVGAQI
AAMTPGELASWLAENLQS
>LFGLNPFC_01072 hypothetical protein
MYERAVNCRHHSPACARLAKSLIESQRPRYVLIEGPADFNDRIDELFLAHQLPVAIYSYC
QYQDGAAPGRGAWTPFAEFSPEWQALQAARRIQAQTYFIDLPCWAQSEEEDDSPDTQDES
QTLLLRDTRMDNSDTLWDHLFEDESQQTALPSALARYFAQLRGDSPGDALNRQREAFMAR
WITWAMQQNNGDVLVVCGGWHAPALANMWRECPQEINKPELSSLADAVTGCYLTPYSEKR
LDVLAGYLSGMPAPVWQNWCWQCGLQQAGEQLLKTVLTRLRQHKLPASTADMAAAHLHAM
ALAQLRGHTLPLRTDWLDAIAGSLIKEALNAPLPWSYRGVIHPDTDPILVTLIDTLAGDG
FGKLAPSTPQPPLPKDVTCELERTGISLPAELTLNRFTPDGLAQSQVLHRLAILEIPGVV
RQQGSTLSLAGNGEECWKLTRPLSQHAALIEAACFGATLQEAARHKLEADMLDAGGIGCI
TTCLSQAALAGLASFSQQLLEQLTLLIAQENQFAEMGQALEVLYALWRLDEISGMQGAQI
LQMTLCAAIDRTLWLCESNGRPDEKEFHTHLHSWQALCHILRDLHSGVNLPGVSLSAAVA
LLERRSQAIHAPALDRGATLGALMRLEHPNASAEAALTMLAQLSPAQSGEALHGLLALAR
HQLACQPVFIAGFSSHLNQLSDADFINALPDLRAAMAWLPPRERGTLAHQVLEHYQLVQL
PVSALQMPLHCPPQDIAHHQQLEQQALASLQHWGVFHV
>LFGLNPFC_01073 putative protein YehL
MSPQNNHLQRPPAAVLYAVELAKLKQNDNAPCPPGWQLSLPAARAFILGDEAQNISRKVV
ISPSAVERMLVTLATGRGLMLVGEPGTAKSLLSELLATAISGDAGLTIQGGASTTEDQIK
YGWNYALLINHGPSTEALVPAPLYQGMRDGKIVRFEEITRTPLEVQDCLLGMLSDRVMTV
PELTGEASQLYAREGFNIIATANTRDRGVNEMSAALKRRFDFETVFPIMDFAQELELVAS
ASARLLAHSGIPHKVPDAVLELLVRTFRDLRTNGEKKTSMDTLTAIMSTAEAVNVAHAVG
VRAWFLANRAGEPADLVDCIAGTIVKDNEEDRARLRRYFEQRVATHKEAHWQAYYQARHR
LP
>LFGLNPFC_01074 hypothetical protein
MDTLTQKLTVLIAVLELLVALLRLIDLLK
>LFGLNPFC_01075 hypothetical protein
MIVQKELVAIYDYEIPVPEEPFSFRLEIHKCSELFTGSVYRLERFRLHPTFHQRDREDAD
H
>LFGLNPFC_01076 hypothetical protein
MDKELPWLADNAQLELKYKKGKTPLSHRNWPGEPVPVITESIIQTLSDELLQKAEKKKNI
VWRYENFSLEWQSAITQAINLIGEHKPSITARTMAALACIAQNDSQQLLDEIVQQEGLEY
ATEVVIARQFIVRCYESDPLVVTLQYQNEDYGYGYRSETYNEFDLRLRKHLSLAEETCWQ
RCADKLIAALPGITKVRRPFIALNLPEKPEIANELVSLECSQTHFRSKEWLKVVADDPKA
VKELARYWSQDIFSDREASYMSHENHFGYAACAALLREQGLAAVPRLAMYAHKEDCGSLL
VQINHPQVIRTLLLVADKNKPSLQRVAKYSKNFPHATLAALAELLALKAPPARPGYPIIE
DKKLPAQQKARDEYWHTLLQTLMASQPQLAEEVMPCLSTQAQAVVNGYLSASPKLAFEST
HSNDLPEILVSPPWRGKKKTALLRLDLVPLELAPKARWQPGERERLAATESARYFSTGSF
TERMERKSGRVVLQELGFGDDVWLFRNYILPGKLDAARKSLVGGQWHYSPRRVEEINNGWH
STEAKSAEQALRSGDVEALINTWENDSYSRYRPEKSVWNLYLLAQLPREMALTFWLRINE
KKHLFAGEDYFLSILGLDTLPGLLLAFSHHPKETFPLILNFGATELALPVARVWRRFAAQ
RDLARQWILQWPEHTATALIPLVFTKPCDNSEAALLALRLLYEQGHGELLQTVANRWQRT
DVWPALEQLLKLGPIEIYPARIPKAPDFWHPAMWSRPRLIKNNQPVTDDALEIIGEMLRF
TQGGRFYGGLEQLKTFCQPQTLAAFAWDLFTAWQQAGAPAKDNWAFLALSLFGDESTARD
LTTQILAWPQEGKSARAVSGLNILTKMNNDMALIQLHHISQRAKSRPLRDNAAEFLQVVA
ENRGLSQEELADRLVPTLGLDDPQALIFDFGPRQFTVRFDENLNPVIFDQQNVRQKSVPR
LRADDDQLKTPEALARLKGLKKDATQVSKNLLPRLETALRTTRRWSLADFHSLFVNHPFT
RLVTQRLIWGVYPANEPRRLLNAFRVAAEGEFCNEQDEPIDLPADALIGIAHPLEMTAEM
RSEFAQLFADYEIMPPFRQLTRRTVLLTPDESASNSLNRWEGKSATVGQLMGMRYKGWES
GYEDAFVYDLGAYRLVLKFSPGFNHYSTDSKALMSFRSLRVYRDNKSVTFAELDVFDLSE
ALSAPDVIFH
>LFGLNPFC_01077 hypothetical protein
MRHFIYQDEKSHKFWAVEQQGNELHISWGKVGTQEQSQVKSFADAVAAEKAELKLIAEKV
KKGYVEQAKDNSLQPSQTVTDSLKVADLSTIIQEQPSFVAETRAADKNTDAVLPWLAKDI
AVVFPPEVVHTTLSHRRFPGVPVQQADKLTQLRRLACSVSQRDNTTATFDFSACSLEWQN
TVAQAISQIDDLKTTQLPSPVMAVLTALEMKCTRYKEREDVMDQIIQEGGLEYATDVIIH
LQQISIEWDYVNNNIVFLSSGISPDYLQQYSSFELRLRKHLSLAEESLWQKCAQKLIAAV
PHIPEWRQPLIALLLPEKPEIAHEIAQRLLGQKKLPSLEWLKIVATDEHILASLEKYHEP
YAIFDDYYCGAIWSATVLQEQGVAALPRFAPYVASDYCADVLRHINHPFALTLLIRVAGH
TKRCHDRMTKACAAFPHAALAALAELLVQKEENSWRIMLMTMLISQPTLAEQVIPWLSTP

AVAVLKSCQQQLTQPSNHASADLLPAIVVSPPWLSKKKKSPIPVLDLAPLNLESICTITD
TEAKEFQTHWDWEPHKPGEGAKNFLYSLGYRRWDFDTYKYIGASDSAIDAWEREDFATLI
QMFKAHHAPYQGEWHLNSLPFLPMQKAIKLWEFLSKEPHTAIKPVMLYLRLAGMSGFLHS
FSRYPQEGFAVANYFAATELAPAVARAFNKLKTLRQDASSWLLKYPEHAITGLLPAALGK
ASEAQDNARAALRMLTENGHQPLLQEIARRYNQPEVTDAVNALLALDPLDNHPTKIPTLP
TFYQPSLWTRPLLKANAQSLPDSALLHLGEMLRFPQEEALYPGLLQVKDACTTDSLAEFA
WDLFTAWQTAGAPSKESWAFTALGVLGNDDTARKLTPLIRAWPGESQHKRATVGLDILAA
IGSDIALMQLNGIAQKLKFKALQERAKEKIADIAESRELTVAELEDRLAPDLGLDDNGSL
LLDFGPRQFTVSFDETLKPFVRDASGSRLKDLPKPNKSDDESQANDAVNRYKLLKKDART
VAAQQVARLESAMCLRRRWSPENFQLFLVEHPLVRHLTHRLIWGVYSAENQLLACFRVAE
DNSYSTADDDLFTLPEGDISIGIPHVLEISPTDAAAFGQLFADYELLPPFRQLDRNSYAL
TEAERNASELTRWTGRKCPSGRVMGLANKGWIKGTPQDAGWIGWMINPLGRWSLIMEIDE
GFAVGMSPAELSAEQLLSKLWLWEGKAESYGWGSNSTQEAQFSVLDAITASELINDIEAL
FE
>LFGLNPFC_01078 Methionine--tRNA ligase
MTQVAKKILVTCALPYANGSIHLGHMLEHIQADVWVRYQRMRGHEVNFICADDAHGTPIM
LKAQQLGITPEQMIGEMSQEHQTDFAGFNISYDNYHSTHSEENRQLSELIYSRLKENGFI
KNRTISQLYDPEKGMFLPDRFVKGTCPKCKSPDQYGDNCEVCGATYSPTELIEPKSVVSG
ATPVMRDSEHFFFDLPSFSEMLQAWTRSGALQEQVANKMQEWFESGLQQWDISRDAPYFG
FEIPNAPGKYFYVWLDAPIGYMGSFKNLCDKRGDSVSFDKYWKKDSTAELYHFIGKDIVY
FHSLFWPAMLEGSNFRKPTNLFVHGYVTVNGAKMSKSRGTFIKASTWLNHFDADSLRYYY
TAKLSSRIDDIDLNLEDFVQRVNADIVNKVVNLASRNAGFINKRFDGVLASELADPQLYK
TFTDAAEVIGEAWESREFGKAIREIMALADLANRYVDEQAPWVVAKQEGRDADLQAICSM
GINLFRVLMTYLKPVLPKLTERAEAFLNTELTWDGIQQPLLGHKVNPFKALYNRIDMKQV
EALVEASKEEVKATAAPVTGPLADDPIQETITFDDFAKVDLRVALIENAEFVEGSDKLLR
LTLDLGGEKRNVFSGIRSAYPDPQALIGRHTIMVANLAPRKMRFGISEGMVMAAGPGGKD
IFLLSPDAGAKPGHQVK
>LFGLNPFC_01079 Iron-sulfur cluster carrier protein
MNEQSQAKSPEALRAMVAGTLANFQHPTLKHNLTTLKALHHVAWMDDTLHVELVMPFVWH
SAFEELKEQCSAELLRITGAKAIDWKLSHNIATLKRVKNQPGINGVKNIIAVSSGKGGVG
KSSTAVNLALALAAEGAKVGILDADIYGPSIPTMLGAENQRPTSPDGTHMAPIMSHGLAT
NSIGYLVTDDNAMVWRGPMASKALMQMLQETLWPDLDYLVLDMPPGTGDIQLTLAQNIPV
TGAVVVTTPQDIALIDAKKGIVMFEKVEVPVLGIVENMSVHICSNCGHHEPIFGTGGAEK
LAEKYHTQLLGQMPLHISLREDLDKGTPTVISRPESEFTAIYRQLADRVAAQLYWQGEVI
PGEISFRAV
>LFGLNPFC_01080 hypothetical protein
MNKYWLSGIIFLAYGLASPAFSSETATLTINGRISPPTCSMAMVNGQPQQHCGQLTYNVD
TRHQVSSPVKGVTTEVIAADSDSKRRIVLNRYD
>LFGLNPFC_01081 putative fimbrial-like protein YehD
MKRSIIAAAVFSSFFMSAGVFAADVDTGTLTIKGNIAESPCKFEAGGDSLSINMPTVPTT
VFEGKAKYSTYDGAVGVTSSMLKISCPKEVAGVKLSLITNDKITGNDKAIASSNDTVGYY
LYLGDNSDVLDVSAPFNIESYKTADGQYAIPFKAKYLKLTDNSVNSGDVLSSLIMRVAQD
>LFGLNPFC_01082 putative fimbrial chaperone YehC
MKGLLALLISSMVLPAHAGIVIYGTRIIYPAEHKEVMVQLMNQGNRSSLMQAWIDDGDTS
LPPEKIQVPFMLTPPVAKIGANSGQQVKIKIMPNKLPTNKESIFYLNVLDIPPNSPEQEG
KNALKFAMQNRIKLFYRPAGIAPVNKVTFKKLLVNRSGNGLVIKNDSANWVTISDVKANN
VKVNYETIMIAPLESQSVNVKSNNANNWHLTIIDDHGNYISDKI
>LFGLNPFC_01083 Outer membrane usher protein YehB
MLRMPPLASAIVALLIGIEAYAAEETFDTHFMIGGMKDQQVSNIRLEDNQPLPGQYDIDI
YVNKQWRGKYEIIVKDNPQETCLSREMIKRLGINTDSFASGKQCLTFKQLIQGGSYTWDI
GVFRLDFSVPQAWVEELESGYVPPENWERGINAFYTSYYMSQYYSDYKASGNSKSTYVRF
NSGLNLLGWQLHSDASFSKTNNNPGVWKSNTLYLERGFAQLLGTLRVGDMYTSSDIFDSV
RFSGVRLFRDMQMLPNSKQNFTPRVQGIAQSNALVTIEQNGFVVYQKEVPPGPFAITDLQ
LAGGGADLDVSVKEADGSVTTYLVPYAAVPNMLQPGVSKYDFAAGRSHIEGASKQSDFVQ
VGHQYGFNNLLTLYGGSMVANNYYAFTLGTGWNTRIGAISVDATKSHSKQDNGDVFDGQS
YQIAYNKFVSQTSTRFGLAAWRYSSRDYRTFNDHVWANNKDNYRRDENDIYDIADYYQND
FGRKNSFSANMSQSLPEGWGSVSLSTLWRDYWGRSGSSKDYQLSYSNNLRRISYTLAASH
AYDENHHEEKRFNIFISIPFDWGDDVTTPRRQIYMSNSTTFDDQGVASNNTGLSGTVGSR
DQFNYGVNLSYQYQGNETTAGANLTWNAPVATVNGSYSQSSAYRQAGASVSGGIVAWSGG
VNLANRLSETFAVMNAPGIKDAYVNGQKYRTTNRNGVVVYDGMTPYRENYLMLDVSQSDS
EAELRGNRKIAAPYRGAVVLVNFDTDQRKPWFIKALRADGQPLTFGYEVNDIHGHNIGVV
GQGSQLFIRTNEVPPSVNVAIDKQQGLSCTITFGKEIDESRNYICQ
>LFGLNPFC_01084 putative fimbrial-like protein YehA
MEIRIMLFIVIMMVMPVSYAACYSELSVQHNLVVQGDFALTQTQMATYEHNFNDSSCVST

NTITPMSPSDIIVGLYNDTIKLNLHFEWTNKNNITLSNNQTSFTSGYSVTVTPAASNAKV
NVSAGGGGSVMINGVATLSSASSSTRGSAAVQFLLCLLGGKSWDACVNSYRNALAQNAGV
YSFNLTLSYNPITTTCKPDDLLITLESIPVSQLPATGNKTTINSKKGDIILRCKNLLGQQ
NQTSRKMQVYLSSSDLLTNSNTILKGAEDNGVGFILESNGTPVTLLNITNSSKGYTNLKE
IAAKSKLTDTTVSIPITASYYVYDTNKVKSGALEATALINVKYD
>LFGLNPFC_01085 Nickel/cobalt homeostasis protein RcnB
MTIKNKMLLGALLLVTSAAWAAPATAGSTNTSGISKYELSSFIADFKHFKPGDTVPEMYR
TDEYNIKQWQLRNLPAPDAGTHWTYMGGAYVLISDTDGKIIKAYDGEIFYHR
>LFGLNPFC_01086 Nickel/cobalt efflux system RcnA
MTEFTTLLQQGNAWFFIPSAILLGALHGLEPGHSKTMMAAFIIAIKGTIKQAVMLGLAAT
ISHTAVVWLIAFGGMVISKRFTAQSAEPWLQLISAVIIISTAFWMFWRTWRGERNWLENM
HEHDHEHHHHDHEDHHDHGHHHHHEHGEYQDAHARAHANDIKRRFDGREVTNWQILLFGL
TGGLIPCPAAITVLLICIQLKALTLGATLVVSFSLGLALTLVTVGVGAAISVQQVAKRWS
GFNTLAKRAPYFSSLLIGLVGVYMGVHGFMGIMR
>LFGLNPFC_01087 Transcriptional repressor RcnR
MSHTIRDKQKLKARASKIQGQVVALKKMLDEPHECAAVLQQIAAIRGAVNGLMREVIKGH
LTEHIVHQGDELKREEDLDVVLKVLDSYIK
>LFGLNPFC_01088 hypothetical protein
MSSARVVNGEKRQFYNRVNKLFDIRIQHLQNDIQIFFSF
>LFGLNPFC_01089 Hydroxyethylthiazole kinase
MQVDLLSSAQSAHALHLFHQHSPLVHCMTNDVVQTFTANTLLALGASPAMVIETEEASQF
AAIASALLINVGTLTQPRAQAMSAAVEQATRSQTPWTLDPVAVGALDYRRRFCVELLSHK
PTAIRGNASEIMALAGVANGGRGVDTTDAAANAIPAAQTLARETGAIVVVTGEVDYVTDG
HRIIGIHGGDPLMTKVVGTGCALSAVVAACCALPGDTLENIASACHWMKQAGERAVARSE
GPGSFVPHFLDALWQLTQEVQA
>LFGLNPFC_01090 Hydroxymethylpyrimidine/phosphomethylpyrimidine kinase
MKRINALTIAGTDPSGGAGIQADLKTFSALGAYGCSVITALVAQNTRGVQSVYRIEPDFV
AAQLDSVFSDVRIDTTKIGMLAETDIVEAVAERLQRYQIQNVVLDTVMLAKSGDPLLSPS
AVATLRSRLLPHVSLITPNLPEAAALLDAPHARTEQEMLEQGRSLLAMGCGAVLMKGGHL
DDEQSPDWLFTREGEQRFTAPRIMTKNTHGTGCTLSAALAALRPRHTNWADTVQEAKSWL
SSALAQADTLEVGHGIGPVHHFHAWW
>LFGLNPFC_01091 hypothetical protein
MQLRITSRKKFTVLLCALGLISIVAIYPRQTVNFFYSTAIQIKDYIHFYGYRPVKSFAIR
IPASYTIHGIDVSRWQERIDWQRVAKMRDNGIRLQFAFIKATEGEKLVDPYFSRNWQLSR
ENGLLRGAYHYFSPSVSASVQARLFLQTVDFSQGDFPAVLDVEERGKLSAKELRKRVSQW
LKMVEKRTGKKPIIYSGAVFYHTNLAGYFNEYPWWVAHYYQRRPDNDGMAWRFWQHSDRG
QVDGINGPVDFNVFNGTVEELQAFVDGIKETP
>LFGLNPFC_01092 HTH-type transcriptional repressor NagR
MEQAHTQLIAQLNERILAADNTPLYIKFAETVKNAVRSGVLEHGNILPGERDLSQLTGVS
RITVRKAMQSLEEEGVVTRSRGYGTQINNIFEYSLKEARGFSQQVVLRGKKPDTLWVNKR
VVKCPEEVAQQLAVEAGSDVFLLKRIRYVDEEAVSIEESWVPAHLIHDVDAIGISLYDYF
RSQHIYPQRTRSRVSARMPDAEFQSHIQLDSKIPVLVIKQVALDQQQRPIEYSISHCRSD
LYVFVCEE
>LFGLNPFC_01093 2-dehydro-3-deoxygluconokinase
MSGARLHTLLPELATRQPVMVVGAAVIDVIADAYALPWRGCDIELKQQSVNVGGCALNIA
VALKRLGIEAGNALPLGQGVWAEIIRNRMAKEDLISLIDNAEGDNGWCLALVEPDGERTF
MSFSGVENQWNRQWLARLTVAPGSLLYFSGYQLASPCGELLVEWLEKLQDVTPFIDFGPR
IGDIPDALLARIMACRPLVSLNRQEAEIAERFALSAEITTLGKQWQEKFAAPLIVRLDK
EGAWYFSNDASGCIPAFPTQVVDTIGAGDSHAGGVLAGLASGLPLADAVLLGNAVASWVV
GHRGGDCAPTREELLLAHKNV
>LFGLNPFC_01094 ADP-ribosylarginine hydrolase Tri1
MKTERILGALYGQALGDAMGMPSELWPRSRVKAHFGWIDRFLPGPKENNAACYFNRAEFT
DDTSMALCLADALLEREGKIDPDLIGRNILDWALRFDAFNKNVLGPTSKIALNAIRDGKP
VAELENNGVTNGAAMRISPLGCLLPARDVDSFIDDVALASSPTHKSDLAVAGAVVIAWAI
SRAIDGESWSAIVNSLPSIARHAQQKRITTFSASLAARLEIALKIVRNADGTESASEQLY
QVVGAGTSTIESVPCAIALVELAQTDPNRCAVLCANLGGDTDTIGAMATAICGALHGVNA
IDPALKAELDAVNQLDFNRYATALAKYRQQREAI
>LFGLNPFC_01095 Putative nucleoside transporter YegT
MKTTAKLSFMMFVEWFIWGAWFVPLWLWLSKSGFSAGEIGWSYACTAIAAILSPILVGSI
TDRFFSAQKVLAVLMFAGAVLMYFAAQQTTFAGFFPLLLAYSLTYMPTIALTNSIAFANV
PDVERDFPRIRVMGTIGWIASGLACGFLPQMLGYADISPTNIPLLITAGSSALLGVFAFF
LPDTPPKSTGKMDIKVMLGLDALILLRDKNFLVFFFCSFLFAMPLAFYYIFANGYLTEVG
MKNATGWMTLGQFSEIFFMLALPFFTKRFGIKKVLLLGLVTAAIRYGFFIYGSADEYFTY
ALLFLGILLHGVSYDFYYVTAYIYVDKKAPVHMRTAAQGLITLCCQGFGSLLGYRLGGVM

MEKMFAYQEPVNGLTFNWAGMWTFGAVMIAIIAVLFMIFFRESDNEITAIKVDDRDIALT
QGEVK
>LFGLNPFC_01096 Fructose-bisphosphate aldolase class 1
MTDIAQLLGKDADNLLQHRCMTIPSDQLYLPGHDYVDRVMIDNNRPPAVLRNMQTLYNTG
RLAGTGYLSILPVDQGVEHSAGASFAANPLYFDPKNIVELALEAGCNCVASTYGVLASVS
RRYAHRIPFLVKLNHNETLSYPNTYDQTLYASVEQAFNMGAVAVGATIYFGSEESRRQIE
EISAAFERAHELGMVTVLWAYLRNSAFKKDGVDYHVSADLTGQANHLAATIGADIVKQKM
AENNGGYKAINYGYTDDRVYSKLTSENPIDLVRYQLANCYMGRAGLINSGGAAGGETDLS
DAVRTAVINKRAGGMGLILGRKAFKKSMADGVKLINAVQDVYLDSKITIA
>LFGLNPFC_01097 D-tagatose-1,6-bisphosphate aldolase subunit GatY
MYVVSTKQMLNNARRGGYAVPAFNIHNLETMQVVVETAASMHAPVIIAGTPGTFTHAGTE
NLMALVSAMAKQYHHPLAIHLDHHTKFDDIAQKVRSGVRSVMIDASHLPFAQNISRVKEV
VDFCHRFDVSVEAELGQLGGQEDDVQVNEADAFYTNPVQAREFAEATGIDSLAVAIGTAH
GMYASAPALDFSRLENIRQWVNLPLVLHGASGLSTKDIQQTIKLGICKINVATELKNAFS
QALKNYLTEYPEATDPRDYLQSAKSAMRDVVSKVIADCGCEGRA
>LFGLNPFC_01098 D-tagatose-1,6-bisphosphate aldolase subunit GatZ
MKTLIARHKAGEHIGICSVCSAHPLVIEAALAFDRNSTRKVLIEATSNQVNQFGGYTGMT
PADFREFVFAIADKVGFARERIILGGDHLGPNCWQQENADAAMEKSVELVKAYVRAGFSK
IHLDASMSCADDSIPLAPETVAERAAVLCLAAESVATDCQREQLNYVIGTEVPVPGGEAS
AIQSVHITQVEDAANTLRTHQKAFIARGLAEALTRVIAIVVQPGVEFDHSNIIHYQAQEA
QALAQWIEKTKMVYEAHSTDYQTQTAYRELVRDHFAILKVGPALTFALREAIFALAQIEQ
ELIAPENRSRCLAVIEEVMLDEPQYWKKYYRTGFNDSLLGIRYSLSDRIRYYWPHSRIKN
SVETMMVNLEGVDIPLGMISQYLPKQFERIQSGELSAIPHQLIMDKIYDVLRAYRYGCAE
>LFGLNPFC_01099 PTS system galactitol-specific EIIA component
MTNLFVRSGISFVDRSEVLTHIGNEMLAKGVVHDTWPQALITREAEFPTGIMLQQHAIAI
PHCEAIHAKSSAIYLLRPTNKVHFQQADDDNDVAVSLVIALIVENPQQQLKLLRCLFGKL
QQPDIVETLITLPETQLKEYFTKYVLDSDE
>LFGLNPFC_01100 PTS system galactitol-specific EIIB component
MKRKIIVACGGAVATSTMAAEEIKELCQSHNIPVELIQCRVNEIETYMDGVHLICTTARV
DRSFGNIPLVHGMPFVSGVGIEALQNKILTILQG
>LFGLNPFC_01101 PTS system galactitol-specific EIIC component
MFSEVMRYILDLGPTVMLPIVIIIFSKILGMKAGDCFKAGLHIGIGFVGIGLVIGLMLDS
IGPAAKAMAENFDLNLHVVDVGWPGSSPMTWASQIALVAIPIAILVNVAMLLTRMTRVVN
VDIWNIWHMTFTGALLHLATGSWMIGMAGVVIHAAFVYKLGDWFARDTRNFFELEGIAIP
HGTSAYLGPIAVLVDAIIEKIPGVNRIKFSADDIQRKFGPFGEPVTVGFVMGLITGILAG
YDVKGVLQLAVKTAAVMLLMPRVIKPIMDGLTPIAKQARSRLQAKFGGQEFLIGLDPALL
LGHTAVVSASLIFIPLTILIAVCVPGNQVLPFGDLATIGFFVAMAVAVHRGNLFRTLISG
VIIMSITLWIATQTIGLHTQLAANAGALKAGGMVASMDQGGSPITWLLIQVFSPQNIPGF
IIIGAIYLTGIFMTWRRARGFIKQEKTVLAE
>LFGLNPFC_01102 Galactitol 1-phosphate 5-dehydrogenase
MKSVVNDTDGIVRVAESVIPEIKHQDEVRVKIASSGLCGSDLPRIFKNGAHYYQITLGHE
FSGYIDAVGSGVDDLHPGDVVACVPLLPCFTCPECLKGFYSQCAKYDFIGSRRDGGLAEY
IVVKRKNVFALPTDMPIEDGAFIEPITVGLHAFHLAQGCENKNVIIIGAGTIGLLAIQCA
VALGAKSVTAIDISSEKLALAKSFGAMQTFNSLEMSAPQMQGVLRELRFNQLILETAGVP
QTVELAVEIAGPHAQLALVGTLHQDLHLTSTTFGKILRKELTVIGSWMNYSSPWPGQEWE
TASRLLTERKLSLEPLIAHRGSFESFAQAVRDIARNAMPGKVLLIP
>LFGLNPFC_01103 Glucitol operon repressor
MTMNSFERRNKIIQLVNEQGTVLVQDLAGVFAASEATIRADLRFLEQKGVVTRFHGGAAK
IMSGNSETETQEVGFKERFQLASAPKNRIAQAAVKMIHEGMTVILDSGSTTMLIAEGLMT
AKNITVITNSLPAAFALSENKDITLVVCGGTVRHKTRSMHGSIAERSLQDINADLMFVGA
DGIDAVNGITTFNEGYSVSGAMVTAANKVIAVLDSSKFNRRGFNQVLPIEKIDIIITDDA
VSEVDKLALQKTRVKLITV
>LFGLNPFC_01104 Lipid kinase YegS
MAEFPASLLILNGKSTDNLPLREAIMLLREEGMTIHVRVTWEKGDAARYVEEARKLGVAT
VIAGGGDGTINEVSTALIQCEGDDIPALGILPLGTANDFATSVGIPEALDKALKLAIAGN
AIAIDMAQVNKQTCFINMATGGFGTRITTETPEKLKAALGGVSYIIHGLMRMDTLQPDRC
EIRGENFHWQGDALVIGIGNGRQAGGGQQLCPNALINDGLLQLRIFTGDEIIPTLVSTLK
SDEDNPNIIEGASSWFDIQAPHEITFNLDGEPLSGQNFHIEILPAALRCRLPPDCPLLR
>LFGLNPFC_01105 hypothetical protein
MKKIAAISLISVFLMSGCAVHNDETSIGKFGLAYKSNIQRKLDNQYYTEAEASLARGRIS
GAENIVKNDAVHFCVTQGKKMQIVDLKTEGAGLHGVARLTFKCGE
>LFGLNPFC_01106 23S rRNA 5-hydroxycytidine synthase
MFKPELLSPAGTLKNMRYAFAYGADAVYAGQPRYSLRVRNNEFNHENLQLGINEAHALGK
KFYVVVNIAPHNAKLKTFIRDLKPVVEMGPDALIMSDPGLIMLVREHFPEMPIHLSVQAN

AVNWATVKFWQQMGLTRVILSRELSLEEIEEIRNQVPDMEIEIFVHGALCMAYSGRCLLS
GYINKRDPNQGTCTNACRWEYNVQEGKEDDVGNIVHKYEPIPVQNVEPTLGIGAPTDKVF
MIEEAQRPGEYMTAFEDEHGTYIMNSKDLRAIAHVERLTKMGVHSLKIEGRTKSFYYCAR
TAQVYRKAIDDAAAGKPFDTSLLETLEGLAHRGYTEGFLRRHTHDDYQNYEYGYSVSDRQ
QFVGEFTGERKGELAAVAVKNKFSVGDSLELMTPQGNINFTLEHMENAKGEAMPVAPGDG
YTVWLPVPQDLELNYALLMRNFSGETTRNPHGK
>LFGLNPFC_01107 hypothetical protein
MTGNIHQNYNARDVINAPEVKTAILQRSLFRQDNETIQKWLLNHFYRWLISDFPMVQQIN
SLAEYSLFNKNDDVIPEWLVSKFNTASVSTATLYYIETDHQQILTKERELVEFLSRKCGT
RLESKLQRITCYVAFRMREEEHEKMLQRREKGWQPSEQNTVKSILDVPDGKIVEFDASHS
NLRREMAYESWHMQHCVGQFDDRKNLTGGYGEYYANQIEQHKLRLFSLRDNNNIPHVTIA
LNVVGDSLEIDQIKGKQNRHPVKKYADDVLSLLQLLSPQAVRHSDCEGMGIVYENTPEYQ
GWKYVTEVYETSFLLSVLHNNFHLLEHFTNPSVELQWLLLHSAPDKLHYLNAIDPIVATS
AEMLFPGAEWHPQFAGQNISNISFEIESLTLQTSHYLPLSEVEK
>LFGLNPFC_01108 hypothetical protein
MSLIIVLLVAIFIFIYRYKKNKPQIDAFIAKEKEKDLIKDKRRATKRRKWALAIGDIVAR
RNGLSVNGFTLDLKLTDDKRQQLALQVKQELGTETYQSNEDFRQQIGVILQRWATGLGNS
PHDFYEQLAAQGQVLDGLAFDCMRTAFLTRCIAGLGWCDENQAWIVLLLNAQRAQDCFAS
WEDYASAYVRARQKWLMIYDTPVATANRDLKEVTAWLKDPSSNWKKLPWNEFKIFEPN
>LFGLNPFC_01109 hypothetical protein
MEQDARLLSAMQKMCDQKMPLNTLERKWQIANIPYMLQEKRYQELDEIYNQVLQESFTSR
QAEKRYFLSWTQMCNYFYDMNTLVDAGTEGLRLIKTWQQARPHSTHAWLAEAQYWNHRAW
LYRSYGWANDTTHAMWLCAGACNEQMVIATLKAIDCDPRQWMAALLTSTNSKVFGQPAWL
AAHLNGDSVAGIPLMIALKNYHRRSPQEVEALMAYSGLSFEHAICPVLPRPNILPEYDDD
GGQKYWLSVCLTIFPHTFYPFVEYIPFRMPRWGGSHKEISELLDSVTCKHLSTEEHDYMD
LLLWWDDYRDVSIEDIAPEEQQYAIDLAENIAQYAQFQECRHNALEWLLACYNKQNDHDK
LWCCIQRAVMEDMKLNNYYTAYAIKFALSYYPDSFWIYNFICQNSQNTTYATPVIYRGFF
QREGILGFEKDEGQGDAWLEKASDIKYNHNWRSAIKDLSWFDLSDYFIPLATIGKQRNIP
AALNLVALEYLDKEDNTKLPYEPSTALEYFRRALKILQDDLNFHSSVSYPLVKNYGYSEH
QQDLQNIYFSIAICYQALNKQEISKETRAIYEKNLLDNLFLAHEAGHEKAWGLFLLNIFE
VKELSLAHLHLQQVQEEANKGTLEAMITLSRLYGNKEDEKLFNMKLSARWTHFAESLYPD
NEIIADCLYHLHFSSLWKRCRYAWYTFRIPASELPGQVNSMV
>LFGLNPFC_01110 hypothetical protein
MLMLKKGMMLVGLIFFSLPANAGCTLEETARKVIMGFQQEDAQLINSLIDKKTGLYVLFH
RGASMDVENLKQINFRQPVPEYFPWPSAGEHVPRDDEFNKKAIPKFNCERGWNKQGYFIS
NSDVHHKVSRSLVFTQMFGSPQNTISDAQIAAARRMEYGAVRVVAAPEESNDGLVFYLSQ
LYSYDSDWYLTVVDQIGDCGA
>LFGLNPFC_01111 hypothetical protein
MAGWFELSKSSDNQFRFVLKAGNGETILTSELYTSKASAEKGIASVRSNSPQEERYEKKT
ASNGKFYFNLKAANHQIIGSSQMYATAQSRETGIASVKVNGTSQTVKDNT
>LFGLNPFC_01112 Transcriptional regulatory protein BaeR
MTELPIDENTPRILIVEDEPKLGQLLIDYLRAASYAPTLISHGDQVLPYVRQTPPDLILL
DLMLPGTDGLTLCREIRRFSDIPIVMVTAKIEEIDRLLGLEIGADDYICKPYSPREVVAR
VKTILRRCKPQRELQQQDAESPLIIDEGRFQASWRGKMLDLTPAEFRLLKTLSHEPGKVF
SREQLLNHLYDDYRVVTDRTIDSHIKNLRRKLESLDAEQSFIRAVYGVGYRWEADACRIV
>LFGLNPFC_01113 Signal transduction histidine-protein kinase BaeS
MKFWRPGITGKLFLAIFATCIVLLITMHWAVRISFERGFIDYIKHGNEQRLQMLGDALGE
QYAQHGNWRFLRNNDRFVFQILRSLEHDNNEDKPGPGMPPHGWRTQFWVVDQNNKVLVGP
RAPVPPDGTRRPIMVNGAEVGAVIASPVERLTRNTDINFDRQQRQTSWLIVALSTLLAAL
ATFPLARGLLAPVKRLVDGTHKLAAGDFTTRVAPTSEDELGRLAEDFNQLASTLEKNQQM
RRDFMADISHELRTPLAVLRGELEAIQDGVRKFTPETLASLQAEVGTLTKLVDDLHQLSM
SDEGALAYQKAPVDLIPLLEVAGGAFRERFASRGLKLQFSLPDSITVFGDRDRLMQLFNN
LLENSLRYTDSGGSLKISAEQHDKTVRLTFADSAPGVSDDQLQKLFERFYRTEGSRNRAS
GGSGLGLAICLNIVEAHNGRIIAAHSPFGGLSITVELPLERDLQREV
>LFGLNPFC_01114 Putative multidrug resistance protein MdtD
MTDLPDSTRWQLWIVAFGFFMQSLDTTIVNTALPSMAQSLGESPLHMHMVIVSYVLTVAV
MLPASGWLADKVGVRNIFFTAIVLFTLGSLFCALSGTLNELLLARALQGVGGAMMVPVGR
LTVMKIVPREQYMAAMTFVTLPGQVGPLLGPALGGLLVEYASWHWIFLINIPVGIIGAIA
TLMLMPNYTMQTRRFDLSGFLLLAVGMAVLTLALDGSKGTGFSPLAIAGLVAVGVVALVL
YLLHAQNNNRALFSLKLFRTRTFSLGLAGSFAGRIGSGMLPFMTPVFLQIGLGFSPFHAG
LMMIPMVLGSMGMKRIVVQVVNRFGYRRVLVATTLGLSLVTLLFMTTALLGWYYVLPFVL
FLQGMVNSTRFSSMNTLTLKDLPDNLASSGNSLLSMIMQLSMSIGVTIAGLLLGLFGSQH
VSVDSGTTQTVFMYTWLSMAFIIALPAFVFARVPSDTHQNVAISRRKRSAQ
>LFGLNPFC_01115 Multidrug resistance protein MdtC

MKFFALFIYRPVATILLSVAITLCGILGFRMLPVAPLPQVDFPVIMVSASLPGASPETMA
SSVATPLERSLGRIAGVSEMTSSSSLGSTRIILQFDFDRDINGAARDVQAAINAAQSLLP
SGMPSRPTYRKANPSDAPIMILTLTSDTYSQGELYDFASTQLAPTISQIDGVGDVDVGGS
SLPAVRVGLNPQALFNQGVSLDDVRTAISNANVRKPQGALEDGTHRWQIQTNDELKTAAE
YQPLIIHYNNGGAVRLGDVATVTDSVQDVRNAGMTNAKPAILLMIRKLPEANIIQTVDSI
RARLPELQSTIPAAIDLQIAQDRSPTIRASLEEVEQTLIISVALVILVVFLFLRSGRATI
IPAVAVPVSLIGTFAAMYLCGFSLNNLSLMALTIATGFVVDDAIVVLENIARHLEAGMKP
LQAALQGTREVGFTVLSMSLSLVAVFLPLLLMGGLPGRLLREFAVTLSVAIGISLLVSLT
LTPMMCGWMLKASKPREQKRLRGFGRMLVALQQGYGKSLKWVLNHTRLVGVVLLGTIALN
IWLYISIPKTFFPEQDTGVLMGGIQADQSISFQAMRGKLQDFMKIIRDDPAVDNVTGFTG
GSRVNSGMMFITLKPRGERSETAQQIIDRLRKKLAKEPGANLFLMAVQDIRVGGRQANAS
YQYTLLSDDLAALREWEPKIRKKLATLPELADVNSDQEDNGAEMNLIYDRDTMARLGIDV
QAANSLLNNAFGQRQISTIYQPMNQYKVVMEVDPRYTQDISALEKMFVINNEGKAIPLSY
FAKWQPANAPLSVNHQGLSAASTISFNLPTGKSLSDASAAIDRAMTQLGVPSTVRGSFAG
TAQVFQETMNSQVILIIAAIATVYIVLGILYESYVHPLTILSTLPSAGVGALLALQLFNA
PFSLIALIGIMLLIGIVKKNAIMMVDFALEAQRHGNLTPQEAIFQACLLRFRPIMMTTLA
ALFGALPLVLSGGDGSELRQPLGITIVGGLVMSQLLTLYTTPVVYLFFDRLRLRFSRKPK
QAVTE
>LFGLNPFC_01116 Multidrug resistance protein MdtB
MQVLPPSSTGGPSRLFIMRPVATTLLMVAILLAGIIGYRALPVSALPEVDYPTIQVVTLY
PGASPDVMTSAVTAPLERQFGQMSGLKQMSSQSSGGASVITLQFQLTLPLDVAEQEVQAA
INAATNLLPSDLPNPPVYSKVNPADPPIMTLAVTSTAMPMTQVEDMVETRVAQKISQISG
VGLVTLSGGQRPAVRVKLNAQAIAALGLTSETVRTAITGANVNSAKGSLDGPSRAVTLSA
NDQMQSAEEYRQLIIAYQNGAPIRLGDVATVEQGAENSWLGAWANKEQAIVMNVQRQPGA
NIISTADSIRQMLPQLTESLPKSVKVTVLSDRTTNIRASVDDTQFELMMAIALVVMIIYL
FLRNIPATIIPGVAVPLSLIGTFAVMVFLDFSINNLTLMALTIATGFVVDDAIVVIENIS
RYIEKGEKPLAAALKGAGEIGFTIISLTFSLIAVLIPLLFMGDIVGRLFREFAITLAVAI
LISAVVSLTLTPMMCARMLSQESLRKQNRFSRASEKMFDRIIAAYGRGLAKVLNHPWLTL
SVALSTLLLSVLLWVFIPKGFFPVQDNGIIQGTLQAPQSSSFANMAQRQRQVADVILQDP
AVQSLTSFVGVDGTNPSLNSARLQINLKPLDERDDRVQKVIARLQTAVDKVPGVDLFLQP
TQDLTIDTQVSRTQYQFTLQATSLDALSTWVPQLMEKLQQLPQLSDVSSDWQDKGLVAYV
NVDRDSASRLGISMADVDNALYNAFGQRLISTIYTQANQYRVVLEHNTENTPGLAALDTI
RLTSSDGGVVPLSSIAKIEQRFAPLSINHLDQFPVTTISFNVPDNYSLGDAVQAIMDTEK
TLNLPVDITTQFQGSTLAFQSALGSTVWLIVAAVVAMYIVLGILYESFIHPITILSTLPT
AGVGALLALMIAGSELDVIAIIGIILLIGIVKKNAIMMIDFALAAEREQGMSPREAIYQA
CLLRFRPILMTTLAALLGALPLMLSTGVGAELRRPLGIGMVGGLIVSQVLTLFTTPVIYL
LFDRLALWTKSRFARHEEEA
>LFGLNPFC_01117 Multidrug resistance protein MdtA
MCPSFRRFPTVFHNSSIFLPYWLATLVSFRETFQEEKLLTMKGSYKSRWVIVIVVVIAAI
AAFWFWQGRNDSQSAAPGATKQAQQSPAGGRRGMRAGPLAPVQAATAVEQAVPRYLTGLG
TITAANTVTVRSRVDGQLMALHFQEGQQVKAGDLLAEIDPSQFKVALAQAQGQLAKDKAT
LANARRDLSRYQQLAKTNLVSRQELDAQQALVSETEGTIKADEASVASAQLQLDWSRITA
PVDGRVGLKQVDVGNQISSGDTTGIVVITQTHPIDLVFTLPESDIATVVQAQKAGKPLVV
EARDRTNSKKLSEGTLLSLDNQIDATTGTIKVKARFNNQDDALFPNQFVNARMLVDTEQN
AVVIPTAALQMGNEGHFVWVLNSENKVSKHLVTPGIQDSQKVVIRAGISAGDRVVTDGID
RLTEGAKVEVVEAQSTTTPEEKATSREYAKKGARS
>LFGLNPFC_01118 hypothetical protein
MSEQITFATSDFASNPEPRCPCILLLDVSGSMSGRPINELNTGLVTFRDELLADSLALKR
VELGIVTFGPVHVEQPFTSAANFFPPILFAQGDTPMGAAITKALDMVEERKREYRANGIS
YYRPWIFLITDGAPTDEWQAAANKVFQGEEDKKFAFFTIGVQGADMKTLAQISVRQPLSL
QGLQFRELFSWLSSSLRSVSRSTPGTEVVLEAPKGWTSV
>LFGLNPFC_01119 Serine/threonine-protein phosphatase 3
MQVAWLNDQQPLLSVFVADGAGSVSQGGEGAMLAVNEAMAYMSQKMQGGELGLNDVLATD
IVLTIRQRLFAEAEAKELAVRDFACTFLGLISSANGTLIMQIGDGGVVVDLGHGLQLPLT
PMVGEYTNMTHFITDEDAVSRLETFTSTERAHKVAAFTDGIQRLALNMLDNSPHVPFFTP
FFNGLASATQEQLDLLPELLKQFLSSPAVNERTDDDKTLALALWLP
>LFGLNPFC_01120 Protein kinase YegI
MKPTLYTATGECVTPGRELGKGGEGAVYDIEEFVDSVAKIYHTPPPALKQDKLAFMAATA
DAQLLNYVAWPQATLHGGRGGKVIGFMMPKVSGKEPIHMIYSPAHRRQSYPHCAWDFLLY
VARNIASSFATVHEHGHVVGDVNQNSFMVGRDSKVVLIDSDSFQINANGTLHLCEVGVSH
FTPPELQTMPSFVGFERTENHDNFGLALLIFHILFGGRHPYSGVPLISDAGNALETDIAH
FRYAYASDNQRRGLKPPPRSIPLSMLPGDVEAMFQQAFTESGVATGRPTAKAWVAALDLL
RQQLKKCTVSAMHVYPAHLTDCPWCALDNQGVIYFIDLGEEVITTGGDFVLARVWAMVMA
SVAPPALQLPLPDHFQPTGRPLPLGLLRREYIILIEIALSALSLLFCGLQAEPSYIILIP

VLAAIWIIGSLTSKAYKAEIQQRREAFNRAKMDYDHLVSQIQQLGGLEGFIAKRARLEKM
KDEILGLPEEEKRDLAALQDTARERQKQKFLEGFFIDVASIPGVGPARKAALRSFGIETA
ADVTRRSVKQVKGFGDHLTQAVIDWKASCERRFVFRPNEAVTPADRQAVMAKMTAKRHRL
ESALTVGATELQRFRLHAPARTMPLMEPLRQAAEKLAQAKADLSRC
>LFGLNPFC_01121 Chaperone protein DnaK
MFIGFDYGTANCSVAVMRDGKPHLLKMENDSTLLPSMLCAPTREAVSEWLYRHHDVPADD
DETQALLRRAIRYNREEDIDVTAKSVQFGLSSLAQYIDDPEEVWFVKSPKSFLGASGLKP
QQVALFEDLVCAMMLHIRQQAQAQLPEAITQAVIGRPINFQGLGSDEANTQAQGILERAA
KRAGFKDVVFQYEPVAAGLDYEATLQEEKRVLVVDIGGGTTDCSLLLMGPQWRSRLDREA
SLLGHSGCRIGGNDLDIALAFKNLMPLLGMGGETEKGIALPILPWWNAVAINDVPAQSDF
YSSANGRLLNDLVRDAREPEKVALLQKVWRQRLSYRLVRSAEESKIALSSVAETRASLPF
ISDELATLISQQGLESALNQPLARILEQVQLALDNAQEKPDVIYLTGGSARSPLIKKALA
EQLPGIPIAGGDDFGSVTAGLARWAEVVFR
>LFGLNPFC_01122 DNA-3-methyladenine glycosylase 2
MYTLNWQPPYDWSWMLGFLAARAVSSVETGADSYYARSLAVGEYRGVVTAIPDIARHTLH
INLSAGLEPVAAECLAKMSCLFDLQCNPQIVNGALGKLGAARPGLRLPGSVDAFEQGVRA
ILGQLVSVAMAAKLTARVAQLYGERLDDFPDYVCFPTPQRLAAADPQALKALGMPLKRAE
ALIHLANAALEGTLPMTIPGDVEQAMKKLQTFPGIGRWTANYFALRGWQAKDVFLPDDYL
IKQRFPGMTPAQIRRYAERWKPWRSYALLHIWYTEGWQPDEA
>LFGLNPFC_01123 putative diguanylate cyclase DgcE
MSKQSQHVLIALPHPLLHLVSLGLVSFIFTLFSLELSQFGTQLAPLWFPTSIMMVAFYRH
AGRMWPGIALSCSLGNIAASILLFSTSSLNMTWTTINIVEAVVGAVLLRKLLPWYNPLQN
LADWLRLALGSAIVPPLLGGVLVILLTPGDDPLRAFLIWVLSESIGALALVPLGLLFKPH
YLLRHRNPRLLFESLLTLAITLTLSWLSMLYLPWPFTFIIVLLMWSAVRLPRMEAFLIFL
TTVMMVSLMMAADPSLLATPRTYLMSHMPWLPFLLILLPANIMTMVMYAFRAERKHISES
ETRFRNAMEYSAIGMALVGTEGQWLQSNKALCQFLGYSQEELRGLTFQQLTWPEDLNKDL
QQVEKLISGEINTYSMEKRYYNRNGDVVWALLAVSLVRHTDGTPLYFIAQIEDINELKRT
EQVNQQLMERITLANEAGGIGIWEWELKPNIFSWDKRMFELYEIPPHIKPNWQVWYECVL
PEDRQHAEKVIRDSLQSRSPFKLEFRITVKDGIRHIRALANRVLNKEGEVERLLGINMDM
TEVKQLNEALFQEKERLHITLDSIGEAVVCIDMAMKITFMNPVAEKMSGWTQEEALGVPL
LTVLHITFGDNGPLMENIYSADTSRSAIEQDVVLHCRSGGSYDVHYSITPLSTLDGSNIG
SVLVIQDVTESRKMLRQLSYSASHDALTHLANRASFEKQLRILLQTVNSTHQRHALVFID
LDRFKAVNDSAGHAAGDALLRELASLMLSMLRSSDVLARLGGDEFGLLLPDCNVESARFI
ATRIISAVNDYHFIWEGRVHRVGASAGITLIDDNNHQAAEVMSQADIACYASKNGGRGRV
TVYEPQQAAAHSERAAISLDEQRRMIKENQLMMIAHGVASPRIPQARNLWLISLKLWSCE
GEIIDEQTFRRSFSDPALSHALDRRVFHDFFQQAAKAIASKGLSIALPLSVAGLSSATLV
NELIEQLENSPLPPRLLHLIIPADAILDHAASVQKLRLAGCRIVFSQVGRDLQIFNSLKA
NMADYLLLDGELCASVQGNLMDEMLITIIQGHAQRLGMKTIAGPVVLPLVMDTLSGIGVD
LIYGDVIVDAQPLDLLVNSSYFAIN
>LFGLNPFC_01124 Uridine kinase
MTDQSHQCVIIGIAGASASGKSLIASTLYRELREQVGDEHIGVIPEDCYYKDQSHLSMEE
RVKTNYDHPSAMDHSLLLEHLQALKRGSAIDLPVYSYVEHTRMKETVTVEPKKVIILEGI
LLLTDARLRDELNFSIFVDTPLDICLMRRIKRDVNERGRSMDSVMAQYQKTVRPMFLQFI
EPSKQYADIIVPRGGKNRIAIDILKAKISQFFE
>LFGLNPFC_01125 dCTP deaminase
MRLCDRDIEAWLDEGRLSINPRPPVERINGATVDVRLGNKFRTFRGHTAAFIDLSGPKDE
VSAALDRVMSDEIVLDESEAFYLHPGELALAVTLESVTLPADLVGWLDGRSSLARLGLMV
HVTAHRIDPGWSGCIVLEFYNSGKLPLALRPGMLIGALSFEPLSGPAARPYNRREDAKYR
NQQGAVASRIDKD
>LFGLNPFC_01126 hypothetical protein
MRRFLTTLMILLVVLVAGLSALVLLVNPNDFRDYMVKQVAARSGYQLQLDGPLRWHVWPQ
LSILSGRMSLTAQGASQPLVRADNMRLDVALLPLLSHQLSVKQVMLKGAVIQLTPQTEAV
RSEDAPVAPRDNTLPDLSDDRGWSFDISSLKMADSVLVFQHEDDEQVTIRNIRLQMEQDP
QHRGSFEFSGRVNRDQRDLTISLNGTVDASDYPHDLTAAIEQINWQLQGADLPKQGIQGQ
GSFQAQWQESHKRLSFNQISLTANDSTLSGQAQVTLTEKPEWQLRLQFPQLNLDNLIPLK
ETANGENGAAQQGQSQSTLPRPVISSRIDEPAYQGLHGFTADILLQASNVRWRGMNFTDV
ATQMTNKSGLLEITQLQGKLNGGQVSLPGTLDATSINPRINFQPRLENVEIGTILKAFNY
PISLTGKMSLAGDFSGADIDADAFRHNWQGQAHVEMTDTRMEGMNFQQMIQQAVERNGGD
VKAAENFDNVTRLDRFTTDLTLKDGVVTLNDMQGQSPVLALTGEGMLNLADQTCDTQFDI
RVIGGWNGESKLIDFLKETPVPLRVYGNWQQLNYSLQVDQLLRKHLQDEAKRRLNDWAER
NKDSRNGKDVKKLLEKM
>LFGLNPFC_01127 hypothetical protein
MKIETFDSVWDAVSDTPEQAENMRIRAELVTIINNWIEQQGFSQAQAASALGVTQPRISE
LARGKIQIFSIDKLITMMAHAGLHIQRIEIQYPHAA

>LFGLNPFC_01128 hypothetical protein
MRKKLAFLDTSLDDLRAFPESSRQEIGYQLDRIQQGLNPYDWKPFSTIGPGVREIRTRDA
DGIYRVMYIAKFEEAVYVLHCFQKKTQTTSQSDIDLDKRRYKELVQERKNEN
>LFGLNPFC_01129 hypothetical protein
MEWIADPSIWAGLITLIVIELVLGIDNLVFIAILAEKLPPKQRDRARVTGLLLAMLMRLL
LLASISWLVTLTQPLFSFRSFTFSARDLIMLFGGFFLLFKATMELNERLEGKDSNNPTQR
KGAKFWGVVTQIVVLDAIFSLDSVITAVGMVDHLLVMMAAVVIAISLMLMASKPLTQFVN
SHPTIVILCLSFLLMIGFSLVAEGFGFVIPKGYLYAAIGFSVMIEALNQLAIFNRRRFLS
ANQTLRQRTTEAVMRLLSGQKEDAELDAETASMLMDHDNQQIFNPQERRMIERVLNLNQR
TVSSIMTSRHDIEHIDLNAPEEEIRQLLERNQHTRLVVTDGDDAEDLLGVVHVIDLLQQS
LRGEPLNLRVLIRQPLVFPETLPLLPALEQFRNARTHFAFVVDEFGSVEGIVTLSDVTET
IAGNLPNEVEEIDARHDIQKNADGSWTANGHMPLEDLVQYVPLPLDEKREYHTIAGLLME
YLQRIPKPGEEVQVGDYLLKTLQVESHRVQKVQIIPLRKDGEMEYEV
>LFGLNPFC_01130 hypothetical protein
MMKSKMKLMPLLVSVTLISGCTVLPGSNMSTMGKDVIKQQDADFDLDKMVNVYPLTPRLI
DQLRPRPNVARPNMTLESEIANYQYRVGPGDVLNVTVWDHPELTTPAGQYRSSSDTGNWV
QPDGTMFYPYIGKVHVVGKTLAEIRSDITGRLATYIADPQVDVNIAAFRSQKAYISGQVN
KSGQQAITNVPLTILDAINAAGGLTDTADWRNVVLTHNGREERISLQALMQNGDLNQNRL
LYPGDILYVPRNDDLKVFVMGEVKKQSTLKMDFSGMTLTEALGNAEGIDMTTSNASGIFV
IRPLKGEGGRNGKIANIYQLDMSDATSLVMATEFRLQPYDVVYVTTAPVSRWNRLINQLL
PTISGVRYMTDTASDIHNW
>LFGLNPFC_01131 Low molecular weight protein-tyrosine-phosphatase Wzb
MFNNILVVCVGNICRSPTAERLLQRYHPELKVESAGLGALVGKGADPTAISVAAEHQLSL
EGHCARQISRSLCRNYDLILTMEKRHIERLCEMAPEMRGKVMLFGHWDNECEIPDPYRKS
RETFAAVYTLLERSARQWAQALNAEQV
>LFGLNPFC_01132 Tyrosine-protein kinase wzc
MTEKVKQHAAPVTGSDEIDIGRLVGTVIEARWWVIGITAVFALCAVVYTFFATPIYSADA
LVQIEQSSGNSLVQDIGSALANKPPASDAEIQLIRSRLVLGKTVDDLDLDIAVSKNTFPI
FGAGWDRLMGRQNETVKVTTFNRPKEMADQVFTLNVLDNKNYTLSSDGGFSARGQAGQML
KKEGVTLMVETIHASPGSEFTVTKYSTLGMINQLQNSLTVTENGKDAGVLSLTYTGEDRE
QIRDILNSIARNYQEQNIERKSAEASKSLAFLAQQLPEVRNRLDVAENKLNAFRQDKDSV
DLPLEAKAVLDSMVNIDAQLNELTFKEAEISKLYTKVHPAYRTLLEKRQALEDEKAKLNG
RVTAMPKTQQEIVRLTRDVESGQQVYMQLLNKEQELKITEASTVGDVRIVDPAITQPGVL
KPKKGLIILGAIILGLMLSIVGVLLRSLFNRGIESPQVLEEHGISVYASIPLSEWQKARD
SVKTIKGVKRYKQSQLLAVGNPTDLAIEAIRSLRTSLHFAMMQAQNNVLMMTGVSPSIGK
TFVCANLAAVISQTNKRVLLIDCDMRKGYTHELLGTNNVNGLSEILIGQGDITTAAKPTS
IAKFDLIPRGQVPPNPSELLMSERFAELVNWASKNYDLVLIDTPPILAVTDAAIVGRHVG
TTLMVARYAVNTLKEVETSLSRFEQNGIPVKGVILNSIFRRASAYQDYGYYEYEYKSDAK
>LFGLNPFC_01133 hypothetical protein
MKDNPLISIYMPTWNRQQLAIRAIKSVLRQDYNNWEMIIVDDCSTSWEQLQQYVTALNDP
RITYIHNDINSGACAVRNQAIMLAQGEYITGIDDDDEWTPNRLSVFLAHKQQLVTHAFLY
ANDYVCQGEVYSQPASLPLYPKSPYSRRLFYKRNIIGNQVFTWAWRFKECLFDTELKAAQ
DYDIFLRMVVEYGEPWKVEEATQILHINHGEMQITSSPKKFSGYFHFYRKHKDKFDRASK
KYQLFTLYQIRNKRMTWRTLLTLLSVRNGKRLADGIRGR
>LFGLNPFC_01134 Serine acetyltransferase
MLEDLRANSWSLRPCCMVLAYRVAHFCSVWRKKNVLNNLWAAPLLVLYRIITECFFGYEI
QAAATIGRRFTIHHGYAVVINKNVVAGDDFTIRHGVTIGNRGADNMACPHIGNGVELGAN
VIILGDITLGNNVTVGAGSVVLDSVPDNALVVGEKARVKVIK
>LFGLNPFC_01135 hypothetical protein
MNILQFNVRLAEGGAAGVALDLHQRALQQGLASHFVYGYGKGGKESVSHQNYPQVIKHTP
RMTAMANIALFRLFNRDLFGNFNELYRTITRTPGPVVLHFHVLHSYWLNLKSVVRFCEKV
KNHKPDVTLVWTLHDHWSVTGRCAFTDGCEGWKTGCQKCPTLNNYPPVKIDRAHQLVAGK
RQLFREMLALGCQFISPSQHVADAFNSLYGPGRCRIINNGIDMATEAILADLPPVRETQG
KPKIAVVAHDLRYDGKTNQQLVREMMALGDKIELHTFGKFSPFTAGNVVNHGFETDKRKL
MSALNQMDALVFSSRVDNYPLILCEALSIGVPVIATHSDAAREVLQKSGGKTVSEEDVLQ
LVQLSKPEIAQAIFGTTLAEFSQRSRAAYSGQQMLEEYVNFYQNL
>LFGLNPFC_01136 hypothetical protein
MSTSIRICSYLLLPLIYLLVNVKIAQLGESFPITIVTFLPVLLLLFLERISVKKLMIALG
IGAGLTAFNYLFGQSLDASKYVTSTMLFVYIVIIIGMVWSIRFKTISPHNHRKILRFFYL
VVGLVVVLAAVEMAQIILTGGSSIMESISKYLIYSNSYVLNFIKFGGKRTTALYFEPAFF
ALALISIWLSIKQFGIKTPKTDAMILAGIILSGSFSGVMTFILFYLLEWAFQYLNKEAIK
KKLPLALISLAVFLVGVVIAFPYISTRLGDLGTEGSSSYYRIVGPLVMVGYSLTHIDGVV
RFGSLYEYVASFGIFNGADVGKTIDNGLYLLIIYFSWFAVFLSLWYMGKVIKMMINAFGD
NRNFRVQLYLFTPVSLFFTGSIFSPEYAFLIVCPFILRKALNIHPK

>LFGLNPFC_01137 hypothetical protein
MLLSIITVAFRNLEGIVKTHASLAHLAQADDISFEWIVVDGGSNDGTREYLENLNGIYDL
RFVSEPDNGIYDAMNKGIEMAQGKFALFLNSGDIFHQDAAYFVRKLKVQKDNVMITGDAL
LDFGDGHKIKRSAKPGWYIYHSLPASHQAIFFPVSGLKKWRYDLEYKVSSDYALAAKMYK
AGYAFKKLNGLVSEFSMGGVSTTNNMELCADAKKVQRQILHVPGFWAELSWHLRQRTTSK
TKALYNKS
>LFGLNPFC_01138 2,3,4,5-tetrahydropyridine-2,6-dicarboxylate N-acetyltransferase
MQDLSGFSVPKGFRGGNAIKVQLWWAVQATIFAWSPQVLYRWRAFLLRLFGAKIGKNVVI
RPSVKITYPWKLTLGDYAWVGDDVNLYTLGEITIGAHSVISQKSYLCTGSHDHASQHFTI
NATPVVIGEKCWLATDVFVAPGVTIGDGTVVGARSSVFKSLPANVVCRGNPAVVIRKRVE
TE
>LFGLNPFC_01139 GDP-mannose 4,6-dehydratase
MSKVALITGVTGQDGSYLAEFLLEKGYEVHGIKRRASSFNTERVDHIYQDPHTCNPKFHL
HYGDLSDTSNLTRILREVQPDEVYNLGAMSHVAVSFESPEYTADVDAMGTLRLLEAIRFL
GLEKKTRFYQASTSELYGLVQEIPQKETTPFYPRSPYAVAKLYAYWITVNYRESYGMYAC
NGILFNHESPRRGETFVTRKITRAIANIAQGLESCLYLGNMDSLRDWGHAKDYVKMQWMM
LQQEQPEDFVIATGVQYSVRQFVEMAAAQLGIKLRFEGTGVEEKGIVVSVTGHDAPGVKP
GDVIIAVDPRYFRPAEVETLLGDPTKAHEKLGWKPEITLREMVSEMVANDLEAAKKHSLL
KSHGYDVAIALES
>LFGLNPFC_01140 GDP-L-fucose synthase
MSRQRIFIAGHRGMVGSAIRRQLEQRGDVELVLRTRDELNLLDSRAVHDFFASERIDQVY
LAAAKVGGIVANNTYPADFIYQNMMIESNIIHAAHQNDVNKLLFLGSSCIYPKLAKQPMA
ESELLQGTLEPTNEPYAIAKIAGIKLCESYNRQYGRDYRSVMPTNLYGPHDNFHPSNSHV
IPALLRRFHEATAQNAPDVVVWGSGTPMREFLHVDDMAAASIHVMELAHEVWLENTQPML
SHINVGTGVDCTIRELAQTIAKVVGYKGRVVFDASKPDGTPRKLLDVTRLHQLGWYHEIS
LEAGLASTYQWFLENQDRFRG
>LFGLNPFC_01141 GDP-mannose mannosyl hydrolase
MMFLRQEDFATVVRSTPLVSLDFIVENSRGEFLLGKRTNRPAQGYWFVPGGRVQKDETLE
AAFERLTMAELGLRLPITAGQFYGVWQHFYDDNFSGSDFTTHYVVLGFRFRVAEDELLLP
DEQHDDYRWLTPDALLASDNVHANSRAYFLAEKRAGVPGL
>LFGLNPFC_01142 hypothetical protein
MKILVYGINYSPELTGIGKYTGEMVEWLAAQGHEVRVITAPPYYPQWQVGENYSAWRYKR
EEGAAMVWRCPLYVPKQPSTLKRLLHLGSFAVSSFFPLMAQRRWKPDRIIGVVPTLFCTP
GMRLLAKLSGARTVLHIQDYEVDAMLGLGLAGKGKGGKVAQLATAFERSGLHNVDNVSTI
SRSMMNKAIEKGVAAENVIFFPNWSEIARFQHVADADVDALRNQLGLPDNKKIILYSGNI
GEKQGLESVIEAADRLRDEPLIFAIVGQGGGKARLEKMAQQRGLRNMQFFPLQSYDALPA
LLKMGDCHLVVQKRGAADAVLPSKLTNILAVGGNAVITAEAHTELGQLCETFPGIAVCVE
PESVEALVAGICQALLLPKHNTVAREYAERTLDKENVLRQFINDIRG
>LFGLNPFC_01143 Mannose-1-phosphate guanylyltransferase 1
MAQSKLYPVVMAGGSGSRLWPLSRVLYPKQFLCLKGDLTMLQTTICRLNGVECESPVVIC
NEQHRFIVAEQLRQLNKLTENIILEPAGRNTAPAIALAALAAKRHSPENDPLMLVLAADH
VIADEDAFRAAVRNAMPYAEAGKLVTFGIVPDLPETGYGYIRRGEVSAGEQDTVAFEVAQ
FVEKPNLETAQAYVASGEYYWNSGMFLFRAGRYLEELKKYRPDILDACEKAMSAVDPDLD
FIRVDEEAFLACPEESVDYAVMERTADAVVVPMDAGWSDVGSWSSLWEISAHTAEGNVCH
GDVINHKTENSYVVYAESGLVTTVGVKDLVVVQTKDAVLIADRNAVQDVKKVVEQIKADGR
HEHRVHREVYRPWGKYDSIDAGDRYQVKRITVKPGEGLSVQMHHHRAEHWVVVAGTAKVT
IDGDIKLLGENESIYIPLGATHCLENPGKIPLDLIEVRSGSYLEEDDVVRFADRYGRV
>LFGLNPFC_01144 Phosphomannomutase/phosphoglucomutase
MKKLTCFKAYDIRGKLGEELNEDIAWRIGRAYGEFLKPKTIVLGGDVRLTSETLKLALAK
GLQDAGVDVLDIGMSGTEEIYFATFHLGVDGGIEVTASHNPMDYNGMKLVREGARPISGD
TGLRDVQRLAEANDFPPVDESKRGRYQQINLRDVYVDHLFGYINVKNLTPLKLVINSGNG
AAGPVVDAIEARFKALGAPVELIKVHNTPDGNFPNGIPNPLLPECRDDTRNAVIKHGADM
GIAFDGDFDRCFLFDEKGQFIEGYYIVGLLAEAFLEKNPGAKIIHDPRLSWNTVDVVTAA
GGTPVMSKTGHAFIKERMRKEDAIYGGEMSAHHYFRDFAYCDSGMIPWLLVAELVCLKGK
TLGELVRDRMSAFPASGEINSKLAQPVEAINRVEQHFSREALAVDRTDGISMTFADWRFN
LRTSNTEPVVRLNVESRGDVPLMEARTRTLLTLLNE
>LFGLNPFC_01145 UDP-glucose:undecaprenyl-phosphate glucose-1-phosphate transferase
MTNLKKRERAKTNASLISMVQRFSDITIMFAGLWLVCEVSGLSFLYMHLLVALITLVVFQ
MLGGITDFYRSWRGVRAATEFALLLQNWTLSVIFSAGLVAFNNDFDTQLKIWLAWYGLTS
IGLVVCRSCIRIGAGWLRNHGYNKRMVAVAGDLAAGQMLMESFRNQPWLGFEVVGVYHDP
KPGGVSNDWAGNLQQLVEDAKAGKIHNVYIAMQMCDGARVKKLVHQLADTTCSVLLIPDV
FTFNILHSRLEEMNGVPVVPLYDTPLSGVNRLLKRAEYIVLATLILLLISPVLCCIALAV
KLSSPGPVIFRQTRYGMDGKPIKVWKFRSMKVMENDKVVTQATQNDPRVTKVGNFLRRTS
LDELPQFINVLTGGMSIVGPRPHAVAHNEQYRQLIEGYMLRHKVKPGITGWAQINGWRGE

TDTLEKMEKRVEFDLEYIREWSVWFDIKIVFLTVFKGFVNKAAY
>LFGLNPFC_01146 Lipopolysaccharide biosynthesis protein WzxC
MSLREKTISGAKWSAIATVIIIGLGLVQMTVLARIIDNHQFGLLTVSLVIIALADTLSDF
GIANSIIQRKEISHLELTTLYWLNVGLGLVVCVAVFLLSDVIGDVLNNPDLAPLIKTLSL
AFVVIPHGQQFRALMQKELEFNKIGMIETSAVLAGFTFTVVSAHFWPLAMTAILGYLVNS
AVRTLLFGYFGRKIYRPGLHFSLASVAPNLRFGAWLTADSIINYLNTNLSTLVLARILGA
GVAGGYNLAYNVAVVPPMKLNPIITRVLFPAFAKIQDDTEKLRVNFYKLLSVVGIINFPA
LLGLMVVSNNFVPLVFGEKWSSIIPVLQLLCVVGLLRSVGNPIGSLLMAKARVDISFKFN
VFKTFLFIPAIIIGGQMAGAIGVTLGFLLVQIINTILSYFVMIKPVLGSSYRQYILSLWL
PFYLSLPTLVVSYALGIVLKGQLALGMLLAVQIAAGVLAFVVMIVLSRHPLVVEVKRQFC
RSEKMKMLLRAG
>LFGLNPFC_01147 hypothetical protein
MKLLILGNHTCGNRGDSAILRGLLDAINILNPHAEVDVMSRYPVSSSWLLNRPVMGDPLF
LQMKQHNSAAGVVGRVKKVLRRRYQHQVLLSRVTDTGKLRNIAIAQGFTDFVRLLSGYDA
IIQVGGSFFVDLYGVPQFEHALCTFMAKKPLFMIGHSVGPFQDEQFNQLAKYVFGRCDAL
ILRESVSLDLMKRSNITTAKVEHGVDTAWLVDHHIEDFTASYAVQHWLDVAAQQKTVAIT
LRELAPFDKRLGTTQQAYEKAFAGVVNRILDEGYQVIALSTCTGIDSYNKDDRMVALNLR
QHISDPARYHVVMDELNDLEMGKILGACELTVGTRLHSAIISMNFATPAIAINYEHKSAG
IMQQLGLPEMAIDIRHLLDGSLQAMVADTLGQLPALNARLNEAVSRERQTGMQMVKSVLE
RIGEAK
>LFGLNPFC_01148 D-inositol-3-phosphate glycosyltransferase
MKVGFFLLKFPLSSETFVLNQITAFIDMGFEVEIIALQKGDTQNTHAAWTKYNLAARTRW
LQDEPAGKVAKLRHRASQTLRGIHRKNTWQALNLKRYGAESRNLILSAICGQVATPFHAD
VFIAHFGPAGVTAAKLRELGVIRGKIATIFHGIDISRREVLNHYTPEYQQLFRRGDLMLP
ISDLWAGRLQKMGCPREKIVVSRMGVDMTRFSPRPVKAPATPLEIISVARLTEKKGLHVA
IEACRQLKEQGVAFRYRILGIGPWERRLRTLIEQYQLEDVVEMPGFKPSHEVKAMLDDAD
VFLLPSVTGADGDMEGIPVALMEAMAVGIPVVSTLHSGIPELVEADKSGWLVPENDARAL
AQRLATFSQLDTDELAPVVKRAREKVEHDFNQQVINRELASLLQAL
>LFGLNPFC_01149 hypothetical protein
MPFKKLSRRTFLTASSALAFLHTPFARALPARQSVNINDYNPHDWIASFKQAFSEGQTVV
VPAGLVCDNINTGIFIPSGKTLHILGSLRGNGRGRFVLQDGSRVTGEGGGSMHNITLDVR
GSDCTIKGLAMSGFGPVTQIYIGGKNKRVMHNLTIDNLTVSHANYAILRQGFHNQIIGAN
ITNCKFSDLQGDAIEWNVAINDSDILISDHLIERINCTNGKINWGIGIGLAGSTYDNNYP
ENQAVKNFVVANITGSDCRQLIHVENGKHFVIRNIKARNITPDFSKKAGIDNATVAIYGC
DNFVIDNIEMINSAGMLIGYGVIKGKYLSIPQNFRVNDIQLDNTHLAYKLRGIQISAGNA
VSFVALTNIEMKRASLELHNKPQHLFMRNINVMQESSVGPALSMNFDMRKDVRGVFMAKR
KTLLSLTNIHAVNEKGQSSVDIDRINHHIVNVEKINFRLPERRE
>LFGLNPFC_01150 UTP--glucose-1-phosphate uridylyltransferase
MTNLKAVIPVAGLGMHMLPATKAIPKEMLPIVDKPMIQYIVDEIVAAGIKEILLVTHASK
NAVENHFDTSYELESLLEQRVKRQLLAEVQSICPPGVTIMNVRQGEPLGLGHSILCARPA
IGDNPFVVVLPDVVIDDASADPLRYNLAAMIARFNETGRSQVLAKRMPGDLSEYSVIQTK
EPLDREGKVSRIVEFIEKPDQPQTLDSDIMAVGRYVLSADIWPELERTQPGAWGRIQLTD
AIAELAKKQAVDAMLMTGDSYDCGKKMGYMQAFVKYGLRNLKEGAKFRKGIEKLLSE
>LFGLNPFC_01151 dTDP-glucose 4,6-dehydratase
MKILVTGGAGFIGSAVVRHIINDTQDSVVNVDKLTYAGNLESLADVSDSERYFFEHADIC
DAAAMARIFAQHQPDAVMHLAAESHVDRSITGPAAFIETNIVGTYVLLEAARNYWSALDG
DKKNSFRFHHISTDEVYGDLPHPDEVNNKEGLPLFTETTAYAPSSPYSASKASSDHLVRA
WKRTYGLPTIVTNCSNNYGPYHFPEKLIPLVILNALEGKGLPIYGKGDQIRDWLYVEDHA
RALYTVVTEGKAGETYNIGGHNEKKNIDVVLTICDLLDEIVPKEKSYREQITYVADRPGH
DRRYAIDAEKIGRELGWKPQETFESGIRKTVEWYLSNTKWVDNVKSGAYQSWIEQNYEGR
H
>LFGLNPFC_01152 dTDP-4-dehydrorhamnose reductase
MNILLFGKTGQVGWELQRALAPLGNLIALDVHSTDYCGDFSNPEGVAETVRSIRPDIIVN
AAAHTAVDKAESEPEFAQLLNATSVEAIAKAANEVGAWVIHYSTDYVFPGTGEIPWQEED
ATAPLNVYGETKLAGEKALQEHCAKHLIFRTSWVYAGKGNNFAKTMLRLAKEREELAVIN
DQFGAPTGAELLADCTAHAIRVALNKPEVAGLYHLVASGTTTWHDYAALVFEEARKAGIP
LALNKLNAVPTTVYPTPARRPHNSRLNTEKFQQNFALVLPDWQVGVKRMLNELFTTTAI
>LFGLNPFC_01153 Glucose-1-phosphate thymidylyltransferase 1
MKTRKGIILAGGSGTRLYPVTMAVSKQLLPIYDKPMIYYPLSTLMLAGIRDILIISTPQD
TPRFQQLLGDGSQWGLNLHYKVQPSPDGLAQAFIIGEEFIGGDDCALVLGDNIFYGHDLP
KLMDAAVNKESGATVFAYHVNDPERYGVVEFDKNGTAISLEEKPLQPKSNYAVTGLYFYD
NYVVEMAKNLKPSARGELEITDINRIYMEQGHLSVAMMGRGYAWLDTGTHQSLIEASNFI
ATIEERQGLKVSCPEEIAYRKGFIDAEQVKVLAEPLKKNAYGQYLLKMIKG
>LFGLNPFC_01154 dTDP-4-dehydrorhamnose 3,5-epimerase

MNVIKTEIPDVLIFEPKVFGDERGFFFESYNQRVFEEAVGRKVEFVQDNHSKSRKGVLRG
LHYQLEPYAQAKLVRCIEGEVFDIAVDIRKSSPFFGKWVGVTLSAENKRQLWIPEGFAHG
FVVISDTAEFVYKTNNYYSQQAERSIIFDDKDLGIAWPLNTHYILSEKDLNAPTFKKISS
NEYFK
>LFGLNPFC_01155 hypothetical protein
MSLIKNSFWNLCGYVLPAIVTLPALGIMGRKLGPELFGVFTLALAVVGYASIFDAGLTRA
VIREVAIEKDNEENKLKIISSATVVIIYLSLAASLLLFFFSGHIALLLNISETFFHNVSV
SLKILAASIPLFLITQIWLSILEGEERFGLLNIYKSITGVILAISPALFILIKPSLMYAI
IGLVLARFLCFILAFIICHDKVLKAKLTIDIPTIKRLFMFGGWITVSNIISPVLSYFDRF
IVSNQLGAANVAFYTAPSEIISRLSIIPGAFSRALFPRLANANNSAERYKTKRLITISLL
IIITPIFCIGVLFSEKIMVLWMGASFFGEPGLVLSILLIGFIFNGLAQVPFASIQSRGHA
KITAFVHLLELFPYLLLLFYLIKAHGVVGAGIAWSVRMIVDYIALSLLDGKYINK
>LFGLNPFC_01156 hypothetical protein
MIYILTLTLLLVIAIMFSLLGTKSRITSPLPLHFLPWLLTLIVGISNYDQFYEFNERSFY
SLLIWFTVIFIFYFIGELVNYKRENINVYYGLSHIKYECKKYWIIVIPISLYTIFEIYMV
GMGGADGFFLNLRLANTLEGYTGKKFILMPAVYPLMMAMFAIVCLTKTSKLNKYSIYFWM
FLYCIGTMGKFSILTPILTYLIIYDFKHRLKVKKTIKFTLLIIILALTLHFTRMAENDHS
TFLSILGLYIYSPIIALGQLNEVNSSHFGEYTFRFIYAITNKIGLIKELPVNTILDYSYV
PVPTNVYTALQPFYQDFGYTGIIFGAVLYGLIYVSLYTAGVRGNNTQALLIYALFSVSSA
TAFFAETLVTNLAGNVKLVLCTILLWRFTVICKPVQ
>LFGLNPFC_01157 Putative glycosyltransferase EpsH
MYHLTMAIYSNMQTSTVTILMATYNGEAFIKNQILSLQQQTFSNWRLFIQDDGSTDNTIS
IIKNFQKSDSRIRLVDDNLKGQGAGKNFLSLIKYSETDYTIYCDQDDIWLENKIFELVKY
ANEIKLNVSDAPSLVYADGYAYMDGEGTIDFSGISNNHADQLKDFLFFNGGYQGCSIMFN
RAMTKFLLNYRGFVYLHDDITTLAAYALGKVYFLPKYLMLYRQHTNAVTGIKTFRNGLTS
KFKSPVNYLLSRKHYQVKKSFFECNSSILSETNKKVFLDFISFCESNNKFTDFFKLWRGG
FRLNNSRTKLLLKFLIRRKFSE
>LFGLNPFC_01158 Undecaprenyl-phosphate 4-deoxy-4-formamido-L-arabinose transferase
MISILTPTFNRQHTLSRLFNSLILQTDKDFEWIIIDDGSIDATAVLVEDFRKKCDFDLIY
CYQENNGKPMALNAGVKACRGDYIFIVDSDDALTPDAIKLIKESIHDCLSEKESFSGVGF
RKAYIKGGIIGNDLNNSSEHIYYLNATEISNLINGDVAYCFKKESLVKNPFPRIEDEKFV
PELYIWNKITDKAKIRFNISKVIYLCEYLDDGLSKNFHNQLKKYPKGFKIYYKDQRKREK
TYIKKTKMLIRYLQCCYYEKIK
>LFGLNPFC_01159 N-acetyl-alpha-D-glucosaminyl L-malate synthase
MKILFVITGLGLGGAEKQVCLLADKLSLSGHHVKIISLGHMSNNKVFPSENNVNVINVNM
SKNISGVIKGCVRIRDVIANFKPDIVHSHMFHANIITRLSVIGIKNRPGIISTAHNKNEG
GYFRMLTYRITDCLSDCCTNVSKEAVDEFLRIKAFNPAKAITMYNGIDTNKFKFDLLARR
EIRDGINIKNDDILLLAAGRLTLAKDYPNLLNAMTLLPEHFKLIIIGDGELRDEINMLIK
KLQLSNRVSLLGVKKNIAPYFSACDIFVLSSRWEGFGLVVAEAMSCERIVVGTDSGGVRE
VIGDDDFLVPISDSTQLASKIEKLSLSQIRDHIGFRNRERILKNFSIDTIIMQWQELYGT
IICSKHER
>LFGLNPFC_01160 D-inositol-3-phosphate glycosyltransferase
MTARTTKVLHLQLLPLLSGVQRVTLNEISALYTDYDYTLVCSKKGPLTKALLEYDVDCHC
IPELTREITVKNDFKALFKLYKFIKKEKFDIVHTHSSKTGILGRVAAKLARVGKVIHTVH
GFSFPAASSKKSYYLYFFMEWIAKFFTDKLIVLNVDDEYIAINKLKFKRDKVFLIPNGVD
TDKFSPLENKIYSSTLNLVMVGRLSKQKDPETLLLAVEKLLNENVNVKLTLVGDGELKEQ
LESRFKRQDGRIIFHGWSDNIVNILKVNDLFILPSLWEGMPLAILEALSCGLPCIVTNIP
GNNSLIEDGYNGCLFEIRDCQLLSQKIMSYVGKPELIAQQSTNARSFILKNYGLVKRNNK
VRQLYDN
>LFGLNPFC_01161 6-phosphogluconate dehydrogenase, decarboxylating
MSKQQIGVVGMAVMGRNLALNIESRGYTVSIFNRSREKTEEVIAENPGKKLVPYYTVKEF
VESLETPRRILLMVKAGAGTDAAIDSLKPYLDKGDIIIDGGNTFFQDTIRRNRELSAQGF
NFIGTGVSGGEEGALKGPSIMPGGQKEAYELVAPILTKIAAVAEDGEPCVTYIGADGAGH
YVKMVHNGIEYGDMQLIAEAYSLLKGGLNLSNEELAQTFTEWNNGELSSYLIDITKDIFT
KKDEDGNYLVDVILDEAANKGTGKWTSQSALDLGEPLSLITESVFARYISSLKDQRVAAS
KVLSGPQAQPAGDKAEFIEKVRRALYLGKIVSYAQGFSQLRAASEEYNWDLNYGEIAKIF
RAGCIIRAQFLQKITDAYAENPQIANLLLAPYFKQIADDYQQALRDVVAYAVQNGIPVPT
FAAAVAYYDSYRAAVLPANLIQAQRDYFGAHTYKRIDKEGVFHTEWLD
>LFGLNPFC_01162 UDP-glucose 6-dehydrogenase
MKITISGTGYVGLSNGLLIAQNHEVVALDILPSRVAMLNDRISPIVDKEIQQFLQSDKIH
FNATLDKNEAYRDADYVIIATPTDYDPKTNYFNTSSVESVIKDVVEINPYAVMVIKSTVP
VGFTAAMHKKYRTENIIFSPEFLREGKALYDNLHPSRIVIGERSERAERFASLLQEGAIK
QNIPTLFTDSTEAEAIKLFANTYLAMRVAYFNELDSYAESLGLNTRQIIEGVCLDPRIGN
HYNNPSFGYGGYCLPKDTKQLLANYQSVPNNLISAIVDANRTRKDFIADAILSRKPQVVG

IYRLIMKSGSDNFRASSIQGIMKRIKAKGVEVIIYEPVMKEDSFFNSRLERDLATFKQQA
DVIISNRMAEELKDVADKVYTRDLFGSD
>LFGLNPFC_01163 Chain length determinant protein
MRVENNNVSGQNHDPEQIDLIDLLVQLWRGKMTIIISVIVAIALAIGYLAVAKEKWTSTA
IITQPDVGQIAGYNNAMNVIYGQAAPKVSDLQETLIGRFSSAFSALAETLDNQEEPEKLT
IESSVKNQQLPLTVSYVGQTAEGAQMKLAQYIQQVDDKVNQELEKDLKDNIVLGRKNLQD
SLRTQEVVAQEQKDLRIRQIQEALQYANQAQVTKPQIQQTQDVTQDTMFLLGSEALESMI
KHEATRPLVFSSNYYQTRQNLLDIDNLDVDKLDIHAYRYVMKPTLPIRRDSPKKAITLIL
AVLLGGMVGAGIVLGRNALRNYNAK
>LFGLNPFC_01164 Histidine biosynthesis bifunctional protein HisIE
MLTEQQRRELDWEKTDGLMPVIVQHAVSGEVLMLGYMNPEALDRTIESGKVTFFSRTKQR
LWTKGETSGNFLNVVSIAPDCDNDTLLVLANPIGPTCHKGTSSCFGDTAHQWLFLYQLEQ
LLAERKSADPETSYTAKLYASGSKRIAQKVGEEGVETALAATVHDRFELTNEASDLMYHL
LVLLQDQDLDLTTVIENLRKRHQ
>LFGLNPFC_01165 Imidazole glycerol phosphate synthase subunit HisF
MLAKRIIPCLDVRDGQVVKGVQFRNHEIIGDIVPLAKRYAEEGADELVFYDITASSDGRV
VDKSWVSRVAEVIDIPFCVAGGIKSLEDAAKILSFGADKISINSPALADPTLITRLADRF
GVQCIVVGIDTWYDAETGKYHVNQYTGDESRTRVTQWETLDWVQEVQKRGAGEIVLNMMN
QDGVRNGYDLKQLKKVREVCHVPLIASGGAGTMEHFLEAFRDADVDGALAASVFHKQIIN
IGELKAYLATQGVEIRIC
>LFGLNPFC_01166 1-(5-phosphoribosyl)-5-[(5-phosphoribosylamino)methylideneamino]
imidazole-4-carboxamide isomerase
MIIPALDLIDGTVVRLHQGDYGKQRDYGNNPLPRLQDYAAQGAEVLHLVDLTGAKDPAKR
QIPLIKTLVAGVNVPVQVGGGVRSEKDVAALLEAGVARVVVGSTAVKSPEMVKGWFERFG
ADALVLALDVRIDEQGNKQVAVSGWQENSGVSLEQLVETYLPVGLKHVLCTDISRDGTLA
GSNVSLYEEVCARYPQVAFQSSGGIGDINDVAALRGTGVRGVIVGRALLEGKFTVKEAIA
CWQNA
>LFGLNPFC_01167 Imidazole glycerol phosphate synthase subunit HisH
MNVVILDTGCANLNSVKSAIARHGYEPKVSRDPDVVLLADKLFLPGVGTAQAAMDQVRER
ELFDLIKACTQPVLGICLGMQLLGRRSEESNGVDLLGIIDEDVPKMTDFGLPLPHMGWNR
VYPQAGNRLFQGIEDGAYFYFVHSYAMPVNPWTIAQCNYGEPFTAAVQKDNFYGVQFHPE
RSGAAGAKLLKNFLEM
>LFGLNPFC_01168 Histidine biosynthesis bifunctional protein HisB
MSQKYLFIDRDGTLISEPPSDFQVDRFDKLAFEPGVIPELLRLQKAGYKLVMITNQDGLG
TQSFPQADFDGPHNLMMQIFTSQGVQFDEVLICPHLPADECDCRKPKVKLVERYLAEQAM
DRANSYVIGDRATDIQLAENMGINGLRYDREILNWPMIGEQLTKRDRYAHVVRNTKETQI
DVQVWLDREGGSKINTGVGFFDHMLDQIATHGGFRMEINVKGDLYIDDHHTVEDTGLALG
EALKIALGDKRGICRFGFVLPMDECLARCALDISGRPHLEYKAEFTYQRVGDLSTEMIEH
FFRSLSYTMGVTLHLKTKGKNDHHRVESLFKAFGRTLRQAIRVEGDTLPSSKGVL
>LFGLNPFC_01169 Histidinol-phosphate aminotransferase
MSTVTITDLARENVRNLTPYQSARRLGGNGDVWLNANEYPTAVEFQLTQQTLNRYPECQP
KVVIENYAQYAGVKPEQVLVSRGADEGIELLIRAFCEPGKDAILYCPPTYGMYSVSAETI
GVECRTVPTLDNWQLDLQGISDKLDGVKVVYVCSPNNPTGQLINPQDFRTLLELTRGKAI
VVADEAYIEFCPQASLAGWLAEYPHLAILRTLSKAFALAGLRCGFTLANEEVINLLMKVI
APYPLSTPVADIAAQALSPQGIVAMRERVAQIITEREYLIAALKEIPCVEQVFDSETNYI
LARFKASSAVFKSLWDQGIILRDQNKQPSLSGCLRITVGTREESQCVIDALRAEQV
>LFGLNPFC_01170 Histidinol dehydrogenase
MSFNTIIDWNSCTAEQQRQLLMRPAISASESITRTVNDILDNVKTRGDEALREYSAKFDK
TTVTALKVSADEIAAASERLSDELKQAMAVAVKNIETFHTAQKLPPVDVETQPGVRCQQV
TRPVASVGLYIPGGSAPLFSTVLMLATPARIAGCKKVVLCSPPPIADEILYAAQLCGVQD
VFNVGGAQAIAALAFGTESVPKVDKIFGPGNAFVTEAKRQVSQRLDGAAIDMPAGPSEVL
VIADSGATPDFVASDLLSQAEHGPDSQVILLTPDADMARRVAEAVERQLAELPRAETARQ
ALNASRLIVTKDLAQCVEISNQYGPEHLIIQTRNARELVDGITSAGSVFLGDWSPESAGD
YASGTNHVLPTYGYTATCSSLGLADFQKRMTVQELSKEGFSTLASTIETLAAAERLTAHK
NAVTLRVNALKEQA
>LFGLNPFC_01171 ATP phosphoribosyltransferase
MTDNTRLRIAMQKSGRLSDDSRELLARCGIKINLHTQRLIAMAENMPIDILRVRDDDIPG
LVMDGVVDLGIIGENVLEEELLNRRAQGEDPRYFTLRRLDFGGCRLSLATPVDEAWDGPL
SLNGKRIATSYPHLLKRYLDQKGISFKSCLLNGSVEVAPRAGLADAICDLVSTGATLEAN
GLREVEVIYRSKACLIQRDGEMEESKQQLIDKLLTRIQGVIQARESKYIMMHAPTERLDE
VIALLPGAERPTILPLAGDQQRVAMHMVSSETLFWETMEKLKALGASSILVLPIEKMME
>LFGLNPFC_01172 Antitoxin YefM
MRTISYSEARQNLSATMMKAVEDHAPILITRQNGEACVLMSLEEYNSLEETAYLLRSPAN
ARRLMDSIDSLKSGKGTEKDIIE

>LFGLNPFC_01173 Toxin YoeB
MKLIWSEESWDDYLYWQETDKRIVKKINEIIKDTRRTPFEGKGKPEPLKHNLSGFWSRRI
TEEHRLVYAVTDDSLLIAACRYHY
>LFGLNPFC_01174 Protein YeeZ
MKKVAIVGLGWLGMPLAMSLSARGWQVTGSKTTQDGVEAARMSGIDSYLLRMEPELVCDS
DDLDALMDADALVITLPARRSGPGDEFYLQAVQELVDSALAHRIPRIIFTSSTSVYGDAQ
GTVKETTPRNPVTNSGRVLEELEDWLHNLPGTSVDILRLAGLVGPGRHPGRFFAGKTAPD
GEHGVNLVHLEDVIGAITLLLQAPKGGHIYNICALAHPARNVFYPQMARLLGLEPPQFRN
SLDSGKGKIIDGSRICNELGFEYQYPDPLVMPLE
>LFGLNPFC_01175 HTH-type transcriptional regulator YhaJ
MHCKGACMKPLLDVLMILDALEKEGSFAAASAKLYKTPSALSYTVHKLESDLNIQLLDRS
GHRAKFTRTGKMLLEKGREVLHTVRELEKQAIKLHEGWENELVIGVDDTFPFSLLAPLIE
AFYQHHSVTRLKFINGVLGGSWDALTQGRADIIVGAMHEPPSSSEFGFSRLGDLEQVFAV
APHHPLAQEEEPLNRRIIKRYRAIVVGDTAQAGASTASQLLDEQEAITVFDFKTKLELQI
SGLGCGYLPRYLAQRFLDSGALIEKKVVAQTLFEPVWIGWNEQTAGLASGWWRDEILANS
AIAGVYAKSDDGKSAI
>LFGLNPFC_01176 Low-affinity putrescine importer PlaP
MSHNVTPNTSRVELRKTLTLVPVVMMGLAYMQPMTLFDTFGIVSGLTDGHVPTAYAFALI
AILFTALSYGKLVRRYPSAGSAYTYAQKSISPTVGFMVGWSSLLDYLFAPMINILLAKIY
FEALVPSIPSWMFVVALVAFMTAFNLRSLKSVANFNTVIVVLQIVLIAVILGMVVYGVFE
GEGAGTLASTRPFWSGDAHVIPMITGATILCFSFTGFDGISNLSEETKDAERVIPRAIFL
TALIGGMIFIFATYFLQLYFPDISRFKDPDASQPEIMLYVAGKAFQVGALIFSTITVLAS
GMAAHAGVARLMYVMGRDGVFPKSFFGYVHPKWRTPAMNIILVGAIALLAINFDLVMATA
LINFGALVAFTFVNLSVISQFWIREKRNKTLKDHFQYLFLPMCGALTVGALWVNLEESSM
VLGLIWAAIGLIYLACVTKSFRNPVPQYEDVA
>LFGLNPFC_01177 Exodeoxyribonuclease I
MMNDGKQQSTFLFHDYETFGTHPALDRPAQFAAIRTDSEFNVIGEPEVFYCKPADDYLPQ
PGAVLITGITPQEARAKGENEAAFAARIHSLFTVPKTCILGYNNVRFDDEVTRNVFYRNF
YDPYAWSWQHDNSRWDLLDVMRACYALRPEGINWPENDDGLPSFRLEHLTKANGIEHSNA
HDAMADVYATIAMAKLVKTRQPRLFDYLFTHRNKHKLMALIDVPQMKPLVHVSRMFGAWR
GNTSWVAPLAWHPENRNAVIMVDLAGDISPLLELDSDTLRERLYTAKADLGDNAAVPVKL
VHINKCPVLAQANTLRPEDADRLGINRQHCLDNLKILRENPQVREKVVAIFAEAEPFTPS
ENVDAQLYNGFFSDADRAAMKIVLETEPRNLPALDITFVDKRIEKLLFNYRARNFPGTLD
YAEQQRWLEHRRQVFTPEFLQGYADELQMLAQQYADDKEKVALLKALWQYAEEIV
>LFGLNPFC_01178 D-alanyl-D-alanine carboxypeptidase DacD
MTGYVVDRAIDSHRITPDDIVTVGRDAWAKDNPVFVGSSLMFLKEGDRVSVRDLSRGLIV
DSGNDACVALADYIAGGQRQFVEMMNNYAEKLHLKDTHFETVHGLDAPGQHSSAYDLAVL
SRAIIHGEPEFYHMYSEKSLTWNGITQQNRNGLLWDKTMNVDGLKTGHTSGAGFNLIASA
VDGQRRLIAVVMGADSAKGREEEARKLLRWGQQNFTTVQILHRGKKVGTERIWYGDKENI
ALGTEQEFWMVLPKAEIPHIKAKYTLDGKELTAPISAHQRVGEIELYDRDKQVAHWPLVT
LESVGEGSMFSRLSDYFHHKD
>LFGLNPFC_01179 DNA gyrase inhibitor
MNYEIKQEDKRTVAGFHLVGPWEQTVKKGFEQLMMWIDSKNIVPKEWVAVYYDNPDETPA
EKLRCDTVVTVPNNFTLPENSEGVILTEISGGQYAVAVARVVGDDFAKPWYQFFNSLLQD
SAYEMLPKPCFEVYLNNGAEDGYWDIEMYVAVQPKHH
>LFGLNPFC_01180 Inner membrane protein YeeA
MRADKSLSPFEIRVYRHYRIVHGTRVALAFLLTFLIIRLFTIPESTWPLVTMVVIMGPIS
FWGNVVPRAFERIGGTVLGSILGLIALQLELISLPLMLVWCAAAMFLCGWLALGKKPYQG
LLIGVTLAIVVGSPTGEIDTALWRSGDVILGSLLAMLFTGIWPQRAFIHWRIQLAKSLTE
YNRVYQSAFSPNLLERPRLESHLQKLLTDAVKMRGLIAPASKETRIPKSIYEGIQTINRN
LVCMLELQINAYWATRPSHFVLLNAQKLRDTQHMMQQILLSLVHALYEGNPQPVFANTEK
LNDAVEELRQLLDNHHDLKVVETPIYGYVWLNMETAHQLELLSNLICRALRK
>LFGLNPFC_01181 hypothetical protein
METTKPSFQDVLEFVRLFRRKNKLQREIQDVEKKIRDNQKRVLLLDNLSDYIKPGMSVEA
IQGIIASMKGDYEDRVDDYIIKNAELSKERRDISKKLKAMGEMKNGEAK
>LFGLNPFC_01182 Protein YeeW
MMTLEADSVNVQALDMGHIVVDIDGVNITELINKAAENGYSLRVVDGRDSTETPATYASP
HQLL
>LFGLNPFC_01183 Cytoskeleton-binding toxin CbtA
MKTLPVLPGQAASSRPSPVEIWQILLSRLLDQHYGLTLNDTPFADERVIEQHIEAGISLC
DAVNFLVEKYALVRTDQPGFSACTRSQLINSIDILRARRATGLMTRDNYRTVNNITLGKY
PEAK
>LFGLNPFC_01184 Cytoskeleton bundling-enhancing antitoxin CbeA
MSDTLPGTTLPDDNHDRPWWGLPCTVTPCFGARLVQEGNRLHYLADRAGIRGLFSDADAY

HLDQAFPLLMKQLELMLTSGELSPRYQHTVTLYAKGLTCEADTLGSCGYVYLAVYPTPEM
KN
>LFGLNPFC_01185 hypothetical protein
MKIITRGEAMRIHQQHPTSRLFPFCTGKYRWHGSAEAYTGREVQDIPGVLAVFAERRKDS
FGPYVRLMSVTLN
>LFGLNPFC_01186 hypothetical protein
MQQISFLPGEMTPGERSHILRALKTLDRHLHEPGVAFTSTRAAREWLILNMAGLEREEFR
VLYLNNQNQLIAGETLFTGTINRTEVHPREVIKRALYHNAAAVVLAHNHPSGEVTPSKAD
RLITERLVQALGLVDIRVPDHLIVGGSQVFSFAEHGLL
>LFGLNPFC_01187 Antirestriction protein KlcA
MKTLSQNTTSSACAPETGLQQLVATIVPDEQRISFWPQHFGLIPQWVTLEPRVFGWMDRL
CENYCGGIWNLYTLNNGGAFIAPEPDEDDGETWILFNAMNGNRAEMSPEAAGIAACLMTY
SHHACRTENYAMTVHYYRLRDYALQHPECSAIMRIID
>LFGLNPFC_01188 hypothetical protein
MSGMNDIRLKFRTAVRVTVPVTDGWPDAIACTQSAQTELSGASGDSGA
>LFGLNPFC_01189 hypothetical protein
MRLASRFGYAANQIRRDRPLTHEELMHHVPGIFGEEKHTSRSQNYTYIPTITVLESLQQE
GFQPFFACQTCVRDPGRRGYTKHMLRLRRNGEINGEHVPEIILLNSHDGTSSYQMLPGYF
RFVCQNGCVCGQSLGEVRVPHRGDVVEKVIEGAYEVVGVFDRIEEKRDAMQSLVLPPPAR
QALAQAALTYRYGDEHQPVTTADILTPRRREDYGKDLWSAYQTIQENMLKGGISGRSAKG
KRIHTRAIHSIDTDIKLNRALWVMAETMLESLR
>LFGLNPFC_01190 hypothetical protein
MKTFIKTLLVAVTILFSVFATAKQVKLPNNIKYVNTTEAFSCTEIDGMNCQTKNPFNYKD
NSYVFVLERGGAWCYDYTVSVLNLKTGKAQMLEYKDNQLCSGSNKPFFEIKNGVPTVGVI
DTSGKPVVVALDKLKT
>LFGLNPFC_01191 hypothetical protein
MQLPVKLLMSLISLVSVIARAGKYKNYIRDEIKYWRYTSYKGGEFPEGFTDEKFSSAIYN
GRIFTMKRLHTLMLFLAVLFTGFNVEAASVKQALSCDPNARAEQPGACPTTYELYEGDAA
YKAALDKALKPVGLSGMFGKGGYMDGPGGNVTPVTINGTVWLQGDGCKANTCGWDFIVTL
YNPKTHEVVGYRYFGLDDPAYLVWFGEIGVHEFAYLVKNYVAAVN
>LFGLNPFC_01192 hypothetical protein
MSTEMKTGLVLSGGGAVGAYQAGVVKALAECGTQISMVSGASIGAFNGAIIAASPDLSEA
AVRLEALWDHLGNNQVLSVNRLVYFSLLKKLFQAMNLCQIPGRAGALLTTLLRHISILNG
FDNLMAQPLLSDEPLTALLDHYLDTDALADGLPLYVSLYPTEGGMQDIIDCIRAELGAGT
TKNAVFQHIQSLPRGQQKEALLASAALPLLFRPREVQGTMFGDGGMGGWRNMQGNTPVTP
LVDAGCNMVIVTHLSDGSLWDRQAFPDTTILEIRPRKRLKYAGDGDNSGGLLSFTLAHTD
AWRQQGYEDTMLTMEHIRKPLAARQVLSRSETVLQKSLEITEEADLALRNAMARIK
>LFGLNPFC_01193 hypothetical protein
MTSPFIQQIADNRVCQVLTCLPEKFVVDFANGIDVAQEHIRTAGERTFFRRLKEGLTGEG
AARQNAINASVAQGLEASLRWLTELTTSLATTNYAITRVNDRVSSLVSDTARLAHYSADT
REQLLILADQVHHKLNHLEEKLHRVDQVQRAQLHLEQIFSWWSAGRYASFSPAGRCYVAL
EELRWGAFGDVIRQGETGQVNQLLDILRHKALTQMAQESGGSATVRLNTLDWLGGQGREQ
ADNEWHDAINWLGDWCSEEQHPVIWSTTQAAEHLPVRMPRLCSAERLSESMVDEIFQKGA
A
>LFGLNPFC_01194 Clamp-binding protein CrfC
MHEKNIALLCDEADRLLQLNINLLRQMVEEPDVLSDSKNENRLLFDKQKALKRIEELEGE
QIKTARREMVLAVVGTMKAGKSTTINAIVGQEILPNRNRPMTSVPTLIRHVPGKTEPVLH
LEHIQPVRNLLITLQEKLATPAGQQVAQTLQQTGDTRELLDILTDDGWLKNEYHGEEEIF
TGLASLNDLVRLAAAMGTEFPFDEYAEVQKLPVIDVEFSHLVGMDACQGTLTLLDTPGPN
EAGQPQMEVMMRDQLQKASAVLAVMDYTQMNSKADEDVRKELNAIADVSTGRLFVLVNKF
DEKDRNGDGADAVRQKVPAMLNSDVLPASRVYPGSSRQAYLANRALHELRKNGTLPVDEA
WVDDFVREAFGRMKKDYVCKDSELATEGATDLWECSLIDQLITEVILSSHSRAAALAVDS
AAAKLMQNAENISEYLSLRHQGLMQSIQSLQAHITSLLEDIREIADCQEQVTADVRMAME
EIDARTRELLTGVCTSLEEELNDYFRSGKRKEQQMLEEENSAQPRERNAFAFFHDIFGTG
NQHDRMRDFDPDSPEIKFSDRREALELMTQIESTVTSLHREAEAQFRPELEKIVSGIETG
FRGTALYATENIAGRINTRLEDEGFTVKISFPAVSQLQTRLAVKTNLSALMEERTETVTR
RRRQSGLWGKICGAFGTSDWGWETYKEDVSRSVININTVRKEVMSLTRAYFGELQASIEQ
DINQPVRQEIDAFFCAFREKVEQLRNTLIQSSEDHKRDQQAQERLTRRLQALNERVPELI
TDSKALREELETML
>LFGLNPFC_01195 hypothetical protein
MNPSDAIEAIEKPLSSLPYPISRHILEHLRKLTRHEPVPGIMGKSGAGKSSLCNALFQGE
VTPVSALMTVLPGHAAIHLMTRLQDELRTESVRTQTREQFTGAVDRIFDTAESVCIASVA
RTVLRAVRDSVVSVARAVWNWIFF
>LFGLNPFC_01196 Ferric-anguibactin receptor FatA

MKTQITFAALLPALASFIPLHAHASSTSEDEMIVTGNTAADTTDSAAGAGFKTNDIDVGP
LGTKSWIETPYSSTTVTKEMIENQQAQSVSEMLKYSPSTQMQARGGMDVGRPQSRGMQGS
VVANSRLDGLNIVSTTAFPVEMLERMDVLNSLTGALYGPASPAGQFNFVAKRPTEETLRK
VTLGYQSRSAFTGHADLGGHFDENKRFGYRVNLLDQEGEGNVDDSTLRRKLVSVALDWNI
QPGTQLQLDASHYEFIQKGYVGSFNYGPNVKLPSAPNPKDKNLALSTAGNDLTTDTISTR
LIHYFNDDWSMNAGVGWQQADRAMRSVSSKILNNQGDISRSMKDSTAAGRFRVLSNTAGL
NGHIDTGSIGHDLSLSTTGYVWSLYSAKGTGSSYSWGTTNMYHPDAIDEQGDGKIRTGGP
RYRSSVNTQQSVTLGDTVTFTPQWSAMFYLSQSWLQTKNYDKHGNQTNQVDENGLSPNAA
LMYKITPNTMAYVSYADSLEQGGTAPTDESVKNAGQTLNPYRSKQYEVGLKSDIGEMNLG
AALFRLERPFAYLDTDNVYKEQGNQVNNGLELTAAGNVWQGLNIYSGVTFLDPKLKDTAN
ASTSNKQVVGVPKVQANLLAEYSLPSIPEWVYSANVHYTGKRAANDTNTSYASSYTTWDL
GTRYTTKVSNVPTTFRVVVNNVFDKHYWASIFPSGTDGDNGSPSAFIGGGREVRASVTFD
F
>LFGLNPFC_01197 Vitamin B12-binding protein
MKLSRLFPLLALLLAASLHAENTHKEVRIASPWPAQNTIIAMLGYGDNIVGTSMIAKRIP
LFRQSLPRIEKVAAVSVNSGHEINPEQIIALGVDMLFVPQNMVVPQQALLKQAGVQVLAF
EANSLRALTQRVQQTAAVLGPDAQQKALAYQRYFDRNVALVTGRLKDLPASQRVSLYHSM
GNPLTTTGRPSLNQDWIDLAGGKNIAENWFGEHQQNRSGEVALEKIVTANPAVIIAMNKR
DADAILSSPQWASVDAVIHHRVYVNPKGMFWWCRETSEEALQFLWLAKTLYPARFADVDI
RKETREFYRQFFGLTLSDAQMSDVLNPPR
>LFGLNPFC_01198 Hemin transport system permease protein HmuU
MTVLESTLSVENRYRQLFRQRLVILAVIFIAIIASLLLDFTLGPSGLPLHSLIKTLMHPS
GATNGMRVIVWDIRLPYALMALLVGMALGLAGAEMQTILNNSLASPFTLGVSSAAAFGAA
LAIVLGIGIPGVPESGFIPANAFLFALLSALLLDSLTRWTRVPTSGVVLFGIALVFTFNA
LVSIMQFVADEDTLQGLVFWTMGSLARASWEKLAVLAAAMAIVLPWSLRRAWQLTALRLG
EERAMSFGIDVRRLRLGSLLRISLLAALSVAFVGPIGFIGLVAPHISRLLLGEDHRFYLP
GSILIGGLVLSLASVASKNIIPGVILPVGIVTSLVGVPFFLSIVMRHRGSMS
>LFGLNPFC_01199 Ferric enterobactin transport ATP-binding protein FepC
MSGLTINALCAGYGKRRIIEHLSISTLPRGEVTVLLGPNGCGKSTLLRALAGLNRASGEA
WLNEENLLSLPFARRAEKVVFLPQSLPQGVHLQVLESVVVAQRASGAGGQNQAQAIALLEE
LGIAHLAMNYLDSLSGGQKQLVGLAQSLIRRPALLLLDEPLSALDLNYQFHVMDVVSRET
RRRNMVTLVVLHDINIALRHAAQVIMLKEGKLIDSGDPQTVIHAESLAQVYGVRGRVERC
AQGRSMVIVDGAIEK
>LFGLNPFC_01200 hypothetical protein
MQHIDRLNVIKALVLLEDEQIVRFNIAANDNASQIHMLVDGLGVGLTHEPVAAGMISHRT
ACHRSRQHDRPAHSYFYYLSAYQVA
>LFGLNPFC_01201 hypothetical protein
MGKELDDICTSCPYIDAIKRHKQQLGAIEEYTQWLKKEPRASYFFLFRLYTRIHNTFFPK
KQQLPFTPGGTHCPEPDVTLRDLTLSPGYHSDYAPQPIPEMDSSAVVPPTNENTSPPEDT
PDNTPAGGNTGQAEKTRNSGLTPIPEKRSGMPPEHLRFATGFPPQPKIAGPKGKPMRTVH
PDKIYREIIWFCSGYLRKSGPEATRTIINSIFCEWASIFNDYSSPFSWVDSRDSEQCDWL
WNAMQVRCVGTPLNPLTPEQKYWFACATFDNWEGWNEQQVQFLLESNPRRNRAKFTQASF
QAPRIQHKAILLDELKSAREQQKRRDERADGSVPLKLSGKIHKQLESIARSRGVLPKKLL
NEMIEQAYQDFVANEQHKTLS
>LFGLNPFC_01202 hypothetical protein
MSEPDLLSEVHPVADLCSHFRDPEPTTPYG
>LFGLNPFC_01203 hypothetical protein
MVTFETVMEIKILHKQGMSSRAIARELGISRNTVKRYLQAKSEPPKYTPRPAVASLLDEY
RDYIRQRIADAHPYKIPATVIAREIRDQGYRGGMTILRGFIRSLSVPQEQEPAVRFETEP
GRQMQVDWGTMRNGRSPLHVFVAVLGYSRMLYIEFTDNMRYDTLETCHRNAFRFFGGVPR
EVLYDNMKTVVLQRDAYQTGQHRFHPSLWQFGKEMGFSPRLCRPFRAQTKGKVERMVQYT
RNSFYIPLMTRLRPMGITVDVETANRHGLRWLHDVANQRKHETIQARPCDRWLEEQQSML
ALPPEKKEYDVHPSENLVNFDKHPLHHPLSIYDSFCRGVA
>LFGLNPFC_01204 IS21 family transposase IS100kyp
MMELQHQRLMALAGQLQLESLISAAPALSQQAVDQEWSYMDFLEHLLHEEKLARHQRKQA
MYTRMAAFPAVKTFEEYDFTFATGAPQKQLQSLRSLSFIERNENIVLLGPSGVGKTHLAI
AMGYEAVRAGIKVRFTTAADLLLQLSTEQRQGRYKTTLQRGVMAPRLLIIDEIGYLPFSQ
EEAKLFFQVIAKRYEKSAMILTSNLPFGQWDQTFAGDAALTSAMLDRILHHSHVVQIKGE
SYRLRQKRKAGVIAEANPE
>LFGLNPFC_01205 hypothetical protein
MKQQYQTRYELLHENYQKWLTGFTRHAVSWGVCHPNIYYFHNLTPGWVSFNGEKPEIAIV
PQSLHRLIYGPDKRSSPSLDDDLVVNLCTSEHLLIHHPMLEGILLSECERLKQHSLANKL
ISLFRQFGGTELRLKLVWLCWLDLMTGNSLDDWTKNLKHKSEKDLEQWIIARQGQSEPLT
NLMDQYVLMAYRTSVDAAHS

>LFGLNPFC_01206 hypothetical protein
MATPVSLMDDQMVDMAFITQLTGLTDKWFYKLIKVGGFPAPIKMGRSSRWLKSEVEAWLQ
ARIAQSRP
>LFGLNPFC_01207 IS110 family transposase ISEc20
MTESSDYESVQVFIGVDVGKDTHHAVAINRSGKRLFDKALPNDENKLRSLISDLKQHGQI
LLVVDQPATIGALPVAVARSEGVLVGYLPGLAMRRIADLHAGEAKTDARDAAIIAEAART
LPHALRTLKLADEQIAELSMLCGFDDDLAAQTTQASNRIRGLLTQIHPALERVLGPRLDH
PAVLDLLQRYPSPEKLASLGEKKLAAQLCKLAPRLGKRLAADIAQALAEQTVVVPGTNAA
AVVLPRLALQLITLRKQRDEVALAVEQRVLAHPLYPVLTSMPGVGVRTAARLLTEVACRA
FASVAHLAAYAGLAPVTRRSGSSIRGEHPSRRGNKALKRALFLSAFAALRDPLSRAYYTR
KMSQGKRHNQALIALARRRCDVLFAMMRDGTFYTPQAS
>LFGLNPFC_01208 hypothetical protein
MVIRKKKCRDCGNAITHNTVCCPYCGSVDPFGYYRNTDRIVTILLALIIVVLLTTVSVSV
YILCSW
>LFGLNPFC_01209 hypothetical protein
MTYKYNPFWQQRIRETVRHALNVHPRLTALRVDLRFPDVPAATDAAVISRFINALKARID
AYQKRKHREGKRVHPTTLHYVWAREFGECKGKKHYHLMLLVNRDTWCRAGDYRAPGSLAG
MIEQAWCSALGVDAGCHATLVHFPAWPAVWLARNDDTGFQQVLERADYLAKEHTKAHCTG
ERNFGCSRG
>LFGLNPFC_01210 hypothetical protein
MYAKSFLALDSNGRLTGARTVQAAPYAHYTCHLCGSALRYHPQYDTELPWFEHTDDRLTE
HGQQCPYVRPERREIQLIKRLQQFVPDALPVVRKASWHCRQCHHDYYGERYCTHCQTGGF
SIPRTTQEEICEF
>LFGLNPFC_01211 hypothetical protein
MRILNCYMANDSKGHFVTAKEAAKHNRQDVLCCVSCGCPLTLQRGNDGQPPWFEHDQMTV
AEKILLRCTWLDPAEKEARRLHLQGMTVPDYTVKVRKWFCVMCDEDYEGEKCCPRCGTGV
YSREGGLQEGNWKDRN
>LFGLNPFC_01212 ISL3 family transposase ISEc38
MLIVNLDTHRPLVLLPGRDQRTLATWFRKYPEIQVVSRDRSGVYATAAREGAPQARQVAD
RWHLLKSIGDEPERMMYRHMPLIRLVVRELSLNKSPEPEISVPVASLRRPERLKQQTRKK
RHQHWTEVMALHNKGCSFREISRITGLSRVTVSRWVRSGTFPEMSTRPPKRGLLDPWREW
LKEQRESGNYNASRIWREMVAQGGTGSETIVRDTVAKWRKGWNPPVTTAARLPSVSRVSR
WLMPWRIIRGEENYASRFISLMCEKEPELKIAQQLVLEFYRILKT
>LFGLNPFC_01213 Phosphotriesterase homology protein
MKDYLQTVTGPVAREDMGLTLPHEHLFNDLSSVVDAPCYPFSQRLVDKKVTAEIQWALKH
DPYCCADNMDRKPIEDVIFEINNFISLGGRTIVDATGSESIGRDAQALREVALKTGLNIV
ASSGPYLEKFESQRIHKTVDELAATIDKELNQGIGDTDIRAGMIGEIGVSPTFTEAEHNS
LRAASLAQINNPHVAMNIHMPGWLRRGDEVLDIVLGEMGVSPNKVSLAHSDPSGKDVAYQ
RKMLDKGVWLEFDMIGLDITFPKEGIAPGVQETADAVAHLIELGYADQLVLSHDVFLKQM
WAKNGGNGWGFVPDVFLAYLAERGVDKTILKKLCIDNPGRLLTA
>LFGLNPFC_01214 hypothetical protein
MNGQLGHWLQDGNACAVISFASGLVVMFFIIMARKETRQQFAAIPSLIKNRKVPLWNWFA
GLCGAMVVFSEGASASALGVATFQTALISALLLSGLLCDRFGIGVDEKKYFTPYRIIGAL
FAVIATIFVVSPQWHSTSFILLAILPFLAGLLAGWQPAGNAKVAEATGSMLVSITWNFIV
GFCVLGTALAIRVALGHVTVQLPDVWWMYLGGPLGLMSIGLMAILVRGLGLLMLGVASTA
GQLLGSVLIDVLIPSLGNTVYLVTIIGTLFALVGAIVTTIPEYRTSKTMKKMEV
>LFGLNPFC_01215 hypothetical protein
MNNTKKSLKVLFIGESWHIHMIHSKGYDSFTSSKYEEGATWLLQCLKNSQVDVTYMPAHT
VQIAFPEDVAQLEQYDAIVISDIGSNTFLLQNDTFYQLRIKPNALELIKEYVNNGGGLLM
IGGYLSFMGIEAKANYKNTVLADVLPVTMLDGDDRVEKPEGVIAQPSQPEHPVIKGFSEY
PFFLGYNRAIAKENAEVVLTINNDPLLVFGNYHNGKIACFMSDCSPHWGTQQFMSWPFYT
ALWVNILTHIAR
>LFGLNPFC_01216 Ribokinase
MNSSKIYKSIIYQTKETQTGTATILVNEGTGDNVIAIYPGANMTMSSDEITIQKEAIINS
DVILLQLETNYTALQQAITLAQKNSIPVIINPAPYNDIVNELIQDVDYITPNETEAGLLS
GIDVHDLESAKRAAEAIHNKGVKNTVITLGSKGSLAFDGKKFIHSPAFPAVVKNTAGAGD
AFNGALASGLAKGKSLESALCYASAFASLAVETSNASDMPEHESVIHRIQSIHYQQTIFT
H
>LFGLNPFC_01217 Ribokinase
MKFERHHEILKRLSKFGSVKVSDLSNSLNVTKETIRSDLNELARLGYLTRCHGGAFIVLD
SLDTIAKNEIAYALENYDTAQGIKKGHSTMKSQVCVIGSFNVDIISYLPRLPTIGESLLA
SNFIFSPGEKAVTKRWQQALPILMFIS
>LFGLNPFC_01218 hypothetical protein
MHSENIAAYVGLDVHKETLAVAIAAPERLGEVRYYGTINNEAQAVRRLFQKLQGLYGNIL

SCYEAGPCGFGLYHQLTAMNIKCQVIAPSRIPKSPTDRIKMIIGTQFLSPGYCGLENLPC
LDP
>LFGLNPFC_01219 IS110 family transposase ISEc45
MKLWDLIRARAAAKRDSRVARQRILSMLLRTDKHYAGKHWTGKHRTWLANQSFSQPSQQI
AFQHYCQSLEQIEDRILQLDQEISRLLPEWSLCNLVCQLQALKGVGQLIAITLVAELGDF
SRFSNPKQLMAFLGLVPGEYSSGNSIRPRGITKVGNSELRRLLYEAAWSYRTPAKVGAWL
IYYRPDSVTQYSKDIAWKAQQRLCSRYRSLTAKGKKSQVAITAVARELTGFMWDIALAAQ
SSFSQQKQN
>LFGLNPFC_01220 hypothetical protein
MHGGEKCHAIQKSRLGEWFSCVLNQWGALCYYSVDGQAEADIMPRKKPFVPSVSEIKTLM
TFVKY
>LFGLNPFC_01221 hypothetical protein
MIKHTLLVPFFFSALPAYAGLTSITAGYDFTDYSGEHGNRNLAYAELVAKVENATLLFNL
SQGRRDYETEHFNATRGQGAVWYKWNNWLTTRTGIAFADNTPVFARQDFRQDINLALLPK
TLFTTGYRYTKYYDDVEVNAWQGGVSLYTGPVITSYRYTHYDSSDAGSSYSNMISVRLKD
PRGAGYTQLWLSRGTGAYTYDWTPETRYGSMKSISLQRIQPLTEQLNLGLTAGKVWFNTP
TDDYNGLQLAAHLIWKF
>LFGLNPFC_01222 Bifunctional protein GlmU
MIIDESAGEVVIGANTRICHGAVIQGPVVIGANCLIGNYAFIRPGTIISNGVKIGFATEI
KNAVIEAEATIGPQCFIADSVVANQAYLGAQVRTSNHRLDEQPVSVRTPEGIIATGCDKL
GCYIGKRSRLGVQVIILPGRIISPNTQLGPRVIVERNLPSGTYSLRQELIRTGD
>LFGLNPFC_01223 Undecaprenyl-phosphate 4-deoxy-4-formamido-L-arabinose transferase
MKTLLFIFMTIAMLLWFLSTLRRKPCQKKGCIDAIIPAYNEGPCLAQSLDNLLRNPYFCR
VICVNDGSTDNTEAVMAEVKRKWGDRFVAVTQKNTGKGGALMHGLKYATCDQVFLSDADT
FVPPDNDGMGYMLAEIERGADAVGGIPSTALKGAGLLPHIRATVKLPMIVMKRTLQQFLG
GAPFIISGACGMFRTDVLRKFGFSDRTKVEDLDLTWTLVANGYRIRQANRCIVYPQECNS
PREEWRRWRRWIVGYAVCMRLHKRLLFSRFGIFSIFPMLLVVLYGVGIYLTTWFNEFITT
GPHGVVLAMFPLIWVGVVCVIGAFSAWFHRCWLLVPLAPLSVVYVLLAYAIWIIYGLIAF
FTGREPQRDKPTRYSALVEASTAYSQPSVTGTEKLSEA
>LFGLNPFC_01224 N-acetyl-alpha-D-glucosaminyl L-malate deacetylase 1
MLNSTTSSGKRTGILAIGAHPDDIELGCGASLSYYSNEKVYIISVVMTPGCCGAEYIDRH
KESQEALNMLGCNKLIPLNFEDTRLYLQIKEMISSLEYIIENETPQDVEITRVYTMNNTD
RHQDHIAVYQASMVACRYIPQILGYETPSTCLSFLPQVFESVDEVHFNKKILALQKHKSQ
IHRRYIQPEYIIAQAKFRGGQVSHSMCEGFVVHKMVL
>LFGLNPFC_01225 Phosphoadenosine phosphosulfate reductase
MGTIQKIRTDVNVLHAAIHRIEWLFETFSSVCLSFSGGKDSTVLLHLTADVARRKKRRFS
VLFIDWEAQYQCTIAHILKMREMYRDVTETFYWVALPLTTVNGVSQFQPEWICWEPGVEW
VRQPPDDAITDMSYFPFYRYAMTFEEFVPAFSSWFAGNRCGVAILTGVRADESLNRFMGL
VSQRKLRYADDKPWTTASPEGFYYTLYPLYDWKARDIWIYNARTRAIYNPLYDLMYRAGV
PLRNMRVCEPFGPEQRKGLWLYHVLEPETWARMCERVSGAASGALYANESGAYFALRKRI
TKPPHHTWRSYAMFLLDVMPERTAEHYRNKIAVYLRWYQTRGFPDDIPDEQENDLGSRDI
PSWRRICKTLIKNDFWCRTLSFSPNKPRHYERYLQRMKERRKEWGIL
>LFGLNPFC_01226 putative protein YbdM
MGDSVTPEVEVLSNMIRQYFSQERSEEETIRALNHLRRVLHEVSPFAQEPVDCVLWVKAD
EVVANDYNPNVMSSSEKKLLKHSLEQDGFTQPVVVSEEKEHYLVVDGFHRQLLGRKADTR
KRLKGWLPVTCINPERKGQASRIAATIRHNRARGKHQITSMSDIVRDLSRLGWTDERIGT
ELGMDQDEVLRLKQISGLTELFQEENFSPSWTVR
>LFGLNPFC_01227 hypothetical protein
MEFLMTKTIVMEFFVLICPLCGQANERIVFVEGFSTLFSIWG
>LFGLNPFC_01228 Colicin I receptor
MLYNIPCRIYILSTLSLCISGIVSTATATSSETKISNEETLVVTTNRSASNLWESPATIQ
VIDQQTLQNSTNASIADNLQDIPGVEITDNSLAGRKQIRIRGEASSRVLILIDGQEVTYQ
RAGDNYGVGLLIDESALERVEVVKGPYSVLYGSQAIGGIVNFITKKGGDKLASGVVKAVY
NSATAGWEESIAVQGSIGGFDYRINGSYSDQGNRDTPDGRLPNTNYRNNSQGVWLGYNSG
NHRFGLSLDRYRLATQTYYEDPDGSYEAFSVKIPKLEREKVGVFYDTDVDGDYLKKIHFD
AYEQTIQRQFANEVKTTQPVPSPMIQALTVHNKTDTHDKQYTQAVTLQSHFSLPANNELV
TGAQYKQDRVSQRSGGMTSSKSLTGFINKETRTRSYYESEQSTVSLFAQNDWRFADHWTW
TMGVRQYWLSSKLTRGDGVSYTAGIISDTSLARESASDHEMVTSTSLRYSGFDNLELRAA
FAQGYVFPTLSQLFMQTSAGGSVTYGNPDLKAEHSNNFELGARYNGNQWLIDSAVYYSEA
KDYIASLICDGSIVCNGNTNSSRSSYYYYDNIDRAKTWGLEISAEYNGWVFSPYISGNLI
RRQYETSTLKTTNTGEPAINGRIGLKHTLVMGGQANIISDVFIRAASSAKDDSNGTETNVP
GWATLNFAVNTEFGNEDQYRINLALNNLTDKRYRTAHETIPAAGFNAAIGFVWNF
>LFGLNPFC_01229 Bifunctional adenosylcobalamin biosynthesis protein CobU
MILVTGGARSGKSRHAEVLIGDSSQVLYIATSQILDDEMAARIEHHRQSRPEHWRTVERW

QHLDELIHADINPNEAVLLECVTTMVTNLLFDYGGDKDPDEWDYQAMEQAINAEIQSLIA
ACQRCPAKVVLVTNEVGMGIVPESRLARHFRDIAGRVNQQLAAAANEVWLVVSGIGVKIK
>LFGLNPFC_01230 Adenosylcobinamide-GDP ribazoletransferase
MSKLFWAMLSFITRLPVPRRWSQGLDFEHYSRGIITFPLIGLLLGAISGLVFMVLQAWCG
VPLAALFSVLVLALMTGGFHLDGLADTCDGVFSARSRDRMLEIMRDSRLGTHGGLALIFV
VLAKILVLSELALRGEPILASLAAACAVSRGTAALLMYRHRYAREEGLGNVFIGKIDGRQ
TCVTLGLAAIFAAVLLPGMHGVAAMVVTMVAIFILGQLLKRTLGGQTGDTLGAAIEFGEL
VFLLALL
>LFGLNPFC_01231 Nicotinate-nucleotide--dimethylbenzimidazole phosphoribosyltransferase
MQILADLLNTIPAINSAAMSRAQRHVDGLLKPVGSLGKLEALAIQLAGMPGLNGIPHVGK
KAVLVMCADHGVWEEGVAISPKEVTAIQAENMTRGTTGVCVLAAQAGANVHVIDVGIDTA
EPIPGLINMRVARGSGNIASAPAMSRRQAEKLLLDVICYTRELAKNGVTLFGVGELGMAN
TTPAAAIVSTITGRDPEEVVGIGANLPTDKLANKIDVVRRAITLNQPNPQDGVDVLAKVG
GFDLVGIAGVMLGAASCGLPVLLDGFLSYAAALAACQMSPAIKPYLIPSHLSAEKGARIA
LSHLGLEPYLNMDMRLGEGSGAALAMSIIEAACAIYNNMGELAASNIVLPGNTTSDLNS
>LFGLNPFC_01232 putative L,D-transpeptidase ErfK/SrfK
MMRRVNILCSFALLFASQNSLAVTYPLPPEGSRLVGGQSLTVTVPDHNTQPLETFAAQYGQ
GLSNMLEANPGADVFLPKPGSQLTIPQQLILPATVRKGIVVNVAEMRLYYYPPDSNTVEV
FPIGIGQAGRETPRNWVTTVERKQEAPTWTPTPNTRREYAKRGESLPAFVPAGPDNPMGL
YAIYIGKLYAIHGTNANFGIGLRVSQGCIRLRNDDIKYLFDNVPVGTRVQIIDQPVKYTT
EPDGSKWLEVHEPLSRNRAEYESDRKVPLPVTPSLRAFINGQEVDVNRANAALQHRSGMP
VQISSGSRQMF
>LFGLNPFC_01233 hypothetical protein
MLINEELVDKIDTKNYGDGSNNVFVIWTLSLKSSTKSECFKFLGFVHLQSVLVEISSSPE
KYYKLRIHQERVMEGMMPEEPKAIVIEDTEWGCIHSQYHYQDKDPAIITCIVNVNNTDLI
ATELVNSVLNTS
>LFGLNPFC_01234 IS3 family transposase IS1351
MHDALVCGRRFRMFNVVDDFNREALSIEIDLNLPAQRVVRVLDRIAANRGYPAMLRLDNG
PEFISLALAEWAEKHAIKLEFIQPGKPTQNAFIERFNRTYRTEILDFYLFRTLNEVREIT
EKWLSKYNCERPHESLNNMTPEEYRQRHYLAGISKSVWN
>LFGLNPFC_01235 IS3 family transposase IS1400
MRKARFTEHQIIAVIKSVEAGRTVKDVCREAGISEATYYNWKSKYGGMEASDIKKIKDLE
DENRRLKQMFADLSLENRALKDVIEKKL
>LFGLNPFC_01236 4'-phosphopantetheinyl transferase Sfp
MRIDILIGHTSFFHQTSRDNFLHYLNEEEIKRYDQFHFVSDKELYILSRILLKTALKRYQ
PDVSLQSWQFSTCKYGKPFIVFPQLAKKIFFNLSHTIDTVAVAISSHCELGVDIEQIRDL
DNSYLNISQHFFTPQEATNIVSLPRYEGQLLFWKMWTLKEAYIKYRGKGLSLGLDCIEFH
LTNKKLTSKYRGSPVYFSQWKICNSFLALASPLITPKITIELFPMQSQLYHHDYQLIHSS
NGQN
>LFGLNPFC_01237 Putative HTH-type transcriptional regulator YhjB
MDKFKEKNPLSLRERQVLRMLAQGDEYSQISHNLNISINTVKFHVKNIKHKIQARNTNHA
IHIANRNEII
>LFGLNPFC_01238 D-alanine--D-alanyl carrier protein ligase
MDNTSGDFPCNKMDTRKQLPLTPSQQGFLFHSLKDKKRSNYHEHFTCIFSQHVDSAHFKW
ALETLFRKHECFRTDYNWEIDERPCQVVKTDVLPDIYVLDCEQEEIRFLLANDDIIIPVP
QDDGIDAIIPQLLQADLKYPFSLKTIPVRAYLIQSTKESAFILSYHHIVMDGWSLSLFIK
QLLQLYGAAVVSGVRDDSAIIPSSLKPLVDTLSARRHTFQHDYWAAYLREGTPTCIVPLS
QYHTDTEAENNSYVNQTNHVEINLSPDVCQKIQTLCSDYRITPAVIFYVAWGILLQRWCY
ADDVLFGATISGRNIPIDGIEETLGLFINTLPLRLRDDGATLLQHLQRMHQTLIAHYSNE
HDALASIQRLVHKEGHAGDLFNTLVVLENYPVDMTLLSCASPVAIRHLSVHEQTHYPLTL
TITQQKGFRFSIAYALNYLTNNMAQALLMHLSYLLEQLVDNPQRPIAALVNLSPCQQAQV
LQPYLERMACRDWDSQSNVIEQFHQVAATSPAQVAVVDELCALTYSELAAQAEQLAAYLV
QQGVMVGDTVGIISERRVNTVVAIIAIMLIGAAYVPISPDYPVGRMQEIIDDSGLALLLV
HGKPLDALNVAQSDLCAFPVAPSVVFPVITPDSRAYVIYSSGSTGKPKGIAVAHRGLLRL
IQGDSPLKVESGETTLLTCPFEFDVSVFEMWSTLLNHGKLVLLSKQALLDINHIRRTIAD
EQVARAWFTSSLFNSYVAEGADFFGMLQHITVGGEAVSAWHVNDVMQKYPHLVVTNGYGP
TENTIFTTAYRFNGLQPARVPIGYAVPGTSLYITDLHGHLLPIGATGELVAGGVGVAIGY
QNNPALSATVFVPDPFIPGGMMYKTGDYARLLDDGCVDCFGRKDGQIKINGQRIETGEIE
QRLLECSGIIEAVVVPYRVRETLHIAAVVCVNDSYDEVEVRGQLADRLPPFAIPESLVVV
TEIAKSHSGKADLAQLRYLLPATQCNAVSTTISEVHSDMEHALHAIWQRVLDRQDIDSNA
SFFALGGTSLDTIRVKGDIKRQLGLEIDITDLFKYPTLTALAHFLDTAVSPEDAIPTRAV
VYSDMPVAIVGMAGRFPGAANIAALWTLVVGGESGLTLFSDEELRAHGVTPDTLKQANYI
KTKGIVDDHEWFDADFFGYTPNEAECMDPQIRLLHQCCWQTLEHAGCDPATFTGAIGIYA
GLLTSPHWLNAVMQDTTDSTALYKASILNIHSVTALIAHALNLTGPAVTLDTACSTSAVA

IHQACIALRNRDCDAALAGGVSIEMPAYRGYEYHEGMINARDGVCRPFDSQASGTVTGDG
LGMLLLKRLDDALADRDCIYGVIKGSAVNNDGNNKIGYTAPSVIGQSTVIRTSLRRAGFD
SDSIGLVEAHGTGTVLGDPIELRALNEVFGPTPVPFCVVSALKSNIGHLNSAAGVAGVIK
TTLALHHQVLPPTAHFRQLNPAIDLSRSALYVNQQVQPWPSTRPRRALVSSFGIGGTNAS
IALEAHQHEDDPSATGVRDSYLLLFSAKTPAALELRVASTLEYVKHGVGVRLPDVAYTLQ
TGRTAFDHRRAYLVSRGSKIDLSCATILQAEIFNGQRTTAEICFMFPGQGSQYHGMASAL
YAHQPMFRQHMDRCFAAFQRYSTVDLKALLFDDEDTRDIDQTQFTQPALFCVEYSLARTL
IDLGITPDSMIGHSLGEYVAACIAGVFTLEDALHVIEARGRLMQSMRPGSMMAVYLSREQ
LTPWLAAERGIELAANNSAHFCVVAGEQAAISRLSTRLVEGGIQHRRLKTSHAFHSAMMT
PMLHDFAQLLGQIPMHAPHKRFISNVSGTWITEEQATSPDYWVQQVRNAVLFSEGAAQLL
VQPTLFIECGPGNTLSTFIQGHNQYSDQPTLLTLRKANAAIDDEHMLHRTLAALWVRGEN
IDWRRFNQTALGKHIPLPDYPFEQTYYYRYGAALSGYRQYPNPLRRPQDEWLQRVLWRMH
DTSLREAFYAPGELIIIISADGDKLQQTLMSSGVDSITMPLPISSEDDVWDNDRILTHFH
DICALLAHKTYRQLHCLYAPGAEAGSSLTQSLSGLYRVARWCMHSTTPLASLTVLTHGAF
RVQEEDNPEPTLAALSGAVNVFAQELHPTEVRLIDIDAQSSDENLNLLTQRLAPKQETVM
ALRQGMLYLRRFIPTRLLAHLPPQTGCIPGNVLWIIGGEKGIGRMIGEALAQREGVRVVL
SSRTGYHHEAVQQDALDVIHCDVTQAEAVRACLATLLERYGRLDGVIFAADATTTLTLHQ
LSESALRDTLTVKERGTANVLHALAQRNLLDERLLLLFCNSLAAVNAEIGQTGYATASAY
LDALAQQLRTRYKVNALSIGLDALREQGMLLDAINGSEYDVLRGLRPLMTGTLLQAYKQQ
GADTSYYARLSPESDWLLDEHRISGIATLPGTGYLALAYEALRHYFVQDQICIDELVFLA
PLTVMDNCSVDVFVDISPNGQGVSVEVKSMTERFSGTLTTHARGRATRLMVDDNVVCDLT
GLMREMHTITPPTKELSSTHFHYGPRWHSVQQLYGNTAQTQVFATLALPTVAANDTIALH
PALLDIASSVVEQLPGFHTDSVPFLYQDLRLYRPLPNTLHVALTVNRHDEEGDSYAFTLY
DMAGEMVARCAAMVKRKVQLHIQDVDDDTRLRVPSADNYQLRLAAEGEGAGKLALCPTPR
LALGDSQVEIEVLATGLNFKDVLFTTGLLRQQPGEAPLQLGLECAGRITRVGKNVTEFAP
GEDVMAVLNGGFVQYARVESDCVVRKPAHCRIEQAAALPIAYLTAYYALVVRANLQPGER
VLIHSAAGGVGLAALHIAKRCGAQIFATAGSEQKRDYLLSLGVHAVADSHDEQFAATLLT
ASDGQGMDVILNSLTGRLLDASLALLAPLGRFLELGSKDIVEDKALPMRFFAQGGTFIPI
NFHAAHGAFSRYLQQIVAWIDDNTLPLLPCKSVPLPEVARAFATLTTPQHIGKVVVTHRT
AAGMDRLNAMIAERRLGGYALSMSNAEVMRQLWPILNTRSPWAQLLLSPRAIDRLARGNR
VDRGVPSAANDTITQQTVKKRPRPEIGVPYSPATREVERVLCQILEEYLGLDRVGIDDNY
AELGATSLDMVQLSGQMARHYPQVSVVSLYNHATVRQLATFCQPPEGESNAPSPQPAVQT
NTRANQIAKRALQIAKNTARSHTSLH
>LFGLNPFC_01239 hypothetical protein
MEYASEMNGMEIAIIGMAVRFPQSRTLHEFWHNIVQGKECVTFFSEEELLAEGVEQSTLD
NPAYVRAKPYIEGICDFDAAFFGYSHKEAQTLDPKSRVLHEVAYHALEDAGYAQRTSDLI
TGVFVGASEDVDWLRRSLSQIGGDALNRFESGIYGHKDLLAHLIAYSLNLNGPVYSLYTS
CSTSLSATHIACRSLLFGECDLALAGGITIDLPQKSGYFCQQGMIHSTDGHCRPFDSQAS
GTLFGDGAGVVVLRRLEDALAAGDRIYAVIRGSAVNNDGKQKIGFVAPGHEGQKAVICAA
CHLAEVSPESIGYVETHGTGTRIGDPIEFAALTEAFDTSHRQYCALGAVKANIGHTHAAA
GVAGLIKTALVLHHRTIPPLANYQMPNSKLDLAHSPFYIPIQPQEWPASRMPPRAGVSSF
GIGGTNVHMILEGLNPAVRDDHDQVRAPVFIPLSAPSFEQLDELTQQLTPLLATLDASTL
AYTQQVARPVFDCRRVIQVENDGTQAMLASLDNLMPDAPWGLHCPDLRTTNDCTYAQWLA
HSAHYQREATALTALLDGMNIPPAYCHAETWAAQANSSLLIRGCQTIAALKTWMNLLPTL
TLLSGAGTGLLPAAAASGMIATQDVLHLLWEMEQKALHLWLPERHEPIPGYVLAWQGNPI
TDAQRNDRGFWSEALLADTRELGEGVHSINWVRLPPEIREDVDVLRYVAQLWCAGINVDW
AVWYGTPLPQRGSASAYPFAHNHYPLPGRVMGSVETQPEAGPETHHPYQARPVLSVPFVA
AHSRGMQYITGLMELLLEISPVGVDDDFFELGGHSLLVTQLTSRLERDFNVHIDLLTLME
NPNPRNIYAHIAAQLGGEDNLEIACQ
>LFGLNPFC_01240 3-hydroxybutyryl-CoA dehydrogenase
MNVAVIGAGVMGTGVAHNMAQYGISTNVVDISQSQLDKCRQMIEANLRLYNFHPQHKKKT
HSTAEIMENIRFTTELDDIVECDLVIENITEDIEKKNALYTRMNTICGASTVFGVNTSAI
SITALSKLMRHPENVVGVHFMNPVPLMHTVELIRGVHTAERTLNIFHHLFAQLNKTGIVV
NDSPGFVTNRAMMIFVNEAIFMVQEQIARAEDIDTLFKTCFGHKMGPLQTADLIGLDTIL
QSLQVLYESFNDDKYRPSFLLKKMVDAGYLGVKSGQGFYRYQQTYAEQ
>LFGLNPFC_01241 D-alanyl carrier protein
MKKQDMKAAIREFLSRSLRGHTLNDDDDIFSLGLVHSLFTVQIILFIEKNFQVELEVSEL
KTEQIATVNKIVELIQRQTGLE
>LFGLNPFC_01242 putative acyl-CoA dehydrogenase fadE25
MCTENYELAQQEAVLFAKQHLALAAQNIERQQFIVPDIISCVAQAGYLGASIPQKYGGRG
YDSYQLCALHEVMAGVHGSLENLITVTGMVSTLLQRVGSAAQKAHYLPKLATGELIGAIA
LTEPNIGSDLVNVETELQQDGDGWRLNGKKKWITLGQIADFFIVLIHCGNQLATVLIDRN
TDGFTITPLNDMLGLRGNMLAEFHFNDCRLKEDALLGPLTPGVPLAVNFALNEGRFTTAC
GSLGLCRAAVDVAARYIRQRKQFKRRLFSHGIVQHLFATMLTQTRSAQLMCFSAAEYRET

LHPAMINQILMAKYVASKAAVDVAGKAVQLLGANGCHADYAVERYYRDAKIMEIIEGTSQ
IHEIQIAMNYMMGSEA
>LFGLNPFC_01243 Malonyl CoA-acyl carrier protein transacylase
MTKDVALMFPGSGSQYVGMARWLYERYPQVRTLFDEASQITERDMAALCLSGTLVQLAEP
TAMALAIYTTSVAHFVAWQQFLAQTGVHVNLRYMLGHSLGEYAALTCSGALSFSQALALV
AMRSRLASEIAREMDASTTIIKQGNQALVAAACEVAERETRQQVGIACFNSPQQFMLSGQ
NSAIIAAEQYLLDHDRQVEVVPLIGGVPYHSPLLKPCGQQLRKALDRCEWRRPCCPVISN
VNAQPYPDTVTPVQWLEQQLSQPVQWQRSLTYLTGHLSPIAIEIGPQSVLKNLLLENRYP
APVYAFDNRHDRAQLALVLGDNMAVKTDPEAVRRQRITLLTNALTATRHHRAADVAASAQ
LKELLSRFFERIQQIEQRGTSSEEDIAFLHELLEQGFQLKGSSQAEIDACHARLASDNGG
QA
>LFGLNPFC_01244 Gramicidin S synthase 2
MEQQGIMRQLPTDDQTIVDYLYRIAGEYGEKAAVLMGDAALSYHDLNARSNQLAHYLRGL
GIGEDRVVAIRLPRGMAMLIAIFAIVKAGGAYLPLAYNAPRSRIENILSNSGAVCLIGTD
DGDRWPIPRVEIDSAAVSAMPTTDLRYRPHARQLAYIIYTSGSTGVPKGVATEHAALLNR
IVWMQNAYPISSQDVLFQKTVYTFDVSVWEMFWWAMYGASVVLLPSGLESDPRTLARLIQ
RHRVSVVHFVPSMLNLFVEYLEMKQDPRLTASLRLVFSSGEKLTVHSVARFYQSVAQGDL
INLYGPTEAAIDVSHHRCLRGYDYDDIPIGQAIDGCRLYVLDDHGNPVADGEEGELYLAG
IGLARGYLNNVALTDRCFTIHPTLRHLGKPERLYKTGDLVWRDGESQQIHYIGRNDFQIK
IRGLRVELGEIEAHAMRFPGVQQAVVVADQDDPDNQLIYAFVVSSVPLNLAALMDALSKN
LPAYMLPNRLLAMSELPLSDNGKCCRKTLLDLARAYSASRVDLRETPAVRYLPLSSAQSS
MWFMQQLAPHTALYNNPTALLLEGELDRTRMDGAIRQLMSRHTLLRAMAETHNGQPVLAV
PQCVSSQALLTIVPLPSVSDDNALQAMINQRAAHPMPLTSGTPLCRFELLTLDDDRSVLL
IHLHHIISDGWSKGVLLRELQAAYNGESLTPEPLLEYADYMEYQEEWRQSDAYQDAMRYW
QNTLAGTLPILDIPTDQPRQKVARYQGAFVAFALSANTCERVLAAARAQRVSLYNYLLTA
FVLLLHRNARQQEYIVGMPIAARLTKEQEHMIAPLVNVLPLRLPLDEAASFSELVQTIRG
ILFAAFRHQRLEFTDIVRAVNVDRSAGHFPIYQCMFQLDNMPLASPTLNGVNVTPLLLDT
SASQVDISLSMQHIDGRITGTFEYDAGLYSADRIQHLVAQWRELLDEASSQPTQLVRDLI
RFTPREHAWLARHNATEVALPPVDNLLALVLPHCQQRPTQVALRHADDAMTYGELQQATM
QMCTWLRAQGVKRGESVALQLPFCFELIIAQLAILSLGASYVPLDGNAPAARNALILAQA
TPCMLLVAQPLESPHGLTIPWVLVPDWRSLLTEIPNLPVSVAPDALDCDAVVIFTSGTTG
QPKGVRLSQRNLVNLTASFISSYQVTHQDVLLPITSVASASFVGEVLPLLAAGGTLVLAQ
KAQSLDSDALIALLASQRVTILSTTPSLSASLSVLAQSMGSLRLFLCGGEALEYEQIAPL
LPHMAVVNGYGLTESGICSTYFPVAKRREQETGALPIGRPIQNTQAYVVDAYNRLVPPGA
CGELCFSGLGISPGYLDARQDPERFVELPEYPGVRVLKTGDRARWATDGMLFYLGRQDRQ
VQIRGYRVELGDIESLLKQHPDIADAWVDVRRNAAATPLLVAFYCSVNGVALDAQQLRVW
LSLRLPLHMLPLLYVPLSAMPLGVNGKIDPQCLPLVDLRQLEGPGEYVPPATELEQRLAE
IWQQLLGLERVGTTTNFFDLGGHSLLLVQMQQYIGQQCGQHVALVDLLRFTTIKRLAEFL
LAPDAAQGTTGDQTQLRAAKQRLAFGHTRWAATTDSHH
>LFGLNPFC_01245 Phthiocerol synthesis polyketide synthase type I PpsE
MAENDFGIAIIGMAGRFPQADTVQAFWENLLASRECISFYSDEELLAMGISPEFVQHPDY
VKAKGEVADIDKFDAAFFGIAPREAELMDPQHRVLLETAWAAFEDAGYVAADYPGDVGIF
AGKSMDSYLMLNLMPHFKRVFSSGSLQAAIGNDKDSITTTIAYHLNLRGPAITVQTSSST
SLVAVCVACQSLLTWQCDMAIAGGVTLGPPAKTGYLSQEGGITAADGHCRAFSDNSSGFV
PGTGAGLVVLKRVDEALRDGDNIYAVIKGFAVNNDGSEKISYTAPSVDAQARAIAQAQRL
AGLTPQDITYVEAHGTGTRLGDPVEFSALSQAFAGASQKQYCALGSVKTNIGHLDTAAGV
AGLIKTALAVQQGIIPATLHFERPNAQIDLTNSPFYINTTCQPWQPESGIRRAGVTSLGM
GGTNAHVVLEQAPAVDLQARAPVPAYSILPFSAKTDSALSSGLARFADFLQHESLPDRRD
LAWTLSQGRKAFAHRAALVTRDLHAAGTLLQQAATAPFARGVAQTQLGLGLLFSGQGSQY
QRMGHQLYQVWPAYADAFDRCATLLEREYQLDIRHELFRAEVSLAQGERLAQTCLTQPLL
FSVEYALAQLWLSWGITPTVMIGHSLGEWVAATLAGVFSLEDALRLVARRAELMHQAPSG
AMLMVALPEAQIRALITAPLAIAAVNAPDYSVIAGPTSEILAVSQRLTEQNIINKRLHTS
HAFHSSMMQDAAQALRQAFENVRLNPPTLTIISTVTGAHVSADTLTTPDYWIEQMLMPVQ
FSAALQEAQATFDVDFLEIGPGATLTQLTNGHALGDRLAFSSLPAGARSSDEHKHILDTV
AALWVRGHNIDLSAFAGEQPRRVSLPTYAFDKIRYWVDSPEEQRSAVTPVADAGSVIPSE
PSVRRQPRPAFSVPYAAPESKTQRGLVAICEALLGIDGLGIDDNFFEAGGHSLMLGMLLA
QVQERFAVTLSFFDVMEDASVRALAQLVEQEQQDDGGSALAVLVNDMINE
>LFGLNPFC_01246 Tyrocidine synthase 3
MTIHHAALARMLPAEKKEKLLRQLAQSGVSPSRIPIIKADPAQAIPLSFNQERLWFLQKY
DSTATNYNLYVVYRLHGVVDMPMLTEALRHVQARHAILRTRIIVRNDRPCQVIDDASSLV
LDTVTLAAQAPTSALDAVIQQVINTRFDLARGPLWGVTQIIQPDQGCHLVFCAHHIIIDG
ISLRLLFDELQQQYARLHAGNETSLPPPPLQYADYAFWQREWFQDTLLANELAYWRARLQ
DAPLLSTFPSLHPRPAQPSTHGSRFSITLDETLSLALKHVARTQETTPFVLMLTAFQLVL
MRYAQQQRLVIGMPVSGRIRPELQSSIGYYASTAVIYTDFNGVEVGREALQRVKASVKET

QGRQQLPFENLVNMLDLPRSLSHSPLFQILYIYHNHVTPRAFTLAGAYWEQVTYHNQTVK
YDMTVEVFQNDATFDVSFEYDLGLYDADVVKQIAEALRQHCLSLTSSLETPIGAIPLHAP
ETATPRRDPLNATNVPWLGPQDVLRIIEQRCVQHPKQLAIQQHDGTLTYAELWARVQFIA
MRFRAHGIQPGDRIGVLLPRHRDVIATMLATWFVGACYVPFDIHQPAARLQRLMQRARLV
CLVVRQPGEWGEIVQLSLPELMQDMSNAIRYSTPCALLPDMQAYLLFTSGSTGEPKGVCV
VHRGLLNLLLDMQRTFAVGSQDRLLSVTTPTFDISFLEYLLPLISGASLYLTEAERAADS
FRMIPLIADYRPTLMQATPSFWHGLLMAGWRGDPELCVLAGGEALPTKVAEELLRCCGSL
WNLYGPTETTIWSLKSQITQAENITLGAPIANTRIYILDNEGHPVPQGVDGELYIAGDGV
AQGYDGQPELNAQFFLSEPGVPGGRMFRTGDLVRSDAQGQLFFVGRKDSQIKLRGYRIEL
GEIERTLARHPHVDAAVVACIERAPLHKALAAFIITSEPPSLFEQLKNELRQQLPDYMVP
TLWQRVADFPNTDNGKIDRKRLAENFVADSSLVSPQTQALSDTEQMLLALWMRYLPIKNV
DPECDFFRLGGHSLLAVTLVAEINRTFHCALTLKDIFHYSTLRALSARIAQQSITDAAAS
QDDWVIVHDPEHRHQPFPLTDVQRAYWLGRQTGATSIATHIYHEFDVEHFNVTRFTHAVN
ALIARHEMLRARVLPDGTQQILAQVPAYQLEQRDLSALSPNARNDALMAIRDRLSHHVHP
ADRWPLFDFSYSACTAQHGRLHFSLDLLIADALSMRTLQQELMMLYREPHVSLPLLPFSF
RDYVQALLVEQASEAYARDQAYWQRALPQLYGPPTLPVQGDLAQLSAIRFVRRRHRLSAH
NWGVLSALAQRTRITKTALLLTVFSQVLARWSLSPTFTLNLTLFNRPQGYPNAEAVIGDF
TAVSLLNVCYDSQHSYAHNAQRIQVQLWEDLEHRRFSGIRASEALIHSGRFHAPMPVVFT
SMLDIDGETTAQDPRDTTRFTLCPDANITQTPQVWLDHQVIELAGELHFNWDAVEQLFDT
TLLDQMFGAYCHALQALVAMPQSWWGVNSSLALPTVSAPVTQAPAPTALLHHGLLRQAAL
TPQETALISPIRELTYRQLSTAADHVARALLALGVQHGDRVAVVMEKGWQQIAAVHGILR
LGAVYLPVDPVLPPQRRQLLLTVGEVRVQVTQPGLTQLEPSLPVLIIDDGMLDTPAAPLP
EVAGDVTDLAYIIFTSGSTGTPKGVMIDHRAAMNTLEDINERFGLNAQDRVFGLSSLSFD
LSVYDAFAPLWWVQRWYCRKQDGKKIRVIGRQLWHTVM
>LFGLNPFC_01247 Tyrocidine synthase 3
MPALMQMLCEYHSGDRMSYPTLRLALLSGDWIPLTLPEQMRERLNETMDIISLGGATECA
IWSVYYPIGEVESTWTSIPYGRGLRNQPVYYVLNAQLEECPVGVEGEICIGGMGLAQGYLN
DAEKTAASFVWREASGERIYRTGDRGRYFADGQVAFLGRNDTQVKVNGYRIELGEVKSHL
EQLDSVGSAAVVCHQGQLYAFITAAENLHPDDTDALLARVRAQLAVQLPYYLLPQHFFLL
KVLPMTGNGKIDQAAMVQEVIQRMSQSTSQKSRALAHASPYEQQVAALWCEVLQREQIGL
NDNFFEAGGGSIQIVLLHRRIEEIFKVTVPIAELFRLTTVKRIAGYLQAMQDNARAVNQT
QQRDASRSRAQQRLVRRHQRQR
>LFGLNPFC_01248 hypothetical protein
MTYSESDIAIVGMNCRYPGVHSVAAFETVLRTGCNILDPKVTPSNGHNHITLNNVYEHMA
EFDANFFGYSRAEAEIMDPQQRVFLTCAWEMFEQSGYNPKQHDARVGLYAGVSTSFYLLT
HLMNNPDKLAQLGGLQIMVGNDKDHLTSQLAYRLNITGPCVTVQASCATSLVAVHLACEG
LLSGQCDMALAGGVTFRMEEQRSYESHGDGLQAEDGLIHTFDAQASGTVYSSGLGMVLLK
RATDAQVQGDNILAVIKGSAINNDGGARSGYTVPGVDGQEAVMIEAHSLAEVTPQQIQYL
ELHGSGTPLGDAIEFAAIKRVFGTPAPNATPWRLGAVKPNVGHVEMASGITSLIKTVLSL
TNRVFYPTLNFQRANPQLGLEDSPFEVVSRLTPWPEGTTPRTAGVSAFGLGGTNAHLVVQ
APLSTPQARAQQMGPCVVVLSAKNHNALEQMQNALLAKLAAHPEIRLQDVAYTLRHGRFS
APVRKCVIAENCTQLARQLRDAPMVEATTGCTIYWRLGHRFVVALETLSDWLACSEVLSQ
AVGQLLEHFPLEPACLQDLSPAQRTFISQYALIALIDERETLNVVLCGDGDGGYAAAVLR
GDCTLEQAWHRLNAGQPFDDVPTNPLLQPDVCSLMLDDAASDANRTALEALGQLWLAGVS
LDWRWVDAAERMLGSQRIALPGTVFTPQRYWVEAVRPATFSHESSNNLLSRATKSDIIAV
VTEIWERTLGVSIDDHHASFFELGGHSLLASTILYDIQQRYGITCTLSAFFADPTIEGLS
CYLLEQGGSETAVSALPDTVFAPDQQHLPFPLTDVQQAYWVGRRKSLGLGNISTHIYVEY
ELQGLDETAFNRALNAVIARHSMLRAIVNDDGMQQILPNVPEYHVAFYTTQCEDAFQQRC
RELRDTLSHQMIDCSRWPLFQMEVVVDPQQKARLHVSIDLLIADAWSLELFIRELAYHYR
HPQAALPTLTYSFRDYVLTLKSYEKTPQFERARDYWRARIETLPPGPRLPLRTDPTKLEN
PTFVRRSYCLSRAIWQRLKTQAGQMSITPTTLLLTGFAQVLARFSSSPHFSLNLTLFNRL
PLHADINHLIGDFTALTLLEIDMSQGETLQARANVIHSQLWRDLDNRLFGGIQVSRLLVQ
THRDPAKSVIPIVFTSLLNQYEASWETDDTLFNQPQDDLYSISQTPQVWLDHQVMERNGE
LHFNWDVVEQLFEPALMDQMFQCYCQLLHALAQRPQLWHETQDVLALPTVSAPVTQAPAP
TALLHHGLLRQAALTPQETALISPIRELTYRQLSTAADHVARALLALGVQHGDRVAVVME
KAGSRLPPYTAFYDWVRSICQWIRCYRHSVASFC
>LFGLNPFC_01249 D-alanine--D-alanyl carrier protein ligase
MLPPQRRQLLLTVGEVRVQVTQPGLTQLEPSLPVLIIDDGMLDTPAAPLPEVAGDVTDLA
YIIFTSGSTGTPKGVMIDHRAAMNTLEDINERFGLNAQDRVFGLSSLSFDLSVYDAFAPF
MVGAALVLPEAGREKDPRHWQTVMAHGHVSVWNAVPALMQMLCEYHSGDRMSYPTLRLAL
LSGDWIPLTLPEQMRERLNETMDIISLGGATECAIWSVYYPIGEVESTWTSIPYGRGLRN
QPVYYVLNAQLEECPVGVEGEICIGGMGLAQGYLNDAEKTAASFVWREASGERIYRTGDRG
RYFADGQVAFLGRNDTQVKVNGYRIELGEIERCIARHPDVEQSVVVAVGNSQHRRLVAFA
KLHDRHQAQALQAKEAEAAALAQGIIVNPAQRLAFKLKEPHIRALDGLGIALTAPADSTR

YIKRRSYRHFSAQKTTLAQLGQLLSGLGQMRLPGLPFAKYAYASAGGLYPVQTYVYLHPD
KIEEGVSGIYYFDPRQSCLMPVAPEVELNSGFHAGPNQSIADRAAFTLFMVADMAVISPF
YGQEAAWHFSVMEAGTLCHLLEEDAPRYGLGLCQLGMADFSAVASHFQLSPHHRYVHCTV
GGAIGQEAASAAALLRDFSTYEKPKETAAPLDMQSYKDAMLRGLRQQLPDYMVPSDLMLA
TDFPLTANGKLDRQKLQLQGEQIAHQRDGVGPIQVDSALQQRLVALWQEVLGVSHVSAED
DFFSLGGSSIELVRIQQALEAIIGQEIPIVDLFRLPTIADVARYLDEQLHNLPAAHDIVL
AQAEVSQVSAARENLALRRKRAQQGEKGDE
>LFGLNPFC_01250 Acylamidase
MSEQSYRSAGTLLAQLASGETTSVALVNHYFSRMAQFNKPLNAVVQQHYALALEAAARAD
RERLEGRARGVLHGLPCTVKESFDVQGWLTTSGAHYLKDNRATQDAPSIARLRAAGAILM
GKTNVPMMTADWQTYNDLYGTTHNLWDRQRSPGGSSGGAAVAVAADFTPVEFGSDLFGSL
RIPAHYTGVYAHRCSLGLMSVRGHVPGGGPQATDEPDLSTAGPMARSAADLRLMMRALST
FWVEPPRIPDFSRYQAKANYRVCTWFSAPHHEIDQQIAQRFQSFIDKLRAQPGVEVDDAM
PADIDPDALFDIAVKLSGRLVSTALNGRQRLTAGLAALGFRLVGKLADVPEGITSYYQGM
LKDSGEQRNTDKLRHEYSRVIETLFARYDVLLTPVSPVLAFAHMQQPVRKRKLIVNGEPQ
DYNEHLFWNMLATVFGLPATVYPLAKTMDELPCGIQIISGHFHDDVTINFAEFCESISGG
FTVPEGY
>LFGLNPFC_01251 Multidrug export protein MepA
MTVNKLEPDNGTPNDELFTVAGMFDGSLYKLLLRMALPMFVGMLTQVTYAIADIFWLSHI
DVTNSGIIAGVGLVFPVGMGLFAIANGIQIGMGSLLSRAIGMQRLDRAQRILSVGIIIAL
FFAIVITVLGYVYAQPLLRSLGATKSIIGYATEFYYYSLLTVFSIMLIGVMMGLFQGAGK
IMVIMKASLLGALVNIMLDPIMIFVFDFGVKGVALASFLAQLSMVAYFIYTLMGLHIGLS
IRIALRPFSWKIYREFLSVGMAQMLMQLIIAVGIVIYNFFIVRLDVNAMAAFTLTGRIDY
FIITPMLAIATALLTVVGQNWGHGNVTRTLNAYWAAVALAFSIVLVLAVMHIVLAPWMYP
LFTRVVAVSDYAVLQTRIMALALPFVAISLLASEYYQAIGKPWYSVLLTLMRHVFISVPV
VYLLAIVLEMRITGVYFGAMSGTFVAALLAWRLLRLSPRLLRWNQEAVRSQHLDMEVAP
>LFGLNPFC_01252 Surfactin synthase subunit 1
MMSGNPLSWPQEQCHIIDQLYPYSAVNIIGGVVTIEGIVDLPRLHAAIQSAIRQFDALRM
WFVMGEESEVVSQVQPYHWRDIRHLTFSPDYDKENLRPAAIETFVDEWFRQPFTLLAHDL
FEFVTFTCGEQYSGYLFKAHHGIADGWSMALLSNHVKRAYEQQDVPDDASPAYSAFLAQQ
QSYQASTRFAVDRGWWRDYIDEYRDCFPDSSPIVTTEGISCSTWLEPAMINRLYRLCNRY
GCTLNTLFIALFALYRARVWGEEKGVIGVPLANRHTREARRCFGMFTNQLPLAYRLVRTE
RFCERVAFFQRELKRGFKHSKYPITLFNQDLAEQGGGKLRAFDYCVNYYNFTYERHIAGA
AQRVESYYSGEQSYKLQIVLQTVNNHKESLRLSLEALRSAFTPHQLTAMKNGLLDLVTAL
DRQPDARLGDLEVYPAPHVALACGSLKPSFTSRFAAQVVEHGDRTALIDNEQSLTYRQLD
DAVERVARYLRQQGIGRGQVVGIIAEHSAQTVMVIYGILRCGAAFLPLNPALPTTRLYAM
CRKAQVAHILYDPAMHELTQALAFPASSLLQALATSALAREPWPAIEPQDLAYVLFTSGS
TGEPKGVQVSHGNLANYLHFAAERYFTAQDRAALYSSLSFDLTITTLFAPLCVGASISVC
RHAESETLLRMAVVDQPNTVIKLTPAHLRLLCAAGISSEQIRTLVVGGEDFKRDLARKAA
ALFPQAVIYNEYGPTEATVGCMIYRYTGQETLPSLPIGMAIDGCQVAICSPWGCPVPEGE
TGELVIYGASVTQGYIDAPQQTAAAYLKDTNGVMIGYRSGDIGYAIAPNTLVYQGRKDDQ
VKINGYRIELCEIEQALLSAPQVESAAVAVIDDVQGQHSGLLACVTPSSVDVATVMQHLR
QQLPTYMQPKQCCAIAQLPLSHNGKVDVRQMVATVRNTAPASGSERLGDAAIRHSVRVCV
EGALEQTEFDDNENLYVLGLDSIKSIQIAAQLRHHGWTMSAVQVMECGTVNAICEFLASH
TTVSQLAQYAHNTRIDLPALRWFTQLALPVPNVYNHVIVLKVLPGCPLEQLHNRLHTLIQ
QQPALHSALDAEGRLLVCDPNVCYPNEVLTEYSTAQWTLAEVIAQCNSMLDVTNGRVFTA
ALLHAPQPASSTLVLCAHHLCVDMHSWYLILSTLDAVSTVNGTSNSGLHRWNDYLASKTV
DSATHESWRTVCQTLPLHFPPVSLPDDSLPRTRAWREDFRHPCVRRLFESSGNTAYSAET
YVLTALALVLRYYSEEPWCRIEMEGMGRGCWPDEPDVADTVGWFTLFYPWAIPLHGDMAT
LLSAIASDLAKRTHGGGDYGLLQMRHAPEDSLAQGIRMNYIGVQAQPSLRYFHIDHFNSD
IYTAPENALGCVLEFNIARSAADGLSFHCRFDPTRIALNDVQLLLARYKNSLTDLDAWLC
QHSATLTGAPTLWTL
>LFGLNPFC_01253 hypothetical protein
MAKDDFTCGSLDIAIIGMSGRFSGAESVPEWWDKLLAGEEFTQPTCTEDDNGNPWIRLRN
IITAPYDFDAAFFNIPPGEALLMDPQQRIFLECCYNALEHAGYIPTQLKRVGVYGATYAN
NYFIDRVYPYLKMSGDHHYLQAQIGNEKDYLCAQVAYKLGFTGPAVSVQTACSSSLVAAY
LACEGLLTFQADVALAGGVTLGFLQAHGYSPQGDKLVSQDGHCAPFSAEATGTVYSSGAG
VVVLKRLEDALRDQDRVYAVIKGGAVNNDGGRRLGFVAPSVEGQVEAINTALAAAEVVPT
DIALIETHGTGTPLGDEIELEALHRVFAPACAPHSIQLGAVKANLGHLGVASGIVSLMKT
ALTLYTGLVPPQINLVNKHKKLLQPASPFYLSDVVTSVPQTKRIHATVSSFGLGGTNAHL
VLQNWCETPAQAVQENERRLFFFSAKTPLALRQQLDAHYHALATYAEADKDRIAYTLAQR
RAHFPYRCALAADSVVALRASLAKLRDADMSFTPINMETTLVFLYPDRDDKLESALTHLL
ACQPNLRQRHQRLSQDVAQICEPADWTPALRQFIQQVSLSEWLIEQSISPVQHIGYLTGA
AAAQYVARIISLENAVQQVIVAETTPEQTLAGNSELSEILANLAVTEGTLMLEIGRAGTF

SILYHQHAQWVGQTVFSPMLNTDTPEDILPLLGTLWQRGVTICLPEMPAVQTIGLPGYSF
DRVRYEIQSSDARENAMLPVSYLSVSDFVEKTWRSLLCIDHYDEHAVIFEYGATSMHVIS
FVDSCNHIYKIGLTAADIYARPAIREHSEFISECVDGIL
>LFGLNPFC_01254 Protein flp
MEHVSIKTLYHLLCCMLLFISAMCALAQEHEPIGAQDERLSTLIHQRMQEAKVPALSVSV
TIKGVRQRFVYGVADVASQKANTLDTVYELGSMSKAFTGLVVQILIQEGRLRQGDDIITY
LPEMRLNYQGKPASLTVADFLYHTSGLPFSTLARLENPMPGSAVAQQLRNENLLFAPGAK
FSYASANYDVLGAVIENVTGKTFTEVIAERLTQPLGMSATVAVKGDEIIVNKASGYKLGF
GKPVLFHAPLARNHVPAAYIHSTLPDMEIWIDAWLHRKALPATLREAMSNSWRGNSDVPL
AADNRILYASGWFIDQNQGPYISHGGQNPNFSSCIALRPDQQIGIVALANMNSNLILQLC
ADIDNYLRIGKYADGAGDAITATDTLFVYLTLLLCFWGAVVVVRGAFRVYRATAHGPGKQ
QRLRLRVRDYIIALAVPGLVAAMLYVAPGILSPGLDWRFILVWGPSSVLAIPFGIILLAF
VLTLNHQIKRILLHNKEWDDE
>LFGLNPFC_01255 Thioesterase PikA5
MSNISLYCLPYSGGSAAMYYKWRSVLSDNITLRPLEPAGRGTRIRQPLCLTMVDAVADLY
QQFVKHYTGGDYAIFGHSLGGIMAFELVHYILDHGHDMPCALFFSGCRPPDRASHEVILH
TLPDQAFMEEIVKLGGTPVDVFRNKELMTIFTPIIKNDYRLYEQYVFQAKARTLTCPIVL
FHGDADNLVMQDELLAWEKFTTRKTRTIIFPAADHFFVDKHFEQVVGYVNQTIESLEIVG
>LFGLNPFC_01256 hypothetical protein
MAVPSSKEELIKAINSNFSLLNKKLESITPQLAFEPLLEGHAKGTTISVANLVSYLIGWG
ELVLHWHDQEAKGKTIIFPEEGFKWNELGRLAQKFYRDYEDITEYEVLLARLKENKQQLV
ALIERFSNDELYGKPWYNKWTRGRMIQFNTASPYKNASGRLNKLQKCLAE
>LFGLNPFC_01257 Prophage integrase IntA
MSLNDAKIRSLKPTDKPFKVSDSHGLYLLVKPGGSRLWYLKYRINGKESRIALGAYPAVS
LSDARQQREGIRKMLALNINPAQQRAAERGSRMQEKMFKSVALEWHSSKKKWSQNTADRV
LARLNRHVFPTIGHLPVTELKSRHFIELLKGIEEKGLLEVASRSRQHLSNIMRYAVHQGL
IEINPAANLDGVTASPARRHYPTLPLERLPELLERIDSYHQGRELTRLAVLLTLHVFIRS
SELRYARWTEINFRNRIWTIPATREAIAGVRYSSRGAKMRTPHIVPLSEQVISILKRIKE
ISGGYELVFPGYHDPYKPMSENTINKALRQMGYNTKQDICGHGFRAMACSALMESGLWSQ
DAVERQMSHQERNTVRLAYIHKAEHMEARMDMMQWWSDYLDMCSEIWVPPYIWSQQNINL
AVT
>LFGLNPFC_01259 putative FMN/FAD exporter YeeO
MNISSALRQVVHGTRWHSKRKSYKVLFWREITPLAVPIFMENACVLLMGVLSTFLVSWLG
KDAMAGVGLADSFNMVIMAFFAAIDLGTTVVVAFSLGKRDRRRARVATRQSLVIMTLFAV
LLATLIHHFGEQIIDFVAGDATTDVKALALTYLELTVLSYPAAAITLIGSGALRGAGNTK
IPLLINGSLNILNIISGILIYGLFSWPGLGFVGAGLGLTISRYIGAVAILWVLAIGFNPA
LRISLKSYFKPLNFSIIWEVMGIGIPASVESVLFTSGRLLTQMFVAGMGTSVIAGNFIAF
SIAALINLPGSALGSASTIITGRRLGVGQIAQAEIQLRHVFWLSTLGLTAIAWLTAPFAG
VMASFYTQDPQVKHVVVILIWLNALFMPIWSASWVLPAGFKGARDARYAMWVSMLSMWGC
RVVVGYVLGIMLGWGVVGVWMGMFADWAVRAVLFYWRMVTGRWLWKYPRSEPQKCEKKPV
VSE
>LFGLNPFC_01261 HTH-type transcriptional regulator cbl
MNFQQLKIIREAARQDYNLTEVANMLFTSQSGVSRHIRELEDELGIEIFVRRGKRLLGMT
EPGKALLVIAERILNEASNVRRLADLFTNDTSGVLTIATTHTQARYSLPEVIKAFRELFP
EVRLELIQGTPQEIATLLQNGEADIGIASERLSNDPQLVAFPWFRWHHSLLVPLDHPLTQ
ITPLTLESIAKWPLITYRQGITGRSRIDDAFARKGLPADIVLSAQDSDVIKTYVALGLGI
GLVAEQSSGEQEEKNLIRLDTRHLFDANTVWLGLKRGQLQRNYVWRFLELCNAGLSVEDI
KRQVMENSEEEIDYQI
>LFGLNPFC_01262 HTH-type transcriptional regulator GltC
MNFRRLKYFVKIVDIGSLTQAAEVLHIAQPALSQQVATLEGELNQQLLIRTKRGVTPTGA
GKILYTHARAILRQCEQAQLAVHNVGQSLSGQVSIGFAPGTAASSITMPLLQAVRAEFPE
VVIYLHENSGAVLNEKLINHQLDMAVIYEHSPVAGVSSQALLREDLFLVGTQDCPGQSVD
VNAIAQMNLFLPSDYSAVRLRVDEAFSLRRLTAKVIGEIESIATLTAAIASGMGVAVLPE
SAARSLCGAVNGWMSRITTPSMSLSLSLNLPARANLSPQAQAVKELLMSVISSPVMEKRQ
WQLVS
>LFGLNPFC_01264 putative transcriptional regulatory protein YeeN
MGRKWANIVAKKTAKDGATSKIYAKFGVEIYAAAKQGEPDPELNTSLKFVIERAKQAQVP
KHVIDKAIDKAKGGGDETFVQGRYEGFGPNGSMIIAETLTSNVNRTIANVRTIFNKKGGN
IGAAGSVSYMFDNTGVIVFKGTDPDHIFEILLEAEVDVRDVTEEEGNIVIYTEPTDLHKG
IAALKAAGITEFSTTELEMIAQSEVELSPEDLEIFEGLVDALEDDDDVQKVYHNVANL
>LFGLNPFC_01265 AMP nucleosidase
MNNKGSGLTPAQALDKLDALYEQSVVALRNAIGKYITSGELPDENARKQGLFVYPSLTVT
WDGSTTNPPKTRAFGRFTHAGSYTTTITRPTLFRSYLNEQLTLLYQDYGAHISVQPSQHE
IPYPYVIDGSELTLDRSMSAGLTRYFPTTELAQIGDETADGIYHPTEFSPLSHFDARRVD

FSLARLRHYTGTPVEHFQPFVLFTNYTRYVDEFVRWGCSQILDPDSPYIALSCAGGNWIT
AETEAPEEAISDLAWKKHQMPAWHLITADGRGITLVNIGVGPSNAKTICDHLAVLRPDVW
LMIGHCGGLRESQAIGDYVLAHAYLRDDHVLDAVLPPDIPIPSIAEVQRALYDATKLVSG
RPGEEVKQRLRTGTVVTTDDRNWELRYSASALRFNLSRAVAIDMESATIAAQGYRFRVPY
GTLLCVSDKPLHGEIKLPGQANRFYEGAISEHLQIGIRAIDLLRAEGDRLHSRKLRTFNE
PPFR
>LFGLNPFC_01266 Fosfomycin resistance protein AbaF
MDSTLISTRPDEGTLSLSRARRAALGSFAGAVVDWYDFLLYGITAALVFNREFFPQVSPA
MGTLAAFATFGVGFLFRPLGGVIFGHFGDRLGRKRMLMLTVWMMGIATALIGILPSFSTI
GWWAPILLVTLRAIQGFAVGGEWGGAALLSVESAPKNKKAFYSSGVQVGYGVGLLLSTGL
VSLISMMTTDEQFLSWGWRIPFLFSIVLVLGALWVRNGMEESAEFEQQQHNQAAAKKRIP
VIEALLRHPGAFLKIIALRLCELLTMYIVTAFALNYSTQNMGLPRELFLNIGLLVGGLSC
LTIPCFAWLADRFGRRRVYITGALIGTLSAFPFFMALEAQSIFWIVFFSIMLANIAHDMV
VCVQQPMFTEMFGASYRYSGAGVGYQVASVVGGGFTPFIAAALITYFAGNWHSVAIYLLA
GCLISAMTALLMKDNQRA
>LFGLNPFC_01267 hypothetical protein
MFLVSLLRRIAFSYYDYKAYNFNIEKTDFVVIHIPDQIGDAMAIFPVIRALELHKIKHLL
IVTSTINLEVFNALKLEQTKLTLVTMTMQDHATLKEIKDLAKNITLQYGTPDLCIEAMRK
KNLKTMIFISQLKAKTNFQVVGLTMKCYSPLCKNASRMDQNLRTPVPMTWAFMMREAGFP
AVRSIYELPLSDDVLDEVREEMRSLGSYIALNLDGSSQERTFSLSIAENLIAKIQSETDI
PIVIVYGPKGEDKARALVDCYNNVYRLSLSPSIKRSAAIIKDAYIAITPDTSILHMASAY
NTPVVAIYADYKTRWPAMADVSESVVVGQKIDNISLDEFAKALKSVLARI
>LFGLNPFC_01268 hypothetical protein
MQSGTNVPYMKISAIDYSQNINGDYKATVTGGGEGIATLIPVLNGVHQAGLSTTIEFISA
ETRPMTGTVSVNSANLPTASFPSQGFTGAYYQLNNDNFAPGKTAADYSFSSSASWVGVDA
TGKVTFKNDGDSNTVIITAPPRSGGAIYQTVPPESRSV
>LFGLNPFC_01269 hypothetical protein
MKANFTLSDGDKAVTDADGKAKVTLKGTKAGAHTVTASMVGGKSEQLVVNFTADTLTAQV
NLNVTEDNFIANNIGMTRLQATVTDGNGNPVEGIKVNFRGTSVTLSSTSVETDDQVFAEI
LVTSTEVGLKTVSASLADKPTEVISRLLNAKVDVNSATITSQEIPEGQVMVAQDIAVKAH
VNDQFGNPVTHQPATFSAAPSSQMIISQNTVSTNTQGVAEVTMTPERNGSYTVKASLANG
ASLEKQLEAIDEKLTLTSSPLIGVNAPKGATLTATLTSANGTPVEGQVINFSVTLEGATL
SGGKVRTNSSGQAPVVLTSNKVGTYTVTASFHNGVTIQTQTTVKVTGNPSTAHVASFIAD
PSTIAATNSDLSTLKATVEDGSGNLIEGLTVYFALKSGSTTLTSLTAVTDQNGIATTSVK
GEITGSVTVSAVTSAGGMQTVDISLVAGPADASQSILKNNQSSLKGDFTDSAELHLVLHD
ISGNPIKVS
>LFGLNPFC_01270 hypothetical protein
MSKKYQPLLITHYMSTWVTITEAVEITTKAIKQKITPSDIYRHALSGNILLSVYFQSPVI
LKKIQTFNGKIKFRQFVGDLLDKLCMLDRDGFIYGQNLRLCTEARYICPVQQIIDTPLLR
KLNQFRTFVRNVRPGDELDVPAQVSEKNLTPPPGNSSGNLEQQIASTSQLIGSLLAEDMN
SEQAANIARGWASSQASGVMTDWLSRFGTARITLGVDEDFSLKNSRDGNPRKWRFDATNS
ELCAERHEC
>LFGLNPFC_01271 Pesticin receptor
MKMTRLYPLALGGLLLPAIANAQTSQQDESTLVVTASKQSSRSASANNVSSTVVSAPELS
DAGVTASDKLPRVLPGLNIENSGNMLFSTISLRGVSSAQDFYNPAVTLYVDGVPQLSTNT
IQALTDVQSVELLRGPQGTLYGKSAQGGIINIVTQQPDSTPRGYIEGGVSSRDSYRSKFN
LSGPIQDGLLYGSVTLLRQVDDGDMINPATGSDDLGGTRASIGNVKLRLAPDDQPWEMGF
AASRECTRATQDAYVGWNDIKGRKLSISDGSPDPYMRRCTDSQTLSGKYTTDDWVFNLIS
AWQQQHYSRTFPSGSLIVNMSQRWNQDVQELRAATLGDARTVDMVFGLYRQNTREKLNSA
YDMPTMPYLSSTGYTTAETLAAYSDLTWHLTDRFDIGGGVRFSHDKSSTQYHGSMLGNPF
GDQGKSNDDQVLGQLSAGYMLTDDWRVYTRVAQGYKPSGYNIVPTAGLDAKPFVAEKSIN
YELGTRYETADVTLQAATFYTHTKDMQLYSGPVGMQTLSNAGKADATGVELEAKWRFAPG
WSWDINGNVIRSEFTNDSELYHGNRVPFVPRYGAGSSVNGVIDTRYGALMPRLAVNLVGP
HYFDGDNQLRQGTYATLDSSLGWQATERMNISVYVDNLFDRRYRTYGYMNGSSAVAQVNM
GRTVGINTRIDFF
>LFGLNPFC_01272 2,3-dihydroxybenzoate-AMP ligase
MNSSFESLIEQYPLPIAEQLRHWAARYASRIAVVDAKGSLTYSELDAQVDELAAGLSSLG
LRSGEHVIVQLPNDNAFVTLLFALLRLGVIPVLAMPSQRALDIDALIELAQPVAYVIHGE
NHAELARQMAHKHACLRHVLVAGETMSDDFTPLFSLHGERQAWPQPDVSTTALLLLSGGT
TGTPKLIPRRHADYSYNFSASAELCGISQQSVYLAVLPVAHNFPLACPGILGTLACGGKV
VLTDSASCDEVMPLIAQERVTHVALVPALAQLWVQAREWEDSDLSSLRVIQAGGARLDPT
LAEQVIATFDCTLQQVFGMAEGLLCFTRLDDPHATILHSQGRPLSPLDEIRIVDQDENDV
APGETGQLLTRGPYTISGYYRAPAHNTQAFTAQGFYRTGDNVRLDEVGNLHVEGRIKEQI
NRAGEKIAAAEVESALLRLAEVQDCAVVAAPDTLLGERICAFIIAQQVPTDYQQLRQQLT

RMGLSAWKIPDQIEFLDHWPLTAVGKIDKKRLTALAVDRYRHSAQ
>LFGLNPFC_01273 Thioesterase PikA5
MTQSAMCIPLWPARNGNTAHLVMCPFAGGSSSAFRHWQAEQLADCALSLVTWPGRDRLRH
LEPLRSITQLAALLANELEASVSPDTPLLLAGHSMGAQVAFETCRLLEQRGLAPQGLIIS
GCHAPHLHSERQLSHRDDADFIAELIDIGGCSPELRENQELMSLFLPLLRADFYATESYH
YDSPDVCPPLRTPALLLCGSHDREASWQQVDAWRQWLSHVTGPVVIDGDHFYPIQQARSF
FTQIVRHFPHAFSAMTAWQKQPSTSER
>LFGLNPFC_01274 hypothetical protein
MMPSASPKQRVLIVGAKFGEMYLNAFMQPPEGLELVGLLAQGSARSRELAHAFGIPLYTS
PEQITRMPDIACIVVRSTVAGGTGTQLARHFLTRGVHVIQEHPLHPDDISSLQTLAQEQG
CCYWVNTFYPHTRAGRTWLRDAQQLRRCLAKTPPVVHATTSRQLLYSTLDLLLLALGVDA
AAVECDVVGSFSDFHCLRLFWPEGEACLLLQRYLDPDDPDMHSLIMHRLLLGWPEGHLSL
EASYGPVIWSSSLFVADHQENAHSLYRRPEILRDLPGLMRSAAPLSWRDCCETVGPEGVS
WLLHQLRSNLAGEHPPAACQSVHQIALSRLWQQILRKTGNAEIRRLTPPHHDRLAGFYND
DDKEAL
>LFGLNPFC_01275 2-methoxy-6-polyprenyl-1,4-benzoquinol methylase, mitochondrial
MDNLRFSSAPTADSIDASIAQHYPDCEPVAVIGYACHFPESPDGETFWQNLLEGRECSRR
FTREELLAVGLDAAIIDDPHYVNIGTVLDNADCFDATLFGYSRQEAESMDPQQRLFLQAV
WHALEHAGYAPGAVPHKTGVFASSRMSTYPGREALNVTEVAQVKGLQSLMGNDKDYIATR
AAYKLNLHGPALSVQTACSSSLVAVHLACESLRAGESDMAVAGGVALSFPQQAGYRYQPG
MIFSPDGHCRPFDASAEGTWAGNGLGCVVLRRLKDALLSGDPIISVILSSAVNNDGNRKV
GYTAPSVAGQQAVIEEALMLAAIDDRQVGYIETHGTGTPLGDAIEIEALRNVYAPRPQDQ
RCALGSVKSNMGHLDTAAGIAGLLKTVLAVSRGQIPPLLNFHTPNPALKLEESPFTIPVS
AQAWQDEMRYAGVSSFGIGGTNCHMIVASLPDALNARLPNTDSGRKSTALLLSAASDSAL
RRLATDYAGALRENADASSLAFTALHARRLDLPFRLAAPLNRETAEALSAWAGEKSGALV
YSGHGASGKQVWLFTGQGSHWRTMGQTMYHHSTAFADTLDRCFSACSEMLTPSLREAMFN
PDSAQLDNMAWAQPAIVAFEIAMAAHWRAEGLKPDFAIGHSVGEFAAAVVCGHYTIEQVM
PLVCRRGALMQQCASGAMVAVFADEDTLMPLARQFELDLAANNGTQHTVFSGPEARLAVF
CATLSQHDINYRRLSVTGAAHSALLEPILDRFQDACAGLHAEPGQIPIISTLTADVIDES
TLNQADYWRRHMRQPVRFIQSIQVAHQLGARVFLEMGPDAQLVACGQREYRDNAYWIASA
RRNKEASDVLNQALLQLYAAGVALPWADLLAGDGQRIAAPCYPFDTERYWKERVSPACEP
ADAALSAGLEVASRAATALDLPRLEALKQCATRLHAIYVDQLVQRCTGYAIENGVDAMTI
MRRGRLLPRYQQLLQRLLNNCVVDGDYRCTDGLYVRARPIEHQQRESLLTELAGYCEGFQ
AIPDTIARAGDRLYEMMSGAEEPVAIIFPQSASDGVEVLYQEFSFGRYFNQIAAGVLRGI
VQTRQPRQPLRILEVGGGTGGTTAWLLPELNGVPALEYHFTDISALFTRRAQQKFADYDF
VKYSELDLEKEAQSQGFQAQSYDLIVAANVIHATRHIGRTLDNLRPLLKPGGRLLMREIT
QPMRLFDFVFGPLVLPLQDLDAREGELFLTTAQWQQQCRHAGFSKVAWLPQDGSPNAGMS
EHIILATLPGQAVSAVTFTAPSEPVLGQALTDNGDYLADWSDCAGQPEQFNARWQEAWRL
LSQRHGDALPVEPPPVAAPEWLGKVRLSWQNEAFSRGQMRVEARHPAGEWLPLSPAAPLP
APQTHYQWRWTPLNVASIDHPLTFSFSAGTLARSDELAQYGIIHDPHASSRLMIVEESED
TLALAEKVIAALTASAAGLIVVTRRAWRVEENEALSASHHALWALLRVAANEQPERLLAA
IDLAENTPWETLHQGLSAVSLSQRWLAARGDTLWLPSLSPNTGCAAELPANVFTGDSRWH
LVTGAFGGLGRLAVNWLREKGARRIALLAPRVDESWLRDVEGGQTRVCRCDVGDAGQLAT
VLDDLAANGGIAGAIHAAGVLADAPLQELDDHQLAAVFAVKAQAASQLLQTLRNHDGRYL
ILYSSAAATLGAPGQSAHALACGYLDGLAQQFSTLDAPKTLSVAWGAWGESGRAATPEML
ATLASRGMGALSDAEGCWHLEQAVMRGAPWRLAMRVFTDKMPPLQQALFNISATEKAATP
VIPPADDNAFNGSLSDETAVMAWLKKRIAVQLRLSDPASLHPNQDLLQLGMDSLLFLELS
SDIQHYLGVRINAERAWQDLSPHGLTQLICSKPEATPAASQPEVLRHDADERYAPFPLTP
IQHAYWLGRTHLIGYGGVACHVLFEWDKRHDEFDLAILEKAWNQLIARHDMLRMVVDADG
QQRILATTPEYHIPRDDLRALSPEEQRIALEKRRHELSYRVLPADQWPLFELVVSEIDDC
HYRLHMNLDLLQFDVQSFKVMMDDLAQVWRGETLAPLAITFRDYVMAEQARRQTSAWHDA
WDYWQEKLPQLPLAPELPVVETPPETPHFTTFKSTIGKTEWQAVKQRWQQQGVTPSAALL
TLFAATLERWSRTTTFTLNLTFFNRQPIHPQINQLIGDFTSVTLVDFNFSAPVTLQEQMQ
QTQQRLWQNMAHSEMNGVEVIRELGRLRGSQRQPLMPVVFTSMLGMTLEGMTIDQAMSHL
FGEPCYVFTQTPQVWLDHQVMESDGELMFSWYCMDNVLEPGAAEAMFNDYCAILQAVIAA
PESLKTLASGIAGHIPRRRWPLNAQADYDLRDIEQATLEYPGIRQARAEITEQGALTLDI
VMADDPSPSAAMPDEHELTQLALPLPEQAQLDELEATWRWLEARALQGIAATLNRHGLFT
TPEIAHRFSAVVQALSAQASHQRLLRQWLQCLTEREWLIREGESWRCRIPLSEIPEPQEA
CPQSQWSQALAQYLETCIARHDALFSGQCSPLELLFNEQHRVTDALYRDNPASACLNRYT
AQIAALCSAERILEVGAGTAATTAPVLKATRNTRQSYHFTDVSAQFLNDARARFHDESQV
SYALFDINQPLDFTAHPEAGYDLIVAVNVLHDASHVVQTLRRLKLLLKAGGRLLIVEATE
RNSVFQLASVGFIEGLSGYRDFRRRDEKPMLTRSAWQEVLVQAGFANELAWPAQESSPLR
QHLLVARSPGVNRPDKKAVSRYLQQRFGTGLPILQIRQREALFTPLHAPSDAPTEPAKPT
PVAGGNPALEKQVAELWQSLLSRPVARHHDFFELGGDSLMATRMVAQLNRRGIARANLQD

LFNHSTLSDFCAHLQAATSGEDNPIPLCQGDGEETLFVFHASDGDISAWLPLANALNRRV
FGLQAKSPQRFATLDQMIDEYVGCIRRQQPHGPYVLAGWSYGAFLAAGAAQRLYAKGKQV
RMVLIDPVCRQDFCCENRAALLRLLAEGQTPLALPEHFDQQTPDSQLADFISLAKTVGMV
SQNLTLQAAETWLDNIAHLLRLLTEHTPGESVPVPCLMVYAAGRPARWTPAETEWQGWIN
NADDAVIEASHWQIMMEAPHVQACAQHITRWLCATSTQPENTL
>LFGLNPFC_01276 D-alanine--D-alanyl carrier protein ligase
MISGAPSQDSLLPDNRHAADYQQLRERLIQELNLTPQQLHEESNLIQAGLDSIRLMRWLH
WFRKNGYRLTLRELYAAPTLAAWNQLMLSRSPENAEEETPPDESSWPNMTESTPFPLTPV
QHAYLTGRMPGQTLGGVGCHLYQEFEGHCLTASQLEQAITTLLQRHPMLHIAFRPDGQQV
WLPQPYWNGVTVHDLRHNDAESRQAYLDALRQRLSHRLLRVEIGETFDFQLTLLPDNRHR
LHVNIDLLIMDASSFTLFFDELNALLAGESLPAIDTRYDFRSYLLHQQKINQPLRDDARA
YWLAKASTLPPAPVLPLACEPATLREVRNTRRRMIVPATRWHAFSNRAGEYGVTPTMALA
TCFSAVLARWGGLTRLLLNITLFDRQPLHPAVGAMLADFTNILLLDTACDGDTVSNLARK
NQLTFTEDWEHRHWSGVELLRELKRQQRYPHGAPVVFTSNLGRSLYSSRAESPLGEPEWG
ISQTPQVWIDHLAFEHHGEVWLQWDSNDALFPPALVETLFDAYCQLINQLCDDESAWQKP
FADMMPASQRAIRERVNATGAPIPEGLLHEGIFRIALQQPQALAVTDMRYQWNYHELTDY
ARRCAGRLIECGVQPGDNVAITMSKGAGQLVAVLAVLLAGAVYVPVSLDQPAARREKIYA
DASVRLVLICQHDASAGSDDIPVLAWQQAIEAEPIANPVVRAPTQPAYIIYTSGSTGTPK
GVVISHRGALNTCCDINTRYQVGPHDRVLALSALHFDLSVYDIFGVLRAGGALVMVMENQ
RRDPHAWCELIQRHQVTLWNSVPALFDMLLTWCEGFADATPENLRAVMLSGDWIGLDLPA
RYRAFRPQGQFIAMGGATEASIWSNACEIHDVPAHWRSIPYGFPLTNQRYRVVDEQGRDC
PDWVPGELWIGGIGVAEGYFNDPLRSEQQFLTLPDERWYRTGDLGCYWPDGTIEFLGRRD
KQVKVGGYRIELGEIESALSQLAGVKQATVLAIGEKEKTLAAYVVPQGEAFCVTDHRNPA
LPQAWHTLAGTLPCCAISPEISAEQVADFLQHRLLKLKPGHTAGADPLPLMNSLAIQPRW
QAVVERWLAFLVTQRRLKPAAEGYQVCAGEEREDEHPHFSGHDLTLSQILRGARNELSLL
NDAQWSPESLAFNHPASAPYIQELATICQQLAQRLQRPVRLLEVGTRTGRAAESLLAQLN
AGQIEYVGLEQSQEMLLSARQRLAPWPGARLSLWNADTLATHAHSADIIWLNNALHRLLP
EDPGLLATLQQLAVPGALLYVMEFRQLTPSALLSTLLLTNGQPEALLHNSADWAALFSAA
GFNCQHGDEVAGLQRFLVQCPDRQVRRDPRQLQAALAGRLPGWMVPQRIVFLDALPLTAN
GKIDYQALKRRHTPEAENPAEADLPQGDIEKQVAALWQQLLSTGNVTRETDFFQQGGDSL
LATRLTGQLHQAGYEAQLSDLFNHPRLADFAATLRKTDVPVEQPFVHSPEDRYQPFALTD
VQQAYLVGRQPGFALGGVGSHFFVEFEIADLDLTRLETVWNRLIARHDMLRAIVRDGQQQ
VLEQTPPWVIPAHTLHTPEEALRVREKLAHQVLNPEVWPVFDLQVGYVDGMPARLWLCLD
NLLLDGLSMQILLAELEHGYRYPQQLLPPLPVTFRDYLQQPSLQSPNPDSLAWWQAQLDD
IPPAPALPLRCLPQEVETPRFARLNGALDSTRWHRLKKRAADAHLTPSAVLLSVWSTVLS
AWSAQPEFTLNLTLFDRRPLHPQINQILGDFTSLMLLSWHPGESWLHSAQSLQQRLSQNL
NHRDVSAIRVMRQLAQRQNVPAVPMPVVFTSALGFEQDNFLARRNLLKPVWGISQTPQVW
LDHQIYESEGELRFNWDFVAALFPAGQVERQFEQYCALLNRMAEDESGWQLPLAALVPPV
KHAGQCAERSPRVCPEHSQPHIAADESTVSLICDAFREVVGESVTPAENFFEAGATSLNL
VQLHVLLQRHEFSTLTLLDLFTHPSPAALADYLAGVATVEKTKRPRPVRRRQRRI
>LFGLNPFC_01277 HTH-type transcriptional activator RhaR
MTESPQTQSEISIHQLVVGKPANDGNIPAQCELLRCSLQEGMDILLWRGHFARPETLQLH
DDLGRINFSCILEGTSRFAIQGLRRHTDWELARNRHYITHTPDCRGSASYCGRFESITLS
FSPETLALWVPDISAVIKNKIDSHCCCQQQRCNAETHLTAQALRHALMRMHGGFSHEQKP
STLWLQGQSLVMLSLVLDEHREDASCLSCHFNPMERQKLLRAKDLLLADLTQAPGVAELA
RESGLSVLKIKRGFRVLFNNSVYGLFQAERMQEARRRLANGNTSVMTVAADLGYANASHF
SAAFQKQFGVTPSTFKRGM
>LFGLNPFC_01278 Vitamin B12 import ATP-binding protein BtuD
MSSQSSNTESLSRFPLWQVITPVRRKVILAMALAGLAALTSLGALLFLAWSLRDIRATPD
AIPAWPLGGVIGCVVLTFVLRLQAFNTSHYAAFHLENILRSRLARKALQLPPGVLQQMGS
GSVAKVMLDDVKSLHIFVADSTPLYARAIIMPLATIVILFWLDWRLAIATLGVLAFGSVV
LVLARQRSENMAQRYHKAREQVSAAVIEFVQAMPVVRTFDSGSTSFLRYQRALEEWVDVL
KTWYRKAGFSARFSFSILNPLPTLFVLIWSGYGLLHYGSFDFIAWVAVLLLGSGMAEAVM
PMMMLNNLVAQTRLSIQRIYQVLAMPELSLPQSDQQPQEASITFEQVSFHYPQARTGAAL
QEVSFHVPAGQIVALVGPSGAGKSTVARLLLRYADPDKGHIRIGGVDLRDMQTDTLMKQL
SFVFQDNFLFADTIANNIRLGAPDTPLEAVIAAARVAQAHDFISALPEGYNTRVGERGVF
LSGGQRQRITIARALLQDRPILVLDEATAFADPENEAALIKALAAAMRGRTVIMVAHRLS
MVTQADVILLFSDGQLREMGNHTQLLAQGGLYQRLWQHYQQAQHWVPGGTQEEVVENERQ
>LFGLNPFC_01279 Putative multidrug export ATP-binding/permease protein
MKDNNPADNLAWRVIWRQLISSVGSQARMLRRSMLALLLAAFMQGIAFACLYPIIDALLR
GDAPQLLNWAMAFSVAAIVTLVLRWYGLGFEYRGHLAQATHELRLRLGEQLRRVPLEKLQ
RGRAGEMNALLLGSVDENLNYVIAIANILLLTIVTPLTASLATLWIDWRLGLVMLLIFPL
LVPFYYWRRPAMRRQMQTLGEAHQRLSGDIVEFAQGMMVLRTCGSDADKSRALLAHFNAL
ENLQTRTHRQGAGATMLIASVVELGLQVVVLSGIVWVVTGTLNLAFLIAAVAMIMRFAEP

MAMFISYTSVVELIASALQRIEQFMAIAPLPVAEQSEMPERYDIRFDNVSYRYEEGDGHA
LNHVSLTFPAASMSALVGASGAGKTTVTKLLMRYADPQQGQISIGGVDIRRLTPEQLNSL
ISVVFQDVWLFDDTLLANIRIARPQATRQEVEEAARAAQCLEFISRLPQGWLTPMGEMGG
QLSGGERQRISIARALLKNAPVVILDEPTAALDIESELAVQKAIDNLVHNRTVIIIAHRL
STIAGAGNILVMEEGQVVEQGTHAQLLSHHGRYQALWQAQMAARVWRDDGGSASGEWVHE
>LFGLNPFC_01280 Anhydromuropeptide permease
MSDVQSNVKPLTLTTGRVIFAIAGVYVTQSLVSALSMQSLPALVRAAGGSLALAGATTLF
MLPWALKFIWAPWIERWRLPPGSQERRSRMLILRGQVALAAILTIAAAIGWFGREGGFPD
TQIVALFVLFMVAGTVASTIDIASDGFCVDQLTRTGYGWGNSVQVGGSYLGMMCGGGVFL
MLSAASGWPVAMLMMAVLIMALSLPLWRITEPTRTATIPHVPALGYALRRKQARLGLLLV
LMLNSGMRFVLPLLAPLLLDHGLSMSALGALFSGGNIAAGIAGTLAGGLLMKYTSPGRAL
LTAYGVQGIALLAVVMTLMMAPGHLLLQILQCLVIVQSISLACALVCLYATLMSLSSPLQ
AGVDFTLFQCTDAAIAILAGVIGGVVAQHFGYAACFLFAGAFTLLAAWVAYIRLHSAREL
MTSAID
>LFGLNPFC_01281 Salicylate synthase
MKISEFLHLALPEEQWLPTISGVLRQFAEEECYVYERQPCWYLGKGCQARLHINADGTQA
TFIDDAGEQKWAVDSIADCARRFMAHPQVKGRRVYGQVGFNFAAHARGIAFNAGEWPLLT
LTVPREELIFEKGNVTVYADSADGCRRLCEWVKEAGTTTQNAPLAVDTALNGEAYKQQVA
RAVAEIRRGEYVKVIVSRAIPLPSRIDMPATLLYGRQANTPVRSFMFRQEGREALGFSPE
LVMSVTGNKVVTEPLAGTRDRMGNPEHNKAKEAELLHDSKEVLEHILSVKEAIAELEAVC
QPGSVVVEDLMSVRQRGSVQHLGSGVSGQLAENKDAWDAFTVLFPSITASGIPKNAALNA
IMQIEKTPRELYSGAILLLDDTRFDAALVLRSVFQDSQRCWIQAGAGIIAQSTPERELTE
TREKLASIAPYLMV
>LFGLNPFC_01282 Prophage integrase IntA
MLALNINPVQQRAAVRGSRTPEKVFKNVALAWHKSNRKWSQNTADRLLASLNNHIFPVIG
NLPVSELKPRHFIDLQKGIEEKGLLEVASRTRQHLSNIIRHAVHQELIDTNPAANLGGVT
TPPVRRHYPALPLERLPELLERIGAYHQGRELTRHAVLLMLHVFIRSSELRFARWSEIDF
TNRVWTIPATREPIIGVHYSGRGAKMRMPHIVPLSEQSIAILKQIKDITGNNELIFPGDH
NPYKPMCENTVNKALRVMGYDTKKDICGHGFRAMACSALMESGLWAKDAVERQMSHQERN
TVRMAYIHKAEHLEARKAMM
>LFGLNPFC_01284 Protein MtfA
MIKWPWKVQESAHQTALPWQEALSIPLLTCLTEQEQSKLVTLAERFLQQKRLVPLQGFEL
NSLRSCRIALLFCLPVLELGLEWLDSFHEVLIYPAPFVVDDEWEDDIGLVHNQRIVQSGQ
SWQQGPIVLNWLDIQDSFDASGFNLIIHEVAHKLDTRNGDRASGVPFIPLREVAGWEHDL
HAAMNNIQEEIELVGENAASIDAYAASDPAECFAVLSEYFFSAPELFAPRFPSLWQRFCQ
FYQQDPLQRLHHANDTDSFSATNVH
>LFGLNPFC_01286 Metal-binding protein ZinT
MAIRLHKLAVALGVFIVSAPAFSHGHHSHGKPLTEVEQKAANGVFDDTNVQNRTLSDWDG
VWQSVYPLLQSGKLDPVFQKKADADKTKTFAEIKDYYRKGYVTDIEMIGIEDGIVEFHRH
NETTSCKYDYDGYKILTYKSGKKGVRYLFECKDPESKAPKYIQFSDHIIAPRKSSHFHIF
MGNDTQQSLLNEMENWPTYYPYQLSSKEVVEEMMSH
>LFGLNPFC_01287 Protein-methionine-sulfoxide reductase heme-binding subunit MsrQ
MRLTAKQVTWLKVCLHLAGLLPFLWLAWAINHGGLGADPVKDIQHFTGRTALKFLLATLL
ITPLARYAKQPLLIRTRRLLGLWCFAWATLHLTSYALLELGVNNLALLGKELITRPYLTL
GIISWVILLALAFTSTQAMQRKLGKHWQQLHNFVYLVAILAPIHYLWSVKIISPQPLIYA
GLAVLLLALRYKKLLSLFNQLRKQVHNKLSL
>LFGLNPFC_01288 Protein-methionine-sulfoxide reductase catalytic subunit MsrP
MKKNQFLKESDVTAESVFFMKRRQVLKALGISAAALSLPHAAHADLLSWFKGNDRPPAPA
GKALEFSKPAAWQNNLPLTPADKVSGYNNFYEFGLDKADPAANAGSLKTDPWTLKISGEV
AKPLTLDHDDLTRRFPLEERIYRMRCVEAWSMVVPWIGFPLHKLLALAEPTSNAKYVAFE
TIYAPEQMPGQQDRFIGGGLKYPYVEGLRLDEAMHPLTLMTVGVYGKALPPQNGAPVRLI
VPWKYGFKGIKSIVSIKLTRERPPTTWNLAAPDEYGFYANVNPHVDHPRWSQATERFIGS
GGILNVQRQPTLLFNGYAEQVASLYRGLDLRENF
>LFGLNPFC_01289 5-hydroxyisourate hydrolase
MLKRYLVLSVVTAAFSLPSLVYAAQQNILSVHILNQQTGKPAADVTVTLEKKADNGWLQL
NTAKTDKDGRIKALWPEQTATTGDYRVVFKTGDYFKKQNLESFFPEIPVEFHINKVNEHY
HVPLLLSQYGYSTYRGS
>LFGLNPFC_01290 Transcriptional regulatory protein HprR
MSFEDSEKTSRVTLQQHYNFVMNQAVSITYDLWHIIFMKILLIEDNQRTQEWVTQGLSEA
GYVIDAVSDGRDGLYLALKDDYALIILDIMLPGMDGWQILQTLRTAKQTPVICLTARDSV
DDRVRGLDSGANDYLVKPFSFSELLARVRAQLRQHHALNSSLEISGLRMDSVSQSVSRDN
ISITLTRKEFQLLWLLASRAGEIIPRTVIASEIWGINFDSDTNTVDVAIRRLRAKVDDPF
PEKLISTIRGMGYSFVAVKK
>LFGLNPFC_01291 Sensor histidine kinase HprS

MKRLSITVRLTLLFILLLFVAGAGIVWTLYNGLASELKWRDDTTLINRTAQIKQLLIDGV
NPDTLPVYFNRMMDVSQDILIIHGDGINKIVNRTNVSNGMLNNIPASETISAAGIYRSII
NDTEIDALRINIDEVSPSLTVTVAKLASARHNMLEQYKINSIIICIVAIILCSVLSPLLI
RTGLREIKKLSGVTEALNYNDSREPVEVSALPRELKPLGQALNKMHQALVKDFERLSQFA
DDLAHELRTPINALLGQNQVTLSQTRSIAEYQKTIAGNIEELENISRLTENILFLARADK
NNVLVKLDSLSLNNEVENLLDYLEYLSDEKEICFKVECNQHIFADKILLQRMLSNLIVNA
IRYSPEKSRIQITSFLDTNGSLNIDIASPGTKIHEPEKLFRRFWRGDNSRHSVGQGLGLS
LVKAIAELHGGSATYHYLSKHNVFRITLPQRN
>LFGLNPFC_01292 Protein/nucleic acid deglycase 1
MTVQKSKNPQVDIAEDNAFFPSEYSLSQYTSPVSDLDGVDYPKPYRGKHKILVIAADERY
LPTDNGKLFSTGNHPIETLLPLYHLHAAGFEFEVATISGLMTKFEYWAMPHKDEKVMPFF
EQHKSLFRNPKKLADVVASLNADSEYAAIFVPGGHGALIGLPESEDVAAALQWAIENDRF
VISLCHGPAAFLALRHGDNPLNGYSICAFPDAADKQTPEIGYMPGHLTWYFGEELKKMGM
NIINDDITGRVHKDRKVLTGDSPFAANALGKLAAQEMLAAYAG
>LFGLNPFC_01293 Outer membrane protein YedS
MKRKVLAMLVPALLVAGAANAAEIYNKDGNKVDFYGKMVGERIWSNTDDNNSENEDTSYA
RFGVKGETQITSELTGFGQFEYNLDASKPEGENQEKTRLTFAGLKYNELGSFDYGRNYGV
AYDAAAYTDMLVEWGGDSWASADNFMNGRTNGVATYRNYDFFGLVDGLDFAIQYQGKNSN
RSTKKQNGDGYALSVDYNINGFGIVGAYSKSDRTNDQVADGNGSNAELWSLAAKYDANNV
YAVVMYGETRNMTPGSIDTGVADREGNTIMRDQLINETQNFEAVVQYQFDFGLRPSLGYV
YSKGKDIKGVPGHRYVDADRVNYIEVGTWYYFNKNMNVYTAYKFNMLDKDDAAITGAAAD
DQFAVGIVYQF
>LFGLNPFC_01294 hypothetical protein
MIVALNGWRDLAHINEGIKKAGIMPAQKI
>LFGLNPFC_01295 Inner membrane protein YedR
MVLSLNFPGYLKMEYGSTKMEERLSRSPGGKLALWAFYTWCGYFVWAMARYIWVMSRIPD
APVSGFESDLGSTAGKWLGALVGFLFMALVGALLGSIAWYTRPRPARSRRYE
>LFGLNPFC_01296 putative protein YedJ
MDLQHWQAQFENWLKNHHQHQDAAHDVCHFRRVWATAQKLAADDDVDMLVILTACYFHDI
VSLAKDHPQRQRSSILAAEETRRLLREEFVQFPAEKIEAVCHAIAAHSFSAQIAPLTTEA
KIVQDADRLEALGAIGLARVFAVSGALGVALFDGEDPFAQHRPLDDKRYALDHFQTKLLK
LPQTMQTARGKQLAQHNAQFLVEFMAKLSAELAGENEGVDHKVIDAFSPAG
>LFGLNPFC_01297 DNA-cytosine methyltransferase
MQENISVTDSYSTGNAAQAMLEKLLQIYDVKTLVAQLNGVGENHWSAAILKRALANDSVW
HRLSEKEFAHLQTLLPKPPAHHPHYAFRFIDLFAGIGGIRRGFESIGGQCVFTSEWNKHA
VRTYKANHYCDPATHHFNEDIRDITLSHKEGVSDEAAAEHIRQHIPEHDVLLAGFPCQPF
SLAGVSKKNSLGRAHGFACDTQGTLFFDVVRIIDARRPAMFVLENVKNLKSHDQGKTFRI
IMQTLDELGYDVADAEDNGPDDPKIIDGKHFLPQHRERIVLVGFRRDLNLKADFTLRDIS
ECFPAQRVTLAQLLDPMVEAKYILTPVLWKYLYRYAKKHQARGNGFGYGMVYPNNPQSVT
RTLSARYYKDGAEILIDRGWDMATGEKDFDDPLNQQHRPRRLTPRECARLMGFEAPGEAK
FRIPVSDTQAYRQFGNSVVVPVFAAVAKLLEPKIKQAVALRQQEAQHGRRSR
>LFGLNPFC_01298 Very short patch repair protein
MVDVHDKATRSKNMRAIATRDTAIEKRLASLLTGQGLAFRVQDASLPGRPDFVVDEYRCV
IFTHGCFWHHHHCYLFKVPATRTEFWLEKIGKNVERDRRDISRLQELGWRVLIVWECALR
GREKLTDAALTERLEEWICGEGASAQIDTQGIHLLA
>LFGLNPFC_01299 putative inner membrane transporter YedA
MRFRQLLPLFGALFALYIIWGSTYFVIRIGVESWPPLMMAGVRFLAAGILLLAFLLLRGH
KLPPLRPLLNAALIGLLLLAVGNGMVTVAEHQNVPSGIAAVVVATVPLFTLCFSRLFGIK
TRKLEWVGIAIGLAGIIMLNSGGNLSGNPWGAILILIGSISWAFGSVYGSRITLPVGMMA
GAIEMLAAGVVLMIASMIAGEKLTALPSLSGFLAVGYLALFGSIIAINAYMYLIRNVSPA
LATSYAYVNPVVAVLLGTGLGGETLSKIEWLALGVIVFAVVLVTLGKYLFPAKPVVAPVI
QDASSE
>LFGLNPFC_01300 Inner membrane protein YedI
MLLAGSSLLTLLDDIATLLDDISVMGKLAAKKTAGVLGDDLSLNAQQVSGVRANRELPVV
WGVAKGSLINKVILVPLALIISAFIPWAITPLLMIGGAFLCFEGVEKVLHMLEARKHKED
PAQSQQRLEKLAAQDPLKFEKDKIKGAIRTDFILSAEIVAITLGIVAEAPLLNQVLVLSG
IALVVTVGVYGLVGVIVKIDDLGYWLAEKSSALMQALGKGLLIIAPWLMKALSIVGTLAM
FLVGGGIVVHGIAPLHHAIEHFAGQQSAVVAMILPTVLNLILGFIIGGIVVLGVKAVAKM
RGQAH
>LFGLNPFC_01301 hypothetical protein
MSFMVSEEVTVKEGGPRMIVTGYSSGMVECR
>LFGLNPFC_01302 putative diguanylate cyclase DgcQ
MQHETKMENQSWLKKLARRLGPGHIVNLCFIVVLLFSTLLTWREVVVLEDAYISSQRNHL
ENVANALDKHLQYNVDKLIFLRNGMREALVAPLDFTSLRNAVTEFEQHRDEHAWQIELNR

RRTLPVNGVSDALVSEGNLLSRENESLDNEITAALEVGYLLRLAHNSSSMVEQAMYVSRA
GFYVSTQPTLFTRNVPTRYYGYVTQPWFIGHSQRENRHRAVRWFTSQPEHASNTEPQVTV
SVPVDSNNYWYGVLGMSIPVRTMQQFLRNAIDKNLDGEYQLYDSKLRFLTSSNPDHPTGN
IFDPRELALLAQAMEHDTRGGIRMNSRYVSWERLDHFDGVLVRVHTLSEGVRGDFGSISI
ALTLLWALFTTMLLISWYVIRRMVSNMYVLQSSLQWQAWHDTLTRLYNRGALFEKARPLA
KLCQTHQHPFSVIQVDLDHFKAINDRFGHQAGDRVLSHAAGLISSSLRAQDVAGRVGGEE
FCVILPGASLTQAAEVAERIRLKLNEKEMLIAKSTTIRISASLGVSSSEETGDYDFEQLQ
SLADRRLYLAKQAGRNRVCASDNA
>LFGLNPFC_01303 Mannosyl-3-phosphoglycerate phosphatase
MLSIQQPLLVFSDLDGTLLDSHSYDWQPAAPWLSRLREANVPVILCSSKTSAEMLYLQKT
LGLQGLPLIAENGAVIQLAEQWQDIDGFPRIISGISHGEISQVLNTLREKEHFKFTTFDD
VDDATIAEWTGLSRSQAALTQLHEASVTLIWRDSDERMAQFTARLNELGLQFMQGARFWH
VLDASAGKDQAANWIIATYQQSSGKRPTTLGLGDGPNDAPLLEVMDYAVIVKGLNREGVH
LHDEDPTRVWRTQREGPEGWREGLDHFFSAR
>LFGLNPFC_01304 hypothetical protein
MKTAKEYSDTAKREVSVDVDALLAAINEISESEVHRSQNDSEHVSVDGREYHTWRELADA
FELDIHDFSVSEVNR
>LFGLNPFC_01305 Protein DsrB
MKVNDRVTVKTDGGPRRPGVVLAVEEFSEGTMYLVSLEDYPLGIWFFNEAGHQDGIFVEK
AE
>LFGLNPFC_01306 Transcriptional regulatory protein RcsA
MSTIIMDLCSYTRLGLTGYLLSRGVKKREINDIETVDDLAIACDSQRPSVVFINEDCFIH
DASNSQRIKLIINQHPNTLFIVFMAIANVHFDEYLLVRKNLLISSKSIKPESLDDILGDI
LKKETTITSFLNMPTLSLSRTESSMLRMWMAGQGTIQISDQMNIKAKTVSSHKGNIKRKI
KTHNKQVIYHVVRLTDNVTNGIFVNMR
>LFGLNPFC_01307 Flagellar biosynthetic protein FliR
MMQVTSDQWLSWLSLYFWPLLRVLALISTAPILSERSVPKRVKLGLAMMITFAIAPSLPA
NDVPVFSFFALWLAVQQILIGIALGFTMQFAFAAVRTAGEIIGLQMGLSFATFVDPASHL
NMPVLARIMDMLALLLFLTFNGHLWLISLLVDTFHTLPIGGEPLNSNAFLALTKAGSLIF
LNGLMLALPLITLLLTLNLALGLLNRMAPQLSIFVIGFPLTLTVGISLMAALMPLIAPFC
EHLFSEMFNLLADIISELPLI
>LFGLNPFC_01308 Flagellar biosynthetic protein FliQ
MTPESVMMMGTEAMKVALALAAPLLLVALVTGLIISILQAATQINEMTLSFIPKIIAVFI
AIIIAGPWMLNLLLDYVRTLFTNLPYIIG
>LFGLNPFC_01309 Flagellar biosynthetic protein FliP
MRRLLSVAPVLLWLVTPLAFAQLPGITSQPLPGGGQSWSLPVQTLVFITSLTFIPAILLM
MTSFTRIIIVFGLLRNALGTPSAPPNQVLLGLALFLTFFIMSPVIDKIYVDAYQPFNEEK
ISMQEALEKGEQPLREFMLRQTREADLGLFARLANTGPLQGPEAVPMRILLPAYVTSELK
TAFQIGFTIFIPFLIIDLVIASVLMALGMMMVPPATIALPFKLMLFVLVDGWQLLVGSLA
QSFYS
>LFGLNPFC_01310 Flagellar protein FliO
MNNHATVQSSAPVSAAPLLQVSGALIAIIALILAAAWLVKRLGFAPKRTGVNGLKISASA
SLGARERVVVVDVEDARLVLGVTAGQINLLHKLPPSAPTEEIPQTDFQSVMKNLLKRSGR
S
>LFGLNPFC_01311 Flagellar motor switch protein FliN
MSDMNNPADDNNGAMDDLWAEALSEQKSTSGKSAADAVFQQFGGGDVSGALQDIDLIMDI
PVKLTVELGRTRMTIKELLRLTQGSVVALDGLAGEPLDILINGYLIAQGEVVVVADKYGV
RITDIITPSERMRRLSR
>LFGLNPFC_01312 Flagellar motor switch protein FliM
MGDSILSQAEIDALLNGDSEVKDEPTASVSGESDIRPYDPNTQRRVVRERLQALEIINER
FARHFRMGLFNLLRRSPDITVGAIRIQPYHEFARNLPVPTNLNLIHLKPLRGTGLVVFSP
SLVFIAVDNLFGGDGRFPTKVEGREFTHTEQRVINRMLKLALEGYSDAWKAINPLEVEYV
RSEMQVKFTNITTSPNDIVVNTPFHVEIGNLTGEFNICLPFSMIEPLRELLVNPPLENSR
NEDQNWRDNLVRQVQHSQLELVANFADISLRLSQILKLKPGDVLPIEKPDRIIAHVDGVP
VLTSQYGTLNGQYALRIEHLINPILNSLNEEQPK
>LFGLNPFC_01313 hypothetical protein
MTDYAISKKSKRSLWIPILVFITLAACASAGYSYWHSHQVAADDKAQQRVVPSPVFYALD
TFTVNLGDADRVLYIGITLRLKDEATRSRLSEYLPEVRSRLLLLFSRQDAAVLATEEGKK
NLIAEIKTTLSTPLVAGQPKQDVTDVLYTAFILR
>LFGLNPFC_01314 Flagellar hook-length control protein
MIRLAPLITANVDTTTLPGGKASDAAQDFLALLSEALAGETTTDKAAPQLLVATDKPTTK
GEPLVSDIVSDAQQADLLIPVDETLPVINDEQSTSTPLTTAQTMTLAAVADKNTTKDEKA
DDLNEDVTASLSALFAMLPGFDNTPKVTDAPSTVLPAEKPTLFTKLTSAQLTTAQPDDAP
GTPAQPLTPLVAEAQSKAEVISTPSPVTADASPLITPHQTQPLPTVAAPVLSAPLGSHEW

QQSLSQHISLFTRQGQQSAELRLHPQDLGEVQISLKVDDNQAQIQMISPHQHVRAALEAA
LPVLRTQLAESGIQLGQSNISGESFSGQQQAASQQQQSQRTANHEPLAGEDDDTLPVPVS
LQGRVTGNSGVDIFA
>LFGLNPFC_01315 Flagellar FliJ protein
MAEHGALATLKDLAEKEVEDAARLLGEMRRGCQQAEEQLKMLIDYQNEYRNNLNSDMSAG
MTSNRWINYQQFIQTLEKAITQHRQQLNQWTQKVDIALNSWREKKQRLQAWQTLQKRQST
AALLAENRLDQKKMDEFAQRAAMRKPE
>LFGLNPFC_01316 Flagellum-specific ATP synthase
MTTRLTRWLTTLDNFEAKMAQLPAVRRYGRLTRATGLVLEATGLQLPLGATCVIERQNGS
ETHEVESEVVGFNGQRLFLMPLEEVEGVLPGARVYAKNISAEGLQSGKQLPLGPALLGRV
LDGSGKPLDGLPSPDTTETGALITPPFNPLQRTPIEHVLDTGVRPINALLTVGRGQRMGL
FAGSGVGKSVLLGMMARYTRADVIVVGLIGERGREVKDFIENILGAEGRARSVVIAAPAD
VSPLLRMQGAAYATRIAEDFRDRGQHVLLIMDSLTRYAMAQREIALAIGEPPATKGYPPS
VFAKLPALVERAGNGISGGGSITAFYTVLTEGDDQQDPIADSARAILDGHIVLSRRLAEA
GHYPAIDIEASISRAMTALISEQHYARVRTFKQLLSSFQRNRDLVSVGAYAKGSDPMLDK
AIALWPQLESYLQQGIFERADWEASLQGLERIFPTVS
>LFGLNPFC_01317 Flagellar assembly protein FliH
MSDNLPWKTWMPDDLAPPQAEFVPMVEPEETIIEEAEPSLEQQLAQLQMQAHEQGYQAGI
AEGRQQGHEQGYQEGLAQGLEQGLAEAKSQQAPIHARMQQLVSEFQTTLDALDSVIASRL
MQMALEAARQVIGQTPTVDNSALIKQIQQLLQQEPLFSGKPQLRVHPDDLQRVDDMLGAT
LSLHGWRLRGDPTLHPGGCKVSADEGDLDASVATRWQELCRLAAPGVV
>LFGLNPFC_01318 Flagellar motor switch protein FliG
MSNLTGTDKSVILLMTIGEDRAAEVFKHLSQREVQTLSAAMANVTQISNKQLTDVLAEFE
QEAEQFAALNINANDYLRSVLVKALGEERAASLLEDILETRDTASGIETLNFMEPQSAAD
LIRDEHPQIIATILVHLKRAQAADILALFDERLRHDVMLRIATFGGVQPAALAELTEVLN
GLLDGQNLKRSKMGGVRTAAEIINLMKTQQEEAVITAVREFDGELAQKIIDEMFLFENLV
DVDDRSIQRLLQEVDSESLLIALKGAEQPLREKFLRNMSQRAADILRDDLANRGPVRLSQ
VENEQKAILLIVRRLAETGEMVIGSGEDTYV
>LFGLNPFC_01319 Flagellar M-ring protein
MNATAAQTKSLEWLNRLRANPKIPLIVAGSAAVAVMVALILWAKAPDYRTLFSNLSDQDG
GAIVSQLTQMNIPYRFSEASGAIEVPADKVHELRLRLAQQGLPKGGAVGFELLDQEKFGI
SQFSEQVNYQRALEGELSRTIETIGPVKGARVHLAMPKPSLFVREQKSPSASVTVNLLPG
RALDEGQISAIVHLVSSAVAGLPPGNVTLVDQGGHLLTQSNTSGRDLNDAQLKYASDVEG
RIQRRIEAILSPIVGNGNIHAQVTAQLDFASKEQTEEQYRPNGDESHAALRSRQLNESEQ
SGSGYPGGVPGALSNQPAPANNAPISTPPANQNNRQQQASTTSNSGPRSTQRNETSNYEV
DRTIRHTKMNVGDVQRLSVAVVVNYKTLPDGKPLPLSNEQMKQIEALTREAMGFSEKRGD
SLNVVNSPFNSSDESGGALPFWQQQVFIDQLLAAGRWLLVLLVAWLLWRKAVRPQLTRRA
EAVKTVQQQAQAREEVEDAVEVRLSKDEQLQQRRANQRLGAEVMSQRIREMSDNDPRVVA
LVIRQWINNDHE
>LFGLNPFC_01320 Flagellar hook-basal body complex protein FliE
MSAIQGIEGVISQLQATAMSARAQESLPQPTISFAGQLHAALDRISDTQTVARTQAEKFT
LGEPGVALNDVMTDMQKASVSMQMGIQVRNKLVAAYQEVMSMQV
>LFGLNPFC_01321 Multidrug transporter EmrE
MNPYIYLGGAILAEVIGTTLMKFSEGFTRLWPSVGTIICYCASFWLLAQTLAYIPTGIAY
AIWSGVGIVLISLLSWGFFGQRLDLPAIIGMMLICAGVLVINLLSRSAPH
>LFGLNPFC_01322 hypothetical protein
MKKLIVSSVLAFITFSAQAAAFQVTSNEIKTGEQLTTSHVFSGFGCEGGNTSPSLTWSGA
PEGTKSFAVTVYDPDAPTGSGWWHWTVANIPATVTYLPTDAGRRDGTKLPTGAVQGRNDF
GYAGFGGACPPKGDKPHHYQFKVWALKTDKIPVDSNSSGALVGYMLNANKIATAEITPVY
EIK
>LFGLNPFC_01323 HTH-type transcriptional activator RhaS
MLAKDKSNLKIEEIRMHKHHEIHRVKPLMPALCRIRQGKKVINWETHSLTVDNNQIILFP
CGYEFYIANYPEAGLYLAEMLYYPIDLIEKFQKFYAITDQIRNTTGFCLPQNPELIYCWE
QLKTSIFRGFSTQIQEHLAMGVLLSLGAHHVNCLLLSDSKQSLISRCYNLMLSEPGTKWT
ANKVARYLYISVSTLHRRLASEGISFQSILDDVRLNNALSAIQTTVKPISEIARENGYKC
PSRFTERFHNRFKITPRELRKASRE
>LFGLNPFC_01324 Outer membrane porin protein OmpD
MKKLTVAISAVAASVLMAMSAQAAEIYNKDSNKLDLYGKVNAKHYFSSNDADDGDTTYVR
LGFKGETQINDQLTGFGQWEYEFKGNRAESQGSSKDKTRLAFAGLKFGDYGSIDYGRNYG
VAYDIGAWTDVLPEFGGDTWTQTDVFMTGRTTGVATYRNNDFFGLVDGLNFAAQYQGKND
RTDVTEANGDGFGFSTTYEYEGFGVGATYAKSDRTNDQVIYGNNSLNASGQNAEVWAAGL
KYDANNIYLATTYSETQNMTVFGNNHIANKAQNFEVVAQYQFDFGLRPSVAYLQSKGKDL
GAWGDQDLVEYIDVGATYYFNKNMSTFVDYKINLIDKSDFTKASGVATDDIVAVGLVYQF
>LFGLNPFC_01325 SOS response-associated protein YedK

MCGRFAQSQTREDYLAFLAEDIERDIPYDPEPIGRYNVAPGTKVLLLSERDEHLHLDPVF
WGYAPGWWDKPPLINARVETAATSRMFKPLWQHGRVICFADGWFEWKKEGDKKQPYFIYR
ADGQPVFIAAIGSTPFERGDEAEGVLIVTAAADQGLVDIHDRRPLVLSPETAREWMRQDI
GGKEASEIATRSCVPANQFIWHPVSRAVGNVKNQGAELIQPV
>LFGLNPFC_01326 Putative sulfur carrier protein YedF
MKNIVPDYRLDMVGEPCPYPAVATLEAMPQLKKGEILEVVSDCPQSINNIPLDARNHGYT
VLDIQQDGPTIRYLIQK
>LFGLNPFC_01327 hypothetical protein
MSWQQFKHAWLIKFWAPIPAVIAAGILSTYYFGITGTFWAVTGEFTRWGGQLLQLFGVHA
EEWGYFKIIHLEGSPLTRIDGMMILGMFGGCFAAALWANNVKLRMPRSRIRIMQAIIGGI
IAGFGARLAMGCNLAAFFTGIPQFSLHAWFFAIATAIGSWFGARFTLLPIFRIPVKMQKV
SAASPLTQKPDQARRRFRLGMLVFFGMLGWALLTAMNQPKLGLAMLFGVGFGLLIERAQI
CFTSAFRDMWITGRTHMAKAIIIGMAVSAIGIFSYVQLGVEPKIMWAGPNAVIGGLLFGF
GIVLAGGCETGWMYRAVEGQVHYWWVGLGNVIGSTILAYYWDDFAPALATDWDKINLLKT
FGPMGGLLVTYLLLFAALMLIIGWEKRFFRRAAPQIAKEIA
>LFGLNPFC_01328 hypothetical protein
MKKLAIAGALMLLAGCAEVENYNNVVKTPAPDWLTGYWQTKGPQRALVSPEAIGSLIVTK
EGDTLDCRQWQRVIAVPGKLTLMSDDLTNVTVKRELYEVERDGNTIEYDGMTMERVDRPT
AECAAALDKAPLPTPLP
>LFGLNPFC_01329 Cytoplasmic alpha-amylase
MRNPTLLQCFHWYYPEGGKLWPELAERADGFNDIGINMVWLPPAYKGASGGYSVGYDSYD
LFDLGEFDQKGCIPTKYGDKAQLLAAIDALKRNDIAVLLDVVVNHKMGADEKEAIRVQRV
NADDRTQIDEEIIECEGWTRYTFPARAGQYSQFIWDFKCFSGIDHIEHPDEDGIFKIVND
YTGEGWNDQVDDELGNFDYLMGENIDFRNHAVTEEIKYWARWVMEQTQCDGFRLDAVKHI
PAWFYKEWIEHVQEVAPKPLFIVAEYWSHEVDKLQTYIDQVEGKTMLFDAPLQMKFHEAS
RMGRDYDMTQIFTGTLVEADPFHAVTLVANHDTQPLQALEAPVEPWFKPLAYALILLREN
GVPSVFYPDLYGAHYEDVGGDGQTYPIDMPIIEQLDELILARQRFAHGVQTLFFDHPNCI
AFSRSGTDEYPGCVVVMSNGDDGEKTIHLGENYGNKTWRDFLGNRQESVVTDENGEATFF
CNGGSVSVWVIEEVI
>LFGLNPFC_01330 Flagellar protein FliT
MNNAPHLYFAWQQLVEKSQLMLRLATEEQWDELIASEMAYVNAVQEIAHLTEEVAPSTTM
QEQLRPMLHLILDNESKVKQLLQIRMDELAKLVGQSSVQKSVLSAYGDQGGFVLAPQDNL
F
>LFGLNPFC_01331 Flagellar secretion chaperone FliS
MYAAKGTQAYAQIGVESAVMSASQQQLVTMLFDGVLSALVRARLFMQDNNQQGKGVSLSK
AINIIENGLRVSLDEESKDELTQNLIALYSYMVRRLLQANLRNDVSAVEEVEALMRNIAD
AWKESLLSPSLIQDPV
>LFGLNPFC_01332 Flagellar hook-associated protein 2
MASISSLGVGSGLDLSSILDSLTAAQKATLTPISNQQSSFTAKLSAYGTLKSALTTFQTA
NTALSKADLFSATSTTSSTTAFSATTAGNAIAGKYTISVTHLAQAQTLTTRTTRDDTKTA
IATSDSKLTIQQGGDKDPITIDISAANSSLSGIRDAINNAKAGVSASIINVGNGEYRLSV
TSNDTGLDNAMTLSVSGDDALQSFMGYDASASSNGMEVSVAAQNAQLTVNNVAIENSSNT
ISNALENITLNLNDVTTGNQTLTITQDTSKAQTAIKDWVNAYNSLIDTFSSLTKYTAVDA
GADSQNSSNGALLGDSTLRTIQTQLKSMLSNTVSSSNYKTLAQIGITTDPSDGKLELDAD
KLTAALKKDASGVGALIVGDGKKTGITTTIGSNLTSWLSTTGIIKAATDGVSKTLNKLTK
DYNAASDRIDAQVARYKEQFTQLDVLMTSLNSTSSYLTQQFENNSNSK
>LFGLNPFC_01333 hypothetical protein
MAQVINTNSLSLITQNNINKNQSALSSSIERLSSGLRINSAKDDAAGQAIANRFTSNIKG
LTQAARNANDGISVAQTTEGALSEINNNLQRIRELTVQASTGTNSDSDLDSIQDEIKSRL
DEIDRVSGQTQFNGVNVLAKDGSMKIQVGANDGETITIDLKKIDSDTLGLNGFNVNGKGT
ITNKAATVSDLTSAGAKLNTTTGLYDLKTENTLLTTDAAFDKLGNGDKVTVGGVDYTYNA
KSGDFTTTKSTAGTGVDAAAQATDSAKKRDALAATLHADVGKSVNGSYTTKDGTVSFETD
SAGNITIGGSQAYVDDAGNLTTNNAGSAAKADMKALLKAASEGSDGASLTFNGTEYTIAK
ATPATTSPVAPLIPGGITYQATVSKDVVLSETKAAAATSSITFNSGVLSKTIGFTAGESS
DAAKSYVDDKGGITNVADYTVSYSVNKDNGSVTVAGYASATDTNKDYAPAIGTAVNVNSA
GKITTETTSAGSATTNPLAALDDAISSIDKFRSSLGAIQNRLDSAVTNLNNTTTNLSEAQ
SRIQDADYATEVSNMSKAQIIQQAGNSVLAKANQVPQQVLSLLQG
>LFGLNPFC_01334 RNA polymerase sigma factor FliA
MNSLYTAEGVMDKHSLWQRYVPLVRHEALRLQVRLPASVELDDLLQAGGIGLLNAVERYD
ALQGTAFTTYAVQRIRGAMLDELRSRDWVPRSVRRNAREVAQAIGQLEQELGRNATETEV
AERLGIDIADYRQMLLDTNNSQLFSYDEWREEHGDSIELVTDDHQRENPLQQLLDSNLRQ
RVMETIETLPEREKLVLTLYYQEELNLKEIGAVLEVGESRVSQLHSQAIKRLRTKLGKL
>LFGLNPFC_01335 Regulator of sigma S factor FliZ
MMVQHLKRRPLSRYLKDFKHSQTHCAHCRKLLDRITLVRDGKIVNKIEISRLDTLLDENG

WQVEQQSWAALCRFCGDLHCKTQSDFFDIIGFKKFLFEQTEMSPGTVREYVVRLRRLGNH
LHEQNISLDQLQDGFLDEILAPWLPSTSTNNYRIALRKYQHYQRQTCTGLVQKSSSQPAS
DIY
>LFGLNPFC_01336 L-cystine-binding protein FliY
MFTNNGSTLQTDITTFGVNMKLAHLGRQALMGVMAVALVAGMSVKSFADEGLLNKVKERG
TLLVGLEGTYPPFSFQGDDGKLTGFEVEFAQQLAKHLGVEASLKPTKWDGMLASLDSKRI
DVVINQVTISDERKKKYDFSTPYTISGIQALVKKGNEGTIKTADDLKGKKVGVGLGTNYE
EWLRQNVQGVDVRTYDDDPTKYQDLRVGRIDAILVDRLAALDLVKKTNDTLAVTGEAFSR
QESGVALRKGNEDLLKAVNDAIAEMQKDGTLQALSEKWFGADVTK
>LFGLNPFC_01337 D-cysteine desulfhydrase
MPLHNLTRFPRLEFIGAPTPLEYLPRFSDYLGREIFIKRDDVTPMAMGGNKLRKLEFLAA
DALREGADTLITAGAIQSNHVRQTAAVAAKLGLHCVALLENPIGTTAENYLTNGNRLLLD
LFNTQIEMCDALTDPNTQLEELATRVEAQGFRPYVIPVGGSNALGALGYVESALEIAQQC
EGAVNISSVVVASGSAGTHAGLAVGLEHLMPESELIGVTVSRSVADQLPKVVNLQQAIAK
ELELTASAEILLWDDYFAPGYGVPNDEGMEAVKLLARLEGILLDPVYTGKAMAGLIDGIS
QKRFKDEGPILFIHTGGAPALFAYHPHV
>LFGLNPFC_01338 L-cystine transport system permease protein YecS
MQESIQLVIDSLPFLLKGAGYTLQLSIGGMFFGLLLGFILALMRLSPIWPVRWLARFYIS
IFRGTPLIAQLFMIYYGLPQFGIELDPIPSAMIGLSLNTAAYAAETLRAAISSIDKGQWE
AAASIGMTPWQTMRRAILPQAARVALPPLSNSFISLVKDTSLAATIQVPELFRQAQLITS
RTLEVFTMYLAASLIYWIMATVLSTLQNHFENQLNRQEREPK
>LFGLNPFC_01339 Arginine transport ATP-binding protein ArtM
MSAIEVKNLVKKFHGQTVLHGIDLEVKPGEVVAIIGPSGSGKTTLLRSINLLEQPEAGTI
TVGDITIDTARSLSQQKSLIRQLRQHVGFVFQNFNLFPHRTVLENIIEGPVIVKGEPKEE
ATARARELLAKVGLAGKETSYPRRLSGGQQQRVAIARALAMRPEVILFDEPTSALDPELV
GEVLNTIRQLAQEKRTMVIVTHEMSFARDVADRAIFMDQGRIVEQGVAKALFADPQQPRT
RQFMEKFLLQ
>LFGLNPFC_01340 Regulatory protein SdiA
MQDTDFFSWLRTMLLRFQRMEAAEEVYHEIELQAQQLEYDYYSLCVRHPVPFTRPKVAFY
TNYPESWVSYYQAKNFLAIDPVLNPENFSQGHLMWNDDLFSEAQPLWEAARAHGLRRGVT
QYLMLPNRALGFLSFSRCSTREIPILSDELQLKMQLLVRESLMALMRLNDEIVMTPEMNF
SKREKEILKWTAEGKTSAEIAMILSISENTVNFHQKNMQKKINAPNKTQVACYAAATGLI
>LFGLNPFC_01341 hypothetical protein
MSTPDFSTAENNQELANEVSCLKAMLTLMLQAMGQADAGRVMLKMEKQLALIEDETQAAV
FSKTVKQIKQAYRQ
>LFGLNPFC_01342 Response regulator UvrY
MINVLLVDDHELVRAGIRRILEDIKGIKVVGEASCGEDAVKWCRANAVDVVLMDMSMPGI
GGLEATRKIARSTADVKIIMLTVHTENPLPAKVMQAGAAGYLSKGAAPQEVVSAIRSVYS
GQRYIASDIAQQMALSQIEPEKTESPFASLSERELQIMLMITKGQKVNEISEQLNLSPKT
VNSYSYRMFSKLNIHGDVELTHLAIRHGLCNAETLSSQ
>LFGLNPFC_01343 UvrABC system protein C
MSDQFDAKAFLKTVTSQPGVYRMYDAGGTVIYVGKAKDLKKRLSSYFRSNLASRKTEALV
AQIQQIDVTVTHTETEALLLEHNYIKLYQPRYNVLLRDDKSYPFIFLSGDTHPRLAMHRG
AKHAKGEYFGPFPNGYAVRETLALLQKIFPIRQCENSVYRNRSRPCLQYQIGRCLGPCVE
GLVSEEEYAQQVEYVRLFLSGKDDQVLTQLISRMETASQNLEFEEAARIRDQIQAVRRVT
EKQFVSNTGDDLDVIGVAFDAGMACVHVLFIRQGKVLGSRSYFPKVPGGTELSEVVETFV
GQFYLQGSQMRTLPGEILLDFNLSDKTLLADSLSELAGRKINVQTKPRGDRARYLKLART
NAATALTSKLSQQSTVHQRLTALASVLKLPEVKRMECFDISHTMGEQTVASCVVFDANGP
LRAEYRRYNITGITPGDDYAAMNQVLRRRYGKAIDDSKIPDVILIDGGKGQLAQAKNVFA
ELDVSWDKNHPLLLGVAKGADRKAGLETLFFEPEGEGFSLPPDSPALHVIQHIRDESHDH
AIGGHRKKRAKVKNTSSLETIEGIGPKRRQMLLKYMGGLQGLRNASVEEIAKVPGISQGL
AEKIFWSLKH
>LFGLNPFC_01344 CDP-diacylglycerol--glycerol-3-phosphate 3-phosphatidyltransferase
MQFNIPTLLTLFRVILIPFFVLVFYLPVTWSPFAAALIFCVAAVTDWFDGFLARRWNQST
RFGAFLDPVADKVLVAIAMVLVTEHYHSWWVTLPAATMIAREIIISALREWMAELGKRSS
VAVSWIGKVKTTAQMVALAWLLWRPNIWVEYAGIALFFVAAVLTLWSMLQYLSAARADLL
DQ
>LFGLNPFC_01348 hypothetical protein
MKTGPLNESELEWLDDILTKYNTDHAILDVAELDGLLTAVLSSPQEIEPAQWLVAVWGGA
DYVPRWASEKEMTRFMNLAFQHMADTAERLNEFPEQFEPLFGLREVDGSELTIVEEWCFG
YMRGVALSDWSTLPDSLKPALEAIALHGTEENFERVEKMSPEAFEESVDAIRLAALDLHA
YWMAHPQEKAVQQPIKAEEKPGRNDPCPCGSGKKFKQCCLH
>LFGLNPFC_01349 Tyrosine-specific transport protein
MKNRTLGSVFIVAGTTIGAGMLAMPLAAAGVGFSVTLILLIGLWALMCYTALLLLEVYQH

VPADTGLGTLAKRYLGRYGGQWLTGFSMMFLMYALTAAYISGAGELLASSISDWTGISMSA
TAGVLLFTFVAGGVVCVGTSLVDLFNRFLFSAKIIFLVVMLVLLLPHIHKVNLLTLPLQQ
GLALSAIPVIFTSFGFHGSVPSIVSYMDGNIRKLRWVFIIGSAIPLVAYIFWQLATLGSI
DSTTFMGLLANHAGLNGLLQALREMVASPHVELAVHLFADLALATSFLGVALGLFDYLAD
LFQRSNTVGGRLQTGAITFLPPLAFALFYPRGFVMALGYAGVALAVLALIIPSLLTWQSR
KHNPQAGYRVKGGRPALVVVFLCGIAVIGVQFLIAAGLLPEVG
>LFGLNPFC_01350 hypothetical protein
MDSIHGHEVLNMMIESGEQYTHASLEAAIKARFGEQARFHTCSAEGMTAGELVAFLAAKG
KFIPSEEGFSTDQSKICRH
>LFGLNPFC_01351 Bacterial non-heme ferritin
MLKPEMIEKLNEQMNLELYSSLLYQQMSAWCSYHTFEGAAAFLRRHAQEEMTHMQRLFDY
LTDTGNLPRINTVESPFAEYSSLDELFQETYKHEQLITQKINELAHAAMTNQDYPTFNFL
QWYVSEQHEEEKLFKSIIDKLSLAGKSGEGLYFIDKELSTLDTQN
>LFGLNPFC_01352 hypothetical protein
MRLLILTLSLITLTGCTVTRQAHVSEVDAATGIVRLVYDQAFLQHASTDRYVSRGIADRA
CQQAGYTHAIPFGQPVGNCSLFAGSLCLNTEFTLSYQCHHSAFPVFL
>LFGLNPFC_01353 hypothetical protein
MSQPLNADQELVSDVVACQLVIKQILDVLDVIAPVEVREKMSSQLKNIDFTNHPAAADPV
TMRAIQKAIALIELKFTPQGESH
>LFGLNPFC_01354 Bacterial non-heme ferritin
MATAGMLLKLNSQMNREFYASNLYLHLSNWCSEQSLNGTATFLRAQAQSNVTQMMRMFNF
MKSVGATPIVKAIDVPGEKLNSLEELFQKTMEEYEQRSSTLAQLADEAKELNDDSTVNFL
RDLEKEQQHDGLLLQTILDEVRSAKLAGMCPVQTDQHVLNVVSHQLH
>LFGLNPFC_01355 L-arabinose-binding periplasmic protein
MHKFTKALAAIGLAAVMSQSAMAENLKLGFLVKQPEEPWFQTEWKFADKAGKDLGFEVIK
IAVPDGEKTLNAIDSLAASGAKGFVICTPDPKLGSAIVAKARGYDMKVIAVDDQFVNAKG
KPMDTVPLVMMAATKIGERQGQELYKEMQKRGWDVKESAVMAITSNELDTARRRTTGSMD
ALKAAGFPEKQIYQVPTKSNDIPGAFDAANSMLVQHPEVKHWLIVGMNDSTVLGGVRATE
GQGFKASDIIGIGINGVDAVSELSKAQATGFYGSLLPSPDVHGYKSSEMLYNWVAKGVEP
TKFTEVTDVVLITRDNFKEELEKKGLGGK
>LFGLNPFC_01356 Arabinose import ATP-binding protein AraG
MQQSTPYLSFRGIGKTFPGVKALTDISFDCYAGQVHALMGENGAGKSTLLKILSGNYAPT
TGSVVINGQEMSFSDTTAALNAGVAIIYQELHLVPEMTVAENIYLGQLPHKGGIVNRSLL
NYEAGLQIKHLGMDIDPDTPLKYLSIGQWQMVEIAKALARNAKIIAFDEPTSSLSAREID
NLFRVIRELRKEGRVILYVSHRMEEIFALSDAITVFKDGRYVKTFTDMQQVDHDALVQAM
VGRDIGDIYGWQPRSYGEERLRLDAVKAPGVRTPISLAVRSGEIVGLFGLVGAGRSELMK
GMFGGTQITAGQVYIDQQPIDIRKPSHAIAAGMMLCPEDRKAEGIIPVHSVRDNINISAR
RKHVLGGCVINNGWEENNADHHIRSLNIKTPGAEQLIMNLSGGNQQKAILGRWLSEEMKV
ILLDEPTRGIDVGAKHEIYNVIYALAAQGVAVLFASSDLPEVLGVADRIVVMREGEIAGE
LLHEQADERQALSLAMPKVSQAVA
>LFGLNPFC_01357 L-arabinose transport system permease protein AraH
MSSVSTSGSGAPKSSFSFGRIWDQYGMLVVFAVLFIACAIFVPNFATFINMKGLGLAISM
SGMVACGMLFCLASGDFDLSVASVIACAGVTTAVVINLTESLWIGVAAGLLLGVLCGLVN
GFVIAKLKINALITTLATMQIVRGLAYIISDGKAVGIEDESFFALGYANWFGLPAPIWLT
VACLIIFGLLLNKTTFGRNTLAIGGNEEAARLAGVPVVRTKIIIFVLSGLVSAIAGIILA
SRMTSGQPMTSIGYELIVISACVLGGVSLKGGIGKISYVVAGILILGTVENAMNLLNISP
FAQYVVRGLILLAAVIFDRYKQKAKRTV
>LFGLNPFC_01358 Trehalose-6-phosphate phosphatase
MTEPLTETPELSAKYAWFFDLDGTLAEIKPHPDQVVVPDTILQGLQLLATASDGALALIS
GRSMVELDALAKPYRFPLAGVHGAERRDINGKTHIVHLPDAIARDISVQLHTVIAQYPGA
ELEAKGMAFALHYRQAPQHEDALMTLAQRITQIWPQMALQQGKCVVEIKPKGTSKGEAIA
AFMQEAPFIGRTPVFLGDDLTDESGFAVVNRLGGMSVKIGTGATQASWRLAGVPDVWSWL
EMITTALQQKRENNRSDDYESFSRSI
>LFGLNPFC_01359 Trehalose-6-phosphate synthase
MSRLVVVSNRIAPPDEHAASAGGLAVGILGALKAAGGLWFGWSGETGNEDQPLKKVKKGN
ITWASFNLSEQDLDEYYNKFSNAVLWPAFHYRLDLVQFQRPAWDGYLRVNALLADKLLPL
LQDDDIIWIHDYHLLPFAHELRKRGVNNRIGFFLHIPFPTPEIFNALPTYDTLLEQLCEY
DLLGFQTENDRLAFLDCLSNLTRVTTRSAKSHTACGKAFRTEVYPIGIEPKEIAKQAAGP
LPPKLAQLKAELKNVQNIFSVERLDYSKGLPERFLAYEALLEKYPQHHGKIRYTQIAPTS
RGDVQAYQDIRHQLENEAGRINGKYGQLGWTPLYYLNQHFDRKLLMKIFRYSDVGLVTPL
RDGMNLVAKEYVAAQDPANPGVLVLSQFAGAANELTSALIVNPYDRDEVAAALDRALTMS
LAERISRHAEMLDVIVKNDINHWQECFISDLKQIVPRSAESQQRDKVATFPKLA
>LFGLNPFC_01360 Universal stress protein C
MSYSNILVAVAVTPESQQLLAKAVSIARPVKGHISLITLASDPEMYNQLAAPMLEDLRNV

MQEETQSFLDKLIQDAGYPVDKTFIAYGELSEHILEVCRKHHFDLVICGNHNHSFFSRAS
CSAKRVITSSEVDVLLVPLTGD
>LFGLNPFC_01361 Flagellar transcriptional regulator FlhD
MHTSELLKHIYDINLSYLLLAQRLIVQDKASAMFRLGINEEMATTLAALTLPQMVKLAET
NQLVCHFRFDSHQTITQLTQDSRVDDLQQIHTGIMLSTRLLNDVNQPEEALRKKRA
>LFGLNPFC_01362 Flagellar transcriptional regulator FlhC
MSEKSIVQEARDIQLAMELITLGARLQMLESETQLSRGRLIKLYKELRGSPPPKGMLPFS
TDWFMTWEQNVHASMFCNAWQFLLKTGLCNGVDAVIKAYRLYLEQCPQAEEGPLLALTRA
WTLVRFVESGLLQLSSCNCCGGNFITHAHQPVGSFACSLCQPPSRAVKRRKLSQNPADII
PQLLDEQRVQAV
>LFGLNPFC_01363 Motility protein A
MLILLGYLVVLGTVFGGYLMTGGSLGALYQPAELVIIAGAGIGSFIVGNNGKAIKGTLKA
LPLLFRRSKYTKAMYMDLLALLYRLMAKSRQMGMFSLERDIENPRESEIFASYPRILADS
VMLEFIVDYLRLIISGHMNTFEIEALMDEEIETHESEAEVPANSLALVGDSLPAFGIVAA
VMGVVHALGSADRPAAELGALIAHAMVGTFLGILLAYGFISPLASVLRQKSAETSKMMQC
VKVTLLSNLNGYAPPIAVEFGRKTLYSSERPSFIELEEHVRAVKNPQQQTTTEEA
>LFGLNPFC_01364 Motility protein B
MKNQAHPIIVVKRRKAKSHGAAHGSWKIAYADFMTAMMAFFLVMWLISISSPKELIQIAE
YFRTPLATAVTGGDRISNSESPIPGGGDDYTQSQGEVNKQPNIEELKKRMEQSRLRKLRG
DLDQLIESDPKLRALRPHLKIDLVQEGLRIQIIDSQNRPMFRTGSADVEPYMRDILRAIA
PVLNGIPNRISLSGHTDDFPYASGEKGYSNWELSADRANASRRELMVGGLNGGKVLRVVG
MAATMRLSDRGPDDAVNRRISLLVLNKHAEQAILHENAESQNEPVSALEKPEVAPQVSVP
TMPSAEPR
>LFGLNPFC_01365 Chemotaxis protein CheA
MSMDISDFYQTFFDEADELLADMEQHLLVLQPEAPDAEQLNAIFRAAHSIKGGAGTFGFS
VLQETTHLMENLLDEARRGEMQLNTDIINLFLETKDIMQEQLDAYKQSQEPDAASFNYIC
QALRQLALEAKGETPSAVTRLSVVAKSEPQDEQSRSQSPRRIILSRLKAGEVDLLEEELG
HLTTLTDVVKGADSLSAILPDDIAEDDITAVLCFVIEADQSTFETVDVSPKISTPPMLKL
AAEQAPIGRVEREKTTRSSESTSIRVAVEKVDQLINLVGELVITQSMLAQRSSELDPVNH
GDLITSMGQLQRNARDLQESVMSIRMMPMEYVFSRYPRLVRDLAGKLGKQVELTLVGSST
ELDKSLIERIIDPLTHLVRNSLDHGIELPEKRLAAGKNSVGNLILSAEHQGGNICIEVTD
DGAGLNRERILAKAASQGLTVSENMSDDEVAMLIFAPGFSTAEQVTDVSGRGVGMDVVKR
NIQEMGGHVEIQSMQGTGTTIRILLPLTLAILDGMSVRVADEVFILPLNAVMESLQPREA
DLHPLAGGERVLEVRGEYLPIVELWKVFNVAGAKTEATQGIVVILQSGGRRYALLVDQLI
GQHQVVVKNLESNYRKVPGISAATILGDGSVALIVDVSALQAINREQRMANTAA
>LFGLNPFC_01366 Chemotaxis protein CheW
MTGMTNVTKLASEPSGQEFLVFTLGDEEYGIDILKVQEIRGYDQVTRIANTPAFIKGVTN
LRGVIVPIVDLRIKFSQVDVDYNDNTVVIVLNLGGQRVVGIVVDGVSDVLSLTAEQIRPAP
EFAVTLSTEYLTGLGALGDRMLILVNIEKLLNSEEMALLDSAASEVA
>LFGLNPFC_01367 Methyl-accepting chemotaxis protein II
MINRIRVVTLLVMVLGVFALLQLISGSLFFSSLHHSQKSFVVSNQLREQQGELTSTWDLM
LQTRINLSRSAVRMMMDSSNQQSNAKVELLDSARKTLAQAATHYKKFKSMAPLPEMVATS
RNIDEKYKNYHTALTELIDYLDYGNTGAYFAQPTQGMQNAMGEAFAQYALSSEKLYRDIV
TDNADDYRFAQWQLAVIALVVVLILLVAWYGIRRMLLTPLAKIIAHIREIAGGNLANTLT
IDGRSEMGDLAQSVSHMQRSLTDTVTHVREGSDAIYAGTREIAAGNTDLSSRTEQQASAL
EETAASMEQLTATVKQNADNARQASQLAQSASDTAQHGGKVVDGVVKTMHEIADSSKKIA
DIISVIDGIAFQTNILALNAAVEAARAGEQGRGFAVVAGEVRNLASRSAQAAKEIKVLIE
DSVSRVDTGSVLVESAGETMNNIVNAVTRVTDIMGEIASASDEQSRGIDQVALAVSEMDR
VTQQNASLVQESAAAAAALEEQASRLTQAVSAFRLAASPLTNKPQTPSRPASEQPPAQPR
LRIAEQDPNWETF
>LFGLNPFC_01368 Methyl-accepting chemotaxis protein IV
MFNRIRISTTLFLILILCGILQIGSNGMSFGHFATICND
>LFGLNPFC_01369 Chemotaxis protein methyltransferase
MTSSLPCGQTSLLLQMTERLALSDAHFRRISQLIYQRAGIVLADHKRDMVYNRLVRRLRA
LGLADFGHYLNLLESNQHSGEWQAFINSLTTNLTAFFREAHHFPLLADHARRRSGEYRVW
SAAASTGEEPYSIAMTLADTLGTAPGRWKVFASDIDTEVLEKARSGIYRHEELKNLTPQQ
LQRYFMRGTGPHEGLVRVRQELANYVDFAPLNLLAKQYTVPGPFDAIFCRNVMIYFDQTT
QQKILRRFVPLLKPDGLLFAGHSENFSHLERRFTLRGQTVYALSKD
>LFGLNPFC_01370 Protein-glutamate methylesterase/protein-glutamine glutaminase
MSKIRVLSVDDSALMRQIMTEIINSHSDMEMVATAPDPLVARDLIKKFNPDVLTLDVEMP
RMDGLDFLEKLMRLRPMPVVMVSSLTGKGSEVTLRALELGAIDFVTKPQLGIREGMLAYS
EMIAEKVRTAAKASLAAHKPLSVPTTLKAGPLLSSEKLIAIGASTGGTEAIRHVLQPLPL
SSPALLITQHMPPGFTRSFADRLNKLCQIGVKEAEDGERVLPGHAYIAPGDRHMELARSG
ANYQIKIHDGPAVNRHRPSVDVLFHSVAKQAGRNAVGVILTGMGNDGAAGMLAMRQAGAW

TLAQNEASCVVFGMPREAINMGGVCEVVDLSQVSQQMLAKISAGQAIRI
>LFGLNPFC_01371 Chemotaxis protein CheY
MADKELKFLVVDDFSTMRRIVRNLLKELGFNNVEEAEDGLDALNKLQAGGYGFVISDWNM
PNMDGLELLKTIRADGAMSALPVLMVTAEAKKENIIAAAQAGASGYVVKPFTAATLEEKL
NKIFEKLGM
>LFGLNPFC_01372 Protein phosphatase CheZ
MMQPSIKPADEHSAGDIIARIGSLTRMLRDSLRELGLDQAIAEAAEAIPDARDRLYYVVQ
MTAQAAERALNSVEASQPHQDQMEKSAKALTQRWDDWFADPIDLADARELVTDTRQFLAD
VPAHTSFTNAQLLEIMMAQDFQDLTGQVIKRMMDVIQEIERQLLMVLLENIPEQESRPKR
ENQSLLNGPQVDTSKAGVVASQDQVDDLLDSLGF
>LFGLNPFC_01373 Flagellar biosynthetic protein FlhB
MSDESDDKTEAPTPHRLEKAREEGQIPRSRELTSLLILLVGVSVIWFGGVSLARRLSGML
SAGLHFDHSIINDPNLILGQIILLIREAMLALLPLISGVVLVAIISPVMLGGLVFSGKSL
QPKFSKLNPLPGIKRMFSAQTGAELLKAILKTILVGSVTGFFLWHHWPQMMRLMAESPIT
AMGNAMDLVGLCALLVVLGVIPMVGFDVFFQIFSHLKKLRMSRQDIRDEFKQSEGDPHVK
GRIRQMQRAAARRRMMADVPKADVIVNNPTHYSVALQYDENKMSAPKVVAKGAGLVALRI
REIGAENNVPTLEAPPLARALYRHAEIGQQIPGQLYAAVAEVLAWVWQLKRWRLAGGQRP
VQPTHLPVPEALDFINEKPTHE
>LFGLNPFC_01374 Flagellar biosynthesis protein FlhA
MSNLAAMLRLPANLKSTQWQILAGPILILLILSMMVLPLPAFILDLLFTFNIALSIMVLL
VAMFTQRTLEFAAFPTILLFTTLLRLALNVASTRIILMEGHTGAAAAGKVVEAFGHFLVG
GNFAIGIVVFVILVIINFMVITKGAGRIAEVGARFVLDGMPGKQMAIDADLNAGLIGEDE
AKKRRSEVTQEADFYGSMDGASKFVRGDAIAGILIMVINVVGGLLVGVLQHGMSMGHAAE
SYTLLTIGDGLVAQIPALVISTAAGVIVTRVSTDQDVGEQMVNQLFSNPSVMLLSAAVLG
LLGLVPGMPNLVFLLFTAGLLGLAWWIRGREQKAPAEPKPVKMAENNTVVEATWNDVQLE
DSLGMEVGYRLIPMVDFQQDGELLGRIRSIRKKFAQEMGFLPPVVHIRDNMDLQPARYRI
LMKGVEIGSGDAYPGRWLAINPGTAAGTLPGEATVDPAFGLNAIWIESALKEQAQIQGYT
VVEASTVVATHLNHLISQHAAELFGRQEAQQLLDRVAQEMPKLTEDLVPGVVTLTTLHKV
LQNLLDEKVPIRDMRTILETLAEHAPIQSDPHELTAVVRVALGRAITQQWFPGKDEVHVI
GLDTPLERLLLQALQGGGGLEPGLADRLLAQTQEALSRQEMLGAPPVLLVNHALRPLLSR
FLRRSLPQLVVLSNLELSDNRHIRMTATIGGK
>LFGLNPFC_01375 Flagellar protein FlhE
MRALLAILLFPLLVQAAGEGMWQASSVGVTLNHRGESMSSAPLSTRQPASGLMTLVAWRY
QLIGPTPAGLRVRLCSQSRCVELEGQSGTTMAFSGIPAAEPLRFIWEVPGGGRLIPPLKI
QRNEVIVNYR
>LFGLNPFC_01376 Arginine--tRNA ligase
MNIQALLSEKVRQAMIAAGAPADCEPQVRQSAKVQFGNYQANGMMAVAKKLGMAPRQLAE
QVLTHLDLNGIASKVEIAGPGFINIFLDPAFLAEHVQQALASDRLGVAMPEKQTIVVDYS
APNVAKEMHVGHLRSTIIGDAAVRTLEFLGHKVIRANHVGDWGTQFGMLIAWLEKQQQEN
AGEMELADLEGFYRDAKKHYDEDEEFAERARNYVVKLQSGDEYFREMWRKLVDITMTQNQ
ITYDRLNVTLTRDDVMGESLYNPMLPGIVADLKAKGLAVESEGATVVFLDEFKNKEGEPM
GVIIQKKDGGYLYTTTDIACAKYRYETLHADRVLYYIDSRQHQHLMQAWAIVRKAGYVPE
SVPLEHHMFGMMLGKDGKPFKTRAGGTVKLADLLDEALERARRLVAEKNPDMPADELEKL
ANAVGIGAVKYADLSKNRTTDYIFDWDNMLAFEGNTAPYMQYAYTRVLSVFRKAEIDEEQ
LAAAPVIIREDREAQLAARLLQFEETLTVVAREGTPHVMCAYLYDLAGLFSGFYEHCPIL
SAENEEVRNSRLKLAQLTAKTLKLGLDTLGIETVERM
>LFGLNPFC_01377 Protein YecM
MANWQSIDELQDIASDLPRFTHALDELSRRLGLDITPLTADHISLRCHQNVTAERWRRGF
EQCGELLSENMINGRPICLFKLHEPVQVAHWQFSIVELPWPGEKRYPHEGWEHIEIVLPG
DPETLNARALALLSDEGLSLPGISVKTSSPKGEHERLPNPTLAVTDGKTTIKFHPWSIEE
IVASEQSA
>LFGLNPFC_01378 Copper homeostasis protein CutC
MALLEICCYSMECALTAQQNGADRVELCAAPKEGGLTPSLGVLKSVRQRVTIPVHPIIRP
RGGDFCYSDGEFAAILEDVRTVRELGFPGLVTGVLEVDGNVDMPRMEKIMAAAGPLAVTF
HRAFDMCANPLNTLNNLTELGITRVLTSGQKSDALQGLSKIMELIAHRDAPIIMAGAGVR
AENLHHFLDAGVLEVHSSAGAWQASPMRYRNQGLSMSSDAHADEYLRYVVDGAAVAEMKG
IIERHQAK
>LFGLNPFC_01379 Cytochrome c-type protein TorY
MRGKKRIGLLFLLIAVVVGGGGLLLAQKALHKTSDTAFCLSCHSMSKPFEEYQGTVHFSN
QKGIRAECADCHIPKSGMDYLFAKLKASKDIYHEFVSGKIDSDDKFEAHRQEMAETVWKE
LKATDSATCRSCHSFDAMDIASQSESAQKMHNKAQKDGETCIDCHKGIAHFPPEIKMDDN
AAHELESQAATSVTNGAHIYPFKTSRIGDLATVTPGTDLTVVDVSGKQPIVRLQGYQMQG
SENTLYLAAGQRLALATLSEEGIKALTVNGEWQADEYGNQWRQASLQGSLIDPALADRKP
LWQYAEKLDDTYCAGCHAPIAADHYTVNAWPSIAKGMGARTSMSENELDILTRYFQYNAK

DITEKQ
>LFGLNPFC_01380 Trimethylamine-N-oxide reductase 2
MTLTRREFIKHSGIAAGTLVVTSAAPLPAWAEEKGGKILTAGRWGAMNVEVKDGKIVSST
GALAKTIPNSLQSTAADQVHTTARIQHPMVRKSYLDNPLQPAKGRGEDTYVQVSWEQALK
LIHEQHDRIRKANGPSAIFAGSYGWRSSGVLHKAQTLLQRYMNLAGGYSGHSGDYSTGAA
QVIMPHVVGSVEVYEQQTSWPLILENSQVVVLWGMNPLNTLKIAWSSTDEQGLEYFHQLK
KSGKPVIAIDPICSETIEFFGDNATWIAPNMGTDVALMLGIAHTLMTQGKHDKVFLEKYT
TGYPQFEEYLTGKSDNTPKSAAWAAEITGVPEAQIVKLAELMAANRTMLMAGWGIQRQQY
GEQKHWMLVTLAAMLGQIGTPGGGFGFSYHYSNGGNPTRVGGVLPEMSAAIAGQASEAAD
DGGITAIPVARIVDALENPGGKYQHNGKEQTYPNIKMIWWAGGGNFTHHQDTNRLIKAWQ
KPEMIVVSECYWTAAAKHADIVLPITTSFERNDLTMTGDYSNQHIVPMKQAVAPQFEARN
DFDVFADLAELLKPGGKEIYTEGKDEMAWLKFFYDAAQKGARAQRVTMPMFNAFWQQNKL
IEMRRSEKNEQYIRYGDFRADPVKNALGTPSGKIEIYSRTLEKFGYKDCPAHPTWLAPDE
WKGTADEKQLQLLTAHPAHRLHSQLNYAELRKKYAVADREPITIHTEDAARFGIANGDLV
RVWNKRGQILTGAVVTDGIKKGVVCVHEGAWLDLENGLCKNGSANVLTADIPSSQLANAC
AGNTALVYIEKYTGNAPKLTAFDQPAVQA
>LFGLNPFC_01381 tRNA U34 carboxymethyltransferase
MIDFGNFYSLIAKNHLSHWLETLPAQIANWQREQQHGLFKQWSNAVEFLPEIKPYRLDLL
HSVTAESEEPLSAGQIKRIETLMRNLMPWRKGPFSLYGVNIDTEWRSDWKWDRVLPHLSD
LTGRTILDVGCGSGYHMWRMIGAGAHLAVGIDPTQLFLCQFEAVRKLLGNDQRAHLLPLG
IEQLPALKAFDTVFSMGVLYHRRSPLEHLWQLKDQLVNEGELVLETLVIDGDENTVLVPG
DRYAQMRNVYFIPSALALKNWLKKCGFVDIRIADVSVTTTEEQRRTELMVTESLADFLDP
HDPGKTVEGYPAPKRAVLIARKP
>LFGLNPFC_01382 Carboxy-S-adenosyl-L-methionine synthase
MSHRDTLFSAPIARLGDWTFDERVAEVFPDMIQRSVPGYSNIISMIGMLAERFVQPGTQV
YDLGCSLGAATLSVRRNIHHDNCKIIAIDNSPAMIERCRRHIDAYKAPTPVDVIEGDIRD
IAIENASMVVLNFTLQFLEPSERQALLDKIYQGLNPGGALVLSEKFSFEDAKVGELLFNM
HHDFKRANGYSELEISQKRSMLENVMLTDSVETHKARLHKAGFEHSELWFQCFNFGSLVA
LKAEDAA
>LFGLNPFC_01383 Inner membrane protein YecN
MVSALYAVLSALLLMKFSFDVVRLRMQYRVAYGDGGFSELQSAIRIHGNAVEYIPIAIML
MLFMEMNGAETWMVHICGIVLLAGRLMHYYGFHHRLFRWRRSGMSATWCALLLMVLANLW
YMPWELVFSLR
>LFGLNPFC_01384 hypothetical protein
MIYIGLPQWSHPKWVRLGITNLEEYARNFNCVEGNTTLYALPKPEVVLRWREQTTDDFRF
CFKFPATISHQAALRHCDDLVTEFLTRMSPLAPRIGQYWLQLPATFGPRELPALWHFLDS
LPGEFNYGVEVRHPQFFAKGEEEQTLNRGLHQRGVNRVILDSRPVHAARPHSEAIRDAQR
KKPKVPVHAVLTAKNPLIRFIGSDDMTQNRELFQVWLQKLAQWHQTTTPYLFLHTPDIAQ
APELVHTLWEDLRKTLPEIGAVPAIPQQSSLF
>LFGLNPFC_01385 Isochorismatase family protein YecD
MLELNAKTTALVVIDLQEGILPFAGGPHTADEVVNRAGKLAAKFRASGQPVFLVRVGWSA
DYAEALKQPVDAPSPAKVLPENWWQHPAALGATDSDIEIIKRQWGAFYGTDLELQLRRRG
IDTIVLCGISTNIGVESTARNAWELGFNLVIAEDACSAASAEQHNNSINHIYPRIARVRS
VEEILHAL
>LFGLNPFC_01386 Aspartate--tRNA ligase
MRTEYCGQLRLSHVGQQVTLCGWVNRRRDLGSLIFIDMRDREGIVQVFFDPDRADALKLA
SELRNEFCIQVTGTVRARDEKNINRDMATGEIEVLASSLTIINRADVLPLDSNHVNTEEA
RLKYRYLDLRRPEMAQRLKTRAKITSLVRRFMDDHGFLDIETPMLTKATPEGARDYLVPS
RVHKGKFYALPQSPQLFKQLLMMSGFDRYYQIVKCFRDEDLRADRQPEFTQIDVETSFMT
APQVREVMEALVRHLWLEVKGVDLGDFPVMTFAEAERRYGSDKPDLRNPMELTDVADLLK
SVEFAVFAGPANDPKGRVAALRVPGGASLTRKQIDEYGNFVKIYGAKGLAYIKVNERAKG
LEGINSPVAKFLNAEIIEAILERTGAQDGDMIFFGADNKKIVADAMGALRLKVGKDLGLT
DESKWAPLWVIDFPMFEDDGEGGLTAMHHPFTSPKDMTAAELKAAPENAVANAYDMVING
YEVGGGSVRIHNGDMQQTVFGILGINEEEQREKFGFLLDALKYGTPPHAGLAFGLDRLTM
LLTGTDNIRDVIAFPKTTAAACLMTEAPSFANPTALAELSIQVVKKAENN
>LFGLNPFC_01387 Dihydroneopterin triphosphate diphosphatase
MAYKRPVSILVVIYAQDTKRVLMLQRRDDPDFWQSVTGSVEEGETAPQAAMREVKEEVTI
DVVAEQLTLIDCQRTVEFEIFSHLRHRYAPGVTRNTESWFCLALPHERQIVFTEHLAYKW
LDASAAAALTKSWSNRQAIEQFVINAA
>LFGLNPFC_01388 putative transcriptional regulatory protein YebC
MAGHSKWANTRHRKAAQDAKRGKIFTKIIRELVTAAKLGGGDPDANPRLRAAIDKALSNN
MTRDTLNRAIARGVGGDDDANMETIIYEGYGPGGTAIMIECLSDNRNRTVAEVRHAFSKC
GGNLGTDGSVAYLFSKKGVISFEKGDEDTIMEAALEAGAEDVVTYDDGAIDVYTAWEEMG
KVRDALEAAGLKADSAEVSMIPSTKADMDAETAPKLMRLIDMLEDCDDVQEVYHNGEISD

EVAATL
>LFGLNPFC_01389 Crossover junction endodeoxyribonuclease RuvC
MAIILGIDPGSRVTGYGVIRQVGRQLSYLGSGCIRTKVDDLPSRLKLIYAGVTEIITQFQ
PDYFAIEQVFMAKNADSALKLGQARGVAIVAAVNQELPVFEYAARQVKQTVVGIGSAEKS
QVQHMVRTLLKLPANPQADAADALAIAITHCHVSQNAMQMSESRLNLARGRLR
>LFGLNPFC_01390 hypothetical protein
MNINYPAEYEIGDIVFTCIGATLFGQISAASNCWSNHVGIIIGHNGEDFLVAESRVPLST
ITTLSRFIKRSANQRYAIKRLDAGLTEQQKQRIVEQVPSRLRKLYHTGFKYESSRQFCSK
FVFDIYKEALCIPVGEIETFGELLNSNPNAKLTFWKFWFLGSIPWERKTVTPASLWHHPG
LVLIHAVGVETPQPELTEAV
>LFGLNPFC_01391 Holliday junction ATP-dependent DNA helicase RuvA
MIGRLRGIIIEKQPPLVLIEVGGVGYEVHMPMTCFYELPEAGQEAIVFTHFVVREDAQLL
YGFNNKQERTLFKELIKTNGVGPKLALAILSGMSAQQFVNAVEREEVGALVKLPGIGKKT
AERLIVEMKDRFKGLHGDLFTPAADLVLTSPASPATDDAEQEAVAALVALGYKPQEASRM
VSKIARPDASSETLIREALRAAL
>LFGLNPFC_01392 Holliday junction ATP-dependent DNA helicase RuvB
MIEADRLISAGTTLPEDVADRAIRPKLLEEYVGQPQVRSQMEIFIKAAKLRGDALDHLLI
FGPPGLGKTTLANIVANEMGVNLRTTSGPVLEKAGDLAAMLTNLEPHDVLFIDEIHRLSP
VVEEVLYPAMEDYQLDIMIGEGPAARSIKIDLPPFTLIGATTRAGSLTSPLRDRFGIVQR
LEFYQVPDLQYIVSRSARFMGLEMSDDGALEVARRARGTPRIANRLLRRVRDFAEVKHDG
TISADIAAQALDMLNVDAEGFDYMDRKLLLAVIDKFFGGPVGLDNLAAAIGEERETIEDV
LEPYLIQQGFLQRTPRGRMATTRAWNHFGITPPEMP
>LFGLNPFC_01393 High-affinity zinc uptake system membrane protein ZnuB
MIELLFPGWLAGIMLACAAGPLGSFVVWRRMSYFGDTLAHASLLGVAFGLLLDVNPFYAV
IAVTLLLAGGLVWLEKRPQLAIDTLLGIMAHSALSLGLVVVSLMSNIRVDLMAYLFGDLL
AVTPEDLISIAIGVVIVVAILFWQWRNLLSMTISPDLAFVDGVKLQRVKLLLMLVTALTI
GVAMKFVGALIITSLLIIPAATARRFARTPEQMAGVAVLVGMVAVTGGLTFSAFYDTPAG
PSVVLCAALLFILSMMKKQAS
>LFGLNPFC_01394 Zinc import ATP-binding protein ZnuC
MTSLVSLENVSVSFGQRRVLSDVSLELKPGKILTLLGPNGAGKSTLVRVVLGLVTPDEGV
IKRNGKLRIGYVPQKLYLDTTLPLTVNRFLRLRPGTHKEDILPALKRVQAGHLINAPMQK
LSGGETQRVLLARALLNRPQLLVLDEPTQGVDVNGQVALYDLIDQLRRELDCGVLMVSHD
LHLVMAKTDEVLCLNHHICCSGTPEVVSLHPEFISMFGPRGAEQLGIYRHHHNHRHDLQG
RIVLRRGNDRS
>LFGLNPFC_01395 High-affinity zinc uptake system protein ZnuA
MKCYNITVLIFITMIGRIMLHKKTLLFAALSAALWGGATQAADAAVVASLKPVGFIASAI
ADGVTETQVLLPDGASEHDYSLRPSDVKRLQNADLVVWVGPEMEAFMQKPVSKLPEAKQV
TIAQLEDVKPLLMKSIHDDDDDHDHAEKSDEDHHHGDFNMHLWLSPEIARATAVAIHGKL
VELMPQSRAKLDANLKDFEAQLASTETQVGNELAPLKGKGYFVFHDAYGYFEKQFGLTPL
GHFTVNPEIQPGAQRLHEIRTQLVEQKATCVFAEPQFRPAVVESVARGTSVRMGTLDPLG
TNIKLGKTSYSEFLNQLANQYASCLKGD
>LFGLNPFC_01396 Murein DD-endopeptidase MepM
MQQIARSVALAFNNLPRPHRVMLGSLTVLTLAVAVWRPYVYHRDATPIVKTIELEQNEIR
SLLPEASEPIDQAAQEDEAIPQDELDDKIAGEAGVHEYVVSTGDTLSSILNQYGIDMGDI
TQLAAADKELRNLKIGQQLSWTLTADGELQRLTWEVSRRETRTYDRTAANGFKMTSEMQQ
GEWVNNLLKGTVGGSFVASARNAGLTSAEVSAVIKAMQWQMDFRKLKKGDEFAVLMSREM
LDGKREQSQLLGVRLRSEGKDYYAIRAEDGKFYDRNGTGLAKGFLRFPTAKQFRISSNFN
PRRTNPVTGRVAPHRGVDFAMPQGTPVLSVGDGEVVVAKRSGAAGYYVAIRHGRSYTTRY
MHLRKILVKPGQKVKRGDRIALSGNTGRSTGPHLHYEVWINQQAVNPLTAKLPRTEGLTG
SDRREFLAQAKEIVPQLRFD
>LFGLNPFC_01397 Lipid A biosynthesis myristoyltransferase
METKKNNSEYIPEFDKSFRHPRYWGAWLGVAAMAGIALTPPKFRDPILARLGRFAGRLGK
SSRRRALINLSLCFPERSEAEREAIVDEMFATAPQAMVMMAELAIRGPEKIQPRVDWQGL
EIIEEMRRNNEKVIFLVPHGWAVDIPAMLMASQGGKMAAMFHNQGNPVFDYVWNTVRRRF
GGRLHARNDGIKPFIQSVRQGYWGYYLPDQDHGPEHSEFVDFFATYKATLPAIGRLMKVC
RARVVPLFPIYDGKTHRLTIQVRPPMDDLLEADDHTIARRMNEEVEIFVGPRPEQYTWIL
KLLKTRKPGEIQPYKRKDLYPIK
>LFGLNPFC_01398 Pyruvate kinase II
MSRRLRRTKIVTTLGPATDRDNNLEKVIAAGANVVRMNFSHGSPEDHKMRADKVREIAAK
LGRHVAILGDLQGPKIRVSTFKEGKVFLNIGDKFLLDANLGKGEGDKEKVGIDYKGLPAD
VVPGDILLLDDGRVQLKVLEVQGMKVFTEVTVGGPLSNNKGINKLGGGLSAEALTEKDKA
DIKTAALIGVDYLAVSFPRCGEDLNYARRLARDAGCDAKIVAKVERAEAVCSQEAMDDII
LASDVVMVARGDLGVEIGDPELVGIQKALIRRARQLNRAVITATQMMESMITNPMPTRAE
VMDVANAVLDGTDAVMLSAETAAGQYPSETVAAMARVCLGAEKIPSINVSKHRLDVQFDN

VEEAIAMSAMYAANHLKGVTAIITMTESGRTALMTSRISSGLPIFAMSRHERTLNLTALY
RGVTPVHFDSANDGVAAASEAVNLLRDKGYLMSGDLVIVTQGDVMSTVGSTNTTRILTVE
>LFGLNPFC_01399 HTH-type transcriptional regulator HexR
MNMLEKIQSQLEHLSKSERKVAEVILASPDNAIHSSIAALALEANVSEPTVNRFCRSMDT
RGFPDFKLHLAQSLANGTPYVNRNVNEDDSVESYTGKIFESAMATLDHVRHSLDKSAINR
AVDLLTQAKKIAFFGLGSSAAVAHDAMNKFFRFNVPVVYSDDIVLQRMSCMNCSDGDVVV
LISHTGRTKNLVELAQLARENDAMVIALTSAGTPLAREATLAITLDVPEDTDIYMPMVSR
LAQLTVIDVLATGFTLRRGTKFRDNLKRVKEALKESRFDKQLLNLSDDR
>LFGLNPFC_01400 hypothetical protein
MTISALKEIEMQFCQILRLCAVLMTKADKKVVIFFHNMISVRFLPNGKR
>LFGLNPFC_01401 Glucose-6-phosphate 1-dehydrogenase
MAVTQTAQACDLVIFGAKGDLARRKLLPSLYQLEKAGQLNPDTRIIGVGRADWDKAAYTK
VVREALETFMKETIDEGLWDTLSARLDFCNLDVNDTAAFSRLGAMLDQKNRITINYFAMP
PSTFGAICKGLGEAKLNAKPARVVMEKPLGTSLATSQEINDQVGEYFEECQVYRIDHYLG
KETVLNLLALRFANSLFVNNWDNRTIDHVEITVAEEVGIEGRWGYFDKAGQMRDMIQNHL
LQILCMIAMSPPSDLSADSIRDEKVKVLKSLRRIDRSNVREKTVRGQYTAGFAQGKKVPG
YLEEEGANKSSNTETFVAIRIDIDNWRWAGVPFYLRTGKRLPTKCSEVVVYFKTPELNLF
KESWQDLPQNKLTIRLQPDEGVDIQVLNKVPGLDHKHNLQITKLDLSYSETFNQTHLADA
YERLLLETMRGIQALFVRRDEVEEAWKWVDSITEAWAMDNDAPKPYQAGTWGPVASVAMI
TRDGRSWNEFE
>LFGLNPFC_01402 Phosphogluconate dehydratase
MNPQLLRVTNRIIERSRETRSAYLARIEQAKTSTVHRSQLACGNLAHGFAACQPEDKASL
KSMLRNNIAIITSYNDMLSAHQPYEHYPEIIRKALHEANAVGQVAGGVPAMCDGVTQGQD
GMELSLLSREVIAMSAAVGLSHNMFDGALFLGVCDKIVPGLTMAALSFGHLPAVFVPSGP
MASGLPNKEKVRIRQLYAEGKVDRMALLESEAASYHAPGTCTFYGTANTNQMVVEFMGMQ
LPGSSFVHPDSPLRDALTAAAARQVTRMTGNGNEWMPIGKMIDEKVVVNGIVALLATGGS
TNHTMHLVAMARAAGIQINWDDFSDLSDVVPLMARLYPNGPADINHFQAAGGVPVLVREL
LKAGLLHEDVNTVAGFGLSRYTLEPWLNNGELDWREGAEKSLDNNVIASFEQPFSHHGGT
KVLSGNLGRAVMKTSAVPVENQVIEAPAVVFESQHDVMPAFEAGLLDRDCVVVVRHQGPK
ANGMPELHKLMPPLGVLLDRCFKIALVTDGRLSGASGKVPSAIHVTPEAYDGGLLAKVRD
GDIIRVNGQTGELTLLVDEAELAAREPHIPDLSASRVGTGRELFSALREKLSGAEQGATC
ITF
>LFGLNPFC_01403 KHG/KDPG aldolase
MKNWKTSAESILTTGPVVPVIVVKKLEHAVPMAKALVAGGVRVLEVTLRTECAVDAIRAI
AKEVPEAIVGAGTVLNPQQLAEVTEAGAQFAISPGLTEPLLKAATEGTIPLIPGISTVSE
LMLGMDYGLKEFKFFPAEANGGVKALQAIAGPFSQVRFCPTGGISPANYRDYLALKSVLC
IGGSWLVPADALEAGDYDRITKLAREAVEGAKL
>LFGLNPFC_01404 Formate-dependent phosphoribosylglycinamide formyltransferase
MTLLGTALRPAATRVMLLGSGELGKEVAIECQRLGVEVIAVDRYADAPAMHVAHRSHVIN
MLDGDALRRVVELEKPHYIVPEIEAIATDMLIQLEEEGLNVVPCARATKLTMNREGIRRL
AAEELQLPTSTYRFADSENLFREAVAAIGYPCIVKPVMSSSGKGQTFIRSAEQLAHAWEY
AQQGGRAGAGRVIVEGVVKFDFEITLLTVSAVDGVHFCAPVGHRQEDGDYRESWQPQQMS
PLALERAQEIARKVVLALGGYGLFGVELFVCGDEVIFSEVSPRPHDTGMVTLISQDLSEF
ALHVRAFLGLPVGGIRQYGPAASAVILPQLTSQNVTFDNVQNAVGADLQIRLFGKPEIDG
SRRLGVALATAESVVDAIERAKHAAGQVKVQG
>LFGLNPFC_01405 hypothetical protein
MAVEVKYVVIREGEEKMSFTSKKEADAYDKMLDTADLLDTWLTNSPVQMEDEQREALSLW
LAEQKDVLSTILKTGKLPSPQVVGAESEEEDASHAA
>LFGLNPFC_01406 Protein YebF
MKKRGAFLGLLLVSACASVFAANNETSKSVTFPKCEGLDAAGIAASVKRDYQQNRVARWA
DDQKIVGQADPVAWVSLQDIQGKDDKWSVPLTVRGKSADIHYQVSVDCKAGMAEYQRR
>LFGLNPFC_01407 hypothetical protein
MYIETKDAQISGAVGRYSKKFAPGKVFTVYKVIRVF
>LFGLNPFC_01408 Inner membrane protein YebE
MANWLNQLQSLLGQSRSSTSSSADQGLGKLLVPGALGGLAGLLVANKSARKLLTKYGTNA
LLVGGGAVAGTVLWNKYKDKIRAAHQDEPQFGAQSTPLDERTERLILALVFAAKSDGHID
AKERAAIDQQLREAGVEEKGRVLIEQAIEQPLDPQRLATGVRNEEEALEIYFLSCAAIDI
DHFMERSYLNALGDALKIPQDVREGIERDLEQQKRTLAE
>LFGLNPFC_01409 Protease 2
MLPKAARIPHAMTLHGDTRIDNYYWLRDDTRSQPEVLDYLQQENSYGHRVMASQQALQDR
ILKEIIDRIPQREVSAPYIKNGYRYRHIYEPGCEYAIYQRQSAFSEEWDEWETLLDANKR
AAHSEFYSMGGMAITPDNTIMALAEDFLSRRQYGIRFRNLETGNWYPELLDNVEPSFVWA
NDSWTFYYVRKHPVTLLPYQVWRHAIGTPASQDKLIYEEKDDTYYVSLHKTTSKHYVVIH
LASSTTSEVRLLDAEMADAEPFVFLPRRKDHEYSLDHYQHRFYLRSNRHGKNFGLYRTRM

RDEQQWEELIPPRDNIMLEGFTLFTDWLVVEERQRGLTSLRQINRKTREVIGIAFDDPAY
VTWIAYNPEPETARLRYGYSSMTTPDTLFELDMDTGERRVLKQTEVPGFDAANYRSEHLW
IVARDGVEVPVSLVYHRKHFRKGHNPLLVYGYGSYGASIDADFSFSRLSLLDRGFVYAIV
HVRGGGELGQQWYEDGKFLKKKNTFNDYLDACDALLKLGYGSPSLCYAMGGSAGGMLMGV
AINQRPELFHGVIAQVPFVDVVTTMLDESIPLTTGEFEEWGNPQDPQYYEYMKSYSPYDN
VTAQAYPHLLVTTGLHDSQVQYWEPAKWVAKLRELKTDDHLLLLCTDMDSGHGGKSGRFK
SYEGVAMEYAFLVALAQGTLPAQSAD
>LFGLNPFC_01410 Exodeoxyribonuclease 10
MLRIIDTETCGLQGGIVEIASVDVIDGKIVNPMSHLVRPDRPISPQAMAIHRITEAMVAD
KPWIEDVIPHYYGSEWYVAHNASFDRRVLPEMPGEWICTMKLARRLWPGIKYSNMALYKT
RKLNVQTPPGLHHHRALYDCYITAALLIDIMNTSGWTAEQMADITGRPSLMTTFTFGKYR
GKAVSDVAERDPGYLRWLFNNLDSMSPELRLTLKHYLENT
>LFGLNPFC_01411 hypothetical protein
MSFWKVAAAQYEPRKTSLTEQVAHHLEFVRAAARQQCQLLVFPSLSLLGCDYSRRALPAP
PDLSLLDPLCYAATTWRMTIIAGLPVEYNDRFIRGIAVFAPWRKTPGIYHQSYGACLGRR
SRTITVVDEQPQGMDMDPTCSLFTTGQCLGEPDLLASARRLQFFSHQYSIAVLMANARGN
SALWDEHGRLIVRADRGSLLLVGQRSSQGWQGDIIPLR
>LFGLNPFC_01412 DNA polymerase III subunit theta
MLKNLAKLDQTEMDKVNVDLAAAGVAFKERYNMPVIAEAVEREQPEHLRSWFRERLIAHR
LASVNLSRLPYEPKLK
>LFGLNPFC_01413 Protein YobA
MASTARSLRYALAILTTSLVTPSVWAHAHLTHQYPAANAQVTAAPQAITLNFSEGVETGF
SGAKITGPKNENIKTLPAKRNEQDQKQLIVPLADSLKPGTYTVDWHVVSVDGHKTKGHYT
FSVK
>LFGLNPFC_01414 Inner membrane protein YebZ
MLAFTWIALRFIHFTSLMLVFGFAMYGAWLAPLTIRRLLTKRFLRLQQHAAVWSLISATA
MLAVQGGLMGTGWSDVFSPNIWLAVLQTQFGGVWLWQIVLALVTLIVALMQPRNMPRLLF
MLTTAQFILLAGVGHATLNEGVTAKIHQTNHAIHLICAAAWFGGLLPVLWCMQLIKGRWR
HQAIQALMRFSWCGHFAVIGVLASGVLNALLITGFPPTLTTYWGQLLLLKAILVMIMVVI
ALANRYVLVPRMRQDEDRAAPWFVWMTKLEWAIGAVVLVIISLLATLEPF
>LFGLNPFC_01415 hypothetical protein
MMRKSILAFLLLTSSAAALAAPQVITVSRFEVGKDKWAFNREEVMLTCRPGNALYVINPS
TLVQYPLNDIAQKEVASGKTKAQPISVIQIDDPNNPGEKMSLAPFIERAEKLC
>LFGLNPFC_01416 Serine/threonine-protein phosphatase 1
MKQPAPVYQRIAGHQWRHIWLSGDIHGCLEQLRRKLWHCRFDPWRDLLISVGDVIDRGPQ
SLRCLQLLEQHWVRAVRGNHEQMAMDALASRQMSLWLINGGDWFIALADNHQKQAKTALE
KCQHLPFILELHSRTGKHVIAHADYPDDVYEWQKDVDLHQVLWSRSRLGERQKGQGIAGA
DHFWFGHTPLRHRVDIGNLHYIDTGAVFGGELTLVQLQ
>LFGLNPFC_01417 hypothetical protein
MAGYLSGLFPRCKISPKLNGTAPHFGDEMFALVLFVCYLDGGCEDIVVDVYNTEQQCLYS
MSDQRIRHGGCFPIEDFIDGFWRPAQEYGDF
>LFGLNPFC_01418 putative protein YebV
MKTSVRIGAFEIDDGELHGESPGDRTLTIPCKSDPDLCMQLDAWDAETSIPALLNGEHSV
LYRTRYDQQSDAWIMRLA
>LFGLNPFC_01419 Ribosomal RNA small subunit methyltransferase F
MLVAQHTVYFPDAFLTQMREAMPSTLSFDDFLAACQRPLRRSIRVNTLKTSVADFLQLTA
PYGWTLTPIPWCEEGFWIERDSEDALPLGSTAEHLSGLFYIQEASSMLPVAALFADGNAP
QRVMDVAAAPGSKTTQIAARMNNKGAILANEFSASRVKVLHANISRCGISNVALTHFDGR
VFGAAVPEMFDAILLDAPCSGEGVVRKDPDALKNWSPESNQEIAATQRELIDSAFHALRL
GGTLVYSTCTLNREENEAVCLWLKETYHDAVEFLPLGDLFPGANKALTEDGFLHVFPQIY
DCEGFFVARLRKTQAIPVLPAPKYKVGNFPFSPVKDREAGQIRQAAASVGLNWDENLRLW
QRDKELWLFPVGIEALIGKVRFSRLGIKLAETHNKGYRWQHEAVIALASPDNVNAFELTP
QEAEEWYRGRDVYPQAAPVADDVLVTFQHQPIGLAKRIGSRLKNSYPRELVRDGKLFTGN
A
>LFGLNPFC_01420 Intermembrane transport protein YebT
MSQETPASTTEAQIKNKRRISPFWLLPFIALMIAGWLIWDSYQDRGNTVTIDFMSADGIV
PGRTPVRYQGVEVGTVQDISLSDDLRKIEVKVSIKSDMKDALREETQFWLVTPKASLAGV
SGLDALVGGNYIGMMPGKGKEQDHFVALDTQPKYRLDNGDLMIHLQAPDLGSLSSGSLVY
FRKIPVGKVYDYAINPNKQGVVIDVLIERRFTDLVKKGSRFWNVSGVDANVSISGAKVKL
ESLAALVNGAIAFDSPEESKPAEAEDTFGLYEDLAHSQRGVIIKLELPGGAGLTADSTPL
MYQGLEVGQLTKLDLNPGGKVTGEMTVDPSVVTLLRKNTRIELRNPKLSLSDANLSALLT
GKTFELVPGDGEPRKEFVVVPGEKALLQEPDVLTLTLTAPESYGIDAGQPLILHGVQVGQ
VIDRKLTSKGVTFTVAIEPQHRELVKGDSKFVVNSRVDVKVGLDGVEFLGASASEWINGG
IRILPGDKGEMKASYPLYANLEKALENSLSDLPTTTVSLSAETLPDVQAGSVVLYRKFEV

GEVITVRPRANAFDIDLHIKPEYRNLLTSNSVFWAEGGAKVQLNGSGLTVQASPLSRALK
GAISFDNLSGASASQRKGDKRILYASETAARAVGGQITLHAFDAGKLAVGMPIRYLGIDI
GQIQTLDLITARNEVQAKAVLYPEYVQTFARGGTRFSVVTPQISAAGVEHLDTILQPYIN
VEPGRGNPRRDFELQEATITDSRYLDGLSIIVEAPEAGSLGIGTPVLFRGLEVGTVTGMT
LGTLSDRVMIAMRISKRYQHLVRNNSVFWLASGYSLDFGLTGGVVKTGTFNQFIRGGIAF
ATPPGTPLAPKAQEGKHFLLQESEPKEWREWGTALPK
>LFGLNPFC_01421 Intermembrane transport protein YebS
MALNTPQITPTKKITVRAIGEELPRGDYQRCPQCDMLFSLPEINSHQSAYCPRCQAKIRD
GRDWSLTRLAAMAFTMLLLMPFAWGEPLLHIWLLGIRIDANVMQGIWQMTKQGDAITGAM
VFFCVIGAPLILVTSIAYLWFGNQLGMNLRPVLLMLERLKEWVMLDIYLVGIGVASIKVQ
DYAHIQAGVGLFSFVALVILTTVTLSHLNVEELWERFYPQRPATRRDEKLRVCLGCHFTG
YPDQRGRCPRCHIPLRLRRRHSLQKCWAALLASIVLLLPANLLPISIIYLNGGRQEDTIL
SGIMSLASSNIAVAGIVFIASILVPFTKVIVMFTLLLSIHFKCQQGLRTRILLLRMVTWI
GRWSMLDLFVISLTMSLINRDQILAFTMGPAAFYFGAAVILTILAVEWLDSRLLWDAHES
GNARFDD
>LFGLNPFC_01422 Free methionine-R-sulfoxide reductase
MLILIGWQFQVRQPSDYIMNKAEFYADLNRDFNALMVGETSFLATLANTSALLYERLTDV
NWAGFYLLEDDTLVLGPFQGKIACVRIPVGRGVCGTAVARNQVQRIEDVHAFDGHIACDA
ASNSEIVLPLVVKNQIIGVLDIDSTVFGRFTDEDEQGLRQLVAQLEKVLATTDYKKFFAS
VAG
>LFGLNPFC_01423 RNA chaperone ProQ
MENQPKLNSSKEVIAFLAERFPHCFSAEGEARPLKIGIFQDLVDRVAGEMNLSKTQLRSA
LRLYTSSWRYLYGVKPGATRVDLDGNPCGELDEQHVEHARKQLEEAKARVQAQRAEQQAK
KREAAAAAGEKEDAPRRERKPRPTTPRRKEGAERKPRSQKPVEKAPKTVKAPREEQHTPV
SDISALTVGQALKVKAGQNAMDATVLEITKDGVRVQLNSGMSLIVRAEHLVF
>LFGLNPFC_01424 Tail-specific protease
MNMFFRLTALAGLLAIAGQTFAVEDITRADQIPVLKEETQHATVSERVTSRFTRSHYRQF
DLDQAFSAKIFDRYLNLLDYSHNVLLESDVEQFAKKKTELGDELRSGKLDVFYDLYNLAQ
KRRFERYQYALSVLEKPMDFTGNDTYNLDRSKAPWPKNEAELNALWDSKVKFDELSLKLT
GKTDKEIRETLTRRYKFAIRRLAQTNSEDVFSLAMTAFAREIDPHTNYLSPRNTEQFNTE
MSLSLEGIGAVLQMDDDYTVINSMVAGGPAAKSKAISVGDKIVGVGQTGKPMVDVIGWRL
DDVVALIKGPKGSKVRLEILPAGKGTKTRTVTLTRERIRLEDRAVKMSVKTVGKEKVGVL
DIPGFYVGLTDDVKVQLQKLEKQNVSSVIIDLRSNGGGALTEAVSLSGLFIPSGPIVQVR
DNNGKVREDSDTDGQVFYKGPLVVLVDRFSASASEIFAAAMQDYGRALVVGEPTFGKGTV
QQYRSLNRIYDQMLRPEWPALGSVQYTIQKFYRVNGGSTQRKGVTPDIIMPTGNEETETG
EKFEDNALPWDSIDAATYVKSGDLTAFEPELLKEHNARIAKDPEFQNIMKDIARFNAMKD
KRNIVSLNYAVREKENNEDDATRLARLNERFKREGKPELKKLDDLPKDYQEPDPYLDETV
NIALDLAKLEKARPAEQPAPVK
>LFGLNPFC_01425 Protease HtpX
MMRIALFLLTNLAVMVVFGLVLSLTGIQSSSVQGLMIMALLFGFGGSFVSLLMSKWMALR
SVGGEVIEQPRNERERWLVNTVATQARQAGIAMPQVAIYHAPDINAFATGARRDASLVAV
STGLLQNMSPDEAEAVIAHEISHIANGDMVTMTLIQGVVNTFVIFISRILAQLAAGFMGG
NRDEGEESNGNPLIYFAVATVLELVFGILASIITMWFSRHREFHADAGSAKLVGREKMIA
ALQRLKTSYEPQEATSMMAFCINGKSKSLSELFMTHPPLDKRIEALRTGEYLK
>LFGLNPFC_01426 Riboflavin transporter RibZ
MPKVQADGLPLPLRYGAILTIVIGISMAVLDGAIANVALPTIATDLHATPASSIWVVNAY
QIAIVISLLSFSFLGDMFGYRRIYKCGLVVFLLSSLFCALSDSLQMLTLARVIQGFGGAA
LMSVNTALIRLIYPQRFLGRGMGINSFIVAVSSAAGPTIAAAILSIASWKWLFLINVPLG
IIALLLAMRFLPPNGSRASKPRFDLPSAVMNALTFGLLITALSGFAQGQSLTLIGAELVV
MVVVGIFFIRRQLSLPVPLLPVDLLRIPLFSLSICTSVCSFCAQMLAMVSLPFYLQTVLG
RSEVETGLLLTPWPLATMVMAPLAGYLIERVHAGLLGALGLFIMAAGLFSLVLLPASPAD
INIIWPMILCGAGFGLFQSPNNHTIITSAPRERSGGASGMLGTARLLGQSSGAALVALML
NQFGDNGTHVSLIAAAILAVIAACVSGLRITQPRSRA
>LFGLNPFC_01427 Transcriptional regulator KdgR
MANADLDKQPDSVSSVLKVFGILQALGEEREIGITELSQRVMMSKSTVYRFLQTMKTLGY
VAQEGESEKYSLTLKLFELGARALQNVDLIRSADIQMREISRLTKETIHLGALDEDSIVY
IHKIDSMYNLRMYSRIGRRNPLYSTAIGKVLLAWRDRDEVMQILEGVEYKRSTERTITST
EALLPVLDQVREQGYGEDNEEQEEGLRCIAVPVFDRFGVVIAGLSISFPTLRFSEERLQE
YVAMLHTAARKISAQMGYHDYPF
>LFGLNPFC_01428 hypothetical protein
MRFIIRTVMLIALVWIGLLLSGYGVLIGSKENAAGLGLQCTYLTARGTSTVQYLHTKSGF
LGITDCPLLRKSNIVVDNG
>LFGLNPFC_01429 hypothetical protein
MNEVVNSGVMNIASLVVSVVVLLIGLILWFFINRASSRTNEQIELLEALLDQQKRQNALL

RRLCEANEPEKADKKTVESQKSVEDEDIIRLVAER
>LFGLNPFC_01430 Cold shock-like protein CspC
MAKIKGQVKWFNESKGFGFITPADGSKDVFVHFSAIQGNGFKTLAEGQNVEFEIQDGQKG
PAAVNVTAI
>LFGLNPFC_01431 23S rRNA (guanine(745)-N(1))-methyltransferase
MSFSCPLCHQPLSREKNSYICPQRHQFDMAKEGYVNLLPVQHKRSRDPGDSAEMMQARRA
FLDAGHYQPLRDAIVGQLRERLDEKAAAVLDIGCGEGYYTHAFADALPEITTFGLDVSKV
AIKAAAKRYPQVTFCVASSHRLPFSDTSMDAIIRIYAPCKAEELVRVVKPGGWVITATPG
PRHLIELKGLIYNEVHLHAPHAEQLKGFTLQQSDELCYLMRLRGDEAVALLQMTPFAWRA
KPEVWQALAAKEVFDCQTDFNIHLWQRSY
>LFGLNPFC_01432 putative manganese efflux pump MntP
MNITATVLLAFGMSMDAFAASIGKGATLHKPKFSEALRTGLIFGAVETLTPLIGWGMGML
ASRFVLEWNHWIAFVLLIFLGGRMIIEGFRGADDEDEEPRRRHGFWLLVTTAIATSLDAM
AVGVGLAFLQVNIIATALAIGCATLIMSTLGMMVGRFIGSIIGKKAEILGGLVLIGIGVQ
ILWTHFHG
>LFGLNPFC_01433 hypothetical protein
MTITDLVLILFIAALLAFAIYDQFIMPRRNGPTLLAIPLLRRGRIDSVIFVGLIVILIYN
NVTNHGALITTWLLSALALMGFYIFWIRVPKIIFKQKGFFFANVWIEYSRIKAMNLSEDG
VLVMQLEQRRLLIRVRNIDDLEKVYKLLVSTQ
>LFGLNPFC_01434 PTS system mannose-specific EIID component
MVDTTQTTTEKKLTQSDIRGVFLRSNLFQGSWNFERMQALGFCFSMVPAIRRLYPENNEA
RKQAIRRHLEFFNTQPFVAAPILGVTLALEEQRANGAEIDDGAINGIKVGLMGPLAGVGD
PIFWGTVRPVFAALGAGIAMSGSLLGPLLFFILFNLVRLATRYYGVAYGYSKGIDIVKDM
GGGFLQKLTEGASILGLFVMGALVNKWTHVNIPLVVSRITDQTGKEHVTTVQTILDQLMP
GLVPLLLTFACMWLLRKKVNPLWIIVGFFVIGIAGYACGLLGL
>LFGLNPFC_01435 PTS system mannose-specific EIIC component
MEITTLQIVLVFIVACIAGMGSILDEFQFHRPLIACTLVGIVLGDMKTGIIIGGTLEMIA
LGWMNIGAAVAPDAALASIISTILVIAGHQSIGAGIALAIPLAAAGQVLTIIVRTITVAF
QHAADKAADNGNLTAISWIHVSSLFLQAMRVAIPAVIVALSVGTSEVQNMLNAIPEVVTN
GLNIAGGMIVVVGYAMVINMMRAGYLMPFFYLGFVTAAFTNFNLVALGVIGTVMAVLYIQ
LSPKYNRVAGAPAQAAGNNDLDNELD
>LFGLNPFC_01436 PTS system mannose-specific EIIAB component
MTIAIVIGTHGWAAEQLLKTAEMLLGEQENVGWIDFVPGENAETLIEKYNAQLAKLDTTK
GVLFLVDTWGGSPFNAASRIVVDKEHYEVIAGVNIPMLVETLMARDDDPSFDELVALAVE
TGREGVKALKAKPVEKAAPAPATAAPKAAPTPAKPMGPNDYMVIGLARIDDRLIHGQVAT
RWTKETNVSRIIVVSDEVAADTVRKTLLTQVAPPGVTAHVVDVAKMIRVYNNPKYAGERV
MLLFTNPTDVERLVEGGVKITSVNVGGMAFRQGKTQVNNAVSVDEKDIEAFKKLNARGIE
LEVRKVSTDPKLKMMDLISKIDK
>LFGLNPFC_01437 hypothetical protein
MEFLMDPSIWAGLLTLVVLEIVLGIDNLVFIAILADKLPPKQRDKARLLGLSLALIMRLG
LLSLISWMVTLTKPLFTVMDFSFSGRDLIMLFGGIFLLFKATTELHERLENRDHDSGHGK
GYASFWVVVTQIVILDAVFSLDAVITAVGMVNHLPVMMAAVVIAMAVMLLASKPLTRFVN
QHPTVVVLCLSFLLMIGLSLVAEGFGFHIPKGYLYAAIGFSIIIEVFNQIARRNFIRHQS
TLPLRARTADAILRLMGGKRQANVQHDADNPMPMPIPEGAFAEEERYMINGVLTLASRSL
RGIMTPRGEISWVDANLGVDEIREQLLSSPHSLFPVCRGELDEIIGIVRAKELLVALEEG
VDVAAIASASPAIIVPETLDPINLLGVLRRARGSFVIVTNEFGVVQGLVTPLDVLEAIAG
EFPDADETPEIITDGDGWLVKGGTDLHALQQALDVEHLADDDDIATVAGLVISANGHIPR
VGDVIDVGPLHITIIEANDYRVDLVRIVKEQPAHDEDE
>LFGLNPFC_01438 putative cyclic di-GMP phosphodiesterase PdeD
MQKAQRIIKTYRRNRMIVCTICALVTLASTLSVRFISQRNLNQQRVVQFANHAVEELDKV
LLPLQAGSEVLLPLIGLPCSVAHLPLRKQAAKLQTVRSIGLVQDGTLYCSSIFGYRNVPV
VDILAELPAPQPLLRLTTDRALIKGSPVLIQWTPAAGSSNAGVMEMINIDLLTAMLLEPQ
LPQISSASLTVDSRHLLYGNGLVDSLPQPENNENYQVSSQRFPFTINVNGPGATALAWHY
LPTQLPLAVLLSLLVGYIAWLATAYRMSFSREINLGLAQHEFELFCQPLLNARSQQCIGV
EILLRWNNPRQGWISPDVFIPIAEEHHLIVPLTRYVMAETIRQRHVFPMSSQFHVGINVA
PSHFHRGVLIKDLNQYWFSAHPIQQLILEITERDALLDVDYRIARELHRKNVKLAIDDFG
TGNSSFSWLETLRPDVLKIDKSFTAAIGSDAVNSTVTDIIIALGQRLNIELVAEGVETQE
QAKYLRRHGVHILQGYLYAQPMPLCDFPKWLAGSQPPPARHNGHITPIMPLR
>LFGLNPFC_01439 L-serine dehydratase 1
MISLFDMFKVGIGPSSSHTVGPMKAGKQFVDDLVEKGLLDSVTRVAVDVYGSLSLTGKGH
HTDIAIIMGLAGNEPATVDIDSIPGFIRDVEERERLLLAQGRHEVDFPRDNGMRFHNGNL
PLHENGMQIHAYNGDEVVYSKTYYSIGGGFIVDEEHFGQDAANEVSVPYPFKSATELLAY
CNETGFSLSGLAMQNELALHSKKEIDEYFAHVWQTMQACIDRGMNTEGVLPGPLRVPRRA
SALRRMLVSSDKLSNDPMNVIDWVNMFALAVNEENAAGGRVVTAPTNGACGIVPAVLAYY

DHFIESVSPDIYTRYFMAAGAIGALYKMNASISGAEVGCQGEVGVACSMAAAGLAELLGG
SPEQVCVAAEIGMEHNLGLTCDPVAGQVQVPCIERNAIASVKAINAARMALRRTSAPRVS
LDKVIETMYETGKDMNAKYRETSRGGLAIKVQCD
>LFGLNPFC_01440 putative Nudix hydrolase NudL
MEYRSLTLDDFLSRFQLLRPQINRETLNHRQAAVLIPIVRRPQPGLLLTQRSIHLRKHAG
QVAFPGGAVDDTDASVIAAALREAEEEVAIPPSAVEVIGVLPPVDSVTGYQVTPVVGIIP
PDLPYRASEDEVSAVFEMPLAQALHLGRYHPLDIYRRGDSHRVWLSWYEQYFVWGMTAGI
IRELALQIGVKP
>LFGLNPFC_01441 Aminodeoxychorismate synthase component 1
MKTLSPAVITLPWRQDAAEFYFSRLSHLPWAMLLHSGYADHPYSRFDIVVAEPICTLTTF
GKETVVSESEKRTTTTDDPLQVLQQVLDRADIRPAHNEDLPFQGGALGLFGYDLGRRFES
LPEIAQQDIVLPDMAVGIYDWALVVDHQRQTVSLLSHNDVNARRAWLESQQFSPQEDFTL
TSDWQSNMTREQYGEKFRQVQEYLHSGDCYQVNLAQRFHATYSGDEWQAFLQLNQANRAP
FSAFLRLEQGAILSLSPERFILCDNSEIQTRPIKGTLPRLPDPQEDSKQAEKLANSAKDR
AENLMIVDLMRNDIGRVAVAGSVKVPELFVVEPFPAVHHLVSTITAQLPEQLHASDLLRA
AFPGGSITGAPKVRAMEIIDELEPHRRNAWCGSIGYLSFCGNMDTSITIRTLTAINGQIY
CSAGGGIVADSQEEAEYQETFDKVNKILRQLEK
>LFGLNPFC_01442 hypothetical protein
MFAGLPSLTHEQQQKAVERIQELMAQGMSSGQAIALVAEELRANHSGERIVARFEDEDE
>LFGLNPFC_01443 hypothetical protein
MPAVIDKALDFIGAMDVSAPTPSSMNESTAKGIFKYLKELGVPASAADITTRADLEGWNP
GFTEKMVGWAKKMESGERIVIKNPEYFSTYMQEELKALV
>LFGLNPFC_01444 2-iminobutanoate/2-iminopropanoate deaminase
MMTIVRIDAEARWSDVVIHNNTLYYTGVPENLDADAFDQTANTLAQIDAVLEKQGSNKSS
ILDATIFLADKNDFAAMNKAWDAWVVAGHAPVRCTVQAGLMNPKYKVEIKIVAAV
>LFGLNPFC_01445 putative ATP-dependent DNA helicase YoaA
MTDDFAPDGQLAKAIPGFKPREPQRQMAVAVTQAIEKGQPLVVEAGTGTGKTYAYLAPAL
RAKKKVIISTGSKALQDQLYSRDLPTVSKALKYTGNVALLKGRSNYLCLERLEQQALAGG
DLPVQILSDVILLRSWSNQTVDGDISTCVSVAEDSQAWPLVTSTNDNCLGSDCPMYKDCF
VVKARKKAMDADVVVVNHHLFLADMVVKESGFGELIPEADVMIFDEAHQLPDIASQYFGQ
SLSSRQLLDLAKDITIAYRTELKDTQQLQKCADRLAQSAQDFRLQLGEPGYRGNLRELLA
NPQIQRAFLLLDDTLELCYDVAKLSLGRSALLDAAFERATLYRTRLKRLKEINQPGYSYW
YECTSRHFTLALTPLSVADKFKELMAQKPGSWIFTSATLSVNDDLHHFTSRLGIELAESL
LLPSPFDYSRQALLCVPRNLPQTNQPGSARQLAAMLRPIIEANNGRCFMLCTSHAMMRDL
AEQFRATMTLPVLLQGETSKGQLLQQFVSAGNALLVATSSFWEGVDVRGDTLSLVIIDKL
PFTSPDDPLLKARMEDCRLRGGDPFDEVQLPDAVITLKQGVGRLIRDADDRGVLVICDNR
LVMRPYGATFLASLPPAPRTRDIARAVRFLAIPSSR
>LFGLNPFC_01446 tRNA threonylcarbamoyladenosine biosynthesis protein TsaB
MRILAIDTATEACSVALWNDGTVNAHFELCPREHTQRILPMVQDILTTSGTSLTDINALA
YGRGPGSFTGVRIGIGIAQGLALGAELPMIGVSTLMTMAQGAWRKNGATRVLAAIDARMG
EVYWAEYQRDENGIWHGEETEAVLKPELVHERMQQLSGEWVTVGTGWQAWPDLGKESGLV
LRDGEELLPAAEDMLPIACQMFAEGKTVAVEHAEPVYLRNNVAWKKLPGKE
>LFGLNPFC_01447 Outer membrane protein Slp
MAVQKNVIKGILAGTFALMLSGCVTVPDAIKGSSPTPQQDLVRVMSAPQLYVGQEARFGG
KVVAVQNQQGKTRLEIATVPLDSGARPTLGEPSRGRIYADVNGFLDPVDFRGQLVTVVGP
ITGAVDGKIGNTPYKFMVMQVTGYKRWHLTQQVIMPPQPIDPWFYGGRGWPYGYGGWGWY
NPGPARVQTVVTE
>LFGLNPFC_01448 Long-chain-fatty-acid--CoA ligase
MLTICLLLGVAMTTNTHFRGEELKKVWLNRYPADVPTEINPDRYQSLVDMFEQSVARYAD
QPAFVNMGEVMTFRKLEERSRAFAAYLQQGLGLKKGDRVALMMPNLLQYPVALFGILRAG
MIVVNVNPLYTPRELEHQLNDSGASAIVIVSNFAHTLEKVVDKTAVQHVILTRMGDQLST
AKGTVVNFVVKYIKRLVPKYHLPDAISFRSALHNGYRMQYVKPELVPEDLAFLQYTGGTT
GVAKGAMLTHRNMLANLEQVNATYGPLLHPGKELVVTALPLYHIFALTINCLLFIELGGQ
NLLITNPRDIPGLVKELAKYPFTAITGVNTLFNALLNNKEFQQLDFSSLHLSAGGGMPVQ
QVVAERWVKLTGQYLLEGYGLTECAPLVSVNPYDIDYHSGSIGLPVPSTEAKLVDDDDNE
VPPGQPGELCVKGPQVMLGYWQRPDATDEIIKNGWLHTGDIAVMDEEGFLRIVDRKKDMI
LVSGFNVYPNEIEDVVMQHPGVQEVAAVGVPSGSSGEAVKIFVVKKDPSLTEESLVTFCR
RQLTGYKVPKLVEFRDELPKSNVGKILRRELRDEARGKVDNKA
>LFGLNPFC_01449 Ribonuclease D
MITTDDALASLCEAVRAFPAIALDTEFVRTRTYYPQLGLIQLFDGEHLALIDPLGITDWS
PLKAILRDPSITKFLHAGSEDLEVFLNVFGELPQPLIDTQILAAFCGRPMSWGFASMVEE
YSGVTLDKSESRTDWLARPLTERQCEYAAADVWYLLPITAKLMVETEASGWLPARWMNAA
>LFGLNPFC_01450 Ribonuclease D
MQMRRQEVVAPEDAWRDITNAWQLRTRQLACLQLLADWRLRKARERDLAVNFVVREEHLW

SVARYMPGSLGELDSLGLSGSEIRFHGKTLLALVEKAQALPEEALPQPMLNLMDMPGYRK
AFKAIKSLITDVSETHKISAELLASRRQINQLLNWHWKLKPQNNLPELISGWRGELMAEA
LHNLLQEYPQ
>LFGLNPFC_01451 Leucine efflux protein
MFAEYGVLNYWTYLVGAIFIVLVPGPNTLFVLKNSVSSGMKGGYLAACGVFIGDAVLMFL
AWAGVATLIKTTPILFNIVRYLGAFYLLYLGSKILYATLKGKNSETKSDEPQYGAIFKRA
LILSLTNPKAILFYVSFFVQFIDVNAPHTGISFFILATTLELVSFCYLSFLIISGAFVTQ
YIRTKKKLAKVGNSLIGLMFVGFAARLATLQS
>LFGLNPFC_01452 putative protein YeaR
MLQIPQNYIHTRSTPFWNKQTAPAGIFERHLDKGTRPGVYPRLSVMHGAVKYLGYADEHS
AEPDQVILIEAGQFAVFPPEKWHNIEAMTDDTYFNIDFFVAPEVLMEGAQQRKVIHNGK
>LFGLNPFC_01453 Protein YoaG
MGKATYTVTVTNNSNGVSVDYETETPMTLLVPEVAAEVIKDLVNTVRSYDTENEHDVCGW
>LFGLNPFC_01454 hypothetical protein
MGILSWIIFGLIAGILAKWIMPGKDGGGFFMTILLGIVGAVVGGWISTLFGFGKVDGFNF
GSFVVAVIGAIVVLFIYRKIKS
>LFGLNPFC_01455 Diguanylate cyclase DgcP
MSDQIIARVSQSLAKEQSLESLVRQLLEMLEMVTDMESTYLTKVDVEARLQHIMFARNSQ
KMHIPENFTVSWDYSLCKRAIDENCFFSDEVPDRWGDCVAARNLGITTFLSTPIHLPDGS
FYGTLCAASSEKRQWSERAEQVLQLFAGLIAQYIQKEALVEQLREANAALIAQSYTDSLT
GLPNRRAIFENLTTLFSLARHLNHKIMIAFIDLDNFKLINDRFGHNSGDLFLIQVGERLN
TLQQNGEVIGRLGGDEFLVVSLNNENADISSLRERIQQIRGEYHLGDVDLYYPGASLGI
VEVDPETTDADSALHAADIAMYQEKKHKQKTPFVTHSALHS
>LFGLNPFC_01456 hypothetical protein
MKIISFVLPCLLVLAGCSTPSQPEAPKPPQIGMANPASVYCQQKGGTLIPVQTAQGVSNN
CKLPGGETIDEWALWRRDHPAGEK
>LFGLNPFC_01457 hypothetical protein
MNIQCKRVYDPAEQSDGYRVLVDRLWPRGIKKTDLALDEWDKEITPSTELRKAFHGEVVD
FATFREQYLAELAQHEQEGKRLADIAKKQPLTLLYSAKNTTQNHALVLADWLRSL
>LFGLNPFC_01458 2-nitroimidazole transporter
MTCSTSLSGKNRIVLIAGILMIATTLRVTFTGAAPLLDTIRSAYSLTTAQTGLLTTLPLL
AFALISPLAAPVARRFGMERSLFAALLLICAGIAIRSLPSPYLLFGGTAVIGGGIALGNV
LLPGLIKRDFPHSVARLTGAYSLTMGAAAALGSAMVAPLALNGFGWQGALLMLMCFPLLA
LFLWLPQWRSQQHANLSTSRALHTRGIWRSPLAWQVTLFLGINSLVYYVIIGWLPAILIS
HGYSEAQAGSLHGLLQLATAAPGLLIPLFLHHVKDQRGIAAFVALMCAVGAAGLCFMPAH
AITWTLLFGFGSGATMILGLTFIGLRASSAHQAAALSGMAQSVGYLLAACGPPLMGKIHD
ANGDWSVPLLGVAILSLLMAIFGLCAGRDKEIR
>LFGLNPFC_01459 HTH-type transcriptional regulator NimR
MHRLNLNGYEPDRHHEAAVAFCIHAGTDELTSPVHQHRKGQLILALHGAITCTVENALWM
VPPQYAVWIPGGVKHSNQVTANAELCFLFIEPSAVTMPTTCCTLKISPLCRELILTLANR
TTTQRAEPMTRRLIQVLFDELPQQPQQQLHLPVSSHLKIRTMVEMMAKEPVEWGSLGQWA
GFFAMSERNLARLIVKETGLSFRQWRQQLQLIMALQGLVKGDTVQKVAHTLGYDSTTAFI
TMFKKGLGQTPGRYIAGLTTVSPQSAKPDPRQ
>LFGLNPFC_01460 hypothetical protein
MFDVTLLILLGLAALGFISHNTTVAVSILVLIIVRVTPLSTFFPWIEKQGLSIGIIILTI
GVMAPIASGTLPPSTLIHSFLNWKSLVAIAVGVIVSWLGGRGVTLMGSQPQLVAGLLVGT
VLGVALFRGVPVGPLIAAGLVSLIVGKQ
>LFGLNPFC_01461 hypothetical protein
MTEMAKGSVTHQRLIALLSQEGANFRVVTHEAVGKCEAVSEIRGTALGQGAKALVCKVKG
NGVNQHVLAILAADQQADLSQLASHIGGLRASLASPAEVDELTGCVFGAIPPFSFHPKLK
LVADPLLFERFDEIAFNAGMLDKSVILKTADYLRIAQPELVNFRRTA
>LFGLNPFC_01462 putative diguanylate cyclase DgcJ
MKLHHRMLRHFIAASVIVLTSSFLIFELVASDRAMSAYLRYIVQRADSSFLYDKYQNQSI
AAHVMRALAAEQSEVSPEQRRAICEAFESANNTHGLNLTAHKYPGLRGTLQTASTDCDTI
VEAAALLPAFDQAVEGNRHQDDYGSGLGMAEEKFHYYLDLNDRYVYFYEPVNVEYFAMNN
WSFLQSGSIGIDRKDIEKVFTGRTVLSSIYQDQRTKQNVMSLLTPVYVAGQLKGIVLLDI
NKNNLRNIFYTHDRPLLWRFLNVTLTDTDSGRDIIINQSEDNLFQYVSYVHDLPGGIRVS
LSIDILYFITSSWKSVLFWILTALILLNMVRMHFRLYQNVSRENISDAMTGLYNRKILTP
ELEQRLQKLVQSGSSVMFIAIDMDKLKQINDTLGHQEGDLAITLLAQAIKQSIRKSDYAI
RLGGDEFCIILVDSTPQIAAQLPERIEKRLQHIAPQKEIGFSSGIYAMKENDTLHDAYKA
SDERLYVNKQNKNSRS
>LFGLNPFC_01463 putative diguanylate cyclase CdgI
MIRSTRISLGLFFNYFLSLTKIDPGQNYISLPSIKSSTHIALLFMVSMGTQKLKAQGFFI
FSLLLTLILFCITTLYNENTNVKLIPQMNYLMVVVALFFLNAVIFLFMLMKYFTNKQILP

TLILSLAFLSGLIYLVETIVIIHKPINGSTLIQTKSNDVSIFYIFRQLSFICLTSLALFC
YGKDNILDNNKKKTGILLLALIPFLVFPLLAHNLSSYNADYSLYVVDYCPDNHTATWGIN
YTKILVCLWAFLLFFIIMRTRLASELWPLIALLCLASLCCNLLLLTLDEYNYTIWYISRG
IEVSSKLFVVSFLIYNIFQELQLSSKLAVHDVLTNIYNRRYFFNSVESLLSRPVVKDFCV
MLVDINQFKRINAQWGHRVGDKVLVSIVDIIQQSIRPDDILARLEGEVFGLLFTELNSAQ
AKIIAERMRKNVELLTGFSNRYDVPEQMTISIGTVFSTGDTHNISLVMTEADKALREAKS
EGGNKVIIHHI
>LFGLNPFC_01464 hypothetical protein
MTWFIDRRLNGKNKSMVNRQRFLRRYKAQIKQSISEAINKRSVTDVDSGESVSIPTEDIS
EPMFHQGRGGLRHRVHPGNDHFVQNDRIERPQGGGGGSGSGQGQASQDGEGQDEFVFQIS
KDEYLDLLFEDLALPNLKQNQQRQLTEYKTHRAGYTANGVPANISVVRSLQNSLARRTAM
TAGKRRELHALEENLAIISNSEPAQLLEEERLRKEIAELRAKIERVPFIDTFDLRYKNYE
KRPDPSSQAVMFCLMDVSGSMDQSTKDMAKRFYILLYLFLSRTYKNVEVVYIRHHTQAKE
VDEHEFFYSQETGGTIVSSALKLMDEVVKERYNPAQWNIYAAQASDGDNWADDSPLCHEI
LAKKILPVVRYYSYIEITRRAHQTLWREYEHLQSTFDNFAMQHIRDQDDIYPVFRELFHK
QNATAKD
>LFGLNPFC_01465 hypothetical protein
MNIFDHYRQRYEAAKDEEFTLQEFLTTCRQDRSAYANAAERLLMAIGEPVMVDTAQEPRL
SRLFSNRVIARYPAFEEFYGMEDAIEQIVSYLKHAAQGLEEKKQILYLLGPVGGGKSSLA
ERLKSLMQLVPIYVLSANGERSPVNDHPFCLFNPQEDAQILEKEYGIPRRYLGTIMSPWA
AKRLHEFGGDITKFRVVKVWPSILQQIAIAKTEPGDENNQDISALVGKVDIRKLEHYAQN
DPDAYGYSGALCRANQGIMEFVEMFKAPIKVLHPLLTATQEGNYNGTEGISALPFNGIIL
AHSNESEWVTFRNNKNNEAFLDRVYIVKVPYCLRISEEIKIYEKLLNHSELTHAPCAPGT
LETLSRFSILSRLKEPENSSIYSKMRVYDGESLKDTDPKAKSYQEYRDYAGVDEGMNGLS
TRFAFKILSRVFNFDHVEVAANPVHLFYVLEQQIEREQFPQEQAERYLEFLKGYLIPKYA
EFIGKEIQTAYLESYSEYGQNIFDRYVTYADFWIQDQEYRDPDTGQLFDRESLNAELEKI
EKPAGISNPKDFRNEIVNFVLRARANNSGRNPNWTSYEKLRTVIEKKMFSNTEELLPVIS
FNAKTSTDEQKKHDDFVDRMMEKGYTRKQVRLLCEWYLRVRKSS
>LFGLNPFC_01466 MltA-interacting protein
MTKLKLLALGVLIATSAGVAHAEGKFSLGAGVGVVEHPYKDYDTDVYPVPVINYEGDNFW
FRGLGGGYYLWNDATDKLSITAYWSPLYFKAKDSGDHQMRHLDDRKSTMMAGLSYAHFTQ
YGYLRTTLAGDTLDNSNGIVWDMAWLYRYTNGGLTVTPGIGVQWNSENQNEYYYGVSRKE
SARSGLRGYNPNDSWSPYLELSASYNFLGDWSVYGTARYTRLSDEVTDSPMVDKSWTGLI
STGITYKF
>LFGLNPFC_01467 Glyoxal reductase
MQQKMIQFSGDVSLPAIGQGTWYMGEDASQRKTEVAALRAGIELGLTLIDTAEMYADGGA
EKVVGEALIGLRDNVFLVSKVYPWNAGGQKAINACEASLRRLNTDYLDLYLLHWSGSFAL
EETVAAMEKLIAQGKIRRWGVSNLDYADMQELWQLPGGNQCATNQVLYHLGSRGIEYDLL
PWCQKQQMPVMAYSPLAQAGRLRNGLLKNAVVNEIAHAHNISAAQVLLAWVISHQGVMAI
PKAATVAHVQQNAAVLEVELSSAELAMLDKAYPAPKGKTALDMV
>LFGLNPFC_01468 Putative glucose-6-phosphate 1-epimerase
MIKKIFALPVIEQISPVLSRRKLDELDLIVVDHPQVKASFALQGAHLLSWKPAGEEEVLW
LSNNTPFKNGVAIRGGIPVCWPWFGPAAQQGLPAHGFARNLPWALKSHREDADGVALTFE
LTQSEETKKFWPHDFTLLAHFHVGKTCEIDLEAHGEFETTSALHTYFNVGDIAKVSVSGL
GDRFIDKVNDAKEDVLTDGIQTFPDRTDRVYLNPQDCSVINDEALNRIIAVGHQHHLNVV
GWNPGPALSVSMGDMPDDGYKTFVCVETAYASETQKVTKEKPAHLAQSIRVAKR
>LFGLNPFC_01469 Glyceraldehyde-3-phosphate dehydrogenase A
MTIKVGINGFGRIGRIVFRAAQKRSDIEIVAINDLLDADYMAYMLKYDSTHGRFDGTVEV
KDGHLIVNGKKIRVTAERDPANLKWDEVGVDVVAEATGLFLTDETARKHITAGAKKVVMT
GPSKDNTPMFVKGANFDKYAGQDIVSNASCTTNCLAPLAKVINDNFGIIEGLMTTVHATT
ATQKTVDGPSHKDWRGGRGASQNIIPSSTGAAKAVGKVLPELNGKLTGMAFRVPTPNVSV
VDLTVRLEKAATYEQIKAAVKAAAEGEMKGVLGYTEDDVVSTDFNGEVCTSVFDAKAGIA
LNDNFVKLVSWYDNETGYSNKVLDLIAHISK
>LFGLNPFC_01470 Peptide methionine sulfoxide reductase MsrB
MANKPSAEELKKNLSEMQFYVTQNHGTEPPFTGRLLHNKRDGVYHCLICDAPLFHSQTKY
DSGCGWPSFYEPLSEESIRYIKDLSHGMQRIEIRCGNCDAHLGHVFPDGPQPTGERYCVN
SASLRFTDGENGEEING
>LFGLNPFC_01471 hypothetical protein
MNLDDIINSMTPEVYQRLSTAVELGKWPDGVTLTEEQKENCLQLVMLWQARHNTEAQHMT
IDTNGQMVMKSKQQLKEDFGISAKPIAMFK
>LFGLNPFC_01472 D-arabitol-phosphate dehydrogenase
MKALARFGKAFGGYKMIDVPQPICGPEDVVIEIKAAAICGADMKHYNVDSGSDEFNSIRG
HEFAGCIAQVGEKVKDWKVGQRVVSDNSGHVCGVCPACEQGDFLCCTEKVNLGLDNNTWG
GGFSKYCLVPGEILKIHRHALWEIPDGVDYEDAAVLDPICNAYKSIAQQAKFLPGQDVVV

IGTGPLGLFSVQMARIMGAVNIVVVGLQEDVAVRFPVAKELGATAVVNGSTEDVVARCQQ
ICGKDNLGLVIECSGANIALKQAIEMLRPNGEVVRVGMGFKPLDFSINDITAWNKSIIGH
MAYDSTSWRNAIRLLASGAIKVKPMITHRIGLSQWREGFDAMVDKTAIKVIMTYDFDE
>LFGLNPFC_01473 Inner membrane metabolite transport protein YdjE
MEQITKPHCGARLDRLPDCRWHSSMFAIVAFGLLVCWSNAVGGLILAQLKALGWTDNSTT
ATFSAITTAGMFLGALVGGIIGDKTGRRNAFILYEAIHIASMVVGAFSPNMDFLIACRFV
MGVGLGALLVTLFAGFTEYMPGRNRGTWSSRVSFIGNWSYPLCSLIAMGLTPLISAEWNW
RVQLLIPAILSLIATALAWRYFPESPRWLESRGRYQEAEKVMRSIEEGVIRQTGKPLPPV
VIADDGKAPQAVPYSALLTGVLLKRVILGSCVLIAMNVVQYTLINWLPTIFMTQGINLKD
SIVLNTMSMFGAPFGIFIAMLVMDKIPRKTMGMGLLILIAVLGYIYSLQTSMLLITLIGF
FLITFVYMYVCYASAVYVPEIWPTEAKLRGSGLANAVGRISGIAAPYAVAVLLSSYGVTG
VFILLGAVSIIVAIAIATIGIETKGVSVESLSIDAVANK
>LFGLNPFC_01474 putative zinc-type alcohol dehydrogenase-like protein YdjJ
MKNSKAILQVPGTMKIISAEIPVPKEDEVLIKVEYVGICGSDVHGFESGPFIPPKDPNQE
IGLGHECAGTVVAVGSRVRKFKPGDRVNIEPGVPCGHCRYCLEGKYNICPDVDFMATQPN
YRGALTHYLCHPESFTYKLPDNMDTMEGALVEPAAVGMHAAMLADVKPGKKIIILGAGCI
GLMTLQACKCLGATEIAVVDVLEKRLTMAEQLGATVVINGAKEDTIARCQQFTEDMGADI
VFETAGSAVTVKQAPYLVMRGGKIMIVGTVPGDSAINFLKINREVTIQTVFRYANRYPVT
IEAISSGRFDVKSMVTHIYDYRDVQQAFEESVNNKRDIIKGVIKISD
>LFGLNPFC_01475 putative protein YdjI
MLADIRYWENDATNKHYAIAHFNVWNAEMLMGVIDAAEEAKSPIIISFGTGFVGNTSFED
FSHMMVSMAQKATVPVITHWDHGRSMEIIHNAWTHGMNSLMRDASAFDFEENIRLTKEAV
DFFHPLGIPVEAELGHVGNETVYEEALAGYHYTDPDQAAEFVERTGCDSLAVAIGNQHGV
YTSEPQLNFEVVKRVRDAVSVPLVLHGASGISDADIKTAISLGIAKINIHTELCQAAMVA
VKENQDQPFLHLEREVRKAIKERALDKIKLFGSDGKAE
>LFGLNPFC_01476 putative sugar kinase YdjH
MDNLDVICIGAAIVDIPLQPVSKNIFDVDSYPLERIAMTTGGDAINEATIISRLGHRTAL
MSRIGKDAAGQFILDHCRKENIDIQSLKQDVSIDTSINVGLVTEDGERTFVTNRNGSLWK
LNIDDVDFARFSQAKLLSLASIFNSPLLDGRALTEIFTQAKARQMIICADMIKPRLNETL
NDICEALSYVDYLFPNFAEAKLLTGKETLDEIADCFLACGVKTVVIKTGKDGCFIKRGDM
TMKVPAVAGITAIDTIGAGDNFASGFIAALLEGKNLRECARFANATAAISVLSIGATTGV
KNRKLVEQLLEEYEG
>LFGLNPFC_01477 NADH-specific methylglyoxal reductase
MKKIPLGTTDITLSRMGLGTWAIGGGPAWNGDLDRQICIDTILEAHRCGINLIDTAPGYN
FGNSEVIVGQALKKLPREQVVVETKCGIVWERKGSLFNKVGDRQLYKNLSPESIREEVKA
SLQRLGIDYIDIYMTHWQSVPPFFTPIAETVAVLNALKAEGKIRAIGAANVDADHIREYL
QHGELDIIQAKYSILDRAMENELLPLCRDNGIVVQVYSPLEQGLLTGTITRDYVPGGARA
NKVWFQRENMLKVIDMLEQWQPLCARYQCTIPTLALAWILKQSDLISILSGATAPEQVRE
NVAALNINLSDADATLMREMAEALER
>LFGLNPFC_01478 putative HTH-type transcriptional regulator YdjF
MAAKDRIQAIKQMVANDKKVTVSNLSGIFQVTEETIRRDLEKLEDEGFLTRTYGGAVLNT
AMLTENIHFYKRASSFYEEKQLIARKALPFIDNKTTMAADSSSTVMELLKLLQDRSDLTL
LTNSAEAIHVLAQSEIKVVSTGGELNKNTLSLQGRITKEIISRYHVDIMVMSCKGLDINS
GALDSNEAEAEIKKTMIRQATEVALLVDHSKFDRKAFVQLADFSHINYIITDKSPGAEWI
AFCKDNNIQLVW
>LFGLNPFC_01479 Inner membrane metabolite transport protein YdjE
MEQYDQIGARLDRLPLARFHYRIFGIISFSLLLTGFLSYSGNVVLAKLVSNGWSNNFLNA
AFTSALMFGYFIGSLTGGFIGDYFGRRRAFRINLLIVGIAATGAAFVPDMYWLIFFRFLM
GTGMGALIMVGYASFTEFIPATVRGKWSARLSFVGNWSPMLSAAIGVVVIAFFSWRIMFL
LGGIGILLAWFLSGKYFIESPRWLAGKGQIAGAESQLREVEQQIEREKRIRLPPLTLNQS
NSKVKVIKGTFWLLFKGEMLRRTLVAITVLIAMNISLYTITVWIPTIFVNSGIDVDKSIL
MTAVIMIGAPVGIFIAALIIDHFPRRLFGSALLIIIAVLGYIYSIQTTEWAILIYGLVMI
FFLYMYVCFASAVYIPELWPTHLRLRGSGFVNAVGRIVAVFTPYGVAALLTHYGSITVFM
VLGVMLVLCALVLSIFGIETRKVSLEEISEVN
>LFGLNPFC_01480 Nicotinamidase
MPPRALLLVDLQNDFCAGGALAVPEGDSTVDVANRLIDWCQSRGEAVIASQDWHPANHGS
FASQHGVEPYTPGQLDGLPQTFWPDHCVQNSEGAQLHPLLKQKAIAAVFHKGENPLVDSY
SAFFDNGRRQKTALDDWLRAHVINELIVMGLATDYCVKFTVLDALQLGYKVNVITDGCRG
VNIQPQDSAHAFMEMSAAGATLYTLADWEETQE
>LFGLNPFC_01481 L-asparaginase 1
MQKKSIYVAYTGGTIGMQRSEQGYIPVSGHLQRQLALMPEFHRPEMPDFTIHEYTPLMDS
SDMTPEDWQHIAEDIKAHYDDYDGFVILHGTDTMAYTASALSFMLENLGKPVIVTGSQIP
LAELRSDGQINLLNALYVAANYPINEVTLFFNNRLYRGNRTTKAHADGFDAFASPNLPPL
LEAGIHIRRLNTPPAPHGEGALIVHPITPQPIGVVTIYPGISADVVRNFLRQPVKALILR

SYGVGNAPQNKAFLQELQEASDRGIVVVNLTQCMSGKVNMGGYATGNALAHAGVIGGADM
TVEATLTKLHYLLSQELDTETIRKAMSQNLRGELTPDD
>LFGLNPFC_01482 Protease 4
MRTLWRFIAGFFKWTWRLLNFVREMVLNLFFIFLVLVGVGIWMQVSGGDSKETASRGALL
LDISGVIVDKPDSSQRFSKLSRQLLGASSDRLQENSLFDIVNTIRQAKDDRNITGIVMDL
KNFAGGDQPSMQYIGKALKEFRDSGKPVYAVGENYSQGQYYLASFANKIWLSPQGVVDLH
GFATNGLYYKSLLDKLKVSTHVFRVGTYKSAVEPFIRDDMSPAAREADSRWIGELWQNYL
NTVAANRQIPAQQVFPGAQGLLEGLTKTGGDTAKYALENKLVDALASSAEIEKTLTKEFG
WSKTDKNYRAISYYDYALKTPADTGDSIGVVFANGAIMDGEETQGNVGGDTTAAQIRDAR
LDPKVKAIVLRVNSPGGSVTASEVIRAELAAARAAGKPVVVSMGGMAASGGYWISTPANY
IVANPSTLTGSIGIFGVITTVENSLDSIGVHTDGVSTSPLADVSITRALPPEAQQMMQLS
IENGYKRFITLVADARHSTPEQIDKIAQGHVWTGQDAKANGLVDSLGDFDDAVAKAAELA
KVKQWHLEYYVDEPTFFDKVMDNMSGSVRAMLPDAFQAMLPAPLASVASTVKSESDKLAA
FNDPQNRYAFCLTCANVR
>LFGLNPFC_01483 Putative NAD(P)H nitroreductase YdjA
MDALELLINRRSASRLAEPAPTGEQLQNILRAGMRAPDHKSMQPWHFFVIEGEGRERFSA
VLEQGAIAAGSDNKAIDKARNAPFRAPLIITVVAKCEENHKVPRWEQEMSAGCAVMAMQM
AAVAQGFGGIWRSGALTESPVVREAFGCREQDKIVGFLYLGTPQLKASTSINVPDPTPFV
TYF
>LFGLNPFC_01484 Selenide, water dikinase
MSENSIRLTQYSHGAGCGCKISPKVLETILHSEQAKFVDPNLLVGNETRDDAAVYDLGNG
TSVISTTDFFMPIVDNPFDFGRIAATNAISDIFAMGGKPIMAIAILGWPINKLSPEIARE
VTEGGRYACRQAGIALAGGHSIDAPEPIFGLAVTGIVPTERVKKNSTAQAGCKLFLTKPL
GIGVLTTAEKKSLLKPEHQGLATEVMCRMNIAGASFANIEGVKAMTDVTGFGLLGHLSEM
CQGAGVQARVDYEAIPKLPGVEEYIKLGAVPGGTERNFASYGHLMGEMPREVRDLLCDPQ
TSGGLLLAVTPEAEDEVKATAAEFGIELSAIGELVSARGGRAMVEIR
>LFGLNPFC_01485 DNA topoisomerase 3
MRLFIAEKPSLARAIADVLPKPHRKGDGFIECGNGQVVTWCIGHLLEQAQPDAYDSRYAR
WNLADLPIVPEKWQLQPRPSVTKQLNVIKRFLHEASEIVHAGDPDREGQLLVDEVLDYLQ
LAPEKRQQIQRCLINDLNPQAVERAIDRLRSNSEFVPLCVSALARARADWLYGINMTRAY
TILGRNAGYQGVLSVGRVQTPVLGLVVRRDEEIENFVAKDFFEVKAHIVTPADERFTAIW
QPSEACEPYQDEEGRLLHRPLAEHVVNRISGQPAIVTSYNDKRESESAPLPFSLSALQIE
AAKRFGLSAQNVLDICQKLYETHKLITYPRSDCRYLPEEHFAGRHAVMNAISVHAPDLLP
QPVVDPDIRNRCWDDKKVDAHHAIIPTARSSAINLTENEAKVYNLIARQYLMQFCPDAVF
RKCVIELDIAKGKFVAKARFLAEAGWRTLLGSKERDEENDGTPLPVVAKGDELLCEKGEV
VERQTQPPRHFTDATLLSAMTGIARFVQDKDLKKILRATDGLGTEATRAGIIELLFKRGF
LTKKGRYIHSTDAGKALFHSLPEMATRPDMTAHWESVLTQISEKQCRYQDFMQPLVGTLY
QLIDQAKRTSVRQFRGIMAPGGREGKKKDSPRKRAPKKSPPSEEAGNGVIT
>LFGLNPFC_01486 NADP-specific glutamate dehydrogenase
MDQTYSLESFLNHVQKRDPNQTEFAQAVREVMTTLWPFLEQNPKYRQMSLLERLVEPERV
IQFRVVWVDDRNQVQVNRAWRVQFSSAIGPYKGGMRFHPSVNLSILKFLGFEQTFKNALT
TLPMGGGKGGSDFDPKGKSEGEVMRFCQALMTELYRHLGADTDVPAGDIGVGGREVGFMA
GMMKKLSNNTACVFTGKGLSFGGSLIRPEATGYGLVYFTEAMLKRHGMGFEGMRVSVSGS
GNVAQYAIEKAMEFGARVITASDSSGTVVDESGFTKEKLARLIEIKASRDGRVADYAKEF
GLVYLEGQQPWSVPVDIALPCATQNELDVDAAHQLIANGVKAVAEGANMPTTIEATELFQ
QAGVLFAPGKAANAGGVATSGLEMAQNAARLGWKAEKVDARLHHIMLDIHHACVEHGGEG
EQTNYVQGANIAGFVKVADAMLSQGVI
>LFGLNPFC_01487 hypothetical protein
MSRALIAVVLAFPLITLANPHYHPDVEVNVPPEVFSSGGQSAQPCTQCCVYQDQNYSEGA
VIKAEGILLQCQRDDKTLSTNPLVWRRVKP
>LFGLNPFC_01488 CTP pyrophosphohydrolase
MKMIEVVAAIIERDGKILLAQRPAQSDQAGLWEFAGGKVEPDESQRQALVRELNEELGIE
ATVGDYVASHQREVSGRIIHLHAWHVPDFHGTLQAHEHQALVWCSPEEALRYPLAPADIP
LLEAFMASRAARPAD
>LFGLNPFC_01489 Inner membrane protein YnjF
MLDRHLHPRIKPLLYQCVRVLDKPGITPDGLTLVGFAIGVLALPFLALGWYLAALIVILL
NRLLDGLDGALARRRGLTDAGGFLDISLDFLFYALVPFGFILAAPEQNALAGGWLLFAFI
GTGSSFLAFAALAAKHQIDNPGYAHKSFYYLGGLTEGSETILLFVLSCLFPGCFPWFAWV
FGALCWMTTFTRVWSGYLTLKSLQRQ
>LFGLNPFC_01490 Thiosulfate sulfurtransferase YnjE
MKRVSQMTALAMALGLACASSWAAELAKPLTLDQLQQQNGKAIDTRPSAFYNGWPQTLNG
PSGHEPAALNLSASWLDKMSTEQLNEWIKQHNLKADAPVALYGNDKDVDAVKTRLQKAGF
THISILSDALSEPSRLQKLPHFEQLVYPQWLHDLQQGKEVTAKPAGDWKVIEAAWGAPKF
YLISHIPGADYIDTNEVESEPLWNKVSDEQLKAMLAKHGIRHDTTVILYGRDVYAAARVA

QIMLYAGVKDVRLLDGGWQTWSDAGLPVERGTPPKVKAEPDFGVKIPAQPQLMLDMEQAR
GLLHRQDASLVSIRSWPEFIGTTSGYSYIKPKGEIAGARWGHAGSDSTHMEDFHNPDGTM
RSADDITAMWKAWNIKPDQQVSFYCGTGWRASETFMYARAMGWNNVSVYDGGWYEWSSDP
KNPVATGERGPDSSK
>LFGLNPFC_01491 Fe(3+) ions import ATP-binding protein FbpC 2
MLCVKNVSLRLPESRLLTNVNFTVDKGDIVTLMGPSGCGKSTLFSWMIGALAGQFSCTGE
LWLNEQRIDMLPTAQRQIGILFQDALLFDQFSVGQNLLLALPATLKGNARRNAVNDALER
SGLDGAFHQDPATLSGGQRARVALLRALLAQPKALLLDEPFSRLDVALRDNFRHWVFSEI
RAMAIPVVQVTHDLQDVPPDSPVLDMAQWSENYNKLR
>LFGLNPFC_01492 Inner membrane ABC transporter permease protein YnjC
MATPLQYALIFLLWAMMAVIYAPLIPAALTLISPALSLTHWQALFADPQLPQALLATLVS
TTIAAVGALLIALLVIVALWPGPKWQRMCARLPWLLAIPHVAFATSALLIFADGGLLYDY
FPYFTPPMDKLGIGLGLTLAVKESAFLLWILAAVLSEKRLLQQLIVLDSLGYSRWQCLNW
LLLPSVASALAMAMLAIVAWSLSVVDVAIILGPGNPPTLAVISWQWLTQGDADQQTKGAL
ASLLLMLLLAAYVLLGYLLWRGWRRTIPRVDGVRKPATPLLPGNTLASFLPLTGVLCVVL
LAILADQSTINSEALINSLTMGLVAAFISLLLLLLWLEWGPQRRQLWLWLPILLPALPLV
AGQYTLALWLNLDGSWTAVVWGHLLWVMPWMLFILQPAWQRIDSRLILIAQTLGWSRAKI
FFYVKCPLMLRPALIAFAVGFSVSIAQYMPTLWLGAGRFPTLTTEAVALSSGGSNGILAA
QALWQLLLPLIIFALTALVAKWVGYVRQGLR
>LFGLNPFC_01493 Protein YnjB
MRHCGWLLGLLSLFSLATHASDWQEIKNEAKGQTVWFNAWGGDTAINRYLDWVSGEMKTH
YAINLKIVRLADAADAVKRIQTEVAAGRKTGGSVDLLWVNGENFRTLKEANLLQTGWAET
LPNWRYVDTQLPVREDFSVPTEGAESPWGGAQLTFIARRDVTPQPPQTPQALLEFAKANP
GTVTYPRPPDFTGTAFLEQLLIMLTPDPTALKEAPDDATFARVTAPLWQYLDALHPYLWR
EGKDFPPSPARMDALLKANTLRLSLTFNPAHAQQKIASGDLPASSYSFGFREGMIGNVHF
VTIPANANASAAAKVVANFLLSPDAQLRKADPAVWGDPSVLDPQKLPDGQRESLQSRMPQ
DLPPVLAEPHAGWVNALEQEWLHRYSTH
>LFGLNPFC_01494 hypothetical protein
MGLPPLSKIPFILRPQTWLHRRHYGEVLSPIRWWGRIPFIFYLVSMFVGWLERKRSPLDP
VVRSLVSARIAQMCLCEFCVDITSMKVAERTGSTDKLLAVADWRQSPLFSDEERLALEYA
EAASVTPPTVDDALRARLATHFDAQALTELTALIGLQNLSARFNSAMDIPAQGLCRIPEK
RS
>LFGLNPFC_01495 TVP38/TMEM64 family inner membrane protein YdjZ
MKMQSRKIWYYRITLIILLFAVLLAWALLPGVHEFINRSVAAFAAVDQQGIERFIQSYGA
LAAVVSFLLMILQAIAAPLPAFLITFANASLFGAFWGGLLSWTSSMAGAALCFFIARVMG
REVVEKLTGKTVLDSMDGFFTRYGKHTILVCRLLPFVPFDPISYAAGLTSIRFRSFFIAT
GLGQLPATIVYSWAGSMLTGGTFWFVTGLFILFALTVVIFMAKKIWLERQKRNA
>LFGLNPFC_01496 hypothetical protein
MLQHYSVSWKKGLAALCLLAVAGLSGCDQKENAAAKVEYDGLSNSQPLRVDANNHTVTML
VQINGRFLTDDTRHGIVFKDGSNGHKSLFMGYATPKAFYEALKEAGGTPGENMTMDNKET
THVTGSKLDISVNWQGAAKAYSFDEVIVDSNGKKLDMRFGGNLTAAEEKKTGCLVCLDSC
PVGIVSNATYTYGAVEKRGEVKFKGNASVLPADNTPATVTFKITE
>LFGLNPFC_01497 TVP38/TMEM64 family inner membrane protein YdjZ
MNAERKFLLACLIFALVIYAIHAFGLFDLLTDLPHLQTLIRQSGLFGYSLYILLFIIATL
FLLPGSILVIAGGIVFGPLLGTLLSLIAATLASSCSFLLARWLGRDLLLKYVGHSHTFQA
IEKGIARNGIDFLILTRLIPLFPYNIQNYAYGLTTIAFWPYTLISALTTLPGIVIYTVMA
SDLANEGITLRFILQLCLAGLALFILVQLAKLYARHKHVDLSASRRSPLSHPKNEG
>LFGLNPFC_01498 Exodeoxyribonuclease III
MKFVSFNINGLRARPHQLEAIVEKHQPDVIGLQETKVHDDMFPLEEVAKLGYNVFYHGQK
GHYGVALLTKETPIAVRRGFPGDDEEAQRRIIMAEIPSPLGNVTVINGYFPQGESRDHPI
KFPAKAQFYQNLQNYLETELKRENPVLIMGDMNISPGDLDIGIGEENRKRWLRTGKCSFL
PEEREWMERLMSWGLVDTFRHANPQTADRFSWFDYRSKGFDDNRGLRIDLLLASQPLAEC
CVETGIDYEIRSMEKPSDHAPVWATFRR
>LFGLNPFC_01499 Succinylornithine transaminase
MSQPITRENFDEWMIPVYAPAPFIPVRGEGSRLWDQQGKEYIDFAGGIAVNALGHAHPEL
REALNEQASKFWHTGNGYTNEPVLRLAKKLIDATFADRVFFCNSGAEANEAALKLARKFA
HDRYGSHKSGIVAFKNAFHGRTLFTVSAGGQPAYSQDFAPLPPDIRHAAYNDINSASALI
DDATCAVIVEPIQGEGGVVPASNAFLQGLRELCDRHNALLIFDEVQTGVGRTGELYACMH
YGVTPDLLTTAKALGGGFPVGALLATEECASVMTVGTHGTTYGGNPLASAVAGKVLDLIN
TPEMLNGVKQRHDWFVERLNSINHHYSLFSEVRGLGLLIGCVLNADYAGQAKQISQEAVK
AGVMVLIAGGNVVRFAPALNVSEEEVTTGLDRFAAACEHFVSRGSS
>LFGLNPFC_01500 Arginine N-succinyltransferase
MMVIRPVERSDVSALMQLASKTGGGLTSLPANEATLSVRIERAIKTWQGELPKSEQGYVF
VLEDSETGTVAGICAIEVAVGLNDPWYNYRVGTLVHASKELNVYNALPTLFLSNDHTGSS

ELCTLFLDPKWRKEGNGYLLSKSRFMFMAAFRDKFNDKVVAEMRGVIDEHGYSPFWQSLG
KRFFSMDFSRADFLCGTGQKAFIAELMPKHPIYTYFLSQEAQDVIGQVHPQTAPARAVLE
KEGFRYRNYIDIFDGGPTLECDIDRVRAIRKSRLVEVAEGQPAQGDFPACLVANENYHHF
RVVLVRTDPATERLILTAAQLDVLKCHAGDRVRLVRLCAEEKTA
>LFGLNPFC_01501 N-succinylglutamate 5-semialdehyde dehydrogenase
MTLWINGDWVTGQGALRVKRNPVSGEVLWQGNDADAAQVGQACRAARAAFPRWARLSFGD
RQVRVERFAGLLESNKAELTAIIARETGKPRWEAATEVTAMINKIAISIKAYHVRTGEQR
SEMPDGAASLRHRPHGVLAVFGPYNFPGHLPNGHIVPALLAGNTIIFKPSELTPWSGDAV
MRLWQQAGLPPGVLNLVQGGRETGQALSALEDLDGLLFTGSANTGYQLHRQLSGQPEKIL
ALEMGGNNPLIIDEVADIDAAVHLTIQSAFVTAGQRCTCARRLFLKSGTQGDAFLARLVA
VSQRLTPGTWDDEPQPFIGGLISEQAAQQVVTAWQELEAMGGRTLLAPRLLQAGTSLLTP
GIIEMTGVTGLPDEEVFGPLLRVWRYDNFDEAIRMANNTRFGLSCGLVSPEREKFDQLLL
EARAGIVNWNKPLTGAASTAPFGGIGASGNHRPSAWYAADYCAWPMASLESDSLTLPATL
NPGLDFSDEVVR
>LFGLNPFC_01502 N-succinylarginine dihydrolase
MNAWEVNFDGLVGLTHHYAGLSFGNEASTRHRFQVSNPRLAAKQGLLKMKKLADAGFPQA
VIPPHERPFIPVLRQLGFRGSDEQVLEKVARQAPHWLSSVSSASPMWVANAATIAPSADT
LDGKVHLTVANLNNKFHRSLEAPVTESLLKAIFNDEEKFSVHSALPQVALLGDEGAANHN
RLGGHYGEPGMQLFVYGREEGNDTRPSRYPARQTREASEAVARLNQVNPQQVIFAQQNPD
VIDQGVFHNDVIAVSNRQVLFCHQQAFARQSQLLANLRARVNGFMAIEVPATQVFVSDAV
STYLFNSQLLSRDDGSMVLVLPQECREHAGVWRYLNELLAADNPISELKVFDLRESMANG
GGPACLRLRVVLTEEERRAVNPAVMMNDTLFNALNDWVDRYYRDRLTAADLADPQLLREG
REALDTLTQLLDLGSVYPFQREGGGNG
>LFGLNPFC_01503 Succinylglutamate desuccinylase
MDNFLALTLTGKKPVITEREINGVRWRWLGDGVLELTPLTPPQGVLVISAGIHGNETAPV
EMLDALLGAISHGEIPLRWRLLVILGNPPALKQGKRYCHSDMNRMFGGRWQLFAESGETC
RARELEQCLEDFYDQGKESVRWHLDLHTAIRGSLHPQFGVLPQRDIPWDEKFLTWLGAAG
LEALVFHQEPGGTFTHFSARHFGALACTLELGKALPFGQNDLRQFAVTASAIAALLSGES
VGIVRTPPLRYRVVSQITRHSPSFEMHMANDTLNFMPFEKGTLLAQDGEERFTVTHDVEY
VLFPNPLVALGLRAGLMLEKIS
>LFGLNPFC_01504 Periplasmic chaperone Spy
MRKLTALFVASTLALGAANLAHAADTTTAAPADAKPMMHHKGKFGPHEDMMFKDLNLTDA
QKQQIREIIKGQRDQMKRPPLEERRAMHDIIASDTFDKAKAEAQIAKMEEQRKANMLAHM
ETQNKIYNILTPEQKKQFNANFEKRLTERPAAKGKMPATAE
>LFGLNPFC_01505 Protein Ves
MEYFDMRKMSVNLWRNAAGETREICTFPPAKRDFYWRASITSIAANGEFSLFPGMERIVT
LLEGGEMFLESADRFNHTLKPLQPFSFAADLVVKAKLTAGQMSMDFNIMTRLDVCKAKVR
IAERTFTTFGSRGGVVFVINGAWQLGDKLLTTDQGACWFDGRHTLRLLQPQGKLLFSEIN
WLAGHSPDQVQ
>LFGLNPFC_01506 Excinuclease cho
MVRRLTSPRLEFEAAAIYEYPEHLRSFLNDLPTRPGVYLFHGESDTMPLYIGKSINIRSR
VLSHLRTPDEAAMLRQSRRISWICTAGEIGALLLEARLIKEQQPLFNKRLRRNRQLCALQ
LNEKRVDVVYAKEVDFSRAPNLFGLFANRRAALQALQTIADEQKLCYGLLGLEPLSRGRA
CFRSALKRCAGACCGKESHDDHALRLRQSLERLRVVCWPWKGAVALKEQHPEMTQYHIIQ
NWLWLGAVNSLKEATTLIRAPAGFDHDGYKILCKPLLSGNYEITELDPVNDQQAS
>LFGLNPFC_01507 NH(3)-dependent NAD(+) synthetase
MTLQQQIIKVLGAKPQINAEEEIRRSIDFLKSYLQTYPFIKSLVLGISGGQDSTLAGKLC
QMAINELRQETGNESLQFIAVRLPYGVQADEQDCQDAIAFIQPDRVLTVNIKGAVLASEQ
ALREAGIELSDFVRGNEKARERMKAQYSIAGMTSGVVVGTDHAAEAITGFFTKYGDGGTD
INPLYRLNKRQGKQLLAALGCPEHLYKKAPTADLEDDRPSLPDEVALGVTYDNIDDYLEG
KNVPEQVARTIENWYLKTEHKRRPPITVFDDFWKK
>LFGLNPFC_01508 Osmotically-inducible putative lipoprotein OsmE
MNKNMAGILSAAAVLTMLAGCTAYDRTKDQFVQPVVKDVKKGMSRAQVAQIAGKPSSEVS
MIHARGTCQTYILGQRDGKAETYFVALDDTGHVINSGYQTCAEYDTDPQATK
>LFGLNPFC_01509 PTS system N,N'-diacetylchitobiose-specific EIIB component
MEKKHIYLFCSAGMSTSLLVSKMRAQAEKYEVPVIIEAFPETLAGEKGQNADVVLLGPQI
AYMLPEIQRLLPNKPVEVIDSLLYGKVDGLGVLKAAVAAIKKAAAN
>LFGLNPFC_01510 PTS system N,N'-diacetylchitobiose-specific EIIC component
MSNVIASLEKVLLPFAVKIGKQPHVNAIKNGFIRLMPLTLAGAMFVLINNVFLSFGEGSF
FYSLGIRLDASTIETLNGLKGIGGNVYNGTLGIMSLMAPFFIGMALAEERKVDALAAGLL
SVAAFMTVTPYSVGEAYAVGANWLGGANIISGIIIGLVVAEMFTFIVHRNWVIKLPDSVP
TSVSRSFSALIPGFIILSVMGIIAWALNTWGTNFHQIIMDTISTPLASLGSVVGWAYVIF
VPLLWFFGIHGALALTALDNGIMTPWALENIATYQQYGSVEAALAAGKTFHIWAKPMLDS
FIFLGGSGATLGLILAIFIASRRADYRQVAKLALPSGIFQINEPILFGLPIIMNPVMFIP

FVLVQPILAAITLAAYYMGIIPPVTNIAPWTMPTGLGAFFNTNGSVAALLVALFNLGIAT
LIYLPFVVVANKAQNAIDKEESEEDIANALKF
>LFGLNPFC_01511 PTS system N,N'-diacetylchitobiose-specific EIIA component
MMDLDNIPDTQTEAEELEEVVMGLIINSGQARSLAYAALKQAKQGDFAAAKTMMDQSRMA
LNEAHLVQTKLIEGDAGEGKMKVSLVLVHAQDHLMTSMLARELITELIELHEKLKA
>LFGLNPFC_01512 HTH-type transcriptional regulator ChbR
MQPVINAPEIATAREQQLFNGKNFHVFIYNKTESISGLHQHDYYEFTLVLTGRYFQEING
KRVLLERGDFVFIPLGSHHQSFYEFGATRILNVGISKRFFEQHYLPLLPYCFVASQVYRT
NNAFLTYVETVISSLNFRETGLEEFVEMVTFYVINRLRHYREEQVIDDIPQWLKSTVEKM
HDKEQFSESALENMVTLSAKSQEYLTRATQRYYGKTPMQIINEIRINFAKKQLEMTNYSV
TDIAFGAGYSSPSLFIKTFKKLTSFTPKSYRKKLTEFNQ
>LFGLNPFC_01513 6-phospho-beta-glucosidase
MSQKLKVVTIGGGSSYTPELLEGFIKRYHELPVSELWLVDVEGGKAKLDIIFDLCQRMID
NAGVPMKLYKTLDRREALKDADFVTTQLRVGQLPARELDERIPLSHGYLGQETNGAGGLF
KGLRTIPVIFDIVKDVEELCPNAWVINFTNPAGMVTEAVYRHTGFKRFIGVCNIPIGMKM
FIRDVLMLKDSDDLSIDLFGLNHMVFIKDVLVNGKSRFAELLDGVASGQLKASGVKNIFD
LPFSEGLIRSLNLLPCSYLLYYFKQKEMLAIEMGEYYKGGARAQVVQKVEKQLFELYKNP
ELNVKPKELEQRGGAYYSDAACEVINAIYNDKQAEHYVNIPHHGHIDNIPADWAVEMTCT
LGRDGATPHPRITHFDDKVMGLIHTIKGFEIAASNAALSGEFNDVLLALNLSPLVHSDRD
AELLAREMILAHEKWLPNFADCIAELKKAH
>LFGLNPFC_01514 Chitooligosaccharide deacetylase ChbG
MERLLIVNADDFGLSKGQNYGIIEACRNGIVTSTTALVNGQAIDHAVQLSRDEPSLAIGM
HFVLTMGKPLTAMPGLTRDGVLGKWIWQLAEEGALPLEEITQELASQYLRFIELFGRKPT
HLDSHHHVHMFPQIFPIVAKFAAEEGIALRIDRQPLSNDGDLPANLRSSQGFSSAFYGEE
ISETLFLQVLDDSSHRGERSLEVMCHPAFVDNTIRQSAYCFPRLTELDVLTSASLKYAIA
ERGYLLGSYHDV
>LFGLNPFC_01515 Catalase HPII
MSQHNEKNPHQHQSPLHDSSEAKPGMDSLAPEDGSHRPAAEPTPPGAQPTAPGSLKAPDT
RNEKLNSLEDVRKGSENYALTTNQGVRIADDQNSLRAGSRGPTLLEDFILREKITHFDHE
RIPERIVHARGSAAHGYFQPYKSLSDITKADFLSDTNKITPVFVRFSTVQGGAGSADTVR
DIRGFATKFYTEEGIFDLVGNNTPIFFIQDAHKFPDFVHAVKPEPHWAIPQGQSAHDTFW
DYVSLQPETLHNVMWAMSDRGIPRSYRTMEGFGIHTFRLINAEGKATFVRFHWKPLAGKA
SLVWDEAQKLTGRDPDFHRRELWEAIEAGDFPEYELGFQLIPEEDEFKFDFDLLDPTKLI
PEELVPVQRVGKMVLNRNPDNFFAENEQVAFHPGHIVPGLDFTNDPLLQGRLFSYTDTQI
SRLGGPNFHEIPINRPTCPYHNFQRDGMHRMGIDTNPANYEPNSINDNWPRETPPGPKRG
GFESYQERVEGNKVRERSPSFGEYYSHPRLFWLSQTPFEQRHIVDGFSFELSKVVRPYIR
ERVVDQLAHIDLTLAQAVAKNLGIELTDDQLNITPPPDVNGLKKDPSLSLYSIPDGDVKG
RVVAILLNDEVRSADLLAILKALKAKGVHAKLLYSRMGEVTADDGTVLPIAATFAGAPSL
TVDAVIVPCGNIADIADNGDANYYLMEAYKHLKPIALAGDARKFKATIKVADQGEEGIAE
ADSADGSFMDELLTLMTAHRVWSRIPKIDKIPA
>LFGLNPFC_01516 Cell division activator CedA
MRLVKPVMKKPLRQQNRQIISYVPRTEPAPPEHAIKMDSFRDVWMLRGKYVAFVLMGESF
LRSPAFTVPESAQRWANQIRQENEVEE
>LFGLNPFC_01517 L-cystine uptake protein TcyP
MNFPLIANIVVFVVLLFALAQTRHKQWSLAKKVLVGLVMGVVFGLALHTIYGSDSQVLKD
SVQWFNIVGNGYVQLLQMIVMPLVFASILSAVARLHNASQLGKISFLTIGTLLFTTLIAA
LVGVLVTNLFGLTAEGLVQGGAETARLNAIESNYVGKVSDLSVPQLVLSFIPKNPFADLT
GANPTSIISVVIFAAFLGVAALKLLKDDAPKGERVLTAIDTLQSWVMKLVRLVMQLTPYG
VLALMTKVVAGSNLQDIIKLGSFVVASYLGLLIMFAVHGLLLGINGVSPLKYFRKVWPVL
TFAFTSRSSAASIPLNVEAQTRRLGVPESIASFAASFGATIGQNGCAGLYPAMLAVMVAP
TVGINPLDPMWIATLVGIVTVSSAGVAGVGGGATFAALIVLPAMGLPVTLLALLISVEPL
IDMGRTALNVSGSMTAGTLTSQWLKQTDKAILDSEDDAELAHR
>LFGLNPFC_01518 Inner membrane protein YdjM
MTAEGHLLFSIACAVFAKNAELTPVLAQGDWWHIVPSAILTCLLPDIDHPKSFLGQRLKW
ISKPIARAFGHRGFTHSLLAVFALLATFYLKVPESWFIPADALQGMVLGYLSHILADMLT
PAGVPLLWPCRWRFRLPILVPQKGNQLERFICMALFVWSVWMPHSLPENSAVRWSSQMIN
TLQIQFHRLIKHQVEY
>LFGLNPFC_01519 Hexitol phosphatase B
MSTPRQILAAIFDMDGLLIDSEPLWDRAELDVMASLGVDISRRNELPDTLGLRIDMVVDL
WYARQPWNGPSRQEVVEQVIARAISLVEETRPLLPGVREAVALCKEQGLLVGLASASPLH
MLEKVLTMFDLRDSFDALASAEKLPYSKPHPQVYLDCAAKLGVDPLTCVALEDSVNGMIA
SKAARMRSIVVPAPEAQNDPRFVLANVKLSSLTELTAKDLLG
>LFGLNPFC_01520 hypothetical protein
MTYQQAGRIAVLKRILGWVIFIPALISTLISLLKFMNTRQENQEGINAVMLDFTHVMIDM

MQANTPFLNVFWYNSPTPNFNGGVNVMFWVIFILIFVGLALQDSGARMSRQARFLREGVE
DQLILEKAKGEEGLTREQIESRIVVPHHTIFLQFFSLYILPLICIAAGYVFFSLLGFI
>LFGLNPFC_01521 putative ketoamine kinase
MWQAISRLLSEQLGEGEIELRNELPGGEVHAAWHLRYAGRDFFVKCDERELLPGFTAEAD
QLELLSRSKTVTVPKVWAVGADRDYSFLVMDYLPPRPLDAHSAFILGQQIARLHQWSDQP
QFGLDFDNALSTTPQPNTWQRRWSTFFAEQRIGWQLELAAEKGIAFGNIDAIVEHIQQRL
ASHQPQPSLLHGDLWSGNCALGPDGPYIFDPACYWGDRECDLAMLPLHTEQPPQIYDGYQ
SVSPLPADFLERQPVYQLYTLLNRARLFGGQHLVIAQQSLDRLLAA
>LFGLNPFC_01522 hypothetical protein
MASGDLVRYVITVMLHEDTLTEINELNNYLTRDGFLLTMTDDDGNIHELGTNTFGLISTQ
SEEEIRELVSGLTQSATGKDPEITITTWEEWNSNRK
>LFGLNPFC_01523 ATP-dependent 6-phosphofructokinase isozyme 2
MVRIYTLTLAPSLDSATITPQIYPEGKLRCTAPVFEPGGGGINVARAIAHLGGSATAIFP
AGGATGEHLVSLLADENVPVATVEAKDWTRQNLHVHVEASGEQYRFVMPGAALNEDEFRQ
LEEQVLEIESGAILVISGSLPPGVKLEKLTQLISAAQKQGIRCIIDSSGEALSAALAIGN
IELVKPNQKELSALVNRELTQPDDVRKAAQEIVNSGKAKRVVVSLGPQGALGVDSENCIQ
VVPPPVKSQSTVGAGDSMVGAMTLKLAENASLEEMVRFGVAAGSAATLNQGTRLCSHDDT
QKIYAYLSR
>LFGLNPFC_01524 hypothetical protein
MKLLKTVPAIVMLAGGMFASLNAAADDSVFTVMDDPASAKKPFEGNLNAGYLAQSGNTKS
SSLTADTTMTWYGQTTAWSLWGNASNTSSNDERSSEKYAAGGRSRFNLTDYDYLFGQASW
LTDRYNGYRERDVLTAGYGRQFLNGPVHSFRFEFGPGVRYDKYTDNASETQPLGYASGAY
AWQLTDNAKFTQGVSVFGAEDTTLNSESALNVAINEHFGLKVAYNVTWNSEPPESAPEHT
DRRTTLSLGYSM
>LFGLNPFC_01525 putative protein YniD
MPTKRFDKKHWKMVVVLLAICGAMLLLRWAAMIWG
>LFGLNPFC_01526 hypothetical protein
MSKNDIIIRTHYESPLRMHIDSDILTPSSEPINQFARQLITLLDTSDLSSMLSYCVTQEF
TASCRKISHNCYTTALFIISFTTSPIHAENTLITLHYKKEIISLFLETTPIKANHLRSIL
DYIEQEQLTAEKRNHCMKLSKKNS
>LFGLNPFC_01527 Threonine--tRNA ligase
MPVITLPDGSQRHYDHAVSPMDVALDIGPGLAKACIAGRVNGELVDACDLIENDAQLSII
TAKDEEGLEIIRHSCAHLLGHAIKQLWPHTKMAIGPVIDNGFYYDVDLDRTLTQEDVEAL
EKRMHELAEKNYDVIKKKVSWHEARETFANRGESYKVSILDENIAHDDKPGLYFHEEYVD
MCRGPHVPNMRFCHHFKLMKTAGAYWRGDSNNKMLQRIYGTAWADKKALNAYLQRLEEAA
KRDHRKIGKQLDLYHMQEEAPGMVFWHNDGWTIFRELEVFVRSKLKEYQYQEVKGPFMMD
RVLWEKTGHWDNYKDAMFTTSSENREYCIKPMNCPGHVQIFNQGLKSYRDLPLRMAEFGS
CHRNEPSGSLHGLMRVRGFTQDDAHIFCTEEQIRDEVNGCIRLVYDMYSTFGFEKIVVKL
STRPEKRIGSDEMWDRAEADLAVALEENNIPFEYQLGEGAFYGPKIEFTLYDCLDRAWQC
GTVQLDFSLPSRLSASYVGEDNERKVPVMIHRAILGSMERFIGILTEEFAGFFPTWLAPV
QVVIMNITDSQSEYVNELTQKLSNAGIRVKADLRNEKIGFKIREHTLRRVPYMLVCGDKE
VESGKVAVRTRRGKDLGSMDVNEVIEKLQQEIRSRSLKQLEE
>LFGLNPFC_01528 Translation initiation factor IF-3
MSLREALEKAEEAGVDLVEISPNAEPPVCRIMDYGKFLYEKSKSSKEQKKKQKVIQVKEI
KFRPGTDEGDYQVKLRSLIRFLEEGDKAKITLRFRGREMAHQQIGMEVLNRVKDDLQELA
VVESFPTKIEGRQMIMVLAPKKKQ
>LFGLNPFC_01529 50S ribosomal protein L35
MPKIKTVRGAAKRFKKTGKGGFKHKHANLRHILTKKATKRKRHLRPKAMVSKGDLGLVIA
CLPYA
>LFGLNPFC_01530 50S ribosomal protein L20
MARVKRGVIARARHKKILKQAKGYYGARSRVYRVAFQAVIKAGQYAYRDRRQRKRQFRQL
WIARINAAARQNGISYSKFINGLKKASVEIDRKILADIAVFDKVAFTALVEKAKAALA
>LFGLNPFC_01531 Phenylalanine--tRNA ligase alpha subunit
MSHLAELVASAKAAISQASDVAALDNVRVEYLGKKGHLTLQMTTLRELPPEERPAAGAVI
NEAKEQVQQALNARKAELESAALNARLAAETIDVSLPGRRIENGGLHPVTRTIDRIESFF
GELGFTVATGPEIEDDYHNFDALNIPGHHPARADHDTFWFDATRLLRTQTSGVQIRTMKA
QQPPIRIIAPGRVYRNDYDQTHTPMFHQMEGLIVDTNISFTNLKGTLHDFLRNFFEEDLQ
IRFRPSYFPFTEPSAEVDVMGKNGKWLEVLGCGMVHPNVLRNVGIDPEVYSGFAFGMGME
RLTMLRYGVTDLRSFFENDLRFLKQFK
>LFGLNPFC_01532 Phenylalanine--tRNA ligase beta subunit
MKFSELWLRGWVNPAIDSDALANQITMAGLEVDGVEPVAGSFHGVVVGEVVECAQHPNAD
KLRVTKVNVGGDRLLDIVCGAPNCRQGLRVAVATIGAVLPGDFKIKAAKLRGEPSEGMLC
SFSELGISDDHNGIIELPADAPIGTDIREYLKLDDNTIEISVTPNRADCLGIIGVARDVA
VSNQLPLVEPEIVPVGATIDDTLPIAVEAPDACPRYLGRVVKGINVKAPTPLWMKEKLRR

CGIRSIDAVVDVTNYVLLELGQPMHAFDKDRIEGGIVVRMAKEGETLVLLDGTEAKLDAD
TLVIADHNKALAMGGIFGGEHSGVNDETQNVLLECAFFSPLSITGRARRHGLHTDASHRY
ERGVDPALQHKAMERATRLLIDICGGEAGPVIDITNEATLPKRATITLRRSKLDRLIGHH
IADEQVTDILRRLGCEVTEGKDEWQAVAPSWRFDMEIEEDLVEEVARVYGYNNIPDEPVQ
ASLIMGTHREADLSLKRVKTLLNDKGYQEVITYSFVDPKVQQMIHPGVEALLLPSPISVE
MSAMRLSLWTGLLATVVYNQNRQQNRVRIFESGLRFVPDTQAPLGIRQDLMLAGVICGNR
YEEHWNLAKETVDFYDLKGDLESVLDLTGKLNEVEFRAEANPALHPGQSAAIYLKGERIG
FVGVVHPELERKLDLNGRTLVFELEWNKLADRVVPQAREISRFPANRRDIAVVVAENVPA
ADILSECKKVGVNQVVGVNLFDVYRGKGVAEGYKSLAISLILQDTSRTLEEEEIAATVAK
CVEALKERFQASLRD
>LFGLNPFC_01533 Integration host factor subunit alpha
MALTKAEMSEYLFDKLGLSKRDAKELVELFFEEIRRALENGEQVKLSGFGNFDLRDKNQR
PGRNPKTGEDIPITARRVVTFRPGQKLKSRVENASPKDE
>LFGLNPFC_01534 Vitamin B12 import system permease protein BtuC
MLTLARQQQRQNIRWLLCLSVLMLLALLLSLCAGEQWISPGDWFTPRGELFVWQIRLPRT
LAVLLVGAALAISGAVMQALFENPLAEPGLLGVSNGAGVGLIAAVLLGQGQLPNWALGLC
AIAGALIITLILLRFARRHLSTSRLLLAGVALGIICSALMTWAIYFSTSVDLRQLMYWMM
GGFGGVDWRQSWLMLALIPVLLWICCQSRPMNMLALGEISARQLGLPLWFWRNVLVAATG
WMVGVSVALAGAIGFIGLVIPHILRLCGLTDHRVLLPGCALAGASALLLADIVARLALAA
AELPIGVVTATLGAPVFIWLLLKAGR
>LFGLNPFC_01535 Thioredoxin/glutathione peroxidase BtuE
MQDSILTTVVKDIDGKVTTLEKYAGNVLLIVNVASKCGLTPQYEQLENIQKAWADRGFVV
LGFPCNQFLEQEPGSDEEIKTYCTTTWGVTFPMFSKIEVNGEGRHPLYQKLIAAAPTAVA
PEESGFYARMVSKGRAPLYPDDILWNFEKFLVGRDGLVIQRFSPDMTPEDPIVMESIKLA
LAK
>LFGLNPFC_01536 Vitamin B12 import ATP-binding protein BtuD
MSIVMQLQDVAESTRLGPLSGEVRAGEILHLVGPNGAGKSTLLARMAGMTSGKGSIQFAG
QPLEAWSATKLALHRAYLSQQQTPPFAMPVWHYLTLHQHDKTRTELLNDVAGALALDDKL
GRSTNQLSGGEWQRVRLAAVVLQITPQANPAGQLLLLDEPMNSLDVAQQSALDKILSALC
QQGLAIVMSSHDLNHTLRHAHRAWLLKGGKMLASGRREEVLTPANLAQAYGMNFRRLDIE
GHRMLISTI
>LFGLNPFC_01537 Murein DD-endopeptidase MepS/Murein LD-carboxypeptidase
MRFCLILITALFLAGCSHHKAPPPNARLSDSITVIAGLNDQLQSWHGTPYRYGGMTRRGV
DCSGFVVVTMRDRFDLQLPRETKQQAAIGTQIDKDELLPGDLVFFKTGSGQNGLHVGIYD
TNNQFIHASTSKGVMRSSLDNVYWQKNFWQARRI
>LFGLNPFC_01538 Putative anti-FlhC(2)FlhD(4) factor YdiV
MKIFLENLYHSDCYFLPIRDNQQDLVGVELITHFSSEDGTVRIPTSRVIAQLTEEQHWQL
FSEQLELLKSCQHFFIQHKLFAWLNLTPHVATLLLDRDNFAGELLKYPFIELLINENYPH
FNEGKDNRDLLSLSQMYPLVLGNLGAGNSTMKAVFDGLFTRVMLDKSFIQQQITHRSFEP
FIRAIQAQISPCCNCIIAGGIDTPEILAQIIPFDFHALQGCLWPAVPINQITTLVQR
>LFGLNPFC_01539 Protein adenylyltransferase SelO
MTLSFITRWRDELPETYTALSPTPLNNARLIWHNTELANTLSIPSSLFKNGAGVWGGENL
LPGMSPLAQVYSGHQFGVWAGQLGDGRGILLGEQLLADGTTMDWHLKGAGLTPYSRMGDG
RAVLRSTIRESLASEAMHYLGIPTTRALSIVTSDSPVYRETVESGAMLMRVAPSHLRFGH
FEHFYYRREPEKVRQLADFAIRHYWSHLDDEEDKYRLWFTDVVARTASLIAQWQTVGFAH
GVMNTDNMSLLGLTLDYGPFGFLDDYEPGFICNHSDHQGRYSFDNQPAVALWNLQRLAQT
LSPFVAVDALNEALDSYQQVLLTHYGQRMRQKLGFMTEQKEDNALLNELFSLMARERSDY
TRTFRMLSLTEQHSAASPLRDEFIDRAAFDDWFARYRGRLQQDEITDSERQQLMQSVNPA
LVLRNWLAQRAIEAAEKDDMTELHRLHEALRNPFSDRDDDYVSRPPDWGKRLEVSCSS
>LFGLNPFC_01540 putative protein YdiE
MRYTDSRKLTPETDASHKTASPQPIRRITSQTLLGPDGKLIIDHDGQEYLLRKTQAGKLL
LTK
>LFGLNPFC_01541 Phospho-2-dehydro-3-deoxyheptonate aldolase, Trp-sensitive
MNRTDELRTARIESLVTPAELALRYPVTPGVATHVTDSRRRIEKILNGEDKRLLVIIGPC
SIHDLTAAMEYATRLQSLRNQYQSRLEIVMRTYFEKPRTVVGWKGLISDPDLNGSYRVNH
GLELARKLLLQVNELGVPTATEFLDMVTGQFIADLISWGAIGARTTESQIHREMASALSC
PVGFKNGTDGNTRIAVDAIRAARASHMFLSPDKNGQMTIYQTSGNPYGHIIMRGGKKPNY
HADDIAAACDTLHEFDLPEHLVVDFSHGNCQKQHRRQLEVCEDICQQIRNGSTAIAGIMA
ESFLREGTQKIVGGQPLTYGQSITDPCLGWEDTERLVEKLASAVDTRF
>LFGLNPFC_01542 Phosphoenolpyruvate synthase regulatory protein
MDNAVDRHVFYISDGTAITAEVLGHAVMSQFPVTISSITLPFVENESRARAVKDQIDAIY
HQTGVRPLVFYSIVLPEIRAIILQSEGFCQDIVQALVAPLQQEMKLDPTPIAHRTHGLNP
NNLNKYDARIAAIDYTLAHDDGISLRNLDQAQVILLGVSRCGKTPTSLYLAMQFGIRAAN
YPFIADDMDNLVLPASLKPLQHKLFGLTIDPERLAAIREERRENSRYASLRQCRMEVAEV

EALYRKNQIPWINSTNYSVEEIATKILDIMGLSRRMY
>LFGLNPFC_01543 Phosphoenolpyruvate synthase
MSNNGSSPLVLWYNQLGMNDVDRVGGKNASLGEMITNLSGMGVSVPNGFATTADAFNQFL
DQSGVNQRIYELLDKTDIDDVTQLAKAGAQIRQWIIDTPFQPELENAIREAYGQLSADDE
NASFAVRSSATAEDMPDASFAGQQETFLNVQGFDAVLVAVKHVFASLFNDRAISYRVHQG
YDHRGVALSAGVQRMVRSDLASSGVMFSIDTESGFDQVVFITSAWGLGEMVVQGAVNPDE
FYVHKQTLAANRPAIVRRTMGSKKIRMVYAPTQEHGKQVKIEDVPQEQRDIFSLTNEEVQ
ELAKQAVQIEKHYGRPMDIEWAKDGHTGKLFIVQARPETVRSRGQVMERYTLHSQGKIIA
EGRAIGHRIGAGPVKVIHDISEMNRIEPGDVLVTDMTDPDWEPIMKKASAIVTNRGGRTC
HAAIIARELGIPAVVGCGDATERMKDGENVTVSCAEGDTGYVYAELLEFSVKSSSVETMP
DLPLKVMMNVGNPDRAFDFACLPNEGVGLARLEFIINRMIGVHPRALLEFDDQEPQLQNE
IREMMKGFDSPREFYVGRLTEGIATLGAAFYPKRVIVRLSDFKSNEYANLVGGERYEPDE
ENPMLGFRGAGRYVSDSFRDCFALECEAVKRVRNDMGLTNVEIMIPFVRTVDQAKAVVEE
LARQGLKRGENGLKIIMMCEIPSNALLAEQFLEYFDGFSIGSNDMTQLALGLDRDSGVVS
ELFDERNDAVKALLSMAIRAAKKQGKYVGICGQGPSDHEDFAAWLMEEGIDSLSLNPDTV
VQTWLSLAELKK
>LFGLNPFC_01544 Medium-chain fatty-acid--CoA ligase
MKVTLTFNEQRRAAYRQQGLWGDASLADYWQQTARAMPDKIAVVDNHGATYTYSALDHAA
SCLANWMLAKGIESGDRIAFQLPGWCEFTVIYLACLKTGAVSVPLLPSWREAELVWVLNK
CQAKMFFAPTLFKQTRPVDLILPLQNQLPQLQQIVGVDKLAPATSSLSLSQILADNTPLT
TAITTHGDELAAVLFTSGTEGLPKGVMLTHNNILASERAYCARLNLTWQDVFMMPAPLGH
ATGFLHGVTAPFLIGARSVLLDIFTPDACLALLEQQRCTCMLGATPFVYDLLNLLEKQPA
DLSALRFFLCGGTTIPKKVARECQQRGIKLLSVYGSTESSPHAVVNLDDPLPRFMHTDGY
AAAGVEIKVVDDARKTLPPGCEGEEASRGPNVFMGYFDEPELTARALDEEGWYYSGDLCR
MDEAGYIKITGRKKDIIVRGGENISSREVEDILLQHPKIHDACVVAMPDERLGERSCAYV
VLKAPHHSLSLEEVVAFFSRKRVAKYKYPEHIVVIEKLPRTVSGKIQKFLLRKDIMRRLT
QDVCEEIE
>LFGLNPFC_01545 Ferredoxin-like protein FixX
MSQNATVNVDIKLGVNKFHVDEGHPHIILAANPDINEFRKLMKACPAGLYKQDDAGNIHF
DSAGCLECGTCRVLCGNTILEQWQYPAGTFGIEFRYG
>LFGLNPFC_01546 D-amino acid dehydrogenase
MSDDKFDAIVVGAGVAGSVAALVMARAGLDVLVIERGDSAGCKNMTGGRLYAHTLEAIIP
GFAASAPIERKVTREKISFLTEESAVTLDFHREQPDVPQHASYTVLRNRLDPWLMEQAEQ
AGAQFIPGVRVDALVREGNKVTGVQAGDDILEANVVILADGVNSMLGRSLGMVSASDPHH
YAVGVKEVIGLTPEQINDRFNVTGEEGAAWLFAGSPSDGLMGGGFLYTNKDSVSLGLVCG
LSDIAHAQKSVPQMLEDFKQHPAIRPLISGGKLLEYSAHMVPEGGLAMVPQLVNDGVMIV
GDAAGFCLNLGFTVRGMDLAIASAQAAATTVIAAKERADFSASSLAQYKRELEQSCVMRD
MQHFRKIPALMENPRLFSQYPRMVADIMNDMFTIDGKPNQPVRKMIMGHAKKIGLINLLK
DGIKGATAL
>LFGLNPFC_01547 Protein FixB
MSQLNSVWVFSDNPEHYAELFGGAQQWGQQVYAIVQNTEQAQAVMPYGPKCIYVLEQNDA
LQRTENYAESIAALLKDKHPAMLLLAATKRGKALAARLSVQLNAALVNDATAVDIVDGHI
CAEHRMYGGLAFAQEKINSPLAIITLAPGVQEPCTRDISHQCPTETVPYVAPRHEILCRE
RRAKAASSVDLSKAKRVVGVGRGLAAQDDLKMVHELAAVLNAEVGCSRPIAEGENWMERE
RYIGVSGVLLKSDLYLTLGISGQIQHMVGGNGAKVIVAINKDKNAPIFNYADYGLVGDIY
KVVPALISQLNR
>LFGLNPFC_01548 Protein FixA
MKIITCFKLVPEEQDIVVTPEYTLNFDNADAKISQFDLNAIEVASQLATDDDEIAALTVG
GSLLQNSKVRKDVLSRGPHSLYMVQDAQLEHALPLDTAKALATAVEKIGFDLLIFGEGSG
DLYAQQVGLLVGELLQLPVINAVSAIQRQGNTLVIERTLEDDVEVIELSVPAVLCVTSDI
NVPRIPSMKAILGAGKKPVNQWQASDIGWSQSAPLAELTGIRVPPQTERKHIILDNDSPE
AIAELAEHLKKALN
>LFGLNPFC_01549 hypothetical protein
MEGKNGLKIVCDSGAGFSTEQAVFLANNLAKKMRSLKQKCNLRQYSYSMTVQSCIQCK
>LFGLNPFC_01550 HTH-type transcriptional activator RhaS
MYQRCFDNASETLFVAGKTPRLSRFAFSDDPKWESGHHVHDNETELIYVKKGVARFTIDS
SLYVAHADDIVVIERGRLHAVASDVNDPATTCTCALYGFQFQGVEENQLLQPHSCPVIAA
GQGKEVIKTLFNELSVILPQSKNSQTSSLWDAFAYTLAILYYENFKNAYRSEQGYIKKDV
LIKDILFYLNNNYREKITLEQLSKKFRASVSYICHEFTKEYRISPINYVIQRRMTEAKWS
LTNTELSQAEISWRVGYENVDHFAKLFLRHVGCSPSDYRRQFKNCFAEQEILSEFPQPVS
LAG
>LFGLNPFC_01551 Crotonobetainyl-CoA reductase
MDFSLTEEQELLLASIRELITTNFPEEYFRTCDQNGTYPREFMRALADNGISMLGVPEEF
GGIPADYVTQMLALMEVSKCGAPAFLITNGQCIHSMRRFGSAEQLRKTAESTLETGDPAY

ALALTEPGAGSDNNSATTTYTRKNGKVYINGQKTFITGAKEYPYMLVLARDPQPKDPKKA
FTLWWVDSSKPGIKINPLHKIGWHMLSTCEVYLDNVEVEESDMVGEEGMGFLNVMYNFEM
ERLINAARSTGFAECAFEDAARYANQRIAFGKPIGHNQMIQEKLALMAIKIDNMRNMVLK
VAWQADQHQSLRTSAALAKLYCARTAMEVIDDAIQIMGGLGYTDEARVSRFWRDVRCERI
GGGTDEIMIYVAGRQILKDYQNK
>LFGLNPFC_01552 Acetate CoA-transferase YdiF
MKPVKPPRINGRVPVLSAQEAVNYIPDEATLCVLGAGGGILEATTLITALADKYKQTQTP
RNLSIISPTGLGDRADRGISPLAQEGLVKWALCGHWGQSPRISDLAEQNKIIAYNYPQGV
LTQTLRAAAAHQPGIISDIGIGTFVDPRQQGGKLNEVTKEELIKLVEFDNKEYLYYKAIA
PDIAFIRATTCDSEGYATFEDEVMYLDALVIAQAVHNNGGIVMMQVQKMVKKATLHPKSV
RIPGYLVDIVVVDPDQTQLYGGAPVNRFISGDFTLDDSTKLSLPLNQRKLVARRALFEMR
KGAVGNVGVGIADGIGLVAREEGCADDFILTVETGPIGGITSQGIAFGANVNTRAILDMT
SQFDFYHGGGLDVCYLSFAEVDQHGNVGVHKFNGKIMGTGGFIDISATSKKIVFCGTLTA
GSLKTEITDGKLNIVQEGRVKKFIRELPEITFSGKIALKRGLDVRYITERAVFTLKEDGL
HLIEIAPGVDLQKDILDKMDFTPVLSPELKLMDERLFIDAAMGFVLPEAAH
>LFGLNPFC_01553 3-dehydroquinate dehydratase
MKTVTVKDLVIGTGAPKIIVSLMAKDIASVKSEALAYREADFDILEWRVDHYADLSNVES
VIAAAKILRETMPEKPLLFTFRSAKEGGEQAISTEAYIALNRAAIDSGLVDMIDLELFTG
DDQVKETVAYAHAHDVKVVMSNHDFHKTPEAEEIIARLRKMQSFDADIPKIALMPQSTSD
VLTLLAATLEMQEQYADRPIITMSMAKTGVISRLAGEVFGSAATFGAVKKASAPGQISVN
DLRTVLTILHQA
>LFGLNPFC_01554 Quinate/shikimate dehydrogenase
MDVTAKYELIGLMAYPIRHSLSPEMQNKALEKAGLPFTYMAFEVDNDSFPAAIEGLKALK
MRGTGVSMPNKQLACEYVDELTPAAKLVGAINTIVNDDGYLRGYNTDGTGHIRAIKESGF
DIKGKTMVLLGAGGASTAIGAQGAIEGLKEIKLFNRRDEFFDKALAFAQRVNENTDCVVT
VTDLADQQAFAEALASADILTNGTKVGMKPLENESLVNDISLLHPGLLVTECVYNPHMTK
LLQQAQQAGCKTIDGYGMLLWQGAEQFTLWTGKDFPLEYVKQVMGFGA
>LFGLNPFC_01555 Inner membrane transport protein YdiN
MSQNKAFSTPFILAVLCIYFSYFLHGISVITLAQNMTSLAEKFSTDNAGIAYLISGIGLG
RLISILFFGVISDKFGRRAVILMAVIMYLLFFFGIPACPNLTLAYCLAVCVGIANSALDT
GGYPALMECFPKASGSAVILVKAMVSFGQMFYPMLVSYMLLNNIWYGYGLIIPGILFVLI
TLMLLKSKFPSQLVDASVANELPQMNSKPLVWLEGVSSVLFGVAAFSTFYVIVVWMPKYA
MAFAGMSEAEALKTISYYSMGSLVCVFIFAALLKKMVRPIWANVFNSALATITAAIIYLY
PSPLVCNAGAFVIGFSAAGGILQLGVSVMSEFFPKSKAKVTSIYMMMGGLANFVIPLITG
YLSNIGLQYIIVLDFTFALLALITAIIVFIRYYRVFIIPENDVRFGEHKFSTRLNTIKHR
G
>LFGLNPFC_01556 Inner membrane transport protein YdiM
MKNPYYPTALGLYFNYLVHGMGVILMSLNMASLETLWQTNAAGVSIVISSLGIGRLSVLL
FAGLLSDRFGRRPFIMLGMCCYMAFFFGILHTNNIIIAYVFGFLAGMANSFLDAGTYPSL
MEAFPRSPGTANILIKAFVSSGQFLLPLIISLLVWAELWFGWSFMIAAGIMFINALFLYR
CTFPPHPGRHLPVIKKTTSSTEHRCSIIDLASYSLYGYISMATFYLVSQWLAQYGQFVAG
MSYTMSIKLLSIYTVGSLLCVFITAPLIRNTVRPTTLLMLYTFISFIALLTVCLHPTFYV
VIIFAFVIGFTSAGGVVQIGLTLMAERFPYAKGKATGIYYSAGSIATFTIPLITAHLSQR
SIADIMWFDTAIAAIGFILALFIGLRSRKETRHHTLKENIAPGG
>LFGLNPFC_01557 hypothetical protein
MNAYELQALRHIFAMTIDECATWIAQTGNSESWRQWENGKCAIPDCVVEQLLAMRQQRKK
HLHAIIEKINNRIGNNTMRFFPDLTAFQQVYPDGNFIDWKIYQSVAAELYAHDLERLC
>LFGLNPFC_01558 Putative transport protein YdiK
MVNVRQPRDVAQILLSVLFLAIMIVACLWIVQPFILGFAWAGTVVIATWPVLLRLQKIMF
GRRSLAVLVMTLLLVMVFIIPIALLVNSIVDGSGPLIKAISSGDMTLPDLAWLNTIPVIG
AKLYAGWHNLLDMGGTAIMAKVRPYIGTTTTWFVGQAAHIGRFMVHCALMLLFSALLYWR
GEQVAQGIRHFATRLAGVRGDAAVLLAAQAIRAVALGVVVTALVQAVLGGIGLAVSGVPY
ATLLTVLMILSCLVQLGPLPVLIPAIIWLYWTGDTTWGTVLLVWSGVVGTLDNVIRPMLI
RMGADLPLILILSGVIGGLIAFGMIGLFIGPVLLAVSWRLFAAWVEEVPPPTDQPEEILE
ELGEIEKPNK
>LFGLNPFC_01559 putative protein
MIPQISQAPGVVQLVLNFLQELEQQGFTGDTATSYADRLTMSTDNSIYQLLPDAVVFPRS
TADVALIARLAAQERYSSLIFTPRGGGTGTNGQALNQGIIVDMSRHMNRIIEINPEEGWV
RVEAGVIKDQLNQYLKPFGYFFAPELSTSNRATLGGMINTDASGQGSLVYGKTSDHVLGV
RAVLLGGDILDTQPLPVELAETLGKSNTTIGRIYNTVYQRCRQQRQLIIDNFPKLNRFLT
GYDLRHVFNDEMTEFDLTRILTGSEGTLAFITEARLDITRLPKVRRLVNVKYDSFDSALR
NAPFMVEARALSVETVDSKVLNLAREDIVWHSVSELITDVPDKEMLGLNIVEFAGDDEAL
IDERVNALCVRLDELIASQQAGVIGWQVCRELAGVERIYAMRKKAVGLLGNAKGAAKPIP
FAEDTCVPPEHLADYIAEFRALLDSHGLSYGMFGHVDAGVLHVRPALDMCDPQQEILMKQ

ISDDVVALTAKYGGLLWGEHGKGFRAEYSPAFFGEELFAELRKVKAAFDPYNRLNPGKIC
PPEGLDAPMMKVDAVKRGTFDRQIPIAVRQQWRGAMECNGNGLCFNFDARSPMCPSMKIT
QNRIHSPKGRATLVREWLRLLADRGVDPLKLEQELPESGVSLRTLIARTRNSWHANKGEY
DFSHEVKEAMSGCLACKACSTQCPIKIDVPEFRSRFLQLYHTRYLRPLRDHLVATVESYA
PLMARAPKTFNFFINQPLVRKLSEKHIGMVDLPLLSVPSLQQQMVGHRSANMTLEQLEAL
NAEQKARTVLVVQDPFTSYYDAQVVADFVRLVEKLGFQPVLLPFSPNGKAQHIKGFLNRF
AKTAKKTADFLNRMAKLGMPMVGVDPALVLCYRDEYKLALGEERGEFNVLLANEWLASAL
DSQPVATVSGESWYFFGHCTEVTALPGAPAQWAAIFARFGAKLENVSVGCCGMAGTYGHE
AKNHKNSLRIYELSWHQAMQRLPRNRCLATGYSCRSQVKRVEGTGVRHPVQALLEIIK
>LFGLNPFC_01560 1,4-dihydroxy-2-naphthoyl-CoA hydrolase
MIWKRKITLEALNAMGEGNMVGLLDIRFEHIGDDTLEATMPVDSRTKQPFGLLHGGASVV
LAESIGSVAGYLCTEGEQKVVGLEINANHVRSAREGRVRGVCKPLHLGSRHQVWQIEIFD
EKGRLCCSSRLTTAIL
>LFGLNPFC_01561 hypothetical protein
MSTQLDPTQLAIEFLRRDQSNLSPAQYLKRLKQLELEFADLLTLSSAELKEEIYFAWRLG
VH
>LFGLNPFC_01562 Protein SufA
MDMHSGTFNPQDFAWQGLTLTPAAAIHIRELVAKQPGMIGVRLGVKQTGCAGFGYVLDSV
SEPDKDDLLFEHDGAKLFVPLQAMPFIDGTEVDFVREGLNQIFKFHNPKAQNECGCGESF
GV
>LFGLNPFC_01563 FeS cluster assembly protein SufB
MSRNTEATDDVKTWTGGPLNYKEGFFTQLATDELAKGINEEVVRAISAKRNEPEWMLEFR
LNAYHAWLEMEEPHWLKAHYDKLNYQDYSYYSAPSCGNCDDNCASEPGAVQQTGANAFLS
KEVEAAFEQLGVPVREGKEVAVDAIFDSVSVATTYREKLAEQGIIFCSFGEAIHDHPELV
RKYLGTVVPGNDNFFAALNAAVASDGTFIYVPKGVRCPMELSTYFRINAEKTGQFERTIL
VADEDSYVSYIEGCSAPVRDSYQLHAAVVEVIIHKNAEVKYSTVQNWFPGDNNTGGILNF
VTKRALCEGENSKMSWTQSETGSAITWKYPSCILRGDNSIGEFYSVALTSGHQQADTGTK
MIHIGKNTKSTIISKGISAGHSQNSYRGLVKIMPTATNARNFTQCDSMLIGANCGAHTFP
YVECRNNSAQLEHEATTSRIGEDQLFYCLQRGISEEDAISMIVNGFCKDVFSELPLEFAV
EAQKLLAISLEHSVG
>LFGLNPFC_01564 putative ATP-dependent transporter SufC
MLSIKDLHVSVEDKAILRGLSLDVRPGEVHAIMGPNGSGKSTLSATLAGREDYEVTSGTV
EFKGKDLLALSPEDRAGEGIFMAFQYPVEIPGVSNQFFLQTALNAVRSYRGQETLDRFDF
QDLMEEKIAPLKMPEDLLTRSVNVGFSGGEKKRNDILQMAVLEPELCILDESDSGLDIDA
LKVVADGVNSLRDGKRSFIIVTHYQRILDYIKPDYVHVLYQGRIVKSGDFTLVKQLEEQG
YGWLTEQQ
>LFGLNPFC_01565 FeS cluster assembly protein SufD
MAGLPNSSNALQQWHHLFEAEGAKRSPQAQQHLQQLLRTGLPTRKHENWKYTPLDGLTNS
QFVSIAGEISPQQRDALALTLDAVRLVFVDGRYVPALSDATEGSGYEVSINDDRQGLPDA
IQAEVFLHLTESLAQSVTHIAVKRGQRPAKPLLLMHITQGVAGEEVNTAHYRHHLDLAEG
AEATVIEHFVSLNDARHFTGARFTINVAANAHLQHIKLAFENPLSHHFAHNDLLLADDAT
AFSHSFLLGGAVLRHNTSTQLNGENSTLRINSLAMPVKNEVCDTRTWLEHNKGFCNSRQL
HKTIVSDKGRAVFNGLINVAQHAIKTDGQMTNNNLLMGKLAEVDTKPQLEIYADDVKCSH
GATVGRIDDEQMFYLRSRGINQQDAQQMIIYAFAAELTEALRDEGLKQQVLARIGQRLPG
GAR
>LFGLNPFC_01566 Cysteine desulfurase
MTFSVDKVRADFPVLSREVNGLPLAYLDSAASAQKPSQVIDAEAEFYRHGYAAVHRGIHT
LSAQATEKMENVRKRASLFINARSAEELVFVRGTTEGINLVANSWGNSNVRAGDNIIISQ
MEHHANIVPWQMLCARVGAELRVIPLNPDGTLQLETLPTLFDEKTRLLAITHVSNVLGTE
NPLAEMITLAHQHGAKVLVDGAQAVMHHPVDVQALDCDFYVFSGHKLYGPTGIGILYVKE
ALLQEMPPWEGGGSMIATVSLSEGTTWTKAPWRFEAGTPNTGGIIGLGAALEYVSALGLN
NIAEYEQNLMHYALSQLESVPDLTLYGPQNRLGVIAFNLGKHHAYDVGSFLDNYGIAVRT
GHHCAMPLMAYYNVPAMCRASLAMYNTHEEVDRLVTGLQRIHRLLG
>LFGLNPFC_01567 Cysteine desulfuration protein SufE
MALLPDKEKLLRNFLRCANWEEKYLYIIELGQRLPELRDEDKSPQNSIQGCQSQVWIVMR
QNAQGIIELHGDSDAAIVKGLIAVVFILYDQMTPQDIVNFDVRPWFEKMALTQHLTPSRS
QGLEAMIRAIRAKAAALS
>LFGLNPFC_01568 putative L,D-transpeptidase YnhG
MKRASLLTLTLIGAFSAIQAAWAVDYPLPSTGSRLVGQNQTYTVQEGDKNLQAIARRFDT
AAMLILEANNTIAPVPKPGTTITIPSQLLLPDAPRQGIIVNLAELRLYYYPPGENIVQVY
PIGIGLQRLETPVMETRVGQKIPNPTWTPTAGIRQRSLERGIKLPPVVPAGPNNPLGRYA
LRLAHGNGEYLIHGTSAPDSVGLRVSSGCIRMNAPDIKALFSSVRTGTPVKVINEPVKYS
VEPNGMRYVEVHRPLSAEEQQNVQTMPYTLPAGFTQFKDNKAVDQKLVDKALYRRAGYPV
AVSSGATPAASNAPSVESAQNGEPEQGNMLRATQ

```
>LFGLNPFC_01569 Major outer membrane prolipoprotein Lpp
MKATKLVLGAVILGSTLLAGCSSNAKIDQLSSDVQTLNAKVDQLSNDVNAMRSDVQAAKD
DAARANQRLDNMATKYRK
>LFGLNPFC_01570 Pyruvate kinase I
MKKTKIVCTIGPKTESEEMLAKMLDAGMNVMRLNFSHGDYAEHGQRIQNLRNVMSKTGKT
AAILLDTKGPEIRTMKLEGGNDVSLKAGQTFTFTTDKSVIGNSEMVAVTYEGFTTDLSVG
NTVLVDDGLIGMEVTAIEGNKVICKVLNNGDLGENKGVNLPGVSIALPALAEKDKQDLIF
GCEQGVDFVAASFIRKRSDVIEIREHLKAHGGENIHIISKIENQEGLNNFDEILEASDGI
MVARGDLGVEIPVEEVIFAQKMMIEKCIRARKVVITATQMLDSMIKNPRPTRAEAGDVAN
AILDGTDAVMLSGESAKGKYPLEAVSIMATICERTDRVMNSRLEFNNDNRKLRITEAVCR
GAVETAEKLDAPLIVVATQGGKSARAVRKYFPDATILALTTNEKTAHQLVLSKGVVPQLV
KEITSTDDFYRLGKELALQSGLAHKGDVVVMVSGALVPSGTTNTASVHVL
>LFGLNPFC_01571 Fumarase D
MGNRTKEDELYREMCRVVGKVVLEMRDLGQEPKHIVIAGVLRTALANKRIQRSELEKQAM
ETVINALVK
>LFGLNPFC_01572 putative ferredoxin-like protein YdhY
MNPVDRPLLNIGLTRLEFLRISGKGLAGLTIAPALLSLLGCKQEDIDSGTVGLINTPKGV
LVTHRARCTGCHRCEISCTNFNDGSVGTFFSRIKIHRNYFFGDNGVGSGGGLYGDLNYTA
DTCRQCKEPQCMNVCPIGAITWQQEEGCITVDHKRCIGCSACTTACPWMMATVNTESKKS
SKCVLCGECANACPTGALKIIEWKDITV
>LFGLNPFC_01573 putative oxidoreductase YdhV
MANGWTGNILRVNLTTGNITLEDSSKFKSFVGGMGFGYKIMYDEVPPGTKPFDEANKLVF
ATGPLTGSGAPCSSRVNITSLSTFTKGNLVVDAHMGGFFAAQMKFAGYDVIIIEGKAKSP
VWLNIKDDKVSLEKADFLWGKGTRATTEEICRLTSPETCVAAIGQAGENLVPLSGMLNSR
NHSGGAGTGAIMGSKNLKAIAVEGTKGVNIADRQEMKRLNDYMMTELIGANNNHVVPSTP
QSWAEYSDPKSRWTARKGLFWGAAEGGPIETGEIPPGNQNTVGFRTYKSVFDLGPAAEKY
TVKMSGCHSCPIRCMTQMNIPRVKEFGVPSTGGNTCVANFVHTTIFPNGPKDFEDKDDGR
VIGNLVGLNLFDDYGLWCNYGQLHRDFTYCYSKGVFKRVLPAEEYAEIRWDQLEAGDVNF
IKDFYYRLAHRVGELSHLADGSYAIAERWNLGEEYWGYAKNKLWSPFGYPVHHANEASAQ
VGSIVNCMFNRDCMTHTHINFIGSGLPLKLQREVAKELFGSEDAYDETKNYTPINDAKIK
YAKWSLLRVCLHNAVTLCNWVWPMTVSPLKSRNYRGDLALEAKFFKAITGEDMTQEKLDL
AAERIFTLHRAYTVKLMQTKDMRNEHDLICSWVFDKDPQIPVFTEGTDKMDRDDMHASLT
MFYKEMGWDPQLGCPTRETLQRLGLEDIAADLAAHNLLPA
>LFGLNPFC_01574 putative protein YdhW
MNHQDELPLAKVSEVDEAKRQWLQGMRHPVDTVTEPEPAEILAEFIRQHSAAGQLVARAV
FLSPPYSVAEEELSVLLENIKQNGDHADIACLTGSQDDYYYSTQAMSENYAAMSLQVVEQ
DICRAIAHAVRFECQTYPRPYKVAMLMQAPYYFQEAQIEAAIAAMDVAPEYADIRQVESS
TAVLYLFSERFMTYGKAYGLCEWFEVEQFQNP
>LFGLNPFC_01575 putative ferredoxin-like protein YdhX
MSFTRRKFVLGMGTVIFFTGSASSLLANTRQEKEVRYAMIHDESRCNGCNICARACRKTN
HVPAQGSRLSIAHIPVTDNDNETQYHFFRQSCQHCEDAPCIDVCPTAASWRDEQGIVRVE
KSQCIGCSYCIGACPYQVRYLNPVTKVADKCDFCAESRLAKGFPPICVSACPGHALIFGR
EDSPEIQAWLQENKYYQYQLPGAGKPHLYRRFGQHLIKKENV
>LFGLNPFC_01576 Putative cytochrome YdhU
MNPSQHAEQFQSQLANYVPLFTPQFWPVWLIIAGLLLVGMWLVLGLHALLRARGVKKSAT
DHGEKVYLYSKAVRLWHWSNALLFVLLLASGLINHFALVGATAVKSLVAVHEVCGFLLLA
CWLGFVLINAVGGNGHHYRIRRQGWLERAAKQTRFYLFGIMQGEEHPFPATTQSKFNPLQ
QVAYVGVMYGLLPLLLLTGLLCLYPQAVGDVFPGVRYWLLQAHFALAFISLFFIFGHLYL
CTTGRTPHETFKSMVDGYHRH
>LFGLNPFC_01577 putative protein YdhT
MYRWFLRHFPRGGSYADIHHALIEEGYTDWAESLVEYAWKKWLADENFAHQEVSSMQKLA
TDPGERLFCSQFARSDDHARIGCCEDNARIATAGYAAQIASMGYSVRIGSVGFNSHIGSS
GARARVAVTGNSSRISSAGDSSRIANTGMRVRVCTLGERCHVASNGDLVQIASFGANARI
ANCGDNVHIIASGENSTVVSTGVVDSIILGPGGCAALAYHDGERVRFAVAIEGENNIRAG
VRYRLNEQHQFVEC
>LFGLNPFC_01578 hypothetical protein
MKKIAIVGAGPTGIYTLFSLLQQQTPLSISIFEQADEAGVGMPYSDDENSKLMLANIASI
EIPPIYCTYLEWLQKQEASHLQRYGVKKETLHDRQFLPRILLGEYFRDQFLRLVDQARQQ
KFAVAVYESCQVTDLQITNAGVMIATNQDLPSETFDLAVIATGHVWPDEEEATRTYFPSP
WSGLMEAKVDACNVGIMGTSLSGLDAAMAVAIQHGSFIEDDKQHVIFHRDNASEKLNITL
MSRTGILPEADFYCPIPYEPLHIVTDQALNAEIQKGEEGLLDRVFRLIVEEIKFADPDWS
QRIALESLNVDSFAQAWFAERKQRDPFDWAEKNLQEVERNKRENHTVPWRYVILRLHEAV
QEIVPHLNEHDHKRFSKGLARVFIDNYAAIPSESIRRLLALREAGIIHILALGEDYEMEI
NESRTVLKTEDNSYSFDVFIDARGQRPLKVKDIPFPGLREQLQKTGDEIPDVGEDYTLQQ
```

PEDIRGRVAFGALPWLMHDQPFVQGLTACAEIGEAMARAVVKPASRARRRLSFD
>LFGLNPFC_01579 Putative monooxygenase YdhR
MATLLQLHFAFNGPFGDAMAEQLKPLAESINQEPGFLWKVWTESEKNHEAGGIYLFINEK
SALAYLEKHTARLKNLGVEEVVAKVFDVNEPLSQINQAKLA
>LFGLNPFC_01582 putative autotransporter
MGSDAKNLMSDGNVQIVKTGEVIGATQLTEGELIVEAGARAENTVVTGAGWLKVATGGIA
KCTQYGNNGTLSVSDGAIATDIVQSEGGAISLSTLATVNGRHPEGEFSVDQGYACGLLLE
NGGNLRVLEGHRAEKIILDQEGGLLVNGTTSAVVVDEGGELLVYPGGEASNCEINQGGVF
MLAGKANDTLLAGGTMNNLGGEDSDTIVENGAIYRLGTDGLQLYSSGKTQNLSVNVGGRA
EVHAGTLENAVIQGGTVILLSPTSADENFVVEEDRAPVELTGSVALLDGASMIIGYGADL
QQSTITVQQGGVLILDGSTVKGDSVTFSVGNINLNGGKLWLITGAATHVQLKVKRLRGEG
AICLQTSAKEISPDFINVKGEVTGDIHVEITDASRQTLCNALKLQPDEDGIGATLQPA
>LFGLNPFC_01583 Multidrug resistance protein MdtK
MQKYISEARLLLALAIPVILAQIAQTAMGFVDTVMAGGYSATDMAAVAIGTSIWLPAILF
GHGLLLALTPVIAQLNGSGRRERIAHQVRQGFWLAGFVSVLIMLVLWNAGYIIRSMQNID
PALADKAVGYLRALLWGAPGYLFFQVARNQCEGLAKTKPGMVMGFIGLLVNIPVNYIFIY
GHFGMPELGGVGCGVATASVYWVMFLAMVSYIKRARSMRDIRNEKGTAKPDPAVMKRLIQ
LGLPIALALFFEVTLFAVVALLVSPLGIVDVAGHQIALNFSSLMFVLPMSLAAAVTIRVG
YRLGQGSTLDAQTAARTGLMVGVCMATLTAIFTVSLREQIALLYNDNPEVVTLAAHLMLL
AAVYQISDSIQVIGSGILRGYKDTRSIFYITFTAYWVLGLPSGYILALTDLVVEPMGPAG
FWIGFIIGLTSAAIMMMLRMRFLQRLPSAIILQRAAR
>LFGLNPFC_01584 Riboflavin synthase
MFTGIVQGTAKLVSIDEKPNFRTHVVELPDHMLDGLETGASVAHNGCCLTVTEINGNHVS
FDLMKETLRITNLGDLKVGDWVNVERAAKFSDEIGGHLMSGHIMTTAEVAKILTSENNRQ
IWFKVQDSQLMKYILYKGFIGIDGISLTVGEVTPTRFCVHLIPETLERTTLGKKKLGARV
NIEIDPQTQAVVDTVERVLAARENAMNQPGTEA
>LFGLNPFC_01585 Cyclopropane-fatty-acyl-phospholipid synthase
MSSSCIEEVSVPDDNWYRIANELLSRAGIAINGSAPADIRVKNPDFFKRVLQEGSLGLGE
SYMDGWWECDRLDMFFSKVLRAGLENQLPHHFKDTLRIAGARLFNLQSKKRAWIVGKEHY
DLGNDLFSRMLDPFMQYSCAYWKDADNLESAQQAKLKMICEKLQLKPGMRVLDIGCGWGG
LAHYMASNYDVSVVGVTISAEQQKMAQERCEGLDVTILLQDYRDLNDQFDRIVSVGMFEH
VGPKNYDTYFAVVDRNLKPEGIFLLHTIGSKKTDLNVDPWINKYIFPNGCLPSVRQIAQS
SEPHFVMEDWHNFGADYDTTLMAWYERFLAAWPEIADNYSERFKRMFTYYLNACAGAFRA
RDIQLWQVVFSRGVENGLRVAR
>LFGLNPFC_01586 Inner membrane transport protein YdhC
MQPGKRFLVWLAGLSVLGFLATDMYLPAFAAIQADLQTPASAVSASLSLFLAGFAAAQLL
WGPLSDRYGRKPVLFIGLTIFALGSLGMLWVENAATLLILRFVQAVGVCAAAVIWQALVT
DYYPSQKVNRIFATIMPLVGLSPALAPLLGSWLLVHFSWQAIFATLFAITVVLILPIFWL
KPTTKARNNSQDGLTFTDLLRSKTYRGNVLIYAACSASFFAWLTGSPFILSEMGYSPAVI
GLSYVPQTIAFLIGGYGCRAALQKWQGKQLLPWLLVLFAVSVIATWAAGFISHVSLVEIL
IPFCVMAIANGAIYPIVVAQALRPFPHATGRAAALQNTLQLGLCFLASLVVSWLISISTP
LLTTTSVMLSTVVLVALGYMMQRCEEVGCQNHGNAEVAHSESH
>LFGLNPFC_01587 putative HTH-type transcriptional regulator YahB
MWSEYSLEVVDAVARNGSFSAAAQELHRVPSAVSYTVRQLEEWLAVPLFERRHRDVELTA
AGAWFLKEGRSVVKKMQITRQQCQQIANGWRGQLAIAVDNIVRPERTRQMIVDFYRHFDD
VELLVFQEVFNGVWDALSDGRVELAIGATRAIPVGGRYAFRDMGMLSWSCVVASHHPLAL
MDGPFSDDTLRNWPSLVREDTSRTLPKRITWLLDNQKRLVVPDWESSATCISAGLCIGMV
PTHFAKPWLNEGKWVALELENPFPDSACCLTWQQNDMSPALTWLLEYLGDSETLNKEWLR
EPEETPATGD
>LFGLNPFC_01588 HTH-type transcriptional repressor PurR
MATIKDVAKRANVSTTTVSHVINKTRFVAEETRNAVWAAIKELHYSPSAVARSLKVNHTK
SIGLLATSSEAAYFAEIIEAVEKNCFQKGYTLILGNAWNNLEKQRAYLSMMAQKRVDGLL
VMCSEYPEPLLAMLEEYRHIPMVVMDWGEAKADFTDAVIDNAFEGGYMAGRYLIERGHRE
IGVIPGPLERNTGAGRLAGFMKAMEEAMIKVPESWIVQGDFEPESGYRAMQQILSQSHRP
TAVFCGGDIMAMGALCAADEMGLRVPQDVSLIGYDNVRNARYFTPALTTIHQPKDSLGET
AFNMLLDRIVNKREEPQSIEVHPRLIERRSVADGPFRDYRR
>LFGLNPFC_01589 putative protein YnhF
MSTDLKFSLVTTIIVLGLIVAVGLTAALH
>LFGLNPFC_01590 Inner membrane transport protein YdhP
MKINYPLLALAIGAFGIGTTEFSPMGLLPVIARGVDVSIPAAGMLISAYAVGVMVGAPLM
TLLLSHRARRSALIFLMAIFTLGNVLSAIAPDYMTLMLSRILTSLNHGAFFGLGSVVAAS
VVPKHKQASAVATMFMGLTLANIGGVPAATWLGETIGWRMSFLATAGLGVISMVSLFFSL
PKGGAGARPEVKKELAVLMRPQVLSALLTTVLGAGAMFTLYTYISPVLQSITHVTPVFVT
AMLVLIGVGFSIGNYLGGKLADRSVNGTLKGFLLLLMVIMLVIPFLARNEFGAAISMVVW

GAATFAVVPPLQMRVMRVASEAPGLSSSVNIGAFNLGNALGAAAGGAVISAGLGYSFVPV
MGAIVAGLALLLVFMSVRKQPETVCVANS
>LFGLNPFC_01591 Superoxide dismutase [Fe]
MSFELPALPYAKDALAPHISAETIEYHYGKHHQTYVTNLNNLIKGTAFEGKSLEEIIRSS
EGGVFNNAAQVWNHTFYWNCLAPNAGGEPTGKVAEAIAASFGSFADFKAQFTDAAIKNFG
SGWTWLVKNSDGKLAIVSTSNAGTPLTTDATPLLTVDVWEHAYYIDYRNARPGYLEHFWA
LVNWEFVAKNLAA
>LFGLNPFC_01592 Murein DD-endopeptidase MepH
MARINRISITLCALLFTTLPLTPMAHASKQARESSATTHITKKADKKKSTATTKKTQKTA
KKAASKSTTKSKTASSVKKSSITASKNAKTRSKHTVNKTASANFTEKCTKRKGYKSHCVK
VKNAASGTLADAHKAKVQKATKVAMNKLMQQIGKPYRWGGSSPRTGFDCSGLVYYAYKDL
VKIRIPRTANEMYHLRDAAPIERSELKNGDLVFFRTQGRGTADHVGVYVGNGKFIQSPRT
GREIQITSLSEDYWQRHYVGARRVMTPKTLR
>LFGLNPFC_01593 Glutaredoxin 4
MSTTIEKIQRQIAENPILLYMKGSPKLPSCGFSAQAVQALAACGERFAYVDILQNPDIRA
ELPKYANWPTFPQLWVDGELVGGCDIVIEMYQRGELQQLIKETAAKYKSEEPDAE
>LFGLNPFC_01594 Ribonuclease T
MSDNAQLTGLCDRFRGFYPVVIDVETAGFNAKTDALLEIAAITLKMDEQGWLMPDTTLHF
HVEPFVGANLQPEALAFNGIDPNDPDRGAVSEYEALHEIFKVVRKGIKASGCNRAIMVAH
NANFDHSFMMAAAERASLKRNPFHPFATFDTAALAGLALGQTVLSKACQTAGMDFDSTQA
HSALYDTERTAVLFCEIVNRWKRLGGWPLPAAEEV
>LFGLNPFC_01595 Lactoylglutathione lyase
MRLLHTMLRVGDLQRSIDFYTNVLGMKLLRTSENPEYKYSLAFVGYGPETEEAVIELTYN
WGVDKYELGTAYGHIALSVDNAAEACEKIRQNGGNVTREAGPVKGGTTVIAFVEDPDGYK
IELIEEKDAGRGLGN
>LFGLNPFC_01596 N-ethylmaleimide reductase
MSSEKLYSPLKVGAITAANRIFMAPLTRLRSIEPGDIPTPLMAEYYRQRASAGLIISEAT
QISAQAKGYAGAPGIHSPEQIAAWKKITAGVHAENGHMAVQLWHTGRISHASLQPGGQAP
VAPSALSAGTRTSLRDENGQAIRVETSMPRALELGEIPGIVNDFRQAIANAREAGFDLVE
LHSAHGYLLHQFLSPSSNHRTDQYGGSVENRARLVLEVVNAGIEEWGADRIGIRISPIGT
FQNTDNGPNEEADALYLIEQLGKRGIAYLHMSEPDWAGGEPYTDAFREKVRARFHGPIIG
AGAYTVEKAETLIGKGLIDAVAFGRDWIANPDLVARLQRKAELNPQRAESFYGGGAEGYT
DYPTL
>LFGLNPFC_01597 HTH-type transcriptional repressor NemR
MNKHTEHDTREHLLATGEQLCLQRGFTGMGLSELLKTAEVPKGSFYHYFRSKEAFGVAML
ERHYAAYHQRLTELLQSGEGNYRDRILAYYQQTLNQFCQHGTISGCLTVKLSAEVCDLSE
DMRSAMDKGARGVIALLSQALENGRENHCLTFCGEPLQQAQVLYALWLGANLQAKISRSF
EPLENALAHVKNIIATPAV
>LFGLNPFC_01598 hypothetical protein
MAEQLEFFPVQSPCRGICQSDERGFCRGCFRSRDERFNWNKMSDGEKQEVLRLCRQRLMR
KLRVNKSAPSDEPEQPSLF
>LFGLNPFC_01599 Oxidoreductase YdhF
MVQRITIAPQGPEFSRFVMGYWRLMDWNMSARQLVSFIEEHLDLGVTTVDHADIYGGYQC
EAAFGEALKLAPHLRERMEIVSKCGIATTACEENVIGHYITDRDHIIKSAEQSLINLATD
HLDLLLIHRPDPLMDADEVADAFKHLHQSGKVRHFGVSNFTPAQFALLQSRLPFTLATNQ
VEISPVHQPLLLDGTLDQLQQLRVRPMAWSCLGGGRLFNDDYFQPLRDELAVVAEELNAG
SIEQVVYAWVLRLPSQPLPIIGSGKIERVRAAVEAETLKMTRQQWFRIRKAALGYDVP
>LFGLNPFC_01600 Superoxide dismutase [Cu-Zn]
MKRFSLAILALVVATGAQAASEKVEMNLVTSQGVGQLIGSVTITETDKGLEFSPDLKALP
PGEHGFHIHAKGSCQPATKDGKASAAESAGGHLDPQNTGKHEGPEGAGHLGDLPALVVNN
DGKATDAVIAPRLKSLDEVKDKALMVHVGGDNMSDQPKPLGGGGERYACGVIK
>LFGLNPFC_01601 p-hydroxybenzoic acid efflux pump subunit AaeB
MNASSWSLRNLPWFRATLAQWRYALRNTIAMCLALTVAYYLNLDEPYWAMTSAAVVSFPT
VGGVISKSLGRIAGSLLGAIAALLLAGHTLNEPWFFLLSMSAWLGFCTWACAHFTNNVAY
AFQLAGYTAAIIAFPMVNITEASQLWDIAQARVCEVIVGILCGGMMMMILPSSSDATALL
TALKNMHARLLEHASLLWQPETTDAIRAAHEGVIGQILTMNLLRIQAFWSHYRFRQQNAR
LNALLHQQLRMTSVISSLRRMLLNWPSPPDATREILEQLLTALASSQTDVYTVARIISPL
RPTNVADYRHVAFWQRLRYFCRLYLQSSQELHRLQSGVDDRARLPRTSGLARHTDNAEAM
WSGLRTFCTLMMIGAWSIASQWDAGANALTLAAISCVLYSAVAAPFKSLSLLMRTLVLLS
LFSFVVKFGLMVQISDLWQFLLFLFPLLATMQLLKLQMPKFAALWGQLIVFMGSFIAVTN
PPVYDFADFLNDNLAKIVGVALAWLAFAILRPGSDARKSRRHIRALRRDFVDQLSRHPTL
SESEFESLTYHHVSQLSNSQDALARRWLLRWGVVLLNCSHVVWQLRDWESRSDPLSRVRD
NCISLLRGVMSERGVQQKSLAATLEELQRICDSLARHHQPAARELAAIVWRLYCSLSQLE
QAPPQGTLAS

>LFGLNPFC_01602 p-hydroxybenzoic acid efflux pump subunit AaeA
MSIKTIKYFSTIIVAVVAVLAGWWLWNYYMQSPWTRDGKIRAEQVSITPQVSGRIIELNI
KDNQLVNAGDLLLTIDKTPFQIAELNAQAQLAKAQSGLAKANNEANRRRHLSQNFISAEE
LDTANLNVKAMQASVDAAQATLKQAQWQLAQTEIRAPVSGWVTNLTTRIGDYADTGKPLF
ALVDSHSFYVIGYFEETKLRHIREGAPAQITLYSDNKTLQGHVSSIGRAIYDQSVESDSS
LIPDVKPNVPWVRLAQRVPVRFTLDKVPGDVTLVSGTTCSIAVGQ
>LFGLNPFC_01603 hypothetical protein
MKFMLNATGLPLQDLVFGASVYFPPFFKAFAFGFVIWLVIHRLLRGWIYAGDIWHPLLMD
LSLFAICVCLALAILIAW
>LFGLNPFC_01604 Transcriptional regulator SlyA
MESPLGSDLARLVRIWRALIDHRLKPLELTQTHWVTLHNIHQLPPDQSQIQLAKAIGIEQ
PSLVRTLDQLEEKGLISRQTCASDRRAKRIKLTEKAEPLISEMEAVINKTRAEILHGISA
EELEQLIKLIAKLEHNIIELQAKG
>LFGLNPFC_01605 Outer membrane lipoprotein SlyB
MIKRVLVVSMVGLSLVGCVNNDTLSGDVYTASEAKQVQNVSYGTIVNVRQVQIQGGDDSN
VIGAIGGAVLGGFLGNTIGGGTGRSLATAAGAVAGGVAGQGVQSAMNKTQGVELEIRKDD
GNTIMVVQKQGNTHFSPGQRVVLASNGSQVTVSPR
>LFGLNPFC_01606 Anhydro-N-acetylmuramic acid kinase
MKSGRFIGVMSGTSLDGVDVVLATIDEHRVAQLASLSWPIPVSLKQAVLDICQGQQLTLS
QFGQLDTQLGRLFADAVNALLKEQNLQARDIVAIGCHGQTVWHEPTGVAPHTLQIGDNNQ
IVARTGITVVGDFRRRDIALGGQGAPLVPAFHHALLAHPTERRMVLNIGGIANLSLLIPG
QPVGGYDTGPGNMLMDAWIWRQAGKPYDKDAEWARAGKVILPLLQNMLSDPYFSQPAPKS
TGREYFNYGWLERHLRHFPGVDPRDVQATLAELTAVTISEQVLLSGGCERLMVCGGGGRN
PLLMARLAALLPGTEVTTTDAVGISGDDMEALAFAWLAWRTLAGLPGNLPSVTGASQETV
LGAIFPANP
>LFGLNPFC_01607 Membrane-bound lysozyme inhibitor of C-type lysozyme
MKKLLLICLPVLLTGCSSFNQLVERMQTDTLEYQCDEKPLTVKLNNPRQEVSFVYDNQLL
HLKQGISASGARYTDGIYVFWSKGEEATVYKRDRIVLSNCQLQNPQR
>LFGLNPFC_01608 Pyridoxine/pyridoxamine 5'-phosphate oxidase
MSDNDELQQIAHLRREYTKGGLRRRDLPADPLTLFERWLSQACEAKLADPTAMVVATVDE
HDQPYQRIVLLKHYDEKGMVFYTNLGSRKAHQIENNPRVSLLFPWHTLERQVMVIGKAER
LSTLEVMKYFHSRPRDSQIGAWVSKQSSRISARGILESKFLELKQKFQQGEVPLPSFWGG
FRVSLEQIEFWQGGEHRLHDRFLYQRENDAWKIDRLAP
>LFGLNPFC_01609 Tyrosine--tRNA ligase
MASSNLIKQLQERGLVAQVTDEEALAERLAQGPIALYCGFDPTADSLHLGHLVPLLCLKR
FQQAGHKPVALVGGATGLIGDPSFKAAERKLNTEETVQEWVDKIRKQVAPFLDFDCGENS
AIAANNYDWFGNMNVLTFLRDIGKHFSVNQMINKEAVKQRLNREDQGISFTEFSYNLLQG
YDFACLNKQYGVVLQIGGSDQWGNITSGIDLTRRLHQNQVFGLTVPLITKADGTKFGKTE
GGAVWLDPKKTSPYKFYQFWINTADADVYRFLKFFTFMSIEEINALEEEDKNSGKAPRAQ
YVLAEQVTRLVHGEDGLQAAKRITECLFSGSLSALSEADFEQLAQDGVPMVEMEKGADLM
QALVDSELQPSRGQARKTIASNAITINGEKQSDPEYFFKEEDRLFGRFTLLRRGKKNYCL
ICWK
>LFGLNPFC_01610 Pyridoxal kinase PdxY
MKNILAIQSHVVYGHAGNSAAEFPMRRLGANVWPLNTVQFSNHTQYGKWTGCVMPPSHLT
EIVQGIAAIDKLHTCDAVLSGYLGSAEQGEHILGIVRQVKAANPQAKYFCDPVMGHPEKG
CIVAPGVAEFHVRHGLPASDIIAPNLVELEILCEHPVNNVEEAVLAARELIAQGPQIVLV
KHLARAGYSRDRFEMLLVTADEAWHISRPLVDFGMRQPVGVGDVTSGLLLVKLLQGATLQ
EALEHVTAAVYEIMVTTKAMQEYELQVVAAQDRIANPEHYFSATKL
>LFGLNPFC_01611 Glutathione S-transferase GstA
MKLFYKPGACSLASHITLRESGKDFTLVSVDLMKKRLENGDNYFAVNPKGQVPALLLDDG
TLLTEGVAIMQYLADSVPDRQLLAPVNSISRYKTIEWLNYIATELHKGFTPLFRPDTPEE
YKSTVRAQLEKKLQYVNEALKDEHWICGQRFTIADAYLFTVLRWAYAVKLNLEGLEHIAA
FMQRMAERPEVQDALSAEGLK
>LFGLNPFC_01612 Dipeptide and tripeptide permease A
MSTANQKPTESVSLNAFKQPKAFYLIFSIELWERFGYYGLQGIMAVYLVKQLGMSEADSI
TLFSSFSALVYGLVAIGGWLGDKVLGTKRVIMLGAIVLAIGYALVAWSGHDAGIVYMGMA
AIAVGNGLFKANPSSLLSTCYEKNDPRLDGAFTMYYMSVNIGSFFSMIATPWLAAKYGWS
VAFALSVVGLLITIVNFAFCQRWVKQYGSKPDFEPINYRNLLLTIIGVVALIAIATWLLH
NQEVARMALGVVAFGIVVIFGKEAFAMKGAARRKMIVAFILMLEAIIFFVLYSQMPTSLN
FFAIRNVEHTILGLAVEPEQYQALNPFWIIIGSPILAAIYNKMGDTLPMPTKFAIGMVMC
SGAFLILPLGAKFASDAGIVSVSWLVASYGLQSIGELMISGLGLAMVAQLVPQRLMGFIM
GSWFLTTAGANLIGGYVAGMMAVPDNVTDPLMSLEVYGRVFLQIGVATAVIAVLMLFTAP
KLHRMTQDDAADKAAKAAVA
>LFGLNPFC_01613 Endonuclease III

MNKAKRLEILTRLRENNPHPTTELNFSSPFELLIAVLLSAQATDVSVNKATAKLYPVANT
PAAMLELGVEGVRTYIKTIGLYNSKAENIIKTCRILLEQHNGEVPEDRAALEALPGVGRK
TANVVLNTAFGWPTIAVDTHIFRVCNRTQFAPGKNVEQVEEKLLKVVPAEFKVDCHHWLI
LHGRYTCIARKPRCGSCIIEDLCEYKEKVDI
>LFGLNPFC_01614 Ion-translocating oxidoreductase complex subunit E
MSEIKDVIVQGLWKNNSALVQLLGLCPLLAVTSTATNALGLGLATTLVLTLTNLTISTLR
HWTPAEIRIPIYVMIIASVVSAVQMLINAYAFGLYQSLGIFIPLIVTNCIVVGRAEAFAA
KKGPALSALDGFSIGMGATCAMFVLGSLREIIGNGTLFDGADALLGSWAKVLRVEIFRTD
SPFLLAMLPPGAFIGLGLMLAGKYLIDEKMKKRRTEAVAERALPNGETGNV
>LFGLNPFC_01615 Ion-translocating oxidoreductase complex subunit G
MLKTIRKHGITLALFAAGSTGLTAAINQMTKTTIAEQASLQQKALFDQVLPAERYNNALA
QSCYLVTAPELGKGEHRVYIAKQDDKPVAAVLEATAPDGYSGAIQLLVGADFNGTVLGTR
VTEHHETPGLGDKIELRLSDWITHFAGKKISGADDANWAVKKDGGDFDQFTGATITPRAV
VNAVKRAGLYAQTLPAQLSQLPACGE
>LFGLNPFC_01616 Ion-translocating oxidoreductase complex subunit D
MVFRIASSPYTHNQRQTSRIMLLVLLAAVPGIAAQLWFFGWGTLVQILLASVSALLAEAL
VLKLRKQSVAATLKDNSALLTGLLLAVSIPPLAPWWMVVLGTVFAVIIAKQLYGGLGQNP
FNPAMIGYVVLLISFPVQMTSWLPPHEIAVNIPGFIDAIQVIFSGHTTSGGDMNTLRLGI
DGISQATPLDTFKTSVRAGHSVEEIMQYPIYSGILAGAGWQWVNLAWLAGGVWLLWQKAI
RWHVPLSFLVTLALCATLGWLFSPDTLAAPQIHLLSGATMLGAFFILTDPVTASTTNRGR
LIFGALAGLLVWMIRSFGGYPDGVAFAVLLANITVPLIDYYTRPRVYGHRKG
>LFGLNPFC_01617 Ion-translocating oxidoreductase complex subunit C
MLKLFSAFRKNKIWDFNGGIHPPEMKTQSNGTPLRQVPLAQRFVIPLKQHIGAEGELCVS
VGDKVLRGQPLTRGRGKMLPVHAPTSGTVTAIAPHSTAHPSALAELSVIIDADGEDCWIP
RDGWADYRSRSREELIERIHQFGVAGLGGAGFPTGVKLQGGGDKIETLIINAAECEPYIT
ADDRLMQDCAAQVVEGIRILAHILQPREILIGIEDNKPQAISMLRAVLADSHDISLRVIP
TKYPSGGAKQLTYILTGKQVPHGGRSSDIGVLMQNVGTAYAVKRAVIDGEPITERVVTLT
GEAIARPGNVWARLGTPVRHLLNDAGFCPSADQMVIMGGPLMGFTLPWLDVPVVKITNCL
LAPSANELGEPQEEQSCIRCSACADACPADLLPQQLYWFSKGQQHDKATTHNIADCIECG
ACAWVCPSNIPLVQYFRQEKAEIAAIRQEEKRAAEAKARFEARQARLEREKAARLERHKS
AAVQPAAKDKDAIAAALARVKEKQAQATQPIVIKAGERPDNSAIIAAREARKAQARAKQA
ELQQTNDAATVADPRKTAVEAAIARAKARKLEQQQANAEPEEQIDPRKAAVEAAIARAKA
RKLEQQQANAEPEEQIDPRKAAVEAAIARAKARKLEQQQANAEPEEQIDPRKAAVEAAIA
RAKARKLEQQQANAEPEEQIDPRKAAVAAAIARVQAKKAAQQKVVNED
>LFGLNPFC_01618 Ion-translocating oxidoreductase complex subunit B
MNAIWIAVAAVSLLGLAFGAILGYASRRFAVEDDPVVEKIDEILPQSQCGQCGYPGCRPY
AEAISCNGEKINRCAPGGEAVMLKIAELLNVEPQPLDGEAQELTPARMVAVIDENNCIGC
TKCIQACPVDAIVGATRAMHTVMSDLCTGCNLCVDPCPTHCISLQPVAETPDSWKWDLNT
IPVRIIPVEHHA
>LFGLNPFC_01619 Ion-translocating oxidoreductase complex subunit A
MTDYLLLFVGTVLVNNFVLVKFLGLCPFMGVSKKLETAMGMGLATTFVMTLASICAWLID
TWILIPLNLIYLRTMAFILVIAVVVQFTEMVVRKTSPVLYRLLGIFLPLITTNCAVLGVA
LLNINLGHNFLQSALYGFSAAVGFSLVMVLFAAIRERLAVADVPAPFRGNAIALITAGLM
SLAFMGFSGLVKL
>LFGLNPFC_01620 Inner membrane protein YdgK
MTTTTPQRIGGWLLGPLAWLLVALLSTTLALLLYTAALSSPQTFQTLGGQALTTQILWGV
SFITAIAMWYYTLWLTIAFFKRRRCVPKHYIIWLLISVLLAVKAFAFSPVEDGIAVRQLL
FTLLATALIVPYFKRSSRVKATFVNP
>LFGLNPFC_01621 OriC-binding nucleoid-associated protein
MTVQDYLLKFRKISSLESLEKLYDHLNYTLTDDQELINMYRAADHRRAELVSGGRLFDLG
QVPKSVWHYVQ
>LFGLNPFC_01622 putative oxidoreductase YdgJ
MSDNIRVGLIGYGYASKTFHAPLIAGTPGLELAVISSSDETKVKADWPTVTVVSEPKHLF
NDPNIDLIVIPTPNDTHFPLAKAALEAGKHVVVDKPFTVTLSQARELDALAKSLGRVLSV
FHNRRWDSDFLTLKGLLAEGVLGEIAYVESHFDRFRPQVRDRWREQGGPGSGIWYDLAPH
LLDQAITLFGLPVSMTVDLAQLRPGAQSTDYFHAILSYPQRRVILHGTMLAAAESARYIV
HGSRGSYVKYGLDPQEERLKNGERLPQEDWGYDMRDGVLTRVEGEERVEETLLTVPGNYP
AYYAAIRDALNGDGENPVPASQAIQVMELIELGIESAKHRATLCLA
>LFGLNPFC_01623 Adenosine deaminase
MIDTTLPLTDIHRHLDGNIRPQTILELGRQYNISLPAQSLETLIPHVQVIANEPDLVSFL
TKLDWGVKVLASLDACRRVAFENIEDAARNGLHYVELRFSPGYMAMAHQLPVAGVVEAVI
DGVREGCRTFGVQAKLIGIMSRTFGEAACQQELEAFLAHRDQITALDLAGDELGFPGSLF
LSHFNRARDAGWHITVHAGEAAGPESIWQAIRELGAERIGHGVKAIEDRALMDFLAEQQI
GIESCLTSNIQTSTVADLAAHPLKTFLEHGIRASINTDDPGVQGVDIIHEYTVAAPAAGL

SREQIRQAQINGLEMAFLSAEEKRALREKVAAK
>LFGLNPFC_01624 Protein MalY
MFDFSKVVDRHGTWCTQWDYVADRFGTADLLPFTISDMDFATAPCIIEALNQRLIHGVFG
YSRWKNDEFLAAIAHWFSTQHYTAIDPQSVVYGPSVIYMVSELIRQWSETGEGVVIHTPA
YDAFYKAIEGNQRTVMPVALEKQPDGWFCDMGKLEAVLAKPECKIMLLCSPQNPTGKVWT
CDELEIMADLCERHGVRVISDEIHMDMVWGEQPHIPWSNVARGDWALLTSGSKSFNIPAL
TGAYGIIENSSSRDAYLSALKGRDGLSSPSVLALTAHIAAYQQGAPWLDALRVYLKDNLT
YIADKMNAAFPELNWQIPQSTYLAWLDLRPLNIDDNALQKALIEQEKIAIMPGYTYGEEG
RGFVRLNAGCPRSKLEKGVAGLINAIRAVG
>LFGLNPFC_01625 PTS system maltose-specific EIICB component
MTAKTAPKVTLWEFFQQLGKTFMLPVALLSFCGIMLGIGSSLSSHDVITLIPVLGNPVLQ
AIFTWMSKIGSFAFSFLPVMFCIAIPLGLARENKGVAAFAGFVGYAVMNLAVNFWLTNKG
ILPTTDAAVLKANNIQSILGIQSIDTGILGAVIAGIIVWMLHERFHNIRLPDALAFFGGT
RFVPIISSLVMGLVGLVIPLVWPIFAMGISGLGHMINSAGDFGPMLFGTGERLLLPFGLH
HILVALIRFTDAGGTQEVCGQTVSGALTIFQAQLSCPTTHGFSESATRFLSQGKMPAFLG
GLPGAALAMYHCARPENRHKIKGLLISGLIACVVGGTTEPLEFLFLFVAPVLYVIHALLT
GLGFTVMSVLGVTIGNTDGNIIDFVVFGILHGLSTKWYMVPVVAAIWFVVYYIIFRFAIT
RFNLKTPGRDSEVASSIEKAVAGAPGKSGYNVPAILEALGGADNIVSLDNCITRLRLSVK
DMSLVNVQALKDNRAIGVVQLNQHNLQVVIGPQVQSVKDEMAGLMHTVQA
>LFGLNPFC_01626 Maltose regulon regulatory protein MalI
MATAKKITIHDVALAAGVSVSTVSLVLSGKGRISTATGERVNAAIEELGFVRNRQASALR
GGQSGVIGLIVRDLSAPFYAELTAGLTEALEAQGRMVFLLHGGKDGEQLAQRFSLLLNQG
VDGVVIAGAAGSSDDLRRIAEEKAIPVIFASRASYLDDVDTVRPDNMQAAQLLTEHLIRN
GHQRIAWLGGQSSSLTRAERVGGYCATLLKFGLPFHSDWVLECTSSQKQAAEAITALLRH
NPTISAVVCYNETIAMGAWFGLLKAGRQSGESGVDRYFEQQVSLAAFTDATPTTLDDIPV
TWASTPARELGTTLADRMMQKITHEETHSSNLIIPARLIAEK
>LFGLNPFC_01627 7-alpha-hydroxysteroid dehydrogenase
MFNSDNLRLDGKCAIITGAGAGIGKEIAITFATAGASVVVSDINADAANHVVNEIQQLGG
QAFACRCDITSEQELSALADFAVSKLGKVDILVNNAGGGGPKPFDMPMADFRRAYELNVF
SFFHLSQLVAPEMEKNGGGVILTITSMAAENKNINMTSYASSKAAASHLVRNMAFDLGEK
NIRVNGIAPGAILTDALKSVITPEIEQKMLQHTPIRRLGQPQDIANAALFLCSPAASWVS
GQILTVSGGGVQELN
>LFGLNPFC_01628 HTH-type transcriptional regulator BetI
MQTEAQPTRTRILNAAREIFSENGFHSASMKAICKSCAISPGTLYHHFISKEALIQAIIL
QDQERALARFREPIEGIHFVDYMVESIVSLTHEAFGQRALVVEIMAEGMRNPQVAAMLKN
KHMTITEFVAQRMRDAQQKGEISPDINTAMTSRLLLDLTYGVLADIEAEDLAREASFAQG
LRAMIGGILTAS
>LFGLNPFC_01629 Beta-glucuronidase
MLRPVETPTREIKKLDGLWAFSLDRENCGIDQRWWESALQESRAIAVPGSFNDQFADADI
RNYVGNVWYQREVFIPKGWAGQRIVLRFDAVTHYGKVWVNNQEVMEHQGGYTPFEADVTP
YVIAGKSVRITVCVNNELNWQTIPPGMVITDENGKKKQSYFHDFFNYAGIHRSVMLYTTP
NTWVDDITVVTHVAQDCNHASVDWQVGANGDVSVELRDADQQVVATGQGTSGTLQVVNPH
LWQPGEGYLYELCVTAKSQTECDIYLLRVGIRSVAVKGEQFLINHKPFYFTGFGRHEDAD
LRGKGFDNVLMVHDHALMDWIGANSYRTSHYPYAEEMLDWADEHGIVVIDETAAVGFNLS
LGIGFEAGNKPKELYSEEAVNGETQQAHLQAIKELIARDKNHPSVVMWSIANEPDTRPQG
AREYFAPLAEATRKLDPTRPITCVNVMFCDAHTDTISDLFDVLCLNRYYGWYVQSGDLET
AEKVLEKELLAWQEKLHQPIIITEYGVDTLAGLHSMYTDMWSEEYQCAWLDMYHRVFDRV
SAVVGEQVWNFADFATSQGILRVGGNKKGIFTRDRKPKSAAFLLQKRWTGMNFGEKPQQG
GKQ
>LFGLNPFC_01630 Glucuronide carrier protein
MNQQLSWRAIVGYSLGDVANNFAFAMGALFLLSYYTDVAGVGAAAAGTMLLLVRVFDAFA
DVFAGRVVDSVNTRWGKFRPFLLFGTAPLMIFSVLVFWVPTDWSHSSKVVYAYLTYMGLG
LCYSLVNIPYGSLATAMTQQPQSRARLGAARGIAASLTFVCLAFLIGPSIKNSSPEEMVS
VYHFWTIVLAIAGMVLYFICFKSTRENVVRIVAQPSLKISLQTLKRNRPLFMLCIGALCV
LISTFAVSASSLFYVRYVLNDTGLFTVLVLVQNLVGTVASAPLVPGMVARIGKKNTFLIG
ALLGTCGYLLFFWVSVWSLPVALVALAIASIGQGVTMTVMWALEADTVEYGEYLTGVRIE
GLTYSLFSFTRKCGQAIGGSIPAFILGLSGYIANQAQTPEVIMGIRTSIALVPCGFMLLA
FVIIWFYPLTDKKFKEIVVEIDNRKKVQQQLINDITS
>LFGLNPFC_01631 Membrane-associated protein UidC
MRKIVAMAVICLTAASGLTSAYAAQPADDEAGLRIRLKNELRRADKPSAGTGRDIYAWVQ
GGLLDFNSGYYSNIIGVEGGAYYVYKLGARADMSTRWYLDGDKSFGFALGTVKIKPSENS
LLKLGRFGTDYSYGSLPYRIPLMAGSSQRTLPTVSEGALGYWALTPNIDLWGMWRSRVFL
WTDSTTGIRDEGVYNSQTGKYDKHRARSFLAASWHDDTSRYSLGASVQKDVSNQIQSILE
KSIPLDPNYTLKGELLGFYAQLEGLSRNTSQPNETALVSGQLTWNAPWGSVFGSGGYLRH

AMNGAVVDTDIGYPFSLSLDRNREGMQSWQLGVNYRLTPQFTLTFAPIVTRGYESSKRDV
RIEGAGILGGMNYRVSEGPLQGMNFFLAADKGREKRDGSTLGDRLNYWDVKMSIQYDFML
K
>LFGLNPFC_01632 Protein YdgA
MNKSLVAVGVIVALGVVWTGGAWYTGKKIETHLEDMVAQANAQLKLTAPESNLEVSYQNY
HRGVFSSQLQLLVKPIAGKVNPWIKSGQSVIFNESVDHGPFPLAQLKKLNLIPSMASIQT
TLVNNEVSKPLFDMAKGETPFEINSRIGYSGDSSSDISLKPLNYEQKDEKVAFSGGEFQL
NADRDGKAISLSGEAQSGRIDAVNEYNQKVQLTFNNLKTDGSSTLASFGERVGNQKLSLE
KMTISVEGKELALLEGMEISGKSDLVNDGKTVNSQLDYSLNSLKVQNQDLGSGKLTLKVG
QIDGEAWHQFSQQYNAQTQALLAQPEIANNPELYQEKVTEAFFSALPLMLKGDPVITIAP
LSWKNSQGESALNLSLFLKDPATTKEAPQTLAQEVDRSVKSLDAKLTIPVDMATELMTQV
AKLEGYQEDQAKKLAKQQVEGASAMGQMFRLTTLQDNTITTSLQYANGQITLNGQKMPLE
DFVGMFAMPALNVPAVPAIPQQ
>LFGLNPFC_01633 Mannose-6-phosphate isomerase
MQKLINSVQNYAWGSKTALTELYGMENPSSQPMAELWMGAHPKSSSRVHDATGDIVSLRD
VIESDKATLLGDAVAKRFGELPFLFKVLCAAQPLSIQVHPNKRNSEIGFAKENAAGIPMD
AAERNYKDPNHKPELVFALTPFLAMNAFREFSEIVSLLQPVAGAHPAIAHFLQQPDAERL
SELFASLLNMQGEEKSRALAILKSALDSQQGEPWQTIRLISEFYPEDSGLFSPLLLNVVK
LNPGEAMFLFAETPHAYLQGVALEVMANSDNVLRAGLTPKYIDIPELVANVKFEAKPANQ
LLTQPVKQGAELDFPIPVDDFAFSLHDLSDKETTISQQSAAILFCVEGDATLCKGSQQLQ
LKPGESAFIAANESPVTVKGHGRLARVYNKL
>LFGLNPFC_01634 Fumarate hydratase class I, aerobic
MSNKPFHYQAPFPLKKDDTEYYLLTSEHVSVSEFEGGQEILKVAPEALTLLARQAFHDASF
MLRPAHQQQVADILRDPEASENDKYVALQFLRNSDIAAKGVLPTCQDTGTAIIVGKKGQR
VWTGGGDEAALARGVYNTYIEDNLRYSQNAPLDMYKEVNTGTNLPAQIDLYAVDGDEYKF
LCIAKGGGSANKTYLYQETKALLTPGKLKNYLVEKMRTLGTAACPPYHIAFVIGGTSAET
NLKTVKLASAKYYDELPTEGNEHGQAFRDVELEKELLIEAQNLGLGAQFGGKYFAHDIRV
IRLPRHGASCPVGMGVSCSADRNIKAKINRQGIWIEKLEHNPGKYIPEELRKAGEGEAVR
VDLNRPMKEILAQLSQYPVSTRLSLNGTIIVGRDIAHAKLKERMDNGEGLPQYIKDHPIY
YAGPAKTPEGYASGSLGPTTAGRMDSYVDQLQAQGGSMIMLAKGNRSQQVTDACKKHGGF
YLGSIGGPAAVLAQGSIKSLECVEYPELGMEAIWKIEVEDFPAFILVDDKGNDFFQQIQL
TQCTRCVK
>LFGLNPFC_01635 Fumarate hydratase class II
MNTVRSEKDSMGAIDVPADKLWGAQTQRSLEHFRISTEKMPTSLIHALALTKRAAAKVNE
DLGLLSEEKASAIRQAADEVLAGQHDDEFPLAIWQTGSGTQSNMNMNEVLANRASELLGG
VRGMERKVHPNDDVNKSQSSNDVFPTAMHVAALLALRKQLIPQLKTLTQTLSEKSRAFAD
IVKIGRTHLQDATPLTLGQEISGWVAMLEHNLKHIEYSLPHVAELALGGTAVGTELNTHP
EYARRVADELAVITCAPFVTAPNKFEALATCDALVQAHGALKGLAASLMKIANDVRWLAS
GPRCGIGEISIPENEPGSSIMPGKVNPTQCEALTMLCCQVMGNDVAINMGGASGNFELNV
FRPMVIHNFLQSVRLLADGMESFNKHCAVGIEPNRERINQLLNESLMLVTALNTHIGYDK
AAEIAKKAHKEGLTLKAAALALGYLSEAEFDRWVRPEQMVGSMKAGG
>LFGLNPFC_01636 DNA replication terminus site-binding protein
MARYDLVDRLNTTFRQMEQELAAFAAHLEQHKLLVARVFSLPEVKKEDEHNPLNRIEVKQ
HLGNDAQSLALRHFRHLFIQQQSENRSSKAAVRLPGVLCYQVDNFSQAALVSHIQHINKL
KTTFEHIVTVESELPSAARFEWVHRHLPGLITLNAYRTLTVLHDPATLRFGWANKHIIKN
LHRDEVLAQLEKSLKSPRSVAPWTREEWQRKLEREYQDIAALPQNAKLKIKRPVKVQPIA
RVWYKGDQKQVQHACPTPLIALINRDNGAGVPDVGELLNYDADNVQHRYKPQAQPLRLII
PRLHLYVAD
>LFGLNPFC_01637 Sensor protein RstB
MKKLFIQFYLLLFVCFLVMSLLVGLVYKFTAERAGKQSLDDLMNSSLYLMRSELREIPPH
DWGKTLKEMDLNLSFDLRVEPLSKYHLDDISMHRLRGGEIVALDDQYTFLQRIPRSHYVL
AVGPVPYLYYLHQMRLLDIALIAFIAISLAFPVFIWMRPHWQDMLKLEAAAQRFGDGHLS
ERIHFDEGSSFERLGVAFNQMADNINALIASKKQLIDGIAHELRTPLVRLRYRLEMSDNL
SAAESQALNRDISQLEALIEELLTYARLDRPQNELHLSEPDLPLWLSTHLADIQAVTPDK
TVRIKTLAQGHYAALDMRLMERVLDNLLNNALRYCHSTVETSLLLSGNRATLIVEDDGPG
IAPENREHIFEPFVRLDPSRDRSTGGCGLGLAIVHSIALAMGGTVNCDTSELGGARFSFS
WPLWHNIPQFTSA
>LFGLNPFC_01638 Transcriptional regulatory protein RstA
MNTIVFVEDDAEVGALIAAYLAKHDMQVTVEPRGDRAEETILRENPDLVLLDIMLPGKDG
MTICRDLRAKWSGPIVLLTSLDSDMNHILALEMGACDYILKTTPPAVLLARLRLHLRQNE
QATVTKGIQETSLTPYKALHFGTLTIDPINRVVSLANTEISLSTADFELLWELATHAGQI
MDRDALLKNLRGVSYDGLDRSVDVAISRLRKKLLDNAAEPYRIKTVRNKGYLFAPHAWE
>LFGLNPFC_01639 Inner membrane protein YdgC
MGLVIKAALGALVVLLIGVLAKTKNYYIAGLIPLFPTFALIAHYIVASERGIEALRATII

FSMWPIIPYFVYLAALWYFTGMMRLPAAFVGSVACWGISAWVLIICWIKLH
>LFGLNPFC_01640 Dihydromonapterin reductase
MGKTQSLPILITGGGRRIGLALAWHFINQKQPVIVSYRTHYPAIDGLIKAGAQCIQADFS
TNDGVMAFADEVLKSTHGLRAILHNASAWMAEKPGAPLTDVLACMMQIHVNTPYLLNHAL
ERLLRGHGHAASDIIHFTDYVVERGSDKHIAYAASKAALDNMTRSFARKLAPEVKVNSIA
PSLILFNEHDDAEYRQQALNKSLMKTAPGEKEVIDLVDYLLTSCFVTGRSFPLDGGRHLR
>LFGLNPFC_01641 Putative arginine/ornithine antiporter
MEKKLGLSALTALVLSSMLGAGVFSLPQNMAAVASPAALLIGWGITGAGILLLAFAMLIL
TRIRPELDGGIFTYAREGFGELIGFCSAWGYWLCAVIANVSYLVIVFSALSFFTDTPELR
LFGDGNTWQSIVGASTLLWIVHFLILRGVQTAASINLVATLAKLLPLGLFVVLAMMMFKL
DTFKLDFTGLALGVPVWEQVKNTMLITLWVFIGVEGAVVVSARARNKRDVGKATLLAVLS
ALGVYLLVTLLSLGVVARPELAEIRNPSMAGLMVEMMGPWGEIIIAAGLIVSVCGAYLSW
TIMAAEVPFLAATHKAFPRIFARQNAQAAPSASLWLTNICVQICLVLIWLTGSDYNTLLT
IASEMILVPYFLVGAFLLKIATRPLHKAVGVGACIYGLWLLYASGPMHLLLSVVLYAPGL
LVFLYARKTHTHDNVLNRQEMVLIGMLLIASVPATWMLVG
>LFGLNPFC_01642 Protein YdgH
MKLKNTLLASALLSATAFSVNAATELTPEQAAAVKPFDRVVVTGRFNAIGEAVKAVSRRA
DKEGAASFYVVDTSDFGNSGNWRVVADLYKADAEKAEETSNRVINGVVELPKDQAVLIEP
FDTVTVQGFYRSQPEVNDAITKAAKAKGAYSFYIVRQIDANQGGNQRITAFIYKKDAKKR
IVQSPDVIPADSEAGRAALAAGGEAAKKVEIPGVATTASPSSEVGRFFETQSSKGGRYTV
TLPDGTKVEELNKATAAMMVPFDSIKFSGNYGNMTEVSYQVAKRAAKKGAKYYHITRQWQ
ERGNNLTVSADLYK
>LFGLNPFC_01643 NAD(P) transhydrogenase subunit alpha
MRIGIPRERLTNETRVAATPKTVEQLLKLGFTVAVESGAGQLASFDDKAFVQAGAEIVEG
NSVWQSEIILKVNAPLDDEIALLNPGTTLVSFIWPAQNPELMQKLAERNVTVMAMDSVPR
ISRAQSLDALSSMANIAGYRAIVEAAHEFGRFFTGQITAAGKVPPAKVMVIGAGVAGLAA
IGAANSLGAIVRAFDTRPEVKEQVQSMGAEFLELDFKEEAGSGDGYAKVMSDAFIKAEME
LFAAQAKEVDIIVTTALIPGKPAPKLITREMVDSMKAGSVIVDLAAQNGGNCEYTVPGEI
FTTENGVKVIGYTDLPGRLPTQSSQLYGTNLVNLLKLLCKEKDGNITVDFDDVVIRGVTV
IRAGEITWPAPPIQVSAQPQAAQKAAPEVKTEEKCACSPWRKYALMALAIILFGWMASVA
PKEFLGHFTVFALACVVGYYVVWNVSHALHTPLMSVTNAISGIIVVGALLQIGQGGWVSF
LSFIAVLIASINIFGGFTVTQRMLKMFRKN
>LFGLNPFC_01644 NAD(P) transhydrogenase subunit beta
MSGGLVTAAYIVAAILFIFSLAGLSKHETSRQGNNFGIAGMAIALIATIFGPDTGNVGWI
LLAMVIGGAIGIRLAKKVEMTEMPELVAILHSFVGLAAVLVGFNSYLHHDAGMAPILVNI
HLTEVFLGIFIGAVTFTGSVVAFGKLCGKISSKPLMLPNRHKMNLAALVVSFLLLIVFVR
TDSVGLQVLALLIMTAIALVFGWHLVASIGGADMPVVVSMLNSYSGWAAAAAGFMLSNDL
LIVTGALVGSSGAILSYIMCKAMNRSFISVIAGGFGTDGSSTGDDQEVGEHREITAEETA
ELLKNSHSVIITPGYGMAVAQAQYPVAEITEKLRARGINVRFGIHPVAGRLPGHMNVLLA
EAKVPYDIVLEMDEINDDFADTDTVLVIGANDTVNPAAQDDPKSPIAGMPVLEVWKAQNV
IVFKRSMNTGYAGVQNPLFFKENTHMLFGDAKASVDAILKAL
>LFGLNPFC_01645 AI-2 transport protein TqsA
MAKPIITLNGLKIVIMLGMLVIILCGIRFAAEIIVPFILALFIAVILNPLVQHMVRWRVP
RVLAVSILMTIIVMAMVLLLAYLGSALNELTRTLPQYRNSIMTPLQAIEPLLQRVGIDVS
VDQLAHYIDPNAAMTLLTNLLTQLSNAMSSIFLLLLTVLFMLLEVPQLPGKFQQMMARPV
EGMAAIQRAIDSVSHYLVLKTAISIITGLVAWAMLAALDVRFAFVWGLLAFALNYIPNIG
SVLAAIPPIAQVLVFNGFYEALLVLAGYLLINLVFGNILEPRIMGRGLGLSTLVVFLSLI
FWGWLLGPVGMLLSVPLTIIVKIALEQTAGGQSIAVLLSDLNKE
>LFGLNPFC_01646 Spermidine export protein MdtJ
MYIYWILLGLAIATEITGTLSMKWASVSEGNGGFILMLVMISLSYIFLSFAVKKIALGVA
YALWEGIGILFITLFSVLLFDESLSLMKIAGLTTLVAGIVLIKSGTRKARKPELEVNHGA
V
>LFGLNPFC_01647 Spermidine export protein MdtI
MAQFEWVHAAWLALAIVLEIVANVFLKFSDGFRRKIFGLLSLAAVLAAFSALSQAVKGID
LSVAYALWGGFGIAATLAAGWILFGQRLNRKGWIGLVLLLAGMIMVKLA
>LFGLNPFC_01648 hypothetical protein
MRTTIAVVLGAISLTSAFVFADKPDVAKSANDEVSTLFFGHDDRVPVNDTTQSPWDAVGQ
LETASGNLCTATLIAPNLALTAGHCLLTPPKGKADKAVALRFVSNKGLWRYEIHDIEGRV
DPTLGKRLKADGDGWIVPPAAAPWDFGLIVLRNPPSGITPLPLFEGDKAALTAALKAAGR
KVTQAGYPEDHLDTLYSHQNCEVTGWAQTSVMSHQCDTLPGDSGSPLMLHTDDGWQLIGV
QSSAPAAKDRWRADNRAISVTGFRDKLDQLSQK
>LFGLNPFC_01649 Acid shock protein
MKKVLALVVAAAMGLSSAAFAAETATTPAPTATTTKAAPAKTTHHKKQHKAAPAQKAQAA
KKHHKNAKAEQKAPEQKAQAAKKHAKKHSHQQPAKPAAQPAA

>LFGLNPFC_01650 Inner membrane transport protein YnfM
MSRTTTVDGAPASDTDKQSISQSNQFIKRGTPQFMRVTLALFSAGLATFALLYCVQPILP
VLSQEFGLTPANSSISLSISTAMLAIGLLFTGPLSDAIGRKPVMVTALLLASICTLLSTM
MTSWHGILIMRALIGLSLSGVAAVGMTYLSEEIHPSFVAFSMGLYISGNSIGGMSGRLIS
GVFTDFFNWRIALAAIGCFALASALMFWKILPESRHFRPTSLRPKTLFINFRLHWRDRGL
PLLFAEGFLLMGSFVTLFNYIGYRLMLSPWHVSQAVVGLLSLAYLTGTWSSPKAGTMTTR
YGRGPVMLFSTGVMLFGLLMTLFSSLWLIFAGMLLFSAGFFAAHSVASSWIGPRAKRAKG
QASSLYLFSYYLGSSIAGTLGGVFWHNYGWNGVGAFIALMLVIALLVGTRLHHRLHA
>LFGLNPFC_01651 Hca operon transcriptional activator HcaR
MNLELRHLRYFVAVAEELHFGRAAARLNISQPPLSQQIQALEQQIGARLLARTNRSVLLT
EAGKQFLVDSRQILSMVDDAAARAERLHQGEAGELRIGFTSSAPFIRAVSDTLSLFRRDY
PDVHLQTREMNTREQIAPLIEGTLDMGLLRNTALPETLEHAVIVHEPLMAMIPHDHPLAN
NPSVTLAELAKEPFVFFDPHVGTGLYDDILGLMRRYNLTPVITQEVGEAMTIIGLVSAGL
GVSILPASFKRVQLNEMRWVPIAEEDAVSEMWLVWPKHHEQSPAARNFRIHLLNALR
>LFGLNPFC_01652 Protein mlc
MVAENQPGHIDQIKQTNAGAVYRLIDQLGPVSRIDLSRLAQLAPASITKIVREMLEAHLV
QELEIKEAGNRGRPAVGLVVETEAWHYLSLRISRGEIFLALRDLSSKLVVEESQELALKD
DSPLLDRIISHIDQFFIRHQKKLERLTSIAITLPGIIDTENGIVHRMPFYEDVKEMPLGE
ALEQHTGVPVYIQHDISAWTMAEALFGASRGARDVIQVVIDHNVGAGVITDGHLLHAGSS
SLVEIGHTQVDPYGKRCYCGNHGCLETIASVDSILELAQLRLNQSMSSMLHGQPLTVDSL
CQAALRGDLLAKDIITGVGAHVGRILAIMVNLFNPQKILIGSPLSKAADILFPVISDSIR
QQALPAYSQHISVESTQFSNQGTMAGAALVKDAMYNGSLLIRLLQG
>LFGLNPFC_01653 ATP-dependent dethiobiotin synthetase BioD 1
MLKRFFITGTDTSVGKTVVSRALLQALASQGKTVAGYKPVAKGSKETPEGLRNKDALVLQ
SVSTIELPYEAVNPIALSEEESSVAHSCPINYTLISNGLANLTDKVDHVVVEGTGGWRSL
MNDLRPLSEWVVQEQLPVLMVVGIQEGCINHALLTAQAIANDGLPLIGWVANRINPGLAH
YAEIIDVLGKKLPAPLIGELPYLPRAEQRELGQYIRLAMLRSVLAVDRVTV
>LFGLNPFC_01654 Voltage-gated ClC-type chloride channel ClcB
MFRRLLIATIVGILAAFAVAGFRHAMLLLEWLFLNNDSGSLVNAATNLSPWRRLLTPALG
GLAAGLLLMGWQKFTQQRPHAPTDYMEALQTDGQFDYAASLVKSLASLLVVTSGSAIGRE
GAMILLAALAASCFAQRLTPRQEWKLWIACGAAAGMAAAYRAPLAGSLFIAEVLFGTMML
ASLGPVIISAVVALLVSNLINHSDALLYSGPLLLTLMNACHRGFVSLKLAPPWQLALGGL
IVGLLSLFTPAVWGNGYSTVQSFLTAPPLLMIIAGIFLCKLCAVLASSGSGAPGGVFTPT
LFIGLAIGMLYGRSLGLWLPDGEEITLLLGLTGMATLLAATTHAPIMSTLMICEMTGEYQ
LLPGLLIACVIASVISRTLHRDSIYRQHTAKHS
>LFGLNPFC_01655 Tat proofreading chaperone DmsD
MTHFSQQDNFSVAARVLGALFYYAPESAEAAPLVAVLTNDSWETQWPLPEASLAPLVTAF
QTQSEETHAQAWQRLFVGPWALPSPPWGSVWLDRESVLFGDSTLALRQWMREKGIQFEMK
QNEPEDHFGSLLLMAAWLAENGRQTECEELLAWHLFPWSTRFLDVFIEKAEHPFYRALGE
LARLTLAQWQSQLLIPVAVKPLFR
>LFGLNPFC_01656 Anaerobic dimethyl sulfoxide reductase chain C
MGNGWHEWPLVIFTVLGQCVVGALIVSGIGWFAAKNNADRQHIIRGMFFLWLLMGIGFIA
SVMHLGSPLRAFNSLNRIGASGLSNEIAAGSIFFAVGGLWWLVAVIGKMPQALGKLWLLV
SMALGIIFVWMMTCVYQIDTVPTWHNGYTTLAFFLTVLLSGPILAATILRAARVTFNTTP
FAIISILALIACAGLIVLQGLSLASIHSSVQQASALVPDYASLQVWRVVLLCAGLGCWLC
PLIRRREPHVAGLVLGLILILGGEMIGRVLFYGLHMTVGMAIAG
>LFGLNPFC_01657 Anaerobic dimethyl sulfoxide reductase chain B
MTTQYGFFIDSSRCTGCKTCELACKDFKDLGPEVSFRRIYEYAGGDWQEDNGVWHQNVFA
YYLSISCNHCDDPACTKVCPSGAMHKREDGFVVVDEDVCIGCRYCHMACPYGAPQYNAEK
GHMTKCDGCYSRVAEGKQPICVESCPLRALEFGPIEELRQKHGTLAAVAPLPRAHFTKPN
IVIKPNANSRPTGDTTGYLANPEEV
>LFGLNPFC_01658 putative dimethyl sulfoxide reductase chain YnfF
MSAEISRRSLMKTSALGSLALASSAFTLPFSQMVRAAQAPVEEKAVWSSCTVNCGSRCLL
RLHVKDDTVYWVESDTTGDDVYGNHQVRACLRGRSIRRRMNHPDRLKYPMKRVGKRGEGK
FERISWDEALDTISDNLRRILKDYGNEAVHVLYGTGVDGGNITNSNVPYRLMNSCGGFLS
RYGSYSTAQISAAMSYMFGANDGNSPDDIANTKLVVMFGNNPAETRMSGGGVTYYVEQAR
ERSNARMIVIDPRYNDTAAGREDEWLPIRPGTDGALACAIAWVLITENMVDQPFLDKYCV
GYDEKTLPANAPRNAHYKAYILGEGPDGIAKTPEWAAKITSIPAEKIIQLAREIGSAKPA
YICQGWGPQRHSNGEQTSRAIAMLSVLTGNVGINGGNSGVREGSWDLGVEWFPMLENPVK
TQISVFTWTDAIDHGTEMTAPRDGVRGKEKLDVPIKFLWCYASNTLINQHGDINHTHEVL
QDDSKCEMIVGIDHFMTASAKYCDILLPDLMPTEQEDLISHESAGNMGYVILAQPATSAK
FERKPIYWMLSEVAKRLGPDVYQTFTEGRSQHEWIKYLHAKTKERNPEMPDYEEMKTTGI
FKKKCPEEHYVAFRAFRVDPQANPLKTPSGKIEIYSERLAKIADTWELKKDEIIHPLPAY
TPGFDGWDDPLRKTYPLQLTGFHYKARTHSSYGNIDVLQQACPQEVWINPIDAQARGIRH

GDTVRVFNNNGEMLIAAKVTPRILPGVTAIGQGAWLKADMFGDRVDHGGSINILTSHRPS
PLAKGNPSHSNLVQIEKV
>LFGLNPFC_01659 Putative dimethyl sulfoxide reductase chain YnfE
MSKNEQMVGISRRTLVKSTAIGSLALAAGGFSLPFTLRSAEAAVQQANEKVVWGACSVNC
GSRCALRLHVKDNEVTWVETDNTGSDEYGNHQVRACLRGRSIRRRINHPDRLNYPMKRVG
KRGEGKFERISWDEALDTIASSLKKTVKQYGNEAVYIQYSSGIVGGNMTRSSPSASAVKR
LMNCYGGSLNQYGSYSTAQISCAMPYTYGSNDGNSTTDIENSKLVVMFGNNPAETRMSGG
GITYLLEKAREKSNAKMIVIDPRYTDTAAGREDEWLPIRPGTDAALVAGIAWVLINENLV
DQPFLDKYCVGYDEKTLPADAPKNGHYKAYILGEGDDKTAKTPQWASQITGIPVDRIIKL
AREIGTAKPAYICQGWGPQRQANGELTARAIAMLPILTGNVGISGGNSGARESTYTITIE
RLPVLDNPVKTSISCFSWTDAIDHGPQMTAIRDGVRGKDKLDVPIKFIWNYAGNTLVNQH
SDINKTHEILQDESKCEMIVVIENFMTSSAKYADILLPDLMTVEQEDIIPNDYAGNMGYL
IFLQPVTSEKFERKPIYWILSEVAKRLGPDVYQKFTEGRTQEQWLQHLYAKMLAKDPALP
SYDELKKMGIYKRKDPNGHFVAYKAFRDDPEANPLKTPSGKIEIYSSKLAEIARTWELEK
DEVISPLPVYASTFEGWDSPERSAFPLQLFGFHYKSRTHSTYGNIDVLKAACRQEVWINP
IDAQKRGIANGDMVRVFNHRGEVRLPAKVTPRILPGVSAMGQGAWHEANMSGDKIDHGGC
VNTLTTLRPSPLAKGNPQHTNLVEIEKI
>LFGLNPFC_01660 putative protein YnfD
MKLSTCCAALLLALASPAVLAAPGSCERIQSDISQRIINNGVPESSFTLSIVPNDQVDQP
DSQVVGHCANDTHKILYTRTTSGNVSAPAQSTQDNAPAEPQ
>LFGLNPFC_01661 hypothetical protein
MIENYLYSQVTVVKYKLLPCLLAILLTGCDRTEVTLSFTPEMASFSNEFDFDPLRGPVKD
FTQTLMDEQGEVTKRVSGTLSEEGCFDSLELLDLENNTLVALVLDANYYRDAETLEKRVR
LQGKCQLAELPSAGVSWETDDNGFVIKASSKQMQMEYRYDDQGYPLGKTTKSNDKTLSVS
ATPSTDPIKKLDYTAVTLLNNQRVGNVKQSCEYDNHANPVDCQLIIVDEGVKPAVERVYT
IKNTIDYY
>LFGLNPFC_01662 Spermidine N(1)-acetyltransferase
MPSAHSVKLRPLEREDLRYVHQLDNNASVMRYWFEEPYEAFVELSDLYDKHIHDQSERRF
VVECDGEKAGLVELVEINHVHRRAEFQIIISPEYQGKGLATRAAKLAMDYGFTVLNLYKL
YLIVDKENEKAIHIYRKLGFTVEGELMHEFFINGQYRNAIRMCIFQHQYLAEHKTPGQTL
LKPTAQ
>LFGLNPFC_01663 hypothetical protein
MKITLSKRIGLLAFLLPCALALSTTVHAETNKLVIESGDSAQSRQRAAMEKEQWNDTRNL
RQKVNKRTEKEWDKADAAFDNRDKCEQSANINAYWEPNTLRCLDRRTGRVIIP
>LFGLNPFC_01664 hypothetical protein
MIKTTLLFFATALCEIIGCFLPWLWLKRNASIWLLLPAGISLALFVWLLTLHPAASGRVY
AAYGGVYVCTALIWLRVVDGVKLTLYDWTGALIALCGMLIIVAGWGRT
>LFGLNPFC_01665 D-galactonate dehydratase family member RspA
MKIVKAEVFVTCPGRNFVTLKITTEDGITGLGDATLNGRELSVASYLQDHLCPQLIGRDA
HRIEDIWQFFYKGAYWRRGPVTMSAISAVDMALWDIKAKAANMPLYQLLGGASREGVMVY
CHTTGHSIDEALDDYARHQELGFKAIRVQCGIPGMKTTYGMSKGKGLAYEPATKGQWPEE
QLWSTEKYLDFMPKLFDAVRNKFGFDEHLLHDMHHRLTPIEAARFGKSIEDYRMFWMEDP
TPAENQECFRLIRQHTVTPIAVGEVFNSIWDCKQLIEEQLIDYIRTTLTHAGGITGMRRI
ADFASLYQVRTGSHGPSDLSPVCMAAALHFDLWVPNFGVQEYMGYSEQMLEVFPHNWTFD
NGYMHPGEKPGLGIEFDEKLAVKYPYEPAYLPVARLEDGTLWNW
>LFGLNPFC_01666 Starvation-sensing protein RspB
MKSILIEKPNQLSIIEREIPTPSAGEVRVKVKLAGICGSDSHIYRGHNPFAKYPRVIGHE
FFGVIDAVGDGVESARVGERVAVDPVVSCGHCYPCSIGKPNVCTTLAVLGVHADGGFSEY
AVVPAKNAWKIPEAVADQYAVMIEPFTIAANVTGHGQPTENDTVLVYGAGPIGLTIVQVL
KGVYNVKNVIVADRIDERLEKAKESGADWAINNSQTPLGESFAEKGIKPTLIIDAACHPS
ILKEAVTLASPAARIVLMGFSSEPSEVIQQGITGKELSIFSSRLNANKFPVVIDWLSKGL
IKPEKLITHTFDFQHVADAISLFEQDQKHCCKVLLTFSE
>LFGLNPFC_01667 Putative transporter YdfJ
MAISKEIGGLIASGFGPILAGIFCTMTESWYPIAIMIMAYSVIGLISALKMPEVKDRDLS
ALEDAAEDQPHVVRAAQPSRSL
>LFGLNPFC_01668 Polyol:NADP oxidoreductase
MGNHLLSAKATLPVYDRNNLAPRIVHLGFGAFHRAHQGVYADILATEHFSDWGYYEVNLI
GGEQQIADLQQQDNLYTVAEMSADAWTARVVGVVKKALHVQIDGLETVLAAMCEPQIAIV
SLTITEKGYFHSPATGQLMLDHPMVVADVQNPHQPKTATGVIVEALARRKAAGLPAFTVM
SCDNMPENGHVMRDVVTSYAQAIDVKLAQWIEDNVTFPSTMVDRIVPAVTEDTLAKIEQL
TGVRDAAGVACEPFRQWVIEDNFVAGRPEWEKAGAELVSDVLPYEEMKLRMLNGSHSFLA
YLGYLAGYQHINDCMEDEHYRHAAYTLMLQEQAPTLKVQGVDLQDYANRLIERYSNPALR
HRTWQIAMDGSQKLPQRMLDSVRWHLAHDSKFDLLALGVAGWMRYVGGVDEQGNPIEISD
PLLPVIQKAVQSSAEGTARVQSLLAIKAIFGDDLPGNSLFTTKVTEAYLSLLAHGAKATV

AKYSVK
>LFGLNPFC_01669 Putative selenoprotein YdfZ
MTTYDRNRNAITTGSRVMVSGTGHTGKILSIDTEGLTAEQIRRGKTVVVEGCEEKLAPLD
LIRLGMN
>LFGLNPFC_01670 HTH-type transcriptional repressor RspR
MTVETQLNPTQPVNQQIYRILRRDIVHCLIAPGTPLSEKEVSVRFNVSRQPVREAFIKLA
ENGLIQIRPQRGSYVNKISMAQVRNGSFIRQAIECAVARRAASMITESQCYQLEQNLHQQ
RIAIERKQLDDFFELDDNFHQLLTQIADCQLAWDTIENLKATVDRVRYMSFDHVSPPEML
LRQHLDIFSALQKRDGDAVERAMTQHLQEISESVRQIRQENSDWFSEE
>LFGLNPFC_01671 NADP-dependent 3-hydroxy acid dehydrogenase YdfG
MIVLVTGATAGFGECITRRFIQQGHKVIATGRRQERLQELKDELGDNLYIAQLDVRNRAA
IEEMLASLPAEWSNIDILVNNAGLALGMEPAHKASVEDWETMIDTNNKGLVYMTRAVLPG
MVERNHGHIINIGSTAGSWPYAGGNVYGATKAFVRQFSLNLRTDLHGTAVRVTDIEPGLV
GGTEFSNVRFKGDDGKAEKTYQNTVALTPEDVSEAVWWVSTLPAHVNINTLEMMPVTQSY
AGLNVHRQ
>LFGLNPFC_01672 Dipeptidyl carboxypeptidase
MTTMNPFLVQSTLPYLAPHFDQIANHHYRPAFDEGIQQKRAEIAAIALNPQTPDFKNTIL
ALEQSGELLTRVTSVFFAMTAAHTNDELQRLDEQFSAELAELANDIYLNGELFARVDAVW
QRRESLGLDSESIRLVEVIHQRFVLAGAKLEQADKAKLKVLNTEAATLTSQFNQRLLAAN
KSGGLVVNDIAQLAGMSEQEIALAAEAAREKGLDNNWLIPLLNTTQQPVLAELRDRATRE
KLFTAGWTRAEKNDANDTRAIIQRLVEIRVQQAKLLGFPHYAAWKIADQMAKTPEAALNF
MREIVPAARQRASDELASIQAVIDKQQGGFSAQPWDWAFYAEQVRREKFDLDESQLKPYF
ELNTVLNEGVFWTANQLFGIKFVERFDIPVYHPDVRVWEIFDHNGVGLALFYGDFFARDS
KSGGAWMGNFVEQSTLNETHPVIYNVCNYQKPAAGEPALLLWDDVITLFHEFGHTLHGLF
ARQRYATLSGTNTPRDFVEFPSQINEHWATHPQVFARYARHYQSGAAMPDELQQKMRNAS
LFNKGYEMSELLSAALLDMRWHCLEENEAMQDVDDFELRALVAENMDLPAIPPRYRSSYF
AHIFGGGYAAGYYAYLWTQMLADDGYQWFVEQGGLTRENGRRFREAILSRGNSEDLERLY
RQWRGKAPQIMPMLQHRGLNV
>LFGLNPFC_01673 Diguanylate cyclase DgcZ
MIKKTTEIDAILLNLNKAIDAHYQWLVSMFRSVVARDASKPEITDNHSYGLCQFGRWIDH
LGPLDNDELPYVRLMDSAHQHMHNCGRELMLAIVENHWQDAHFDAFQEGLLSFTAALTDY
KIYLLTIRSSMDVLTGLPGRRVLDESFDHQLRNAEPLNLYLMLLDIDRFKLVNDTYGHLI
GDVVLRTLATYLASWTRDYETVYRYGGEEFIIIVKATNDEEACRAGVRICQLVDNHAITH
SEGHINITVTAGVSRAFPEEPLDVVIGRADRAMYEGKQTGRNRCMFIDEQNVINRV
>LFGLNPFC_01674 Small protein MgtS
MLGNMNVFMAVLGIILFSGFLAAYFSHKWDD
>LFGLNPFC_01675 Na(+), Li(+), K(+)/H(+) antiporter
MNLSLRRSTSALLASSLLLTIGRGATLPFMTIYLSRQYSLSVDLIGYAMTIALTIGVIFS
LGFGILADKFDKKRYMLLAITAFASGFIAIPLVNNVTLVVLFFALINCAYSVFATVLKAW
FADNLSSNSKTKIFSINYTMLNIGWTIGPPLGTLLVMQSINLPFWLAAICSAFPMLFIQI
WVKRSEKIIATETGSVWSPKVLLQDKALLWFTCSGFLASFVSGAFASCISQYVMVIADGD
FAEKVVAVVLPVNAAMVVTLQYSVGRRLNPANIRALMTAGTLCFVIGLVGFIFSGNSLLL
WGMSAAVFTVGEIIYAPGEYMLIDHIAPPGMKASYFSAQSLGWLGAAINPLVSGIVLTSL
PPFSLFIILALVIVVAWVLMLKGIRARPWGQPALC
>LFGLNPFC_01676 putative amino-acid metabolite efflux pump
MSRKDGVLALLVVVVWGLNFVVIKVGLHNMPPLMLAGLRFMLVAFPAIFFVARPKVPLNL
LLGYGLTISFAQFAFLFCAINFGMPAGLASLVLQAQAFFTIVLGVFTFGERLHGKQLAGI
ALAIFGVLVLIEDSLNGQHVAMLGFMLTLAAAFSWACGNIFNKKIMSHATRPAVMSLVIW
SALIPIIPFFVASLILDGSATMIHSLVTIDMTTILSLMYLAFVATIVGYGIWGTLLGRYE
TWRVAPLSLLVPVVGLASAALLLDERLTGLQFFGAVLIMTGLYINVFGLRWRKAVKVRG
>LFGLNPFC_01677 Mannosyl-D-glycerate transport/metabolism system repressor MngR
MIFQKIARLLKSEINGNSWHVGDLLPSEAELAVRYNVSRNTLRKALSLLEGEGIIHRKHG
SGTYIQKKNFVAHIDHMNSFSEIAHKSGKEAGSQIMKFEVQDASPTIATELNLVTGEQVY
YIKRLRFIEDNAAQLEETWMSVARFPDLTVSHMQKSKFSYIENECGIKIIGTFETFSPTF
PTPEIASILRISPRDPILKIQTQAVDSNSIPLDYSLLYSNIFEFQVKYFFPR
>LFGLNPFC_01678 PTS system cellobiose-specific EIIB component
MKKILLVCAAGMSTSMLVKRMIDHATAISLEVNISALAIAEAKGKIKNNEVDVVLLGPQV
RFQKPEIEAVAQGKMPVAVIEMKDYGTMNGQAVLEFAMKLLQE
>LFGLNPFC_01679 PTS system N,N'-diacetylchitobiose-specific EIIC component
MGLMASFERGMERFLVPVAIKLNSQKHVAAVRDGFVFTFPIIMASSLIILINFAILSPDG
FIAGLLHLNSIFPNLEKAQAIFTPVMNGSVNIMSIMIAFLVARNVAISYEQDDLLCGLTA
IGAFFIVYTPYQMIDGQAFLTTKYLGAQGLFVAVIVALITSEIFCRLARNPKITITMPAA
VPPAVARSFKVLLPIFFVMVFFSALNYCLTLISPAGLNDLIYTLIQTPLKHMGTNIFAVI
ILGAVGNFLWVLGIHGPNTTSAIRETVFSEANLENLSWAAQHGTTWGAPYPITWTSINDA

FANCGGSGMTLGLLLAIFIASKRAEYRDLAKMSFIPGIFNINEPIMFGLPIVLNPIMMVP
FIMVPIVNCAIGYFFVSMEIIPPVAYAVPWTTPGPLIAFLGTGGNWLALLVGFLCLGVAI
MIYLPFVIAANKVNNMATNG
>LFGLNPFC_01680 PTS system N,N'-diacetylchitobiose-specific EIIA component
MFADEELVMELLINAGQARSDAMEAIRCAGQKDWQGATKLMASSESACLQAHKIQTALIS
QDEGCGKIEVNLILIHAQDHLMNAILCQDLAREIISLRKELHA
>LFGLNPFC_01681 Cryptic outer membrane porin BglH
MNIKTLNVSLLSISIITALFPLNAMATKLTIEQRLELLENELSQNKQELKATQNELGVYK
FRLSTLQKSITENKYQSASLAEISATSPVADNIKNENGEQNSFAAAHTINGSQQIAVIES
KGDKTTIESVTLKDISKYIKDDIGFSYQGYFRSGWGTGNHGSPQTYAAGSLGRFGNEMSG
WFDLTLNQRVYNQDGRTANAVVTYDGNVGQQYNDAWFGDSANENIMQFSDIYLTTRGFLP
FAREAEFWVGKHKLPQYEIQMLDWKTLTTDVAAGVGIENWALGVGLFDMSLSRDDVDVYS
RDFTRTSQMNTNSVDVRYRNIPLWDDATLSLMAKYSAPNKTDQQQDNENDDSYFEMKDSW
MLASVLRQNLQRDTFNEFTLQVANNSYASSFASFSDASNTMAHGRYYYGDHTNGIAWRLI
SQGEMYLTDNIIMANALVYSHGEDVYSYESGAHSDFDSIRTVIRPAWIWNTWNQTGLELG
WFKQQNKTQQGVTLNESAYKTTLWHALKVGESILGSRPEIRFYGTYINILDNELSNFKFN
ENSKDEFMAGIQAEVWW
>LFGLNPFC_01682 Aryl-phospho-beta-D-glucosidase BglA
MSGFKEDFLWGGAVAAHQLEGGWNEGGKGISIADVMTAGAHGVPREVTEGVIDGLNYPNH
EAIDFYHRYKTDIQLFAGMGFKCFRTSIAWTRIFPQGDEQEPNEEGLQFYDDLFDECLKQ
GMEPVVTLSHFEMPYHLVTKYGGWRNRKLIDFFIHFASTVFTRYKAKVKYWMTFNEINNQ
VNFSESLCPFTNSGILYSPEEDLNEREQIMYQAVHYELVASALAVQTGKLINPEFNIGCM
IAMCPIYPLTCAPNDMMMATKAMHRRYWFTDVHARGYYPQHMLNYFARKGFNLDITPDDN
AILARGCVDFIGFSYYMSFTTQFSPDNPQLDYVEPRDLVSNPYIDTSEWGWQIDPAGLRY
SLNWFWDHFQLPLFIVENGFGAVDQRQADGTVNDHYRIDYFSSHIREMKKAVVEDGVDLI
GYTPWGCIDLVSAGTGEMKKRYGMIYVDKDNEGKGTLERIRKASFYWYRDLIANNGENI
>LFGLNPFC_01683 hypothetical protein
MKPLLSAIATALILFSAQGVAEQTTQPVVTSCGNVVVVPTSQEQPPFDLNHMGTGSDKSD
ALGVPYYNQHAM
>LFGLNPFC_01684 Multiple antibiotic resistance protein MarA
MSRRNTDAITIHSILDWIEDNLESPLSLEKVSERSGYSKWHLQRMFKKETGHSLGQYIRS
RKMTEIAQKLKESNEPILYLAERYGFESQQTLTRTFKNYFDVPPHKYRMTNMQGESRFLH
PLNHYNN
>LFGLNPFC_01685 Multiple antibiotic resistance protein MarR
MKNTSDLFNEIIPLGRLIHMVNQKKDRLLNEYLSPLDITAAQFKVLCSIRCAACITPVEL
KKVLSVDLGALTRMLDRLVCKGWVERLPNPNDKRGVLVKLTTSGAAICEQCHQLVGQDLH
QELTKNLTADEVATLEHLLKKVLP
>LFGLNPFC_01686 hypothetical protein
MLDLFKAIGLGLVVLLPLANPLTTVALFLGLAGNMSSAERNRQSLMASVYVFAIMMVAYY
AGQLVMDTFGISIPGLRIAGGLIVAFIGFRMLFPQQKAIDSPEAKSKSEELEDEPSANIA
FVPLAMPSTAGPGTIAMIISSASTVRQSSTFADWVLMVAPPLIFFLVAVILWGSLRSSGA
IMRLVGKGGIEAISRLMGFLLVCMGVQFIINGILEIIKTYH
>LFGLNPFC_01687 Sugar efflux transporter
MTTNTVSRKVAWLRVVTLAVAAFIFNTTEFVPVGLLSDIAHSFHMQTAQVGIMLTIYAWV
VALMSLPFMLMTSQVERRKLLICLFVVFIASHVLSFLSWSFTVLVISRIGVAFAHAIFWS
ITASLAIRMAPAGKRAQALSLIATGTALAMVLGLPLGRIVGQYFGWRMTFFAIGIGALIT
LLCLIKLLPLLPSEHSGSLKSLPLLFRRPALMSIYLLTVVVVTAHYTAYSYIEPFVQNIA
GFSANFATALLLLLGGAGIIGSVIFGKLGNQYASALVSTAIALLLVCLALLLPAANSEIH
LGVLSIFWGIAMMIIGLGMQVKVLALAPDATDVAMALFSGIFNIGIGAGALVGNQVSLHW
SMSMIGYVGAVPAFAALIWSIIIFRRWPVTLEEQTQ
>LFGLNPFC_01688 HTH-type transcriptional regulator GltR
MDLTQLEMFNAVAEAGSITQAAAKVHRVPSNLTTRLRQLETELGVDLFIRENQRLRLSPA
GHNFLRYSQQILTLVDEARSVVAGDEPQGLFSLGSLESTAAVRIPATLAEFNRRYPKIQF
SLSTGPSGTMLEGVLEGKLNAAFIDGPINHTAIDGIPVYREELMIVTPQGHAPVTRASQV
NGSNIYAFRANCSYRRHFESWFHADGAAPGTIHEMESYHGMLACVVAGAGIALIPRSMLE
SMPGHHQVEAWPLAEQWRWLTTWLVWRRGAKTRPLEAFIQLLDAPDSAKQGYQ
>LFGLNPFC_01689 Succinate semialdehyde dehydrogenase [NAD(P)+] Sad
MTITPATHAISINPATGEQLSVLPWAGVDDIENALQLAAAGFRDWRETNIDYRAEKLRDI
GKALRARSEEMAQMITREMGKPINQARAEVAKSANLCDWYAEHGPAMLKAEPTLVENQQA
VIEYRPLGTILAIMPWNFPLWQVMRGAVPIILAGNGYLLKHAPNVMGCAQLIAQVFKDAG
IPQGVYGWLNADNDGVSLMIKDSRIAAVTVTGSVRAGAAIGAQAGAALKKCVLELGGSDP
FIVLNDADLELAVKAAVAGRYQNTGQVCAAAKRFIIEEGIASAFTERFVAAAAALKMGDP
RDEENALGPMARFDLRDELHHQVEKTLAQGARLLLGGEKIAGAGNYYPPTVLANVTPEMT
AFREEMFGPVAAITIAKDAEHALELANDSEFGLSATIFTTDETQARQMAARLECGGVFIN

GYCASDARVAFGGVKKSGFGRELSHFGLHEFCNIQTVWKDRN
>LFGLNPFC_01690 Glutaminase 2
MAVAMDNAILENILRQVRPLIGQGKVADYIPALATVDGSRLGIAICTVDGQLFQAGDAQE
RFSIQSISKVLSLVVAMRHYSEEEIWQRVGKDPSGSPFNSLVQLEMEQGIPRNPFINAGA
LVVCDMLQGRLSAPRQRMLEVVRGLSGVSDISYDTVVARSEFEHSARNAAIAWLMKSFGN
FHHDVTTVLQNYFHYCALKMSCVELARTFVFLANQGKAIHIDEPVVTPMQARQINALMAT
SGMYQNAGEFAWRVGLPAKSGVGGGIVAIVPHEMAIAVWSPELDDAGNSLAGIAVLEQLT
KQLGRSVY
>LFGLNPFC_01691 hypothetical protein
MQSLDPLFARLSRSKFRSRFRLGVKERQYCLEKGAPVIEQHAADFVAKRLAPALPANDGK
QTPMRGHPVFIAQHATATCCRGCLAKWHNIPQGEALSEEQQRYIVTVIYHWLVIQMNQP
>LFGLNPFC_01692 hypothetical protein
MHVQPISTFRLFQEGHLLRNSIAIFVLTTLFYFIGAELRLVHELSLFWPLNGVMAGVFAR
YVWLNRLHYYAISYVAMLVYDAITTEWGLVSLAINFSNMMFIVTVALLVARDKSLGKNKY
EPVSALRLFNYCLLAALLCAIVGAIGSVSIDSLDFWPLLADWFSEQFSTGVLIVPCMLTL
AIPGVLPRFKAEQMMPAIALIVSVIASVVIGGAGSLAFPLPALIWCAVRYTPQVTCLLTF
VTGAVEVVLVANSVIDISVGSPFSIPQMFSARLGIATMAICPIMVSFSVAAIHSLMKQVA
LRADFDFLTQVYSRSGLYEALKSPSLKQTQHLTVMLLDIDYFKSINDNYGHECGDKVLSV
FAQHIQKIVGDKGLVARMGGEEFAVAVPSVNPVDGLLMAEKIRKGVELQPFTWQQKTLYL
TVSIGVGSGCASYRTLTDDFNKLMVEADTCLYRSKKDGRNRTSTMRYGEEVV
>LFGLNPFC_01693 hypothetical protein
MQYVLPLYDPRKGLFPRLNHDPRHTFLIKLLINHQELIKTGVRRIKVC
>LFGLNPFC_01694 Altronate oxidoreductase
MKTLNRRDFPGAQYPERIIQFGEGNFLRAFVDWQIDLLNEHTDLNSGVVVVRPIETSFPP
SLSTQDGLYTTIIRGLNEKGEAVSDARLIRSVNREISVYSEYDEFLKLAHNPEMRFVFSN
TTEAGISYHAGDKFDDAPAVSYPAKLTRLLFERFSHFNGALDKGWIIIPCELIDYNGDAL
RELVLRYAQEWALPEAFIQWLDQANSFCSTLVDRIVTGYPRDEVAKLEEELGYHDGFLDT
AEHFYLFVIQGPKSLATELRLDKYPLNVLIVDDIKPYKERKVAILNGAHTALVPVAFQAG
LDTVGEAMNDAEICAFVEKAIYEEIIPVLDLPRDELESFASAVTGRFRNPYIKHQLLSIA
LNGMTKFRTRILPQLLAGQKANGTLPARLTFALAALIAFYRGERNGETYPVQDDAHWLER
YQQLWSQYRDRVIGTQELVAIVLAEKDHWEQDLTQVPGLVEQVANDLDAILEKGMREAVR
PLC
>LFGLNPFC_01695 hypothetical protein
MIVRPQQHWLRRIFVWHGSVLSKISSRLLLNFLFSIAVIFMLPWYTHLGIKFTLAPFSIL
GVAIAIFLGFRNNAGYARYVEARKLWGQLMIASRSLLREVKTTLPDSASVREFARLQIAF
AHCLRMTLRKQPQAEVLAQYLKTEDLQRVLASNSPANRILLIMGEWLAVQRRNGQLSDIL
FISLNDRLNDISAVLAGCERIAYTPIPFAYTLILHRTVYLFCIMLPFALVVDLHYMTPFI
SVLISYTFISLDCLAEELEDPFGTENNDLPLDAICNAIEIDLLQMNDEAEIPAKILPDRH
YQLT
>LFGLNPFC_01696 Trans-aconitate 2-methyltransferase
MSDWNPSLYLHFAAERSRPAVELLARVSLENIEYIADLGCGPGNSTALLHQRWPAARITG
IDSSPAMIAEARSALPDCLFVEADIRNWQPEQALDLIFANASLQWLPDHYELFPHLVSLL
SPLGVLAVQMPDNWLEPTHVLMREVAWEQNYPDRGREPLAGVHAYYDILSEAGCEVDIWR
TTYYHQMPSHQAIIDWVTATGLRPWLQDLTESEQQHFLTRYHQMLEEQYPLQENGQILLA
FPRLFIVARRTE
>LFGLNPFC_01697 Antitoxin HipB
MMSFQKIYSPMQLANAMKLVRQQNGWTQSELAKKIGIKQATISNFENNPNNTSLTTFFKI
LQSLELSMTLCDAKNASPEATEQQDLEW
>LFGLNPFC_01698 Serine/threonine-protein kinase toxin HipA
MPKLVTWMNNQRVGELTKLANGAHTFKYAPEWLASRYARPLSLSLPLQRRNITSDAVFNF
FDNLLPDSPIVRDRIVKRYHAKSRQPFDLLSEIGRDSVGAVTLIPEDETVMCPIMAWEKL
TEARLEEVLTAYKADIPLGMIREENDFRISVAGAQEKTALLRIGNDWCIPKGITPTTHII
KLPIGEIRQPNATLDLSQSVDNEYYCLLLAKELGLNVPDAEIIKAGRVRALAVKRFDRRW
NTERTVLLRLPQEDMCQTFGLPSSVKYESDGGPGIAQIMAFLMGSSEALKDRYDFMKFQV
FQWLIGATDGHAKNFSVFIQAGGSYRLTPFYDIISAFPVLGGTGIHISDLKLAMGLNASK
GKKTAIDKIYPRHFLATAKVLKFPEVQMHEILSDFARMIPAALDNVKTSLPTDFPENVVT
AVETNVLRLHGRLSREYGIK
>LFGLNPFC_01699 hypothetical protein
MNALLGKIAGAVATFFVALILNKFFSTFRIRQLYLAYEHILEHTTQSITGYTVMFTVTNK
GKEKEKNVTITIPKSKSISLISANNVSIECKDNKIIIDRILPKENITLIVLVKNGGVLSK
SNLPILKSEDANGKCYDSIKKIPPSLGPAFFSFSLFIAFLVLMGGAIYKGYDPFEVTYTQ
YFKMRFTPFYDRGFENNSFQNNSLTKQYDTSKSDFPIDLKSLNVKDGKIEYVFIIDNKTN
YNLSADMKFIVRDENQYYDELGKVLNIRNDADRRQALHELRDKYHVAEDTTGATYSISEW
IDKGQRKEIKMTRQLSKGIDYKDLGVDIRIRNDANDDNYYGDYQFKPERSAIAKNKLDAA

IATQRFPLK
>LFGLNPFC_01700 hypothetical protein
MSRNNETSGVELVVVGIFAFCLAVVAWLMKTFDVEWQTALETAPSLIVWLLVVGAGIFFG
IKMETGLVRWGAPLAIALLIPVFKPILKEAAGVRETGGLVFDDMVSWYGTGWGMSLMFFG
ILIIGYGLLYWWHRRNSYHW
>LFGLNPFC_01701 hypothetical protein
MGNNEHEVMADKQRAERIKKVILEHSTYEELAEKTGISVSTLVRIASGKTEPKFSDIIQI
AKITGADLNTLAYGYALDVKEEATERKLITSADGYTDEETTNAHNFIVWNIRTLEKQDIL
ALARQVSALSSYTYSTKMFMKKAVSGEE
>LFGLNPFC_01702 hypothetical protein
MIDWFTGILPCTHRPLPAGSVVSVNADGAVEWETVKRLTVRGSHESTMKVRSVGSDGEGR
ATHLYIDGNPSKFLQGHSVIGSDDLQGLVLTAYARILALLHIPHDLPSYRQVMEGQFKIS
RIDINYMYSLSTLENVRAWLYAAEFKAKTRHGRACGKGGTVYLGKNSRRWSLKFYSKYDE
HTSGKKGHQMADEFVKAGLLDWSKDKLRIELTLRTTELIDLNLTLGNSWNIETPNKLFSD
YVGRIEMNQNTILTDEK
>LFGLNPFC_01703 hypothetical protein
MKEMLPKPTFYRHRKELLSFGIDINFYCESPDSNNVVPLVRTLEAKPAKIPSWVYEKGLI
FDYNRISHASNWH
>LFGLNPFC_01704 hypothetical protein
MSNYGLFVKGKMLGARQRNKVNGQGYYNEIGVGLEIPDGFGGTKQDQIIIRVSQALVNAG
VMNQANNFIGKLVQIPVYVRVWSMEGREGVTYNISSDGGITEIKG
>LFGLNPFC_01705 hypothetical protein
MDTSGFDIQFDNHIPENGYRIEGYLCNANNAKECQAIMVRSEPFHQIDYSAMGNYWTLGF
GSVLLLWLFSVGVGQVIKMVRTA
>LFGLNPFC_01706 hypothetical protein
MFKKIVSFLAVPALMAVSGATFAAEGAASSGVDLSPLTNSIDFSTVLVAIMAVAASLVTL
YAGVAGVRWVLRTVKSA
>LFGLNPFC_01707 hypothetical protein
MEIGFPFMGDRLSLCRYSWAKRLTVYSFIFSFLWVSFPRYSYSFVPAIAAVAARAVIPKV
VGRVLVRRFAANDAIYTASQLTATVCFVGRAAANAAEYLPAASSYKMSGVATWAGIAAAV
SSFVPSSLSSSDGSVMVMTNGKKISDNLYEVTYSGQSGESKTITVNFEPQELSPVILHVS
RNNVDAGSPVVGVETGYSTPDNALYYYQDSKELIYYYGDNPTEIARNYLNDYNSRTYTET
LTNFERTVTNKVVNSNGDVSFTEHNYKFTYPSSFYEIPEITHLYSNPSASSFPAGIPMYE
NVAGLPMYYSVAYLTAGKQYQYHNTPCKTTNQSNGGYSTICAVPEKEDYTAKDIDEKSEL
TIWTNTKYKAMTEVLEAGNIESMIDYLEYLDSVSVSPALLADMINELWSEAAVNADYNGL
PFKEVSPAEVTSAMSELRLSPTLLDMLSPVSDSAGADVNIDITINNNSGSDTGNNGNIAL
GEDPGVKEPELEETPTARDILTPIINLLPFTNEFNIGSRSASCPVVEFSVFNHQYRIDSH
CPLIEQNRGAVETIFLIIWGFVALRIILSA
>LFGLNPFC_01708 hypothetical protein
MFGILISALNTLLGFVFRSLIIKFVVFFALYFVVQGFVEILVELLPDSSNLSSLFANLSD
GFWYFINLSKLPQGISMIISAMATRFIIRRIPVIG
>LFGLNPFC_01709 hypothetical protein
MDNDDVLKTDFFPFKGGEGSFCQFGDLIVIDEAWRIFGSDKDMTAEKKSFIAEHRHFTHP
ETGISCDLVIVNQSLSNIARFLKDKIETTYRMRKLKALGLNNHYCIDVYSGHKIYKSNLV
TSYRNKYNPDIFELYKSYEGNNGNEKQTDKRQSIWNSGKVRFFLVLFPLMFIGSGWLIYS
FFSTFGRSDPRQIWLQQMYVMRPCFVLPLLLQHQILPQNQLNRHFQPSGVYQGE
>LFGLNPFC_01710 hypothetical protein
MTSEGRAFVILVNGAGVLRAVPASSFNYKGMLMSGIIDGERVTLYTGKK
>LFGLNPFC_01711 Type 3 secretion system secretin
MLDPQLVNDERMITFRLTPDIDEREFVTRYLGNMNIRIWTKKGVDFIAPYTPKEPVKPRY
TWTYTPQYRSVAYLSDILGGYVSGSFNNSGAVISDDSLKGSSGASNYINRTGDILVYYGT
KEDIAILKTLVTSLDTMSDEVVVSGYVFEVQTSQSDGSGILLAAKILSDKFNISVGAAGL
DNFINIRTGSIDAIFNLLKTDSRFTVVSAPRLRVKNNASASFSVGSDVPVLGSVTVNNNT
TTQSVEYRSSGVLFNVTPSIKSRTMDLKIQQQLSNFVTTETGVNNSPTLIKRDVTTEVSL
ADGDIILLGGLAEQKDSKASSGWSFFGSRTSESNKTDIMVMLQVRKVDRSRATPRSAARS
GELFGTT
>LFGLNPFC_01712 hypothetical protein
MTHQELKVMLLKDPAFRAAYEAESQNPQSGYQIIRHHGDGTEEVVFDSRVSGTDLPTNWM
NRPDW
>LFGLNPFC_01713 hypothetical protein
MNALLGKIAGAVATFFVALILNKFFSTFRIRQLYLAYEHILEHTTQSITGYTVMFTVTNK
GKEKEKNVTITIPKSKSISLISANNVSIECKDNKIIIDRILPKENITLIVLVKNGGVLSK
SNLPILKSEDANGKCYDSIKKIPPSLGPAFFSFSLFIAFLVLMGGAIYKGYDPFEVTYTQ
YFKMRFTPFYDRGFENNSFQNNSLTKQYDTSKSDFPIDLKSLNVKDGKIEYVFIIDNKTN

YNLSADMKFIVRDENQYYDELGKVLNIRMTLIDVKLYTN
>LFGLNPFC_01714 hypothetical protein
MSRNNETSGVELVVVGIFAFCLAVVAWLMKTFDVEWQTALETAPSLIVWLLVVGAGIFFG
IKMETGLVRWGAPLAIALLIPVFKPILKEAAGVRETGGLVFDDMVSWYGTGWGMSLMFFG
ILIIGYGLLYWWHRRNSYHW
>LFGLNPFC_01715 hypothetical protein
MGNNEHEVMADKQRAERIKKVILEHSTYEELAEKTGISVSTLVRIASGKTEPKFSDIIQI
AKITGADLNTLAYGYALDVKEEATERKLITSADGYTDEETTNAHNFIVWNIRTLEKQDIL
ALARQVSALSSYTYSTKMFMKKAVSGEE
>LFGLNPFC_01716 hypothetical protein
MIDWFTGILPCTHRPLPAGSVVSVNADGAVEWETVKRLTVRGSHESTMKVRSVGSDGEGR
ATHLYIDGNPSKFLQGHSVIGSDDLQGLVLTAYARILALLHIPHDLPSYRQVMEGQFKIS
RIDINYMYSLSTLENVRAWLYAAEFKAKTRHGRACGKGGTVYLGKTPSLEPEILFKI
>LFGLNPFC_01717 hypothetical protein
MADEFVKAGLLDWSKDKLRIELTLRTTELIDLNLTLGNSWNIETPNKLFSDYVGRIEMNQ
NTILTDEKIINLPRKIQSTYLLWKQGANMKEMLPKPTFYRHRKELLSFGIDINFYCESPD
SNNVVPLVRTLEAKPAKIPSWVYEKGLIFDYNRISHASNWH
>LFGLNPFC_01718 hypothetical protein
MLGARQRNKVNGQGYYNEIGVGLEIPDGFGGTKQDQIIIRVSQALVNAGVMNQANNFIGK
LVQIPVYVRVWSMEGREGVTYNISSDGGITEIKG
>LFGLNPFC_01719 hypothetical protein
MDTSGFDIQFDNHIPENGYRIEGYLCNANNAKECQAIMVRSEPFHQIDYSAMGNYWTLGF
GSVLLLWLFSVGVGQVIKMVRTA
>LFGLNPFC_01720 hypothetical protein
MFKKIVSFLAVPALMAVSGATFAAEGAASSGVDLSPLTNSIDFSTVLVAIMAVAASLVTL
YAGVAGVRWVLRTVKSA
>LFGLNPFC_01721 hypothetical protein
MEIGFPFMGDRLSLCRYSWAKRLTVYSFIFSFLWVSFPRYSYSFVPAIAAVAARAVIPKV
VGRVLVRRFAANDAIYTASQLTATRVFVGRAAANAAEYLPAASSYKMSGVATWAGIAAAV
SSFVPSSLSSSDGSVMVMTNGKKISDNLYEVTYSGQSGESKTITVNFEPQELSPVILHVS
RNNVDAGSPVVGVETGYSTPDNALYYYQDSKELIYYYGDNPTEIARNYLNDYNSRTYTET
LTNFERTVTNKVVNSNGDVSFTEHNYKFTYPSSFYEIPEITHLYSNPSASSFPAGIPMYE
NVAGLPMYYSVAYLTAGKQYQYHNTPCKTTNQSNGGYSTICAVPEKEDYTAKDIDEKSEL
TIWTNTKYKAMTEVLEAGNIESMIDYLEYLDSVSVSPALLADMINELWSEAAVNADYNGL
PFKEVSPAEVTSAMSELRLSPTLLDMLSPVSDSAGADVNIDITINNNSGSDTGNNGNIAL
GEDPGVKEPELEETPTARDILTPIINLLPFTNEFNIGSRSASCPLLNSACLITSTELTPI
VR
>LFGLNPFC_01722 hypothetical protein
MFGILISALNTLLGFVFRSLIIKFVVFFALYFVVQGFVEILVELLPDSSNLSSLFANLSD
GFWYFINLSKLPQGISMIISAMATRFIIRRIPVIG
>LFGLNPFC_01723 hypothetical protein
MAISAYIGIPGSGKSYEAVCNVIIPAFTSGRRVVTNIYGLQKDKITERYPDATGEIIVVD
NDDVLKTDFFPFKGGEGSFCQFGDLIVIDEAWRIFGSDKDMTAEKKSFIAEHRHFTHPET
GISCDLVIVNQSLSNIARFLKDKIETTYRMRKLKALGLNNHYCIDVYSGHKIYKSNLVTS
YRNKYNPDIFELYKSYEGNNGNEKQTDKRQSIWNSGKVRFFLVLFPLMFIGSGWLIYSFF
STFGRSDPSPDLATTDVRDAAMFRSSAATPAPDTPSEPAEPPLSTEWRISGRMTSEGRAF
VILVNGAGVLRAVPASSFNYKGMLMSGIIDGERVTLYTGKK
>LFGLNPFC_01724 Type 3 secretion system secretin
MKKILLALTLLFSSCAFAGSELELNKVKLPEAISLIYSEVLKVPYMLDPQLVNDERMITF
RLTPDIDEREFVTRYLGNMNIRIWTKKGVDFIAPYTPKEPVKPRYTWTYTPQYRSVAYLS
DILGGYVSGSFNNSGAVISDDSLKGSSGASNYINRTGDILVYYGTKEDIAILKTLVTSLD
TMSDEVVVSGYVFEVQTSQSDGSGILLAAKILSDKFNISVGAAGLDNFINIRTGSIDAIF
NLLKTDSRFTVVSAPRLRVKNNASASFSVGSDVPVLGSVTVNNNTTTQSVEYRSSGVLFN
VTPSIKSRTMDLKIQQQLSNFVTTETGVNNSPTLIKRDVTTEVSLADGDIILLGGLAEQK
DSKASSGWSFFGSRTSESNKTDIMVMLQVRKVDRSRATPRSAARSGELFRDNLN
>LFGLNPFC_01725 hypothetical protein
MSVKNKARDRLPGGRLKSYRRVGSHFASCARWFDKSPSWYRNMMMTRPERREVRRLLNQV
MRGHDADGIAFPVSHRPFVYWW
>LFGLNPFC_01726 hypothetical protein
MTHQELKVMLLKDPAFRAAYEAESQNPQSGYQIIRHHGDGTEEVVFDSRVSGTDLPTNWM
NRPDW
>LFGLNPFC_01727 hypothetical protein
MRFEGNASTRKSIQQRLKQASDARAQEVAGSEVLYSGDANLTDEQIAKLPFALYRQGYEY
YWKTHAHPNIFRSVRDIVPSKRWLL

>LFGLNPFC_01728 hypothetical protein
MRILVAGATGSIGIHVVNTAIAMGHQPVTLVRNRRKIKLLPRGTDIFYGDVSIPETLTDL
PKDIDAIIFTLGSDGQGRIGARAIDYGGVRNILRIFMDTPVRIALMTTIGVTERLSSWNQ
RTEVHDWKRRSERLVRASGHIYTIVRPGWFDYNNDDEHRIVMLQGDRRHAGTPEDGVISR
EQIAQVLVTALSNDAAKNKTFELVAERGEAQQDLTPLFAELRNDNPQKNDGVFDIDNMPL
TEEPECVINDLNLYSKNSKI
>LFGLNPFC_01729 hypothetical protein
MKALLITALFMEFISVNTASAAEEIHHADSKVDDQVLIAE
>LFGLNPFC_01730 Type-1 fimbrial protein, A chain
MKLKHVGIIVVSVLAMSSAAVSAAEGDESVTTTVNGGVIHFKGEVVNAACAIDSESMNQT
VELGQVRSSRLAKAGDLSSAVGFNIKLNDCDTNVSSNAAVAFLGTTVTSNDDTLALQSSA
AGSAQNVGIQILDRTGEVLVLDGATFSAKTDLIDGTNILPFQARYIALGQSVAGTANADA
TFKVQYL
>LFGLNPFC_01731 Chaperone protein FocC
MQTTRTPYSVSFMATVLLLLLFACHSTIANAAVALGATRVIYPANQKQVLLPVTNNDPAS
VYLIQSWIENAGDQKDTQFVITPPLFSMQGKKENTLRIINATNHQLPGDRESLFWVNVKA
IPAMEKDQKNENTLQLAIISRIKMFYRPTHLAMAPEEAPAMLRFRRSGSKLTLINPTPYF
ITVTNMKAGNSNLPNTMVPPKGEVSVDIPHAVTGDISFQTINDYGALTPRIKATMQ
>LFGLNPFC_01732 Outer membrane usher protein FimD
MTAFRAAFKAYRMHQVLILPRFARLTFALGLATAVFPVDAEYYFNPRFLSNDLAESVDLS
AFTKGREAPPGTYRVDIYLNDEFMASRDITFIADDNNADLIPCLSTDLLVSLGIKKSALL
DNKEHSADKHVPDNSACTPLQDRLADASSEFDVGQQHLSLSVPQIYVGRMAHGYVSPDLW
EEGINAGLLNYSFNGNSINNRSNHNAGKSNYAYLNLQSGINIGSWRLRDNSTWSYNSGSS
NSSDSNKWQHINTSAERDIIPLRSRLTVGDSYTDGDIFDSVNFRGLKINSTEAMLPDSQH
GFAPVIHGIARGTAQVSVKQNGYDVYQTTVPPGPFTIDDINSAANGGNLQVTIKEADGSI
QTLYVPYSSVPVLQRAGYTRYALAMGEYRSGNNLQSTPKFVQASLMHGLKGNWTPYGGMQ
IAEDYQAFNLGIGKDLGLFGAFSFDITQANTTLADDTRHSGQSVKFVYSKSFYQTGTNIQ
VAGYRYSTQGFYNLSDSAYSRMSGYTDYFVINRLSD
>LFGLNPFC_01733 Protein FimG
MVTPITLSLTDCPIETSAVTAIVTGSTDNTGYYKNEGTAENIQIELRDDQDATLKNGDSK
TVIVDVITRNAQFPLKARAITVNGNASQGTIEALINVIYTWQ
>LFGLNPFC_01734 Type 1 fimbrin D-mannose specific adhesin
MGKTFSIKVLFGIYLLLMAGKVFAFSCNVDGGSSIGAGTTSVYVNLDPVIQPGQNLVVDL
SQHISCWNDYGGWYDTDHINLVQGSAFAGSLQSYKGSLYWNNVTYPFPLTTNTNVLDIGD
KTPMPLPLKLYITPVGAAGGVVIKAGEVIARIHMYKIATLGSGNPRNFTWNIISNNSVVM
PTGGCTVDSRNVTVNLPDFPGSAEIPLGVYCSSEQKLSFYLSGATTDSARQVFANAAPDA
TKASGVGVSLIRNGKILATGENVSLGTVNKSKVPLGLSATYGQTGNKVAAGAVQSVIGVT
FIYE
>LFGLNPFC_01735 Protein YdeP
MKKKIESYQGAAGGWGAVKSVANAVRKQMDIRQDVIAMFDMNKPEGFDCPGCAWPDPKHS
ASFDICENGAKAIAWEVTDKQVNASFFAENTVQSLLTWGDHELEAAGRLTQPLKYDDVSD
CYKPLSWQQAFDEIGARLQSYSDPNQVEFYTSGRTSNEAAFLYQLFAREYGSNNFPDCSN
MCHEPTSVGLAASIGVGKGTVLLEDFEKCDLVICIGHNPGTNHPRMLTSLRALVKRGAKM
IAINPLQERGLERFTAPQNPFEMLTNSETQLASAYYNVRIGGDMALLKGMMRLLIERDDA
ASAAGRPSLLDDEFIQTHTVGFDELRRDVLNSEWKDIERISGLSQTQIAELADAYAAAER
TIICYGMGITQHEHGTQNVQQLVNLLLMKGNIGKPGAGICPLRGHSNVQGDRTVGITEKP
SAEFLDRLCERYGFTPPHAPGHAAIASMQAICTGQARALICMGGNFALAMPDREASAVPL
TQLDLAVHVATKLNRSHLLTARHSYILPVLGRSEIDMQKSGAQAVTVEDSMSMIHASRGV
LKPAGVMLKSECAVVAGIAQAALPQSVVAWEYLVEDYDRIRNDIEAVLPEFADYNQRIRH
PGGFHLINAAAERRWMTSSGKANFITSKGLLEDPSSAFNSKLVMATVRSHDQYNTTIYGM
DDRYRGVFGQRDVVFMSAKQAKICRVKNGERVNLIALTPDGKRSSRRMDRLKVVIYPMAD
RSLVTYFPESNHMLTLDNHDPLSGIPGYKSIPVELEPSN
>LFGLNPFC_01736 Two-component-system connector protein SafA
MHATTVKNKITQRDNYKEIMSVIVVVLLLTLTLIAIFSAIDQLGISEMGRMARDLTHFII
NSLLD
>LFGLNPFC_01737 HTH-type transcriptional regulator YdeO
MSLVCSIIFIHHAFNANILDKDYAFSDGEILMVDNAVRTHFEPYERHFKEIGFNENTIKK
YLQCTNIQTVTMPVPAKFLRASNVPTGLLNEMIAYLNSEERNHHNFSELLLFSCLSIFAA
CKGFITLLTNGVLSVSGKVRNIVNMKLAHPWKLKDICDCLYISESLLKKKLKQEQTTFSQ
ILLDARMQHAKNLIRVEGSVNKIAEQCGYASTSYFIYAFRKHFGNSPKRVSKEYRCQRHT
GMNTDNTMNALAI
>LFGLNPFC_01738 hypothetical protein
MKSALKKSVVSTSISLILASGMAAFAAHAADDVKLKATKTNVAFSDFTPTEYSTKGKPNI
IVLTMDDLGYGQLPFDKGSFDPKTMENREVVDTYKIGIDKAIEAAQKSTPTLLSLMDEGV

RFTNGYVAHGVSGPSRAAIMTGRAPARFGVYSNTDAQDGIPLTETFLPELFQNHGYYTAA
VGKWHLSKISNVPVPEDKQTRDYHDNFTTFSAEEWQPQNRGFDYFMGFHAAGTAYYNSPS
LFKNRERVPAKGYISDQLTDEAIGVVDRAKTLDQPFMLYLAYNAPHLPNDNPAPEQYQKQ
FNTGSQTADNYYASVYSVDQGVKRILEQLKKNGQYDNTIILFTSDNGAVIDGPLPLNGAQ
KGYKSQTYPGGTHTPMFMWWKGKLQPGNYDKLISAMDFYPTALDAADISIPKDLKLDGVS
LLPWLQNKKQGEPHKNLTWITSYSHWFDEENIPFWDNYHKFVRHQSDDYPHNPNTEDLSQ
FSYTVRNNDYSLVYTVENNQLGLYKLTDLQQKDNLAAANPQVVKEMQGVVREFIDNSQPP
LSEVNQEKFNNIKKALSEAK
>LFGLNPFC_01739 Anaerobic sulfatase-maturating enzyme
MHVTAKPSSFQCNLKCDYCFYLEKESQFTHEKWMDDSTLKEFIKQYIAASGNQVYFTWQG
GEPTLAGLDFFRKVIHYQQRYAGQKRIFNALQTNGILLNNEWCAFLKEHEFLVGISIDGP
QELHDCYRRSNSGNGTFAKVIAAIERLKSYQIEFNTLTVINNINVHYPLEVYHFLKSIGS
KHMQFIELLETGTPNIDFSGHSENTFRIIDFSVPPTAYGKFMSTIFMQWVKNDVGEIFIR
QFESFVSRFLGNGHTSCIFQESCKDNLVVESNGDIYECDHFVYPQYKIGNINKSELKTMN
SVQLTAQKKRISAKCQQCVYKPICNGGCPKHRITKVNNETVSYFCEGYKILFSTMVPYMN
AMVELAKNRVPLYHIMDVAKQMENN
>LFGLNPFC_01740 Inner membrane ABC transporter ATP-binding protein YddA
MLIAKYLCLLKPFWLRKNNKTSVLLIIIILAMILGVVKIQVWLNDWNNDFFNALSQKETD
KLWQLVLWFPALLGIFVLISVNKTWLIKLLTIRWREWLTDYYLNRWFADKNYYFTQIYGE
HKNTDNPDQRIAEDILLLISKTLSLSFGFIQSLSMLITFTVILWQSAGTLSFTVGGTEWS
IQGYMVYTVVLIVIGGTLFTHKVGKRIRPLNVEKQRSEATFRTNLVQHNKQAELIALSNA
ESLQRQELSENFHTIKENWHRLMNRQRWLDYWQNIYSRSLSVLPYFLLLPQFISGQINLG
GLMKSRQAFMLVSNNLSWFIYKYDELAELAAVIDRLYEFHQLTEQRPTNKPKNCQHAVQV
ADASIRTPDNKIILENLNFHVSPGKWLLLKGYSGAGKTTLLKTLSHCWPWFKGDISSPAD
SWYVSQTPLIKTGLLKEIICKALPLSVDDKSLSEVLHQVGLGKLAARIHDHDRWGDILSS
GEKQRIALARLILRRPKWIFLDETTSHLEEQEAIRLLRLVREKLPTSGVIMVTHQPGVWN
LVDDICDISAVI
>LFGLNPFC_01741 hypothetical protein
MKRVLIPGVILCGADVAQAVDDKNMYMHVFEEMTVYAPVPVPVNGNTHYTSESIERLPTG
NGNISDLLRTNPAVRMDSTQSTSLNQGDIRPEKISIHGASPYQNAYLIDGISATNNLNPA
NESDASSATNISGMSQGYYLDVSLLDNVTLYDSFVPVEFGRFNGGVIDAKIKRFNADDSS
VKLGYRTTRSDWLTSHIDENNKSAFNQGSSGSTYYSPDFKKNFYTLSFNQELADNFGVTA
GLSRRQSDITRADYVSNDGIVAGRAQYKNVIDTALSKFTWFASDRFTHDLTLKYTGSSRD
YNTSTFPESDREMGNKSYGLAWDMDTQLAWAKLRTTVGWDHISDYTRHDHDIWYTELSCT
YGDISGRCTRGGLGHISQAVDNYTFKTRLDWQKFAVGNVSHQPYFGAEYIYSDAWTERHN
QSESYVINAAGKKTNHTIYHKGKGSLGIDNYTLYMADRISWRNVSLMPGVRYDYDNYLSN
HNISPRFMTEWDIFADQTSMITAGYNRYYGGNILDMGLRDIRNSWTESVSGNKTLTRYQD
LKTPYNDELAMGLQQKIGKNVIARANYVYREAHDQISKSSRTDSATKTTITEYNNDGKTK
THSFNLSFELAEPLHISQVDINPQIVFSYIKSKGNLSLNNGYEESNTGDNRVVYNGNLVS
YDSVPVADFNNPLKISLNMDFTHQPSGLVWANTLTWQEARKARIILGKTNAQYISEYSDY
KQYVDEKLDSSLTWDTRLSWTPQFLKQQNLTISADILNVLDSKTAVDTTNTGVATYASGR
TFWLDVSMKF
>LFGLNPFC_01742 hypothetical protein
MRNLCFLLTLVATLLLPGRLIAAALPQDEKLITGQLDNGLRYMIYPHAQPKDQVNLWLQI
HTGSLQEEDNERGVAHFVEHMMFNGTKTWPGNKVIETFESMGLRFGRDVNAYTSYDETVY
QVSLPTTQKQNLQQVMAIFSEWSNAATFEKLEVDAERGVITEEWRAHQDAKWRTSQARRP
FLLANTRNLDREPIGLMDTVATVTPAQLRQFYQRWYQPNNMTFIVVGDIDSKEALALIKD
NLSKLPANKAAENRVWPTKAENHLRFNIINDKENRVNGIALYYRLPMVQVNDEQSFIEQA
EWSMLVQLFNQRLQERIQSGELKTISGGTARSVKIAPDYQSLFFRVNARDDNMQDAANAL
MAELATIDQHGFSAEELDDVKSTRLTWLKNAVDQQAERDLRMLTSRLASSSLNNTPFLSP
EETYQLSKRLWQQITVQSLAEKWQQLRKNQDAFWEQMVNNEVAAKKALSPAAILALEKEY
ANKKLAAYIFPGRNLSLTVDADPQAEISSKETLAENLTSLTLSNGARVILAKSAGEEQKL
QITAVSNKGDLSFPAQQKSLIALANKAVSGSGVGELSSSSLKRWSAENSVTMSSKVSGMN
TLLSVSARTNNPEPGFQLINQRINHSTINDNIWASLQNAQIQALKTLDQRPAEKFAQQMY
ETRYADGRTKLPQENQIAQFTAADALAADRQLFSSPADITFVIVGNVAEDKLVALITRYL
GSIKHSDSPLAAGKPLTRATDNASVTVKEQNEPVAQVSQWKRYNSRTPVNLATRMALDAF
NVALAKDLRINIREQASGAYSVSSRLSVDPQAKDISHLLAFTCQPERHDELLTLANEVMV
KRLAKGISEQELNEYQQNVQRSLDIQQRSVQQLANTIVNSLIQYDDPAAWTEQEQLLKQM
TVENVNTAVKQYLSHPVNTYTGVLLPK
>LFGLNPFC_01743 hypothetical protein
MFYVVILCVFYRLLNYHVKIRPYNIIISTRLFTLNTFINYYIKIANIDKIPANDVLTGL
>LFGLNPFC_01744 Glutamate decarboxylase beta
MDKKQVTDLRSELLDSRFGAKSISTIAESKRFPLHEMRDDVAFQIINDELYLDGNARQNL
ATFCQTWDDDNVHKLMDLSINKNWIDKEEYPQSAAIDLRCVNMVADLWHAPAPKNGQAVG

TNTIGSSEACMLGGMAMKWRWRKRMEAAGKPTNKPNLVCGPVQICWHKFARYWDVELREI
PMRPGQLFMDPKRMIEACDENTIGVVPTFGVTYTGNYEFPQPLHDALDKFQADTGIDIDM
HIDAASGGFLAPFVAPDIVWDFRLPRVKSISASGHKFGLAPLGCGWVIWRDEEALPQELV
FNVDYLGGQIGTFAINFSRPAGQVIAQYYEFLRLGREGYTKVQNASYQVAAYLADEIAKL
GPYEFICTGRPDEGIPAVCFKLKDGEDPGYTLYDLSERLRLRGWQVPAFTLGGEATDIVV
MRIMCRRGFEMDFAELLLEDYKASLKYLSDHPKLQGIAQQNSFKHT
>LFGLNPFC_01745 putative glutamate/gamma-aminobutyrate antiporter
MATSVQTGKAKQLTLLGFFAITASMVMAVYEYPTFATSGFSLVFFLLLGGILWFIPVGLC
AAEMATVDGWEEGGVFAWVSNTLGPRWGFAAISFGYLQIAIGFIPMLYFVLGALSYILKW
PALNEDPITKTIAALIILWALALTQFGGTKYTARIAKVGFFAGILLPAFILIALAAIYLH
SGAPVAIEMDAKTFFPDFSKVGTLVVFVAFILSYMGVEASATHVNEMSNPGRDYPLAMLL
LMVAAICLSSVGGLSIAMVIPGNEINLSAGVMQTFTVLMSHVAPEIEWTVRVISALLLLG
VLAEIASWIVGPSRGMYVTAQKNLLPAAFAKMNKNGVPVTLVISQLVITSIALIILTNTG
GGNNMSFLIALALTVVIYLCAYFMLFIGYIVLVLKHPDLKRTFNIPGGKGVKLVVAIVGL
LTSIMAFIVSFLPPDNIQGDSTDMYVELLVVSFLVVLALPFILYAVHDRKSKANTGVTLE
PINSQNAPKGHFFLHPRARSPHYIVMNDKKH
>LFGLNPFC_01746 hypothetical protein
MDICSRNEKLAIRRPAILVALALLLCSCKSTPPESMVTPPAGSKPPATTQQSSQPMRGIW
LATVSRLDWPPVSSVNISNPTSRARVQQQAMIDKLDHLQRLGINTVFFQVKPDGTALWPS
KILPWSDLMTGKIGENPGYDPLQFMLDEAHKRGMKVHAWFNPYRVSVNTKPGTIRELNST
LSQQPASVYVQHRDWIRTSGDRFVLDPGIPEVQDWITSIVAEVVSRYPVDGVQFDDYFYT
ESPGSRLNDNETYRKYGGAFASKADWRRNNTQQLIAKVSHTIKSIKPEVEFGVSPAGVWR
NRSHDPLGSDTRGAAAYDESYADTRRWVEQGLLDYIAPQIYWPFSRSAARYDVLAKWWAD
VVKPTRTRLYIGIAFYKVGEPSKIEPDWMINGGVPELKKQLDLNDAVPEISGTILFREDY
LNKPQTQQAVSYLQSRWGS
>LFGLNPFC_01747 Oxygen sensor protein DosP
MKLTDADTAADGIFFPALEQNMMGAVLINENDEVMFFNPAAEKLWGYKREEVIGNNIDML
IPRDLRPAHPEYIRHNREGGKARVEGMSRELQLEKKDGSKVWTRFALSKVSAEGKVYYLA
LVRDASVEMAQKEQTRQLIIAVDHLDRPVIVLDPERHIVQCNRAFTEMFGYCINEASGMQ
PDTLLNIPEFPADNRIRLQQLLWKTARDQDEFLLLTRTGEKIWIKASISPVYDVLAHLQN
LVMTFSDITEERQIRQLEGNILAAMCSSPPFHEMGEIICRNIESVLNESHVSLFALRNGM
PIHWASSSHGAEVQNAQSWSATIRQRDGAPAGILQIKTSSGAETSAFIERVADISQHMAA
LALEQEKSRQHIEQLIQFDPMTGLPNRNNLHNYLDDLVDKAVSPVVYLIGVDHIQDVIDS
LGYAWADQALLEVVNRFREKLKPDQYLCRIEGASFVLVSLENDVSNITQIADELRNVVSK
PIMIDDKPFPLTLSIGISYDEGKNRDYLLSTAHNAMDFIRKNGGNGWQFFSPAMNEMVKE
RLVLGAALKEAISNNQLKLVYQPQIFAETGELYGIEALARWYDPLHGHVPPSRFIPLAEE
IGEIENIGRWVIAEACRQLAEWRSQNIHIPALSVNLSALHFRSNQLPNQVSDAMHAWGID
GHQLTVEITESMMMEHDTEIFKRIQILRDMGIGLSVDDFGTGFSGLSRLVSLPVTEIKID
KSFVDRCLTEKRILALLEAITSIGQSLNLTVVAEGIETKEQFEMLRKIHCRVIQGYFFSR
PLPAEEIPGWMSSVLPLKI
>LFGLNPFC_01748 Peroxiredoxin OsmC
MTIHKKGQAHWEGDIKRGKGTVSTESGVLNQQPYGFNTRFEGEKGTNPEELIGAAHAACF
SMALSLMLGEAGFTPTSIDTTADVSLDRVDAGFAITKIALKSEVAVPGIDASTFDGIIQK
AKAGCPVSQVLKAEITLDYQLKS
>LFGLNPFC_01749 Protein bdm
MFTYYQAENSTAEPALVNAIEQGLRAEHGVVTEDDILMELTKWVEASDNDILSDIYQQTI
NYVVSGQHPTL
>LFGLNPFC_01750 Stationary-phase-induced ribosome-associated protein
MKSNRQARHILGLDHKISNQRKIVTEGDKSSVVNNPTGRKRPAEK
>LFGLNPFC_01751 NAD-dependent malic enzyme
MEPKTKKQRSLYIPYAGPVLLEFPLLNKGSAFSMEERRNFNLLGLLPEVVETIEEQAERA
WIQYQGFKTEIDKHIYLRNIQDTNETLFYRLVNNHLDEMMPVIYTPTVGAACERFSEIYR
RSRGVFISYQNRHNMDDILQNVPNHNIKVIVVTDGERILGLGDQGIGGMGIPIGKLSLYT
ACGGISPAYTLPVVLDVGTNNQQLLNDPLYMGWRNPRITDDEYYEFVDEFIQAVKQRWPD
VLLQFEDFAQKNAMPLLNRYRNEICSFNDDIQGTAAVTVGTLIAASRAAGGQLSEKKIVF
LGAGSAGCGIAEMIIAQTQREGLSEEAARQKVFMVDRFGLLTDKMPNLLPFQTKLVQKRE
NLSDWDTDSDVLSLLDVVRNVKPDILIGVSGQTGLFTEEIIREMHKHCPRPIVMPLSNPT
SRVEATPQDIIAWTEGNALVATGSPFNPVVWKDKIYPIAQCNNAFIFPGIGLGVIASGAS
RITDEMLMSASETLAQYSPLVLNGEGLVLPELKDIQKVSRAIAFAVGKMAQQQGVAVKTS
AEALQQAIDDNFWHAEYRDYRRTSI
>LFGLNPFC_01752 Alcohol dehydrogenase, propanol-preferring
MKAAVVTKDHHVDVTDKTLRSLKHGEALLKMECCGVCHTDLHVKNGDFGDKTGVILGHEG
IGVVAEVGPGVTSLKPGDRASVAWFYEGCGHCEYCNSGNETLCRSVKNAGYSVDGGMAEE
CIVVADYAVKVPDGLDSAAASSITCAGGTTYKAVKLSKIRPGQWIAIYGLGGLGNLALQY

AKNVFNAKVIAIDVNDEQLKLATEMGADLAINSRTEDAARIVQEKAGGAHAAVVTAVAKA
AFNSAVDAVRAGGRVVAVGLPPESMSLDIPRLVLDGIEVVGSLVGTRQDLTEAFQFAAEG
KVVPKVALRPLADINTIFTEMEEGKIRGRMVIDFRR
>LFGLNPFC_01753 hypothetical protein
MGEEKKKSEIAESVSQNRLRPEHGSRSKDLLATEINHHTATDFAFFHLGKYGVDVR
>LFGLNPFC_01754 Endoribonuclease HigB
MIMNFRHKGLRDLFLLGKTSGVIPTQVKRLRHRLAVIDAACCLADIDMPGYRLHPLSGDR
DGIWAISVSGNWRITFEFVNGDAYILDYEDYH
>LFGLNPFC_01755 putative HTH-type transcriptional regulator YddM
MKMANHPRPGDIIQESLDELNVSLREFARAMEIAPSTASRLLTGKAALTPEMAIKLSVVI
GSSPQMWLNLQNAWSLAEAEKTVDVSRLRRLVTQ
>LFGLNPFC_01756 Formate dehydrogenase, nitrate-inducible, cytochrome b556(Fdn) subunit
MSKSKMIVRTKFIDRACHWTVVICFFLVALSGISFFFPTLQWLTQTFGTPQMGRILHPFF
GIAIFVALMFMFVRFVHHNIPDKKDIPWLLNIVEVLKGNEHKVADVGKYNAGQKMMFWSI
MSMIFVLLVTGVIIWRPYFAQYFPIQVVRYSLLIHAAAGIILIHAILIHMYMAFWVKGSI
KGMIEGKVSRRWAKKHHPRWYREIEKAEAKKESEEGL
>LFGLNPFC_01757 Formate dehydrogenase, nitrate-inducible, iron-sulfur subunit
MAMETQDIIKRSATNSITPPSQVRDYKAEVAKLIDVSTCIGCKACQVACSEWNDIRDEVG
HCVGVYDNPADLSAKSWTVMRFSETEQNGKLEWLIRKDGCMHCEDPGCLKACPSAGAIIQ
YANGIVDFQSENCIGCGYCIAGCPFNIPRLNKEDNRVYKCTLCVDRVSVGQEPACVKTCP
TGAIHFGTKKEMLELAEQRVAKLKARGYEHAGVYNPEGVGGTHVMYVLHHADQPELYHGL
PKDPKIDTSVSLWKGALKPLAAAGFIATFAGLIFHYIGIGPNKEVDDDEEGNHE
>LFGLNPFC_01758 Formate dehydrogenase, nitrate-inducible, major subunit
MTNHWVDIKNANVVMVMGGNAAEAHPVGFRWAMEAKNNNDATLIVVDPRFTRTASVADIY
APIRSGTDITFLSGVLRYLIENNKINAEYVKHYTNASLLVRDDFAFEDGLFSGYDAEKRQ
YDKSSWNYQFDENGYAKRDDTLTHPRCVWNLLKAHVSRYTPDVVENICGTPKADFLKVCE
VLASTSAPDRTTTFLYALGWTQHTVGAQNIRTMAMIQLLLGNMGMAGGGVNALRGHSNIQ
GLTDLGLLSTSLPGYLTLPSEKQVDLQSYLEANTPKATLADQVNYWSNYPKFFVSLMKSF
YGDAAQKENNWGYDWLPKWDQTYDVIKYFNMMDEGKVTGYFCQGFNPVASFPDKNKVVSC
LSKLKYMVVIDPLVTETSTFWQNHGESNDVDPASIQTEVFRLPSTCFAEEDGSIANSGRW
LQWHWKGQDAPGEARNDGEILAGIYHHLRELYQAEGGKGVEPLMKMSWNYKQPHEPQSDE
VAKENNGYALEDLYDANGVLIAKKGQLLSSFAHLRDDGTTASSCWIYTGSWTEQGNQMAN
RDNSDPSGLGNTLGWAWAWPLNRRVLYNRASADINGKPWDPKRMLIQWNGSKWTGNDIPD
FGNAAPGTPTGPFIMQPEGMGRLFAINKMAEGPFPEHYEPIETPLGTNPLHPNVVSNPVV
RLYEQDALRMGKKEQFPYVGTTYRLTEHFHTWTKHALLNAIAQPEQFVEISETLAAAKGI
ANGDRVTVSSKRGFIRAVAVVTRRLKPLNVNGQQVETVGIPIHWGFEGVARKGYIANTLT
PNVGDANSQTPEYKAFLVNIEKA
>LFGLNPFC_01759 Formate dehydrogenase, nitrate-inducible, major subunit
MDVSRRQFFKICAGGMAGTTVAALGFAPKQALAQARNYKLLRAKEIRNTCTYCSVGCGLL
MYSLGDGAKNAREAIYHIEGDPDHPVSRGALCPKGAGLLDYVNSENRLRYPEYRAPGSDK
WQRISWEEAFSRIAKLMKADRDANFIEKNEQGVTVNRWLSTGMLCASGASNETGMLTQKF
ARSLGMLAVDNQARV
>LFGLNPFC_01760 Aromatic amino acid exporter YddG
MVGLIRGVSEGLGPVGGAAAIYSLSGLLLIFTVGFPRIRQIPKGYLLAGSLLFVSYEICL
ALSLGYAATRHQAIEVGMVNYLWPSLTILFAILFNGQKTNWLIVPGLLLALVGVCWVLGG
DNGLHYDQIINNITTSPLSYFLAFIGAFIWAAYCTVTNKYARGFNGITVFVLLTGASLWV
YYFLTPQPEMVFSTPVMIKLISAAFTLGFAYAAWNVGILHGNVTIMAVGSYFTPVLSSAL
AAVLLSAPLSFSFWQGALMVCGGSLLCWLATRRG
>LFGLNPFC_01761 Nitrate/nitrite transporter NarU
MALQNEKNSRYLLRDWKPENPAFWENKGKHIARRNLWISVSCLLLAFCVWMLFSAVTVNL
NKIGFNFTTDQLFLLTALPSVSGALLRVPYSFMVPIFGGRRWTVFSTAILIIPCVWLGIA
VQNPNTPFGIFIVIALLCGFAGANFASSMGNISFFFPKAKQGSALGINGGLGNLGVSVMQ
LVAPLVIFVPVFAFLGVNGVPQADGSVMSLANAAWIWVPLLAIATIAAWSGMNDIASSRA
SIADQLPVLQRLHLWLLSLLYLATFGSFIGFSAGFAMLAKTQFPDVNILRLAFFGPFIGA
IARSVGGAISDKFGGVRVTLINFIFMAIFSALLFLTLPGTGSGNFIAFYAVFMGLFLTAG
LGSGSTFQMIAVIFRQITIYRVKMKGGSDEQAQREAVTETAAALGFISAIGAVGGFFIPQ
AFGMSLNMTGSPVGAMKVFLIFYIVCVLLTWLVYGRRKFSQK
>LFGLNPFC_01762 Respiratory nitrate reductase 2 alpha chain
MSKLLDRFRYFKQKGETFADGHGQVMHSNRDWEDSYRQRWQFDKIVRSTHGVNCTGSCSW
KIYVKNGLVTWEIQQTDYPRTRPDLPNHEPRGCPRGASYSWYLYSANRLKYPLIRKRLIE
LWREALKQHSDPVLAWASIMNDPQKSLSYKQVRGRGGFIRSNWQELNQLIAAANVWTIKT
YGPDRVAGFSPIPAMSMVSYAAGTRYLSLLGGTCLSFYDWYCDLPPASPMTWGEQTDVPE
SADWYNSSYIIAWGSNVPQTRTPDAHFFTEVRYKGTKTIAITPDYSEVAKLCDQWLAPKQ
GTDSALAMAMGHVILKEFHLDNPSDYFINYCRRYSDMPMLVMLEPRDDGSYVPGRMVRAS

DLVDGLGESNNPQWKTVAVNTAGELVVPNGSIGFRWGEKGKWNLESIAAGTETELSLTLL
GQHDAVAGVAFPYFGGIENPHFRSVKHNPVLVRQLPVKNLTLADGSTCPVVSVYDLVLAN
YGLDRGLEDENSAKDYAEIKPYTPAWGEQITGVPRQYIETIAREFADTAHKTHGRSMIIL
GAGVNHWYHMDMNYRGMINMLIFCGCVGQSGGGWAHYVGQEKLRPQTGWLPLAFALDWNR
PPRQMNSTSFFYNHSSQWRYEKVTAQELLSPLADASKYSGHLIDFNVRAERMGWLPSAPQ
LGRNPLSLKAEADKAGLSPAEFTVQALKSGELRMACEQPDNGSNHPRNLFVWRSNLLGSS
GKGHEYMQKYLLGTKSGIQGEELGPTEGIQPEEVEWQTAAIEGKLDLLVTLDFRMSSTCL
FSDIVLPTATWYEKDDMNTSDMHPFIHPLSAAVDPAWESRSDWEIYKGIAKAFSQVCIGH
LGKETDVVLQPLLHDSPAELSQPCEVLDWRKGECDLIPGKTAPNIVAVERDYPATYERFT
SLGPLMDKLGNGGKGISWNTQDEIDFLGKLNYTKRDGPAKGRPLIDTAIDASEVILALAP
ETNGHVAVKAWQALGEITGREHTHLALHKEDEKIRFRDIQAQPRKIISSPTWSGLESDHV
SYNAGYTNVHELIPWRTLSGRQQLYQDHPWMRAFGESLVAYRPPIDTRSVSEMRQIPPNG
FPEKALNFLTPHQKWGIHSTYSENLLMLTLSRGGPIVWLSETDARELTIVDNDWVEVFNA
NGALTARAVVSQRVPPGMTMMYHAQERIMNIPGSEVTGMRGGIHNSVTRVCPKPTHMIGG
YAQLAWGFNYYGTVGSNRDEFIMIRKMKNVNWLDDEGRDQVQEAKK
>LFGLNPFC_01763 Respiratory nitrate reductase 2 beta chain
MKIRSQVGMVLNLDKCIGCHTCSVTCKNVWTGREGMEYAWFNNVETKPGIGYPKNWEDQE
EWQGGWVRDVNGKIRPRLGSKMGVITKIFANPVVPQIDDYYEPFTFDYEHLHSAPEGKHI
PTARPRSLIDGKRMDKVIWGPNWEELLGGEFEKRARDRNFEAMQKEMYGQFENTFMMYLP
RLCEHCLNPSCVATCPSGAIYKREEDGIVLIDQDKCRGWRLCISGCPYKKIYFNWKSGKS
EKCIFCYPRIESGQPTVCSETCVGRIRYLGVLLYDADRIEEAASTEREVDLYERQCEVFL
DPHDPSVIEEALKQGIPQNVIDAAQRSPVYKMAMDWKLALPLHPEYRTLPMVWYVPPLSP
IQSYADAGGLPKSEGVLPAIESLRIPVQYLANMLSAGDTGPVLRALKRMMAMRHYMRSQT
VEGVTDTRAIDEVGLSVAQVEEMYRYLAIANYEDRFVIPTSHREMAGDAFAERNGCGFTF
GDGCHGSDSKFNLFNSSRIDAINITEVRDKAEGE
>LFGLNPFC_01764 putative nitrate reductase molybdenum cofactor assembly chaperone NarW
MQILKVIGLLMEYPDELLWECKDDALALIRRDAPMLTDFALDLLNAPLLDKQAEWCEVFD
RGRTTSLLLFEHVHAESRDRGQAMVDLLAEYEKVGLQLNCRELPDYLPLYLEYLSVLPDD
QAKEGLLNVAPILALLGGRLKQREAPWYALFDALLQLAGSPLSSDSVIKQIRSEERDDTH
QALDAVWEEEQVKFIEDNATACDSSPLNQYQRRFSQDVAPQYVDISAGGGK
>LFGLNPFC_01765 Respiratory nitrate reductase 2 gamma chain
MIQYLNVFFYDIYPYLCGTVFILGSWLRYDHGQYTWRAASSQMLDKRGMVLWSNLFHIGI
LGIFFGHLFGMLTPHWMYAWFLPVAVKQQMAMILGGICGVLTLIGGAGLLVRRLTNPRIR
ATSSGADILILAILLIQCILGLTTIPFSAQHPDGSEMMKLVGWAQSIVTFQGGASTHLDG
VAFIFRVHLVLGMTIFLLFPFTRLVHVWSAPFEYFTRRYQIVRSRR
>LFGLNPFC_01766 putative isomerase YddE
MKPQVYHVDAFTSQPFRGNSAGVVFPADNLSEAQMQLIARELGHSETAFLLHSDDSDVRI
RYFTPTVEVPICGHATVAAHYVRAKVLGLGNCTVWQTSLAGKHRVTIEKQNDDYRISLEQ
GTPGFEPPLTGETRAAIINALHLTEDDILQGLPIQVATTGHSKVMIPLKPEVDIDALSPD
LAALTAISKQIGCNGFFPFQIRPGKSETDGRMFSPAIGIVEDPVTGNANGPMGAWLVHHN
VLSHDGNVLRVKGHQGRALGRDGVIDVTVTIRDNQPEKVTISGAAVILFHAEWAIEL
>LFGLNPFC_01767 Arylamine N-acetyltransferase
MTPILNHYFARINWLGAAAANIDTLRALHLKHNCTIPFENLDVLLPREIQLDDQSLEDKL
VIARRGGYCFEQNGVFERVLRELGFNVRSLLGRVVLSNPPALPPRTHRLLLVELEGEKWI
ADVGFGGQTLTAPIRLMPDIMQTTPHGEYRLLQEGDDWVLQFNHHQHWQSMYRFDLCEQQ
QSDYVMGNFWSAHWPQSHFRHHLLMCRHLPDGGKLTLTNFHFTHYENGHAVEQRNLPDVA
SLYAVMQEQFGLGVDDAKHGFTVDELALVMAAFDTHPEAGK
>LFGLNPFC_01768 hypothetical protein
MSQFIPVELHHASRLLNHGPTVMITSFDEQSQRRNIMAAAWSMPVEFEPPRVAIVVDKST
WTRELIERNGKFGIVIPGVAATNWTWAVGSVSGREEDKFNCYGIPVVRGPVLGLPLVEEK
CLAWMECRLLPATSAQEKYDTLFGEVVSAAADARVFVEGRWQFDDDKLNTLHHLGAGTFV
TSGKRVTAG
>LFGLNPFC_01769 hypothetical protein
MFIRPVKPSDVEPLMDMLLDRDQFDQDGLHHVQKTLTHYFSGQSADLWFSAEHLGLAGIA
YCASEMMTNDV
>LFGLNPFC_01770 hypothetical protein
MLQAIKKIIIMTIVNIIFFYSFQSTADEMVLIKKYGFGLERDIKGRPLIYPIENYDECKK
KCNHMNYIADVNAQLAMSKKNNRIFANITFTNNSSTTYFFSKYYLPMKVKDGKGDEYDAI
CKKPFRINSGNITLDYLGGSCPFEINTNPERWNTIKPGRSFLLSITLNDMYAFIPGDNYY
FIKSSGYKFVNDKWFTLKSINNIFLYFLNLKINCADDFFDINNFTTCNNLLLDEKNTFLL
ISYGQLRMKKILFTSPLMKFL
>LFGLNPFC_01771 hypothetical protein
MPYIINIGAKFEYRDGKKNPVTGKDSHVATLCHEMSHIQWYYGDNKKGGMWSQDYTTTDK
YSTCKEDEVSYDEHIRIATKLISKQKDQIFENAYNIERYFEIRLIESEIDSIDDEILSNS

VKKK
>LFGLNPFC_01772 IS66 family transposase ISCro1
MYGSCPEKIHDVHARAPTYITTEALQRIGELYAIEAEVRGCSAEQRLAARKARAAPLMQS
LYDWIQQQMKTLSRHSDTAKAFAYLLKQWDALNVYCSNGWVEIDNNIAENALRGVAVGRK
NWMFAGSDSGGEHAAVLYSLIGTCRLNNVEPEKWLRYVIEHIQDWPANRVRDLLPWKVDL
SSQ
>LFGLNPFC_01773 hypothetical protein
MRPYRAGTSTFKTHCTQLCRSGASGPCCHREICRPSAVIPPVRNIPSSGVELSRATLGRW
TGAVAELLEPLYDVLRQYVLMPGKVHADDIPVPVQEPGSGKTRQPGCGSTSVMTVTPVHR
CPRRSGSRTVRTGKVSIHKITWPVTAVCFRPMLTVVTGRYTNPAE
>LFGLNPFC_01774 IS66 family transposase ISCro1
MDTSLAHENARLRALLQTQQDTIRQMAEYNRLLSQRVAAYASEINRLKALVAKLQRMQFG
KSSEKLRAKTERQIQEAQERISALQEEMAETLGEQYDPVLPSALRQSSARKPLPASLPRE
TRVIRPEEECCPACGGELSSLGCDVRATGAYQQRL
>LFGLNPFC_01775 hypothetical protein
MISLPAGSRIWLVAGITDMRNGFNGLASKVQNVLKDDPFSGHLFIFRGRRGDQIKVLWAD
SDGLCLFTKRLERGRFVWPVTRDGKVHLTPAQLSMLLEGINWKHPKRTERAGIRI
>LFGLNPFC_01776 hypothetical protein
MKHRTWITEALRLHFEEHLPRVVAGRRLGVPKSTVCSMFVRFRRAGLSWPLPAGMSEQEL
DACLYGQFSTVPVVRPESTVISEAPVVKKRPRRPNFPYEFKIALVEQSLQPGACVAQIAR
ENGINDNLLFNWRHQYRKGGLLPSGKMPALLPVTLTPEPDNKIPAPAQEPEQINTPSDSL
CCELVLPAGTLRLKGKLTPALLQTLIREIKGSSH
>LFGLNPFC_01777 hypothetical protein
MDDNNQTSGQPKPEPEECVKEQKITDHFKIMIDKARKAQKLVLIKRADDLLRWGAQEEYD
FSKIFGVKGNKEVNIRKYGHNTGRRMNARFLMMDGVRRLMIIANDLTMSSFIN
>LFGLNPFC_01778 Actin cross-linking toxin VgrG1
MSLKGLRFTLEVDGQEPDTFAVVSFRLIQNQSYPFVMSVDVASDSFMQTAEMLLEKNATL
TIWQGVIPLRYVTGVVAGFGMQENNGWQMRYHLRIEPPLWRCGLRRNFRIFQQQDIRTIS
ATLLNENGVTEWTPLFYEDHPAREFCVQYGESDLAFLARLWAEEGIFFFERFAADSPEQK
LTLCDDVAGLSQAGELPFNPDTSAGAETECVSMFRYEAHVRPSSVQSQDYTFKVPDWPGM
YEQQGESLNGQLEQYEIFDYPGRYKDEQHGKDFTLYRMESLRSDAEKATGQSNSPKLWPG
TWFTLTGHPQKMLNREWQVVQSILSGDQPQALHGSQGRGTTLGNQLEVIPADRTWRPRLQ
SKPKVDGPQSAIVTGPAGEEIFCDEHGRVRVKFHWDRYHGMTEESSCWVRVSQAWAGPGF
GNLAIPRVGQEVIVDFLNGDPDQPLVMGRTYHEDNRSPGDLPGTKTQMTIRSKTYKGSGF
NELRFEDATDKEQVYIHAQKNMDTEVLNDRTTDVKHDHTETIGNDQKITVVKGQTVQVGT
RKEGGHDQSITVANDRCITVRNDQTLQVTNDRTVSVSNDDGLYVRNDRKVTVEGKQEHKT
TGNHVSLVEGKHSLVVKGDLARKVSGALGIKVDGDIVLESSSRISLKVGGSFVVIHSGGV
DIVGPKISLNSGGSPGTPVPALQPTVLKTLGDEKSGDGSDSGEENEDSGGNCVTGSGGDD
RGDDEDEPEKYTLQFHFTDDDGIPYSETRYIAFFEDGTQTHGETDEEGYTERFFVSSKHE
IKVKLLFANDDFLSMEGHYGR
>LFGLNPFC_01779 hypothetical protein
MPLAAKLTDKGTQHDGYYETVITAGSSTVFIDGLPAARQGDPLTPHAKPKHPPHPRKIAR
GSSTVFIDGLPAARTGDAIDCGGVVIGGGTVNIG
>LFGLNPFC_01780 hypothetical protein
MTETAAIALMVLDRRPDLAPPLGRAERQQFQRLLVWLVANVYPTFTFADYPKRWASDAPV
IEYRKSLYIWLNSQLTAEPYVFGEQLTLVDCYLCTMRTWGPGHEWFQDNAPNINAIADAV
CQIPKLQEVLKRNVII
>LFGLNPFC_01781 hypothetical protein
MIKVYGVPGWGSTISELMLTLADIPYQFVDVSGFDHEGTSRDLLKTLNPLCQVPTLAL
>LFGLNPFC_01782 L-asparagine permease 2
MSKHNTDTSDQHAAKRRWLNAHEEGYHKAMGNRQVQMIAIGGAIGTGLFLGAGARLQMAG
PALALVYLICGLFSFFILRALGELVLHRPSSGSFVSYAREFLGEKAAYVAGWMYFINWAM
TGIVDITAVALYMHYWGAFGGVPQWVFALAALTIVGTMNMIGVKWFAEMEFWFALIKVLA
IVTFLVVGTVFLGSGQPLDGNTTGFHLITDNGGFFPHGLLPALVLIQGVVFAFASIEMVG
TAAGECKDPQTMVPKAINSVIWRIGLFYVGSVVLLVMLLPWSAYQAGQSPFVTFFSKLGV
PYIGSIMNIVVLTAALSSLNSGLYCTGRILRSMAMGGSAPSFMAKMSRQHVPYAGILATL
VVYVVGVFLNYLVPSRVFEIVLNFASLGIIASWAFIIICQMRLRKAIKEGKAADVSFKLP
GAPFTSWLTLLFLLSVLVLMAFDYPNGTYTIAALPIIGILLVIGWFGVRKRVAEIHSTAP
VVEEDEEKQEIVFKPETSS
>LFGLNPFC_01783 putative protein YncE
MHLRHLFSSRLRGSLLLGSLLVASSFSTQAAEEMLRKAVGKGAYEMAYSQQENALWLATS
QSRKLDKGGVVYRLDPVTLEVTQAIHNDLKPFGATINNTTQTLWFGNTVNSAVTAIDAKT
GEVKGRLVLDDRKRTEEVRPLQPRELVADDATNTVYISGIGKESVIWVVDGENIKLKTAI
QNTGKMSTGLALDSKGKRLYTTNADGELITIDTADNKILSRKKLLDDGKEHFFINISLDT

ANERAFITDSKAAEVLVVDTRNGNILAKVAAPESLAVLFNPARNEAYVTHRQAGKVSVID
AKSYKVVKTFDTPTHPNSLALSADGKTLYVSVKQKSTKQQEATQPDDVIRIAL
>LFGLNPFC_01784 Metal-pseudopaline receptor CntO
MKIFSVRQTVLPALLVLSPVVFAADEQTMIVSAAPQVVSELDTPAAVSVVDGEEMRLATP
RINLSESLTGVPGLQVQNRQNYAQDLQLSIRGFGSRSTYGIRGIRLYVDGIPATMPDGQG
QTSNIDLSSVQNVEVLRGPFSALYGNASGGVMNVTTQTGQQPPTIEASSYYGSFGSWRYG
LKATGATGDGTQPGDVDYTVSTTRFTTHGYRDHSGAQKNLANAKLGVRIDDASKLSLIFN
SVDIKADDPGGLTEAEWKANPQQAPRAEQYDTRKTIKQTQAGLRYERSLSAQDDMSVMMY
AGERETTQYQSIPMAPQLNPSHAGGVITLQRHYQGIDSRWTHRGELGVPVTFTTGLNYEN
MSENRKGYNNFRLNRGVPEYGQKGELRRDERNLMWNVDPYLQTQWQLTDKLSLDAGVRYS
SVWFDSNDHYVTPGNGDDSGDASYHKWLPAGSLKYAMTDAWNVYLAAGRGFETPTINELS
YRADGQSGMNFGLKPSTNDTIEIGSKTRIGDGLLSLALFQTDTDDEIVVDSSSGGRTTYK
NAGKTRRQGAELAWDQRFAGDFRVKASWTWLDATYRSNVCNEQDCNGNRMPGIARNMAFA
SIGYIPEDGWYAGTEARYMGDIMADDENTAKAPSYTLVGLFTGYKYNYHNLTVDLFGRVD
NLFDKEYVGSVIVNESNGRYYEPAPGRNYGVGINIAWRFE
>LFGLNPFC_01785 hypothetical protein
MLGSVLASAWRAGSLRLVSVNVLFVAPAQAFTVPEVGKRQLNEINRIRYELELMAVALAV
ENLTMQKNVAMLCQQYSR
>LFGLNPFC_01786 L-amino acid N-acyltransferase MnaT
MSIRFARKADCAAIAEIYNHAVLYTAAIWNDQTVDADNRIAWFEARTIAGYPVLVSEEDG
VVTGYASFGDWRSFDGFRHTVEHSVYVHPDHQGKGLGRKLLSRLIDEARDCGKHVMVAGI
ESQNQASLHLHQSLGFVVTAQMPQVGTKFGRWLDLTFMQLQLDERTEPDAIG
>LFGLNPFC_01787 Inner membrane protein YdcZ
MNQSLTLAFLIAAGIGLVVQNTLMVRITQTSSTILIAMLLNSLVGIVLFVSILWFKQGMA
GFGELVSSVRWWTLIPGLLGSFFVFASISGYQNVGAATTIAVLVASQLIGGLVLDIFRSH
GVPLRALIGPICGAILLVVGAWLVARRSF
>LFGLNPFC_01788 putative protein YdcY
MSHLDEVIARVDAAIEESVIAHMNELLIALSDDAELSREDRYTQQQRLRTAIAHHGRKHK
EDMEARHEQLTKGGTIL
>LFGLNPFC_01789 Orphan toxin OrtT
MSLYQHMLVFYAVMAAIAFLITWFLSHDKKRIRFLSAFLVGATWPMSFPVALLFSLF
>LFGLNPFC_01790 Gamma-aminobutyraldehyde dehydrogenase
MQHKLLINGELVSGEGEKQPVYNPATGDVLLEIAEASAEQVNAAVRAADAAFAEWGQTTP
KARAECLLKLADVIEENGQVFAELESRNCGKPLHSAFNDEIPAIVDVFRFFAGAARCLNG
LAAGEYLEGHTSMIRRDPLGVVASIAPWNYPLMMAAWKLAPALAAGNCVVLKPSEITPLT
ALKLAELAKDIFPAGVINVLFGRGKTVGDPLTGHPKVRMVSLTGSIATGEHIISHTAPSI
KRTHMELGGKAPVIVFDDADIEAVVEGVRTFGYYNAGQDCTAACRIYAQKGIYDTLVEKL
GAAVATLKSGSPDDESTELGPLSSLAHLERVSKAVEEAKATGHIKVITGGEKRKGNGYYY
APTLLAGALQDDAIVQKEVFGPVVSVTLFDNEEQVVNWANDSQYGLASSVWTKDVGRAHR
VSARLQYGCTWVNTHFMLVSEMPHGGQKLSGYGKDMSLYGLEDYTVVRHVMVKH
>LFGLNPFC_01791 Inner membrane ABC transporter permease protein YdcV
MHSERAPFFLKLAAWGGVVFLHFPILIIAAYAFNTEDAAFSFPPQGLTLRWFSVAAQRSD
ILDSVTLSLKVAALATLIALVLGTLAAAALWRRDFFGKNAISLLLLLPIALPGIVTGLAL
LTAFKTINLEPGFFTIVVGHATFCVVVVFNNVIARFRRTSWSLVEASMDLGANGWQTFRY
VVLPNLSSALLAGGMLAFALSFDEIIVTTFTAGHERTLPLWLLNQLGRPRDVPVTNVVAL
LVMLVTTLPILGAWWLTREGDNGQ
>LFGLNPFC_01792 Inner membrane ABC transporter permease protein YdcU
MAMNVLQSPSRPGLGKVPGFFWRNPGLGLFLLLLGPLMWFGIVYFGSLLTLLWQGFYTFD
DFTMSVTPELTLANIRALFNPANYDIIVRTLTMAVAVTIASAILAFPMAWYMARYTSGKM
KAFFYIAVMLPMWASYIVKAYAWTLLLAKDGVAQWFLQHLGLEPLLTAFLTLPAVGGNTL
STSGLGRFLVFLYIWLPFMILPVQAALERLPPSLLQASADLGARPRQTFRYVVLPLAIPG
IAAGSIFTFSLTLGDFIVPQLVGPPGYFIGNMVYSQQGAIGNMPMAAAFTLVPIVLIALY
LAFVKRLGAFDAL
>LFGLNPFC_01793 Spermidine/putrescine import ATP-binding protein PotA
MTYAVEFDNVSRLYGDVRAVNGVSIAIKDGEFFSMLGPSGSGKTTCLRLIAGFEQLSGGA
ISIFGTPASNLPPWERDVNTVFQDYALFPHMSILDNVAYGLMVKGVNKKQRHAMAQEALE
KVALGFVQQRKPSQLSGGQRQRVAIARALVNEPRVLLLDEPLGALDLKLREQMQLELKKL
QQSLGITFIFVTHDQGEALSMSDRVAVFNNGRIEQVDSPRDLYMRPRTPFVAGFVGTSNV
FDGLMAEKLCGMTGSFALRPEHIRLNTPGEMQANGTIQAVQYQGAATRFELTLSGGEKLL
VSQANMTGEELPATLTPGQQVMVSWSRDVMVALVEER
>LFGLNPFC_01794 Bifunctional polyhydroxybutyrate synthase / ABC transporter periplasmic binding protein
MSKTFARSSLCALSMTIMTAHAAEPPTNLENPEGRLDIIAWPGYIERGQTDKQYDWVTQF
EKETGCAVNVKTAATSDEMVSLMVKGGYDLVTASGDASLRLIMGKRVQPINTALIPNWKA

LDPRVVKGDWFNVGGKVYGTPYQWGPNLLMYITKTFPTPPDSWQVVFVEQNLPDGKSNKG
RVQAYDGPIYIADAALFVKATQPQLGISDPYQLTEEQYQAVLKVLRDQHSLIHRYWHDTT
VQMSDFKNEGVVASSAWPYQANALKAEGQPVATVFPKEGVTGWADTTMLHSEAKHPVCAY
KWMNWSLTPKVQGDVAAWFGSLPVVPQGCKASPLLGEKGCETNGFNYFDKIAFWKTPIAE
GGKFVPYSRWTQDYIAIMGGR
>LFGLNPFC_01795 2-aminoadipate transaminase
MKKYQQLAEQLREQIASGIWQPGDRLPSLRDQVALSGMSFMTVSHAYQLLESQGYIIARP
QSGYYVAPQAIKMPKATVIPVTRDEAVDINTYIFDMLQASRDPSVVPFASAFPDPRLFPL
QQLNRSLAQVSKTATAMSVIENLPPGNVELRQAIARRYALQGITISPDEIVITAGALEAL
NLSLQAVTEPGDWVIVENPCFYGALQALERLRLKALSVATDIKEGIDLQALELALQEYPV
KACWLMTNSQNPLGFTLTPQKKVRLVALLNQYNVTLIEDDVYSELYFGREKPLPAKAWDR
HDGVLHCSSFSKCLVPGFRIGWVAAGKHARKIQRLQLMSTLSTSSPMQLALVDYLSTRRY
DAHLRRLRRQLAERKQRAWQALLRYLPAEVKIHHNDSGYFLWLELPEPLDAGELSLVALT
HHISIAPGNMFSTGENWSRFFRFNTAWQWGEREEQAVKQLGKLIQERL
>LFGLNPFC_01796 hypothetical protein
MAGHKGHEFMWVKNVDHQLRHEADSDELRAVAEESAEGLREHFYWQKSRKPEAGQR
>LFGLNPFC_01797 23S rRNA 5-hydroxycytidine synthase
MAKIAAIFQLLDKKVTVSSHRLELLSPARDAAIAREAILHGADAVYIGGPGFGARHNASN
SLKDIAELVPFAHRYGAKIFVTLNTILHDDELEPAQRLITDLYQTGVDALIVQDMGILEL
DIPPIELHASTQCDIRTVEKAKFLSDVGFTQIVLARELNLEQIRAIHQATDATIEFFIHG
ALCVAYSGQCYISHAQTGRSANRGDCSQACRLPYTLKDDQGRVVSYEKHLLSMKDNDQTA
NLGALIDAGVRSFKIEGRYKDMSYVKNITAHYRQMLDAIIEERGDLARASSGRTEHFFVP
STEKTFHRGSTDYFVNARKGDIGAFDSPKFIGLPVGEVLKVAKDHIDVAVTEPLANGDGL
NVMIKREVVGFRANTVEKTGENQYRVWPNEMPADLHKIRPHHPLNRNLDHNWQQALTKTS
SERRVAVDIELGGWQEQLILTLTSEEGVSITHTLDGQFDEANNAEKAMNNLKDGLAKLGQ
TIYYARNVQINLPGALFVPNSLLNQFRREAADMLDAARLASYQRGSRKPVADPAPVYPQT
HLSFLANVYNQKAREFYHRYGVQLIDAAYEAHEEKGEVPVMITKHCLRFAFNLCPKQAKG
NIKSWKATPMQLVNGDEVLTLKFDCRPCEMHVIGKIKNHILKMPLPGSVVASVSPDDLLK
TLPKRKG
>LFGLNPFC_01798 HTH-type transcriptional regulator SutR
MENLARFLSTTLKQLRQQRGWSLSRLAEATGVSKAMLGQIERNESSPTVATLWKIATGLN
VPFSTFISPPQSATPSVYDPQRQAMVITSLFPYDPQLCFEHFSIQMAPGAISESTPHEKG
VIEHVVVIDGQLDLCVDGEWQSLNCGEGVRFAADVTHVYRNGGEQTVHFHSLIHYPRS
>LFGLNPFC_01799 Inner membrane protein YdcO
MRLFSIPPPTLLAGFLAVLIGYASSAAIIWQAAIVAGATTAQISGWMTALGLAMGVSTLA
LTLWYRVPVLTAWSTPGAALLVTGLQGLTLNETIGVFIVTNVLIVLCGITGLFARLMRII
PHSLAAAMLAGILLRFGLQAFASLDGQFTLCGSMLLAWLATRAVAPRYAVIAAMIIGIVI
VIAQGDVVTTDVVFKPVLPTYIPPDFSFAHSLSVALPLFLVTMASQNAPGIAAMKAAGYS
APVSPLIVFTGLLALVFSPFGVYSVGIAAITAAICQSPEAHPDKDQRWLAAAVAGIFYLI
AGLFGSAITGMMAALPVSWIQMLAGLALLSTIGGSLYQALHNERERDAAVVAFLVTASGL
TLFGIGSAFWGLIAGGVCYVVLNLIADRNR
>LFGLNPFC_01800 hypothetical protein
MRTTSFAKVAALCGLLALSGCASKITQPDKYSGFLNNYSDLKETTSATGKPVLRWVDPSF
DQSKYDSIVWNPITYYPVPKPSTQVGQKVLDKILNYTNTEMKEAIAQRKPLVTTAGPRSL
IFRGAITGVDTSKEGLQFYEVVPVALVVAGTQMATGHRTMDTRLYFEGELIDAATNKPVI
KVVRQGEGKDLNNESTPMAFENIKQVIDDMATDATMFDVNKK
>LFGLNPFC_01801 Tellurite methyltransferase
MIIRDENYFTDKYELTRTHSEVLEAVKVVKPGKTLDLGCGNGRNSLYLAANGYDVDAWDK
NAMSIANVERIKSIENLDNLHTRVVDLNNLTFDGQYDFILSTVVLMFLEAKTIPGLIANM
QRCTKPGGYNLIVAAMDTADYPCTVGFPFAFNEGELRRYYEGWERVKYNEDVGELHRTDA
NGNRIKLRFATMLARKK
>LFGLNPFC_01802 Tellurite resistance protein TehA
MQSDKVLNLPAGYFGIVLGTIGMGFAWRYASQLWQVSHWLGDGLVILAMIIWGLLTGAFI
TRLIRFPHSVLAEVRHPVLSSFVSLFPATTMLVAIGFVPWFRPLAVCLFSFGVVVQLAYA
AWQTAGLWRGSHPEEATTPGLYLPTVANNFISAMACGALGYTDAGLVFLGAGVFSWLSLE
PVILQRLRSSGELPTALRTSLGIQLAPALVACSAWLSVNGGEGDTLAKMLFGYGLLQLLF
MLRLMPWYLSQPFNASFWSFSFGVSALATTGLHLGSGSDNGFFHTLAVPLFIFTNFIIAI
LLIRTFALLMQGKLLVRTERAVLMKAEDKE
>LFGLNPFC_01803 UDP-3-O-(3-hydroxymyristoyl)glucosamine N-acyltransferase
MRKYRLSEEQRAFSYQEDGTKKSVLLRQIIAMSDFNDVIAGSAGGWIDHETVLAQEGNCW
IYDQNAIAFGGTVISGNTRITGTSVLWGEVYATDNVWIDNSEISQGAYISDSVTIRDSLV
CGQCRIFGHALIDQHSMIVAAQGLTPDHQLLLQIYDRAKVSASRIVHQAQIYGDAVVRYA
FIEHRAEVFDFASVEGNEENNVWLCDCAKVYGHAQVKAGIEEDAIPTIHYSSQVAEYAIV
EGNCVLKHHVLIGGNAVVRGGPILLDEHVVIQGESRITGAVIIENHVELTDHAVVEAFDG

DTIHVRGPKVINGEERITRTLLAGLL
>LFGLNPFC_01804 Ribosomal-protein-serine acetyltransferase
MTETIKVSESLELHAVAESHVTPLYQLICKNKTWLQQSLNWPQFVQSEEDTRKTVQGNVM
LHQRGYAKMFMIFKEDELIGVISFNRIEPLNKTAEIGYWLDESHQGQGIISQALQALIHH
YAQSGELRRFVIKCRVDNPQSNQVALRNGFILEGCLKQAEFLNDAYDDVNLYARIIDSQ
>LFGLNPFC_01805 hypothetical protein
MFPEYRDLISRLKNENPRFMSLFDKHNKLDHEIARKEGSDGRGYNAEVVRMKKQKLQLKD
EMLKILQQVSVKEV
>LFGLNPFC_01806 Glucans biosynthesis protein D
MAAVCGTSGIASLFSQAAFAADSDIADGQTQRFDFSILQSMAHDLAQTAWRGAPRPLPDT
LATMTPQAYNSIQYDAEKSLWHNVENRQLDAQFFHMGMGFRRRVRMFSVDPATHLAREIH
FRPELFKYNDAGVDTKQLEGQSDLGFAGFRVFKAPELARRDVVSFLGASYFRAVDDTYQY
GLSARGLAIDTYTDSKEEFPDFTAFWFDTVKPGATTFTVYALLDSASITGAYKFTIHCEK
NQVIMDVENHLYARKDIKQLGIAPMTSMFSCGTNERRMCDTIHPQIHDSDRLSMWRGNGE
WICRPLNNPQKLQFNAYTDNNPKGFGLLQLDRDFSHYQDIMGWYNKRPSLWVEPRNKWGK
GTIGLMEIPTTGETLDNIVCFWQPEKAVKAGDEFAFQYRLYWSAQPPVHCPLARVMATRT
GMGGFPEGWAPGEHYPEKWARRFAVDFVGGDLKAAAPKGIEPVITLSSGEAKQIEILYIE
PIDGYRIQFDWYPTSDSTDPVDMRMYLRCQGDAISETWLYQYFPPAPDKRQYVDDRVMS
>LFGLNPFC_01807 putative protein
MANSITADEIREQFSQAMSAMYQQEVPQYGTLLELVADVNLAVLENNPQLHEKMVNADEL
ARLNVERHGAIRVGTAQELATLRRMFAIMGMYPVSYYDLSQAGVPVHSTAFRPIDDASLA
RNPFRVFTSLLRLELIENEILRQKAAEILRQRDIFTPRCRQLLEEYEQQGGFNETQAQEF
VQEALETFRWHQSATVDEETYRALHNENRLIADVVCFPGCHINHLTPRTLDIDRVQSMMP
ECGIEPKVLIEGPPRREVPILLRQTSFKALEETVLFAGQKQGTHTARFGEIEQRGVALTP
KGRQLYDDLLRNAGTGQDNLTHQMHLQETFRTFPDSEFLMRQQGLGWFRYRLTPSGEAHR
QVIHPGDDPQPLIERGWVVAQPITYEDFLPVSAAGIFQSNLGNETRACNHGDASREAFEQ
ALGCPVLDEFQLYQEAEERSKRRCGLL
>LFGLNPFC_01808 HTH-type transcriptional regulator CatM
MEKNSLFSQRIRLRHLHTFVAVAQQGTLGRAAETLNLSQPALSKTLNELEQLTGARLFER
GRQGAQLTLPGEQFLTHAVRVLDAINTAGQSLHRKEGLNNDVVRVGALPTAALGILPSVI
GQFHQQQKETTLQVATMSNPMILAGLKTGEIDIGIGRMSDPELMNGLNYELLFLESLKLV
VRPNHPLLQENVTLSRVLEWPVVVSPEGTAPRQHSDALVQSQGCKIPSGCIETLSASLSR
QLTVEYDYVWFVPSGAVKDDLRHATLVALPVPGHGAGEPIGILTRVDATFSSGCQLMINA
IRKSMPF
>LFGLNPFC_01809 Methyl-accepting chemotaxis protein III
MLLSQGPGFAVVASEVRTLASRSAQAAKEIEGLISESVRLIDLGSDEVATAGKIMCTIVD
AVASVTHIMQEIATASGEQSRGITQVSQAISEMDKVTQQNASLVEEASTAAVSLEEQAAR
LTEAVDVFRLNKHSVSAEPRGACEPVSFATV
>LFGLNPFC_01810 hypothetical protein
MKKLALILVMGTLVSFYADAGRKPCSGSKGGISHCTAGGKFVCNDGSISASKKTCTN
>LFGLNPFC_01811 Cytochrome b561
MGNKYSRLQISIHWLVFLLVIVAYCAMEFRGFFPRSDRPLINMIHVSCGISILVLMVVRL
LLRLKCPTPPIIPKPKPMMTGLAHLGHLVIYLLFIALPVIGLVMMYNRGNPWFAFGLTMP
YASEANFERVDSLKSWHETLANLGYFVIGLHAAAALAHHYFWKDNTLLRMMPRKRS
>LFGLNPFC_01812 Glyceraldehyde-3-phosphate dehydrogenase
MSKVGINGFGRIGRLVLRRLLEVKSNIDVVAINDLTSPKILAYLLKHDSNYGPFPWSVDY
TEDSLIVNGKSIAVYAEKEAKNIPWKAKGAEIIVECTGFYTSAEKSQAHLDAGAKKVLIS
APAGEMKTIVYNVNDDTLDGNDTIVSVASCTTNCLAPMAKALHDSFGIEVGTMTTIHAYT
GTQSLVDGPRGKDLRASRAAAENIIPHTTGAAKAIGLVIPELSGKLKGHAQRVPVKTGSV
TELVSILGKKVTAEEVNNALKKATNNNESFGYTDEEIVSSDIIGSHFGSVFDATQTEITA
VGDLQLVKTVAWYDNEYGFVTQLIRTLEKFAKL
>LFGLNPFC_01813 Lactaldehyde dehydrogenase
MSVPVQHPMYIDGQFVTWRGDAWIDVVNPATEVVISRIPDGQAEDARKAIDAAERAQPEW
EALPAIERASWLRKISAGIRERASEISALIVEEGGKIQQLAEVEVAFTADYIDYMAEWAR
RYEGEIIQSDRPGENILLFKRALGVTTGILPWNFPFFLIARKMAPALLTGNTIVIKPSEF
TPNNAIAFAKIVDEIGLPRGVFNLVLGRGETVGQELAGNPKVAMVSMTGSVSAGEKIMAT
AAKNITKVCLELGGKAPAIVMDDADLELAVKAIVDSRVINSGQVCNCAERVYVQKSIYDQ
FVNRLGEAMQAVQFGNPAERNDIAMGPLINAAALERVEQKVARAVEEGARVALGGKAVEG
KGYYYPPTLLLDVRQEMSIMHEETFGPVLPVVAFDTLEEAISMANDSDYGLTSSIYTQNL
NVAMKAIKGLKFGETYINRENFEAMQGFHAGWRKSGIGGADGKHGLHEYLQTQVVYLQS
>LFGLNPFC_01814 Protein YdcF
MNITPFPTLSPATIDAINVIGQWLAQDDFSGEVPYQADCVILAGNAVMPTIDAACKIARD
QQIPLLISGGIGHSTTFLYSAIAQHPHYNTIRTTGRAEATILADIAHQFWHIPHEKIWIE
DQSTNCGENARFSIALLNQAVERVHTAIVVQDPTMQRRTMATFRRITGDNPDAPRWLSYP

GFVPQLGNNADSVIFVNPLQGLWPVERYLSLLTGELPRLRDDSDGYGPRGRDFIVHVDFP
AEVIQAWQTLKHDAVLIEAMESRSLR
>LFGLNPFC_01815 ATP-dependent RNA helicase DbpA
MTEQQKLTFTALQQRLDSLMLRDRLRFSRRLHGVKKVKNPDAQQAIFQEMAKEIDHAAGK
VLLREAARPEITYPDNLPVSQKKQDILEAIRDHQVVIVAGETGSGKTTQLPKICMELGRG
IKGLIGHTQPRRLAARTVANRIAEELKTEPGGCIGYKVRFSDHVSDNTMVKLMTDGILLA
EIQQDRLLMQYDTIIIDEAHERSLNIDFLLGYLKELLPRRPDLKIIITSATIDPERFSRH
FNNAPIIEVSGRTYPVEVRYRPIVEEADDTERDQLQAIFDAVDELSQESPGDILIFMSGE
REIRDTADALNKLNLRHTEILPLYARLSNSEQNRVFQSHSGRRIVLATNVAETSLTVPGI
KYVIDPGTARISRYSYRTKVQRLPIEPISQASANQRKGRCGRVSEGICIRLYSEDDFLSR
PEFTDPEILRTNLASVILQMTALGLGDIAAFPFVEAPDKRNIQDGVRLLEELGAITTDEQ
ASAYKLTPLGRQLSQLPVDPRLARMVLEAQKHGCVREAMIITSALSIQDPRERPMDKQQA
SDEKHRRFHDKESDFLAFVNLWNYLGEQQKALSSNAFRRLCRTDYLNYLRVREWQDIYTQ
LRQVVKELGIPVNSEPADYREIHIALLTGLLSHIGMKDADKQEYTGARNARFSIFPGSGL
FKKPPKWVMVAELVETSRLWGRIAARIDPEWVEPVAQHLIKRTYSEPHWERAQGAVMATE
KVTVYGLPIVAARKVNYSQIDPALCRELFIRHALVEGDWQTRHAFFRENLKLRAEVEELE
HKTRRRDILVDDETLFEFYDQRISHDVISARHFDSWWKKVSRETPDLLNFEKSMLIKEGA
EKISKLDYPNFWHQGNLKLRLSYQFEPGADADGVTVHIPLPLLNQVEESGFEWQIPGLRR
ELIIALIKSLPKPVRRNFVPAPNFAEAFLGRVTPLELPLLDSLERELRRMTGVTVDREDW
HWDQVPDHLKITFRVVDDKNKKLKEGRSLQDLKDALKGKVQETLSAVADDGIEQSGLHIW
SFGQLPESYEQKRGNYKVKAWPALVDERDSVAIKLFDNPLEQKQAMWSGLRRLLLLNIPS
PIKYLHEKLPNKAKLGLYFNPYGKVLELIDDCISCGVDKLIDANGGPVWTEEGFAALHEK
VRAELNDTVVDIAKQVEQILTAVFNINKRLKGRVDMTMALGLSDIKAQMGGLVYRGFVTG
NGFKRLGDTLRYLQAIEKRLEKLAIDPHRDRAQMLKVENVQQAWQQWINKLPPARREDED
VKEVRWMIEELRVSYFAQQLGTPYPISDKRILQAMEQISG
>LFGLNPFC_01816 FMN-dependent NADH-azoreductase
MSKVLVLKSSILAGYSQSNQLSDYFVEQWREKHSADEITVRDLAANPIPVLDGELVGALR
PSDAPLTPRQQEALALSDELIAELKAHDVIVIAAPMYNFNISTQLKNYFDLVARAGVTFR
YTENGPEGLVTGKKAIVITSRGGIHKDGPTDLVTPYLSTFLGFIGITDVKFVFAEGIAYG
PEMAAKAQSDAKAAIDSIVAA
>LFGLNPFC_01817 putative protein YnbD
MLLAPFFFFTYGFLNQFTATQDLNNHDIPSQVFGWETAIPFLPWTIVPYWSLDLLYGFSL
FICSSTFEQRRLVHRLILATVMACCGFFLYPLKFSFIRPEVSGVTGWLFSQLELFDLPYN
QSPSLHIILCWLLWRHFRQHLAVRWRKVCGGWFLLIAISTLTTWQHHFIDVITGLAVGML
IDWMVPVDRRWNYQKPDQRRIKIALPYVAGACSCIVLMELMIMLQLWWSVWLCWPVLSLF
IIGRGYGGLGAITTGKDSQGKLPPAVYWLTLPWRIGMWLSMRWFCLRLEPVSKITAGVYL
GAFPRHIPAQNAVLDVTFEFPRGRATKDRLYFCVPMLDRVVPEEGELRQAVAMLETLREE
QGSVLVHCALGLSRSALVVAAWLLCYGHCKTVDEAISYIRARRSRIVLKEEHKAMLKLWE
NR
>LFGLNPFC_01818 putative protein YnbC
MENSRIPGEHFFTTSDNTALFYRHWPTLLPGAKKVIVLFHRGHEHSGRLQHIVDELAMPD
TAFYAWDARGHGQTSGPRGYSPSLARSVQDVDEFVRFAASDSQVGLEEVVVIAQSVGAVM
VATWVHDYAPAIRGLVLASPAFKVKLYVPLARPGLALWHRLRGLFFINSYVKGRYLTHDR
QRVASFNNDPLITRAIAVNILLDLYKTSERIVSDAAAITLPTQLLISGDDYVVHRQPQID
FYQRLRSPLKELHLLPGFYHDTLGEENRAQAFEKMQSFISRLYANKSQKFDYQHEDRTGP
SADRWRLLSGGPVPLSPVDLAYRFMRKAMKLFGAHSAGLHLGMSTGFDSGSSLDYVVYQNQ
PQGSNAFGRFIDKIYLNSVGWRGIRQRKTHLQMLIKQAVAHLHAKGLAVRVVDIAAGHGR
YVLDALANEPAVSDILLRDYSELNVAQGQEMIAQRGMSGRVRFEQGDAFNLEELSALTPR
PTLAIVSGLYELFPENEQVKNSLAGLANAIDPGGILIYTGQPWHPQLELIAGVLTSHKDG
KPWVMRVRSQGEMDSLVHDAGFDKCTQRIDVWGIFTVSMAVRRDN
>LFGLNPFC_01819 hypothetical protein
MLEKSLAILFALLILATLINRFLVWRLPERKGDEVTLRIRTWWGIVICFSLVISGPRWMT
LTFFALISFLALKEYCTLISVHFPRWLYWVIPLNYLLIGFNCFELFLLFIPLAGFLILAT
WRVFVGDPSGFLHTVSAIFCGWIMTVFTLSHAAWLLMLPTINIQGGALLVLFLLALTESN
DIAQYLWGKFCGRRKVVPKVSPGKTLEGLVGGVITTMIASLIIGPLLTPLNTLQVLLAGL
LIGISGFCGDVVMSATKRDVGVKDSGKLLPGHGGLLDRIDSLIFTAPVFFYFIRYCCY
>LFGLNPFC_01820 Inner membrane protein YnbA
MTLYQIKPLFQSLLRPTMFWLYKHHVTANHITLAALALSLLTGLLLVLVAQPILFLLLPI
VLFIRMTLNALDGMLARECNQKTRLGAILNETGDVISDIALYLPFLFLPESNASLVILML
FCTILTEFCGLLAQTINGIRSYVGPFGKSDRALIFGLWGLAIAIYPQWMQWNNLLWSIAS
ILLLWTAINRCRSVLLMSAER
>LFGLNPFC_01821 Pyridoxine 4-dehydrogenase
MSSNTFTLGTKSVNRLGYGAMQLAGPGVFGPPRDRHVAITVLREALALGVNHIDTSDFYG
PHVTNQIIREALYPYSDDLTIVTKIGARRGEDASWLPAFSPAELQKAVHDNLRNLGLDVL

DVVNLRVMMGDGHGPAEGSIEASLTVLAEMQQQGLVKHIGLSNVTPTQVAEARKIAEIVC
VQNEYNIAHRADDAMIDALARDGIAYVPFFPLGGFTPLQSSTLSDVATSLGATPMQVALA
WLLQRSPNILLIPGTSSVAHLRENMAAEKLHLSEKVLSTLDGISRE
>LFGLNPFC_01822 hypothetical protein
MQRKTLLSACIALALSGQGWAADITEIETTTGEKKNTNVTCPADPGKLSPEELKRLPSEC
SSVVEQNLMPWLVTGAATALITTLAIVELNDDDDHHRNNSPLPPTPPDDDSDDTPVPPTP
GGDEIIPDDGPDDTPTPPKPIAFNNDVILDKTEKTLTIRDSVFSYTENADGTISLQDSNG
RKATINLWQIDETNNTVALEGMSADGATKWQYNHNGELVITGDNTTVNNTGKTIVDGKGA
TGTEIAGNNAVVNQDGELDVSGGGHGIDITGDSATVDNKGGMTVTDPDSIGIQIDGDKAV
VNKDGDSAISNGGTGTQVNGDEATVNNNGSTTVDGKDSTGTEINGDKAIVNNDGDSTILD
GGTGTRITGDDATANNSGNTTVDGQGSTGTEIAGNNAVVNQDGTLDVSGGGHGIDITGDS
ATVDNKGGMTVTDPDSIGIQRDGDKAVVNNDGDNAISNGGTGTQVNGDEATVNNNGSTTV
DGKDSTGTEINGDKAIVNNDGDSTILDGGTGTRITGDDATANNSGNTTVDGQGSTGTEIA
GNNAVVNQDGELDVSGGGHGIDITGDSATVDNKGGMTVTDPDSIGIQIDGDKAVVNNDGG
SAISNGGTGTQINGDEATVNNNGNTTVDGQGSTGTEIAGNNAVVNQDGELDVSGGGHGID
ITGDSATVDNKGGMTVTDPDSIGIQIDGDKAVVNNDGDSAISNGGTGTQVNGDEATVNNN
GKTTVDGKDSTGTEINGDKAIVNNDGDSTILDGGTGTRITGDDATANNSGNTTVDGQGST
GTEIAGNNAVVNQDGELDVSGGGHGIDITGDSATVDNKGGMTVTDPDSIGIQIDGDKAVV
NNDGDNAISNGGTGTQVNGDEATVNNNGNTTVDGKDSTGTEINGDKAIVNNDGDSTILDG
GTGTRITGDDATANNSGNTTVDGQGSTGTEIAGNNAVVNQDGELDVSGGGHGIDITGDSA
TVDNKGGMTVTDPDSIGIQIDGDKAVVNNDGDSAISNGGTGTQVNGDEATVNNNGNTTVD
GKDSTGTEINGDKAIVNNDGDSTILDGGTGTRITGDDATANNSGNTTVDGQGSTGTEIAG
NNAVVNQDGELDVSGGGHGIDITGDSATVDNKGGMTVTDPDSIGIQIDGDKAVVNNDGDN
AISNGGTGTQVNGDEATVNNNGSTTVDGQGSTGTEIAGNNAVVNQDGTLDVSGGGHGIDI
TGDSATVDNKGGMTVTDPDSIGIQIDGDKAVVNNEGDNAISNGGTGTQVNGDEATVNNNG
KTTVDGKDSTGTEINGDKAIVNNDGDSTILDGGTGTRITGDDATANNSGNTTVDGQGSTG
TEIAGNNAVVNQDGLLDVSGGGHGIDITGDSATVINKGNITVTDKDSVGVLINGDRATFA
NTGHIDVNNSATGMSITTSEGAISQAGSMNVGDFSTGMALSGNNNSVTLAAKDLNVIGQK
ATGVNISGDNNAVDITGNILVDKDQTATNAVDYFYEPSIGVNVSGNSNTVSLDGKLTVVA
DSELTSRIYADFDGSQENISGLVVSGDDNTVYLNGGIQLVGEENQLTDGSTVASNRNGYG
KTPVITVDGKSSVYLNGDSTINGDLPLAYSGMIRLKNSAMIEIGADATINMQVDIYDHYA
RSESQMIFVESGAELVNKGDIDTRNIGFAAISGENSTGSNSGNITLSQYNYGLLANAGVG
YFTTKGGSAVNNGTITAKVMEQESVINLGASLGLNEANTFYSDANSMMGLDAFDHGYVSN
ESGGSIEMYGRGNVGMLAIDESTAENAGQITLDALWVDADDTTTLRSNIGNDARSYGVGM
AVGTNTYSGPRKNATAVNKQGGVITVYNAGIGMAAYGASNTVINEGIINLEKNANYDSSL
GADSLIGMAAYKSGTAINEQSGVININADNGQAFYSDGSGTILNYGTICVNTNCLTGNDY
NETDSYTSLLYTGGDVITAQNETQNLTQKASINDKKEGNVVNSGSLSGADIAISSGELVN
TSTGTINNAIIINDGELSNEGSVAKVTLNAGTFGNTGTVNSRMFQTGGTFNNQQGGVVQN
GANLSKTAITNNEGTWYLGASSSSDSNNASMMEIYNTAVFNNSGDFILNNSRNAIHLYQS
GSFYNTGHMLISGANYSGNAINYWNANNNGRFINSGTVDVTAKALATSGVDASTNHAYFW
NQNSGIVNFDKDSGVAVKFTHSNYVAQNDGTMNISGNNAIAMEGNKNAQLINNGTINLGA
QGTTDTGMIGMQLDSSATADAVIENNGTINIYANNSFAFSMLGSVGHLVNNGTVTIADGV
TGSGLIKQGNSVNIEGVNGNNGNNSEVHYANYTLPDVPGSSVFVSTDNVSDNGGQNNLNG
YVVGTSSDGSAGKLKVSNASLKGVSVNTGFTSGTSATSVTFDNVVQGNNLTDADTITSTS
VVWSAQGNTDANGNVDVTMTKNAYTDVVTDSSVNNVAQVLDTGYTNNDLYTSLNVGTTAE
LNSALKQISGSQATTVFNEARVLSNRFSMLSDAAPEVANGLAFNVVAKGDPRAELGNDTQ
YDMMALRKSLTLTEHQNLSLEYGIARLEGNGSDTAGDNGVTGGYSQFFGLKHQMAFDNGM
NWNNALRYDVHQLDSSRSIAYGDVNKTADANVKQQYLEFRSEGAKTFELREGLNVTPYAG
VKLRHTLEGGYQERNAGDFNLSMNSGSETAVDSIVGLKLDYAGKEGWSANATLEGGPNLS
YVKSQRTASISGAGSQRFNIDDGQSGGGFNSLATMGVKYSSQESALQVDAFHWKEDGISD
KGVMLNFKKTF
>LFGLNPFC_01823 hypothetical protein
MKRTLLLCAFLVGLVSSNVMALTLDEARTQGRVGETFYGYLVALKTDAETEKLVTDINAE
RKASYQQLAKQNNVSVDDIAKLAGQKLVERAKPGEYVQGINGKWVRKF
>LFGLNPFC_01824 hypothetical protein
MKILLAALTSSFMLVGCTPRIEVAAPKEPITINMNVKIEHEIIIKADKDVEELLETRSDL
F
>LFGLNPFC_01825 hypothetical protein
MLGKYKAVLALLLLIILVPLTLLMTLGLWVPTLAGIWLPLGTRIALDESPRITRKGLIIP
DLRYLVGDCQLAHITNASLSHPSRWLLSVGTVELDSACLAKLPQTEQSPAAPKTLAQWQS
MLPNTWINIDKLIFSPWQEWQGKLSLALTSDIQQLCYQGEKVKFQGQLKGQQLTVSELDV
AAFENQPPVKLVGEFTMPLVPDGLPVSGHATATLNLPQEPSLVDAELDWQENSGQLIVLA
RDNGDPLLDLPWQITRQQLTVSDGRWSWPYAGFPLSGRLGVKVDNWQAGLENALVSGRLS
VLTQGQAGKGNAVLNFGPGKLSMDNSQLPLQLTGEAKQADLILYARLPAQLSGSLSDPTL

TFEPGALLRSKGRVIDSLDIDEIRWPLAGVKVTQRGVDGRLQAILQAHENELGDFVLHMD
GLANDFLPDAGRWQWRYWGKGSFTPMNATWDVAGKGEWHDSTITLTDLSTGFDQLQYGTM
TVEKPRLILDKPVVWGRDAQHPSFSGALSLDAGQTLFTGGSVLPPSTLKFSVDGRDPTYF
LFKGDLHAGEIGPVRVNGRWDGIRLRGNAWWPKQSLTVFQPLVPPDWKMNLRDGELYAQV
AFSAAPEQGFRAGGHGVLKGGSAWMPDNQVNGVDFVLPFRFADGAWHLGTRGPVTLRIAE
VINLVTAKNITADLQGRYPWTEEEPLLLTDVSVDVLGGNVLMKQLRMPQHDPALLRLNNL
SSSELVSAVNPKQFAMSGAFSGALPLWLNNEKWIVKDGWLANSGPMTLRLDKDTADAVVK
DNMTAGSAINWLRYMEISRSSTKINLDNLGLLTMQANITGTSRVDGKSGTVNLNYHHEEN
IFTLWRSLRFGDNLQAWLEQNARLPGNDCPQGKECEDKQ
>LFGLNPFC_01826 D-lactate dehydrogenase
MKLAVYSTKQYDKKYLQQVNESFGFELEFFDFLLTEKTAKTANGCEAVCIFVNDDGSRPV
LEELKKHGVKYIALRCAGFNNVDLDAAKELGLKVVRVPAYDPEAVAEHAIGMMMTLNRRI
HRAYQRTRDANFSLEGLTGFTMYGKTAGVIGTGKIGVAMLRILKGFGMRLLAFDPYPSAA
ALELGVEYVDLPTLFSESDVISLHCPLTPENYHLLNEAAFDQMKNGVMIVNTSRGALIDS
QAAIEALKNQKIGSLGMDVYENERDLFFEDKSNDVIQDDVFRRLSACHNVLFTGHQAFLT
AEALTSISQTTLQNLSNLEKGETCPNELV
>LFGLNPFC_01827 Heat shock protein HslJ
MKKVAALVALSLLMAGCVSSDKIAVTPEQLQHHRFVLESVNGKPVTSDKNPPEISFGEKM
MISGSMCNRFSGEGKLSNGELTAKGLAMTRMMCVNPQLNELDNTISEMLKEGAQVDLTAN
QLTLATAKQTLTYKLADLMN
>LFGLNPFC_01828 hypothetical protein
MRAAFWVGCAALLLSACSSEPVQQATAAHVAPGLKASMSSSGEANCAMIGGSLSVARQLD
GTAIGMCALPNGKRCSEQSLAAGSCGSY
>LFGLNPFC_01829 Pyruvate-flavodoxin oxidoreductase
MITIDGNGAVASVAFRTSEVIAIYPITPSSTMAEQADAWAGNGLKNVWGDTPRVVEMQSE
AGAIATVHGALQTGALSTSFTSSQGLLLMIPTLYKLAGELTPFVLHVAARTVATHALSIF
GDHSDVMAVRQTGCAMLCAASVQEAQDFALISHIATLKSRVPFIHFFDGFRTSHEINKIV
PLADDTILELMPQAEIDAHRARALNPEHPVIRGTSANPDTYFQSREATNPWYNAVYDHVE
QAMNDFAAATGRHYQPFEYYGHPQAERVIILMGSAIGTCEEVVDELLTRGEKVGVLKVRL
YRPFSAKHLLQALPGSVRNVAVLDRTKEPGAQAEPLYLDVMTALAEAFNNGERETLPRVI
GGRYGLSSKEFGPDCVLAVFAELNAAKPKARFTVGIYDDVTNLSLPLPENTLPNSAKLEA
LFYGLGSDGSVSATKNNIKIIGNSTPWYAQGYFVYDSKKAGGLTVSHLRVSEQPIRSAYL
ISQADFVGCHQLQFIDKYQMAERLKPGGIFLLNTPYSAAEVWSRLPQEVQAVLNQKKARF
YVINAAKIARECGLAARINTVMQMAFFHLTQILPGDSALAELQGAIAKSYSSKGQDLVER
NWQALALARESVEEVPLQPVNPHSANRPPVVSDAAPDFVKTVTAAMLAGLGDALPVSALP
PDGTWPMGTTRWEKRNIAEEIPIWKEELCTQCNHCVAACPHSAIRAKVVPPEAMENAPAS
LHSLDVKSRDMRGQKYVLQVAPEDCTGCNLCVEVCPAKDRQNPEIKAINMMSRLEHVEEE
KINYDFFLNLPEIDRSKLERIDIRTSQLITPLFEYSGACSGCGETPYIKLLTQLYGDRML
IANATGCSSIYGGNLPSTPYTTDANGRGPAWANSLFEDNAEFGLGFRLTVDQHRVRVLRL
LDQFADKIPTELLTALKSDATPEVRRAQVAALRQQLNDVAEAHELLRDADALVEKSIWLI
GGDGWAYDIGFGGLDHVLSLTENVNILVLDTQCYSNTGGQASKATPLGAVTKFGEHGKRK
ARKDLGVSMMMYGHVYVAQISLGAQLNQTVKAIQEAEAYPGPSLIIAYSPCEEHGYDLAL
SHDQMRQLTATGFWPLYRFDPRRADEGKLPLALDSRPPSEALEETLLHEQRFRRLNSQQP
EVAEQLWKDAAADLQKRYDFLAQMAGKAEKSNTD
>LFGLNPFC_01830 Outer membrane porin N
MKSKVLALLIPALLAAGAAHAAEVYNKDGNKLDLYGKVDGLHYFSDNSAKDGDQSYARLG
FKGETQINDQLTGYGQWEYNIQANNTESSTNQSWTRLAFAGLKFADYGSFDYGRNYGVMY
DIEGWTDMLPEFGGDSYTNADNFMTGRANGVATYRNTDFFGLVNGLNFAVQYQGNNEGAS
NGQEGTNNGRDVRHENGDGWGLSTTYDLGMGFSAGAAYTSSDRTNDQVNHTAAGGDKADA
WTAGLKYDANNIYLATMYSETRNMTPFGDSDYAVANKTQNFEVTAQYQFDFGLRPAVSFL
MSKGRDLHAAGGADNPAGVDDKDLVKYADVGATYYFNKNMSTYVDYKINLLDEDDSFYAA
NGISTDDIVALGLVYQF
>LFGLNPFC_01831 Universal stress protein F
MNRTILVPIDISDSELTQRVISHVEAEAKIDDAEVHFLTVIPSLPYYASLGLAYSAELPA
MDDLKAEAKSQLEEIIKKFKLPTDRVHVHVEEGSPKDRILELAKKIPAHMIIIASHRPDI
TTYLLGSNAAAVVRHAECSVLVVR
>LFGLNPFC_01832 hypothetical protein
MPLGIGAFIVGLFCAIATGYLKDNSIIKKDTIIGIVFSGIFVLTINRHKSADYRYHYYIT
VYSAWYRLPFLPH
>LFGLNPFC_01833 tRNA-cytidine(32) 2-sulfurtransferase
MQENQQITKKEQYNLNKLQKRLRRNVGEAIADFNMIEEGDRIMVCLSGGKDSYTMLEILR
NLQQSAPINFSLVAVNLDQKQPGFPEHVLPEYLETLGVEYKIVEENTYGIVKEKIPEGKT
TCSLCSRLRRGILYRTATELGATKIALGHHRDDILQTLFLNMFYGGKMKGMPPKLMSDDG
KHIVIRPLAYCREKDIQRFADAKAFPIIPCNLCGSQPNLQRQVIADMLRDWDKRYPGRIE

TMFSAMQNVVPSHLCDTNLFDFKGITHGSEVVNGGDLAFDREEIPLQPSGWQPEEDENQL
DELRLNVVEVK
>LFGLNPFC_01834 ATP-dependent RNA helicase DbpA
MTAFSTLNVLPPAQLTNLNELGYLTMTPVQDAALPAILAGKDVRVQAKTGSGKTAAFGLG
LLQQIDASLFQTQALVLCPTRELADQVAGELRRLARFLPNTKILTLCGGQPFGMQRDSLQ
HAPHIIVATPGRLLDHLQKGTVSLDALNTLVMDEADRMLDMGFSDAIDDVIRFAPASRQT
LLFSATWPEAIAAISGRVQRDPLAIEIDSTDALPPIEQQFYETSSKGKIPLLQRLLSLHQ
PSSCVVFCNTKKDCQSVCDVLNEVGQSALSLHGDLEQRDRDQTLVRFANGSARVLVATDV
AARGLDIKSLELVVNFELAWDPEVHVHRIGRTARAGNSGLAISFCAPEEAQRANIISDML
QIKLNWQTLPANSSIVPLEAEMATLCIDGGKKAKMRPGDVLGALTGDIGLDGADIGKIAV
HPAHVYVAVRQAVAHKAWKQLQGGKIKGKTCRVRLLK
>LFGLNPFC_01835 Protein YnaL
MTTLIYLQIPVPEPIPGDPVPVPDPIPRPQPMPDPPPDEEPIKLSHRERRSARIRAC
>LFGLNPFC_01836 Zinc transport protein ZntB
MEAIKGSDVNVPDAVFAWMLDGRGGVKPLENTDVIDEAHPCWLHLNYVHHDSAQWLATTP
LLPNNVRDALAGESTRPRVSRLGEGTLITLRCINGSTDERPDQLVAMRVYMDGRLIVSTR
QRKVLALDDVVSDLEEGTGPTDCGGWLVDVCDALTDHSSEFIEQLHDKIIDLEDNLLDQQ
IPPRGFLALLRKQLIVMRRYMAPQRDVYARLASERLPWMSDDQRRRMQDIADRLGRGLDE
IDACIARTGVMADEIAQVMQENLARRTYTMSLMAMVFLPSTFLTGLFGVNLGGIPGGGWQ
FGFSIFCILLVVLIGGVALWLHRSKWL
>LFGLNPFC_01837 Diguanylate cyclase DgcM
MITHNFNTLDLLTSPVWIVSPFEEQLIYANSAARLLMQDLTFSQLRTGPYSVSSQKELPK
YLSDLQNQHDIIEILTVQRKEEETALSCRLVLRKLTEAEPVIIFEGIEAPATLGLKASRS
ANYQRKKQGFYARFFLTNSAPMLLIDPSRDGQIVDANLAALNFYGYNHETMCQKHTWEIN
MLGRRVMPIMHEISHLPGGHKPLNFVHKLADGSTRHVQTYAGPIEIYGDKLMLCIVHDIT
EQKRLEEQLEHAAHHDAMTGLLNRRQFYHITEPGQMQHLAIAQDYSLLLIDTDRFKHIND
LYGHSKGDEVLCALARTLESCARKGDLVFRWGGEEFVLLLPRTPLDTALSLAETIRVSVA
KVSISSLPRFTVSIGVAHHEGNESIDELFKRVDDALYRAKNDGRNRVLAA
>LFGLNPFC_01838 putative DNA endonuclease SmrA
MNLDDKSLFLDAMEDVQPLKRATDVHWHPTRNQRAPQRIDTLQLDNFLTTGFLDIIPLSQ
PLEFRREGLQHGVLDKLRSGKYPQQASLNLLRQPVEECRKMMFSFIQQALADGLRNVLII
HGKGRDDKSHANIVRSYVARWLTEFDDVQAYCTALPHHGGSGACYVALRKTAQAKQENWE
RHAKRSR
>LFGLNPFC_01839 Colistin resistance protein EmrB
MSMRKHIAFASMCIGLFIAQLDIQIVSSSLNEIGGGLSAGKDEMAWLQTSYLIAEIIVIP
LSGWLSRVFSTRWLFTLSAGIFTLMSIACGLAWNIQIMIFFRALQGVAGASMIPLVFTTA
FIYYQGKELGLAAAVVSALASLSPTLGPTLGGWITDNLDWRWLFYINILPGIYLVLSIPF
LVNFDKPDLSLLKVADYPSIILLAMTLGCLEYTLEEGARWGWLDDNTILLTSVLALVSFI
LFAARTLKISNPIMDLHAFKDKYFTLGCFFSFSGGVGIFSTVYLIPVFLGQVRGLNAEEI
GFAVCTTGIFQLFSVPFYFWLSKKINLQWLLMAGLGGFVFSMYLFTPITHEWGWQELLFP
QAIRGISQQFAMAPIVTLTLGGIPKERLKLASGVFNLTRNLGGASGIALCGSILNNRTNF
HFSRMGEKMVSVPHTMNDFISRSALFFNRSGSDQTSEILASTKLLSQLMLREAQTMAFSD
TFLLISGLLFIAFLLVPAMNKSS
>LFGLNPFC_01840 Colistin resistance protein EmrA
MIISKKQLIGVVAIGILLAGVVFFIWWVSKGRFIQTTDDAYIGGNITTVASKVSGYISAI
EVRDNQSVKKGDIILRLDDRDYRANVARLEAKIKSSKANLESIQATIAMQQSIIQSASET
WQAVKHEEQKRLRDTERYEKLAQSAAISQQIIDNARFDYQQVAAKERKAANDFLVEKQRL
AVLSAQEENVRASIEEVQAALTQALLDLEYTLVRAPIDGIVANRSAHTGSWVEGGTSLVS
LVPVSELWVDANYKENQIAGMKPGMKAEIRADILKGEVFHGHIESLSPATGASFSLIPIE
NATGNFTKIVQRVPVRIAFDDAKELKQLLRPGLSVTVSVDER
>LFGLNPFC_01841 HTH-type transcriptional regulator NimR
MTITLQPKASPGHHIIGLDSEHLNGGTVPWHKHLYAQLLYPAEGVVRVWAGESVWLVHAS
SALWLPPQMPHKFVATGNVLLKTVLVSEAESETLGKVCFMTGISPLLRELLIAINQLPPS
QSTTDKQQLRFSALETLILQEIKMGVKMSLELPWPNDERLQQLCENLLNNQGYLPTLDNL
ADKINVSSRTLMRLFVKETGLTFRHWVQQMHVISAVTLLDDGYSLTKIAHRLGYASAESF
GNMFKRRTGYSPGKFTRRLTMHDYAITRQMI
>LFGLNPFC_01842 Methylated-DNA--protein-cysteine methyltransferase
MLRLLEEKIATPLGPLWVICDEQFRLRAVEWEEYSERMVQLLDIHYRKEGYERISATNPG
GLSDKLREYFAGNLSIIDTLPTATGGTPFQREVWKTLRTIPCGQVMHYGQLAEQLGRPGA
ARAVGAANGSNPISIVVPCHRVIGRNGTMTGYAGGVQRKEWLLRHEGYLLL
>LFGLNPFC_01843 Fumarate and nitrate reduction regulatory protein
MIPEKRIIRRIQSGGCAIHCQDCSISQLCIPFTLNEHELDQLDNIIERKKPIQKGQTLFK
AGDELKSLYAIRSGTIKSYTITEQGDEQITGFHLAGDLVGFDAIGSGHHPSFAQALETSM
VCEIPFETLDDLSGKMPNLRQQMMRLMSGEIKGDQDMILLLSKKNAEERLAAFIYNLSRR

FAQRGFSPREFRLTMTRGDIGNYLGLTVETISRLLGRFQKSGMLAVKGKYITIENNDALA
QLAGHTRNVA
>LFGLNPFC_01844 Universal stress protein E
MAMYQNMLVVIDPNQDDQPALRRAVYLHQRIGGKIKAFLPIYDFSYEMTTLLSPDERTAM
RQGVISQRTAWIHEQAKYYLNAGVPIEIKVVWHNRPFEAIIQEVISGGHDLVLKMAHQHD
RLEAVIFTPTDWHLLRKCPSPVWMVKDQPWPEGGKALVAVNLASEEPYHNALNEKLVKET
IELAEQVNHTEVHLVGAYPVTPINIAIELPEFDPSVYNDAIRGQHLLAMKALRQKFGINE
NMTHVEKGLPEEVIPDLAEHLQAGIVVLGTVGRTGISAAFLGNTAEQVIDHLRCDLLVIK
PDQYQTPVELDDEEDD
>LFGLNPFC_01845 hypothetical protein
MIMAKLKSAKGKKFLFGLLAVFIIAASVVTRATIGGVIEQYNIPLSEWTTSMYVIQSSMI
FVYSLVFTVLLAIPLGIYFLGGEEQ
>LFGLNPFC_01846 Low conductance mechanosensitive channel YnaI
MIAELFTNNALNLVIIFGSCAALILMSFWFRRGNRKRKGFLFHAVQFLIYTIIISAVGSI
INYVIENYKLKFITPGVIDFICTSLIAVILTIKLFLLINQFEKQQIKKGRDITSARIMSR
IIKITIIVVLVLLYGEHFGMSLSGLLTFGGIGGLAVGMAGKDILSNFFSGIMLYFDRPFS
IGDWIRSPDRNIEGTVTEIGWRITKIKTFDNRPLYVPNSLFSSISVENPGRMTNRRITTT
IGLRYEDAAKVGVIVEAVREMLKNHPAIDQRQTLLVYFNQFADSSLNIMVYCFTKTTVWA
EWLAAQQDVYLKIIDIVQSHGADFAFPSQTLYMDNITPPDQGR
>LFGLNPFC_01847 Periplasmic murein peptide-binding protein
MKHSVSVTCCALLVSSISLSYAAEVPSGTVLAEKQELVRHIKDEPASLDPAKAVGLPEIQ
VIRDLFEGLVNQNEKGEIVPGVATQWKSNDNRIWTFTLRDNAKWADGTPVTAQDFVYSWQ
RLVDPKTLSPFAWFAALAGINNAQAIIDGKAAPDQLGVTAVDAHTLKIQLDKPLPWFVNL
TANFAFFPVQKANVESSKEWTKPGNLIGNGAYVLKERVVNEKLVVVPNTHYWDNAKTVLQ
KVTFLPINQESAATKRYLAGDIDITESFPKNMYQKLLKDIPGQVYTPPQLGTYYYAFNTQ
KGPTADQRVRLALSMTIDRRLMTEKVLGTGEKPAWHFTPDVTAGFTPEPSPFEQMSQEEL
NAQAKTLLSAAGYGPQKPLKLTLLYNTSENHQKIAIAVASMWKKNLGVDVKLQNQEWKTY
IDSRNTGNFDVIRASWVGDYNEPSTFLTLLTSTHSGNISRFNNPAYDKVLAQASTENTVK
ARNADYNAAEKILMEQAPIAPIYQYTNGRLIKPWLKGYPINNPEDVAYSRTMYIVKH
>LFGLNPFC_01848 HTH-type transcriptional regulator PgrR
MKREEIADLMAFVVVAEERSFTRAAARLSMAQSALSQIVRRIEERLGLRLLTRTTRSVVP
TEAGEHLLSVLGPMLHDIDSALTSLSDLQNRPSGTIRITTVEHAAKTILLPAMRTFLKSH
PEIDIQLTIDYGLTDVVSERFDAGVRLGGEMDKDMIAIRIGPDIPMAIVGSPDYFSRRSA
PTSVSQLIDHQAINLYLPTSGTANRWRLIRGGREVRVRMEGQLLLNTIDLIIDAAIDGHG
LAYLPYDQVERAIKGKKLIRVLDKFTPDLPGYHLYYPHRRHAGSAFSLFIDRLKYKGAV
>LFGLNPFC_01849 putative protein YcjY
MMNNKVSFTNSNNPTISLSAVIYFPPKFDETRQYPAIVVSHPGGGVKEQTAGTYAEKLAE
KGFVTIAYDASYQGESGGEPRQLENPYIRTEDVSAVIDYLTTLSYVDNTRIGAMGICAGA
GYTANAAIQDRRIKAIGTVSAVNIGSMFRNGWENNVKSIDALPYVDAGSNARTSDISSGE
YATMPLAPMKESDAPNEELRQAWEYYHTPRAQYPTAPGYATLRSLNQIITYDAYHMAEVY
LTQPIQIVAGSQAGSKCMSDDLYDRASSQDKRYHIVEGANHMDLYDGKVYVAEAISVLAP
FFEETL
>LFGLNPFC_01850 hypothetical protein
MNNVLILGAGGQIARHVINQLADKQTIKQTLFARQPAKIHKPYPTNSKIIMGDVLNHAAL
KQAMQGQDVVYANLTGEDLDIQANSVIAAMKACDVKRLIFVLSLGIYDEVPGKFGEWNNA
VIGEPLKPFRRAADAIEASGLEYTILRPAWLTDEDIIDYELTSRNEPFKGTIVSRKSVAA
LITDIIDKPEKHIGENIGINQPGTDGDKPFFM
>LFGLNPFC_01851 Murein peptide amidase A
MTVTRPRAERGAFPPGTEHYGRSLLGAPLIWFPAPAASHESGLILAGTHGDENSSIVTLS
CALRTLTPSLRRHHVVLCVNPDGCQLGLRANANGVDLNRNFPAANWKEGETVYRWNSAAE
ERDVVLLTGDKPGSEPETQALCQLIHRIQPAWVVSFHDPLACIEDPRHSELGEWLAQAFE
LPLVTSVGYETPGSFGSWCADLNLHCITAEFPPISSDEASEKYLFAMANLLRWHPKDAIR
PS
>LFGLNPFC_01852 L-Ala-D/L-Glu epimerase
MRTVKVFEEAWPLHTPFVIARGSRSEARVVVVELEEEGIKGTGECTPYPRYGESDASVMA
QIMSVVPQLEKGLTREELQKILPAGAARNALDCALWDLAARKQQQSLADLIGITLPETVT
TAQTIVIGTPDQMANSASTLWQAGAKLLKVKLDNHLISERMVAIRTAVPDATLIVDANES
WRAEGLAARCQLLADLGVAMLEQPLPAQDDAALENFIHPLPICADESCHTRSNLKALKGC
YEMVNIKLDKTGGLTEALALATEARAQGFRLMLGCMLCTSRAISAALPLVPQVSFADLDG
PTWLAVDVEPALQFTTGELHL
>LFGLNPFC_01853 Thiol peroxidase
MSQTVHFQGNPVTVANSIPQAGSKAQTFTLVAKDLSDVTLGQFAGKRKVLNIFPSIDTGV
CAASVRKFNQLATEIDNTVVLCISADLPFAQSRFCGAEGLNNVITLSTFRNAEFLQAYGV
AIADGPLKGLAARAVVVIDENDNVIFSQLVDEITTEPDYEAALAVLKA

>LFGLNPFC_01854 Transcriptional regulatory protein TyrR
MRLEVFCEDRLGLTRELLDLLVLRGIDLRGIEIDPIGRIYLNFAELEFESFSSLMAEIRR
IAGVTDVRTVPWMPSEREHLALSALLEALPEPVLSVDMKSKVDMANPASCQLFGQKLDRL
RNHTAAQLINGFNFLRWLESEPQDSHNEHVVINGQNFLMEITPVYLQDENDQHVLTGAVV
MLRSTIRMGRQLQNVAAQDVSAFSQIVAVSPKMKHVVEQAQKLAMLSAPLLITGDTGTGK
DLFAYACHQASPRASKPYLALNCASIPEDAVESELFGHAPEGKKGFFEQANGGSVLLDEI
GEMSPRMQAKLLRFLNDGTFRRVGEDHEVHVDVRVICATQKNLVELVQKGVFREDLYYRL
NVLTLNLPPLRDCPQDIMPLTELFVARFADEQGVPRPKLAADLNTVLTRYAWPGNVRQLK
NAIYRALTQLDGYELRPQDILLPDYDAATVAVGEDAMEGSLDEITSRFERSVLTQLYRNY
PSTRKLAKRLGVSHTAIANKLREYGLSQKKNEE
>LFGLNPFC_01855 hypothetical protein
MTEPLKPRIDFDGPLEVDQNPKFRAQQTFDENQAQNFAPATLDEAQEEEGQVEAVMDAAL
RPKRSLWRKMVMGGLALFGASVVGQGVQWTMNAWQTQDWVALGGCAAGALIIGAGVGSVV
TEWRRLWRLRQRAHERDEARDLLHSHGTGKGRAFCEKLAQQAGIDQSHPALQRWYASIHE
TQNDREVVSLYAHLVQPVLDAQARREISRSAAESTLMIAVSPLALVDMAFIAWRNLRLIN
RIATLYGIELGYYSRLRLFKLVLLNIAFAGASELVREVGMDWMSQDLAARLSTRAAQGIG
AGLLTARLGIKAMELCRPLPWIDDDKPRLGDFRRQLIGQVKETLQKGKTPSEK
>LFGLNPFC_01856 putative protein YcjX
MKRLKNELNALVNRGVDRHLRLAVTGLSRSGKTAFITAMVNQLLNIHAGARLPLLSAVRE
ERLLGVKRIPQRDFGIPRFTYDEGLAQLYGDPPAWPTPTRGVSEIRLALRFKSNDSLLRH
FKDTSTLYLEIVDYPGEWLLDLPMLAQDYFSWSRQMTGLLNGQRGEWSAKWRMMCEGLDP
LAPADENRLADIAAAWTDYLHHCKQQGLHFIQPGRFVLPGDMAGAPALQFFPWPDVDAWG
ESKLAQADKHTNAGMLRERFNYYCEKVVKGFYKNHFLRFDRQIVLVDCLQPLNSGPQAFN
DMRLALTQLMQSFHYGQRTLFRRLFSPVIDKLLFAATKADHVTIDQHANMVSLLQQLIQD
AWQNAAFEGISMDCLGLASVQATTSGIIDVNGEKIPALRGNRLSDGAPLTVYPGEVPARL
PGQAFWDKQGFQFEAFRPQVMDVDKPLPHIRLDAALEFLIGDKLR
>LFGLNPFC_01857 Catabolite control protein A
MSPTIYDIARVAGVSKSTVSRVLNKQTNISPEAREKVLRAIEELQYQPNKLARALTSSGF
DAIMVISTRSTKTTAGNPFFSEVLHAITAKAEEEGFDVILQTSHNLAEDLHKCESKIKQK
MIKGIIMLSSPADESFFAQLDKYDIPVVVIGKVEGQYSHVYSVDTDNYGDSIALTDALIE
SGHKNIACLHAPLDVHVSVDRVNGYKQSLATHNIAVRDEWIVDGGYTHETALKAARELLS
QSPLPEAVFATDSLKLMSIYRAAAEKNIAIPQQLAVVGYSNETLSFILTPAPGGIDVPTQ
ELGQRSCELLFQLIAGKPSPQNITVATHMSLK
>LFGLNPFC_01858 Outer membrane porin G
MDDSRNNGEFSTQEIRAYLPLTLGNHSVTPYTRIGLDRWSNWDWQDDIEREGHDFNRVGL
FYGYDFQNGLSVSLEYAFEWQDHDEGDSDKFHYAGVGVNYSF
>LFGLNPFC_01859 Outer membrane porin G
MKKLLLCTALVMCAGMACAQAEEKNDWHFNIGAMYEIENVEGYGEDMDGLAEPSVYFNAA
NGPWRISLAYYQEGPVDYSAGKRGTWFDRPELEVHYQFLESDDFSFGLTGGFRNYGYHYV
DEPGKDTANMQRWKIAPDWDVKLTDDLRFNGWLSMYKFANDLNTTGYADTRVETETGLQY
TFNETLALRVNLLSRAWFQYG
>LFGLNPFC_01860 Oligosaccharides import ATP-binding protein MsmX
MAQLSLQHIQKIYDNQVHVVKDFNLEIVDKEFIVFVGPSGCGKSTTLRMIAGLEEISGGD
LLIDGKRMNDVPAKARNIAMVFQNYALYPHMTVYDNMAFGLKMQKIAREVIDERVNWAAQ
ILGLREYLKRKPGALSGGQRQRVALGRAIVREAGVFLMDEPLSNLDAKLRVQMRAEISKL
HQKLNTTMIYVTHDQTEAMTMATRIVIMKDGIVQQVGAPKTVYNQPANMFVAGFIGSPAM
NFIRGTIDGDKFVTETLKLTIPEEKLAVLKTQESLHKPIVMGIRPEDIHPDAQEENNISA
KISVAELTGAEFMLYTTVGGHELVVRAGALNDYHAGENITIHFDMTKCHFFDAETEIAIC
>LFGLNPFC_01861 Beta-phosphoglucomutase
MKLQGVIFDLDGVITDSAHLHFQAWQQIAAEIGISIDAQFNEFLKGISRDESLRRILQHG
GKEGDFNPQERAQLAYRKNLLYVHSLRELTVNAVLPGIHNLLVDLRAQQIPVGLASVSLN
APTILAALELREFFTFCADASQLKNSKPDPEIFLAACAGLGVPPQACIGIEDAQAGIEAI
NASGMRSVGIGAGLTGAQLLLPSTDSLTWPRLSAFWQNV
>LFGLNPFC_01862 Kojibiose phosphorylase
MIRPVTLTEPHFSQHTLNKYASLMAQGNGYLGLRASHEEDYTRQTRGMYLAGLYHRAGKG
EINELVNLPDVVGMEIAINGEIFSLSREAWQRELNFASGELRRNVVWRTSNGAGYTITSR
RFVSADQLPLIALEITITPLDADALVLISTGIDATQTNHGRQHLDETQVRVFGQHLMQGI
YTTQDGRSDVAISCCCKVSGDVQQCYTAKERRLLQHTCAQLHAGETLTLQKRVWIDWRDD
RQAALDEWGSASLRQLEMCAQQSYDQLLAASTENWRQWWQKRRITVNGGDAHDQQALDYA
LYHLRIMTPAHDERSSIAAKGLTGEGYKGHVFWDTEVFLLPFHLFSDPTVARSLLRYRWH
NLPGAQEKARRNGWQGALFPWESARSGEEETPEFAAINIRTGLRQKVASAQAEHHLVADI
AWAVIQYWRTTGDESFIAHEGMALLLETAKFWISRAVRVNDRLEIHDVIGPDEYTEHVNN
NAFTSYMAYYNVQQALNIARQFGCSDDAFIHRAEMYLKELLLPEIQPDGVLPQDDSFMAK
PVINLAKYKAAAGKQTILLDYSRAEVNEMQILKQADVVMLNYMLPEQFSAASCLANLQFY

EPRTIHDSSLSKAIHGIVAARCGLLTQSYQFWREGTEIDLGADPHSCDDGIHAAATGAIW
LGAIQGFAGVSVRDGELHLNPALPEQWQQLSFPLFWQGCELQVTLDAQRIAIRTSAPVSL
RLNGQLIYVAEESVFCLGDFILPFNGTATTHQEGE
>LFGLNPFC_01863 Myo-inositol 2-dehydrogenase
MKSAMTSSPLRVAIIGAGQVADKVHASYYCTRNDLELVAVCDSRLSQAQALAEKYGNASV
WDDPQAMLLAVKPDVVSVCSPNRFHYEHTLMALEAGCHVMCEKPPAMTAEQAREMCDTAR
KQGKVLAYDFHHRFALDTQQLREQVTNGVLGEIYVTTARALRRCGVPGWGVFTNKELQGG
GPLIDIGIHMLDAAMYVLGFPAVKSVTAHSFQKIGTQKSCGQFGEWDPATYSVEDSLFGT
IEFHNGGILWLETSFALNIREQSIMNVSFCGDKAGATLFPAHIYTDNNGELMTLMQREMA
DDNRHLRSMEAFINHVQGKPVMIADAEQGYIIQQLVAALYQSAETGTRVEL
>LFGLNPFC_01864 Inosose dehydratase
MKIGTQNQAFFPENILEKFRYIKEMGFDGFEIDGKLLVNNLEEVKAAIKETGLPVTTACG
GYDGWIGDFIEERRLNGLKQIERILEALAEVGGKGIVVPAAWGMFTFRLPPMTSPRSLDG
DRKMVSDSLRVLEQVAARTGTVVYLEPLNRYQDHMINTLADARRYIVENDLKHVQIIGDF
YHMNIEEDNLAQALHDNRDLLGHVHIADNHRYQPGSGTLDFHALFEQLRADNYQGYVVYE
GRIRAENPAQAYRDSLAWLRTC
>LFGLNPFC_01865 L-threonine 3-dehydrogenase
MKKLVATAPRVAALVEYEERAILANEVKIRVRFGAPKHGTEVVDFRAASPFINDDFNGEW
QMFTPRPADAPRGIEFGKFQLGNMVVGDIIECGSDVNDYAVGDSVCGYGPLSETVIINAV
NNYKLRKMPQGSSWKNAVCYDPAQFAMSGVRDANVRVGDFVVVVGLGAIGQIAIQLAKRA
GASVVIGVDPIAHRCDIARRHGADFCLNPIGADVGKEIKTLTGKQGADVIIETSGYADAL
QSALRGLAYGGTISYVAFAKPFAEGFNLGREAHFNNAKIVFSRACSEPNPDYPRWSRKRI
EETCWELLMNGYLNCEDLIDPVVTFANSPESYMQYVDQHPEQSIKMGVTF
>LFGLNPFC_01866 Inner membrane ABC transporter permease protein YcjP
MATNKRTLSRIGFYCGLALFLIITLFPFFVMLMTSFKSAKEAISLHPTLLPQQWTLEHYV
DIFNPVIFPFVDYFRNSMVVSVVSSVVAVFLGILGAYALSRLRFKGRMTINASFYTVYMF
SGILLVVPLFKIITALGIYDTEMALIITMVTQTLPTAVFMLKSYFDTIPDEIEEAAMMDG
LNRLQIIFRITVPLAMSGLISVFVYCFMVAWNDYLFASIFLSSASNFTLPVGLNALFSTP
DYIWGRMMAASLVTALPVVIMYALSEHFIKSGLTAGGVKG
>LFGLNPFC_01867 Inner membrane ABC transporter permease protein YcjO
MNRLFSGRSDMPFALLLLAPSLLLLGGLVAWPMVSNIEISFLRLPLNPNIEATFVGVSNY
VRILSDPGFWHSLWMTVWYTALVVAGSTVLGLAVAMFFNREFRLRKTARSLVILSYVTPS
ISLVFAWKYMFNNGYGIVNYLGVDLLHLYEQAPLWFDNPGSSFVLVVLFAIWRYFPYAFI
SFLAILQTIDKSLYEAAEMDGANAWQRFRIVTLPAIMPVLATVVTLRTIWMFYMFADVYL
LTTKVDILGVYLYKTAFAFNDLGKAAAISVVLFIIIFAVILLTRKRVNLNGNK
>LFGLNPFC_01868 hypothetical protein
MIKSKIVLLSALVSCTLISGCKEENKNNVSIEFMHSSVEQERQAVISKLIARFEKENPGI
TVKQVPVEEDAYNTKVITLSRSGSLPEVIETSHDYAKVMDKEQLIDRQAVATVISNVGEG
AFYDGVLRIVRTEDGSAWTGVPVSAWIGGIWYRKDVLAKAGLEEPKNWQQLLDVAQKLND
PANKKYGIALPTAESVLTEQSFSQFALSNQANVFNAEGKITLDTPEMMQALTYYRNLAAN
TMPGSNDIMEVKDAFMNGTAPMAIYSTYILPAVIKEGDPKNVGFVVPTEKNSAVYGMLTS
LTITAGQKAEETEAAEKFVTFMEQADNIADWVMMSPGAALPVNKAVVTTATWKDNDVIKA
LGELPNQLISELPNIQVFGAVGDKNFTRMGDVTGSGVVSSMVHNVTVGKADLPGTLQASQ
KKLDELIEQH
>LFGLNPFC_01869 Glucosylglycerate phosphorylase
MKQKITDYLDEIYGGTFTATHLQKLVTRLESAKRLITQRRKKHWDESDVVLITYADQFHS
NDLKPLPTFNQFYPQWLQRIFSHVHLLPFYPWSSDDGFSVIDYHQVASEAGEWQDIQQLG
ECSHLMFDFVCNHMSAKSEWFKNYLQQQPGFEDFFIAVDPKTDLSAVTRPRALPLLTPFQ
MRDHSMRHLWTTFSDDQIDLNYRSPEVLLAMVDVLLCYLEKGAEYVRLDAVGFMWKEPGT
NCIHLEKTHLIIKLLRSIIDNVAPGTVIITETNVPHKDNIAYFGEGDDEAHMVYQFSLPP
LVLHAVQKQNVEALCAWAQNLTLPSSNTTWFNFLASHDGIGLNPLRGLLPENEILALVEV
LQQEGALVNWKNNPDGTRSPYEMNVTYMDALSRRECSDEERCARFILAHAILLSFPGVPA
IYIQSILGSRNDYAGVEKLGYNRAINRKKYSSKEITTELNNKATLSYSVYYKLSHLITLR
RSHKEFHPDNNFTIDAINSFVMRIQRSNADGDCLTGLFNVSENIQHINITDLHGRDLISE
VDILGNEITLHPWQVMWIK
>LFGLNPFC_01870 Thiosulfate sulfurtransferase PspE
MFKKGLLALTLVFSLPVFAAEHWIDVRVPEQYQQEHVQGAINIPLKEVKERIATAVPDKN
DTVKVYCNAGRQSGQAKEILSEMGYTHVENAGGLKDIAMPKVKG
>LFGLNPFC_01871 Phage shock protein D
MNTRWQQAGQKVKPGFKLAGKLVLLTALRYGPAGVAGWAIKSVARRPLKMLLAVALEPLL
SRAANKLAQRYKR
>LFGLNPFC_01872 Phage shock protein C
MAGINLNKKLWRIPQQGMVRGVCAGIANYFDVPVKLVRILVVLSIFFGLALFTLVAYIIL
SFALDPMPDNMVFGEQLPSSSELLDEVDRELAASETRLREMERYVTSDTFTLRSRFRQL

>LFGLNPFC_01873 Phage shock protein B
MSALFLAIPLTIFVLFVLPIWLWLHYSNRSGRSELSQSEQQRLAQLADEAKRMRERIQAL
ESILDAEHPNWRDR
>LFGLNPFC_01874 Phage shock protein A
MGIFSRFADIVNANINALLEKAEDPQKLVRLMIQEMEDTLVEVRSTSARALAEKKQLTRR
IEQASAREIEWQEKAELALLKDREDLARAALIEKQKLTDLIKSLEHEVTLVDDTLVRMKK
EIGELENKLSETRARQQALMLRHQAANSSRDVRRQLDSGKLDEAMARFESFERRIDQMEA
EAESHSFGKQKSLDDQFAELKADDAISEQLAQLKAKMKQDNQ
>LFGLNPFC_01875 Psp operon transcriptional activator
MAEYKDNLLGEANSFLEVLEQVSHLAPLDKPVLIIGERGTGKELIASRLHYLSSRWQGPF
ISLNCAALNENLLDSELFGHEAGAFTGAQKRHPGRFERADGGTLFLDELATAPMMVQEKL
LRVIEYGELERVGGSQPLQVNVRLVCATNADLPAMVNEGTFRADLLDRLAFDVVQLPPLR
ERESDIMLMAEHFAIQMCREIKLPLFPGFTEHARETLLNYRWPGNIRELKNVVERSVYRH
GTSDYPLDDIIIDPFKRRPSEEAIAVSENTSLPTLPLDLREFQMQQEKELLQLSLQQGKY
NQKRAAELLGLTYHQFRALLKKHQI
>LFGLNPFC_01876 hypothetical protein
MNHDIPLKYFDIADEYATECAEPVADAERTPLAHYFQLLLTRLMNNEEISEEAQHEMAAE
AGINPVRIDEIAEFLNQWGNE
>LFGLNPFC_01877 Peptide transport periplasmic protein SapA
MRQVLSSLLVIAGLVSGQAIAAPESPPHADIRDSGFVYCVSGQVNTFNPSKASSGLIVDT
LAAQFYDRLLDVDPYTYRLMPELAESWEVLDNGATYRFHLRRDVPFQKTDWFTPTRKMNA
DDVVFTFQRIFDRNNPWHNVNGSNFPYFDSLQFADNVKSVRKLDNHTVEFRLAQPDASFL
WHLATHYASVMSAEYAGKLEKEDRQEQLDRQPVGTGPYQLSEYRAGQYIRLQRHDDFWRG
NPLMPQVVVDLGSGGTGRLSKLLTGECDVLAWPAASQLSILRDDPRLRLTLRPGMNVAYL
AFNTAKPPLNNPAVRHALALAINNQRLMQSIYYGTAETAASILPRASWAYDNEAKITEYN
PAKSREQLKALGLENLTLKLWVPTRSQAWNPSPLKTAELIQADMAQVGVKVVIVPVEGRF
QEARLMDMSHDLTLSGWATDSNDPDSFFRPLLSCAAIHSQTNLAHWCDPKFDSVLRKALS
SQQLAARIEAYDEAQSILAQELPILPLASSLRLQAYRYDIKGLVLSPFGNASFAGVYREK
QDEVKKP
>LFGLNPFC_01878 Putrescine export system permease protein SapB
MIIFTLRRILLLIVTLFLLTFVGFSLSYFTPHAPLQGASLWNAWVFWFNGLIHWDFGVSS
INGQPIAEQLKEVFPATMELCILAFGFALIVGIPVGMIAGITRHKWQDNLINAIALLGFS
IPVFWLALLLTLFCSLTLGWLPVSGRFDLLYEVKPITGFALIDAWLSNSPWRDEMVMSAI
RHMILPVITLSVAPTTEVIRLMRISTIEVYDQNYVKAAATRGLSRFTILRRHVLHNALPP
VIPRLGLQFSTMLTLAMITEMVFSWPGLGRWLINAIRQQDYAAISAGVMVCGSLVIIVNV
ISDILGAMANPLKHKEWYALR
>LFGLNPFC_01879 Putrescine export system permease protein SapC
MPYDSVYSEKRPPGTLRTAWRKFYSDASAMVGLYGCAGLAVLCIFGGWFAPYGIDQQFLG
YQLLPPSWSRYGEVSFFLGTDDLGRDVLSRLLSGAAPTVGGAFVVTLAATICGLVLGTFA
GATHGLRSAVLNHILDTLLAIPSLLLAIIVVAFAGPSLSHAMFAVWLALLPRMVRSIYSM
VHDELEKEYVIAARLDGASTLNILWFAVMPNITAGLVTEITRALSMAILDIAALGFLDLG
AQLPSPEWGAMLGDALELIYVAPWTVMLPGAAIMISVLLVNLLGDGVRRAIIAGVE
>LFGLNPFC_01880 Putrescine export system ATP-binding protein SapD
MPLLDIRNLTIEFKTGDEWVKAVDRVSMTLTEGEIRGLVGESGSGKSLIAKAICGVNKDN
WRVTADRMRFDDIDLLRLSARERRKLVGHNVSMIFQEPQSCLDPSERVGRQLMQNIPAWT
YKGRWWQRFGWRKRRAIELLHRVGIKDHKDAMRSFPYELTEGECQKVMIAIALANQPRLL
IADEPTNSMEPTTQAQIFRLLTRLNQNSNTTILLISHDLQMLSQWADKINVLYCGQTVET
APSKELVTMPHHPYTQALIRAIPDFGSAMPHKSRLNTLPGAIPLLEQLPIGCRLGPRCPY
AQRECIVTPRLTGAKNHLYACHFPLNMEKE
>LFGLNPFC_01881 Putrescine export system ATP-binding protein SapF
MIETLLEVRNLSKTFRYRTGWFRRQTVEAVKPLSFTLREGQTLAIIGENGSGKSTLAKML
AGMIEPTSGELLIDDHPLHFGDYSFRSQRIRMIFQDPSTSLNPRQRISQILDFPLRLNTD
LEPEQRRKQIIETMRMVGLLPDHVSYYPHMLAPGGKQRLGLARALILRPKVIIADEALAS
LDMSMRSQLINLMLELQEKQGISYIYVTQHIGMMKHISDQVLVMHQGEVVERGSTADVLA
SPLHELTKRLIAGHFGEALTADAWRKDR
>LFGLNPFC_01882 Bicyclomycin resistance protein
MARVSLSWALILGLLAGIGPMCTDLYLPALPEMSEQLAATTTITQLTLTASLIGLGVGQL
LFGPLSDKIGRKRPLILSLLLFIVSSILCATTNNIYWLVVWRFIQGIAGAGGSVLSRSIA
RDKYQGVTLTQFFALLMTVNGLAPVLSPVLGGYIVSTFDWRTLFWVMAEISTVLLLGCLL
FINETLPENKRGSSLLLTGRSVVQNRRFMRFCLIQSFMLAGLFAYIGSSSFVLQKEFGFS
PMQFSLVFGLNGIGLIIASWIFSRLARRINAMTLLRGGLIAAILCALLTVLCAWVQLPIP
ALVALFFTIAFCSGIGTVGGAEAMSAVGTQESGTASALMGMSMFVFGGIAAPLSGIGGET
LLKMSLAITVCYTLALLVALTRIDNQK
>LFGLNPFC_01883 putative efflux pump outer membrane protein TtgC

MLRRSLIFLVLLSAGCVSLDPHYSTPESPIPATLPGAQGQGKAISHDWQQVIHDPRLQQV
VTIALNSNRDVQKAIADIDSARALYGQTNASLFPTVNAALSSTRSRSLANGTGTTAEADG
TVSSYTLDLFGRNQSLSRAARETWLASEFTAQNTRLTLIAEISTAWLTLAADNSNLALAK
ETMASAENSLKIIQRQQQVGTAAATDVSEAMSVYQQARASVASYQTQVMQDKNALNLLAG
TTLAENLLPGTLESLPEQMISLVPAGVSSDVLLRRPDIQEAEHNLKSANADIGAARANFF
PTISLTASAGVGSDALSSLFSHGMQIWSFAPSVTLPLFTGGSNLAQLRYAEAQKRGLIAT
YEKTVQSAFKDVANALARRTTLEEQLDAQRQYVKAEQQTVDVGLRRYQAGVGDYLTVLTA
QRSLWSAQQELLALQLTDFTNRITLWQSLGGGMSSLK
>LFGLNPFC_01884 Multidrug resistance protein MexB
MFSRFFVRRPVFAWVIAILIMLAGILAIRTLPVAQYPDVAPPTIKISATYTGASAETLEN
SVTQVIEQQLTGLDNLLYFSSTSSSDGSVSINVTFEQGTDPDTAQVQVQNKIQQAESRLP
SEVQQTGVTVEKSQSNFLLIAAVYDTTDKASSSDIADWLVSNVQDPLARVEGVGSLQVFG
AEYAMRIWLDPAKLASYSLMPSDVQSAIEAQNVQVTAGKIGALPSPNTQQLTATVRAQSR
LQTVDQFKNIIVKSQSDGAVVRIKDVARVEMGSEDYTAIGKLNGHPSAGVAVMLSPGANA
LNTATLVKDKIAEFQRNMPQGYDIAYPKDSTEFIKISVEDVIQTLFEAIVLVVCVMYLFL
QNLRATLIPALAVPVVLLGTFGVLALFGYSINTLTLFAMVLAIGLLVDDAIVVVENVERI
MRDEGLPAREATEKSMGEISGALVAIALVLSAVFLPMAFFGGSTGVIYRQFSITIISAML
LSVVVALTLTPALCGSVLQHVPPHKKGFFGAFNRFYRRTEDKYQRGVIYVLRRAARTMGL
YVVLGGGMALMMWKLPGSFLPTEDQGEIMVQYTLPAGATAARTAEVNRQIVDWFLINEKA
NTDVIFTVDGFSFSGSGQNTGMAFVSLKNWSQRKGAENTAQAIALRATKELGTIRDATVF
AMTPPAVDGLGQSNGFTFELLANGGADRETLLQMRNQLIEKANQSPELHSVRANDLPQMP
QLQVDIDSNKAVSLGLSLNDVTDTLSSAWGGTYVNDFIDRGRVKKVYIQGDSEFRSAPSD
LGKWFVRGSDNAMTPFSAFATTRWLYGPERLVRYNGSAAYEIQGENATGFSSGDAMTKME
ELANSLPAGTTWAWSGLSLQEKLASGQALSLYAVSILVVFLCLAALYESWSVPFSVILVI
PLGLLGAALAAWMRDLNNDVYFQVALLTTIGLSSKNAILIVEFAEAAVAEGYSLSRAALR
AAQTRLRPIIMTSLAFIAGVMPLAIATGAGANSRIAIGTGIIGGTLTATLLAIFFVPLFF
VLVKRLFAGKPRRQE
>LFGLNPFC_01885 Multidrug resistance protein MexA
MKYIATSVVAMLLLSGCDNTQSNNSSPSETEVGVVTLKSQPVSVVSELTGRTSAALSAEV
RPQVGGIIQKRLFKEGDLVKAGQPLYQIDAASYQAAWNEARAALQQAQALVKADCQKAQR
YTRLVKENGVSQQDADDAQSTCAQDKASVEAKKAALETARINLDWTTVTAPISGRIGISS
VTPGALVTASQDTALTTIRGLDTMYVDLTRSSVDLLRLRKQSLATNSDTMSVSLILEDGT
TYSEKGRLELTEVAVDESTGSVTLRAIFPNPQQQLLPGMFVRARVDEGVMEDAILAPQQG
VTRDAKGNATALVVNKDNKVEQRTLETGETYGDKWLVLNGLHNGDRLIVEGSAKVTSGQT
VKAVEVQANGGNA
>LFGLNPFC_01886 HTH-type transcriptional regulator BetI
MTSKLEIRHKQRQDEIINAARRCFRRCGFHAASMSQIASEAQLSVGQIYRYFANKDAIIE
EMVRRIIDFRIAQMDIDARTDHFPEVLALRKSLNEDDDALMLEVAAEATRNPRVMAMLEE
ADARMFANGCAHMKRMHPHLSDEHIRCCVEVFATMMEGTVYRRLTPQKSDPQHLQEIYQD
IVSMLINK
>LFGLNPFC_01887 Enoyl-[acyl-carrier-protein] reductase [NADH] FabI
MGFLSGKRILVTGVASKLSIAYGIAQAMHREGAELAFTYQNDKLKGRVEEFAAQLGSDIV
LQCDVAEDASIDTMFAELGKVWPKFDGFVHSIGFAPGDQLDGDYVNAVTREGFKIAHDIS
SYSFVAMAKACRSMLNPGSALLTLSYLGAERAIPNYNVMGLAKASLEANVRYMANAMGPE
GVRVNAISAGPIRTLAASGIKDFRKMLAHCEAVTPIRRTVTIEDVGNSAAFLCSDLSAGI
SGEVVHVDGGFSIAAMNELELK
>LFGLNPFC_01888 hypothetical protein
MEQRHITGKSHWYHETQSSTAEYDVLPLVPEAAKVSDPFLLDVILDEETLAPFLSWLVPA
RVLAVELFPDPLTVTRSQTFTAYERLSTALTVAQVCGVQRLCNYYSARLTPLPGPDSSRE
SNHRLAQITQYARQLASSPSIIDNRSRQHLNDVGLTVCDCVIINQIIGFIGFQARTIATF
QAYLGHPVRWLPGLEIQNYADASLFADESLRWRSSYEVEKLPEEHTKSSTAELCQLANTL
SLHPISLSLLEKLLNSTRVNTQPGNLLAALLCARINGSPACFAACMNSSNEYKKISPLLR
KGENEINRWADRHSVERATVQAIQWLTRAPDRFSAAQFSPLLEHEKSSTQIINLLVWSGL
CGWINRLKIALGETY
>LFGLNPFC_01889 Exoribonuclease 2
MFQDNPLLAQLKQQLHSQTPRAEGVVKATEKGFGFLEVDAQKSYFIPPPQMKKVMHGDRI
IAVIHSEKERESAEPEELVEPFLTRFVGKVQGKNDRLAIVPDHPLLKDAIPCRAARGLNH
EFKEGDWAVAEMRRHPLKGDRSFYAELTQYITFGDDHFVPWWVTLARHNLEKEAPDGVAT
EMLDEGLVREDLTALDFVTIDSASTEDMDDALFAKALPDGKLQLIVAIADPTAWIAEGSK
LDKAAKIRAFTNYLPGFNIPMLPRELSDDLCSLRANEVRPVLACRMTFSTDGTIEDNIEF
FAATIESKAKLVYDQVSDWLENTGDWQPESEAIAEQVRLLAQICQRRGEWRHNHALVFKD
RLDYRFILGEKGEVLDIVAEPRRIANRIVEEAMIAANICAARVLRDKLGFGIYNVHMGFD
PANADALAALLKTHGLHVDAEEVLTLDGFCKLRRELDAQPTGFLDSRIRRFQSFAEISTE
PGPHFGLGLEAYATWTSPIRKYGDMINHRLLKAVIKGETATRPQDEITVQMAERRRLNRM

AERDVGDWLYARFLKDKAGTDTRFAAEIVDISRGGMRVRLVDNGAIAFIPAPFLHAVRDE
LVCSQENGTVQIKGETAYKVTDVIDVTIAEVRMETRSIIARPVA
>LFGLNPFC_01890 Cyclic di-GMP phosphodiesterase PdeR
MKTVRESTTLYNFLGSHNPYWRLTESSDVLRFSTTEITEPDRILQLSAEQAARIREMTVI
TSSLMMSLTVDESDLSVHLVGRKINKREWGGNASAWHDTPAVARDLSHGLSFAEQVVSEA
HSAIVILDSRGNIQRFNRLCEDYTGLKEHDVIGQSVFKLFMSRREAAASRRNNRVFFRSG
NAYEVELWIPTRKGQRLFLFRNKFVHSGSGKNEIFLICSGTDITEERRAQERLRILANTD
SITGLPNRNAMQELIDHAINQADNNKVGVVYLDLDNFKKVNDAYGHLFGDQLLRDVSLAI
LSCLEHDQVLARPGGDEFLVLASNTSQSALEALASRILTRLRLPFRIGLIEVYTSCSVGI
SLSPEHGSDSAAIIRHADTAMYTAKEGGRGQFCVFTPEMNQRVFEYLWLDTNLRKALEND
QLVIHYQPKITWRGEVRSLEALVRWQSPERGLIPPLDFISYAEESGLIVPLGRWVILDVV
RQVAKWRDKGINLRVAVNISARQLADQTIFTALKQVLQELNFEYCPIDVELTESCLIEND
ELALSVIQQFSRLGAQVHLDDFGTGYSSLSQLARFPIDAIKLDQVFVRDIHKQPVSQSLV
RAIVAVAQALNLQVIAEGVESAKEDAFLTKNGINERQGFLFAKPMPAVAFERWYKRYLKR
T
>LFGLNPFC_01891 hypothetical protein
MSEFDAQRVAERIDIVLDILVAGDYHSAIHNLEILKAELLRQVAESTPDIPKAPWEI
>LFGLNPFC_01892 Glucitol operon repressor
MNSRQQTILQMVIDQGQVSVTDLAKATGVSEVTIRQDLNTLEKLSYLRRAHGFAVSLDSD
DVETRMMSNYTLKRELAEFAASLVQPGETIFIENGSSNALLARTLGEQKKNVTIITVSSY
IAHLLKDAPCEVILLGGVYQKKSESTVGPLTRQCIQQVHFSKAFIGIDGWQPETGFTGRD
MMRTDVVNAVLEKECEAIVLTDSSKFGAVHSYSIGPVERFNRVITDSKIRASDLMHLEQS
KLTVHVVDI
>LFGLNPFC_01893 Osmotically-inducible lipoprotein B
MFVTSKKMTAAVLAITLAMSLSACSNWSKRDRNTAIGAGAGALGGAVLTDGSTLGTLGGA
AVGGVIGHQVGK
>LFGLNPFC_01894 putative protein YciH
MSDSNSRLVYSTETGRIDEPKAAPVRPKGDGVVRIQRQTSGRKGKGVCLITGVALDDAEL
TKLAAELKKKCGCGGAVKDGIIEIQGDKRDLLKSLLEAKGMKVKLAGG
>LFGLNPFC_01895 Orotidine 5'-phosphate decarboxylase
MLAVYLFRATSGAHHQEGLVMTLTASSSSRAVTNSPVVVALDYHNRDAAMAFVDKIDPRD
CRLKVGKEMFTLFGPQFVRELQQRGFDIFLDLKFHDIPNTAAHAVAAAADLGVWMVNVHA
SGGARMMTAAREALVPFGKDAPLLIAVTVLTSMEASDLADLGVTLSPADYAERLAALTQK
CGLDGVVCSAQEAVRFKQVFGQEFKLVTPGIRPQGSDAGDQRRIMTPEQALAAGVDYMVI
GRPVTQSVDPAQTLKAINASLQRSA
>LFGLNPFC_01896 Lipopolysaccharide assembly protein B
MLELLFLLLPVAAAYGWYMGRRSAQQNKQDEANRLSRDYVAGVNFLLSNQQDKAVDLFLD
MLKEDTGTVEAHLTLGNLFRSRGEVDRAIRIHQTLMESASLTYEQRLLAIQQLGRDYMAA
GLYDRAEDMFNQLTDETDFRIGALQQLLQIYQATSEWQKAIDVAERLVKLGKDKQRVEIA
HFYCELALQHMASDDLDRAMTLLKKGAAADKNSARVSIMMGRVFMAKGEYAKAVESLQRV
ISQDRELVSETLEMLQTCYQQLGKTAEWAEFLQRAVEENTGADAELMLADIIEARDGSEA
AQVYITRQLQRHPTMRVFHKLMDYHLNEAEEGRAKESLMVLRDMVGEKVRSKPRYRCQKC
GFTAYTLYWHCPSCRAWSTIKPIRGLDGL
>LFGLNPFC_01897 Lipopolysaccharide assembly protein A
MKYLLIFLLVLAIFVISVTLGAQNDQQVTFNYLLAQGEYRISTLLAVLFAAGFAIGWLIC
GLFWLRVRVSLARAERKIKRLENQLSPATDVAVAPHSSASKE
>LFGLNPFC_01898 Phosphatidylglycerophosphatase B
MRSIARRTAVGAALLLVMPVAVWLSGWRWQPGEQSWLLKAAFWVTETVTQPWGVITHLIL
FGWFLWCLRFRIKAAIMLFAILAAAILMGQGVKSWIKDKVQEPRPFVIWLEKTHHIPVDE
FYTLKRAERGNLVKEQLAEEKNIPQYLRSHWQKETGFAFPSGHTMFAASWALLAVGLLWP
RRRTLTIAILLVWATGVMGSRLLLGMHWPRDLVVATLISWALVAAATWLAQRICGPLTPP
AEENREIAQREQES
>LFGLNPFC_01899 GTP cyclohydrolase-2
MQLKRVAEAKLPTPWGDFLMVGFEELATGHDHVALVYGDISGHTPVLARVHSECLTGDAL
FSLRCDCGFQLEAALTQIAEEGRGILLYHRQEGRNIGLLNKIRAYALQDQGYDTVEANHQ
LGFAADERDFTLCADMFKLLGVNEVRLLTNNPKKVEILTEAGINIIERVPLIVGRNPNNE
HYLDTKAEKMGHLLNK
>LFGLNPFC_01900 Aconitate hydratase A
MSSTLREASKDTLQAKDKTYHYYSLPLAAKSLGDITRLPKSLKVLLENLLRWQDGKSVTE
EDIHALAGWLKNAHADREIAYRPARVLMQDFTGVPAVVDLAAMREAVKRLGGDTAKVNPL
SPVDLVIDHSVTVDRFGDDEAFEENVRLEMERNHERYVFLKWGKQAFSRFSVVPPGTGIC
HQVNLEYLGKAVWSELQDGEWIAYPDTLVGTDSHTTMINGLGVLGWGVGGIEAEAAMLGQ
PVSMLIPDVVGFKLTGKLREGITATDLVLTVTQMLRKHGVVGKFVEFYGDGLDSLPLADR
ATIANMSPEYGATCGFFPIDAVTLDYMRLSGRSEDQVELVEKYAKAQGMWRNPGDEPIFT

SVLELDMNDVEASLAGPKRPQDRVALPDVPKAFAASRELEVNATHKDRLPVDYVMNGHQY
QLPDGAVVIAAITSCTNTSNPSVLMAAGLLAKKAVTLGLKRQPWVKASLAPGSKVVSDYL
AKAKLTPYLDELGFNLVGYGCTTCIGNSGPLPDPIETAIKKGDLTVGAVLSGNRNFEGRI
HPLVKTNWLASPPLVVAYALAGNMNINLASEPIGHDRKGEPVYLKDIWPSAQEIARAVDQ
VSTEMFRKEYAEVFEGTAEWKEINVTRSDTYGWQEDSTYIRLSPFFDEMQATPAPVEDIH
GARILAMLGDSVTTDHISPAGSIKPDSPAGRYLQGRGVERKDFNSYGSRRGNHEVMMRGT
FANIRIRNEMVPGVEGGMTCHLPDSDVVSIYDAAMRYKQEQTPLAVIAGKEYGSGSSRDW
AAKGPRLLGIRVVIAESFERIHRSNLIGMGILPLEFPQGVTRKTLELTGEEKIDIGDLQN
LQPGATVPVTLTRADGSQEVVPCRCRIDTATELTYYQNDGILHYVIRNMLK
>LFGLNPFC_01901 hypothetical protein
MVGQEQLESSPLCQHSDNEPEAKRECSVVIPDDWQLTSQQQAFIELFAEDDQPKQ
>LFGLNPFC_01902 Protein YmiA
MPSGNQEPRRNPELKRKAWLAVFLGSALFWVVVALLIWKLWG
>LFGLNPFC_01903 HTH-type transcriptional regulator CysB
MKLQQLRYIVEVVNHNLNVSSTAEGLYTSQPGISKQVRMLEDELGIQIFSRSGKHLTQVT
PAGQEIIRIAREVLSKVDAIKSVAGEHTWPDKGSLYIATTHTQARYALPNVIKGFIERYP
RVSLHMHQGSPTQIADAVSKGNADFAIATEALHLYEDLVMLPCYHWNRAIVVTPDHPLAG
KKAITIEELAQYPLVTYTFGFTGRSELDTAFNRAGLTPRIVFTATDADVIKTYVRLGLGV
GVIASMAVDPVADPDLVRVDAHDIFSHSTTKIGFRRSTFLRSYMYDFIQRFAPHLTRDVV
DAAVALRSNEEIEVMFKDIKLPEK
>LFGLNPFC_01904 DNA topoisomerase 1
MGKALVIVESPAKAKTINKYLGSDYVVKSSVGHIRDLPTSGSTAKKSADSTSTKTAKKPK
KDERGALVNRMGVDPWHNWEAHYEVLPGKEKVVSELKQLAEKADHIYLATDLDREGEAIA
WHLREVIGGDDARYSRVVFNEITKNAIRQAFNKPGELNIDRVNAQQARRFMDRVVGYMVS
PLLWKKIARGLSAGRVQSVAVRLVVEREREIKAFVPEEFWEVDASTTTPSGEALALQVTH
QNDKPFRPVNKEQTQAAVSLLEKARYSVLEREDKPTTSKPGAPFITSTLQQAASTRLGFG
VKKTMMMAQRLYEAGYITYMRTDSTNLSQDAVNMVRGYISDNFGKKYLPESPNQYASKEN
SQEAHEAIRPSDVNVMAESLKDMEADAQKLYQLIWRQFVACQMTPAKYDSTTLTVGAGDF
RLKARGRILRFDGWTKVMPALRKGDEDRILPAVDKGDALTLVELTPAQHFTKPPARFSEA
SLVKELEKRGIGRPSTYASIISTIQDRGYVRVENRRFYAEKMGEIVTDRLEENFRELMNY
DFTAQMENSLDQVANHEAEWKAVLDHFFSDFTQQLDKAEKDPEEGGMRPNQMVLTSIDCP
TCGRKMGIRTASTGVFLGCSGYALPPKERCKTTINLVPENEVLNVLEGEDAETNALRAKR
RCPKCGTAMDSYLIDPKRKLHVCGNNPTCDGYEIEEGEFRIKGYDGPIVECEKCGSEMHL
KMGRFGKYMACTNEECKNTRKILRNGEVAPPKEDPVPLPELPCEKSDAYFVLRDGAAGVF
LAANTFPKSRETRAPLVEELYRFRDRLPEKLRYLADAPQQDPEGNKTMVRFSRKTKQQYV
SSEKDGKATGWSAFYVDGKWVEGKK
>LFGLNPFC_01905 Protein YciN
MNKETQPIDRETLLKEANKIIREHEDTLAGIEATGVTQRNGVLVFTGDYFLDEQGLPTAK
STAVFNMFKHLAHVLSEKYHLVD
>LFGLNPFC_01906 putative protease SohB
MELLSEYGLFLAKIVTVVLAIAAIAAIIVNVAQRNKRQRGELRVNNLSEQYKEMKEELAA
ALMDTHQQKQWHKAQKKKHKQEAKAAKAKAKLGEVVTDSKPRVWVLDFKGSMDAHEVNSL
REEITAVLAAFKPQDQVVLRLESPGGMVHGYGLAASQLQRLRDKNIPLTVTVDKVAASGG
YMMACVADKIVSAPFAIVGSIGVVAQMPNFNRFLKSKDIDIELHTAGQYKRTLTLLGENT
EEGREKFREELNETHQLFKDFVKRMRPSLDIEQVATGEHWYGQQAVEKGLVDEINTSDEV
ILSLMEGREVVNVRYMQRKRLIDRFTGSAAESADRLLLRWWQRGQKPLM
>LFGLNPFC_01907 putative oxidoreductase YciK
MHYQPKQDLLNDRIILVTGASDGIGREAAMTYARYGATVILLGRNEEKLRQVASHINEEI
GRQPQWFILDLLTCTSEDCQQLAQRIAVNYPRLDGVLHNAGLLGDVCPMSEQNPQVWQDV
MQVNVNATFMLTQALLPLLLKSDAGSLVFTSSSVGRQGRANWGAYAASKFATEGMMQVLA
DEYQQRLRVNCINPGGTRTAMRASAFPTEDPQKLKTPADIMPLYLWLMGDDSRRKTGMTF
DAQPGRKPGISQ
>LFGLNPFC_01908 Corrinoid adenosyltransferase
MSDEHYQQRQQRVKEKVDARVAQAQDERGIIIVFTGNGKGKTTAAFGTATRAVGHGKKVG
VVQFIKGTWPNGERNLLEPHGVEFQVMATGFTWDTQNRESDTAACREVWQHAKRMLADSS
LDMVLLDELTYMVAYDYLPLEEVVQALNERPHQQTVIITGRGCHRDILELADTVSELRPV
KHAFDAGVKAQIGIDY
>LFGLNPFC_01909 Ribosomal large subunit pseudouridine synthase B
MSEKLQKVLARAGHGSRREIESIIEAGRVSVDGKIAKLGDRVEITPGLKIRIDGHLISVR
ESAEQICRVLAYYKPEGELCTRNDPEGRPTVFDRLPKLRGARWIAVGRLDVNTCGLLLFT
TDGELANRLMHPSREVEREYAVRVFGQVDDAKLRDLSRGVQLEDGPAAFKTIKFSGGEGI
NQWYNVTLTEGRNREVRRLWEAVGVQVSRLIRVRYGDIPLPKGLPRGGWTELDLAQTNYL
RELVELPPETSSKVAVEKDRRRMKANQIRRAVKRHSQVSGGRRSGGRNNNG
>LFGLNPFC_01910 hypothetical protein

MMAGIYRCILLLIVGLFFSSLSYAKNTEIPSYEEGISLFDVEATLQPNGVLDIKENIHFQ
ARNQQIKHGFYRDLPRLWMQPDGDAALLNYHIVGVTRDGIPEPWHLDWHIGLMSIVVGDK
QRFLPQGDYHYQIHYQVKNAFLREGDSDLLIWNVTGNHWSFEIYKTLFSLKLPDIAGNPF
SEIDLFTGEEGDTYRNGRILEDGRIESRDPFYREDFTVLYRWPHALLGNAPAPQTTNIFS
HLLLPSTSSLLICFPSLFLACGWLYLWKRRPQFTPVDVIETDVIPPDYTPGMLRLDAKLV
YDDKGFCADIVNLIVKGKIHLEDHYDKNQQILIRVNEGATRNNAVLLPAEQLLLEALFRK
GDKVVLTGRRNRVLRKAFLRMQKFYLPRKKSSFYRPDAFLQWGGMAILAVILYGNLSPVG
WAGMSLVGDMFIMICWLLTFLFCSLDLLFARDDDKPCVNRVIITLFLPLICSGVAFYSLY
INVGDVFFYWYMPAGYFSAVFLTGYLTGMGYIFLPKFTQNGQQRYAHGEAIVNYLARKEA
ATHSGRRRKGETRKLDYALLGWAVSANLGREWAARITPSLTAAVRAPEIARSGVLFSLQM
HLSLGANTSLLGRSYSGGGAGGGAGGGGGGGW
>LFGLNPFC_01911 putative protein YciO
MSQFFYIHPDNPQQRLINQAVEIVRKGGVIVYPTDSGYALGCKIEDKNAMERICRIRQLP
DGHNFTLMCRDLSELSTYSFVDNVAFRLMKNNTPGNYTFILKGTKEVPRRLLQEKRKTIG
MRVPSNPIAQALLEALGEPMLSTSLMLPGSEFTESDPEEIKDRLEKQVDLIIHGGYLGQK
PTTVIDLTDDTPVVVREGVGDVKPFL
>LFGLNPFC_01912 5'-3' exoribonuclease
MSDTNYAVIYDLHSHTTASDGCLTPEALVHRAVEMRVGTLAITDHDTTAAIAPAREEISR
SGLALNLIPGVEISTVWENHEIHIVGLNIDITHPLMCEFLAQQTERRNQRAQLIAERLEK
AQIPGALEGAQRLAQGGAVTRGHFARFLVECGKASSMADVFKKYLARGKTGYVPPQWCTI
EQSIDVIHHSGGKAVLAHPGRYNLSAKWLKRLVAHFAEHHGDAMEVAQCQQSPNERTQLA
TLARQHHLWASQGSDFHQPCPWIELGRKLWLPAGVEGVWQLWEQPQNTTEREL
>LFGLNPFC_01913 Anthranilate synthase component 1
MQTQKPTLELLTCKGAYRDNPTALFHQLCGDRPATLLLESADIDSKDDLKSLLLVDSALR
ITALSDTVTIQALSGNGEALLTLLDNALPAGVENEQSPNCRVLRFPPVSPLLDEDARLCS
LSVFDAFRLLQNLLNVPKEEREAMFFGGLFSYDLVAGFENLPQLSAENSCPDFCFYLAET
LMVIDHQKKSTRIQASLFAPNEEEKQRLTARLNELRQQLTEAAPPLPVVSVPHMRCECNQ
SDEEFGGVVRLLQKAIRAGEIFQVVPSRRFSLPCPSPLAAYYVLKKSNPSPYMFFMQDND
FTLFGASPESSLKYDATSRQIEIYPIAGTRPRGRRADGSLDRDLDSRIELEMRTDHKELS
EHLMLVDLARNDLARICTPGSRYVADLTKVDRYSYVMHLVSRVVGELRHDLDALHAYRAC
MNMGTLSGAPKVRAMQLIAEAEGRRRGSYGGAVGYFTAHGDLDTCIVIRSALVENGIATV
QAGAGVVLDSVPQSEADETRNKARAVLRAIATAHHAQETF
>LFGLNPFC_01914 Bifunctional protein TrpGD
MADILLLDNIDSFTYNLADQLRSNGHNVVIYRNHIPAQTLIERLATMSNPVLMLSPGPGV
PSEAGCMPELLTRLRGKLPIIGICLGHQAIVEAYGGYVGQAGEILHGKASSIEHDGQAMF
AGLTNPLPVARYHSLVGSNIPAGLTINAHFNGMVMAVRHDADRVCGFQFHPESILTTQGA
RLLEQTLAWAQQKLEPTNTLQPILEKLYQAQTLSQQESHQLFSAVVRGELKPEQLAAALV
SMKIRGEHPNEIAGAATALLENAAPFPRPDYLFADIVGTGGDGSNSINISTASAFVAAAC
GLKVAKHGNRSVSSKSGSSDLLAAFGINLDMNADKSRQALDELGVCFLFAPKYHTGFRHA
MPVRQQLKTRTLFNVLGPLINPAHPPLALIGVYSPELVLPIAETLRVLGYQRAAVVHSGG
MDEVSLHAPTIVAELHDGEIKSYQLTAEDFGLTPYHQEQLAGGTPEENRDILTRLLQGKG
DAAHEAAVAANVAMLMRLHGHEDLQANAQTVLEVLRSGSAYDRVTALAARG
>LFGLNPFC_01915 Tryptophan biosynthesis protein TrpCF
MQTVLAKIVADKAIWVEARKQQQPLASFQNEIQPSTRHFYDALQGARTAFILECKKASPS
KGVIRDDFDPARIATVYRNYASAISVLTDEKYFQGSFDFLPIVSQIAPQPILCKDFIIDP
YQIYLARYYQADACLLMLSVLDDEQYRQLAAVAHSLEMGVLTEVSNEEELERAIALGAKV
VGINNRDLRDLSIDLNRTRELAPKLGHNVTVISESGINTYAQVRELSHFANGFLIGSALM
AHDDLNAAVRRVLLGENKVCGLTRPQDARAAYDAGAIYGGLIFVTTSPRCVSVEQAQEVM
AAAPLQYVGVFRNHDIADVVDKAKVLSLAAVQLHGNEDQLYIDTLREALPAHVAIWKALS
VGETLPARDFQHIDKYVFDNGQGGSGQRFDWSLLNGQSLGNVLLAGGLGADNCVEAAQTG
CAGLDFNSAVESQPGIKDARLLASVFQTLRAY
>LFGLNPFC_01916 Tryptophan synthase beta chain
MTTLLNPYFGEFGGMYVPQILMPALRQLEEAFVSAQKDPEFQAQFNDLLKNYAGRPTALT
KCQNITAGTNTTLYLKREDLLHGGAHKTNQVLGQALLAKRMGKTKIIAETGAGQHGVASA
LASALLGLKCRIYMGAKDVERQSPNVFRMRLMGAEVIPVHSGSATLKDACNEALRDWSGS
YETAHYMLGTAAGPHPYPTIVREFQRMIGEETKAQILEREGRLPDAVIACVGGGSNAIGM
FADFINETDVGLIGVEPGGHGIETGEHGAPLKHGRVGIYFGMKAPMMQTEDGQIEESYSI
SAGLDFPSVGPQHAYLNSTGRADYVSITDDEALEAFKTLCLHEGIIPALESSHALAHALK
MMRENPEKEQLLVVNLSGRGDKDIFTVHDILKARGEI
>LFGLNPFC_01917 Tryptophan synthase alpha chain
MERYESLFTQLKERKEGAFVPFVTLGDPGIEQSLKIIDTLIEAGADALELGIPFSDPLAD
GPTIQNATLRAFAAGVTPAQCFEVLALIRQKHPTIPIGLLMYANLVFNKGIDEFYAECEK
VGVDSVLVADVPVEESAPFRQAALRHNVAPIFICPPNADDDLLRQIASYGRGYTYLLSRA
GVTGAENRAALPLNHLVAKLKEYNAAPPLQGFGISAPDQVKAAIDAGAAGAISGSAIVKI

IEQHINEPEKMLAALKAFVQPMKAATRS
>LFGLNPFC_01918 hypothetical protein
MTEHRGGSGNFAEDREKASNAGRKGGQHSGGNFKNDPQRASEAGKKGGQQSGDNKSGKS
>LFGLNPFC_01919 Protein YciF
MNMKTIEDVIIHLLSDTYSAEKQLTRALAKLARATSNEKLSQAFHAHLEETHGQIERIDQ
VVESESNLKIKRMKCVAMEGLIEEANEVIENTEKNEVRDAALIAAAQKVEHYEIASYGTL
ATLAEQLGYRKAAKLLKETLEEEKATDIKLTDLALNNVNKKAENKA
>LFGLNPFC_01920 Protein YciE
MNRIEHYHDWLRDAHAMEKQAESMLESMASRIDNYPELRARIEQHLSETKNQIVQLETIL
DRNDISRSVIKDSMSKIAALGQSIGGIFPSDEIVKGSISGYVFEQFEIACYTSLLAAAKN
AGDTASIPIIEAILNDEKHMADWLIQHIPQTTEKFLIRSETDGVEAKK
>LFGLNPFC_01921 hypothetical protein
MVRLPPVVMSAKSATARTTIAREDAEDHQVKLSAQKLEELLASMVKDEVDRNDGIY
>LFGLNPFC_01922 hypothetical protein
MLPQHSDIEIAWYASIQQEPNGWKTVTTQFYIQEFSEYIAPLQDAVDLEIATEEERSLLE
AWNKYRVLLNRVDTSTAPDIEWPANPVRE
>LFGLNPFC_01923 hypothetical protein
MKKNTDDGAKIYTPLTLKLYDWWVLGVSNRLAWGCPTKEHLLPHFLEHVGNNHLDIGVGT
GFYLTHVPESSLISLMDLNEASLNAASTRAGESKIKHKISHDVFEPYPAALHGQFDSISM
FYLLHCLPGNISTKSCVIRNAAQALTDDGTLYGATILGDGVVHNSFGQKLMRIYNQKGIF
SNTKDSEEGLTHILSEHFENVKTKVQGTVVMFSASGKK
>LFGLNPFC_01924 Prophage tail fiber assembly protein TfaE
MAFRMSEQARTIKIYNLLAGTNEFIGEGDAYIPPHTGLPANSTDIAPPDIPAGFVAVFNS
DEASWHLVEDHRGKTVYDVASGDELFISELGPLPENVTWLSPEGEFQKWNGTAWVKDTEA
EKMFRIREAEETKNNLMQVASEHIAPLQDAADLEIATEEETSLLEAWKKYRVLLNRVDTS
TAPDIEWPTNPVRE
>LFGLNPFC_01925 hypothetical protein
MTVKISGVLKDGTGKPVQNCTIVLKARRTSSTVVVNTVASENPDEAGRYSMDVEHGQYSV
TLLVEGFPPSHAGTITVYEGSRPGTLNDFLGAMTEDDVRPEALRRFEQMVEEVSRNASAV
AQNTAAAKKSASDASASASEAATHATDAAASARAASTSAGQAASSAQSASSSAGTASTKA
REAAKSAAAAESSKSAAATSASAAKTSETNAAASQQSAATSASTATTKASEAATSARDAS
ASKEAAKSSETNAASSASSAASSATAAANSAKAAKTSETNARSSETAAGQSASAAADSKT
AAALSASAASTSAGQASASATAAGKSAESAASSASTATTKAGEAAVQASAAARSASAAKT
SKTNAKASETSAESSKTAAASSASSAASSASSASASKDEATRQASAAKGSATTASTKATE
AAGSATAAAQSKSTAESAATRAETAAKRAEDIASAVALEDASTTKKGIVQLSSATNSTSE
SLAATPKAVKAAYELANGKYTAQDATTAQKGIVQLSNATNSTSEMLAATPKSVKAAYDLA
NGKYTAQDATTAQKGIVQLSSATNSTSEMLAATPKSVKAAYDLANGKYTAQDATTAQKGI
VQLSSATNSASETLAATPKAVKAANNNANGRVPSARKVNGKALSADITLTPKDIGTLNST
TMSFSGGAGWFKLATVTMPQASSVVSITLIGGAGFNVGSPQQAGISELVLRAGNGNPKGI
TGALWQRTSTGFTNFAWVNTSGDTYDIYVAIGNYATGVNIQWDYTSNASVTIHTSPAYSA
NKPEGLTDGTVYSLYTPSEQFYPPGAPIPWPSDTVPSGYALMQGQTFDKSAYPKLAAAYP
SGVIPDMRGWTIKGKPASGRAVLSQEQDGIKSHTHSASASSTDLGTKNTSSFDYGTKSTN
NTGAHTHSLSGSTGSAGDHTHGNGIRWPGGGGSALAFYDGGGFTYVQDSQYQVSPGTSSR
RSYYQRIQTQSAGAHTHSLSGTAASSGAHAHTVGIGAHTHSVAIGSHGHTITVNAAGNAE
NTVKNIAFNYIVRLA
>LFGLNPFC_01926 Outer membrane protein X
MRKVCAVILSAAICLSVSGAPAWASEHQSTLSAGYLHARTNAPGSDNLNGINVKYRYEFT
DALGLITSFSYANAEDEQKTHYSDTRWHEDSVRNRWFSVMAGPSVRVNEWFSAYSMAGVA
YSRVSTFSGDYLRVTDNKGKTHDVLTGSDDGRHSNTSLAWGAGVQFNPTESVTIDLAYEG
SGSGDWRTDAFIVGIGYRF
>LFGLNPFC_01927 hypothetical protein
MGKGSSKGHTPREARDNLKSTQLLSVIDAISEGPVEGPVDGLKSVLLNSTPVLDSEGNTN
ISGVTVVFRAGEQEQTPPEGFESSGSETVLGTEVKYDTPITRTITSANIDRLRFTFGVQA
LVETTSKGDRNPSEVRLLVQIQRNGGWVTEKDITIKGKTTSQYLASVVVDNLPPRPFNIR
MRRMTPDSTTDQLQNKTLWSSYTEIIDVKQCYPNTALVGVQVDSEQFGSQQVSRNYHLRG
RILQVPSNYNPQTRQYSGIWDGTFKPAYSNNMAWCLWDMLTHPRYGMGKRLGAADVDKWA
LYVIGQNCDQSVPDGFGGTEPRITCNAYLTTQRKAWDVLSDFCSAMRCMPVWNGQTLTFV
QDRPSDKVWTYNRSNVVMPDDGAPFRYSFSALKDRHNAVEVNWIDPDNGWETATELVEDT
QAIARYGRNVTKMDAFGCTSRGQAHRAGLWLIKTELLETQTVDFSVGAEGLRHVPGDVIE
ICDDDYAGISTGGRVLAVNSQTRTLTLDREITLPSSGTTLISLVDGQGNPVSVEVQSVTD
GVKVKVSRVPDGVAEYSVWGLKLPTLRQRLFRCVSIRENDDGTYAITAVQHVPEKEAIVD
NGAHFDGDQSGTVNGVTPPAVQHLTAEVTADSGEYQVLARWDTPKVVKGVSFLLRLTVTA
DDGSERLVSTARTTETTYRFTQLALGNYRLTVRAVNARGQQGDPASVSFRIAAPAAPSRI
ELTPGYFQITATPHLAVYDPTVQFEFWFSEKRITDIRQVETTARYLGTALYWIAASINIK

PGHDYYFYVRSVNTVGKSAFVEAVGRASDDAEGYLDFFKGEIGKTHLAQELWTQIDNGQL
APDLAEIRTSITNVSNEITQTVNKKLEDQSAAIQQIQKVQVDTNNNLNSMWAVKLQQMKD
GRLYIAGIGAGIENTPAGMQSQVLLAADRIAMINPANGNTKPMFVGQGDQIFMNDVFLKR
LTAPTITSGGNPPAFSLTPDGRLTAKNADISGNVNANSGTLNNVTINENCRVLGKLSANQ
IEGDLVKTVGKAFPRDSRAPERWPSGTITVRVYDDQPFDRQIVIPAVAFSGAKHERENND
IYSSCRLIVRKNGAEIYNRTALDNTLIYSGVIDMPAGRGHMTLEFSVSAWLVNNWYPTAS
ISDLLVVVMKKATAGISIS
>LFGLNPFC_01928 hypothetical protein
MEGNSLKNIDELSGCISRQWAGNGTPITSLPIENGVSLLVPQAMGGYDVVLDIKKAGNGS
SFTLYERVPALTPKIFADSVNACK
>LFGLNPFC_01929 hypothetical protein
MAATHTLPLASPGMARICLYGDLQRFGRRIDLRVKTGAEAIRALATQLPAFRQKLSDGWY
QVRIAGRDAGETELSSRLNEPLANGAVIHIVPRLAGAKSGGVFQAVLGAALIATAIWMPG
ISIAFSDILFSMGAAMTLGGVAQMLAPKARTPRTQTTDNGKQNTYFSSLDNMVAQGNVLP
VLYGEMRVGSRVASQEISTADEGDGGQVVVIGR
>LFGLNPFC_01930 hypothetical protein
MAPEDWLQAEMQGEIVALVHSHPGGLPWLSEADRRLQVQSDLPWWLVCRGAIHKFRCVPH
LTGRRFEHGVTDCYTLFRDAYHLAGIEMPDFHREDDWWRNGQNLYLDNLEATGLYQVPLS
ARSRAMCCCAALVHRCRIMPLFTVVTASCCTIFLNN
>LFGLNPFC_01931 hypothetical protein
MQDIRQETLNECTRAEQSASVVLWEIDLTEVGGERYFFCNEQNEKGEPVTWQGRQYQPYP
IQGSSFELNGKGTSTRPTLTVSNLYGMVTGMVEDLQSLVGGTVVRRKVYARFLDAVNFVN
GNSDADPEQEVISRWRIEQCSELSAVSASFVLSTPTETDGAVFPGRIMLANTCTWTYRGD
ECGYHGPAVADEYDQPTSDITKDKCSKCLSGCKFRNNVGNFGGFLSINKLSQ
>LFGLNPFC_01932 hypothetical protein
MKTFRWKVKPGMDVTSAPSVREVRFGDGYSQRAPAGLNADLKTYSVTLSVSREEATALES
FLAEHGGWKAFLWTPPYGYRQIKVTCAKWSSRVSMLRVEFSAEFEQVVN
>LFGLNPFC_01933 hypothetical protein
MSQPVGDLVIDLSLDAVRFDEQMSRVRRHFSGLDTDARKTASAVEQGLSRQALAAQKAGI
SVGQYKAAMRTLPAQFTDIATQLAGGQNPWLILLQQGGQVKDSFGGMIPMFRGLAGAITL
PMVGVTSLAVATGALAYAWYQGDATLSEFNKTLVLSGNQAGLTADRMLTLSRAGQAAGLT
FNQARESLAALVNAGVRGGEQFDAINQSVARFASASGVEVDKVAEAFGKLTTDPTSGLMA
MARQFRNVTAEQIAYVAQLQRSGDEAGALQAANDIATKGFDEQTRRLKENMGTLETWADK
TGKAFKSMWDAILDIGRPESSADMLASAQKAFDEADKKWQWYQSRSQRRGKTSSFRANLQ
GAWDDRENARLGLAAATLQSDMEKAGELAARDRAERESSQLKYTGEAQKAYERLQTPLDK
YTARQKELNKALKDGKILQADYNTLMASAKKDYESTLKKPSGVKVSAGERQEDRAHAALL
ALETELRTLEKHSGVNEKISQQRRDLWEAESQYVVLKEAATKRQLSEQEKSLLAHEKETL
EYKRQLAELGDKIEHQKRLNELAQQAARFEQQQSAKQAAISAKARGLTDRQAQRESEEQR
LREVYGDNPAALAKATSALKNTWSAEEQLRGSWMAGMKSGWGEWAESATDSMSQVKSAAT
QTFDGIAQNMAAMLTGSEQNWRGFTRSVLSMMTEILLKQAMVGIVGSIGSAIGGAVGGGA
SASGGTAIQAAAAKFHFATGGFTGTGGKYEPAGIVHRGEFVFTKEATSRIGVGNLYRLMR
GYATGGYVGTPGSLADSRSQASGTFEQNNHVVINNDGTNGQIGPAALKAVYDMARKGARD
EIQAQMRDGGLFSGGGR
>LFGLNPFC_01934 hypothetical protein
MGRPDWRAMLAGMTSTEYADWHRFYRTHYFQDTQLDMHFSGLTYAVLSLFFCDPDMHPSD
FSLLVPRHEEEQVERPDEDKMLMQKAAGLAGGVRFGGDGGRDILSSADVADVMVDDAALM
MASAGIPGGVRYVPAGW
>LFGLNPFC_01935 hypothetical protein
MFLKTEQFEYNGVSVTLSELSALQRIEHLALLKRRAEQAESCGNLQVSVEELVRTGAFLV
AMSLWHNHPQKTQSPSMNEAVMKIEQEVLTTWPADAIARAEDVVLCLSGMIEAVRPDTDI
TEVAKNNTLTDDDFSAGKSSTAS
>LFGLNPFC_01936 hypothetical protein
MATPNPLEPVKGAGTTLWVYNGKGDAYANPLSDDDWQRLAKVKDLTPGEMTAESYDDNYL
DDEDADWTATGQGQKSAGDTSFTLAWKPGEEGQKGLIGWFESGDVRAYKIRFPNGTVDVF
RGWVSSIGKAVTAKEVITRTVKVTNVGKPSVAEERSKITPVSAIKVTPTSGTVAKGKTTT
LTVSFEPESATDKTFRAVSADPSKATISVKDMTITVNGVATGKVQIPVVSGNGQFAAVAE
VTVTEAGAAG
>LFGLNPFC_01937 hypothetical protein
MKHTDIRAAVLDALEQHEHGATLFDGRPVVFDEEDFPAIAVYLTDAEYTGEELDADTWRA
TLHIEVFLPAQVPDSELDQWMESRIYPAMTAIPALAGLITTMVTQGYEYRRDDDMALWSS
ADLTYSITYEM
>LFGLNPFC_01938 hypothetical protein
MAIKGLDQAIDNLSRVRKNAIPAASAMAINRVATTAINQSSSQVARETKVRRKLVKERSR
LKRATVRNPNARIIVNRGDLPVIKLGIRMLGRRPNSILKAGQHRYQRAFIQRLKNGRWHV

MQRVAGKNRYPIDVVKIPMAAPLKQAFDENVDRIRRERLPKELAYALKQQLRIAIKR
>LFGLNPFC_01939 hypothetical protein
MRDFQNAFDAALAGVDSTIVEVMGLCAQFTSGAQCGSEVQGVFDDPESLGFAGGGVRIEG
SSPSLFVRTDTVRAVRRGDTLTINGEIFWVDRVSPDDGGSCYLWLNRGQPPAVNRRR
>LFGLNPFC_01940 hypothetical protein
MATKEENLNRLRQLADLLGREADMSGSAADIAQRVSEWEEELAVSPEGIMHSDESGADQN
HTDDGEQLNNTDAPDDVKAVRVRKCLQVMGYCPETGRPVELALRGMRVLVPSSLATAMIQ
HGTAEYA
>LFGLNPFC_01941 hypothetical protein
MGLFTTRQLLGYTEQKVKFRALFLELFFRRTVNFHTEEVMLDKITGKTPVAAYVSPIVEG
KVLRHRGGETRVLRPGYVKPKHEFNYQQAVERLPGEDPAQLNDPAYRRLRIITDNLKQEE
HAIVQVEEMQAVNAVLYGKYTMEGDQFDTVEVDFGRSEGNNIEQADGKKWSEQDRDTFDP
THDIDLYCDQASGLVNIAIMDGTVWRLLNGFKLFREKLDTRRGSNSQLETAVKDLGAVVS
FKGYYGDLAIVVAKTSYVAEDGTEKRYLPEGMLVLGNTAAEGIRCYGAIKDAQALSEGVV
ASSRYPKHWLTVGDPSCEFTMTQSAPLMVLPDPDEFVVVQVK
>LFGLNPFC_01942 hypothetical protein
MVTKTITEQRAEVRIFAGNDPAHTATGSSGISSATPALTPLMLDEATGKLVVWDGQKAGS
AVGILVLPLEGTETVLTYYKSGTFATEAIRWPDSVDEHKKANAFAGTALSHAALP
>LFGLNPFC_01943 hypothetical protein
MRRNLSHIIAAAFNEPLLLEPAYARVFFCALGREMGAASLSVPQQQVQLDAPGMLAETDE
YMAGGKRPARVYRVVNGIAVLPVTGTLVHRLGGMRPFSGMTGYDGIVACLQQAMADSQVR
GILLDIDSPGGQAAGAFDCADMIYRLRQQKPVWALCNDTACSAAMLLASACSRRLVTQTS
RIGSIGVMMSHVSYAGHLAQAGVDITLIYSGAHKVDGNQFEALPAEVRQDMQQRIDAARR
MFAEKVAMFTGLSVDAVTGTEAAVFEGQSGIEAGLADELINASDAISVMATALNSNVRGG
TMPQLTATEAAVQENQRVMGILTCQEAKGREQLATMLAGQQGMSVEQARAILAAAAPQQP
VASAQSEADRIMACEEANGREQLAATLAAMPEMTVEKARPILAAAPLADAGPSLRDQIMA
LDEAKGAEAQAEKLAACPGMTVENARAVLAAGSGKAEPVSASTTALFEHFMANHSPAAVR
GGVSQTSADGDADVKMLMAMP
>LFGLNPFC_01944 hypothetical protein
MFGEIDVEGKRTFTEFIREGVGVHAFNGEIFVQPVWDTETTQLFRTRFKAVSPKRVDTPG
HGMGNRFLRAGVEVDRYGRAVAYHICEDDFPFSGSGRWERIPRELPTGRPAMLHIFEPVE
DGQTRGANQFYSVMERLKMLDSLQATQLQSAIVKAMYAATIESELDTEKAFEYIAGAPQG
QKDNPLINILDKFSTWYDTNSVTLGGVKIPHLFPGDDLKLQTAQDSDNGFSALEQALLRY
IAAGLGVSYEQLSRDYSKVSYSSARASANESWRYFMGRRKFIASRLATQMFSCWLEEALL
RGIIRPPRARFDFYQARSAWSRAEWIGAGRMAIDGLKEVQESVMRIEAGLSTYEKELALM
GEDYQDIFRQQVRESAEREKAGLSRPVWIAQAYQQQIAESRRPEEETTPRET
>LFGLNPFC_01945 hypothetical protein
MVTVAELQALRQARLDLLTGKRVVSVQKDGRRIEYTAASLDELNRAINDAESVLGTTRRR
RRPLGVRL
>LFGLNPFC_01946 hypothetical protein
MISDAQKAANAAGAIATGLLSLIIPVPLTTVQWANKHYYLPKESSYTPGRWETLPFQVGI
MNCMGNDLIRTVNLIKSARVGYTKMLLGVEAYFIEHKSRNSLLFQPTDSAAEDFMKSHVE
PTIRDVPALLELAPWFGRKHRDNTLTLKRFSSGVGFWCLGGAAAKNYREKSVDVVCYDEL
SSFEPDVEKEGSPTLLGDKRIEGSVWPKSIRGSTPKIKGSCQIEKAANESAHFMRFYVPC
PHCGEEQYLKFGDDASPFGLKWEKNKPESVFYLCEHHGCVIHQSELDQSNGRWICENTGM
WTRDGLTFFSAADNEIPPPRSITFHIWTAYSPFTTWVQIVYDWLDALKDPNGLKTFVNTT
LGETWEEAVGEKLDHQVLMDKVVRYTAAVPARVVYLTAGIDSQRNRFEMYVWGWAPGEEA
FLVDKIIIMGRPDEEETLLRVDVAINKKYRHADGTEMTISRVCWDTGGIDGEIVYQRSKK
HGVFRVLPVKGASVYGKPVITMPKTRNQRGVYLCEVGTDTAKEILYARMKADPTPADEAT
SYAIRFPDDPEIFSQTEAQQLVAEELVEKWEKGKMRLLWDNKKRRNEALDCLVYAYAALR
VSVQRWQLDLAVLAKSREEETTRPTLKELAAKLSGGVNGYSR
>LFGLNPFC_01947 Prophage DNA-packing protein NohA
MKVNKKRLAEIFNVDPRTIERWQSQGLPCVSKGSKGIESVFDTAMAIQWYAQRETDIENE
KLRKELADLRAAAESDLQPGTIDYERYRLTKAQADAQELKNAREDGVVLETELFTFILQR
VAQEISGILVRVPLTLQRKYPDISPSHLDVVKTEIAKASNVAAKAGENVGGWIDDFRRTE
GS
>LFGLNPFC_01948 putative protein YdfO
MAQIAIFKQIFDKVRNNLNYHWFYSELKRHNVSHYIYYLATENIHLVLENDNTVLIKGQG
KVVNVRFSKNKCLIEATLKGFKSGELSFYEYRKNLATAGVFRWITNIHENKRYYYTFDNS
LLFTENIQNTTQIFPH
>LFGLNPFC_01949 hypothetical protein
MNRVLCVVIIALLVACGALSLGLNHYRDNAITYKAQRDKNVRELKLANAAITDMQMRQRD
VAALDAKYTKELADAKAENDALRDDVAAGRRRLHIKAVCQSVREATTASGVDNAASPRLA
DTAERDYFTLRARLIIMQKQLEGAQLYIREQCLR

>LFGLNPFC_01950 Lysozyme RrrD
MNAKIKYGLSAAVLALIGAGASAPQILDQFLDEKEGNHTTAYRDGSGNWTICRGATMVDG
KPVFPGMKLSKEKCDQVNAIERDKALAWVEKNIKVPLSEPQKAGIASFCPYNIGPGKCFP
STFYKRINAGDRRGACEAIRWWIKDGGRDCRIRSNNCYGQVSRRDQESALACWGIDR
>LFGLNPFC_01951 hypothetical protein
MDNSTISLQELLDCISRLRDDVNALTVAFSHLALSIPREQMLPTLASIHFESRNPKWSQE
QQNSFKWLAALLEENYAGKTTISAESSGNQ
>LFGLNPFC_01952 hypothetical protein
MTQNYELIVKGIRNFENKVTVTLALQDKERFDGEIFDLDISLDRVEGAALEFYEAAARRS
IRQVFLDVAAGLCEGDEQSPEKRPVILEAQNVWITYKGKLPGRITGSLKTPPKW
>LFGLNPFC_01953 hypothetical protein
MDQMEKITTGVSYTTSAVGTGYWFLQLLDRVSPSQWAAIGVLGSLLFGLLTYLTNLYFKI
REDRRKAARGE
>LFGLNPFC_01954 hypothetical protein
MAFKHYDVVRAVSPSDLAKRLTQKLKEGWQPFGSPVAITPYTLMQAIAAEGDVVVSGATE
PDWYYVIVLARHSRP
>LFGLNPFC_01958 hypothetical protein
MLNTVKISSCELINADCLEFIRSLPENSVDLIVTDPPYFKVKPEGWDNQWKGDDDYLKWL
DQCLAQFWRVLKPAGSLYLFCGHRLASDIEIMMRERFSVLNHIIWAKPSGRWNGCNKESL
RAYFPATERILFAEHYQGPYRPKDAGYAAKGSALKQHVMAPLISYFRDARAALGITAKQI
ADATGKKNMVSHWFSASQWQLPNESDYLKLQALFARVAEEKHRRGELEKLHHQLVDTYTS
LNRQYAELLSEYKHLRRYFGVTVQVPYTDVWTHKPVQFYPGKHPCEKPAEMLQQIISASS
RPGDLVADFFMGSGSTVKAAMALGRRAIGVELETGRFEQTVREVQDLIV
>LFGLNPFC_01959 hypothetical protein
MLKQQDMTETARVVFNELSVTDPATVGEIAQNTYLSRERCQLILTQLVMAGLADYQFGCY
RRLPQ
>LFGLNPFC_01960 hypothetical protein
MKLEDLPKYYSPKSPGLTDASASTSKDALSITDVMAAQGMTQNRAEMGFSAFLGKMGISM
NDRARATELLADYALSRCDRVAALRKLPAEIKPVVMRIMASYAFEDYARSAASKKQCPCC
HGKKFIESEVFTNKIQYPDGKPPVWAKCTKGVYPSYWEEWKKVREVVKVACPECGGKGEV
STACKDCRGRGVAIHREESVKRGMPVIRDCQRCGGRGCERLPSTEAFNAICKVTSAITLD
TWKKSVKRFYDTLVVRFDIEEAWAERQLKRVTR
>LFGLNPFC_01961 Crossover junction endodeoxyribonuclease RusA
MLIDLVLPYPPTVNTYWRRRGSTYFISEEGKRYRRAVALIVRQQRLKLSLSGRLAIKVIA
EPPDKRRRDLDNILKAPLDALTHAGVLMDDEQFDEINIVRGQPVSGGRMGVKIYPIMHEE
QVKK
>LFGLNPFC_01962 hypothetical protein
MRVLLRPVLVPELGLVIVKPGRESMPVFHNTRVLVEPEPKSMRNLPSGVVPAVRQPLVED
KTLLPFFSNARVIRAAGGAGALSDWLLRHIKSCQWPHGDYHHSETVIHRYGTGAMVLCWH
CDNQLRDQTSESLEQLAHQNLSAWMIDVILHAMNGSQERELSLAELSWWAVRNQVADALP
EAVLRRSLGLRAEKIRSMYRESDIVPGEQTATSILKQRTKNLAPLPHAHQQNPPQEETVV
SIAVDPESPESFMKRPKRRRWVNEKYTRWVKTQPCACCGKPADDPHHLIGHGQGGMGTKS
HDIFTLPLCREHHNELHADPLAFEEKHGSQVDLIFRFLDHAFATGVLG
>LFGLNPFC_01963 hypothetical protein
MNGKSRLASYVPKGKEKQAMKQQKAMLIALIVICLTVIVTALVTRKDLCEVRIRTGQTEV
AVFTVYEPEE
>LFGLNPFC_01964 hypothetical protein
MDIRTRKTKFLESLDSTEVIRKAVSLAIDCIIDNHNSNEDTPLVITSYDDLCRIQVLNYV
QEFCEAAFPDMDEYYFSPNILRINGKTSEEACINLIKLLRSTKGMLFWSDAPSWFASLPD
GLFHVVNIDQKIVTRGLNKKNSKPTIINKDYSVDTLLSELFLNGAHMEQPNVHNVSEGNM
KFYDECHAGLIRPIPAPIGASYDEEITINSPDWQKLACVALRRYQSKECHDGMQWDTTDH
GWTDVIAYPFVEEIQSMDNSGYRQCLVGLVTINNSNANSPYLSTVWIHPFYRRRGLLSKL
WPKLQELYGSNFEIERPNENMKAFLKSAKHADY
>LFGLNPFC_01965 hypothetical protein
MLCISLWYNSCAIDTRERYIVKIVFEVNGKKVPLKSLKYISKKDQLEVMKNWFFENFEDP
ANACPYESREGGYAYIYGGPYDASEELQSIFGQYVKSEYIEELVDELQTQCFDWSGNSNN
IDDWYDDDIYDAVTSSGNPYLKFIDNIDKIKKLAKDKTEQQQKNHLLSLLYTNVITALET
LYVELFINSIEKDDVYIANCIEKGKTEFKVSKDIAALPFKGEPIEKIRGELIRSIKEHLI
SASWHSTKKVIDRYEATFDIKVQKDCPIEAIELATLNRNHLVHRGGKDKEGNLVVITDQD
LETLIENASNLAIMLYNSLNVATNKTTILQPDDKPFIHEF
>LFGLNPFC_01966 hypothetical protein
MAKVFTQEEREKIKGQVVELVRQSGRETLRQLEVKTGATRYLMSVLARELVASGDVCNSG
YGLFPSEQARKDWQNARKKLSRAKLKKPSAVDPDLIWSLPDGEIRRYDRRQNIICRECRK
SEVMQRILSFYQGDVRYLLK

>LFGLNPFC_01967 hypothetical protein
MANAWLRLWHDMPNDPKWRTIARVSGQPIATVMAVYIHLLVSASRNVTRGHIDVTTEDLA
SALDVTEEVIDSILQTMQGRVLDGDLITGWEKRQVLKEDNGNISQTAKSPAERKRAQRER
ERKREQNGDCHGASRNVTHMSRRVTTDKDTDKDTDQEDQNTMVHGVKNATNQAGDVQTVN
LGQPAGTTPEADSAYALKADSGAVQQVMTARPEQSHQLQQPEADSAIQREADRVVPENTG
QSVGRVDYPDVFEQVWREYPLRAGANPKKSAFSAWKARLREGVPPEAMLDGVRRYARYLA
ATGKTGTEFVQRATTFFGPDRNFENPWLLPVSGTNNQRCVNHISEPDNEIPPGFRG
>LFGLNPFC_01968 hypothetical protein
MKIKHEHIRMAMNAWARPDGEKVPAAGITQAYFELGMTFPELYDDSHPEALARNTQKIFR
WIEKDTPDAVEKIQALLPAIEKAMPPLLVARMRSHSSAYFRELVETRERLVRDADDFVAV
AIAGFNQMNRGGPAGNAVAVH
>LFGLNPFC_01969 hypothetical protein
MKKSEVLGYFGGVVKTAAALGTSKTTVSMWGEEVPWKWALLIQAVTAGALKYELHIPTVV
IPGSDHNPPSNQGGIHENQA
>LFGLNPFC_01970 LexA repressor
MKNSSITSCVQLVGEIPANTFAVVLESDSMSTSGGGVSIPNGSTVFVDPDRTVQPGNIVL
ALPKGTTTPVIRKLEIEGPDILLVPTNPRYPSIMLDDLSCILGVCFKIQQNI
>LFGLNPFC_01971 hypothetical protein
MTAGFNFNNYAAGFCSATPALRGNEVSMDTIDLGNSESLVCGVFPNQDGTFTAMTYTKSK
TFKTENGARRWLERNSGE
>LFGLNPFC_01972 putative protein YdfB
MDFDTIMEKAYEEYFEGLAEGEEALSFSEFKQALSSSAKSNG
>LFGLNPFC_01973 hypothetical protein
MQKREPVIIAPDYTNDELYEWMRQKINAAQDLKWANEARAKQAENLSALEQDITNLEKAA
ALSIARMITYPRE
>LFGLNPFC_01974 hypothetical protein
MNQLPRRALSRYGLTFQGNILSVNCQCRMLTRVRRTHSTSPVENSLITNLSFVG
>LFGLNPFC_01975 hypothetical protein
METLLPNVNTSEGCFEIGVTISNPVFTEDAINKRKHERELLNKICILSMLARLRPIQKGC
WQ
>LFGLNPFC_01976 hypothetical protein
MNTAFALVLTVFLVSGEPVDTAVSVHRTMQECVTAATEQKIPGNCYPVDKVIHQDNNEIP
AGL
>LFGLNPFC_01977 hypothetical protein
MSKVFICAAIPDEQAIKEEGAVAVATAIEAGDERRARAKFHWQFLEHYPAAQDCAYKFLV
CEDKPGIPRPALDSWDAEYMQENRWDEASASFVPVETESDPMNVTFDKLAPEVQNAVMVK
FDTCENITVDMVISAQELLQEDMATFDGHIVEALMKMPEVNAMYPELKLHAIGWVKHKCI
PGAKWPEIQAEMRIWKKRREGERKETGKYTSVVDLARARANQQYTENSTGKISPVIAAIH
REYKQTWKTLDDELAYALWPGDVDAGNIDGSIHRWAKKEVIDNDREDWKRISASMRKQPD
ALRYDRQTIFGLVRERPIDIHKDPVALNKYICEYLTTKGVFENEETDLGTVDVLQSSETQ
TDAVETEVSDIPKNETAPEAEPSVEREGPFYFLFADKDGEKYGRANKLSGLDKALAAGAT
EITKEEYFARKNGTYTGLPQNVDTAEDSEQPEPIKVTADEVNKIMQAANISQPDADKLLA
ASRGEFVEGISDPNDPKWIKGIQTRDSVSQNQHESERNYQKAEQNSPNALQNEPETKQPE
PVAHQEVEKACTACGQTGGGNCPDCGAVMGDATYQETFDEEYQVEVQEDDPEEMEGAEHP
HKENTGGNQHHNSDNETGETADHPIKVNGHHEITSTSRTCDHLMIDLETMGKNPDAPIIS
IGAIFFDPQTGDMGPEFSKTIDLETAGGVIDRDTIKWWLKQSREAQSAIMTDEIPLDDAL
LQLREFIDENSGEFFVQVWGNGANFDNTILRRSYERQGIPCPWRYYNDRDVRTIVELGKA
IDFDARTAIPFEGERHNALDDARYQAKYVSAIWQKLIPNQADF
>LFGLNPFC_01978 hypothetical protein
MARPRKYKTDVPGLSPYFDKRNNKVYWRYRHPITGKNHGLGSIDQKLAETIAAEANSRLA
RQQMEQMLSLQEKIISDTGGSSTVTIFLNNYRKIQQERYENGEIKLNTLKQKAAPLRVFD
ERFGTRPLDAITVKDVVSVLEEYKARGHNRMGQIFRKVLIDVFREAQQTGDVPPGFNPAE
SAKKPQVRISRQRLTFDEWMMIYNAAEKDGYFLQRGMLLALMTGQRLSDICKMQFSDIRD
GYLHVEQQKTGTRIAIPLALRCDKLNLTLDDVVSSCRDCVLSPWLLHHHHAKGTAKRGGM
VKPATLTVAFKKARDSVDYNWRANGTPPSFHEQRSLSERLFREQGVDTKILLGHSNQKMT
DIYNDARGKEWKKLVI
>LFGLNPFC_01979 Outer membrane protein W
MKKLTVAALAVATLLSGSAFAHEAGEFFMRAGSATVRPTEGAGGTLGSLGGFSVTNNTQL
GLTFTYMATDNIGVELLAATPFRHKIGTRATGDIATVHHLPPTLMAQWYFGDASSKFRPY
VGAGINYTTFFDNGFNDHGKEAGLSDLSLKDSWGAAGQVGVDYLINRDWLVNMSVWYMDI
DTTAKYKSGVTTVKDSVRLDPWVFMFSAGYRF
>LFGLNPFC_01980 hypothetical protein
MSITAQSVYRDTGNFFRNQFMTILLVSLLCAFITVVLGHVFSPSDAQLAQLNDGVPVSGS
SGLFDLVQNMSPEQQQILLQASAASTFSGLIGNAILAGGVILIIQLVSAGQRVSALRAIG

ASAPILPKLFILIFLTTLLVQIGIMLVVVPGIIMAILLALAPVMLVQDKMGVFASMRSSM
RLTWANMRLVAPAVLSWLLAKTLLLLFASSFAALTPEIGAVLANTLSNLISAVLLIYLFR
LYMLIRQ
>LFGLNPFC_01981 putative intracellular septation protein A
MKQFLDFLPLVVFFAFYKIYDIYAATAALIVATAIVLIYSWVRFRKVEKMALITFVLVVV
FGGLTLFFHNDEFIKWKVTVIYALFAGALLVSQWVMKKPLIQRMLGKELTLPQSVWSKLN
LAWAVFFILCGLANIYIAFWLPQNIWVNFKVFGLTALTLIFTLLSGIYIYRHMPQEDKS
>LFGLNPFC_01982 putative acyl-CoA thioester hydrolase
MSTTHNVPQGDLVLRTLAMPADTNANGDIFGGWLMSQMDIGGAILAKEIAHGRVVTVRVE
GMTFLRPVAVGDVVCCYARCVQKGTTSVSINIEVWVKKVASEPIGQRYKATEALFKYVAV
DPEGKPRALPVE
>LFGLNPFC_01983 Protein TonB
MTLDLPRRFPWPTLLSVCIHGAVVAGLLYTSVHQVIELPAPAQPISVTMVAPADLEPPQA
VQPPPEPVVEPEPEPEPIPEPPKEAPVVIEKPKPKPKPKPVKKVEQPKRDVKPVESRP
ASPFENTAPARPTSSTATAATSKPVTSVASGPRALSRNQPQYPARAQALRIEGQVKVKFD
VTPDGRVDNVQILSAKPANMFEREVKNAMRRWRYEPGKPGSGIVVNILFKINGTTEIQ
>LFGLNPFC_01984 Protein YciI
MLYVIYAQDKADSLEKRLSVRPAHLARLQLLHDEGRLLTAGPMPAVDSNDPGAAGFTGST
VIAEFESLEAAQAWADADPYVAAGVYEHVSVKPFKKVF
>LFGLNPFC_01985 Voltage-gated potassium channel Kch
MSHWTTFKQTATKLWVTLRHDILALAVFLNGLLIFKTIYGMSVNLLDIFHIKAFSELDLS
LLANAPLFMLGVFLVLNSIGLLFRAKLAWAISIILLLIALIYTLHFYPWLKFSIGFCIFT
LVFLLILRKDFSHSSAAAGTIFAFISFTTLLFYSTYGALYLSEGFNPRIESLMTAFYFSI
ETMSTVGYGDIVPVSESARLFTISVIISGITVFATSMTSIFGPLIRGGFNKLVKGNNHTM
HRKDHFIVCGHSILAINTILQLNQRGQNVTVISNLPEDDIKQLEQRLGDNADVIPGDSND
SSVLKKAGIDRCRAILALSDNDADNAFVVLSAKDMSSDVKTVLAVSDSKNLNKIKMVHPD
IILSPQLFGSEILARVLNGEEINNDMLVSMLLNSGHGIFSDNDEQETKADSKESAQK
>LFGLNPFC_01986 Cardiolipin synthase A
MTTVYTLVSWLAILGYWLLIAGVTLRILMKRRAVPSAMAWLLIIYILPLVGIIAYLAVGE
LHLGKRRAERARAMWPSTAKWLNDLKACKHIFAEENSSVAAPLFKLCERRQGIAGVKGNQ
LQLMTESDDVMQALIRDIQLARHNIEMVFYIWQPGGMADQVAESLMAAARRGIHCRLMLD
SAGSVAFFRSPWPELMRNAGIEVVEALKVNLMRVFLRRMDLRQHRKMIMIDNYIAYTGSM
NMVDPRYFKQDAGVGQWIDLMARMEGPIATAMGIIYSCDWEIETGKRILPPPPDVNIMPF
EQASGHTIHTIASGPGFPEDLIHQALLTAAYSAREYLIMTTPYFVPSDDLLHAICTAAQR
GVDVSIILPRKNDSMLVGWASRAFFTELLAAGVKIYQFEGGLLHTKSVLVDGELSLVGTV
NLDMRSLWLNFEITLAIDDKGFGADLAAVQDDYISRSRLLDARLWLKRPLWQRVAERLFY
FFSPLL
>LFGLNPFC_01987 hypothetical protein
MDMDLNNRLTEDETLEQAYDIFLELAADNLDPADVLLFNLQFEERGGAELFDPAEDWLEH
VDFDLNPDFFAEVVIGLADSEDGEINDVFARILLCREKDHKLCHIIWRE
>LFGLNPFC_01988 Vitamin B12 import ATP-binding protein BtuD
MNAVTEGRKVLLEIADLKVHFEIKDGKQWFWQPPKTLKAVDGVTLRLYEGETLGVVGESG
CGKSTFARAIIGLVKATDGHVAWLGKELLGMKPDEWRAVRSDIQMIFQDPLASLNPRMTI
GEIIAEPLRIYHPKMSRQEVRERVKAMMLKVGLLPNLINRYPHEFSGGQCQRIGIARALI
LEPKLIICDEPVSALDVSIQAQVVNLLQQLQREMGLSLIFIAHDLAVVKHISDRVLVMYL
GHAVELGTYDEVYHNPLHPYTRALMSAVPIPDPDLEKNKTIQLLEGELPSPINPPSGCVF
RTRCPIAGPECAKTRPVLEGSFRHAVSCLKVDPL
>LFGLNPFC_01989 Oligopeptide transport ATP-binding protein OppD
MSVIETATVPLAQQQADALLNVKDLRVTFSTPDGDVTAVNDLNFSLRAGETLGIVGESGS
GKSQTAFALMGLLAANGRIGGSATFNGREILNLPERELNKLRAEQISMIFQDPMTSLNPY
MRVGEQLMEVLMLHKNMSKAEAFEESVRMLDAVKMPEARKRMKMYPHEFSGGMRQRVMIA
MALLCRPKLLIADEPTTALDVTVQAQIMTLLNELKREFNTAIIMITHDLGVVAGICDKVL
VMYAGRTMEYGNARDVFYQPVHPYSIGLLNAVPRLDAEGETMLTIPGNPPNLLRLPKGCP
FQPRCPHAMDICSSAPPLEEFTPGRLRACFKPVEELL
>LFGLNPFC_01990 Oligopeptide transport system permease protein OppC
MMLSKKNSETLENFSEKLEVEGRSLWQDARRRFMHNRAAVASLIVLVLIALFVILAPMLS
QFAYDDTDWAMMSSAPDMESGHYFGTDSSGRDLLVRVAIGGRISLMVGVAAALVAVVVGT
LYGSLSGYLGGKVDSVMMRLLEILNSFPFMFFVILLVTFFGQNILLIFVAIGMVSWLDMA
RIVRGQTLSLKRKEFIEAAQVGGVSTPGIVIRHIVPNVLGVVVVYASLLVPSMILFESFL
SFLGLGTQEPLSSWGALLSDGANSMEVSPWLLLFPAGFLVVTLFCFNFIGDGLRDALDPK
DR
>LFGLNPFC_01991 Oligopeptide transport system permease protein OppB
MLKFILRRCLEAIPTLFILITISFFMMRLAPGSPFTGERTLPPEVMANIEAKYHLNDPIM
TQYFSYLKQLAHGDFGPSFKYKDYSVNDLVASSFPVSAKLGAAAFFLAVILGVSAGVIAA

LKQNTKWDYTVMGLAMTGVVIPSFVVAPLLVMIFAIILHWLPGGGWNGGALKFMILPMVA
LSLAYIASIARISRGSMIEVLHSNFIRTARAKGLPMRRIILRHALKPALLPVLSYMGPAF
VGIITGSMVIETIYGLPGIGQLFVNGALNRDYSLVLSLTILVGALTILFNAIVDVLYAVI
DPKIRY
>LFGLNPFC_01992 Periplasmic oligopeptide-binding protein
MLVNTSNYNEGVQKTMTNITKRSLVAAGVLAALMAGNVALAADVPAGVTLAEKQTLVRNN
GSEVQSLDPHKIEGVPESNISRDLFEGLLVSDLDGHPAPGVAESWDNKDAKVWTFHLRKD
AKWSDGTPVTAQDFVYSWQRSVDPNTASPYASYLQYGHIAGIDEILEGKKPITDLGVKAI
DDHTLEVTLSEPVPYFYKLLVHPSTSPVPKAAIEKFGEKWTQPGNIVTNGAYTLKDWVVN
ERIVLERSPTYWNNAKTVINQVTYLPIASEVTDVNRYRSGEIDMTYNNMPIELFQKLKKE
IPDEVHVDPYLCTYYYEINNQKPPFNDVRVRTALKLGMDRDIIVNKVKAQGDMPAYGYTP
PYTDGAKLTQPEWFGWSQEKRNEEAKKLLAEAGYTADKPLTINLLYNTSDLHKKLAIAAS
SLWKKNIGVNVKLVNQEWKTFLDTRHQGTFDVARAGWCADYNEPTSFLNTMLSNSSMNTA
HYKSPAFDSIMAETLKVTDEAQRTALYTKAEQQLDKDSAIVPVYYYVNARLVKPWVGGYT
GKDPLDNTYTRNMYIVKH
>LFGLNPFC_01993 hypothetical protein
MSLFSDYSSSSEMHNNLTIDYYLALSSTKGSGITNIISIILQQAQDYDVAKIT
>LFGLNPFC_01994 hypothetical protein
MLRLLRHYFYFVPAQLSKAKGIGKNILLKLQLVIGISLTASPHHHVIFATS
>LFGLNPFC_01995 hypothetical protein
MIQTLFDFPVYFKFFIGLFALVNPVGIIPVFISMTSYQTAAARNKTNLTANLSVAIILWI
SLFLGDTILQLFGISIDSFRIAGGILVVTIAMSMISGKLGEDKQNKQEKSETAVRESIGV
VPLALPLMAGPGAISSTIVWGTRYHSISYLFGFFVAIALFALCCWGLFRMAPWLVRVLRQ
TGINVITRIMGLLLMALGIEFIVTGIKGIFPGLLN
>LFGLNPFC_01996 Aldehyde-alcohol dehydrogenase
MAVTNVAELNALVERVKKAQREYASFTQEQVDKIFRAAALAAADARIPLAKMAVAESGMG
IVEDKVIKNHFASEYIYNAYKDEKTCGVLSEDDTFGTITIAEPIGIICGIVPTTNPTSTA
IFKSLISLKTRNAIIFSPHPRAKDATNKAADIVLQAAIAAGAPKDLIGWIDQPSVELSNA
LMHHPDINLILATGGPGMVKAAYSSGKPAIGVGAGNTPVVIDETADIKRAVASVLMSKTF
DNGVICASEQSVVVVDSVYDAVRERFATHGGYLLQGKELKAVQDVILKNGALNAAIVGQP
AYKIAELAGFSVPENTKILIGEVTVVDESEPFAHEKLSPTLAMYRAKDFEDAVEKAEKLV
AMGGIGHTSCLYTDQDNQPARVSYFGQKMKTARILINTPASQGGIGDLYNFKLAPSLTLG
CGSWGGNSISENVGPKHLINKKTVAKRAENMLWHKLPKSIYFRRGSLPIALDEVITDGHK
RALIVTDRFLFNNGYADQITSVLKAAGVETEVFFEVEADPTLSIVRKGAELANSFKPDVI
IALGGGSPMDAAKIMWVMYEHPETHFEELALRFMDIRKRIYKFPKMGVKAKMIAVTTTSG
TGSEVTPFAVVTDDTTGQKYPLADYALTPDMAIVDANLVMDMPKSLCAFGGLDAVTHAME
AYVSVLASEFSDGQALQALKLLKEYLPASYHEGSKNPVARERVHSAATIAGIAFANAFLG
VCHSMAHKLGSQFHIPHGLANALLICNVIRYNANDNPTKQTAFSQYDRPQARRRYAEIAD
HLGLSAPGDRTAAKIEKLLAWLETLKAELGIPKSIREAGVQEADFLANVDKLSEDAFDDQ
CTGANPRYPLISELKQILLDTYYGRDYVEGETAAKKEAAPAKAEKKAKKSA
>LFGLNPFC_01997 hypothetical protein
MLIGQALDLVSRYDSLRNPLTSLGDYLAPKLISRCLAESGTVTLRKRRLPLEMMVWCIVG
MALERKEPLHQIVNRLDIMLPGNRPFVAPSAVIQTRQRLGIEAVRRVFTKTAQLWHNATP
HPHWCGLTLLAIDGVFWRTPDTPENDAAFPRQTHAGNPALYPQVKMVCQMELTSHLLTTA
AFGTMKNSENELAEQLIEQTGDNTLTLMDKGYYSPGLLNAWSLAGEHRHWMIPLRKGAQY
EEIRKLGKGDHLVKLNISPQARKKWPGLGNEVTARLLTVTRKGKVCHLLTSMTDAIRFTG
TYTGADARSCKYGTTSEITDKKGKGLPESGKGEALEIPHSPEKEPVSCLTDWHYPFWGRF
LFSQNNFSINRQHCPSSYLRRCITPTHSDIVLYRLQVNVGV
>LFGLNPFC_01998 Thymidine kinase
MAQLYFYYSAMNAGKSTALLQSSYNYQERGMRTVVYTAEIDDRFGAGKVSSRIGLSSPAK
LFNQNSSLFDEIRAEHEQQAIHCVLVDECQFLTRQQVYELSEVVDQLDIPVLCYGLRTDF
RGELFIGSQYLLAWSDKLVELKTICFCGRKASMVLRLDQAGRPYNEGEQVVIGGNERYVS
VCRKHYKEALEVGSLTAIQERHRHD
>LFGLNPFC_01999 DNA-binding protein H-NS
MSEALKILNNIRTLRAQARECTLETLEEMLEKLEVVVNERREEESAAAAEVEERTRKLQQ
YREMLIADGIDPNELLNSLAAVKSGTKAKRAQRPAKYSYVDENGETKTWTGQGRTPAVIK
KAMDEQGKSLDDFLIKQ
>LFGLNPFC_02000 UTP--glucose-1-phosphate uridylyltransferase
MAAINTKVKKAVIPVAGLGTRMLPATKAIPKEMLPLVDKPLIQYVVNECIAAGITEIVLV
THSSKNSIENHFDTSFELEAMLEKRVKRQLLDEVQSICPPHVTIMQVRQGLAKGLGHAVL
CAHPVVGDEPVAVILPDVILDEYESDLSQDNLAEMIRRFDETGHSQIMVEPVADVTAYGV
VDCKGVELAPGESVPMVGVVEKPKADVAPSNLAIVGRYVLSADIWPLLAKTPPGAGDEIQ
LTDAIDMLIEKETVEAYHMKGKSHDCGNKLGYMQAFVEYGIRHNTLGTEFKAWLEEEMGI
KK

>LFGLNPFC_02001 Regulator of RpoS
MTQPLVGKQILIVEDEQVFRSLLDSWFSSLGATTVLAADGVDALELLGGFTPDLMICDIA
MPRMNGLKLLEHIRNSGDQTPVLVISATENMADIAKALRLGVEDVLLKPVKDLNRLREMV
FACLYPSMFNSRVEEEERLFRDWDAMVDNPAAAAKLLQELQPPVQQVISQCRVNYRQLVA
ADKPGLVLDIAALSENDLAFYCLDVTRAGHNGVLAALLLRALFNGLLQEQLAHQNQRLPE
LGALLKQVNHLLRQANLPGQFPLLVGYYHRELKNLILVSAGLNATLNTGEHQVQISNGVP
LGTLGNAYLNQLSQRCDAWQCQIWGTGGRLRLMLSAE
>LFGLNPFC_02002 putative NTE family protein
MATIAFQGNLAGIMRKIKIGLALGSGAARGWSHIGVINALKKVGIEIDIVAGCSIGSLVG
AAYACDRLSALEDWVTSFSYWDVLRLMDLSWQRGGLLRGERVFNQYREIMPETEIENCSR
RFAAVATNLSTGRELWFTEGDLHLAIRASCSIPGLMAPVAHNGYWLVDGAVVNPIPISLT
RALGADIVIAVDLQHDAHLMQQDLLSFNVSEENSENGDSLPWHARLKERLGSITTRRAVT
APTATEIMTTSIQVLENRLKRNRMAGDPPDILIQPVCPQISTLDFHRAHAAIAAGQLAVE
KKMDELLPLVRTNI
>LFGLNPFC_02003 hypothetical protein
MSQLCPCGSAVEYSLCCHPYVSGEKVAPDPEHLMRSRYCAFVMQDADYLIKTWHPSCGAA
ALRAELIAGFAHTEWLGLTVFEHCWQDGGNIGFVSFVARFTEGGKTGAIIERSRFLKENG
QWYYIDGTRPQFGRNDPCPCGSGKKFKKCCGQ
>LFGLNPFC_02004 Formyltetrahydrofolate deformylase
MHSLQRKVLRTICPDQKGLIARITNICYKHELNIVQNNEFVDHRTGRFFMRTELEGIFND
STLLADLDSALPEGSVRELNPAGRRRIVILVTKEAHCLGDLLMKANYGGLDVEIAAVIGN
HDTLRSLVERFDIPFELVSHEGLSRNEHDQKMADAIDAYQPDYVVLAKYMRVLTPEFVAR
FPNKIINIHHSFLPAFIGARPYHQAYERGVKIIGATAHYVNDNLDEGPIIMQDVIHVDHT
YTAEDMMRAGRDVEKNVLSRALYKVLAQRVFVYGNRTIIL
>LFGLNPFC_02007 IS4 family transposase ISSfl1
MPARQVCQNFFRGALAPFHKYRQNALLDATIALINGASLTLTSIGRYLPGNAQVKNKIKR
VDRLLGNESLHHDIPLIFRNIISMLTSKLSLCVIAVDWSGYPSQEYHVLRASLICYGHSI
PLLSWIVPSEKQQNAKIQQAFLNTLSEAVNPKARVIIVTDAGFQNAWFRHIKSLGWDFIG
RIRGNKQLHLARKGECWFRRQELQASNKPEYLGPGTLSRAEYARCDGHFYLHKKEPKGRR
NKRSRCGIARPSQIKDARSAAKEPWLIFSSTDDFKPREIMKLYSRRMQIEQNFRDEKSER
FGFGLRASYSCSAGRMLVLSLLATLSTIVLWLIGYHAENQGLHLRYQANSIKTRRVISYL
TLAENVLRHSPLILKRTVLSTILNHLTRTYQNMVLVYYR
>LFGLNPFC_02008 Respiratory nitrate reductase 1 gamma chain
MQFLNMFFFDIYPYIAGAVFLIGSWLRYDYGQYTWRAASSQMLDRKGMNLASNLFHIGIL
GIFVGHFFGMLTPHWMYEAWLPIEVKQKMAMFAGGASGVLCLIGGVLLLKRRLFSPRVRA
TTTGADILILSLLVIQCALGLLTIPFSAQHMDGSEMMKLVGWAQSVVTFHGGASQHLDGV
AFIFRLHLVLGMTLFLLFPFSRLVHIWSVPVEYLTRKYQLVRARH
>LFGLNPFC_02009 Nitrate reductase molybdenum cofactor assembly chaperone NarJ
MIELVIVSRLLEYPDAALWQHQQEMFEAIAASKNLPKEDAHALGIFLRDLTTMDPLDAQA
QYSELFDRGRATSLLLFEHVHGESRDRGQAMVDLLAQYEQHGLQLNSRELPDHLPLYLEY
LAQLPQSEAVEGLKDIAPILALLSARLQQRESRYAVLFDLLLKLANTAIDSDKVAEKIAD
EARDDTPQALDAVWEEEQVKFFADKGCGDSAITAHQRRFAGAVAPQYLNITTGGQH
>LFGLNPFC_02010 Respiratory nitrate reductase 1 beta chain
MKIRSQVGMVLNLDKCIGCHTCSVTCKNVWTSREGVEYAWFNNVETKPGQGFPTDWENQE
KYKGGWIRKINGKLQPRMGNRAMLLGKIFANPHLPGIDDYYEPFDFDYQNLHTAPEGSKS
QPIARPRSLITGERMAKIEKGPNWEDDLGGEFDKLAKDKNFDNIQKAMYSQFENTFMMYL
PRLCEHCLNPACVATCPSGAIYKREEDGIVLIDQDKCRGWRMCITGCPYKKIYFNWKSGK
SEKCIFCYPRIEAGQPTICSETCVGRIRYLGVLLYDADAIERAASTENEKDLYQRQLDVF
LDPNDPKVIEQAIKDGIPLSVIEAAQQSPVYKMAMEWKLALPLHPEYRTLPMVWYVPPLS
PIQSAADAGELGSNGILPDVESLRIPVQYLANLLTAGDTKPVLRALKRMLAMRHYKRAET
VDGKVDTRALEEVGLTEAQAQEMYRYLAIANYEDRFVVPSSHRELAREAFPEKNGCGFTF
GDGCHGSDTKFNLFNSRRIDAIDVTSKTEPHP
>LFGLNPFC_02011 Respiratory nitrate reductase 1 alpha chain
MSKFLDRFRYFKQKGETFADGHGQLLNTNRDWEDGYRQRWQHDKIVRSTHGVNCTGSCSW
KIYVKNGLVTWETQQTDYPRTRPDLPNHEPRGCPRGASYSWYLYSANRLKYPMMRKRLMK
MWREAKALHSDPVEAWASIIEDADKAKSFKQARGRGGFVRSSWQEVNELIAASNVYTIKN
YGPDRVAGFSPIPAMSMVSYASGARYLSLIGGTCLSFYDWYCDLPPASPQTWGEQTDVPE
SADWYNSSYIIAWGSNVPQTRTPDAHFFTEVRYKGTKTVAVTPDYAEIAKLCDLWLAPKQ
GTDAAMALAMGHVMLREFHLDNPSQYFTDYVRRYTDMPMLVMLEERDGYYAAGRMLRAAD
LVDALGQENNPEWKTVAFNTNGEMVAPNGSIGFRWGEKGKWNLEQRDGKTGEETELQLSL
LGSQDEIAEVGFPYFGGDGTEHFNKVELENVLLHKLPVKRLQLADGSTALVTTVYDLTLA
NYGLERGLNDVNCATSYDDVKAYTPAWAEQITGVSRSQIIRIAREFADNADKTHGRSMII
VGAGLNHWYHLDMNYRGLINMLIFCGCVGQSGGGWAHYVGQEKLRPQTGWQPLAFALDWQ
RPARHMNSTSYFYNHSSQWRYETVTAEELLSPMADKSRYTGHLIDFNVRAERMGWLPSAP

QLGTNPLTIAREAEKAGMNPVDYTVKSLKEGSIRFAAEQPENGKNHPRNLFIWRSNLLGS
SGKGHEFMLKYLLGTEHGIQGKDLGQQGGVKPEEVDWQDNGLEGKLDLVVTLDFRLSSTC
LYSDIILPTATWYEKDDMNTSDMHPFIHPLSAAVDPAWEAKSDWEIYKAIAKKFSEVCVG
HLGKETDIVTLPIQHDSAAELAQPLDVKDWKKGECDLIPGKTAPHIMVVERDYPATYERF
TSIGPLMEKIGNGGKGIAWNTQSEMDLLRKLNYTKAEGPAKGQPMLNTAIDAAEMILTLA
PETNGQVAVKAWAALSEFTGRDHTHLALNKEDEKIRFRDIQAQPRKIISSPTWSGLEDEH
VSYNAGYTNVHELIPWRTLSGRQQLYQDHQWMRDFGESLLVYRPPIDTRSVKEVMGQKSN
GNPEKALNFLTPHQKWGIHSTYSDNLLMLTLGRGGPVVWLSEADAKELGIADNDWIEVFN
SNGALTARAVVSQRVPAGMTMMYHAQERIVNLPGSEITQQRGGIHNSVTRITPKPTHMIG
GYAHLAYGFNYYGTVGSNRDEFVVVRKMKNIDWLDGEGNDQVQESVK
>LFGLNPFC_02012 Nitrate/nitrite transporter NarK
MSHSSAPERATGAVITDWRPEDPAFWQQRGQRIASRNLWISVPCLLLAFCVWMLFSAVAV
NLPKVGFNFTTDQLFMLTALPSVSGALLRVPYSFMVPIFGGRRWTAFSTGILIIPCVWLG
FAVQDTSTPYSVFIIISLLCGFAGANFASSMANISFFFPKQKQGGALGLNGGLGNMGVSV
MQLVAPLVVSLSIFAVFGSQGVKQPDGTELYLANASWIWVPFLAIFTIAAWFGMNDLATS
KASIKEQLPVLKRGHLWIMSLLYLATFGSFIGFSAGFAMLSKTQFPDVQILQYAFFGPFI
GALARSAGGALSDRLGGTRVTLVNFILMAIFSGLLFLTLPTDGQGGSFMAFFAVFLALFL
TAGLGSGSTFQMISVIFRKLTMDRVKAEGGSDERAMREAATDTAAALGFISAIGAIGGFF
IPKAFGSSLALTGSPVGAMKVFLIFYIACVVITWAVYGRHSKK
>LFGLNPFC_02013 hypothetical protein
MSNNLNECDDTFWNGSILGYWLKYTHTRKELLLICRVVSRFTSVRVGILQHREKSHNFYE
ITVLTMDNDKYQ
>LFGLNPFC_02014 Nitrate/nitrite sensor protein NarX
MLKRCLSPLTLVNQVALIVLLSTAIGLAGMAVSGWLVQGVQGSAHAINKAGSLRMQSYRL
LAAVPLSEKDKPLIKEMEQTAFSAELTRAAERDGQLAQLQGLQDYWRNELIPALMRAQNR
ETVSADVSQFVAGLDQLVSGFDRTTEMRIETVVLVHRVMAVFMALLLVFTIIWLRARLLQ
PWRQLLAMASAVSHRDFTQRANISGRNEMAMLGTALNNMSAELAESYAVLEQRVQEKTAG
LEHKNQILSFLWQANRRLHSRAPLCERLSPVLNGLQNLTLLRDIELRVYDTDDEENHQEF
TCQPDMTCDDKGCQLCPRGVLPVGDRGTTLKWRLADTHTQYGILLATLPQGRHLSHDQQQ
LVDTLVEQLTATLALDRHQERQQLIVMEERATIARELHDSIAQSLSCMKMQVSCLQMQG
DALPESSRELLSQIRNELNASWVQLRELLTTFRLQLTEPGLRPALEASCEEYSAKFGFPV
KLDYQLPPRLVPSHQAIHLLQIAREALSNALKHSQASEVVVTVAQNDNQVKLTVQDNGCG
VPENAIRSNHYGMIIMRDRAQSLRGDCRVRRRESGGTEVVVTFIPEKTFTDVQGDTHE
>LFGLNPFC_02015 Nitrate/nitrite response regulator protein NarL
MSNQEPATILLIDDHPMLRTGVKQLISMAPDITVVGEASNGEQGIELAESLDPDLILLDL
NMPGMNGLETLDKLREKSLSGRIVVFSVSNHEEDVVTALKRGADGYLLKDMEPEDLLKAL
HQAAAGEMVLSEALTPVLAASLRANRATTERDVNQLTPRERDILKLIAQGLPNKMIARRL
DITESTVKVHVKHMLKKMKLKSRVEAAVWVHQERIF
>LFGLNPFC_02016 Invasin
MSRFVPRIIPFYLLLLAAGGTANAQSTFEQKAANPFDNNNDGLPDLGMAPENHDGEKHFA
EIVKDFGETSMNDNGLDTGEQAKAFALGKVRDALSQQVNQHVESWLSPWGNASVDVKVDN
EGHFTGSRGSWFVPLQDNDRYLTWSQLGLTQQDDGLVSNVGVGQRWARGSWLVGYNTFYD
NLLDENLQRAGFGAEAWGEYLRLSANFYQPFAAWHEQTATQEQRMARGYDLTARMRMPFY
QHLNTSVSVEQYFGDRVDLFNSGTGYHNPVALSLGLNYTPVPLVTVTAQHKQGESGENQN
NLGLNLNYRFGVPLKKQLSAGEVAESQSLRGSRYDNPQRNNLPTLEYRQRKTLTVFLATP
PWDLKPGETVPLKLQIRSRYGIRQLIWQGDTQILSLTPGAQANSEEGWTLIMPDWQNGEG
ASNHWRLSVVVEDNQGQRVSSNEITLTLVEPFDALSNDELRWEP
>LFGLNPFC_02017 Protein YchN
MQKIVIVANGAPYGSESLFNSLRLAIALREQENNLDLRLFLMSDAVTAGLRGQKPGEGYN
IQQMLEILTAQNVPVKLCKTCTDGRGISTLPLIDGVEIGTLVELAQWTLSADKVLTF
>LFGLNPFC_02018 Glutathione-specific gamma-glutamylcyclotransferase
MQWRRCKVITRDFLMNADCKTAFGAIEESLLWSAEQRAASLAATLACRPDEGPVWIFGYG
SLMWNPALEFTESCTGTLVGWHRAFCLRLTAGRGTAHQPGRMLALKEGGRTTGVAYRLPE
ETLEQELTLLWKREMITGCYLPTWCQLDLDDGRTVNAIVFIMDPRHPEYESDTRAQVIAP
LIAAASGPLGTNAQYLFSLEQELIKLGMQDDGLNELLVSVKKLLAENYPDGVLRPGFA
>LFGLNPFC_02019 Putative cation transport regulator ChaB
MPYKTKSDLPESVKHVLPSHAQDIYKEAFNSAWDQYKDKEDRRDDASREETAHKVAWAAV
KHEYAKGDDDKWHKKS
>LFGLNPFC_02020 Sodium-potassium/proton antiporter ChaA
MSNAQEAVKTRHKETSLIFPVLALVVLFLWGGSQTLPVVIAINLLALIGILSSAFSVVRH
ADVLAHRLGEPYGSLILSLSVVILEVSLISALMATGDAAPTLMRDTLYSIIMIVTGGLVG
FSLLLGGRKFATQYMNLFGIKQYLIALFPLAIIVLVFPMALPAANFSTGQALLVALISAA
MYGVFLLIQTKTHQSLFVYEHEDDSDDDDPHHGKPSAHSSLWHAIWLIVHLIAVIAVTKM
NASPLETLLDSMNAPVAFTGFLVALLILSPEGLGALKAVLNNQVQRAMNLFFGSVLATIS

LTVPVVTLIAFMTGNELQFALGAPEMVVMVASLVLCHISFSTGRTNVLNGAAHLALFAAY
LMTIFA
>LFGLNPFC_02021 Small toxic polypeptide LdrA
MTLAQFAMIFWHDLAAPILAGIITAAIVGWWRNRK
>LFGLNPFC_02022 2-dehydro-3-deoxyphosphooctonate aldolase
MKQKVVSIGDINVANDLPFVLFGGMNVLESRDLAMRICEHYVTVTQKLGIPYVFKASFDK
ANRSSIHSYRGPGLEEGMKIFQELKQTFGVKIITDVHEPSQAQPVADVVDVIQLPAFLAR
QTDLVEAMAKTGAVINVKKPQFVSPGQMGNIVDKFKEGGNEKVILCDRGANFGYDNLVVD
MLGFSIMKKVSGNSPVIFDVTHALQCRDPFGAASGGRRAQVAELARAGMAVGLAGLFIEA
HPDPEHAKCDGPSALPLAKLEPFLKQMKAIDDLVKGFEELDTSK
>LFGLNPFC_02023 hypothetical protein
MRSLADFEFNKAPLCEGMILACEAIRRDFPSQDVYDELERLVSLAKEEISQLLPLEEQLE
KLIALFYGEWGFKASRGVYRLSDALWLDQVLKNRQGSAVSLGAVLLWVANRLDLPLLPVI
FPTQLILRIECPDGEIWLINPFNGESLSEHMLDVWLKGNISPSAELFYEDLDEADNIEVI
RKLLDTLKASLMEENQMELALRTSEALLQFNPEDPYEIRDRGLIYAQLDCEHVALNDLSY
FVEQCPEDPISEMIRAQINNIAHKHIVLH
>LFGLNPFC_02024 Protein YchQ
MTSFSTLLSVHLISIALSVGLLTLRFWLRYQKHPQAFARWTRIVPPVVDTLLLLSGIALM
AKAHIQPFSGQAQWLTEKLFGVIIYIVLGFIALDYRRMHSQQARIIAFPLALVVLYIIIK
LATTKVPLLG
>LFGLNPFC_02025 Release factor glutamine methyltransferase
MEYQHWLREAISQLQASESPRRDAEILLEHVTGRGRTFILAFGETQLTDEQCQQLDALLT
RRRDGEPIAHLTGVREFWSLPLFVSPATLIPRPDTECLVEQALARLPEQPCRILDLGTGT
GAIALALASERPDCEITAVDRMPDAVSLAQRNAQHLAIKNIHILQSDWFSALAGQQFAMI
VSNPPYIDEQDPHLQQGDVRFEPLTALVAADSGMADIVHIIEQSRNALVSGGFLLLEHGW
QQGEAVRQAFIFAGYHDVETCRDYGDNERVTLGRYYQ
>LFGLNPFC_02026 Peptide chain release factor RF1
MKPSIVAKLEALHERHEEVQALLGDAQTIADQERFRALSREYAQLSDVSRCFTDWQQVQE
DIETAQMMLDDPEMREMAQDELREAKEKSEQLEQQLQVLLLPKDPDDERNAFLEVRAGTG
GDEAALFAGDLFRMYSRYAEARRWRVEIMSASEGEHGGYKEIIAKISGDGVYGRLKFESG
GHRVQRVPATESQGRIHTSACTVAVMPELPDAELPDINPADLRIDTFRSSGAGGQHVNTT
DSAIRITHLPTGIVVECQDERSQHKNKAKALSVLGARIHAAEMAKRQQAEASTRRNLLGS
GDRSDRNRTYNFPQGRVTDHRINLTLYRLDEVMEGKLDMLIEPIIQEHQADQLAALSEQE
>LFGLNPFC_02027 Glutamyl-tRNA reductase
MILRILYDASRLTLSTLVLFPADMTLLALGINHKTAPVSLRERVSFSPDKLDQALDSLLA
QPMVQGGVVLSTCNRTELYLSVEERDDLQEALIRWLCDYHNLNEDDLRNSLYWHQDNDAV
SHLMRVASGLDSLVLGEPQILGQVKKAFADSQKGHMKASELERMFQKSFSVAKRVRTETD
IGASAVSVAFAACTLARQIFESLSTVTVLLVGAGETIELVARHLREHKVQKMIIANRTRE
RAQILADEVGAEVIALSDIDERLREADIIISSTASPLPIIGKGMVERALKSRRNQPMLLV
DIAVPRDVEPEVGKLANAYLYSVDDLQSIISHNLAQRKAAAVEAETIVAQEASEFMAWLR
AQSASETIRDYRSQAEQVRDELTAKALAALEQGGDAQTIMQDLAWKLTNRLIHAPTKSLQ
QAARDGDNERLNILRDSLGLE
>LFGLNPFC_02028 Outer-membrane lipoprotein LolB
MPLPDFRLIRLLPLAALVLTACSVTTPKGPGKSPDSPQWRQHQQDVRNLNQYQTRGAFAY
ISDQQKVYARFFWQQTGQDRYRLLLTNPLGSTELELNAQPGNVQLVDNKGQRYTSDDAEE
MIGKLTGMPIPLNSLRQWILGLPGDATDYKLDDQYRLSEITYSQNGKNWKVVYGGYDTKT
QPAMPANMELTDGGQRIKLKMDNWIVK
>LFGLNPFC_02029 4-diphosphocytidyl-2-C-methyl-D-erythritol kinase
MRTQWPSPAKLNLFLYITGQRADGYHTLQTLFQFLDYGDTISIELRDDGDIRLLTPVEGV
EHEDNLIVRAARLLMKTAADSGRLSTGSGANISIDKRLPMGGGLGGGSSNAATVLVALNH
LWQCGLSMDELAEMGLTLGADVPVFVRGHAAFAEGVGEILTPVDPPEKWYLVAHPGVSIP
TPVIFKDPELPRNTPKRSIETLLKCEFSNDCEVIARKRFREVDAVLSWLLEYAPSRLTGT
GACVFAEFDTESEARQVLEQAPEWLNGFVAKGVNLSPLHRAML
>LFGLNPFC_02030 Ribose-phosphate pyrophosphokinase
MPGPHSFRQILSTNGRMPEVLLVPDMKLFAGNATPELAQRIANRLYTSLGDAAVGRFSDG
EVSVQINENVRGGDIFIIQSTCAPTNDNLMELVVMVDALRRASAGRITAVIPYFGYARQD
RRVRSARVPITAKVVADFLSSVGVDRVLTVDLHAEQIQGFFDVPVDNVFGSPILLEDMLQ
LNLDNPIVVSPDIGGVVRARAIAKLLNDTDMAIIDKRRPRANVSQVMHIIGDVAGRDCVL
VDDMIDTGGTLCKAAEALKERGAKRVFAYATHPIFSGNAANNLRNSVIDEVVVCDTIPLS
DEIKSLPNVRTLTLSGMLAEAIRRISNEESISAMFEH
>LFGLNPFC_02031 C4-dicarboxylic acid transporter DauA
MPFRALIDACWKEKYTAARFTRDLIAGITVGIIAIPLAMALAIGSGVAPQYGLYTAAVAG
IVIALTGGSRFSVSGPTAAFVVILYPVSQQFGLAGLLVATLLSGIFLILMGLARFGRLIE
YIPVSVTLGFTSGIGITIGTMQIKDFLGLQMAHVPEHYLQKVGALFMALPTINVGDAAIG

IVTLGILVFWPRLGIRLPGHLPALLAGCAVMGIVNLLGGHVATIGSQFHYVLADGSQGNG
IPQLLPQLVLPWDLPNSEFTLTWDSIRTLLPAAFSMAMLGAIESLLCAVVLDGMTGTKHK
ANSELVGQGLGNIIAPFFGGITATAAIARSAANVRAGATSPISAVIHSILVILALLVLAP
LLSWLPLSAMAALLLMVAWNMSEAHKVVDLLRHAPKDDIIVMLLCMSLTVLFDMVIAISV
GIVLASLLFMRRIARMTRLAPVVVDVPDDVLVLRVIGPLFFAAAEGLFTDLESRLEGKRI
VILKWDAVPVLDAGGLDAFQRFVKRLPEGCELRVCNLEFQPLRTMARAGIQPIPGRLAFF
PNRRAAMADL
>LFGLNPFC_02032 hypothetical protein
MKRKNASLLGNVLMGLGLVVMVVGVGYSILNQLPQFNMPQYFAHGAVLSIFVGAILWLAG
ARVGGHEQVCDRYWWVRHYDKRCRRSDNRRHS
>LFGLNPFC_02033 Peptidyl-tRNA hydrolase
MTIKLIVGLANPGAEYAATRHNAGAWFVDLLAERLRAPLREEAKFFGYTSRVTLGGEDVR
LLVPTTFMNLSGKAVAAMASFFRINPDEILVAHDELDLPPGVAKFKLGGGHGGHNGLKDI
ISKLGNNPNFHRLRIGIGHPGDKNKVVGFVLGKPPVSEQKLIDEAIDEAARCTEMWFTDG
LTKATNRLHAFKAQ
>LFGLNPFC_02034 Ribosome-binding ATPase YchF
MGFKCGIVGLPNVGKSTLFNALTKAGIEAANFPFCTIEPNTGVVPMPDPRLDQLAEIVKP
QRTLPTTMEFVDIAGLVKGASKGEGLGNQFLTNIRETEAIGHVVRCFENDNIIHVSGKVN
PADDIEVINTELALADLDTCERAIHRVQKKAKGGDKDAKAELAVLEKCLPQLENAGMLRA
LDLSAEEKAAVRYLSFLTLKPTMYIANVNEDGFENNPYLDQVREIAAKEGSVVVPVCAAV
EADIAELDDEERDEFMQELGLEEPGLNRVIRAGYKLLNLQTYFTAGVKEVRAWTIPVGAT
APQAAGKIHTDFEKGFIRAQTISFEDFITYKGEQGAKEAGKMRAEGKDYIVKDGDVMNFL
FNV
>LFGLNPFC_02035 hypothetical protein
MKKTFIFSLLAIVFASLLSACVPHHHHRHNDGPRGPAASGKPMPPSNGGPNHGSHGRY
>LFGLNPFC_02036 PTS-dependent dihydroxyacetone kinase operon regulatory protein
MSGAFNNDGRGISPLIATSWERCNKLMKRETWNVPHQAQGVTFASIYRRKKAMLTLGQAA
LEDAWEYMASRECALLILDETACILSRNGDPQTLQQLSTLGFNDGTYCAEGIIGTCALSL
AAISGQAVKTMADQHFKQALWNWAFCATPLFDSKGRLTGTIALACPVEQTTAADLPLTLA
IAREVGNLLLTDSLLAETNRHLNQLNALLESMDDGVISWDEQGNLQFINAQAARVLRLDA
TASQGRAITELLTLPAVLQQAIKQSHPLKHVEATFESQHQFIDAVITLKPIIETQGTSFI
LLLHPVEQMRQLMTSQLGKVSHTFAHMPQDDPQTRRLIHFGRQAARSSFPVLLCGEEGVG
KALLSQAIHNESERAAGPYIAVNCELYGDAALAEEFIGGDRTDSENGRLSRLELAHGGTL
FLEKIEYLAVELQSALLQVIKQGVITRLDARRLIPIDVKVIATTTADLAMLVEQNRFSRQ
LYYALHAFEITIPPLRMRRGSIPALVNNKLRSLEKRFSTRLKIDDDALARLVSCAWPGND
FELYSVIENLALSSDNGRIRVSDLPEHLFTEQATDDVSATRLSTSLSFAEVEKEAIINAA
QVTGGRIQEMSALLGIGRTTLWRKMKQHGIDVSQFKRRG
>LFGLNPFC_02037 PEP-dependent dihydroxyacetone kinase, dihydroxyacetone-binding subunit DhaK
MKKLINDVQDVLDEQLAGLAKAHPSLTLHQDPVYVTRADAPVAGKVALLSGGGSGHEPMH
CGYIGQGMLSGACPGEIFTSPTPDKIFECAMQIDGGEGVLLIIKNYTGDILNFETATELL
HDSGVKVTTVVIDDDVAVKDSLYTAGRRGVANTVLIEKLVGAAAERGDSLDACAELGRKL
NNQGHSIGIALGACTVPAAGKPSFTLADNEMEFGVGIHGEPGIDRRPFSSLDQTVDEMFD
TLLENGSYHRTLRFWDYQQGSWQEEPQTKQPLQSGDRVIALVNNLGATPLSELYGVYNRL
TTRCQQAGLTIERNLIGAYCTSLDMTGFSITLLKVDDETLAFWDAPVHTPALNWGK
>LFGLNPFC_02038 PEP-dependent dihydroxyacetone kinase, ADP-binding subunit DhaL
MSLSRTQIVNWLTRCGDIFSTESEYLTGLDREIGDADHGLNMNRGFSKVVEKLPAIADKD
IGFILKNTGMTLLSSVGGASGPLFGTFFIRAAQTTQARQSLTLEELYQMFRDGADGVISR
GKAEPGDKTMCDVWVPVVESLRQSCEQNLPVPAALDAASSIAESAAQSTITMQARKGRAS
YLGERSIGHQDPGATSVMFMMQMLALAAKE
>LFGLNPFC_02039 PEP-dependent dihydroxyacetone kinase, phosphoryl donor subunit DhaM
MVNLVIVSHSSRLGEGVGELARQMLMSDSCKIAIAAGIDDPHNPIGTDAVKVMEAIESVA
DADHVLVMMDMGSALLSAETALELLAPEIAAKVRLCAAPLVEGTLAATVSAASGADIDKV
IFDAMHALEAKREQLGLPSSDTEISDTCPPYDEEARSLSVVIKNRNGLHVRPASRLVYTL
STFNADMLLEKNGKCVTPDSINQIALLQVRYNDTLRLIAKGPEAEEALIAFRQLAEDNFG
ETEEVAPPTLRPVPPVSGKAFYYQPVLCTVQAKSTLTVEEEQERLRQAIDFTLLDLMTLT
AKAETCGLDDIAAIFSGHHTLLDDPELQAAASELLQHEHCTAEYAWQQVLKELSQQYQQL
DDEYLQARYIDVDDLLHRTLVHLTQTKEELPQFNSPTILLAENIYPSTVLQLDPAVVKGI
CLSAGSPLSHSALIARELGIGWICQQGEKLYAIQPEETLTLDVKTQRFNRQG
>LFGLNPFC_02040 Periplasmic trehalase
MKSPAPSRPQKMALIPACIFLCFAALSVQAEETSVTPQPPDILLGPLFNDVQNAKLFPDQ
KTFADAVPNSDPLMILADYRMQQNQSGFDLRHFVNVNFTLPKEGEKYVPPEGQSLREHID
GLWPVLTRSTENTEKWDSLLPLPKPYVVPGGRFREVYYWDSYFTMLGLAESGHWDKVADM
VANFAHEIDNYGHIPNGNRSYYLSRSQPPFFALMVELLAQHEGDAALKQYLPQMQKEYAY

WMDGVENLQAGQQEKRVVKLQDGTLLNRYWDDRDTPRPESWVEDIATAKSNPNRPATEIY
RDLRSAAASGWDFSSRWMDNPQQLNTLRTTSIVPVDLNSLMFKMEKILARASKAIGDNAM
ANQYETLANARQKGIEKYLWNDQQGWYADYDLKSHKVRNQLTAAALFPLYVNAAAKDRAS
KMATATKTHLLQPGGLNTTSVKSGQQWDAPNGWAPLQWVATEGLQNYGQKEVAMDISWHF
LTNVQHTYDREKKLVEKYDVSTTGTGGGGGEYPLQDGFGWTNGVTLKMLDLICPKEQPCD
NVPATRPLSESTTQPLKQKEAEPTP
>LFGLNPFC_02041 hypothetical protein
MSLTRRRFTQILASTLFLHHLPSFAQSVKFWASRTLPEAQNITRIVSAGAPADLLLLAVA
PEKMVGFSSFDFARQALIPLPEHIRQFPRLGRLAGRASTLSLEGLMALHPDLVVDCGNTD
ETWISQARQVSEQTQIPWLLLNGKLEQSAEQLTTLGKTLGEEHRAAEQANLASRFVGEAQ
AFATSPAANLSFYAARGPRGLETGLQGSLHTEAAELLGLHNVAQIADRHGLTQVSMENLL
RWQPDIILVQEAVTADFIRRDPLWQGVKAVAEQRILFLSGLPFGWLDAPPGINRLLGLRR
LHAWLDPAINRQFKSDIQHYAQLFWHCSLSDADYQKLVAS
>LFGLNPFC_02042 putative ABC transporter permease protein
MRTVNGCILLLAAISITFAAVSGAYHLDMQQLLALILRQENVPVQEQIVFWQIRVPRILA
ALFLGAALAGAGTTYQGMFRNPLVSPDILGVSAGAGLGACAAILWGLSIVYIQLYAFCGG
LMVVAGVWLITRRVTRHDPILTLVLVGIALGTLCGAGISLIKTLADPYTQLPSITFWLLG
GLSTVTLRDLCYAAPIILIGSLPLFFLRWRMNLLTLSDDEARSLGLNVTRLRFGLIVCAT
LITASTVAIAGIIGWVGLVVPHIARLLTGHNHQQLLPMAMCTGAILLLLTDTLARSIGTT
EIPLGILTAFVGAPFFLFLLLRGGRQ
>LFGLNPFC_02043 putative ABC transporter ATP-binding protein
MTLLAVRHASLGYSRHPVLRDVSFTLSQGTICCLLGANGCGKTTLMRSILGVIPLLKGEI
LLDSLPVQTLSHRQRAQAIAWVPQAHDGIFAFSVLDMVLMGLAPTIGAFSVPGKQERLKA
TEQLEKLGILHLAARRWNTLSGGERQLALIARALVQQPRLLLLDEPASSLDFGHQIQLLD
TLAQLKNNGMTMLMSTHHPLHANAIADSIIQVEPDGRVTQGLPTEQLTTKKLAALYRVSA
DQIHHHLSAIKH
>LFGLNPFC_02044 2-methoxy-6-polyprenyl-1,4-benzoquinol methylase, mitochondrial
MLIDDIDFADLYLQQLRLAHRTEKTPDHWDQRAEKMAENCASPTDSYLQQLTSKIDLQGA
QTLFDMGCGPGTVSLALADKLTTIYGVDYSQGMLNVAARRAAALKADNVHWIQRAWEEDW
SDLPRCDIAVASRSTLVADMRQAMSKLNNQARLRVYTTHLVSTSFVSPAIQRAAGREVIE
LPNYIFALNVLYQMGIYAHVDFIRGQNCQQDNSTWERFEQNVSWSLGALNDDERERLYRW
YQQQDARALAPASRDWALIWWDSVPQETLR
>LFGLNPFC_02045 Nicotinate-nucleotide pyrophosphorylase [carboxylating]
MIFLSQAQIDALLLEDIQGGDLTTRALNIGHQHGYIEFFLRQGGCVSGISVACKMLTTLG
LTIDDAVSDGSQANAGQRLIRAQGNAAALHQGWKAVQNVLEWSCGVSDYLAQMLALLRER
YPDGNIACTRKAIPGTRLLASQAILAAGGLIHRAGCAETILLFANHRHFLHDNQDWSGAI
NQLRRHAPEKKIVVEADTPKEAIAALRAQPDVLQLDKFSPQQATEIAQIAPSLAPHCTLA
LTGGINLTTLKNYLDCGIRLFITSAPYYAAPADIKVSLQPAASI
>LFGLNPFC_02046 Colicin I receptor
MRLKKRYLCTVLTLAFTQQAVAAQESDTLTVWSSPVSSTTTTVLDQPTMKALDKQNVAQA
LSVVPGVVLQKSGSRNEEQVKVRGFDSRQVPVYFDGVPIYVPYDGNLDLARILTNNLGAV
EVSKGYSSLLQGPNQMGGAINITTQKPTKPLEASLGYRQGWSRSQDNAYDMHASFAASSD
LGYLQVSGSQLKQDFLGLPHGVNNDIAGKHGKMINSSADDKRGIVKLGFTPRENDEYTLT
YIKQDGEKDNPPYSGNSGQKSRYWQWPEYDKESFYYQGTTQLNDRFTLKSRLYRDTFENT
LMMYNSLADLKNKKGSYSHYSDYSDGAGLQLAADVRENDLLSFAVNWKDDVHREKGAPHA
AYDRYEDRTWSLASEYQWAAADNVDVVAGISYDWRDSVEAKKHEKDGSITHYDDNNQSAF
NWQVMGKYHFANEDTLALSYYDRTRFPTLKERYTTSKPAYNQIAIVNPQLKPERARGVDL
TWNGAFTHDWGFEVSVYYNRVSDAILSHNIDADTIQNQNSGTVDYSGLDAGIKGKISNIL
DVGLSYALIHADAKRKDIGKITDLPTQTMTAWMTLKPWEPLSVTLSEEARSSSYSNSDGS
QKAAGFAVTHIRADYTLGHGFSVNASVNNLFDTKYAYSEGFIEEGRNFWAGIEYTF
>LFGLNPFC_02047 hypothetical protein
MGIIAWIIFGLIAGIIAKLIMPGRDGGGFFLTCILGIVGAVVGGWLATMFGIGGSISGFN
LHSFLVAVVGAILVLGVFRLLRRE
>LFGLNPFC_02048 Flagellar brake protein YcgR
MSHYHEQFLKQNLLAVLGVLRDLHKAAIPLRISWNGGQLISKILVITPDKLVLDFGSQAE
DNNAVLKAQHITITAETQGAKVEFTVEQLQQSEYLQLPAFITVPPPTLWFVQRRRYFRIS
APLHPPYFCQTKLADNSTLRFRLHDLSLGGMGALLETAKPAELHEGMRFAQIEVNMGQWG
VFHFDAQLISISERKVIDGKNETITTPRLSFRFLNVSPTVERQLQRIIFSLEREAREKAD
KVRD
>LFGLNPFC_02049 Endo-type membrane-bound lytic murein transglycosylase A
MKLRWFAFLIVLLAGCSSKHDYTNPPWNAKVPVQRAMQWMPISQKAGAAWGVDPQLITAI
IAIESGGNPNAVSKSNAIGLMQIKASTSGRDVYRRMGWSGEPTTSELKNPERNISMGAAY
LNILETGPLAGIEDPKVLQYALVVSYANGAGALLRTFSSDRKKAISKINDLDADEFLDHV
ARNHPAPQAPRYIYKLEQALDAM

>LFGLNPFC_02050 Murein tetrapeptide carboxypeptidase
MSLFHLIAPSGYCIKQHAALRGIQRLTDAGHQVNNVEVIARRCERFAGTETERLEDLNSL
ARLTTPNTIVLSVRGGYGASRLLADIDWQALVARQQHDPLLICGHSDFTAIQCGLLAQGN
VITFSGPMLVANFGADELNAFTEHHFWLALRNKTFTIEWQGEGPTCQTEGTLWGGNLAML
ISLIGTPWMPKIENGILVLEDINEHPFRVERMLLQLYHAGILPRQKAIILGSFSGSTPND
YDAGYNLESVYAFLRSRLSIPLITGLDFGHEQRTVTLPLGAHAILNNTQEGTQLTISGHP
VLKM
>LFGLNPFC_02051 K(+)/H(+) antiporter NhaP2
MDATTIISLFILGSILVTSSILLSSFSSRLGIPILVIFLAIGMLAGVDGVGGIPFDNYPF
AYMVSNLALAIILLDGGMRTQASSFRVALGPALSLATLGVLITSGLTGMMAAWLFNLDLI
EGLLIGAIVGSTDAAAVFSLLGGKGLNERVGSTLEIESGSNDPMAVFLTITLIAMIQQHE
SSVSWMFVVDILQQFGLGIVIGLGGGYLLLQMINRIALPAGLYPLLALSGGILIFALTTA
LEGSGILAVYLCGFLLGNRPIRNRYGILQNFDGLAWLAQIAMFLVLGLLVNPSDLLPIAI
PALILSAWMIFFARPLSVFAGLLPFRGFNLRERVFISWVGLRGAVPIILAVFPMMAGLEN
ARLFFNVAFFVVLVSLLLQGTSLSWAAKKAKVVVPPVGRPVSRVGLDIHPENPWEQFVYQ
LSADKWCVGAALRDLHMPKETRIAALFRDNQLLHPTGSTRLREGDVLCVIGRERDLPALG
KLFSQSPPVALDQRFFGDFILEASAKYADVALIYGLEDGREYRDKQQTLGEIVQQLLGAA
PVVGDQVEFAGMIWTVAEKEDNEVLKIGVRVAEEEAES
>LFGLNPFC_02052 Alanine racemase, catabolic
MTRPIQASLDLQALKQNLSIVRQAAPRARVWSVVKANAYGHGIERIWSAIGATDGFALLN
LEEAITLRERGWKGPILMLEGFFHAQDLEIYDQHRLTTCVHSNWQLKALQNARLKAPLDI
YLKVNSGMNRLGFQPDRVLTVWQQLRAMANVGEMTLMSHFAEAEHPDGISGAMARIEQAA
EGLECRRSLSNSAATLWHPEAHFDWVRPGIILYGASPSGQWRDIANTGLRPVMTLSSEII
GVQTLKAGERVGYGGRYTARDEQRIGIVAAGYADGYPRHAPTGTPVLVDGVRTMTVGTVS
MDMLAVDLTPCPQAGIGTPVELWGKEIKIDDVAAAAGTVGYELMCALALRVPVVTV
>LFGLNPFC_02053 D-amino acid dehydrogenase
MRVVILGSGVVGVASAWYLNQAGHEVTVIDREPGAALETSAANAGQISPGYAAPWAAPGV
PLKAIKWMFQRHAPLAVRLDGTQFQLKWMWQMLRNCDTSHYMENKGRMVRLAEYSRDCLK
ALRAETNIQYEGRQGGTLQLFRTEQQYENATRDIAVLEDAGVPYQLLESSRLAEVEPALA
EVAHKLTGGLQLPNDETGDCQLFTQNLARMAEQAGVKFRFNTPVDQLLCDGEQIYGVKCG
DEVIKADAYVMAFGSYSTAMLKGIVDIPVYPLKGYSLTIPIAQEDGAPVSTILDETYKIA
ITRFDNRIRVGGMAEIVGFNTELLQPRRETLEMVVRDLYPRGGHVEQATFWTGLRPMTPD
GTPVVGRTRFKNLWLNTGHGTLGWTMACGSGQLLSDLLSGRTPAIPYEDLSVARYSRGFT
PLRPGHLHGAHS
>LFGLNPFC_02054 hypothetical protein
MATIDSMNKDTTRLSDGPDWTFDLLDVYLAEIDRVAKLYRLDTYPHQIEVITSEQMMDAY
SSVGMPINYPHWSFGKKFIETERLYKHGQQGLAYEIVINSNPCIAYLMEENTITMQALVM
AHACYGHNSFFKNNYLFRSWTDASSIVDYLIFARKYITECEERYGVDEVERLLDSCHALM
NYGVDRYKRPQKISLQEEKARQKSREEYLQSQVNMLWRTLPKREEEKTVAEARRYPSEPQ
ENLLYFMEKNAPLLESWQREILRIVRKVSQYFYPQKQTQVMNEGWATFWHYTILNHLYDE
GKVTERFMLEFLHSHTNVVFQPPYNSPWYSGINPYALGFAMFQDIKRICQSPTEEDKYWF
PDIAGSDWLETLHFAMRDFKDESFISQFLSPKVMRDFRFFTVLDDDRHNYLEISAIHNEE
GYREIRNRLSSQYNLSNLEPNIQIWNVDLRGDRSLTLRYIPHNRAPLDRGRKEVLKHVHR
LWGFDVMLEQQNEDGSVELLERCPPRMGNL
>LFGLNPFC_02055 Fatty acid metabolism regulator protein
MVIKAQSPAGFAEEYIIESIWNNRFPPGTILPAERELSELIGVTRTTLREVLQRLARDGW
LTIQHGKPTKVNNFWETSGLNILETLARLDHESVPQLIDNLLSVRTNISTIFIRTAFRQH
PDKAQEVLATANEVADHADAFAELDYNIFRGLAFASGNPIYGLILNGMKGLYTRIGRHYF
ANPEARSLALGFYHKLSALCSEGAHDQVYETVRRYGHESGEIWHRMQKNLPGDLAIQGR
>LFGLNPFC_02056 Na(+)/H(+) antiporter NhaB
MEISWGRALWRNFLGQSPDWYKLALIIFLIVNPLIFLISPFVAGWLLVAEFIFTLAMALK
CYPLLPGGLLAIEAVFIGMTSAEHVREEVAANLEVLLLLMFMVAGIYFMKQLLLFIFTRL
LLSIRSKMLLSLSFCVAAAFLSAFLDALTVVAVVISVAVGFYGIYHRVASSRTEDTDLQD
DSHIDKHYKVVLEQFRGFLRSLMMHAGVGTALGGVMTMVGEPQNLIIAKAAGWHFGDFFL
RMSPVTVPVLICGLLTCLLVEKLRWFGYGETLPEKVREVLQQFDDQSRLQRTRQDKIRLI
VQAIIGVWLVTALALHLAEVGLIGLSVIILATSLTGVTDEHAIGKAFTESLPFTALLTVF
FSVVAVIIDQQLFSPIIQFVLQASEHAQLSLFYIFNGLLSSISDNVFVGTIYINEAKAAM
KSGAITLKQYELLAVAINTGTNLPSVATPNGQAAFLFLLTSALAPLIRLSYGRMVWMALP
YTLVLTLVGLLCVEFTLAPVTEWFMQMGWIATL
>LFGLNPFC_02057 Disulfide bond formation protein B
MLRFLNQCSQGRGAWLLMAFTALALELTALWFQHVMLLKPCVLCIYERCALFGVLGAALI
GAIAPKTPLRYVAMVIWLYSAFRGVQLTYEHTMLQLYPSPFATCDFMARFPEWLPLDKWV
PQVFVASGDCAERQWEFLGLEMPQWLLGIFIAYLIVAVLVVISQPFKAKKRDLFGR
>LFGLNPFC_02058 Protein UmuC

MFALCDVNAFYASCETVFRPDLWGKPVVVLSNNDGCVIARNAEAKALGVKMGDPWFKQKD
LFRRCGVVCFSSNYELYADMSNRVMSTLEELSPRVEIYSIDEAFCDLTGVHNCRDLTDFG
REICATVLQRTHLTVGVGIAQTKTLAKLANHAAKKWQRQTGGVVDLSNLERQRKLMSALP
VDEVWGIGRRISKKLDAMGIKTVLDLADSDIRFIRKHFNVVLERTVRELRGEPCLQLEEF
APTKQEIICSRSFGERITDYPSMRQAICSYAARAAEKLRSEHQYCRFISTFIKTSPFAFN
EPYYGNSASVKLLTPTQDSRDIINAATRSLDAIWQAGHCYQKAGVMLGDFFSQGVAQLNL
FDDNAPRPGSEQLMAVMDTLNAKEGRGTLYFAGQGIQQQWQMKRAMLSPCYTTRSSDLLR
VK
>LFGLNPFC_02059 Protein UmuD
MLFIKPADLREIVTFPLFSDLVQCGFPSPAADYVEQRIDLNQLLIQHPSATYFVKASGDS
MIDGGISDGDLLIVDSAITASHGDIVIAAVDGEFTVKKLQLRPTVQLTPMNSAYSPITIS
SEDTLDVFGVVIHVVKAMR
>LFGLNPFC_02060 hypothetical protein
MSDVPFWQSKTLDEMSDAEWESLCDGCGQCCLHKLMDEDTDEIYFTNVACRQLNIKTCQC
RNYERRFEFEPDCIKLTRENLPTFEWLPMTCAYRLLAEGKGLPAWHPLLTGSKAAMHGER
ISVRHIAVKESEVIDWQDHILNKPDWAQ
>LFGLNPFC_02061 putative protein YcgM
MYQHHNWQGALLDYPVSKVVCVGSNYAKHIKEMGSAVPEEPVLFIKPETALCDLRQPLAI
PSDFGSVHHEVELAVLIGATLRQATEEHVRKAIAGYGVALDLTLRDVQGKMKKAGQPWEK
AKAFDNSCPLSGFIPAAEFTGDPQNTTLGLSVNGEQRQQGTTADMIHKIVPLIAYMSKFF
TLKAGDVVLTGTPDGVGPLQSGDELTVTFDGHSLTTRVL
>LFGLNPFC_02062 Protein YcgL
MFCVIYRSSKRDQTYLYVEKKDDFSRVPEELMKGFGQPQLAMILPLDGRKKLVNADIEKV
KQALTEQGYYLQLPPPPEDLLKQHLSVMGQKTDDTNK
>LFGLNPFC_02063 Inhibitor of g-type lysozyme
MKIKGISKAVLLLALLTSTSFAAGKNVNVEFRKGHSSAQYSGEIKGYDYDTYTFYAKKGQ
KVHVSISNEGADTYLFGPGIDDSVDLSRYSPELDSHGQYSLPASGKYELRVLQTRNDARK
NKTKKYNVDIQIK
>LFGLNPFC_02064 hypothetical protein
MNMMRIFYIGLSGVGMMFSSMASGNDAGGLQSPACGVVCDPYICVSSDGISPELTRKYLG
EKAAENLQSLQGYDPSEFTFSNGVFCDVKEKLCRDDRYFGVDGKRSGKINQTTTKMLFMC
RE
>LFGLNPFC_02065 Septum site-determining protein MinC
MSNTPIELKGSSFTLSVVHLHEAEPKVIHQALEDKIAQAPAFLKHAPVVLNVSALEDPVN
WSAMHKAVSATGLRVIGVSGCKDAQLKAEIEKMGLPILTEGKEKAPRPAPAPQAPAQNTT
PVTKTRLIDTPVRSGQRIYAPQCDLIVTSHVSAGAELIADGNIHVYGMMRGRALAGASGD
RETQIFCTNLMAELVSIAGEYWLSDQIPAEFYGKAARLQLVENALTVQPLN
>LFGLNPFC_02066 Septum site-determining protein MinD
MARIIVVTSGKGGVGKTTSSAAIATGLAQKGKKTVVIDFDIGLRNLDLIMGCERRVVYDF
VNVIQGDATLNQALIKDKRTENLYILPASQTRDKDALTREGVAKVLDDLKAMDFEFIVCD
SPAGIETGALMALYFADEAIITTNPEVSSVRDSDRILGILASKSRRAENGEEPIKEHLLL
TRYNPGRVSRGDMLSMEDVLEILRIKLVGVIPEDQSVLRASNQGEPVILDINADAGKAYA
DTVERLLGEERPFRFIEEEKKGFLKRLFGG
>LFGLNPFC_02067 Cell division topological specificity factor
MALLDFFLSRKKNTANIAKERLQIIVAERRRSDAEPHYLPQLRKDILEVICKYVQIDPEM
VTVQLEQKDGDISILELNVTLPEAEELK
>LFGLNPFC_02068 putative autotransporter
MESKSVDTRSIYRELGATLSYNMRLGNGMEIEPWLKAAVRKEFVDDNRVKVNSDGNFIND
LSGRRGIYQAGIKASFSSTLSGHLGVGYSRGAGVESPWNAVVGVNWSF
>LFGLNPFC_02069 hypothetical protein
MADMNRTTKGALLGAGVGLLTGNGVNGVLKGAAVGAGVGAVTEKGRDGKNARKGAKVGAA
VGAVTGVLTGNGLEGAIKGAVIGGTGGAILGKMK
>LFGLNPFC_02070 putative protein YmgD
MKKFALLAGLFVFAPMTWAQDYNIKNGLPSETYITCAEANEMAKTDSAQVAEIVAVMGNA
SVASRDLKIEQSPELSAKVVEKLNQVCAKDPQMLLITAIDDTMRAIGKK
>LFGLNPFC_02071 hypothetical protein
MRLNDRADETRYIDPVTEQERSSRFWLRQIGGHNAWRDSNGQLRTTSAQAIWMGVDHNVH
TEANGSRIENDANNNIQTRLGFHTFIRTQEKNSGPHGDDFEPFVEMNGIHNSKDFAVSIN
GVKVEQAGARNLGEIKHGVNGNLNPAASVWGNVGVQLGDNGYNDNAMMVGLKYKF
>LFGLNPFC_02072 hypothetical protein
MGKNAVGVLACSSPGESRTCVDAVDDEVCDSNSYEVISRADLKMNGGSITTNGINSYGAY
ANGKKAYINLDYVVLETVADGSYAVAIRQGNIDIKKFYYNKWH
>LFGLNPFC_02073 hypothetical protein
MKLKKLPGFSLGLIALAVGNAYVTQLLDIYSIISYITDEESPIEIKNNTSTSNGEYLIT

>LFGLNPFC_02074 IS1 family transposase IS1A
MASVSISCPSCSATDGVVRNGKSTAGHQRYLCSHCRKTWQLQFTYTASQPGTYQKIIDMA
MNGVGCRATARIMGVGLNTILRHLKKLRPQSVTSRIQPGSDVIVCAEMDEQWGYVGAKSR
QRWLFYAYDRLRKTVVAHVFGERTMATLGRLMSLLSPFDVVIWMTDGWPLYESRLKGKLH
VISKRYTQRIERHNLNLRQHLARLGRKSLSFSKSVELHDKVIGHYLNIKHYQ
>LFGLNPFC_02075 hypothetical protein
MKTLSGKEFRKLFNRYETGFYLSEFEYLKDFDPLFPECVGKELKLGYKRKVDVWCEEDAA
LYAEFIATKERLKRGRLILKINQVWSRVYPAYIYAIEG
>LFGLNPFC_02076 putative periplasmic iron-binding protein
MHSIKKVTMLLGGLALTCSIAFQASATEKFKVITTFTIIADMAKNVAGDAAEVSSITKPG
AEIHEYQPTPGDIKRAQGAQLILANGMNLELWFQRFYQHLNGVPEVIVSSGVTPVGITEG
PYEGKPNPHAWMSPDNALIYVDNIRDALIKYDPANAQTYQRNADTYKAKITQTLAPLRKQ
ITELPENQRWMVTSEGAFSYLARDLGLKELYLWPINADQQGTPQQVRKVVDIVKKNHIPA
VFSESTISDKPARQVARETGAHYGGVLYVDSLSTENGPVPTYIDLLKVTTSTLVQGIKAG
KREK
>LFGLNPFC_02077 Manganese transport system ATP-binding protein MntB
MMQSAGIVVNDVTVTWRNGHTALRDASFTVPSGSIAALVGVNGSGKSTLFKAIMGFVRLT
SGKISVLGIPTRQALQKNLVAYVPQSEDVDWSFPVLVEDVVMMGRYGHMGMLRIAKKRDR
QIVTDALERVDMVDFRHRQIGELSGGQKKRVFLARAIAQQGDVILLDEPFTGVDVKTEAK
IISLLRELRAEGKTMLVSTHNLGSVTTFCDYTVMVKGTVLASGPTDTTFTAENLELAFSG
VLRHVTLNGSEESIITDDERPFVAHRPSAVQREER
>LFGLNPFC_02078 Manganese transport system membrane protein MntB
MNVLLEPFSYEYMLNAMWVSAMVGGLCAFLSCYLMLKGWSLIGDALSHSIVPGVAGAYML
GLPFSLGAFFSGGLAAGSMLFLNQRTRLKEDAIIGLIFSSFFGLGLFMVSLNPTSVNIQT
IVLGNILAIDPADILQLTIIGILSIIVLFFKWKDLMVTFFDENHARAIGLHPGRLKLIFF
TLLSVSTVAALQTVGAFLVICLVVTPGATAWLLTDRFPRLLMIAVTIGSVTSFLGAWVSY
FLDGATGGIIVVAQTLLFLLAFVFAPTHGLLANRRRAHKALEDRS
>LFGLNPFC_02079 Manganese transport system membrane protein MntB
MMALLLEPLQFTFMSHALLISLVVSIPCALLSVFLVLKGWALMGDAMSHAVFPGIVLAWI
LGLPLATGAFVAGVFCAVATGYLKDNSRIKQDTVMGIVFSGMFAAGLILYIAVKPDVHLD
HILFGDMLGITIGDIIQTMIIAGLVTLVISVKWRDFLLFSFDYQQAQVSGLHTRWLHYGL
LCMVSLTIVATLKAVGIILSISLLIAPGAIAVLLTQRFHIALLLATGISVIVSMTGVWLS
FFIDSAPAPTIVVLFAVLFIMTFAVTSINARKKGNSHTQDLLSPN
>LFGLNPFC_02080 hypothetical protein
MDISPLLHALCAVAAQILVGLFTGNWAYGAIAGCTFFIAREHTQAEYRWIEMFGHGKRMN
MPWWGGFDPRAWDVASLMDFAVPVVACLLVWLLVNRG
>LFGLNPFC_02081 hypothetical protein
MKDLTLKFHDKLQFKAFLSSLDWEEDEDLQNKLLVDEIGFTYTETGVTEEGEPVCVRNDG
YFVNIRILDDLFDVSVFSDYVVELETPLREWS
>LFGLNPFC_02082 hypothetical protein
MAVQISGVLKDGAGKPIQNCTIQLKAKRNSTTVVVNTVASENPDEAGRYSMDVEYGQYSV
ILLVEGFPPSHAGAITVYEDSKPGTLNDFLGAATEDDVRPEALYRFEKMVEEVARNAEAA
SQSAAAAKKSETAAASSRNAAKTSETNAGNSAKAAASSKTAAQNAATAAERSETNARASE
EASADSEEASRRNAESAAENAGVATTKAREAAADATKAGQKKDEALSAATRAEKAADRAE
VAAEVTAEPYANIVPPLPDVWIPFNDSLDMIAGFSPGYKKIAIGDDVVQVASDKQVNFSR
ASTATYINKSGELKTAEINEPRFECDGLLIEGQRTNYMLNSESPASWGKSSNMDVPETGT
DSFGFTYGKFVCNDSLVGQTSAINMASIAATKSVDVSGDNKYVTTSCRFKTERQVRLRIR
FDKYDGSATTFLGDAYIDTQTLEINMTGGAAGRITARVRKDKTTGWIFAEATIQAIDGEL
KIGSQIQYSPERGGATVSGDYIYLATPQVENGPCVSSFIISGGSATTRASDLVSIPTRNN
LYKLPFTFLLEIHKNWDIAPNAAPRVWDIAAANTGQSAIAAINRGSGKLYMSLSNPSGLY
VNSAATDVFAEKTTFGCIAKADGHFHVVTNGKAVNEVYCEYNGVTADKNIRFGGQTNTGE
RHLFGHIRNFRIWHKELNDRQLKEVV
>LFGLNPFC_02083 hypothetical protein
MGKGSSKGHTPREAKDNLKSSQMLSVIDAISEGPVEGPVDGLKSVLLNSTPVLDSEGNTN
IFGVTVVFRAGEQEQTPPEGFESSGSETVLGTEVKYDTPITRTITSANIDRLRFTFGVQA
LVETTSKGDRNPSEVRLLVQIQRNGGWVTEKDITIKGKTTSQYLASVVVDNLPPRPFNIR
MRRMTPDSTTDQLQNKTLWSSYTEIIDVKQCYPNTALVGVQVDSEQFGSQQVSRNYHLRG
RILQVPSNYNPQTRQYSGIWDGTFKPAYSNNMAWCLWDMLTHPRYGMGKRLGAADVDKWA
LYVIGQNCDQSVPDGFGGTEPRITCNAWLTTQRKAWDVLSDFCSAMRCMPVWNGQTLTFV
QDRPSDKVWTYNRSNVVMPDDGAPFRYSFSALKDRHNAVEVNWIDPDNGWETATELVEDT
QAIARYGRNVTKMDAFGCTSRGQAHRAGLWLIKTELLETQTVDFSVGAEGLRHVPGDVIE
ICDDDYAGISTGGRVLAVNSQTRTLTLDREITLPSSGTTLISLVDGQGNPVSVEVQSVTD
GVKVKVSRVPDGVAEYSVWGLKLPTLRQRLFRCVSIRENDDGTYAITAVQHVPEKEAIVD
NGAHFDGDQSGTVNGVTPPAVQHLTAEVTADSGEYQVLARWDTPKVVKGVSFMLRLTVAA

DDGSERLVSTARTTETTYRFRQLALGNYRLTVRAVNAWGQQGDPASVSFRIAAPAAPSQI
ELTPGYFQITATPHLAVYDPTVQFEFWFSEKRIADIRQVETTARYLGTALYWIAASINIK
PGHDYYFYIRSVNTVGKSAFVEAVGQPSDDASGYLNFFKGEIGKTHLAQELWTQIDNGQL
APDLAEIRTSITGVSNEITQTVNKKLEDQSAAIQQIQKVQVDTNNNLNSMWAVKLQQMQD
GRLYIAGIGAGVENTPDGMQSQVLLAADRIAMINPANGNTKPMFVGQGDQIFMNEVFLKY
LTAPTITSGGNPPTFSLTPDGRLSAKNADISGNVNANSGTLNNVTINQNCRILGKLSANQ
IEGDIVKTVGKAFPRNGSYASGTITVTVYDDQAFDRQIVVPPVLFRGGKHENFNSNNQQS
YWYSTCKLQVLKNGQEIFQQPATDVSRVFSSVIDMPAGHGHVTLTFNVSSYGANNWTPTT
SISDLLVVVMKKSTAGISIS
>LFGLNPFC_02084 hypothetical protein
MKTGAEAIRALATQLPVFRQKLSDGWYQVRIAGRDVSTSGLTAQLHETLPDGAVIHIVPR
VAGAKSGGVFQIVLGAAAIAGSFFTAGATLAAWGAAIGAGGMTGILFSLGASMVLGGVAQ
MLAPKARTPRTQTTDNGKQNTYFSSLDNMVAQGNVLPVLYGEMRVGSSVVSQEISTADEG
DGGQVVVIGR
>LFGLNPFC_02085 hypothetical protein
MAPEDWLQAEMQGEIVALVHSHPGGLPWLSEADRRLQVQSDLPWWLVCRGAIHKFRCVPH
LTGRRFEHGVTDCYTLFRDAYHLAGIEMPDFHREDDWWRNGQNLYLDNLEATGLYQVPLS
AAQPGDVLLCCFGSSVPNHAAIYCGDGELLHHIPEQLSKRERYTDKWQRRTHSLWRHRAW
RASAFTGIYNDLAAASTFV
>LFGLNPFC_02086 hypothetical protein
MVTGMVEDLQSLVGGTVVRRKVYARFLDAVNFVNGNSDADPEQEVISRWRIEQCSELSAV
SASFVLSTPTETDGAVFPGRIMLANTCTWTYRGDECGYHGPAVADEYDQPTSDITKDKCS
KCLSGCKFRNNVGNFGGFLSINKLSQ
>LFGLNPFC_02087 hypothetical protein
MQDIRQETLNECTRAEQSASVVLWEIDLTEVGGERYFFCNEQNEKVSRSPGRGDSISRIP
FRGVVLN
>LFGLNPFC_02088 hypothetical protein
MKTFRWKVKPGMDVTSAPSVREVRFGDGYSQRAPAGLNADLKTYSVTLSVSREEATALES
FLAEHGGWKAFLDAALWLQADKGDLRKMVVAGQYAAC
>LFGLNPFC_02089 hypothetical protein
MAEPVGDLVVDLSLDAARFDEQMARVRRHFSGTESDAKKTAAVVEQSLSRQALAAQKAGI
SVGQYKAAMRMLPAQFTDVATQLAGGQSPWLILLQQGGQVKDSFGGMIPMFRGLAGAITL
PMVGATSLAVATGALAYAWYQGNSTLSDFNKTLVLSGNQSGLTADRMLVLSRAGQAAGLT
FNQTSESLSALVKAGVSGEAQIASISQSVARFSSASGVEVDKVAEAFGKLTTDPTSGLTA
MARQFHNVTAEQIAYVAQLQRSGDESGALQAANEAATKGFDDQTRRLKENMGTLETWADR
TARAFKSMWDAVLDIGRPDTAQEMLIKAEAAFKKADDIWNLRKDDYFVNDEARARYWDDR
EKARLALEAARKKAEQQSQQDKNAQQQSDTEASRLKYTEEAQKAYERLQTPLEKYTARQE
ELNKALKDGKILQADYNTLMAAAKKDYEATLKKPKQSGVKVSAGDRQEDSAHAALLTLQA
ELRTLEKHAGANEKISQQRRDLWKAESQFAVLEEAAQRRQLSAQEKSLLAHKDETLEYKR
QLAALGDKVTYQERLNALAQQADKFAQQQRAKRAAIDAKSRGLTDRQAEREATEQRLKEQ
YGDNPLALNNVMSEQKKTWAAEDLLRGNWMAGLKSGWSEWEESATDSMSQVKSAATQTFD
GIAQNMAAMLTGSEQNWRSFTRSVLSMMTEILLKQAMVGIVGSIGSAIGGAVGGGASASG
GTAIQAAAAKLHFATGGFTGTGGKYEPAGIVHRGEFVFTKEATSRIGVGNLYRLMRGYAT
GGYVGGTGSPAQMRRSEGIRFEQNNNVVIQNDGTNGLPGPQMMKAVYDMARKGARDEIQA
QMRDGGLFSGGGR
>LFGLNPFC_02090 hypothetical protein
MFDGELSFALKLAREMGRPDWRAMLAGMSSTEYADWHRFYSTHYFHDVLLDMHFSGLTYT
VLSLFFSDPDMHPLDFSLLNRREDDEEPEDDVLMQKAAGLTGGVRFDPDGNEVIPASPDM
AGMTEDDVMLMTVSEGIAGGVRYG
>LFGLNPFC_02091 hypothetical protein
MFLKTESFEHNGVTVTLSELSALQRIEHLALMKQQAEQAESDSNRQVTVEDAIRTGAFVV
AMSLWHNHPQKTKQPSMNEAVKQIEQEVLTTWPAEAISHAENVVYRLSGMYGFVVNDAPD
QAEDSGPAEPVSAGKCSTVS
>LFGLNPFC_02092 hypothetical protein
MPTPNPLAPVKGAGTTLWVYNGSGDPYANPLSDNDWSRLAKIKDLTPGELTAESYDDSYL
DDEDADWTATGQGQKSAGDTSFTLAWMPGEQGGQQALLAWFNEGDTRAYKIRFPNGTVDVF
RGWVSSIGKAVTAKEVITRTVKVTNVGRPSMAEDRSTVTAATGMTVTPASSSVVKGQSTT
LTVAFQPEGATDKSFRAVSADKTKATVSVSGMTITVNGVAAGKVNIPVVSGNGELAAVAE
ITVTDS
>LFGLNPFC_02093 hypothetical protein
MKHTELRAAVLDALEKHDTGATLFDGRPAVFDEEDFPAIAVYLTGAEYTGEELDSDTWQA
ELHIEVFLPAQVPDSELDSWMESRIYPVMSDIPALSDLITSMVASGYDYRRDDDAGLWSS
ADLTYVITYEM
>LFGLNPFC_02094 hypothetical protein

MAIKGLEQAVENLSRISKTAVPGAAAMAINRVASSAISQSASLVARETKVRRKLVKERAR
LKRATVKNPQARIKVNRGDLPVIRLGNARVVLSRRRRRKKGQRSSLKGGGSVLVVGNRRI
PGAFIQQLKNGRWHVMQRVAGKNRYPIDVVKIPMAVPLTTAFKQNIERIRRERLPKELGY
ALQHQLRMVIKR
>LFGLNPFC_02095 hypothetical protein
MRDFQNAFDAALAGVDSTIVEVMGISAQFTSGAQRGGEVHGVFDDPESLGFASSGIRIEG
SNPSLFVLTDTVCAVRRGDTLTINGEMFWVDRVSPDDGGSCYLWLNRGQPPAASRRR
>LFGLNPFC_02096 hypothetical protein
MATKEQNLKRLDELALILGREPDISGSAAEIAQRVAEWEEEMQSSGDDVQVMNMDIRERE
TAAHDVREETSGALTRIRVLTCLHLCGVDGETGESVELADVGRVILIMSSDAKTHVDGGM
AVYA
>LFGLNPFC_02097 hypothetical protein
MGLFTTRQLLGYTEQKVKFRALFLELFFRRTVNFHTEEVMLDKITGKTPVAAYVSPVVEG
KVLRHRGGETRVLRPGYVKPKHEFNYQQAVERLPGEDPSQLNDPAYRRLRIITDNLKQEE
HAIVQVEEMQAVNAVLYGKYTMEGDQFEKIEVDFGRSTKNNITQGSGKEWSKQDRDTFDP
THDIDLYCDLASGLVNIAIMDGTVWRLLNGFKLFREKLDTRRGSNSQLETAVKDLGAVVS
FKGYYGDLAIVVAKTSYIAEDGIEKRYLPDGMLVLGNTAADGIRCYGAIQDAQALSEGVV
ASSRYPKHWLTVGDPAREFTMTQSAPLMVLPDPDEFVVVQVK
>LFGLNPFC_02098 hypothetical protein
MVTKTITEQRAEVRIFAGNDPAHTATGSSGISSPTPALTPLMLDEATGKLVVWDGQKAGS
AVGILVLPLEGTETALTYYKSGTFATEAIHWPESVDEHKKANAFAGSALSHAALP
>LFGLNPFC_02099 hypothetical protein
MRRNLSHIIAAAFNEPLLLEPAYARVFFCALGREMGAASLSVPQQQVQFDAPGMLAETDE
YMAGGKRPARVYRVVNGIAVLPVTGTLVHRLGGMRPFSGMTGYDGIVACLQQAMADSQVR
GVLLDIDSPGGQAAGAFDCADMIYRLRQQKPVWALCNDTACSAAMLLASACSRRLVTQTS
RIGSIGVMMSHVSYAGHLAQAGVDITLIYSGAHKVDGNQFEALPAEVRQDMQQRIDAARR
MFAEKVAMYTGLSVDAVTGTEAAVFEGQSGIEAGLADELINASDAISVMATALNSNVRGG
TMPQLTATEAAAQENQRVMGILTCQEAKGREQLATMLAGQQGMSVEQARAILAAAAPQQP
VASTQSEADRIMVCEEANGREQLAATLAAMPEMTVEKARPILAASPQADAGPSLRDQIMA
LDEAKGAEAQAEQLAACPGMTVESARAVLAAGSGKAEPVSASTTAMFEHFMANHSPAAVQ
GGVAQTSADGDADVKMLMAMP
>LFGLNPFC_02100 hypothetical protein
MKRTPVLIDVNGVPLRESLSYNGGGAGFGGQMAEWLPPAQSADAALLPALRLGNARADDL
VRNNGIAANAVALHKDHIVGHMFLISYRPNWRWLGMRETAAKSFVDEVEAAWSEYAEGMF
GEIDVEGKRTFTEFIREGVGVHAFNGEIFVQPVWDTETTQLFRTRFKAVSPKRVDTPGHG
MGNRFLRAGVEVDRYGRAVAYHICEDDFPFSGSGRWERIPRELPTGRPAMLHIFEPVEDG
QTRGANQFYSVMERLKMLDSLQATQLQSAIVKAMYAATIESELDTEKAFEYIAGAPQEQK
DNPLINILEKFSSWYDTNNVTLGGVKIPHLFPGDDLKLQTAQDSDNGFSALEQALLRYIA
AGLGVSYEQLSRDYSKVSYSSARASANESWRYFMGRRKFIAARLATQMFSCWLEEALLRG
IIRPPRARFDFYQARSAWSRAEWIGAGRMAIDGLKEVQESVMRIEAGLSTYEKELALMGE
DYQDIFRQQVRESAERQKAGLSRPVWIEQAYQQQIAESRRPEEETTPRET
>LFGLNPFC_02101 hypothetical protein
MVTVAELQALRQARLDLLTGKRVVSVQKDGRRIEYTAASLDELNRAINDAESVLGTTRCR
RRPLGVRL
>LFGLNPFC_02102 hypothetical protein
MISDAQKAANAAGAIATGLLSLIIPVPLTTVQWANKHYYLPKESSYTPGRWETLPFQVGI
MNCMGNDLIRTVNLIKSARVGYTKMLLGVEAYFIEHKSRNSLLFQPTDSAAEDFMKSHVE
PTIRDVPALLELAPWFGRKHRDNTLTLKRFSSGVGFWCLGGAAAKNYREKSVDVVCYDEL
SSFEPDVEKEGSPTLLGDKRIEGSVWPKSIRGSTPKIKGSCQIEKAANESAHFMRFYVPC
PHCGEEQYLKFGDDASPFGLKWEKNKPESVFYLCEHHGCVIHQSELDQSNGRWICENTGM
WTRDGLMFFSARGDEIPPPRSITFHIWTAYSPFTTWVQIVYDWLDALKDPNGLKTFVNTT
LGETWEEAVGEKLDHQVLMDKVVRYTAAVPARVVYLTAGIDSQRNRFEMYVWGWAPGEEA
FLVDKIIIMGRPDEEETLLRVDAAINKKYRHADGTEMTISRVCWDIGGIDGEIVYQRSKK
HGVFRVLPVKGASVYGKPVITMPKTRNQRGVYLCEVGTDTAKEILYARMKADPTPVDEAT
SYAIRFPDDPEIFSQTEAQQLVAEELVEKWEKGKMRLLWDNKKRRNEALDCLVYAYAALR
VSVQRWQLDLAVLAKSREEETTRPTLKELAAKLSGGVNGYSR
>LFGLNPFC_02103 Prophage DNA-packing protein NohA
MKVNKKRLAEIFNVDPRTIERWQSQGLPCASKGSKGIESVFDTAMAIQWYAQRETDIENE
KLRKELDDLRAAAESDLQPGTIDYERYRLTKAQADAQELKNAREDGVVLETELFTFILQR
VAQEISGILVRVPLTLQRKYPDISPSHLDVVKTEIAKASNVAAKAGENVGGWIDDFRRAE
GS
>LFGLNPFC_02104 hypothetical protein
MPHTVHYPEQPSQGHVTPPPSRDAPVRFVLCCVAKTDNIRIYLVFHDEFTQRLIEEGKMV
SKSKAHCRRMLQALQQTRAGIFDQLENCQHTLPEYIAISSETSATLIHRVPPEKKKK

>LFGLNPFC_02105 hypothetical protein
MKRRLLLLFLLSVLAVGCSQQKADEPRQLVTVYPRYPEYAAANYIKGLVEVKFDIGADGT
VTRIVFLRSEPHNLFRDEVVKAMAKWRFEKNRPCQGVKRQFIFTPSRP
>LFGLNPFC_02106 hypothetical protein
MKKMLFSAALAMLITGCAQQTFTVGNKPTAVTPKETITHHFFVSGIGQKKTVDAAKICGG
AENVVKTETQQTFVNGLLGFITLGIYTPLEARVYCSQ
>LFGLNPFC_02107 hypothetical protein
MNRVTAIISALVICIIVCLSWAVNHYRDNAITYKAQRDKNARELTLANAVITDIQMRQRD
VAALDAKYTKELADAKAENDALRDDVAAGRRRLHIKAVCQSVREATTASGVDNAASPRLA
DTAERDYFTLRERLITMQKQLEGTQKYINEQCR
>LFGLNPFC_02108 Lysozyme RrrD
MPPSLRKAVAAAIGGGAIAIASVLITGPSGNDGLEGVSYIPYKDIVGVWTVCHGHTGKDI
MLGKTYTKAECKALLNKDLATVARQINPYIKVDIPETMRGALYSFVYNVGAGNFRTSTLL
RKINQGDIKGACDQLRRWTYAGGKQWKGLMTRREIEREICLWGQQ
>LFGLNPFC_02109 hypothetical protein
MKSMDKLTTGVAYGTSAGSAGYWFLQLLDKVTPSQWAAIGVLGSLVFGLLTYLTNLYFKI
KEDKRKAARGE
>LFGLNPFC_02110 Outer membrane porin protein OmpD
MKKLTVAISAVAASVLMAMSAQAAEIYNKDSNKLDLYGKVNAKHYFSSNDADDGDTTYAR
LGFKGETQINDQLTGFGQWEYEFKGNRAESQGSSKDKTRLAFAGLKFGDYGSIDYGRNYG
VAYDIGAWTDVLPEFGGDTWTQTDVFMTGRTTGVATYRNNDFFGLVDGLNFAAQYQGKND
RTDVTEANGDGFGFSTTYEYEGFGVGATYAKSDRTDGQVAYGKSKFNASGKNAEVWAAGL
KYDANNIYLATTYSETQNMTVFGNNHIANKAQNFEAVAQYQFDFGLRPSVAYLQSKGKDL
GVHGDRDLVKYVDVGATYYFNKNMSTFVDYKINLIDDSKFTKTAGIDTDDIVAVGLVYQF
>LFGLNPFC_02111 hypothetical protein
MRDIQMVLERWGAWAANNHEDVTWSSIAAGFKGLIPSKVKSRPQCCDDDAMIICGCMARL
KKNNSDLHDLLVDYYVCGMTFMSLASKHCCSDGYIGKRLQKAEGIIEGMLMALDIRLDMD
IVANNSN
>LFGLNPFC_02112 putative protein YlcG
MFEFNMAELLRHRWGRLRLYRFLGSVLTDYRILKNYAKTLTGTGV
>LFGLNPFC_02113 Crossover junction endodeoxyribonuclease RusA
MNTYSITLPWPPSNNRYYRHNRGRTHVSAEGQAYRDNVARIIKNAMLDIGLAMPVKIRIE
CHMPDRRRRDLDNLQKAAFDALTKAGFWLDDAQVVDYRVVKMPVTKGGRLELTITEMGNE
>LFGLNPFC_02114 hypothetical protein
MDYSQLSDFEINRMVGDIIFKGLWASKPETSGNNTNKWYYGNADTTFEPLNHLPDYCNDP
SASWPIIEKYRISILDQLTEWCVDAKGVSPIFDTRPLRAAMIVFLLMQEANNA
>LFGLNPFC_02115 hypothetical protein
MTIKSNTPAHDKDCWQTPLWLFDALDIEFGFWLDSAASDKNALCAHWLTEDDDALNSEWV
SHGAIWNNPPYSNIRPWVEKAAEQCIQQRQTVVMLVPEDMSVGWFSKALESVDEVRIITD
GRINFIEPSTGLEKKGNSKGSMLLIWRPFISPRRMFTTVSKAALMAIGQGVRRAA
>LFGLNPFC_02116 hypothetical protein
MKKLTFEIRSPAHQQNAIHAVQQILPDPTKPIVVTIQERNRSLDQNRKLWACLGDVSRQV
EWHGRWLDAESWKCVFTAALKQQDVVPNLAGNGFVVIGQSTSRMRVSEFAELLELIQAFG
TERGVKWSDEARLALEWKARWGDRAA
>LFGLNPFC_02117 hypothetical protein
MTGKEAIIHYLGTHNSFCAPDVAALTGATVTSINQAAAKMARAGLLVIEGKVWRTVYYRF
ATKEEREGKMSTNLIFKECRQSATMKRILAVYGVKR
>LFGLNPFC_02118 hypothetical protein
MKNIAAQMVNFDREQMRRIANNMPEQYDEKPQVQQVAQIINGVFSQLLATFPASLANRDQ
NELNEIRRQWVLAFRENGITTMEQVNAGMRVARRQNRPFLPSPGQFVAWCREEASVIAGL
PNVSELVDMVYEYCRKRGLYPDAESYPWKSNAHYWLVTNLYQNMRANALTDAELRRKAAD
ELTCMTARINRGETIPEPVKQLPVMGGRPLNRVQALAKIAEIKAKLGLKGASV
>LFGLNPFC_02119 hypothetical protein
MTNTAKILNFCRGNFAKQERNVADLDDGYARLSNMLLEAYSGADLTKRQFKVLLAILRKT
YGWNKPMDRITDSQLSEITKLPVKRCNEAKLELVRMNIIKQQGGMFGPNKNISEWCIPQN
EGKSPKTRDKTSLKLGDCYPSKQGDTKDTITKEKRKDYSSENSGESSDQPENDLSVVKPD
AAIQSGSKWGTAEDLTAAEWMFDMVKTIAPSARKPNFAGWANDIRLMRERDGRNHRDMCV
LFRWACQDNFWSGNVLSPAKLRDKWTQLEINRNKQQAGVTACKPKLDLTNTDWIYGVDL
>LFGLNPFC_02120 hypothetical protein
MERTSYSKLSQRDVDRAETDLLINLSAITQRGLAKMIGCHESKISRTDWRFIASVLCAFG
MASDISPISRAFKYALDGLTNKKRPAATERSEQIQMEF
>LFGLNPFC_02121 hypothetical protein
MSNLRKYRESLNISQTTLAKAVGCTQGAIGHWESGRRFPDLKTCRALVACLNKLGAKVSL
DDVFPPEHKAA

>LFGLNPFC_02122 putative HTH-type transcriptional regulator
MKWYELARSRMKELGITQEKLAEELGMTQGGIGHWLRGSRHPSLSDIGVVFKYLGIDNIS
FNHDGTFSPVGEYSSAPVKKQYEYPVFSHVQAGMFSPELRTFTKGDAERLVSTTKKASDS
AFWLEVEGNSMTAPTGSKPSFPDGMLILVDPEQAVEPGDFCIARLGGDEFTFKKLIRDSG
QVFLQPLNPQYPMIPCNESCSVVGKVIASQWPEETFG
>LFGLNPFC_02123 hypothetical protein
MAKIDDYQPSQVEVDKVLYCKKIVNFSGVKWKQKPSRSDMWLQAHIIPLDEDCIPIQGLK
FELKWKPDQDSEPDDPISYPKINIIAFYHNKRVFAVDTYHFDKHTNSYKVDHPKYQDIIY
GAHYHVYYEEAGYYSDRIAFPIEDDINPDDLVGYWNYFCKHLNITYSGRIPLPLEDESGQ
MGFGI
>LFGLNPFC_02124 hypothetical protein
MCSTVISQLGFECHPIGKTLRIISPFTYCDDGEHVGAFIREVNGRYLVSDRCDALMNMEA
RGISLTKKRLDEIRQLLLKEGAELNARGEIIAWATEKDVGAITSNIIRAGILASTLSLDW
YQPVQAEKFESMVIDYLYHTELRDALSLRENVYGLSGHQITVPVTIKTDIPKYVFTSSVK
HGGSWNSAYSLLGKLIDLKASSEEYNNRFVVIDSEAIGDQMQQLSLLFHESSQVLPFSKR
ETWVKRLAA
>LFGLNPFC_02125 hypothetical protein
MSRKTEFKGTAASRRRARRANLQSQEAISSDKLHRPTPSRVVLQCKLKPAMRAEVITLTT
LTRKYEGSTCLPNVALYAAGYRKSKQLTAR
>LFGLNPFC_02126 hypothetical protein
MNNSWWQELMHFFLQGMTLKQLIHMLIILIILIIVMPVSVKEWINLHNPEILPHYWMYYI
LLFCVSYVLNGVVNSAYHAVTERIEVFAAQKRKSKEEKYVQDLFDSLTLGERAYLAFAVA
ANNQLQTEKGAHESISLLKKGLLVRRPPAVGYPDTDRFVIPESYRHECYIRFAGKADSLM
DELIAQDKHGKNK
>LFGLNPFC_02127 hypothetical protein
MTTTIDTNQWCGQFKRCNGCKLQSECMVKPEEMFPVMEDGKYVDKWAIRTTAMIARELGK
QNNKAA
>LFGLNPFC_02128 hypothetical protein
MSNIKKYIIDYDWKASIEIEIDHDVMTEEKLHQINNFWSDSEYRLNKHGSVLNAVLIMLA
QHALLIAISSDLNAYGVVCEFDWNDGNGQEGWPPMDGSEGIRITDIDTSGIFDSDDMTIK
AA
>LFGLNPFC_02129 hypothetical protein
MQYAIAGWPVAGCPSESLLERITRKLRDGWKRLIDILNQPGVPKNGSNNYGYPD
>LFGLNPFC_02130 hypothetical protein
MDQTIMAIQTKFTIATFIGDEKMFREAVDAYKKWILILKLRSSKSIH
>LFGLNPFC_02131 hypothetical protein
MNAYYIQDRLEAQSWARHYQQIAREEKEAELADDMEKGLPQHLFESLCIDHLQRHGASKK
AITRAFDDDVEFQERMAEHIRYIVETIAHHQADIDSEV
>LFGLNPFC_02132 hypothetical protein
MSTALATLAGKLAERVGMDSVDPQELITTLRQTAFKGDASDAQFIALLIVANQYGLNPWT
KEIYAFPDKQNGIVPVVGVDGWSRIINENQQFDGMDFEQDNESCTCRIYRKDRNHPICVT
EWMDECRREPFKTREGREITGPWQSHPKRMLRHKAMIQCARLAFGFAGIYDKDEAERIVE
NTAYTAERQPERDITPVNDETMQEINTLLIALDKTWDDDLLPLCSQIFRRDIRASSELTQ
AEAVKALGFLKQKATEQKVAA
>LFGLNPFC_02133 hypothetical protein
MTPDIILQRTGIDVRAVEQGDDAWHKLRLGVITASEVHNVIAKPRSGKKWPDMKMSYFHT
LLAEVCTGVAPEVNAKALAWGKQYENDARTLFEFTSGVNVTESPIIYRDESMRTACSPDG
LCSDGNGLELKCPFTSRDFMKFRLGGFEAIKSAYMAQVQYSMWVTRKDAWYFANYDPRMK
REGLHYVVVERDEKYMASFDEMVPEFIEKMDEALAEIGFVFGEQWR
>LFGLNPFC_02134 hypothetical protein
MTHPHDNIRVGAITFVYSVTKRGWVFHGLSVIRNPLKAQRLAEEINNKRGAVCTKHLLLS
>LFGLNPFC_02135 hypothetical protein
MANLQLAVKSEYFDAMIRGEKTEEYRLCNDYWNKRIMFREYDRLIITKGYPKRDDSSRRI
DVPYDGYEVKTITHPHFGDKPVKVFAIKVNISTEYQSAQHKVKNVQSD
>LFGLNPFC_02136 hypothetical protein
MFRVIDPNTWYVDHHGTPCKILRSTHNKVHYIRKGRTCIASMFRFNHDFEPVNKADADRI
AEEIETAEHIKKLRDMRRK
>LFGLNPFC_02137 hypothetical protein
MSRLITLQDWAKEEFGDLAPSERVLKKYAQGKMMAPPAIKVGRYWMIDRNSRFVGTLAEP
QLPINANPKLQRIIADGC
>LFGLNPFC_02138 hypothetical protein
MAARPRSHKISIPNLYCKLDKRTGKVYWQYKHPLSGRFHSLGTDENEAKQVATEANTIIA
EQRTRQILSVNERLERMKGRRSDITVTEWLDKYISIQEDRLQHNELRPNSYRQKGKPIRL
FREHCGMQHLKDITALDIAEIIDAVKAEGHNRMAQVVRMVLIDVFKEAQHAGHVPPGFNP

AQATKQPRNRVNRQRLSLPEWQAIFDSVSRRQPYLKCGMLLALVTGQRLGDICNLKFSDI
WDDMLHITQEKTGSKLAIPLNLKCDALNITLREVISQCRDAVVSKYLVHYRHTTSQANRG
DQVSANTLTTAFKKAREKCGIKWEQGTAPTFHEQRSLSERLYREQGLDTQKLLGHKSRKM
TDRYNDDRGKDWIIVDIKTA
>LFGLNPFC_02139 Isocitrate dehydrogenase [NADP]
MESKVVVPAQGKKITLQNGKLNVPENPIIPYIEGDGIGVDVTPAMLKVVDAAVEKAYKGE
RKISWMEIYTGEKSTQVYGQDVWLPAETLDLIREYRVAIKGPLTTPVGGGIRSLNVALRQ
ELDLYICLRPVRYYQGTPSPVKHPELTDMVIFRENSEDIYAGIEWKADSADAEKVIKFLR
EEMGVKKIRFPEHCGIGIKPCSEEGTKRLVRAAIEYAIANDRDSVTLVHKGNIMKFTEGA
FKDWGYQLAREEFGGELIDGGPWLKVKNPNTGKEIVIKDVIADAFLQQILLRPAEYDVIA
CMNLNGDYISDALAAQVGGIGIAPGANIGDECALFEATHGTAPKYAGQDKVNPGSIILSA
EMMLRHMGWTEAADLIVKGMEGAINAKTVTYDFERLMEGAKLLKCSEFGDAIIKNM
>LFGLNPFC_02140 Ribosomal large subunit pseudouridine synthase E
MRQFIISENTMQKTSFRNHQVKRFSSQRSTRRKPENQPTRVILFNKPYDVLPQFTDEAGR
KTLKEFIPVQGVYAAGRLDRDSEGLLVLTNNGALQARLTQPGKRTGKIYYVQVEGIPTQD
ALEALRNGVTLNDGPTLPAGAELVEEPAWLWPRNPPIRERKSIPTSWLKITLYEGRNRQV
RRMTAHVGFPTLRLIRYAMGDYSLDNLANGEWRDATD
>LFGLNPFC_02141 Phosphatase NudJ
MFKPHVTVACVVHAEGKFLVVEETINGKALWNQPAGHLEADETLVEAAARELWEETGISA
QPQHFIRMHQWIAPDKTPFLRFLFAIELEQICPTQPHDSDIDCCRWVSAEEILQASNLRS
PLVAESIRCYQSGQRYPLEMIGDFNWPFTKGVI
>LFGLNPFC_02142 tRNA-specific 2-thiouridylase MnmA
MSETAKKVIVGMSGGVDSSVSAWLLQQQGYQVEGLFMKNWEEDDGEEYCTAAADLADAQA
VCDKLGIELHTVNFAAEYWDNVFELFLAEYKAGRTPNPDILCNKEIKFKAFLEFAAEDLG
ADYIATGHYVRRADVDGKSRLLRGLDSNKDQSYFLYTLSHEQIAQSLFPVGELEKPQVRK
IAEDLGLVTAKKKDSTGICFIGERKFREFLGRYLPAQPGKIITVDGDEIGEHQGLMYHTL
GQRKGLGIGGTKDGTEEPWYVVDKDVENNILIVAQGHEHPRLMSVGLIAQQLHWVDREPF
TGTMRCTVKTRYRQTDIPCTVKALDADRIEVIFDEPVAAVTPGQSAVFYNGEVCLGGGII
EQRLPLPV
>LFGLNPFC_02143 High frequency lysogenization protein HflD
MAKNYYDITLALAGICQSARLVQQLAHQGHCDGDALHVSLNSIIDMNPSSTLAVFGGSEA
NLRVGLETLLGVLNASSRQGLNAELTRYTLSLMVLERKLSSAKGALDTLGNRINGLQRQL
EHFDLQSETLMSAMAAIYVDVISPLGPRIQVTGSPAVLQSPQVQAKVRATLLAGIRAAVL
WHQVGGGRLQLMFSRNRLTTQAKQILAHLTPEL
>LFGLNPFC_02144 Adenylosuccinate lyase
MELSSLTAVSPVDGRYGDKVSALRGIFSEYGLLKFRVQVEVRWLQKLAAHAAIKEVPAFA
ADAIGYLDAIVASFSEEDAARIKTIERTTNHDVKAVEYFLKEKVADIPELHAVSEFIHFA
CTSEDINNLSHALMLKTARDEVILPYWRQLIDGIKDLAVQYRDIPLLSRTHGQPATPSTI
GKEMANVAYRMERQYRQLNQVEILGKINGAVGNYNAHIAAYPEVDWHQFSEEFVTSLGIQ
WNPYTTQIEPHDYIAELFDCVARFNTILIDFDRDVWGYIALNHFKQKTIAGEIGSSTMPH
KVNPIDFENSEGNLGLSNAVLQHLASKLPVSRWQRDLTDSTVLRNLGVGIGYALIAYQST
LKGVSKLEVNRDHLLDELDHNWEVLAEPIQTVMRRYGIEKPYEKLKELTRGKRVDAEGMK
QFIDGLALPEEEKARLKAMTPANYIGRAITMVDELK
>LFGLNPFC_02145 Transcriptional regulatory protein PhoP
MRVLVVEDNALLRHHLKVQIQDAGHQVDDAEDAKEADYYLNEHLPDIAIVDLGLPDEDGL
SLIRRWRSNDVSLPILVLTARESWQDKVEVLSAGADDYVTKPFHIEEVMARMQALMRRNS
GLASQVISLPPFQVDLSRRELSINDEVIKLTAFEYTIMETLIRNNGKVVSKDSLMLQLYP
DAELRESHTIDVLMGRLRKKIQAQYPQEVITTVRGQGYLFELR
>LFGLNPFC_02146 Sensor protein PhoQ
MKKLLHLFFPLSLRVRFLLATAAVVLVLSLAYGMVALIGYSVSFDKTTFRLLRGESNLFY
TLAKWENNKLHVELPENIDKQSPTMTLIYDENGQLLWAQRDVPWLMKMIQPDWLKSNGFH
EIEADVNDTSLLLSGDHSIQQQLQEVREDDDDAEMTHSVAVNVYPATSRMPKLTIVVVDT
IPVELKSSYMVWSWFIYVLSANLLLVIPLLWVAAWWSLRPIEALAKEVRELEEHNRELLN
PATTRELTSLVRNLNRLLKSERERYDKYRTTLTDLTHSLKTPLAVLQSTLRSLRSEKMSV
SDAEPVMLEQISRISQQIGYYLHRASMRGGTLLSRELHPVAPLLDNLTSALNKVYQRKGV
NISLDISPEISFVGEQNDFVEVMGNVLDNACKYCLEFVEISARQTDEHLYIVVEDDGPGI
PLSKREVIFDRGQRVDTLRPGQGVGLAVAREITEQYEGKIVAGESMLGGARMEVIFGRQH
SAPKDE
>LFGLNPFC_02147 50S ribosomal protein L16 3-hydroxylase
MEYQLTLNWPDFLERHWQKRPVVLKRGFNNFIDPISPDELAGLAMESEVDSRLVSHQDGK
WQVSHGPFESYDHLGETNWSLLVQAVNHWHEPTAALMRPFRELPDWRIDDLMISFSVPGG
GVGPHLDQYDVFIIQGTGRRRWRVGEKLQMKQHCPHPDLLQVDPFEAIIDEELEPGDILY
IPPGFPHEGYALENAMNYSVGFRAPNTRELISGFADYVLQRELGGNYYSDPDVPPRAHPA
DVLPQEMDKLREMMLELINQPEHFKQWFGEFISQSRHELDIAPPEPPYQPDEIYDALKQG

DVLVRLGGLRVLRIGDDVYANGEKIDSPHRPALDALASNIALTAENFGDALEDPSFLAML
AALVNSGYWFFEG
>LFGLNPFC_02148 Peptidase T
MDKLLERFLNYVSLDTQSKAGVRQVPSTEGQWKLLHLLKEQLEEMGLINVTLSEKGTLMA
TLPANVPGDIPAIGFISHVDTSPDCSGKNVNPQIVENYRGGDIALGIGDEVLSPVMFPVL
HQLLGQTLITTDGKTLLGADDKAGIAEIMTALAVLQQKNIPHGDIRVAFTPDEEVGKGAK
HFDVDAFDARWAYTVDGGGVGELEFENFNAASVNIKIVGNNVHPGTAKGVMVNALSLAAR
IHAEVPADESPEMTEGYEGFYHLASMKGTVERADMHYIIRDFDRKQFEARKRKMMEIAKK
VGKGLHPDCYIELVIEDSYYNMREKVVEHPHILDIAQQAMRDCDIEPELKPIRGGTDGAQ
LSFMGLPCPNLFTGGYNYHGKHEFVTLEGMEKAVQVIVRIAELTAQRK
>LFGLNPFC_02149 Spermidine/putrescine import ATP-binding protein PotA
MGQSKKLNKQPNSLSPLVQLAGIRKCFDGKEVIPQLDLTINNGEFLTLLGPSGCGKTTVL
RLIAGLETVDSGRIMLDNEDITHVPAENRYVNTVFQSYALFPHMTVFENVAFGLRMQKTP
AAEITPRVMEALRMVQLETFAQRKPHQLSGGQQQRVAIARAVVNKPRLLLLDESLSALDY
KLRKQMQNELKALQRKLGITFVFVTHDQEEALTMSDRIVVMRDGRIEQDGTPREIYEEPK
NLFVAGFIGEINMFNATVIERLDEQRVRANVEGRECNIYVNFAVEPGQKLHVLLRPEDLR
VEEINDDNHAEGLIGYVRERNYKGMTLESVVELENGKMVMVSEFFNEDDPDFDHSLDQKM
AINWVESWEVVLADEEHK
>LFGLNPFC_02150 Spermidine/putrescine transport system permease protein PotB
MKNTSKFQNVVIVTIVGWLVLFVFLPNLMIIGTSFLTRDDASFVKMVFTLDNYTRLLDPL
YFEVLLHSLNMALIATLACLVLGYPFAWFLAKLPHKVRPLLLFLLIVPFWTNSLIRIYGL
KIFLSTKGYLNEFLLWLGVIDTPIRIMFTPSAVIIGLVYILLPFMVMPLYSSIEKLDKPL
LEAARDLGASKLQTFIRIIIPLTMPGIIAGCLLVMLPAMGLFYVSDLMGGAKNLLIGNVI
KVQFLNIRDWPFGAATSITLTIVMGLMLLVYWRASRLLNKKVELE
>LFGLNPFC_02151 Inner membrane ABC transporter permease protein YdcV
MIGRLLRGGFMTAIYAYLYIPIIILIVNSFNSSRFGINWQGFTTKWYSLLMNNDSLLQAA
QHSLTMAVFSATFATLIGSLTAVALYRYRFRGKPFVSGMLFVVMMSPDIVMAISLLVLFM
LLGIQLGFWSLLFSHITFCLPFVVVTVYSRLKGFDVRMLEAAKDLGASEFTILRKIILPL
AMPAVAAGWVLSFTLSMDDVVVSSFVTGPSYEILPLKIYSMVKVGVSPEVNALATILLVL
SLVMVIASQLIARDKTKGNTGDVK
>LFGLNPFC_02152 Spermidine/putrescine-binding periplasmic protein
MKKWSRHLLAAGALALGMSAAHADDNNTLYFYNWTEYVPPGLLEQFTKETGIKVIYSTYE
SNETMYAKLKTYKDGAYDLVVPSTYYVDKMRKEGMIQKIDKSKLSNFSNLDPDMLNKPFD
PNNDYSIPYIWGATAIGVNGDAVDPKSVTSWADLWKPEYKGSLLLTDDAREVFQMALRKL
GYSGNTTDPKEIEAAYNELKKLMPNVAAFNSDNPANPYMEGEVNLGMIWNGSAFVARQAG
TPIDVVWPKEGGIFWMDSLAIPANAKNKEGALKLINFLLRPDVAKQVAETIGYPTPNLAA
RKLLSPEVANDKTLYPDAETIKNGEWQNDVGSASSIYEEYYQKLKAGR
>LFGLNPFC_02153 Inner membrane protein YmfA
MSQDSRVFFRIFFGIGLVLILISVVIFYNQFTYSKDAIHTEGVIVDTVWHSSHSHRTGKN
GSWYPVVVFRPTPDYTLIFNSSIGSDFYEDSEGDKVNVYYSPGHPEKAEINNPWVNFFKW
GFIGIMGVIFIAVGLLISMPSSKKTRRKRKSRP
>LFGLNPFC_02154 Inner membrane protein YcfZ
MKKIIILLSLLILLPLTATSNPLIPIMKTLFTDVTGTVPDAEEIAHKAELFRQQTGVAPF
IVVLPDINNEASLRQNGKAMLAHAASSMSNVKGSVLLLFTTREPRLIMITNGQVESSMDD
KHLGLLVENHTLAYLHADLWYQGINNALAVLQAQILKQPTPPLTYYPHPGQQHENDPPGS
TTTLGLFAWAVAFIVFAAFFNYTTRLYYALKFAVAMAVANMGYQALCLYIDNSFAITRIS
PLWAGLIGVCTFIAALLWTSKR
>LFGLNPFC_02155 NAD-dependent protein deacylase
MLSRRGHRLSRFRKNKRRLRERLRQRIFFRDKVVPEAMEKPRVLVLTGAGISAESGIRTF
RAADGLWEEHRVEDVATPEGFDRDPELVQTFYNARRRQLQQPEIQPNAAHLALAKLQDAL
GDRFLLVTQNIDNLHERAGNTNVIHMHGELLKVRCSQSGQVLDWTGDVTPEDKCHCCQFP
APLRPHVVWFGEMPLGMDEIYMALSMADIFIAIGTSGHVYPAAGFVHEAKLHGAHTVELN
LEPSQVGNEFAEKYYGPASQVVPEFVEKLLKGL
>LFGLNPFC_02156 N-acetyl-D-glucosamine kinase
MYYGFDIGGTKIALGVFDSGRQLQWEKRVPTPRDSYDAFLDAVCELVAEADRRFGCKGSV
GIGIPGMPETEDGTLYAANVPAASGKPLRADLSARLDRDVRLDNDANCFALSEAWDDEFT
QYPLVMGLILGTGVGGGLIFNGKPITGKSYITGEFGHMRLPVDALTMMGLDFPLRRCGCG
QHGCIENYLSGRGFAWLYQHYYHQPLQAPEIIALYDQGDEQARAHVERYLDLLAVCLGNI
LTIVDPDLVVIGGGLSNFPAITTQLAERLPRHLLPVARVPRIERARHGDAGGMRGAAFLH
LTD
>LFGLNPFC_02157 Lipoprotein-releasing system transmembrane protein LolE
MPLSLLIGLRFSRGRRRGGMVSLISVISTIGIALGVAVLIVGLSAMNGFERELNNRILAV
VPHGEIEAVDQPWTNWQEALDNVQKVPGIAAAAPYINFTGLVESGANLRAIQVKGVNPQQ
EQRLSALPSFVQGDAWRNFKAGEQQIIIGKGVADALKVKQGDWVSIMIPNSNPEHKLMQP

KRVRLHIAGILQLSGQLDHSFAMIPLADAQQYLDMGSSVSGIALKMTDVFNANKLVRDAG
EVTNSYVYIKSWIGTYGYMYRDIQMIRAIMYLAMVLVIGVACFNIVSTLVMAVKDKSGDI
AVLRTLGAKDGLIRAIFVWYGLLAGLFGSLCGVIIGVVVSLQLTPIIEWIEKLIGHQFLS
SDIYFIDFLPSELHWLDVFYVLVTALLLSLLASWYPARRASNIDPARVLSGQ
>LFGLNPFC_02158 Lipoprotein-releasing system ATP-binding protein LolD
MNKILLQCDNLCKRYQEGSVQTDVLHNVSFSVGEGEMMAIVGSSGSGKSTLLHLLGGLDT
PTSGDVIFNGQPMSKLSSAAKAELRNQKLGFIYQFHHLLPDFTALENVAMPLLIGKKKPA
EINSRALEMLKAVGLEHRANHRPSELSGGERQRVAIARALVNNPRLVLADEPTGNLDARN
ADSIFQLLGELNRLQGTAFLVVTHDLQLAKRMSRQLEMRDGRLTAELSLMGAE
>LFGLNPFC_02159 Lipoprotein-releasing system transmembrane protein LolC
MYQPVALFIGLRYMRGRAADRFGRFVSWLSTIGITLGVMALVTVLSVMNGFERELQNNIL
GLMPQAILSSEHGSLNPQQLPETAVKLDGVNRVAPITTGDVVLQSARSVAVGVMLGIDPA
QKDPLTPYLVNVKQTDLEPGKYNVILGEQLASQLGVNRGDQIRVMVPSASQFTPMGRIPS
QRLFNVIGTFAANSEVDGYEMLVNIEDASRLMRYPAGNITGWRLWLDEPLKVDSLSQQKL
PEGSKWQDWRDRKGELFQAVRMEKNMMGLLLSLIVAVAAFNIITSLGLMVMEKQGEVAIL
QTQGLTPRQIMMVFMVQGASAGIIGAILGAALGALLASQLNNLMPIIGVLLDGAALPVAI
EPLQVIVIALVAMAIALLSTLYPSWRAAATQPAEALRYE
>LFGLNPFC_02160 Inner membrane protein YcfT
MKQKELWINQIKGLCICLVVIYHSVITFYPHLTTFQHPLSEVLSKCWIYFNLYLAPFRMP
VFFFISGYLIRRYIDSVPWGNCLDKRIWNIFWVLALWGVVQWLALSALNQWLAPERNLSN
ASNAAYADSTGEFLHGMITASTSLWYLYALIVYFVICKIFNRLALPLFVLFILMSVAVNF
VPTPWWGMNSVSRNLPYYSLGAWFGATLMTCVKAVPLRRHLLMASLLAVLAVGAWLFNIS
LLLSLVSIVVIMKLFYQYEQRFGMRSTSLLNVIGSNTIAIYTTHRILVEIFSLTLLAQMN
AARWSPQVELTLLLVYPFVSLFICTVAGLLVRKLSQRAFSDLLFSPPSLPAAVSYSR
>LFGLNPFC_02161 Transcription-repair-coupling factor
MPPYVEAYPNENLTTVMPEQYRYTLPVKAGEQRLLGELTGAACATLVAEIAERHAGPVVL
IAPDMQNALRLHDEISQFTDQMVMNLADWETLPYDSFSPHQDIISSRLSTLYQLPTMQRG
VLIVPVNTLMQRVCPHSFLHGHALVMKKGQRLSRDALRTQLDSAGYRHVDQVMEHGEYAT
RGALLDLFPMGSELPYRLDFFDDEIDSLRVFDVDSQRTLEEVEAINLLPAHEFPTDKAAI
ELFRSQWRDTFEVKRDPEHIYQQVSKGTLPAGIEYWQPLFFSEPLPPLFSYFPANTLLVN
TGDLENSAERFQADTLARFENRGVDPMRPLLPPQSLWLRVDELFSELKNWPRVQLKTEHL
PTKAANANLGFQKLPDLAIQAQQKAPLDALRKFLETFDGPVVFSVESEGRREALGELLAR
IKIAPQRIMRLDEASDRGRYLMIGAAEHGFVDTMRNLALICESDLLGERVARRRQDSRRA
INPDTLIRNLAELHIGQPVVHLEHGVGRYAGMTTLEAGGITGEYLMLTYANDAKLYVPVS
SLHLISRYAGGAEENAPLHKLGGDAWSRARQKAAEKVRDVAAELLDIYAQRAAKEGFAFK
HDREQYQLFCDSFPFETTPDQAQAINAVLSDMCQPLAMDRLVCGDVGFGKTEVAMRAAFL
AVDNHKQVAVLVPTTLLAQQHYDNFRDRFANWPVRIEMISRFRSAKEQTQILAEVAEGKI
DILIGTHKLLQSDVKFKDLGLLIVDEEHRFGVRHKERIKAMRANVDILTLTATPIPRTLN
MAMSGMRDLSIIATPPARRLAVKTFVREYDSLVVREAILREILRGGQVYYLYNDVENIQK
AAERLAELVPEARIAIGHGQMRERELERVMNDFHHQRFNVLVCTTIIETGIDIPTANTII
IERADHFGLAQLHQLRGRVGRSHHQAYAWLLTPHPKAMTTDAQKRLEAIASLEDLGAGFA
LATHDLEIRGAGELLGEEQSGSMETIGFSLYMELLENAVDALKAGREPSLEDLTSQQTEV
DLRMPSLLPDDFIPDVNTRLSFYKRIASAKTENELEEIKVELIDRFGLLPDPARTLLDIA
RLRQQAQKLGIRKLEGNEKGGVIEFAEKNHVNPAWLIGLLQKQPQHYRLDGPTRLKFIQD
LSERKTRIEWVRQFMRELEENAIA
>LFGLNPFC_02162 putative L,D-transpeptidase YcfS
MIKTHFSRWLTFFTFAAAVALALPAKANTWPLPQAGSRLVGENKFHVVENDGGSLEAIAK
KYNVGFLALLQANPGVDPYVPRAGSVLTIPLQTLLPDAPREGIVINIAELRLYYYPPGKN
SVTVYPIGIGQLGGDTLTPTMVTTVSDKRANPTWTPTANIRARYKAQGIELPAVVPAGPD
NPMGHHAIRLAAYGGVYLLHGTNADFGIGMRVSSGCIRLRDDDIKTLFSQVTPGTKVNII
NTPIKVSAEPNGARLVEVHQPLSEKIDDDPQLLPITLNSAMQSFKDAAQTDAEVMQHVMD
VRSGMPVDVRRHQVSPQTL
>LFGLNPFC_02163 Multiple stress resistance protein BhsA
MKNVKNLIAAAILSSMSFASFAAVEVQSTPEGQQKVGTISANAGTNLGSLEEQLAQKADE
MGAKSFRITSVTGPNTLHGTAVIYK
>LFGLNPFC_02164 HTH-type transcriptional repressor ComR
MATDSTQCVKKSRGRPKVFDRDAALDKAMKLFWQHGYEATSLADLVEATGAKAPTLYAEF
TNKEGLFRAVLDRYIDRFAAKHEAQLFCEEKSVESALADYFAAIANCFTSKDTPAGCFMI
NNCTTLSPDSGDIANTLKSRHAMQERTLQQFLCQRQARGEIPTHCDVTHLAEFLNCIIQG
MSISAREGASLEKLMQIARTTLRLWPELLK
>LFGLNPFC_02165 hypothetical protein
MNKSMLAGIGIGVAAALGVAAVASLNVFERGPQYAQVVSATPIKETVKTPRQECRNVTVT
HRRPVQDENRITGSVLGAVAGGVIGHQFGGGRGKDVATVVGALGGGYAGNQIQGSLQESD
TYTTTQQRCKTVYDKSEKMLGYDVTYKIGDQQGKIRMDRDPGTQIPLDSNGQLILNNKA

>LFGLNPFC_02166 NADH dehydrogenase
MTTPLKKIVIVGGGAGGLEMATQLGHKLGRKKKAKITLVDRNHSHLWKPLLHEVATGSLD
EGVDALSYLAHARNHGFQFQLGSVIDIDREAKTITIAELRDEKGELLVPERKIAYDTLVM
ALGSTSNDFNTPGVKENCIFLDNPHQARRFHQEMLNLFLKYSANLGANGKVNIAIVGGGA
TGVELSAELHNAVKQLHSYGYKGLTNEALNVTLVEAGERILPALPPRISAAAHSELTKLG
VRVLTQTMVTSADEGGLHTKDGEYIEADLMVWAAGIKAPDFLKDIGGLETNRINQLVVEP
TLQTTRDPDIYAIGDCASCPRPEGGFVPPRAQAAHQMATCAMNNILAQMNGKPLKNYQYK
DHGSLVSLSNFSTVGSLMGNLTRGSMMIEGRIARFVYISLYRMHQIALHGYFKTGLMMLV
GSINRVIRPRLKLH
>LFGLNPFC_02167 hypothetical protein
MIIYLHGFDSNSPGNHEKVLQLQFIDPDVRLISYSTRHPKHDMQHLLKEVDKMLQLNVDE
RPLICGVGLGGYWAERIGFLCDIRQVIFNPNLFPYENMEGKIDRPEEYADIATKCVTNFR
EKNRDRCLVILSRNDEALNSQRTSEELHHYYEIVWDEEQTHKFKNISPHLQRIKAFKTLG
>LFGLNPFC_02168 Beta-hexosaminidase
MGPVMLDVEGYELDAEEREILAHPLVGGLILFTRNYHDPAQLRELVRQIRAASRNHLVVA
VDQEGGRVQRFREGFTRLPAAQSFAALLGMEEGGKLAQEAGWLMASEMIAMDIDISFAPV
LDVGHISAAIGERSYHADPQKALAIASRFIDGMHEAGMKTTGKHFPGHGAVTADSHKETP
CDPRPQAEIRAKDMSVFSSLIRENKLDAIMPAHVIYSDVDPRPASGSPYWLKTVLRQELG
FDGVIFSDDLSMEGAAIMGSYAERGQASLDAGCDMILVCNNRKGAVSVLDNLSPIKAERV
TRLYHKGSFSRQELMDSARWKAISTRLNQLHERWQEEKAGH
>LFGLNPFC_02169 Thiamine kinase
MPFRSNNPLTRDELLSRFFPQFHPVTTFNSGLSGGSFLIEHQGQRFVVRQPHDPDAPQSA
FLRQYRALSQLPACIAPKPHLYLRDWMVVDYLPGEVKTYLPDTNELAGLLYYLHQQPRFG
WRITLLPLLELYWQQSDPARRTVGWLRMLKRLRKAREPRLLRLSPLHMDVHAGNLVHSAS
GLKLIDWEYAGDGDIALELAAVWVENIDQHRQLVNDYATRAKIYPAQLWRQVRRWFPWLL
MLKAGWFEYRWRQTGDQQFIRLADDTWRQLLIKQ
>LFGLNPFC_02170 Penicillin-binding protein activator LpoB
MTKMSRYALITALAMFLAGCVGQREPAPVEEVKPAPEQPAEPQQPVPTVPSVPTIPQQPG
PIEHEDQTAPPAPHIRHYDWNGAMQPMVSKMLGADGVTAGSVLLVDSVNNRTNGSLNAAE
ATETLRNALANNGKFTLVSAQQLSMAKQQLGLSPQDSLGTRSKAIGIARNVGAHYVLYSS
ASGNVNAPTLQMQLMLVQTGEIIWSGKGAVSQQ
>LFGLNPFC_02171 hypothetical protein
MSLALLVLVGCRSHPEIPVNDEQSLVMESSLLAAGISAEKPVLSTSDIQPSASSTLYNER
QEPVTVHYRFYWYDARGLEMHPLERPRSVTIPAHSAVTLYGSANFLGAHKVRLYLYL
>LFGLNPFC_02172 Purine nucleoside phosphoramidase
MAEETIFSKIIRREIPSDIVYQDDLVTAFRDISPQAPTHILIIPNILIPTVNDVSAEHEQ
ALGRMITVAAKIAEQEGIAEDGYRLIMNTNRHGGQEVYHIHMHLLGGCPLGPMLAHKGL
>LFGLNPFC_02173 FhuE receptor
MLSTQFNRDNQHQAIIKPSLLAGCIALALLPSAAFAAPVTEETVIVEGSATAPDDGENDY
NVTSTSAGTKMQMTQRDIPQSVTIVSQQRMEDQQLQTLGEVMENTLGISKSQADSDRALY
YSRGFQIDNYMVDGIPTYFESRWNLGDALSDMALFERVEVVRGATGLMTGTGNPSAAINM
VRKHATSREFKGDVSAEYGSWNKERYVADLQSPLTEDGKIRARIVGGYQNNDSWLDRYNS
EKTFFSGIVDADLGDLTMLSAGYEYQRIDVNSPTWGGLPRWNTDGSSNSYDRARSTAPDW
AYNDKEINKVFMTLKQRFADTWQATLNATHSEVEFDSKMMYVDAYVNKADGMLVGPYSNY
GPGFDYVGGTGWNSGKRKVDALDLFADGSYELFGRQHNLMFGGSYSKQNNRYFSSWANIF
PDEIGSFYNFNGNFPQTDWSPQSLAQDDTTHMKSLYAATRVTLADPLHLILGARYTNWRV
DTLTYSMEKNHTTPYAGLVFDINDNWSTYASYTSIFQPQNDRDSSGKYLTPITGNNYELG
LKSDWMNSRLTTTLAIFRIEQDNVAQSTGTPIPGSNGETAYKAVDGTVSKGVEFELNGAI
TDNWQLTFGATRYIAEDNEGNAVNPNLPRTTVKMFTSYRLPVMPELTVGGGVNWQNRVYT
DTVTPYGTFRAEQGSYALVDLFTRYQVTKNFSLQGNVNNLFDKTYDTNVEGSIVYGAPRN
FSITGTYQF
>LFGLNPFC_02174 PTS system glucose-specific EIICB component
MFKNAFANLQKVGKSLMLPVSVLPIAGILLGVGSANFSWLPAVVSHVMAEAGGSVFANMP
LIFAIGVALGFTNNDGVSALAAVVAYGIMVKTMAVVAPLVLHLPAEEIASKHLADTGVLG
GIISGAIAAYMFNRFYRIKLPEYLGFFAGKRFVPIISGLAAIFTGVVLSFIWPPIGSAIQ
TFSQWAAYQNPVVAFGIYGFIERCLVPFGLHHIWNVPFQMQIGEYTNAAGQVFHGDIPRY
MAGDPTAGKLSGGFLFKMYGLPAAAIAIWHSAKPENRAKVGGIMISAALTSFLTGITEPI
EFSFMFVAPILYIIHAILAGLAFPICILLGMRDGTSFSHGLIDFIVLSGNSSKLWLFPIV
GIGYAIVYYTIFRVLIKALDLKTPGREDATEDAKATGTSEMAPALVAAFGGKENITNLDA
CITRLRVSVADVSKVDQAGLKKLGAAGVVVAGSGVQAIFGTKSDNLKTEMDEYIRNH
>LFGLNPFC_02175 putative metal-dependent hydrolase YcfH
MFLVDSHCHLDGLDYESLHKDVDDVLAKAAARDVKFCLAVATTLPGYLHMRDLVGERDNV
VFSCGVHPLNQNDPYDVEDLRRLAAEEGVVALGETGLDYYYTPETKVRQQESFIHHIQIG
RELNKPVIVHTRDARADTLAILREEKVTDCGGVLHCFTEDRETAGKLLDLGFYISFSGIV

TFRNAEQLRDAARYVPLDRLLVETDSPYLAPVPHRGKENQPAMVRDVAEYMAVLKGVAVE
ELAQVTTDNFARLFHIDASRLQSIR
>LFGLNPFC_02176 DNA polymerase III subunit delta'
MRWYPWLRPDFEKLVASYQAGRGHHALLIQALPGMGDDALIYALSRYLLCQQPQGHKSCG
HCRGCQLMQAGTHPDYYTLTPEKGKNALGIDAVREVTEKLNEHARLGGAKVVWVTDAALL
TDAAANALLKTLEEPPAETWFFLATREPERLLATLRSRCRLHYLAPPPEQYAVTWLSREV
TMSQAALLAALRLSAGSPGAALALFQGDNWQARETLCQALAYSVQSGDWYSLLAALNHEQ
APARLHWLATLLMDALKRHHGAAQVTNVDVPGLVVELANHLSPSRLQAILGDVCHIREQL
MSVTGINRELLITDLLLRIEHYLQPGVVLPVPHL
>LFGLNPFC_02177 Thymidylate kinase
MRSKYIVIEGLEGAGKTTARNVVVETLEQLGIRDMVFTREPGGTQLAEKLRSLVLDIKSV
GDEVITDKAEVLMFYAARVQLVETVIKPALANGTWVIGDRHDLSTQAYQGGGRGIDQHML
ATLRDAVLGGFRPDLTLYLDVTPEVGLKRARARGELDRIEQESFDFFNRTRARYLELAAQ
DKSIHTIDATQPLEAVMDAIRTTVTNWVKELDA
>LFGLNPFC_02178 Endolytic murein transglycosylase
MKKVLLIILLLLVVLGIAAGVGVWKVRHLADSKLLIKEETIFTLKPGTGRLALGEQLYAD
KIINRPRVFQWLLRIEPDLSHFKAGTYRFTPQMTVREMLKLLESGKEAQFPLRLVEGMRL
SDYLKQLREAPYIKHTLSDDKYATVAQALELENPEWIEGWFWPDTWMYTANTTDVALLKR
AHKKMVKAVDSAWEGRADGLPYKDKNQLVTMASIIEKETAVASERDQVASVFINRLRIGM
RLQTDPTVIYGMGERYNGKLSRADLETPTAYNTYTITGLPPGAIATPGADSLKAAAHPAK
TPYLYFVADGKGGHTFNTNLASHNKSVQDYLKVLKEKNAQ
>LFGLNPFC_02179 Aminodeoxychorismate lyase
MFLINGHKQESLAVSDRATQFGDGCFTTARVIDGKVSLLSAHIQRLQDACQRLMISCDFW
PQLEQEMKTLAAEQQNGVLKVVISRGSGGRGYSTLNSGPATRILSVTAYPAHYDRLRNEG
MTLALSPVRLGRNPHLAGIKHLNRLEQVLIRSHLEQTNADEALVLDSEGWVTECCAANLF
WRKGNVVYTPRLDQAGVNGIMRQFCIRLLAQSSYQLVEVQASLEEALQADEMVICNALMP
VMPVRACGDVSFSSATLYEYLAPLCERPN
>LFGLNPFC_02180 3-oxoacyl-[acyl-carrier-protein] synthase 2
MSKRRVVVTGLGMLSPVGNTVESTWKALLAGQSGISLIDHFDTSAYATKFAGLVKDFNCE
DIISRKEQRKMDAFIQYGIVAGVQAMQDSGLEITEENATRIGAAIGSGIGGLGLIEENHT
SLMNGGPRKISPFFVPSTIVNMVAGHLTIMYGLRGPSISIATACTSGVHNIGHAARIIAY
GDADVMVAGGAEKASTPLGVGGFGAARALSTRNDNPQAASRPWDKERDGFVLGDGAGMLV
LEEYEHAKKRGAKIYAELVGFGMSSDAYHMTSPPENGAGAALAMANALRDAGIEASQIGY
VNAHGTSTPAGDKAEAQAVKTIFGEAASRVLVSSTKSMTGHLLGAAGAVESIYSILALRD
QAVPPTINLDNPDEGCDLDFVPHEARQVSGMEYTLCNSFGFGGTNGSLIFKKI
>LFGLNPFC_02181 Acyl carrier protein
MSTIEERVKKIIGEQLGVKQEEVTNNASFVEDLGADSLDTVELVMALEEEFDTEIPDEEA
EKITTVQAAIDYINGHQA
>LFGLNPFC_02182 3-oxoacyl-[acyl-carrier-protein] reductase FabG
MNFEGKIALVTGASRGIGRAIAETLAARGAKVIGTATSENGAQAISDYLGANGKGLMLNV
TDPASIESVLEKIRAEFGEVDILVNNAGITRDNLLMRMKDEEWNDIIETNLSSVFRLSKA
VMRAMMKKRHGRIITIGSVVGTMGNGGQANYAAAKAGLIGFSKSLAREVASRGITVNVVA
PGFIETDMTRALSDDQRAGILAQVPAGRLGGAQEIANAVAFLASDEAAYITGETLHVNGG
MYMV
>LFGLNPFC_02183 Malonyl CoA-acyl carrier protein transacylase
MTQFAFVFPGQGSQTVGMLADMAASYPIVEETFAEASAALGYDLWALTQQGPAEELNKTW
QTQPALLTASVALYRVWQQQGGKAPAMMAGHSLGEYSALVCAGVIDFADAVRLVEMRGKF
MQEAVPEGTGAMAAIIGLDDASIAKACEEAAEGQVVSPVNFNSPGQVVIAGHKEAVERAG
AACKAAGAKRALPLPVSVPSHCALMKPAADKLAVELAKITFNAPTVPVVNNVDVKCETNG
DAIRDALVRQLYNPVQWTKSVEYMAAQGVEHLYEVGPGKVLTGLTKRIVDTLTASALNEP
SAMAAALEL
>LFGLNPFC_02184 3-oxoacyl-[acyl-carrier-protein] synthase 3
MYTKIIGTGSYLPEQVRTNADLEKMVDTSDEWIVTRTGIRERHIAAPNETVSTMGFEAAT
RAIEMAGIEKDQIGLIVVATTSATHAFPSAACQIQSMLGIKGCPAFDVAAACAGFTYALS
VADQYVKSGAVKYALVVGSDVLARTCDPTDRGTIIIFGDGAGAAVLAASEEPGIISTHLH
ADGSYGELLTLPNADRVNPENSIHLTMAGNEVFKVAVTELAHIVDETLAANNLDRSQLDW
LVPHQANLRIISATAKKLGMSMDNVVVTLDRHGNTSAASVPCALDEAVRDGRIKPGQLVL
LEAFGGGFTWGSALVRF
>LFGLNPFC_02185 Phosphate acyltransferase
MGGDFGPSVTVPAALQALNSNSQLTLLLVGNPDAITPLLAKADFEQRSRLQIIPAQSVIA
SDARPSQAIRASRGSSMRVALELVKEGRAQACVSAGNTGALMGLAKLLLKPLEGIERPAL
VTVLPHQQKGKTVVLDLGANVDCDSTMLVQFAIMGSVLAEEVVEIPNPRVALLNIGEEEV
KGLDSIRDASAVLKTIPSINYIGYLEANELLTGKTDVLVCDGFTGNVTLKTMEGVVRMFL
SLLKSQGEGKKRSWWLLLLKRWLQKSLTRRFSHLNPDQYNGACLLGLRGTVIKSHGAANQ

RAFAVAIEQAVQAVQRQVPQRIAARLESVYPAGFELLDGGKSGTLR
>LFGLNPFC_02186 50S ribosomal protein L32
MAVQQNKPTRSKRGMRRSHDALTAVTSLSVDKTSGEKHLRHHITADGYYRGRKVIAK
>LFGLNPFC_02187 Large ribosomal RNA subunit accumulation protein YceD
MQKVKLPLTLDPVRTAQKRLDYQGIYTPDQVERVAESVVSVDSDVECSMSFAIDNQRLAV
LNGDAKVTVTLECQRCGKPFTHQVYTTYCFSPVRSDEQAEALPEAYEPIEVNEFGEIDLL
AMVEDEIILALPVVPVHDSEHCEVSEADMVFGELPEEAQKPNPFAVLASLKRK
>LFGLNPFC_02188 7-methyl-GTP pyrophosphatase
MAIINTLYLMEKNMPKLILASTSPWRRALLEKLQISFECAAPEVDETPRSDESPRQLVLR
LAQEKAQSLASRYPDHLIIGSDQVCVLDGEITGKPLTEENARLQLRKASGNIVTFYTGLA
LFNSANGHLQTEVEPFDVHFRHLSEAEIDNYVRKEHPLHCAGSFKSEGFGITLFERLEGR
DPNTLVGLPLIALCQMLRREGKNPLMG
>LFGLNPFC_02189 Ribosomal large subunit pseudouridine synthase C
MKTETPSVKIVAITADEAGQRIDNFLRTQLKGVPKSMIYRILRKGEVRVNKKRIKPEYKL
EAGDEVRIPPVRVAEREEEAVSPHLQKVAALADVILYEDDHILVLNKPSGTAVHGGSGLS
FGVIEGLRALRPEARFLELVHRLDRDTSGVLLVAKKRSALRSLHEQLREKGMQKDYLALV
RGQWQSHVKSVQAPLLKNILQSGERIVRVSQEGKPSETRFKVEERYAFATLVRCSPVTGR
THQIRVHTQYAGHPIAFDDRYGDREFDRQLTEAGTGLNRLFLHAAALKFTHPGTGEVMRI
EAPMDEGLKRCLQKLRNAR
>LFGLNPFC_02190 Ribonuclease E
MKRMLINATQQEELRVALVDGQRLYDLDIESPGHEQKKANIYKGKITRIEPSLEAAFVDY
GAERHGFLPLKEIAREYFPANYSAHGRPNIKDVLREGQEVIVQIDKEERGNKGAALTTFI
SLAGSYLVLMPNNPRAGGISRRIEGDDRTELKEALASLELPEGMGLIVRTAGVGKSAEAL
QWDLSFRLKHWEAIKKAAESRPAPFLIHQESNVIVRAFRDYLRQDIGEILIDNPKVLELA
RQHIAALGRPDFSSKIKLYTGEIPLFSHYQIESQIESAFQREVRLPSGGSIVIDSTEALT
AIDINSARATRGGDIEETAFNTNLEAADEIARQLRLRDLGGLIVIDFIDMTPVRHQRAVE
NRLREAVRQDRARIQISHISRFGLLEMSRQRLSPSLGESSHHVCPRCSGTGTVRDNESLS
LSILRLIEEEALKENTQEVHAIVPVPIASYLLNEKRSAVNAIETRQDGVRCVIVPNDQME
TPHYHVLRVRKGEETSTLSYMLPKLHEEAMALPSEEEFAERKRPEQPALATFAMPDVPPA
PTPAEPAAPVVAPAPKAATATPASPAQPGLLSRFFGALKALFSGGEETKPAEQSAPKAEA
KPERQQDRRKPRQNNRRDRNERRDTRSERTEGSDNREENRRNRRQAQQQTAETRESRQQA
EVTEKARTTDEQQAPRRERSRRRNDDKRQAQQEAKALNVEEQSVQETEQEERVRPVQPRR
KQRQLNQKVRYEQSVAEEAVVAPVVEETVAAEPIVQEAPAPRTELVKVPLPVVAQAAPEQ
QEENNADNRDNGGMPRRSRRSPRHLRVSGQRRRRYRDERYPTQSPMPLTVACASPELASG
KVWIRYPIVRPQDVQVEEQHEQEEVQVQPMVTEVPVAAAVKPVVSAPVVEEVAEVVEAPV
QVAEPQPEVVETTHPEVIAAAVTEQPQVITESDVAVAQEVAEHAEPVVEPQEETADIEEV
AETAEVVVAEPEVVAQPAAPVVAEVAAEVETVAAVEPEVTVEHNHATAPMTRAPAPEYVP
EAPRHSDWQRPTFAFEGKGAAGGHTATHHASAAPARPQPVE
>LFGLNPFC_02191 Flagellar hook-associated protein 3
MRFSTQMMYQQNMRGITNSQAEWMKYGEQMSTGKRVVNPSDDPIAASQAVVLSQAQAQNS
QYTLARTFATQKVSLEESVLSQVTTAIQNAQEKIVYASNGTLSDDDRASLATDIQGLRDQ
LLNLANTTDGNGRYIFAGYKTETAPFSEADGDYVGGTESIKQQVDASRSMVIGHTGDKIF
DSITSNAVAEPDGSASETNLFAMLDSAIAALKTPVADSEADKETAAAALDKTNRGLKNSL
NNVLTVRAELGTQLNELESLDSLGSDRALGQTQQMSDLVDVDWNATISSYIMQQTALQAS
YKAFTDMQGLSLFQLNK
>LFGLNPFC_02192 Flagellar hook-associated protein 1
MSSLINNAMSGLNAAQAALNTASNNISSYNVAGYTRQTTIMAQANSTLGAGGWVGNGVYV
SGVQREYDAFITNQLRAAQTQSSGLTARYEQMSKIDNMLSTSTSSLATQMQDFFTSLQTL
VSNAEDPAARQALIGKSEGLVNQFKTTDQYLRDQDKQVNIAIGASVDQINNYAKQIASLN
DQISRLTGVGAGASPNNLLDQRDQLVSELNQIVGVEVSVQDGGTYNITMANGYSLVQGST
ARQLAAVPSSADPSRTTVAYVDRTAGNIEIPEKLLNTGSLGGILTFRSQDLDQTRNTLGQ
LALAFAEAFNTQHKAGFDANGDAGEDFFAIGKPAVLQNTKNKGDVAIGATVTDASAVLAT
DYKISFDNNQWQVTRLASNTTFTVTPDANGKVAFDGLELTFTGTPAVNDSFTLKPVSDAI
VNMDVLITDEAKIAMASEEDAGDSDNRNGQALLDLQSNSKTVGGAKSFNDAYASLVSDIG
NKTATLKTSSATQGNVVTQLSNQQQSISGVNLDEEYGNLQRFQQYYLANAQVLQTANAIF
DALINIR
>LFGLNPFC_02193 Peptidoglycan hydrolase FlgJ
MISDSKLLASAAWDAQSLNELKAKAGEDPAANIRPVARQVEGMFVQMMLKSMRDALPKDG
LFSSERTRLYTSMYDQQIAQQMTAGKGLGLAEMMVKQMTPEQPLPEESTPAAPMKFPLET
VVRYQNQALSQLVQKAVPRNYDDSLPGNSKAFLAQLSLPAQLASQQSGVPHHLILAQAAL
ESGWGQRQIRRENGEPSYNLFGVKASGNWKGPVTEITTTEYENGEAKKVKAKFRVYSSYL
EALSDYVGLLTRNPRYAAVTTAVSAEQGAQALQDAGYATDPHYARKLTNMIQQMKSISDK
VSKTYSMNIDNLF
>LFGLNPFC_02194 Flagellar P-ring protein

MIKFLSALILLLVTTAAQAERIRDLTSVQGVRQNSLIGYGLVVGLDGTGDQTTQTPFTTQ
TLNNMLSQLGITVPTGTNMQLKNVAAVMVTASLPPFGRQGQTIDVVVSSMGNAKSLRGGT
LLMTPLKGVDSQVYALAQGNILVGGAGASAGGSSVQVNQLNGGRITNGAVIERELPSQFG
VGNTLNLQLNDEDFSMAQQIADTINRVRGYGSATALDARTIQVRVPSGNSSQVRFLADIQ
NMQVNVTPQDAKVVINSRTGSVVMNREVTLDSCAVAQGNLSVTVNRQANVSQPDTPFGGG
QTVVTPQTQIDLRQSGGSLQSVRSSASLNNVVRALNALGATPMDLMSILQSMQSAGCLRA
KLEII
>LFGLNPFC_02195 Flagellar L-ring protein
MQKNAAHTYAISSLLVLSLTGCAWIPSTPLVQGATSAQPVPGPTPVANGSIFQSAQPINY
GYQPLFEDRRPRNIGDTLTIVLQENVSASKSSSANASRDGKTNFGFDTVPRYLQGLFGNA
RADVEASGGNTFNGKGGANASNTFSGTLTVTVDQVLVNGNLHVVGEKQIAINQGTEFIRF
SGVVNPRTISGSNTVPSTQVADARIEYVGNGYINEAQNMGWLQRFFLNLSPM
>LFGLNPFC_02196 Flagellar basal-body rod protein FlgG
MISSLWIAKTGLDAQQTNMDVIANNLANVSTNGFKRQRAVFEDLLYQTIRQPGAQSSEQT
TLPSGLQIGTGVRPVATERLHSQGNLSQTNNSKDVAIKGQGFFQVMLPDGSSAYTRDGSF
QVDQNGQLVTAGGFQVQPAITIPANALSITIGRDGVVSVTQQGQAAPVQVGQLNLTTFMN
DTGLESIGENLYTETQSSGAPNESTPGLNGAGLLYQGYVETSNVNVAEELVNMIQVQRAY
EINSKAVSTTDQMLQKLTQL
>LFGLNPFC_02197 Flagellar basal-body rod protein FlgF
MDHAIYTAMGAASQTLNQQAVTASNLANASTPGFRAQLNALRAVPVEGLSLPTRTLVTAS
TPGADMTPGKMDYTSRPLDVALQQDGWLAVQTADGSEGYTRNGSIQVDPTGQLTIQGHPV
VGEAGPIAVPEGAEITIAADGTISALNPGDPANTVAPVGRLKLVKATGSEVQRGDDGIFR
LSAESQATRGPVLQADPTLRVMSGVLEGSNVNAVAAMSDMIASARRFEMQMKVISSVDDN
AGRANQLLSMS
>LFGLNPFC_02198 Flagellar hook protein FlgE
MAFSQAVSGLNAAATNLDVIGNNIANSATYGFKSGTASFADMFAGSKVGLGVKVAGITQD
FTDGTTTNTGRGLDVAISQNGFFRLVDSNGSVFYSRNGQFKLDENRNLVNMQGLQLTGYP
ATGTPPTIQQGANPTNISIPNTLMAAKTTTTASMQINLNSSDSLPSVNAFDASNADSYNK
KGSVTVFDSQGNAHDMSVYFVKTGDNNWQVYTQDSSDPNSIAKTATTLVFNANGVLTSNP
TANISTGAINGADPATFSLSFLNSMQQNTGANNIVATTQNGYKPGDLVSYQINDDGTVVG
NYSNEQTQLLGQIVLANFANNEGLASEGDNVWSATQSSGVALLGTAGTGNFGTLTNGALE
ASNVDLSKELVNMIVAQRNYQSNAQTIKTQDQILNTLVNLR
>LFGLNPFC_02199 Basal-body rod modification protein FlgD
MSIAVTTTDPTNTGVSTTSSSSLTGSNAADLQSSFLTLLVAQLKNQDPTNPMENNELTSQ
LAQISTVSGIEKLNTTLGSISGQIDNSQSLQASNLIGHGVMIPGTTVLAGTGSEEGAVTT
TTPFGVELQQAADKVTATITDKNGAVVRTIDIGELTAGVHSFTWDGTLTDGSTAPNGSYN
VAISASNGGTQLVAQPLQFALVQGVIRGNNGNTLDLGTYGTTTLDEVRQII
>LFGLNPFC_02200 Flagellar basal-body rod protein FlgC
MALLNIFDIAGSALTAQSQRLNVAASNLANADSVTGPDGQPYRAKQVVFQVNAAPGAATG
GVKVADVIESQAPDKLVYEPGNPLADAKGYVKMPNVDVVGEMVNTMSASRSYQANVEVLN
TVKSMMLKTLTLGQ
>LFGLNPFC_02201 Flagellar basal body rod protein FlgB
MLDKLDAALRFQQEALNLRAQRQEVLAANIANADTPGYQARDIDFASELKKVMQRGRDAT
SVVALTMTSTKHIPAQALTPPSAELQYRIPDQPSLDGNTVDMDRERTQFADNSLQYQMSL
SALSGQIKGMMNVLQSGN
>LFGLNPFC_02202 Flagella basal body P-ring formation protein FlgA
MLTIKRSVAIIAILFSPLSAASNLTSQLHTFFSAQLAGVSDEVRVSIRTAPNLLPPCEQP
LLSMSNNSRLWGNVNVLARCGNDKRYLQVNVQATGNYVVAAMPIVRGGKLEAGNVKLKRG
RLDTLPPRTVLDINQLVDAVSLRDLSPDQPIQLTHFRQAWRVKAGQRVNVIASGDGFSAN
AEGQALNNAAVAQNARVRMISGQVVSGVVDADGNILINL
>LFGLNPFC_02203 Negative regulator of flagellin synthesis
MSIDRTSPLKPVSTVQPRETTDAPVTNTRAAKTTASTSTSVTLSDAQAKLMQPGSSDINL
ERVEALKLAIRNGELKMDTGKIADALINEAQQDLQSN
>LFGLNPFC_02204 Flagella synthesis protein FlgN
MTRLAEILDQMSAVLNDLKTVMDQEQQHLSMGGQINGSQLQWITEQKSSLLATLDYLEQLR
RKEPNTANSVDISQRWQEITGKTQQLRQLNQHNGWLLEGQIERNQQALEMLKPHQEPTLY
GANGQTSTTHRGSKKISI
>LFGLNPFC_02205 Lipid II flippase MurJ
MNLLKSLAAVSSMTMFSRVLGFARDAIVARIFGAGMATDAFFVAFKLPNLLRRIFAEGAF
SQAFVPILAEYKSKQGEDATRVFVSYVSGLLTLALAVVTVAGMLAAPWVIMVTAPGFADT
ADKFALTSQLLKITFPYILLISLASLVGAILNTWNRFSIPAFAPTLLNISMIGFALFAAP
YFNPPVLALAWAVTVGGVLQLVYQLPHLKKIGMLVLPRINFHDAGAMRVVKQMGPAILGV
SVSQISLIINTIFASFLASGSVSWMYYADRLMEFPSGVLGVALGTILLPSLSKSFASGNH
DEYNRLMDWGLRLCFLLALPSAVALGILSGPLTVSLFQYGKFTAFDALMTQRALIAYSVG

LIGLIVVKVLAPGFYSRQDIKTPVKIAIVTLILTQLMNLAFIGPLKHAGLSLSIGLAACL
NASLLYWQLRKQKIFTPQPGWMAFLLRLVVAVLVMSGVLLGMLHIMPEWSLGTMPWRLLR
LMAVVLAGIAAYFAALAVLGFKVKEFARRTV
>LFGLNPFC_02206 Putative oxidoreductase YceM
MKKLRIGVVGLGGIAQKAWLPVLAAASDWTLQGAWSPTRAKALPIGDSWRIPYADSLSSL
AASCDAVFVHSSTASHFDVVSTLLNAGVHVCVDKPLAENLRDAERLVELAARKKLTLMVG
FNRRFAPLYGELKTQLATAASLRMDKHRSNSVGPHDLYFTLLDDYLHVVDTALWLSGGKA
SLDGGTLLTNDAGEMLFAEHHFSAGPLQITTCMHRRAGSQRETVQAVTDGALIDITDMRE
WREERGQGVVNKPIPGWQSTLEQRGFVGCARHFIECVQNQTVPQTAGEQAVLAQRIVDKI
WRDAMSE
>LFGLNPFC_02207 hypothetical protein
MKYQLTALEARVIGCLLEKQVTTPEQYPLSVNGVVTACNQKTNREPVMNLSESEVQEQLD
NLVKRHYLRTVSGFGNRVTKYEQRFCNSEFGDLKLSAAEVALITTLLLRGAQTPGELRSR
AARMYEFSDMAEVESTLEQLANREDGPFVVRLAREPGKRESRYMHLFSGEVEDQPAVMDM
SNAVDGDLQARVEALEIEVAELKQRLDSLLAHLGD
>LFGLNPFC_02208 [Ribosomal protein S5]-alanine N-acetyltransferase
MFGYRSNVPKVRLTTDRLVVRLVHDRDAWRLADYYAENRHFLKPWEPVRDESHCYPSGWQ
ARLGMINEFHKQGSAFYFGLFDPDEKEIIGVANFSNVVRGSFHACYLGYSIGQKWQGKGL
MFEALSAAIRYMQRTQHIHRIMANYMPHNKRSGDLLARLGFEKEGYAKDYLLIDGQWRDH
VLTALTTPDWTPGR
>LFGLNPFC_02209 Multidrug resistance protein MdtH
MSRVSQARNLGKYFLLIDNMLVVLGFFVVFPLISIRFVDQMGWAAVMVGIALGLRQFIQQ
GLGIFGGAIADRFGAKPMIVTGMLMRAAGFATMGIAHEPWLLWFSCLLSGLGGTLFDPPR
SALVVKLIRPQQRGRFFSLLMMQDSAGAVIGALLGSWLLQYDFRLVCATGAVLFVLCAAF
NAWLLPAWKLSTVRTPVREGMTRVMRDKRFVTYVLTLAGYYMLAVQVMLMLPIMVNDVAG
APSAVKWMYAIEACLSLTLLYPIARWSEKHFRLEHRLMAGLLIMSLSMMPVGMVSGLQQL
FTLICLFYIGSIIAEPARETLSASLADARARGSYMGFSRLGLAIGGAIGYIGGGWLFDLG
KSAHQPELPWMMLGIIGIFTFLALGWQFSQKRAARRLLERDA
>LFGLNPFC_02210 Glutaredoxin 2
MKLYIYDHCPYCLKARMIFGLKNIPVELHVLLNDDAETPTRMVGQKQVPILQKDDSRYMP
ESMDIVHYVDKLDGKPLLTGKRSPAIEEWLRKVNGYANKLLLPRFAKSAFDEFSTPAARK
YFVDKKEASAGNFADLLAHSDGLIKNISDDLRALDKLIVKPNAVNGELSEDDIQLFPLLR
NLTLVAGINWPSRVADYRDNMAKQTQINLLSSMAI
>LFGLNPFC_02211 putative lipoprotein YceB
MNKFLFAAALIVSGLLVGCNQLTQYTITEQEINQSLAKHNNFSKDIGLPGVADAHIVLTN
LTSQIGREEPNKVTLTGDANLDMNSLFGSQKATMKLKLKALPVFDKEKGAIFLKEMEVVD
ATVQPEKMQTVMQTLLPYLNQALRNYFNQQPAYVLREDGSQGEAMAKKLAKGIEVKPGEI
VIPFTD
>LFGLNPFC_02212 Dihydroorotase
MTAPSQVLKIRRPDDWHLHLRDGDMLKTVVPYTSEIYGRAIVMPNLAPPVTTVEAAVAYR
QRILDAVPAGHDFTPLMTCYLTDSLDPNELERGFNEGVFTAAKLYPANATTNSSHGVTSV
DAIMPVLERMEKIGMPLLVHGEVTHADIDIFDREARFIESVMEPLRQRLTALKVVFEHIT
TKDAADYVRDGNERLAATITPQHLMFNRNHMLVGGVRPHLYCLPILKRNIHQQALRELVA
SGFNRVFLGTDSAPHARHRKESSCGCAGCFNAPTALGSYATVFEEMNALQYFEAFCSVNG
PQFYGLPVNDTFIELVREEQQVAESIALTDDTLVPFLAGETVRWSVKQ
>LFGLNPFC_02213 DNA damage-inducible protein I
MRIEVTIAKTSPLPAGAIDALAGELSRRIQYAFPDNEGHVSVRYAAANNLSVIGATKEDK
QRISEILQETWESADDWFVSE
>LFGLNPFC_02214 Biofilm regulator BssS
MEKNNEVIQTHPLVGWDISTVDSYDALMLRLHYQTPNKSEQEGTEVGGQTLWLTTDVARQF
ISILEAGIAKIESGDFQVNEYRRH
>LFGLNPFC_02215 N-methyl-L-tryptophan oxidase
MKYDLIIIGSGSVGAAAGYYATRAGLNVLMTDAHMPPHQHGSHHGDTRLIRHAYGEGEKY
VPLVLRAQMLWDELSRHNEDDPIFVRSGVINLGPADSAFLANVAHSAEQWQLNVEKLDAQ
GIMARWPEIRVPDNYIGLFETDSGFLRSELAIKTWIQLAKEAGCAQLFNCPVTAIRHDDD
GVTIETADGEYQAKKAIVCAGTWVKDLLPELPVQPVRKVFAWYQADGRYSVKNKFPAFTG
ELPNGDQYYGFPAENDALKIGKHNGGQVIHSADERVPFAEVVSDGSEAFPFLRNVLPGIG
CCLYGAACTYDNSPDEDFIIDTLPGHDNTLLITGLSGHGFKFASVLGEIAADFAQDKKSD
FDLTPFRLSRFQ
>LFGLNPFC_02216 putative protein YceO
MRRLLHYLINNIREHLMLYLFLWGLLAIMDLIYVFYF
>LFGLNPFC_02217 Cytochrome b561
MSFTNTPERYGVISAALHWLSAIIVYGMFALGLWMVTLSYYDGWYHQAPELHKSIGILLM
MGLVIRVLWRVISPPPGPLPSYSPMTRLAAKAGHLALYLLLFAIGISGYLISTADGKPIS

VFGWFDVPATLSDAGAQADFAGALHFWLAWSVVVLSVMHGFMALKHHFIDKDDTLKRMLG
KSSSDYGV
>LFGLNPFC_02218 Protein YceI
MKKSLLGLTFASLMFSAGSAVAADYKIDKEGQHAFVNFRIQHLGYSWLYGTFKDFDGTFT
FDEKNPAADKVNVTINTTSVDTNHAERDKHLRSADFLNTAKYPQATFTSTSVKKDGDELD
ITGDLTLNGVTKPVTLEAKLIGQGDDPWGGKRAGFEAEGKIKLKDFNIKTDLGPASQEVD
LIISVEGVQQK
>LFGLNPFC_02219 hypothetical protein
MPVLHNRISNDALKAKMLAESEPRTTISFYKYFHIADPKVTRDALYQLFTALNVFGRVYL
AHEGINAQISVPASNVETFRAQLYAFDPALEGLRLNIALDDDGKSFWVLRMKVRDRIVAD
GIDDPHFDASNVGEYLQAAEVNAMLDDPDALFIDMRNHYEYEVGHFENALEIPADTFREQ
LPKAVEMMQAHKDKKIVMYCTGGIRCEKASAWMKHNGFNKVWHIEGGVIEYARKAREQGL
PVRFIGKNFVFDERMGERISDEIIAHCHQCGAPCDSHTNCKNDGCHLLFIQCPVCAEKYK
GCCSEICCEESALPPDEQRRRRAGRENGNKIFNKSRGRLNTTLGIPDPTE
>LFGLNPFC_02220 Lipid A biosynthesis lauroyltransferase
MTNLPKFSTALLHPRYWLTWLGIGVLWLVVQLPYPVIYRLGCGLGKLALRFMKRRAKIVH
RNLELCFPEMSEQERRKMVVKNFESVGMGLMETGMAWFWPDRRIARWTEVIGMEHIRDVQ
AQKRGILLVGIHFLTLELGARQFGMQEPGIGVYRPNDNPLIDWLQTWGRLRSNKSMLDRK
DLKGMIKALKKGEVVWYAPDHDYGPRSSVFVPLFAVEQAATTTGTWMLARMSGACLVPFV
PRRKPDGKGYQLIILPPECSPPLDDAETTAAWMNKVVEKCIMMAPEQYMWLHRRFKTRPE
GVPSRY
>LFGLNPFC_02221 Staphyloferrin B transporter
MSPCENDTPINWKRNLIVAWLGCFLTGAAFSLVMPFLPLYVEQLGVTGHSALNMWSGIVF
SITFLFSAIASPFWGGLADRKGRKLMLLRSALGMGIVMVLMGLAQNIWQFLILRALLGLL
GGFVPNANALIATQVPRNKSGWALGTLSTGGVSGALLGPMAGGLLADSYGLRPVFFITAS
VLILCFFVTLFCIREKFQPVSKKEMLHMREVVTSLKNPKLVLSLFVTTLIIQVATGSIAP
ILTLYVRELAGNVSNVAFISGMIASVPGVAALLSAPRLGKLGDRIGPEKILITALIFSVL
LLIPMSYVQTPLQLGILRFLLGAADGALLPAVQTLLVYNSSNQIAGRIFSYNQSFRDIGN
VTGPLMGAAISANYGFRAVFLVTAGVVLFNAVYSWNSLRRRRIPQISN
>LFGLNPFC_02222 hypothetical protein
MTMYATLEEAIDAAREEFLADNPGIDAENANVQQFNAQKYVLQDGDIMWQVEFFADEGEE
GECLPMLSGEAAQSVFDGDYDEIEIRQEWQDENTLHEWDEGEFQLEPPLDTEEGRTAADE
WDER
>LFGLNPFC_02223 hypothetical protein
MRLIVMSIMVTLLSGCGSIISRTIPGQGHGNQYYPGVQWDVRDSAWRYVTILDLPFSLVF
DTLLLPIDVHHGPYE
>LFGLNPFC_02224 Glucans biosynthesis glucosyltransferase H
MPIAASEKAALPKTDIRAVHQALDAEHRTWAREDDSPQGSVKARLEQAWPDSLADGQLIK
DDEGRDQLKAMPEVKRSSMFPDPWRTNPVGRFWDRLRGRDVTPRYLARLTKEEQESEQKW
RTVGTIRRYILLILTLAQTVVATWYMKTILPYQGWALINPMDMVGQDVWVSFMQLLPYML
QTGILILFAVLFCWVSAGFWTALMGFLQLLIGRDKYSISASTVGDEPLNPEHRTALIMPI
CNEDVNRVFAGLRATWESVKATGNAKHFDVYILSDSYNPDICVAEQKAWMELIAEVGGEG
QIFYRRRRRRVKRKSGNIDDFCRRWGSQYSYMVVLDADSVMTGDCLCGLVRLMEANPNAG
IIQSSPKASGMDTLYARCQQFATRVYGPLFTAGLHFWQLGESHYWGHNAIIRVKPFIEHC
ALAPLPGEGSFAGSILSHDFVEAALMRRAGWGVWIAYDLPGSYEELPPNLLDELKRDRRW
CHGNLMNFRLFLVKGMHPVHRAVFLTGVMSYLSAPLWFMFLALSTALQVVHALTEPQYFL
QPRQLFPVWPQWRPELAIALFASTMVLLFLPKLLSILLIWCKGTKEYGGFWRVTLSLLLE
VLFSVLLAPVRMLFHTVFVVSAFLGWEVVWNSPQRDDDSTSWGEAFKRHGSQLLLGLVWA
VGMAWLDLRFLFWLAPIVFSLILSPFVSVISSRATVGLRTKRWKLFLIPEEYSPPQVLVD
TDRFLEMNRQCSLDDGFMHAVFNPSFNALATAMATARHRASKVLEIARDRHVEQALNETP
EKLNRDRRLVLLSDPVTMARLHFRVWNSPERYSSWVSYYEGIKLNPLALRKPDAASQ
>LFGLNPFC_02225 Glucans biosynthesis protein G
MLTLYTSSSWAFSIDDVAKQAQSLAGKGYEAPKSNLPSVFRDMKYADYQQIQFNHDKAYW
NNLKTPFKLEFYHQGMYFDTPVKINEVTATAVKRIKYSPDYFTFGDVQHDKDTVKDLGFA
GFKVLYPINSKDKNDEIVSMLGASYFRVIGAGQVYGLSARGLAIDTALPSGEEFPRFKEF
WIERPKPTDKRLTIYALLDSPRATGAYKFVVMPGRDTVVDVQSKIYLRDKVGKLGVAPLT
SMFLFGPNQPSPANNYRPELHDSNGLSIHAGNGEWIWRPLNNPKHLAVSSFSMENPQGFG
LLQRGRDFSRFEDLDDRYDLRPSAWVTPKGEWGKGSVELVEIPTNDETNDNIVAYWTPDQ
LPEPGKEMNFKYTITFSRDEDKLHAPDNAWVQQTRRSTGDVKQSNLIRQPDGTIAFVVDF
TGAEMKKLPEDTPVTAQTSIGDNGEIVESTVRYNPVTKGWRLVMRVKVKDAKKTTEMRAA
LVNADQTLSETWSYQLPANE
>LFGLNPFC_02226 Glucans biosynthesis protein C
MNPVPAQREYFLDSIRAWLMLLGIPFHISLIYSSHTWHVNSAEPSLWLTLFNDFIHSFRM
QVFFVISGYFSYMLFLRYPLKKWWKVRVERVGIPMLTAIPLLTLPQFIMLQYVKGKAESW

PGLSLYDKYNTLAWELISHLWFLLVLVVMTTLCVWIFKRIRNNLENSDKTNKKFSMVKLS
VIFLCLGIGYAVIRRTIFIVYPPILSNGMFNFIVMQTLFYLPFFILGALAFIFPHLKALF
TTPSRGCTFAAALAFVAYLLNQRYGSGDAWMYETESVITMVLGLWMVNVVFSFGHRLLNF
QSARVTYFVNASLFIYLVHHPLTLFFGAYITPHITSNWLGFLCGLIFVVGIAIILYEIHL
RIPLLKFLFSGKPVVKRENDKAPAR
>LFGLNPFC_02227 Cardiolipin synthase C
MMKKTPTSTKDSLLNKEMNDLPRLASAVLPLCSQHPGQCGLFPLEKSLDAFAARYRLAEM
AEHTLDVQYYIWQDDMSGRLLFSALLAAAKRGVRVRLLLDDNNTPGLDDILRLLDSHPRI
EVRLFNPFSFRLLRPLGYITDFSRLNRRMHNKSFTVDGVVTLVGGRNIGDAYFGAGEEPL
FSDLDIMAIGPVVEDVADDFARYWYCKSVSPLQQVLDVPEGEMADRIELPASWHDDAMTH
RYLRKMESSPFVNHLVDGTLPLIWAKTRLLSDDPAKGEGKAKRHSLLPQRLFDIMGSPSE
RIDIISSYFVPTRAGVAQLLRMVRKGVKIAILTNSLAANDVAVVHAGYARWRKKLLRYGV
ELYELKPTREQSSTLHDRGITGNSGASLHAKTFSIDGKTVFIGSFNFDPRSTLLNTEMGF
VIESETLAQLIDKRFIQSQYDAAWQLRLDRWGRINWVDRHAKKEIVLKKEPATSFWKRVM
VRLASILPVEWLL
>LFGLNPFC_02228 O-acetyl-ADP-ribose deacetylase
MKTRIHVVQGDITKLAVDVIVNAANPSLMGGGGVDGAIHRAAGPALLDACLKVRQQQGDC
PTGHAVITLAGDLPAKAVVHTVGPVWRGGEQNEDQLLQDAYLNSLRLVAANSYTSVAFPA
ISTGVYGYPRAAAAEIAVKTVSEFITRHALPEQVYFVCYDEENAHLYERLLTQQGDE
>LFGLNPFC_02229 putative protein YmdA
MFRPFLDSLMLGSMFFPFIAIAGSTAQGGVIHFYGQIVEPACDVSTQSTPVEMNCPQNGS
VPGRTYSSKALMSGNVKNAQIASVKVQYLDKQKKLAVMNIEYN
>LFGLNPFC_02230 Curli assembly protein CsgC
MNALLLLAALSSQITFNTTQQGDMYTIIPEVTLTQSCLCRVQILSLREGSSGQSQTKQEK
TLSLPANQPIALTKLSLNISPDDRVKIVVTVSDGQSLHLSQQWPPSSEKS
>LFGLNPFC_02231 Major curlin subunit
MKLLKVAAIAAIVFSGSALAGVVPQYGGGGNHGGGGNNSGPNSELNIYQYGGGNSALALQ
ADARNSDLTITQHGGGNGADVGQGSDDSSIDLTQRGFGNSATLDQWNGKDSTMTVKQFGG
GNGAAVDQTASNSSVNVTQVGFGNNATAHQY
>LFGLNPFC_02232 Minor curlin subunit
MKNKLLFMMLTILGAPGIAAAAGYDLANSEYNFAVNELSKSSFNQAAIIGQAGTNNSAQL
RQGGSKLLAVVAQEGSSNRAKIDQTGDYNLAYIDQAGNANDASISQGAYGNTAMIIQKGS
GNKANITQYGTQKTAIVVQRQSQMAIRVTQR
>LFGLNPFC_02233 CsgBAC operon transcriptional regulatory protein
MFNEVHSIHGHTLLLITKPSLQATALLQHLKQSLAITGKLHNIQRSLDDISSGSIILLDM
MEADKKLIHYWQDTLSRKNNNIKILLLNTPEDYPYRDIENWPHINGVFYAMEDQERVVNG
LQGVLRGECYFTQKLASYLITHSGNYRYNSTESALLTHREKEILNKLRIGASNNEIARSL
FISENTVKTHLYNLFKKIAVKNRTQAVSWANDNLRR
>LFGLNPFC_02234 Curli production assembly/transport component CsgE
MKRYLRWIVAAEFLFAAGNLHAVEVEVPGLLTDHTVSSIGHDFYRAFSDKWESDYTGNLT
INERPSARWGSWITITVNQDVIFQTFLFPLKRDFEKTVVFALIQTEEALNRRQINQALLS
TGDLAHDEF
>LFGLNPFC_02235 Curli production assembly/transport component CsgF
MRVKHAVVLLMLISPLSWAGTMTFQFRNPNFGGNPNNGAFLLNSAQAQNSYKDPSYNDDF
GIETPSALDNFTQAIQSQILGGLLSNINTGKPGRMVTNDYIVDIANRDGQLQLNVTDRKT
GQTSTIQVSGLQNNSTDF
>LFGLNPFC_02236 Curli production assembly/transport component CsgG
MQRLFLLVAVMLLSGCLTAPPKEAARPTLMPRAQSYKDLTHLPAPTGKIFVSVYNIQDET
GQFKPYPASNFSTAVPQSATAMLVTALKDSRWFIPLERQGLQNLLNERKIIRAAQENGTV
AINNRIPLQSLTAANIMVEGSIIGYESNVKSGGVGARYFGIGADTQYQLDQIAVNLRVVN
VSTGEILSSVNTSKTILSYEVQAGVFRFIDYQRLLEGEVGYTSNEPVMLCLMSAIETGVI
FLINDGIDRGLWDLQNKAERQNDILVKYRHMSVPPES
>LFGLNPFC_02237 Inner membrane protein YcdZ
MAAFSAIMRGMNILLSIAITTGILSGIWGWVAVSLGLLSWAGFLGCTAYFACPQGGLKGL
AISAATLLSGVVWAMVIIYGSALAPHLEILGYVITGIVAFLMCIQAKQLLLSFVPGTFIG
ACATFAGQGDWKLVLPSLALGLVFGYAMKNSGLWLAARSTKTAHREQQIKNKA
>LFGLNPFC_02238 Chaperone protein YcdY
MNEFSILCRVLGSLYYRQPQDPLLVPLFTLIREGKLAANWPLEQDELLTRLQKSCDMAQV
SADYNALFIGDECAVPPYRSAWVEGATEAEVRAFLSERGMPLADTPADHIGTLLLAASWL
EDQSTEDESEALETLFSEYLLPWCGAFLGKVEAHATTPFWRTMAPLTRDAISAMWDELEE
DSEE
>LFGLNPFC_02239 putative phosphatase YcdX
MYPVDLHMHTVASTHAYSTLSDYIAQAKQKGIKLFAITDHGPDMEDAPHHWHFINMRIWP
RVVDGVGILRGIEANIKNVDGEIDCSGKMFDSLDLIIAGFHEPVFAPHDKATNTQAMIAT

IASGNVHIISHPGNPKYEIDVKAVAEAAAKHQVALEINNSSFLHSRKGSEDNCRAVAAAV
RDAGGWVALGSDSHTAFTMGEFEECLKILDAVDFPPERILNVSPRRLLNFLESRGMAPIA
EFADL
>LFGLNPFC_02240 Glyoxylate/hydroxypyruvate reductase A
MDIIFYHPTFDTQWWIEALRKAIPQARVRAWKSGDNDSADYALVWHPPVEMLAGRDLKAV
FALGAGVDSILSKLQAHPEMLKPSVPLFRLEDTGMGEQMQEYAVSQVLHWFRRFDDYRIQ
QNSSHWQPLPEYHREDFTIGILGAGVLGSKVAQSLQTWRFPLRCWSRTRKSWPGVQSFAG
REELSAFLSQCRVLINLLPNTPETVGIINQQLLEKLPDGAYLLNLARGVHVVEDDLLAAL
DSGKVKGAMLDVFNREPLPPESPLWQHPRVTITPHVAAITRPAEAVEYISRTIAQLEKGE
RLCGQVDRARGY
>LFGLNPFC_02242 hypothetical protein
MSDITISRLEVVNGHTDIICSTSVSHILAVRKTTLLQIDTLIRQLTEISAMTESIGGKTA
LDWAMKQDFRCGCWLMDKPDTAMKAITRNLDREIWRDLMQCSGMLSLMDAQARDTWYRSL
EYDNFPEISEENILSTFEQLHQNKDEVFERGVINLFRGLSWNYKTNCPCKFGNKIIVNNL
VRWDRWGLHLITGQQADRLADLERMLHLFSGKPIPDNRENITIHLDEHIRSLQGKECYED
EMFIIKYFKKGSAHITFRKPELVDRLNDIIAKHYPDMLAV
>LFGLNPFC_02243 hypothetical protein
MPAGFTHFRLVTMNNMKSLTTETALDILIAWLQDNIDCESGIIFDNDEDKTDSAALLPCI
EQAREGIRTLCQLQLLHQNR
>LFGLNPFC_02244 hypothetical protein
MMKLALTLEADSVNVQALNMGRIVVDVDGIELAELINVVCDNGHSLRVVDESDRASTDCT
PPFAALTGIRCSTAHITAKDNAWLYSLSHQTNDTGESEWIHFTGSGYLLRTDAWSYPVLR
LKRLGLSKTFRRLVVTLIRRYGVSLIHLDASAECLPGLPTFDW
>LFGLNPFC_02245 Cytoskeleton-binding toxin CbtA
MNTLPDTHVREASGCPSPVTIWQTLLTRLLDQHYGLTLNDTPFADERVIEQHIEAGISLC
DAVNFLVEKYALVRTDQPGFSACTRSQLINSIDILRARRATGLMTRDNYRTVNNITLGKH
PEAK
>LFGLNPFC_02246 Cytoskeleton bundling-enhancing antitoxin CbeA
MSDTLPGTTLPDDNHDRPWWGLPCTVTPCFGARLVQEGNRLHYLADRTGIRGRFSNADAY
HLDQAFPLLMKQLELMLTSGELNPRHQHTVTLYAKGLTCEADTLGSCGYVYLAVYPTPAA
PAITV
>LFGLNPFC_02247 hypothetical protein
MSRIITTTVYTLHELSSTAQEKARDWYRQHHADSNWYENVYEDFREVCGIFGIDLRQRVF
RLSNGRFMEEPCIWFSGFCSQGDGACFEGRWHWQPATPRKIREYAPQDRELHRIADALQA
VQKRNFWQLQAEISHRGRYCHPYSMDITVTRNSPTGQALTADAEAAVSEALRDLAFWLYR
QLENEYDWLTSDAAVDEAIHINAYTFTEAGLHAG
>LFGLNPFC_02248 hypothetical protein
MKIITRGEAMRIHQQHPASRLFPFCTGKYRWHGSTETYTGREVQDIPGVLAVFAERRKDS
FGPYVRLMSVTLN
>LFGLNPFC_02249 hypothetical protein
MQQLSFLPGEMTPGERSLILRALKTLDRHLHEPGVAFTSTRAAREWLILNMAGLEREEFR
VLYLNNQNQLIAGETLFTGTINRTEVHPREVIKRALYHNAAAVVLAHNHPSGEVTPSKAD
RLITERLVQALGLVDIRVPDHLIVGGSQVFSFAEHGLL
>LFGLNPFC_02250 Antirestriction protein KlcA
MKTLSQNTTSSACAPETGLQQLVATIVPDEQRISFWPQHFGLIPQWVTLEPRVFGWMDRL
CENYCGGIWNLYTLNNGGAFMAPEPDDYDDETWVLFNAMNGNRAEMSPEAAGIAACLMTY
SHHACRTECYAMTVHYYRLRDYALQHPECSAIMRIID
>LFGLNPFC_02251 hypothetical protein
MQHVPEGVIQAPESPDAPEALPGCRSISDDDLCAWCTRLLYRPGEISLCRLSTGNGIWPS
VCDRNGYAYGCPEFQPNIIHP
>LFGLNPFC_02252 hypothetical protein
MRLASRFGYAANQIRRDRPLTHEELMHYVPSIFGKDRHTSRSKRYAYIPTITVLESLQRE
GFQPFFACQTCVRDPGRRGYTKHMLRLRRNGEINGEHVPEIILLNSHDGTSSYQMLPGYF
RFVCQNGCVCGQSLGEVRVPHRGNVVDRVIEGAYEVVGVFDRIEEKRDAMQSLILPPPAR
QALAQAALTYRYGDEHRPVTTADILTPRRREDYGKDLWSTYQTIQENMLKGGISGRSAKG
KRIHTRAIHSIDTDIKLNRALWVMAETLLESLR
>LFGLNPFC_02253 Antigen 43
MKRHLNTSYRLVWNHITGTLVVASELARSRGKGAGVAVALSLAAVTSVPALAADTVVQAG
ETVNGGTLTNHDNQIVLGTANGMTISTGLEYGPDNEANTGGQWIQNGGIANNTTVTGGGL
QRVNAGGSVSDTVISAGGGQSLQGQAVNTTLNGGEQWVHEGGIATGTVINEKGWQAVKSG
AMATDTVVNTGAEGGPDAENGDTGQTVYGDAVRTTINKNGRQIVAAEGTANTTVVYAGGD
QTVHGHALDTTLNGGYQYVHNGGTASDTVVNSDGWQIIKEGGLADFTTVNQKGKLQVNAG
GTATNVTLTQGGALVTSTAATVTGSNRLGNFTVENGNADGVVLESGGRLDVLEGHSAWKT
LVDDGGTLAVSAGGKATDVTMTSGGALIADSGATVEGTNASGKFSIDGISGQASGLLLEN

GGSFTVNAGGLASNTTVGHRGTLTLAAGGSLSGRTQLSKGASMVLNGDVVSTGDIVNAGE
IRFDNQTTPDAALSRAVAKGDSPVTFHKLTTSNLTGQGGTINMRVRLDGSNASDQLVING
GQATGKTWLAFTNVGNSNLGVATSGQGIRVVDAQNGATTEEGAFALSRPLQAGAFNYTLN
RDSDEDWYLRSENAYRAEVPLYASMLTQAMDYDRILAGSRSHQSGVSGENNSVRLSIQGG
HLGHDNNGGIARGATPESNGSYGFVRLEGDLLRTEVAGMSLTTGVYGAAGHSSVDVKDDD
GSRAGTVRDDAGSLGGYLHLVHTSSGLWADIVAQGTRHSMKASSDNNDFRARGWGWLGSL
ETGLPFSITDNLMLEPQLQYTWQGLSLDDGQDNAGYVKFGHGSAQHVRAGFRLGSHNDMN
FGKGTSSRDTLHDSAKHSVRELPVNWWVQPSVIRTFSSRGDMSMGTAAAGSNMTFSPSRN
GTSLDLQAGLEARVRENITLGVQAGYAHSVSGSSAEGYNGQATLNVTF
>LFGLNPFC_02254 GTPase Era
MNPSDAIEAIEKPLSSLPYSLSRHILEHLRKLTSHEPVIGIMGKSGAGKSSLCNALFQGE
VTPVSDVHAGTREVRRFRLSGHGHNMVITDLPGVGESRDRDAEYEALYRDILPELDLVLW
LIKADDRALSVDEYFWRHILHRGHQQVLFVVTQADKTEPCHEWDMAGIQPSPAQAQNIRE
KTEAVFRLFRPVHPVVAVSVRTGWELDTLVSALMTALPDHAASPLMTRLQDELRTESVRA
QAREQFTGAVDRIFDTAESVCIASVACTVLRAVRDTVVSVARAVWNWIFF
>LFGLNPFC_02255 hypothetical protein
MSQMPLYFLNTQKKLTAHYEWLQINLTDTYELVKRLMPIPSLDVVVKVGKLVLPEKGHLG
FYPEAGVVYRTVAPENP
>LFGLNPFC_02256 hypothetical protein
MLDVELSVEEIACYLMVRSGRQLVDAPEIDLKYQIPKSNE
>LFGLNPFC_02257 hypothetical protein
MAIPERMSPVKVSSKKIRELAQKAKEILAAIDSGASEEDAFLKDMIDDWNSQVVCPCEFS
DFRDYSSWTNANEFTRIAFNLEKFYEDFTWEELVQTISCVCDPKSNESEKILLFCCLKRI
FMEIHLILYSGPMNGFRIQIC
>LFGLNPFC_02258 Secretory immunoglobulin A-binding protein EsiB
MGKIKYWLIVGFIILFAIFYIAISDRDSTLSRLKSAGENGDVEAQYALGLMYLYGEILDV
DYQQAKIWYEKAADQNDPRAQAKLGVMYANGLGVNQDYQQSKLWYEKAAAQNDVDAQFLL
GEMYDDGLGVSQDYQHAKMWYEKAAAQNDERAQVNLAVLYAKGNGVEQDYRQAKSWYEKA
AAQNSPDAQFALGILYANANGVEQDYQQAKDWYEKAAEQNFANAQFNLGMLYYKGEGVKQ
NFRQAREWFEKAASQNQLNAQYNLGQIYYYGQGVTQSYRQAKYWFEKAAEKGHVDAQYNL
GVIYENGEGVSQNYQQAKAWYEKAASQNDAQAQFELGVMNELGQGESIDLKQARHYYERS
CNNGLKKGCERLKELLDK
>LFGLNPFC_02259 IS66 family transposase IS682
MFFGNDHGGERSALLYGLIGACRLNGIDPEAYLRHILNVLPEWPSNRVDELLPWNVVLTQ
>LFGLNPFC_02260 Putative metal chaperone YciC
MAWSQFPVILGITLTHRCPVNMNRIPVIIINGFLGAGKTTLMKNLLTQANRNHLAVSVIV
NDMSDLDVDGVLIANTEIVNIANNNFVSITSDSISSQSGIKTVDKAIQNMLRHYLPDVIF
VETSGGSHPLPLIKYLQTHERLQLKSLLSLVDTVMLNDDYNGGKALIPAFQENLFHGKRR
IENLLAEQIMFCSTLLLTKKDRLSFDIVTDVAKAIHPLNPYVNVIAVSWGNLKLAELLTL
PDYDFNRVGLLIRELEDTITVEEAKISAQNGEIISRVIKDDRPFHPQRLWETYHYFMGMG
IYRSKGFFWLPGRPDMALLWNQAAGSINLEFISYWNSGVLADPDNHLTHEERSVLQKKVN
KTMGRFGDRRCHLTIIGRSDEVHDFTSALINCFLSEEEIVWWQSGGVFADPWPVNISRLN
>LFGLNPFC_02261 putative protein
MMKNTGYILALCLTASGHVLAHDVWITGKQSENNVTAEIGYGHNFPSKGTIPDRRDFFEN
PRLYNGKETITLKPASTDYVYKTESASKDNGYVLSTYMKPGYWSRTSSGWKPVSREGRND
VAYCEFVTKYAKSFILGEQQMPAQLYQSPTGHELEIIPLSDISRFSENVKLKVLYKTSPL
AGAIMELDSVSYLTSSRHTHAVEHKHPVHKAELTFVTNEDGIVTVPSLHIGQWLAKVQNK
KSFQDKSLCDETVDVATLSFSRN
>LFGLNPFC_02262 Hemin receptor
MYMNVIRTVICTLIILPVGLQAATSHSSMVKDTITIVATGNQNTVFETPSMVSVVTNDTP
WSQNAVTSAGMLKGVAGLSQTGAGRTNGQTFNLRGYDKSGVLVLVDGVRQLSDMAKSSGT
YLDPALVKRIEVVRGPNSSLYGSGGLGGVVDFRTADAADFLPPGETNGLSLWGNIASGDH
STGSGLTWFGKTGKTDALLSVIMRKRGNIYQSDGERAPNKEKPAALFAKGSVGITDSNKA
GASLRLYRNNTTEPGNSTQTHGDSGLRDRKTVQNDVQFWYQYAPVDNSLINVKSTLYLSD
ITIKTNGHNKTAEWRNNRTSGVNVVNRSHTLIFPGAHQLSYGAEYYRQQQKPEGSATLYP
EGNIDFTSLYFQDEMTMKSYPVNIIVGSRYDRYKSFNPRAGELKAERLSPRAAISVSPTD
WLMMYGSISSAFRAPTMAEMYRDDVHFYRKGKPNYWVPNLNLKPENNITREIGAGIQLDG
LLTDNDRLQLKGGYFGTDARNYIATRVDMKRMRSYSYNVSRARIWGWDMQGNYQSDYVDW
MLSYNRTESMDASSREWLGSGNPDTLISDISIPFGHRGVYAGWRAELSASATHVKKGDPH
QAGYTIHSFSLSYKPVSVKGFEASVTLDNAFNKLAMNGKGVPLSGRTVSLYTRYQW
>LFGLNPFC_02263 IS4 family transposase ISEc13
MVLSDCYSWDNEQSGHARLGDPRRTRRLVSLTSSLAQHAGLSIVKSSQSTAQVEGAYRLI
HNPSVSPQAIAEAGFTATVRVCEAHPLLLALEDTTTINFSHSTASDDQGNTTTNPKTRGL
LAHSVLMYAPDSALPVGFIEQQRWSRVTDTYGVKHQRKERPCEEKESYRWQQASERMAER

LGEIQKRVITVCDREADIWHYLHYKVSHGQRFVVRAAQNSRLEEAPGKLFELPEVLATAG
SHTLNVMQKGGRAARQARMFIRYSEVSIKIATTAARRSRSRMSVAGSRQRTVPAGIC
>LFGLNPFC_02264 IS4 family transposase ISEc13
MQTRDNLERMVVIKAFIAVRVLGLRQEGISEETQNDSCKKILTPTEWKLLWVKLEGKQLP
SQTPTLKWACLKLGRWHDSKRTGRPGWVVMWDGWFRLQDMVEGYLVMKSLDQEI
>LFGLNPFC_02265 hypothetical protein
MLNYSQLRERSVVLLVGNLLKESSMVNLPVRLSLDQIS
>LFGLNPFC_02267 hypothetical protein
MKDLSFIRFFLAILWLLVPPALEFPVIHRHITPGALTLCVITALIITLLASLGGRLVSVK
CISEISFIRRHRIECMMAGFMIYFWTFSLIAGWYKPQFKKEYQTLHYENAGYYVLARYDG
RLVLSQSYRNGSRKFVIINGGHRL
>LFGLNPFC_02268 IS4 family transposase IS4
MQLSRLTLRSKKPELVEQELWGVLLAYNLVRYQMIKMAEHLKGYWPNQLSFSESCGMVMR
MLMTLQGASPGRIPELMRDLASMGQLVKLPTRRERAFPRVVKERP
>LFGLNPFC_02269 Elloramycin glycosyltransferase ElmGT
MRILFVGPPLYGLLYPVLSLAQAFRVNGHEVLIASGGQFAQKAAEAGLVVFDAAPGLDSE
AGYRHHEAQRKKSNIGTQMGNFSFFSEEMADHLVEFAGHWRPDLIIYPPLGVIGPLIAAK
YDIPVVMQTVGFGHTPWHIKGVTRSLTDAYRRHNVGATPRDMAWIDVTPPSMSILENDGE
PIIPMQYVPYNGGAVWEPWWERRPERKRLLVSLGTVKPMVDGLDLIAWVMDSASEVDAEI
ILHISANARSDLRSLPSNVRLVDWIPMGVFLNGADGFIHHGGAGNTLTALHAGIPQIVFG
QGADRPVNARVVAERGCGIIPGDVGLSSNMINAFLNNRSLRKASEEVAAEMAAQPCPGEV
AKSLITMVQKG
>LFGLNPFC_02270 Multidrug efflux ATP-binding/permease protein
MPANHTPTPAQSWIVRLARVCWERKKLSVIVVVASVSTILLAALTPLLTRQAVNDALAGN
PARLPWLACGLLLIAFFDFIGNYVRRGYAGMLSLWVQHTLRGRVFDSIQKLDGAGQDALR
TGQVISRTNSDLQQVHTLLQMCPVPLAVFTYYIAGIAVMLWMSPAMTLIVVCVLVCLAIT
ALRARRRVFAQTGLASDQLANLTEHIREVLAQISVVKSCVAEMRETHWLDRQSRQIVRVR
IGAVISQAMPGATMLALPVLGQIVLLCYGGWSVMHGRIDLGTFVAFASFLAMLTGPTRVL
ASFLVIAQRTQASVERVFALIDTRSQMEDGTESINSQVVGLELENMSFDYHHGDRHILSN
ISFSLRAGETVAVVGASGSGKSTLLMLLARFYDPCSGKIWLNTSEGRQNLRDIRLEALRR
RVGIVFEDAFLFAGTVAENIAYGHPQATADDIRRAAAAAGASDFINALPKGFDSLLTERG
TNLSGGQRQRIALARALITAPDVLILDDTTSAVDAVTEAEINTALGRYADEGHMLLVIAR
RRSTLQLASRVVVLDKGRMVDTGTPAELEARCPAFRALMTGDSDFLATSHNSHNELWPAE
PATQDDVTDTGDKGFVARMTRVPENAVQQALAGKGRKVTSLLKPVAWMFVIAALLIALDS
AAGVGVLILLQHGIDSGVAAGDMSIIGLCALLALCLVIVGWCSYSLQTVFAARAAESVQH
SVRLRSFGHMLRLGLPWHEKHADSRLTRMTVDVDSLARFLQNGLAGAATSLVTMFAIAAT
MFWLDPFLALTALSAVPVAALATMIYRRLSTPAYAQARLEIGKVNSTLQEKVSGLRVVQS
HGQQELEGARLRALSERFRATRVRAQKYLAVYFPFLTFCTEASYAAVLLVGASQVAAGEM
TAGVLAAFFLLLGQFYGPVQQLSGIVDAWQQATASGKHIDELLATEGTENLGSSSVLPVT
GALHLDEVTFSYPDSHEPALNKLTLTIPEGMVVAVVGRSGAGKSTLIKLIAGLYFPTHGN
IRIGVQMLDDASLTEYRRQIGLVDQDVALFSSDIAENIRYSRPSATNEDVEIASQRAGLY
EMVCNLPQGFRTPVNNGGADLPAGQRQLIALARAQLANAHILLLDEATSCLDRTSEERLM
SSLTDVVHAGKHSALIVAHRLTTAQRCDLIAVIDKGLLAEYGTHEQLLSAGGLYTRLWHD
SVSSTALHRQHNMKEETPG
>LFGLNPFC_02271 Enterochelin esterase
MLNMQQHLSAIASLRNQLAAGHIANLTDFWREAESLNVPLVTPVEGAEDEREVTFLWRAR
HPLQGVYLRLNRVTDKEHVEKGMMSALPETDIWTLTLRLPASYCGSYSLLEIPPGTTAET
IALSGGRFATLAGKADPLNKMPEINVRGNAKESVLTLDKAPALSEWNGGFHTGQLLTSMR
IIAGKSRQVRLYIPDVDISQPLGLVVLPDGETWFDHLGVCAAIDAAINNGRIVPVAVLGI
DNINEHERTEILGGRSKLIKDIAGHLLPMIRAEQPQRQWADRSRTVLAGQSLGGISALMG
ARYAPETFGLVLSHSPSMWWTPERTSRPGLFSETDTSWVSEHLLSAPPQGVRISLCVGSL
EGSTVPHVQQLHQRLITAGVESHCAIYTGGHDYAWWRGALIDGIGLLQG
>LFGLNPFC_02272 hypothetical protein
MYAREYRSTRPHKAIFFHLSCLTLICSAQVYAKPDMRPLGPNIADKGSVFYHFSVTSFDS
VDGTRHYRVWTAVPNTTAPASGYPILYMLDGNAVMDRLDDELLKQLSEKTPPVIVAVGYQ
TNLPFDLNSRAYDYTPAAESRKTDLHSGRFSRKSGGSNNFRQLLETRIAPKVEQGLNIDR
QRRGLWGHSYGGLFVLDSWLSSSYFRSYYSASPSLGRGYDALLSRVTAVEPLQFCAKHLA
IMEGSATQGDNRETHAVGVLSKIHTTLTILKDKGVNAVFWDFPNLGHGPMFNASFRQALL
DISGENANYTAGCHELSH
>LFGLNPFC_02273 Ferric enterobactin receptor
MRINKILWSLTVLLVGLNSQVSVAKSSDDDNDETLVVEATAEQVLKQQPGVSVITSEDIK
KTPPVNDLSDIIRKMPGVNLTGNSASGTRGNNRQIDIRGMGPENTLILIDGVPVTSRNSV
RYSWRGERDTRGDTNWVPPEQVERIEVIRGPAAARYGSGAAGGVVNIITKRPTNDWHGSL
SLYTNQPESSDEGATRRANFSLSGPLAGNALTTRLYGNLNKTDADSWDINSPVGTKNAAG

HEGVRNKDINGVVSWKLNPQQILDFEAGYSRQGNIYAGDTQNSSSSAVTESLAKSGKETN
RLYRQNYGITHNGIWDWGQSRFGVYYEKTNNTRMNEGLSGGGEGRILAGEKFTTNRLSSW
RTSGELNIPLNVMVDQTLTVGAEWNRDKLDDPSSTSLTVNDSDISGISGSAADRSSKNHS
QISALYIEDNIEPVPGTNIIPGLRFDYLSDSGGNFSPSLNLSQELGDYFKVKAGVARTFK
APNLYQSSEGYLLYSKGNGCPKDITSGGCYLIGNKDLDPEISVNKEIGLEFTWEDYHASV
TYFRNDYQNKIVAGDNVIGQTASGAYILKWQNGGKALVDGIEASMSFPLVKDRLNWNTNA
TWMITSEQKDTGNPLSVIPKYTINNSLNWTITQAFSASVNWTLYGRQKPRTHAETRSEDT
GGLSGKELGAYSLVGTNFNYDINKNLRLNVGVSNILNKQIFRSSEGANTYNEPGRAYYAG
VTASF
>LFGLNPFC_02274 Transcriptional repressor MprA
MNNTDTLEKIIRHQKNKDPVYPFREHLLMQLCIRANKRMQDNISEFLGAYGINHSVYMVL
TTLFTAESHCLSPSEISQKLQFTRTNITRITDFLEKTGYVKRTDSREDRRAKKISLTSEG
MFFIQRLTLAQSMYLKEIWGYLTHDEQELFEVINKKLLAHLDDVSS
>LFGLNPFC_02275 Anti-FlhC(2)FlhD(4) factor YdiV
MSGAVNSILEVKSGRTEYILSPVFEPVWDQTGKIFAFEMLSDIRSAKDGRKICASVFFRS
ASPDIQYKILISQLKTAETLHKWCLQKKIMLSVNISRVVALYLRKHGIPGRPGTHIRLEV
SEDFPAVALKPGDDPLLRFLSERFTLWLDDFGSGNAGLMWGLSGMFERVKISHEFFHYAL
KNRCAMPLLHAVADTVACYNRGVILEGVENEALFRIARDMNVQGCQGWLYRRVEADELSA
IIELHG
>LFGLNPFC_02276 S-fimbrial protein subunit SfaH
MRKYYPLFKKSTVLKGGVLFTLIMAYSQPSFALLCRNNQTGQVFNSGDTSFRVNVSPVVQ
YDKSISVLDLSQLVSCQNEDSTGQNYDYLKILKGSGFSPALDTKTYGRLDFTSRPTGYAR
QLPLQFDLQVTEAFYQYGVWKPFPAKLYLYPAPGVFGKVINNGDLLATLYVNKFSTKGQE
AGERNFTWRFYATNDVYIQTGTCRVSSNNVKVDLPSYPGGPVTVPLTVRCDQTQSVSYTL
SGPVTGSGNTVFANTAASGSGGVGIQLSDNVGPVPAGQPRSLGQVGSSPVSLGLKASYAL
TGQASPTPGAVQSVINVTFSYN
>LFGLNPFC_02277 S-fimbrial adhesin protein SfaS
MKLKAIILATGLINCIVFSAQAVDTTITVTGNVLQRTCNVPGNVDVSLGNLYVSDFPNAG
SGSPWVNFDLSLTGCQNMNTVRATFSGTADGQTYYANTGNAGGIKIEIQDRDGSNASYHN
GMFKTLNVQNNNATFNLKARAVSKGQVTPGNISSVITVTYTYA
>LFGLNPFC_02278 S-fimbrial protein subunit SfaG
MVKDIIKTVTFSCMLAGSMFVTCHVCAAGSVVNITGNVQDNTCDVDINSRNFDVSLGSYD
SRQFTAAGDTTPASVFHVGLTSCGSAVRAVKLTFTGTPDNQEAGLIQINSINGARGVGIQ
LLDKDKHELKINVPTTIALMPGTQTIAFYARLKATYLPVKAGNVDAVVNFVLDYQ
>LFGLNPFC_02279 Outer membrane usher protein FimD
MFSGDGGQLLSDKSLTGSAGGGNNRMKFNILPLAFFIGIIVSPARAELYFNPRFLSDDPD
AVADLSAFTQGQELPPGVYRVDIYLNDTYISTRDVQFQMSQDGKQLAPCLSPEHMSAMGV
NRYAVPGMERLPADTCTSLNSMIQGATFRFDVGQQRLYLTVPQLYMSNQARGYIAPEYWD
NGITAALLNYDFSGNRVRDSYGGTSDYAYLNLKTGLNIGSWRLRDNTSWSYSAGKGYSQN
NWQHINTWLERDIVPLRSRLTMGDSYTRGDIFDGVNFRGIQLASDDNMVPDSQRGYAPTI
HGISRGTSRISIRQNGYEIYQSTLPPGPFEINDIYPAGSGGDLQVTLQEADGSVQRFNVP
WSSVPVLQREGHLKYALSAGEFRSGGHQQDNPRFAEGTLKYGLPAGWTVYGGAWIAERYR
AFNLGMGKNMGWLGAVSLDATRANARLPDESRHDGQSYRFLYNKSLTETGTNIQLIGYRY
STRGYFSFADTAWKKMSGYSVLTQDGVIQIQPKYTDYYNLAYNKRGRVQVSISQQTGESS
TLYLSGSHQSYWGTDRTDRQLNAGFNSSVNDISWSLNYSLSRNAWQHETDRILSFDVSIP
FSHWMRSDSTSAWRNASARYSQTLEAHGQAASTAGLYGTSLGDNNLGYSIQSGYTRGGYE
GSSKTGYASLNYRGGYGNASAGYSHSGGYRQLYYGLSGGILAHANGLTLSQPLGDTLILV
RAPGASDTRIENQTGVSTDWRGYAVLPYATDYRENRVALDTNTLADNVDIENTVVSVVPT
HGAVVRADYKTRVGVKVLMTLMRNGKAVPFGSVVTARNGGSSIAGENGQVYLSGMPLSGQ
VSVKWGSQTTDQCTADYKLPKESAGQILSHVTVSCR
>LFGLNPFC_02280 Chaperone protein FocC
MMKHMRIWAVLASFLVFFYIPQSYAGVALGATRVIYPEGQKQVQLAVTNNDDKSSYLIQS
WIENVEGKKDARFVITPPLFSMQGKKENTLRIIDATNGQMPEDRESLFWVNVKAIPAMDK
AKTGENYLQFAIVSRIKLLYRPQGLVIPPEQAPGKLEFTRENGGLTLFNPTPYYLTVTDL
KAGNKSLENTMVPPQGKVTVNIPGGYTGGDITYKTINDYGALTEQVRGVVK
>LFGLNPFC_02281 putative major fimbrial subunit LpfA
MRFQGKIIAEACSLALSDRQMTVDMGQLSSNRFHAAGEYGDPVGFDIHLQDCSTVVSQRV
GISFYGVSDIHEPELLSVEEENDASDGIAIALFNESGELVKLNQPPENWVHLTRGDMKLH
MQARYKATHYPVTGGKANGQVWFSLTYL
>LFGLNPFC_02282 S-fimbrial protein subunit SfaA
MKLKFISMAVFSALTLGVATSASAAPATVNGGTVHFKGEVVNAACAVNMNSVDQTVLLGQ
VRTKKLANLDDVSGPVGFNIQLDECDSTTSGSVKILFSGTPVAGKNNALAIQSSASGAAT
NVGIQILDSSGNPVTLNSDQSAVYTLTDGTNNIPFQARYIATGQSTAGTANADATFKVQY
Q

>LFGLNPFC_02283 hypothetical protein
MQCEKYTQAAFMTLSPDNIFRITDRNLLYR
>LFGLNPFC_02284 Major pilu subunit operon regulatory protein PapB
MAQHEVITRGGDAFLLKLRESALSSGSMSEEQFFLLIGISSIHSDRVILAMKDYLVSGHS
RKDVCEKYQMNNGYFSTTLGRLTRLNVLVARLAPYYTDSVSAIAETASL
>LFGLNPFC_02285 P fimbrial regulatory protein KS71A
MQNEIMGFLSRHNGGKTAEIAEALAVTDYQARYYLLLLEKEGMVQRSPLRRGMATYWFLK
GEMQAGQSCSSTT
>LFGLNPFC_02286 hypothetical protein
MDYVTSFELPFRLLLTRTSQLIVTLREAWDISQKNVVFNDKRFGCVYSLKASLSGVPDIF
RYHLSHRIRRMVGSENTSSPWQQIAREVKAPRERLKYALETGLQVIALNGLFWSDSQRLV
VDILRLSANRRDTGNRPHPHLPSYITGADTESMVVRDSVWQPREQRPEVIDRSLAPGCVC
SPRTDSLSS
>LFGLNPFC_02287 hypothetical protein
MQITEALISEPGDIRRFVQQAVDHWPNLLAFHFTLYSAEGNINGQQIHAFCTAFYRQVQE
HITERNHTASPAPPVVLRWLREQHGGATIRCLLLLSQASICHLRVSATVDEECSQVVDLL
QQAWRGINAGGQCRVERCFRVTRPDTSEQYVALKTAVQSLMPLVIATIIR
>LFGLNPFC_02288 Galactarate dehydratase (L-threo-forming)
MPEPLPPLEDYTFEGYRNADGSVGTKNLLGITTSVHCMADVVNYVVKIIERNLLPKYPSI
DGVVDLNHLYGCGVAINAPTAVVPIRTIHNIALNPNFGGEVMVVGLGCEKLQP
>LFGLNPFC_02289 Galactarate dehydratase (L-threo-forming)
MHCGGSDAFSGVTANPAVGYASDLLVRCGATVMFSEVTDVHDAIHLLTPRAINEEVGRCL
LEEMA
>LFGLNPFC_02290 Galactarate dehydratase (L-threo-forming)
MGKTDRSANPSPGNKKGGLANVVKKALGSIAKSGKTAIVEVLSPGQHPTKRGLIYAATPA
SDFICGTQQVASGITVQVFTTGRGTPYGLMAVPVIKMATRTGQANRWFDLMDINAGTIAT
GEETIEEVGWKLFHFILDVASGKKKTFSDQWGLRNQLAVFNPAPVV
>LFGLNPFC_02291 Glucitol operon repressor
MLFLGVDAIDLECGISTHNEDEARLNRRMCEVAERIIVVTDSSKFNRSSLHKIIDTQRID
MIIVDEGIPADSLEGLRKAGVEVILVGELASSL
>LFGLNPFC_02292 hypothetical protein
MSNTDASGEKRVTGTSERREQIIQRLRQQGSVQVNDLSALYGVSTVTIRNDLAFLEKQGI
AVRAYGGALICDSTTPSVAIFLR
>LFGLNPFC_02293 D-tagatose-1,6-bisphosphate aldolase subunit KbaZ
MKHLTEMVRQHKAGKTNGIYAVCSAHPLVLEAAIRYASANQTPLLIEATSNQVDQFSGYT
GMTPADFRGFVCQLADSLNFPQDALILGGDHLRPKSLVDSETLIVVYISSHPYTRQYDLG
LLTELRRDRQAMRVIAIAVETDAIIEAGPHILLPPSRSFIDMEQAFCFLMYAQVFALAQS
IHVGNTPDLPSASGTINRVVQGVIIHP
>LFGLNPFC_02294 D-tagatose-1,6-bisphosphate aldolase subunit KbaY
MSIISTKYLLQDAQANAYAVPTFNIHNAETIQAILEVCSEMRSPVILAGPPRLVQTYCPG
RDLRPVQCVFDHLRHAASTASRSSRIVG
>LFGLNPFC_02295 Cystathionine beta-lyase PatB
MFDFDKIIERQSDKCRKWDHAFVCSRFGDVPESFIPLWIADMDFTSPPAVIDGFRRIVEH
GTFGYTWCFDEFYDAVIAFQRKRHQVEVEKSWITLTYGTVSTLHYTIQAYCKPGDSVMMN
TPVYDPFAMAAQRQGVQVLANPLRVEENRYQLDFNLIEEQLKTYRPTLWFFCSPHNPSGR
IWREEEIRQVSDLCQRYSTILVVDEVHAEHILDGKFVSCLTSGCAAQDNLIVLTSPNKAL
KLGGLKTSYSMIPDDSLRQRFRQQLEKNSITSPNLFGVWGIILAYQHGLPWLDALNGYLQ
GNARYLADALQTYFPAWKMMTPESSYLAWIDVSADESSATQLTQHFARQAGVVIEDGSHY
VQNGENYLQINFGSQRYWLERSTNRMQ
>LFGLNPFC_02296 hypothetical protein
MKKVLTLSLLALCVSHSAVAANYTFNNDNIALSFDDTNSTIVLKDRRTNHPITPQELFFL
TLPDETKIHTADFKIKHIKKQDNAIVIDFTRPDFNVTVQLNLVKGKYASIDYTIAAVGQP
RDVAKITFFPTKKQFQAPYVDGAITSSPIIADSFFILPNKPIVNTYAYEATTNLNVELKT
PIQPETPVSFTTWFGTFPETSQLRRSVNQFINAVRPRPYKPYLHYNSWMDIGFFTPYTEQ
DVLGRMDEWNKEFISGRGVALDAFLLDDGWDDLTGRWLFGPAFSNGFSKVREKADSLHSS
VGLWLSPWGGYNKPRDVRVSHAKEYGFETVDGKLALSGANYFKNFNEQIINLIKNEHITS
FKLDGMGNASSHIKGSPFASDFDASIALLHNMRRANPNLFINLTTGTNASPSWLFYADSI
WRQGDDINLYGPGTPVQQWITYRDAETYRSIVRKGPLFPLNSLMYHGIVSAENAYYGLEK
VQTDSDFADQVWSYFATGTQLQELYITPSMLNKVKWDTLAKAAKWSKENASVLVDTHWIG
GDPTALAVYGWASWSKDKAILGLRNPSDKPQTYYLDLAKDFEIPAGNAAQFSLKAVYGSN
KTVPVEYKNATVITLQPLETLVFEAVPIN
>LFGLNPFC_02297 Ribosomal RNA small subunit methyltransferase I
MAPLNDALYRYVMNTRLGTIHGMSVGELLAWIKEDENPSKGEMVLIIEGHKAQDDELPAD
ALRTLALL

>LFGLNPFC_02298 hypothetical protein
MYNANPNYEMDFMILKDVNEHMEGLFQRFSKLLPFRIDFAYRKDTPSFGHSCRHSMCIEM
HRLLSETQTMLAGYYWVMEYTSNKGLHIHFIGYLDGGQRHKKSYRISRQLGDIWRRITEGE
GYFHLCRAKDKYPVRIDHVIHYSDKSAVDGLRYALSYLAKQDQKEHGIILRRSRLSEKSN
RGRPRLNSILPGICSQL
>LFGLNPFC_02299 hypothetical protein
MLRIQKRRTTALINEVTSSTDNLIKLFRITSLQ
>LFGLNPFC_02300 hypothetical protein
MCLLAPENPYPIYALPPLVRNAIIETQKNTQAPLAMVATSALTATSIACQNQVDVCRPGN
LRGPVNLYSLILADSGERKTTVDKVFMKAFYLRDEALAEEYAKLVENYSTEKEIWEQKQK
ALESKLHKEIRAGKGCKATESELKRHLNKCPVPPQIRRTIFNETTIEGMLKYYSDSNRSF
ALVSSEGGIIFDGRAMSKLGILNSLWDGGSLFIDRKSSPGIILKDPRLTVSVMIQPDVYQ
KGFCTRKKELVKTSGHHARFLMCQPTSTQGTRIITGDNYSSQYQDLFEQRINELIDESLA
MSGERRCLHFSPQAARIWTDYYNDVESKLGGLGPLRHCREYAAKNAEYMARLAGLIHHSS
GEEGDISPYTAEMARELAIWYGNEYVRLSNPLTFDNPALTVPVRLIPEELELFNWIKSYC
IEKGIPCMKKNDILQRGPNRFRKKDKINWLLDLLYEQNRVVPVIEGKTLCVAPNFDL
>LFGLNPFC_02301 hypothetical protein
MTKTYLFMVISEESVCRTSSLTPLTADLAGTVCKVSHLSIADERQPTEPLPRRTGLGKVA
S
>LFGLNPFC_02302 hypothetical protein
MYNFITIMYDVFSCFGVLAKNQNTRDIRNIKNFSSHQHSLGDMFDELINIIDKEQVLSTE
QRKVIFRRYEDLYVKLMHYSVFTDKTHQIIKQKYFNDIVPMILALDIRNTYRPDNEMAFY
YHIHSFLTQIPDNEDDIYHAARTYLRNYVKLCLSGYTPANAHFKDIFDGVYEFIRNIRKN
STPGKTKLIATINTCKETCKHLLYLSNEDKEKIISDLDKIQVACYYLTILLAFERRTSLT
STLATLYKMLISEREVSEYECQLLYLTNPIDVMNILNKYIYYFPNENSPFYTLKIDSALS
WDAIDAIRDYSISDIYLYPEQKTINCVVEIENIVFGGYIYTLNNGVTLQNIENTLKDSSC
HYVLNGYTEFVNCLRQLTSGKTESVHRTINKLNYEKLPFGFIIAAFAILKIAFKIKFSKN
HVNIRALLNDINYFMTYQGESINLISLDHEYPESCLQNDTNTYLLGRVIFLYNSMIYKFI
NCQEHETNNIHSAMINNLLQEVDIALGKINDIIDSRNISAPHELANILTREKILTTREKK
GNLISLFDGFTLFHCVGMITFLIHYLRTPEEKVENIFMLYGADKNNKLRRRLIYDALGII
QSQQE
>LFGLNPFC_02303 Prophage integrase IntA
MAVLTDTKARHIKPDDKPLPHGGITGLTLHPSSVKGRGKWVFRYVSLVTQKRRNAGLGTY
PEVSIAEAARTARIMREQLAAGDDPLEIKKAESEKVVIPTFADAARRVHAELSPGWENPK
HVRQWLSTLENYAFPQLGAKTLDSITAADVAETLRPVWLTLSETASRVKQRIHVVMQWGW
AHGFCVANPVDVVDHLLPQQTRGRDEHQPAMPWRQLPLFVATSVYTDEPYNVTRALLLMV
ILTATRSGEARGMRWAEIDFHKRVWTIPAERMKARLQHRVPLSRQAIYILENIRGLHDEL
VFPSPRKQQILSDMVLTSFLRKKKAVSDIPGRVATAHGFRSTFRDWCSEQGYSRDLAERA
LAHTLKNKVEAAYHRTDLLEQRVPMMQAWADYVMSQIVNK
>LFGLNPFC_02304 hypothetical protein
MSLSLVVDLRLGLLIPPVYAIYEKKARTFVRALL
>LFGLNPFC_02305 putative diguanylate cyclase DgcT
MEKDYLGISSTVLVSLLFGLALVLVNSWFNQPTVEEVVPRSTYLMVMIALFFIDTVAFIF
MQLYFIYDRRQFSNCILSLAFLSCLIYFIKTVIIIQQIIEGRLTSSVVQNDIAIYYLFRQ
MSLCILIFLALVNKVSENTKQRNLFSKKMTLCISLFFVVGGPIVAHILSSHYESYNLHIA
ELTNENDQVVWKTSYVTIMIFMWLTLLSVNLYFNGLRCDIWNGVTVIAFCAVLYNVSLLF
MSRYSVSIWYISRTIEVVSKLTVMVIFMCHIFSALRVTKDIAHRDSLTNIFNRNYFFNEL
TVQSASAKKTPYCVMIMDIDHFKKVNDTWGHPVGDQVIKTVVSIIGKSIRPDDLFARVGG
EEFGVLLTDIDTERAKALAERIRENVERLTGDNPEYAIPQKVTISIGAVVTQKNELNPKE
IYQLADNALYEAKETGRNKVVVKEAEILINRKDDE
>LFGLNPFC_02306 Poly-beta-1,6-N-acetyl-D-glucosamine export protein
MYSSSRKRCPKTKWALKLLTAAFLAASPAAKSAVNNAYDALIIEARKGNTQPALLWFAQK
SALSNNQIADWLQIALWAGQDKQVITVYNRYRHQQLPARGYAAVAVAYRNLQQWQNSLTL
WQKALSLESQNKDYQRGQILTLADAGHYDSALVKLKQLNSGAPDKANLLAEAYIYKLAGR
HEDELRAMTGSLPENALTQQYPTEYVQALRNNQLAAAIDDANLTPDIRADIHAELVRLSF
MPTRSESERYAIADRALAQYAALEILWHDNSDRTAQYQRIQVDHLGALLTRDRYKDVISH
YQRLKNTRQIIPPWAQYWVASAYLKEQQPKKAQSIMTELFYHKETIAPDLSDEELADLFY
SHLESENYPGALTVTQHTINTSPPFLRLMGTPTSIPNDTWLQGHSFLSTVAKYSNDLPQA
EMIARELAYNAPGNQGLRIDYASVLQARGWHRAAENELKKAEVIEPRNINLEVEQAWTAL
TLQEWQQAAVLTHDVVEREPQDPGVVRLKRAVDVHNLAELRIAGSTGIDAEGPDSGKHDV
DLTTIVYSPPLKDNWRGFAGFGYADGQFSEGKGIVRDWLAGVEWRSRNIWLEAEYAERVF
NHEHKPGARLSGWYDFNDNWRIGSQLERLSHRVPLRAMKNGVTGNSAQAYVRWYQNERRK
YGVSWAFTDFSDSNQRQEVSLEGGQERIWSSPYLIVDFLPNLYYEQNTEHDTPYYNPIKTF
DIVPAFEASHLLWRSYENSWEQIFSAGVGASWQKHYGTDVVTQLGYGQRISWNDVIDAGA

TLRWEKRPYDGDREHNLYVEFDMTFRF
>LFGLNPFC_02307 Poly-beta-1,6-N-acetyl-D-glucosamine N-deacetylase
MLRNGNKYILMLVSIIMLTACISQSRTSFIPPQDRESLLAEQPWPHNGFVAISWHNVEDE
AADQRFMSVRTSALREQFAWLRENGYQPVSIAQIREAHRGGKPLPEKAVVLTFDDGYQSF
YTRVFPILQAFQWPAVWAPVGSWVDTPADEQVKFGDEMVDREYFATWQQVREVARSRLVE
VASHTWNSHYGIQANATGSLLPVYVNRAYFTDHARYETAAEYRERIRLDAVKMTEYLRTK
AKVNPHVFVWPYGEANGIAIEELKKLGYDMFFTLESGLANASQLDSIPRVLIANNPSLKE
FAQQIITVQEKSPQRIMHIDLDYVYDENHQQMDRNIDVLIQRVKDMQISTVYLQAFADPD
GDGLVKEVWFPNRLLPMKADIFSRVAWQLRTRSGVNIYAWMPVLSWDLDPTLTRVKYLPT
GEKKAQIHPEQYRRLSPFDDRVRAQVGMLYEDLAGHAAFDGILFHDDALLSDYEDASAPA
ITAYQQAGFSGSLSEIRQNPEQFKQWTRFKSRALTDFTLELSARVKAIRGPHVITARNIF
ALPVIQPESEAWFAQNYADFLKSYDWTAIMAMPYMEGVAEKSADQWLIQLTNQIKNIPQA
KDKSILELQAQNWQKNGQHQAISSQQLAHWMSLLQLNGGKNYGYYPDNFLHNQPEIDVIR
PEFSTAWYPKND
>LFGLNPFC_02308 Poly-beta-1,6-N-acetyl-D-glucosamine synthase
MINRIVSFFILCLVLCIPLCVAYFHSGELMMRFVFFWPFFMSIMWIVGGVYFWVYRERHW
PWGENAPAPQLKDNPSISIIIPCFNEEKNVEETIHAALAQRYENIEVIAVNDGSTDKTRA
ILDRMAAQIPHLRVIHLAQNQGKAIALKTGAAAAKSEYLVCIDGDALLDRDAAAYIVEPM
LYNPRVGAVTGNPRIRTRSTLVGKIQVGEYSSIIGLIKRTQRIYGNVFTVSGVIAAFRRS
ALAEVGYWSDDMITEDIDISWKLQLNQWAIFYEPRALCWILMPETLKGLWKQRLRWAQGG
AEVFLKNMTRLWRKENFRMWPLFFEYCLTTIWAFTCLVGFIIYAVQLAGVPLNIELTHIA
ATHTAGILLCTLCLLQFIVSLMIENRYEHNLTSSLFWIIWFPVIFWMLSLATTLVSFTRV
MLMPKKQRARWVSPDRGILRG
>LFGLNPFC_02309 Biofilm PGA synthesis protein PgaD
MNNLIITTRQSPVRLMVDYVATTILWTLFALFIFLFAMDLLTGYYWQSEARSRLQFYFLL
AVANAVVLIVWALYNKLRFQKQQHHAAYQYTPQEYAESLAIPDELYQQLQKSHRMSVHFT
SQGQIKMVVSEKALVRA
>LFGLNPFC_02310 Protein PhoH
MGRQKAVIKARREAKRVLRRDSRSHKQREEESVTSLVQMSGVEAIGMARDSRDTSPILAR
NEAQLHYLKAIESKQLIFATGEAGCGKTWISAAKAAEALIHKDVDRIIVTRPVLQADEDL
GFLPGDIAEKFAPYFRPVYDVLVRRLGASFMQYCLRPEIGKVEIAPFAYMRGRTFENAVV
ILDEAQNVTAAQMKMFLTRLGENVTVIVNGDITQCDLPRGVRSGLSDALERFEEDEMVGI
VRFGKEDCVRSALCQRTLHAYS
>LFGLNPFC_02311 hypothetical protein
MVTSCSGQCFVKQRSTTCGDFSSGSNFVTLYFSHFLSLLVLPTPQTVCVAVFRHFIQKQT
MAYKEANPLMFVRIIALPTARVMNTNNSRCSPWEDKKQ
>LFGLNPFC_02312 Deferrochelatase/peroxidase EfeB
MQYEDENGVNEPSRRRLLKGIGALALAGSCPVAHAQKTQSAPGTLSPDARNEKQPFYGEH
QAGILTPQQAAMMLVAFDVLASDKADLERLFRLLTQRFAFLTQGGAAPETPNPRLPPLDS
GILGGYIAPDNLTITLSVGHSLFDERFGLAPQMPKKLQKMTRFPNDSLDAALCHGDVLLQ
ICANTQDTVIHALRDIIKHTPDLLSVRWKREGFISDHAARSKGKETPINLLGFKDGTANP
DSQNDKLMQKVVWVTADQQEPAWTIGGSYQAVRLIQFRVEFWDRTPLKEQQTIFGRDKQT
GAPLGMLHEHDVPDYASDPEGKVIALDSHIRLANPRTAESESSLMLRRGYSYSLGVTNSG
QLDMGLLFVCYQHDLEKGFLTVQKRLNGEALEEYVKPIGGGYFFALPGVKDANDYLGSAL
LRV
>LFGLNPFC_02313 Iron uptake system component EfeO
MTINFRRNALQLSVAALFSSAFMANAADIPQVKVTVTDKQCEPMTITVNAGKTQFIIQNH
SQKALEWEILKGVMVVEERENIAPGFSQKMTANLQPGEYDMTCGLLTNPKGKLIVKGEAT
ADAAQSDALLSLGGAITAYKAYVMAETTQLVTDTKAFTDAIKAGDIEKAKALYAPTRQHY
ERIEPIAELFSDLDGSIDAREDDYEQKAADPKFTGFHRLEKALFGDNTTKGMDKYADQLY
TDVVDLQKRISELAFPPSKVVGGAAGLIEEVAASKISGEEDRYSHTDLWDFQANVEGSQK
IVDLLRPQLQKANPELLAKVDANFKKVDTILAKYRTKDGFENYDKLTDADRNALKGPITA
LAEDLAQLRGVLGLD
>LFGLNPFC_02314 Ferrous iron permease EfeU
MFVPFLIMLREGLEAALIVSLIASYLKRTQRGRWIGVMWIGVLLAAALCLGLGIFINETT
GEFPQKEQELFEGIVAVIAVVILTWMVFWMRKVSRNVKVQLEQAVDSALQRGNHHGWALV
MMVFFAVAREGLESVFFLLAAFQQDVGIWPPLGAMLGLATAVVLGFLLYWGGIRLNLGAF
FKWTSLFILFVAAGLAAGAIRAFHEAGLWNHFQEIAFDMSAVLSTHSLFGTLMEGIFGYQ
EAPSVSEVAVWFIYLIPALVAFVLPPRAGATASRSV
>LFGLNPFC_02315 Sodium/proline symporter
MAISTPMLVTFCVYIFGMILIGFIAWRSTKNFDDYILGGRSLGPFVTALSAGASDMSGWL
LMGLPGAVFLSGISESWIAIGLTLGAWINWKLVAGRLRVHTEYNNNALTLPDYFTGRFED
KSRILRIISALVILLFFTIYCASGIVAGARLFESTFGMSYETALWAGAAATILYTFIGGF
LAVSWTDTVQASLMIFSLILTPVIVIISVGGFGDSLEVIKQKSIENVDMLKGLNFVAIIS

LMGWGLGYFGQPHILARFMAADSHHSIVHARRISMTWMILCLAGAVAVGFFGIAYFNEHP
AVAGAVNQNAERVFIELAQILFNPWIAGILLSAILAAVMSTLSCQLLVCSSAITEDLYKA
FLRKQASQKELVWVGRVMVLVVALVAIALAANPENRVLGLVSYAWAGFGAAFGPVVLFSV
MWSRMTRNGALAGMIIGALTVIVWKQFGWLGLYEIIPGFIFGSIGIVVFSLLGKAPSAAM
QKRFAEADAHYHSAPPSRLQEG
>LFGLNPFC_02316 Bifunctional protein PutA
MGTTTMGVKLDDATRERIKSAATRIDRTPHWLIKQAIFSYLEQLENSDTLPELPALLSGA
ANESDEAPTPAEEPHQPFLDFAEQILPQSVSRAAITAAYRRPETEAVSMLLEQARLPQPV
AEQAHKLAYQLADKLRNQKNASGRAGMVQGLLQEFSLSSQEGVALMCLAEALLRIPDKAT
RDALIRDKISNGNWQSHIGRSPSLFVNAATWGLLFTGKLVSTHNEASLSRSLNRIIGKSG
EPLIRKGVDMAMRLMGEQFVTGETIAEALANARKLEEKGFRYSYDMLGEAALTAADAQAY
MVSYQQAIHAIGKASNGRGIYEGPGISIKLSALHPRYSRAQYDRVMEELYPRLKSLTLLA
RQYDIGINIDAEEADRLEISLDLLEKLCFEPELAGWNGIGFVIQAYQKRCPLVIDYLIDL
ATRSRRRLMIRLVKGAYWDSEIKRAQMDGLEGYPVYTRKVYTDVSYLACAKKLLAVPNLI
YPQFATHNAHTLAAIYQLAGQNYYPGQYEFQCLHGMGEPLYEQVTGKVADGKLNRPCRIY
APVGTHETLLAYLVRRLLENGANTSFVNRIADTSLPLDELVADPVTAVEKLAQQEGQTGL
PHPKIPLPRDLYGHGRDNSAGLDLANEHRLASLSSALLNSALQKWQSLPMLEQSVAAGEM
SPVINPAEPKDIVGYVREATPREVEQALESAVNNAPIWFATPPAERAAILHRAAVLMESQ
MQQLIGILVREAGKTFSNAIAEVREAVDFLHYYAGQVRDDFANETHRPLGPVVCISPWNF
PLAIFTGQIAAALAAGNSVLAKPAEQTPLIAAQGIAILLEAGVPPGVVQLLPGRGETVGA
QLTGDDRVRGVMFTGSTEVATLLQRNIASRLDAQGRPIPLIAETGGMNAMIVDSSALTEQ
VVIDVLASAFDSAGQRCSALRVLCLQDEIADHTLKMLRGAMAECRMGNPGRLTTDIGPVI
DSEAKANIERHIQTMRSKGRQVFQAVRENSEDAREWQSGTFVAPTLIELDDFAELQKEVF
GPVLHVVRYNRNQLPELIEQINASGYGLTLGVHTRIDETIAQVTGSAQVGNLYVNRNMVG
AVVGVQPFGGEGLSGTGPKAGGPLYLYRLLANRPESALAVTLARQDAEYPVDAQLKAALT
QPLNALREWAANRPELQALCTQYGELAQAGTQRLLPGPTGERNTWTLLPRERVLCIADDE
QDALTQLAAVLAVGSQMLWPDDALHRQLVKALPSAVSERIQLAKAENITAQPFDAVIFHG
DSDQLRALCEAVAARDGAIVSVQGFARGESNILLERLYIERSLSVNTAAAGGNASLMTIG
>LFGLNPFC_02317 HTH-type transcriptional regulator RutR
MTQGAVKTTGKRSRAVSAKKKAILSAALDTFSQFGFHGTRLEQIAELAGVSKTNLLYYFP
SKEALYIAVLRQILDIWLAPLKAFREDFAPLAAIKEYIRLKLEVSRDYPQASRLFCMEML
AGAPLLMDELTGDLKALIDEKSALIAGWVKSGKLAPIDPQHLIFMIWASTQHYADFAPQV
EAVTGVTLRDEVFFNQTVENVQRIIIEGIRPR
>LFGLNPFC_02318 Pyrimidine monooxygenase RutA
MKIGVFVPIGNNGWLISTHAPQYMPTFELNKAIVQKAEHYHFDFALSMIKLRGFGGKTEF
WDHNLESFTLMAGLAAVTSRIQIYATAATLTLPPAIVARMAATIDSISGGRFGVNLVTGW
QKPEYEQMGIWPGDDYFSRRYDYLTEYVQVLRDLWGSGKSDFKGDFFTMDDCRVSPQPSV
PMKVICAGQSDAGMAFSARYADFNFCFGKGVNTPTAFAPTAARMKQAAEQTGRDVGSYVL
FMVIADETDDAARAKWEHYKAGADEEALSWLTEQSQKDTRSGTDTNVRQMADPTSAVNIN
MGTLVGSYASVARMLDEVASVPGAEGVLLTFDDFLSGIENFGERIQPLMQCRAHLPALTQ
EVA
>LFGLNPFC_02319 Peroxyureidoacrylate/ureidoacrylate amidohydrolase RutB
MMTTLTARPEAITFDPQQTALIVVDMQNAYATPGGYLDLAGFDVSTTRPVIANIQTAVTA
ARTAGMLIIWFQNGWDEQYVEAGGPGSPNYHKSNALKTMRNQPLLQGKLLAKGSWDYQLV
DELVPQPGDIVLPKPRYSGFFNTPLDSILRSRGIRHLVFTGIATNVCVESTLRDGFFLEY
FGVVLEDATHQAGPEFAQKAALFNIETFFGWVSDVETFCDALSSTSFARIA
>LFGLNPFC_02320 Putative aminoacrylate peracid reductase RutC
MPKSVIIPAGSSAPLAPFVPGTLADGVVYVSGTLAFDQHNNVLFADDPKAQTRHVLETIR
KVIETAGGTMADVTFNSIFITDWKNYAAINEIYAEFFPGDKPARFCIQCGLVKPDALVEI
ATIAHIAK
>LFGLNPFC_02321 Putative aminoacrylate hydrolase RutD
MKLSLSPPPYADAPVVVLISGLGGSGSYWLPQLAVLVQEYQVVCYDQRGTGNNPDTLAED
YSIAQMAAELHQALVAAGIERYAVVGHALGALVGMQLALDYPASVTVLVSVNGWLRINAH
TRRCFQVREQLLHSGGAQAWVEAQPLFLYPADWMAARAPRLEAEDALALAHFQGKNNLLR
RLNALKRADFSHHADRIRCPVQIICASDDLLVPTACSSELHAALPDSQKMVMRYGGHACN
VTDPETFNALLLNGLASLLHHREAAL
>LFGLNPFC_02322 putative malonic semialdehyde reductase RutE
MNEAVSPGALSTLFTDARTHNGWRETPVSDETLREIYALMKWGPTSANCSPARIVFIRTA
EGKERLRPALSSGNLQKTLTAPVTAIVAWDSEFYERLPQLFPHGDARSWFTSSPQLAEET
AFRNSSMQAAYLIFACRALGLDTGPMSGFDRQYVDDAFFAGSTLKSNLLINIGYGDSSKL
FARLPRLSFEEACGLL
>LFGLNPFC_02323 FMN reductase (NADH) RutF
MNIVDQQTFRDAMSCMGAAVNIITTDGPAGRAGFTASAVCSVTDTPPTLLVCLNRGASVW
PVFNENRTLCVNTLSAGQEPLSNLFGGKTPMELRFAAARWQTGVTGCPQLEEALVSFDCR

ISQVVSVGTHDILFCAIEAIHRHATPYGLVWFDRSYHALMRPAC
>LFGLNPFC_02324 Putative pyrimidine permease RutG
MAMFGFPHWQLKSTSTESGVVAPDERLPFAQTAVMGVQHAVAMFGATVLMPILMGLDPNL
SILMSGIGTLLFFFITGGRVPSYLGSSAAFVGVVIAATGFNGQGINPNISIALGGIIACG
LVYTVIGLVVMKIGTRWIERLMPPVVTGAVVMAIGLNLAPIAVKNVSASAFDSWMA
>LFGLNPFC_02325 Putative pyrimidine permease RutG
MTNVLGLGKAVDFTLVSHAAWFGLPHFSTPAFNGQAMMLIAPVAVILVAENLGHLKAVAG
MTGRNMDPYMGRAFVGDGLATMLSGSVGGSGVTTYAENIGVMAVTKVYSTLVFVAAAVIA
MLLGFSPKFGALIHTIPAAVIGGASIVVFGLIAVAGARIWVQNSVDLSQNGNLIMVAVTL
VLGAGDFALTLGGFTLGGIGTATFGAILLNALLSRRLVDVPPPEVVHQKP
>LFGLNPFC_02326 hypothetical protein
MANHRGGSGNFAEDRERASEAGKKGGQHSGGNFKNDPQRASEAGKKGGKSSHGKSDN
>LFGLNPFC_02327 NAD(P)H dehydrogenase (quinone)
MAKVLVLYYSMYGHIETMARAVAEGASKVDGAEVVVKRVPETMPPQLFEKAGGKTQTAPV
ATPQELADYDAIIFGTPTRFGNMSGQMRTFLDQTGGLWASGALYGKLASVFSSTGTGGGQ
EQTITSTWTTLAHHGMVIVPIGYAAQELFDVSQVRGGTPYGATTIAGGDGSRQPSQEELS
IARYQGEYVAGLAVKLNG
>LFGLNPFC_02328 putative protein YccJ
MPTQEAKAHHVGEWASLRNTSPEIAEAIFEVAGYDEKMAEKIWEEGSDEVLVKAFAKTDK
DSLFWGEQTIERKNV
>LFGLNPFC_02329 Glucose-1-phosphatase
MNKTLIAATVAGIVLLASNAQAQTVPEGYQLQQVLMMSRHNLRAPLANNGSVLEQSTPNK
WPEWDVPGGQLTTKGGVLEVYMGHYMREWLAEQGMVKSGECPPPDTVYAYANSLQRTVAT
AQFFITGAFPGCDIHVHHQEKMGTMDPTFNPVITDDSAAFSEQAVAAMEKELSKLQLTDS
YKLLEQIVNYKDSPACKEKQQCSLVDGKNTFSAKYQQEPGVSGPLKVGNSLVDAFTLQYY
EGFPMDQVAWGEIKSDQQWKVLSKLKNGYQDSLFTSPEVARNVAKPLVSYIDKALVTDRT
SAPKITVLVGHDSNIASLLTALDFKPYQLHDQNERTPIGGKIVFQRWHDSKANRDLMKIE
YVYQSAEQLRNADALTLQAPAQRVTLELSGCPIDANGFCPMDKFDSVLNEAVK
>LFGLNPFC_02330 Curved DNA-binding protein
MELKDYYAIMGVKPTDDLKTIKTAYRRLARKYHPDVSKEPDAEARFKEVAEAWEVLSDEQ
RRAEYDQMWQHRNDPQFNRQFHHGDGQSFNAEDFDDIFSSIFGQHARQSRQRPATRGHDI
EIEVAVFLEETLTEHKRTISYNLPVYNAFGMIEQEIPKTLNVKIPAGVGNGQRIRLKGQG
TPGENGGPNGDLWLVIHIAPHPLFDIVGQDLEIVVPVSPWEAALGTKVTVPTLKESILLT
IPPGSQAGQRLRVKGKGLVSKKQTGDLYAVLKIVMPPKPDENTAALWQQLADAQSSFDPR
KDWGKA
>LFGLNPFC_02331 Chaperone modulatory protein CbpM
MANVTVTFTITEFCLHTGISEEELNEIVGLGVVEPSEIQETTWVFDDHAAIVVQRAVRLR
HELALDWPGIAVALTLMDDIAHLKQENRLLRQRLSRFVAHP
>LFGLNPFC_02332 Chaperone protein TorD
MTTLTVQQIACVYAWLAQLFSRELDDEQLTQIASAQMAEWFSLLKSEPPLTAAVNGLENS
IATLTVRDDARLELAADFCGLFLMTDKQAALPYASAYKQDEQEIKRLLVEAGMETSGNFN
EPADHLAIYLELLSHLHFSLGEGTVPARRIDGLRQKTLTALREWLPEFAARCRQYDSFGF
YAALSQLLLVLVECDYQKR
>LFGLNPFC_02333 Trimethylamine-N-oxide reductase 1
MNNNDLFQASRRRFLAQLGGLTVAGMLGPSLLTPRRATATQAATEAVISKEGILTGSHWG
AIRATVKDGRFVAAKPFELDKYPSKMIAGLPDHVHNAARIRYPMVRVDWLRKRHLSDTSQ
RGDNRFVRVSWDEALDMFYEELERVQKTHGPSALLTASGWQSTGMFHNASGMLAKAIALH
GNSVGTGGDYSTGAAQVILPRVVGSMEVYEQQTSWPLVLQNSKTIVLWGSDLLKNQQANW
WCPDHDVYEYYEQLKAKVAAGEIEVISIDPVVTSTHEYLGREHVKHIAVNPQTDVPLQLA
LAYTLYSENLYDKNFLANYCVGFEQFLPYLLGEKDGQPKDAAWAEKLTGIDAETIRGLAR
QMAANRTQIIAGWCVQRMQHGEQWAWMIVVLAAMLGQIGLPGGGFGFGWHYNGAGTPGRK
GVILSGFSGSTSIPPVHDNSDYKGYSSTIPIARFIDAILEPGKVINWNGKSVKLPPLKMC
IFAGTNPFHRHQQINRIIEGWRKLETVIAIDNQWTSTCRFADIVLPATTQFERNDLDQYG
NHSNRGIIAMKQVVPPQFEARNDFDIFRELCRRFNREEAFTEGLDEMGWLKRIWQEGVQQ
GKGRGVHLPAFDDFWNNKEYVEFDHPQMFVRHQAFREDPDLEPLGTPSGLIEIYSKTIAD
MNYDDCQGHPMWFEKIERSHGGPGSQKYPLHLQSVHPDFRLHSQLCESETLRQQYTVAGK
EPVFISPQDASARGIRHGDVVRVFNVRGQVLAGAVVSDRYAPGVARIHEGAWYDPDKGGE
PGALCKYGNPNVLTIDIGTSQLAQATSAHTTLVEIEKYNGTVEQVTAFNGPVEMVAQCEY
IPASQVKS
>LFGLNPFC_02334 Cytochrome c-type protein TorC
MRKLWNALRRPSARWSVLALVATGIVIGIALIVLPHVGIKVTSTTEFCVSCHSMQPVYEE
YKQSVHFQNASGVRAECHDCHIPPDIPGMVKRKLEASNDIYQTFIAHSIDTPEKFEAKRA
ELAEREWARMKENNSATCRSCHNYDAMDHAKQHPEAARQMKVAAKDNQSCIDCHKGIAHQ
LPDMSSGFRKQFDELRASANDSGDTLYSIDIKPIYAAKGDKEASGSLLPASEVKVLKRDG

DWLQIEITGWTESAGRQRVLTQFPGKRIFVASIRGDVQQQVKTLEKTTVADTNTEWSKLQ
ATAWMKKGDMVNDIKPIWAYADSLYNGTCNQCHGAPEISHFDANGWIGTLNGMIGFTSLD
KREERTLLKYLQMNASDTAGKAHGDKKEEK
>LFGLNPFC_02335 TorCAD operon transcriptional regulatory protein TorR
MPHHIVIVEDEPVTQARLQSYFTQEGYTVSVTASGAGLREIMQNQPVDLILLDINLPDEN
GLMLTRALRERSTVGIILVTGRSDRIDRIVGLEMGADDYVTKPLELRELVVRVKNLLWRI
DLARQAQPHTQDNCYRFAGYCLNVSRHTLERDGEPIKLTRAEYEMLVAFVTNPGEILSRE
RLLRMLSARRVENPDLRTVDVLIRRLRHKLSADLLVTQHGEGYFLAADVC
>LFGLNPFC_02336 Periplasmic protein TorT
MRVLLFLLLSLFMLSAFSADNLLRWHDAQHYTVQASTPLKAKRAWKLCALYPSLKDSYWL
SLNYGMQEAARRYGVDLKVLEAGGYSQLATQQAQIDQCKQWGAEAILLGSSTTSFPDLQK
QVANLPVIELVNAIDAPQVKSRVGVPWFQMGYQPGRYLVQWAHGKPLNVLLMPGPDNAGG
SKEMVEGFRAAIAGSPVRIVDIALGDNDIEIQRNLLQEMLERHPEIDVVAGTAIAAEAAM
GEGRNLKTPLTVVSFYLSHQVYRGLKRGRVIMAASDQMVWQGELAVEQAIRQLQGQSVSD
NVSPPILVMTPKNADREHIRRSLSPGGFRPVYFYQHTSAAKK
>LFGLNPFC_02337 Sensor protein TorS
MALLTLTSTLVGWYNLRFISQVEKDNTQALIPTMNMARQLSEASAWELFAAQNLTSADNE
KMWQAQGRMLTAQSLKINALLQALREQGFDTTAIEQQEQEISRSLRQQGELVGQRLQLRQ
QQQQLSQQIVAAADEIARLAQGQANNAATSAGATQAGIYDLIEQHQRQAAESALDRLIDI
DLEYVNQMNELRLSALRVQQMVMNLGLEQIQKNAPTLEKQLNNAVKILQRRQIRIEDPGV
RTQVATTLTTVSQYSDLLALYQQDSEISNHLQTLAQNNIAQFAQFSSEVSQLVDTIELRN
QHGLAHLEKASARGQYSLLLLGLVSLCALILILWRVVYRSVTRPLAEQTQALQRLLDGDI
DSPFPETAGVRELDTIGRLMDAFRSSVHALNRHREQLAAQVKARTAELQELVIEHRQARA
EAEKASQAKSAFLAAMSHEIRTPLYGILGTAQLLADNPALNAQRDDLRAITDSGESLLTI
LNDILDYSAIEAGGKNVSVSDEPFEPRPLLESTLQLMSGRVKGRPIRLATEIADDVPTAL
MGDPRRIRQVITNLLSNALRFTDEGHIILRSRTDGEQWLVEVEDSGCGIDPAKLAEIFQP
FVQVSSKRGGTGLGLTISSRLAQAMGGELSATSTPEVGSCFCLRLPLRIATAPVPKTVNQ
AVRLDDLRLLLIEDNPLTQRITVEMLNTSGAQVVAVGNAAQAIEALQNSEPFAAALVDFD
LPDIDGITLARQLAQQYPSLVLIGFSAHVIDETLRQRTSSLFRGIIPKPVPREVLGQLLA
HYLQLQVNNDQPLDVSQLNEDAHLMGAEKIHEWLILFKQHALPLLDEIDIARASQDNEKI
KRAAHQLKSSCSSLGMRSASQQCAQLEQQPLSAPLPHEEITRSVAALEAWLIRKT
>LFGLNPFC_02338 Putative electron transport protein YccM
MAEKKRTRWQRRPGTTGGKLPWNDWRNATTWRKATQLLLLAINIYIAITFWYWVRYYETA
GSTTFVTRPGGIEGWLPIAGLMNLKYSLATGQLPSVHAAAMLLLVAFIVISLLLKKAFCS
WLCPVGTLSELIGDLGNKLFGRQFVLPRWLDIPLRGVKYLLLSFFLYIALLMPAQAIHYF
MLSPYSVVMDVKMLDFFRHMGTATLISMIVLLIASLFIRHAWCRYLCPYGALMSVVSLLS
PFKIRRNAESCIDCGKCAKSCPSRIPVDKLTQVRTVECTGCMTCVESCPVASTLTFSLQK
PAANKKAFALSGWLMTLLVMGIMFAATGYAMYAGVWQSPVPEELYQRLIPQSPMIGH
>LFGLNPFC_02339 Protein GnsA
MNIEELKKQAETEIADFIAQKIAELNKNTGKEVSEIRFTAREKMTGLESYDVKIKIM
>LFGLNPFC_02340 Cold shock-like protein CspG
MSNKMTGLVKWFNADKGFGFITPDDGSKDVFVHFTAIQSNEFRTLNENQKVEFSIEQGQR
GPAAANVVTL
>LFGLNPFC_02341 Periplasmic AppA protein
MKAILIPFLSLLIPLTPKFAFAQSEPELKLESVVIVSRHGVRAPTKATQLMQDVTPDAWP
TWPVKLGWLTPRGGELIAYLGHYQRQRLVADGLLAKEGCPQPGQVAVIADVDERTRKTGE
AFAAGLAPDCAITVHTQADTSSPDPLFNPLKTGVCQLDNANVTDAILSRAGGSIADFTGH
RQTAFRELERVLNFPQSNLCLNRKKQDESCSLTQALPSELKVSADNVSLTGAVSLASMLT
EIFLLQQAQGMPEPGWGRITDSHQWNTLLSLHNAQFYLLQRTPEVARSRATPLLDLIMTA
LAPHPPQKQVYGVTLPTSVLFIAGHDTNLANLGGALELNWTLPGQPDNTPPGGELVFERW
RRLSDNSQWIQVSLVFQTLQQMRDKTPLSLNTPPGEVKLTLAGCEERNARGMCSLAGFTQ
IVNEARIPACALHQDK
>LFGLNPFC_02342 Putative cytochrome bd-II ubiquinol oxidase subunit AppX
MWYLLWFVGILLMCSLSTLVLVWLDPRLKS
>LFGLNPFC_02343 Cytochrome bd-II ubiquinol oxidase subunit 2
MFDYETLRFIWWLLIGVILVVFMISDGFDMGIGCLLPLVARNDDERRIVINSVGAHWEGN
QVWLILAGGALFAAWPRVYAAAFSGFYVAMILVLCSLFFRPLAFDYRGKIADARWRKMWD
AGLVIGSLVPPVVFGIAFGNLLLGVPFAFTPQLRVEYLGSFWQLLTPFPLLCGLLSLGMV
ILQGGVWLQLKTVGVIHLRSQLATKRAALLVMLCFLLAGYWLWVGIDGFVLLAQDANGPS
NPLMKLVAVLPGAWMNNFVESPVLWIFPLLGFFCPLLTVMAIYRGRPGWGFLMASLMQFG
VIFTAGITLFPFVMPSSVSPISSLTLWDSTSSQLTLSIMLVIVLIFLPIVLLYTLWSYYK
MWGRMTTETLRRNENELY
>LFGLNPFC_02344 Cytochrome bd-II ubiquinol oxidase subunit 1
MWDVIDLSRWQFALTALYHFLFVPLTLGLIFLLAIMETIYVVTGKTIYRDMTRFWGKLFG

INFALGVATGLTMEFQFGTNWSFYSNYVGDIFGAPLAMEALMAFFLESTFVGLFFFGWQR
LNKYQHLLVTWLVAFGSNLSALWILNANGWMQYPTGAHFDIDTLRMEMTSFSELVFNPVS
QVKFVHTVMAGYVTGAMFIMAISAWYLLRGRERDVALRSFAIGSVFGTLAIISTLQLGDS
SAYEVAQVQPVKLAAMEGEWQTEPAPAPFHVVAWPEQDQERNAFAIKIPALLGILATHSL
DKPVPGLKNLMAETYPRLQRGRMAWLLMQEISQGNREPHVLQAFRELEGDLGYGMLLSRY
APDMNHVTAAQYQAAMRGAIPQVAPVFWSFRIMVGCGSLLLLVMLIALVQTLRGKIDQHR
WVLKMALWSLPLPWIAIEAGWFMTEFGRQPWAIQDILPTYSAHSALTTGQLAFSLIMIVG
LYTLFLIAEVYLMQKYARLGPSAMQSEQPTQQQG
>LFGLNPFC_02345 hypothetical protein
MSETFFHLLGPGTQPNDDSFSMNPLPITCRVNGEPSMAALEQCAHSPQVIALLNELQHQL
SERQPPLGEVLAVDLLNLNADDRHFINTLLGEGEVSVRIQQADDSESEIQEAIFCGLWRV
RRRHGEQLLEDKLEAGCAPLALWQAATQNVLPTDSLLPPPIDGLMNGLPLAHELLAHVRN
PDAQPHSINMTQLPISEADRLFLSRLCGPGNIQIRTIGYGESYINATGLRHVWHLRCTDT
LKGPLLESYEICPIPEVVLAAPEDLVDSAQRLSEVCQWLAEGAPT
>LFGLNPFC_02346 Hydrogenase-1 operon protein HyaE
MSNDTPFNALWQRMLARGWTPVSECRLDDWLTQAPDGVVLLSSDPKRTPEVSDNPVMIGE
LLREFPDYTWQVAIADLEQSEAIGDRFGVFRFPATLVFTGGNYRGVLNGIHPWAELINLM
RGLVEPQQERAS
>LFGLNPFC_02347 Hydrogenase 1 maturation protease
MSEQRVVVMGLGNLLWADEGFGVRVAERLYAHYHWPEDVEIVDGGTQGLNLLGYVESAGH
LLILDAIDYGLEPGTLRTYAGERIPAYLSAKKMSLHQNSFSEVLALADIRGHLPAHIALV
GLQPAMLDDYGGSLSELAREQLPAAEQAALAQLAAWGIVPQPANESRCLNYDCLSMENYE
GVRLRQYRMRLEEQG
>LFGLNPFC_02348 putative Ni/Fe-hydrogenase 1 B-type cytochrome subunit
MQQKSDNVVSHYVFEAPVRIWHWLTVLCMAVLMVTGYFIGKPLPSVSGEATYLFYMGYIR
LIHFSAGMIFTVVLLMRIYWAFVGNHYSRELFIVPVWRKSWWQGVWYEIRWYLFLAKRPS
ADIGHNPIAQAAMFGYFLMSVFMIITGFALYSEHSQYAIFAPFRYVVEFFYWTGGNSMDI
HSWHRLGMWLIGAFVIGHVYMALREDIMSDDTVISTMVNGYRSHKFGKISNKERS
>LFGLNPFC_02349 Hydrogenase-1 large chain
MSTQYETQGYTINNAGRRLVVDPITRIEGHMRCEVNINDQNVITNAVSCGTMFRGLEIIL
QGRDPRDAWAFVERICGVCTGVHALASVYAIEDAIGIKVPDNANIIRNIMLATLWCHDHL
VHFYQLAGMDWIDVLDALKADPRKTSELAQSLSSWPKSSPGYFFDVQNRLKKFVEGGQLG
IFRNGYWGHPQYKLPPEANLMGFAHYLEALDFQREIVKIHAVFGGKNPHPNWIVGGMPCA
INIDESGAVGAVNMERLNLVQSIITRTADFINNVMIPDALAIGQFNKPWSEIGTGLSDKC
VLSYGAFPDIANDFSEKSLLMPGGAVINGDFNNVLPVDLVDPQQVQEFVDHAWYRYPNDQ
VGRHPFDGITDPWYNPGDVKGSDTNIQQLNEQERYSWIKAPRWRGNAMEVGPLARTLIAY
HKGDAATVESVDRMMSALKLPLSGIQSTLGRILCRAHEAQWAAGKLQYFFDKLMTNLKNG
NLATASTEKWEPATWPTECRGVGFTEAPRGALGHWAAIRDGKIDLYQCVVPTTWNASPRD
PKGQIGAYEAALMNTKMAIPEQPLEILRTLHSFDPCLACSTHVLGDDGSELISVQVR
>LFGLNPFC_02350 Hydrogenase-1 small chain
MNNEETFYQAMRRQGVTRRSFLKYCSLAATSLGLGAGMAPKIAWALENKPRIPVVWIHGL
ECTCCTESFIRSAHPLAKDVILSLISLDYDDTLMAAAGTQAEEVFEDIIAQYNGKYILAV
EGNPPLGEQGMFCISSGRPFIEKLKRAAAGASAIIAWGTCASWGCVQAARPNPTQATPID
KVITDKPIIKVPGCPPIPDVMSAIITYMVTFDRLPDVDRMGRPLMFYGQRIHDKCYRRAH
FDAGEFVQSWDDDAARKGYCLYKMGCKGPTTYNACSSTRWNDGVSFPIQSGHGCLGCAEN
GFWDRGSFYSRVVDIPQMGTHSTADTVGLTALGVVAAAVGVHAAASAVDQRRRHNQQPTE
TEHQPGNEDKQA
>LFGLNPFC_02352 Modulator of FtsH protease YccA
MDRIVSSSHDRTSLLSTHKVLRNTYFLLSLTLAFSAITATASTVLMLPSPGLILTLVGMY
GLMFLTYKTANKPTGIISAFAFTGFLGYILGPILNTYLSAGMGDVIAMALGGTALVFFCC
SAYVLTTRKDMSFLGGMLMAGIVVVLIGMVANIFLQLPALHLAISAVFILISSGAILFET
SNIIHGGETNYIRATVSLYVSLYNIFVSLLSILGFASRD
>LFGLNPFC_02353 Sulfurtransferase TusE
MLIFEGKEIETDTEGYLKESSQWSEPLAVVIAENEGIALSPEHWEVVRFVRDFYLEFNTS
PAIRMLVKAMANKFGEEKGNSRYLYRLFPKGPAKQATKIAGLPKPVKCI
>LFGLNPFC_02354 Acylphosphatase
MSKVCIIAWIYGRVQGVGFRYTTQYEAKKLGLTGYAKNLDDGSVEVVACGDEGQVEKLIQ
WLKSGGPRSARVERVLSEPHHPSGELTDFRIR
>LFGLNPFC_02355 Ribosomal RNA large subunit methyltransferase I
MLAKGREKSLLRRHPWVFSGAVARMEGKANLGETIDIVDHQGKWLARGAYSPASQIRARV
WTFDPSESIDIAFFTRRLQQAQKWRDWLAQKDGLDSYRLIAGESDGLPGITIDRFGNFLV
LQLLSAGAEYQRAALISALQTLYPECAIYDRSDVAVRKKEGMELTQGPITGELPPALLPI
EEHGMKLLVDIQHGHKTGYYLDQRDSRLATRRYVENKRVLNCFSYTGGFAVSALMGGCSQ
VVSVDTSHEALDIARQNVELNKLDLSKAEFVRDDVFKLLRTYRDRGEKFDVIVMDPPKFV

ENKSQLMGACRGYKDINMLAIQLLNEGGILLTFSCSGLMTSDLFQKIIADAAIDAGRDVQ
FIEQFRQAADHPVIATYPEGLYLKGFACRVM
>LFGLNPFC_02356 Heat shock protein HspQ
MIASKFGIGQQVRHSLLGYLGVVVDIDPVYSLSEPSPDELAVNDELRAAPWYHVVMEDDN
GLPVHTYLAEAQLSSELQDEHPEQPSMDELAQTIRKQLQAPRLRN
>LFGLNPFC_02357 putative protein YccU
MKETDIAGILTSTHTIALVGASDKPDRPSYRVMKYLLDQGYHVIPVSPKVAGKTLLGQQG
YGTLADVPEKVDMVDVFRNSEAAWGVAQEAIAIGAKTLWMQLGVINEQAAVLARDAGLNV
VMDRCPAIEIPRLGLAK
>LFGLNPFC_02358 hypothetical protein
MKTGIVTTLIALCLPVSVFATTLRLSTDVDLLVLDGKKVSSSLLRGADSIELDNGPHQLV
FRVEKTIHLSNSEERLYISPPLVVSFNTQLINQVNFRLPRLENEREANHFDAAPRLELLD
GDATPIPVKLDILAITSTAKTIDYEVEVERYNKSAKRASLPQFATMMADDSTLLSGVSEL
DAIPPQSQVLTEQRLKYWFKLADPQTRNTFLQWAEKQPSS
>LFGLNPFC_02359 Methylglyoxal synthase
MELTTRTLPSRKHIALVAHDHCKQMLMSWVERHQPLLEQHVLYATGTTGNLISRATGMNV
NAMLSGPMGGDQQVGALISEGKIDVLIFFWDPLNAVPHDPDVKALLRLATVWNIPVATNV
ATADFIIQSPHFNDAVDILIPDYQRYLADRLK
>LFGLNPFC_02360 DNA helicase IV
MELKATTLGKRLAQHPYDRAVILNAGIKVSGDRHEYLIPFNQLLAIHCKRGLVWGELEFV
LPDEKVVRLHGTEWGETQRFYHHLDAHWRRWSGEMSEIASGVLRQQLDLIATRTGENKWL
TREQTSGVQQQIRQALSALPLPVNRLEEFDNCREVWRKCQAWLKDIEGARLQHNQAYTEA
MLTEYADFFRQVESSPLNPAQARAVVNGEHSLLVLAGAGSGKTSVLVARAGWLLARGEAS
PEQILLLAFGRKAAEEMHERIRERLHTEDITARTFHALALHIIQQGSKKVPIVSKLENDT
AARHELFIAEWRKQCSEKKAQAKGWRQWLTEEMQWSVPEGNFWDDEKLQRRLASRLDRWV
SLMRMHGGAQAEMIASAPEEIRDLFSKRIKLMAPLLKAWKGALKAENAVDFSGLIHQAIV
ILEKGRFISPWKHILVDEFQDISPQRAALLAALRKQNSQTTLFAVGDDWQAIYRFSGAQM
SLTTAFHENFGEGDRCDLDTTYRFNSRIGEVANRFIQQNPGQLKKPLNSLTNGDKKAVTL
LDEGQLDALLDKLSGYAKPEERILILARYHHMRPASLEKAATRWPKLQIDFMTIHASKGQ
QADYVIIVGLQEGSDGFPAAARESIMEEALLPPVEDFPDAEERRLMYVALTRARHRVWAL
FNKENPSPFVDILKNLDVPVARKP
>LFGLNPFC_02361 Inner membrane protein YccF
MRTVLNILNFVLGGFATTLGWLLATLVSIVLIFTLPLTRSCWEITKLSLVPYGNEAIHVD
ELNPAGKNVLLNTGGTVLNIFWLIFFGWWLCLMHIATGIAQCISIIGIPVGIANFKIAAI
ALWPVGRRVVSVETAQAAREANARRRFQ
>LFGLNPFC_02362 Inner membrane protein YccS
MLSPLLKRYTWNSAWLYYARIFIALCGTTAFPWWLGDVKLTIPLTLGMVAAALTDLDDRL
AGRLRNLIITLFCFFIASASVELLFPWPWLFAIGLTLSTSGFILLGGLGQRYATIAFGAL
LIAIYTMLGTSLYEHWYQQPMYLLAGAVWYNVLTLIGHLLFPVRPLQDNLARCYEQLARY
LELKSRMFDPDIEDESQAPLYDLALANGQLMATLNQTKLSLLTRLRGDRGQRGTRRTLHY
YFVAQDIHERASSSHIQYQTLREHFRHSDVLFRFQRLMSMQGQACQQLSRCILLRQPYQH
DPHFERAFTHIDAALERMRDNGAPADLLKTLGFLLNNLRAIDAQLATIESEQAQALPHNN
DENELADDSPHGLSDIWLRLSRHFTPESALFRHAVRMSLVLCFGYAIIQITGMHHGYWIL
LTSLFVCQPNYNATRHRLKLRIIGTLVGIAIGIPVLWFVPSLEGQLVLLVITGVLFFAFR
NVQYAHATMFITLLVLLCFNLLGEGFEVALPRVIDTLIGCAIAWAAVSYIWPDWKFRNLP
RMLERATEANCRYLDAILEQYHQGRDNRLAYRIARRDAHNRDAELASVVSNMSSEPNVTP
QIREAAFRLLCLNHTFTSYISALGAHREQLTNPEILAFLDDAVCYVDDALHHQPADEERV
NQALAGLKQRMQQLEPRADSKEPLVVQQVGLLIALLPEIGRLQRQITQVPQETPVSA
>LFGLNPFC_02363 Protein Sxy
MKSLSYKRIYKSQEYLATLGTIEYRSLFGSYSLTVDDTVFAMVSDGELYLRACEQSAPYC
VKHPPVWLTYKKCGRSVTLNYYRVDESLWRNQLKLVRLSKYSLDAALKEKSTRNIRERLK
DLPNMSFHLEAILGEVGIKDVRALRILGAKMCWLRLRQQNSLVTEKILFMLEGAIIGIHE
AALPVARRQELAEWADSLTPKQEFPAELE
>LFGLNPFC_02364 Cell division inhibitor SulA
MYTAGYAHRDSSFSSTASKIARVSTENTTAGLISEVVYREDQPMMTQLLLLPLLQQLGQQ
SRWQLWLTPQQKLSREWVQASGLPLTKVMQISQLSPCHTVESMVRALRTGNYSVVIGWLA
DDLTEEEHAELVDAANEGNAMGFIMRPVSASSHATRQLSGLKIHSNLYH
>LFGLNPFC_02365 Outer membrane protein A
MKKTAIAIAVALAGFATVAQAAPKDNTWYTGAKLGWSQYHDTGFINNNGPTHENQLGAGA
FGGYQVNPYVGFEMGYDWLGRMPYKGSVENGAYKAQGVQLTAKLGYPITDDLDVYTRLGG
MVWRADTKSNVYGKNHDTGVSPVFAGGVEYAITPEIATRLEYQWTNNIGDAHTIGTRPDN
GMLSLGVSYRFGQGEAAPVVAPAPAPAPEVQTKHFTLKSDVLFTFNKATLKPEGQAALDQ
LYSQLSNLDPKDGSVVVLGYTDRIGSDAYNQALSERRAQSVVDYLISKGIPADKISARGM
GESNPVTGNTCDNVKQRAALIDCLAPDRRVEIEVKGIKDVVTQPQA

>LFGLNPFC_02366 Macrodomain Ter protein
MKYQQLENLESGWKWKYLVKKHREGELITRYIEASAAQEAVDELLSLENEPVLVNGWIDK
HMNPELVNRMKQTIRARRKRHFNAEHQHTRKKSIDLEFIVWQRLAGLAQRRGKTLSETIV
QLIEDAENKEKYANKMSSLKQDLQALLGKE
>LFGLNPFC_02367 Lon protease
MTITKLAWRDLVPDTDSYQEIFAQPHLIDENDPLFSDTQPRLQFALEQLLHTRASSSFML
AKAPEESEYLNLIADAARTLQSDAGQLVGGHYEVSGHTIRLRHAVSADDNFATLTQVVAA
DWVEAEQLFGCLRQFNGDITLQPGLVHQANGGILIISLRTLLAQPLLWMRLKNIVNRERF
DWVAFDESRPLPVSVPSMPLKLKVILVGERESLADFQEMEPELSEQAIYSEFEDTLQIVD
AESVSQWCRWVTFTARHNHLPAPGADAWPVLIREAARYTGEQETLPLSPQWILRQCQEVA
SLCDGDTFSGEQLNLMLQQREWREGFLAERMQDEILQEQILIETEGERIGQINALSVIEF
PGHPRAFGEPSRISCVVHIGDGEFTDIERKAELGGNIHAKGMMIMQAFLMSELQLEQQIP
FSASLTFEQSYSEVDGDSASMAELCALISALADVPVNQSIAITGSVDQFGRAQPVGGLNE
KIEGFFAICQQRELTGKQGVIIPTANVRHLSLHSELVKAVEEDKFTIWAVDDVTDALPLL
LNLVWDGEGQTTLMQTIQERIAQASQQEGRHRFPWPLRWLNWFIPN
>LFGLNPFC_02368 3-hydroxydecanoyl-[acyl-carrier-protein] dehydratase
MAITLAELVYSELIGLVQRTRVSYPACFNKIRLTENMVDKRESYTKEDLLASGRGELFGA
KGPQLPAPNMLMMDRVVKMTETGGNFDKGYVEAELDINPDLWFFGCHFIGDPVMPGCLGL
DAMWQLVGFYLGWLGGEGKGRALGVGEVKFTGQVLPTAKKVTYRIHFKRIVNRRLIMGLA
DGEVLVDGRLIYTANDLKVGLFQDTSAF
>LFGLNPFC_02369 Ribosome modulation factor
MKRQKRDRLERAHQRGYQAGIAGRSKEMCPYQTLNQRSQWLGGWREAMADRVVMA
>LFGLNPFC_02370 Intermembrane transport lipoprotein PqiC
MKKWLVTIAALWLAGCSSGEINKNYYQLPVVQSGTQSTASQGNRLLWVEQVAVPDYLAGN
GVVYQTSDVKYVIANNNLWASPLDQQLRNTLVANLSTQLPGWVVASQPLGSAQDTLNVTV
TEFNGRYDGKVIVSGEWLLNHQGQLIKRPFRLEGVQTQDGYDEMVKVLASVWSQEAASIA
QEIKRLP
>LFGLNPFC_02371 Intermembrane transport protein PqiB
MESNNGEAKIQKVKNWSPVWIFPIVTALIGAWVLFYHYSHQGPEVTLITANAEGIEGGKT
TIKSRSVDVGVVESATLADDLTHVEIKARLNSGMEKLLHKDTVFWVVKPQIGREGISGLG
TLLSGVYIELQPGAKGSKMDKYDLLDSPPLAPPDAKGIRVLLDSKKAGQLSPGDPVLFRG
YRVGSVETSTFDTQKRNISYQLFINAPYDRLVTSNVRFWKDSGIAVDLTSAGMRVEMGSL
TTLLSGGVSFDVPEGLDLGQPVAPKTAFVLYDDQKSIQDSLYTDHIDYLMFFKDSVRGLQ
PGAPVEFRGIRLGTVSKVPFFAPNMRQTFNDDYRIPVLIRIEPERLKMQLGENADVVEHL
GELLKRGLRGSLKTGNLVTGALYVDLDFYPNTPAITGIREFNGYQIIPTVSGGLAQIQQR
LMEALDKINKLPLNPMIEQATSTLSESQRTMKNLQTTLDSMNKILASQSMQQLPTDMQST
LRELNRSMQGFQPGSAAYNKMVADMQRLDQVLRELQPVLKTLNEKSNALVFEAKDKKDPE
PKRAKQ
>LFGLNPFC_02372 Intermembrane transport protein PqiA
MCEHHHAAKHILCSQCDMLVALPRLEHGQKAACPRCGTTLTVAWDAPRQRPTAYALAALF
MLLLSNLFPFVNMNVAGVTSEITLLEIPGVLFSEDYASLGTFFLLFVQLVPAFCLITILL
LVNRAELPVRLKEQLARVLFQLKTWGMAEIFLAGVLVSFVKLMAYGSIGVGSSFLPWCLF
CVLQLRAFQCVDRRWLWDDIAPMPELRQPLKPGVTGIRQGLRSCSCCTAILPADESVCPR
CSTKGYVRRRNSLQWTLALLVTSIMLYLPANILPIMVTDLLGSKMPSTILAGVILLWSEG
SYPVAAVIFLASIMVPTLKMIAIAWLCWDAKGHGKRDSERMHLIYEVVEFVGRWSMIDVF
VIAVLSALVRMGGLMSIYPAMGALMFALVVIMTMFSAMTFDPRLSWDRQPESEHEES
>LFGLNPFC_02373 ABC transporter ATP-binding protein uup
MSLISMHGAWLSFSDAPLLDNAELHIEDNERVCLVGRNGAGKSTLMKILNREQGLDDGRI
IYEQDLIVARLQQDPPRNVEGSVYDFVAEGIEEQAEYLKRYHDISRLVMNDPSEKNLNEL
AKVQEQLDHHNLWQLENRINEVLAQLGLDPNVALSSLSGGWLRKAALGRALVSNPRVLLL
DEPTNHLDIETIDWLEGFLKTFNGTIIFISHDRSFIRNMATRIVDLDRGKLVTYPGNYDQ
YLLEKEEALRVEELQNAEFDRKLAQEEVWIRQGIKARRTRNEGRVRALKAMRRERGERRE
VMGTAKMQVEEASRSGKIVFEMEDVCYQVDGKQLVKDFSAQVLRGDKIALIGPNGCGKTT
LLKLMLGQLQADSGRIHVGTKLEVAYFDQHRAELDPDKTVMDNLAEGKQEVMVNGKPRHV
LGYLQDFLFHPKRAMTPVRALSGGERNRLLLARLFLKPSNLLILDEPTNDLDVETLELLE
ELIDSYQGTVLLVSHDRQFVDNTVTECWIFEGGGKIGRYVGGYHDARGQQEQYVALKQPA
VKKIEEAAAPKAETVKRSSSKLSYKLQRELEQLPQLLEDLEAKLEALQTQVADASFFSQP
HEQTQKVLADMAAAEQELEQAFERWEYLEALKNGG
>LFGLNPFC_02374 Ribosomal RNA large subunit methyltransferase K/L
MNSLFASTARGLEELLKTELENLGAVECQVVQGGVHFKGDTRLVYQSLMWSRLASRIMLP
LGECKVYSDLDLYLGVQAINWTEMFNPGATFAVHFSGLNDTIRNSQYGAMKVKDAIVDAF
TRKNLPRPNVDRDAPDIRVNVWLHKETASIALDLSGDGLHLRGYRDRAGMAPIKETLAAA
IVMRSGWQPGTPLLDPMCGSGTLLIEAAMLATDRAPGLHRGRWGFSGWTQHDEAIWQEVK
AEAQTRARKGLAEYSSHFFGSDSDARVIQRARTNARLAGIGELITFEVNDVAQLANPLPK

GPYGTVLSNPPYGERLDSEPALIALHSLLGRIMKNQFGGWNLSLFSASPDLLSCLQLRAD
KQYKAKNGPLDCVQKNYHVAESTPDSKPVMAAEDYANRLRKNLKKFEKWARQEGIECYRL
YDADLPEYNVAVDRYADWVVVQEYAPPKTIDAHKARQRLFDIIAATISVLGIAPNKLVLK
TRERQKGKNQYQKLGEKGEFLEVTEYNAHLWVNLTDYLDTGLFLDHRIARRMLGQMSKGK
DFLNLFSYTGSATVHAGLGGARSTTTVDMSRTYLEWAERNLRLNGLTGRAHRLIQADCLA
WLREANEQFDLIFIDPPTFSNSKRMEDAFDVQRDHLALMKDLKRLLRAGGTIMFSNNKRG
FRMDLDGLAKLGLKAQEITQKTLSQDFARNRQIHNCWLITAA
>LFGLNPFC_02375 putative protein YcbX
MATLTRLFIHPVKSMRGIGLTHTLADVSGLAFDRIFMITETDGTFITARQFPLMVRFTPS
PVHDGLHLTAPDGSSAYVRFADFATQDAPTEVWGTHFTARIAPDAINKWLSGFFSREVQL
RWVGPQMTRRVKRHNTVPLSFADGYPYLLANEASLRDLQQRCPASVKMEQFRPNLVVSGA
SAWEEDSWKVIRIGDVVFDVVKPCSRCIFTTVSPEKGQKHPAGEPLKTLQSFRTAQDNGD
VDFGQNLIVRNSGVIRVGDEVEILATAPAKIYGAGAADDTANITQQPDANVDIDWQGQAF
RGNNQQVLLEQLENQGIRIPYSCHAGICGSCRVQLLEGEVTPLKKSAIGDDGTILCCSCV
PKTALKLAR
>LFGLNPFC_02376 Cell division protein ZapC
MRIKPDDNWRWYYDEEHDRMMLDLANGMLFRSRFARKMLTPDAFSPAGFCVDDAALYFSF
EEKCRDFNLSKEQKAELVLNALVAIRYLKPQMPKSWHFVSHGEMWMPMPGDAAYVWLSDT
HEQVNLLVVESGENAALCLLAQPCVVIAGRAMQLGDAIKIMNDRLKPQVNVDSFSLEQAV
>LFGLNPFC_02377 Dihydroorotate dehydrogenase (quinone)
MYYPFVRKALFQLDPERAHEFTFQQLRRITGTPFEALVRQKVPAKPVNCMGLTFKNPLGL
AAGLDKDGECIDALGAMGFGSIEIGTVTPRPQPGNDKPRLFRLVDAEGLINRMGFNNLGV
DNLVENVKKAHYDGVLGINIGKNKDTPVEQGKDDYLICMEKIYAYAGYIAINISSPNTPG
LRTLQYGEALDDLLTAIKNKQNDLQVMHHKYVPIAVKIAPDLSEEELIQVADSLVRHNID
GVIATNTTLDRSLVQGMKNCDQTGGLSGRPLQLKSTEIIRRLSQELNGRLPIIGVGGIDS
VIAAREKIAAGASLVQIYSGFIFKGPPLIKEIVTHI
>LFGLNPFC_02378 FMN reductase (NADPH)
MRVITLAGSPRFPSRSSSLLEYAREKLNGLDVEVYHWNLQNFVPEDLLYARFDSPALKTF
TEQLQQADGLIVATPVYKAAYSGALKTLLDLLPERALQGKVVLPLATGGTVAHLLAVDYA
LKPVLSTLKAQEILHGVFADDSQVIDYQHKPQFTPNLQTRLDTALETFWQALHRRDVQVP
DLLSLRGNAHA
>LFGLNPFC_02379 Putative aliphatic sulfonates-binding protein
MRKIIKLALAGLLSVSTLAVAAESSPEALRIGYQKGSIGMVLAKSHQLLEKRYPQTKISW
VEFPAGPQMLEALNVGSIDLGSTGDIPPIFAQAAGADLVYVGVEPPKPKAEVILVAENSP
IKTVADLKGHKVAFQKGSSSHNLLLRALRQAGLKFTDIQPTYLTPADARAAFQQGNVDAW
AIWDPYYSAALLQGGVRVLKDGTDLNQTGSFYLAARPYAEKNGAFIQGVLTTFSEADALT
RSQREQSIALLAKTMGLPAPVIASYLDHRPPTTIKPLSAEVAALQQQTADLFYENRLVPK
KVDIRQRIWQPTQLEGKQL
>LFGLNPFC_02380 Alkanesulfonate monooxygenase
MSLNMFWFLPTHGDGHYLGTEEGSRPVDHGYLQQIAQAADRLGYTGVLIPTGRSCEDAWL
VAASMIPVTQRLKFLVALRPSVTSPTVAARQAATLDRLSNGRALFNLVTGSDPQELAGDG
VFLDHSERYEASAEFTQVWRRLLLGETVDFNGKHIHVRGAKLLFPPIQQPYPPLYFGGSS
DVAQELAAEQVDLYLTWGEPPELVKEKIEHVRAKAAAHGRKIRFGVRLHVIVRETNDEAW
QAAERLISRLDDETIAKAQAAFARTDSVGQQRMAALHNGKRDNLEISPNLWAGVGLVRGG
AGTALVGDGPTVAARINEYAALGIDSFVLSGYPHLEEAYRVGELLFPHLDVAIPEIPQPQ
PLNPQGEAVANDFIPRNVAQS
>LFGLNPFC_02381 Putative aliphatic sulfonates transport permease protein SsuC
MMATPVKKWLLRVAPWFLPVGIVAVWQLASSVGWLSTRILPSPEGVVMAFWTLSASGELW
QHLAISSWRALIGFSIGGSLGLILGLISGLSRWGERLLDTSIQMLRNVPHLALIPLVILW
FGIDESAKIFLVALGTLFPIYINTWHGIRNIDRGLVEMARSYGLSGIPLFIHVILPGALP
SIMVGVRFALGLMWLTLIVAETISANSGIGYLAMNAREFLQTDVVVVAIILYALLGKLAD
VSAQLLERLWLRWNPAYHLKEATV
>LFGLNPFC_02382 Aliphatic sulfonates import ATP-binding protein SsuB
MNTARLNQGTPLLLNAVSKHYAENIVLNQLDLHIPAGQFVAVVGRSGGGKSTLLRLLAGL
ETPTAGDVLAGTTPLAEIQDDTRMMFQDARLLPWKSVIDNVGLGLKGQWRDAARQALAAV
GLENRAGEWPAALSGGQKQRVALARALIHRPGLLLLDEPLGALDALTRLEMQDLIVSLWQ
EHGFTVLLVTHDVSEAVAMADRVLLIEEGKIGLDLTVDIPRPRRLGSVRLAELEAEVLQR
VMQRGHSEQPIRRHG
>LFGLNPFC_02383 Aminopeptidase N
MTQQPQAKYRHDYRAPDYQITDIDLTFDLDAQKTVVTAVSQAVRHGASDVPLRLNGEDLK
LVSVYINDEPWTAWKEEEGALVISNLPERFTLKIVNEISPAANTALEGLYQSGDALCTQC
EAEGFRHITYYLDRPDVLARFTTKIIADKTKYPFLLSNGNRVAQGELENGRHWVRWQDPF
PKPCYLFALVAGDFDVLRDTFTTRSGREVALELYVDRGNLDRAPWAMTSLKNSMKWDEER
FGLEYDLDIYMIVAVDFFNMGAMENKGLNIFNSKYVLARTDTATDKDYLDIERVIGHEYF

HNWTGNRVTCRDWFQLSLKEGLTVFRDQEFSSDLGSRAVNRINNVRTMRGLQFAEDASPM
AHPIRPDMVIEMNNFYTLTVYEKGAEVIRMIHTLLGEENFQKGMQLYFERHDGSAATCDD
FVQAMEDASNVDLSHFRRWYSQSGTPVVTVKDDYNPETEQYTLTISQRTPATPDQAEKQP
LHIPFAVELYDNEGKVIPLQKGGHPVNSVLNVTQAEQTFVFDNVYFQPVPALLCEFSAPV
KLEYKWSDQQLTFLMRHARNDFSRWDAAQSLLATYIKLNVARHQQGQPLSLPVHVADAFR
AVLLDEKIDPALAAEILTLPSVNEMAELFDIIDPIAIAEVREALTRTLATELADELLAIY
NANYQSEYRVEHEDIAKRTLRNACLRFLAFGETHLADVLVSKQFHEANNMTDALAALSAA
VAAQLPCRDALMQEYDDKWHQDGLVMDKWFILQATSPAANVLETVRGLLQHRSFTMSNPN
RIRSLIGAFAGSNPAAFHAEDGSGYQFLVEMLTDLNSRNPQVASRLIEPLIRLKRYDAKR
QEKMRAALEQLKGLENLSGDLYEKITKALA
>LFGLNPFC_02384 Nicotinate phosphoribosyltransferase
MTQFASPVLHSLLDTDAYKLHMQQAVFHHYYDVHVAAEFRCRGDDLLGIYADAIREQVQA
MQHLRLQDDEYQWLSALPFFKADYLNWLREFRFNPEQVTVSNDNGKLDIRLSGPWREVIL
WEVPLLAVISEMVHRYRSPQADVAQALDTLENKLVDFSALTAGLDMSRFHLMDFGTRRRF
SREVQETIVKRLQQESWFVGTSNYDLARRLSLTPMGTQAHEWFQAHQQISPDLANSQRAA
LAAWLEEYPDQLGIALTDCITMDAFLRDFGVEFASRYQGLRHDSGDPVEWGEKAIAHYEK
LGIDPQSKTLVFSDNLDLRKAVELYRHFSSRVQLSFGIGTRLTCDIPQVKPLNIVIKLVE
CNGKPVAKLSDSPGKTICHDKAFVRALRKAFDLPHIKKAS
>LFGLNPFC_02385 Asparagine--tRNA ligase
MSVVPVADVLQGRVAVDSEVTVRGWVRTRRDSKAGISFLAVYDGSCFDPVQAVINNSLPN
YNEDVLRLTTGCSVIVTGKVVASPGQGQQFEIQASKVEVAGWVEDPDTYPMAAKRHSIEY
LREVAHLRPRTNLIGAVARVRHTLAQALHRFFNEQGFFWVSTPLITASDTEGAGEMFRVS
TLDLENLPRNDQGKVDFDKDFFGKESFLTVSGQLNGETYACALSKIYTFGPTFRAENSNT
SRHLAEFWMLEPEVAFANLNDIAGLAEAMLKYVFKAVLEERADDMKFFAERVDKDAVSRL
ERFIEADFAQVDYTDAVTILENCGRKFENPVYWGVDLSSEHERYLAEEHFKAPVVVKNYP
KDIKAFYMRLNEDGKTVAAMDVLAPGIGEIIGGSQREERLDVLDERMLEMGLNKEDYWWY
RDLRRYGTVPHSGFGLGFERLIAYVTGVQNVRDVIPFPRTPRNASF
>LFGLNPFC_02386 Outer membrane porin F
MMKRNILAVIVPALLVAGTANAAEIYNKDGNKVDLYGKAVGLHYFSKGNGENSYGGNGDM
TYARLGFKGETQINSDLTGYGQWEYNFQGNNSEGADAQTGNKTRLAFAGLKYADVGSFDY
GRNYGVVYDALGYTDMLPEFGGDTAYSDDFFVGRVGGVATYRNSNFFGLVDGLNFAVQYL
GKNERDTARRSNGDGVGGSISYEYEGFGIVGAYGAADRTNLQEESSLGKGKKAEQWATGL
KYDANNIYLAANYGETRNATPITNKFTNTSGFANKTQDVLLVAQYQFDFGLRPSIAYTKS
KAKDVEGIGDVDLVNYFEVGATYYFNKNMSTYVDYIINQIDSDNKLGVGSDDTVAVGIVY
QF
>LFGLNPFC_02387 Aspartate aminotransferase
MFENITAAPADPILGLADLFRADERPGKINLGIGVYKDETGKTPVLTSVKKAEQYLLENE
TTKNYLGIDGIPEFGRCTQELLFGKGSALINDKRARTAQTPGGTGALRVAADFLAKNTSV
KRVWVSNPSWPNHKSVFNSAGLEVREYAYYDAENHTLDFDALINSLNEAQAGDVVLFHGC
CHNPTGIDPTLEQWQTLAQLSVEKGWLPLFDFAYQGFARGLEEDAEGLRAFAALHKELIV
ASSYSKNFGLYNERVGACTLVAADSETVERAFSQMKAAIRANYSNPPAHGASVVATILSN
DALRAIWEQELTDMRQRIQRMRQLFVNTLQEKGANRDFSFIIKQNGMFSFSGLTKEQVLR
LREEFGVYAVASGRVNVAGMTPDNMAPLCEAIVAVL
>LFGLNPFC_02388 Hydroxyacylglutathione hydrolase GloC
MNYRIIPVTAFSQNCSLIWCEQTRLAALVDPGGDAEKIKQEVDASGLTLMQILLTHGHLD
HVGAAAELAQHYGVPVFGPEKEDEFWLQGLPAQSRMFGLEECQPLTPDRWLNEGDTISIG
NVTLQVLHCPGHTPGHVVFFDDRAKLLISGDVIFKGGVGRSDFPRGDHNQLISSIKDKLL
PLGDDVTFIPGHGPLSTLGYERLHNPFLQDEMPVW
>LFGLNPFC_02389 hypothetical protein
MDKFDANRRKLLALGGVALGAAILPTPAFATLSTPRPRILTLNNLHTGESIKAEFFDGRG
YIQEELAKLNHFFRDYRANKIKSIDPGLFDQLYRLQGLLGTRKPVQLISGYRSIDTNNEL
RARSRGVAKKSYHTKGQAMDFHIEGIALSNIRKAALSMRAGGVGYYPRSNFVHIDTGPAR
HW
>LFGLNPFC_02390 putative L,D-transpeptidase YcbB
MLLNMMCGRRLSAISLCLAVTFAPLFNAQADEPEVIPGDSPVAVSEQGEALPQAQATAIM
AGIQPLPEGAAEKARTQIESQLPAGYKPVYLNQLQLLYAARDMQPMWENRDAVKAFQQQL
AEVAIAGFQPQFNKWVELLTDPGVNGMTRDVVLSDAMMGYLHFIANIPVKGTRWLYSSKP
YALSTPPLSVINQWQLALDQGQLPTFVAGLAPQHPQYAAMHESLLALLSDTKPWPQLTGK
ATLRPGQWSNDVPALREILQRTGMLDGGPKITLPGDDTPTDAVVSPSAVTVETVETKLMD
KQTTSRSKPAPAVRAAYDNELVEAVKRFQAWQGLGADGAIGPATRDWLNVTPAQRAGVLA
LNIQRLRLLPTELSTGIMVNIPAYSLVYYQNGNQVLDSRVIVGRPDRKTPMMSSALNNVV
VNPPWNVPPTLARKDILPKVRNDPGYLESHGYTVMRGWNSREAIDPWQVDWSTITASNLP
FRFQQAPGPRNSLGRYKFNMPSSEAIYLHDTPNHNLFKRDTRALSSGCVRVNKASDLANM
LLQDAGWNDKRISDALKQGDTRYVNIRQSIPVNLYYLTAFVGADGRTQYRTDIYNYDLPA

RSSSQIVSKAEQLIR
>LFGLNPFC_02391 Chromosome partition protein MukB
MIERGKFRSLTLINWNGFFARTFDLDELVTTLSGGNGAGKSTTMAAFVTALIPDLTLLHF
RNTTEAGATSGSRDKGLHGKLKAGVCYSMLDTINSHHQRVVVGVRLQQVAGRDRKVDIKP
FAIQGLPMSVQPTQLVTETLNERQARVLPLNELKDKLEAMEGVQFKQFNSITDYHSLMFD
LGIIARRLRSASDRSKFYRLIEASLYGGISSAITRSLRDYLLPENSGVRKAFQDMEAALR
ENRMTLEAIRVTQSDRDLFKHLISEATNYVAADYMRHANERRVHLDKALEFRRELHTSRK
QLAAEQYKHVDMARELAEHNGAEGDLEADYQAASDHLNLVQTALRQQEKIERYEADLDEL
QIRLEEQNEVVAEAIERQEENEARAEAAELEVDELKSQLADYQQALDVQQTRAIQYNQAI
AALNRAKELCHLPDLTADSAAEWLETFQAKELEATEKMLSLEQKMSMAQTAHSQFEQAYQ
LVVAINGPLARNEAWDVARELLREGVDQRHLAEQVQPLRMRLSELEQRLREQQEAERLLA
DFCKRQGKNFDIDELEALHQELEARIASLSDSVSNAREERMALRQEQEQLQSRIQSLMQR
APVWLAAQNSLNQLSEQCGEEFTSSQDVTEFLQQLLEREREAIVERDEVGARKNAVDEEI
ERLSQPGGSEDQRLNALAERFGGVLLSEIYDDVSLEDAPYFSALYGPSRHAIVVPDLSQV
TEHLEGLTDCPEDLYLIEGDPQSFDDSVFSVDELEKAVVVKIADRQWRYSRFPEVPLFGR
AARESRIESLHAEREVLSERFATLSFDVQKTQRLHQAFSRFIGSHLAVAFESDPEAEIRQ
LNSRRVELERALSNHENDNQQQRIQFEQAKEGVTALNRILPRLNLLADDSLADRVDEIRE
RLDEAQEAARFVQQFGNQLAKLEPIVSVLQSDPEQFEQLKEDYAYSQQMQRDARQQAFAL
TEVVQRRAHFSYSDSAEMLSGNSDLNEKLRERLEQAEAERTRAREALRGHAAQLNQYNQV
LASLKSSYDTKKELLNDLQRELQDIGVRADSGAEERARIRRDELHAQLSNNRSRRNQLEK
ALTFCEAEMDNLTRKLRKLERDYFEMREQVVTAKAGWCAVMRMVKDNGVERRLHRRELAY
LSADDLRSMSDKALGALRLAVADNEHLRDVLRMSEDPKRPERKIQFFVAVYQHLRERIRQ
DIIRTDDPVEAIEQMEIELSRLTEELTSREQKLAISSRSVANIIRKTIQREQNRIRMLNQ
GLQNVSFGQVNSVRLNVNVRETHAMLLDVLSEQHEQHQDLFNSNRLTFSEALAKLYQRLN
PQIDMGQRTPQTIGEELLDYRNYLEMEVEVNRGSDGWLRAESGALSTGEAIGTGMSILVM
VVQSWEDESRRLRGKDISPCRLLFLDEAARLDARSIATLFELCERLQMQLIIAAPENISP
EKGTTYKLVRKVFQNTEHVHVVGLRGFAPQLPETLPGSDEAPSQAS
>LFGLNPFC_02392 Chromosome partition protein MukE
MSSTNIEQVMPVKLAQALANPLFPALDSALRSGRHIGLDELDNHAFLMDFQEYLEEFYAR
YNVELIRAPEGFFYLRPRSTTLIPRSVLSELDMMVGKILCYLYLSPERLANEGIFTQQEL
YDELLTLADEAKLLKLVNNRSTGSDVDRQKLQEKVRSSLNRLRRLGMVWFMGHDSSKFRI
TESVFRFGADVRAGDDPREAQRRLIRDGEAMPIENHLQLNDETEESQPDSGEEE
>LFGLNPFC_02393 Chromosome partition protein MukF
MSEFSQTVPELVAWARKNDFSISLPVDRLSFLLAVATLNGERLDGEMSEGELVDAFRHVS
DAFEQTSETIGVRANNAINDMVRQRLLNRFTSEQAEGNAIYRLTPLGIGITDYYIRQREF
STLRLSMQLSIVAGELKRAADAAEEGGDEFHWHRNVYAPLKYSVAEIFDSIDLTQRLMDE
QQQQVKDDIAQLLNKDWRAAISSCELLLSETSGTLRELQDTLEAAGDKLQANLLRIQDAT
MTHDDLHFVDRLVFDLQSKLDRIISWGQQSIDLWIGYDRHVHKFIRTAIDMDKNRVFAQR
LRQSVQTYFDEPWALTYANADRLLDMRDEEMVLRDEEVTGELPEDLEYEEFNEIREQLAA
IIEEQLAVYKTRQVPLDLGLVVREYLSQYPRARHFDVARIVIDQAVRLGVAQADFTGLPA
KWQPINDYGAKVQAHVIDKY
>LFGLNPFC_02394 tRNA 5-carboxymethoxyuridine methyltransferase
MQDRNFDDIAEKFSRNIYGTTKGQLRQAILWQDLDRVLAEMGPQKLRVLDAGGGEGQTAI
KMAERGHQVILCDLSVQMIDRAKQAAEAKGVSDNMQFIHCAAQDVASHLETPVDLILFHA
VLEWVADPRSVLQTLWSVLRPGGVLSLMFYNAHGLLMHNMVAGNFDYVQAGMPKKKKRML
SPDYPRDPAQVYLWLEEAGWQIMGKTGVRVFHDYLREKHQQRDCYEALLELETRYCRQEP
YITLGRYIHVTARKPQSKDKV
>LFGLNPFC_02395 hypothetical protein
MLFTLKKVIGNMLLPLPLMLLIIGAGLALLWFSRFQKTGKIFISIGWLALLLLSLQPVAD
RLLRPIESTYPTWNNSQKVDYIVVLGGGYTWNPQWAPSSNLINNSLPRLNEGIRLWRENP
GSKLIFTGGVAKTNTVSTAEVGARVAQSLGVPREQIITLDLPKDTEEEAAAVKQAIGDAP
FLLVTSASHLPRAMIFFQQEGLNPLPAPANQLAIDSPLNPWERAIPSPVWLMHSDRVGYE
TLGRIWQWLKGSSGEPRQE
>LFGLNPFC_02396 hypothetical protein
MEQLRAELSHLLGEKLSRIECVNEKADTALWALYDSQGNPMPLMARSFSAPGKARQLAWK
TTMLARSGTVRMPTIYGVMTHEEHPGPDVLLLERMRGVSVEAPARTPERWEQLKDQIVEA
LLAWHRQDSRGCVGAVDNTQENFWPSWYRQHVEVLWTTLNQFNNTGLTMQDKRILFRTRE
CLPALFEGFNDNCVLIHGNFCLRSMLKDSRSDQLLAMVGPGLMLWAPREYELFRLMDNSL
AEDLLWSYLQRAPVAESFIWRRWLYVLWDEVAQLVNTGRFSRRNFDLASKSLLPWLA
>LFGLNPFC_02397 3-deoxy-manno-octulosonate cytidylyltransferase
MSFVVIIPARYASTRLPGKPLVDINGKPMIVHVLERARESGAERIIVATDHEDVARAVEA
AGGEVCMTRADHQSGTERLAEVVEKCAFSDDTVIVNVQGDEPMIPATIIRQVADNLAQRQ
VGMATLAVPIHNAEEAFNPNAVKVVLDAEGYALYFSRATIPWDRDRFAKDLETVGDNFLR
HLGIYGYRAGFIRRYVTWQPSPLEHIEMLEQLRVLWYGEKIHVAVAQEVPGTGVDTPEDL

KRVRAEMR
>LFGLNPFC_02398 hypothetical protein
MDHRLLEIIACPVCNGKLWYNQEKQELICKLDNLAFPLRDGIPVLLETEARVLTADESKS
>LFGLNPFC_02399 hypothetical protein
MSLPHLSLADARNLHLAAQGLLNKPRRRASLEDIPATISRMSLLQIDTINIVVRSPYLVL
FSRLGDYPAQWLDESLARGELMEYWAHEACFMPRSDFRLIRHRMLAPEKMGWKYKDAWMQ
EHAAEIALLIQHIHDKGPVRSADFEHPRKGASGWWEWKPHKRHLEGLFTAGKVMVIERRN
FQRVYDLTHRVMPDWDDERDLVSQAEAEIIMLDNSARSLGIFREQWLADYYRLKRPALAA
WREARAEQQQIIAVHVEKLGNLWLHADLLPLLERALAGKLTATHSAVLSPFDPVVWDRKR
AEQLFDFSYRLECYIPAPKRQYGYFVLPLLHRGQLVGRMDAKMHRQTGILEVISLWLQEG
IKPTTTLQKGLRQAITDFANWQQATRVTLGRCPQGLFTDCRAGWEIDPVA
>LFGLNPFC_02400 Tetraacyldisaccharide 4'-kinase
MIEKIWSGESPLWRLLLPLSWLYGLVSGAIRLCYKLKLKRAWRAPVPVVVVGNLTAGGNG
KTPVVVWLVEQLQQRGIRVGVVSRGYGGKAESYPLLLSADTTTAQAGDEPVLIYQRTGAP
VAVSPVRSDAVKAILAQHPDVQIIVTDDGLQHYRLARDVEIVVIDGVRRFGNGWWLPAGP
MRERAGRLKSVDAVIVNGGVPRSGEIPMHLLPGQAVNLRTGTRCDVAQLEHVVAIAGIGH
PPRFFATLKMCGVQPEKCVPLADHQSLNHADVSALVSAGQTLVMTEKDAVKCRAFAEENW
WYLPVDAQLSGDEPAKLLAQLTSLASGH
>LFGLNPFC_02401 Lipid A export ATP-binding/permease protein MsbA
MHNDKDLSTWQTFRRLWPTIAPFKAGLIVAGVALILNAASDTFMLSLLKPLLDDGFGKTD
RSVLMWMPLVVIGLMILRGITSYISSYCISWVSGKVVMTMRRRLFGHMMGMPVSFFDKQS
TGTLLSRITYDSEQVASSSSGALITVVREGASIIGLFIMMFYYSWQLSIILIVLAPIVSI
AIRVVSKRFRNISKNMQNTMGQVTTSAEQMLKGHKEVLIFGGQEVETKRFDKVSNRMRLQ
GMKMVSASSISDPIIQLIASLALAFVLYAASFPSVMDSLTAGTITVVFSSMIALMRPLKS
LTNVNAQFQRGMAACQTLFTILDSEQEKDEGKRVIERATGDVEFRNVTFTYPGRDVPALR
NINLKIPAGKTVALVGRSGSGKSTIASLITRFYDIDEGEILMDGHDLREYTLASLRNQVA
LVSQNVHLFNDTVANNIAYARTEQYSREQIEEAARMAYAMDFINKMDNGLDTVIGENGVL
LSGGQRQRIAIARALLRDSPILILDEATSALDTESERAIQAALDELQKNRTSLVIAHRLS
TIEKADEIVVVEDGVIVERGTHNDLLEHRGVYAQLHKMQFGQ
>LFGLNPFC_02402 hypothetical protein
MKITTVGVCIICGIFPLLILPQLPGTVTLAFLTLFACVLAFIPVKTVRYIALTLLFFVWG
ILAAKQILWAGETLTGATQDAIVEIIATDGMTTHYGQITHLQGRRIFPAPGLVLYGEYLP
QAVCAGQVWSMKLKVRAVHGQLNDGGFDSQRYAIAQHQPLTGRFLQASVIEPNCSLRAQY
LASLQTTLQPYMWNAVILGLGIGERLSVPKEIKNIMRDTGTAHLMAISGLHIAFAALLAA
GLIRGGQVFLPGCWIHWQMPLIGGICCAAFYAWLTGMQPPALRTVVALAIWGMLKLSGRQ
WSGWDVWICCLAAILLMDPVAILSQSLWLSAAAVAALIFWYQWFPCPEWQLPPVLRAVVS
LIHLQLGITLLLMPVQIVIFHGISLTSFIANLFAIPLVTFITVPLILAAMVVHLSGPFIL
EQGLWFLADRSLALLFWGLKSLPEGWINIAERWQWLTFSPWFLLVVWRLNAWRTLPAMCV
AVGLLMCLPLWQKPRPDEWQVYMLDVGQGLAMVIARNGKAILYDTGLAWPEGDSGQQLII
PWLHWHNLEPEGVILSHEHLDHRGGLDSILHTWPMLWIRSPLNWEHHQPCVRGEAWQWQG
LRFSAHWPLQGSNDKGNNHSCVVKIDDGTNSILLTGDIEAPAEQKMLSRYWQQMQATLLQ
VPHHGSNTSSSLPLIQRVNGKVALASASRYNAWRLPSSKVKHRYQQQGYKWLDTPHQGQI
TVNFSAQGWRISSLREQILPRWYHQWFGVPVDNG
>LFGLNPFC_02403 Integration host factor subunit beta
MTKSELIERLATQQSHIPAKTVEDAVKEMLEHMASTLAQGERIEIRGFGSFSLHYRAPRT
GRNPKTGDKVELEGKYVPHFKPGKELRDRANIYG
>LFGLNPFC_02404 30S ribosomal protein S1
MTESFAQLFEESLKEIETRPGSIVRGVVVAIDKDVVLVDAGLKSESAIPAEQFKNAQGEL
EIQVGDEVDVALDAVEDGFGETLLSREKAKRHEAWITLEKAYEDAETVTGVINGKVKGGF
TVELNGIRAFLPGSLVDVRPVRDTLHLEGKELEFKVIKLDQKRNNVVVSRRAVIESENSA
ERDQLLENLQEGMEVKGIVKNLTDYGAFVDLGGVDGLLHITDMAWKRVKHPSEIVNVGDE
ITVKVLKFDRERTRVSLGLKQLGEDPWVAIAKRYPEGTKLTGRVTNLTDYGCFVEIEEGV
EGLVHVSEMDWTNKNIHPSKVVNVGDVVEVMVLDIDEERRRISLGLKQCKANPWQQFAET
HNKGDRVEGKIKSITDFGIFIGLDGGIDGLVHLSDISWNVAGEEAVREYKKGDEIAAVVL
QVDAERERISLGVKQLAEDPFNNWVALNKKGAIVTGKVTAVDAKGATVELADGVEGYLRA
SEASRDRVEDATLVLSVGDEVEAKFTGVDRKNRAISLSVRAKDEADEKDAIATVNKQEDA
NFSNNAMAEAFKAAKGE
>LFGLNPFC_02405 Cytidylate kinase
MTAIAPVITIDGPSGAGKGTLCKAMAEALQWHLLDSGAIYRVLALAALHHHVDVASEDAL
VPLASHLDVRFVSTNGNLEVILEGEDVSGEIRTQEVANAASQVAAFPRVREALLRRQRAF
RELPGLIADGRDMGTVVFPDAPVKIFLDASSEERAHRRMLQLQEKGFSVNFERLLAEIKE
RDDRDRNRAVAPLVPAADALVLDSTTLSIEQVIEKALQYARQKLALA
>LFGLNPFC_02406 Metalloprotease YcaL
MKNTKLLLAIATSAALLTGCQNTHGINTDLAISSGLNAYKAATLSDADAKAIANQGCVEM

DSGNKVASKSSKYGKRLAKIAKALGNNINGTPVNYKVYMTSDVNAWAMANGCVRVYSGLM
DMMNDNEIEGVLGHELGHVALGHSLAEMKASYAIVAARDAISATSGVASQLSRSQLGDIA
EGAINAKYSRDKESEADDFSFDLLKKRGISTQGLVGSFEKLASLDGGRTQSMFDSHPPST
ERAQHIRDRIASGK
>LFGLNPFC_02407 3-phosphoshikimate 1-carboxyvinyltransferase
MESLTLQPIARVDGTINLPGSKSVSNRALLLAALAHGKTVLTNLLDSDDVRHMLNALTAL
GVSYTLSADRTRCEIIGNGGPLHAESARELFLGNAGTAMRPLAAALCLGSNDIVLTGEPR
MKERPIGHLVDALRQGGAKITYLEQENYPPLRLQGGFTGGNVDVDGSVSSQFLTALLMTA
PLAPEDTVIRIKGDLVSKPYIDITLNLMKTFGVEIENQHYQQFVVKGGQSYQSPGTYLVE
GDASSASYFLAAAAIRGGTVKVTGIGRNSMQGDIRFADVLEKMGATICWGDDYISCTRGE
LNAIDMDMNHIPDAAMTIATAALFAKGTTTLRNIYNWRVKETDRLFAMATELRKVGAEVE
EGHDFIRITPPEKLKFAEIATYNDHRMAMCFSLVALSDTPVTILDPKCTAKTFPDYFEQL
ARISQPG
>LFGLNPFC_02408 Phosphoserine aminotransferase
MAQIFNFSSGPAMLPAEVLEQAQQELRDWNGLGTSVMEVSHRGKEFIQVAEEAEKDFRDL
LNVPSNYKVLFCHGGGRGQFAAVPLNILGDKTTADYVDAGYWAASAIKEAKKYCTPNVFD
AKVTVDGLRAVKPMSEWQLSDNAAYMHYCPNETIDGIAIDETPDFGKDVVVAADFSSTIL
SRPIDVSRYGVIYAGAQKNIGPAGLTIVIVREDLLGKANIACPSILDYSILNDNDSMFNT
PPTFAWYLSGLVFKWLKANGGVAAMDKINQQKAELLYGVIDNSDFYRNDVAKANRSRMNV
PFQLADSALDKLFLEESFAAGLHALKGHRVVGGMRASIYNAMPLEGVKALTDFMVEFERR
HG
>LFGLNPFC_02409 hypothetical protein
MKAFDLHRIAFDKVPFDFLGEVALRSLYTFVLVFLFLKMTGRRGVRQMSLFEVLIILTLG
SAAGDVAFYDDVPMVPVLIVFITLALLYRLVMWLMAHSEKLEDLLEGKPVVIIEDGELAW
SKLNNSNMTEFEFFMELRLRGVEQLGQVRLAILETNGQISVYFFEDDKVKPGLLILPSDC
TQRYKVVPESADYACIRCSEIIHMNAGEKQLCPRCANPEWTKASRAKRVT
>LFGLNPFC_02410 Ribosomal protein S12 methylthiotransferase accessory factor YcaO
MTQTFIPGKDAALEDSIARFQQKLSDLGFQIEEASWLNPVPNVWSVHIRDKECALCFTNG
KGATKKAALASALGEYFERLSTNYFFADFWLGETIANGPFVHYPNEKWFPLTENDDVPEG
LLDDRLRAFYDPENELTGSMLIDLQSGNEDRGICGLPFTRQSDNQTIYIPMNIIGNLYVS
NGMSAGNTRNEARVQGLSEVFERYVKNRIIAESISLPEIPADVLARYPAVVEAIETLEAE
DFPIFAYDGSLGGQYPVICVVLFNPANGTCFASFGAHPDFGVALERTVTELLQGRGLKDL
DVFTPPTFDDEEVAEHTNLETHFIDSSGLISWDLFKQDADYPFVDWSFSGTTEEEFATLM
AIFKKEDKEVYIADYEHLGVYACRIIVPGMSDIYPAEDLWLANNSMGSHLRETILSLPGS
EWEKEDYLNLIEQLDEEGFDDFTRVRELLGLATGSDNGWYTLRIGELKAMLALAGGDLEQ
ALVWAEWTMEFNSSVFSPERANYYRCLQTLLLLAQEEDRQPLQYLNAFVRMYGADAVEAA
SAAMSGEAAFYGLQPVDSDLHAFAAHQSLLKAYEKLQRAKAAFWAK
>LFGLNPFC_02411 hypothetical protein
MPRFTYICINKIIVIIRLNIGNLYLINTIDIVTLFARCSLLINCFSGGCGKNSTHLLIFK
IYFYLDNQIFTPYLHKNHASYGPISQARYDLYQFLIYNALLVSRRRLNKERVSVKADNPF
DLLLPAAMAKVAEEAGVYKATKHPLKTFYLAITAGVFISIAFVFYITATTGTGTMPFGMA
KLVGGICFSLGLILCVVCGADLFTSTVLIVVAKASGRITWGQLAKNWLNVYFGNLVGALL
FVLLMWLSGEYMTANGQWGLNVLQTADHKVHHTFIEAVCLGILANLMVCLAVWMSYSGRS
LMDKAFIMVLPVAMFVASGFEHSIANMFMIPMGIVIRDFASPEFWTAVGSAPENFSHLTV
MNFITDNLIPVTIGNIIGGGLLVGLTYWVIYLRENDHH
>LFGLNPFC_02412 Formate acetyltransferase 1
MSELNEKLATAWEGFTKGDWQNEVNVRDFIQKNYTPYEGDESFLAGATEATTTLWDKVME
GVKLENRTHAPVDFDTAVASTITSHDAGYINKQLEKIVGLQTEAPLKRALIPFGGIKMIE
GSCKAYNRELDPMIKKIFTEYRKTHNQGVFDVYTPDILRCRKSGVLTGLPDAYGRGRIIG
DYRRVALYGIDYLMKDKLAQFTSLQADLENGVNLEQTIRLREEIAEQHRALGQMKEMAAK
YGYDISGPATNAQEAIQWTYFGYLAAVKSQNGAAMSFGRTSTFLDVYIERDLKAGKITEQ
EAQEMVDHLVMKLRMVRFLRTPEYDELFSGDPIWATESIGGMGLDGRTLVTKNSFRFLNT
LYTMGPSPEPNMTILWSEKLPLNFKKFAAKVSIDTSSLQYENDDLMRPDFNNDDYAIACC
VSPMIVGKQMQFFGARANLAKTMLYAINGGVDEKLKMQVGPKSEPIKGDVLNYDEVMERM
DHFMDWLAKQYITALNIIHYMHDKYSYEASLMALHDRDVIRTMACGIAGLSVAADSLSAI
KYAKVKPIRDEDGLAIDFEIEGEYPQFGNNDPRVDDLAVDLVERFMKKIQKLHTYRDAIP
TQSVLTITSNVVYGKKTGNTPDGRRAGAPFGPGANPMHGRDQKGAVASLTSVAKLPFAYA
KDGISYTFSIVPNALGKDDEVRKTNLAGLMDGYFHHEASIEGGQHLNVNVMNREMLLDAM
ENPEKYPQLTIRVSGYAVRFNSLTKEQQQDVITRTFTQSM
>LFGLNPFC_02413 Pyruvate formate-lyase 1-activating enzyme
MSVIGRIHSFESCGTVDGPGIRFITFFQGCLMRCLYCHNRDTWDTHGGKEVTVEDLMKEV
VTYRHFMNASGGGVTASGGEAILQAEFVRDWFRACKKEGIHTCLDTNGFVRRYDPVIDEL
LEVTDLVMLDLKQMNDEIHQNLVGVSNHRTLEFAKYLANKNVKVWIRYVVVPGWSDDDDS
AHRLGEFTRDMGNVEKIELLPYHELGKHKWVAMGEEYKLDGVKPPKKETMERVKGILEQY

GHKVMF
>LFGLNPFC_02414 putative MFS-type transporter YcaD
MSTYTRPVMLLLSGLLLLTLAIAVLNTLVPLWLAQEHMSTWQVGVVSSSYFTGNLVGTLL
TGYVIKRIGFNRSYYLASFIFAAGCAGLGLMIGFWSWLAWRFVAGIGCAMIWVVVESALM
CSGTSRNRGRLLAAYMMVYYVGTFLGQLLVSKVSTELMSVLPWVTGLTLAGILPLLFTHV
LNQQAENHDSTSITSMLKLRQARLGVNGCIISGIVLGSLYGLMPLYLNHKGVSNASIGFW
MAVLVSAGILGQWPIGRLADKFGRLLVLRVQVFVVILGSIAMLSQAAMAPALFILGAAGF
TLYPVAMAWACEKVEHHQLVAMNQALLLSYTVGSLLGPSFTAMLMQNFSDNLLFIMIASV
SFIYLLMLLRNAGHTPKPVAHV
>LFGLNPFC_02415 putative hydrolase YcaC
MTKPYVRLDKNDAAVLLVDHQAGLLSLVRDIEPDKFKNNVLALGDLAKYFNLPTILTTSF
ETGPNGPLVPELKAQFPDAPYIARPGNINAWDNEDFVKAVKATGKKQLIIAGVVTEVCVA
FPALSAIEEGFDVFVVTDASGTFNEITRHSAWDRMSQAGAQLMTWFGVACELHRDWRNDI
EGLATLFSNHIPDYRNLMTSYDTLTKQK
>LFGLNPFC_02416 Anaerobic dimethyl sulfoxide reductase chain C
MGSGWHEWPLMIFTVFGQCVAGGFIVLALALLKGDLRAEAQQRVIACMLGLWVLMGIGFI
ASMLHLGSPMRAFNSLNRVGASALSNEIASGSIFFAVGGIGWLLAMLKKLSPALRTLWLV
VTMVLGVVFVWMMVRVYNSIDTVPTWYSIWTPMGFFLTMFMGGPLLGYLLLSLAGVDGWA
MRLLPAISVLALVVSGVMSVMQGAELANIHSSVQQAAALVPDYGALMSWRIVLLAVALCL
WIAPQLKGYQPAVPLLSVSFILLLAGELIGRGVFYGLHMTVGMAVAS
>LFGLNPFC_02417 Anaerobic dimethyl sulfoxide reductase chain B
MTTQYGFFIDSSRCTGCKTCELACKDYKDLTPEVSFRRIYEYAGGDWQEDNGVWHQNVFA
YYLSISCNHCEDPACTKVCPSGAMHKREDGFVVVDEDVCIGCRYCHMACPYGAPQYNETK
GHMTKCDGCYDRVAEGKKPICVESCPLRALDFGPIDELRKKHGDLAAVAPLPRAHFTKPN
IVIKPNANSRPTGDTTGYLANPKEV
>LFGLNPFC_02418 Dimethyl sulfoxide reductase DmsA
MKTKIPDAVLAAEVSRRGLVKTTAIGGLAMASSALTLPFSRIAHAVDSAIPTKSDEKVIW
SACTVNCGSRCPLRMHVVDGEIKYVETDNTGDDNYDGLHQVRACLRGRSMRRRVYNPDRL
KYPMKRVGARGEGKFERISWEEAYDIIATKMQRLIKEYGNESIYLNYGTGTLGGTMTRSW
PPGNTLVARLMNCCGGYLNHYGDYSSAQIAEGLNYTYGGWADGNSPSDIENSKLVVLFGN
NPGETRMSGGGVTYYLEQARQKSNARMIIIDPRYTDTGAGREDEWIPIRPGTDAALVNGL
AYVMITENLVDQEFLDKYCVGYDEKTLPASAPKNGHYKAYILGEGPDGVAKTPEWASQIT
GVPADKIIKLAREIGSTKPAFISQGWGPQRHANGEIATRAISMLAILTGNVGINGGNSGA
REGSYSLPFVRMPTLENPIQTSISMFMWTDAIERGPEMTALRDGVRGKDKLDVPIKMIWN
YAGNCLINQHSEINRTHEILQDDKKCELIVVIDCHMTSSAKYADILLPDCTASEQMDFAL
DASCGNMSYVIFNDQVIKPRFECKTIYEMTSELAKRLGVEQQFTEGRTQEEWMRHLYAQS
REAIPELPTFEEFRKQGIFKKRDPQGHHVAYKAFREDPQANPLTTPSGKIEIYSQALADI
AATWELPEGDVIDPLPIYTPGFESYQDPLNKQYPLQLTGFHYKSRVHSTYGNVDVLKAAC
RQEMWINPLDAQKRGINNGDKVRIFNDRGEVHIEAKVTPRMMPGVVALGEGAWYDPDTKR
VDKGGCINVLTTQRPSPLAKGNPSHTNLVQVEKV
>LFGLNPFC_02419 Serine--tRNA ligase
MLDPNLLRNEPDAVAEKLARRGFKLDVDKLGALEERRKVLQVKTENLQAERNSRSKSIGQ
AKARGEDIEPLRLEVNKLGEELDAAKAELDALQAEIRDIALTIPNLPADEVPVGKDENDN
VEVSRWGTPREFDFEVRDHVTLGEMHSGLDFAAAVKLTGSRFVVMKGQIARMHRALSQFM
LDLHTEQHGYSENYVPYLVNQDTLYGTGQLPKFAGDLFHTRPLEEEADTSNYALIPTAEV
PLTNLVRGEIIDEDDLPIKMTAHTPCFRSEAGSYGRDTRGLIRMHQFDKVEMVQIVRPED
SMAALEEMTGHAEKVLQLLGLPYRKIILCTGDMGFGACKTYDLEVWIPAQNTYREISSCS
NVWDFQARRMQARCRSKSDKKTRLVHTLNGSGLAVGRTLVAVMENYQQADGRIEVPEVLR
PYMNGLEYIG
>LFGLNPFC_02420 Replication-associated recombination protein A
MSNLSLDFSDNTFQPLAARMRPENLAQYIGQQHLLAVGKPLPRAIEAGHLHSMILWGPPG
TGKTTLAEVIARYANADVERISAVTSGVKEIREAIERARQNRNAGRRTILFVDEVHRFNK
SQQDAFLPHIEDGTITFIGATTENPSFELNSALLSRARVYLLKSLSTEDIEQVLTQAMED
KTRGYGGQDIVLPDETRRAIAELVNGDARRALNTLEMMADMAEVDDSGKRVLKPELLTEI
AGERSARFDNKGDRFYDLISALHKSVRGSAPDAALYWYARIITAGGDPLYVARRCLAIAS
EDVGNADPRAMQVAIAAWDCFTRVGPAEGERAIAQAIVYLACAPKSNAVYTAFKAALADA
RERPDYDVPVHLRNAPTKLMKEMGYGQEYRYAHDEANAYAAGEVYFPPEIAQTRYYFPTN
RGLEGKIGEKLAWLAEQDQNSPIKRYR
>LFGLNPFC_02421 Outer-membrane lipoprotein carrier protein
MKKIAITCALLSSLVASSVWADAASDLKSRLDKVSSFHASFTQKVTDGSGAAVQEGQGDL
WVKRPNLFNWHMTQPDESILVSDGKTLWFYNPFVEQATATWLKDATGNTPFMLIARNQSS
DWQQYNIKQNGDDFVLTPKASNGNLKQFTINVGRDGTIHQFSAVEQDDQRSSYQLKSQQN
GAVDAAKFTFTPPQGVTVDDQRK
>LFGLNPFC_02422 DNA translocase FtsK

MSQEYTEDKEVTLTKLSSGRRLLEALLILIVLFAVWLMAALLSFNPSDPSWSQTAWHEPI
HNLGGMPGAWLADTLFFIFGVMAYTIPVIIVGGCWFAWRHQSSDEYIDYFAVSLRIIGVL
ALILTSCGLAAINADDIWYFASGGVIGSLLSTTLQPLLHSSGGTIALLCVWAAGLTLFTG
WSWVTIAEKLGGWILNILTFASNRTRRDDTWVDEDEYEDDEEYEDENHGKQHESRRARIL
RGALARRKRLAEKFINPMGRQTDAALFSGKRMDDEEEITYTARGVAADPDDVLFSGNRAT
QPEYDEYDPLLNGAPITEPVAVAAAATTATQSWAAPVEPVTQTPPVASVDVPPTQPTVAW
QPVPGPQTGEPVIAPAPEGYPQQSQYAQPAVQYNEPLQQPVQPQQPYYAPAAEQPVQQPY
YAPAPEQSAQQPYYAPAPEQPVAGNAWQAEEQQSTFAPQSTYQTEQTYQQPAAQEPLYQQ
PQPVEQQPVVEPEPVVEETTPTRPPLYYFEEVEEKRAREREQLAAWYQPIPEPVKEPEPI
KSSLKAPSVAAVPPVEAAAAVSPLASGVKKATLATGAAATVAAPVFSLANGGGPRPQVKE
GIGPQLPRPKRIRVPTRRELASYGIKLPSQRAAEEKAREAQRNQYDSGDQYNDDEIDAMQ
QDELARQFAQTQQQRYGEQYQHDVPVNTEDADAAAEAELARQFAQTQQQRYSGEQPAGAN
PFSLDDFEFSPMKALLDDGPHEPLFTPIVEPVQQPQQPVAPQQQYQQPQQPVAPQQQYQQ
PQQPVAPQPQYQQPQYQQPQQPVAQQPQYQQPQQPVAQQPQYQQPQQPVVSQPQDTLLHP
LLMRNGDSRPLHKPTTPLPSLDLLTPPPSEVEPVDTFALEQMARLVEARLADFRIKADVV
NYSPGPVITRFELNLAPGVKAARISNLSRDLARSLSTVAVRVVEVIPGKPYVGLELPNKK
RQTVYLREVLDNAKFRDNPSPLTVVLGKDIAGEPVVADLAKMPHLLVAGTTGSGKSVGVN
AMILSMLYKAQPEDVRFIMIDPKMLELSVYEGIPHLLTEVVTDMKDAANALRWCVNEMER
RYKLMSALGVRNLAGYNEKIAEADRMMRPIPDPYWKPGDSMDAQHPVLKKEPYIVVLVDE
FADLMMTVGKKVEELIARLAQKARAAGIHLVLATQRPSVDVITGLIKANIPTRIAFTVSS
KIDSRTILDQAGAESLLGMGDMLYSGPNSTLPVRVHGAFVRDQEVHAVVQDWKARGRPQY
VDGITSDSESEGGVGGFDGAEELDPLFDQAVQFVTEKRKASISGVQRQFRIGYNRAARII
EQMEAQGIVSEQGHNGNREVLAPPPFD
>LFGLNPFC_02423 Leucine-responsive regulatory protein
MVDSKKRPGKDLDRIDRNILNELQKDGRISNVELSKRVGLSPTPCLERVRRLERQGFIQG
YTALLNPHYLDASLLVFVEITLNRGAPDVFEQFNTAVQKLEEIQECHLVSGDFDYLLKTR
VPDMSAYRKLLGETLLRLPGVNDTRTYVVMEEVKQSNRLVIKTR
>LFGLNPFC_02424 Thioredoxin reductase
MLPIAQIVNKIVILFFYVCKFPTILPLSANNYGDLMGTTKHSKLLILGSGPAGYTAAVYA
ARANLQPVLITGMEKGGQLTTTTEVENWPGDPNDLTGPLLMERMHEHATKFETEIIFDHI
NKVDLQNRPFRLTGDSGEYTCDALIIATGASARYLGLPSEEAFKGRGVSACATCDGFFYR
NQKVAVIGGGNTAVEEALYLSNIASEVHLIHRRDGFRAEKILIKRLMDKVENGNIILHTN
RTLEEVTGDQMGVTGVRLRDTQNSDNIESLDVAGLFVAIGHSPNTAIFEGQLELENGYIK
VQSGIHGNATQTSIPGVFAAGDVMDHIYRQAITSAGTGCMAALDAERYLDGLADAK
>LFGLNPFC_02425 ATP-binding/permease protein CydD
MNKSRQKELTRWLKQQSVISQRWLNISRLLGFVSGILIISQAWFMARILQHMIMENIPRE
ALLLPFTLLFLTFVLRAWVVWLRERVGYHAGQHIRFAIRRQVLDRLQQAGPAWIQGKPAG
SWATLVLEQIDDMHDYYARYLPQMALAVSVPLLIVVAIFPSNWAAALILLGTAPLIPLFM
ALVGMGAADANRRNFLALARLSGHFLDRLRGMETLRIFGRGEAEIESIRSASEDFRQRTM
EVLRLAFLSSGILEFFTSLSIALVAVYFGFSYLGELDFGHYDTGVTLAAGFLALILAPEF
FQPLRDLGTFYHAKAQAVGAADSLKTFMETPLAHPQRGEAELASTDPVTIEAEDLFITSP
EGKTLAGPLNFTLPAGQRAVLVGRSGSGKSSLLNALSGFLSYQGSLRINGIELRDLSPES
WRKHLSWVGQNPQLPAATLRDNVLLARPDASEQELQTALDNAWVSEFLPLLPQGIDSPVG
DQAARLSVGQAQRVAVARALLNPCSLLLLDEPAASLDAHSEQRVMEALNTASLRQTTLMV
THQLEDLADWDVIWVMQDGQIIEQGRYAELSVAGGPFATLLAHRQEEI
>LFGLNPFC_02426 Lipid A export ATP-binding/permease protein MsbA
MRALLPYLALYKRHKWMLSLGIVLAIVTLLASIGLLTLSGWFLSASAVAGVAGLYSFNYM
LPAAGVRGAAITRTAGRYFERLVSHDATFRVLQHLRIYTFSKLLPLSPAGLARYRQGELL
NRVVADVDTLDHLYLRVISPLVGAFVVIMVVTIGLSFLDFTLAFTLGGIMLLTLFLMPPL
FYRAGKSTGQNLTHLRGQYRQQLTAWLQGQAELTIFGASDRYRTQLENTEIQWLEAQRRQ
SELTALSQAIMLLIGALAVILMLWMASGGVGGNAQPGALIALFVFCALAAFEALAPVTGA
FQHLGQVIASAVRITDLTDQKPEVTFPDTQTRVADRVSLTLRDVQFTYPEQSQQALKGIY
LQVNAGEHIAILGRTGCGKSTLLQLLTRAWDPQQGEILLNDSPIASLNEAALRQTISVVP
QRVHLFSATLRDNLLLASPGSSDEALAEILRRVGLEKLLEDAGLNSWLGEGGRQLSGGEL
RRLAIARALLHDAPLVLLDEPIEGLDATTESQILELLAEMMCEKTVLMVTHRLRGLSRFQ
QIIVMDNGQIIEQGTHAELLARQGRYYQFKQGL
>LFGLNPFC_02427 Leucyl/phenylalanyl-tRNA--protein transferase
MRLVQLSRHSIAFPSPEGALREPNGLLALGGDLSPARLLMAYQRGIFPWFSPGDPILWWS
PDPRAVLWPESLHISRSMKRFHKRSPYRVTMNYAFGQVIEGCASDREEGTWITRGVVEAY
HRLHELGHAHSIEVWREDELVGGMYGVAQGTLFCGESMFSRMENASKTALLVFCDEFIRH
GGKLIDCQVLNDHTASLGACEIPRRDYLNYLNQMRLGRLPNNFWVPRCLFSPQE
>LFGLNPFC_02428 Translation initiation factor IF-1
MAKEDNIEMQGTVLETLPNTMFRVELENGHVVTAHISGKMRKNYIRILTGDKVTVELTPY
DLSKGRIVFRSR

>LFGLNPFC_02430 CRISPR-associated endonuclease Cas6/Csy4
MDHYLEIRVLPDPEFSSEMLMAALFAKLHRVLGARGQGDIGVSFPDVNVMPGARLRLHGS
AQALQALEASTWRKGLTDYCQCSPVTPVPEIKGWRVVSRVQVKSNPQRLLRRSVKKGWLT
EEQAIERLATQAEQRTDLPFLNMKSLSSQQLFKLFIRHGDLLKEPVKGEFSSYGLSATAT
IPWF
>LFGLNPFC_02431 CRISPR-associated protein Csy3
MAKAPVAIKTASVLAFERKLATSDAVMYAGNWQGNEWQPIEIQEKAVRGTISNRLKNAIV
SDPTKLDAEIQKANLQRVDVASLPVDTDTLKVVFTLRVLGNLSTPSVCNDMAYQEALSGV
IEGYISEHGFKELALRYALNLANGRFLWRNRIGAEQIQVKVTANETSWTFNSHDFSLRQF
DQGQAAVTQLAAIIEQGLSAKEWVMLTVEAQVRLGAGQEVFPSQELVLDSNSSKSRVLYQ
VAGIAGIHSQKIGNALRTIDTWHPKVDELGAIAVEPYGSVTSRGVACRQPKEKLDFYTLL
DNWVTKGMKPDVEQQHYVMAVLIRGGVFGEKSE
>LFGLNPFC_02432 CRISPR-associated protein Csy2
MHLTVSLLIECNGEITNGEYGRKVLCDYLKMLCQSHKLAGGSIVSMRDPQLFHAPEDEKQ
LRKIVWRLMPGYALYDRSEWLAEHHQQHPDISLLDAWLDFAAIKYQAESPAEDNSAKWVY
QPKPIPGFLVPLMCGYQRISPVYAPGEVENARDTVTPFAFAEAVYGIGEWRGLHRTTDLQ
ALMWRYRTTDTGYYCSATPVVDDFTFNEYDDLE
>LFGLNPFC_02433 CRISPR-associated protein Csy2
MSYLLLLPHIRIENANAVSGLTWGFPSMTHFLGYVHALSRKVVDEFGVSFDGCAVVSHEQ
HIQAYSSGRDF
>LFGLNPFC_02434 CRISPR-associated protein Csy1
MEKTALIQFITEYIASRRQPKIDAFEKEAAKRVEQGEDASVIAQERQELEARYLPRNWLT
DAAKRAGQIKLVTHGAKFSHGDSKASSFYLETSANESYLNTASLANVATDAIGNAAALDV
AKLLQTDVNGDSLLASLKRGDYQALSTFAEDKVQLELWVAGFSQAWTTGRPSSHKLAKQI
YFPVADGYHLLCPLFSSSLAQAMYEKLTAVRFSEESKAIRDARKAGKWHSQPDIWFPNLA
EMHFGGTKPQNISLLNSVRGGRVWLLPSMPPTWATLDRAPQNMRSIFSLRGDFNRAASGI
VAQMTYLLKVDINNVHIRTVRAKYIDELIDLLFMQASAFQQEKWQGWSAQSPDLPRHQQL
WLDPWRSLSDETFKQEREKNDWQVTVADDFARWLNYRLKKSSFDVGAVEQKEWRSQSLFA
QRMREMEAVLQEALK
>LFGLNPFC_02435 CRISPR-associated nuclease/helicase Cas3 subtype I-F/YPEST
MNVLIISRCTKNARVESCRIIDQFAERTGDAAWQTVITLEGVNTLRRLLRKTARRNTAVA
CHWLKKNGQTELMWIVGNIRRFNAQGRVPTNRTTQISLVNKEENRWQCAESIALLAAIAG
LFHDFGKSGRSFQQSLTQKNTRSYQPYRHEWISLRLFQTFVGERPDDVWLAKLGELTADD
ESAILERLQKDTPQFSNSPFSSLQPLAKTVGWLILSHHRMPENLSPQSEQSCKGCESWLE
KQLNADWNAVNHRRDDWSQQDFAHVWQFPSGTPLQSASWREKARQIARRASNALGLKTYG
QMEQLFPLHMARFCLMLADHYYSSSDAQQKWQDPDYIVWANSDRQSGTLKQRVDEHLVGV
AHHAYLLGRQLMHTRASLPAIARHKGFSQRATDVNYRWQDKAWDVARALRERSREEGFFG
VNMASTGCGKTFANARIMYALADEREGCRFTVALGLRTLTLQTGDALRSRLGLGEDELAV
LIGSAAVNQLWQQEKIDNDCGSASQESPAEEQQFVKYEGSLHTGALEKWLKDDSKLKELV
SAPILVSTIDHLISATEGVRGGKQLPAMLRLLTSDLVLDEPDDFDIADLHAVCRLMNWAG
MLGTRVLLSSATLPPGLIQALFAAYLAGRKMWQASCGINGRPVNICCAWFDEKDADATQI
YDGPGFRDAHAKFVARRAVMLAEKERLHFGRVASVSSASGAIQDVTESVAQTVHTQMLKL
HQAHRQRHESGKTVSLGLVRFANINPLVAVTKALIAIPSPEDVCIHYCVYHSRHPLAVRS
DIEKRLDRAFTRHNELDFWNNEDIADALHNRPESHHLFVVLGTSVIEVGRDWDADWGIIE
PSSMRSIIQFAGRIQRHRRNVPTSENLVILRSNIKSLQKKAPAFCKPGFETKEHLLNSHD
ICELIPIADYRTINALPRILSANNANKLAELEHSRLSAELMSSKSKHVVAAQWWRAPLSW
NGWMQRQTPFRYSPLPEAVFFLHMDDEESEARFYSRTGDNERKAQGNFRREEVRCASGVA
CWGVMDYQHVLLNLADKQSCEIANVGEKFAEVRVPVSEEDAVDDWRYHPWLGVFRNM
>LFGLNPFC_02436 CRISPR-associated endonuclease Cas1
MSSNYLTPSDLKTILHSKRANIYYLEKCRVQVNGGRVEYVTSEGKESYYWNIPIANTTAL
ILGMGTSVTQAAMREFAHAGVMVGFCGTDGTPLYSANEVDVDVSWLSPQSEYRPTEYLQQ
WVSFWFVEDKRLAAAKRFQLIRLTHIDKHWSSSKMLREHAFQPDVNALHTLLNRTCEEID
AAENHTQLMLVEAKLTKALYKMVSQTVGYGDFTRAKRGGGIDMANRFLDQGNYLAYGLAA
VAAWVTGIPHGLAVMHGKTRRGGLVFDLADLIKDALVMPQAFIAAMAGEDAQEFRQRCVN
IFQQADALDVMITSLQETAQALAKADQ
>LFGLNPFC_02437 ATP-dependent Clp protease ATP-binding subunit ClpA
MLNQELELSLNMAFARAREHRHEFMTVEHLLLALLSNPSAREALEACSVDLVALRQELEA
FIEQTTPVLPASEEERDTQPTLSFQRVLQRAVFHVQSSGRNEVTGANVLVAIFSEQESQA
AYLLRKHEVSRLDVVNFISHGTRKDEPTQSSDPGSQPNSEEQAGGEERMENFTTNLNQLA
RVGGIDPLIGREKELERAIQVLCRRRKNNPLLVGESGVGKTAIAEGLAWRIVQGDVPEVM
ADCTIYSLDIGSLLAGTKYRGDFEKRFKALLKQLEQDTNSILFIDEIHTIIGAGAASGGQ
VDAANLIKPLLSSGKIRVIGSTTYQEFSNIFEKDRALARRFQKIDITEPSIEETVQIING
LKPKYEAHHDVRYTAKAVRAAVELAVKYINDRHLPDKAIDVIDEAGARARLMPVSKRKKT
VNVADIESVVARIARIPEKSVSQSDRDTLKNLGDRLKMLVFGQDKAIEALTEAIKMARAG

LGHEHKPVGSFLFAGPTGVGKTEVTVQLSKALGIELLRFDMSEYMERHTVSRLIGAPPGY
VGFDQGGLLTDAVIKHPHAVLLLDEIEKAHPDVFNILLQVMDNGTLTDNNGRKADFRNVV
LVMTTNAGVRETERKSIGLIHQDNSTDAMEEIKKIFTPEFRNRLDNIIWFDHLSTDVIHQ
VVDKFIVELQVQLDQKGVSLEVSQEARNWLAEKGYDRAMGARPMARVIQDNLKKPLANEL
LFGSLVDGGQVTVALDKEKNELTYGFQSAQKHKAEAAH
>LFGLNPFC_02438 ATP-dependent Clp protease adapter protein ClpS
MGKTNDWLDFDQLAEEKVRDALKPPSMYKVILVNDDYTPMEFVIDVLQKFFSYDVERATQ
LMLAVHYQGKAICGVFTAEVAETKVAMVNKYARENEHPLLCTLEKA
>LFGLNPFC_02439 Cold shock-like protein CspD
MEKGTVKWFNNAKGFGFICPEGGGEDIFAHYSTIQMDGYRTLKAGQSVQFDVHQGPKGNH
ASVIVPVEVEAAVA
>LFGLNPFC_02440 Macrolide export ATP-binding/permease protein MacB
MTPLLELKDIRRSYPAGDEQVEVLKGISLDIYAGEMVAIVGASGSGKSTLMNILGCLDKA
TSGTYRVAGQDVATLDADALAQLRREHFGFIFQRYHLLSHLTAEQNVEVPAVYAGLERKQ
RLLRAQELLQRLGLEDRTEYYPAQLSGGQQQRVSIARALMNGGQVILADEPTGALDSHSG
EEVMAILHQLRDRGHTVIIVTHDPQVAAQAERVIEIRDGEIVRNPPAVEKVNATGGTEPV
VNTASGWRQFVSGFNEALTMAWRALAANKMRTLLTMLGIIIGIASVVSIVVVGDAAKQMV
LADIRSIGTNTIDVYPGKDFGDDDPQYQQALKYDDLIAIQKQPWVASATPAVSQNLRLRY
NNVDVAASANGVSGDYFNVYGMTFSEGNTFNQEQLNGRAQVVVLDSNTRRQLFPHKADVV
GEVILVGNMPARVIGVAEEKQSMFGSSKVLRVWLPYSTMSGRVMGQSWLNSITVRVKEGF
DSAEAEQQLTRLLSLRHGKKDFFTWNMDGVLKTVEKTTRTLQLFLTLVAVISLVVGGIGV
MNIMLVSVTERTREIGIRMAVGARASDVLQQFLIEAVLVCLVGGALGITLSLLIAFTLQL
FLPGWEIGFSPLALLLAFLCSTVTGILFGWLPARNAARLDPVDALARE
>LFGLNPFC_02441 Macrolide export protein MacA
MKKRKTVKKRYVIAMVIVIAGLITLWRILNAPVPTYQTLIVRPGDLQQSVLATGKLDALR
KVDVGAQVSGQLKTLSVAIGDKVKKDQLLGVIDPEQAENQIKEVEAMLMELRAQRQQAEA
ELKLARVTYSRQQRLAQTQAVSQQDLDNAATEMAVKQAQIGTIDAQIKRNQASLDTAKTN
LDYTRIVAPMAGEVTQITTLQGQTVIAAQQAPNILTLADMSTMLVKAQVSEADVIHLKPG
QKAWFTVLGDPLTRYEGQIKDVLPTPEKINDAIFYYARFEVPNPNGLLRLDMTAQVHIQL
TDVKNVLTIPLSALGDPVGDNRYKVKLLRNGETREREVTIGARNDTDVEIVKGLEAGDEV
VIGEAKPGAAQ
>LFGLNPFC_02442 hypothetical protein
MVKSTSCITIDFMNMSQLTERTFTPSESLSSLSLFLSLARGQCRPGKFWHRRSFRQKFLL
RSLIMPRLSVEWMNELSHWPNLNVLLTRQPRLPVRLHRPYLAANLSRKQLLEALRYHYAL
LRGCMSAEEFSLYLNTPGLQLAKLEGKNGEQFTLELTMMISMDKEGDSTILFRNSEGIPL
AELTFTLCEYQGKRTMFIGGLQGAKWEIPHQEIQNATKACHGLFPKRLVMEAACLFAQRL
QVEQIIAVSNETHIYRSLRYRDKEGKIHADYNAFWESVGGVCDAERHYRLPAQIARKEIA
EIASKKRAEYRRRYEMLDAIQPQMATMFCS
>LFGLNPFC_02443 DNA replication and repair protein RecF
MILERVEIVGFRGINRLSLMLEQNNVLIGENAWGKSSLLDALTLLLSPESDLYHFERDDF
WFPPGDINGREHHLHIILTFRESLPGRHRVRRYRPLEACWTPCTDGYHRIFYRLEGESAE
DGSVMTLRSFLDKDGHPIDVEDINDQARHLVRLMPVLRLRDARFMRRIRNGTVPNVPNVE
VTARQLDFLARELSSHPQNLSDGQIRQGLSAMVQLLEHYFSEQGAGQARYRLMRRRASNE
QRSWRYLDIINRMIDRPGGRSYRVILLGLFATLLQAKGTLRLDKDARPLLLIEDPETRLH
PIMLSVAWHLLNLLPLQRIATTNSGELLSLTPVEHVCRLVRESSRVAAWRLGPSGLSTED
SRRISFHIRFNRPSSLFARCWLLVEGETETWVINELARQCGHHFDAEGIKVIEFAQSGLK
PLVKFARRMGIEWHVLVDGDEAGKKYAATVRSLLNNDREAEREHLTALPALDMEHFMYRQ
GFSDVFHRVAQIPENIPMNLRKVISKAIHRSSKPDLAIEVAMEAGRRGVDSVPTLLKKMF
SRVLWLARGRAD
>LFGLNPFC_02444 Aquaporin Z
MFRKLAAECFGTFWLVFGGCGSAVLAAGFPELGIGFAGVALAFGLTVLTMAFAVGHISGG
HFNPAVTIGLWAGGRFPAKEVVGYVIAQVVGGIVAAALLYLIASGKTGFDAAASGFASNG
YGEHSPGGYSMLSALVVELVLSAGFLLVIHGATDKFAPAGFAPIAIGLALTLIHLISIPV
TNTSVNPARSTAVAIFQGGWALEQLWFFWVVPIVGGIIGGLIYRTLLEKRD
>LFGLNPFC_02445 Lysine exporter LysO
MFSGLLIILVPLIVGYLIPLRQKAALRVINQLLSWMVYLILFFMGISLAFLDNLASNLLA
ILHYSAVSITVILLCNIAALMWLERGLPWRNHHHQEKLPSRIAMALESLKLCGVVVIGFA
IGLSGLAFLQHATEASEYTLILLLFLVGIQLRNNGMTLKQIVLNRRGMIVAVVVVASSLI
GGLINAFILDLPINTALAMASGFGWYSLSGILLTESFGPVIGSAAFFNDLARELIAIMLI
PGLIRRSRSTALGLCGATSMDFTLPVLQRTGGLDMVPAAIVHGFILSLLVPILIAFFSA
>LFGLNPFC_02446 Hydroxylamine reductase
MFCVQCEQTIRTPAGNGCSYAQGMCGKTAETSDLQDLLIAALQGLSAWAVKAREYGIINH
DVDSFAPRAFFSTLTNVNFDSPRIVGYAREAIALREALKAQCLAVDANARVDNPMADLQL
VSDDLGELQRQAAEFTPNKDKAAIGENILGLRLLCLYGLKGAAAYMEHAHVLGQYDNDIY

AQYHKIMAWLGTWPADMNALLECSMEIGQMNFKVMSILDAGETGKYGHPTPTQVNVKATA
GKCILISGHDLKDLYNLLEQTEGTGVNVYTHGEMLPAHGYPELRKFKHLVGNYGSGWQNQ
QVEFARFPGPIVMTSNCIIDPTVGAYDDRIWTRSIVGWPGVRHLDGEDFSAVIAQAQQMA
GFPYSEIPHLITVGFGRQTLLGAADTLIDLVSREKLRHIFLLGGCDGARGERHYFTDFAT
SVPDDCLILTLACGKYRFNKLEFGDIEGLPRLVDAGQCNDAYSAIILAVTLAEKLGCGVN
DLPLSLVLSWFEQKAIVILLTLLSLGVKNIVTGPTAPGFLTPDLLAVLNEKFGLRSITTV
EEDMKQLLSA
>LFGLNPFC_02447 NADH oxidoreductase HCR
MTMPTNQCPWRMQVHHITQETPDVWTISLICHDYYPYRAGQYALVSVRNSAETLRAYTIS
STPGVSEYITLTVRRIDDGVGSRWLTRDVKRGDYLWLSDAMGEFTCDDKAEDKFLLLAAG
CGVTPIMSMRRWLAKNRPQADVQVIYNVRTPQDVIFADEWRNYPVTLVAENNVTEGFIAG
RLTRELLTRVPDLASRTVMTCGPAPYMDWVEQEVKALGVTRFFKEKFFTPVAEAATSGLK
FTKLQPAREFYAPVGTTLLEALESNNVPVVAACRAGVCGCCKTKVISGEYTVSSTMTLTD
AEIAEGYVLACSCHPQGDLVLA
>LFGLNPFC_02448 Pyruvate dehydrogenase [ubiquinone]
MKQTVAAYIAKTLESAGVKRIWGVTGDSLNGLSDSLNRMGTIEWMSTRHEEVAAFAAGAE
AQLSGELAVCAGSCGPGNLHLINGLFDCHRNHVPVLAIAAHIPSSEIGSGYFQETHPQEL
FRECSHYCELVSSPEQIPQVLAIAMRKAVLNRGVSVVVLPGDVALKPAPEGATTHWYHAP
QPVVTPEEEELRKLAQLLRYSSNIALMCGSGCAGAHKELVEFAGKIKAPIVHALRGKEHV
EYDNPYDVGMTGLIGFSSGFHTMMNADTLVLLGTQFPYRVFYPTDAKIIQIDINPASIGA
HSKVDMALVGDIKSTLRALLPLVEEKTERKFLDKALEDYRDARKGLDDLAKPSEKAIHPQ
YLAQQISHFAADDAIFTCDVGTPTVWAARYLKMNGKRRLLGSFNHGSMANAMPQALGAQA
TEPERQVVAMCGDGGFSMLMGDFLSVVQMKLPVKIIVFNNSVLGFVAMEMKAGGYLTDGT
ELHDTNFARIAEACGITGIRVEKASEIDEALQRAFSIDGPVLVDVVVAKEELAIPPQIKL
EQAKGFSLYMLRAIISGRGDEVIELAKTNWLR
>LFGLNPFC_02449 Low specificity L-threonine aldolase
MIDLRSDTVTRPSRAMLEAMMAAPVGDDVYGDDPTVNALQDYAAELSGKEAAIFLPTGTQ
ANLVALLSHCERGEEYIVGQAAHNYLFEAGGAAVLGSIQPQPIDAAADGTLPLDKVAMKI
KPDDIHFARTKLLSLENTHNGKVLPREYLKEAWEFTRKRNLALHVDGARIFNAVVAYGCE
LKEITQYCDSFTICLSKGLGTPVGSLLVGNRDYIKRAIRWRKMTGGGMRQSGILAAAGMY
ALKNNVARLQEDHDNATWMAEQLREAGTDVMRQDTNMLFVRVGEENAAALGEYMKARNVL
INASPIVRLVTHLDVSRAQLAEVAAHWRAFLAR
>LFGLNPFC_02450 hypothetical protein
MPQRILVLGASGYIGQHLVRTLSQQGHQLLAAARHVDRLAKLQLANVSCHKVDLSWPDNL
PALLQDIDTVYFLVHSMGEGGDFIAQERQVALNVRDALREVPVKQLIFLSSLQAPPHEQS
DHLRARQATADILREAGVPVTELRAGIIVGAGSAAFEVMRDMVYNLPVLTPPRWVRSRTT
PIALENLLHYLVALLDHPASEHRIFEAAGPEVLSYQQQFEHFMAVSGKRRWLLPIPFPTR
WISVWFLNVITSVPPTTARALIQGLKHDLLADDTALRALIPQPLIAFDDAVRSTLKEEEK
LVNSSDWGYDAQAFARWRPEYGYFAKQAGFTVKTSARLQALWRVVNQIGGKERYFFGNIL
WQTRALMDLAIGHKLAKGRPEREYLQTGDAVDSWKVIVVEPEKQLTLLFGMKAPGLGRLC
FTLEDKGDYRTIDVRAFWHPHGMPGLFYWLLMIPAHLFIFRGMAKRIARLAEQSTD
>LFGLNPFC_02451 2-alkyl-3-oxoalkanoate reductase
MKVLVTGATSGLGRNAVEFLCQKGISVRATGRNEAMGKLLEKMGAEFVPADLTELVSSQA
KVMLAGIDTLWHCSSFTSPWGTQQAFDLANVRATRRLGEWAVAWGVRNFIHISSPSLYFD
YHHHRDIKEDFRPHRFANEFARSKAASEEVINMLSQANPQTRFTILRPQSLFGPHDKVFI
PRLAHMMHHYGSILLPHGGSALVDMTYYENAVHAMWLASQEACDKLPSGRVYNITNGEHR
TLRSIVQKLIDELNIDCRIRSVPYPMLDMIARSMERLGRKSAKEPPLTHYGVSKLNFDFT
LDITRAQEELGYQPVLTLDEGIEKTAAWLRDHGKLPR
>LFGLNPFC_02452 N-acetylmuramoyl-L-alanine amidase AmiD
MRRVFWLVAAALLLAGCTGEKGIVEKEGYQLDTRRQAQAAYPRIKVLVIHYTADDFDSSL
ATLTDKQVSSHYLVPAVPPRYNGKPRIWQLVPEQELAWHAGISAWRGATRLNDTSIGIEL
ENRGWQKSAGVKYFAPFEPAQIQALIPLAKDIIARYYIKPENVVAHADIAPQRKDDPGPL
FPWKQLAQQGIGAWPDAQRVNFYLAGRAPHTPVETASLLELLARYGYDVKPDMTPREQRR
VIMAFQMHFRPTLYNGEADAETQAIAEALLEKYGQD
>LFGLNPFC_02453 hypothetical protein
MQFSTTPTLEGQTIVEYCGVVTGEAILGANIFRDFFAGIRDIVGGRSGAYEKELRKAREI
AFEELGSQARALGADAVVGIDIDYETVGQNGSMLMVSVSGTAVKTRR
>LFGLNPFC_02454 putative lipoprotein YbjP
MRYSKLTMLIPCALLLSACTTVTPAYKDNGPRTGSCVQGGPDSVAQQFYDYRIQHRGNDI
TALRPYLSDKLATLLSDASRDNSHRELLSSDPFSSRTTLPDSAHVASASTIPNRDARNIP
LRVDLKQGDQGWQDEVLMIQEGQCWVIDDVRYLGGSVHATAGTLRQSIENR
>LFGLNPFC_02455 Arginine transport ATP-binding protein ArtP
MSIQLNGINCFYGAHQALFDITLDCPQGETLVLLGPSGAGKSSLLRVLNLLEMPRSGTLN
IAGNHFDFTKTPSDKAIRDLRRNVGMVFQQYNLWPHLTVQQNLIEAPCRVLGLSKDQALA

RAEKLLERLRLKPYSDRYPLHLSGGQQQRVAIARALMMEPQVLLFDEPTAALDPEITAQI
VSIIRELAETNITQVIVTHEVEVARKTASRVVYMENGHIVEQGDASCFTEPQTEAFKNYL
SH
>LFGLNPFC_02456 Putative ABC transporter arginine-binding protein 2
MKKVLIAALIAGFSLSATAAETIRFATEASYPPFESIDANNQIVGFDVDLAQALCKEIDA
TCTFSNQAFDSLIPSLKFRRVEAVMAGMDITPEREKQVLFTTPYYDNSALFVGQQGKYTS
VDQLKGKKVGVQNGTTHQKFIMDKHPEITTVPYDSYQNAKLDLQNGRIDSVFGDTAVVTE
WLKDNPKLAAVGDKVTDKDYFGTGLGIAVRQGNTELQQKLNTALEKVKKDGTYETIYNKW
FQK
>LFGLNPFC_02457 Arginine ABC transporter permease protein ArtQ
MNEFFPLASAAGMTVGLAVCALIVGLALAMFFAVWESAKWRPVAWAGSALVTILRGLPEI
LVVLFIYFGSSQLLLTLSDGFTINLGFVQIPVQMDIENFDVSPFLCGVIALSLLYAAYAS
QTLRGALKAVPVGQWESGQALGLSKSAIFFRLVMPQMWRHALPGLGNQWLVLLKDTALVS
LISVNDLMLQTKSIATRTQEPFTWYIVAAVIYLVITLLSQYILKRIDLRATRFERRPS
>LFGLNPFC_02458 Arginine ABC transporter permease protein ArtM
MFEYLPELMKGLHTSLTLTVASLIVALILALIFTIILTLKTPVLVWLVRGYITLFTGTPL
LVQIFLIYYGPGQFPTLQEYPALWHLLSEPWLCALIALSLNSAAYTTQLFYGAIRAIPEG
QWQSCSALGMSKKDTLAILLPYAFKRSLSSYSNEVVLVFKSTSLAYTITLMEVMGYSQLL
YGRTYDVMVFGAAGIIYLVVNGLLTLMMRLIERKALAFERRN
>LFGLNPFC_02459 ABC transporter arginine-binding protein 1
MKKLVLAALLASFTFGASAAEKINFGVSATYPPFESIGANNEIVGFDIDLAKALCKQMQA
ECTFTNHAFDSLIPSLKFRKYDAVISGMDITPERSKQVSFTTPYYENSAVVIAKKDTYKT
FADLKGKRIGMENGTTHQKYIQDQHPEVKTVSYDSYQNAFIDLKNGRIDGVFGDTAVVNE
WLKTNPQLGVATEKVTDPQYFGTGLGIAVRPDNKALLEKLNNALAAIKADGTYQKVNDQW
FPQ
>LFGLNPFC_02460 23S rRNA (uracil(747)-C(5))-methyltransferase RlmC
MQCALYDAGRCRSCQWITQPIPEQLSAKTVDLKNLLADFPVEEWCAPVSGPEQGFRNKAK
MVVSGSVEKPLLGMLHRDGTPEDLCDCPLYPASFAPVFAALKPFIARAGLTPYNVARKRG
ELKYILLTESQSDGGMMLRFVLRSDTKLAQLRKALPWLQEQLPQLKVITVNIQPVHMAIM
EGETEIYLTEQQALAERFNDVPLWIRPQSFFQTNPAVASQLYATARDWVRQLPVKHMWDL
FCGVGGFGLHCATPDMQLTGIEIAPEAIACAKQSAAELGLTRLQFQALDSTQFATAQGEV
PELVLVNPPRRGIGKPLCDYLSTMAPRFIIYSSCNAQTMAKDIRELPGYRIERVQLFDMF
PHTAHYEVLTLLVKQ
>LFGLNPFC_02461 Inner membrane protein YbjO
MEDETLGFFKKTSSSHARLNVPALVQVAALAIIMIRGLDVLMIFNTLGVRGIGEFIHRSV
QTWSLTLVFLSSLVLVFIEIWCAFSLVKGRRWARWLYLLTQITAASYLWAASLGYGYPEL
FSIPGESKREIFHSLMLQKLPDMLILMLLFVPSTSRRFFQLQ
>LFGLNPFC_02462 Inner membrane ABC transporter permease protein YdcV
MNNLPVVRSPWRIVILLLGFTFLYAPMLMLVIYSFNSSKLVTVWAGWSTRWYGELLRDDA
MMSAVGLSLTIAACAATAAAILGTIAAVVLVRFGRFRGSNGFAFMITAPLVMPDVITGLS
LLLLFVALAHAIGWPADRGMLTIWLAHVTFCTAYVAVVISSRLRELDRSIEEAAMDLGAT
PLKVFFVITLPMIMPAIISGWLLAFTLSLDDLVIASFVSGPGATTLPMLVFSSVRMGVNP
EINALATLILGAVGIVGFIAWYLMARAEKQRIRDIQRARRG
>LFGLNPFC_02463 Putrescine transport system permease protein PotH
MSTLEPAAQSKPPGGFKLWLSQLQMKHGRKLVIALPYIWLILLFLLPFLIVFKISLAEMA
RAIPPYTELMEWADGQLSITLNLGNFLQLTDDPLYFDAYLQSLQVAAISTICCLLIGYPL
AWAVAHSKPSTRNILLLLVILPSWTSFLIRVYAWMGILKNNGVLNNFLLWLGVIDQPLTI
LHTNFAVYIGIVYAYVPFMVLPIYTALIRIDYSLVEAALDLGARPLKTFFTVIMPLTKGG
IIAGSMLVFIPAVGEFVIPELLGGPDSIMIGRVLWQEFFNNRDWPVASAVAIIMLLLLIV
PIMWFHKHQQKSVGEHG
>LFGLNPFC_02464 Spermidine/putrescine import ATP-binding protein PotA
MNDAIPRPQAKTRKALTPLLEIRNLTKSYDGQHAVDDVSLTIYKGEIFALLGASGCGKST
LLRMLAGFEQPSAGQIMLDGVDLSQVPPYLRPINMMFQSYALFPHMTVEQNIAFGLKQDK
LPKAEIASRVNEMLGLVHMQEFAKRKPHQLSGGQRQRVALARSLAKRPKLLLLDEPMGAL
DKKLRDRMQLEVVDILERVGVTCVMVTHDQEEAMTMAGRIAIMNRGKFVQIGEPEEIYEH
PTTRYSAEFIGSVNVFEGVLKERQEDGLVLYSPGLVHPLKVDADASVVDNVPVHVALRPE
KIMLCEEPPANGCNFAVGEVIHIAYLGDLSVYHVRLKSGQMISAQLQNAHRHRKGLPTWG
DEVRLCWEVDSCVVLTV
>LFGLNPFC_02465 Putrescine-binding periplasmic protein
MTALNKKWLSGLVAGALMAVSVSTLAAEQKTLHIYNWSDYIAPDTVANFEKETGIKVVYD
VFDSNEVLEGKLMAGSTGFDLVVPSASFLERQLTAGVFQPLDKSKLPEWKNLDPELLKLV
AKHDPDNKFAMPYMWATTGIGYNVDKVKAVLGENAPVDSWDLILKPENLEKLKSCGVSFL
DAPEEVFATVLNYLGKDPNSTKADDYTGPATDLLLKLRPNIRYFHSSQYINDLANGDICV
AIGWAGDVWQASNRAKEAKNGVNVSFSIPKEGAMAFFDVFAMPADAKNKDEAYQFLNYLL

RPDVVAHISDHVFYANANKAATPLVSAEVRDNPGIYPPADVRAKLFTLKVQDPKIDRVRT
RAWTKVKSGK
>LFGLNPFC_02466 putative protein YbjN
MTSLVVPGLDTLRQWLDDLGMSFFECDNCQALHLPHMQNFDGVFDAKIDLIDNTILFSAM
AEVRPSAVLPLAADLSAINASSLTVKAFLDMQDDNLPKLVVCQSLSVMQGVTYEQFAWFV
RQSEEQISMVILEANAHQLLLPTDDEGQNNVTENYFLH
>LFGLNPFC_02467 Ribosomal protein S6--L-glutamate ligase
MKIAILSRDGTLYSCKRLREAAIQRGHLVEILDPLSCYMNINPAASSIHYKGRKLPHFDA
VIPRIGTAITFYGTAALRQFEMLGSYPLNESVAIARARDKLRSMQLLARQGIDLPVTGIA
HSPDDTSDLIDMVGGAPLVVKLVEGTQGIGVVLAETRQAAESVIDAFRGLNAHILVQEYI
KEAQGCDIRCLVVGDEVVAAIERRAKEGDFRSNLHRGGAASVASITPQEREIAIKAARTM
ALDVAGVDILRANRGPLVMEVNASPGLEGIEKTTGIDIAGKMIRWIERHATTEYCLKTGG
>LFGLNPFC_02468 Oxygen-insensitive NADPH nitroreductase
MTPTIELICGHRSIRHFTDEPISEAQREAIINSARATSSSSFLQCSSIIRITDKALREEL
VTLTGGQKHVAQAAEFWVFCADFNRHLQICPDAQLGLAEQLLLGVVDTAMMAQNALTAAE
SLGLGGVYIGGLRNNIEAVTELLKLPQHVLPLFGLCLGWPADNPDLKPRLPASILVHENS
YQPLDKDALAQYDEQLAEYYLTRGSNNRRDTWSDHIRRTIIKESRPFILDYLHKQGWATR
>LFGLNPFC_02469 hypothetical protein
MRAIGKLPKSVLILEFIGMMLLAVALLSVSDSLSLPEPFSRPEVQILMIFLGVLLMLPAA
VVVILQVAKRLAPQLMNHPPQYSRSEREKDNDANH
>LFGLNPFC_02470 Glutaredoxin 1
MQTVIFGRPGCPYCVRAKDLAEKLSNERDDFQYQYVDIRAEGITKEDLQQKAGKPVETVP
QIFVDQQHIGGYTDFAAWAKENLDA
>LFGLNPFC_02471 Inner membrane protein YbjM
MKHKQRWAGAICCFVLFIVVCLFLATHMKGAFRAAGHPEIGLLFFILPGAVASFFSHRRE
VLKPLFGAMLAAPCSMLIMRLFFSPTRSFWQELAWLLSAVFWCALGALCFLFISSLFKPQ
HRKNQ
>LFGLNPFC_02472 Aspartate/alanine antiporter
MNINVAELLNGNYILLLFVVLALGLCLGKLRLGSIQLGNSIGVLVVSLLLGQQHFSINTD
ALNLGFMLFIFCVGVEAGPNFFSIFFRDGKNYLMLALVMVGSALVIALGLGKLFGWDIGL
TAGMLAGSMTSTPVLVGAGDTLRHSGMESRQLSLALDNLSLGYALTYLIGLVSLIVGARY
LPKLQHQDLQTSAQQIARERGLDTDANRKVYLPVIRAYRVGPELVAWTDGKNLRELGIYR
QTGCYIERIRRNGILANPDGDAVLQMGDEIALVGYPDAHARLDPSFRNGKEVFDRDLLDM
RIVTEEVVVKNHNAVGKRLAQLKLTDHGCFLNRVIRSQIEMPIDDNVVLNKGDVLQVSGD
ARRVKTIADRIGFISIHSQVTDLLAFCAFFVIGLMIGMITFQFSTFSFGMGNAAGLLFAG
IMLGFMRANHPTFGYIPQGALSMVKEFGLMVFMAGVGLSAGSGINNGLGAIGGQMLIAGL
IVSLVPVVICFLFGAYVLRMNRALLFGAMMGARTCAPAMEIISDTARSNIPALGYAGTYA
IANVLLTLAGTIIVMVWPGLG
>LFGLNPFC_02473 HTH-type transcriptional regulator RcdA
MRRANDPQRREKIIQATLEAVKLYGIHAVTHRKIATLAGVPLGSMTYYFSGIDELLLEAF
SRFTEIMSRQYQAFFSDVSDAPGACQAITDMIYSSQVATPDNMELMYQLYALASRKPLLK
TVMQNWMQRSQQTLEQWFEPGTARALDAFIEGMTLHFVTDRKPLSREEILRMVERIAG
>LFGLNPFC_02474 Inner membrane protein YbjJ
MASWATRTPAIRDILSVSIAEMGGVLFGLSIGSMSGILCSAWLVKRFGTRNVILVTMSCA
LIGMMILSLALWLTSPLLFAVGLGVFGASFGSAEVAINVEGAAVEREMNKTVLPMMHGFY
SLGTLAGAGVGMALTAFGVPATVHILLAALVGIAPIYIAIQAIPDGTGKNAADGTQHGEK
GVPFYRDIQLLLIGVVVLAMAFAEGSANDWLPLLMVDGHGFSPTSGSLIYAGFTLGMTVG
RFTGGWFIDRYSRVAVVRASALMGALGIGLIIFVDSAWVAGVSVVLWGLGASLGFPLTIS
AASDTGPDAPTRVSVVATTGYLAFLVGPPLLGYLGEHYGLRSAMLVVLALVILAAIVAKA
VAKPDTKTQTAMENS
>LFGLNPFC_02475 5-amino-6-(5-phospho-D-ribitylamino)uracil phosphatase YbjI
MSIKLIAVDMDGTFLSDQKTYNRERFMAQYQQMKAQGIRFVVASGNQYYQLISFFPEIAN
EIAFVAENGGWVVSEGKDVFNGELSKDAFTTVVEHLLTRPEVEIIACGKNSAYTLKKYDD
AMKTVAEMYYHRLEYVDNFDNLEDIFFKFGLNLSDELIPQVQKALHEAIGDIMVPVHTGN
GSIDLIIPGVHKANGLRQLQKLWGIDDSEVVVFGDGGNDIEMLRQAGFSFAMENAGSAVV
AAAKYRAGSNNREGVLDVIDKVLKHEAPFNQ
>LFGLNPFC_02476 hypothetical protein
MKNCLLLGALLMGFTGVAMAQSVTVDVPSGYKVVVVPDSVSVATVPQTVYVAPAPAPAYR
PHPYVRHLASVGEGMVIEHQIDDHHH
>LFGLNPFC_02477 Multidrug transporter MdfA
MQNKLASGARLGRQALLFPLCLVLYEFSTYIGNDMIQPGMLAVVEQYQAGIDWVPTSMTA
YLAGGMFLQWLLGPLSDRIGRRPVMLAGVVWFIVTCLAILLAQNIEQFTLLRFLQGISLC
FIGAVGYAAIQESFEEAVCIKITALMANVALIAPLLGPLVGAAWIHVLPWEGMFVLFAAL
AAISFFGLQRAMPETATRIGEKLSLKELGRDYKLVLKNGRFVAGALALGFVSLPLLAWIA

QSPIIIIITGEQLSSYEYGLLQVPIFGALIAGNLLLARLTSRRTVRSLIIMGGWPIMIGLL
VAAAATVISSHAYLWMTAGLSIYAFGIGLANAGLVRLTLFASDMSKGTVSAAMGMLQMLI
FTVGIEISKHAWLNGGNGLFNLFNLVNGILWLSLMVIFLKDKQMGNSHEG
>LFGLNPFC_02478 Putative undecaprenyl-diphosphatase YbjG
MLENLNLSLFSLINATPDSAPWMISLAIFIAKDLITVVPLLAAVLWLWGLTAQRQLVIKI
AIALAVSLFVSWTMGHLFPHDRPFVENIGYNFLHHAADDSFPSDHGTVIFTFALAFLCWH
RLWSGSLLMVLAVVIAWSRVYLGVHWPLDMLGGLLAGMIGCLSAQIIWQAMGHKLYQRLQ
SWYRFCFALPIRKGWVRD
>LFGLNPFC_02479 Deoxyribose operon repressor
METRREERIGQLLQELKRSDKLHLKDAAALLGVSEMTIRRDLNNHSAPVVLLGGYIVLEP
RSASHYLLSDQKSRLVEEKRRAAKLAATLVEPDQTLFFDCGTTTPWIIEAIDNEIPFTAV
CYSLNTFLALKEKPHCRAFLCGGEFHASNAIFKPIDFQQTLNNFCPDIAFYSAAGVHVSK
GATCFNLEELPVKHWAMSMAQKHVLVVDHSKFGKVRPARMGDLKRFDIVVSDCCPEGEYV
KYAQTQRIKLMY
>LFGLNPFC_02480 D-alanyl-D-alanine carboxypeptidase DacC
MTQYSSLLRGLAAGSAFLFLFAPTAFAAEQTVEAPSVDARAWILMDYASGKVLAEGNADE
KLDPASLTKIMTSYVVGQALKADKIKLTDMVTVGKDAWATGNPALRGSSVMFLKPGDQVS
VADLNKGVIIQSGNDACIALADYVAGSQESFIGLMNGYAKKLGLTNTTFQTVHGLDAPGQ
FSTARDMALLGKALIHDVPEEYAIHKEKEFTFNKIRQPNRNRLLWSSNLNVDGMKTGTTA
GAGYNLVASATQGDMRLISVVLGAKTDRIRFNESEKLLTWGFRFFETVTPIKPDATFVTQ
RVWFGDKSEVNLGAGEAGSVTIPRGQLKNLKASYTLMEPQLTAPLKKGQVVGTIDFQLNG
KSIEQRPLIVMENVEEGGFFGRMWDFVMMKFHQWFGSWFS
>LFGLNPFC_02481 Glutathione S-transferase GstB
MITLWGRNNSTNVKKVLLTLEELELPYEQILAGREFGINHDADFLAMNPNGLVPLLRDDE
SDLILWESNAIVRYLAAQYGQKRLWIDSPARRAEAEKWMDWANQTLSNAHRGILMGLVRT
PPEERDQAAIDASCKECDALFALLDAELAKVKWFSGDEFGVGDIAIAPFIYNLFNVGLTW
TPRPNLQRWYQQLTERPAVRKVVMIPVS
>LFGLNPFC_02482 Aldose sugar dehydrogenase YliI
MHRQSFFLVPLICLSSALWAAPATVNVEVLQDKLDHPWALAFLPDNHGMLITLRGGELRH
WQAGKGLSAPLSGVPDVWAHGQGGLLDVVLAPDFAQSRRIWLSYSEVGDDGKAGTAVGYG
RLSDDLSKVTDFRTVFRQMPKLSTGNHFGGRMVFDGKGYLFIALGENNQRPTAQDLDKLQ
GKLVRLTDLGEIPDDNPFIKESGVRAEIWSYGIRNPQGMAMNPWSNALWLNEHGPRGGDE
INIPQKGKNYGWPLATWGINYSGFKIPEAKGEIVAGTEQPVFYWKDSPAVSGMAFYNSDK
FPQWQQKLFIGALKDKDVIVMSVNGDKVTEDGRILTDKGQRIRDVRTGPDGYLYVLTDES
SGELLKVSPRN
>LFGLNPFC_02483 hypothetical protein
MFVDRQRIDLLNRLIDARVDLAAYVQLRKAKGYMSVSESNHLRDNFFKLNRELHDKSLRL
NLHLDQEEWSALHHAEEALATAAVCLMSGHHDCPTVITVNADKLENCLMSLTLSIQSLQK
HAMLEKA
>LFGLNPFC_02484 Ribosomal protein S12 methylthiotransferase RimO
MSKVTPQPKIGFVSLGCPKNLVDSERILTELRTEGYDVVPSYDDADMVIVNTCGFIDSAV
QESLEAIGEALNENGKVIVTGCLGAKEDQIREVHPKVLEITGPHSYEQVLEHVHHYVPKP
KHNPFLSLVPEQGVKLTPRHYAYLKISEGCNHRCTFCIIPSMRGDLVSRPIGEVLSEAKR
LVDAGVKEILVISQDTSAYGVDVKHRTGFHNGEPVKTSMVSLCEQLSKLGIWTRLHYVYP
YPHVDDVIPLMAEGKILPYLDIPLQHASPRILKLMKRPGSVDRQLARIKQWRKICPELTL
RSTFIVGFPGETEEDFQMLLDFLKEARLDRVGCFKYSPVEGADANALPDQVPEEVKEERW
NRFMQLQQQISAERLQEKVGREILVIIDEVDEEGAIGRSMADAPEIDGAVYLNGETNVKP
GDILRVKVEHADEYDLWGSRV
>LFGLNPFC_02485 hypothetical protein
MSRINKFVLTVSLLIFIMISAVACGIYTQMVKERVYGLKQSVIDTAFAVANIAEYRRSVA
IDLINTLNPTEEQLLVGLRTAYADSVSPSYLYDVGPYLISSDECIQVKEFEKNYCADIMQ
VVKYRHVKNTGFISFDGKTFVYYLYPVTHNRSLIFLLGLERFSLLSKSLAMDSENLMFSL
FKNGKSVTGDEYNAKNAIFTVSEAMEHFSYLPTGLYVFAYKKDVYFQVCTLIIFFAALVA
VISGASCLYLVRRVINRGIVEKEAIINNHFERVLDGGLFFSAADVKKLYSMYNSAFLDDL
TKAMGRKSFDEDLKALPEKGGYLCLFDVDKFKNINDTFGHLLGDEVLMKVVKILKSQIPV
DKGKVYRFGGDEFAVIYTGGTLEELLSILKEIVHFQVGSINLSTSIGVAHSNECTTVERL
KMLADERLYKSKKNGRAQISWQ
>LFGLNPFC_02486 putative cyclic di-GMP phosphodiesterase PdeI
MLSLYEKIKIRLIILFLLAALSFIGLFFIINYQLVSERAVKRADSRFELIQKNVGYFFKD
IERSALTLKDSLYLLKNTEEIQRAVILKMEMMPFLDSVGLVLDDNKYYLFSRRANDKIVV
YHQEQVNGPLVDESGRVIFADFNPSKRPWSMASDDSNNSWNPAYNCFDRPGKKCISFTLH
INGKDHDLLAVDKIHVDLNWRYLNEYLDHISANDEVLFLKQGHEIIAKNQLAREKLIIYN
SEGNYNIIDSVDTEYIEKTSAVPNNALFEIYFYYPGGNLLNASDKLFYLPFAFIIIVLLV
VYLMTTRVFRRQFSEMTDLVNTLAFLPDSTDQIEALKIREGDAKEIISIKNSIAEMKDAE

IERANKLLSLISYDQESGFIKNMAIIESNNNQYLAVGIIKLCGLEAVEAVFGVDERNKIV
RKLCQRIAEKYAQCCDIVTFNADLYLLLCRENVQTFTRKIATVNDFDSSFGYRNLRIHKS
AICEPLQGENAWSYAEKLKLAISSIRNHMFSEFIFCDDAKLNEIEENIWIARNIRHAMEI
GELFLVYQPIVDINTRTILGAEALCRWVSAERGIISPLKFITIAEDIGFINELGYQIIKT
AMGEFRHFSQRASLKDDFLLHINVSPWQLNEPHFHERFTTIMKENGLKANSLCVEITETV
IERINEHFYLNIEQLRKQGVRVSIDDFGTGLSNLKRFYEINPDSIKVDSQFTGDIFGTAG
KIVRIIFDLARYNRIPVIAEGVESEDVARELIKLGCVQAQGYLYQKPMPFSAWDKSGKLV
KE
>LFGLNPFC_02487 Glutathione transport system permease protein GsiD
MRLFNWRRQAVLNAMPLVKPDQVRTPWHEFWRRFRRQHMAMTAALFVILLIVVAIFARWI
APYDAENYFDYDNLNNGPSLQHWFGVDSLGRDIFSRVLVGAQISLAAGVFAVFIGAAIGT
LLGLLAGYYEGWWDRLSMRICDVLFAFPGILLAIAVVAVLGSGIANVIIAVAIFSIPAFA
RLVRGNTLVLKQQTFIESARSIGASDMTILLRHILPGTVSSIVVFFTMRIGTSIISAASL
SFLGLGAQPPTPEWGAMLNEARADMVIAPHVAVFPALAIFLTVLAFNLLGDGLRDALDPK
IKG
>LFGLNPFC_02488 Glutathione transport system permease protein GsiC
MLNYVIKRLLGLIPTLFIVSVLVFLFVHMLPGDPARLIAGPEADAQVIELVRQQLGLDQP
LYHQFWHYISNAVQGDFGLSMVSRRPVADEIASRFMPTLWLTITSMVWAVIFGMAAGIIA
AVWRNRWPDRLSMTIAVSGISFPAFALGMLLIQVFSVELGWLPTVGADSWQHYILPSLTL
GAAVAAVMARFTRASFVDVLSEDYMRTARAKGVSETWVVLKHGLRNAMIPVVTMMGLQFG
FLLGGSIVVEKVFNWPGLGRLLVDSVEMRDYPVIQAEILLFSLEFILINLVVDVLYAAIN
PAIRYK
>LFGLNPFC_02489 Glutathione-binding protein GsiB
MARAVHRSGLVALGIATALMASCAFAAKEVVVAVGSNFTTLDPYDANDTLSQAVAKSFYQ
GLFGLDKEMKLKNVLAESYTVSDDGLTYTVKLREGIKFQDGTDFNAAAVKANLDRASDPA
NHLKRYNLYKNIAKTEAIDPTTVKITLKQPFSAFINILAHPATAMISPAALEKYGKEIGF
HPVGTGPYELDTWNQTDFVKVKKFAGYWQPGLPKLDSITWRPVADNNTRAAMLQTGEAQF
AFPIPYEQAALLEKNKNIELMASPSIMQRYISMNVTQKPFDNPKVREALNYAINRPALVK
VAFAGYATPATGVVPPSIAYAQSYKPWPYDPVKARELLKEAGYPNGFSTTLWSSHNHSTA
QKVLQFTQQQLAQVGIKAQVTAMDAGQRAAEVEGKGQKESGVRMFYTGWSASTGEADWAL
SPLFASQNWPPTLFNTAFYSNKQVDDFLAQALKTNDPAEKTRLYKAAQDIIWQESPWIPL
VVEKLVSAHSKNLTGFWIMPDTGFSFEDADLQ
>LFGLNPFC_02490 Glutathione import ATP-binding protein GsiA
MPHSDELDAGDVLAVENLNIAFMQEQHKIAAVRNLSFSLQRGETLAIVGESGSGKSVTAL
ALMRLLEQAGGLVQCDKMLLQRRSREVIELSEQSAAQMRHVRGADMAMIFQEPMTSLNPV
FTVGEQIAESIRLHQNASREEAMVEAKRMLDQVRIPEAQTILSRYPHQLSGGMRQRVMIA
MALSCRPAVLIADEPTTALDVTIQAQILQLIKVLQKEMSMGVIFITHDMGVVAEIADRVL
VMYQGEAVETGSVEQIFHAPQHPYTRALLAAVPQLGAMKGLDYPRRFPLISLEHPAKQEP
PIEQKTVVDGEPVLRVRNLVSRFPLRSGLLNRVTREVHAVEKVSFDLWPGETLSLVGESG
SGKSTTGRALLRLVESQGGEIIFNGQRIDTLSPGKLQALRRDIQFIFQDPYASLDPRQTI
GDSILEPLRVHGLLPGKEAAARVAWLLERVGLLPEHAWRYPHEFSGGQRQRICIARALAL
NPKVIIADEAVSALDVSIRGQIINLLLDLQRDFGIAYLFISHDMAVVERISHRVAVMYLG
QIVEIGPRRAVFENPQHPYTRKLLAAVPVAEPSRQRPQRVLLSDDLPSNIHLRGEEVAAV
SLQCVGPGHYVAQPQSEYAFMRR
>LFGLNPFC_02491 Isoaspartyl peptidase
MGKAVIAIHGGAGAISRAQMSLQQELRYIEALSAIVETGQKMLVAGESALDVVTEAVRLL
EECPLFNAGIGAVFTRDETHELDACVMDGNTLKAGAVAGVSHLRNPVLAARLVMEQSPHV
MMIGEGAENFAFAHGMECVSPEIFSTPLRYEQLLAAREEGATVLDHSGAPLDEKQKMGTV
GAVALDLDGNLAAATSTGGMTNKLPGRVGDSPLVGAGCYANNASVAVSCTGTGEVFIRAL
AAYDIAALMDYGGLSLAEACERVVMEKLPALGGSGGLIAIDHEGNVALPFNTEGMYRAWG
YAGDTPTTGIYREKGDTVATQ
>LFGLNPFC_02492 Molybdopterin molybdenumtransferase
MEFTTGLMSLDTALNEMLSRVTPLTAQETLPLVQCFGRILASDVVSPLDVPGFDNSAMDG
YAVRLADIASGQPLPVAGKSFAGQPYHGEWPAGTCIRIMTGAPVPEGCEAVVMQEQTEQT
DNGVRFTAEARSGQNIRRRGEDISAGAVVFPAGTRLTTAELPVIASLGIAEVPVIRKVRV
ALFSTGDELQLPGQPLGDGQIYDTNRLAVHLMLEQLGCEVINLGIIRDDPHALRAAFIEA
DSQADVVISSGGVSVGEADYTKTILEELGEIAFWKLAIKPGKPFAFGKLSNSWFCGLPGN
PVSATLTFYQLVQPLLAKLSGNTASGLPARQRVRTASRLKKTPGRLDFQRGVLQRNADGE
LEVTTTGHQGSHIFSSFSLGNCFIVLERERGNVDVGEWVEVEPFNALFGGL
>LFGLNPFC_02493 Molybdopterin-synthase adenylyltransferase
MAELSDQEMLRYNRQIILRGFDFDGQEALKDSRVLVVGLGGLGCAASQYLASAGVGNLTL
LDFDTVSLSNLQRQTLHSDATVGQPKVESARDALTRINPHIAITPVNALLDDAELAAMIA
KHDLVLDCTDNVAVRNQLNAGCFAAKVPLVSGAAIRMEGQITVFTYQDGEPCYHCLSRLF
GENALTCVEAGVMAPLIGVIGSLQAMEAIKLLAGYGKPASGKIVMYDAMTCQFREMKLMR

NPGCEVCGQ
>LFGLNPFC_02494 Fructose-6-phosphate aldolase 1
MELYLDTSDVVAVKALSRIFPLAGVTTNPSIIAAGKKPLEVVLPQLHEAMGGQGRLFAQV
MATTAEGMVNDARKLRSIIADIVVKVPVTAEGLAAIKMLKAEGIPTLGTAVYGAAQGLLS
ALAGAEYVAPYVNRIDAQGGSGIQTVTDLHQLLKMHTPQAKVLAASFKTPRQALDCLLAG
GESITLPLDVAQQMISYPAVDAAVAKFEQDWQGAFGRTSI
>LFGLNPFC_02495 Choline trimethylamine-lyase activating enzyme
MIFNIQRYSTHDGPGIRTVVFLKGCSLGCRWCQNPESRARTQDLLYDPRLCLEGCELCAK
TAPEVIERALNGLLIHREKLTPEHFTALTDCCPTQALTVCGEVKSVEEIMATVLRDKPFY
DRSGGGLTLSGGEPFMQPEMAMGLLQASHEAGIHTAVETCLHVPWKYIAPSLSYIDLFLA
DLKHVADAPFKQWTDGNAARVLDNLKKLAAAGKKIIIRVPLIQGFNADETSVKAITDFAA
DELHVSEIHFLPYHTLGINKYHLLNLPYDAPEKPLDAPELLDFAQQYACQKGLTATLRG
>LFGLNPFC_02496 Trans-4-hydroxy-L-proline dehydratase
MTTLKLDTLSDRIKAHKNALVHIVKPPVCTERAQHYTEMYQQHLDKPIPVRRALALAHHL
ANRTIWIKHDELIIGNQASEVRAAPIFPEYTVSWIEKEIDDLADRPGAGFAVSEENKRVL
HEVCPWWRGQTVQDRCYGMFTDEQKGLLATGIIKAEGNMTSGDAHLAVNFPLLLEKGLDG
LREKVAERRSRINLTVLEDLHGEQFLKAIDIVLVAVSEHIERFAALAREMAATETRESRR
DELLTIAENCDLIAHQPPQTFWQALQLCYFIQLILQIESNGHSVSFGRMDQYLYPYYRRD
VELNQTLDREHAIEMLHSCWLKLLEVNKIRSGSHSKASAGSPLYQNVTIGGQNLVDGQPM
DAVNPLSYAILESCGRLRSTQPNLSVRYHAGMSNDFLDACVQVIRCGFGMPAFNNDEIVI
PEFIKLGIEPQDAYDYAAIGCIETAVGGKWGYRCTGMSFINFARVMLAALEGGRDATSGK
VFLPQEKALSAGNFNNFDEVMDAWDTQIRYYTRKSIEIEYVVDTMLEENVHDILCSALVD
DCIERAKSIKQGGAKYDWVSGLQVGIANLGNSLAAVKKLVFEQGAIGQQQLAAALADDFD
GLTHEQLRQRLINGAPKYGNDDDTVDTLLARAYQTYIDELKQYHNPRYGRGPIGGNYYAG
TSSISANVPFGAQTMATPDGRKAHTPLAEGASPASGTDHLGPTAVIGSVGKLPTAAILGG
VLLNQKLNPATLENESDKQKLMILLRTFFEVHKGWHIQYNIVSRETLLEAKKHPDQYRDL
VVRVAGYSAFFTALSPDAQDDIIARTEHML
>LFGLNPFC_02497 Sugar phosphatase YbiV
MSVKVIVTDMDGTFLNDAKTYNQPRFMAQYQELKKRGIEFVVASGNQYYQLISFFPELKD
EISFVAENGALVYEHGKQLFHGELTRHESRIVIGELLKDKQLNFVACGLQSAYVSENAPE
AFVALMAKHYHRLKPVKDYQEIDDVLFKFSLNLPDEQIPLVIDKLHIALDGIMKPVTSGF
GFIDLIIPGLHKANGISRLLKRWDLSPQNVVAIGDSGNDAEMLKMARYSFAMSNAAENIK
QIARYATDDNNHEGALNVIQAVLENKPPFNL
>LFGLNPFC_02498 putative ABC transporter ATP-binding protein YbiT
MLVSSNVTMQFGSKPLFENISVKFGGGNRYGLIGANGSGKSTFMKILGGDLEPTLGNVSL
DPNERIGKLRQDQFAFEEFTVLDTVIMGHKELWEVKQERDRIYALPEMSEEDGYKVADLE
VKYGEMDGYSAEARAGELLLGVGIPVEQHYGPMSEVAPGWKLRVLLAQALFADPDILLLD
EPTNNLDIDTIRWLEQVLNERDSTMIIISHDRHFLNMVCTHMADLDYGELRVYPGNYDEY
MTAATQARERLLADNAKKKAQIAELQSFVSRFSANASKSRQATSRARQIDKIKLEEVKAS
SRQNPFIRFEQDKKLFRNALEVEGLTKGFDNGPLFKNLNLLLEVGEKLAVLGTNGVGKST
LLKTLVGDLQPDSGTVKWSENARIGYYAQDHEYEFENDLTVFEWMSQWKQEGDDEQAVRS
ILGRLLFSQDDIKKPAKVLSGGEKGRMLFGKLMMQKPNILIMDEPTNHLDMESIESLNMA
LELYQGTLIFVSHDREFVSSLATRILEITPERVIDFSGNYEDYLRSKGIE
>LFGLNPFC_02499 putative L,D-transpeptidase YbiS
MNMKLKTLFAAAFAVVGFCSTASAVTYPLPTDGSRLVGQNQVITIPEGNTQPLEYFAAEY
QMGLSNMMEANPGVDTFLPKGGTVLNIPQQLILPDTVHEGIVINSAEMRLYYYPKGTNTV
IVLPIGIGQLGKDTPINWTTKVERKKAGPTWTPTAKMHAEYRAAGEPLPAVVPAGPDNPM
GLYALYIGRLYAIHGTNANFGIGLRVSHGCVRLRNEDIKFLFEKVPVGTRVQFIDEPVKA
TTEPDGSRYIEVHNPLSTTEAQFEGQEIVPITLTKSVQTVTGQPDVDQVVLDEAIKNRSG
MPVRLN
>LFGLNPFC_02500 Inner membrane protein YbiR
MSLPFLRTLQGDRFFQLLILVGIGLSFFVPFAPKSWPAAIDWHTIITLSGLMLLTKGVEL
SGYFDVLGRKMVRRFATERRLAMFMVLAAALLSTFLTNDVALFIVVPLTITLKRLCEIPV
NRLIIFEALAVNAGSLLTPIGNPQNILIWGRSGLSFAGFIAQMAPLAGAMMLTLLLLCWC
CFPGKALQYHTGVQTPEWKPRLVWSCLGLYIVFLTALELKQELWGLVIVAAGFALLARRV
VLSVDWTLLLVFMAMFIDVHLLTQLPVLQGVLSNVSHLSEPGLWLTAIGLSQVISNVPST
ILLLNYVPPSLLLAWAVNVGGFGLLPGSLANLIALRMANDRRIWWRFHLYSIPMLLWAAL
VGYVLLVMIPAG
>LFGLNPFC_02501 Transcriptional regulator MntR
MSRRAGTPTAKKVTQLVNVEEHVEGFRQVREAHRRELIDDYVELISDLIREVGEARQVDM
AARLGVSQPTVAKMLKRLATMGLIEMIPWRGVFLTAEGEKLAQESRERHQIVENFLLVLG
VSPEIARRDAEGMEHHVSEETLDAFRLFTQKHGAK
>LFGLNPFC_02502 Small protein MntS
MNEFKRCMRVFSHSPFKVRLMLLSMLCDMVNNKPQQDKPSDK

>LFGLNPFC_02503 Phosphoethanolamine transferase OpgE
MNLTLKESLVTRSRVFSPWTAFYFLQSLLINLGLGYPFSLLYTAAFTAILLLLWRTLPRV
QKVLVGISSLVAACYFPFAQAYGAPNFNTLLALHSTNMEESTEILTIFPWYSYLVGIFIF
ALGVIAIRRKKESEKARWNTFDSLCLVFSVATFFVAPVQNLAWGGVFKLKDTGYPVFRFA
KDVIVNNNEVIEEQERMAKLSGMKDTWTVTAVKPKYQTYVVVIGESARRDALGAFGGHWD
NTPFASSVNGLIFADYIAASGSTQKSLGLTLNRVVDGKPQFQDNFVTLANRAGFQTWWFS
NQGQIGEYDTAIASIAKRADEVYFLKEGNFEADKNTKDEALLDMTAQVLAQEHSQPQLIV
LHLMGSHPQACDRTQGKYETFVQSKETSCYLYTMTQTDNLLRKLYDQLRNSGSSFSLVYF
SDHGLAFKERGKDVQYLAHDDKYQQNFQVPFMVISSDDKAHRVIKARRSANDFLGFFSQW
TGIKAKEINIKYPFISEKKAGPIYITNFQLQKVDYNHLGTDIFDPKP
>LFGLNPFC_02504 Outer membrane protein X
MKKIACLSALAAVLAFTAGTSVAATSTVTGGYAQSDAQGQMNKMGGFNLKYRYEEDNSPL
GVIGSFTYTEKSRTASSGDYNKNQYYGITAGPAYRINDWASIYGVVGVGYGKFQTTEYPT
YKHDTSDYGFSYGAGLQFNPMENVALDFSYEQSRIRSVDVGTWIAGVGYRF
>LFGLNPFC_02505 Threonine/homoserine exporter RhtA
MPGSLRKMPVWLPIVILLVAMASIQGGASLAKSLFPLVGAPGVTALRLALGTLILIAFFK
PWRLRFAKEQRLPLLFYGVSLGGMNYLFYLSIQTVPLGIAVALEFTGPLAVALFSSRRPV
DFVWVVLAVLGLWFLLPLGQDVSHVDLTGCALALGAGACWAIYILSGQRAGAEHGPATVA
IGSLIAALIFVPIGALQAGEALWHWSVIPLGLAVAILSTALPYSLEMIALTRLPTRTFGT
LMSMEPALAAVSGMIFLGETLTPIQLLALGAIIAASMGSTLTVRKESKIKELDIN
>LFGLNPFC_02506 DNA protection during starvation protein
MSTAKLVKSKATNLLYTRNDVSDSEKKATVELLNRQVIQFIDLSLITKQAHWNMRGANFI
AVHEMLDGFRTALIDHLDTMAERAVQLGGVALGTTQVINSKTPLKSYPLDIHNVQDHLKE
LADRYAIVANDVRKAIGEAKDDDTADILTAASRDLDKFLWFIESNIE
>LFGLNPFC_02507 Glutamine-binding periplasmic protein
MKSVLKVSLAALTLAFAVSSHAADKKLVVATDTAFVPFEFKQGDKYVGFDVDLWAAIAKE
LKLDYELKPMDFSGIIPALQTKNVDLALAGITITDERKKAIDFSDGYYKSGLLVMVKANN
NDVKSVKDLDGKVVAVKSGTGSVDYAKANIKTKDLRQFPNIDNAYMELGTNRADAVLHDT
PNILYFIKTAGNGQFKAVGDSLEAQQYGIAFPKGSDELRDKVNGALKTLRENGTYNEIYK
KWFGTEPK
>LFGLNPFC_02508 Glutamine transport system permease protein GlnP
MQFDWSAIWPAIPLLIEGAKMTLWISVLGLAGGLVIGLLAGFARTFGGWIANHVALVFIE
VIRGTPIVVQVMFIYFALPMAFNDLRIDPFTAAVVTIMINSGAYIAEITRGAVLSIHKGF
REAGLALGLSRWETIRYVILPLALRRMLPPLGNQWIISIKDTSLFIVIGVAELTRQGQEI
IAGNFRALEIWSAVAVFYLIITLVLSFILRRLERRMKIL
>LFGLNPFC_02509 Glutamine transport ATP-binding protein GlnQ
MIEFKNVSKHFGPTQVLHNIDLNIAQGEVVVIIGPSGSGKSTLLRCINKLEEITSGDLIV
DGLKVNDPKVDERLIRQEAGMVFQQFYLFPHLTALENVMFGPLRVRGANKEEAEKLAREL
LAKVGLAERAHHYPSELSGGQQQRVAIARALAVKPKMMLFDEPTSALDPELRHEVLKVMQ
DLAEEGMTMVIVTHEIGFAEKVASRLIFIDKGRIAEDGDPQVLIKNPPSQRLQEFLQHVS
>LFGLNPFC_02510 Moderate conductance mechanosensitive channel YbiO
MRWILFILFCLLGAPAHAVSIPGVTTTTTTDSTTEPAPEPDIEQKKAAYGALADVLDNDT
SRKELIDQLRTVAATPPAEPVPKIVPPTLVEEQTVLQKVTEVSRHYGEALSARFGQLYRN
ITGSPHKPFNPQTFSNALTHFSMLAVLVFGFYWLIRLCALPLYRKMGQWARQKNRERSNW
LQLPAMIIGAFIIDLLLLALTLFVGQVLSDNLNAGSRTIAFQQSLFLNAFALIEFFKAVL
RLIFCPNVAELRPFTIHDETARYWSRRLSWLSSLIGYGLIVAVPIISNQVNVQIGALANV
IIMLCMTVWALYLIFRNKKEITQHLLNFAEHSLAFFSLFIRAFALVWHWLASAYFIVLFF
FSLFDPGNSLKFMMGATVRSLAIIGIAAFVSGMFSRWLAKTITLSPHTQRNYPELQKRLN
GWLSAALKTARILTVCVAVMLLLSAWGLFDFWNWLQNGAGQKTVDILIRIALILFFSAVG
WTVLASLIENRLASDIHGRPLPSARTRTLLTLFRNALAVIISTITIMIVLSEIGVNIAPL
LAGAGALGLAISFGSQTLVKDIITGVFIQFENSMNTGDLVTIGPLTGTVERMSIRSVGVR
QDTGAYHIIPWSSITTFANFVRGIGSVVANYDVDRHEDADKANQALKDAVAELMENEEIR
GLIIGEPNFAGIVGLSNTAFTLRVSFTTLPLKQWTVRFALDSQVKKHFDLAGVRAPVQTY
QVLPAPGATPAEPLPPGEPTL
>LFGLNPFC_02511 Ribosomal RNA large subunit methyltransferase F
MSAQKPGLHPRNRHHSRYDLATLCQVNPELRQFLTLTPAGEQSVDFANPLAVKALNKALL
AHFYAVANWDIPDGFLCPPVPGRADYIHHLADLLAEASGTIPANASILDIGVGANCIYPL
IGVHEYGWRFTGSETSSQALSSAQAIISANPGLNRAIRLRRQKESGAIFNGIIHKNEQYD
ATLCNPPFHDSAAAARAGSERKRRNLGLNKDDALNFGGQQQELWCEGGEVAFIKKMIEES
KGFAKQVMWFTSLVSRGENLPPLYRALTDVGAVKVVKKEMAQGGQKQSRFIAWTFMNDEQR
RRFVNRQR
>LFGLNPFC_02512 putative protein McbA
MKKCLTLLIATVLSGISLTAYAAQPMSNLDSGQLRPAGTVSATGASNLSDLEDKLAEKAR
EQGAKGYVINSAGGNDQMFGTAIIYK

>LFGLNPFC_02513 Catecholate siderophore receptor Fiu
MENNRNFPARQFHSLTFFAGLCIGITPVAQALAAEGQANADDTLVVEASTPSLYAPQQSA
DPKFSRPVADTTRTMTVISEQVIKDQGATNLTDALKNVPGVGAFFAGENGNSTTGDAIYM
RGADTSNSIYIDGIRDIGSVSRDTFNTEQVEVIKGPSGTDYGRSAPTGSINMISKQPRND
SGIDASASIGSAWFRRGTLDVNQVIGDTTAVRLNVMGEKTHDAGRDKVKNERYGVAPSVA
FGLGTANRLYLNYLHVTQHNTPDGGIPTIGLPGYSAPSAGTAALNHSGKVDTHNFYGTDS
DYDDSTTDTATMRFEHDINDNTTIRNTTRWSRVKQDYLMTAIMGGASNITQPTSDVNSWT
WSRTANTKDVSNKILTNQTNLTSTFYTGAIGHDVSTGVEFTRETQTNYGVNPVTLPAVNI
YHPDSSIHPGGLTRNGANANGQTDTFAIYAFDTLQITRDFELNGGIRLDNYHTEYDSATA
CGGSGRGAITCPAGVAKGSPVTTVDTAKSGNLVNWKAGALYHLTENGNIYINYAVSQQPP
GGNNFALAQSGSGNSANRTDFKPQKANTSEIGTKWQVLDKRLLLTAALFRTDIENEVEQN
DDGTYSQYGKKRVEGYEISVAGNITPAWQMIGGYTQQKATIKNGKDVAQDGSSSLPYTPE
HAFTLWSQYQATDDISVGAGARYIGSMHKGSDGAVGTPAFTEGYWVADAKLGYRVNRNLD
FQLNVYNLFDTDYVASINKSGYRYHPGEPRTFLLTANMHF
>LFGLNPFC_02514 PKHD-type hydroxylase
MYHIPGVLSPQDVARFREQLEQAEWVDGRVTTGAQGAQVKNNQQVDTRSALYAALQNEVL
NAVNQHALFFAAALPRTLSTPLFNRYQNNETYGFHVDGAVRSHPQNGWMRTDLSATLFLS
DPESYDGGELVVNDTFGQHRVKLPAGDLVLYPSSSLHCVTPVTRGVRVASFMWIQSMIRD
DKKRAMLFELDNNIQSLKSRYGESEEILSLLNLYHNLLREWSEI
>LFGLNPFC_02515 putative protein YbiI
MASGWANDDAVNEQINSTIEDAIARARGEIPRGESLYECEECGAPIPQARREAIPGVRLC
IHCQQEKDLQKPAYTGYNRRGSKDSQLR
>LFGLNPFC_02516 Multiple stress resistance protein BhsA
MKTINTVVAAMALSTLSFGVFAAEPVTASQAQNMNKIGVVSADGASTLDALEAKLAEKAA
AAGASGYSITSATNNNKLSGTAVIYK
>LFGLNPFC_02517 Hydroxycarboxylate dehydrogenase B
MVSGHRFDAQTLHSFIQAVFRQMGSEEQEAKLVADHLIAANLAGHDSHGIGMIPSYVRSW
SQGHLQINHHAKVVKEAGAAVTLDGDRAFGQVAAHEAMALGIEKARQHGIAAVALHNSHH
IGRIGYWAEQCAAAGFVSIHFVSVVGIPMVAPFHGRDSRFGTNPFCVVFPRKDNFPLLLD
YATSAIAFGKTRVAWHKGVPVPPGCLIDVNGVPTTNPAVMQESPLGSLLTFAEHKGYALA
AMCEILGGALSGGKTTHQETLQTSPDAILNCMTTIIINLELFGAPDCSAQTEAFAEWVKA
SPHDDDKPILLPGEWEVNTRRERQEQGIPLDAGSWQAICDAARQIGMPEETLQAFCQQLA
S
>LFGLNPFC_02518 putative protein YbiB
MDYRKIIKEIGRGKNHARDLDRDTARGLYAHMLNSEVPDLELGGVLIALRIKGEGEAEML
GFYEAMQNHTIKLTPPAGKPMPIVIPSYNGARKQANLTPLLAILLHKLGFPVVVHGVSED
PTRVLTETIFELMGITPTLHGGQAQAKLDEHQPVFMPVGAFCPPLEKQLAMRWRMGVRNS
AHTLAKLATPFAEGEALRLSSVSHPEYIGRVAKFFSDIGGRALLMHGTEGEVYANPQRCP
QINLIDREGMRVLYEKQDTAGSELLPQAKDPETTAQWIERCLAGSEPIPESLKIQMACCL
MATGEAATISDGLARVNQAF
>LFGLNPFC_02519 ATP-dependent DNA helicase DinG
MALTAALKAQIAAWYKALQEQIPDFIPRAPQRQMIADVAKTLAGEEGRHLAIEAPTGVGK
TLSYLIPGIAIAREEQKTLVVSTANVALQDQIYSKDLPLLKKIIPDLKFTAAFGRGRYVC
PRNLTALASTEPTQQDLLAFLDDELTPNNQEEQKRCAKLKGDLDTYKWDGLRDHTDIAID
DDLWRRLSTDKASCLNRNCYYYRECPFFVARREIQEAEVVVANHALVMAAMESEAVLPDP
KNLLLVLDEGHHLPDVARDALEMSAEITAPWYRLQLDLFTKLVATCMEQFRPKTIPPLAI
PERLNAHCEELYELIASLNNILNLYMPAGQEAEHRFAMGELPDEVLEICQRLAKLTEMLR
GLAELFLNDLSEKTGSHDIVRLHRLILQMNRALGMFEAQSKLWRLASLAQSSGAPVTKWA
TREEREGQLHLWFHCVGIRVSDQLERLLWRSIPHIIVTSATLRSLNSFSRLQEMSGLKEK
AGDRFVALDSPFNHCEQGKIVIPRMRVEPSIDNEEQHIAEMAAFFREQVESKKHLGMLVL
FASGRAMQRFLDYVTDLRLMLLVQGDQPRYRLVELHRKRVANGERSVLVGLQSFAEGLDL
KGDLLSQVHIHKIAFPPIDSPVVITEGEWLKSLNRYPFEVQSLPSASFNLIQQVGRLIRS
HGCWGEVVIYDKRLLTKNYGKRLLDALPVFPIEQPEVPEGIVKKKEKTKSPRRRRR
>LFGLNPFC_02520 N-glycosidase YbiA
MRKALRAKFEQHAELRTLLRATASAKLVEHTQNDAYWGDGGNGQGKNRLGYLLMALRGQL
AAEK
>LFGLNPFC_02521 ATP-dependent RNA helicase RhlE
MSFDSLGLSPDILRAVAEQGYREPTPIQQQAIPAVLEGRDLMASAQTGTGKTAGFTLPLL
QHLITRQPHAKGRRPVRALILTPTRELAAQIGENVRDYSKYLNIRSLVVFGGVSINPQMM
KLRGGVDVLVATPGRLLDLEHQNAVKLDQVEILVLDEADRMLDMGFIHDIRRVLTKLPAK
RQNLLFSATFSDDIKALAEKLLHNPLEIEVARRNTASDQVTQHVHFVDKKRKRELLSHMI
GKGNWQQVLVFTRTKHGANHLAEQLNKDGIRSAAIHGNKSQGARTRALADFKSGDIRVLV
ATDIAARGLDIEELPHVVNYELPNVPEDYVHRIGRTGRAAATGEALSLVCVDEHKLLRDI
EKLLKKEIPRIAIPGYEPDPSIKAEPIQNGRQQRGGGRGQGGGRGQQQPRRTESGAKSGN

AKPAEKPSRRLGDAKPAGEQQRRRRPRKPAAAQ
>LFGLNPFC_02522 HTH-type transcriptional dual regulator CecR
MNNPAMTIKGEQAKKQLIAAALAQFGEYGMNATTREIAAQAGQNIAAITYYFGSKEDLYL
ACAQWIADFIGEQFRPHAEEAERLFAQPQPDRAAIRELILRACRNMIKLLTQDDTVNLSK
FISREQLSPTAAYHLVHEQVISPLHSHLTRLIAAWTGCDASDTRMILHTHALIGEILAFR
LGKETILLRTGWTAFDEEKTELINQTVTCHIDLILQGLSQRSL
>LFGLNPFC_02523 hypothetical protein
MKKPVVIGLAVVVLAAVVAGGYWWYQSRQDNGLTLYGNVDIRTVNLSFRVGGRVESLAVD
EGDAIKAGQVLGELDHKPYEIALMQAKAGVSVAQAQYDLMLAGYRDEEIAQAAAAVKQAQ
AAYDYAQNFYNRQQGLWKSRTISANDLENARSSRDQAQATLKSAQDKLRQYRSGNREQDI
AQAKASLEQAQAQLAQAELNLQDSTLIAPSDGTLLTRAVEPGTVLNEGGTVFTVSLTRPV
WVRAYVDERNLDQAQPGRKVLLYTDGRPNKPYHGQIGFVSPTAEFTPKTVETPDLRTDLV
YRLRIVVTDADDALRQGMPVTVQFGDEAGHE
>LFGLNPFC_02524 putative multidrug ABC transporter ATP-binding protein YbhF
MNDAVITLNGLEKRFPGMDKPAVAPLDCTIHAGYVTGLVGPDGAGKTTLMRMLAGLLKPD
SGSATVIGFDPIKNDAALHAVLGYMPQKFGLYEDLTVMENLNLYADLRSVTGEARKQTFA
RLLEFTSLGPFTGRLAGKLSGGMKQKLGLACTLVGEPKVLLLDEPGVGVDPISRRELWQM
VHELAGEGMLILWSTSYLDEAEQCRDVLLMNEGELLYQGEPTALTQTMAGRSFLMTSPHE
GNRKLLQRALKLPQVSDGMIQGKSVRLILKKETTPDDIRHADGMPEIDINETTPRFEDAF
IDLLGGAGTSESPLGAILHTVEGTPGETVIEAKELTKKFGDFAATDHVNFAVKRGEIFGL
LGPNGAGKSTTFKMMCGLLVPTSGQALVLGMDLKESSGKARQHLGYMAQKFSLYGNLTVE
QNLRFFSGVYGLRGRAQNEKISRMSEAFGLKSIASHATDELPLGFKQRLALACSLMHEPD
ILFLDEPTSGVDPLTRREFWLHINSMVEKGVTVMVTTHFMDEAEYCDRIGLVYRGKLIAS
GTPDDLKAQSANDEQPDPTMEQAFIQLIHDWDKEHSNE
>LFGLNPFC_02525 putative multidrug ABC transporter permease YbhS
MSNPILSWRRVRALCVKETRQIVRDPSSWLIAVVIPLLLLFIFGYGINLDSSKLRVGILL
EQRSEAALDFTHTMTGSPYIDATISDNRQELIAKMQAGKIRGLVVIPVDFAEQMERANAT
APIQVITDGSEPNTANFVQGYVEGIWQIWQMQRAEDNGQTFEPLIDVQTRYWFNPAAISQ
HFIIPGAVTIIMTVIGAILTSLVVAREWERGTMEALLSTEITRTELLLCKLIPYYFLGML
AMLLCMLVSVFILGVPYRGSLLILFFISSLFLLSTLGMGLLISTITRNQFNAAQVALNAA
FLPSIMLSGFIFQIDSMPAVIRAVTYIIPARYFVSTLQSLFLAGNIPVVLIVNVLFLIAS
AVMFIGLTWLKIKRRLD
>LFGLNPFC_02526 putative multidrug ABC transporter permease YbhR
MFHRLWTLIRKELQSLLREPQTRAILILPVLIQVILFPFAATLEVTNATIAIYDEDNGEH
SVELTQRFARASAFTHVLLLKSPQEIRPTIDTQKALLLVRFPADFSRKLDTFQTAPLQLI
LDGRNSNSAQIAANYLQQIVKNYQQELLEGKPKPNNSELVVRNWYNPNLDYKWFVVPSLI
AMITTIGVMIVTSLSVAREREQGTLDQLLVSPLTTWQIFIGKAVPALIVATFQATIVLAI
GIWAYQIPFAGSLALFYFTMVIYGLSLVGFGLLISSLCSTQQQAFIGVFVFMMPAILLSG
YVSPVENMPVWLQNLTWINPIRHFTDITKQIYLKDASLDIVWNSLWPLLVITATTGSAAY
AMFRRKVM
>LFGLNPFC_02527 Inner membrane protein YbhQ
MKWQQRVRVATGLSCWQIMLHLLVVALLVVGWMSKTLVHVGVGLCALYCVTVVMMLVFQR
HPEQRWREVADVLEELTTTWYFGAALIVLWLLSRVLENNFVLAIAGLAILAGPAVVSLLA
KDKKLHHLTSKHRVRR
>LFGLNPFC_02528 hypothetical protein
MPDQTQQFSFKVLTINIHKGFTAFNRRFILPELRDAVRTVSADIVCLQEVMGAHEVHPLH
VENWPDTSHYEFLADTMWSDFTYGRNAVYPEGHHGNAVLSRYPIEHYENRDVSVDGAEKR
GVLYCRIVPPMTGKAIHVMCVHLGLREAHRQAQLAMLAEWVNELPDGEPVLVAGDFNDWR
QKANHPLKVQAGLDEIFTRAHGRPARTFPVQFPLLRLDRIYVKNASASAPTALPLRTWRH
LSDHAPLSAEIHL
>LFGLNPFC_02529 Cardiolipin synthase B
MKCSWREGNKIQLLENGEQYYPAVFKAIGEAQERIILETFIWFEDDVGKQLHAALLAAAQ
RGVKAEVLLDGYGSPDLSDEFVNELTAAGVVFRYYDPRPRLFGMRTNVFRRMHRKIVVID
ARIAFIGGLNYSAEHMSSYGPEAKQDYAVRLEGPIVEDILQFELENLPGQSAARRWWRRH
HKAEENRQPGEAQVLLVWRDNEEHRDDIERHYLKMLTQARREVIIANAYFFPGYRFLHAL
RKAARRGVRIKLIIQGEPDMPIVRVGAHLLYNYLVKGGVQVFEYRRRPLHGKVALMDDHW
ATVGSSNLDPLSLSLNLEANVIIHDRNFNQTLRDNLNGIIAADCQQVDETMLPKRTWWNL
TKSVLAFHFLRHFPALVGWLPAHTPRLAQVGPPAQPTMETQDRVETENTGVKP
>LFGLNPFC_02530 Inner membrane protein YbhN
MSKSHPRWRLAKKLLTWLFFIAVIVLLVVYAKKVDWEEVWKVIRDYNRVALLSAVGLVVV
SYLIYGCYDLLARFYCGHKLAKRQVMLVSFICYAFNLTLSTWVGGIGMRYRLYSRLGLPG
STITRIFSLSITTNWLGYILLAGVIFTAGVVELPDHWYVDQTTLRILGIGLLMIIAVYLW
FCAFAKHRHMTIKGQKLVLPSWKFALAQMLISSVNWMVMGAIIWLLLGQSVSYFFVVGVL
LVSSIAGVIVHIPAGIGVLEAVFIALLAGEHTSKGTIIAALLAYRVLYYFIPLLLALICY

LLLESQAKKLRAKNEAAM
>LFGLNPFC_02531 hypothetical protein
MESYSQNSNKVDFQHEARILNGIWLITALGLVATAGLAWGAKYLEITATKYDSPQMYVAI
GLLLLSMYGLSKDINKINTAIAGVIYLFLISLVAIVVASLAPVSAIIVVFSTAGSMFLIS
MLAGLLFKFDPGSHRFIIMMTLTGLALVIIVNATLMSERPIWVISCLMIVLWSGIISHGR
NKLLELTGKSHSEELWSPVRCAFVGALTLYYYFIGFFGILATIAITLVWQRHTRFFH
>LFGLNPFC_02532 Inner membrane protein YbhL
MDRFPRSDSIVQPRAGLQTYMAQVYGWMTVGLLLTAFVAWYAANSAAVMELLFTNRVFLI
GLIIAQLALVIVLSAMIQKLSAGVTTMLFMLYSALTGLTLSSIFIVYTAASIASTFVVTA
GMFGAMSLYGYTTKRDLSGFGNMLFMALIGIVLASLVNFWLKSEALMWAVTYIGVIVFVG
LTAYDTQKLKNMGEQIDTRDTSNLRKYSILGALTLYLDFINLFLMLLRIFGNRR
>LFGLNPFC_02533 Molybdopterin synthase catalytic subunit
MAETKIVVGPQPFSVGEEYPWLAERDEDGAVVTFTGKVRNHNLGDSVKALTLEHYPGMTE
KALAEIVDEARNRWPLGRVTVIHRIGELWPGDEIVFVGVTSAHRSSAFEAGQFIMDYLKT
RAPFWKREATPEGDRWVEARESDQQAAKRW
>LFGLNPFC_02534 Molybdopterin synthase sulfur carrier subunit
MINVLFFAQVRELVGTDATEVAADFPTVEALRQHLAAQSDRWALALEDGKLLAAVNQTLV
SFDHSLTDGDEVAFFPPVTGG
>LFGLNPFC_02535 Cyclic pyranopterin monophosphate synthase
MSQLTHINAAGEAHMVDVSAKAETVREARAEAFVTMRSETLAMIIDGRHHKGDVFATARI
AGIQAAKRTWDLIPLCHPLMLSKVEVNLQAEPEHNRVRIETLCRLTGKTGVEMEALTAAS
VAALTIYDMCKAVQKDMVIGPVRLLAKSGGKSGDFKVEADD
>LFGLNPFC_02536 Molybdenum cofactor biosynthesis protein B
MSQVSTEFIPTRIAILTVSNRRGEEDDTSGHYLRDSAQEAGHHVVDKAIVKENRYAIRAQ
VSAWIASDDVQVVLITGGTGLTEGDQAPEALLPLFDREVEGFGEVFRMLSFEEIGTSTLQ
SRAVAGVANKTLIFAMPGSTKACRTAWENIIAPQLDARTRPCNFHPHLKK
>LFGLNPFC_02537 GTP 3',8-cyclase
MASQLTDAFARKFYYLRLSITDVCNFRCTYCLPDGYKPSGVTNKGFLTVDEIRRVTRAFA
SLGTEKVRLTGGEPSLRRDFTDIIAAVRENDAIRQIAVTTNGYRLERDVANWRDAGLTGI
NVSVDSLDARQFHAITGQDKFNQVMAGIDAAFEAGFEKVKVNTVLMRDVNHHQLDTFLNW
IQHRPIQLRFIELMETGEGIELFRKHHISGQVLRNELLRRGWIHQLRQRSDGPAQVFCHP
DYAGEIGLIMPYEKDFCATCNRLRVSSIGKLHLCLFGEGGVNLRDLLEDDTQQQALEARI
SAALREKKQTHFLHQNNTGITQNLSYIGG
>LFGLNPFC_02538 Putative gluconeogenesis factor
MRNRTLADLDRVVALGGGHGLGRVLSSLSSLGSRLTGIVTTTDNGGSTGRIRRSEGGIAW
GDMRNCLNQLITEPSVASAMFEYRFGGNGELSGHNLGNLMLKALDHLSVRPLEAINLIRN
LLKVDAHLIPMSEHPVDLMAIDDQGHEVYGEVNIDQLTAPIQELLLTPNVPATREAVHAI
SEADLIIIGPGSFYTSLMPILLLKEIAQALRRTPAPMVYIGNLGRELSLPAANLKLESKL
AIMEQYVGKKVIDAVIVGPKEDVSAVKERIVIQEVLEASDIPYRHDRQLLHSALEKALQA
LG
>LFGLNPFC_02539 UvrABC system protein B
MSKPFKLNSAFKPSGDQPEAIRRLEEGLEDGLAHQTLLGVTGSGKTFTIANVIADLQRPT
MVLAPNKTLAAQLYGEMKEFFPENAVEYFVSYYDYYQPEAYVPSSDTFIEKDASVNEHIE
QMRLSATKAMLERRDVVVVASVSAIYGLGDPDLYLKMMLHLTVGMIIDQRAILRRLAELQ
YARNDQAFQRGTFRVRGEVIDIFPAESDDIALRVELFDEEVERLSLFDPLTGQIVSTIPR
FTIYPKTHYVTPRERIVQAMEEIKEELAARRKVLLENNKLLEEQRLTQRTQFDLEMMNEL
GYCSGIENYSRFLSGRGPGEPPPTLFDYLPADGLLVVDESHVTIPQIGGMYRGDRARKET
LVEYGFRLPSALDNRPLKFEEFEALAPQTIYVSATPGNYELEKSGGDVVDQVVRPTGLLD
PIIEVRPVATQVDDLLSEIRQRAAINERVLVTTLTKRMAEDLTEYLEEHGERVRYLHSDI
DTVERMEIIRDLRLGEFDVLVGINLLREGLDMPEVSLVAILDADKEGFLRSERSLIQTIG
RAARNVNGKAILYGDKITPSMAKAIGETERRREKQQKYNEEHGITPQGLNKKVVDILALG
QNIAKTKAKGRGKSRPIVEPDNVPMDMSPKALQQKIHELEGLMMQHAQNLEFEEAAQIRD
QLHQLRELFIAAS
>LFGLNPFC_02540 ATP-dependent dethiobiotin synthetase BioD 1
MSKCYFVTGTDTEVGKTVASCALLQAAKAAGYRTAGYKPVASGSEKTPKGLRNSDALALQ
RNSSLQLDYATVNPYTFAEPTSPHIISAQEGRPIEALVMSAGLRALEQQADWVLVEGAGG
WFTPLSDTFTFADWVTQEQLPVILVVGVKLGCINHAMLTAQAILHAGLTLAGWVANDVTP
PGKRHAEYMTTLTRMIPAPLLGEIPWLAENPENAATGKYINLALM
>LFGLNPFC_02541 Malonyl-[acyl-carrier protein] O-methyltransferase
MATVNKQAIAAAFGRAAAHYEQHADLQRQSADALLAMLPQRKYTRVLDAGCGPGWMSRRW
RERHAQVTALDLSPPMLVQARQKDAADHYLAGDIESLPLATATFDLAWSNLAVQWCGNLS
TALRELYRVVRPGGVVAFTTLVQGSLPELHQAWQAVDERPHANRFLPPDEIEQSLNAVHY
QHYIQPITLWFDDALSAMRSLKGIGATHLHEGRDPRILTRSQLQRLQLAWPQQQGRYPLT
YHLFLGVIARE

>LFGLNPFC_02542 8-amino-7-oxononanoate synthase
MIWQEKIDAALDARRVADALRRRYPVAQGAGRWLVADDCQYLNFSSNDYLGLSHHPQIIR
AWQQGADQFGVGSGGSGHVSGYSVAHQVLEEELAEWLGYSRALLFISGFAANQAVIAAMM
AKEDRIVADRLSHASLLEAASLSPSPLRRFAHNDVTHLARLLASPCPGQQLVVTEGVFSM
DGDSAPLEEIQQVTQQHDGWLMVDDAHGTGVIGEQGRGSCWLQKVKPELLVVTFGKGFGV
SGAAVLCSNTVADYLLQFARHLIYSTSMPPAQAQALRASLAVIRSDEGDARREKLAALIT
RFRAGVQDLPFTLAGSCSAIQPLIVGDNSRALQLAEKLRQQGCWVTAIRPPTVPAGTARL
RLTLTAAHEMQDIDRLLEVLHGNG
>LFGLNPFC_02543 Biotin synthase
MAHRPRWTLSQVTELFEKPLLDLLFEAQQVHRQHFDPRQVQVSTLLSIKTGACPEDCKYC
PQSSRYKTGLEAERLMEVEQVLESARKAKAAGSTRFCMGAAWKNPHERDMPYLEQMVQGV
KAMGLEACMTLGTLSESQAQRLANAGLDYYNHNLDTSPEFYGNIITTRTYQERLDTLEKV
REAGIKVCSGGIVGLGETVKDRAGLLLQLANLPTPPESVPINMLVKVKGTPLADNDDVDA
FDFIRTIAVARIMMPTSYVRLSAGREQMNEQTQAMCFMAGANSIFYGCKLLTTPNPEEDK
DLQLFRKLGLNPQQTAVLAGDNEQQQRLEQALMTPDTDEYYNAAAL
>LFGLNPFC_02544 Adenosylmethionine-8-amino-7-oxononanoate aminotransferase
MTTDDLAFDQRHIWHPYTSMTSPLPVYPVASAEGCELILSDGRRLVDGMSSWWAAIHGYN
HPQLNAAMKSQIDAMSHVMFGGITHAPAIELCRKLVAMTPQPLECVFLADSGSVAVEVAM
KMALQYWQAKGEARQRFLTFRNGYHGDTFGAMSVCDPDNSMHSLWKGYLPENLFAPAPQS
RMDGEWDERDMVGFARLMAAHRHEIAAVIIEPIVQGAGGMRMYHPEWLKRIRKMCDREGI
LLIADEIATGFGRTGKLFACEHAEIAPDILCLGKALTGGTMTLSATLTTREVAETISNGE
AGCFMHGPTFMGNPLACAAANASLAILESGDWQHQVAAIEAQLREQLAPACDAEMVADVR
VLGAIGVVETTRPVNMAALQKFFVEQGVWIRPFGKLIYLMPPYIILPQQLQRLTAAVNRA
VQDETFFCQ
>LFGLNPFC_02545 hypothetical protein
MKLISNDLRDGDKLPHRHVFNGMGYDGDNISPHLAWDDVPVGTKSFVVTCYDPDAPTGSG
WWHWVVVNLPADTRVLPQGFGSGLVAMPDGVLQTRTDFGKTGYDGAAPPKGETHRYIFTV
HALDVERIDVDEGASGAMVGFNVHFHSLASASITAMFS
>LFGLNPFC_02546 Putative acyl-CoA thioester hydrolase YbhC
MNTFSVSRLALALAFGVTLTACSSTPPDQRPSDQTAPGTSSRPILSAKEAQNFDAQHYFA
SLTPGAAAWNPSPITLPAQPDFVVGPAGTQGVTHTTIQAAVDAAIIKRTNKRQYIAVMPG
EYQGTVYVPAAPGGITLYGTGEKPIDVKIGLSLDGGMSPADWRHDVNPRGKYMPGKPAWY
MYDSCQSKRSDSIGVLCSAVFWSQNNGLQLQNLTIENTLGDSVDAGNHPAVALRTDGDKV
QINNVNILGRQNTFFVTNSGVQNRLETNRQPRTLVTNSYIEGDVDIVSGRGAVVFDNTEF
RVVNSRTQQEAYVFAPATLSNIYYGFLAVNSRFNASGDGVAQLGRSLDVDANTNGQVVIR
DSAINEGFNTAKPWADAVISNRPFAGNTGSVDDNDEVQRNLNDTNYNRMWEYNNRGVGSK
VVAEAKK
>LFGLNPFC_02547 3-isopropylmalate dehydratase large subunit
MIKLSEKGVFLASNNEIIAEEHFTGEIKKEEAKKGTIAWSILSSHNTSGNMDKLKIKFDS
LASHDITFVGIVQTAKASGMERFPLPYVLTNCHNSLCAVGGTINGDDHVFGLSAAQRYGG
IFVPPHIAVIHQYMREMMAGGGKMILGSDSHTRYGALGTMAVGEGGGELVKQLLNDTWDI
DYPGVVAVHLTGKPAPYVGPQDVALAIIGAVFKNGYVKNKVMEFVGPGVAALSTDFRNSV
DVMTTETTCLSSVWQTDEEVHNWLALHGRGQDYCQLNPQPMAYYDGSISVDLSAIKPMIA
LPFHPSNVYEIDTLNQNLTDILREIEIESERVAHGKAKLSLLDKVENGRLKVQQGIIAGC
SGGNYENVIAAANALRGQSCGNDTFSLAVYPSSQPVFMDLAKKGVVADLIGAGAIIRTAF
CGPCFGAGDTPINNGLSIRHTTRNFPNREGSKPANGQMSAVALMDARSIAATAANGGYLT
SASELDCWDNVPEYAFDVTPYKNRVYQGFVKGATQQPLIYGPNIKDWPELGALTDNIVLK
VCSKILDEVTTTDELIPSGETSSYRSNPIGLAEFTLSRRDPGYVGRSKATAELENQRLAG
NVSELADMFARIKQIAGQEHVDPLQTEIGSMVYAVKPGDGSAREQAASCQRVIGGLANIA
EEYATKRYRSNVINWGMLPLQMAEVPNFDVGDYIYIPGIKAALDNPGTTFKGYVIHEDAP
VTEITLYMESLTAEEREIIKAGSLINFNKNRQM
>LFGLNPFC_02548 Inner membrane protein YbhI
MNKKSLWKLILILAIPCIIGFMPAPAGLSELAWVLFGIYLAAIVGLVIKPFPEPVVLLIA
VAASMVVVGNLSGGEFKTTAVLSGYSSGTTWLVFSAFTLSAAFVTTGLGKRIAYLLIGKI
GSTTLGLGYVTVFLDLVLAPATPSNTARAGGIVLPIINSVAVALGSEPEKSPRRVGHYLM
MSIYMVTKTTSYMFFTAMAGNILALKMINDILHLQISWGGWALAAGLPGIIMLLVTPLVI
YTMYPPEIKKVDNKTIAKAGLAELGPMKIREKMLLGVFVLALLGWIFSKSLGVDESTVAI
VVMATMLLLGIVTWEDVVKNKGGWNTLIWYGGIIGLSSLLSKVKFFEWLAEVFKNNLAFD
GHGNVAFFVIIFLSIIVRYFFASGSAYIVAMLPVFAMLANVSGAPLMLTALALLFSNSYG
GMVTHYGGAAGPVIFGVGYNDIKSWWLVGAVLTILTFLVHITLGVWWWNMLIGWNML
>LFGLNPFC_02549 Putative isomerase YbhH
MKKIPCVMMRGGTSRGAFLLAEHLPEDQTQRDKILMAIMGSGNDLEIDGIGGGNPLTSKV
AIISRSSDPHADVDYLFAQVIVHEQRVDTTPNCGNMLSGVGAFAIENGLIAATSPVTRVR
IRNVNTGTFIEADVQTPNGVVEYEGSARIDGVPGTAAPVALTFLNAAGTKTGKVFPTDNQ

IDYFDDVPVTCIDMAMPVVIIPAEYLGKTGYELPAELDADKALLARIESIRLQAGKAMGL
GDVSNMVIPKPVLISPAQKGGAINVRYFMPHSCHRALAITGAIAISSSCALEGTVTRQIV
PSVGYGNINIEHPSGALDVHLSNEGQDATTLRASVIRTTRKIFSGEVYLP
>LFGLNPFC_02550 Hydrogen peroxide-inducible genes activator
MKHELSSMKAFVILAESSSFNNAAKLLNITQPALTRRIKKMEEDLHIQLFERTTRKVTLT
KAGKRLLPEARELIKKFDETLFNIRDMNAYHRGMVTLACIPTAVFYFLPLAIGKFNELYP
NIKVRILEQGTNNCMESVLCNESDFGINMNNVTNSSIDFTPLVNEPFVLACRRDHPLAKK
QLVEWQELVGYKMIGVRSSSGNRLLIEQQLADKPWKLDWFYEVRHLSTSLGLVEAGLGIS
ALPGLAMPHAPYSSIIGIPLVEPVIRRTLGIIRRKDAVLSPAAERFFALLINLWTDDKDN
LWTNIVERQRHALQEIG
>LFGLNPFC_02551 6-phosphogluconolactonase
MKQTVYIASPESQQIHVWNLNHEGALTLTQVVDVPGQVQPMVVSPDKRYLYVGVRPEFRV
LAYRIAPDDGALTFAAESALPGSPTHISTDHLGQFVFVGSYNAGNVSVTRLEDGLPVGVV
DVVEGLDGCHSANISPDNRTLWVPALKQDRICLFTVSDDGHLVAQDPAEVTTVEGAGPRH
MVFHPNEQYAYCVNELNSSVDVWELKDPHGNIECVQTLDMMPENFSDTRWAADIHITPDG
RHLYACDRTASLITVFSVSEDGSVLSKEGFQPTETQPRGFNVDHSGKYLIAAGQKSHHIS
VYEIVGEQGLLHEKGRYAVGQGPMWVVVNAH
>LFGLNPFC_02552 Pyridoxal phosphate phosphatase YbhA
MTTRVIALDLDGTLLTPKKTLLPSSIEALARAREAGYQLIIVTGRHHVAIHPFYQALALD
TPAICCNGTYLYDYHAKTVLEADPMPVNKALQLIEMLNEHHIHGLMYVDDAMVYEHPTGH
VIRTSNWAQTLPPEQRPTFTQVASLAETAQQVNAVWKFALTHDDLPQLQHFGKHVEHELG
LECEWSWHDQVDIARGGNSKGKRLTKWVEAQGWSMENVVAFGDNFNDISMLEAAGTGVAM
GNADDAVKARANIVIGDNTTDSIAQFIYSHLI
>LFGLNPFC_02553 Vitamin B12 import ATP-binding protein BtuD
MLELNFSQTLGNHCLTINETLPANGITAIFGVSGAGKTSLINAISGLTRPQKGRIVLNGR
VLNDAEKGICLSPEKRRVGYVFQDARLFPHYKVRGNLRYGMAKSMVNQFDKLVALLGIEP
LLDRLPGSLSGGEKQRVAIGRALLTAPELLLLDEPLASLDIPRKRELLPYLQRLTREINI
PMLYVSHSLDEILHLADRVMVLENGQVKAFGALEEVWGSSVMNPWLPKEQQSSILKVTVL
EHHPHYAMTALALGDQHLWVNKLDEPLQAALRIRIQASDVSLVLQPPQQTSIRNVLRAKV
VNSYDDNGQVEVELEVGGKTLWARISPWARDELAIKPGLWLYAQIKSVSITA
>LFGLNPFC_02554 Molybdenum transport system permease protein ModB
MILTDPEWQAVLLSLKVSSLAVLFSLPFGIFFAWLLVRCTFPGKALLDSVLHLPLVLPPV
VVGYLLLVSMGRRGFIGERLYDWFGLSFAFSWRGAVLAAAVMSFPLMVRAIRLALESVDI
KLEQAARTLGAGRWRVFFTITLPLTLPGIIVGTVLAFARSLGEFGATITFVSNIPGETRT
IPSAMYTLIQTPGGESGAARLCIISIALAMISLLISEWLARISRERAGR
>LFGLNPFC_02555 Molybdate-binding protein ModA
MARKWLNLFTGAALSFAVAGNALADEGKITVFAAASLTNAMQDIATQYKKEKGVDVVSSF
ASSSTLARQIEAGAPADLFISADQKWMDYAVDKKAIDTASRQTLLGNSLVVVAPKASEQK
DFTIDSKTNWTSLLNGGRLAVGDPEHVPAGIYAKEALQKLGAWDTLSPTLAPAEDVRGAL
ALVERNEAPLGIVYGSDAVASKGVKVVATFPEDSHKKVEYPVAVVEGHNNATVKAFYDYL
KGPQAAEIFKRYGFTTK
>LFGLNPFC_02556 Multidrug efflux pump accessory protein AcrZ
MLELLKSLVFAVIMVPVVMAIILGLIYGLGEVFNIFSGVGKKDQPGQNH
>LFGLNPFC_02557 DNA-binding transcriptional dual regulator ModE
MQAEILLTLKLQQKLFADPRRISLLKHIALSGSISQGAKDAGISYKSAWDAINEMNQLSE
HILVERATGGKGGGGAVLTRYGQRLIQLYDLLAQIQQKAFDVLSDDDALPLNSLLAAISR
FSLQTSARNQWFGTITARDHDDVQQHVDVLLADGKTRLKVAVTAQSGARLGLDEGKEVLI
LLKAPWVGITQDDAVAQNADNQLPGIISHIERGAEQCEVLMALPDGQTLCATVPVNEATS
LQQGQNVTAYFNADSVIIATLC
>LFGLNPFC_02558 ABC transporter ATP-binding protein ModF
MSSLQILQGTFRLGDTKTLQLPRLTLNAGDSWAFVGSNGSGKSALARALSGELPLLKGER
QSQFSHITRLSFEQLQKLVSDEWQRNNTDMLSPGEDDTGRTTAEIIQDEVKDAPRCMQLA
QQFGITALLDRRFKYLSTGETRKTLLCQALMSEPDLLILDEPFDGLDVASRQHLAELLAS
LHQSGITLVLVLNRFDEIPEFVQFAGVLADCTLAETGAKEELLQQALVAQLAHSELLEGV
QMPEPDEPSARHALPANEPRIVLNNGVVSYNDRPILNNLSWQVNPGEHWQIVGPNGAGKS
TLLSLITGDHPQGYSNDLTLFGRRRGSGETIWDIKKHIGYVSSSLHLDYRVSTTVRNVIL
SGYFDSIGIYQAVSDRQQKLVQQWLDILGIDKRTADAPFHSLSWGQQRLALIVRALVKHP
TLLILDEPLQGLDPLNRQLIRRFVDVLISEGKTQLLFVSHHAEDAPACITHRLEFVPDGD
FYRYALTKIN
>LFGLNPFC_02559 UDP-glucose 4-epimerase
MRVLVTGGSGYIGSHTCVQLLQNGHDVIILDNLCNSKRSVLPVIERLGGKHPTFVEGDIR
NEALMTEILHDHAIDTVIHFAGLKAVGESVQKPLEYYDNNVNGTLRLISAMRAANVKNFI
FSSSATVYGDQPKIPYVESFPTGTPQSPYGKSKLMVEQILTDLQKAQPDWSIALLRYFNP
VGAHPSGDMGEDPQGIPNNLMPYIAQVAVGRRDSLAIFGNDYPTEDGTGVRDYIHVMDLA

DGHVVAMEKLANKPGVHIYNLGAGVGSSVLDVVNAFSKACGKPVNYHFAPRREGDLPAYW
ADASKANHELNWRVTRTLDEMAQDTWHWQSRHPQGYPD
>LFGLNPFC_02560 Galactose-1-phosphate uridylyltransferase
MTQFNPVDHPHRRYNPLTGQWILVSPHRAKRPWQGAQETPAKQVLPAHDPDCFLCAGNVR
VTGDKNPDYTGTYVFTNDFAALMSDTPDAPESNDPLMRCQSARGTSRVICFSPDHSKTLP
ELSVAALTEIVKTWQEQTAELGETYPWVQVFENKGAAMGCSNPHPHGQIWANSFLPNEAE
REDRLQKEYFAAQKSPMLVDYVQRELADGSRTVVETEHWLAVVPYWAAWPFETLLLPKAH
VLRITDLTDAQRSDLALALKKLTSRYDNLFQCSFPYSMGWHGAPFNGEENQHWQLHAHFY
PPLLRSATVRKFMVGYEMLAETQRDLTAEQAAERLRAVSDIHFRESGV
>LFGLNPFC_02561 Galactokinase
MSLKEKTQSLFANAFGYPATHTIQAPGRVNLIGEHTDYNDGFVLPCAIDYQTVISCAPRD
DRKVRVMAADYENQLDEFSLDAPIVAHENYQWANYVRGVVKHLQLRNNSFGGVDMVISGN
VPQGAGLSSSASLEVAVGTVLQQLYHLPLDGAQIALNGQEAENQFVGCNCGIMDQLISAL
GKKDHALLIDCRSLGTKAVSMPKGVAVVIINSNFKRTLVGSEYNTRREQCETGARFFQQP
ALRDVTIEEFNAVAHELDPIVAKRVRHILTENARTVEAASALEQGDLKRMGELMAESHAS
MRDDFEITVPQIDTLVEIVKAVIGDKGGVRMTGGGFGGCIVALFPEELVPAVQQAVAEQY
EAKTGIKETFYVCKPSQGAGQC
>LFGLNPFC_02562 Aldose 1-epimerase
MLNETPALAPDGQPYRLLTLRNNAGMVVTLMDWGATLLSARIPLSDGSVREALLGCASPE
CYQDQAAFLGASIGRYANRIANSRYTFDGETVTLSPSQGVNQLHGGPEGFDKRRWQIVNQ
NDRQVLFALSSDDGDQGFPGNLGATVQYRLTDDNRISITYRATVDKPCPVNMTNHVYFNL
DGEQSDVRNHKLQILAEEYLPVDEGGIPHDGLKSVAGTSFDFRNAKIIASEFLADDDQRK
VKGYDHAFLLQAKGDGKKVAAHVWSADEKLQLKVYTTAPALQFYSGNFLGGTPSRGTEPY
ADWQGLALESEFLPDSPNHPEWPQPDCFLRPGEEYSSLTEYQFIAQ
>LFGLNPFC_02563 2,3-bisphosphoglycerate-dependent phosphoglycerate mutase
MAVTKLVLVRHGESQWNKENRFTGWYDVDLSEKGVSEAKAAGKLLKEEGYSFDFAYTSVL
KRAIHTLWNVLDELDQAWLPVEKSWKLNERHYGALQGLNKAETAEKYGDEQVKQWRRGFA
VTPPELTKDDERYPGHDPRYAKLSEKELPLTESLALTIDRVIPYWNETILPRMKSGERVI
IAAHGNSLRALVKYLDNMSEEEILELNIPTGVPLVYEFDENFKPLKRYYLGNADEIAAKA
AAVANQGKAK
>LFGLNPFC_02564 Phospho-2-dehydro-3-deoxyheptonate aldolase, Phe-sensitive
MNYQNDDLRIKEIKELLPPVALLEKFPATENAANTVAHARKAIHKILKGNDDRLLVVIGP
CSIHDPVAAKEYATRLLALREELKDELEIVMRVYFEKPRTTVGWKGLINDPHMDNSFQIN
DGLRIARKLLLDINDSGLPAAGEFLDMITPQYLADLMSWGAIGARTTESQVHRELASGLS
CPVGFKNGTDGTIKVAIDAINAAGAPHCFLSVTKWGHSAIVNTSGNGDCHIILRGGKEPN
YSAKHVAEVKEGLNKAGLPAQVMIDFSHANSSKQFKKQMDVCADVCQQIAGGEKAIIGVM
VESHLVEGNQSLESGEPLAYGKSITDACIGWDDTDALLRQLANAVNARRG
>LFGLNPFC_02565 hypothetical protein
MKMTKLATLFLTATLSLASGAALAADSGAQSNNGQANAAADAGQVAPDARENVAPNNVDN
NGVNTGSDGTMLHPDGSSMNNDGMTKDEEHKNTMCKDGRCPDINKKVQTGDGINNDVDTK
TDGTTQ
>LFGLNPFC_02566 Zinc transporter ZitB
MAHSHSHASSHLPEDNNARRLLYAFGVTAGFMLVEVIGGFLSGSLALLADAGHMLTDTAA
LLFALLAVQFSRRPPTIRHTFGWLRLTTLAAFVNAIALVVITILIVWEAIERFRTPRPVE
GGMMMAIAVAGLLANILSFWLLHHGSEEKNLNVRAAALHVLGDLLGSVGAIIAALIIIWT
GWTPADPILSILVSLLVLRSAWRLLKDSVNELLEGAPVSLDIAELKRRLCREIPEVRNVH
HVHVWMVGEKPVMTLHVQVIPPHDHDALLDQIQHYLMDHYQIEHATIQMEYQPCHGPDCH
LNEGVSGHSHHHH
>LFGLNPFC_02567 Nicotinamide riboside transporter PnuC
MDFFSVQNILVHIPIGAGGYDLSWIEAVGTIAGLLCIGLASLEKISNYFFGLINVTLFGI
IFFQIQLYASLLLQVFFFAANIYGWYAWSRQTSQNEAELKIRWLPLPKALSWLAVCVVSI
GLMTVFINPVFAFLTRVAVMIMQALGLQVAMPELQPDAFPFWDSCMMVLSIVAMILMTRK
YVENWLLWVIINVISVVIFALQGVYAMSLEYIILTFIALNGSRMWINSARERGSRALSH
>LFGLNPFC_02568 Quinolinate synthase A
MSVMFDPDTAIYPFPPKPTPLSIDEKAYYREKIKRLLKERNAVMVAHYYTDPEIQQLAEE
TGGCISDSLEMARFGAKHPASTLLVAGVRFMGETAKILSPEKTILMPTLQAECSLDLGCP
VEEFNAFCDAHPDRTVVVYANTSAAVKARADWVVTSSIAVELIDHLDSLGEKIIWAPDKH
LGCYVQKQTGADILCWQGACIVHDEFKTQALTRLQEEYPDAAILVHPESPQAIVEMADAV
GSTSQLIAAAKTLPHQRLIVATDRGIFYKMQQAVPDKELLEAPTAGEGATCRSCAHCPWM
AMNGLQAIAEALELEGSNHEVYVDERLLERALVPLNRMLDFAATLRG
>LFGLNPFC_02576 Cell division coordinator CpoB
MSSNFRHQLLSLSLLVGIAAPWAAFAQAPISSVGSGSVEDRVTQLERISNAHSQLLTQLQ
QQLSDNQSDIDSLRGQIQENQYQLNQVVERQKQILLQIDSLSSGGAAAQSTSGDQSGVAA
STTPTADAGTANAGAPVKSGDANTDYNAAIALVQDKSRQDDAMVAFQNFIKNYPDSTYLP

NANYWLGQLNYNKGKKDDAAYYFASVVKNYPKSPKAADAMFKVGVIMQDKGDTAKAKAVY
QQVISKYPGTDGAKQAQKRLNAM
>LFGLNPFC_02577 Peptidoglycan-associated lipoprotein
MQLNKVLKGLMIALPVMAIAACSSNKNASNDGSEGMLGAGTGMDANGGNGNMSSEEQARL
QMQQLQQNNIVYFDLDKYDIRSDFAQMLDAHANFLRSNPSYKVTVEGHADERGTPEYNIS
LGERRANAVKMYLQGKGVSADQISIVSYGKEKPAVLGHDEAAYAKNRRAVLVY
>LFGLNPFC_02578 Tol-Pal system protein TolB
MKQALRVAFGFLILWASVLHAEVRIVIDSGVDSGRPIGVVPFQWAGPGAAPEDIGGIVAA
DLRNSGKFNPLDRARLPQQPGSAQEVQPAAWSALGIDAVVVGQVTPNPDGSYNVAYQLVD
TGGAPGTVLAQNSYKVNKQWLRYAGHTASDEVFEKLTGIKGAFRTRIAYVVQTNGGQFPY
ELRVSDYDGYNQFVVHRSPQPLMSPAWSPDGSKLAYVTFESGRSALVIQTLANGAVRQVA
SFPRHNGAPAFSPDGSKLAFALSKTGSLNLYVMDLASGQIRQVTDGRSNNTEPTWFPDSQ
NLAFTSDQAGRPQVYKVNINGGAPQRITWEGSQNQDADVSSDGKFMVMVSSNGGQQHIAK
QDLATGGVQVLSSTFLDETPSLAPNGTMVIYSSSQGMGSVLNLVSTDGRFKARLPATDGQ
VKFPAWSPYL
>LFGLNPFC_02579 hypothetical protein
MSKATEQNDKLKRAIIISAVLHVILFAALIWSSFDENIEASAGGGGGSSIDAVMVDSGAV
VEQYKRMQSQESSAKRSDEQRKMKEQQAAEELREKQAAEQERLKQLEKERLAAQEQKKQA
EEAAKQAELKQKQAEEAAAKAAADAKAKAEADAKAAEEAAKKAAADAKKKAEAEAAKAAV
EAQKKAEAAAAALKKKAEAAEAAAAEARKKAATEAAEKAKAEAEKKAAAEKAAADKKAAA
DKKAAEKAAAEKAAADKKAAAEKAAADKKAAAAKAAAEKAAAAKAAAEADDIFGELSSGK
NAPKTGGGAKGNNASPAGSGNTKNNGASGADINNYAGQIKSAIESKFYDASSYAGKTCTL
RIKLAPDGMLLDIKPEGGDPALCQAALAAAKLAKIPKPPSQAVYEVFKNAPLDFKP
>LFGLNPFC_02580 Tol-Pal system protein TolR
MARARGRGRRDLKSEINIVPLLDVLLVLLLIFMATAPIITQSVEVDLPDATESQAVSSND
NPPVIVEVSGIGQYTVVVEKDRLERLPPEQVVAEVSSRFKANPKTVFLIGGAKDVPYDEI
IKALNLLHSAGVKSVGLMTQPI
>LFGLNPFC_02581 Tol-Pal system protein TolQ
MTDMNILDLFLKASLLVKLIMLILIGFSIASWAIIIQRTRILNAAAREAEAFEDKFWSGI
ELSRLYQESQGKRDNLSGSEQIFYSGFKEFVRLHRANSHAPEAVVEGASRAMRISMNREL
ENLETHIPFLGTVGSISPYIGLFGTVWGIMHAFIALGAVKQATLQMVAPGIAEALIATAI
GLFAAIPAVMAYNRLNQRVNKLELNYDNFMEEFTAILHRQAFTVSESNKG
>LFGLNPFC_02582 Acyl-CoA thioester hydrolase YbgC
MNTTLFRWPVRVYYEDTDAGGVVYHASYVAFYERARTEMLRHHHFSQQALMAERVAFVVR
KMTVEYYAPARLDDMLEIQTEITSMRGTSLVFTQRIVNAENTLLNEAEVLVVCVDPLKMK
PRALPKSIVAEFKQ
>LFGLNPFC_02583 hypothetical protein
MSKIIATLYAVMDKRPLRALSFVMALLLAGCMFWDPSRFAAKTSELEIWHGLLLMWAVCA
GVIHGVGFRPQKVLWQGIFCPLLADIVLIVGLIFFFF
>LFGLNPFC_02584 Cytochrome bd-I ubiquinol oxidase subunit X
MWYFAWILGTLLACSFGVITALALEHVESGKAGQEDI
>LFGLNPFC_02585 Cytochrome bd-I ubiquinol oxidase subunit 2
MIDYEVLRFIWWLLVGVLLIGFAVTDGFDMGVGMLTRFLGRNDTERRIMINSIAPHWDGN
QVWLITAGGALFAAWPMVYAAAFSGFYVAMILVLASLFFRPVGFDYRSKIEETRWRNMWD
WGIFIGSFVPPLVIGVAFGNLLQGVPFNVDEYLRLYYTGNFFQLLNPFGLLAGVVSVGMI
ITQGATYLQMRTVGELHLRTRATAQVAALVTLVCFALAGVWVMYGIDGYVVKSTMDHYAA
SNPLNKEVVREAGAWLVNFNNTPILWAIPALGVVLPLLTILTARMDKAAWAFVFSSLTLA
CIILTAGIAMFPFVMPSSTMMNASLTMWDATSSQLTLNVMTWVAVVLVPIILLYTAWCYW
KMFGRITKEDIERNTHSLY
>LFGLNPFC_02586 Cytochrome bd-I ubiquinol oxidase subunit 1
MLDIVELSRLQFALTAMYHFLFVPLTLGMAFLLAIMETVYVLSGKQIYKDMTKFWGKLFG
INFALGVATGLTMEFQFGTNWSYYSHYVGDIFGAPLAIEGLMAFFLESTFVGLFFFGWDR
LGKVQHMCVTWLVALGSNLSALWILVANGWMQNPIASDFNFETMRMEMVSFSELVLNPVA
QVKFVHTVASGYVTGAMFILGISAWYMLKGRDFAFAKRSFAIAASFGMAAVLSVIVLGDE
SGYEMGDVQKTKLAAIEAEWETQPAPAAFTLFGIPDQEEETNKFAIQIPYALGIIATRSV
DTPVIGLKELMVQHEERIRNGMKAYSLLEQLRSGSTDQAVRDQFNSMKKDLGYGLLLKRY
TPNVADATEAQIQQATKDSIPRVAPLYFAFRIMVACGFLLLAIIALSFWSVIRNRIGEKK
WLLRAALYGIPLPWIAVEAGWFVAEYGRQPWAIGEVLPTAVANSSLTAGDLIFSMVLICG
LYTLFLVAELFLMFKFARLGPSSLKTGRYHFEQSSTTTQPAR
>LFGLNPFC_02587 hypothetical protein
MHCSIINSLFTMPFYKNESGIIFFNFICLLVFLVKLSIIHCFIILRMMEIKGFLMIIKHI
LMLFNHEFFNMIMYQYVILPDVLLNYRLLL
>LFGLNPFC_02588 Photosystem I assembly protein Ycf3
MGVNSDVYAADVNIDILSATVKDKRIEGVSVTLQRNGAQSVSGTTNASGSVNLGSTFADD

QDALLIVKKEGYSNLVVKCSCAGMTYAISPAMTSLDGMRVVLSWGEKPFDLDSHLIFPGG
HIYFDSKEGTDANLDVDDTDSYGSETVTISKKHFGESYIYAVQDYSNKGLPNSNYLSASK
AKVFVYVGSSLVRSYSVPAGKRGNIWTVFKLNPNGEFEDINSVTSANFNDTTLGVRDLAT
VIMPATGSSTPASPAMQNSGDTQLARKYNREGEAVYKTGQLEQAIQLFQQATELDGNYGQ
AFSNLGLAYQKNGNIAEAIWANRKAISLASGANAATTRANSYYNIAKIYETAGQDADALQ
HYQLALHDAIL
>LFGLNPFC_02589 Succinate--CoA ligase [ADP-forming] subunit alpha
MSILIDKNTKVICQGFTGSQGTFHSEQAIAYGTKMVGGVTPGKGGTTHLGLPVFNTVREA
VAATGATASVIYVPAPFCKDSILEAIDAGIKLIITITEGIPTLDMLTVKVKLDEAGVRMI
GPNCPGVITPGECKIGIQPGHIHKPGKVGIVSRSGTLTYEAVKQTTDYGFGQSTCVGIGG
DPIPGSNFIDILEMFEKDPQTEAIVMIGEIGGSAEEEAAAYIKEHVTKPVVGYIAGVTAP
KGKRMGHAGAIIAGGKGTADEKFAALEAAGVKTVRSLADIGEALKTVLK
>LFGLNPFC_02590 Succinate--CoA ligase [ADP-forming] subunit beta
MNLHEYQAKQLFARYGLPAPVGYACTTPREAEEAASKIGAGPWVVKCQVHAGGRGKAGGV
KVVNSKEDIRAFAENWLGKRLVTYQTDANGQPVNQILVEAATDIAKELYLGAVVDRSSRR
VVFMASTEGGVEIEKVAEETPHLIHKVALDPLTGPMPYQGRELAFKLGLEGKLVQQFTKI
FMGLATIFLERDLALIEINPLVITKQGDLICLDGKLGADGNALFRQPDLREMRDQSQEDP
REAQAAQWELNYVALDGNIGCMVNGAGLAMGTMDIVKLHGGEPANFLDVGGGATKERVTE
AFKIILSDDKVKAVLVNIFGGIVRCDLIADGIIGAVAEVGVNVPVVVRLEGNNAELGAKK
LADSGLNIIAAKGLTDAAQQVVAAVEGK
>LFGLNPFC_02591 Dihydrolipoyllysine-residue succinyltransferase component of
2-oxoglutarate dehydrogenase complex
MSSVDILVPDLPESVADATVATWHKKPGDAVVRDEVLVEIETDKVVLEVPASADGILDAV
LEDEGTTVTSRQILGRLREGNSAGKETSAKSEEKASTPAQRQQASLEEQNNDALSPAIRR
LLAEHNLDASAIKGTGVGGRLTREDVEKHLAKAPAKESAPAAPAPAAQPALAARSEKRVP
MTRLRKRVAERLLEAKNSTAMLTTFNEVNMKPIMDLRKQYGEAFEKRHGIRLGFMSFYVK
AVVEALKRYPEVNASIDGDDVVYHNYFDVSMAVSTPRGLVTPVLRDVDTLGMADIEKKIK
ELAVKGRDGKLTVEDLTGGNFTITNGGVFGSLMSTPIINPPQSAILGMHAIKDRPMAVNG
QVEILPMMYLALSYDHRLIDGRESVGFLVTIKELLEDPTRLLLDV
>LFGLNPFC_02592 2-oxoglutarate dehydrogenase E1 component
MQNSALKAWLDSSYLSGANQSWIEQLYEDFLTDPDSVDANWRSTFQQLPGTGVKPDQFHS
QTREYFRRLAKDASRYSSTISDPDTNVKQVKVLQLINAYRFRGHQHANLDPLGLWQQDKV
ADLDPSFHDLTEADFQETFNVGSFASGKETMKLGELLEALKQTYCGPIGAEYMHITSTEE
KRWIQQRIESGRATFNSEEKKRFLSELTAAEGLERYLGAKFPGAKRFSLEGGDALIPMLK
EMIRHAGNSGTREVVLGMAHRGRLNVLVNVLGKKPQDLFDEFAGKHKEHLGTGDVKYHMG
FSSDFQTDGGLVHLALAFNPSHLEIVSPVVIGSVRARLDRLDEPSSNKVLPITIHGDAAV
TGQGVVQETLNMSKARGYEVGGTVRIVINNQVGFTTSNPLDARSTPYCTDIGKMVQAPIF
HVNADDPEAVAFVTRLALDFRNTFKRDVFIDLVCYRRHGHNEADEPSATQPLMYQKIKKH
PTPRKIYADKLEQEKVATLEDATEMVNLYRDALDAGDCVVAEWRPMNMHSFTWSPYLNHE
WDEEYPNKVEMKRLQELAKRISTVPEAVEMQSRVAKIYGDRQAMAAGEKLFDWGGAENLA
YATLVDEGIPVRLSGEDSGRGTFFHRHAVIHNQSNGSTYTPLQHIHNGQGAFRVWDSVLS
EEAVLAFEYGYATAEPRTLTIWEAQFGDFANGAQVVIDQFISSGEQKWGRMCGLVMLLPH
GYEGQGPEHSSARLERYLQLCAEQNMQVCVPSTPAQVYHMLRRQALRGMRRPLVVMSPKS
LLRHPLAVSSLEELANGTFLPAIGEIDELDPKGVKRVVMCSGKVYYDLLEQRRKNNQHDV
AIVRIEQLYPFPHKAMQEVLQQFAHVKDFVWCQEEPLNQGAWYCSQHHFREVIPFGASLR
YAGRPASASPAVGYMSVHQKQQQDLVNDALNVE
>LFGLNPFC_02593 Succinate dehydrogenase iron-sulfur subunit
MRLEFSIYRYNPDVDDAPRMQDYTLEAEEGRDMMLLDALIQLKEKDPSLSFRRSCREGVC
GSDGLNMNGKNGLACITPISALNQPGKKIVIRPLPGLPVIRDLVVDMGQFYAQYEKIKPY
LLNNGQNPPAREHLQMPEQREKLDGLYECILCACCSTSCPSFWWNPDKFIGPAGLLAAYR
FLIDSRDTETDSRLDGLSDAFSVFRCHSIMNCVSVCPKGLNPTRAIGHIKSMLLQRNA
>LFGLNPFC_02594 Succinate dehydrogenase flavoprotein subunit
MKLPVREFDAVVIGAGGAGMRAALQISQSGQTCALLSKVFPTRSHTVSAQGGITVALGNT
HEDNWEWHMYDTVKGSDYIGDQDAIEYMCKTGPEAILELEHMGLPFSRLDDGRIYQRPFG
GQSKNFGGEQAARTAAAADRTGHALLHTLYQQNLKNHTTIFSEWYALDLVKNQDGAVVGC
TALCIETGEVVYFKARATVLATGGAGRIYQSTTNAHINTGDGVGMAIRAGVPVQDMEMWQ
FHPTGIAGAGVLVTEGCRGEGGYLLNKHGERFMERYAPNAKDLAGRDVVARSIMIEIREG
RGCDGPWGPHAKLKLDHLGKEVLESRLPGILELSRTFAHVDPVKEPIPVIPTCHYMMGGI
PTKVTGQALTVNEKGEDVVVPGLFAVGEIACVSVHGANRLGGNSLLDLVVFGRAAGLHLQ
ESIAEQGALRDASESDVEASLDRLNRWNNNRNGEDPVAIRKALQECMQHNFSVFREGDAM
AKGLEQLKVIRERLKNARLDDTSSEFNTQRVECLELDNLMETAYATAVSANFRTESRGAH
SRFDFPDRDDENWLCHSLYLPESESMTRRSVNMEPKLRPAFPPKIRTY
>LFGLNPFC_02595 Succinate dehydrogenase hydrophobic membrane anchor subunit
MVSNASALGRNGVHDFILVRATAIVLTLYIIYMVGFFATSGELTYEVWIGFFASAFTKVF

TLLALFSILIHAWIGMWQVLTDYVKPLALRLMLQLVIVVALVVYVIYGFVVVWGV
>LFGLNPFC_02596 Succinate dehydrogenase cytochrome b556 subunit
MWALFMIRNVKKQRPVNLDLQTIRFPVTAIASILHRVSGVITFVAVGILLWLLGTSLSSP
EGFEQASAIMGSFFVKFIMWGILTALAYHVVVGIRHMMMDFGYLEETFEAGKRSAKISFV
ITVVFSLLAGVLVW
>LFGLNPFC_02597 Protein YbgU
MRKSYEVGISPKINLCNSVEVLTNSFGTVISGRQV
>LFGLNPFC_02598 Citrate synthase
MADTKAKLTLNGDTAVELDVLKGTLGQDVIDIRTLGSKGVFTFDPGFTSTASCESKITFI
DGDEGILLHRGFPIDQLATDSNYLEVCYILLNGEKPTQEQYDEFKTTVTRHTMIHEQITR
LFHAFRRDSHPMAVMCGITGALAAFYHDSLDVNNPRHREIAAFRLLSKMPTMAAMCYKYS
IGQPFVYPRNDLSYAGNFLNMMFSTPCEPYEVNPILERAMDRILILHADHEQNASTSTVR
TAGSSGANPFACIAAGIASLWGPAHGGANEAALKMLEEISSVKHIPEFVRRAKDKNDSFR
LMGFGHRVYKNYDPRATVMRETCHEVLKELGTKDDLLEVAMELENIALNDPYFIEKKLYP
NVDFYSGIILKAMGIPSSMFTVIFAMARTVGWIAHWSEMHSDGMKIARPRQLYTGYEKRD
FKSDIKR
>LFGLNPFC_02599 hypothetical protein
MIDLGKFNLLDIRRHTMSKTFSIKTTKSQNDQCTDGFKVSSSFYTEKTLVEEDKALLIGN
GLKLRILDENASPYTFNKYAEYADFTSDMLIYEKTYTAELSSTPGTPIAAGPFDTVVLFK
INYN
>LFGLNPFC_02600 hypothetical protein
MPVLQWGMLCVLSLLLSIGFLALHLPAALLLGPMIAGIIFSMRGVTLQLPRSAFLAAQAI
LGCMIAQNLTGSILTTLAVNWPIVLAILLVTLLSSAIVGWLLVRYSSLPGNTGAWGSSPG
GAAAMVAMAQDYGADIRLVAFMQYLRVLFVAGAAVLVTRMMLGDNAEAVNQQIVWFPPVS
INLLLTILLAVVAGTAGCLLRLPSGTMLIPMLAGAVLQSGQLITIELPEWLLAMAYMAIG
WRIGLGFDKQILLRALRPLPQILLSIFALLAICAGMAWGLTRFMHIDFMTAYLATSPGGL
DTVAVIAAGSNADMALIMAMQTLRLFSILLTGPAIARFISTYAPKRSA
>LFGLNPFC_02601 Endonuclease 8
MPEGPEIRRAADNLEAAIKGKPLTDVWFAFPQLKSYQSRLIGQHVTHVETRGKALLTHFS
NDLTLYSHNQLYGVWRVVDTGEEPQTTRVLRVKLQTADKTILLYSASDIEMLTPEQLTTH
PFLQRVGPDVLDPNLTPEVVKERLLSPRFRNRQFAGLLLDQAFLAGLGNYLRVEILWQVG
LTGNHKAKDLNAAQLDALAHALLDTPRLSYATRGQVDENKYHGALFRFKVFHRDGEPCER
CGGIIEKTTLSSRPFYWCPGCQH
>LFGLNPFC_02602 5-oxoprolinase subunit A
MKIDLNADLGEGCASDAELLTLVSSANIACGFHAGDAQTMQACVREAIKNGVAIGAHPSF
PDRENFGRSAMQLPPETVFAQTLYQIGALAAITRAQGGVMCHVKPHGMLYNQAAKEAQLA
DAIARAVYACDPALILVGLAGSELIRAGERYGLVTREEVFADRGYQADGSLVPRSQPGAL
IENEEQALAQTLEMVQYGRVKSITGEWAMVTAQTVCLHGDGEHALAFARRLRATFAEKGI
VVAA
>LFGLNPFC_02603 5-oxoprolinase subunit C
MLKIIRAGMYTTVQDGGRHGFRQSGISHCGALDMPALRIANLLVGNDANAPALEITLGQL
TVEFETDGWFALTGAGCEARLDDNAVWTGWRLPMRAGQRLTLKRPQHGMRSYLAVAGGID
VPPVMGSCSTDLKVGIGGLEGRLLRDGDRLPIGKAKRDFMEAQGVKQLLWGNRIRALPGP
EYHEFDRASQDAFWRSPWQLSSQSNRMGYRLQGQILKRTTDRELLSHGLLPGVVQVPHNG
QPIVLMNDAQTTGGYPRIACIIEADMYHLAQIPLGQPIHFVQCSLEEALKARQDQQRYFE
QLAWRLHNEN
>LFGLNPFC_02604 5-oxoprolinase subunit B
MQRARCYLIGETAVVLELEPPVTLASQKRIWRLAQRLVDMPNVVEAIPGMNNITVILRNP
ESLALDAIERLQRWWEESEALEPESRFIEIPVVYGGAAGPDLAVVAAHCGLSEKQVVELH
SSVEYVVWFLGFQPGFPYLGSLPEQLHTPRRAEPRLHVPAGSVGIGGPQTGVYPLATPGG
WQLIGHTSLSLFDPERDEPILLRPGDSVRFVPQKEGVC
>LFGLNPFC_02605 GTP cyclohydrolase 1 type 2
MKNTELEQLINEKLNSAAISDYAPNGLQVEGKETVQKIVTGVTASQALLDEAVRLGADAV
IVHHGYFWKGESPVIRGMKRNRLKTLLANDINLYGWHLPLDAHPELGNNAQLAALLGITV
MGEIEPLVPWGELTMPVPGLELASWIEARLGRKPLWCGDTGPEVVQRVAWCTGGGQSFID
SAARFGVDAFITGEVSEQTIHSAREQGLHFYAAGHHATERGGIRALSEWLNENTDLDVTF
IDIPNPA
>LFGLNPFC_02606 Dipeptide permease D
MNKHASQPRAIYYVVALQIWEYFSFYGMRALLILYLTNQLKYSDNHAYELFSAYCSLVYV
TPILGGFLADKVLGNRMAVMLGALLMAIGHVVLGASEIHPSFLYLSLAIIVCGYGLFKSN
VSCLLGELYEPTDPRRDGGFSLMYAAGNVGSIIAPIACGFAQEEYSWAMGFGLAAVGMIA
GLVIFLCGNRHFTHTRGVNKKVLRATNFLLPNWGWLLVLLVATPALITVLFWKEWSVYAL
IVATIIGLGVLAKIYRKAENQKQRKELGLIVTLTFFSMLFWAFAQQGGSSISLYIDRFVN
RDMFGYTVPTAMFQSINAFAVMLCGVFLAWVVKESVAGNRTVRIWGKFALGLGLMSAGFC

ILTLSARWSAMYGHSSLPLMVLGLAVMGFAELFIDPVAMSQITRIEIPGVTGVLTGIYML
LSGAIANYLAGVIADQTSQASFDASGAINYSINAYIEVFDQITWGALACVGLVLMIWLYQ
ALKFRNRALALES
>LFGLNPFC_02607 Deoxyribodipyrimidine photo-lyase
MTTHLVWFRQDLRQHDNLALAAACRNSSARVLALYIATPRQWAAHNVSPRQAELINTQLN
ALQNALAEKGIPLLFREVDDFAASVETVKQVCAENRITHLFYNYQYEVNERARDVQAERT
LRNVVCEGFDDSVILPPGAVMTGNHEMYKVFTPFKNAWLKRLREGMPECVAAPKFRSSGS
IEPAPSITLNYPRQSFDTAHFPVEEKAAIAQLRQFCQNGAGEYEQQRDFPAVEGTSRLSA
SLATGGLSPRQCLHRLLAEQPQVLEGGPGSVWLNELIWREFYRHLMTYYPSLCKHRPFIA
WTDRVQWQSNPAHLKAWQEGKTGYPIVDAAMRQLNSTGWMHNRLRMITASFLVKDLLIDW
REGERYFMSQLIDGDLAANNGGWQWAASTGTDAAPYFRIFNPTTQGEKFDREGEFIRQWL
PELRDVPGKAVHEPWKWAEKTGVTLDYPQPIVDHKEARLRTLAAYEEARKGA
>LFGLNPFC_02608 hypothetical protein
MNQQLFDDSTLIRIFALHELHNLKDRGLTRGALLDYHSRYKLVFLAHSQPEYRKLGPFVA
DIHRWQSLDDYYNQYRQRVIVLLSHPANRRDHTNVLMHVQGYFRPHIDSTERQQLAALID
SYRRGEQPLLAPLMRIKHYMALYPDAWLSGQRYFELWPRVINLRHAGVL
>LFGLNPFC_02609 hypothetical protein
MELYKEYPAWLIFLRRTCAVAAGVLALPFMLFWKDRARFYSYLHRVWSKTSDKPVWMDQA
EKATGDFY
>LFGLNPFC_02610 Potassium-transporting ATPase potassium-binding subunit
MAAQGFLLIATFLLVFMVLARPLGSGLARLINDIPLPGTTGVERVLFSALGVSDREMNWK
QYLSAILGLNILGLAVLFFMLLGQHYLPLNPQQLPGLSWDLALNTAVSFVTNTNWQSYSG
ETTLSYFSQMAGLTVQNFLSAASGIAVIFALIRAFTRQSMNTLGNAWVDLLRITLWVLTP
VALLIALFFIQQGALQNFLPYQAVTTIEGAQQLLPMGPVASQEAIKMLGTNGGGFFNANS
SHPFENPTALTNFVQMLAIFLIPTALCFAFGEVAGDRRQGRMLLWAMSVIFVICVGVVMW
AEVQGNPHLLALGADSSINMEGKESRFGVLVSSLFAVVTTAASCGAVIAMHDSFTALGGM
VPMWLMQIGEVVFGGVGSGLYGMMLFVLLAVFIAGLMIGRTPEYLGKKIDVREMKLTALA
ILVTPTLVLMGAALAMMTDAGRSAMLNPGPHGFSEVLYAVSSAANNNGSAFAGLSANSPF
WNCLLALCMFVGRFGVIIPVMAIAGSLVSKKSQPASSGTLPTHGPLFVGLLIGTVLLVGA
LTFIPALALGPVAEYLS
>LFGLNPFC_02611 Potassium-transporting ATPase ATP-binding subunit
MSRKQLALFEPTLVVQALKEAVKKLNPQAQWRNPVMFIVWIGSLLTTCISIAMASDVMPG
NALFSAAISGWLWVTVLFANFAEALAEGRSKAQANSLKGVKKTAFARKLREPKYGAAADK
VPADQLRKGDIVLVEASDIIPCDGEVIEGGASVDESAITGESAPVIRESGGDFASVTGGT
RILSDWLVIECSVNPGETFLDRMIAMVEGAQRRKTPNEIALTILLIALTIVFLLATATLW
PFSAWGGNAVSVTVLVALLVCLIPTTIGGLLSAIGVAGMSRMLGANVIATSGRAVEAAGD
VDVLLLDKTGTITLGNRQASEFIPAQGVEEKALADAAQLASLADETPEGRSIVILAKQRF
NLRERDVQSLHATFVPFTAQSRMSGINIDNRMIRKGSVDAIRRHVEANGGHFPADVDQKV
DQVARQGATPLVVVEGSRVLGVIALKDIVKGGIKERFAQLRKMGIKTVMITGDNRLTAAA
IAAEAGVDDFLAEATPEAKLALIRQYQAEGRLVAMTGDGTNDAPALAQADVAVAMNSGTQ
AAKEAGNMVDLDSNPTKLIEVVHIGKQMLMTRGSLTTFSIANDVAKYFAIIPAAFAATYP
QLNALNIMRLHSPDSAILSAVIFNALIIVFLIPLALKGVSYKPLTASAMLRRNLWIYGLG
GLLVPFIGIKVIDLLLTVCGLV
>LFGLNPFC_02612 Potassium-transporting ATPase KdpC subunit
MRGLRPALSTFLFLLLITGGVYPLLTTALGQWWFPWQANGSLIREGDTVRGSALIGQNFT
GNGYFHGRPSATAEMPYNPQASGGSNLAVSNPELDKQIAARVAALRAANPDASTNVPVEL
VTASASGLDNNITPQAAAWQIPRVAKARNLSVEQLTQLIAKYSQQPLVKYIGQPVVNIVE
LNLALDRLDE
>LFGLNPFC_02613 Sensor protein KdpD
MNNEPLRPDPDRLLEQAAAPHRGKLKVFFGACAGVGKTWAMLAEAQRLRAQGLDIVVGVV
ETHGRKDTAAMLKGLAVLPLKRQAYRGRHISEFDLDAALARRPALILMDELAHSNAPGSR
HPKRWQDIEELLEAGIDVFTTVNVQHLESLNDVVSGVTGIQVRETVPDPFFDAADDVVLV
DLPPDDLRQRLKEGKVYIAGQAERAIEHFFRKGNLIALRELALRRTADRVDEQMRAWRGH
PGEEKVWHTRDAILLCIGHNTGSEKLVRAAARLASRLGSVWHAVYVETPALHRLPEKKRR
AILSALRLAQELGAETATLSDPAEEKAVVRYAREHNLGKIILGRPASRRWWRRETFADRL
ARIAPDLDQVLVALDEPPARTINNAPDSRSFKDKWRVQIQGCVVAAALCAVITLIAMQWL
IAFDAANLVMLYLPGVVVVALFYGRWPSVVATVINVVSFDLFFIAPRGTLAVSDVQYLLT
FAVMLTVGLVIGNLTAGVRYQARVARYREQRTRHLYEMSKALAVGRSSQDIAATSEQFIA
STFHARSQVLLPDDNGKLQPLTHPQGMTPWDDAIAQWSFDKGLPAGAGTDTLPGVPYQIL
PLKSGEKTYGLVVVEPGNLRQLMIPEQQRLLETFTLLVANALERLTLTASEEQARMASER
EQIRNALLAALSHDLRTPLTVLFGQAEILTLDLASEGSPHARQASEIRQHVLNTTRLVNN
LLDMARIQSGGFNLKKEWLTLEEVVGSALQMLEPGLSSPINLSLPEPLTLIHVDGPLFER
VLINLLENAVKYAGAQAEIGINAHVEGENLQLDVWDNGPGLPPGQEQTIFDKFARGNKES
AVPGVGLGLAICRAIVDVHGGTITAFNRPEGGACFRVTLPQQTAPELEEFHEDM

>LFGLNPFC_02614 KDP operon transcriptional regulatory protein KdpE
MTNVLIVEDEQAIRRFLRTALEGDGMRVFEAETLQRGLLEAATRKPDLIILDLGLPDGDG
IEFIRDLRQWSAVPVIVLSARSEESDKIAALDAGADDYLSKPFGIGELQARLRVALRRHS
ATTTPDPLVKFSDVTVDLAARVIHRGEEEVHLTPIEFRLLAVLLNNAGKVLTQRLLLNQV
WGPNAVEHSHYLRIYMGHLRQKLEQDPTRPRHFITETGIGYRFMP
>LFGLNPFC_02615 Inducible ornithine decarboxylase
MSELKIAVSRSCPDCFSTHRECVNIDKSNYIDVAAIILSVNDVERGKLDEIDATGYGIPV
FIATENEERVPAEYLPRISGVFEHCESRKEFYGRQLETAASHYETQLRPPFFRALVDYVN
QGNSAFDCPGHQGGEFFRRHPAGNQFVEYFGEMLFRSDLCNADVAMGDLLIHEGAPCIAQ
QHAAKVFNADKTYFVLNGTSSSNKVVLNALLTPGDLVLFDRNNHKSNHHGALLQAGATPV
YLETARNPYGFIGGIDAHCFEESYLRELITEVAPQRAKEARPFRLAVIQLGTYDGTIYNA
RQVVDKIGHLCDYILFDSAWVGYEQFIPMMADCSPLLLELNENDPGILVTQSVHKQQAGF
SQTSQIHKKDSHIKGQQRYVPHKRMNNAFMMHASTSPFYPLFAALDINAKMHEGVSGRNM
WMDCVVNGINARKLILDNCQHIRPFVPELVDGKPWQSYETAQIAVDLRFFKFVPGEHWHS
FEGYAENQYFVDPCKLLLTTPGIDARNGEYEAFGVPATILANFLRENGVVPEKCDLNSIL
FLLTPAEDMAKLQQLVALLVRFEKLLEADAPLAEVLPSIYKQHEERYAGYTLRQLCQEMH
DLYARHNVKQLQKEMFRKEHFPRVSMNPQEANYAYLRGEVELVRLPDAEGRIAAEGALPY
PPGVLCVVPGEIWGGAVLRYFSALEEGINLLPGFAPELQGVYIEEHDGRKQVWCYVIKPR
DAQSALLKGEKL
>LFGLNPFC_02616 Putrescine transporter PotE
MSQAKSNKMGVVQLTILTMVNMMGSGIIMLPTKLAEVGTISIISWLVTAVGSMALAWAFA
KCGMFSRKSGGMGGYAEYAFGKSGNFMANYTYGVSLLIANVAIAISAVGYGTELLGASLS
PVQIGLATIGVLWICTVANFGGARITGQISSITVWGVIIPVVGLCIIGWFWFSPTLYVDS
WNPHHAPFFSAVGSSIAMTLWAFLGLESACANTDVVENPERNVPIAVLGGTLGAAVIYIV
STNVIAGIVPNMELANSTAPFGLAFAQMFTPEVGKVIMALMVMSCCGSLLGWQFTIAQVF
KSSADEGYFPKIFSRVSKVDAPVQGMLTIVIIQSGLSLMTISPSLNSQFNVLVNLAVVTN
IIPYILSMAALVIIQKVANVPPSKAKVANFVAFVGAMYSFYALYSSGEEAMLYGSIVTFL
GWTLYGLVSPRFELKNKHG
>LFGLNPFC_02617 Phosphoglucomutase
MAIHNRAGQPAQQSDLINVAQLTAQYYVLKPEAGNAEHAVKFGTSGHRGSAARHSFNEPH
ILAIAQAIAEERAKNGITGPCYVGKDTHALSEPAFISVLEVLAANGVDVIVQENNGFTPT
PAISNAILVHNKKGGPLADGIVITPSHNPPEDGGIKYNPPNGGPADTNVTKVVEDRANAL
LADGLKGVKRISLDEAMASGHVKEQDLVQPFVEGLADIVDMAAIQKAGLTLGVDPLGGSG
IEYWKRIGEYYNLNLTIVNDQVDQTFRFMHLDKDGAIRMDCSSECAMAGLLALRDKFDLA
FANDPDYDRHGIVTPAGLMNPNHYLAVAINYLFQHRPQWGKDVAVGKTLVSSAMIDRVVN
DLGRKLVEVPVGFKWFVDGLFDGSFGFGGEESAGASFLRFDGTPWSTDKDGIIMCLLAAE
ITAVTGKNPQEHYNELAKRFGAPSYNRLQAAATSAQKAALSKLSPEMVSASTLAGDPITA
RLTAAPGNGASIGGLKVMTDNGWFAARPSGTEDAYKIYCESFLGEEHRKQIEKEAVEIVS
EVLKNA
>LFGLNPFC_02618 Negative modulator of initiation of replication
MKTIEVDDELYSYIASHTKHIGESASDILRRMLKFTAASQPAAPVTKEVRVASPAIVEAK
PVKTIKDKVRAMRELLLSDEYAEQKRAVNRFMLLLSTLYSLDAQAFAEATESLHGRTRVY
FAADEQTLLKNGNQTKPKHVPGTPYWVITNTNTGRKCSMIEHIMQSMQFPAELIEKVCGT
I
>LFGLNPFC_02619 Esterase YbfF
MKLNIRAQTAQNQHNNSPIILVHGLFGSLDNLGVLARDLVNDHNIIQVDMRNHGLSPRDP
VMNYPAMAQDLVDTLDALQIDKATFIGHSMGGKAVMALTALAPDRIDKLVAIDIAPVDYH
VRRHDEIFAAINAVSESDAQTRQQAAAIMRQHLNEEGVIQFLLKSFVDGEWCFNVPVLWD
QYPHIVGWEKIPAWDHPTLFIPGGNSPYVSEQYRDDLLAQFPQARAHVIAGAGHWVHAEK
PDAVLRAIRRYLND
>LFGLNPFC_02620 putative protein YbfE
MYYGALSIRAEAWLIVSPEVTKIMAKEQTDRTTLDLFAHERRPGRPKTNPLSRDEQLRIN
KRNQLKRDKVRGLKRVELKLNAEAVEALNELAESRNMSRSELIEEMLMQQLAALRSQGIV
>LFGLNPFC_02621 Flavodoxin 1
MAITGIFFGSDTGNTENIAKMIQKQLGKDVADVHDIAKSSKEDLEAYDILLLGIPTWYYG
EAQCDWDDFFPTLEEIDFNGKLVALFGCGDQEDYAEYFCDALGTIRDIIEPRGATIVGHW
PTAGYHFEASKGLADDDHFVGLAIDEDRQPELTAERVEKWVKQISEELHLDEILNA
>LFGLNPFC_02622 Ferric uptake regulation protein
MTDNNTALKKAGLKVTLPRLKILEVLQEPDNHHVSAEDLYKRLIDMGEEIGLATVYRVLN
QFDDAGIVTRHNFEGGKSVFELTQQHHHDHLICLDCGKVIEFSDDSIEARQREIAAKHGI
RLTNHSLYLYGHCAEGDCREDEHAHEGK
>LFGLNPFC_02623 putative lipoprotein ChiQ
MKKLILIAMMASGLVACAQSTAPQEDSRLKEAYSACINTAQGSPEKIEACQSVLNVLKKE
KQHQQFAEQESVRVLDYQQCIQATQTGNDQAVKADCDKVWQEIRSNNK

>LFGLNPFC_02624 Chitoporin
MRTFSGKRSTLALAIAGVTAMSGFMAIPEARAEGFIDDSTLTGGIYYWQRERDRKDVTDG
DKYKTNLSHSTWNANLDFQSGYAADMFGLDIAAFTAIEMAENGDSSHPNEIAFSKSNKAY
DEDWSGDKSGISLYKAAAKFKYGPVWARAGYIQPTGQTLLAPHWSFMPGTYQGAEAGANF
DYGDAGALSFSYMWTNEYKAPWHLEMDEFYQNDKTTKVDYLHSLGAKYDFKNNFVLEAAF
GQAEGYIDQYFAKASYKFDIAGSPLTTSYQFYGTRDKVDDRSVNDLYDGTAWLQALTFGY
RAADVVDLRLEGTWVKADGQQGYFLQRMTPTYASSNGRLDIWWDNRSDFNANGEKAVFFG
AMYDLKNWNLPGFAIGASYVYAWDAKPATWQSNPDAYYDKNRTIEESAYSLDAVYTIQDG
RAKGTMFKLHFTEYDNHSDIPSWGGGYGNIFQDERDVKFMVIAPFTIF
>LFGLNPFC_02625 Glutamine--tRNA ligase
MSEAEARPTNFIRQIIDEDLASGKHTTVHTRFPPEPNGYLHIGHAKSICLNFGIAQDYKG
QCNLRFDDTNPVKEDIEYVDSIKNDVEWLGFHWSGNVRYSSDYFDQLHAYAIELINKGLA
YVDELTPEQIREYRGTLTQPGKNSPYRDRSVEENLALFEKMRTGGFEEGKACLRAKIDMA
SPFIVMRDPVLYRIKFAEHHQTGNKWCIYPMYDFTHCISDALEGITHSLCTLEFQDNRRL
YDWVLDNITIPVHPRQYEFSRLNLEYTVMSKRKLNLLVTDKHVEGWDDPRMPTISGLRRR
GYTAASIREFCKRIGVTKQDNTIEMASLESCIREDLNENAPRAMAVIDPVKLVIENYQGE
GEMVTMPNHPNKPEMGSRQVPFSGEIWIDRADFREEANKQYKRLVLGKEVRLRNAYVIKA
ERVEKDAEGNITTIFCTYDADTLSKDPADGRKVKGVIHWVSAAHALPVEIRLYDRLFSVP
NPGAADDFLSVINPESLVIKQGFAEPSLKDAVAGKAFQFEREGYFCLDSRHSTAEKPVFN
RTVGLRDTWAKVGE
>LFGLNPFC_02626 HTH-type transcriptional repressor GlcR
MKGYNRLEQIMDYLKSHNLVTVDELVAVTNASPATIRRDLIKLDEQGVISRTHGGVTLNR
FIPTQPTTHEKMQRSLAEKHAIASAAASMVKAGDSVVLDAGTTMIELARQITHLPLRVIT
SDLHIALFLAEFKQIEVTIIGGRIDDSSQSCIGEHGRKLLQNTWPDVAFLSCNSWDLEKG
ITAPTEEKAALKRDLIAHASRKILLADSSKYGSWSLFNIAHLNELTEIITDSQLDGQTRR
TLASLSTRLIIAD
>LFGLNPFC_02627 D-threonate 4-phosphate dehydrogenase
MNNIIAVTMGDPAGIGPEIIIKSLSEAGLAGAPVVVVGCVRTLRRIMAMNITPQAELRVI
QKVCDAHFAPGVINVMDEPLANPDALTPGVVQAAAGDLAYRCIKRATALALSGEVKAIAT
APLNKEALHLGGHNYPGHTELLAHLTGSKEYAMVLYTDKLKVIHISTHISLRKFLDTLNG
ERVKTVIRVANHFLKRVGIERPRIAVAGVNPHAGEHGLFGTEEIEIIAPAIEAMQAEDID
VTGPCPPDTVFMQCHEGLFDMVVAMYHDQGHIPLKLLGFYDGVNITAGLPFIRTSADHGT
AFDIAWTGKAKSESMAVSIQLAMQISRE
>LFGLNPFC_02628 D-threonate kinase
MEKGITKPSILVVADDFTGANDAGVSLAQVGHTVDVAFEMHYRGDASVWVINSDSRAMDP
KLAAMKITSLMSHLPLANNPPLVIKKIDSTLRGNIGAEIEALMKACGITGAVVAPAFPQA
GRTTVAGECWVNGVRITETEFASDPKTPVLSARIADIIRLQTAIPCQPVTVSQLSHLSYE
QPWIGVIDAQTDSDLDRIAAAVMQAKQPLLLVGSAGICDAVARRSAIMSPPTVLAIIGSM
SEIAQRQIATLHSHPRITQIYVDVEHILAGNASDYDARIVQALQKGDHCIVHTCNDSVAR
HQIDTLCQRWQMSRAALGEKICRFLGELTRQVLLRTMPDALYLSGGDVAMATASALGATG
FRITGKVAQCVPYGHFLGGVWSRSVMTKAGGFGDETTLHQVLNFIEEKCSE
>LFGLNPFC_02629 Long-chain-alcohol dehydrogenase 1
MVIINSTILSGAGAIPSLTSLLPDIRKMLLVTDRNIAQLDGVQQIRALLEKHCPQVNVID
NVPAEPTHHDVRQLMDALGDASFDVVVGIGGGSVLDVAKLLSVLCHPQSPGLDALLAGEK
PTQRVQSWLIPTTAGTGSEATPNAILAIPEQSTKVGIISQVLLPDYVALFPELTTSMPAH
IAASTGIDALCHLLECFTATVANPVSDNAALTGLSKLFRHIQPAVNDPQDLRAKLEMLWA
SYYGGVAITHAGTHLVHALSYPLGGKYHLPHGVANAILLAPCMAFVRPWAVEKFARVWDC
IPDAETALSAEEKSHALVTWLQALVNQLKLPNNLADLGVPPEDIASLSEAALNVKRLMNN
VPCQLDLQDVQAIYQTLFPQHQFKE
>LFGLNPFC_02630 4-hydroxy-tetrahydrodipicolinate synthase
MPKTIEGVLTAIVTPFTATGSLNIPALKVQVNRQLEAGNAIFCGGTNGEFFVLNEQEKLS
VTQTCVDEVAGRAPVVAHIGEISTRETIRLGQQIEKLGVDAVSVIAPYFVPLKQEELIAH
YSAIADALSIPMFLYNIPARTGNTIQPETARALASHPNIIGIKDSAGSDESLKGFLDAVR
DIDGFNVLNGPDSLIHKGFVEGCSACISGLANIAPREINAIWSRFHAGDIEGSYEAQESV
TGLRTDLYKVAFSPAAVKKALQLMGHDVGDSRYAVNFTPEQISEIQQIIRHYNIN
>LFGLNPFC_02631 hypothetical protein
MFLWFMQPDIWLTLITLTFLEIILGVDNIIFLSLVVAKLPLSQQNMARKLGLSCAMIMRI
LLLISIAWLSHITQPLFTVLTLEISFRTLILLGAGCS
>LFGLNPFC_02632 hypothetical protein
MLLDIVFSLDSVITAVGLSQHIFIMIAAVMIAVGVMMFAAKTIGDFVNATPSIKILALTF
LLFVGVLLVADSLNIHIAKEYLYFAIFFSLSVETLNIIRERRKFRH
>LFGLNPFC_02633 hypothetical protein
MSVALIRDGVIRPSENDSHVLTAMLPSSCPQNHAANILPLPDGALMCVWFAGTQEGIADI
SVWGSRLPAGGMQWSDAVKLSHDDTRSEQNPVLFLAPDNVLWLLWTAQISGNQDTAIVRY

RKSDDLGQTWGEIATLLDKPGTFIRQPITVLDNGNWLLPVFYCRTQPGEKWVGNDDISAV
KISADGGHSWRDVEVPQSLGCVHMNITMLHDGTLVALFRSRWADNIYISHSVDNGESWSV
PQATELPNNNSSIQVTTLASGELALVYNAMSAAGAVERRASLYDEIVDGDDSRKEPTAVG
RSAFWGAPRAPVTVAISADGGKSWPWRRNLDEGDGYCMTNNSLEKLNREFSYPSIKQSPD
GTLHIAYTWWRQAIKYVRISPEWVKGQAS
>LFGLNPFC_02634 Toxin-antitoxin biofilm protein TabA
MIIGNLNHLSLAGLPAWVRNILLRPECSLSALSTREDGRWQPEGCRWFCTLGTSDTQPAE
LRHTEYHHLWADIQVVITGCEGINAGTRPIARENDEERKPDLFIAPSPENSVAITLHAGD
FAVFMPEEPHQALCAIGTATSIRKAVFKVPRDMLEA
>LFGLNPFC_02635 hypothetical protein
MTMEMILALGILVLMIVLIMSDKMPFGAPPLLACLLLVVSGLSTVQQAFAGFVNPSVVMI
AGFMVVMAALQKTRLISNVKSAMISLVNKGSYRSYGLLLVIVMLGASLAGTGATGYYVLI
LSLVSTIPYSKKLPTSKLMMPLGFATNHPLLPINLALLFGVTATVLETAGFHQEISMGRF
ALVNLIMSAAFLAWSLIAYRFLPDHPIADASEDALAAREETFNALPAWKEYCTIAAFAVS
VIGMMLMNILGNIAFVIPGLAGAFVLMIDVLDFKEVRDHMGAPVILMMAGVIGIADALAG
TGFTAMVGDAVAGVLGSGVSPFVFIVAFALLTSTCATFTGSNMGSVYIFAPIAIAACTSL
GLNPTAAAIAVVISGWNGGYMPIDGMPAMILGMGKYKLPEFWVFSVPMYLIRILALCAGA
VFIFPM
>LFGLNPFC_02636 PTS system N-acetylglucosamine-specific EIICBA component
MNILGFFQRLGRALQLPIAVLPVAALLLRFGQPDLLNVAFIAQAGGAIFDNLALIFAIGV
ASSWSKDSAGAAALAGAVGYFVLTKAMVTINPEINMGVLAGIITGLVGGAAYNRWSDIKL
PDFLSFFGGKRFVPIATGFFCLVLAAIFGYVWPPVQHAIHAGGEWIVSAGALGSGIFGFI
NRLLIPTGLHQVLNTIAWFQIGEFTNAAGTVFHGDINRFYAGDGTAGMFMSGFFPIMMFG
LPGAALAMYFAAPKERRPMVGGMLLSVAVTAFLTGVTEPLEFLFMFLAPLLYLLHALLTG
ISLFVATLLGIHAGFSFSAGAIDYALMYNLPAASQNVWMLLVMGVVFFAIYFVVFSLVIR
MFNLKTPGREDKEDEIVTEEANSNTEEGLNQLATNYIAAVGGTDNLKAIDACITRLRLTV
VDSARVNDAMCKRLGASGVVKLNKQTIQVIVGAKAESIGDAMKKVVARGPVAAASAEATP
ATAAPVAKPQAVPNAVSIAELVSPITGDVVALDQVPDEAFASKAVGDGVAVKPTDKIVVS
PAAGTIVKIFNTNHAFCLETEKGAEIVVHMGIDTVALEGKGFKRLVEEGAQVSAGQPILE
MDLDYLNANARSMISPVVCSNIDDFSGLIIKAQGHVVAGQTPLYEIKK
>LFGLNPFC_02637 Glucosamine-6-phosphate deaminase
MRLIPLTTAEQVGKWAARHIVNRINAFKPTADRPFVLGLPTGGTPMTTYKALVEMHKAGQ
VSFKHVVTFNMDEYVGLPKEHPESYYSFMHRNFFDHVDIPAENINLLNGNAPDIDAECRQ
YEEKIRSYGKIHLFMGGVGNDGHIAFNEPASSLASRTRIKTLTHDTRVANSRFFDNDVNQ
VPKYALTVGVGTLLDAEEVMILVLGSQKALALQAAVEGCVNHMWTISCLQLHPKAIMVCD
EPSTMELKVKTLRYFNELEAENIKGL
>LFGLNPFC_02638 N-acetylglucosamine-6-phosphate deacetylase
MYALTQGRIFTGHEFLDDHAVVIADGLIKSVCPVAELPPEIEQRSLNGAILSPGFIDVQL
NGCGGVQFNDTAEAVSVETLEIMQKANEKSGCTNYLPTLITTSDELMKQGVRVMREYLAK
HPNQALGLHLEGPWLNLVKKGTHNPNFVRKPDAALVDFLCENADVITKVTLAPEMVPAEV
ISKLANAGIVVSAGHSNATLKEAKAGFRAGITFATHLYNAMPYITGREPGLAGAILDEAD
IYCGIIADGLHVDYANIRNAKRLKGDKLCLVTDATAPAGANIEQFIFAGKTIYYRNGLCV
DENGTLSGSSLTMIEGVRNLVEHCGIALDEVLRMATLYPARAIGVEKRLGTLAAGKVANL
TAFTPDFKITRTIVNGNEVVTQ
>LFGLNPFC_02639 N-acetylglucosamine repressor
MTPGGQAQIGNVDLVKQLNSAAVYRLIDQYGPISRIQIAEQSQLAPASVTKITRQLIERG
LIKEVDQQASTGGRRAISIVTETRNFHAIGVRLGRHDATITLFDLSSKVLAEEHYPLPER
TQQTLEHALLNAIAQFIDSYQRKLRELIAISVILPGLVDPDSGKIHYMPHIQVENWGLVE
ALEERFKVTCFVGHDIRSLALAEHYFGASQDCEDSILVRVHRGTGAGIISNGRIFIGRNG
NVGEIGHIQVEPLGERCHCGNFGCLETIAANAAIEQRVLNLLKQGYQSRVPLDDCTIKTI
CKAANKGDSLASEVIEYVGRHLGKTIAIAINLFNPQKIVIAGEITEADKVLLPAIESCIN
TQALKAFRTNLPVVRSELDHRSAIGAFALVKRAMLNGILLQHLLEN
>LFGLNPFC_02640 Ribonucleotide monophosphatase NagD
MTIKNVICDIDGVLMHDNVAVPGAAEFLHGIMDKGLPLVLLTNYPSQTGQDLANRFATAG
VDVPDSVFYTSAMATADFLRRQEGKKAYVVGEGALIHELYKAGFTITDVNPDFVIVGETR
SYNWDMMHKAAYFVANGARFIATNPDTHGRGFYPACGALCAGIEKISGRKPFYVGKPSPW
IIRAALNKMQAHSEETVIVGDNLRTDILAGFQAGLETILVLSGVSSLDDIDSMPFRPSWI
YPSVAEIDVI
>LFGLNPFC_02641 Asparagine synthetase B [glutamine-hydrolyzing]
MCSIFGVFDIKTDAVELRKKALELSRLMRHRGPDWSGIYASDNAILAHERLSIVDVNAGA
QPLYNQQKTHVLAVNGEIYNHQALRAEYGDRYQFQTGSDCEVILALYQEKGPEFLDDLQG
MFAFALYDSEKDAYLIGRDHLGIIPLYMGYDEHGQLYVASEMKALVPVCRTIKEFPAGSY
LWSQDGEIRSYYHRDWFDYDAVKDNVTDKNELRQALEDSVKSHLMSDVPYGVLLSGGLDS
SIISAITKKYAARRVEDQERSEAWWPQLHSFAVGLPGSPDLKAAQEVANHLGTVHHEIHF

TVQEGLDAIRDVIYHIETYDVTTIRASTPMYLMSRKIKAMGIKMVLSGEGSDEVFGGYLY
FHKAPNAKELHEETVRKLLALHMYDCARANKAMSAWGVEARVPFLDKKFLDVAMRINPQD
KMCGNGKMEKHILRECFEAYLPASVAWRQKEQFSDGVGYSWIDTLKEVAAQQVSDQQLET
ARFRFPYNTPTSKEAYLYREIFEELFPLPSAAECVPGGPSVACSSAKAIEWDEAFKKMDD
PSGRAVGVHQSAYK
>LFGLNPFC_02649 3-demethoxyubiquinol 3-hydroxylase
MTNQPTEIAIVGGGMVGGALALGLAQHGFAVTVIEHAEPAPFVADSQPDVRISAISAASV
SLLKGLGVWDAVQAMRCHPYRRLETWEWETAHVVFDAAELKLPLLGYMVENTVLQQALWQ
ALEAHPKVTLRVPGSLIALHRHNDLQELELKGGETILAKLVIGADGANSQVRQMAGIGVH
AWQYAQSCMLISVQCENDPGDSTWQQFTPDGPRAFLPLFDNWASLVWYDSPARIRQLQNM
NMAQLQAEIAKHFPSRLGYVTPLAAGAFPLIRRHALQYVQPGLALVGDAAHTIHPLAGQG
VNLGYRDVDALIDVLVNARSYGEAWASYPVLKRYQMRRMADNFMMQSGMDLFYAGFSNNL
PPLRFVRNLGLMAAERAGVLKRKALKYALGL
>LFGLNPFC_02650 tRNA-2-methylthio-N(6)-dimethylallyladenosine synthase
MTKKLHIKTWGCQMNEYDSSKMADLLDATHGYQLTDVAEEADVLLLNTCSIREKAQEKVF
HQLGRWKLLKEKNPDLIIGVGGCVASQEGEHIRQRAHYVDIIFGPQTLHRLPEMINSVRG
DRSPVVDISFPEIEKFDRLPEPRAEGPTAFVSIMEGCNKYCTYCVVPYTRGEEVSRPSDD
ILFEIAQLAAQGVREVNLLGQNVNAWRGENYDGTTGSFADLLRLVAAIDGIDRIRFTTSH
PIEFTDDIIEVYRDTPELVSFLHLPVQSGSDRILNLMGRTHTALEYKAIIRKLRAARPDI
QISSDFIVGFPGETTDDFEKTMKLIADVNFDMSYSFIFSARPGTPAADMVDDVPEEEKKQ
RLYILQERINQQAMAWSRRMLGTTQRILVEGTSRKSIMELSGRTENNRVVNFEGTPDMIG
KFVDVEITDVYPNSLRGKVVRTEDEMGLRVAETPESVIARTRKENDLGVGYYQP
>LFGLNPFC_02651 PhoH-like protein
MFSFRRHTDKRNGLNIDTREITLEPADNARLLSLCGPFDDNIKQLERRLGIEINRRDNHF
KLTGRPICVTAAADILRSLYVDTAPMRGQIQDIEPEQIHLAIKEARVLEQSAESVPEYGK
AVNIKTKRGVIKPRTPNQAQYIANILDHDITFGVGPAGTGKTYLAVAAAVDALERQEIRR
ILLTRPAVEAGEKLGFLPGDLSQKVDPYLRPLYDALFEMLGFEKVEKLIERNVIEVAPLA
YMRGRTLNDAFIILDESQNTTIEQMKMFLTRIGFNSKAVITGDVTQIDLPRNTKSGLRHA
IEVLADVEEISFNFFHSEDVVRHPVVARIVNAYEAWEEAEQKRKAALAAERKREEQEQK
>LFGLNPFC_02652 Endoribonuclease YbeY
MSQVILDLQLACEDNSGLPEESQFQTWLNAVIPQFQEESEVTIRVVDTAESHSLNLTYRG
KDKPTNVLSFPFEVPPGMEMSLLGDLVICRQVVEKEAQEQGKPLEAHWAHMVVHGSLHLL
GYDHIEDDEAEEMEALETEIMLALGYEDPYIAEKE
>LFGLNPFC_02653 Magnesium and cobalt efflux protein CorC
MSDDNSHSSDTISNKKGFFSLLLSQLFHGEPKNRDELLALIRDSGQNDLIDEDTRDMLEG
VMDIADQRVRDIMIPRSQMITLKRNQTLDECLDVIIESAHSRFPVISEDKDHIEGILMAK
DLLPFMRSDAEAFSMDKVLRQAVVVPESKRVDRMLKEFRSQRYHMAIVIDEFGGVSGLVT
IEDILELIVGEIEDEYDEEDDIDFRQLSRHTWTVRALASIEDFNEAFGTHFSDEEVDTIG
GLVMQAFGHLPARGETIDIDGYQFKVAMADSRRIIQVHVKIPDDSPQPKLDE
>LFGLNPFC_02654 Apolipoprotein N-acyltransferase
MAFASLIERQRIRLLLALLFGACGTLAFSPYDVWPAAIISLMGLQALTFNRRPLQSAAIG
FCWGFGLFGSGINWVYVSIATFGGMPGPVNVFLVVLLAAYLSLYTGLFAGVLSRLWSKTT
WLRVAIAAPALWQMTEFLRGWVLTGFPWLQFGYSQIDGPLKGLAPIMGVEAINFLLMMVS
GLLALALVKRNWRPLVVAVVLFALPFPLRYIQWFTPQPEKTIQVSMVQGDIPQSLKWDEG
QLLNTLKIYYNATAPLMGKSSLIIWPESAITDLEINQQPFLKALDGELRDKGSSLVTGIV
DARLNKQNRYDTYNTIITLGKGAPYSYESADRYNKNHLVPFGEFVPLESILRPLAPFFDL
PMSSFSRGPYIQPPLSVNGIELTAAICYEIILGEQVRDNFRPDTDYLLTISNDAWFGKSI
GPWQHFQMARMRALELARPLLRSTNNGITAVIGPQGEIQAMIPQFTREVLTTNVTPTTGL
TPYARTGNWPLWVLTALFGFAAVLMSLRQRRKR
>LFGLNPFC_02655 Rhomboid protease GlpG
MSASSVKPLNVQLPAITLILFALCVGIFCYLAQWMSYEEVDQSALIHLGANVASLTLSDE
SWRLLSSVFLHSSFSHLLMNMFALLVVGTVAERILGKWRLLIIWLFSGIFGGLISACYTL
RESEQIVISIGASGAIMGIAGAAIATQLASGAGTHHKNQRRVFPLLGMVALTLLYGTRQT
GIDNACHIGGLIAGGALGWLSARLVGQNRLVTEGGIIVAVTLLLTGTIWFVQQQIDESVL
QVGQSLREAFYPQEIEQERRQKKQQLVEERNALRETLSAPVSREQASGDLLAEIADIHDM
AISRDGNTLYAAIENTNSIVVFDLGQKKILHTFTAPIAKEKSVKHCGGCKDQGVRSLALS
LDEKLIYATSFEANALSVINVATGEIIQSITTGAHPDSFILSRDGTKAWVMNRTSNSVSA
IDLVAYQHVADIPLEKYDGTGMSGKPGAWVMALSPDEKTLLVPGAGRGNIVRINTITHQK
EDFPAGNARGVVSAMGFRPKNGEIIFADSQGISRIRAEDQQASIMTQWCSRSVYSVEGIS
PDGQYLALVSYGLQGYVILLNINAGQIIGVYPASYVNHLRFSADDRKIFVMAKNRLIQMD
RTRSLDPQAIIRHPQYGDVACIPEP
>LFGLNPFC_02656 Glutamate/aspartate import solute-binding protein
MQLRKPATAILALALSAGLAQADDAAPAAGSTLDKIAKNGVIVVGHRESSVPFSYYDNQQ
KVVGYSQDYSNAIVEAVKKKLNKPDLQVKLIPITSQNRIPLLQNGTFDFECGSTTNNAER

QKQAAFSDTIFVVGTRLLTKKGGDIKDFADLKGKAVVVTSGTTSEVLLNKLNEEQKMNMR
IISAKDHGDSFRTLESGRAVAFMMDDALLAGERAKAKKPDNWEIVGKPQSQEAYGCMLRK
DDPQFKKLMDDTIAQVQTSGEAEKWFDKWFKNPIPPKNLNMNFELSDEMKALFKEPNDKA
LN
>LFGLNPFC_02657 Glutamate/aspartate import permease protein GltJ
MSIDWNWGIFLQQAPFGNTTYLGWIWSGFQVTIALSICAWIIAFLVGSFFGILRTVPNRF
LSGLGTLYVELFRNVPLIVQFFTWYLVIPELLPEKIGMWFKAELDPNIQFFLSSMLCLGL
FTAARVCEQVRAAIQSLPRGQKNAALAMGLTLPQAYRYVLLPNAYRVIVPPMTSEMMNLV
KNSAIASTIGLVDMAAQAGKLLDYSAHAWESFTAITLAYVLINAFIMLVMTLVERKVRLP
GNMEGK
>LFGLNPFC_02658 Glutamate/aspartate import permease protein GltK
MYEFDWSSIVPSLPYLLDGLVITLKITVTAVVIGILWGTMLAVMRLSSFAPVAWFAKAYV
NVFRSIPLVMVLLWFYLIVPGFLQNVLGLSPKNDIRLISAMVAFSMFEAAYYSEIIRAGI
QSISRGQSSAALALGMTHWQSMKLIILPQAFRAMVPLLLTQGIVLFQDTSLVYVLSLADF
FRTASTIGERDGTQVEMILFAGFVYFVISLSASLLVSYLKRRTA
>LFGLNPFC_02659 Glutamine transport ATP-binding protein GlnQ
MITLKNVSKWYGHFQVLTDCSTEVKKGEVVVVCGPSGSGKSTLIKTVNGLEPVQQGEITV
DGIVVNDKKTDLAKLRSRVGMVFQHFELFPHLSIIENLTLAQVKVLKRNKAPAREKALKL
LERVGLSAHANKFPAQLSGGQQQRVAIARALCMDPIAMLFDEPTSALDPEMINEVLDVMV
ELANEGMTMMVVTHEMGFARKVANRVIFMDEGKIVEDSPKDAFFDDPKSDRAKDFLAKIL
H
>LFGLNPFC_02660 Pyrimidine-specific ribonucleoside hydrolase RihA
MALPILLDCDPGHDDAIAIVLALASPELDVKAITSSAGNQTPEKTLRNVLRMLTLLNRTD
IPVASGAVKPLMRNLIIADNVHGESGLDGPALPEPTFAPQNCTAVELMAKTLRESEEPVT
IVSTGPQTNVALLLNSHPELHSKIARIVIMGGAMGLGNWTPAAEFNIYVDPEAAEIVFQS
GIPVVMAGLDVTHKAQIHVEDTERFRAIGNPVSTIVAELLDFFLEYHKDEKWGFVGAPLH
DPCTIAWLLKPELFTTVERWVGVETQGKYTQGMTVVDYYYLTGNKPNATVMVDVDRQGFV
DLLADRLKFYA
>LFGLNPFC_02661 hypothetical protein
MNKVAQYYRELVASLSERLRNGERDIDALVEQARERVIKTGELTRTEVDELTRAVRRDLE
EFAMSYEESLKEESDSVFMRVIKESLWQELADITDKTQLEWREVFQDLNHHGVYHSGEVV
GLGNLVCEKCHFHLPIYTPEVLTLCPKCGHDQFQRRPFEP
>LFGLNPFC_02662 Leucine--tRNA ligase
MQEQYRPEEIESKVQLHWDEKRTFEVTEDESKEKYYCLSMLPYPSGRLHMGHVRNYTIGD
VIARYQRMLGKNVLQPIGWDAFGLPAEGAAVKNNTAPAPWTYDNIAYMKNQLKMLGFGYD
WSRELATCTPEYYRWEQKFFTELYKKGLVYKKTSAVNWCPNDQTVLANEQVIDGCCWRCD
TKVERKEIPQWFIKITAYADELLNDLDKLDHWPDTVKTMQRNWIGRSEGVEITFNVKDYD
NTLTVYTTRPDTFMGCTYLAVAAGHPLAQKAAENNPELAAFIDECRNTKVAEAEMATMEK
KGVDTGFKAVHPLTGEEIPVWAANFVLMEYGTGAVMAVPGHDQRDYEFASKYGLNIKPVI
LAADGSEPDLSQQALTEKGVLFNSGEFNGLDHEAAFNAIADKLTEMGVGERKVNYRLRDW
GVSRQRYWGAPIPMVTLEDGTVMPTPDDQLPVILPEDVVMDGITSPIKADPEWAKTTVNG
MPALRETDTFDTFMESSWYYARYTCPEYKEGMLDSKAANYWLPVDIYIGGIEHAIMHLLY
FRFFHKLMRDAGMVNSDEPAKQLLCQGMVLADAFYYVGENGERNWVSPVDAIVERDEKGR
IVKAKDAAGHELVYTGMSKMSKSKNNGIDPQVMVERYGADTVRLFMMFASPADMTLEWQE
SGVEGANRFLKRVWKLVYEHTAKGDVAALNVDALTEDQKALRRDVHKTIAKVTDDIGRRQ
TFNTAIAAIMELMNKLAKAPTDGEQDRALMQEALLAVVRMLNPFTPHICFTLWQELKGEG
DIDNAPWPVADEKAMVEDSTLVVVQVNGKVRAKITVPVDATEEQVRERAGQEHLVAKYLD
GVTVRKVIYVPGKLLNLVVG
>LFGLNPFC_02663 LPS-assembly lipoprotein LptE
MRYLATLLLSLAVLITAGCGWHLRDTTQVPSTMKVMILDSGDPNGPLSRAVRNQLRLNGV
ELLDKETTRKDVPSLRLGKVSIAKDTASVFRNGQTAEYQMIMTVNATVLIPGRDIYPISA
KVFRSFFDNPQMALAKDNEQDMIVKEMYDRAAEQLIRKLPSIRAADIRSDEEQTSTTTDT
PATPARVSTTLGN
>LFGLNPFC_02664 DNA polymerase III subunit delta
MIRLYPEQLRAQLNEGLRAAYLLLGNDPLLLQESQDAVRQVAAAQGFEEHHTFSIDPNTD
WNAIFSLCQAMSLFASRQTLLLLLPENGPNAAINEQLLTLTGLLHDDLLLIVRGNKLSKA
QENAAWFTALANRSVQVTCQTPEQAQLPRWVAARAKQLNLELDDAANQVLCYCYEGNLLA
LAQALERLSLLWPDGKLTLPRVEQAVNDAAHFTPFHWVDALLMGKSKRALHILQQLRLEG
SEPVILLRTLQRELLLLVNLKRQSAHTPLRALFDKHRVWQNRRGMMSEALNRLSQSQLRQ
AVQLLTRTELTLKQDYGQSVWAELEGLSLLLCHKPLADVFIDG
>LFGLNPFC_02665 Nicotinate-nucleotide adenylyltransferase
MKSLQALFGGTFDPVHYGHLKPVETLANLIGLTRVTIIPNNVPPHRPQPEANSMQRKHML
ELAIADKPLFTLDERELKRNAPSYTAQTLKEWRQEQGPDVPLAFIIGQDSLLTFPTWYEY
ETILDNAHLIVCRRPGYPLEMAQPQYQQWLEDHLTHNPEDLHLQPAGKIYLAETPWFNIS

ATIIRERLQNGESCEDLLPEPVLTYINQQGLYR
>LFGLNPFC_02666 Adenosylcobalamin/alpha-ribazole phosphatase
MRLWLIRHGETQANVDGLYSGHAPTPLTARGIEQAQNLHTLLHDVSFDLVLCSELERAQH
TARLVLSDRQHPVHIIPELNEMFFGDWEMRHHRDLMQEDAENYSAWCNDWQHAIPTNGEG
FQAFSQRVERFIARLSEYQHYQNILVVSHQGVLSLLIARLIGMPAESMWHFRVDQGCWSA
IDINQKFATLRVLNSRAIGVENA
>LFGLNPFC_02667 Ribosomal silencing factor RsfS
MQGKALQDFVIDKIDDLKGQDIIALDVQGKSSITDCMIICTGTSSRHVMSIADHVVQESR
AAGLLPLGVEGENSADWIVVDLGDVIVHVMQEESRRLYELEKLWS
>LFGLNPFC_02668 Ribosomal RNA large subunit methyltransferase H
MKLQLVAVGTKMPDWVQTGFTEYLRRFPKDMPFELIEIPAGKRGKNADIKRILDKEGEQM
LAAAGKNRIVTLDIPGKPWDTPQLAAELERWKLDGRDVSLLIGGPEGLSPACKAAAEQSW
SLSALTLPHPLVRVLVAESLYRAWSITTNHPYHRE
>LFGLNPFC_02669 Peptidoglycan D,D-transpeptidase MrdA
MKLQNSFRDYTAESALFVRRALVAFLGILLLTGVLIANLYNLQIVRFTDYQTRSNENRIK
LVPIAPSRGIIYDRNGIPLALNRTIYQIEMMPEKVDNVQQTLDALRSVVDLTDDDIAAFR
KERARSHRFTSIPVKTNLTEVQVARFAVNQYRFPGVEVKGYKRRYYPYGSALTHVIGYVS
KINDKDVERLNNDGKLANYAATHDIGKLGIERYYEDVLHGQTGYEEVEVNNRGRVIRQLK
EVPPQAGHDIYLTLDLKLQQYIETLLAGSRAAVVVTDPRTGGVLALVSTPSYDPNLFVDG
ISSKDYSALLNDPNTPLVNRATQGVYPPASTVKPYVAVSALSAGVITRNTTLFDPGWWQL
PGSEKRYRDWKKWGHGRLNVTRSLEESADTFFYQVAYDMGIDRLSEWMGKFGYGHYTGID
LAEERSGNMPTREWKQKRFKKPWYQGDTIPVGIGQGYWTATPIQMSKALMILINDGIVKV
PHLLMSTAEDGKQVPWVQPHEPPVGDIHSGYWELAKDGMYGVANRPNGTAHKYFASAPYK
IAAKSGTAQVFGLKANETYNAHKIAERLRDHKLMTAFAPYNNPQVAVAMILENGGAGPAV
GTLMRQILDHIMLGDNNTDLPAENPAVAAAEDH
>LFGLNPFC_02670 Peptidoglycan glycosyltransferase MrdB
MTDNPNKKTFWDKVHLDPTMLLILLALLVYSALVIWSASGQDIGMMERKIGQIAMGLVIM
VVMAQIPPRVYEGWAPYLYIICIILLVAVDAFGAISKGAQRWLDLGIVRFQPSEIAKIAV
PLMVARFINRDVCPPSLKNTAIALVLIFMPTLLVAAQPDLGTSILVALSGLFVLFLSGLS
WRLIGVAVVLVAAFIPILWFFLMHDYQRQRVMMLLDPESDPLGAGYHIIQSKIAIGSGGL
RGKGWLHGTQSQLEFLPERHTDFIFAVLAEELGLVGILILLALYILLIMRGLWIAARAQT
TFGRVMAGGLMLILFVYVFVNIGMVSGILPVVGVPLPLVSYGGSALIVLMAGFGIVMSIH
THRKMLSKSV
>LFGLNPFC_02671 Endolytic peptidoglycan transglycosylase RlpA
MRKQWLGICIAAGMLAACTSDDSQQQTVSVPQPAVCNGPIVEISGADPRFEPLNATANQD
YQRDGKSYKIVQDPSRFSQAGLAAIYDAEPGSNLTASGEAFDPTQLTAAHPTLPIPSYAR
ITNLANGRMIVVRINDRGPYGNDRVISLSRAAADRLNTSNNTKVRIDPIIVAQDGSLSGP
GMACTTVAKQTYALPAPPDLSGGAGTSSVPVPQGDILPVSNSTLKSEDPTGAPVTSSGFL
GAPTTLAPGVLEGSEPTPAPQPVVTAPSTTPATSPAMVTPQAASQSASGNFMVQVGAVSD
QARAQQYQQQLGQKFGVPGRVTQNGAVWRIQLGPFASKAEASTLQQRLQTEAQLQSFITT
AQ
>LFGLNPFC_02672 D-alanyl-D-alanine carboxypeptidase DacA
MNTIFSARIMKRLALTTALCTAFISAAHADDLNIKTMIPGVPQIDAESYILIDYNSGKVL
AEQNADVRRDPASLTKMMTSYVIGQAMKAGKFKETDLVTIGNDAWATGNPVFKGSSLMFL
KPGMQVPVSQLIRGINLQSGNDACVAMADFAAGSQDAFVGLMNSYVNALGLKNTHFQTVH
GLDADGQYSSARDMALIGQALIRDVPNEYSIYKEKEFTFNGIRQLNRNGLLWDNSLNVDG
IKTGHTDKAGYNLVASATEGQMRLISAVMGGRTFKGREAESKKLLTWGFRFFETVNPLKV
GKEFASEPVWFGDSDRASLGVDKDVYLTIPRGRMKDLKASYVLNSSELHAPLQKNQVVGT
INFQLDGKTIEQRPLVVLQEIPEGNFFGKIIDYIKLMFHHWFG
>LFGLNPFC_02673 hypothetical protein
MKTKLNELLEFPTPFTYKVMGGQALPELVDQVVEVVQRHAPGDYTPTVKPSSKGNYHSVSI
TINATHIEQVETLYEELGKIDIVRMVL
>LFGLNPFC_02674 Octanoyltransferase
MYQDKILVRQLGLQPYEPISQAMHEFTDTRDDSTLDEIWLVEHYPVFTQGQAGKAEHILM
PGDIPVIQSDRGGQVTYHGPGQQVMYVLLNLKRRKLGVRELVTLLEQTVVNTLAELGIEA
HPRADAPGVYVGEKKICSLGLRIRRGCSFHGLALNVNMDLSPFLRINPCGYAGMEMAKIS
QWKPEATTNNIAPRLLENILALLNNPDFEYITA
>LFGLNPFC_02675 Lipoyl synthase
MSKPIVMERGVKYRDADKMALIPVKNVATEREALLRKPEWMKIKLPADSTRIQGIKAAMR
KNGLHSVCEEASCPNLAECFNHGTATFMILGAICTRRCPFCDVAHGRPVAPDANEPVKLA
QTIADMALRYVVITSVDRDDLRDGGAQHFADCITAIREKSPQIKIETLVPDFRGRMDRAL
DILTATPPDVFNHNLENVPRIYRQVRPGADYNWSLKLLERFKEAHPEIPTKSGLMVGLGE
TNEEIIEVMRDLRRHGVTMLTLGQYLQPSRHHLPVQRYVSPDEFDEMKAEALAMGFTHAA
CGPFVRSSYHADLQAKGMEVK

>LFGLNPFC_02676 Sec-independent protein translocase protein TatE
MGEISITKLLVVAALVVLLFGTKKLRTLGGDLGAAIKGFKKAMNDDDAAAKKGADVDLQA
EKLSHKE
>LFGLNPFC_02677 Deaminated glutathione amidase
MLVAAGQFAVTSVWEKNAEICGSLMAQAAENDVSLFVLPEALLARDDHDADLSVKSAQLL
EGEFLGRLRRESKRNMMTTILTIHVPSTPGRAWNMLVALQAGNIVARYAKLHLYDAFAIQ
ESRHVDAGNEIAPLLEVEGMKVGLMTCYDLRFPELALAQALQGAEILVLPAAWVRGPLKE
HHWSTLLAARALDTTCYMVAAGECGNKNIGQSRIIDPFGVTIAAASEMPALIMAEVTPER
VRQVRAQLPVLNNRRFAPPQLL
>LFGLNPFC_02678 Putative fluoride ion transporter CrcB
MLQLLLAVFIGGGTGSVARWLLSMRFNPLHQAIPLGTLAANLIGAFIIGMGFAWFSRMTN
IDPVWKVLITTGFCGGLTTFSTFSAEVVFLLQEGRFGWALLNVFVNLLGSFAMTALAFWL
FSASTAH
>LFGLNPFC_02679 Cold shock-like protein CspE
MSKIKGNVKWFNESKGFGFITPEDGSKDVFVHFSAIQTNGFKTLAEGQRVEFEITNGAKG
PSAANVIAL
>LFGLNPFC_02680 Lipid A palmitoyltransferase PagP
MNVSKYVAIFFFVFIQLISVGKVFANADEWMTTFRENIAQTWQQPEHYDLYIPAITWHAR
FAYDKEKTDRYNERPWGGGFGQSRWDEKGNWHGLYAMAFKDSWNKWEPIAGYGWESTRRP
LADENFHLGLGFTAGVTARDNWNYIPLPVLLPLASVGYGPATFQMTYIPGTYNNGNVYFA
WMRFQF
>LFGLNPFC_02681 Putative cryptic C4-dicarboxylate transporter DcuD
MLTFIELLIGVVVIVGVARYIIKGYSATGVLFVGGLLLLIISAIMGHKVLPSSQASTGYS
ATDIVEYVKILLMSRGGDLGMMIMMLCGFAAYMTHIGANDMVVKLASKPLQYINSPYLLM
IAAYFVACLMSLAVSSATGLGVLLMATLFPVMVNVGISRGAAAAICASPAAIILAPTSGD
VVLAAQASEMSLIDFAFKTTLPISIAAIIGMAIAHFFWQRYLDKKEHISHEMLDVSEITT
TAPAFYAILPFTPIIGVLIFDGKWGPQLHIITILVICMLIASILEFIRSFNTQKVFSGLE
VAYRGMADAFANVVMLLVAAGVFAQGLSTIGFIQSLISIATSFGSASIILMLVLVILTML
AAVTTGSGNAPFYAFVEMIPKLAHSSGINPAYLTIPMLQASNLGRTLSPVSGVVVAVAGM
AKISPFEVVKRTSVPVLVGLVIVIVATELMVPGTAAAVTGK
>LFGLNPFC_02682 Transcriptional regulatory protein DpiA
MTAPLTLLIVEDETPLAEMHAEYIRHIPGFSQILLAGNLAQARMMIERFKPGLILLDNYL
PDGRGINLLHELVQAHYPGDVVFTTAASDMETVSEAVRCGVFDYLIKPIAYERLGQTLTR
FRQRKHMLESIDSASQKQIDEMFNAYARGEPKDELPTGIDPLTLNAVRKLFKEPGVQHTA
ETVAQALTISRTTARRYLEYCASRHLIIAEIVHGKVGRPQRIYHSG
>LFGLNPFC_02683 Sensor histidine kinase DpiB
MLQLKENKQFAFFQRLAFPLRIFLLILVFSIFVIAALAQYFTASFEDYLTLHVRDMAMNQ
AKIIASNDSIISAVKTRDYKRLATIADKLQRDTDFDYVVIGDRHSIRLYHPNPEKIGYPM
QFTKPGALEKGESYFITGKGSIGMAMRAKTPIFDDDGKVIGVVSIGYLVSKIDSWRAEFL
LPMAGVFVVLLGILMLLSWFLAAHIRRQMMGMEPKQIARVVRQQEALFSSVYEGLIAVDP
HGYITAINRNARKMLGLSSPGRQWLGKPIAEVVRPADFFTEQIDEKRQDVVANFNGLSVI
ANREAIRSGDDLLGAIISFRSKDEISTLNAQLTQIKQYVESLRTLRHEHLNWMSTLNGLL
QMKEYDRVLAMVQGESQAQQQLIDSLREAFADRQVAGLLFGKVQRARELGLKMVIVPGSQ
LSQLPPGLDRTEFAAIVGNLLDNAFEASLRSDEGNKIVELFLSDEGDDVVIEVADQGCGV
PESLRDKIFEQGVSTRADEPGEHGIGLYLIASYVTRCGGVITLEDNDPCGTLFSIYIPKV
KPNDSSINPIDR
>LFGLNPFC_02684 [Citrate [pro-3S]-lyase] ligase
MFGNDIFTRVKRSENKKMAEIAQFLHENDLSVDTTVEVFITVTRDEKLIACGGIAGNIIK
CVAISESVRGEGLALTLATELINLAYERHSTHLFIYTKTEYEALFRQCGFSTLTSVPGVM
VLMENSATRLKRYAESLKKFRHPGNKIGCIVMNANPFTNGHRYLIQQAAAQCDWLHLFLV
KEDSSRFPYEDRLDLVLKGTADIPRLTVHRGSEYIISRATFPCYFIKEQSVINHCYTEID
LKIFRQYLAPALGVTHRFVGTEPFCRVTAQYNQDMRYWLETPTISAPPIELVEIERLRYQ
EMPISASRVRQLLAKNDLTAIAPLVPAVTLHYLQNLLEHSRQDAAARQKTPA
>LFGLNPFC_02685 Citrate lyase acyl carrier protein
MKINQPAVAGTLESGDVMIRIAPLDTQDIDLQINSSVEKQFGDAIRTTILDVLARYNVRG
VQLNVDDKGALDCILRARLEALLARASGIPALPWEDCQ
>LFGLNPFC_02686 Citrate lyase subunit beta
MISASLQQRKTRTRRSMLFVPGANAAMVSNSFIYPADALMFDLEDSVALREKDTARRMVY
HALQHPLYRDIETIVRVNALDSEWGVNDLEAVVRGGADVVRLPKTDTAQDVLDIEKEILR
IEKACGREPGSTGLLAAIESPLGITRAVEIAHASERLIGIALGAEDYVRNLRTERSPEGT
ELLFARCSILQAARSAGIQAFDTVYSDANNEAGFLQEAAHIKQLGFDGKSLINPRQIDLL
HNLYAPTQKEVDHARRVVEAAEAAAREGLGVVSLNGKMVDGPVIDRARLVLSRAELSGIR
EE
>LFGLNPFC_02687 Citrate lyase alpha chain

MTQKIEQSQRQERVAAWNRRAECDLAAFQNSPKQTYQAEKARDRKLCANLEEAIRRSGLQ
DGMTVSFHHAFRGGDLTVNMVMDVIAKMGFKNLTLASSSLSDCHAPLVEHIRQGVVTRIY
TSGLRGPLAEEISRGLLAEPVQIHSHGGRVHLVQSGELNIDVAFLGVPSCDEFGNANGYS
GKACCGSLGYAMVDADNAKQVVMLTEELLPYPHNPASIEQDQVDLIVKVDRVGDAAKIGA
GATRMTTNPRELLIARSAADVIVNSGYFKEGFSMQTGTGGASLAVTRFLEDKMRSRDIRA
DFALGGITATMVDLHEKGLIRKLLDVQSFDSHAAQSLARNPNHIEISANQYANWGSKGAA
VDRLDVVVLSALEIDTQFNVNVLTGSDGVLRGASGGHCDTAIASALSIIVAPLVRGRIPT
LVDNVLTCITPGSSVDILVTDHGIAVNPARPELAERLQEAGIKVVSIEWLRERARLLTGE
PQPIEFTDRVVAVVRYRDGSVIDVVHQVKE
>LFGLNPFC_02688 Apo-citrate lyase phosphoribosyl-dephospho-CoA transferase
MHLLPEFASHHAVSIPELLVSRDERQARQHAWLKRHPVPLVSFTVVAPGPIKDSEVTRRI
FNHGVTALRALATKQGWQIQEQAALVSASGPEGMLSIAAPARDLKLATIELEHSHPLGRL
WDIDVLTPEGDILSRRDYSLPPRRCLLCEQSAAVCARGKTHQLTDLLNRMEALLNDVDAC
NVN
>LFGLNPFC_02689 2-(5''-triphosphoribosyl)-3'-dephosphocoenzyme-A synthase
MSMPATSTKTTKLATSLIDEYALLGWRAMLTEVNLSPKPGLVDRINCGAHKDMALEDFHR
SALAIQGWLPRFIEFGACSAEMAPEEVLHGLRPIGMACEGDMFRATAGVNTHKGSIFSLG
LLCAAIGRLLQLNQSVTPITICATAASFCRGLTDRELRTNNSQLTAGQRLYQQLGLTGAR
GEAEAGYPLVINHALPHYLTLLDQGLDPELALLDTLLLLMATNGDTNVASRGGEGGLRWL
QREAQTLLNNGGIRTPADLDYLRQFDRECIERNISPGGSADLLILTWFLAQI
>LFGLNPFC_02690 L-tartrate/succinate antiporter
MSLAKDNIWKLLAPLVVMGVMFLIPVPDGMPPQAWHYFAVFVAMIVGMILEPIPATAISF
IAVTICVIGSNYLLFDAKELADPAFNAQKQALKWGLAGFSSTTVWLVFGAFIFALGYEVS
GLGRRIALFLVKFMGKRTLTLGYAIVIIDILLAPFTPSNTARTGGTVFPVIKNLPPLFKS
FPNDPSARRIGGYLMWMMVISTSLSSSMFVTGAAPNVLGLEFVSKIAGIQISWLQWFLCF
LPVGVILLIIAPWLSYVLYKPEITHSEEVATWAGDELKTMGALTRREWTLIGLVLLSLGL
WVFGSEVINATAVGLLAVSLMLALHVVPWKDITRYNSAWNTLVNLATLVVMANGLTRSGF
IDWFANTMSTHLEGFSPNATVIVLVLVFYFAHYLFASLSAHTATMLPVILAVGKGIPGVP
MEQLCILLVLSIGIMGCLTPYATGPGVIIYGCGYVKSKDYWRLGAIFGVIYISMLLLVGW
PILAMWN
>LFGLNPFC_02691 Ribonuclease I
MKAFWRNAALLAVSLLPFSSANAVALQAKQYDDFDRYVLALSWQTGFCQSQYDRNRNERD
ECRLQTETTNKADFLTVHGLWPGLPKSVAARGVDERRWMRFGCATRPIPNLPEARASRMC
SSPETGLSLETAAKLSEVMPGAGGRSCLERYEYAKHGACFGFDPDAYFGTMVRLNQEIKE
SEAGKFLADNYGKTVSRRDFDAAFAKSWGKENVKAVKLTCQGNPAYLTEIQISIKADAIN
APLSANSFLPQPHPGNCGKTFVIDKAGY
>LFGLNPFC_02692 Regulator of nucleoside diphosphate kinase
MSRPTIIINDLDAERIDILLEQPAYAGLPIADALNAELDRAQMCSPEEMPHDVVTMNSRV
KFRNLSDGEVRVRTLVYPAKMTDSNTQLSVMAPVGAALLGLRVGDSIHWELPGGVATHLE
VLELEYQPEAAGDYLL
>LFGLNPFC_02693 hypothetical protein
MSEALGITAFYIFIAAIIVAAVLYFEQRW
>LFGLNPFC_02694 Universal stress protein UP12
MYKTIIMPVDVFEMELSDKAVRHAEFLAQDDGVIHLLHVLPGSASLSLHRFAADVRRFEE
HLQHEAEERLQTMVSHFTIDPSRIKQHVRFGSVRDEVNELAKELDADVVVIGSRNPSIST
HLLGSNASSVIRHANLPVLVVR
>LFGLNPFC_02695 Alkyl hydroperoxide reductase subunit F
MMMFKAQEINMLDTNMKTQLKAYLEKLTKPVELIATLDDSAKSAEIKELLAEIAELSDKV
TFKEDNSLPVRKPSFLITNPGSNQGPRFAGSPLGHEFTSLVLALLWTGGHPSKEAQSLLE
QIRHIDGDFEFETYYSLSCHNCPDVVQALNLMSVLNPRIKHTAIDGGTFQNEITDRNVMG
VPAVFVNGKEFGQGRMTLTEIVAKIDTGAEKRAAEELNKRDAYDVLIVGSGPAGAAAAIY
SARKGIRTGLMGERFGGQILDTVDIENYISVPKTEGQKLAGALKVHVDEYDVDVIDSQSA
SKLIPAAVEGGLHQIETASGAVLKARSIIVATGAKWRNMNVPGEDQYRTKGVTYCPHCDG
PLFKGKRVAVIGGGNSGVEAAIDLAGIVEHVTLLEFAPEMKADQVLQDKLRSLKNVDIIL
NAQTTEVKGDGSKVVGLEYRDRVSGDIHNIELAGIFVQIGLLPNTNWLEGAVERNRMGEI
IIDAKCETNVKGVFAAGDCTTVPYKQIIIATGEGAKASLSAFDYLIRTKTA
>LFGLNPFC_02696 Alkyl hydroperoxide reductase C
MSLINTKIKPFKNQAFKNGEFIEITEKDTEGRWSVFFFYPADFTFVCPTELGDVADHYEE
LQKLGVDVYAVSTDTHFTHKAWHSSSETIAKIKYAMIGDPTGALTRNFDNMREDEGLADR
ATFVVDPQGIIQAIEVTAEGIGRDASDLLRKIKAAQYVASHPGEVCPAKWKEGEATLAPS
LDLVGKI
>LFGLNPFC_02697 Thiol:disulfide interchange protein DsbG
MLKKILLLALLPAIAFAEELPAPVKAIEKQGITIIKTFDAPGGMKGYLGKYQDMGVTIYL
TPDGKHAISGYMYNEKGENLSNTLIEKEIYAPAGREMWQRMEQSHWLLDGKKDAPVIVYV

FADPFCPYCKQFWQQARPWVDSGKVQLRTLLVGVIKPESPATAAAILASKDPAKTWQQYE
ASGGKLKLSVPANVSTEQMKVLSDNEKLMDDLGANVTPAIYYMSKENTLQQAVGLPDQKT
LNIIMGNK
>LFGLNPFC_02698 putative HTH-type transcriptional regulator YbdO
MANLYDLKKFDLNLLVIFECIYQHLSISKAAESLYITPSAVSQSLQRLRAQFNDPLFIRS
GKGIAPTTTGLNLHHHLEKNLRGLEQTINIVNKSELKKNFIIYGPQLISCTNNNMLIRCL
RQDTSIDIECHDILLSAENAEELLVQRKTDLAITLQPVISRSVICMPLHTIRNSLICSNK
HPRITDTSNYEQIIAEEFTLLISKSAGIDEIQMDIDERFMNRKVSFRSSSLMTIINSISV
TDLLGIVPTELYDLHRDFLKLKEIKLEQPLPAVKLYISYNKASLNNLVFSRFIDRLNDSF
>LFGLNPFC_02699 Phosphoadenosine phosphosulfate reductase
MSIYKIPLPLNILEAAKERITWTLNTRPRICVSFSGGKDSGLMLHLTAEIARQMGKKICV
LFIDWEAQFSCTINYVQSLREFYADVIEEFYWVALPLTTQNSLSQYQPEWQCWEPDVEWV
RQPPQDAITDPDFFSFYQPGMTFEQFVREFAEWFSQKRPAAMMIGIRADESYNRFVAIAS
LNKQRFADDKPWTTAAPGGHSWYIYPIYDWKVADIWTWYANHQQLCNPLYNIMYQAGVPL
RHMRICEPFGPEQRQGLWLYHVIEPDRWAAMCARVSGVKSGGIYAGHDNHFYGHRKILKP
EHLDWQEYALLLLNSMPEKTAEHYRNKIAIYLHWYQKKGIEVPQTQQGDIGAKDIPSWRR
ICKVLLNNDYWCRALSFSPTKAKNYQRYNERIKGKRQEWGILCNND
>LFGLNPFC_02700 putative protein YbdM
MGDTMQQRLTQDLTQFLASLPEDDRIKAINEIRMAIHQVSPFREEPVDCVLWVKNSQLMP
NDYNPNNVAPPEKKLLKKSIEIDGFTQPIVVTHTDKNALEIVDGFHRHEIGKGSSSLKLR
LKGYLPVTCLEGTRNQRIAATIRHNRARGRHQITAMSEIVRELSQLGWDDNKIGKELGMD
SDEVLRLKQINGLQELFADRQFSRAWTVK
>LFGLNPFC_02701 Methionine aminotransferase
MTNNPLIPQSKLPQLGTTIFTRMSALAQQHQAINLSQGFPDFDGPRYLQERLAYHVAQGA
NQYAPMTGVQALREAIAQKTERLYGYQPDVDSNITVTAGATEALYAAITALVRNGDEVIC
FDPSYDSYAPAIALSGGIVKRIALQPPHFRVDWQEFAALLSERTRLVILNTPHNPSATVW
QQTDFAALWQAIAGHEIFVISDEVYEHINFSQQGHASVLAHPQLRERAVAVSSFGKTYHM
TGWKVGYCVAPAPISAEIRKVHQYLTFSVNTPAQLALADMLRAEPEHYLALPDFYRQKRD
ILVNALNESRLEILPCEGTYFLLVDYSAVSTMDDVEFCQWLTREHGVAAIPLSVFCADPF
PHKLIRLCFAKKESTLLAAAERLRQL
>LFGLNPFC_02702 Hydroxycarboxylate dehydrogenase A
MPHNPIRVVVGPANYFSHPGSFNHLHDFFTDEQLSRAVWIYGERAIAAAQTKLPPAFELP
GVKHILFRGHCSESDVQQLAAESGDDRSVVIGVGGGALLDTAKALARRLGLPFVAVPTIA
ATCAAWTPLSVWYNDAGQALHYEIFDDANFMVLLEPEIILNAPQEYLLAGIGDTLAKWYE
AVVLAPQPETLPLTVRLGINNAQAIRDVLLNSSEQALADQQNQQLTQSFCDVVDAIIAGG
GMVGGLGDRFTRVAAAHAVHNGLTVLPQTEKFLHGTKVAYGILVQSALLGQDDVLAQLTG
AYQRFHLPTTLAELEVDINNQVEIDKMIAHTLRPVESIHYLPVTLTPDTLRAAFEKVESF
KA
>LFGLNPFC_02703 hypothetical protein
MFDSLAKAGKYLGQAAKLMIGMPDYDNYVEHMRVNHPDQTPMTYEEFFRERQDARYGGKG
GARCC
>LFGLNPFC_02704 Peptide transporter CstA
MNKSGKYLVWTVLSVMGAFALGYIALNRGEQINALWIVVASVCIYLIAYRFYGLYIAKNV
LAVDPTRMTPAVRHNDGLDYVPTDKKVLFGHHFAAIAGAGPLVGPVLAAQMGYLPGMIWL
LAGVVLAGAVQDFMVLFVSTRRDGRSLGELVKEEMGPTAGVIALVACFMIMVIILAVLAM
IVVKALTHSPWGTYTVAFTIPLALFMGIYLRYLRPGRIGEVSVIGLVFLIFAIISGGWVA
ESPTWAPYFDFTGVQLTWMLVGYGFVAAVLPVWLLLAPRDYLSTFLKIGTIVGLAVGILI
MRPTLTMPALTKFVDGTGPVWTGNLFPFLFITIACGAVSGFHALISSGTTPKMLANEGQA
CFIGYGGMLMESFVAIMALVSACIIDPGVYFAMNSPMAVLAPAGTADVVASAAQVVSSWG
FAITPDTLNQIASEVGEQSIISRAGGAPTLAVGMAYILHGALGGMMDVAFWYHFAILFEA
LFILTAVDAGTRAARFMLQDLLGVVSPGLKRTDSLPANLLATALCVLAWGYFLHQGVVDP
LGGINTLWPLFGIANQMLAGMALMLCAVVLFKMKRQRYAWVALVPTAWLLICTLTAGWQK
AFSPDAKVGFLAIANKFQAMIDSGNIPSQYTESQLAQLVFNNRLDAGLTIFFMVVVVVLA
LFSIKTALAALKEPKPTAKETPYEPMPENVEEIVAQAKGAH
>LFGLNPFC_02705 Proofreading thioesterase EntH
MIWKRHLTLDELNATSDNTMVAHLGIVYTRLGDDVLEAEMPVDTRTHQPFGLLHGGASAA
LAETLGSMAGFMMTRDGQCVVGTELNATHHRPVSEGKVRGVCQPLHLGRQNQSWEIVVFD
EQGRRCCTCRLGTAVLG
>LFGLNPFC_02706 2,3-dihydro-2,3-dihydroxybenzoate dehydrogenase
MDFSGKNVWITGAGKGIGYATALAFVEAGAKVTGFDQAFTQEQYPFATEVMDVADAAQVA
QVCQRLLAETERLDVLVNAAGILRMGATDQLSKEDWQQTFAVNVGGAFNLFQQTMNQFRR
QRGGAIVTVASDAAHTPRIGMSAYGASKAALKSLALSVGLELAGSGVRCNVVSPGSTDTD
MQRTLWVSDDAEEQRIRGFGEQFKLGIPLGKIARPQEIANTILFLASDLASHITLQDIVV
DGGSTLGA

>LFGLNPFC_02707 Enterobactin synthase component B
MAIPKLQAYALPESHDIPQNKVDWAFEPQRAALLIHDMQDYFVSFWGENCPMMEQVIANI
AALRDYCKQHNIPVYYTAQPKEQSDEDRALLNDMWGPGLTRSPEQQKVVDRLTPDADDTV
LVKWRYSAFHRSPLEQMLKESGRNQLIITGVYAHIGCMTTATDAFMRDIKPFMVADALAD
FSRDEHLMSLKYVAGRSGRVVMTEELLPAPIPASKAALREVILPLLDESDEPFDDDNLID
YGLDSVRMMALAARWRKVHGDIDFVMLAKNPTIDAWWKLLSREVK
>LFGLNPFC_02708 Enterobactin synthase component E
MSIPFTRWPEEFARRYREKGYWQDLPLTDILTRHAASDSIAVIDGERQLSYRELNQAADN
LACSLRRQGIKPGETALVQLGNVAELYITFFALLKLGVAPVLALFSHQRSELNAYASQIE
PALLIADRQHALFSGDDFLNTFVAEHSSIRVVQLLNDSGEHNLQDAINHPAEDFTATPSP
ADEVAYFQLSGGTTGTPKLIPRTHNDYYYSVRRSVEICQFTQQTRYLCAIPAAHNYAMSS
PGSLGVFLAGGTVVLAADPSATLCFPLIEKHQVNVTALVPPAVSLWLQALAEGESRAQLA
SLKLLQVGGARLSATLAARIPAEIGCQLQQVFGMAEGLVNYTRLDDSAEKIIHTQGYPMC
PDDEVWVADAEGNPLPQGEVGRLMTRGPYTFRGYYKSPQHNASAFDANGFYCSGDLISID
PEGYITVQGREKDQINRGGEKIAAEEIENQLLRHPAVIYAALVSMEDELMGEKSCAYLVV
KEPLRAVQVRRFLREQGIAEFKLPDRVECVDSLPLTAVGKVDKKQLRQWLASRTSA
>LFGLNPFC_02709 Isochorismate synthase EntC
MDTSLAEEVQQTMATLAPNRFFFMSPYRSFTTSGCFARFDEPAVNGDSPDSPFQQKLAAL
FADAKAQGIKNPVMVGAIPFDPRQPSSLYIPESWQSFSRQEKQASARRFTRSQSLNVVER
QAIPEQTTFEQMVARAAALTATPQVDKVVLSRLIDITTDAAIDSGVLLERLIAQNPVSYN
FHVPLADGGVLLGASPELLLRKDGERFSSIPLAGSARRQPDEVLDREAGNRLLASEKDRH
EHELVTQAMKEVLRKRSSELHVPSSPQLITTPTLWHLATPFEGKANSQENALTLACLLHP
TPALSGFPHQAATQVIAELEPFDRELFGGIVGWCDSEGNGEWVVTIRCAKLRENQVRLFA
GAGIVPASSPLGEWRETGVKLSTMLNVFGLH
>LFGLNPFC_02710 Ferrienterobactin-binding periplasmic protein
MRLAPLYRNALLLTGLLLSGIAAVQAADWPRQITDSRGTHTLESQPQRIVSTSVTLTGSL
LAIDAPVIASGATTPNNRVADDQGFLRQWSKVAKERKLQRLYIGEPSAEAVAAQMPDLIL
ISATGGDSALALYDQLSTIAPTLIINYDDKSWQSLLTQLGEITGHEKQAAERIAQFDKQL
AAAKEQIKLPPQPVTAIVYTAAAHSANLWTPESAQGQMLEQLGFTLAKLPAGLNASQSQG
KRHDIIQLGGENLAAGLNGESLFLFAGDQKDADAIYANPLLAHLPAVQNKQVYALGTETF
RLDYYSAMQVLDRLKALF
>LFGLNPFC_02711 Enterobactin exporter EntS
MNKQSWLLNLSLLKTHPAFRAVFLARFISIVSLGLLGVAVPVQIQMMTHSTWQVGLSVTL
TGGAMFVGLMVGGVLADRYERKKVILLARGTCGIGFIGLCLNALLPEPSLLAIYLLGLWD
GFFASLGVTALLAATPALVGRENLMQAGAITMLTVRLGSVISPMIGGLLLATGGVAWNYG
LAAAGTFITLLPLLSLPALPPPPQPREHPLKSLLAGFRFLLASPLVGGIALLGGLLTMAS
AVRVLYPALADNWQMSAAQIGFLYAAIPLGAAIGALTSGKLAHSVRPGLLMLLSTLGAFL
AIGLFGLMPMWILGVVCLALFGWLSAVSSLLQYTMLQTQTPEAMLGRINGLWTAQNVTGD
AIGAALLGGLGAMMTPVASASASGFGLLIIGVLLLLVLVELRRFRQTPPQVTASDS
>LFGLNPFC_02712 Ferric enterobactin transport system permease protein FepD
MSGSVAVTRAIAVPGLLLLLIIATALSLLIGAKSLPASVVLEALSGTCQSADCTIVLDAR
LPRTLAGLLAGGALGLAGALMQTLTRNPLADPGLLGVNAGASFAIVLGAALFGYSSAQEQ
LAMAFAGALVASLIVAFTGSQGGGQLSPVRLTLAGVALAAVLEGLTSGIALLNPDVYDQL
RFWQAGSLDIRNLHTLKVVLIPVLTAGATALLLSRALNSLSLGSDTATALGSRVARTQLI
GLLAITVLCGSATAVVGPIAFIGLMMPHMARWLVGADHRWSLPVTLLATPALLLFADIIG
RVIVPGELRVSVVSAFIGAPVLIFLVRRKTRGGA
>LFGLNPFC_02713 Ferric enterobactin transport system permease protein FepG
MIYVSRRLIITCLLLLIACVMAGVWGLRSGAVTLETSQVFAALMGDTPRSMTMVVTEWRL
PRVLMALLIGAALGVSGAIFQSLMRNPLGSPDVMGFNTGAWSGVLVAMVLFGQDLTAIAL
AAMVGGIVTSLLVWLLAWRNGIDTFRLIIIGIGVRAMLVAFNTWLLLKASLETALTAGLW
NAGSLNGLTWAKTSPSAPIIILMLIAAALLVRRMRLLEMGDDTACALGVSVERSRLLMML
VAVVLTAAATALAGPISFIALVAPHIARRISGTARWGLTQAALCGALSLLVADLCAQQLF
MPYQLPVGVVTVSLGGIYLIVLLIQESRKK
>LFGLNPFC_02714 Ferric enterobactin transport ATP-binding protein FepC
MTESVARLRGEQLTLGYGKYTVAENLTVEIPDGHFTAIIGPNGCGKSTLLRTLSRLMTPA
HGHVWLDGEHIQHYASKEVARRIGLLAQNATTPGDITVQELVARGRYPHQPLFTRWRKED
EDAVTKAMQATGITHLADQSVDTLSGGQRQRAWIAMVLAQETAIMLLDEPTTWLDISHQI
DLLELLSELNREKGYTLAAVLHDLNQACRYASHLIALREGKIVAQGAPKEIVTAELIERI
YGLRCMIIDDPVAGTPLVVPLGRTAPSTAKI
>LFGLNPFC_02715 Ferric enterobactin transport protein FepE
MSSLNIKQGSEAHFPEYPLASPSNNEIDLLSLIEVLWRAKKTVMAVVFAFACAGLLISFI
LPQKWTSSAVITPAEAIQWQDLEKTFTKLRVLDLDINIDRGGAFNLFIKRFQSVSLLEEY
LRSSPYVMDQLKEAKIDELDLHRAIVALSEKMKAVDDNASKKKDEPSLYTSWTLSFTAPT
SEEAQKVLAGYIDYISALVVKESIENVRNKLEIKTQFEKEKLAQDRIKTKNQLDANIQRL

NYSLDIANAAGIKKPVYSNGQAVKDDPDFSISLGADGIERKLEIEKAVTDVAELNGELRN
RQYLVEQLTKANINDVNFTPFKYQLRPSLPVKKDGPGKSIIVILSALIGGMVACGGVLLR
HAMASRKQDAMMADHLV
>LFGLNPFC_02716 Enterobactin synthase component F
MSQHLPLVAAQPGIWMAEKLSDLPSAWSVAHYVELTGEVDAPLLARAVVAGLAQADTLRM
RFTEDNGEVWQWVDDAQTFELPEIIDLRTNIDPHGTARALMQADLQQDLRVDSGKPLVFH
QLIQVADNRWYWYQRYHHLLVDGFSFPAITRQIANIYCALLRGEPTPASPFTPFADVVEE
YQQYRESEAWQRDAAFWAEQRRQLPPPASLSPAPLPGRSASADILRLKLEFTNGEFRQLA
TQLSGVQRTDLALALAALWLGRLCNRMDYAAGFIFMRRLGSAALTATGPVLNVLPLGIHI
AAQETLPQLATRLAAQLKKMRRHQRYDAEQIVRDSGRAAGEEPLFGPVLNIKVFDYQLDI
PGVQAQTHTLATGPVNDLELALFPDEHGDLSIEILANKQRYDEPTLIQHAERLKMLIAQF
AADPSLLCGDVDIMLPGEYAQLAQINATQVEIPETTLSALVAEQAAKTPDAPALADARYQ
FSYREMREQVVALANLLRERGVKPGDSVAVALPRSVFLTLALHAIVEAGAAWLPLDTGYP
DDRLKMMLEDARPSLLITTDDQLPRFSDIPNLTSLCYNAPLTPQGSAPLQLSQPHHTAYI
IFTSGSTGRPKGVMVGQTAIVNRLLWMQNHYPLTGEDVVAQKTPCSFDVSVWEFFWPFIA
GAKLVMAEPEAHRDPLAMQQFFAEYGVTTTHFVPSMLAAFVASLTPQTARQSCATLKQVF
CSGEALPADLCREWQQLTGAPLHNLYGPTEAAVDVSWYPAFGEELAEVRGSSVPIGYPVW
NTGLRILDAMMHPVPPGVVGDLYLTGIQLAQGYLGRPDLTASRFIADPFAPGERMYRTGD
VARWLDNGAVEYLGRSDDQLKIRGQRIELGEIDRVMQALPDVEQAVTHACVINQAAATGG
DARQLVGYLVSQSGLPLDTSALQAQLRETLPPHMVPVVLLQLPQLPLSANGKLDRKALPL
PELKAQAPGRAPKAGSETIIAAAFSSLLGCDVQDADADFFALGGHSLLAMKLAAQLSRQF
ARQVTPGQVMVASTVAKLATIIDGEEDSTQRMGFETILPLREGNGPTLFCFHPASGFAWQ
FSVLSRYLDPQWSIIGIQSPRPHGPMQTSANLDEVCEAHLATLLEQQPHGPYYLLGYSLG
GTLAQGIAARLRARGEQVAFLGLLDTWPPETQNWQEKEANGLDPEVLAEINREREAFLAA
QQGSTSTELFTTIEGNYADAVRLLTTAHSVPFDGKATLFVAERTLQEGMTPEQAWAPWIA
GLDIYRQDCAHVDIISPVAFEKIGPIIRATLNK
>LFGLNPFC_02717 Enterobactin biosynthesis protein YbdZ
MAFSNPFDDPQGAFYILRNAQGQFSLWPQQCALPAGWDVVCQPQSQASCQQWLEAHWRTL
TPANFTQLQEAQ
>LFGLNPFC_02718 Enterochelin esterase
MTALKVGSESWWQSKHGPEWQRLNDEMFEVTFWWRDPQGSEEYSTIKRVWVYITGVTDHH
QNSQPRSMQRIAGTDVWQWTTQLNANWRGSYCFIPTERDDIFSAPSPDRLELREGWRKLL
PQAIADPLNPQSWKGGRGHAVSALEMPQASLQPGWDCPQAPETPAKEIIWKSERLKNSRR
VWIFTTGDATAEERPLAVLLDGEFWAQSMPVWPALTSLTHRRQLPPAVYVLIDAIDTTHR
AHELPCNADFWLAVQQELLPQVKAIAPFSDRADRTVVAGQSFGGLSALYAGLHWPERFGC
VLSQSGSYWWPHRGGHQEGMLLEQLNTGEVSAEGLRIVLEAGVREPMIMQANQALYAQLH
PLKESIFWRQVDGGHDALCWRGGLMQGLIDLWQPLFHDRS
>LFGLNPFC_02719 Ferrienterobactin receptor
MNKKIHSLALLVNLGIYGVAQAQEPTDTPVSHDDTIVVTAAEQNLQAPGVSTITADEIRK
NPVARDVSEIIRTMPGVNLTGNSTSGQRGNNRQIDIRGMGPENTLILIDGKPVSSRNSVR
QGWRGERDTRGDTSWVPPEMIERIEVLRGPAAARYGNGAAGGVVNIITKKGSGEWHGSWD
AYFNAPEHKEEGATKRTNFSLTGPLGDEFSFRLYGNLDKTQADAWDINQGHQSARAGTYA
TTLPAGREGVINKDINGVVRWDFAPLQSLELEAGYSRQGNLYAGDTQNTNSDAYTRSKYG
DETNRLYRQNYSLTWNGGWDNGVTTSNWVQYEHTRNSRIPEGLAGGTEGKFNEKAAQDFV
DIDLDDVMLHSEVNLPIDFLVNQTLTLGTEWNQQRMKDLSSNTQALTGTNTGGAIDGVSA
TDRSPYSKAEIFSLFAENNMELTDSTIVTPGLRFDHHSIVGNNWSPALNISQGLGDDFTL
KMGIARAYKAPSLYQTNPNYILYSKGQGCYASAGGCYLQGNDDLKAETSINKEIGLEFKR
DGWLAGVTWFRNDYRNKIEAGYVAVGQNAVGTDLYQWDNVPKAVVEGLEGSLNVPVSETV
MWTNNITYMLKSENKTTGDRLSIIPEYTLNSTLSWQAREDLSMQTTFTWYGKQQPKKYNY
KGQPAVGPETKEISPYSIVGLSATWDVTKNVSLTGGVDNLFDKRLWRAGNAQTTGDLAGA
NYIAGAGAYTYNEPGRTWYMSINTHF
>LFGLNPFC_02720 Enterobactin synthase component D
MNALSGLQKSCQFNILQDHVGLISVAHQAVLRLSSFSNMVDMKTTHTSLPFAGHTLHFVE
FDPASFREQDLLWLPHYAQLQHAGRKRKTEHLAGRIAAIYALREYGYKCVPAIGELRQPV
WPAGVYGSISHCGTTALAVVSRQPIGIDIEEIFSAQTARELTDNIITPAEHKRLADCGLA
FPLALTLAFSAKESAFKASEIQAAQGFLDYQIISWNKQQIIIRLEDEQFAVHWQIKEKIV
ITLCQHD
>LFGLNPFC_02721 Toxic protein HokE
MLTKYALVAVIVLCLTVLGFTLLVGDSLCEFTVKERNIEFKAVLAYEPKK
>LFGLNPFC_02722 hypothetical protein
MFLFFGDGELMVTEAIWLLLQYDQLRYSWLAAKNFDD
>LFGLNPFC_02723 Putative glutamate--cysteine ligase 2
MPLPDFHVSEPFTLGIELEMQVVNPPGYDLSQDSSMLIDAVKNQITAGEVKHDITESMLE
LATDVCRDINQAAGQFSAMQKVVLQAAADHHLEICGGGTHPFQKWQRQEVCDNERYQRTL

ENFGYLIQQATVFGQHVHVGCASGDDAIYLLHGLSRFVPHFIALSAASPYMQGTDTRFAS
SRPNIFSAFPDNGPMPWVSNWQQFEALFRCLSYTTMIDSIKDLHWDIRPSPHFGTVEVRV
MDTPLTLSHAVNMAGLIQATAHWLLTERPFKHQEKDYLLYKFNRFQACRYGLEGVITDPH
TGDRRSLTEATLRLLEKIAPSAHKIGASSAIEALHRQVVSGLNEAQLMRDFVADGGSLIG
LVKKHCEIWAGE
>LFGLNPFC_02724 hypothetical protein
MVFYLTFNNVMKHPLETLTTAAGILLMAFLSCLLLPAPALGLTLAQKLVTTFHLMDLSQL
YTLLFCLWFLVLGAIEYFVLRFIWRRWFSLAD
>LFGLNPFC_02725 hypothetical protein
MDKQSLHETAKRLALELPFVELCWPFGPEFDVFKIGGKIFMLSSELRGVPFINLKSDPQK
SLLNQQIYPSIKPGYHMNKKHWISVYPGEEISEALLRDLINDSWNLVVDGLAKRDQKRVR
PDSTVNQ
>LFGLNPFC_02726 Oxygen-insensitive NAD(P)H nitroreductase
MDIISVALKRHSTKAFDASKKLTPEQAEQIKTLLQYSPSSTNSQPWHFIVASTEEGKARV
AKSAADNYVFNERKMLDASHVVVFCAKTAMDDAWLKLVVDQEDADGRFATPEAKAANDKG
RKFFADMHRKDLHDDAEWMAKQVYLNVGNFLLGVAALGLDAVPIEGFDAAILDEEFGLKE
KGYTSLVVVPVGHHSVEDFNATLPKSRLPQNITLTEV
>LFGLNPFC_02727 Miniconductance mechanosensitive channel YbdG
MQDLISQVEDLAGIEIDHTTSMVMIFGIIFLTAVVVHIFLHWVVLRTFEKRAIASSRLWL
QIITQNKLFHRLAFTLQGIIVNIQAVFWLQKGTEAADILTTCAQLWIMMYALLSVFSLLD
VILNLAQKFPAASQLPLKGIFQGIKLIGAILVGILMISLLIGQSPAILISGLGAMAAVLM
LVFKDPILGLVAGIQLSANDMLKLGDWLEMPKYGADGAVIDIGLTTVKVRNWDNTITTIP
TWSLVSDSFKNWSGMSASGGRRIKRSISIDVTSIRFLDEDEMQRLNKAHLLKPYLTSRHQ
EINEWNRQQGSTESVLNLRRMTNIGTFRAYLNEYLRNHPRIRKDMTLMVRQLAPGDNGLP
LEIYAFTNTVVWLEYESIQADIFDHIFAIVEEFGLRLHQSPTGNDIRSLAGAFKQ
>LFGLNPFC_02728 Phenylalanine-specific permease
MKNASTVSEDTASNQEPTLHRGLHNRHIQLIALGGAIGTGLFLGIGPAIQMAGPAVLLGY
GVAGIIAFLIMRQLGEMVVEEPVSGSFAHFAYKYWGPFAGFLSGWNYWVMFVLVGMAELT
AAGIYMQYWFPDVPTWIWAAAFFIIINAVNLVNVRLYGETEFWFALIKVLAIIGMIGFGL
WLLFSGHGGEKASIDNLWRYGGFFATGWNGLILSLAVIMFSFGGLELIGITAAEARDPEK
SIPKAVNQVVYRILLFYIGSLVVLLALYPWVEVKSNSSPFVMIFHNLDSNVVASALNFVI
LVASLSVYNSGVYSNSRMLFGLSVQGNAPKFLTRVSRRGVPINSLMLSGAITSLVVLINY
LLPQKAFGLLMALVVATLLLNWIMICLAHLRFRAAMRRQGRETQFKALLYPFGNYLCIAF
LGMILLLMCTMDDMRLSAILLPVWIVFLFVAFKTLRRK
>LFGLNPFC_02729 Cation efflux system protein CusA
MIEWIIRRSVANRFLVLMGALFLSIWGTWTIINTPVDALPDLSDVQVIIKTSYPGQAPQI
VENQVTYPLTTTMLSVPGAKTVRGFSQFGDSYVYVIFEDGTDPYWARSRVLEYLNQVQGK
LPAGVSAELGPDATGVGWIYEYALVDRSGKHDLADLRSLQDWFLKYELKTIPDVAEVASV
GGVVKEYQVVIDPQRLAQYGISLAEVKSALDASNQEAGGSSIELAEAEYMVRASGYLQTL
DDFNHIVLKASENGVPVYLRDVAKVQVGPEMRRGIAELNGEGEVAGGVVILRSGKNAREV
IAAVKDKLETLKSSLPEGVEIVTTYDRSQLIDRAIDNLSGKLLEEFIVVAVVCALFLWHV
RSALVAIISLPLGLCIAFIVMHFQGLNANIMSLGGIAIAVGAMVDAAIVMIENAHKRLEE
WQHQHPDATLDNKTRWQVITDASVEVGPALFISLLIITLSFIPIFTLEGQEGRLFGPLAF
TKTYAMAGAALLAIVVIPILMGYWIRGKIPPESSNPLNRFLIRVYHPLLLKVLHWPKTTL
LVAALSVLTVLWPLNKVGGEFLPQINEGDLLYMPSTLPGISAAEAASMLQKTDKLIMSVP
EVARVFGKTGKAETATDSAPLEMVETTIQLKPQDQWRPGMTMDKIIEELDNTVRLPGLAN
LWVPPIRNRIDMLSTGIKSPIGIKVSGTVLADIDTMAEQIEEVARTVPGVASALAERLEG
GRYINVEINREKAARYGMTVADVQLFVTSAVGGAMVGETVEGIARYPINLRYPQSWRDSP
QALRQLPILTPMKQQITLADVADVKVSTGPSMLKTENARPTSWIYIDARDRDMVSVVHDL
QKAIAEKVQLKPGTSVAFSGQFELLERANHKLKLMVPMTLMIIFVLLYLAFRRVGEALLI
ISSVPFALVGGIWLLWWMGFHLSVAMGTGFIALAGVAAEFGVVMLMYLRHAIEAEPSLNN
PQTFSEQKLDEALYHGAVLRVRPKAMTVAVIIAGLLPILWGTGAGSEVMSRIAAPMIGGM
ITAPLLSLFIIPAAYKLMWLHRHRVRK
>LFGLNPFC_02730 Cation efflux system protein CusB
MKKIALIIGSMIAGGIISAAGFTWFAKAELPAEKTSTAERKVLFWYDPMYPNTRFDKPGK
SPFMDMDLVPKYADEESSASGVRIDPTQTQNLGVKTATVTRGPLTFAQSFPANVSYNEYQ
YAIVQARAAGFIDKVYPLTVGDKVQKGAPLLDLTIPDWVEAQSEYLLLRETGGTATQTEG
ILERLRLAGMPEADIRRLIATQKIQTRFTLKAPIDGVITAFDLRAGMNIAKDNVVAKIQG
MDPVWVTAAIPESIAWLVKDASQFTLTVPARPDKTLTIRKWTLLPGVDAATRTLQLRLEV
DNADEALKPGMNAWLQLNTASEPMLLIPSQALIDTGNEQRVITVDADGRFVPKRVAVFQA
SQGVTALRSGLAEGEKVVSSGLFLIDSEANISGALERMRSESATHAH
>LFGLNPFC_02731 Cation efflux system protein CusF
MKKALQVAMFSLFTVIGFNAQANEHHHETMSEAQPQVISATGVVKGVDLESKKITIHHDP
IAAVNWPEMTMRFTITPQTKMSEIKTGDKVAFNFVQQGSLSLLQDIKVSQ

>LFGLNPFC_02732 Cation efflux system protein CusC
MSPCKLLPFCVALALTGCSLAPDYQRPAMPVPQQFSLSQNGLVNAADNYQNAGWRTFFVD
NQVKTLISEALVNNRDLRMAALKVQEARAQYRLTDADRYPQLNGEDSGSWSGNLKGDSAT
TREFSTGLNASFDLDFFGRLKNMSEAERQNYLATEEAQRAVHILLVSNVAQSYFNQQLAY
AQLQIAEETLRNYQQSYAFVEKQLLTGSSNVLALEQARGVIESTRSDIAKRQGELAQANN
ALQLLLGSYGKLPQAQTVNSDSLQSVKLPAGLPSQILLQRPDIMEAEHALMAANANIGAA
RAAFFPSISLTSGISTASSDLSSLFNASSGMWNFIPKIEIPIFNAGRNQANLDIAEIRQQ
QSVVNYEQKIQNAFKEVADALALRQSLNDQISAQQRYLASLQITLQRARALYQHGAVSYL
EVLDAERSLFATRQTLLDLNYARQVNEISLYTALGGGWQQ
>LFGLNPFC_02733 Transcriptional regulatory protein CusR
MKLLIVEDEKKTGEYLTKGLTEAGFVVDLADNGLNGYHLAMTGDYDLIILDIMLPDVNGW
DIVRMLRSANKGMPILLLTALGTIEHRVKGLELGADDYLVKPFAFAELLARVRTLLRRGA
AVIIESQFQVADLMVDLVSRKVTRSGTRITLTSKEFTLLEFFLRHQGEVLPRSLIASQVW
DMNFDSDTNAIDVAVKRLRGKIDNDFEPKLIQTVRGVGYMLEVPDGQ
>LFGLNPFC_02734 Sensor histidine kinase CusS
MVSKPFQRPFSLATRLTFFISLATIAAFFAFAWIMIHSVKVHFAEQDINDLKEISATLER
VLNHPDETQARRLMTLEDIVSGYSNVLISLADSHGKTVYHSPGAPDIREFTRDAIPDKDA
QGGEVYLLSGPTMMMPGHGHGHMEHSNWRMINLPVGPLVDGKPIYTLYIALSIDFHLHYI
NDLMNKLIMTASVISILIVFIVLLAVHKGHAPIRSVSRQIQNITSKDLDVRLDPQTVPIE
LEQLVLSFNHMIERIEDVFTRQSNFSADIAHEIRTPITNLITQTEIALSQSRSQKELEDV
LYSNLEELTRMAKMVSDMLFLAQADNNQLIPEKKMLNLADEVGKVFDFFEALAEDRGVEL
RFVGDECQVAGDPLMLRRALSNLLSNALRYTPTGETIVVRCQTVDHLVQVTVENPGTPIA
PEHLPRLFDRFYRVDPSRQRKGEGSGIGLAIVKSIVVAHKGTVAVTSDVRGTRFVIILPA
>LFGLNPFC_02735 hypothetical protein
MDWLLDVFATWLYGLKVIAITLAVIMFISGLDDFFIDVVYWVRRIKRKLSVYRRYPRMSY
RELYKPDEKPLAIMVPAWNETGVIGNMAELAATTLDYENYHIFVGTYPNDPDTQRDVDEV
CARFPNVHKVVCARPGPTSKADCLNNVLDAITQFERSANFAFAGFILHDAEDVISPMELR
LFNYLVERKDLIQIPVYPFEREWTHFTSMTYIDEFSELHGKDVPVREALAGQVPSAGVGT
CFSRRAVTALLADGDGIAFDVQSLTEDYDIGFRLKEKGMTEIFVRFPVVDEAKEREQRKF
LQHARTSNMICVREYFPDTFSTAVRQKSRWIIGIVFQGFKTHKWTSSLTLNYFLWRDRKG
AISNFVSFLAMLVMIQLLLLLAYESLWPDAWHFLSIFSGSAWLMTLLWLNFGLMINRIVQ
RVIFVTGYYGLTQGLLSVLRLFWGNLINFMANWRALKQVLQHGDPRRVAWDKTTHDFPSV
TGDTRSLRPLGQILLENQVITEEQLDAALRNRVEGLRLGGSMLMQGLISAEQLAQALAEQ
NGVAWESIDAWQIPSSLIAEMPASVALHYAVLPLRLDNDELIVGSEDGIDPVSLAALTRK
VGRKVRYVIVLRGQIVTGLRHWYARRRGHDPRAMLYNAVQHQWLTEQQAGEIWRQYVPHQ
FLFAEILTTLGHINRSAINVLLLRHERSSLPLGKFLVTEGVISQETLDRVLTIQRELQVS
MQSLLLKAGLNTEQVAQLESENEGE
>LFGLNPFC_02736 Bacteriophage adsorption protein A
MKENNLNRVIGWSGLLLTSLLSTSALADNIGTSAEELGLSDYRHFVIYPRLDKALKAQKN
NDEATAIREFEYIHQQVPDNIPLTLYLAEAYRHFSHDDRERLLLEDQLKRHPGDDRLERS
LVAIPVEVKSVTTVEELLAQQKACDAAPTLRCRSEVGQNALRLAQLPVARAQLNDATFAA
SPEGKTLRTDLLQRAIYLKQWSQADTLYNEARQQNTLSAAERRQWFDVLLAAQLDDRILA
LQSQGIFTDPQSYITYATALAYRGEKARLQHYLIENTPLFTTDAQEKSWLYLLSKYSGNP
VQALANYTVQFADNRQYVVGATLPVLLKEGQYGAAQKLLATLPANEMLEERYAVSVATRN
KAEALRLARLLYQQEPANLTRLDQLTWQLMQNEQSREAADLLLQRYPFQGDARVSQTLMA
RLASLLESHPYLATPAKVAILSKPLPLAEQRQWQSQLPGIADNCPAIVRLLGDMSPSYDA
AAWNRLAKCYRDTLPGVALYAWLQAEQRQPDAWQHRAVAYQAYQVEDYATALAAWQKISL
HDMSNEDLLAAANTAQAAGNGAARDRWLQQAEQRGLGNNALYWWLHAQRYIPGQPELALN
DLTRSINIAPSANAYVARATIYRQRHNVPAAVSDLRAALELEPNNSNTQAALGYALWDSG
DIAQSREMLEQAHKGLPDDPALIRQLAYVNQRLDDMPATQHYARLVIDDIDNQALITPLT
PEQNQQRFNFRRLHEEVGRRWTFSFDSSIGLRSGAMSTANNNVGGAAPGKSYRSYGQLEA
EYRLGRNMLLEGDLLSVYSRVFADTGENGVMMPVKNPMSGTGLRWKPLRDQIFFLAVEQQ
LPLNGQNGASDTMLRASASFFNGGKYSDEWHPNGSGWFAQNLYLDAAQYVRQDIQAWTAD
YRVSWHQKVANGQTIEPYAHLQDNGYRDKGTQGAQLGGVGVRWNIWTGETHYDAWPHKVS
LGVEYQHTFKAINQRNGERNNAFLTIGVHW
>LFGLNPFC_02737 hypothetical protein
MRKFIFVLLTLLLVSPFSFAMKGIIWQPQNRDSQVTDTQWQGLMSQLRLQGFDTLVLQWT
RYGDAFTQPEQRALLFKRAAAAQQAGLKLIVGLNADPEFFMHQKQSSAALESYLNRLLAA
DLQQARLWSAVPGVTPDGWYISAEIDDLNWCSEAARQPLLTWLNNAQRLISDVSAKPVYI
SSFFAGNMSPDGYRQLLEQVKATGVNVWVQDGSGVDKLTAEQRERYLQASADCQSSAPAS
GIVYELFVAGKGKTFTAKPKPDAEIASLLAKRSSCGKDTLYFSLRYLPVAQSILEY
>LFGLNPFC_02738 Protease 7
MRAKLLGIVLTPPIAISSFASTETLSFTPDNINADISLGTLSGKTKERVYLAEEGGRKVS
QLDWKFNNAAIIKGAINWDLMPQISIGAAGWTTLGSRGGNMVDQDWMDSSNPGTWTDESR

HPDTQLNYANEFDLNIKGWLLNEPNYRLGLMAGYQESRYSFTARGGSYIYSSEEGFRDDI
GSFPNGERAIGYKQRFKIPYIGLTGSYRYEDFELGGTFKYSGWVEASDNDEHYDPGKRIT
YRSKVKDQNYYSVAVNAGYYVTPNAKVYVEGAWNRVTNKKGNTSLYDHNDNTSDYSKNGA
GIENYNFITTAGLKYTF
>LFGLNPFC_02739 hypothetical protein
MKKNTDDGAKIYTPLTLKLYDWWVLGVSNRLAWGCPTKEHLLPHFLEHLGNNHLDIGVGT
GFYLTHVPESSLISLMDLNEASLNAASTRAGESKIKHKISHDVFDPYPAALHGQFDSISM
FYLLHCLPGNISTKSCVIRNAAQALTDDGTLYGATILGDGVVHNSFGQKLMRIYNQKGIF
SNTKDSEEGLTHILSEHFENVKTKVKGTVVMFSASGKK
>LFGLNPFC_02740 hypothetical protein
MRWNGTRDLQKNQEKRLIDESPKPLKSVVKFALVTVLRKSNIINLEWQQIDMQRQVARVN
PEDSKSNRAIGVALNDTASKVLHDQTGKHHKCGCTYQGG
>LFGLNPFC_02742 Bifunctional protein FolD protein
MAAKIIDGKTIAQQVRSEVAQKVQARIAAGLRAPGLAVVLVGSNPASQIYVASKRKACEE
VGFVSRSYDLPETTSEAELLELIDALNADNTIDGILVQLPLPAGIDNVKVLERIHPDKDV
DGFHPYNVGRLCQRAPRLRPCTPRGIVTLLERYNIDTFGLNAVVIGASNIVGRPMSMELL
LAGCTTTVTHRFTKNLRHHVENADLLIVAVGKPGFIPGDWIKEGAIVIDVGINRLENGKV
VGDVVFEDAAKRASYITPVPGGVGPMTVATLIENTLQACVEYHDPQGE
>LFGLNPFC_02743 putative protein YbcJ
MATFSLGKHPHVELCDLLKLEGWSESGAQAKIAIAEGQVKVDGAVETRKRCKIVAGQTVS
FAGHSVQVVA
>LFGLNPFC_02744 Inner membrane protein YbcI
MPTVITHAAVPLCIGLGLGSKAIPPRLLFAGIILAMLPDADVLSFKFGVAYGNVFGHRGF
THSLVFAFVVPLLCVLIGRRWFRAGLIRCWLFLTVSLLSHSLLDSVTTGGKGVGWLWPWS
DERFFAPWQVIKVAPFALSRYTTPYGHQVIISELMWVWLPGMLLMGMLWWRRR
>LFGLNPFC_02745 Cysteine--tRNA ligase
MLKIFNTLTRQKEEFKPIHAGEVGMYVCGITVYDLCHIGHGRTFVAFDVVARYLRFLGYK
LKYVRNITDIDDKIIKRANENGESFVALVDRMIAEMHKDFDALNILRPDMEPRATHHIAE
IIELTEQLIAKGHAYVADNGDVMFDVPTDPTYGVLSRQDLDQLQAGARVDVVDDKRNPMD
FVLWKMSKEGEPSWPSPWGAGRPGWHIECSAMNCKQLGNHFDIHGGGSDLMFPHHENEIA
QSTCAHDGQYVNYWMHSGMVMVDREKMSKSLGNFFTVRDVLKYYDAETVRYFLMSGHYRS
QLNYSEENLKQARAALERLYTALRGTDKTVAPAGGEAFEARFIEAMDDDFNTPEAYSVLF
DMAREVNRLKAEDMAAANAMASHLRKLSAVLGLLEQEPEAFLQSGAQADDSEVAEIEALI
QQRLDARKAKDWAAADAARDRLNEMGIVLEDGPQGTTWRRK
>LFGLNPFC_02746 Peptidyl-prolyl cis-trans isomerase B
MVTFHTNHGDIVIKTFDDKAPETVKNFLDYCREGFYNNTIFHRVINGFMIQGGGFEPGMK
QKATKDPIKNEANNGLKNTRGTLAMARTQAPHSATAQFFINVVDNDFLNFSGESLQGWGY
CVFAEVVEGMDVVDKIKGVATGRSGMHQDVPKEDVIIESVTVSE
>LFGLNPFC_02747 UDP-2,3-diacylglucosamine hydrolase
MATLFIADLHLCVEEPAITAGFLRFLAGEARKADALYILGDLFEAWIGDDDPNPLHHQMA
AAIKAVSDSGVPCYFIHGNRDFLLGKRFARESGMTLLPEEKVLELYGRRVLIMHGDTLCT
DDAGYQAFRAKVHKPWLQTLFLALPLFVRKRIAARMRANSKEANSSKSLAIMDVNQNAVV
SAMEKHQVQWLIHGHTHRPAVHELIANQQPAFRVVLGAWHTEGSMVKVTADDVELIHFPF
>LFGLNPFC_02748 hypothetical protein
MQRCNGFLISGIALTAGAPLCISWFGLVGFYFGALSLVAAAVISWIVYFWLIYLVLTRPH
NQRFVERFILIVGICFFPWSIVLIWAFFASSARAHNQHAEQIKNSTTDG
>LFGLNPFC_02749 N5-carboxyaminoimidazole ribonucleotide mutase
MSSRNNPARVAIVMGSKSDWATMQFAAEIFEILNVPHHVEVVSAHRTPDKLFSFAESAEE
NGYQVIIAGAGGAAHLPGMIAAKTLVPVLGVPVQSAALSGVDSLYSIVQMPRGIPVGTLA
IGKAGAANAALLAAQILATHDKELHQRLNEWRKAQTDEVLENPDPRGAA
>LFGLNPFC_02750 N5-carboxyaminoimidazole ribonucleotide synthase
MKQVCVLGNGQLGRMLRQAGEPLGIAVWPVGLDAEPAAVPFQQSVITAEIERWPETALTR
ELARHPAFVNRDVFPIIADRLTQKQLFDKLHLPTAPWQLLSEHSEWPAVFDRLGELAIVK
RRTGGYDGRGQWRLRADETEQLPAECYGECIVEQGINFSGEVSLVGARGFDGSTVFYPLT
HNLHQNGILRTSVAFPQANAQQQAQAEEMLSAIMQELSYVGVMAMECFVTPQGLLINELA
PRVHNSGHWTQNGASISQFELHLRAITDLPLPQPVVNNPSVMINLIGSDLNYDWLKLPLV
HLHWYDKEVRPGRKVGHLNLTDSDTSRLTATLEALIPLLPPEYASGVMWAQSKFS
>LFGLNPFC_02751 Carbamate kinase 1
MKTLVVALGGNALLQRGEALTAKNQYRNIASAVPALARLARSYRLAIVHGNGPQVGLLAL
QNLAWKEVEPYPLDVLVAESQGMIGYMLAQQGLSAQPQMPPVTTVLTRIEVSPDDPAFLQP
EKFIGPVYQPEEQEALEAAYGWQMKRDGKYLRRVVASPQPRKILDSEAIELLLKEGHVVI
CSGGGGVPVAEDGAGSEAVIDKDLAAALLAEQINADGLVILTDADAVYENWGTPQQRAIR
RATPDELAPFAKADGSMGPKVTAVSGYVRSRGKPAWIGALSRIEETLAGEAGTCISL
>LFGLNPFC_02752 hypothetical protein

MTIIHPLLASRSAPNYRQSWRLAGVWRRAINLMTESGELLTLHRQGSGFGPGGWVLRRAQ
FDALCGGLCGNERPQVVAQGIRLGRFTVKQPQRYCLLRITPPAHPQPLAVAWMQRAEETG
LFGPLALAASDPLPAELRQFRHCFQAALNGVKTDWRHWLGKGPGLTPSHDDTLSGMLLAA
WYYGALDARSGRPFFACSDNLQLVTTAVSVSYLRYAAQGYFASPLLHFVHALSCPKRTAA
AIDSLLALGHTSGADTLLGFWLGQQLLQGKP
>LFGLNPFC_02753 hypothetical protein
MFTSVAQANAAVIEQIRRARPHWLDVQPASSLISELNKGKTLLHAGPPMRWQEMTGPMKG
ACVGACLFEGWAKDEAQAQAMLEQGEVNFIPCHHVNAVGPMGGITSASMPMLVVENVTDG
NRAYCNLNEGIGKVMRFGAYGEDVLTRHRWMRDVLMPVLSAALGRMERGIDLTAMMAQGI
TMGDEFHQRNIASSALLMRALAPQIARLDHDKQHIAEVMDFLSVTDQFFLNLAMAYCKAA
MDAGAMIRAGSIVTAMTRNGNMFGIRVSGLGERWFTAPVNTPQGLFFTGFSQEQANPDMG
DSAITETFGIGGAAMIAAPGVTRFVGAGGMEAARAVSEEMAEIYLERNMQLQIPGWDFQG
ACLGLDIRRVVETGITPLINTGIAHKEAGIGQIGAGTVRAPLACFEQALEALAESMGIG
>LFGLNPFC_02754 Protein FdrA
MIHAFIKKGCFQDSVSLMIISRKLSESENVDDVSVMMGTPANKALLDTTGFWHDDFNHAT
PNDICVAIRSEAADAGIAQAVMQQLEEALKQLAQGSGSSQALTQVRRWDSASQKLPDANL
ALISVAGEYAAELANQALDRNLNVMMFSDNVTLEDEIQLKTRAREKGLLVMGPDCGTSMI
AATPLAFANVMPEGNIGVIGASGTGIQELCSQIALAGEGITHAIGLGGRDLSREVGGISA
LTALEMLSADEKSEVLAFVSKPPAETVRLKIVNAMKATGKPTVALFLGYTPAVARDENVW
FASSLDEAARLACLLSRVTARRNAIAPVSSGFICGLYTGGTLAAEAAGLLAGHLGVEADD
THQHGMMLDADGHQILDLGDDFYTVGRPHPMIDPTLRNLLIADLGAKPQVRVLLLDVVIG
FGATADPAASLVSAWQKACAARSDNQPLYAIATVTGTERDPQCRSQQIATLEDAGIAVVS
SLPEATLLAAALIHPLPSATQQHTPSLLENVAVINIGLRSFALELQSASKPVVHYQWSPV
AGGNKKLARLLERLQ
>LFGLNPFC_02755 Ureidoglycolate dehydrogenase (NAD(+))
MKISRETLHQLIENKLCQAGLKREHAATVAEVLVYADARGIHSHGAVRVEYYAERISKGG
TNREPEFRLEETGPCSAILHADNAAGQVAAKMGMEHAIKTAQQKGVAVVGISRMGHSGAI
SYFVQQAARAGLIGISLCQSDPMVVPFGGAEIYYGTNPLAFAAPGEGDEILTFDMATTVQ
AWGKVLDARSRNMSIPDTWAVDKNGAPTTDPFAVHALLPAAGPKGYGLMMMIDVLSGVLL
GLPFGRQVSSMYDDLHAGRNLGQLHIVINPNFFSSSELFRQHLSQTMRELNTITPAPGFN
QVYYPGQDQDIKQRKAAVEGIEIVDDIYQYLISDALYNTSYETKNPFAQ
>LFGLNPFC_02756 Allantoate amidohydrolase
MITHFRQAIEETLPWLSSFGADPAGGMTRLLYSPEWLETQQQFKKRMAASGLETRFDEVG
NLYGRLSGTEYPQEVVLSGSHIDTVVNGGNLDGQFGALAAWLAIDWLKTQYGAPLRTVEV
VAMAEEEGSRFPYVFWGSKNIFGLANPDDVRNICDAKGNSFVDAMKACGFTLPNAPLTPR
QDIKAFVELHIEQGCVLESNGQSIGVVNAIVGQRRYTVTLNGESNHAGTTPMGYRRDTVY
AFSRICHQSVEKAKKMGDPLVLTFGKVEPRPNTVNVVPGKTTFTIDCRHTDATVLRDFTQ
QLENDMRAICDEMDIGIDIDLWMDEEPVPMNKELVATLTELCESEKLNYRVMHSGAGHDA
QIFAPRVPTCMIFIPSINGISHNPAERTNITDLAEGVKTLALMLYQLAWQK
>LFGLNPFC_02757 (S)-ureidoglycine aminohydrolase
MGYLNNVTGYREDLLANRAIVKHGNFALLTPDGLVKNIIPGFENCDATILSTPKLGASFV
DYLVTLHQNGGNQQGFGGEGIETFLYVISGNITAKAEGKTFVLSEGGYLYCPPGSLMTFV
NAQAEDSQIFLYKRRYIPVEGHAPWLVSGNASELERIHYEGMDDVILLDFLPKELGFDMN
MHILSFAPGASHGYIETHVQEHGAYILSGQGVYNLDNNWIPVKKGDYIFMGAYSLQAGYG
VGRGEAFSYIYSKDCNRDVEI
>LFGLNPFC_02758 Glycerate 3-kinase
MKIVIAPDSFKESLSAEKCCQAIKAGFSTIFPDAHYICLPIADGGEGTVEAMVAATGGNI
VTLEVCGPMGETVNAFYGLTGDGKTAVIEMAAASGLMLVAPEKRNPLLASSFGTGELIRH
ALDNGIRHIILGIGGSATVDGGMGMAQALGVRFLDADGQVLAANGGNLARVASIEMDECD
PRLANCHIEVACDVDNPLVGARGAAAVFGPQKGATPEMVEELEQGLQNYARVLQQQTEIN
VCQMAGGGAAGGMGIAAAVFLNADIKPGIEIVLNAVNLAQAVQGAALVITGEGRIDSQTA
GGKAPLGVASVAKQFNVPVIGIAGVLGDGVEVVHQYGIDAVFSILPRLAPLAEVLASGET
NLFNSARNIACAIKIGQGIKN
>LFGLNPFC_02759 Putative purine permease YbbY
MFNFAVGRESLLSGFQWFFFIFCNTVVVPPTLLSAFQLPQSSLLTLTQYAFLATALACFA
QAFCGHRRAIMEGPGGLWWGTILTITLGEASRGTPINDIATSLAVGIALSGVLTMLIGFS
GLGHRLARLFTPSVMVLFMLMLGAQLTTIFFKGMLGLPFGIADPNFKIQLPPFALSVAVM
CLVLAMIIFLPQRFARYGLLVGTITGWLLWYFCFPSSHSLSGELHWQWFPLGSGGALSPG
IILTAVITGLVNISNTYGAIRGTDVFYPQQGAGNTRYRRSFVATGFMTLITVPLAVIPFS
PFVSSIGLLTQTGDYTRRSFIYGSVICLLVALVPALTRLFCSIPLPVSSAVMLVSYLPLL
FSALVFSQQITFTARNIYRLALPLFVGIFLMALPPVYLQDLPLTLRPLLSNGLLVGILLA
VLMDNLIPWERIE
>LFGLNPFC_02760 Allantoinase
MSFDLIIKNGTVILENEARVVDIAVKDGKIAAIGQDLGDAKDVMDASGLVVSPGMVDAHT

HISEPGRSHWEGYETGTRAAAKGGITTMIEMPLNQLPATVDRASIELKFDAAKGKLTIDA
AQLGGLVSYNIDRLHELDEVGVVGFKCFVATCGDRGIDNDFRDVNDWQFFKGAQKLGELG
QPVLVHCENALICDALGEEAKREGRVTAHDYVASRPVFTEVEAIRRVLYLAKVAGCRLHV
CHVSSPEGVEEVTRARQEGQDVTCESCPHYFVLDTDQFEEIGTLAKCSPPIRDLENQKGM
WEKLFNGEIDCLVSDHSPCPPEMKAGNIMKAWGGIAGLQSCMDVMFDEAVQKRGMSLPMF
GKLMATNAADIFGLQQKGRIAPGKDADFVFIQPNSSYVLTNDDLEYRHKVSPYVGRTIGA
RITKTILRGDVIYDIEQGFPVAPKGQFILKHQQ
>LFGLNPFC_02761 Putative allantoin permease
MEHQRKLFQQRGYSEDLLPKTQSQRTWKTFNYFTLWMGSVHNVPNYVMVGGFFILGLSTF
SIMLAIILSAFFIAAVMVLNGAAGSKYGVPFAMILRASYGVRGALFPGLLRGGIAAIMWF
GLQCYAGSLACLILIGKIWPGFLTLGGDFTLLGLSLPGLITFLLFWLVNVGIGFGGGKVL
NKFTAILNPCIYIVFGGMAIWAISLVGIGPIFDYIPGGIQKAENSGFLFLVVINAVVAVW
AAPAVSASDFTQNAHSFREQALGQTLGLVVAYILFAVAGVCIIAGASIHYGADTWNVLDI
VQRWDSLFASFFAVLVILMTTISTNATGNIIPAGYQIAAIAPTKLTYKNGVLIASIISLL
ICPWKLMENQDSIYLFLDIIGGMLGPVIGVMMAHYFVVMRGQINLDELYTAPGDYKYYDN
GFNLTAFSVTLVAVILSLGGKFIPFMEPLSRVSWFVGVIVAFAAYALLKKRTTAEKTGEQ
KTIG
>LFGLNPFC_02762 2-hydroxy-3-oxopropionate reductase
MKLGFIGLGIMGTPMAINLARAGHQLHVTTIGPVADELLSLGAVSVETARQVTDASDIIF
IMVPDTPQVEDVLFGENGCTKASLKGKTIVDMSSISPIETKRFARQVNELGGDYLDAPVS
GGEIGAREGTLSIMVGGDEAVFERVKPLFELLGKNITLVGGNGDGQTCKVANQIIVALNI
EAVSEALLFASKAGADPVRVRQALMGGFASSRILEVHGERMIKRTFNPGFKIALHQKDLN
LALQSAKALALNLPNTATCQELFNTCAANGGSQLDHSALVQALELMANHKLA
>LFGLNPFC_02763 Hydroxypyruvate isomerase
MLRFSANLSMLFGEYDFLARFEKAAQCGFRGVEFMFPYDYDIEELKQVLASNKLEHTLHN
LPAGDWAAGERGIACIPGREEEFRDGVAAAIRYARALGNKKINCLVGKTPTGFSSEQIHT
TLVENLRYAANMLMKEDILLLIEPINHFDIPGFHLTGTRQALKLIDDVGCCNLKIQYDIY
HMQRMEGELTNTMTQWADKIGHLQIADNPHRGEPGTGEINYEYLFTVIENSDYNGWVGCE
YKPQTTTEAGLRWMDPYR
>LFGLNPFC_02764 Glyoxylate carboligase
MAKMRAVDAAMYVLEKEGITTAFGVPGAAINPFYSAMRKHGGIRHILARHVEGASHMAEG
YTRATAGNIGVCLGTSGPAGTDMITALYSASADSIPILCITGQAPRARLHKEDFQAVDIE
AIAKPVSKMAVTVREAALVPRVLQQAFHLMRSGRPGPVLVDLPFDVQVAEIEFDPDMYEP
LPVYKPAASRMQIEKAVEMLIQAERPVIVAGGGVINADAAALLQQFAELTSVPVIPTLMG
WGCIPDDHELMAGMVGLQTAHRYGNATLLASDMVFGIGNRFANRHTGSVEKYTEGRKIVH
IDIEPTQIGRVLCPDLGIVSDAKAALTLLVEVAQEMQKAGRLPCRKEWVADCQQRKRTLL
RKTHFDNVPVKPQRVYEEMNKAFGRDVCYVTTIGLSQIAAAQMLHVFKDRHWINCGQAGP
LGWTIPAALGVCAADPERNVVAISGDFDFQFLIEELAVGAQFNIPYIHVLVNNAYLGLIR
QSQRAFDMDYCVQLAFENINSSEVNGYGVDHVKVAEGLGCKAIRVFKPEDIAPAFEQAKI
LMAQYRVPVVVEVILERVTNISMGSELDNVMEFEDIADNAADAPTETCFMHYE
>LFGLNPFC_02765 HTH-type transcriptional repressor AllR
MTEVRRRGRPGQAEPVAQKGAQALERGIAILQYLEKSGGSSSVSDISLNLDLPLSTTFRL
LKVLQAADFVYQDSQLGWWHIGLGVFNVGAAYIHNRDVLSVAGPFMRRLMLLSGETVNVA
IRNGNEAVLIGQLECKSMVRMCAPLGSRLPLHASGAGKALLYPLAEEELMSIILQTGLQQ
FTPTTLVDMPTLLKDLEQARELGYTVDKEEHVVGLNCIASAIYDDVGSVVAAISISGPSS
RLTEDRFVSQGELVRDTARDISTALGLKAHL
>LFGLNPFC_02766 Ureidoglycolate lyase
MKLQVLPLSQEAFSAYGDVIETQKRDFFHINNGLVERYHDLALVEILEQDRTLISINRAQ
PANLPLTIHELERHPLGTQAFIPMKGEVFVVVVALGDDKPDLSTLRAFITNGEQGVNYHR
NVWHHPLFAWQRVTDFLTIDRGGSDNCDVESIPEQELCFA
>LFGLNPFC_02767 HTH-type transcriptional activator AllS
MFDPETLRTFIAVAETGSFSKAAERLCKTTATISYRIKLLEENTGVALFFRTTRSVTLTA
AGEHLLCQARDWLGWLESMPSELQQVNDGVERQVNIVINNLLYNPQAVARLLAWLNERYP
FTQFHISRQIYMGVWDSLLYEGFSLAIGVTGTEALANTFSLDPLGSVQWRFVMAADHPLA
NVEEPLTEAQLRRFPAVNIEDSARTLTKRVAWRLPGQKEIIVPDMETKIAAHLAGVGIGF
LPKSLCQSMLDNQQLVSRVIPTMRPPSPLSLAWRKFGSGKAVEDIVTLFTQRRPEISGFL
EIFGNPRS
>LFGLNPFC_02768 tRNA 2-selenouridine synthase
MQERHTEQDYRALLIADTPIIDVRAPIEFEQGAMPAAINLPLMNNDERAAVGICYKQQGS
DAALALGHKLVAGEIRQQRMDAWRAACLQNPHGILCCARGGQRSHIVQRWLHDAGIDYPL
VEGGYKALRQTAIQATIELSQKPIVLIGGCTGCGKTLLVQQQPNGVDLEGLARHRGSAFG
RTLQPQLSQASFENLLAAEMLKTDARQNLRLWVLEDESRMIGSNHLPECLRERMTQATIA
VVEDPFEIRLERLNEEYFLRMHHDFTHAYGDEQGWQEYCEYLHHGLSAIKRRLGLQRYNE
LAARLDAALTTQLTTGSTDGHLAWLVPLLEEYYDPMYRYQLEKKAEKVVFRGEWAEVAEW

VKAQ
>LFGLNPFC_02769 hypothetical protein
MIARWFWREWRSPSLLIVWLALSLAVACVLALGNISDRMEKGLSQQSREFMAGDRALRSS
REVPQAWLEEAQKRGLKVGKQLTFATMTFAGDTPQLANVKAVDDIYPMYGDLQTNPPGLK
PQAGSVLLAPRLMALLNLKTGDTIDVGDATLRIAGEVIQEPDSGFNPFQIAPRLMMNLAD
VDKTGAVQPGSRVTWRYKFGGSENQLDGYEKWLLPQLKPEQRWYGLEQDEGALGRSIERS
QQFLLLSALLTLLLAVAAVAVAMNHYCRSRYDLVAILKTLGAGRAQLRKLIVGQWLMVLV
LSAVTGGAIGLLFENVLMVLLKPVLPAALPPASLWPWLWALGTMTVISLLVGLRPYRLLL
ATQPLRVLRNDVVANVWPLKFYLPIVSVVVVLLLAGLMGGSMLLWAVLAGAVVLALLCGV
LGWMLLNVLRRMTLKSLPLRLAVSRLLRQPWSTLSQLSAFSLSFMLLALLLVLRGDLLDR
WQQQLPPESPNYFLINIATEQVTPLKAFLAEHQIVPESFYPVVRARLTAINDKPTEGNGD
EALNRELNLTWQNARPDHNPIVAGNWPPKADEVSMEEGLAKRLNVALGDTVTFMGDTQEF
RAKVTSLRKVDWESLRPNFYFIFPEGALDGQPQSWLTSFRWENGNGMLTQLNRQFPTISL
LDIGAILKQVGQVLEQVSRALEVMVVLVTACGMLLLLAQVQVGMRQRHQELVVWRTLGAG
KKLLRTTLWCEFAMLGFVSGLVAAIGAETALAVLQSKVFDFPWEPDWRLWIVLPCSGALL
LSLCGGWLGARLLKGKALFRQFAG
>LFGLNPFC_02770 Lipoprotein-releasing system ATP-binding protein LolD
MPAENIVEVHHLKKSVGQGEHELSILTGVELVVKRGETIALVGESGSGKSTLLAILAGLD
DGSSGEVRLVGQPLHNMDEEARAKLRAKHVGFVFQSFMLIPTLNALENVELPALLRGESS
AESRNGAKALLEQLGLCKRLDHLPAQLSGGEQQRVALARAFNGRPDVLFADEPTGNLDRQ
TGDKIADLLFSLNREHGTTLIMVTHDLQLAARCDRCLRLVNGQLQEEA
>LFGLNPFC_02771 Thioesterase 1/protease 1/lysophospholipase L1
MPFLFLVLLTFRAAAADTLLILGDSLSAGYRMSASAAWPALLNDKWQSKTSVVNASISGD
TSQQGLARLPALLKQHQPRWVLVELGGNDGLRGFQPQQTEQTLRQILQDVKAANAEPLLM
QIRLPANYGRRYNEAFSAIYPKLAKEFDVPLLPFFMEEVYLKPQWMQDDGIHPNRDAQPF
IADWMAKQLQPLVNHDS
>LFGLNPFC_02772 hypothetical protein
MQKSVLITGCSSGIGLESALELKRQGFHVLAGCRKPDDVERMNSMGFTGVLIDLDLPESV
DRAADEVIALTDNCLYGIFNNAGFGMYGPLSTISRAQMEQQFSANFFGAHQLTMRLLPAM
LPHGEGRIVMTSSVMGLISTPGRGAYAASKYALEAWSDALRMELRYSGIKVSLIEPGPIR
TRFTDNVNQTQSDKPVENPGIAARFTLGPEAVVDKVRHAFISEKPKMRYPVTLVTWAVMV
LKRLLPGRVMDKILQG
>LFGLNPFC_02773 Chaperedoxin
MSVENIVNINESNLQQVLEQSMTTPVLFYFWSERSQHCLQLTPILESLAAQYNGQFILAK
LDCDAEQMIAAQFGLRAIPTVYLFQNGQPVDGFQGPQPEEAIRALLDKVLPREEELKAQQ
AMQLMQEGNYTDALPLLKDAWQLSNQNGEIGLLLAETLIALNRSEDAEAVLKTIPLQDQD
TRYQGLVAQIELLKQAADTPEIQQLQQQVAENPEDAALATQLALQLHQVGRNEEALELLF
GHLRKDLTAADGQTRKTFQEILAALGTGDALASKYRRQLYALLY
>LFGLNPFC_02774 putative iron export permease protein FetB
MNSHNITNESLALALMLVVVAILISHKEKLALEKDILWSVGRAIIQLIIVGYVLKYIFSV
DDASLTLLMVLFICFNAAWNAQKRSKYIAKAFISSFIAITVGAGITLAVLILSGSIEFIP
MQVIPIAGMIAGNAMVAVGLCYNNLGQRVISEQQQIQEKLSLGATPKQASAILIRDSIRA
ALIPTVDSAKTVGLVSLPGMMSGLIFAGIDPVKAIKYQIMVTFMLLSTASLSTIIACYLT
YRKFYNSRHQLVVTQLKKK
>LFGLNPFC_02775 putative iron export ATP-binding protein FetA
MQENSPLLQLQNVGYLAGDTKILNNINFSLRAGEFKLITGPSGCGKSTLLKIVASLISPT
SGTLLFEGEDVSTLKPEIYRQQVSYCAQTPTLFGDTVYDNLIFPWQIRNQQPDPAIFLDF
LERFALPDSILTKNIAELSGGEKQRISLIRNLQFMPKVLLLDEITSALDESNKHNVNEMI
HRYVREQNIAVLWVTHDKDEINHADKVITLQPHAGEMQEARYELA
>LFGLNPFC_02776 Protein QmcA
MLIFIPILIFVALVIVGAGVKIVPQGYQWTVERFGRYTKTLQPGLSLVVPFMDRIGRKIN
MMEQVLDIPSQEVISKDNANVTIDAVCFIQVIDAPRAAYEVSNLELAIINLTMTNIRTVL
GSMELDEMLSQRDSINSRLLHIVDEATNPWGIKVTRIEIRDVRPPAELISSMNAQMKAER
TKRAYILEAEGIRQAEILKAEGEKQSQILKAEGERQSAFLQAEARERSAEAEARATKMVS
EAIASGDIQAVNYFVAQKYTEALQQIGSSSNSKVVMMPLEASSLMGSIAGIAELVKDSAN
KRTQP
>LFGLNPFC_02777 Inner membrane protein YbbJ
MMELMVVHPHIFWLSLGGLLLAAEMLGGNGYLLWSGVAAVITGLVVWLLPLGWEWQGVMF
AVLTLLAAWLWWKWLSRRVREQKHSDSHLNQRGQQLIGRRFVLESPLVNGRGHMRVGDSS
WPVSASEDLGAGTHVEVIAIEGITLIIRAVIA
>LFGLNPFC_02778 hypothetical protein
MNEFEKIFNEMNLDRALLPILFRSNRSTVWKYLSGDSTAPASAMSLIMLLQLIQKRNPDL
LAEWLTLSDFTIPPEVYLDQPDYWKGWVYTQHKVNKNVLEYLKKHYPDEDQKSMGKGREE
>LFGLNPFC_02779 Chromosome partition protein Smc

MKTVNVALLALIISATSSPVVLAGDTIEAAATELSAINSGMSQSEIEQKITRFLERTDNS
PAAYTYLTEHHYIPSETPDTTQTPPVQTDPDAGQKTVAATGVVQIPARYQSMINARQSAV
TDAQQTQITEQQAQIVATQKTLAATGDTQNTAHYQEMINARLAAQNEANQRTTTEQGQKM
NALTTDVAAQQQKERAQYDKQMQSLAQKSVQAHEQIESLRQDSAQTQQQLTNTQKRVADN
SQQINTLNNHFDSLKNEVEDNRKEANAGTASAIAIASQPQVKTGDVMMVSAGAGTFNGES
AVSVGTSFNAGTHTVLKAGISADTQSDFGAGVGVGYSF
>LFGLNPFC_02780 hypothetical protein
MNSSIKSFSLLAVILLTGCSSPTSRIADCQAQGVSHDTCYLAEQQRQTAILSASEAQAFK
NAEAAQHAQAAKKAIYKGFGMTFRMSSKNFAYLNDSLCAIDEDNKDATVYQSGLYNVIVY
HHTGKVALMKEGQFVGYLK
>LFGLNPFC_02781 hypothetical protein
MKEQRKIPLTHIMIIGAFIFAFLQVVLLASLVHAVNVNNEIQEGLFQSGRIMVESLQHIL
SVQTEIN
>LFGLNPFC_02782 HTH-type transcriptional regulator CueR
MNISDVAKITGLTSKAIRFYEEKGLVTPPMRSENGYRTYTQQHLNELTLLRQARQVGFNL
EESGELVNLFNDPQRHSADVKRRTLEKVAEIERHIEELQSMRNQLLALANACPGDDSADC
PIIENLSGCCHHRAG
>LFGLNPFC_02783 Inner membrane transport protein YbaT
MMNTEGNNGNKPLGLWNVVSIGIGAMVGAGIFALLGQAALLMEASTWVAFAFGGIVAMFS
GYAYARLGASYPSNGGIIDFFRRGLGNGVFSLALSLLYLLTLAVSIAMVARAFGAYAVQF
LHEGSQEEHLILLYALGIIAVMTLFNSLSNHAVGRLEVILVGIKMMILLLLIIAGVWSLQ
PAHISVSAPPSSGAFFSCIGITFLAYAGFGMMANAADKVKDPEVIMPRAFLVAIGVTTLL
YISLALVLLSDVSALELEKYADTAVAQAASPLLGHVGYVIVVIGALLATASAINANLFAV
FNIMDNMGSERELPKLMNKPLWRQSTWGNIIVVVLIMLMTAALNLGSLASVASATFLICY
LAVFVVAIRLRHDIHASLPILIVGTLVMLLVIVGFIYSLWSQGSRALIWIIGALLLSLIV
AMVMKRNKTV
>LFGLNPFC_02784 Glutaminase 1
MLDANKLQQAVDQTYTQFHSLNGGQNADYIPFLANVPGQLAAVAIVTSDGNVYSAGDSDY
RFALESISKVCTLALALEDVGPQAVQDKVGADPTGLPFNSVIALELHGGKPLSPLVNAGA
IATTSLINAENTEQRWQRILHIQQQLAGEQVALSDEVNQSEQTTNFHNRAIAWLLYSAGY
LYCDAMEACDVYTRQCSTLINTIELATLGATLAAGGVNPLTHKRVLQADNVPYILAEMMM
EGLYGRSGDWAYRVGLPGKSGVGGGILAVVPGVMGIAAFSPPLDEEGNSVRGQKMVASVA
KQLGYNVFKG
>LFGLNPFC_02785 Copper-exporting P-type ATPase
MSQTIDLTLDGLSCGHCVKRVKESLEQRPDVEQADVSITEAHVTGTASAEQLIETIKQAG
YDASVSHPKAKPLAESSIPSEALTAVSEALPAATADDDDSQQLLLSGMSCASCVTRVQNA
LQSVPGVTQARVNLAERTALVMGSASPQDLVQAVEKAGYGAEAIEDDAKRRERQQETAVA
TMKRFRWQAIVALAVGIPVMVWGMIGDNMMVTADNRSLWLVIGLITLAVMVFAGGHFYRS
AWKSLLNGAATMDTLVALGTGVAWLYSMSVNLWPQWFPMEARHLYYEASAMIIGLINLGH
MLEARARQRSSKALEKLLDLTPPTARLVTDEGEKNVPLADVQPGMLLRLTTGDRVPVDGE
ITQGEAWLDEAMLTGEPIPQQKGEGDSVHAGTVVQDGSVLFRASAVGSHTTLSRIIRMVR
QAQSSKPEIGQLADKISAVFVPVVVVIALISAAIWYFFGPAPQIVYTLVIATTVLIIACP
CALGLATPMSIISGVGRAAEFGVLVRDADALQRASTLDTVVFDKTGTLTEGKPQVVAVKT
FADVDEAQALRLAAALEQGSSHPLARAILDKAGDMQLPQVNGFRTLRGLGVSGEAEGHAL
LLGNQALLNEQQVDTKAIEAEITAQASQGATPVLLAVDGKAVALLAVRDPLRSDSVAALQ
RLHKAGYRLVMLTGDNPTTANAIAKEAGIDEVIAGVLPDGKAEAIKRLQSEGRQVAMVGD
GINDAPALAQADVGIAMGGGSDVAIETAAITLMRHSLMGVADALAISRATLRNMKQNLLG
AFIYNSIGIPVAAGILWPFTGTLLNPVVAGAAMALSSITVVSNANRLLRFKPKE
>LFGLNPFC_02786 putative HTH-type transcriptional regulator YbaQ
MKQATRKPTTPGDILLYEYLEPLDLKINELAELLHVHRNSVSALINNNRKLTTEMAFRLA
KVFDTTVDFWLNLQAAVDLWEVENNMRTQEELGRIETVAEYLARREERAKKVA
>LFGLNPFC_02787 Toxin HigB-1
MAQKKNIRSFRDAWLADFFVHSTPHRKIPAEIHTTLSRKLDIINAATSHWDLRSPPGNRY
EELSGKLQEYSSIRVNKQYRLIFKWVNGKAEELFLDPHNY
>LFGLNPFC_02788 hypothetical protein
MDLLYRVKTLWAALRGNHYTWPAIDITLPGNRHFHLIGSIHMGSHDMAPLPTRLLKKLKN
ADALIVEADVSTSDTPFANLPACEALEERISEEQLQNLQHISQEMGISPSLFSTQPLWQI
AMVLQATQAQKLGLRAEYGIDYQLLQAAKQQHKPVIELEGAENQIAMLLQLPDKGLALLD
DTLTHWHTNARLLQQMMSWWLNAPPQNNEITLPNTFSQSLYDVLMHQRNLAWRDKLRAMP
PGRYVVAVGALHLYGEGNLPQMLR
>LFGLNPFC_02789 Cys-tRNA(Pro)/Cys-tRNA(Cys) deacylase YbaK
MTPAVKLLEKNKISFQIHTYEHDPAETNFGDEVVKKLGLNPDQVYKTLLVAVNGDMKHLA
VAVTPVAGQLDLKKVAKALGAKKVEMADPMVAQRSTGYLVGGISPLGQKKRLPTIIDAPA
QEFATIYVSGGKRGLDIELAASDLAKILDAKFADIARRD

>LFGLNPFC_02790 hypothetical protein
MERITETLSIACADEIPDIALLRQTSREKVLHLHFASEAERYREEFGLVLKAQLDEFVVG
MHSADPVIRIARLISLEEINRHQDFFEHCAKEYRKLATELIFALAEQLNVEMAENNPLVT
FAPFKCNRKRKGKMGKWQYCFHGFHCTFENKKTEQNIEVPLAYGFAFGDLDPYFFSGFIK
STPAWQPLPVAIYDDYHDGSRIIQQLLALGKLQKIPSPIPQYTGVAAVDRSNVDIANFRS
TLESRLHRCKLRWLLKHVKNSQRSER
>LFGLNPFC_02791 hypothetical protein
MKFDHLVDVSFHNFVFNGKFDFVELGQTMAWLEQNFVEPDSKNDMGNGFYIWLFGNVEFH
FENNKLYMIWCDYLSQIHLGKAIRFDKGILNNVAELNVTRVFNMLVNEGADLQIRRQSPH
TLLIHVNRSGVTLWFEADEEQPGDPQNYMLMAFGLTHRDYDARFQYS
>LFGLNPFC_02792 Protein UshA
MKLLQRGVALALLTTFTLASETALAYEQDKTYKITVLHTNDHHGHFWRNEYGEYGLAAQK
TLVDGIRKEVAAEGGSVLLLSGGDINTGVPESDLQDAEPDFRGMNLVGYDAMAIGNHEFD
NPLTVLRQQEKWAKFPLLSANIYQKSTGERLFKPWALFKRQDLKIAVIGLTTDDTAKIGN
PEYFTDIEFRKPADEAKLVIQELQQTEKPDIIIAATYMGHYDNGEHGSNAPGDVEMVRAL
PAGSLAMIVGGHSQDPVCMAAENKKQVDYVPGTPCKPDQQNGIWIVQAHEWGKYVGRADF
EFRNGEMKMVNYQLIPVNLKKKVTWEDGKSERVLYTPEIAENQQMISLLSPFQNKGKAQL
EVKIGETNGRLEGDRDKVRFVQTNMGRLILAAQMDRTGADFAVMSGGGIRDSIEAGDISY
KNVLKVQPFGNVVVYADMTGKEVIDYLTAVAQMKPDSGAYPQFANVSFVAKDGKLNDLKI
KGEPVDPAKTYRMATLNFNATGGDGYPRLDNKPGYVNTGFIDAEVLKAYIQKSSPLDVSV
YEPKGEVSWQ
>LFGLNPFC_02793 Fosmidomycin resistance protein
MAMSEQTQPVAGAAASTTKARTSFGILGAISLSHLLNDMIQSLILAIYPLLQSEFSLTFM
QIGMITLTFQLASSLLQPVVGYWTDKYPMPWSLPIGMCFTLSGLVLLALAGSFGAVLLAA
ALVGTGSSVFHPESSRVARMASGGRHGLAQSIFQVGGNFGSSLGPLLAAVIIAPYGKGNV
AWFVLAALLAIVVLAQISRWYSAQHRMNKGKPKATIINPLPRNKVVLAVSILLILIFSKY
FYMASISSYYTFYLMQKFGLSIQNAQLHLFAFLFAVAAGTVIGGPVGDKIGRKYVIWGSI
LGVAPFTLILPYASLHWTGVLTVIIGFILASAFSAILVYAQELLPGRIGMVSGLFFGFAF
GMGGLGAAVLGLIADHTSIELVYKICAFLPLLGILTIFLPDNRHKD
>LFGLNPFC_02794 Putative cation/proton antiporter YbaL
MHHATPLITTIVGGLVLAFILGMLANKLRISPLVGYLLAGVLAGPFTPGFVADTKLAPEL
AELGVILLMFGVGLHFSLKDLMAVKSIAIPGAIAQIAVATLLGMALSAVLGWSLMTGIVF
GLCLSTASTVVLLRALEERQLIDSQRGQIAIGWLIVEDLVMVLTLVLLPAVAGMMEQGDV
GFATLAVDMGITIGKVIAFIAIMMLVGRRLVPWIMARSAATGSRELFTLSVLALALGIAF
GAVELFDVSFALGAFFAGMVLNESELSHRAAHDTLPLRDAFAVLFFVSVGMLFDPLILIQ
QPLAVLATLAIILFGKSVAALFLVRLFGHSQRTALTIAASLAQIGEFAFILAGLGMALNL
LPQAGQNLVLAGAILSIMLNPVLFALLEKYLAKTETLEEQTLEEAIEEEKQIPVDICNHA
LLVGYGRVGSLLGEKLLASDIPLVVIETSRTRVDELRERGVRAVLGNAANEEIMQLAHLE
CAKWLILTIPNGYEAGEIVASARAKNPDIEIIARAHYDDEVAYITERGANQVVMGEREIA
RTMLELLETPPAGEVVTR
>LFGLNPFC_02795 Inosine-guanosine kinase
MKFPGKRKSKHYFPVNARDPLLQQFQPENETSAAWVVGIDQTLVDIEAKVDDEFIERYGL
SAGHSLVIEDDVAEALYQELKQKNLITHQFAGGTIGNTMHNYSVLADDRSVLLGVMCSNI
EIGSYAYRYLCNTSSRTDLNYLQGVDGPIGRCFTLIGESGERTFAISPGHMNQLRAESIP
EDVIAGASALVLTSYLVRCKPGEPMPEATMKAIEYAKKYNVPVVLTLGTKFVIAENPQWW
QQFLKDHVSILAMNEDEAEALTGESDPLLASDKALDWVDLVLCTAGPIGLYMAGFTEDEA
KRKTQHPLLPGAIAEFNQYEFSRAMRHKDCQNPLRVYSHIAPYMGGPEKIMNTNGAGDGA
LAALLHDITANSYHRSNVPNSSKHKFTWLTYSSLAQVCKYANRVSYQVLNQHSPRLTRGL
PEREDSLEESYWDR
>LFGLNPFC_02796 Acetyl esterase
MKPENKLPVLDLISAEMKTVVNTLQPDLPPWPATGAIAEQRQYYTLERRFWNVGAPEMAT
RAYRVPTKYGQVKTRLFYPQPDSPATLFYLHGGGFILGNLDTHDRIMRLLASYSQCTVIG
IDYTLSPEARFPQAIEEIVAACCYFHQQAEDYQINMSRIGFAGDSAGAMLALASALWLRD
KQIDCGKVAGVLLWYGLYGLRDSVTRRLLGGVWDGLTQQDLQMYEEAYLSNDADRESPYY
CLFNNDLTREVPPCFIAGAEFDPLLDDSCLLYQTLAAHQQPCEFKLYSGMLHAFLHYSRM
MKTADEALRDGAQFFTAQL
>LFGLNPFC_02797 Ferrochelatase
MRQTKTGILLANLGTPDAPTPEAVKRYLKQFLSDRRVVDTSRLLWWPLLRGVILPLRSPR
VAKLYASVWMEGGSPLMVYSRQQQQALAQRLPETPVALGMSYGSPSLESAVDELLAEHVD
HIVVLPLYPQYSCSTVGAVWDELARILARKRSIPGISFIRDYADNHDYINALANSVRASF
AKHGEPDLLLLSYHGIPQRYADEGDDYPQRCRTTTRELASALEMAPEKVMMTFQSRFGRE
PWLMPYTDETLKMLGEKGVGHIQVMCPGFAADCLETLEEIAEQNREVFLGAGGKKYEYIP
ALNATPEHIEMMANLVAAYR
>LFGLNPFC_02798 Adenylate kinase

MRIILLGAPGAGKGTQAQFIMEKYGIPQISTGDMLRAAVKSGSELGKQAKDIMDAGKLVT
DELVIALVKERIAQEDCRYGFLLDGFPRTIPQADAMKEAGINVDYVLEFDVPDELIVDRI
VGRRVHAPSGRVYHVKFNPPKVEGKDDVTGEELTTRKDDQEETVRKRLVEYHQMTAPLIG
YYSKEAEAGNTKYAKVDGTKPVAEVRAALEKILG
>LFGLNPFC_02799 Chaperone protein HtpG
MKGQETRGFQSEVKQLLHLMIHSLYSNKEIFLRELISNASDAADKLRFRALSNPDLYEGD
GELRVRVSFDKDKRTLTISDNGVGMTRDEVIDHLGTIAKSGTKSFLESLGSDQAKDSQLI
GQFGVGFYSAFIVADKVTVRTRAAGEKPENGVFWESAGEGEYTVADITKEDRGTEITLHL
REGEDEFLDDWRVRSIISKYSDHIALPVEIEKREEKDGETVISWEKINKAQALWTRNKSE
ITDEEYKEFYKHIAHDFNEPLTWSHNRVEGKQEYTSLLYIPSQAPWDMWNRDHKHGLKLY
VQRVFIMDDAEQFMPNYLRFVRGLIDSSDLPLNVSREILQDSTVTRNLRNALTKRVLQML
EKLAKDDAEKYQTFWQQFGLVLKEGPAEDFANQEAIAKLLRFASTHTDSSAQTVSLEDYV
SRMKEGQEKIYYITADSYAAAKSSPHLELLRKKGIEVLLLSDRIDEWMMNYLTEFDGKPF
QSVSKVDESLEKLADEVDESAKEAEKALTPFIDRVKALLGERVKDVRLTHRLTDTPAIVS
TDADEMSTQMAKLFAAAGQKVPEVKYIFELNPDHVLVKRAADTEDEAKFSEWVELLLDQA
LLAERGTLEDPNLFIRRMNQLLVS
>LFGLNPFC_02800 Recombination protein RecR
MQTSPLLTQLMEALRCLPGVGPKSAQRMAFTLLQRDRSGGMRLAQALTRAMSEIGHCADC
RTFTEQEVCNICSNPRRQENGQICVVESPADIYAIEQTGQFSGRYFVLMGHLSPLDGIGP
DDIGLDRLEQRLAEEKITEVILATNPTVEGEATANYIAELCAQYDVEASRIAHGVPVGGE
LEMVDGTTLSHSLAGRHKIRF
>LFGLNPFC_02801 Nucleoid-associated protein YbaB
MFGKGGLGNLMKQAQQMQEKMQKMQEEIAQLEVTGESGAGLVKVTINGAHNCRRVEIDPS
LLEDDKEMLEDLVAAAFNDAARRIEETQKEKMASVSSGMQLPPGFKMPF
>LFGLNPFC_02802 DNA polymerase III subunit tau
MSYQVLARKWRPQTFADVVGQEHVLTALANGLSLGRIHHAYLFSGTRGVGKTSIARLLAK
GLNCETGITATPCGVCDNCREIEQGRFVDLIEIDAASRTKVEDTRDLLDNVQYAPARGRF
KVYLIDEVHMLSRHSFNALLKTLEEPPEHVKFLLATTDPQKLPVTILSRCLQFHLKALDV
EQIRHQLEHILNEEHIAHEPRALQLLARAAEGSLRDALSLTDQAIASGDGQVSTQAVSAM
LGTLDDDQALSLVEAMVEANGERVMALINEAAARGIEWEALLVEMLGLLHRIAMVQLSPA
ALGNDMAAIELRMRELARTIPPTDIQLYYQTLLIGRKELPYAPDRRMGVEMTLLRALAFH
PRMPLPEPEVPRQSFAPVAPTAVMTPTQVPPQPQSAPQQAPTVPLPETTSQVLAARQQLQ
RVQGATKAKKSEPAAATRARPVNNAALERLASVTDRVQARPVPSALEKAPAKKEAYRWKA
TTPVMQQKEVVATPKALKKALEHEKTPELAAKLAAEAIERDPWAAQVSQLSLPKLVEQVA
LNAWKEESDNAVCLHLRSSQRHLNNRGAQQKLAEALSTLKGSTVELTIVEDDNPAVRTPL
EWRQAIYEEKLAQARESIIADNNIQTLRRFFDAELDEESIRPI
>LFGLNPFC_02803 Adenine phosphoribosyltransferase
MTATAQQLEYLKNSIKSIQDYPKPGILFRDVTSLLEDPKAYALSIDLLVERYKNAGITKV
VGTEARGFLFGAPVALGLGVGFVPVRKPGKLPRETISETYDLEYGTDQLEIHVDAIKPGD
KVLVVDDLLATGGTIEATVKLIRRLGGEVADAAFIINLFDLGGEQRLEKQGITSYSLVPF
PGH
>LFGLNPFC_02804 Inner membrane protein YbaN
MQRIILIIIGWLAVVLGTLGVVLPVLPTTPFILLAAWCFARSSPRFHAWLLYRSWFGSYL
RFWQKHHAMPRGAKPRAILLILITFTISLWFVQMPWVRIMLLVILACLLFYMWRIPVIDE
KQEKH
>LFGLNPFC_02805 Primosomal replication protein N''
MKTALLLEKLEGQLATLRQRCAPVAQFATLSARFNRHLFQTRATTLQACLDEAGDNLAAL
RHAVEQQQLPQVAWLAEHLAAQLEAIAREATAWSLREWDSAPPKIARWQRKRIQHQDFER
RLREMVAERRARLARATDLVEQQTLHREVEAYEARLARCRHALEKIENRLARLTR
>LFGLNPFC_02806 hypothetical protein
MSLENAPDDVKLAVDLIVLLEENQIPARTVLRALDIVKRDYEKKLTRDDEAEK
>LFGLNPFC_02807 Mechanosensitive channel MscK
MTMFQYYKRSRHFVFSAFIAFVFVLLCQNTAFARASSNGDLPTKADLQAQLDSLNKQKDL
SAQDKLVQQDLTDTLATLDKIDRVKEETVQLRQKVAEAPEKMRQATAALTALSDVDNDEE
TRKILSTLSLRQLETRVAQALDDLQNAQNDLASYNSQLVSLQTQPERVQNAMYNASQQLQ
QIRSRLDGTDVGETALRPSQKVLMQAQQALLNAEIDQQRKSLEGNTVLQDTLQKQRDYVT
ANSARLEHQLQLLQEAVNSKRLTLTEKTAQEAVSPDEAARIQANPLVKQELEINQQLSQR
LITATENGNQLMQQNIKVKNWLERALQSERNIKEQIAVLKGSLLLSRILYQQQQTLPSAD
ELENMTNRIADLRLEQFEVNQQRDALFQSDAFVSKLEEGHTNEVNSEVHDALLQVVDMRR
ELLDQLNKQLGNQLMMAINLQINQQQLMSVSKNLKSILTQQIFWVNSNRPMDWDWIKAFP
QTLKDEFKSMKITVNWEKAWPAVFIAFLAGLPLLLIAGLIHWRLGWLKAYQQKLASAVGS
LRNDSQLNTPKAILIDLIRALPVCLIILAVGLILLTMQLNISELLWSFSKKLAIFWLVFG
LCWKVLEKNGVAVRHFGMPEQQTSHWRRQIVRISLALLPIHFWSVVAELSPLHLMDDVLG
QAMIFFNLLLIAFLVWPMCRESWRDKESHTMRLVTITVLSIIPIALMVLTATGYFYTTLR

LSGRWIETVYLVIIWNLLYQTVLRGLSVAARRIAWRRALARRQNLVKEGAEGAEPPEEPT
IALEQVNQQTLRITMLLMFALFGVMFWAIWSDLITVFSYLDSITLWHYNATEAGAAVVKN
VTMGSLLFAIIASMVAWALIRNLPGLLEVLVLSRLNMRQGASYAITTILNYIIIAVGAMT
VFGSLGVSWDKLQWLAAALSVGLGFGLQEIFGNFVSGLIILFERPVRIGDTVTIGSFSGT
VSKIRIRATTITDFDRKEVIIPNKAFVTERLINWSLTDTTTRLVIRLGVAYGSDLEKVRK
VLLKAATEHPRVMHEPMPEVFFTAFGASTLDHELRLYVRELRDRSRTVDELNRTIDQLCR
ENDINIAFNQLEVHLHNEKGDEVTEVKRDYKGDDPTPAVG
>LFGLNPFC_02808 HTH-type transcriptional regulator AcrR
MARKTKQEAQETRQHILDVALRLFSQQGVSSTSLGEIAKAAGVTRGAIYWHFKDKSDLFS
EIWELSESNIGELELEYQAKFPGDPLSVLREILIHVLESTVTEERRRLLMEIIFHKCEFV
GEMAVVQQAQRNLCLESYDRIEQTLKHCIEAKMLPADLMTRRAAIIMRGYISGLMENWLF
APQSFDLKKEARDYVAILLEMYLLCPTLRNPATNE
>LFGLNPFC_02809 Multidrug efflux pump subunit AcrA
MNKNRGFTPLAVVLMLSGSLALTGCDDKQAQQGGQQMPAVGVVTVKTEPLQITTELPGRT
SAYRIAEVRPQVSGIILKRNFKEGSDIEAGVSLYQIDPATYQAAYDSAKGDLAKAQAAAN
IAQLTVNRYQKLLGTQYISKQEYDQALADAQQANAAVTAAKAAVETARINLAYTKVTSPI
SGRIGKSNVTEGALVQNGQATALATVQQLDPIYVDVTQSSNDFLRLKQELANGTLKQENG
KAKVSLITSDGIKFPQDGTLEFSDVTVDQTTGSITLRAIFPNPDHTLLPGMFVRARLEEG
LNPNAILVPQQGVTRTPRGDATVLVVGADDKVETRPIVASQAIGDKWLVTEGLKAGDRVV
ISGLQKVRPGVQVKAQEVTADNNQQAASGAQPEQSKS
>LFGLNPFC_02810 Multidrug efflux pump subunit AcrB
MPNFFIDRPIFAWVIAIIIMLAGGLAILKLPVAQYPTIAPPAVTISASYPGADAKTVQDT
VTQVIEQNMNGIDNLMYMSSNSDSTGTVQITLTFESGTDADIAQVQVQNKLQLAMPLLPQ
EVQQQGVSVEKSSSSFLMVVGVINTDGTMTQEDISDYVAANMKDAISRTSGVGDVQLFGS
QYAMRIWMNPNELNKFQLTPVDVITAIKAQNAQVAAGQLGGTPPVKGQQLNASIIAQTRL
TSTEEFGKILLKVNQDGSRVLLRDVAKIELGGENYDIIAEFNGQPASGLGIKLATGANAL
DTAAAIRAELAKMEPFFPSGLKIVYPYDTTPFVKISIHEVVKTLVEAIILVFLVMYLFLQ
NFRATLIPTIAVPVVLLGTFAVLAAFGFSINTLTMFGMVLAIGLLVDDAIVVVENVERVM
AEEGLPPKEATRKSMGQIQGALVGIAMVLSAVFVPMAFFGGSTGAIYRQFSITIVSAMAL
SVLVALILTPALCATMLKPIAKGDHGEGKKGFFGWFNRMFEKSTHHYTDSVGGILRSTGR
YLVLYLIIVVGMAYLFVRLPSSFLPDEDQGVFMTMVQLPAGATQERTQKVLNEVTHYYLT
KEKNNVESVFAVNGFGFAGRGQNTGIAFVSLKDWADRPGEENKVEAITMRATRAFSQIKD
AMVFAFNLPAIVELGTATGFDFELIDQAGLGHEKLTQARNQLLAEAAKHPDMLTSVRPNG
LEDTPQFKIDIDQEKAQALGVSINDINTTLGAAWGGSYVNDFIDRGRVKKVYVMSEAKYR
MLPDDIGDWYVRAADGQMVPFSAFSSSRWEYGSPRLERYNGLPSMEILGQAAPGKSTGEA
MELMEQLASKLPTGVGYDWTGMSYQERLSGNQAPSLYAISLIVVFLCLAALYESWSIPFS
VMLVVPLGVIGALLAATFRGLTNDVYFQVGLLTTIGLSAKNAILIVEFAKDLMDKEGKGL
IEATLDAVRMRLRPILMTSLAFILGVMPLVISTGAGSGAQNAVGTGVMGGMVTATVLAIF
FVPVFFVVVRRRFSRKNEDIEHSHTVDHH
>LFGLNPFC_02811 Hha toxicity modulator TomB
MDEYSPKRHDIAQLKFLCETLYHDCLANLEESNHGWVNDPTSAINLQLNELIEHIATFAL
NYKIKYNEDNKLIEQIDEYLDDTFMLFSSYGINMQDLQKWRKSGNRLFRCFVNATKENPA
SLSC
>LFGLNPFC_02812 Hemolysin expression-modulating protein Hha
MSEKPLTKTDYLMRLRRCQTIDTLERVIEKNKYELSDNELAVFYSAADHRLAELTMNKLY
DKIPSSVWKFIR
>LFGLNPFC_02813 Maltose O-acetyltransferase
MSTEKEKMIAGELYHSADGALSRDRLRARRLIHRYNHSLVEEHTLRQQILADLFGQVTEA
YIEPTFRCDYGYNIFLGNNFFANFDCVMLDVCPIRIGDNCMLAPGVHIYTATHPIDPVAR
NSGAELGKPVTIGNNVWIGGRAVINPGVTIGDNVVVASGAVVTKDVPDNVVVGGNPARII
KKL
>LFGLNPFC_02814 Inner membrane protein YlaC
MTEIQRLLTETIESLNTREKRDNKPRFSISFIRKHPGLFIGMYVAFFATLAVMLQSETLS
GSVWLLVVLFILLNGFFFFDVYPRYRYEDIDVLDFRVCYNGEWYNTRFVPAALVEAILNS
PRVADVHKEQLQKMIVRKGELSFYDIFTLARAESTS
>LFGLNPFC_02815 putative cyclic di-GMP phosphodiesterase PdeB
MRTRHLVGLISGVLILSVLLPVGLSIWLAHQQVETSFIEELNTYSSRVAIRANKVATQGK
DALQELERWQGAACSEAHLMEMRRVSYSYRYIQEVVYIDNNVPQCSSLEHESPPDTFPEP
GKISKDGYRVWLTSHNDLGIIRYMVAMGTAHYVVMIDPASFIDVIPYSSWQIDAAIIGNA
HNVVITSSDELAQGIITRLQKTPGEHIENNGIIYDILPLPEMNISIITWASTKMLQKGWH
RQVFIWLPLGLVIGLLAAMFVLRILRRIQSPHHRLQDAIENRDICVHYQPIVSLANGKIV
GAEALARWPQTDGSWLSPDSFIPLAQQTGLSEPLTLLIIRSVFEDMGDWLRQHPQQHISI
NLESTVLTSEKIPQLLREMINHYQVNPRQIALELTEREFADPKTSAPIISRYREAGHEIY
LDDFGTGYSSLSYLQDLDVDILKIDKSFVDALEYKNVTPHIIKMAKTLKLKMVAEGIETS

KQEEWLRQHGVHYGQGWLYSKALPKEDFLRWAEQHL
>LFGLNPFC_02816 putative protein YbaA
MKYVDGFVVAVPADKKDAYREMAAKAAPLFKEFGALRIVECWASDVPDGKVTDFRMAVKA
EENEEVVFSWIEYPSKEVRDAANQKMMSDPRMKEFGESMPFDGKRMIYGGFESIIDE
>LFGLNPFC_02817 DNA base-flipping protein
MEKEDSFPQRVWQIVAAIPEGYVTTYGDVAKLAGSPRAARQVGGVLKRLPEGSTLPWHRV
VNRHGTISLTGPDLQRQRQALLAEGVMVSGSGQIDLQLYRWNY
>LFGLNPFC_02818 putative lipoprotein YbaY
MKLVHMASGLAVAIALAACADKSADIQTPAPAANTSISATQQPAIQQPNVSGTVWIRQKV
ALPPDAVLTVTLSDASLADAPSKVLAQKAVRTEGKQSPFSFVLPFNPADVQPNARILLSA
AITVNDKLVFITDTVQPVINQGGTKADLTLVPVQQTAVPVQASGGATTTVPSTSPTQVNP
SSAVPAPTQY
>LFGLNPFC_02819 Acyl-CoA thioesterase 2
MSQALKNLLTLLNLEKIEEGLFRGQSEDLGLRQVFGGQVVGQALYAAKETVPDERLVHSF
HSYFLRPGDSKKPIIYDVETLRDGNSFSARRVSAIQNGKPIFYMTASFQAPEAGFEHQKT
MPSAPAPDGLPSETQIAQSLAHLLPPVLKDKFICDRPLEVRPVEFHNPLKGHVAEPHRQV
WIRANGSVPDDLRVHQYLLGYASDLNFLPVALQPHGIGFLEPGIQIATIDHSMWFHRPFN
LNEWLLYSVESTSASSARGFVRGEFYTQDGVLVASTVQEGVMRNHN
>LFGLNPFC_02820 Ammonia channel
MKIATIKTGLASLAMLPGLVMAAPAVADKADNAFMMICTALVLFMTVPGIALFYGGLIRG
KNVLSMLTQVTVTFALVCILWVVYGYSLAFGEGNNFFGNINGLMLKNIELTAVMGSIYQY
IHVAFQGSFACITVGLIVGALAERIRFSAVLIFVVVWLTLSYIPIAHMVWGGGLLASHGA
LDFAGGTVVHINAAIAGLVGAYLIGKRVGFGKEAFKPHNLPMVFTGTAILYIGWFGFNAG
SAGTANEIAALAFVNTVVATAAAILGWIFGEWALRGKPSLLGACSGAIAGLVGVTPACGY
IGVGGALIIGVVAGLAGLWGVTMLKRLLRVDDPCDVFGVHGVCGIVGCIMTGIFAASSLG
GVGFAEGVTMGHQLLVQLESIAITIVWSGVVAFIGYKLADLTVGLRVPEEQEREGLDVNS
HGENAYNA
>LFGLNPFC_02821 Nitrogen regulatory protein P-II 2
MKLVTVIIKPFKLEDVREALSSIGIQGLTVTEVKGFGRQKGHAELYRGAEYSVNFLPKVK
IDVAIADDQLDEVIDIVSKAAYTGKIGDGKIFVAELQRVIRIRTGEADEAAL
>LFGLNPFC_02822 Multidrug resistance-like ATP-binding protein MdlB
MRSFSQLWPTLKRLLAYGSPWRKPLGIAVLMMWVAAAAEVSGPLLISYFIDNMVAKNNLP
LKVGAGLAAAYVGLQLFAAGLHYAQSLLFNRAAVGVVQQLRTDVMDAALRQPLSEFDTQP
VGQVISRVTNDTEVIRDLYVTVVATVLRSAALVGAMLVAMFSLDWRMALVAIMIFPVVLV
VMVIYQRYSTPIVRRVRAYLADINDGFNEIINGMSVIQQFRQQARFGERMGEASRSHYMA
RMQTLRLDGFLLRPLLSLFSSLILCGLLMLFGFSASGTIEVGVLYAFISYLGRLNEPLIE
LTTQQAMLQQAVVAGERVFELMDGPRQQYGNDDRPLQSGTIDVDNVSFAYRDDNLVLKNI
NLSVPSRNFVALVGHTGSGKSTLASLLMGYYPLTEGEIRLDGRPLSTLSHSALRQGVAMV
QQDPVVLADTFLANVTLGRDISEERVWQALEIVQLAELARSMSDGIYTPLGEQGNNLSVG
QKQLLALARVLVETPQILILDEATASIDSGTEQAIQHALAAVREHTTLVVIAHRLSTIVD
ADTILVLHRGQAVEQGTHQQLLAAQGRYWQMYQLQLAGEELAASVREEESLSA
>LFGLNPFC_02823 putative multidrug resistance ABC transporter ATP-binding/permease protein YheI
MRLFAQLSWYFRREWRRYLGAVALLVIIAMLQLVPPKVVGIVVDGVTEQHFTTGQILMWI
ATMVLIAVVVYLLRYVWRVLLFGASYQLAVELREDYYRQLSRQHPEFYLRHRTGDLMARA
TNDVDRVVFAAGEGVLTLVDSLVMGCAVLIMMSTQISWQLTLFALLPMPVMAIMIKRNGD
ALHERFKLAQAAFSSLNDRTQESLTSIRMIKAFGLEDRQSALFAADAEDTGKKNMRVARI
DARFDPTIYIAIGMANLLAIGGGSWMVVQGSLTLGQLTSFIMYLGLMIWPMLALAWMFNI
VERGSAAYSRIRTMLAEAPVVIDGSDKVPEGRGELDVNIRQFTYPQTDHPALENVNFALK
PGQMVGICGPTGSGKSTLLSLIQRHFDVSEGDIRFHDIPLTKLQLDSWRSRLAVVSQTPF
LFSDTVANNIALGCPNAIQQEIEHVARLASVHDDILRLPQGYDTEVGERGVMLSGGQKQR
ISIARALLVNAEILILDDALSAVDGRTEHQILHNLRQWGQGRTVIISAHRLSALTEASEI
IVMQHGHIGQRGNHDVLVQQSGWYRDMYRYQQLEAALDDAPEIREEAIDA
>LFGLNPFC_02824 DNA-binding transcriptional activator DecR
MLDKIDRKLLALLQQDCTLSLQALAEAVNLTTTPCWKRLKRLEDDGILIGKVALLDPEKI
GLGLTAFVLIKTQHHSSEWYCRFVTVVTEMPEVLGFWRMAGEYDYLMRVQVADMKRYDEF
YKRLVNSVPGLSDVTSSFAMEQIKYTTSLPIE
>LFGLNPFC_02825 HMP-PP phosphatase
MARLAAFDMDGTLLMPDHHLGEKTLSTLARLRERDITLTFATGRHALEMQHILGALSLDA
YLITGNGTRVHSLEGELLHRDDLPADVAELVLYQQWDTRASMHIFNDDGWFTGKEIPALL
QAFVYSGFRYQIIDVKKMPLGSVTKICFCGDHDDLTRLQIQLYEALGERAHLCFSATDCL
EVLPVGCNKGAALTVLTQHLGLSLRDCMAFGDAMNDREMLGSVGSGFIMGNAMPQLRAEL
PHLPVIGHCRNQAVSHYLTHWLDYPHLPYSPE
>LFGLNPFC_02826 HTH-type transcriptional regulator SgrR

MRLLNRLNQYQRLWQPSAGKPQTVTVSELAERCFCSERHVRTLLRQAQEAGWLEWQAQSG
RGKRGQLRFLVTPESLRNAMMEQALETGKQQDVLELAQLAPGELRTLLQPFMGGQWQNDT
PTLRIPYYRPLEPLQPGFLPGRAEQHLAGQIFSGLTRFDNNTQRPIGDLAHHWETSTDGL
RWDFYLRSTLHWHNGDAVKASHLHQRLLMLLQLPALDQLFISVKRIEVTHPQCLTFFLHR
PDYWLAHRLASYCSHLAHPQFPLIGTGPFRLTQFTAELVRLESHDYYHLRHPLLKAVEYW
ITPPLFEKDMGTSCRHPVQITIGKPEELQRVSQVSSGISLGFCYLTLRKSLRLSLWQARK
VISIIHQSGLLQTLEVGENLITASHALLPGWTIPHWQVPDEVKLPKTLTLVYHLPIELHT
MAERLQATLAAEGCELTIIFHNAKNWDDTTLLAHADLMMGDRLIGEAPEYTLEQWLRCDP
LWPHVFDAPAYAHLQSTLDAVQVMPDEENRFNALKAVFSQLMADATLTPLFNYHYRISAP
PGVNGVRLTPRGWFEFTEAWLPAPSQ
>LFGLNPFC_02827 7-cyano-7-deazaguanine synthase
MKRAVVVFSGGQDSTTCLVQALQQYDEVHCVTFDYGQRHRAEIDVARELALKLGARAHKV
LDVTLLNELAVSSLTRDSIPVPDYEPEADGIPNTFVPGRNILFLTLAAIYAYQVKAEAVI
TGVCETDFSGYPDCRDEFVKALNHAVSLGMAKDIRFETPLMWIDKAETWALADYYGKLDL
VRNETLTCYNGIKGDGCGHCAACNLRANGLNHYLADKPTVMAAMKQKTGLK
>LFGLNPFC_02828 Long-chain acyl-CoA thioesterase FadM
MQTQIKVRGYHLDVYQHVNNARYLEFLEEARWHGLENSDSFHWMTAHNIAFVVVNININY
RRPAVLSDLLTITSQLQQLNGKSGILSQVITLEPEGQVVADALITFVCIDLKTQKALALE
GELREKLEQMVK
>LFGLNPFC_02829 putative protein
MKHGIKALLITLSLACAGMSHSALAAASVAKPTTVETKAEAPAAQSKAAVPAKASDEEGT
RVSINNASAEELARAMNGVGLKKAQAIVSYREEYGPFKTVEDLKQVPGMGNSLVERNLAV
LTL
>LFGLNPFC_02830 Peptidyl-prolyl cis-trans isomerase D
MMDSLRTAANSLVLKIIFGIIIVSFILTGVSGYLIGGGNNYAAKVNDQEISRGQFENAFN
SERNRMQQQLGDQYSELAANEGYMKTLRQQVLNRLIDEALLDQYARELKLGISDEQVKQA
IFATPAFQVDGKFDNSRYNGILNQMGMTADQYAQALRNQLTTQQLINGVAGTDFMLKGET
DELAALVAQQRVVREATIDVNALAAKQPVTEQEIASYYEQNKNNFMTPEQFRVSYIKLDA
ATMQQPVSDADIQSYYDQHQDQFTQPQRTRYSIIQTKTEDEAKAVLDELNKGGDFAALAK
EKSADIISARNGGDMGWLEDATIPDELKNAGLKEKGQLSGVIKSSVGFLIVRLDDIQPAK
VKSLDEVRDDIAAKVKHEKALDAYYALQQKVSDAASNDTESLAGAEQAAGVKATQTGWFS
KDNLPEELNFKPVADAIFNGGLVGENGAPGINSDIITVDGDRAFVLRISEHKPEAVKPLA
DVQEQVKALVQHNKAEQQAKVDAEKLLVDLKAGKGAEAMQAAGLKFGEPKTLSRSGRDPI
SQAAFALPLPAKDKPSYGMATDMQGNVVLLALDEVKQGSMPEDQKKAMVQGITQNNAQIV
FEALMSNLRKEAKIKIGDALEQQ
>LFGLNPFC_02831 DNA-binding protein HU-beta
MNKSQLIDKIAAGADISKAAAGRALDAIIASVTESLKEGDDVALVGFGTFAVKERAARTG
RNPQTGKEITIAAAKVPSFRAGKALKDAVN
>LFGLNPFC_02832 Lon protease
MNPERSERIEIPVLPLRDVVVYPHMVIPLFVGREKSIRCLEAAMDHDKKIMLVAQKEAST
DEPGVNDLFTVGTVASILQMLKLPDGTVKVLVEGLQRARISALSDNGEHFSAKAEYLESP
TIDEREQEVLVRTAISQFEGYIKLNKKIPPEVLTSLNSIDDPARLADTIAAHMPLKLADK
QSVLEMSDVNERLEYLMAMMESEIDLLQVEKRIRNRVKKQMEKSQREYYLNEQMKAIQKE
LGEMDDAPDENEALKRKIDAAKMPKEAKEKAEAELQKLKMMSPMSAEATVVRGYIDWMVQ
VPWNARSKVKKDLRQAQEILDTDHYGLERVKDRILEYLAVQSRVNKIKGPILCLVGPPGV
GKTSLGQSIAKATGRKYVRMALGGVRDEAEIRGHRRTYIGSMPGKLIQKMAKVGVKNPLF
LLDEIDKMSSDMRGDPASALLEVLDPEQNVAFSDHYLEVDYDLSDVMFVATSNSMNIPAP
LLDRMEVIRLSGYTEDEKLNIAKRHLLPKQIERNALKKGELTVDDSAIIGIIRYYTREAG
VRGLEREISKLCRKAVKQLLLDKSLKHIEINGDNLHDYLGVQRFDYGRADNENRVGQVTG
LAWTEVGGDLLTIETACVPGKGKLTYTGSLGEVMQESIQAALTVVRARAEKLGINPDFYE
KRDIHVHVPEGATPKDGPSAGIAMCTALVSCLTGNPVRADVAMTGEITLRGQVLPIGGLK
EKLLAAHRGGIKTVLIPFENKRDLEEIPDNVIADLDIHPVKRIEEVLTLALQNEPSGMQV
VTAK
>LFGLNPFC_02833 ATP-dependent Clp protease ATP-binding subunit ClpX
MTDKRKDGSGKLLYCSFCGKSQHEVRKLIAGPSVYICDECVDLCNDIIREEIKEVAPHRE
RSALPTPHEIRNHLDDYVIGQEQAKKVLAVAVYNHYKRLRNGDTSNGVELGKSNILLIGP
TGSGKTLLAETLARLLDVPFTMADATTLTEAGYVGEDVENIIQKLLQKCDYDVQKAQRGI
VYIDEIDKISRKSDNPSITRDVSGEGVQQALLKLIEGTVAAVPPQGGRKHPQQEFLQVDT
SKILFICGGAFAGLDKVISHRVETGSGIGFGATVKAKSDKASEGELLAQVEPEDLIKFGL
IPEFIGRLPVVATLNELSEEALIQILKEPKNALTKQYQALFNLEGVDLEFRDEALDAIAK
KAMARKTGARGLRSIVEAALLDTMYDLPSMEDVEKVVIDESVIDGQSKPLLIYGKPEAQQ
ASGE
>LFGLNPFC_02834 ATP-dependent Clp protease proteolytic subunit
MSYSGERDNFAPHMALVPMVIEQTSRGERSFDIYSRLLKERVIFLTGQVEDHMANLIVAQ

MLFLEAENPEKDIYLYINSPGGVITAGMSIYDTMQFIKPDVSTICMGQAASMGAFLLTAG
AKGKRFCLPNSRVMIHQPLGGYQGQATDIEIHAREILKVKGRMNELMALHTGQSLEQIER
DTERDRFLSAPEAVEYGLVDSILTHRN
>LFGLNPFC_02835 Trigger factor
MQVSVETTQGLGRRVTITIAADSIETAVKSELVNVAKKVRIDGFRKGKVPMNIVAQRYGA
SVRQDVLGDLMSRNFIDAIIKEKINPAGAPTYVPGEYKLGEDFTYSVEFEVYPEVELQGL
EAIEVEKPIVEVTDADVDGMLDTLRKQQATWKEKDGAVEAEDRVTIDFTGSVDGEEFEGG
KASDFVLAMGQGRMIPGFEDGIKGHKAGEEFTIDVTFPEEYHAENLKGKAAKFAINLKKV
EERELPELTAEFIKRFGVEDGSVEGLRAEVRKNMERELKSAIRNRVKSQAIEGLVKANDI
DVPAALIDSEIDVLRRQAAQRFGGNEKQALELPRELFEEQAKRRVVVGLLLGEVIRTNEL
KADEERVKGLIEEMASAYEDPKEVIEFYSKNKELMDNMRNVALEEQAVEAVLAKAKVTEK
ETTFNELMN
>LFGLNPFC_02836 DNA-binding transcriptional regulator BolA
MIRERIEEKLRAAFQPVFLEVVDESYRHNVPAGSESHFKVVLVSDRFTGERFLNRHRMIY
STLAEELSTTVHALALHTYTIKEWEGLQDTVFASPPCRGAGSIA
>LFGLNPFC_02837 hypothetical protein
MFKKILFPLVALFMLAGCAKPPTTIEVSPTITLPQQDPSLMGVTVSINGADQRTDQALAK
VTRDNQIVTLTASRDLRFLLQEVLEKQMTARGYMVGPNGPVNLQIIVSQLYADVSQGNVR
YNIATKADIAIIATAQNGNKMTKNYRASYNVEGAFQASNKNIADAVNSVLTDTIADMSQD
TSIHEFIKQNAR
>LFGLNPFC_02838 Anhydromuropeptide permease
MSSQYLRIFQQPRSAILLILGFASGLPLALTSGTLQAWMTVENIDLKTIGFFSLVGQAYV
FKFLWSPLMDRYTPPFFGRRRGWLLATQILLLVAIAAMGFLEPGTQLRWMAALAVVIAFC
SASQDIVFDAWKTDVLPAEERGAGAAISVLGYRLGMLVSGGLALWLADKWLGWQGMYWLM
AALLIPCIIATLLAPEPTDTIPVPKTLEQAVVAPLRDFFGRNNAWLILLLIVLYKLGDAF
AMSLTTTFLIRGVGFDAGEVGVVNKTLGLLATIVGALYGGILMQRLSLFRALLIFGILQG
ASNAGYWLLSITDKHLYSMGAAVFFENLCGGMGTSAFVALLMTLCNKSFSATQFALLSAL
SAVGRVYVGPVAGWFVEAHGWSTFYLFSVAAAVPGLILLLVCRQTLEYTRVNGNFISRTE
YPAGYAFAMWTLAAGISLLAVWLLLLTMDALDLTHFSFLPALLEVGVLVALSGVVLGGLL
DYLALRKTHLT
>LFGLNPFC_02839 Cytochrome bo(3) ubiquinol oxidase subunit 2
MRLRKYNKSLGWLSLFAGTVLLSGCNSALLDPKGQIGLEQRSLILTAFGLMLIVVIPAIL
MAVGFAWKYRASNKDAKYSPNWSHSNKVEAVVWTVPILIIIFLAVLTWKTTHALEPSKPL
AHDEKPITIEVVSMDWKWFFIYPEQGIAAVNEIAFPANTPVYFKVTSNSVMNSFFIPRLG
SQIYAMAGMQTRLHLIANEPGTYDGISASYSGPGFSGMKFKAIATPDRAAFDQWVAKAKQ
SPNTMSDMATFEKLAAPSEYNQVEYFSNVKPDLFADVINKFMAHGKSMDMTQPEGEHSAH
EGMEGMDMSHAESAH
>LFGLNPFC_02840 Cytochrome bo(3) ubiquinol oxidase subunit 1
MFGKLSLDAVPFHEPIVMVTIAGIILGGLALVGLITYFGKWTYLWKEWLTSVDHKRLGIM
YIIVAIVMLLRGFADAIMMRSQQALASAGEAGFLPPHHYDQIFTAHGVIMIFFVAMPFVI
GLMNLVVPLQIGARDVAFPFLNNLSFWFTVVGVILVNVSLGVGEFAQTGWLAYPPLSGIE
YSPGVGVDYWIWSLQLSGIGTTLTGINFFVTILKMRAPGMTMFKMPVFTWASLCANVLII
ASFPILTVTVALLTLDRYLGTHFFTNDMGGNMMMYINLIWAWGHPEVYILILPVFGVFSE
IAATFSRKRLFGYTSLVWATVCITVLSFIVWLHHFFTMGAGANVNAFFGITTMIIAIPTG
VKIFNWLFTMYQGRIVFHSAMLWTIGFIVTFSVGGMTGVLLAVPGADFVLHNSLFLIAHF
HNVIIGGVVFGCFAGMTYWWPKAFGFKLNETWGKRAFWFWIIGFFVAFMPLYALGFMGMT
RRLSQQIDPQFHTMLMIAASGAVLIALGILCLVIQMYVSIRDRDQNRDLTGDPWGGRTLE
WATSSPPPFYNFAVVPHVHERDAFWEMKEKGEAYKKPDHYEEIHMPKNSGAGIVIAAFST
IFGFAMIWHIWWLAIVGFAGMIITWIVKSFDEDVDYYVPVAEIEKLENQHFDEITKAGLK
NGN
>LFGLNPFC_02841 Cytochrome bo(3) ubiquinol oxidase subunit 3
MATDTLTHATAHAHEHGHHDAGGTKIFGFWIYLMSDCILFSILFATYAVLVNGTAGGPTG
KDIFELPFVLVETFLLLFSSITYGMAAIAMYKNNKSQVVSWLALTWLFGAGFIGMEIYEF
HHLIVNGMGPDRSGFLSAFFALVGTHGLHVTSGLIWMAVLMVQIARRGLTSTNRTRIMCL
SLFWHFLDVVWICVFTVVYLMGAM
>LFGLNPFC_02842 Cytochrome bo(3) ubiquinol oxidase subunit 4
MSHSNASSGASHGSVKTYMTGFILSIILTVIPFWMVMTGAASPAVILGTILAMAVVQILV
HLVCFLHMNTKSDEGWNMTAFVFTVLIIAILVVGSIWIMWNLNYNMMMH
>LFGLNPFC_02843 Protoheme IX farnesyltransferase
MIFKQYLQVTKPGIIFGNLISVIGGFLLASKGSIDYPLFIYTLVGVSLVVASGCVFNNYI
DRDIDRKMERTKNRVLVKGLISPAVSLVYATLLGIAGFMLLWFGANPLACWLGVMGFVVY
VGVYSLYMKRHSVYGTLIGSLSGAAPPVIGYCAVTGEFDSGAAILLAIFSLWQMPHSYAI
AIFRFKDYQAANIPVLPVVKGISVAKNHITLYIIAFAVATLMLSLGGYAGYKYLVVAAAV
SVWWLGMALRGYKVADDRIWARKLFGFSIIAITALSVMMSVDFMVPDSHTLLAAVW

>LFGLNPFC_02844 Inner membrane transport protein YajR
MNDYKMTPGERRATWGLGTVFSLRMLGMFMVLPVLTTYGMALQGASEALIGIAIGIYGLT
QAVFQIPFGLLSDRIGRKPLIVGGLAVFAAGSVIAALSDSIWGIILGRALQGSGAIAAAV
MALLSDLTREQNRTKAMAFIGVSFGITFAIAMVLGPIITHKLGLHVLFWMIAILATTGIA
LTIWVVPNSSTHVLNRESGMVKGSFSKVLAEPRLLKLNFGIMCLHILLMSTFVALPGQLA
DAGFPAAEHWKVYLATMLIAFGSVVPFIIYAEVKRKMKQVFVFCVGLIVVAEIVLWNAQT
QFWQLVVGVQLFFVAFNLMEALLPSLISKESPAGYKGTAMGVYSTSQFLGVAIGGSLGGW
IDGMFDGQGVFLAGAMLAAVWLAVASTMKEPPYVSSLRIEIPANIAANEALKVRLLETEG
IKEVLIAEEEHSAYVKIDSKVTNRFEVEQAIRQA
>LFGLNPFC_02845 hypothetical protein
MPSFDIVSEVDLQEARNAVDNASREVESRFDFRNVEASFELNDASKTIKVLSESDFQVNQ
LLDILRAKLLKRGIEGSSLDVPENIVHSGKTWFVEAKLKQGIESATQKKIVKMIKDSKLK
VQAQIQGDEIRVTGKSRDDLQAVMAMVRGGDLGQPFQFKNFRD
>LFGLNPFC_02846 2-dehydropantoate 2-reductase
MKITVLGCGALGQLWLTALCKQGHEVQGWLRVPQPYCSVNLVETDGSIFNESLTANDPDF
LATSDLLLVTLKAWQVSDAVKSLASTLPVTTPILLIHNGMGTIEELQNIQQPLLMGTTTH
AARRDGNVIIHVANGITHIGPARQQDGDYSYLADILQTVLPDVAWHNNIRAELWRKLAVN
CVINPLTATWNCPNGELRHHPQEIMQICEEVAAVIEREGHHTSAEDLRDYVMQVIDATAE
NISSMLQDIRALRHTEIDYINGFLLRRARAHGIAVPENTRLFEMVKRKESEYERIGTGLP
RPW
>LFGLNPFC_02847 Protein/nucleic acid deglycase 3
MSASALVCLAPGSEETEAVTTIDLLVRGGIKVTTASVASDGNLAITCSRGVKLLADAPLV
EVADGEYDVIVLPGGIKGAECFRDSTLLVETVKQFHRSGRIVAAICAAPATVLVPHDIFP
IGNMTGFPTLKDKIPAEQWQDKRVVWDARVKLLTSQGPGTAIDFGLKIIDLLVGREKAHE
VASQLVMAAGIYNYYE
>LFGLNPFC_02848 tRNA sulfurtransferase
MKFIIKLFPEITIKSQSVRLRFIKILTGNIRNVLKHYDETLAVVRHWDNIEVRAKDENQR
LAIRDALTRIPGIHHILEVEDVPFTDMHDIFEKALVQYRDQLEGKTFCVRVKRRGKHDFS
SIDVERYVGGGLNQHIESARVKLTNPEVTVHLEVEDDRLLLIKGRYEGIGGFPIGTQEDV
LSLISGGFDSGVSSYMLMRRGCRVHYCFFNLGGAAHEIGVRQVAHYLWNRFGSSHRVRFV
AINFEPVVGEILEKIDDGQMGVILKRMMVRAASKVAERYGVQALVTGEALGQVSSQTLTN
LRLIDNVSDTLILRPLISYDKEHIINLARQIGTEDFARTMPEYCGVISKSPTVKAVKSKI
EAEEEKFDFSILDKVVEEANNVDIREIAQQTEQEVVEVETVNGFGPNDVILDIRSIDEQE
DKPLKVEGIDVVSLPFYKLSTKFGDLDQSKTWLLWCERGVMSRLQALYLREQGFNNVKVY
RP
>LFGLNPFC_02849 Exodeoxyribonuclease 7 small subunit
MPKKNEAPASFEKALSELEQIVTRLESGDLPLEEALNEFERGVQLARQGQAKLQQAEQRV
QILLSDNEDASLTPFTPDNE
>LFGLNPFC_02850 Farnesyl diphosphate synthase
MDFPQQLEACVKQANQALSRFIAPLPFQNTPVVETMQYGALLGGKRLRPFLVYATGHMFG
VSTNTLDAPAAAVECIHAYSLIHDDLPAMDDDDLRRGLPTCHVKFGEANAILAGDALQTL
AFSILSDADMPEVSDRDRISMISELASASGIAGMCGGQALDLDAEGKRVPLDALERIHRH
KTGALIRAAVRLGALSAGDKGRRALPVLDKYAESIGLAFQVQDDILDVVGDTATLGKRQG
ADQQLGKSTYPALLGLEQARKKARDLIDDARQSLKQLAEQSLDTSALEALADYIIQRNK
>LFGLNPFC_02851 1-deoxy-D-xylulose-5-phosphate synthase
MSFDIAKYPTLALVDSTQELRLLPKESLPKLCDELRRYLLDSVSRSSGHFASGLGTVELT
VALHYVYNTPFDQLIWDVGHQAYPHKILTGRRDKIGTIRQKGGLHPFPWRGESEYDVLSV
GHSSTSISAGIGIAVAAEKEGKNRRTVCVIGDGAITAGMAFEAMNHAGDIRPDMLVVLND
NEMSISENVGALNNHLAQLLSGKLYSSLREGGKKVFSGVPPIKELLKRTEEHIKGMVVPG
TLFEELGFNYIGPVDGHDVLGLITTLKNMRDLKGPQFLHIMTKKGRGYEPAEKDPITFHA
VPKFDPSSGCLPKSSGGLPSYSKIFGDWLCETAAKDNKLMAITPAMREGSGMVEFSRKFP
DRYFDVAIAEQHAVTFAAGLAIGGYKPIVAIYSTFLQRAYDQVLHDVAIQKLPVLFAIDR
AGIVGADGQTHQGAFDLSYLRCIPEMVIMTPSDENECRQMLYTGYHYNDGPSAVRYPRGN
AVGVELTPLEKLPIGKGIVKRRGEKLAILNFGTLMPEAAKVAESLNATLVDMRFVKPLDE
ALILEMAASHEALVTVEENAIMGGAGSGVNEVLMAHRKPVPVLNIGLPDFFIPQGTQEEM
RAELGLDAAGMEAKIKAWLA
>LFGLNPFC_02852 1-deoxyxylulose-5-phosphate synthase YajO
MQYNPLGKTDLRVSRLCLGCMTFGEPDRGNHAWTLPEESSRPIIKRALEGGINFFDTANS
YSDGSSEEIVGRALRDFARREDVVVATKVFHRVGDLPEGLSRAQILRSIDDSLRRLGMDY
VDILQIHRWDYNTPIEETLEALNDVVKAGKARYIGASSMHASQFAQALELQKQHGWAQFV
SMQDHYNLIYREEEREMLPLCYQEGVAVIPWSPLARGRLTRPWGETTARLVSDEVGKNLY
QESNENDAQIVERLTGVSEELGATRAQVALAWLLSKPGIAAPIIGTSREEQLDELLNAVD
ITLKPEQIAELETPYKPHPVVGFK
>LFGLNPFC_02853 Phosphatidylglycerophosphatase A

MTILPRHKDVAKSRLKMSNPWHLLAVGFGSGLSPIVPGTMGSLAAIPFWYLMTFLPWQLY
SLVVMLGICIGVYLCHQTAKDMGVHDHGSIVWDEFIGMWITLMALPTNDWQWVTAGFVIF
RILDMWKPWPIRWFDRNVHGGMGIMIDDIVAGVISAGILYFIGHHWPLGILS
>LFGLNPFC_02854 Thiamine-monophosphate kinase
MACGEFSLIARYFDRVRSSRLDVELGIGDDCALLNIPEKQTLAISTDTLVAGNHFLPDID
PADLAYKALAVNLSDLAAMGADPAWLTLALTLPDVDEAWLESFSDSLFDLLNYYDMQLIG
GDTTRGPLSMTLGIHGFVPMGRALTRSGAKPGDWIYVTGTPGDSAAGLAILQNRLQVADA
KDADYLIKRHLRPSPRILQGQALRDLANSAIDLSDGLISDLGHIVKASDCGARIDLALLP
FSDALSRHVEPEQALRWALSGGEDYELCFTVPELNRGALDVALGHLGVPFTCIGQMTADI
EGLCFIRDGEPVTLDWKGYDHFATP
>LFGLNPFC_02855 Transcription antitermination protein NusB
MKPAARRRARECAVQALYSWQLSQNDIADVEYQFLAEQDVKDVDVLYFRELLAGVATNTA
YLDGLMKPYLSRLLEELGQVEKAVLRIALYELSKRSDVPYKVAINEAIELAKSFGAEDSH
KFVNGVLDKAAPVIRPNKK
>LFGLNPFC_02856 6,7-dimethyl-8-ribityllumazine synthase
MNIIEANVATPDARVAITIARFNNFINDSLLEGAIDALKRIGQVKDENITIVWVPGAYEL
PLAAGALAKTGKYDAVIALGTVIRGGTAHFEYVAGGASNGLAHVAQDSEIPVAFGVLTTE
SIEQAIERAGTKAGNKGAEAALTALEMINVLKAIKA
>LFGLNPFC_02857 Riboflavin biosynthesis protein RibD
MQDEYYMARALKLAQRGRFTTHPNPNVGCVIVKDGEIVGEGYHQRAGEPHAEVQALRMAG
EKAKGATAYVTLEPCSHHGRTPPCCDALIAAGVARVVAAMQDPNPQVAGRGLYRLQQAGI
DVSHGLMMSEAEQLNKGFLKRMRTGFPYIQLKLGASLDGRTAMASGESQWITSPQARRDV
QRLRAQSHAILTSSATVLADDPALTVRWSELDEQTQGLYPQQNLRQPVRIVIDSQNRVTP
EHRIVQQPGETWFARTQEDSREWPETVRTLLIPEHKGHLDLVVLMMQLGKQQINSIWVEA
GPTLAGALLQAGLVDELIVYIAPKLLGNDARGLCTLPGLEKLADAPQFKFKEIRHVGPDV
CLHLVGA
>LFGLNPFC_02858 Transcriptional repressor NrdR
MHCPFCFAVDTKVIDSRLVGEGSSVRRRRQCLVCNERFTTFEVAELVMPRVVKSNDVREP
FNEEKLRSGMLRALEKRPVSSDDVEMAINHIKSQLRATGEREVPSKMIGNLVMEQLKKLD
KVAYIRFASVYRSFEDIKEFGEEIARLED
>LFGLNPFC_02859 putative lipoprotein YajI
MNTNVFRLLLLGSLFSLSACVQQSEVRQMKHSVSTLNQEMTQLNKETVKITQQNRLNAKS
SSGVYLLPGAKTPARLESQIGTLRMSLVNITPDTDGTTLTLRIQGESNDPLPAFSGTVEY
GQIQGTIDNFQEINVQNQLINAPASVLAPSDVDIPLQLKGMSVDQLGFVRIHDIQPVMQ
>LFGLNPFC_02860 Nucleoside-specific channel-forming protein Tsx
MKKTLLAAGAVLALSSSFTVNAAENDKPQYLSDWWHQSVNVVGSYHTRFGPQLRNDTYLE
YEAFAKKDWFDFYGYADAPVFFGGNSDAKGIWNHGSPLFMEIEPRFSIDKLTNTDLSFGP
FKEWYFANNYIYDMGRNKDGRQSTWYMGLGTDIDTGLPMSLSMNVYAKYQWQNYGAANEN
EWDGYRFKVKYFVPITDLWGGQLSYIGFTNFDWGSDLGDDSGYANNGIKTRTNNSIASSH
ILALNYDHWHYSVVARYWHNGGQWNDDAELNFGNGNFNVRSTGWGGYLVVGYNF
>LFGLNPFC_02861 hypothetical protein
MAIIPKNYARLESDYREKALKIYPWVCGRCSREFVYSNLRELTVHHIDHDHTNNPEDGSN
WELLCLYCHDHEHSKYTEADQYGTTVIAGEDAQKDVGEAKYNPFADLKAMMNKKK
>LFGLNPFC_02862 Protein translocase subunit SecF
MAQEYTVEQLNHGRKVYDFMRWDYWAFGISGLLLIAAIVIMGVRGFNWGLDFTGGTVIEI
TLEKPAEIDVMRDALQKAGFEEPMLQNFGSSHDIMVRMPPAEGETGGQVLGSQVLKVINE
STNQNAAVKRIEFVGPSVGADLAQTGAMALMAALLSILVYVGFRFEWRLAAGVVIALAHD
VIITLGILSLFHIEIDLTIVASLMSVIGYSLNDSIVVSDRIRENFRKIRRGTPYEIFNVS
LTQTLHRTLITSGTTLMVILMLYLFGGPVLEGFSLTMLIGVSIGTASSIYVASALALKLG
MKREHMLQQKVEKEGADQPSILP
>LFGLNPFC_02863 Protein translocase subunit SecD
MLIVVIVIGLLYALPNLFGEDPAVQITGARGVAASEQTLIQVQKTLQEEKITAKSVALEE
GAILARFDSTDTQLRAREALMGVMGDKYVVALNLAPATPRWLAAIHAEPMKLGLDLRGGV
HFLMEVDMDTALGKLQEQNIDSLRSDLREKGIPYTTVRKENNYGLSITFRDAKARDEAIA
YLSKRHPDLVISSQGSNQLRAVMSDARLSEAREYAVQQNINILRNRVNQLGVAEPVVQRQ
GADRIVVELPGIQDTARAKEILGATATLEFRLVNTNVDQAAAASGRVPGDSEVKQTREGQ
PVVLYKRVILTGDHITDSTSSQDEYNQPQVNISLDSAGGNIMSNFTKDNIGKPMATLFVE
YKDSGKKDANGRAVLVKQEEVINIANIQSRLGNSFRITGINNPSEARQLSLLLRAGALIA
PIQIVEERTIGPTLGMQNIEQGLEACLAGLLVSILFMIIFYKKFGLIATSALIANLILIV
GIMSLLPGATLSMPGIAGIVLTLAVAVDANVLINERIKEELSNGRTVQQAIDEGYRGAFS
SIFDANITTLIKVIILYAVGTGAIKGFAITTGIGVATSMFTAIVGTRAIVNLLYGGKRVK
KLSI
>LFGLNPFC_02864 Sec translocon accessory complex subunit YajC
MSFFISDAVAATGAPAQGSPMSLILMLVVFGLIFYFMILRPQQKRTKEHKKLMDSIAKGD

EVLTNGGLVGRVTKVAENGYIAIALNDTTEVVIKRDFVAAVLPKGTMKAL
>LFGLNPFC_02865 Queuine tRNA-ribosyltransferase
MKFELDTTDGRARRGRLVFDRGVVETPCFMPVGTYGTVKGMTPEEVEATGAQIILGNTFH
LWLRPGQEIMKLHGDLHDFMQWKGPILTDSGGFQVFSLGDIRKITEQGVHFRNPINGDPI
FLDPEKSMEIQYDLGSDIVMIFDECTPYPADWDYAKRSMEMSLRWAKRSRERFDSLGNKN
ALFGIIQGSVYEDLRDISVKGLVDIGFDGYAVGGLAVGEPKADMHRILEHVCPQIPADKP
RYLMGVGKPEDLVEGVRRGIDMFDCVMPTRNARNGHLFVTDGVVKIRNAKYKSDTGPLDP
ECDCYTCRNYSRAYLHHLDRCNEILGARLNTIHNLRYYQRLMAGLRKAIEEGKLESFVTD
FYQRQGREVPPLNVD
>LFGLNPFC_02866 S-adenosylmethionine:tRNA ribosyltransferase-isomerase
MRVTDFSFELPESLIAHYPMPERSSCRLLSLDGPTGALTHGTFTDLLDKLNPGDLLVFNN
TRVIPARLFGRKASGGKIEVLVERMLDEKRILAHIRASKAPKPGAELLLGDDESINATMT
ARHGALFEVEFNDDRSVLDILNSIGHIPLPPYIDRPDEDADRELYQTVYSEKPGAVAAPT
AGLHFDEPLLEKLRAKGVEMAFVTLHVGAGTFQPVRVDTIEDHIMHSEYAEVPQDVVDAV
LAAKARGNRVIAVGTTSVRSLESAAQAAKNDLIEPFFDDTQIFIYPGFQYKVVDALVTNF
HLPESTLIMLVSAFAGYQHTMNAYKAAVEEKYRFFSYGDAMFITYNPQAINERVGE
>LFGLNPFC_02867 Acyl carrier protein phosphodiesterase
MNFLAHLHLAHLAESSLSGNLLADFVRGNPEESFPPDVVAGIHMHRRIDVLTDNLPEVRE
AREWFRNETRRVAPITLDVMWDHFLSRHWSQLSPDFPLQEFTCYAREQVMTILPDSPPRF
INLNNYLWSEQWLVRYRDMDFIQSVLNGMASRRPRLDALRDSWYDLDAHYDALETRFWQF
YPRMMEQASRKAL
>LFGLNPFC_02868 Maltodextrin glucosidase
MMLNAWHLPVPPFVKQSKDQLLITLWLTGEDPPQRIMLRTEHDNEEMSVPMHKQRSQPQP
GVTAWRAAIELSCGQPRRRYSFKLLWHDRQRWFTPQGFSRMPPARLEQFAIDVPDIGPQW
AADQIFYQIFPDRFARNLPREAEQDHVYYHHAAGGQEIILRDWDEPVTAQAGGSTFYGGDL
DGISEKLPYLKKLGVTALYLNPVFKAPSVHKYDTEDYRHVDPQFGGDGALLRLRHNTQQL
GMRLVLDGVFNHSGDSHAWFDRHNRGTGGACHNPESPWRDWYSFSDDGTALDWLGYASLP
KLDYQSESLVNEIYRGEDSIVRHWLKAPWNMDGWRLDVVHMLGEAGGARNNLQHVAGITE
AAKETQPDAYIVGEHFGDARQWLQADVEDAAMNYRGFTFPLWGFLANTDISYDPQQIDAQ
TCMVWMDNYRAGLSHQQQLRMFNQLDSHDTARFKTLLGRDVARLPLAVVWLFTWPGVPCI
YYGDEVGLDGKNDPFCRKPFPWQVEKQDSALFALYQRMIALRKKSQALRRGGCQVLYAED
NVVVFVRVLNQQRVLVAINRGEACEVVLPASPLLNVAQWQRKEGHGQLTDGILALPAISA
TVWMN
>LFGLNPFC_02869 Proline-specific permease ProY
MESKNKLKRGLSTRHIRFMALGSAIGTGLFYGSADAIKMAGPSVLLAYIIGGIAAYIIMR
ALGEMSVHNPAASSFSRYAQENLGPLAGYITGWTYCFEILIVAIADVTAFGIYMGVWFPT
VPHWIWVLSVVLIICAVNLMSVKVFGELEFWFSFFKVATIIIMIVAGFGIIIWGIGNGGQ
PTGIHNLWSNGGFFSNGWLGMVMSLQMVMFAYGGIEIIGITAGEAKDPEKSIPRAINSVP
MRILVFYVGTLFVIMSIYPWNQVGTAGSPFVLTFQHMGITFAASILNFVVLTASLSAINS
DVFGVGRMLHGMAEQGSAPKIFSKTSRRGIPWVTVLVMTTALLFAVYLNYIMPENVFLVI
ASLATFATVWVWIMILLSQIAFRRRLPPEEVKALKFKVPGGVATTIGGLIFLLFIIGLIG
YHPDTRISLYVGFAWIVVLLIGWMFKRRHDRQLAENQ
>LFGLNPFC_02870 Branched-chain amino acid transport system 2 carrier protein
MTHQLRSRDIIALGFMTFALFVGAGNIIFPPMVGLQAGEHVWTAAFGFLITAVGLPVLTV
VALAKVGGGVDSLSTPIGKVAGVLLATVCYLAVGPLFATPRTATVSFEVGIAPLTGDSAL
PLFIYSLVYFAIVILVSLYPGKLLDTVGNFLAPLKIIALVILSVAAIIWPAGSISTATEA
YQNAAFSNGFVNGYLTMDTLGAMVFGIVIVNAARSRGVTEARLLTRYTVWAGLMAGVGLT
LLYLALFRLGSDSASLVDQSANGAAILHAYVQHTFGGGGSFLLAALIFIACLVTAVGLTC
ACAEFFAQYVPLSYRTLVFILGGFSMVVSNLGLSQLIQISVPVLTAIYPPCIALVVLSFT
RSWWHNSSRVIAPPMFISLLFGILDGIKASAFSDILPSWAQRLPLAEQGLAWLMPTVVMV
VLAIIWDRAAGRQVTSSAH
>LFGLNPFC_02871 Phosphate regulon sensor protein PhoR
MLERLSWKRLVLELLLCCLPAFILGAFFGYLPWFLLASVTGLLIWHFWNLLRLSWWLWVD
RSMTPPPGRGSWEPLLYGLHQMQLRNKKRRRELGNLIKRFRSGAESLPDAVVLTTEEGGI
FWCNGLAQQILGLRWPEDNGQNILNLLRYPEFTQYLKTRDFSRPLNLVLNTGRHLEIRVM
PYTHKQLLMVARDVTQMHQLEGARRNFFANVSHELRTPLTVLQGYLEMMDEQPLEGAVRE
KALHTMREQTQRMEGLVKQLLTLSKIEAAPTHLLNEKVDVPMMLRVVEREAQTLSQKKQT
FTFEIDNGLKVSGNEDQLRSAISNLVYNAVNHTPEGTHITVRWQRVPHGAEFSVEDNGPG
IAPEHIPRLTERFYRVDKARSRQTGGSGLGLAIVKHAVNHHESRLNIESTVGKGTRFSFV
IPERLIAKNSD
>LFGLNPFC_02872 Phosphate regulon transcriptional regulatory protein PhoB
MARRILVVEDEAPIREMVCFVLEQNGFQPVEAEDYDSAVNQLNEPWPDLILLDWMLPGGS
GIQFIKHLKRESMTRDIPVVMLTARGEEEDRVRGLETGADDYITKPFSPKELVARIKAVM
RRISPMAVEEVIEMQGLSLDPTSHRVMAGEEPLEMGPTEFKLLHFFMTHPERVYSREQLL

NHVWGTNVYVEDRTVDVHIRRLRKALEPGGHDRMVQTVRGTGYRFSTRF
>LFGLNPFC_02873 Nuclease SbcCD subunit D
MRILHTSDWHLGQNFYSKSREAEHQAFLDWLLETAQAHQVDAIIVAGDVFDTGSPPSYAR
TLYNRFVVNLQQTGCHLVVLAGNHDSVATLNESRDIMAFLNTTVVASAGHAPQILPRRDG
TPGAVLCPIPFLRPRDIITSQAGLNGIEKQQHLLAAITDYYQQHYADACKLRGAQPLPII
TTGHLTTVGASKSDAVRDIYIGTLDAFPAQNFPPADYIALGHIHRAQIIGGMEHVRYCGS
PIPLSFDECGKSKYVHLVTFSNGKLESVENLNVPVTQPMAVLKGDLASITAQLEQWRDIS
QEPPVWLDIEITTDEYLHDIQRKIQALTESLPVEVLLVRRSREQRERVLASQQRETLSEL
SVEEVFNRRLALEELDESQQQRLQHLFATTLHSLAGEHEA
>LFGLNPFC_02874 Nuclease SbcCD subunit C
MKILSLRLKNLNSLKGEWKIDFTREPFASNGLFAITGPTGAGKTTLLDAICLALYHETPR
LSNVSQSQNDLMTRDTAECLAEVEFEVKGEAYRAFWSQNRARNQPDGNLQVPRVELARCA
DGKILADKVKDKLELTATLTGLDYGRFTRSMLLSQGQFAAFLNAKPKERAELLEELTGTE
IYGQISAMVFEQHKSARTELEKLQAQASGVALLTPEQVQSLTASLQVLTDEEKQLITAQQ
QEQQSLNWLTRLDELQQEGSRRQQALQQALAEEEKAQPQLAALSLAQPARNLRPHWERIA
EYSTALAHTRQQIEEVNTRLQSTMALRASIRHHAAKQSAELQQQQQSLNAWLQEHDRLRQ
WNNELAGWRAQFSQQTSDREHLRQWQQQLTHAEQKLNALAAITLTLTADEVASALAQHAE
QRPLRQRLVALHGQIVPQQKRLAQLQVAIQNVTLEQTQRNAALNEMRHRYKEKTQQLADV
KTICEQETRIKTLEAQRAQLQAGQPCPLCGSTSHPAVEAYQALEPGVNQARLLTLEKEVK
KLGEEGATLRGQLDALTKQLQRDENEAQSLRQDEQALTQQWQAVTASLNITLQPQDDIQP
WLDAQDEHERQLRLLSQRHELQGQIAAHNQQIIQYQQQIEQRQQQLLTALAGYALTLPQE
DEEESWLATRQQEAQSWQHRQNELTALQNRIQQLTPILETLPQSDELPHSEETVALENWR
QVHEQCLALHSQQQTLQQQDVLAAQSLQKAQAQFDTALQASVFDDQQAFLAALMDEQTLT
QLEQLKQNLENQRRQAQTLVTQTAETLTQHQQHRPGGLSLTVTVEQIQQELAQTHQKLRE
NTTSQGEIRQQLKQDADNRQQQQTLMQQIAQMTQQVEDWGYLNSLIGSKEGDKFRKFAQG
LTLDNLVHLANQQLTRLHGRYLLQRKASEALEVEVVDTWQADAVRDTRTLSGGESFLVSL
ALALALSDLVSHKTRIDSLFLDEGFGTLDSETLDTALDALDALNASGKTIGVISHVEAMK
ERIPVQIKVKKINGLGYSKLESAFAMK
>LFGLNPFC_02875 Putative transporter AraJ
MASLVVILQSITLLATVIGSRSGGCDGGMKKVILSLALGTFGLGMAEFGIMGVLTELAHN
VGISIPAAGHMISYYALGVVVGAPIIALFSSRYSLKHILLFLVALCVIGNAMFTLSSSYL
MLAIGRLVSGFPHGAFFGVGAIVLSKIIKPGKVTAAVAGMVSGMTVANLLGIPLGTYLSQ
EFSWRYTFLLIAVFNIAVMASVYFWVPDIRDEAKGKLREQFHFLRSPAPWLIFAATMFGN
AGVFAWFSYVKPYMMFISGFSETAMTFIMMLVGLGMVLGNMLSGRISGRYSPLRIAAVTD
FIIVLALLMLFFFGGMKTTSLIFAFICCAGLFALSAPLQILLLQNAKGGELLGAAGGQIA
FNLGSAVGAYCGGMMLTLGLAYNYVALPAALLSFAAMSSLLLYGRYKRQQAADSSVLAKP
LG
>LFGLNPFC_02876 Fructokinase
MRIGIDLGGTKTEVIALGDAGEQLYRHRLPTPRDDYRQTIETIATLVDMAEQATGQRGTV
GMGIPGSISPYTGVVKNANSTWLNGQPFDKDLSARLQREVRLANDANCLAVSEAVDGAAA
GAQTVFAVIIGTGCGAGVAFNGRAHIGGNGTAGEWGHNPLPWMDEDELRYREEVPCYCGK
QGCIETFISGTGFATDYRRLSGHALKGSEIIRLVEESDPVAELALRRYELRLAKSLAHVV
NILDPDVIVLGGGMSNVDRLYQTVPQLIKQFVFGGECETPVRKAKHGDSSGVRGAAWLWP
QE
>LFGLNPFC_02877 Recombination-associated protein RdgC
MQGRRQFVIMPAKFNDKAVEIIMLWFKNLMVYRLSREISLRAEEMEKQLASMAFTPCGSQ
DMAKMGWVPPMGSHSDALTHVANGQIVICARKEEKILPSPVIKQALEAKIAKLEAEQARK
LKKTEKDSLKDEVLHSLLPRAFSRFSQTMMWIDTVNGLIMVDCASAKKAEDTLALLRKSL
GSLPVVPLSMENPIELTLTEWVRSGSAAQGFQLLDEAELKSLLEDGGVIRAKKQDLTSEE
ITNHIEAGKVVTKLALDWQQRIQFVMCDDGSLKRLKFCDELRDQNEDIDREDFAQRFDAD
FILMTGELAALIQNLIEGLGGEAQR
>LFGLNPFC_02878 hypothetical protein
MSLTTTPGYVRCAMPFGANDHCDFAAMHATIHHLIYLLSSPVPQQYLEIFADIYINLDEL
>LFGLNPFC_02879 Pyrimidine/purine nucleoside phosphorylase
MLQSNEYFSGKVKSIGFSSSSTGRASVGVMVEGEYTFSTAEPEEMTVISGALNVLLPDAT
DWQVYEAGSVFNVPGHSEFHLQVAEPTSYLCRYL
>LFGLNPFC_02880 hypothetical protein
MSASLAILTIGIVPMQEVLPLLTEYIDEDNISHHSLLGKLSREEVMAEYAPEAGEDTILT
LLNDNHLAHVSRRKVERDLQGVVEVLDNQGYDVIILMSTANISSMTARNTIFLEPSRILP
PLVSSIVEDHQVGVIVPVEELLTVQAQKWQILQKPPVFSLGNPIHDSEQKIIDAGKELLA
KGADVIMLDCLGFNQRHRDLLQKQLDVPVLLSNVLIARLAAELLM
>LFGLNPFC_02881 putative protein YaiA
MPTKPPYPREAYIVTIDKGKPGQTVTWYQLRADHPKPDSLISEHPTAQEAMDAKKRYEDP
DKE

>LFGLNPFC_02882 Shikimate kinase 2
MTQPLFLIGPRGCGKTTVGMALADSLNRRFVDTDLWLQSQLNMTVAEIVEREEWAGFRAR
ETAALEAVTAPSTVIATGGGIILTEFNRHFMQNNGIVVYLCAPVSVLVNRLQAAPEEDLR
PTLTGKPLSEEVQEVLEERDALYREVAHIIIDATNEPSQVISEIRSALAQTINC
>LFGLNPFC_02883 hypothetical protein
MTIWVDADACPNVIKEILYRAAERMQMPLVLVANQSLRVPPSRFIRTLRVAAGFDVADNE
IVRQCEAGDLVITADIPLAAEAIEKGAAALNPRGERYTPATIRERLTMRDFMDTLRASGI
QTGGPDSLSQRDRQAFAAELEKWWLEVQRSRG
>LFGLNPFC_02884 Pyrroline-5-carboxylate reductase
MEKKIGFIGCGNMGKAILGGLIASGQVLPGQIWVYTPSPDKVTVLHDQFGINAAESAQEV
AQIADIIFAAVKPGIMIKVLSEITSSLNKDSLVVSIAAGITLDQLARALGHDRKIIRAMP
NTPALVNAGMTSVTPNALVTPEDTADVLNIFRCFGEAEVIAEPMIHPVVGVSGSSPAYVF
MFIEAMADAAVLGGMPRAQAYKFAAQAVMGSAKMVLETGEHPGTLKDMVCSPGGTTIEAV
RVLEEKGFRAAVIEAMTKCMEKSEKLSKS
>LFGLNPFC_02885 putative diguanylate cyclase DgcC
MFPKIMNDENFFKKAAAHGEEPPLTPQNEHQRSGLRFARRVRLPRAVGLAGMFLPIASTL
VSHPSPGWWWLLLVGWAFVWPHLAWQIASRAVDPLSREIYNLKTDAVLAGMWVGVMGVNA
LPSTAMLMIMCLNLMGAGGPRLFVAGLVLMVVSCLVTLELTGITVSFNSAPLEWWLSLPI
IVIYPLLFGWVSYQTATKLAEHKRRLQVMSTRDGMTGVYNRRHWETMLRNEFDNCRRHNR
DATLLIIDIDHFKSINDTWGHDVGDEAIVALTRQLQITLRGSDVIGRFGGDEFAVIMSGT
PAESAITAMLRVHEGLNTLRLPNTPQVTLRISVGVAPLNPQMSHYREWLKSADLALYKAK
KAGRNRTEVAA
>LFGLNPFC_02886 Phosphate starvation-inducible protein PsiF
MKITLLVTLLFGLVFLTTVGAAERTLTPQQQRMTSCNQQATAQALKGDARKTYMSDCLKN
SKSAPGEKSLTPQQQKMRECNNQATQQSLKGDDRNKFMSACLKKAA
>LFGLNPFC_02887 Alkaline phosphatase
MKQSTIALALLPLLFTPVTKARTPEMPVLENRAAQGDITAPGGARRLTGDQTAALRDSLS
DKPAKNIILLIGDGMGDSEITAARNYAEGAGGFFKGIDALPLTGQYTHYALNKKTGKPDY
VTDSAASATAWSTGVKTYNGALGVDIHEKDHPTILEMAKAAGLATGNVSTAELQDATPAA
LVAHVTSRKCYGPSATSEKCPGNALEKGGKGSITEQLLNARADVTLGGGAKTFAETATAG
EWQGKTLREQAQARGYQLVSDTASLNSVTEANQQKPLLGLFADGNMPVRWQGPKATYHGN
IDKPAVTCTPNPQRNDSVPTLAQMTDKAIELLSKNEKGFFLQVEGASIDKQDHAANPCGQ
IGETVDLDEAVQRALEFAKKDGNTLVIVTADHAHASQIVAPDTKAPGLTQALNTKDGAVM
VMSYGNSEEDSQEHTGSQLRIAAYGPHAANVVGLTDQTDLFYTMKAALGLK
>LFGLNPFC_02888 Anti-adapter protein IraP
MKNLIAELLFKLAQKEEESKELCAQVEALEIIVTAMLRNMAQNDQQRLIDQVEGALYEVK
PDASIPDDDTELLRDYVKKLLRHPRQ
>LFGLNPFC_02889 D-alanine--D-alanine ligase A
MEKLRVGIVFGGKSAEHEVSLQSAKNIVDAIDKSRFDVVLLGIDKQGQWHVSDASNYLLN
ADDPAHIALRPSATSLAQVPGKHEHQLIDAQNGQPLPTVDVIFPIVHGTLGEDGSLQGML
RVANLPFVGSDVLASAACMDKDVTKRLLRDAGLNIAPFITLTRANRHNISFAEVESKLGL
PLFVKPANQGSSVGVSKVTSEEQYTIAVDLAFEFDHKVIVEQGIKGREIECAVLGNDNPQ
ASTCGEIVLTSDFYAYDTKYIDEDGAKVVVPAAIAPEINDKIRAIAVQAYQTLGCAGMAR
VDVFLTPENEVVINEINTLPGFTNISMYPKLWQASGLGYTDLITRLIELALERHAADNAL
KTTM
>LFGLNPFC_02890 hypothetical protein
MNLPVKIRRDWHYYAFAIGLIFILNGVVGLLGFEAKGWQTYAVGLVTWVISFWLAGLIIR
RRDEETENAQ
>LFGLNPFC_02891 Inner membrane protein YaiY
MADFTLSKSLFSGKYRNASSTPGNIAYALFVLFCFWAGAQLLNLLVHAPGVYERLMQVQE
TGRPRVEIGLGVGTIFGLIPFLVGCLIFAVVALWLHWRHRRQ
>LFGLNPFC_02892 hypothetical protein
MSRVNPLSSLSLLAVLILAGCSSQAPQPLKKGEKAIDVASVVRQKMPASVKDRDAWAKDL
ATTFESQGLAPTLENVCSVLAVAQQESNYQADPAVPGLSKIAWQEIDRRAERMHIPAFLV
HTALKIKSPNGKSYSERLDSVRTEKQLSAIFDDLISMVPMGQTLFGSLNPVRTGGPMQVS
IAFAEQHTKGYPWKMDGTVRQEVFSRRGGLWFGTYHLLNYPASYSAPIYRFADFNAGWYA
SRNAAFQNAVSKASGVKLALDGDLIRYDSKEPGKTELATRKLAGKLGMSDSEIRRQLEKG
DSFSFEETALYKKVYQLAEAKTGKSLPREMLPGIQLESPKITRNLTTAWFAKRVDERRAR
CMKQ
>LFGLNPFC_02893 Peptide antibiotic transporter SbmA
MFKSFFPKPGTFFLSAFVWALIAVIFWQAGGGDWVARITGASGQIPISAARFWSLDFLIF
YAYYIVCVGLFALFWFIYSPHRWQYWSILGTALIIFVTWFLVEVGVAVNAWYAPFYDLIQ
TALSSPHKVTIEQFYREVGVFLGIALIAVVISVLNNFFVSHYVFRWRTAMNEYYMANWQQ
LRHIEGAAQRVQEDTMRFASTLENMGVSFINAIMTLIAFLPVLVTLSAHVPELPIVGHIP

YGLVIAAIVWSLMGTGLLAVVGIKLPGLEFKNQRVEAAYRKELVYGEDDATRATPPTVRE
LFSAVRKNYFRLYFHYMYFNIARILYLQVDNVFGLFLLFPSIVAGTITLGLMTQITNVFG
QVRGAFQYLINSWTTLVELMSIYKRLRSFEHELDGDKIQEVTHTLS
>LFGLNPFC_02894 hypothetical protein
MQALVSRKKIVFTRRYVFNTIIALIRPLQSINE
>LFGLNPFC_02895 D-alanyl-D-alanine-carboxypeptidase/endopeptidase AmpH
MKRSLLFSAVLYAASLTSVHAAQPITEPEFASDIVDRYADHIFYGSGATGMALVVIDGNQ
RVFRSYGETRPGNNVRPQLDSVIRIASLTKLMTSEMLVKLLDQGTVKLNDPLSKYAPPGA
RVPTYNGTPITLVNLATHTSALPREQPGGAAHRPVFVWPTREQRWKYLSTAKLKAAPGSQ
AAYSNLAFDLLADALANASGKPYTQLFEEQITRPLGMKDTTYTPSPDQCRRLMVAERGAS
PCNNTLAAIGSGGVYSTPGDMMRWMQQYLSSDFYQRSNQADRMQTLIYQRAQFTKVIGMD
VPGKADALGLGWVYMAPKEGRPGIIQKTGGGGGFITYMAMIPQKNIGAFVVVTRSPLTRF
KNMSDGINDLVTELSGNKPLVIPAS
>LFGLNPFC_02896 Inhibitor of hydrogen peroxide resistance
MLSVVKPLQEFGKLDKCLSRYGTRFEFNNEKQVIFSSNVNSEDTFVILEGVISLRREENV
LIGITQAPYIMGLADGLMKNDIPYKLISEGNCTGYHLPAKQTITLIEQNQLWRDAFYWLA
WQNRILELRDVQLIGHNSYEQIRATLLSMIDWNEELRSRIGVMNYIHQRTRISRSVVAEV
LAALRKGGYIEMNKGKLVAINRLPSEY
>LFGLNPFC_02897 hypothetical protein
MHSWKKKLVVSQLALACTLAITSQANAANYDTWGYHDNTTTDLVWPYDTDNDGIADAGGM
DPMGSDGNYVTYNGFVYYNNANGDFDTVFKGDTVNGTISTYYLNHDYASGSVNQVDISNS
VIHGAITSELPFGYYSITPAEVNAKGEVVTPQTYNGYNFYNGITTDLDYNWYDGDVFTLN
VSNSTIDDDYEALYFTDTYLDGDKSKSTNETFYTALGVAVNLDVESNINITNNSRVAGIA
LSEGNTSNTTYTSEYHQWDNNINVSNSTVTSGSQTPLEESNGFFGKSAEPSDYAGNGGEN
DVALSFSDNAGSNYSMKNNVNFDHSTLLGDVEFTSHWNNDGVFFYSTGHDSNGDGVLDTN
GGWVDDAQNVDELNITLNNGSKWVGSANMSAEVIAPADMYDVAPNSLTPGATIEANDWGR
IIDNKVFQSGVFNVALNNGSEWNTVNSSVIDTLAVNNGSQVNVTDSSLVSDTIGLTNGSS
LNIGEDGLVATDHLTVDSYSTVNLTESTGWNNYSNLYANIITVTNGGVLDVNVDQFDTEA
FRTDKLELTSGNIADHNGNVVSGVFDINSSDYVLNADLVNDRTWDTAQANYGYGVVAMNS
DGHLTINGNGDVDNGTELDNSSVDNVVAATGNYKVRIDNATGAGAIADYKDKEIIYVNDV
NSNATFSAANKADLGAYTYQAEQRGNTVVLQQMELTDYANMALSIPSANTNIWNLEQDTV
GTRLTNSRHGLADNGGAWVSYFGGNFNGDNGTINYDQDVNGIMVGVDTKIDGNNAKWIVG
AAAGFAKGDMNDRSGQVDQDSQTAYIYSSAHFANNVFVDGSLSYSHFNNDLSATMSNGTY
VDGSTNSDAWGFGLKAGYDFKLGDAGYVTPYGSVSGLFQSGDDYQLSNDMKVDGQSYDSM
RYELGVDAGYTFTYSEDQALTPYFKLAYVYDDSNNDNDVNGDSIDNGTEGSAVRVGLGTQ
FSFTKNFSAYTDANYLGGGDVDQDWSANVGVKYTW
>LFGLNPFC_02898 Delta-aminolevulinic acid dehydratase
MTDLIQRPRRLRKSPALRAMFEETTLSLNDLVLPIFVEEEIDDYKAVEAMPGVMRIPEKH
LAREIERIANAGIRSVMTFGISHHTDETGSDAWREDGLVARMSRICKQTVPEMIVMSDTC
FCEYTSHGHCGVLCEHGVDNDATLENLGKQAVVAAAAGADFIAPSAAMDGQVQAIRQALD
AAGFKDTAIMSYSTKFASSFYGPFREAAGSALKGDRKSYQMNPMNRREAIRESLLDEAQG
ADCLMVKPAGAYLDIVRELRERTELPIGAYQVSGEYAMIKFAALAGAIDEEKVVLESLGS
IKRAGADLIFSYFALDLAEKKILR
>LFGLNPFC_02899 Alpha-ketoglutarate-dependent taurine dioxygenase
MSERLSITPLGPYIGAQITGADLTRPLSDNQFEQLYHAVLRHHVVFLRDQAITPQQQRAL
AQRFGELHIHPVYPHAEGVDEIIVLDTHNDNPPDNDNWHTDVTFIQTPPAGAILAAKELP
STGGDTLWASGIAAYEALSVPFRQLLSGLRAEHDFRKSFPEYKYRKTEAEHQRWREAVAK
NPPLLHPVVRTHPVTGKQALFVNEGFTTRIVDVSEKESEALLGFLFAHITKPEFQVRWRW
QPNDIAIWDNRVTQHYANADYLPQRRIMHRATILGDIPFYRAG
>LFGLNPFC_02900 Putative aliphatic sulfonates transport permease protein SsuC
MSVLINEKLHSHRLKWRWPLSRQVTLSIGTLAVLLTVWWAVAALQLISPLFLPPPQQVLA
KLLTIAGPQGFMDATLWQHLAASLTRIVLALLAAVLIGIPVGIAMGLSPTVRGILDPIIE
LYRPVPPLAYLPLMVIWFGIGETSKILLIYLAIFAPVAMSALAGVKSAQQVRIRAAQSLG
ASRAQVLWFVILPGALPEILTGLRIGLGVGWSTLVAAELIAATRGLGFMVQSAGEFLATD
VVLAGIAVIAIIAFLLELGLRALQRRLTPWHGEVQ
>LFGLNPFC_02901 Taurine import ATP-binding protein TauB
MLQISHLYADYGGKPALEDINLTLESGELLVVLGPSGCGKTTLLNLIAGFVPYQHGSIQL
AGKGIQGPGAERGVVFQNEGLLPWRNVQDNVAFGLQLAGVEKMQRLEIAHQMVKKVGLEG
AEKRYIWQLSGGQRQRVGIARALAANPQLLLLDEPFGALDAFTRDQMQTLLLKLWQETGK
QVLLITHDIEEAVFMATELVLLSPGPGRVLERLPLNFARRFVAGESSRSIKSDPQFIAMR
EYVLSRVFEQREAFS
>LFGLNPFC_02902 Taurine-binding periplasmic protein
MVISSRNTFLAALAFIAFQAQAVNVTVAYQTSAEPAKVAQADNTFAKESGATVDWRKFDS
GASIVRALASGDVQIGNLGSSPLAVATSQQVPIEVFLLASKLGNSEALVVKKTISKPEDL

IGKRIAVPFISTTHYSLLAALKHWGIKPGQVEIVNLQPPAIIAAWQRGDIDGAYVWAPAV
NALEKDGKVLTDSEQVGQWGAPTLDVWVVRKDFAEKHPEVVKAFAKSAIDAQQPYIANPD
AWLKQPENISKLARLSGVPEGDVPGLVKGNTYLTPQQQTAELTGPVNKAIIDTAQFLKEQ
GKVPAVANDYSQYVTSRFVQ
>LFGLNPFC_02903 N-acetyl-alpha-D-glucosaminyl L-malate deacetylase 1
MDKVLDSAILSSANKRKGILAIGAHPDDIELGCGASLARLAQKGIYIATVVMTTGNSGVD
GIIDRHEESRNALKILGCHQTIHLNFADTRAHLQLNDMISALENIIKNQIPSDVEIIRVY
TMHDADRHQDHLAVYQASMVACRAIPQILGYETPSTWLSFMPQVFESVKEEYFSLKLTAL
KKHKSQSQRDYMRPERLRAVAQFRGQQVNSDLGEGFVIHKMIL
>LFGLNPFC_02904 Undecaprenyl-phosphate 4-deoxy-4-formamido-L-arabinose transferase
MKTWIFICMAVAILLWFLSTLRRKPSQKKGCIDAIIPAYNEGPCLAQSLDNLLRNPYFCR
VICVNDGSMDNTEAVMAEVKRKWGDRFVAVTQKNTGKGGALMNGLNYATCDQVFLSDVDT
YVPPDQDGMGYMLAEIERGADAVGGIPSTALKGAGLLPHIRATVKLPMIVMKRTLQQLLG
GAPFIISGACGMFRTDVLRKFGFSDRTKVEDLDLTWTLVANGYRIRQANRCIVYPQECNS
PREEWRRWRRWIVGYAVCMRLHKRLLFSRFGIFSIFPMLLVVLYGVGIYLTTWFNEFITT
GPHGVVLAMFRLSGSA
>LFGLNPFC_02905 Bifunctional protein GlmU
MDLLPFLLDANLSATNPPAIPHWWKRQPLIPTLLSQELKNYLKLNVKEKNIQIADQVIID
ESAGEVVIGANTRICHGAVIQGPVVIGANCLIGNYAFIRPGTIISNGVRIGFATEIKNAV
IEAEATIGPQCFIADSVVANQAYLGAQVRTSNHRLDEQPVSVRTPDGIIATGCDKLGCYI
GQRSRLGVQVIILPGRIISPNTQLGPRVIVERNLPTGTYSLRQELIRTGD
>LFGLNPFC_02906 hypothetical protein
MIKRTLLAAAIFSALPVYAGLTSITAGYDFTDYSGDHGNRNLAYAELVAKVENATLLFNL
SQGRRDYETEHFNATRGQGAVWYKWNNWLTTRTGIAFADNTPVFARQDFRQDINLALLPK
TLFTTGYRYTKYYDDVEVDAWQGGISLYTGPVITSYRYTHYDSSDAGGSYSNMISVRLND
PRGTGYTQLWLSRGTGAYTYDWTPETRYGSMKSISLQRIQPLTEQLNLGLTAGKVWYDTP
TDDYNGLQLAAHLTWKF
>LFGLNPFC_02907 Transcriptional repressor FrmR
MPSTPEEKKKVLTRVRRIRGQIDALERSLEGDAECRAILQQIAAVRGAANGLMAEVLESH
IRETFDRNDCYSREVSQSVDDTIELVRAYLK
>LFGLNPFC_02908 S-(hydroxymethyl)glutathione dehydrogenase
MKSRAAVAFAPGKPLEIVEIDVAPPKKGEVLIKVTHTGVCHTDAFTLSGDDPEGVFPVVL
GHEGAGVVVEVGEGVTSVKPGDHVIPLYTAECGECEFCRSGKTNLCVAVRETQGKGLMPD
GTTRFSYNGQPLYHYMGCSTFSEYTVVAEVSLAKINPEANHEHVCLLGCGVTTGIGAVHN
TAKVQPGDSVAVFGLGAIGLAVVQGARQAKAGRIIAIDTNPKKFELARRFGATDCINPND
YDKPIKDVLLDINKWGIDHTFECIGNVNVMRAALESAHRGWGQSVIIGVAGSGQEISTRP
FQLVTGRVWKGSAFGGVKGRSQLPGMVEDAMKGDIDLEPFVTHTMSLDEINDAFDLMHEG
KSIRTVIRY
>LFGLNPFC_02909 S-formylglutathione hydrolase FrmB
MELIEKHASFGGWQNVYRHYSQSLKCEMNVGVYLPPKAENEKLPVLYWLSGLTCNEQNFI
TKSGMQRYAAEHNIIVVAPDTSPRGSHVADADRYDLGQGAGFYLNATQAPWNEHYKMYDY
IRNELPNLVMHHFPATARKSISGHSMGGLGALVLALRNPDEYVSVSAFSPIVSPSQVPWG
QQAFAAYLGENKDAWLDYDPVSLISQGQRVAEIMVDQGLSDDFYAEQLRTPNLEKICQEM
NIKTLIRYHEGYDHSYYFVSSFIGEHIAYHANKLNMR
>LFGLNPFC_02910 hypothetical protein
MAKLTLQEQLLKAGLVTSKKAAKVERTAKKSRVQAREARAAVEENKKAQLERDKQLSEQQ
KQAALAKEYKAQVKQLIEMNRITIANGDIGFNFTDGNLIKKIFVDKLTQAQLINGRLAIA
RLLVDNNSEGEYAIIPASVADKIAQRDASSIVLHSALSAEEQDEDDPYADFKVPDDLMW
>LFGLNPFC_02911 Lactose operon repressor
MVNVKPVTLYDVAEYAGVSYQTVSRVVNQACHVSAKTREKVEAAMAELNYIPNRVAQQLA
GKQSLLIGVATSSLALHAPSQIVAAIKSRADQLGASVVVSMVERSGVEACKAAVHNLLAQ
RVSGLIINYPLDDQDAIAVEAACANVPALFLDVSDQTPINSIIFSHEDGTRLGVEHLVAL
GHQQIALLAGPLSSVSARLRLAGWHKYLTRNQIQPIAEREGDWSAMSGFQQTMQMLNEGI
VPTAMLVANDQMALGAMRAITESGLRVGADISVVGYDDTEDSSCYIPPLTTIKQDFRLLG
QTSVDRLLQLSQGQAVKGNQLLPVSLVKRKTTLPPNTQTASPRALADSLMQLARQVSRLE
SGQ
>LFGLNPFC_02912 Beta-galactosidase
MTMITDSLAVVLQRRDWENPGVTQLNRLAAHPPFASWRNSEEARTDRPSQQLRSLNGEWR
FAWFPAPEAVPESWLECDLPDADTVVVPSNWQMHGYDAPIYTNVTYPITVNPPFVPAENP
TGCYSLTFNIDESWLQEGQTRIIFDGVNSAFHLWCNGRWVGYGQDSRLPSEFDLSAFLHA
GENRLAVMVLRWSDGSYLEDQDMWRMSGIFRDVSLLHKPTTQISDFQVTTRFNDDFSRAV
LEAEVQMYGELRDELRVTVSLWQGETQVASGTAPFGGEIIDERGGYADRVTLRLNVENPE
LWSAEIPNLYRAVVELHTADGTLIEAEACDVGFREVRIENGLLLLNGKPLLIRGVNRHEH
HPLHGQVMDEQTMVQDILLMKQNNFNAVRCSHYPNHPLWYTLCDRYGLYVVDEANIETHG

MVPMNRLTDDPRWLPAMSERVTRMVQRDRNHPSVIIWSLGNESGHGANHDALYRWIKSVD
PSRPVQYEGGGADTTATDIICPMYARVDEDQPFPAVPKWSIKKWLSLPGEMRPLILCEYA
HAMGNSLGGFAKYWQAFRQYPRLQGGFVWDWVDQSLIKYDENGNPWSAYGGDFGDTPNDR
QFCMNGLVFADRTPHPALTEAKHQQQFFQFRLSGRTIEVTSEYLFRHSDNEFLHWMVALD
GKPLASGEVPLDVGPQGKQLIELPELPQPESAGQLWLTVRVVQPNATAWSEAGHISAWQQ
WRLAENLSVTLPSASHAIPQLTTSGTDFCIELGNKRWQFNRQSGFLSQMWIGDEKQLLTP
LRDQFTRAPLDNDIGVSEATRIDPNAWVERWKAAGHYQAEAALLQCTADTLADAVLITTA
HAWQHQGKTLFISRKTYRIDGHGEMVINVDVAVASDTPHPARIGLTCQLAQVSERVNWLG
LGPQENYPDRLTAACFDRWDLPLSDMYTPYVFPSENGLRCGTRELNYGPHQWRGDFQFNI
SRYSQQQLMETSHRHLLHAEEGTWLNIDGFHMGIGGDDSWSPSVSAEFQLSAGRYHYQLV
WCQK
>LFGLNPFC_02913 Lactose permease
MYYLKNTNFWMFGLFFFFYFFIMGAYFPFFPIWLHDINHISKSDTGIIFAAISLFSLLFQ
PLFGLLSDKLGLRKYLLWIITGMLVMFAPFFIFIFGPLLQYNILVGSIVGGIYLGFCFNA
GAPAVEAFIEKVSRRSNFEFGRARMFGCVGWALCASIVGIMFTINNQFVFWLGSGCALIL
AVLLFFAKTDAPSSATVANAVGANHSAFSLKLALELFRQPKLWFLSLYVIGVSCTYDVFD
QQFANFFTSFFATGEQGTRVFGYVTTMGELLNASIMFFAPLIINRIGGKNALLLAGTIMS
VRIIGSSFATSALEVVILKTLHMFEVPFLLVGCFKYITSQFEVRFSATIYLVCFCFFKQL
AMIFMSVLAGNMYESIGFQGAYLVLGLVALGFTLISVFTLSGPGPLSLLRRQVNEVA
>LFGLNPFC_02914 Galactoside O-acetyltransferase
MIAMNMSMTERIKAGKLFTDMCEGLPEKRLRGKTLMYEFNHSHPSEVDKRESLIKEMFAT
VGENAWVEPPVYFSYGSNIYIGRNFYANFNLTIVDDYTVTIGDNVLIAPNVTLSVTGHPV
HHELRKNGEMYSFPITIGNNVWIGSHVVINPGVTIGDNSVIGAGSVVTKDIPPNVVAAGV
PCRVIREINDRDKQYYFKDFKVESSV
>LFGLNPFC_02915 Cytosine deaminase
MSNNALQTIINAQLPGKEGLWQIHLHDGKISAIDAQSGVMPVTENSLDAEQGLVLPPFVE
PHIHLDTTQTAGQPNWNQSGTLFEGIERWAERKALLTHDDVKQRAWQTLKWQIANGIQHV
RTHVDVSDATLTALKAMLEVKQEVAPWIDLQIVAFPQEGILSYPNGEALLEEALRLGADV
VGAIPHFEFTREYGVESLHKTFALAQKYDRLIDVHCDEIDDEQSRFVETVAALAHREGMG
ARVTASHTTAMHSYNGAYTSRLFRLLKMSGINFVANPLVNIHLQGRFDTYPKRRGITRVK
EMLESGINVCFGHDDVFDPWYPLGTANMLQVLHMGLHVCQLMGYGQINDGLNLITHHSAR
TLNLQDYGIAAGNSANLIILPAENGFDALRRQVPVRYSVRGGKVIASTQPAQTTVYLEQP
EAIDYKR
>LFGLNPFC_02916 Cytosine permease
MSQDNNFSQGPVPQSARKGVLALTFVMLGLTFFSASMWTGGTLGTGLSYHDFFLAVLIGN
LLLGIYTSFLGYIGAKTGLTTHLLARFSFGVKGSWLPSLLLGGTQVGWFGVGVAMFSIPV
GKATGLDINLLIAVSGLLMTVTVFFGISALTVLSVIAVPAIACLGGYSVWLAVNGMGGLD
VLKAVVPAQPLDFNVALALVVGSFISAGTLTADFVRFGRNAKLAVLVAMVAFFLGNSLMF
IFGAAGAAALGMADISDVMIAQGLLLPAIVVLGLNIWTTNDNALYASGLGFANITGMSSK
TLSVINGIIGTVCALWLYNNFVGWLTFLSAAIPPVGGVIIADYLMNRRRYEHFATTRMMS
VNWVAILAVALGIAAGHWLPGIVPVNAVLGGALSYLILNPILNRKTTAAMTHVEANSVE
>LFGLNPFC_02917 Acetyl-coenzyme A synthetase
MSFSEFYQRSINEPEQFWAEQARRIDWQTPFTQTLDHSNPPFARWFCEGRTNLCHNAIDR
WLEKQPEALALIAVSSETEEERTFTFRQLHDEVNAVASMLRSLGVQRGDRVLVYMPMIAE
AHITLLACARIGAIHSVVFGGFASHSVAARIDDAKPVLIVSADAGARGGKIIPYKKLLDD
AISQAQHQPRHVLLVDRGLAKMARVSGRDVDFASLRHQHIGARVPVAWLESNETSCILYT
SGTTGKPKGVQRDVGGYAVALATSMDTIFGGKAGGVFFCASDIGWVVGHSYIVYAPLLAG
MATIVYEGLPTWPDCGVWWKIVEKYQVSRMFSAPTAIRVLKKFPTAEIRKHDLSSLEVLY
LAGEPLDEPTASWVSNTLDVPVIDNYWQTESGWPIMAIARGLDDRPTRLGSPGVPMYGYN
VQLLNEVTGEPCGVNEKGMLVVEGPLPPGCIQTIWGDDDRFVKTYWSLFSRPVYATFDWG
IRDADGYHFILGRTDDVINVAGHRLGTREIEESISSHPGVAEVAVVGVKDALKGQVAVAF
VIPKESDSLEDREVAHLQEKAIMALVDSQIGNFGRPAHVWFVSQLPKTRSGKMLRRTIQA
ICEGRDPGDLTTIDDPASLDQIRQAMEE
>LFGLNPFC_02918 2-methylcitrate dehydratase
MSAQINNIRPEFDREIVDIVDYVMNYEISSRVAYDTAHYCLLDTLGCGLEALEYPACKKL
LGPIVPGTVVPNGVRVPGTQFQLDPVQAAFNIGAMIRWLDFNDTWLAAEWGHPSDNLGGI
LATADWLSRNAIASGKAPLTMKQVLTGMIKAHEIQGCIALENSFNRVGLDHVLLVKVAST
AVVAEMLGLTREEILNAVSLAWVDGQSLRTYRHAPNTGTRKSWAAGDATSRAVRLALMAK
TGEMGYPSALTAPVWGFYDVSFKGESFRFQRPYGSYVMENVLFKISFPAEFHSQTAVEAA
MTLYEQMQAAGKTAADIEKVTIRTHEACIRIIDKKGPLNNPADRDHCIQYMVAIPLLFGR
LTAADYEDNVAQDKRIDALREKINCFEDPAFTADYHDPEKRAIANAITLEFTDGTRFEEV
VVEYPIGHARRRQDGIPKLVDKFKINLARQFPTRQQQRILEVSLDRTRLEQMPVNEYLDL
YVI
>LFGLNPFC_02919 2-methylcitrate synthase

MSDTTILQNSTHVIKPKKSVALSGVPAGNTALCTVGKSGNDLHYRGYDILDLAAHCEFEE
VAHLLIHGKLPTRDELAAYKTKLKALRGLPANVRTVLEALPAASHPMDVMRTGVSALGCT
LPEKEGHTVSGARDIADKLLASLSSILLYWYHYSHNGERIQPETDDDSIGGHFLHLLHGE
KPSPSWEKAMHISLVLYAEHEFNASTFTSRVIAGTGSDMYSAIIGAIGALRGPKHGGANE
VSLEIQQRYETPDEAEADIRKRVENKEVVIGFGHPVYTIADPRHQVIKRVAKQLSQEGGS
LKMYNIADRLETVMWESKKMFPNLDWFSAVSYNMMGVPTEMFTPLFVIARVTGWAAHIIE
QRQDNKIIRPSANYVGPEDRQFVALDKRQ
>LFGLNPFC_02920 2-methylisocitrate lyase
MSLHSPGKAFRAALSKENPLQIVGTINANHALLAQRAGYQAIYLSGGGVAAGSLGLPDLG
ISTLDDVLTDIRRITDVCSLPLLVDADIGFGSSAFNVARTVKSMIKAGAAGLHIEDQVGA
KRCGHRPNKAIVSKEEMVDRIRAAVDAKTDPDFVIMARTDALAVEGLDAAIERAQAYVEA
GAEMLFPEAITELAMYRQFADAVQVPILANITEFGATPLFTTDELRSAHVAMALYPLSAF
RAMNRAAEHVYNVLRQEGTQKSVIDTMQTRNELYESINYYQYEEKLDDLFARNQAK
>LFGLNPFC_02921 Propionate catabolism operon regulatory protein
MAHPPRLNDDKPVIWTVSVTRLFELFRDISLEFDHLANITPIQLGFEKAVTYIRKKLASE
RCDAIIAAGSNGAYLKSRLSVPVILIKPSGYDVLQALAKAGKLTSSIGVVTY
>LFGLNPFC_02922 Propionate catabolism operon regulatory protein
MAFQKTFNLRLDQRSYITEEDARGQINELKANGTEAVVGAGLITDLAEEAGMTGIFIYSA
ATVRQAFSDALDMTRMSLRHNTHDATRNALRTRYVLGDMLGQSPQMEQVRQTILLYARSS
AAVLIEGETGTGKELAAQAIHREYFARHDARQGKKSHPFVAVNCGAIAESLLEAELFGYE
EGAFTGSRRGGRAGLFEIAHGGTLFLDEIGEMPLPLQTRLLRVLEEKEVTRVGGHQPVPV
DVRVISATHCNLEEDMRQGQFRRDLFYRLSILRLQLPPLRERVTDILPLAESFLKVSLAA
LSAPFSAALRQGLQASETVLVHYDWPGNIRELRNMMERLALFLSVEPTPDLTPQFLQLLL
PELARESAKTPAPRLLTPQQALEKFKGDKTAAANYLGISRTTFWRRLKN
>LFGLNPFC_02923 hypothetical protein
MKIISKMLVGALAFAVTNVYAAELMTKAEFEKVESQYEKIGDISTSNEMSTADAKEDLIK
KADEKGADVLVLTSGQTDNKIHGTANIYKKK
>LFGLNPFC_02924 Threonine efflux protein
MDPLHAVYLTVGLFVITFFNPGANLFVVVQTSLASGRRAGVLTGLGVALGDAFYSGLGLF
GLATLITQCEEIFSLIRIVGGAYLLWFAWCSMRRQSTPQMSTLQQPISAPWYVFFRRGLI
TDLSNPQTVLFFISIFSVTLNAETPTWARLMAWAGIVLASIIWRVFLSQAFSLPAVRRAY
GRMQRVASRVIGAIIGVFALRLIYEGVTQR
>LFGLNPFC_02926 Aldehyde reductase YahK
MKIKAVGAYSAKQPLEPMDITRREPGPHDVKIEIAYCGVCHSDIHQVRSEWAGTVYPCVP
GHEIVGRVVAVGDQVEKYAPGDLVGVGCIVDSCKHCEECEDGLENYCDHMTGTYNSPTPD
EPGHTLGGYSQQIVVHERYVLRIRHPQEQLAAVAPLLCAGITTYSPLRHWLAGPGKKVGV
VGIGGLGHMGIKLAHAMGAHVVAFTTSESKREAAKALGADEVVNSRNADEMVAHVKSFDF
ILNTVAAPHNLDDFTTLLKRDGTITLVGAPATPHKSPEVFNLIMKRRAIAGSMIGGIPET
QEMLDFCAEHGIVADIEMIRADQINEAYERMLRGDVKYRFVIDNRTLTD
>LFGLNPFC_02927 Autoinducer 2 import system permease protein LsrD
MKKSWRNNVEFYLIGLLVLTVAAFSITMPEIFWSISNFQSVASQMPVLGILALAMAVTML
CGGINLSIIATANACSLVMAWVATQYPPGIATVVATLLAGAGAAVIIGLCNGVLIAGIRV
SPILATLGMMTLLKGVNILVTGGSAIANYPSWVLWLNHAQWFGIPLPMWLFTAVALGLWI
LLEKTPLGKSIYLIGSNERATLYSGINTRRVLIWVYVISALLCAVAAFLMMSKLNSAKAS
YGESYLLVSILAAVLGGVNPDGGSGRIIGMVLALFLLQIIESGFNILGISPYLTMALWGT
LLLCFIQARGMLGLDRVV
>LFGLNPFC_02928 Ribose import permease protein RbsC
MAELKKRHEFWLALLIVVLFVGLAWRSDEFLTFGNLYDLANNYAMLTILACGLFVVLISG
GIDISFPAMTIIAQYGMVLLLQKIGGNFAVAFALAGGIGILLGLINALLVNRLRVPSIII
TISTLNIFYGLLLWLSKGVWLYDFPPWFEQGVMLFKYTDADGYDYGLGLPLIAMITVVLL
TAFIMNFTSVGRKIYALGGNRESASRIGFSVLKLQLFVYGYMGLMSGAAGVVQSWTVMTV
APDSLLGYELTVLAAVVLGGTSLLGGRGTLTGTLLGVVLLAVMQNGLNLLGVSSYWQTLI
TGIIIVASISATAWSQHQNRSLL
>LFGLNPFC_02929 Fructose import ATP-binding protein FruK
METFLSLRHINKTFHATRALRDVSLDFMSGEVHCLAGQNGCGKSTLIKIMSGVYRPDEGA
EITLGGKNWSKLTPAASVAQGIQVIYQDLSLFPNLSVWENIAVNHYHHGLFVNRRRLREV
AQAAMTSINVTLPLDTLVSELSIARCQLVAICRALAQDARLIVMDEPTASLTHQEVQGLL
QVVHQLRERGICVVFVSHRLEEVMEVSDRISVLKDGELVGTFPAAEMTTKQLGFLMTGQE
FEYQVRELWQGKSSTPVLEVRNLSRHGEYLNINLRVEAGEVVSIVGLLGAGRTELCLSLF
GMTRPDAGEILINGQLVTLHSNQDAIRHGIGYVSEDRMSRGLVMAQSIEDNIISTVFHKV
KDRFGFLSEAKVCDLVDRLIKALTIKVSDPHLPVNTLSGGNAQRVSIAKWLAIGPRLLIL
DSPTVGVDIANKAGIYGIISDLAAHGIAVLMICDEIEEAWYQSHRILVMQKGQITHSFLP
DSSSQARIAEVVNG
>LFGLNPFC_02930 Autoinducer 2-binding protein LsrB

MMNKRFVINMVSSLLLGAALISAPLQAAEKVVVNISKVDGMPWFNRMGEGVVEAGKAFGV
NASQVGPSSTDAPQQVKIIEDLIARKVNAITIVPNDANVLEPVFKKARDAGIVVLTNESP
GQPSANWDIEIIDNEKFAAEYVEHMAKRMGGKGGYVIYVGSLTVPQHNLWADLLVKYQKE
HYPDMHEVTRRMPVAESVDDSRRTTLDLMKTYPDLKAVVSFGSNGPIGAGRAVKEKRAKN
KVAVYGMMIPSQAASLIKSGDITEGITYDPASAGYALAAVASTLLKGEEIKPGLEMQNLG
KADVDMDKRIIRFHKVLLVNKDNIDSLY
>LFGLNPFC_02931 hypothetical protein
MTIKEKISQKYPHASFCTFGDSAALADHLATLIATGVKTASCGSLAGCIEDNAFPMIGEY
KIVENSRGEPVCVIRVIGLHLLRFSDVTAELARKEGEGDLSLEYWRNEHRRFFQAEGCYS
PEMDVIFEEYALIDVV
>LFGLNPFC_02932 5'-deoxyadenosine deaminase
MKENNSRREFLSQSGKMVTAAALFGPSVPLAHAGVAGTLNCEANNTMKITDPYYYLDNVL
LETGFDYENGVAVQTRTARQTVEIQNGKIVALRENKQHPDATLPHYDAGGKLMLPTTRDM
HIHLDKTFYGGPWRSLNRPAGTTIQDMIKLEQKMLPELQPYTQERAEKLIDLLQSKGTTI
ARSHCNIEPVSGLKNLQNLQAVLARRQAGFECEIVAFPQHGLLLSKSEPLMREAMQAGAH
YVGGLDPTSVDGAMEKSLDTMFQIALDYDKGVDIHLHETTPAGVAAINYMVETVEKTPQL
KGKLTISHAFALATLNEQQVDELAHRMAAQQISIASTVPIGTLHMPLKQLHDKGVKVMTG
TDSVIDHWSPYGLGDMLEKANLYAQLYIRPNEQNLSRSLFLATGDVLPLNEKGERVWPKA
QDDASFVLVDASCSAEAVARISPRTATFHKGQLVWGSVAG
>LFGLNPFC_02933 Carbamate kinase 2
MKELVVVAIGGNSIIKDNASQSIEHQAEAVKAVADTVLEMLASDYDIVLTHGNGPQVGLD
LRRAEIAHEREGLPLTPLANCVADTQGGIGYLIQQALNNRLARHGEKKAVTVVTQVEVDK
NDPGFAHPIKPIGAFFSESQRDELQKANPDWRFVEDAGRGYRRVVASPEPKRIVEAPAIK
ALIQQGFVVIGAGGGGIPVVRTDAGDYQSVDAVIDKDLSTALLAREIHADILVITTGVEK
VCIHFGKPQQQALDRVDIATMTRYMQEGHFPPGSMLPKIIASLTFLEQGGKEVIITTPEC
LPAALRGETGTHIIKT
>LFGLNPFC_02934 hypothetical protein
MSQSLFSQPLNVINVGIAMFSDDLKKQHVEVTQLDWTPPGQGNMQVVQALDNIADSPLAD
KIASANQQALERIIQSHPVLIGFDQAINVVPGMTAKTILHAGPPVTWEKMCGAMKGAVTG
ALVFEGLAKDLDEAAELAASGEITFSPCHEHDCVGSMAGVTSASMFMHIVKNKTYGNIAY
TNMSEQMAKILRMGANDQSVIDRLNWMRDVQGPMLRDAMKIIGEIDLRLMLAQALHMGDE
CHNRNNAGTTLLIQALTPGIIQAGYSVEQQREVFEFVASCDYFSGPTWMAMCKAAMDAAH
GIEYSTVVTTMARNGVEFGLRVSGLPGQWFTGPAQQVIGPMFAGYKPEDSGLDIGDSAIT
ETYGIGGFAMATAPAIVALVGGTVEEAIDFSRQMREITLGENPNVTIPLLGFMGVPSAID
ITRVGSSGILPVINTAIAHKDAGIGMIGAGIVHPPFACFEKAIFGWCERYGV
>LFGLNPFC_02935 Protein FdrA
MSVKIVIKPNTYFDSVSLMSISTRANKLDGVEQAFVAMATEMNKGVLKNLGLLTPELEQA
KNGDLMIVINGKSGVDNEQLLAEIEELFNTKAQSGSHEARYATIASAKKHIPESNLAVIS
VNGLFAAREARQALQNDLNVMLFSDNVSVEDELALKQLAHEKGLLMMGPDCGTAIINGAA
LCFGNAVRRGNIGIVGASGTGSQELSVRIHEFGGGVSQLIGTGGRDLSEKIGGLMMLDAI
GMLENDPQTEIIVLISKPPAPAVARKVLERARACRKPVVACFLGRGETPVDEQGLQFARG
SKEAALKAVMLSGVKQEHLDLHTLNQPLIADVRARLQPQQKYIRGLFCGGTLCDETLFAV
MEKHGDVYSNIQPDPEFRLQDINRSIKHTFLDFGDDDFTNGKPHPMIDPTNRISRVIEEA
RDPEVAVIVMDFVLGFGSHEDPVGSTIEAIKEAKAIAAAEGRELIILAYVLGTDLDTPSL
EQQSQMLLDAGVILASSSTNTGLLAREFICKGEEA
>LFGLNPFC_02936 hypothetical protein
MWALTADADFLAQRGQGQVEQVFARAVNIALPARQQLLTLLCEEYDNAPNSCRLALTHFN
GLFRHGDKVQFDDQGITVGQHLHIEMSHCQRWLSPTLQMTALNFHLIAWQQWHDIIHQHL
GENETLFNYRGDNPFYQALNKELHIKRRAVIQAVNDKQNIAVAVASMMGLGIGLTPSADD
YLTGLALILFLPGHPAEKYKEEFYLGLQRGRNNTTLLSAITLEAALQQRCRENIHRFIHN
IIYDIPGNATQAIEKIKHIGSSSGCDMLYGMADGCALSQTYGGNYVS
>LFGLNPFC_02937 hypothetical protein
MTIKNLPADYLLAAQQGDIDKVKICLALGVDINTCDRQGKTAITLASLYQQYACVQALID
AGANINKQDHTCLNPFLISCLNDDLTLLRIILPAKPDLNCVTRFGGVGLTPACEKGHLSI
VKELLAHTEINVNQTNHVGWTPLLEAIVLNDGGIKQQAIVQLLLEHGASPHLTDKYGKTP
LELARERGFEEIAQLLIAAGA
>LFGLNPFC_02938 hypothetical protein
MNGLTATGVTVGICAGLWQLVSSHVGLSQGWELLGTIGFVAFCSFYAAGGGKSGFIKSLA
VNYSGMVWAFFAALAAGWLAPVSGLSAFWASVITTVPFSAVVVWQGRFWLLSFIPGGFLG
MTLFFASGMNWTVTLLGFLAGNCVGIISEYGGQKLSEATTKSDGY
>LFGLNPFC_02939 putative HTH-type transcriptional regulator YahB
MNSIFTEENLLAFTTAARFGSFSKAAEELGLTTSAISYTIKRMETGLDVVLFTRSTRSIE
LTESGRYFFRKATDLLNDFHAIKRSIDTISQGIEARVRICINQLLYTPKHTARLLQVLKK
QFPTCQITVTTEVYNGVWDAIINNQANIAIGAPDTLLDGGGIDYTEIGAIRWAFAIAPDH

PLAFVPEPIAESQLRLYPNIMVEDTAHTINKKVGWLLHGQESILVPDFNTKCQCQILGEG
IGFLPDYMVREAMAQSLLVTRQIHNPRQDSRMLLATQHSATGQVTQWIKKQFAPNGILTG
IYQDLLHREN
>LFGLNPFC_02940 Cyclic di-GMP phosphodiesterase PdeL
MNSCDFRVFLQEFGTTVHLSLPGSVSEKERLLLKLLMQGMSVTEISQYRNRSAKTISHQK
KQLFEKLGIQSDITFWRDIFFQYNPEIISATGNNSHKYINDNHYHHIVTPEAISLALENH
EFKPWIQPVFCAQTGVLTGCEVLVRWEHPQTGMIPPDQFIPLAESSGLIVIMTRQLMKQT
ADILMPVKHLLPDNFHIGINVSAGCFLAAGFEKECLNLVKKLGNDKIKLVLELTERNPIP
VTPEARAIFDSLHQHNITFALDDFGTGYATYRYLQAFPVDFIKIDKSFVQMASVDEISGH
IVDNIVELARKPGLSIVAEGVETQEQADLMIGKGVHFLQGYLYSPPVPGNKFISEWVMKA
GG
>LFGLNPFC_02941 High-affinity choline transport protein
MTDLSHSREKDKINPVVFYTSAGLILLFSLTTILFRDFSALWIGRTLDWVSKTFGWYYLL
AATLYIVFVVCIACSRFGSVKLGPEQSKPEFSLLSWAAMLFAAGIGIDLMFFSVAEPVTQ
YMQPPEGAGQTIEAARQAMVWTLFHYGLTGWSMYALMGMALGYFSYRYNLPLTIRSALYP
IFGKRINGPIGHSVDIAAVIGTIFGIATTLGIGVVQLNYGLSVLFDIPDSMAAKAALIAL
SVIIATISVTSGVDKGIRVLSELNVALALGLILFVLFMGDTSFLLNALVLNVGDYVNRFM
GMTLNSFAFDRPVEWMNNWTLFFWAWWVAWSPFVGLFLARISRGRTIRQFVLGTLIIPFT
FTLLWLSVFGNSALYEIIHGGAAFAEEAMVHPERGFYSLLAQYPAFTFSASVATITGLLF
YVTSADSGALVLGNFTSQLKDINSDAPGWLRVFWSVAIGLLTLGMLMTNGISALQNTTVI
MGLPFSFVIFFVMAGLYKSLKVEDYRRESANRDTAPRPLGLQDRLSWKKRLSRLMNYPGT
RYTKQMMETVCYPAMEEVAQELRLRGAYVELKNLPPEEGQQLGHLDLLVHMGEEQNFVYQ
IWPQQYSVPGFTYRARSGKSTYYRLETFLLEGSQGNDLMDYSKEQVITDILDQYERHLNF
IHLHREAPGHSVMFPDS
>LFGLNPFC_02942 HTH-type transcriptional regulator BetI
MPKLGMQSIRRRQLIDATLEAINEVGMHDATIAQIARRAGVSTGIISHYFRDKNGLLEAT
MRDITSQLRDAVLNRLHALPQGSAELRLQAIVGGNFDETQVSSAAMKAWLAFWASSMHQP
MLYRLQQVSSRRLLSNLVSEFRRELPRQQAQEAGYGLAALIDGLWLRAALSGKALDKPLA
HSLTRHFITQHLPTD
>LFGLNPFC_02943 NAD/NADP-dependent betaine aldehyde dehydrogenase
MSRMAEQQLYIHGGYTSATSGRTFETINPANGNVLATVQAAGREDVDRAVKSAQQGQKIW
AAMTAMERSRILRRAVDILRERNDELAKLETLDTGKAYSETSTVDIVTGADVLEYYAGLI
PALEGSQIPLRETSFVYTRREPLGVVAGIGAWNYPIQIALWKSAPALAAGNAMIFKPSEV
TPLTALKLAEIYSEAGLPDGVFNVLPGVGAETGQYLTDHPGIAKVSFTGGVASGKKVMAN
SAASSLKEVTMELGGKSPLIVFDDADLDLAADITMMANFFSSGQVCTNGTRVFVPTKCKA
AFEQKILARVERIRAGDVFDPQTNFGPLVSFPHRDNVLRYIAKGIEEGARVLCGGDVLKG
DSFDNGAWVAPTVFTDCSDDMTIVREEIFGPVMSILTYESEDEVIRRANDTDYGLAAGIV
TADLNLAHRVIHQLEAGICWINTWGESPAEMPVGGYKHSGIGRENGVMTLQSYTQVKSIQ
VEMAKFQSIF
>LFGLNPFC_02944 Oxygen-dependent choline dehydrogenase
MQFDYIIIGAGSAGNVLATRLTEDPNTTVLLLEAGGPDYRFDFRTQMPAALAFPLQGKRY
NWAYETEPEPFMNNRRMECGRGKGLGGSSLINGMCYIRGNALDLDNWAQEPGLENWSYLD
CLPYYRKAETRDVGENDYHGGDGPVSVTTSKPGVNPLFEAMIEAGVQAGYPRTDDLNGYQ
QEGFGPMDRTVTPHGRRASTARGYLDQAKSRPNLTIRTHAMTDHIFFDGKRAVGVEWLEG
DSTIPTRAAANKEVLLCAGAIASPQILQRSGVGNAELLAEFDIPLVHELPGVGENLQDHL
EMYLQYECKEPVSLYPALQWWNQPKIGAEWLFGGTGVGASNHFEAGGFIRSREEFAWPNI
QYHFLPVAINYNGSNAVKEHGFQCHVGSMRSPSRGHVRIKSRDPHQHPAILFNYMSHEQD
WQEFRDAIRITREIMHQPALDQYRGREISPGVECQTDEQLDEFVRNHAETAFHPCGTCKM
GYDEMAVVDAEGRVHGLEGLRVVDASIMPQIITGNLNATTIMIGEKMADMIRGKDALPRS
TARYFVANGMPVRAKKMSRDVN
>LFGLNPFC_02945 hypothetical protein
MLTVSGNEQVTIARNISGVILKLSGISSVVYLCMHSTFISQIEQVSLEFAGEIVTMKHES
YLSVLYKDSVLIVER
>LFGLNPFC_02946 ISKra4 family transposase ISCep1
MTRKYLTQDEVYRLMDAAQSMSFPERNRCLIMMAFIHGFRASELLDLRLSDIDASGKQLN
IRRIKNGFSTTHPLLPDEYNLIKLWLKQRKLIENGVEGDWLFLSRKRRPISRQHFFSIIR
EAGKRAGLAVKAHPHMLRHACGFALADNGVDTRLLQDYLGHRNIQHTVRYTASNAARFKG
VWKKKPR
>LFGLNPFC_02947 hypothetical protein
MKKPLVIISACQFTRLALESLIPTDRYIVRVYSNVTTEVEFVLKTSCGYLLADIPSLPSG
ELVLLACLARLRSDAGQWQVCLTGDSDWFDNAPAASLYNGFPRIGTMLTASRYQNQIIRW
LLRPLPGDMSDFWLLTARELSVLKILMQGMSFSEIARYEQRSSKTLHAIATRALMKLGLG
TLSDFRLLYTGCGDSRVKRNIRLHAKYIGQHSAHRLTQYVQNMVSGILYPEKVAVGESIN
SDAISGKVLSGQRKNISKKRPGLL

>LFGLNPFC_02948 hypothetical protein
MTQQRCYYHPLRRIRTRYSVGAKIFLLFWAAGLGGTLYSLYSVLAY
>LFGLNPFC_02949 hypothetical protein
MRFVLFCPSGIVPAQFAALSTGSVGNVTCIRTEEELRNKLRHRPQSVVISAGRPAECAEM
WFRFYRDHSFVVVLCVAPFFLPPDVSISGVLKNLRLLKPGMSVERVISIANASGGFSGLK
HAEILPVMDSYSVFMKEVNNRTKTIVMSERFPEKQKKVLSLLLAGHSWEYSAQFLKTGIR
QIWLAEQSLKKRWGIPDSMSLREALLLSSNNFHGDGNALETTNAMTLRENGNTNRYSVVV
NAGTQALSLHKYK
>LFGLNPFC_02950 hypothetical protein
MKNSKAFYRSALATAIVMALSAPAFATDSTVSTDPVTLNTEKTTLDQDVVINGDNKITAV
TIETSDSDKDLNVTFGGHDITAASTVNQDFVEGVKVSGNKNVVINATDSTITAQGEGTYV
RTAMVIDSTGDVVVNGGNFVAKNEKGSATGISLEATTGNNLTLNGTTINAQGNKSYSNGS
TAIFAQKGNLLQGFDGDATDNITLADSNIINGGIETIVTAGNKTGIHTVNLNIKDGSVIG
AANNKQTIYASASAQGAGSATQNLNLSVADSTIYSDVLALSESENSASTTTNVNMNVARS
YWEGNAYTFNSGDKAGSDLDINLSDSSVWKGKVSGAGDASVSLQNGSVWNVTGSSTVDAL
AVKDSTVNITKATVNTGTFASQNGTLIVDASSENTLDISGKASGDLRVYSAGSLDLINEQ
TAFISTGKDSTLKATGTTEGGLYQYDLTQGADGNFYFVKNTHKASNASSVIQAMAAAPAN
VANLQADTLSARQDAVRLSENDKGGVWIQYFGGKQKHTTAGNASYDLDVNGVMLGGDTRF
MTEDGSWLAGVAMSSAKGDMTTMQSKGDTEGYSFHAYLSRQYNNGIFIDTAAQFGHYSNT
ADVRLMNGGGTIKADFNTNGFGAMVKGGYTWKDGNGLFIQPYAKLSALTLEGVDYQLNGV
DVHSDSYNSVLGEAGTRVGYDFAVGNATVKPYLNLAALNEFSDGNKVRLGDESVNASIDG
AAFRVGAGVQADITKNMGAYASLDYTKGDDIENPLQGVVGINVTW
>LFGLNPFC_02951 hypothetical protein
MNEQIKQDIDLIEILFYLKKKIRVILFIIAICMVMVLLFLYINKDNIKVTYSLKINQTTP
GILVSCDSNNNFACQTTMTEDVIQRITTFFQTSPDVKNREIKLEWSGNKRDLPTAEAEIS
RVQASIIKWYASEYHNGRQVLDEIQTPSAINSELYTKMIYLTRNWSLYPNGDGCVTISSP
EIKNKYPAAICLALGFFLSIVISVMFCLVKKMVDEYQQNSGQ
>LFGLNPFC_02952 Lactate utilization protein C
MDNRSKFLNNVAQALGRPLRLEPQAEDAPLNNYANERLTQLTQQQRCDAFIQFASDVMLT
RCELTSEAKAAEAAIRLCKELGDQSVVISGDTRLEELGISERLQQECNAVVWDPAKGIEN
ISQAEQAKVGVVYAEYGLTESGGVVLFSAAGRGRSLSLLPESSLFILRKSTILPRVAQLA
EKLHQKAQAGERMPSCINIISGPSSTADIELIKVVGVHGPVKAVYLIIEDC
>LFGLNPFC_02953 Lactate utilization protein B
MSIKTSNTDFKTRIRQQIEDPIMRKAVANAQQRIGANRQKMVDELGHWEEWRDRAAQIRD
HVLSNLDAYLYQLSEKVTQNGGHVYFAKTKEDATRYILQVAQRKNAWKVVKSKSMVTEEI
GVNHVLQDAGIQVIETDLGEYILQLDQDPPSHVVVPAIHKDRHQIRRVLHEHLGYEGPET
PEAMTLFIRQKIREDFLSAEIGITGCNFAVAETGSVCLVTNEGNARMCTTLPKTHIAVMG
MERIAPTFAEVDVLITMLARSAVGARLTGYNTWLTGPREAGHVDGPEEFHLVIVDNGRSE
VLASEFRDVLRCIRCGACMNTCPAYRHIGGHGYGSIYPGPIGAVISPLLGGYKDFKDLPY
ACSLCTACDSVCPVRIPLSKLILRHRRVMAEKGITAKAEQRAIKMFAYANSHPGLWKVGM
MAGAHAASWFINGGKTPLKFGAISDWMEARDLPEADGESFRSWFKKHQAQEKKNG
>LFGLNPFC_02954 Lactate utilization protein A
MNVNFFVTCIGDALKSRMARDSVLLLEKLGCRVNFPEKQGCCGQPAINSGYIKEAIPGMK
NLIAALEDNDDPIISPAGSCTYAVKSYPTYLADEPEWASRAEKVAARMQDLTSFIVNTLG
VVDVGASLQGRAVYHPSCSLARKLGVKDEPLTLLKNVRGLELLTFAEQDTCCGFGGTFSV
KMAEISGEMVKEKVAHLMEVRPEYLIGADVSCLLNISGRLQREGQKVKVMHIAEVLMSR
>LFGLNPFC_02955 RCS-specific HTH-type transcriptional activator RclR
MDALSRLLMLNAPQGTIDKNCVLGSDWQLPHGAGELSVIRWHALTQGAAKLEMPTGEIFT
LRPGNVVLLPQNSAHRLSHVDNESTCIVCGTLRLQHSARYFLTSLPETLFVAPVNHSVEY
NWLREAIPFLQQESRSAMPGVDALCSQICATFFTLAVREWIAQVNTEKNILSLLLHPRLG
AVIQQMMEMPGHAWTVESLASIAHMSRASFAQLFRDVSGTTPLAVLTKLRLQIAAQMFSR
EMLPVVVIAESVGYASESSFHKAFVREFGCTPGEYRERVRQLAP
>LFGLNPFC_02956 putative pyridine nucleotide-disulfide oxidoreductase RclA
MNKYQAVIIGFGKAGKTLAVTLAKAGWRVALIEQSNAMYGGTCINIGCIPTKTLVHDAQQ
HTDFVRAIQRKNEVVNFLRNKNFHNLADMPNIDVIDGQAEFINNHSLRVHRPGRNLEIHG
EKIFINTGAQAVVPPIPGITTTSGVYDSTGLLNLKELPGHLGILGGGYIGVEFASMFANF
GSKVTILEAASLFLPREDRDIADNIATILRDQGVDIILNAHVERISHHENQVQVHSEHAQ
LAVDALLIASGRQPATASLHPENAGIAVNERGAIVVDKQLHTTADNIWAMGDVTGGLQFT
YISLDDYRIVRDELLGEGRRSTDDRKNVPYSVFMTPPLSRVGMTEEQARESGADIQVVTL
PVAAIPRARVMNDTRGVLKAIVDNKTQRILGASLLCVDSHEMINIVKMVMDAGLPYSILR
DQIFTHPSMSESLNDLFSLVK
>LFGLNPFC_02957 Inner membrane protein RclC
MERYLHLLSRGDKIGLTLIRLSIAIVFMWIGLLKFVPYEADSITPFVANSPLMSFFYEHP
EDYKQHLTHEGEYKPEARAWQTANNTYGFSNGLGVVEVIIALLVLANPVNRWLGLLGGLM

AFTTPLVTLSFLITTPEAWVPALGDAHHGFPYLSGAGRLVLKDTLMLAGAVMIMADSARD
ILKQRSNESSSTLKTEY
>LFGLNPFC_02958 putative oxidoreductase YtbE
MEFSVLSNNLKMPMMGFGVFQVTDKDVCKQSVLNAIRTGYRLIDTAAVYGNEDAVGEAVR
EAISEGLCTREELFITSKLWVQDMLNQDIAAAGIEASLKKSGLEYFDLYLLHQAMRDYFS
AWRALEDAYEEGKLKAIGVSNFYPHVLANFCETVRVKPMVNQVELHPYFAQPEALATMKY
YNVQPEAWAPLGGGRHKPFENNLLQSIADAHQKSISQVILRWNIQRGVVVIPKSTHQQRI
EENFAIWDFSLTEKEMAQISSLDLGYVGESVKHFNPEFVRGCLAVKIHD
>LFGLNPFC_02959 Right origin-binding protein
MIRQKILQQLLEWIECNLEHPISIEDIAQKSGYSRRNIQLLFRNFMHVPLGEYIRKRRLC
RAAILVRLSAKSMLDIALSLHFDSQQSFSREFKKLFGCSPREYRHRDYWDLANIFPSFLI
RQQQKTECRLVNFPETPIFGNSFKYDIEVSNKLPDEEVKLRRHHLVRCMKNFKTDIYFVS
TFEPSTKSVDLLTVETFAGTVCKQTDSEMPKEWTINRGLYASFRYEGEWEHYPEWARNLY
LMELPARGLARVNGSDIERFYYNENFVEIDSNNIVCEIFIPVRPV
>LFGLNPFC_02960 hypothetical protein
MSRYKTDNKQPRFRYSVLARCVAWANISVQVLFPLAVTFTPVMAARAQHAVQPRLSMENT
TVTADNNVEKNVASLAANAGTFLSSQPDSDATRNFITGMATAKANQEIQEWLGKYGTARV
KLNVDKNFSLKDSSLEMLYPIYDTPTNMLFTQGAIHRTDDRTQSNIGFGWRHFSENDWMA
GVNTFIDHDLSRSHTRIGVGAEYWRDYLKLSANGYIRASGWKKSPDVEDYQERPANGWDI
RAEGYLPAWPQLGASLMYEQYYGDEVGLFGKDKRQKDPHAITAEVNYTPVPLLTLSAGHK
QGKSGENDTRFGLEVNYRIGEPLEKQLDTDSIRERRMLAGSRYDLVERNNNIVLEYRKSE
VIRIALPERIEGKGGQTVSLGLVVSKATHGLKNVQWEAPSLLAAGGKITGQGNQWQVTLP
AYQAGKDNYYAISAIAYDNKGNASKRVQTEVVISGAGMSADRTALTLDGQSRIQMLANGN
EQKPLVLSLRDAEGQPVTGMKDQIKTELTFKPAGNIVTRTLKATKSQAKPTLGEFTETEA
GVYQSVFTTGTQSGEATITVSVDDMSKTVTAELRATMMDVSNSTLSANEPSGDVVADGQQ
AYTLTLTAVDSEGNPVTGEASRLRLVPQDTNGVTVGAISEIKPGVYSATVSSTRAGNVVV
RAFSEQYQLGTLQQTLKFVAGPLDAAHSSITLNPDKPVVGGTVTAIWTAKDANDNPVTGL
NPDAPSLSGAAAAGSTASGWTDNGDGTWTAQISLGTTAGELDVMPKLNGQDAAANAAKVT
VVADALSSNQSKVSVAEDHVKAGESTTVTLVAKDAHGNAISGLSLSASLTGTASEGATVS
SWTEKGDGSYVATLTTGGKTGELRVMPLFNGQPAATEAAQLTVIAGEMSSANSTLVADNK
TPTVKTTTELTFTMKDAYGNPVTGLKPDAPVFSGAASTGSERPSAGNWTEKGNGVYVVSTL
TLGSAAGQLSVMPRVNGQNAVAQPLVLNVAGDASKAEIRDMTVKVNNQLANGQSANQITL
TVVDSYGNPLQGQEVTLTLPQGVTSKTGNTVTTNAAGKVDIELMSTVAGELEIEASVKNS
QKTVKVKFKADFSTGQASLEVDAAAQKVANGKDAFTLTATVKDQYGNLLPGAVVVFNLPR
GVKPLADGNIMVNADKEGKAELKVVSVTAGTYEITASAGNDQPSNAQSVTFVADKTTATI
SSIEVIGNRAVADGKTKQTYKVTVTDANNNLLKDSEVTLTASPENLVLTPNGTATTNEQG
QAIFTATTTVAATYTLTAKVEQADGQESTKTAESKFVADDKNAVLAASPERVDSLVADGK
TTATLTVTLMSGVNPVGGTMWVDIEAPEGVTEADYQFLPSKNDHFASGKITRTFSTNKPG
TYTFTFNSLTYGGYEMKPVTVTINAVPADTEGAEEK
>LFGLNPFC_02961 Glyoxal reductase
MQKVKLNNGIEMPLLGFGVFQMTDAAECERAVIDAINSGYRLIDTAASYQNEIQVGNALK
QSGIARNELFVTTKLWLQDTSYEGAKAQFERSLNRLQLDYVDLYLIHQPYGDVHGAWRAM
EELQQAGKIRAIGVSNFHPDRLADLIAFNNVVPAVNQVEVNPFNQQLQAVPWMQSRGIQP
EAWAPFAEGKNGLFQHPVLTAIGEKYGKSVGQVVLRWIYQRGIVSLAKSVRKERMEENIN
ILDFELSPEDMLQITALDTATSAFFSHRDPAMVEWLTGRKLDV
>LFGLNPFC_02962 Aldo-keto reductase IolS
MQKRYLGKSGLEVSALGLGCMGLSHGYGPATDTRQAIELIRAAIERGVTFFDTAEVYGPY
LNEEVVGEALKPFRDRVVIATKFGFTFGDDNKQQILNSRPEHIREAVEGSLRRLKTDVID
LLYQHRVDPDVPIEDVAGTVKDLIAEGKVKHFGLSEAGAQTIRRAHAVQPVTALQSEYSM
WWREPEQEILPLLEELGIGFVPFSPLGKGFLTGAIKPGTTFGKDDYRSTVPRFAAQAIEA
NEKLVTLLGELAAEKGVTSAQIALAWLLAQKPWIVPIPGTTKLHRLEENLAAADIVLSQK
DTQQISEALETIKIVGERYSPEHQARVGR
>LFGLNPFC_02963 HTH-type transcriptional regulator PgrR
MLKENFNELQIFLVVARERSFTKAAGKLGVSQSALSHAMKALEERLNIRLLTRTTRSVAP
TEAGERIIACLEPRIDELEQELESLIQLNGTPSGNIRLSAGEHAARSLVWPKLKPFLREY
PEINVELVVDNGFVDIVEGRFDAGIRLGENVDKDMVAVRIGPDMRMAVVGAPAYFAANPA
PETPHELQNHRCINMRLPTAGGLYHWEFEREGKPLRVKVDGQLTCSPLPERIDAALSGFG
IACVPEDMVQEYIESGKLIQVLQEWCPTFPGYYLYYPSRKQHPPAFALLIDALRYTE
>LFGLNPFC_02964 HTH-type transcriptional regulator PgrR
MMKIEPSILPSLAWFALIVRAGSFSRAASEMGITRAALSQNLKSLEERLNTKLIYRTTRN
MSLTEEGQHLYEVLVSALGQIDDALKDVGDTQLEPTGLLRINSSRVAARMLVEPHIGEFL
TRYPKTKIELIMDDGLSNIIADGCDVGIRLEQGLDEHMTAVPVSPLIKLVTVASPDYLKE
HGIPETPQELSNHNCLRLRHKSSGALSAWEFSNVVGGNEEFEIEVSGKYISNDDESMIRM
ALNGTGIIQHLDFAIAEHINAGKLQPILEDWAVSFPGFYIYVSSRVRMPSKVRAFIDFMV

EKRVKIES
>LFGLNPFC_02965 putative protein YcjY
MKTVSIKHTYWDIAADIYFPDDFNSEKKYPAIISAHPIGSCKEQTSGSVYGAALAKAGFI
VIAFDASFQGSSGGEPRYLEDPTMRVKDFSIVVDYLTTLPYVDAGRIGVLGICGGGGYAI
NAAMTERRIKAIGTVTGANYGRLMREGFTAFNPIGALEAMAQQRTDEANGAKLRVDDLLP
SSPEAALEAGLTEIDLFEATEYYRSPRGCAPNGVNRSLFSHQTVAVGWDAFHLAEVLLTQ
PLMVVVGDRVGAFGAYRDGCEIIGRAASKHKELVVVEGYSHYDLYDKPEPVKQALDKLIP
FYKTHL
>LFGLNPFC_02966 NADH oxidase
MTNKHPSLFSPFMLTEKIKLRNRIVMAPMTTWSANPDGTISEQELEFYKRRSQNVGLVIT
GCTYVTPSGIGFTHEFAAYDDRFINSLEKLAAAAKSGGAPAILQIFHAGNKAIPELVPNN
DVISASASSVKSGDFMKRVVQSREMTENEIQETIRAFGDVTKRAIKAGFDGVELHGAHGF
LLQNFFSPLFNQRNDRWGGDLEGRMRFPLAVLQEVKNVVYEYATKPFAIGYRISPEESVT
GGLRIEDTYKLLDRLISSGISYIHTSLVSINDSYPVESPNGPRTIELILNHIAGRVPVIA
AGKIRTPSQAQEAISAGLPLVAIGKGLVINPEWVTLAESGRSHEIQTALNPQRVPELTIP
DKLWDQIQASKGTGWFPLMD
>LFGLNPFC_02967 putative membrane protein YkgR
MKKNKVQQISHKLINIVVFVAIVEYAYLFLHFY
>LFGLNPFC_02968 50S ribosomal protein L31 type B
MMKPNIHPEYRTVVFHDTSVDEYFKIGSTIKTDREIELDGVTYPYVTIDVSSKSHPFYTG
KLRTVASEGNVARFTQRFGRFVSTKKGA
>LFGLNPFC_02969 50S ribosomal protein L36 2
MKVLNSLRTAKERHPDCQIVKRKGRLYVICKSNPRFKAVQGRKKKR
>LFGLNPFC_02970 HTH-type transcriptional regulator EcpR
MECQNRSDKYIWSPHDAYFYKGLSELIVDIDRLIYLSLEKIRKDFVFINLNTDSLTEFIN
RDNEWLSAVKGKQVVLIAARKSEALANYWYYNSNIRGVVYAGLSRDIRKELAYVINGRFL
RKDIKKDKITDREMEIIRMTAQGMLPKSIARIENCSVKTVYTHRRNAEAKLYSKLYKLVQ
>LFGLNPFC_02971 Common pilus major fimbrillin subunit EcpA
MSSIQLGKKSNEKKVLAIALVTVFTGTGVAQAADVTAQAVATWSATAKKDTTSKLVVTPL
GSLAFQYAEGIKGFNSQKGLFDVAIEGDSTATAFKLTSRLITNTLTQLDTSGSTLNVGVD
YNGATVEKTGDTVMIDTANGVLGGNLSPLANGYNASNRTTAQDGFTFSIISGTTNGTTAV
TDYSTLPEGIWSGDVSVQFDATWTS
>LFGLNPFC_02972 putative fimbrial chaperone EcpB
MKKHLLPLALLFSGISPAQALDVGDISSFMNSDSSTLSKTIQNSTDSGRLINIRLERLSS
PLDDGQVIAMDKPDELLLTPASLLLPAQASEVIRFFYKGPADEKERYYRIVWFDQALSDA
QRDNANRSAVATASARIGTILVVAPRQANYHFQYANGSLTNTGNATLRILAYGPCLKAAN
GKECKENYYLMPGKSRRFTRVDTADNKGRVALWQGDKFIPVK
>LFGLNPFC_02973 putative outer membrane usher protein EcpC
MPLRRFSPGLKAQFAFGMVFLFVQPDASAADISAQQIGGVIIPQAFSQALQDGMSVPLYI
HLAGSQGRQDDQRIGSAFIWLDDGQLRIRKIQLEESEDNASVSEQTRQQLMTLANAPFNE
ALTIPLTDNAQLDLSLRQLLQLVVKREALGTVLRSRSEDIGQSSVNTLSSNLSYNFGVYN
NQLRNGGSNTSSYLSLNNVTALREHHVVLDGSLYGIGSGQQDSELYKAMYERDFAGHRFA
GGMLDTWNLQSLGPMTAISAGKIYGLSWGNQASSTIFDSSQSATPVIAFLPAAGEVHLTR
DGRLLSVQNFTMGNHEVDTRGLPYGIYDVEVEVIVNGRVISKRTQRVNKLFSRGRGVGAP
LAWQIWGGSFHMDRWSENGKKTRPAKESWLAGASTSGSLSTFSWAATGYGYDNQAVGETR
LTLPLGGAINVNLQNMLASDSSWSNIASISATLPGGFSSLWVNQEKTRIGNQLRRSDADN
RAIGGTLNLNSLWSKLGTFSISYNDDRRYNSHYYTADYYQSVYSGTFGSLGLRAGIQRYN
NGDSSANTGKYIALDLSLPLGNWFSAGMTHQNGYTMANLSARKQFDEGTIRTVGANLSRA
ISGDTGDDKTLSGGAYAQFDARYASGTLNVNSAADGYINTNLTANGSVGWQGKNIAASGR
TDGNAGVIFDTGLENDGQISAKINGRIFPLNGKRNYLPLSPYGRYEVELQNSKNSLDSYD
IVSGRKSHLTLYPGNVAVIEPEVKQMVTVSGRIRAEDGTLLANARINNHIGRTRTDENGE
FVMDVDKKYPTIDFRYSGNKTCEVALELNQARGAVWVGDVVCSGLSSWAAVTQTGEENES
>LFGLNPFC_02974 Fimbria adhesin EcpD
MRVNLLIAMIIFALIWPATALRAAVSKTTWADAPAREFVFVENNSDDNFFVTPGGALDPR
LTGANRWTGLKYNGSGTIYQQSLGYIDNGYNTGLYTNWKFDMWLENSPVSSPLTGLRCIN
WYAGCNMTTSLILPQTTDASGFYGATVTSGGAKWMHGMLSDAFYQYLQQMPVGSSFTMTI
NACQTSVNYDANSGARCKDQASGNWYVRNVTHTKAANLRLINTHSLAEVFINSDGVPTLG
EGNADCRTQTIGSRSGLSCKMVNYTLQTNGLSNTSIHIFPAIANSSLASAVGAYDMQFSL
NGSSWKPVSNTAYYYTFNEMKSADSIYVFFSSNFFKQMVNLGISDINTKDLFNFRFQNTT
SPESGWYEFSTSNTLIIKPRDFSISIISDEYTQTPSREGYVGSGESALDFGYIVTTSGKT
AADEVLIKVTGPAQVIGGRSYCVFSSDDGKAKVPFPATLSFITRNGATKTYDAGCDDSWR
DMTDALWLTTPWTDISGEVGQMDKTTVKFSIPMDNAISLRTVDDNGWFGEVSASGEIHVQ
ATWRNIN
>LFGLNPFC_02975 putative fimbrial chaperone EcpE

MFRRRGVTLTKALLTVVCMLAAPLTQAISVGNLTFSLPSETDFVSKRVVNNNKSARIYRI
AISAIDSPGSSELRTRPVDGELLFAPRQLALQAGESEYFKFYYHGPRDNRERYYRVSFRE
VPTRNLTKRSPSGGEVSTEPVVVMDTILVVRPRQVQFKWSFDQVTGTVSNTGNTWFKLLI
KPGCDSTEEEGDAWYLRPGDVVHQPELRQPGNHYLVYNDKFIKISDSCPAKPPSAD
>LFGLNPFC_02976 Inner membrane protein YagU
MNIFEQTPPNRRRYGLAAFIGLIAGVVSAFVKWGAEVPLPPRSPVDMFNAACGPESLIRA
AGQIDCSRNFLNPPYIFLRDWLGLTDPNSAVYTFAGHVFNWVGVTHIIFSIVFAVGYCVV
AEVFPKIKLWQGLLAGALAQLFVHMISFPLMGLTPPLFDLPWYENVSEIFGHLVWFWSIE
IIRRDLRNRITHEPDPEIPLGSNR
>LFGLNPFC_02977 hypothetical protein
MATVTVHRPRCNSDKVYRHGRSCSQHERFRCRSCKRVFQLTYSYEARKPGFKELIVEMAH
NGTGAVISPEY
>LFGLNPFC_02978 Temperature-sensitive hemagglutinin tsh autotransporter
MNKIYALKYCYITNTVKVVSELARRVCKGSTRRGKRLSVLTSLALSALLPTVAGASTVGG
NNPYQTYRDFAENKGQFQAGATNIPIFNNKGELVGHLDKAPMVDFSSVNVSSNPGVATLI
NPQYIASVKHNKGYQSVSFGDGQNSYHIVDRNEHSSSDLHTPRLDKLVTEVAPATVTSSS
TADILNPSKYSAFYRAGSGSQYIQDSQGKRHWVTGGYGYLTGGILPTSFFYHGSDGIQLY
MGGNIHDHSILPSFGEAGDSGSPLFGWNTAKGQWELVGVYSGVGGGTNLIYSLIPQSFLS
QIYSEDNDAPVFFNASSGAPLQWKFDSSTGTGSLKQGSDEYAMHGQKGSDLNAGKNLTFL
GHNGQIDLENSVTQGAGSLTFTDDYTVTTSNGSTWTGAGIIVDKDASVNWQVNGVKGDNL
HKIGEGTLVVQGTGVNEGGLKVGDGTVVLNQQADSSGHVQAFSSVNIASGRPTVVLADNQ
QVNPDNISWGYRGGGSGC
>LFGLNPFC_02979 Hemoglobin-binding protease hbp autotransporter
MDGSANMSGTFTQENGRLTIQGHPVIHASTSQSIANTVSSLGDNSVLTQPTSFTQDDWEN
RTFSFGSLVLKDTDFGLGRNATLNTTIQADNSSVTLGDSRVFIDKKDGQGTAFTLEEGTS
VATKDADKSVFNGTVNLDNQSVLNINEIFNGGIQANNSTVNISSDSAVLENSTLTSTALN
LNKGANVLASQSFVSDGPVNISDATLSLNSRPDEVSHTLLPVYDYAGSWNLKGDDARLNV
GPYSMLSGNINVQDKGTVTLGGEGELSPDLTLQNQMLYSLFNGYRNTWSGSLNAPDATVS
MTDTQWSMNGNSTAGNMKLNRTIVGFNGGTSSFTTLTTDNLDAVQSAFVMRTDLNKADKL
VINKSATGHDNSIWVNFLKKPSDKDTLDIPLVSAPEATADNLFRASTRVVGFSDVTPTLS
VRKEDGKKEWVLDGYQVARNDGQGKAAATFMHISYNNFITEVNNLNKRMGDLRDINGEAG
TWVRLLNGSGSADGGFTDHYTLLQMGADRKHELGSMDLFTGVMATYTDTDASAGLYSGKT
KSWGGGFYASGLFRSGAYFDLIAKYIHNENKYDLNFAGAGKQNFRSHSLYAGAEVGYRYH
LTDTTFVEPQAELVWGRLQGQTFNWNDSGMDVSMRRNSVNPLVGRTGVVSGKTFSGKDWS
LTARAGLHYEFDLTDSADVHLKDAAGEHQINGRKDGRMLYGVGLNARFGDNTRLGLEVER
SAFGKYNTDDAINANIRYSF
>LFGLNPFC_02980 Transcriptional repressor MprA
MSFLLPCGGIMNNIDLLKTITNYKKIKNPAYPAQESLLIHLYIRVNDKIQSIIENELSEY
RINTSTFMVLVSLYMSDDYCQSPSDIYKELQFSKTNITHIIDKLEKKNIAKRINNKNDRR
SKSICLTPDGVTLAQKLINTQNVMLKKIWSGLSDDEMKTFELANKKLLSNLNVN
>LFGLNPFC_02981 Prophage integrase IntS
MKLNARQIDTAKPKEKAYKLADGGGLYLLVKPGGGEYWRLKYRVAGKEKLLALGVYPEVT
LADAPAKLEEAKRGISGGIDLMEVKREEKIARETQLNNTFKDIALEWHSNKL
>LFGLNPFC_02983 Gamma-glutamyl phosphate reductase
MLEQMGIAAKQASYKLAQLSSREKNRVLEKIADELEAQSESILNANAQDVADARANGLSE
AMLDRLALTPARLKGIADDVRQVCNLADPVGQVIDGGVLDSGLRLERRRVPLGVIGVIYE
ARPNVTVDVASLCLKTGNAVILRGGKETCRTNAATVAVIQDALKSCGLPAGAVQAIDNPD
RALVSEMLRMDKYIDMLIPRGGAGLHKLCREQSTIPVITGGIGVCHIYVDESAEIAEALK
VIVNAKTQRPSTCNTVETLLVNKNIADSFLPALSKQMAESGVTLHADAAALAQLQAGPAK
VVAVKAEEYDDEFLSLDLNVKIVSDLDDAIAHIREHGTQHSDAILTRDMRNAQRFVNEVD
SSAVYVNASTRFTDGGQFGLGAEVAVSTQKLHARGPMGLEALTTYKWIGIGDYTIRA
>LFGLNPFC_02984 Glutamate 5-kinase
MSDSQTLVVKLGTSVLTGGSRRLNRAHIVELVRQCAQLHAAGHRIVIVTSGAIAAGREHL
GYPELPATIASKQLLAAVGQSRLIQLWEQLFSIYGIHVGQMLLTRADMEDRERFLNARDT
LRALLDNNIVPVINENDAVATAEIKVGDNDNLSALAAILAGADKLLLLTDQKGLYTADPR
SNPQAELIKDVYGIDDALRAIAGDSVSGLGTGGMSTKLQAADVACRAGIDTIIAAGSKPG
VIGDVMEGISVGTLFHAQATPLENRKRWIFGAPPAGEITVDEGATAAILERGSSLLPKGI
KSVTGNFSRGEVIRICNLEGRDIAHGVSRYNSDALRRIAGHHSQEIDAILGYEYGPVAVH
RDDMITR
>LFGLNPFC_02985 Outer membrane porin PhoE
MKKSTLALVVMGIVASVSVQAAEIYNKDGNKLDVYGKVKAMHYMSDNDSKDGDQSYIRFG
FKGETQINVQLTGYGRWEAEFAGNKAESDTAQQKTRLAFAGLKYKDLGSFDYGRNLGALY
DVEAWTDMFPEFGGDSSAQTDNFMTKRASGLATYRNTDFFGVIDGLNLTLQYQGKNENRD
VKKQNGDGFGTSLTYDFGGSDFAISGAYTNSDRTNEQNLQSRGTGKRAEAWATGLKYDAN

NIYLATFYSETRKMTPISGGFANKTQNFEAVAQYQFDFGLRPSLGYVLSKGKDIEGIGDE
DLVNYVDVGATYYFNKNMSAFVDYKINQLDSDNKLNINNDDIVAVGMTYQF
>LFGLNPFC_02986 Sigma factor-binding protein Crl
MTLPSGHPKSRLVKKFTALGPYIREGKCEDNRFFFDCLAVCVNVKPAPEVREFWGWWMEL
EAQESRFTYSYQFGLFDKAGDWTSVQIKDAEVVERLEHTLREFHEKLRELLATLNLKLEP
ADDFRDEPVKLTA
>LFGLNPFC_02987 hypothetical protein
MTQANLSETLFKPRFKHPETSTLVRRFSHGAQPPVQSALDGKTIPHWYRMINRLMWIWRG
IDPREILDVQARIVMSDAERTDDDLYDTVIGYRGGNWIYEWATQAMVWQQKACAEEDPQL
SGRHWLHAATLYNIAAYPHLKGDDLAEQAQALSNRAYEEAAQRLPGTMRQMEFTVPGGAP
ITGFLHMPKGDGPFPTVLMCGGLDAMQTDYYSLYERYFAPRGIAMLTIDMPSVGFSSKWK
LTQDSSLLHQHVLKALPNVPWVDHTRVAAFGFRFGANVAVRLAYLESPRLKAVACLGPVV
HTLLSDFKCQQQVPEMYLDVLASRLGMHDASDEALRVELNRYSLKVQGLLGRRCPTPMLS
GYWKNDPFSPEEDSRLITSSSADGKLLEIPFNPVYRNFDKGLQEITDWIEKRLC
>LFGLNPFC_02988 Xanthine phosphoribosyltransferase
MSEKYIVTWDMLQIHARKLASRLMPSEQWKGIIAVSRGGLVPGALLARELGIRHVDTVCI
SSYDHDNQRELKVLKRAEGDGEGFIVIDDLVDTGGTAVAIREMYPKAHFITIFAKPAGRP
LVDDYVVDIPQNTWIEQPWDMGVVFVPPISGR
>LFGLNPFC_02989 Cytosol non-specific dipeptidase
MSELSQLSPQPLWDIFAKICSIPHPSYHEEQLAEYIVGWAKEKGFHVERDQVGNILIRKP
ATAGMENRKPVVLQAHLDMVPQKNNDTVHDFTKDPIQPYIDGEWVKARGTTLGADNGIGM
ASALAVLADENVVHGPLEVLLTMTEEAGMDGAFGLQSNWLQADILINTDSEEEGEIYMGC
AGGIDFTSNLHLDREAVPAGFETFKLTLKGLKGGHSGGEIHVGLGNANKLLVRFLAGHAE
ELDLRLIDFNGGTLRNAIPREAFATIAVAADKVDALKSLVDTYQEILKNELAEKEKNLAL
LLDSVANDKAALIAKSRDTFIRLLNATPNGVIRNSDVAKGVVETSLNVGVVTMTDNNVEI
HCLIRSLIDSGKDYVVSMLDSLGKLAGAKTEAKGAYPGWQPDANSPVMHLVRETYQRLFN
KTPNIQIIHAGLECGLFKKPYPEMDMVSIGPTITGPHSPDEQVHIESVGHYWTLLTELLK
EIPAK
>LFGLNPFC_02990 Peptide chain release factor 1
MILLQLSSAQGPEECCLAVKKALDRLIKEATRQDVAVTVLETETGRYSDTLRSALISLDG
DNAWALSESWCGTIQWICPSPYRPHHGRKNWFLGIGRFTADEQEQSDAIRYETLRSSGPG
GQHVNKTDSAVRATHLASGISVKVQSERSQHANKRLARLLIAWKLEQQQQENSAALKSQR
RMFHHQIERGNPRRTFTGMAFIEG
>LFGLNPFC_02991 RNA-splicing ligase RtcB
MGKYIRPLSDAVFTIASDDLWIESSAIQQLHTTANLPNMQRVVGMPDLHPGRGYPIGAAF
FSVGRFYPALVGNDIGCGMALWQTDILARKYNADKFEKRLSDLDDVAEESWLEENLPSAF
AQHPWRNSLGSIGGGNHFAELQQIDQIIDAELFALAGLDAQHLQLLVHSGSRGLGQSILQ
RHIASFSHHGLPEGSDDALRYIAEHDDALAFARINRQLIALRIMQQVKATGSPVLDVAHN
FVSACQIGDQQGWLHRKGATPDDNGLVIIPGSRGDYSWLVKPVANEKTLHSLAHGAGRKW
GRTECKGRLAAKYTATQLSRTELGSRVICRDKQLIFEEAPQAYKSAESVVQCLVLAGLII
PVARLRPVLTLKNSGGKKG
>LFGLNPFC_02992 putative N-acetyltransferase YafP
MTASQHYSPQQIAAWAQIDESRWKEKLAKSQVRVAVINAQPVGFITCIGHYIDMLFVEPE
YTRRGVASALLKPLIKSESELTVDASITAKPFFERYGFQTVKQQRVECRGTWFTNFYMRY
KPQH
>LFGLNPFC_02993 mRNA interferase toxin YafO
MRVFKTKLIRLQLTAEELEALTADFISYKRDGVLPDIFGRDALYDDSFTWPLIKFERVAH
IHLANVNNPFPPQLRQFSRTNDAAHLVYCQGAFDEQAWLLIAILKPEPHKLARDNNQMHK
IGKMAEAFRMRF
>LFGLNPFC_02994 Antitoxin YafN
MHRILAEKSVNITELRKNPAKYFIDQPVAVLSNNRPAGYLLSASAFEALMDMLAEQEEKK
PIKARFRPSAARLEEITRRAEQYLNDMTDDDFNDFKE
>LFGLNPFC_02995 DNA polymerase IV
MRKIIHVDMDCFFAAVEMRDNPALRDIPIAIGGSRERRGVISTANYPARKFGVRSAMPTG
MALKLCPHLTLLPGRFDAYKEASNHIREIFSRYTSRIEPLSLDEAYLDVTDSVHCHGSAT
LIAQEIRQTIFNELQLTASAGVAPVKFLAKIASDMNKPNGQFVITPAEVPAFLQTLPLAK
IPGVGKVSAAKLEAMGLRTCGDVQKCDLVTLLKRFGKFGRILWERSQGIDERDVNSERLR
KSVGVERTMAEDIHHWSECEAIIERLYPELERRLAKVKPDLLIARQGVKLKFDDFQQTTQ
EHVWPRLNKSDLIATARKTWDERRGGRGVRLVGLHVTLLDPQMERQLVLGL
>LFGLNPFC_02996 hypothetical protein
MSPLNKISPSHLPKPATVAVPEETEKKARDVNEKTALLKKKSATELGELATSINTIARDA
HMEANLEMEIVPQGLRVLIKDDQNRNMFERGSAKIMPFFKTLLVELAPVLDSLDNKIIIT
GHTDAMAYKNNIYNNWNLSGDRALSARRVLEEAGMPENKVMQVSAMADQMLLDAKNPQSA
GNRRIEIMVLTKSASDTLYQYFGGQHGDKVVQPLVQKLDKQQVLSQRAR

>LFGLNPFC_02997 Flagellar biosynthesis protein FlhA
MLSRSDLLTLLTINFIVVTKGAERISEVSARFTLDAMPGKQMAIDADLNAGLINQTQAQT
RRKDVASEADFYGAMDGASKFVRGDAIAGMMILAINLIGGVCIGIFKYNLSADAAFQQYV
LMTIGDGLVAQIPSLLLSTAAAIIVTRVSDNGDIAHDVRNQLLASPSVLYTATGIMFVLA
VVPGMPHFPFLMFSALLGFTGWRMSKRPQAAEAEEKSLETLTRTMTETSEQQVSWETIPL
IEPISLSLGYKLVALVDKAQGNPLTQRIRGVRQVISDGNGVLLPEIRIRENFRLKPSQYA
IFINGIKADEADIPADKLMALPSSETYGEIDGVLGNDPAYGMPVTWIQPAQKAKALNMGY
QVIDSASVIATHVNKIVRSYIPDLFNYDDITQLHNRLSSMAPRLAEDLSAALNYSQLLKV
YRTLLTEGVSLRDIVTIATVLVASSAVTKDHILLAADVRLVLRRSITHPFVRKQELTVYT
LNNELENLLTNVVNQAQQAGKVMLDSVPVDPNMLNQFQITMPQVKEQMKAAGKDPVLLVP
PQLRPLLARYARLFAPGLHVLSYNEVLDELELKIMGALM
>LFGLNPFC_02998 REP-associated tyrosine transposase
MSEYRRYYIKGGTWFFTVNLQNRRNQLLTTQFQTLRNVIIKVKRDRPFEINAWVVLPEHM
HCIWTLPEGDDDFSSRWREIKKQFTHACGLKNIWQPRFWEHAIRNTKDYRHHVDYIYINP
VKHGWVKQVSDWPFSTFHRDVAKGLYPIDWAGDVTDFSAGERIIL
>LFGLNPFC_02999 hypothetical protein
MSFMSSFLHGRFLHPGVFSLCVLLPLLASATTSHISFSYAARQRMQNRARLLKQYQTHLK
KQASYIVQGNAKSRRALRQHNREQIKQHPEWFPAPLKASDRRWQALVENNHFLSSDHLHN
ITEVAIHRLEQQLGKPYIWGGTRPDKGFDCSGLVFYAYNKILEAKLPRTANEMYHYRRAT
IVANNDLRRGDLLFFHIHSREIADHMGVYLGDGQFIESPRTGETIRVSRLAEPFWQDHFL
GARRILTEETIL
>LFGLNPFC_03000 hypothetical protein
MRKIALILAMLLIPCVSFAGLLGSSSSTTPVSKEYKQQLMGSPVYIQIFKEERTLDLYVK
MGEQYQLLDSYKICKYSGGLGPKQRQGDFKSPEGFYSVQRNQLKPDSRYYKAINIGFPNA
YDRAHGYEGKYLMIHGDCVSIGCYAMTNQGIDEIFQFVTGALVFGQPSVQVSIYPFRMTD
ANMKRHKYSNFKDFWEQLKPGYDYFEQTRKPPTVSVVNGRYVVSKPLSHEVVQPQLASNY
TLPEAK
>LFGLNPFC_03001 Putative glutamine amidotransferase YafJ
MCELLGMSANVPTDICFSFTGLVQRGGGTGPHKDGWGITFYEGKGCRTFKDPQPSFNSPI
AKLVQDYPIKSCSVLAHIRQANRGEVALENTHPFTRELWGRNWTYAHNGQLTGYKSLETG
NFRPVGETDSEKAFCWLLHKLTQRYPRTPGNMAAVFKYIASLADELRQKGVFNMLLSDGR
YVMAYCSTNLHWITRRAPFGVATLLDQDVEIDFSSQTTPNDVVTVIATQPLTGNETWQKI
MPGEWRLFCLGECVV
>LFGLNPFC_03002 Phosphoheptose isomerase
MYQDLIRNELNEAAETLANFLKDDANIHAIQRAAVLLADSFKAGGKVLSCGNGGSHCDAM
HFAEELTGRYRENRPGYPAIAISDVSHISCVGNDFGFNDIFSRYVEAVGREGDVLLGIST
SGNSANVIKAIAAAREKGMKVITLTGKDGGKMAGTADIEIRVPHFGYADRIQEIHIKVIH
ILIQLIEKEMVK
>LFGLNPFC_03003 Acyl-coenzyme A dehydrogenase
MMILSILATVVLLSALFYHRVSLFISSLILLAWTAALGVAGLWSAWVLVPLAIILVPFNF
APMRKSMISAPVFRGFRKVMPPMSRTEKEAIDAGTTWWEGDLFQGKPDWKKLHNYPQPRL
TAEEQAFLDGPVEEACRMANDFQITHELADLPPELWAYLKEHRFFAMIIKKEYGGLEFSA
YAQSRVLQKLSGVSGILAITVGVPNSLGPGELLQHYGTDEQKNHYLPRLARGQEIPCFAL
TSPEAGSDAGAIPDTGIVCMGEWQGQQVMGMRLTWNKRYITLAPIATVLGLAFKLSDPEK
LLGGAEDLGITCALIPTTTPGVEIGRRHFPLNVPFQNGPTRGKDVFVPIDYIIGGPKMAG
QGWRMLVECLSVGRGITLPSNSTGGVKSVALATGAYAYIRRQFKISIGKMEGIEEPLARI
AGNAYVMDAAASLITYGIMLGEKPAVLSAIVKYHCTHRGQQSIIDAMDITGGKGIMLGQS
NFLARAYQGAPIAITVEGANILTRSMMIFGQGAIRCHPYVLEEMEAAKNNDVNAFDKLLF
KHIGHVGSNKVRSFWLGLTRGLTSSTPTGDATKRYYQHLNRLSANLALLSDVSMAVLGGS
LKRRERISARLGDILSQLYLASAVLKRYDDEGRNEADLPLVHWGVQDALYQAEQAMDDLL
QNFPNRVVAGLLNVVIFPTGRHYLAPSDKLDHKVAKILQVPNATRSRIGRGQYLTPSEHN
PVGLLEEALVDVIAADPIHQRICKELGKNLPFTRLDELAHNALAKGLIDKDEAAILVKAE
ESRLRSINVDDFDPEELATKPVKLPEKVRKVEAA
>LFGLNPFC_03004 Inhibitor of vertebrate lysozyme
MFKAITTVAALVIATSAMAQDDLTISSLAKGETTKAAFNQMVQGHKLPAWVMKGGTYTPA
QTVTLGDETYQVMSACKPHDCGSQRIAVMWSEKSNQMTGLFSTIDEKTSQEKLTWLNVND
ALSIDGKTVLFAALTGSLENHPDGFNFK
>LFGLNPFC_03005 Omega-amidase YafV
MPGLKITLLQQPLVWMDGPANLRHFDRQLEGITGRDVIVLPEMFTSGFAMEAAASSLAQD
DVVNWMTAKAQQCNALIAGSVALQTESGSVNRFLLVEPGGTVHFYDKRHLFRMADEHLHY
KAGNARVIVEWRGWRILPLVCYDLRFPVWSRNLNDYDLALYVANWPAPRSLHWQALLTAR
AIENQAYVAGCNRVGSDGNGCHYRGDSRVINPQGEIIATADAHQATRIDAELSMMALREY
REKFPAWRDADEFRLW
>LFGLNPFC_03006 hypothetical protein

MLKMSLYVIILLFSLQFSAAITGKESEVVSPLLMDVNPSLTMENISELSTSSEPSQQGVF
PVICTRLHPGSVMKRQLLTGWGPVFIIGDDPFSLRWMSEHLEILKSLNALGLVVNVESVE
RMEVLQQRADGLLLLPVICDNFVQALQLNAYPVLITEMEISQ
>LFGLNPFC_03007 hypothetical protein
MVDIVEADKTDIYFIQESVYGKIGLPAFGNTIGPSAQQVVKKVFAVVKERDKTHAKQRLL
LEYNGNKLWMNAIDGSEAILPTEFSKRYELSLFNTTNFGEDPFPDVNLYNNMKSSFFVRF
GGTSHPEAWAIYNASTKEVKYIETAREIDKIFSDFNLSGTLPIHIGQ
>LFGLNPFC_03008 hypothetical protein
MRDCHSSDDKDVIAIDGKTLRHSYDKSRRKGAIYVIKIRLHIICDVPDELIDFTFEWKEL
KKLCMAVSFRSIITEQKKEPEMTDRYYSISADLTAEKFATANRNHWYVENKLHWHLDVVM
NKDDCKIRRGNAAELFSGIRKIAINILTKDKILKAGARCKM
>LFGLNPFC_03009 hypothetical protein
MELKKLMEHISIIPDYRQAWKVEHKLPDILSVNYLRRYFWCIMLGRYRGFGETHLDFLK
>LFGLNPFC_03010 hypothetical protein
MGIVFTNHNIDLLSVEFDEITKNCNYTFSVDGETAIFTARISIIRNIKGIKYSEELDKFI
MSIMPLQPKVSKILGGVTWDCICGKEVGFPVRLIGK
>LFGLNPFC_03011 hypothetical protein
MGNYHIGDGECRFRVVCSSPDVCEVGGYKVPFDSYQTLDSERQYSSTVWARGCRALNVGS
VIAGTQSNAGKGVISGTSQGTGDCVILTGSPTVTIEGKPVAYHGSVVGINNHNCLGKLYT
KIKSPMISVIDRTFNYERTAEVIHDLLLLKDLLSVGNIFDGDISPEVKNDLFQIKDPDQS
WGEFFSIKNIRESLRNGIEGDKSQIREWFGENTLTQMGNGAITTLHGVADLALVTFDALL
DTATATVACPIGEDGLCEQANINLNEKEQALFNISNSLINGQAWDALKKMIMDTNNGDQI
ALEHFASFLWGFMIPAKIPEENISGKVFVEPVVLEGGAGGNWTVFDEVLDSNVIKQLTLT
GCGAACGEMLLRDRYIFVTQNVIGTELTSMTSLANKLNKFDVGWEGNAVSESSLYALSNT
GSWGAMMWDSGSKVGHWVLVKGVDDAGNVIIYDPYQGSRYLMTEQEFKEVWNGHSVYKP
>LFGLNPFC_03012 hypothetical protein
MIEMKNNTPFPLLSFEKYGRYGLLFDVIAIKMSLRIKNGFYADLAEFQKELSMSDEYYGE
SETSSLKSETDLVLCKRNTDIHVTGSAHAPSGDKSQWKACVRVNSFSKELSLSGVRYLQY
ERNRWQMSLPDKIINVPLRYELAYGGIWQPDGMEKLVFSANPVGCGYYPDISQLNTSCQY
KLPQITSSALSENATIFNGESDFFQGVGPVSRWWKSRLQYAGTYNEVWREKRYPYLPDDF
DERFYNSAHPDMIYTGFLSGDENISLEGFFKFEQVVKTKLPGIRPVLILKTKHNTSHMFL
PVADTMVIDLSRQEIYLTWRLTIPDFFGMKEGVLSCIIPECIGKKYYG
>LFGLNPFC_03013 Actin cross-linking toxin VgrG1
MSLKGLRFTLEVDGQEPDTFAVVNFRLIQNQSYPFVMSVDVASDSFMQTAEMLLEKNATL
TIWQGVIPLRYVTGVVAGFGMQENNGWQMRYHLRIEPPLWRCGLRRNFRIFQQQDIRTIS
ATLLNENGVTEWTPLFYEDHPAREFCVQYGESDLAFLARLWAEEGIFFFERFAADSPEQK
LTLCDDVAGLSQAGELPFNPDTSAGAETECVSMFRYEAHVRPSSVQSQDYTFKVPDWPGM
YEQQGESLNGQLEQYEIFDYPGRYKDEQHGKDFTLYRMESLRSDAEKATGQSNSPKLWPG
TWFTLTGHPQKMLNREWQVVQSILSGDQPQALHGSQGRGTTLGNQLEVIPADRTWRPRLQ
SKPKVDGPQSAIVTGPAGEEIFCDEHGRVRVKFHWDRYNPATEASSCWVRVSQAWAGPGF
GNLAIPRVGQEVIVDFLNGDPDQPIIMGRTYHEDNRSPGSLPGTKTQMTIRSKTYKGSGF
NELRFEDATGGEQVYIHAQKNMDTEVLNNRTTDVKADHTETIGNDQKITVGLGQTVNVGS
KKEGGHDQKVTVANDQHLTIKNDRHKVVNNNQTSKVTGTDTEEVVKKQSIKIGDNYELKV
EHGTNIISGDSIELICGQGESGTCSIKLEKTGKIIIRGTEFLFEATGPVDIKGKDIHLNG
>LFGLNPFC_03014 hypothetical protein
MREMVDAGNPGGSVPENVKMSVTLHWHPLLHGGNHLVRE
>LFGLNPFC_03015 Major exported protein
MPTPCYISITGQTQGNITAGAFTADSVGNIYVQGHEDEMLVQEFLHNVTVPTDPQSGQPS
GQRAHKPFIFTVALNKAVPLLYNALASGEMLPKVELHWWRTSVEGKQEHYFTTRLTDATI
VDMNLHMPHCQDPAQREFTQLLAVSLAYRKVEWEHIKSGTSGADDWRAPLEA
>LFGLNPFC_03016 hypothetical protein
MSKMNNNGGSVAPKERISVRYTPKVDGVAADIELPLNLLITGNLKGKPDNTPLDERTAIA
INRYNLNAVISEADIEREFVVPAELSDAPNEQMYVNLKVKSMDDLSPDHIASQVPEIKRL
LELREALVALKSPLGNIPAFRAQLQALLENEDTREQLIQELGIAVQK
>LFGLNPFC_03017 hypothetical protein
MSVKEEIAPVSASQTAAPPSLLDEIMAQTRVQPKSESYDITRQGVSAFIAAMLQGDSSAE
PINILAVDAMIADIDARTSRQMDAIIHAPEFQELESLLRSLKLLVERADTRENIKVHFLN
VTQEELLDDFEFAPEITQSAYYKHVYSSGYGQFGGEPVAAVIGNFAFKNTTPDMKLLKYI
SQVSAMAHSPFLSSVSSEFFGLDSWTELPGIKEPGAIFEGPAYSRWRALRESEDSRYLGL
TAPRFLLRHPYSPDENPVKTFRYHEDVSQSHESYLWGNTSFLLAANLAESFAKYRWCPNI
IGPSSGGAVKDLPVHLYESMGQMQAKIPTEVLITDRREYELAEEGFITLTMRKGSDNACF
FSANSVQKPKTFPKTPEGKAAETNYKLGTQLPYLFVISRLAHYIKVIQREQLGSWKERSD
LERELNTWIRQYVADQENPPAEVRSHRPLRQAKIEVLDVDGEPGWYQVAISVRPHFKYMG
ASFDLSLVGRLDKE

```
>LFGLNPFC_03018 hypothetical protein
MLRRDSGPTGSLFERIREAANPPSYQNPKEALIRSIRRNLRQVLNTRSGSCYGSPELGIT
DLNDESLASSDFRREIRKSISQCILHYEPRITDVVVTAAAPDEYAPVELCFHIVATVDVS
ETRGVFEFDILLDNHQRYCVE
>LFGLNPFC_03019 hypothetical protein
MAFEERYYREELDYLRQLGKLLAQEKPHLAHFLAEKEGDPDVERLMEAFAFMSGSLRQKL
EDEFPEFTHGLIRMLWANYLRPVPAMTVIAYEPKTDQLKVPVQVCRNELIRSRSEKSSLT
AQKVLPDNHDKIVSSVACHFTLARDIWLQPLRILDTRNASSLKEGIIDISFTADNNVSPA
MLDLNKITFWLGNEDDYTRHQLYLWFCECLMDAELIAGEYRLPLPDLWLEAAGFDNRDAL
LPWPKNVHSGYRVLQEYFCYPEAFFFFHLRDVAPLPDDFPINAFTLRLHFNRPLPADIKL
RQDSLRLHCTPAINLFTHYAEPVRPDGRMAEYPLRASHKHPDAYDIFQVSTVTSKVKVSG
TEISPGSQARVWPEFESFQHQMEYSRQREVVYWHHRTKTSLLHHGLEHSIAFVHADGSIP
GSSRFNDDVITTSLICTNRMIPARLHTGDICVAVNKNPAVASFRNVTRPTLPLWPVTDGD
MHWSLISAMNLNYLSLLDRETLIQILRTFDLPGAHHPQRARLSRQKLDAIEKLETKPVDR
LFKGVPVRGLATTLWIRPDPFICEGEIYLLGTVLSHFFALYASINSYHCLKIINTESQES
WEWQEKMGQHALI
>LFGLNPFC_03020 hypothetical protein
MAGENGPARPDIATKPPLPADVRNCNFYVLMEALYRRHGAPGQDISLRTEPAREIVRFSS
DASISFPGTDLSALSRSQNGQYVLQTRFLGFSGSQSPLPGYYLDQMAQESAQNEDGLKEF
LDLFSHRWTQFAYHAWRKYRYYICFRSGGTDTFSQRMYALVGLGNQSVRDRLAINHSKML
AYAGILATPGRAPEVICNLVSHCFDLPDVTIERWQLRKVAIDPAHQNRLGVRNPKGKTAG
HIPGRSILGVNFTLGARVPDRSGKFLLQIGGLSRERYLSFLPDGENHLPLTMFLSFILRD
QFAWDLRLCLAPQQAKGMRLSDPASSRLGRTSFIGQPKVPPAITIRIRE
>LFGLNPFC_03021 hypothetical protein
MAEEKQQSRQASLTLQVMNGNELESGRAAKCLFSENGGDIGHTPECHWQVQDRAGSIAAR
ACTVIRHDGAYCLRCLTPGLMINLAPASSDALIRLRQGDEIQLGALALKVFLHDGAAVTY
DERMATPETIVMNRDSLADTLLTTEGQPAYPGMPFQHQLAPTVAHGFSSDPLQALQTESL
MVADDPIAPRVSRPVAPASDLTGANGINTPFMDLPPGNARHEDGDEFSAMAQYHLAVTPL
LRGMECPLTLHNSQDADEFLEEAGRALQAAIKGLLSLQQQQNSLSDKHLRPLEDNPLRLD
MDYATALNVMFAEGKSPVHLAAPAAIAESLRNIRHHEEANRAAIVEALRVMLDAFSPGNL
MRRFAQYRRSHELRQKMDDAWAWQMYSNYYDELASSRQQGFEMLFNEVYAQVYDRVLREK
QREPEA
>LFGLNPFC_03022 hypothetical protein
MNGKAFLACVLMSVVLTGCETAKKISQVIRNPDIQVGKLMDQSTELTVTLLTEPDSNLTA
DGEAAPVDVQLVYLSDDSKFHAADYDQVATTALPDVLGKNYIDHQDFNLLPDTVKTLPPI
KLDEKTGYIGVIAYFSDDQATEWKQIESVESIGHHYRLLVHIRASAIEMKKEEN
>LFGLNPFC_03023 hypothetical protein
MATMKNKVIWQEGLFALPQHFQQQRHCEYLLTRRLDALGDFFWGFTELSINTELLAQGK
IMIDRAAGCMPDGTVFSIPDQDLLPEPFQPGTLSSKESHNIYLALPVISDVINEIQGLHS
AGQGTERYRLTHTRVRDFHTDEGDEQPVGLGQLIPRIVSGADDLSAMVTLPLCRILNKNA
TGALVLDNTFIPTIQAVRVSGLLGAFSGEVQGLLATRAADLAGRIGSPEQSGIADVAEFM
MLQMLNRYQMQFTHRSQLHTLHPEAFYRDLVGLLGELMTFTEGNRLPCTVEPYNHRDLTA
TFTRTVIPELRRALNTVLVPRAQNLPLFFSEGTWIATINDPTLLQSCKLVLAVRARMPYD
QVQRQFIQQSKVAATDKIRSVVSVQVPGIPLHTLTAAPRQLPYHEGYVYFELEKGTPAWQ
DVIKAGALALHISGSFPDLNLQLWAIRG
>LFGLNPFC_03024 hypothetical protein
MSDMSEASVFPVEPESGTPGRQYRLALRGNSLNPMIDAATPLLGMVMRLSTMNSQAMPEH
LFTQVVTDVQAVEQLLQEQGYEPGVIVSFRYILCTFIDEAALGNGWSNKNEWIKQSLLVH
FHNEAWGGEKVFILLERLIREPVRYQDLLEFLYLCFSLGFRGRYKVAIQQQDEFERIYQR
LHHALHKLRGDAPFPLLHQKNNIQGGRYQLIRRLTIKHVLCGGLVVLTVFYLFYLLRLDS
QTQDILHQLNKLLR
>LFGLNPFC_03025 Protein ClpV1
MIQIDLATLVKRLNPFAKQALEMAASECMSQQASEITVAHVLLQMLAIPRNDVRVIAERT
GISAEDLRQALTVESYPGGRSAEGYPSFSPMLIEWLKESWLLASAQMQHSELRSGVLLLT
LLHSPLRYIPPAAARLLTAINRDQLQQDFAAWTKESAESVDLAGGQTPRATETGDTLLAR
YAKNMTADARNGRLDPVLCRNYEIDLMIDILCRRRKNNPVVVGEAGVGKSALIEGLALRI
VAGQVPDKLKNTDIMTLDLGALQAGASVKGEFEKRFKGLMAEVIFSPVPVILFIDEAHTL
IGAGNQQGGLDISNLLKPALARGELKTIAATTWSEYKKYFEKDAALSRRFQLVKVSEPNA
AEATIILRGLSAVYEQSHGVLIDDDALQAAATLSERYLSGRQLPDKAIDVLDTACARVAI
NLSSPPKQISALTTLSHQQEAEIRQLERELRIGLRTDTSRMTEVLEQYDETLSALDELEV
AWQQQTLVQEIIALRQQLLGVAEDDVASLPDADAVEDTPPEAEQDSTDAESADDAGSVQP
EETAETVSPVQRLAQLTAELDALHNDQLLVSPHVDKKQIAAVIAEWTGVPLNRLSQNEMS
VITDLPVWLGGTIKGQDLAIASLHKHLLTARADLRRPGRPLGAFLLAGPSGVGKTETVLQ
LAELLYGGRQYLTTINMSEFQEKHTVSRLIGSPPGYVGYGEGGVLTEAIRQKPYSVVLLD
```

EVEKAHPDVLNLFYQAFDKGEMADGEGRLIDCKNIVFFLTSNLGYQVIVEHADDPETMQE
ALYPVLADFFKPALLARMEVVPYLPLSKETLATIIAGKLARLDNVLRSRFGADVIIGPEV
TDEIMSRVTRAENGARMLESVIDGDMLPPLSLLLLQKMAANTAIARIRLSAADGAFTADV
EDALPDDAVTPQTEDETVL
>LFGLNPFC_03026 hypothetical protein
MSRLCVFRQTGWFIAGLMAGLPASAAPTETSSVAGVAVAVATATPPDATATLQAMQSCRQ
ETAALERLDCYDRILAPEQAGFGGAALVKARYQGEAWARATEQEKRRQGNTTELLVTQVP
GERPTVVITTPAIGHVPPRPVLMFSCVDNITRMQVALMHPLDVHDIAVTLNADSRALRSH
WFVRENGTLLESSRGLSGIDEIKQLFGAKTLTVDTGTDNAAGKLTFNIDGLARVIAPLRD
ACHWAGE
>LFGLNPFC_03027 hypothetical protein
MAMDLRNPDVWLAHLLENLPEDKLSAALDDGNADWEFVDSEIVKLGSLAHSQLDIPELQR
RGLMLLASETKDFRLLAHLLRTLQHAGDILLASRMLAQYTEHYWTCAAPQNMAHKKRFAA
QVIKRFESAVQDFAGNAATIQRDALLGELAKLAQCWQAHNASELAKATDDLFSLFQRAFR
DVAPEMTSSARSAAIAPQATTGYVPDMSPHTVAAQPPVAATPIPQVTVENHDDKAWRDTL
LKVAAILCERQPESPQGYRLRRHALWQTITSTPQAESDGRTPLAAFPVDMMDDYLARLNN
ADMALWQQVEKSLLLAPYWLDGHYLSAQTALRLGYKQVADAIRDEVTDFLARLPALINLL
FNDRTPFVSEQTKQWLASSGSVNQTVPVVQTDEELQAAKACFDENGLEAALRYLENLPEG
DPRHQFHRQFFGAQLLEEAGMVQLAQQQYRMLFRTGLHMMLSEWEPSLLKALEQKLTAEQ
>LFGLNPFC_03028 hypothetical protein
MFKFPTSRLFSTLKSALRPAMPRFKVSATWLLTLAWIFLLVWIWWQGPKWTLYEQHWLAP
LANRWLATAVWGLIALVWLTWRVMKRLQKLEKQQKQQREEEKDPLTVELHRQQQYLDHWL
LRLRRHLDNRRYLWQLPWYMVIGPAGSGKSTLLREGFPSDIVYTPESIRGVEYHPLITPR
VGNQAVIFDVDGVLTTPGGDDLLRRRLREHWLGWLMQTRARQPLNGLILTLDLPDLLTAD
KSRRETLVQNLRQQLQEIRQSLHCRLPVYVVLTRLDLLNGFAALFHSLDKKDRDAILGVT
FTRRAHESDGWRSELGAFWQTWVQQVNLALSDLVLAQTGAAPRSAVFSFSRQMQGTGEIV
TALLAALLDGENMDVMLRGVWLTSSLQRGQVDDIFTQSAARQYGLGNSSLATWPLVETTP
YFTRRLFPEVLLAEPNLAGENSVWLNSSRRRLTAFSTCGAALAALMVGSWHHYYNQNWQS
GVNVLAQAKAFMDVPPPQGTDEFGNLQLPLLNPVRDATLAYGDYRDHGFLADMGLYQGAR
VGPYVEQTYIQLLEQRYLPSLMNGLIRDLNIAPPESEEKLAVLRVVRMMEDKSGRNNEAV
KQYMARRWSNEFHGQRDIQAQLMVHLDYALEHTDWHAQRQSSDSDAVSRWTPYDKPIINA
QQELSKLPIYQRVYQTLRTKALSVLPADLNLRDQVGPTFDNVFVAGNDEKLVIPQFLTRY
GLQSYFVKQREGLVELTALDSWVLNLTQSVAYSEADREEIQRHITEQYISDYTATWRAGM
DNLNVRDYEAMSALTDALEQIISGDQPFQRALTALRDNTHALTLSGKLDDKAREAAINEM
DYRLLSRLGHEFAPENSALEEQKDKASTLQAVYQQLTELHRYLLAIQNSPVPGKSALKAV
QLRLDQNSSDPIFATRQMAKTLPAPLNRWVGKLADQAWHVVMVEAVRYMEVDWRDNVVKP
FNEQLADNYPFNPRATQDASLDSFERFFKPDGILDNFYKNNLRLFLENDLTFGDDGRVLI
REDIRQQLDTAQKIRDIFFSQQNGLGAQFAVETVSLSGNKRRSVLNLDGQLVDYSQGRNY
TAHLVWPNNMREGNESKLTLIGTSGRAPRSIAFSGPWAQFRLFGAGQLTNVTSDTFNVRF
NVDGGAMVYQVHVDTEDNPFTGGLFSLFRLPDTLY
>LFGLNPFC_03029 hypothetical protein
MSNYALTQTIVTGSDPRALPEFSAIREEINKANHPSQPELNWKLVESLALSIFKANGVDL
HTATYYTLARTRNQGLAGFCEGAELLAAMIIHEWDKFWPQSGPARTEMLDWFNTRTGNIL
RQQVSFSENDLSLLYRTERALQLICDKLQQVELKRQPRVENLLYFVQNTRKRFEPQPRNR
TDTAAQTMVRTLVYAPEGTASATAETMPPLPDLPEMRVGVRGVADNADKAKQGDTVKGFV
AGAACTAVIASALWWWQAYPMQQQLTQVRDTTQGAATVWLASPVLKEYEQYLQQLLNAPP
LQPLETGMQMMRTADTLWPESLQQQEASRMWSNTLRNRAQASPQMKGWQQARQNLRDFAD
LMMKKETEKQGFTLSYIKTVTWQAERLLNQETPLEYLLTQYQETRTQKQDTQALEKEINE
RLDGLLSRWLLLKNTGQDMATDNRTEPVHPTH
>LFGLNPFC_03030 Major exported protein
MANPVYLTLNGELQGLISSGCSSMPSIGNKAQIAHQDQIMVTSLSHGLSRAQNVNHQELT
ITKPVDKSSPLLGKAISENECLTCDFVFYRTNRFGINEPYYKLKLANARLSNIGLTVPHT
INDSPGQAEESVSFTYESINWEHSVAGTSAYSLWSERIF
>LFGLNPFC_03031 hypothetical protein
MNLKKVCCICVLFSLLAGCASESPIDEKKKKTQTTQISINKNVPQQLTDKDLYGNETTLA
VSEEDIQAALEGDEFRVPLNSPVILVQSGSRAPETIMQEEMRKYYTVATFSGIPDRKKTV
TCNKDKNKDESLDIVDAENMNWMQALRFVAAKGHQKAIIVFQDTLQTGKYDSGLKSIVWT
DYKNQKLTDTMSLRYLVRFTLVDVATGEWATWSPVNYESKVIFPQIGNKNSDSLDVAEQQ
ISQLKQKTYAAVVKDLVNRYQ
>LFGLNPFC_03033 DNA polymerase III subunit epsilon
MTAMSTAITRQIVLDTETTGMNQIGAHYEGHKIIEIGAVEVVNRRLTGNNFHVYLKPDRL
VDPEAFGVHGIADEFLLDKPTFAEVADEFMDYIRGAELVIHNAAFDIGFMDYEFSLLKRD
IPKTNTFCKVTDSLAVARKMFPGKRNSLDALCARYEIDNSKRTLHGALLDAQILAEVYLA
MTGGQTSMAFAMEGETQQQQGETTIQRIVRQASKLRVVFATDEELAAHEARLDLVQKKGG

SCLWRA
>LFGLNPFC_03034 Ribonuclease HI
MLKQVEIFTDGSCLGNPGPGGYGAILRYRGREKTFSAGYTRTTNNRMELMAAIVALEALK
EHCEVILSTDSQYVRQGITQWIHNWKKRGWKTADKKPVKNVDLWQRLDAALGQHQIKWEW
VKGHAGHPENERCDELARAAAMNPTLEDTGYQVEV
>LFGLNPFC_03035 2-methoxy-6-polyprenyl-1,4-benzoquinol methylase, mitochondrial
MKPARVPQTVVAPDCWGDLPWGELYRKALERQLNPWFTKMYGFHLLKIGNLSAEINCEAC
AVSHQVNVSAQGMPVQVQADPLHLPFADKSVDVCLLAHTLPWCTDPHRLLREADRVLIDD
GWLVISGFNPISLMGLRKLVPVLRKTSPYNSRMFTLMRQLDWLSLLNFEVLHASRFHVLP
WNKHGGKLLNAHIPALGCLQLIVARKRTIPLTLNPMKQSKNKPRIRQAVGATRQCRKPQA
>LFGLNPFC_03036 Hydroxyacylglutathione hydrolase GloB
MNLNSIPAFDDNYIWVLNDEAGRCLIVDPGDAEPVLNAISANNWQPEAIFLTHHHHDHVG
GVKELVEKFPQIVVYGPQETQDKGTTQVVKDGETAFVLGHEFSVIATPGHTLGHICYFSK
PYLFCGDTLFSGGCGRLFEGTPSQMYQSIKKLSALPDDTLVCCAHEYTLSNMKFALSILP
HDLSINDYYRKVKELRAKNQITLPVILKNERQINVFLRTEDIDLINVINEETLLQQPEER
FAWLRSKKDRF
>LFGLNPFC_03037 Membrane-bound lytic murein transglycosylase D
MDDGTSIAPDGDLWAFIGDELKMGIPENDRIREQKQKYLRNKSYLHDVTLRAEPYMYWIA
GQVKKRNMPMELVLLPIVESAFDPHATSGANAAGIWQIIPSTGRNYGLKQTRNYDARRDV
VASTTAALNMMQRLNKMFDGDWLLTVAAYNSGEGRVMKAIKTNKARGKSTDFWSLPLPQE
TKQYVPKMLALSDILKNSKRYGVRLPTTDESRALARVHLSSPVEMAKVADMAGISVSKLK
TFNAGVKGSTLGASGPQYVMVPKKHADQLRESLASGEIAAVQSTLVADNTPLNSRVYTVR
SGDTLSSIASRLGVSTKDLQQWNKLRGSKLKPGQSLTIGAGSSAQRLANNSDSITYRVRK
GDSLSSIAKRHGVNIKDVMRWNSDTANLQPGDKLTLFVKNNNMPDS
>LFGLNPFC_03038 putative methyltransferase YcgJ
MTTQSHHDHVEKQFSSQASEYLTSTVHASGRDLQRLAVRLADYPDASVLDMGCGAGHASF
VAAQNVSAVVAYDLSAQMLDVVAQAAEARQLKNITTRQGYAESLPFADNAFDIVISRYSA
HHWHDVGAALREVNRILKPGGRLIVMDVMSPGHPVRDIWLQTVEALRDTSHVRNYASGEW
LRLINEANLIVDNLITDKLPLEFSSWVARMRTPEALVDAIRIYQQSASTEVKTYFALQND
GSFTSDIIMVEAHKAA
>LFGLNPFC_03039 hypothetical protein
MIEVSKSKVRKNTYAMRYVAGQPAERILPPGSFASIGQALPPGEPLSTEERIRILVWNIY
KQQRAEWLSVLKNYGKDAHLVLLQEAQTTPELVQFATANYLAADQVPAFVLPQHPSGVMT
LSAAHPVYCCPLREREPILRLAKSALVTVYPLPDTRLLMVVNIHAVNFSLGVDVYSKQLL
PIGDQIAHHSGPVIMAGDFNAWSRRRMNALYRFAREMSLRQVRFTDDQRRRAFGRPLDFV
FYRGLNVSEASVLVTRASDHNPLLVEFSPGKPDK
>LFGLNPFC_03040 HTH-type transcriptional regulator DmlR
MKATSEELAIFVSVVESGSFSRAAEQLGQANSAVSRAVKKLEMKLGVSLLNRTTRQLSLT
EEGERYFHRVQSILQEMAAAESEIMETRNTPRGLLRIDAATPVVLHFLMPLIKPFRERYP
EVTLSLVSSETIINLIERKVDVAIRAGTLTDSSLRARPLFNSYRKIIASPDYISCYGKPE
TIDDLKQHICLRFTEPASLNTWPIACSDGGQLHEVKYGLSSNSGETLKQLCLSGNGIACLS
DYMIDKEIARGELVELMADKVLPVEMPFSAVYYSDRAVSTRIRAFIDFLSGHVKTAPGGA
VREA
>LFGLNPFC_03041 2,5-diketo-D-gluconic acid reductase B
MAIPAFGLGTFRLKDDVVISSVKTALELDYRAIDTAQIYDNEAAVGQAIAESGVPRHELY
ITTKIWIENLSKDKLIPSLKESLQKLRTDYVDLTLIHWPSPSDEVSAEEFMQALLEAKKQ
GLTREIGISNFTIPLMEKAIAAVGAENIATNQIELSPYLQNRKVVAWAKQHGIHITSYMT
LAYGNALKDKVIARIAAKHNATPAQVILAWAMGEGYSVIPSSTKRENLESNLKAQNLQLD
AEDKKAIAALDCNDRLVSPEGLAPEWD
>LFGLNPFC_03048 D-glycero-beta-D-manno-heptose-1,7-bisphosphate 7-phosphatase
MAKSVPAIFLDRDGTINVDHGYVHEIDNFEFIDGVIDAMRELKKMGFALVVVTNQSGIAR
GKFTEAQFETLTEWMDWSLADRDVDLDGIYYCPHHPQGSVEEFRQVCDCRKPHPGMFLSA
RDYLHIDMAASYMVGDKLEDMQAAAAASVGTKVLVRTGKPITPEAENAADWVLNSLADLP
QAIKKQQKPA
>LFGLNPFC_03049 Methionine import ATP-binding protein MetN
MIKLSNITKVFHQGTRTIQALNNVSLHVPAGQIYGVIGASGAGKSTLIRCVNLLERPTEG
SVLVDGQELTTLSESELTKARRQIGMIFQHFNLLSSRTVFGNVALPLELDNTPKDEIKRR
VTELLSLVGLGDKHDSYPSNLSGGQKQRVAIARALASNPKVLLCDEATSALDPATTRSIL
ELLKDINRRLGLTILLITHEMDVVKRICDCVAVISNGELIEQDTVSEVFSHPKTPLAQKF
IQSTLHLDIPEDYQERLQAEPFTDCVPMLRLEFTGQSVDAPLLSETARRFNVNNNIISAQ
MDYAGGVKFGIMLTEMHGTQQDTQAAIAWLQEHHVKVEVLGYV
>LFGLNPFC_03050 D-methionine transport system permease protein MetI
MSEPMIWLLVRGVWETLAMTFVSGFFGFVIGLPVGVLLYVTRPGQIIANAKLYRTISAIV
NIFRSIPFIILLVWMIPFTRVIVGTSIGLQAAIVPLTVGAAPFIARMVENALLEIPTGLI

EASRAMGATPMQIVRKVLLPEALPGLVNAATITLITLVGYSAMGGAVGAGGLGQIGYQYG
YIGYNATVMNTVLVLLVILVYLIQFAGDRIVRAVTRK
>LFGLNPFC_03051 D-methionine-binding lipoprotein MetQ
MAFKFKTFAAVGALIGSLALVGCGQDEKDPNHIKVGVIVGAEQQVAEVAQKVAKDKYGLD
VELVTFNDYVLPNEALSKGDIDANAFQHKPYLDQQLKDRGYKLVAVGNTFVYPIAGYSKK
IKSLDELQDGSQVAVPNDPTNLGRSLLLLQKVGLIKLKDGVGLLPTVLDVVENPKNLKIV
ELEAPQLPRSLDDAQIALAVINTTYASQIGLTPAKDGIFVEDKESPYVNLIVTREDNKDA
ENVKKFVQAYQSDEVYEAANKVFNGGAVKGW
>LFGLNPFC_03052 Outer membrane lipoprotein RcsF
MRALPICLVALMLSGCSMLSRSPVEPVQSTAPQPKAEPAKPKAPRATPVRIYTNAEELVG
KPFRDLGEVSGDSCQASNQDSPPSIPTARKRMQINASKMKANAVLLHSCEVTSGTPGCYR
QAVCIGSALNITAK
>LFGLNPFC_03053 tRNA (adenine(37)-N6)-methyltransferase
MSSFQFEQIGVIRSPYKEKFAVPRQPGLVKSANGELHLIAPYNQADAVRGLEAFSHLWIL
FVFHQTMEGGWRPTVRPPRLGGNARMGVFATRSTFRPNPIGMSLVELKEVVCHKDSVILK
LGSLDLVDGTPVVDIKPYLPFAESLPDASASYAQSAPAAEMAVCFTAEVEKQLLTLEKRY
PQLTLFIREVLAQDPRPAYRKGEETGKTYAVWLHDFNVRWRVTDAGFEVFALEPR
>LFGLNPFC_03054 Proline--tRNA ligase
MRTSQYLLSTLKETPADAEVISHQLMLRAGMIRKLASGLYTWLPTGVRVLKKVENIVREE
MNNAGAIEVLMPVVQPSELWQESGRWEQYGPELLRIADRGDRPFVLGPTHEEVITDLIRN
ELSSYKQLPLNFYQIQTKFRDEVRPRFGVMRSREFLMKDAYSFHTSQESLQETYDAMYAA
YSKIFSRMGLDFRAVQADTGSIGGSASHEFQVLAQSGEDDVVFSDTSDYAANIELAEAIA
PKEPRAAATQEMTLVDTPNAKTIAELVEQFNLPIEKTVKTLLVKAVEGSSFPLVALLVRG
DHELNEVKAEKLPQVASPLTFATEEEIRAVVKAGPGSLGPVNMPIPVVIDRTVAAMSDFA
AGANIDGKHYFGINWDRDVATPEIADIRNVVAGDPSPDGQGTLLIKRGIEVGHIFQLGTK
YSEALKASVQGEDGRNQILTMGCYGIGVTRVVAAAIEQNYDERGIVWPDAIAPFQVAILP
MNMHKSFRVQELAEKLYSELRAQGIEVLLDDRKERPGVMFADMELIGIPHTIVLGDRNLD
NDDIEYKYRRNGEKQLIKTGDIVDYLVKQIKG
>LFGLNPFC_03055 hypothetical protein
MDKPKAYCRLLLPCFLLLSACTVDISQPDSSATVVNAEAKTWAVKFQHQSSFTEQSIKEI
TEPDLKPGDLLFSSSLGVTSFGIRVFSTSSVSHVAIYLGDNNVAEATGAGVQIVSLKKAI
KHSDKLFVLRVPDLTPQQATEITAFANKIKDSGYNYRGIVEFIPFMVTRQMCSLNPFSED
FRQQCVSGLAKAQLSSVGEGDKKSWFCSEFVTDAFAKAGHPLTLAQSGWISPADLMHMRS
GDVSAFKPETQLQYIGHLKPGIYIKASRFVGLTQ
>LFGLNPFC_03056 Lipoprotein NlpE
MVKKAIVTAMAVISLFTLMGCNNRAEVDTLSPAQAAELKPMPQSWRGVLPCADCEGIETS
LFLEKDGTWVMNERYLGAREEPSSFASYGTWARTADKLVLTDSKGEKSYYRAKGDALEML
DREGNPIESQFNYTLEPAQSSLPMTPMTLRGMYFYMADAATFTDCATGKRFMVANNAELE
RGYLAARGNSEKPVLLSVEGHFTLEANPDTGAPTKVLAPDTAGKFYPNQDCSSLGL
>LFGLNPFC_03057 Peptidyl-tRNA hydrolase ArfB
MIVISRHVAIPDGEFEITAIRAQGAGGQHVNKTSTAIHLRFDIRASSLPEYYKERLLAAS
HHLISSDGVIVIKAQEYRNQELNREAALARLVAVIKDLTTEQKARRPTRPTRASKERRLA
SKAQKSSVKAMRGKVRSGRE
>LFGLNPFC_03058 putative protein YaeQ
MALKATIYKATVNVADLDRNQFLDASLTLARHPSETQERMMLRLLAWLKYADERLQFTRG
LCADDEPEAWLRNDHLGIDLWIELGLPDERRIKKACTQAAEVALFAYNSRAAQIWWQQNQ
SKCAQFANLSVWYLDDEQLAKVSAFADRTMTLQATIQDGVIWLSDDKNNLEVNLTVWQQP
S
>LFGLNPFC_03059 hypothetical protein
MEKYCELIRKRYAEIASGDLGYVPDALGCVLKVLNEMAADDALSEAVREKAAYAAANLLV
SDYVNK
>LFGLNPFC_03060 Protein rof
MSINDTYQPINCDDYDNLELACQHHLMLTLELKDGEKLQAKASDLVSRKNVEYLVVEAAG
ATRELRLDKITSFSHPEIGTVVVSES
>LFGLNPFC_03061 tRNA(Ile)-lysidine synthase
MTLTLNRQLLSSRQILVAFSGGLDSTVLLHQLVQWRTENPGVTLRAIHVHHGLSANADAW
VKHCENICQQWQVPLVVERVQLAQEGLGIEAQARQARYQAFARTLLPGEVLVTAQHLDDQ
CETFLLALKRGSGPAGLSAMAEVSEFAGTQLIRPLLARTRGELVQWALAHGLRWIEDESN
QDDSYDRNFLRLRVVPLLQQRWPHFAEATARSAALCAEQESLLDELLADDLAHCQTPQGT
LQIAPMLAMSDARRAAIIRRWLAGQNAPMPSRDALVRIWQEVALAREDASPCLRLGAFEI
RRYQSQLWWIKFVTGQSETIVPWQTWLQPLELPAGLGSVQLTAGGDIRPPRADEAVSVRF
KAPGLLHIVGRNGGRKLKKIWQELGVPPWLRDTTPLLFYGETLIAAAGVFVTQEGVAVGE
NGVSFVWKKTLS
>LFGLNPFC_03062 2-epi-5-epi-valiolone epimerase

MLGLKQVHHIAIIATDYAMSKAFYCDILGFTLQSEVYREARDSWKGDLALNGQYVIELFS
FPFPPERPSRSEACGLRHLAFSVDDIDAAVAHLESHNVKCEAIRVDPYTQKRFTFFNDPD
GLPLELYEQ
>LFGLNPFC_03063 Constitutive lysine decarboxylase
MNIIAIMGPHGVFYKDEPIKELESALVAQGFQIIWPQNSVDLLKFIEHNPRICGVIFDWD
EYSLDLCSDINQLNEYLPLYAFINTHSTMDVSVQDMRMALWFFEYALGQAEDIAIRMRQY
TNEYLDNITPPFTKALFTYVKERKYTFCTPGHMGGTAYQKSPVGCLFYDFFGGNTLKADV
SISVTELGSLLDHTGPHLEAEEYIARTFGAEQSYIVTNGTSTSNKIVGMYAAPSGSTLLI
DRNCHKSLAHLLMMNDVVPVWLKPTRNALGILGGIPRGEFTRDSIEEKVAATTQAQWPVH
AVITNSTYDGLLYNTDWIKQTLDVPSIHFDSAWVPYTHFHPIYQGKSGMSGERVAGKVIF
ETQSTHKMLAALSQASLIHIKGEYDEEAFNEAFMMHTTTSPSYPIVASVETAAAMLRGNP
GKRLINRSVERALHFRKEVQRLREESDGWFFDIWQPPQVDEAECWPVAPGEQWHGFSDAD
ANHMFLDPVKVTILTPGMDEHGNMSEEGIPAALVAKFLDERGIVVEKTGPYNLLFLFSIG
IDKTKAMGLLRGLTEFKRSYDLNLRIKNMLPDLYAEDPDFYRNMRIQDLAQGIHKLIRKH
DLPGLMLRAFDTLPEMIMTPHQAWQRQIKGEVETIALEQLVGRVSANMILPYPPGVPLLM
PGEMLTEESRTVLDFLLMLCSVGGQHYPGFETDIHGAKQDEDGVYRVRVLKMAG
>LFGLNPFC_03064 Acetyl-coenzyme A carboxylase carboxyl transferase subunit alpha
MSLNFLDFEQPIAELEAKIDSLTAVSRQDEKLDINIDEEVHRLREKSVELTRKIFADLGA
WQIAQLARHPQRPYTLDYVRLAFDEFDELAGDRAYADDKAIVGGIARLDGRPVMIIGHQK
GRETKEKIRRNFGMPAPEGYRKALRLMQMAERFKMPIITFIDTPGAYPGVGAEERGQSEA
IARNLREMSRLGVPVVCTVIGEGGSGGALAIGVGDKVNMLQYSTYSVISPEGCASILWKS
ADKAPLAAEAMGIIAPRLKELKLIDSIIPEPLGGAHRNPEAMAASLKAQLLTDLADLDVL
STEDLKNRRYQRLMSYGYA
>LFGLNPFC_03065 DNA polymerase III subunit alpha
MSEPRFVHLRVHSDYSMIDGLAKTAPLVKKAAALGMPALAITDFTNLCGLVKFYGAGHGA
GIKPIVGADFNVQCDLLGDELTHLTVLAANNTGYQNLTLLISKAYQRGYGTAGPIIDRDW
LIELNEGLILLSGGRMGDVGRSLLRGNSALVDECVAFYEEHFPDRYFLELIRTGRPDEES
YLHAAVELAEARGLPVVATNDVRFIDSSDFDAHEIRVAIHDGFTLDDPKRPRNYSPQQYM
RSEEEMCELFADIPEALANTVEIAKRCNVTVRLGEYFLPQFPTGDMSTEDYLVKRAKEGL
EERLAFLFPDEEERLKRRPEYDERLDTELQVINQMGFPGYFLIVMEFIQWSKDNGVPVGP
GRGSGAGSLVAYALKITDLDPLEFDLLFERFLNPERVSMPDFDVDFCMEKRDQVIEHVAD
MYGRDAVSQIITFGTMAAKAVIRDVGRVLGHPYGFVDRISKLIPPDPGMTLAKAFEAEPQ
LPEIYEADEEVKALIDMARKLEGVTRNAGKHAGGVVIAPTKITDFAPLYCDEEGKHPVTQ
FDKSDVEYAGLVKFDFLGLRTLTIINWALEMINKRRAKNGEPPLDIAAIPLDDKKSFDML
QRSETTAVFQLESRGMKDLIKRLQPDCFEDMIALVALFRPGPLQSGMVDNFIDRKHGREE
ISYPDVQWQHESLKPVLEPTYGIILYQEQVMQIAQVLSGYTLGGADMLRRAMGKKKPEEM
AKQRSVFAEGAEKNGINAELAMKIFDLVEKFAGYGFNKSHSAAYALVSYQTLWLKAHYPA
EFMAAVMTADMDNTEKVVGLVDECWRMGLKILPPDINSGLYHFHVNDDGEIVYGIGAIKG
VGEGPIEAIIEARNKGGYFRELFDLCARTDTKKLNRRVLEKLIMSGAFDRLGPHRAALMN
SLGDALKAADQHAKAEAIGQADMFGVLAEEPEQIEQSYASCQPWPEQVVLDGERETLGLY
LTGHPINQYLKEIERYVGGVRLKDMHPTERGKVITAAGLVVAARVMVTKRGNRIGICTLD
DRSGRLEVMLFTDALDKYQQLLEKDRILIVSGQVSFDDFSGGLKMTAREVMDIDEAREKY
ARGLAISLTDRQIDDQLLNRLRQSLEPHRSGTIPVHLYYQRADARARLRFGATWRVSPSD
RLLNDLRGLIGSEQVELEFD
>LFGLNPFC_03066 Ribonuclease HII
MIEFVYPHTQLVAGVDEVGRGPLVGAVVTAAVILDPARPIAGLNDSKKLSEKRRLVLCEE
IKEKALSWSLGRAEPHEIDELNILHATMLAMQRAVAGLHIAPEYVLIDGNRCPKLPMPSM
AVVKGDSRVPEISAASILAKVTRDAEMAALDIVFPQYGFAQHKGYPTAFHLEKLAEHGAT
EHHRRSFGPVKRALGLAS
>LFGLNPFC_03067 Lipid-A-disaccharide synthase
MTEQRPLTIALVAGETSGDILGAGLIRALKERVPNARFVGVAGPRMQAEGCEAWYEMEEL
AVMGIVEVLGRLRRLLHIRADLTKRFGELKPDVFVGIDAPDFNITLEGNLKKQGIKTIHY
VSPSVWAWRQKRVFKIGRATDLVLAFLPFEKAFYDKYNVPCRFIGHTMADAMPLDPDKNG
ARDVLGIPYDAHCLALLPGSRGAEVEMLSADFLKTAQLLRQTYPDLEIVVPLVNAKRREQ
FERIKAAVAPDLSVHLLDGMGREAMVASDAALLASGTAALECMLAKCPMVVGYRMKPFTF
WLAKRLVKTDYVSLPNLLAGRELVKELLQEECEPQKLAAALLPLLANGKTSHAMHDTFRE
LHQQIRCNADEQAAQAVLELAQ
>LFGLNPFC_03068 Acyl-[acyl-carrier-protein]--UDP-N-acetylglucosamine O-acyltransferase
MIDKSAFVHPTAIVEEGASIGANAHIGPFCIVGPHVEIGEGTVLKSHVVVNGHTKIGRDN
EIYQFASIGEVNQDLKYAGEPTRVEIGDRNRIRESVTIHRGTVQGGGLTKVGSDNLLMIN
AHIAHDCTVGNRCILANNATLAGHVSVDDFAIIGGMTAVHQFCIIGAHVMVGGCSGVAQD
VPPYVIAQGNHATPFGVNIEGLKRRGFSREAITAIRNAYKLIYRSGKTLDEVKPEIAELA
ETYPEVKAFTDFFARSTRGLIR
>LFGLNPFC_03069 3-hydroxyacyl-[acyl-carrier-protein] dehydratase FabZ

MTTNTHTLQIEEILELLPHRFPFLLVDRVLDFEEGRFLRAVKNVSVNEPFFQGHFPGKPI
FPGVLILEAMAQATGILAFKSVGKLEPGELYYFAGIDEARFKRPVVPGDQMIMEVTFEKT
RRGLTRFKGVALVDGKVVCEATMMCARSREA
>LFGLNPFC_03070 UDP-3-O-(3-hydroxymyristoyl)glucosamine N-acyltransferase
MPSIRLADLAQQLDAELHGDGDIVITGVASMQSAQTGHITFMVNPKYREHLGLCQASAVV
MTQDDLPFAKSAALVVKNPYLTYARMAQILDTTPKPAQNIAPSAVIDETAKLGNNVSIGA
NAVIESGVELGDNVIIGAGCFVGKNSKIGAGSRLWANVTIYHEIQIGQNCLIQSGTVVGA
DGFGYANDRGNWVKIPQIGRVIIGDRVEIGACTTIDRGALDDTVIGNGVIIDNQCQIAHN
VVIGDNTAVAGGVIMAGSLKIGRYCMIGGASVINGHMEICDKVTVTGMGMVMRPITEPGV
YSSGIPLQPNKVWRKTAALVMNIDDMSKRLKSLERKVNQQD
>LFGLNPFC_03071 Chaperone protein Skp
MKKWLLAAGLGLALATSAQAADKIAIVNMGSLFQQVAQKTGVSNTLENEFKGRASELQRM
ETDLQAKMKKLQSMKAGSDRTKLEKDVMAQRQTFAQKAQAFEQDRARRSNEERGKLVTRI
QTAVKSVANSQDIDLVVDANAVAYNSSDVKDITADVLKQVK
>LFGLNPFC_03072 Outer membrane protein assembly factor BamA
MAMKKLLIASLLFSSATVYGAEGFVVKDIHFEGLQRVAVGAALLSMPVRTGDTVNDEDIS
NTIRALFATGNFEDVRVLRDGDTLLVQVKERPTIASITFSGNKSVKDDMLKQNLEASGVR
VGESLDRTTIADIEKGLEDFYYSVGKYSASVKAVVTPLPRNRVDLKLVFQEGVSAEIQQI
NIVGNHAFTTDELISHFQLRDEVPWWNVVGDRKYQKQKLAGDLETLRSYYLDRGYARFNI
DSTQVSLTPDKKGIYVTVNITEGDQYKLSGVEVSGNLAGHSAEIEQLTKIEPGELYNGTK
VTKMEDDIKKLLGRYGYAYPRVQSMPEINDADKTVKLRVNVDAGNRFYVRKIRFEGNDTS
KDAVLRREMRQMEGAWLGSDLVDQGKERLNRLGFFETVDTDTQRVPGSPDQVDVVYKVKE
RNTGSFNFGIGYGTESGVSFQAGVQQDNWLGTGYAVGINGTKNDYQTYAELSVTNPYFTV
DGVSLGGRLFYNDFQADDADLSDYTNKSYGTDVTLGFPINEYNSLRAGLGYVHNSLSNMQ
PQVAMWRYLYSMGEHPSTSDQDNSFKTDDFTFNYGWTYNKLDRGYFPTDGSRVNLTGKVT
IPGSDNEYYKVTLDTATYVPIDDDHKWVVLGRTRWGYGDGLGGKEMPFYENFYAGGSSTV
RGFQSNTIGPKAVYFPHQASNYDPDYDYECATQDGAKDLCKSDDAVGGNAMAVASLEFIT
PTPFISDKYANSVRTSFFWDMGTVWDTNWDSSQYSGYPDYSDPSNIRMSAGIALQWMSPL
GPLVFSYAQPFKKYDGDKAEQFQFNIGKTW
>LFGLNPFC_03073 Regulator of sigma-E protease RseP
MLSFLWDLASFIVALGVLITVHEFGHFWVARRCGVRVERFSIGFGKALWRRTDKLGTEYV
IALIPLGGYVKMLDERAEPVVPELRHHAFNNKSVGQRAAIIAAGPVANFIFAIFAYWLVF
IIGVPGVRPVVGEIAANSIAAEAQIAPGTELKAVDGIETPDWDAVRLQLVDKIGDESTTI
TVAPFGSDQRRDVKLDLRHWAFEPDKEDPVSSLGIRPRGPQIEPVLENVQPNSAASKAGL
QAGDRIVKVDGQPLTQWVTFVMLVRDNPGKSLALEIERQGSPLSLTLIPESKPGKGKAIG
FVGIEPKVIPLPDEYKVVRQYGPFNAIVEATDKTWQLMKLTVSMLGKLITGDVKLNNLSG
PISIAKGAGMTAELGVVYYLPFLALISVNLGIINLFPLPVLDGGHLLFLAIEKIKGGPVS
ERVQDFCYRIGSILLVLLMGLALFNDFSRL
>LFGLNPFC_03074 Phosphatidate cytidylyltransferase
MLAAWEWGQLSGFTTRSQRVWLAVLCGLLLALMLFLLPEYHRNIHQPLVEISLWASLGWW
IVALLLVLFYPGSAAIWRNSKTLRIIFGVLTIVPFFWGMLALRAWHYDENHYSGAIWLLY
VMILVWGADSGAYMFGKLFGKHKLAPKVSPGKTWQGFIGGLVTAAVISWGYGMWVNLDVA
PVTLLICSIVAALASVLGDLTESMFKREAGIKDSGHLIPGHGGILDRIDSLTAAVPVFAC
LLLLVFRTL
>LFGLNPFC_03075 hypothetical protein
MGVAFAEVSPDICFCVNTRRHRGVVSVAAGGVRHCNAGGLYAGSVGMGTA
>LFGLNPFC_03076 Ditrans,polycis-undecaprenyl-diphosphate synthase ((2E,6E)-farnesyl-diphosphate specific)
MLSATQPLSEKLPAHGCRHVAIIMDGNGRWAKKQGKIRAFGHKAGAKSVRRAVSFAANNG
IEALTLYAFSSENWNRPAQEVSALMELFVWALDSEVKSLHRHNVRLRIIGDTSRFNSRLQ
ERIRKSEALTAGNTGLTLNIAANYGGRWDIVQGVRQLAEKVQQGNLQPDQIDEEMLNQHV
CMHELAPVDLVIRTGGEHRISNFLLWQIAYAELYFTDVLWPDFDEQDFEGALNAFANRER
RFGGTEPGDETA
>LFGLNPFC_03077 1-deoxy-D-xylulose 5-phosphate reductoisomerase
MKQLTILGSTGSIGCSTLDVVRHNPEHFRVVALVAGKNVARMVEQCLEFSPRYAVMDDEA
SAKLLKTMLQQQGSRTEVLSGQQAACDMAALEDVDQVMAAIVGAAGLLPTLAAIRAGKTI
LLANKESLVTCGRLFMDAVKQSKAQLLPVDSEHNAIFQSLPQPIQHNLGYADLEQNGVVS
ILLTGSGGPFRETPLRDLATMTPDQACRHPNWSMGRKISVDSATMMNKGLEYIEARWLFN
ASASQMEVLIHPQSVIHSMVRYQDGSVLAQLGEPDMRTPIAHTMAWPNRVNSGVKPLDFC
KLSALTFAAPDYDRYPCLKLAMEAFEQGGQAATTALNAANEITVAAFLAQQIRFTDIAALN
LSVLEKMDMREPQCVDDVLSVDANAREVARKEVMRLAS
>LFGLNPFC_03078 Ribosome-recycling factor
MISDIRKDAEVRMDKCVEAFKTQISKIRTGRASPSLLDGIVVEYYGTPTPLRQLASVTVE
DSRTLKINVFDRSMSPAVEKAIMASDLGLNPNSAGSDIRVPLPPLTEERRKDLTKIVRGE

AEQARVAVRNVRRDANDKVKALLKDKEISEDDDRRSQDDVQKLTDAAIKKIEAALADKEA
ELMQF
>LFGLNPFC_03079 Uridylate kinase
MATNAKPVYKRILLKLSGEALQGTEGFGIDASILDRMAQEIKELVELGIQVGVVIGGGNL
FRGAGLAKAGMNRVVGDHMGMLATVMNGLAMRDALHRAYVNARLMSAIPLNGVCDSYSWA
EAISLLRNNRVVILSAGTGNPFFTTDSAACLRGIEIEADVVLKATKVDGVFTADPAKDPT
ATMYEQLTYSEVLEKELKVMDLAAFTLARDHKLPIRVFNMNKPGALRRVVMGEKEGTLIT
E
>LFGLNPFC_03080 Elongation factor Ts
MAEITASLVKELRERTGAGMMDCKKALTEANGDIELAIENMRKSGAIKAAKKAGNVAADG
VIKTKIDGNYGIILEVNCQTDFVAKDAGFQAFADKVLDAAVAGKITDVEVLKAQFEEERV
ALVAKIGENINIRRVAALEGDVLGSYQHGARIGVLVAAKGADEELVKHIAMHVAASKPEF
IKPEDVSAEVVEKEYQVQLDIAMQSGKPKEIAEKMVEGRMKKFTGEVSLTGQPFVMEPSK
TVGQLLKEHNAEVTGFIRFEVGEGIEKVETDFAAEVAAMSKQS
>LFGLNPFC_03081 30S ribosomal protein S2
MATVSMRDMLKAGVHFGHQTRYWNPKMKPFIFGARNKVHIINLEKTVPMFNEALAELNKI
ASRKGKILFVGTKRAASEAVKDAALSCDQFFVNHRWLGGMLTNWKTVRQSIKRLKDLETQ
SQDGTFDKLTKKEALMRTRELEKLENSLGGIKDMGGLPDALFVIDADHEHIAIKEANNLG
IPVFAIVDTNSDPDGVDFVIPGNDDAIRAVTLYLGAVAATVREGRSQDLASQAEESFVEA
E
>LFGLNPFC_03082 Methionine aminopeptidase
MAISIKTPEDIEKMRVAGRLAAEVLEMIEPYVKPGVSTGELDRICNDYIVNEQHAVSACL
GYHGYPKSVCISINEVVCHGIPDDAKLLKDGDIVNIDVTVIKDGFHGDTSKMFIVGKPTI
MGERLCRITQESLYLALRMVKPGINLREIGAAIQKFVEAEGFSVVREYCGHGIGRGFHEE
PQVLHYDSRETNVVLKPGMTFTIEPMVNAGKKEIRTMKDGWTVKTKDRSLSAQYEHTIVV
TDNGCEILTLRKDDTIPAIISHDE
>LFGLNPFC_03083 Bifunctional uridylyltransferase/uridylyl-removing enzyme
MNTLPEQYANTALPTLSGQPQNPCAWPRDELTVGGIKAHIDTFQRWLGDAFDNGISAEQL
IEARTEFIDQLLQRLWIEAGFSQIADLALVAVGGYGRGELHPLSDIDLLILSRKKLPDDQ
AQKVGELLTLLWDVKLEVGHSVRTLEECMLEGLSDLTVATNLIESRLLIGDVALFLELQK
HIFSEGFWPSDKFYAAKVEEQNQRHQRYHGTSYNLEPDIKSSPGGLRDIHTLQWVARRHF
GATSLDEMVGFGFLTSAERAELNECLHILWRIRFALHLVVSRYDNRLLFDRQLSVAQRLN
YSGEGNEPVERMMKDYFRVTRRVSELNQMLLQLFDEAILALPADEKPRPIDDEFQLRGTL
IDLRDETLFMRQPEAILRMFYTMVRNSAITGIYSTTLRQLRHARRHLQQPLCNIPEARKL
FLSILRHPGAVRRGLLPMHRHSVLGAYMPQWSHIVGQMQFDLFHAYTVDEHTIRVMLKLE
SFASEETRQRHPLCVDVWPRLPSTELIFIAALFHDIAKGRGGDHSILGAQDVVHFAELHG
LNSRETQLVAWLVRQHLLMSVTAQRRDIQDPEVIKQFAEEVQTENRLRYLVCLTVADICA
TNETLWNSWKQSLLRELYFATEKQLRRGMQNTPDMRERVRHHQLQALALLRMDNIDEEAL
HQIWSRCRANYFVRHSPNQLAWHARHLLQHDLSKPLVLLSPQATRGGTEIFIWSPDRPYL
FAAVCAELDRRNLSVHDAQIFTTRDGMAMDTFIVLEPDGSPLSADRHEVIRFGLEQVLTQ
SSWQPPQPRRQPAKLRHFTVETEVTFLPTHTDRKSFLELIALDQPGLLARVGKIFADLGI
SLHGARITTIGERVEDLFIIATADRRALNNELQQEVHQRLTEALNPNDKG
>LFGLNPFC_03084 2,3,4,5-tetrahydropyridine-2,6-dicarboxylate N-succinyltransferase
MQQLQNIIETAFERRAEITPANADTVTREAVNQVIALLDSGALRVAEKIDGQWVTHQWLK
KAVLLSFRINDNQVIEGAESRYFDKVPMKFANYDEARFQKEGFRVVPPAAVRQGAFIARN
TVLMPSYVNIGAYVDEGTMVDTWATVGSCAQIGKNVHLSGGVGIGGVLEPLQANPTIIED
NCFIGARSEVVEGVIVEEGSVISMGVYIGQSTRIYDRETGEIHYGRVPAGSVVVSGNLPS
KDGKYSLYCAVIVKKVDAKTRGKVGINELLRTID
>LFGLNPFC_03085 hypothetical protein
MYDNLKSLGITNPEEIDRYSLRQEANNDILKIYFQKDKGEFFAKSVKFKYPRQRKTVVAD
GVGQGYKEVQEISPNLRYIIDELDQICQRDRSEVDLKRKILDDLRHLESVVTNKISEIEA
DLEKLTRK
>LFGLNPFC_03086 Carbohydrate diacid regulator
MARTMRIIDTNINVMDARGRIIGSGDRERIGELHEGALLVLSQGRVVDIDDAVARHLHGV
RQGINLPLRLEGEIVGVIGLTGEPENLRKYGELVCMTAEMMLEQSRLMHLLAQDSRLREE
LVMNLIQAEENTPALTEWAQRLGIDLNQPRVVAIVEVDSGQLGVDSAMAELQQLQNALTT
PERNNLVAIVSLTEMVVLKPALNSFGRWDAEDHRKRVEQLITRMKEYGQLRFRVSLGNYF
TGPGSIARSYRTAKTTMVVGKQRMPESRCYFYQDLMLPVLLDSLRGDWQANELARPLARL
KAMDNNGLLRRTLAAWFRHNVQPLATSKALFIHRNTLEYRLNRISELTGLDLGNFDDRLL
LYVALQLDEER
>LFGLNPFC_03087 Periplasmic serine endoprotease DegP
MKKTTLALSALALSLGLALSPLSATAAETSSATTAQQMPSLAPMLEKVMPSVVSINVEGS
TTVNTPRMPRNFQQFFGDDSPFCQEGSPFQSSPFCQGGLGGNGGGQQQKFMALGSGVIID
ADKGYVVTNNHVVDNATVIKVQLSDGRKFDAKMVGKDPRSDIALIQIQNPKNLTAIKMAD

SDALRVGDYTVAIGNPFGLGETVTSGIVSALGRSGLNAENYENFIQTDAAINRGNSGGAL
VNLNGELIGINTAILAPDGGNIGIGFAIPSNMVKNLTSQMVEYGQVKRGELGIMGTELNS
DLAKAMKVDAQRGAFVSQVLPNSSAAKAGIKAGDVITSLNGKPISSFAALRAQVGTMPVG
SKLTLGLLRDGKQVNVNLELQQSSQNQVDSSTIFNGIEGAEMSNKGKDQGVVVNNVKTGT
PAAQIGLKKGDVIIGANQQAVKNIAELRKVLDSKPSVLALNIQRGDSTIYLLMQ
>LFGLNPFC_03088 Deoxyguanosinetriphosphate triphosphohydrolase
MAQIDFRKKINWHRRYRSPQGVKTEHEILRIFESDRGRIINSPAIRRLQQKTQVFPLERN
AAVRTRLTHSMEVQQVGRYIAKEILSRLKELKLLEAYGLDELTGPFESIVEMSCLMHDIG
NPPFGHFGEAAINDWFRQRLYPEDAESQPLTDDRCSVAALRLRDGEEPLNALRRKIRQDL
CHFEGNAQGIRLVHTLMRMNLTWAQVGGILKYTRPAWWRGETPETHHYLMKKPGYYLSEE
AYIARLRKELNLALYSRFPLTWIMEAADDISYCVADLEDAVEKRIFTVEQLYHHLHEAWG
QHEKGSLFSLVVENAWEKSRSNSLSRSTEDQFFMYLRVNTLNKLVPYAAQRFIDNLPAIF
AGTFNHALLEDASECSDLLKLYKNVAVKHVFSHPDVEQLELQGYRVISGLLEIYRPLLNL
PLSDFTELVEKERVKRFPIETRLFHKLSTRHRLAYVEAVSKLPSDSPEFPLWEYYYRCRL
LQDYISGMTDLYAWDEYRRLMAVEQ
>LFGLNPFC_03089 5'-methylthioadenosine/S-adenosylhomocysteine nucleosidase
MKIGIIGAMEEEVTLLRDKIENRQTISLGGCEIYTGQLNGTEVALLKSGIGKVAAALGAT
LLLEHCKPDVIINTGSAGGLAPTLKVGDIVVSDEARYHDADVTAFGYEYGQLPGCPAGFK
ADDKLIAAAEACIAELNLNAVRGLIVSGDAFINGSVGLAKIRHNFPQAIAVEMEATAIAH
VCHNFNVPFVVVRAISDVADQQSHLSFDEFLAVAAKQSSLMVESLVQKLAHG
>LFGLNPFC_03090 Vitamin B12-binding protein
MAKSLFRALVALSFLAPLWLNAAPRVITLSPANTELAFAAGITPVGVSSYSDYPPQAQKI
GQVSTWQGMNLERIVALKPDLVIAWRGGNAERQVDQLASLGIKVMWVDATSIEQIANALR
QLAPWSPQPDKAEQAAQSLLDQYAQLKAQYADKPKKRVFLQFGINPPFTSGKESIQNQVL
EVCGGENIFKDSRVPWPQVSREQVLARSPQAIVITGGPDQIPKIKQYWGEQLKIPVIPLT
SDWFERASPRIILAAQQLCNALSQVD
>LFGLNPFC_03091 hypothetical protein
MLVYWLDIVGTAVFAISGVLLAGKLRMDPFGVLVLGVVTAVGGGTIRDMALDHGPVFWVK
DPTDLVVAMVTSMLTIVLVRQPRRLPKWMLPVLDAVGLAVFVGIGVNKAFNAEAGPLIAV
CMGVITGVGGGIIRDVLAREIPMILRTEIYATACIIGGIVHATAYYTFSVPLETASMMGM
VVTLLIRLAAIRWHLKLPTFALDENGR
>LFGLNPFC_03092 Iron-sulfur cluster insertion protein ErpA
MSDDVALPLEFTDAAANKVKSLIADEDNPNLKLRVYITGGGCSGFQYGFTFDDQVNEGDM
TIEKQGVGLVVDPMSLQYLVGGSVDYTEGLEGSRFIVTNPNAKSTCGCGSSFSI
>LFGLNPFC_03093 H(+)/Cl(-) exchange transporter ClcA
MKTDTPSLETPQAARLRRRQLIRQLLERDKTPLAILFMAAVVGTLVGLAAVAFDKGVAWL
QNQRMGALVHTADNYPLLLTVAFLCSAVLAMFGYFLVRKYAPEAGGSGIPEIEGALEDQR
PVRWWRVLPVKFFGGLGTLGGGMVLGREGPTVQIGGNIGRMVLDVFRLKGDEARHTLLAT
GAAAGLAAAFNAPLAGILFIIEEMRPQFRYTLISIKAVFIGVIMSTIMYRIFNHEVALID
VGKLSDAPLNTLWLYLILGIIFGIFGPIFNKWVLGMQDLLHRVHGGNITKWVLMGGAIGG
LCGLLGFVAPATSGGGFNLIPIATAGNFSMGMLVFIFVARVITTLLCFSSGAPGGIFAPM
LALGTVLGTAFGMVAVELFPQYHLEAGTFAIAGMGALLAASIRAPLTGIILVLEMTDNYQ
LILPMIITGLGATLLAQFTGGKPLYSAILARTLAKQEAEQLARSKAASARENT
>LFGLNPFC_03094 Glutamate-1-semialdehyde 2,1-aminomutase
MSKSENLYSAARELIPGGVNSPVRAFTGVGGTPLFIEKADGAYLYDVDGKAYIDYVGSWG
PMVLGHNHPAIRNAVIEAAERGLSFGAPTEMEVKMAQLVTELVPTMDMVRMVNSGTEATM
SAIRLARGFTGRDKIIKFEGCYHGHADCLLVKAGSGALTLGQPNSPGVPADFAKHTLTCT
YNDLASVRAAFEQYPQEIACIIVEPVAGNMNCVPPLPEFLPGLRALCDEFGALLIIDEVM
TGFRVALAGAQDYYGVEPDLTCLGKIIGGGMPVGAFGGRRDVMDALAPTGPVYQAGTLSG
NPIAMAAGFACLNEVAQPGVHETLDELTTRLAEGLREAAEEAGIPLVVNHVGGMFGIFFT
DAESVTCYQDVMACDVERFKRFFHMMLDEGVYLAPSAFEAGFMSVAHSMEDINNTIDAAR
RVFAKL
>LFGLNPFC_03095 Iron(3+)-hydroxamate import system permease protein FhuB
MSKRIALFPVLLLALLVVAAAALTWMNFSQALPRSQWAQAAWSPDIDVIEQMIFHYSLLP
RLAISLLVGAGLGLVGVLFQQVLRNPLAEPTTLGVATGAQLGITVTTLWAIPGAMASQFA
ALAGACVVGLIVFGVAWGKRLSPVTLILAGLVVSLYCGAINQLLVIFHHDQLQSMFLWST
GTLTQTDWGGVERLWPQLLGGVMLTLLLLRPLTLMGLDDGVARNLGLALSLARLAALSLA
IVISALLVNAVGIIGFIGLFAPLLAKMLGARRLLPRLMLASLIGALILWLSDQIILWLTR
VWMEVSTGSVTALIGAPLLLWLLPRLRSISAPDMKVNDRVATERQHVLAFALAGGVLLLM
AVVVALSFGRDAHGWTWASGALLEDLMPWRWPRIMAALFAGVMLAVAGCIIQRLTGNPMA
SPEVLGISSGAAFGVVLMLFLVPGNAFGWLLPAGSLGAAVTLLIIMIAAGRGGFSPHRML
LAGMALSTAFTMLLMMLQASGDPRMAQVLTWISGSTYNATDAQVWRTGIVMVILLAITPL
CRRWLTILPLGGDTARAVGMALTPTRIALLLLAACLTATATMTIGPLSFVGLMAPHIARM
MGFRRTMPHIVISALVGGLLLVFADWCGRMVLFPFQIPAGLLSTFIGAPYFIYLLRKQSR

>LFGLNPFC_03096 Iron(3+)-hydroxamate-binding protein FhuD
MSGLPLISRRRLLTAMALSPLLWQMNTAHAAVIDPNRIVALEWLPVELLLALGIVPYGVA
DTINYRLWVSEPPLPDSVIDVGLRTEPNLELLTEMKPSFMVWSAGYGPSPEMLARIAPGR
GFNFSDGKQPLAMARKSLTEMADLLNLQSAAETHLAHYEDFIRSMKPRFVKRGARPLLLT
TLIDPRHMLVFGPNSLFQEILDEYGIPNAWQGETNFWGSTAVSIDRLAAYKDVDVLCFDH
DNSKDMDALMATPLWQAMPFVRAGRFQRVPAVWFYGATLSAMHFVRILDNAIGGKA
>LFGLNPFC_03097 Iron(3+)-hydroxamate import ATP-binding protein FhuC
MQEYTNHSDTTFALRNISFRVPGRTLLHPLSLTFPAGKVTGLIGHNGSGKSTLLKMLGRH
QPPSEGEILLDAQPLESWSSKAFARKVAYLPQQLPPAEGMTVRELVAIGRYPWHGALGRF
GAADREKVEEAISLVGLKPLAHRLVDSLSGGERQRAWIAMLVAQDSRCLLLDEPTSALDI
AHQVDVLALVHRLSQERGLTVIAVLHDINMAARYCDYLVALRGGEMIAQGTPAEIMRGET
LEMIYGIPMGILPHPAGAAPVSFVY
>LFGLNPFC_03098 Ferrichrome outer membrane transporter/phage receptor
MAPSKTAQPKHSLRKIAVVVATAVSGMSVYAQAAVEPKEDTITVTAAPAPQESAWGPAAT
IAARQSATGTKTDTPIQKVPQSISVVTAEEMALHQPKSVKEALSYTPGVSVGTRGASNTY
DHLIIRGFAAEGQSQNNYLNGLKLQGNFYNDAVIDPYMLERAEIMRGPVSVLYGKSSPGG
LLNMVSKRPTTEPLKEVQFKAGTDSLFQTGFDFSDALDDDGVYSYRLTGLARSANAQQKG
SEEQRYAIAPAFTWRPDDKTNFTFLSYFQNEPETGYYGWLPKEGTVEPLPNGKRLPTDFN
EGAKNNTYSRNEKMVGYSFDHEFNDTFTVRQNLRFAENKTSQNSVYGYGVCSDPANAYSK
QCAALAPADKGHYLARKYVVDDEKLQNFSVDTQLQSKFATGDIDHTLLTGVDFMRMRNDI
NAWFGYDDSVPLLDLYNPVNTDFDFNAKDPDNSGPYRILNKQKQTGVYVQDQAQWDKVLV
TLGGRYDWADQESLNRVAGTTDKRDDKQFTWRGGVNYLFDNGVTPYFSYSESFEPSSQVG
KDGNIFAPSKGKQYEVGVKYVPEDRPIVVTGAVYNLTKTNNLMADPEGSFFSVEGGEIRA
RGVEIEAKAALSASVNVVGSYTYTDAEYTTDTTYKGNTPAQVPKHMASLWADYTFFDGPL
SGLTLGTGGRYTGSSYGDPANSFKVGSYTVVDALVRYDLARVGMAGSNVALHVNNLFDRE
YVASCFNTYGCFWGAERQVVATATFRF
>LFGLNPFC_03099 Penicillin-binding protein 1B
MAGNDREPIGRKGKPTRPVKQKVSRRRYEDDDDYDDYDDYEDEEPMPRKGKGKGKGRKPR
GKRGWLWLLLKLAIVFAVLIAIYGVYLDQKIRSRIDGKVWQLPAAVYGRMVNLEPDMTIS
KNEMVKLLEATQYRQVSKMTRPGEFTVQANSIEMIRRPFDFPDSKEGGQVRARLTFDGDHL
ATIVNMENNRQFGFFRLDPRLITMISSPNGEQRLFVPRSGFPDLLVDTLLATEDRHFYEH
DGISLYSIGRAVLANLTAGRTVQGASTLTQQLVKNLFLSSERSYWRKANEAYMALIMDAR
YSKDRILELYMNEVYLGQSGDNEIRGFPLASLYYFGRPVEELSLDQQALLVGMVKGASIY
NPWRNPKLALERRNLVLRLLQQQQIIDQELYDMLSARPLGVQPRGGVISPQPAFMQLVRQ
ELQAKLGDKVKDLSGVKIFTTFDSVAQDAAEKAAVEGIPALKKQRKLSDLETAIVVVDRF
SGEVRAMVGGSEPQFAGYNRAMQARRSIGSLAKPATYLTALSQPKIYRLNTWIADAPIAL
RQPNGQVWSPQNDDRRYSESGRVMLVDALTRSMNVPTVNLGMALGLPAVTETWIKLGVPK
DQLNPVPAMLLGALNLTPIEVAQAFQTIASGGNRAPLSALRSVIAEDGKVLYQSFPQAER
AVPAQAAYLTLWTMQQVVQRGTGRQLGAKYPNLHLAGKTGTTNNNVDTWFAGIDGSTVTI
TWVGRDNNQPTKLYGASGAMSIYQRYLANQTPTPLNLVPPEDIADMGVDYDGNFVCSGGM
RVLPVWTSDPQSLCQQSEMQQQPSGNPFDQSSQPQQQPQQQPAQQEQKDSDGVAGWIKDM
FGSN
>LFGLNPFC_03100 ATP-dependent RNA helicase HrpB
MSSLPVAAVLPELLAALDGASQVLLSAPTGAGKSTWLPLQLLAHPGINGKIILLEPRRLA
ARNVAQRLAELLNEKPGDTVGYRMRAQNCVGPNTRLEVVTEGVLTRMIQRDPELSGVGLV
ILDEFHERSLQADLALALLLDVQQGLRDDLKLLIMSATLDNDRLQQMLPEAPVVISEGRS
FPVERRYLPLPAHQRFDEAVAVATAEMLRQESGSLLLFLPGVGEIQRVQEQLASRIGSDV
LLCPLYGALSLNDQRKAILPAPQGMRKVVLATNIAETSLTIEGIRLVVDCAQERVARFDP
RTGLTRLVTQRVSQASMTQRAGRAGRLEPGICLHLIAKEQAERAAAQSEPEILQSDLSGL
LMELLQWGCSDPAQMSWLDQPPTVNLLAAKRLLRMLGALDGERLSAQGQKMATLGNDPRL
AAMLVSAKNDDEAATAAKIAAILEEPPRMGNSDLGVAFSRNQPAWQQRSQQLLKRLNVRG
GEADSSLIAPLLARAYADRIARRRGQDERYQLANGMGAMLDADDALSRHEWLIAPLLLQG
SASPDARILLALPVDIDELVQRCPQLVQQSDTVEWDDAQGTLKAWRRLQIGGLMVKVQPL
AKPSEDELHQAMLNGIRDKGLSVLNWTAEAEQLRLRLLCAAKWLPEYDWPAVDDESLLAT
LETWLLPHMTGVHSLRGLKSLDIYQALRGLLDWVMQQRLDSELPAHYTVPTGSRIAIRYH
EDNPPALAVRMQEMFGEATNPTIAQGRVPLVLELLSPAQRPLQITRDLSAFWKGAYREVQ
KEMKGRYPKHVWPDDPANTAPTRRTKKYS
>LFGLNPFC_03101 RNA 2',3'-cyclic phosphodiesterase
MSEPQRLFFAIDLPAEIREQIIRWRATHFPPEAGRPVAADNLHLTLAFLGEVSAEKEKAL
SLLAGRIRQPGFTLTLDDAGQWLRSRVVWLGMRQPPRGLIQLANMLRSQAARSGCFQSNR
PFHPHITLLRDASEAVTIPPPGFNWSYTVTEFTLYASSFARGRTRYTPLKRWALTQ
>LFGLNPFC_03102 Sugar fermentation stimulation protein A
MEFSPPLQRATLIQRYKRFLADVITPDGRELTLHCPNTGAMTGCATPGDTVWYSTSDNTK
RKYPHTWELTQSQSGAIICVNTLWANRLTKEAILNESISELAGYSSLKSEVKYGAERSRI

DFMLQADSRPDCYIEVKSVTLAENEQGYFPDAVTERGQKHLRELMSVAAEGQRAVIFFAV
LHSAITRFSPARHIDEKYAQLLSEAQQRGVEILAYKAELSAEGMALKKSLPVTL
>LFGLNPFC_03103 RNA polymerase-binding transcription factor DksA
MQEGQNRKTSSLSILAIAGVEPYQEKPGEEYMNEAQLAHFRRILEAWRNQLRDEVDRTVT
HMQDEAANFPDPVDRAAQEEEFSLELRNRDRERKLIKKIEKTLKKVEDEDFGYCESCGVE
IGIRRLEARPTADLCIDCKTLAEIREKQMAG
>LFGLNPFC_03104 Glutamyl-Q tRNA(Asp) synthetase
MLPPYFLFKEMTDTHYIGRFAPSPSGELHFGSLIAALGSYLQARARQGRWLVRIEDIDPP
REVPGAAETILRQLEHYGLHWDGDVLWQSQRHHAYREALAWLHEQGLSYYCTCTRARIQS
IGGIYDGHCRDLHHGPDNAAVRIRQQHPVTQFTDLLRGIIHADEKLAREDFIIHRRDGLF
AYNLAVVVDDHFQGVSEIVRGADLIEPTVRQISLYQLFGWKVPDYIHLPLALNPQGAKLS
KQNHAPALPKGDPRPVLIAALHFLGQQVETHWQDFSVEQILQSAVKNWTLTAVPESAIVN
STFSNASC
>LFGLNPFC_03105 Poly(A) polymerase I
MLSREESEAEQAVARPQVTVIPREQHAISRKDISENALKVMYRLNKAGYEAWLVGGGVRD
LLLGKKPKDFDVTTNATPEQVRKLFRNCRLVGRRFRLAHVMFGPEIIEVATFRGHHEGNV
SDRTTSQRGQNGMLLRDNIFGSIEEDAQRRDFTINSLYYSVADFTVRDYVGGMKDLKDGV
IRLIGNPETRYREDPVRMLRAVRFAAKLGMRISPETAEPIPRLATLLNDIPPARLFEESL
KLLQAGYGYETYKLLCEYHLFQPLFPTITRYFTENGDSPMERIIEQVLKNTDTRIHNDMR
VNPAFLFAAMFWYPLLETAQKIAQESGLTYHDAFALAMNDVLDEACRSLAIPKRLTTLTR
DIWQLQLRMSRRQAKRAWKLLEHPKFRAAYDLLALRAEVERNAELQRLVKWWGEFQVSAP
PDQKGMLNELDEEPSPRRRTRRPRKRAPRREGTA
>LFGLNPFC_03106 2-amino-4-hydroxy-6-hydroxymethyldihydropteridine pyrophosphokinase
MTVAYIAIGSNLASPLEQVNAALKALGDIPESRILAVSSFYRTPPLGPQDQPDYLNAAVA
LKTTLAPEGLLNHTQRIELQQGRVRKAERWGPRTLDLDIMLFGNEVINTERLTVPHYDMK
NRGFMLWPLFEIAPELVFPDGLSLVEALQAKGFNELDKW
>LFGLNPFC_03107 putative fimbrial-like protein YadN
MSKKLGFALSGIMLAMAAGTAFAANDMDGGQLNISGLVVDNTCEARVDGGNKDGLILLQT
ATVAEITDGVLDTTVGAKAKPFSITIDCSKANPAPGTTAKMTFGSVFFGNSKGTLNNDMS
INTPSDGVNIALHNIEGSTIKQVQVNNPGDVYSKTLDSTSKSATYDFKASYVRADASKAA
TAGYVKTNSAYTITYQ
>LFGLNPFC_03108 putative fimbrial chaperone YadV
MFFNTKHTAALCLASCMAFSSSAIADIVISGTRVVYKSEQKSVNVRLENKGNNPLLVQSW
LDTGDDNAEPGSITVPFTATPPVSRIDAKRGQTIKLMYTASSALPKDRESVFWFNVLEVP
PKPDAAKAANQSLLQLAFRTRIKLFYRPEGLSGISSDAPLALKWSWATSEGKAALRVDNP
TPYYVSFSSGDLEANGKRYPLDMTMIAPFSNDVIKVNGMSGRTSSAKVHFFAINDFGGSI
EGNANL
>LFGLNPFC_03109 Outer membrane usher protein HtrE
MTIKSTNHLTHIATFCALLYSNSALCAELVEYDHTFLMGKDASNIDLSRYTEGNPTLPGI
YDVSVYVNDQPIMSQSIAFAVIEGKKNAQACITQKNLLQFHISSPDKNSEKAILLKRDDD
LGDCLNLAEMIPQSSIRYDVNDQRLDIDVPQAWIMKNYQNYVDPSLWENGINAAMLSYNL
NGYHSESPGRTNDSIYAAFNGGINLGAWRLRASGNYNWMTNVHSDYDFQNRYLQRDLASL
RSQLVIGESYTTGETFDSVRIRGIRLYSDSRMLPPVLASFAPIIHGVANTNAKVTVMQNG
YKIYETTVPPGAFAIDDLSPSGYGSDLIVTIEEADGTKRTFSQPFSSVVQMLRPGVGRWD
ISAGQVLKDSIQDEPNLFQASYYYGLNNYLTGYTGIQLTDNNYTAGLLGLGMNTPVGAFS
VDVTHSNVSIPDDKTYQGQSYRISWNKLFENTSTSLNIAAYRYSTQHYLGLNDALTLIDE
VEHPEQDLEPKSMRNYSRMKNQVTVSINQPLKFEKKDYGSFYLSGSWSDYWASGQNSTNY
SIGYSNSASWGSYSISAQRSLNEDGGQTDDSIYLSFTIPIENLLGTEHRSSGFQSIDTQLN
SDFKGNNQLNISSSGYSDTNRISYSVNTGYMMNKSSDDLSYIGGYASYESPWGTLSGSAS
ASSDNSRQFSLNTDGGFVLHSGGLTFSNDSFSDSDTLAVIQAPGAKGARINYGNSTVDRW
GYGVTSALSPYHENRIALDINDLENDVELKSTSTVAVPRQGAVVFADFETVQGQSAIMNI
VRSDGKNIPFAADIYDEQNNIIGNVGQGGQAFVRGIGQEGNIRITWIEEGKPVSCFAHYQ
QNTTSEKIAQSIILNGLRCQIQ
>LFGLNPFC_03110 putative fimbrial-like protein YadM
MIKITPHKITILMGLLLSPSVFATDVNVDFTATVKATTCNITLTGTNVTDNGKDKYTLVI
PSMGMDKIANKTAQSEANFKLVANGCSSGISWIDTTLTGNQSGSSPALIIPLASDTTSTT
SYIGMGFKRKATSGDTFLKPNSAEYIRWSASEISTDGLEMTVALRETSVGKGVPGKFRAL
ATFNFSYQ
>LFGLNPFC_03111 putative fimbrial-like protein YadL
MMILKKLCYGLSGSLFLASSLLTISSHAATDSIGLTVTTDVEMGTCTSTLTDDGTTKIST
INFGDVYISEINAETKIKTFKLQFKDCAGIPGKKAKIKLTTRALCEGNSNNGPGFANAST
ATAKAAAVAVEVWSTSTPGKNGAKQFSCVTPATEEVSIANATGSNVVDYPMSAVLVVAKD
KTVTNVTAGDFTAPATFTVTYN
>LFGLNPFC_03112 putative fimbrial-like protein YadK

MRPTQRNLMKKSILLLSLLFSATSPVVAGQDVDIVANIKNNTCQSGISNNGNIDLGVVGV
GDFTGNISAENYHPGGKEFTITVQDCTLQGTGNVLNQLHINFRALSGVMATGSSQIFANE
DNAGAKNVGVVIFSIQDPANTFNVLSATGSSRSVYPVMSSALNNSSWKFSTRMQKIDPAL
NVTSGPLISHVLVDIYYE
>LFGLNPFC_03113 putative fimbrial-like protein YadC
MKIFFRYFLFLALCSCCYIASAGTDDHVGYVVGNNYGVGPSGQKWRETGPNGDVTVKFRY
GSVTNNLVFYKPTQLGPTGVSLKWAQLDSASGGGFLYCNRSGSTSGGPMHIENKMVDSGK
SYGGHKLFKTSVPGLYYTLAISNIWSTYTYTDINPSGMYIGDSTSQSFNWRGESEQTLYW
SCNNANTKNKYWAVGGVMQTLTIEFYTDTDFNPTTNQRVTLSRTDNYLYSFKAYGAGIGI
NDYSYFLKIDFDLTDIVLTNPTCFTAALSGSSVSGSTVKMGDYTPAQIKNGATAVPFDIT
LQNCIRVRNIETKLKSNKVGSVSKELLANTLTGNDAAKGVGVLIEGLKNTKSAQMVLKPN
DATSIYKDYETEDDTTGGIYPDKGNGTSQPLHFQATLKQDGNIAIEPGDFKATSTFQVTY
P
>LFGLNPFC_03114 3-methyl-2-oxobutanoate hydroxymethyltransferase
MKPTTIASLQKCKQDKKRFATITAYDYSFAKLFAEEGLNVMLVGDSLGMTVQGHDSTLPV
TVADIAYHTAAVRRGAPNCLLLADLPFMAYATPEQAFENAATVMRAGANMVKIEGGEWLV
ETVQMLTERAVPVCGHLGLTPQSVNIFGGYKVQGRGDEAGDRLLSDALALEAAGAQLLVL
ECVPVELAKRITEALAIPVIGIGAGNVTDGQILVMHDAFGITGGHIPKFAKNFLAETGDI
RAAVRQYMAEVESGVYPGEEHSFH
>LFGLNPFC_03115 Pantothenate synthetase
MLIIETLPLLRQQIRRLRMEGKRVALVPTMGNLHDGHMKLVDEAKARADVVVVSIFVNPM
QFDRPEDLARYPRTLQEDCEKLNKRKVDLVFAPSVKEIYPNGTETHTYVDVPGLSTMLEG
ASRPGHFRGVSTIVSKLFNLVQPDIACFGEKDFQQLALIRKMVADMGFDIEIVGVPIMRA
KDGLALSSRNGYLTAEQRKIAPGLYKVLSSIADKLQAGERDLDEIIAIAGQELNEKGFRS
DDIQIRDADTLLEVSENSKRAVILVAAWLGDARLIDNKLVELA
>LFGLNPFC_03116 ISNCY family transposase ISRor2
MDAPSTTPHDAVFKQFLMHAETARDFLDIHLPAELRELCDLDTLHLESGSFIEESLKGHS
TDVLYSVQMQGSTGYLHVVIEHQSKPDKKMAFRMMRYSIAAMHRHLEADHDKLPLVVPIL
FYQGEATPYPLSMCWFDMFYSPELARRVYNSPFPLVDITITPDDEIMQHRRIAILELLQK
HIRQRDLMLLLEQLVTLIDEGYTSGSQLVAMQNYMLQRGHTEQADLFYGVVRDRETGGES
MMTLAQWFEEKGIQQGRQEVSQEFALRLLSKGMSREDVAEMANLPLAEIDKMIN
>LFGLNPFC_03117 Aspartate 1-decarboxylase
MIRTMLQGKLHRVKVTHADLHYEGSCAIDQDFLDAAGILENEAIDIWNVTNGKRFSTYAI
AAERGSRIISVNGAAAHCASVGDIVIIASFVTMPDEEARTWRPNVAYFEGDNEMKRTAKA
IPVQVA
>LFGLNPFC_03118 hypothetical protein
MYKQAVILLLMLFTASVSAALPARYMQTIENAAVWAQIGDKMVTVGNIRAGQIIAVEPTA
ASYYVFNFGFGKGFIDKGHLEPVQGRQKVEDGLGDLNKPLSNQNLITRKDTPVYNAPSVG
SAPFGVLADNLRYPILHKLKDRLNQTWYQIRIGDRLAYISALDAQPDNGLPVLTYHHILR
DEENTRFRHTSTTTSVRAFNNQMAWLRDRGYATLSMAQLEGYVKNKINLPARAVVITFDD
GLKSVSRYAYPVLKQYGMKATAFIVTSRIKRHPQKWNPKSLQFMSVSELNEIRDVFDFQS
HTHFLHRIDGYRRPILLSRSEHNILFDFARSRRALAQFNPHVLYLSYPFGGFNDKAVKAA
KEAGFHLAVTTMKGKVKPGDNPLLLKRLYILRTDSLETMSRLVSNQPQG
>LFGLNPFC_03119 hypothetical protein
MLGWVITCHDDRAQEILDALEKKHGALLQCRAVNFWRGLSSNMLSRMMCDALHETDSGEG
VIFLTDIAGAPPYRVASLLSHKHSRCEVISGVTLPLIEQMMACRETMTSSAFREHIVELG
APEVSSLWHQQQKNPPFVLKHNLYEY
>LFGLNPFC_03120 Inner membrane transport permease YadH
MMHLYWVALKSIWAKEIHRFMRIWVQTLVPPVITMTLYFIIFGNLIGSRIGDMHGFSYMQ
FIVPGLIMMSVITNAYANVASSFFGAKFQRNIEELLVAPVPTHVIIAGYVGGGVARGLFV
GILVTAISLFFVPFQVHSWVFVALTLVLTAVLFSLAGLLNGVFAKTFDDISLVPTFVLTP
LTYLGGVFYSLTLLPPFWQGLSHLNPIVYMISGFRYGFLGINDVPLVTTFGVLVVFIVAF
YLICWSLIQRGRGLRS
>LFGLNPFC_03121 putative ABC transporter ATP-binding protein YadG
MTIALELQQLKKTYPGGVQALRGIDLQVEAGDFYALLGPNGAGKSTTIGIISSLVNKTSG
RVSVFGYDLEKDVVNAKRQLGLVPQEFNFNPFETVQQIVVNQAGYYGVERKEAYIRSEKY
LKQLDLWGKRNERARMLSGGMKRRLMIARALMHEPKLLILDEPTAGVDIELRRSMWGFLK
DLNDKGTTIILTTHYLEEAEMLCRNIGIIQHGELVENTSMKALLAKLKSETFILDLAPKS
PLPKLDGYQYRLVDTATLEVEVLREQGINSVFTQLSEQGIQVLSMRNKANRLEELFVSLV
NEKQGDRT
>LFGLNPFC_03122 Carbonic anhydrase 2
MKDIDTLISNNALWSKMLVEEDPGFFEKLAQAQKPRFLWIGCSDSRVPAERLTGLEPGEL
FVHRNVANLVIHTDLNCLSVVQYAVDVLEVEHIIICGHYGCGGVQAAVENPELGLINNWL
LHIRDIWFKHSSLLGEMPQERRLDTLCELNVMEQVYNLGHSTIMQSAWKRGQKVTIHGWA

YGIHDGLLRDLDVTATNRETLEQRYRHGISNLKLKHINHK
>LFGLNPFC_03123 Hypoxanthine phosphoribosyltransferase
MVIDMKHTVEVMIPEAEIKARIAELGRQITERYKDSGSDMVLVGLLRGSFMFMADLCREV
QVSHEVDFMTASSYGSGMSTTRDVKILKDLDEDIRGKDVLIVEDIIDSGNTLSKVREILS
LREPKSLAICTLLDKPSRREVNVPVEFIGFSIPDEFVVGYGIDYAQRYRHLPYIGKVILL
DE
>LFGLNPFC_03124 Quinoprotein glucose dehydrogenase
MAINNTGSRRLLVTLTALFAALCGLYLLIGGGWLVAIGGSWYYPIAGLVMLGVAWMLWRS
KRAALWLYAALLLGTMIWGVWEVGFDFWALTPRSDILVFFGIWLILPFVWRRLVIPASGA
VAALVVALLISGGILTWAGFNDPQEISGTLSADTTPAEAISPVADQDWPAYGRNQEGQRF
SPLKQINTDNVHKLKEAWVFRTGDVKQPNDPGEITNEVTPIKVGDTLYLCTAHQRLFALD
AASGKEKWHYDPELKTNESFQHVTCRGVSYHEAKAETASPEVMADCPRRIILPVNDGRLI
AINAENGKLCETFANKGVLNLQSNMPDTKPGLYEPTSPPIITDKTIVMAGSVTDNFSTRE
TSGVIRGFDVNTGELLWAFDPGAKDPNAIPSDEHTFTFNSPNSWAPAAYDAKLDLVYLPM
GVTTPDIWGGNRTPEQERYASSILALNATTGKLAWSYQTVHHDLWDMDLPAQPTLADITV
NGQKVPVIYAPAKTGNIFVLDRRNGELVVPAPEKPVPQGAAKGDYVTPTQPFSELSFRPT
KDLSGADMWGATMFDQLVCRVMFHQMRYEGIFTPPSEQGTLVFPGNLGMFEWGGISVDPN
REVAIANPMALPFVSKLIPRGPGNPMEQPKDAKGTGTESGIQPQYGVPYGVTLNPFLSPF
GLPCKQPAWGYISALDLKTNEVVWKKRIGTPQDSMPFPMPVPVPFNMGMPMLGGPISTAG
NVLFIAATADNYLRAYNMSNGEKLWQGRLPAGGQATPMTYEVNGKQYVVISAGGHGSFGT
KMGDYIVAYALPDDVK
>LFGLNPFC_03125 Blue copper oxidase CueO
MQRRDFLKYSVALGVASALPLWSRAVFAAERPTLPIPDLLTTDARNRIQLTIGAGQSTFG
GKTATTWGYNGNLLGPAVKLQRGKAVTVDIYNQLTEETTLHWHGLEVPGEVDGGPQGIIP
PGGKRSVTLNVDQPAATCWFHPHQHGKTGRQVAMGLAGLVVIEDDEILKLMLPKQWGIDD
VPVIVQDKKFNADGQIDYQLDVMTAAVGWFGDTLLTNGAIYPQHAAPRGWLRLRLLNGCN
ARSLNFATSDNRPLYVIASDGGLLPEPVKVSELPVLMGERFEVLVEVNDNKPFDLVTLPV
SQMGMAIAPFDKPHPVMRIQPIAISASGALPDTLSSLPALPSLEGLTVRKLQLSMDPMLD
MMGMQMLMEKYGDQAMAGMDHSQMMGHMGHGNMNHMNHSGKFDFHHANKINGQAFDMNKP
MFAAAKGQYERWVISGVGDMMLHPFHIHGTQFRILSENGKPPATHRAGWKDTVKVEGNVS
EVLVKFNHDAPKEHAYMAHCHLLEHEDTGMMLGFTV
>LFGLNPFC_03126 hypothetical protein
MAVCANSYALSESEAEDMADLTAVFVFLKNDCGYQNLPNGQIRRALVFFAQQNQWDLSNY
DTFDMKALGEDSYRDLSGIGIPVAKKCKALARDSLSLLAYVK
>LFGLNPFC_03127 Polyamine aminopropyltransferase
MAEKKQWHETLHDQFGQYFAVDNVLYHEKTDHQDLIIFENAAFGRVMALDGVVQTTERDE
FIYHEMMTHVPLLAHGHAKHVLIIGGGDGAMLREVTRHKNVESITMVEIDAGVVSFCRQY
LPNHNAGSYDDPRFKLVIDDGVNFVNQTNQTFDVIISDCTDPIGPGESLFTSAFYEGCKR
CLNPGGIFVAQNGVCFLQQEEAIDSHRKLSHYFSDVGFYQAAIPTYYGGIMTFAWATDND
ALRHLSTEIIQARFLASGLKCRYYNPAVHTAAFALPQYLQDALASQPS
>LFGLNPFC_03128 S-adenosylmethionine decarboxylase proenzyme
MKKLKLHGFNNLTKSLSFCIYDICYAKTTEERDGYIAYIDELYNANRLTEILSETCSIIG
ANILNIARQDYEPQGASVTILVSEEPVDPKLIDKTEHPGPLPETVVAHLDKSHICVHTYP
ESHPEGGLCTFRADIEVSTCGVISPLKALNYLIHQLESDIVTIDYRVRGFTRDINGMKHF
IDHEINSIQNFMSEDMKALYDMVDVNVYQENIFHTKMLLKEFDLKHYMFHTKPEDLTDSE
RQEITAALWKEMREIYYGRNMPAV
>LFGLNPFC_03129 hypothetical protein
MDYEFLRDITGVVKVRMSMGHEVVGHWFNEEVKENLALLDEVEQAAHALKGSERSWQRAG
HEYTLWMDGEEVMVRANQLEFAGDEMEEGMNYYDEESLSLCGVEDFLQVVAAYRNFVQQK
>LFGLNPFC_03130 Aconitate hydratase B
MLEEYRKHVAERAAEGIAPKPLDANQMAALVELLKNPPAGEEEFLLDLLTNRVPPGVDEA
AYVKAGFLAAVAKGEAKSPLLTPEKAIELLGTMQGGYNIHPLIDALDDAKLAPIAAKALS
HTLLMFDNFYDVEEKAKAGNEYAKQVMQSWADAEWFLNRPALAEKLTVTVFKVTGETNTD
DLSPAPDAWSRPDIPLHALAMLKNAREGIEPDQPGVVGPIKQIEALQQKGFPLAYVGDVV
GTGSSRKSATNSVLWFMGDDIPHVPNKRGGGLCLGGKIAPIFFNTMEDAGALPIEVDVSN
LNMGDVIDVYPYKGEVRNHETGELLATFELKTDVLIDEVRAGGRIPLIIGRGLTTKAREA
LGLPHSDVFRQAKDVAESDRGFSLAQKMVGRACGVKGIRPGAYCEPKMTSVGSQDTTGPM
TRDELKDLACLGFSADLVMQSFCHTAAYPKPVDVNTHHTLPDFIMNRGGVSLRPGDGVIH
SWLNRMLLPDTVGTGGDSHTRFPIGISFPAGSGLVAFAAATGVMPLDMPESVLVRFKGKM
QPGITLRDLVHAIPLYAIKQGLLTVEKKGKKNIFSGRILEIEGLPDLKVEQAFELTDASA
ERSAAGCTIKLNKEPIIEYLNSNIVLLKWMIAEGYGDRRTLERRIQGMEKWLANPELLEA
DADAEYAAVIDIDLADIKEPILCAPNDPDDARPLSAVQGEKIDEVFIGSCMTNIGHFRAA
GKLLDAHKGQLPTRLWVAPPTRMDAAQLTEEGYYSVFGKSGARIEIPGCSLCMGNQARVA
DGATVVSTSTRNFPNRLGTGANVFLASAELAAVAALIGKLPTPEEYQTYVAQVDKTAVDT

YRYLNFDQLSQYTEKADGVIFQTAV
>LFGLNPFC_03131 hypothetical protein
MKMTLPFKPHVLALICSAGLCAASAGLYIKSRTVEAPVEAQSTQQTAPDISAVTLPATVS
APPVTPAVVKSAFSTAQIDQWVAPVALYPDSLLSQVLMASTYPANVAQAVQWSHDNPLKQ
GDAAIQAVSDQPWDASVKSLVAFPQLMALMGENPQWVQNLGDAFLAQPQDVMDSVQRLRQ
LAQQTGSLKSSTEQKIIATTKKVVPVNQPANAPATQSNTVSTSSPVVAEPAPTVITIEPA
NPDVVYIPNYNPNVVYGSWANTAYPPVYLPPPAGEPFVDSFVRGFGYSMGVATTYALFSS
IDWDDDDHDHHHHDDDDYHHHDGGHRDGNDWQHNGDNINIDVNNFNRITGEHLTDKNMAW
RHNPNYRNGVTYHDQDMAKRFHQTDVNGGMSATQLPAPTRDSQRHAAASQFQQRTHAAPV
ITRDTQRQAAAQRFNEAEHYGSYDDFRDFSRRQPLTQQQKDAARQRYQSASPEQRQAVRE
KIQANPQNQQRREAARQRIQSASPEQRQAVREKMQTNPQNQQQRDAARQRIQSASPEQRQ
VFREKVQESRPQRLNDSNHTARLNNEQRSAVRERLSERGARRLER
>LFGLNPFC_03132 Dihydrolipoyl dehydrogenase
MSTEIKTQVVVLGAGPAGYSAAFRCADLGLETVIVERYNTLGGVCLNVGCIPSKALLHVA
KVIEEAKALAEHGIVFGEPKTDIDKIRTWKEKVINQLTGGLAGMAKGRKVKVVNGLGKFT
GANTLEVEGENGKTVINFDNAIIAAGSRPIQLPFIPHEDPRIWDSTDALELKEVPERLLV
MGGGIIGLEMGTVYHALGSQIDVVEMFDQVIPAADKDIVKVFTKRISKKFNLMLETKVTA
VEAKEDGIYVTMEGKKAPAEPQRYDAVLVAIGRVPNGKNLDAGKAGVEVDDRGFIRVDKQ
LRTNVPHIFAIGDIVGQPMLAHKGVHEGHVAAEVIAGKKHYFDPKVIPSIAYTEPEVAWV
GLTEKEAKEKGISYETATFPWAASGRAIASDCADGMTKLIFDKESHRVIGGAIVGTNGGE
LLGEIGLAIEMGCDAEDIALTIHAHPTLHESVGLAAEVFEGSITDLPNPKAKKK
>LFGLNPFC_03133 Dihydrolipoyllysine-residue acetyltransferase component of pyruvate dehydrogenase complex
MAIEIKVPDIGADEVEITEILVKVGDKVEAEQSLITVEGDKASMEVPSPQAGIVKEIKVS
VGDKTQTGALIMIFDSADGAADAAPAQAEEKKEAAPAAAPAAAAAKDVNVPDIGSDEVEV
TEILVKVGDKVEAEQSLITVEGDKASMEVPAPFAGTVKEIKVNVGDKVSTGSLIMVFEVA
GEAGAVAPAAKQEAAPAAAPASAAGVKEVNVPDIGGDEVEVTEVMVKVGDKVAAEQSLIT
VEGDKASMEVPAPFAGVVKELKVNVGDKVKTGSLIMIFEVEGAAPAAAPAKQEAAAPAPA
AKAEAPAAAPAAKAEGKSEFAENDAYVHATPLIRRLAREFGVNLAKVKGTGRKGRILRED
VQAYVKEAIKRAEAAPAATGGGIPGMLPWPKVDFSKFGEIEEVELGRIQKISGANLSRNW
VMIPHVTHFDKTDITELEAFRKQQNEEAAKRKLDVKITPVVFIMKAVAAALEQMPRFNSS
LSEDGQRLTLKKYINIGVAVDTPNGLVVPVFKDVNKKGIIELSRELMTISKKARDGKLTA
GEMQGGCFTISSIGGLGTTHFAPIVNAPEVAILGVSKSAMEPVWNGKEFVPRLMLPISLS
FDHRVIDGADGARFITIINNTLSDIRRLVM
>LFGLNPFC_03134 Pyruvate dehydrogenase E1 component
MSERFPNDVDPIETRDWLQAIESVIREEGVERAQYLIDQLLAEARKGGVNVAAGTGISNY
INTIPVEEQPEYPGNLELERRIRSAIRWNAIMTVLRASKKDLELGGHMASFQSSATIYDV
CFNHFFRARNEQDGGDLVYFQGHISPGVYARAFLEGRLTQEQLDNFRQEVHGNGLSSYPH
PKLMPEFWQFPTVSMGLGPIGAIYQAKFLKYLEHRGLKDTSKQTVYAFLGDGEMDEPESK
GAITIATREKLDNLVFVINCNLQRLDGPVTGNGKIINELEGIFEGAGWNVIKVMWGSRWD
ELLRKDTSGKLIQLMNETVDGDYQTFKSKDGAYVREHFFGKYPETAALVADWTDEQIWAL
NRGGHDPKKIYAAFKKAQETKGKATVILAHTIKGYGMGDAAEGKNIAHQVKKMNMDGVRH
IRDRFNVPVSDADIEKLPYITFPEGSEEHTYLHAQRQKLHGYLPSRQPNFTEKLELPSLQ
DFGALLEEQSKEISTTIAFVRALNVMLKNKSIKDRLVPIIADEARTFGMEGLFRQIGIYS
PNGQQYTPQDREQVAYYKEDEKGQILQEGINELGAGCSWLAAATSYSTNNLPMIPFYIYY
SMFGFQRIGDLCWAAGDQQARGFLIGGTSGRTTLNGEGLQHEDGHSHIQSLTIPNCISYD
PAYAYEVAVIMHDGLERMYGEKQENVYYYITTLNENYHMPAMPEGAEEGIRKGIYKLETL
EGSKGKVQLLGSGSILRHVREAAEILAKDYGVGSDVYSVTSFTELARDGQDCERWNMLHP
LETPRVPYIAQVMNDAPAVASTDYMKLFAEQVRTYVPADDYRVLGTDGFGRSDSRENLRH
HFEVDASYVVVAALGELAKRGEIDKKVVADAIAKFNIDADKVNPRLA
>LFGLNPFC_03135 Pyruvate dehydrogenase complex repressor
MAYSKIRQPKLSDVIEQQLEFLILEGTLRPGEKLPPERELAKQFDVSRPSLREAIQRLEA
KGLLLRRQGGGTFVQSSLWQSFSDPLVELLSDHPESQYDLLETRHALEGIAAYYAALRST
DEDKERIRELHHAIELAQQSGDLDAESNAVLQYQIAVTEAAHNVVLLHLLRCMEPMLAQN
VRQNFELLYSRREMLPLVSSHRTRIFEAIMAGKPEEAREASHRHLAFIEEILLDRSREES
RRERSLRRLEQRKN
>LFGLNPFC_03136 Colicin-E7 immunity protein
MHDLKYKIYFYTEGEFLEMLEEIVNATSKDKSLKGKKLEKYLDTLVDHFIKITEHPKKGD
LIFYPNSPEDGKPENILKIVKEWRRSQGLPLFKDSE
>LFGLNPFC_03137 Colicin-E7 immunity protein
MYNFKDEIEDYTEREFIELLGEFTNPTGDNAQLKGEELDKYWDDLEEHLTRITQHPLMSD
LIYYPAKKGDDEPENILKIVKEWRRSQGLPLFKDSK
>LFGLNPFC_03138 Colicin-E7 immunity protein
MFKEKLQDYTEDEFLNFLGGLRSSMKDGKSLKGKELEMYWDSLVDHFIEITQHPSGSDLI

FYPKSQGDDKPENILKIVKEWRRSQGLPLFKDSK
>LFGLNPFC_03139 hypothetical protein
MGDIVYLRIIGEKQGDISSGCGTYASVGNRWQVGHEDEIFAFALTNAITSTGKGVNLQGL
QFCKLIDKSSPLLSNAINQNERLFIEIDLYRINKSGRWERYYYIQLRNASLTAIHVNISD
NNLPTECVNVNYDYILCKHLIANTEFDWLAFPAGYNSLFIPPKNPPASNLNPEPLPVVNL
PLSPPAVKPVYAKSCLKEKGCTDAGTAEEPAENFGQVAIFALPVVDDCCGYHHPEANDVG
QPAEAQTMLLFPGSVLAAQIWGKWSLSGILSATCGSYIGALASALYIPSAGEGSARVPGR
DEFWYEEELRQKALAGSTATTRVRFFWGTDIHGKPQVYGVHTGEGTPYENVRVANMQWNE
QTQRYEFTPAHDVDGPLITWTPENPEHGYVPGHTGNDRPPLEQPTILVTPIPDGTDTYTT
PPFPVPDPKEFNDYILVFPAGSGIKPIYVYLKEDPRKLPGVVTGRGVPLSPGTRWLDMSV
SNNGNGAPIPAHIADKLRGREFKTFDEFREALWLEVSQDPELIAQFSSGNQTRIKQGLTA
KAPIDGWYYGPKEIVKKFQIHHRVAVEYGGSVYDIDNLRIVTPRLHDEIHYRR
>LFGLNPFC_03140 Aromatic amino acid transport protein AroP
MEGQQHGEQLKRGLKNRHIQLIALGGAIGTGLFLGSASVIQSAGPGIILGYAIAGFIAFL
IMRQLGEMVVEEPVAGSFSHFAYKYWGSFAGFASGWNYWVLYVLVAMAELTAVGKYIQFW
YPEIPTWVSAAVFFVVINAINLTNVKVFGEMEFWFAIIKVIAVVAMIIFGAWLLFSGNGG
PQASVSNLWDQGGFLPHGFTGLVMMMAIIMFSFGGLELVGITAAEADNPEQSIPKATNQV
IYRILIFYIGSLAVLLSLMPWTRVTADTSPFVLIFHELGDTFVANALNIVVLTAALSVYN
SCVYCNSRMLFGLAQQGNAPKALASVDKRGVPVNTILVSALVTALCVLINYLAPESAFGL
LMALVVSALVINWAMISLAHMKFRRAKQEQGVVTRFPALLYPLGNWICLLFMAAVLVIML
MTPGMAISVYLIPVWLIVLGIGYLFKEKTAKAVKAH
>LFGLNPFC_03141 Protein AmpE
MTLFTTLLVLIFERLFKLGEHWQLDHRLEAFFRRVKHFSLGRTLCMTIIAMGVTFLLLRA
LQGVLFNVPTLLVWLLIGLLCIGAGKVRLHYHAYLTAASRNDSHARATMAGELTMIHGVP
AGCDEREYLRELQNALLWINFRFYLAPLFWLIVGGTWGPVTLMGYAFLRAWQYWLARYQT
PHHRLQSGIDAVLHVLDWVPVRLAGVVYALIGHGEKALPAWFASLGDFHTSQYQVLTRLA
QFSLAREPHVDKVETPKAAVSMAKKTSFVVVVVIALLTIYGALV
>LFGLNPFC_03142 1,6-anhydro-N-acetylmuramyl-L-alanine amidase AmpD
MLLEQGWLAGARRVPSPHYDCRPDDETPTLLVVHNISLPPGEFGGPWIDALFTGTIDPQA
HPFFAEIAHLRVSAHCLIRRDGEIVQYVPFDKRAWHAGVSQYQGRERCNDFSIGIELEGT
DTLAYTDAQYQQLAAVTRALIDRYPDIANNMTGHCDIAPDRKTDPGPAFDWARFRALVSK
ETT
>LFGLNPFC_03143 Nicotinate-nucleotide pyrophosphorylase [carboxylating]
MPPRRYNPDTRRDELLERINLDIPGAVAQALREDLGGTVDANNDITAKLLPENSRSHATV
ITRENGVFCGKRWVEEVFIQLAGDDVTIIWHVDDGDVINANQPLFELEGPSRVLLTGERT
ALNFVQTLSGVASKVRHYVELLEGTNTQLLDTRKTLPGLRSALKYAVLCGGGANHRLGLS
DAFLIKENHIIASGSVRQAVEKASWLHPDAPVEVEVENLEELDEALKAGADIIMLDNFET
EQMREAVKRTNGKALLEVSGNVTDKTLREFAETGVDFISVGALTKHVQALDLSMRFR
>LFGLNPFC_03144 hypothetical protein
MDKQRGFTLIELMVVIGIIAILSAIGIPAYQNYLRKAALTDMLQTFVPYRTAVELCALEH
GGLNTCDGGSNGIPSPTTTRYVSAMSVAKGVVSLTGQESLNGLSVVMTPGWDNANGVTGW
TRNCNIQSDSALQQACEDVFRFDDAN
>LFGLNPFC_03145 Type II secretion system protein E
MNIPQLTALCLRYQGVLLDASEEVVHVAVVDAPSHELLDALHFATTKRIEITCWTRQQME
GHASRTQQTLPVAVQEKHQPKAELLTRTLQSALEQRASDIHIEPADNAYRIRLRIDGVLH
PLPDVSPDAGVALTARLKVLGNLDIAEHRLPQDGQFTVELAGNAVSFRIATLACRGGEKV
VLRLLQQVNQALDVNTLGMQPSQLVDFAHALQQPQGLVLVTGPTGSGKTVTLYSALQMLN
TADINICSVEDPVEIPIAGLNQTQIHSRAGLTFQGVLRALLRQDPDVIMIGEIRDGETAE
IAIKAAQTGHLVLSTLHTNSTCETLVRLQQMGVARWMLSSALTLVIAQRLVRKLCPHCRR
QQGEPIHIPDNVWPSPLPHWQAPGCVHCYHGFYGRTALFEVLPITPVIRQLISANTDVES
LETHARQAGMRTLFENGCLAVEQGLTTFEELIRVLGMPHGE
>LFGLNPFC_03146 Type II secretion system protein F
MASKQLWRWHGITGDGNAQDGMLWAESRALLLMALQQQMVTPLSLKRITINSTQWRGDIS
AEVIHQLATLLKAGLTLSEGLALLAEQHPSKQWQALLQSLAHDLEQGVAFSNALLPWSEV
FPPLYQAMIRTGELTGKLDECCFELARQQKAQRQLTDKVKSALRYPIIILAMAIMVVVAM
LHFVLPEFAAIYKTFNTPLPALTQGIMTLASFSGEWGWLLVLFGFLLAIANKLLMRHPTR
LIARQKLLLRIPIMGSLMRGQKLTQIFTILTLTQSAGISFLQGVESVRETMRCPYWVQLL
TQIQHDISNGHPIWLALKNAGEFSPLCLQLVRTGEASGSLDLMLDNLAHHHRDNTMALAD
NLAALLEPALLIITGGIIGTLVVAMYLPIFHLGDAMSGMG
>LFGLNPFC_03147 GMP reductase
MRIEEDLKLGFKDVLIRPKRSTLKSRSDVELERQFTFKHSGQSWSGVPIIAANMDTVGTF
SMASALASFDILTAVHKHYSVEEWQAFINNSSADVLKHVMVSTGTSDADFEKTKQILDLN
PALNFVCIDVANGYSEHFVQFVAKAREAWPTKTICAGNVVTGEMCEELILSGADIVKVGI
GPGSVCTTRVKTGVGYPQLSAVIECADAAHGLGGMIVSDGGCTTPGDVAKAFGGGADFVM

LGGMLAGHEESGGRIVEENGEKFMLFYGMSSESAMKRHVGGVAEYRAAEGKTVKLPLRGP
VENTARDILGGLRSACTYVGASRLKELTKRTTFIRVQEQENRIFNNL
>LFGLNPFC_03148 Dephospho-CoA kinase
MRYIVALTGGIGSGKSTVANAFANLGINVIDADIIARQVVEPGAPALHAIADHFGANMIA
ADGTLQRRALRERIFANPEEKNWLNALLHPLIQQETQHQIQQATSPYVLWVVPLLVENSL
YKKANRVLVVDVSPETQLKRTMQRDDVTREHVEQILAAQATREARLAVADDVIDNNGAPD
AIASDVARLHAHYLQLASQFVSQEKP
>LFGLNPFC_03149 Cell division protein ZapD
MQTQVLFEHPLNEKMRTWLRIEFLIQQLTVNLPIVDHAGALHFFRNVSELLDVFERGEVR
TELLKELDRQQRKLQTWIGVPGVDQSRIEALIQQLKAAGSVLISAPRIGQFLREDRLIAL
VRQRLSIPGGCCSFDLPTLHIWLHLPQAQRDSQVETWIASLNPLTQALTMVLDLIRQSAP
FRKQTSLNGFYQDNGGDADLLRLNLSLDSQLYPQISGHKSRFAIRFMPLDSENGQVPERL
DFELACC
>LFGLNPFC_03150 DNA gyrase inhibitor YacG
MSETITVNCPTCGKTVVWGEISPFRPFCSKRCQLIDLGEWAAEEKRIPSSGDLSESDDWS
EEPKQ
>LFGLNPFC_03151 8-oxo-dGTP diphosphatase
MKKLQIAVGIIRNENNEIFITRRAADAYMANKLEFPGGKVEMGETPEQAVVRELQEEVGI
TPQHFSLFEKLEYEFPDRHITLWFWLVESWEGEPWGKEGQPGEWMSLVGLNADDFPPANE
PVIAKLKRVYAG
>LFGLNPFC_03152 Protein translocase subunit SecA
MLIKLLTKVFGSRNDRTLRRMRKVVNIINAMEPEMEKLSDEELKGKTAEFRARLEKGEVL
ENLIPEAFAVVREASKRVFGMRHFDVQLLGGMVLNERCIAEMRTGEGKTLTATLPAYLNA
LTGKGVHVVTVNDYLAQRDAENNRPLFEFLGLTVGINLPGMPAPAKREAYAADITYGTNN
EYGFDYLRDNMAFSPEERVQRKLHYALVDEVDSILIDEARTPLIISGPAEDSSEMYKRVN
KIIPHLIRQEKEDSETFQGEGHFSVDEKSRQVNLTERGLVLIEELLVKEGIMDEGESLYS
PANIMLMHHVTAALRAHALFTRDVDYIVKDGEVIIVDEHTGRTMQGRRWSDGLHQAVEAK
EGVQIQNENQTLASITFQNYFRLYEKLAGMTGTADTEAFEFSSIYKLDTVVVPTNRPMIR
KDLPDLVYMTEAEKIQAIIEDIKERTAKGQPVLVGTISIEKSELVSNELTKAGIKHNVLN
AKFHANEAAIVAQAGYPAAVTIATNMAGRGTDIVLGGSWQAEVAALENPTAEQIEKIKAD
WQVRHDAVLAAGGLHIIGTERHESRRIDNQLRGRSGRQGDAGSSRFYLSMEDALMRIFAS
DRVSGMMRKLGMKPGEAIEHPWVTKAIANAQRKVESRNFDIRKQLLEYDDVANDQRRAIY
SQRNELLDVSDVSETINSIREDVFKATIDAYIPPQSLEEMWDIPGLQERLKNDFDLDLPI
AEWLDKEPELHEETLRERILAQSIEVYQRKEEVVGAEMMRHFEKGVMLQTLDSLWKEHLA
AMDYLRQGIHLRGYAQKDPKQEYKRESFSMFAAMLESLKYEVISTLSKVQVRMPEEVEEL
EQQRRMEAERLAQMQQLSYQDDDSAAAAALAAQTGERKVGRNDPCPCGSGKKYKQCHGRL
Q
>LFGLNPFC_03153 Secretion monitor
MLWTSGFNDKICALNTFEYDRDGNNVSGILTRWRQFGKRYFWPHLLLGMVAASLGLPALS
NAAEPNAPAKATTRNHEPSAKVNFGQLALLEANTRRPNSNYSVDYWHQHAIRTVIRHLSF
AMAPQTLPVAEESLPLQAQHLALLDTLSALLTQEGTPSEKGYRIDYAHFTPQAKFSTPVW
ISQAQGIRAGPQRLS
>LFGLNPFC_03154 UDP-3-O-acyl-N-acetylglucosamine deacetylase
MIKQRTLKRIVQATGVGLHTGKKVTLTLRPAPANTGVIYRRTDLNPPVDFPADAKSVRDT
MLCTCLVNEHDVRISTVEHLNAALAGLGIDNIVIEVNAPEIPIMDGSAAPFVYLLLDAGI
DELNCAKKFVRIKETVRVEDGDKWAEFKPYNGFSLDFTIDFNHPAIDSSNQRYAMNFSAD
AFMRQISRARTFGFMRDIEYLQSRGLCLGGSFDCAIVVDDYRVLNEDGLRFEDEFVRHKM
LDAIGDLFMCGHNIIGAFTAYKSGHALNNKLLQAVLAKQEAWEYVTFQDDAELPLAFKAP
SAVLA
>LFGLNPFC_03155 Cell division protein FtsZ
MFEPMELTNDAVIKVIGVGGGGGNAVEHMVRERIEGVEFFAVNTDAQALRKTAVGQTIQI
GSGITKGLGAGANPEVGRNAADEDRDALRAALEGADMVFIAAGMGGGTGTGAAPVVAEVA
KDLGILTVAVVTKPFNFEGKKRMAFAEQGITELSKHVDSLITIPNDKLLKVLGRGISLLD
AFGAANDVLKGAVQGIAELITRPGLMNVDFADVRTVMSEMGYAMMGSGVASGEDRAEEAA
EMAISSPLLEDIDLSGARGVLVNITAGFDLRLDEFETVGNTIRAFASDNATVVIGTSLDP
DMNDELRVTVVATGIGMDKRPEITLVTNKQVQQPVMDRYQQHGMAPLTQEQKPVAKVVND
NAPQTAKEPDYLDIPAFLRKQAD
>LFGLNPFC_03156 Cell division protein FtsA
MIKATDRKLVVGLEIGTAKVAALVGEVLPDGMVNIIGVGSCPSRGMDKGGVNDLESVVKC
VQRAIDQAELMADCQISSVYLALSGKHISCQNEIGMVPISEEEVTQEDVENVVHTAKSVR
VRDEHRVLHVIPQEYAIDYQEGIKNPVGLSGVRMQAKVHLITCHNDMAKNIVKAVERCGL
KVDQLIFAGLASSYSVLTEDERELGVCVVDIGGGTMDIAVYTGGALRHTKVIPYAGNVVT
SDIAYAFGTPPSDAEAIKVRHGCALGSIVGKDESVEVPSVGGRPPRSLQRQTLAEVIEPR
YTELLNLVNEEILQLQEKLRQQGVKHHLAAGIVLTGGAAQIEGLAACAQRVFHTQVRIGA

PLNITGLTDYAQEPYYSTAVGLLHYGKESHLNGEAEVEKRVTASVGSWIKRLNSWLRKEF
>LFGLNPFC_03157 Cell division protein FtsQ
MSQAALNTRNSEEEVSSRRNNGTRLAGILFLLTVLTTVLVSGWVVLGWMEDAQRLPLSKL
VLTGERHYTRNDDIRQSILALGEPGTFMTQDVNIIQTQIEQRLPWIKQVSVRKQWPDELK
IHLVEYVPIARWNDQHMVDAEGNTFSVPPDRTSKQVLPMLYGPEGSANEVLQGYREMGQM
LAKDRFTLKEAAMTARRSWQLTLNNDIKLNLGRGDTMKRLARFVELYPVLQQQAQTDGKR
ISYVDLRYDSGAAVGWAPLPPEESTQQQNQAQAEQQ
>LFGLNPFC_03158 D-alanine--D-alanine ligase B
MTDKIAVLLGGTSAEREVSLNSGAAVLAGLREGGIDAYPVDPKEVDVTQLKSMGFQKVFI
ALHGRGGEDGTLQGMLELMGLPYTGSGVMASALSMDKLRSKLLWQGAGLPVAPWVALTRA
EFEKGLNDKQLAEISSLGLPVIVKPSREGSSVGMSKVVAENALQDALRLAFQHDEEVLIE
KWLSGPEFTIAILGEEILPSIRIQPAGTFYDYEAKYLSDETQYFCPAGLEASQEANLQAL
VLKAWTTLGCKGWGRIDVMLDSDGQFYLLEANTSPGMTSHSLVPMAARQAGMSFSQLVVR
ILELAD
>LFGLNPFC_03159 UDP-N-acetylmuramate--L-alanine ligase
MNTQQLAKLRSIVPEMRRVRHIHFVGIGGAGMGGIAEVLANEGYQISGSDLAPNPVTQQL
MNLGATIYFNHRPENVRDASVVVVSSAISADNPEIVAAHEARIPVIRRAEMLAELMRFRH
GIAIAGTHGKTTTTAMVSSIYAEAGLDPTFVNGGLVKAAGVHARLGHGRYLIAEADESDA
SFLHLQPMVAIVTNIEADHMDTYQGDFENLKQTFINFLHNLPFYGRAVMCVDDPVIRELL
PRVGRQTTTYGFSEDADVRVEDYQQIGPQGHFTLLRQDKEPMRVTLNAPGRHNALNAAAA
VAVATEEGIDDEAILRALESFQGTGRRFDFLGEFPLEPVNGKSGTAMLVDDYGHHPTEVD
ATIKAARAGWPDKNLVMLFQPHRFTRTRDLYDDFANVLTQVDTLLMLEVYPAGEAPIPGA
DSRSLCRTIRGRGKIDPILVPDPAQVAEMLAPVLTGNDLILVQGAGNIGKIARSLAEIKL
KPQTPEEEQHD
>LFGLNPFC_03160 UDP-N-acetylglucosamine--N-acetylmuramyl-(pentapeptide) pyrophosphoryl-undecaprenol N-acetylglucosamine transferase
MSGQGKRLMVMAGGTGGHVFPGLAVAHHLMAQGWQVRWLGTADRMEADLVPKHGIEIDFI
RISGLRGKGIKALIAAPLRIFNAWRQARAIMKAYKPDVVLGMGGYVSGPGGLAAWSLGIP
VVLHEQNGIAGLTNKWLAKIATKVMQAFPGAFPNAEVVGNPVRTDVLALPLPQQRLAGRE
GPVRVLVVGGSQGARILNQTMPQVAAKLGDSVTIWHQSGKGSQQSVEQAYAEAGQPQHKV
TEFIDDMAAAYAWADVVVCRSGALTVSEIAAAGLPALFVPFQHKDRQQYWNALPLEKAGA
AKIIEQSQLSVDAVANTLAGWSREILLTMAERARAASIPDATERVANEVSRAARA
>LFGLNPFC_03161 putative peptidoglycan glycosyltransferase FtsW
MRLSLPRLKMPRLPGFSILVWISTALKGWVMGSREKDTDSLIMYDRTLLWLTFGLAAIGF
IMVTSASMPIGQRLTNDPFFFAKRDGVYLILAFILAIITLRLPMEFWQRYSATMLLGSII
LLMIVLVVGSSVKGASRWIDLGLLRIQPAELTKLSLFCYIANYLVRKGDEVRNNLRGFLK
PMGVILVLAVLLLAQPDLGTVVVLFVTTLAMLFLAGAKLWQFIAIIGMGISAVVLLILAE
PYRIRRVTAFWNPWEDPFGSGYQLTQSLMAFGRGELWGQGLGNSVQKLEYLPEAHTDFIF
AIIGEELGYVGVVLALLMVFFVAFRAMSIGRKALEIDHRFSGFLACSIGIWFSFQALVNV
GAAAGMLPTKGLTLPLISYGGSSLLIMSTAIMMLLRIDYETRLEKAQAFVRGSR
>LFGLNPFC_03162 UDP-N-acetylmuramoylalanine--D-glutamate ligase
MADYQGKNVVIIGLGLTGLSCVDFFLARGVTPRVMDTRMTPPGLDKLPEAVERHTGGLND
EWLMAADLIVASPGIALAHPSLSAAADAGIEIVGDIELFCREAQAPIVAITGSNGKSTVT
TLVGEMAKAAGVNVGVGGNIGLPALMLLDDECELYVLELSSFQLETTSSLQAVAATILNV
TEDHMDRYPFGLQQYRAAKLRIYENAKVCVVNADDALTMPIRGADERCVSFGVNMGDYHL
NHQQGETWLRVKGEKVLNVKEMKLSGQHNYTNALAALALADAAGLPRASSLKALTTFTGL
PHRFEVVLEHNGVRWVNDSKATNVGSTEAALNGLHVDGTLHLLLGGDGKSADFSPLARYL
NGDNVRLYCFGRDGAQLAALRPEVAEQTETMEQAMRLLAPRVQPGDMVLLSPACASLDQF
KNFEQRGNEFARLAKELG
>LFGLNPFC_03163 Phospho-N-acetylmuramoyl-pentapeptide-transferase
MLVWLAEHLVKYYSGFNVFSYLTFRAIVSLLTALFISLWMGPRMIAHLQKLSFGQVVRND
GPESHFSKRGTPTMGGIMILTAIVISVLLWAYPSNPYVWCVLVVLVGYGVIGFVDDYRKV
VRKDTKGLIARWKYFWMSVIALGVAFALYLAGKDTPATQLVVPFFKDVMPQLGLFYILLA
YFVIVGTGNAVNLTDGLDGLAIMPTVFVAGGFALVAWATGNMNFASYLHIPYLRHAGELV
IVCTAIVGAGLGFLWFNTYPAQVFMGDVGSLALGGALGIIAVLLRQEFLLVIMGGVFVVE
TLSVILQVGSFKLRGQRIFRMAPIHHHYELKGWPEPRVIVRFWIISLMLVLIGLATLKVR
>LFGLNPFC_03164 UDP-N-acetylmuramoyl-tripeptide--D-alanyl-D-alanine ligase
MISVTLSQLTDILNGELQGADITLDAVTTDTRKLTPGCLFVALKGERFDAHDFADQAKAG
GAGALLVSRPLDIDLPQLIVKDTRLAFGELAAWVRQQVPARVVALTGSSGKTSVKEMTAA
ILSQCGNTLYTAGNLNNDIGVPMTLLRLTPEYDYAVIELGANHQGEIAWTVSLTRPEAAL
VNNLAAAHLEGFGSLAGVAKAKGEIFSGLPENGIAIMNADNNDWLNWQSVIGSRKVWRFS
PNAANSDFTATNIHVTSHGTEFTLQTPTGSVDVLLPLPGRHNIANALAAAALSMSVGATL
DAIKAGLANLKAVPGRLFPIKLAENQLLLDDSYNANVGSMTAAVQVLAEMPGYRVLVVGD
MAELGAESEACHVQVGEAAKAAGIDCVLSVGKQSHAISTASGVGEHFSDKTALIARLKSL

IAEQQVITILVKGSRSAAMEEVVRALQENGTC
>LFGLNPFC_03165 UDP-N-acetylmuramoyl-L-alanyl-D-glutamate--2,6-diaminopimelate ligase
MADRNLRDLLAPWVPDAPSRALREMTLDSRVAAAGDLFVAVVGHQADGRRYIPQAIAQGV
AAIIAEAKDEATDGEIREMHGVPVIYLSQLNERLSALAGRFYHEPSDNLRLVGVTGTNGK
TTTTQLLAQWSQLLGETSAVMGTVGNGLLGKVIPTENTTGSAVDVQHELAGLVDQGATFC
AMEVSSHGLVQHRVAALKFAASVFTNLSRDHLDYHGDMEHYEAAKWLLYSEHHCGQAIIN
ADDEVGRRWLAKLPDAVAVSMEDHINPNCHGRWLKATDVNYHDSGATIRFSSSWGDGEIE
SRLMGAFNVSNLLLALATLLALGYPLADLLKTAARLQPVCGRMEVFTAPGKPTVVVDYAH
TPDALEKALQAARLHCAGKLWCVFGCGGDRDKGKRPLMGAIAEEFADVAVVTDDNPRTEE
PRAIINDILAGMLDAGYAKVMEGRAEAVTCAVMQAKENDVVLVAGKGHEDYQIVGNQRLD
YSDRVTVARLLGVIA
>LFGLNPFC_03166 Peptidoglycan D,D-transpeptidase FtsI
MKAAAKTQKPKRQEEHANFISWRFALLCGCILLALAFLLGRVAWLQVISPDMLVKEGDMR
SLRVQQVSTSRGMITDRSGRPLAVSVPVKAIWADPKEVHDAGGISVGDRWKALANALNIP
LDQLSARINANPKGRFIYLARQVNPDMADYIKKLKLPGIHLREESRRYYPSGEVTAHLIG
FTNVDSQGIEGVEKSFDKWLTGQPGERIVRKDRYGRVIEDISSTDSQAAHNLTLSIDERL
QALVYRELNNAVAFNKAESGSAVLVDVNTGEVLAMANSPSYNPNNLSGTPKEAMRNRTIT
DVFEPGSTVKPMVVMTALQRGVVRENSVLNTVPYRINGHEIKDVARYSELTLTGVLQKSS
NVGVSKLALAMPSSALVDTYSRFGLGKATNLGLVGERSGLYPQKQRWSDIERATFSFGYG
LMVTPLQLARVYATIGSYGIYRPLSITKVDPPVPGERVFPESIVRTVVHMMESVALPGGG
GVKAAIKGYRIAIKTGTAKKVGPDGRYINKYIAYTAGVAPASQPRFALVVVINDPQAGKY
YGGAVSAPVFGAIMGGVLRTMNIEPDALTTGDKNEFVINQGEGTGGRS
>LFGLNPFC_03167 Cell division protein FtsL
MISRVTEALSKVKGSMGSHERHALPGVIGDDLLRFGKLPLCLFICIILTAVTVVTTAHHT
RLLTAQREQLVLERDALDIEWRNLILEENALGDHSRVERIATEKLQMQHVDPSQENIVVQ
K
>LFGLNPFC_03168 Ribosomal RNA small subunit methyltransferase H
MMENYKHTTVLLDEAVNGLNIRPDGIYIDGTFGRGGHSRLILSQLGEEGRLLAIDRDPQA
IAVAKTIDDPRFSIIHGPFSALGEYVAERDLIGKIDGILLDLGVSSPQLDDAERGFSFMR
DGPLDMRMDPTRGQSAAEWLQTAEEADIAWVLKTYGEERFAKRIARAIVERNREQPMTRT
KELAEVVAAATPVKDKFKHPATRTFQAVRIWVNSELEEIEQALKSSLNVLAPGGRLSIIS
FHSLEDRIVKRFMRENSRGPQVPAGLPMTEEQLKKLGGRQLRALGKLMPGEEEVAENPRA
RSSVLRIAERTNA
>LFGLNPFC_03169 Transcriptional regulator MraZ
MFRGATLVNLDSKGRLSVPTRYREQLLENAAGQMVCTIDIHHPCLLLYPLPEWEIIEQKL
SRLSSMNPVERRVQRLLLGHASECQMDGAGRLLIAPVLRQHAGLTKEVMLVGQFNKFELW
DETTWHQQVKEDIDAEQLATGDLSERLQDLSL
>LFGLNPFC_03170 Catabolite repressor/activator
MKLDEIARLAGVSRTTASYVINGKAKQYRVSDKTVEKVMAVVREHNYHPNAVAAGLRAGR
TRSIGLVIPDLENTSYTRIANYLERQARQRGYQLLIACSEDQPDNEMRCIEHLLQRQVDA
IIVSTSLPPEHPFYQRWANDPFPIVALDRALDREHFTSVVGADQDDAEMLAEELRKFPAE
TVLYLGALPELSVSFLREQGFRTAWKDDPREVHFLYANSYEREAAAQLFEKWLETHPMPQ
ALFTTSFALLQGVMDVTLRRDGKLPSDLAIATFGDNELLDFLQCPVLAVAQRHRDVAERV
LEIVLASLDEPRKPKPGLTRIKRNLYRRGVLSRS
>LFGLNPFC_03171 Acetolactate synthase isozyme 3 small subunit
MRRILSVLLENESGALSRVIGLFSQRGYNIESLTVAPTDDPTLSRMTIQTVGDEKVLEQI
EKQLHKLVDVLRVSELGQGAHVEREIMLVKIQASGYGRDEVKRNTEIFRGQIIDVTPSLY
TVQLAGTSDKLDAFLASIRDVAKIVEVARSGVVGLSRGDKIMR
>LFGLNPFC_03172 Acetolactate synthase isozyme 3 large subunit
MEMLSGAEMVVRSLIDQGVKQVFGYPGGAVLDIYDALHTVGGIDHVLVRHEQAAVHMADG
LARATGEVGVVLVTSGPGATNAITGIATAYMDSIPLVVLSGQVATSLIGYDAFQECDMVG
ISRPVVKHSFLVKQTEDIPQVLKKAFWLAASGRPGPVVVDLPKDILNPANKLPYVWPESV
SMRSYNPTTSGHKGQIKRALQTLVAAKKPVVYVGGGAITAGCHLQLKEAVEVLNLPVVSS
LMGLGAFPATHRQALGMLGMHGTYEANMTMHNADVIFAVGVRFDDRTTNNLAKYCPNATV
LHIDIDPTSISKTVTADIPIVGDARQVLEQMLELLSQESAHQPLDEIRDWWQQIEQWRAR
QCLKYDTHSEKIKPQAVIETLWRLTHGDAYVTSDVGQHQMFAALYYPFDKPRRWINSGGL
GTMGFGLPAALGVKMALPEETVVCVTGDGSIQMNIQELSTALQYELPVLVVNLNNRYLGM
VKQWQDMIYSGRHSQSYMQSLPDFVRLAEAYGHVGIQISHPQELESKLSEALEQVRNNRL
VFVDVTVDGSEHVYPMQIRGGGMDEMWLSKTERT
>LFGLNPFC_03173 HTH-type transcriptional regulator LeuO
MPEVQTDHPETAELSKPQLRMVDLNLLTVFDAVMQEQNITRAAHALGMSQPAVSNAVARL
KVMFNDELFVRYGRGIQPTARAFQLFGSVRQALQLVQNELPGSGFEPASSERVFHLCVCS
PLDSILTSQIYNHIEQIAPNIHVMFKSSLNQNTEHQLRYQETEFVISYEDFHRPEFTSVP
LFKDEMVLVASKNHPTIKGPLLKHDVYNEQHAAVSLDRFASFSQTWYDTVDKQASIAYQG

MAMMSVLSVVSQTHLVAIAPRWLAEEFAESLELQVLPLPLKQNSRTCYLSWHEAAGRDKG
HQWMEEQLVSICKR
>LFGLNPFC_03174 2-isopropylmalate synthase
MSQQVIIFDTTLRDGEQALQASLSVKEKLQIALALERMGVDVMEVGFPVSSPGDFESVQT
IARQVKNSRVCALARCVEKDIDVAAESLKVAEAFRIHTFIATSPMHIATKLRSTLDEVIE
RAIYMVKRARNYTDDVEFSCEDAGRTPIADLARVVEAAINAGATTINIPDTVGYTMPFEF
AGIISGLYERVPNIDKAIISVHTHDDLGLAVGNSLAAVHAGARQVEGAMNGIGERAGNCS
LEEVIMAIKVRKDILNVHTAINHQEIWRTSQLVSQICNMPIPANKAIVGSGAFAHSSGIH
QDGVLKNRENYEIMTPESIGLNQIQLNLTSRSGRAAVKHRMDEMGYKESEYNLDNLYDAF
LKLADKKGQVFDYDLEALAFIGKQQEEPEHFRLDYFSVQSGSNDIATAAVKLACGEEVKA
EAANGNGPVDAVYQAINRITDYNVELVKYSLTAKGHGKDALGQVDIVANYNGRRFHGVGL
ATDIVESSAKAMVHVLNNIWRAAEVEKELQRKAQHNENNKETV
>LFGLNPFC_03175 3-isopropylmalate dehydrogenase
MSKNYHIAVLPGDGIGPEVMTQALKVLDAVRNRFAMRITTSHYDVGGAAIDNHGQPLPPA
TVEGCEQADAVLFGSVGGPKWEHLPPDQQPERGALLPLRKHFKLFSNLRPAKLYQGLEAF
CPLRADIAANGFDILCVRELTGGIYFGQPKGREGSGQYEKAFDTEVYHRFEIERIARIAF
ESARKRRHKVTSIDKANVLQSSILWREIVNEIATEYPDVELAHMYIDNATMQLIKDPSQF
DVLLCSNLFGDILSDECAMITGSMGMLPSASLNEQGFGLYEPAGGSAPDIAGKNIANPIA
QILSLALLLRYSLDADDAASAIERAINRALEEGIRTGDLARGAAAVSTDEMGDIIARYVA
EGV
>LFGLNPFC_03176 3-isopropylmalate dehydratase large subunit
MAKTLYEKLFDAHVVYEAENETPLLYIDRHLVHEVTSPQAFDGLRAHGRPVRQPGKTFAT
MDHNVSTQTKDINACGEMARIQMQELIKNCKEFGVELYDLNHPYQGIVHVMGPEQGVTLP
GMTIVCGDSHTATHGAFGALAFGIGTSEVEHVLATQTLKQGRAKTMKIEVQGKAAPGITA
KDIVLAIIGKTGSAGGTGHVVEFCGEAIRDLSMEGRMTLCNMAIEMGAKAGLVAPDETTF
NYVKGRLHAPKGKDFDDAVAYWKTLQTDEGATFDTVVTLQAEEISPQVTWGTNPGQVISV
NDNIPDPASFADPVERASAEKALAYMGLKPGIPLTEVAIDKVFIGSCTNSRIEDLRAAAE
IAKGRKVAPGVQALVVPGSGPVKAQAEAEGLDKIFIEAGFEWRLPGCSMCLAMNNDRLNP
GERCASTSNRNFEGRQGRGGRTHLVSPAMAAAAAVTGHFADIRNIK
>LFGLNPFC_03177 3-isopropylmalate dehydratase small subunit
MAEKFIKHTGLVVPLDAANVDTDAIIPKQFLQKVTRTGFGAHLFNDWRFLDEKGQQPNPD
FVLNFPQYQGASILLARENFGCGSSREHAPWALTDYGFKVVIAPSFADIFYGNSFNNQLL
PVKLSDAEVDELFALVKANPGIHFDVDLEAQEVKAGEKTYRFTIDAFRRHCMMNGLDSIG
LTLQHDDAIASYEEKQPAFMR
>LFGLNPFC_03178 Putative inhibitor of glucose uptake transporter SgrT
MRQFYQHYFTATAKLCWLRWLSVPQRLTMLEELMQWEGSHSDY
>LFGLNPFC_03179 HTH-type transcriptional regulator SgrR
MPSARLQQQFIRLWQCCEGKSQETTLNELAALLSCSRRHMRTLLNTMQDRGWLTWEAEVG
RGKRSRLTFLYTGLALQQQRAEDLLEQDRIDQLVQLVGDKATVRQMLVSHLGRSFRQGRH
ILRVLYYRPLRNLLPGSALRRSETHIARQIFSSLTRINEENGELEADIAHHWQQISPLHW
RFFLRPGVHFHHGRELEMDDVIASLKRINTLPLYSHIANIVSPTPWTLDIHLTQPDRWLP
LLLGQVPAMILPREWETLSNFASHPIGTGPYAVIRNSTNQLKIQAFDDFFGYRALIDEVN
VWVLPEIADEPAGGLMLKGPQGEEKEIESRLEEGCYYLLFDSRTHRGANQQVRDWVSYVL
SPTNLVYFAEEQYQQLWFPAYGLLPRWHHARTIKSEKPAGLESLTLTFYQDHSEHRVIAG
IMQQILASHQVTLEIKEISYDQWHEGEIESDIWLNSANFTLPLDFSLFAHLCEVPLLQHC
IPIDWQADAARWRNGEMNLANWCQQLVASKAMVPLIHHWLIIQGQRSMRGLRMNTLGWFD
FKSAWFAPPDP
>LFGLNPFC_03180 Thiamine-binding periplasmic protein
MLKKCLPLLLLCTAPVFAKPVLTVYTYDSFAADWGPGPVVKKAFEADCNCELKLVALEDG
VSLLNRLRMEGKNSKADVVLGLDNNLLDAASKTELFAKSGVAAEAVNVPGGWNNDTFVPY
DYGYFAFVYDKNKLKNPPQSLKELVESDQNWRVIYEDPRTSTPGLGLLLWMQKVYGDNAP
QAWQKLAKKTVTVTKGWSEAYGLFLKGESDLVLSYTTSPAYHILEEKKDNYAATNFIEGH
YLQVEVAARTAASKQPELAQKFLQFMVSPAFQNAIPTGNWMYPVANVTLPAGFEQLTKPA
TTLEFTPAEVAAQRQAWISEWQRAVSR
>LFGLNPFC_03181 hypothetical protein
MATRRQPLIPGWLIPGVSAATLVVAVALAAFLALWWNAPQGDWVAVWQDSYLWHVVRFSF
WQAFLSALLSVVPAIFLARALYRRRFPGRQMLLRLCAMTLILPVLVAVFGILSVYGRQGW
LASLWQSLGLEWTFSPYGLQGILLAHVFFNLPMASRLLLQALENIPGEQRQLAAQLGMHG
WHFFRFVEWPWLRRQIPPVAALIFMLCFASFATVLSLGGGPQATTIELAIYQALSYDYDP
ARAAMLALIQMVCCLGLVLLSQRLSKAIAPGTTLLQGWRDPDDRLHSRICDTALIVLALL
LLLPPLLAVIVDGVNRQLPEVLAQPVLWQALWTSLRIALAAGVLCVVLTMMLLWSSRELR
ARQKMLAGQALEMSGMLILAMPGIVLATGFFLLLNNTIGLPQSADGIVIFTNALMAIPYA
LKVLENPMRDITARYSMLCQSLGIEGWSRLKVVELRALKRPLAQALAFACVLSIGDFGVV
ALFGNDDFRTLPFYLYQQIGSYRSQDGAVTALILLLLCFLLFTVIEKIPGRNVKTD

>LFGLNPFC_03182 Thiamine import ATP-binding protein ThiQ
MLKLTDITWLYHHLPMRFSLTVERGEQVAILGPSGAGKSTLLNLIAGFLTPASGLLTIDD
VDHTTTPPSRRPVSMLFQENNLFSHLTVAQNIGLGLNPGLKLNAAQQKKMHAIAHQMGID
NLMARLPGELSGGQRQRVALARCLVREQPILLLDEPFSALDPALRQEMLTLVSSSCQQQK
MTLLMVSHSVEDAARIATRSVVVADGRIAWQGKTDELLSGKASASALLGIKG
>LFGLNPFC_03183 Inner membrane protein YabI
MQALLEHFITQSTVYSLMAVVLVAFLESLALVGLILPGTVLMAGLGALIGSGELSFWYAW
LAGIIGCLMGDWISFWLGWRFKKPLHRWSFLKKNKALLDKTEHALHQHSMFTILVGRFVG
PTRPLVPMVAGMLDLPVAKFITPNIIGCLLWPQFYFLPGILAGAAIDIPAGMQSGEFKWL
LLATAVFLWVGGWLCWRLWRSGKATDRLSHYLSRGRLLWLTPLISAIGVVALVVLIRHPL
MPVYIDILRKVVGV
>LFGLNPFC_03184 hypothetical protein
MSKYIYILLSFLVLFFIFFYAYISLMSKEHHYTQHELSPFFLYTPESLRNLPNISNVAEY
SYYYNVDDMQTRVIVTWRNIDNIFLQKAKLIDFLKRMGPSLQNDCIWFFHDKSDYANNFQ
RYCIIEHRDSLQVEYFETIE
>LFGLNPFC_03185 hypothetical protein
MSKRNDITDGIFATTKKYGLVYTEELGWIDLGHAQGQDARILKRKLEQEHFSTYYDEFHD
WYFPVDYHQEMGIRKKILGVDLTFHTGVYTKVMVRSCLSPTLKARVALTLMYGTAKRFEA
WQNSFIFNWYTDSGFSAEDLVSDLIGFYRVFGTGPDPLLLAKPLSYTKALQIWDTYGAPG
NFKNTEFTPFLFTTHPPFKKIN
>LFGLNPFC_03186 Arabinose operon regulatory protein
MAEAQNDPLLPGYSFNAHLVAGLTPIEANGYLDFFIDRPLGMKGYILNLTIRGQGVVKNQ
GREFVCRPGDILLFPPGEIHHYGRHPEAHEWYHQWVYFRPRAYWHEWLNWPSIFANTGFF
RPDEAHQPHFSDLFGQIINAGQGEGRYSELLAINLLEQLLLRRMEAINESLHPPMDNRVR
EACQYISDHLADSNFDIASVAQHVCLSPSRLSHLFRQQLGISVLSWREDQRISQAKLLLS
TTRMPIATVGRNVGFDDQLYFSRVFKKCTGASPSEFRAGCE
>LFGLNPFC_03187 Ribulokinase
MAIAIGLDFGSDSVRALAVDCATGEEIATSVEWYPRWQKGQFCDAPNNQFRHHPRDYIES
MEAALKTVLAELSAEQRAAVVGIGVDTTGSTPAPIDADGNVLALRPEFAENPNAMFVLWK
DHTAVEEAEEITRLCHAPGNVDYSRYIGGIYSSEWFWAKILHVTRQDSAVAQSAASWIEL
CDWVPALLSGTTRPQDIRRGRCSAGHKSLWHESWGGLPPASFFDELDPILNRHLPSPLFT
DTWTADIPVGTLCPEWAQRLGLPESVVISGGAFDCHMGAVGAGAQPNALVKVIGTSTCDI
LIADKQSVGERAVKGICGQVDGSVVPGFIGLEAGQSAFGDIYAWFGRVLSWPLEQLAAQH
PELKEQINASQKQLLPALTEAWAKNPSLDHLPVVLDWFNGRRTPNANQRLKGVITDLNLA
TDAPLLFGGLIAATAFGARAIMECFTDQGIAVNNVMALGGIARKNQVIMQACCDVLNRPL
QIVASDQCCALGAAIFAAVAAKVHADIPSAQQKMASAVEKTLQPRSEQAQRFEQLYRRYQ
QWAMSAEQHYPPTSAPAQAAQAVPTL
>LFGLNPFC_03188 L-arabinose isomerase
MTIFDNYEVWFVIGSQHLYGPETLRQVTQHAEHVVNALNTEAKLPCKLVLKPLGTTPDEI
TAICRDANYDDRCAGLVVWLHTFSPAKMWINGLTMLNKPLLQFHTQFNAALPWDSIDMDF
MNLNQTAHGGREFGFIGARMRQQHAVVTGHWQDKQAHERIGSWMRQAVSKQDTRHLKVCR
FGDNMREVAVTDGDKVAAQIKFGFSVNTWAVGDLVQVVNSISDGDVNALVDEYESCYTMT
PATQIHGEKRQNVLEAARIELGMKRFLEQGGFHAFTTTFEDLHGLKQLPGLAVQRLMQQG
YGFAGEGDWKTAALLRIMKVMSTGLQGGTSFMEDYTYHFEKGNDLVLGSHMLEVCPSIAV
EEKPILDVQHLGIGGKDDPARLIFNTQTGPAIVASLIDLGDRYRLLVNCIDTVKTPHSLP
KLPVANALWKAQPDLPTASEAWILAGGAHHTVFSHALNLNDMRQFAEMHDIEITVIDNDT
RLPAFKDALRWNEVYYGFRR
>LFGLNPFC_03189 L-ribulose-5-phosphate 4-epimerase AraD
MLEDLKRQVLEANLALPKHNLVTLTWGNVSAVDRGRGVLVIKPSGVDYSTMTADDMVVVS
IETGEVVEGTKKPSSDTPTHRLLYQAFPSIGGIVHTHSRHATIWAQAGQSIPATGTTHAD
YFYGTIPCTRKMTDAEINGEYEWETGNVIVETFEKQGIDAAQMPGVLVHSHGPFAWGKNA
EDAVHNAIVLEEVAYMGIFCRQLAPQLPDMQQTLLDKHYLRKHGAKAYYGQ
>LFGLNPFC_03190 DNA polymerase II
MAQAGFILTRHWRDTPQGTEVSFLLATDNGPLQVTLAPQESVAFIPADHVPRAQHILQGE
QGFRLTPLALKDFHRQPVYGLYCRAHRQLMNYEKRLREGGVTVYEADVRPPERYLMERFI
TSPVWVEGDMRNGAIVNARLKPHPDYRPPLKWVSIDIETTRHGELYCIGLEGCGQRIVYM
LGPENGDASALDFELEYVASRPLLLEKLNAWFATHDPDVIIGWNVVQFDLRMLQKHAERY
RIPLRLGRDNSELEWREHGFKNDVFFAQAKGRLIIDGIEALKSAFWNFSSFSLETVAQEL
LGEGKSIDNPWDRMDEIDRRFAEDKPALATYNLKDCELVTQIFHKTEIMPFLLERATVNG
LPVDRHGGSVAAFGHLYFPRMHRAGYVAPNLGEVPPHASPGGYVMDSRPGLYDSVLVLDY
KSLYPSIIRTFLIDPVGLVEGMAQPDPEHSTEGFLDAWFSREKHCLPEIVTNIWHGRDEA
KRQGNKPLSQALKIIMNAFYGVLGTTACRFFDPRLASSITMRGHQIMRQTKTLIEAQGYD
VIYGDTDSTFVWLKGAHSEEEAAKIGRALVQHVNAWWAETLQKQRLTSALELEYETHFCR
FLMPTIRGADTGSKKRYAGLIQEGDKQRMVFKGLETVRTDWTPLAQQFQQELYLRIFRNE

PYQEYVRETIDKLMAGELDTRLVYRKRLRRPLSEYQRNVPPHVRAARLADEENHKRGRPL
QYQNRGTIKYVWTTNGPEPLDYQRSPLDYEHYLTRQLQPVAEGILPFIEDNFATLMTGQL
GLF
>LFGLNPFC_03191 RNA polymerase-associated protein RapA
MPFTLGQRWISDTESELGLGTVVAVDARTVTLLFPSTGENRLYARSDSPVTRVMFNPGDT
ITSHDGWQMQVEEVKEENGLLTYIGTRLDTEESGVALREVFLDSKLVFSKPQDRLFAGQI
DRMDRFALRYRARKYSSEQFRMPYSGLRGQRTSLIPHQLNIAHDVGRRHAPRVLLADEVG
LGKTIEAGMILHQQLLSGAAERVLIIVPETLQHQWLVEMLRRFNLRFALFDDERYAEAQH
DAYNPFDTEQLVICSLDFARRSKQRLEHLCEAEWDLLVVDEAHHLVWSEDAPSREYQAIE
QLAEHVPGVLLLTATPEQLGMESHFARLRLLDPNRFHDFAQFVEEQKNYRPVADAVAMLL
AGNKLSNDELNMLGEMIGEQDIEPLLQAANSDSEDAQSARQELVSMLMDRHGTSRVLFRN
TRNGVKGFPKRELHTIKLPLPTQYQTAIKVSGIMGARKSAEDRARDMLYPERIYQEFEGD
NATWWNFDPRVEWLMGYLTSHRSQKVLVICAKAATALQLEQVLREREGIRAAVFHEGMSI
IERDRAAAWFAEEDTGAQVLLCSEIGSEGRNFQFASHMVMFDLPFNPDLLEQRIGRLDRI
GQAHDIQIHVPYLEKTAQSVLVRWYHEGLDAFEHTCPTGRTIYDSVYNDLINYLASPDQT
EGFDDLIKNCREQHEALKAQLEQGRDRLLEIHSNGGEKAQALAESIEEQDDDTNLIAFAM
NLFDIIGINQDDRGDNMIVLTPSDHMLVPDFPGLSEDGITITFDREVALAREDAQFITWE
HPLIRNGLDLILSGDTGSSTISLLKNKALPVGTLLVELIYVVEAQAPKQLQLNRFLPPTP
VRMLLDKNGNNLAAQVEFETFNRQLNAVNRHTGSKLVNAVQQDVHAILQLGEAQIEKSAR
ALIDAARNEADEKLSAELSRLEALRAVNPNIRDDELTAIESNRQQVMESLDQAGWRLDAL
RLIVVTHQ
>LFGLNPFC_03192 Dual-specificity RNA pseudouridine synthase RluA
MGMENYNPPQDPWLVVLYQDDHIMVVNKPSGLLSVPGRLEEHKDSVMTRIQRDYPQAESV
HRLDMATSGVIVVALTKAAERELKRQFREREPKKQYVARVWGHPSPAEGLVDLPLICDWP
NRPKQKVCYETGKPAQTEYEVVEYAADNTARVVLKPITGRSHQLRVHMLALGHPILGDRF
YASPEARAMAPRLLLHAEMLTITHPAYGNSMTFKAPADF
>LFGLNPFC_03193 Co-chaperone protein DjlA
MGKSAFILWEIALISVCRFGEYMQYWGKIIGVAVALIMGGGFWGVVLGLLIGHMFDKARS
RKMAWFANQRERQALFFATTFEVMGHLTKSKGRVTEADIHIASQLMDRMNLHGASRTAAQ
NAFRVGKSDNYPLREKMRQFRSVCFGRFDLIRMFLEIQIQAAFADGSLHPNERAVLYVIA
EELGISRAQFDQFLRMMQGGAQFGGGYQQQSGGGNWQQAQRGPTLEDACNVLGVKPTDDA
TTIKRAYRKLMSEHHPDKLVAKGLPPEMMEMAKQKAQEIQQAYELIKQQKGFK
>LFGLNPFC_03194 LPS-assembly protein LptD
MKKRIPTLLATMIATALYSQQGLAADLASQCMLGVPSYDRPLVQGDTNDLPVTINADHAK
GDYPDDAVFTGSVDIMQGNSRLQADEVQLHQKEAPGQPEPVRTVDALGNVHYDDNQVILK
GPKGWANLNTKDTNVWEGDYQMVGRQGRGKADLMKQRGENRYTILDNGSFTSCLPGSDTW
SVVGSEIIHDREEQVAEIWNARFKVGPVPIFYSPYLQLPVGDKRRSGFLIPNAKYTTTNY
FEFYLPYYWNIAPNMDATITPHYMHRRGNIMWENEFRYLSQAGAGLMELDYLPSDKVYKD
EHPNDDSSRRWLFYWNHSGVMDQVWRFNVDYTKVSDPSYFNDFDNKYGSSTDGYATQKFS
VGYAVQNFNATVSTKQFQVFSEQNTSSYSAEPQLDVNYYQNDVGPFDTRIYGQAVHFVNT
RDDMPEATRVHLEPTINLPLSNNWGSINTEAKLLATHYQQTNLDWYNSRNTTKLAESANR
VMPQFKVDGRMVFERDMEMLAPGYTQTLEPRAQYLYVPYRDQSKIYNYDSSLLQSDYSGL
FRDRTYGGLDRIASANQVTTGVTSRIYDDAAVERFNISVGQIYYFTESRTGDDNITWEND
DKTGSLVWAGDTYWRISDRWGLRGGIQYDTRLDNVATSNSSIEYRRDEDRLVQLNYRYAS
PEYIQATLPKYYSTAEQYKNGISQVGAVASWPIADRWSIVGAYYYDTNANKQADSMLGVQ
YSSCCYAIRVGYERKLNGWDNDKQHAVYDNAIGFNIELRGLSSNYGLGTQEMLRSNILPY
QNTL
>LFGLNPFC_03195 Chaperone SurA
MKNWKTLLLGIAMIANTSFAAPQVVDKVAAVVNNGVVLESDVDGLMQSVKLNAAQARQQL
PDDATLRHQIMERLIMDQIILQMGQKMGVKISDEQLDQAIANIAKQNNMTLDQMRSRLAY
DGLNYNTYRNQIRKEMIISEVRNNEVRRRITILPQEVESLAQQVGNQNDASTELNLSHIL
IPLPENPTSDQVNEAESQARAIVDQARNGADFGKLAIAHSADQQALNGGQMGWGRIQELP
GIFAQALSTAKKGDIVGPIRSGVGFHILKVNDLRGESKNISVTEVHARHILLKPSPIMTD
EQARVKLEQIAADIKSGKTTFAAAAKEFSQDPGSANQGGDLGWATPDIFDPAFRDALTRL
NKGQMSAPVHSSFGWHLIELLDTRNVDKTDAAQKDRAYRMLMNRKFSEEAASWMQEQRAS
AYVKILSN
>LFGLNPFC_03196 4-hydroxythreonine-4-phosphate dehydrogenase
MVKTQRVVITPGEPAGIGPDLIVQLAQREWPVELVVCADATLLTDRAAMLGLPLTLRPYS
PNSPAQPQTAGTLTLLPVALRESVTVGQLAVENGHYVVETLARACDGCLNGEFAALITGP
VHKGVINDAGIPFTGHTEFFEERSQAKKVVMMLATEELRVALATTHLPLRDIADAITPAL
LHEVIAILHHDLRTKFGIAEPRILVCGLNPHAGEGGHMGTEEIDTIIPVLNELREQGMKL
NGPLPADTLFQPKYLDNADAVLAMYHDQGLPVLKYQGFGRGVNITLGLPFIRTSVDHGTA
LELAGRGKADVGSFITALNLAIKMIVNTQ
>LFGLNPFC_03197 Ribosomal RNA small subunit methyltransferase A

MNNRVHQGHLARKRFGQNFLNDQFVIDSIVSAINPQKGQAMVEIGPGLAALTEPVGERLD
QLTVIELDRDLAARLQTHPFLGPKLTIYQQDAMTFNFGELAAKMGQPLRVFGNLPYNIST
PLMFHLFSYTDAIADMHFMLQKEVVNRLVAGPNSKAYGRLSVMAQYYCNVIPVLEVPPSA
FTPPPKVDSAVVRLVPHATMPHPVKDVRVLSRITTEAFNQRRKTIRNSLGNLFSVEVLTG
MGIDPAMRAENISVAQYCQMANYLAENAPLQES
>LFGLNPFC_03198 Protein ApaG
MINSPRVCIQVQSVYIEAQSSPDNERYVFAYTVTIRNLGRAPVQLLGRYWLITNGNGRET
EVQGEGVVGVQPLIAPGEEYQYTSGAIIETPLGTMQGHYEMIDENGVPFSIDIPVFRLAV
PTLIH
>LFGLNPFC_03199 Bis(5'-nucleosyl)-tetraphosphatase [symmetrical]
MATYLIGDVHGCYDELIALLHKVEFTPGKDTLWLTGDLVARGPGSLDVLRYVKSLGDSVR
LVLGNHDLHLLAVFAGISRNKPKDRLTPLLEAPDADELLNWLRRQPLLQIDKEKKLVMAH
AGITPQWDLQTAKECARDVEAVLSSDSYPFFLDAMYGDMPNNWSPELRGLGRLRFITNAF
TRMRFCFPNGQLDMYSKESPEEAPAPLKPWFAIPGPVAEEYNIAFGHWASLEGKGTPEGI
YALDTGCCWGGTLTCLRWEDKQYFVQPSNRHKDLSEGEAVAS
>LFGLNPFC_03200 hypothetical protein
MQFTVYRSRGRNAAFPFVIDVTSDIVGEINRRIVIPLTPI
>LFGLNPFC_03201 Antitoxin CcdA
MTAKRTTQSVTVTVDRELVNRARDAGLNMSATLTVALNAELKKHAATRWREENAEAIAAL
NQLADETGCFSDEYRSF
>LFGLNPFC_03202 Dihydrofolate reductase
MDSPAEYKIFLNIILAPVDDGLRFTYSGDNFFYREISMISLIAALAVDRVIGMENAMPWN
LPADLAWFKRNTLNKPVIMGRHTWESIGRPLPGRKNIILSSQPGTDDRVTWVKSVDEAIA
ACGDVPEIMVIGGGRVYEQFLPKAQKLYLTHIDAEVEGDTHFPDYEPDDWESVFSEFHDA
DAQNSHSYCFEILERR
>LFGLNPFC_03203 Glutathione-regulated potassium-efflux system protein KefC
MDSHTLIQALIYLGSAALIVPIAVRLGLGSVLGYLIAGCIIGPWGLRLVTDAESILHFAE
IGVVLMLFIIGLELDPQRLWKLRAAVFGGGALQMVICGGLLGLFCMLLGLRWQVAELIGM
TLALSSTAIAMQAMNERNLMVTQMGRSAFAVLLFQDIAAIPLVAMIPLLAASSASTTMGA
FVLSALKVAGALALVVLLGRYVTRPALRFVARSGLREVFSAVALFLVFGFGLLLEEVGLS
MAMGAFLAGVLLASSEYRHALESDIEPFKGLLLGLFFIGVGMSIDFGTLIENPLRIVILL
LGFLIIKIAMLWLIARPLQVPNKQRRWFAVLLGQGSEFAFVVFGAAQMANVLEPEWAKSL
TLAVALSMAATPILLVILNRLEQSSTEEAREADEIDEEQPRVIIAGFGRFGQITGRLLLS
SGVKMVVLDHDPDHIETLRKFGMKVFYGDATRMDLLESAGAAKAEVLINAIDDPQTNLQL
TEMVKEHFPHLQIIARARDVDHYIRLRQAGVEKPERETFEGALKTGRLALESLGLGPYEA
RERADVFRRFNIQMVEEMAMVENDTKARAAVYKRTSAMLSEIITEDREHLSLIQRHGWQG
TEEGKHTGNMADEPETKPSS
>LFGLNPFC_03204 Glutathione-regulated potassium-efflux system ancillary protein KefF
MILIIYAHPYPHYSHANKRMLEQARTLEGVEIRSLYQLYPDFNIDIAAEQEALSRADLIV
WQHPMQWYSIPPLLKLWIDKVFSHGWAYGHGGTALHGKHLLWAVTTGGGESHFEIGAHPG
FDVLSQPLQATAIYCGLNWLPPFAMHCTFICDDETLEGQARHYKQRLLEWQEAHHG
>LFGLNPFC_03205 Inner membrane metabolite transport protein YgcS
MQPSRNFDDLKFSSIHRRILLWGSGGPFLDGYVLVMIGVALEQLTPALKLDADWIGLLGA
GTLAGLFVGTSLFGYISDKVGRRKMFLIDIIAIGVISVATMFVSSPVELLVMRVLIGIVI
GADYPIATSMITEFSSTRQRAFSISFIAAMWYVGATCADLVGYWLYDVEGGWRWMLGSAA
IPCLLILIGRFELPESPRWLLRKGRVKECEEMMIKLFGEPVAFEEEQPQQTRFRDLFNRR
HFPFVLFVAAIWTCQVIPMFAIYTFGPQIVGLLGLGVGKNAALGNVVISLFFMLGCIPPM
LWLNTAGRRPLLIGSFAMMTLALAVLGLIPDMGIWLVVMAFAVYAFFSGGPGNLQWLYPN
ELFPTDIRASAVGVIMSLSRIGTIVSTWALPIFINNYGISNTMLMGAGISLFGLLISVAF
APETRGMSLAQTSNMTIRGQRMG
>LFGLNPFC_03206 Ferredoxin-like protein FixX
MTSPVNVDVKLGVNKFNVDEEHPHIVVKADADKQALELLVKACPAGLYKKQDDGSVRFDY
AGCLECGTCRILGLGSALEQWEYPRGTFGVEFRYG
>LFGLNPFC_03207 Thiamine thiazole synthase
MSEDIFDAIIVGAGLAGSVAALVLAREGAQVLVIERGNSAGAKNVTGGRLYAHSLEHIIP
GFADSAPVERLITHEKLAFMTEKSAMTMDYCNGDETSPSQRSYSVLRSKFDAWLMEQAEE
AGAQLITGIRVDNLVQRDGKVVGVEADGDVIEAKTVILADGVNSILAEKLGMAKRVKPTD
VAVGVKELIELPKSVIEDRFQLQGNQGAACLFAGSPTDGLMGGGFLYTNENTLSLGLVCG
LHHLHDAKKSVPQMLEDFKQHPAVAPLIAGGKLVEYSAHVVPEAGINMLPELVGDGVLIA
GDAAGMCMNLGFTIRGMDLAIAAGEAAAKTVLSAMKSDDFSKQKLAEYRQHLESGPLRDM
RMYQKLPAFLDNPRMFSGYPELAVGVARDLFTIDGSAPELMRKKILRHGKKVGFINLIKD
GMKGVTVL
>LFGLNPFC_03208 Protein FixB
MNTFSQVWVFSDTPSRLPELMNGAQALANQINTFVLNDADGTQAIQLGANHVWKLSGKPD

ERMIEDYAGVMADTIRQHGADGLVLLPNTRRGKLLAAKLGYRLNAAVSNDASAVSVQDGK
ATVKHMVYGGLAIGEERIATPYAVLTISSGTFDAAQPDASRTGETHTVEWQAPAVAITRT
ATQARQSNSVDLDKARLVVSVGRGIGSKENIALAEQLCKAIGAELACSRPVAENEKWMEH
ERYVGISNLMLKPELYLAVGISGQIQHMVGANASQTIFAINKDKNAPIFQYADYGIVGDA
VKILPALTVALAR
>LFGLNPFC_03209 Protein FixA
MKIITCYKCVPDEQDIAVNNADGSLDFSKADAKISQYDLNAIEAACQLKQQAAEAQVTAL
SVGGKALTNAKGRKDVLSRGPDELIVVIDDQFEQALPQQTASVLAAAAQKAGFDLILCGD
GSSDLYAQQVGLLVGEILNIPAVNGVSKIISLTADTLTVERELEDETETLSIPLPAVVAV
STDINSPQIPSMKAILGAAKKPVQVWSAADIGFNAEAAWSEQQVAAPKQRERQRIVIEGD
GEEQIAAFAENLRKVI
>LFGLNPFC_03210 L-carnitine/gamma-butyrobetaine antiporter
MKNEKRKTGIEPKVFFPPLIIVGILCWLTVRDLDAANVVINAVFSYVTNVWGWAFEWYMV
VMLFGWFWLVFGPYAKKRLGNEPPEFSTASWIFMMFASCTSAAVLFWGSIEIYYYISTPP
FGLEPNSTGAKELGLAYSLFHWGPLPWATYSFLSVAFAYFFFVRKMEVIRPSSTLVPLVG
EKHAKGLFGTIVDNFYLVALIFAMGTSLGLATPLVTECMQWLFGIPHTLQLDAIIITCWI
ILNAICVACGLQKGVRIASDVRSYLSFLMLGWVFIVSGASFIMNYFTDSVGMLLMYLPRM
LFYTDPIAKGGFPQGWTVFYWAWWVIYAIQMSIFLARISRGRTVRELCFGMVLGLTASTW
ILWTVLGSNTLLLIDKNIINIPNLIEQYGVARAIIETWAALPLSTATMWGFFILCFIATV
TLVNACSYTLAMSTCREVRDGEEPPLLVRIGWSILVGIIGIVLLALGGLKPIQTAIIAGG
CPLFFVNIMVTLSFIKDAKQNWKD
>LFGLNPFC_03211 Crotonobetainyl-CoA reductase
MDFNLNDEQELFVAGIRELMASENWEAYFAECDRDSVYPERFVKALADMGIDSLLIPEEH
GGLDAGFVTLAAVWMELGRLGAPTYVLYQLPGGFNTFLREGTQEQIDKIMAFRGTGKQMW
NSAITEPGAGSDVGSLKTTYTRRNGKIYLNGSKCFITSSAYTPYIVVMARDGASPDKPVY
TEWFVDMSKPGIKVTKLEKLGLRMDSCCEITFDDVELDEKDMFGREGNGFNRVKEEFDHE
RFLVALTNYGTAMCAFEDAARYANQRVQFGEAIGRFQLIQEKFAHMAIKLNSMKNMLYEA
AWKADNGTITSGDAAMCKYFCANAAFEVVDSAMQVLGGVGIAGNHRISRFWRDLRVDRVS
GGSDEMQILTLGRAVLKQYR
>LFGLNPFC_03212 L-carnitine CoA-transferase
MDHLPMPKFGPLAGLRVVFSGIEIAGPFAGQMFAEWGAEVIWIENVAWADTIRVQPNYPQ
LSRRNLHALSLNIFKDEGREAFLKLMETTDIFIEASKGPAFARRGITDEVLWQHNPKLVI
AHLSGFGQYGTEEYTNLPAYNTIAQAFSGYLIQNGDVDQPMPAFPYTADYFSGLTATTAA
LAALHKVRETGKGESIDIAMYEVMLRMGQYFMMDYFNGGEMCPRMTKGKDPYYAGCGLYK
CADGYIVMELVGITQIAECFKDIGLAHLLGTPEIPEGTQLIHRIECPYGPLVEEKLDAWL
AAHTIAEVKERFAELNIACAKVLTVPELESNPQYVARESITQWQTMDGRTCKGPNIMPKF
KNNPGQIWRGMPSHGMDTAAILKNIGYSENDIQELVSKGLAKVED
>LFGLNPFC_03213 Crotonobetaine/carnitine--CoA ligase
MDIIGGQHLRQMWDDLADVYGHKTALICESSGGVVNRYSYLELNQEINRTANLFYTLGIR
KGDKVALHLDNCPEFIFCWFGLAKIGAIMVPINARLLREESTWILQNSQACLLVTSAQFY
PMYQQIQQEDASQLRHICLIDMALPADDGVSSFTQLKNQQPATLCYAPPLSTDDTAEILF
TSGTTSRPKGVVITHYNLRFAGYYSAWQCALRDDDVYLTVMPAFHIDCQCTAAMAAFSAG
ATFVLVEKYSARAFWGQVQKYRATITECIPMMIRTLMVQPPSANDRQHRLREVMFYLNLS
EQEKDAFCERFGVRLLTSYGMTETIVGIIGDRPGDKRRWPSIGRAGFCYEAEIRDDHNRP
LPAGELGEICIKGVPGKTIFKEYFLNPKATAKVLEADGWLHTGDTGYRDEEGFFYFVDRR
CNMIKRGGENVSCVELENIIATHPKIQDIVVVGIKDSIRDEAIKAFVVLNEGETLSEEEF
FCFCEQNMAKFKVPSYLEIRKDLPRNCSGKIIRKNLK
>LFGLNPFC_03214 Carnitinyl-CoA dehydratase
MSESLHLTRNGSILEITLDRPKANAIDAKTSFEMGEVFLNFRDDPQLRVAIITGAGEKFF
SAGWDLKAAAEGEAPDADFGPGGFAGLTEIFNLDKPVIAAVNGYAFGGGFELALAADFIV
CADNASFALPEAKLGIVPDSGGVLRLPKILPPAIVNEMVMTGRRMGAEEALRWGVVNRVV
SQAELMDNARELAQQLVNSAPLAIAALKEIYRTTSEMPVEEAYRYIRSGVLKHYPSVLHS
EDAIEGPLAFAEKRDPVWKGR
>LFGLNPFC_03215 Protein YrdA
MSYYAFEGLIPVVHPTAFVHPSAVLIGDVIVGAGVYIGPLASLRGDYGRLIVQAGANIQD
GCIMHGYCDTDTIVGENGHIGHGAILHGCVIGRDALVGMNSVIMDGAVIGEESIVAAMSF
VKAGFRGEKRQLLMGTPARAVRSVSDDELHWKRLNTKEYQDLVGRCHAALHETQPLRQME
ENRPRLQGTTDVTPKR
>LFGLNPFC_03216 hypothetical protein
MCEGCVEKPLYLLIAEWMMAENRWVIAREISIHFDIEHSKAVNTLTYILSEVTEISCEVK
MIPNKLEGRGCQCQRLVKVVDIDEQIYARLRNNSRDKLVGVRKTPRIPAVPLTELNREQK
WQMMLSKSMRR
>LFGLNPFC_03217 IS200/IS605 family transposase IS200C
MGNEKSLAHTRWNCKYHIVFAPKYRRQVFYREKRRAIGCILRKLCEWKSVRILEAECCAD

HIHMLVEIPPKMSVSGFMGYLKGKSSLMPYEQFGDLKFKYRNREFWCRGYYVDTVGKNTA
KIQDYIKHQLEEDKMGEQLSIPYPGSPFTGRK
>LFGLNPFC_03218 Carbamoyl-phosphate synthase large chain
MPKRTDIKSILILGAGPIVIGQACEFDYSGAQACKALREEGYRVILVNSNPATIMTDPEM
ADATYIEPIHWEVVRKIIEKERPDAVLPTMGGQTALNCALELERQGVLEEFGVTMIGATA
DAIDKAEDRRRFDVAMKKIGLETARSGIAHSMEEALAVAAEVGFPCIIRPSFTMGGSGGG
IAYNREEFEEICARGLDLSPTKELLIDESLIGWKEYEMEVVRDKNDNCIIVCSIENFDAM
GIHTGDSITVAPAQTLTDKEYQIMRNASMAVLREIGVETGGSNVQFAVNPKNGRLIVIEM
NPRVSRSSALASKATGFPIAKVAAKLAVGYTLDELMNDITGGRTPASFEPSIDYVVTKIP
RFNFEKFAGANDRLTTQMKSVGEVMAIGRTQQESLQKALRGLEVGATGFDPKVSLDDPEA
LTKIRRELKDAGAERIWYIADAFRAGLSVDGVFNLTNIDRWFLVQIEELVRLEEKVAEVG
ITGLHAEFLRQLKRKGFADARLAKLAGVREAEIRKLRDQYDLHPVYKRVDTCAAEFATDT
AYMYSTYEEECEANPSTDREKIMVLGGGPNRIGQGIEFDYCCVHASLALREDGYETIMVN
CNPETVSTDYDTSDRLYFEPVTLEDVLEIVRIEKPKGVIVQYGGQTPLKLARALEAAGVP
VIGTSPDAIDRAEDRERFQHAVERLKLKQPANATVTTIEMAVEKAKEIGYPLVVRPSYVL
GGRAMEIVYDEADLRRYFQTAVSVSNDAPVLLDHFLDDAVEVDVDAICDGEMVLIGGIME
HIEQAGVHSGDSACSLPAYTLSQEIQDVMRQQVQKLAFELQVRGLMNVQFAVKNNEVYLI
EVNPRAARTVPFVSKATGVPLAKVAARVMAGKSLAEQGVTKEVIPPYYSVKEVVLPFNKF
PGVDPLLGPEMRSTGEVMGVGRTFAEAFAKAQLGSNSTMKKHGRALLSVREGDKERVVDL
AAKLLKQGFELDATHGTAIVLGEAGINPRLVNKVHEGRPHIQDRIKNGEYTYIINTTSGR
RAIEDSRVIRRSALQYKVHYDTTLNGGFATAMALNADATEKVISVQEMHAQIK
>LFGLNPFC_03219 Carbamoyl-phosphate synthase small chain
MIKSALLVLEDGTQFHGRAIGATGSAVGEVVFNTSMTGYQEILTDPSYSRQIVTLTYPHI
GNVGTNDADEESSQVHAQGLVIRDLPLIASNFRNTEDLSSYLKRHNIVAIADIDTRKLTR
LLREKGAQNGCIIAGDNPDAALALEKARAFPGLNGMDLAKEVTTAEPYSWTQGSWTLTGG
LPEAKKEDELPYHVVAYDFGAKRNILRMLVDRGCRLTIVPAQTSAEDVLKMNPDGIFLSN
GPGDPAPCDYAITAIQKFLETDIPVFGICLGHQLLALASGAKTVKMKFGHHGGNHPVKDV
EKNVVMITAQNHGFAVDEATLPANLRVTHKSLFDGTLQGIHRTDKPAFSFQGHPEASPGP
HDAAPLFDHFIALIEQYRKTAK
>LFGLNPFC_03220 4-hydroxy-tetrahydrodipicolinate reductase
MHDANIRVAIAGAGGRMGRQLIQAALALEGVQLGAALEREGSSLLGSDAGELAGAGKTGV
TVQSSLDAIKDDFDVFIDFTRPEGTLNHLAFCRQHGKGMVIGTTGFDEAGKQAIRDAAAD
IAIVFAANFSVGVNVMLKLLEKAAKVMGDYTDIEIIEAHHRHKVDAPSGTALAMGEAIAH
ALDKDLKDCAVYSREGHTGERVPGTIGFATVRAGDIVGEHTAMFADIGERLEITHKASSR
MTFANGAVRSALWLSGKESGLFDMRDVLDLNNL
>LFGLNPFC_03221 hypothetical protein
MTYGEAYLEGWKNIFNYEGVSNRFEFWSFMIGSGVICLLPLLCWWLAVTINNDYGVFIFF
ALPASFILTLIFAIPAIALAVRRMHDIGYSGWWVTIVVLIPVTGVILLILCCLPSKSQDQ
A
>LFGLNPFC_03222 Non-specific ribonucleoside hydrolase RihC
MRLPIFLDTDPGIDDAVAIAAAIFAPELDLQLMTTVAGNVSVEKTTRNALQLLHFWNVDI
PLAQGAAVPLVRAPRDAASVHGESGMAGYDFVEHNRQPLGIPAFLAIRDALMRAPEPVTL
VAIGPLTNIALLLSQCPECKPYIRRLVIMGGSAGRGNCTPNAEFNIAADPEAAACVFRSG
IEIVMCGLDVTNQAILTPDYLATLPELNRTGKMLHALFSHYRSGSMQSGLRMHDLCAIAW
LVRPELFTLKPCFVAVETQGEFTSGTTVVDIDGCLGKPANVQVALDLDVKGFQQWVAEVL
ALAL
>LFGLNPFC_03223 4-hydroxy-3-methylbut-2-enyl diphosphate reductase
MQILLANPRGFCAGVDRAISIVENALAIYGAPIYVRHEVVHNRYVVDSLRERGAIFIEQI
SEVPDGAILIFSAHGVSQAVRNEAKSRDLTVFDATCPLVTKVHMEVARASRRGEESILIG
HAGHPEVEGTMGQYSNPEGGMYLVESPDDVWKLTVKNEEKLSFMTQTTLSVDDTSDVIDA
LRKRFPKIVGPRKDDICYATTNRQEAVRALAEQAEVVLVVGSKNSSNSNRLAELAQRMGK
RAFLIDDATDIQEEWVKEAKCVGVTAGASAPDILVQNVVARLQQLGGGEAIPLEGREENI
VFEVPKELRVDIREVD
>LFGLNPFC_03224 FKBP-type 16 kDa peptidyl-prolyl cis-trans isomerase
MSESVQSNSAVLVHFTLKLDDGTTAESTRNNGKPALFRLGDASLSEGLEQHLLGLKVGDK
TTFSLEPDAAFGVPSPDLIQYFSRREFMDAGEPEIGAIMLFTAMDGSEMPGVIREINGDS
ITVDFNHPLAGQTVHFDIEVLEIDPALEA
>LFGLNPFC_03225 Lipoprotein signal peptidase
MSQSICSTGLRWLWLVVVVLIIDLGSKYLILQNFALGDTVPLFPSLNLHYARNYGAAFSF
LADSGGWQRWFFAGIAIGISVILAVMMYRSKATQKLNNIAYALIIGGALGNLFDRLWHGF
VVDMIDFYVGDWHFATFNLADTAICVGAALIVLEGFLPSKAKKQ
>LFGLNPFC_03226 Isoleucine--tRNA ligase
MSDYKSTLNLPETGFPMRGDLAKREPGMLARWTDDDLYGIIRAAKKGKKTFILHDGPPYA
NGSIHIGHSVNKILKDIIKSKGLSGYDSPYVPGWDCHGLPIELKVEQEYGKPGEKFTAA

EFRAKCREYAATQVDGQRKDFIRLGVLGDWSHPYLTMDFKTEANIIRALGKIIGNGHLHK
GAKPVHWCVDCRSALAEAEVEYYDKTSPSIDVAFLAVDQDALKTKFGVSNVNGPISLVIW
TTTPWTLPANRAISIAPDFDYALVQIDGQAVILAKDLVESVMQRIGVSDYTILGTVKGAE
LELLRFTHPFMDFDVPAILGDHVTLDAGTGAVHTAPGHGPDDYVIGQKYGLETANPVGPD
GTYLPGTYPTLDGVNVFKANDIVIALLQEKGALLHVEKMQHSYPCCWRHKTPIIFRATPQ
WFVSMDQKGLRAQSLKEIKGVQWIPDWGQARIESMVANRPDWCISRQRTWGVPMSLFVHK
DTEELHPRTLELMEEVAKRVEVDGIQAWWDLDAKEILGDEADQYVKVPDTLDVWFDSGST
HSSVVDVRPEFAGHAADMYLEGSDQHRGWFMSSLMISTAMKGKAPYRQVLTHGFTVDGQG
RKMSKSIGNTVSPQDVMNKLGADILRLWVASTDYTGEMAVSDEILKRAADSYRRIRNTAR
FLLANLNGFDPAKDMVKPEEMVVLDRWAVGCAKAAQEDILKAYEAYDFHEVVQRLMRFCS
VEMGSFYLDIIKDRQYTAKADSVARRSCQTALYHIAEALVRWMAPILSFTADEVWGYLPG
EREKYVFTGEWYEGLFGLADSEAMNDAFWDELLKVRGEVNKVIEQARADKKVGGSLEAAV
TLYAEPELAAKLTALGDELRFVLLTSGATVADYNDAPADAQQSEVLKGLKVALSKAEGEK
CPRCWHYTQDVGKVAEHAEICGRCVSNVAGDGEKRKFA
>LFGLNPFC_03227 Bifunctional riboflavin kinase/FMN adenylyltransferase
MKLIRGIHNLSQAPQEGCVLTIGNFDGVHRGHRALLQGLQEEGRKRNLPVMVMLFEPQPL
ELFATDKAPARLTRLREKLRYLAECGVDYVLCVRFDRRFAALTAQNFISDLLVKHLRVKF
LAVGDDFRFGAGREGDFLLLQKAGMEYGFDITSTQTFCEGGVRISSTAVRQALADDNLAL
AESLLGHPFAISGRVVHGDELGRTIGFPTANVPLRRQVSPVKGVYAVEVLGLGEKPLPGV
ANIGTRPTVAGIRQQLEVHLLDVAMDLYGRHIQVVLRKKIRNEQRFASLDELKAQIARDE
LTAREFFGLTKPA
>LFGLNPFC_03228 30S ribosomal protein S20
MANIKSAKKRAIQSEKARKHNASRRSMMRTFIKKVYAAIEAGDKAAAQKAFNEMQPIVDR
QAAKGLIHKNKAARHKANLTAQINKLA
>LFGLNPFC_03229 Transcriptional activator protein NhaR
MSHINYNHLYYFWHVYKEGSVVGAAEALYLTPQTITGQIRALEERLQGKLFKRKGRGLEP
SELGELVYRYADKMFTLSQEMLDIVNYRKESNLLFDVGVADALSKRLVSSVLDAAVVEDE
QIHLRCFESTHEMLLEQLSQHKLDMIISDCPIDSTQQEGLFSMKIGECGVSFWCTNPLPE
KPFPACLEERRLLIPGRRSMLGRKLLNWFNSQGLNVEILGEFDDAALMKAFGATHNAIFV
APSLYANDFYNDDSVVEIGRVENVMEEYHAIFAERMIQHPAVQRICNTDYSALFTPASK
>LFGLNPFC_03230 Na(+)/H(+) antiporter NhaA
MKHLHRFFSSDASGGIILIIAAVLAMIMANSGATSGWYHDFLETPVQLRVGTLEINKNML
LWINDALMAVFFLLVGLEVKRELMQGSLASLRQAAFPVIAAIGGMIVPALLYLAFNYADP
ITREGWAIPAATDIAFALGVLALLGSRVPLALKIFLMALAIIDDLGAIIIIALFYTNDLS
MASLGVAAVAIAVLVVLNLCGVRRTGVYILVGVVLWTAVLKSGVHATLAGVIVGFFIPLK
EKHGRSPAKRLEHVLHPWVAYLILPLFAFANAGVSLQGVTLEGLTSILPLGIIAGLLIGK
PLGISLFCWLALRLKLAHLPEGTTYQQIMAVGILCGIGFTMSIFIASLAFGSVDPELINW
AKLGILVGSISSAVIGYSWLRVRLRPSV
>LFGLNPFC_03231 hypothetical protein
MFTNVNVDCCKTPGCKNLGLLNSQDYVAQGKNILCRECGYLFPVISEQSLNIYRNIVNHS
WRGLICQCSTCGGTSLKKYGYSAQGQRRMYCHHCEKTFITLEHVITTPRGALLALMIEQG
EALADIRKSLLLNSTGLSRELLKLAREANYKESRQCFPASDITLSTRAFRVKYNGSNNSL
YALVTAEEQSGRVVAISTNYSPSAVEQHYQYTSNYEERMSPGTLAHHVQRKELLTMRRDT
LFDIDYGPAVLHQNDPGMLVKPVLPAYRHFELVRILTDEHSNNVQHYLDHECFILGGCLM
ANLQHIHQGRCHISFVKERGVAPATIDFPPRLFLSGGVRNNVWRAFSNRNYSMAVCNLTG
SKKVREMRHATLNSATRFIHFVENHPFLISLNRMSPANVVSTLDILKHLWNKKLEHGTI
>LFGLNPFC_03232 N-acetylgalactosamine-6-O-sulfatase
MQKTLMASLIGLAVCTGNAFNPVVAAETKQPNLVIIMADDLGYGDLATYGHQIVKTPNID
RLAQEGVKFTDYYAPAPLSSPSRAGLLTGRMPFRTGIRSWIPTGKDVALGRNELTIANLL
KAQGYDTAMMGKLHLNAGGDRTDQPQAKDMGFDYSLVNTAGFVTDATLDNAKERPRYGMV
YPTGWLRNGQPTPRADKMSGEYVSSEVVNWLDNKKDSKPFFLYVAFTEVHSPLASPKKYL
DMYSQYMSAYQKQHPDLFYGDWADKPWRGVGEYYANISYLDAQVGKVLDKIKAMGEEDNT
IVIFTSDNGPVTREARKVYELNLAGETDGLRGRKDNLWEGGIRVPAIIKYGKHLPQGMVS
DTPVYGLDWMPTLAKMMNFKLPTDRTFDGESLVPVLEQKALKREKPLIFGIDMPFQDDPT
DEWAIRDGDWKMIIDRNNKPKYLYNLKSDRYETLNLIGKKPDIEKQMYGKFLKYKTDIDN
DSLMKARGDKPEAVTWG
>LFGLNPFC_03233 hypothetical protein
MSAKRRLLIACALTTIFYHLPAYSSLEYKGSFGSINAGYADWNSGFINTHRGEVWKVTAD
FGVNFKEAEFYSFYESNVLNHAVAGRNHTVSAMTHVRLFDSNMTFFGKIYGQWDNSWGDD
LDMFYGLGYLGWNGSWGFFKPYIGLHNQSGDYVSAKYGQTNGWNGYVVGWTAVLPFTLFD
EKFVLSNWNEIELDRNDAYAEQQFGRNGLNGGLTVAWKFYPRWKASVTWRYFDNKLGYDG
FGDQMIYMLGYDF
>LFGLNPFC_03234 Toxic protein HokC
MKQHKAMIVALIVICITAVVAALVTRKDLCEVHIRTGQTEVAVFTAYESE

>LFGLNPFC_03235 Chaperone protein DnaJ
MAKQDYYEILGVSKTAEEREIKKAYKRLAMKYHPDRNQGDKEAEAKFKEIKEAYEVLTDS
QKRAAYDQYGHAAFEQGGMGGGGFGGGADFSDIFGDVFGDIFGGGRGRQRAARGADLRYN
MELTLEEAVRGVTKEIRIPTLEECDVCHGSGAKPGTQPQTCPTCHGSGQVQMRQGFFAVQ
QTCPHCQGRGTLIKDPCNKCHGHGRVERSKTLSVKIPAGVDTGDRIRLAGEGEAGEHGAP
AGDLYVQVQVKQHPIFEREGNNLYCEVPINFAMAALGGEIEVPTLDGRVKLKVPGETQTG
KLFRMRGKGVKSVRGGAQGDLLCRVVVETPVGLNEKQKQLLQELQESFGGPTGEHNSPRS
KSFFDGVKKFFDDLTR
>LFGLNPFC_03236 Chaperone protein DnaK
MGKIIGIDLGTTNSCVAIMDGTTPRVLENAEGDRTTPSIIAYTQDGETLVGQPAKRQAVT
NPQNTLFAIKRLIGRRFQDEEVQRDVSIMPFKIIAADNGDAWVEVKGQKMAPPQISAEVL
KKMKKTAEDYLGEPVTEAVITVPAYFNDAQRQATKDAGRIAGLEVKRIINEPTAAALAYG
LDKGTGNRTIAVYDLGGGTFDISIIEIDEVDGEKTFEVLATNGDTHLGGEDFDSRLINYL
VEEFKKDQGIDLRNDPLAMQRLKEAAEKAKIELSSAQQTDVNLPYITADATGPKHMNIKV
TRAKLESLVEDLVNRSIEPLKVALQDAGLSVSDIDDVILVGGQTRMPMVQKKVAEFFGKE
PRKDVNPDEAVAIGAAVQGGVLTGDVKDVLLLDVTPLSLGIETMGGVMTTLIAKNTTIPT
KHSQVFSTAEDNQSAVTIHVLQGERKRAADNKSLGQFNLDGINPAPRGMPQIEVTFDIDA
DGILHVSAKDKNSGKEQKITIKASSGLNEDEIQKMVRDAEANAEADRKFEELVQTRNQGD
HLLHSTRKQVEEAGDKLPADDKTAIESALTALETALKGEDKAAIEAKMQELAQVSQKLME
IAQQQHAQQQTAGADASANNAKDDDVVDAEFEEVKDKK
>LFGLNPFC_03237 hypothetical protein
MKSVITISASLAISLMLCCTAQANDHKILGVIAMPRNETNDLALKLPVCRIVKRIQLSAD
HGDLQLSGASIYFKATRSASQTLNIPSEIKEEQTTDWININSDNDNKRCVSKITFSGHTV
NSSDMATLKIIGDD
>LFGLNPFC_03238 hypothetical protein
MNVNYLNDSDLDFLQHCSEEQLANFARLLTHNEKGKTRLSSVLMRNELFKSMEGHPERHR
RNWQLIAGELQHFGGDSIANKLRGHGKLYRAILLDVSKRLKLKADKEMSTFEIEQQLLEQ
FLRNTWKKMDEEHKQEFLHAVDARVNELEELLPLLMKDKLLAKGVSHLLSSQLTRILRTH
AAMSVLGHGLLRGAGLGGPVGAALNGVKAVSGSAYRVTIPAVLQIACLRRMVSATQV
>LFGLNPFC_03239 Succinate-acetate/proton symporter SatP
MGNTKLANPAPLGLMGFGMTTILLNLHNVGYFALDGIILAMGIFYGGIAQIFAGLLEYKK
GNTFGLTAFTSYGSFWLTLVAILLMPKLGLTDAPNAQFLGVYLGLWGIFTLFMFFGTLKG
ARVLQFVFFSLTVLFALLAIGNIAGNAAIIHFAGWIGLICGASAIYLAMGEVLNEQFGRT
VLPIGESH
>LFGLNPFC_03240 Molybdopterin adenylyltransferase
MNTLRIGLVSISDRASSGVYQDKGIPALEEWLTSALTTPFELETRLIPDEQAIIEQTLCE
LVDEMSCHLVLTTGGTGPARRDVTPDATLAVADREMPGFGEQMRQISLHFVPTAILSRQV
GVIRKQALILNLPGQPKSIKETLEGVKDAEGNVVVHGIFASVPYCIQLLEGPYVETAPEV
VAAFRPKSARREVSE
>LFGLNPFC_03241 Transaldolase B
MTDKLTSLRQYTTVVADTGDIAAMKLYQPQDATTNPSLILNAAQIPEYRKLIDDAVAWAK
QQSNDRAQQIVDATDKLAVNIGLEILKLVPGRISTEVDARLSYDTEASIAKAKRLIKLYN
DAGISNDRILIKLASTWQGIRAAEQLEKEGINCNLTLLFSFAQARACAEAGVFLISPFVG
RILDWYKANTDKKEYAPAEDPGVVSVSEIYQYYKEHGYETVVMGASFRNIGEILELAGCD
RLTIAPALLKELAESEGAIERKLSYTGEVKARPARITESEFLWQHNQDPMAVDKLAEGIR
KFAVDQEKLEKMIGDLL
>LFGLNPFC_03242 Amino-acid carrier protein AlsT
MPDFFSFINSVLWGSVMIYLLFGAGCWFTFRTGFVQFRYIRQFGKSLKNSIHPQPSGLTS
FQSLCTSLAARVGSGNLAGVALAITAGGPGAVFWMWVAAFIGMATSFAECSLAQLYKERD
ANGQFRGGPAWYMARGLGMRWMGVLFAVFLLIAYAIIFSGVQANAVARALSFSFDFPPLV
TGIILAVFALLAITRGLHGVARLMQGFVPLMAIIWVLTCLVICVMNIGQLPHVIWSIFES
AFGWQEAAGGAAGYTLSQAITNGFQRSMFSNEAGMGSTPNAAAAAASWPPHPAAQGIVQM
IGIFIDTLVICTASAMLILLAGNGTTYMPLEGIQLIQKAMRVLMGSWGAEFVTLVVILFA
FSSIVTNYIYAENNLFFLRLNNPKAIWCLRICTFATVIGGTLLSLPLMWQLADIIMACMA
ITNLTAILLLSPVVHTIASDYLRQRKLGVRPVFDPLRYPEIGRQLSPDAWDDVSQE
>LFGLNPFC_03243 Peroxide stress resistance protein YaaA
MLILISPAKTLDYQSPLTTTRYTLPELLDNAQQLIHEARKLTPPQISSLMRISDKLAGIN
AARFHDWQPNFTPENARQAILAFKGDVYTGLQAETFSEDDFDFAQQHLRMLSGLYGVLRP
LDLMQPYRLEMGIRLENARGKDLYQFWGDIITNKLNEALAAQGDNVVINLASDEYFKSVK
PKKLNAEIIKPVFLDEKNGKFKIISFYAKKARGLMSRFIIENRLTKPEQLTGFNSEGYFF
DEASSSNGELVFKRYEQR
>LFGLNPFC_03244 hypothetical protein
MKKMQSIVLALSLVLVAPMAAQAAEITLVPSVKLQIGDRDNRGYYWDGGHWRDHGWWKQH
YEWRGNRWHPYGPPPSPRHNKHNDHRGDHRPEPDKHHR

>LFGLNPFC_03245 Threonine synthase
MKLYNLKDHNEQVSFAQAVTQGLGKNQGLFFPHDLPEFSLTEIDEMLKLDFVTRSAKILS
AFIGDEIPQEILEERVRAAFAFPAPVANVESDVGCLELFHGPTLAFKDFGGRFMAQILTH
IAGDKPVTILTATSGDTGAAVAHAFYGLPNVKVVILYPRGKISPLQEKLFCTLGGNIETV
AIDGDFDACQALVKQAFDDEELKVALGLNSANSINISRLLAQICYYFEAVAQLPQEARNQ
LVVSVPSGNFGDLTAGLLAKSLGLPVKRFIAATNVNDTVPRFLHDGQWSPKATQATLSNA
MDVSQPNNWPRVEELFRRKIWQLKELGYAAVDDETTQQTMRELKELGYTSEPHAAVAYRA
LRDQLNPGEYGLFLGTAHPAKFKESVEAILGETLDLPKELAERADLPLLSHNLPADFAAL
RKLMMNHQ
>LFGLNPFC_03246 Homoserine kinase
MVKVYAPASSANMSVGFDVLGAAVTPVDGALLGDVVTVEAAETFSLNNLGRFADKLPSEP
RENIVYQCWERFCQELGKQIPVAMTLEKNMPIGSGLGSSACSVVAALMAMNEHCGKPLND
TRLLALMGELEGRISGSIHYDNVAPCFLGGMQLMIEENDIISQQVPGFDEWLWVLAYPGI
KVSTAEARAILPAQYRRQDCIAHGRHLAGFIHACYSRQPELAAKLMKDVIAEPYRERLLP
GFRQARQAVAEIGAVASGISGSGPTLFALCDKPDTAQRVADWLGKNYLQNQEGFVHICRL
DTAGARVLEN
>LFGLNPFC_03247 Bifunctional aspartokinase/homoserine dehydrogenase 1
MRVLKFGGTSVANAERFLRVADILESNARQGQVATVLSAPAKITNHLVAMIEKTISGQDA
LPNISDAERIFAELLTGLAAAQPGFPLAQLKTFVDQEFAQIKHVLHGISLLGQCPDSINA
ALICRGEKMSIAIMAGVLEARGHNVTVIDPVEKLLAVGHYLESTVDIAESTRRIAASRIP
ADHMVLMAGFTAGNEKGELVVLGRNGSDYSAAVLAACLRADCCEIWTDVDGVYTCDPRQV
PDARLLKSMSYQEAMELSYFGAKVLHPRTITPIAQFQIPCLIKNTGNPQAPGTLIGASRD
EDELPVKGISNLNNMAMFSVSGPGMKGMVGMAARVFAAMSRARISVVLITQSSSEYSISF
CVPQSDCVRAERAMQEEFYLELKEGLLEPLAVTERLAIISVVGDGMRTLRGISAKFFAAL
ARANINIVAIAQGSSERSISVVVNNDDATTGVRVTHQMLFNTDQVIEVFVIGVGGVGGAL
LEQLKRQQSWLKNKHIDLRVCGVANSKALLTNVHGLNLENWQEELAQAKEPFNLGRLIRL
VKEYHLLNPVIVDCTSSQAVADQYADFLREGFHVVTPNKKANTSSMDYYHQLRYAAEKSR
RKFLYDTNVGAGLPVIENLQNLLNAGDELMKFSGILSGSLSYIFGKLDEGMSFSEATTLA
REMGYTEPDPRDDLSGMDVARKLLILARETGRELELADIEIEPVLPAEFNAEGDVAAFMA
NLSQLDDLFAARVAKARDEGKVLRYVGNIDEDGVCRVKIAEVDGNDPLFKVKNGENALAF
YSHYYQPLPLVLRGYGAGNDVTAAGVFADLLRTLSWKLGV
>LFGLNPFC_03248 tRNA (guanosine(18)-2'-O)-methyltransferase
MRITIILVAPARAENIGAAARAMKTMGFSELRIVDSQAHLEPVARWVAHGSGDIIDNIKV
FKTLAESLHDVDFTVATTARSRAKYHYYATPVELVPLLEEKSSWMSHAALVFGREDSGLT
NEELALADVLTGVPMVADYPSLNLGQAVMVYCYQLATLIQQPAKSDTTADQHQLQALRER
AMTLLTTLAVADDIKLVDWLQQRLGLLEQRDTAMLHRLLHDIEKNITK
>LFGLNPFC_03249 Aerobic respiration control protein ArcA
MQTPHILIVEDELVTRNTLKSIFEAEGYDVFEATDGAEMHQILSEYDINLVIMDINLPGK
NGLLLARELREQANVALMFLTGRDNEVDKILGLEIGADDYITKPFNPRELTIRARNLLSR
TMNLGTVSEERRSVESYKFNGWELDINSRSLIGPDGEQYKLPRSEFRAMLHFCENPGKIQ
SRAELLKKMTGRELKPHDRTVDVTIRRIRKHFESTPDTPEIIATIHGEGYRFCGDLED
>LFGLNPFC_03250 Inner membrane protein CreD
MLKSPLFWKMTTLFGAVLLLLIPIMLIRQVIVERADYRSDVEDVIRQSTSGPQKLVGPLI
AIPVTELYTVQEDDKTVERKRSFIHFWLPESLMVDGNQNVEERKIGIYTGQVWHSDLTLK
ADFDVSRLSELDAPNITLGKPFIVISVGDARGIGVVKAPEVNGTALTIEPGTGLEQGGQG
VHIPLPEGDWRKQNLKLNVALNLSGTGDLSVVPGGRYSEMTLTSNWPHPSFLGDFLPAKR
EVSESGFQAQWQSSWFANNLGERFASGNDTGWENFPAFSVAVTTPADQYQLTDRATKYAI
LLIALTFMAFFVFETLTAQRLHPMQYLLVGLSLVMFYLLLLALSEHTGFTVAWIIASLIG
ALMNGIYLQAVLKGWRNSMLFTLALLLLDGVMWGLLNSADSALLLGTSVLVVALAGMMFV
TRNIDWYAFSLPKMKASKEVTMDDELRIWK
>LFGLNPFC_03251 Sensor protein CreC
MRIGMRLLLGYFLLVAVAAWFVLAIFVKEVKPGVRRATEGTLIDTATLLAELARPDLLSG
DPTHGQLAQAFNQLQHRPFRANIGGINKVRNEYHVYMTDAQGKVLFDSANKAVGQDYSRW
NDVWLTLRGQYGARSTLQNPADPESSVMYVAAPIMDGSRLIGVLSVGKPNAAMAPVIKRS
ERRILWASAILLGIALVIGAGMVWWINRSIARLTRYADSVTDNKPVPLPDLGSSELRKLA
QALESMRVKLEGKNYIEQYVYALTHELKSPLAAIRGAAEILREGPPPEVVARFTDNILTQ
NARMQALVETLLRQARLENRQEVVLTAVDVAALFRRVSEARTVQLAEKNITLHVMPTEVN
VASEPALLEQALGNLLDNAIDFTPESGCITLSAEVDQEYVTLKVLDTGSGIPDYALSRIF
ERFYSLPRANGQKSSGLGLAFVSEVARLFNGEVTLRNVQEGGVLASLRLHRHFT
>LFGLNPFC_03252 Transcriptional regulatory protein CreB
MQRETVWLVEDEQGIADTLVYMLQQEGFDVEVFERGLPVLDKARQQVPDVMILDVGLPDI
SGFELCRQLLALHPALPVLFLTARSEEVDRLLGLEIGADDYVAKPFSPREVCARVRTLLR
RVKKFSTPSPVIRIGHFELNEPAAQISWFDTPLTLTRYEFLLLKTLLKSPGRVWSRQQLM
DSVWEDAQDTYDRTVDTHIKTLRAKLRAINPDLSPINTHRGMGYSLRGL

>LFGLNPFC_03253 hypothetical protein
MKYKHLILSLSLIMLGPLAHAEEIGSVDTVFKMIGPDHKIVVEAFDDPDVKNVTCYVSRA
KTGGIKGGLGLAEDTSDAAISCQQVGPIELSDRIKNGKAQGEVVFKKRTSLVFKSLQVVR
FYDAKRNALAYLAYSDKVVEGSPKNAISAVPVMPWRQ
>LFGLNPFC_03254 Right origin-binding protein
MDQAGIIRDLLIWLEGHLDHPLSLDNVAAKAGYSKWHLQRMFKDVTGHAIGAYIRARRLS
KSAVALRLTARPILDIALQYRFDSQQTFTRAFKKQFAQTPALYRRSPEWSAFGIRPPLRL
GEFTMPEHKFVTLEDTPLIGVTQSYSCSLEQISDFRHEMRYQFWHDFLGNSPTIPPVLYG
LNETRPSQDKDDEQEVFYTTALAQDQADGYVLTGHPVMLQGGEYVMFTYEGLGTGVQEFI
LTVYGTCMPMLNLTRRKGQDIERYYPAEDAKAGDRPINLRCELLIPIRR
>LFGLNPFC_03255 Putative phosphoserine phosphatase 2
MLQVYLVRHGETQWNAERRIQGQSDSPLTAKGEQQAMQVATRAKELGITHIISSDLGRTR
RTAEIIAQACGCDIIFDSRLRELNMGVLETRNIDSLTEEEENWRRQLVNGTVDGRIPEGE
SMQELSDRVNAALESCRDLPQGSRPLLVSHGIALGCLVSTILGLPAWAERRLRLRNCSIS
RVDYQESLWLASGWVVETAGDISHLDAPALDELQR
>LFGLNPFC_03256 Inosine/xanthosine triphosphatase
MHQVVCATTNPAKIQAILQAFHEIFGEGSCHIASVAVESGVPEQPFGSEETRAGARNRVA
NARRLLPEADFWVAIEAGIDGDSTFSWVVIENTSQRGEARSATLPLPAVILEKVREGEAL
GPVMSRYTGIDEIGRKEGAIGVFTAGKLTRTSVYHQAVILALSPFHNAVYQPLQA
>LFGLNPFC_03257 Trp operon repressor
MAEQRHQEWLRFVDLLKNAYQNDLHLPLLNLMLTPDEREALGTRVRIVEELLRGEMSQRE
LKNELGAGIATITRGSNSLKAAPVELRQWLEDVLLKSD
>LFGLNPFC_03258 Soluble lytic murein transglycosylase
MEKAKHVTWRLLAVGVCLLTVSSVARADSLDEQRSRYAQIKQAWDNRQMDVVEQMMPGLK
DYPLYPYLEYRQITDDLMNQPAVTVTNFVRANPTLPPARTLQSRFVNELARREDWRGLLA
FSPEKPGTTEAQCNYYYAKWNTGQSEEAWQGAKELWLTGKSQPNACDKLFSVWRASGKQD
PLAYLERIRLAMKAGNTGLVTVLAGQMPADYQTIASAIISLANNPNTVLTFARTTGATDF
TRQMAAVAFASVARQDAENARLMIPSLAQAQQLNEDQIQELRDIVAWRLMGNDVTDEQAK
WRDDAIMRSQSTSLIERRVRMALGTGDRRGLNTWLARLPMEAKEKDEWRYWQADLLLERG
REAEAKEILHQLMQQRGFYPMVAAQRIGEEYELKIDKAPQNVDSALTQGPEMARVRELMY
WNLDNTARSEWANLVKSKSKTEQAQLARYAFNNQWWDLSVQATIAGKLWDHLEERFPLAY
NDLFKRYTSGKEIPQSYAMAIARQESAWNPKVKSPVGASGLMQIMPGTATHTVKMFSIPG
YSSPGQLLDPETNINIGTSYLQYVYQQFGNNRIFSSAAYNAGPGRVRTWLGNSAGRIDAV
AFVESIPFSETRGYVKNVLAYDAYYRYFMGDKPTLMSATEWGRRY
>LFGLNPFC_03259 Energy-dependent translational throttle protein EttA
MAQFVYTMHRVGKVVPPKRHILKNISLSFFPGAKIGVLGLNGAGKSTLLRIMAGIDKDIE
GEARPQPDIKIGYLPQEPQLNPEHTVRESIEEAVSEVVNALKRLDEVYALYADPDADFDK
LAAEQGRLEEIIQAHDGHNLNVQLERAADALRLPDWDAKIANLSGGERRRVALCRLLLEK
PDMLLLDEPTNHLDAESVAWLERFLHDFEGTVVAITHDRYFLDNVAGWILELDRGEGIPW
EGNYSSWLEQKDQRLAQEASQEAARRKSIEKELEWVRQGTKGRQSKGKARLARFEELNST
EYQKRNETNELFIPPGPRLGDKVLEVSNLRKSYGDRLLIDSLSFSIPKGAIVGIIGPNGA
GKSTLFRMISGQEQPDSGTITLGETVKLASVDQFRDSMDNSKTVWEEVSGGLDIMKIGNT
EMPSRAYVGRFNFKGVDQGKRVGELSGGERGRLHLAKLLQVGGNMLLLDEPTNDLDIETL
RALENALLEFPGCAMVISHDRWFLDRIATHILDYQDEGKVEFFEGNFTEYEEYKKRTLGA
DALEPKRIKYKRIAK
>LFGLNPFC_03260 Antitoxin HigA-2
MSFFDELKTSLEEAVEIKQGLKEPARVTRYEIADVKAIREQLNVSQSEMAKALGTSVDTI
KSWESKRRNPTGLAAKVLATIKENPAFFRELASH
>LFGLNPFC_03261 Toxin HigB-2
MGKAIIFIETPMFTRQIKQIATDDELKELQKELIGTPDKGDLIQQTGGLRKVRMAAGSQG
KSGSVRIIYFLATEEIIYLIMAYPKNAKDSLTDTEKAQLKKLTKLLKGEI
>LFGLNPFC_03262 Trifunctional NAD biosynthesis/regulator protein NadR
MSSFDYLKTAIKQQGCTLQQVADASGMTKGYLSQLLNAKIKSPSAQKLEALHRYLGLEFP
RQKKTIGVVFGKFYPLHTGHIYLIQRACSQVDELHIIMGFDDTRDRALFEDSAMSQQPTV
PDRLRWLLQTFKYQKNIRIHAFNEEGMEPYPHGWDVWSNGIKKFMAEKGIQPDLIYTSEE
ADAPQYMEHLGIETVLVDPKRTFMSISGAQIRENPFRYWEYIPTEVKPFFVRTVAILGGE
SSGKSTLVNKLANIFNTTSAWEYGRDYVFSHLGGDEIALQYSDYDKIALGHAQYIDFAVK
YANKVAFIDTDFVTTQAFCKKYEGREHPFVQALIDEYRFDLVILLENNTPWVADGLRSLG
SSVDRKEFQNLLVEMLEENNIEFVRVEEDDYDSRFLRCVELVREMMGEQR
>LFGLNPFC_03263 DNA repair protein RadA
MAKAPKRAFVCNECGADYPRWQGQCSACHAWNTITEVRLAASPTVARNERLSGYAGSAGV
AKVQKLSDISLEELPRFSTGFKEFDRVLGGGVVPGSAILIGGNPGAGKSTLLLQTLCKLA
QQMKTLYVTGEESLQQVAMRAHRLGLPTDNLNMLSETSIEQICLIAEEEQPKLMVIDSIQ
VMHMADVQSSPGSVAQVRETAAYLTRFAKTRGVAIVMVGHVTKDGSLAGPKVLEHCIDCS

VLLDGDADSRFRTLRSHKNRFGAVNELGVFAMTEQGLREVSNPSAIFLSRGDEVTSGSSV
MVVWEGTRPLLVEIQALVDHSMMANPRRVAVGLEQNRLAILLAVLHRHGGLQMADQDVFV
NVVGGVKVTETSADLALLLAMVSSLRDRPLPQDLVVFGEVGLAGEIRPVPSGQERISEAA
KHGFRRAIVPAANVPKKAPEGMQIFGVKKLSDALSVFDDL
>LFGLNPFC_03264 Phosphoserine phosphatase
MPNITWCDLPEDVSLWPGLPLSLSGDEVMPLDYHAGRSGWLLYGRGLDKQRLTQYQSKLG
AAMVIVAAWCVEDYQVIRLAGSLTARATRLAHEAQLDVAPLGKIPHLRTPGLLVMDMDST
AIQIECIDEIAKLAGTGERVAEVTERAMRGELDFTASLRSRVATLKGADANILQQVRENL
PLMPGLTQLVLKLETLGWKVAIASGGFTFFAEYLRDKLRLTAVVANELEIMDGKFTGNVI
GDIVDAQYKAKTLTRLAQEYEIPLAQTVAIGDGANDLPMIKAAGLGIAYHAKPKVNEKTE
VTIRHADLMGVFCILSGSLNQK
>LFGLNPFC_03265 putative inner membrane protein Smp
MARTKLKFRLHRAVIVLFCLALLVALMQQGASWFSQNHQRQRNPQLEELARTLARQVTLNV
APRMRTDSPDEKRIQAILDQLTDESRILDAGVYDEQGDLIARSGESVDVRDRLALDGKKA
GGYFNQQIVEPIAGKNGPLGYLRLTLDTHTLATEAQQVDNTTNILRLMLLLSLAIGVVLT
RTLLQGKRTRWQQSPFLLTASKPVPEEEESEKKE
>LFGLNPFC_03266 Lipoate-protein ligase A
MSTLRLLISDSYDPWFNLAVEECIFRQMPATQRVLFLWRNADTVVIGRAQNPWKECNTRR
MEEDNVRLARRSSGGGAVFHDLGNTCFTFMAGKPEYDKTISTSIVLNALNALGVSAEASG
RNDLVVKTAEGDRKVSGSAYRETKDRGFHHGTLLLNSDLSRLANYLNPDKKKLAAKGITS
VRSRVTNLTELLPGITHEQVCEAITEAFFAHYGERVEAEIISPDKTPDLPNFAETFARQS
SWEWNFGQAPAFSHLLDERFTWGGVELHFDVEKGHITRAQVFTDSLNPAPLEALAGRLQG
CLYRADMLQQECEALLVDFPEQEKELRELSAWIAGAVR
>LFGLNPFC_03267 Toxin YjjJ
MSELTDLLLQGPRSAPELRQRLAISQATFSRLVAREDRVIRFGKARATRYALLRPYRGIE
RIPVWRVDDAGKAHKFADIRLCWPQGSCLVTGADGDERWFDGLPWYLTDLRPQGFLGRAW
GRKLAAQLNLTEDIRLWQEEDVLYALTVFSGEYTGGWLVGEGNYQRWITAQRPAAIPLDQ
KLTHYEQLASDALAGEIVGSSAGGEQPKFTCYAQTPSGNKHVLVKFTVPQQTAVSQRWGD
LLIAESIAAQILRDGGIHAIESTVLVTSNRQVFLEAERFDCKGNDGRLPIVSLEAVQSEF
ISSPGSWPEAMRRLCEQQLVTHQSVAQTEVIWAFGRLIANSDMHAGNLSFYLSEPPFALT
PVYDMLPMAYAPNSAGMLRDAAIEVRFDLNVSKSAWLTAIPLAQQFWQTVAGDPRISEAF
RHIAQEMPEKIRQIEGKVARMGG
>LFGLNPFC_03268 Purine nucleoside phosphorylase DeoD-type
MATPHINAEMGDFADVVLMPGDPLRAKYIAETFLEDAREVNNVRGMLGFTGTYKGRKISV
MGHGMGIPSCSIYTKELITDFGVKKIIRVGSCGAVLPHVKLRDVVIGMGACTDSKVNRIR
FKDHDFAAIADFDMVRNAVDAAKALGVDARVGNLFSADLFYSPDGEMFDVMEKYGILGVE
MEAAGIYGVAAEFGAKALTICTVSDHIRTHEQTTAAERQTTFNDMIKIALESVLLGDKE
>LFGLNPFC_03269 Phosphopentomutase
MKRAFIMVLDSFGIGATEDAERFGDVGADTLGHIAEACAKGEADHGRKGPLNLPNLTRLG
LAKAHEGSTGFIPAGMDGNAEVIGAYAWAHEMSSGKDTPSGHWEIAGVPVLFEWGYFSDH
ENSFPQELLDKLVERANLPGYLGNCHSSGTVILDQLGEEHMKTGKPIFYTSADSVFQIAC
HEETFGLDKLYELCEIAREELTNGGYNIGRVIARPFIGDKAGNFQRTGNRHDLAVEPPAP
TVLQKLVDEKHGQVVSVGKIADIYANCGITKKVKATGLDALFDATIKEMKEAGDNTIVFT
NFVDFDSSWGHRRDVAGYAAGLELFDRRLPELMSLLRDDDILILTADHGCDPTWTGTDHT
REHIPVLVYGPKVKPGSLGHRETFADIGQTLAKYFGTSDMEYGKAMF
>LFGLNPFC_03270 Thymidine phosphorylase
MFLAQEIIRKKRDGHALSDEEIRFFINGIRDNTISEGQIAALAMTIFFHDMTMPERVSLT
MAMRDSGTVLDWKSLHLNGPIVDKHSTGGVGDVTSLMLGPMVAACGGYIPMISGRGLGHT
GGTLDKLESIPGFDIFPDDNRFREIIKDVGVAIIGQTSSLAPADKRFYATRDITATVDSI
PLITASILAKKLAEGLDALVMDVKVGSGAFMPTYELSEALAEAIVGVANGAGVRTTALLT
DMNQVLASSAGNAVEVREAVQFLTGEYRNPRLFDVTMALCVEMLISGKLAKNDAEARAKL
QAVLDNGKAAEVFGRMVAAQKGPTDFVENYAKYLPTAMLTKAVYADTEGFVSEMDTRALG
MTVVAMGGGRRQASDTIDYSVGFTDMARLGDQVDGQRPLAVIHAKDENSWQEAAKAVKAA
IKLADKAPESTPTVYRRISE
>LFGLNPFC_03271 Deoxyribose-phosphate aldolase
MTDLKASSLRALKLMDLTTLNDDDTDEKVIALCHQAKTPVGNTAAICIYPRFIPIARKTL
KEQGTPEIRIATVTNFPHGNDDIEIALAETRAAIAYGADEVDVVFPYRALMAGNEQVGFD
LVKACKEACAAANVLLKVIIETGELKDEALIRKASEISIKAGADFIKTSTGKVAVNATPE
SARIMMEVIRDMGVEKIVGFKPAGGVRTAEDAQKYLAIADELFGADWADARHYRFGASSL
LASLLKALGHGDGKSASSY
>LFGLNPFC_03272 putative protein YjjI
MPTSHENALQQRCQQIVTSPVLSPEQKRHFLALEAENNLPYPQLPAEARRALDEGVICDM
FEGHAPYKPRYVLPDYARFLANGSEWLELEGAKDLDDALSLLTILYHHVPSVTSMPVYLG
QLDALLQPYVRILTQDEIDIRIKRFWRYLDRTLPDAFMHANIGPSDSPITRAILRADAEL

KQVSPNLTFIYDPDITPDDLLLEVAKNICECSKPHIANGPVHDKIFTKGGYGIVSCYNSL
PLAGGGSTLVRLNLKAIAERSESLEDFFTRTLPHYCQQQIAIIDARCEFLYQQSHFFENS
FLVKEGLINPERFVPMFGMYGLAEAVNLLCEKEGIAARYGKEAAANEVGYRISAQLAEFV
ANTPVKYGWQKRAMLHAQSGISSDIGTTPGARLPYGDEPDPITHLQTVAPHHAYYYSGIS
DILTLDETIKRNPQALVQLCLGAFKAGMREFTANVSGNDLVRVTGYMVRLSDLEKYRAEG
SRTNTTWLGEEAARNTRILERQPRVISHEQQMRFSQ
>LFGLNPFC_03273 Putative glycyl-radical enzyme activating enzyme YjjW
MPQCPHQALQIVDGKVAWSAAVCEQCDTCLKMCPQHATPMAQSMSVDEVLNHVRKAVLFI
EGITVSGGEATTQLPFVVALFTAIKNDPQLCHLTCLVDSNGMLSETGWEKLLPVCDGAML
DLKAWGSECHQQLTGRDNQQIKRSICLLAERGKLAELRLLVIPGQVDYLQHIEELAALIK
GLGDVPVRLNAFHAHGVYGEAQSWPSATPEDVEQLADALRERGVSRLIFPALYL
>LFGLNPFC_03274 putative metal-dependent hydrolase YjjV
MICRFIDTHCHFDFPPFSGDEEASLQRAAQAGVGKIIVPATEAENFARVQALAEKYQPLY
AALGLHPGMLEKHSDVSLDQLQQALERHPAKVVAVGEIGLDLFGDDPQFERQQWLLDEQL
KLAKRYDLPVILHSRRTHDKLAMHLKRHDLPCTGVVHGFSGSLQQAERFVQLGYKIGVGG
TITYPRASKTRDVIAKLPLASLLLETDAPDMPLNGFQGQPNRPEQAARVFAVLCELRPEP
ADEIAEVLLNNTYTLFNVP
>LFGLNPFC_03275 hypothetical protein
MGQRIPVTLGNIAPLSLRPFQPGRIALVCEGGGQRGIFTAGVLDEFMRAQFNPFDLYLGT
SAGAQNLSAYICNQPGYARKVIMRYTTKREFFDPLRFVRGGNLIDLDWLVEATASQMPLQ
MDTAARLFDSGKSFYMCACRQDDYAPNYFLPTKQNWLDVIRASSAIPGFYRSGVSLEGIN
YLDGGISDAIPVKEAVRQGAKTLVVIRTVPSQMYYTPQWFKRMERWLGDSSLQPLVNLVQ
HHETSYREIQQFIEKPPGQLRIFEIYPPKPLHSIALGSRIPALREDYKLGRLCGRYFLAT
VGKLLTEKAPLSRHLVPVVTPESIVIPPAPVANDTLVAEVSDAPQANDPTFNNEDLA
>LFGLNPFC_03276 hypothetical protein
MFRWGIIFLVIALIAAALGFGGLAGTAAGAAKIVFVVGIILFLVSLFMGRKRP
>LFGLNPFC_03277 Osmotically-inducible protein Y
MTMTRLKISKTLLAVMLTSAVATGSAYAENNAQTTNESAGQKVDSSMNKVGNFMDDSAIT
AKVKAALVDHDNIKSTDISVKTDQKVVTLSGFVESQAQAEEAVKVAKGVEGVTSVSDKLH
VRDAKEGSVKGYAGDTATTSEIKAKLLADDIVPSRHVKVETTDGVVQLSGTVDSQAQSDR
AESIAKAVDGVKSVKNDLKTK
>LFGLNPFC_03278 Peptide chain release factor RF3
MTSLLAAEVAKRRTFAIISHPDAGKTTITEKVLLFGQAIQTAGTVKGRGSNQHAKSDWME
MEKQRGISITTSVMQFPYHDCLVNLLDTPGHEDFSEDTYRTLTAVDCCLMVIDAAKGVED
RTRKLMEVTRLRDTPILTFMNKLDRDIRDPMELLDEVENELKIGCAPITWPIGCGKLFKG
VYHLYKDETYLYQSGKGHTIQEVRIVKGLNNPDLDAAVGEDLAQQLRDELELVKGASNEF
DKELFLAGEITPVFFGTALGNFGVDHMLDGLVEWAPAPMPRQTDTRTVEASEDKFTGFVF
KIQANMDPKHRDRVAFMRVVSGKYEKGMKLRQVRTAKDVVISDALTFMAGDRSHVEEAYP
GDILGLHNHGTIQIGDTFTQGEMMKFTGIPNFAPELFRRIRLKDPLKQKQLLKGLVQLSE
EGAVQVFRPISNNDLIVGAVGVLQFDVVVARLKSEYNVEAVYESVNVATARWVECADAKK
FEEFKRKNESQLALDGGDNLAYIATSMVNLRLAQERYPDVQFHQTREH
>LFGLNPFC_03279 DNA damage-inducible protein I
MRIEICIAKEKMNKMPNGAVDALKEELTRRISKRYDDVEVIVKATSNDGISVTRTADKDS
TKTFVQETLKDTWESADEWFVH
>LFGLNPFC_03280 hypothetical protein
MEDAIQTYEINDIDDIGNIFDRVISGEEIHIENLKLNFLESIDFKFFGDEDKYNGTLPAG
LAQGICEFQTEMYKVFTLIKYKTSNLQKLTAEDREAAELVFTIKPGCTEIITSVKELIDS
FGNAFEKVTQGMSPRQKTMCFLFAVLVLGGAWVGTSYLDHQTQVETKTIELQQEEAKQRA
ESERMTILRDGMLSAIKAHEGIDTIERAEGIQEHTAKAYTGVLKGASDADKVTIRGASNI
ELSQGQVQEIIKNPIEKAKSEQQTLEVVIDSIKRSADKLTLSCHEPTGDDSFPIYVDTSF
INDPDEIGLIFDAMKNNKTVNILGSYKIRSGVIEQGNASTISPP
>LFGLNPFC_03281 hypothetical protein
MLTEKQFFELIKALQSSNFSTTEILGLSFAIIIVALIVNFIVSFITEKAKISATNANYEI
LRKQLALNTTTIKDIEKKITSELWISQQIWQKKYDMYEYIYTQLLSIKKWADNEFEIIEI
HMMPTYVANSYQGYFNQEQEKQFWDEVQQAHEDRDKALNDEDLKLKNKELQQKLSLAFTA
LTEMMLTKAVLLNKEVTVILNELIENIGTNPSPQEYEEPDDYGYRIKGAMDKALEKIRIN
ALSDLEIKNPEC
>LFGLNPFC_03282 hypothetical protein
MDITPFLHALCAAAAQLLIGLFTGNWAYGAIAGCTFFIAREHTQAEYRWIEMFGHGKRMN
MPWWGGFDPRAWDVASLMDFSVPVVACLLVWLLVNRG
>LFGLNPFC_03283 hypothetical protein
MATSTVIPDDIKTLKSDVSKLKNDQGSYATKSYVDSKDETVGDWSASWYQQVLPTSGAIF
GRKLRSTHRTAGVEDAYCELYLKKWIDSPGNAMARLNLNDNGTNICWDFTNLYGGTMIFP
GDSGYLKMGNCLMSYSKRGSNALIKFDYTDTLQIKYANHGSTMTLNTQGTAHSGATTSLW

GNSTRPVVYEVGADGGAYMFYAQKNTDNTYMLSVNGACHATAFNQHSDRDLKDNIQVIDN
ATDRIRKMNGYTYTLKENGMPYAGVIAQEALEAIPEVVGSAMKYQDGAGGSEGEEGERYY
TVDYSGVTGLLVQVARESDDRITALEEENAELRQRLSAIEAALASK
>LFGLNPFC_03284 hypothetical protein
MKDLTLKFADRADFSAFMESIGYYDDESMQDDILIDVIGNVYKENGELNEDGEPVCVKDD
GYFVNVRIINDVKTPSIFDEYVVAVEHQLRGWM
>LFGLNPFC_03285 hypothetical protein
MAAVKISGVLKDGAGKPIQNCTIQLKAKRNSTTVLVNTVASENPDEAGRYSMDVEYGQYS
VILLVEGFPPSHAGTITVYEGSRPGTLNDFLGAMTEDDVMPEALRRFEEMVEEAARNAEA
ASQSAAAAKKSETAAASSKNAAKTSETNAANSAQAAATSQTASANSATAAKKSETNAKNS
ETAAKTSETNAKSSQTAAKTSETNAKASETAAKNSQVAAAQSESAAAGSATSAAGSATAA
ANSQKAAKTSETNAKSSQTAAKTSETNAKASETAAKSSQDAAAQSESAAASSASEAAASA
TASANSQKAAKTSETNAKASETTAANSAKASAASQTAAKASEDAAREYASQAADPYKYVL
QPLPDVWIPFNDSLDMITGFSPSYKKIVIGDDEITMPGDKIVKFKRASKATYINKSGVLT
EAAIDEPRFERDGLLIEGKRTNYMLNSENPASWGRSSNMDVPETGTDSFGFTYGKFVCND
SLIGQTSAINMASIAATKSVDVSGDNKYVTTSCRFKTELQVRLRIRFDKYDGSATTFLGD
AYIDTQTLEINMTGGAASRITARVRKDEATGWIFAEATIQAIDGELKIGSQIQYSPKQGG
ATVSGDYIYLATPQVENGPCVSSFIISGTTAATRASDIVTVPIKNNLYNLPFTVLCEVHK
NWYKTPNAAPRVFDTGGHQTGAAIILGFGSSADYDGFPYCDIGGANRRVNENALLEKMVM
GMRVKSDQSTCSVSNGRISSETKTTWSCIQNTAIIRIGGQTTAGLRHLFGHVRNFRIWHK
ALTDAQVGESI
>LFGLNPFC_03286 Outer membrane protein X
MRKLCAVILSAVVWLVAAGTPASAAEHQSTLSAGYLQTHTYMPGSDDLKGINVKYRYEFT
DTLGLVTSFSYAGYKNRQLTRYSDTRWHKDSVRNRWFSVMAGPSVRVNEWFSAYAMAGMA
YSRVSTFSGDYLRVTDNKGKTHDVLTGSDDGRHSNTSLAWGAGVQFNPTESVAIDIAYEG
SGSGDWRTDGFIVGVGYKF
>LFGLNPFC_03287 hypothetical protein
MGKGSSKGHTPREAKDNLKSSQMLSVIDAISEGPVEGPVDGLKSVLLNSTPVLDSEGNTN
IFGVTVVFRAGEQEQTPPEGFESSGSETVLGTEVKYDTPITRTITSANIDRLRFTFGVQA
LVETTSKGDRNPSEVRLLVQIQRNGGWVTEKDITIKGKTTSQYLASVVVDNLPPRPFNIR
MRRMTPDSTTDQLQNKTLWSSYTEIIDVKQCYPNTALVGVQVDSEQFGSQQVSRNYHLRG
RILQVPSNYNPQTRQYSGIWDGTFKPAYSNNMAWCLWDMLTHPRYGMGKRLGAADVDKWA
LYVIGQNCDQSVPDGFGGTEPRITCNAWLTTQRKAWDVLSDFCSAMRCMPVWNGQTLTFV
QDRPSDKVWTYNRSNVVMPDDGAPFRYSFSALKDRHNAVEVNWIDPNNGWETATELVEDT
QAIARYGRNVTKMDAFGCTSRGQAHRAGLWLIKTELLETQTVDFSVGAEGLRHVPGDVIE
ICDDDYAGISIGGRVLAVNSQTRTLTLDREITLPSSGTTLISLVDGSGNPVSVEVQSVTD
GVKVKVSRVPDGVAEYSVWGLKLPTLRQRLFRCVSIRENDDGTYAITAVQHVPEKEAIVD
NGAHFDGDQSGTVNGVTPPAVQHLTAEVTSDSGEYQVLARWDTPKVVKGVSFMLRLTVAA
DDGSERLVSTARTTETTYRFRQLALGRYTLTVRAVNAWGQQGDPASVSFRIAAPAAPSRI
ELTPGYFQITATPHLAVYDPTVQFEFWFSEKRITDIRQVETTARYLGTALYWIAASINIK
PGHDYYFYIRSVNTVGKSAFVEAVGQPSDDAEGYLDFFKGQITESHLGKELLEKVELTED
NASKLEEFSKEWKDANDKWNAMWGVKIEQTEDGRHYVAGLGLSMEDTEEGKLSQFLVAAN
RIAFIDPANGNETPMFVAQGNQIFMNEVFLKYLTAPTITSGGNPPVFSLTPDGRLTAKNA
DISGNVNANSGTLNNVTINENCRVLGKLSANQIEGDLVKTVGKAFPRDSRAPERWPSGTI
TVRVYDDQSFDRQIVIPAVAFCGARHERENSDTYSSCRLIVKKNGAEIYNRTALDNTLIY
TGVIDMPAGSGVMTLEFSVSAWWVNGWYPTASISDLLVVVMKKATAGISIS
>LFGLNPFC_03288 hypothetical protein
MKTGAEAIRALATQLPSFRQKLNEGWYQVRIAGRDAGENELSARLNEPLANGAVIHIVPR
LAGAKSGGVFQAVLGAALIAVAWWNPVGWLGAAAVSGMYAAGASMILGGVAQMLAPKART
PRTQTTDNGKQNTYFSSLDNMVAQGNVLPVLYGEMRVGSRVVSQEISTADEGDGGQVVVI
GR
>LFGLNPFC_03289 hypothetical protein
MAPEDWLQAEMQGEIVALVHSHPGGLPWLSEADRRLQVQSDLPWWLVCRGAIHKFRCVPH
LTGRRFEHGVTDCYTLFRDAYHLAGIEMPDFHREDDWWRNGQNLYLDNLEATGLYQVPLS
AAQPGDVLLCCFGSSVPNHAAIYCGDGELLHHIPEQLSKRERYTDKWQRRTHSLWRHRAW
HASAFTGIYNDLAAASTFV
>LFGLNPFC_03290 hypothetical protein
MQDIPQETHHETTRLTQSAQVVLWEIDLTEVGGERYFFCNEQNEKGEPVTWQGRQYQAYP
IQGTGFELNGKGSAARPTLTVSNLHGMVTGMAEDLQSLVGGTVVRRKVYARFLDAVNFVN
GNSDADPEQEVISRWRIEQCSELSAVSASFVLSTPTETDGAVFPGRIMLANTCTWTYRGD
ECGYHGPAVADEYDQPTTDITKDKCSKCLSGCKFRNNVGNFGGFLSINKLSQ
>LFGLNPFC_03291 hypothetical protein
METFHWKVRPDMNVVSEPKVVTVKLGDGYEQRRAAGLNNQLSTYSVTIRVRKCEHPSLKA
FLERHGGVRAFQWTPPYDWKPIRVVCRKWSASVGALWVTITADFEQVVA

>LFGLNPFC_03292 hypothetical protein
MDQIANLVIDLGIDAAEFKNEIPRIKNLLNGAASDAERSSARMQRFMERQTQAARQTTQA
ASSAATAASVHAQTVEKNAQAHERMAREVEKTRQRMEALSQKMREEQAQAMALAEAQDKA
AAAFYRQIDSVKQASAGLQELQRIQQQIRQARNSGGIGQQDYLALISEVTAKTRVLTQAE
EEATRQKVAFIRQLKEQATRQNLSSSELLRAKAAQLGVSSAAEVYIRKMEQAGKATHSLG
LKSAAARQEIGVLIGELARGNLGALRGSGITLANRAGWIDTLMSPKGMMLGGVIGGIAAA
VYGLGKAWYDGQKEGEEFNRQLSLTGHYAGVTAGQLWTLSRAISGNGITQHAAAGALAQV
VGSGAFRGNDIGMVARAAAQMERSVGQSVSDTISQFKRLKDDPVNAAKALDNELHFLAAT
QLEQIRVLGEQGRSSDAARIAMSALAEETGRRTADIDNNLNALGSTLKYLSDLWSRFWDA
AMNIGREDSLDEQISALQEKVSRAKRLPWTASSSQVEYDQQRLNELQEKKRQKDLQDAKE
QAERNYQEQQKRRNAENAALNRMNETEAARHQREIARINAMQYADQTVRDAAIQRENERY
EKALASGKKKTRETRNDEATRLLLQYSQQQAQVEGQIAAARQSAGIATERMTEAHKQLLA
LQQRISDLDGKKLTEDEKSVLARKDELIQALTLLDVKQQELQKQTALNELKKKTIQLTSQ
LAEEERAQRQQHNLDIATVGMGDQQRQRYQVQLSLRQKYQQQLEQLRRDSEQKGTYNTDD
YRKAEQALTESLNRQLNENRRYWQQLEIAQGNWKNGVLRALQNVTENADNTAWTVEQLFT
SAFSSMSDWLATFCTTGKLNFKSFTSSVLSDMSRIMAQIALMKAVKGIASALPFDFVANA
DGGVYQSADLSRYSGTVVNRPTFFAFAKGAGVMGEAGPEAILPLRRGADGKLGVVADIGG
SGMAMFAPQYNIEINNDGTNGQIGPAALKVVYDLGKKAAADFMQQQARDGGRLSGAYR
>LFGLNPFC_03293 hypothetical protein
MRLAREFRRADWRRMLSEMSATELGEWGDYFRMQSFSDVWMDAQFASLKALIVRMVSGSS
DAAVADFSLLPEENGIPERTDEELMHLGEGISGGVRYGPDSQPGH
>LFGLNPFC_03294 hypothetical protein
MFLKQGTFNYEKQSVVLSELSGLQRIEYLAFVQQRTAKFDAGEGELPEAERQIAFLRMGM
DINAWLVSRSLWNADQSKDVETLCASVITTWSYDALGAGAEMVLSLSGMGAIDNAGDDEH
EALTPEKS
>LFGLNPFC_03295 hypothetical protein
MTTPNPLAKTKGAGTTFWMYTGNGDAFANPLSDTDWLRLAMVKDLQPGEMTADAEDDTYL
DDEDADWKTTTQGQKSVGDTSATLAWRPGDSGQKKLVQLFDSGEVCAFRIKYPNGTVDVF
RGWLSSLGKTIASKDVMTRTVKISGVGRPYLAEEGTEIVSVTGLTVAPASASVKVGATTT
LTFTVKPDGASDKAISVHSSDPQTATVTLNGLVATVKGVKQGSVSIVGMTADGNFVAVVA
VTVSAAG
>LFGLNPFC_03296 hypothetical protein
MNRHTQIRQAVLARLREQCGDSATFFDGLPAFIDAQELPAVAVWLSDAQYTGKMTDEDDW
QAVLHIAVFIRAQAPDSELDMWMESTIFPALNDIPALSGLIDTLIPLGFNYQRDNEMATW
AMAEITYQITYTN
>LFGLNPFC_03297 hypothetical protein
MKGLENAIRNLNSLDTRMVPQASAWAINRVAQKAVSVATRQVAGNTVAGDNQVKGIPLKL
VRQRVRVFKASPSGKMTARIRVNRGNLPAIKLNTTRRRAGEGLRVGKYFFRRAFVQQLAN
GRWHVLRRLPEARFATGHDHQGRPRKNRLPVEVVKIPLSGPLTQAFEDARDRIIAAEMPK
QLGYALKQQLRLWLTR
>LFGLNPFC_03298 hypothetical protein
MSDPFSRLAARMDAITVRKMGKTASINDVDMTVIPGETLAELNALSGPAVSLVVFSSGYR
PRRGDRVVYDGQQWTVTRHERFNGKPMIFIE
>LFGLNPFC_03299 hypothetical protein
MAKNFVEEGKTVAIVASAAISSGDLVQVGDVFAVALTDIPQGETGDGMTEGVFMLPKLKT
DDMKTGKKVYLKSGKVQLTNSGSDPLVGVVWADAGTSAEEVPVKLNV
>LFGLNPFC_03300 ATP-dependent Clp protease proteolytic subunit
MPQRNDRSRSTPTTSPKNNSWFRMQAGHQSDADIYIYDEIGFWGVTAKQFISDLNALGDI
THINLHINSPGGDVFEGIAIFNALKTHGASITVYVDGVAASMASVIAMVGNPVIMPENTF
MMIHKPFGFTGGDAEDMRTYADLLDKVEAVLLPAYAQKTGKTTDEIAAMLADETWMSGAE
CLAHGFADQVTPAVKAMACIQSKRTEEFKKMPESIRNMITPPRNSAPRVQDDEPAASRTP
VQAAAPVVDENSIRAQVLAEQKARVNGINDLFAMFGGRYQTLQAQCLADPECSLEQAREK
LLNEMGRESTPSNKNTPAHIYAGNGNFVGDGIRQALMARAGFEKTERDNVYNGMTLREYA
RMSLTERGIGVSSYNPMQMVGAAFTHSTSDFGNILLDVANKAILQGWEDAPETYEQWTRK
GQLSDFKIAHRVGMGGFSALRQVREGAEYKYVTTGDKQATIALATYGELFSITRQAIIND
DLNMLTDVPMKLGRAAKSTIADLVYAILTSNPKISTDNVSLFDKAKHANVLESAAMDVAS
LDKARQLMRVQKEGERHLNIRPAFVLVPTAMESVANQVIRSSSVKGADINAGIINPVKDF
ATVIAEPRLDDNSQTTFYLAASKGSDTIEVAYLNGVDTPYIDQMEGFSVDGVTTKVRIDA
GVAPVDHRGLVKCTA
>LFGLNPFC_03301 hypothetical protein
MAILDDVIGVFSPGWKAARLRSRAVIQAYEAVKTTRTHKARRENRTADQLSQYGAVSLRE
QARYLDNNHDLVIGVFDKLEERVVGKNGIIVEPHPVLRNGAIARDLAAEIRTRWSEWSVS
PEVTGQFTRPMLERLMLRTWLRDGEVFAQMVSGRINSLTPSAGVHFWLEALEPDFIPMTS
DESNRLNQGVFVDDWGRPEKYLVYKSRPVSGRQMETKEVDAERMLHLKFVRRLHQMRGTS

LLSGVLIRLSALKEYEDSELTAARIAAALGMYIRKGDGQSYEPDGNGSKENERELTIQPG
IIYDDLKPGEEIGMVKSDRPNPNLETFRNGQLRAVAAGSRLSFSSTARNYNGTYSAQRQE
LVESTDGYLILQDWFIGAVTRPMYRAWLKQAVASGVIRLPRDLDRSSLYTAVYSGPVMPW
IDPVKEAEAWKIQIRGGAATESDWVRAGGRNPDDVKRRRKAEIDENRKLDLVFDTDPASD
KGGSSAATKRQEPQHTDDQSEE
>LFGLNPFC_03302 hypothetical protein
MNQNDIEAMIQRYTEAEMAVLDGKSVTFNGQQMTMENLSEIRQGRQEWERRLAALITRRR
GHPGYRLARF
>LFGLNPFC_03303 hypothetical protein
MLNQETAKAARTDSGYILRAPRRMRVADAVAQYMRVPMGAGNSVPWDPLVAPYVIEPMNC
LASREYDAVIFVGPARTGKTIGLIDGWVIYNVICDPADMLIIQMTEEKAREHSKKRLART
FRVSPEVVSRLSPNKNDNNVYDRTFLAGNYLKIGWPSVNIMSSSDYKCVALTDYDRFPED
IDGEGDAFSLASKRTTTFMSSGMTLVESSPGRDVKDVKWRRTSPHEAPPTTGILSLYNRG
DRRRWYWPCPHCGEYFQPCGDVVAGFRDIADPVLASEAAYIQCPSCSGRIMPEQKRELNG
RGVWLRDGESINADGSRYGDPRRSRIASFWMEGPAAAYQTLSQLVYKLLTAEQEYETTGS
EETLKTVINTDWGLPYLPRASMEQRKSELLEQRAEPVPSRSVPDGVNFLVATVDVQAGRH
RRFVVQVTGYGSRGERWIIDRYNITQSLRGDSDGESQRIDPASYPEDWDVLLTDVFHKSW
PLASDPSQQMRLMAMAVDSGGEDGVTDNAYKFWRRCRRDGLGKRIYLFKGDSIRRAKLIT
RTFPDNTGRTGRRAQAAGDVPLWLLQTDALKDRVNNALWRDSPGPGYVHFPDWLGSWFYD
ELTYEERSSDGKWSKPGRGANEAFDLMVYAEALVILHGYEKIRWPDAPGWASRETWLECV
PDSTEPSPSPEPVSTPVKKQKRKKTVTDDVNPWLTSGGWL
>LFGLNPFC_03304 hypothetical protein
MDHELKKLVLNINQLAALSGLHRQTVVARLKNIRPAGGHDKLKLYRLTDILTEFMGLPPP
VAEGEMDPHERKAWYQSERERLKFEQETAQLIPASDVRREFAIWAKAVVQVLETLPDILE
RDCGLQPAAVSRVQSIIDDLRDQIALRVTEAGADDEEELQQEE
>LFGLNPFC_03305 hypothetical protein
MRDKPANHKRGKNPVCIVFDYSRTLAQKEFPVGLRSLLIREYGDDTAHDVSGLNTFIR
>LFGLNPFC_03306 hypothetical protein
MPSMIAIILLIILHIWLCRQDGDHFWSESRLNISLLMLDIEHLARGKGLR
>LFGLNPFC_03307 hypothetical protein
MSRVTAIISALVICIIVCLSWAVNHYRDNAIAYKEQRDKKVSELKQATATITDMQQRQRS
ADALDAKYTKELADAKAENDALRRKLDNGGRVFVKGKCPVPSSAETSSASGMGNDATVEL
SPVAGRNVLGIRDGIIRDQTALRTLQEYIRTQCLR
>LFGLNPFC_03308 Lysozyme RrrD
MPPSLRKAVAAAIGGGAIAIASVLITGPSGDDGLEGVSYIPYKDIVGVWTVCHGHTGKDI
MLGKTYTEAECKALLNKDLATVARQINPYIKVDIPETTRGALYSFVYNVGAGNFRTSTLL
RKINQGDIKGACDQLRRWIYAGGKQWKGLMTRREIEREVCLWGQQ
>LFGLNPFC_03309 hypothetical protein
MKSMDKLTTGVAYGTSAGSAGYWFLQLLDKVTPSQWAAIGVLGSLVFGLLTYLTNLYFKI
KEDKRKAARGE
>LFGLNPFC_03310 hypothetical protein
MKNTVRINSVDLINADCLHFIQSLPDDSIDLIVTDPPYFKVKPNGWDNQWKGDEDYLKWL
DHCLAQFWRVLKPAGSLYLFCGHRLASDIEVMMRERFNVLNHIIWAKPSGRWNGCNKESL
RAYFPATERVLFAEHYQGPYRGKSDGYAAKERELKQHIMAPLISYFRDARAELGITAKQI
AEATGKKNMVSHWFGASQWQLPNEADYRKLQALFSRIAAEKFQEQQLEQPHHQLVASYDS
LNRKYSELLDEFKSLRRYFSVSVSVPYTDVWMHKPVQFYPGKHPCEKPADMLRQIINASS
RPGDLVADFFMGSGSTIKAAMALGRRALGVELESERFNQTVKEINELVGK
>LFGLNPFC_03311 hypothetical protein
MLKQQDMTETAAAVLHFLPADKWVTPRMMTRTTGVSEAQCQLILTQLVLAGLAKDNGGYG
NKFRRCQ
>LFGLNPFC_03312 hypothetical protein
MNKITVLLLSATIISGCTSSVPLIKKTQSGKPEGVYQNTTKDKVKDALVNYCNSRGLIIY
NADNSSVICGKELEGGSAVFGQMLIGNAYSTTPVSKVRFTIAQVNNDTKVWADMWMETQM
AMGQVQQMAITDNASKNTIQQRLDELKP
>LFGLNPFC_03313 hypothetical protein
MRDIQMVLEHWGAWVANNHEDVTWSSIAAGFKGLIPSKVKSRPQCCDDDAMIICGCMARL
KKNNSDLHDLLVDYYVGGMTFMALARKHGRSDCWVGRLLQKAEGVVDGMLMMLEIELEMD
R
>LFGLNPFC_03314 hypothetical protein
MRALLTPEIAPRMGIVLFRPGSELMPLFMQGRVLLEPEPERYSSFASGAVPAASQPLADD
PAVRAVFRNEAVIRRAGGVECLESWLLREKGCQWPHSDWHSENMTTMRHAPGAIRLCWHC
DNQLRDQFTERLESMATDNCARWVLSVVRRDLGFDDSHVVTMPELCWWLIRNDLADALPE
SAARKALRLPKPVVPSVTRESDLVPSVPATSIIQDKAKKVLALKVDPESPESYMLRPKRR
RWVNEKYTRWVKTQPCACCGKPADDPHHLIGHGQGGMGTKAHDLFVLPLCRKHHDELHAD

TVAFEEKYGSQLELIFRFIDRALAIGVLA
>LFGLNPFC_03315 hypothetical protein
MNNLMVIDGIEVRRDAYGRYSLNDLHRAAGSLDKHKPAFWLRNEQTEHLISELQICNSVN
IEPVNVIRGGNNQGTYVCKELVYAYAMWISPSFHLKVIRTFDMVTSVPEKLSGQAADKMQ
AGVILLDFMRRELNLSNSSVLGACQKLQEAVGLPNLAPRYAIDAPADAPDGSSRPTLSLS
ALLKQYGIRLTANQAYHQMAKLGIVEQRERYSRTEINNIKKFWSLTAKGCMFGKNITSPA
NPRETQPHFFESRFPELLKLLDTVH
>LFGLNPFC_03316 Crossover junction endodeoxyribonuclease RusA
MKLILPFPPSVNTYWRHPNKGAFAGKSLISAAGRKFQSAACAAIVEQLRRLPKPTSAPAS
VEIVLFPPDNRIRDLDNYNKALFDALTHAGVWEDDSQVKRMLVEWGPVIPKGKVEITISK
YEKPAGAAA
>LFGLNPFC_03317 LexA repressor
MTTLTQCQQQVLDMLISYQKERGFPPTNQEVATMLGYRSVNAAVEHLRALEKKGVITIKR
GVARGITLHTAVKDDDSEAVGIIRSLLAGEENARLRATHWLHERGLKV
>LFGLNPFC_03318 hypothetical protein
MSNKYCQALVELRNKPAHELKEVGDQWRTPDNIFWGINTLFGPFVLDLFTDGDNAKCAAY
YTAEDNALAHDWSERLAELKGAAFGNPPYSRASQHEGQYITGMRYIMKHASAMRDKGGRY
VFLIKAATSEVWWPEDADHIAFIRGRIGFELPAWFIPKDEKQVPTGAFFAGAIAVFDKTW
KGPAISYIGRDELEACGEAFLAQVRQQAEKLVREMAA
>LFGLNPFC_03319 hypothetical protein
MSLLNEVQKFIEAHPGCTSGDIADAFAGYSRQRVLQSASKLRQSGRVAHRCEGDTRRHFP
RLTERAQEPEPQSVRETRPVRNFYVGTNDPRVILCLTRLAEELESRGLFRRAATVWMEAF
RESHSQPERNNFLAHRERCLRKSSKRAASGEEWYLSGNYVGA
>LFGLNPFC_03320 hypothetical protein
MSMELMVKAMKIRVGNPLRKLVLIKLADNASDQGECWPSYQHIADQCEISKRSVMNHIAA
LCESGLVKKVTRKGEKGNSSNIYLLHLDGAGDSLGGSANNSLSGAANSPGSAGVAPGGSA
GDSPRTSHSFEPVKEPVNEPIAVGASADESVRVRSNRPEYSPEFEQAWLAYPKRAGGNSK
SAAFKAWKARLNEGVNPETMLEGVKRYAGWVSAMGNSGTQFVKQAVTFFGPDRHFEESWE
VPAVSAARREDPYFKASYDNVDYSQIPAGFRG
>LFGLNPFC_03321 hypothetical protein
MIRNIFKRFTNQTFRCPRPGQWYTTPAGHVLRVSLVDRECQKVICEPLGRNYRVSMPLIA
FRSGKNMKHLGGAA
>LFGLNPFC_03322 hypothetical protein
MNSLTANNRLSQQLVVSVAAHLLLRHECRLPNHLAVSNHRELYLTVGGELCRNLTAGFVT
EEDFMFMLFVGSQKHSALSIFAKTTRMSALVLCGNSGVILLSVKDHQHIDSAIPGRYTVQ
APYKAGAGRGNPEFTKAHNRALAVFLCHEQHYAQIMVGRAGPTSVGPGSLVTGISTPVRL
TTNKVVESLGGELLKITKEAAIMATIPTLTQPEIAIVDGQAVTSSLAVANFFSKRHDDVL
KKIRTLECSASFTARNFSVSDYTDCTGRKLPCYQITRDGFAFLAMGFTGKRAAQFKEAYI
NAFNQMEKQLSKPAVPSDVAHNASVLCSYISSIHQVWLQQLYPMLAKAESPLAVSLYDYI
NDASALACLINLSLNPSEVRGRK
>LFGLNPFC_03323 hypothetical protein
MGNHHWKVEKQPEWYVKAVRKTIAALPGGYAEAAEWLDVTENALFNRLRADGDQIFPLGW
AMILQRAAGTHYIADAVAQSAGGVFVSLPEIEEVENADINQRLLEVIEQIGSYSKQIRSA
IEDGVVEPHEQTAINDELYLSISKLQEHAALVYKIFCAPEKSDARECAAPGVVAFCVCGE
TNA
>LFGLNPFC_03324 hypothetical protein
MQSPLRNVRKAHGFTLQHVAAGVQVNPATLSRIERLEQIPSIDLAERLANFFKGEISEMQ
ILYPARFQSSQNQNGFKPQEQEVSRG
>LFGLNPFC_03325 putative HTH-type transcriptional regulator
MKIADLAEAIGVDAANISRLETGKQKQFTEQALSNIARSLGVDIADLFTSDFKSNTVCKN
SISEDVAQVKDVFRIEMLDVSASAGNGLIQGGDVIDVIHAIEYRTDNAVSMFGGRPANHI
KVINVRGDSMCPTIEPGDLIFVDVSINQFDGDGIYVFGFDDKIYVKRLQMIPDKLLVISD
NQIYREWGITSENEHRFMVFGKVLISQSQTLKRHN
>LFGLNPFC_03326 hypothetical protein
MASERSTDVQAFIGELDGGVFETKIGAVLSEVASGVMNTKTKGKVSLNLEIEPFDENRVK
IKHKLSYVRPTNRGKISEEDTTETPMYVNRGGRLTILQEDQGQLLTLAGEPDGKLRAAGR
>LFGLNPFC_03327 hypothetical protein
MSQNLDATAINQIHALISAQGVNEIISKIGADAVALPENFRIHDLEKFNLNRFRFRGALS
TASIDDFTRYSKDLADEGTRCFIDADNMRAVSVLNLGTIDEPGHADNTATLKLKKTAPFS
ALLSVNGERNSQKSLAEWIEDWADYLVSFDANGDTIQATKAAAAVRKITIEANQTADFED
NDFSGKRS
>LFGLNPFC_03328 hypothetical protein
MESVEAKTKDIMPVAFEFKCVPFEGLKERPFKLRLSIITGDRPVLVLRIIQLEAMQEEMA
NEFRDLLVEKFKDSKVETFIGTFTA

>LFGLNPFC_03329 hypothetical protein
MSFIKTFSGKHFYYDKINKDDIVINDIAVSLSNICRFAGHLSHFYSVAQHAVLCSQLVPQ
EFAFEALMHDATEAYCQDIPAPLKRLLPDYKRMEEK
>LFGLNPFC_03330 hypothetical protein
MKYADLIMLATERRDLGLDDGSFWPVLEGIPATEMFKVIPLSPGHAYGMFMERFNELSEL
RKCA
>LFGLNPFC_03331 hypothetical protein
MRMNVFEMEGFLRGKCVPRDLKVNETNAEYLVRKFDALEAKCAALENKIMPVSAELPPAN
ESVLLFDANGEGWLIGWRSLWYTWGQKETGEWQWTFQVGDLENVNITHWAVMPKAPEAGA
>LFGLNPFC_03332 hypothetical protein
MTTFTDKELIKEIKERIGSLDVRDNIERRAYEIALASLEAEPVAECIIEDGCMCVDGVGE
YVGHSLPDGTHQLYTAPPALVVPDKLPREYIRGWPLAYSDYAEGWNDCREAMLQGKGE
>LFGLNPFC_03333 hypothetical protein
MALTKKQRAELRMKFGGRCAYCGCELGEKWHADHVKPVIRFDGNMLHQERDDISNMVPAC
HPCNLHKHCSSLEDYRRIISDGRREFLASGKGKALVRMGLVEMKSDPVVFWFEKYQEGAT
A
>LFGLNPFC_03334 hypothetical protein
MITISKERLQWLANISGRDDIDDIDGGEIRELALIALASLEAEPVAVNDDMAYAFHHALS
DSSLGSDEIEEIKTGLRAAFANVTVQPVLIVPDEIEPDDSNTFDYVDGWNACRAAMLHGA
EPVSQTYKSQHTQFEQVADLYEMQFDDGRTCAFHTDAQKAAQWLQACDGNRVQEYVKLER
LRNALSGNSPVTPDGWISCSERMPNDKQYVWCWGKSYGWTECDTFEGYYDWSRNKWWAVT
DDVEEPASKVTHWMPLPEPPQEVK
>LFGLNPFC_03335 hypothetical protein
MKDLFGLLIYIIKKYGFIIVVAIIMVSPVMNFVLKTSLSDIQLESSSIVNLINEKKEFIL
FTATALVFVGSFVAIMTSYLNKGIGGRDIENTRYIFHKEIVKLNKKLSEIENRTSSGVSI
EITESEREKLISDAKKRIIGNTILLADNSLKSDISDFKKSYSLHKLHTDMVLRLESEIDR
LNRRGGLNLAIGTIIALTGILSLAYFLYSAPDIVDGVGFFIHHLPKLSFVIVVELFAYFF
LRLYKNGFDEIKYFQNEITNIEMKVMSLKYAQEFKNEDMIKELAMLLMKTERNFILEKGQ
TTVSIEKDRLQNLSDSKLTTMISEIIKLKQK
>LFGLNPFC_03336 Prophage integrase IntA
MALSDAWLRSVVGKERDKVLVKSDRDGLSVRVSPKGRVVFQYRYQWAGKGERLDIGTYPA
TGLKEAREEVIRLRGELESNRNPRLVKQAEKRKATEAMTVESVIRAWYEAYCVKNKKGSE
QILRSFELHLFSKIGNIPHDAATLHDWLEVLEPLSTKTPAIADRLLINAKQAHVWAYKRK
LIETRPLSDITGKDMDIRKGQKKRFLTHDEIKILYAAIDGSRMVPKYRAFIKLLLHFGCR
SSELITARVDDFDFINKVWTVPPERHKTGEITGEPLKRPIIEPVEELIKYAISMNNGSDM
LFTKEGSREPVGRTSLQSLPYNLMQYAWRRLGYQFPHWSLHDLRRTARTNFSDLTAPHIA
EIMLGHKLPGVWQVYDKSDYLEEQRKAYQAWWERVESIVTCTGS
>LFGLNPFC_03337 Pyrimidine 5'-nucleotidase YjjG
MKWDWIFFDADETLFTFDSFTGLQRMFLDYSVTFTAEDFQDYQAVNKPLWVDYQNGAITS
LQLQHGRFESWAERLNVEPGKLNEAFINAMAEICTPLPGAVSLLNAIRGNAKIGIITNGF
SALQQVRLERTGLRDYFDLLVISEEVGVAKPNKKIFDYALEQAGNPDRSRVLMVGDTAES
DILGGINAGLATCWLNAHNREQPEGIAPTWTVSSLHELEQLLCKH
>LFGLNPFC_03338 [Ribosomal protein S18]-alanine N-acetyltransferase
MNTISSLETTDLPAAYHIEQRAHAFPWSEKTFASNQGERYLNFQLTQNGKMAAFAITQVV
LDEATLFNIAVDPDYQRQGLGRALLEHLIDELEKRGVATLWLEVRASNAAAIALYESLGF
NEATIRRNYYPTTDGREDAIIMALPISM
>LFGLNPFC_03339 DNA polymerase III subunit psi
MTSRRDWQLQQLGITQWSLRRPGALQGEIAIAIPPHVRLVMVANDLPALTDPLVSDVLRA
LTVSPDQVLQLTPEKIAMLPQGSRCNSWRLGTDEPLSLEGAQVASPALTELRANPTARAA
LWQQICTYEHDFFPRND
>LFGLNPFC_03340 Ribosomal RNA small subunit methyltransferase C
MSAFTPASEVLLRHSDDFEQSRILFAGDLQDDLPARLDTAASRAHTQQFHHWQVLSRQMG
DNARFSLVATANDVADCDTLIYYWPKNKPEAQFQLMNLLSLLPVGTDIFVVGENRSGVRI
AEQMLADYAPLNKVDSARRCGLYFGRLEKQPVFDANKFWGEYSVDGLTVKTLPGVFSRDG
LDVGSQLLLSTLTPHTKGKVLDVGCGAGVLSVAFARHSPKIRLTLCDVSAPAVEASRATL
ATNGVEGEVFASNVFSEVKGRFDMIISNPPFHDGMQTSLDAAQTLIRGAVRHLNSGGELR
IVANAFLPYPDVLDETFGFHEVIAQTGRFKVYRAIMTRQAKKG
>LFGLNPFC_03341 hypothetical protein
MSPHTVSDAAYTPYLTDKRIIHNPLMDIFPAITPLSIINY
>LFGLNPFC_03345 hypothetical protein
MLQRTLGSGWGVLLPGLLIAGLMYADLSPDQWRIVILMGLVLTPVMLYHKQLRHYVLLPS
CLALIAGIMLMIMNLNQG
>LFGLNPFC_03346 Ferric iron reductase protein FhuF
MAYRSAPLYEDVIWRTHLQPQDAGLAQAVRATIAEHREHLLEFIRLDEPAPLNAMTLAQW

SSPNALSSLLAVYSDHIYRNQPLMIRENKPLISLWAQWYIGLMVPPLMLALLTQEKALDV
SPEHVHVEFHETGRAACFWVDVCEDKNATLHSPQQRMETLISQALVPVLQALEATGEING
KLIWSNTGYLINWYLTEMKQLLGEATVESLRYALFFEKTLTTGEDNPLWRTVVLRDGLLV
RRTCCQRYRLPDVQQCGDCTLK
>LFGLNPFC_03347 Transcriptional activator protein BglJ
MEHSRIKKRNVALIEKCVMSRIGIESLFRKFAGNPYKLHTYTSQESFQDAMSRISFAAVI
FSFSAMRSARREGLSCLTELAIKFPRTRRLVIADDDIEARLIGSLSPSPLDGVLSKASTL
EIFHQELFLSLNGVRQATDRLNNQWYINQSRTLSPTEREILRFMSRGYSMTQIAEQLKRN
IKTIRAHKFNVMSKLGVSSDAGLLEAADILLCMRHCETSNVLHPY
>LFGLNPFC_03348 Putative transcription factor YjjQ
MLPGCCKNGIVISKIPVMQAGLKEVMRTHFPEYEIISSASAEDLTLLQLRRSGLVIADLA
GESEDPRSVCEHYYSLISQYREIHWVFMVSRSWYSQAVELLMCPTATLLSDVEPIENLVK
TVRSGNTHSERISAMLTSPAMTETHDFSYRSVILTLSERKVLRLLGKGWGINQIASLLKK
SNKTISAQKNSAMRRLAIHSNAEMYAWINSAQGARELNLPSVYGDAAEWNTAELRREMSH
S
>LFGLNPFC_03349 Inner membrane protein YjjP
MQTEQQRAVTRLCIQCGLFLLQHGAESALVDELSSRLGRALGMDSVESSISSNAIVLTTI
KDGQCLTSTRKNHDRGINMHVVTEVQHIVILAEHHLLDYKGVEKRFSQIQPLRYPRWLVA
LMVGLSCACFCKLNKGGWDGAVITFLASTAAMYIRQLLAQRHLHPQINFCLTAFAATTIS
GLLLQLPTFSNTPTIAMAASVLLLVPGFPLINAVADMFKGHINTGLARWAIASLLTLATC
VGVVMALTIWGLRGWV
>LFGLNPFC_03350 hypothetical protein
MGVIEFLLALAQDMILAAIPAVGFAMVFNVPVRALRWCALLGAIGHGSRMILMTSGLNIE
WSTFMASMLVGTIGIQWSRWYLAHPKVFTVAAVIPMFPGISAYTAMISAVKISQLGYSEP
LMITLLTNFLTASSIVGALSIGLSIPGLWLYRKRPRV
>LFGLNPFC_03351 Primosomal protein 1
MSSRVLTPDVVGIDALVHDHQTVLAKAEGGVVAVFANNAPAFYAITPARLAELLALEEKL
ARPGSDVALDDQLYQEPQTAPVAVPMGKFAMYPDWQPDADFIRLAALWGVALRESVTAEE
LASFIAYWQAEGKVFHHVQWQQKLARSLQIGRASNGGLPKRDVNTVSEPDSQIPPGFRG
>LFGLNPFC_03352 DNA replication protein DnaC
MKNVGDLMQRLQKMMPAHIKPAFKTGEELLAWQKEQGAIRSAALERENRAMKMQRTFNRS
GIRPLHQNCSFENYRVECEGQMNALSKARQYVEEFDGNIASFIFSGKPGTGKNHLAAAIC
NELLLRGKSVLIITVADIMSAMKDTFRNSGTSEEQLLNDLSNVDLLVIDEIGVQTESKYE
KVIINQIVDRRSSSKRPTGMLTNSNMEEMTKLLGERVMDRMRLGNSLWVIFNWDSYRSRV
TGKEY
>LFGLNPFC_03353 hypothetical protein
MMKTIKHLLCCAIAASALISTGVHAASWKDALSSAASELGNQNNTTQEGGWSLASLTNLL
SNGNQALSADNMNNAAGILQYCAKQKLASVTDAENIKNQVLEKLGLNSEEQKEDTNYLDG
IQGLLKTKDGQQLNLDNIGTTPLAEKVKTKACDLVLKQGLNFIS
>LFGLNPFC_03354 Phosphoglycerol transferase I
MSELLSFALFLASVLIYAWKAGRNTWWFAATLTVLGLFVVLNITLFASDYFTGDGINDAV
LYTLTNSLTGAGVSKYILPGIGIVLGLAAVFGALGWILRRRRHHPHHFGYSLLALLLALG
SVDASPAFRQITELVKSQSRDGDPDFAAYYKEPSKTIPDPKLNLVYIYGESLERTYFDNE
AFPDLTPELGALKNEGLDFSHTQQLPGTDYTIAGMVASQCGIPLFAPFEGNASASVSSFF
PQNICLGDIMKNSGYQNYFVQGANLRFAGKDVFLKSHGFDHLYGSEELKSVVADPHYRND
WGFYDDTVLDEAWKKFEELSRSGQRFSLFTLTVDTHHPDGFISRTCNRKKYDFDGKPNQS
FSAVSCSQENIATFINKIKASPWFKDTVIVVSSDHLAMNNTAWKYLNKQDRNNLFFVIRG
DKPQQETLAVKRNTMDNGATVLDILGGDNYLGLGRSSLSGQSMSEIFLNIKEKTLAWKPD
IIRLWKFPKEMKEFTIDQQKNMIAFSGSHFRLPLLLRVSDKRVEPLPESEYSAPLRFQLA
DFAPRDNFVWVDRCYKMAQLWAPELALSTDWCVSQGQLGGQQIVQHVDKAIWKGKTAFKD
TVIDMARYKGNVDTLKIVDNDIRYKADSFIFNVAGAPEEVKQFSGISRPESWGRWSNAQL
GDEVKIEYKHPLPKKFDLVITAKAYGNNASRPIPVRVGNEEQTLVLGNEVTTTTLHFDNP
TDADTLVIVPPEPVSTNEGNILGHSPRKLGIGMVEIKVVEREG
>LFGLNPFC_03355 L-galactonate-5-dehydrogenase
MSTMKVLICQEIKNLVWEVRERPVPGDNEALIKIKTVGICGTDIHAWAGNQPFFSYPRVL
GHEICGEIIESGKNIHNFNIGQQVAVIPYISCQQCPACKSGRTNCCEKISVIGVHQDGGF
SEYLSVPVANILPADGIDPQAAALIEPFAISAHAVRRAAIAPSEQVLVVGAGPIGLGAAA
IAKADGAQVVVADTSPARREHVATRLELPVLDPSAEDFDAQLRAQFGGSLAQKVIDATGN
QHAMNNTVNLIRHGGTVVFVGLFKGELQFSDPEFHKKETTMMGSRNATPEDFAKVGRLMA
EGKITADMMLTHRYPFATLAETYERDVINNRELIKGVITF
>LFGLNPFC_03356 putative HTH-type transcriptional regulator LgoR
MSRSQNLRHSVINQVIEDMARGNIPSPLPSQSGLAEMYNISRTTVRHILQHLSACGVLTL
VGKNYVIARKPEQNDGFDCITASLKEQNRLFEQAFFTMINLRQLRAGESFSELQLARAAS
VSPVVVREYLLKFGRYNLIQSEKRGQWSMKQFDQSYAEQLFELREMLETHSLQHFLNLPD

DDPRWLQAKTMLERHRTLRDSIGSNFRMFSQLDRDFHAMLLSAADNIFFNQSLEIISVIF
HFHYQWDESDLKQRNIIAVDEHMTILSALICRSDLDAITALRNHLDTAKQSMIRSIRQHH
R
>LFGLNPFC_03357 Sodium, potassium, lithium and rubidium/H(+) antiporter
MEILLTILILTLVVSLTSVLTRLSPVQIPLPLIQIAAGAVLAQPIFGLHVEFNPELFLLL
FIPPLLFAESSKIQPKELIKHSREIISLALVLVLITIFGVGYVIHLLLPNVPLIAAFALA
AVLSPTDAVALLGIVGKGRISKNIQEVLEGEALMNDASGLVALKFAVAVTMGTMEFSVHG
ATIAFFVVALGGIAVGIAVTWLYGKGLLLISRYAHDEPSIQIVLMLLLPFIVYLVAEHFG
LSGILAAVAAGLTTSRIGVVHHAPIRLRIKAKSSWSMLEYVFNGMVFLILGLQLPGIISE
SLIRAENDPLVDTGSLLLDVIWIYLALMVVRLGWLWLMRLYSRKRPGKHPMLFAGYKTRH
MLLATFAGVRGAITLAGVLSIPLLLPDGTPFPGRYLLVFLATGVILFSLVCGIIILPLLL
GKNVGIFSDNRQKEEEAADLFMAKMAILSVQKTEQRLLRARTENLDNELIMETGARVIGY
MRHSEEDMSPNDNSLHMQRAKLERTFNLAALNAKRAAIYQMRARQEVSDETLAKIVSELD
VLETLIMDSR
>LFGLNPFC_03358 Sialic acid-binding periplasmic protein SiaP
MSKIIAMLITVSTLFLPSIIQAKPISIKVAYENNPGEPLDVVMRYWADLLNKKSNGEITL
ALYPSSQLGSKQDVTEQAMMGMNVITLSDVAFLADYEPDLGILFGPYLTDDPQKLFKIYE
SDWFKQKNESLKKKGIHVVMNNYLYGTRQIISKKPIRTVDDLAGLKIRVPNNVMQIKAIQ
AMGATPTPMPLGEVYPALTQGVIDGVENPISVLQGQKLYEQARYLTMVNYLTNTSVWIGG
EAFFSTLSPEQLEMIHQTGYEAGVYSQKLTLERDAEMLKTMQAAGVEVIYPDTGPFQKKA
REVYSQFPEWTPGLYETIQQQLQ
>LFGLNPFC_03359 hypothetical protein
MRLFGKVAEFSAAFALFVLVVVTIGAVFMRYFIGQPLQWTEEMSGMLMIWVVMLGGVVAE
RDRAHLTIPFLMEMLPGKLRRVIAVLVALLSIALLLYMAWLGYRLAEMAQFKVTQILKVS
WFWIDLAVPVGALATAIYTLYWLINDTKQSDSGDKMQ
>LFGLNPFC_03360 C4-dicarboxylate TRAP transporter large permease protein DctM
MSWLLIIPLMIVCLFLNIRVYLAMFIGIIAYFLFFSSVPIEIAVQRLIAPTQSPSLLAIP
FFILLGTLMSYTGIAERILHIANVLVGKMRGGLGVANILVSTMMGGVSASNLADAAMLSR
MMVPEMERKGYNRAFAAAITAGGSLVTPIIPPGIALIIYGLVADVSIGKMFIAGLVPGLL
CAVVLMFTVYLVARKTNAKPSRESWPTGKEVVFSLGGQAWPALFLIFAVVGGIRANIFTPT
EAGAVAVLIVLAIGFFIYREMRISHVVKALGETARATASVMLVIMASAALGWIFSMEHAG
VAVANFVTSLTENKYMFLLIINILLLTLGMFLEGNAILLILVPLLKPVVEHFGIDPVHFG
IIMIFNLSIGAFTPPVGTVMLLVCNITQVSVGNFFKQSLPLLAALLLMLMLVTYIPAISL
FLV
>LFGLNPFC_03361 Methyl-accepting chemotaxis protein I
MLKRIKIVTSLLLVLAVFGLLQLTSGGLFFNALKNDKENFTVLQTIRQQQSTLNGSWVAL
LQTRNTLNRAGIRYMMDQNNIGSGSTVAELMQSASISLKQAEKNWADYEALPRDPRQSTA
AAAEIKRNYDIYHNALAELIQLLGAGKINEFFDQPTQGYQDGFEKQYVAYMEQNDRLYDI
AVSDNNASYSQAMWILVGVMIVVLAVILAVWFGIKASLVAPMNRLIDSIRHIAGGDLVKP
IEVDGSNEMGQLAESLRHMQGELMRTVGDVRNGANAIYSGASEIATGNNDLSSRTEQQAA
SLEETAASMEQLTATVKQNAENARQASHLALSASETAQRGGKVVDNVVQTMRDISTSSQK
IADIISVIDGIAFQTNILALNAAVEAARAGEQGRGFAVVAGEVRNLAQRSAQAAREIKSL
IEDSVGKVDVGSTLVESAGETMAEIVSAVTRVTDIMGEIASASDEQSRGIDQVGLAVAEM
DRVTQQNAALVEESAAAAAALEEQASRLTEAVAVFRIQQQQREASAVVKTVTPATPRKMA
VADSEENWETF
>LFGLNPFC_03362 Pyruvate/proton symporter BtsT
MDTKKIFKHIPWVILGIIGAFCLAVVALRRGEHVSALWIVVASVSVYLVAYRYYSLYIAQ
KVMKLDPTRATPAVINNDGLNYVPTNRYVLFGHHFAAIAGAGPLVGPVLAAQMGYLPGTL
WLLAGVVLAGAVQDFMVLFISSRRNGASLGEMIKEEMGPVPGTIALFGCFLIMIIILAVL
ALIVVKALAESPWGVFTVCSTVPIALFMGIYMRFIRPGRVGEVSVIGIVLLVASIYFGGV
IAHDPYWGPALTFKDTTITFALIGYAFVSALLPVWLILAPRDYLATFLKIGVIVGLALGI
VVLNPELKMPAMTQYIDGTGPLWKGALFPFLFITIACGAVSGFHALISSGTTPKLLANET
DARFIGYGAMLMESFVAIMALVAASIIEPGLYFAMNTPPAGLGITMPNLHEMGGENAPII
MAQLKDVTAHAAATVSSWGFVISPEQILQTAKDIGEPSVLNRAGGAPTLAVGIAHVFHKV
LPMADMGFWYHFGILFEALFILTALDAGTRSGRFMLQDLLGNFIPFLKKTDSLVAGIIGT
AGCVGLWGYLLYQGVVDPLGGVKSLWPLFGISNQMLAAVALVLGTVVLIKMKRTQYIWVT
VIPAVWLLICTTWALGLKLFSTNPQMEGFFYMASQYKEKIANGTDLTAQQIANMNHIVVN
NYTNAGLSILFLIVVYSIIFYGFKTWLKVRNSDKRTDKETPYVPIPEGGVKISSHH
>LFGLNPFC_03363 hypothetical protein
MFGNLGQAKKYLGQAAKMLIGIPDYDNYVEHMKTNHPDKPYMSYEEFFRERQNARYGGDG
KGGMRCC
>LFGLNPFC_03364 P-loop guanosine triphosphatase YjiA
MNPIAVTLLTGFLGAGKTTLLRHILNEQHGYKIAVIENEFGEVSVDDQLIGDRATQIKTL
TNGCICCSRSNELEDALLDLLDNLDKGNIQFDRLVIECTGMADPGPIIQTFFSHEILCQR

YLLDGVIALVDAVHADEQMNQFTIAQSQVGYADRILLTKTDVAGEAEKLRERLARINARA
PVYTVTHGDIDLGLLFNTNGFMLEENVVSTKPRFHFIADKQNDISSIVVELDYPVDISEV
SRVMENLLLESADKLLRYKGMLWIDGEPNRLLFQGVQRLYSADWDRPWGDEKPHSTMVFI
GIQLPEDEIRAAFAGLRK
>LFGLNPFC_03365 Mrr restriction system protein
MTVPTYDKFIEPVLRYLATKPEGAAARDVHEAAADALGLDDSQRSKVITSGQLVYKNRAG
WAHDRLKRAGLSQSLSRGKWCLTPAGFDWVASHPQPMTEQEMNHLAFNFVNVKLKSRPDA
VDLDPKADSPDHEELAKSSPDDRLDQALKELRDAVADEVLENLLQVSPSRFEVIVLDVLH
RLGYGGHRDDLQRVGGTGDGGIDGVISLDKLGLEKVYVQAKRWQNTVGRPELQAFYGALA
GQKAKRGVFITTSGFTSQARDFAQSVEGMVLVDGERLVHLMIENEVGVSSRLLKVPKLDM
DYFE
>LFGLNPFC_03366 hypothetical protein
MMNKSNFEFLKGVNDFTYAIACAAENNYPDDPNTTLIKMRMFGEATAKHLGLLLNIPPCE
NQHDLLRELGKIAFVDDNILSVFHKLRRIGNQAVHEYHNDLDDAQMCLRLGFRLAVWYYR
LVTKDYDFPVPVFVLPERGENLYHQEVLTLKQQLEQQAREKAQTQAEVEAQQQKLVALNG
YIAILEGKQQETEAQTQARLAALEAQLAEKNAELAKQTEQERKAYHKEITDQAIKRTLNL
SEEESRFLIDAQLRKAGWQADSKTLRFSKGARPEPGVNKAIAEWPTGKDETGNQGFADYV
LFVGLKPIAVVEAKRKNIDVPGKLNESYRYSKCFDNGFLRETLLEHYSPDEVHEAVPEYE
TSWQDTSGQQRFKIPFCYSTNGREYRAAMKTKSGIWYRDVRDTRNMSKALPEWHRPEELL
EMLGSEPQKQNQWFADNPGMSELGLRYYQEDAVRAVEKAIVKGQQEILLAMATGTGKTRT
AIAMMFRLIQSQRFKRILFLVDRRSLGEQALGAFEDTRINGDTFNSIFDIKGLTDKFPED
STKIHVATVQSLVKRTLQSDEPMPVARYDCIVVDEAHRGYILDKEQTEGELQFRSQLDYV
SAYRRILDHFDAVKIALTATPALHTVQIFGEPVYRYTYRTAVIDGFLIDQDPPIQIITRN
AQEGVYLSKGEQVERISPQGEVINDTLEDDQDFEVADFNRGLVIPAFNRAVCNELTNYLD
PTGSQKTLVFCVTNAHADMVVEELRTAFKKKYPQLEHDAIIKITGDADKDARKVQTMITR
FNKERLPNIVVTVDLLTTGVDIPSICNIVFLRKVRSRILYEQMKGRATRLCPDVNKTSFK
IFDCVDIYSTLESVDTMRPVVVRPKVELQTLVNEITDSETYKITEADGRSFAEHSHEQLV
AKLQRIIGLATFNRDRSETIDKQVRRLDELCQDAAGVNFNGFASRLREKGPHWSAEVFNK
LPGFIARLEKLKTDINNLNDAPIFLDIDDEVVSVKSLYGDYDTPQDFLEAFDSLVQRSPN
AQPALQAVINRPRDLTRKGLVELQEWFDRQHFEESSLRKAWKETRNEDIAARLIGHIRRA
AVGDALKPFEERVDHALTRIKGENDWSSEQLSWLDRLAQALKEKVVLDDDVFKTGNFHRR
GGKAMLQRTFDDNLDNLLDKFSDYIWDELA
>LFGLNPFC_03367 Type I restriction enzyme EcoKI M protein
MNNNDLVAKLWKLCDNLRDGGVSYQNYVNELASLLFLKMCKETGQEAEYLPEGYRWDDLK
SRIGQEQLQFYRKMLVHLGEDDKKLVQAVFHNVSTTITEPKQITALVSNMDSLDWYNGAH
GKSRDDFGDMYEGLLQKNANETKSGAGQYFTPRPLIKTIIHLLKPQPREVVQDPAAGTAG
FLIEADRYVKSQTNDLDDLDGDTQDFQIHRAFIGLELVPGTRRLALMNCLLHDIEGNLDH
GGAIRLGNTLGSDGENLPKAHIVATNPPFGSAAGTNITRTFVHPTSNKQLCFMQHIIETL
HPGGRAAVVVPDNVLFEGGKGTDIRRDLMDKCHLHTILRLPTGIFYAQGVKTNVLFFIKG
TVANPNQDKNCTDDVWVYDLRTNMPSFGKRTPFTDEHLQPFERVFGEDPHGLSPRTEGEW
SFNAEETEIADSEENKNTDQHLATSRWRKFSREWIRTAKSDSLDISWLKDKDSIDADNLP
EPDVLAAEAMGELVQALSELDALMRELGASDEADAQRQLLEEAFGGVKE
>LFGLNPFC_03368 Type-1 restriction enzyme EcoKI specificity protein
MSAGKLPEGWEQIEIGDIADVISGGTPKSGVAENFAPSGEGVAWLTPADLSGYKEKYISH
GARDLTTLGYSSCSAKLMPKGTILFSSRAPIGYVAIAANEIATNQGFKSFAFPSDIFPDY
AYYFLRNIRHIAEEMGTGTTFKEISGSSAKTLPFVLVPFAEQKIIAEKLDTLLAQVDSTK
ARLEQIPQILKRFRQAVLGAAVRGKLTEDWRDNSSLSGWREGKLGEFIKKPSYGTSSKSN
KEGLIPVLRMGNLQGGKLDWTDLVYTSDTIEIEKYKLEYNDVLFNRTNSPELVGKTAIYK
SEQPAIYAGYLIRVQCLPDLNPDYLNYHLNSILGRQYCYSVKSDGVSQSNINAQKLIAYP
ITVPPLPEQAEIVRRVEQLFAYADTIEKQVNNALARVNNLTQSILAKAFRGELTAQWRAE
NPELISGENSAAALLEKIKAERAASGGKKASRKKS
>LFGLNPFC_03369 Toxic protein SymE
MTDTHSIAQPFEAEVSPANNRQLTVSYASRYPDYSRIPAITLKGQWLEAAGFATGTAIDV
KVMEGCIVLTAQPPAAEESELMQSLRQVCKLSARKQRQVQEFIGVIAGKQKVA
>LFGLNPFC_03370 hypothetical protein
MSPRQIRGYYCEIIASRKDVRMKATEARLLDFLKRSQQFVITIYQRTYSWTEQQCRQLWD
DIIRAGKRDDISAHFIGSVVYIEQGLYQVSGISPLLVIEGQQRLTTAMLLIEALSRHLGE
DEVFDGFSAMKLRNYYLLNPYESGEKGFKLLLTETDKDSLLALIKQRPMPENYSHRIMEN
FTFFDEQITKLGDDLIPLCRGLAKLLIVDVALNRGQDNPQLIFESMNSTGKALSQADLVR
NFILMGLEPEHQTRLYEDHWRPMEVAFGQQGYSEYFDSFMRHYLTVKTGEIPRTDEVYEA
FKLHARSQSVAEKGVDRLVEDIHIYAEYYCAMALGKESDKSLATAFQDLRELKVDVAYPF
LLALYHDYKNGVLSHEDFLSIIRLIESYVFRRAVCAIPTNSLNKTFATFYKVINKEKYLE
SIQVHFLNLPSYRRFPNDDEFKRELKVRDLYNFRSRSYWLRRLENDKRRERVEEFTIEHI
MPQNENLSAKWREELGSDWQRIHKELLHTLGNLTLTRYNSRYSDRPFAEKRDIEDGFKHS

PLYLNIGLGQCEKWDEAAIRARADRLADLAVQVWQAPALPEEVLAVYRAQPENKTSYSLS
DYPFLADGSHSRVLFDHLRDEVMRLDAGITQEVLKLYIAFKAETNFVDVVPQKSRLRLSL
NMQFHELVDPKGIAKDVTNVGRWGNGDVEIGFSDLAQLPYIMGLIRQAFEKQMESALV
>LFGLNPFC_03371 ISNCY family transposase ISRor2
MTNFTTSTPHDALFKSFLTHSDTARDFMEIHLPKDLRELCDLDSLKLESASFVDEKLRAL
HSDILWSVKTREGDGYIYVVIEHQSREDIHMAFRLMRYSMAVMQRHIEHDKRRQLPLVIP
MLFYHGSRSPYPWSLCWLDEFADPTTARKLYTAAFPLVDITVVPDDEIVQHRRVALLELI
QKHIRQRDLMGLIDQLVVLLVTECANDSQITALLNYILLTGDEARFNEFISEITRRLPQQ
RERIMTIAERIHNDGYIKGEQRILRLFLQNGVDPEWIQKITGLSAEQIQALEQPLPEREP
YSWLKS
>LFGLNPFC_03372 hypothetical protein
MNKLIELRRAKMLALSLLLIAAATFVVTLFLPPNFWVSGVKAIAEAAMVGALADWFAVVA
LFRRVPIPIISRHTAIIPRNKDRIGENLGQFVQEKFLDTQSLVALIRRHEPALLIGNWFS
QPENARRVGQHLLQIMSGFLELTDDARIQRLLKRAVHRAIDKVDLSGTSALMLESMTKND
RHQVLLDTLIAQLIALLQRDKSRKFIAQQIVRWLESEHPLKAKILPTEWLGEHSAELVSD
AVNSLLDDISRDRAHQIRHAFDHATFALIDKLKNDPEMAARADAVKSYLKEDEAFNRYLS
ELWGDLREWLKADINSEDSRVKERIARAGQWFGETLIADDALRASLNGHLEQAAHRVAPE
FSAFLTRHISDTVKSWDARDMSRQIELNIGKDLQFIRVNGTLVGGCIGLILYLLSQLPAL
FPLGNL
>LFGLNPFC_03373 (R)-phenyllactyl-CoA dehydratase beta subunit
MSLVTDLPAIFDQFSEARQKGFLTVMDLKERGIPLVGTYCTFMPQEIPMAAGAVVVSLCS
TSDETIEEAEKDLPRNLCPLIKSSYGFGKTDKCPYFYFSDLVVGETTCDGKKKMYEYMAE
FKPVHVMQLPNSVKDDASRALWKAEMLRLQKTVEERFGHEISEDALRDAIALKNRERRAL
ANFYHLGQLNPPALSGSDILKVVYGATFRFDKEALINELDAMTARVRQQWEEGQRLDPRP
RILITGCPIGGAAEKVVRAIEENGGWVVGYENCTGAKATEQCVAETGDVYDALADKYLAI
GCSCVSPNDQRLQMLSQMVEEYQVDGVVDVILQACHTYAVESLAIKRHVRQQHNIPYIAI
ETDYSTSDVGQLSTRVAAFIEML
>LFGLNPFC_03374 (R)-phenyllactate dehydratase activator
MTYSIGIDSGSTTTKGILLADGVITRRFLVPTPFRPATAITEAWETLREGLETTPFLTLT
GYGRQLVDFADKQVTEISCHGLGARFLEPATRAVIDIGGQDSKVIQLDDDGNLCDFLMND
KCAAGTGRFLEVISRTLGTSVEQLDSITENVTPHAITSMCTVFAESEVISLRSAGVAPEA
ILAGVINAMARRSANFIARLSCEAPILFTGGVSHCQTFARMLESHLGMPVNTHPDAQFAG
AIGAAVIGQRQRKRA
>LFGLNPFC_03375 hypothetical protein
MKEYLFLFHSTVGVIQTRKALQAAGMTFRVSDIPRDLRGGCGLCIWLTCPPGEEIQWVIP
GHTESVYCQQDGGWRCIAHYGISPR
>LFGLNPFC_03376 putative protein YjiK
MVAGAALHITGFLRVKDSLRSLRILLDILLICLKGITVTKSISLSKRIFVIVILFVIVAV
CTFFVQSCARKSNHAASFQNYHATIDGKEIAGITNNISSLTWSAQSNTLFSTINKPAAIV
EMTTNGDLIRTIPLDFVKDLETIEYIGDNQFVISDERDYAIYVISLTPNSEVKILKKIKI
PLQESPTNCGFEGLAYSRQDHTFWFFKEKNPIEVYKVNGLLSSNELHISKDKALQRQFTL
DDVSGAEFNQQKNTLLVLSHESRALQEVTLVGEVIGEMSLTKGSRGLSHNIKQAEGVAMD
ASGNIYIVSEPNRFYRFTPQSSH
>LFGLNPFC_03377 hypothetical protein
MPSPSHPVERFSFSTALFGMLVLTLGMGLGRFLYTPMLPVMMAEGSFSFSQLSWIASGNY
AGYLAGSLLFSFGAFHQPSRLRPFLLASALASGLLILAMAWLLPFILVLLIRVLAGVASA
GMLIFGSTLIMQHTRHPFVLAALFSGVGIGIALGNEYVLAGLHFDLSSQTLWQGAGALSG
MMLIALTLLMPSKKHAITPMPLAKTEQQIMSWWLLAILYGLAGFGYIIVATYLPLMAKDA
GSPLLTAHLWTLVGLSIVPGCFGWLWAAKRWGALPCLTANLLVQAISVLLTLASDSPLLL
IISSLGFGGTFMGTTSLVMTIARQLSVPGNLNLLGFVTLIYGIGQILGPALTSMLGNGTS
ALASATLCGAAALFIAALISTAQLFKLQVVTS
>LFGLNPFC_03378 RNA 2'-phosphotransferase
MHLSADEATARKVGARHGSPVILTVKAQEMAKRGIPFWQAENGVWLTSTVAVEFLEW
>LFGLNPFC_03379 putative RNA 2'-phosphotransferase
MAKYNDKELAETSKFLSFVLRHKPEAIGIVLDREGWADIDKLILCAQKAGKRLTRALLDI
VVATSDKKRFSYSSDGTCIRAVQGHLTSQVAIGFIEKTPPQFLYHGTASRFLDEIKNKG
>LFGLNPFC_03380 hypothetical protein
MTQQGDAVAGELATEKVGVKGYLAFFLTIIFFSGVFSGTDSWWRVFDFSVLNGSFGQLPG
ANGATTSFRGAGGTGAKDGFLFALELAPSVILSLGIISITDGLGGLRAAQQLMTPVLKPL
LGIPGICSLALIANLQNTDAAAGMTKELAQEGEITERDKVIFAAYQTSGSAIITNYFSSG
VAVFAFLGTSVIVPLAVILVFKFVGANILRVWLNFEERRNPTQGAQA
>LFGLNPFC_03381 Inner membrane protein YjiG
MTTQVRKNVMDMFIDGARRGFTIATTNLLPNVVMAFVIIQALKITGLLDWVGHICEPVMA
LWGLPGEAATVLLAALMSMGGAVGVAASLATAGALTGHDVTVLLPAMYLMGNPVQNVGRC

LGTAEVNAKYYPHIITVCVINALLSIWVMQLIV
>LFGLNPFC_03382 Isoaspartyl dipeptidase
MIDYSAAGFTLLQGAHLYAPEDRGICDVLIANGKIIAVASNIPSDIVPDCTVVDLSGQIL
CPGFIDQHVHLIGGGGEAGPTTRTPEVALSRLTEAGVTSVVGLLGTDSISRHPESLLAKT
RALNEEGISAWMLTGAYHVPSRTITGSVEKDVAIIDRVIGVKCAISDHRSAAPDVYHLAN
MAAESRVGGLLGGKPGVTVFHMGDSKKALQPVYDLLENCDVPISKLLPTHVNRNVPLFEQ
ALEFARKGGTIDITSSIDEPVAPAEGIARAVQAGIPLARVTLSSDGNGSQPFFDDEGNLT
HIGVAGFETLLETVQVLVKDYDFSISDALRPLTSSVAGFLNLTGKGEILPGNDADLLVMT
PELRIEQVYARGKLMVKDGKACVKGTFETA
>LFGLNPFC_03383 HTH domain-truncated transcriptional regulator QseD
MTPLQLSEQGKIFHSQIRHLLQQLESNLAELRGGSDYAQRKIKIAAAHSLSLGLLPSIIS
QMPPLFTWAIEAIDVDEAVDKLREGQSDCIFSFHDEDLLEAPFDHIRLFESQLFPVCASD
EHGEALFNLAQPHFPLLNYSRNSYMGRLINRTLTRHSELSFSTFFVSSMSELLKQVALDG
CGIAWLPEYAIQQEIRSGQLVVLNRDELVIPIQAYAYRMNTRMNPVAERFWRELRELEIV
LS
>LFGLNPFC_03384 Malate-2H(+)/Na(+)-lactate antiporter
MREKPSFYVALTPIIFMMIVLVVGIGVMGWPASVCLLISAAFSCIIAMAKLKYTWDEIQG
FIIDKISAVMAPVLIVIFVGFMVATWSYAGTLPMLVYYGMLLVAPAWLYAIAFFLNAVLS
YVSGASWGSVASIGVALMGIGSGLHADLPILAAAIVTGAYFGDKLSPLSDTTNLTSAVTK
TKLYDLIKYLLWTTIPSTIISLLFFTWLGMIHTSTGSISPESMQMLVQLKELYKFGLLPL
LPMLIVLCFTFLRLPTVPALLSSAFVAVLVGWLYQGFPLAEGIKATMSGFRLTMITDSTT
LLPSVKELLEQGGLNNQGGFLSFIICAMMFAGILTGTGMMDVTLQSAANKIKSAFGAILA
SGVLAVIINLLTGSDGLNKIVISELMMKKFEDLNLSPLVLARTLEDFGTMSAPIIPWSAA
GLYMATTLGVPTFSYLPYCVFCFCSMIFALIYASTGFRLLRFNEAKA
>LFGLNPFC_03385 Thiamine thiazole synthase
MEFYLEPARNIPVLATTEVLVVGGGPSGIAAAMSAAREGAATMLIERFGCFGGMMTTAGV
ESIAWWRHENTVESGGLAREIEETAKSMGASSPEPQSNSQAINAERFKLVADAMLEQAGV
RRVLHITAVDVIKQGNNLLGVITESKSGRQAILANVIIDCTGDADIAWFAGAPFIKRERE
ELMCMTTVFSCANINKNAFMQNINSTEPKYGDWGADEENKNWSYDVHESCRDMFSPYLGK
VFAKGKSAGIIPKDVTLGGSWSTVTEYGDANYLNVVSIPAVDCTDVFDLTRAEIEGRKQA
MQAIEALRQFQPGFEQAQLKNFGMTVGTRESRHIIGRVQLTENDICNEGRHADSIGVFPE
FIDGNGHLKLPLEANYFQIPYGVMIPQQVENLLVCGRAIDADNFAYATIRNMGCCIVTGE
GAGTAAAIAIKNNTTVSQVDIQTVQERLQQNGVKVF
>LFGLNPFC_03386 PTS-dependent dihydroxyacetone kinase operon regulatory protein
MNKESYHNDLKNKWKMFVKHGWVATNSTNHVMLRSWQKCLKHCDPRHWNTPVKASGQTLQ
TIFSRNEEFIRISQRVVEDHFTLAGDDRLAFLIIDPHGWVLSLNAAGDYSSQLRELGIES
GMSWAEDGIGTNVYSLCRETNLYTQLEGAEHFSEQLHCYAMSAAPVIDYYGNIHGYIVCI
IETTAELVKLKTSYSCATEIANYIYIENEQKLINKVLCQHNAVIECMDDGFICWNSHSLI
TMVNSQAQTLLNIDKESLIGQNIRKGFVFPPILNEAITQRNKLSQKQIVLECRGEFIELM
VTLRPLSDGSFLLFLHPLDKIRKIAQQQISTNANFTFDSLHAASGGMKQVLLIARRAIKS
ISPILINGEEGVGKLSLAMAIHNESEQRDGPFISVDCQMLSPENILHELLGSDVGPSPSK
FELAHNGTLYLDKVEYLSGEVQSVLLKVLKTGLVTRSDSHRLIPVRFRLITCTSSSLREY
VQQGAFSRQLYYEISMNEIEIPPLRKRREDLKQMIDDIIDKYQERTRKKMTITPDANSVL
LEYRWPGNISEFKNRMEKVFINCNRLVLGLENIPLDIRQNNSSGDDDIPHLTSLAELEMQ
AIEHTCRVCEWNLTKAAEVLKIGRTTLWRKLKIYNLYPNVEHAD
>LFGLNPFC_03387 Glyoxylate carboligase
MARMRAIEAAVEILKKEGISVAFGVPGAAINPLYAAMKKSGGIDHILARHVEGASHMAEG
YTRSQNGNIGVCIGTSGPAGTDMITGLYSASADSIPILCITGQAPVGKLHKEDFQAVDIE
AIATPVTKMARTILEAGQLPGIFQKAFWEMRSGRPGPVLLDLPFDVQMTEIEFDIDLYQP
LIPWQPKATRAQAVRALEMLNDAEKPVIIAGGGIINAEASELLREFVELTGVPVIQTLRG
WGALSDDHPLMIGRMGCQAGHRYGNASYLASDFVFGIGNRWANRHTGAIETYTEGRKFIH
VDIEPAQIGRIFAPDLGIVSDAESALTLFIQVARDMKSRGELKDRSRWIAECAERKRTML
RRSDFDCNPIKPQRVYHEMNKVFGPETRYISTIGLAQIAANQFLHVYRPRHWINACQAGP
LGWTMPAALGAVKADPSVPVVAISGDYDFQFLIEELAVGAQFNLPYIHILLNNAYLGLIR
QSQRAFDIDYCVQLSFENINAPEINGYGVDHKAVVEGLGCKAIRVFASQDIAPALQEAQR
LRDEFHVPVVVEIITERVTNIAMGPDINKVTEFEEILDL
>LFGLNPFC_03388 Hydroxypyruvate isomerase
MAKFAANLSMLFTEHPFIERFAQASHAGFHGVEYLFPYDFSTDELASQLHQHNLTQVLFN
LPAGNWQEGERGIACHPSRAKEFQEGVCRAIEYAQALNCSQVNCLAGKHPGGYSHEQCHE
TLVENLRYAVDKLASHGIKLVLEAINTKDIPGFFVNNTRQALNIIHDVNHPDFRYQYDIY
HMQIMEGNIATTIKNNLNNIEHIQLADNPGRHEPGTGEINYPWLLNYIDQIGYQGWIGCE
YVPSTTTTESLRWLKNETQF
>LFGLNPFC_03389 2-hydroxy-3-oxopropionate reductase
MNIGFIGTGIMGKPMAYNLQQAGHTLYFSAHFEPAPQEFIGERGIVCSTPREVAQECEVI

ITMLPDTPHVEDVLFHPNYGVIHGLSHGKIVIDMSSISPVATKAFAQRIIAGGAEYIDAP
VSGGEVGAKAGTLSIMVGGCEEVYLQIKPILELMGKNITLVGNVGDGQTCKVANQIIVAL
NIEAVAEALLFASKSGADPARVREALMGGFASSRVLEVHGERMIKGTFEPGFRISLHQKD
LNLALENARLLNTPLPNTATTQQLFSACAALGGKEWDHSALIRALETQANFTIRK
>LFGLNPFC_03390 Glycerate 3-kinase
MKIVISPDSFKECLPAWKVAEALATGWRKVLPGSQLVCLPVADGGEGTLETLIHATDGTF
YTKKVTGPLGESIHAQYGILGNQTTAVIELAQASGLELVSPVQRSPLYTTSFGTGELILA
ALEHNIDTVILCLGGSATNDGGIGLMSALGASFTDAEGLSVSVNGMGLAAIHHIDLQHLD
PRLKNVKFIAACDVTNPLTGDNGATRVFAQQKGASANDLEQLEQGMKNYARCIYRCCGKD
VDTIPGSGAAGGVGAALMAFLDARLQPGISLVLEAIQYTQHLKYAALAIVGEGKLDSQSL
NGKAPVGAAKVAQMMGVPVIAIAGYIDDQLDLNELRQCGIEACFSVVNGPCNLPTALSQG
ENNLIRLGENLAGYFRAILS
>LFGLNPFC_03391 L-carnitine/gamma-butyrobetaine antiporter
MHYLKKRFSLIELNVFIPAILFIAVIILCLTIYPQDTSRYINKIHHFLTWEMGGIFLVMT
FLVVLCCLWLAFSRYGDILLGQSGEKPDFSLLTWLGLIFTSGTGGSLLYLASVEWIWIIQ
QPPFGATAGSAQAARWASVYGMFHWGPSAWAWYLICAVPIGWFMHVKKTNSLKVSDLCRG
CLGARADGFCGHCVNFFYMFGLLGGAVTSLALGTPMISAVFCHVFHLDPAGQFINVMVIF
IWTLVPLFILFFGLKKGVAWASNWNIRADILMLLAILICGPTAFILNQSIDGLGLMLQNF
VAMSLSTDAIGRSGFPQMWTVFYFSWWVVYAIPFGLFIARISKGRTIRQLIVCGTLAGSL
GCMVFYMVLANFGLSLQTTHVIDFVPILNEQGRGVVVSRLLEQLPASQVFLVAFGAIALI
SYITGHCTVGYALGFATQKRADSESEPAFWNVAFWLIMTGIVAITLYLLDAQSLQPLQTV
SILAGLPLCGVVFILLKSFLTQLAAEEKNARDE
>LFGLNPFC_03392 hypothetical protein
MRDDDLEHLSANYWITTVLCGLFLGIFLFMLQQHQRGLALYCIAALSLICAFFSLRLSRQ
LQHMKKERRNIRPH
>LFGLNPFC_03393 Thiamine thiazole synthase
MVDMINESARQTPVIAQTDVLVIGGGPAGLSAAIAAGRLGARTMIVERYGSLGGVLTQVG
VESFAWYRHPGTEDCEGICREYEGRARALGFTRPEPQSISEVIDTEGFKVVADQMITESG
VEPLYHSWVVDVIKDGDTLCGVIVENKSGRGAILAKRIVDCTGDADIAARAGAPWTKRSK
DQLMGVTVMFSCAGVDVARFNRFVAEELKPTYADWGKNWTIQTTGKEDPMFSPYMEDIFT
RAQQDGVIPGDAQAIAGTWSTFSESGEAFQMNMVYAFGFDCTDVFDLTKAEIAGRQQALW
AIDALRHYVPGFENVRLRNFGATLGTRESRLIEGEIRIADDYVLNQGRCSDSVGIFPEFI
DGSGYLILPTTGRFFQIPYGCLVPQKVENLLVAGRCISAGVVAHTSMRNMMCCAVTGEAA
GTAAVVSLQQNCTVRQVAIPDLQNTLQQQGVRLA
>LFGLNPFC_03394 Inner membrane protein YihN
MTSNWKRWITLALCGIAGSAIYKLPYLRETYYDAMQQATGATNAELGFLMTAYGLVNFLL
YLPGGWAADRFSARKLMTFSLISTGISGFYYATFPSYTMICLLHALWAVTTVFTFWAVCV
RIIRTLGTSEEQGRLYGYWFLGKGLTSIVLGFLSVPVFAKFGEGVDGLRATIIFYSVVTI
LAGVLAWFVCQDETHSEDKANFRLADMAFVLKMPTVWLAGVVTFCMWSIYIGFGMVTPYL
TQILHMGESEVAVASILRAYVLFAMGGLIGGQLADRCASRTRFMIYAFIGMIVFTTVYFF
LPGESRYVTIALANMVALGVFIYSANAVFFSIIDEVRIPAKVTGTAAGLISLLTYFPEIY
CYTMVGNMVDRKPGIAGYQDVFLFMLVCAFIGLIAALVLQRVNRKSKNQVNEITQNNADC
AS
>LFGLNPFC_03395 Glycerol dehydrogenase
MDKIIISPAKYIQGNGSLDNIATYAASLGTEPLIIADEFVTGLVGDRVSQSFARENIIAD
FDVFCGECSQNEISRIRKKFNQRKYNVVIGIGGGKTLDTAKAVAYYQKIPVVVVPTIAST
DAPTSSLAVIYTPDGQFSEYLFFPKNPDMVIMDTGVISAAPVRLLVAGMGDALSTWFEAR
ANQASGKATMAGGASTLAALAIARLCYTTLLEDGYKAKVAVEQGVSTKAVENIIEANTYL
SGIGFESSGLAAAHAIHNGMTQLEECHHCYHGEKVAFGVLVQLVLENAPQEEIETVLNFC
HSVGLPTNLHMLGVKEINKDKLRAVAKAATAEGETIHNMPFVVTAQDVLCAILTAHHLGL
>LFGLNPFC_03396 PEP-dependent dihydroxyacetone kinase, dihydroxyacetone-binding subunit DhaK
MKKLINQIDSVVTEQMEGLIATWPHLQANYAPRYVWCKQTDNAVALISGGGSGHEPLHAG
FVGMGMLTGACPGEIFTSPTPDQMIECAKAVDNGSGVLFFIKNYTGDILNFETAVEMLHE
EGIAVGTVIIDDDVAVKDSLYTAGRRGVAGTVFVEKIVGAAALQGYNLGQCEQLGKDVNN
ATRSFGIALSACTVPAAGKPSFELADNEIEFGVGIHGEPGIERRTLQDLNTLIDSVIAQL
LDNTPWRRTLRHWDRHAGGWIDASSMNESFDQNAEYIVLINGLGSTPESELYGVARVFMC
AAQRQGIKISRQLVGNYCTSLDMAGFSISLLKCTPEFLQLWDAPVNTPALRWGC
>LFGLNPFC_03397 PEP-dependent dihydroxyacetone kinase, ADP-binding subunit DhaL
MKIQNKHVIAWLESCAAHLTEQQDFLTALDRDIGDADHGLNMNRGFSAVKATLPDIERQH
IGNILKNTGMKLLSSVGGASGPLYGTLFIRASAAVGARTELTLEEWLACLEEGIAGVIAR
GKAEQGDKTLCDVWVPVLHEAKKNLQAGMSPSLLLNTMVQDAATAVDNTINMQAKKGRAS
YLGARSVGHQDPGATSSWLMIKAMQEGFAG
>LFGLNPFC_03398 hypothetical protein

MVAIVIVSHSLRLAQGVEELALQMSGGDVPLAIAAGIDDPQNPIGTDAIAIMSAIESVWS
PDGVLVLMDMGSALLSTEMALELLSEEQRSAIYLLAAPVVKGAMSAVTSAAAGLSVTEII
AEVDLALCAKQQQLTPSSTAGEVIPVISPVNHCDEWQTFCWTIRNPHGIHARPAASILKV
SAQYSANIIVIKGDKRASTRSLNELAMLGVRCGDEIIFQAEGTDASDALKAIETLAKNHF
GEAHLLASSEIPLTETPVVSAPVDGAISGLSVQNGIAIGPVKWFTCERPEITQRTVDSPQ
EELSRIESAIDIVVCELADKAAGPEGDIFAAHKMMLEDPEINRQLQQRLAKGKQAEFAWL
EVMQALAEQYCQAETLYLREREADIRDLTRQVLNQLCGVSEQHFITTAPCILLANDLLPS
QITSLNKAHILGICLHNGGTTSHTAILARAMGIPAIVKAAITPQNVRDNDTVILDGETGR
LWLQPDEVTRLDLLQRAEAWRQQRDRQLADAMLPAVTQGGRKISVLANIGDLQDIEAALS
HGAEGVGLLRTEFLFHESATLPDEEEQFRVYCSVAQAFGDKPVTIRTLDIGGDKPLPSYP
LPAEDNPFLGLRGIRLCLAHPQIFIPQLRALLRAGKEYPTLQIMLPMVSTLEEVNAVKTL
IQTQAKLLGLTAENLPALGIMIEVPAAVMIAEKLASEVDFFSIGTNDLTQYIMAADRGNS
TVAKLVDYRNDAVINAIAMVCQAGRNNEIPVSMCGEMAGDTQQTARLLTMGIDKLSASPS
RLPALKAAIRASH
>LFGLNPFC_03399 Anti-adapter protein IraD
MMRQSVQTVLPESTGNNTLSLRDSVCRDLFQLFSSPHSPLPILLVSGMPEWQGHNQSDKL
LQSWYCRQLRSALLFHEPRIAALQVNLKEAYCHELAISLEMMLYHDDEPLTFDLVWQKGN
WHRTMPQ
>LFGLNPFC_03400 Pyruvate dehydrogenase complex repressor
MKSATSAQRPYQEVGAMIRDLIIKTPYNPGERLPPEREIAEMLDVTRTVVREALIMLEIK
GLVEVRRGAGIYVLDSSGSHNADSPDANVCNDAGPFELLQARQLLESNIAEFAALQATRE
DIVKMRKALQLEERELASSAPGSSESGDMQFHLAIAEATHNSMLVELFRQSWQWRENNPM
WIQLHSHLDDSLYRKEWLGDHKQILAALIKKDARAAKLAMWQHLENVKQRLLEFSNVDDI
YFDGYLFDSWPLDKVDA
>LFGLNPFC_03401 Polyol:NADP oxidoreductase
MTTIVDSNLPVARPSWDHSRLESRIVHLGCGAFHRAHQALYTHHLLESTDSDWGICEVNL
MPGNDRVLIENLKKQQLLYTVAEKGAESTELKIIGSMKEALHPEIDGCEGILNAMARPQT
AIVSLTVTEKGYCADAASGQLDLNNPLIKHDLENPTAPKSAIGYIVEALRLRREKGLKAF
TVMSCDNVRENGHVAKVAVLGLALARDPQLAAWIEENVTFPCTMVDRIVPAATPETLQEI
ADQLGVYDPCAIACEPFRQWVIEDNFVNGRPDWDKVGAQFVADVVPFEMMKLRMLNGSHS
FLAYLGYLGGYETIADTMTNPAYRKAAFALMMQEQAPTLSMPEGTDLNAYATLLIERFSN
PSLRHRTWQIAMDGSQKLPQRLLDPVRLHLQNGGSWRHLALGVAGWMRYTQGVDEQGNAI
DVVDPMMAEFQKINAQYQGADRVKALLGLSGIFADDLPQNADFVGAVTAAYQQLCERGAR
ECVAAL
>LFGLNPFC_03402 Mannonate dehydratase
MEQTWRWYGPNDPVSLADVRQAGATGVVTALHHIPNGEVWSVEEILKRKAIVEDAGLVWS
VVESVPIHEDIKTHTGNYEQWIANYQQTLRNLAQCGIRTVCYNFMPVLDWTRTDLEYVLP
DGSKALRFDQIEFAAFEMHILKRPGAEADYTEEEIAQAAERFATMSDEDKARLTRNIIAG
LPGAEEGYTLDQFRKHLELYKDIDKAKLRENFAVFLKAIIPVAEEVGVRMAVHPDDPPRP
ILGLPRIVSTIEDMQWMVDTVNSMANGFTMCTGSYGVRADNDLVDMIKQFGPRIYFTHLR
STMREDNPKTFHEAAHLNGDVDMYEVVKAIVEEEHRRKAEGKEDLIPMRPDHGHQMLDDL
KKKTNPGYSAIGRLKGLAEVRGVELAIQRAFFSR
>LFGLNPFC_03403 hypothetical protein
MHVLNILWVVFGIGLMLVLTMTAGSDTFPTLAMPLRLL
>LFGLNPFC_03404 Type 1 fimbrin D-mannose specific adhesin
MKRVITLFAVLLMGWSVNAWSFACKTANGTAIPIGGGSANVYVNLAPAVNVGQNLVVDLS
TQIFCHNDYPETITDYVTLQRGAAYGGVLSSFSGTVKYNGSSYPFPTTSETPRVVYNSRT
DKPWPVALYLTPVSSAGGVAIKAGSLIAVLILRQTNNYNSDDFQFVWNIYANNDVVVPTG
GCDVSARDVTVTLPDYPGSVPIPLTVYCAKSQNLGYYLSGTTADAGNSIFTNTASFSPAQ
GVGVQLTRNGTIIPANNTVSLGAVGTSAVSLGLTANYARTGGQVTAGNVQSIIGVTFVYQ
>LFGLNPFC_03405 Protein FimG
MKWCKRGYLLAAMLAFASATIQAADVTITVNGKVVAKPCTVSTTNATVDLGDLYSFSLMS
AGAASAWHDVALELTNCPVGTSRVTASFSGAADSTGYYKNQGTAQNIQLELQDDSGNTLN
TGATKTVQVDDSSQSAHFPLQVRALTVNGGATQGTIQAVISITYTYS
>LFGLNPFC_03406 Protein FimF
MRNKPFYLLCAFLWLAVSHALAADSTITIRGYVRDNGCSVAAESTNFTVDLMENAAKQFN
NIGATTPVVPFRILLSPCGNAVSAVKVGFTGVADSHNANLLALENTVSAAAGLGIQLLNE
QQNEIPLNAPSSAISWTTLTPGKPNTLNFYARLMATQVPVTAGHINATATFTLEYQ
>LFGLNPFC_03407 Outer membrane usher protein FimD
MSYLNLRLYQRNTQCLHIRKHRLAGFFVRLFVACAFAAQAPLSSAELYFNPRFLADDPQA
VADLSRFENGQELPPGTYRVDIYLNNGYMATRDVTFNTGDSEQGIVPCLTRAQLASMGLN
TASVSGMNLLADDACVPLTSMIHDATAQLDVGQQRLNLTIPQAFMSNRARGYIPPELWDP
GINAGLLNYNFSGNSVQNRIGGNSHYAYLNLQSGLNIGAWRLRDNTTWSYNSSDSSSGSK
NKWQHINTWLERDIIPLRSRLTLGDGYTQGDIFDGINFRGAQLASDDNMLPDSQRGFAPV

IHGIARGTAQVTIKQNGYDIYNSTVPPGPFTINDIYAAGNSGDLQVTIKEADGSTQIFTV
PYSSVPLLQREGHTRYSITAGEYRSGNAQQEKPRFFQSTLLHGLPAGWTIYGGTQLADRY
RAFNFGIGKNMGALGALSVDMTQANSTLPDDSQHDGQSVRFLYNKSLNESGTNIQLVGYR
YSTSGYFNFADTTYSRMNGYNIETQDGVIQVKPKFTDYYNLAYNKRGKLQLTVTQQLGRT
STLYLSGSHQTYWGTSNVDEQFQAGLNTAFEDINWTLSYSLTKNAWQKGRDQMLALNVNI
PFSHWLRSDSKSQWRHASASYSMSHDLNGRMTNLAGVYGTLLEDNNLSYSVQTGYAGGGD
GNSGSTGYATLNYRGGYGNANIGYSHSDDIKQLYYGVSGGVLAHANGVTLGQPLNDTVVL
VKAPGAKDAKVENQTGVRTDWRGYAVLPYATEYRENRVALDTNTLADNVDLDNAVANVVP
TRGAIVRAEFKARVGIKLLMTLTHNNKPLPFGAMVTSESSQSSGIVADNGQVYLSGMPLA
GKVQVKWGEEENAHCVANYQLPPESQQQLLTQLSAECR
>LFGLNPFC_03408 Chaperone protein FimC
MSNKNVNVRKSQEITFCLLAGILMFMAMVVAGRAEAGVALGATRVIYPAGQKQVQLAVTN
NDENSTYLIQSWVENADGVKDGRFIVTPPLFAMKGKKENTLRILDATNNQLPQDRESLFW
MNVKAIPSMDKSKLTENTLQLAIISRIKLYYRPAKLALPPDQAAEKLRFRRSANSLTLIN
PTPYYLTVTELNAGTRVLENALVPPMGESAVKLPSDAGSNITYRTINDYGALTPKMTGVM
E
>LFGLNPFC_03409 putative major fimbrial subunit LpfA
MFALAGNKWNTTLPGGNMQFQGVIIAEACRIEAGDKQMTVNMGQISSNRFHAVGEDSSPV
PFVIHLRECSTVVSERVGVAFHGVADGKNPDVLSVGEGPGIATNIGVALFDDEGNLVPIN
RPPANWKRLYSGSTSLHFIAKYRATGRRVTGGIANAQAWFSLTYQ
>LFGLNPFC_03410 Type-1 fimbrial protein, A chain
MKIKTLAIVVLSALSLSSTAALADTTPTTVNGGTVHFKGEVVNAACAVDAGSVDQTVQLG
QVRTATLKQAGATSSAVGFNIQLNDCDTTVATKAAVAFLGTAIDSTHPKVLALQSSAAGS
ATNVGVQILDRTGNELTLDGATFSAETTLNNGTNTIPFQARYFATGAATPGAANADATFK
VQYQ
>LFGLNPFC_03411 ISKra4 family transposase ISCep1
MSKRRYLTGKEVQAMMQAVCYGATGARDYCLILLAYRHGMRISELLDLHYQDLDLNEGRI
NIRRLKNGFSTVHPLRFDEREAVERWTQERANWKGADRTDAIFISRRGSRLSRQQAYRII
RDAGIEAGTVTQTHPHMLRHACGYELAERGADTRLIQDYLGHRNIRHTVRYTASNAARFA
GLWERNNLINEKLKREEA
>LFGLNPFC_03412 ISKra4 family transposase ISCep1
MKNKADNKKRNFLTHSEIESLLKAANTGPHAARNYCLTLLCFIHGFRASEICRLRISDID
LKAKCIYIHRLKKGFSTTHPLLNKEIQALKNWLSIRTSYPHAESEWVFLSRKGNPLSRQQ
FYHIISTSGGNAGLSLEIHPHMLRHSCGFALANMGIDTRLIQDYLGHRNIRHTVWYTASN
AGRFYGIWDRARGRQRHAVL
>LFGLNPFC_03413 putative N-acetylneuraminic acid outer membrane channel protein NanC
MKKAKILSGVLLLCFSSPLISQAATLDVRGGYRSGSHAYETRLKVSEGWQNGWWASMESN
TWNTIHDNKKENAALNDVQVEVNYAIKLDDQWTVRPGMLTHFSSNGTRYGPYVKLSWDAT
KDLNFGIRYRYDWKAYRQQDLSGDMSRDNVHRWDGYVTYHINSDFTFAWQTTLYSKQNDY
RYANHKKWATENAFVLQYHMTPDITPYIEYDYLDRQGVYNGRDNLSENSYRIGVSFKL
>LFGLNPFC_03414 N-acetylneuraminate epimerase
MNKTITALTIIMASFATNASVLPETPVPFKSGTGAIDNDTVYIGLGSAGTAWYKLDTQAK
DKKWTALAAFPGGPRDQATSAFIDGNLYVFGGIGKNSEGLTQVFNDVHKYNPKTNSWVKL
MSHAPMGMAGHVTFVHNGKAYVTGGVNQNIFNGYFEDLNEAGKDSTTIDKINAHYFDKKA
EDYFFNKFLLSFDPSTQQWSYAGESPWYGTAGAAVVNKGDKTWLINGEAKPGLRTDAVFE
LDFTGNNLKWNKLAPVASPDGVAGGFAGMSNDSLIFAGGAGFKGSRENYQNGKNYAHEGL
KKSYSADIHLWHNGKWDKSGELSQGRAYGVSLPWNNSLLIIGGETAGGKAVTDSVLISVK
DNKVTVQN
>LFGLNPFC_03415 putative 9-O-acetyl-N-acetylneuraminic acid deacetylase
MNAIISPDYYYVLTVAGQSNAMAYGEGLPLPDKEDAPHPRIKQLARFAHTHPGGPSCHFN
DIIPLTHCPHDVQDMQGYHHPLATNHQTQYGTVGQALHIARKLLPFIPDNAGVLIVPCCR
GGSAFTAGSEGTYSEPHGASHDACRWGSDTPLYQDLVSRTRAALAKNPQNKFLGVCWMQG
EFDLMTSDYASHPQHFNHMVDAFRRALKQYHSQLNKITDAPWFCGDTTWYWKENFPHAYE
AIYGNYQNNVLANIIFVDFQQQGERGLTNVPDEDPDDLSTGYYGSAYRSPENWTTALRSS
HFSAAARRGIISNRFVEAILQFWRER
>LFGLNPFC_03416 hypothetical protein
MASSKSLQQAIANIKIWHKGEQRAPHKPLLLLYVLAGYLNGHPRLFDYGSEIYEPLHSLL
ERFGPQRSQYRPDMPFWRLQGDGFWQLRNAELCSTAGSSRQPPVKELTEHRVAGGFDEQH
YALVTGNKKLINTLAQQILEAHFTESIQEEIADELGFDLQQIRKQRDPLFRKNVLRAYKY
QCAICGFNMRHDDTTVALEAAHIKWKQHGGPCEIPNGLALCAIHHKAFDKGSIGLDENMR
VLVSDAVNGGGIVERLFWDFDGKTITLPQVRKNYPFEGFVEWHRKEVFRG
>LFGLNPFC_03417 hypothetical protein
MHDINNSFFAATKCAGIWRKNTKKSHALILNLIMFCEMFLSSLSSLVVVNDPKKMKRIFP
LFIVSENINIHAEPDIECFRVIDRAFDEVANYTGRIFSYLRTHGPLKLTSCVNKQTLLSM

GGYLNEWNVFDSLSRVRDFFRLSSAVFTKLDNNIYSLEVDSFCLYRDYEIARNRLMMRAS
HLYSEVHEFSNKHFHLNSWVKDHMPSYLNSDGVFSSFHLSELENMSPDDLHEEYGNISLF
NWVHAYQCLVELSKEEMSKRFSSTKPIPLQLDRWLIIKSRESWLSFFQRKGIAADAAKKL
IDYFTFNSKSHDLNDCPFIPCMDGLCLMPALIANSSVTRSLMSLFGSKKISQASKGRFHE
QQFIKRVRDAGIKASPIDAHANYQCDCVILLDDCLIFTELKSNGQPIYYGKYYQQVCNIV
GDSSLIHDHNNKFMRSYFQQINRISEHYLNHLDVIIKEFELPSTWQPKGVYKLIVTTTML
GGKYHVDDTYVADKYALSSFFQRIPGVIYQTNENGKMAKNIIDGFECCEGEITIDKFIDY
LSSLPSINAVRKNIKKLTYSVRFNEKLVHHPYYDSWAFGPYIRKGND
>LFGLNPFC_03418 hypothetical protein
MFESVLRTTPYANRVAQERLFLCLKSPISVQYIGLPEKQFSILHFVVDGPKFALAGSCIV
HITWAGWFRYAT
>LFGLNPFC_03419 hypothetical protein
MMSDTTLSRPEVVSGHTDVIYSTSVCHILAVRKSTLLPIDTIIRHQVSDIS
>LFGLNPFC_03420 hypothetical protein
MKSLTTETALDILIAWLQDNIDCESGIIFDNDEYRTDSAALLPCIEQAREDVRTLRHLQL
LHQNR
>LFGLNPFC_03421 hypothetical protein
MKLALTLEADSINVQALNMGRIVVDVDGVTLAELINVVCDNGYSLRVVDESDRTSAERTP
PSAALTGIRCSTAHITEKDNAWLYSLSHQTSDFGESEWIHFTGSGYLLRTDAWSYPVLRL
KRLGLSKTFRRLVVTLIQRYGVSLIHLDASAECLPDLPTFDW
>LFGLNPFC_03422 Cytoskeleton-binding toxin CbtA
MNTLPDTHIREASHCQSPVTIWQTLLTRLLDQHYGLTLNDTPFADERVIEQHIEAGISLC
DAVNFLVEKYALVRTDQPGFSACTRSQLINSIDILRARRATGLMARDNYRTVNNITLGKH
PGAKQ
>LFGLNPFC_03423 Cytoskeleton bundling-enhancing antitoxin CbeA
MSDTLPGTTHPDDNNDRPWWGLPCTVTPCFGARLVQEGNRLHYLADRAGIRGRFSDADAY
HLDQAFPLLMKQLELMLTSGELNPRHQHTVTLYAKRLTCEADTLGSCGYVYMAVYPTPET
KK
>LFGLNPFC_03424 hypothetical protein
MKIITRGEAMRIHRQHPASRLFPFCTGKYRWHGSTDTYTGREVQDIPGVLAVFAERRKDS
FGPYVRLMSVTLN
>LFGLNPFC_03425 hypothetical protein
MQQISFLPGEMTPGERSHILRALKTLDRHLHEPGVAFTSTRAAREWLILNMAGLEREEFR
VLYLNNQNQLIAGETLFTGTINRTEVHPREVIKRALYHNAAAVVLAHNHPSGEVTPSKAD
RLITERLVQALGLVDIRVPDHLIVGGSQVFSFAEHGLL
>LFGLNPFC_03426 Antirestriction protein KlcA
MTTSSHNSTTPSVSVAAASGNNQSQLVATPVPDEQRISFWPQHFGLIPQWVTLEPRVFGW
MDRLCENYCGGIWNLYTLNNGGAFIAPEPDEDDGETWILFNAMNGNRAEMSPEAAGIAAC
LMTYSHHACRTENYAMTVHYYRLRDYALQHPECSAIMRIID
>LFGLNPFC_03427 hypothetical protein
MQLASRFGHVNQIRRERPLTREELMYHVPSIFGEDRHTSRSERYAYIPTITVLENLQREG
FQPFFACQTRVRDQSRREYTKHMLRLRRAGQITGQHVPEIILLNSHDGSSSYQMLPGYFR
AICTNGLVCGQSLGEVRVPHRGNVVDRVIEGAYEVVGVFDLIEEKRDAMQSLVLPPPARQ
ALAQAALTYRYGDEHQPVTTTDILTPRRREDYGKDLWSAYQTIQENMLKGGISGRSARGK
RIHTRAIHSIDTDIKLNRALWVMAETLLESLR
>LFGLNPFC_03428 hypothetical protein
MSDCHPVLLPEGPFSREQAVAVTTAYRNVLIEDDQGTHFRLVIRNAGGQLRWRCWNFEPD
AGKQLNSYLASEGILRQ
>LFGLNPFC_03429 hypothetical protein
MNTRALFPLLFTVASFSASAGNWAVKNGWCQTMTEDGQALVMLKNGTIGITGLMQGCPNG
VQTLLGSRISINGNLIPTSQMCNQQTGFRAVEVEIGQAPEMVKKAVHSIAERDVSVLQAF
GVRMEFTRGDMLKVCPKFVTSLAGFSPKQTTTINKDSVLQAARQAYAREYDEETTETADF
GSYEVKGNKVEFEVFNPEDRAYDKVTVTVGADGNATGASVEFIGK
>LFGLNPFC_03430 hypothetical protein
MSDNRSRHDRLAVRLSLIISRLMAGESLSLKTLSDEFGVTERTLQRDFHQRLVHLDLEYR
NGRYSLRRQSSPGAIPEMLSFIQNTGIARILPLRNGRLITCLTDNQEPSPCLIWLPAPDI
TATFPECFSQLILAIRQCIHISLMTERWYPSLEPCRLIYYSGSWYLIALQKGKLQVFPLA
DIKSVSLTSERFERRGHIHSLVAEERFISALPHFSFIHKLINTFNL
>LFGLNPFC_03431 hypothetical protein
MQQKKYSFNYLIFKQDFIKRRMLVNPATHPVGVSVSEQTLPQCDQQVDNAVPGFTQKL
>LFGLNPFC_03432 GTPase Der
MSFSHFSLSAQVKSYLTFLPEEIRQKILEHLHGVIHYEPVIGIMGKSGTGKSSLCNAIFQ
SRICATHPLNGCTRQAHRLTLQLGERRMTLVDLPGIGETPQHDQEYQALYRQLLPELDLI
IWILRSDERAYAADIAMHQFLLNEGADPSRFLFVLSHADRMFPAEEWNATEKCPSRHQEL

SLATVTARVATLFPSSFPVLPVAAPAGWNLPALVSLMIHALPPQATSAVYSHIRGENRSE
QARKHAQQTFGDAIGKSFDDAVARFSFPAWMLQLLRKARDRIIHLLITLWERLF
>LFGLNPFC_03433 hypothetical protein
MAKNKGICRNKKCSNPVSEFDEANALRLCDDHYKLWKRNKENRRVRNICRIISRYDKSLD
WEIFYRVRDAAKNKTGKRIIEDTKILHQWRASKQVRKSSHKGVLRSREWLNTQELLSRKH
ISVTGELFKFYIKDIIRLYCYKFLYSREYLNENAYKKRYQRDIHPRKVNVPILQLEVAWI
NNNCRSSDSSTDIIILPRMIRRMVVFNYTKINKQLEKVLGGIARKQTSERNKGGLYTRIK
KESDFLSMDRFMVEIENIIRPERFPTSIYPVFSHSFIERELPLLNLARSQYRRAISKRVA
NSIFYTLQKTNVALWLYLEMFALVAFIKLYTGKGFEFIKNMCVFIDYEKGIIKSTHEELL
GEIKRNIQVNLKVNPDNFPELIDIYNSCFSENILSYDIQDGDFKWRS
>LFGLNPFC_03434 hypothetical protein
MNQPIHNAYWLTRFDSILDSALAQHRAISLIRVDLRFPEYMPATIMDTDLDSAVISRFFE
SLKAKIQAYQRKKRRANKRVRATTLHYFWCREFGKEKGRKHYHVILLLNKDTWCSPGDFT
VPSSLATLIKLAWCSALHLEPWQGNGLVHFSRRTPFRKPTSSDARPSSDDTPLSGGCSET
RKASDKKPGGSAVLWVKRGDVEAMQKARNRASYLVKYETKQHDGSGQRNYGCSRGLGRLL
DGR
>LFGLNPFC_03435 hypothetical protein
MKLKMQDLRLFNIVFESDPGWILDFSNRTLSAFFDEELNIDIDDERYQKEGTSKAKRVRC
LLKQVDRETALRVLGALWQYKTESMPEQAEQSRNDYLALISRLENADTDEAKGVKPVQAW
HGVDWHSLIAEMNEMKSLPPHPRGFRFEAWLAELFSIFKLAPRSSFRNTGEQIDGSFRLN
DEFYLMEAKWHQKRTSAADLHVFEGKLSTKATWTRGVFISWMGFTPEGLTAFGKGKRVIC
VSGYDLYHSLNHRIPLPDLLDAKTRHAAETGEPYAEFGRLYALKDKQFGTLK
>LFGLNPFC_03436 hypothetical protein
MDMQKVDSARSLIAGVMHWAVTTGGFNSETVQDVMPGAVERRFGNELPASPVEWLTDNGS
CYRANETRQFARMLGLESKNTAVRSPESNRIAESFVKTIKRDYIVFMPKPDPVHDSV
>LFGLNPFC_03437 hypothetical protein
MIDVLGPEKRRRRTTQEKIAIVQQSFEPGMTVSLVARQHDVAASYFSGASNTRREVLLR
>LFGLNPFC_03438 Malate-2H(+)/Na(+)-lactate antiporter
MNNNNTPRSPTLVESLLPILAMILLLGFGYAAFDLPPEPLMVLSTVIAALLVKRLGYGYN
AILESISQKIAKTMPALLILISVGLLIGTWMIGGTIPLMIYYGLNLINPSMIYVTALLVT
AIVSVCTGTSWGSAGTVGVAFMGVAIGMEANLAATAGAIVAGAYFGDKLSPLSGDTNLAA
MAAQIDLYEHIAHLLYTTLPSLILSAFVMTLYGMNSELGGSTIPEKIQLITEGLKGIYHF
NLLLLLPIAIVLYGSITKKPTIPIMLVSALVAMINASVIQGFALHDIVKSAVDGFNVSML
TDKDVNPLLGNLLNRGGMNSMMSTLLICFCALSFAGTLALSGALEVIVHNLLKLVHSTGT
MILATIACGLTMISVTCNGQISILIPIEMLRSAYIERGLHPKNLARTVEDSATIFEPILP
WTAAGAYMAGTLGVATLSYLPWAILCWSGIFFATLWGFTGFGIARLSKEKQQQLMMETIN
ESK
>LFGLNPFC_03439 Cystathionine beta-lyase PatB
MKVYNFDKVISRDGTYSAKYNNKGHDIIPLSVADMDIPVADFIVSELSVANQKGIYGYTL
LSDDWQQVAAQWYQRHYSWKVNPEHIVFCPRVVQAVSLFIQNFTQPGDAIVSLTPAYHPI
SHAVEVNHRVLLESALLYRDHHYEIDFADLEDKFKMACCFILISPHNPTGTIWSEDNLRK
IASLAEKYNIFIISDDVHADFNFTQKQHQTISSISSYVAQNSFICTSPAKTFNMAGLEIA
NIVIANDKYREQFKLALIAAGIHNPGYFSVPAFLCAYRHGDSWLAALKDYLVENRSWVQS
FCQAHFPDWFVASGGGTYMLWIDYRAMNISEEQLRHWFVSLAGVEMSWGSDFGDEGLGFF
RVNIATPRSNLTQAFERIYQSIPFTTGVSLHE
>LFGLNPFC_03440 IS3 family transposase ISEc25
MTKKTRFSPEVRQRAVRMVLESQGEYDSQWAAICSIAPKTGCTPETLRVWVRQYERDTGG
GDGGLTTAERQRLKELERENRELRRSNNILRQASAYFAKAEFDRLWKK
>LFGLNPFC_03441 IS3 family transposase IS629
MALLDKLREQYGVRPVCSELHIAPSTYYHCQQQRHHPDKRSAHAQRDDWLKREIQRVYDE
NHQVYGVRKVWRQLLREGIRVARCTVARLMAVMGLSGVLRGKKVRTTVSRKTVAAGDRVN
RQCVAERPDQLWVADFTYVSTWQGFVYVAFIIDVFAGYIVGWRVSSSMETTFVLDALEQA
LWARRPSGTIHHSDKGSQYVSLAYTERLKEAKLLASTGSTGDSYGSAMAESINGLYKAEV
IHRKSWKNRAEVELATLTWVDWYNNRRLLGRLRHTPPAEAEKAYYASIGNDELAA
>LFGLNPFC_03442 HTH-type transcriptional regulator BetI
MLLPMNTETQHHKKDPIRLYQQLLESAAMIAGRDGIAALSLNAVAREAGVSKGGLLHHFP
NKQALIYALFARLLAIMEEAIAALMQKDNISYGRFTRAYVNYLSALTDTQESRQLMVLSL
AMPDEPVLRKCWRDWMLGHLANGDELDNSPTGTLVRYTADGIWLSELTEGITMSPEHRQA
LVDSLNKMTLPA
>LFGLNPFC_03443 hypothetical protein
MRYNINARFIYDATDGTLTLPQSTEPDCQLSITSCALLNFFLHHTDIVSREEVLTKVWDN
NGLTSSNSNLNQYLSMLRKTFRHYGIDNIIITVARGYLQLNPNVSIEPLDEAPAPPAIIE
PSIIPDVDVPTSSEDTESSPPTLAPVTHRHDAYWYFAGACLLTISILLVAFNLFGISEAR
PIALTQLSHSQCELLASDEMRRSVSVKAYEKNYDEVRKRLNIACKPSERFLFFYGDRLET

NGLGRVFLAHCAMHENNPFSYCDNYFYYSWKL
>LFGLNPFC_03444 hypothetical protein
MRIAPRTFFAISALAFIVASGFSFWRLSPAENTGIMSCSTKGIMRFENMEKENVNGNIHF
NFGSQGKGSMVLEGYTDSAAGWLYLQRYVKFTYTSKRVSATERHYRISQWESSASSIDES
PDVIFDYFMREMSDSHDGLFLNAQKLNDKAILLSSINSPLWICTLKSGSKLD
>LFGLNPFC_03445 Propanediol utilization protein PduA
MGDALGLIETKGLVACIAAADAMCKSANVELIGYENIGSGLVTVMVKGDVGAVKASVDSG
LESAQHIGEVVTSLVIARPHNDINKIVIKHKA
>LFGLNPFC_03446 Propanediol utilization protein PduA
MGDALGLIETKGLVACIEAADAMCKAANVELIGYENVGSGLVTAMVKGDVGAVKAAVDSG
VESAQRIGEVVTSLVIARPHNDINKIVSHYKITD
>LFGLNPFC_03447 Propanediol utilization protein PduA
MREALGLIETKGLVACIEAADAMCKAANVELIGYENVGSGLVTAMVKGDVGAVNAAVDSG
VEAARRIGEVVTSRVIARPHNDIEKIASQHKA
>LFGLNPFC_03448 Aldehyde-alcohol dehydrogenase
MIELDTDLRSRQNARVLVRNAKKAQAIMATFSQQQIDAIVKNVAQEAAHHAEALAKMAAE
ETGFGNWQDKVLKNRFASLRVYDAIKDMKTVGIIHDDQQQKVMDVGVPLGVICALVPSTN
PTSTIFYKTLIALKAGNAIIFSPHPGARQCSWKAIEVVKRAAEAAGAPAGIVDGVTELTL
EATQELMHSKDVSLILATGGEGMVRAAYASGTPTISGGPGNGPAFIERSADIHHAVKDII
TSKTFDNGVICASEQSIIVERCIYNEVHRELEAQGAYFMNEDEAAKMAALLLRPNGTINP
KVVGKTALYLSQMAGFCVPASTRVLIAEQTTVSHKNPYSREKLCPVLGLYIEEDWKAACH
RVVELLTNEGLGHTLVIHTRNQDVIRQFCLEKPVNRILINTPAALGGIGATTNITPALTL
GCGAVGGGSSSDNVGPMNLLNIRKVGYGVRSIDELRSPGSRAEPQPAIAAPIVDPHRSIL
DDARFTSPAPATTSADDRFTVATTDPEGEINEQNVERVIRQVLERLGK
>LFGLNPFC_03449 Carbon dioxide concentrating mechanism protein CcmL
MILAKVTGHVVATQKCDELRGSNLLLITQLDDNQQLMKDRSWVAVDSVGAGVHDVVLAEE
YFALNKDRYKAMSVVAIVEKVFRDA
>LFGLNPFC_03450 Aldehyde-alcohol dehydrogenase
MSEFLLKPRICFGQDALSVLDELTACHVLLVTDQAMVKFGLAERVTQRLCARGIDWQVWD
DVVADPDIATVVRGMKLMDSCYPDLVIALGGGSVIDAAKAVIFALAQTRPEAGRERPCFV
AIPTTSGTGSEVTSFSVVKAHAEKLVLVDSSLLPDIAILDPSLVASVPPAITADTGMDVL
CHALEAYVSLAASDFSDALAEKVVQQVFSYLPGCWRNGQDLQAREKMHNASCMAGMAFTN
ASLGITHSLAHALGGVFRVPHGRANALLMAHVVAWNADYHGQCDTLAARKYARLAHLLDL
PASTPREGVTSLLVAIQTLKDEMNMPRGINDTGVNAADFDQRLAEMVAQALRDSCTPTNP
REPDARALTELYRQAWSGNIAQCH
>LFGLNPFC_03451 Choline trimethylamine-lyase
MANYNLTPRVKVLAERLLAHPSTLCVEHAGILSGLDGDIAGIPAAVKPARRFYELMRQLP
LAVSPDELIVGNQTHRPHGAIFHDESTAHRPSVFQFLNLNSDLDAPDYKLVIEKGVLAIK
QQLEEKTRSLGSAVSRSGMDEVNACRAAIYACDALMQLAQNLATSAEKLAATETNAYRKA
ELSESAAILHHIPARPARSFKEACQAFYLFQLALQLDNGSYAVNPEGADKALLAYYQHDI
ANGLLTEAQAYEIVECLWFKLAELSEVRAACAIDGYPMFDALLHGASLENAVINPLSEMF
LNAQRNLSALNLPIRLFHGAHKTVTTLCAACNETPVLEGLTPRIQRLRNHYLTVRPSVSI
YRALAFTEVVKANPGMPTILLRAKAFRHACETAPILIQDDELIVGHPCGKPRAGAFSPDI
AWRWVRDELDTMSTRPQDPFEISEEDKKTIREEIVPFWEGRSLDEICEAQYREAGVWSFS
GETFVSDLSYHQVNGGGDTCPGYDVLLFTKGMNGIKADAEAHLAELSMENPEDIDRIYYY
KAAIDTCEGVINYAHRIAARARELAAVEQNAQRRAELLTIAEVNQNVPANPPKTLQEALQ
SIWTVESLFEIEENQTGLSLGRVDQYCYPMFEADIREGRLTHEGALELMQAFIIKCAELM
WMSSELGAKYFAGYQPFINLTVGGQKRSGGDACNDLTYLIMDAVRFVKVYQPSLACRIHN
QSPQKYMEKIVDVVKAGMGFPACHFDDSHIKMMLRKGFDFEDARDYCLMGCVEPQKSGRI
YQWTSTGYTQWPIAIEFVLNRGRMVLFDSYQGLDTGDLKDLRTFEDFDAAVKKQVAHIIR
LSAIGTVISQRVHRDVAPKPLMSLLVEGCMEKGKDVSAGGAMVNHGPGLIFSGLATYVDS
MAAIRKLVYEDKKYTLEQIRDALLANFEGYEGLRRDCLNAPKYGNDDNYVDQYALDITEW
TERECRKYKMLYSTLSHGTLSISNNTPIGELTNATPNGRLAWMPLSDGISPTQGADKHGP
TAIIKSVSKMNVETMNIGMVHNFKFLKGLLDTPEGRHGLITLLRTASILGNGQMQFSYVD
NEVLKKAQQEPEKYRDLIVRVAGYSAYFVELCKEVQDEIISRTVIEKF
>LFGLNPFC_03452 Choline trimethylamine-lyase activating enzyme
MSANKELSGRIFNIQKYSIYDGDGIRTLIFFKGCNLRCPWCANPEGLSSQFQVMFSQDKC
INCGDCVNVCPAGIHYRAEVNGEMKHFVNRNKDCIGCRKCEEICTQNALDIMGKDVTVSE
LMEIIMQDYDFYVSSGGGVTIGGGEMSLQTDFAVALFSECKKMMINTAVETQGTTPLANY
QKLAPVTDTFLFDIKQIDSNHHKTLFGIGNEGVRRNLEWLVDSGANVIVRMPLIRGYNDS
WEAITGAIDYVQKLAKRGNIRRIDILPYHQLGRKKYERLEMPYPIVEDPSYSVEELDKLE
AFFAQFDFDIRLVRH
>LFGLNPFC_03453 hypothetical protein
MNSLGVIETRGLVAAIQAVDAACKAAGVTCIGYRKVGSGLVTVCFDGEISAVYTAIERGI

AVASATDHQAKSLVIARPERCVVEALSNLKGHPPRVESGQKPHAELPTVAEMAVCEETAE
VSTDTETAMPVPEKEVQAPIVEVKSHGHKKGKKA
>LFGLNPFC_03454 Phosphate propanoyltransferase
MIDALLQEKITTRLIDTAPTIPVGVSNRHVHLSQQDVEALFGVGYQLTPFKPLRQPGQFA
AEECVTVVGPKGSLTHVRVLGPVRPTSQLEISRADCFTLGIKAPVRESGQLENAGDALLV
GPKGHVELHSQVICAWRHIHMSPQDARLLNVSNGQKVSVRSHGERQLTFDEVVVRVRNDF
ALELHIDTEEANAAGLKNGAQVTLIR
>LFGLNPFC_03455 hypothetical protein
MTEQQIDTIVNLILQRLQPAVLVMVTSADGYRDLIHQRLARCGERLHLALDETISDSERW
QQIGDVIPAKTWQHKLPSTPYKALLLPFLSYPLAVDIVNGTLQSPVAQRVHDALLAGIPV
LALRYYCDPHSELNALRGTVHSDYAAHLSATLTGLSECGITLCSMNEMLEKLATDVSSQS
PVSHHRRYLTVTDIVNNPALAKSPDAVLTDAAVDFLKAQKK
>LFGLNPFC_03456 Spermidine export protein MdtJ
MSSKTKCWLWMLLVILSETSATSTLKMFDNSEGMTKTLLLALIVVLYCICYYSLSRAVKD
IPVGLAYATWSGTGILMVSTLGILFYGQHPDTAAIIGMVIIASGIIIMNLFSKMGSEEAE
ETPVTNLDKKIAN
>LFGLNPFC_03457 Multidrug transporter EmrE
MFNIGFLWLALSIGSEITGTSMIKKTNGFRRLWPSVLVVCAYSTCYFALTRAMSTIPVGV
AYSLWCGFGIVGVTIFSMILYKQKPDLPAIFSMLLIISGGIIMNVFSNM
>LFGLNPFC_03458 HTH-type transcriptional regulator DsdC
MEPLREIRNRLLNGWQLSKLHTFEVAARHQSFALAAEELSLSPSAVSHRINQLEEELGIQ
LFVRSHRKVELTHEGKRVYWALKSSLDTLNQEILDIKNQELSGTLTLYSRPSIAQCWLVP
ALGDFTRRYPSISLTVLTGNDNVNLQRAGIDLAIYFDDAPSAQLTHHFLMDEEILPVCSP
EYAQRHALTNQVINLRHCTLLHDRQAWSNDSGTDEWHSWAQHYAVNLPTSSGIGFDRSDL
AVIAAMNHIGVAMGRKRLVQKRLISGELVAPFGDMTVKCHQHYYITTLPGRQWPKIEAFI
TWLREQVCQSCQYQ
>LFGLNPFC_03459 D-serine transporter DsdX
MHSQIWVVSTLLISIVLIVLTIVKFKFHPFLALLLASFFVGTMMGMGPLDMVNAIESGIG
GTLGFLAAVIGLGTILGKMMEVSGAAERIGLTLQRCRWLSADVIMVLVGLICGITLFVEV
GVVLLIPLAFSIAKKTNTSLLKLAIPLCTALMAVHCVVPPHPAALYVANKLGADIGSVIV
YGLLVGLMASLIGGPLFLKFLGQRLPFKPVPTEFADLKVRDEKTLPSLGATLFTILLPIA
LMLVKTIAELNMARESGFYTLLEFIGNPITAMFIAVFVAYYVLGIRQHMSMGTMLTHTEN
GFGSIANILLIIGAGGAFNAILKSSSLADTLAVILSNMHMHPILLAWLVALILHAAVGSA
TVAMMGATAIVAPMLPLYPDISPEIIAIAIGSGAIGCTIVTDSLFWLVKQYCGATLNETF
KYYTTATFIASVIALAGTFLLSFII
>LFGLNPFC_03460 D-serine dehydratase
MENAKMNSLIAQYPLVKDLVALKETTWFNPGTTSLAEGLPYVGLTEQDVQDAHARLSRFA
PYLAKAFPETAATGGIIESELVAIPAMQKRLEKEYQQPISGQLLLKKDSHLPISGSIKAR
GGIYEVLAHAEKLALEAGLLTLEDDYSKLLSPEFKQFFSQYSIAVGSTGNLGLSIGIMSA
RIGFKVTVHMSADARAWKKAKLRSHGVTVVEYEQDYGVAVEEGRKAAQSDPNCFFIDDEN
SRTLFLGYSVAGQRLKAQFAQQGRIVDADNPLFVYLPCGVGGGPGGVAFGLKLAFGDHVH
CFFAEPTHSPCMLLGVHTGLHDQISVQDIGIDNLTAADGLAVGRASGFVGRAMERLLDGF
YTLSDQTMYDMLGWLAQEEGIRLEPSALAGMAGPQRVCASVSYQQMHGFSAEQLRNATHL
VWATGGGMVPEEEMEQYLAKGH
>LFGLNPFC_03461 hypothetical protein
MPKSYTPNWFFTALLDNHINQMMARYSCLRALRMDFFYRKDTPDFLQPDHRWLELQLRML
LEQVEQFENIVGFFWVIEWTADHGFHAHVVFWIDRQRVKKIYPFAERIAECWQAITYNCG
SAHRCTYQPHYAYNINIPVRHNDPESIDNIRGALHYLAKEEQKDGLCAYGCNEVPERPAA
GRPRKPHF
>LFGLNPFC_03462 hypothetical protein
MNAIPYFDYSLAPFWPSYQNKVIGVLERALREQSGSRIRRILLRLPWEHDNAFNSRQTWF
GMDFIETVSALMNAKPGRDLCWLLTRHPEKPEYHVVLCVRQEYFDGPELDRLILDAWSNV
LGFASPGEAKPYQKQITRDVVLDRRSPDCEALFKDLIWAFSDFARDRRGVCDPEARCLAG
NPGWQC
>LFGLNPFC_03463 hypothetical protein
MLTTTSHDSVWLRADDPLIDMNYITSFTGMTDKWFYRLISEGHFPKPIKLGRSSRWYKSE
VEQWMQQRIEESRGAAA
>LFGLNPFC_03464 hypothetical protein
MQITEALISEPGDIRRFIQHAVDHWPRLLAVHFILHSTEGNIYGQQIHAFCTSFYRQLHE
RITESNHTASPSSSVVLRWLREQHGGATIRCLLLLSQTSICHPRASVTVDEQCSQVVDLL
QHSWQVISAGGQCRVERCFRVARGDTSGQYVALKTVALSLGLPVVTAITHRPVQRCTLIT
AQ
>LFGLNPFC_03465 hypothetical protein
MRTRIDYLADKYSFTERNESPRLRRQWQDVLEECRLTEAGPEERLRIALLNVDYVTSFEL

PFRLLLTRTPQLIAALREEWGLSQKNVVFNDKRFGCVYSLKASLSGVPDTFRYHLSHRIR
RMVGNENTSSPYQQIAREVKVPRERLKYALEAGLLVTALDGLFWSGSQRIAADILRLRKS
GMPVVTTSVEASDNLTGTTRKIPAYHL
>LFGLNPFC_03466 hypothetical protein
MARSDYDFINLSLGHELNEWLAERGYAGQADNRNRLAEVVTRKLRDSFYADVSWDALNVA
YSEHPEWFSELASGDED
>LFGLNPFC_03467 Hemolysin transporter protein ShlB
MQHRQDNLLANRNLLPGMVSGQYAFRIRTLSQVVRYFSLLPCLCILSFSSPAAMLSPGDR
SAIQQQQQQLLDENQRQRDALERSAPLTITPSPETSAGTEGPCFTVSRIVVSGATRLTSA
ETDRLVAPWVNQCLNITGLTAVTDAVTDGYIRRGYITSRAFLTEQDLSGGVLHITVMEGR
LQQIRAEGADLPGRTLKMVFPGMEGKVLNLRDIEQGMEQINRLRTESVQIEISPGDREGW
SVVTLTALPEWPVTGSVGIDNSGQKNTGTGQLNGVLSFNNPLGLADNWFVSGGRSSDFSV
SHDARNFAAGVSLPYGYTLVDYTYSWSDYLSTIDNRGWLWRSTGDLQTHRLGLSHVLFRN
GNMKTALTGGLQHRIIHNYLDDVLLQGSSRKLTSFSVGLNHTHKFLWGVGTLNPVFTRGM
PWFGAESDHGKRGDLPVNQFRKWSVSASFQRPVTDRVWWLTSAYAQWSPDRLHGVEQLSL
GGESSVRGFKEQYISGNNGGYLRNELSWSLFSLPYVGTVRAVTALDGGWLHSDRDDPYSS
GTLWGAAAGLSTTSGHVSGSFTAGLPLVYPDWLAPDHLTVYWRVAVAF
>LFGLNPFC_03468 tRNA nuclease CdiA
MHQPPVRFTYRLLSYLISTIIAGQPLLPAVGAVITPQNGAGMDKAANGVPVVNIATPNGA
GISHNRFTDYNVGKEGLILNNATGKLNPTQLGGLIQNNPNLKAGGEAKGIINEVTGGNRS
LLQGYTEVAGKAANVMVANPYGITCDGCGFINTPHATLTTGKPVMNADGSLQALEVTEGS
ITINGAGLDGTRSDAVSIIARATEVNAALHAKDLTVTAGANRITADGRVSALKGEGDVPK
VAVDTGALGGMYARRIHLTSTESGVGVNLGNLYAREGDIILNSAGKLVLKNSLAGGNTTV
TGTNVSLSGDNKAGGNLSVTGTTGLTLNQSRLVTDKNLVLSSSGQIVQNGGELTAGQNAM
LSAQHLNQTSGTVNAAENVTLTTTDDTTLKGRSVAGKTLTVSSGSLNNGGTLVAGRDATV
KTGTFSNTGAVQGNGLKVTATDLTSTGSIKSGSTLDISVRNATLSGDAGAKDSARVTVSG
TLENRGRLVSDDVLTLSATQINNSGTLSGAKELVVSADTLTTTEKSVTNSDGNLMLNSAS
STLAGETSAGGTVSVKGNSLKTTTTAQTQGNSVSVDVQNAQLDGTQAARDILTLNASEKL
THSGKSSAPSLSLSAPELTSSGVLVASALNTQSQTLTNSGLLQGEASLTVNTQRLDNQQN
GTLYSAADLTLDIPDIRNSGLITGDNGLTLNTASLSNPGKITADTLNVRATTLDGDGLLQ
GAGALALAGDTLSQGRNGRWLTAGDLSLRGKTLNTAGTTQGQNLTVQADNWANSGSVLAT
GNLTASATGQLTSTGDIMSQGDTTLKAATTDNRGSLLSAGTLSLNGNSLDNSGTVQGNHI
TIRQNSVTNSGTLTGIAALTLAARMDMASPQPALMNNGGSLLTSGDLTITAGSITSSGHW
QGKRVLITADSLANSGAIQAADSLTARLTGELVSAAGSKVTSNGEMALSALNLSNSGQWI
AKNLTLKANSLTSAGDITGVDALTLTVNQTLHNQTNGKLLSAGVLTLKADSATNDGQLQG
NATTITAGQLTNGGHLQGETLTLTASGGVNNRSGGVLMSRNALNVSTATLSNQGTIQGGG
GVSLNATDRLQNDGKILSGSNLTLTAQVLANTGSGLVQAATLLLDVVNTVNGGRVLATGS
ADVKGTTLNNTGTLQGAELLVNYHTFSNSGTLLGTSGLGVKGSSLLQNGTGRLYSAGNLL
LDAQDFSGQGQVVATGDVTLKLIAALTNHGTLAAGKTLSVTSQNAITNGGVMQGDAMVLG
AGEAFTNNGMLTAGKGNSVFSAQRLFLNAPGSLQAGGDVSLNSRSDITISGFTGTAGSLT
MNVAGTLLNSALIYAGNNLKLFTDRLHNQHGDILAGNSLWVQKDASGGANTEIINTSGNI
ETHQGDIVVRTGHLLNQREGFSATTTTRTNPSSIQGMGNALVDIPLSLLPDGSYGYFTRE
VENQHGTPCNGHGACNITMDTLYYYAPFADSATQRFLSSQNITTVTGADNPAGRIASGRN
LSAEAERLENRASFILANGDIALSGRELSNQSWQTGTENEYLVYRYDPKTFYGSYATGSL
DKLPLLSPEFENNTIRFSLDGREKDYTPGKTYYSVIQAGGDVKTRFTSSINNGTTTAHAG
SVSPVVSAPVLNTLSQQTGGDSLTQTALQQYEPVVVGSPQWHDELAGALKNIAGGSPLTG
QTGISDDWPLPSGNNGYLVPSTDPDSPYLITVNPKLDGLGQVDSHLFAGLYELLGAKPGQ
APRETAPSYTDEKQFLGSSYFLDRLGLKPEKDYRFLGDAVFDTRYVSNAVLSRTGSRYLN
GLGSDTEQMRYLMDNAARQQKGLGLEFGVALTAEQIAQLDGSILWWESATINGQTVMVPK
LYLSPEDITLHNGSVISGNNVQLAGGNITNSGGSINAQNGLSLDSTGYIDNLNAGLISAG
GSLDLSAIGDISNISSVISGKTVQLESVSGNISNITRRQQWNAGSDSRYGGVHLSGTDTG
PVATIKGTDSLSLDAGKNIDITGATVSSGGTLGMSAGNDINIAANLISGSKSQSGFWHTD
DNSASSTTSQGSSISAGGNLAMAAGHNLDVTASSVSAGHSALLSAGNDLSLNAVRESKNS
RNGRSESHESHAAVSTVTAGDNLLLVAGRDVASQAAGVAAENNVVIRGGRDVNLVAESAG
AGDSYTSKKKKEINETVRQQGTEIASGGDTTVNAGRDITAVASSVTATGNISVNAGRDVA
LTTATESDYHYLETKKKSGGFLSKKTTHTISEDSASREAGSLLSGNRVTVNAGDNLTVEG
SDVVADQDVSLAAGNHVDVLAATSTDTSWRFKETKKSGLMGTGGIGFTIGSSKTTHDRRE
AGTTQSQSASTIGSTAGNVSITAGKQAHISGSDVIANRDISITGDSVVVDPGHDRRTVDE
KFEQKKSGLTVALSGTVGSAINNAVTSAQETKESSDSRLKALQATKTALSGVQAGQAAAM
ATATGDPNATGVSLSLTTQKSKSQQHSESDTVSGSTLNAGNNLSVVATGKNRGDNRGDIV
IAGSQLKAGGNTSLDAANDVLLSGAANTQKTTGRNSSSGGGVGVSIGAGGNGAGISVFAS
VNAAKGSEKGNGTEWTETTTDSGKTVTINSGRDTVLNGAQVNGNRIIADVGHDLLISSQQ
DTSKYDSKQTSVAAGGSFTFGSMTGSGYIAASRDKMKSRFDSVAEQTGMFSGDGGFDITV
GNHTQLDGAVIASTATADKNSLDTGTLGFSDIHNEADYKVSHSGISLSGGGSFGDKFQGN

MPGGMISAGGHSGHAEGTTQAAVADGTITIRDRDNQKQNLANLSRDPAHANDSISPIFDK
EKEQRRLQTVGLISDIGSQVADIARTQGELNALKAAQDKYGPVPADATEEQRQAYLAKLR
DTPEYKKEQEKYGTGSDMQRGIQAATAALQGLVGGNMAGALAGASAPELANIIGHHAGID
DNTAAKAIAHAILGGVTAALQGNSAAAGAIGAGTGEVIASAIAKSLYPGVDPSKLTEDQK
QTVSTLATLSAGMAGGIASGDVAGAAAGAGAGKNVVENNALSLVARGCAVAAPCRTKVAE
QLLEIGAKAGMAGLAGAAVKDMADRMTSDELEHLITLQMMGNDEITTKYLSSLHDKYGSG
AASNPNIGKDLTDAEKVELGGSGSGTGTPPPSENDPKQQNEKTVDKLNQKQESAIKKIDN
TIKNALKDHDIIGTLKDMDGKPVPKENGGYWDHMQEMQNTLRGLRNHADTLKNVNNPEAQ
AAYGRATDAINKIESALKGYGI
>LFGLNPFC_03469 Immunity protein CdiI
MITLRKLIGNINMTKEPEQQSPLELWFERIIDVPLEKLTVEDLCRAIRQNLCIDQLMPRV
LEVLTKEPLAGEYYDGELIAALSTIKGEDLKDQKSTFTQIRQLINQLEPSDINDDLRKDI
LKINQIIV
>LFGLNPFC_03470 hypothetical protein
MCAGHVQQLGGESPLSSLMVAKAFAYALNQWPALTYYANDGWVEIDNNIAENALRAVSLG
RKNFLFFGSDHGGERGALLYSLIGTCKLNDVDPESYLRHVLAVIADWPVNRVSELLPWRI
ALPAE
>LFGLNPFC_03471 hypothetical protein
MDNWQKSVRSRALPEEAMTGWNEGMIRLQQLAERLNRQDEQRGKYMTVSELKTEVFGIMQ
AFNRHIPAEEQLRRYGEVRNQNGSEQQQKQAEMALNQLINRYQMIRAGKQ
>LFGLNPFC_03472 hypothetical protein
MQANNSGSHNAGAKPESGRVILTEFGFLAILVMVGCAWLAEQAFSDHALSPHSAWPYSAS
RDAGLADTGAGGYPTCKQRWADDTVGLKARLLQLPALDIWTAFKKIDQSQVVYEEAVLRS
RVSERNMQVSQNGRVYPSYGGNVDGTVANAATRLASGARNILGSIAACTAFDSVR
>LFGLNPFC_03473 hypothetical protein
MVQAQLQIALVICIPLITLCSAWDVKVVMTLTFVQFALFFLTFWWELARWLDSWLLDVLY
NSDTHSSWNLAGIQNTQDDVIINLVMRLMFLVLPTFWLGAMTWAGVRVGVALNGALAG
>LFGLNPFC_03474 putative BsuMI modification methylase subunit YdiO
MIVIDFFCGCGGASEGLRQAGFDIELGLDIDQQASETFKANFPDAKFIQDDIRKIEPQDI
SDIIDIKAKRPLLLSACAPCQPFSQQNKNKTSDDSRRNLLNETHRFIRELLPEYIMLENV
PGMQKIDEEKEGPFQEFIKLLKELEYNYISFIANAENYGIPQRRKRLVLLASRVGKVTLP
EITHGKNKIPFKTVRDYIQDFTKLCSGETDPKDPLHRAGTLSPLNLKRIMHTPEGGDRRN
WPEELVNKCHKNYDGHTDTYGRMSWDKPAPTLTTKCNSYSNGRFGHPDPTQHRAISIREA
SRLQTFPLSYVFKGSLNSMAKQIGNAVPCELARLFGLHLIENCTNKDS
>LFGLNPFC_03475 hypothetical protein
MLGRQQIAGIPTALSELFKNAHDAYADNVEVDFFRKENLLILRDDGLGMTTDEFEERWLT
IGTSSKLIDDDAINKPAVDSNKAFRPIMGEKGIGRLSIAAIGPQVLVLTRAKRDNELKPL
VAAFVNWSLFAIPSLDLDDIEIPIRTIINDECFTKKTLDEMIEQARNNLDSLSHKISKSK
VSQINTQLSSFEFDPILWEKKLGGLRLSGDGHGTHFIIMPTEEILIDDISTSDSNKTSEQ
SSRLEKALLGFTNTMYSDSNPPIIARFRDYLEDGECIDRISESIFFTPQEFNLADHHIEG
WFNEFGQFSGTVSVYGEEPIHHVVTWKNNNQLTQCGPFKIKLAYIHGRLRDSRLPMELWA
PLKEKTDRYGGLYIYRDGLRILPYGDSDTDFLKIEKRRTLSASEYFFSYRRLFGAIELTK
ENNASLVEKAGREGFIENKPYKQFKEMLENFFIEIARDFFKDDGDMSELFVETKQRRNEE
HDLLSKRSKQTKAKKDRLKKDLYDFFDKLDNDYWNIEINKLINKNEEYFSSTEITDTNID
YVYNKIKEQNDAIIKNLRNSVDIKKPSGVGLTKELSNLWDRYQIERQKILLSLNELKDNV
DRKLIELDNKNNDFLNLRKRLEDSLNLQQSYYEKELTKLYNDAKNALKDVQSKANRLISD
NKKKHKSELKNISYEFQSTNLNGKDTAYILDVKRNLESKIENTSNEVINEIRKLTDQIAI
ISDSTTSENLSSAQVTEAIETELEHLRDQQANNAELILLGMALSVVHHEFNGNIRAIRSA
LRELKAWADRNPKLDIIYQKIRTSFDHLDGYLKTFTPLTRRLSRSKTNITGTAILEFIRD
VFDDRLEKEGIELFTTSKFVNQEIVTYTSTIYPVFINLIDNAIYWLGKTTGEKRLILDAT
ETGFVIGDTGPGVSTRDRDIIFDMGFTRKTGGRGMGLFISKECLSRDGFTIRLDDYTPEQ
GAFFIIEPSEETSE
>LFGLNPFC_03476 hypothetical protein
MTSSTDFHKLSEDCVRRFLHSVVAVDDNMSFGAGSDTFPTDEDINALVDPDDDPTPIITA
SASPRIESTKSKAKVKNHPFDYQALAEAFAKDGIACCGLLAKSFNVEERDIITASSHKAD
ITILDWDMQSDSGQFAIEIIKSIIVSDINSGGRLRLLSIYTGEHVTAVITKLNNELKKTY
RSVIKNDDSIFIEDNYALEQWCIVVISKDVYEKDLPNVLIKKFTNLTAGLLSNAALSCIS
EIREKTHGILTKYNNKLDTAYVSHILNLIKSKESRAYAYENAHDYAVDLISEEIRSILQI
SENLKKSLSKNSLSHWPIFHYAKNGCKNFLLTGKKQKDLSVEHLRNILSADSLEEIQHAI
EHASLGKKEYLSQDGEEDKKLMQLCSLEITRRSLRYHSHIDNVSLKQGTLLLDAYNFVYL
CIQPLCDSVRLHEKADFLFLRGTLDDNNYNLLIEDEYGGFYKIKMPAKASNIISFSFGVE
NGNGVIIGKKNNLVNTDYISFVPLLVEKISTPKVLKWIGEIKTTYAQKITTDIVANLSRI
GLDQHEWLRIKSKDI
>LFGLNPFC_03477 hypothetical protein

MKHRTWITEALRLHFEEHLPQVVVGRRLGVPKSTACGMFVRFRKAGFSWPLPAGMSEREL
DGRLYGSTSTVPVVLCSGSVIQDTSKSC
>LFGLNPFC_03478 hypothetical protein
MIKTRRTKRTFSPEFKLEAFEQVVVKYQRDVREVAQALELNPDHLRKWIRLYKQELQGIE
PAGNAITPEQREIQQLKAQIKRVEMEKEILKQAAVLMSEIPGKLSR
>LFGLNPFC_03479 hypothetical protein
MLSVVAQPGSRAISQMLRQSGVDAGRWLA
>LFGLNPFC_03480 Adaptive-response sensory-kinase SasA
MNCSLTLSQRLSLVFTVVLLFCAAVTCGVHIYSSNLYGNAMVQRLSAGLAQQIVITESLL
DNRGQVNHRTLKSLFERLMTLNPSVELYIVSPEGRLLVEAAPPGHIKRRYINIAPLKKFL
SGAVWPVYGDDPRSVNKKKVFSTAPLYLRDDLKGYLYIILQGEELNALTDAAWTKALWNA
LYWSLFLVVICGLLSGMLVWYWVTRPIQQLTENVSGIEQDSISAIKQLAIQRPATPPSNE
VEILHNAFIELARKISCQWDQLSESDQQRREFIANISHDLRTPLTSLLGYLETLSMKSDS
LSSEDCHKYLTTALRQGHKVRHLSCQLFELARLEHGAIKPQLEQFSVCELIQDVAQKFEL
SIETRRLQLRIMMSHSLPLIRADISMIERVITNLLDNAVRHTPPEGSIRLKVWQEDNRLH
VEVADSGPGLTEDMRTHLFRRASVLCHEPSEEPRGGLGLLIVRRMLVLHGGDIRLTDSTT
GACFRFFLPL
>LFGLNPFC_03481 Alkaline phosphatase synthesis transcriptional regulatory protein PhoP
MNNVKKILLMEDDYDIAALLRLNLQDEGYQIVHEADGARARLLLDKQTWDAVILDLMLPN
VNGLEICRYIRQMTRYLPVIIISARTSETHRVLGLEMGADDYLPKPFSIPELIARIKALF
RRQEAMGQNILLAGGLICCHGLCINPFSREVHLHNKQVDLTPREFDLLLWFARHPGEVFS
RLSLLDNVWGYQHEGYEHTVNTHINRLRAKIEQDAAEPKMIQTVWGKGYRFSVDNAGMR
>LFGLNPFC_03482 Putative protein-methionine-sulfoxide reductase subunit YedZ1
MQPLPLKQIHPLWLRCCHWINAVTVAGMICSGWRIYNASPLFSFNYPAALTLGGWLGGAL
LWHFALMWILLINATIYLTLGICSGRFRSKFFPLSWTSLKKSLTQALYGKLHHTNMQEYN
MVQKVAYILIIILDGAALLLSGLVLWKPVQFSLLTILFGGYDTARYIHFLCMNIMVLFIII
HLVMVLLVPRALLSMIRGH
>LFGLNPFC_03483 Putative protein-methionine-sulfoxide reductase subunit YedZ1
MKKKRFLSDSDAHYVVKDAEKILAPDRRRLLQQGLTLGGIMMLTGCDISNNDDIEKALFR
ISRFNDRIQSFLFSKNSVAPEYLPEDITTPFPFNAFYGEEEIPVIDGQDYKLEIRGLVRD
NRNWFLSELYNMQHVSQITRHICVEGWSAIGKWGGIPFRHFLTLIGADMRAKYVSFRCVD
GYYTSIDMATALHSQTLLTLTWNNRVLPKEYGYPIKIRIPTKLGYKNPKHVQVIEVTNHF
PGGYWEDQGYNWFGGC
>LFGLNPFC_03484 hypothetical protein
MKNIISATVLSALFMFNAQAADTMDKTHEVKGSMSSAHDKMCKDGMCKNAMDKEKMEKEH
MSKTDGMKNDHMGKKDNMSQ
>LFGLNPFC_03485 Hemolysin-activating lysine-acyltransferase HlyC
MNMNNPLEVLGHVSWLWASSPLHRNWPVSLFAINVLPAIRANQYALLTRDNYPVAYCSWA
NLSLENEIKYLNDVTSLVAEDWTSGDRKWFIDWIAPFGDNGALYKYMRKKFPDELFRAIR
VDPKTHVGKVSEFHGGKIDKQLANKIFKQYHHELITEVKNKTDFNFSLTG
>LFGLNPFC_03486 Hemolysin, chromosomal
MPTITTAQIKSTLQSAKQSAANKLHSAGQSTKDALKKAAEQTRNAGNRLILLIPKDYKGQ
GSSLNDLVRTADELGIEVQYDEKNGTAITKQVFGTAEKLIGLTERGVTIFAPQLDKLLQK
YQKAGNKLGGSAENIGDNLGKAGSVLSTFQNFLGTALSSMKIDELIKKQKSGSNVSSSEL
AKASIELINQLVDTAASINNNVNSFSQQLNKLGSVLSNTKHLNGVGNKLQNLPNLDNIGA
GLDTVSGILSAISASFILSNADADTGTKAAAGVELTTKVLGNVGKGISQYIIAQRAAQGL
STSAAAAGLIASVVTLAISPLSFLSIADKFKRANKIEEYSQRFKKLGYDGDSLLAAFHKE
TGAIDASLTTISTVLASVSSGISAAATTSLVGAPVSALVGAVTGIISGILEASKQAMFEH
VASKMADVIAEWEKKHGKNYFENGYDARHAAFLEDNFKILSQYNKEYSVERSVLITQQHW
DMLIGELASVTRNGDKTLSGKSYIDYYEEGKRLERRPKEFQQQIFDPLKGNIDLSDSKSS
TLLKFVTPLLTPGEEIRERRQSGKYEYITELLVKGVDKWTVKGVQDKGSVYDYSNLIQHA
SVGNNQYREIRIESHLGDGDDKVFLSAGSANIYAGKGHDVVYYDKTDTGYLTIDGTKATE
AGNYTVTRVLGGDVKVLQEVVKEQEVSVGKRTEKTQYRSYEFTHINGKNLTETDNLYSVE
ELIGTTRADKFFGSKFTDIFHGADGDDHIEGNDGNDRLYGDKGNDTLRGGNGDDQLYGGD
GNDKLIGGTGNNYLNGGDGDDELQVQGNSLAKNVLSGGKGNDKLYGSEGADLLDGGEGND
LLKGGYGNDIYRYLSGYGHHIIDDEGGKDDKLSLADIDFRDVAFKREGNDLIMYKAEGNV
LSIGHKNGITFKNWFEKESDDLSNHQIEQIFDKDGRVITPDSLKKAFEYQQSNNKVSYVY
GHDASTYGSQDNLNPLINEISKIISAAGNFDVKEERSAASLLQLSGNASDFSYGRNSITL
TASA
>LFGLNPFC_03487 Alpha-hemolysin translocation ATP-binding protein HlyB
MDSCHKIDYGLYALEILAQYHNVSVNPEEIKHRFDTDGTGLGLTSWLLAAKSLELKVKQV
KKTIDRLNFISLPALVWREDGRHFILTKVSKEANRYLIFDLEQRNPRVLEQSEFEALYQG
HIILIASRSSVAGKLAKFDFTWFIPAIIKYRRIFIETLVVSVFLQLFALITPLFFQVVMD
KVLVHRGFSTLNVITVALSVVVVFEIILSGLRTYIFAHSTSRIDVELGAKLFRHLLALPI

SYFESRRVGDTVARVRELDQIRNFLTGQALTSVLDLLFSFIFFAVMWYYSPKLTLVILFS
LPCYAAWSVFISPILRRRLDDKFSRNADNQSFLVESVTAINTIKAMAVSPQMTNIWDKQL
AGYVAAGFKVTVLATIGQQGIQLIQKTVMIINLWLGAHLVISGDLSIGQLIAFNMLAGQI
VAPVIRLAQIWQDFQQVGISVTRLGDVLNSPTESYHGKLALPEINGDITFRNIRFRYKPD
SPVILDNINLSIKQGEVIGIVGRSGSGKSTLTKLIQRFYIPENGQVLIDGHDLALADPNW
LRRQVGVVLQDNVLLNRSIIDNISLANPGMSVEKVIYAAKLAGAHDFISELREGYNTIVG
EQGAGLSGGQRQRIAIARALVNNPKILIFDEATSALDYESEHIIMRNMHKICKGRTVIII
AHRLSTVKNADRIIVMEKGKIVEQGKHKELLSEPESLYSYLYQLQSD
>LFGLNPFC_03488 Hemolysin secretion protein D, chromosomal
MKTWLMGFSEFLLRYKLVWSETWKIRKQLDTPVREKDENEFLPAHLELIETPVSRRPRLV
AYFIMGFLVIAVILSVLGQVEIVATANGKLTLSGRSKEIKPIENSIVKEIIVKEGESVRK
GDVLLKLTALGAEADTLKTQSSLLQTRLEQTRYQILSRSIELNKLPELKLPDEPYFQNVS
EEEVLRLTSLIKEQFSTWQNQKYQKELNLDKKRAERLTILARINRYENLSRVEKSRLDDF
RSLLHKQAIAKHAVLEQENKYVEAANELRVYKSQLEQIESEILSAKEEYQLVTQLFKNEI
LDKLRQTTDSIELLTLELEKNEERQQASVIRAPVSGKVQQLKVHTEGGVVTTAETLMVIV
PEDDTLEVTALVQNKDIGFINVGQNAIIKVEAFPYTRYGYLVGKVKNINLDAIEDQKLGL
VFNVIVSVEENDLSTGNKHIPLSSGMAVTAEIKTGMRSVISYLLSPLEESVTESLHER
>LFGLNPFC_03489 hypothetical protein
MGNQWQQKYLLEYNELVSNFPSPERVVSDYIKNCFKTDLPWFSRIDPDNAYFICFSQNRS
NSRSYTGWDHLGKYKTEVLTLTQAALINIGYRFDVFDDANSSTGIYKTKSADVFNEENEE
KMLPSEYLHFLQKCDFAGVYGKTLSDYWSKYYDKFKLLLKNYYISSALYLYKNGELDERE
YNFSMNALNRSDNISLLFFDIYGYYASDIFVAKNNDKVMLFIPGAKKPFLFKKNIADLRL
TLKELIKDSDNKQLLSQHFSLYSRQDGVSYAGVNSVLHAIENDGNFNESYFLYSNKTLSN
KDVFDAIAISVKKRSFSDGDIVIKSNSEAQRDYALTILQTILSMTPIFDIVVPEVSVPLG
LGIITSSMGISFDQLINGDTYEERRSAIPGLATNAVLLGLSFAIPLLISKAGINQEVLSS
VINNEGRTLNETNIDIFLKEYGIAEDSISSTNLLDVKLKSSGQHVNIVKLSDEDNQIVAV
KGSSLSGIYYEVDIETGYEILSRRIYRTEYNNEILWTRGGGLKGGQPFDFESLNIPVFFK
DEPYSAVTGSPLSFINDDSSLLYPDTNPKLPQPTSEMDIVNYVKGSGSFGDRFVTLMRGA
TEEEAWNIASYHTAGGSTEELHEILLGQGPQSSLGFTEYTSNVNSADAASRRHFLVVIKV
HVKYITNNNVSYVNHWAIPDEAPVEVLAVVDRRFNFPEPSTPPDISTIRKLLSLRYFKES
IESTSKSNFQKLSRGNIDVLKGRGSISSTRQRAIYPYFEAANADEQQPLFFYIKKDRFDN
HGYDQYFYDNTVGLNGIPTLNTYTGEIPSDSSSLGSTYWKKYNLTNETSIIRVSNSARGA
NGIKIALEEVQEGKPVIITSGNLSGCTTIVARKEGYIYKVHTGTTKSLAGFTSTTGVKKA
VEVLELLTKEPIPRVEGIMSNDFLVDYLSENFEDSLITYSSSEKKPDSQITIIRDNVSVF
PYFLDNIPEHGFGTSATVLVRVDGNVVVRSLSESYSLNADASEISVLKVFSKKF
>LFGLNPFC_03490 IS481 family transposase ISErsp1
MGVSRDTFYRYRELVDEGGVDALINRSRRAPNLKNRTDEATEQAVVDYAVAFPAHGQHRT
SNKLRKQGVFISGSGVRSVWLRHNLENFKKRLKALEEKVARDGIELTDSQIAALERKASD
DEACGEIETAHPGYLGSQDTFYVGNLKGVA
>LFGLNPFC_03491 IS3 family transposase ISEc16
MTKPVSISKKPRKQHTPEFRNEALKLAERIGVAAAARELSLYESQLYAWRSKQQQQMSSS
ERESELAAENVRLKRQLAEQAEELAILQKAATYFAKRLK
>LFGLNPFC_03492 IS3 family transposase ISEc16
MKYVFIENHRAEFSIKAMCRVLRVARSGWYVWLRRRHQMSLRQQFRLTCDAAVHKAFFEA
KQRYGAPRLADEMLEFNI
>LFGLNPFC_03493 hypothetical protein
MLPALLTKCWSSIFKTIAASLRRQGLRAKASRKFSPVSYRAHGLPVLENLLEQDFSASGP
NQKWAGDITYLRTDEGWLYLAVVIDLWSRAVIGWSMSPRMTAQLACDALQMALWRRRRPE
CQRQ
>LFGLNPFC_03494 hypothetical protein
MVKLASQPGASVARIAREHDINDNLLFKWLRLWQNEGRISRRLPVTTSSGAGVELLPVEI
TPDEQKEPVAALTPLLSTPSQSTVSASSCKVEFRHGNMTLENPSPELLTVLIRELTGRGR
>LFGLNPFC_03495 hypothetical protein
MISLPSDTRIWLVAGVTDMRKSFNGLGEQVQHVLDENPFSGHLFIFRGRRGDTIKILWAD
ADGLCLFTKRLEEGQFIWPAVRDGKVSITRSQLAMFLDKLDWRQPKTSRLNALTML
>LFGLNPFC_03496 IS66 family transposase IS682
MSRKYLIRITELERLLSEQAEALRQKDQQLSLVEETEAFLRSALARAEEKIEEEERETEH
LRAQIEKLRRMLFGTRSEKLRREVEQAEALLNQRRQDSDRYSGWEDDPQVPRQLRQSRHR
RPLPAHLPREIHRLESEESCCPECGSELDYLGEVSAEQLELVSSALKVIRTVRVKKACTK
CDCIVEAPAPSRPIERGIAGSGLLARVLTGKYCEHLPLYRQSEIFARQGAELSRALLSNW
VDACCQLMTPLNDALYRYVMNTRKVHTDDIPVKVLAPGRKKAKTGRIWTYVRDDRNAGSS
EPPAVWFAYSPDRQGKHPVQHLRPFRGILQADAFSGYDRLFSAEREGGALTEVACWAHAR
RKIHDVYISSKSTTAEEALKRISELYAIEDEIRGLPESERLAARQQRSKALLTSLHEWMV
EKNGTLSKKSRLGEAFSYVLNQWDALCYYSDDGLAEADNNTAERALRAVCLGKKNSYDLC

QILSPKRPYAAPGASLYRGLKNLKQSDCILDFTNSYSGILAP
>LFGLNPFC_03497 IS110 family transposase ISEc45
MHSENIAAYVGLDVHKETLAVAIAAPERLGEVRYYGTINNEAQAVRRLFQKLQGLYGNIL
SCYEAGPCGFRLYHQLTAMNIKCQVIAPSRIPKSPTDRIKNDHRDAISLARLLRAGELTP
VWIPDLTHEAMRDLIRARAAAKRDSRVARQRILSMLLRTDKHYAGKHWTGKHRTWLANQS
FSQPSQQIAFQHYCQSLEQIEDRILQLDQEISRLLPEWSLCNLVCQLQALKGVGQLIAIT
LVAELGDFSRFSNPKQLMAFLGLVPGEYSSGNSIRPRGITKVGNSELRRLLYEAAWSYRT
PAKVGAWLIYYRPDSVTQYSKDIAWKAQQRLCSRYRSLTAKGKKSQVAITAVARELTGFM
WDIALAAQSSFSQQKQN
>LFGLNPFC_03498 IS66 family transposase IS682
MFFGNDHGGERGALLCGLIGSCRLNGIDPEAYLRHILSVLPEWPSNRVDELLPWNVVFTD
K
>LFGLNPFC_03499 HTH-type transcriptional regulator BetI
MPANMNRPHHARKTRSCSPEKTARTRQQIARAALEEFSAQGFARASISNISKRAGVAKGT
VYNYFPTKELLFEAVLKEFIATVRTELESSPRRNGETVKAYLLRVMLPAIRKIDDASTGR
ARIAHLVMTEGSRFPVIAQAYLREIHQPLQQAMTQLIQEAASAGELKAEQLLCFPCLLLA
PNWFGMVYNEVLNPAAPVSTGDLFEAGIGAFFR
>LFGLNPFC_03500 hypothetical protein
MKHFIATAVMASSLLFGSNALAASSSAAQKESSPEQSGGTFKYSTDSKIATEQKNVYDTI
IKYQNALNAGDTKTILGLFADESYSQWNDKLTADNTEKRQGQYDDLFKREKFETDFAFDS
IWINGDTAVVRTHHHVGSVVTNFKEQKTIIDLNREVFVLSKINGEWKIFLYTFNTNPLQG
VA
>LFGLNPFC_03501 4-hydroxymandelate oxidase
MPLSRRNFIQNAVLGISAAGLSAAPALAKNISSSTAHIISKTSGHADTSTSKSLHIISLD
RLETSAKDVMTEAAYAYIAHGAGDEWTYHENRRAFSDYPLLPHRLSGVAAHSIDIRTDLL
GHHLEHPLLIAPMGAHMFVHPEGEVIAAAGAEKAGALYESSGASNRSLEDIAKASKGPKW
FQLYFNADAGVTRSLLERAKAAGYSAIIITADALGPGTSDAFLSMSSPFPAGATFGNHDP
RYGGKGDFFNQKVELTPADIEFVKKITGLPVIVKGILRGEDAVVAIDAGADAIQVSNHGG
RQIDGVPSAISQLQEVAARVGHKVPVIFDSGIRRGIDVVRAISLGATAVAVGRPVLYGIA
AGGVGGVAGVIEHLKTELRTAMLLSGARTLKDLAQGFIRNKETEH
>LFGLNPFC_03502 Inner membrane protein YphA
MGNYTSALLLLARLMLACLYMISGVPKLLSFTGTIDKMASLGLIFPAITAAIVVLVEIVG
ALMIVFGFFTRPVSIILCLYTVLASFLGHAFWTMPAELAHGNMIHFYKNICISGGFLALV
VSGPGRISVDRR
>LFGLNPFC_03503 Major fimbrial subunit SMF-1
MKIKVIALATFVSAVFAGSAMAYDGTITFTGKVVAQTCTVNTSDKDLAVTLPTVATSSLK
DNAATSGLTPFAIRLTGCATGMNSAQNVKAYFEPSSNIDLATHNLKNTATPTKADNVQIQ
LLNSNGTSTILLGEADNGQDVQSETIGSDGSATLRYMAQYYATGQSTAGDVKATVHYTIA
YE
>LFGLNPFC_03504 putative fimbrial chaperone YadV
MLRFVLFFFTLSVSAVVQGSVVIMGTRVVYPATQKSISIRLNNDNESPALVQSWLDDGDA
AAPPESVHVPFIITPPIFRMDSKSGQTVRIVYTGESLPKDRESLFYLNVLDIPAKPRAKK
DSDNSEQRNNNYLQLAIRSRIKFFFRPAHLKLTPNDAYLKVTWHQEEGRIKAVNPTPYYI
TYNKIAVDQNKHLTPVEHGGMIPPFSSTVFALKGRTAPASKVSWVIVNDYGGYQQGESVL
E
>LFGLNPFC_03505 Outer membrane usher protein HtrE
MNANNLSCLIYCRCSLLLFAALGLTVTNHSFAAEEAEFDSEFLHLDKGINAIDIRRFSHG
NPVPEGRYYSDIYVNNVWKGKADLQYLRTANTGAPTLCLTPELLSLIDLVKDTMSGNTSC
FPASTGLSSARINFDLSTLRLNIEIPQALLNTRPRGYISPAQWQSGVPAAFINYDANYYQ
YSSSGTSNEQTYLGLKAGFNLWGWALRHRGSESWNNSYPAGYQNIETSIMHDLAPLRAQF
TLGDFYTNGELMDSLSLRGVRLASDERMLPGSLRGYAPAVRGIANSNAKVTIYQNAHILY
ETTVPAGPFVINDLYPSGYAGDLLVKITESNGQTRMFTVPFAAVAQLIRPGFSRWQMSVG
KYRYANKTYNDLIAQGTYQYGLTNDITLNSGLTTASGYTAGLAGLAFNTPLGAIASDITL
SRTAFRYSGVTRKGYSLHSSYSINIPASNTNITLAAYRYSSKDFYHLKDALSANHNAFID
DVSVKSTAFYRPRNQFQISINQELGEKWGGMYLTGTTYNYWGHKGSRNEYQMGYSNFWKQ
LGYQIGLSQSRDNEQQRRDDRFYINFTLPLGGSVQSPVFSTVLNYSKEEKNSIQTSISGT
GGEDNQFSYGISGNSQENGPSGYAMNGGYRSPYVNITTTVGHDTQNNNQRSFGASGAVVA
HPYGVTLSNDLSDTFAIIHAEGAQGAVINNASGSRLDFWGNGVVPYVTPYEKNQISIDPS
NLDLNVELSATEQEIIPRANSATLVKFDTKTGRSLLFDIRMSTGNPPPMASEVLDEHGQL
AGYVAQAGKVFTRGLPEKGHLSVVWGPDNKDRCSFVYHVAHNKDDMQSQLVPVLCIQHPN
QEKT
>LFGLNPFC_03506 hypothetical protein
MKRIFFIPLFLILLPKLAVAGPDDYVPSQIAVNTSTLPGVVIGPADAHTYPRVIGELAGT
SNQYVFNGGAIALMRGKFTPALPKIGSITYTFHQGNSRDSSDFDIYDIGVSGLGIIIGMA

GYWPATPLVPINSSGIYIDPVGANTNPNTYNGATASFGARLFVAFVATGRLPNGYITIPT
RQLGTILLEAKRTSLNNKGLTAPVMLNGGRIQVQSQTCTMGQKNYVVPLNTVYQSQFTSL
YKEIQGGKIDIHLQCPDGIDVYATLTDASQPVNRTDILTLSSESTAKGFGIRLYKDSDVT
AISYGEDSPVKGNGSQWHFSDYRGEVNPHINLRANYIKIADATTPGSVKAIATITFSYQ
>LFGLNPFC_03507 hypothetical protein
MHPDVAPLLLVRECMPDQFASLGTAACVIDKAGNGMALSSWSASDATGAVTVGVVAKGTH
QNSMAQGEFSCTTRENEVYIRYDSGVTNPVSPRGPDKIRGPGGISDGAWDTEAATIRQLN
PLTDEVYSGISGRITA
>LFGLNPFC_03508 hypothetical protein
MLNYRIRSVQLFIVNGRADRAQVRLECDDEAVFECYLLAEGEGELKELSLSELEERALMY
AADSFRYE
>LFGLNPFC_03509 hypothetical protein
MLIIIMTRDRYLEYGLMRILSGYQVTTGRELFNAGKQRQSLPEDSYVILCDRNLERLTYS
MFCGRRFLVIPVSSVRCLTDIRQTIRRGAWLFGHTARPLTRTEMVVVFGVVFHDYGFTFL
ADRLGITMKTVCAHLYNAMEKNGMRGVSIKYLCNTIDR
>LFGLNPFC_03510 hypothetical protein
MADSADIAYENEQFSMSIRLKNRIRNRLPETGFCYNCGEPVKTGLFCDGDCREDYEKRER
FGQINTDNV
>LFGLNPFC_03511 hypothetical protein
MAESGFISLELSRSQAPVTISKTAILFAEADNDTPESTHLHCVGGITHTVNGVFNDIAEL
LPRFIKTHRRDNGQPAVAIHDWNVSYIEKGNDASAVVYFTESGSSITVRESYEDIIAKFH
AL
>LFGLNPFC_03512 hypothetical protein
MTGALRRDAYVLPGQTVSVRRIYKLLLLMLPGKGVTKKQPPLPDSKEAEWSLQDLLFYNV
WPCSKTGQVLLSCEVF
>LFGLNPFC_03513 P fimbrial regulatory protein KS71A
MKNEILEFLNRHNGGKTAEIAEALAVTDYQARYYLLLLEKEGMVQRSPLRRGMATYWFLK
GEMQAGQNCSSTT
>LFGLNPFC_03514 Pap fimbrial major pilin protein
MIKSVIAGAVAMAVVSFGVNAAPTTPQGQGRVTFNGTVVDAPCSISQKSADQSIDFGQLS
KSFLQAGGVSKPMNLDIELVNCDITSFKGVGGAPAAKKGTVKLAFSGPRVSGHNEELDTS
GGTGTAIVVQAAGKNVSFDGTEGDANTLKDGDNVLHYTAIVKKSSANNAQVTEGAFSAVA
TFNLSYQ
>LFGLNPFC_03515 PAP fimbrial minor pilin protein
MSLPEYWGEEHVWWDGRAAFHGEVVRPACTLAMEDAWQIIDMGESPVRDLQNGFSGPERK
FSLRLRNCEFNSQGGNLFSDSRIRVTFDGVRGETPDKFNLSGQAKGINLQIADVRGNIAR
AGKVMPAIPLTGNEEALDYTLRIVRNGKKLEAGNYFAVLGFRVDYE
>LFGLNPFC_03516 Outer membrane usher protein PapC
MRGMKDRIPFAVNNITCVILLSLFCNAASAVEFNTDVLDAADKKNIDFTRFSEAGYVLPG
QYLLDVIVNGQSISPASLQISFVEPALSGDKAEKKLPQACLTSDMVRLMGLTAESLDKVV
YWHDGQCADFHGLPGVDIRPDTGAGVLRINMPQAWLEYSDATWLPPSRWDDGIPGLMLDY
NLNGTVSRNYQGGDSHQFSYNGTVGGNLGPWRLRADYQGSQEQSRYNGEKTTNRNFTWSR
FYLFRAIPRWRANLTLGENNINSDIFRSWSYTGASLESDDRMLPPRLRGYAPQITGIAET
NARVVVSQQGRVLYDSMVPAGPFSIQDLDSSVRGRLDVEVIEQNGRKKTFQVDTASVPYL
TRPGQVRYKLVSGRSRGYGHETEGPVFATGEASWGLSNQWSLYGGAVLAGDYNALAAGAG
WDLGMPGTLSADITQSVARIEGERTFQGKSWRLSYSKRFDNADADITFAGYRFSERNYMT
MEQYLNARYRNDYSSREKEMYTVTLNKNVADWNTSFNLQYSRQTYWDIRKTDYYTVSVNR
YFNVFGLQGVAVGLSASRSKYLGRDNDSAYLRISVPLGTGTASYSGSMSNDRYVNMAGYT
DTFNDGLDSYSLNAGLNSGGGLTSQRQINAYYSHRSPLANLSANIASLQKGYTSFGVSAS
GGATITGKGAALHAGGMSGGTRLLVDTDGVGGVPVDGGQVVTNRWGTGVVTDISSYYRNT
TSVDLKRLPDDVEATRSVVESALTEGAIGYRKFSVLKGKRLFAILRLADGSQPPFGASVT
SEKGRELGMVADEGLAWLSGVTPGETLSVNWDGKIQCQVNVPETAISDQQLLLPCTPQK
>LFGLNPFC_03517 Chaperone protein PapD
MIRKKILMAAIPLFVISGADAAVSLDRTRAVFDGSEKSMTLDISNDNKQLPYLAQAWIEN
ENQEKIITGPVIATPPVQRLEPGAKSMVRLSTTPDISKLPQDRESLFYFNLREIPPRSEK
ANVLQIALQTKIKLFYRPAAIKTRPNEVWQDQLILNKVSGGYRIENPTPYYVTVIGLGGS
EKQAEEGEFETVMLSPRSEQTVKSANYNTPYLSYINDYGGRPVLSFICNGSRCSVKKEK
>LFGLNPFC_03518 hypothetical protein
MVVNKTTAVLYLIALSLSGFIHTFLRAEERGIYDDVFTADELHHYRINERGGRTGSLAVS
GALLSSPCTLVSNEVPLSLRPENHSASAGAPLMLRLAGCGDGGALQPGKRGVAMTVSGSL
VTGPGTGSALLPDRKLSGCDHLVIHDGDTFLLCRPDRRQEEMLAAWRKRATQEGEYSDAR
SNPAMLRLSIKYE
>LFGLNPFC_03519 Fimbrial adapter PapK
MIKSTGALLLFAALSAGQAMASDVAFRGNLLDRPCHVSGDSLNKHVVFKTRASRDFWYPP

GRSPTESFVIRLENCHATAVGKIVTLTFKGTEEAALPGHLKVTGVNAGRLGIALLDTDGS
SLLKPGASHNKGQGEKVTGNSLELPFGAYVVATPEALRTKSVVPGDYEATATFELTYR
>LFGLNPFC_03520 Fimbrial protein PapE
MKKIRGLCLPVMLGAVLMSQHVHAADNLTFKGKLIIPACTVQNAEVNWGDIEIQNLVQSG
GNQKDFTVDMNCPYSLGTMKVTITSNGQTGNSILVPNTSTASGDGLLIYLYNSNNSGIGN
AVTLGSQFTPGKITGTAPARKITLYAKLGYKGNMQSLQAGTFSATATLVASYS
>LFGLNPFC_03521 Minor fimbrial protein PrsF
MIRLSLFISLLLTSVAVLADVQINIRGNVYIPPCTINNGQNIVVDFGNINPEHVDNSRGE
VTKTISISCPYKSGSLWIKVTGNTMGGGQNNVLATNITHFGIALYQGKGMSTPLTLGNGS
GNGYRVTAGLDTARSTFTFTSVPFRNGSGILNGGDFRTTASMSMIYN
>LFGLNPFC_03522 Fimbrial adhesin PapG
MKKWLPAFLFLSLSGCNDALAANQSTMFYSFNDNIYRPQLSVKVTDIVQFIVDINSASST
ATLSYVACNGFTWTHGLYWSEYFAWLVVPKHVSYNGYNIYLELQSRGSFSLDAEDNDNYY
LTKGFAWDEVNSSGRVCFDIGEKRSLAWSFGGVTLNARLPVDLPKGDYTFPVKFLRGIQR
NNYDYIGGRYKIPSSLMKTFPFNGTLNFSIKNTGGCRPSAQSLEINHGDLSINSANNHYA
AQTLSVSCDVPTNIRFFLLSNTTPAYSHGQQFSVGLGHGWDSIVSINGVDTGETTMRWYR
AGTQNLTIGSRLYGESSKIQPGVLSGSATLLMILP
>LFGLNPFC_03523 Transcriptional repressor MprA
MNNTDTLEKIIRHQKNKDPAYPFREHLLMQLCIRTNKRMQDNISEFLGAYGINHSAYMVL
TTLFAAESHCLSPSEISQKLQFTRTNITRITDFLEKAGYVKRTDSREDRRAKKISLTSEG
MFFIQRLTLAQSMYLKEIWDYLTHDEQELFEVINKKLLAHFSDASS
>LFGLNPFC_03524 hypothetical protein
MALLWNQAAGSINLEFISYWNSGVLAAPDNHLTHEERSALQKLWGGLETGDVTIIGRSDE
VHDFTSALINCFLSEEEIVWWQSGGIFPDPWPANISRLN
>LFGLNPFC_03525 hypothetical protein
MSAQEKTVQIDSFDAKFLDINTLSKLIYERKSFVIENVSDVSETVKRVEHEIEKTKLSCR
VYTEYRSTALAGSLWSPTVILGVASAVAIGVHNLSTWNPDYEIGKNYIKRRLSVKYKKAE
VL
>LFGLNPFC_03526 Outer membrane protein PagN
MNKVFVVSVVAAACVFAVNAGAKEGKSGFYLTGKAGASVMSLSDQRFLSGDEEETSKYKG
GDDHDTVFSGGIAVGYDFYPQFSIPVRTELEFYARGKADSKYNVDKDSWSGGYWRDDLKN
EVSVNTLMLNAYYDFRNDSAFTPWVSAGIGYARIHQKTTGISTWDYEYGSSGRESLSRSG
SADNFAWSLGAGVRYDVTPDIALDLSYRYLDAGDSSVSYKDEWGDKYKSEVDVKSHDIML
GMTYNF
>LFGLNPFC_03527 Phosphoethanolamine transferase OpgE
MNIRKLFCPGNTPRILLFLFFFVVSAITTIACGYTEKNATGNVLLLFLLLLLAHRNTLTS
ITALLFLFCCALYAPAGMTYGKINNSFIVALLQTTTDEAAEFTGMIPVYHFLVSAAILVF
MVIFWRTHHRGHRNWLALLLFVLCSVNSWPLRMVKGIVVGTTDTLREMQRYKQLNQHGAD
NWKILPGVPLYDTIVIVTGESVRRDYMSVYGYPVPTTPWLNTAPGLFIDGYTSAAASTVP
SLSRTLIYDYEQNPDSGNNVVALAAKAGYSTWWISNQGKLGEHDTRISVIASDAEHTVFL
KKGSFASRKTDDMLLLQETERALADKSSPKVIFLHMMGSHPNPCDRLHSWPNHYLEQYPR
KVACYLASISKLDNFLGQLDGILRRHSRHFAMLYFSDHGLSVSDSANPVHHDGHVQGGYS
VPLIITASDITSHQSVSRKISARHFAGIFQWLTGIRTENIPPFNPLTDEDNEPVMVFNGE
RNVPADSLKPQPLILPDRR
>LFGLNPFC_03528 ATP-dependent RecD-like DNA helicase
MDENALGFTSYWRNSLADAESGKGSFERKDAKNFTHWHGIAAGRLDEAIVSKFFKGEKDD
VETVDVILRPKVYFRLLQHGKDRSAGAPDIVTPIVTPALLSREGFLYPTPATSIPRDLLE
PLPKGAFSIGEIGQYDKYKTTHTTFSINFDDSVDKTAETDEEREARYAALQQEWRQYLYD
SERLLKSVAGDWIEKPEQYELAEHGYIVKTAQSGGASFHILSLYDHLLVCNKDVPLFNRF
ASREVHAAESLLAPGAKFSDRLGHSGDKFPLAKAQRDALSHFLDARHGDILAVNGPPGTG
KTTLVLSIIATQWARAALEKSEPPVIIATSTNNQAVTNIIEAFGKDFSQGSGAMAGRWLP
GLKSFGAYFPSSSRKAEAAKKYQTEDFFNQVESKEYVEDALLFYLEKAKAAFPEKECSSP
EKVIELLHGQLAAKSEQLIRLNATWQTLSQVWAARELIANDIEQYLDNLNKLLSGQEQKI
TLLKSAKTEWKKYRAGESLIYSLFSWLPAVRSKRQYQIQLFLEDKLGALIAGNQWSDPET
IERNIDGLLNSAEREQTTYRQQIDSAHEIILKEQQAVQEWQRLALDLGYEGDEELSFSQA
DELADTQIRFPAFLLTTHYWEGRWLMDMASIDDLQEEKKKKGAKGVTARWQRRMKLTPCV
VMTCYMLPGNMQISEHKGQRKFEKSYLYDFADLLIVDEAGQVLPEVAAASFALAKKALVI
GDTEQLPPIWSIAPAIDVGNMLAEKILSGSTQEEITEKYTAIADLGKSAASGSVMKIAQF
ASRYQYDPELARGMYLYEHRRCYDNIIGYCNTLCYHGKLLPKRGREESNLMPEMGYLHID
GKGELASSGSRYNLLEAETIAVWLAENQQNIEAHYGKSLHEVVGIVTPFSAQVSTIKQVL
GKQGISTGANEKSLTVGTVHSLQGAERAIVIFSPVYSKHEDGGFIDSDNSMLNVAVSRAK
DSFLVFGDMDLFEVQPASSPRGLLAKYLFESEKNALSFDYKERKDLKTAGTKIYTLHGVE
QHDNFLNQTFENTSKHIMIVSPWLTWQRLEQTGFLDSMIAACSRGVNVTIVTDRSYNTEH
NDFEKRKEKQQNFKAALEKLNALGIATKLVNRVHSKIVIGDDGLLCVGSFNWFSATREAR

YERYDTSMVYCGDNLKGEVEAIYNSLERRQV
>LFGLNPFC_03529 Prophage integrase IntA
MALTDLKVRTAKPADKQYKLTDGGGMHLLVHPNGSKYWRLQYRYEGKQKMLALGVYPEIT
LADARVRRDEARKLLANGVDPGDKKKNDKVEQSKARTFKEVAIEWYGTNKKWSEDHAHRV
LKSLEDNLFAALGERNIAELKTRDLLAPIKAVEMSGRLEVAARLQQRTTAIMRYAVQSGL
IDYNPAQEMAGAVASCNRQHRPALELKRIPELLTKIDSYTGRPLTRWATELTLLIFIRSS
ELRFARWSEIDFEASIWTIPPEREPIPGVKHSHRGSKMRTTHLVPLSRQALAILKQIKQF
CGAHDLIFIGDHDSHKPMSENTVNSALRVMGYDTKVEVCGHGFRTMACSSLIESGLWSRD
AVERQMSHMERNSVRAAYIHKAEHLEERRLMLQWWADFLDANREKGISPFDYAKINNPLK
>LFGLNPFC_03531 Aldehyde reductase Ahr
MSMIKSYAAKEAGGELEVYEYDPGELRPQDVEVQVDYCGICHSDLSMIDNEWGFSQYPLI
AGHEVIGRVVALGSAAQDKGLQVGQRVGIGWTARSCGHCDACISGNQINCEQGAVPTIMN
RGGFAEKLRADWQWVIPLPENIDIESAGPLLCGGITVFKPLLMHHITATSRVGVIGIGGL
GHIAIKLLHAMGCEVTAFSSNPAKEQEVLAMGADKVVNSRDPQALKTLAGQFDLIINTVN
VSLDWQPYFEALTYGGNFHTVGAVLTPLPVPAFTLIAGDRSVSGSATGTPYELRKLMRFA
ARSKVAPTTELFPMSKINDAIQHVRDGKARYRVVLKADF
>LFGLNPFC_03532 Thermoresistant gluconokinase
MAGESFILMGVSGSGKTLIGSKVAALLSAKFIDGDDLHPAKNIDKMSQGIPLSDEDRLPW
LERLNDASYSLYKKNETGFIVCSSLKKQYRDILRKGSPHVHFLWLDGDYETILARMQRRA
GHFMPVALLKSQFEALERPQDDEQDIVRIDINHDIANVTEQCRQAVLAIRQNRICAKEGC
ASDQRCE
>LFGLNPFC_03533 L-idonate 5-dehydrogenase (NAD(P)(+))
MQVKTQSCVVAGKKTVAVTEQTIDWNNNGTLVQITRGGICGSDLHYYQEGKVGNFMIKAP
MVLGHEVIGKVIHSDSSKLHEGQTVAINPSKPCGHCKYCIEHNENQCTEMRFFGSAMYFP
HVDGGFTRYKMVETSQCVPYPAKADEKVMAFAEPLAVAIHAAHQAGELQGKRVFISGVGP
IGCLIVSAVKTLGAAEIVCADVSPRSLSLGKEMGADVLVNPQNDDMDHWKAEKGYFDVSF
EVSGHPSSVNTCLEVTRARGVMVQVGMGGAMAEFPMMTLIGKEISLKGSFRFTSEFNTSV
SWLANGVINPLPLLSAEYPFTDLEEALRFAGDKTQAAKVQLVF
>LFGLNPFC_03534 5-keto-D-gluconate 5-reductase
MNDLFSLAGKNILITGSAQGIGFLLATGLGKYGAQIIINDITAERAELAVEKLHQEGIQA
VAAPFNVTHKHEIDAAVEHIEKDIGPIDVLVNNAGIQRRHPFTEFPEQEWNDVIAVNQTA
VFLVSQAVTRHMVERKAGKVINICSMQSELGRDTITPYAASKGAVKMLTRGMCVELARHN
IQVNGIAPGYFKTEMTKALVEDEAFTAWLCKRTPAARWGDPQELIGAAVFLSSKASDFVN
GHLLFVDGGMLVAV
>LFGLNPFC_03535 Gnt-II system L-idonate transporter
MPLIIIAAGVALLLILMIGFKVNGFIALVLVAAVVGFAEGMDAQAVLHSIQNGIGSTLGG
LAMILGFGAMLGKLISDTGAAQRIATTLIATFGKKRVQWALVITGLVVGLAMFFEVGFVL
LLPLVFTIVASSGLPLLYVGVPMVAALSVTHCFLPPHPGPTAIATIFEANLGTTLLYGFI
ITIPTVIVAGPLFSKLLTRFEKAPPEGLFNPHLFSEEEMPSFWNSIFAAVIPVILMAIAA
VCEITLPKTNTVRLFFEFVGNPAVALFIAIVIAIFTLGRRNGRTIEQIMDIIGDSIGAIA
MIVFIIAGGGAFKQVLVDSGVGQYISHLMTGTTLSPLLMCWTVAALLRIALGSATVAAIT
TAGVVLPIINVTHADPALMVLATGAGSVIASHVNDPGFWLFKGYFNLTVGETLRTWTVME
TLISIMGLLGVLAINAVLH
>LFGLNPFC_03536 HTH-type transcriptional regulator GntR
MRNHRISLQDIATLAGVTKMTVSRYIRSPKKVAKETGERIAKIMEEINYIPNRAPGMLLN
AQSYTLGILIPSFQNQLFADILAGIESVTSVHNYQTIIANYNYDRDSEEESVINLLSYNI
DGIILSEKYHTIRTVKFLRSATIPVVELMDVQGERLDMEVGFDNRQAAFDMVCTMLDKRV
RRKILYLGSKDDTRDEQRYQGYCDAMMLHNLSPLRMNPRAISSIHLGMQLMRDALSANPD
LDGVFCTNDDIAMGALLLCRERNLAVPEQISIAGFHGLEIGRQMIPSLASVITPRFDIGR
MAAQMLLSKIKNNDHNHNTVDLGYQIYHGNTL
>LFGLNPFC_03537 hypothetical protein
MSEPLLIARTPDTELFLLPGMANRHGLITGATGTGKTVTLQKLAESLSEIGVPVFMADVK
GDLTGVAEEGTSSEKLLARLKNIGVNDWQPHTNPVVVWDIFGEKGHPVRATVSDLGPLLL
ARLLNLNDVQSGVLNIIFRIADDQGLLLLDFKDLRAITQYIGDNAKSFQNQYGNISSASV
GAIQRGLLSLEQQGAAHFFGEPMLDIKDWMRTDANGKGVINILSAEKLYQMPKLYAASLL
WMLSELYEQLPEAGDLEKPKLVFFFDEAHLLFNDAPQVLLDKIEQVIRLIRSKGVGVWFV
SQNPSDIPDNVLGQLGNRVQHALRAFTPKDQKAVKAAAQTMRANPTFDTEKAIQELGTGE
ALISFLDAKGSPSVVERAMVIAPCSRMGPVTEDERNGLINHSPVYGKYEDDVDRESAYEM
LQKGFQASIEQQNNPPAKGKEVAVDDGILGGLKDILFGTTGPRGGKKDGVVQTMAKSAAR
QVTNQIVRGMLGSLLGGRRR
>LFGLNPFC_03538 Lipopolysaccharide export system permease protein LptG
MQPFGVLDRYIGKTIFTTIMMTLFMLVSLSGIIKFVDQLKKAGQGSYDALGAGMYTLLSV
PKDVQIFFPMAALLGALLGLGMLAQRSELVVMQASGFTRMQVALSVMKTAIPLVLLTMAI
GEWVAPQGEQMARNYRAQAMYGGSLLSTQQGLWAKDGNNFVYIERVKGDEELGGISIYAF

NENRRLQSVRYAAAAKFDPEHKVWRLSQVDESDLTNPKQITGSQTVSGTWKTNLTPDKLG
VVALDPDALSISGLHNYVKYLKSSGQDAGRYQLNMWSKIFQPLSVAVMMLMALSFIFGPL
RSVPMGVRVVTGISFGFVFYVLDQIFGPLTLVYGIPPIIGALLPSASFFLISLWLLMRKS
>LFGLNPFC_03539 Lipopolysaccharide export system permease protein LptF
MRETLKSQLAILFILLLIFFCQKLVRILGAAVDGDIPANLVLSLLGLGVPEMAQLILPLS
LFLGLLMTLGKLYTESEITVMHACGLSKAVLVKAAMILAVFTAIVAAVNVMWAGPWSSRH
QDEVLAEAKANPGMAALAQGQFQQATNGSSVLFIESVDGSDFKDVFLAQIRPKGNARPSV
VVADSGHLTQLRDGSQVVTLNQGTRFEGTALLRDFRITDFQDYQAIIGHQAVALDPNDTD
QMDMRTLWNTDTDRARAELNWRITLVFTVFMMALMVVPLSVVNPRQGRVLSMLPAMLLYL
LFFLIQTSLKSNGGKGKLDPTLWMWTVNLIYLALAIVLNLWDTVPVRRLRASFSRKGAV
>LFGLNPFC_03540 Cytosol aminopeptidase
MEFSVKSGSPEKQRSACIVVGVFEPRRLSPIAEQLDKISDGYISALLRRGELEGKPGQTL
LLHHVPNVLSERILLIGCGKERELDERQYKQVIQKTINTLNDTGSMEAVCFLTELHVKGR
NNYWKVRQAVETAKETLYSFDQLKTNKSEPRRPLRKMVFNVPTRRELTSGERAIQHGLAI
AAGIKAAKDLGNMPPNICNAAYLASQARQLADSYSKNVITRVIGEQQMKELGMHSYLAVG
QGSQNESLMSVIEYKGNASEDARPIVLVGKGLTFDSGGISIKPSEGMDEMKYDMCGAAAV
YGVMRMVAELQLPINVIGVLAGCENMPGGRAYRPGDVLTTMSGQTVEVLNTDAEGRLVLC
DVLTYVERFEPEAVIDVATLTGACVIALGHHITGLMANHNPLAHELIAASEQSGDRAWRL
PLGDEYQEQLESNFADMANIGGRPGGAITAGCFLSRFTRKYNWAHLDIAGTAWRSGKAKG
ATGRPVALLAQFLLNRAGFNGEE
>LFGLNPFC_03541 DNA polymerase III subunit chi
MKNATFYLLDNDTTVDGLSAVEQLVCEIAAERWRSGKRVLIACEDEKQAYRLDEALWARP
AESFVPHNLAGEGPRGGAPVEIAWPQKRSSSPRDILISLRTSFADFATAFTEVVDFVPYE
DSLKQLARERYKAYRVAGFNLNTATWK
>LFGLNPFC_03542 Valine--tRNA ligase
MEKTYNPQDIEQPLYEHWEKQGYFKPNGDESQESFCIMIPPPNVTGSLHMGHAFQQTIMD
TMIRYQRMQGKNTLWQVGTDHAGIATQMVVERKIAAEEGKTRHDYGREAFIDKIWEWKAE
SGGTITRQMRRLGNSVDWERERFTMDEGLSNAVKEVFVRLYKEDLIYRGKRLVNWDPKLR
TAISDLEVENRESKGSMWHIRYPLADGAKTADGKDYLVVATTRPETLLGDTGVAVNPEDP
RYKDLIGKYVILPLVNRRIPIVGDEHADMEKGTGCVKITPAHDFNDYEVGKRHALPMINI
LTFDGDIRESAQVFDTKGNESDVYSSEIPAEFQKLERFAARKAVVAAVDALGLLEEIKPH
DLTVPYGDRGGVVIEPMLTDQWYVRADVLAKPAVEAVENGDIQFVPKQYENMYFSWMRDI
QDWCISRQLWWGHRIPAWYDEAGNVYVGRNEDEVRKENNLGADVALRQDEDVLDTWFSSA
LWTFSTLGWPENTDALRQFHPTSVMVSGFDIIFFWIARMIMMTMHFIKDENGKPQVPFHT
VYMTGLIRDDEGQKMSKSKGNVIDPLDMVDGISLPELLEKRTGNMMQPQLADKIRKRTEK
QFPNGIEPHGTDALRFTLAALASTGRDINWDMKRLEGYRNFCNKLWNASRFVLMNTEGQD
CGFNGGEMTLSLADRWILAEFNQTIKAYREALDSFRFDIAAGILYEFTWNQFCDWYLELT
KPVMNGGTEAELRGTRHTLVTVLEGLLRLAHPIIPFITETIWQRVKVLCGITADTIMLQP
FPQYDASQVDEAALADTEWLKQAIVAVRNIRAEMNIAPGKPLELLLRGCSADAERRVNEN
RGFLQTLARLESITVLPADDKGPVSVTKIVDGAELLIPMAGLINKEDELARLAKEVAKIE
GEISRIENKLANEGFVARAPEAVIAKEREKLEGYAEAKAKLIEQQAVIAAL
>LFGLNPFC_03543 Inner membrane protein YjgN
MNDVNIGKDNSRHSFVFTGKGGEYFLICLVNFSLTIITLGIYGPWALIKCRRYIYQHVTL
KGQPFSYKGTGGAIFVSMLLIVVVYLLSISCFAGQHFALGLFLFALLICGIPCMAVKSLQ
YQANMTSLNDIRFGFNCSMMRAWWVMLGLPVLLALVFWFALYLIAQVTTSIGGLFFNLVA
LSLLSAIGLGVVHGITYSKWMPLLGNNATFGIHKFSIQVNVKECIKGCMLAILTMVPFII
VIGIMIAPVFQQLMMMTMLGRSDAGSEFVLQYYPQIMASYFLYFVAILVFASYLYVTLRN
LFLNNLTLANGTIRFHSSVTAIGMLLRMLAVLMGSSITCGLAYPWLKMWMVSWIANNTHV
QGDLDSLELTNDDKPQDSGSLMWISRGIMPYVPFI
>LFGLNPFC_03544 hypothetical protein
MNVVASPALRLRKLTVADNPAIAHVIRQVSAEYGLTADKGYTVADPNLDELYQVYSQPGH
AYWVVEYEGEVVGGGGIAPLTGSESDICELQKMYFLPAIRGKGLAKKLALMAMEQAREMG
FKRCYLETTAFLKEAIALYEHLGFEHIDYALGCTGHVDCEVRMLRKL
>LFGLNPFC_03545 Regulator of ribonuclease activity B
MANPEQLEEQREETRLIIEELLEDGSDPDALYTIEHHLSADDLETLEKAAVEAFKLGYEV
TDPEELEVEDGDIVICCDILSECALNADLIDAQVEQLMTLAEKFDVEYDGWGTYFEDPNG
EDGDDEDFVDEDDDGIRH
>LFGLNPFC_03546 Ornithine carbamoyltransferase subunit I
MSGFYHKHFLKLLDFTPAELNSLLQLAAKLKADKKSGKEEARLTGKNIALIFEKDSTRTR
CSFEVAAYDQGARVTYLGPSGSQIGHKESIKDTARVLGRMYDGIQYRGYGQEIVETLAEY
AGVPVWNGLTNEFHPTQLLADLLTMQEHLPGKAFNEMTLVYAGDARNNMGNSMLEAAALT
GLDLRLVAPQACWPEAALVAECSALAQKHGGKITLTEDIASGVKGADFIYTDVWVSMGEP
KEKWAERIALLRDYQVNSKMMALTGNSQVKFLHCLPAFHDEQTTLGKKMAAEFGLYGGME
VTDEVFESPASIVFDQAENRMHTIKAVMVATLAK

>LFGLNPFC_03547 Toxin-antitoxin biofilm protein TabA
MIVGNIHHLQSWLPEELREAIEYIKSHVSDETAKGKHAIDGDRLFYLISEDTTEPGELRR
AEYHARYLDIQIVLKGQEGMTFSTQPAGVPETDWLADKDIAFIGQGIDEKTVILNEGDFV
VFYPGEVHKPLCAVGAPAQVRKAVVKLLKS
>LFGLNPFC_03548 Arginine deiminase
MEKHYVGSEIGQLRSVMLHRPNLSLKRLTPSNCQELLFDDVLSVERAGEEHDIFANTLRQ
QGIEVLLLTDLLTQTLDIPEAKSWLLETQISDYRLGPTFATDVRTWLAEMSHRDLARHLS
GGLTYSEIPASIKNMVVDTHDINDFIMKPLPNHLFTRDTSCWIYNGVSINPMAKPARQRE
TNNLRAIYRWHPQFAGGEFIKYFGDENINYDHATLEGGDVLVIGRGAVLIGMSERTTPQG
IEFLAQALFKHRQAERVIAVELPKHRSCMHLDTVMTHIDIDTFSVYPEVVRPDVNCWTLT
PDGHGGLKRTQESTLLHAIEKALGIDQVRLITTGGDAFEAEREQWNDANNVLTLRPGVVV
GYERNIWTNEKYDKAGITVLPIPGDELGRGRGGARCMSCPLHRDGI
>LFGLNPFC_03549 Carbamate kinase 1
MENKPTLVIALGGNALLKRGEPLEAEIQRKNIDLAAKTIAQLTQHWRVVLVHGNGPQVGL
LALQNSAYAHVAPYPLDILGAESQGMIGYMLQQALKNQLPQREISVLLTQVEVDANDPAF
SNPTKYIGPIYDHAQTQVLQAEKGWVFKADGHSFRRVVPSPQPKRIVERDAIQTLIAHDH
LVICNGGGGVPVVEKADGYHGIEAVIDKDLSAALLASQIHADALLILTDADAVYLDWGKP
TQRPLAQVTPELLNEMQFDAGSMGPKVTACAKFVSQCRGIAGIGSLADGPEILAGDKGTL
IRLDTPITTLDPFL
>LFGLNPFC_03550 Ornithine carbamoyltransferase
MATSLKNRNFLKLLDYTPAEIQYLIDLAINLKAAKKSGNEKQTLVGKNIALIFEKSSTRT
RCAFEVAAFDQGAQVTYIGPSGSQIGHKESMKDTARVLGRMYDGIEYRGYGQNIVEELGE
FAGVPVWNGLTNEFHPTQILADLMTMLEHAPGKTLPELSFAYLGDARNNMGNSLMVGAAK
MGMDIRLVAPKSFWPDEALVTQCREIASVTGARITLTEDVEEGVYDVDFLYTDVWVSMGE
PKEAWAERVSLMTPYQINQQVITATRNPEVKFMHCLPAFHNEHTTVGREIEMAYGLKGLE
VTDEVFESAHSIVFDEAENRMHTIKAVMVATLGD
>LFGLNPFC_03551 hypothetical protein
MGKFKFPTAYTILFVLIALVAAMTWIVPAGKYQMATNAALGKEVPVAGTYAPVEAQPQGI
TAVLLAPIDGLYNHQTYTAGAIDVALFVLIIGGFLGVVNKTGAIDAGIERVTTRLNGREE
WMIPILMALFAAGGTIYGMAEESLPFYTLLVPVMMAARFDPLVAAATVLLGSGIGTLGST
INPFATVIAANAAGIPFTQGILLRVILLLVGYVICVYWVMRYARKVRNSPESSIVADKMA
ENQAHFLGNRSETMLEFTPTRKAILILFAASFAFMIYGVAVLGWWMAEISAVFLAAAVIV
GVIARMGEETFTSTFIDGARDLLGVALIIGIARGIVVVMDNGMITHTILHSAENLVSGLS
TTVFINVTYWLEVLLSFLVPSSSGLAVLTMPIMAPLADFAHVQRDLVVTAYQSASGVVNL
ITPTSAVVMGGLAIARVPWVRYLRWVAPLLLILTMLNMIVLSIAAMI
>LFGLNPFC_03552 Arginine repressor
MKEYDDYSAKEKKQLAVCQRLITEKSYLSQEEIRRDLQNHGFDSISQSTVSRLLKLLGVI
KIRNTKGQKIYSVNPQLLPTPDAGRSVAEMVLSVEHNGEFILIHTVAGYGRAVARILDFH
ALPEILGVIAGSNIVWVAPRVVKRTALVHKQINYLLKLNIYS
>LFGLNPFC_03553 Aspartate carbamoyltransferase catalytic subunit
MANPLYQKHIISINDLSRDDLNLVLATAAKLKANPQPELLKHKVIASCFFEASTRTRLSF
ETSMHRLGASVVGFSDSANTSLGKKGETLADTISVISTYVDAIVMRHPQEGAARLATEFS
GNVPVLNAGDGSNQHPTQTLLDLFTIQETQGRLDNLHVAMVGDLKYGRTVHSLTQALAKF
DGNRFYFIAPDALAMPQYILDMLDEKGIAWSLHSSIEEVMAEVDILYMTRVQKERLDPSE
YANVKAQFVLRASDLHNAKANMKVLHPLPRVDEIATDVDKTPHAWYFQQAGNGIFARQAL
LALVLNRDLVL
>LFGLNPFC_03554 Aspartate carbamoyltransferase regulatory chain
MTHDNKLQVEAIKRGTVIDHIPAQIGFKLLSLFKLTETDQRITIGLNLPSGEMGRKDLIK
IENTFLSEDQVDQLALYAPQATVNRIDNYEVVGKSRPSLPERIDNVLVCPNSNCISHAEP
VSSSFAVRKRANDIALKCKYCEKEFSHNVVLAN
>LFGLNPFC_03555 2-iminobutanoate/2-iminopropanoate deaminase
MSKTIATENAPAAIGPYVQGVDLGNMIITSGQIPVNPKTGEVPADVAAQARQSLDNVKAI
VEAAGLKVGDIVKTTVFVKDLNDFATVNATYEAFFTEHNATFPARSCVEVARLPKDVKIE
IEAIAVRR
>LFGLNPFC_03556 Magnesium-transporting ATPase, P-type 1
MFKEIFTRLIRHLPSRLVHRDPLPGAQQTVNAAVPPSLSAHCLKMAVMPEEELWKTFDTH
PEGLNLAEVESAREQHGENKLPAQQPSPWWVHLWVCYRNPFNILLTILGAISYATEDLFA
AGVIALMVAISTLLNFIQEARSTKAADALKAMVSNTATVLRVINDKGENGWLEIPIDQLV
PGDIIKLAAGDMIPADLRILQARDLFVAQASLTGESLPVEKAATTRQPEHSNPLECDTLC
FMGTTVVSGTAQAMVIATGANTWFGQLAGRVSEQESEPNAFQQGISRVSMLLIRFMLVMA
PVVLLINGYTKGDWWEAALFALSVAVGLTPEMLPMIVTSTLARGAVKLSKQKVIVKHLDA
IQNFGAMDILCTDKTGTLTQDKIVLENHTDISGKTSERVLHSAWLNSHYQTGLKNLLDTA
VLEGTDEESARSLASRWQKIDEIPFDFERRRMSVVVAENTEHHQLVCKGALQEILNVCSQ
VRHNGEIVPLDDTMLRKIKRVTDTLNRQGLRVVAVATKYLPAREGDYQRADESDLILEGY

IAFLDPPKETTAPALKALKASGITVKILTGDSELVAAKVCHEVGLDAGEVVIGSDIETLS
DDELANLAQRTTLFARLTPMHKERIVTLLKREGHVVGFMGDGINDAPALRAADIGISVDG
AVDIAREAADIILLEKSLMVLEEGVIEGRRTFANMLKYIKMTASSNFGNVFSVLVASAFL
PFLPMLPLHLLIQNLLYDVSQVAIPFDNVDDEQIQKPQRWNPADLGRFMVFFGPISSIFD
ILTFCLMWWVFHANTPETQTLFQSGWFVVGLLSQTLIVHMIRTRRVPFIQSCASWPLMIM
TVIVMIVGIALPFSPLASYLQLQALPLSYFPWLVAILAGYMTLTQLVKGFYSRRYGWQ
>LFGLNPFC_03557 HTH-type transcriptional regulator TreR
MQNRLTIKDIARLSGVGKSTVSRVLNNESGVSQRTRERVEAVMNQHGFSPSRSARAMRGQ
SDKVVAIIVTRLDSLSENLAVQTMLPAFYEQGYDPIMMESQFSPQLVAEHLGVLKRRNID
GVVLFGFTGITEEMLAHWQSSLVLLARDAKGFASVCYDDEGAIKILMQRLYDQGHRNISY
LGVPHSDVTTGKRRHEAYLAFCKAHKLHPVAALPGLAMKQGYENVAKVITPETTALLCAT
DTLALGASKYLQEQRIDTLQLASVGNTPLMKFLHPEIVTVDPGYAEAGRQAACQLIAQVT
GRSEPQQIIIPATLS
>LFGLNPFC_03558 PTS system trehalose-specific EIIBC component
MMSKINQTDIDRLIELVGGRGNIATVSHCITRLRFVLNQPANARPKEIEQLPMVKGCFTN
AGQFQVVIGTNVGDYYQALIASTGQAQVDKEQVKKAARQNMKWHEQLISHFAEIFFPLLP
ALISGGLILGFRNVIGDLPMSNGQTLAQMYPSLQTIYDFLWLIGEAIFFYLPVGICWSAV
KKMGGTPILGIVLGVTLVSPQLMNAYLLGQQLPEVWNFGMFSIAKVGYQAQVIPALLAGL
ALGVIETRLKRIVPDYLYLVVVPVCSLILAVFLAHALIGPFGRMIGDGVAFAVRHLMTGS
FAPIGAALFGFLYAPLVITGVHQTTLAIDLQMIQSMGGTPVWPLIALSNIAQGSAVIGII
ISSRKHNEREISVPAAISAWLGVTEPAMYGINLKYRFPMLCAMIGSGLAGLLCGLNGVMA
NGIGVGGLPGILSIQPSYWQVFALAMAIAIIIPIVLTSFIYQRKYRLGTLDIV
>LFGLNPFC_03559 Trehalose-6-phosphate hydrolase
MTHLPHWWQNGVIYQIYPKSFQDTTGSGTGDLRGVIQRLDYLHKLGVDAIWLTPFYVSPQ
VDNGYDVANYTAIDPTYGTLDDFDELVTQAKSRGIRIILDMVFNHTSTQHAWFREALNKE
SPYRQFYIWRDGEPETPPNNWRSKFGGSAWRWHAESEQYYLHLFAPEQADLNWENPAVRA
ELKKVCEFWANRGVDGLRLDVVNLISKDPRYPEDLDGDGRRFYTDGPRAHEFLHEMNRDV
FTPRGLMTVGEMSSTSLEHCQRYAALTGSELSMTFNFHHLKVDYPGGEKWTLAKPDFVAL
KTLFRHWQQGMHNVAWNALFWCNHDQPRIVSRFGDEGEYRVPAAKMLAMVLHGMQGTPYI
YQGEEIGMTNPHFTRITDYRDVESLNMFAELRNDGRDADELLAILASKSRDNSRTPMQWT
NGDNAGFTAGEPWIGLGDNYQEINVEAALADESSVFYTYQKLIALRKQEAVLTWGDYQDL
LPNSPVLWCYRREWKGQTLLVIANLSRETQPWQPGKMLGNWQLVMHNYEEASPQPCAMTL
RPFEAVWWLQK
>LFGLNPFC_03560 hypothetical protein
MTGATAICKFFNEFFDYKINKNNMLTGKTPE
>LFGLNPFC_03561 Anaerobic ribonucleoside-triphosphate reductase
MTPHVMKRDGCKVPFKSERIKEAILRAAKAAEVDDADYCATVAAVVSEQMQGRNQVDINE
IQTAVENQLMSGPYKQLARAYIEYRHDRDIEREKRGRLNQEIRGLVEQTNSSLLNENANK
DSKVIPTQRDLLAGIVAKHYARQHLLPRDVVQAHERGDIHYHDLDYSPFFPMFNCMLIDL
KGMLTQGFKMGNAEIEPPKSISTATAVTAQIIAQVASHIYGGTTINRIDEVLAPFVTASY
NKHRKTAEEWNIPDAEGYANSRTIKECYDAFQSLEYEVNTLHTANGQTPFVTFGFGLGTS
WESRLIQESILRNRIAGLGKNRKTAVFPKLVFAIRDGLNHKKGDPNYDIKQLALECASKR
MYPDILNYDQVVKVTGSFKTPMGCRSFLGVWENENGEQIHDGRNNLGVISLNLPRIALEA
KGDEATFWKLLDERLVLARKALMTRIARLEGVKARVAPILYMEGACGVRLNADDDVSEIF
KNGRASISLGYIGIHETINALFGGEHVYDNEQLRAKGIAIVERLRQAVDQWKEETGYGFS
LYSTPSENLCDRFCRLDTAEFGVVPGVTDKGYYTNSFHLDVEKKVNPYDKIDFEAPYPPL
ANGGFICYGEYPNIQHNLKALEDVWDYSYQHVPYYGTNTPIDECYECGFTGEFECTSKGF
TCPKCGNHDTSRVSVTRRVCGYLGSPDARPFNAGKQEEVKRRVKHLGNGQIG
>LFGLNPFC_03562 Anaerobic ribonucleoside-triphosphate reductase-activating protein
MNYHQYYPVDIVNGPGTRCTLFVSGCVHECPGCYNKSTWRVNSGQPFTKAMEDQIINDLN
DTRIKRQGISLSGGDPLHPQNVPDILKLVQRIRAECPGKDIWVWTGYKLDELNAAQMQVV
DLINVLVDGKFVQDLKDPSLIWRGSSNQVVHHLR
>LFGLNPFC_03563 Soluble cytochrome b562
MRKSLLAILAVSSLVFSSASFAADLEDNMETLNDNLKVVEKADNAAQVKDALTKMRAAAL
DAQKATPPKLEGKSPDSPEMKDFRHGFDILVGQIDDALKLANEGKVKEAQAAAEQLKTTR
NAYHQKYR
>LFGLNPFC_03564 Metalloprotease PmbA
MALAMKVISQVEAQRKILEEAVSTALELASGKSDGAEVAVSKTTGISVSTRYGEVENVEF
NSDGALGITVYHQNRKGSASSTDLSPQAIARTVQAALDIARYTSPDPYAGVADKELLAFD
APDLDLFHPADVSPDEAIELAARAEQAALQADKRITNTEGGSFNSHYGVKVFGNSHGMLQ
GYCSTRHSLSSCVIAEENGDMERDYAYTIGRAMSDLQTPEWVGADCARRTLSRLSPRKLS
TMKAPVIFANEVATGLFGHLVGAIAGGAVYRKSTFLLDSLGTQILPDWLTIEEHPHLLKG
LASTPFDSEGVRTERRDIVKDGILTQWLLTSYSARKLGLKSTGHAGGIHNWRIAGQGLSF
EQMLKEMGTGLVVTELMGQGVSAITGDYSRGAAGFWVENGEIQYPVSEITIAGNLKDMWR

NIVTVGNDIETRSNIQCGSVLLPEMKIAGQ
>LFGLNPFC_03565 hypothetical protein
MTKQPEDWLDDVPGDDIEDEDDEIIWVSKSEIKRDAEELKRLGAEIVDLGKNALDKIPLD
ADLRAAIELAQRIKMEGRRRQLQLIGKMLRQRDVEPIRQALDKLKNRHNQQVVLFHKLEN
LRDRLIDQGDDAIAEVLNLWPDADRQQLRTLIRNAKKEKEGNKPPKSARQIFQYLRELAE
NEG
>LFGLNPFC_03566
UDP-N-acetylmuramate--L-alanyl-gamma-D-glutamyl-meso-2,6-diaminoheptandioate ligase
MRIHILGICGTFMGGLAMLARQLGHEVTGSDANVYPPMSTLLEKQGIELIQGYDASQLDP
QPDLVIIGNAMTRGNPCVEAVLEKNIPYMSGPQWLHDFVLRDRWVLAVAGTHGKTTTAGM
ATWILEQCGYKPGFVIGGVPGNFEVSARLGESDFFVIEADEYDCAFFDKRSKFVHYCPRT
LILNNLEFDHADIFDDLKAIQKQFHHLVRIVPGQGRIIWPENDINLKQTMAMGCWSEQEL
VGEQGHWQAKKLTTDASEWEVLLDGEKVGEVKWSLVGEHNMHNGLMAIAAARHVGVAPAD
AANALGSFINARRRLELRGEANGVTVYDDFAHHPTAILATLAALRGKVGGTARIIAVLEP
RSNTMKMGICKDDLAPSLGRADEVFLLQPAHIPWQVAEVAEACVQPAHWSGDVDTLADMV
VKTAQPGDHILVMSNGGFGGIHQKLLDGLAKKAEAAQ
>LFGLNPFC_03567 Fructose-1,6-bisphosphatase class 1
MKTLGEFIVEKQHEFSHATGELTALLSAIKLGAKIIHRDINKAGLVDILGASGAENVQGE
VQQKLDLFANEKLKAALKARDIVAGIASEEEDEIVVFEGCEHAKYVVLMDPLDGSSNIDV
NVSVGTIFSIYRRVTPVGTPVTEEDFLQPGNKQVAAGYVVYGSSTMLVYTTGCGVHAFTY
DPSLGVFCLCQERMRFPEKGKTYSINEGNYIKFPNGVKKYIKFCQEEDKSTNRPYTSRYI
GSLVADFHRNLLKGGIYLYPSTASHPDGKLRLLYECNPMAFLAEQAGGKASDGKERILDI
IPETLHQRRSFFVGNDHMVEDVERFIREFPDA
>LFGLNPFC_03568 Inner membrane ABC transporter permease protein YjfF
MIKRNLPLMITIGVFVLGYIYCLTQFPGFASTRVICNILTDNAFLGIIAVGMTFVILSGG
IDLSVGSVIAFTGVFLAKVIGDFGLSPLLAFPLVLVMGCAFGAFMGLLIDALKIPAFIIT
LAGMFFLRGVSYLVSEESIPINHPVYDTLSSLAWKIPGGGRLSAMGLLMLAVVVIGILLA
HRTRFGNQVYAIGGNATSANLMGISTRSTTIRIYMLSTGLATLAGIVFSIYTQAGYALAG
VGVELDAIASVVIGGTLLSGGVGTVLGTLFGVAIQGLIQTYINFDGTLSSWWTKIAIGIL
LFIFIALQRGLTVLWENRQSSPVTRVNIAQR
>LFGLNPFC_03569 Inner membrane ABC transporter permease protein YtfT
MPQSLPDTTPPKRRFRWPTGMPQLAALLLVLLVDSLVAPHFWQVVLQDGRLFGSPIDILN
RAAPVALLAIGMTLVIATGGIDLSVGAVMAIAGATTAAMTVAGFSLPIVLLSALGTGILA
GLWNGILVAILKIQPFVATLILMVAGRGVAQLITSGQIVTFNSPDLSWFGSGSLLFLPTP
VIIAVLTLLLFWLLTRKTALGMFIEAVGINIRAAKNAGVNTRIIVMLTYVLSGLCAAIAG
IIVAADIRGADANNAGLWLELDAILAVVIGGGSLMGGRFNLLLSVVGALIIQGMNTGILL
SGFPPEMNQVVKAVVVLCVLIVQSQRFISLIKGVRNRDKT
>LFGLNPFC_03570 Fructose import ATP-binding protein FruK
MTTDQHQEILRTEGLSKFFPGVKALDNVDFSLRRGEIMALLGENGAGKSTLIKALTGVYH
VDRGTIWLEGQAISPKNTAHAQQLGIGTVYQEVNLLPNMSVADNLFIGREPKRFGLLRRK
EMEKRATELMASYGFSLDVREPLNRFSVAMQQIVAICRAIDLSAKVLILDEPTASLDTQE
VELLFGLMRQLRDRGVSLIFVTHFLDQVYQVSDRITVLRNGSFVGCRETRELPQIELVKM
MLGRELDTHALQRAGRTLLSDKPVAAFKNYGKKGTIAPFDLEVRPGEIVGLAGLLGSGRT
ETAEVIFGIKPADSGTALIKGKLQTLRSPHQASVLGIGFCPEDRKTDGIIAAASVRENII
LALQAQRGWLRPISRKEQQEIAERFIRQLGIRTPSTEQPIEFLSGGNQQKVLLSRWLLTR
PQFLILDEPTRGIDVGAHAEIIRLIETLCADGLALLVISSELEELVGYADRVIIMRDRKQ
VAEIPLAELSVPAIMNAIAA
>LFGLNPFC_03571 ABC transporter periplasmic-binding protein YtfQ
MWKRLLVVSAVSAAMSSMALAAPLTVGFSQVGSESGWRAAETNVAKSEAEKRGITLKIAD
GQQKQENQIKAVRSFVAQGVDAIFIAPVVATGWEPVLKEAKDAEIPVFLLDRSIDVKDKS
LYMTTVTADNILEGKLIGDWLVKEVNGKPCNVVELQGTVGASVAIDRKKAFAEAIKNAPN
IKIIRSQSGDFTRSKGKEVMESFIKAENNGKNICMVYAHNDDMVIGAIQAIKEAGLKPGK
DILTGSIDGVPDIYKAMIDGEANASVELTPNMAGPAFDALEKYKKDGTMPEKLTLTKSTL
YLPDTAKEELEKKKNMGY
>LFGLNPFC_03572 Inorganic pyrophosphatase
MSLLNVPAGKDLPEDIYVVIEIPANADPIKYEIDKESGALFVDRFMSTAMFYPCNYGYIN
HTLSLDGDPVDVLVPTPYPLQPGSVIRCRPVGVLKMTDEAGEDAKLIAVPHTKLSKEYDH
IKDVNDLPELLKAQIAHFFEHYKDLEKGKWVKVEGWENAEAAKAEIVASFERAKNK
>LFGLNPFC_03573 Gamma-glutamylcyclotransferase family protein YtfP
MRIFVYGSLRHKQGNSHWMTNAQLLGDFSIDNYQLYSLGHYPGAVPGNGTVHGEVYRIDN
ATLAELDALRTRGGEYARQLIQTPYGSAWMYVYQRPVDGLKLIESGDWLDRDK
>LFGLNPFC_03574 Translocation and assembly module subunit TamB
MSLWKKISLGVVIVILLLLGSVAFLVGTTSGLHLVFKAADRWVPGLDIGKVTGGWRDLTL
SDVRYEQPGVAVKAGNLHLAVGLECLWNSSVCINDLALKDIQVNIDSKKMPPSEQVEEEE

DSGPLDLSTPYPITLTRVALDNVNIKIDDTTVSVMDFTSGLNWQEKTLTLKPTSLKGLLI
ALPKVAEAAQEEVVEPKIENPQPEEKPLGETLKDLFSRPVLPEMTDVHLPLNLNIEEFKG
EQLRVTGDTDITVSTMLLKVSSIDGNTKLDALDIDSSQGIVNASGTAQLSDNWPVDITLN
STLNVEPLKGEKVKLKVGGALREQLEIGVNLSGPVDMDLRAQTRLAEAGLPLNVEVNSKQ
LYWPFTGEKQYQADDLKLKLTGKMTDYTLSMRTAVKGQEIPPATITLDAKGNEQQVNLDK
LTVAALEGKTELKALLDWQQAISWRGELTLNGINTAKEIPEWPSKLNGLIKTRGSLYGGT
WQMEVPELKLTGNVKQNKVNVDGTLKGNSYMQWMIPGLHLELGPNSAEVKGELGVKDLNL
DATINAPGLDNALPGLGGTAKGLVKVRGTVEAPQLLADITARGLRWQELSVAQVRVEGDI
KSTDQIAGKLDVRVEQISQPDVNINLVTLNAKGSEKQHELQLRIQGEPVSGQLNLAGSFD
RKEERWKGTLSNTRFQTPVGPWSLTRDIALDYRNKEQKISIGPHCWLNPNAELCVPQTID
AGAEGRVVVNLNRFDLAMLKPFMPETTQASGIFTGKADVAWDTTKEGLPQGSITLSGRNV
QVTQTVNDAALPVAFQTLNLTAELRNNRAELGWTIRLTNNGQFDGQVQVTDPQGRRNLGG
NVNIRNFNLAMINPIFTRGEKAAGMVSANLRLGGDVQSPQLFGQLQVTGVDIDGNFMPFD
MQPSQLAVNFNGMRSTLAGTVRTQQGEIYLNGDADWSQIENWRARVTAKGSKVRITVPPM
VRMDVSPDVVFEATPNLFTLDGRVDVPWARIVVHDLPESAVGVSSDVVMLNDNLQPEEPK
TASIPINSNLTVHVGNNVRIDAFGLKARLTGDLNVVQDKQGLGLNGQINIPEGRFHAYGQ
DLIVREGELLFSGPPDQPYLNIEAIRNPDATEDDVIAGVRVTGLADEPKAEIFSDPAMSQ
QAALSYLLRGQGLESDQSDSAAMTSMLIGLGVAQSGQIVGKIGETFGVSNLALDTEGVGD
SSQVVVSGYVLPGLQVKYGVGIFDSIATLTLRYRLMPKLYLEAVSGVDQALDLLYQFEF
>LFGLNPFC_03575 Translocation and assembly module subunit TamA
MRYIRQLCCVSLLCLSGSAVAANVRLQVEGLSGQLEKNVRAQLSTIESDEVTPDRRFRAR
VDDAIREGLKALGYYQPTIEFDLRPPPKKGRQVLIAKVTPGVPVLIGGTDVVLRGGARTD
KDYLKLLDTRPAIGTVLNQGDYENFKKSLTSIALRKGYFDSEFTKAQLGIALGLHKAFWD
IDYNSGERYRFGHVTFEGSQIRDEYLQNLVPFKEGDEYESKDLAELNRRLSATGWFNSVV
VAPQFDKARETKVLPLTGVVSPRTENTIETGVGYSTDVGPRVKATWKKPWMNSYGHSLTT
STSISAPEQILDFSYKMPLLKNPLEQYYLVQGGFKRTDLNDTESDSTTLVASRYWDLSSG
WQRAINLRWSFDHFTQGEITNTTMLFYPGVMISRTRSRGGLMPTWGDSQRYSIDYSNTAW
GSDVDFSVFQAQNVWIRTLYDRHRFVTRGTLGWIETGDFDKVPPDLRFFAGGDRSIRGYK
YKSIAPKYANGDLKGASKLITGSLEYQYNVTGKWWGAVFVDSGEAVSDIRRSDFKTGTGV
GVRWESPVGPIKLDFAVPVADKDEHGLQFYIGLGPEL
>LFGLNPFC_03576 Peptide methionine sulfoxide reductase MsrA
MSLFDKKHLVSPADALPGRNTPMPVATLHAVNGHSMTNVPDGMEIAIFAMGCFWGVERLF
WQLPGVYSTAAGYTGGYTPNPTYREVCSGDTGHAEAVRIVYDPSVISYEQLLQVFWENHD
PAQGMRQGNDHGTQYRSAIYPLTPEQDAAARASLERFQAAMLAADDDRRITTEIANATPF
YYAEDDHQQYLHKNPYGYCGIGGIGVCLPPEA
>LFGLNPFC_03577 hypothetical protein
MLNSILVILCLIAVSAFFSMSEISLAASRKIKLKLLADEGNINAQRVLNMQENPGMFFTV
VQIGLNAVAILGGIVGDAAFSPAFHSLFSRYMSAELSEQLSFILSFSLVTGMFILFADLT
PKRIGMIAPEAVALRIINPMRFCLYVCTPLVWFFNGLANIIFRIFKLPMVRKDDITSDDI
YAVVEAGALAGVLRKQEHELIENVFELESRTVPSSMTPRENVIWFDLHEDEQSLKNKVAE
HPHSKFLVCNEDIDHIIGYVDSKDLLNRVLANQSLALNSGVQIRNTLIVPDTLTLSEALE
SFKTAGEDFAVIMNEYALVVGIITLNDVMTTLMGDLVGQGLEEQIVARDENSWLIDGGTP
IDDVMRVLDIDEFPQSGNYETIGGFMMFMLRKIPKRTDSVKFAGYKFEVVDIDNYRIDQL
LVTRIDSKATALSPKLPDAKDKEESVA
>LFGLNPFC_03578 hypothetical protein
MKIFQRYNPLQVAKYVKILFRGRLYIKDVGAFEFDKGKILIPKVKDKLHLSVMSEVNRQV
MRLQTEMA
>LFGLNPFC_03579 putative protein YtfJ
MTLRKILALTCLLLPMMASAHQFETGQRVPPIGITDRGELVLDKDQFSYKTWNSAQLVGK
VRVLQHIAGRTSAKEKNATLIEAIKSAKLPHDRYQTTTIVNTDDAIPGSGMFVRSSLESN
KKLYPWSQFIVDSNGVARGAWQLDEKSSAVVVLDKDGRVQWAKDGALTQEEVQQVMDLLH
KLINK
>LFGLNPFC_03580 3'(2'),5'-bisphosphate nucleotidase CysQ
MLDQVCQLARNAGDAIMQVYDGTKPMDVVSKADNSPVTAADIAAHTVIMDGLRTLTPDIP
VLSEEDPPGWEVRQHWQRYWLVDPLDGTKEFIKRNGEFTVNIALIDHGKPILGVVYAPVM
NVMYSAAEGKAWKEECGVRKQIQVRDARPPLVVISRSHADAELKEYLQQLGEHQTTSIGS
SLKFCLVAEGGQAQLYPRFGPTNIWDTAAGHAVAAAAGAHVHDWQGKPLDYTPRESFLNPG
FRVSIY
>LFGLNPFC_03581 2',3'-cyclic-nucleotide 2'-phosphodiesterase/3'-nucleotidase
MIKFSATLLATLIAASVNAATVDLRIMETTDLHSNMMDFDYYKDTATEKFGLVRTASLIN
DARNEVKNSVLVDNGDLIQGSPLADYMSAKGLKAGDIHPVYKALNTLDYTVGTLGNHEFN
YGLDYLKNALAGAKFPYVNANVIDARTKQPMFTPYLIKDTEVVDKDGKKQTLKIGYIGVV
PPQIMGWDKANLSGKVTVNDITETVRKYVPEMREKGADVVVVLAHSGLSADPYKVMAENS
VYYLSEIPGVNAIMFGHAHAVFPGKDFADIEGADIAKGTLNGVPAVMPGMWGDHLGVVDL

QLSNDSGKWQVTQAKAEARPIYDIANKKSLAAEDSKLVETLKADHDATRQFVSKPIGKSA
DNMYSYLALVQDDPTVQVVNNAQKAYVEHYIQGDPDLAKLPVLSAAAPFKVGGRKNDPAS
YVEVEKGQLTFRNAADLYLYPNTLIVVKASGKEVKEWLECSAGQFNQIDPNSTKPQSLIN
WDGFRTYNFDVIDGVNYQIDVTQPARYDGECQMINANAERIKNLTFNGKPIDPNAMFLVA
TNNYRAYGGKFAGTGDSHIAFASPDENRSVLAAWIADESKRAGEIHPAADNNWRLAPIAG
DKKLDIRFETSPSDKAAAFIKEKGQYPMNKVATDDIGFAIYQVDLSK
>LFGLNPFC_03582 hypothetical protein
MSQVSLSQQLKEGNLFAEQCPSREVLKHVTSRWGVLILVALREGTHRFSDLRRKIGGVSE
KMLAQSLQALEQDGFLNRIAYPVVPPHVEYSLTPLGEQVSEKVAALADWIELNLPEVLAV
RDERAA
>LFGLNPFC_03583 Quinone oxidoreductase 2
MIAITGATGQLGHYVIESLMKTVPASQIVAIVRNPAKAQVLTAQGITVRQADYGDEAALT
SALQGVEKLLLISSSEVGQRAPQHRNVINAAKAADVKFIAYTSLLHADSSPLGLADEHIE
TEKMLADSGIVYTLLRNGWYTENYLASAPAALEHGVFIGAAGDGKIASATRADYAVAAAR
VISEAGHEGKVYELAGDSAWTLTQLAAELTKQSGKPITYQNLSEADFAAALKSVGLPDGL
ADMLADSDVGASKGGLFDDSKTLSKLIGRPTTTLAESVSHLFNVNN
>LFGLNPFC_03584 Inner membrane protein YtfF
MISGVLYALLAGLMWGLIFVGPLIVPEYPAMLQSMGRYLALGLIALPIAWLGRVRLRQLA
RRDWLTALMLTMMGNLIYYFCLASAIQRTGAPVSTMIIGTLPVVIPVFANLLYSQRDGKL
AWGKLAPALVCIGIGLACVNIAELNHGLPDFDWARYTSGIVLALVSVVCWAWYALRNARW
LRENPDKHPMMWATAQALVTLPVSLIGYLVACYWLNMQTPDFSLPFGPRPLVFISLMVAI
AVLCSWIGALCWNVASQRLPTVILGPLIVFETLAGLLYTFLLRQQMPPLMTLSGIALLVI
GVVIAVRAKPEKPLTESVSES
>LFGLNPFC_03585 Iron-sulfur cluster repair protein YtfE
MAYRDQPLGELALSIPRASALFRKYDMDYCCGGKQTLARAAARKELDVDVIEAELAKLAE
QPIEKDWRSAPLAEIIDHIIVRYHDRHREQLPELILQATKVERVHADKPSVPKGLTKYLT
MLHEELSSHMMKEEQILFPMIKQGMGSQAMGPISVMESEHDEAGELLEVIKHTTNNVTPP
PEACTTWKAMYNGINELIDDLMEHISLENNVLFPRALAGE
>LFGLNPFC_03586 D-serine/D-alanine/glycine transporter
MVDQVKVVADDQAPAEQSLRRNLTNRHIQLIAIGGAIGTGLFMGSGKTISLAGPSIIFVY
MIIGFMLFFVMRAMGELLLSNLEYKSFSDFASDLLGPWAGYFTGWTYWFCWVVTGMADVV
AITAYAQFWFPGLSDWVASLSVIILLLVLNLATVKMFGEMEFWFAMIKIVAIVSLIVVGL
VMVAMHFQSPTGVEASFAHLWNDGGWFPKGLSGFFAGFQIAVFAFVGIELVGTTAAETKD
PEKSLPRAINSIPIRIIMFYVFSLIVIMSVTPWSSVVPEKSPFVELFVLVGLPAAASVIN
FVVLTSAASSANSGVFSTSRMLFGLAQEGVAPKAFAKLSKRAVPAKGLTFSCICLLGGVV
MLYVNPSVIGAFTMITTVSAILFMFVWTIILCSYLVYRKQRPHLHEKSIYKMPLGKLMCW
VCMAFFVFVLVLLTLEDDTRQALLVTPLWFIALGLGWLFIGKKRAAELRK
>LFGLNPFC_03587 FKBP-type 22 kDa peptidyl-prolyl cis-trans isomerase
MTTPTFDTIEAQASYGIGLQVGQQLSESGLEGLLPEALVAGIADALEGKHPAVPVDVVHR
ALREIHERADAVRRQRFQAMAAEGVKYLEENAKKEGVNSTESGLQFRVINQGEGAIPART
DRVRVHYTGKLIDGTVFDSSVARGEPAEFPVNGVIPGWIEALTLMPVGSKWELTIPQELA
YGERGAGASIPPFSTLVFEVELLEIL
>LFGLNPFC_03588 hypothetical protein
MPGRFELKPTLEKVWHAPDNFRFMDPLPPMHRRGIIIAAIVLVVGFLLPSDDTPNAPVVT
REAQLDIQSQSQPPTEEQLRAQLVTPQNDPDQVAPVAPEPIQEGQPEEQPQTTHTQPFQP
DSGIDNQWRSYRVEPGKTMAQLFRDHGLPATDVYAMAQVEGADKPLSNLQNGQMVKIRQN
ASGVVTGLTIDTGNNQQVLFTRQPDGSFIRAR
>LFGLNPFC_03589 HTH-type transcriptional repressor NicS
MYLDTSNVQSLKEKLLLCAVNEFAEYGYEGARVDNIVKAAGCSKQTVYHHFGNKENLFIE
VLEYTWNDIRQKEKALDFSDLPPQKAIEKIIDFTWDYYISNPWFLKIVHSENQSKGVHYA
KSQRLLEINHAHLQLMESLLDEGKKQNIFKPDIDPLQVNINIAALGGYYLINQHTLGLVY
HISMVSPQALEARRKVIKETILSWLLVDPSSTARE
>LFGLNPFC_03590 3-oxoacyl-[acyl-carrier-protein] reductase FabG
MKSTRPVAVITGAARGIGKGCALELARGGFNLLINDRPDADSVEKLHATQQECLAEGVEV
ICFPADVGDLSLHEEMLDAAQNQWGRLDCLLNNAGISVKKRGDLLDLEPDSFDQNIAINT
RAPFFLAQAFSKRLLAQPKPEAELPHRSIIFVSSINAIMLAMNRGEYTIAKTAVSAAARL
FAARLCNEQIGVYEVRPGLIKTDMTIPATAYYDELIAKGLVPWGRWGYPADIASTVRAMA
EGKLIYTCGQAVAIDGGLSMPRF
>LFGLNPFC_03591 hypothetical protein
MRDLLQRPDLFSINTATLGYKTPLPAIIDACAARGIGAIAPWRRELQSEDLQQIARQLAA
SNMNVSGLCRSTYYTAPTLAERKLAIDDNRRALDDAAVLNAACYMQVVGGLPMGTKDLYE
AREQVKQGIRQLLPHSKEVGVPIALEPLHPMTAADRSCLCTLRQALDWCDELDPDGEFGL
GVAVDVYHVWWDPDLASQILRAGKRILAFHVSDWLVPTTDLVNDRECREMVSSIFRQFVG
WLKTPDLMALLNWKYSHRTGGRKILTARWILASIASHIIVKE

>LFGLNPFC_03592 Short-chain-enoyl-CoA hydratase
MSDQPVLFSRAAASCRLTLNREDKCHAINEEMIESLDHYLNEIENDTALRLVELTATGDK
FFCAGGDIKSWSAYSPLDMGRKWIKRGNDVFNRLRNLPQLTVANLNGHTIGGGIELALCC
DIRIARPGAKFSNPEVMLGMVPGWMGIERVLNQVGPVVGRQMLMLGKRLTAQEAQVANLI
DEVVEKEQVESWMANQLAQLEKCGPVALAHIKQLILALENKHADYSHQLLAGLMSATQDC
QQATHAFAEKAGVSFHNQ
>LFGLNPFC_03593 Acetate CoA-transferase YdiF
MRKLSTAETLAAQIQDGATIAISGNGGGMVEADHIMAAIEERFLQTGHPRDLTLIHSLGI
GDRDSKGTNRFAHAEMLKRIIAGHFTWSPKMQELVKSNAIEAYCFPGGVIQALLREIGAG
RPGLFTHVGLGSFVDPRNGGGKSNECTTEDLVELIEIDGETKLRYRPFKVDYAILRGTYA
DPRGNVSLEEEAIDMDSYSMALAAHNSGGKVFVQVRDVLEAGTIEPRKVKLPGILVDGIV
EHREQPQTYLGGYDLTISGQHRRLSSNDAIELVSHPVRRLIARRAARELAAGASTNFGFG
IPGGIPGVALREGVPYQSLWLSVEQGVHNGMMLDDALFGCARNADAIIPSLDQFEFYSGG
GIDITFLGMGEMDQYGNVNVSHLNGNLIGPGGFLEIAQNARKVVFCGTFDAKGSKIDVTP
DGLHIAQSGQIPKLVTQVEKITFSAAYAQQSGQEVLYITERAVFQLTAEGVELIEIAPGV
EIERDILPFMAFRPIIKHPRLMESSLFTPMEDA
>LFGLNPFC_03594 hypothetical protein
MSQLDEAILDALTHVTFPKGFAQAEPAWVVTVDGVDYPLWKTDALVVGSGAAGLRAAVEL
KRRQQNVLIATAGLYMGTSACSGSDKQTLFTAATAGNGDNFAKLAEALASGGAMDHDTAY
VEAVGSLHTLGGLQYLGLELPEDRYGAILRYQTDHDEAGRATSCGPRTSRLMVKVLLEEV
QRLAIPMLTSATVIKLLHQRDENGEDRVAGAIIATGHRAHNPWGLAIVTAPNVVLATGGP
GELYRDSVYPHKCFGSLGLALEEGLTLTNLTESQFGIGTPRSTFPWNLSGTYVQVIPYIY
SVDAGGNEYNFLADYYRTTQELASNIFRKGYQWPFHATRVMDFGSSLLDMAVAQEQQSGR
LVFMDFNRNPEPVPGDLPFSLDRLDDDVRAYLENNDALAPSPIERLQQMNPLSISLYKMH
GYDLITQPLQFAMNNQHMNGGIEVDIWGQTSLPGCFAVGEVAGTHGVTRPGGAALNAGQV
FAVRLARFIGCTQKRNIDGDIAQLAAPALASIREIITQAHDNGSGMPLSVVREKIQARMS
DYAGFICHADKVRRATRDALLLNEFVQRHGLAIKHVGEVAELFMWRHMALTSAAVLTQLT
HYIDAGGGSRGARMVIDPQGKCLPQTRRGVKEEWRFRSELAEDKNHKLTIQYSQGSFITE
VKSLRMQPCINGIYFEKNWPDFLKGDIYTQ
>LFGLNPFC_03595 IS1 family transposase IS1A
MASVSISCPSCSATDGVVRNGKSTAGHQRYLCSHCRKTWQLQFTYTASQPGTHQKIIDMA
MNGVGCRVTARIMGVGLNTILRHLKNSGRSR
>LFGLNPFC_03596 IS1 family transposase IS1A
MDEQWGYVGAKSRQRWLFYAYDSLRKTVVAHVFGERTMATLGRLMSLLSPFDVVIWMTDG
WPLYESRLKGKLHVISKRYTQRIERHNLNLRQHLARLGRKSLSFSKSVELHDKVIGHYLN
IKHYQ
>LFGLNPFC_03597 Hexuronate transporter
METTGITKEQYSWIVSAFQLAYTLGQPIMGFFIDTVGLKLSFAICAAIWGLATMGHALTG
TWSGLAFMRALMGFSEASAIPAGVKTASTWFPAKERGVATGVFNMGTSLGAMLAPPLIAW
CIMFHSWQFAFIVSGSLALLAALFWFFCYKDPKDAKRLSDEERHYIESGQEQHLKTDKKE
KTSIKHILSQRNFWGIGIARFLADPAWGTINFWVPIFFVETLHFSLKEIAMFVWLPFLLG
DLGCLASGFVAKFFHDRGVSLINSRRITFTIAAVIMMTIGLVSIVENPYIAVLLISIGAF
SHQCLSTVAATLGGDLFKKDEVATAVGMAGACAWSGQLIFNLFIGAFVHIIGFAPFFIAL
AFFDIIGAIALWMLIKVKDEEPQVQLATS
>LFGLNPFC_03598 hypothetical protein
MALIGVYADWEGLDGPERIGYLHSRRTRTREIFEFEYDKKALADPSLNFIQLDPEIMLYE
GAQYPIPPKDKFGAFSDSCPDRWGRMLMKRRFERDIRDGLCDKDSHLYESDYLLGVHDLY
RVGALRYKREDAGEFLDNRIDVAAPPFTEIASLERASRAIEEDPDNKELMGQKWLRMLIA
PGGSLGGARPKASVVDEAGHLYIAKFPSVKDEYDVGGWEMVVNALAVGCGLNVAPAQAHK
FASNYHCFMVRRFDRTNAGRRLHFASAMTLTRHQDGEDASTGVSYLELADVLIRHGAQTN
TDLKELWSRIVFNILVSNTDDHLRNHGYILIPGKGWRLSEAYDLNPVARSDGLKLNITEN
DNALDLELAREVAEYFRLGLTEADDIIANFRGIVSQWRIIAERLRLPGREQELMAEAFRG
AI
>LFGLNPFC_03599 hypothetical protein
MGKKTAPLLPYTNQMLSEFGERLKLARLRRRLTAKQVAERAGMSPITLRSLESGSAGVTI
GAYLSVMQVLGLETDLNKLAAEDDLGRQLQDSRLIKKRGLSRSSGKNEEGIQTDPFQTRY
EKEGLLTTDLTALAEGSTPSITGETSTSLAALLKPIKKPLKGN
>LFGLNPFC_03600 50S ribosomal protein L9
MQVILLDKVANLGSLGDQVNVKAGYARNFLVPQGKAVPATKKNIEFFEARRAELEAKLAE
VLAAANARAEKINALETVTIASKAGDEGKLFGSIGTRDIADAVTAAGVEVAKSEVRLPNG
VLRTTGEHEVSFQVHSEVFAKVIVNVVAE
>LFGLNPFC_03601 30S ribosomal protein S18
MARYFRRRKFCRFTAEGVQEIDYKDIATLKNYITESGKIVPSRITGTRAKYQRQLARAIK
RARYLSLLPYTDRHQ

>LFGLNPFC_03602 Primosomal replication protein N
MTNRLVLSGTVCRTPLRKVSPSGIPHCQFVLEHRSVQEEAGFHRQAWCQMPVIVSGHENQ
AITHSITVGSRITVQGFISCHKAKNGLSKMVLHAEQIELIDSGD
>LFGLNPFC_03603 30S ribosomal protein S6
MRHYEIVFMVHPDQSEQVPGMIERYTAAITGAEGKIHRLEDWGRRQLAYPINKLHKAHYV
LMNVEAPQEVIDELETTFRFNDAVIRSMVMRTKHAVTEASPMVKAKDERRERRDDFANET
ADDAEAGDSEE
>LFGLNPFC_03604 hypothetical protein
MFSRVLALLAVLLLSANTWAAIEINNHQARNMDDVQSLGVIYINHNFATESEARQALNEE
TDAQGATYYHVILMREPGSNGNMHASADIYR
>LFGLNPFC_03605 L-ribulose-5-phosphate 4-epimerase UlaF
MQKLKQQVFEANMELPRYGLVTFTWGNVSAIDRERGLVVIKPSGVAYETMKADDMVVVDM
SGNVVEGEYRPSSDTATHLELYRRYPSLGGIVHTHSTHATAWAQAGLAIPALGTTHADYF
FGDIPCTRGLSKEEVQGEYELNTGKVIIETLGDAEPLHTPGIVVYQHGPFAWGKDAHDAV
HNAVVMEEVAKMAWIARSINPQLNHIDSFLMNKHFMRKHGPNAYYGQK
>LFGLNPFC_03606 L-ribulose-5-phosphate 3-epimerase UlaE
MLSKQIPLGIYEKALPAGECWLERLQLAKTLGFDFVEMSVDETDDRLSRLDWSREQRLAL
VNAIVETGVRVPSMCLSAHRRFPLGSEDDAVRAQGLEIMRKAIQFAQDVGIRVIQLAGYD
VYYQEANNETRRRFRDGLKESVEMASRAQVTLAMEIMDYPLMNSISKALGYAHYLNNPWF
QLYPDIGNLSAWDNDVQMELQAGIGHIVAVHVKDTKPGVFKNVPFGEGVVDFERCFETLK
QSGYCGPYLIEMWSETAEDPAAEVAKARDWVKARMAKAGMVEAA
>LFGLNPFC_03607 3-keto-L-gulonate-6-phosphate decarboxylase UlaD
MSLPMLQVALDNQTMDSAYETTRLIAEEVDIIEVGTILCVGEGVRAVRDLKALYPHKIVL
ADAKIADAGKILSRMCFEANADWVTVICCADINTAKGALDVAKEFNGDVQIELTGYWTWE
QAQQWRDAGIGQVVYHRSRDAQAAGVAWGEADITAIKRLSDMGFKVTVTGGLALEDLPLF
KGIPIHVFIAGRSIRDAASPVEAARQFKRSIAELWG
>LFGLNPFC_03608 Ascorbate-specific PTS system EIIA component
MKLRDSLAENKSIRLQAEAETWQDAVKIGVDLLVAADVVEPRYYQAILDAVEQHGPYFVL
APGLAMPHGRPEEGVKKTGFALVTLKKPLEFNHEDNDPVDILITMAAVDANTHQEVGIMQ
IVNLFEDEENFDRLRACRTEQEVLDLIDRTNAAA
>LFGLNPFC_03609 Ascorbate-specific PTS system EIIB component
MTVRILAVCGNGQGSSMIMKMKVDQFLTQSNIDHTVNSCAVGEYKSELSGADIIIASTHI
AGEITVTGNKYVVGVRNMLSPADFGPKLLEVIKEHFPQDVK
>LFGLNPFC_03610 Ascorbate-specific PTS system EIIC component
MEILYNIFTVFFNQVMTNAPLLLGIVTCLGYILLRKSVSVIIKGTIKTIIGFMLLQAGSG
ILTSTFKPVVAKMSEVYGINGAISDTYASMMATIERMGDAYSWVGYAVLLALALNICYVL
LRRITGIRTIMLTGHIMFQQAGLIAVTLFIFGYSMWTTIICTAILVSLYWGITSNMMYKP
TQEVTDGCGFSIGHQQQFASWIAYKVAPFLGKKEESVEDLKLPGWLNIFHDNIVSTAIVM
TIFFGAILLSFGIDTVQAMAGKVNWTVYILQTGFSFAVAIFIITQGVRMFVAELSEAFNG
ISQRLIPGAVLAIDCAAIYSFAPNAVVWGFMWGTIGQLIAVGILVACGSSILIIPGFIPM
FFSNATIGVFANHFGGWRAALKICLVMGMIEIFGCVWAVKLTGMSAWMGMADWSILAPPM
MQGFFSIGIAFMAVIIVIALAYMFFAGRALRAEEDAEKQLAEQSA
>LFGLNPFC_03611 putative L-ascorbate-6-phosphate lactonase UlaG
MSKVKSITRESWILSTFPEWGSWLNEEIEQEQVAPGTFAMWWLGCTGIWLKSEGGANVCV
DFWCGTGKQSHGNPLMKQGHQMQRMAGVKKLQPNLRTTPFVLDPFAIRQIDAVLATHDHN
DHIDVNVAAAVMQNCADDVPFIGPKTCVDLWIGWGVPKERCIVVKPGDVVKVKDIEIHAL
DAFDRTALITLPADQKAAGVLPDGMDDRAVNYLFKTPGGTLYHSGDSHYSNYYAKHGNEH
QIDVALGSYGENPRGITDKMTSADILRMGEALNAKVVIPFHHDIWSNFQADPQEIRVLWE
MKKDRLKYGFKPFIWQVGGKFTWPLDKDNFEYHYPRGFDDCFTIEPDLPFKSFL
>LFGLNPFC_03612 HTH-type transcriptional regulator UlaR
MTEAQRHQILLEMLAQLGFVTVEKVVERLGISPATARRDINKLDESGKLKKVRNGAEAIT
QQRPRWTPMNLHQAQNHDEKVRIAKAASQLVNPGESVVINCGSTAFLLGREMCGKPVQII
TNYLPLANYLIDQEHDSVIIMGGQYNKSQSITLSPQGSENSLYAGHWMFTSGKGLTAEGL
YKTDMLTAMAEQKMLSVVGKLVVLVDSSKIGERAGMLFSRADQIDMLITGKNANPEILQQ
LEAQGVSILRV
>LFGLNPFC_03613 2-hydroxy-6-oxo-6-phenylhexa-2,4-dienoate hydrolase
MIEIESRELADIPVLHAYPVGGQKDTPLPCVIFYHGFTSSSLVYSYFAVALAQAGLRVIMP
DAPDHGSRFSGDAARRLNQFWQILLQSMQEFTTLRAAIAEENWLLDDRLAVGGASMGAMT
ALGITARHPTIKCIASMMGSGYFTSLARSLFQPLIPETAAQQNEFNNIVAPLAEWEATNH
LEQLGDRPLLLWHGLDDDVVPADESLRLQQALSETGRDKLLTCSWQPGVRHRITPEALDA
AVTFFRQHL
>LFGLNPFC_03614 Lipoprotein BsmA
MVSRKRNSVIYRFASLLLVLMLSACSALQGTPQPAPPVTDHPQEIRRDQTQGLQRIGSVS
TMVRGSPDDALAEIRAKAVAAKADYYVVVMVDETIVTGQWYSQAILYRK

>LFGLNPFC_03615 hypothetical protein
MKQLLASPSLQLVTYPASATAQSAEFASADCVTGLNEIGQISVSNISGDPQDVERIVALK
ADEQGASWYRIITMYEDQQPDNWRVQAILYA
>LFGLNPFC_03616 Putative acyl-CoA dehydrogenase AidB
MHWQTHTVFNQPIPLNNSNLYLSDGALCEAVTREGAGWDSDFLASIGQQLGTAESLELGR
LANVNPPELLRYDAQGRRLDDVRFHPAWHLLMQALCTNRVHNLAWEEDARSGAFVARAAR
FMLHAQVEAGSLCPITMTFAATPLLLQMLPAPFQDWTTPLLSDRYDSHLLPGGQKRGLLI
GMGMTEKQGGSDVMSNTTRAERLDDGSYRLVGHKWFFSVPQSDAHLVLAQTAGGLSCFFV
PRFLPDGQRNAIRLERLKDKLGNRSNASCEVEFQDAIGWLLGQEGEGIRLILKMGGMTRF
DCALGSHAMMRRAFSLAIYHAHQRHVFGNPLIQQPLMRHVLSRMALQLEGQTALLFRLAR
AWDRRADAKEALWARLFTPAAKFVICKRGIPFVAEAMEVLGGIGYCEESELPRLYREMPV
NSIWEGSGNIMCLDVLRVLNKQAGVYDLLSEAFVEVKGQDRYFDRAVRRLQQQLRKPAEE
LGREITHQLFLLGCGAQMLKYAFPPMAQAWCKVMLDTRGGVRLSEQIQNDLLLRATGGVC
L
>LFGLNPFC_03617 Putative acid--amine ligase YjfC
MLRHNVPVRRDLDRIAADNGFDFHIIDNEIYWDESRAYRFTLRQIEEQIEKPTAELHQMC
LEVVDRAVKDEEILTQLAIPPLYWDVIAESWRARDPSLYGRMDFAWCGNAPVKLLEYNAD
TPTSLYESAYFQWLWLEDARRSGVIPRDADQYNAIQERLSSRFSELYSREPFYFCCCQDT
DEDRSTVLYLQDCAEQAGQESRFIYIEELGLGVGGVLTDLDDNVIQRAFKLYPLEWMMRD
DNGPLLRKRREQWVEPLWKSILSNKGLMPLLWRFFPGHPNLLASWFDGEKPQIAAGESYV
RKPIYSREGGNVTIFDGQNNVVDHADGDYADEPMIYQAFQPLPRFGDSYTLIGSWIVDDE
ACGMGIREDNTLITKDTSRFVPHYIAG
>LFGLNPFC_03618 hypothetical protein
MARKRKSRNNKNIGHGAISRIGRPHNPFEPRRNRYAQKYLTLALMGGAAFFVLKGCGDSG
DVDNDGDGTFYATAQDCIDDGNNSDICAHGWNNAKAAFYADVPKNMTQHNCQSKYENCYY
DNVEQSWIPVVSGFLLSRVIRKDRDEPFVYNSGGSSFASRPVWRNTSGDYSWRSGSGKKE
SYSSGGFTTKKASTVSRGGYGRSSSARGHWGG
>LFGLNPFC_03619 hypothetical protein
MHILDSLLAFSAYFFIGVAMVIIFLFIYSKITPHNEWQLIKNNNTAAALAFSGTLLGYVI
PLSSAAINAVSIPDYFAWGGIALVIQLLVFAGVRLYMPALSEKIINHNTAAGMFMGTAAL
AGGIFNAACMTW
>LFGLNPFC_03620 hypothetical protein
MSGFFQRLFGKDNKPAIARGPLGLHLNSGFTLDTLAFRLLEDALLIELPGEEYTVAAVSC
IDLGGGSQIFRYYTSGDEFLQINTTGGEDIDDIDDIKLFVYEESYGISKESHWREAINAK
AMGAMTLNWQEKRWQRFFNSEEPGNIEPVYMLEKVENQNHAKWEVHNFSMGYQRQVTEDT
YEYLLLNGEESFNDLGEPEWLFSRALGVDIPITSLHIIG
>LFGLNPFC_03621 hypothetical protein
MGILKSLFTLGKSFISQAEESIEETQGVRMLEQHIRDAKAELDKAGKSRVDLLARVKLSH
DKLKDLRERKASLEARALEALSKNVNPSLINEVAEEIARLENLITAEEQVLSNLEVSRDG
VEKAVTATAQRIAQFEQQMEVVKATEAMQRAQQAVTTSTVGASSSVSTAAESLKRLQTRQ
AERQARLDAAAQLEKVADGRDLDEKLAEAGIGGSNKSSAQDVLARLQRQQGE
>LFGLNPFC_03622 hypothetical protein
MTWNPLALATALQTVPEQNIDVTNSESALIIKMNDYGDLQINILFTSRQMIIETFICPVS
SISNPDEFNTFLLRNQKLMPLSSVGISSVQQEEYYIVFGALSLKSSLEDILLEITSLVDN
ALDLAEITEEYSH
>LFGLNPFC_03623 23S rRNA (guanosine-2'-O-)-methyltransferase RlmB
MSEMIYGIHAVQALLERAPERFQEVFILKGREDKRLLPLIHALESQGVVIQLANRQYLDE
KSDGAVHQGIIARVKPGRQYQENDLPDLIASLDQPFLLILDGVTDPHNLGACLRSADAAG
VHAVIVPKDRSAQLNATAKKVACGAAESVPLIRVTNLARTMRMLQEENIWIVGTAGEADH
TLYQSKMTGRLALVMGAEGEGMRRLTREHCDELISIPMAGSVSSLNVSVATGICLFEAVR
QRS
>LFGLNPFC_03624 Ribonuclease R
MSQDPFQEREAEKYANPIPSREFILEHLTKREKPASRDELAVELHIEGEEQLEGLRRRLR
AMERDGQLVFTRRQCYALPERLDLVKGTVIGHRDGYGFLRVEGRKDDLYLSSEQMKTCIH
GDQVLAQPLGADRKGRREARIVRVLVPKTSQIVGRYFTEAGVGFVVPDDSRLSFDILIPP
DQIMGARMGFVVVVELTQRPTRRTKAVGKIVEVLGDNMGTGMAVDIALRTHEIPYIWPQA
VEQQVAGLKEEVPEEAKVGRVDLRDLPLVTIDGEDARDFDDAVYCEKKRGGGWRLWVAIA
DVSYYVRPPTPLDREARNRGTSVYFPSQVIPMLPEVLSNGLCSLNPQVDRLCMVCEMTVS
SKGRLTGYKFYEAVMSSHARLTYTKVWHILQGDQDLREQYAPLVKHLEELHNLYKVLDKA
REERGGISFESEEAKFIFNAERRIERIEQTQRNDAHKLIEECMILANISAARFVEKAKEP
ALFRIHDKPSTEAITSFRSVLAELGLELPGGNKPEPRDYAELLESVADRPDAEMLQTMLL
RSMKQAIYDPENRGHFGLALQSYAHFTSPIRRYPDLTLHRAIKYLLAKEQGHQGNTTETG
GYHYSMEEMLQLGQHCSMAERRADEATRDVSDWLKCDFMLDQVGNVFKGVISSVTGFGFF
VRLDDLFIDGLVHVSSLDNDYYRFDQVGQRLMGESSGQTYRLGDRVEVRVEAVNMDERKI

DFSLISSERAPRNVGKTAREKAKKGDAGKKGGKRRQVGKKVNFEPDSAFRGEKKPKPKAA
KKDARKAKKPSVKTQKIAAATKAKRAAKKKVAE
>LFGLNPFC_03625 HTH-type transcriptional repressor NsrR
MQLTSFTDYGLRALIYMASLPEGRMTSISEVTDVYGVSRNHMVKIINQLSRAGYVTAVRG
KNGGIRLGKPASAIRIGDVVRELEPLSLVNCSSEFCHITPACRLKQALSKAVQSFLTELD
NYTLADLVEENQPLYKLLLVE
>LFGLNPFC_03626 Adenylosuccinate synthetase
MGNNVVVLGTQWGDEGKGKIVDLLTERAKYVVRYQGGHNAGHTLVINGEKTVLHLIPSGI
LRENVTSIIGNGVVLSPAALMKEMKELEDRGIPVRERLLLSEACPLILDYHVALDNAREK
ARGAKAIGTTGRGIGPAYEDKVARRGLRVGDLFDKETFAEKLKEVMEYHNFQLVNYYKAE
AVDYQKVLDDTMAVADILTSMVVDVSDLLDQARQRGDFVMFEGAQGTLLDIDHGTYPYVT
SSNTTAGGVATGSGLGPRYVDYVLGILKAYSTRVGAGPFPTELFDETGEFLCKQGNEFGA
TTGRRRRTGWLDTVAVRRAVQLNSLSGFCLTKLDVLDGLKEVKLCVAYRMPDGREVTTTP
LAADDWKGVEPIYETMPGWSESTFGVKDRSGLPQAALNYIKRIEELTGVPIDIISTGPDR
TETMILRDPFDA
>LFGLNPFC_03627 hypothetical protein
MNSTIWLALALVLVLEGLGPMLYPKAWKKMISAMTNLPDNILRRFGGGLVVAGVVVYYML
RKTIG
>LFGLNPFC_03628 Modulator of FtsH protease HflC
MRKSVIAIIIIVLVVLYMSVFVVKEGERGITLRFGKVLRDDDNKPLVYEPGLHFKIPFIE
TVKMLDARIQTMDNQADRFVTKEKKDLIVDSYIKWRISDFSRYYLATGGGDISQAEVLLK
RKFSDRLRSEIGRLDVKDIVTDSRGRLTLEVRDALNSGSAGTEDEVTTPAADNAIAEAAE
RVTAETKGKVPVINPNSMAALGIEVVDVRIKQINLPTEVSEAIYNRMRAEREAVARRHRS
QGQEEAEKLRATADYEVTRTLAEAERQGRIMRGEGDAEAAKLFADAFSKDPDFYAFIRSL
RAYENSFSGNQDVMVMSPDSDFFRYMKTPTSATR
>LFGLNPFC_03629 Modulator of FtsH protease HflK
MAWNQPGNNGQDRDPWGSSKPGGNSEGNGNKGGRDQGPPDLDDIFRKLSKKLGGLGGGKG
TGSGGGSSSQGPRPQLGGRVVTIAAAAIVIIWAASGFYTIKEAERGVVTRFGKFSHLVEP
GLNWKPTFIDEVKPVNVEAVRELAASGVMLTSDENVVRVEMNVQYRVTDPEKYLYSVTSP
DDSLRQATDSALRGVIGKYTMDRILTEGRTVIRSDTQRELEETIRPYDMGITLLDVNFQA
ARPPEEVKAAFDDAIAARENEQQYIREAEAYTNEVQPRANGQAQRILEEARAYKAQTILE
AQGEVARFAKLLPEYKAAPEITRERLYIETMEKVLGNTRKVLVNDKGGNLMVLPLDQMLK
SGNAPAAKSDNGASNLLRLPPASSSTTSGASNTSSTSQGDIMDQRRANAQRNDYQRQGE
>LFGLNPFC_03630 GTPase HflX
MFDRYDAGEQAVLVHIYFTQDKDMEDLQEFESLVSSAGVEALQVITGSRKAPHPKYFVGE
GKAVEIAEAVKATGASVVLFDHALSPAQERNLERLCECRVIDRTGLILDIFAQRARTHEG
KLQVELAQLRHLATRLVRGWTHLERQKGGIGLRGPGETQLETDRRLLRNRIVQIQSRLER
VEKQREQGRQSRIKADVPTVSLVGYTNAGKSTLFNRITEARVYAADQLFATLDPTLRRID
VADVGETVLADTVGFIRHLPHDLVAAFKATLQETRQATLLLHVIDAADVRVQENIEAVNT
VLEEIDAHEIPTLLVMNKIDMLDDFEPRIDRDEENKPIRVWLSAQTGAGIPQLFQALTER
LSGEVAQHTLRLPPQEGRLRSRFYQLQAIEKEWMEEDGSVSLQVRMPIVDWRRLCKQEPA
LIDYLI
>LFGLNPFC_03631 RNA-binding protein Hfq
MAKGQSLQDPFLNALRRERVPVSIYLVNGIKLQGQIESFDQFVILLKNTVSQMVYKHAIS
TVVPSRPVSHHSNNAGGGTSSNYHHGSSAQNTSAQQDSEETE
>LFGLNPFC_03632 tRNA dimethylallyltransferase
MSDISKASLPKAIFLMGPTASGKTALAIELRKILPVELISVDSALIYKGMDIGTAKPNAE
ELLAAPHRLLNIRDPSQAYSAADFRRDALAEMADITAAGRIPLLVGGTMLYFKALLEGLS
PLPSADPEVRARIEQQAAEQGWESLHRQLQEVDPVAAARIHPNDPQRLSRALEVFFISGK
TLTELTQTSGDALPYQVHQFAIAPASRELLHQRIEQRFHQMLASGFEAEVRALFARGDLH
TDLPSIRCVGYRQMWSYLEGEISYDEMVYRGVCATRQLAKRQITWLRGWEGVHWLDSEKP
EQARDEVLQVVGAIAG
>LFGLNPFC_03633 DNA mismatch repair protein MutL
MPIQVLPPQLANQIAAGEVVERPASVVKELVENSLDAGATRIDIDIERGGAKLIRIRDNG
CGIKKDELALALARHATSKIASLDDLEAIISLGFRGEALASISSVSRLTLTSRTAEQQEA
WQAYAEGRDMDVTVKPAAHPVGTTLEVLDLFYNTPARRKFLRTEKTEFNHIDEIIRRIAL
ARFDVTISLSHNGKIVRQYRAVPEGGQKERRLGAICGTAFLEQALAIEWQHGDLTLRGWV
ADPNHTTPALAEIQYCYVNGRMMRDRLINHAIRQACEDKLGADQQPAFVLYLEIDPHQVD
VNVHPAKHEVRFHQSRLVHDFIYQGVLSVLQQQLETPLPLDDEPQPAPRAIPENRVAAGR
NHFAEPAVREPVAPRYSPAPASGSRPAASWPNAQPGYQKQQGEVYRQLLQTPAPMQKPKA
PEPQEPALAANSQSFGRVLTIVHSDCALLERDGNISLLSLPVAERWLRQAQLTPGEVPVC
AQPLLIPLRLKVSGEEKSALEKAQSALAELGIDFQSDAQHVTIRAVPLPLRQQNLQILIP
ELIGYLAKQSVFEPGNIAQWIARNLMSEHAQWSMAQAITLLADVERLCPQLVKTPPGGLL
QSVDLHPAIKALKDE

>LFGLNPFC_03634 N-acetylmuramoyl-L-alanine amidase AmiB
MMYRIRNWLVATLLLLCAQVGAATLSDIQVSNGNQQARITLSFIGDPDYAFSHQSKRTVA
LDIKQTGVIQGLPLLFSGNNLVKAIRSGTPKDAQTLRLVVDLTENGKTEAVKRQNGSNYT
VVFTINADVPPPPPPPPMVAKRVETPAVVAPRVSEPARNPFKTESNRTTGVISSNTVTRP
AARATANTGDKIIIAIDAGHGGQDPGAIGPGGTREKNVTIAIARKLRTLLNNDPMFKGVL
TRDGDYFISVMGRSDVARKQNANFLVSIHADAAPNRSATGASVWVLSNRRANSEMASWLE
QHEKQSELLGGAGDVLANSQSDPYLSQAVLDLQFGHSQRVGYDVATSMISQLQRIGEIHK
RRPEHASLGVLRSPDIPSVLVETGFISNNSEERLLASDDYQQQLAEAIYKGLRNYFLAHP
MQSAPQGATAQTASTVTTPDRTLPN
>LFGLNPFC_03635 tRNA threonylcarbamoyladenosine biosynthesis protein TsaE
MMNRVIPLPDEQATLDLGERVAKACDGATVIYLYGDLGAGKTTFSRGFLQALGHQGNVKS
PTYTLVEPYMLDNLMVYHFDLYRLADPEELEFMGIRDYFANDAICLVEWPQQGTGVLPDP
DVEIHIDYQAQGREARVSAVSSAGELLLARLAG
>LFGLNPFC_03636 Bifunctional NAD(P)H-hydrate repair enzyme Nnr
MKKNPVSIPHTVWYADDIRRGEREAADALGLTLYELMLRAGEAAFQVCRSAYPDARHWLV
LCGHGNNGGDGYVVARLAKAIGIDVTLLAQESDKPLPEEAALAREAWLNAGGEIHASNIV
WPESVDLIVDALLGTGLQQAPRESISQLIDHANTHPAPIVAVDIPSGLLAETGATPGAVI
NADHTITFIALKPGLLTGKARDVTGQLHFDSLGLDSWLAGQETKIQRFSAEQLSQWLIPR
RPTSHKGDHGRLVIIGGDHGTAGAIRMTGEAALRAGAGLVRVLTRSENIAPLLTARPELM
VHELTMDSLTESLEWADVVVIGPGLGQQEWGKKALQKVENFRKPMLWDADALNLLAINPD
KRHNRVITPHPGEAARLLGCSVAEIESDRLHCTQRLVQRYGGVAVLKGAGTVVAAHPDAL
GIIDAGNAGMASGGMGDVLSGIIGALLGQKMSPYDAACAGCVAHGAAADVLAARFGTRGM
LATDLFSTLQRIVNPEVTDKNHDESSNSAP
>LFGLNPFC_03637 Epoxyqueuosine reductase
MSEPLDLNQLAQNIKQWGLELGFQQVGITDTDLSASEPKLQAWLDKQYHGEMDWMARHGM
LRARPHELLPGTLRVISVRMNYLPANAAFASTLKNPKLGYVSRYALGRDYHKLLRNRLKK
LGEMIQQHCVSLNFRPFVDSAPILERPLAEKAGLGWTGKHSLILNREAGSFFFLGELLVD
IPLPVDQPVEEGCGKCVACMTICPTGAIVEPYTVDARRCISYLTIELEGAIPEELRPLMG
NRIYGCDDCQLICPWNRYSQLTTEDDFSPRKPLHAPELIELFAWSEEQFLKVTEGSAIRR
IGHLRWLRNIAVALGNAPWDETILTALESRKGEHPLLDEHIAWAIAQQIERRNACIVEVQ
LPKKQRLVRVIEKGLPRDA
>LFGLNPFC_03641 Oligoribonuclease
MSANENNLIWIDLEMTGLDPERDRIIEIATLVTDANLNILAEGPTIAVHQSDEQLALMDD
WNVRTHTASGLVERVKASTMGDREAELATLEFLKQWVPAGKSPICGNSIGQDRRFLFKYM
PELEAYFHYRYLDVSTLKELARRWKPEILDGFTKQGTHQAMDDIRESVAELAYYREHFIK
L
>LFGLNPFC_03642 Small ribosomal subunit biogenesis GTPase RsgA
MSKNKLSKGQQRRVNANHQRRLKTSKEKPDYDDNLFGEPDEGIVISRFGMHADVESADGD
VHRCNIRRTIRSLVTGDRVVWRPGKPAAEGVNVKGIVEAVHERTSVLTRPDFYDGVKPIA
ANIDQIVIVSAILPELSLNIIDRYLVACETLQIEPIIVLNKIDLLDDEGMEFVNEQMDIY
RNIGYRVLMVSSHTQDGLKPLEEALTGRISIFAGQSGVGKSSLLNALLGLQKEILTNDVS
DNSGLGQHTTTAARLYHFPHGGDVIDSPGVREFGLWHLEPEQITQGFVEFHDYLGLCKYR
DCKHDTDPGCAIREAVEEGKIAETRFENYHRILESMAQVKTRKNFSDTDD
>LFGLNPFC_03643 Phosphatidylserine decarboxylase proenzyme
MLNSFKLSLQYILPKLWLTRLAGWGASKRAGWLTKLVIDLFVKYYKVDMKEAQKPDTASY
RTFNEFFVRPLRDEVRPIDTDPNVLVMPADGVISQLGKIEEDKILQAKGHNYSLEALLAG
NYLMADLFRNGTFVTTYLSPRDYHRVHMPCNGILREMIYVPGDLFSVNHLTAQNVPNLFA
RNERVICLFDTEFGPMAQILVGATIVGSIETVWAGTITPPREGIIKRWTWPAGENDDSVA
LLKGQEMGRFKLGSTVINLFAPGKVNLVEQLESLSVTKIGQPLAVSTETFVTPDAEPAPL
PAEEIEAEHDASPLVDDKKDQV
>LFGLNPFC_03644 Miniconductance mechanosensitive channel MscM
MRLIITFLMAWCLSWGAYAATAPDSKQITQELEQAKAAKPAQPEVVEALQSALNALEERK
GSLERIKQYQEVIDNYPKLSATLRAQLNNMRDEPRSVSPGMSNDALNQEILQISSQLLDK
SRQAQQEQERAREIADSLNQLPQQQTDARRQLNEIERRLGTLTGNTPLNQAQNFALQSDS
ARLKALVDELELAQLSANNRQELARLRSELAEKESQQLDAYLQALRNQLNSQRQQEAERA
LESTEQLAESSADLPKDIVAQFKINRELSAALNQQAQRMDLVASQQRQAASQTLQVRQAL
NTLREQSQWLGSSNLLGEALRAQVARLPEMPKPQQLDTEMAQLRVQRLRYEDLLNKQPLL
RQIHQADGQPLTAEQNRILEAQLRTQRELLNSLLQGGDTLLLELTKLKVSNGQLEDALKE
VNEATHRYLFWTSDVRPMTIAWPLEIAQDLRRLISLDTFSQLGKASVMMLTSKETILPLF
GALILVGCSIYSRRYFTRFLERSAAKVGKVTQDHFWLTLRTLFWSILVASPLPVLWMTLG
YGLREAWPYPLAVAIGDGVTATVPLLWVVMICATFARPNGLFIAHFGWPRERVSRGMRYY
LMSIGLIVPLIMALMMFDNLDDREFSGSLGRLCFILICGALAVVTLSLKKAGIPLYLNKE
GSGDNITNHMLWNMMIGAPLVAILASAVGYLATAQALLARLETSVAIWFLLLVVYHVIRR
WMLIQRRRLAFDRAKHRRAEMLAQRARGEEEAHHHSSPEGAIEVDESEVDLDAISAQSLR

LVRSILMLIALLSVIVLWSEIHSAFGFLENISLWDVTSTVQGVESLEPITLGAVLIAILV
FIITTQLVRNLPALLELAILQHLDLTPGTGYAITTITKYLLMLIGGLVGFSMIGIEWSKL
QWLVAALGVGLGFGLQEIFANFISGLIILFEKPIRIGDTVTIRDLTGSVTKINTRATTIS
DWDRKEIIVPNKAFITEQFINWSLSDSVTRVVLTIPAPADANSEEVTEILLTAARRCSLV
IDNPAPEVFLVDLQQGIQIFELRIYAAEMGHRMPLRHEIHQLILAGFHAHGIDMPFPPFQ
MRLESLNGKQTGRTLTSAGKGRQAGSL
>LFGLNPFC_03645 Inner membrane protein YjeO
MFILCCIWFIVAFLWITITSALDKEWMIDGRGINNVCDVLMYLEDDDTRDVGVIMTLPLF
FPFLWFALWRKKRGWFMYATALAIFGYWLWQFFLRYQFCL
>LFGLNPFC_03646 hypothetical protein
MDDASRDPVITEDEIRELQFSAGDVAEIEQTVLSFVDTRHTRKVAMVVGNTINTLKERDG
PRWGNLPDIYCAYLIRCLVFRGELVGYGDLFRMRYSEIKRPIIS
>LFGLNPFC_03647 Inner membrane transporter YjeM
MIFTSVFGFANSPSAYYLMGYSAIPFYIFSALLFFIPFALMMAEMGAAYRKEEGGIYSWM
NNSVGPRFAFIGTFMWFSSYIIWMVSTSAKVWVPFSTFLYGSDMTQHWHIAGLEPTQVVG
LLAVAWMILVTVVASKGINKIARITAVGGIAVMCLNLVLLLVSITILLLNGGHFAQDINF
LASPNPGYQSGLAMLSFVVFAIFAYGGIEAVGGLVDKTENPEKNFAKGIVFAAIVISIGY
SLAIFLWGVSTNWQQVLSNGSVNLGNITYVLMKSLGVTLGNALHLSPEASLSLGVWFARI
TGLSMFLAYTGAFFTLCYSPLKAIIQGTPKALWPEPMTRLNAMGMPSIAMWMQCGLVTVF
ILLVSFGGGTASAFFNKLTLMANVSMTLPYLFLALAFPFFKARQDLDRPFVIFKTHMSAM
IATVVVVLVVTFANVFTIIQPVVEAGDWDSTLWMIGGPVFFSLLAMAIYQNYCSRMANKP
ELALD
>LFGLNPFC_03648 Elongation factor P--(R)-beta-lysine ligase
MSETASWQPSASIPNLLKRAAIMAEIRRFFADRGVLEVETPCMSQATVTDIHLVPFETRF
VGPGHSQGMNLWLMTSPEYHMKRLLVAGCGPVFQLCRSFRNEEMGRYHNPEFTMLEWYRP
HYDMYRLMNEVDDLLQQVLDCPAAESLSYQQAFLRYLEIDPLSADKTQLREVAAKLDLSN
VADTEEDRDTLLQLLFTFGVEPNIGKEKPTFVYHFPASQASLAQISTEDHRVAERFEVYY
KGIELANGFHELTDAREQQQRFEQDNRKRAARGLPQHPIDQNLIEALKVGMPDCSGVALG
VDRLVMLALGAETLAEVIAFSVDRA
>LFGLNPFC_03649 Fumarate reductase flavoprotein subunit
MQTFQADLAIVGAGGAGLRAAIAAAQANPNAKIALISKVYPMRSHTVAAEGGSAAVAQDH
DSFEYHFHDTVAGGDWLCEQDVVDYFVHHCPTEMTQLELWGCPWSRRPDGSVNVRRFGGM
KIERTWFAADKTGFHMLHTLFQTSLQFPQIQRFDEHFVLDILVDDGHVRGLVAMNMMEGT
LVQIRANAVVMATGGAGRVYRYNTNGGIVTGDGMGMALSHGVPLRDMEFVQYHPTGLPGS
GILMTEGCRGEGGILVNKNGYRYLQDYGMGPETPLGEPKNKYMELGPRDKVSQAFWHEWR
KGNTISTPRGDVVYLDLRHLGEKKLHERLPFICELAKAYVGVDPVKEPIPVRPTAHYTMG
GIETDQNCETRIKGLFAVGECSSVGLHGANRLGSNSLAELVVFGRLAGEQATERAATAGN
GNEAAIEAQAAGVEQRLKDLVNQDGGENWAKIRDEMGMAMEEGCGIYRTPELMQKTIDKL
AELQERFKRVRITDTSSVFNTDLLYTIELGHGLNVAECMAHSAMARKESRGAHQRLDEGC
TERDDVNFLKHTLAFRDADGTTRLEYSDVKITTLPPAKRVYGGEADAADKAEAANKKEKA
NG
>LFGLNPFC_03650 Fumarate reductase iron-sulfur subunit
MAEMKNLKIEVVRYNPEVDTAPHSAFYEVPYDATTSLLDALGYIKDNLAPDLSYRWSCRM
AICGSCGMMVNNVPKLACKTFLRDYTDGMKVEALANFPIERDLVVDMTHFIESLEAIKPY
IIGNARTADQGTNIQTPAQMAKYHQFSGCINCGLCYAACPQFGLNPEFIGPAAITLAHRY
NEDSRDHGKKERMAQLNSQNGVWSCTFVGYCSEVCPKHVDPAAAIQQGKVESSKDFLIAT
LKPR
>LFGLNPFC_03651 Fumarate reductase subunit C
MTTKRKPYVRPMTSTWWKKLPFYRFYMLREGTAVPAVWFSIELIFGLFALKNGPEAWAGF
VDFLQNPVIVIINLITLAAALLHTKTWFELAPKAANIIVKDEKMGPEPIIKSLWAVTVVA
TIVILFVALYW
>LFGLNPFC_03652 Fumarate reductase subunit D
MINPNPKRSDEPVFWGLFGAGGMWSAIIAPVMILLVGILLPLGLFPGDALSYERVLAFAQ
SFIGRVFLFLMIVLPLWCGLHRMHHAMHDLKIHVPAGKWVFYGLAAILTVVTLIGVVTI
>LFGLNPFC_03653 Beta-lactamase
MFKTTLCTLLITASCSTFAAPQQINDIVHRTITPLIEQQKIPGMAVAVIYQGKPYYFTWG
YADIAKKQPVTQQTLFELGSVSKTFTGVLGGDAIARGEIKLSDPTTKYWPELTAKQWNGI
TLLHLATYTAGGLPLQVPDEVKSSSDLLRFYQNWQPAWAPGTQRLYANSSIGLFGALAVK
PSGLSFEQAMKTRVFQPLKLNHTWINVPSAEEKNYAWGYREGKAVHVSPGALDAEAYGVK
STIEDMARWVQSNLKPFDINEKILQQGIQLAQSRYWQTGDMYQGLGWEMLDWPVNPDIII
NGSDNKIALAARPVKPITPPTPAVRASWVHKTGATGGFGSYVAFIPEKELGIVMLANKNY
PNPARVTAAWQILNTLQ
>LFGLNPFC_03654 Outer membrane lipoprotein Blc
MRLLPLVAAATAAFLVVACSSPTPPRGVTVVNNFDAKRYLGTWYEIARFDHRFERGLEKV

TATYSLRDDGGLNVINKGYNPDREMWQQSEGKAYFTGAPTRAALKVSFFGPFYGGYNVIA
LDREYRHALVCGPDRDYLWILSRTPTISDEVKQEMLAVATREGFDVSKFIWVQQPGS
>LFGLNPFC_03655 Guanidinium exporter
MSWIILVIAGLLEVVWAVGLKYTHGFSRLTPSVITVTAMIVSLALLAWAMKSLPVGTAYA
VWTGIGAVGAAITGIVLLGESANPMRLASLALIVLGIIGLKLSTH
>LFGLNPFC_03656 hypothetical protein
MVKKTIAAIFSVLVLSTVLTACNTTRGVGEDISDGGNAISGAATKAQQ
>LFGLNPFC_03657 hypothetical protein
MMKRLIVLVLLASTLLTGCNTARGFGEDIKHLGNSISRAAS
>LFGLNPFC_03658 Elongation factor P
MATYYSNDFRAGLKIMLDGEPYAVEASEFVKPGKGQAFARVKLRRLLTGTRVEKTFKSTD
SAEGADVVDMNLTYLYNDGEFWHFMNNETFEQLSADAKAIGDNAKWLLDQAECIVTLWNG
QPISVTPPNFVELEIVDTDPGLKGDTAGTGGKPATLSTGAVVKVPLFVQIGEVIKVDTRS
GEYVSRVK
>LFGLNPFC_03659 L-lysine 2,3-aminomutase
MAHIVTLNTPSREDWLTQLADVVTDPDELLRLLNIDADEKLLAGRSAKKLFALRVPRSFI
DRMEKGNPNDPLLRQVLTSQDEFVVAPGFSTDPLEEQHSVVPGLLHKYHNRALLLVKGGC
AVNCRYCFRRHFPYAENQGNKRNWQTALEYVAAHPELDEMIFSGGDPLMAKDHELDWLLT
QLEAIPHIKRLRIHSRLPIVIPARITDALVERFSHSTLQILLVNHINHANEVDETFRQAM
AKLRRVGVTLLNQSVLLRGVNDNAQTLANLSNALFDAGVMPYYLHVLDKVQGAAHFMVSD
DEARQIMRELLTLVSGYLVPKLAREIGGEPSKTPLDLQLRQQ
>LFGLNPFC_03660 hypothetical protein
MAISIKGVNTGVIRKSNNFIALALKIKEPRNKESLFFMSAMELRDLLIALESRLHQKHKL
DAAARLQYEQARDKVIKKMAENIPEILVDELKNADINRRVNTLELTDDQGENLTFVLTLH
DGSKCELVVNELQIEMLARAIIHAINNAEMRELALRITSLLDFLPLYDVDCQENGNLEYD
TYSQPEWKHNLFNHYLAVLYRFKDESGNEQFSGAVVKTREATPGKEVEAITRRMLDFSPR
LKKLAGVPCQVYVRTVAANNAQPLTQDQCLRALHHLRVQSTSKTAPQAK
>LFGLNPFC_03661 hypothetical protein
MHVKYLAGIVGAALLMAGCSSSNELSAAGQSVRIVDEQPGAECQLIGTATGKQSNWLSGQ
HGEEGGSMRGAANDLRNQAAAMGGNVIYGISSPSQGMLSSFVPTDSQIIGQVYKCPN
>LFGLNPFC_03662 60 kDa chaperonin
MAAKDVKFGNDARVKMLRGVNVLADAVKVTLGPKGRNVVLDKSFGAPTITKDGVSVAREI
ELEDKFENMGAQMVKEVASKANDAAGDGTTTATVLAQAIITEGLKAVAAGMNPMDLKRGI
DKAVTAAVEELKALSVPCSDSKAIAQVGTISANSDETVGKLIAEAMDKVGKEGVITVEDG
TGLQDELDVVEGMQFDRGYLSPYFINKPETGAVELESPFILLADKKISNIREMLPVLEAV
AKAGKPLLIIAEDVEGEALATLVVNTMRGIVKVAAVKAPGFGDRRKAMLQDIATLTGGTV
ISEEIGMELEKATLEDLGQAKRVVINKDTTTIIDGVGEEAAIQGRVAQIRQQIEEATSDY
DREKLQERVAKLAGGVAVIKVGAATEVEMKEKKARVEDALHATRAAVEEGVVAGGGVALI
RVASKLADLRGQNEDQNVGIKVALRAMEAPLRQIVLNCGEEPSVVANTVKGGDGNYGYNA
ATEEYGNMIDMGILDPTKVTRSALQYAASVAGLMITTECMVTDLPKNDAADLGAAGGMGG
MGGMGGMM
>LFGLNPFC_03663 10 kDa chaperonin
MNIRPLHDRVIVKRKEVETKSAGGIVLTGSAAAKSTRGEVLAVGNGRILENGEVKPLDVK
VGDIVIFNDGYGVKSEKIDNEEVLIMSESDILAIVEA
>LFGLNPFC_03664 L-methionine/branched-chain amino acid exporter YjeH
MSGLKQELGLAQGIGLLSTSLLGTGVFAVPALAALVAGNNSLWAWPVLIILVFPIAIVFA
ILGRHYPSAGGVAHFVGMAFGSRLERVTGWLFLSVIPVGLPAALQIAAGFGQAMFGWHSE
QLLLAELGTLALVWYIGTRGASSSANLQTVIAGLIVALIVAIWWAGDIKPANIPFPAPGN
IELTGLFAALSVMFWCFVGLEAFAHLASEFKNPERDFPRALMIGLLLAGLVYWGCTVVVL
HFDAYGEQMAAAASLPKIVVQLFGVGALWIACVIGYLACFASLNIYIQSFARLVWSQAQH
NPDHYLARLSSRHIPNNALNAVLGCCVVSTLVIHALEINLDALIIYANGIFIMIYLLCML
AGCKLLQGRYRLLAVVGGLLCVLLLAMVGWKSLYALIMLAGLWLFLPKRKTPGNGITT
>LFGLNPFC_03665 hypothetical protein
MRWLPFIAIFLYVYIEISIFIQVAHVLGVLLTLVLVIFTSVIGMSLVRNQGFKNFVLMQQ
KMAAGENPAAEMIKSVSLIIAGLLLLLPGFFTDFLGLLLLLPPVQKHLTVKLMPHLRFSR
MPGGGFSAGTGSGNTFDGEYQRKDDDRDRLDHKDDRQD
>LFGLNPFC_03666 Aspartate ammonia-lyase
MSNNIRIEEDLLGTREVPADAYYGVHTLRAIENFYISNNKISDIPEFVRGMVMVKKAAAM
ANKELQTIPKSVANAIIAACDEVLNNGKCMDQFPVDVYQGGAGTSVNMNTNEVLANIGLE
LMGHQKGEYQYLNPNDHVNKCQSTNDAYPTGFRIAVYSSLIKLVDAINQLREGFERKAVE
FQDILKMGRTQLQDAVPMTLGQEFRAFSILLKEEVKNIQRTAELLLEVNLGATAIGTGLN
TPKEYSPLAVKKLAEVTGFPCVPAEDLIEATSDCGAYVMVHGALKRLAVKMSKICNDLRL
LSSGPRAGLNEINLPELQAGSSIMPAKVNPVVPEVVNQVCFKVIGNDTTVTMAAEAGQLQ
LNVMEPVIGQAMFESVHILTNACYNLLEKCINGITANKEVCEGYVVYNSIGIVTYLNPFIG

HHNGDIVGKICAETGKSVREVVLERGLLTEAELDDIFSVQNLMHPAYKAKRYTDESEQ
>LFGLNPFC_03667 Anaerobic C4-dicarboxylate transporter DcuA
MLVVELIIVLLAIFLGARLGGIGIGFAGGLGVLVLAAIGVKPGNIPFDVISIIMAVIAAI
SAMQVAGGLDYLVHQTEKLLRRNPKYITILAPIVTYFLTIFAGTGNISLATLPVIAEVAK
EQGVKPCRPLSTAVVSAQIAITASPISAAVVYMSSVMEGHGISYLHLLSVVIPSTLLAVL
VMSFLVTMLFNSKLSDDPIYRKRLEEGLVELRGEKQIEIKSGAKTSVWLFLLGVVGVVIY
AIINSPSMGLVEKPLMNTTNAILIIMLSVATLTTVICKVDTDNILNSSTFKAGMSACICI
LGVAWLGDTFVSNNIDWIKDTAGEVIQGHPWLLAVIFFFASALLYSQAATAKALMPMALA
LNVSPLTAVASFAAVSGLFILPTYPTLVAAVQMDDTGTTRIGKFVFNHPFFIPGTLGVAL
AVCFGFVLGSFML
>LFGLNPFC_03668 Divalent-cation tolerance protein CutA
MLDEKSSNTTSVVVLCTAPDEATAQDLAAKVLAEKLAACATLIPGATSLYYWEGKLEQEY
EVQMILKTTVSHQQALLECLKSHHPYQTPELLVLPVTHGDTDYLSWLNASLR
>LFGLNPFC_03669 Thiol:disulfide interchange protein DsbD
MAQRIFTLILLLCSTSVFAGLFDAPGRSQFVPADQAFAFDFQQNQHDLNLTWQIKDGYYL
YRKQIRITPEHAKIADVQLPQGVWHEDEFYGKSEIYRDRLTLPVTINQASAGATLTVTYQ
GCADAGFCYPPETKTVPLSEVVANNEASQPVSVPQQEQPTAQLPFSALWALLIGIGIAFT
PCVLPMYPLISGIVLGGKQRLSTARALLLTFIYVQGMALTYTALGLVVAAAGLQFQAALQ
HPYVLIGLAIVFTLLAMSMFGLFTLQLPSSLQTRLTLMSNRQQGGSPGGVFIMGAIAGLI
CSPCTTAPLSAILLYIAQSGNMWLGGGTLYLYALGMGLPLMLITVFGNRLLPKSGPWMEQ
VKTAFGFVILALPVFLLERVIGDIWGLRLWSALGVAFFGWAFITSLQAKRGWMRVVQIIL
LAAALVSVRPLQDWAFGETHTAQTQTHLNFTQIKTVDELNQALVEAKGKPVMLDLYADWC
VACKEFEKYTFSDPQVQKALADTVLLQANVTANDAQDVALLKHLNVLGLPTILFFDGQGQ
EHPQARVTGFMDAETFSAHLRDRQP
>LFGLNPFC_03670 hypothetical protein
MQREDVLGEALKLLELQGIANTTLEMVAERVDYPLDELRRFWPDKEAILYDALRYLSQQI
DVWRRQLMLDETQTAEQKLLARYQALSECVKNNRYPGCLFIAACTFYPDPGHPIHQLADQ
QKSAAYDFTHELLTTLEVDDPAMVAKQMELVLEGCLSRMLVNRSQADVDTAHRLAEDILR
FARCRQGGALT
>LFGLNPFC_03672 Transcriptional activator CadC
MQQPVVRVGEWLVTPSINQISRNGRQLTLEPRLIDLLVFFAQHSGEVLSRDELIDNVWKR
SIVTNHVVTQSISELRKSLKDNDEDSPVYIATVPKRGYKLMVPVIWYSEEEGEEIMLSSP
PPIPEAVPATDSPSHSLNIQNTTTPPEQSPVKSKRFTTFWVWFFFLLSLGICVALVAFSS
LETRLPMSKSRILLNPRDIDINMVNKSCNSWSSPYQLSYAIGVGDLVATSLNTFSTFMVH
DKINYNIDEPSSSGKTLSIAFVNQRQYRAQQCFMSVKLVDNADGSTMLDKRYVITNGNQL
AIQNDLLQSLSKALNQPWPQRMQEMLQQILPHRGALLTNFYQAHDYLLHGDDKSLDRASE
LLGEIVQSSPEFTYARAEKALVDIVRHSQHPLDEKQLAALNTEIDNIVTLPELNNLSIIY
QIKAVSALVKGKTDESYQAINTGIDLEMSWLNYVLLGKVYEMKGMNREAADAYLTAFNLR
PGANTLYWIENGIFQTSVPYVVPYLDKFLASE
>LFGLNPFC_03673 putative cadaverine/lysine antiporter
MSSAKKIGLFACTGVVAGNMMGSGIALLPANLASIGGIAIWGWIISIIGAMSLAYVYARL
ATKNPQQGGPIAYAGEISPAFGFQTGVLYYHANWIGNLAIGITAVSYLSTFFPVLNDPVP
AGIACIAIVWVFTFVNMLGGTWVSRLTTIGLVLVLIPVVMTAIVGWHWFDAATYAANWNT
ADTTDGHAIIKSILLCLWAFVGVESAAVSTGMVKNPKRTVPLATMLGTGLAGIVYIAATQ
VLSGMYPSSVMAASGAPFAISASTILGNWAAPLVSAFTAFACLTSLGSWMMLVGQAGVRA
ANDGNFPKVYGEVDSNGIPKKGLLLAAVKMTALMILITLMNSAGGKASDLFGELTGIAVL
LTMLPYFYSCVDLIRFEGVNIRNFVSLICSVLGCVFCFIALMGASSFELAGTFIVSLIIL
MFYARKMHERQSHSMDNHTASNAH
>LFGLNPFC_03674 Inducible lysine decarboxylase
MNVIAILNHMGVYFKEEPIRELHRALERLNFQIVYPNDRDDLLKLIENNARLCGVIFDWD
KYNLELCEEISKMNENLPLYAFANTYSTLDVSLNDLRLQISFFEYALGAADDIANKIKQT
TDEYINTILPPLTKALFKYVREGKYTFCTPGHMGGTAFQKSPVGSLFYDFFGPNTMKSDI
SISVSELGSLLDHSGPHKEAEQYIARVFNADRSYMVTNGTSTANKIVGMYSAPAGSTILI
DRNCHKSLTHLMMMSDVTPIYFRPTRNAYGILGGIPQSEFQHATIAKRVKETPNATWPVH
AVITNSTYDGLLYNTDFIKKTLDVKSIHFDSAWVPYTNFSPIYEGKCGMSGGRVEGKVIY
ETQSTHKLLAAFSQASMIHVKGDVNEETFNEAYMMHTTTSPHYGIVASTETAAAMMKGNA
GKRLINGSIERAIKFRKEIKRLRTESDGWFFDVWQPDHIDTTECWPLRSDSTWHGFKNID
NEHMYLDPIKVTLLTPGMEKDGTMSDFGIPASIVAKYLDEHGIVVEKTGPYNLLFLFSIG
IDKTKALSLLRALTDFKRAFDLNLRVKNMLPSLYREDPEFYENMRIQELAQNIHKLIVHH
NLPDLMYRAFEVLPTMVMTPYAAFQKELHGMTEEVYLDEMVGRINANMILPYPPGVPLVM
PGEMITEESRPVLEFLQMLCEIGAHYPGFETDIHGAYRQADGRYTVKVLKEESKK
>LFGLNPFC_03675 Dipeptide and tripeptide permease C
MKTPSQPRAIYYIVAIQIWEYFSFYGMRALLILYLTHQLGFDDNHAISLFSAYASLVYVT
PILGGWLADRLLGNRTAVIAGALLMTLGHVVLGIDTNSTFSLYLALAIIICGYGLFKSNI

SCLLGELYDENDHRRDGGFSLLYAAGNIGSIAAPIACGLAAQWYGWHVGFALAGGGMFIG
LLIFLSGHRHFQSTRSMDKKALTSVKFALPVWSWLVVMLCLAPVFFTLLLENDWSGYLLA
IVCLIAAQIIARMMIKFPEHRRALWQIVLLMFVGTLFWVLAQQGGSTISLFIDRFVNRQA
FNIEVPTALFQSVNAIAVMLAGVVLAWLASPESRGNSALRVWLKFAFGLLLMACGFMLLA
FDARHAAAGGQASMGVMISGLALMGFAELFIDPVAIAQITRLKMSGVLTGIYMLATGAVA
NWLAGVVAQQTTESQISGMAIAAYQRFFSQMGEWTLACVAIIVVLAFATRFLFSTPTNMI
QESND
>LFGLNPFC_03676 Lysine--tRNA ligase, heat inducible
MSERETRGANEAIDFNDELRNRREKLAALRQQGVAFPNDFRRDHTSDQLHEEFDAKDNQE
LESLNIEVSVAGRMMTRRIMGKASFVTLQDVGGRIQLYVARDSLPEGVYNDQFKKWDLGD
IIGARGTLFKTQTGELSIHCTELRLLTKALRPLPDKFHGLQDQEVRYRQRYLDLIANDKS
RQTFVVRSKILAAIRQFMVARGFMEVETPMMQVIPGGASARPFITHHNALDLDMYLRIAP
ELYLKRLVVGGFERVFEINRNFRNEGISVRHNPEFTMMELYMAYADYHDLIELTESLFRT
LAQEVLGTTKVTYGEHVFDFGKPFEKLTMREAIKKYRPETDMADLDNFDAAKALAESIGI
TVEKSWGLGRIVTEIFDEVAEAHLIQPTFITEYPAEVSPLARRNDVNPEITDRFEFFIGG
REIGNGFSELNDAEDQAERFQEQVNAKAAGDDEAMFYDEDYVTALEYGLPPTAGLGIGID
RMIMLFTNSHTIRDVILFPAMRPQK
>LFGLNPFC_03677 Toxin GhoT
MALFSKILIFYVIGVNISFVIIWFISHEKTHIRLLSAFLVGITWPMSLPVALLFSLF
>LFGLNPFC_03678 Endoribonuclease antitoxin GhoS
MEGKNKFNTYVVSFDYPSSYSSVFLRLRSLMYDMNFSSIVADEYGIPRQLNENSFAITTS
LAASEIEDLIRLKCLDLPDIDFDLSIMTVDDYFRQFYK
>LFGLNPFC_03679 putative protein YjdJ
MEIREGHNKFYINDEQGKQIAEIVFVPTGENLAIIEHTDVDESLKGQGIGKQLVAKVVEK
MRREKRKIIPLCPFAKHEFDKTREYDDILS
>LFGLNPFC_03680 hypothetical protein
MDRALLDGGYRCYTGENIDVYFNTAICQHSGNCVRGNGKLFNLKRKPWIMPDEVDVATVV
KVIDTCPSGALKYRHK
>LFGLNPFC_03681 Sensor histidine kinase DcuS
MRHSLPYRMLRKRPMKLSTTVILMVSAVLFSVLLVVHLIYFSQISDMTRDGLANKALAVA
RTLADSPEIRQGLQKKPQESGIQAIAEAVRKRNDLLFIVVTDMQSLRYSHPEAQRIGQPF
KGDDILKALNGEENVAINRGFLAQALRVFTPIYDENHKQIGVVAIGLELSRVTQQINDSR
WSIIWSVLFGMLVGLIGTCILVNVLKKILFGLEPYEISTLFEQRQAMLQSIKEGVVAVDD
RGEVTLINDAAQELLNYRKSQDDEKLSTLSHSWSQVVDVSEVLRDGTPRRDEEITIKDRL
LLINTVPVRSNGVIIGAISTFRDKTEVRKLMQRLDGLVNYADALRERSHEFMNKLHVILG
LLHLKSYKQLEDYILKTANNYQEEIGSLLGKIKSPVIAGFLISKINRATDLGHTLILNSE
SQLPDSGSEDQVATLITTLGNLIENALEALGPEPGGEISVTLHYRHGWLHCEVNDDGPGI
APDKIDHIFDKGVSTKGSERGVGLALVKQQVENLGGSIAVESEPGIFTQFFVQIPWDGER
SNR
>LFGLNPFC_03682 Transcriptional regulatory protein DcuR
MINVLIIDDDAMVAELNRRYVAQIPGFQCCGTASTLEKAKEIIFNSDTPIDLILLDIYMQ
KENGLDLLPVLHNARCKSDVIVISSAADAVTIKDSLHYGVVDYLIKPFQASRFEEALTGW
RQKKMALEKHQYYDQAELDQLIHGSSSNEQDPRRLPKGLTPQTLRTLCQWIDAHQDYEFS
TDELANEVNISRVSCRKYLIWLVNCHILFTSIHYGVTGRPVYRYRIQAEHYSLLKQYCQ
>LFGLNPFC_03683 Anaerobic C4-dicarboxylate transporter DcuB
MLFTIQLIIILICLFYGARKGGIALGLLGGIGLVILVFVFHLQPGKPPVDVMLVIIAVVA
ASATLQASGGLDVMLQIAEKLLRRNPKYVSIVAPFVTCTLTILCGTGHVVYTILPIIYDV
AIKNNIRPERPMAASSIGAQMGIIASPVSVAVVSLVAMLGNVTFDGRHLEFLDLLAITIP
STLIGILAIGIFSWFRGKDLDKDEEFQKFISVPENREYVYGDTATLLDKKLPKSNWLAMW
IFLGAIAVVALLGADSDLRPSFGGKPLSMVLVIQMFMLLTGALIIILTKTNPASISKNEV
FRSGMIAIVAVYGIAWMAETMFGAHMSEIQGVLGEMVKEYPWAYAIVLLLVSKFVNSQAA
ALAAIVPVALAIGVDPAYIVASAPACYGYYILPTYPSDLAAIQFDRSGTTHIGRFVINHS
FILPGLIGVSVSCVFGWIFAAMYGFL
>LFGLNPFC_03684 Fumarate hydratase class I, anaerobic
MSNKPFIYQAPFPMGKDNTEYYLLTSDYVSVADFDGETILKVEPEALTLLAQQAFHDASF
MLRPAHQKQVAAILHDPEASENDKYVALQFLRNSEIAAKGVLPTCQDTGTAIIVGKKGQR
VWTGGGDEEALSKGVYNTYIEDNLRYSQNAPLDMYKEVNTGTNLPAQIDLYAVDGDEYKF
LCVAKGGGSANKTYLYQETKALLTPGKLKNFLVEKMRTLGTAACPPYHIAFVIGGTSAET
NLKTVKLASAHYYDELPTEGNEHGQAFRDIQLEQELLEEAQKLGLGAQFGGKYFAHDIRV
IRLPRHGASCPVGMGVSCSADRNIKAKINREGIWIEKLEHNPGQYIPEELRQAGEGEAVK
VDLNRPMKEILAQLSQYPVSTRLSLTGTIIVGRDIAHAKLKELIDAGKELPQYIKDHPIY
YAGPAKTPAGYPSGSLGPTTAGRMDSYVDLLQSHGGSMIMLAKGNRSQQVTDACHKHGGF
YLGSIGGPAAVLAQQSIKHLECVAYPELGMEAIWKIEVEDFPAFILVDDKGNDFFQQIVN
KQCANCTK

>LFGLNPFC_03685 Inner membrane protein YjdF
MTRTLKPLILNTGALALTLILIYTGISAHDKLTWLMEVTPVIIVVPLLLATAKRYPLTPL
LYTLIFFHAIILMVGGQYTYAKVPVGFEVQEWLGLSRNPYDKLGHFFQGLVPALVAREIL
VRGMYVRGRKMVAFLVCCVALAISAMYELIEWWAALAMGQGADDFLGTQGDPWDTQSDMF
CALLGALTTVIFLARFHCRQLRRFGLITRAPEAITP
>LFGLNPFC_03686 Alpha-galactosidase
MMSAPKITFIGAGSTIFVKNILGDVFHREALKTAHIALMDIDPTRLEESHIVVRKLMDSA
GASGKITCHTQQKEALQDADFVVVAFQIGGYEPCTVTDFEVCKRHGLEQTIADTLGPGGI
MRALRTIPHLWQICEDMTEVCPDATMLNYVNPMAMNTWAMYARYPHIKQVGLCHSVQGTA
EELARDLNIDPATLRYRCAGINHMAFYLELERKTADGSYVNLYPELLAAYEAGQAPKPNI
HGNTRCQNIVRYEMFKKLGYFVTESSEHFAEYTPWFIKPGREDLIERYKVPLDEYPKRCV
EQLANWHKELEEYKNASRIDIKPSREYASTIMNAIWTGEPSVIYGNVRNDGLIDNLPQGC
CVEVACLVDANGIQPTKAGTLPSHLAALMQTNINVQTLLTEAILTENRDRVYHAAMMDPH
TAAVLGIDEIYALVDDLIAAHGDWLPGWLHR
>LFGLNPFC_03687 Melibiose operon regulatory protein
MNTDTFMCSSDEKQTRSPLSLYSEYQRMEIEFRAPHIMPTSHWHGQVEVNVPFDGDVEYL
INNEKVNINQGHITLFWACTPHQLTDTGTCQSMAIFNLPMHLFLSWPLDKDLINHVTHGM
VIKSLATQQLSPFEVRRWQQELNSQNEQIRQLAIDEIGLMLKRFSLSGWEPILVNKTSRT
HKNSVSRHAQFYVSQMLGFIAENYDQALTINDVAEHVKLNANYAMGIFQRVMQLTMKQYI
TAMRINHVRALLSDTDKSILDIALTAGFRSSSRFYSTFGKYVGMSPQQYRKLSQQRRQTF
PG
>LFGLNPFC_03688 Biodegradative arginine decarboxylase
MMKVLIVESEFLHQDTWVGNAVERLADALSQQNVTVIKSTSFDDGFAILSSNEAIDCLMF
SYQMEHPDEHQNVRQLIGKLHERQQNVPVFLLGDREKALAAMDRDLLELVDEFAWILEDT
ADFIAGRAVAAMTRYRQQLLPPLFSALMKYSDIHEYSWAAPGHQGGVGFTKTPAGRFYHD
YYGENLFRTDMGIERTSLGSLLDHTGAFGESERYAARVFGADRSWSVVVGTSGSNRTIMQ
ACMTDNDVVVVDRNCHKSIEQGLMLTGAKPVYMVPSRNRYGIIGPIYPQEMQPETLQKKI
SESPLTKDKVGQKPSYCVVTNCTYDGVCYNAKEAQDLLEKTSDRLHFDEAWYGYARFNPI
YADHYAMRGEPGDHNGPTVFATHSTHKLLNALSQASYIHVREGRGAINFSRFNQAYMMHA
TTSPLYAICASNDVAVSMMDGNSGLSLTQEVIDEAVDFRQAMARLYKEFTADGSWFFKPW
NKEVVTDPQTGKTYDFADAPTKLLTTVQDCWVMHPGESWHGFKDIPDNWSMLDPIKVSIL
APGMGEDGELEETGVPAALVTAWLGRHGIVPTRTTDFQIMFLFSMGVTRGKWGTLVNTLC
SFKRHYDANTPLAQVMPELVEQYPDTYANIGIHDLGDTMFAWLKENNPGARLNEAYSGLP
VAEITPREAYNAIVDNNVELVSIENLPGRIAANSVIPYPPGIPMLLSGENFGDKNSPQVS
YLRSLQSWDHHFPGFEHETEGTEIIDGIYHVMCVKA
>LFGLNPFC_03689 HTH-type transcriptional regulator GadX
MRICSDQPCIVLLTEKDVWIRVNGKEPISLKANHMALLNCENNIIDVSSLNNTLVAHISH
DIIKDYLRFLNKDLSQIPVWQRSATPILTLPCLTPDVFRVAAQHSMMPAETESEKERTRA
LLFTVLSRFLDSKKFLSLMMYMLRNCVSDSVYQIIESDIHKDWNLSMVASCLCLSPSLLK
KKLKSENTSYSQIITTCRMRYAVNELMMDGKNISQVSQSCGYNSTSYFISVFKDFYGMTP
LHYVSQHRERTVA
>LFGLNPFC_03690 Arginine/agmatine antiporter
MSSDADAHKVGLIPVTLMVSGNIMGSGVFLLPANLASTGGIAIYGWLVTIIGALGLSMVY
AKMSFLDPSPGGSYAYARRCFGPFLGYQTNVLYWLACWIGNIAMVVIGVGYLSYFFPILK
DPLVLTITCVVVLWIFVLLNIVGPKMITRVQAVATVLALIPIVGIAVFGWFWFRGETYMA
AWNVSGLGTFGAIQSTLNVTLWSFIGVESASVAAGVVKNPKRNVPIATIGGVLIAAVCYV
LSTTAIMGMIPNAALRVSASPFGDAARMALGDTAGAIVSFCAAAGCLGSLGGWTLLAGQT
AKAAADDGLFPPIFARVNKAGTPVAGLIIVGILMTIFQLSSISPNATKEFGLVSSVSVIF
TLVPYLYTCAALLLLGHGHFGKARPAYLAVTTIAFLYCIWAVVGSGAKEVMWSFVTLMVI
TAMYALNYNRLHKNPYPLDAPISKD
>LFGLNPFC_03691 Phosphoethanolamine transferase EptA
MLKRLLKRPSLNLLAWLLLAAFYISICLNIAFFKQVLQALPLDSLHNVLVFLSMPVVAFS
VINIVLTLSSFLWLNRPLACLFILVGAAAQYFIMTYGIVIDRSMIANIIDTTPAESYALM
TPQMLLTLGFSGVLAALIACWIKIKPTTSRLRSVLFRGANILISVLLILLVAALFYKDYA
SLFRNNKELVKSLSPSNSIVASWSWYSHQRSANLPLIRIGEDAHRNPLMQNGKRKNLTIL
IVGETSRAENFSLNGYPRETNPRLAKDNVVYFPNTASCGTATAVSVPCMFSDMPREHYKE
ELAQHQEGVLDIIQRAGINVLWNDNDGGCKGVCDRVPHQNVTALNLPGQCINGECYDEVL
FHGLEEYINNLQSDGLIVLHTIGSHGPTYYNRYPPQFRKFTPTCDTNEIQTCTQEQLVNT
YDNTLVYVDYIVDKAINLLKEHQDKFTTSLVYLSDHGESLGENGIYLHGLPYAIAPDSQK
QVPMLLWLSEDYQKRYQVDQNCLQKQAQTQHYSQDNLFSTLLGLTGVETKYYQAADDILQ
TCRRVSE
>LFGLNPFC_03692 Transcriptional regulatory protein BasR
MKILIVEDDTLLLQGLILAAQTEGYACDGVSTARMAEQSLEAGHYSLVVLDLGLPDEDGL
HFLARIRQKKYTLPVLILTARDTLTDKIAGLDVGADDYLVKPFALEELHARIRALLRRHN

NQGESELNVGNLTLNMGRRQVWMSGEELILTPKEYALLSRLMLKAGSPVHREILYNDIYN
WDNEPSTNTLEVHIHNLRDKVGKARIRTVRGFGYMLVANEEN
>LFGLNPFC_03693 Sensor protein BasS
MNLMRFLRRPISLRQRLILTIGAILLVFELISVFWLWHESTEQIQLFEQALRDNRNNDRH
IMREIREAVASLIVPGVFMVSLTLFICYQAVRRITRPLAELQKELEARTADNLTPIAIHS
ATLEIDAVVSALNDLVSRLTSTLDNERLFTADVAHELRTPLAGVRLHLELLAKTHHIDVA
PLVARLDQMMESVSQLLQLARAGQSFSSGNYQHVKLLEDVILPSYDELSTMLDQRQQTLL
LPESAADITVQGDATLLRMLLRNLVENAHRYSPQGSNIMIKLQEDGGAVMAVEDEGPGID
ESKCGELSKAFVRMDSRYGGIGLGLSIVSRITQLHHGQFFLQNRQETSGTRAWIRLKKDQ
YVANQI
>LFGLNPFC_03694 hypothetical protein
MKNRVYESLTTVFSVLVVSSFLYIWFATY
>LFGLNPFC_03695 Proline/betaine transporter
MLKRKKVKPITLRDVTIIDDGKLRKAITAASLGNAMEWFDFGVYGFVAYALGKVFFPGAD
PSVQMVAALATFSVPFLIRPLGGLFFGMLGDKYGRQKILAITIVIMSISTFCIGLIPSYD
TIGIWAPILLLICKMAQGFSVGGEYTGASIFVAEYSPDRKRGFMGSWLDFGSIAGFVLGA
GVVVLISTIVGEENFLDWGWRIPFFIALPLGIIGLYLRHALEETPAFQQHVDKLEQGDRE
GLQDGPKVSFKEIATKHWRSLLTCIGLVIATNVTYYMLLTYMPSYLSHNLHYSEDHGVLI
IIAIMIGMLFVQPVMGLLSDRFGRRPFVLLGSVALFVLAIPAFILINSNVIGLIFAGLLM
LAVILNCFTGVMASTLPAMFPTHIRYSALAAAFNISVLVAGLTPTLAAWLVESSQNLMMP
AYYLMVVAVVGLITGVTMKETANRPLKGATPAASDIQEAKEILVEHYDNIEQKIDDIDHE
IADLQAKRTRLVQQHPRIDE
>LFGLNPFC_03696 hypothetical protein
MTKTLLDGPGRVLESVHPRFLVDLAQGDDARLPQAHQQQFRERLMQELLARVQLQTWTNG
GMLNAPLSLRLTLVEKLASMLDPGHLALTQIAQHLALLQKMDHRQHSAFPELPQQIVDLY
EWFSARCRWKEKALTQRGLLVQAGEQSEQIFTRWRAGAYNAWSLPGRCFIVLEELRWGAF
GDACRLGRPQAVALLLGDLRVKATQHLAESINAAPTTRHYYHQWFASSTVSTGGEHADFL
SWLGKWSTADKQPVCWSVTQRWRTVALGMPRLCSAQRLAGAMVEEIFSVNLV
>LFGLNPFC_03697 Clamp-binding protein CrfC
MYTQTLYELSQEAERLLQLSRQQLQLLEKMPLSVPGDDAPQLALPWSQPNIAERHAMLNN
ELRKISRLEMVLAIAGTMKAGKSTTINAIVGTEVLPNRNRPMTALPTLIRHTPGQKEPVL
HFSHVAPIDHLIQQLQQRLRDCDIKHLTDVLEIDKDMRALMQRIENGIAFEKYYLGAQPI
FHCLKSLNDLVRLAKALDVDFPFSAYAAIEHIPVIEVEFVHLAGLESYPGQLTLLDTPGP
NEAGGQPHLQKMLNQQLARASAVLAVLDYTQLKSISDEEVREAILAVGQSVPLYVLVNKFD
QQDRNSDDADQVRALISGTLMKGCITPQQIFPVSSMWGYLANRARHELANNGKLPPPEQQ
RWVEDFAHAALGRRWRHADLADLEHIRHAADQLWEDSLFAQPIQALLHAAYANASLYALR
SAAHKLLNYAQQAREYLDFRAHGLNVACEQLQQNIHQVEESLQLLQLNQAQVSGEIKHEI
ELALTSTNHFLRQQQDAVNAQLAALFQDDSESLSEMRTRCETLLQTAQNTISRDFTLRFA
ELESTLCRVLTDVIRPIEQQVKMELSESGFRPGFHFPVFHGVVPHFNTRQLFSDAISRQE
ATDEQITRFGVVREIFSRWLNQPDWGRGNEKFPTETVDYSVLQRALSAEVDLYCQQMAKV
MAEQVDESVTAGMNTFFAEFASCLTELQTRLRESLALRQQNESVVRLMQQQLQQTVMTHG
WIYTDAQLLRDDIQTLFTAERY
>LFGLNPFC_03698 hypothetical protein
MSLPHCPKCNSEYTYEDNGMYICPECAYEWNDAEPAQESDELIVKDANGNLLADGDSVTI
IKDLKVKGSSSMLKIGTKVKNIRLVEGDHNIDCKIDGFGPMKLKSEFVKKN
>LFGLNPFC_03699 hypothetical protein
MPLSPYLSFAGNCSDAIAYYQRTLGAELLYKISFGEMPKSAQDSAENCPSGMQFPDTAIA
HANVRIAGSDIMMSDAIPSGKASYSGFTLVLDSQQVEEGKRWFDNLAANGKIEMAWQETF
WAHGFGKVTDKFGVPWMINVVKQQPTQ
>LFGLNPFC_03700 Phosphate-import ATP-binding protein PhnC
MQTIIRVEKLAKTFNQHQALHAVDLNIHHGEMVALLGPSGSGKSTLLRHLSGLITGDKSV
GSHIELLGRTVQREGRLARDIRKSRAHTGYIFQQFNLVNRLSVLENVLIGALGSTPFWRT
CFSYFTREQKQRALQALTRVGMVHFAHQRVSTLSGGQQQRVAIARALMQQAKVILADEPI
ASLDPESARIVMDTLRDINQNDGITVVVTLHQVDYALRYCERIVALRQGHVFYDGCSQQF
DNERFDHLYRSINRVEENAKAA
>LFGLNPFC_03701 hypothetical protein
MNAKIIASLAFTSMFSLSTLLNPAYAEEQEKALNFGIISTESQQNLKPQWTPFLQDMEKK
LGVKVNAFFAPDYAGIIQGMRFNKVDIAWYGNLSAMEAVDRANGQVFAQTVAADGSPGYW
SVLIVNKDSPINNLNDLLAKRKDLTFGNGDPNSTSGFLVPGYYVFAKNNISASDFKRTVN
AGHETNALAVANKQVDVATNNTENLDKLKTSAPEKLKELKVIWKSPLIPGDPIVWRKNLS
ETTKDKIYDFFMNYGKTPEEKAVLERLGWAPFRASSDLQLVPIRQLALFKEMQSVKDNKG
LNEQDKLAKTTAIQAQLDDLDRLNNALSAMSSVSKAVQ
>LFGLNPFC_03702 Phosphate-import permease protein PhnE
MQTITIAPPKRSWFSLLSWAVVIAVLVVSWQGAEMAPLTLIKDGGNMATFAADFFPPDFS

QWQDYLTEMAVTLQIAVWGTALAVVLSIPFGLMSAENLVPWWVYQPVRRLMDACRAINEI
VFAMLFVVAVGLGPFAGVLALFIHTTGVLSKLLSEAVEAIEPGPVEGIRATGANKLEEIL
YGVLPQVMPLLISYSLYRFESNVRSATVVGMVGAGGIGVTLWEAIRGFQFQQTCALMVLI
IVTVSLLDFLSQRLRKHFI
>LFGLNPFC_03703 putative transcriptional regulator PhnF
MAMHLSTHPTSYPTRYQEIAAKLEQELRQHYRCGDYLPAEQQLAARFEVNRHTLRRAIDQ
LVEKGWVQRRQGVGVLVLMRPFDYPLNAQARFSQNLLDQGSHPTSEKLLSVLRPASGHVA
DALGITEGENVIHLRTLRRVNGVALCLIDHYFADLTLWPTLQRFDSGSLHDFLREQTGIT
LRRSQTRISARRAQAKECQRLEIPNMSPLLCVRTLNHRDGESSPAEYSVSLTRADMIEFT
MEH
>LFGLNPFC_03704 Alpha-D-ribose 1-methylphosphonate 5-triphosphate synthase subunit PhnG
MHADTATRQHWMSVLAHSQPAELAARLNALNINADYEVIRAAETGLVQIQARMGGTGERF
FAGDATLTRAAVRLTDGTLGYSWVLGRDKQHAERCALIDALMQQNRHFQNLSETLITPLD
ADRMARIAARQAEVNASRVDFFTMVRGDNA
>LFGLNPFC_03705 Alpha-D-ribose 1-methylphosphonate 5-triphosphate synthase subunit PhnH
MTLETAFMLPVQDAQHSFRRLLKAMSEPGVIVALHQLKRGWQPLNIATTSVLLTLADNDT
PVWLAAPLSNDIVNQSLRFHTNAPLVNQPKQATFAVTDEAISSEQLNALSTGTAVAPEAG
ATLILQVTSLSGGRMLRLTGAGIAEERMIAPQLPKCILHELTERPHPFPLGIDLILTCGE
RLLAIPRTTHVEVC
>LFGLNPFC_03706 Alpha-D-ribose 1-methylphosphonate 5-triphosphate synthase subunit PhnI
MYVAVKGGEKAIDAAHALQESRRRGDTDLPELSVAQIEQQLNLAVDRVMTEGGIADRELA
ALALKQASGDNVEAIFLLRAYRTTLAKLAVSEPLDTTGMRLERRISAIYKDIPGGQLLGP
TYDYTHRLLDFTLLANGETPTLTTADSEQQPSPHVFSLLARQGLAKFEEDSGAQPDDITR
TPPVYPCSRSSRLQQLMRGDEGYLLALAYSTQRGYGRNHPFAGEIRSGYIDIFIVPEELG
FAVNVGELLMTECEMVNGFIDPPDEPPHFTRGYGLVFGMSERKAMAMALVDRALQAPEYG
EHATGPAQDEEFVLAHADNVEAAGFVSHLKLPHYVDFQAELELLKRLQQEQNHG
>LFGLNPFC_03707 Alpha-D-ribose 1-methylphosphonate 5-phosphate C-P lyase
MANLSGYNFAYLDEQTKRMIRRAILKAVAIPGYQVPFGGREMPMPYGWGTGGIQLTASVI
GESDVLKVIDQGADDTTNAVSIRNFFKRVTGVNTTERTDDATLIQTRHRIPETPLTEDQI
IIFQVPIPEPLRFIEPRETETRTMHALEEYGVMQVKLYEDIARFGHIATTYAYPVKVNGR
YVMDPSPIPKFDNPKMDMMPALQLFGAGREKRIYAVPPFTRVESLDFDDHPFTVQQWDEP
CAICGSTHSYLDEVVLDDAGNRMFVCSDTDYCRQQNEAKSQ
>LFGLNPFC_03708 Putative phosphonates utilization ATP-binding protein PhnK
MNQPLLSVNNLTHLYAPGKGFSDVSFDLWPGEVLGIVGESGSGKTTLLKSISARLTPQQG
EIRYENRSLYAMSEADRRRLLRTEWGVVHQHPLDGLRRQVSAGGNIGERLMATGARHYGD
IRATAQKWLEEVEIPANRIDDLPTTFSGGMQQRLQIARNLVTHPKLVFMDEPTGGLDVSV
QARLLDLLRGLVMELNLAVVIVTHDLGVARLLADRLLVMKQGKVVESGLTDRVLDDPHHP
YTQLLVSSVLQN
>LFGLNPFC_03709 Alpha-D-ribose 1-methylphosphonate 5-triphosphate synthase subunit PhnL
MINVQNVSKTFILHQQNGVRLPVLQGASLTVNAGECVVLHGHSGSGKSTLLRSLYANYLP
DEGQIQIKHGDEWVDLVTAPARKVVEIRKTTVGWVSQFLRVIPRISALEVVMQPLLDTGV
PREACAAKAARLLTHLNVPERLWHLAPSTFSGGEQQRVNIARGFIVDYPILLLDEPTASL
DAKNSAAVVELIREAKARGAAIVGIFHDETVREQVADRLHPMGVSS
>LFGLNPFC_03710 Alpha-D-ribose 1-methylphosphonate 5-triphosphate diphosphatase
MIINNVKLVLENEVVHGSLEMQDGEIRAFAESQSHLSEAMDGEGGWLLPGLIELHTDNLD
KFFTPRPKVDWPAHSAMSSHDALMVASGITTVLDAVAIGDVRDGGDRLENLEKMINAIEE
TQKRGVNRAEHRLHLRCELPHHTTLPLFEKLVQRELVTLVSLMDHSPGQRQFANREKYRE
YYQGKYSLTDAQMQQYEEEQLALAARWSQPNRETIAAMCRARHIALASHDDATHAHVAES
HQLGSVIAEFPTTFEAAEASRKHGMNVLMGAPNIVRGGSHSGNVAASELAQLGLLDILSS
DYYPASLLDAAFRVADDESKRADLVLAHRKGNHIHIDHVWRQGKRVF
>LFGLNPFC_03711 Ribose 1,5-bisphosphate phosphokinase PhnN
MTGKLIWLMGASGSGKDSLLTELRQREQTQLLVAHRYITRDASAGSENHIALSEQEFFTR
AGQNLLALSWHANGLYYGVGIEIDLWLHAGFDVVVNGSRAHLPQARARYQSALLPICLQV
SPEILRQRLENRGRENASEINARLARAARYTPQDCHTLNNDGSLRQSVDTLLTLIHQKEK
HHACL
>LFGLNPFC_03712 Aminoalkylphosphonate N-acetyltransferase
MPACELRPATQYDTDAVYALICELKQAEFDHQAFRVGFNANLRDPNMRYHLALLDGEVVG
MIGLHLQFHLHHVNWIGEIQELVVMPQARGLNVGSKLLAWAEEEARQAGAEMTELSTNVK
RHDAHRFYLREGYEQSHFRFTKAL
>LFGLNPFC_03713 Phosphoribosyl 1,2-cyclic phosphate phosphodiesterase
MSLTLTLTGTGGAQGVPAWGCECAACARARRSPQYRRQPCSGVVKFNDAITLIDAGLHDL
ADRWSPGSFQQFLLTHYHMDHVQGLFPLRWGVGDTIPVYGPPDEQGCDDLFKHPGLLDFS
HTVEPFVVFDLQGLQVTPLPLNHSKLTFGYLLETAHSRVAWLSDTAGLPEKTLKFLRNNQ
PQVMVIDCSHPPREDAPRNHCDLNTVLALNQVIRSPRVILTHISHQFDAWLMENALPSGF

EAGFDGMDIEVA
>LFGLNPFC_03714 hypothetical protein
MKRFPLFLLFTLLTLSTVPAQADIIDDTIGNIQQAINDAYNPDRGRDYEDSRDDGWQRQV
SDDRRKQYNDRRRQFEDRRRQLDDRQRQLDQERRQLEDEERRMEDEYGR
>LFGLNPFC_03715 Ribose-5-phosphate isomerase B
MKKIAFGCDHVGFILKHEIVAHLVERDVEVIDKGTWSLERTDYPHYASQVALAVAGGEAD
GGILICGTGVGISIAANKFAGIRAVVCSEPYSAQLSRQHNDTNVLAFGSRVVGLELAKMI
VDAWLGAQYEGGRHQQRVEAITAIEQQRN
>LFGLNPFC_03716 HTH-type transcriptional regulator RpiR
MSQSEFDSALPNGIGLAPYLRMKQEGMTENESRIVEWLLKPGNLSCAPAIKDVAEALAVS
EAMIVKVSKLLGFSGFRNLRSALEDYFSQSEQVLPSELAFDEAPQDVVNKVFNITLRTIM
EGQSIVNVDEIHRAARFFYQARQRDLYGAGGSNAICADVQHKFLRIGVRCQAYPDAHIMM
MSASLLQEGDVVLVVTHSGRTSDVKAAVELAKKNGAKIICITHSYHSPIAKLADYIICSP
APETPLLGRNASARILQLTLLDAFFVSVAQLNIEQANINMQKTGAIVDFFSPGALK
>LFGLNPFC_03717 D-allose-binding periplasmic protein
MNKYLKYFSGTLVGLMLSTSAFAAAEYAVVLKTLSNPFWVDMKKGIEDEAKTLGVSVDIF
ASPSEGDFQSQLQLFEDLSNKNYKGIAFAPLSSVNLVMPVARAWKKGIYLVNLDEKIDMD
NLKKAGGNVEGFVTTDNVAVGAKGASFIIDKLGAEGGEVAIIEGKAGNASGEARRNGATE
AFKKASQIKLVASQPADWDRIKALDVATNVLQRNPNIKAIYCANDTMAMGVAQAVANAGK
TGKVLVVGTDGIPEARKMVEAGQMTATVAQNPADIGATGLKLMVDAEKSGKVIPLDKAPE
FKLVDSILVTQ
>LFGLNPFC_03718 Ribose import ATP-binding protein RbsA
MATPYISMAGIGKSFGPVHALKSVNLTVYPGEIHALLGENGAGKSTLMKVLSGIHEPTKG
TITINNINYNKLDHKLAAQLGIGIIYQELSVIDELTVLENLYIGRHLTKKICGVNIIDWR
EMRVRAAMMLLRVGLKVDLDEKVANLSISHKQMLEIAKTLMLDAKVIIMDEPTSSLTNKE
VDYLFLIMNQLRKEGTAIVYISHKLAEIRRICDRYTVMKDGSSVCSGMVSDVSNDDIVRL
MVGRELQNRFNAMKENVSNLAHDTVFEVRNVTSRDRKKVRDISFSVCRGEILGFAGLVGS
GRTELMNCLFGVDKRAGGEIRLNGKDISPRSPLDAVKKGMAYITESRRDNGFFPNFSIAQ
NMAISCSLKDGGYKGAMGLFHEVDEQRTAENQRELLALKCHSVNQNITELSGGNQQKVLI
SKWLCCRPEVIIFDEPTRGIDVGAKAEIYKVMRQLADDGKVILMVSSELPEIITVCDRIA
VFCEGRLTQILTNRDDMSEEEIMAWALPHE
>LFGLNPFC_03719 D-allose transport system permease protein AlsC
MGFTTRVKSEASEKKPFNFALFWDKYGTFFILAIIVAIFGSLSSEYFLTTNNITQIFVQS
SVTVLIGMGEFFAILVAGIDL
>LFGLNPFC_03720 D-allose transport system permease protein AlsC
MVTAKLMLAGIDPFLAALIGGVLVGGALGAINGCLVNWTGLHPFIITLGTNAIFRGITLV
ISDANSVYGFSFDFVNFFAASVIGIPVPVIFSLIVALILWFLTTRMRLGRNIYALGGNKN
SAFYSGIDVKFHILVVFIISGVCAGLAGVVSTARLGAAEPLAGMGFETYAIASAIIGGTS
FFGGKGRIFSVVIGGLIIGTINNGLNILQVQTYYQLVVMGGLIIAAVALDRLISK
>LFGLNPFC_03721 D-allulose-6-phosphate 3-epimerase
MKISPSLMCMDLLKFKEQIEFIDSHADYFHIDIMDGHFVPNLTLSPFFVSQVKKLASKPL
DCHLMVTRPQDYIAQLARAGADFITLHPETINGQAFRLIDEIRRHGMKVGLILNPETPVE
AMKYYIHKADKITVMTVDPGFAGQPFIPEMLDKVAELKAWREREGLEYEIEVDGSCNQAT
YEKLMAAGADVFIVGTSGLFNHAENIDEAWRIMTAQILAAKSEVQPHAKTA
>LFGLNPFC_03722 D-allose kinase
MQKQHNVVAGVDMGATHIRFCLRTAEGETLHCEKKRTAEVIAPDLVSGIGEMIDEQLRRF
NARCHGLVMGFPALVSKDKRTIISTPNLPLTAADLYDLADKLENTLNCPVEFSRDVNLQL
SWDVVENRLTQQLVLAAYLGTGMGFAVWMNGAPWTGAHGVAGELGHIPLGDMTQHCACGN
PGCLETNCSGMALRRWYEQQPRNYPLSDLFVHAENAPFVQSLLENAARAIATSINLFDPD
AVILGGGVMDMPAFPPETLIAMTQKYLRRPLPYQVVRFIAASSSDFNGAQGAAILAHQRF
LPQSCAKVP
>LFGLNPFC_03723 Putative alkyl/aryl-sulfatase YjcS
MNNSRLFRLSRIVIAFTAASGMMINTAYATDEAKAATQYTQQVNQNYAKSLPFGDRQDFD
DAQRGFIAPLLDEGILRDANGKIYYRANDYKFDINAAAPETVNPSLWRQSQINGISGLFK
VTDKMYQVRGQDISNITFVEGEKGIIVIDPLVTPPAAKAALDLYFQHRPQKPIVAVIYTH
SHADHYGGVKGIISEADVKSGKVQVIAPAGFMDEAISENVLAGNIMSRRALYSYGLLLPH
NAQGNVGNGLGVTLATGDPSIIAPTKTIVRTGEKMIIDGLEFDFLMTPGSEAPAEMHFYI
PALKALCTAENATHTLHNFYTLRGAKTRDTSKWTEYLNETLDMWGNDAEVLFMPHTWPVW
GNKHINDYIGKYRDTIKYIHDQTLHLANQGYTMNEIGDMIKLPPALANNWASRGYYGSVS
HNARAVYNFYLGYYDGNPANLHPYGQVEMGKRYVQALGGSARVINLAQEANKQGDYRWSA
ELLKQVIAANPGDQVAKNLQANNFEQLGYQAESATWRGFYLTGAKELREGVHKFSHGTTG
SPDTIRGMSVEMLFDFMSVRLDSVKAAGKNISLNFNMSNGDNLNLTLNDSVLNYRKTLQP
QADASFYISREDLHAVLTGQAKMADQVKAKKAKIIGNGAKLEEIIACLDNFDLWVNIVTP
N

>LFGLNPFC_03724 hypothetical protein
MPTVLSRMAMQLKKTAWIIPVFMVSGCSLSPAIPVIGAYYPGWFFCAIASLILTLITRRI
IQRTNINLAFVGIIYTALFALYAMLFWLAFF
>LFGLNPFC_03725 Multidrug resistance protein MdtN
MESTPKKAPRSKFPALLVVALALVALVFVIWRVDSAPSTNDAYASADTIDVVPEVSGRIV
ELAVTDNQAVKQGDLLFRIDPRPYEANLAKAEASLAALDKQIMLTQRSVDAQQFGADSVN
ATVEKARAAAKQASDTLRRTEPLLREGFVSAEEVDRARTAQRAAEADLNAVLLQAQSAAS
SVSGVDALVAQRAAVEADIALTKLHLEMATVRAPFDGRVISLKTSVGQFASAMRPIFTLI
DTRHWYVIANFRETDLKNIRSGTLATIRLMSDSGKTFEGKVDSIGYGVLPDDGGLVLGGL
PKVSRSINWVRVAQRFPVKIMVDKPDPEMFRIGASAVANLEPQ
>LFGLNPFC_03726 Multidrug resistance protein MdtO
MSALNSLPLPVVRLLAFFHEELSERRPGRVPQTMQLWVGCLLVILISMTFEIPFVALSLA
VLFYGIQSNAFYTKFVAILFVVATVLEIGSLFLIYKWSYGEPLIRLIIAGPILMGCMFLM
RTHRLGLVFFAVAIVAIYGQTFPAMLDYPEVVVRLTLWCIVVGLYPTLLMTLIGVLWFPS
RAITQMHQALNDRLDDAISHLTDSLAPLPETRIEREALALQKLNVFCLADDANWRTQSAW
WQSCVATVTYIYSTLNRYDPTSFADSQAIIEFRQKLVSEINKLQHTIAEGQCWQSDWRIS
ESEAMAARECNLENICQTLLQLGQMDPNTPPTPAAKPPSMVADAFTNPDYMRYAVKTLLA
CLICYTFYSGVDWEGIHTCMLTCVIVANPNVGSSYQKMVLRFGGAFCGAILALLFTLLVM
PWLDNIVELLFVLAPIFLLGAWIATSSERSSYIGTQMVVTFALATLENVFGPVYDLVEIR
DRALGIIIGTVVSAVIYTFVWPESEARTLPQKLAGALGMLSKVMRIPRQQEVTALRTYLQ
IRIGLHAAFNACEEMCQRVVLERQLDSEERALLIERSQTVIRQGRDILHAWDATWNSAQA
LDNALQPDRAGQFADALEKYAAGLATALSRSPQITLEETPASQAILPTLLKQEQHVCQLF
ARLPDWTAPALTPATEQAQGATQ
>LFGLNPFC_03727 Cation efflux system protein CusC
MINRQLSRLLLCSILGSTTLISGCALVRKDSAPHQQLKPEQIKLADDIHLASSGWPQAQW
WKQLNDPQLDALIQRTLSGSHTLAEAKLREEKAQSQADLLDAGSQLQVAALGMLNRQRVS
ANGFLSPYAMDAPALGMDGPYYTEATVGLFAGLDLDLWGVHRSAVAAAIGAHNAALAETA
AVELSLTTGVAQLYYSMQASYQMLDLLEQTRDVIDYAVKAHQSKVAHGLEAQVPFHGARA
QILAVDKQIAAVKGQITETRESLRALIGAGASDMPEIKPVALPRVQTGIPATLSYELLAR
RPDLQAMRWYVQASLDQVDSARALFYPSFDIKAFFGLDSIHLDTLFKKTSRQFNFIPGLK
LPLFDGGRLNANLEGTRAASNMMIERYNQSVLNAVRDVAVNGTRLQTLNDEREMQAERVE
ATRFTQRAAEAAYQRGLTSRLQATEARLPVLAEEMSLLMLDSRRVIQSIQLMKSLGGGYQ
AAPIVEKK
>LFGLNPFC_03728 Formate dehydrogenase H
MKKVVTVCPYCASGCKINLVVDNGKIVRAEAAQGKTNQGTLCLKGYYGWDFINDTQILTP
RLKTPMIRRQRGGKLEPVSWDEALNYVAERLSAIKEKYGPDAIQTTGSSRGTGNETNYVM
QKFARAVIGTNNVDCCARV
>LFGLNPFC_03729 Formate dehydrogenase H
MSNAINEIDNTDLVFVFGYNPADSHPIVANHVINAKRNGAKIIVCDPRKIETARIADMHI
ALKNGSNIALLNAMGHVIIEENLYDKAFVASRTEGFEEYRKIVEGYTPESVEDITGVSAS
EIRQAARMYAQAESAAILWGMGVTQFYQGVETVRSLTSLAMLTGNLGKPHAGVNPVRGQN
NVQGACDMGALPDTYPGYQYVKDPANREKFAKAWGVESLPAHTGYRISELPHRVAHGEVR
AAYIMGEDPLQTDAELSAVRKAFEDLELVIVQDIFMTKTASAADVILPSTSWGEHEGVFT
AADRGFQRFFKAVEPKWDLKTDWQIISEIATRMGYPMHYNNTQEIWDELRHLCPDFYGAT
YEKMGELGFIQWPCRDTSDADQGTSYLFKEKFDTPNGLAQFFTCDWVAPIDKLTDEYPMV
LSTVREVGHYSCRSMTGNCAALAALADEPGYAQINTEDAKRLGIEDEALVWVHSRKGKII
TRAQVSDRPNKGAIYMTYQWWIGACNELVTENLSPITKTPEYKYCAVRVEPIADQRAAEQ
YVIDEYNKLKTRLREAALA
>LFGLNPFC_03730 putative D,D-dipeptide-binding periplasmic protein DdpA
MIKKLLPLLVLSTISATTVAATPPNTLVVAQGLDDIVSLDPAEANELSSIQTVPSLYQRV
VQPDRNDPTKITPILAESWQADPAAKTLTIKLKSDAKFASGNPVRPEDVIYSYVRAVTLN
KSGAFILNVLGWQPENIASQLKKIDDHTVQVQWSADVSPALALNILSTPIASIVDEKLVA
PNAKNNDFGNEWLKMHSAGSGAFKMRTYQPHQAIVMEANASSPTGAPKLKNVIIKNVPDP
ATRRLLIQQGDADMARDLGADQIDALQGKPGVKVMSIASAEQNYLAFNTGNKDNPLMSNP
AFWEAARWLVDYDGITKNLLKGQYFTHQSFLPVGFPGALEETPFTFNPAKAKEILAKAGI
KDPHFTLDVENKPPFITIAQSIQASFAQGGVKVDLLPAAGSQVYSRVRAHQHQGAIRMWL
PDYFDAHSNASSFAYNDGKSSTVAGLNGWKIPELNKETLAAIAEPDSTKRLDLYKKMQQE
LQRSSPYVFIDQGKTQIVMRDNVQGYQQGLNADMVWFDQVTK
>LFGLNPFC_03731 Dipeptide transport system permease protein DppB
MSVVFSTRARWRGKRPLLALLQGLFTVALTLLGLLLITFALSALSPVDRVLQIVGDHASQ
STYDQVRHQLGLDQPLAVQFWHYLMNLAHGDLGIASSTGQPVLQDLLSVFPATIELATLA
LIVGAVVGVIAGVLCARYVGSPLDFTVRTLTLLGNSVPVFWLGLLMLALFYARLQWSAGP
GRLDDIYQFTIEPRSGFALIDTWLSGDREAFTNAINHLILPVLLLAYFSLASITRLTRSA
CLGEMNKEYILLARAKGASEMTILLRHVLPNIRGTLLTVIALAYTSMLEGAVLTETVFSW

PGIGRYLTTALFAGDTTAIMGGTLLIGVCFVLINNLTDLLVRLTDPRLR
>LFGLNPFC_03732 putative D,D-dipeptide transport system permease protein DdpC
MPMFLFLRRLRHSPAAFCGLIIILLLMLIALFAPWLAPHDPNWQDAAARLQAPSRQHWLG
TDSYGRDLLSRLIYGSRPALGLVALVTIITLPVGLLIGILAGYYGGWLERVLMRFTDVVM
SMPRLILAFAFVAMLGPGLVNGALALALTTWPAYARQARSEIQRLRHSDYLAAAEMMGIR
GRRLLAGHILPLCLPSAIVRLALDLAGIILAAAGLGFLGLGARPPMAEWGAMIADGMQVI
FDQWWIAAAPGAIILIASLAFNLLGDGLRDVLEPQHD
>LFGLNPFC_03733 Dipeptide transport ATP-binding protein DppD
MTDSRLSINNLCVDYPESRVVNNVSFTLGNERLALVGESGSGKSMTARALMGLVRKPGVV
TADTLNILGRDALTLNARGWQQLRGNDIAMVLQDPRYALNPVKTVKAQLEEALTLHQRLN
RREKEEKINTAIQAVGLNTRVLQSYPRELSGGMGQRVMIAIALINDPRVLIADEPTSALD
VRLRNQILELLVTQCEQRQMAMLLISHDLPLVAQFCHRVLVMYQGNKVDEMHAAALPTAT
HPYTRTLWTCRPNAQTYGQMLPTLDRTAMTPEKYHDDC
>LFGLNPFC_03734 Oligopeptide transport ATP-binding protein OppF
MTIVEINHLQVTFAEKTAVSAASFSVNAGETFSLIGESSCGKSTILRVIAGLQRDWHGQV
SLFGHTIKPGMRFQGDLRRNVQMVFQDPYASLHPNHTLWRTLAEPLKIRGEREIEKRVQT
ALEQVGLPFDAARRYPHQLSGGQRQRVVIARALLLRPQLLLLDEPTSALDMSVQAEILNL
LNQLKLEHQMTYLLVSHDADVIAHMSDRAALMSEGKIQRFFDRDAMEKGEHRMD
>LFGLNPFC_03735 hypothetical protein
MKKIITLMLFLTFFAHANDSEPGSQYLKAAEAGDRRAQYFLADSWFSSGDLSKAEYWAQK
AADSGDADACALLAQIKITNPVSLDYPQAKVLAEKAAQAGSKEGEVTLAHILVNTQAGKP
DYPKAISLLENASEDLENDSAVDAQMLLGLIYANGVGIKADDDKATWYFKRSSAISRTGY
SEYWAGMMFLNGEEGFIEKNKQKALHWLNLSCMEGFDTGCEEFEKLTNG
>LFGLNPFC_03736 Proton/glutamate-aspartate symporter
MKNIKFSLAWQILFAMVLGILLGSYLHHHSDSRDWLVVNLLSPAGDIFIHLIKMIVVPIV
ISTLVVGIAGVGDAKQLGRIGAKTIIYFEVITTVAIILGITLANVFQPGAGVDMSQLATV
DISKYQSTTEAVQSSSHGIMGTILSLVPTNIVASMAKGEMLPIIFFSVLFGLGLSSLPAT
HREPLVTVFRSISETMFKVTHMVMRYAPVGVFALIAVTVANFGFSSLWPLAKLVLLVHFA
ILFFALVVLGIVARLCGLSVWILIRILKDELILAYSTASSESVLPRIIEKMEAYGAPASI
TSFVVPTGYSFNLDGSTLYQSIAAIFIAQLYGIDLSIWQEIILVLTLMVTSKGIAGVPGV
SFVVLLATLGSVGIPLEGLAFIAGVDRILDMARTALNVVGNALAVLVIAKWEHKFDRKKA
LAYEREVLGKFDKTADQ
>LFGLNPFC_03737 Formate-dependent nitrite reductase complex subunit NrfG
MKQPQIPVKMLTTLTILMVFLCVGSYLLSPKWQAVRTEYQRQRDPLHQFASQQTPEAQLQ
ALQDKIRANPQNSEQWALLGEYYLWQNDYSNSLLAYRQALQLRGENAELYAALATVLYYQ
ASQHMTVQTRAMIDRALALDSNEITALMLLASDAFMQANYAQAIELWQKVMDLNSPRINR
TQLVESINMAKLLQRRSD
>LFGLNPFC_03738 Cytochrome c-type biogenesis protein CcmH
MNKGLLTLLLLFTCFARAQVVDTWQFANPQQQQALNIASQLRCPQCQNQNLLESNAPVA
VSMRHQVYTMVEEGKSEVEIIDWMTERYGDFVRYNPPLTGQTLVLWALPVVLLLLMALIL
WRVRAKR
>LFGLNPFC_03739 Cytochrome c-type biogenesis protein CcmF
MRWPAMMRLTCIGILAQFALLLLAFGVLTYCFLISDFSVIYVAQHSYSLLSWELKLAAVW
GGHEGSLLLWVLLLSAWSVLFAWHYRQQTDPLFPLTLTVLSLILAALLLFVVGWSDPFVR
IFPPAIEGRDLNPMLQHPGLIFHPPLLYLGYGGLMVAASVALASLLRGEFGGTCARICWR
WALPGWSALTAGIILGSWWAYCELGWGGWWFWDPVENASLLPWLSATALLHSLSLTRQRG
IFRHWSLLLAIVTLMLSLLGTLIVRSGILVSVHAFALDNVRAVPLFSLFALISLASLALY
GWRARDGGPAVRFSGLSREMLILATLLLFCAVLLIVLVGTLYPMIYGLLGWGRLSVGAPY
FNRATLPFALLMLVVIVLATFVSGKRAQLPALLAHAGVLLFAAGIVVSSVSRQEISLNLQ
PGQQVTLAGYTFRFERLDLQAKGNYTSEKAIVALFDHQQRIGELTPERRFYEARRQQMME
PSIRWNGIHDWYAVMGEKTGADRYAFRLYVQSGVRWIWGGGLLMIAGALLSGWRGRKRDE
>LFGLNPFC_03740 hypothetical protein
MTQTSAFHFESLVWDWPIAIYLFLIGISAGLVTLAVLLRRVYPQAGGADSTLLRTTLIVG
PGAVILGLLILVFHLTRPWTFWKLMFHYSFTSVMSMGVMLFQLYMVVLVLWLAKIFEHDL
LALQQRWLPKLGIVQKVLSLLTPVHRGLETLMLVLAVLLGAYTGFLLSALKSYPFLNNPI
LPVLFLFSGISSGAAVALIAMAIRQRSNPHSTEAHFVHRMEIPVVWGEIFLLVAFFVGLA
LGDDGKVRALVAALGGGFWTWWFWLGVAGLGLIVPMLLKPWVNRSSGIPAVLAACGASLV
GVLMLRFFILYAGQLTVA
>LFGLNPFC_03741 putative ferredoxin-like protein YdhX
MTWSRRQFLTGVGVLAAVSGTAGRVVAKTLNINGVRYGMVHDESLCIGCTACMDACREVN
KVPEGVSRLTIIRSEPQGEFPDVKYRFFRKSCQHCDHAPCVDVCPTGASFRDAASGIVDV
NPDLCVGCQYCIAACPYRVRFIHPVTKTADKCDFCRKTHLQAGKLPACVEACPTKALTFG
NLDDPSSEISQLLRQKPTYRYKLALGTKPKLYRVPFKYGEVSQ
>LFGLNPFC_03742 Cytochrome c-type protein NrfB

MSVLRSLLTAGVLASGLLWSLNGITATPAAQASGDRYEVTQQRNPDAACLDCHKPDTEGM
HGKHASVINPNNKLPVTCTNCHGQPSPQHREGVKDVMRFNEPMYKVGEQNSVCMSCHLPE
QLQKAFWPHDVHVTKVACASCHSLHPQQDTMQTLSDKGRIKICVDCHSDQRTNPNFNPAS
VPLLKEQP
>LFGLNPFC_03743 Cytochrome c-552
MTRIKINARRIFSLLIPFFFFTSVHAEQTAAPATPVTVEAKNETFAPQHPDQYLSWKATS
EQSERVDALAEDPRLVILWAGYPFSRDYNKPRGHAFAVTDVRETLRTGAPKNAEDGPLPM
ACWSCKSPDVARLIQKDGEDGYFHGKWARGGPEIVNNLGCADCHNTASPEFAKGKPELTL
SRPYAARAMEAIGKPFEKAGRFDQQSMVCGQCHVEYYFDGKNKAVKFPWDDGMKVENMEQ
YYDKIAFSDWTNSLSKTPMLKAQHPEYETWTAGIHGKNNVTCIDCHMPKVQNAEGKLYTD
HKIGNPFDNFAQTCANCHTQDKAALQKVVAERKQSINDLKIKVEDQLVHAHFEAKAALDA
GATEAEMKPIQDDIRHAQWRWDLAIASHGIHMHAPEEGLRMLGTAMDKAADARTKLARLL
ATKGITHEIQIPDISTKEKAQQAIGLNMEQIKAEKQDFIKTVIPQWEEQARKNGLLSQ
>LFGLNPFC_03744 Acetyl-coenzyme A synthetase
MSQIHKHTIPANIADRCLINPQQYEAMYQQSINAPDTFWGEQGKILDWITPYQKVKNTSF
APGNVSIKWYEDGTLNLAANCLDRHLQENGDRTAIIWEGDDASQSKHISYKELHRDVCRF
ANTLLELGIKKGDVVAIYMPMVPEAAVAMLACARIGAVHSVIFGGFSPEAVAGRIIDSSS
RLVITSDEGVRAGRSIPLKKNVDDALKNPNVTSVEHVVVLKRTGGKIDWQEGRDLWWHDL
VEQASDQHQAEEMNAEDPLFILYTSGSTGKPKGVLHTTGGYLVYAALTFKYVFDYHPGDI
YWCTADVGWVTGHSYLLYGPLACGATTLMFEGVPNWPTPARMAQVVDKHQVNILYTAPTA
IRALMAEGDKAIEGTDRSSLRILGSVGEPINPEAWEWYWKKIGNEKCPVVDTWWQTETGG
FMITPLPGATELKAGSATRPFFGVQPALVDNEGNPLEGATEGSLVITDSWPGQARTLFGD
HERFEQTYFSTFKNMYFSGDGARRDEDGYYWITGRVDDVLNVSGHRLGTAEIESALVAHP
KIAEAAVVGIPHNIKGQAIYAYVTLNHGEEPSPELYAEVRNWVRKEIGPLATPDVLHWTD
SLPKTRSGKIMRRILRKIAAGDTSNLGDTSTLADPGVVEKLLEEKQAIAMPS
>LFGLNPFC_03745 Inner membrane protein YjcH
MNGTIYQRIEDNAHFRELVEKRQRFATILSIIMLAVYIGFILLIAFAPGWLGTPLNPNTS
VTRGIPVGVGVIVISFVLTGIYIWRANGEFDRLNNEVLHEVQAS
>LFGLNPFC_03746 Cation/acetate symporter ActP
MKRVLTALAATLPFAANAADAISGAVERQPTNWQAIIMFLIFVVFTLGITYWASKRVRSR
NDYYTAGGNITGFQNGLAIAGDYMSAASFLGISALVFTSGYDGLIYSLGFLVGWPIILFL
IAERLRNLGRYTFADVASYRLKQGPIRILSACGSLVVVALYLIAQMVGAGKLIELLFGLN
YHIAVVLVGVLMMMYVLFGGMLATTWVQIIKAVLLLFGASFMAFMVMKHVGFSFNNLFSE
AMAVHPKGVDIMKPGGLVKDPISALSLGLGLMFGTAGLPHILMRFFTVSDAREARKSVFY
ATGFMGYFYILTFIIGFGAIMLVGANPEYKDAAGHLIGGNNMAAVHLANAVGGNLFLGFI
SAVAFATILAVVAGLTLAGASAVSHDLYANVFKKGATEREELRVSKITVLILGVIAIILG
VLFENQNIAFMVGLAFAIAASCNFPIILLSMYWSKLTTRGAMLGGWLGLITAVVLMILGP
TIWVQILGHEKAIFPYEYPALFSISVAFLGIWLFSATDNSAEGARERELFRAQFIRSQTG
FGVEQGRAH
>LFGLNPFC_03747 Inner membrane protein YohK
MANFQLSMLCLAITLVIYFLNKRLYRRFRKLPLMPLVLTPTLLVMLLVFGNISWQNYIGE
AHGLLWLLGPATIAFAVPVYDNRAIIKRHWMSLTAGVLTATVVAVTSSVWLARLFTLPDE
IQRSLAVRSVTTPFALAASQSLGGQPDLVALFVVVTGVFGMAIGDVLFLRLSIREGMAKG
AGFGAASHGAGTARSYELGQQEGVVASLVMMLSGVLMVLVSPLVARMMF
>LFGLNPFC_03748 Holin-like protein CidA
MPVALRRITPAVVQSIQVFFQVVLYAGLFIFAQYLVSWLHLPLPANLVGMILMLALIVCR
ILPLQWVRAGARWLLAEMLLFFVPAVVAVVNYAQLLLVDGWRIFAVIALSTVMVLGTTAW
VVEKVYRYEMRRLNRG
>LFGLNPFC_03749 Hydrogen peroxide-inducible genes activator
MDIRTLRYFVEVVRQQSFTRAAEKLFVTQPTISKMLKNLEDELNCILLIRDGRRLLLTDT
GRVVFERGLALLAEFRQLEAELSDINQLKTGVLRLGIPPMVGMLMAGPIGLFRQRYPGVE
LKISEFGGLTVQQAVMNGELDMALTALPVEESGLTTLSLFSHPLCVLTPHSGKWLECDAV
SPETLAEHPLLIYNEDFALSKQLMKLFDEHGVKPRIAVRSGQWDFLAAMVQAGVGIAILP
EPICQRLDKNTLKWLPLESELSWKLGMIWREGVYLSHSAHAWLSCCEGYWLTPE
>LFGLNPFC_03750 Sodium, potassium, lithium and rubidium/H(+) antiporter
MEIFFTILIMTLVVSLSGVVTRVMPFQIPLPLMQIAIGALLAWPTFGLHVEFDPELFLVL
FIPPLLFADGWKTPTREFLEHGREIFGLALALVVVTVVGIGFLIYWVVPGIPLIPAFALA
AVLSPTDAVALSGIVGEGRIPKKIMGILQGEALMNDASGLVSLKFAVAVAMGTMIFTVGG
ATVEFMKVAIGGILAGFVVSWLYGRSLRFLSRWGGDEPATQIVLLFLLPFASYLIAEHIG
VSGILAAVAAGMTITRSGVMRRAPLAMRLRANSTWAMLEFVFNGMVFLLLGLQLPGILET
SLMAAEIDPNVEIWMLFTDIILIYAALMLVRFGWLWTMKKFSNRFLKKKPMEFGSWTTRE
ILIASFAGVRGAITLAGVLSIPLLLPDGNVFPARYELVFLAAGVILFSLFVGVVMLPILL
QHIEVADHSQQLKEERIARAATAEVAIVAIQKMEERLAADTEENIDNQLLTEVSSRVIGN
LRRRADGRNDVESSILEENLERRFRLAALRSERAELYHLRATREISNETLQKLLHDLDLL

EALLIEENQ
>LFGLNPFC_03751 Guanine/hypoxanthine permease GhxP
MSTPSARTGGSLDAWFKISQRGSTVRQEVVAGLTTFLAMVYSVIVVPGMLGKAGFPPAAV
FVATCLVAGLGSIVMGLWANLPLAIGCAISLTAFTAFSLVLGQHISVPVALGAVFLMGVL
FTVISATGIRSWILRNLPHGVAHGTGIGIGLFLLLIAANGVGLVIKNPLDGLPVALGDFT
TFPVMMSLVGLAVIIGLEKLKVPGGILLTIIGISIVGLIFDPNVHFSGVFAMPSLSDENG
NSLIGSLDIMGALNPVVLPSVLALVMTAVFDATGTIRAVAGQANLLDKDGQIIDGGKALT
TDSMSSVFSGLVGAAPAAVYIESAAGTAAGGKTGLTAITVGVLFLLILFLSPLSYLVPGY
ATAPALMYVGLLMLSNVAKIDFADFVDAMAGLVTAVFIVLTCNIVTGIMIGFATLVIGRL
VSGEWRKLNIGTVVIAVALLTFYAGGWAI
>LFGLNPFC_03752 Redox-sensitive transcriptional activator SoxR
MEKKLPRIKALLTPGEVAKRSGVAVSALHFYESKGLITSIRNSGNQRRYKRDVLRYVAII
KIAQRIGIPLATIREAFGVLPEGHTLSAKEWKQLSSQWREELDRRIHTLVALRDELDGCI
GCGCLSRSDCPLRNPGDRLGEEGTGARLLEDEQN
>LFGLNPFC_03753 Regulatory protein SoxS
MSHQKIIQDLIAWIDEHIDQPLNIDVVAKKSGYSKWYLQRMFRTVTHQTLGDYIRQRRLL
LAAVELRTTERPIFDIAMDLGYVSQQTFSRVFRRQFDRTPSDYRHRL
>LFGLNPFC_03754 putative cyclic di-GMP phosphodiesterase PdeC
MSHRARHQLLAFPGIIFLVLFPIILSLWIAFFWAKSEVNNQLRTFAQLALDKSELVIRQA
DLVSDAAERYQGQVCTPAHQKRMLNIIRGYLYINELIYARDNHFLCSSLIASVNGYTIAP
ADYKREPNVSIYYYRDTPFFSGYKMTYMQRGNYVAVINPLFWSEVMSDDPTLQWGVYDTV
TKTFFSLSNEASAATFSPLIHLNDLTVQRNGYLYATVYSTKRPIAAIVATSYQRLITHFY
NHLIFALPAGILGSLVLLLLWLRIRQNYLSPKRKLQRALEKHQLCLYYQPIIDIKTEKCI
GAEALLRWPGEQGQVMNPAEFIPLAEKEGMIEQVTDYVIDNVFRDLGAYLATHTDRYVSI
NLSASDFHTSRLIARTNQKTEQHAVRPQQIKFEVTEHAFLDVDKMTPIILAFRKAGYEVA
IDDFGIGYSNLHNLKSLNVDILKIDKSFVETLTTHKTSHLIAEHIIELAHSLGLKTIAEG
VETEAQVNWLRKRGVRYCQGWFFAKAMPPQVFMQWMEQLPARELTRGQ
>LFGLNPFC_03755 hypothetical protein
MATLTTGVVLLRWQLLSAVMMFLASTLNIRFRRADYVGLAVISSGLGVVSACWFAMGLLG
ITMADITAIWHNIESVMIEEMNQTPPQWPMILT
>LFGLNPFC_03756 Single-stranded DNA-binding protein
MASRGVNKVILVGNLGQDPEVRYMPNGGAVANITLATSESWRDKATGEMKEQTEWHRVVL
FGKLAEVASEYLRKGSQVYIEGQLRTRKWTDQSGQDRYTTEVVVNVGGTMQMLGGRQGGG
APAGGNIGGGQPQGGWGQPQQPQGGNQFSGGAQSRPQQSAPAAPSNEPPMDFDDDIPF
>LFGLNPFC_03757 UvrABC system protein A
MDKIEVRGARTHNLKNINLVIPRDKLIVVTGLSGSGKSSLAFDTLYAEGQRRYVESLSAY
ARQFLSLMEKPDVDHIEGLSPAISIEQKSTSHNPRSTVGTITEIHDYLRLLYARVGEPRC
PDHDVPLAAQTVSQMVDNVLSQPEGKRLMLLAPIIKERKGEHTKTLENLASQGYIRARID
GEVCDLSDPPKLELQKKHTIEVVVDRFKVRDDLTQRLAESFETALELSGGTAVVADMDDP
KAEELLFSANFACPICGYSMRELEPRLFSFNNPAGACPTCDGLGVQQYFDPDRVIQNPEL
SLAGGAIRGWDRRNFYYFQMLKSLADHYKFDVEAPWGSLSANVHKVVLYGSGKENIEFKY
MNDRGDTSIRRHPFEGVLHNMERRYKETESSAVREELAKFISNRPCASCEGTRLRREARH
VYVENTPLPAISDMSIGHAMEFFNNLKLAGQRAKIAEKILKEIGDRLKFLVNVGLNYLTL
SRSAETLSGGEAQRIRLASQIGAGLVGVMYVLDEPSIGLHQRDNERLLGTLIHLRDLGNT
VIVVEHDEDAIRAADHVIDIGPGAGVHGGEVVAEGPLEAIMAVPESLTGQYMSGKRKIEV
PKKRVPANPEKVLKLTGARGNNLKDVTLTLPVGLFTCITGVSGSGKSTLINDTLFPIAQR
QLNGATIAEPAPYRDIQGLEHFDKVIDIDQSPIGRTPRSNPATYTGVFTPVRELFAGVPE
SRARGYTPGRFSFNVRGGRCEACQGDGVIKVEMHFLPDIYVPCDQCKGKRYNRETLEIKY
KGKTIHEVLDMTIEEAREFFDAVPALARKLQTLMDVGLTYIRLGQSATTLSGGEAQRVKL
ARELSKRGTGQTLYILDEPTTGLHFADIQQLLDVLHKLRDQGNTIVVIEHNLDVIKTADW
IVDLGPEGGSGGGEILVSGTPETVAECEASHTARFLKPML
>LFGLNPFC_03758 putative protein YjbR
MTISELLQYCMAKPGAEQSVHNDWKATQIKVEDVLFAMVKEVENRPAVSLKTSPELAELL
RQQHSDVRPSRHLNKAHWSTVYLDGSLPDSQIYYLVDASYQQAVNLLPEEKRKLLVQL
>LFGLNPFC_03759 hypothetical protein
MWYQKTLTLSAKSRGFHLVTDEILNQLADMPRVNIGLLHLLLQHTSASLTLNENCDPTVR
HDMERFFLRAVPDNGNYEHDYEGADDMPSHIKSSMLGTSLVLPVHKGRIQTGTWQGIWLG
EHRIHGGSRRIIATLQGE
>LFGLNPFC_03760 Class B acid phosphatase
MRKITQALSAVCLLFALNSSAVALASSPSPLNPGTNVAKLAEQAPIHWVSVAQIENSLAG
RPPMAVGFDIDDTVLFSSPGFWRGKKTFSPESEDYLKNPVFWEKMNNGWDEFSIPKEVAR
QLIDMHVRRGDAIFFVTGRSPTKTETVSKTLADNFHIPATNMNPVIFAGDKPGQNTKSQW
LQDKNIRIFYGDSDNDITAARDVGARGIRILRASNSTYKPLPQAGAFGEEVIVNSEY
>LFGLNPFC_03761 C4-dicarboxylate transport sensor protein DctB

MKISWNYIFKNKWRFHITSISLFLIMLAVSIAFLHLRFNTLSGTDKMRLEMYKSTLYSTI
EQFYVLPYMLSTDHIIRQAVITPDDMTSSELNQRIAHFNTQLKTAAIFILDTQGKAIASS
NWQDPGSYVGQNYSYRPYYKHAMSGLNGRFYGIGSTTNTPGFFLSTSIKDKGKIVGVVVV
KISLNEIEKAWAEGPENIIVNDEHGIIFLSSKSPWRMRTLQPLPVQAKQKLQSTRQYSLD
NLLPADYYPCYTVSNFTFLKDKKEQLCLFPQYYTQQIAIPEFNWKMTIMVPLDNLYWSWA
ISLVITLIIYLLFLLFIKYWRMRSHAQQLLTLANETLEKQVKERTSALELINQKLIQEIK
ERSQAEQVLQITRSELAESSKLAALGQMATEIAHEQNQPLAAIHALTDNARTMLKKEMYP
QVEQNLKHIISVIERMTQLISELKAFASRHRVPKGSADVIKVMYSAVALLNHSMEKNNIE
RRIKAPSMPLFVNCDELGLEQIFSNLISNALDSMEGSSYKRLDIAIRQANNKVIITIKDS
GGGFAPEVVDRIFEPFFTTKRRGMGLGLAIVSEIVRNSNGALHASNHPEGGAVMTLTWPE
WGEEHE
>LFGLNPFC_03762 C4-dicarboxylate transport transcriptional regulatory protein DctD
MNNKSSEITIVYIEDSDDVRFACEQTLTLAGYRVISCCDAEHSIPLIQSQANIIILTDVR
LPGISGLELLSYINEMDSKIPVILITGHGDVEMAVDAMRNGAFDFIEKPSSSDKLLSIIA
RAVEKRRLVLENQQLLANLQQENGPVLIGRSPQMQQLRKMILNVADTGADVLIYGETGCG
KEVVARMLHHWSTRRQGQFVALNCAGLPETLFESEIFGHEAGAFTGAVKKRIGKIEHANG
GTLFLDEIEGMPSGMQVKLLRVLQERTIERLGANQLIPVNCRVIAATKEDLLRRSEEHLF
RLDLYYRLNVVSLNIPPLRQRREDIPELFYWFASQAAQKYNRPLPDISPMLLAWLQSQSW
PGNVRELKHNAERFVLGLLTHHQPVPMTQQEESGLTACIDAFEKKLIEDMLRQTEGQVSL
TARLLQLPRKTLYDKLNKHQIQPQVYRPESSS
>LFGLNPFC_03763 L-lactate dehydrogenase
MSLQIAVEKVRWLAAGLLELNGCDADIAQDVAEHMIEAERYGFASHGVTLLPKYLENIAR
GDVTANARPECLTSEGNLQRFHAHNGFGQHAGKVAVNAAIEQAKHRGFCLLTLCHAHHLG
RMGHYGGQMVADQGLAMLAMSNVTGRPALVAPWGGAEPRMTTNPFCFAWPFSDGRPPILVD
FATSSMALNKARVMSSTGKQAAPGQLIDAQGNPSNDPGVLFSNPPGALLPFGEHKGFGLA
LMIEMMAGILSGGDTIAEDHQTDGSAHNHLFALIIDPDQFTDLAGEKGQFFADYLLATQP
QPGGNPVIYPGMPEAANRERNAKMITIPVSFWSWIVEHYARKGIDLGLHPQESALAG
>LFGLNPFC_03764 Inner membrane protein YbhI
MSSISHGAPQKRRIIPNPGLWLAIIAGIIITLLPLGDTLPVAGQNMIAILVFAIIVWISE
AMDYTASAIVISALIIFMVGFAPDMNHPDTILGTAKALKMTLSGFSNSALALVAAAMFIA
AAMTITGLDKRIALFTMSKIGASSRSIIIGAIVVTIVLSLVVPSATARTACVVPIMMGVI
AAFKVDKHSRLAASMMIVIAQATSIWNVGIQTSAAQNLLSIGFINKTFGAGHSVSWLDWL
LAGAPWSLTMSVLLYFLARKLLPPETEAVEGGSEAIKKALAELGPTTGKEKRLIGISLLL
LLFWSTGGKLHSIDTTSVTLAGLAIMLLPGIGVMSWKEVEKRVQWGTLLMFGIGISLGST
LLDTQAASWMANYVVKGFGLDGLPSLAIFAILAAFLIIIHLGFASATALTAALLPILISL
LSSLPPELGVNPVGMTILLAFSVSFGFILPINAPQNMVCMGTDTFTPRQFTRVGLYLTVI
GYLLLLLFAATWWKILGLM
>LFGLNPFC_03765 Succinate--CoA ligase [ADP-forming] subunit alpha
MSVLINKHTRVLVQGITGKNGTFHTEQAIAYGTQIVGGVTPGKGGQRHLDRPVFDSMKEA
MRQTEADASVIYVPAPFVLDSVVEAIEAGVKLVVVITEGVPTLDMVKVRRLLDCHPDVRL
IGPNCPGVITPGECKIGIMPGEIHRPGRIGIVSRSGTLTYEAVAQTTALGLGQSTCIGIG
GDPVPGTNFIDALRLFENDPQTDAVIMIGEIGGNAEERAAEFIRHEMNKPVVGYIAGVTA
PKGKRMGHAGAIISGGSGSAEDKFRAFDKAGMAWTRNPALLGETLWNVIK
>LFGLNPFC_03766 Succinate--CoA ligase [ADP-forming] subunit beta
MNLHEYQAKSLLAGMGMPCPKEIAIQQISQLADAWQHIACPSKGAVLKAQVHAGGRGKAG
GVKVLKQLPEAQAFVQQMLGSQLVTYQTGPEGQYVSSILLCENIYPVRQELYFGMVVDRE
SQRVTFIVSPEGGVEIEKVAHETPEKISSVSIDPLTGVQPCHIREMFAVLQLEHGLFATF
SRLVNQAWKAFNELDFALLEINPLVLRETGEFMCADAKVSLDDNALYRHPELQVLRDETQ
EDPRESQAAKLDLNYVSLDGNIGCMVNGAGLAMATMDIIKLYGEQPANFLDVGGGATQER
VSEAFRLIVSDSKVKAILVNIFGGIVRCDMIARAIIHALNEARITLPVVVRLSGNNAAEG
QRLLAESGLTVEAVNSLDDAAKRIIALLN
>LFGLNPFC_03767 Dihydrolipoyl dehydrogenase
MSTIFDVAVMGGGPGGYVAALRAAQNGLSVVCIDDGVNAQGGEPSPGGTCLNVGCIPSKSL
LQSSELYAQVQHEASIHGVNVEGVSFNAAAMIQRKDAIVSRLTMGISLLFKKNKVKHLCG
LATLERAQDEIWQLRVNDQHIHARNVVIATGSQPRQLPGVTIDNQQILDNRGALALSEVP
PRLGVIGAGVIGLELGSVWNRVGSDVTLLEMAPTFLPALEARLSNEVRKAMIASGMKMQL
AVEIEAIEQRDDGVHVRWRQGEKREESRFDKLILAIGRVPRLSGVDLVQLGLEADNRGGI
AVDNLCRTGKAGLWAIGDVVRGPMLAHKAMAEGVVVADQIAGLAVEPINFALIPSVIYTQ
PEVAWVGENEASLKAAGRVFNKGNSLFAGNGRALALGQEGGRCTLYSDKHTDRVLGGAIV
GPQASELINEIALAMTFSASGEDIACAIHAHPTLSEVIHEAAMALNNKALHG
>LFGLNPFC_03768 Dihydrolipoyllysine-residue succinyltransferase component of
2-oxoglutarate dehydrogenase complex
MIEITVPVLPESVTEGTLTTWCKQEGEHVKRDDVIAELETDKVILEIPAPHDGVLSNIIV
SEGSTVTSAQLLAHLKPQAVIEETVTPVTETLAMPSARLEAQRSGVELADVAGSGRNGRI

LKEDVQRVTPAPVIQPERVAEIAPAKPLTPGARQERREPMSRLRQRIAERLLASQQNNAI
LTTFNEVNMQSVMDLRTRWKDRFAEKHGVKLGFMSFFVKAVTRALERFPVVNASVDGNEI
IWRDYCDIGIAVSSNRGLVVPVLRNAQSLSLVEIERQIAEYATQARNGKLPLEALQGGTF
SITNGGTFGSMMSTPIINPPQSAILGMHAITPRPVAENGQVVIRPMMYLALSYDHRIIDG
QEAVQTLVAIRELLESPEQLLLDL
>LFGLNPFC_03769 2-oxoglutarate dehydrogenase E1 component
MENMTSPGTLLSGDNATWLEEYYQTWLRTPEQLPEDWRRFFLSPELTVQSVSGDNNVSGA
TLKKQAAVIQLINAWRTQGHLRAKLDPLGLNPPADVPSLQPGFWGLSEEDLLQEFSVTFG
AHTTQMPLKQLLNLLEQAWAGSQAYELAHLENREEINWLLSRIESSNAPQADAQTCIARF
EKLMAAETLERYLHTRYVGQKRFSLEGGESAIPALDTLTKRLRAQGVEEMVIGMAHRGRL
NVLVNLLNKDPAQLFAEFEGKQTIGSGSGDVKYHMGYSSNLETPAGSLHVALAYNPSHLE
IVNPVVLGQVRARQERRGEDGQAKVVGVLIHGDSALGGLGVNQTTFNLSQTQGYGTGGTL
HLVINNQIGFTTSRLQDMRSSRYCTDIAKMVAAPIIHVNGDDVDAVCQVMELACEWRDTF
RRDIIIDICCFRKHGHNESDEPRLTQPQMYQAVDAHPGTLARYGESLARRGLLTQAQQDE
MTARYRDWLDSCQKREPQPLKPAIHSFSANWYGLTNPHWSAPVSTALPRQKLAAYGEIIS
TLPPDVVAHPTIKRQLALRQDMAAGTQPIDWGMAEMLAYASLVDAGVGVRLSGEDSGRGT
FSHRHAVVHHQTEARRYLPLQHIRAGQASFDVYDSVLNEEALLAFEYGYSTSAPQQLVIW
EAQFGDFANGAQVAIDQFISSGETKWDRYSGLTILLPHGYDGQGPEHSSARPERWLQLCA
ENNMQVVMPSESAQMFHLLRGQALRPMRKPLVIMMSKRLLRFKGAMSELSEFTDGAYKPV
VTDPQLHQSQKVKRVILCSGQVYYDVLEARKQRECEDEVAIVRLEQLYPFPVAELNDVLA
SWPNCCEWIWLQEEPENQGAWRQIRHELAALKINTPYWQYAGRPAVAAPATGYGRVHKQQ
IDEFLAAAFADIQP
>LFGLNPFC_03770 Aromatic-amino-acid aminotransferase
MFQKVDAYAGDPILTLMERFKEDPRSDKVNLSIGLYYNEDGIIPQLKAVADAEARLNAQP
HGASLYLPMEGLNSYRHAIAPLLFGADHPVLQQQRVATIQTLGGSGALKVGADFLKRYFP
ESGVWVSDPTWENHVAIFAGAGFEVSTYPWYDEATNGVRFNDLLATLKTLPARSIVLLHP
CCHNPTGADLTNDQWDSVIEILKARELIPFLDIAYQGFGAGMEEDAYAIRAIASAGLPAL
VSNSFSKIFSLYGERVGGLSVLCEDAEAAGRVLGQLKATVRRNYSSPPNFGAQVVAAVLN
DEALKASWLAEVEEMRTRILAMRQELVKVLSTEMPERNFDYLLNQRGMFSYTGLRTAQVD
RLREEFGVYLIASGRMCVAGLNAQNVHRVAKAFAAVM
>LFGLNPFC_03771 Trifunctional NAD biosynthesis/regulator protein NadR
MKPFATGLVVGKFAPLHCGHEKLINTALAQCEELFIISYSVPEMPDCEPEKRLTWLQVRF
PQATILVLTPELVARYNLPAIPHNDADIHRHYVATLCLQILRCRPHAVFTAEDYGDGFAN
VLARRFAQPVEHVRMARPVGDEAPSGTLIRSDVHRYRYMLANDVYYSFVRRICLLGGEST
GKSTLSKALADGLDTVYVAEFGRDYWEEKNGILTADDLLHIACEQVRREQQAEANHYLIC
DTSPLTTLFYALDQYGHAPQELHQLAEREYSLVVLCGAEFPFVQDGTRQGEVFRARQQVW
YEQELSRRNIPYLSVSGSLQERLGQILHRLPD
>LFGLNPFC_03772 hypothetical protein
MSYSEIAACLAYAVSVWLAARNNVHTWWIGIIGSILYGWVFWSVQLYADVTLQLFFIATS
ITGWIHWLKGQGGDILPVRRTQASHFFLLLLCAVVVAGGYGFLLHTFTNAWAPWLDSLIL
TFSVLAQFMLMGRRIENWFVWLAVNTLAVPLYMTRGLNLTAGLYFLFWINAWHGLYQWRK
ELQTS
>LFGLNPFC_03773 Alanine racemase, biosynthetic
MQAATVVINRRALRHNLQRLRELAPASKMVAVVKANAYGHGLLETARTLPDADAFGVARL
EEALRLRAGGITKPVLLLEGFFDARDLPTISAQHFHTAVHNEEQLAALEEASLDEPVTVW
MKLDTGMHRLGVRPEQAEAFYHRLTQCKNVRQPVNIVSHFARADEPKCGATEKQLAIFNT
FCEGKPGQRSIAASGGILLWPQSHFDWVRPGIILYGVSPLEDRSTGADFGCQPVMSLTSS
LIAVREHKAGEPVGYGGTWVSERDTRLGVVAMGYGDGYPRAAPSGTPVLVNGREVPIVGR
VAMDMICVDLGPQAQDKAGDPVILWGEGLPVERIAEMTKVSAYELITRLTSRVAMKYVD
>LFGLNPFC_03774 Replicative DNA helicase
MAGNRPFNKQQTDNRERDPQVAGLKVPPHSIEAEQSVLGGLMLDNERWDDVAERVVADDF
YTRPHRHIFTEMARLQESGSPIDLITLAESLERQGQLDSVGGFAYLAELSKNTPSAANIS
AYADIVRERAVVREMISVANEIAEAGFDPQGRTSEDLLDLAESRVFKIAESRANKDEGPK
NIADVLDATVARIEQLFQQPHDGVTGVNTGYDDLNKKTAGLQPSDLIIVAARPSMGKTTF
AMNLVENAAMLQDKPVLIFSLEMPSEQIMMRSLASLSRVDQTKIRTGQLDDEDWARISGT
MGILLEKRNIYIDDSSGLTPTEVRSRARRIAREHGGIGLIMIDYLQLMRVPALSDNRTLE
IAEISRSLKALAKELNVPVVALSQLNRSLEQRADKRPVNSDLRESGSIEQDADLIMFIYR
DEVYHENSDLKGIAEIIIGKQRNGPIGTVRLTFNGQWSRFDNYAGPQYDDE
>LFGLNPFC_03775 HTH-type transcriptional repressor CytR
MAKSDTGRKRVTLTDVARAAGVSKSTVSLVLNDSSLIKKETQQKVQQAIEQLGYVYNRFA
ANLRSQKSLTIGVVIDDLINPFFAEFTMGLEMTLAEHGFITVMSNTSQRSDRQKQVLDTL
LEHHVAGIVLCPVNSTSEADLQRYANSSTPLLITMRPLDWQQLPVDYVGVDSHAGVREAT
EYLIQQGHRDIVFIGGLTHHMRYQGYLEAMNHHGLQPWSTDAFSLRAEPTQANGYQLMQQ
LLDMPSPPTAVICYNDLMAFGAESALGERGLFAGEDISLIGNDGVAACAYSNPPLTTIAV

EPLALGKQAAQQILRRIAQPDAPLSHYIYRPTLQIRASTGKRGR
>LFGLNPFC_03776 Caffeate CoA-transferase
MCSVRTITAPEAADLIVDGAVVTVSSSSGMGCPDAVLAAIGERFQRAGHPQNITSIHPIA
AGDMYGVKGIDHLAQPGLLAKVIAGSYPSGPSSMAPPLIWQMINDNAIPARNLPSGILFD
MHREAAAHRPGVLTQTGMDTYVDPLLQGGAMNEKAAQQSLVERVRFANDDWLFFPSIVPQ
VAIIRATTADERGNLTYEHEGAYLGPLEQATAVRNNGGIIIAQVKRQVAAGSLKPKEVRI
PGVLVDYIVIAPEQTQTTQTQYEPAISGEISRPLSAFRYMEHGPARVIAQRVAQELQSGD
AVNIGFGISANVPRILLEQGRHGDVTWLLEQGAIGGVPLLEFQFGCASNAEAFLPSPQQF
TYFQGGGFDLTLMSFLQIGADGSVNVSHLPARPHVTAGCGGFIDITSHAKRIIFSGFFNA
GAQLQLEEGQLRIIKEGKAKKLVQDVAHVTFSGKRAIRLGQQVQYITERCVLELTPDGLL
VTEIAPGIDPERDILAQSNAPLRLADDLKLMNPHYFQQGRHHE
>LFGLNPFC_03777 Short-chain-enoyl-CoA hydratase
MNNGTIDLQRNGFIATLVINRPAKLNALTPEMLEQLLAHCHTLETDSEIRVVLLTSASEK
AFCVGADIHRWTSLSALDMWRNWIRRGHQAFDALAQLPQPVIALLHGLAYGGGLELAATT
DIRICSAEAQMALPETGLATVPGWSGTQRLTALLGRSLVKELVFTGEPLDAQRALSCGLV
NRVVAREALHDAGLQLAQRIAQRAPVAVQIAKQVIDAGEGIAVASTLEALAGAFSATTED
AHEGSQAFTEKRQPHFSAR
>LFGLNPFC_03778 Putative transporter YdfJ
MQPSQPAEKANVSSADLRRAAWTCSLGSALEYYDFALYSLASAIIFGPLFFPNQEPGMAL
IASFGTYFLGFAVRPVGGIIFGSLGDRLGRKFVLLATVLLMGIASTLIGVLPTYETAGIW
APIMLIALRLLQGLGAGAEQAGAAVLMTEYAPDGKRGYYAALPFLGIQIGTFMASAVYFI
LLHNVTNLAETWWWRLPFLSSVVIIAVAIYIRLHLKESPSFAKLEARQQVTESPLRNLLK
TSRRNVFVGIGLRLAENGGSSIYQALAISYIVGVVGLDKSVGTLCLMSAVVVGAIMVPIA
GWLTDRFGRVLVYRSLAIFQLLITFPVWWSFSHGNVPVTIVSLSLALGIGAWGMFGAQGA
FLPELFGARHRYIGVALAREVSAVIAGGIAPLVGSAIISWVIHTNDPSGIAAWMPIAIYL
SLLTVGTIIATFFAPETRDRDLDDLADAETISVSQHNQTTGTSHDA
>LFGLNPFC_03779 hypothetical protein
MTHNVSESIIDSLTDVKMPHYAPLAQVSGSIDLNGTILPEYRCNTLVLGSGAAGWRAAVE
LKRQNVDVMVASSKAFWGTSACSGSDKQTLHTANTRANGDHFLTLSNTLAAGGAMDHDTA
YVEAVGSVNTCEVLKYLGLDLPEDLFGATLRYQTDHDEFGRATSCGPRTSRLMVKVLAQE
AMRLNIPLCDHTTAIRILTSGEGENRRVEGVIAIDKACRDNPWRMVVIRCQHLVLATGGP
GELYRDSVYPVNCFSALGMALEAGITLVNLTESQFGIGTPRNQFPWNLSGTYMQAIPRIY
SQDHSGRQYNFLATYYPTTRMLASAIFRKGYQWPFHAERMLEYGSSLLDVAVYEETQKGR
DVFLDFLREPEPAADGTLFNLRELDDDVIDYLQQNHALLAQPLARLTQMNPLAIRLYQMH
GHDLTASPLKFTLNNQHLNGGIEVDIWARTSLTGCYAAGENAGTHGVTRPGGAALNAGQV
FARRCALHIAHQKSQDRPLERQQILSTINEAQQHLNQGLLRDDVRETIQNTMSHHAAILC
HTQGINTAAQTLATLCEDIRREGIQCDENSLAQSFQWRQSAQLALAVLRSLQCYVENGGG
SRGARAIYDGEAGIAPQTPGGPLLAWRFRPENQDARQYMICVCREANDYQTWTRPCRERG
VLTLTNFERQWQNWIAATPFQY
>LFGLNPFC_03780 Quinone oxidoreductase 1
MAIRIEFHKHGGPEVLQAVEFTPADPAENEIQVENKAIGINFIDTYIRSGLYPPPSLPSG
LGTEAAGIVSKVGSGVKHIKAGDRVVYAQSALGAYSSVHNINADKAAILPAAISFEQAAA
SFLKGLTVYYLLRKTYEIKPDEQFLFHAAAGGVGLIACQWAKALGAKLIGTVGTAQKAQS
ALKAGAWQVINYREENLVERLKEITGGKKVRVVYDSVGRDTWERSLDCLQRRGLMVSFGN
SSGAVTGVNLGILNQKGSLYVTRPSLQGYITTREELTEASNELFSLIASGVIKVDVAEQQ
KYPLKDARRAHEILESRATQGSSLLIP
>LFGLNPFC_03781 Phage shock protein G
MLELLFVIGFFVMLMVTGVSLLGIIAALIVATAFMFLGGMLALMIKLLPWLLLAVAVVWV
IRAIKAPKVPKYQRYDRWRY
>LFGLNPFC_03782 tRNA-dihydrouridine(20/20a) synthase
MHDNPETQKTNQTSVMPEKTGAYWSSRFSIAPMLDWTDRHCRYFLRLLSRNTLLYTEMVT
TGAIIHGKGDYLAYSEEEHPVALQLGGSDPAALAQCAKLAEARGYDEINLNVGCPSDRVQ
NGMFGACLMGNAQLVADCVKAMRDVVSIPVTVKTRIGIDDQDSYEFLCDFINTVSGKGEC
EMFIIHARKAWLSGLSPKENREIPPLDYPRVYQLKRDFPHLTMSINGGIKSLEEAKAHLQ
HMDGVMVGREAYQNPGILAAVDREIFGSSDTDADPVAVVRAMYPYIERELSQGTYLGHIT
RHMLGLFQGIPGARQWRRYLSENAHKAGADINVLEHALKLVADKR
>LFGLNPFC_03783 hypothetical protein
MAYYNIEKRLKSDGTPRYRCNVIIKEKGVITYRESKTFPKHAHAKTWGTQKVMELDLYGI
PSSNAVDGLTVRDLLHKYLNDPNAGGKAGRTKRYVLELLMDSDISAIKLSELTENDVIEH
CRLRNNAGAGPATVSHDVSYLGSVLDAAKPVYGINYTSNPAKSARPYLLKLGLIGKSNRR
NRRPASDELDMLIEGLQQRSTHKCSKIPFVDILKFSVWSCMRIGEVCRLRWEDLDQEQKS
ILVRDRKDPRKKEGNHMKVALLGEAWDIVQRQPKKSEFIFPYNSTSVTAGFQRVRSKLGI
KDLRYHDLRREGASRLFEAGFSIEEVAQVTGHRSLNVLWQVYTELYPKSLHNRFEELQKS
RNKTS

>LFGLNPFC_03784 DNA damage-inducible protein I
MRIEICIAKEKMTKMPTGAVDALKEELTRRISKRYDDVEVIVKATSNDGLSVTRTADKDS
AKTFVQETLKDTWESADEWFVH
>LFGLNPFC_03785 hypothetical protein
MDISPLLHALCAVAAQILVGLFTGNWAYGAIAGCTFFIAREHTQAEYRWIEMFGHGKRMN
MPWWGGFDPRAWDVASLMDFAVPVVACLLIWMLIR
>LFGLNPFC_03786 hypothetical protein
MKDLTLKFHDKLQFKAFLSSLGWVEDEDLQNKLLVDEIGFTYTETGVTEEGEPVCIRNDG
YFVNIRILDDLFDVSVFSDYVVELETPLREWS
>LFGLNPFC_03787 hypothetical protein
MASIAATKSVDVSGDNKYVTTSCRFKTERQVRLRIRFDKYDGSATTFLGDAYIDTQTLEI
NMTGGAAGRITARVRKDKTTGWIFAEATIQAIDGELKIGSQIQYSPERGGATVSGDYIYL
ATPQVENGPCVSSFIISGGSATTRASDLVSIPTRNNLYKLPFTFLLEIHKNWDIAPNAAP
RVWDIAAANTGQSAIAAINRGSGKLYMSLSNPSGSYVNSAATDVFTEKTTFGCIAKADGH
FHVVTNGKAVNEVYCEYNGVTADKNIRFGGQTNTGERHLFGHIRNFRIWHKELNDRQLKE
VV
>LFGLNPFC_03788 hypothetical protein
MAAVKISGVLKDGAGKPIQNCTIQLKAKRNSTTVLVNTVASENPDEAGRYSMDVEYGQYS
VTLLVEGFPPSHAGTITVYEGSRPGTLNDFLGAMTEDDVMPEALRRFEAMVEEVARNAEA
ASQSAAAAKKSETAAASSRNAAKTSETNAGNSAKAAASSKTAAQNAATAAERSETNARAS
EEASADSEEASRRNAESAAENAGVATTKAREAAADATKAGQKKDEALSAATRAEKAADRA
EVAAEVTAEPYANIVPPLPDVWIPFNDSLDMIAGFSPGYKKIAIGDDVVQVASDKQVNFS
RASTATYINKSGELKTAEINEPRFECDGLLIEGQRTNYMLNSESPASWGSHQTWMCPKPG
QIVLVLLMESLSATILWLGKLRLLIWHQLLQQSQLMSQAITST
>LFGLNPFC_03789 Outer membrane protein X
MRKLCAVILSAVVWLVAAGTPASAAEHQSTLSAGYLQAHTDMPGSDDLKGINVKYRYEFT
DTLGLVTSFSYANAKDEQKTHYSDTRWHEDSVRNRWFSMMAGPSVRVNEWFSAYAMAGVA
YSRVSTFSGDYLRVTDNKGKTHDVLTGSDDDRHSNTSLAWGAGVQFNPTESVAIDIAYEG
SGSGDWRTDGFIVGVGYKF
>LFGLNPFC_03790 hypothetical protein
MGKGSSKGHTPREAKDNLKSTQLLSVIDAISEGPVEGPVDGLKSVLLNSTPVLDSEGNTN
ISGVTVVFRAGEQEQTPPEGFESSGSETVLGTEVKYDTPITRTITSANIDRLRFTFGVQA
LVETTSKGDRNPSEVRLLVQIQRNGGWVTEKDITIKGKTTSQYLASVVVDNLPPRPFNIR
MRRMTPDSTTDQLQNKTLWSSYTEIIDVKQCYPNTALVGVQVDSEQFGSQQVSRNYHLRG
RILQVPSNYNPQTRQYSGIWDGTLKPAYSNNMAWCLWDMLTHPRYGMGKRLGAADVDKWA
LYVIGQNCDQSVPDGFGGTEPRITCNAYLTTQRKAWDVLSDFCSAMRCMPVWNGQTLTFV
QDRPSDKVWTYNRSNVVMPDDGAPFRYSFSALKDRHNAVEVNWIDPDNGWETATELVEDT
QAIARYGRNVTKMDAFGCTSRGQAHRAGLWLIKTELLETQTVDFSVGAEGLRHVPGDVIE
ICDDDYAGISTGGRVLAVNSQTRTLTLDREITLPSSGTTLISLVDGSGNPVSVEVQSVTD
GVKVKVSRVPDGVAEYSVWGLKLPTLRQRLFRCVSIRENDDGTYAITAVQHVPEKEAIVD
NGAHFDGDQSGTVNGVTPPAVQHLTAEVTADSGEYQVLARWDTPKVVKGVSFLLRLTVAA
DDGSERLVSTARTTETTYRFRQLALGNYRLTVRAVNAWGQQGDPASVSFRIAAPAAPSRI
ELTPGYFQITATPHLAVYDPTVQFEFWFSETRITDIRQVETTARYLGTALYWIAASINIK
PGHDYYFYIRSVNTVGKSAFVEAVGQPSDDASGYLDFFKGEIGKSHLAQELWTQIDNGQL
APDLAEIRTSITDVSNEITQTVNKKLEDQSAAIQQIQKVQVDTNNNLNSMWAVKLQQMQD
GRLYIAGIGAGIENTPDGMQSQVLLAADRIAMINPANGNTKPMFVGQGDQIFMNEVFLKY
LTAPTITSGGNPPAFSLTPDGRLTAKNADISGNVNANSGTLNNVTINENCRVLGKLSANQ
IEGDLVKTVGKAFPRDSRAPERWPSGTITVRIYDDQPFDRQIVIPAVAFSGAKHEREHTD
IYSSCRLIVRKNGAEIYNRTALDNTMIYSGVIDMPAGHGHMTLEFSVSAWLVNGWYPTAS
ISDLLVVVMKKATAGISIS
>LFGLNPFC_03791 hypothetical protein
MKTGAEAIRALATQLPAFRQKLSDGWYQVRIAGRDVSTSGLTAQLHETLPDGAVIHIVPR
VAGAKSGGVFQIVLGAAAIAGSFFTAGATLAAWGAAIGAGGMTGILFSLGASMVLGGVAQ
MLAPKARTPRTQTTDNGKQNTYFSSLDNMVAQGNVLPVLYGEMRVGSRVASQEISTADEG
DGGQVVVIGR
>LFGLNPFC_03792 hypothetical protein
MSPEDWLQAEMQGEIVALVHSHPGGLPWLSEADRRLQVQSDLPWWLVCRGAIYKFRCVPH
LTGRRFEHGVTDCYTLFRDAYHLAGIEMPDFHREDDWWRNGQNLYLDNLEATGLYQVPLS
AAQLGDVLLCCFGSSVPNHAAIYCGDGELLHHIPEQLSKRERYTDKWQRRTHSLWRHRAW
HASAFTGIYNDLAAASIFV
>LFGLNPFC_03793 hypothetical protein
MQNIRQETLNECTRAEQSACVVLWEIDLTEVGGERYFFCNEQNEKGEPVTWQGRQYQAYP
IQGSGFELNGKGTSTRPTLTVSNLYGMVTGMAEDLQSLVGGTVVRRKVYARFLDAVNFVN
GNSDADPEQEVISRWRIEQCSELSAVSASFVLSTPTETDGAVFPGRIMLANTCTWTYRGD

ECGYNGPAVADEYDQPTSDITKDKCSKCLSGCKFRNNVGNFGGFLSINKLSQ
>LFGLNPFC_03794 hypothetical protein
MKTFRWKVKPGMDVASAPSVIKVRFGDGYSQRAPAGLNADLKTYSVTLSVPREEATALES
FLAEHGGWKAFLWTPPYGYRQIKVTCAKWSSRVSMLRVEFSAEFEQVVN
>LFGLNPFC_03795 hypothetical protein
MDQIANLVIDLGIDAAEFKNEIPRIKNLLNGAASDAERSSARMQRFMERQTQAARQTTQA
ASSAATAASVHAQTVEKNAQAHERMAREVEKTRQRMEALSQKMREEQAQAMALAEAQDKA
AAAFYRQIDSVNQASAGLQELQRIQQQIRQARNSGGIGQQDYLALISEVTAKTRVLTQAE
EESTRQKVAFIRQLKEQATRQNLSSSELLRAKAAQLGVSSAAEVYIRKMEQAGKATHSLG
LKSAAARQEIGVLIGELARGNLGALRGSGITLANRAGWIDTLMSPKGMMLGGVIGGIAAA
VYGLGKAWYDGQKEGEEFNRQLSLTGHYAGVTAGQLWTLSRAISGNGITQHAAAGALAQV
VGSGAFRGNDIGMVARAAAQMERSVGQSVSDTISQFKRLKDDPVNAAKALDNELHFLTAT
QLEQIRVLGEQGRSSDAARIAMSALAEETDRRTADIDNNLNALGSTLQTLSDWWKQFWDA
AMNIGREDSLDEQIAALQEKVSRAKRLPWTASSSQVEYDQQRLNDLQEKKRQKDLQDAKE
QAERNYQEQQKRRNAENAALNRMNETEAARHQREIARINAMQYADQAVRDAAIQRENERY
EKALASGKKKTRETRNDEATRLLLQYSQQQAQVEGQIAAARQSAGIATDRMTEAHKQLLA
LQQRISDLDGKKLTADEKSLLARKDELIQALTLLDVKQQELQKQTALNDLKKKTIQLTSQ
LAEEERAQRQQHDLDIATVGMGDQQRQRYQVQLSLRQKYQQQLEQLRRDSEQKGTYNTDD
YRKAEQALTESLNRQLNENRRYWQQLEIAQGNWKNGAMRAFQNFTADADNAAGTAEQMLT
AAFNSAGNALATFCTTGKLNFKSFTASLLSDLAKIMAQMSMMQAVKGIGSAFGWGSAATA
SVTPNADGGVYQSADLSRYSGTVVNRPTFFAFAKGAGVMGEAGPEAILPLRRGADGKLGV
VADIGGSGMAMFAPQYNIEINNDGTNGQIGPAALKVVYDLGKKAAADFMQQQSRDGGLFS
GGGR
>LFGLNPFC_03796 hypothetical protein
MRLAREFRRADWRRMLSEMSATELGEWGDYFRMQSFSDVWMDAQFASLKALIVRMVSGSS
DAAVADFSLLPEENGIPERTDEELMHLGEGISGGVRYGPDSQPGH
>LFGLNPFC_03797 hypothetical protein
MFLKQGTFNYEKQSVVLSELSGLQRIEYLAFVQQRTAKFDAEEGELPEAERQIAFLRMGM
DINAWLVSRSLWNADQSKDVETLCASVITTWSYDALGAGAEMVLSLSGMGTIDNAGDDEH
EALTPEKS
>LFGLNPFC_03798 hypothetical protein
MTTPNPLAKTKGAGTTFWMYTGNGDAFANPLSDTDWLRLAMVKDLQPGEMTADAEDDTYL
DDEDADWKTTTQGQKSVGDTSATLAWRPGDSGQKKLVQLFDSGEVCAFRIKYPNGTVDVF
RGWLSSLGKTIASKDVMTRTVKISGVGRPYLAEEGTETVGVTGLTVAPASASVKVGATTT
LTFTVKPDGASDKAISVHSTDPQTATVTLNGLVATVKGVKQGSVSIVGMTSDGDFVAVAT
VAVSAAG
>LFGLNPFC_03799 hypothetical protein
MNRHTQIRQVVLARLREQCGDSATFFDGLPAFIDAQELPAVAVWLSDAQYTGKMTDEDDW
QAVLHIAVFIRAQAPDSELDMWMESTIFPALNDVPALSGLIDTLNPLGFNYQRDNEMATW
AMAEITYQITYTN
>LFGLNPFC_03800 hypothetical protein
MKGLENAIRNLNSLDTRMVPQASAWAINRVAQKAVSVATRQVAGNTVAGDNQVKGIPLKL
VRQRVRVFKASPSGKMTARIRVNRGNLPAIKLNTTRRRAGEGLRVGKYFFRGAFVQQLAN
GRWHVLRRLPEARFATGHDHQGRPRKNRLPVEVVKIPLSGPLTQAFEDARDRIIAAEMPK
QLGYALKQQLRLWLTR
>LFGLNPFC_03801 hypothetical protein
MSDPFSRLAARMDAITVRKMGKTASINDVDMTVIPGETLVELNALSGPAVSLVVFSSGYR
PRRGDRVVYDGQQWTVTRHERFNGKPMIFIE
>LFGLNPFC_03802 hypothetical protein
MAKNFVEEGKTVAIVASAAISSGDLVQVGDVFAVALTDIPQGETGDGMTEGVFMLPKLKT
DDMKTGKKVYLKSGKVQLTNSGSDPLVGVVWADAGTSAEEVPVKLNV
>LFGLNPFC_03803 ATP-dependent Clp protease proteolytic subunit
MPQRNDRSRSTPTTSPKNNSWFRMQAGHQSDADIYIYDEIGFWGVTAKQFISDLSALGDI
THINLHINSPGGDVFEGIAIFNALKTHGASITVYVDGVAASMASVIAMVGNPVIMPENTF
MMIHKPFGFTGGDAEDMRTYADLLDKVEAVLLPAYAQKTGKTTDEIAAMLADETWMSGAE
CLAHGFADQVTPAVKAMACIQSKRTEEFKKMPESIRNMITPPRNSAPRVQDNEPEASRTP
VQAAAPVVDENSIRAQVLAEQKARVNGINDLFAMFGGRYQTLQAQCLADPECSLEQAREK
LLNEMGRESTPSNKNTPAHIYAGNGNFVGDGIRQALMARAGFEKTERDNVYNGMTLREYA
RMSLTERGIGVSGYNPMQMVGAAFTHSTSDFGNILLDVANKAILQGWEDAPETYEQWTRK
GQLSDFKIAHRVGMGGFSALRQVREGAEYKYVTTGDKQATIALATYGELFSITRQAIIND
DLNMLTDVPMKLGRAAKSTIADLVYAILTSNPKISTDNVSLFDKAKHANVLESAAMDVAS
LDKARQLMRVQKEGERHLNIRPAFVLVPTAMESVANQVIRSSSVKGADINAGIINPVKDF
ATVIAEPRLDDNSQTTFYLAASKGSDTIEVAYLNGVDTPYVDQMEGFSVDGVTTKVRIDA
GVAPVDHRGLVKCTA

>LFGLNPFC_03804 hypothetical protein
MAILDDVIGVFSPGWKAARLRSRAVIQAYEAVKTTRTHKARRENRTADQLSQYGAVSLRE
QARYLDNNHDLVIGVFDKLEERVVGKNGIIVEPHPVLRNGAIARDLAAEIRTRWSEWSVS
PEVTGQFTRPMLERLMLRTWLRDGEVFAQMVSGRINSLTPSAGVHFWLEALEPDFIPMTS
DESNRLNQGVFVDDWGRPEKYLVYKSRPVSGRQMETKEVDAERMLHLKFVRRLHQMRGTS
LLSGVLIRLSALKEYEDSELTAARIAAALGMYIRKGDGQSYEPDGNGCKENERELTIQPG
IIYDDLKPGEEIGMVKSDRPNPNLETFRNGQLRAVAAGSRLSFSSTARNYNGTYSAQRQE
LVESTDGYLILQDWFIGAVTRPMYRAWLKQAVASGVIRLPRDLDRSSLYTAVYSGPVMPW
IDPVKEAEAWKIQIRGGAATESDWVRAGGRNPDDVKRRRKAEIDENRKLDLVFDTDPASD
KGGSSAATKRQEPQHTDDQSEE
>LFGLNPFC_03805 hypothetical protein
MNQNDIEAMIQRYTEAEMAVLDGKSVTFNGQQMTMENLSEIRQGRQEWERRLAALITRRR
GHPGYRLARF
>LFGLNPFC_03806 hypothetical protein
MAMAVDSGGEDGVTDNAYKFWRRCRRDGLGKRIYLFKGDSIRRAKLITRTFPDNTGRTGR
RAQAAGDVPLWLLQTDALKDRVNNALWRDSPGPGYVHFPDWLGSWFYDELTYEERSSDGK
WSKPGRGANEAFDLMVYAEALVILHGYEKIRWPDTPEWASRETWLECVPDSTEPSPSPEP
VSTPVKKQKRKKTVTDDVNPWLTSGGWL
>LFGLNPFC_03807 hypothetical protein
MLNQETAKAARTDSGYILRAPRRMRVADAVAQYMRVPMGAGNSVPWDPLVAPYVIEPMNC
LASREYDAVIFVGPARTGKTIGLIDGWVIYNVICDPADMLIIQMTEEKAREHSKKRLART
FRVSPEVVSRLSPNKNDNNVYDRTFLAGNYLKIGWPSVNIMSSSDYKCVALTDYDRFPED
IDGEGDAFSLASKRTTTFMSSGMTLVESSPGRDVKDVKWRRTSPHEAPPTTGILSLYNRG
DRRRWYWPCPHCGEYFQPCGDVVAGFRDIADPVLASEAAYIQCPSCSGRIMPEQKRELNG
RGVWLRDGESINADGSRYGDPRRSRIASFWMEGPAAAYQTLSQLVYKLLTAEQEYETTGS
EETLKTVINTDWGLPYLPRASMEQRKSELLEQRAEPVPSRSVPDGVNFLVATVDVQAGRH
RRFVVQVTGYGSRGERWIIDRYNITQSLRGDSDGESQRIDPASYPEDWDVLLTDVFHKAG
RWPPILLNKCD
>LFGLNPFC_03808 hypothetical protein
MDRELKNLTLNISQLAALSGVHRQTAAARLQNLPVAGGHESNLKLYRVVDIVSAFLALPP
PVAEGEMDAHERKAWYQSERERLKFEQETAQLIPASDVRREFAIWAKAVVQVLETLPDIL
ERDCGLQPAAVSRVQSIIDDLRDQIALRVTEAGADDEEELQQEE
>LFGLNPFC_03809 hypothetical protein
MRDKPANHKRGKNPVCIVFDYSRTLAQKEFPVGLRSLLIREYGDDTAHDVSGLNTFIR
>LFGLNPFC_03810 hypothetical protein
MPSMIAIILLIILHIWLCRQGGDHFWSESRLNISLLMLDIEHLARGKGLRWDKSQSLQIP
GFSLAFAGLL
>LFGLNPFC_03811 hypothetical protein
MSRVAAIIYALVICIIVCLSWAVNHYRDNAITYKAQRDKNARELEKANATITDMQQRQRA
ADALDAKYTKELADAKAENDALRRKLDNGGRVFVKGKCPVPTSAEASSASGMGNDATVEL
SPVAGRNVLGIRDGIISDQTALRTLQEYIRTQCLR
>LFGLNPFC_03812 Lysozyme RrrD
MSAKIKYGLSAAVLALIAAGASAPQILDQFLDEKEGNHTTAYRDGSGIWTICRGATMVDG
KPVIPGMKLSKEKCDQVNAIERDKALAWVERNIKVPLTEPQKAGIASFCPYNIGPGKCFP
STFYKRLNAGDRKGACEAIRWWIKDGGRDCRIRSNNCYGQVIRRDQESALACWGIDQ
>LFGLNPFC_03813 putative protein YdfR
MTQNYEVIVKGIRNFENKVTVTLALRDKKRFDGEIFDLDISLDRVEGAALEFYEAAARRS
IRQVFLDVAAGLCEGDELLPETRPCSKARYTIKINSSDNSITGC
>LFGLNPFC_03814 hypothetical protein
MKSMDKLTTGVAYGTSAGSAGYWFLQLLDKVTPSQWAAIGVLGSLVFGLLTYLTNLYFKI
KEDKRKAARGE
>LFGLNPFC_03815 hypothetical protein
MKNTVKINSVDLINADCLHFIQSLPDDSIDLIVTDPPYFKVKPNGWDNQWKGDEDYLKWL
DHCLAQFWRVLKPAGSLYLFCGHRLASDIEIMMRERFNVLNHIIWAKPSGRWNGCNKESL
RAYFPATERVLFAEHYQGPYRGKSDGYAAKERELKQHIMAPLISYFRDARAELGITAKQI
AEATGKKNMVSHWFGASQWQLPNEADYRKLQALFSRIAAEKFQEQQLEQPLEQPHHQLVA
SYDSLNRKYSELLDEFKSLRRYFSVSVSVPYTDVWMHKPVQFYPGKHPCEKPADMLRQII
NASSRPGDLVADFFMGSGSTIKAAMALGRRALGVELESERFNQTVKEINELVGK
>LFGLNPFC_03816 hypothetical protein
MLKQQDMTETAAAVLHFLPADKWVTPRMMTRTTGVSEARCQLILTQLVLAGLAKDNGGYG
NKFRRCQ
>LFGLNPFC_03817 hypothetical protein
MDKNVEHVLVDAIENKQSLTVVYLGGSQPGTLRNISPISINGDKLRARCHSSGAVKVFNL
GKIQLPSDSCAVSMHYGDLEVKAYETMQSVNDNFHALYPEGRWGVDFNEHRFALFDFFKN

GKRKKTAFMAIEFRERDEEKIITGVTIDIGISGTVISEKSRIPKRRPWVVVGPEHGEYST
YSTLDKAATAFFERLSLIASGLEDN
>LFGLNPFC_03818 hypothetical protein
MRDIQMVLERWGAWVANNHEDVEWSSVAAGFKGLIPSKVKSRPQCSDDDGLIISSAMTVL
KKKEPYQYELLEMYYVYGVTLRALGVKLGISLNQVVIRLQKAEGFIDGCLAMLGVSLEID
CYI
>LFGLNPFC_03819 hypothetical protein
MRALLTPEIAPRMGIVLFRPGSELMPLFMQGRVLLEPEPERYSSFASGAVPAASQPLADD
PAVRAVFRNEAVIRRAGGVECLESWLLREKGCQWPHSDWHSENMTTMRHATGAIRLCWHC
DNQLRDQFTERLESMATDNCARWVLSVVRRDLGFDDSHVVTMPELCWWLIRNDLADVLPE
SAARKALRLPKPVVPSVTRESDLVPSVPATSIIQDKAKKVLALKVDPESPESFMLRPKRR
RWVNEKYTRWVKTQPCACCGKPADDPHHLIGHGQGGMATKAHDLFVLPLCRKHHDELHAD
TVAFEEKYGSQLELIFRFIDRALAIGVLA
>LFGLNPFC_03820 hypothetical protein
MNNLMVIDGIEVRRDAYGRYSLNDLHRAAVASGANARTKEPGKFLSSQQTVELVHELTNT
QNLGVDPVSVIHGGNERGTYVCKELVYAYAMWISPSFHLKVIRTFDMVTSAPEKLSGQAA
DKMQAGVILLDFMRRELNLSNSSVLGACQKLQEAVGLPNLAPRYAIDAPADAPDGSSRPT
LSLSALLKQYGIRLTANQAYHQMVKLGIVEQRERYSRTAINNIKKFWSLTAKGCMFGKNI
TSPANPRETQPHFFESRFPELLKLLDTVH
>LFGLNPFC_03821 Crossover junction endodeoxyribonuclease RusA
MKLILPFPPSVNTYWRHPNKGAFAGKSLISEAGRKFQSAACAAIVEQLRRLPKPTSAPAS
VEIVLFPPDNRIRDLDNYNKALFDALTHAGVWEDDRQVKRMLVEWGPVIPKGKVEITISK
YEKPAGAAA
>LFGLNPFC_03822 LexA repressor
MTTLTQCQQQVLDMLISYQQERGFPPTNQEVATMLGYRSVNAAVEHLRALEKKGVITIKR
GVARGITLHTAVKDDDSEAVGIIRALLAGEENARLRAAHWLHERGLKV
>LFGLNPFC_03823 hypothetical protein
MSNKYCQALVELRNKPAHELKEVGDQWRTPDNIFWGINTLFGPFVLDLFTDGDNAKCAAY
YTAEDNALAHDWSERLAELKGAAFGNPPYSRASQHEGQYITGMRYIMKHASAMRDKGGRY
VFLIKAATSEVWWPEDADHIAFIRGRIGFELPAWFIPKDGKQVPTGAFFAGAIAVFDKTW
KGPAISYIGRDELEACGEAFLAQVRLQAEKLVREMAA
>LFGLNPFC_03824 hypothetical protein
MSLLMTSQPIVINRDLACRIGLNEAIVLQQLHYWLNETNSGTEHGGIRWVYNTTEQWLEQ
FPFWSESTLKRTFASLKSLGVLRREQLNKSKRDMTNFYTINYESELLEEVKVNESIRSKC
TSPSGQSDLMDGRKMTRSIGSKRHAVIGSKWPNDLTENTTEITTENKTSSRPDASQPDTQ
TAEQEFLTRHPDAVVFSPKKRQWGTQDDLTCAQWLWKKIIALYEQAAECDGEVVRPKEPN
WTAWANEIRLMCVQDGRTHKQICEMYSRVSRDPFWCRNVLSPSKLREKWDELSLRLSPSV
STHTEKREDPYFKASYDNVDYSQIPAGFRG
>LFGLNPFC_03825 hypothetical protein
MNSLTTHYRRSQLIALPVPGGKAKVEYCYAVNVPGDREIVTHSFAEWAVGDFNRQKETVL
CDKLTAGSKITTECPSESFVGSRKHNGLSTSVKAMNMNASVRSNSFVVNSGK
>LFGLNPFC_03826 hypothetical protein
MGKEPEWKVDKQPAWLVAAIRRTIADLPHGYEEAAEILGLYKSDDITPAKDQLHNRLRSG
GDQIFPLEWAMVLQDASGTRHVTDAIARRSNGVFVPLVVIDDIDNGDINQRLMESIEWIG
KHSQYLRKATADGVIDQAEREQIEENSYQVMAKWQEHLTLLFRVFCAPEKSNARECAAPG
VVASIASGCGETNA
>LFGLNPFC_03827 hypothetical protein
MRKSDVINYFGGVCKTAEALGIKHPSVSEWPEIIPEGRAYQLEKITNGKLKVDVSLYQKT
NSAAA
>LFGLNPFC_03828 putative HTH-type transcriptional regulator
MMNMSDRIRQRRKELNLTQQALADLTGVNRVTVTGWEKDDYQPNGANLQALANALKCDPL
WLVSGKGSPEPKINLKPEIFAVKKVPLISWVQAGSWTMTEPGVRKEDAEEWVYTTALVSE
MAFALRVRGDSMTNPLGSPSIPEGSIVIVEPDIIDTECINGKIVVAHINGGQEATLKKFV
EDWPNRYLVPLNPNYKTIECGENCRIVGLVKQVIMDF
>LFGLNPFC_03829 hypothetical protein
MASERSTDVQAFIGELDGGVFETKIGAVLSEVASGVMNTKTKGKVSLNLEIEPFDENRVK
IKHKLSYVRPTNRGKISEEDTTETPMYVNRGGRLTILQEDQGQLLTLAGEPDGKLRAAGH
>LFGLNPFC_03830 hypothetical protein
MSQNLDATAINQIHALISAQGVNEIISKIGADAVALPEHFRIHDLEKFNLNRFRFRGALS
TASIDDFTRYSKDLADEGTRCFIDADNMRAVSVLNLGTIDEPGHADNTATLKLKKTAPFS
ALLSVNGERNSQKSLAEWIEDWADYLVSFDANGDTIQATKAAAAVRKITIEANQTADFED
NDFSGKRSLMESVEAKTKDIMPVAFEFKCVPFEGLKERPFKLRLSIITGDRPVLVLRIIQ
LEAMQEEMANEFRDLLVEKFKDSKVETFIGTFTA
>LFGLNPFC_03831 hypothetical protein

MSFIKTFSGKHFYYDKINKDDIVINDIAVSLSNICRFAGHLSHFYSVAQHAVLCSQLVPQ
EFAFEALMHDATEAYCQDIPAPLKRLLPDYKRMEEKIDAVIREKYGLPPVMSTPVKYADL
IMLATERRDLGLDDGSFWPVLEGIPATEMFKVIPLSPGHAYGMFMERFNELSELRKCA
>LFGLNPFC_03832 hypothetical protein
MRMNVFEMEGFLRGKCVPRDLKVNETNAEYLVRKFDALEAKCAALENKIMPVSAELPPAN
ESVLLFDANGEGWLIGWRSLWYTWGQKETGEWQWTFQVGDLENVNITHWAVMPKAPENKK
>LFGLNPFC_03833 hypothetical protein
MSVIKTHTGIVITRDGPQVKKLHQTKRMWVVGKNEFYHKETGRRHFAENTRRRLLIDTIK
PIEVKHV
>LFGLNPFC_03834 hypothetical protein
MFKQNEKSIAQIAEYIPRACRGMQLQEAKARLEKKIALYIDDGCDAAVLNAAFAPALNSH
TRESFFSCIAAQIRKGGNQ
>LFGLNPFC_03835 hypothetical protein
MSESKCQINGNKIEPCAALAKSLEHDAEYTARKGLLIYKIWNESLTRGPDLVMLRSGEFS
KSPVRVSFCPFCGESLKTWENRNE
>LFGLNPFC_03836 hypothetical protein
MNEIKEIPVVRDEYGCWTHPEYEKFCDGREHISTEEFNAWMEGNNLQWTIRTMDEDDFNL
DADGPDIASWKPERPEGEGWFIGSIHDTEDGPVCVWLRNKVEA
>LFGLNPFC_03837 hypothetical protein
MAELTKWLQNTITGIETVVDDKSFVCDEIVFKIDVVKNVLTAFKVALASLEAEPVTWRYR
YVRKGVTNFQEKPWVGDWKYVPTKEDCNDRPNYEIQALFTAQPVPLTPEGLIKAVRFYEQ
VKSENPPVETGAWKDAVDWVLKEACQAVNIGIKGE
>LFGLNPFC_03838 hypothetical protein
MATLQELIDLTPEQEKAWNRLVKAVKDFRAAGGKFYSVLDTLSAYNGEHVASIDNDKGYH
TASVYMPSIDAPGLTSWADDWHGITLKDGVEVDED
>LFGLNPFC_03839 hypothetical protein
MTTFTDKELIKEIRERIGSLDVRDNIERLAYEIALASLEREQIRHEHAKWSDSTFGCVGP
IGPLKHLSKEALEAAAEPEDLSEWADMQFLLWDAQRRAGISDAEITAAMEDKLKINMERQ
WPEPKDGEPRLHIKEPGNSPVIPDGWISCSDRMPEKGQNVLISVNFDSSLVEPLICSARY
TGSTFRRGDATIKPGNGIEQATHWMPLPEPPQEVK
>LFGLNPFC_03840 hypothetical protein
MNNLMIDLETMGKNKDAPIVSIGAVFFTPETGDIGQEFYAVVSLDSAMKQGATPDGDTIL
WWLKQSPEARAAICIDDTLSISDALSELNHFINRHADNTKYLKVWGNGATFDNVILRGAY
ERAGQICPWAYWNDHDVRTIVTLGRSIGFDPKMDMPFDGERHNALADARHQAKYVSAIWQ
KLIPATSTEL
>LFGLNPFC_03841 hypothetical protein
MNTLFLLMAEFNTPNIELSAVSQKYFGMSPATAEAKANACKLPVPTYRIGTSQKAKRCIN
IQDLAEYIDKRREEGRIEWERVRTDKQKSKEHH
>LFGLNPFC_03842 hypothetical protein
MPLKASLNGKEIHSFEFNSQEWEYLKQTYKSQSLFMSCCGQLGIPKTSKLNNYYFAHKSK
SDCLYAKESAEHLYLKFLIAKLASDAGWSVTTEKQGVTPTGEQWIADVFCTRNNAKLVFE
VQLSPQKDDEFKERQKRYIASGVRALWLRKLRKGSKEHGGNIYHSYDLPVFGVRQNENGD
LYLPQFGISVREFIAGVFARKLLWFPKVGDVLTAKIESRVTKCWRCKKRTGIIRGISVYS
KSVFVKFLSFRDSDVKELIATHVDNKKLWAAGIGTVKDRDSDCYLSNCCIHCYSLIGDFF
LLEVFDYPNDKQKIMIEFTFTYASNMKILSSEWVFDGKKAEMFF
>LFGLNPFC_03843 hypothetical protein
MEDQKATKPQLKFDTMKAFAGMGAAVEVLMKAAPNAFTHVTVSGKEQQGKLRRRKAV
>LFGLNPFC_03844 hypothetical protein
MTDYQPYRKGTVLAPTGPCNHLHVICNDPVYYPVNDCYCVLVVNISSIKDGVPHDPSCVL
NSGDHRFIKHPSYVVYAEAIIWRVDNMVRKQRSGEISVHDDMPEATFNRILDGFDISDEV
TPKNLKFKNKYCVSSIDDE
>LFGLNPFC_03845 hypothetical protein
MFCEEKVAQMAAYLLLKRGGRMAYLKLMKLLYLSNRKSILKHGRMIGEDSLYSMKFGPVM
SNTLNLIRGKAEGIGDYWYNLIETNGHDVLLRSDPREMDADEVFDELSRADIRILDEIYS
LYGHMNRFDLANMTHLESVCPEWHNPGNSRKPIDLKEMLISEGKSEDEANRIIGKMEESQ
KLKEFSLQLS
>LFGLNPFC_03846 Zinc uptake regulation protein
MEKTTTQELLAQAEKICAQRNVRLTPQRLEVLRLMSLQDGAISAYDLLDLLREAEPQAKP
PTVYRALEFLLEQGFVHKVESTNSYVLCHLFDQPTHTSAMFICDRCGAVKEECAEGVEDI
MHTLAAKMGFALRHNVIEAHGLCSACVEVEACRHPEQCHHDHSIQVKKKPR
>LFGLNPFC_03847 hypothetical protein
MNKDEAGGNWKQFKGKVKEQWGKLTDDDMTIIEGKRDQLVGKIQERYGYQKDQAEKEVDS
WEKRHDYRW
>LFGLNPFC_03848 DNA damage-inducible protein F

MAFLTSSDKALWHLALPMIFSNITVPLLGLVDTAVIGHLDSPVYLGGVAVGATATSFLFM
LLLFLRMSTTGLTAQAYGAKNPQALARALVQPLLLALGAGALIALLRTPIIDLALHIVGG
SEAVLEQARRFLEIRWLSAPASLANLVLLGWLLGVQYARAPVILLVVGNILNIVLDVWLV
MGLHMNVQGAALATVIAEYATLLIGLLMVRKILKLRGISGEMLKTAWRGNFRHLLALNRD
IMLRSLLLQLCFGAITVLGARLGSDIIAVNAVLMTLLTFTAYALDGFAYAVEAHSGQAYG
ARDGSQLLDVWRAACRQSGIVALLFSVVYLLAGEHIIALLTSLTQIQQLADRYLIWQVIL
PLVGVWCYLLDGMFIGATRAAEMRNSMAVAAAGFALTLLTLPWLGNHGLWLALTVFLALR
GLSLAAIWRRHWRNDTWFVAT
>LFGLNPFC_03849 LexA repressor
MKALTARQQEVFDLIRDHISQTGMPPTRAEIAQRLGFRSPNAAEEHLKALARKGVIEIVS
GASRGIRLLQEEEEGLPLVGRVAAGEPLLAQQHIEGHYQVDPSLFKPNADFLLRVSGMSM
KDIGIMDGDLLAVHKTQDVRNGQVVVARIDDEVTVKRLKKQGNKVELLPENSEFKPIVVD
LRQQSFTIEGLAVGVIRNGDWL
>LFGLNPFC_03850 Diacylglycerol kinase
MANNTTGFIRIIKAAGYSWKGLRAAWINEAAFRQEGVAVLLAVVIACWLDVDAITRVLLI
SSVMLVMIVEILNSAIEAVVDRIGSEYHELSGRAKDMGSAAVLIAIIVAVITWCILLWSH
FG
>LFGLNPFC_03851 Glycerol-3-phosphate acyltransferase
MSGWPRIYYKLLNLPLSILVKSKSIPADPAPELGLDTSRPIMYVLPYNSKADLLTLRAQC
LAHDLPDPLEPLEIDGTLLPRYVFIHGGPRVFTYYTPKEESIKLFHDYLDLHRSNPNLDV
QMVPVSVMFGRAPGREKGEVNPPLRMLNGVQKFFAVLWLGRDSFVRFSPSVSLRRMADEH
GTDKTIAQKLARVARMHFARQRLAAVGPRLPARQDLFNKLLASRAIAKAVEDEARSKKIS
HEKAQQNAIALMEEIAANFSYEMIRLTDRILGFTWNRLYQGINVHNAERVRQLAHDGHEL
VYVPCHRSHMDYLLLSYVLYHQGLVPPHIAAGINLNFWPAGPIFRRLGAFFIRRTFKGNK
LYSTVFREYLGELFSRGYSVEYFVEGGRSRTGRLLDPKTGTLSMTIQAMLRGGTRPITLI
PIYIGYEHVMEVGTYAKELRGATKEKESLPQMLRGLSKLRNLGQGYVNFGEPMPLMTYLN
QHVPDWRESIDPIEAVRPAWLTPTVNNIAADLMVRINNAGAANAMNLCCTALLASRQRSL
TREQLTEQLNCYLDLMRNVPYSTDSTVPSASASELIDHALQMNKFEVEKDTIGDIIILPR
EQAVLMTYYRNNIAHMLVLPSLMAAIVTQHRHISRDVLMEHVNVLYPMLKAELFLRWDRD
ELPDVIDALANEMQRQGLITLQDDELHINPAHSRTLQLLAAGARETLQRYAITFWLLSAN
PSINRGTLEKESRTVAQRLSVLHGINAPEFFDKAVFSSLVLTLRDEGYISDSGDAEPAET
MKVYQLLAELITSDVRLTIESATQGEG
>LFGLNPFC_03852 4-hydroxybenzoate octaprenyltransferase
MEWSLTQNKLLAFHRLMRTDKPIGALLLLWPTLWALWVATPGVPQLWILAVFVAGVWLMR
AAGCVVNDYADRKFDGHVKRTANRPLPSGAVTEKEARALFVVLVLISFLLVLTLNTMTIL
LSIAALALAWVYPFMKRYTHLPQVVLGAAFGWSIPMAFAAVSESVPLSCWLMFLANILWA
VAYDTQYAMVDRDDDVKIGIKSTAILFGQYDKLIIGIFQIGVLALMAIIGELNGLGWGYY
WSILVAGALFVYQQKLIANREREACFKAFMNNNYVGLVLFLGLAMSYWHF
>LFGLNPFC_03853 Chorismate pyruvate-lyase
MSHPALTQLRALRYFTEIPALEPQLLDWLLLEDSMTKRFEQQGKTVSVTMIREGFVEQNE
IPEELPLLPKESRYWLREILLCADGEPWLAGRTVVPVSTLSGPELALQKLGKTPLGRYLF
TSSTLTRDFIEIGRDAGLWGRRSRLRLSGKPLLLTELFLPASPLY
>LFGLNPFC_03854 hypothetical protein
MKMNKSLIALCLSAGLLASAPGISLADVNYVPQNTSDAPAIPSAALQQLTWTPVDQSKTQ
TTQLSTGGQQLNVPGISGPVAAYSVPANIGELTLTLTSEVNKQTSVFAPNVLILDQNITP
SAFFPSSYFTYQEPGVMSADRLEGVMRLTPALGQQKLYVLVFTTEKDLQQTTQLLDPAKA
YAKGVGNSIPDIPDPVARHTTDGLLKLKVKTNSSSSVLVGPLFGSSAPAPVTVGNTAAPA
VAAPAPAPVKKSEPMLNDTESYFNTAIKNAVAKGDVDKALKLLDEAERLGSTSARSTFIS
SVKGKG
>LFGLNPFC_03855 Maltoporin
MITLRKLPLAVAVAAGVMSAQAMAVDFHGYARSGIGWTGSGGEQQCFQTTGAQSKYRLGN
ECETYAELKLGQEVWKEGDKSFYFDTNVAYSVAQQNDWEATDPAFREANVQGKNLIEWLP
GSTIWAGKRFYQRHDVHMIDFYYWDISGPGAGLENIDVGFGKLSLAATRSSEAGGSSSFA
SNNIYDYTNETANDVFDVRLAQMEINPGGTLELGVDYGRANLRDNYRLVDGASKDGWLFT
AEHTQSVLKGFNKFVVQYATDSMTSQGKGLSQGSGVAFDNEKFAYNINNNGHMLRILDHG
AISMGDNWDMMYVGMYQDINWDNDNGTKWWTVGIRPMYKWTPIMSTVMEIGYDNVESQRT
GDKNNQYKITLAQQWQAGDSIWSRPAIRVFATYAKWDEKWGYDYTGSSSTNPYYGKAVSA
DFNGGSFGRGDSDEWTFGAQMEIWW
>LFGLNPFC_03856 Maltose/maltodextrin import ATP-binding protein MalK
MASVQLQNVTKAWGEVVVSKDINLDIHEGEFVVFVGPSGCGKSTLLRMIAGLETITSGDL
FIGEKRMNDTPPAERGVGMVFQSYALYPHLSVAENMSFGLKLAGAKKEVINQRVNQVAEV
LQLAHLLDRKPKALSGGQRQRVAIGRTLVAEPSVFLLDEPLSNLDAALRVQMRIEISRLH
KRLGRTMIYVTHDQVEAMTLADKIVVLDAGRVAQVGKPLELYHYPADRFVAGFIGSPKMN
FLPVKVTATAIDQVQVELPMPNRQQVWLPVESRDVQVGANMSLGIRPEHLLPSDIADVIL

EGEVQVVEQLGNETQIHIQIPSIRQNLVYRQNDVVLVEEGATFAIGLPPERCHLFREDGT
ACRRLHKEPGV
>LFGLNPFC_03857 Maltose/maltodextrin-binding periplasmic protein
MKIKTGARILALSALTTMMFSASALAKIEEGKLVIWINGDKGYNGLAEVGKKFEKDTGIK
VTVEHPDKLEEKFPQVAATGDGPDIIFWAHDRFGGYAQSGLLAEITPDKAFQDKLYPFTW
DAVRYNGKLIAYPIAVEALSLIYNKDLLPNPPKTWEEIPALDKELKAKGKSALMFNLQEP
YFTWPLIAADGGYAFKYENGKYDIKDVGVDNAGAKAGLTFLVDLIKNKHMNADTDYSIAE
AAFNKGETAMTINGPWAWSNIDTSKVNYGVTVLPTFKGQPSKPFVGVLSAGINAASPNKE
LAKEFLENYLLTDEGLEAVNKDKPLGAVALKSYQDELAKDPRIAATMDNAQKGEIMPNIP
QMSAFWYAVRTAVINAASGRQTVDAALKDAQTRITK
>LFGLNPFC_03858 Maltose/maltodextrin transport system permease protein MalF
MDVIKKKHWWQSDALKWSVLGLLGLLVGYLVVLMYAQGEYLFAITTLILSSAGLYIFANR
KAYAWRYVYPGMAGMGLFVLFPLVCTIAIAFTNYSSTNQLTFERAQEVLLDRSWQAGKIY
NFGLYPAGDEWQLALSDGETGKNYLSDAFKFGGEQKLQLKETTAQPEGERANLRVITQNR
QALSDITAILPDGNKVMMSSLRQFSGTQPLYTLDGDGTLTNNQSGVKYRPNNQIGFYQSI
TADGHWGDEKLSPGYTVTTGWKNFTRVFTDEGIQKPFLAIFVWTVVFSLITVFLTVAVGM
VLACLVQWEALRGKAVYRVLLILPYAVPSFISILIFKGLFNQSFGEINMMLSALFGVKPA
WFSDPTTARTMLIIVNTWLGYPYMMILCMGLLKAIPDDLYEASAMDGAGPFQNFFKITLP
LLIKPLTPLMIASFAFNFNNFVLIQLLTNGGPDRLGTTTPAGYTDLLVNYTYRIAFEGGG
GQDFGLAAAIATLIFLLVGALAIVNLKATRMKFD
>LFGLNPFC_03859 Maltose/maltodextrin transport system permease protein MalG
MAMVQPKSQKARLFITHLLLLLFIAAIMFPLLMVVAISLRQGNFATGSLIPEQISWDHWK
LALGFSVEQADGRITPPPFPVLLWLWNSVKVAGISAIGIVALSTTCAYAFARMRFPGKAT
LLKGMLIFQMFPAVLSLVALYALFDRLGEYIPFIGLNTHGGVIFAYLGGIALHVWTIKGY
FETIDSSLEEAAALDGATPWQAFRLVLLPLSVPILAVVFILSFIAAITEVPVASLLLRDV
NSYTLAVGMQQYLNPQNYLWGDFAAAAVMSALPITIVFLLAQRWLVNGLTAGGVKG
>LFGLNPFC_03860 Protein PsiE
MTSLSRPRVEFISTILQTVLNLGLLCLGLILVVFLGKETVHLADVLFAPEQASKYELVEG
LVVYFLYFEFIALIVKYFQSGFHFPLRYFVYIGITAIVRLIIVDHKSPLDVLIYSAAILL
LVITLWLCNSKRLKRE
>LFGLNPFC_03861 hypothetical protein
MGDFFGLCVLPDATLMGDAHLIELNKTYTKNIDLRHIFALYRKLATVKEVLL
>LFGLNPFC_03862 hypothetical protein
MKKRHLLSLLALGISTACYGEIYPAPIGPSQSDFGGVGLLQTPTARMAREGELSLNYRDN
DQYRYYSASVQLFPWLETTLRYTDVRTRQYSSVEAFSGDQTYKDKAFDLKLRLWEESYWL
PQVAVGARDIGGTGLFDAEYLVASKAWGPFDFTLGLGWGYLGTSGNVKNPLCSASDKYCY
RDNSYKQAGSIDGSQMFHGPASLFGGVEYQTPWQPLRLKLEYEGNNYQQDFAGKLEQKSK
FNVGAIYRVTDWADVNLSYERGNTFMFGVTLRTNFNDLRPSYNDNARPQYQPQPQDAILQ
HSVVANQLTLLKYNAGLADPQIQAKGDTLYVTGEQVKYRDSREGIIRANRIVMNDLPDGI
KTIRITENRLNMPQVTTETDVASLKNHLGGEPLGHETTLAQKRVEPVVPKSTEQGWYIDK
SRFDFHIDPVLNQSVGGPENFYMYQLGVMGTADLWLTDHLLTTGSLFANLANNYDKFNYT
NPPQDSHLPRVRTHVREYVQNDVYYVNNLQANYFQHLGNGFYGQVYGGYLETMFGGAGAEV
LYRPLDSNWAFGLDANYVKQRDWRSAKDMMKFTDYSVKTGHLTAYWTPSFAQDVLVKASV
GQYLAGDKGGTLEIAKRFDSGVVVGGYATITNVSKEEYGEGDFTKGVYVSVPLDLFSSGP
TRSRAAIGWTPLTRDGGQQLGRKFQLYDMTSDRSVNFR
>LFGLNPFC_03863 hypothetical protein
MIKQTIVALILSVGASSVFAAGTVKVFSNGSSEAKTLTGAEHLIDLVGGQPRLANSWWPGA
VISEELATAAALRQQQALLTRLAELAADSSADDAAAINALRQQIQALKVTGRQKINLDPD
IVRVAERGNPPLQGNYTLWVGTPPSTVTLFGLISRPGKQSFTPGRDVASYLSDQSLLSGA
DRSYAWVVYPSGRTQKAPVAYWNKRHVEPMPGSIIYVGLADSVWSETPDALNADILQTLT
QRIPQ
>LFGLNPFC_03864 putative lipoprotein GfcB
MKRPALILICLLLQACSATTKELGNSLWDSLFGTPGVQLTDDDIQNMPYASQYMQLNGGP
QLFVVLAFAEDGQQKWVTQDQATLVTQHGRLVKTLLGGDNLIEVNNLATDPLIKPAQIVD
GATWTRTMGWTEYQQVRYATARSVFKWDGTDTVKVGSDETPVRVLDEEVSTDQARWHNRY
WIDSEGQIRQSEQYLGADYFPVKTTLIKAAKQ
>LFGLNPFC_03865 hypothetical protein
MKKVLYGIFAISALAATSAWAAPVQVGEAAGSAATSVSAGSSSATSVSTVSSAVGVALAA
TGGGDGSNTGTTTTTTTSTQ
>LFGLNPFC_03866 Glucose-6-phosphate isomerase
MKNINPTQTAAWQALQKHFDEMKDVTIADLFAKDGDRFSKFSATFDDQMLVDYSKNRITE
ETLAKLQDLAKECDLAGAIKSMFSGEKINRTENRAVLHVALRNRSNTPILVDGKDVMPEV
NAVLEKMKTFSEAIISGEWKGYTGKAITDVVNIGIGGSDLGPYMVTEALRPYKNHLNMHF
VSNVDGTHIAEVLKKVNPETTLFLVASKTFTTQETMTNAHSARDWFLKAAGDEKHVAKHF

AALSTNAKAVGEFGIDTANMFEFWDWVGGRYSLWSAIGLSIVLSIGFDNFVELLSGAHAM
DKHFSTTPAEKNLPVLLALIGIWYNNFFGAETEAILPYDQYMHRFAAYFQQGNMESNGKY
VDRNGKVVDYQTGPIIWGEPGTNGQHAFYQLIHQGTKMVPCDFIAPAITHNPLSDHHQKL
LSNFFAQTEALAFGKSREVVEQEYRDQGKDPATLDYVVPFKVFEGNRPTNSILLREITPF
SLGALIALYEHKIFTQGVILNIFTFDQWGVELGKQLANRILPELKDDKEISSHDSSTNGL
INRYKAWRG
>LFGLNPFC_03867 Lysine-sensitive aspartokinase 3
MSEIVVSKFGGTSVADFDAMNRSADIVLSDANVRLVVLSASAGITNLLVALAEGLEPGER
FEKLDAIRNIQFAILERLRYPNVIREEIERLLENITVLAEAAALATSPALTDELVSHGEL
MSTLLFVEILRERDVQAQWFDVRKVMRTNDRFGRAEPDVAALAELAALQLLPRLNEGLVI
TQGFIGSENKGRTTTLGRGGSDYTAALLAEALHASRVDIWTDVPGIYTTDPRVVSAAKRI
DEIAFAEAAEMATFGAKVLHPATLLPAVRSDIPVFVGSSKDPRAGGTLVCNKTENPPLFR
ALALRRNQTLLTLHSLNMLHSRGFLAEVFGILARHNISVDLITTSEVSVALTLDTTGSTS
TGDTLLTQSLLMELSALCRVEVEEGLALVALIGNDLSKACGVGKEVFGVLEPFNIRMICY
GASSHNLCFLVPGEDAEQVVQKLHFNLFE
>LFGLNPFC_03868 hypothetical protein
MALPRITQKEMTEREQRELKTLLDRARIAHGRVLTNSETNSIKKEYIDKLMVEREAEAKK
ARQLKKKQAYKPDPEASFSWSANTSTRGRR
>LFGLNPFC_03869 Dual-specificity RNA pseudouridine synthase RluF
MLPDSSVRLNKYISESGICSRREADRYIEQGNVFLNGKRATIGDQVKPGDVVKVNGQLIE
PRESEDLVLIALNKPVGIVSTTEDGERDNIVDFVNHSKRVFPIGRLDKDSQGLIFLTNHG
DLVNKILRAGNDHEKEYLVTVDKPITDEFIRGMGAGVPILGTVTKKCKVKKEAPFVFRIT
LVQGLNRQIRRMCEHFGYEVKKLERTRIMNVSLSGIPLGEWRDLTDDELIDLFKLIENSS
SEAKPKAKAKPKTVGIKRPVVKMEKTAEKGGRPASNGKRFTSPGRKKKGR
>LFGLNPFC_03870 Sorbitol operon regulator
MENSDDIRLIVKIAQLYYEQDMTQAQIARELGIYRTTISRLLKRGRDQGIVTIAINYDYN
ENLWLEQQVKQKFGLKDVVVVSGNDEDEDIQLAMMGLHGAQLLDRLLEPGDIVGFSWGRA
VSALVENLPQAGQSRQLICVPIIGGPSGKLESRYHVNTLTYSAAAKLKGESHLADFPALL
DNPLIRNGIMQSQHFKTISAYWDNLDVALVGIGSPAIRDGANWHAFYGGEESDDLNARQV
AGDICSRFFDIHGAMVETNMSEKTLSIEMNKLKQARYSIGIAMSEEKYSGIVGALRGKYI
NCLVTNSSTAELLLK
>LFGLNPFC_03871 Dihydroanticapsin 7-dehydrogenase
MQTWLNLQDKIIIVTGGASGIGLAIVEELLAQGANVQMVDIHGGDGQYESHKGYQFWPTD
ISSAKEINHTVAEIIQRFGRIDGLVNNAGVNFPRLLVDEKAPAGQYELNEAAFEKMVNIN
QKGVFLMSQAVARQMVKQHDGVIVNVSSESGLEGSEGQSCYAATKAALNSFTRSWSKELG
KHGIRVVGIAPGILEKTGLRTPEYEEALAWTRNITVEQLREGYTKNAIPIGRAGRLAEVA
DFVCYLLSERASYITGVTTNIAGGKTRG
>LFGLNPFC_03872 PTS system sorbose-specific EIIA component
MVNAIFCAHGKLACAMLESVQMVYGDARVEAVEFVPGENAGDIVAKLEKLVSIHNHDEWL
IAVDLQCGSPWNAAAMLAMRNPRLRVISGLSLPLALELVDNQDSMNVDELCEHLTQIAKQ
TCVVWRQVATAEEDF
>LFGLNPFC_03873 PTS system sorbose-specific EIIB component
MNITLARIDDRLIHGQVTTVWSKVANAQRIIICNDEVYNDEVRRTLLRQAAPPGMKVNVV
NIEKAVAVYHNPQYQDETVFYLFTRPQDALAMVRQGVKIGTLNIGGMAWRPGKKQLTKAV
SLDDDDINAFHELNNLGVILDLRVVASDPSINIIDKINEQLIAN
>LFGLNPFC_03874 PTS system sorbose-specific EIIC component
MEISTLQIIAIFLFSCIAGMGSVLDEFQTHRPLIACTVIGLILGDLKTGIMLGGTLELIA
LGWMNVGAAQSPDSALASIISAILVIVGQQSIATGIAIALPVAAAGQVLTVFARTITVVF
QHAADKAAEEARFRTLDILHVSALGVQALRVAIPALIVSLFVSADMVSNMLSAIPEFVTR
GLQIAGGFIVVVGYAMVLRMMGVKYLMPFFFLGFLAGGYLDLSLLAFGGVGVIMALLYIQ
LNPQWRKAEPHPQTTTITALDQLDD
>LFGLNPFC_03875 PTS system sorbose-specific EIID component
MEQRKITRSDLVSMFLRSNLQQASFNFERIHGLGFCYDMIPAIKRLYPLKEDQVAALRRH
LVFFNTTPAVCGPVIGVTAAMEEARANGAEIDDGTINGIKVGLMGPLAGVGDPLVWGTLR
PITAVLGASLALSGNILGPLLFFFIFNAVRLAMKWYGLQLGFRKGVNIVSDMGGNLLQKL
TEGASILGLFVMGVLVTKWTSINVPLVVSQTPAADGATVTITVQNILDQLCPGLLALGLT
LLMVRLLNKKINPVWLIFALFGLGIIGNALGFLS
>LFGLNPFC_03876 Mannitol-1-phosphate 5-dehydrogenase
MKTTALRLYGKRDLRLETFDLPEMQEDEILATVVTDSLCLSSWKEANLGENHKKVPDDVA
TNPIIIGHEFCGDILAVGKKWQHKFQPGQRYVIQANLQLPDRPDCPGYSFPWVGGEATHV
VIPNEVMEQDCLLAYDGETYFEGSLVEPLSCVIGAFNANYHLQEGSYNHTMGIRPQGRTL
ILGGTGPMGLLAIDYALHGPVNPSLLVITDTDNDKLSYARKHYPSEPQTLIHYLNAADAA
FDTLMALSGGHGFDDIFVFVPNEGLVTLASSLLATDGCLNFFAGPQDKHFSAPINFYDVH
YAFTHYVGTSGGNTDDMRAAVKLIEEKKVQAAKVVTHILGLNAAGETTLELPAVGGGKKL

VYTGKYLPLTSLTQIQDQALAAILARHQGIWSGEAEQYLLAHAEAISHD
>LFGLNPFC_03877 Peptidase E
MELLLLSNSTLPGKAWLEHALPLIAEQLQGRRSAVFIPFAGVTQTWDDYTAKTAAVLAPL
GVSVTGIHSVVDPVAAIENAEIVIVGGGNTFQLLKQCRERGLLAPITDVVKRGALYIGWS
AGANLACPTIRTTNDMPIVDPQGFDALNLFPLQINPHFTNALPEGHKGETREQRIRELLV
VAPELTIIGLPEGNWITVSKGHATLGGPNTTYVFKAGEEAVPLEAGHRF
>LFGLNPFC_03878 hypothetical protein
MLTLLHLLSAVALLVWGTHIVRTGVMRVFGARLRTVLSRSVEKKPLAFCAGIGVTALVQS
SNATTMLVTSFVAQDLVALAPALVIVLGADVGTALMARILTFDLSWLSPLLIFIGVIFFL
GRKQSRAGQLGRVGIGLGLILLALELIVQAVTPITQANGVQVIFASLTGDILLDALIGAM
FAIISYSSLAAVLLTATLTAAGIISFPVALCLVIGANLGSGLLAMLNNSAANAAARRVAL
GSLLFKLVGSLIILPFVHLLAETMGKLPLPKAELVIYFHVFYNLVRCLVMLPFVDPMARF
CKTIIRDEPELDTQLRPKHLDVSALDTPTLALANAARETLRIGDAMEQMMEGLNKVMHGE
PRQEKELRKLADDINVLYTAIKLYLARMPKEELAEEESRRWAEIIEMSLNLEQASDIVER
MGSEIADKSLAARRAFSLDGLKELDALYEQLLSNLKLAMSVFFSGDVTSARRLRRSKHRF
RILNRRYSHAHVDRLHQQNVQSIETSSLHLGLLGDMQRLNSLFCSVAYSVLEQPDEDEGR
DEY
>LFGLNPFC_03879 hypothetical protein
MQRQYKKYILLFWGVMDVIAIATYLFYSLGNGRIPFYSDIVHSLSLLKDIGVEGGFYAYA
VATVTLQIVLLLSLFFSAQCFLRQKLFSFSFLAIQEFMRFATVSCSISVIPLLLNYFYSQ
DLVLNISLFIFSEVTKVTTIIWCKRQGKV
>LFGLNPFC_03880 hypothetical protein
MIKIDEIDFFAFEGCSESEVTELLGKKPTGYNDWMINIKTGVEWVFYDGRYWLDFRKSKE
GIRCISCSILYHDKSKGRIDVLFSEGIADRGLKNTGGVLIQKRPQL
>LFGLNPFC_03881 Methionine synthase
MSSKVEQLRAQLNERILVLDGGMGTMIQSYRLNEADFRGERFADWPCDLKGNNDLLVLSK
PEVIAAIHNAYFEAGADIIETNTFNSTTIAMADYQMESLSAEINFAAAKLARACADEWTA
RTPEKPRYVAGVLGPTNRTASISPDVNDPAFRNITFDQLVAAYRESTKALVEGGADLILI
ETVFDTLNAKAAVFAVKTEFEALGVELPIMISGTITDASGRTLSGQTTEAFYNSLRHAEA
LTFGLNCALGPDELRQYVQELSRIAECYVTAHPNAGLPNAFGEYDLDADTMAKQIREWAE
AGFLNIVGGCCGTTPQHIAAMSRAVEGLAPRKLPEIPVACRLSGLEPLNIGEDSLFVNVG
ERTNVTGSAKFKRLIKEEKYSEALDVARQQVENGAQIIDINMDEGMLDAEAAMVRFLNLI
AGEPDIARVPIMIDSSKWDVIEKGLKCIQGKGIVNSISMKEGVDAFIHHAKLLRRYGAAV
VVMAFDEQGQADTRARKIEICRRAYKILTEEVGFPAEDIIFDPNIFAVATGIEEHNNYAQ
DFIGACEDIKRELPHALISGGVSNVSFSFRGNDPVREAIHAVFLYYAIRNGMDMGIVNAG
QLAIYDDLPTELRDAVEDVILNRRDDGTERLLELAEKYRGSKTDDTANTQQAEWRSWEVN
KRLEYSLVKGITEFIEQDTEEARQQATRPIEVIEGPLMDGMNVVGDLFGEGKMFLPQVVK
SARVMKQAVAYLEPFIEASKEQGKTNGKMVIATVKGDVHDIGKNIVGVVLQCNNYEIVDL
GVMVPAEKILRTAKEVNADLIGLSGLITPSLDEMVNVAKEMERQGFTIPLLIGGATTSKA
HTAVKIEQNYSGPTVYVQNASRTVGVVAALLSDTQRDDFVARTRKEYETVRIQHGRKKPR
TPPVTLEAARDNDFAFDWQAYTPPVAHRLGVQEVEASIETLRNYIDWTPFFMTWSLAGKY
PRILEDEVVGVEAQRLFKDANDMLDKLSAEKMLNPRGVVGLFPANRVGDDIEIYRDETRT
HVINVSHHLRQQTEKTGFANYCLADFVAPKLSGKADYIGAFAVTGGLEEDALADAFEAQH
DDYNKIMVKALADRLAEAFAEYLHERVRKVYWGYAPNENLSNEELIRENYQGIRPAPGYP
ACPEHTEKATIWELLEVEKHTGMKLTESFAMWPGASVSGWYFSHPDSKYYAVAQIQRDQV
EDYARRKGMSVTEVERWLAPNLGYDAE
>LFGLNPFC_03882 Transcriptional repressor IclR
MVAPIPAKRGRKPAVATAPATGQVQSLTRGLKLLEWIAESNGSVALTELAQQAGLPNSTT
HRLLTTMQQQGFVRQVGELGHWAIGAHAFMVGSSFLQSRNLLAIVHPILRNLMEESGETV
NMAVLDQSNHEAIIIDQVQCTHLMRMSAPIGGKLPMHASGAGKAFLAQLSEEQVTKLLHR
KGLHAYTHATLVSPVHLKEDLAQTRKRGYSFDDEEHALGLRCLAACIFDEHREPFAAISI
SGPISRITDDRVTEFGAMVIKAAKEVTLSYGGIR
>LFGLNPFC_03883 Isocitrate dehydrogenase kinase/phosphatase
MPRGLELLIAQTILQGFDAQYGRFLEVTSGAQQRFEQADWHAVQQAMKNRIHLYDHHVGL
VVEQLRCITNGQSTDAAFLLRVKEHYTRLLPDYPRFEIAESFFNSVYCRLFDHRSLTPER
LFIFSSQPERRFRTIPRPLAKDFHPDHGWESLLMRVISDLPLRLRWQNKSRDIHYIVRHL
TETLGTDNLAESHLQVANELFYRNKAAWLVGKLITPSGTLPFLLPIHQTDDGELFIDTCL
TTTAEASIVFGFARSYFMVYAPLPAALVEWLREILPGKTTAELYMAIGCQKHAKTESYRE
YLVYLQGCNEQFIEAPGIRGMVMLVFTLPGFDRVFKVIKDRFAPQKEMSAAHVRACYQLV
KEHDRVGRMADTQEFENFVLEKRHISPALMELLLQEAAEKITDLGEQIVIRHLYIERRMV
PLNIWLEQVEGQQLRDAIEEYGNAIRQLAAANIFPGDMLFKNFGVTRHGRVVFYDYDEIC
YMTEVNFRDIPLPRYPEDELASEPWYSVSPGDVFPEEFRHWLCADPRIGPLFEEMHADLF
RADYWRALQNRIREGHVEDVYAYRRRQRFSVRFV
>LFGLNPFC_03884 Isocitrate lyase

MKTRTQQIEELQKEWTQPRWEGITRPYSAEDVVKLRGSVNPECTLAQLGAAKMWRLLHGE
SKKGYINSLGALTGGQALQQAKAGIEAVYLSGWQVAADANLAASMYPDQSLYPANSVPAV
VERINNTFRRADQIQWSAGIEPGDPRYVDYFLPIVADAEAGFGGVLNAFELMKAMIEAGA
AAVHFEDQLASVKKCGHMGGKVLVPTQEAIQKLVAARLAADVTGVPTLLVARTDADAADL
ITSDCDPHDSEFITGERTSEGFFRTHAGIEQAISRGLAYAPYADLVWCETSTPDLELARR
FAQAIHAKYPGKLLAYNCSPSFNWQKNLDDKTIASFQQQLSDMGYKFQFITLAGIHSMWF
NMFDLANAYAQGEGMKHYVEKVQQPEFAAAKDGYTFVSHQQEVGTGYFDKVTTIIQGGTS
SVTALTGSTEESQF
>LFGLNPFC_03885 Malate synthase A
MTEQATTIDELAFTRPYGEQEKQILTAEAVEFLTELVTHFTPQRNKLLAARIQQQQDIDN
GMLPDFISETASIRDADWKIRGIPSDLEDRRVEITGPVERKMVINALNANVKVFMADFED
SLAPDWNKVIDGQINLRDAVNGTISYSNEAGKIYQLKPNPAVLICRVRGLHLPEKHVTWR
GETIPGSLFDFALYFFHNYQALLAKGSGPYFYLPKTQSWQEAAWWSEVFSYAEDRFNLPR
GTIKATLLIETLPAVFQMDEILHALRDHIVGLNCGRWDYIFSYIKTLKNYPDRVLPDRQA
VTMDKPFLNAYSRLLIKTCHKRGAFAMGGMAAFIPSKDEERNNQVLDKVKADKSLEANNG
HDGTWIAHPGLADTAMAVFNDILGSRKNQLEVMREQDAPITADQLLAPCDGERTEEGMRA
NIRVAVQYIEAWISGNGCVPIYGLMEDAATAEISRTSIWQWIHHQKTLSNGKPVTKALFR
QMLGEEMKVIASELGEERFSHGRFDDAARLMEQITTSDELIDFLTLPGYRLLA
>LFGLNPFC_03886 Homoserine O-succinyltransferase
MPIRVPDELPAVNFLREENVFVMTTSRASGQEIRPLKVLILNLMPKKIETENQFLRLLSN
SPLQVDIQLLRIDSRESRNTPAEHLNNFYCNFEDIQEQNFDGLIVTGAPLGLVEFNDVAY
WPQIKQVLEWSKDHVTSTLFVCWAVQAALNILYGIPKQTRTDKLSGVYEHHILHPHALLT
RGFDDSFLAPHSRYADFPAALIRDYTDLEILAETEEGDAYLFASKDKRIAFVTGHPEYDA
QTLAQEYFRDVEAGLDPDVPYNYFPHNDPQNTPRASWRSHGNLLFTNWLNYYVYQITPYD
LRHMNPTLD
>LFGLNPFC_03887 Peptidyl-lysine N-acetyltransferase YjaB
MVISIRRSRHEEGEELVAIWCRSVDATHDFLSAEYRAELEELVHSFLPEAPLWVAVNERD
RPVGFMLLSGQHMDALFIDPDVRGCGVGRMLVKHALSMAPELTTNVNEQNEQAVGFYKKV
GFKVTGRSEVDDLGKPYPLLNLAYVGV
>LFGLNPFC_03888 hypothetical protein
MSVLYIQIRRNQITVRDLESKREVSGDAAFSNQRLLIANFFVAEKVLQDLVLQLHPRSTW
HSFLPAKRMDIVVSALEMNEGGLSQVEERILHEVVAGATLMKYRQFHIHAQSVVLSDSAV
LAMFKQK
>LFGLNPFC_03893 Bifunctional purine biosynthesis protein PurH
MQQRRPVRRALLSVSDKAGIVEFAQALSARGVELLSTGGTARLLAEKGLPVTEVSDYTGF
PEMMDGRVKTLHPKVHGGILGRRGQDDTIMEEHQIQPIDMVVVNLYPFAQTVAREGCSLE
DAVENIDIGGPTMVRSAAKNHKDVAIVVKSSDYDAIIKEIDANEGSLTLETRFDLAIKAF
EHTAAYDSMIANYFGSMVPAYHGESKEAAGRFPRTLNLNFIKKQDMRYGENSHQQAAFYI
EENVKEASVATATQVQGKALSYNNIADTDAALECVKEFAEPACVIVKHANPCGVAIGNSI
LDAYDRAYKTDPTSAFGGIIAFNRELDAETAQAIISRQFVEVIIAPSASEEALKITAAKQ
NVRVLTCGQWGERVPGLDFKRVNGGLLVQDRDLGMVGAEELRVVTKRQPTEQELRDALFC
WKVAKFVKSNAIVYAKNNMTIGIGAGQMSRVYSAKIAGIKAADEGLEVKGSSMASDAFFP
FRDGIDAAAAAGVTCVIQPGGSIRDDEVIAAADEHGIAMLFTDMRHFRH
>LFGLNPFC_03894 Phosphoribosylamine--glycine ligase
MKVLVIGNGGREHALAWKAAQSPLVETVFVAPGNAGTALEPALQNVAIGVTDIPALLDFA
QNEKVDLTIVGPEAPLVKGVVDTFRAAGLKIFGPTAGAAQLEGSKAFTKDFLARHNIPTA
EYQNFTEVEPALAYLREKGAPIVIKADGLAAGKGVIVAMTLEEAEAAVRDMLAGNAFGDA
GHRIVIEEFLDGEEASFIVMVDGEHVLPMATSQDHKRVGDKDTGPNTGGMGAYSPAPVVT
DEVHQRTMERIIWPTVKGMAAEGNTYTGFLYAGLMIDKQGNPKVIEFNCRFGDPETQPIM
LRMKSDLVELCLAACEGKLDEKTSEWDERASLGVVMAAGGYPGDYRTGDVIHGLPLEEVA
DGKVFHAGTKLADDEQVVTSGGRVLCVTALGHTVAEAQKRAYALMTDIHWDDCFCRKDIG
WRAIEREQN
>LFGLNPFC_03895 Transcriptional regulatory protein ZraR
MFGHEKGAFTGADKRREGRFVEADGGTLFLDEIGDISPMMQVRLLRAIQEREVQRVGSNQ
TISVDVRLIAATHRDLAAEVNAGRFRQDLYYRLNVVAIEVPSLRQRREDIPLLAGHFLQR
FAERNRKAVKGFTPQAMDLLIHYDWPGNIRELENAVERAVVLLTGEYISERELPLAIAST
PIPLVQSQDIQPLVEVEKEVILAALEKTGGNKTEAARQLGITRKTLLAKLSR
>LFGLNPFC_03896 Transcriptional regulatory protein ZraR
MTHDNIDILVVDDDISHCTILQALLRGWGYNVALANSGRQALEQVREQVFDLVLCDVRMA
EMDGIATLKEIKTLNPAIPVLIMTAYSSIETAVEALKTGALDYLIKPLDFDNLQSTLEKA
LAHTHSVDAETPAVSASQFGMVGKSPAMQHLLSEIALVAPSEATVLIHGDSGTGKELVAR
AIHASSARSENRW
>LFGLNPFC_03897 Sensor protein ZraS
MRFMQRSKDSLAKWLSAILPVVIVGLVGLFAVTVIRDYGRETAAARQTLLEKGSVLIRAL

ESGSRVGMGMRMHHAQQQALLEEMAGQPGVRWFAVTDEQGTIVMHSNSGMVGKQLYSPQE
MQQLHPGNEEAWRRIDSADGEPVLEIYRQFQPMFATGMHRMRHMQQYAATPQAIFIAFDA
SNIVSAEDREQRNTLIILFALATVLLASVLSFFWYRRYLRSRQLLQDEMKRKEKLVALGH
LAAGVAHEIRNPLSSIKGLAKYFAERAPAGGEAHQLAQVMAKEADRLNRVVSELLELVKP
THLALQAVELNTLINHSLQLVSQDANSREIQLRFTANDTLPEIQADPDRLTQVLLNLYLN
AIQAIGQHGVISVTASESGAGVKISVTDSGKGIAADQLEAIFTPYFTTKAEGTGLGLAVV
HNIVEQHGGTIQVASQEGKGATFTLWLPVNITRKDPQG
>LFGLNPFC_03898 hypothetical protein
MTRYPQQASHFYSFIMPDYPSYQRFAVGTEDAIPEVRQPLED
>LFGLNPFC_03899 Zinc resistance-associated protein
MKRNTKIALVMMALSAMAMGSTSAFAHGGHGMWQQNAAPLTSEQQTAWQKIHNDFYAQSS
ALQQQLVTKRYEYNALLAANPPDSSKINALAKEMENLRQSLDELRVKRDIAMAEAGIPRG
TGMGYGGCGGGGHMGMGHW
>LFGLNPFC_03900 hypothetical protein
MLAGALFLTACSHNSSLPPFTASGFAEDQGAVRIWRKDSGDNVHLLAVFSPWRSGDTTTR
EYRWQGDNLTLININVYSKPPVNIRARFDDRGDLSFMQRESDGEKQQLSNDQIDLYRYRA
DQIRQISDALRQGRVVLRQGRWHAMEQTVTTCEGQTIKPDLDSQAIAHIERRQSRSSVDV
SVAWLEAPEGSQLLLVANSDFCRWQPNEKTF
>LFGLNPFC_03901 DNA-binding protein HU-alpha
MNKTQLIDVIAEKAELSKTQAKAALESTLAAITESLKEGDAVQLVGFGTFKVNHRAERTG
RNPQTGKEIKIAAANVPAFVSGKALKDAVK
>LFGLNPFC_03902 putative protein
MLQNPIHLRLERLESWQHVTFMACLCERMYPNYAVFCQQTGFGDGQIYRRILDLIWETLT
VKDAKVNFDSQLEKFEEAIPSADDFDLYGVYPAIDACVALSELVHSRLSGETLEHAVEVS
KTSITTVAMLEMTQAGREMSDEELKKNPAVEQEWDIQWEIFRLLAECEERDIELIKGLRA
DLREAGESNIGIIFQQ
>LFGLNPFC_03903 Endonuclease V
MDLASLRAQQIELASSVIREDRLDKDPPDLIAGADVGFEQGGEVTRAAMVLLKYPSLELV
EYKVARIATTMPYIPGFLSFREYPALLAAWEMLSQKPDLVFVDGHGISHPRRLGVASHFG
LLVDVPTIGVAKKRLCGKFEPLSSEPGALAPLMDKGEQLAWVWRSKARCNPLFIATGHRV
SVDSALAWVQRCMKGYRLPEPTRWADAVASERPAFVRYTANQP
>LFGLNPFC_03904 Uroporphyrinogen decarboxylase
MTELKNDRYLRALLRQPVDVTPVWMMRQAGRYLPEYKATRAQAGDFMSLCKNAELACEVT
LQPLRRYPLDAAILFSDILTVPDAMGLGLYFEAGEGPRFTSPVTCKADVDKLPIPDPEDE
LGYVMNAVRTIRRELKGEVPLIGFSGSPWTLATYMVEGGSSKAFTVIKKMMYADPQALHA
LLDKLAKSVTLYLNAQIKAGAQAVMIFDTWGGVLTGRDYQQFSLYYMHKIVDGLLRENDG
RRVPVTLFTKGGGQWLEAMAETGCDALGLDWTTDIADARRRVGNKVALQGNMDPSMLYAP
PARIEEEVASILAGFGHGEGHVFNLGHGIHQDVPPEHAGVFVEAVHRLSEQYHR
>LFGLNPFC_03905 NADH pyrophosphatase
MDRIIEKLDHGWWVVSHEQKLWLPKGELPYGEAANFDLVGQRALQIGEWQGEPVWLVQLQ
RRHDMGSVRQVIDLDVGLFQLAGRGVQLAEFYRSHKYCGYCGHEMYPSKTEWAMLCSHCR
ERYYPQIAPCIIVAIRRDDSILLAQHTRHRNGVHTVLAGFVEVGETLEQAVAREVMEESG
IKVKNLRYVTSQPWPFPQSLMTAFMAEYDSGEIVIDPKELLEAHWYRYDDLPLLPPPGTV
ARRLIEDTVAMCRAEYE
>LFGLNPFC_03906 Regulator of sigma D
MLNQLDNLTERVRGSNKLVDRWLHVRKHLLVAYYNLVGIKPGKESYMRLNEKALDDFCQS
LVDYLSAGHFSIYERILHKLEGNGQLARAAKIWPQLEANTQQIMDDYDSSLETAIDHDNY
LEFQQVLSDIGEALEARFVLEDKLILLVLDAARVKYPA
>LFGLNPFC_03907 Phosphomethylpyrimidine synthase
MSATKLTRREQRAQAQHFIDTLEGSAFPNSKRIYITGTHPGVRVPMREIQLSPTLIGGSK
EQPQYEENEAITVYDTSGPYGDPQIAINVQQGLAKLRQPWIDARGDTEELTVRSSDYTKA
RLADDGLDELRFSGVLTPKRAKAGHRVTQLHYARKGIITPEMEFIAIRENMGRERIRSEV
LRHQHPGMSFGARLPENITAEFVRDEVAAGRAIIPANINHPESEPMIIGRNFLVKVNANI
GNSAVTSSIEEEVEKLVWSTRWGADTVMDLSTGRYIHETREWILRNSPVPIGTVPIYQAL
EKVNGIAEDLTWEVFRDTLLEQAEQGVDYFTIHAGVLLRYVPMTAKRLTGIVSRGGSIMA
KWCLSHHQENFLYQRFREICEICAAYDVSLSLGDGLRPGSIQDANDEAQFAELHTLGELT
KIAWEYDVQVMIEGPGHVPMQMIRRNMTEELEHCHEAPFYTLGPLTTDIAPGYDHFTSGI
GAAMIGWFGCAMLCYVTPKEHLGLPNKEDVKQGLITYKIAAHAADLAKGHPGAQIRDNAM
SKARFEFRWEDQFNLALDPFTARAYHDETLPQESGKVAHFCSMCGPKFCSMKISQEVRDY
AAAQTIEVGMADMSENFRARGGEIYLRKEEA
>LFGLNPFC_03908 Thiamine-phosphate synthase
MYQPEFPPVPFRLGLYPVVDSVQWIERLLDAGVRTLQLRIKDRRDEEVEADVVAAIALGR
RYNARLFINDYWRLAIKHQAYGVHLGQEDLQATDLSAIRAAGLRLGVSTHDDMEIDVALA
ARPSYIALGHVFPTQTKQMPSAPQGLEQLARHVERLADYPTVAIGGISLARAPAVIATGV

GSIAVVSAITQAADWRLATAQLLEIAGVGDE
>LFGLNPFC_03909 Sulfur carrier protein ThiS adenylyltransferase
MNDRDFMRYSRQILLDDIALDGQQKLLDSQVLIIGLGGLGTPAALYLAGAGVGTLVLADD
DDVHLSNLQRQILFTTEDIDRPKSQVSQQRLTQLNPDIQLMALQQRLTGETLKDAVAQAD
VVLDCTDNMATRQEINATCVALNTPLITASAVGFGGQLMVLTPPWEQGCYRCLWPDNQEP
ERNCRTAGVVGPVVGVMGTLQALEAIKLLSGIETPAGELRLFDGKSSQWRSLALRRASGC
PVCGGSNADPV
>LFGLNPFC_03910 Sulfur carrier protein ThiS
MQILFNDQPMQCAAGQTVHELLEQLDQQQAGAALAINQQIVPREQWAQHIVQDGDQILLF
QVIAGG
>LFGLNPFC_03911 Thiazole synthase
MLRIADKTFDSHLFTGTGKFASSQLMMEAIRACGSQLVTLAMKRVNLRQHNDAILEPLIA
AGVTLLPNTSGAKTAEEAIFAAHLAREALGTNWLKLEIHPDARWLLPDPIETLKAAEKLV
QQGFVVLPYCGADPVLCKRLEEVGCAAVMPLGAPIGSNQGLETRAMLEIIIQQATVPVVV
DAGIGVPSHAAQALEMGADAVLVNTAIAVADDPVNMAKAFRLAVEAGLLARQSGPGSRSH
FAHATSPLTGFLEASE
>LFGLNPFC_03912 2-iminoacetate synthase
MKTFSDRWRQLDWDDIRLRINGKTAADVERALNASQLTRDDMMALLSPAASGYLEPLAQR
AQRLTRQRFGNVVSFYVPLYLSNLCANDCTYCGFSMSNRIKRKTLDEADIARESAAIREM
GFEHLLLVTGEHQAKVGMDYFRRHLPALREQFSSLQMEVQPLAEAEYAELKQLGLDGVMV
YQETYHEATYAHHHLKGKKQDFFWRLETPDRLGRAGIDKIGLGALIGLSDSWRVDCYMVV
EHLLWLQQHYWQSRYSVSFPRLRPCTGGIEPASIMDERQLVQAICAFRLLAPEIELSLST
RESPWFRDRVIPLAINNVSAFSKTQPGGYADNHPELEQFSPHDDRRPEAVAAALTAQGLQ
PVWKDWDSYLGRPSQRP
>LFGLNPFC_03913 DNA-directed RNA polymerase subunit beta'
MKDLLKFLKAQTKTEEFDAIKIALASPDMIRSWSFGEVKKPETINYRTFKPERDGLFCAR
IFGPVKDYECLCGKYKRLKHRGVICEKCGVEVTQTKVRRERMGHIELASPTAHIWFLKSL
PSRIGLLLDMPLRDIERVLYFESYVVIEGGMTNLERQQILTEEQYLDALEEFGDEFDAKM
GAEAIQALLKSMDLEQECEQLREELNETNSETKRKKLTKRIKLLEAFVQSGNKPEWMILT
VLPVLPPDLRPLVPLDGGRFATSDLNDLYRRVINRNNRLKRLLDLAAPDIIVRNEKRMLQ
EAVDALLDNGRRGRAITGSNKRPLKSLADMIKGKQGRFRQNLLGKRVDYSGRSVITVGPY
LRLHQCGLPKKMALELFKPFIYGKLELRGLATTIKAAKKMVEREEAVVWDILDEVIREHP
VLLNRAPTLHRLGIQAFEPVLIEGKAIQLHPLVCAAYNADFDGDQMAVHVPLTLEAQLEA
RALMMSTNNILSPANGEPIIVPSQDVVLGLYYMTRDCVNAKGEGMVLTGPKEAERLYRSG
LASLHARVKVRITEYEKDANGELVAKTSLKDTTVGRAILWMIVPKGLPYTIVNQALGKKA
ISKMLNTCYRILGLKPTVIFADQIMYTGFAYAARSGASVGIDDMVIPEKKHEIISEAEAE
VAEIQEQFQSGLVTAGERYNKVIDIWAAANDRVSKAMMDNLQTETVINRDGQEEKQVSFN
SIYMMADSGARGSAAQIRQLAGMRGLMAKPDGSIIETPITANFREGLNVLQYFISTHGAR
KGLADTALKTANSGYLTRRLVDVAQDLVVTEDDCGTHEGIMMTPVIEGGDVKEPLRDRVL
GRVTAEDVLKPGTADILVPRNTLLHEQWCDLLEENSVDAVKVRSVVSCDTDFGVCAHCYG
RDLARGHIINKGEAIGVIAAQSIGEPGTQLTMRTFHIGGAASRAAAESSIQVKNKGSIKL
SNVKSVVNSSGKLVITSRNTELKLIDEFGRTKESYKVPYGAVLAKGDGEQVAGGETVANW
DPHTMPVITEVSGFVRFTDMIDGQTITRQTDELTGLSSLVVLDSAERTAGGKDLRPALKI
VDAQGNDVLIPGTDMPAQYFLPGKAIVQLEDGVQISSGDTLARIPQESGGTKDITGGLPR
VADLFEARRPKEPAILAEISGIVSFGKETKGKRRLVITPVDGSDPYEEMIPKWRQLNVFE
GERVERGDVISDGPEAPHDILRLRGVHAVTRYIVNEVQDVYRLQGVKINDKHIEVIVRQM
LRKATIVNAGSSDFLEGEQVEYSRVKIANRELEANGKVGATYSRDLLGITKASLATESFI
SAASFQETTRVLTEAAVAGKRDELRGLKENVIVGRLIPAGTGYAYHQDRMRRRAAGEAPA
APQVTAEDASASLAELLNAGLGGSDNE
>LFGLNPFC_03914 DNA-directed RNA polymerase subunit beta
MVYSYTEKKRIRKDFGKRPQVLDVPYLLSIQLDSFQKFIEQDPEGQYGLEAAFRSVFPIQ
SYSGNSELQYVSYRLGEPVFDVQECQIRGVTYSAPLRVKLRLVIYEREAPEGTVKDIKEQ
EVYMGEIPLMTDNGTFVINGTERVIVSQLHRSPGVFFDSDKGKTHSSGKVLYNARIIPYR
GSWLDFEFDPKDNLFVRIDRRRKLPATIILRALNYTTEQILDLFFEKVIFEIRDNKLQME
LVPERLRGETASFDIEANGKVYVEKGRRITARHIRQLEKDDVKLIEVPVEYIAGKVVAKD
YIDESTGELICAANMELSLDLLAKLSQSGHKRIETLFTNDLDHGPYISETLRVDPTNDRL
SALVEIYRMMRPGEPPTREAAESLFENLFFSEDRYDLSAVGRMKFNRSLLREEIEGSGIL
SKDDIIDVMKKLIDIRNGKGEVDDIDHLGNRRIRSVGEMAENQFRVGLVRVERAVKERLS
LGDLDTLMPQDMINAKPISAAVKEFFGSSQLSQFMDQNNPLSEITHKRRISALGPGGLTR
ERAGFEVRDVHPTHYGRVCPIETPEGPNIGLINSLSVYAQTNEYGFLETPYRKVTDGVVT
DEIHYLSAIEEGNYVIAQANSNLDEEGHFVEDLVTCRSKGESSLFSRDQVDYMDVSTQQV
VSVGASLIPFLEHDDANRALMGANMQRQAVPTLRADKPLVGTGMERAVAVDSGVTAVAKR
GGVVQYVDASRIVIKVNEDEMYPGEAGIDIYNLTKYTRSNQNTCINQMPCVSLGEPVERG
DVLADGPSTDLGELALGQNMRVAFMPWNGYNFEDSILVSERVVQEDRFTTIHIQELACVS

RDTKLGPEEITADIPNVGEAALSKLDESGIVYIGAEVTGGDILVGKVTPKGETQLTPEEK
LLRAIFGEKASDVKDSSLRVPNGVSGTVIDVQVFTRDGVEKDKRALEIEEMQLKQAKKDL
SEELQILEAGLFSRIRAVLVAGGVEAEKLDKLPRDRWLELGLTDEEKQNQLEQLAEQYDE
LKHEFEKKLEAKRRKITQGDDLAPGVLKIVKVYLAVKRRIQPGDKMAGRHGNKGVISKIN
PIEDMPYDENGTPVDIVLNPLGVPSRMNIGQILETHLGMAAKGIGDKINAMLKQQQEVAK
LREFIQRAYDLGADVRQKVDLSTFSDEEVMRLAENLRKGMPIATPVFDGAKEAEIKELLK
LGDLPTSGQIRLYDGRTGEQFERPVTVGYMYMLKLNHLVDDKMHARSTGSYSLVTQQPLG
GKAQFGGQRFGEMEVWALEAYGAAYTLQEMLTVKSDDVNGRTKMYKNIVDGNHQMEPGMP
ESFNVLLKEIRSLGINIELEDE
>LFGLNPFC_03915 50S ribosomal protein L7/L12
MSITKDQIIEAVAAMSVMDVVELISAMEEKFGVSAAAAVAVAAGPVEAAEEKTEFDVILK
AAGANKVAVIKAVRGATGLGLKEAKDLVESAPAALKEGVSKDDAEALKKALEEAGAEVEV
K
>LFGLNPFC_03916 50S ribosomal protein L10
MALNLQDKQAIVAEVSEVAKGALSAVVADSRGVTVDKMTELRKAGREAGVYMRVVRNTLL
RRAVEGTPFECLKDAFVGPTLIAYSMEHPGAAARLFKEFAKANAKFEVKAAAFEGELIPA
SQIDRLATLPTYEEAIARLMATMKEASAGKLVRTLAAVRDAKEAA
>LFGLNPFC_03917 50S ribosomal protein L1
MAKLTKRMRVIREKVDATKQYDINEAIALLKELATAKFVESVDVAVNLGIDARKSDQNVR
GATVLPHGTGRSVRVAVFTQGANAEAAKAAGAELVGMEDLADQIKKGEMNFDVVIASPDA
MRVVGQLGQVLGPRGLMPNPKVGTVTPNVAEAVKNAKAGQVRYRNDKNGIIHTTIGKVDF
DADKLKENLEALLVALKKAKPTQAKGVYIKKVSISTTMGAGVAVDQAGLSASVN
>LFGLNPFC_03918 50S ribosomal protein L11
MAKKVQAYVKLQVAAGMANPSPPVGPALGQQGVNIMEFCKAFNAKTDSIEKGLPIPVVIT
VYADRSFTFVTKTPPAAVLLKKAAGIKSGSGKPNKDKVGKISRAQLQEIAQTKAADMTGA
DIEAMTRSIEGTARSMGLVVED
>LFGLNPFC_03919 Transcription termination/antitermination protein NusG
MSEAPKKRWYVVQAFSGFEGRVATSLREHIKLHNMEELFGEVMVPTEEVVEIRGGQRRKS
ERKFFPGYVLVQMVMNDASWHLVRSVPRVMGFIGGTSDRPAPISDKEVDAIMNRLQQVGD
KPRPKTLFEPGEMVRVNDGPFADFNGVVEEVDYEKSRLKVSVSIFGRATPVELDFSQVEK
A
>LFGLNPFC_03920 Protein translocase subunit SecE
MSANTEAQGSGRGLEAMKWVVVVALLLVAIVGNYLYRDIMLPLRALAVVILIAAAGGVAL
LTTKGKATVAFAREARTEVRKVIWPTRQETLHTTLIVAAVTAVMSLILWGLDGILVRLVS
FITGLRF
>LFGLNPFC_03921 Elongation factor Tu 2
MSKEKFERTKPHVNVGTIGHVDHGKTTLTAAITTVLAKTYGGAARAFDQIDNAPEEKARG
ITINTSHVEYDTPTRHYAHVDCPGHADYVKNMITGAAQMDGAILVVAATDGPMPQTREHI
LLGRQVGVPYIIVFLNKCDMVDDEELLELVEMEVRELLSQYDFPGDDTPIVRGSALKALE
GDAEWEAKILELAGFLDSYIPEPERAIDKPFLLPIEDVFSISGRGTVVTGRVERGIIKVG
EEVEIVGIKETQKSTCTGVEMFRKLLDEGRAGENVGVLLRGIKREEIERGQVLAKPGTIK
PHTKFESEVYILSKDEGGRHTPFFKGYRPQFYFRTTDVTGTIELPEGVEMVMPGDNIKMV
VTLIHPIAMDDGLRFAIREGGRTVGAGVVAKVLS
>LFGLNPFC_03926 Pantothenate kinase
MTPYLQFDRNQWAALRDSVPMTLSEDEIARLKGINEDLSLEEVAEIYLPLSRLLNFYISS
NLRRQAVLEQFLGTNGQRIPYIISIAGSVAVGKSTTARVLQALLSRWPEHRRVELITTDG
FLHPNQVLKERGLMKKKGFPESYDMHRLVKFVSDLKSGVPNVTAPVYSHLIYDVIPDGDK
TVVQPDILILEGLNVLQSGMDYPHDPHHVFVSDFVDFSIYVDAPEDLLQTWYINRFLKFR
EGAFTDPDSYFHNYAKLTKEEAIKTAMTLWKEINWLNLKQNILPTRERASLILTKSANHA
VEEVRLRK
>LFGLNPFC_03927 Bifunctional ligase/repressor BirA
MKDNTVPLKLIALLANGEFHSGEQLGETLGMSRAAINKHIQTLRDWGVDVFTVPGKGYSL
PEPIQLLNAEKILSQLDDGSVAVLPVIDSTNQYLLDRIGELKSGDACVAEYQHAGRGRRG
RKWFSPFGANLYLSMFWRLEQGPAAAIGLSLVIGIVMAEVLRKLGADKVRVKWPNDLYLQ
DRKLAGILVELTGKTGDAAQIVIGAGINMAMRRVEESVVNQGWITLQEAGINLDRNTLAA
MLIRELRAALELFEQEGLTPYLSRWEKLDNFINRPVKLIIGDKEIFGISRGIDKQGALLL
EQDGIIKPWMGGEISLRSAEK
>LFGLNPFC_03928 UDP-N-acetylenolpyruvoylglucosamine reductase
MNHSLKPWNTFGIDHNAQHIVCAEDEQQLLNAWQHATAEGQPVLILGEGSNVLFLEDYRG
TVIINRIKGIEIHDEPDAWYLHVGAGENWHRLVKYTLQEGMPGLENLALIPGCIGSSPIQ
NIGAYGVELQRVCAYVDCVELATGKQVRLTAKECRFGYRDSIFKHEYQDRFAIVAVGLRL
PKEWQPVLTYGDLTRLDPSTVTPQQVFDAVCHMRTTKLPDPKVNGNAGSFFKNPVVSAET
ANALLAQFPTAPHYPQVDGSVKLAAGWLIDQCQLKGTQIGGAAVHRQQALVLINEHDAKS
EDVVQLAHHVRQKVGEKFNVWLEPEVRFIGASGEVSAVETIS

>LFGLNPFC_03933 Glutamate racemase
MATKLQDGNTPCLAATPSEPRPTVLVFDSGVGGLSVYDEIRHLLPDLHYIYAFDNVAFPY
GEKSEVFIVERVVEIVTAVQERYPLALAVVACNTASTVSLPALREKFDFPVVGVVPAIKP
AARLTANGIVGLLATRGTVKRSYTHELIARFANECQIEMLGSAEMVELAEAKLHGEDVSL
DALKRILRPWLRMKEPPDTVVLGCTHFPLLQEELLQVLPEGTRLVDSGAAIARRTAWLLE
HEAPDAKSADANIAFCMAMTPEAEQLLPVLQRYGFETLEKLAVLG
>LFGLNPFC_03934 Vitamin B12 transporter BtuB
MIKKASLLTACSVTAFSAWAQDTSPDTLVVTANRFEQPRSTVLAPTTVVTRQDIDRWQST
SVNDVLRRLPGVDITQNGGSGQLSSIFIRGTNASHVLVLIDGVRLNLAGVSGSADLSQFP
IALVQRVEYIRGPRSAVYGSDAIGGVVNIITTRDHPGTEISAGWGSNSYQNYDVSTQQQL
GDKTRVTLLGDYAHTHGYDVVAYGNTGTQAQPDNDGFLSKTLYGALEHNFTDVWSGFVRG
YGYDNRTNYDAYYSPGLPLVDTRKLYSQSWDAGLRYNGELIKSQLITSYSHSKDYNYDPH
YGRYDSSATLDEMKQYTVQWANNIIIGHGNIGAGVDWQKQSTAPGTAYVEDGYDQRNTGI
YLTGLQQVGDFTFEGAGRSDDNSQFGRHGTWQTSAGWEFIEGYRFIASYGTSYKAPNLGQ
LYGTYGNPNLNPEKSKQWEGAFEGLTAGVNWRISGYRNDVSDLIDYDDHTLKYYNEGKAR
IKGVEATANFDTGPLTHTVSYDYVDARNAITDTPLLRRAKQQVKYQLDWQLYDFDWGITY
QYLGTRYDKDYSSYPYQTVKMGGVSLWDLAVAYPVTSHLTVRGKIANLFDKDYETVYGYQ
TAGREYTLSGSYTF
>LFGLNPFC_03935 tRNA/tmRNA (uracil-C(5))-methyltransferase
MTPEHLPTEQYEAQLAEKVVRLQSMMAPFSDLVPEVFRSPVSHYRMRAEFRIWHDGDDLY
HIIFDQQTKSRIRVDSFPAASELINQLMTAMIAGVRNNPILRHKLFQIDYLTTLSNQAVV
SLLYHKKLDDEWRQQAEALRDALRAQNLNVHLIGRATKTKIALDQDYIDERLPIAGKEMI
YRQVENSFTQPNAAMNIQMLEWALDVTKGSKGDLLELYCGNGNFSLALARNFDRVLATEI
AKPSVAAAQYNIAANHIDNVQIIRMAAEEFTQAMNGVREFNRLQGIDLKSYQCETIFVDP
PRSGLDSETEKMVQAYPRILYISCNPETLCKNLETLSQTHKVERLALFDQFPYTHHMECG
VLLTAK
>LFGLNPFC_03936 Inner membrane protein YijD
MKQANQDRGTLLLALVAGLSINGTFAALFSSIVPFSVFPIISLVLTVYCLHQRYLNRTMP
VGLPGLAAACFILGVLLYSTVVRAEYPDIGSNFFPAVLSVIMVFWIGAKMRNRKQEVAE
>LFGLNPFC_03937 HTH-type transcriptional repressor FabR
MFILWYSASSTFGKDSDIVMGVRAQQKEKTRRSLVEAAFSQLSAERSFASLSLREVAREA
GIAPTSFYRHFRDVDELGLTMVDESGLMLRQLMRQARQRIAKGGSVIRTSVSTFMEFIGN
NPNAFRLLLRERSGTSAAFRAAVAREIQHFIAELADYLELENHMPRAFTEAQAEAMVTIV
FSAGAEALDVGVEQRRQLEERLVLQLRMISKGAYYWYRREQEKTTIIPGNVKDE
>LFGLNPFC_03938 Alpha-ketoglutarate permease
MTAIVNETVSVKKKPSGRRVIFASAFGNALEFFDFGVYNFFVVYISTLFFPPSADHNVAL
LLAFATFGVSFFMRPLGGIIVGAWADRFGRKPAMVFTIALMSLGTLMIGIAPTYETAGYW
GTATLVLARLIQGVAAGGEVGASMSLLVESAPANRRGFYSSWSLATQGLATTFGGVVALG
LSAWLPFATGSETVMAEWGWRVPFFIGVLLAPIGCWLRLSLENDVPEPVRNKKAATSESA
FSLLLQHKATIVNGVLLAIGSTVATYISLFYYGTWAAKYLAMPQHYSHAAMLLAGVITFV
GALLVGMLCDSVGRKKLILISRVMVLLCSWPSFWLLVNYPSPGMLLTVVFVMVSFTTLGG
VPVMLLISELLPKRIRALGFALVYSIGVAIFGGFAQYFATQSIVLLDSLTAPAWYLGGGT
LLSMLALLYVKEPAKELQ
>LFGLNPFC_03939 Hippurate hydrolase
MIEHYIRGFEEELREIRHQIHENPELGLQEFKTSALVAEKLRQWGYEVEQGLATTGVVAT
LKVGDGEKSIGLRADMDALPIYENSGKPWASKHPGLMHACGHDGHTTILLGAARYFAETR
RFNGTLRLIFQPAEEMINGGEIMVKEGLFDRFPCDVIFGMHNMPGLPVGKFFFQPGALMA
SMDQFHITVRGCGGHGAIPHKAIDPVLVAAHITTALQSIVSRNVDPLEAAVITVGSIVAG
EAANVIPDSAEMKISVRSLSRDTRQLLLTRIPALAQAQAASFGATAEVTHVNGTPVLVND
EEMARFAWQVACKTFGEDRAEFGIKPLMGSEDFSFMLEAQPKGGFLLFGNGDVGEGSCMV
HNPGYDFNDASLVPASSYWGALVEAWLQ
>LFGLNPFC_03940 Soluble pyridine nucleotide transhydrogenase
MPHSYDYDAIVIGSGPGGEGAAMGLVKQGARVAVIERYQNVGGGCTHWGTIPSKALRHAV
SRIIEFNQNPLYSDHSRLLRSSFADILNHADNVINQQTRMRQGFYERNHCEILQGNARFV
DEHTLALDCPDGSVETLTAEKFVIACGSRPYHPTDVDFTHPRIYDSDSILSMHHEPRHVL
IYGAGVIGCEYASIFRGMDVKVDLINTRDRLLAFLDQEMSDSLSYHFWNSGVVIRHNEEY
EKIEGCDDGVIMHLKSGKKLKADCLLYANGRTGNTDSLALQNIGLETDSRGQLKVNSMYQ
TAQPHVYAVGDVIGYPSLASAAYDQGRIAAQALVKGEATAHLIEDIPTGIYTIPEISSVG
KTEQQLTAMKVPYEVGRAQFKHLARAQIVGMNVGTLKILFHRETKEILGIHCFGERAAEI
IHIGQAIMEQKGGGNTIEYFVNTTFNYPTMAEAYRVAALNGLNRLF
>LFGLNPFC_03941 Hydrogen peroxide-inducible genes activator
MNIRDLEYLVALAEHRHFRRAADSCHVSQPTLSGQIRKLEDELGVMLLERTSRKVLFTQA
GMLLVDQARTVLREVKVLKEMASQQGETMSGPLHIGLIPTVGPYLLPHIIPMLHQTFPKL
EMYLHEAQTHQLLAQLDSGKLDCVILALVKESEAFIEVPLFDEPMLLAIYEDHPWANREC

VPMADLAGEKLLMLEDGHCLRDQAMGFCFEAGADEDTHFRATSLETLRNMVAAGSGITLL
PALAVPPERKRDGVVYLPCIKPEPRRTIGLVYRPGSPLRSRYEQLAEAIRARMDGHFDKV
LKQAV
>LFGLNPFC_03942 putative L-galactonate transporter
MDVDVSTSVAGNKPQRIRRIQTVTLVLLFMAGIVNFLDRSSLSVAGEAIRGELGLSATEF
GVLLSAFSLSYGFSQLPSGILLDRFGPRIVLGAGLIFWSLMQALTGMVNSFSHFILMRIG
LGIGEAPFMPAGVKSITDWYAQKERGTALGIFNSSTVIGQAIAPPALVLMQLAWGWRTMF
VIIGVAGILVGICWYAWYRNRAQFVLTEEERTYLSAPVKPRPQLQFSEWLALFKHRTTWG
MILGFSGVNYTGWLYIAWLPGYLQAEQGFSLAKTGWVAAIPFLAAAVGMWVNGIVVDRLA
KKGYDLAKTRKTAIVCGLMMSALGTLLVVQSSSPAQAVAFISMALFCVHFAGTSAWGLVQ
VMVSETKVASIAGIQNFGSFVFASFAPIVTGWVVDTTHSFNLALVIAACVTFTGALCYFF
IVKDRIE
>LFGLNPFC_03943 Starvation-sensing protein RspA
MKIIAVDVFVTCPGRNFVTLKITTESGLCGLGDATLNGRELSVASYLKDHLCPQLIGRDA
SRIEDIWQFFYKGAYWRRGPVTMSAISAIDMALWDIKAKAANMPLYQLLGGASREGVMVY
CHTTGRTIDEVLEDYAKHQQMGFKAIRVQCGVPGMQTTYGLAKGKGLAYEPATKGLWPEE
QLWSSEKYLDFTPKLFEAVRNKFGFNEHLLHDMHHRLTPIEAARFGRSIEDYRLFWMEDP
TPAENQECFRLIRQHTVTPIAVGEVFNSIWDCKQLIEEQLIDYIRTTITHAGGITGMRRI
VDFASLYQVRTGSHGPSDLSPVCHAAALHFDLWVPNFGVQEYMGYSEQMFEVFPHSWRFD
EGYMHPGDEPGLGISFDEKLAAKYPYDPAYLPVARLEDGTLWNW
>LFGLNPFC_03944 Argininosuccinate lyase
MALWGGRFTQAADQRFKQFNDSLRFDYRLAEQDIVGSVAWSKALVTVGVLTAEEQAQLEE
ALNVLLEDVRARPQQILESDAEDIHSWVEGKLIDKVGQLGKKLHTGRSRNDQVATDLKLW
CKDTVSELLTANRQLQSALVETAQNNQDAVMPGYTHLQRAQPVTFAHWCLAYVEMLARDE
SRLQDALKRLDVSPLGCGALAGTAYEIDREQLAGWLGFASATRNSLDSVSDRDHVLELLS
AAAIGMVHLSRFAEDLIFFNTGEAGFVELSDRVTSGSSLMPQKKNPDALELIRGKCGRVQ
GALTGMMMTLKGLPLAYNKDMQEDKEGLFDALDTWLDCLHMAALVLDGIQVKRPRCQEAA
QQGYANATELADYLVAKGVPFREAHHIVGEAVVEAIRQGKALEDLPLSELQKFSQVIGED
VYPILSLQSCLDKRAAKGGVSPQQVAQAIAFAQARLG
>LFGLNPFC_03945 Acetylglutamate kinase
MMNPLIIKLGGVLLDSEEALERLFSALVNYRESHQRPLVIVHGGGCVVDELMKGLNLPVK
KKNGLRVTPADQIDIITGALAGTANKTLLAWAKKHQIAAVGLFLGDGDSVKVTQLDEELG
HVGLAQPGSPKLINSLLENGYLPVVSSIGVTDEGQLMNVNADQAATALAATLGADLILLS
DVSGILDGKGQRIAEMTAAKAEQLIEQGIITDGMIVKVNAALDAARTLGRPVDIASWRHA
EQLPALFNGMPMGTRILA
>LFGLNPFC_03946 N-acetyl-gamma-glutamyl-phosphate reductase
MLNTLIVGASGYAGAELVTYVNRHPHMNITALTVSAQSNDAGKLISDLHPQLKGIVDLPL
QPMSDISEFSPGVDVVFLATAHEVSHDLAPQFLEAGCVVFDLSGAFRVNDVAFYEKYYGF
THQYPELLEQAAYGLAEWCGNKLKEANLIAVPGCYPTAAQLALKPLIDADLLDLNQWPVI
NATSGVSGAGRKAAISNSFCEVSLQPYGVFTHRHQPEIATHLGADVIFTPHLGNFPRGIL
ETITCRLKPGVSQVQVAQALQQAYAHKPLMRLYDKGVPALKNVVGLPFCDIGFAVQGEHL
IIVATEDNLLKGAAAQAVQCANIRFGYAETQSLI
>LFGLNPFC_03947 Acetylornithine deacetylase
MKNKLPPFIEIYRALIATPSISATEEALDQSNADLITLLADWFKDLGFNVEVQPVPGTRN
KFNMLASCGQGAGGLLLAGHTDTVPFDDGRWTRDPFTLTEHDGKLYGLGTADMKGFFAFI
LDALRDVDVTKLAKPLYILATADEETSMAGARYFAETTALRPDCAIIGEPTSLQPVRAHK
GHISNAIRIQGQSGHSSDPARGVNAIELMHDAIGHILQLRDNLKERYHYDAFTVPYPTLN
LGHIHGGDASNRICACCELHMDIRPLPGMTLNELNGLLNDALAPVSERWPGRLTVDELHP
PIPGYECPPNHQLVEVVEKLLGAKTEVVNYCTEAPFIQTLCPTLVLGPGSINQAHQPDEY
LETRFIKPTRELITQVIHHFCWH
>LFGLNPFC_03948 Phosphoenolpyruvate carboxylase
MNEQYSALRSNVSMLGKVLGETIKDALGEHILERVETIRKLSKSSRAGNDANRQELLTTL
QNLSNDELLPVARAFSQFLNLANTAEQYHSISPKGEAASNPEVIARTLRKLKNQPELSED
TIKKAVESLSLELVLTAHPTEITRRTLIHKMVEVNACLKQLDNKDIADYEHNQLMRRLRQ
LIAQSWHTDEIRKLRPSPVDEAKWGFAVVENSLWQGVPNYLRELNEQLEENLGYKLPVEF
VPVRFTSWMGGDRDGNPNVTADITRHVLLLSRWKATDLFLKDIQVLVSELSMVEATPELL
ALVGEEGAAEPYRYLMKNLRSRLMATQAWLEARLKGEELPKPEGLLTQNEELWEPLYACY
QSLQACGMGIIANGDLLDTLRRVKCFGVPLVRIDIRQESTRHTEALGELTRYLGIGDYES
WSEADKQAFLIRELNSKRPLLPRNWQPSAETREVLDTCQVIAEAPQGSIAAYVISMAKTP
SDVLAVHLLLKEAGIGFAMPVAPLFETLDDLNNANDVMTQLLNIDWYRGLIQGKQMVMIG
YSDSAKDAGVMAASWAQYQAQDALIKTCEKAGIELTLFHGRGGSIGRGGAPAHAALLSQP
PGSLKGGLRVTEQGEMIRFKYGLPEITVSSLSLYTGAILEANLLPPPEPKESWRRIMDEL
SVISCDVYRGYVRENKDFVPYFRSATPEQELGKLPLGSRPAKRRPTGGVESLRAIPWIFA
WTQNRLMLPAWLGAGTALQKVVEDGKQNELEAMCRDWPFFSTRLGMLEMVFAKADLWLAE

YYDQRLVDKALWPLGKELRNLQEEDIKVVLAIANDSHLMADLPWIAESIQLRNIYTDPLN
VLQAELLHRSRQAEKEGGQEPDPRVEQALMVTIAGIAAGMRNTG
>LFGLNPFC_03949 Phosphoethanolamine transferase EptC
MHSTEVQAKPLFSWKALGWALLYFWFFSTLLQAIIYISGYSGTNGIRDSLLFSSLWLIPV
FLFPKRIKIIAAVIGVVLWAASLAALCYYVIYGQEFSQSVLFVMFETNTNEASEYLSQYF
SLKIVLIALAYTAVAVLLWTRLRPVYIPKPWRYVVSFALLYGLILHPIAMNTFIKNKPFE
KTLDNLASRMEPAAPWQFLTGYYQYRQQLNSLTKLLNENNALPPLANFKDESGNEPRTLV
LVIGESTQRGRMSLYGYPRETTPELDALHKTDPNLTVFNNVVTSRPYTIEILQQALTFAN
EKNPDLYLTQPSLMNMMKQAGYKTFWITNQQTMTARNTMLTVFSRQTDKQYYMNQQRTQS
AREYDTNVLKPFQEVLKDPAPKKLIIVHLLGTHIKYKYRYPEDQGKFDGNTEHVPPGLNA
EELESYNDYDNANLYNDHVVASLIKDFKATDPNGFLVYFSDHGEEVYDTPPHKTQGRNED
NPTRHMYTIPFLLWTSEKWQATHPRDFSQDVDRKYSLAELIHTWSDLAGLSYDGYDPTRS
VVNPQFKETTRWIGNPYKKNALIDYDTLPYGDQVGNQ
>LFGLNPFC_03950 HTH-type transcriptional activator RhaS
MYHDVSYLLSRLINGPLSLRQIYFASSNGPVPDLAYQVDFPRLEIVLEGEFIDTGAGAAL
VPGDVLYVPAGGWNFPQWQAPATTFSVLFGKQQLGFSVVQWDGKQYQNLAKQHVARRGPR
IGSFLLQTLNEMQMQSQEQQTARLIVASLLSHCRDLLGSQIQTASRSQALFEAIRDYIDE
RYASALTRESVAQAFYISPNYLSHLFQKTGAIGFNEYLNHTRLEHAKTLLKGYDLKVKEV
AHACGFVDSNYFCRLFRKNTERSPSEYRRQYHSQLTEKPTTPE
>LFGLNPFC_03951 PTS system fructose-like EIIB component 3
MAYLVAVTACVSGVAHTYMAAERLEKLCQLEKWGVSIETQGALGTENRLADEDIRRADVA
LLITDIELAGAERFEHCRYVQCSIYAFLREPQRVMSAVRKVLSAPQQTHLILE
>LFGLNPFC_03952 Choline trimethylamine-lyase activating enzyme
MTSSAGQRISCNVVETRRNDVARIFNIQRYSLNDGEGIRTVVFFKGCPHLCPWCANPESI
SGKIQTVRREAKCLHCAKCLRDADECPSGAFERIGRDISLDALEREVMKDDIFFRTSGGG
VTLSGGEVLMQAEFATRFLQRLRLWGVSCAIETAGDAPASKLLPLAKLCDEVLFDLKIMD
AAQARDVVKMNLQRVLENLRLLVSEGVNVIPRLPLIPGFTLSRENMQQALDVLIPLNIKQ
IHLLPFHQYGEPKYRLLGKTWSMKEVSAPSSADVATMREMAERAGFQVTVGG
>LFGLNPFC_03953 Choline trimethylamine-lyase
MTNRISRLKTALFANTREISLERALLYTASHRQTEGEPVIMRRAKATAYILEHVEISIRD
EELIAGNRTVKPRAGIMSPEMDPYWLLKELDQFPTRPQDRFAISEEDKRIYREELFPYWE
KRSMKDFINGQMTDEVKAATSTQIFSINQTDKGQGHIIIDYPRLLNHGLGELVAQMQQHC
QQQPENHFYQAALLLLEASQKHILRYAELAETMAASCTDGQRREELLTIAEISRHNAEHK
PQTFWQACQLFWYMNIILQYESNASSLSLGRFDQYMLPFYQTSLTQGEDPAFLKELLESL
WVKCNDIVLLRSTSSARYFAGFPTGYTALLGGLTENGRSAVNVLSFLCLDAYQSVQLPQP
NLGVRTNALIDTPFLMKTAETIRLGTGIPQIFNDEVVVPAFLNRGVSLEDARDYSVVGCV
ELSIPGRTYGLHDIAMFNLLKVMEICLHENEGNAALTYEGLLEQIRAKISHYITLMVEGS
NICDIGHRDWAPVPLLSSFISDCLEKGRDITDGGARYNFSGVQGIGIANLSDSLHALKGM
IFDQQRLSFDELLSVLKANFATPEGEKVRARLINRFEKYGNDIDEVDNISAELLRHYCKE
VEKYQNPRGGYFTPGSYTVSAHVPLGSVVGATPDGRFAGEQLADGGLSPMLGQDAQGPTA
VLKSVSKLDNTLLSNGTLLNVKFTPATLEGEAGLRKLADFLRAFTQLKLQHIQFNVVNAD
TLREAQQRPQDYAGLVVRVAGYSAFFVELSKEIQDDIIRRTAHQL
>LFGLNPFC_03954 PTS system fructose-like EIIB component 1
MTKIIAVTACPSGVAHTYMAAEALESAAKAKGWEVKVETQGSIGLENELTAEDVASADMV
ILTKDIGIKFEERFAGKTIVRVNISDAVKRADAIMSKIEAHLAQTA
>LFGLNPFC_03955 PTS system mannose-specific EIIBCA component
MNELVQILKNTRQHLMTGVSHMIPFVVSGGILLAVSVMLYGKGAVPDAVADPNLKKLFDI
GVAGLTLMVPFLAAYIGYSIAERSALAPCAIGAWVGNSFGAGFFGALIAGIIGGIVVHYL
KKIPVHKVLRSVMPIFIIPIVGTLITAGVMMWGLGEPVGALTNSLTQWLQGMQQGSIVML
AVIMGLMLAFDMGGPVNKVAYAFMLICVAQGVYTVVAIAAVGICVPPLGMGLATLIGRKN
FSAEERETGKAALVMGCVGVTEGAIPFAAADPLRVIPSIMVGSVCGAVTAALVGAQCYAG
WGGLIVLPVVEGKLGYIAAVAVGAVVTAVCVNVLKSLARKNGSSTEEKEDDLDLDFEIN
>LFGLNPFC_03956 Multiphosphoryl transfer protein 1
MALIVEFICELPNGVHARPASHVETLCNTFSSQIEWHNLRTDRKGNAKSALALIGTDTLV
GDNCQLLISGADEQEAYQRLSQWLRDEFPHCDAPLAEVKSDELEPLPVSLTNLNPQIIRA
RTVCSGSAGGILTPISSLDLNALGNLPAAKGVDAEQSALENGLTLVLKNIEFRLLDSDGA
TSAILEAHRSLAGDTSLREHLLAGVSAGLSCAEAIVASANHFCEEFARSSSSYLQERALD
VRDVCFQLLQQIYGEQRFPAPGKLTQPAICMADELTPSQFLELDKNHLKGLLLKSGGTTS
HTVILARSFNIPTLVGVDIAALTPWQHQTIYIDGNAGAIVVEPGESVARYYQQEARVQDA
LREQQRVWLTQQARTADGIRIEIAANIAHSVEAQAAFGNGAEGVGLFRTEMLYMDRTSAP
GESELYNIFCQALESANGRSIIVRTMDIGGDKPVDYLNIPAEANPFLGYRAVRIYEEYAS
LFTTQLRSILRASAHGSLKIMIPMISSMEEILWVKEKLAEAKQQLRNEHIPFDEKIQLGI
MLEVPSVMFIIDQCCEEIDFFSIGSNDLTQYLLAVDRDNAKVTRHYNSLNPAFLRALDYA
VQAVHRQGKWIGLCGELGAKGSVLPLLVGLGLDELSMSAPSIPAAKARMAQLDSRECRQL

LNQAMACRTSLEVEHLLAQFRMTQQDAPLVTAECITLESDWRSKEEVLKGMTDNLLLAGR
CRYPRKLEADLWAREAVFSTGLGFSFAIPHSKSEHIEQSTISVARLQAPVRWGDDEAQFI
IMLTLNKHAAGDQHMRIFSRLARRIMHEEFRNALVNAASADAIASLLQHELEL
>LFGLNPFC_03957 Fructose-6-phosphate aldolase 2
MELYLDTANVAEVERLARIFPIAGVTTNPSIIAASKESIWEVLPRLQKAIGDEGILFAQT
MSRDAQGMVKEAKHLRDAIPGIVVKIPVTSEGLAAIKMLKKEGITTLGTAVYSAAQGLLA
ALAGAKYVAPYVNRVDAQGGDGIRTVQELQALLEMHAPESMVLAASFKTPRQALDCLLAG
CESITLPLDVAQQMLNTPAVESAIEKFEHDWNAAFDTTHL
>LFGLNPFC_03958 Glycerol dehydrogenase
MDRIIQSPGKYIQGADVINRLGEYLKPLAERWLVVGDKFVLGFAQSTVEKSFKDAGLVVE
IAPFGGECSQNEIDRLRGIAETAQCGAILGIGGGKTLDTAKALAHFMGVPVAIAPTIAST
DAPCSALSVIYTDEGEFDRYLLLPNNPNMVIVDTKIVAGAPARLLAAGIGDALATWFEAR
ACSRSGATTMAGGKCTQAALALAELCYNTLLEEGEKAMLAAEQHVVTPALERVIEANTYL
SGVGFESGGLAAAHAVHNGLTAIPDAHHYYHGEKVAFGTLTQLVLENASVEEIETVAALS
HAVGLPITLAQLDIREDVPAKMRIVAEAACAEGETIHNMPGGATPDQVYAALLVADQYGQ
RFLQEWE
>LFGLNPFC_03959 hypothetical protein
MKASLALFSLLTLFTSYSLKSPAVPPTVVQIQANTNLAIADGARLQIGSTLFYDPAYVQL
TYPGGDVPQERGVCSDVVIRALRSQKVDLQKLVHEDMAKNFAAYPQKWQLKRPDSNIDHR
RVPNLETWFTRHDKTRPTSKNPSDYQAGDIVSWRLDNGLAHIGVVSDGFARDGTPLVIHN
IGAGAQEEDVLFSWQMVGHYRYFAK
>LFGLNPFC_03960 putative cystine transporter YijE
MSAAGKSNPLAISGLVVLTLIWSYSWIFMKQVTSYIGAFDFTALRCIFGALVLFIVLLLR
GRGMRPTPFKYTLAIALLQTCGMVGLAQWALVSGGAGKVAILSYTMPFWVVIFAALFLGE
RLRRGQYFAILIAAFGLFLVLQPWQLDFSSMKSAMLAILSGVSWGASAIVAKRLYARHPR
VDLLSLTSWQMLYAALVMSVVALLVPQREIDWQPTVFWALAYSAILATALAWSLWLFVLK
NLPASIASLSTLAVPVCGVLFSWWLLGENPGAVEGSGIVLIVLALALVSRKKKEAVSVKR
I
>LFGLNPFC_03961 Catalase-peroxidase
MSTSDDIHNTTATGKCPFHQGGHDQSAGGGTTTRDWWPNQLRVDLLNQHSNRSNPLGEDF
DYRKEFSKLDYYGLKKDLKALLTESQPWWPADWGSYAGLFIRMAWHGAGTYRSIDGRGGA
GRGQQRFAPLNSWPDNVSLDKARRLLWPIKQKYGQKISWADLFILAGNVALESSGFRTFG
FGAGREDVWEPDLDVNWGDEKAWLTHRHPEALAKAPLGATEMGLIYVNPEGPDHSGEPLS
AAAAIRATFGNMGMNDEETVALIAGGHTLGKTHGAGPTSNVGPDPEAAPIEEQGLGWAST
YGSGVGADAITSGLEVVWTQTPTQWSNYFFENLFKYEWVQTRSPAGAIQFEAVDAPEIIP
DPFDPSKKRKPTMLVTDLTLRFDPEFEKISRRFLNDPQAFNEAFARAWFKLTHRDMGPKS
RYIGPEVPKEDLIWQDPLPQPIYNPTEQDIIDLKFAIADSGLSVSELVSVAWASASTFRG
GDKRGGANGARLALMPQRDWDVNAAAVRALPVLEKIQKESGKASLADIIVLAGVVGVEKA
ASAAGLSIHVPFAPGRVDARQDQTDIEMFELLEPIADGFRNYRARLDVSTTESLLIDKAQ
QLTLTAPEMTALVGGMRVLGANFDGSKNGVFTDRVGVLSNDFFVNLLDMRYEWKATDESK
ELFEGRDRETGEVKYTASRADLVFGSNSVLRAVAEVYASSDAHEKFVKDFVAAWVKVMNL
DRFDLL
>LFGLNPFC_03962 5,10-methylenetetrahydrofolate reductase
MSFFHASQRDALNQSLAEVQGQINVSFEFFPPRTSEMEQTLWNSIDRLSSLKPKFVSVTY
GANSGERDRTHSIIKGIKDRTGLEAAPHLTCIDATPAELRIIARDYWNNGIRHIVALRGD
LPPGSGKPEMYASDLVTLLKEVADFDISVAAYPEVHPEAKSAQADLLNLRRKVDAGANRA
ITQFFFDVESYLRFRDRCVSAGIDVEIIPGILPVSNFKQAKKFADMTNVRIPAWMAQMFD
GLDDDAETRKLVGANIAMDMVKILSREGVKDFHFYTLNRAEMSYAICHTLGVRPGL
>LFGLNPFC_03963 Trifunctional nucleotide phosphoesterase protein YfkN
MKIKILAAGIALTLPFWACAKDVTIIYTNDLHAHVEPYKVPWIADGKRDIGGWANITTLV
KQEKAKNKATWFFDAGDYFTGPYISSLTKGKAIIDIMNTMPFDAVTIGNHEFDHGWDNTL
LQLSQAKFPIVQGNIFYQNSSKSFWDKPYTIIEKDGVKIGVIGLHGVFAFNDTVSAATRV
GIEARDEIKWLQRYIDELKGKVDLTVALIHEGVPARQSSMGGTDVRRALDKDIQTASQVK
GLDILITGHAHVGTPEPIKVGNTLILSTDSGGIDVGKLVLDYKEKPHNFTVKNFELKTIY
ADEWKPDPQTKQVIDGWNKKLDEVVQQTVAQSPVELKRAYGESASLGNLAADALLAAAGK
NTQLALTNSGGIRNEIPAGAITMGGVISTFPFPNELVTMELTGKQLRSLMEHGASLSNGV
LQVSKGLEMKYDSSKPVGQRVITLTLNGKPIEDATVYHIATQSFLADGGDGFTAFTEGKA
RNITGGYYVYHAVVDYFKAGNTITDEQLNGMRVKDIK
>LFGLNPFC_03964 hypothetical protein
MSTLFTINACKTFGCRNLGLASSEDYSWPDYKLGYPALHCRACGSYPPLFDEQQFRDWLS
VHLSTYAIEKGHFCPVCYGTDMICYGHNPQGSQRIQCRNCKKVWTPKKYQKEITHPQAIE
TVQLFIPFQGASAVQKLYVLVSLDATRGNILHLSTNYTQHQTGDSLRYSYKGNTEPTMHH
RDIVQKVDMREAQFLRRSQFDEIQYGSAVLKRNGKGAILRPVITAHGHFRILKIRYPDVK
THIISHECFLRGAIITAWADQFRQQQGELWFVEEEISDSNADTPWHFKGTTYHGWWQNQW

QRWEQGNNCKMVCLLTGASLERGANVSLATSRCFITWLTDQHDFTQSALLSAGRVTQMLT
SLALKYNESLTPSC
>LFGLNPFC_03965 Mannosylglucosyl-3-phosphoglycerate phosphatase
MKTVFTTLSVIFGVIFSHSVLAQDVTIYYTNDIHAHVNPAKILAVDKNRLIGGMANIAGI
VNEAKKKSKDVFFFDAGDYFTGPYISTLTKGEAIIDIMNTMPFDAVSVGNHEFDHGVPNM
VSQLSKAKFPILLGNIYYTDTNKPVWDHPWTIIEKDGLKIGVIGLHGAFAFYDTVAAKAR
EGVEARDEIKYLNKALAELKGKVDITVLLIHEGVPARQSSFGSKDVERLLQADIETAKKV
NGVDVLITGHAHVGTPQPIKVNNTLIVSTDAYGTNIGKLVLDYNPKTKKIDSYNGELITI
FADQFKPDTIVQNTIDKWSAKLNKITQEVVGHSPVVLTREYGSSSSTGNLILDAMMEKTP
DAVAGFQNSGGMRADFPKGDITLGDVISTFPFNNDLIEMDLTGRDLKSLMTHATNLTNGV
LQVSKSVAVVYDSKKPLNQRLISFTINGKPVEDNQTYRIATHSFCASGGDGFEAFLNGKN
VKTIPGTTSAESIIDYFKNHKPVTPDLTKRVMDVAK
>LFGLNPFC_03966 Mannosylglucosyl-3-phosphoglycerate phosphatase
MKTAQRTLFAATLLTIFSTSVMAQDVTIIYTNDLHAHVDSYKVPYVADGKRDIGGFANIS
TLVKQEKAKNKATFYFDAGDYFTGPYISSLTKGEAIIDIMNTMPFDAVSIGNHEFDHGWD
NALRQLSKANFPVLLGNVYHKESEKPFWNKPYTILEKDGIKIGIVGLHGVFAFNDTVSEL
SLQGLDNDNNNRFDKSSATLKNQGIEARDEVKYLQHYIDELRDKVDLTVALVHEGVPARQ
SSIGNTDVRRALDKDIQTASKVKGLDILITGHAHVGTPEPIKVGNTLILSTDSGGIDIGK
LILDVNPTARTHKVKSFELKTVYADEWIPDPTTQKVINSWNKKLADLVRQPVGESSIALT
RAYGESSQLGNLFTDAMLVAAPTAQIALINSGSLRADINAGTITFGDITSTFPFKNELTE
MDLSGKDLRNLLEHGASLTNGILQMSKGAEMRYTLQKPVGQRIVSFKINGEEIVDTNIYH
VATTTFLALGGDGFLAFKEGKNVQVRAGNNMSNVVIDYFKKGHKITPAQVNEMRVEVSK
>LFGLNPFC_03967 Nucleoside-specific channel-forming protein Tsx
MYIKKHWIALSILLIPCIGNAQEIKIDESWLHQSLNVIGRTDSRFGPRLTNDLYPEYTVA
GRKDWFDFYGYVDLPKFFGVGSHYDVGIWDEGSPLFTEIEPRFSIDKLTGLNLAFGPFKE
WFIANNYVYDMGDNQSSRQSTWYMGLGTDIDTGLPIKLSANIYAKYQWQNYGAANENEWD
GYRFKIKYSIPLTNLFGGRLVYNSFTNFDFGSDLADKSHNNKRTSNAIASSHILSLLYEH
WKFAFTLRYFHNGGQWNAGEKVNFGDGPFELKNTGWGTYTTIGYQF
>LFGLNPFC_03968 Bifunctional aspartokinase/homoserine dehydrogenase 2
MSVIAQAGAKGRQLHKFGGSSLADVKCYLRVAGIMAEYSQPDDMMVVSAAGSTTNQLINW
LKLSQTDRLSAHQVQQTLRRYQCDLISGLLPAEEADNLISAFVSDLEHLAALLDSGINDA
VYAEVVGHGEVWSARLMSAVLNQQGLPAAWLDAREFLRAERAAQPQVDEGLSYPLLQQLL
VQHPGKRLVVTGFISRNNAGETVLLGRNGSDYSATQIGALAGVSRVTIWSDVAGVYSADP
RKVKDACLLPLLRLDEASELARLAAPVLHARTLQPVSGSEIDLQLRCSYTPDQGSTRIER
VLASGTGARIVTSHDDVCLIEFQVPASQDFKLAHKEIDQILKRAQVRPLAVGVHNDRQLL
QFCYTSEVADSALKILDEAGLPGELRLRQGLALVAMVGAGVTRNPLHCHRFWQQLKGQPV
EFTWQSDDGISLVAVLRTGPTESLIQGLHQSVFRAEKRIGLVLFGKGNIGSRWLELFARE
QSTLSARTGFEFVLAGVVDSRRSLLSYDGLDASRALAFFNDEAVEQDEESLFLWMRAHPY
DDLVVLDVTASQQLADQYLDFASHGFHVISANKLAGASDSNKYRQIHDAFEKTGRHWLYN
ATVGAGLPINHTVRDLIDSGDTILSISGIFSGTLSWLFLQFDGSVPFTELVDQAWQQGLT
EPDPRDDLSGKDVMRKLVILAREAGYNIESDQVRVESLVPAHCEGGSIDHFFENGDELNE
QMVQRLEAAREMGLVLRYVARFDANGKARVGVEAVREDHPLASLLPCDNVFAIESRWYRD
NPLVIRGPGAGRDVTAGAIQSDINRLAQLL
>LFGLNPFC_03969 Cystathionine gamma-synthase
MTRKQATIAVRSGLNDDEQYGCVVPPIHLSSTYNFTGFNEPRAHDYSRRGNPTRDVVQRA
LAELEGGAGAVLTNTGMSAIHLVTTVFLKPGDLLVAPHDCYGGSYRLFDSLAKRGCYRVL
FVDQGDEQALRAALAENPKLVLVESPSNPLLRVVDIAKICHLAREVGAVSVVDNTFLSPA
LQNPLALGADLVLHSCTKYLNGHSDVVAGVVIAKDPDVVTELAWWANNIGVTGGAFDSYL
LLRGLRTLVPRMELAQRNAQAIVKYLQTQPLVKKLYHPSLPENQGHEIAARQQKGFGAML
SFELDGDEHTLRRFLGELSLFTLAESLGGVESLISHAATMTHAGMAPEARAAAGISETLL
RISTGIEDGEDLIADLENGFRAANKG
>LFGLNPFC_03970 Met repressor
MAEWSGEYISPYAEHGKKSEQVKKITVSIPLKVLKILTDERTRRQVNNLRHATNSELLCE
AFLHAFTGQPLPDDADLRKERSDEIPEAAKEIMREMGINPETWEY
>LFGLNPFC_03971 hypothetical protein
MKIRLLIPGLLVSVPAFAWQPQTGDIIFQVSRSSQSKAIQLATHSDYSHTGMLVIRNKKP
YVFEAVGPVKYTPLKQWIAHGEKGKYVVRRVEGGLSVEQQQKLAQTAKRYLGKPYDFSFS
WSDDRQYCSEVVWKVYQNALGMRVGEQQKLKEFDLSNPLVQAKLKERYGKNIPLEETVVS
PQAVFDAPQLTTVAKEWPLFSW
>LFGLNPFC_03972 50S ribosomal protein L31
MKKDIHPKYEEITASCSCGNVMKIRSTVGHDLNLDVCSKCHPFFTGKQRDVATGGRVDRF
NKRFNIPGSK
>LFGLNPFC_03973 Primosomal protein N'
MPVAHVALPVPLPRTFDYLLPEGMTVKAGSRVRVPFGKQQERIGVVVSVSDVSELPLNEL

KAVVEVLDVEPVFTHSVWRLLLWAADYYHHPIGDVLFHALPILLRQGRPAANAPMWYWFA
TEQGQAVDLNSLKRSPKQQQALAALRQRKIWRDQVAELEFNDAALQALRKKGLCDLASET
PEFSDWRTNYAVSGERLRLNTEQATAVGAIHSAADTFSAWLLAGVTGSGKTEVYLSVLEN
VLAQGKQALVMVPEIGLTPQTIARFRERFNAPVEVLHSGLNDSERLSAWLKAKNGEAAIV
IGTRSALFTPFKNLGVIVIDEEHDSSYKQQEGWRYHARDLAVYRAHSEQIPIILGSATPA
LETLCNVQQKKYRLLRLTRRAGNARPAIQHVLDLKGQKVQAGLAPALITRMRQHLQADNQ
VILFLNRRGFAPALLCHDCGWIAECPRCDHYYTLHQAQQHLRCHHCDSQRPVPRQCPSCG
STHLVPVGLGTEQLEQTLAPLFPGVPISRIDRDTTSRKGALEQQLAEVHRGGARILIGTQ
MLAKGHHFPDVTLVALLDVDGALFSADFRSAERFAQLYTQVAGRAGRAGKQGEVVLQTHH
PEHPLLQTLLYKGYDAFAEQALAERRMMQLPPWTSHVIVRAEDHNNQHAPLFLQQLRNLI
LASPLADEKLWVLGPVPALAPKRGGRWRWQILLQHPSRVRLQHIINGTLALINTIPDSRK
VKWVLDVDPIEG
>LFGLNPFC_03974 HTH-type transcriptional repressor CytR
MKAKKQETAATMKDVALKAKVSTATVSRALMNPDKVSQATRNRVEKAAREVGYLPQPMGR
NVKRNESRTILVIVPDICDPFFSEIIRGIEVTAANHGYLVLIGDCAHQNQQEKTFIDLII
TKQIDGMLLLGSRLPFDASIEEQRNLPPMVMANEFAPELELPTVHIDNLTAAFDAVNYLY
EQGHKRIGCIAGPEEMPLCHYRLQGYVQALRRCGIMVDPQYIARGDFTFEAGSKAMQQLL
DLPQPPTAVFCHSDVMALGALSQAKRQGLKVPEDLSIIGFDNIDLTQFCDPPLTTIAQPR
YEIGREAMLLLLDQMQGQHVGSGSRLMDCELIIRGSTRALP
>LFGLNPFC_03975 Cell division protein FtsN
MVAIAAAVLVTFIGGLYFITHHKKEESETLQSQKVTGNGLPPKPEERWRYIKELESRQPG
VRAPTEPSAGGEVKTPEQLTPEQRQLLEQMQADMRQQPTQLVEVPWNEQTPEQRQQTLQR
QRQAQQLAEQQRLAQQSRTTEQSWQQQTRTSQAAPVQAQPRQSKPASTQQPYQDLLQTPA
HTTAQSKPQQAAPVARAADAPKPTAEKKDERRWMVQCGSFRGAEQAETVRAQLAFEGFDS
KITTNNGWNRVVIGPVKGKENADSTLNRLKMAGHTNCIRLAAGG
>LFGLNPFC_03976 ATP-dependent protease subunit HslV
MTTIVSVRRNGHVVIAGDGQATLGNTVMKGNVKKVRRLYNDKVIAGFAGGTADAFTLFEL
FERKLEMHQGHLIKAAVELAKDWRTDRMLRKLEALLAVADETASLIITGNGDVVQPENDL
IAIGSGGPYAQAAARALLENTELSAREIAEKALDIAGDICIYTNHFHTIEELSYKA
>LFGLNPFC_03977 ATP-dependent protease ATPase subunit HslU
MSEMTPREIVSELDKHIIGQDNAKRSVAIALRNRWRRMQLNEELRHEVTPKNILMIGPTG
VGKTEIARRLAKLANAPFIKVEATKFTEVGYVGKEVDSIIRDLTDAAVKMVRVQAIEKNR
YRAEELAEERILDVLIPPAKNNWGQTEQQQEPSAARQAFRKKLREGQLDDKEIEIDLAAA
PMGVEIMAPPGMEEMTSQLQSMFQNLGGQKQKARKLKIKDAMKLLIEEEAAKLVNPEELK
QDAIDAVEQHGIVFIDEIDKICKRGESSGPDVSREGVQRDLLPLVEGCTVSTKHGMVKTD
HILFIASGAFQIAKPSDLIPELQGRLPIRVELQALTTSDFERILTEPNASITVQYKALMA
TEGVNIEFTDSGIKRIAEAAWQVNESTENIGARRLHTVLERLMEEISYDASDLSGQTITI
DADYVSKHLDALVADEDLSRFIL
>LFGLNPFC_03978 1,4-dihydroxy-2-naphthoate octaprenyltransferase
MTEQQISRTQAWLESLRPKTLPLAFAAIIVGTALAWWQGHFDPLVALLALITAGLLQILS
NLANDYGDAVKGSDKPDRIGPLRGMQKGVITQQEMKRALIITVVLICLSGLALVAVACHT
LADFVGFLILGGLSIIAAITYTVGNRPYGYIGLGDISVLVFFGWLSVMGSWYLQAHTLIP
ALILPATACGLLATAVLNINNLRDINSDRENGKNTLVVRLGAVNARRYHACLLMGSLVCL
ALFNLFSLHSLWGWLFLLAAPLLVKQARYVMREMDPVAMRPMLERTVKGALLTNLLFVLG
IFLSQWAA
>LFGLNPFC_03979 Regulator of ribonuclease activity A
MKYDTSELCDIYQEDVNVVEPLFSNFGGRASFGGQIITVKCFEDNGLLYDLLEQNGRGRV
LVVDGGGSVRRALVDAELARLAVQNEWEGLVIYGAVRQVDDLEELDIGIQAMAAIPVGAA
GEGIGESDVRVNFGGVTFFSGDHLYADNTGIILSEDPLDIE
>LFGLNPFC_03980 Cell division protein ZapB
MSLEVFEKLEAKVQQAIDTITLLQMEIEELKEKNNSLSQEVQNAQHQREELERENNHLKE
QQNGWQERLQALLGRMEEV
>LFGLNPFC_03981 Glycerol uptake facilitator protein
MSQTSTLKGQCIAEFLGTGLLIFFGVGCVAALKVAGASFGQWEISVIWGLGVAMAIYLTA
GVSGAHLNPAVTIALWLFACFDKRKVIPFIVSQVAGAFCAAALVYGLYYNLFFDFEQTHH
IVRGSVESVDLAGTFSTYPNPHINFVQAFAVEMVITAILMGLILALTDDGNGVPRGPLAP
LLIGLLIAVIGASMGPLTGFAMNPARDFGPKVFAWLAGWGNVAFTGGRDIPYFLVPLFGP
IVGAIVGAFAYRKLIGRHLPCDICVVEEKETTTPSEQKASL
>LFGLNPFC_03982 Glycerol kinase
MTEKKYIVALDQGTTSSRAVVMDHDANIISVSQREFEQIYPKPGWVEHDPMEIWATQSST
LVEVLAKADISSDQIAAIGITNQRETTIVWEKETGKPIYNAIVWQCRRTAEICEHLKRDG
LEDYIRSNTGLVIDPYFSGTKVKWILDHVEGSRERARRGELLFGTVDTWLIWKMTQGRVH
VTDYTNASRTMLFNIHTLDWDDKMLEVLDIPREMLPEVRRSSEVYGQTNIGGKGGTRIPI
SGIAGDQQAALFGQLCVKEGMAKNTYGTGCFMLMNTGEKAVKSENGLLTTIACGPTGEVN

YALEGAVFMAGASIQWLRDEMKLINDAYDSEYFATKVQNTNGVYVVPAFTGLGAPYWDPY
ARGAIFGLTRGVNANHIIRATLESIAYQTRDVLEAMQADSGIRLHALRVDGGAVANNFLM
QFQSDILGTRVERPEVREVTALGAAYLAGLAVGFWQNLDELQEKAVIEREFRPGIETTER
NYRYAGWKKAVKRAMAWEEHDE
>LFGLNPFC_03983 Fructose-1,6-bisphosphatase 1 class 2
MRRELAIEFSRVTESAALAGYKWLGRGDKNTADGAAVNAMRIMLNQVNIDGTIVIGEGEI
DEAPMLYIGEKVGTGRGDAVDIAVDPIEGTRMTAMGQANALAVLAVGDKGCFLNAPDMYM
EKLIVGPGAKGTIDLNLPLADNLRNVAAALGKPLSELTVTILAKPRHDAVIAEMQQLGVR
VFAIPDGDVAASILTCMPDSEVDVLYGIGGAPEGVVSAAVIRALDGDMNGRLLARHDVKG
DNEENRRIGEQELARCKAMGIEAGKVLRLGDMARSDNVIFSATGITKGDLLEGISRKGNI
ATTETLLIRGKSRTIRRIQSIHYLDRKDPEMQVHIL
>LFGLNPFC_03984 Flavodoxin/ferredoxin--NADP reductase
MADWVTGKVTKVQNWTDALFSLTVHAPVLPFTAGQFTKLGLEIEGERVQRAYSYVNSPDN
PDLEFYLVTVPDGKLSPRLAALKPGDEVQVVSEAAGFFVLDEVPDCETLWMLATGTAIGP
YLSILQLGKDLERFKNMVLVHAARYAADLSYLPLMQELEKRYEGKLRIQTVVSRETAAGS
LTGRLPALIESGELESAVGLPINKETSHVMLCGNPQMVRDTQQLLKETRQMTKHLRRRPG
HMTAEHYW
>LFGLNPFC_03985 Universal stress protein D
MAYKHIGVAISGNEEDALLVNKALELARHNDAHLTLIHIDDGLSELYPGIYFPATEDILQ
LLKNKSDNKLYKLTKNIQWPKTKLRIERGEMPETLLEIMQKEQCDLLVCGHHHSFINRLM
PAYRGMINKLSADLLIVPFIDK
>LFGLNPFC_03986 hypothetical protein
MKDVVDKCSTKGCAIDIGTVIDNDNCTSKFSRFFATREEAESFMTKLKELAAAASSADEG
ASVAYKIKDLEGQVELDAAFTFSCQAEMIIFELSLRSLA
>LFGLNPFC_03987 hypothetical protein
MTIQQWLFSFKGRIGRRDFWIWIGLWFAGMLVLFSLAGKNLLDIQTAAFCLVCLLWPTAA
VTVKRLHDRGRSGAWAFLMIVAWMLLAGNWAILPGVWQWAVGRFVPTLILVMMLIDLGAF
VGTQGENKYGKDTQDVKYKADNKSSN
>LFGLNPFC_03988 hypothetical protein
MKPGCTLFFLLCSALTVTTTAHAQTPDTATTAPYLLAGAPTFDLSISQFREDFNSQNPSL
PLNEFRAIDSSPDKANLTRAASKINENLYASTALERGTLKIKSIQITWLPIQGPEQKAAK
AKAQAYMAAVIRTLTPLMTKTQSQKKLQSLLTAGKNKRYYTETEGALRYVVADNGEKGLT
FAVEPIKLALSESLEGLNK
>LFGLNPFC_03989 Triosephosphate isomerase
MRHPLVMGNWKLNGSRHMVHELVSNLRKELAGVAGCAVAIAPPEMYIDMAKREAEGSHIM
LGAQNVDLNLSGAFTGETSAAMLKDIGAQYIIIGHSERRTYHKESDELIAKKFAVLKEQG
LTPVLCIGETEAENEAGKTEEVCARQIDAVLKTQGAAAFEGAVIAYEPVWAIGTGKSATP
AQAQAVHKFIRDHIAKVDANIAEQVIIQYGGSVNASNAAELFAQPDIDGALVGGASLKAD
AFAVIVKAAEAAKQA
>LFGLNPFC_03990 CDP-diacylglycerol pyrophosphatase
MKKAGLLFLVMIVIAVVATGIGYWKLTGEESDTLRKIVLEQCLPNQQENQNPSPCAEVKP
NAGYVVLKDRHGPLQYLLMPTYRINGTESPLLTDPSTPNFFWLAWQARDFMSQKYGQPVP
DRAVSLAINSRTGRTQNHFHIHISCIRPDVREQLDNNLANISSRWLPLPGGLRGHEYLAR
RVTESELVQRSPFMMLAEEVPEAREHMGSYGLAMVRQSDNSFVLLATQRNLLTLNRASAE
EIQDHQCEILR
>LFGLNPFC_03991 Sulfate-binding protein
MKKWGVGLTFLLAATSVMAKDIQLLNVSYDPTRELYEQYNKAFSAHWKQQTGDNVVIRQS
HGGSGKQATSVINGIEADVVTLALAYDVDAIAERGRIDKEWIKRLPDNSAPYTSTIVFLV
RKGNPKQIHDWNDLIKPGVSVITPNPKSSGGARWNYLAAWGYALHHNNNDQVKAQDFVRA
LYKNVEVLDSGARGSTNTFVERGIGDVLIAWENEALLAANELGKDKFEIVTPSESILAEP
TVSVVDKVVEKKGTKEVAEAYLKYLYSPEGQEIAAKNYYRPRDAEVAKKYENAFPKLKLF
TIDEEFGGWTKAQKEHFANGGTFDQISKR
>LFGLNPFC_03992 ATP-dependent 6-phosphofructokinase isozyme 1
MIKKIGVLTSGGDAPGMNAAIRGVVRSALTEGLEVMGIYDGYLGLYEDRMVQLDRYSVSD
MINRGGTFLGSARFPEFRDENIRAVAIENLKKRGIDALVVIGGDGSYMGAMRLTEMGFPC
IGLPGTIDNDIKGTDYTIGFFTALSTVVEAIDRLRDTSSSHQRISVVEVMGRYCGDLTLA
AAIAGGCEFVVVPEVEFSREDLVNEIKAGIAKGKKHAIVAITEHMCDVDELAHFIEKETG
RETRATVLGHIQRGGSPVPYDRILASRMGAYAIDLLLAGYGGRCVGIQNEQLVHHDIIDA
IENMKRPFKGDWLDCAKKLY
>LFGLNPFC_03993 Ferrous-iron efflux pump FieF
MNQSYGRLVSRAAIAATAMASLLLLIKIFAWWYTGSVSILAALVDSLVDIGASLTNLLVV
RYSLQPADDNHSFGHGKAESLAALAQSMFISGSALFLFLTGIQHLVSPTPMTDPGVGVIV
TIVALICTIILVSFQRWVVRRTQSQAVRADMLHYQSDVMMNGAILLALGLSWYGWHRADA
LFALGIGIYILYSALRMGYEAVQSLLDRALPDEERQEIIDIVTSWPGVSGAHDLRTRQSG

PTRFIQIHLEMEDSLPLVQAHMVADQVEQAILRRFPGSDVIIHQDPCSVVPREGKRSMLS
>LFGLNPFC_03994 Periplasmic protein CpxP
MRIVTAAVMASTLAVSSLSHAAEVGSGDNCHPGEELTQRSTQSHMFDGISLTEHQRQQMR
DLMQQARHEQPPVNVSELETMHRLVTAENFDENAVRAQAEKMANEQIARQVEMAKVRNQM
YRLLTPEQQAVLNEKHQQRMEQLRDVTQWQKSSSLKLLSSSNSRSQ
>LFGLNPFC_03995 Transcriptional regulatory protein CpxR
MNKILLVDDNRELTSLLKELLEMEGFNVIVAHDGEQALDLLDDSIDLLLLDVMMPKKNGI
DTLKALRQTHQTPVIMLTARGSELDRVLGLELGADDYLPKPFNDRELVARIRAILRRSHW
SEQQQNNDNGSPTLEVDALSLNPGRQEATFDGQTLELTGTEFTLLYLLAQHLGQVVSREH
LSQEVLGKRLTPFDRAIDMHISNLRRKLPDRKDGHPWFKTLRGRGYLMVSAS
>LFGLNPFC_03996 Sensor histidine kinase CpxA
MIGSLTARIFAIFWLTLALVLMLVLMLPKLDSRQMTELLDSEQRQGLMIEQHVEAELAND
PPNDLMWWRRLFRAIDKWAPPGQRLLLVTTEGRVIGAERSEMQIIRNFIGQADNADHPQK
KKYGRVELVGPFSVRDGEDNYQLYLIRPASSSQSDFINLLFDRPLLLLIVTMLVSTPLLL
WLAWSLAKPARKLKNAADEVAQGNLRQHPELEAGPQEFLAAGASFNQMVTALERMMTSQQ
RLLSDISHELRTPLTRLQLGTALLRRRSGESKELERIETEAQRLDSMINDLLVMSRNQQK
NALVSETIKANQLWSEVLDNAAFEAEQMGKSLTVNFPPGPWPLYGNPNALESALENIVRN
ALRYSHTKIEVGFAVDKDGITITVDDDGPGVSPEDREQIFRPFYRTDEARDRESGGTGLG
LAIVETAIQQHRGWVKAEDSPLGGLRLVIWLPLYKRS
>LFGLNPFC_03997 Protein YiiM
MRYPVDVYTGKIQAYPEGKPSAIAKIQVDGELMLTDLGLEGDEQAEKKIHGGPDRALCHY
PREHYLYWAREFPEQAELFVAPAFGENLSTDGLTESNVYIGDIFRWGEALIQVSQPRSPC
YKLNYHFDISDIAQLMQNTGKVGWLYSVIAPGLVSADAPLELVSRVSDVTVQEAAAIAWH
MPFDDDQYHRLLSAAGLSKSWTRTMQKRRLSGKIEDFSRRLWGK
>LFGLNPFC_03998 2-keto-3-deoxygluconate permease
MQIKRSIEKIPGGMMLVPLFLGALCHTFSPGAGKYFGSFTNGMITGTVPILAVWFFCMGA
SIKLSATGTVLRKSGTLVVTKIAVAWVVAAIASRIIPEHGVEVGFFAGLSTLALVAAMDM
TNGGLYASIMQQYGTKEEAGAFVLMSLESGPLMTMIILGTAGIASFEPHVFVGAVLPFLV
GFALGNLDPELREFFSKAVQTLIPFFAFALGNTIDLTVIAQTGLLGILLGVAVIIVTGIP
LIIADKLIGGGDGTAGIAASSSAGAAVATPVLIAEMVPAFKPMAPAATSLVATAVIVTSI
LVPILTSIWSRKVKARAAKIEILGTVK
>LFGLNPFC_03999 Superoxide dismutase [Mn]
MSYTLPSLPYAYDALEPHFDKQTMEIHHTKHHQAYVNNANAALESLPEFANLPVEELITK
LDQLPADKKTVLRNNAGGHANHSLFWKGLKKGTTLQGDLKAAIERDFGSVDNFKAEFEKA
AASRFGSGWAWLVQKGDKLAVVSTANQDSPLMGEAISGASGFPILGLDVWEHAYYLKFQN
RRPDYIKEFWNVVNWDEAAARFAAKK
>LFGLNPFC_04000 L-rhamnose-proton symporter
MSNAITMGIFWHLIGAASAACFYAPFKKVKKWSWETMWSVGGIVSWIILPWAISALLLPN
FWAYYSSFSLSTLLPVFLFGAMWGIGNINYGLTMRYLGMSMGIGIAIGITLIVGTLMTPI
INGNFDVLINTEGGRMTLLGVLVALIGVGIVTRAGQLKERKMGIKAEEFNLKKGLVLAVM
CGIFSAGMSFAMNAAKPMHEAAAALGVDPLYVALPSYVVIMGGGAIINLGFCFIRLAKVK
DLSLKADFSLAKPLIIHNVLLSALGGLMWYLQFFFYAWGHARIPAQYDYISWMLHMSFYV
LCGGIVGLVLKEWNNAGRRPVTVLSLGCVVIIVAANIVGIGMAN
>LFGLNPFC_04001 HTH-type transcriptional activator RhaR
MAFCNNANLLNVFVRHIANNQLRSLAEVATVAHQLKLLKDDFFASDQQAVAVADRYPQDV
FAEHTHDFCELVIVWRGNGLHVLNDRPYRITRGDLFYIHADDKHSYASVNDLVLQNIIYC
PERLKLNLDWQGAIPGFNASAGQPHWRLGSVGMAQARQVIGQLEHESSQHVPFANEMAEL
LFGQLVMLLNRHRYTSDSLPPTSSETLLDKLITRLAASLKSPFALDKFCDEASCSERVLR
QQFRQQTGMTINQYLRQVRVCHAQYLLQHSRLLISDISTECGFEDSNYFSVVFTRETGMT
PSQWRHLNSQKD
>LFGLNPFC_04002 HTH-type transcriptional activator RhaS
MTVLHSVDFFPSGNASVAIEPRLPQADFPEHHHDFHEIVIVEHGTGIHVFNGQPYTITGG
TVCFVRDHDRHLYEHTDNLCLTNVLYRSPDRFQFLAGLNQLLPQEQDGQYPSHWRVNHSV
LQQVRQLVAQMEQQEEENDLPSTASREILFMQLLLLLRKSSLQENLENSASRLNLLLAWL
EDHFADEVNWDAVADQFSLSLRTLHRQLKQKTGLTPQRYLNRLRLMKARHLLRHSEASVT
DIAYRCGFSDSNHFSTLFRREFNWSPRDIRQGRDGFLQ
>LFGLNPFC_04003 Rhamnulokinase
MTFRNCVAVDLGASSGRVMLARYERECRSLTLREIHRFNNGLHSQNGYVTWNVDSLESAI
RLGLNKVCEEGIRIDSIGIDTWGVDFVLLDQQGQRVGLPVAYRDSRTNGLMAQAQQQLGK
RDIYQRSGIQFLPFNTIYQLRALTEQQPELIPHIAHALLIPDYFSYRLTGKMNWEYTNAT
TTQLVNINSDDWDESLLAWSGANKAWFGRPTHPGNVIGHWICPQGNEIPVVAVASHDTAS
AVIASPLNGSRAAYLSSGTWSLMGFESQTPFTNDTALAANITNEGGAEGRYRVLKNIMGL
WLLQRVLQERQINDLPALIAATQALPACRFIINPNDDRFINPDEMCSEIQAACRETAQPI
PESDAELARCIFDSLALLYADVLHELAQLRGEDFSQLHIVGGGCQNTLLNQLCADACGIR

VIAGPVEASTLGNIGIQLMTLDELNNVDDFRQVVSTTANLTTFTPNPDSEIAHYVAQIHS
TRQTKELCA
>LFGLNPFC_04004 L-rhamnose isomerase
MTTQLEQAWELAKQRFAAVGIDVEEALRQLDRLPVSMHCWQGDDVSGFENPEGSLTGGIQ
ATGNYPGKARNASELRADLEQAMRLIPGPKRLNLHAIYLESDTPVSRDQIKPEHFKNWVE
WAKANQLGLDFNPSCFSHPLSADGFTLSHADDRIRQFWIDHCKASRRVSAYFGEQLGTPS
VMNIWIPDGMKDITVDRLAPRQRLLAALDEVISEKLNPAHHIDAVESKLFGIGAESYTVG
SNEFYLGYATSRQTALCLDAGHFHPTEVISDKISAAMLYVPQLLLHVSRPVRWDSDHVVL
LDDETQAIASEIVRHDLFDRVHIGLDFFDASINRIAAWVIGTRNMKKALLRALLEPTAEL
RKLEAAGDYTARLALLEEQKSLPWQAVWEMYCQRHDTPAGSEWLENVRTYEKEILSRRG
>LFGLNPFC_04005 Rhamnulose-1-phosphate aldolase
MQNITQSWFVQGMIKATTDAWLKGWDERNGGNLTLRLDDADIAPYHDNFHQQPRYIPLSQ
PMPLLANTPFIVTGSGKFFRNVQLDPAANLGIVKVDSDGAGYHILWGLTNEAVPTSELPA
HFLSHCERIKATNGKDRVIMHCHATNLIALTYVLENDTAVFTRQLWEGSTECLVVFPDGV
GILPWMVPGTDEIGQATAQEMQKHSLVLWPFHGVFGSGPTLDETFGLIDTAEKSAQILVK
VYSMGGMKQTISREELIALGQRFGVTPLASALAL
>LFGLNPFC_04006 L-rhamnose mutarotase
MIRKAFVMQVNPDAHEEYQRRHNPIWPELEAVLKSHGAHNYAIYLDKAHNLLFATVEIES
EERWNAVASTDVCQRWWKYMTDVMPANADNSPVSSELQEVFYLP
>LFGLNPFC_04007 hypothetical protein
MKKSTLSLAIGLLLACSTGMAKTQHLTLEQRMALLEERLEAAEMRAAKAEGQVKQLQTQQ
AAEIREIKTAQGNTPVNGQSTTESEKKNATPPNLLLSGYGDLKIYGDVEFNMDAESNHGL
LAMTNADVNSDPTNEQWNLNGRILLGFDGMRKLDNGYFAGFSAQPLGDMHGSVNIDDAVF
FFGKENDWKVKVGRFEAYDMFPLNQDTFVEHSGNTANDLYDDGSGYIYMMKEGRGRSNAG
GNFLVSKQLDNWYFELNTLLEDGTSLYNDGNYHGRDMEQQKNVAYLRPVIAWSPTEEFTV
SAAMEANVVNNAYGYTDSKGNFVDQSDRTGYGMSMTWNGLKTDPENGIVVNLNTAYLDAN
NEKDFTAGINALWKRFELGYIYAHNKIDEFSGVVCDNDCWIDDEGTYNIHTIHASYQFAN
VMDMENFNIYLGTYYSILDSDGDKIHGDDSDDRYGARVRFKYFF
>LFGLNPFC_04008 hypothetical protein
MISAKQINNLISQDKFDAEAAMKKVSELETLVAQAKEADKSGMNFSFINSAGQYQLEAKK
YVRRIRDKVPYSDWDKEQLQDANSSWMAEDSFPRALCDYNEMVDEIFQLIVIAGRVCDEH
GYVTKS
>LFGLNPFC_04009 hypothetical protein
MALTPALQPIDGVAVSYIDAAVALGNTINEMDKYYTQENYKDDAFAKGKTLHQTFLKIWK
PLNL
>LFGLNPFC_04010 Sulfur carrier protein FdhD
MKKTQQKEIENVTNITGVRQIELWRRDDLQHPRLDEVAEEVPVALVYNGISHVVMMASPK
DLEYFALGFSLSEGIIESPRDIFGMDVVPSCNGLEVQIELSSRRFMGLKERRRALAGRTG
CGVCGVEQLNDIGKPVQPLPFTQTFDLNKLDDALRHLNDFQPVGQLTGCTHAAAWMLPSG
ELVGGHEDVGRHVALDKLLGRRSQEGESWQQGAVLVSSRASYEMVQKSAMCGVEILFAVS
AATTLAVEVAERCNLTLVGFCKPGRATVYTHPQRLSN
>LFGLNPFC_04011 Formate dehydrogenase-O major subunit
MQVSRRQFFKICAGGMAGTTAAALGFAPSVALAETRQYKLLRTRETRNTCTYCSVGCGLL
MYSLGDGAKNAKASIFHIEGDPDHPVNRGALCPKGAGLVDFIHSESRLKFPEYRAPGSDK
WQQISWEEAFDRIAKLMKEDRDANYIAQNAEGVTVNRWLSTGMLCASASSNETGYLTQKF
SRALGMLAVDNQARV
>LFGLNPFC_04012 Formate dehydrogenase-O major subunit
MTNHWVDIKNANLVVVMGGNAAEAHPVGFRWAMEAKIHNGAKLIVIDPRFTRTAAVADYY
APIRSGTDIAFLSGVLLYLLNNEKFNREYTEAYTNASLIVREDYGFEDGLFTGYDAEKRK
YDKSTWTYELDENGFAKRDTTLQHPRCVWNLLKQHVSRYTPDVVENICGTPKDAFLKVCE
YIAETSAHDKTASFLYALGWTQHSIGAQNIRTMAMIQLLLGNMGMAGGGVNALRGHSNIQ
GLTDLGLLSQSLPGYMTLPSEKQTDLQTYLTANTPKPLLEGQVNYWGNYPKFFVSMMKAF
FGDKATAENSWGFDWLPKWDKGYDVLQYFEMMKEGKVNGYICQGFNPVASFPNKNKVIGC
LSKLKFLVTIDPLNTETSNFWQNHGELNEVDSSKIQTEVFRLPSTCFAEENGSIVNSGRW
LQWHWKGADAPGIALTDGEILSGIFLRLRKMYAEQGGANPDQVLNMTWNYAIPHEPSSEE
VAMESNGKALADITDPATGAVIVKKGQQLSSFAQLRDDGTTSCGCWIFAGSWTPEGNQMA
RRDNADPSGLGNTLGWAWAWPLNRRILYNRASADPQGNPWDPKRQLLKWDGTKWTGWDIP
DYSAAPPGSGVGPFIMQQEGMGRLFALDKMAEGPFPEHYEPFETPLGTNPLHPNVISNPA
ARIFKDDAEALGKADKFPYVGTTYRLTEHFHYWTKHALLNAILQPEQFVEIGESLANKLG
IAQGDTVKVSSNRGYIKAKAVVTKRIRTLKANGKDIDTIGIPIHWGYEGVAKKGFIANTL
TPFVGDANTQTPEFKSFLVNVEKV
>LFGLNPFC_04013 Formate dehydrogenase-O iron-sulfur subunit
MAYQSQDIIRRSATNGLTPAPQARDFQEEVAKLIDVTTCIGCKACQVACSEWNDIRDTVG
NNIGVYDNPNDLSAKSWTVMRFSEVEQNDKLEWLIRKDGCMHCSDPGCLKACPAEGAIIQ

YANGIVDFQSEQCIGCGYCIAGCPFDIPRLNPEDNRVYKCTLCVDRVVVGQEPACVKTCP
TGAIHFGTKESMKTLASERVAELKTRGYDNAGLYDPAGVGGTHVMYVLHHADKPNLYHGL
PENPEISETVKFWKGIWKPLAAVGFAATFAASIFHYVGVGPNRADEEENNLHEEKDEERK
>LFGLNPFC_04014 Formate dehydrogenase, cytochrome b556(fdo) subunit
MKRRDTIVRYTAPERINHWITAFCFILAAVSGLGFLFPSFNWLMQIMGTPQLARILHPFV
GVVMFASFIIMFFRYWHHNLINRDDIFWAKNIRKIVVNEEVGDTGRYNFGQKCVFWAAII
FLVLLLVSGVIIWRPYFAPAFSIPVIRFALMLHSFAAVALIVVIMVHIYAALWVKGTITA
MVEGWVTSAWAKKHHPRWYREVRKTTEKKAE
>LFGLNPFC_04015 Protein FdhE
MSIRIIPQDELGSSEKRTADMIPPLLFPRLKNLYNRRAERLRELAENNPLGDYLRFAALI
AHAQEVVLYDHPLEMDLTTRIKEASAQGKPPLDIHVLPRDKHWQKLLMALIAELKPEMSG
PALAVIENLEKASTQELEDMASALFASDFSSVSSDKAPFIWAALSLYWAQMANLIPGKAR
AEYGEQRQYCPVCGSMPVSSMVQIGTTQGLRYLHCNLCETEWHVVRVKCSNCEQSGKLHY
WSLDDEQAAIKAESCDDCGTYLKILYQEKEPKVEAVADDLASLVLDARMEQEGYARSSIN
PFLFPGEGE
>LFGLNPFC_04016 hypothetical protein
MAMNTVFLHLSEEAIKRLNKLRGWRKVSRSAILREAVEQYLERQQFPVRKAKGGRQRDEA
VGVEELCKQHKE
>LFGLNPFC_04017 hypothetical protein
MSTFNTRKDRDRNGKAKPHKHSGIPTLTGKWRHPSMDCWVNYSTPGDHEWKRLYTTLRRR
ADDRRQCHRVMQGDDPEGIVWFPDCYPQIYYY
>LFGLNPFC_04018 hypothetical protein
MTRNRAERRHHMVRLKKIRCRYRTAGDGSKKASGICFRTPCICSCWMCGHRRYHNGPRVS
EIRAKARYAN
>LFGLNPFC_04019 Antitoxin HigA-2
MDKALFERLTHSMAQMNEIIEGTRQPSRTFEVDAMKIKEIRRASGLSQSKFADLISVSVD
TLRNWEQGRRSPTGPAKALLRAIANDPQHVLQALNR
>LFGLNPFC_04020 Toxin HigB-2
MLFIETEIFTEDVQKLLNDDEFSRFQFFLALNPDYGEVIPETGGLRKVRWVSGGKGKRAG
VRVIYFHQVKHYEIRLLLIYRKGIKDDLSPQEKAMLRLLNTRW
>LFGLNPFC_04021 Carboxylesterase NlhH
MALEKGIAELVEEFIAAGRPSSRQQSITQRREGYIASAVLAGETETRVDIQTIELEGMTL
RIVSPLNAPTLLPTIIYYYGGCFVSGGFATHDNQLRQLAYYGQCRIIAVQYRLAPEHTFP
AAHDDAQRGAELVRQHAERLGVDKQRITLAGDSAGGHLALVTALRLKRAGEWQPAQLILI
YPMLDATAHFESYIRNGHDYIITRDTLLSGFEMYLPGIERRHPEASPIWRNDFNGLPPVH
IITAEYDPLCDEGEALYHRMTGQGVQCTCQRYLGVIHGFFQLGGISEAARSALRDVAWRA
GR
>LFGLNPFC_04022 Amino-acid acetyltransferase
MYHLRVPQTEEELERYYQFRWEMLRKPLHQPKGSERDAWDAMAHHQMVVDEQGNLVAVGR
LYINADNEASIRFMAVHPDVQDKGLGTLMAMTLESVARQEGVKRVTCSAREDAVEFFAKL
GFVNQGEITTPTTTPIRHFLMIKPVATLDDILHRGDWCAQLQQAWYEHIPLSEKMGVRIQ
QYTGQKFITTMPETGNQNPHHTLFAGSLFSLATLTGWGLIWLMLRERHLGGTIILADAHI
RYSKPISGKPHAVADLGALSGDLDRLARGRKARVQMQVEIFGDETPGAVFEGTYIVLPAK
PFGPYEEGGNEEE
>LFGLNPFC_04023 D-aminoacyl-tRNA deacylase
MIALIQRVTRASVTVEGEVTGEIGAGLLVLLGVEKDDDEQKANRLCERVLGYRIFSDAEG
KMNLNVQQAGGSVLVVSQFTLAADTERGMRPSFSKGASPDRAEALYDYFVERCRQQEMNT
QTGRFAADMQVSLVNDGPVTFWLQV
>LFGLNPFC_04024 hypothetical protein
MLKTIQDKARHRTRPLWAWLKLLWQRIDEDNMTTLAGNLAYVSLLSLVPLVAVVFALFAA
FPMFSDVSIQLRHFIFANFLPATGDVIQRYIEQFVANSNKMTAVGACGLIVTALLLMYSI
DSALNTIWRSKRARPKIYSFAVYWMILTLGPLLAGASLAISSYLLSLRWASDLNTVIDNV
LRIFPLLLSWISFWLLYSIVPTIRVPNRDAIVGAFVAALLFEAGKKGFALYITMFPSYQL
IYGVLAVIPILFVWVYWTWCIVLLGAEITVTLGEYRKLKQAAEQEEDDEP
>LFGLNPFC_04025 Alpha-D-glucose 1-phosphate phosphatase YihX
MLYIFDLGNVIVDIDFNRVLGAWSDLTRVPLATLKKSFHMGEAFHQHERGEISDEAFAEA
LCHEMALPLSYEQFSHGWQAVFVALRPEVIAIMHKLREQGHRVVVLSNTNRLHTTFWPEE
YPEIRDAADHIYLSQDLGMRKPEARIYQHVLQAEGFSPDDTVFFDDNADNIEGANQLGIT
SILVKDKTTIPDYFAKVLC
>LFGLNPFC_04026 putative protein YphB
MKTERKNEHYLALQQAFDAPWPGPVGELVTLEKGNIHLQIYPHDGARITSLKAFGSEVLR
QWQPQRRAFQYGCFPMVPWAGRLGNATLNAGGQCYSLPANKPPHALHGMACYSTWEIIDK
TADSLTLRMPLASPWPWQGEVIQTFLLENDALVLQLEVHSYADTFPASAGWHPWFAKKLT
PQNTESLQVLFDADWQEEAGSDELPTGNRISPQVGPWDDCFGFYDGVKVKLLWPGKLAMT

MTSSANSLVVFDKQPDATCINPLTQAPNAINLTPEFVTPDKPLVIETRWQFTPES
>LFGLNPFC_04027 Fosfomycin resistance protein AbaF
MSEKLPAPREGLSGKAMRRVVMGSFAGALMEWYDFFIFGTAAGLVFAPLFYPDSDPFIGL
IASFATFGVGFLTRPLGGIVFGHFGDKIGRKITLIWTLAIVGCSTFLIGFIPTYQEIGIW
APLVLMVLRLIQGFGLGGEYGGAALMTIESAPESRRGFLGSLPQTAASVGIMLATGIFAL
CNHFLTSEQFLSWGWRIPFWLSAVMLIVGLFIRLHTEETLDFQKQKTTNNKEKSVPPLIE
LFKKHPRNILLALGARLAESVSSNIINAFGIVYISSQLALSRDIPLTGMLIASAIGIFSC
PLVGWLSDRIGQKSLYLSGAGFCVLFAFPFFLLLDSKSTLIIWCSMILGYNLGPTMMFAV
QPTLFTRMFGTKVRYTGLSFAYQFSAILGGLSPLIASSLLALGGGKPWYVALFLFAVSVL
SFVCVWLIEPTDEQETASYRYIREQSHEN
>LFGLNPFC_04028 Inner membrane metabolite transport protein YgcS
MSQITSPATYSISRPQDVIDIVNKNSAINTSIGVIFIALGGILIDAYQAAMVGFGNKYIA
AQFGISPGLAATVNASVLIAALIGGLLANRVINRFGQKRAFIIGMGLCTIGAAAVAIAPS
IWWVLVCRVIMGFGLGIDFPLATNAVAELRGSTSKKTGTSVNLWQMAWYYVSTTVVYLVLL
PLLLSGIAEEQLWRYGIFIGAIFAVIFMILRYFFIGESAMWAARVGRYQEACDILGKRYG
VQAHVAASGTTEAKFSEKAENKYSGGYGILFNDRYRKRTILGCVVATMQAWQYNAVGVYL
PLTLAGIISGGLTGALTGSAVVNALCGVTGGMIGSFILQRLGTRRQSMYGFAVVTLALLS
LGALATTNPWLSLGLLGSIIFFHSAGPGGLGMTIATLSYPPAIRPTGVGFARAIMRTGAI
AGLIFWPMLWGALKTEAFYWLAIVPFLGFLTCVLINWEPLGANVDAEDAEVLAELKK
>LFGLNPFC_04029 putative zinc-type alcohol dehydrogenase-like protein YjmD
MDKITQVLFSDIGKVTTQYVEVPHQQLKPHEVRIAPVFYGICGSDLHVLKGGHPFAKPPV
VPGHEIAARVTEVGSDVKNVQPGDHVVVDPIMACMECRACKAGRFNLCEPPQVAGFRAPG
FARSQHIVPARNCHVAPASLPLKVLAFAEPAACARHCVNRMPKASLESVLVIGAGTIGLS
IVQALRIMGAGKITVIEPDAAKRALALKLGAAEVWAPGELAADVRFTGAIDVVAAQATLN
DACTRVYAGGTVVCMGVPSGPREIPLPMMQRFERDLLNSGMYIPEDFDAVIEWLADGRFD
TSELVTDLFAIEDAAAAFERAQQNDSIKVMLQFAPE
>LFGLNPFC_04030 Sulfofructosephosphate aldolase
MSSHFTPFTLKDISRPGGGFAMLAVDQREAMRLMFAAAGQPKPIADSVLTDFKVAATRIL
SPYASAVLADKQFCLEQIVEQGAVANSCGLIVAADLFIPGNGIPVDSVEIDMSVDPHKAR
EMGAKAMKLLVLWREDEPAEERLAMVDKFVRRCRSAGLVSIIEPVVRPPRRGWDFDRESA
IVAAAAELGGTEADLYKAEMPLGGKGDEKTLLAACQQLNDQMKMPWVILSSGVDADIFGR
AVSIAMKGGASGFLAGRAVWASVVGAQDPQTMLRDVSVPRLQRLAEIVDEGIAQR
>LFGLNPFC_04031 3-sulfolactaldehyde reductase
MATIAFLGLGQMGSPMANNLLQKGHSLQVFDVNTQAVDALVTQGATAAQTPAEAAASAEF
IITMLPNGDIVRQVLLGEKGVCETVSSDALVIDMSTIHPLQTDALIRELQEKGINMMDAP
VGRTSVNAIDGTLLILAGGTDEQIARARPILMCMGNELVEAGGPGMGIRVKLINNYMSIA
LNALSSEAAVLCESLGLNLDVAIKVMSGTAAGKGHFTTTWPGKVLKGDLSPAFMVDLALK
DLRIAVDVARKTGAPLNMGIAAESYYAAASQDGKGRQDWSALLNQVRQQAGL
>LFGLNPFC_04032 Sulfofructose kinase
MTRIACVGITVQDRIYSLPTLPEGGGKYQANHYLEIGGGPAATAAVAIAKLGVEVDFIGR
VGDDSCGNTLLAELEGWGVNTAFCRRYPHARSSQSAILVDQHGERIIVNYPSPDLGTDAE
WLEAIDFSRYDLILADVRWHSGTEKAFSLARLAGVTTLLDADMTPQDISPLVALADHAVF
STPGLKRMTGLQSPEEGLFQATTQTAGKVYVTLGSEGSLWIEDGHLCQQEAFSVNVVDTT
GAGDVFHGALAVALAEKMPTKEAIRFASAVAAMKCTQPGGRAGIPNREQTESFLSLYA
>LFGLNPFC_04033 HTH-type transcriptional repressor CsqR
MSIIEVTGNPRHDQLVHLIAERGYMNIEELAQLLDVSTQTVRRDIRKLSEQGLITRHHGG
AGRVSSVMNTAFEQRELSLTAEKRAIAEAVADYLPERCTVFITIGTTVEAVARALLNRRD
LRIITNSLRVAQILYKNQDIEVMVPGGTLRAHNGGIIGPGAVDFIEGFRADYLITSIGAI
EHDGTLLEFDVNEALVARTMIKHARNTLLVADHTKFAASAAVSIGNARNVRAFFTDAPPP
NSFCQLLSEENVELVVAEQEVS
>LFGLNPFC_04034 GTP-binding protein TypA/BipA
MIEKLRNIAIIAHVDHGKTTLVDKLLQQSGTFDSRAETQERVMDSNDLEKERGITILAKN
TAIKWNDYRINIVDTPGHADFGGEVERVMSMVDSVLLVVDAFDGPMPQTRFVTKKAFAYG
LKPIVVINKVDRPGARPDWVVDQVFDLFVNLDATDEQLDFPIVYASALNGIAGLDHEDMA
EDMTPLYQAIVDHVPAPDVDLDGPFQMQISQLDYNSYVGVIGIGRIKRGKVKPNQQVTII
DSEGKTRNAKVGKVLGHLGLERIETDLAEAGDIVAITGLGELNISDTVCDTQNVEALPAL
SVDEPTVSMFFCVNTSPFCGKEGKFVTSRQILDRLNKELVHNVALRVEETEDADAFRVSG
RGELHLSVLIENMRREGFELAVSRPKVIFREIDGRKQEPYENVTLDVEEQHQGSVMQALG
ERKGDLKNMNPDGKGRVRLDYVIPSRGLIGFRSEFMTMTSGTGLLYSTFSHYDDVRPGEV
GQRQNGVLISNGQGKAVAFALFGLQDRGKLFLGHGAEVYEGQIIGIHSRSNDLTVNCLTG
KKLTNMRASGTDEAVVLVPPIRMTLEQALEFIDDDELVEVTPTSIRIRKRHLTENDRRRA
NRAPKDD
>LFGLNPFC_04035 Glutamine synthetase
MSAEHVLTMLNEHEVKFVDLRFTDTKGKEQHVTIPAHQVNAEFFEEGKMFDGSSIGGWKG

INESDMVLMPDASTAVIDPFFADSTLIIRCDILEPGTLQGYDRDPRSIAKRAEDYLRSTG
IADTVLFGPEPEFFLFDDIRFGSSISGSHVAIDDIEGAWNSSTQYEGGNKGHRPAVKGGY
FPVPPVDSAQDIRSEMCLVMEQMGLVVEAHHHEVATAGQNEVATRFNTMTKKADEIQIYK
YVVHNVAHRFGKTATFMPKPMFGDNGSGMHCHMSLSKNGVNLFAGDKYAGLSEQALYYIG
GVIKHAKAINALANPTTNSYKRLVPGYEAPVMLAYSARNRSASIRIPVVSSPKARRIEVR
FPDPAANPYLCFAALLMAGLDGIKNKIHPGEAMDKNLYDLPPEEAKEIPQVAGSLEEALN
ELDLDREFLKAGGVFTDEAIDAYIALRREEDDRVRMTPHPVEFELYYSV
>LFGLNPFC_04036 Sensory histidine kinase/phosphatase NtrB
MATGTQPDAGQILNSLINSILLIDDNLAIHYANPAAQQLLAQSSRKLFGTPLPELLSYFS
LNIELMQESLEAGQGFTDNEVTLVIDGRSHILSVTAQRMPDGMILLEMAPMDNQRRLSQE
QLQHAQQVAARDLVRGLAHEIKNPLGGLRGAAQLLSKALPDPSLLEYTKVIIEQADRLRN
LVDRLLGPQLPGTRITESIHKVAERVVTLVSMELPNNVRLIRDYDPSLPELAHDPDQIEQ
VLLNIVRNALQALGPEGGEIILRTRTAFQLTLHGERYRLAARIDVEDNGPGIPPHLQDTL
FYPMVSGREGGTGLGLSIARNLIDQHSGKIEFTSWPGHTEFSVYLPIRK
>LFGLNPFC_04037 DNA-binding transcriptional regulator NtrC
MQRGIVWVVDDDSSIRWVLERALAGAGLTCTTFENGAEVLEALASKTPDVLLSDIRMPGM
DGLALLKQIKQRHPMLPVIIMTAHSDLDAAVSAYQQGAFDYLPKPFDIDEAVALVERAIS
HYQEQQQPRNVQLNGPTTDIIGEAPAMQDVFRIIGRLSRSSISVLINGESGTGKELVAHA
LHRHSPRAKAPFIALNMAAIPKDLIESELFGHEKGAFTGANTIRQGRFEQADGGTLFLDE
IGDMPLDVQTRLLRVLADGQFYRVGGYAPVKVDVRIIAATHQNLEQRVQEGKFREDLFHR
LNVIRVHLPPLRERREDIPRLARHFLQVAARELGVEAKLLHPETEAALTRLAWPGNVRQL
ENTCRWLTVMAAGQEVLIQDLPGELFESNVPESTSHMQPDSWATLLAQWADRALRSGHQN
LLSEAQPELERTLLTTALRHTQGHKQEAARLLGWGRNTLTRKLKELGME
>LFGLNPFC_04038 putative protein YshB
MLESIINLVSSGAVDSHTPQTAVAAVLCAAMIGLFS
>LFGLNPFC_04039 Oxygen-independent coproporphyrinogen III oxidase
MSVQQIDWDLALIQKYNYSGPRYTSYPTALEFSEDFGEQAFLQAVARYPERPLSLYVHIP
FCHKLCYFCGCNKIVTRQQHKADQYLDALEQEIVHRAPLFAGRHVSQLHWGGGTPTYLNK
AQISRLMKLLRENFQFNADAEISIEVDPREIELDVLDHLRAEGFNRLSMGVQDFNKEVQR
LVNREQDEEFIFALLNHAREIGFTSTNIDLIYGLPKQTPESFAFTLKRVAELNPDRLSVF
NYAHLPTIFAAQRKIKDADLPSPQQKLDILQETIAFLTQSGYQFIGMDHFARPDDELAVA
QREGVLHRNFQGYTTQGDTDLLGMGVSAISMIGDCYAQNQKELKQYYQQVDEQGNALWRG
IALTRDDCIRRDVIKSLICNFRLDYAPIEQQWDLLFADYFAEDLKLLAPLAKDGLVDVDE
KGIQVTAKGRLLIRNICMCFDTYLRQKARMQQFSRVI
>LFGLNPFC_04040 Der GTPase-activating protein YihI
MKPSSSNSRSKGHAKARRKTREELDQEARDRKRQKKRRGHAPGSRAAGGNTTSGSKGQNA
PKDPRIGSKTPIPLGVAEKVTKQHKPKSEKPMLSPQAELELLETDERLDALLERLEAGET
LSAEEQSWVDVKLDRIDELMQKLGLSYDDDEEEEEDEKQEDMMRLLRGN
>LFGLNPFC_04041 putative GTP-binding protein EngB
MTNLNYQQTHFVMSAPDIRHLPSDTGIEVAFAGRSNAGKSSALNTLTNQKSLARTSKTPG
RTQLINLFEVADGKRLVDLPGYGYAEVPEEMKRKWQRALGEYLEKRQSLQGLVVLMDIRH
PLKDLDQQMIEWAVDSNIAVLVLLTKADKLASGARKAQLNMVREAVLAFNGDVQVETFSS
LKKQGVDKLRQKLDTWFSEMQPVEETQDGE
>LFGLNPFC_04042 DNA polymerase I
MVQIPQNPLILVDGSSYLYRAYHAFPPLTNSAGEPTGAMYGVLNMLRSLIMQYKPTHAAV
VFDAKGKTFRDELFEHYKSHRPPMPDDLRAQIEPLHAMVKAMGLPLLAVSGVEADDVIGT
LAREAEKTGRPVLISTGDKDMAQLVTPNITLINTMTNTILGPEEVVNKYGVPPELIIDFL
ALMGDSSDNIPGVPGVGEKTAQALLQGLGGLDTLYAEPEKIAGLSFRGAKTMAAKLEQNK
EVAYLSYQLATIKTDVELELTCEQLEVQPPAAEELLGLFKKYEFKRWTADVEAGKWLQAK
GAKPAARPQETSVADEAPEVTATVISYDNYVTILDEETLKEWIAKLEKAPVFAFDTETDS
LDNISANLVGLSFAIEPGVAAYIPVAHDYLDAPDQISRERALELLKPLLEDEKALKVGQN
LKYDRGILANYGIELRGIAFDTMLESYILNSVAGRHDMDSLAERWLKHKTITFEEIAGKG
KNQLTFNQIALEEAGRYAAEDADVTLQLHLKMWPDLQKHKGPLNVFENIEMPLVPVLSRI
ERNGVKIDPKVLHNHSEELTLRLAELEKKAHEIAGEEFNLSSTKQLQTILFEKQGIKPLK
KTPGGAPSTSEEVLEELALDYPLPKVILEYRGLAKLKSTYTDKLPLMINPKTGRVHTSYH
QAVTATGRLSSTDPNLQNIPVRNEEGRRIRQAFIAPEDYVIVSADYSQIELRIMAHLSRD
KGLLTAFAEGKDIHRATAAEVFGLPLETVTSEQRRSAKAINFGLIYGMSAFGLARQLNIP
RKEAQKYMDLYFERYPGVLEYMERTRAQAKEQGYVETLDGRRLYLPDIKSSNGARRAAAE
RAAINAPMQGTAADIIKRAMIAVDAWLQAEQPRVRMIMQVHDELVFEVHKDDVDAVAKQI
HQLMENCTRLDVPLLVEVGRGENWDQAH
>LFGLNPFC_04043 hypothetical protein
MSFSPLFVKIFCRSSGSLANRHVDNFVHKLFTSLLIYYHAFYSMVFVFVFHAVKNKAIPN
N
>LFGLNPFC_04044 putative acyltransferase YihG

MANLLNKFIMTRILAAITLLLSIVLTILVTIFCSVPIIIAGIVKLLLPVPVIWRKVSRFC
DFMMYCWCEGLAVLLHLNPHLQWEVHGLEGLSKKNWYLLICNHRSWADIVVLCVLFRKHI
PMNKYFLKQQLAWVPFLGLACWALDMPFMKRYSRAYLLRHPERRGKDVETTRRSCEKFRL
HPTTIVNFVEGSRFTQEKHQQTHSSFQNLLPPKAAGIAMALNVLGKQFDKLLNVTLCYPD
NNRQPFFDMLSGKLTRIVVHVDLQPIADELHGDYINDKSFKRHFQQWLNSLWQEKDRLLT
SLMSSQRQEK
>LFGLNPFC_04045 Protein YdgA
MIRKSATGAIVALAVIWGGGTWYTGTQIQPGVEKFIKGFNDAKKKGEHAYDMTLSYKNFD
KGFFNSRFQMQMTFDNGAPDLNIKPGQKIVFDVDVEHGPLPITMLMHGNVIPALAAAKVN
LVNNELTQPLFIAAKNKSPVEATLRFAFGGPFSTTLDVAPAEYGKFSFGEGQFTFNGDDS
SLSNLDIEGKVEDIVLQFSPMNKVTAKSFTIDSLTRLEEKKFPVGESESKFNQVNIINQG
EVVAQIDAFVAKTRLDRVKDKDYINVNLTYELDKLTKGNQQLGSGEWSLIAESIDPSAVR
QFIIQYNIAMQKQLAAHPELANDEVALQEVNAALFKEYLPLLQKSEPTIKQPVRWKNALG
ELNANLDISIADPAKSSSSTNKDIKSLNFDMKLPLNVATETAKQLNLSEGMDAEKAQKRA
DKQISGMMTLGLMLQLITIDNNTASLQLRYTPGKVVFNGQEMSEEEFMSRAGRFVH
>LFGLNPFC_04046 Thiol:disulfide interchange protein DsbA
MKKIWLALAGLVLAFSASAAQYEDGKQYTTLEKPVAGAPQVLEFFSFFCPHCYQFEEVLH
ISDNVKKKLPEGVKMTKYHVNFMGGDLGKELTQAWAVAMALGVEDKVTVPLFEGVQKTQT
IRSASDIRDVFINAGIKGEEYDAAWNSFVVKSLVAQQEKAAADVQLRGVPAMFVNGKYQL
NPQGMDTSNMDVFVQQYADTVKYLSEKK
>LFGLNPFC_04047 Stress response kinase A
MNNSAFTFQTLHPDTIMDALFEHGIRVDSGLTPLNSYENRVYQFQDEDRRRFVVKFYRPE
RWTADQILEEHQFALQLVNDEVPVAAPVAFNGQTLLNHQGFYFAVFPSVGGRQFEADNID
QMEAVGRYLGRMHQTGRKQLFIHRPTIGLNEYLIEPRKLFEDATLIPSGLKAAFLKATDE
LIAAVTAHWREDFTVLRLHGDCHAGNILWRDGPMFVDLDDARNGPAVQDLWMLLNGDKAE
QRMQLETIIEAYEEFSEFDTAEIGLIEPLRAMRLVYYLAWLMRRWADPAFPKNFPWLTGE
DYWLRQTVTFIEQAKVLQEPPLQLTPMY
>LFGLNPFC_04048 Protein YihD
MKCKRLNEVIELLQPAWQKEPDLNLLQFLQKLAKESGFDGELADLTDDILIYHLKMRDSA
KDAVIPGLQKDYEEDFKTALLRARGVIKE
>LFGLNPFC_04049 Molybdenum cofactor guanylyltransferase
MNLMTMITGVVLAGGKARRMGGVDKGLLELNGKPLWQYVVADALMTQLSHVVINANRHQEI
YQVSGLKVIEDSLADYPGPLAGMLSVMQQEAGEWFLFCPCDTPYIPHDLAARLTHQRKDA
PVVWVHDGERDHPTIALVNRAIEPLLLEYLQAGERRVMAFMRLAGGHAVDFSDRKEAFIN
VNTPEELARWQEKR
>LFGLNPFC_04050 Molybdopterin-guanine dinucleotide biosynthesis adapter protein
MAGKTMIPLLAFAAWSGTGKTTLLKKLIPALCARGIRPGLIKHTHHDMDVDKPGKDSYEL
RKAGAAQTIVASQQRWALMTETPDEEELDLHFLASRMDTSKLDLILVEGFKHEEIAKIVL
FRDGAGHRPEELVIDRHVIAVASDVPLNLDVALLDINDVEGLADFVVEWMQKQDG
>LFGLNPFC_04056 Protoporphyrinogen IX dehydrogenase [menaquinone]
MKTLILFSTRDGQTREIASYLASELKELGIQADVANVHRIEEPQWENYDRVVIGASIRYG
HYHSAFQEFVKKHATRLNSMPSAFYSVNLVARKPEKRTPQTNSYARKFLMNSQWRPDRCA
VIAGALRYPRYRWYDRFMIKLIMKMSGGETDTRKEVVYTDWEQVANFAREIAHLTDKPTL
K
>LFGLNPFC_04057 Trk system potassium uptake protein TrkH
MHFRAITRIVGLLVILFSGTMIIPGLVALIYRDGAGRAFTQTFFVALAIGSMLWWPNRKE
KGELKSREGFLIVVLFWTVLGSVGALPFIFSESPNLTITDAFFESFSGLTTTGATTLVGL
DSLPHAILFYRQMLQWFGGMGIIVLAVAILPILGVGGMQLYRAEMPGPLKDNKMRPRIAE
TAKTLWLIYVLLTVACALALWFAGMDAFDAIGHSFATIAIGGFSTHDASIGYFDSPTINT
IIAIFLLISGCNYGLHFSLLSGRSLKVYWRDPEFRMFIGVQFSLVVICTLVLWFHNVYSS
ALMTINQAFFQVVSMATTAGFTTDSIARWPLFLPVLLLCSAFIGGCAGSTGGGLKVIRIL
LLFKQGNRELKRLVHPNAVYSIKLGNRALPERILEAVWGFFSAYALVFIVSMLAIIATGV
DDFSAFASVVATLNNLGPGLGVVADNFTSMNPVAKWILIANMLFGRLEVFTLLVLFTPTF
WRE
>LFGLNPFC_04058 IMPACT family member YigZ
MESWLIPAAPVTVVEEIKKSRFITLLAHTDGVEAAKAFVESVRAEHPDARHHCVAWVAGA
PDDSQQLGFSDDGEPAGTAGKPMLAQLMGSGVGEITAVVVRYYGGILLGTGGLVKAYGGG
VNQALRQLTTQRKTPLTEYTLQCEYSQLTGIEALLGQCDGKIINSDYLAFVLLRVALPAA
KVAEFSAKLADFSRGSLQLLAIEE
>LFGLNPFC_04059 Xaa-Pro dipeptidase
MESLASLYKNHIATLQERTRDALARFKLDALLIHSGELFNVFLDDHPYPFKVNPQFKAWV
PVTQVPNCWLLVDGVNKPKLWFYLPVDYWHNVEPLPNSFWTEDVEVIALPKADGIGSLLP
AARGNIGYIGPVPERALQLGIEASNINPKGVIDYLHYYRSFKTEYELACMREAQKMAVNG
HRAAEEAFRSGMSEFDINIAYLTATGHRDTDVPYSNIVALNEHAAVLHYTKLDHQAPEEM

RSFLLDAGAEYNGYAADLTRTWSAKSDNDYAQLVKDVNDEQLALIATMKAGVSYVDYHIQ
FHQRIAKLLRKHQIITDMSEEAMVENDLTGPFMPHGIGHPLGLQVHDVAGFMQDDSGTHL
AAPAKYPYLRCTRILQPGMVLTIEPGIYFIESLLAPWREGQFSKHFNWQKIEALKPFGGI
RIEDNVVIHENNVENMTRDLKLA
>LFGLNPFC_04060 Fatty acid oxidation complex subunit alpha
MLYKGDTLYLDWLEDGIAELVFDTPGSVNKLDTATVASLGEAIGVLEQQSDLKGLLLRSN
KAAFIVGADITEFLSLFLVPEEQLSQWLHFANSVFNRLEDLPVPTIAAVNGYALGGGCEC
VLATDYRLATPDLRIGLPETKLGIMPGFGGSVRMPRMLGADSALEIIAAGKDVGADQALK
IGLVDGVVKAEKLVEGAIAILRQAINGDLDWKAKRQPKLEPLKLSKIEAAMSFTIAKGMV
AQTAGKHYPAPITAVKTIEAAARFGREEALNLENKSFVPLAHTNEARALVGIFLNDQYVK
GKAKKLTKDVETPKQAAVLGAGIMGGGIAYQSAWKGVPVVMKDINDKSLTLGMTEAAKLL
NKQLERGKIDGLKLAGVISTIHPTLDYAGFDRVDVVVEAVVENPKVKKAVLAETEQKVRP
DTVLASNTSTIPISELANALERPENFCGMHFFNPVHRMPLVEIIRGEKSSDETIAKVVAW
ASKMGKTPIVVNDCPGFFVNRVLFPYFAGFSQLLRDGAGFRKIDKVMEKQFGWPMGPAYL
LDVVGIDTAHHAQAVMAAGFPQRMQKDYRDAIDALFDANRFGQKNGLGFWRYKEDSKGKP
KKEEDVVVDDLLAKVSQPKRDFSEEEIIARMMIPMVNEVVRCLEEGIIATPAEADMALVY
GLGFPPFHGGAFRWLDTLGSAKYLDMAQQYQHLGPLYEVPEGLRNKARHNEPYYPPVEPA
RPVGDLKTA
>LFGLNPFC_04061 3-ketoacyl-CoA thiolase FadA
MEQVVIVDAIRTPMGRSKGGAFRNVRAEDLSAHLMRSLLARNPALEAAALDDIYWGCVQQ
TLEQGFNIARNAALLAEVPHSVPAVTVNRLCGSSMQALHDAARMIMTGDAQACLVGGVEH
MGHVPMSHGVDFHPGLSRNVAKAAGMMGLTTEMLARMHGISREMQDAFAARSHARAWAAT
QSGAFKNEIIPTGGHDADGVLKQFNYDEVIRPETTVEALATLRPAFDPVSGTVTAGTSSA
LSDGAAAMLVMSESRARELGLKPRARVRSMAVVGCDPSIMGYGPVPASKLALKKAGLSAS
DIGVFEMNEAFAAQILPCIKDLGLMEQIDEKINLNGGAIALGHPLGCSGARISTTLLNLM
EHKDVQFGLATMCIGLGQGIATVFERV
>LFGLNPFC_04062 NAD(P)H-flavin reductase
MTTLSCKVTSVEAITDTVYRVRIVPDAAFSFRAGQYLMVVMDERDKRPFSMASTPDEKGF
IELHIGASEINLYAKAVMDRILKDHQIVVDIPHGEAWLRDDEERPMILIAGGTGFSYARS
ILLTALARNPNRDITIYWGGREEQHLYDLCELEALSLKHPGLQVVPVVEQPEAGWRGRTG
TVLTAVLQDHGTLAEHDIYIAGRFEMAKIARDLFCSERNAREDRLFGDAFAFI
>LFGLNPFC_04063 3-octaprenyl-4-hydroxybenzoate carboxy-lyase
MDAMKYNDLRDFLTLLEQQGELKRITLPVDPHLEITEIADRTLRAGGPALLFENPKGYSM
PVLCNLFGTPKRVAMGMGQEDVSALREVGKLLAFLKEPEPPKGFRDLFDKLPQFKQVLNM
PTKRLRGAPCQQKIVSGDDVDLNRIPIMTCWPEDAAPLITWGLTVTRGPHKERQNLGIYR
QQLIGKNKLIMRWLSHRGGALDYQEWCAAHPGERFPVSVALGADPATILGAVTPVPDTLS
EYAFAGLLRGTKTEVVKCISNDLEVPASAEIVLEGYIEQGETAPEGPYGDHTGYYNEVDS
FPVFTVTHITQREDAIYHSTYTGRPPDEPAVLGVALNEVFVPILQKQFPEIVDFYLPPEG
CSYRLAVVTIKKQYAGHAKRVMMGVWSFLRQFMYTKFVIVCDDDVNARDWNDVIWAITTR
MDPARDTVLVENTPIDYLDFASPVSGLGSKMGLDATNKWPGETQREWGRPIKKDPDVVAH
IDAIWDELAIFNNGKSA
>LFGLNPFC_04064 Transcription antitermination protein RfaH
MQSWYLLYCKRGQLQRAQEHLERQAVNCLAPMITLEKIVRGKRTAVSEPLFPNYLFVEFD
PEVIHTTTINATRGVSHFVRFGASPAIVPSAVIHQLSVYKPKDIVDPSTPYPGDKVIITE
GAFEGFQAIFTEPDGEARSMLLLNLINKEIKHSVKNTEFRKL
>LFGLNPFC_04065 3'-5' ssDNA/RNA exonuclease TatD
MFDIGVNLTSSQFAKDRDDVVARAFDAGVNGLLITGTNLRESQQAQKLARQYSSCWSTAG
VHPHDSSQWQAVTEEAIIELAAQPEVVAIGECGLDFNRNFSTPEEQERAFVAQLRIAAEL
NMPVFMHCRDAHERFMTLLEPWLDKLPGAVLHCFTGTREEMQACVACGIYIGITGWVCDE
RRGLELRELLPLIPAEKLLIETDAPYLLPRDLTPKPSSRRNEPAHLPHILQRIAHWRGED
AAWLAATTDANVKTLFGIAF
>LFGLNPFC_04066 Sec-independent protein translocase protein TatC
MSVEDTQPLITHLIELRKRLLNCIISVIVIFLCLVYFANDIYHLVSAPLIKQLPQGSTMI
ATDVASPFFTPIKLTFMVSLILSAPVILYQVWAFIAPALYKHERRLVVPLLVSSSLLFYI
GMAFAYFVVFPLAFGFLANTAPEGVQVSTDIASYLSFVMALFMAFGVSFEVPVAIVLLCW
MGITSPEDLRKKRPYVLVGAFVVGMLLTPPDVFSQTLLAIPMYCLFEIGVFFSRFYVGKG
RNREEENDAEAESEKTEE
>LFGLNPFC_04067 Sec-independent protein translocase protein TatB
MFDIGFSELLLVFIIGLVVLGPQRLPVAVKTVAGWIRALRSLATTVQNELTQELKLQEFQ
DSLKKVEKASLTNLTPELKASMDELRQAAESMKRSYVANDPEKASDEAHTIHNPVVKDNE
TAHEGVTPAAAQTQASSPEQKPETTPEPVVKPAADAEPKTAAPSPSSSDKP
>LFGLNPFC_04068 Sec-independent protein translocase protein TatA
MGGISIWQLLIIAVIVVLLFGTKKLGSIGSDLGASIKGFKKAMSDDEPKQDKTSQDADFT
AKTIADKQADTNQEQAKIEDAKRHDKEQV

>LFGLNPFC_04069 putative protein kinase UbiB
MTPGEVRRLYFIIRTFLSYGLDELIPKMRITLPLRLWRYSLFWMPNRHKDKPLGERLRLA
LQELGPVWIKFGQMLSTRRDLFPPHIADQLALLQDKVAPFDGKLAKQQIEAAMGGLPVEA
WFDDFEIKPLASASIAQVHTARLKSNGKEVVIKVIRPDILPVIKADLKLIYRLARWVPRL
LPDGRRLRPTEVVREYEKILIDELNLLRESANAIQLRRNFEDSPMLYIPEVYPDYCSEGM
MVMERIYGIPVSDVATLEKNGTNMKLLAERGVQVFFTQVFRDSFFHADMHPGNIFVSYEH
PENPKYIGIDCGIVGSLNKEDKRYLAENFIAFFNRDYRKVAELHVDSGWVPPDTNVEEFE
FAIRTVCEPIFEKPLAEISFGHVLLNLFNTARRFNMEVQPQLVLLQKTLLYVEGVGRQLY
PQLDLWKTAKPFLESWIKDQVGIPALVRAFKEKAPFWVEKMPELPELVYDSLRQGKYLQH
SVDKIARELQSNHVRQGQSRYFLGIGATLVLSGTFLLVSRPEWGLMPGWLMAGGLIAWFV
GWRKTR
>LFGLNPFC_04070 Ubiquinone biosynthesis accessory factor UbiJ
MPFKPLVTAGIESLLNTFLYRSPALKTARSRLLGKVLRVEVKGFSTSLILVFSERQVDVL
GEWAGDADCTVIAYASVLPKLRDRQQLAALIRSGELEVQGDIQVVQNFVALADLAEFDPA
ELLAPYTGDIAAEGISKALRGGAKFLHHGIKRQQCYVAEAITEEWRMAPGPLEVAWFAEE
TAAVERAVDALTKRLEKLEAK
>LFGLNPFC_04071 Ubiquinone/menaquinone biosynthesis C-methyltransferase UbiE
MVDKSQETTHFGFQTVAKEQKADMVAHVFHSVASKYDVMNDLMSFGIHRLWKRFTIDCSG
VRRGQTVLDLAGGTGDLTAKFSRLVGETGKVVLADINESMLKMGREKLRNIGVIGNVEYV
QANAEALPFPDNTFDCITISFGLRNVTDKDKALRSMYRVLKPGGRLLVLEFSKPIIEPLS
KAYDAYSFHVLPRIGSLVANDADSYRYLAESIRMHPDQDTLKTMMQDAGFESVDYYNLTA
GVVALHRGYKF
>LFGLNPFC_04072 hypothetical protein
MDFSIMVYAVIALVGVAIGWLFASYQHAQQKAEQLAEREEMVAELSAAKQQITQSEHWRA
ECELLNNEVRSLQSINTSLEADLREVTTRMEAAQQHADDKIRQMINSEQRLSEQFENLAN
RIFEHSNRRVDEQNRQSLNSLLSPLREQLDGFRRQVQDSFGKEAQERHTLTHEIRNLQQL
NAQMAQEAINLTRALKGDNKTQGNWGEVVLTRVLEASGLREGYEYETQVSIENDARSRMQ
PDVIVRLPQGKDVVIDAKMTLVAYERYFNAEDDYTRESALQEHIASVRNHIRLLGRKDYQ
QLPGLRTLDYVLMFIPVEPAFLLALDRQPELITEALKNNIMLVSPTTLLVALRTIANLWR
YEHQSRNAQKIADRASKLYDKMRLFVDDMSAIGQSLDKAQDNYRQAMKKLSSGRGNVLAQ
AEAFRGLGVEIKREINPDLAEQAVSQDEEYRLRSVPEQPNDEAYQRDDEYNQQSR
>LFGLNPFC_04073 HTH-type transcriptional regulator XynR
MAETSSTTAGAQTLLRGLAVLNAVYNGCHDLKSIGEFTGTTRSTTHRLVTVLVEQRYLRH
VPTQGYQLGAKLIEFGARALESTSLYEIAQPVLQRLARYTLDTVHLGIVEGDEVLYLEKI
NSQRGLEMRSRPGHRMPLAITGIGKALILNRTEEEWRTLFKTCGDETKLGAFIQNMRRYA
ASGFAFDLEENEPTIRCVAAPVYNARDEIVAAISVASTTTYMSLARLEELAPYVKSCAEE
ISAELGWGKHVRKDK
>LFGLNPFC_04074 putative 2-dehydro-3-deoxygalactonokinase DgoK1
MPGAPSFIALDWGTSSLRAWRFGESPNPQEKREFPWGIMKLPSQAATREDTFHDTFLRVC
SDWLAQTPCPVLACGMLGSAQGWQPAAYLPCPVTLEGLAKQLTPVIHQQQTMLHIIPGVI
KEGEMPEVMRGEETQIFGAISMEPALQNAIHQGMPVLIGLPGTHAKWAVVENNTITDFRT
FMTGELFDVLSRHSILGATMHPGDEPHWDAFTHGLTAAQEHHQTGLLSTLFSTRSRLLTS
NLTSSSQGDYLSGLLIGHELCGLASSLLRDLPATTPIALIGSANLNSRYSQAFSHVFPDR
QIHAIPNATEQGLWRIAHAAGLLSTNARECTHAI
>LFGLNPFC_04075 2-dehydro-3-deoxy-6-phosphogalactonate aldolase
MPFNTLLQKTGLVAILRGVKPDEIVAIGEKLYAAGFRLIEIPMNSPEALQSISLLRDALP
KDCLVGAGTVLSVEQVVAVKEAGGQIIVMPHCDTAVIRRARALGMYCAPGVATPTEAFAA
IEHGANAIKLFPAEQITPEVTKAWRAVIPQSVPMLPVGGITPETMARYLSHGANGFGLGS
ALYRPGMTPEQVYENAVLFMNAWNNLNSN
>LFGLNPFC_04076 hypothetical protein
MIKHLTLPKKMAAMVLLAGAALSIAPVQAASYPTKQIELVVPYAAGGGTDLVARAFADAA
KNHLPVSIGVINKPGGGGAIGLSEIAAARPNGYKIGLGTVELTTLPSLGMVRFKTSDFKP
IARLNADPAAITVRADAPWNSYEEFMTYAKANPGKVRIGNSGTGAIWHLAAAALEDKTGA
KFSHVPYDGAAPAITGLLGGHIEAVSVSPGEVINHVNGGKLKTLVVMADERMKTMPDVPT
LKEKGVDLSIGTWRGLIVSQKTQQDVVDVLAKAAKETAEEPAFQDALQKLNLNYAWLDAA
SFQTQISEQEKYFDELLTRLGLKK
>LFGLNPFC_04077 hypothetical protein
MNTKQSVAQLAVPHRKRLSSTMVVALLLCVVAGAVMINAADFPATAIETDPGASAFPTFY
ACALIVLAVLLVIRDLLQAKPASCANAQEKPAFRKTATGIAATAFYIVAMSYCGYLITTP
VFLIVIMTLMGYRRWVLTPGIALLLTAILWLLFVEALQVPLPVGTFFE
>LFGLNPFC_04078 hypothetical protein
MFESELLTQGFSTLLNNPQALLFATFGVMLGIVIGALPGLTATMGVAILLPFTYGMEPVS
GLLMICGVFFGGVYGGSITAILLKIPGTPAAAATAIDGYELTKQGKAGLALSAATFSSFS
GGTLSIIVLMFLSPVLASWALKFSASESFALATFGLSIIASISGESLIKGLIAGVGGLLI

ATIGLDPMGGFPRFTGGFVELMNVPFIPVMIGLFAASEAFRSMEQNQQIRRGAKVAIGSL
LLPWQTLRRIALTILRSSGLGVFIGMIPGAGADIAAFVAYNETRRFSKTPENFGKGEIKA
VASCEAGANGCTGGALLPMLTLGIPGDAVTAIMLGALTLQGMQPGPLMFTDHGDMVYTLF
VGMIFCYFMLLVLGLLSLKVIGNVVKIPGNILTPMILALCVVGTYALNNSLFDVGIMLIA
GVVGYFMQKGGYPASPVVLALIMGPMAESNFRRALSLSGGSLDFLYTRPITLALLTLAAF
TLLTPIIRKIMRLRRQ
>LFGLNPFC_04079 Uridine phosphorylase
MSKSDVFHLGLTKNDLQGATLAIVPGDPDRVEKIAALMDKPVKLASHREFTTWRAELDGK
PVIVCSTGIGGPSTSIAVEELAQLGIRTFLRIGTTGAIQPHINVGDVLVTTASVRLDGAS
LHFAPLEFPAVADFECTTALVEAAKSIGATTHVGVTASSDTFYPGQERYDTYSGRVVRHF
KGSMEEWQAMGVMNYEMESATLLTMCASQGLRAGMVAGVIVNRTQQEIPNAETMKQTESH
AVKIVVEAARRLLK
>LFGLNPFC_04080 Carboxymethylenebutenolidase
MATTQQSGFAPAASPLASTIVQTPDDAIVAGFTSIPSQGDNMPAYHARPKQSDGPLPVVI
VVQEIFGVHEHIRDICRRLALEGYLAIAPELYFREGDPNDFADIPTLLSGLVAKVPDSQV
LADLDHVASWASRNGGDVHRLMITGFCWGGRITWLYAAHNPQLKAAVAWYGKLTGDKSLN
SPKQPVDIATDLNAPVLGLYGGQDNSIPQESVETMRQALRAANAKAEIIVYPDAGHAFNA
DYRPSYHAESAKDGWQRMLEWFTQYGVKK
>LFGLNPFC_04081 hypothetical protein
MTNVKGVKEWHTATNFLMLYGFEIKKIVEIV
>LFGLNPFC_04082 Peroxyureidoacrylate/ureidoacrylate amidohydrolase RutB
MTQSIFQAQPFELPFDPRTTALVMIDMQRDFVEAGGFGEALGNDVSLVRTAIAPCTEVLA
AARQKGIMVIHTREGHRADLSDCPPAKLTRGGKTFIGEPGPMGRILVRGEAGHDIIPELY
PVAGEPVIDKPGKGAFYQTDLHLILQNHGIKTLIVCGVTTEVCVTTTVREANDRGYECII
PEDCVGSYFPEFQKYALEMIKAQGAIFGWVTDSKAIIAGLEG
>LFGLNPFC_04083 hypothetical protein
MVQVTLCASSLGYLFPQNETQDLRLHSAFKHAVNLYSDAGTFITLLCAQTYLNLPDAARV
WLPECWDWRREIAHSDPIQLTQGLLRTPRFCVALENATLWQSPFVGRMLTLEAFPLVFQH
YPTMASQRLLFCLEHNVQSTLHLPDSLTHQGLAIMEHPDALERQVPQLIGFGKGLTPDGD
DYLLGYLAALWLWQLPAPLADHQYRLQQAIDQHAHNTTDISRHYLERALQGHFSEPICQL
LAQLVGSASAMTIASCAEQVMQFGATSGVDCLAGMLHGFRTLNTMN
>LFGLNPFC_04084 Protein FdrA
MKVKYKVFSNLYQDSVSLMQISAQISKLPGIQQASVVMGTPNNLEQLRDAGLGNEINASP
NDLVIAVMGEEDICNEALVLAQQRLTSKPDDETDSGIKTPEKVSLEMALEAEPEANLALI
SVPGDYAAAEAIKALNLGMNVMMFSDNVSIGQEKSIKTLARERQRIVMGPDCGTAIVNGI
PLGFANVVKRGAIGVIGASGTGLQEVTCRIDQLGAGISQALGTGGHDLSEEIGGISMLFA
LDALAQDDETRVIVLISKPPSPIVAQTILERAEACGKPVVVNFLGANPHDLARPNITAAT
TLASAANIAVALLNAQPLPAVETEISCADLTMLQNACQRLPAHRQAIRGVFAGGTFCYEA
QLICQQKGFSAASNTPVAGNRALANIWQSEDHTLIDMGDDDFTRGKPHPMIDPTLRNQRL
LNELNDSHTAVVLFDLVLGYGASTAPASELLDQLSHIDMNNAPLLIAHVCGTEADPQIRS
QQIRALQNAGVIIASSNAQAALWASTVAQTQLQKKGLNA
>LFGLNPFC_04085 hypothetical protein
MGQHRCTDSVAEKGAKRMNTLFNQPLKVVNAGLHSFADNIQHAGGSAIALNWQPPAQGDI
DAGLDLASLLRHPLVENANQIAMTRYLEAQPMLVDVMLAKEAIPAMAEQKRILHSGPPIA
WEEMCGPVKGAIIGAMLYEGWATSQQDAENQINAGEIDLAPCHHYHAVGPMAGIISPSMP
LWVVENKTNGHRTFSNFNEGLGKVLRFGANNDEVLNRLAWMRDELAPAMKAAIAQHGELE
LKPLMAQALHMGDEVHNRNAAATGLLIKRLLPALLACSLPQEHIQRVVAFITGNDHFFLN
LSMAACKAMMDAAANVPFSSMVTVMARNGVSFGIRLSGTSDRWFQAPANAVEGLFFPGFG
VDDAAADLGDSAITETAGMGGFAMASSPAIVKFVGGTPADATNNSRRMQAITLGGNPAFT
LPALNFAPTAAGIDARKVTDRGILPVINTGIAHKQAGVGQIGAGITTAPMACFVAAVRAL
AEIVAKENHHG
>LFGLNPFC_04086 Carbamate kinase 1
MVKPLAVVAVGGNALIQDEQRNSIPDQYVAVMESVQHIVDMVEAGWDLVLTHGNGPQVGF
ILRRSELASNEVSPVPLDYAVGDTQGAIGYMFQKALHNELARRGINKPVIALVTQTRVSP
HDDAFASPSKPIGAFLDEATAQQRQQQLGWTLMEDAGRGWRRTVPSPAPLEIIEHDTIAH
LVRQGYLVIACGGGGIPVVRDGQQLKGVEAVIDKDLASALLASQLGADLLVIPTGVEKVA
INFGTPQQQWLDAISVAEAQTLLREGQFGVGSMQPKVEAIVDFINASQQQGKQASGLITS
PQTIKAALAHQSGTWITL
>LFGLNPFC_04087 Allophanate hydrolase
MTTTLLAVNGTLMRGLELNPNMQKAGGIFVREDRTDAHYRLWSINDRHPGMIRVNEGGTH
VDVEIWQLPLASFAALLMSEPAGLAIGKIKLADGSEVLGVLAENWLTEGGQREITELGSWR
KYTGHFHTV
>LFGLNPFC_04088 Cytosine permease
MRKKEENLNTASGLRIAMILLGIAVTPVLLSSSSLGNQLSSGSLISVVLLGGVILTLLSA

ITISVGEKARLPTYGIVKYSFGEKGAIAINILMAISLFGWIAVTANMFGHSVHDLLAQHG
LEVPLALLVAAGCVIFVASTAFGFAVLGKIAQVAVPVIALVLCYILYVATHTEVAVPAAI
VEMNTGVAVSTVVGTIIVLVATLPDFGSFVHNRKHALIAAGVTFLVAYPLLYWAGATPSA
ISGQGSLLGAMAVFGAVLPAALLLIFACVTGNAGNMFQGTLVVSTLLTRFPKWQITVALG
ILSAIVVSMDIMAWFIPFLLFLGIATPPVAGIYIADFFLYRRNGYQESVLAQESQIKVLT
FAAWIIGAAVGFMTVKGLFTLTTIPSVDSILVACIAYAILSRASQHR
>LFGLNPFC_04089 putative HTH-type transcriptional regulator YahB
MFISDETLRVIHLVARHQSITTAAEQLNKVPSAISYTIKKAEESLGVELFIRKGRYIELS
PAGSYFIEHSKTILGDLEALKRNTVLIHDGIERELTIAVNNIIPGDLLVAFIRDFERQFT
STTLTVDLEVYNGCWDALYSKRADLVYGAPHAVPSSEGIISEPVGQMEWDFVVSPLHPLA
AKRHPLENSELRHYPAVCIRDTSVNFPPMQAWLLEGQKPIFVPDFATAIALIEQNVGIGY
IPHHLALPLLNSGKLLKKPMREHKHATKLFLAARSDGMGKACQWCIEYLRNPQLMTQFVF
N
>LFGLNPFC_04090 putative HTH-type transcriptional regulator YbbH
MNNITVKSLIQSNYPTLHQAEKKVADYILSHAHEVVNYSVTELSEKSHASEATIVRTCKK
LGYQGYYHLKIALAKEVINPDNSYPDNTDFTDITSLATFLLKKQAEDLIQSTQFFNAEVL
ESILKLLANCDTIFFFAAGNSNPLAVYGAYKFSQLGLKTVVHVSPEMQINAAYSMGKRDL
AIGISNSGSTNLMVDIFKVVKERGAKSVCITNYIKSPLSKLSTHQLNTAVSDKIFFEAFD
STRVPAMGVIDMLVLLFMYKNKAHYEKYRTREEFLGSRFKG
>LFGLNPFC_04091 Transketolase 1
MNSQLLANAIRMLSVDAIQKANSGHPGAPMGMADIAEVVWRRHLRHNPKNPQWFNRDRYI
QSNGHGSMLIYALLHLTGYDLSMDDIRDFRQLHSRTPGHPEYGYTPGVETTTGPLGQGVA
NAVGMAIAEKALAAEFNKPGFNIVDHHTWLFLGDGCLMEGISHEACGLAGTLKLGNLIAI
WDDNGISIDGHVEGWFAEDTAARFRAYGWHVIEGVDGHDPEEVDAAVREAKSVTDKPSLL
CCKTIIGFGSPNKANSHDCHGSALGADEVALVRERLQWPYAPFEIPGEIYAAWDATEKGA
QVQQKWDALFADYAKQWPELAAEFTRRMKGDLPAGWAENMQKYVHDLQSHPAALATRQVS
QKCLNHFADMLPELMGGSADLSPSNLTRHQKSVDFTGENPAGNYISYGVREFGMSAIMNG
LALHGGFIPYGGTFLMFMEYARNALRMAALMKIRSVFVYTHDTIGLGEDGPTHQPVEQLA
SLRLTPNMETWRGCDQVEVAVAWQQAIERKDGPTSLVLTRQPLAQQPRTAAQLAEIARGG
YVLSDCDGQPEMILISAGSEIELVVSAAKALTEEGRKVRVVSMPCTERFDNQDAAYKESV
LPKAVRKRLAVEASIAGFWERYVGLDGKVIGMTSFGESAPANVLFKHFGFTPENVLAQAR
ELLNS
>LFGLNPFC_04092 PTS system glucose-specific EIICBA component
MSKKITSLFNFKKLQKFSSALMLPIAILPAAGLILAIGISFNIELLANCGGLIFANLPLL
FCVGIVIGLTGDGSSSLSAIISYMIMNATMGTQLGITAESVAASNGDYAMILGVPTLQTG
VFGGLIVAIFVYLLYTRFHNIELPQFLGFFGGKRFVPIITAVAAIIVGYLLPWLWIPVQK
GLASLSTLVTSGHNANFAAFIYAVGERSLIPFGLHHIWNVPFYYNFGDYMTKSGQLVTGD
IPVFFAQLRDGVPLTAGLFMTGRFPIMMFALPAAALAMYQEAKPERKALIKGLLLSGALT
TFVTGITEPLEYAFLFAAPVLYIIHIFLYATSFVLMNILNVHIGHPFAGGLIDFVLNGVM
PNRTPWYLVFLAGAGYAAIYYTVFRVLIRKLNLKTPGREDEEIAVATTLTREERPYQIIE
AVGGFDNIEDVDACATRLRLALVDDKKVNEKRLKELGAAGLVKLGDGGVQVIFGGKSQIL
RDEIKTVMSRPRPTEMTVACAN
>LFGLNPFC_04093 3-hexulose-6-phosphate isomerase
MDAKLYITEIINELTFVKDSIREDDCTKLIDAIQQSQRVFCYGLGRAGFSMKAFTMRLMH
MGKEVYFLTETITPNFGPGDLFIVSSASGETAQLVALAKKARQFGGAVAVLTTNRHATIT
EFVDVIVQINAPSKNQKDSVFRSAQPMASLYEQALLVIADALVMKMAAESGAPESELFKR
HANLE
>LFGLNPFC_04094 Major exported protein
MSDEIYLTITGEQQGCISSRCGTSASIGNRWQIGHEDEIFAFSLSNSITNTGKGSQLHGL
SFCKLIDKSSPLLINAINNNEQLFMEFDFYRINRFGR
>LFGLNPFC_04095 hypothetical protein
MRFPDLESFEHFGARGAVSGRPFNPDLAGGPIENLTIDGVTINREGIAIVEKHISRFDHD
PANDVMISRLKKIANKELLPEKYDLNYYTHECREYQRYCNLGWETGEPNGLDGYELWNNA
HTATLEDFKIKDTDLFHPDAKK
>LFGLNPFC_04096 hypothetical protein
MTITIVEFNVLKVMAEKDIDWSWMVLDRTLAIRNIPGFGNVANIVTKLVNHGLVDIVNGE
GNSKPRYRVSQYGLNLIKEQQDNLF
>LFGLNPFC_04097 5-methyltetrahydropteroyltriglutamate--homocysteine methyltransferase
MTILNHTLGFPRVGLRRELKKAQESYWAGNSTREELLAVGRELRARHWDQQKQAGIDLLP
VGDFAWYDHVLTTSLLLGNVPQRHQNNDGSVDIDTLFRIGRGRAPTGEPAAAAEMTKWFN
TNYHYMVPEFVKGQQFKLTWTQLLEEVDEALALGHKVKPVLLGPITYLWLGKVKGEQFDR
LSLLNDILPVYQQVLAELAKRGIEWVQIDEPALVLELPQAWLNAYKPAYDALQGQVKLLL
TTYFEGVTPNLDTITALPVQGLHVDLVHGKDDVAELHKRLPSDWLLSAGLINGRNVWRAD
LTEKYAQIKDIVGKRDLWVASSCSLLHSPIDLSVETRLDAEVKSWFAFALQKCHELALLR

DALNSGDTAALAEWSAPIQARRHSTRVHNPAVEKRLAAITAQDSQRANVYEVRAEAQRAR
FKLPAWPTTTIGSFPQTTEIRTLRLDFKKGNLDANNYRTGIAEHIKQAIVEQERLGLDVL
VHGEAERNDMVEYFGEHLDGFVFTQNGWVQSYGSRCVKPPIVIGDVSRPAPITVEWAKYA
QSLTDKPVKGMLTGPVTILCWSFPREDVSRETIAKQIALALRDEVADLEAAGIGIIQIDE
PALREGLPLRRSDWDAYLQWGVEAFRINAAVAKDDTQIHTHMCYCEFNDIMDSIAALDAD
VITIETSRSDMELLESFEEFDYPNEIGPGVYDIHSPNVPSVEWIEALLKKAAKRIPAERL
WVNPDCGLKTRGWPETRAALANMVQAAQNLRRG
>LFGLNPFC_04098 HTH-type transcriptional regulator MetR
MIEVKHLKTLQALRNCGSLAAAAATLHQTQSALSHQFSDLEQRLGFRLFVRKSQPLRFTP
QGEILLQLANQVLPQISQALQACNEPQQTRLRIAIECHSCIQWLTPALENFHKNWPQVEM
DFKSGVTFDPQPALQQGELDLVMTSDILPRSGLHYSPMFDYEVRLVLAPDHPLAAKTRIT
PEDLASETLLIYPVQRSRLDVWRHFLQPAGVSPSLKSVDNTLLLIQMVAARMGIAALPHW
VVESFERQGLVVTKTLGEGLWSRLYAAVRDGEQRQPITEAFIRSARNHACDHLPFVKSAE
RPTYDAPTVRPGSPARL
>LFGLNPFC_04099 Biotin transporter
MALLIITTILWAFSFSFYGEYLAGHVDSYFAVLVRVGLAALVFLPFLRTRGNSLKTVGLY
MLVGAMQLGVMYMLSFRAYLYLTVSELLLFTVLTPLYITLIYDIMSKRRLRWGYAFSALL
AVIGAGIIRYDQVTDHFWTGLLLVQLSNITFAIGMVGYKRLMETRPMPQHNAFAWFYLGA
FLVAVIAWFLLGNAQKMPQTTLQWGILVFLGVVASGIGYFMWNYGATQVDAGTLGIMNNM
HVPAGLLVNLAIWHQQPHWPTFITGALVILASLWVHRKWVAPRSSQTADDRRRDCALNE
>LFGLNPFC_04100 Pyridoxal phosphate phosphatase YigL
MYQVVASDLDGTLLSPDHTLSPYAKETLKLLTARGINFVFATGRHHVDVGQIRDNLEIKS
YMITSNGARVHDLDGNLIFAHNLDRDIASDLFGVVNDNPDIITNVYRDDEWFMNRHRPEE
MRFFKEAVFKYALYEPGLLEPEGVSKVFFTCDSHEKLLPLEQAINARWGDRVNVSFSTLT
CLEVMAGGVSKGHALEAVAKKLGYSLKDCIAFGDGMNDAEMLSMAGKGCIMGSAHQRLKD
LHPELEVIGTNAEDAVPHYLRKLYLS
>LFGLNPFC_04101 Lysophospholipase L2
MFQQQKDWETRENAFAAFTMGPLTDFWRQRDEAEFTGVDDIPVRFVRFRAQHHDRVVVIC
PGRIESYVKYAELAYDLFHLGFDVLIIDHRGQGRSGRLLGDPHLGHVNRFNDYVDDLAAF
WQQEVQPGPWRKRYILAHSMGGAISTLFLQRHPGVCDAIALTAPMFGIVIRMPSFMARQI
LNWAEAHPRFRDGYAIGTGRWRALPFAINVLTHSRQRYRRNLRFYADDPTIRVGGPTYHW
VRESILAGEQVLAGAGDDATPTLLLQAEEERVVDNRMHDRFCELRTAAGHPVEGGRPLVI
KGAYHEILFEKDAMRSVALHAIVDFFNRHNSPSGNRSTEV
>LFGLNPFC_04102 Homoserine/homoserine lactone efflux protein
MTLEWWFAYLLTSIILSLSPGSGAINTMTTSLNHGYRGAVASIAGLQTGLAIHIVLVGVG
LGTLFSRSVIAFEVLKWAGAAYLIWLGIQQWRAAGAIDLKSLASTQSRRHLFQRAVFVNL
TNPKSIVFLAALFPQFIMPQQPQLMQYIVLGVTTIVVDIIVMIGYATLAQRIALWIKGPK
QMKALNKIFGSLFMLVGALLASARHA
>LFGLNPFC_04103 Threonine efflux protein
MLMLFLTVAMVHIVALMSPGPDFFFVSQTAVSRSRKEAMMGVLGITCGVMVWAGIALLGL
HLIIEKMAWLHTLIMVGGGLYLCWMGYQMLRGALKKEVVSAPAPQVELAKSGRSFLKGLL
TNLANPKAIIYFGSVFSLFVGDNVGTTERWGIFALIIVETLAWFTVVASLFALPQMRRGY
QRLAKWIDGFAGALFAGFGIHLIISR
>LFGLNPFC_04104 ATP-dependent DNA helicase RecQ
MNVAQAEVLNLESGAKQVLQETFGYQQFRPGQEEIIDTVLSGRDCLVVMPTGGGKSLCYQ
IPALLLNGLTVVVSPLISLMKDQVDQLQANGVAAACLNSTQTREQQLEVMTGCRTGQIRL
LYIAPERLMLDNFLEHLAHWNPVLLAVDEAHCISQWGHDFRPEYAALGQLRQRFPTLPFM
ALTATADDTTRQDIVRLLGLNDPLIQISSFDRPNIRYMLMEKFKPLDQLMRYVQEQRGKS
GIIYCNSRAKVEDTAARLQSKGISAAAYHAGLENNVRADVQEKFQRDDLQIVVATVAFGM
GINKPNVRFVVHFDIPRNIESYYQETGRAGRDGLPAEAMLFYDPADMAWLRRCLEEKPQG
QLQDIERHKLNAMGAFAEAQTCRRLVLLNYFGEGRQEPCGNCDICLDPPKQYDGSTDAQI
ALSTIGRVNQRFGMGYVVEVIRGANNQRIRDYGHDKLKVYGMGRDKSHEHWVSVIRQLIH
LGLVTQNIAQHSALQLTEAARPVLRGESSLQLAVPRIVALKPKAMQKSFGGNYDRKLFAK
LRKLRKSIADESNVPPYVVFNDATLIEMAEQMPITASEMLSVNGVGMRKLERFGKPFMAL
IRAHVDGDDEE
>LFGLNPFC_04105 Phospholipase A1
MRTLQGWLLPVFMLPMAVYAQEATVKEVHDAPAVRGSIIANMLQEHDNPFTLYPYDTNYI
IYTQTSDLNKEAIASYDWAENARKDEVKFQLSLAFPLWRGILGPNSVLGASYTQKSWWQL
SNSDESSPFRETNYEPQLFLGFATDYNFAGWTLRDVEMGYNHDSNGRSDPTSRSWNRLYT
RLMAENGNWLVEVKPWYVVGNTDDNPDITKYMGYYQLKIGYHLGDAVLSAKGQYNWNTGY
GGAELGLSYPITKHVRLYTQVYSGYGESLIDYNFNQTRVGVGVMLNDLF
>LFGLNPFC_04106 hypothetical protein
MSAVLTAEQALKLVGEMFVYHIPFNRALGMELERYEKEFAQLAFKNQPMMVGNWAQSILH
GGVIASALDVAAGLVCVGSTLTRHETISEDELRQRLSRMGTIDLRVDYLRPGRGERFTAT

SSLLRAGNKVAVARVELHNEEQLYIASATATYMVG
>LFGLNPFC_04107 Protein RarD
MDAKQTRQGVLLALAAYFIWGIAPAYFKLIYYVPADEILTHRVIWSFFFMVVLMSICRQW
SYLKTLIQTPQKIFMLAVSAVLIGGNWLLFIWAVNNHHMLEASLGYFINPLVNIVLGMIF
LGERFRRMQWLAVILAICGVLVQLWTFGSLPIIALGLAFSFAFYGLVRKKIAVEAQTGML
IETMWLLPVAAIYLFAIADSSTSHMGQNPMSLNLLLIAAGIVTTVPLLCFTAAATRLRLS
TLGFFQYIGPTLMFLLAVTFYGEKPGADKMVTFTFIWVALAIFVMDAIYTQRRTSK
>LFGLNPFC_04108 hypothetical protein
MPCTLFETITLFDDFSADDMQYGDMEEQDFLSLGLSDISAKVDPYRLIKYHFPGPNSTYG
AFSVPTSGTKISQSECIDILFAEMKDLAKMFSFFGQYKTLIQDLIDHFRYENGNSFHSQE
LNLSFHERINKYDYNSPIRVIKECIENDISSTPTIGYRPLLLQKIKTELLSSRLNKFNDF
KDNFNGLGISIHDISAQKISLLNFQKYPMGWSATIHFIAQDHFGLDVTDIKNKIYNKYRF
FRIWFFLQRHKDFAFKPFFTNFNTIERIENYL
>LFGLNPFC_04109 hypothetical protein
MKRKLQKIILYLFLLCCIYNLWTLRPVQILYTYSDAGNSVFLVVDHLPWTDSDKINWYLK
HQDEIKSQNPLPEDSWHTWYVIDIGNGFTDYKKYIEGPYEDLYCFPTIKSNDNCIVKNYL
MVINEYPYRNTHFFIADNNEYQLTQENKIERVFNPHDFEKDNFQQ
>LFGLNPFC_04110 Magnesium transport protein CorA
MLSAFQLENNRLTRLEVEESQPLVNAVWIDLVEPDDDERLRVQSELGQSLATRPELEDIE
ASARFFEDDDGLHIHSFFFFEDAEDHAGNSTVAFTIRDGRLFTLRERELPAFRLYRMRAR
SQSMVDGNAYELLLDLFETKIEQLADEIENIYSDLEQLSRVIMEGHQGDEYDEALSTLAE
LEDIGWKVRLCLMDTQRALNFLVRKARLPGGQLEQAREILRDIESLLPHNESLFQKVNFL
MQAAMGFINIEQNRIIKIFSVVSVVFLPPTLVASSYGMNFEFMPELKWSFGYPGAIIFMI
LAGLAPYLYFKRKNWL
>LFGLNPFC_04111 hypothetical protein
MAHRLFIGKGMITLNLKRIFLTLTLLPLFAVAADDCAPSDPTLTVQAYTVNPQTERVKMY
WQKANGEAWGTLHALLADINSQGQVQMAMNGGIYDESYAPLGLYIENGQQKVALNLASGE
GNFFIRPGGVFYVAGDKVGIVRLDAFKTSKEIQFAVQSGPLLMENGVINPRIHPNVASRK
IRNGVGINKHGNAVFLLSQQATNFYDFACYAKAKLNVEQLLYLDGTISHMYMKGEAIPWQ
RYPFVTMISVEQKG
>LFGLNPFC_04112 Adenosyl-chloride synthase
MKKTLATLFLLTCLGSSSAYADNALILQTDFSLKDGAVSAMKGVAFGVDHNLKIFDLTHE
IPPYNIWEGAYRLYQTASYWPQGSVFVSVVDPGVGTDRKSVVLKTKNGQYFVSPDNGTLT
LVAESLGIESVRQIDEKTNRLKGSEKSYTFHGRDVYAYTGARLASGAITFEQVGPELPAK
VVELSYQKAKATKGEVKGNIPILDIQYGNVWSNISDELLNQAGIKLNDTLCVTISEGSQQ
KYAGKMPYVASFGDVPEGQPMVYLNSLLNVSVALNMDNFAQKHQVASGADWNIDVKKCAK
>LFGLNPFC_04113 DNA helicase II
MDVSYLLDSLNDKQREAVAAPRSNLLVLAGAGSGKTRVLVHRIAWLMSVENCSPYSIMAV
TFTNKAAAEMRHRIGQLMGTSQGGMWVGTFHGLAHRLLRAHHMDANLPQDFQILDSEDQL
RLLKRLIKAMNLDEKQWPPRQAMWYINSQKDEGLRPHHIQSYGNPVEQTWQKVYQAYQEA
CDRAGLVDFAELLLRAHELWLNKPHILQHYRERFTNILVDEFQDTNNIQYAWIRLLAGDT
GKVMIVGDDDQSIYGWRGAQVENIQRFLNDFPGAETIRLEQNYRSTSNILSAANALIENN
NGRLGKKLWTDGADGEPISLYCAFNELDEARFVVNRIKTWQDNGGALAECAILYRSNAQS
RVLEEALLQASMPYRIYGGMRFFERQEIKDALSYLRLIANRNDDAAFERVVNTPTRGIGD
RTLDVVRQTSRDRQLTLWQACRELLQEKALAGRAASALQRFMELIDALAQETADMPLHVQ
TDRVIKDSGLRTMYEQEKGEKGQTRIENLEELVTATRQFSYNEEDEDLMPLQAFLSHAAL
EAGEGQADTWQDAVQLMTLHSAKGLEFPQVFIVGMEEGMFPSQMSLDEGGRLEEERRLAY
VGVTRAMQKLTLTYAETRRLYGKEVYHRPSRFIGELPEECVEEVRLRATVSRPVSHQRMG
TPMVENDSGYKLGQRVRHAKFGEGTIVNMEGSGEHSRLQVAFQGQGIKWLVAAYARLETV
>LFGLNPFC_04114 5-amino-6-(5-phospho-D-ribitylamino)uracil phosphatase YigB
MRFYRPLGRISALTFDLDDTLYDNRPVILRTEREALTFVQNYHPALRSFQNEDLQRLRQA
VREAEPEIYHDVTRWRFRSIEQAMLDAGLSAEEASAGAHAAMINFAKWRSRIDVPQQTHD
TLKQLAKKWPLVAITNGNAQPELFGLGHYFEFVLRAGPHGRSKPFSDMYFLAAEKLNVPI
GEILHVGDDLTTDVGGAIRSGMQACWIRPENGDLMQTWDSRLLPHLEISRLASLTSLI
>LFGLNPFC_04115 Tyrosine recombinase XerC
MTDLHTDVERYLRYLSVERQLSPITLLNYQRQLEAIIHFASENGLQSWQQCDVTMVRNFA
VRSRRKGLGAASLALRLSALRSFFDWLVSQNELKANPAKGVSAPKAPRHLPKNIDVDDMN
RLLDIDINDPLAVRDRAMLEVMYGAGLRLSELVGLDIKHLDLESGEVWVMGKGSKERRLP
IGRNAVAWIEHWLDLRDLFGSEDDALFLSKLGKRISARNVQKRFAEWGIKQGLNNHVHPH
KLRHSFATHMLESSGDLRGVQELLGHANLSTTQIYTHLDFQHLASVYDAAHPRAKRGK
>LFGLNPFC_04116 hypothetical protein
MKQPGEELQETLTELDDRAVVDYLIKNPEFFIRNARAVEAIRVPHPVRGTVSLVEWHMAR
ARNHIHVLEENMALLMEQAIANEGLFYRLLYLQRSLTAASSLDDMLMRFHRWARDLGLAG
ASLRLFPDRWRLGAPSNHTHLALSRQSFEPLRIQRLGQEQHYLGPLNGPELLVVLPEAKA

VGSVAMSMLGSDADLGVVLFTSRDASHYQQGQGTQLLHEIALMLPELLARWIERV
>LFGLNPFC_04117 Diaminopimelate epimerase
MQFSKMHGLGNDFMVVDAVTQNVFFSPELIRRLADRHLGVGFDQLLVVEPPYDPELDFHY
RIFNADGSEVAQCGNGARCFARFVRLKGLTNKRDIRVSTANGRMVLTVTDDDLVRVNMGE
PNFEPSAVPFRANKAEKTYIMRAAEQTILCGVVSMGNPHCVIQVDDVDTAAVETLGPVLE
SHERFPERANIGFMQVVKREHIRLRVYERGAGETQACGSGACAAVAVGIQQGLLAEEVRV
ELPGGRLDIAWKGPGHPLYMTGPAVHVYDGFIHL
>LFGLNPFC_04118 hypothetical protein
MKNVFKALTVLLTLFSLTGCGLKGPLYFPPADKNAPPPTKPVETQTQSTVPDKNDRATGD
GPSQVNY
>LFGLNPFC_04119 hypothetical protein
MHYRNVVCLLSCSLFLSSAWGCRLDEPEHNIYQKQGKGVVYLRPYEKTNLSLPQINYKRL
RLLPNLLIDPTKLEDWETVPPATNLMTDVVYSGANATLPHYSYYSDGRAILYAGEIVQNP
PGTPPVDISSFQAWGDFAADKYSLYYEGKRTDSNQQLNRRTLRQVEFNPQWKPDWLGLIL
RDKHYLYANGQRLDDPDTFTVLAQKSWDQRGKFSTAFNPCLPAPFGPWDTLARTQTKIMI
NGEQLDADPDTFSVVRWMPGSLFTWRDKNGLQRKVLDKENLAWDEDLTKHCLDFSLQEKK
VFWRKGPACKQEELSGLDPEQFHPISDAVAQYQDSLYTIIETESGDRKLEIVKLDDPNLI
INKRFNAGKRHGYLLTRAEGWVNHSSLHVFESDGPLILLDNRSPDEREAHLNDHPFLRRW
YARDNRYVYSFDGAQLWRYRTADPKQVRLIWKEQHSG
>LFGLNPFC_04120 IS1 family transposase IS1A
MDEQWGYVGAKSRQRWLFYAYDSLRKTVVAHVFGERTMATLGRLMSLLSPFDVVIWMTDG
WPLYESRLKGKLHVISKRYTQRIERHNLNLRQHLARLGRKSLSFSKSVELHDKVIGHYLN
IKHYQ
>LFGLNPFC_04121 IS1 family transposase IS1A
MASVSISCPSCSATDGVVRNDKSTTGHQRYLCSHCRKTWQLQFTYTASQPGTHQKIIDMA
MNGVGCRATACIMGVGLNTILRHLKNSGRSR
>LFGLNPFC_04122 hypothetical protein
MNGQIITVALILLGNTFLTPPVQAIGFDYYNDHSVMSYGKGYWGDKKIIAQFPEMNRADL
RLVKNISGKTGYIPSDEISIENEEYHWVTDGHVILWRGKIVSNPPGTPTVDIASFQAMGR
FAVDKYSLYFDGQRTESNSGASRVDLATLKAIEGNSTTLVDSKNLYLSGRRQGSSSNVTV
LEKRWWGINPRLMSVNRNLYSNDLLIRSGQNIYLNGAHLTANADSFEIIRWIPHSLLVFR
DNKGLHRYPFGQLSGKAIPVDDDVSFEVGESRVRWRKQLTPDRQWSKWIDLPGIEPEQFH
LITDNIAQYKDRLYVTKLSTFGEDQLEIIPLDTPDLVIDDSFNSGKQHAYFIRQLRSRKS
VQIIPVNGPLTKNDRFAYDDRNVYTWTDTEVRITPSHCPAKTRVREENVREIHNSDIIIP
VTDESCRNAAADGQTLKP
>LFGLNPFC_04123 Iron-sulfur cluster assembly protein CyaY
MNDSEFHRLADQLWLTIEEHLDDWDGDSDIDCEINGGVLTITFENGSKIIINRQEPLHQV
WLATKQGGYHFDLKGDEWICDRSGETFWDLLEQAATQQAGETVSFR
>LFGLNPFC_04124 Adenylate cyclase
MYLYIETLKQRLDAINQLRVDRALAAMGPAFQQVYSLLPTLLHYHHPLMPGYLDGNVPKG
ICLYTPDETQRHYLNELELYRGMSVQDPPKGELPITGVYTMGSTSSVGQSCSSDLDIWVC
HQSWLDSEERQLLQRKCSLLESWAASLGVEVSFFLIDENRFRHNESGSLGGEDCGSTQHI
LLLDEFYRTAVRLAGKRILWNMVPCDEEEHYDDYVMTLYAQGVLTPNEWLDLGGLSSLSA
EEYFGASLWQLYKSIDSPYKAVLKTLLLEAYSWEYPNPRLLAKDIKQRLHDGEIVSFGLD
PYCMMLERVTEYLTAIEDFTRLDLVRRCFYLKVCEKLSRERACVGWRREVLSQLVKEWEW
DDARLAMLDNRANWKIDQVREAHNELLDAMMQSYRNLIRFARRNNLSVSASPQDIGVLTR
KLYAAFEALPGKVTLVNPQISPDLSEPNLTFIYVPPGRANRSGWYLYNRAPNIESIISHQ
PLEYNRYLNKLVAWAWFNGLLTSRTRLYIKGNGIVDLPKLQEMVADVSHHFPLRLPAPTP
KALYSPCEIRHLAIIVNLEYDPTAAFRNQVVHFDFRKLDVFSFGENQNCLVGSVDLLYRN
SWNEVRTLHFNGEQSMIEALKTILGKMHQDAAPPDSVEVFCYSQHLRGLIRTRVQQLVSE
CIELRLSSTRQETGRFKALRVSGQTWGLFFERLNVSVQKLENAIEFYGAISHNKLHGLSV
QVETNHVKLPAVVDGFASEGIIQFFFEETQDENGFNIYILDESNRVEVYHHCEGSKEELV
RDVSRFYSSSHDRFTYGSSFINFNLPQFYQIVKVDGREQVIPFRTKSIGNMPPANQEHDA
PLLQQYFS
>LFGLNPFC_04125 Porphobilinogen deaminase
MIMTVTSMLDNVLRIATRQSPLALWQAHYVKDKLMASHPGLVVELVPMVTRGDVILDTPL
AKVGGKGLFVKELEVALLENRADIAVHSMKDVPVEFPQGLGLVTICKREDPRDAFVSNTY
DSLDALPAGSIVGTSSLRRQCQLAERRPDLIIRSLRGNVGTRLSKLDNGEYDAIILAVAG
LKRLGLESRIRAALPPEISLPAVGQGAVGIECRLDDARTRELLAALNHHETALRVTAERA
MNTRLEGGCQVPIGSYAELIDGEIWLRALVGAPDGSQIIRGERRGAPQDAEQMGISLAEE
LLNNGAREILAEVYNGDAPA
>LFGLNPFC_04126 Uroporphyrinogen-III synthase
MSILVTRPSPAGEELVSRLRTLGQVAWHFPLIEFSPGRQLPQLADQLAALGESDLLFALS
QHAVAFAQSQLHQQDRKWPRLPDYFAIGRTTALALHTVSGQKILYPQDREISEVLLQLPE

LQNIAGKRALILRGNGGRELIGDSLTARGAEVTFCECYQRCAIHYDGAEEAMRWQSREVT
TVVVTSGEMLQQLWSLIPQWYREHWLLRCRLLVVSERLAKLARELGWQDIKVADNADNDA
LLRALQ
>LFGLNPFC_04127 Protein HemX
MTEQEKTSAVVEETREAVDTTSQPVATEKKSKNNTALILSAVAIAIALAAGVGLYGWGKQ
QAVNQTATSDALANQLTALQKAQESQKAELEGIIKQQAVQLEQANRQQETLAKQLDEVQQ
KVATISGSDAKTWLLAQADFLVKLAGRKLWSDQDVTTAAALLKSADASLADMNDPSLITV
RRAITDDIASLSAVSQVDYDGIILKLNQLSNQVDNLRLADNDSDGSPMDSDGEELSSSIS
EWRINLQKSWQNFMDNFITIRRRDDTAVPLLAPNQDIYLRENIRSRLLVAAQAVPRHQEE
TYRQALENVSTWVRAYYDTDDATTKAFLDEVDQLSQQNISMDLPETLQSQAMLEKLMQTR
VRNLLAQPAAGTTEAKPAPAPAPAPAPQADAPAAAPQGE
>LFGLNPFC_04128 Protein HemY
MLKVLLLFVLLIAGIVVGPMIAGHQGYVLIQTDNYNIETSVTGLAIILILAMVVLFAIEW
LLRRIFRTGAHTRGWFVGRKRRRARKQTEQALLKLAEGDYQQVEKLMAKNADHAEQPVVN
YLLAAEAAQQRGDEARANQHLERAAELAGNDTIPVEITRVRLQLARNENHAARHGVDKLL
EVTPRHPEVLRLAEQAYIRTGAWSSLLDIIPSMAKAHVGDEEHRAMLEQQAWIGLMDQAR
ADNGSEGLRNWWKNQSRKTRHQVALQVAMAEHLIECDDHDTAQQIIIDGLKRQYDDRLLL
PIPRLKTNNPEQLEKVLRQQIKNVGDRPLLWSTLGQSLMKHGEWQEASLAFRAALKQRPD
AYDYAWLADALDRLHKPEEAAAMRRDGLMLTLQNNPPQ
>LFGLNPFC_04129 N-acetylgalactosamine-6-O-sulfatase
MEFSFSPKLLVVAVAAALPLIASAADTPSTATARKGFAGYDHPNQYLVKPATTIADNMMP
VMQHPAQDKETQQKLAELEKKTGKKPNVVVFLLDDVGWMDVGFNGGGVAVGNPTPDIDAV
ASQGLILTSAYSQPSSSPTRATILTGQYSIHHGILMPPMYGQPGGLQGLTTLPQLLHDQG
YVTQAIGKWHMGENKESQPQNVGFDDFRGFNSVSDMYTEWRDVHVNPEVALSPARSEYIQ
KLPFSKDDVHAVRGGEQEAIADITPKYMEDLDQRWMEYGVKFLDKMAKSDKPFFLYYGTR
GCHFDNYPNAKYAGSSPARTSYGDCMVEMNDIFANLYKALEKNGQLDNTLIVFTSDNGPE
AEVPPHGRTPFRGAKGSTWEGGVRVPTFVYWKGMIQPRKSDGIVDLADLFPTALDLAGHP
GAKVANLVPKTTFIDGVDQTSFFLGTNGQSNRKAEHYFLNGKLSAVRMDEFKYHVLIQQP
YAYTQSGYQGGFTGTVMQTAGSSVFNLYTDPQESDSIGVRHIPMGVPLQTEMHAYMEILK
KYPPRAQIKSD
>LFGLNPFC_04130 Anaerobic sulfatase-maturating enzyme
MQQQVPTRAFHVMAKPSGSDCNLNCDYCFYLEKQSLYHEKPVTHMDDDTLEAYVRHYIAA
SETQNEVAFTWQGGEPTLLGLDFYRRAVALQAKYGAGRKISNSFQTNGVLLDDEWCAFLA
ENHFLVGLSLDGPAEIHNQYRVTKGGRPTHKLVMRALTLLQKHHVDYNVLVCVNRTSALQ
PLQVYDFLCDAGVEFIQFIPVVERLADETAAHAGLKLHAPGDIQGELTEWSVCPQEFGEF
LVAIFDHWIKRDVGKVFVMNIEWAFANFVGAPGAVCHHQPTCGRSVIVEHNGDVYACDHY
VYPQYRLGNMLQQTIAEMIDSPQQQAFGEDKFKQLPAQCRSCNVLKACWGGCPKHRFMLD
ASGKPGLNYLCAGYQRYFRHLPPYLKAMADLLAHGRPASDIMQAHLMVVNK
>LFGLNPFC_04135 putative transport protein YifK
MADNKPELQRGLEARHIELIALGGTIGVGLFMGAASTLKWAGPSVLLAYIIAGLFVFFIM
RSMGEMLFLEPVTGSFAVYAHRYMSPFFGYLTAWSYWFMWMAVGISEITAIGVYVQFWFP
EMAQWIPALIAVALVALANLAAVRLYGEIEFWFAMIKVTTIIVMIVIGLGVIFFGFGNGG
QSIGFSNLTEHGGFFAGGWKGFLTALCIVVASYQGVELIGITAGEAKNPQVTLRSAVGKV
LWRILIFYVGAIFVIVTIFPWNEIGSNGSPFVLTFAKIGITAAAGIINFVVLTAALSGCN
SGMYSCGRMLYALAKNRQLPAAMAKVSRHGVPVAGVAVSIAILLIGSCLNYIIPNPQRVF
VYVYSASVLPGMVPWFVILISQLRFRRAHKAAIASHPFRSILFPWANYVTMAFLICVLIG
MYFNEDTRMSLFVGIIFMLVVTAIYKVFGLNRHGKAHKLEE
>LFGLNPFC_04136 UDP-N-acetyl-D-mannosaminuronic acid transferase
MNNNTTAPTYTLRGLQLIGWRDMQHALDYLFADGQLKQGTLVAINAEKMLTIEDNAEVRE
LINAAEFKYADGISVVRSVRKKYPQAQVSRVAGADLWEELMARAGKEGTQVFLVGGKPEV
LAQTEAKLRNQWNVNIVGSQDGYFKPEQRQALFERIHASGAQIVTVAMGAPKQEIFMRDC
RLVHPDALYMGVGGTYDVFTGHVKRAPKIWQTLGLEWLYRLLSQPSRIKRQLRLLRYLRW
HYTGNL
>LFGLNPFC_04137 putative ECA polymerase
MSLLQFSGLFVVWLLCTLFIATLTWFEFRRVRFNFNVFFSLLFLLTFFFGFPLTSVLVFR
FDVGVAPPEILLQALLSAGCFYAVYYVTYKTRLRKRVADAPRRPLFTMNRVETNLTWVIL
MGIALVSVGIFFMHNGFLLFRLNSYSQIFSSEVSGVALKRFFYFFIPAMLVVYFLRQDSK
AWLFFLVSTVAFGLLTYMIVGGTRANIIIAFAIFLFIGIIRGWISLWMLAAAGVLGIVGM
FWLALKRYGMNVSGDEAFYTFLYLTRDTFSPWENLALLLQNYDNIDFQGLAPIVRDFYVF
IPSWLWPGRPSMVLNSANYFTWEVLNNHSGLAISPTLIGSLVVMGGALFIPLGAIVVGLI
IKWFDWLYELGNRETNRYKAAILHSFCFGAIFNMIVLAREGLDSFVSRVVFFIVVFGACL
MIAKLLYWLFESAGLIHKRTKSSLRTQVEG
>LFGLNPFC_04138 TDP-N-acetylfucosamine:lipid II N-acetylfucosaminyltransferase
MTVLIHVLGSDIPHHNRTVLRFFNDALAATSGHAREFMVAGKDDGLSDSCPALSVQFFPG

KKSLAEAVIAKAKANRQQRFFFHGQFNPKLWLALLSGGIKPSQFFWHIWGADLYELSSGL
RYKLFYPLRRLAQKRVGCVFATRGDLSFFAKTHPKVRGELLYFPTRMDPSLNTMANDRQR
EGKMTILVGNSGDRSNEHIAALRAVHQQFGDTVKVVVPMGYPPNNEAYIEEVRQAGLELF
SEENLQVLSEKLEFDAYLTLLRQCDLGYFIFARQQGIGTLCLLIQAGIPCVLNRENPFWQ
DMTEQHLPVLFTTDDLNEDIVREAQRQLASVDKNTIAFFSPNYLQGWQRALAIAAGEVA
>LFGLNPFC_04139 Lipid III flippase
MSLAKASLWTAASTLVKIGAGLLVGKLLAVSFGPAGLGLAANFRQLITVLGVLAGAGIFN
GVTKYVAQYHDNPQQLRRVVGTSSAMVLGFSTLMALVFVLAAALISQGLFGNTDYQGLVR
LVALVQMGIAWGNLLLALMKGFRDAAGNALSLIVGSLIGVLAYYVSYRLGGYEGALLGLA
LIPALVVIPAVIMLIKRGVIPLSYLKPSWDNGLAGQLSKFTLMALITSVTLPVAYIMMRK
LLAAQYSWDEVGIWQGVSSISDAYLQFITASFSVYLLPTLSRLTEKRDITREVVKSLKFV
LPAVAAASFTVWLLRDFAIWLLLSNKFTAMRDLFAWQLVGDVLKVGAYVFGYLVIAKASL
RFYILAEISQFTLLMVFAHWLIPAHGALGAAQAYMATYIVYFSLCCGVFLLWRRWA
>LFGLNPFC_04140 dTDP-4-amino-4,6-dideoxygalactose transaminase
MIPFNAPPVVGTELDYMQSAMGSGKLCGDGGFTRRCQQWLEQRFGSAKVLLTPSCTASLE
MAALLLDIQPGDEVIMPSYTFVSTANAFVLRGAKIVFVDVRPDTMNIDETLIEAAITDKT
RVIVPVHYAGVACEMDTIMALAKKHNLFVVEDAAQGVMSTYKGRALGTIGHIGCFSFHET
KNYTAGGEGGATLINDKALIERAEIIREKGTNRSQFFRGQVDKYTWRDIGSSYLMSDLQA
AYLWAQLEAADRINQQRLALWQNYYDALAPLAKNGRIELPSIPDGCVQNAHMFYIKLRDI
DDRSALINFLKEAEIMAVFHYIPLHGCPAGERFGEFHGEDRYTTKESERLLRLPLFYNLS
PVNQRTVIATLLNYFS
>LFGLNPFC_04141 dTDP-fucosamine acetyltransferase
MPVRASIEPLTWENAFFGVNSAIVRITSEAPLLTPDALAPWSRVQAKIAASNTGELDALQ
QLGFSLVEGEVDLALPVNNVSDSGAVVAQETDIPALRQLASAAFAQSRFRAPWYAPDASG
RFYAQWIENAVRGTFDHQCLILRAASGDIRGYVSLRELNATDARIGLLAGRGAGAELMQT
ALNWAYARGKTTLRVATQMGNTAALKRYIQSGANVESTAYWLYR
>LFGLNPFC_04142 Glucose-1-phosphate thymidylyltransferase 2
MKGIILAGGSGTRLHPITRGVSKQLLPIYDKPMIYYPLSVLMLAGIREILIITTPEDKGY
FQRLLGDGSEFGIQLEYAEQPSPDGLAQAFIIGETFLNGEPSCLVLGDNIFFGQGFSPKL
RHVAARTEGATVFGYQVMDPERFGVVEFDDNFRAISLEEKPKQPKSNWAVTGLYFYDSKV
VEYAKQVKPSERGELEITSINQMYLEAGNLTVELLGRGFAWLDTGTHDSLIEASTFVQTV
EKRQGFKIACLEEIAWRNGWLDDEGVKRAASSLAKTGYGQYLLELLRARPRQY
>LFGLNPFC_04143 dTDP-glucose 4,6-dehydratase 2
MRKILITGGAGFIGSALVRYIINETSDAVVVVDKLTYAGNLMSLAPVAQSERFAFEKVDI
CDRAELARVFTEYQPDCVMHLAAESHVDRSIDGPAAFIETNIVGTYTLLEAARAYWTALT
EDKKSAFRFHHISTDEVYGDLHSTDDFFTETTPYAPSSPYSASKASSDHLVRAWLRTYGL
PTLITNCSNNYGPYHFPEKLIPLMILNALAGKPLPVYGNGQQIRDWLYVEDHARALYCAA
TTGKVGETYNIGGHNERKNLDVVETICELLEELAPNKPHGVAHYRDLITFVADRPGHDLR
YAIDASKIARELGWLPQETFESGMRKTVQWYLANESWWKQVQDGSYQGERLGLKG
>LFGLNPFC_04144 UDP-N-acetyl-D-mannosamine dehydrogenase
MSFATISVIGLGYIGLPTAAAFASRQKQVIGVDINQHAVDTINRGEIHIVEPDLASVVKT
AVEGGFLRASTTPVEADAWLIAVPTPFKGDHEPDMTYVESAARSIAPVLKKGALVILEST
SPVGSTEKMAEWLAEMRPDLTCPQQVGELADVNIAYCPERVLPGQVMVELIKNDRVIGGM
TPVCSARASELYKIFLEGECVVTNSRTAEMCKLTENSFRDVNIAFANELSLICADQGINV
WELIRLANRHPRVNILQPGPGVGGHCIAVDPWFIVAQNPQQARLIRTAREVNDHKPFWVI
DQVKAAVADCLAATDKRVSELKIACFGLAFKPNIDDLRESPAMEIAELIAQWHSGETLVV
EPNIHELPKKLTGLCTLAQLDEALATADVLVMLVDHSQFKVINGDNVHQQYVVDAKGVWR
>LFGLNPFC_04145 UDP-N-acetylglucosamine 2-epimerase
MKVLTVFGTRPEAIKMAPLVHALAKDPFFEAKVCVTAQHREMLDQVLKLFSIVPDYDLNI
MQPGQGLTEITCRILEGLKPILAEFKPDVVLVHGDTTTTLATSLAAFYQRIPVGHVEAGL
RTGDLYSPWPEEANRTLTGHLAMYHFSPTETSRQNLLRENVADSRIFITGNTVIDALLWV
RDQVMSSDTLRSELAANYPFIDPDKKMILVTGHRRESFGRGFEEICQALADIATTHQDIQ
IVYPVHLNPNVREPVNRILGHVKNVILIDPQEYLPFVWLMNHAWLILTDSGGIQEEAPSL
GKPVLVMRDTTERPEAVTAGTVRLVGTDKQRIVEEVTRLLKDENEYQAMSRAHNPYGDGQ
ACSRILEALKNNRISL
>LFGLNPFC_04146 ECA polysaccharide chain length modulation protein
MTQPMPGKPAEDAENELDIRGLFRTLWAGKLWIIGMGLAFALIALAYTFFARQEWSSTAI
TDRPTVNMLGGYYSQQQFLRNLDVRSNMASADQPSVMDEAYKEFVMQLASWDTRREFWLQ
TDYYKQRMVGNSKADAALLDEMINNIVFIPGDFTRAVNDSVKLIAETAPDANNLLRQYVA
FASQRAASHLNDELKGAWAARTIQMKAQVKRQEEVAKAIYDRRMNSIEQALKIAEQHNIS
RSATDVPAEELPDSEMFLLGRPMLQARLENLQAVGPAFDLDYDQNRAMLNTLNVGPTLDP
RFQTYRYLRTPEEPVKRDSPRRAFLMIMWGIVGGLIGAGVALTRRCSK
>LFGLNPFC_04147 Undecaprenyl-phosphate alpha-N-acetylglucosaminyl 1-phosphate transferase

MNLLTVSTDLISIFLFTTLFLFFARKVAKKVGLVDKPNFRKRHQGLIPLVGGISVYAGIC
FTFGIVDYYIPHASLYLACAGVLVFIGALDDRFDISVKIRATIQAAVGIVMMVFGKLYLS
SLGYIFGSWEMVLGPFGYFLTLFAVWAAINAFNMVDGIDGLLGGLSCVSFAAIGMILWFD
GQTSLAIWCFAMIAAILPYIMLNLGILGRRYKVFMGDAGSTLIGFTVIWILLETTQGKTH
PISPVTALWIIAIPLMDMVAIMYRRLRKGMSPFSPDRQHIHHLIMRAGFTSRQAFVLITL
AAALLASIGVLAEYSHFVPEWVMLVLFLLAFFLYGYCIKRAWKVARFIKRVKRRLRRNRG
GSPNLTK
>LFGLNPFC_04148 Transcription termination factor Rho
MNLTELKNTPVSELITLGENMGLENLARMRKQDIIFAILKQHAKSGEDIFGDGVLEILQD
GFGFLRSADSSYLAGPDDIYVSPSQIRRFNLRTGDTISGKIRPPKEGERYFALLKVNEVN
FDKPENARNKILFENLTPLHANSRLRMERGNGSTEDLTARVLDLASPIGRGQRGLIVAPP
KAGKTMLLQNIAQSIAYNHPDCVLMVLLIDERPEEVTEMQRLVKGEVVASTFDEPASRHV
QVAEMVIEKAKRLVEHKKDVIILLDSITRLARAYNTVVPASGKVLTGGVDANALHRPKRF
FGAARNVEEGGSLTIIATALIDTGSKMDEVIYEEFKGTGNMELHLSRKIAEKRVFPAIDY
NRSGTRKEELLTTQEELQKMWILRKIIHPMGEIDAMEFLINKLAMTKTNDDFFEMMKRS
>LFGLNPFC_04149 Thioredoxin 1
MSDKIIHLTDDSFDTDVLKADGAILVDFWAEWCGPCKMIAPILDEIADEYQGKLTVAKLN
IDQNPGTAPKYGIRGIPTLLLFKNGEVAATKVGALSKGQLKEFLDANLA
>LFGLNPFC_04150 ATP-dependent RNA helicase RhlB
MSKTHLTEQKFSDFALHPKVVEALEKKGFHNCTPIQALALPLTLAGRDVAGQAQTGTGKT
MAFLTSTFHYLLSHPAIADRKVNQPRALIMAPTRELAVQIHADAEPLAEATGLKLGLAYG
GDGYDKQLKVLESGVDILIGTTGRLIDYAKQNHINLGAIQVVVLDEADRMYDLGFIKDIR
WLFRRMPPANQRLNMLFSATLSYRVRELAFEQMNNAEYIEVEPEQKTGHRIKEELFYPSN
EEKMRLLQTLIEEEWPDRAIIFANTKHRCEEIWGHLAADGHRVGLLTGDVAQKKRLRILD
EFTRGDLDILVATDVAARGLHIPAVTHVFNYDLPDDCEDYVHRIGRTGRAGASGHSISLA
CEEYALNLPAIETYIGHSIPVSKYNPDALMTDLPKPLRLTRPRTGNGPRRTGAPRNRRRS
G
>LFGLNPFC_04151 Guanosine-5'-triphosphate,3'-diphosphate pyrophosphatase
MGSTSSLYAAIDLGSNSFHMLVVREVAGSIQTLTRIKRKVRLAAGLNSENALSNEAMERG
WQCLRLFAERLQDIPPSQIRVVATATLRLAVNAGDFIAKAQEILGCPVQVISGEEEARLI
YQGVAHTTGGADQRLVVDIGGASTELVTGTGAQTTSLFSLSMGCVTWLERYFADRNLGQE
NFDAAEKAAREVLRPVADELRYHGWKVCVGASGTVQALQEIMMAQGMDERITLEKLQQLK
QRAIHCGRLEELEIDGLTLERALVFPSGLAILIAIFTELNIQCMTLAGGALREGLVYGML
HLTVEQDIRSRTLRNIQRRFMIDIDQAQRVAKVAANFFDQVENEWHLEAISRDLLISACQ
LHEIGLSVDFKQAPQHAAYLVRNLDLPGFTPAQKKLLATLLLNQTNPVDLSSLHQQNAVP
PRVAEQLCRLLRLAIIFASRRRDDLVPEMTLQANHELLTLTLPQGWLTQHPLGKEIIDQE
SQWQSYVHWPLEVH
>LFGLNPFC_04152 ATP-dependent DNA helicase Rep
MRLNPGQQQAVEFVTGPCLVLAGAGSGKTRVITNKIAHLIRGCGYQARHIAAVTFTNKAA
REMKERVAQTLGRKEARGLMISTFHTLGLDIIKREYAALGMKSNFSLFDDTDQLALLKEL
TEGLIEDDKVLLQQLISTISNWKNDLKTPAQAAAEAKGERDRIFAHCYGLYDAHLKACNV
LDFDDLILLPTLLLQRNEEVRERWQNKIRYLLVDEYQDTNTSQYELVKLLVGSRARFTVV
GDDDQSIYSWRGARPQNLVLLSQDFPALKVIKLEQNYRSSGRILKAANILIANNPHVFEK
RLFSELGYGTELKVLSANNEEHEAERVTGELIAHHFVNKTQYKDYAILYRGNHQSRVFEK
FLMQNRIPYKISGGTSFFSRPEIKDLLAYLRVLTNPDDDSAFLRIVNTPKREIGPATLKK
LGEWAMTRNKSMFTASFDMGLSQTLSGRGYEALTRFTHWLAEIQRLAEREPIAAVRDLIH
GMDYESWLYETSPSPKAAEMRMKNVNQLFSWMTEMLEGSELDEPMTLTQVVTRFTLRDMM
ERGESEEELDQVQLMTLHASKGLEFPYVYMVGMEEGFLPHQSSIDEDNIDEERRLAYVGI
TRAQKELTFTLCKERRQYGELVRPEPSRFLLELPQDDLIWEQERKVVSAEERMQKGQSHL
ANLKAMMAAKRGK
>LFGLNPFC_04153 Peptidyl-prolyl cis-trans isomerase C
MAKTAAALHILVKEEKLALDLLEQIKNGADFGKLAKKHSICPSGKRGGDLGEFRQGQMVP
AFDKVVFSCPVLEPTGPLHTQFGYHIIKVLYRN
>LFGLNPFC_04154 Ketol-acid reductoisomerase (NADP(+))
MANYFNTLNLRQQLAQLGKCRFMGRDEFADGASYLQGKKVVIVGCGAQGLNQGLNMRDSG
LDISYALRKEAIAEKRASWRKATENGFKVGTYEELIPQADLVVNLTPDKQHSDVVRSVQP
LMKDGAALGYSHGFNIVEVGEQIRKDITVVMVAPKCPGTEVREEYKRGFGVPTLIAVHPE
NDPKGEGMAIAKAWAAATGGHRAGVLESSFVAEVKSDLMGEQTILCGMLQAGSLLCFDKL
VEEGTDPAYAEKLIQFGWETITEALKQGGITLMMDRLSNPAKLRAYALSEQLKEIMAPLF
QKHMDDIISGEFSSGMMADWANDDKKLLTWREETGKTAFETAPQYEGKIGEQEYFDKGVL
MIAMVKAGVELAFETMVDSGIIEESAYYESLHELPLIANTIARKRLYEMNVVISDTAEYG
NYLFSYACVPLLKPFMAELQPGDLGKAIPEGAVDNAQLRDVNEAIRSHAIEQVGKKLRGY
MTDMKRIAVAG
>LFGLNPFC_04155 HTH-type transcriptional regulator HdfR

MTGNLLRKLIYSQRHIAISATSTLRAAPLDLRDLKTFLHLAESRHFGRSARAMHVSPSTL
SRQIQRLEDDLGQPLFVRDNRTVTLTEAGEELRVFAQQTLLQYQQLRHTIDQQGPSLSGE
LHIFCSVTAAYSHLPPILDRFRAEHPSVEIKLTTGDAADAMEKVVTGEADLAIAGKPETL
PGAVAFSMLENLAVVLIAPALPCPVRNQVSAEKPDWSTVPFIMADQGPVRRRIELWFRRN
KISNPMIYATVGGHEAMVSMVALGCGVALLPEVVLENSPEPVRNRVMILERSDEKTPFEL
GVCAQKKRLHEPLIEAFWKILPNH
>LFGLNPFC_04156 L-threonine dehydratase biosynthetic IlvA
MMADSQPLSGAPEGAEYLRAVLRAPVYEAAQVTPLQKMEKLSSRLDNVILVKREDRQPVH
SFKLRGAYAMMAGLTEEQKAHGVITASAGNHAQGVAFSSARLGVKALIVMPTATADIKVD
AVRGFGGEVLLHGANFDEAKAKAIELSQQQGFTWVPPFDHPMVIAGQGTLALELLQQDAH
LDRVFVPVGGGGLAAGVAVLIKQLMPQIKVIAVEAEDSACLKAALDAGHPVDLPRVGLFA
EGVAVKRIGDETFRLCQEYLDDIITVDSDAICAAMKDLFEDVRAVAEPSGALALAGMKKY
IAQHNIRGERLAHILSGANVNFHGLRYVSERCELGEQREALLAVTIPEEKGSFLKFCQLL
GGRSVTEFNYRFADAKNACIFVGVRLSRGLEERKEILQMLNDGGYSVVDLSDDEMAKLHV
RYMVGGRPSHPLQERLYSFEFPESPGALLRFLNTLGTHWNISLFHYRSHGTDYGRVLAAF
ELGDHEPDFETRLNELGYDCHDETNNPAFRFFLAG
>LFGLNPFC_04157 Dihydroxy-acid dehydratase
MPKYRSATTTHGRNMAGARALWRATGMTDADFGKPIIAVVNSFTQFVPGHVHLRDLGKLV
AEQIEAAGGVAKEFNTIAVDDGIAMGHGGMLYSLPSRELIADSVEYMVNAHCADAMVCIS
NCDKITPGMLMASLRLNIPVIFVSGGPMEAGKTKLSDQIIKLDLVDAMIQGADPKVSDSQ
SDQVERSACPTCGSCSGMFTANSMNCLTEALGLSQPGNGSLLATHADRKQLFLNAGKRIV
ELTKRYYEQDDESALPRNIASKAAFENAMTLDIAMGGSTNTVLHLLAAAQEAEIDFTMSD
IDKLSRKVPQLCKVAPSTQKYHMEDVHRAGGVIGILGELDRAGLLNRDVKNVLGLTLPQT
LEQYDIIVTQDDAVKNMFRAGPAGIRTTQAFSQDCRWDTLDDDRSNGCIRSLEHAYSKDG
GLAVLYGNFAENGCIVKTAGVDDSILKFTGPAKVYESQDDAVEAILGGKVVAGDVVVIRY
EGPKGGPGMQEMLYPTSFLKSMGLGKACALITDGRFSGGTSGLSIGHVSPEAASGGSIGL
IEDGDLIAIDIPNRGIQLQVSDAELAARREAQEARGDKAWTPKNRERQVSFALRAYASLA
TSADKGAVRDKSKLGG
>LFGLNPFC_04158 Branched-chain-amino-acid aminotransferase
MTTKKADYIWFNGEMVRWEDAKVHVMSHALHYGTSVFEGIRCYDSHKGPVVFRHREHMQR
LHDSAKIYRFPVSQSIDELMEACRDVIRKNNLTSAYIRPLIFVGDVGMGVNPPAGYSTDV
IIAAFPWGAYLGAEALEQGIDAMVSSWNRAAPNTIPTAAKAGGNYLSSLLVGSEARRHGY
QEGIALDVNGYISEGAGENLFEVKDGVLFTPPFTSSALPGITRDAIIKLAKELGIEVHEQ
VLSRESLYLADEVFMSGTAAEITPVRSVDGIQVGEGRCGPVTKRIQQAFFGLFTGETEDK
WGWLDQVNQ
>LFGLNPFC_04159 Acetolactate synthase isozyme 2 small subunit
MMQHQVNVSARFNPETLERVLRVVRHRGFHVCSMNMAAASDAQNINIELTVASPRSVDLL
FSQLNKLVDVAHVAICQSTTTSQQIRA
>LFGLNPFC_04160 Acetolactate synthase isozyme 2 large subunit
MNGAQWVVHALRAQGVNTVFGYPGGAIMPVYDALYDGGVEHLLCRHEQGAAMAAIGYARA
TGKTGVCIATSGPGATNLITGLADALLDSIPVVAITGQVSAPFIGTDAFQEVDILGLSLA
CTKHSFLVQSLEELPRIMAEAFDVASSGRPGPVLVDIPKDIQLASGDLEPWFTTVENEVT
FSHAEVEQARQMLAKAQKPMLYVGGGVGMAQAVSALREFLAATKMPATCTLKGLGAVEAD
YPYYLGMLGMHGTKAANFAVQECDLLIAVGARFDDRVTGKLNTFAPHASVIHMDIDPAEM
NKLRQAHVALQGDLNALLPALQQPLNIDDWQQHCAQLRDEHAWRYDHPGDAIYAPLLLKQ
LSDRKSADCIVTTDVGQHQMWAAQHIVHTRPENFITSSGLGTMGFGLPAAVGAQVARPND
TVVCISGDGSFMMNVQELGTVKRKQLPLKIVLLDNQRLGMVRQWQQLFFQERYSETTLTD
NPDFLMLASAFGIPGQHITHKDQVEAALDTMLNSDGPYLLHVSIDELENVWPLVPPGASN
SEMLEKLS
>LFGLNPFC_04161 Competence protein ComM
MSLSIVHTRAALGVNAPPITVEVHISKGLPGLTMVGLPETTVKEARDRVRSAIINSGYEY
PAKKITINLAPADLPKEGGRYDLPIAIALLAASEQLTANKLDEYELVGELALTGALRGVP
GAISSATEAIKSGRKIIVAKDNEDEVGLINGEGCLIADHLQAVCAFLEGKHALEHPKPTN
AVSRALQHDLSDVVGQEQGKRGLEITAAGGHNLLLIGPPGTGKTMLASRINGLLPDLSNE
EALESAAILSLVNAESVQKQWRQRPFRSPHHSASLTAMVGGGAIPGPGEISLAHNGVLFL
DELPEFERRTLDALREPIESGQIHLSRTRAKITYPARFQLVAAMNPSPTGHYQGNHNRCT
PEQTLRYLNRLSGPFLDRFDLSLEIPLPPPGILSKTVVPGENSTTVKQRVMAARERQFKR
QNKLNAWLDNPEIRQFCKLESEDAQWLEETLIHLGLSIRAWQRLLKVARTIADIDQSDII
TRQHLQEAVSYRAIDRLLIHLQKLLT
>LFGLNPFC_04162 hypothetical protein
MAESFTTTNRYFDNKHYPRGFSRHGDFTIKEAQLLERHGYAFNELDLGKREPVTEEEKLF
VAVCRGEREPVTEAERVWSKYMTRIKRPKRFHTLSGGKPQVEGAEDYTDSDD
>LFGLNPFC_04163 HTH-type transcriptional regulator HdfR
MDTELLKTFLEVSRTRHFGRAAESLYLTQSAVSFRIRQLENQLGVNLFTRHRNNIRLTAA

GEKLLPYAETLMSTWQAARKEVAHTSRHNEFSIGASASLWECMLNQWLGRLYQNQDVHTG
LQFEARIAQRQSLVKQLHERQLDLLITTEAPKMDEFCSQLLGYFTLALYTSAPSKLKGDL
NYLRLEWGPDFQQHEAGLIGADEVPILTTSSAELAQQQIAMLNGCTWLPVSWARKKGGLH
TVVDSTTLSRPLYAIWLQNSDKNALIRDLLKINVLDEVY
>LFGLNPFC_04170 HTH-type transcriptional repressor NanR
MPLSAQQLAAQKNLSYVLAEKLAQRILKGEYEPGTILPGEIELGEQFGVSRTAVREAVKT
LTAKGMVLPRPRIGTRVMPQSNWNFLDQELLTWWMTEENFHQVIDHFLVMRICLEPQACL
LAAKVGTAEQKAHLNTLMAEMAALKENFRRERWIEVDMAWHEHIYEMSANPFLTSFASLF
HSVYHTYFTSITSDTVIKLDLHQAIVDAIIQSDGDAAFKACQALLRSPDK
>LFGLNPFC_04171 putative transport protein HsrA
MSDKKKRSMAGLPWIAAMAFFMQALDATILNTALPAIAHSLNRSPLAMQSAIISYTLTVA
MLIPVSGWLADRFGTRRIFTLAVSLFTLGSLACALSNSLPQLVVFRVIQGIGGAMMMPVA
RLALLRAYPRNELLPVLNFVAMPGLVGPILGPVLGGVLVTWATWHWIFLINIPIGIAGLL
YAHKHMPNFITARRRFDTTGFLLFGLSLVLFSSGIELFGEKIVASWIALTVIVTSIGLLL
LYILHARRTPNPLISLDLFKTRTFSIGIVGNIATRLGTGCVPFLMPLMLQVGFGYQAFIA
GCMMAPTALGSIIAKSMVTQVLRRLGYRHTLVGITVIIGLMIAQFSLQSPAMAIWMLILP
LFILGMAMSTQFTAMNTITLADLTDDNASSGNSVLAVTQQLSISLGVAVSAAVLRVYEGM
EGTTTVEQFHYTFITMGIITVASAAMFMLLKTTDGNNLIKRQRKSKPNHVPSESE
>LFGLNPFC_04172 Ribose operon repressor
MATMKDVARLAGVSTSTVSHVINKDRFVSEAITAKVEAAIKELNYAPSALARSLKLNQTH
TIGMLITASTNPFYSELVRGVERSCFERGYSLVLCNTEGDEQRMNRNLETLMQKRVDGLL
LLCTETHQPSREIMQRYPTVPTVMMDWAPFDGDSDLIQDNSLLGGDLATQYLIDKGHTRI
ACITGPLDKTPARLRLEGYRAAMKRAGLNIPDGYEVTGDFEFNGGFDAMRQLLSHPLRPQ
AVFTGNDAMAVGVYQALYQAELQVPQDIAVIGYDDIELASFMTPPLTTIHQPKDELGELA
IDVLIHRITQPALQQQRLQLTPILMERGSA
>LFGLNPFC_04173 Ribokinase
MQNAGSLVVLGSINADHILNLQSFPTPGETVTGNHYQVAFGGKGANQAVAAGRSGANIAF
IACTGDDSIGESVRQQLATDNIDISPVSVIKGESTGVALIFVNGEGENVIGIHAGANAAL
SPALVEAQRERIANASALLMQLESPLESVMAAAKIAHQNKTIVALNPAPARELPDELLAL
VDIITPNETEAEKLTGIRVENDEDAAKAAQVLHEKGIRTVLITLGSRGVWASVNGEGQRV
PGFRVQAVDTIAAGDTFNGALITALLEEKPLPEAIRFAHAAAAIAVTRKGAQPSVPWREE
IDAFLDRQR
>LFGLNPFC_04174 Ribose import binding protein RbsB
MNMKKLATLVSAVALSATVSANAMAKDTIALVVSTLNNPFFVSLKDGAQKEADKLGYNLV
VLDSQNNPAKELANVQDLTVRGTKILLINPTDSDAVGNAVKMANQANIPVITLDRQATKG
EVVSHIASDNVLGGKIAGDYIAKKAGEGAKVIELQGIAGTSAARERGEGFQQAVAAHKFN
VLASQPADFDRTKGLNVMQNLLTAHPDVQAVFAQNDEMALGALRALQTAGKSDVMVVGFD
GTPDGEKAVNDGKLAATIAQLPDQIGAKGVETADKVLKGEKVQAKYPVDLKLVVKQ
>LFGLNPFC_04175 Ribose import permease protein RbsC
MTTQTVSGRRYFTKAWLMEQKSLIALLVLIAIVSTLSPNFFTINNLFNILQQTSVNAIMA
VGMTLVILTSGIDLSVGSLLALTGAVAASIVGIEVNALVAVAAALALGAAIGAVTGVIVA
KGRVQAFIATLVMMLLLRGVTMVYTNGSPVNTGFTENADLFGWFGIGRPLGVPTPVWIMG
IVFLAAWYMLHHTRLGRYIYALGGNEAATRLSGINVNKIKIIVYSLCGLLASLAGIIEVA
RLSSAQPTAGTGYELDAIAAVVLGGTSLAGGKGRIVGTLIGALILGFLNNGLNLLGVSSY
YQMIVKAVVILLAVLVDNKKQ
>LFGLNPFC_04176 Ribose import ATP-binding protein RbsA
MEALLQLKGIDKAFPGVKALSGAALNVYPGRVMALVGENGAGKSTMMKVLTGIYTRDAGT
LLWLGKETTFTGPKSSQEAGIGIIHQELNLIPQLTIAENIFLGREFVNRFGKIDWKTMYA
EADKLLAKLNLRFKSDKLVGDLSIGDQQMVEIAKVLSFESKVIIMDEPTDALTDTETESL
FRVIRELKSQGRGIVYISHRMKEIFEICDDVTVFRDGQFIAEREVASLTEDSLIEMMVGR
KLEDQYPHLNKAPGDIRLKVDNLCGPGVNDVSFTLRKGEILGVSGLMGAGRTELMKVLYG
ALPRTSGYVTLDGHEVVTRSPQDGLANGIVYISEDRKRDGLVLGMSVKENMSLTALRYFS
RAGGSLKHADEQQAVSDFIRLFNVKTPSMEQAIGLLSGGNQQKVAIARGLMTRPKVLILD
EPTRGVDVGAKKEIYQLINQFKADGLSIILVSSEMPEVLGMSDRIIVMHEGHLSGEFTRE
QATQEVLMAAAVGKLNRVNQE
>LFGLNPFC_04177 D-ribose pyranase
MKKGTVLNSDISSVISRLGHTDTLVVCDAGLPIPKSTTRIDMALTQGVPSFMQVLGVVTN
EMQVEAVIIAEEIKQHNPQLHETLLTHLEQLQKHQGNTIEIRYTTHEQFKQQTAGSQAVI
RSGECSPYANIILCAGVTF
>LFGLNPFC_04178 Low affinity potassium transport system protein kup
MSTDNKQSLPAITLAAIGVVYGDIGTSPLYTLRECLSGQFGFGVERDAVFGFLSLIFWLL
IFVVSIKYLTFVMRADNAGEGGILTLMSLAGRNTSARTTSMLVIMGLIGGSFFYGEVVIT
PAISVMSAIEGLEIVAPQLDTWIVPLSIIVLTLLFMIQKHGTAMVGKLFAPIMLTWFLIL
AGLGLRSIIANPEVLHALNPMWAVHFFLEYKTVSFIALGAVVLSITGVEALYADMGHFGK

FPIRLAWFTVVLPSLTLNYFGQGALLLKNPEAIKNPFFLLAPDWALIPLLIIAALATVIA
SQAVISGVFSLTRQAVRLGYLSPMRIIHTSEMESGQIYIPFVNWMLYVAVVIVIVSFEHS
SNLAAAYGIAVTGTMVLTSILSTTVARQNWHWNKYFVALILIAFLCVDIPLFTANLDKLL
SGGWLPLSLGTVMFIVMTTWKSERFRLLRRMHEHGNSLEAMIASLEKSPPVRVPGTAVYM
SRAINVIPFALMHNLKHNKVLHERVILLTLRTEDAPYVHNVRRVQIEQLSPTFWRVVASY
GWRETPNVEEVFHRCGLEGLSCRMMETSFFMSHESLILGKRPWYLRLRGKLYLLLQRNAL
RAPDQFEIPPNRVIELGTQVEI
>LFGLNPFC_04179 ATPase RavA
MAHPHLLAERISRLSSSLEKGLYERSHAIRLCLLAALSGESVFLLGPPGIAKSLIARRLK
FAFQNARAFEYLMTRFSTPEEVFGPLSIQALKDEGRYERLTSGYLPEAEIVFLDEIWKAG
PAILNTLLTAINERQFRNGALVEKIPMRLLVAASNELPEADSSLEALYDRMLIRLWLDKV
QDKANFRSMLTSQQDENDNPVPASLQITDEEYERWQKEIGEITLPDHVFELIFMLRQQLD
KLPDAPYVSDRRWKKAIRLLQASAFFSGRSAVAPVDLILLKDCLWYDAQSLNLIQQQIDV
LMTGHAWQQQGMLTRLGAIVQRHLQLQQQQSDKTALTVIRLGGIFSRRQQYQLPVNVTAS
TLTLLLQKPLKLHDMEVVHISFERSALEQWLSKGGEIRGKLNGIGFAQKLNLEVDSAQHL
VVRDVSLQGSTLALPGSSAEGLPGEIKQQLEELESDWRKQHALFSEQQKCLFIPGDWLGR
IEASLQDVGAQIRQAQQC
>LFGLNPFC_04180 Protein ViaA
MLTLDTLNVMLAVSEEGLIEEMIIALLASPQLAVFFEKFPRLKAAITDDVPRWREALRSR
LKDARVPPELTEEVMCYQQSQLLSTPQFIVQLPQILDLLHRLNSPWAEQARQLVDANSTI
TSALHTLFLQRWRLSLIVQATTLNQQLLEEEREQLLSEVQERMTLSGQLEPILADNNTAA
GRLWDMSAGQLKRGDYQLIVKYGEFLNEQPELKRLAEQLGRSREAKSIPRNDAQMETFRT
MVREPATVPEQVDGLQQSDDILRLLPPELATLGITELEYEFYRRLVEKQLLTYRLHGESW
REKMIERPVVHKDYDEQPRGPFIVCVDTSGSMGGFNEQCAKAFCLALMRIALAENRRCYI
MLFSTEIVRYELSGPQGIEQAIRFLSQQFRGGTDLASCFRAIMERLQSREWFDADAVVIS
DFIAQRLPDDVTSKVKELQRVHQHRFHAVAMSAHGKPGIMRIFDHIWRFDTGMRSRLLRR
WRR
>LFGLNPFC_04181 Aspartate--ammonia ligase
MKTAYIAKQRQISFVKSHFSRQLEERLGLIEVQAPILSRVGDGTQDNLSGCEKAVQVKVK
ALPDAQFEVVHSLAKWKRQTLGQHDFSAGEGLYTHMKALRPDEDRLSPLHSVYVDQWDWE
RVMGDGERQFSTLKSTVEAIWEGIKATEAAVSEEFGLAPFLPDQIHFVHSQELLSRYPEL
DAKGRERAIAKDLGAVFLVGIGGKLSDGHRHDVRAPDYDDWSTPSELGHAGLNGDILVWN
PVLEDAFELSSMGIRVDADTLKHQLALTGDEDRLELEWHQALLRGEMPQTIGGGIGQSRL
TMLLLQLPHIGQVQCGVWPAAVRESVPSLL
>LFGLNPFC_04182 Regulatory protein AsnC
MENYLIDNLDRGILEALMGNARTAYAELAKQFGVSPGTIHVRVEKMKQAGIITGARIDVS
PKQLGYDVGCFIGIILKSAKDYPSALAKLESLDEVTEAYYTTGHYSIFIKVMCRSIDALQ
HVLINKIQTIDEIQSTETLIVLQNPIMRTIKP
>LFGLNPFC_04183 Protein MioC
MADITLISGSTLGGAEYVAEHLAEKLEETGLTTETLHGPLLEDLSASGIWLVISSTHGAG
DIPDNLFPFYEALQEQKPDLSAVRFGAIGIGSREYDTFCGAIDKIEAELKNSGAKQTGET
LKINILDHDIPEDPAEEWLGSWINLLK
>LFGLNPFC_04184 tRNA uridine 5-carboxymethylaminomethyl modification enzyme MnmG
MFYPDPFDVIIIGGGHAGTEAAMAAARMGQQTLLLTHNIDTLGQMSCNPAIGGIGKGHLV
KEVDALGGLMAKAIDQAGIQFRILNASKGPAVRATRAQADRVLYRQAVRTALENQPNLMI
FQQAVEDLIVENDRVVGAVTQMGLKFRAKAVVLTVGTFLDGKIHIGLDNYSGGRAGDPPS
IPLSRRLRELPLRVGRLKTGTPPRIDARTIDFSVLAQQHGDNPMPVFSFMGNASQHPQQV
PCYITHTNEKTHDVIRSNLDRSPMYAGVIEGVGPRYCPSIEDKVMRFAERNQHQIFLEPE
GLTSNEIYPNGISTSLPFDVQMQIVRSMQGMENAKIVRPGYAIEYDFFDPRDLKPTLESK
FIQGLFFAGQINGTTGYEEAAAQGLLAGLNAARLSADKEGWAPARSQAYLGVLVDDLCTL
GTKEPYRMFTSRAEYRLMLREDNADLRLTEIGRELGLVDDERWARFNEKLENIERERQRL
KSTWVTPSAEAAAEVNAHLTAPLSREASGEDLLRRPEMTYEKLTTLTPFAPALTDEQAAE
QVEIQVKYEGYIARQQDEIEKQLRNENTLLPATLDYRQVSGLSNEVIAKLNDHKPASIGQ
ASRISGVTPAAISILLVWLKKQGMLRRSA
>LFGLNPFC_04185 Ribosomal RNA small subunit methyltransferase G
MLNKLSLLLKDAGISLTDHQKNQLIAYVNMLHKWNKAYNLTSVRDPNEMLVRHILDSIVV
APYLQGERFIDVGTGPGLPGIPLSIVRPEAHFTLLDSLGKRVRFLRQVQHELKLENIEPV
QSRVEEFPSEPPFDGVISRAFASLNDMVSWCHHLPGEQGRFYALKGQMPEDEIALLPEEY
QVESVVKLQVPALDGERHLVVIKANKI
>LFGLNPFC_04186 ATP synthase protein I
MSVSLVSRNVARKLLLVQLLVVIASGLLFSLKDPFWGVSAISGGLAVFLPNVLFMIFAWR
HQAHTPAKGRVAWTFAFGEAFKVLAMLVLLVVALAVLKAVFLPLIVTWVLVLVVQILAPA
VINNKG
>LFGLNPFC_04187 ATP synthase subunit a

MASENMTPQDYIGHHLNNLQLDLRTFSLVDPHNPPATFWTINIDSMFFSVVLGLLFLVLF
RSVAKKATSGVPGKFQTAIELVIGFVNGSVKDMYHGKSKLIAPLALTIFVWVFLMNLMDL
LPIDLLPYIAEHVLGLPALRVVPSADVNVTLSMALGVFILILFYSIKMKGIGGFTKELTL
QPFNHWAFIPVNLILEGVSLLSKPVSLGLRLFGNMYAGELIFILIAGLLPWWSQWILNVP
WAIFHILIITLQAFIFMVLTIVYLSMASEEH
>LFGLNPFC_04188 ATP synthase subunit c
MENLNMDLLYMAAAVMMGLAAIGAAIGIGILGGKFLEGAARQPDLIPLLRTQFFIVMGLV
DAIPMIAVGLGLYVMFAVA
>LFGLNPFC_04189 ATP synthase subunit b
MNLNATILGQAIAFVLFVLFCMKYVWPPLMAAIEKRQKEIADGLASAERAHKDLDLAKAS
ATDQLKKAKAEAQVIIEQANKRRSQILDEAKAEAEQERTKIVAQAQAEIEAERKRAREEL
RKQVAILAVAGAEKIIERSVDEAANSDIVDKLVAEL
>LFGLNPFC_04190 ATP synthase subunit delta
MSEFITVARPYAKAAFDFAVEHQSVERWQDMLAFAAEVTKNEQMAELLSGALAPETLAES
FIAVCGEQLDENGQNLIRVMAENGRLNALPDVLEQFIHLRAVSEATAEVDVISAAALSEQ
QLAKISAAMEKRLSRKVKLNCKIDKSVMAGVIIRAGDMVIDGSVRGRLERLADVLQS
>LFGLNPFC_04191 ATP synthase subunit alpha
MQLNSTEISELIKQRIAQFNVVSEAHNEGTIVSVSDGVIRIHGLADCMQGEMISLPGNRY
AIALNLERDSVGAVVMGPYADLAEGMKVKCTGRILEVPVGRGLLGRVVNTLGAPIDGKGP
LDHDGFSAVEAIAPGVIERQSVDQPVQTGYKAVDSMIPIGRGQRELIIGDRQTGKTALAI
DAIINQRDSGIKCIYVAIGQKASTISNVVRKLEEHGALANTIVVVATASESAALQYLAPY
AGCAMGEYFRDRGEDALIIYDDLSKQAVAYRQISLLLRRPPGREAFPGDVFYLHSRLLER
AARVNAEYVEAFTKGEVKGKTGSLTALPIIETQAGDVSAFVPTNVISITDGQIFLETNLF
NAGIRPAVNPGISVSRVGGAAQTKIMKKLSGGIRTALAQYRELAAFSQFASDLDDATRKQ
LDHGQKVTELLKQKQYAPMSVAQQSLVLFAAERGYLADVELSKIGSFEAALLAYVDRDHA
PLMQEINQTGGYNDEIEGKLKGILDSFKATQSW
>LFGLNPFC_04192 ATP synthase gamma chain
MAGAKEIRSKIASVQNTQKITKAMEMVAASKMRKSQDRMAASRPYAETMRKVIGHLAHGN
LEYKHPYLEDRDVKRVGYLVVSTDRGLCGGLNINLFKKLLAEMKTWTDKGVQCDLAMIGS
KGVSFFNSVGGNVVAQVTGMGDNPSLSELIGPVKVMLQAYDEGRLDKLYIVSNKFINTMS
QVPTISQLLPLPASDDDDLKHKSWDYLYEPDPKALLDTLLRRYVESQVYQGVVENLASEQ
AARMVAMKAATDNGGSLIKELQLVYNKARQASITQELTEIVSGAAAV
>LFGLNPFC_04193 ATP synthase subunit beta
MATGKIVQVIGAVVDVEFPQDAVPRVYDALEVQNGNERLVLEVQQQLGGGIVRTIAMGSS
DGLRRGLDVKDLEHPIEVPVGKATLGRIMNVLGEPVDMKGEIGEEERWAIHRAAPSYEEL
SNSQELLETGIKVIDLMCPFAKGGKVGLFGGAGVGKTVNMMELIRNIAIEHSGYSVFAGV
GERTREGNDFYHEMTDSNVIDKVSLVYGQMNEPPGNRLRVALTGLTMAEKFRDEGRDVLL
FVDNIYRYTLAGTEVSALLGRMPSAVGYQPTLAEEMGVLQERITSTKTGSITSVQAVYVP
ADDLTDPSPATTFAHLDATVVLSRQIASLGIYPAVDPLDSTSRQLDPLVVGQEHYDTARG
VQSILQRYQELKDIIAILGMDELSEEDKLVVARARKIQRFLSQPFFVAEVFTGSPGKYVS
LKDTIRGFKGIMEGEYDHLPEQAFYMVGSIEEAVEKAKKL
>LFGLNPFC_04194 ATP synthase epsilon chain
MAMTYHLDVVSAEQQMFSGLVEKIQVTGSEGELGIYPGHAPLLTAIKPGMIRIVKQHGHE
EFIYLSGGILEVQPGNVTVLADTAIRGQDLDEARAMEAKRKAEEHISSSHGDVDYAQASA
ELAKAIAQLRVIELTKKAM
>LFGLNPFC_04195 Bifunctional protein GlmU
MLNNAMSVVILAAGKGTRMYSDLPKVLHTLAGKAMVQHVIDAANELGAAHVHLVYGHGGD
LLKQALKDDNLNWVLQTEQLGTGHAMQQAAPFFADDEDILMLYGDVPLISVETLQRLRDA
KPQGGIGLLTVKLDDPTGYGRITRENGKVTGIVEHKDATDEQRQIQEINTGILIANGADM
KRWLAKLTNNNAQGEYYITDIIALAYQEGREIVAVHPQRLSEVEGVNNRLQLSRLERVYQ
SEQAEKLLLAGVMLRDPARFDLRGTLTHGRDVEIDTNVIIEGNVTLGHRVKIGTGCVIKN
SVIGDDCEISPYTVVEDANLAAACTIGPFARLRPGAELLEGAHVGNFVEMKKARLGKGSK
AGHLTYLGDAEIGDNVNIGAGTITCNYDGANKFKTIIGDDVFVGSDTQLVAPVTVGKGAT
IAAGTTVTRNVGENALAISRVPQTQKEGWRRPVKKK
>LFGLNPFC_04196 Glutamine--fructose-6-phosphate aminotransferase [isomerizing]
MCGIVGAIAQRDVAEILLEGLRRLEYRGYDSAGLAVVDAEGHMTRLRRLGKVQMLAQAAE
EHPLHGGTGIAHTRWATHGEPSEANAHPHVSEHIVVVHNGIIENHEPLREELKARGYTFV
SETDTEVIAHLVNWELKQGGTLREAVLRAIPQLRGAYGTVIMDSRHPDTLLAARSGSPLV
IGLGMGENFIASDQLALLPVTRRFIFLEEGDIAEITRRSVNIFDKTGAEVKRQDIESNLQ
YDAGDKGIYRHYMQKEIYEQPNAIKNTLTGRISHGQVDLSELGPNADELLSKVEHIQILA
CGTSYNSGMVSRYWFESLAGIPCDVEIASEFRYRKSAVRRNSLMITLSQSGETADTLAGL
RLSKELGYLGSLAICNVPGSSLVRESDLALMTNAGTEIGVASTKAFTTQLTVLLMLVAKL
SRLKGLDASIEHDIVHGLQALPSRIEQMLSQDKRIEALAEDFSDKHHALFLGRGDQYPIA
LEGALKLKEISYIHAEAYAAGELKHGPLALIDADMPVIVVAPNNELLEKLKSNIEEVRAR

GGQLYVFADQDAGFVSSDNMHIIEMPHVEEVIAPIFYTVPLQLLAYHVALIKGTDVDQPR
NLAKSVTVE
>LFGLNPFC_04197 Phosphate-binding protein PstS
MKVMRTTVATVVAATLSMSAFSVFAEASLTGAGATFPAPVYAKWADTYQKETGNKVNYQG
IGSSGGVKQIIANTVDFGASDAPLSDEKLAQEGLFQFPTVIGGVVLAVNIPGLKSGELVL
DGKTLGDIYLGKIKKWDDEAIAKLNPGLKLPSQNIAVVRRADGSGTSFVFTSYLAKVNEE
WKNNVGTGSTVKWPIGLGGKGNDGIAAFVQRLPGAIGYVEYAYAKQNNLAYTKLISADGK
PVSPTEENFANAAKGADWSKTFAQDLTNQKGEDAWPITSTTFILIHKDQKKPEQGTEVLK
FFDWAYKTGAKQANDLDYASLPDSVVEQVRAAWKTNIKDSSGKPLY
>LFGLNPFC_04198 Phosphate transport system permease protein PstC
MAATKPAFNPPGKKGDIIFSVLVKLAALIVLLMLGGIIVSLIISSWPSIQKFGLAFLWTK
EWDAPNDIYGALVPIYGTLVTSFIALLIAVPVSFGIALFLTELAPGWLKRPLGIAIELLA
AIPSIVYGMWGLFIFAPLFAVYFQEPVGNIMSNIPIVGALFSGPAFGIGILAAGVILAIM
IIPYIAAVMRDVFEQTPVMMKESAYGIGCTTWEVIWRIVLPFTKNGVIGGIMLGLGRALG
ETMAVTFIIGNTYQLDSASLYMPGNSITSALANEFAEAESGLHVAALMELGLILFVITFI
VLAASKFMIMRLAKNEGAR
>LFGLNPFC_04199 Phosphate transport system permease protein PstA
MAMVEMQTTAALAESRRKMQARRRLKNRIALTLSMATMAFGLFWLIWILMSTITRGIDGM
SLALFTEMTPPPNTEGGGLANALAGSGLLILWATVFGTPLGIMAGIYLAEYGRKSWLAEV
IRFINDILLSAPSIVVGLFVYTIVVAQMEHFSGWAGVIALALLQVPIVIRTTENMLKLVP
DSLREAAYALGTPKWKMIAAITLKASVSGIMTGILLAIARIAGETAPLLFTALSNQFWST
DMMQPIANLPVTIFKFAMSPFAEWQQLAWAGVLIITLCVLLLNILARVVFAKNKHG
>LFGLNPFC_04200 Phosphate import ATP-binding protein PstB
MSMVETAPSKIQVRNLNFYYGKFHALKNINLDIAKNQVTAFIGPSGCGKSTLLRTFNKMF
ELYPEQRAEGEILLDGDNILTNSQDIALLRAKVGMVFQKPTPFPMSIYDNIAFGVRLFEK
LSRADMDERVQWALTKAALWNETKDKLHQSGYSLSGGQQQRLCIARGIAIRPEVLLLDEP
CSALDPISTGRIEELITELKQDYTVVIVTHNMQQAARCSDHTAFMYLGELIEFSNTDDLF
TKPAKKQTEDYITGRYG
>LFGLNPFC_04201 Phosphate-specific transport system accessory protein PhoU
MDSLNLNKHISGQFNAELESIRTQVMTMGGMVEQQLSDAITAMHNQDSDLAKRVIEGDKN
VNMMEVAIDEACVRIIAKRQPTASDLRLVMVISKTIAELERIGDVADKICRTALEKFSQQ
HQPLLVSLESLGRHTIQMLHDVLDAFARMDIDEAVRIYREDKKVDQEYEGIVRQLMTYMM
EDSRTIPSVLTALFCARSIERIGDRCQNICEFIFYYVKGQDFRHVGGDELDKLLAEKDSD
K
>LFGLNPFC_04202 Cryptic beta-glucoside bgl operon antiterminator
MNMQITKILNNNVVVVIDDQQREKVVMGRGIGFQKRPGERINSSGIEKEYALSSHELNGR
LSELLSHMPLEVMATCDRIISLAQERLGKLQDSIYISLTDHCQFAIKRFQQNVLLPNPLL
WDIQRLYPKEFQLGEEALTIIDKRLGVQLPKDEVGFIAMHLVSAQMSGNMEDVAGVTQLM
REMLQLIKFQFSLNYQEESLSYQRLVTHLKFLSWRILEHASINDSDESLQQAVKQNYPQA
WQCAERIAIFIGLQYQRKISPAEIMFLAINIERVRKEH
>LFGLNPFC_04203 PTS system beta-glucoside-specific EIIBCA component
MTELARTIVAGVGGADNIVSLMHCATRLRFKLKDESKAQAEVLKKTPGIIMVVESGGQFQ
VVIGNHVADVFLAVNSVAGLGEKAQQAPENDDKDNLLNRFFYVISGIFTPLIGLMAATGI
LKGMLALALTFQWATEQSGTYLILFSASDALFWFFPIILGYTAGKRFSGNPFTAMVIGGA
LVHPLILTAFENGQKVDALGLDFLGIPVTLLNYSSSVIPIIFSAWLCSILERRLNTWLPS
AIKNFFTPLLCLMVITPITFLLVGPLSTWISELIAAGYLWLYQAVPAFAGAVMGGFWQIF
VMFGLHWGLIPLIINNFTVLGYDTMIPLLMPAIMAQVGAALGVFLCERDAQKKVVAGSAA
LTGLFGITEPAVYGVNLPRKYPFVIACISGALGATIIGYAQTKVYSFGLPGIFTFMQTIP
STGIDFTVWASVIGGVIAIGCAFVGTVMFHFITAKRQPAQVAQQEKTPEVITPEQGGICS
PMTGEIVPLIHVADTTFASGLLGKGIAILPSVGEVRSPVAGQIASLFATLHAIGIESDDG
VEILIHVGIDTVKLDGKFFSAHVNVGDKVNTGDRLISFDIPAIREAGFDLTTPVLISNSD
DFTDVLPHGTAQINAGEPLLSIIR
>LFGLNPFC_04204 6-phospho-beta-glucosidase BglB
MKAFPETFLWGGATAANQVEGAWQEDGKGITTSDLQPHGVMGKMEPRILGKENIKDVAID
FYHRYPEDIALFAEMGFTCLRISIAWARIFPQGDEAEPNEAGLAFYERLFDEMAQAGIKP
LVTLSHYEMPYGLVKNYGGWANRAVIDHFEHYARTVFTRYQHKVALWLTFNEINMSLHAP
FTGVGLAEESGEAEVYQAIHHQLVASARAVKACHSLIPEAKIGNMLLGGLVYPLTCQPQD
MLQAMEENRRWMFFGDVQARGQYPGYMQRFFRDHNITIEMTESDAEDLKHTVDFISFSYY
MTGCVSHDESINKNAQGNILNMIPNPHLKSSEWGWQIDPVGLRILLNTLWDRYQKPLFIV
ENGLGAKDSVEVDGSIQDDYRIAYLNDHLVQVNEAIADGVDIMGYTSWGPIDLVSASHSQ
MSKRYGFIYVDRDDNGEGSLTRTRKKSFGWYAEVIKTRGLSLKK
>LFGLNPFC_04205 Cryptic outer membrane porin BglH
MFRRNIITSAILLMAPLAFSAQSLAESLTVEQRLELLEKALRETQSELKKYKDEEKKKYT
PATVNRSVSTNDQGYAANPFPTSRAAKPDAVLVKNEEKNASETGSIYSSMTLKDFSKFVK

DEIGFSYNGYYRSGWGTASHGSPKSWAIGSLGRFGNEYSGWFDLQLKQRVYNENGKRVDA
IVMMDGNVGQQYSTGWFGDNAGGENFMQFSDMYVTTKGFLPFAPEADFWVGKHGAPKIEI
QMLDWKTQRTDAAAGVGLENWKVGPGKIDIALVREDVDDYDRSLQNKQQINTNTIDLRYK
DIPLWDKVTLMVSGRYVTANESASEKDNQDNNGYYDWKDTWMFGTSLTQKFDKGGFNEFS
FLVANNSIASNFGRYAGASPFTTFNGRYYGYHTGGTAVRLTSQGEAYIGDHFIVANAIVY
SFGNDIYSYETGAHSDFESIRAVVRPAYIWDQYNQTGVELGYFTQQNKDANSNKFNESGY
KTTLFHTFKVNTSMLTSRPEIRFYATYIKALENELDGFTFEDNKDDQFAVGAQAEIWW
>LFGLNPFC_04206 Carbohydrate acetyl esterase/feruloyl esterase
MNIKIAALTLAIASGISAQWAIAADMPASPAPTIPVKQYVTQVNADNSVTFRYFAPGAKN
VSVVVGVPVPDNIHPMTKDEAGVWSWRTPVLKGNLYEYFFNVDGVRSIDTGTAMTKPQRQ
VNSSMILVPGSYLDTRSVAHGDLITITYHSSALQSERQMYVWTPPGYSGMGEPLPVLYFY
HGFGDTGRSAIDQGRIPQIMDNLLAEGKIKPMLVVIPDTETDAKGIIPEDFVPQERRKVF
YPLNAKAADRELMNDIIPLISKRFNVRKDADGRALAGLSQGGYQALVSGMNHLESFGWLA
TFSGVTTTTVPDEGVAARLNEPAAINQQLRNFTVVVGDKDVVTGKDIVGLKTELEQKKIK
FDYQEYPGLNHEMDVWRPAYAAFVQKLFK
>LFGLNPFC_04207 putative glucosamine-6-phosphate deaminase 2
MKLIITEDYQEMSRVAAQHLLGYMSKTRRVNLAITAGSTPKGMYEYLITLVKGKPWYDNC
YFYNFDEIPFRGKEGEGVTITNLRNLFFTPAGIKEENIQKLTIDNYREHDQKLAREGGLD
LVVLGLGVDGHFCGNLPNTTHFHEQTVEFPIQGEMVDIVAHGELGGDFSLVPDSYVTMGP
KSIMAAKNLLIIVSGAGKAQALKNVLQGPVTEDVPASVLQLHPSLMVIADKAAAAELALG
>LFGLNPFC_04208 Inner membrane protein CbrB
MSVSRRVIHHGLYFAVLGPLIGVLFLVLYIFFAKEPLILLVIIQVLPLFLLMSITTGAIP
AMLTGVMVACLPEKIGSQKRYRCLVGGIGGVVITEIYCAVIVHIKDMASSALFENILSGE
NLVVRIIPALLAGVVMSRIITHLPGLDISCPETDSLS
>LFGLNPFC_04209 6-phosphogluconate phosphatase
MFHAAKAMNVNVENCILVDDSSAGAQSGIDAGMEVFYFCADPHNKPIVHPKVTTFTHLSQ
LPELWKARGWDITA
>LFGLNPFC_04210 6-phosphogluconate phosphatase
MSQIEAVFFDCDGTLVDSEVICSRAYVTMFREFGIHVDLEEIFTRFKGVKLYEIIDIISK
EQGVTMVQADAEHVYRAEVARLFDTELEDIAGANELLASINVPMCVVSNGPVSKMQHSLG
KLNMLHYFPEKLFCSAAMISSVGSPIRH
>LFGLNPFC_04211 Adenine permease AdeP
MSQQHTTQASGQGMLERVFKLREHGTTARTEVIAGFTTFLTMVYIVFVNPQILGVAGMDT
SAVFVTTCLIAAFGSIMMGLFANLPVALAPAMGLNAFFAFVVVQAMGLPWQVGMGAIFWG
AIGLLLLTIFRVRYWMIANIPVSLRVGITSGIGLFIGMMGLKNAGVIVANPETLVSIGNL
TSHSVLLGILGFFIIAILASRNIHAAVLVSIVVTTLLGWMLGDVHYNGIVSAPPSVMTVV
GHVDLAGSFNLGLAGVIFSFMLVNLFDSSGTLIGVTDKAGLADEKGKFPRMKQALYVDSI
SSVTGSFIGTSSVTAYIESSSGVSVGGRTGLTAVVVGLLFLLVIFLSPLAGMVPGYAAAG
ALIYVGVLMTSSLARVNWQDLTESVPAFITAVMMPFSFSITEGIALGFISYCVMKIGTGR
LRDLSPCVIIVALLFILKIVFIDAH
>LFGLNPFC_04212 Quinone reductase
MSEKLQVVTLLGSLRKGSFNGMVARTLPKIAPASMEINALPSIADIPLYDADVQQEDGFP
ATVEALAEQIRQADGVVIVTPEYNYSVPGGLKNAIDWLSRLPDQPLAGKPVLIQTSSMGV
IGGARCQYHLRQILVFLDTMVMNKPEFMGGVIQNKVDPQTGEVIDQGTLDHLTGQLTAFG
EFIQRVKI
>LFGLNPFC_04213 hypothetical protein
MATHFARGILTEGHLISVRLPSLCHQEARNIPPHRQSRFLASRGLLAELMFMLYGIGELP
EIVTLPKGKPVFSDKNLPSFSVSYAGNMVGVALTTEGECGLDMELQRATRGFHSPHAPDN
HTFSSNESLWISKQNDPNEARAQLITLRRSVLKLTGDVLNDDPRDLQLLPIAGRLKCAHV
NHVEALCDAEDVLVWSVAVTPTIEKLSVWELDGKHGWKSLPDIHSRANNPTSRMMRFAQL
STVKAFSPN
>LFGLNPFC_04214 HTH-type transcriptional regulator YidZ
MKKSITTLDLNLLLCLQLLMQERSVTKAAKRMNVTPSAVSKSLAKLRAWFDDPLFVNSPL
GLSPTPLMVSMEQNLAEWMQMSNQLLDKPLHETPRGLKFELAAESPLMMIMLNALSKRIY
QRYPQATIKLRNWDYDSLDAITRGEVDIGFSGRESHPRSRELLSSLPLAIDYEVLFSDVP
CVWLRKDHPALHEAWNLDTFLRYPHISICWEQSDTWALDNVLQELGRERTIAMSLPEFEQ
SLFMAAQPDNLLLATAPRYCQYYNQLHQLPLVALPLPFDESQQKKLEVPFTLLWHKRNSR
NPKIVWLRETIKNLYASMA
>LFGLNPFC_04215 Multidrug resistance protein MdtL
MSRFLICSFALVLLYPAGIDMYLVGLPRIAADLNASEAQLHIAFSVYLAGMAAAMLFAGK
VADRSGRKPVAIPGAALFIIASVFCSLAETSALFLAGRFLQGLGAGCCYVVAFAILRDTL
DDRRRAKVLSLLNGITCIIPVLAPVLGHLIMLKFPWQSLFWTMATMGIALLMLSLFILKE
TRPAAPTTSDKPRENSESLLNRFFLSRVVITTLSVSVILTFVNTSPVLLMEIMGFERGEY
ATIMALTAGVSMTVSFSTPFALGIFKPRTLMITSQVLFLAAGITLAVSPSHAVSLFGITL

ICAGFSVGFGVAMSQALGPFSLRAGVASSTLGIAQVCGSSLWIWLAAVVGIGAWNMLIGI
LIACSIVSLLLIMFVAPGRPVAAHEEIHHHA
>LFGLNPFC_04216 Low affinity tryptophan permease
MTDQSEKKHSAFWGVMVIAGTVIGGGMFALPVDLAGAWFFWGAFILIIAWFSMLHSGLLL
LEANLNYPVGSSFNTITKDLIGNTWNIISGITVAFVLYILTYAYISANGAIISETISMNL
GYHANPRIVGICTAIFVASVLWISSLAASRITSLFLGLKIISFVIVFGSFFFQVDYSILR
DSTSTTAGTSYFPYIFMALPVCLASFGFHGNIPSLIICYGKRKDKLIKSVVFGSLLALVI
YLFWLYCTMGNIPRESFKAIISSGGNVDSLVKSFLGTKQHGIIEFCLLVFSNLAVASSFF
GVTLGLFDYLADLFKIDNSHAGRFKTVLLTFLPPALLYLIFPNGFIYGIGGAGLCATIWA
VIIPAVLAIKARKKFPNQMFTVWGGNLIPAIVILFGITVILCWFGNVFNVLPKFG
>LFGLNPFC_04217 Tryptophanase
MENFKHLPEPFRIRVIEPVKRTTRAYREEAIIKSGMNPFLLDSEDVFIDLLTDSGTGAVT
QSMQAAMMRGDEAYSGSRSYYALAESVKNIFGYQYTIPTHQGRGAEQIYIPVLIKKREQE
KGLDRSKMVAFSNYFFDTTQGHSQINGCTVRNVYIKEAFDTGVRYDFKGNFDLEGLERGI
EEVGPNNVPYIVATITSNSAGGQPVSLANLKAMYSIAKKYDIPVVMDSARFAENAYFIKQ
REAEYKDWTIEQITRETYKYADMLAMSAKKDAMVPMGGLLCMKDDSFFDVYTECRTLCVV
QEGFPTYGGLEGGAMERLAVGLYDGMNLDWLAYRIAQVQYLVDGLEEIGVVCQQAGGHAA
FVDAGKLLPHIPADQFPAQALACELYKVAGIRAVEIGSFLLGRDPKTGKQLPCPAELLRL
TIPRATYTQTHMDFIIEAFKHVKENAANIKGLTFTYEPKVLRHFTAKLKEV
>LFGLNPFC_04218 tRNA modification GTPase MnmE
MSDNDTIVAQATPPGRGGVGILRISGLKAREVAETVLGKLPKPRYADYLPFKDADGSVLD
QGIALWFPGPNSFTGEDVLELQGHGGPVILDLLLKRILTIPGLRIARPGEFSERAFLNDK
LDLAQAEAIADLIDASSEQAARSALNSLQGAFSARVNHLVEALTHLRIYVEAAIDFPDEE
IDFLSDGKIEAQLNNVIADLDAVRAEARQGSLLREGMKVVIAGRPNAGKSSLLNALAGRE
AAIVTDIAGTTRDVLREHIHIDGMPLHIIDTAGLREASDEVERIGIERAWQEIEQADRVL
FMVDGTTTDAVDPAEIWPEFIARLPAKLPITVVRNKADITGETLGMSEVNGHALIRLSAR
TGEGVDVLRNHLKQSMGFDTNMEGGFLARRRHLQALEQAAEHLQQGKAQLLGAWAGELLA
EELRLAQQNLSEITGEFTSDDLLGRIFSSFCIGK
>LFGLNPFC_04219 Membrane protein insertase YidC
MDSQRNLLVIALLFVSFMIWQAWEQDKNPQPQAQQTTQTTTTAAGSAADQGVPASGQGKL
ISVKTDVLDLTINTRGGDVEQALLPAYPKELNSTQPFQLLETSPQFIYQAQSGLTGRDGP
DNPANGPRPLYNVEKDAYVLAEGQNELQVPMTYTDAAGNTFTKTFVLKRGDYAVNVNYNV
QNAGEKPLEISTFGQLKQSITLPPHLDTGSSNFALHTFRGAAYSTPDEKYEKYKFDTIAD
NENLNISSKGGWVAMLQQYFATAWIPHNDGTNNFYTANLGNGIAAIGYKSQPVLVQPGQT
GAMNSTLWVGPEIQDKMAAVAPHLDLTVDYGWLWFISQPLFKLLKWIHSFVGNWGFSIII
ITFIVRGIMYPLTKAQYTSMAKMRMLQPKIQAMRERLGDDKQRISQEMMALYKAEKVNPL
GGCFPLLIQMPIFLALYYMLMGSVELRQAPFALWIHDLSAQDPYYILPILMGVTMFFIQK
MSPTTVTDPMQQKIMTFMPVIFTVFFLWFPSGLVLYYIVSNLVTIIQQQLIYRGLEKRGL
HSREKKKS
>LFGLNPFC_04220 Ribonuclease P protein component
MLTPSQFTFVFQQPQRAGTPQITILGRLNSLGHPRIGLTVAKKNVRRAHERNRIKRLTRE
SFRLRQHELPAMDFVVVAKKGVADLDNRALSEALEKLWRRHCRLARGS
>LFGLNPFC_04221 50S ribosomal protein L34
MKRTFQPSVLKRNRSHGFRARMATKNGRQVLARRRAKGRARLTVSK
>LFGLNPFC_04222 Chromosomal replication initiator protein DnaA
MSLSLWQQCFARLQDELPATEFSMWIRPLQAELSDNTLALYAPNRFVLDWVRDKYLNNIN
GLLTSFCGADAPQLRFEVGTKPVTQTPQAAVTSNVAAPAQVAQTQPQRAAPSTRSGWDNV
PAPAEPTYRSNVNVKHTFDNFVEGKSNQLARAAARQVADNPGGAYNPLFLYGGTGLGKTH
LLHAVGNGIMARKPNAKVVYMHSERFVQDMVKALQNNAIEEFKRYYRSVDALLIDDIQFF
ANKERSQEEFFHTFNALLEGNQQIILTSDRYPKEINGVEDRLKSRFGWGLTVAIEPPELE
TRVAILMKKADENDIRLPGEVAFFIAKRLRSNVRELEGALNRVIANANFTGRAITIDFVR
EALRDLLALQEKLVTIDNIQKTVAEYYKIKVADLLSKRRSRSVARPRQMAMALAKELTNH
SLPEIGDAFGGRDHTTVLHACRKIEQLREESHDIKEDFSNLIRTLSS
>LFGLNPFC_04223 Beta sliding clamp
MKFTVEREHLLKPLQQVSGPLGGRPTLPILGNLLLQVADGTLSLTGTDLEMEMVARVALV
QPHEPGATTVPARKFFDICRGLPEGAEIAVQLEGERMLVRSGRSRFSLSTLPAADFPNLD
DWQSEVEFTLPQATMKRLIEATQFSMAHQDVRYYLNGMLFETEGEELRTVATDGHRLAVC
SMPIGQSLPSHSVIVPRKGVIELMRMLDGGDNPLRVQIGSNNIRAHVGDFIFTSKLVDGR
FPDYRRVLPKNPDKHLEAGCDLLKQAFARAAILSNEKFRGVRLYVSENQLKITANNPEQE
EAEEILDVTYSGAEMEIGFNVSYVLDVLNALKCENVRMMLTDSVSSVQIEDAASQSAAYV
VMPMRL
>LFGLNPFC_04224 DNA replication and repair protein RecF
MSLTRLLIRDFRNIETADLALSPGFNFLVGANGSGKTSVLEAIYTLGHGRAFRSLQIGRV
IRHEQEAFVLHGRLQGEERETAIGLTKDKQGDSKVRIDGTDGHKVAELAHLMPMQLITPE

GFTLLNGGPKYRRAFLDWGCFHNEPGFFTAWSNLKRLLKQRNAALRQVTRYEQLRPWDKE
LIPLAEQISTWRAEYSAGIAADMADTCKQFLPEFSLTFSFQRGWEKETEYAEVLERNFER
DRQLTYTAHGPHKADLRIRADGAPVEDTLSRGQLKLLMCALRLAQGEFLTRESGRRCLYL
IDDFASELDDERRGLLASRLKATQSQVFVSAISAEHVIDMSDENSKMFTVEKGKITD
>LFGLNPFC_04225 DNA gyrase subunit B
MSNSYDSSSIKVLKGLDAVRKRPGMYIGDTDDGTGLHHMVFEVVDNAIDEALAGHCKEII
VTIHADNSVSVQDDGRGIPTGIHPEEGVSAAEVIMTVLHAGGKFDDNSYKVSGGLHGVGV
SVVNALSQKLELVIQREGKIHRQIYEHGVPQAPLAVTGETEKTGTMVRFWPSLETFTNVT
EFEYDILAKRLRELSFLNSGVSIRLRDKRDGKEDHFHYEGGIKAFVEYLNKNKTPIHPNI
FYFSTEKDGIGVEVALQWNDGFQENIYCFTNNIPQRDGGTHLAGFRAAMTRTLNAYMDKE
GYSKKAKVSATGDDAREGLIAVVSVKVPDPKFSSQTKDKLVSSEVKSAVEQQMNELLAEY
LLENPTDAKIVVGKIIDAARAREAARRAREMTRRKGALDLAGLPGKLADCQERDPALSEL
YLVEGDSAGGSAKQGRNRKNQAILPLKGKILNVEKARFDKMLSSQEVATLITALGCGIGR
DEYNPDKLRYHSIIIMTDADVDGSHIRTLLLTFFYRQMPEIVERGHVYIAQPPLYKVKKG
KQEQYIKDDEAMDQYQISIALDGATLHTNASAPALAGEALEKLVSEYNATQKMINRMERR
YPKAMLKELIYQPTLTEADLSDEQTVTRWVNALVSELNDKEQHGSQWKFDVHTNAEQNLF
EPIVRVRTHGVDTDYPLDHEFITGGEYRRICTLGEKLRGLLEEDAFIERGERRQPVASFE
QALDWLVKESRRGLSIQRYKGLGEMNPEQLWETTMDPESRRMLRVTVKDAIAADQLFTTL
MGDAVEPRRAFIEENALKAANIDI
>LFGLNPFC_04226 hypothetical protein
MGLFDDVVGAFLKGDAGKYQAILSWVEEQGGIQVLLEKLQSGGLGAILSTWLSNQQSNQS
VSGEQVESALGTNAVSDLGQKLGVDTSTASSLLAEQLPKIIDALSPQGEVSPQAHNDLLS
AGMELLKGKLFR
>LFGLNPFC_04227 Sugar phosphatase YidA
MAIKLIAIDMDGTLLLPDHTISPAVKNAIAAARARGVNVVLTTGRPYAGVHNYLKELHME
QPGDYCITYNGALVQKAADGSTVAQTALSYDDYRFLEKLSREVGSHFHALDRTTLYTANR
DISYYTVHESFVATIPLVFCEAEKMDPNTQFLKVMMIDEPAILDQAIARIPQEVKEKYTV
LKSAPYFLEILDKRVNKGTGVKSLADVLGIKPEEIMAIGDQENDIAMIEYAGVGVAMDNA
IPSVKEVANFVTKSNLEDGVAFAIEKYVLN
>LFGLNPFC_04228 hypothetical protein
MKLNLKGFFKAASLCSLAFALTGCITWGLVSNTASRPHAEQWRSDTIKGLSLAEDSNGTK
GYVFVGESLDYLLTTGGDEVVKMLNDPAIHGERITVSDNAKFILSSSNKNFSGAITLYYD
WNNEEDKALATQYGFICDTRRCTWMLDGLKGSIHQKNKKADYSNVMVFHQPFTVGFYEYK
ATDGVPHGLVNALLPVTLTLDIVTSPLQFLILCTTRNC
>LFGLNPFC_04229 HTH-type transcriptional regulator LutR
MTLNKTDRIVITLGKQIVHGKYVPGSPLPAEAELCEEFATSRNIIREVFRSLMAKRLIEM
KRYRGAFVAPRNQWNYLDTDVLQWVLENDYDPRLISAMSEVRNLVEPAIARWAAERATSS
DLAQIESALNEMIANNQDREAFNEADIRYHEAVLQSVHNPVLQQLSIAISSLQRAVFERT
WMGDEANMPQTLQEHKALFDAIRHQDGDAAEQAALTMIASSTRRLKEIT
>LFGLNPFC_04230 putative 2-dehydro-3-deoxygalactonokinase DgoK1
MTARYIAIDWGSTNLRAWLYQGDHCLDSRQSEAGVTRLNGKSPAAVLAEVTTDWREENTP
VVMAGMVGSNVGWKVAPYLSVPARFSSIGEQLTSAGDNIWIIPGLCVSHDDNHNVMRGEE
TQLIGARTLAPSSLYVMPGTHCKWVQADSQQINDFRTVMTGELHHLLLNHSLIGAGLPPQ
ENAADAFAAGLERGLNAPDILPQLFEVRASHVLGTLPREQVSEFLSGLLIGAEVASMRDY
VTHQQVITLVAGTSLTVRYQQAFQAMGCDVTAVAGDTAFQAGIRSIAHAVAN
>LFGLNPFC_04231 2-dehydro-3-deoxy-6-phosphogalactonate aldolase
MQWQTKLPLIAILRGITPDEALAHVGAVIDAGFDAVEIPLNSPQWEQSIPAIVDAYGDKA
LIGAGTVLKPEQVDALARMGCQLIVTPNIHSEVIRRAVGYGMTVCPGCATATEAFTALEA
GAQALKIFPSSAFGPQYIKALKAVLPPDIAVFAVGGVTPENLAQWIDAGCAGAGLGSDLY
RAGQSVERTAQQAAAFVKAYREAVQ
>LFGLNPFC_04232 D-galactonate dehydratase
MKITKITTYRLPPRWMFLKIETDEGVVGWGEPVIEGRARTVEAAVHELSDYLIGQDPSRI
NDLWQVMYRAGFYRGGPILMSAIAGIDQALWDIKGKVLNAPVWQLMGGLVRDKIKAYSWV
GGDRPADVIDGIKTLREIGFDTFKLNGCEELGLIDNSRAVDAAVNTVAQIREAFGNQIEF
GLDFHGRVSAPMAKVLIKELEPYRPLFIEEPVLAEQAEYYPKLAAQTHIPLAAGERMFSR
FDFKRVLEAGGISILQPDLSHAGGITECYKIAGMAEAYDVTLAPHCPLGPIALAACLHID
FVSYNAVLQEQSMGIHYNKGAELLDFVKNKEDFSMAGGFFKPLTKPGLGVEIDEAKVIEF
SKNAPDWRNPLWRHEDNSVAEW
>LFGLNPFC_04233 D-galactonate transporter
MDIPVNAAKPGRRRYLTLVMIFITVVICYVDRANLAVASAHIQEEFGITKAQMGYVFSAF
AWLYTLCQIPGGWFLDRVGSRVTYFIAIFGWSVATLFQGFATGLMSLIGLRAITGIFEAP
AFPTNNRMVTSWFPEHERASAVGFYTSGQFVGLAFLTPLLIWIQEMLSWHWVFIVTGGIG
IIWSLIWFKVYQPPRLTKGISKAELDYIRDGGGLVDGDAPVKKEARQPLTAKDWKLVFHR
KLIGVYLGQFAVASTLWFFLTWFPNYLTQEKGITALKAGFMTTVPFLAAFIGVLLSGWVA

DLLVRKGFSLGFARKTPIICGLLISTCIMGANYTNDPMMIMCLMALAFFGNGFASITWSL
VSSLAPMRLIGLTGGVFNFAGGLGGITVPLVVGYLAQGYGFAPALVYISAVALIGALSYI
LLVGDVKRVG
>LFGLNPFC_04234 Protein CbrA
MEHFDVAIIGLGPAGSALARKLAGKMQVIALDKKHQHGTEGFSKPCGGLLAPDAQRSFIR
DGLTLPVDVIANPQIFSVKTVDVAASLTRNYQRSYININRHAFDLWMKSLIPASVEVYHD
SLCRKIWREDDKWHVIFRADGWEQHITARYLVGADGANSMVRRHLYPDHQIRKYVAIQQW
FAEKHPVPFYSCIFDNAITDCYSWSISKDGYFIFGGAYPMKDGQTRFTTLKEKMSAFQFQ
FGKAVKSEKCTVLFPSRWQDFVCGKDNAFLIGEAAGFISASSLEGISYALDSAEILRSVL
LKLPEKLNTAYWRATRKLRLKLFGKIVKSRCLTAPALRKWIMRSGVAHIPQLKDYPTRFT
SPTSRM
>LFGLNPFC_04235 hypothetical protein
MKQITFAPRNHLLTNTNTWTPDSQWLVFDVRPSGASFTGETIERVNIHTGEVEVIYRASQ
GAYVGVVTVHPKSEKYVFIHGPENPDETWYYDFHHRRGVIVESGKVSNLDAMDITAPYTP
GALRGGSHVHVFSPNGERVSFTYNDHVMHELDPALDLRNVGVAAPFGPVNVQKQHPREYS
GSHWCVLVSKTTPTPQPGSDEINRAYEEGWVGNHALAFIGDTLSPKGEKVPELFIVELPK
DEAGWKAAGDVPLSGTETTLPAPPRGVVQRRLTFTHHRIYPGLVNIPRHWVRCNPQGTQI
AFLMRDDNGIVQLWLISPQGGEPRQLTHNKTDIQSAFNWHPSGEWLGFVLDNRIACAHAQ
SGEVEYLTENHANPPSADAVVFSPDGQWLAWMEDGQLWITETDR
>LFGLNPFC_04236 hypothetical protein
MSHTGGKEGTYPGTRASATMIGDDETNWGTKSLAILDMPFTAVLDTILLPWDVFRKDSSV
RSRVEKSEANAQETNNVIPPAKMPAN
>LFGLNPFC_04237 Small heat shock protein IbpA
MRNFDLSPLYRSAIGFDRLFNHLENNQSQSNGGYPPYNVELVDENHYRIAIAVAGFAESE
LEITAQDNLLVVKGAHADEQKERTYLYQGIAERNFERKFQLAENIHVRGANLVNGLLYID
LERVIPEAKKPRRIEIN
>LFGLNPFC_04238 Small heat shock protein IbpB
MRNFDLSPLMRQWIGFDKLANALQNAGESQSFPPYNIEKSDDNHYRITLALAGFRQEDLE
IQLEGTRLSVKGTPEQPKEEKKWLHQGLMNQPFSLSFTLAENMEVSGATFVNGLLHIDLI
RNEPEPIAAQRIAISERPALNS
>LFGLNPFC_04239 Aspartate/alanine antiporter
MSDIALTVSILALVAVVGLFIGNVKFRGVGLGIGGVLFGGIIVGHFVSQAGMTLSSDMLH
VIQEFGLILFVYTIGIQVGPGFFASLRVSGLRLNLFAVLIVIIGGLVTAILHKLFDIPLP
VVLGIFSGAVTNTPALGAGQQILRDLGTPMAMVDQMGMSYAMAYPFGICGILFTMWMLRV
IFRVNVETEAQQHESTRTNGGALIRTINIRVENPNLHNLAIKDVPILNGDKVICSRLKRE
ETLKVPSPETVIQLGDLLHLVGQPADLHNAQLVIGQEVDTSLSTKGTDLRVARVVVTNEN
VLGKRIRDLHFKERYDVVISRLNRAGVELVASSDISLQFGDILNLVGRPSAIDAVANVLG
NAQQKLQQVQMLPVFIGIGLGVLLGSIPVFVPGFPAALKLGLAGGPLIMALILGRIGSIG
KLYWFMPPSANLALRELGIVLFLSVVGLKSGGDFIHTLVDGEGLSWIGYGALITAVPLIT
VGILARMLAKMNYLTMCGMLAGSMTDPPALAFANNLHPTSGAAALSYATVYPLVMFLRII
TPQLLAVLFWSIG
>LFGLNPFC_04240 HTH-type transcriptional activator RhaR
MNGKLQSSDVKNETPYNIPLLINENVISSGISLISLWHTYADEHYRVIWPRDKKKPLIAN
SWVAVYTVQGCGKILLKNDEQITLHGNCIIFLKPMDIHSYHCEGLVWEQYWMEFTPTSMM
DIPVGQQSVIYNGEVYNQELTEVAELITSPEAIKNNLAVAFLTKIIYQWICLMHTVGKKD
PQRRQIEKLIATLHANLQQRWSVADMAATIPCSEAWLRRLFLRYTGKTPKEYYLDARLDL
ALSLLKQQGNSVGEVADTLNFFDSFHFSKAFKHKFGYAPSAVLKNTDR
>LFGLNPFC_04241 putative symporter YidK
MNSLQILSFVGFTLLVAVITWWKVRKTDTGSQQGYFLAGRSLKAPVIAASLMLTNLSTEQ
LVGLSGGQAYKSGMSVMGWEVTSAVTLIFLALIFLPRYLKRGIATIPDFLEERYDKTTRII
IDFCFLIATGVCFLPIVLYSGALALNSLFHVGESLQLSDGAAIWLLVILLGLAGILYAVI
GGLRAMAVADSINGIGLVIGGLMVPVFGLIAMGKGSFMQGIEQLTTVHAEKLNSVGGPTD
PLPIGAAFTGLILVNTFYWCTNQGIVQRTLASKSLAEGQKGALLTAVLKMLDPLVLVLPG
LIAFHLYQDLPKADMAYPTLVNNVLPVPLVGFFGAVLCGAVISTFNGFLNSASTLFSMGI
YRRIINQNAEPQQLVTVGRKFGFFIAVVSVMVAPWIANAPQGLYSWMKQLNGIYNVPLVT
IIIMGFLFPRIPALAAKVAMGIGIISYITINYLVKFDFHFLYVLACTFCINVVVMLVIGF
IKPRATPFTFKDAFAVDMKPWKNVKIASIGILFAMIGVYAGLAEFGGYGTRWLAMISYFI
AAVVIVYLIFDSWRHRHDPAVTFTPDAKDSL
>LFGLNPFC_04242 Ulvan-active sulfatase
MKRPNFLFIMTDTQATNMVGCYSGKPLNTQNIDSLAAEGIRFNSAYTCSPVCTPARAGLF
TGIYANQSGPWTNNVAPGKNISTMGRYFKDAGYHTCYIGKWHLDGHDYFGTGECPPEWDA
DYWFDGANYLSELTEKEISLWRNGLNSVEDLQANHIDETFTWAHRISNRAVDFLQQPARA
DEPFLMVISYDEPHHPFTCPVEYLEKYTDFYYELGEKAEDDLANKPEHHRLWAQAMPSPV
GDDGLYHHPLYFACNDFVDDQIGRVINALTPEQRENTWVIYTSDHGEMMGAHKLISKGAA

MYDDITRIPLIIRSPQGERRQVDTPVSHIDLLPTMMALADIEKPEILPGEISLP
>LFGLNPFC_04243 hypothetical protein
MEFNRYEIEHDSFGGFIPVRCWVTDDFKLVLNLFTSDELYDRRNDPNEMHNLIDDIHFAD
VRSKMHDALLDYMDKIRDPFRSYQWNLRPWRKDAQPRWMGAFRPRPQDGYSPVVRDYDTG
LPTQGVKVEEKKQKF
>LFGLNPFC_04244 Inner membrane protein YidH
MKISRLGEAPDYRFSLANERTFLAWIRTALGFLAAGVGLDQLAPDFATPVIRELLALLLC
LFSGGLAMYGYLRWLRNEKAMRLKEDLPYTNSLLIISLILMVVAVIVMGLVLYAG
>LFGLNPFC_04245 Inner membrane protein YidG
MPDSRKARRIADPGLQPERTSLAWFRTMLGYGALMALAVKHNWHQAGMLFWISIGILAIV
ALILWHYTRNRNLMDVTNSDFSQFHVVRDKFLISLAVLSLAILFAVTHIHQLIVFIERVA
>LFGLNPFC_04246 hypothetical protein
MTGSQVIDAEEDRHKLVVEYKDALQPADFYHNFKQLGIRSVQLIPHLEFDDRGDLTAASV
TAELWGKFLIALFECWVRADISRISIELFDTTLQKWCGSEKSQPRRDCQACDWHRLCPHA
REDKPDNVLCAGYQAFYSYSAPHMRVMRDLIKQHRSPMELMTMLR
>LFGLNPFC_04247 Multidrug resistance protein D
MKRHRNVNLLLMLVLLVAVGQMAQTIYIPAIADMARDLNVREGAVQSVMGAYLLTYGVSQ
LFYGPISDRVGRRPVILVGMSIFMLATLVAVTTSSLMVLIAASAMQGMGTGVGGVMARTL
PRDLYERTQLRHANSLLNMGILVSPLLAPLIGGLLDTMWNWRACYLFLLVLCAGVTFSMA
RWMPETRPVDAPRTRLLTSYKTLFGNSGFNCYLLMLIGGLAGIAAFEACSGVLMGAVLGL
SSMTVSILFILPIPAAFFGAWFAGRPNKRFSTLMWQSVICCLLAGLLMWIPDWFGVMNVW
TLLIPAALFFFGAGMLFPLATSGAMEPFPFLAGTAGALVGGLQNIGSGVLASLSAMLPQT
GQGSLGLLMTLMGLLIVLCWLPLATRMSHQGQPV
>LFGLNPFC_04248 Acetolactate synthase isozyme 1 large subunit
MASSGTTSTRKRFTGAEFIVHFLEQQGIKIVTGIPGGSILPVYDALSQSTQIRHILARHE
QGAGFIAQGMARTDGKPAVCMACSGPGATNLVTAIADARLDSIPLICITGQVPASMIGTD
AFQEVDTYGISIPITKHNYLVRHIEELPQVMSDAFRIAQSGRPGPVWIDIPKDVQTAVFE
IETQPAMAEKAAAPAFSEESIRDAAAMINAAKHPVLYLGGGVINAPARVRELAEKAQLPT
TMTLMALGMLPKAHPLSLGMLGMHGVRSTNYILQEADLLIVLGARFDDRAIGKTEQFCPN
AKIIHVDIDRAELGKIKQPHVAIQADVDDVLAQLIPLVEAQPRAEWHQLVADLQREFPCP
IPKACDPLSHYGLINAVAACVDDNAIITTDVGQHQMWTAQAYPLNRPRQWLTSGGLGTMG
FGLPAAIGAALANPDRKVLCFSGDGSLMMNIQEMATASENQLDVKIILMNNEALGLVHQQ
QSLFYKQGVFAATYPGKINFMQIAAGFGLETCDLNNEADPQAALQEIINRPGPALIHVRI
DAEEKVYPMVPPGAANTEMVGE
>LFGLNPFC_04249 Acetolactate synthase isozyme 1 small subunit
MQNTTHDNVILELTVRNHPGVMTHVCGLFARRAFNVEGILCLPIQDSDKSHIWLLVNDDQ
RLEQMISQIDKLEDVVKVQRNQSDPTMFNKIAVFFQ
>LFGLNPFC_04250 Transcriptional regulatory protein UhpA
MITVALIDDHLIVRSGFAQLLGLEPDLQVVAEFGSGREALAGLPGCGVQVCICDISMPDI
SGLELLSQLPKGMATIMLSVHDSPALVEQALNAGARGFLSKRCSPDELIAAVHTVATGGC
YLTPDIAIKLASGRQDPLTKRERQVAEKLAQGMAVKEIAAELGLSPKTVHVHRANLMEKL
GVSNDVELARRMFDGW
>LFGLNPFC_04251 Signal transduction histidine-protein kinase/phosphatase UhpB
MKTLFSRLITVIACFFIFSAAWFCLWSISLHLVERPDMAVLLFPFGLRLGLMLQCPRGYW
PVLLGAEWLLIYWLMQAVGLTHFSLLMIGSLLTLLPVALISRYRHQRDWRTLLLQGAALT
AAALLQSLPWLWHGKESWNALLLTLTGGLTLAPICLVFWHYLANNTWLPLGPSLVSQPIN
WRGRHLVWYLLLFVISLWLQLGLPDELSRFTPFCLALPIIALAWHYGWQGALIATLMNAI
ALIASQTWRDHPVDLLLSLLVQSLTGLLLGAGIQRLRELNQSLQKELARNQHLAERLLET
EESVRRDVARELHDDIGQTITAIRTQAGIVQRLAADNASVKQSGQLIEQLSLGVYDAVRR
LLGRLRPRQLDDLTLEQAIRSLMREMELEGRGIVSHLEWRIDESALSENQRVTLFRVCQE
GLNNIVKHADASAVTLQGWQQDERLMLVIEDDGSGLPPDSGQHGFGLTGMRERVTALGGT
LTISCLHGTRVSVSLPQRYV
>LFGLNPFC_04252 Membrane sensor protein UhpC
MLPFLKAPADAPLMTDKHEIDARYRYWRRHILLTIWLGYALFYFTRKSFNAAVPEILANG
VLSRSDIGLLATLFYITYGVSKFVSGIVSDRSNARYFMGIGLIATGIINILFGFSTSLWA
FAVLWVLNAFFQGWGSPVCARLLTAWYSRTERGGWWALWNTAHNVGGALIPIVMAASALH
YGWRAGMMIAGCMAIVVGIFLCWRLRDRPQALGLPAVGEWRHDALEIAQQQEGAGLTRKE
ILTKYVLLNPYIWLLSFCYVLVYVVRAAINDWGNLYMSEMLGVDLVTANTAVTMFELGGF
IGALVAGWGSDKLFNGNRGPMNLIFAAGILLSVGSLWLMPFASYVMQATCFFTIGFFVFG
PQMLIGMAAAECSHKEAAGAATGFVGLFAYLGASLAGWPLAKVLDTWHWSGFFVVIAIAA
GISALLLLPFLNAQTPREA
>LFGLNPFC_04253 Hexose-6-phosphate:phosphate antiporter
MLAFLNQVRKPTLDLPLEVRRKMWFKPFMQSYLVVFIGYLTMYLIRKNFNIAQNDMISTY
GLSMTQLGMIGLGFSITYGVGKTLVSYYADGKNTKQFLPFMLILSAICMLGFSASMGSGS

VSLFLMIAFYALSGFFQSTGGSCSYSTITKWTPRRKRGTFLGFWNISHNLGGAGAAGVAL
FGANYLFDGHVIGMFIFPSIIALIVGFIGLRYGSDSPESYGLGKAEELFGEEISEEDKET
ESTDMTKWQIFVEYVLKNKVIWLLCFANIFLYVVRIGIDQWSTVYAFQELKLSKAVAIQG
FTLFEAGALVGTLLWGWLSDLANGRRGLVACIALALIIATLGVYQHASNEYIYLASLFAL
GFLVFGPQLLIGVAAVGFVPKKAIGAADGIKGTFAYLIGDSFAKLGLGMIADGTPVFGLT
GWAGTFAALDIAAIGCICLMAIVAVMEERKIRREKKIQQLTVA
>LFGLNPFC_04254 Adenine deaminase
MNNSINHKFHHISRTEYQELLAVSRGDAVADYIIDNVSILDLINGGEISGPIVIKGRYIA
GVGAEYADAPALQRIDARGATAVPGFIDAHLHIESSMMTPVTFETATLPRGLTTVICDPH
EIVNVMGEAGFAWFARCAEQARQNQYLQVSSCVPALEGCDVNGASFTLEQMLAWRDHPQV
TGLAEMMDYPGVISGQNALLDKLDAFRHLTLDGHCPGLGGKELNAYIAAGIENCHESYQL
EEGRRKLQLGMSLMIREGSAARNLNALAPLINEFNSPQCMLCTDDRNPWEIAHEGHIDAL
IRRLIEQHNVPLHVAYRVASWSTARHFGLNHLGLLAPGKQADIVLLSDARKVTVQQVLVK
GEPIDAQTLQAEESARLAQSAPPYGNTIDRQPVSASDFALQFTPGKRYRVIEVIHNELIT
HSRSSVYSENGFDRDDVCFIAVLERYGQRLAPACGLLGGFGLNEGALAATVSHDSHNIVV
IGRSAEEMALAVNQVIQDGGGLCVVRNGQVQSHLPLPIAGLMSTDTAQSLAEQIDALKAA
ARECGPLPDEPFIQMAFLSLPVIPALKLTSQGLFDGEKFAFTTLEFTE
>LFGLNPFC_04255 Adenine permease AdeQ
MNNDNTDYVSNESGTLSRLFKLSQHGTTVRTELIAGMTTFLTMVYIVFVNPQILGAAQMD
PKVVFVTTCLIAGIGSIAMGVFANLPVALAPAMGLNAFFAFVVVGAMGISWQTGMGAIFW
GTVGLFLLTLFRIRYWMISNIPLSLRIGITSGIGLFIALMGLKNTGVIVANKDTLVMIGD
LSSHGVLLGILGFFIITVLSSRHFHAAVLVSIVVTSCCGLFFGDVHFSGVYSIPPDISGV
IGEVDLSGALSLELAGIIFSFMLINLFDSSGTLIGVTDKAGLIDSNGKFPNMNKALYVDS
VSSVAGAFIGTSSVTAYIESTSGVAVGGRTGLTAVVVGVMFLLVMFFSPLVAMVPPYATA
GALIFVGVLMTSSLARVNWDDFTESVPAFITTVMMPFTFSITEGIALGFMSYCIMKVCTG
RWRDLNLCVVVVATLFALKIILVD
>LFGLNPFC_04256 hypothetical protein
MIWIMLATLAVVFVVGFRVLTSGARKAIRRLSDRLNIDVVPVESMVDQMGKSAGDEFLRY
LHRPDESHLQNAAQVLLIWQIVIVDGSEQNLLQWHRILQKARLAAPITDAQVRLALGFLR
ETEPEMQDINAFQMRYNAFFQPAEGVHWLH
>LFGLNPFC_04257 Purine ribonucleoside efflux pump NepI
MLKWSAFPLKHATGNTMSEFIAENRGADAITRPNWSAVFSVAFCVACLIIVEFLPVSLLT
PMAQDLGISEGVAGQSVTVTAFVAMFASLFITQTIQATDRRYVVILFAVLLTLSCLLVSF
ANSFSLLLIGRACLGLALGGFWAMSASLTMRLVPPRTVPKALSVIFGAVSIALVIAAPLG
SFLGELIGWRNVFNAAAAMGVLCIFWIIKSLPSLPGEPSHQKQNTFRLLQRPGVMAGMIA
IFMSFAGQFAFFTYIRPVYMNLAGFGVDGLTLVLLSFGIASFVGTSLSSFILKRSVKLAL
AGAPFVLALSALVLTLWGSDKIVATGVAIIWGLTFALIPVGWSTWITRSLADQAEKAGSI
QVAVIQLANTCGAAIGGYALDNIGLTSPLMLSGTLMLLTALLVTAKVKMKKS
>LFGLNPFC_04258 hypothetical protein
MKPTMLLMITVFLIFPAISQAESPFSSLQSAKEKTTVLQDLRKICTPQASLSDEAWEKLM
LSDENNKQHIREAIVAMERNNQSNYWEALGKVECPDM
>LFGLNPFC_04259 Lipoprotein 28
MKLTTHHLRAGAALLLAGVLLAGCDQSSSDEKHIKVGVINGAEQDVAEVAKKVAKEKYGL
DVELVGFSGSLLPNDATNHGELDANVFQHRPFLEQDNQAHGYKLVAVGNTFVFPMAGYSK
KIKTVAQIKEGATVAIPNDPTNLGRALLLLQKEKLITLKEGKGLLPTALDITDNPRHLQI
MELEGAQLPRVLDDPKVDVAIISTTYIQQTGLSPVNDSVFIEDKNSPYVNILVAREDNKN
AENVKEFLQSYQSPEVAKAAETIFNGGAVPGW
>LFGLNPFC_04260 putative inner membrane transporter YicL
MGSTRKGMLNVLIAAVLWGSSGVCAQYIMEQSQMSSQFLTMTRLIFAGLILLTLSFIHGD
KIFSIINNHKDAISLLIFSVVGALTVQLTFLLTIEKSNAATATVLQFLSPTIIVAWFSLV
RKSRPGILVFCAILTSLIGTFLLVTHGNPTSLSISPAALFWGIASAFAAAFYTTYPSTLI
ARYGTLPVVGWSMLIGGLILLPFYARQGTNFVVNGSLILAFFYLVVIGTSLTFSLYLKGA
QLIGGPKASILSCAEPLSSALLSLLLLGITFTLPDWLGTLLILSSVILISMDSRRRARKI
NRPARHE
>LFGLNPFC_04261 hypothetical protein
MGHGTCKAAKSARHLIESKEKNRSSRVKVFSTHINLSHPAASWLATQTCANNIFNLLQNN
NAIYLFKNDNKIELRMGYDSFFSIETIDKINKENIKEIKLKNDTTYVSLPNPTILITNDI
NT
>LFGLNPFC_04262 hypothetical protein
MSDKDLTQPPAGEFIMFASGDGRVRVECRFESDTIWLSQAAMAELYDKDVRTINEHLINI
FSEGELVQNSTIRKFRIVRQEGKRQVSREIDHYNLEAILAVGYRVRSPRGTQFRQWATQT
LQEYLIKGFVMDDERLKNPPVGSSVVPDYFDEMLERIRDIRASERRVYLRVREIFALAAD
YQPSLKETTQFFQTIQNKLHFACTGHTAAELIHKRADASQPHMGLTSYKGEEVRKGDVTV
AKNYLTQDEVSELNRVVNMWLDFAEDQARRRQQVFLRDWQDKLDQFLQFNDREVLQGAGK

ISKKMADEKAQAEYVQFAEQQRRLKEAEGEKDIAGLLQWNKESKK
>LFGLNPFC_04264 Inner membrane symporter YicJ
MKSEVLSVKEKIGYGMGDAASHIIFDNVMLYMMFFYTDIFGIPAGFVGTMFLVARALDAI
SDPCMGLLADRTRSRWGKFRPWVLFGALPFGIVCVLAYSTPDLSMNGKMIYAAITYTLLT
LLYTIVNIPYCALGGVITNDPTQRISLQSWRFVLATAGGMLSTVLMMPLVNLIGGDNKPL
GFQGGIAVLSVVAFMMLAFCFFTTKERVEAPPTTTSMRDDLRDIWQNDQWRIVGFLTIFN
ILAVCVRGGAMMYYVTWILGTPEVFVAFLTTYCVGNLIGSALAKPLTDWKCKVTIFWWTN
ALLAVISLAMFFVPMQASITMFVFIFVIGVLHQLVTPIQWVMMSDTVDYGEWCNGKRLTG
ISFAGTLFVLKLGLAFGGALIGWMLAYGGYDAAEKAQNSVTISIIIALFTIVPAICYLLS
AIIAKRYYSLTTHNLKTVMEQLAQGKRRCQQQFTSQEVQN
>LFGLNPFC_04265 Alpha-xylosidase
MKISDGNWLIQPGLNLIHPLQVFEVEQQDNEMVVYAAPRDVRERTWQLDTPLFTLRFFSP
QEGIVGVRIEHFQGALNNGPHYPLNILQDVKVTIENTERYAEFKSGNLSARVSKGEFWSL
DFLRNGERITGSQVKNNGYVQDTNNQRNYMFERLDLGVGETVYGLGERFTALVRNGQTVE
TWNRDGGTSTEQAYKNIPFYMTNRGYGVLVNHPQCVSFEVGSEKVSKVQFSVESEYLEYF
VIDGPTPKAVLDRYTRFTGRPALPPAWSFGLWLTTSFTTNYDEATVNSFIDGMAERNLPL
HVFHFDCFWMKAFQWCDFEWDPLTFPDPEGMIRRLKAKGLKICVWINPYIGQKSPVFKEL
QEKGYLLKRPDGSLWQWDKWQPGLAIYDFTNPDACKWYADKLKGLVAMGVDCFKTDFGER
IPTDVQWFDGSDPQKMHNHYAYIYNELVWNVLKDTVGEEEAVLFARSASVGAQKFPVHWG
GDCYANYESMAESLRGGLSIGLSGFGFWSHDIGGFENTAPAHVYKRWCAFGLLSSHSRLH
GSKSYRVPWAYDDESCDVVRFFTQLKCRMMPYLYREAARANARGTPMMRAMMMEFPDDPA
CDYLDRQYMLGDNVMVAPVFTEAGDVQFYLPEGRWTHLWHNDELDGSRWHKQQHSFLSLP
VYVRDSTLLALGNNDQRPDYAWHEGTAFHLFNLQDGHEAVCEVPDADGSVIFTLKAARTG
NTITVTGTGEAKNWTLCLRNIVKVNGLQGGSQAESEQGLVVTPQGNALTITL
>LFGLNPFC_04266 Transcriptional regulator ManR
MQMITSRQNRLLRFLLPRREYTTIVTIAGYLNVSEKTIQRDLRLLEQWLGQWRINVEKRA
GAGVMLSAENIADLLHLDHLLVAECEEIDGVMNNARRVKIASQLLSETPNETSISKLSER
YFISGASIVNDLRVIESWLAPLGLSLIRSPSGTHIEGSEGQVRQAMALLINGIINHNEPQ
GVVYSRLDPGSYKALVHYFGEEEVLFVQSLLLDMENELSWSLGEPYYVNIFTHILIMMYR
NTHGNALSREEDQTRQYDENIFNVASQMIHKIEQRIAHTLPDDEVWFIYQYIISSGVAID
GQKDVSIISHMQASNEARLITWRLITVFSDIVDCDFSEDSALYDGLLVHIKPLINRLNYR
IHIRNPLLEDIKAELADVWRLTQYVVNQVFKTWGENAVSEDEVGYLTVHFQAAMERQIAR
KRVLLVCSTGIGTSHLLKSRILRAFPEWTIVDVISAANLSQVLPDNIELIISTINLPTVT
MPVAYVTAFFNDADIKRVTEMVITEKLHHATSRVVEI
>LFGLNPFC_04267 PTS system fructose-specific EIIABC component
MDITKILNTNRVILDMQATNKAEAIEELTNLLKKDGAIDCRETFIKDVWQREAEGSTGFE
NHIAIPHGKSSAVINTTLAIGRTRQDIPWETLDGSSVRCIILFAVRLEDQNTTHIRLLSQ
VAGALADDDIIEQLLVETSPQKIIDLFSQYAQSDVF
>LFGLNPFC_04268 PTS system fructose-like EIIB component 3
MNIVCVAACTAGIAHTYIAREKLIKGAKALGHTIKVETQGTIGTENELQAADISAADVVI
LAVDVKIKGEERFTNKRIVRVKTEIVIKSPVQFLEKVEKSLGNK
>LFGLNPFC_04269 PTS system fructose-specific EIIB'BC component
MKENKIPVSQEIKKHLLTGISWMIPLIVAAGICIALGQVLGGTDVGEKTGTIPWMLNQIG
GWGMGLIVPLISAAIAYSIADRPGFAPGLIVGFLCGQIHTGFIGGMLGGFLVGYTILLLK
RYIRLPQSMQGLMPIMVLPVLSTIIGGLLMMTLIGKPIAWLQEALIHLLESMQGGSRFLM
GAILGAMATFDFGGPVNKTMSLFSDGLLVSGVYGPEAVKFVGSIIPPFGITLSFLLTRHK
YTRAEREALKAAFPMGICMITEGVIPIAARDLLRVVGSCVVASAVAGGLIMVWGVESPVP
HGGMFVVPLFTHPLLFCLALGIGTVICGVMLSLWKKPVTERDEEFDELSDQKLKDEEITF
TLE
>LFGLNPFC_04270 D-tagatose-1,6-bisphosphate aldolase subunit KbaY
MFADMKSMVMKAWHEHYALLAINCMNLESAHAAIRVAEKNRAPIILNLYQGHLAHFPAPV
AAAVVKTLAESASVPVALALDHGKNPDCIRQAFRAGFSGLMIDASAFPLEENVRQTRAVV
ELAASAQLCVEGELGHLADAPRYDQAANADLMTQPTDVEPFIAQTGIDLLAVSVGTAHGM
YAPGVVPAIDFQRLAEISRCSSVPLALHGGSGTPFDQLQLCTTLGVAKINVGAAIFERGK
SALLHTLCHDISIELVDALKAMELAFEEAITPYLQASGSIGKA
>LFGLNPFC_04271 putative fructose-bisphosphate aldolase
MLVSMKELLQPTRQHGFAIGAFNVADNCFLRAVVDEAEATNTPAIIAIHPSEHDFVGDAF
FAHVREITQRSPVPFVLHLDHGASVEHVLRAIRCGFTSVMIDGSLLPYEENVALTAEVVR
LAHAVGVSVEGELGTIGQTGTSVEGGVSQVTYTDPAQAADFIARTGADTLAVAIGTAHGI
YPKGMQPKLQMDILRDIAGRIDIPLVLHGGSANPDAEIAESVTLGVGKINISSDMKYAYF
QKVREILAKESWWDPNVIYPDAINAAREVIRHKMKLFGSLGKASLY
>LFGLNPFC_04272 Fructokinase
MHYLGLDIGGTKIAAVVMDAHGWEIRRYRCPTQKSTYQQFVSCVVALIEQIRRDVQRPML
TGIALPGSISPLTGLIKNANIQVINGHALQADLQQLLGQPVVIANDGNCFALSEACDGAG

QDYDVVFGITLGTGCGGGIAIKQRPFIGAWGNAAECGHITLPGYTEQEDGPSVSCYCGKH
NCVESFVSGSGFSERYQQMTGNLLTPAAIVTLAQRGDACAMQQVARFRQQLARTLATIVN
VVDPGVIVIGGGLSNVELLITDLNAEVAPLVFTDQFTTPIVKAQHGDSSGMRGAAWLAMR
NGEANETFTN
>LFGLNPFC_04273 hypothetical protein
MKRSQINYVIDKAHAIAQTFRVCLPEFAYFTVDDWLQRERDNWQEVVDLQLGWDITDFGR
GDFNQTGLTLLTMRNGALGSAAYPKPYAEKMLQIQQDQQTPWHFHTHKMEDILNRGGGDL
CMRLAWANEANLCDDQRVITVSVDGQRRTMKPGETLVLKPGQGICLPPRLYHRFWAEKAF
VLGWEISMVNDDQHDNYFLEPGGRFPAIEEDEPVKWLLCGEYGILR
>LFGLNPFC_04274 hypothetical protein
MKFIGKLLLYILIALLVVIAGLYFLLQTRWGAEHISAWVSENSDYHLAFGAMDHRFSAPS
HIMLENVTFGRDGQPATLVAKSVDIALSSRQLTEPRHVDTILLENGTLNLTDQTAPLPFK
ADRLQLRDMAFNSPNSEWKLSAQRVNGGVVPWSPEAGKVLGTKAQIQFSAGSLSLNDVPA
TNVLIEGSIDNDRVTLTNLGADIARGTLTGNAQRNADGSWQVENLRMADIRLQSEKSLTD
FFAPLRSVPSLQIGRLEVIDARLQGPDWAVADLDLSLRNMTFSKDDWQTQEGKLSMNASE
FIYGSLHLFDPIINAEFSPQGVALRQFTSRWEGGMVRTSGNWQRDGKTLILDDAAIAGLE
YTLPKNWQQLWMETTPGWLNSLQLKRFSASRNLIIDIDPDFPWQLTALDGYGANLTLVTD
HKWGVWSGSANLNAAAATFNRVDVRRPSLALTANSSTVNISELSAFTEKGILEATASVSQ
TPQRQTHISLNGRGVPVNILQHWGWPELPLTGDGNIQLTASGDIQVNVPLKPTVSGQLHA
VNAAKQQVTQTMNAGVVSSGEVTSTEPVQ
>LFGLNPFC_04275 Xanthine permease XanP
MSVSTLESENAQPVAQTQNSELIYRLEDRPPLPQTLFAACQHLLAMFVAVITPALLICQA
LGLPAQDTQHIISMSLFASGVASIIQIKAWGPVGSGLLSIQGTSFNFVAPLIMGGTALKT
GGADVPTMMAALFGTLMLASCTEMVISRVLHLARRIITPLVSGVVVMIIGLSLIQVGLTS
IGGGYAAMSDNTFGAPKNLLLAGVVLALIILLNRQRNPYLRVASLVIAMAAGYALAWFMG
MLPESNEPMTQELIMVPTPLYYGLGIEWSLLLPLMLVFMITSLETIGDITATSDVSEQPV
SGPLYMKRLKGGVLANGLNSFVSAVFNTFPNSCFGQNNGVIQLTGVASRYVGFVVALMLI
VLGLFPAVSGFVQHIPEPVLGGATLVMFGTIAASGVRIVSREPLNRRAILIIALSLAVGL
GVSQQPLILQFAPEWLKNLLSSGIAAGGITAIVLNLIFPPEKQ
>LFGLNPFC_04276 Sodium/glutamate symporter
MFHLDTLATLVAATLTLLLGRKLVHSVSFLKKYTIPEPVAGGLLVALALLILKKSMGWEV
NFDMSLRDPLMLAFFATIGLNANLASLRAGGRVVGVFLIVVVGLLVMQNAIGIGMASLLG
LDPLMGLLAGSITLSGGHGTGAAWSKLFIERYGFTNATEVAMACATFGLVLGGLIGGPVA
RYLVKHSTTPNGIPDDQEVPTAFEKPDVGRMITSLVLIETIALIAICLTVGKIVAQLLAG
TAFELPTFVCVLFVGVILSNGLSMMGFYRVFERAVSVLGNVSLSLFLAMALMGLKLWELA
SLALPMLAILVVQTIFMALYAIFVTWRMMGKNYDAAVLAAGHCGFGLGATPTAIANMQAI
TERFGPSHMAFLVVPMVGAFFIDIVNALVIKLYLMLPIFAG
>LFGLNPFC_04277 ATP-dependent DNA helicase RecG
MKGRLLDAVPLSSLTGVGAALSNKLAKINLHTVQDLLLHLPLRYEDRTHLYPIGELLPGV
YATVEGEVLNCNISFGGRRMMTCQISDGSGILTMRFFNFNAAMKNSLATGRRVLAYGEAK
RGKYGAEMIHPEYRVQGDLSTPELQETLTPVYPTTEGVKQATLRKLTDQALDLLDTCAIE
ELLPPELSQGMMTLPEALRTLHRPPPTLQLSDLETGQHPAQRRLILEELLAHNLSMLALR
AGAQRFHAQPLSANDALKNKLLAALPFKPTGAQARVVAEIERDMALDVPMMRLVQGDVGS
GKTLVAALAALRAVAHGKQVALMAPTELLAEQHANNFRNWFAPLGIEVGWLAGKQKGKAR
LAQQEAIASGQVQMIVGTHAIFQEQVQFNGLALVIIDEQHRFGVHQRLALWEKGQQQGFH
PHQLIMTATPIPRTLAMTAYADLDTSVIDELPPGRTPVTTVAIPDTRRTDIIDRVRHACM
TEGRQAYWVCTLIEESELLEAQAAEATWEELKLALPELNVGLVHGRMKPAEKQAVMASFK
QGELHLLVATTVIEVGVDVPNASLMIIENPERLGLAQLHQLRGRVGRGAVASHCVLLYKT
PLSKTAQIRLQVLRDSNDGFVIAQKDLEIRGPGELLGTRQTGNAEFKVADLLRDQAMIPE
VQRLARHIHERYPQQAKALIERWMPETERYSNA
>LFGLNPFC_04278 tRNA (guanosine(18)-2'-O)-methyltransferase
MLARRQPDLTVCMEQVHKPHNVSAIIRTADAVGVHEVHAVWPGSRMRTMASAAAGSNSWV
QVKTHRTIGDAVAHLKGQGMQILATHLSDNAVDFREIDYTRPTCILMGQEKTGITQEALA
LADQDIIIPMIGMVQSLNVSVASALILYEAQRQRQNAGMYLRENSMLPEAEQQRLLFEGG
YPVLAKVAKRKGLPYPHVNQQGEIEADADWWSTMQAAG
>LFGLNPFC_04279 Bifunctional (p)ppGpp synthase/hydrolase SpoT
MYLFESLNQLIQNYLPEDQIKRLRQAYLVARDAHEGQTRSSGEPYITHPVAVACILAEMK
LDYETLMAALLHDVIEDTPATYQDMEQLFGKSVAELVEGVSKLDKLKFRDKKEAQAENFR
KMIMAMVQDIRVILIKLADRTHNMRTLGSLRPDKRRRIARETLEIYSPLAHRLGIHHIKT
ELEELGFEALYPNRYRVIKEVVKAARGNRKEMIQKILSEIEGRLQEAGIPCRVSGREKHL
YSIYCKMVLKEQRFHSIMDIYAFRVIVNDSDTCYRVLGQMHSLYKPRPGRVKDYIAIPKA
NGYQSLHTSMIGPHGVPVEVQIRTEDMDQMAEMGVAAHWAYKEHGETSTTAQIRAQRWMQ
SLLELQQSAGSSFEFIESVKSDLFPDEIYVFTPEGRIVELPAGATPVDFAYAVHTDIGHA
CVGARVDRQPYPLSQPLTSGQTVEIITAPGARPNAAWLNFVVSSKARAKIRQLLKNLKRD

DSVSLGRRLLNHALGGSRKLNEIPQENIQRELDRMKLATLDDLLAEIGLGNAMSVVVAKN
LQHGDASIPPATQSHGHLPIKGADGVLITFAKCCRPIPGDPIIAHVSPGKGLVIHHESCR
NIRGYQKEPEKFMAVEWDKETAQEFITEIKVEMFNHQGALANLTAAINTTTSNIQSLNTE
EKDGRVYSAFIRLTARDRVHLANIMRKIRVMPDVIKVTRNRN
>LFGLNPFC_04280 DNA-directed RNA polymerase subunit omega
MARVTVQDAVEKIGNRFDLVLVAARRARQMQVGGKDPLVPEENDKTTVIALREIEEGLIN
NQILDVRERQEQQEQEAAELQAVTAIAEGRR
>LFGLNPFC_04281 Guanylate kinase
MAQGTLYIVSAPSGAGKSSLIQALLKTQPLYDTQVSVSHTTRQPRPGEVHGEHYFFVNHD
EFKEMISRDAFLEHAEVFGNYYGTSREAIEQVLATGVDVFLDIDWQGAQQIRQKMPHARS
IFILPPSKIELDRRLRGRGQDSEEVIAKRMAQAVAEMSHYAEYDYLIVNDDFDTALTDLK
TIIRAERLRMSRQKQRHDALISKLLAD
>LFGLNPFC_04282 DNA ligase B
MKVWMAILISILCWQSSAWAVCPAWSPARAQEEISRLQQQIKQWDDDYWKEGKSEVEDGV
YDQLSARLTQWQRCFGNETRDVMMPPLNGAVMHPVAHTGVRKMADKNALSLWMRERSDLW
VQPKVDGVAVTLVYRDGKLNKAISRGNGLKGEDWTQKVRLISAVPQTVSGPLANSTLQGE
IFLQREGHIQQQMGGINARAKVAGLMMRQGNSDTLNSLAVFVWAWPDGPHLMTDRLKDLA
TAGFTLTQTYTRAVKNADEVAHVRNEWWKAKLPFVTDGVVVRAAKEPESRHWLPGQAEWL
VAWKYQPVAQVAEVKAIQFAVGKSGKISVVASLAPVMLDDKKVQRVNIGSVRRWQEWDIA
PGDQILVSLAGQGIPRIDDVVWRGAERTKPTPPENRFNSLTCYFASDVCQEQFISRLVWL
GSKQVLGLDGIGEAGWRALHQTHRFEHIFSWLLLTPEQLQNTPGIAKSKSAQLWHQFNLA
RQQPFTRWVMAMGIPLTRAALNASDERSWSQLLFSTEQFWQQQPGTGSGRARQVIEWKEN
AQIKKLGSWLAAQQITGFEP
>LFGLNPFC_04283 hypothetical protein
MLLHILYLVGITAEAMTGALAAGRRRMDTFGVIIIATATAIGGGSVRDILLGHYPLGWVK
HPEYVIIVATAAVLTTIVAPVMPYLRKVFLVLDALGLVVFSIIGAQVALDMGHGPIIAVV
AAVTTGVFGGVLRDMFCKRIPLVFQKELYAGVSFASAVLYIALQHYVSNHDVVIISTLVF
GFFARLLALRLKLGLPVFYYSHEGH
>LFGLNPFC_04284 hypothetical protein
MNEHHQPFEEIRHYGTEGQEFWSARELAPLLDYRDWRNFQKVLARATQACEASNQAASDH
FVETTKMVVLGSGAQRELEDVHLSRYACYLVVQNGDPAKPVIAAGQTYFAIQTRRQELAD
DEAFRQLREDEKRLFLRNELKEHNKQLVEAAQQAGVATAIDFAIFQNHGYRGLYGGLDQK
AIHQRKGLKKSQKILDHMGSTELAANLFRATQTEEKLKRDGVNSKQQANTTHFDVGRKVR
QTIQELGGTMPEELPTPQVSIKQLENSVKITEKK
>LFGLNPFC_04285 hypothetical protein
MIRSMTAYARREIKGEWGSATWEMRSVNQRYLETYFRLPEQFRSLEPVVRERIRSRLTRG
KVECTLRYEPDVSAQGELILNEKLAKQLVTAANWVKMQSDEGEINPVDILRWPGVMAAQE
QDLDAIAAEILAALDGTLDDFIIARETEGQALKVLIEQRLEGVTAEVVKVRAHMPEILQW
QRERLVAKLEDAQVQLENNRLEQELVLLAQRIDVAEELDRLEAHVKETYNILKKKEAVGR
RLDFMMQEFNRESNTLASKSINAEVTNSAIELKVLIEQMREQIQNIE
>LFGLNPFC_04286 Ribonuclease PH
MRPAGRSNNQVRPVTLTRNYTKHAEGSVLVEFGDTKVLCTASIEEGVPRFLKGQGQGWIT
AEYGMLPRSTHTRNAREAAKGKQGGRTMEIQRLIARALRAAVDLKALGEFTITLDCDVLQ
ADGGTRTASITGACVALADALQKLVENGKLKTNPMKGMVAAVSVGIVNGEAVCDLEYVED
SAAETDMNVVMTEDGRIIEVQGTAEGEPFTHEELLTLLALARGGIESIVATQKAALAN
>LFGLNPFC_04287 Orotate phosphoribosyltransferase
MKPYQRQFIEFALSKQVLKFGEFTLKSGRKSPYFFNAGLFNTGRDLALLGRFYAEALVDS
GIEFDLLFGPAYKGIPIATTTAVALAEHHDLDLPYCFNRKEAKDHGEGGNLVGSALQGRI
MLVDDVITAGTAIRESMEIIQANGATLAGVLISLDRQERGRGEISAIQEVERDYNCKVIS
IITLKDLIAYLEEKPEMAEHLAAVKAYREEFGV
>LFGLNPFC_04288 Nucleoid occlusion factor SlmA
MAEKQTAKRNRREEILQSLALMLESSDGSQRITTAKLAASVGVSEAALYRHFPSKTRMFD
SLIEFIEDSLITRINLILKDEKDTTARLRLIVLLLLGFGERNPGLTRILTGHALMFEQDR
LQGRINQLFERIEAQLRQVLREKRMREGEGYATDETLLASQILAFCEGMLSRFVRSEFKY
RPTDDFDARWPLIAAQLQ
>LFGLNPFC_04289 Deoxyuridine 5'-triphosphate nucleotidohydrolase
MMKKIDVKILDPRVGKEFPLPTYATSGSAGLDLRACLDDAVELAPGDTTLVPTGLAIHIA
DPSLAAMMLPRSGLGHKHGIVLGNLVGLIDSDYQGQLMISVWNRGQDNFTIQPGERIAQM
IFVPVVQAEFNLVEDFDATDRGEGGFGHSGRQ
>LFGLNPFC_04290 Coenzyme A biosynthesis bifunctional protein CoaBC
MSLAGKKIVLGVSGGIAAYKTPELVRRLRDRGADVRVAMTEAAKAFITPLSLQAVSGYPV
SDSLLDPAAEAAMGHIELGKWADLVILAPATADLIARVAAGMANDLVSTICLATPAPVAV
LPAMNQQMYRAAATQHNLEVLASRGLLIWGPDSGSQACGDIGPGRMLDPLTIVDMAVAHF
SPVNDLKHLNIMITAGPTREPLDPVRYISNHSSGKMGFAIAAAAARRGANVTLVSGPVSL

PTPPFVNRVDVMTALEMEAAVNASVQQQNIFIGCAAVADYRAATVAPEKIKKQATQGDEL
TIKMVKNPDIVAGVAALKDHRPYVVGFAAETNNVEEYARQKRIRKNLDLICANDVSQPTQ
GFNSDNNALHLFWQDGDKVLPLERKELLGQLLLDEIVTRYDEKNRR
>LFGLNPFC_04291 hypothetical protein
MKNNAQLLMPREKMLKFGISALTDVELLALFLRTGTRGKDVLTLAKEMLENFGSLYGLLT
SEYEQFSGVHGIGVAKFAQLKGIAELARRYYNVRMREESPLLSPEMTREFLQSQLTGEER
EIFMVIFLDSQHRVITHSRLFSGTLNHVEVHPREIIREAIKINASALILAHNHPSGCAEP
SKADKLITERIIKSCQFMDLRVLDHIVIGRGEYVSFAERGWI
>LFGLNPFC_04292 50S ribosomal protein L28
MSRVCQVTGKRPVTGNNRSHALNATKRRFLPNLHSHRFWVESEKRFVTLRVSAKGMRVID
KKGIDTVLAELRARGEKY
>LFGLNPFC_04293 50S ribosomal protein L33
MAKGIREKIKLVSSAGTGHFYTTTKNKRTKPEKLELKKFDPVVRQHVIYKEAKIK
>LFGLNPFC_04294 Formamidopyrimidine-DNA glycosylase
MPELPEVETSRRGIEPHLVGATILHAVVRNGRLRWPVSEEIYRLSDQPVLSVQRRAKYLL
LELPEGWIIIHLGMSGSLRILPEELPPEKHDHVDLVMSNGKVLRYTDPRRFGAWLWTKEL
EGHNVLAHLGPEPLSDDFNGEYLHQKCAKKKTAIKPWLMDNKLVVGVGNIYASESLFAAG
IHPDRLASSLSLAECELLARVIKAVLLRSIEQGGTTLKDFLQSDGKPGYFAQELQVYGRK
GEPCRVCGTPIVATKHAQRATFYCRQCQK
>LFGLNPFC_04295 Phosphopantetheine adenylyltransferase
MQKRAIYPGTFDPITNGHIDIVTRATQMFDHVILAIAASPSKKPMFTLEERVELAQQATA
HLGNVEVVGFSDLMANFARNQHATVLIRGLRAVADFEYEMQLAHMNRHLMPELESVFLMP
SKEWSFISSSLVKEVARHQGDVTHFLPENVHQALMAKLA
>LFGLNPFC_04296 3-deoxy-D-manno-octulosonic acid transferase
MLELLYTALFYLIQPLIWIRLWVRGRKAPAYRKRWGERYGFYRHPLKPGGIMLHSVSVGE
TLAAIPLVRALRHRYPDLPITVTTMTPTGSERVQSAFGKDVQHVYLPYDLPDALNRFLNK
VDPKLVLIMETELWPNLIAALHKRKIPLVIANARLSARSAAGYAKLGKFVRRLLRRITLI
AAQNEEDGARFVALGAKNNQVTVTGSLKFDISVTPQLAAKAVTLRRQWAPHRPVWIATST
HEGEESVVIAAHQALLQQFPNLLLILVPRHPERFPDAINLVRQAGLSYITRSSGEVPSTS
TQVVVGDTMGELMLLYGIADLAFVGGSLVERGGHNPLEAAAHAIPVLMGPHTFNFKDICA
RLEQASGLITVTDATTLAKEVSSLLTDADYRSFYGRHAVEVLYQNQGALQRLLQLLEPYL
PPKTH
>LFGLNPFC_04297 Lipopolysaccharide core heptosyltransferase RfaQ
MDKPFRRILLIKMRFHGDMLLTTPVISSLKKNYPDAKIDVLLYQDTIPILSENPEINALY
GIKNKKAKASEKIANFFHLIKVLRANKYDLIVNLTDQWMIAILVRLLNARVKISQDYHHR
QSAFWRNSFTHLVPLQGGNVVESNLSVLTPLGLESLVKQTTMSYPPASWKRMRRELDHAG
VGQNYVVIQPTARQIFKCWDNAKFSAVIDALHARGYEVVLTSGPDKDDLACVNEIAQGCQ
TLPVTALAGKVTFPELGALIDHAQLFIGVDSAPAHIAAAVNTPLISLFGATDHIFWRPWS
NNMIQFWAGDYREMPTRDQRDRNEMYLSVIPAADVIAAVDKLLPSSTTGTSL
>LFGLNPFC_04298 Lipopolysaccharide core biosynthesis protein RfaG
MIVAFCLYKYFPFGGLQRDFMRIAQTVAARGHHVRVYTQSWEGECPDVFELIKVPVKSHT
NHGRNAEYFAWVQKHLREHPVDKVVGFNKMPGLDVYYAADVCYAEKVAQEKGFFYRLTSR
YRHYAAFERATFEQGKPTQLLMLTDKQIADFQKHYQTEAERFHILPPGIYPDRKYSQQPA
NSREIFRKKNGITEQQYLLLQVGSDFTRKGVDRSIEALASLPDSLRHNTLLYVVGQDKPR
KFEALAEKRGVRSNVHFFSGRNDVSELMAAADLLLHPAYQEAAGIVLLEAITAGLPVLTT
AVCGYAHYIVDANCGEAIAEPFRQETLNEILRKALTQSSLRQAWAENARHYADTQDLYSL
PEKAADIITGGLDG
>LFGLNPFC_04299 Lipopolysaccharide core heptose(I) kinase RfaP
MVELKEPFATLWRGKDPFEEVKTLQGEVFRELETRRTLRFEMAGKSYFLKWHRGTTLKEI
IKNLLSLRMPVLGADREWNAIHRLRDVGVDTMYGVAFGEKGINPLTRTSFIITEDLTPTI
SLEDYCADWATNPPDVRVKRMLIKRVATMVRDMHAAGINHRDCYICHFLLHLPFSGKEEV
LKISVIDLHRAQIRAKVPRRWRDKDLIGLYFSSMNIGLTQRDIWRFMKVYFVAPLKDIIK
QEQGLLSQAEEKATKIRERTIRKSL
>LFGLNPFC_04300 hypothetical protein
MSAHYFNPQEMINKTIIFDESPEASVASSFHVAYGIDQNFLFGCGVSITSVLLHNNDVSF
VFHVFIDDIPEADIQRLSQLAKSYHTCIQIHLVNCERLKALPTTKNWSIAMYFRFVIADY
FIDQQDKILYLDADIACQGNLKPLITMDLANNVAAVVTERDANWWSLRGQSLQCNELEKG
YFNSGVLLINTLAWAQESVSAKAMSMLADKAIVSRLTYMDQDILNLILLGKVKFIDAKYN
TQFSLNYELKKSFVCPINDETVLIHYVGPTKPWHYWAGYPSAQPFIKAKEASPWKNEPLM
RPVNSNYARYCAKHNFKQNKPINGIMNYIYYFYLKIIK
>LFGLNPFC_04301 hypothetical protein
MNEFIKERFSYLADNKKENAPELNVSYGIDKNFLYGAGVSISSVLINNSDINFVFHVFTD
YVDDDYLKSFNETAKQFNTSIIVYLIDPKYFADLPTSQFWSYATYFRVLSFEYLSESIST
LLYLDADVVCKGSLKPLTKIIFKDEFAAVIPDNDSTQAACAKRLNIPEMNGRYFNAGVIY

VNLKKWHEANLTPYLLTLLRGETKYGSLKYLDQDALNIAFNMNNIYLAKDFDTIYTLKNE
LHDRSHRKYQQTITDKTVLIHYTGITKPWHSWAGYPSASYFNIAREQSPWKKYPLKEART
VAEMQKQYKHLFAHGEYIKGITSLIKYKLKK
>LFGLNPFC_04302 Lipopolysaccharide core heptose(II) kinase RfaY
MITSIRYRGFSFYYKDNDNKYKEIFDEILAYNFKTVKVLRNIDDTKVSLIDTKYGRYVFK
VFAPKTKRNERFLKSFVKGDYYQNLIVETDRVRSAGLTFPNDFYFLAERKIFNYASVFIM
LIEYVEGVELNDMPIIPEDVKAEIKASMEKLHALNMLSGDPHRGNFIVSKDGVRIIDLSG
KSCTAERKARDRLAMERHLGIANEIKDYGYYSVIYRTKLRKFIKKLKGKA
>LFGLNPFC_04303 General stress protein A
MDLLAESITEVAVSGEIANTDRVLNIAYGIDRNFLFGAAVSMQSVVMHNPDLAVKFHLFT
DYIDEDYLQRVNAFTSKNANVEVRIYKVSSAFIDIFPSLKQWSYATFFRLVAFQYLSETI
ENLLYIDADVICKGSLAGLLDINFDGDKFAAVIKDVPFMQEKPAKRLAIEGLPGNYFNAG
VVYLQLEAWAKNDFMNKAIAMLASDPQHTKYKCLDQDILNILFFSHCIFISGDYDCFYGI
DYELKNKSDEDYKKTITDDTKLIHYVGVTKPWNDWTNYPCQKYFNEAYQVSCWNDVAFIP
ATNEKQYQVKYQHAKKNGDTFNAFIYFIKFKLNKYKRKLFGQ
>LFGLNPFC_04304 Undecaprenyl-phosphate 4-deoxy-4-formamido-L-arabinose transferase
MSNDYPLVSIIIPTYNSSDYITETLTKLEKQTYPNFEIVIVNDGSKDNTSSVLREYGLTH
SRLIIINKENGGVSSARNTGIRKAQGQFICFMDDDDEIDPNYLLKMYSRQHETGGDAIYC
GLYGHHIKNGVTYSPINTDFNEGSLLFDFFYKKVRFHIGCLFIRKQLLEDNNLFFDEDLR
LGEDLDFIYRLLITCDMYAVPYYMYKHNYRENSLMNSCRTITHYRHESFAHERIYSSVMQ
LYKGNRKEEIHTLLSKNRTYHKTRYLWNVLLNGDFELLNQLVESNEKELKDCNLLGKRDK
RRAKILASKNYILWRMVRLVNRKKNKR
>LFGLNPFC_04305 O-antigen ligase
MSFCWNEINSGVKSLILILCMLSLMTLSLWDDVATKFLHAAGIISALYFLATPKKTITNN
PTLLIFISLCLLGIVNIIWYSHYKVSGSVYTNAYRGPMETGKIALCSAFIFLVLFAKDEM
RTKIKFGKLILFASLATQLLFFAHAMWQHFYLNVDRVALSASHATTAGYIILFPSLLASI
LILKSDFRHKTTLYTINFMLSLCAVIVTETRAAILVFPFFALLLIVMDSYINKRINYKLY
CFIAIALLAGVFSFKDTLLTRMNDLNRDLVNYSHDNTRTSVGARLAMYEVGLKTYSPIGQ
SLEKRAEKIHELEEKEPRLSGALPFVDSHLHNDLIDTLSTRGIPGVALTILAFSAIFIYA
LRTAKEPYILILLFSLLVVGLSDVILFSKPVPTAVFVTIILLCAYFKVQSDQCLLDK
>LFGLNPFC_04306 Lipopolysaccharide heptosyltransferase 1
MRVLIVKTSSMGDVLHTLPALTDAQQAIPGIKFDWVVEEGFAQIPSWHAAVERVIPVAIR
RWRKAWFSAPIKAERKAFREALQAKNYDAVIDAQGLVKSAALVTRLAHGVKHGMDWQTAR
EPLASLFYNRKHHIAKQQHAVERTRELFAKSLGYSKPQTQGDYAIAQHFLTNLPTDAGEY
AVFLHATTRDDKHWPEEHWRELIGLLADSGIRIKLPWGAPHEEERAKRLAEGFAYVEVLP
KMSLEGVARVLAGAKFVVSVDTGLSHLTAALDRPNITVYGPTDPGLIGGYGKNQMVCRAP
GNELSQLTANAVKQFIEENAEKAAMI
>LFGLNPFC_04307 ADP-heptose--LPS heptosyltransferase 2
MKILVIGPSWVGDMMMSQSLYRTLQARYPQAIIDVMAPAWCRPLLSRMPEVNEAIPMPLG
HGALEIGERRKLGHSLREKRYDRAYVLPNSFKSALVPFFAGIPHRTGWRGEMRYGLLNDV
RVLDKEAWPLMVERYVALAYDKGIMRTAQDLPQPLLWPQLQVSEGEKSYTCNQFSLSSER
PMIGFCPGAEFGPAKRWPHYHYAELAKQLIDEGYQVVLFGSAKDHEAGNEILAALNTEQQ
AWCRNLAGETQLDQAVILIAACKAIVTNDSGLMHVAAALNRPLVALYGPSSPDFTPPLSH
KARVIRLITGYHKVRKGDAAEGYHQSLIDITPQRVLEELNALLLQEEA
>LFGLNPFC_04308 ADP-L-glycero-D-manno-heptose-6-epimerase
MIIVTGGAGFIGSNIVKALNDKGITDILVVDNLKDGTKFVNLVDLDIADYMDKEDFLIQI
MAGEEFGDVEAIFHEGACSSTTEWDGKYMMDNNYQYSKELLHYCLEREIPFLYASSAATY
GGRTSDFIESREYEKPLNVYGYSKFLFDEYVRQILPEANSQIVGFRYFNVYGPREGHKGS
MASVAFHLNTQLNNGESPKLFEGSENFKRDFVYVGDVADVNLWFLENGVSGIFNLGTGRA
ESFQAVADATLAYHKKGQIEYIPFPDKLKGRYQAFTQADLTNLRAAGYDKPFKTVAEGVT
EYMAWLNRDA
>LFGLNPFC_04309 2-amino-3-ketobutyrate coenzyme A ligase
MRGEFYQQLTNDLETARAEGLFKEERIITSAQQADITVADGSHVINFCANNYLGLANHPD
LIAAAKAGMDSHGFGMASVRFICGTQDSHKELEQKLAAFLGMEDAILYSSCFDANGGLFE
TLLSAEDAIISDALNHASIIDGVRLCKAKRYRYANNDMQELEARLKEAREAGARHVLIAT
DGVFSMDGVIANLKGVCDLADKYDALVMVDDSHAVGFVGENGRGSHEYCDVMGRVDIITG
TLGKALGGASGGYTAGRKEVVEWLRQRSRPYLFSNSLAPAIVAASIKVLEMVEAGSELRD
RLWANARQFREQMSAAGFTLAGADHAIIPVMLGDAVVAQKFARELQKEGIYVTGFFYPVV
PKGQARIRTQMSAAHTPEQITRAVEAFTRIGKQLGVIA
>LFGLNPFC_04310 L-threonine 3-dehydrogenase
MKALSKLKAEEGIWMTDVPVPELGHNDLLIKIRKTAICGTDVHIYNWDEWSQKTIPVPMV
VGHEYVGEVVGIGQEVKGFKIGDRVSGEGHITCGHCRNCRGGRTHLCRNTIGVGVNRPGC
FAEYLVIPAFNAFKIPDNISDDLASIFDPFGNAVHTALSFDLVGEDVLVSGAGPIGIMAA
AVAKHVGARNVVITDVNEYRLELARKMGITRAVNVAKENLNDVMTELGMTEGFDVGLEMS

GAPPAFRTMLDTMNHGGRIAMLGIPPSDMSIDWTKVIFKGLFIKGIYGREMFETWYKMAA
LIQSGLDLSPIITHRFSIDDFQKGFDAMRSGQSGKVILSWD
>LFGLNPFC_04311 Undecaprenyl-phosphate 4-deoxy-4-formamido-L-arabinose transferase
MMNSTNKLSVIIPLYNAGDDFRTCMESLITQTWTALEIIIINDGSTDNSVEIAKHYAENY
PHVRLLHQANSGASVARNRGIEVATGKYVAFVDADDEVYPTMYETLMTMALEDDLDVAQC
NADWCFRETGETWQSIPTDRLRSTGVLTGPDWLRMGLSSRRWTHVVWMGVYRRDVIVKNN
IKFIAGLHHQDIVWTTEFMFNALRARYTEQSLYKYYLHNTSVSRLHRQGNKNLNYQRHYI
KITRLLEKLNRNYADKITIYPEFHQQITYEALRVCHAVRKEPDIITRQRMIAEIFTSGMY
KRLITNVRSVKVGYQALLWSFRLWQWRDKTRSHHRITRSAFNLR
>LFGLNPFC_04312 putative protein
MFPFRRNVLAFAALLALSSPVIAGKLAIVIDDFGYRPHNENQVLAMPSAISVAVLPDSPH
AREMATKAHNSGHEVLIHLPMAPLSKQPLEKNTLRPEMSSDEIERIIRSAVNNVPYAVGI
NNHMGSKMTSNLFGMQKVMQALERYNLYFLDSVTIGNTQAMRAAQGTGVKVIKRKVFLDD
SQNEADIRMQFNRAIDLARRNGSTIAIGHPHPSTVRVLQQMVYNLPPDITLVKASSLLNE
PQVDTSTPPKNTVPDTPRNPFRGVKLCKPKKPIEPVYANRFFEVLSESISQSTLIVYFQH
QWQGWGKQPESAKLNASAN
>LFGLNPFC_04313 Murein hydrolase activator EnvC
MTRAVKPRRFAIRPIIYASVLSAGVLLCAFSAHADERDQLKSIQADIAAKERAVRQKQQQ
RASLLAQLKKQEEAISEATRKLRETQNTLNQLNKQIDEMNASIAKLEQQKAAQERSLAAQ
LDAAFRQGEHTGIQLILSGEESQRGQRLQAYFGYLNQARQETIAQLKQTREEVAMQRAEL
EEKQSEQQTLLYEQRAQQAKLTQALSERKKTLAGLESSIQQGQQQLSELRANESRLRNSI
ARAEAAAKARAEREAREAQAVRDRQKEATRKGTTYKPTESEKSLMSRTGGLGAPRGQAFW
PVRGPTLHRYGEQLQGELRWKGMVIGASEGTEVKAIADGRVILADWLQGYGLVVVVEHGK
GDMSLYGYNQSALVSVGSQVRAGQPIALVGSSGGQGRPSLYFEIRRQGQAVNPQPWLGR
>LFGLNPFC_04314 2,3-bisphosphoglycerate-independent phosphoglycerate mutase
MSVSKKPMVLVILDGYGYREEQQDNAIFSAKTPVMDALWANRPHTLIDASGLEVGLPDRQ
MGNSEVGHVNLGAGRIVYQDLTRLDVEIKDRAFFANPVLTGAVDKAKNAGKAVHIMGLLS
AGGVHSHEDHIMAMVELAAERGAEKIYLHAFLDGRDTPPRSAESSLKKFEEKFAALGKGR
VASIIGRYYAMDRDNRWDRVEKAYDLLTLAQGEFQADTAVAGLQAAYARDENDEFVKATV
IRAEGQPDAAMEDGDALIFMNFRADRAREITRAFVNADFDGFARKKVVNVDFVMLTEYAA
DIKTAVAYPPASLVNTFGEWMAKNDKTQLRISETEKYAHVTFFFNGGVEESFKGEDRILI
NSPKVATYDLQPEMSSAELTEKLVAAIKSGKYDTIICNYPNGDMVGHTGVMEAAVKAVEA
LDHCVEEVAKAVESVGGQLLITADHGNAEQMRDPATGQAHTAHTNLPVPLIYVGDKNVKA
VEGGKLSDIAPTMLSLMGMEIPQEMTGKPLFIVE
>LFGLNPFC_04315 putative protein YibN
MQEIMQFVGRHPILSIAWIALLVAVLVTTFKSLTSKVKVITRGEATRLINKEDAVVVDLR
QRDDFRKGHIAGSINLLPSEIKANNVGELEKHKDKPVIVVDGSGMQCQEPANALTKAGFA
QVFVLKEGVAGWAGENLPLVRGK
>LFGLNPFC_04316 Glutaredoxin 3
MANVEIYTKETCPYCHRAKALLSSKGVSFQELPIDGNAAKREEMIKRSGRTTVPQIFIDA
QHIGGCDDLYALDARGGLDPLLK
>LFGLNPFC_04317 Protein-export protein SecB
MSEQNNTEMTFQIQRIYTKDISFEAPNAPHVFQKDWQPEVKLDLDTASTQLADDVYEVVL
RVTVTASLGEETAFLCEVQQGGIFSIAGIEGTQMAHCLGAYCPNILFPYARECITSMVSR
GTFPQLNLAPVNFDALFMNYLQQQAGEGTEEHQDA
>LFGLNPFC_04318 Glycerol-3-phosphate dehydrogenase [NAD(P)+]
MNQRNASMTVIGAGSYGTALAITLARNGHEVVLWGHDPEHIATLERDRCNAAFLPDVPFP
DTLHLESDLATALAASRNILVVVPSHVFGEVLRQIKPLMRPDARLVWATKGLEAETGRLL
QDVAREALGDQIPLAVISGPTFAKELAAGLPTAISLASTDQTFADDLQQLLHCGKSFRVY
SNPDFIGVQLGGAVKNVIAIGAGMSDGIGFGANARTALITRGLAEMSRLGAALGADPATF
MGMAGLGDLVLTCTDNQSRNRRFGMMLGQGMDVQSAQEKIGQVVEGYRNTKEVRELAHRF
GVEMPITEEIYQVLYCGKNAREAALTLLGRARKDERSSH
>LFGLNPFC_04319 Serine acetyltransferase
MSCEELEIVWNNIKAEARTLADCEPMLASFYHATLLKHENLGSALSYMLANKLSSPIMPA
IAIREVVEEAYAADPEMIASAACDIQAVRTRDPAVDKYSTPLLYLKGFHALQAYRIGHWL
WNQGRRALAIFLQNQVSVTFQVDIHPAAKIGRGIMLDHATGIVVGETAVIENDVSILQSV
TLGGTGKSGGDRHPKIREGVMIGAGAKILGNIEVGRGAKIGAGSVVLQPVPPHTTAAGVP
ARIVGKPDSDKPSMDMDQHFNGINHTFEYGDGI
>LFGLNPFC_04320 tRNA (cytidine(34)-2'-O)-methyltransferase
MLNIVLYEPEIPPNTGNIIRLCANTGFRLHIIEPMGFAWDDKRLRRAGLDYHEFTAVTRH
HDYRAFLEAENPQRLFALTTKGTPAHSAVSYQDGDYLMFGPETRGLPASILDALPAEQKI
RIPMVPDSRSMNLSNAVSVVVYEAWRQLGYPGAVLRD
>LFGLNPFC_04321 L-lactate dehydrogenase
MIISAASDYRAAAQRILPPFLFHYMDGGAYSEYTLRRNVEDLSEVALRQRILKNMSDLSL

ETTLFNEKLSMPVALGPVGLCGMYARRGEVQAAKAADAHGIPFTLSTVSVCPIEEVAPAI
KRPMWFQLYVLRDRGFMRNALERAKAAGCSTLVFTVDMPTPGARYRDAHSGMSGPNAAMR
RYLQAVTHPQWAWDVGLNGRPHDLGNISAYLGKPTGLEDYIGWLANNFDPSISWKDLEWI
RDFWDGPMVIKGILDPEDARDAVRFGADGIVVSNHGGRQLDGVLSSARALPAIADAVKGD
IAILADSGIRNGLDVVRMIALGADTVLLGRAFLYALATAGQAGVANLLNLIEKEMKVAMT
LTGAKSISEITQDSLVQVLGKELPAALAPMAKGNAA
>LFGLNPFC_04322 Putative L-lactate dehydrogenase operon regulatory protein
MIVLPRRLSDEVADRVRALIDEKNLEAGMKLPAERQLAMQLGVSRNSLREALAKLVSEGV
LLSRRGGGTFIRWRHDTWSEQNIVQPLKTLMADDPDYSFDILEARYAIEASTAWHAAMRA
TPGEKEKIQLCFEATLSEDPDLASQADVRFHLAIAEASHNIVLLQTMRGFFDVLQSSVKH
SRQRMYLVPPVFSQLTEQHQAVIDAIFAGDADGARKAMMAHLSFVHTTMKRFDEDQARHA
RITRLPGDHNEHSREKNA
>LFGLNPFC_04323 L-lactate permease
MNLWQQNYDPAGNIWLSSLIASLPILFFFFALIKLKLKGYVAASWTVAIALAVALLFYKM
PVANALASVVYGFFYGLWPIAWIIIAAVFVYKISVKTGQFDIIRSSILSITPDQRLQMLI
VGFCFGAFLEGAAGFGAPVAITAALLVGLGFKPLYAAGLCLIVNTAPVAFGAMGIPILVA
GQVTGIDSFEIGQMVGRQLPFMTIIVLFWIMAIMDGWRGIKETWPAVVVAGGSFAIAQYL
SSNFIGPELPDIISSLVSLLCLTLFLKRWQPVRVFRFGDLGASQVDMTLAHTGYTAGQVL
RAWTPFLFLTATVTLWSIPPFKALFASGGALYEWVINIPVPYLDKLVARMPPVVSEATAY
AAVFKFDWFSATGTAILFAALLSIVWLKMKPSDAISTFGSTLKELALPIYSIGMVLAFAF
ISNYSGLSSTLALALAHTGHAFTFFSPFLGWLGVFLTGSDTSSNALFAALQATAAQQIGV
SDLLLVAANTTGGVTGKMISPQSIAIACAAVGLVGKESDLFRFTVKHSLIFTCMVGVITT
LQAYVLTWMIP
>LFGLNPFC_04324 Autotransporter adhesin EhaG
MNKIFKVIWNPATGSYTVASETAKSRGKKSGRSKLLISALVAGGLLSSFSVWANAGNDDG
QGIGTGTGWVAIGESAKATAYTDSNGASTAVGYKSNAQGVWSSAIGALTSALGDSSMAFG
VNAVSTGQRSIAMGASSSSLGEYSIALGRYASSGGMFSLAQGDRSNAAGLNATAVGSNSN
AAGAKAIALGNAAKATEIMSIALGDTANASKEYSMALGVSSAASAANAIAVGRNSAAAGV
DSLALGRLSVASAANAIAMGAESEAAENATAIGNNAHAKGVNSIAMGSGSIADKVNTIAL
GNGSQSLADNAIAIGQGNKANGTDAIALGNASLSSGLNSIALGKTSVVTGDNSLALGSNT
NANGINSVALGADSIADQDNSVSVGSSSLQRKIVNVKNGAIKADSHDAINGSQLYAISDS
IAKRLGGGSSVNPDDGTVNAPTYNLKNGNKNNVGSALTVLDENTLQWDQIKGKYSAVHGS
STTSVITDVANGTISAASKDAVNGSQLYDLQQDALLWNGTAFSAAHGTEATSKITNVTAG
NLTASSTDAVNGSQLKTTNDNVTTNTTNIATNTTNITNLTDAVDSLGDDSLLWNKTAGAF
SAAHGTDATSKITNVKAGDLTAGSTDAVNGSQLKTTNDNVSTNTTNIATNTTNITNLTDS
VGDLKDDSLLWNKAAGAFSAAHGTEATSKITNLLAGKISSNSTDAINGSQLYGVADSFTS
YLGGGADISDTGVLSGPTYTIGGTDYTNVGDALAAINTSFSTSLGDALLWDATAGKFSAK
HGINNAPSVITDVANGAVSSTSSDAINGSQLYGVSDYIADALGGNAVVNTDGSITTPTYA
IAGGSYNNVGDALEAIDTTLDDALLWDTTANGGNGAFSAAHGKDKTASVITNVANGAVSA
TSSDAINGSQLYSTNKYIADALGGDAEVNADGTITAPTYTIANTDYNNVGEALDALDNNA
LLWDEDAGAYNASHDGNASKITNVAAGDLSTTSTDAVNGSQLNATNILVTQNSQMINQLA
GNTSETYIEENGAGINYVRTNDTGLTFTDASAAGIGSTAVGYNTVAKGDSSVAMGYNSFA
KGDSSVAIGQGSYSGVDTGIALGSSSVSSRVIVKGSRNTSVSEEGVVIGYDTTDGELLGA
LSIGDDGKYRQIINVADGSEAHDAVTVRQLQNAIGAVATTPTKYYHANSTAEDSLAVGED
SLAMGAKTIVNGNAGIGIGLNTLVLADAINGIAIGSNARANHADSIAMGNGSQTTRGAQT
NYTAYNMDAPQNSVGEFSVGSEDGQRQITNVAAGSADTDAVNVGQLKVTDAQVSQNTQSI
TNLNTQVTNLDTRVTNIENGIGDIVTTGSTKYFKTNTDGADANAQGKDSVAIGSGSIAAA
DNSVALGTGSVADEENTISVGSSTNQRRITNVAAGVNATDAVNVSQLKSSEAGGVRYDTK
ADGSIDYSNITLGGGNSGTTRISNVSAGVNNNDAVNYAQLKQSVQETKQYTDQRMVEMDN
KLSQTESKLSGGIASAMAMTGLPQAYTPGASMASIGGGTYNGESAVALGVSMVSANGRWV
YKLQGSTNSQGEYSAALGAGIQW
>LFGLNPFC_04325 Inner membrane lipoprotein SadB
MVKAVKDKQDEFVFTEFNPAQTQYFILNNGSVGLAGKILSIDAVENGSVIRISLVNLLSV
PVSNMGFYATWGGEKPTDINALAKWQQLLFSTSMNSSLKLLPGQWQDINLTLKGVSPNNL
KYLKLAINMANIQFDRLQPAESPQRKNKK
>LFGLNPFC_04326 hypothetical protein
MNLKKTLLSVLMILQLCLLVGCDYIEKASKVDDLVTQQELQKSKIEALEKQQ
>LFGLNPFC_04327 putative protein YibL
MKEVEKNEIKRLSDRLDAIRHQQADLSLVEAADKYAELEKEKATLEAEIARLREVHSQKL
SKEAQKLMKMPFQRAITKKEQADMGKLKKSVRGLVVVHPMTALGREMGLQEMTGFSKTAF
>LFGLNPFC_04328 hypothetical protein
MGKLGENVPLLIDKAVDFMASSQAFREYLKKLPPRNAIPSGIPDESVPLYLQRLEYYRQL
YRPKQVEEK
>LFGLNPFC_04329 Mannitol operon repressor

MVDQAQDTLRPNNRLSDMQATMEQTQAFENRVLERLNAGKTVRSFLITAVELLTEAVNLL
VLQVFRKDDYAVKYAVEPLLDGDGPLGDLSVRLKLIYGLGVINRQEYEDAELLMALREEL
NHDGNEYAFTDDEILGPFGELHCVAALPPPPQFEPADSSLYAMQIQRYQQAVRSTMVLSL
TELISKISLKKAFQK
>LFGLNPFC_04330 Mannitol-1-phosphate 5-dehydrogenase
MKALHFGAGNIGRGFIGKLLADAGIQLTFADVNQVVLDALNARHSYQVHVVGETEQVDTV
SGVDAVSSIGDDVVDLIAQVDLVTTAVGPVVLERIAPAIAKGLVKRKEQGNESPLNIIAC
ENMVRGTTQLKGHVMNALPEDAKAWVEEHVGFVDSAVDRIVPPSASATNDPLEVTVETFS
EWIVDKTQFKGTLPNIPGMELTDNLMAFVERKLFTLNTGHAITAYLGKLAGHQTIRDAIL
DEKIRAVVKGAMEESGAVLIKRYDFDADKHAAYIQKILGRFENPYLKDDVERVGRQPLRK
LSAGDRLIKPLLGTLEYGLPHKNLIEGIAAAMHFRSEDDPQAQELAALIADKGPQAALAQ
ISGLDANSEVVSEAVTAYKAMQ
>LFGLNPFC_04331 PTS system mannitol-specific EIICBA component
MSSDIKIKVQSFGRFLSNMVMPNIGAFIAWGIITALFIPTGWLPNETLAKLVGPMITYLL
PLLIGYTGGKLVGGERGGVVGAITTMGVIVGADMPMFLGSMIAGPLGGWCIKHFDRWVDG
KIKSGFEMLVNNFSAGIIGMILAILAFLGIGPVVEALSKMLAAGVNFMVVHDMLPLASIF
VEPAKILFLNNAINHGIFSPLGIQQSHELGKSIFFLIEANPGPGMGVLLAYMFFGRGSAK
QSAGGAAIIHFLGGIHEIYFPYVLMNPRLILAVILGGMTGVFTLTILGGGLVSPASPGSI
LAVLAMTPKGAYFANIAGVCAAMAVSFVVSAILLKTSKVKEEDDIEAATRRMQDMKAESK
GASPLSAGDVTNDLSHVRKIIVACDAGMGSSAMGAGVLRKKIQDAGLSQISVTNSAINNL
PPDVDLVITHRDLTERAMRQVPQAQHISLTNFLDSGLYTSLTERLVAAQRHTENEVKVKD
SLKDSFDDSSANLFKLGAENIFLGRKAATKEEAIRFAGEQLVKGGYVEPEYVQAMLDREK
LTPTYLGESIAVPHGTVEAKDRVLKTGVVFCQYPEGVRFGEEEDDIARLVIGIAARNNEH
IQVITSLTNALDDESVIERLAHTTSVDEVLELLAGRK
>LFGLNPFC_04332 Inner membrane protein YiaW
MFLNYFALGVLIFVFLVIFYGIIAIHDIPYLIAKKRNHPHADAIHTAGWVSLFTLHVIWP
FLWIWATLYQPERGWGMQSHVTPQEKTTDAEIAALSDRISRLEHQLAAEKKTDYSTFPEI
>LFGLNPFC_04333 Inner membrane protein YibH
MDLLIVLTYVALAWAVFKIFRIPVNQWTLATAALGGVFLVSGLILLMNYNHPYTFTAQKA
VIAIPITPQVTGIVTEVTDKNNQLIQKGEVLFKLDPVRYQARVDRLQADLMTATHNIKTL
RAQLTEAQANTTQVSAERDRLFKNYQRYLKGSQAAVNPFSERDIDDARQNFLAQDALVKG
SVAEQAQIQSQLDSMVNGEQSQIVSLRAQLTEAKYNLEQTVIRAPSNGYVTQVLIRPGTY
AAALPLRPVMVFIPEQKRQIVAQFRQNSLLRLKPGDDAEVVFNALPGQVFHGKLTSILPV
VPGGSYQAQGVLQSLTVVPGTDGVLGTIELDPNADIDALPDGIYAQVAVYSDHFSHVSVM
RKVLLRMTSWMHYLYLDH
>LFGLNPFC_04334 putative GST-like protein YibF
MKLVGSYTSPFVRKLSILLLEKGITFEFINELPYNADNGVAQFNPLGKVPVLVTEEGECW
FDSPIIAEYIELMNVAPAMLPRDPLESLRVRKIEALADGIMDAGLVSVREQARPAAQQSE
DELLRQREKINRSLDVLEGYLVDGTLKTDTVNLATIAIACAVGYLNFRRVAPGWCVDRPH
LVKLVENLFSRESFARTEPPKA
>LFGLNPFC_04335 L-seryl-tRNA(Sec) selenium transferase
MTTETRSLYSQLPAIDRLLRDSSFLSLRDTYGHTRVVELLRQMLDEAREVIRDSQTLPAW
CENWAQEVDARLTKEAQSALRPVINLTGTVLHTNLGRALQAEAAVEAVTKAMRSPVTLEY
DLDDAGRGHRDRALAQLLCRITGAEDACIVNNNAAAVLLMLAATASGKEVVVSRGELVEI
GGAFRIPDVMRQAGCTLHEVGTTNRTHANDYRQAVNENTALLMKVHTSNYSIQGFTKAID
EAELVALGKELDVPVVTDLGSGSLVDLSQYGLPKEPMPQELIAAGVSLVSFSGDKLLGGP
QAGIIVGKKEMIARLQSHPLKRALRADKMTLAALEATLRLYLHPEALSEKLPTLRLLTRS
AEVIQIQAQRLQAPLAAHYGAEFAVQVMPCLSQIGSGSLPVDRLPSAALTFTPHDGRGSH
LESLAARWRELPVPVIGRIYDGRLWLDLRCLEDEQRFLEMLLK
>LFGLNPFC_04336 Selenocysteine-specific elongation factor
MIIATAGHVDHGKTTLLQAITGVNADRLPEEKKRGMTIDLGYAYWPQPDGRVPGFIDVPG
HEKFLSNMLAGVGGIDHALLVVACDDGVMAQTREHLAILQLTGNPMLTVALTKADRVDEA
RVDEVERQVKEVLREYGFAEAKLFITAATEGRGIDALREHLLQLPEREHASQHSFRLAID
RAFTVKGAGLVVTGTALSGEVKVGDSLWLTGVNKPMRVRALHAQNQPTETAHAGQRIALN
IAGDAEKEQINRGDWLLADVPPEPFTRVIVELQTHTPLTQWQPLHIHHAASHVTGRVSLL
EDNLAELVFDTPLWLADNDRLVLRDISARNTLAGARVVMLNPPRRGKRKPEYLQWLASLA
RAQSDADALSVHLERGAVNLADFAWARQLNGEGMRELLQQPGYIQAGYSLLNAPVAARWQ
RKILDTLATYHEQHRDEPGPGRERLRRMALPMEDDALVLLLIEKMRESGDIHSHHGWLHL
PDHKAGFSEEQQAIWQKAEPLFGDEPWWVRDLAKETGTDEQAMRLTLRQAAQQGIITAIV
KDRYYRNDRIVEFANMIRDLDQECGSTCAADFRDRLGVGRKLAIQILEYFDRIGFTRRRG
NDHLLRDALLFPEK
>LFGLNPFC_04337 Alcohol dehydrogenase 2
MAASTFFIPSVNVIGADSLNDAMNMMADYGFTRTLIVTDNMLTKLGMAGDVQKALEERNI
FSVIYDGTQPNPTTENVAAGLKLLKENNCDSVISLGGGSPHDCAKGIALVAANGGDIRDY

EGVDRSAKPQLPMIAINTTAGTASEMTRFCIITDEARHIKMAIVDKHVTPLLSVNDSSLM
IGMPKSLTAATGMDALTHAIEAYVSIAATPITDACALKAVTMIAENLPLAVEDGSNAKAR
EAMAYAQFLAGMAFNNASLGYVHAMAHQLGGFYNLPHGVCNAVLLPHVQVFNSKVAAARL
RDCAAAMGVNVTGKNDAEGAEACINAIRELAKKVDIPAGLRDLNVKEEDFAVLATNALKD
ACGFTNPIQATHEEIVAIYRAAM
>LFGLNPFC_04338 hypothetical protein
MAIKSPPGLIPLSHLSGEELLAHLRFNRVTDEKGRYLPFDELQYRIKKGENVDVAWTLTR
LARNAAIQRINYCNEAGEQAGFNITPVIAEACELVDKRATALALKDQTERLRGAGAELSQ
LRLEEPITSSQLEGANTTTLVARKMLETGRSPRTEDEHMIAGNARLMAEIPHLLAEPLTP
ALIRQLHAIGMGGINDAKYRPGEFRETDDVVIADYDGNIVHQPSAAALLPERLEKVCQWL
NSHEGYIHPLVRACILHFMLAHEHPFRDGNGRTSRALFYWYMLKSGYDVFKYISISSLLH
AAPVKYAASYQYTESDGMDLTYFLEYQAGVIKRALQNWQQHIDEITQRSAKLDSVLFSSG
VLKRLNPRQVTLLNVMLANPGKEYTVAEISVSLSVSDNTARTDLRTIVKEGFAQEKRINN
QQAVYVAHYPL
>LFGLNPFC_04339 Aldehyde dehydrogenase B
MTNNPPSAQIKPGEYGFPLKLKARYDNFIGGEWVAPADGEYYQNLTPVTGQLLCEVASSG
KRDIDLALDAAHKVKDKWAHTSVQDRAAILFKIADRMEQNLELLATAETWDNGKPIRETS
AADVPLAIDHFRYFASCIRAQEGGISEVDSETVAYHFHEPLGVVGQIIPWNFPLLMASWK
MAPALAAGNCVVLKPARLTPLSVLLLMEIVGDLLPPGVVNVVNGAGGEIGEYLATSKRIA
KVAFTGSTEVGQQIMQYATQNIIPVTLELGGKSPNIFFADVMDEEDAFFDKALEGFALFA
FNQGEVCTCPSRALVQESIYERFMERAIRRVESIRSGNPLDSVTQMGAQVSHGQLETILN
YIDIGKKEGADVLTGGRRKLLEGELKDGYYLEPTILFGQNNMRVFQEEIFGPVLAVTTFK
TMEEALELANDTQYGLGAGVWSRNGNLAYKMGRGIQAGRVWTNCYHAYPAHAAFGGYKQS
GIGRETHKMMLEHYQQTKCLLVSYSDKPLGLF
>LFGLNPFC_04340 L-ribulose-5-phosphate 4-epimerase SgbE
MLEQLKADVLAANLALPVHHLVTFTWGNVSAIDETRQWMVIKPSGVEYEVMTADDMVVVE
IASGKVVEGSKKPSSDTPTHLALYRRFAEIGGIVHTHSRHATIWSQVGLDLPAWGTTHAD
YFYGAIPCTRLMTAEEINGEYEYQTGEVIIKTFEERGLNPAQIPAVLVHSHGPFAWGKNA
ADAVHNAVVLEECAYMGLFSRQLAPQLPAMQNELLDKHYLRKHGANAYYGQ
>LFGLNPFC_04341 L-ribulose-5-phosphate 3-epimerase UlaE
MRNHPLGIYEKALAKDLSWPERLVLAKSCGFDFVEMSVDETDERLSRLDWSAAQRASLVT
AMIETGVAIPSMCLSAHRRFPFGSRDDAVRQRAREIMSKAIRLARDLGIRTIQLAGYDVY
YEDHDEGTQQRFAEGLAWAVEQAAASQVMLAVEIMDTAFMNSISKWKKWDEMLASPWFTV
YPDVGNLSAWGNDVPAELKLGIDRIAAIHLKDTQPVTEHSPGQFRDVPFGEGCVDFVGIF
KTLHELNYRGSFLIEMWTEKAKEPVLEIIQARRWIEARMQEAGFIC
>LFGLNPFC_04342 3-keto-L-gulonate-6-phosphate decarboxylase SgbH
MSRPLLQLALDHSSLEAAQRDVTRLKDSVDIVEAGTILCLNEGLGAVKALREQCPDKIIV
ADWKVADAGETLAQQAFGAGANWMTIICAAPLATVEKGHAMAQRCGGEIQIELFGNWTLD
DARDWHRIGVRQAIYHRGRDAQASGQQWGEADLARMKALSDIGLELSITGGITPADLPLF
KEIRVKAFIAGRALAGAANPAQVAGDFHAQIDSIWGGKRA
>LFGLNPFC_04343 L-xylulose/3-keto-L-gulonate kinase
MTQYWLGLDCGGSWLKAGLYDREGREAGVQRLPLCALSPQPGWAERDMAELWQCCTAVIR
ALLTHSGVSGEQIVGIGISAQGKGLFLLDKNDKPLGNAILSSDRRAMEIVRRWQKDGIPE
KLYPLTRQTLWTGHPVSLLRWLKEHKPERYAQIGCVMMTHDYLRWCLTGVKGCEESNISE
SNLYNMGRGEYDPYLTDWLGIAEINHALPPVVGSAEICGEITAQTAVLTGLKAGTPVVGG
LFDVVSTALCAGLEDEFTLNAVMGTWAVTSGITHGLRDGEAHPYVYGRYVNDGQFIVHEA
SPTSSGNLEWFTAQWGEISFAEINQAVASLPKAGGDLFFLPFLYGSNAGLEMTSGFYGMQ
AIHTRAHLLQAIYEGVVFSHMTHLNRMRERFTDVHTLRVTGGPAHSDVWMQMLADVSGLR
IELPQVEETGCFGAALAARVGTGVYRDFSEAQRDLRHPVRTLLPDMAAHQLYQQKYQRYQ
HLITTLQGYHARIKEHTL
>LFGLNPFC_04344 2,3-diketo-L-gulonate-binding periplasmic protein YiaO
MKLRSVTYALLIAGVATFSTSSLAAQSLRFGYETSQTDSQHIAAKKFNELLQEKTKGELK
LKLFPDSTLGNAQAMISGVRGGTIDMEMSGSNNFTGLSPVINLLDVPFLFRDTAHAHKTL
DGKVGDDLKVSLEGKGLKVLAYWENGWRDVTNSRAPVKTPADLKGLKIRTNNSPMNIAAF
KVFGANPIPMPFAEVYTGLETRTIDAQEHPINVVWSAKFYEVQKYLSLTHHAYSPLLVVI
NKAKFDGLTPEFQQALISSAQEAGSYQRKLVAEDQQKIIDGMKEAGVEVITDLDRKAFSD
ALGTQVRDMFVKDVPQGADLLKAVDEVQ
>LFGLNPFC_04345 Sialic acid TRAP transporter large permease protein SiaM
MTILIFIVSLLGAIAIGVPIAWALLLCGISLMFWMDIFDVQILAQTLVNGADSFSLLAIP
FFVLAGEIMNAGGLSQRIVDLPMKLVGHRPGGLGYVGVLAAMIMASLSGSAVADTAAVAA
LLVPMMRQANYPVNRAAGLIGSGGIIAAIIPPSIPLIIFGVSSGLSISKLFMAGIAPGIM
MGVTLMVTWWWQAKRLNLPCQPKASLREVWQSLVSGIWALFLPIIIIGGFRSGLFTPTEA
GAVAAFYALFVSVVVYREMTFSTLYHVLINAAKTTSVVMFLVASAAVSAWLITIAELPMM
VSELLQPLVDSPRLLFIVIMLAIMVISTVMDLTPTVLILTPVLMPLVKEAGIDPVYFGIM

FIINCSISLITPPVGNVLNVVCGVAKLKFDDAVKGVAPYVMVLFMLLALFIFIPELITAP
LKWMS
>LFGLNPFC_04346 2,3-diketo-L-gulonate TRAP transporter small permease protein YiaM
MKRILEGILAIIIAILSCIIFINIILRYGFHTSILSIDELSRLLFVWLTFIGAIVAYMDN
SHVQVTFLVEKLSPANQQRVSLLTHTLILLLCLGLAWGAIEKTAQDWSNLSPILGVPIGL
MYAAAIPTSLIIALLELRHLYRQFTNTPSRNQQGA
>LFGLNPFC_04347 putative protein YiaX1
MKTNNAGYIIGAYPCAPSFHQRSEEEEKDFWRQLSETPDILGLEQPCLENFHPLGDQWLL
RHTPESWKFVVTAVMETMRRRSENSGFGLASSDEEQRQACVAYYRHLFNKINSLQANKVL
ALELQAAPLATNPNVMQATDAFARSLKEIASWDWPCKLVLEHCDAMTSSSPRKGFLPLEN
VLEVITDYDISICINWARSAIEGRNTTLPLTHTQMAKQAGKLGALMFSGTTLNGAYGEWQ
DLHAPFAPFCAESLMTTDHVRELFNVAESSTLHFAGIKLLEINATADVHHRIEILRNGIH
SLNESR
>LFGLNPFC_04348 putative protein
MIFGHIAQPNPCRLPAAIEKALDFLRTTDFNALEPGVVEIDGKNIYAQIIDLTTREAVEN
RPEVHRRYIDIQFLAWGEEKIGIAIDTGNNKVSESLLEQRDIIFYHDSEHESFIEMIPGS
YAIFFPQDVHRPACNKNATTAIRKIVVKVALAAL
>LFGLNPFC_04349 2,3-diketo-L-gulonate reductase
MKVTFEQLKAAFNRVLISRGVDNETADACAEMFARTTESGVYSHGVNRFPRFIQQLENGD
IIPDAQPKRITSLGAIEQWDAQRSIGNLTAKKMMDRAIELAADHGIGLVALRNANHWMRG
GSYGWQAAEKGYIGICWTNSIAVMPPWGAKECRIGTNPLIVAIPSTPITMVDMSMSMFSY
GMLEVNRLAGRQLPVDGGFDDEGNLTKEPGVIEKNRRILPMGYWKGSGMSIVLDMIATLL
SDGASVAEVTEDNSDEYGISQIFIAIEVDKLIDGPTRDAKLQRIMDYVTSAERADENQAI
RLPGHEFTTLLAENRRNGITVDDSVWAKIQAL
>LFGLNPFC_04350 DNA-binding transcriptional repressor YiaJ
MGKEVMGKKENEMAQEKERPAGSQSLFRGLMLIEILSNYPNGCPLAHLSELAGLNKSTVH
RLLQGLQSCGYVTTAPAAGSYRLTTKFIAVGQKALSSLNIIHIAAPHLEALNIATGETIN
FSSREDDHAILIYKLEPTTGMLRTRAYIGQHMPLYCSAMGKIYMAFGHPDYVKSYWESHQ
HEIQPLTRNTITELPAMFDELAHIRESGAAMDREENELGVSCIAVPVFDIHGRVPYAVSI
SLSTSRLKQVGEKNLLKPLRETAQAISNELGFTVRDDQGAIT
>LFGLNPFC_04351 Hydrogenase-4 component A
MNRFIIADATKCIGCRTCEVACAVSHQENQDCAALSPDEFISRIRVIKDHTYTTAVACHQ
CEDAPCANVCPVDAISREHGHIFVEQSRCIGCKSCMLACPFGAMEVVSSRKKARAIKCDL
CWHRATGPACVEACPTKALQCVDVEKVQRHRLRQQPV
>LFGLNPFC_04352 Valine--pyruvate aminotransferase
MTFSLFGDKFTRHSGITLLMEDLNDGLRTPGAIMLGGGNPAQIPEMQDYFQTLLTDMLES
GKATDALCNYDGPQGKTELLTLLAGMLREKLGWDIEPQNIALTNGSQSAFFYLFNLFAGR
RADGRVKKVLFPLAPEYIGYADAGLEEDLFVSARPNIELLPEGQFKYHVDFEHLHIGEET
GMICVSRPTNPTGNVITDEELLKLDALANQHGIPLVIDNAYGVPFPGIIFSEARPLWNPN
IVLCMSLSKLGLPGSRCGIIIANEKIITAITNMNGIISLAPGGIGPAMMCEMIKRNDLLR
LSETVIKPFYYQRVQETIAIIRRYLPEDRCLIHKPEGAIFLWLWFKDLPITTEQLYQRLK
ARGVLMVPGHNFFPGLDKPWPHTHQCMRMNYVPEPEKIEAGVKILAEEIERAWAESH
>LFGLNPFC_04353 Periplasmic alpha-amylase
MKLAACFLTLLPGFAVAASWTSPGFPAFSEQGTGTFVSHAQLPKGTRPLTLNFDQQCWQP
ADAIKLNQMLSLQPCSNTPPQWRLFRDGEYTLQLDTRSGTPTLMISLQNTVEPVASLVRE
CPKWDGLPLTLDVSATFAEGAAVRDYYSQQIAIVKNGQITLQPAATSNGLLLLERAETDT
SAPFDWHNATVYFVLTDRFENGDPSNDQSYGRHKDGMAEIGTFHGGDLRGLTNKLDYLQQ
LGVNALWISAPFEQIHGWVGGGTKGDFPHYAYHGYYTQDWTNLDANMGSEADLRTLVDSA
HQRGIRILFDIVMNHTGYATLADMQEYQFGALYLSGDELKKTLGERWSDWKPAAGQTWHS
FNDYINFSDKTGWDKWWGKNWIRTDIGDYDNPGFDDLTMSLAFLPDIKTESTTASGLPVF
YKNKTDTHAKVIDGFTPRDYLTHWLSQWVRDYGIDGFRVDTAKHVELPAWQQLKTEASAA
LREWKKANPDKALDDKPFWMTGEAWGHGVMQSDYYRHGFDAMINFDYQEQAAKAVDCLAQ
MDTTWQQMAEKLQDFNVLSYLSSHDTRLFREGGDKAAELLLLAPGAVQIFYGDESSRPFG
PTGSDPLQGTRSDMNWQDVSGKSAASVAHWQKISQFRARHPAIGAGKQTTLSLKQGYGFV
REHGDDKVLVIWAGQQ
>LFGLNPFC_04354 hypothetical protein
MILTPIRRYGAMILMLLTLVFSSEVLAKTHTTTASQKSHLTKASNKQVSSKQEYSRNSAK
SSSLPDLRKYPSGTPRKKAFLRTVMPYITSQNAAITAERNWLISKQYQGQWSPAERARLK
DIAKRYKVKWSGNTRKIPWNTLLERVDIIPTSMVATMAAAESGWGTSKLARNNNNLFGMK
CMKGRCTNAPGKVKGYSQFSSVKESVSAYVTNLNTHPAYSSFRKSRAQLRKADQEVTATA
MIHKLKGYSTKGKSYNNYLFAMYQDNQRLIAAHM
>LFGLNPFC_04355 Xylose operon regulatory protein
MFTKRHRITLLFNANKAYDRQVVEGVGEYLQASQSEWDIFIEEDFRARIDKIKDWLGDGV
IADFDDKQIEQALADVDVPIVGVGGSYHLAESYPPVHYIATDNYALVESAFLHLKEKGVN

RFAFYGLPESSGKRWATEREYAFRQLVAEEKYRGVVYQGLETAPENWQHAQNRLADWLQT
LPPQTGIIAVTDARARHILQVCEHLHIPVPEKLCVIGIDNEELTRYLSRVALSSVAQGAR
QMGYQAAKLLHRLLDKEEMPLQRILVPPVRVIERRSTDYRSLTDPAVIQAMHYIRNHACK
GIKVDQVLDAVGISRSNLEKRFKEEVGETIHAMIHAEKLEKARSLLISTTLSINEISQMC
GYPSLQYFYSVFKKAYDTTPKEYRDVNSEVML
>LFGLNPFC_04356 Xylose transport system permease protein XylH
MSKSNPSEVKLAVPTSGGFSGLKSLNLQVFVMIAAIIAIMLFFTWTTDGAYLSARNVSNL
LRQTAITGILAVGMVFVIISAEIDLSVGSMMGLLGGVAAICDVWLGWPLPLTIIVTLVLG
LLLGAWNGWWVAYRKVPSFIVTLAGMLAFRGILIGITNGTTVSPTSAAMSQIGQSYLPAS
TGFIIGALGLMAFVGWQWRGRMRRQALGLQSPASTAVVGRQALTAIIVLGAIWLLNDYRG
VPTPVLLLTLLLLGGMFMATRTAFGRRIYAIGGNLEAARLSGINVERTKLAVFAINGLMV
AIAGLILSSRLGAGSPSAGNIAELDAIAACVIGGTSLAGGVGSVAGAVMGAFIMASLDNG
MSMMDVPTFWQYIVKGAILLLAVWMDSATKRRS
>LFGLNPFC_04357 Xylose import ATP-binding protein XylG
MPYLLEMKNITKTFGSVKAIDNVSLRLNAGEIVSLCGENGSGKSTLMKVLCGIYPHGSYE
GEIIFAGEEIQASHIRDTERKGIAIIHQELALVKELTVLENIFLGNEITHNGIMDYDLMT
LRCQKLLAQVSLSISPDTRVGDLGLGQQQLVEIAKALNKQVRLLILDEPTASLTEQETSV
LLDIIRDLQQHGIACIYISHKLNEVKAISDTICVIRDGQHIGTRDAAGMSEDDIITMMVG
RELTALYPNEPHTTGDEILRIEHLTAWHPVNRHIKRVNDVSFSLKRGEILGIAGLVGAGR
TETIQCLFGVWPGQWEGKIYIDGKQVDIRNCQQAIAQGIAMVPEDRKRDGIVPVMAVGKN
ITLAALNKFTGGISQLDDAAEQKCILESIQQLKVKTSSSDLAIGRLSGGNQQKAILARCL
LLNPRILILDEPTRGIDIGAKYEIYKLINQLVQQGIAVIVISSELPEVLGLSDRVLVMHE
GKLKANLINHNLTQEQVMEAALRSEHHVEKQSV
>LFGLNPFC_04358 D-xylose-binding periplasmic protein
MKIKNILLTLCTSLLLTNVAAHAKEVKIGMAIDDLRLERWQKDRDIFVKKAESLGAKVFV
QSANGNEETQMSQIENMINRGVDVLVIIPYNGQVLSNVVKEAKQEGIKVLAYDRMINDAD
IDFYISFDNEKVGELQAKALVDIVPQGNYFLMGGSPVDNNAKLFRAGQMKVLKPYVDSGK
IKVVGDQWVDGWLPENALKIMENALTANNNKIDAVVASNDATAGGAIQALSAQGLSGKVA
ISGQDADLAGIKRIAAGTQTMTVYKPITLLANTAAEIAVELGNGQEPKADTTLNNGLKDV
PSRLLTPIDVNKNNIKDTVVKDGFHKESEL
>LFGLNPFC_04359 Xylose isomerase
MQAYFDQLDRVRYEGSKSSNPLAFRHYNPDELVLGKRMEEHLRFAACYWHTFCWNGADMF
GVGAFNRPWQQPGEALALAKRKADVAFEFFHKLHVPFYCFHDVDVSPEGASLKEYINNFA
QMVDVLAGKQEESGVKLLWGTANCFTNPRYGAGAATNPDPEVFSWAATQVVTAMEATHKL
GGENYVLWGGREGYETLLNTDLRQEREQLGRFMQMVVEHKHKIGFQGTLLIEPKPQEPTK
HQYDYDAATVYGFLKQFGLEKEIKLNIEANHATLAGHSFHHEIATAIALGLFGSVDANRG
DAQLGWDTDQFPNSVEENALVMYEILKAGGFTTGGLNFDAKVRRQSTDKYDLFYGHIGAM
DTMALALKIAARMIEDGELDKRIAQRYSGWNSELGQQILKGQMSLADLAKYAQEHNLSPV
HQSGRQEQLENLVNHYLFDK
>LFGLNPFC_04360 Xylulose kinase
MYIGIDLGTSGVKVILLNEQGEVVAAQTEKLTVSRPHPLWSEQDPEQWWQATDRAMKALG
VQHSLQDVKALGIAGQMHGATLLDAQQRVLRPAILWNDGRCAQECTLLEARVPQSRVITG
NLMMPGFTAPKLLWVQRHEPEIFRQIDKVLLPKDYLRLRMTGEFASDMSDAAGTMWLDVA
KRDWSDLMLQACHLSRDQMPALYEGSEITGALLPEVAKAWGMAAVPVIAGGGDNAAGAVG
VGMVDANQAMLSLGTSGVYFAVSEGFLSKPESAVHSFCHALPQRWHLMSVMLSAASCLDW
AAKLTGLSNVPALITAAQQADESAEPVWFLPYLSGERTPHNNPQAKGGFFGLTHQHGPNE
LARAVLEGVGYALADGMDVVHACGIKPQSVTLIGGGARSEYWRQMLADISGQQLDYRTGG
DVGPALGAARLAQIAANPEKSLIELLPQLPLEQSHLPDAQRYAAYQPRRETFRRLYQQLL
PLMA
>LFGLNPFC_04361 Inner membrane protein YiaB
MKTSKTVAKLLFVVGALVYLVGLWISCPLLSGKGYFLGVLMTATFGNYAYLRAEKLGQLD
DFFIHVCQLVALITIGLLFIGVLNAPINAYEMVIYPIAFFVCLFGQMRLFRSA
>LFGLNPFC_04362 Inner membrane protein YiaA
MDNKISTYSPAFSIVSWVALIGGIVTYLLGLWNAEMQLNEKGYYFAVLVLGLFSAASYQK
TVRDKYEGIPTTPIYYMTCLTVFIISVALLLVGLWNATLLLSEKGFYGLAFFLSLFGAVA
VQKNIHDAGINPPKETQVTQEEYSE
>LFGLNPFC_04363 O-acetyltransferase WecH
MQPKIYWIDNLRGIACLMVVMIHTTTWYVTNAHSVSPITWDIANVLNSASRVSVPLFFMI
SGYLFFGERSAQSRHFLRIGLCLFFYSAIALLYIALFTSINVELALKNLLQKPVFYHLWF
FFAIAVIYLVSPLIQVKNVGGKMLLVLMVVIGIIANPNTVPQKIDGFEWLPINLYINGDT
FYYILYGMLGRAIGMMDTQHKALSWVSAALFATGVFIISRGTLYELQWRGNFADTWYLYC
GPMVFICAIALLTLVKNTLYTRTICGLGLISRHSLGIYGFHALIIHALRTRGIELKNWPI
LDIIWIFCATLAVSLLLSMLVQRIDRNRLVS
>LFGLNPFC_04364 hypothetical protein

MMMNAFFPAMALIVLVGCSTPPPVQKAQRVKVDPLRSLNMEALCKDQAAKRYNTGEQKID
VTAFEQFQGSYEMRGYTFRKEQFVCSFDADGHFLHLSMR
>LFGLNPFC_04365 Glycine--tRNA ligase alpha subunit
MQKFDTRTFQGLILTLQDYWARQGCTIVQPLDMEVGAGTSHPMTCLRALGPEPMAAAYVQ
PSRRPTDGRYGENPNRLQHYYQFQVVIKPSPDNIQELYLGSLKELGMDPTIHDIRFVEDN
WENPTLGAWGLGWEVWLNGMEVTQFTYFQQVGGLECKPVTGEITYGLERLAMYIQGVDSV
YDLVWSDGPLGKTTYGDVFHQNEVEQSTYNFEYADVDFLFTCFEQYEKEAQQLLALENPL
PLPAYERILKAAHSFNLLDARKAISVTERQRYILRIRTLTKAVAEAYYASREALGFPMCN
KDK
>LFGLNPFC_04366 Glycine--tRNA ligase beta subunit
MSEKTFLVEIGTEELPPKALRSLAESFAANFTAELDNAGLAHGTVQWFAAPRRLALKVAN
LAEAQPDREIEKRGPAIAQAFDAEGKPSKAAEGWARGCGITVDQAERLTTDKGEWLLYRA
HVKGESTEALLPNMVATSLAKLPIPKLMRWGASDVHFVRPVHTVTLLLGDKVIPATILGI
QSDRVIRGHRFMGEPEFTIDNADQYPEILRERGKVIADYEERKAKIKADAEEAARKIGGN
ADLSESLLEEVASLVEWPVVLTAKFEEKFLAVPSEALVYTMKGDQKYFPVYANDGKLLPN
FIFVANIESKDPQQIISGNEKVVRPRLADAEFFFNTDRKKRLEDNLPRLQTVLFQQQLGT
LRDKTDRIQALAGWIAEQIGADVNHATRAGLLSKCDLMTNMVFEFTDTQGVMGMHYARHD
GEAEDVAVALNEQYQPRFAGDDLPSNPVACALAIADKMDTLAGIFGIGQHPKGDKDPFAL
RRAALGVLRIIVEKNLNLDLQTLTEEAVRLYGDKLTNANVVDDVIDFMLGRFRAWYQDEG
YTVDTIQAVLARRPTRPADFDARMKAVSHFRTLEAAAALAAANKRVSNILAKSDEVLSDR
VNASTLKEPEEIKLAMQVVVLRDKLEPYFAEGRYQDALVELAELREPVDAFFDKVMVMVD
DKELRINRLTMLEKLRELFLRVADISLLQ
>LFGLNPFC_04367 hypothetical protein
MHRHHKVSLLLVGNQHRGLGMPQKYRLLSLIVICFTLLFFTWMIRDSLCELHIKQESYEL
AAFLACKLKE
>LFGLNPFC_04368 Cold shock protein CspA
MSGKMTGIVKWFNADKGFGFITPDDGSKDVFVHFSAIQNDGYKSLDEGQKVSFTIESGAK
GPAAGNVTSL
>LFGLNPFC_04369 hypothetical protein
MEYKDPMHELLSSLEQIVFKDETQKITLTHRTTSCTEIEQLRKGTGLKIDDFARVLGVSV
AMVKEWESRRVKPSSAELKLMRLIQANPALSKQLME
>LFGLNPFC_04370 hypothetical protein
MATGKSCSRWFAPLAALLMVVSLSGCFDKEGDQRKAFIDFLQNTVMRSGERLPTLTADQK
KQFGPFVSDYAILYGYSQQVNQAMDSGLRPVVDSVNAIRVPQDYVTQSGPLREMNGSLGV
LAQQLQNAKLQADAAHSALKQSDDLKPVFDQAFTKVVTTPADALQPLIPAAQTFTQQLVM
VGDYIAQQGTQVSFVANGIQFPTSQQASEYNKLIAPLPAQHQAFNQAWTTAVTATQ
>LFGLNPFC_04371 Glyoxylate/hydroxypyruvate reductase B
MKPSVILYKALPDDLLQRLQEHFTVHQVANLSPQTVEQNAAIFAEAEGLLGSNENVDAAL
LEKMPKLRATSTISVGYDNFDVDALTARKILLMHTPTVLTETVADTLMALVLSTARRVVE
VAERVKAGEWTASIGPDWYGTDVHHKTLGIVGMGRIGMALAQRAHFGFNMPILYNARRHH
KEAEERFNARYCDLDTLLQESDFVCLILPLTDETHHLFGAEQFAKMKSSAIFINAGRGPV
VDENALIAALQKGEIHAAGLDVFEQEPLSVDSPLLSMANVVAVPHIGSATHETRYGMAAC
AVDNLIDALQGKVEKNCVNPHVAD
>LFGLNPFC_04372 putative lipoprotein YiaD
MKKRVYLIAAVVSGALAVSGCTTNPYTGEREAGKSAIGAGLGSLVGAGIGALSSSKKDRG
KGALIGAAAGAALGGGVGYYMDVQEAKLRDKMRGTGVSVTRSGDNIILNMPNNVTFDSSS
ATLKPAGANTLTGVAMVLKEYPKTAVNVIGYTDSTGSHDLNMRLSQQRADSVASALITQG
VDASRIRTQGLGPANPIASNSTAEGKAQNRRVEITLSPL
>LFGLNPFC_04373 Biotin sulfoxide reductase
MANSSSRYSVLTAAHWGPMLVETDGETVFSSRGALATGMENSLQSAVRDQVHSNTRVRFP
MVRKGFLASPENPQGIRGQDEFVRVSWDEALELIHHQHKRIREAYGPASIFAGSYGWRSN
GVLHKASTLLQRYMALAGGYTGHLGDYSTGAAQAIMPYVVGGSEVYQQQTSWPLVLEHSD
VVVLWSANPLNTLKIAWNASDEQGLSYFSALRDSGKKLICIDPMRSETIDFFGDKMEWVA
PHMGTDVALMLGIAHTLVENGWHDEAFLARCTTGYAVFASYLLGESDGIAKNAEWAAEIC
GVGAAKIRELAAIFHQNTTMLMAGWGMQRQQFGEQKHWMLVTLAAMLGQIGTPGGGFGLS
YHFANGGNPTRRAAVLSSMQGSLPGGTDAVDKIPVARIVEALENPGGAYQHNGMDRHFPD
IRFIWWAGGANFTHHQDTNRLIRAWQKPELVVISECFWTAAAKHADIVLPATTSFERNDL
TMTGDYSNQHLVPMKQVVPPRYEARNDFDVFADLSERWQKGGYARFTEGKSELQWLETFY
NVARQRGASQQVELPPFAEFWQANQLIEMPENPDSERFIRFADFRRDPQAHPLKTASGKI
EIFSQRIADYAYPDCPGHPMWLAPDEWQGSAEPEQLQVLSAHPAHRLHSQLNYSSLRELY
AVANREPVTIHPDDAQTRGIQDGDTVRLWNSRGQILAGAVISEGIKPGVICIHEGAWPDL
DLTAGGICKNGAVNVLTKDLPSSRLGNGCAGNTALAWLEKYTGSELTLTAFEPPASS
>LFGLNPFC_04374 Peptidyl-lysine N-acetyltransferase YiaC
MIREVQRSELPAILQLWLESTTWGHPFIKSSYWRDCIPLVRDAYLANAQNWVWEEDGKLL

GFVSIMEGRFLAAMFVAPKAVRRGIGKALMQYVQQRHPHLMLEVYQKNQPAIDFYHAQGF
HIVDCAWQDETQLPTWIMSWPVVQTL
>LFGLNPFC_04375 DNA-3-methyladenine glycosylase 1
MERCGWVSQDPLYIAYHDNEWGVPETDSKKLFEMICLEGQQAGLSWITVLKKRENYRACF
HQFDPVKVAAMQEEDIERLVQDAGIIRHRGKIQAIIGNARAYLQMEQNGEPFADFVWSFV
NHQPQVTQATTLSEIPTSTPASDALSKALKKRGFKFVGTTICYSFMQACGLVNDHVVGCC
CHPGNKP
>LFGLNPFC_04376 hypothetical protein
MIIKKSGGRWQLSLLASVVISAFFLNTAYAWQQEYIVDTQPGHSTERYTWDSDHQPDYND
ILSQRIQSSQRALGLEVNLAEETPVDVTSSMSMGWNFPLYEQVTTGPVAAFHYDGTTTSM
YNEFGDSTTTLADPLWHASVSTLGWRVDSRLGDLRPWAQISYNQQFGENIWKAQSGLSRM
TATNQNGNWLDVTVGADMLLNQNIAAYAALSQAENTTNNSDYLYTMGVSARF
>LFGLNPFC_04377 putative MFS-type transporter YhjX
MTPSNYQRTRWLTLIGTIITQFALGSVYTWSLFNGALSAKLGAPVSQVAFSFGLLSLGLA
ISSSVAGKLQERFGVKRVTMASGILLGLGFFLTAHSNNLMMLWLSAGVLVGLADGAGYLL
TLSNCVKWFPERKGLISAFAIGSYGLGSLGFKFIDTHLLETVGLEKTFVIWGAIVLVMIV
FGATLMKDAPKQEVKTSNGVVEKDYTLAESMRKPQYWMLAVMFLTACMSGLYVIGVAKDI
AQSLAHLDAISAANAVTVISIANLSGRLVLGILSDKIARIRVITIGQVIALVGMAALLFA
PLNAVTFFAAIACVAFNFGGTITVFPSLVSEFFGLNNLAKNYGVIYLGFGIGSICGSIIA
SLFGGFYVTFYVIFALLILSLALSTTIRQPEQKMLREAHGSL
>LFGLNPFC_04378 Kdo(2)-lipid A phosphoethanolamine 7''-transferase
MRYIKSITQQKLSFLLAIYIGLFMNGAVFYRRFGSYAHDFTVWKGISAVVELAATVLVTF
FLLRLLSLFGRRSWRILASLVVLFSAGASYYMTFLNVVIGYGIIASVMTTDIDLSKEVVG
LNFILWLIAVSALPLILIWNNRCRYTLLRQLRTPGQRIRSLAVVVLAGIMVWAPIRLLDI
QQKKVERATGVDLPSYGGVVANSYLPSNWLSALGLYAWARVDESSDNNSLLNPAKKFTYQ
APQNVDDTYVVFIIGETTRWDHMGIFGYERNTTPKLAQEKNLAAFRGYSCDTATKLSLRC
MFVRQGGAEDNPQRTLKEQNIFAVLKQLGFSSDLYAMQSEMWFYSNTMADNIAYREQIGA
EPRNRGKPVDDMLLVDEMQQSLGRNPDGKHLIILHTKGSHFNYTQRYPRSFAQWKPECIG
VDSGCTKAQMINSYDNSVTYVDHFISSVIDQVRDKKAIVFYAADHGESINEREHLHGTPR
ELAPPEQFRVPMMVWMSDKYLENPVNAQAFAQLKKEADMKVPRRHVELYDTIMGCLGYTS
PDGGINENNNWCHIPQAKEASAN
>LFGLNPFC_04380 Periplasmic dipeptide transport protein
MRISLKKSGMLKLGLSLVAMTVAASVQAKTLVYCSEGSPEGFNPQLFTSGTTYDASSVPL
YNRLVEFKIGTTEVIPGLAEKWEVSEDGKTYTFHLRKGVKWHDNKEFKPTRELNADDVVF
SFDRQKNAQNPYHKVSGGSYEYFEGMGLPELISEVKKVDDNTVQFVLTRPEAPFLADLAM
DFASILSKEYADAMMKAGTPEKLDLNPIGTGPFQLQQYQKDSRIRYKAFDGYWGTKPKID
TLVFSITPDASVRYAKLQKNECQVMPYPNPADIARMKQDKSINLMEMPGLNVGYLSYNVQ
KKPLDDVKVRQALTYAVNKDAIIKAVYQGAGVSAKNLIPPTMWGYNDDVQDYTYDPEKAK
ALLKEAGLEKGFSIDLWAMPVQRPYNPNARRMAEMIQADWAKVGVQAKIVTYEWGEYLKR
AKDGEHQTVMMGWTGDNGDPDNFFATLFSCAASEQGSNYSKWCYKPFEDLIQPARATDDH
NKRVELYKQAQVVMHDQAPALIIAHSTVFEPVRKEVKGYVVDPLGKHHFENVSIE
>LFGLNPFC_04381 hypothetical protein
MTRSLELRVCIAHKSIPALVEQGMKYCCRMALHAEAQRHRVWLLIIR
>LFGLNPFC_04382 Dipeptide transport system permease protein DppB
MLQFILRRLGLVIPTFIGITLLTFAFVHMIPGDPVMIMAGERGISPERHAQLLAELGLDK
PMWQQYLHYIWGVMHGDLGISMKSRIPVWEEFVPRFQATLELGVCAMIFATAVGIPVGVL
AAVKRGSIFDHTAVGLALTGYSMPIFWWGMMLIMLVSVHWNLTPVSGRVSDMVFLDDSNP
LTGFMLIDTAIWGEDGNFIDAVAHMILPAIVLGTIPLAVIVRMTRSSMLEVLGEDYIRTA
RAKGLTRMRVIIVHALRNAMLPVVTVIGLQVGTLLAGAILTETIFSWPGLGRWLIDALQR
RDYPVVQGGVLLVATMIILVNLLVDLLYGVVNPRIRHKK
>LFGLNPFC_04383 Dipeptide transport system permease protein DppC
MSQVTENKVISAPVPMTPLQEFWHYFKRNKGAVVGLVYVVIVLFIAIFANWIAPYNPAEQ
FRDALLAPPAWQEGGSMAHLLGTDDVGRDVLSRLMYGARLSLLVGCLVVVLSLIMGVILG
LIAGYFGGLVDNIIMRVVDIMLALPSLLLALVLVAIFGPSIGNAALALTFVALPHYVRLT
RAAVLVEVNRDYVTASRVAGAGAMRQMFINIFPNCLAPLIVQASLGFSNAILDMAALGFL
GMGAQPPTPEWGTMLSDVLQFAQSAWWVVTFPGLAILLTVLAFNLMGDGLRDALDPKLKQ
>LFGLNPFC_04384 Dipeptide transport ATP-binding protein DppD
MALLNVDKLSVHFGDESAPFRAVDRISYSVKQGEVVGIVGESGSGKSVSSLAIMGLIDYP
GRVMAEKLEFNGQDLQRISEKERRNLVGAEVAMIFQDPMTSLNPCYTVGFQIMEAIKVHQ
GGNKSTRRQRAIDLLNLVGIPDPASRLDVYPHQLSGGMSQRVMIAMAIACRPKLLIADEP
TTALDVTIQAQIIELLLELQQKENMALVLITHDLALVAEAAHKIIVMYAGQVVETGDAHA
IFHAPRHPYTQALLRALPEFAQDKERLASLPGVVPGKYDRPNGCLLNPRCPYATDKCRAE
EPALNMLADGRQSKCHYPLDDAGRPTL
>LFGLNPFC_04385 Oligopeptide transport ATP-binding protein OppF

```
MSTHEATSQQPLLQAIDLKKHYPVKKGMFAPERLVKALDGVSFNLERGKTLAVVGESGCG
KSTLGRLLTMIETPTGGELYYQGQDLLKHDPQAQKLRRQKIQIVFQNPYGSLNPRKKVGQ
ILEEPLLINTSLSKEQRREKALSMMAKVGLKTEHYDRYPHMFSGGQRQRIAIARGLMLDP
DVVIADEPVSALDVSVRAQVLNLMMDLQQELGLSYVFISHDLSVVEHIADEVMVMYLGRC
VEKGTKDQIFNNPRHPYTQALLSATPRLNPDDRRERIKLTGELPSPLNPPPGCAFNARCR
RRFGPCIQLQPQLKDYGGQLVACFAVDQDENPQR
>LFGLNPFC_04386 Inner membrane transport protein YhjV
MQHNTLPKHDQKLPFTRYDFGWVLLCIGMAIGAGTVLMPVQIGLKGIWVFITAAIIAYPA
TWVVQDIYLKTLSESDSCNDYTDIISHYLGKNWGIFLGVIYFLMIIHGIFIYSLSVVFDS
ASYLKTFGLTDADLSQSLFYKVAIFAVLVAIASGGERLLFKISGPMVVVKVGIIVVFGFA
MIPHWNFANITAFPQASDFFRDVLLTIPFCFFSAVFIQVLNPMNIAYRKREADKVLATRL
ALRTHRISYITLIAVILFFAFSFTFSISHEEAVSAFEQNISALALAAQVIPGHIIHITST
VLNIFAVLTAFFGIYLGFHEAIKGIILNLLSRIIDTRKINSRMLTLAICTFIVITLTIWV
SFRVSVLVFFQLGSPLYGIVSCLIPFFLIYKVSQLEKLRGFKAWMILLYGILLCLSPLLK
LIE
>LFGLNPFC_04387 Small toxic polypeptide LdrD
MTLAELGMAFWHDLAAPVIAGILASMIVSWLNKRK
>LFGLNPFC_04388 Small toxic polypeptide LdrD
MTLAELGMAFWHDLAAPVIAGILASMIVNWLNKRK
>LFGLNPFC_04389 Cellulose biosynthesis protein BcsG
MTQFTQNTAMPSSLWQYWRGLSGWNFYFLVKFGLLWAGYLNFHPLLNLVFAAFLLMPIPR
YSLHRLRHWIALPIGFALFWHDTWLPGPESIMSQGSQVAGFSTDYLIDLVTRFINWQMIG
AIFVLFVAWLFLSQWIRITVFVVAILLWLNVLTLAGPSFSLWPAGQPTTTVTTTGGNAAA
TVAATGGAPVVGDIPAQTAPPTTANLNAWLNNFYNAEAKRKSIFPSSLPADAQPFELLVI
NICSLSWSDIEAAGLMSHPLWSHFDIEFKNFNSATSYSGPAAIRLLRASCGQTSHTNLYQ
PANNDCYLFDNLSKLGFTQHLMMGHNGQFGGFLKEVRENGGMQSELMDQTNLPVILLGFD
GSPVYDDTAVLNRWLDVTEKDKNSRSATFYNTLPLHDGNHYPGVSKTADYKARAQKFFDE
LDAFFTELEKSGRKVMVVVVPEHGGALKGDRMQVSGLRDIPSPSITDVPVGVKFFGMKAP
HQGAPIVIDQPSSFLAISDLVVRVLDGKIFSEDNVDWKKLTSGLPQTAPVSENSNAVVIQ
YQDKPYVRLNGGDWVPYPQ
>LFGLNPFC_04390 hypothetical protein
MMTISDIIEIIVVCALIFFPLGYLARHSLRRIRDTLRLFFAKPRYVKPAGTLRRTEKARA
TKK
>LFGLNPFC_04391 Cyclic di-GMP binding protein BcsE
MDPVFSIGISSLWNELRHMPAGGVWWFNVDRHEDAISLANQTIASQAETAHVAVISMDSD
PAKIFQLDDSQGPEKITLFSMLNHEKGLYYLARDLQCSIDPHNYLFILVCANNAWQNIPA
ERLRSWLDKMNKWSRLNHCSLLVINSGNNNDKQFSLLLEEYRSLFGLASLRFQGDQHLLD
IAFWCNEKGVSARQQLSVQQQNGCWTLVQHQEAEIQPRSDEKRILSNVAVLEGAPPLSEH
WQLFNNNEVLFNEARTAQAATVVFSLQQNAQIEPLARSIHTLRRQRGSAMKILVRENTAS
LRATDERLLLACGANMVIPWNAPLSRCLTMIESVQGQKFSRYVPEDITTLLSMTQPLKLR
GFQKWDVFCNAVNNMMNNPLLPAHGKGVLVALRPVPGIRVEQALTLCRPNRTGDIMTIGG
NRLVLFLSFCRINDLDTALNHIFPLPTGDIFSNRMVWFEDDQISAELVQMRLLAPEQWGM
PLPLAQSSKPVINAEHDGRHWRRIPEPMRLLDDAVERSS
>LFGLNPFC_04392 Protein YhjR
MNNNEPDTLPDPAIGYIFQNDILALKQAFSLPDIDYADISQREQLAAALKRWPLLAEFAQ
QK
>LFGLNPFC_04393 Cellulose biosynthesis protein BcsQ
MAVLGLQGVRGGVGTTTITAALAWSLQMLGENVLVVDACPDNLLRLSFNVDFTHRQGWAR
AMLDGQDWRDAGLRYTSQLDLLPFGQLSIEEQENPQHWQTRLSDICTGLQQLKASGRYQW
ILIDLPRDASQITHQLLSLCDHSLAIVNVDANCHIRLHQQALPDGAHILINDFRIGSQVQ
DDIYQLWLQSQRRLLPMLIHRDEAMAECLAAKQPVGEYRSDALAAEEILTLANWCLLNYS
GLKMPVGSAS
>LFGLNPFC_04394 Cellulose synthase catalytic subunit [UDP-forming]
MSILTRWLLIPPVNARLIGRYRDYRRHGASAFSATLGCFWMILAWIFIPLEHPRWQRIRA
EHKNLYPHINASRPRPLDPVRYLIQTCWLLIGASRKETPKPRRRAFSGLQNIRGRYHQWM
NELPERVSHKTQHLDEKKELGHLSAGARRLILGIIVTFSLILALICVTQPFNPLAQFIFL
MLLWGVALIVRRMPGRFSALMLIVLSLTVSCRYIWWRYTSTLNWDDPVSLVCGLILLFAE
TYAWIVLVLGYFQVVWPLNRQPVPLPKDMSLWPSVDIFVPTYNEDLNVVKNTIYASLGID
WPKDKLNIWILDDGGREEFRQFAQNVGVKYIARTTHEHAKAGNINNALKYAKGEFVSIFD
CDHVPTRSFLQMTMGWFLKEKQLAMMQTPHHFFSPDPFERNLGRFRKTPNEGTLFYGLVQ
DGNDMWDATFFCGSCAVIRRKPLDEIGGIAVETVTEDAHTSLRLHRRGYTSAYMRIPQAA
GLATESLSAHIGQRIRWARGMVQIFRLDNPLTGKGLKFAQRLCYVNAMFHFLSGIPRLIF
LTAPLAFLLFHAYIIYAPALMIALFVLPHMIHASLTNSKIQGKYRHSFWSEIYETVLAWY
IAPPTLVALINPHKGKFNVTAKGGLVEEEYVDWVISRPYIFLVLLNLVGVAVGIWRYFYG
```

PPTEMLTVVVSMVWVFYNLIILGGAVAVSVESKQVRRSHRVEMTMPAAIAREDGHLFSCT
VQDFSDGGLGIKINGQAQILEGQKVNLLLKRGQQEYVFPAQVARVMGNEVGLKLMPLTTQ
QHIDFVQCTFARADTWALWQDSYPEDKPLESLLDILKLGFRGYRHLAEFAPSSVKGIFRV
LTSLVSWVVSFIPRRPERSETAQPSDQALAQQ
>LFGLNPFC_04395 Cyclic di-GMP-binding protein
MALGMSAFPSFMTQATPATQPLINAEPAVTAQAEQNPQVGQVMPGVQGADAPVVAQNGPS
RDVKLTFAQIAPPPGSMVLRGINPNGSIEFGMRSDEVVTKAMLNLEYTPSPSLLPVQSQL
KVYLNDELMGVLPVTKEQLGKKTLAQMPINPLFITDFNRVRLEFVGHYQDVCENPASTTL
WLDVGRSSGLDLTYQTLNVKNDLSHFPVPFFDPRDNRTNTLPMVFAGAPDVELQQASAIV
ASWFGSRSGWRGQNFPVLYNQLPDRNAIVFATNDKRPDFLRDHPAVKAPVIEMINHPQNP
YVKLLVVFGRDDKDLLQAAKGIAQGNILFRGESVVVNEVKPLLPRKPYDAPNWVRTDRPV
TFGELKTYEEQLQSSGLEPAAINVSLNLPPDLYLMRSTGIDMDINYRYTMPPVKDSSRMD
ISLNNQFLQSFNLSSKQEANRLLLRIPVLQGLLDGKTDVSIPALKLGATNQLRFDFEYMN
PMPGGSVDNCITFQPVQNHVVIGDDSTIDFSKYYHFIPMPDLRAFANAGFPFSRMADLSQ
TITVMPKTPNEAQMETLLNTVGFIGAQTGFPAINLTVTDDGSTIQGKDADIMIVGGIPDK
LKDDKQIDLLVQATESWVKTPMRQTPFPGIVPDESDRAAETQSTLTSSGAMAAVIGFQSP
YNDQRSVIALLADSPRGYEMLNDAVNDSGKRATMFGSVAVIRESGINSLRVGDVYYVGHL
PWFERLWYALANHPILLAVLAAISVILLAWVLWRLLRIISRRRLNPDNE
>LFGLNPFC_04396 Endoglucanase
MNVLRSGLVTMLLLAAFSVQAACTWPAWEQFKKDYISQEGRVIDPSDARKITTSEGQSYG
MFFALAANDRAAFDNLLDWTQNNLAQGSLKEHLPAWLWGKKENSKWEVLDSNSASDGDVW
MAWSLLEAGRLWKEQRYTDIGSALLKRIAREEVVTVPGLGSMLLPGKVGFAEDNSWRFNP
SYLPPTLAQYFTRFGAPWTTLRETNQRLLLETAPKGFSPDWVRYEKDKGWQLKAEKTLIS
SYDAIRVYMWVGMMPDSDPQKARMLNRFKPMATFTEKNGYPPEKVDVATGKAQGKGPVGF
SAAMLPFLQNRDAQAVQRQRVADNFPGSDAYYNYVLTLFGQGWDQHRFRFSTKGELLPDW
GQECANSH
>LFGLNPFC_04397 Cellulose synthase operon protein C
MRKFTLNIFTLSLGLAVMPMVEAAPTAQQQLLEQVRLGEATHREDLVQQSLYRLELIDPN
NPDVIAARFRSLLRQGDIDGAQKQLDRLSQLAPSSNAYKSSRTTMLLSTPDGRQALQQAR
LQATTGHAEEAVASYNKLFNGAPPEGDIAVEYWSTVAKIPARRGEAINQLKRINADTPGN
TGLQNNLALLLFSSDRRDEGFAVLEQMAKSNAGREGASKIWYGQIKDMPVSDASVQALKK
YLSIFSDGDSVAAAQSQLAEQQKQLADPAFRARAQGLAAVDSGMAGKAIPELQQAVRANP
KDSEALGALGQAYSQKGDRANAVVNLEKALALDPHNSNNDKWNSLLKVNRYWLAIQQGDA
ALKANNPDRAERLFQQARNVDNTDSYAVLGLGDVAMARKDYPAAERYYQQTLRMDSGNTN
AVRGLANIYRQQSPEKAEAYIASLSASQRRSIDDIERSLQNDRLAQQAEALENQGKWAQA
AALQRQRLALDPGSVWITYRLSQDLWQAGQRSQADTLMRNLAQQKPNDPEQVYAYGLYLS
GHDQDRAALAHINSLPRGQWNSNIQELVNRLQNDQVLETANRLRENGKEAEAEAMLRQQP
PSSRIDLTLADWAQQRRDYTAARAAYQNVLTREPTNADAILGLTEVDIAAGDKAAARSQL
AKLPAIDNASLNTQRRVALAQAQLGDTAAAQQTFNKLIPQAKSQPPSMESAMVLRDGAKF
EALAGDPTQALETYKDAMVASGVTTTRPQDNDTFTRLTRNDEKDDWLKRGVRSDTADLYR
QQDLNVTLEHDYWGSSGTGGYSDLKAHTTMLQVDAPYSDGRMFFRSDFVNMNVGSFSTNA
DGKWDDNWGTCTLQDCSGNRSQSDSGASVAVGWRNDVWSWDIGTTPMGFNVVDVVGGISY
SDDIGPLGYTVNAHRRPISSSLLAFGGQKDSPSNTGKKWGGVRADGVGLSLSYDKGEANG
VWASLSGDQLTGKNVEDNWRVRWMTGYYYKVINQNNRRVTIGLNNMIWHYDKDLSGYSLG
QGGYYSPQEYLSFAIPVMWRERTENWSWELGASGSWSHSRTKTMPRYPLMNLIPTDWQEE
AARQSNDGGSSQGFGYTARALLERRVTSNWFVGTAIDIQQAKDYAPSHFLLYVRYSAAGW
QGDMDLPPQPLIPYADW
>LFGLNPFC_04398 putative cyclic di-GMP phosphodiesterase PdeK
MVAAVVLVFVFIFCTVLLFHLVQQNRYNTATQLESIARSVREPLSSAILKGDIPEAEAIL
ASIKPAGVVSRADVVLPNQFQALRKSFIPERPVPVMVTRLFELPVQISLGVYSLERPANP
QPIAYLVLQADSFRMYKFVMSTLSTLVTIYLLLSLILTVAISWCINRLILHPLRNIAREL
NAIPPQELVGHQLALPRLHQDDEIGMLVRSYNLNQQLLQRHYEEQNENAMRFPVSDLPNK
ALLMEMLEQVVARKQTTALMIITCETLRDTAGVLKEAQREILLLTLVEKLKSVLSPRMIL
AQISGYDFAVIANGVQEPWHAITLGQQVLTIMSERLPIERIQLRPHCSIGVAMFYGDLTA
EQLYSRAISAAFTARHKGKNQIQFFDPQQMEAAQQRLTEESDILNALENHQFAIWLQPQV
EMTSGKLVSAEVLLRIQQPDGSWDLPDGLIDRIECCGLMVTVGHWVLEESCRLLAAWQER
GIMLPLSVNLSALQLMHPNMVADMLELLTRYRIQPGTLILEVTESRRIDDPHAAVAILRP
LRNAGVRVALDDFGMGYAGLRQLQHMKSLPIDVLKIDKMFVEGLPEDSSMIAAIIMLAQS
LNLQMIAEGVETEAQRDWLAKAGVGIAQGFLFARPLPIEIFEESYLEEK
>LFGLNPFC_04399 Aerobic C4-dicarboxylate transport protein
MKTSLFKSLYFQVLTAIAIGILLGHFYPEIGEQMKPLGDGFVKLIKMIIAPVIFCTVVTG
IAGMESMKAVGRTGAVALLYFEIVSTIALIIGLIIVNVVQPGAGMNVDPATLDAKAVAVY
ADQAKDQGIVAFIMDVIPASVIGAFASGNILQVLLFAVLFGFALHRLGSKGQLIFNVIES
FSQVIFGIINMIMRLAPIGAFGAMAFTIGKYGVGTLVQLGQLIICFYITCILFVVLVLGS

IAKATGFSIFKFIRYIREELLIVLGTSSSESALPRMLDKMEKLGCRKSVVGLVIPTGYSF
NLDGTSIYLTMAAVFIAQATNSQMDIVHQITLLIVLLLSSKGAAGVTGSGFIVLAATLSA
VGHLPVAGLALILGIDRFMSEARALTNLVGNGVATIVVAKWVKELDHKKLDDVLNNRAPD
GKTHELSS
>LFGLNPFC_04400 Protein YhjJ
MQGTKIRLLAGGLLMMATAGYVQADALQPDPAWQQGTLSNGLQWQVLTTPQRPSDRVEIR
LLVNTGSLAESTQQSGYSHAIPRIALTQSGGLDAAQARSLWQQGIDPKRPMPPVIVSYDT
TLFNLSLPNNRNDLLKEALSYLANATGKLTITPETINHALQSQDMVATWPADTKEGWWRY
RLKGSTLLGHDPADPLKQPVEAEKIKDFYQKWYTPDAMTLLVVGNVDARSVVDQINKTFG
ELKGKRETPAPVPTLSPLRAEAVSIMTDAVRQDRLSIMWDTPWQPIRESAALLRYWRADL
AREALFWHVQQALSASNSKDIGLGFDCRVLYLRAQCAINIESPNDKLNSNLNLVARELAK
VRDKGLPEEEFNALVAQKKLELQKLFAAYARADTDILMGQRMRSLKNQVVDIAPEQYQKL
RQDFLNSLTVEMLNQDLRQQLSNDMALILLQPKGEPEFNMKALQAAWDQIMAPSTAAAAT
SVATDDVHPEVTDIPPVQ
>LFGLNPFC_04401 2-dehydro-3-deoxygluconokinase
MSKKIAVIGECMIELSEKGADVKRGFGGDTLNTSVYIARQVDPAALTVHYVTALGTDSFS
QQMLDAWRGENVDTSLTQRMENRLPGLYYIETDSTGERTFYYWRNEAAAKFWLESEQSAA
ICEELANFDYLYLSGISLAILSPTSREKLLSLLRECRANGGKVIFDNNYRPRLWASKEET
QQVYQQMLECTDIAFLTLDDEDALWGQQPVEDVIARTHNAGVKEVVVKRGADSCLVSIAG
EGLVDVPAVKLPKEKVIDTTAAGDSFSAGYLAVRLTGGIAEDAAKRGHLTASTVIQYRGA
IIPREAMPA
>LFGLNPFC_04402 Cyclic di-GMP phosphodiesterase PdeH
MIRQVIQRISNPEASIESLQERRFWLQCERAYTWQPIYQTCGRLMAVELLTVVTHPLNPS
QRLPPDRYFTEITVSHRMEVVKEQIDLLAQKADFFVEHGLLASVNIDGPTLIALRQQPKI
LRQIEDLPWLRFELVEHIRLPKDSTFASMCEFGPLWLDDFGTGMANFSALSEVRYDYIKI
ARELFVMLRQSPEGRTLFSQLLHLMNRYCRGVIVEGVETPEEWRDVQNSPAFAAQGWFLS
RPAPIETLNTAVLAL
>LFGLNPFC_04403 hypothetical protein
MSKAGKITAAISGAFLLLIVVAIILIATFDWNRLKPTINQKVSAELNRPFAIRGDLGVVW
ERQKQETGWRSWVPWPHVHAEDIILGNPPDIPEVTMVHLPRVEATLAPLALLTKTVWLPW
IKLEKPDARLIRLSEKNNNWTFNLANDDNKDANAKPSAWSFQLDNILFDQGRIAIDDKVS
KADLEIFVDPLGKPLPFSEVTGSKGKADKEKVGDYVFGLKAQGRYNGEPLTGTGKIGGML
ALRGEGTPFPVQADFRSGNTRVAFDGVVNDPMKMGGVDLRLKFSGDSLGDLYELTGVLLP
DTPPFETDGRLVAKIDTEKSSVFDYRGFNGRIGDSDIHGSLIYTTGKPRPKLEGDVESRQ
LRLADLGPLIGVDSGKGAEKSKRSEQKKGEKSVQPAGKVLPYDRFETDKWDVMDADVRFK
GRRIEHGSSLPISDLSTHIILKNADLRLQPLKFGIAGGSIAANIHLEGDKKPMQGRADIQ
ARRLKLKELMPDVELMQKTLGEMNGDAELRGSGNSVAALLGNSNGNLKLLMNDGLVSRNL
MEIVGLNVGNYIVGAIFGDDEVRVNCAAANLDIANGVARPQVFAFDTENALINVTGTASF
ASEQLDLTIDPESKGIRIITLRSPLYVRGTFKNPQAGVKAGPLIARGAVAAALATLVTPA
AALLALISPSEGEANQCRTILSQMKK
>LFGLNPFC_04404 Inner membrane metabolite transport protein YhjE
MQATATTLDHEQEYTPINSRNKVLVASLIGTAIEFFDFYIYATAAVIVFPHIFFPQGDPT
AATLQSLATFAIAFVARPIGSAVFGHFGDRVGRKATLVASLLTMGISTVVIGLLPGYATI
GIFAPLLLALARFGQGLGLGGEWGGAALLATENAPPRKRALYGSFPQLGAPIGFFFANGT
FLLLSWLLTDEQFMSWGWRVPFIFSAVLVIIGLYVRVSLHESPVFEKVAKAKKQVKIPLG
TLLTKHVRVTVLGTFIMLATYTLFYIMTVYSMTFSTAAAPVGLGLPRNEVLWMLMMAVIG
FAVMVPLAGLLADAFGRRKSMVVITTLIILFALFAFNPLLGSGNPALVFVFLLLGLSLMG
LTFGPMGALLPELFPTEVRYTGASFSYNVSSILGASVAPYIAAWLQTNYGLGAVGLYLAA
MAGLTLIALLLTHETRHQSL
>LFGLNPFC_04405 Inner membrane protein YhjD
MTQENEIKRPTQDLEHEPIKQLDNSEKGGKVSQALETVTTTAEKVQRQPVIAHLIRATER
FNDRLGNQFGAAITYFSFLSMIPILMVSFAAGGFVLASHPMLLQDIFDKILQNISDPTLA
ATLKNTINTAVQQRTTVGLVGLAVALYSGINWMGNLREAIRAQSRDVWERSPQDQEKFWV
KYLRDFISLIGLLIALIVTLSITSVAGSAQQMIISALHLNSIEWLKPTWRLIGLAISIFA
NYLLFFWIFWRLPRHRPRKKALIRGTFLAAIGFEVIKIVMTYTLPSLMKSPSGAAFGSVL
GLMAFFYFFARLTLFCAAWIATAEYKDDPRMPGKTQP
>LFGLNPFC_04406 HTH-type transcriptional regulator DmlR
MLEKTINNAICALLFRCEQQSVKEMDKIHAMQLFIKVAELESFSRAADFFALPKGSVSRQ
IQALEHQLGTQLLQRTTRRVKLTPEGITYYQRAKDVLSNLSELDGLFQQDATSISGKLRI
DIPSGIAKSLLLPRLSEFLYLHPGIELELSSHDRPVDILHDGFDCVIRTGALPEDGVIAR
SLGKLTMVNCASPHYLTRFGYPQSPDDLTSHAIVRYTPHLGVHPLGFEVASVNGVQWFKS
GGMLTVNSSENYLAAGIAGLGIIQIPRIAVREALRAGRLIEVLPGYRAEPLSLSLVYPQR
RELSRRVNLFMQWLAGVMKEHLD
>LFGLNPFC_04407 Putative HTH-type transcriptional regulator YhjB

MQIVMFDRQSIFIHGMKISLQHRIPGVSIQGASQADELWQKLESYPEALVMLDGDQDGEF
CYWLLQKTAVQFPEVKVLITATDCNKRWLQEVIHFNVLAIVPRDSTVETFALAVNSAAMG
MMFLPGDWRTTPEKDIKDLKSLSARQREILTMLAAGESNKEIGRALNISTGTVKAHLESL
YRRLEVKNRTQAAMMLNISS
>LFGLNPFC_04408 Cytoplasmic trehalase
MLNQKIQNPNPDELMIEVDLCYELDPYELKLDEMIEAEPEPEMIEGLPASDALTPADRYL
ELFEHVQSAKIFPDSKTFPDCAPKMDPLDILIRYRKVRRHRDFDLRKFVENHFWLPEVYS
SEYVSDPQNSLKEHIDQLWPVLTREPQDHIPWSSLLALPQSYIVPGGRFSETYYWDSYFT
MLGLAESGREDLLKCMADNFAWMIENYGHIPNGNRTYYLSRSQPPVFALMVELFEEDGVR
GARRYLDHLKMEYAFWMDGAESLIPNQAYRHVVRMPDGSLLNRYWDDRDTPRDESWLEDV
ETAKHSGRPPNEVYRDLRAGAASGWDYSSRWLRDTGRLASIRTTQFIPIDLNAFLFKLES
AIANISALKGEKETEALFRQKASARRDAVNRYLWDDENGIYRDYDWRREQLALFSAAAIV
PLYVGMANHEQADRLANAVRSRLLTPGGILASEYETGEQWDKPNGWAPLQWMAIQGFKMY
GDDLLGDEIARNWLKTVNQFYLEQHKLIEKYHIADGVPREGGGGEYPLQDGFGWTNGVVR
RLIGLYGEP
>LFGLNPFC_04409 hypothetical protein
MKMVSRITAIGLAGVAICYLGLSGYVWYHDNKRSKQADVQASAVSENNKVLGFLREKGCD
YCHTPSAELPAYYYIPGAKQLMDYDIKLGYKSFNLEAVRAALLANKPVSQSDLNKIEWVM
QYETMPPTRYTALHWAGKVSDEERAEILAWIAKQRAEYYASNDTAPEHRNEPVQPIPQKL
PTDAQKVALGFALYHDPRLSADSTISCAHCHALNAGGVDSRKTSIGVGGAVGPINAPTVF
NSVFNVEQFWDGRAATLQDQAGGPPLNPIEMASKSWDEIIAKLEKDPQLKAQFLEVYPQG
FSGENITDAIAEFEKTLITPDSPFDKWLRGDENALTAQQKKGYQLFKDNKCATCHGGIIL
GGRSFEPLGLKKDFNFGEITAADIGRMNVTKEERDKLRQKVPGLCNVALTAPYFHRGDVP
TLDGAVKLMLRYQVGKELPQEDVDDIVAFLHSLNGVYTPYMQDKQ
>LFGLNPFC_04410 Glutamate decarboxylase alpha
MDQKLLTDFRSELLDSRFGAKAISTIAESKRFPLHEMRDDVAFQIINDELYLDGNARQNL
ATFCQTWDDENVHKLMDLSINKNWIDKEEYPQSAAIDLRCVNMVADLWHAPAPKNGQAVG
TNTIGSSEACMLGGMAMKWRWRKRMEAAGKPTNKPNLVCGPVQICWHKFARYWDVELREI
LCAPVSCLWTRNA
>LFGLNPFC_04411 Glutamate decarboxylase beta
MHIDAASGGFLAPFVAPDIVWDFRLPRVKSISASGHKFGLAPLGCGWVIWRDEEALPQEL
VFNVDYLGGQIGTFAINFSRPAGQVIAQYYEFLRLGREGYTKVQNASYQVAAYLADEIAK
LGPYEFICTGRRTKASRRFASN
>LFGLNPFC_04412 HTH-type transcriptional regulator GadX
MQPLHGNCLIAYARHKYILTMVNGEYRYFNGGDLVFADASQIQVDKCVENFVLVSRDTLS
LFLPMLKEEALKLHVHKKVPSLLVHHCTRDIPVFQEVAQLSQNKNLRYAEMLRKRALIFA
LLSVFLEDTQFIPLLLNVLQPNMRTRVCTVINNNIAHEWTLARIASELLMSPSLLKKKLR
EEGTSYSQLLTECRMQRALQLIVIYGVSIKRVAVSCGYHSVSYFIYVFRNYYGMTPTEYQ
ERSAQELPNCGSAASMAAQGIFYGTDRSAEGIRL
>LFGLNPFC_04413 HTH-type transcriptional regulator GadW
MAHVCSVILVRRSFDIHHEQQKISLHNESILLLDKNLADDFAFCSLDTRRLDIEELTVCH
YLQNIRQLPRNLGLHSKDRLLINQSPPIQLVTAIFDSFNDPRVNSPILSKMLYLSCLSMF
SHKKELIPLLFNSISTVSGKVERLISFDIAKRWYLRDIAERMYTSESLIKKKLQDENTCF
SKILLASRMSMARRLLELRQIPLHTIAEKCGYSSTSYFINTFRQYYGVTPHQFSQHSPGT
FS
>LFGLNPFC_04414 hypothetical protein
MFGIIKLTIHTITGMWVSIVLFKLMTNGWSGFYFQCCVLSLVFLTVSWLLSGEWLAGKSK
AEPSRSTLLSFTRYAFLKRAKRCSTTTKKTGTK
>LFGLNPFC_04415 Multidrug resistance protein MdtF
MANYFIDRPVFAWVLAIIMMLAGGLAIMNLPVAQYPQIAPPTITISATYPGADAQTVEDS
VTQVIEQNMNGLDGLMYMSSTSDAAGNASITLTFETGTSPDIAQVQVQNKLQLAMPSLPE
AVQQQGISVDKSSSNILMVAAFISDNGSLNQYDIADYVASNIKDPLSRTAGVGSVQLFGS
EYAMRIWLDPQKLNKYNLVPSDVISQIKVQNNQISGGQLGGMPQAADQQLNASIIVQTRL
QTPEEFGKILLKVQQDGSQVLLRDVARVELGAEDYSTVARYNGKPAAGIAIKLATGANAL
DTSRAVKEELNRLSAYFPASLKTIYPYDTTPFIKISIQEVFKTLVEAIILVFLVMYLFLQ
NFRATIIPTIAVPVVILGTFAILSAVGFTINTLTMFGMVLAIGLLVDDAIVVVENVERVI
AEDKLPPKEATHKSMGQIQRALVGIAVVLSAVFMPMAFMSGATGEIYRQFSITLISSMLL
SVFVAMSLTPALCATILKAAPEGGHKPNALFARFNTLFEKSTQHYTDSTRSLLRCTGRYM
VVYLLICAGMAVLFLRTPTSFLPEEDQGVFMTTAQLPSGATMVNTTKVLQQVTDYYLTKE
KNNVQSVFTVGGFGFSGQGQNNGLAFISLKPWSERVGEENSVTAIIQRAMIALSSINKAV
VFPFNLPAVAELGTASGFDMELLDNGNLGHEKLTQARNELLSLAAQSPNQVIGVRPNGLE
DTPMFKVNVNAAKAEAMGVALSDINQTISTAFGSSYVNDFLNQGRVKKVYVQAGTPFRML
PDNINQWYVRNASGTMAPLSAYSSTEWTYGSPRLERYNGIPSMEILGEAAAGKSTGDAMK
FMADLVAKLPAGVGYSWTGLSYQEALSSNQAPALYAISLVVVFLALAALYESWSIPFSVM

LVVPLGVVGALLATDLRGLSNDVYFQVGLLTTIGLSAKNAILIVEFAVEMMQKEGKTPVE
AIIEAARMRLRPILMTSLAFILGVLPLVISHGAGSGAQNAVGTGVMGGMFAATVLAIYFV
PVFFVVVEHLFARFKKA
>LFGLNPFC_04416 Multidrug resistance protein MdtE
MNRRRKLLIPLLFCGAMLTACDDKSAENAAAMTPEVGVVTLSPGSVNVLSELPGRTVPYE
VAEIRPQVGGIIIKRNFIEGDKVNQGDSLYQIDPAPLQAELNSAKGSLAKALSTASNARI
TFNRQASLLKTNYVSRQDYDTARTQLNEAEANVTVAKAAVEQATINLQYANVTSPITGVS
GKSSVTVGALVTANQADSLVTVQRLDPIYVDLTQSVQDFLRMKEEVASGQIKQVQGSTPV
QLNLENGKRYGQTGTLKFSDPTVDETTGSVTLRAIFPNPNGDLLPGMYVTALVDEGSRQN
VLLVPQEGVTHNAQGKATALILDKDDVVQLREIEASKAIGDQWVVTSGLQAGDRVIVSGL
QRIRPGIKARAISSSQENASTESKQ
>LFGLNPFC_04417 hypothetical protein
MRDLQTSGIVGLSASKMGCKHSDRHGEVNDKKNVAVLPTVSASMRATKGSTGGYTYQGNK
VVFVSPSIITGGIEISICIPRHASHQFQLLMSLLKIRCDTQGDDVAHTS
>LFGLNPFC_04418 Transcriptional regulator GadE
MIFLMTKDSFLLQGFWQLKDNHEMIKINSLSEIKKVGNKPFKVIIDTYHNHILDEEAIKF
LEKLDAERIIVLAPYHISKLKAKAPIYFVSRKESIKNLLEITYGKHLPHKNSQLCFSHNQ
FKIMQLILKNKNESNITSTLNISQQTLKIQKFNIMYKLKLRRMSDIVTLGITSYF
>LFGLNPFC_04419 Protein HdeD
MLYIDKATILKFDLEMLKKHRRAIQFIAVLLFIVGLLCISFPFVSGDILSTVVGALLICS
GIALIVGLFSNRSHNFWPVLSGFLVAVAYLLIGYFFIRAPELGIFAIAAFIAGLFCVAGV
IRLMSWYRQRSMKGSWLQLVIGVLDIVIAWIFLGATPMVSVTLVSTLVGIELIFSAASLF
SFASLFVKKQ
>LFGLNPFC_04420 Acid stress chaperone HdeA
MKKVLGVILGGLLLLPVVSNAADAQKAADNKKPVNSWTCEDFLAVDESFQPTAVGFAEAL
NNKDKPEDAVLDVQGIATVTPAIVQACTQDKKASFKDKVKGEWDKIKKDM
>LFGLNPFC_04421 Acid stress chaperone HdeB
MGYKMNISSLRKAFIFMGAVAALSLVNAQSALAANESAKDMTCQEFIDLNPKAMTPVAWW
MLHEETVYKGGDTVTLNETDLTQIPKVIEYCKKNPQKNLYTFKNQASNDLPN
>LFGLNPFC_04422 Protein SapB
MRGKGAGLRTHVLIGMGSALFMIVSKYGFADVLSLGHVGLDPSRIAAQVVTGVGFIGAGN
ILVRNQNIVGLTTAADIWVTAAIGMVIGSGMYELGIYGSVMTLLVLEVFHQLTFRLMNKN
YHLQLTLVNGNTVSMLDWFKQQKIKTDLVSLQENEDHEVVAIDIQLHATTSIEDLLRLLK
GMAGVKGVSIS
>LFGLNPFC_04423 Hemin import ATP-binding protein HmuV
MISAQNLVYSLQGRRLTDNVSLTFPGGEIVAILGPNGAGKSTLLRQLTGYLQPDSGECRL
FNKPLNEWSITELAKHRAVMRQNSHMAFPFSVQEVIQMGRHPHRTGNQDNETAQIMALCD
CQALANRNYRQLSGGEQQRVQLARLLVQLWEPTPSPKWLFLDEPTSALDIHHQQHLFRLL
RQLVHERQFNVCCVLHDLNLAARYADRVVLMQKGKVIANGKPQDVLTQQALTMLYGADIT
VLKDPANHSPLIVLDH
>LFGLNPFC_04424 Hemin transport system permease protein HmuU
MGLSLLLLALVLFGASQGALKISFDALFDEEYRDIWLNIRLPRVLLAVLVGAALATAGVI
MQGLFRNPMADPGLLGVSSGSALMVGVAIVLPFSFPVVLVLYEQMVFAIAGSLVVCTIIF
LITQRHRDGSMMQLLLAGIAINALCGAAIGILSYIGDEQQLRQLTLWMMGNLGQAQWPTL
LVASSFILPAIIATTCLAGTLNLLQLGDEEAHYLGVNVKRKRQQLLLVSSLLVGAAVSVS
GIIGFIGLVIPHLIRMTTGANHRWLIPCSALAGACLLLMADTLARTLVQPAEMPVGLLTS
LLGGPYFMWLILRNRRIT
>LFGLNPFC_04425 hypothetical protein
MTPWLLFGAGGKGVGARTLELALAEQRPVVAVIRHADAATKLAQQGVQVFTGDACDASVV
AAACRATGPDALIISTMGGAQDYLAHRTVIDEAEKAGISRMILVTSLGCGDSWPFLSERA
KAAFGQAVREKTLAESWLQTSQLDYAILRPGGLLDGAATGKAQRIQNQECHGFVNRADVA
AHIHELANAPALNQQVYSLIEPDLKPA
>LFGLNPFC_04426 Intracellular heme transport protein HutX
MSHVSLQEFLKTEPDGTLEAIAEQYNTTLLEVVKNLPSPTVVSGDKFDTVWDTVCEWGNV
TTLVHTTDVILEFSGELPSGFHRHGYFNLRGKHGMSGHIKAENCTHIALIERKFMGMDTA
SILFFNKEGSAMLKIFLGRDDHRQLLSEQVSAFHALAASLKEHA
>LFGLNPFC_04427 Anaerobilin synthase
MNTNNTLDLTPHFALDGDQPFKDRRAMMPFRGAIPVAKEQLAQTWQEMINQTVSPRKRLV
YLHIPFCATHCTFCGFYQNRFNEDACAHYTDALIREIEMEADSVLHQSAPIHAVYFGGGT
PSALSAHDLARIITTLREKLPLAPDCEITIEGRVLNFDAERIDACLDAGANRFSIGIQSF
NSKIRKKMARTSDGPTAIAFMESLVKRDRAAVVCDLLFGLPGQDAQTWGEDLAIARDIGL
DGVDLYALNVLPNTPLGKAVENGRTTVPSPAERRDLYLQGCDFMDDAGWRCISNSHWGRT
TRERNLYNLLIKQGADCLAFGSGAGGSINGYSWMNERNLQTWHESVAAGKKPLMMIMRNA
ERNAQWRHTLQSGIETARVPLDELTPHAEKLAPLLAQWHQKGLSRDASTCLRLTNEGRFW

ASNILQSLNELIQVLNAPAIALEKP
>LFGLNPFC_04428 Hemin-binding periplasmic protein HmuT
MPRIITRPFLFSPLTLCISAVASAAKTMVKRKKLFTALLALSWTFSVTAAERIVVAGGSL
TELIYAMGAGKRVVGVDETTSYPPETAKLPHIGYWKQLSSEGILSLRPDSVITWQDAGPQ
IVLDQLRAQKVNVVTLPRVPATLEQMYANIRQLAKTLQVPEQGEALVTQINQRLERVQQN
VAAKKAPVKAMFILSAGGSAPQVAGKGSVADAILSLAGAENVATHQQYKSYSAESLIAAN
PEVIVVTSQMVDGDINRLRSIAGITHTAAWKNQRIITVDQNLILGMGPRIADVVESLHQQ
LWPQ
>LFGLNPFC_04429 Hemin receptor
MSRPQFTSLRLSLLALAVSATLPTFAFATETMTVTATGNARSSFEAPMMVSVIDTSAPEN
QTATSATDLLRHVPGITLDGTGRTNGQDVNMRGYDHRGVLVLVDGVRQGTDTGHLNGTFL
DPALIKRVEIVRGPSALLYGSGALGGVISYDTVDAKDLLQEGQSSGFRVFGTGGTGDHSL
GLGASAFGRTENLDGIVAWSSRDRGDLRQSNGETAPNDESINNMLAKGTWQIDSAQSLSG
LVRYYNNDAREPKNPQTVEASDSSNPMVDRSTIQRDAQLSYKLAPQGNDWLNADAKIYWS
EVRINAQNTGSSGEYREQITKGARLENRSTLFADSFASHLLTYGGEYYRQEQHPGGATTG
FPQAKIDFSSGWLQDEITLRDLPITLLGGTRYDSYRGSSDGYKDVDADKWSSRAGMTINP
TNWLMLFGSYAQAFRAPTMGEMYNDSKHFSIGRFYTNYWVPNPNLRPETNETQEYGFGLR
FDDLMLSNDALEFKASYFDTKAKDYISTTVDFAAATTMSYNVPNAKIWGWDVMTKYTTDL
FSLDVAYNRTRGKDTDTGEYISSINPDTVTSTLNIPIAHSGFSVGWVGTFADRSTHISSS
YSKQPGYGVNDFYVSYQGQQALKGVTTTLVLGNAFDKEYWSPQGIPQDGRNGKIFVSYQW
>LFGLNPFC_04430 Hemin transport protein HemS
MNHYTRWLELKEQNPGKYARDIAGLMNISEAELAFARVTHDAWRMRGDIREILAALESVG
ETKCICRNEYAVHEQVGSFTNQHLNGHAGLILNPRALDLRLFLNQWASVFHIKENTARGE
RQSIQFFDHQGDALLKVYATDNTDMAAWSELLARFITDENTPLELKAVDAPVVQTRADAS
VVEQEWRAMTDVHQFFTLLKRHNLTRQQAFNLVADDLACKVSNSALAQILESAQQDGNEI
MVFVGNRGCVQIFTGVVEKVVPMKGWLNIFNPTFTLHLLEESIAEAWVTRKPTSDGHVTS
LELFAHDGTQIAQLYGQRTEGEQEQAQWRKQIASLIPEGVTA
>LFGLNPFC_04431 HTH-type transcriptional regulator DctR
MFLIITRDTMFFTAMKNILSKGNVVHIQNEEEIDVMLHQNAFVIIDTLMNNVFHSNFLTQ
IERLKPVHVIIFSPFNIKRCLGKVPVTFVPRTIAIIDFVALINGSYCSVPEADVSLSRKQ
HQVLSCIANQMTTEDILEKLKISLKTFYCHKHNIMMILNLKRINELVRHQHINYLV
>LFGLNPFC_04432 Outer membrane protein Slp
MNMTKGALILSLSFLLAACSSIPQNIKGNNQPDIQKSFVAVHNQPGLYVGQQARFGGKVI
NVINGKTDTLLEIAVLPLDSYAKPDIEANYQGRLLARQSGFLDPVNYRNHFVTILGTIQG
EQPGFINKVPYNFLEVNMQGIQVWHLREVVNTTYNLWDYGYGAFWPEPGWGAPYYTNAVS
QVTPELVK
>LFGLNPFC_04433 hypothetical protein
MSIDFTPGVINTYNGDIYNCTTNTDNVKTPDTPKWPCDNREEQHPINAPFSGEWIHL
>LFGLNPFC_04434 Arsenate reductase
MSNITIYHNPACGTSRNTLEMIRNSGTEPTIIYYLETPPTRDELVKLIADMGITVRALLR
KNVEPYEELGLAEDKFTHDQLIDFMLQHPILINRPIVVTPLGTRLCRPSEVVLEILPDAQ
KGAFTKEDGEKVVDEAGNRLK
>LFGLNPFC_04435 hypothetical protein
MHPLTHPLPVTAHVSLLDDDYLTPARASVNGTTRTSDQDFESVYAHCQSENVSELTG
>LFGLNPFC_04436 Glutathione reductase
MTKHYDYIAIGGGSGGIASINRAAMYGQKCALIEAKELGGTCVNVGCVPKKVMWHAAQIR
EAIHMYGPDYGFDTTINKFNWETLIASRTAYIDRIHTSYENVLGKNNVDVIKGFARFVDA
KTIEVNGETITADHILIATGGRPSHPDIPGVEYGIDSDGFFALPALPERVAVVGAGYIAV
ELAGVINGLGAKTHLFVRKHAPLRSFDPMLSETLVEVMNAEGPQLHTNAIPKAVVKNADG
SLTLELEDGRSETVDCLIWAIGREPANDNINLEAAGVKTNEKGYIVVDKYQNTNVEGIYA
VGDNTGAVELTPVAVAAGRRLSERLFNNKPDEHLDYSNIPTVVFSHPPIGTVGLTEPQAR
EQYGDDQVKVYKSSFTAMYTAVTTHRQPCRMKLVCVGPEEKIVGIHGIGFGMDEMLQGFA
VALKMGATKKDFDNTVAIHPTAAEEFVTMR
>LFGLNPFC_04437 Ribosomal RNA large subunit methyltransferase J
MLSYRHSFHAGNHADVLKHTVQSLIIESLKEKDKPFLYLDTHAGAGRYQLGSEHAERTGE
YLEGIARIWQQDDLPAELEAYINVVKHFNRSGQLRYYPGSPLIARQLLREQDSLQLTELH
PSDYPLLRSEFQKDSRARVEKADGFQQLKAKLPPVSRRGLILIDPPYEMKTDYQAVVSGI
AEGYKRFATGTYALWYPVVLRQQIKRMIHDLEATGIRKILQIELAVLPDSDRRGMTASGM
IVINPPWKLEQQMNNVLPWLHSKLVPAGTGHATVSWIVPE
>LFGLNPFC_04438 Oligopeptidase A
MTNPLLTPFELPPFSKILPEHVVPAVTKALNDCRENVERVVAQGAPYTWENLCQPLAEVD
DVLGRIFSPVSHLNSVKNSPELREAYEQTLPLLSEYSTWVGQHEGLYKAYRDLRDGDHYA
TLNTAQKKAVDNALRDFELSGIGLPKEKQQRYGEIATRLSELGNQYSNNVLDATMGWTKL
VTDEAELAGMPESALAAAKAQAEAKELEGYLLTLDIPSYLPVMTYCDNQALREEMYRAYS

TRASDQGPNAGKWDNSKVMEEILALRHELAQLLGFENYAFKSLATKMAENPQQVLDFLTD
LAKRARPQGEKELAQLRAFAKAEFGVDELQPWDIAYYSEKQKQHLYSISDEQLRPYFPEN
KAVNGLFEVVKRIYGITAKERKDVDVWHPDVRFFELYDENDELRGSFYLDLYARENKRGG
AWMDDCVGQMRKADGSLQKPVAYLTCNFNRPVNGKPALFTHDEVITLFHEFGHGLHHMLT
RIETAGVSGISGVPWDAVELPSQFMENWCWEPEALAFISGHYETGEPLPKELLDKMLAAK
NYQAALFILRQLEFGLFDFRLHAEFRPDQGAKILETLAEIKKLVAVVPSPSWGRFPHAFS
HIFAGGYAAGYYSYLWADVLAADAFSRFEEEGIFNRETGQSFLDNILSRGGSEEPMELFK
RFRGREPQLDAMLEHYGIKG
>LFGLNPFC_04439 Ribosomal RNA small subunit methyltransferase J
MKICLIDETGAGDGALSVLAARWGLEHDEDNLMALVLTPEHLELRKRDEPKLGGIFVDFV
GGAMAHRRKFGGGRGEAVAKAVGIKGDYLPDVVDATAGLGRDAFVLASVGCRVRMLERNP
VVAALLDDGLARGYADAEIGGWLQERLQLIHASSLTALSDITPRPQVVCLDPMFPHKQKS
ALVKKEMRVFQSLVGPDLDADGLLEPARLLATKRVVVKRPDYAPPLANVATPNAVVTKGH
RFDIYAGTPV
>LFGLNPFC_04440 Dipeptide and tripeptide permease B
MNTTTPMGMLQQPRPFFMIFFVELWERFGYYGVQGVLAVFFVKQLGFSQEQAFVTFGAFA
ALVYGLISIGGYVGDHLLGTKRTIVLGALVLAIGYFMTGMSLLKPDLIFIALGTIAVGNG
LFKANPASLLSKCYPPKDPRLDGAFTLFYMSINIGSLIALSLAPVIADKFGYSVTYNLCG
AGLIIALLVYIACRGMVKDIGSEPDFRPMSFSKLLYVLLGSVVMIFVCAWLMHNVEVANL
VLIVLSIVVTIIFFRQAFKLDKTGRNKMFVAFVLMLEAVVFYILYAQMPTSLNFFAINNV
HHEILGFSINPVSFQALNPFWVVLASPILAGIYTHLGNKGKDLSMPMKFTLGMFMCSLGF
LTAAAAGMWFADAQGLTSPWFIVLVYLFQSLGELFISALGLAMIAALVPQHLMGFILGMW
FLTQAAAFLLGGYVATFTAVPDNITDPLETLPVYTNVFGKIGLVTLGVAVVMLLMVPWLK
RMIATPESH
>LFGLNPFC_04441 Universal stress protein A
MAYKHILIAVDLSPESKVLVEKAVSMARPYNAKVSLIHVDVNYSDLYTGLIDVNLGDMQK
RISEETHHALTELSTNAGYPITETLSGSGDLGQVLVDAIKKYDMDLVVCGHHQDFWSKLM
SSARQLINTVHVDMLIVPLRDEEE
>LFGLNPFC_04442 Universal stress protein B
MISTVALFWALCVVCIVNMARYFSSLRALLVVLRNCDPLLYQYVDGGGFFTSHGQPNKQV
RLVWYIYAQRYRDHHDDEFIRRCERVRRQFILTSALCGLVVVSLIALMIWH
>LFGLNPFC_04443 Low-affinity inorganic phosphate transporter 1
MLHLFAGLDLHTGLLLLLALAFVLFYEAINGFHDTANAVATVIYTRAMRSQLAVVMAAVF
NFLGVLLGGLSVAYAIVHMLPTDLLLNMGSSHGLAMVFSMLLAAIIWNLGTWYFGLPASS
SHTLIGAIIGIGLTNALMTGTSVVDALNIPKVLSIFGSLIVSPIVGLVFAGGLIFLLRRY
WSGTKKRARIHLTPAEREKKDGKKKPPFWTRIALILSAIGVAFSHGANDGQKGIGLVMLV
LIGVAPAGFVVNMNATGYEITRTRDAINNVEAYFEQHPALLKQATGADQLVPAPEAGATQ
PAEFHCHPSNTINALNRLKGMLTTDVESYDKLSLDQRSQMRRIMLCVSDTIDKVVKMPGV
SADDQRLLKKLKSDMLSTIEYAPVWIIMAVALALGIGTMIGWRRVATTIGEKIGKKGMTY
AQGMSAQMTAAVSIGLASYTGMPVSTTHVLSSSVAGTMVVDGGGLQRKTVTSILMAWVFT
LPAAVLLSGGLYWLSLQFL
>LFGLNPFC_04444 putative protein
MERFDAIIIGAGAAGMFCSALAGQAGRRVLLIDNGKKPGRKILMSGGGRCNFTNLYVEPG
AYLSQNPHFCKSALARFTQWDFIDLVNKHGIAWHEKTLGQLFCDDSAQQIVDMLVDECEK
GNVAFRLRSEVLSVAKDDTGFTLELNGMTVGCEKLVIATGGLSMPGLGASPFGYKIAEQF
GLNVLPTRAGLVPFTLHKPLLEELQVLAGVAVPSVITAENGIVFRENLLFTHRGLSGPAV
LQISSYWQPGEFVSINLLPDVDLETFLNEQRNAHPNQSLKNTLAVHLPKRLVERLQQLGQ
IPDVSLKQLNVRDQQTLISTLTDWRVQPNGTEGYRTAEVTLGGVDTNELSSRTMEARKVP
GLYFIGEVMDVTGWLGGYNFQWAWSSAWACAQDLVAE
>LFGLNPFC_04445 Inner membrane protein YhiM
MGFKMNIYIGWLFKLIPLLMGIICIALGEFVLTGSGQSEYFVAGHVLISLSAICLALFTT
AFIIISQLTHGMNKFYNRLFPVIGYAGSATTMIWGWSLLASNNVMADEFVAGHVIFGVGM
IAACVSTVAASSGHFLLIPKNASGSKSDGTPLQAYSSTIGNCLIAVPVLLTLFGFIWSVV
LLRSADITPHYVAGHVLMGLTAICACLIGLVATIVHQTRNTFSVKEHWLWCYWVILLGSL
TIIFGIYVLISSDASARLAPGIILICLGMICYSIFSKVWLLALVWRRTCSLANRIPMIPV
FTCLFCLFLAAFLAEIAQTDMAYFIPSRVLVGLGAVCFTLFSIVSILEAGSAKK
>LFGLNPFC_04446 p-hydroxybenzoic acid efflux pump subunit AaeA
MDKSKRHLAWWGVGALAVAAVVAWWLLRPAGVPEGFAVSNGRIEATEVDIASKIAGRIDT
ILVKEGQFVREGEVLAKMDTRVLQEQRLEAIAQIKEAQSAVAAAQALLEQRQSETRAAQS
LVNQRQAELDSVAKRHTRSRSLAQRGAISAQQLDDDRAAAESARAALESAKAQVSASKAA
IEAARTNIIQAQTRVEAAQATERRIAADIDDSELKAPRDGRVQYRVAEPGEVLAAGGRVL
NMVDLSDVYMTFFLPTEQAGTLKLGGEARLILDAAPDLRIPATISFVASVAQFTPKTVET
SDERLKLMFRVKARIPPELLQQHLEYVKTGLPGVAWVRVNEELPWPDDLVVRLPQ
>LFGLNPFC_04447 Ribosome-associated ATPase

MTHLELVPVPPVAQLAGVSQHYGKTVALNNITLDIPARCMVGLIGPDGVGKSSLLSLISG
ARVIEQGSVMVLGGDMRDPKHRRDVCPRIAWMPQGLGKNLYHTLSVYENVDFFARLFGHD
KAEREVRINELLTSTGLAPFRDRPAGKLSGGMKQKLGLCCALIHDPELLILDEPTTGVDP
LSRAQFWDLIDSIRQRQSNMSVLVATAYMEEAERFDWLVAMNAGKVLATGSAEELRQQTQ
SATLEEAFINLLPQAQRQAHQAVVIPPYQPENAEIAIEARDLTMRFGSFVAVDHVNFRIP
RGEIFGFLGSNGCGKSTTMKMLTGLLPASEGEAWLFGQPVDPKDIDTRRRVGYMSQAFSL
YNELTVRQNLELHARLFHIPEAEIPARVAEMSERFKLNDVEDVLPESLPLGIRQRLSLAV
AVIHRPEMLILDEPTSGVDPVARDMFWQLMVDLSRQDKVTIFISTHFMNEAERCDRISLM
HAGKVLASGTPPELVEKRGAASLEEAFIAYLQEAAGQSNEAEAPPVVHDTTHAPRQGFSL
RRLFSYSRREALELRRDPVRSTLALMGTVILMLIMGYGISMDVENLRFAVLDRDQTVSSQ
AWTLNLSGSRYFIEQPPLTSYDELDRRMRAGDITVAIEIPPNFGRDIARGTPVELGVWID
GAMPSRAETVKGYVQAMHQSWLQDVASRQSTPASQSGLMNIETRYRYNPDVKSLPAIVPA
VIPLLLMMIPSMLSALSVVREKELGSIINLYVTPTTRSEFLLGKQLPYIALGMLNFFLLC
ALSVFVFGVPHKGSFLTLTLAALLYIIIATGMGLLISTFMKSQIAAIFGTAIITLIPATQ
FSGMIDPVASLEGPGRWIGEVYPTSHFLTIARGTFSKALDLTDLWQLFIPLLIAIPLVMG
LSILLLKKQEG
>LFGLNPFC_04448 Inner membrane transport permease YhhJ
MRHLRNIFNLGIKELRSLLGDKAMLTLIVFSFTVSVYSSATVTPGSLNLAPIAIADMDQS
QLSNRIVNSFYRPWFLPPEMITADEMDAGLDAGRYTFAINIPPNFQRDVLAGRQPDIQVN
VDATRMSQAFTGNGYIQNIINGEVNSFVARYRDNSEPLVSLETRMRFNPNLDPAWFGGVM
AIINNITMLAIVLTGSALIREREHGTVEHLLVMPITPFEIMMAKVWSMGLVVLVVSGLSL
VLMVKGVLGVPIEGSIPLFMLGVALSLFATTSIGIFMGTIARSMPQLGPLVILVLLPLQM
LSGGSTPRESMPQMVQDIMLTMPTTHFVSLAQAILYRGAGFEIVWPQFLTLMAIGGAFFT
IALLRFRKTIGTMA
>LFGLNPFC_04449 D-tagatose-1,6-bisphosphate aldolase subunit KbaY
MPLVNGRILLDRIQEKRVLAGAFNTTNLETTISILNAIERSGLPNFIQIAPTNAQLSGYD
YIYEIVKRHADKMDVPVSLHLDHGKTQEDVKQAVRAGFTSVMIDGAAFSFEENIAFTQEA
VDFCKSYGVPVEAELGAILGKEDDHVSEADCKTEPEKVKTFVERTGCDMLAVSIGNVHGL
DDIPRIDIPLLKRIAEVSPVPLVIHGGSGIAPEILRSFVNYRVAKVNIASDLRKAFITAV
GKAYVNNHNEANLARVMASAKNAVEEDVYSKILMMNEGHRLGK
>LFGLNPFC_04450 Phosphocarrier protein HPr
MLSKTVEVRNSTGLHARPAACLAKAAKKYSCKVTLHYEGNDINATSMMNIMRAGIKGGKT
VEIRCEGDDENEAIQTLTTLFRDRFGEAE
>LFGLNPFC_04451 Xylulose kinase
MPDNSAAIVIDIGTTNCKVTCFSCLDATTLGAHKFVTAKQISPQGNVDFDIDALWQEVRQ
AIAQLNAASPLPVRRISIASFGESGVFLDKHGEILTPMLAWYDRRGEEYLATLSEADSAA
LYDICGLPLHSNYSAFKMRWLLEHYPLRNRRGLRWLHAPEVLLWRLTGEQRTDITLASRT
LCLDVRKGEWSAKAAALLHVPCSAFAPLLQPGEHAGWVSESLCKTLGFSQSVSVTLAGHD
HMVGARALQMMPGDILNSTGTTEGILQLDTQPTLDEQAKRDKLANGCYSLANQFTLFASL
PVGGFALEWLRNTFRLTDEEIAVSLNRGYADYLAGNWSLDDIPVFIPHLRGSGSPYKNRH
TRGLFYGLGDTLNINMLIASVSLGLTMEFANCFACFNVPGTSALKVIGPATHNPLWLQLK
ADILQRPVEAIAFNEAVSVGALLTAAPDIPPPQVTIAQRLLPNRARYHQLQRYQHKWKSW
YQLKLQQEGVMPLHHREEHYVE
>LFGLNPFC_04452 PTS system galactitol-specific EIIC component
MNDIAHTLYNIVQYILGFGPTVMLPLVLFILALFFKVKPAKALRSSLTVGIGFVGIYAIF
DILTSNVGPAAQAMVERTGINLPVVDLGWPPLSAITWGSPIAPFVIPLTILINVAMLALN
KTRTVDVDMWNYWHFALAGTLVYYSTGSLFFGLLAAAIAAVVVLKLADWSAPLVQKYFGL
EGISLPTLSSVVFFPVGLLVDKIIDHIPGLNRIHIDPETVQKKFGIFGEPMMVGTILGIL
LGVIAGYDFKKVLLLGISIGGVMFILPRMVRILMEGLLPLSEAIKKYLNAKYPDRDDLYI
GLDIAVAVGNPAIISTALLLTPISVFIAFVLPGNEVLPLGDLANLAVMASMIALASRGNI
FRTVLAAIPVIIADLWIATKIAPFITGMAKDVNFKFAEGSSGQVSSFLDGGNPFRFWLLE
IFNGNLIAIGLVPVIALVLYGIFRMTRSTVYA
>LFGLNPFC_04453 PTS system galactitol-specific EIIB component
MSQTILFVCATGIATSTAVTEKVMEYCKEHGLNVNYSQTNVASLPGNTDGVALVVSTTKV
PYELDVPVVNGLPIITGIGEEKVLAQIVSILKK
>LFGLNPFC_04454 hypothetical protein
MQGIQFQENFIQRLPAGLSVEQIIHQLAQPLVTAELVVPDFADHVLEREATYPTGLPTEP
PCVAIPHTDHKHVRHNAIAVGILPEPVVFADMGGDSDPVPVRVIFLLALGESNKQLNALG
WIMEMIQDTPFMRALLTMETTEIHTAILNKMKERGEI
>LFGLNPFC_04455 Mannosyl-D-glycerate transport/metabolism system repressor MngR
MKSLSKSSQIPLYQQAVEWIRESIYTGDLVEDDRIPSEYQIMDMLEVSRGTVKKAVAQLV
KEGVLIQVQGKGTFVKKENVAYPLGEGLLSFAESLESQKIHFTTEVITSRIEPANRYVAE
KLRITPGQDILYLERLRSIGDEKAMLIENRINIELCPGIVEIDFNQHNLFPTIESLSQRK
IRYSESRYAARLIGNERGHFLDISEDAPVLHLEQLVFFSRGLPVEFGNVWLKGNKYYLGT

VLQRREVS
>LFGLNPFC_04456 hypothetical protein
MDNFKRIVGEQAKKRIEASHILGDQIVINKM
>LFGLNPFC_04457 Nickel-responsive regulator
MQRVTITLDDDLLETLDSLSQRRGYNNRSEAIRDILRSALAQEATQQHGTQGFAVLSYVY
EHEKRDLASRIVSTQHHHHDLSVATLHVHINHDDCLEIAVLKGDMGDVQHFADDVIAQRG
VRHGHLQCLPKED
>LFGLNPFC_04458 Nickel import ATP-binding protein NikE
MTLLNVSDLSHHYAHGGFSGKHQHQAVLNNVSLALKSGETVALLGRSGCGKSTLARLLVG
LESPSQGNISWRGEPLAKLNRAQRKAFRRDIQMVFQDSISAVNPRKTVREILREPMRHLL
SLKKAEQLARASEMLKAVDLDDSVLDKRPPQLSGGQLQRVCLARALAVEPKLLILDEAVS
NLDLVLQAGVIRLLKKLQQQFGTACLFITHDLRLVERFCQRVMVMDNGQIVETQVVGDKL
TFSSDAGRVLQNAVLPAFPVRRRTTEKV
>LFGLNPFC_04459 Nickel import ATP-binding protein NikD
MPQQIELRNIALQAAQPLVHGVSLTLQRGRVLALVGGSGSGKSLTCAATLGILPAGVRQT
AGEILADGKPVSPCALRGIKIATIMQNPRSAFNPLHTMHTHARETCLALGKPADDATLTA
AIEAVGLENAARVLKLYPFEMSGGMLQRMMIAMAVLCESPFIIADEPTTDLDVVAQARIL
DLLESIMQKQAPGMLLVTHDMGVVARLADDVAVMSHGKIVEQGDVETLFNAPKHAVTRSL
VSAHLALYGMELAS
>LFGLNPFC_04460 Nickel transport system permease protein NikC
MNFFLSSRWSVRLALIIIALLALIALTSQWWLPYDPQAIDLPSRLLSPDAQHWLGTDHLG
RDIFSRLMAATRVSLGSVMACLLLVLTLGLIIGGSAGLIGGRVDQATMRVADMFMTFPTS
ILSFFMVGVLGTGLTNVIIAIALSHWAWYARMVRSLVISLRQREFVLASRLSGAGHVRVF
IDHLAGAVIPSLLVLATLDIGHMMLHVAGMSFLGLGVTAPTAEWGVMINDARQYIWTQPL
QMFWPGLALFISVMAFNLVGDALRDHLDPHLVTEHAH
>LFGLNPFC_04461 Nickel transport system permease protein NikB
MLRYVLRRFLLLIPMVLAASVIIFLMLRLGTGDPALDYLRLSNLPPTPEMLASTRTMLGL
DQPLYVQYGTWLWKALHLDFGISFASQRPVLDDMLNFLPATLELAGAALVLILLTSVPLG
IWAARHRDRLPDFAVRFIAFLGVSMPNFWLAFLLVMAFSVYLQWLPAMGYGGWQHIILPA
VSIAFMSLAINARLLRASMLGVAGQRHVTWARLRGLSDKQTERRHILRNASLPMITAVGM
HIGELIGGTMIIENIFAWPGVGRYAVSAIFNRDYPVIQCFTLMMVVVFVVCNLIVDLLNA
ALDPRIRRHEGAHA
>LFGLNPFC_04462 Nickel-binding periplasmic protein
MFSTLRRTLFALLACASFIVHAAAPDEITTAWPVNVGPLNPHLYTPNQMFAQSMVYEPLV
KYQADGSVIPWLAKSWTHSEDGKTWTFTLRDDVKFSNGEPFDAEAAAENFRAVLDNRQRH
AWLELANQIVDVKALSKNELQITLKSAYYPFLQELALPRPFRFIAPSQFKNHETMNGIKA
PIGTGPWVLQESKLNQYDVFVRNENYWGEKPAIKKITFNVIPDPTTRAVAFETGDIDLLY
GNEGLLPLDTCARFSQNPAYHTQLSQPIETVMLALNTAKAPTNELAVREALNYAVNKKSL
IDNALYGTQQVADTLFAPSVPYANLGLKPRQYDPQKAKALLEKAGWTLPAGKDIREKNGQ
PLRIELSFIGTDALSKSMAEIIQADMRQIGADVSLIGEEESSIYARQRDGRFGMIFHRTW
GAPYDPHAFLSSMRVPSHADFQAQQGLADKPLIDKEIGEVLATHDETQRQALYRDILTRL
HDEAVYLPISYISMMVVSKPELGNIPYAPIATEIPFEQIKPVKP
>LFGLNPFC_04463 hypothetical protein
MYRIVLGKVSTLSAAPLPPGLREQAPQGPRRERWLAGRALLSHTLSPLPEIIYGEQGKPA
FAPETPLWFNLSHSGDDIALLLSDEGEVGCDIEVIRPRANWRWLANAVFSLGEHAEMDAV
HPDQQLEMFWRIWTRKEAIVKQRGGSAWQIVSVDSTYNSSLSVSHCQLENLSLAICTPTP
FTLTADSVQWIDSVN
>LFGLNPFC_04464 Putative transport protein YhhT
METPQPDKTGMHILLKLASLVVILAGIHAAADIIVQLLLALFFAIVLNPLVTWFIRRGVQ
RPVAITIVVVVMLIALTALVGVLAASFNEFISMLPRFNKELTRKLFKLQEMLPFLNLHMS
PERILQRMDSEKVVTFTTALMTGLSGAMASVLLLVMTVVFMLFEVRHVPYKMRFALNNPQ
IHIAGLHRALKGVSHYLALKTLLSLWTGIIVWLGLELMGVQFALMWAVLAFLLNYVPNIG
AVISAVPPMIQVLLFNGIYECILVGALFLVVHMVIGNILEPRMMGHRLGMSTMVVFLSLL
IWGWLLGPVGMLLSVPLTSVCKIWMETTKGGSKLAILLGPGRPKSRLPG
>LFGLNPFC_04465 putative MFS-type transporter YhhS
MVKMKHCCKNVVILMPEPVAEPALNGLRLNLRIVSIVMFNFASYLTIGLPLAVLPGYVHD
VMGFSAFWAGLVISLQYFATLLSRPHAGRYADLLGPKKIVVFGLCGCFLSGLGYLTAGLT
ASLPVISLLLLCLGRVILGIGQSFAGTGSTLWGVGVVGSLHIGRVISWNGIVTYGAMAMG
APLGVVFYHWGGLQALALIIMGVALVAILLAIPRPTVKASKGKPLPFRAVLGRVWLYGMA
LALASAGFGVIATFITLFYDAKGWDGAAFALTLFSCAFVGTRLLFPNGINRIGGLNVAMI
CFSVEIIGLLLVGVATMPWMAKIGVLLAGAGFSLVFPALGVVAVKAVPQQNQGAALATYT
VFMDLSLGVTGPLAGLVMSWAGVPVIYLAAAGLVAIALLLTWRLKKRPPVEIPEAASSS
>LFGLNPFC_04466 hypothetical protein
MRNLVKYVGIGLLVMGLAACDDKDTNATAQGSVAESNATGNPVNLLDGKLSFSLPADMTD

QSGKLGTQANNMHVWSDATGQKAVIVIMGDDPKEDLAVLAKRLEDQQRSRDPQLQVVTNK
AIELKGHKMQQLDSIISAKGQTAYSSVILGNVGNQLLTMQITLPADNQQKAQTTAENIIN
TLVIQ
>LFGLNPFC_04467 Queuosine precursor transporter
MNVFSQTQRYKALFWLSLFHLLVITSSNYLVQLPVSIFGFHTTWGAFSFPFIFLATDLTV
RIFGAPLARRIIFAVMIPALLISYVISSLFYMGSWQGFGALAHFNLFVARIATASFMAYA
LGQILDVHVFNRLRQSRHWWLAPTASTLFGNVSDTLAFFFIAFWRSPDAFMAEHWMEIAL
VDYCFKVLISIVFFLPMYGVLLNMLLKRLADKSEINALQAS
>LFGLNPFC_04468 Sulfur carrier protein TusA
MTDLFSSPDHTLDALGLRCPEPVMMVRKTVRNMQPGETLLIIADDPATTRDIPGFCTFME
HELVAKETDGLPYRYLIRKGG
>LFGLNPFC_04469 Zinc/cadmium/lead-transporting P-type ATPase
MSTPDNHGKKAPQFAAFKPLTTVQNTNDCCCDGACSSTPTLSENVSGTRYSWKVSGMDCA
ACARKVENAVRQLAGVNQVQVLFATEKLVVDADNDIRAQVESAVQKAGYSLRDEQASDEP
QESRLKENLPLITLIVMMAISWGLEQFNHPFGQLAFIATTLVGLYPIARQALRLIKSGSY
FAIETLMSIAAIGALFIGATAEAAMVLLLFLIGERLEGWAASRARQGVSALMALKPETAT
RLRNGEREEVAINSLRPGDVIEVAAGGRLPADGKLLSPFASFDESALTGESIPVERATGD
KVPAGATSVDRLVTLEVLSEPGASAIDRILKLIEEAEERRAPIERFIDRFSRIYTPAIMA
VALLVTLVPPLLFAASWQEWIYKGLTLLLIGCPCALVISTPAAITSGLAAAARRGALIKG
GAALEQLGRVTQVAFDKTGTLTVGKPRVTAIHPATGISESELLTLAAAVEQGATHPLAQA
IVREAQVAELAIPTAQSQRALVGSGIEAQVNGERVLICAAGKHPADAFAGLINELESAGQ
TVVLVVRNDDVLGVIALQDTLRADAATAISELNALGVKGVILTGDNPRAAAAIAGELGLE
FKAGLLPEDKVKAVTELNQHAPLAMVGDGINDAPAMKAAAIGIAMGSGTDVALETADAAL
THNHLRGLVQMIELARATHANIRQNITIALGLKGVFLVTTLLGMTGLWLAVLADTGATVL
VTANALRLLRRK
>LFGLNPFC_04470 hypothetical protein
MLWSFIAVCLSAWLSVDASYRGPTWQRWVFKPLTLLLLLLLAWQAPMFDAISYLVLAGLC
ASLLGDTLTLLPRQRLMYAIGAFFLSHLLYTIYFASQMTLSFFWPLPLVLLVLGALLLAI
IWTRLEEYRWPICTFIGMTLVMVWLAGELWFFRPTAPALSAFVGASLLFISNFVWLGSHY
RRRFRADNAIAAACYFAGHFLIVRSLYL
>LFGLNPFC_04471 hypothetical protein
MSKPPLFFIVIIGLIIVAASFRFMQQRREKADNDMAPLQQKLVVVSNKREKPINDRRSRQ
QEVTPAGTSMRYEASFKPQSGGMEQTFRLDAQQYHALTVGDKGTLSYKGTRFVSFVGEQ
>LFGLNPFC_04472 hypothetical protein
MLINIGRLLMLCVWGFLILNLVHPFPRPLNIFVNVALIFTVLMHGMQLALLKSTLPKDGP
QMTTAEKVRIFLFGVFELLVWQKKFKVKK
>LFGLNPFC_04473 Ribosomal RNA small subunit methyltransferase D
MKKPNHSGSGQIRIIGGQWRGRKLPVPDSPGLRPTTDRVRETLFNWLAPVIVDAQCLDCF
AGSGALGLEALSRYAAGATLIEMDRAVSQQLIKNLATLKAGNARVVNSNAMSFLAQKGTP
HNIVFVDPPFRRGLLEETINLLEDNGWLADEALIYVESEVENGLPTVPANWSLHREKVAG
QVAYRLYQREAQGESDAD
>LFGLNPFC_04474 Signal recognition particle receptor FtsY
MAKEKKRGFFSWLGFGQKEQTPEKETEVQNEQPVVEEIVQAQEPVKASEHAVEEQPQAHT
EAEAETFAANVVEVTEQVAESEKAQPEAEVVAQPESVVEETPEPVAIEREELPLPEDVNA
EAVSPEEWQAEAETVEIVEAAEEEAAKEEITDEEPEAQALAAEVAEEAVMVVSPAEEEQP
VEEIAQEQEKPTKEGFFARLKRSLLKTKENLGSGFISLFRGKKIDDDLFEELEEQLLIAD
VGVETTRKIITNLTEGASRKQLRDAEALYGLLKEEMGEILAKVDEPLNVEGKTPFVILMV
GVNGVGKTTTIGKLARQFEQQGKSVMLAAGDTFRAAAVEQLQVWGQRNNIPVIAQHTGAD
SASVIFDAIQAAKARNIDVLIADTAGRLQNKSHLMEELKKIVRVMKKLDVEAPHEVMLTI
DASTGQNGVSQAKLFHEAVGLTGITLTKLDGTAKGGVIFSVADQFGIPIRYIGVGERIED
LRPFKADDFIEALFARED
>LFGLNPFC_04475 Cell division ATP-binding protein FtsE
MIRFEHVSKAYLGGRQALQGVTFHMQPGEMAFLTGHSGAGKSTLLKLICGIERPSAGKIW
FSGHDITRLKNREVPFLRRQIGMIFQDHHLLMDRTVYDNVAIPLIIAGASGDDIRRRVSA
ALDKVGLLDKAKNFPIQLSGGEQQRVGIARAVVNKPAVLLADEPTGNLDDALSEGILRLF
EEFNRVGVTVLMATHDINLISRRSYRMLTLSDGHLHGGVGHE
>LFGLNPFC_04476 Cell division protein FtsX
MNKRDAINHIRQFGGRLDRFRKSVGGSGDGGRNAPKRAKSSPKPVNRKTNVFNEQVRYAF
HGALQDLKSKPFATFLTVMVIAISLTLPSVCYMVYKNVNQAATQYYPSPQITVYLQKTLD
DDAAAGVVAQLQAEQGVEKVNYLSREDALGEFRNWSGFGGALDMLEENPLPAVAVVIPKL
DFQGTESLNTLRDRITQINGIDEVRMDDSWFARLAALTGLVGRVSAMIGVLMVAAVFLVI
GNSVRLSIFARRDSINVQKLIGATDGFILRPFLYGGALLGFSGALLSLILSEILVLRLSS
AVAEVAQVFGTKFDINGLSFDECLLLLLVCSMIGWVAAWLATVQHLRHFTPE
>LFGLNPFC_04477 RNA polymerase sigma factor RpoH

MTDKMQSLALAPVGNLDSYIRAANAWPMLSADEERALAEKLHYHGDLEAAKTLILSHLRF
VVHIARNYAGYGLPQADLIQEGNIGLMKAVRRFNPEVGVRLVSFAVHWIKAEIHEYVLRN
WRIVKVATTKAQRKLFFNLRKTKQRLGWFNQDEVEMVARELGVTSKDVREMESRMAAQDM
TFDLSSDDDSDSQPMAPVLYLQDKSSNFADGIEDDNWEEQAANRLTDAMQGLDERSQDII
RARWLDEDNKSTLQELADRYGVSAERVRQLEKNAMKKLRAAIEA
>LFGLNPFC_04478 4-hydroxy-tetrahydrodipicolinate synthase
MKELKGIITAMVTPFDETEKMDITAAKKMARWLVDNGVHGLFICGTNGEFHLLSDDEKVE
LTKAVVEEVGNEVTICAGAGCCSTKQTIELTKRLVDAGADYISVVTPYYLVPNQEDLYRH
YYDIAFSTTAPIILYNLPGQTGLSIEYETANRLADIKNIVAIKDSSGKFEVQKQYLEIAK
HKNFKVLNGSDSLMLDAFKEGSVAAVAATSNVLPTIEVDLYNYFMAGEMEKAQEERNKMD
ALRMTIKKMTAPAVMKEALNLMGVKAGITRRPIHMPNDAIVEDIKEMLKGYNFL
>LFGLNPFC_04479 Hydroxypyruvate reductase
MKKIIITPRPFVGKGKVYIDKLKSAGYQVECNNSGGRYSKEELIEKIKDANAIITGNDPL
SREVIDQAKNLKVISKYGVGLDNIDVDYANSKDIVVHKALNANSISVAEMTILMMLSSSR
KYVEIESQARNGKDIRLVGYELYQKNLGLIGLGAIGQHVAHIAHSMGMTITAHDPHIDKS
KVPSYIELKSPDEIYQYSDVISLHLPLLDSTRNIINDSVFEKMKSSAILINTARGGLVDE
KSLYTALSNQKIAFASEDIELRERSKELTELKNYSITPHAASFTDEADHNTMQISIKNVL
QELEKE
>LFGLNPFC_04480 PTS system mannose-specific EIID component
MDNIIESGYSESSPITKRDVQIVWLKWHAFSETSLNFERLQALAFCNAMTGVLAKLYPDE
KDLAKALQRHLTMFNTQANWGSVIAGISIALEEKAAQESEEQRESTTQLVTGLKTGLMGP
VSGIGDTLDFGTLRPIVIGVCIPFVIQGYVIAALLPLIYQVSYMFFASRFALNIGYKKGK
ESILEILHSGSIHRIIEGAGMFGLLMMGALSATYVKIKTPLHITTGGGNTIVLQDMLDKI
VPNMLPLIAVLGIYFYIQKCGPHFLRILVTILVLSLAFSFFGLL
>LFGLNPFC_04481 PTS system sorbose-specific EIIC component
MSIFTAAMIALVYWISQAKVWYGFSIMRMPLSIAPIMGLIFNDMPTALSVGATLQMIYIG
SIAPGGNPPADEGLASCIAIPIALTAGIKPEIAISLAIPLGLLGVVLENVRKTLNTTFIH
MADRYAEKGDIKGIQRAATIYPLLLAFPMRFVPVFIACLYGPDAIASFVNLLPAWSTNGL
AIAGNILPALGFAITIIVIGKKQYIPLFIIGFFLVTYSGLNTIGISIFGLCAVLLYMQSQ
NTKGTKNG
>LFGLNPFC_04482 PTS system mannose-specific EIIAB component
MSIVHARIDYRLIHGQVITKWLKRSDANKIIVIDDPLSRDPFLAEVYKMAAPSGVEVIMT
SIEDTLQRWNSNSFYEGKLLILFKSIDSALKTIQGGLMLEELQVGGVENTPGRKIVFNQI
SLNHEDADKLQIIEDKNIKVYFQTIPEEDPASLQKIKNKLP
>LFGLNPFC_04483 PTS system mannose-specific EIIAB component
MNQFNIIILTHGEAGNALLASSEMIVGKTPHTTAISLMPGMSPEALMQQVKTILNPDVET
IIFTDIYGGTPSNIAYLLSREFPIRCISGVNLAMLIEANMLQDSDEEQSFDEYIQHIHDA
GKEMIQTYCFNKG
>LFGLNPFC_04484 Leu/Ile/Val-binding protein
MNIKGKALLAGCIALAFSNMALAEDIKVAVVGAMSGPVAQYGDQEFTGAEQAVADINAKG
GIKGNKLQIVKYDDACDPKQAVAVANKVVNDGIKYVIGHLCSSSTQPASDIYEDEGILMI
TPAATAPELTARGYQLILRTTGLDSDQGPTAAKYILEKVKPQRIAIVHDKQQYGEGLARA
VQDGLKKGNANVVFFDGITAGEKDFSTLVARLKKENIDFVYYGGYHPEMGQILRQARAAG
LKTQFMGPEGVANVSLSNIAGESAEGLLVTKPKNYDQVPANKPIVDAIKAKKQDPSGAFV
WTTYAALQSLQAGLNQSDDPAEIAKYLKANSVDTVMGPLTWDEKGDLKGFEFGVFDWHAN
GTATDAK
>LFGLNPFC_04485 PanD regulatory factor
MKLTIIRLENFSDQDRIDLQKIWPEYSPSSLQVDDNHRIYAARFNERLLAAVRVTLSGTE
GALDSLRVREVTRRRGVGQYLLEEVLRNNPGVSCWWMADAGVEDRGVMTAFMRALGFTAQ
QGGWEKR
>LFGLNPFC_04486 Leucine-specific-binding protein
MKRNAKTIIAGMIALTISHTAMADDIKVAVVGAMSGPIAQWGDMEFNGARQAIKDINAKG
GIKGDKLVGVEYDDACDPKQAVAVANKIVNDGIKYVIGHLCSSSTQPASDIYEDEGILMI
SPGATNPELTQRGYQYIMRTAGLDSSQGPTAAKYILETVKPQRIAIIHDKQQYGEGLARS
VQDGLKAANANVVFFDGITAGEKDFSALIARLKKENIDFVYYGGYYPEMGQMLRQARSVG
LKTQFMGPEGVGNASLSNIAGDAAEGMLVTMPKRYDQDPTNQGIVDALKADKKDPSGPYV
WITYAAVQSLATALERTGSDEPLALVKDLEANGANTVIGPLNWDEKGDLKGFDFGVFQWH
ADGSSTAAK
>LFGLNPFC_04487 High-affinity branched-chain amino acid transport system permease protein LivH
MSEQFLYFLQQMFNGVTLGSTYALIAIGYTMVYGIIGMINFAHGEVYMIGSYVSFMIIAA
LMMLGIDTGWLLVVAGFVGAIVIASAYGWSIERVAYRPVRNSKRLIALISAIGMSIFLQN
YVSLTEGSRDVALPSLFNGQWVVGHSENFSASITTMQAVIWIVTFLAMLALTIFIRYSRM
GRACRACAEDLKMASLLGINTDRVIALTFVIGAAMAAVAGVLLGQFYGVINPYIGFMAGM

KAFTAAVLGGIGSIPGAMIGGLILGIAEALSSAYLSTEYKDVVSFALLILVLLVMPTGIL
GRPEVEKV
>LFGLNPFC_04488 hypothetical protein
MKPMHIAMALLSAAMFFVLAGVFMGVQLELDGTKLVVDTASDIRWQWVFIGTAVVFFFQL
LRPAFQKGLKSVSGPKFILPAIDGSTVKQKLFLVALLVLAVAWPFMVSRGTVDIATLTMI
YIILGLGLNVVVGLSGLLVLGYGGFYAIGAYTFALLNHYYGLGFWTCLPIAGLMAAAAGF
LLGFPVLRLRGDYLAIVTLGFGEIVRILLLNNTEITGGPNGISQIPKPTLFGLEFSRTAR
EGGWDTFSNFFGLKYDPSDRVIFLYLVALLLVVLSLFVINRLLRMPLGRAWEALREDEIA
CRSLGLSPRRIKLTAFTISAAFAGFAGTLFAARQGFVSPESFTFAESAFVLAIVVLGGMG
SQFAVILAAVLLVVSRELMRDFNEYSMLMLGGLMVLMMIWRPQGLLPMTRPQLKLKNGAA
KGEQA
>LFGLNPFC_04489 Lipopolysaccharide export system ATP-binding protein LptB
MSQPLLSVNGLMMRFGGLLAVNNVNLELYPQEIVSLIGPNGAGKTTVFNCLTGFYKPTGG
TILLRDQHLEGLPGQQIARMGVVRTFQHVRLFREMTVIENLLVAQHQQLKTGLFSGLLKT
PSFRRAQSEALDRAATWLERIGLLEHANRQASNLAYGDQRRLEIARCMVTQPEILMLDEP
AAGLNPKETKELDELIAELRNHHNTTILLIEHDMKLVMGISDRIYVVNQGTPLANGTPEQ
IRNNPDVIRAYLGEA
>LFGLNPFC_04490 High-affinity branched-chain amino acid transport ATP-binding protein LivF
MEKVMLSFDKVSAHYGKIQALHEVSLHINQGEIVTLIGANGAGKTTLLGTLCGDPRATSG
RIVFDDKDITDWQTAKIMREAVAIVPEGRRVFSRMTVEENLAMGGFFAERDQFQERIKWV
YELFPRLHERRVQRAGTMSGGEQQMLAIGRALMSNPRLLLLDEPSLGLAPIIIQQIFDTI
EQLREQGMTIFLVEQNANQALKLADRGYVLENGHVVLSDTGDALLANEAVRSAYLGG
>LFGLNPFC_04491 sn-glycerol-3-phosphate-binding periplasmic protein UgpB
MKPLRYTASALALGLALMANAQAVTTIPFWHSMEGELGKEVDSLAQRFNAENPDYKIVPT
YKGNYEQNLSAGIAAFRTGNAPAILQVYEVGTATMMASKAIKPVYDVFKEAGIQFDESQF
VPTVSGYYSDSKTGHLLSQPFNSSTPVLYYNKDAFKKAGLDPEQPPKTWQDLADYAAKLK
ASGMKCGYASGWQGWIQLENFSAWNGLPFASKNNGFDGTDAVLEFNKPEQVKHIAMLEEM
NKKGDFSYVGRKDESTEKFYNGDCAMTTASSGSLANIREYAKFNYGVGMMPYDADAKDAP
QNAIIGGASLWVMQGKDKETYTGVAKFLDFLAKPENAAEWHQKTGYLPITKAAYDLTREQ
GFYEKNPGADIATRQMLNKPPLPFTKGLRLGNMPQIRVIVDEELESVWTGKKTPQQALDT
AVERGNQLLRRFEKSTKS
>LFGLNPFC_04492 sn-glycerol-3-phosphate transport system permease protein UgpA
MSSSRPVFRSRWLPYLLVAPQLIITVIFFIWPAGEALWYSLQSVDPFGFSSQFVGLDNFV
TLFHDSYYLDAFWTTIKFSTFVTVSGLLVSLFFAALVEYIVRGSRFYQTLMLLPYAVAPA
VAAVLWIFLFNPGRGLITHFLAEFGYDWNHAQNSGQAMFLVVFASVWKQISYNFLFFYAA
LQSIPRSLIEAAAIDGAGPIRRFFKIALPLIAPVSFFLLVVNLVYAFFDTFPVIDAATSG
GPVQATTTLIYKIYREGFTGLDLASSAAQSVVLMFLVIVLTVVQFRYVESKVRYQ
>LFGLNPFC_04493 Lactose transport system permease protein LacG
MIENRPWLTIFSHTMLILGIAVILFPLYVAFVAATLDKQEVYAAPMTLIPGTHLLENIHN
IWVNGVGTNSAPFWRMLLNSFVMAFSITLGKITVSMLSAFAIVWFRFPLRNLFFWMIFIT
LMLPVEVRIFPTVEVIANLKMLDSYAGLTLPLMASATATFLFRQFFMTLPDELVEAARID
GASPMRFFCDIVFPLSKTNLAALFVITFIYGWNQYLWPLLIITDVDLGTTVAGIKGMIAT
GEGTTEWNSVMAAMLLTLIPPVVIVLVMQRAFVRGLVDSEK
>LFGLNPFC_04494 sn-glycerol-3-phosphate import ATP-binding protein UgpC
MAGLKLQAVTKSWDGKTQVIKPLTLDVADGEFIVMVGPSGCGKSTLLRMVAGLERVTTGD
IWIDRKRVTEMEPKDRGIAMVFQNYALYPHMSVEENMAWGLKIRGMGKQQIAERVKEAAR
ILELDGLLKRRPRELSGGQRQRVAMGRAIVREPAVFLFDEPLSNLDAKLRVQMRLELQQL
HRRLKTTSLYVTHDQVEAMTLAQRVMVMNGGVAEQIGTPVEVYEKPASLFVASFIGSPAM
NLLAGRVNNEGTHFELDGGITLPLNGGYRQYAGRKMTLGIRPEHIALSSQAEGGVPLVMD
TLEILGADNLAHGRWGEQKLVVRLAHQERPTAGSTLWLHLPENQLHLFDGETGQRV
>LFGLNPFC_04495 Glycerophosphodiester phosphodiesterase, cytoplasmic
MSNWPYPRIVAHRGGGKLAPENTLAAIDVGAKYGHKMIEFDAKLSKDGEIFLLHDDNLER
TSNGWGVAGELNWQDLLRVDAGSWYSKAFKGEPLPLLSQVAERCREHGMMANIEIKPTTG
TGPLTGKTVALAARELWTGMTPPLLSSFEIDALEAAQQAAPELPRGLLLDEWRDDWRELT
ARLGCVSIHLNHKLLDKARVMQLKDAGLRILVYTVNKPQRAAELLRWGVDCICTDAIDVI
GPNFTAQ
>LFGLNPFC_04496 putative protein YhhA
MKRLLLLTALLPFVGFAQPINTLNNPNQPGYQIPSQQRMQTQMQTQQIQQKGMLNQQLKT
QTQLQQQHLENQINNNSQRVLQSQPGERNPARQQMLPNTNGGMLNSNRNPDSSLNQQHML
PERRNGDMLNQPSTPQPDIPLKTIGP
>LFGLNPFC_04497 Glutathione hydrolase proenzyme
MIKPTFLRRVAIAALLTGSCFSTVAAPPVSYGVEEDVFHPVRAKQGMVASVDATATQVGV
DILKEGGNAVDAAVAVGYALAVTHPQAGNLGGGGFMLIRSKNGNTTAIDFREMAPAKATR

DMFLDDQGNPDSKKSLTSHLASGTPGTVAGFSLALDKYGTMPLNKVVQPAFKLARDGFIV
NDALADDLKTYGSEVLPNHENSKAIFWKEGEPLKKGDKLVQANLAKSLEMIAENGPDEFY
KGTIAEQIAQEMQKNGGLISKEDLAAYKAVERTPISGDYRGYQVYSMPPPSSGGIHIVQI
LNILENFDMQKYGFGSADAMQIMAEAEKYAYADRSEYLGDPDFVKVPWQALTNKAYAKSI
ADQIDINKAKPSSEIRPGKLAPYESNQTTHYSVVDKDGNAVAVTYTLNTTFGTGIVAGES
GILLNNQMDDFSAKPGVPNVYGLVGGDANAVGPNKRPLSSMSPTIVVKDGKTWLVTGSPG
GSRIITTVLQMVVNSIDYGMNVAEATNAPRFHHQWLPDELRVEKGFSPDTIKLLEAKGQK
VALKEAMGSTQSIMIGPDGELYGASDPRSVDDLTAGY
>LFGLNPFC_04498 hypothetical protein
MITYHDAFAKANNYLDDANLPVVITLHGRFSQGWYFCFEAREFLETGDEAARLAGNAPFI
VDKGSGEIHSLGTAKPLEEYLQDYEIKKATFGLP
>LFGLNPFC_04499 hypothetical protein
MMTKTQINKLKKMMNDLDYPFEAPLKESFIESIIQIKFNSNSTNCLEKLCNEVSILFNNQ
PDYLTFLRAMDGFEVNGLRLFSLSIPEPSVKNLFAVNEFYRDNDDFINPDLQERLVIGND
SISIFTYDIKSNFFEIRDNIGTENIFSSFSDFSSFLNEIMDSCS
>LFGLNPFC_04500 hypothetical protein
MSNIVYLTVTGEQQGSISAGCGTTESIGNRWQSGHEDEIFTFSLLNSITNTGLGSQFHGI
TFCKLIDKSTPLFINSINNNEQLFMEFDFYRINRFGRWEKYYYIQLRGAFLSAIHHQIFE
NQLDTETITVSYEFILCQHLITNTEFSYLALPENYNRLFLPNSKNKTNNGLKTLNSEAVG
RLLAAGGVYNGNIEGFRDTAEKLGGDAIKGYDQILNEKTAGIAIATASILLTKRSNVDTY
TEINSYLGKLRGQQKLLDSIDIIEII
>LFGLNPFC_04501 L-amino acid N-acetyltransferase AaaT
MSEIVIRHAETRDHEAIRQIHAQPEVYYNTLQVPHPSEQMWLERLTARPGIKQLVACIDG
IVVGHLTIDVQQRPRRSHVADFGICVDSRWKNRGVASALMREMIDMCDNWLRVDRIELTV
FVDNAPAIKVYKKFGFEIEGTGKKYALRNGEYVDAYYMARVK
>LFGLNPFC_04502 putative oxidoreductase YhhX
MVINCAFIGFGKSTTRYHLPYVLNRKDSWHVAHIFRRHAKPEEQAPIYSHIHFTSDLDEV
LNDPDVKLVVVCTHADSHFEYAKRALEAGKNVLVEKPFTPTIAQAKELFALAKSKGLIVT
PYQNRRFDSCFLTAKKAIESGKLGEIVEVESHFDYYRPVAETKPGLPQDGAFYGLGVHTM
DQIISLFGRPDHVAYDIRSLRNKANPDDTFEAQLFYGDLKAIVKTSHLVKIDYPKFIVHG
KKGSFIKYGIDQQETSLKANIMPGEPGFAADDSVGVLEYVNDEGVTVREEMKPEMGDYGR
VYDALYQTITNGAPNYVKESEVLTNLEILERGFEQSSPSAVTLAR
>LFGLNPFC_04503 Quercetin 2,3-dioxygenase
MIYLRKANERGHANHGWLDSWHTFSFANYYDPNFMGFSALRVINDDVIEAGQGFGTHPHK
DMEILTYVLEGTVEHQDSMGNKEQVPAGEFQIMSAGTGIRHSEYNPSSTERLHLYQIWIM
PEENGITPRYEQRRFDAVQGKQLVLSPDARDGSLKVHQDMELYRWALVKDEQSVHQIAAE
RRVWIQVVKGNVTINGVKASTSDGLAIWDEQAISIHADSDSEILLFDLPPV
>LFGLNPFC_04504 HTH-type transcriptional regulator GntR
MKKKRPVLQDVADRVGVTKMTVSRFLRNPEQVSVALRGKIAAALDELGYIPNRAPDILSN
ATSRAIGVLLPSLTNQVFAEVLRGIESVTDAHGYQTMLAHYGYKPEMEQERLESMLSWNI
DGLILTERTHTPRTLKMIEVAGIPVVELMDSKSPCLDIAVGFDNFEAARQMTTAIIARGH
RHIAYLGARLDERTIIKQKGYEQAMLDAGLVPYSVMVEQSSSYSSGIELIRQARREYPQL
DGVFCTNDDLAVGAAFECQRLGLKVPDDMAIAGFHGHDIGQVMEPRLASVLTPRERMGSI
GAERLLARIRGESVIPKMLDLGFTLSPGGSI
>LFGLNPFC_04505 Thermoresistant gluconokinase
MSTTNHDHHIYVLMGVSGSGKSAVASEVAHQLHAAFLDGDFLHPRCNIEKMASGEPLNDD
DRKPWLQALNDAAFAMQRTNKVSLIVCSALKKHYRDLLREGNPNLSFIYLKGDFDVIESR
LKARKGHFFKTQMLVTQFETLQEPGADETDVLVVDIDQPLEGVVASTIEVIKKGK
>LFGLNPFC_04506 Low-affinity gluconate transporter
MTTLTLVLTAVGSVLLLLFLVMKARMHAFLALMVVSMGAGLFSGMPLDKIAATMEKGMGG
TLGFLAVVVALGAMFGKILHETGAVDQIAVKMLKSFGHSRVHYAIGLAGLVCALPLFFEV
AIVLLISVAFSMARHTGTNLVKLVIPLFAGVAAAAAFLVPGPAPMLLASQMNADFGWMIL
IGLCAAIPGMIIAGPLWGNFISRYVELHIPDDISEPHLGEGKMPSFGFSLSLILLPLVLV
GLKTIAARFVPEGSTAYEWFEFIGHPFTAILVACLVAIYGLAMRQGMPKDKVMEICGHAL
QPAGIILLVIGAGGVFKQVLVDSGVGPALGEALTGMGLPIAITCFVLAAAVRIIQGSATV
ACLTAVGLVMPVIEQLNYSGAQMAALSICIAGGSIVVSHVNDAGFWLFGKFTGATEAETL
KTWTMMETILGTVGAIVGMIAFQLLS
>LFGLNPFC_04507 hypothetical protein
MNEIISATVLLILIMDPLGNLPIFMSVLKHTEPKRRRAIMVRELLIALLVMLVFLFAGEK
ILAFLSLRAETVSISGGIILFLIAIKMIFPSASGNSSGLPAGEEPFIVPLAIPLVAGPTI
LATLMLLSHQYPNQMGHLVIALLLAWGGTFVILLQSSLFLRLLGEKGVNALERLMGLILV
MMATQMFLDGIRMWMKG
>LFGLNPFC_04508 Aspartate-semialdehyde dehydrogenase
MKNVGFIGWRGMVGSVLMQRMVEERDFDAIRPVFFSTSQLGQAAPSFGGTTGTLQDAFDL

EALKALDIIVTCQGGDYTNEIYPKLRESGWQGYWIDAASSLRMKDDAIIILDPVNQDVIT
DGLNNGIRTFVGGNCTVSLMLMSLGGLFANDLVDWVSVATYQAASGGGARHMRELLTQMG
HLYGHVADELANPSSAILDIERKVTTLTRSGELPVDNFGVPLAGSLIPWIDKQLDNGQSR
EEWKGQAETNKILNTSSVIPVDGLCVRVGALRCHSQAFTIKLKKDVSIPTVEELLAAHNP
WAKVVPNDREITMRELTPAAVTGTLTTPVGRLRKLNMGPEFLSAFTVGDQLLWGAAEPLR
RMLRQLA
>LFGLNPFC_04509 1,4-alpha-glucan branching enzyme GlgB
MSDRIDRDVINALIAGHFADPFSVLGMHKTTAGLEVRALLPDATDVWVIEPKTGRKLAKL
ECLDSRGFFSGVIPRRKNFFRYQLAVVWHGQQNLIDDPYRFGPLIQEMDAWLLSEGTHLR
PYETLGAHADTMDGVTGTRFSVWAPNARRVSVVGQFNYWDGRRHPMRLRKESGIWELFIP
GAHNGQLYKYEMIDANGNLRLKSDPYAFEAQMRPETASLICGLPEKVVQTEERKKANQFD
APISIYEVHLGSWRRHTDNNFWLSYRELADQLVPYAKWMGFTHLELLPINEHPFDGSWGY
QPTGLYAPTRRFGTRDDFRYFIDAAHAAGLNVILDWVPGHFPTDDFALAEFDGTNLYEHS
DPREGYHQDWNTLIYNYGRREVSNFLVGNALYWIERFGIDALRVDAVASMIYRDYSRKEG
EWIPNEFGGRENLEAIEFLRNTNRILGEQVSGAVTMAEESTDFPGVSRPQDMGGLGFWYK
WNLGWMHDTLDYMKLDPIYRQYHHDKLTFGMLYNYTENFVLPLSHDEVVHGKKSILDRMP
GDAWQKFANLRAYYGWMWAFPGKKLLFMGNEFAQGREWNHDASLDWHLLEGGDNWHHGVQ
RLVRDLNHTYRHHKAMHELDFDPYGFEWLVVDDKERSVLIFVRRDKEGNEIIVASNFTPV
PRHDYRFGINQPGKWREILNTDSMHYHGSNAGNGGAVHSDEIASHGRQHSLSLTLPPLAT
IWLVREAE
>LFGLNPFC_04510 Glycogen debranching enzyme
MTQLAIGKPTPLGAHYDGQGVNFTLFSAHAERVELCVFDANGQEHRYDLPGHSGDIWHGY
LPDARPGLRYGYRVHGPWQPAEGHRFNPAKLLIDPCARQIDGEFKDNPLLHAGHNEPDYR
DNASIAPKCVVVVDHYDWEDDAPPRMPWGCTIIYEAHVKGLTYLHPEIPVEIRGTYKALG
HPVMINYLKQLGITALELLPVAQFASEPRLQRMGLSNYWGYNPVAMFALHPAYACSPETA
LDEFRDAIKALHKAGIEVILDIVLNHSAELDLDGPLFSLRGIDNRSYYWIREDGDYHNWT
GCGNTLNLSHPAVVDYASACLRYWVETCHVDGFRFDLAAVMGRTPEFRQDALLFTAIQNC
PVLSQVKLIAEPWDIAPGGYQVGNFPPLFAEWNDHFRDAARRFWLHYDLPLGAFAGRFAA
SSDVFKRNGRLPSAAINLVTAHDGFTLRDCVCFNHKHNEANGEENRDGTNNNYSNNHGKE
GLGGTLDLVERRRDSIHALLTTLLLSQGTPMLLAGDEHGHSQRGNNNAYCQDNQLTWLDW
SQASSGLTAFTAALIHLRKRIPALMENRWWEEGDGNVRWLNRYAQPLSTDEWQNGPKQLQ
ILLSDRFLIAINATLEVTEIVLPAGEWHAIPPFAGEDNPVITAVWQGPAHGLCVFQR
>LFGLNPFC_04511 Glucose-1-phosphate adenylyltransferase
MVSLEKNDHLMLARQLPLKSVALILAGGRGTRLKDLTNKRAKPAVHFGGKFRIIDFALSN
CINSGIRRMGVITQYQSHTLVQHIQRGWSFFNEEMNEFVDLLPAQQRMKGENWYRGTADA
VTQNLDIIRRYKAEYVVILAGDHIYKQDYSRMLIDHVEKGARCTVACMPVPIEEASAFGV
MAVDENDKIIEFVEKPANPPSMPNDPGKSLASMGIYVFDADYLYELLEEDDRDENSSHDF
GKDLIPKITEAGLAYAHPFPLSCVQSDPDAEPYWRDVGTLEAYWKANLDLASVVPELDMY
DRNWPIRTYNESLPPAKFVQDRSGSHGMTLNSLVSGGCVISGSVVVQSVLFSRVRVNSFC
NIDSAVLLPEVWVGRSCRLRRCVIDRACVIPEGMVIGENAEEDARRFYRSEEGIVLVTRE
MLRKLGHKQER
>LFGLNPFC_04512 Glycogen synthase
MQVLHVCSEMFPLLKTGGLADVIGALPAAQIADGVDARVLLPAFPDIRRGVTDAQVVSRR
DTFAGHITLLFGHYNGVGIYLIDAPHLYDRPGSPYHDTNLFAYTDNVLRFALLGWVGAEM
ASGLDPFWRPDVVHAHDWHAGLAPAYLAARGRPAKSVFTVHNLAYQGMFYAHHMNDIQLP
WSFFNIHGLEFNGQISFLKAGLYYADHITAVSPTYAREITEPQFAYGIEGLLQQRHREGR
LSGVLNGVDEKIWSPETDLLLASRYTRDTLEDKAENKRQLQIAMGLKVDDKVPLFAVVSR
LTSQKGLDLVLEALPGLLEQGGQLALLGAGDPVLQEGFLAAAAEYPGQVGVQIGYHEAFS
HRIMGGADVILVPSRFEPCGLTQLYGLKYGTLPLVRRTGGLADTVSDCSLENLADGVASG
FVFEDSNAWSLLRAIRRAFVLWSRPSLWRFVQRQAMAMDFSWQVAAKSYRELYYRLK
>LFGLNPFC_04513 Maltodextrin phosphorylase
MNAPFTYSSPTLSVEALKHSIAYKLMFTIGKDPVVANKHEWLNATLFAVRDRLVERWLRS
NRAQLSQETRQVYYLSMEFLIGRTLSNAMLSLGIYEDVQGALEAMGLNLEELIDEENDPG
LGNGGLGRLAACFLDSLATLGLPGRGYGIRYDYGMFKQNIVNGSQKESPDYWLEYGNPWE
FKRHNTRYKVRFGGRIQQEGKKTRWIETEEILGVAYDQIIPGYDTDATNTLRLWSAQASS
EINLGKFNQGDYFAAVEDKNHSENVSRVLYPDDSTYSGRELRLRQEYFLVSSTIQDILSR
HYQLHKTYDNLADKIAIHLNDTHPVLSIPEMMRLLIDEHQFSWDDAFEVCCQVFSYTNHT
LMSEALETWPVDMLGKILPRHLQIIFEINDYFLKTLQEQYPNDTDLLGRASIIDESNGRR
VRMAWLAVVVSHKVNGVSELHSNLMVQSLFADFAKIFPGRFTNVTNGVTPRRWLAVANPS
LSAVLDEHLGRNWRTDLSLLNDLQQHCDFPMVNHAVHQAKLENKKRLAEYIAQQLNVVVN
PKALFDVQIKRIHEYKRQLMNVLHVITRYNRIKADPDAKWVPRVNIFGGKAASAYYMAKH
IIHLINDVAKVINNDPQIGDKLKVVFIPNYSVSLAQLIIPAADLSEQISLAGTEASGTSN
MKFALNGALTIGTLDGANVEMLDHVGADNIFIFGNTAEEVEELRRQGYKPREYYEKDEEL
HQVLTQIGSGVFSPEDPGRYRDLVDSLINFGDHYQVLADYRSYVDCQDKVDELYERQEEW

TAKAMLNIANMGYFSSDRTIKEYADHIWHIDPVRL
>LFGLNPFC_04514 hypothetical protein
MKFNLSNLSAVLLASGMLMSTAVTAAPGDATQFGGADTDWSTVDYPRLTDMDDNVDSMGG
KIRFTGRVVKATCKVATDSKQIEVVLPVVPSNLFTGIDVEAQGVSNQTDFNINLTECSNT
DDQKIEFRFTGTADSANKTLANEVEGSTDADNSGNAGATGVGIRIYSKGTTNNGLINLNT
TAAEGSASTAAYTIPGNATTHDFSAAFTAGYAQNGSTVAPGVVKSTASFVVLYE
>LFGLNPFC_04515 Chaperone protein FimC
MKCIIGNKKWVCGFRDELKVSQNIIRSGILFLLSILFINNASAGGFGLGVTRLIYPAESK
QITLSVQNSGDNTSYLIQSWVDDSKTEKKSQDFVITPPLFMLPTRKEASLRIMFLGKTNL
PTDRETLYWMNVKAIPPTDEKNTQKNTLQLALQNKIKLFYRPENLPVQPGKARNMLRFKY
EGKQLKVINPSPYYLTLTGIKVQGSKLPNMFVPPKSDITVSSPVTLAGEITYQTINDFGA
TTERQKGIMQ
>LFGLNPFC_04516 putative outer membrane usher protein ElfC
MNLKLKRCEYWMAAQKQMKRVVPLLLVIMPACSIAGMRFNPAFLSGDTEAVADLSRFEKG
MTYLPGSYEVEVWVNDSPLLSRTVTFKADDANQLIPCLSLADLLSLGINKNALPEQALAS
SENSCLDLRIWFPDVHYMPELDAQRLKLTFPQAIIKRDARGYIPPEQWDNGITAFLLNYD
FSGNNDRGDYSSNNYYLNLRAGINIGAWRFRDYSTWSRGSNSAGKLEHISSTLQRVIIPF
RSELTLGDTWSSSDVFDSVSIRGIKLESDENMLPDSQSGFAPTVRGIAKSRAQVTIKQNG
YVIYQTYMPPGPFEISDLNPTSSAGDLEVTIKESDNSETVYTVPYAAVPILQREGHSKYS
TTVGQYRSNSYNQKSPYIFQGELIWGLPWDITAYGGAQFSEDYRALALGLGLNLGVFGAT
SFDVTQANSSLVDGSKHQGQSYRFLYSKSLVQTGTAFHIIGYRYSTQGFYTLSDTTYQQM
SGTVVDPKTLDDKDYVYNWNDFYNLRYSKRGKFQASVSQPFGNYGSMYLSASQQTYWNTD
KKDSLYQVGYNTSIKGIYLNVAWNYSKSPGTNADKIVSLNVSLPISNWLSSTNDGRSSSN
AMTATYGYSQDNHGQVNQYTGVSGSLLEQHNLSYNIQHGFANQDNSSSGSVGVNYRGAYG
SLNSAYSYDNEGNQQINYGISGALVVHENGLTLSQPLGETNVLIKAPGANNVDVQRGTGI
STDWRGYAVVPYATEYRRNNISLDPMSMNMHTELDITSTEVIPGKGALVRAEFAAHIGIR
GLFTVRYRNKSVPFGATASAQIKNSSQITGIVGDNGQLYLSGLPLEGVINIQWGDGVQQK
CQANYKLPETELDNPVSYATLECR
>LFGLNPFC_04517 putative major fimbrial subunit LpfA
MNLIFSKYSVFFILFAFFAFPLSGQANDSVVVTFTGILLAKSCDITTGSKDQSVKMGTYD
ANEFLNVGDVSPSQTFTINVQGCPTAKSTVYSSGVSANVRFTGDADAINSALLRLSAGAD
SATGLGIEILDNNDVAIAINGESGFRDLVLNENGDANLTFKLRYKSTQENVHAGQANALL
YFDIDYQ
>LFGLNPFC_04518 putative fimbrial-like protein YcbV
MPGKQMLCCILISIISEGDMKIFISLFLFIISTNSFADDITHAGVVRIEGLITEKTCIIS
DESKNFTVNMPDVPSSSVRSAGDVTEKVYFSITLTRCGSDVGNAYIKFTGNTVSEDASLY
KLEDGSVEGLALTIFDKNKGSISNDVKSMGFSLTSSVDNILHFFAAYKALKNNVQPGDAN
ASVSFIVTYD
>LFGLNPFC_04519 putative fimbrial chaperone YcbF
MRQYRLLSPMIKVVIVLFSLLFSTAQAGLVIGGTRFAYPENQSSISVELKNTSDRDMLVK
VAVSPDDARQLTGTVESLPSYSETVFIATPPLFVLKPDKNTKIRINRVGGSLSADRESLF
ILNVAALPTLENSHSVKTDNQLQIAVRNRMKIFYRPSNLSEDPNMSYQKLRWARKNEIVT
VYNPGPRYVTLYNLHVDGKVIDGGMVAPFSHRQQSWCKSQGVCEIAWQTLDVYNNVLPAW
KVKMNLLQMDVSGLQVKTD
>LFGLNPFC_04520 putative minor fimbrial subunit LpfD
MITYTGYKLKVIFIIFLFLGYHVVAYAEMGRDREFCYPGSPENNTTPAVFYYNFGTTIIS
DINNNIPGTILPEKNWKIGLYKAYCNSISNYEVYFSGVSGIDPSGTSGIQQGSDIFVPVT
HEISISTHVKLYNKNGTQTDKTVPFYNYSTNYPTDRSKPSNWSSGTEGYIKIKLDKKIIS
DISLNNVLLVSLYASQRQTEHGPVPLFNAWISNLDIQVPQGCTINEVRVLLLICRMCGPV
N
>LFGLNPFC_04521 putative fimbrial-like protein SfmH
MPDVWASELSRAGAGAKPAGVTPVATTIPINCTNKDTDAVMTLVFDGNISATRDTNGKQS
IIQAQDNPDVGIMIMDSQQNSVDLNALATSVGVPFRLVENQTASAQTADVTFLTLPVSTT
GKVPAAGRYNALAILRVEYQ
>LFGLNPFC_04522 hypothetical protein
MELVDTLFANFSGTDPFTGVDITIAHCKSTYWDEGIVQQLINQVLDEGEKFAGAAGLEGL
SRYDVTLNIGLTSSNVWPGFSLDTATISRLCACGADFGFALYISDVPDVQCDLNTTNDFT
VQFTAMLNPDERVIIAKRPLKKCDAWIEDVYIFQVFKEAWQFQNDNSLRGFRDKQAELKL
YARHYSVENCTEESCWDCNYCIRPSFSLSRSAIIRLNAANARFVYQPFTRDQRARG
>LFGLNPFC_04523 hypothetical protein
MPPTPAMQALIEQIYHIFRRYPAPKQFVVCCEYCLSQQEQKALRNTSLRAIPYSLINAWN
SSPGPDPQNSDEVRYFLPRLLEFVAQGQFDNIHEVFSLRRINLASKENWREDEREILQQF
ACQYMTDWVSGDEAVELQYKLEMFFRADIALSPLLDAIISVPGFWSAASLACLLNTYRDG
YIRDNQDDIDKAITAQINAWAFNNQSILKERARQAIENPLKQPEQGTQYQIWEDDWMIDE

CLCAMYDASSESPGK
>LFGLNPFC_04524 hypothetical protein
MIKFRLYIPPVILGFVIVPLLVWPTVIALAVLIFTLTFLAEIIFSFPLLVVRISLQELQL
ELLVEYALFFSVMGGIGWQFSRRTPPELKNRLHCWLVFSPVYFWLILSNFILYISPEKSA
LLENIRNFFLTFVWLPLNFSPFWPQPWTDFVGPISAQLGFALGYYCQWRSKNRSHRKKWG
DWVTCLSLAILALGPLFNYLQ
>LFGLNPFC_04525 Aerobic glycerol-3-phosphate dehydrogenase
METKDLIVIGGGINGAGIAADAAGRGLSVLMLEAQDLACATSSASSKLIHGGLRYLEHYE
FRLVSEALAEREVLLKMAPHIAFPMRFRLPHRPHLRPAWMIRIGLFMYDHLGKRTSLPGS
TGLRFGANSVLKPEIKRGFEYSDCWVDDARLVLANAQMVVRKGGEVLTRTRATSARRENG
LWIVEAEDIDTGKKYTWQARGLVNATGPWVKQFFDDGMHLPSPYGIRLIKGSHIVVPRVH
TQKQAYILQNEDKRIVFVIPWMDEFSIIGTTDVEYKGDPKAVKIEESEINYLLKVYNTHF
KKQLSRDDIIWTYSGVRPLCDDESDSPQAITRDYTLDIHDEDGKAPLLSVFGGKLTTYRK
LAEHALEKLTPYYQGIGPAWTKESVLPGGAIEGDRDDYAARLRRRYPFLTESLARHYART
YGSNSELLLGNAGAISDLGENFGHEFYEAELKYLVDHEWVRRADDALWRRTKQGMWLNAD
QQSRVSQWLMEYTQQRLSLAS
>LFGLNPFC_04526 Thiosulfate sulfurtransferase GlpE
MDQFECINVADAHQKLQEKEAVLVDIRDPQSFAMGHATQAFHLTNDTLGAFMRDNDFDTP
VMVMCYHGNSSKGAAQYLLQQGYDVVYSIDGGFEAWQRQFPAEVAYGA
>LFGLNPFC_04527 Rhomboid protease GlpG
MLMITSFANPRVAQAFVDYMATQGVILTIQQHNQSDVWLADESQAERVRAELARFLENPA
DPRYLAASWQAGHTGSGLHYRRYPFFAALRERAGPVTWVMMIACVVVFIAMQILGDQEVM
LWLAWPFDPTLKFEFWRYFTHALMHFSLMHILFNLLWWWYLGGAVEKRLGSGKLIVITLI
SALLSGYVQQKFSGPWFGGLSGVVYALMGYVWLRGERDPQSGIYLQRGLIIFALIWIVAG
WFDLFGMSMANGAHIAGLAVGLAMAFVDSLNARKRK
>LFGLNPFC_04528 Glycerol-3-phosphate regulon repressor
MKQTQRHNGIIELVKQQGYVSTEELVEHFSVSPQTIRRDLNELAEQNLILRHHGGAALPS
SSVNTPWHDRKATQTEEKERIARKVAEQIPNGSTLFIDIGTTPEAVAHALLNHSNLRIVT
NNLNVANTLMVKEDFRIILAGGELRSRDGGIIGEATLDFISQFRLDFGILGISGIDSDGS
LLEFDYHEVRTKRAIIENSRHVMLVVDHSKFGRNAMVNMGSISMVDAVYTDTMPPASVMQ
VLKDHHIQLELC
>LFGLNPFC_04529 Anaerobic nitric oxide reductase transcription regulator NorR
MRKTVAFGFVGTVLDYAGRGSQRWSKWRPTLCLCQQESLVIDRLELLHDARSRSLFETLK
RDIASVSPETEVVGVEIELHNPWDFEEVYACLHDFARGYAFQADKEDYLIHITTGTHVAQ
ICWFLLAEARYLPARLIQSSPPRKKERPDSPGAVTIIDLDLSRYNAIASRFAEERQQTLD
FLKSGIATRNSHFNRMIEQIEKVAIKSRAPILLNGPTGAGKSFLARRIFELKQARHQFSG
AFVEVNCATLRGDTAMSTLFGHVKGAFTGARESREGLLRSANGGMLFLDEIGELGADEQA
MLLKAIEEKTFYPFGSDRQVSSDFQLIAGTVRDLRQLVAEGKFREDLYARINLWTFTLPG
LRQRQEDIEPNLDYEVERHASLTGDSVRFNTEARRAWLAFATSPQATWRGNFRELSASVT
RMATFATSGRITLDTVEDEINRLRYNWQESRPSTLTALLGAEAENIDLFDRMQLEHVIAI
CRQAKSLSAAGRELFDISRQGKASVNDADRLRKYLARFGLTWEAMQDQHSSS
>LFGLNPFC_04530 RNA-splicing ligase RtcB
MNYELLTTENAPVKMWTKGVPVEADARQQLINTAKMPFIFKHIAVMPDVHLGKGSTIGSV
IPTKGAIIPAAVGVDIGCGMNALRTALTAADLPENLAELRQAIETAVPHGRTTGRCKRDK
GAWENPPVNVDAKWAELEAGYQWLTQKYPRFLNTNNYKHLGTLGTGNHFIEICLDESDQV
WIMLHSGSRGIGNAIGTYFIDLAQKEMQETLETLPSRDLAYFMEGTEYFDDYLKAVAWAQ
LFASLNRDAMMENVVTALQSVTQKTVKQPQTLAMEEINCHHNYVQKEQHFGEEIYVTRKG
AVSARAGQYGIIPGSMGAKSFIVRGLGNEESFCSCSHGAGRVMSRTKAKKLFSVEDQIRA
TAHVECRKDAEVIDEIPMAYKDIDAVMAAQSDLVEVIYTLRQVVCVKG
>LFGLNPFC_04531 RNA 3'-terminal phosphate cyclase
MKRMIALDGAQGEGGGQILRSALSLSMITGQPFTITGIRAGRAKPGLLRQHLTAVKAAAE
ICRATVEGAELGSQRLVFRPGTVRGGDYRFAIGSAGSCTLVLQTVLPALWFADGPSRVEV
SGGTDNPSAPPADFIRRVLEPLLAKIGVHQQTTLLRHGFYPAGGGVVATEVSPVASFNSL
QLGERGNIVQMRGEVLLAGVPRHVAEREIATLAGSFSLHEQNIHNLPRDQGPGNTVSLEV
ESENITERFFVVGEKRVSAEVVAAQLVKEVKRYLASPAAVGEYLADQLVLPMALAGTGEF
TVAHPSCHLLTNIAVVERFLPVRFGLIETDGVTRVSIE
>LFGLNPFC_04532 HTH-type transcriptional regulator MalT
MLIPSKLSRPVRLDHTVVRERLLAKLSGANNFRLALITSPAGYGKTTLISQWAAGKNDIG
WYSLDEGDNQQERFASYLIAAVQQATNGHCAICETMAQKRQYASLTSLFAQLFIELAEWH
SPLYLVIDDYHLITNPVIHESMRFFIRHQPENLTLVVLSRNLPQLGIANLRVRDQLLEIG
SQQLAFTHQEAKQFFDCRLSSPIEAAESSRICDDVSGWATALQLIALSARQNTHSAHKSA
RRLAGINASHLSDYLVDEVLDNVDLATRHFLLKSAILRSMNDALITRVTGEENGQMRLEE
IERQGLFLQRMDDTGEWFCYHPLFGNFLRQRCQWELAAELPEIHRAAAESWMAQGFPSEA
IHHALAAGDALMLRDILLNHAWSLFNHSELSLLEESLKALPWDSLLENPQLVLLQAWLMQ

SQHRYGEVNTLLARAEHEIKDIREGTMHAEFNALRAQVAINDGNPDEAERLAKLALEELP
PGWFYSRIVATSVLGEVLHCKGELTRSLALMQQTEQMARQHDVWHYALWSLIQQSEILFA
QGFLQTAWETQEKAFQLINEQHLEQLPMHEFLVRIRAQLLWAWARLDEAEASARSGIEVL
SSYQPQQQLQCLAMLIQCSLARGDLDNARSQLNRLENLLGNGKYHSDWISNANKVRVIYW
QMTGDKAAAANWLRHTAKPEFANNHFLQGQWRNIARAQILLGEFESAEIVLEELNENARS
LRLMSDLNRNLLLLNQLYWQAGRKSDAQRVLLDALKLANRTGFISHFVIEGEAMAQQLRQ
LIQLNTLPELEQHRAQRILREINQHHRHKFAHFDENFVERLLNHPEVPELIRTSPLTQRE
WQVLGLIYSGYSNEQIAGELEVAATTIKTHIRNLYQKLGVAHRQAAVQHAQKLLKMMGYG
V
>LFGLNPFC_04533 Maltodextrin phosphorylase
MSQPIFNDKQFQEALSRQWQRYGLNSAAEMTPRQWWLAVSEALAEMLRAQPFAKPVANQR
HVNYISMEFLIGRLTGNNLLNLGWYQDVQDSLKAYDINLTDLLEEEIDPALGNGGLGRLA
ACFLDSMATVGQSATGYGLNYQYGLFRQSFVDGKQVEAPDDWHRGNYPWFRHNEALDVQV
GIGGKVTKDGRWEPEFTITGQAWDLPVVGYRNGVAQPLRLWQATHAHPFDLTKFNDGDFL
RAEQQGINAEKLTKVLYPNDNHTAGKKLRLMQQYFQCACSVADILRRHHLAGRKLHELAE
YEVIQLNDTHPTIAIPELLRVLIDEHQMSWDDAWAITSKTFAYTNHTLMPEALERWDVKL
VKGLLPRHMQIINEINTRFKTLVEKTWPGDEKVWAKLAVVHDKQVHMANLCVVGGFAVNG
VAALHSDLVVKDLFPEYHQLWPNKFHNVTNGITPRRWIKQCNPALAALLDKSLKKEWTND
LDQLINLEKFADDAKFRQQYREIKQANKVRLAEFVKVRTGIEINPQAIFDIQIKRLHEYK
RQHLNLLHILALYKEIRENPQADRVPRVFLFGAKAAPGYYLAKNIIFAINKVADVINNDP
QVGDKLKVVFLPDYCVSAAEKLIPAADISEQISTAGKEASGTGNMKLALNGALTVGTLDG
ANVEIAEKVGEENIFIFGHTVEQVKAILAKGYDPVKWRKKDKVLDAVLKELESGKYSDGD
KHAFDQMLHSIGKQGGDPYLVMADFAAYVEAQKQVDVLYRDQEAWTRAAILNTARCGMFS
SDRSIRDYQARIWQAKR
>LFGLNPFC_04534 4-alpha-glucanotransferase
MESKRLDNAALAAGISPNYINAHGKPQSISAETKRRLLDAMHQRTATKVAVTPVPNVMVY
TSGKKMPMVVEGSGEYSWLLTTEEGTQYKGHVTGGKAFNLPTKLPEGYHTLTLTQDDQRA
HCRVIVAPKRCYEPQALLNKQKLWGACVQLYTLRSEKNWGIGDFGDLKAMLEDVAKRGGS
FIGLNPIHALYPANPESASPYSPSSRRWLNVIYIDVNAVEDFHLSEEAQAWWQLPTTQQT
LQQARDADWVDYSTVTTLKMTALRMAWKGFAQRDDEQMTAFRQFVAEQGDSLFWQAAFDA
LHAQQVKEDEMRWGWPAWPEMYQNVDSPEVRQFCEEHRDDVDFYLWLQWLAYSQFAACWE
ISQGYEMPIGLYRDLAVGVAEGGAETWCDRELYCLKASVGAPPDILGPLGQNWGLPPMDP
HIITARAYEPFIELLRANMQNCGALRIDHVMSMLRLWWIPYGETADQGAYVHYPVDDLLS
ILALESKRHRCMVIGEDLGTVPVEIVGKLRSSGVYSYKVLYFENDHEKTFRSPKAYPEQS
MAVAATHDLPTLRGYWESGDLTLGKTLGLYPDEVVLRGLYQDRELAKQGLLDALHKYGCL
PKRAGHKASLMSMTPTLNRGLQRYIADSNSALLGLQPEDWLDMAEPVNIPGTSYQYKNWR
RKLSATLETMFADDGVNKLLKDLDRRRRAAAKKK
>LFGLNPFC_04535 High-affinity gluconate transporter
MPLVIVAIGVILLLLLMIRFKMNGFIALVLVALAVGLMQGMPLDKVIGSIKAGVGGTLGS
LALIMGFGAMLGKMLADCGGAQRIATTLIAKFGKKHIQWAVVLTGFTVGFALFYEVGFVL
MLPLVFTIAASANIPLLYVGVPMAAALSVTHGFLPPHPGPTAIATIFNADMGKTLLYGTI
LAIPTVILAGPVYARVLKGIDKPIPEGLYSAKTFSEEEMPSFGVSVWTSLVPVVLMAMRA
IAEMILPKGHAFLPVAEFLGDPVMATLIAVLIAMFTFGLNRGRSMDQINDTLVSSIKIIA
MMLLIIGGGGAFKQVLVDSGVDKYIASMMHETNISPLLMAWSIAAVLRIALGSATVAAIT
AGGIAAPLIATTGVSPELMVIAVGSGSVIFSHVNDPGFWLFKEYFNLTIGETIKSWSMLE
TIISVCGLVGCLLLNMVI
>LFGLNPFC_04536 Fe/S biogenesis protein NfuA
MIRISDAAQAHFAKLLANQEEGTQIRVFVINPGTPNAECGVSYCPPDAVEATDTALKFDL
LTAYVDELSAPYLEDAEIDFVTDQLGSQLTLKAPNAKMRKVADDAPLMERVEYMLQSQIN
PQLAGHGGRVSLMEITEDGYAILQFGGGCNGCSMVDVTLKEGIEKQLLNEFPELKGVRDL
TEHQRGEHSYY
>LFGLNPFC_04537 Putative ribose-phosphate pyrophosphokinase
MLTVPGLCWLCRMPLALGHWGICSVCSRAARTDKTLCPQCGLPATHSHLPCGRCLQKPPP
WQRLVTVADYAPPLSPLIHQLKFSRRSEIASALSRLLLLEVLHARRTTGLQLPDRIISVP
LWQRRHWRRGFNQSDLLCQPLSRWLHCRWDSEAVTRTRATATQHFLSARLRKRNLKNAFR
LELPVQGRHMVIVDDVVTTGSTVAEIAQLLLRNGAATVQVWCLCRTL
>LFGLNPFC_04538 Pimeloyl-[acyl-carrier protein] methyl ester esterase
MNNIWWQTKGQGNVHLVLLHGWGLNAEVWRCIDEELSSHFTLHLVDLPGFGRSRGFGALS
LADMAEAVLRQAPDKAIWLGWSLGGLVASQIALTHPERVQALVTVASSPCFSARDEWPGI
KPDVLAGFQQQLSDDFQRTVERFLALQTMGTETARQDARALKKTVLALPMPEVDVLNGGL
EILKTVDLRLPLQNVSMPFLRLYGYLDGLVPRKVVPMLDKLWPHSESYIFAKAAHAPFIS
HPVEFHHLLVALKQRV
>LFGLNPFC_04539 hypothetical protein
MTDAEREYLIIYLIGYLLGRRLEALDIAKRML

>LFGLNPFC_04540 ISNCY family transposase ISSen7
MSKKQSSTPHDALFKLFLRQPETARDFLAFHLPAPIHALCDMKTLKLESSSFIDDDLRES
YSDVLWSVKTEQGPGYIYCLIEHQSTSNKLIAFRMMRYAIAAMQNHLDAGYKTLPMVVPL
LFYHGIESPYPYSLCWLDCFADLNLARQLYASAFPLIDVTVMPDDEIMQHRRMALLELIQ
KHIRQRDLMGLVEQMACLLSSGYANDRQIKGLFNYILQTGDAVRFNDFIDGVAEHSPKHK
ENLMTIAERLRQEGFQKGIRTNALNIAKTMLDAGVSLEDVLRFTSLTVEDLAEINHQ
>LFGLNPFC_04541 putative [Fe-S]-dependent transcriptional repressor FeoC
MASLIQVRDLLALRGRMEAAQISQTLNTPQPMINAMLKQLESMGKAVRIQEEPDGCLSGS
CKSCPEGKACLHEWWALR
>LFGLNPFC_04542 Fe(2+) transporter FeoB
MKKLTIGLIGNPNSGKTTLFNQLTGSRQRVGNWAGVTVERKEGQFSTTDHQVTLVDLPGT
YSLTTISSQTSLDEQIACHYILSGDADLLINVVDASNLERNLYLTLQLLELGIPCIVALN
MLDIAEKQNIRIEIDALSARLGCPVIPLVSTRGRGIEALKLAIDRYKANENVELVHYAQP
LLNEADSLAKVMPSDIPLKQRRWLGLQMLEGDIYSRAYAGEASQHLDAALARLRNEMDDP
ALHIADARYQCIAAICDVVSNTLTAEPSRFTTAVDKIVLNRFLGLPIFLFVMYLMFLLAI
NIGGALQPLFDVGSVALFVHGIQWIGYTLHFPDWLTIFLAQGLGGGINTVLPLVPQIGMM
YLFLSFLEDSGYMARAAFVMDRLMQALGLPGKSFVPLIVGFGCNVPSVMGARTLDAPRER
LMTIMMAPFMSCGARLAIFAVFAAAFFGQNGALAVFSLYMLGIVMAVLTGLMLKYTIMRG
EATPFVMELPVYHVPHVKSLVIQTWQRLKGFVLRAGKVIIIVSIFLSAFNSFSLSGKIVD
NINDSALASVSRVITPVFKPIGVHEDNWQATVGLFTGAMAKEVVVGTLNTLYTAENIQDE
EFNPAEFNLGEELFSAVDETWQSLKDTFSLSVLMNPIEASKGDGEMGTGAMGVMDQKFGS
AAAAYSYLIFVLLYVPCISVMGAIARESSRGWMGFSILWGLNIAYSLATLFYQVASYSQH
PTYSLVCILAVILFNIVVIGLLRRARSRVDVELLATRKSVSSCCAASTTGDCH
>LFGLNPFC_04543 Fe(2+) transport protein A
MQYTPDTAWKITGFSREISPAYRQKLLSLGMLPGSSFNVVRVAPLGDPIHIETRRVSLVL
RKKDLALLEVEAVSC
>LFGLNPFC_04544 Protein YhgF
MMNDSFCRIIAGEIQARPEQVDAAVRLLDEGNTVPFIARYRKEITGGLDDTQLRNLETRL
SYLRELEERRQAILKSISEQGKLTDDLANAINATLSKTELEDLYLPYKPKRRTRGQIAIE
AGLEPLADLLWSDPSHTPEVAAAQYIDADKGVADTKAALDGARYILMERFAEDAALLAKV
RDYLWKNAHLVSTVVSGKEEEGAKFRDYFDHHEPLSTVPSHRALAMFRGRNEGILQLSLN
ADPQFEEPPKESYCEQIIMEHLGLRLNNAPADSWRKGVVSWTWRIKVLMHLETELMGTVR
ERAEDEAINVFARNLHDLLMAAPAGLRATMGLDPGLRTGVKVAVVDATGKLVATDTIYPH
TGQAAKAAMTVAALCEKHNVELVAIGNGTASRETERFYLDVQKQFPKVTAQKVIVSEAGA
SVYSASELAAQEFPDLDVSLRGAVSIARRLQDPLAELVKIDPKSIGVGQYQHDVSQTQLA
RKLDAVVEDCVNAVGVDLNTASVPLLTRVAGLTRMMAQNIVAWRDENGQFQNRQQLLKVS
RLGPKAFEQCAGFLRINHGDNPLDASTVHPEAYPVVERILAATQQALKDLMGNSSELRNL
KASDFTDEKFGVPTVTDIIKELEKPGRDPRPEFKTAQFADGVETMNDLQPGMILEGAVTN
VTNFGAFVDIGVHQDGLVHISSLSNKFVEDPHTVVKAGDIVKVKVLEVDLQRKRIALTMR
LDEQPGETNARRGGGNERPQNNRPAAKPRGREAQPAGNSAMMDALAAAMGKKR
>LFGLNPFC_04545 Transcription elongation factor GreB
MKTPLVTREGYEKLKQELNYLWREERPEVTKKVTWAASLGDRSENADYQYNKKRLREIDR
RVRYLTKCMENLKIVDYSPQQEGKVFFGAWVEIENDDGVTHRFRIVGYDEIFGRKDYISI
DSPMARALLKKEVGDLAVVNTPAGEASWYVNAIEYVKP
>LFGLNPFC_04546 Transcriptional regulatory protein OmpR
MQENYKILVVDDDMRLRALLERYLTEQGFQVRSVANAEQMDRLLTRESFHLMVLDLMLPG
EDGLSICRRLRSQSNPMPIIMVTAKGEEVDRIVGLEIGADDYIPKPFNPRELLARIRAVL
RRQANELPGAPSQEEAVIAFGKFKLNLGTREMFREDEPMPLTSGEFAVLKALVSHPREPL
SRDKLMNLARGREYSAMERSIDVQISRLRRMVEEDPAHPRYIQTVWGLGYVFVPDGSKA
>LFGLNPFC_04547 Osmolarity sensor protein EnvZ
MRRLRFSPRSSFARTLLLIVTLLFVSLVTTYLVVLNFAILPSLQQFNKVLAYEVRMLMTD
KLQLEDGTQLVVPPAFRREIYRELGISLYSNEAAEEAGLRWAQHYEFLSHQMAQQLGGPT
EVRVEVNKSSPVVWLKTWLSPNIWVRVPLTEIHQGDFSPLFRYTLAIMLLAIGGAWLFIR
IQNRPLVDLEHAALQVGKGIIPPPLREYGASEVRSVTRAFNHMAAGVKQLADDRTLLMAG
VSHDLRTPLTRIRLATEMMSEQDGYLAESINKDIEECNAIIEQFIDYLRTGQEMPMEMAD
LNAVLGEVIAAESGYEREIETALYPGSIEVKMHPLSIKRAVANMVVNAARYGNGWIKVSS
GTEPNRAWFQVEDDGPGIAPEQRKHLFQPFVRGDSARTISGTGLGLAIVQRIVDNHNGML
ELGTSERGGLSIRAWLPVPVTRAQGMTKEG
>LFGLNPFC_04548 Phosphoenolpyruvate carboxykinase (ATP)
MRVNNGLTPQELEAYGISDVHDIVYNPSYDLLYQEELDPSLTGYERGVLTNLGAVAVDTG
IFTGRSPKDKYIVRDDTTRDTFWWADKGKGKNDNKPLSPETWQHLKGLVTKQLSGKRLFV
VDAFCGANPDTRLSVRFITEVAWQAHFVKNMFIRPSDEELAGFKPDFIVMNGAKCTNPQW
KEQGLNSENFVAFNLTERMQLIGGTWYGGEMKKGMFSMMNYLLPLKGIASMHCSANVGEK
GDVAVFFGLSGTGKTTLSTDPKRRLIGDDEHGWDDDGVFNFEGGCYAKTIKLSKEAEPEI

YNAIRRDALLENVTVREDGTIDFDDGSKTENTRVSYPIYHIENIVKPVSKAGHATKVIFL
TADAFGVLPPVSRLTADQTQYHFLSGFTAKLAGTERGITEPTPTFSACFGAAFLSLHPTQ
YAEVLVKRMQAAGAQAYLVNTGWNGTGKRISIKDTRAIIDAILNGSLDNAETFTLPMFNL
AIPTELPGVDTKILDPRNTYASPEQWQEKAETLAKLFIDNFDKYTDTPAGAALVAAGPKL
>LFGLNPFC_04549 hypothetical protein
MDNVELSPATRWGMIATGLLQGVVCYLLIAWLAGKNLSWIVYGVPATVAFSSVLLFSVIS
FKQNRLWGWLALVFIATLGMSGWLKWQTDGMTPWRAEKALWDFGCYLLLMAMLLLPWIQQ
SLRIRNDSSRYRYFYQSVWHNVLILLVIFLANGLTWLVLLLWSELFKLVGITFFKTLFFA
TDWFIYLTLGLVTALAVILARTQSRLIDSIQKLFTLIATGLLPLVSLLTLMFIITLPFTG
LSAISRHISAAGLLLTLAFLQLILMAIVLDPQKASLPWTGPLRCLIKTALLVAPLYVFAA
AWALWLRVAQYGWTADRLQGALAVLVLLVWSLGYFVSIVWRKGQNPLDLQGKVNLVVSLL
VLVILVLLNSPVLDSMRISVNSHMARYQSGKNTPDQVTIYMLEQSGRYGRAALESLKSDA
EYMKDPKRARDLLMALDGEQHLQEQVSEKVLADNVLIAPGSGKPDATFWSALIQDRYNVM
TCIEKDACVLVEQDLNSDGQAERILFAFNDDRVIVYGFDSARKEWDALDMSLLPRQITKE
KLLAAAKDGKLGTRPKAWRDLVVDGERLDVNLNE
>LFGLNPFC_04550 33 kDa chaperonin
MPQHDQLHRYLFENFAVRGELVTVSETLQQILENHDYPQPVKNVLAELLVATSLLTATLK
FDGDITVQLQGDGPMNLAVINGNNNQQMRGVARVQGEIPENADLKTLVGNGYVVITITPS
EGERYQGVVGLEGDTLAACLEDYFMRSEQLPTRLFIRTGDVDGKPAAGGMLLQVMPAQNA
QQDDFDHLATLTETIKTEELLTLPANEVLWRLYHEEEVTVYDPQDVEFKCTCSRERCADA
LKTLPDEEVDSILAEDGEIDMHCDYCGNHYLFNAMDIAEIRNNASPADPQVH
>LFGLNPFC_04551 Heat shock protein 15
MKEKPAVEVRLDKWLWAARFYKTRALAREMIEGGKVHYNGQRSKPSKIVELNATLTLRQG
NDERTVIVKAITEQRRPASEAALLYEETAESVEKREKMALARKLNALTMPHPDRRPDKKE
RRDLLRFKHGDSE
>LFGLNPFC_04552 GMP/IMP nucleotidase YrfG
MHINIAWQDVDTVLLDMDGTLLDLAFDNYFWQKLVPETWGAKNGVTPQEAMEYMRQQYHD
VQHTLNWYCLDYWSEQLGLDICAMTTEMGPRAVLREDTIPFLEALKASGKQRILLTNAHP
HNLAVKLEHTGLDAHLDLLLSTHTFGYPKEDQRLWHAVAEATGLKAERTLFIDDSEAILD
AAAQFGIRYCLGVTNPDSGIAEKQYQRHPSLNDYRRLIPSLM
>LFGLNPFC_04553 Putative membrane protein IgaA
MSTIVIFLAALLACSLLAGWLIKVRSRRRQLPWTNAFVDAQTRKLTSEERSAVENYLESL
TQVLQVPGPTGASAAPISLALNAESNNVMMLTHAITRYGISTDDPNKWRYYLDSVEVHLP
PFWEQYINDENTVELIHTDSLPLVISLNGHTLQEYMQEIRGYALQPVPSTQASIRGEESE
QIELLNIRKETHEEYALSRPRGLREALLIVASFLMFFFCLITPDVFVPWLAGGALLLLGA
GLWGLFAPPAKSSLREIHCLRGTPRRWGLFGENDQEQINNISLGIIDLVYPAHWQPYIAQ
DLGQQTDIDIYLDRHVVRQGRYLSLHDEVKNFPLQHWLRSTIIAAGSLLVLFMLLFWIPL
DMPLKFTLSWMKGAQTIEATSVKQLADAGVRVGDTLRISGTGMCNIRTSGTWSAKTNSPF
LPFDCSQIIWNDARSLPLPESELVNKATALTEAVNRQLHPKPEDESRVSASLRSAIQKSG
MVLLDDFGDIVLKTADLCSAKDDCVRLKNALVNLGNSKDWDALVKRANAGKLDGVNVLLR
PVSAESLDNLVATSTAPFITHETARAAQSLNSPAPGGFLIVSDEGSDFVDQPWPSASLYD
YPPQEQWNAFQKLAQMLMHTPFNAEGIVTKIFTDANGTQHIGLHPIPDRSGLWRYLSTTL
LLLTMLGSAIYNGVQAWRRYQRHRTRMMKIQAYYESCLNPQLITPSESLIE
>LFGLNPFC_04554 ADP compounds hydrolase NudE
MSKSLQKPTILNVETVARSRLFTVESVDLEFSNGVRRVYERMRPTNREAVMIVPIVDDHL
ILIREYAVGTESYELGFSKGLIDPGESVFEAANRELKEEVGFGANDLTFLKKLSMAPSYF
SSKMNIVVAQDLYPESLEGDEPEPLPQVRWPLAHMMDLLEAPDFNEARNVSALFLVREWL
KGQGRV
>LFGLNPFC_04555 Penicillin-binding protein 1A
MKFVKYFLILAVCCILLGAGSIYGLYRYIEPQLPDVATLKDVRLQIPMQIYSADGELIAQ
YGEKRRIPVTLDQIPPEMVKAFIATEDSRFYEHHGVDPVGIFRAASVALFSGHASQGAST
ITQQLARNFFLSPERTLMRKIKEVFLAIRIEQLLTKDEILELYLNKIYLGYRAYGVGAAA
QVYFGKTVDQLTLNEMAVIAGLPKAPSTFNPLYSMDRAVARRNVVLSRMLDEGYITQQQF
DQTRTEAINANYHAPEIAFSAPYLSEMVRQEMYNRYGESAYEDGYRIYTTITRKVQQAAQ
QAVRNNVLDYDMRHGYRGPANVLWKVGESAWDNNKITDTLKALPTYGPLLPAAVTSANPQ
EATAMLADGSTVVLSMEGVRWARPYRSDTQQGPTPRKVTDVLQTGQQIWVRQVDDAWWLA
QVPEVNSALVSINPQNGAVMALVGGFDFNQSKFNRATQALRQVGSNIKPFLYTAAMDKGL
TLASMLNDVPISRWDAGAGSDWQPKNSPPQYAGPIRLRQGLGQSKNVVMVRAMRAMGVDY
AAEYLQRFGFPAQNIVHTESLALGSASFTPMQVARGYAVMANGGFLVDPWFISKIENDQG
GVIFEAKPKVACPECDIPVIYGDTQKSNVLENNDVEDVAISREQQNVSVPMPQLEQANQA
LVAKTGAQEYAPHVINTPLAFLIKSALNTNIFGEPGWQGTGWRAGRDLQRHDIGGKTGTT
NSSKDAWFSGYGPGVVTSVWIGFDDHRRNLGHTTASGAIKDQISGYEGGAKSAQPAWDAY
MKAVLEGVPEQPLTPPPGIVTVNIDRSTGQLANGGNSREEYFIEGTQPTQQAVHEVGTTI
IDNGEAQELF

>LFGLNPFC_04556 hypothetical protein
MAFKNWQIGLHLQQQEAVAVAIVRSAKECLLHRWWRLPLENDIIKDGRIVDVQRLANTLL
PWSRELPQRHHIMLAFPASRTLQRSFPRPSMSLGEREQMAWLSGTMARELDMDPDSLRFD
YSEDALSPAYNVTAAQSKELATLLTLAERLRVHVSAITPDASALQRFLPFLPSHQQCLAW
RDNEQWLWATRYSWGRKLAVGMTSAKELAAALSVDPESVALCGEGGFDPWEAVSVRQPPL
PPPGGDFAIALGLALGKVY
>LFGLNPFC_04557 hypothetical protein
MNPPINFLPWRQQRRTAFLRFWLLMFVAPLLLAVGITLILRLTSNAEARVNAVLLQAEQQ
LARSLQITKPRLLERQQLREQRLQRQRQRQFTRDWQSALEALAALLPEHAWLTTISWQQG
TLEIKGLTTSITALNALETSLRQDASFHLNQRGATQQDAQGRWQFEYQLTRKVSDEHVL
>LFGLNPFC_04558 hypothetical protein
MNMFFDWWFATSLRLRQFCWAVWLLVLVTLIFLSLTHHEESDALIRLRANHHQQWAALYR
LVDTTSFSEEKTLPFSPLDFQLSGAQLVYWHPSAQGGELALKTLWEAVPSAFTRLAERNV
SVSRFLLSVEGDDLLFTLQLETPHEG
>LFGLNPFC_04559 hypothetical protein
MRVKRWLLAGIALCLLTGMRDPFKPPEDLCRISELSQWRYQGMVGRGERIIGVIKDGQKK
WRRVQQNDVLENGWTILQLTPDALTLGTGTNCEPPHWLWQRQGDTNEAMDSRTTVDAYTR
RTGGKAAKSDADGG
>LFGLNPFC_04560 Type IV pilus biogenesis and competence protein PilQ
MLIPGVQAAKPQKVTLMVDDVPVAQVLQVLAEQEKLNLVVSPDVSGTVSLHLTDVPWKQA
LQTVVKSAGLITRQEGNILSVHSVAWQNDNIARQEAEQTRAQANLPLENRNITLQYADAG
ELAKAGEKLLSAKGSMTVDKRTNRLLLRDNKTALSTLEQWVSQMDLPVGQVELSAHIVTI
NEKSLRELGVKWTLADAQQAGGVGQVTTLGSDLSVATATTHIGFNIGRINGRLLDLELSA
LEQKQQLDIIASPRLLASHLQPASIKQGSEIPYQVSSGESGATSVEFKEAVLGMEVTPTV
LQKGRIRLKLHISQNVPGQVLQQADGEVLAIDKQEIETQVEVKSGETLALGGIFTRKNKS
GQDSVPLLGDIPWFGQLFRHDGKEDERRELVVFITPRLVSSE
>LFGLNPFC_04561 Shikimate kinase 1
MAEKRNIFLVGPMGAGKSTIGRQLAQQLNMEFYDSDQEIEKRTGADVGWVFDLEGEEGFR
DREEKVINELTEKQGIVLATGGGSVKSRETRNRLSARGVVVYLETTIEKQLARTQRDKKR
PLLHVETPPREVLEALANERNPLYEEIADVTIRTDDQSAKVVANQIIHMLESN
>LFGLNPFC_04562 3-dehydroquinate synthase
MERIVVTLGERSYPITIASGLFNEPASFLPLKSGEQVMLVTNETLAPLYLDKVRGVLEQA
GVNVDSVILPDGEQYKSLAVLDTVFTALLQKPHGRDTTLVALGGGVVGDLTGFAAASYQR
GVRFIQVPTTLLSQVDSSVGGKTAVNHPLGKNMIGAFYQPASVVVDLDCLKTLPPRELAS
GLAEVIKYGIILDGAFFNWLEENLDALLRLDGPAMAYCIRRCCELKAEVVAADERETGLR
ALLNLGHTFGHAIEAEMGYGNWLHGEAVAAGMVMAARTSERLGQFSSAETQRIITLLTRA
GLPVNGPREMSAQAYLPHMLRDKKVLAGEMRLILPLAIGKSEVRSGVSHELVLNAIADCQ
SA
>LFGLNPFC_04563 Cell division protein DamX
MDEFKPEDELKPDPSDRRTGRSRQSSERSERTERGEPQINFDDIELDDTDDRRPTRAQKA
RNEEPEIEEEIDESEDETVDEERVERRPRKRKKAASKPASRQYMMMGVGILVLLLLIIGI
GSALKAPSTSSSDQTASGEKSIDLAGNATDQANGVQPAPGTTSAENTQQDVSLPPISSTP
TQGQTPVATDGQQRVEVQGDLNNALTQPQNQQQLNNVAVNSTLPTEPATVAPVRNGNASR
DTAKTQTAERPATTRPARQQAVIEPKKPQATVKTEPKPVAQTPKRTEPAAPVASTKAPAA
TSTPAPKETATTAPVQTASPAQTTATPAAGGKTAGNVGSLKSAPSSHYTLQLSSSSNYDN
LNGWAKKENLKNYVVYETTRNGQPWYVLVSGVYASKEEAKKAVSTLPADVQAKNPWAKPL
RQVQADLK
>LFGLNPFC_04564 DNA adenine methylase
MKKNRAFLKWAGGKYPLLDDIKRHLPKGECLVEPFVGAGSVFLNTDFSRYILADINSDLI
SLYNIVKMRTDEYVQAARELFVPETNCAEVYYQFREEFNKSQDPFRRAVLFLYLNRYGYN
GLCRYNLRGEFNVPFGRYKKPYFPEAELYHFAEKAQNAFFYCESYADSMARADDASVVYC
DPPYAPLSATANFTAYHTNSFTLEQQAHLAEIAEGLVDRHIPVLISNHDTMLTREWYQRA
KLHVVKVRRSISSNGGTRKKVDELLALYKPGVVSPAKK
>LFGLNPFC_04565 Ribulose-phosphate 3-epimerase
MKQYLIAPSILSADFARLGEDTAKALAAGADVVHFDVMDNHYVPNLTIGPMVLKSLRNYG
ITAPIDVHLMVKPVDRIVPDFAAAGASIITFHPEASEHVDRTLQLIKENGCKAGLVFNPA
TPLSYLDYVMDKLDVILLMSVNPGFGGQSFIPQTLDKLREVRRRIDESGFDIRLEVDGGV
KVNNIGEIAAAGADMFVAGSAIFDQPDYKKVIDEMRSELAKVSHE
>LFGLNPFC_04566 Phosphoglycolate phosphatase
MNKFEDIRGVAFDLDGTLVDSAPGLAAAVDMALYALELPVAGEERVITWIGNGADILMER
ALTWARQERATLRKTMGKPPVDDDIPAEEQVRILRKLFDRYYGEVAEEGTFLFPHVADTL
GALQAKGLPLGLVTNKPTPFVAPLLEALDIAKYFSVVIGGDDVQNKKPHPDPLLLVAERM
GIAPQQMLFVGDSRNDIQAAKAAGCPSVGLTYGYNYGEAIDLSQPDVIYQSLNDLLPALG
LPHSENQESKND

>LFGLNPFC_04567 Tryptophan--tRNA ligase
MTKPIVFSGAQPSGELTIGNYMGALRQWVNMQDDYHCIYCIVDQHAITVRQDAQKLRKAT
LDTLALYLACGIDPEKSTIFVQSHVPEHAQLGWALNCYTYFGELSRMTQFKDKSARYAEN
INAGLFDYPVLMAADILLYQTNLVPVGEDQKQHLELSRDIAQRFNALYGDIFKVPEPFIP
KSGARVMSLLEPTKKMSKSDDNRNNVIGLLEDPKSVVKKIKRAVTDSDEPPVVRYDVQNK
AGVSNLLDILSAVTGQSIPELEKQFEGKMYGHLKGEVADAVSGMLTELQERYHRFRNDEA
FLQQVMKDGAEKASAHASRTLKAVYEAIGFVAKP
>LFGLNPFC_04568 hypothetical protein
MRRTFIKKEGVVITTLARYLLGEKCGNRLKTIDELATECRSSVGLTQAALKTLESSGAIR
IERRGRNGSYLVEMDNKALLTHVDINNVVCAMPLPYTRLYEGLASGLKAQFDGIPFYYAH
MRGADIRVECLLNGVYDMAVVSRLAAESYLTQKGLCLALELGPHTYVGEHQLICRKGESA
NVKRVGLDNRSADQKIMTDVFFGDSDVERVDLSYHESLQRIVKGDVDAVIWNVVAENELT
MLGLEATPLTDDPRFLQATEAVILTRADDYPMQQLLRAVVDKHALLAHQQRVVSGEQEPS
Y
>LFGLNPFC_04569 hypothetical protein
METRLNLLCDAGVIDKDICKGMMQVVNVLETECHLPVRSEQGTMAMTHMASALMRSRRGE
EIEPLDDELLAELAQSSHWQAVVQLHQVLLKEFALEVNPCEEGYLLANLYGLWMAANEEV
>LFGLNPFC_04570 putative protein YhfX
MFVEALKRQNPALISAALSLWQQGKIAPDSWVIDVDQVLENGKRLIETARLYGIELYLMT
KQFGRNPWLAEKLLALGYSGIVAVDYKEARVMRRAGLPVAHQGHLVQIPCHQVSDAVEQG
TDVITVFTLDKAREISAAAVKTGRVQSVLLKVYSDDDFLYPGQESGFVQHSLHEVVAEIQ
NLPGLHLAGLTHFPCLLWDEAAGKVLPTPNLHTLVQARDQLAKSGIAIEQLNAPSATSCT
SLPLLAEYGVTHTEPGHALTGTIPANQQGDQPERIAMLWLSEISHHFRGDSYCYGGGYYR
RGHAQHALVFTPENQRITETYLNAVDDSSIDYTLPLAGEHPVSSAVVLCFRTQIFITRSD
VVLVSGIHRGEPEIVGRYDSLGNPLEA
>LFGLNPFC_04571 Phosphopentomutase
MARFVVLVIDSFGVGAMKDVTLVRPQDAGANTCGHILSQLPHLQLPTLETLGLINALGYA
PGDMQPSDSSTWGVAELQHEGGDTFMGHQEILGTRPLPPLRMPFRDVIDRVEQALVSAGW
QVERRGDDLQFLWVNQAVAIGDNLEADLGQVYNITANLSVISFDDAIKMGRIVREQVQVG
RVITFGGLLPDSQRILDAAESKEGRFIGINAPRSGAYDNGFQVVHMGYGVDEKVQVPQKL
YEAGVPTVLVGKVADIVSNPYGVSWQNLVDSQRIMDITLNEFNTYPTAFICTNIQETDLA
GHAEDVARYAERLQVVDRNLARLVEAMQPDDCLVVMADHGNDPTIGHSHHTREVVPVLVY
QQGLVATQLGVRTTLSDVGATVCEFFRAPPPQNGRSFLSSFRFAGDTL
>LFGLNPFC_04572 Phosphotriesterase homology protein
MSFDPTGYTLAHEHLHIDLSGFKNNVDCRLDQYAFICQEMNDLMARGVRNVIEMTNRYMG
RNVQFMLDVMRETGINVVACTGYYQDAFFPEHVATRSVQELAQEMVDEIEQGIDGTDLKA
GIIAEIGSSEGKITPLEEKVFIAAALAHNQTGRPISTHTSFSTMGLEQLALLQAHGVDLS
RVTVGHCDLKDNLDNILKMIDLGAYVQFDTIGKNSYYPDEKRIAMLHALRDRGLLNRVML
SMDITRRSHLKANGGYGYDFLLTTFIPQLRQSGFSQADVDVMLRENPSQFFQ
>LFGLNPFC_04573 hypothetical protein
MKKIGVAGLQREQIKKTIEATAPGCFEVFIHNDMEAAMKVKSGQLDYYIGACNTGAGAAL
SIAIAVIGYNKSCTIAKPGIKAKDEHIAKMIAEGKVAFGLSVEHVEHAIPMLINHLK
>LFGLNPFC_04574 hypothetical protein
MDLYIQIIVVACLTGMTSLLAHRSAAVFHDGIRPILPQLIEGYMNRREAGSIAFGLSIGF
VASVGISFTLKNGLLNAWLLFLPTDILGVLAINSLMAFGLGAIWGILILTCLLPVNQLLT
ALPVDVLGSLGELSSPVVSAFALFPLVAIFYQFGWKQSLIAAVVVLMTRVVVVRYSPHLN
PESIEIFIGMVMLLGIAITHDLRHRDENDIDASGMSVFEERTSRIIKNLPYIAIVGALIA
AVASMKIFAGSEVSIFTLEKAYSAGVTPEQSQTLINQAALAEFMRGLGFVPLIATTALAT
GVYAVAGFTFVYAVGYLSPNPMVAAVLGAVVISAEVLLLRSIGKWLGRYPSVRNASDNIR
NAMNMLMEVALLVGSIFAAIKMAGYTGFSIAVAIYFLNESLGRPVQKMAAPVVAVMITGI
LLNVLYWLGLFVPA
>LFGLNPFC_04575 putative protein YhfS
MKTFPLQSLTLIEAQQKQFALVDTICRHFPGCEFLTCGDLGLTPGLNQPRITQRVEQVLA
DAFHAQAAALVQGAGTGAIRAALAALLKSGQRLLVHDAPVYPTTRVIIEQMGLTLITADF
NDLSALKQVVDEQQPDAALVQHTRQQPQDRYVLADVLATLRAAGVPALTDDNYAVMKVAR
IGCECGANVSTFSCFKLFGPEGVGAVVGDADVISRIRATLYSGGSQIQGAQALEVLRGLV
LAPVMHAVQAGVSERLLALLNGGAVAEVKSAVIANAQSKVLIVEFHQPIAARVLEEAQKL
GALPYPVGAESKYEIPPLFYRLSGTFRQANPQLEHCAIRINPNRSGEETILRILRESIAS
I
>LFGLNPFC_04576 hypothetical protein
MNKFIKVALVGAVLATLTACTGHIENRDKNCSYDYLLHPAISISKIIGGCGPTAQ
>LFGLNPFC_04577 Siroheme synthase
MDHLPIFCQLRDRDCLIVGGGDVAERKARLLLDAGARLTVNALAFIPQFTAWADAGMLTL
VEGPFDESLLDTCWLAIAATDDDALNQRVSEAAESRRIFCNVVDAPKAASFIMPSIIDRS

PLMVAVSSGGTSPVLARLLREKLESLLPLHLGQVAKYAGQLRGRVKQQFATMGERRRFWE
KLFVNDRLAQSLANNDQKAITETTEQLINEPLDHRGEVVLVGAGPGDAGLLTLKGLQQIQ
QADVVVYDRLVSDDIMNLIRRDADRVFVGKRAGYHCVPQEEINQILLREAQKGKRVVRLK
GGDPFIFGRGGEELETLCNAGIPFSVVPGITAASGCSAYSGIPLTHRDYAQSVRLITGHL
KTGGELDWENLAAEKQTLVFYMGLNQAATIQQKLIEYGMPGEMPVAIVENGTAVTQRVID
GTLTQLGELAQQMNSPSLIIIGRVVGLRDKLNWFSNH
>LFGLNPFC_04578 Nitrite transporter NirC
MFTDTITKCAANAARIARLSANNPLGFWVSSAMAGAYVGLGIILIFTLGNLLDPSVRPLV
MGATFGIALTLVIIAGSELFTGHTMFLTFGVKAGTISHGQMWAILPQTWLGNLVGSVFVA
MLYSWGGGSLLPVDTSIVHSVALAKTTAPAMVLFFKGALCNWLVCLAIWMALRTEGAAKF
IAIWWCLLAFIASGYEHSIANMTLFALSWFGNHSEAYTLAGIGHNLLWVTLGNTLSGAVF
MGLGYWYATPKANRPVADKFNQTETAAG
>LFGLNPFC_04579 Nitrite reductase (NADH) small subunit
MSQWKDICKIDDILPETGVCALLGDEQVAIFRPYHSDQVFAISNIDPFFESSVLSRGLIA
EHQGELWVASPLKKQRFRLSDGLCMEDEQFSVKHYDARVKDGVVQLRG
>LFGLNPFC_04580 Nitrite reductase [NAD(P)H]
MSKVRLAIIGNGMVGHRFIEDLLDKSDAANFDITVFCEEPRIAYDRVHLSSYFSHHTAEE
LSLVREGFYEKHGIKVLVGERAITINRQEKVIHSSAGRTVFYDKLIMATGSYPWIPPIKG
SDTQDCFVYRTIEDLNAIESCARRSKRGAVVGGGLLGLEAAGALKNLGIETHVIEFAPML
MAEQLDQMGGEQLRRKIESMGVRVHTSKNTLEIVQEGVEARKTMRFADGSELEVDFIVFS
TGIRPRDKLATQCGLDVAPRGGIVINDSCQTSDPDIYAIGECASWNNRVFGLVAPGYKMA
QVAVDHILGSENAFEGADLSAKLKLLGVDVGGIGDAHGRTPGARSYVYLDESKEIYKRLI
VSEDNKTLLGAVLVGDTSDYGNLLQLVLNAIELPENPDSLILPAHSGSGKPSIGVDKLPD
SAQICSCFDVTKGDLIAAINKGCHTVAALKAETKAGTGCGGCIPLVTQVLNAELAKQGIE
VNNNLCEHFAYSRQELFHLIRVEGIKTFEELLAKHGKGYGCEVCKPTVGSLLASCWNEYI
LKPEHTPLQDSNDNFLANIQKDGTYSVIPRSPGGEITPEGLMAVGRIAREFNLYTKITGS
QRLAMFGAQKDDLPEIWRQLIEAGFETGHAYAKALRMAKTCVGSTWCRYGVGDSVGLGVE
LENRYKGIRTPHKMKFGVSGCTRECSEAQGKDVGIIATEKGWNLYVCGNGGMKPRHADLL
AADIDRETLIKYLDRFMMFYIRTADKLTRTAPWLENLEGGIDYLKAVIIDDKLGLNAHLE
EEMARLREAVVCEWTETVNTPSAQTRFKHFINSDKRDPNVQMVPEREQHRPATPYERIPV
TLVEDNA
>LFGLNPFC_04581 Protein TsgA
MTNSNRIKLTWISFLSYALTGALVIVTGMVMGNIADYFNLPVSSMSNTFTFLNAGILISI
FLNAWLMEIVPLKTQLRFGFLLMVLAVAGLMFSHSLALFSAAMFILGVVSGITMSIGTFL
VTQMYEGRQRGSRLLFTDSFFSMAGMIFPMIAAFLLARSIEWYWVYACIGLVYVAIFILT
FGCEFPALGKHAPKTDAPVEKEKWGIGVLFLSVAALCYILGQLGFISWVPEYAKGLGMSL
NDAGTLVSNFWMSYMVGMWAFSFILRFFDLQRILTVLAGLAAILMYVFNTGTPAHMAWSI
LALGFFSSAIYTTIITLGSQQTKVPSPKLVNFVLTCGTIGTMLTFVVTGPIVEHSGPQAA
LLTANGLYAVVFVMCFLLGFVSRHRQHNTLTSH
>LFGLNPFC_04582 Peptidyl-prolyl cis-trans isomerase A
MFKSTLAAMAAVFALSALSPAAMAAKGDPHVLLTTSAGNIELELDKQKAPVSVQNFVDYV
NSGFYNNTTFHRVIPGFMIQGGGFTEQMQQKKPNPPIKNEADNGLRNTRGTIAMARTADK
DSATSQFFINVADNAFLDHGQRDFGYAVFGKVVKGMDVADKISQVPTHDVGPYQNVPSKP
VVILSAKVLP
>LFGLNPFC_04583 putative protein YhfG
MKKLTDKQKSRLWELQRNRNFQASRRLEGVEMPLVTLTAAEALARLEALRRHYER
>LFGLNPFC_04584 putative protein adenylyltransferase Fic
MSDKFGEGRDPYLYPGLDIMRNRLNIRQQQRLEQAAYEMTALRAATIELGPLVRGLPHLR
TLHRQLYQDIFDWAGQLREVDIYQGDTPFCHFAYLEKEGNALMQDLEEEKYLTSLEKDKF
VERLAHYYCEINVLHPFRVGSGLAQRIFFEQLAIHAGYQLSWQGIEKEAWNQANQNGAMG
DLTALQMIFSKVVSEAGESE
>LFGLNPFC_04585 Aminodeoxychorismate synthase component 2
MILLIDNYDSFTWNLYQYFCELGADVLVKRNDALTLADIDALKPQKIVISPGPCTPDEAG
ISLDVIRHYAGRLPILGVCLGHQAMAQAFGGKVVRAAKVMHGKTSPITHNGVGVFKGLAN
PLTVTRYHSLVVEPDSLPECFEVTAWSETREIMGIRHRQWDLEGVQFHPESILSEQGHQL
LANFLHR
>LFGLNPFC_04586 Acetylornithine/succinyldiaminopimelate aminotransferase
MAIEQTAITRATFDEVILPIYAPAEFIPVKGQGSRIWDQQGKEYVDFAGGIAVTALGHCH
PALVNALKTQGETLWHISNVFTNEPALRLGRKLIEATFAERVVFMNSGTEANETAFKLAR
HYACVRHSPFKTKIIAFHNAFHGRSLFTVSVGGQPKYSDGFGPKPADIIHVPFNDLHAVK
AVMDDHTCAVVVEPIQGEGGVTAATPEFLQGLRELCDQHQALLVFDEVQCGMGRTGDLFA
YMHYGVTPDILTSAKALGGGFPVSAMLTTAEIASAFHPGSHGSTYGGNPLACAVAGAAFD
IINTPEVLEGIQAKRQRFVDHLQKIDQQYDVFSDIRGMGLLIGAELKPQYKGRARDFLYA
GAEEGVMVLNAGPDVMRFAPSLVVEDADIDEGMHRFAHAVAKVIGA

>LFGLNPFC_04587 hypothetical protein
MWRRLIYHPDINYALRQTLVLCLPVAVGLMLGELRFGLLFSLVPACCNIAGLDTPHKRFF
KRLIIGASLFATCSLLTQVLLAKDVPLPFLLTGLTLVLGVTAELGPLHAKLLPASLLAAI
FTLSLAGYMPVWEPLLIYALGTLWYGLFNWFWFWIWREQPLRESLSLLYRELADYCEAKY
SLLTQHTDPDKALPPLLVRQQKAVDLITQCYQQMHMLSAQNNTDYKRMLRIFQEALDLQE
HISVSLHQPEEVQKLVERSHAEEVIRWNAQTVAARLRVLADDILYHRLPTRFTMEKQIGA
LEKIARQHPDNPVGQFCYWHFSRIARVLRTQKPLYARDLLADKQRRMPLLPALKSYLSLK
SPALRNAGRLSVMLSVASLMGTALHLPKSYWILMTVLLVTQNGYGATRLRIVNRSVGTVV
GLIIAGVTLHFKIPEGYTLTLMLITTLASYLILRKNYGWATVGFTITAVYTLQLLWLNGE
QYILPRLIDTIIGCLIAFGGTVWLWPQWQSGLLRKNAHDALEAYQDAIRLILSEDPQPTP
LAWQRMRVNQAHNTLYNSLNQAMQEPAFNSHYLADMKLWVTHSQFIVEHINAMTTLAREH
RALPPELAQEYLQSCEIAIQRCQQRLEYDEPGSSGDANIMDAPEMQPHEGAAGTLEQHLQ
RVIGHLNTMHTISSMAWRQRPHHGIWLSRKLRDSKA
>LFGLNPFC_04588 cAMP-activated global transcriptional regulator CRP
MVLGKPQTDPTLEWFLSHCHIHKYPSKSTLIHQGEKAETLYYIVKGSVAVLIKDEEGKEM
ILSYLNQGDFIGELGLFEEGQERSAWVRAKTACEVAEISYKKFRQLIQVNPDILMRLSAQ
MARRLQVTSEKVGNLAFLDVTGRIAQTLLNLAKQPDAMTHPDGMQIKITRQEIGQIVGCS
RETVGRILKMLEDQNLISAHGKTIVVYGTR
>LFGLNPFC_04589 Protein YhfA
MQARVKWVEGLTFLGESASGHQILMDGNSGDKAPSPMEMVLMAAGGCSAIDVVSILQKGR
QDVVDCEVKLTSERREEAPRLFTHINLHFIVTGRDLKDAAVARAVDLSAEKYCSVALMLE
KAVNITHSYEVVAA
>LFGLNPFC_04590 Phosphoribulokinase, plasmid
MSAKHPVIAVTGSSGAGTTTTSLAFRKIFAQLNLHAAEVEGDSFHRYTRPEMDMAIRKAR
DAGRHISYFGPEANDFGLLEQTFIEYGQSGKGKSRKYLHTYDEAVPWNQVPGTFTPWQSL
PEPTDVLFYEGLHGGVVTPQHNVAQHVDLLVGVVPIVNLEWIQKLIRDTSERGHSREAVM
DSVVRSMEDYINYITPQFSRTHLNFQRVPTVDTSNPFAAKGIPSLDESFVVIHFRNLEGI
DFPWLLAMLQGSFISHINTLVVPGGKMGLAMELIMLPLVQRLMEGKKIE
>LFGLNPFC_04591 hypothetical protein
MLIPWQDLSPETLENLIESFVLREGTDYGEHERTLEQKVSDVKRQLQCGEAVLVWSELHE
TVNIMPRSQFRE
>LFGLNPFC_04592 hypothetical protein
MAQITTTDANEFSSSAKFTPMRGFSNCHLQTMLPRLFRRKVKFTPYWQRLELPDGDFVDL
AWSEDPAQARHKPRLVVFHGLEGSLNSPYAHGLVEAAQKRGWLGVVMHFRGCSGEPNRMH
RIYHSGETEDASWFLRWLQREFGHAPTAAVGYSLGGNMLACLLAKEGNDLPIDAAVIVSA
PFMLEACSYHMEKGFSRIYQRYLLNLLKANAARKLAAYPGTLPINLTQLKSVRRIREFDD
LITARIHGYADAIDYYRQCSAMPMLNRIAKPTLIIHAKDDPFMDHQVIPKPESLPPQVEY
QLTEHGGHVGFIGGTLLHPQMWLESRIPDWLTTYLEAKSC
>LFGLNPFC_04593 putative ABC transporter ATP-binding protein YheS
MIVFSSLQIRRGVRILLDNATATINPGQKVGLVGKNGCGKSTLLALLKNEISADGGSYTF
PVSWQLAWVNQETPALPQAALEYVIDGDREYRQLEAQLHDANERNDGHAIATIHGKLDAI
DAWSIRSRAASLLHGLGFSNEQLERPVSDFSGGWRMRLNLAQALICRSDLLLLDEPTNHL
DLDAVIWLEKWLKSYQGTLILISHDRDFLDPIVDKIIHIEQQSMFEYTGNYSSFEVQRAT
RLAQQQAMYESQQERVAHLQSYIDRFRAKATKAKQAQSRIKMLERMELIAPAHVDNPFRF
SFRAPESLPNPLLKMAKVSAGYGDRIILDSIKLNLVPGSRIGLLGRNGAGKSTLIKLLAG
ELAPVSGEIGLAKGIKLGYFAQHQLEYLRADESPIQHLARLAPQELEQKLRDYLGGFGFQ
GDKVTEETRRFSGGEKARLVLALIVWQRPNLLLLDEPTNHLDLDMRQALTEALIEFEGAL
VVVSHDRHLLRSTTDDLYLVHDRKVEPFDGDLEDYQQWLSDVQKQENQTDEAPKENANSA
QARKDQKRREAELRAQTQPLRKEIARLEKEMEKLNAQLAQAEEKLGDSELYDQSRKAELT
ACLQQQASAKSGLEECEMAWLEAQEQLEQMLLEGQSN
>LFGLNPFC_04594 General stress protein 14
MSQPAKVLLLYAHPESQDSVANRVLLKPATQLSNVTVHDLYAHYPDFFIDIPREQALLRE
HEVIVFQHPLYTYSCPALLKEWLDRVLSRGFASGPGGNQLAGKYWRSVITTGEPESAYRY
DALNRYPMSDVLRPFELAAGMCRMHWLSPIIIYWARRQSAQELASHARAYGDWLANPLSP
GGC
>LFGLNPFC_04595 Glutathione-regulated potassium-efflux system protein KefC
MEGSDFLLAGVLFLFAAVAAVPLASRLGIGAVLGYLLAGIAIGPWGLGFISDVDEILHFS
ELGVVFLMFIIGLELNPSKLWQLRRSIFGVGAAQVLLSAALLAGLLMLTHFSWQAAVVGG
IGLAMSSTAMALQLMREKGMNRSESGQLGFSVLLFQDLAVIPALALVPLLAGSADEHFDW
MKIGMKVLAFVGMLIGGRYLLRPVFRFIAASGVREVFTAATLLLVLGSALFMDALGLSMA
LGTFIAGVLLAESEYRHELETAIDPFKGLLLGLFFISVGMSLNLGVLYTHLLWVVISVVV
LVAVKILVLYLLARLYGVRSSERMQFAGVLSQGGEFAFVLFSTASSQRLFQGDQMALLLV
TVTLSMMTTPLLMKLVDKWLSRQFNGPEEEDEKPWVNDDKPQVIVVGFGRFGQVIGRLLM
ANKMRITVLERDISAVNLMRKYGYKVYYGDATQVDLLRSAGAEAAESIVITCNEPEDTMK

LVEICQQHFPHLHILARARGRVEAHELLQAGVTQFSRETFSSALELGRKTLVTLGMHPHQ
AQRAQLHFRRLDMRMLRELIPMHADTVQISRAREARRELEEIFQREMQQERRQLDGWDEF
E
>LFGLNPFC_04596 hypothetical protein
MAIRKRFIAGAKCPACQAQDSMAMWRENNIDIVECVKCGHQMREADKEARDHVRKDEQVI
GIFHPD
>LFGLNPFC_04597 FKBP-type peptidyl-prolyl cis-trans isomerase SlyD
MKVAKDLVVSLAYQVRTEDGVLVDESPVSAPLDYLHGHGSLISGLETALEGHEVGDKFDV
AVGANDAYGQYDENLVQRVPKDVFMGVDELQVGMRFLAETDQGPVPVEITAVEDDHVVVD
GNHMLAGQNLKFNVEVVAIREATEEELAHGHVHGAHDHHHDHDHDGCCGGHGHDHGHEHG
GEGCCGGKGNGGCGCH
>LFGLNPFC_04598 Protein SlyX
MQDLSLEARLAELESRLAFQEITIEELNVTVTAHEMEMAKLRDHLRLLTEKLKASQPSNI
ASQAEETPPPHY
>LFGLNPFC_04599 FKBP-type peptidyl-prolyl cis-trans isomerase FkpA
MKSLFKVTLLATTMAVALHAPITFAAEAAKPATTADSKAAFKNDDQKSAYALGASLGRYM
ENSLKEQEKLGIKLDKDQLIAGVQDAFADKSKLSDQEIEQTLQAFEARVKSSAQAKMEKD
AADNEAKGKEYREKFAKEKGVKTSSTGLVYQVVEAGKGEAPKDSDTVVVNYKGTLIDGKE
FDNSYTRGEPLSFRLDGVIPGWTEGLKNIKKGGKIKLVIPPELAYGKAGVPGIPPNSTLV
FDVELLDVKPAPKADAKPEADAKAADSAKK
>LFGLNPFC_04600 Transcriptional regulator DauR
MSRSLLTNETSELDLLDQRPFDQTDFDILKSYEAVVDGLAMLIGSHCEIVLHSLQDLKCS
AIRIANGEHTGRKIGSPITDLALRMLHDMTGADSSVSKCYFTRAKSGVLMKSLTIAIRNR
EQRVIGLLCINMNLDVPFSQIMSTFVPPETPDVGSSVNFASSVEDLVTQTLEFTIEEVNA
DRNVSNNAKNRQIVLNLYEKGIFDIKDAINQVADRLNISKHTVYLYIRQFKSGDFQGQDK
>LFGLNPFC_04601 Sulfurtransferase TusD
MRFAIVVTGPAYGTQQASSAFQFAQALIAEGHELSSVFFYREGVYNANQLTSPASDEFDL
VRSWQQLNMQHGVALNICVAAALRRGVVDETEAGRLGLASSNLQTGFTLSGLGALAEASL
TCDRVVQF
>LFGLNPFC_04602 Protein TusC
MKRIAFVFSTVPHGTAAGREGLDALLATSALTDDLAVFFIADGVFQLLPGQKPDAVLARD
YIATFKLLGLYDIEQCWVCAASLRERGLDPQTPFVVEATPLEADALRRELANYDVILRF
>LFGLNPFC_04603 Protein TusB
MLHTLHRSPWLTDFAALLRLLSEGDELLLLQDGVTAAVDGNRYLESLRNAPIKVYALNED
LIARGLTGRISNDIIPIDYTDFVRLTVKHSSQMAW
>LFGLNPFC_04604 30S ribosomal protein S12
MATVNQLVRKPRARKVAKSNVPALEACPQKRGVCTRVYTTTPKKPNSALRKVCRVRLTNG
FEVTSYIGGEGHNLQEHSVILIRGGRVKDLPGVRYHTVRGALDCSGVKDRKQARSKYGVK
RPKA
>LFGLNPFC_04605 30S ribosomal protein S7
MPRRRVIGQRKILPDPKFGSELLAKFVNILMVDGKKSTAESIVYSALETLAQRSGKSELE
AFEVALENVRPTVEVKSRRVGGSTYQVPVEVRPVRRNALAMRWIVEAARKRGDKSMALRL
ANELSDAAENKGTAVKKREDVHRMAEANKAFAHYRW
>LFGLNPFC_04606 Elongation factor G
MARTTPIARYRNIGISAHIDAGKTTTTERILFYTGVNHKIGEVHDGAATMDWMEQEQERG
ITITSAATTAFWSGMAKQYEPHRINIIDTPGHVDFTIEVERSMRVLDGAVMVYCAVGGVQ
PQSETVWRQANKYKVPRIAFVNKMDRMGANFLKVVNQIKTRLGANPVPLQLAIGAEEHFT
GVVDLVKMKAINWNDADQGVTFEYEDIPADMVELANEWHQNLIESAAEASEELMEKYLGG
EELTEAEIKGALRQRVLNNEIILVTCGSAFKNKGVQAMLDAVIDYLPSPVDVPAINGILD
DGKDTPAERHASDDEPFSALAFKIATDPFVGNLTFFRVYSGVVNSGDTVLNSVKAARERF
GRIVQMHANKREEIKEVRAGDIAAAIGLKDVTTGDTLCDPDAPIILERMEFPEPVISIAV
EPKTKADQEKMGLALGRLAKEDPSFRVWTDEESNQTIIAGMGELHLDIIVDRMKREFNVE
ANVGKPQVAYRETIRQKVTDVEGKHAKQSGGRGQYGHVVIDMYPLEPGSNPKGYEFINDI
KGGVIPGEYIPAVDKGIQEQLKAGPLAGYPVVDMGIRLHFGSYHDVDSSELAFKLAASIA
FKEGFKKAKPVLLEPIMKVEVETPEENTGDVIGDLSRRRGMLKGQESEVTGVKIHAEVPL
SEMFGYATQLRSLTKGRASYTMEFLKYDEAPSNVAQAVIEARGK
>LFGLNPFC_04607 Elongation factor Tu 1
MSKEKFERTKPHVNVGTIGHVDHGKTTLTAAITTVLAKTYGGAARAFDQIDNAPEEKARG
ITINTSHVEYDTPTRHYAHVDCPGHADYVKNMITGAAQMDGAILVVAATDGPMPQTREHI
LLGRQVGVPYIIVFLNKCDMVDDEELLELVEMEVRELLSQYDFPGDDTPIVRGSALKALE
GDAEWEAKILELAGFLDSYIPEPERAIDKPFLLPIEDVFSISGRGTVVTGRVERGIIKVG
EEVEIVGIKETQKSTCTGVEMFRKLLDEGRAGENVGVLLRGIKREEIERGQVLAKPGTIK
PHTKFESEVYILSKDEGGRHTPFFKGYRPQFYFRTTDVTGTIELPEGVEMVMPGDNIKMV
VTLIHPIAMDDGLRFAIREGGRTVGAGVVAKVLG

>LFGLNPFC_04608 putative bifunctional chitinase/lysozyme
MKLNIFTKSMIGMGLVCSALPALAMEAWNNQQGGNKYQVIFDGKIYENAWWVSSTNCPGK
AKANDATNPWRLKRTATAAEISQFGNTLSCEKSGSSSSSTSNTPASNTPANGASATPAQG
TVPSNSSVVAWNKQQGGQTWYVVFNGAVYKNAWWVASSNCPGDAKGNDASNPWRYVRAAT
ATEISETSNPQSCTSAPQPAPDVKPAPDVKPAPDVQPAPADKSNDNYAVVAWKGQEGSST
WYVIYNGGIYKNAWWVGAANCPGDAKENDASNPWRYVRAATATEITQYGNPASCSVKPDN
NGGAVTPVDPTPETPETPVTPTPDNNEPSTPADSSNDYSLQAWSGQEGSEIYHVIFNGNV
YQNAWWVGSEDCPRGTSVENSNNPWRLVRTATAAEMSQYGNPTTCEIDNGGVIIADGFQA
SKAYSANSIVDYNDAHYKTSVDQDAWGFVPGGDNPWKKYEPAKAWSASTVYVKGDRVVVD
GQAYEALFWTQSDNPALIANQNATGSNSRPWKPLGKTQSYSNEELNNAPQFNPETLYASD
TLIRFNGENYISQSKVQKVSPSDSNPWRVFVDWTGTKERVGTPKKAWPKHVYAPYVDFTL
NTIPDLAALAKSHNVNHFTLAFVVSKDANTCLPTWGTAYGMQNYAQYSKIKALREAGGDV
MLSIGGANNAPLAASCKNVDDLMQHYYDIVDNLNLRVLDFDIEGTWVADQASIERRNLAV
KKVQDKWKSEGKDIAIWYTLPILPTGLTPEGMNVLSDAKAKGVELAGVNVMTMDYGNAIC
QSANTEGQNIHGKCATSAIANLHSQLKGLHPNKSDAEIDAMMGTTPMVGVNDVQGEVFYL
SDARLVMQDAQKRNLGMVGIWSIARDLPGGTNLSPEFHGLTKEQAPKYAFSEIFAPFTKQ
>LFGLNPFC_04609 Bacterioferritin-associated ferredoxin
MYVCLCNGISDKKIRQAVRQFSPHSFQQLKKFIPVGNQCGKCVRAAREVMEDELMQLPEF
KESA
>LFGLNPFC_04610 Bacterioferritin
MKGDTKVINYLNKLLGNELVAINQYFLHARMFKNWGLKRLNDVEYHESIDEMKHADRYIE
RILFLEGLPNLQDLGKLNIGEDVEEMLRSDLALELDGAKNLREAIGYADSVHDYVSRDMM
IEILRDEEGHIDWLETELDLIQKMGLQNYLQAQIREEG
>LFGLNPFC_04611 Type 4 prepilin-like proteins leader peptide-processing enzyme
MTMLLPLFILVGFIAGYFVNVMAYHLSPLEDKTALTFRQVLVHFWQKKYAWHDTVPLILC
VAAAIACALAPFTPIVTGALFLYFCFALTLSVIDFRTQLLPDKLTLPLLWLGLVFNAQSG
LIDLHDAVYGAVAGYGVLWCVYWGVWLVCHKEGLGYGDFKLLAAAGAWCGWQTLPMILLI
ASLGGIGYAIVSQLLQRRTITTIAFGPWLALGSMINLGYLAWISY
>LFGLNPFC_04612 Putative type II secretion system protein M
MIKSWWAEKSTSEKQIVAALAVLSLGVFCWLAVIKPIDTYIEEHQSHAQKIKKDIKWMQD
QASTHGLLGHPALTQPIKNILLEEAKRENLTITLENGPDNTLTINPVTAPLENVSRWLTT
AQITYGIVIEDLQFTLAGNEEITLKHLSFREQQ
>LFGLNPFC_04613 Putative type II secretion system protein L
MPESLMVIRSFSTLRKHWEWMTFSADSVSSVHTLTDDLPLESLADQPGAGNVHLLIPPEG
LLYRSLTLPNAKYKLTAQTLQWLAEETLPDASQNWHWMVVDKQNESVEVIGIQSEKLSRY
LERLHTAGLNVTRVLPDGCYLPWEVDSWTLVNQQTSWLIRSAAHAFNELDEHWLQHLANQ
FPPENMRCYGVAPHGVAAANPLIQHPEIPSLSLYSADIAFQRYDMLHGVFRKQKTVSKSG
KWLARLAVSCLVLAILSFVGSRGIAFWQTLKIEDQLQQQQETWQRYFPQIKHTHNFRFY
FKQQLAQQYPEAVPLLYHLQTLLLEHPELQLMEANYSQRQKSLTLKMSAKSEANIDRFCE
LTQSWLPMEKTEKDPVSGVWTVRNSGK
>LFGLNPFC_04614 Putative type II secretion system protein K
MNNEQRGVALLIVLMLLALMAALAADMTISFHGQLHRTRQVNHHLQRQYDIELAEKLALA
SLTQDVKDNDRQTTLQQYWAQPQQLQLENGNTVKWQLRDAQHCFNLNALAKISDAPLASP
DFPAQVFSALLINAGIDRGNTDEIVQSIADYIDADDSPRFHGAEDNFYQSQTPPRHSANQ
MLFLTGELRQIKGITENIYQRLIPYVCVLPTSELSINLNMLTENDIPLFRALFLNNITDA
DARVLLQKRPREGWLTTDAFLYWAQQDFSGVKPLVAQVKGHLFPYSRYFTLSTESISDEQ
SQGWQSHIFFNRKQQSAQIYRRTLQLY
>LFGLNPFC_04615 Putative type II secretion system protein J
MINRQQGFTLLEVMAALAIFSMLSVLAFMIFSQASELHQRSQKEIQQFNQLQRTITILDN
DLLQLVARRNRSTDKIMVLGEEAIFTTQSRDPLAPLSEAQTLLTVHWYLRNHTLYRAVRT
SVDGRKDQPAQAMLEHVESFLLESNSGESQELPLSVTLHLKTQQYGALQRRFALPEQLAR
EESPAQTQAGNNNHE
>LFGLNPFC_04616 Putative type II secretion system protein I
MNKQSGMTLLEVLLAMSIFTAVALTLMSSMQGQRTAIERMRNETLALWIADNQLQSQDSF
DEENTSSSGKELINGEELINGEEWNWRSDIHSSKDGTLLEHTITVTLPSGQTTSLTRYQS
INNKSGQAQDD
>LFGLNPFC_04617 Putative type II secretion system protein H
MNQQRGFTLLEMMLVLALVAITASVVLFTYGREDAASTRARETAARFTAALELAIDRATL
SGQPVGIHFSDSAWRIMVPGKTPSAWRWVPLQEDAADESKNDWGEELSIQLQPFKPDDSN
QPQVVILADGQITPFSLLMANAGTSKPLLTLVSSGSWPLDQTLARDTRP
>LFGLNPFC_04618 Putative type II secretion system protein G
MRATDKQRGFTLLEIMVVIVIIGVLASLVVPNLMGNKEKADKQKAVSDIVALENALDMYK
LDNHRYPTTNQGLESLVEAPTLPPLAANYNKEGYIKRLPADPWGNDYVLVNPGEHGAYDL
LSAGPDGEMGTEDDITNWGLSKKKK

>LFGLNPFC_04619 Putative type II secretion system protein F
MNYRYRAMTQDGQKLQGIIDANDERQARLRLREEGLFLLDIRPQKSSGVKTRRPRISHSE
LTLFTRQLATLSAAALPLEESLAVIGQQSSNNRLADVLNQVRSAILEGHPLSDALQHFPT
LFDSLYRTLVKAGEKSGLLAPVLEKLADYNENRQKIRSKLIQSLIYPCMLTTVAIVVVII
LLTAVVPKITEQFVHMKQQLPLSTRILLGLSDTLQRTGPTLLATVFIVAVGFWLWLKRGN
NRHRFHAMLLRVALIGPLICAINSARYLRTLSILQSSGVPLLDGMNLSTESLNNLEIRQR
LANAAENVRQGNSIHLSLEQTAIFPPMMLYMVASGEKSGQLGTLMVRAADNQETLQQNRI
ALTLSIFEPALIITMALIVLFIVVSVLQPLLQLNSMIN
>LFGLNPFC_04620 Putative type II secretion system protein E
MRIHSPYPASWALAQRIGYLYSEGEIIYLADTPFERLLDIQRQVGQCQTMTSLSQADFEA
RLEAVFHQNTGESQQIAQDIDQSVDLLSLSEEMPANEDLLNEDSAAPVIRLINAILSEAI
KETASDIHIETYEKTMSIRFRIDGVLRTILQPNKKLAALLISRIKVMARLDIAEKRIPQD
GRISLRIGRRNIDVRVSTLPSIYGERAVLRLLDKNSLQLSLNNLGMTAADKQDLENLIQL
PHGIILVTGPTGSGKSTTLYAILSALNTPGRNILTVEDPVEYELEGIGQTQVNTRVDMSF
ARGLRAILRQDPDVVMVGEIRDTETAQIAVQASLTGHLVLSTLHTNSASGAVTRLRDMGV
ESFLLSSSLAGIIAQRLVRRLCPQCRQFTPVSPQQAQMFKHHQLAVTTIGTPVGCPHCHQ
SGYQGRMAIHEMMVVTPELRAAIHENVDEQALERLVRQQHNALIKNGLQKVIRGDTSWDE
VMRVASATLENEA
>LFGLNPFC_04621 Putative secretin GspD
MKGLNKITCCLLAALLMPCAGHAENEQYGANFNNADIRQFVEIVGQHLGKTILIDPSVQG
TISVRSNDTFSQQEYYQFFLSILDLYGYSVITLDNGFLKVVRSANVKTSPGMIADSSRPG
VGDELVTRIVPLENVPARDLAPLLRQMMDAGSVGNVVHYEPSNVLILTGRASTINKLIEV
IKRVDVIGTEKQQIIHLEYASAEDLAEILNQLISESHGKSQMPALLSAKIVADKRTNSLI
ISGPEKARQRITSLLKSLDVEESEEGNTRVYYLKYAKATNLVEVLTGVSEKLKDEKGNSR
KPSSTSAMDNVAITADEQTNSLVITADQSVQEKLATVIARLDIRRAQVLVEAIIVEVQDG
NGLNLGVQWANKNVGAQQFTNTGLPVFNAAQGVADYKKNGGITSANPAWDMFSAYNGMAA
GFFNGDWGVLLTALASNNKNDILATPSIVTLDNKLASFNVGQDVPVLSGSQTTSGDNVFN
TVERKTVGTKLKVTPQVNEGDAVLLEIEQEVSSVDSSSNSTLGPTFNTRTIQNAVLVKTG
ETVVLGGLLDDFSKEQVSKVPLLGDIPLVGQLFRYTSTERAKRNLMVFIRPTIIRDDDVY
RSLSKEKYTRYRQEQQLRIDGKSKALIGSEDLPVLDENTFNSHAPAPSSR
>LFGLNPFC_04622 Putative type II secretion system protein C
MPTLPFPFHLANHNKDAAINILIIFISIGLIIFNVNYFHTTIVKNEQIINQPTNAFQSEF
SLAALWRNENHAGVKDANPVAVNQETPKLSIALNGIVLTSNDETSFVLINEGNEQKRYSL
NEALESAPGTFIRKINKTSVVFETHGHYEKVTLHPGLPDIIKQPDSENQNVLADYIIATP
IRDGEQIYGLRLNPRKGLNAFTTSLLQPGDIALRINNLSLTHPDEVSQALSLLLTQQSAQ
FTIRRNGVPRLINVSVAELTGMNGQRNEGTQ
>LFGLNPFC_04623 Putative general secretion pathway protein A
MSTRREVILSWLREKRQTWRLCYLLGEAGSGKTWLAQQLQKDKHRRVITLSLVVSWQGKA
AWIVTDDNAAEQGCRDSAWTRDEMAGQLLHALHRTDSRCPLIIIENAHLNHRRILDDLQR
AISLIPDGQFLLIGRPDRKVERDFKKQGIELVSTGRLTEHELKASILEGQNIDQPDLLLT
ARVLKRIALLCRGDRRKLALAGETISLLQQAEQTRVFTAKQWRMIYRVLGDKRPRKMQLA
VVMSGTILALTCGWLLLSSFTAPLPVPAWLIPVTPVVKQDMTKDIAHVVMRDSEALSVLY
GVWGYEVPADSAWCDQAVRAGLVCKSGNASLQTLVDQNLPWIASLKVGDKKLPVVVVRVG
DATVDVLVGQQTWTLTHKWFELVWTGDYLLLWKMSPEGESTIMRDSSEEEILWLETMLNR
ALHISTESSAEWRPLLVEKIKQFQKSHHLKTDGVVGFSTLVHLWQVAGESAYLYRDEANI
SPETTVKGK
>LFGLNPFC_04624 Putative general secretion pathway protein B
MFEFYIAAREQKETGHPGIFSRQKHSTIIYVICLLLICLWFAGMVLVGGYARQLWALWIV
KAEVTVDAETPAFKQSTQHYFFKKQPLPVVESVEEEDEPGVAVENAPSSSEDEENTVEES
DEKAGLRERVKNALNELER
>LFGLNPFC_04625 30S ribosomal protein S10
MQNQRIRIRLKAFDHRLIDQATAEIVETAKRTGAQVRGPIPLPTRKERFTVLISPHVNKD
ARDQYEIRTHLRLVDIVEPTEKTVDALMRLDLAAGVDVQISLG
>LFGLNPFC_04626 50S ribosomal protein L3
MIGLVGKKVGMTRIFTEDGVSIPVTVIEVEANRVTQVKDLANDGYRAIQVTTGAKKANRV
TKPEAGHFAKAGVEAGRGLWEFRLAEGEEFTVGQSISVELFADVKKVDVTGTSKGKGFAG
TVKRWNFRTQDATHGNSLSHRVPGSIGQNQTPGKVFKGKKMAGQMGNERVTVQSLDVVRV
DAERNLLLVKGAVPGATGSDLIVKPAVKA
>LFGLNPFC_04627 50S ribosomal protein L4
MELVLKDAQSALTVSETTFGRDFNEALVHQVVVAYAAGARQGTRAQKTRAEVTGSGKKPW
RQKGTGRARSGSIKSPIWRSGGVTFAARPQDHSQKVNKKMYRGALKSILSELVRQDRLIV
VEKFSVEAPKTKLLAQKLKDMALEDVLIITGELDENLFLAARNLHKVDVRDATGIDPVSL
IAFDKVVMTADAVKQVEEMLA
>LFGLNPFC_04628 50S ribosomal protein L23

MIREERLLKVLRAPHVSEKASTAMEKSNTIVLKVAKDATKAEIKAAVQKLFEVEVEVVNT
LVVKGKVKRHGQRIGRRSDWKKAYVTLKEGQNLDFVGGAE
>LFGLNPFC_04629 50S ribosomal protein L2
MAVVKCKPTSPGRRHVVKVVNPELHKGKPFAPLLEKNSKSGGRNNNGRITTRHIGGGHKQ
AYRIVDFKRNKDGIPAVVERLEYDPNRSANIALVLYKDGERRYILAPKGLKAGDQIQSGV
DAAIKPGNTLPMRNIPVGSTVHNVEMKPGKGGQLARSAGTYVQIVARDGAYVTLRLRSGE
MRKVEADCRATLGEVGNAEHMLRVLGKAGAARWRGVRPTVRGTAMNPVDHPHGGGEGRNF
GKHPVTPWGVQTKGKKTRSNKRTDKFIVRRRSK
>LFGLNPFC_04630 30S ribosomal protein S19
MPRSLKKGPFIDLHLLKKVEKAVESGDKKPLRTWSRRSTIFPNMIGLTIAVHNGRQHVPV
FVTDEMVGHKLGEFAPTRTYRGHAADKKAKKK
>LFGLNPFC_04631 50S ribosomal protein L22
METIAKHRHARSSAQKVRLVADLIRGKKVSQALDILTYTNKKAAVLVKKVLESAIANAEH
NDGADIDDLKVTKIFVDEGPSMKRIMPRAKGRADRILKRTSHITVVVSDR
>LFGLNPFC_04632 30S ribosomal protein S3
MGQKVHPNGIRLGIVKPWNSTWFANTKEFADNLDSDFKVRQYLTKELAKASVSRIVIERP
AKSIRVTIHTARPGIVIGKKGEDVEKLRKVVADIAGVPAQINIAEVRKPELDAKLVADSI
TSQLERRVMFRRAMKRAVQNAMRLGAKGIKVEVSGRLGGAEIARTEWYREGRVPLHTLRA
DIDYNTSEAHTTYGVIGVKVWIFKGEILGGMAAVEQPEKPAAQPKKQQRKGRK
>LFGLNPFC_04633 50S ribosomal protein L16
MLQPKRTKFRKMHKGRNRGLAQGTDVSFGSFGLKAVGRGRLTARQIEAARRAMTRAVKRQ
GKIWIRVFPDKPITEKPLAVRMGKGKGNVEYWVALIQPGKVLYEMDGVPEELAREAFKLA
AAKLPIKTTFVTKTVM
>LFGLNPFC_04634 50S ribosomal protein L29
MKAKELREKSVEELNTELLNLLREQFNLRMQAASGQLQQSHLLKQVRRDVARVKTLLNEK
AGA
>LFGLNPFC_04635 30S ribosomal protein S17
MTDKIRTLQGRVVSDKMEKSIVVAIERFVKHPIYGKFIKRTTKLHVHDENNECGIGDVVE
IRECRPLSKTKSWTLVRVVEKAVL
>LFGLNPFC_04636 50S ribosomal protein L14
MIQEQTMLNVADNSGARRVMCIKVLGGSHRRYAGVGDIIKITIKEAIPRGKVKKGDVLKA
VVVRTKKGVRRPDGSVIRFDGNACVLLNNNSEQPIGTRIFGPVTRELRSEKFMKIISLAP
EVL
>LFGLNPFC_04637 50S ribosomal protein L24
MAAKIRRDDEVIVLTGKDKGKRGKVKNVLSSGKVIVEGINLVKKHQKPVPALNQPGGIVE
KEAAIQVSNVAIFNATTGKADRVGFRFEDGKKVRFFKSNSETIK
>LFGLNPFC_04638 50S ribosomal protein L5
MAKLHDYYKDEVVKKLMTEFNYNSVMQVPRVEKITLNMGVGEAIADKKLLDNAAADLAAI
SGQKPLITKARKSVAGFKIRQGYPIGCKVTLRGERMWEFFERLITIAVPRIRDFRGLSAK
SFDGRGNYSMGVREQIIFPEIDYDKVDRVRGLDITITTTAKSDEEGRALLAAFDFPFRK
>LFGLNPFC_04639 30S ribosomal protein S14
MAKQSMKAREVKRVALADKYFAKRAELKAIISDVNASDEDRWNAVLKLQTLPRDSSPSRQ
RNRCRQTGRPHGFLRKFGLSRIKVREAAMRGEIPGLKKASW
>LFGLNPFC_04640 30S ribosomal protein S8
MSMQDPIADMLTRIRNGQAANKAAVTMPSSKLKVAIANVLKEEGFIEDFKVEGDTKPELE
LTLKYFQGKAVVESIQRVSRPGLRIYKRKDELPKVMAGLGIAVVSTSKGVMTDRAARQAG
LGGEIICYVA
>LFGLNPFC_04641 50S ribosomal protein L6
MSRVAKAPVVVPAGVDVKINGQVITIKGKNGELTRTLNDAVEVKHADNTLTFGPRDGYAD
GWAQAGTARALLNSMVIGVTEGFTKKLQLVGVGYRAAVKGNVINLSLGFSHPVDHQLPAG
ITAECPTQTEIVLKGADKQVIGQVAADLRAYRRPEPYKGKGVRYADEVVRTKEAKKK
>LFGLNPFC_04642 50S ribosomal protein L18
MDKKSARIRRATRARRKLQELGATRLVVHRTPRHIYAQVIAPNGSEVLVAASTVEKAIAE
QLKYTGNKDAAAAVGKAVAERALEKGIKDVSFDRSGFQYHGRVQALADAAREAGLQF
>LFGLNPFC_04643 30S ribosomal protein S5
MAHIEKQAGELQEKLIAVNRVSKTVKGGRIFSFTALTVVGDGNGRVGFGYGKAREVPAAI
QKAMEKARRNMINVALNNGTLQHPVKGVHTGSRVFMQPASEGTGIIAGGAMRAVLEVAGV
HNVLAKAYGSTNPINVVRATIDGLENMNSPEMVAAKRGKSVEEILGK
>LFGLNPFC_04644 50S ribosomal protein L30
MAKTIKITQTRSAIGRLPKHKATLLGLGLRRIGHTVEREDTPAIRGMINAVSFMVKVEE
>LFGLNPFC_04645 50S ribosomal protein L15
MRLNTLSPAEGSKKAGKRLGRGIGSGLGKTGGRGHKGQKSRSGGGVRRGFEGGQMPLYRR
LPKFGFTSRKAAITAEVRLSDLAKVEGGVVDLNTLKAANIIGIQIEFAKVILAGEVTTPV
TVRGLRVTKGARAAIEAAGGKIEE

>LFGLNPFC_04646 Protein translocase subunit SecY
MAKQPGLDFQSAKGGLGELKRRLLFVIGALIVFRIGSFIPIPGIDAAVLAKLLEQQRGTI
IEMFNMFSGGALSRASIFALGIMPYISASIIIQLLTVVHPTLAEIKKEGESGRRKISQYT
RYGTLVLAIFQSIGIATGLPNMPGMQGLVINPGFAFYFTAVVSLVTGTMFLMWLGEQITE
RGIGNGISIIIFAGIVAGLPPAIAHTIEQARQGDLHFLVLLLVAVLVFAVTFFVVFVERG
QRRIVVNYAKRQQGRRVYAAQSTHLPLKVNMAGVIPAIFASSIILFPATIASWFGGGTGW
NWLTTISLYLQPGQPLYVLLYASAIIFFCFFYTALVFNPRETADNLKKSGAFVPGIRPGE
QTAKYIDKVMTRLTLVGALYITFICLIPEFMRDAMKVPFYFGGTSLLIVVVVIMDFMAQV
QTLMMSSQYESALKKANLKGYGR
>LFGLNPFC_04647 50S ribosomal protein L36
MKVRASVKKLCRNCKIVKRDGVIRVICSAEPKHKQRQG
>LFGLNPFC_04648 30S ribosomal protein S13
MARIAGINIPDHKHAVIALTSIYGVGKTRSKAILAAAGIAEDVKISELSEGQIDTLRDEV
AKFVVEGDLRREISMSIKRLMDLGCYRGLRHRRGLPVRGQRTKTNARTRKGPRKPIKK
>LFGLNPFC_04649 30S ribosomal protein S11
MAKAPIRARKRVRKQVSDGVAHIHASFNNTIVTITDRQGNALGWATAGGSGFRGSRKSTP
FAAQVAAERCADAVKEYGIKNLEVMVKGPGPGRESTIRALNAAGFRITNITDVTPIPHNG
CRPPKKRRV
>LFGLNPFC_04650 30S ribosomal protein S4
MARYLGPKLKLSRREGTDLFLKSGVRAIDTKCKIEQAPGQHGARKPRLSDYGVQLREKQK
VRRIYGVLERQFRNYYKEAARLKGNTGENLLALLEGRLDNVVYRMGFGATRAEARQLVSH
KAIMVNGRVVNIASYQVSPNDVVSIREKAKKQSRVKAALELAEQREKPTWLEVDAGKMEG
TFKRKPERSDLSADINEHLIVELYSK
>LFGLNPFC_04651 DNA-directed RNA polymerase subunit alpha
MQGSVTEFLKPRLVDIEQVSSTHAKVTLEPLERGFGHTLGNALRRILLSSMPGCAVTEVE
IDGVLHEYSTKEGVQEDILEILLNLKGLAVRVQGKDEVILTLNKSGIGPVTAADITHDGD
VEIVKPQHVICHLTDENASISMRIKVQRGRGYVPASTRIHSEEDERPIGRLLVDACYSPV
ERIAYNVEAARVEQRTDLDKLVIEMETNGTIDPEEAIRRAATILAEQLEAFVDLRDVRQP
EVKEEKPEFDPILLRPVDDLELTVRSANCLKAEAIHYIGDLVQRTEVELLKTPNLGKKSL
TEIKDVLASRGLSLGMRLENWPPASIADE
>LFGLNPFC_04652 50S ribosomal protein L17
MRHRKSGRQLNRNSSHRQAMFRNMAGSLVRHEIIKTTLPKAKELRRVVEPLITLAKTDSV
ANRRLAFARTRDNEIVAKLFNELGPRFASRAGGYTRILKCGFRAGDNAPMAYIELVDRSE
KAEAAAE
>LFGLNPFC_04653 hypothetical protein
MWLLDQWAERHIAEAQAKGEFDNLVGSGEPLILDDDSHVPPELRAGYRLLKNAGCLPPEL
EHRREAIQLLDILKGIRHDDPQYQEVSRRLSLLELKLRQAGLSTDFLRGDYADKLLNKIN
DN
>LFGLNPFC_04654 HTH-type transcriptional regulator ZntR
MYRIGELAKMAEVTPDTIRYYEKQQMMEHEVRTEGGFRLYTESDLQRLKFIRHARQLGFS
LESIRELLSIRIDPEHHTCQESKGIVQERLQEVEARIAELQSMQRSLQRLNDTCCGTAHS
SVYCSILEALEQGASGVKSGC
>LFGLNPFC_04655 Alternative ribosome-rescue factor A
MSRYQHTKGQIKDNAIEALLHDPLFRQRVEKNKKGKGSYMRKGKHGNRGNWEASGKKVNH
FFTTGLLLSDAC
>LFGLNPFC_04656 Large-conductance mechanosensitive channel
MSIIKEFREFAMRGNVVDLAVGVIIGAAFGKIVSSLVADIIMPPLGLLIGGIDFKQFAVT
LREAQGDIPAVVMHYGVFIQNVFDFLIVAFAIFMAIKLINKLNRKKEEPAAATPAPTKEE
VLLTEIRDLLKEQNNRS
>LFGLNPFC_04657 Trk system potassium uptake protein TrkA
MKIIILGAGQVGGTLAENLVGENNDITVVDTNGERLRTLQDKFDLRVVQGHGSHPRVLRE
AGADDADMLVAVTSSDETNMVACQVAYSLFNTPNRIARIRSPDYVRDADKLFHSDAVPID
HLIAPEQLVIDNIYRLIEYPGALQVVNFAEGKVSLAVVKAYYGGPLIGNALSTMREHMPH
IDTRVAAIFRHDRPIRPQGSTIVEAGDEVFFIAASQHIRAVMSELQRLEKPYKRIMLVGG
GNIGAGLARRLEKDYSVKLIERNQQRAAELAEKLQNTIVFFGDASDQELLAEEHIDQVDL
FIAVTNDDEANIMSAMLAKRMGAKKVMVLIQRRAYVDLVQGSVIDIAISPQQATISALLS
HVRKADIVGVSSLRRGVAEAIEAVAHGDESTSRVVGRVIDEIKLPPGTIIGAVVRGNDVM
IANDNLRIEQGDHVIMFLTDKKFITDVERLFQPSPFFL
>LFGLNPFC_04658 Ribosomal RNA small subunit methyltransferase B
MKKQRNLRSMAAQAIEQVVEQGQSLSNILPPLQQKVSDKDKALLQELCFGVLRTLSQLDW
LINKLMARPMTGKQRTVHYLIMVGLYQLLYTRIPPHAALAETVEGAVAIKRPQLKGLING
VLRQFQRQQDELLAEFNASDARYLHPSWLLKRLQKAYPEQWQSIVEANNQRPPMWLRVNR
THHSRDSWLALLDEAGMKGFPHADYPDAVQLETPAPVHALPGFEEGWVTVQDASAQGCMT
WLAPQNGEHILDLCAAPGGKTTHILEVAPEAQVLAVDIDEQRLSRVYDNLKRLGMKATVK

QGDGRYPSQWCGEQQFDRILLDAPCSATGVIRRHPDIKWLRRDRDIPELAQLQSEILDAI
WSHLKSGGTLVYATCSMLPEENSLQIKAFLQRTADAELCETGTPEQPGKQNLPGAEEGDG
FFYAKLIKK
>LFGLNPFC_04659 Methionyl-tRNA formyltransferase
MSESLRIIFAGTPDFAARHLDALLSSGHNIVGVFTQPDRPAGRGKKLMPSPVKVLAEDKG
LPVFQPVSLRPQENQQLVADLQADVMVVVAYGLILPKAVLEMPRLGCINVHGSLLPRWRG
AAPIQRSLWAGDAETGVTIMQMDVGLDTGDMLYKLSCPITAEDTSGTLYDKLAELGPQGL
ITTLKQLADGTAKPEVQDETLVTYAEKLSKEEARIDWSLSAAQLERCIRAFNPWPMSWLE
IEGQPVKVWKASVIDTTTKAAPGTILEANKQGIQVATGDGILNLLSMQPAGKKAMSVQDL
LNSRREWFVPGNRLA
>LFGLNPFC_04660 Peptide deformylase
MSVLQVLHIPDERLRKVAKPVEEVNAEIQRIVDDMFETMYAEEGIGLAATQVDIHQRIIV
IDVSENRDERLVLINPELLEKSGETGIEEGCLSIPEQRALVPRAEKVKIRALDRDGKPFE
LEADGLLAICIQHEMDHLVGKLFMDYLSPLKQQRIRQKVEKLDRLKARA
>LFGLNPFC_04661 hypothetical protein
MVDTEIWLRLISISSLYGDDMVRIAHWLAKQSHIDAVVLQQTGLTLRQAQRFLSFPRKSI
ESSLCWLEQPNHHLIPADSEFYPPQLLATTDYPGALFVEGELHALHSFQLAVVGSRAHSW
YGERWGRLFCETLATRGVTITSGLARGIDGVAHKAALQVNGVSIAVLGNGLNTIHPRRHA
PLAASLLEQGGALVSEFPLDVPPLAYNFPRRNRIISGLSKGVLVVEAALRSGSLVTARCA
LEQGREVFALPGPIGNPGSEGPHWLIKQGAILVTEPEEILENLQFGLHWLPDAPENSFYS
PDQEDVALPFPELLANVGDEVTPVDVVAERAGQPVPEVVTQLLELELAGWIAAVPGGYVR
LRRACHVRRTNVFV
>LFGLNPFC_04662 Protein Smg
MFDVLMYLFETYIHTEAELRVDQDKLEQDLTDAGFDREDIYNALLWLEKLADYQEGLAEP
MQLASDPLSMRIYTPEECERLDASCRGFLLFLEQIQVLNLETREMVIERVLALDTAEFDL
EDLKWVILMVLFNIPGCENAYQQMEELLFEVNEGMLH
>LFGLNPFC_04663 DNA topoisomerase 1
MAKSALFTVRNNESCPKCGAELVIRSGKHGPFLGCSQYPACDYVRPLKSSADGHIVKVLE
GQVCPACGANLVLRQGRFGMFIGCSNYPECEHTELIDKPDETAITCPQCRTGHLVQRRSR
YGKTFHSCDRYPECQFAINFKPIAGECPECHYPLLIEKKTAQGVKHFCASKQCGKPISAE
>LFGLNPFC_04664 Threonylcarbamoyl-AMP synthase
MNNNLQGDAIAAAIDVLNEERVIAYPTEAVFGVGCDPDSETAVMRLLELKQRPVDKGLIL
IAANYEQLKPYIDDTMLTDAQRETIFSRWPGPVTFVFPAPATTPRWLTGRFDSLAVRVTD
HPLVVALCQAYGKPLVSTSANLSGLPPCRTVDEVRAQFGAAFPVVPGETGGRLNPSEIRD
ALTGELFRQG
>LFGLNPFC_04665 Shikimate dehydrogenase (NADP(+))
METYAVFGNPIAHSKSPFIHQQFAQQLNIEHPYGRVLAPINDFINTLNAFFSAGGKGANV
TVPFKEEAFARADELTERAALAGAVNTLKRLEDGRLLGDNTDGIGLLSDLERLSFIRPGL
RILLIGAGGASRGVLLPLLSLDCAVTITNRTVSRAEELTKLFAHTGSIQALGMDELEGHE
FDLIINATSSGISGDIPAIPSSLIHPGIYCYDMFYQKGKTPFLAWCEQRGSKRNADGLGM
LVAQAAHAFLLWHGVLPDVEPVIKLLQQELSA
>LFGLNPFC_04666 hypothetical protein
MNQAIQFPDREEWDENKRCVCFPALVNGMQLTCAISGESLAYRFTGDTPEQWLASFRQHR
WDLEEEAENLIQEQSEDDQGWVWLP
>LFGLNPFC_04667 Protein YrdA
MSDVLRPYRDLFPQIGQRVMIDDSSVVIGDVRLADDVGIWPLVVIRGDVHYVQIGARTNI
QDGSMLHVTHKSSYNPDGNPLTIGEDVTVGHKVMLHGCTIGNRVLVGMGSILLDGAIVED
DVMIGAGSLVPQNKRLESGYLYLGSPVKQIRPLSDEEKAGLRYSANNYVKWKDEYLDQGN
QTQP
>LFGLNPFC_04675 Glutamine transport ATP-binding protein GlnQ
MSQILLQPANAMITLENVNKWYGQFHVLKNINLIVQPGERIVLCGPSGSGKSTTIRCINH
LEEHQQGRIVVDGIELNEDIRNIERVRQEVGMVFQHFNLFPHLTVLQNCTLAPIWVRKMP
KKEAEALAMHYLERVRIAEHAQKFPGQISGGQQQRVAIARSLCMKPKIMLFDEPTSALDP
EMVKEVLDTMIGLAQSGMTMLCVTHEMGFARTVADRVIFMDRGEIVEQAAPNEFFAHPKS
ERTRTFLSQVIH
>LFGLNPFC_04676 Inner membrane amino-acid ABC transporter permease protein YhdY
MTKVLLSHPPRLASHNSSRAMVWVRKNLFSSWSNSLLTIGCIWLMWELIPPLLNWVFLQA
NWVGSTRADCTKSGACWVFIHERFGQFMYGLYPHDQRWRINLALLIGLVSIAPMFWKILP
HRSRYIAVWAVIYPLVVWWLMYGGFLGLDRVETRQWGGLTLTLIIASVGIAGALPWGILL
ALGRRSHMPIVRILSVIFIEFWRGVPLITVLFMSSVMLPLFMAEGTSIDKLIRALVGVIL
FQSAYVAEVVRGGLQALPKGQYEAAESLALGYWKTQGLVILPQALKLVIPGLVNTIIALF
KDTSLVIIIGLFDLFSSVQQATVDPAWLGMSTEGYVFAALIYWIFCFSMSRYSQHLEKRF
NTGRTPH
>LFGLNPFC_04677 hypothetical protein

MSHRRSTVKGSLSFANPTVRAWLFQILAVVAVVGIIGWLFHNTVTNLSNRGITSGFAFLD
RGAGFGIVQHLIDYQQGDTYGRVFIVGLLNTLLVSALCIVFASVLGFFIGLARLSDNWLL
RKLSTIYIEIFRNIPPLLQIFFWYFAVLRNLPGPRQAVSALDLVFLSNRGLYIPSPQLGD
GFLAFILAVVIAIVLSVGLFRFNKTHQIKTGQLRRTWPIAAVLIIGLPLLAQWLFGAALH
WDVPALRGFNFRGGMVLIPELAALTLALSIYTSAFIAEIIRAGIQAVPYGQHEAARSLGL
PNPVTLRRVIIPQALRVIIPPLTSQYLNIVKNSSLAAAIGYPDMVSLFAGTVLNQTGQAI
ETIAMTMSVYLIISLTISLLMNIYNRRIAIVER
>LFGLNPFC_04678 Putative amino-acid ABC transporter-binding protein YhdW
MKKMMIATLAAASVLLAVANQAHAGATLDAVQKKGFVQCGISDGLPGFSYADADGKFSGI
DVDVCRGVAAAVFGDDTKVKYTPLTAKERFTALQSGEVDLLSRNTTWTSSRDAGMGMAFT
GVTYYDGIGFLTHDKAGLKSAKELDGATVCIQAGTDTELNVADYFKANNMKYTPVTFDRS
DESAKALESGRCDTLASDQSQLYALRIKLSNPAEWIVLPEVISKEPLGPVVRRGDDEWFS
IVRWTLFAMLNAEEMGINSQNVDEKAANPATPDMAHLLGKEGDYGKDLKLDNKWAFNIIK
QVGNYSEIFERNVGSESPLKIKRGQNNLWNNGGIQYAPPVR
>LFGLNPFC_04679 hypothetical protein
MKRLIPVTLLTALLAGCAHDSPCVPVYDDQGRLVHTNTCMKGTTQDNWETAGAIAGGAAA
VAGLTMGIIALSK
>LFGLNPFC_04680 hypothetical protein
MTIYVNCKNSEGSGNYRKFLKSEKKNILLIFMGFTENKKGA
>LFGLNPFC_04681 Multidrug export protein AcrF
MANFFIRRPIFAWVLAIILMMAGALAILQLPVAQYPTIAPPAVSVSANYPGADAQTVQDT
VTQVIEQNMNGIDNLMYMSSTSDSAGSVTITLTFQSGTDPDIAQVQVQNKLQLATPLLPQ
EVQQQGISVEKSSSSYLMVAGFVSDNPGTTQDDISDYVASNVKDTLSRLNGVGDVQLFGA
QYAMRIWLDADLLNKYKLTPVDVINQLKVQNDQIAAGQLGGTPALPGQQLNASIIAQTRL
KNPEEFGKVTLRVNSDGSVVRLKDVARVELGGENYNVIARINGKPAAGLGIKLATGANAL
DTAKAIKAKLAELQPFFPQGMKVLYPYDTTPFVQLSIQEVVKTLFEAIMLVFLVMYLFLQ
NMRATLIPTIAVPVVLLGTFAILAAFGYSINTLTMFGMVLAIGLLVDDAIVVVENVERVM
MEDKLPPREATEKSMSQIQGALVGIAMVLSAVFIPMAFFGGSTGAIYRQFSITIVSAMAL
SVLVALILTPALCATLLKPVSAEHHENKGGFFGWFNTTFDHSVNHYTNSVGKILGSTGRY
LLIYALIVAGMVVLFLRLPSSFLPEEDQGVFLTMIQLPAGATQERTQKVLDQVTDYYLKN
EKANVESVFTVNGFSFSGQAQNAGMAFVSLKPWEERSGDENSAEAVIHRAKMELGKIRDG
FVIPFNMPAIVELGTATGFDFELIDQAGLGHDALTQARNQLLGMAAQHPASLVSVRPNGL
EDTAQFKLEVDQEKAQALGVSLSDINQTISTALGGTYVNDFIDRGRVKKVYVQADAKFRM
LPEDVDKLYVRSANGEMVPFSAFTTSHWVYGSPRLERYNGLPSMEIQGEAAPGTSSGDAM
ALMENLASKLPAGIGYDWTGMSYQERLSGNQAPALVAISFVVVFLCLAALYESWSIPVSV
MLVVPLGIVGVLLAATLFNQKNDVYFMVGLLTTIGLSAKNAILIVEFAKDLMEKEGKGVV
EATLMAVRMRLRPILMTSLAFILGVLPLAISNGAGSGAQNAVGIGVMGGMVSATLLAIFF
VPVFFVVIRRCFKG
>LFGLNPFC_04682 Multidrug export protein AcrE
MTKHARFFLLPSFILISAALIAGCNDKGEEKAHVGEPQVTVHIVKTAPLEVKTELPGRTN
AYRIAEVRPQVSGIVLNRNFTEGSDVQAGQSLYQIDPATYQASYDSAKGELAKSEAAAAI
AHLTVKRYVPLVGTKYISQQEYDQAIADARQADAAVIAAKATVESARINLAYTKVTAPIS
GRIGKSTVTEGALVTNGQTTELATVQQLDPIYVDVTQSSNDFMRLKQSVEQGNLHKENAT
SNVELVMENGQTYPLKGTLQFSDVTVDESTGSITLRAVFPNPQHTLLPGMFVRARIDEGV
QPDAILIPQQGVSRTPRGDATVLIVNNKSQVEARPVVASQAIGDKWLISEGLKSGDQVIV
SGLQKARPGEQVKATTDTPADTASK
>LFGLNPFC_04683 HTH-type transcriptional regulator AcrR
MAKRTKAEALKTRQELIETAIAQFAQHGVSKTTLNDIADAANVTRGAIYWHFENKTQLFN
EMWLQQPSLRELIQDHLTAGLEHDPFQQLREKLIVGLQYIAKIPRQQALLKILYHKCEFN
DEMLAEGVIREKMGFNPQTLREVLQACQQQGCVANNLDLDVVMIIIDGAFSGIVQNWLMN
MAGYDLYKQAPALVDNVLRMFMPDENITKLIHKTNELSVI
>LFGLNPFC_04684 hypothetical protein
MIRKYWWLVVFAVFVFLFDTLLMQWIELLATETDKCRNMNSVNPLKLVNCDELNFQDRM
>LFGLNPFC_04685 DNA adenine methyltransferase YhdJ
MRTGCEPTRFGNEAKTIIQGDALTELKKLPAESVDLIFADPPYNIGKNFDGLIEAWKEDL
FIDWLFEVIAECHRVLKKQGSMYIMNSTENMPFIDLQCRKLFTIKSRIVWSYDSSGVQAK
KHYGSMYEPILMMVKDAKNYTFNGDAILVEAKTGSQRALINYRKNPPQPYNHQKVPGNVW
DFPRVRYLMDEYENHPTQKPEALLKRIILASSNPGDIVLDPFAGSFTTGAVAVASGRKFI
GIEINSEYIKMGLRRLDVASHYSAEELAKVKKRKTGNLSKRSRLSEVDPDLIAK
>LFGLNPFC_04686 DNA-binding protein Fis
MFEQRVNSDVLTVSTVNSQDQVTQKPLRDSVKQALKNYFAQLNGQDVNDLYELVLAEVEQ
PLLDMVMQYTRGNQTRAALMMGINRGTLRKKLKKYGMN
>LFGLNPFC_04687 tRNA-dihydrouridine synthase B
MRIGQYQLRNRLIAAPMAGITDRPFRTLCYEMGAGLTVSEMMSSNPQVWESDKSRLRMVH

IDEPGIRTVQIAGSDPKEMADAARINVESGAQIIDINMGCPAKKVNRKLAGSALLQYPDV
VKSILTEVVNAVDVPVTLKIRTGWAPEHRNCEEIAQLAEDCGIQALTIHGRTRACLFNGE
AEYDSIRAVKQKVSIPVIANGDITDPLKARAVLDYTGADALMIGRAAQGRPWIFREIQHY
LDTGELLPPLPLAEVKRLLCAHVRELHDFYGPAKGYRIARKHVSWYLQEHAPNDQFRRTF
NAIEDASEQLEALEAYFENFA
>LFGLNPFC_04688 Ribosomal protein L11 methyltransferase
MPWIQLKLNTTGANAEDLSDALMEAGAVSITFQDTHDTPVFEPLPGETRLWGDTDVIGLF
DAETDMNDVVAILENHPLLGAGFAHKIEQLEDKDWEREWMDNFHPMRFGERLWICPSWRD
VPDENAVNVMLDPGLAFGTGTHPTTSLCLQWLDSLDLTGKTVIDFGCGSGILAIAALKLG
AAKAIGIDIDPQAIQASRDNAERNGVSDRLELYLPKDQPEEMKADVVVANILAGPLRELA
PLISVLPVSGGLLGLSGILASQAESVCEAYADSFALDPVVEKEEWCRITGRKN
>LFGLNPFC_04689 Sodium/pantothenate symporter
MQLEVILPLVAYLVVVFGISVYAMRKRSTGTFLNEYFLGSRSMGGIVLAMTLTATYISAS
SFIGGPGAAYKYGLGWVLLAMIQLPAVWLSLGILGKKFAILARRYNAVTLNDMLFARYQS
RLLVWLASLSLLVAFVGAMTVQFIGGARLLETAAGIPYETGLLIFGISIALYTAFGGFRA
SVLNDTMQGLVMLIGTVVLLIGVVHAAGGLSNAVDTLQTIDPQLVTPQGADDILSPAFMT
SFWVLVCFGVIGLPHTAVRCISYKDSKAVHRGIIIGTIVVAILMFGMHLAGALGRAVIPD
LTVPDLVIPTLMVKVLPPFAAGIFLAAPMAAIMSTINAQLLQSSATIIKDLYLNIRPDQM
QNETRLKRMSAVITLVLGALLLLAAWKPPEMIIWLNLLAFGGLEAVFLWPLVLGLYWERA
NAKGALSAMIVGGVLYAVLAKLNIQYLGFHPIVPSLLLSLLAFLVGNRFGTSVPQATVLT
TDK
>LFGLNPFC_04690 putative membrane protein YhdT
MDTRFVQAHKEARWALGLTLLYLAVWLVAAYLPGVAPGFTGFPRWFEMACILTPLLFIGL
CWAMVKFIYRDIPLEDDDAA
>LFGLNPFC_04691 Tagatose kinase
MTITICAMGELLAEFLSRNPHQKFTQPGEFIGPFPSGAPAIFAAQVAKLSHRAIFFGCVG
NDDFARLIIERLRHEGVITDGIHVMNNAVTGTAFVSYQNPQQRDFVFNIPNSACGLFTAE
HIDKDLLKQCNHLHIVGSSLFSFRMIDVMRKAITTIKSAGGTVSFDPNIRKEMLSIPEMA
QALDYLIEYTDIFIPSESELPFFARHKNLSEEQIVSDLLHGGVKHVAIKRAQRGASYYKL
KNGTLHAQHVAGHDIEIIDPTGAGDCFGATFITLFLSGFPAHKALQYANASGALAVMRQG
PMEGISSLADIEDFLQQH
>LFGLNPFC_04692 Glucitol operon repressor
MPRKSSNLLKRRLKIAEIVAAQGEIKVDDLSAQLGVSGVTIRGDLSYLEQQGYLKRSFGG
AIATPLSSEPEAIQPIAPLSLAQQMEIARHCARLIRDRDTLFLGHGTICRKIIPLLSEVK
KLRLITNDPEHALLANQFIDGEIILAGSEMLRPDTALAGKPLEQALQHFTITHSILEISH
LDADGTLNINVPRLAAAWQLCFDHAQNKTVIVAADPAPSTAAIGHLHQAENVIVSRSINE
QYQQQLQSADFVILYTSNECFTWSNRERLQE
>LFGLNPFC_04693 D-tagatose-1,6-bisphosphate aldolase subunit GatY
MYIISSKNMLKKAQSEGYAVPAFNIHNLETLQVVVESAAQLRSPVMLAGTPGTYRYGGVG
SLISLVQSLAREYNLPLVLHLDHHEESDDIFNKVRAGIRSVMIDGSHLPFEDNIARVQEV
TTFCHRFDVSVEAELGRLGGQEDDLRVDEKDSAYTSPAAAAEFVQRTQIDSLAVAIGTAH
GLYAHEPRLDFVRLEQIRQRVEIPLVLHGASGLSEQDVQHCIRRGICKVNVATELKIAFA
DALKGYFYNNPAANDPRHYMQPAKAAMKEVVIRIIGVCGSEGKI
>LFGLNPFC_04694 Ribose import binding protein RbsB
MKNIKLLGSAMLVSLALFSQSSLAKEYKIGASLLTQQHPFYIDLANAMKEEAKKDNVNLS
VSIANQDLNKQLSDVEDFITKKVDAIIVSPVDSKGVQAAIIKADKAGIPVITVDVAAEGV
PVVSHVATDNYAGGVEAGKLMGKLLHGKGTVAIISYPALQSVVARVDGFKKGLSDTPDIK
IVAEQPGITRAEALTTAQNIMQANANLNGLFGFGDDAALAAVIAAKSAHNDNIKIIGFDG
MKEACDAVDSEKTFAAVIRQYPDQMGAKAIDAAVDHLNGKPVEKMIPVQPGVYTGK
>LFGLNPFC_04695 Ribose import ATP-binding protein RbsA
MTDVILDVSHIAKTFGHVQALKDITLSLRKGRVHTLLGENGAGKSTLMKILAGVYPPTQG
TITLRGETITINNPQHSRQLGIAIIFQELSLSNNMTVAENIYANNEPRRFGIINDKKMLA
DCQNLLADLGIPLDPLEMVGNMSMAHRQLVEIAKALSYAADVVIMDEPTSSLSDNEAEIL
FNIIEKLKQRGCAVIYISHRMDEIMRISDDISVIRDGEYIATHEKKNSDIQHLIAQMVGR
EMKNIWPARLGEKPDENVPAKLEVKNLSHPSLFKEVSFAVRPGEVLGFFGLVGAGRSDVM
KALFGLVSYHGTVLIDGKEVRIANPKQAIDHGIAFVTENRKEEGLVLMHDVNMNTHHVAF
QYNASRMGLINHRQEEAKTLQSIARMNTKVSSVHQAVGALSGGNQQKIVLSKWLEKTPRI
LLLDEPTRGVDVGAKFEIYNVIRQLAAAGTAIILVSSELPEVMALSDRLVVMRNKTIADI
YSCENLTQIQVMTAATGVR
>LFGLNPFC_04696 Ribose import permease protein RbsC
MTEKTLASPKPSEIPIANNFKKVFRQYGGILSGMVVLIILFSFINDSFFTANNITNIILQ
VSIIAITAYGMTYVLLLGDIDLSVGSTIALIGTFAALGASWGIPFVLLVPLSIIAALTLG
MINGGLTAIAGIPSFIVTVATMGIFRGIAYIVTDGMPIMIKDDAFLALGNGEFLYIPIPI
WILIIILLLINHFILTKTTFGRKIYITGGNKEAAIYSGINVTRLKIKVFMITAVLAGISGM

ILASRLYSGQPNSALSYELDAIAAAVLGGTSLNGGYGTVVGTVIGALTIGVINNGMNLMN
VPYFYQMVVKGLVILVAVYFDVRNKRKRS
>LFGLNPFC_04697 Tagatose kinase
MSKVFTIGEILVEIMASKIGQPFDQPGIWNGPYPSGAPAIFIDQVTRLGVPCGIISCVGN
DGFGDINIHRLAADGVDIRGISVLPLEATGSAFVTYHNSGDRDFIFNIKNAACGKLSAQH
VDENILKDCTHFHIMGSSLFSFHMVDAVKKAVTIVKANGGVISFDPNIRKEMLDIPEMRD
ALHFVLELTDIYMPSEGEVLLLSPHSTPERAIAGFLEEGVKEVIVKRGNQGASYYSANEQ
FHVESYPVEEVDPTGAGDCFGGAWIACRQLGFDAHRALQYANACGALAVTRRGPMEGTSR
LMEIETFIQRHDMSIREAAQ
>LFGLNPFC_04698 hypothetical protein
MKNEIWSWIGDLSQVAGIKHYELRSGRAKGTEAFDVRTGAGLAFTVVKDRALDIAWASYK
DTALSFITPNGVVAPAFFESQGNGFLRSFYAGLLTTCGLSYIGTPCEDEGETLGLHGRLA
ATPAEEVGYRTERTDDSIEFVINGKVRETRLFGENLTLERTIRCRYGENVMRIEDKVTNH
GFTRQPLQILYHFNYGWPLLSPQAEILLSAKSITPRTPHAAEGLASHLEICTPQPGFDEQ
VYYLTLNSDSQGMSKVALVNAELGWGIYEKFDTMQLPNFIQWKNLGAGEYVMGLEVSNSF
PDGRDKERAQGRLPFIEPGETKTYCFELGIVDGDAEISALKAEIASYR
>LFGLNPFC_04699 Biotin carboxylase
MLDKIVIANRGEIALRILRACKELGIKTVAVHSSADRDLKHVLLADETVCIGPAPSVKSY
LNIPAIISAAEITGAVAIHPGYGFLSENANFAEQVERSGFIFIGPKAETIRLMGDKVSAI
AAMKKAGVPCVPGSDGPLGDDMDKNRAIAKRIGYPVIIKASGGGGGRGMRVVRSDAELAQ
SISMTRAEAKAAFSNDMVYMEKYLENPRHVEIQVLADGQGNAIYLAERDCSMQRRHQKVV
EEAPAPGITPELRRYIGERCAKACVDIGYRGAGTFEFLFENGEFYFIEMNTRIQVEHPVT
EMITGVDLIKEQLRIAAGQPLSIKQEEVHVRGHAVECRINAEDPNTFLPSPGKITRFHAP
GGFGVRWESHIYAGYTVPPYYDSMIGKLICYGENRDVAIARMKNALQELIIDGIKTNVDL
QIRIMNDENFQHGGTNIHYLEKKLGLQEK
>LFGLNPFC_04700 Biotin carboxyl carrier protein of acetyl-CoA carboxylase
MDIRKIKKLIELVEESGISELEISEGEESVRISRAAPAASFPVMQQAYAAPMMQQPAQSN
AAAPATVPSMEAPAAAEISGHIVRSPMVGTFYRTPSPDAKAFIEVGQKVNVGDTLCIVEA
MKMMNQIEADKSGTVKAILVESGQPVEFDEPLVVIE
>LFGLNPFC_04701 putative acrylyl-CoA reductase AcuI
MQALLLEQQDGKTLASVQTLDESRQPEGDVTVDVHWSSLNYKDALAITGKGKIIRNFPMI
PGIDFAGTVRTSEDPRFHAGQEVLLTGWGVGENHWGGLAEQARVKGDWLVAMPQGLDARK
AMIIGTAGFTAMLCVMALEDAGVRPQDGEIVVTGASGGVGSTAVALLHKLGYQVVAVSGR
ESTHEYLKSLGASRILPRDEFAESRPLEKQVWAGAIDTVGDKVLAKVLAQMNYGGCVAAC
GLAGGFTLPTTVMPFILRNVRLQGVDSVMTPPARRAQAWQRLVADLPESFYTQAAKEISL
AEAPKFAEAIINNQIQGRTLVKVN
>LFGLNPFC_04702 RNase E specificity factor CsrD
MRLTTKFSAFVTLLTGLTIFVTLLGCSLSFYNAIQYKFSHRVQAVATAIDTHLVSNDFST
LRPQITELMMSADIVRVDLLHGDKQVYTLARNGSYRPVGTNDLFRELSVPLIKHPGMSLR
LVYQDPMGNYFHSLMTTAPLTGAIGFIILMLFLAVRWLQRQLAGQELLETRATRILNGER
GSNVLGTIYEWPPRTSSALDTLLCEIQNAREQHSRLDTLIRSYAAQDMKTGLNNRLFFDN
QLATLLEDQEKVGTHGIVMMIRLPDFNMLSDTWGHSQVEEQFFSLTNLLSTFMMRYPGAL
LARYHRSDFAALLPHRTLKEAESIASQLIKAVDTLPNNKMLDRDDMIHIGICAWRSGQDT
EQVMEHAESATRNAGLQGGNSWAIYDDSLPEKGRGNVRWRTLIEQMLSRGGPRLYQKPAV
TREGRVHHRELMCRIFDGNEEVSSAEYMPMVLQFGLSEEYDRLLISRLIPLLRYWPEENL
AIQVTVESLIRPRFQRWLRDTLMQCEKSQRRRIIIELAEADVGQHISRLQPVIRLVNALG
VRVAVNQAGLTLVSTSWIKELNVELLKLHPGLVRNIEKRTENQLLVQSLVEACSGTSTQV
YATGVRSRSEWQTLIQRGVTGGQGDFFASSQPLDTNVKKYSQRYSV
>LFGLNPFC_04703 Cell shape-determining protein MreB
MLKKFRGMFSNDLSIDLGTANTLIYVKGQGIVLNEPSVVAIRQDRAGSPKSVAAVGHDAK
QMLGRTPGNIAAIRPMKDGVIADFFVTEKMLQHFIKQVHSNSFMRPSPRVLVCVPVGATQ
VERRAIRESAQGAGAREVFLIEEPMAAAIGAGLPVSEATGSMVVDIGGGTTEVAVISLNG
VVYSSSVRIGGDRFDEAIINYVRRNYGSLIGEATAERIKHEIGSAYPGDEVREIEVRGRN
LAEGVPRGFTLNSNEILEALQEPLTGIVSAVMVALEQCPPELASDISERGMVLTGGGALL
RNLDRLLMEETGIPVVVAEDPLTCVARGGGKALEMIDMHGGDLFSEE
>LFGLNPFC_04704 Cell shape-determining protein MreC
MKPIFSRGPSLQIRLILAVLVALGIIIADSRLGTFSQIRTYMDTAVSPFYFVSNAPRELL
DGVSQTLASRDQLELENRALRQELLLKNSELLMLGQYKQENARLRELLGSPLRQDEQKMV
TQVISTVNDPYSDQVVIDKGSVNGVYEGQPVISDKGVVGQVVAVAKLTSRVLLICDATHA
LPIQVLRNDIRVIAAGNGCTDDLQLEHLPANTDIRVGDVLVTSGLGGRFPEGYPVAVVSS
VKLDTQRAYTVIQARPTAGLQRLRYLLLLWGADRNGANPMTPEEVHRVANERLMQMMPQV
LPSPDAMGPKLPEPATGITQPTPQQPATGNAVTAPAAPTQPAANRSPQRATPPQSGAQPP
ARAPGGQ
>LFGLNPFC_04705 Rod shape-determining protein MreD

MASYRSQGRWVIWLSFLIALLLQIMPWPDNLIVFRPNWVLLILLYWILALPHRVNVGTGF
VMGAILDLISGSTLGVRVLAMSIIAYLVALKYQLFRNLALWQQALVVMLLSLVVDIIVFW
AEFLVINVSFRPEVFWSSVVNGVLWPWIFLLMRKVRQQFAVQ
>LFGLNPFC_04706 dTTP/UTP pyrophosphatase
MTSLYLASGSPRRQELLAQLGVTFERIVTGIEEQRQPQESAQQYVVRLAREKAQAGVAQT
AQDLPVLGADTIVILNGEVLEKPRDAEHAAQMLRKLSGQTHQVMTAVALADSQHILDCLV
VTDVTFRTLTDEDIAGYVASGEPLDKAGAYGIQGLGGCFVRKINGSFHAVVGLPLVETYE
LLSNFNALREKRDKHDG
>LFGLNPFC_04707 Ribonuclease G
MTAELLVNVTPSETRVAYIDGGILQEIHIEREARRGIVGNIYKGRVSRVLPGMQAAFVDI
GLDKAAFLHASDIMPHTECVAGEEQKQFTVRDISELVRQGQDLMVQVVKDPLGTKGARLT
TDITLPSRYLVFMPGASHVGVSQRIESESERERLKKVVAEYCDEQGGFIIRTAAEGVGEA
ELASDAAYLKRVWTKVMERKKRPQTRYQLYGELALAQRVLRDFADAELDRIRVDSRLTYE
ALLEFTSEYIPEMTSKLEHYTGRQPIFDLFDVENEIQRALERKVELKSGGYLIIDQTEAM
TTVDINTGAFVGHRNLDDTIFNTNIEATQAIARQLRLRNLGGIIIIDFIDMNNEDHRRRV
LHSLEQALSKDRVKTSVNGFSALGLVEMTRKRTRESIEHVLCNECPTCHGRGTVKTVETV
CYEIMREIVRVHHAYDSDRFLVYASPAVAEALKGEESHSLAEVEIFVGKQVKVQIEPLYN
QEQFDVVMM
>LFGLNPFC_04708 hypothetical protein
MRRLPGILLLTGAALVVIAALLVSGLRIALPHLDAWRPEILNKIESATGMPVEASQLSAS
WQNFGPTLEAHDIRAELKDGGEFSVKRVTLALDVWQSLLHMRWQFRDLTFWQLRFRTNTP
ITSGGGNDSLEASHISDLFLRQFDHFDLRDSEVSFLTPSGQRAELAIPQLTWLNDPRRHR
AEGLVSLSSLTGQHGVMQVRMDLRDDEGLLSNGRVWLQADDIDLKPWLGKWMQDNIALET
AQFSLEGWMTIDKGDVTGGDVWLKQGGASWLGEKETHTLSVDNLTAHITRENPGWQFSIP
DTRITMDGKPWPSGALTLAWIPEQDVGGKDNKRSDELRIRASNLELAGLEGVRPLVAKLS
PALGDVWRSTQPSGKINTLALDIPLQAADKTRFQASWSDLAWKQWKLLPGAEHFSGTLSG
SVENGLLTASMKQAKMPYETVFRAPLEIADGQATISWLNNDKGFQLDGRNIDVKAKAVHA
RGGFRYLQPANDEPWLGILAGISTDDGSQAWRYFPENLMGKDLVDYLSGAIQGGEADNAT
LVYGGNPQLFPYKHNEGQFEVLVPLRNAKFAFQPDWPALTNLDIELDFINDGLWMKTDGV
NLGGVRASNLTAVIPDYSKEKLLIDADIKGPGKAVGPYFDETPLKDSLGATLQELQLDGD
VNARLHLDIPLNGELVTAKGEVTLRNNSLFIKPLDSTLKNLSGKFSFINGDLQSEPLTAS
WFNQPLNVDFSTKEGAKAYQVAVNLNGNWQPAKTGVLPVAVNEALSGSVAWDGKVGIDLP
YHAGATYNVELNGDLKNVSSHLPSPLAKPAGEPLAVNVKVDGNLNSFELTGQAGADNHFN
SRWLLGQKLTLDRAIWAADSKTLPPLPEQSGVELNMPPMNGAEWLALFQKGAAESVGGAA
SFPQHITLRTPMLSLGNQQWNNLSIVSQPTANGTLVEAQGREINATLAMRNNAPWLANIK
YLYYNPSVAKTRGDSTQSSPFPTTERINFRGWPDAQIRCAECWFWGQKFGRIDSDITISG
DTLTLTNGLIDTGFSRLTADGEWVNNPGNERTSLKGKLRGQKIDAAAEFFGVTTPIRQSS
FNVDYDLHWRKAPWQPDEATLNGIIHTQLGKGEITEINTGHAGQLLRLLSVDALMRKLRF
DFRDTFGEGFYFDSIRSTAWIKDGVMHTDDTLVDGLEADIAMKGSVNLVRRDLNMEAVVA
PEISATVGVAAAFAVNPIVGAAVFAASKVLGPLWSKVSILRYHISGPLDDPQINEVLRQP
RKEKAQ
>LFGLNPFC_04709 Metalloprotease TldD
MSLNLVSEQLLAANGLKHQDLFAILGQLAERRLDYGDLYFQSSYHESWVLEDRIIKDGSY
NIDQGVGVRAISGEKTGFAYADQISLLALEQSAQAARTIVRDSGDGKVQTLGAVEHSPLY
TSVDPLQSMSREEKLDILRRVDKVAREADKRVQEVTASLSGVYELILVAATDGTLAADVR
PLVRLSVSVLVEEDGKRERGASGGGGRFGYEFFLADLDGEVRADAWAKEAVRMALVNLSA
VAAPAGTMPVVLGAGWPGVLLHEAVGHGLEGDFNRRGTSVFSGQVGELVASELCTVVDDG
TMVDRRGSVAIDDEGTPGQYNVLIENGILKGYMQDKLNARLMGMTPTGNGRRESYAHLPM
PRMTNTYMLPGKSTPQEIIESVEYGIYAPNFGGGQVDITSGKFVFSTSEAYLIENGKVTK
PVKGATLIGSGIETMQQISMVGNDLKLDNGVGVCGKEGQSLPVGVGQPTLKVDNLTVGGT
A
>LFGLNPFC_04710 HTH-type transcriptional regulator DmlR
MERLKRMSVFAKVVEFGSFTAAARQLQMSVSSISQTVSKLEDELQVKLLNRSTRSIGLTE
AGRIYYQGCRRMLHEVQDVHEQLYAFNNTPIGTLRIGCSSTMAQNVLAGLTAKMLKEYPG
LSVNLVTGIPAPDLIADGLDVVIRVGALQDSSLFSRRLGAMPMVVCAAKSYLTQYGIPEK
PADLSSHSWLEYSVRPDNEFELIAPEGISTRLIPQGRFVTNDPMTLVRWLTAGAGIAYVP
LMWVINEINRGELEILLPRYQSDPRPVYALYTEKDKLPLKVQVVINSLTDYFVEVGKLFQ
EMHGRGKEK
>LFGLNPFC_04711 Protein AaeX
MSLFPVIVVFGLSFPPIFFELLLSLAIFWLVRRVLVPTGIYDFVWHPALFNTALYCCLFY
LISRLFV
>LFGLNPFC_04712 p-hydroxybenzoic acid efflux pump subunit AaeA
MKTLIRKFSRTAITVVLVILAFIAIFNAWVYYTESPWTRDARFSADVVAIAPDVSGLITQ
VNVHDNQLVKKGQVLFTIDQPRYQKALEEAQADVAYYQVLAQEKRQEAGRRNRLGVQAMS

REEIDQANNVLQTVLHQLAKAQATRDLAKLDLERTVIRAPADGWVTNLNVYTGEFITRGS
TAVALVKQNSFYVLAYMEETKLEGVRPGYRAEITPLGSNKVLKGTVDSVAAGVTNASSTR
DDKGMATIDSNLEWVRLAQRVPVRIRLDNQQENIWPAGTTATVVVTGKQDRDESQDSFFR
KMAHRLREFG
>LFGLNPFC_04713 p-hydroxybenzoic acid efflux pump subunit AaeB
MGIFSIANQHIRFAVKLATAIVLALFVGFHFQLETPRWAVLTAAIVAAGPAFAAGGEPYS
GAIRYRGFLRIIGTFIGCIAGLVIIIAMIRAPLLMILVCCIWAGFCTWISSLVRIENSYA
WGLAGYTALIIVITIQPEPLLTPQFAVERCSEIVIGIVCAIMADLLFSPRSIKQEVDREL
ESLLVAQYQLMQLCIKHGDGEVVDKAWGDLVRRTTALQGMRSNLNMESSRWARANRRLKA
INTLSLTLITQSCETYLIQNTRPELITDTFREFFDTPVETAQDVHKQLKRLRRVIAWTGE
RETPVTIYSWVAAATRYQLLKRGVISNTKINATEEEILQGEPEVKVESAERHHAMVNFWR
TTLSCILGTLFWLWTGWTSGSGAMVMIAVVTSLAMRLPNPRMVAIDFIYGTLAALPLGLL
YFLVIIPNTQQSMLLLCISLAVLGFFLGIEVQKRRLGSMGALASTINIIVLDNPMTFHFS
QFLDSALGQIVGCVLAFTVILLVRDKSRDRTGRVLLNQFVSAAVSAMTTNVARRKENHLP
ALYQQLFLLMNKFPGDLPKFRLALTMIIAHQRLRDAPIPVNEDLSAFHRQMRRTADHVIS
ARSDDKRRRYFGQLLEELEIYQEKLRIWQAPPQVTEPVHRLTGMLHKYQHALTDS
>LFGLNPFC_04714 putative protein YhcO
MNIYTFDFDEIESQEDFYRDFSQTFGLAKDKVRDLDSLWDVLMNDVLPLPLEIEFVHLGE
KTRRRFGALILLFDEAEEELEGHLRFNVRH
>LFGLNPFC_04715 Multiple stress resistance protein BhsA
MKIKTTVAALSVLSVLSFGAFAADSIDAAQAQNREAIGTVSVSGVASSPMDMREMLNKKA
EEKGATAYQITEARSGDTWHATAELYK
>LFGLNPFC_04716 Arginine repressor
MRSSAKQEELVKAFKALLKEEKFSSQGEIVAALQEQGFDNINQSKVSRMLTKFGAVRTRN
AKMEMVYCLPAELGVPTTSSPLKNLVLDIDYNDAVVVIHTSPGAAQLIARLLDSLGKAEG
ILGTIAGDDTIFTTPANGFTVKELYEAILELFDQEL
>LFGLNPFC_04717 Malate dehydrogenase
MKVAVLGAAGGIGQALALLLKTQLPSGSELSLYDIAPVTPGVAVDLSHIPTAVKIKGFSG
EDATPALEGADVVLISAGVARKPGMDRSDLFNVNAGIVKNLVQQVAKTCPKACIGIITNP
VNTTVAIAAEVLKKAGVYDKNKLFGVTTLDIIRSNTFVAELKGKQPGEVEVPVIGGHSGV
TILPLLSQVPGVSFTEQEVADLTKRIQNAGTEVVEAKAGGGSATLSMGQAAARFGLSLVR
ALQGEQGVVECAYVEGDGQYARFFSQPLLLGKNGVEERKSIGTLSAFEQNALEGMLDTLK
KDIALGEEFVNK
>LFGLNPFC_04718 Serine endoprotease DegS
MFVKLLRSVAIGLIVGAILLVAMPSLRSLNPLSTPQFDSTDETPASYNLAVRRAAPAVVN
VYNRGLNTNSHNQLEIRTLGSGVIMDQRGYIITNKHVINDADQIIVALQDGRVFEALLVG
SDSLTDLAVLKINATGGLPTIPINARRVPHIGDVVLAIGNPYNLGQTITQGIISATGRIG
LNPTGRQNFLQTDASINHGNSGGALVNSLGELMGINTLSFDKSNDGETPEGIGFAIPFQL
ATKIMDKLIRDGRVIRGYIGIGGREIAPLHAQGGGIDQLQGIVVNEVSPDGPAANAGIQV
NDLIISVDNKPAISALETMDQVAEIRPGSVIPVVVMRDDKQLTLQVTIQEYPATN
>LFGLNPFC_04719 Periplasmic pH-dependent serine endoprotease DegQ
MKKQTQLLSALALSVGLTLSASFQAVASIPGQVADQAPLPSLAPMLEKVLPAVVSVRVEG
TASQGQKIPEEFKKFFGDDLPDQPAQPFEGLGSGVIINANKGYVLTNNHVINQAQKISIQ
LNDGREFDAKLIGSDDQSDIALLQIQNPSKLTQIAIADSDKLRVGDFAVAVGNPFGLGQT
ATSGIISALGRSGLNLEGLENFIQTDASINRGNSGGALLNLNGELIGINTAILAPGGGSV
GIGFAIPSNMARTLAQQLIDFGEIKRGLLGIKGTEMSADIAKAFNLDVQRGAFVSEVLPG
SGSAKAGVKAGDIITSLNGKPLNSFAELRSRIATTEPGTKVKLGLLRNGKPLEVEVTLDT
STSSSASAEMITPALEGATLSDGQLKDGGKGIKIDEVVKGSPAAQAGLQKDDVIIGVNRD
RVNSIAEMRKVLAAKPAIIALQIVRGNESLYLLMR
>LFGLNPFC_04720 Inner membrane protein YhcB
MTWEYALIGLVVGIIIGAVAMRFGNRKLRQQQALQYELEKNKAELDEYREELVSHFARSA
ELLDTMAHDYRQLYQHMAKSSSSLLPELSAEANPFRNRLAESEASNDQAPVQMPRDYSEG
ASGLLRTGAKRD
>LFGLNPFC_04721 Cell division protein ZapE
MQSVTPTSQYLKALNEGSHQPDDVQKEAVSRLEIIYQELINSTPPAPRTSGLMARVGKLW
GKREDTKHMPVRGLYMWGGVGRGKTWLMDLFYQSLPGERKQRLHFHRFMLRVHEELTALQ
GQTDPLEIIADRFKAETDVLCFDEFFVSDITDAMLLGGLMKALFARGITLVATSNIPPDE
LYRNGLQRARFLPAIDAIKRHCDVMNVDAGVDYRLRTLTQAHLWLSPLNDETRAQMDKLW
LALAGAKRENSPTLEINHRPLATMGVENQTLAVSFTTLCVDARSQHDYIALSRLFHTVML
FDVPVMTRLMESEARRFIALVDEFYERHVKLVVSAEVPLYEIYQGERLKFEFQRCLSRLQ
EMQSEEYLKREHLAG
>LFGLNPFC_04722 50S ribosomal protein L13
MKTFTAKPETVKRDWYVVDATGKTLGRLATELARRLRGKHKAEYTPHVDTGDYIIVLNAD
KVAVTGNKRTDKVYYHHTGHIGGIKQATFEEMIARRPERVIEIAVKGMLPKGPLGRAMFR

KLKVYAGNEHNHAAQQPQVLDI
>LFGLNPFC_04723 30S ribosomal protein S9
MAENQYYGTGRRKSSAARVFIKPGNGKIVINQRSLEQYFGRETARMVVRQPLELVDMVEK
LDLYITVKGGGISGQAGAIRHGITRALMEYDESLRSELRKAGFVTRDARQVERKKVGLRK
ARRRPQFSKR
>LFGLNPFC_04724 Stringent starvation protein A
MAVAANKRSVMTLFSGPTDIYSHQVRIVLAEKGVSFEIEHVEKDNPPQDLIDLNPNQSVP
TLVDRELTLWESRIIMEYLDERFPHPPLMPVYPVARGESRLYMHRIEKDWYTLMNTIING
SASEADAARKQLREELLAIAPVFGQKPYFLSDEFSLVDCYLAPLLWRLPQLGIEFSGPGA
KELKGYMTRVFERDSFLASLTEAEREMRLGRS
>LFGLNPFC_04725 Stringent starvation protein B
MDLSQLTPRRPYLLRAFYEWLLDNQLTPHLVVDVTLPGVQVPMEYARDGQIVLNIAPRAV
GNLELANDEVRFNARFGGIPRQVSVPLAAVLAIYARENGAGTMFEPEAAYDEDASIMNDE
EASADNETVMSVIDGDKPDHDDETHPDDEPPQPPRGGRPALRVVK
>LFGLNPFC_04726 HTH-type transcriptional repressor NanR
MNAFDPQAEDSTTTIGRNLRSRPLARKKLSEMVEEELEQMIRRREFGEGEQLPSERELMA
FFNVGRPSVREALAALKRKGLVQINNGERARVSRPSADTIIGELSGMAKDFLSHPGGIAH
FEQLRLFFESSLVRYAAEHATDEQIDLLAKALEINSQSLDNNAAFIRSDVDFHRVLAEIP
GNPIFMAIHVALLDWLIAARPTVADQALHEHNNVSYQQHIAIVDAIRRHDPDEADRALQS
HLNSVSATWHAFGQTTNKKK
>LFGLNPFC_04727 N-acetylneuraminate lyase
MATNLRGVMAALLTPFDQQQALDKASLRRLVQFNIQQGIDGLYVGGSTGEAFVQSLSERE
QVLEIVAEEAKGKIKLIAHVGCVSTAESQQLAASAKRYGFDAVSAVTPFYYPFSFEEHCD
HYRAIIDSADGLPMVVYNIPALSGVKLTLDQINTLVTLPGVGALKQTSGDLYQMEQIRRE
HPDLVLYNGYDEIFASGLLAGADGGIGSTYNIMGWRYQGIVKALKEGDIQTAQKLQTECN
KVIDLLIKTGVFRGLKTVLHYMDVVSVPLCRKPFGPVDEKYLPELKALAQQLMQERG
>LFGLNPFC_04728 Sialic acid transporter NanT
MSTTTQNIPWYRHLNRAQWRAFSAAWLGYLLDGFDFVLIALVLTEVQGEFGLTTVQAASL
ISAAFISRWFGGLMLGAMGDRYGRRLAMVTSIVLFSAGTLACGFAPGYITMFIARLVIGM
GMAGEYGSSATYVIESWPKHLRNKASGFLISGFSVGAVVAAQVYSLVVPVWGWRALFFIG
ILPIIFALWLRKNIPEAEDWKEKHGGKAPVRTMVDILYRGEHRIANIVMTLAAATALWFC
FAGNLQNAAIVAVLGLLCAAIFISFMVQSTGKRWPTGVMLMVVVLFAFLYSWPIQALLPT
YLKTDLAYDPHTVANVLFFSGFGAAVGCCVGGFLGDWLGTRKAYVCSLLASQLLIIPVFA
IGGANVWVLGLLLFFQQMLGQGIAGILPKLIGGYFDTDQRAAGLGFTYNVGALGGALAPI
LGALIAQRLDLGTALASLSFSLTFVVILLIGLDMPSRVQRWLRPEALRTHDAIDGKPFSG
AVPFGSAKNDLVKTKS
>LFGLNPFC_04729 Putative N-acetylmannosamine-6-phosphate 2-epimerase
MSLLAQLDQKIAANGGLIVSCQPVPDSPLDKPEIVAAMALAAEQAGAVAIRIEGVANLQA
TRAVVSVPIIGIVKRDLEDSPVRITAYIEDVDALAQAGADIIAIDGTDRPRPVPVETLLA
RIHHHGLLAMTDCSTPEDGLACQKLGAEIIGTTLSGYTTPETPEEPDLALVKTLSDAGCR
VIAEGRYNTPAQAADAMRHGAWAVTVGSAITRLEHICQWYNTAMKKAVL
>LFGLNPFC_04730 N-acetylmannosamine kinase
MTTLAIDIGGTKLAAALIGADGQIRDRRELPTPASQTPQALRDALSALVSPLQAHAQRVA
IASTGIIRDGSLLALNPHNLGGLLHFPLVKTLEQLTNLPTIAINDAQAAAWAEYQALDGD
ITDMVFITVSTGVGGGVVSGGKLRTGPGGLAGHIGHTLADPHGPACGCGRTGCVEAIASG
RGIATAAQGELAGANAKTIFTRAGQGDEQAQQLIHRSARTLARLIADIKATTDCQCVVVG
GSVGLAEGYLALVETYLAQEPAAFHVDLLAAHYRHDAGLLGAALLAQGEIL
>LFGLNPFC_04731 putative protein YhcH
MMMGEVQSLPSAGLHPALQDALTLALAARPQEKAPGRYELQGDNIFMNVMTFNTQSPVEK
KAELHEQYIDIQLLLNGEERILFGMAGTARQCEEFHHEDDYQLCSAIENEQAIILKPGMF
AVFMPGEPHKPGCVVGEPGEIKKVVVKIKADLMA
>LFGLNPFC_04732 Glutamate synthase [NADPH] small chain
MSQNVYQFIDLQRVDPPKKPLKIRKIEFVEIYEPFSEGQAKAQADRCLSCGNPYCEWKCP
VHNYIPNWLKLANEGRIFEAAELSHQTNTLPEVCGRVCPQDRLCEGSCTLNDEFGAVTIG
NIERYINDKAFEMGWRPDMSGVKQTGKKVAIIGAGPAGLACADVLTRNGVKAVVFDRHPE
IGGLLTFGIPAFKLEKEVMTRRREIFTGMGIEFKLNTEVGRDVQLDDLLSDYDAVFLGVG
TYQSMRGGLENEDADGVYAALPFLIANTKQLMGFGETNDEPFVSMEGKRVVVLGGGDTAM
DCVRTSVRQGAKHVTCAYRRDEENMPGSRREVKNAREEGVEFKFNIQPLGIEVNGNGKVS
GVKMVRTEMGEPDAKGRRRAEIVAGSEHIVPADAVIMAFGFRPHNMEWLAKHSVELDSQG
RIIAPEGSDNAFQTSNPKIFAGGDIVRGSDLVVTAIAEGRKAADGIMNWLEV
>LFGLNPFC_04733 Glutamate synthase [NADPH] large chain
MTRKPRRHALTVPVRSGSEVGFPQSLGEVHDMLYDKSLERDNCGFGLIAHIEGEPSHKVV
RTAIHALARMQHRGAILADGKTGDGCGLLLQKPDRFFRIVAQERGWRLAKNYAVGMLFLN
KDPELAAAARRIVEEELQRETLSIVGWRDVPTNEGVLGEIALSSLPRIEQIFVNAPAGWR

PRDMERRLFIARRRIEKRLEADKDFYVCSLSNLVNIYKGLCMPADLPRFYLDLADLRLES
AICLFHQRFSTNTVPRWPLAQPFRYLAHNGEINTITGNRQWARARTYKFQTPLIPDLHDA
APFVNETGSDSSSMDNMLELLLAGGMDIIRAMRLLVPPAWQNNPDMDPELRAFFDFNSMH
MEPWDGPAGIVMSDGRFAACNLDRNGLRPARYVITKDKLITCASEVGIWDYQPDEVVEKG
RVGPGELMVIDTRSGRILHSAETDDDLKSRHPYKEWMEKNVRRLVPFEDLPDEEVGSREL
DDDTLASYQKQFNYSAEELDSVIRVLGENGQEAVGSMGDDTPFAVLSSQPRIIYDYFRQQ
FAQVTNPPIDPLREAHVMSLATSIGREMNVFCEAEGQAHRLSFKSPILLYSDFKQLTTMK
EDHYRADTLDITFDVTKTTLEATVKELCDKAEKMVRSGTVLLVLSDRNIAKDRLPVPAPM
AVGAIQTRLVEQSLRCDANIIVETASARDPHHFAVLLGFGATAIYPYLAYETLGRLVDTH
AIAKDYRTVMLNYRNGINKGLYKIMSKMGISTIASYRCSKLFEAVGLHDDVVGLCFQGAV
SRIGGASFEDFQQDLLNLSKRAWLARKPISQGGLLKYVHGGEYHAYNPDVVRTLQQAVQS
GEYSDYQEYAKLVNERPATTLRDLLAITPGENAVNIADVEPASELFKRFDTAAMSIGALS
PEAHEALAEAMNSIGGNSNSGEGGEDPARYGTNKVSRIKQVASGRFGVTPAYLVNADVIQ
IKVAQGAKPGEGGQLPGDKVTPYIAKLRYSVPGVTLISPPPHHDIYSIEDLAQLIFDLKQ
VNPKAMISVKLVSEPGVGTIATGVAKAYADLITIAGYDGGTGASPLSSVKYAGCPWELGL
VETQQALVANGLRHKIRLQVDGGLKTGVDIIKAAILGAESFGFGTGPMVALGCKYLRICH
LNNCATGVATQDDKLRKNHYHGLPFKVTNYFEFIARETRELMAQLGVTRLVDLIGRTDLL
KELDGFTAKQQKLALSKLLETAEPHPGKALYCTENNPPFDNGLLNAQLLQQAKPFVDERQ
SKTFWFDIRNTDRSVGASLSGYIAQTHGDQGLAADPIKAYFNGTAGQSFGVWNAGGVELY
LTGDANDYVGKGMAGGLIAIRPPVGSAFRSHEASIIGNTCLYGATGGRLYAAGRAGERFG
VRNSGAITVVEGIGDNGCEYMTGGIVCILGKTGVNFGAGMTGGFAYVLDESGDFRKRVNP
ELVEVLSVDDLAIHEEHLRGLITEHVQHTGSQRGEEILANWSTFATKFALVKPKSSDVKA
LLGHRSRSAAELRVQAQ
>LFGLNPFC_04734 hypothetical protein
MQLQKLVNMFGGDLTRRYGQKVHKLTLHGGFSCPNRDGTIGRGGCTFCNVASFADEAQQH
RSIAEQLAHQANLVNRAKRYLAYFQAYTSTFAEVQVLRSMYQQAVSQANIVGLCVGTRPD
CVPDAVLDLLCEYKDQGYEVWLELGLQTAHDKTLHRINRGHDFACYQRTTQLARERGLKV
CSHLIVGLPGEGQAECLQTLERVVETGVDGIKLHPLHIVKGSIMAKAWEAGRLNGIELED
YTLTAGEMIRHTPPEVIYHRISASARRPTLLAPLWCENRWTGMVELDRYLNEHGVQGSAL
GRPWLPPTA
>LFGLNPFC_04735 Aerobic respiration control sensor protein ArcB
MKQIRLLAQYYVDLMMKLGLVRFSMLLALALVVLAIVVQMAVTMVLHGQVESIDVIRSIF
FGLLITPWAVYFLSVVVEQLEESRQRLSRLVQKLEEMRERDLSLNVQLKDNIAQLNQEIA
VREKAEAELQETFGQLKIEIKEREETQIQLEQQSSFLRSFLDASPDLVFYRNEDKEFSGC
NRAMELLTGKSEKQLVHLKPADVYSPEAAAKVIETDEKVFRHNVSLTYEQWLDYPDGRKA
CFEIRKVPYYDRVGKRHGLMGFGRDITERKRYQDALERASRDKTTFISTISHELRTPLNG
IVGLSRILLDTELTAEQEKYLKTIHVSAVTLGNIFNDIIDMDKMERRKVQLDNQPVDFTS
FLADLENLSALQAQQKGLRFNLEPTLPLPHQVITDGTRLRQILWNLISNAVKFTQQGQVT
VRVRYDEGDMLHFEVEDSGIGIPQDELDKIFAMYYQVKDSHGGKPATGTGIGLAVSRRLA
KNMGGDITVTSEQGKGSTFTLTIHAPSVAEEVDDAFDEDDMPLPALNVLLVEDIELNVIV
ARSVLEKLGNSVDVAMTGKAALEMFKPGEYDLVLLDIQLPDMTGLDISRELTKRYPREDL
PPLVALTANVLKDKQEYLNAGMDDVLSKPLSVPALTAMIKKFWDTQDDEESTVTTEENSK
SEALLDIPMLEQYLELVGPKLITDGLAVFERMMPGYVSVLESNLTAQDKKGIVEEGHKIK
GAAGSVGLRHLQQLGQQIQSPDLPAWEDNVGEWIEEMKEEWRHDVEVLKAWVAKATKK
>LFGLNPFC_04736 Glyoxalase ElbB
MKKIGVILSGCGVYDGSEIHEAVLTLLAISRSGAQAVCFAPDKQQVDVINHLTGEAMTET
RNVLIEAARITRGEIRPLAQADAAELDALIVPGGFGAVKNLSNFASLGSECTVDRELKAL
AQAMHQAGKPLGFMCIAPAILPKIFDFPLRLTIGTDIDTAEVLEEMGAEHVPCPVDDIVV
DEDNKIVTTPAYMLAQNIAEAASGIDKLVSRVLVLAE
>LFGLNPFC_04737 Biosynthetic peptidoglycan transglycosylase
MSKSRLTVFSFVRRFLLRLMVVLAIFWGGGIALFSVAPVPFSAVMVERQVSAWLHGNFRY
VAHSDWVSMDQISPWMGLAVIAAEDQKFPEHWGFDVASIEQALAHNERNENRIRGASTIS
QQTAKNLFLWDGRSWVRKGLEAGLTLGIETVWSKKRILTVYLNIAEFGDGVFGVEAAAQR
YFHKPASKLTRSEAALLAAVLPNPLRFKVSAPSGYVRSRQAWILRQMYQLGGEPFMQQHQ
LD
>LFGLNPFC_04738 putative protein YrbL
MIRLSEQSPLGTGRHRKCYAHPEDAQRCIKIVYHRGDGGDKEIRRELKYYAHLGRRLKDW
SGIPRYHGTVETDCGTGYVYDVIADFDGKPSITLTEFAEQCRYEEDIAQLRQLLKQLKRY
LQDNRIVTMSLKPQNILCHRISESEVTPVVCDNIGESTLIPLATWSKWCCLRKQERLWKR
FIAQPALAIALQKDLQPRESKTLALTSREA
>LFGLNPFC_04739 Phosphocarrier protein NPr
MTVKQTVEITNKLGMHARPAMKLFELMQGFDAEVLLRNDEGTEAEANSVIALLMLDSAKG
RQIEVEATGPQEEEALAAVIALFNSGFDED
>LFGLNPFC_04740 RNase adapter protein RapZ

MVLMIVSGRSGSGKSVALRALEDMGFYCVDNLPVVLLPDLARTLADREISAAVSIDVRNM
PESPEIFEQAMSNLPDAFSPQLLFLDADRNTLIRRYSDTRRLHPLSSKNLSLESAIDKES
DLLEPLRSRADLIVDTSEMSVHELAEMLRTRLLGKRERELTMVFESFGFKHGIPIDADYV
FDVRFLPNPHWDPKLRPMTGLDKPVAAFLDRHTEVHNFIYQTRSYLELWLPMLETNNRSY
LTVAIGCTGGKHRSVYIAEQLADYFRSRGKNVQSRHRTLEKRKP
>LFGLNPFC_04741 Nitrogen regulatory protein
MTNNDTTLQLSSVLNRECTRSRVHCQSKKRALEIISELAAKQLSLAPQVVFEAILTREKM
GSTGIGNGIAIPHGKLEEDTLRAVGVFVQLETPIAFDAIDNQPVDLLFALLVPADQTKTH
LHTLSLVAKRLADKTICRRLRAAQSDEELYQIITDTEGTPDEA
>LFGLNPFC_04742 Ribosome hibernation promoting factor
MQLNITGNNVEITEALREFVTAKFAKLEQYFDRINQVYVVLKVEKVTHTSDATLHVNGGE
IHASAEGQDMYAAIDGLIDKLARQLTKHKDKLKQH
>LFGLNPFC_04743 RNA polymerase sigma-54 factor
MKQGLQLRLSQQLAMTPQLQQAIRLLQLSTLELQQELQQALESNPLLEQIDTHEEIDTRE
TQDSETLDTADALEQKEMPEELPLDASWDTIYTAGTPSGTSGDYIDDELPVYQGETTQTL
QDYLMWQVELTPFSDTDRAIATSIVDAVDDTGYLTVPLEDILESMGDEEIDIDEVEAVLK
RIQRFDPVGVAAKDLRDCLLIQLSQFDKTTPWLEEARLIISDHLDLLANHDFRTLMRVTR
LKEDVLKEAVNLIQSLDPRPGQSIQTGEPEYVIPDVLVRKHNGHWTVELNSDSIPRLQIN
QHYASMCNNARNDGDSQFIRSNLQDAKWLIKSLESRNDTLLRVSRCIVEQQQAFFEQGEE
YMKPMVLADIAQAVEMHESTISRVTTQKYLHSPRGIFELKYFFSSHVNTEGGGEASSTAI
RALVKKLIAAENPAKPLSDSKLTSLLSEQGIMVARRTVAKYRESLSIPPSNQRKQLV
>LFGLNPFC_04744 Lipopolysaccharide export system ATP-binding protein LptB
MATLTAKNLAKAYKGRRVVEDVSLTVNSGEIVGLLGPNGAGKTTTFYMVVGIVPRDAGNI
IIDDDDISLLPLHARARRGIGYLPQEASIFRRLSVYDNLMAVLQIRDDLSAEQREDRANE
LMEEFHIEHLRDSMGQSLSGGERRRVEIARALAANPKFILLDEPFAGVDPISVIDIKRII
EHLRDSGLGVLITDHNVRETLAVCERAYIVSQGHLIAHGTPTEILQDEHVKRVYLGEDFR
L
>LFGLNPFC_04745 Lipopolysaccharide export system protein LptA
MKFKTNKLSLNLVLASSLLAASIPAFAVTGDTDQPIHIESDQQSLDMQGNVVTFTGNVIV
TQGTIKINADKVVVTRPGGEQGKEVIDGYGKPATFYQMQDNGKPVEGHASQMHYELAKDF
VVLTGNAYLQQVDSNIKGDKITYLVKEQKMQAFSDKGKRVTTVLVPSQLQDKNNKGQTPA
QKKGN
>LFGLNPFC_04746 Lipopolysaccharide export system protein LptC
MSKARRWVIIVLSLAVLVMIGINMAEKDDTAQVVVNNNDPTYKSEHTDTLVYNPEGALSY
RLIAQHVEYYSDQAVSWFTQPVLTTFDKDKIPTWSVKADKAKLTNDRMLYLYGHVEVNAL
VPDSQLRRITTDNAQINLVTQDVTSEDLVTLYGTTFNSSGLKMRGNLRSKNAELIEKVRT
SYEIQNKQTQP
>LFGLNPFC_04747 3-deoxy-D-manno-octulosonate 8-phosphate phosphatase KdsC
MSKAGASLATCYGPVSADVMAKAENIRLLILDVDGVLSDGLIYMGNNGEELKAFNVRDGY
GIRCALTSDIEVAIITGRKAKLVEDRCATLGITHLYQGQSNKLIAFSDLLEKLAIAPENV
AYVGDDLIDWPVMEKVGLSVAVADAHPLLIPRADYVTRIAGGRGAVREVCDLLLLAQGKL
DEAKGQSI
>LFGLNPFC_04748 Arabinose 5-phosphate isomerase KdsD
MSHVELQPGFDFQQAGKEVLAIERECLAELDQYINQNFTLACEKMFWCKGKVVVMGMGKS
GHIGRKMAATFASTGTPSFFVHPGEAAHGDLGMVTPQDVVIAISNSGESSEITALIPVLK
RLHVPLICITGRPESSMARAADVHLCVKVAKEACPLGLAPTSSTTATLVMGDALAVALLK
ARGFTAEDFALSHPGGALGRKLLLRVNDIMHTGDEIPHVKKTASLRDALLEVTRKNLGMT
VICDDNMMIEGIFTDGDLRRVFDMGVDVRQLSIADVMTPGGIRVRPGILAVEALNLMQSR
HITSVMVADGDHLLGVLHMHDLLRAGVV
>LFGLNPFC_04749 Inner membrane protein YrbG
MLLATALLIVGLLLVVYSADRLVFAASILCRTFGIPPLIIGMTVVSIGTSLPEIIVSLAA
SLHEQRDLAVGTALGSNIINILLILGLAALVRPFTVHSDVLRRELPLMLLVSVVAGSVLY
DGQLSRSDGIFLLFLAVLWLLFIVKLARQAERQGTDSLTREQLAELPREGGLPVAFLWLG
IALIIMPVATRMVVDNATVLANYFAISELTMGLTAIAIGTSLPELATAIAGVRKGENDIA
VGNIIGANIFNIVIVLGLPALITPGEIDPLAYSRDYSVMLLVSIIFALLCWRRSPQPGRG
VGVLLTGGFIVWLAMLYWLSPILVE
>LFGLNPFC_04750 Intermembrane phospholipid transport system ATP-binding protein MlaF
MEQSVANLVDMRDVSFTRGNRCIFDNISLTVPRGKITAIMGPSGIGKTTLLRLIGGQIAP
DHGEILFDGENIPAMSRSRLYTVRKRMSMLFQSGALFTDMNVFDNVAYPLREHTQLPAPL
LHSTVMMKLEAVGLRGAAKLMPSELSGGMARRAALARAIALEPDLIMFDEPFVGQDPITM
GVLVKLISELNSALGVTCVVVSHDVPEVLSIADHAWILADKKIVAHGSAQALQANPDPRV
RQFLDGIADGPVPFRYPAGDYHADLLPGS
>LFGLNPFC_04751 Intermembrane phospholipid transport system permease protein MlaE
MLLNALASLGHKGIKTLRTFGRAGLMLFNALVGKPEFRKHAPLLVRQLYNVGVLSMLIIV

VSGVFIGMVLGLQGYLVLTTYSAETSLGMLVALSLLRELGPVVAALLFAGRAGSALTAEI
GLMRATEQLSSMEMMAVDPLRRVISPRFWAGVISLPLLTVIFVAVGIWGGSLVGVSWKGI
DSGFFWSAMQNAVDWRMDLVNCLIKSVVFAITVTWISLFNGYDAIPTSAGISRATTRTVV
HSSLAVLGLDFVLTALMFGN
>LFGLNPFC_04752 Intermembrane phospholipid transport system binding protein MlaD
MQTKKNEIWVGIFLLAALLAALFVCLKAANVTSIRTEPTYTLYATFDNIGGLKARSPVSI
GGVVVGRVADITLDPKTYLPRVTLEIEQRYNHIPDTSSLSIRTSGLLGEQYLALNVGFED
PELGTAILKDGDTIQDTKSAMVLEDLIGQFLYGSKGDDNKNSGDAPAAAPGNNETTEPVG
TTK
>LFGLNPFC_04753 Intermembrane phospholipid transport system binding protein MlaC
MFKRLMMVALLVIAPLSAATAADQTNPYKLMDEAAQKTFDRLKNEQPQIRANPDYLRTIV
DQELLPYVQVKYAGALVLGQYYKSATPAQRDAYFAAFREYLKQAYGQALAMYHGQTYQIA
PEQPLGDKIIVPIRVTIIDPNGRPPVRLDFQWRKNSQTGNWQAYDMIAEGVSMITTKQNE
WGTLLRTKGIDGLTAQLKSISQQKITLEEKK
>LFGLNPFC_04754 Intermembrane phospholipid transport system binding protein MlaB
MSESLSWMQTGDTLALSGELDQDVLLPLWEIREEAVKGITSIDLSRVSRVDTGGLALLLH
LIDLAKKQGNNVTLQGVNDKVYTLAKLYNLPADVLPR
>LFGLNPFC_04755 Acid stress protein IbaG
MIEDPMENNEIQSVLMNALSLQEVHVSGDGSHFQVIAVGELFDGMSRVKKQQTVYGPLME
YIADNRIHAVSIKAYTPAEWARDRKLNGF
>LFGLNPFC_04756 UDP-N-acetylglucosamine 1-carboxyvinyltransferase
MDKFRVQGPTKLQGEVTISGAKNAALPILFAALLAEEPVEIQNVPKLKDVDTSMKLLSQL
GAKVERNGSVHIDARDVNVFCAPYDLVKTMRASIWALGPLVARFGQGQVSLPGGCTIGAR
PVDLHISGLEQLGATIKLEEGYVKASVDGRLKGAHIVMDKVSVGATVTIMCAATLAEGTT
IIENAAREPEIVDTANFLITLGAKISGQGTDRIVIEGVERLGGGVYRVLPDRIETGTFLV
AAAISRGKIICRNAQPDTLDAVLAKLRDAGADIEVGEDWISLDMHGKRPKAVNVRTAPHP
AFPTDMQAQFTLLNLVAEGTGFITETVFENRFMHVPELSRMGAHAEIESNTVICHGVEKL
SGAQVMATDLRASASLVLAGCIAEGTTVVDRIYHIDRGYERIEDKLRALGANIERVKGE
>LFGLNPFC_04757 hypothetical protein
MESNFIDWHPADIIAGLRKKETSMAAESRRNGLSSSTLANALSRPWPKGEMIIAKALGTD
PWVIWPSRYHDPQTHEFIDRTQLMRSYTKPKK
>LFGLNPFC_04758 Octaprenyl diphosphate synthase
MNLEKINELTAQDMAGVNAAILEQLNSDVQLINQLGYYIVSGGGKRIRPMIAVLAARAVG
YEGNAHVTIAALIEFIHTATLLHDDVVDESDMRRGKATANAAFGNAASVLVGDFIYTRAF
QMMTSLGSLKVLEVMSEAVNVIAEGEVLQLMNVNDPDITEENYMRVIYSKTARLFEAAAQ
CSGILAGCTPEEEKGLQDYGRYLGTAFQLIDDLLDYNADGEQLGKNVGDDLNEGKPTLPL
LHAMHHGTPEQAQMIRTAIEQGNGRHLLEPVLEAMNACGSLEWTRQRAEEEADKAIAALQ
VLPDTPWREALVGLAHIAVQRDR
>LFGLNPFC_04759 50S ribosomal protein L21
MYAVFQSGGKQHRVSEGQTVRLEKLDIATGETVEFAEVLMIANGEEVKIGVPFVDGGVIK
AEVVAHGRGEKVKIVKFRRRKHYRKQQGHRQWFTDVKITGISA
>LFGLNPFC_04760 50S ribosomal protein L27
MAHKKAGGSTRNGRDSEAKRLGVKRFGGESVLAGSIIVRQRGTKFHAGANVGCGRDHTLF
AKADGKVKFEVKGPKNRKFISIEAE
>LFGLNPFC_04761 putative inner membrane transporter YhbE
MKQQAGIGILLALTTAICWGALPIAMKQVLEVMEPPTIVFYRFLMASIGLGAILAVKKRL
PPLRVFRKPRWLILLAVATAGLFGNFILFSSSLQYLSPTASQVIGQLSPVGMMVASVFIL
KEKMRSTQVVGALMLLSGLVMFFNTSLVEIFTKLTDYTWGVIFGVGAATVWVSYGVAQKV
LLRRLASPQILFLLYTLCTIALFPLANPGVIAQLSHWQLACLIFCGLNTLVGYGALAEAM
ARWQAAQVSAIITLTPLFTLFFSDLLSLAWPDFFARPMLNLLGYLGAFVVVAGAMYSAIG
HRIWGGLRKHTTVVSQPRAGE
>LFGLNPFC_04762 GTPase ObgE/CgtA
MKFVDEASILVVAGDGGNGCVSFRREKYIPKGGPDGGDGGDGGDVWMEADENLNTLIDYR
FEKSFRAERGGQNGASRDCTGKRGKDVTIKVPVGTRVIDQGTGETMGDMTKHGQRLLVAKG
GWHGLGNTRFKSSVNRTPRQKTNGTPGDKRELLLELMLLADVGMLGMPNAGKSTFIRAVS
AAKPKVADYPFTTLVPSLGVVRMDNEKSFVVADIPGLIEGAAEGAGLGIRFLKHLERCRV
LLHLIDIDPIDGTDPVENARIIISELEKYSQDLAAKPRWLVFNKIDLLDKVEAEEKAKAI
AEALGWEDKYYLISAASGLGVKDLCWDVMTFIIENPVVQAEEAKQPEKVEFMWDDYHRQQ
LEEIAEEDDEDWDEDDEEGVEFIYKR
>LFGLNPFC_04763 D-alanyl-D-alanine carboxypeptidase DacB
MRFSRFIIGLTSCIAFSVQAANVDEYITQLPAGANLALMVQKVGASAPAIDYHSQQMALP
ASTQKVITALAALIQLGPDFRFTTTLETKGNVENGVLKGDLVARFGADPTLRRQDIRNMV
ATLKKSGVNQIDGNVLIDTSIFASHDKAPGWPWNDMTQCFSAPPAAAIVDRNCFSISLYS
APKPGDMAFIRVASYYPVTMFSQVRTLPRGSAEAQYCELDVVPGDLNRFTLTGCLPQRSE

PLPLAFAVQDGASYAGAILKDELKQAGITWSGTLLRQTQVNEPGTVVASKQSAPLHDLLK
IMLKKSDNMIADTVFRMIGHARFNVPGTWRAGSDAVRQILRQQAGVDIGNTIIADGSGLS
RHNLIAPATMMQVLQYIAQHDNELNFISMLPLAGYDGSLQYRAGLHQAGVDGKVSAKTGS
LQGVYNLAGFITTVSGQRMAFVQYLSGYAVEPADQRNRRIPLVRFESRLYKDIYQNN
>LFGLNPFC_04764 Transcription elongation factor GreA
MQAIPMTLRGAEKLREELDFLKSVRRPEIIAAIAEAREHGDLKENAEYHAAREQQGFCEG
RIKDIEAKLSNAQVIDVTKMPNNGRVIFGATVTVLNLDSDEEQTYRIVGDDEADFKQNLI
SVNSPIARGLIGKEEDDVVVIKTPGGEVEFEVIKVEYL
>LFGLNPFC_04765 RNA-binding protein YhbY
MNLSTKQKQHLKGLAHPLKPVVLLGSNGLTEGVLAEIEQALEHHELIKVKIATEDRETKT
LIVEAIVRETGACNVQVIGKTLVLYRPTKERKISLPR
>LFGLNPFC_04766 Ribosomal RNA large subunit methyltransferase E
MTGKKRSASSSRWLQEHFSDKYVQQAQKKGLRSRAWFKLDEIQQSDKLFKPGMTVVDLGA
APGGWSQYVVTQIGGKGRIIACDLLPMDPIVGVDFLQGDFRDELVMKALLERVGDSKVQV
VMSDMAPNMSGTPAVDIPRAMYLVELALEMCRDVLAPGGSFVVKVFQGEGFDEYLREIRS
LFTKVKVRKPDSSRARSREVYIVATGRKP
>LFGLNPFC_04767 ATP-dependent zinc metalloprotease FtsH
MSDMAKNLILWLVIAVVLMSVFQSFGPSESNGRKVDYSTFLQEVNNDQVREARINGREIN
VTKKDSNRYTTYIPVQDPKLLDNLLTKNVKVVGEPPEEPSLLASIFISWFPMLLLIGVWI
FFMRQMQGGGGKGAMSFGKSKARMLTEDQIKTTFADVAGCDEAKEEVAELVEYLREPSRF
QKLGGKIPKGVLMVGPPGTGKTLLAKAIAGEAKVPFFTISGSDFVEMFVGVGASRVRDMF
EQAKKAAPCIIFIDEIDAVGRQRGAGLGGGHDEREQTLNQMLVEMDGFEGNEGIIVIAAT
NRPDVLDPALLRPGRFDRQVVVGLPDVRGREQILKVHMRRVPLAPDIDAAIIARGTPGFS
GADLANLVNEAALFAARGNKRVVSMVEFEKAKDKIMMGAERRSMVMTEAQKESTAYHEAG
HAIIGRLVPEHDPVHKVTIIPRGRALGVTFFLPEGDAISASRQKLESQISTLYGGRLAEE
IIYGPEHVSTGASNDIKVATNLARNMVTQWGFSEKLGPLLYAEEEGEVFLGRSVAKAKHM
SDETARIIDQEVKALIERNYNRARQLLTDNMDILHAMKDALMKYETIDAPQIDDLMARRD
VRPPAGWEEPGASNNSGDNGSPKAPRPVDEPRTPNPGNTMSEQLGDK
>LFGLNPFC_04768 Dihydropteroate synthase
MKLFAQGTSLDLSHPHVMGILNVTPDSFSDGGTHNSLLDAVKHANLMINAGATIIDVGGE
STRPGAAEVSVEEELQRVIPVVEAIAQRFEVWISVDTSKPEVIRESAKVGAHIINDIRSL
SEPGALEAAAETGLPVCLMHMQGNPKTMQEAPKYDDVFAEVNRYFIEQIARCEQAGIAKE
KLLLDPGFGFGKNLSHNYSLLARLAEFHHFNLPLLVGMSRKSMIGQLLNVGPSERLSGSL
ACAVIAAMQGAHIIRVHDVKETVEAMRVVEATLSAKENKRYE
>LFGLNPFC_04769 Phosphoglucosamine mutase
MSNRKYFGTDGIRGRVGDAPITPDFVLKLGWAAGKVLARHGSRKIIIGKDTRISGYMLES
ALEAGLAAAGLSALFTGPMPTPAVAYLTRTFRAEAGIVISASHNPFYDNGIKFFSIDGTK
LPDAVEEAIEAEMEKEISCVDSAELGKASRIVDAAGRYIEFCKATFPNELSLSELKIVVD
CANGATYHIAPNVLRELGANVIAIGCEPNGVNINAEVGATDVRALQARVLAEKADLGIAF
DGDGDRVIMVDHEGNKVDGDQIMYIIAREGLRQGQLRGGAVGTLMSNMGLELALKQLGIP
FARAKVGDRYVLEKMQEKGWRIGAENSGHVILLDKTTTGDGIVAGLQVLAAMARNHMSLH
DLCSGMKMFPQILVNVRYTAGSGDPLEHESVKAVTAEVEAALGSRGRVLLRKSGTEPLIR
VMVEGEDEAQVTEFAHRIADAVKAV
>LFGLNPFC_04770 Protein-export membrane protein SecG
MYEALLVVFLIVAIGLVGLIMLQQGKGADMGASFGAGASATLFGSSGSGNFMTRMTALLA
TLFFIISLVLGNINSNKTNKGSEWENLSAPAKTEQTQPAAPAKPTSDIPN
>LFGLNPFC_04772 Putative transferase YhbX
MVQRLLFFVLTILVVKRISSLPLRLLIAVPFVLLTAADMSISLYSWCIFGTTFNDGFAIS
VLQSDPDEVVKMLGMYIPYLCAFAFLSLLFFAVIIKYDVSLPTKKVTGILLLIVISGSLF
SACQFAYKDAKNKEAFSPYILASRFATYTPFFNLNYFALAAKEHQRLLSIANTVPYFQLS
VRDTGIDTYVLIVGESVRVDNMSLYGYTRSTTPQVEAQRKQIKLFNQAISGAPYTALSVP
LSLTADSVLSHDIHNYPDNIINMANQAGFQTFWLSSQSAFRQNGTAVTSIAMRAMETVYV
RGFDELLLPHLSQALQQKTQQKKLIVLHLNGSHEPACSAYPQSSAVFQPQDDQDACYDNS
IHYTDSLLGQVFELLKDRRASVMYFADHGLERDPTKKNVYFHGGREASQQAYHVPMFIWY
SPVLGDGVDRTTENDIFSTAYNNYLINAWMGVTKPEQPQTLEEVIAHYKGDSRVVDANHD
VFDFVMLRKEFTEDKQGNPTPEGQG
>LFGLNPFC_04773 Argininosuccinate synthase
MTTILKHLPVGQRIGIAFSGGLDTSAALLWMRQKGAVPYAYTANLGQPDEEDYDAIPRRA
MEYGAENARLIDCRKQLVAEGIAAIQCGAFHNTTGGLTYFNTTPLGRAVTGTMLVAAMKE
DGVNIWGDGSTYKGNDIERFYRYGLLTNAELQIYKPWLDTDFIDELGGRHEMSEFMIACG
FDYKMSVEKAYSTDSNMLGATHEAKDLEYLNSSVKIVNPIMGVKFWDESVKIPAEEVTVR
FEQGHPVALNGKTFSDDVEMLLEANRIGGRHGLGMSDQIENRIIEAKSRGIYEAPGMALL
HIAYERLLTGIHNEDTIEQYHAHGRQLGRLLYQGRWFDSQALMLRDSLQRWVASQITGEV
TLELRRGNDYSILNTVSENLTYKPERLTMEKGDSVFSPDDRIGQLTMRNLDITDTREKLF

GYAKTGLLSSSATSGVPQVENLENKGQ
>LFGLNPFC_04775 Ribosome maturation factor RimP
MSTLEQKLTEMITAPVEALGFELVGIEFIRGRTSTLRIYIDSEDGINVDDCADVSHQVSA
VLDVEDPITVAYNLEVSSPGLDRPLFTAEHYARFVGEEVTLVLRMAVQNRRKWQGVIKAV
DGEMITVTVEGKDEVFALSNIQKANLVPHF
>LFGLNPFC_04776 Transcription termination/antitermination protein NusA
MNKEILAVVEAVSNEKALPREKIFEALESALATATKKKYEQEIDVRVQIDRKSGDFDTFR
RWLVVDEVTQPTKEITLEAARYEDESLNLGDYVEDQIESVTFDRITTQTAKQVIVQKVRE
AERAMVVDQFREHEGEIITGVVKKVNRDNISLDLGNNAEAVILREDMLPRENFRPGDRVR
GVLYSVRPEARGAQLFVTRSKPEMLIELFRIEVPEIGEEVIEIKAAARDPGSRAKIAVKT
NDKRIDPVGACVGMRGARVQAVSTELGGERIDIVLWDDNPAQFVINAMAPADVASIVVDE
DKHTMDIAVEAGNLAQAIGRNGQNVRLASQLSGWELNVMTVDDLQAKHQAEAHAAIDTFT
KYLDIDEDFATVLVEEGFSTLEELAYVPMKELLEIEGLDEPTVEALRERAKNALATIAQA
QEESLGDNKPADDLLNLEGVDRDLAFKLAARGVCTLEDLAEQGIDDLADIEGLTDEKAGA
LIMAARNICWFGDEA
>LFGLNPFC_04777 Translation initiation factor IF-2
MTDVTIKTLAAERQTSVERLVQQFADAGIRKSADDSVSAQEKQTLIDHLNQKNSGPDKLT
LQRKTRSTLNIPGTGGKSKSVQIEVRKKRTFVKRDPQEAERLAAEEQAQREAEEQARREA
EESAKREAQQKAEREAAEQAKREAAEQAKREAAEKDKVSNQQDDMTKNAQAEKARREQEA
AELKRKAEEEARRKLEEEARRVAEEARRMAEENKWTDNAEPTEDSSDYHVTTSQHARQAE
DESDREVEGGRGRGRNAKAARPKKGNKHAESKADREEARAAVRGGKGGKRKGSSLQQGFQ
KPAQAVNRDVVIGETITVGELANKMAVKGSQVIKAMMKLGAMATINQVIDQETAQLVAEE
MGHKVILRRENELEEAVMSDRDTGAAAEPRAPVVTIMGHVDHGKTSLLDYIRSTKVASGE
AGGITQHIGAYHVETENGMITFLDTPGHAAFTSMRARGAQATDIVVLVVAADDGVMPQTI
EAIQHAKAAGVPVVVAVNKIDKPEADPDRVKNELSQYGILPEEWGGESQFVHVSAKAGTG
IDELLDAILLQAEVLELKAVRKGMASGAVIESFLDKGRGPVATVLVREGTLHKGDIVLCG
FEYGRVRAMRNELGQEVLEAGPSIPVEILGLSGVPAAGDEVTVVRDEKKAREVALYRQGK
FREVKLARQQKSKLENMFANMTEGEVHEVNIVLKADVQGSVEAISDSLLKLSTDEVKVKI
IGSGVGGITETDATLAAASNAILVGFNVRADASARKVIEAESLDLRYYSVIYNLIDEVKA
AMSGMLSPELKQQIIGLAEVRDVFKSPKFGAIAGCMVTEGVVKRHNPIRVLRDNVVIYEG
ELESLRRFKDDVNEVRNGMECGIGVKNYNDVRTGDVIEVFEIIEIQRTIA
>LFGLNPFC_04778 30S ribosome-binding factor
MAKEFGRPQRVAQEMQKEIALILQREIKDPRLGMMTTVSGVEMSRDLAYAKVYVTFLNDK
DEDAVKAGIKALQEASGFIRSLLGKAMRLRIVPELTFFYDNSLVEGMRMSNLVTSVVKHD
EERRVNPDDSKED
>LFGLNPFC_04779 tRNA pseudouridine synthase B
MSRPRRRGRDINGVLLLDKPQGMSSNDALQKVKRIYNANRAGHTGALDPLATGMLPICLG
EATKFSQYLLDSDKRYRVIARLGQRTDTSDADGQIVEERPVTFSAEQLAAALDTFRGDIE
QIPSMYSALKYQGKKLYEYARQGIEVPREARPITVYELLFIRHEGNELELEIHCSKGTYI
RTIIDDLGEKLGCGAHVIYLRRLAVSKYPVERMVTLEHLRELVEQAEQQDIPAAELLDPL
LMPMDSPASDYPVVNLPLTSSVYFKNGNPVRTSGAPLEGLVRVTEGENGKFIGMGEIDDE
GRVAPRRLVVEYPA
>LFGLNPFC_04780 30S ribosomal protein S15
MSLSTEATAKIVSEFGRDANDTGSTEVQVALLTAQINHLQGHFAEHKKDHHSRRGLLRMV
SQRRKLLDYLKRKDVARYTQLIERLGLRR
>LFGLNPFC_04781 Polyribonucleotide nucleotidyltransferase
MLNPIVRKFQYGQHTVTLETGMMARQATAAVMVSMDDTAVFVTVVGQKKAKPGQDFFPLT
VNYQERTYAAGRIPGSFFRREGRPSEGETLIARLIDRPIRPLFPEGFVNEVQVIATVVSV
NPQVNPDIVAMIGASAALSLSGIPFNGPIGAARVGYINDQYVLNPTQDELKESKLDLVVA
GTEAAVLMVESEAELLSEDQMLGAVVFGHEQQQVVIQNINELVKEAGKPRWDWQPEPVNE
ALNARVAALAEARLSDAYRITDKQERYAQVDVIKSETIATLLAEDETLDENELGEILHAI
EKNVVRSRVLAGEPRIDGREKDMIRGLDVRTGVLPRTHGSALFTRGETQALVTATLGTAR
DAQVLDELMGERTDTFLFHYNFPPYSVGETGMVGSPKRREIGHGRLAKRGVLAVMPDMDK
FPYTVRVVSEITESNGSSSMASVCGASLALMDAGVPIKAAVAGIAMGLVKEGDNYVVLSD
ILGDEDHLGDMDFKVAGSRDGISALQMDIKIEGITKEIMQVALNQAKGARLHILGVMEQA
INAPRGDISEFAPRIHTIKINPDKIKDVIGKGGSVIRALTEETGTTIEIEDDGTVKIAAT
DGEKAKHAIRRIEEITAEIEVGRVYNGKVTRIVDFGAFVAIGGGKEGLVHISQIADKRVE
KVTDYLQMGQEVPVKVLEVDRQGRIRLSIKEATEQSQPAAAPEAPAAEQGE
>LFGLNPFC_04782 Lipoprotein NlpI
MKPFLRWCFVATALTLAGCSNTSWRKSEVLAVPLQPTLQQEVILARMEQILASRALTDDE
RAQLLYERGVLYDSLGLRALARNDFSQALAIRPDMPEVFNYLGIYLTQAGNFDAAYEAFD
SVLELDPTYNYAHLNRGIALYYGGRDKLAQDDLLAFYQDDPNDPFRSLWLYLAEQKLDEK
QAKEVLKQHFEKSDKEQWGWNIVEFYLGNISEQTLMERLKADATDNTSLAEHLSETNFYL
GKYYLSLGDLDSATALFKLAVANNVHNFVEHRYALLELSLLGQDQDDLAESDQQ

>LFGLNPFC_04783 ATP-dependent RNA helicase DeaD
MAEFETTFADLGLKAPILEALNDLGYEKPSPIQAECIPHLLNGRDVLGMAQTGSGKTAAF
SLPLLQNLDPELKAPQILVLAPTRELAVQVAEAMTDFSKHMRGVNVVALYGGQRYDVQLR
ALRQGPQIVVGTPGRLLDHLKRGTLDLSKLSGLVLDEADEMLRMGFIEDVETIMAQIPEG
HQTALFSATMPEAIRRITRRFMKEPQEVRIQSSVTTRPDISQSYWTVWGMRKNEALVRFL
EAEDFDAAIIFVRTKNATLEVAEALERNGYNSAALNGDMNQALREQTLERLKDGRLDILI
ATDVAARGLDVERISLVVNYDIPMDSESYVHRIGRTGRAGRAGRALLFVENRERRLLRNI
ERTMKLTIPEVELPNAELLGKRRLEKFAAKVQQQLESSDLDQYRALLSKIQPTAEGEELD
LETLAAALLKMAQGERTLIVPPDAPMRPKREFRDRDDRGPRDRNDRGPRGDREDRPRRER
RDVGDMQLYRIEVGRDDGVEVRHIVGAIANEGDISSRYIGNIKLFASHSTIELPKGMPGE
VLQHFTRTRILNKPMNMQLLGDAQPHTGGERRGGGRGFGGERREGGRNFSGERREGGRGD
GRRFSGERREGRAPRRDDSTGRGRFGGDA
>LFGLNPFC_04784 Tryptophan-specific transport protein
MATLTTTQTSPSLLGGVVIIGGTIIGAGMFSLPVVMSGAWFFWSMAALIFTWFCMLHSGL
MILEANLNYRIGSSFDTITKDLLGKGWNVVNGISIAFVLYILTYAYISASGSILHHTFAE
MSLNVPARAAGFGFALLVAFVVWLSTKAVSRMTAIVLGAKVITFFLTFGSLLGHVQPATL
FNVAESNASYAPYLLMTLPFCLASFGYHGNVPSLMKYYGKDPKTIVKCLVYGTLMALALY
TIWLLATMGNIPRPEFIGIAEKGGNIDVLVQALSGVLNSRSLDLLLVVFSNFAVASSFLG
VTLGLFDYLADLFGFDDSAMGRLKTALLTFAPPVVGGLLFPNGFLYAIGYAGLAATIWAA
IVPALLARASRKRFGSPKFRVWGGKPMIMLILVFGVGNALVHILSSFNVLPVYQ
>LFGLNPFC_04785 F420-dependent glucose-6-phosphate dehydrogenase
MTDKTIAFSLLDLAPIPEGSSARDAFSHSLDLARLAEKRGYHRYWLAEHHNMTGIASAAT
SVLIGYLAANTTTLHLGSGGVMLPNHSPLVIAEQFGTLNTLYPGRIDLGLGRAPGSDQRT
MMALRRHMSGDIDNFPRDVAELVDWFDARDPNPHVRPVPGYGEKIPVWLLGSSLYSAQLA
AQLGLPFAFASHFAPDMLFQALHLYRSNFKPSARLEKPYAMVCINIIAADSNRDAEFLFT
SMQQAFVKLRRGETGQLPPPIQNMDQFWSPSEQYGVQQALSMSLVGDKAKVRHGLQSILR
ETDADEIMVNGQIFDHQARLHSFELAMDVKEELLG
>LFGLNPFC_04786 hypothetical protein
MKYSLGPVLWYWPKETLEEFYQQAAASSADVIYLGEAVCSKRRATKVGDWLEMAKSLAGS
GKQIVLSTLALVQASSELGELKRYVENGEFLIEASDLGVVNMCAERKLPFVAGHALNCYN
AVTLKILLKQGMMRWCMPVELSRDWLVNLLNQCDELGIRNQFEVEVLSYGHLPLAYSARC
FTARSEDRPKDECETCCIKYPNGRNVLSQENQQVFVLNGIQTMSGYVYNLGNELASMQGL
VDVVRLSPQGTDTFAMLDAFRANENGAAPLPLTANSDCNGYWRRLAGLELQA
>LFGLNPFC_04787 putative protease YhbU
MELLCPAGNLPALKAAIENGADAVYIGLKDDTNARHFAGLNFTEKKLQEAVSFVHQHRRK
LHIAINTFAHPDGYARWQRAVDMAAQLGADALILADLAMLEYAAERYPHIERHVSVQASA
TNEEAINFYHRHFDVARVVLPRVLSIHQVKQLARVTPVPLEVFAFGSLCIMSEGRCYLSS
YLTGESPNTVGACSPARFVRWQQTPQGLESRLNEVLIDRYQDGENAGYPTLCKGRYLVNG
ERYHALEEPTSLNTLELLPELMAANIASVKIEGRQRSPAYVSQVAKVWRQAIDRCKADPQ
NFIPQSAWMETLGSMSEGTQTTLGAYHRKWQ
>LFGLNPFC_04788 Ubiquinone biosynthesis accessory factor UbiJ
MLDKLRSRIVHLGPSLLSVPVKLTPFALKRQVLEQVLSWQFRQALDDGELEFLEGRWLSI
HVRDIDLQWFTSVVNGKLVVSQNAQADVSFSADASDLLMIAARKQDPDTLFFQRRLVIEG
DTELGLYVKNLMDAIELEQMPKALRMMLLQLADFVEAGMKTAPETKQTSVGEPC
>LFGLNPFC_04789 N-acetyltransferase Eis
MLIRVEIPIDAPGIDALLRRSFESDAEAKLVHDLREDGFLTLGLVATDDEGQVIGYVAFS
PVDVQGEDLQWVGMAPLAVDEKYRGQGLARQLVYEGLDSLNEFGYAAVVTLGDPALYSRF
GFELAAHHDLRCRWPGTESAFQVHRLADDALNGVTGLVEYHEHFNRF
>LFGLNPFC_04790 hypothetical protein
MTPWFLYLIRTADNKLYTGITTDVERRYQQHQSGKGAKALRGKGELTLAFSAPVGDRSLA
LRAEYRVKQLTKRQKERLVAEGAVFAELLSSLQTPEIKSD
>LFGLNPFC_04791 hypothetical protein
METLTAISRWLAKQHVVTWCVQQEGELWCANAFYLFDAQKVAFYILTEEKTRHAQMSGPQ
AAIAGTVNGQPKTVALIRGVQFKGEIRRLEGEESDLARKAYNRRFPVARMLSAPVWEIRL
DEIKFTDNTLGFGKKMIWLRNSGTEQA
>LFGLNPFC_04792 Protein/nucleic acid deglycase 2
MSKKIAVLITDEFEDSEFTSPADEFRKAGHEVITIEKQAGKTVKGKKGEASVAIDKSIDE
VTPAEFDALLLPGGHSPDYLRGDNRFVTFTRDFVNSGKPVFAICHGPQLLISADVILGRK
LTAVKPIVIDVKNAGAEFYDQEVVVDKDQLVTSRTPDDLPAFNREALRLLGA
>LFGLNPFC_04793 hypothetical protein
MSQVLITGATGLVGGHLLRMLINEPKVNAIAAPTRRPLGDMPGVFNPHDPQLTDALAQVT
DPIDIVFCCLGTTRREAGSKEAFIHADYTLVVDTALTGRRLGAQHMLVVSAMGANAHSPF
FYNRVKGEMEEALIAQNWPKLTIARPSMLLGDRSKQRMNETLFAPLFRLLPGNWKSIDAR
DVARVMLAEAMRPEHEGVTILSSSELRKRAE

>LFGLNPFC_04794 hypothetical protein
MTGQSSSQAATPIQWWKPALFFLVVIAGLWYVKWQPYYGKAFTAAETHSIGKSILAQADA
NPWQAALNYAMIYFLAVWKAAVLGVILGSLIQVLIPRDWLLRTLGQSRFRGTLLGTLFSL
PGMMCTCCAAPVAAGMRRQQASMGGALAFWMGNPLLNPATLVFMGFVLGWDFAAIRLVAG
LVMVLLIATLVQKWVRETPQTQAPVEIDIPEAQGGFFSRWGRALWTLFWSTIPVYILAVL
VLGAARVWLFPHADGAVDNSLMWVVAMAVAGCLFVIPTAAEIPIVQTMMLAGMGTAPALA
LLMTLPAVSLPSLIMLRKAFPTKALWLTGAMVAVSGVIVGGLALLV
>LFGLNPFC_04795 Osmotically-inducible protein Y
MKALSPIAVLISALLLQGCVAAAVVGTAAVGTKAATDPRSVGTQVDDGTLEVRVNSALSK
DEQIKKEARINVTAYQGKVLLVGQSPNAELSARAKQIAMGVDGANEVYNEIRQGQPIGLG
EASNDTWITTKVRSQLLTSDLVKSSNVKVTTENGEVFLMGLVTEREAKAAADIASRVSGV
KRVTTAFTFIK
>LFGLNPFC_04796 DnaA initiator-associating protein DiaA
MQERIKACFTESIQTQIAAAEALPDAISRAAMTLVQSLLNGNKILCCGNGTSAANAQHFA
ASMINRFETERPSLPAIALNTDNVVLTAIANDRLHDEVYAKQVRALGHAGDVLLAISTRG
NSRDIVKAVEAAVTRDMTIVALTGYDGGELAGLLGPQDVEIRIPSHRSARIQEMHMLTVN
CLCDLIDNTLFPHQDV
>LFGLNPFC_04797 hypothetical protein
MATVPTRSGSPRQLTTKQTGDAWEAQARRWLEGKGLRFIAANVNERGGEIDLIMREGRTT
VFIEVRYRRSALYGGAAASVTRSKQHKLLQTARLWLARHNGSFDTVDCRFDVVAFTGNEV
EWIKDAFNDHS
>LFGLNPFC_04798 Penicillin-binding protein activator LpoA
MVPSTFSRLKAARCLPVVLAALIFAGCGTHTPDQSTAYMQGTAQADSAFYLQQMQQSSDD
TRINWQLLAIRALVKEGKTGQAVELFNQLPQELNDTQRREKTLLAAEIKLAQKDFAGAQN
LLAKITPADLEQNQQARYWQAKIDASQGRPSIDLLRALIAQEPLLGAKEKQQNIDATWQA
LSSMTQEQANTLVINADENILQGWLDLQRVWFDNRNDPDMMKAGIADWQKRYPNNPGAKM
LPTQLVNVKAFKPASTNKIALLLPLNGQAAVFGRTIQQGFEAAKNIGTQPVVAQVAAAPA
ADVAEQPQPQTVDGVASPAQASVSDLTGEQPAAQSVPVSAPATSTAAVSAPANPSAELKI
YDTSSQPLSQILSQVQQDGASIVVGPLLKNNVEELLKSNTPLNVLALNQPENIENRVNIC
YFALSPEDEARDAARHIRDQGKQAPLVLIPRSSLGDRVANAFAQEWQKLGGGTVLQQKFG
STSELRAGVNGGSGIALTGSPITPRETTDSGMTTNNPTLQTTPTDDQFTNNGGRVDAVYI
VATPGEIAFIKPMIAMRNGSQSGATLYASSRSAQGTAGPDFRLEMEGLQYSEIPMLAGGN
LPLMQQALSAVNNDYSLARMYAMGVDAWSLANHFSQMRQVQGFEINGNTGSLTANPDCVI
NRKLSWLQYQQGQVVPAS
>LFGLNPFC_04799 Ribosomal RNA small subunit methyltransferase I
MKQHQSADNSQGQLYIVPTPIGNLADITQRALEVLQAVDLIAAEDTRHTGLLLQHFGINA
RLFALHDHNEQQKAETLLAKLQEGQNIALVSDAGTPLINDPGYHLVRTCREAGIRVVPLP
GPCAAITALSAAGLPSDRFCYEGFLPAKSKGRRDALKAIEAEPRTLIFYESTHRLLDSLE
DIVAVLGESRYVVLARELTKTWETIHGAPVGELLAWVKEDENRRKGEMVLIVEGHKAQEE
DLPADALRTLALLQAELPLKKAAALAAEIHGVKKNALYKYALEQQGE
>LFGLNPFC_04800 Glucosamine-6-phosphate deaminase
MQTLQQVENYTALSERASEYLLAVIRRKPDAVICLATGATPLLTYHYLVEKIHQQQVDVS
QLTFVKLDEWVDLPLTMPGTCETFLQQHIVQPLGLREDQLISFHSEEINETECERVTNLI
ARKGGLDLCVLGLGKNGHLGLNEPGESLQPACHISQLDARTQQHEMLKTADRPVTRGITL
GLKDILNAREVLLLVTGEGKQDATERFLTAKVSTAIPASFLWLHNYFTCMIDEMCRR
>LFGLNPFC_04801 PTS system mannose-specific EIID component
MGSEISKKDITRLGFRSSLLQASFNYERMQAGGFTWAMLPILKKIYKDDKPGLSAAMKDN
LEFINTHPNLVGFLMGLLISMEEKGENRDTIKGLKVALFGPIAGIGDAIFWFTLLPIMAG
ICSSFASQGNLLGPILFFAVYLLIFFLRVGWTHVGYSVGVKAIDKVRENSQMIARSATIL
GITVIGGLIASYVHINVVTSFAIDSTHSVALQQDFFDKVFPNILPMAYTLLMYYFLRVKK
AHPVLLIGVTFVLSIVCSAFGIL
>LFGLNPFC_04802 N-acetylgalactosamine permease IIC component 1
MHEITLLQGLSLAALVFFLGIDFWLEALFLFRPIIVCTLTGAILGDIQTGLITGGLTELA
FAGLTPAGGVQPPNPIMAGLMTTVIAWSTGVDAKTAIGLGLPFSLLMQYVILFFYSAFSL
FMTKADKCAKEADTAAFSRLNWATMLIVASAYAVIAFLCTYLAQGAMQALVKAMPAWLTH
GFEVAGGILPAVGFGLLLRVMFKAQYIPYLIAGFLFVCYIQVSNLLPVAVLGAGFAVYEF
FNAKSRQQAQPQPVASKNEEEDYSNGI
>LFGLNPFC_04803 PTS system sorbose-specific EIIB component
MSSPNILLTRIDNRLVHGQVGVTWTSTIGANLLVVVDDVVANDDIQQKLMGITAETYGFG
IRFFTIEKTINVIGKAAPHQKIFLICRTPQTVRKLVEGGIDLKDVNVGNMHFSEGKKQIS
SKVYVDDQDLTDLRFIKQRGVNVFIQDVPGDQKEQIPD
>LFGLNPFC_04804 D-tagatose-1,6-bisphosphate aldolase subunit KbaY
MSIISTKYLLQDAQANGYAVPAFNIHNAETIQAILEVCSEMRSPVILAGTPGTFKHIALE
EIYALCSAYSTTYNMPLALHLDHHESLDDIRRKVHAGVRSAMIDGSHFPFAENVKLVKSV

VDFCHSQDCSVEAELGRLGGVEDDMSVDAESAFLTDPQEAKRFVELTGVDSLAVAIGTAH
GLYSKTPKIDFQRLAEIREVVDVPLVLHGASDVPDEFVRRTIELGVTKVNVATELKIAFA
GAVKAWFAENPQGNDPRYYMRVGMDAMKEVVRNKINVCGSANRISA
>LFGLNPFC_04805 Putative D-galactosamine-6-phosphate deaminase AgaS
MPENYTPAAAATGTWTEEEIRHQPRAWIRSLTNIDALRSALNNFLEPLLRKENLRIILTG
AGTSAFIGDIIAPWLASHTGKNFSAVPTTDLVTNPMDYLNPAHPLLLISFGRSGNSPESV
AAVELANQFVPECYHLPITCNEAGALYQNAINSDNAFALLMPAETHDRGFAMTSSITTMM
ASCLAVFAPETINSQTFRDVADRCQAILTSLGDFSEGVFGYAPWKRIVYLGSGGLQGAAR
ESALKVLELTAGKLAAFYDSPTGFRHGPKSLVDNETLVVVFVSSHPYTRQYDLDLLAELR
RDNQAMRVIAIAAESNDVIAAGPHIILPPSRHFIDVEQAFCFLMYAQTFALMQSLHMGNT
PDTPSASGTVNRVVQGVIIHPWQA
>LFGLNPFC_04806 N-acetylgalactosamine-6-phosphate deacetylase
MTHVLRARRLLTEEGWLDDHQLRIADGVIAAIEPIPVGVTERDAELLCPAYIDTHVHGGA
GVDVMDDAPDVLDKLAMHKAREGVGSWLPTTVTAPLNTIHAALERIAQRCQRGGPGAQVL
GSYLEGPYFTPQNKGAHPPELFRELEIAELDQLIAVSQHTLRVVALAPEKEGALQAIRHL
KQQNVRVMLGHSAATWQQTRAAFDAGADGLVHCYNGMTGLHHREPGMVGAGLTDKRAWLE
LIADGHHVHPAAMSLCCCCAKERIVLITDAMQAAGMPDGHYTLCGEEVQMRGGVVRTASG
GLAGSTLSVDAAVRNMVELTGVTPAEAIHMASLHPARMLGVDGVLGSLKPGKRASVVALD
SGLHVQQIWIQGQLASF
>LFGLNPFC_04807 PTS system mannose-specific EIIAB component
MLSIILTGHGGFASGMEKAMKQILGEQSQFIAIDFPETSSTALLTSQLEEAIAQLDCEDG
IVFLTDLLGGTPFRVASTLAMQKPGCEVITGTNLQLLLEMVLEREGLSGEEFRVQALECG
HRGLTSLVDELGRCHEECPVEEGI
>LFGLNPFC_04808 PTS system mannose-specific EIID component
MASNQTTLPNVSENEETLLTGVNENVYEDQSIGAELTKKDINRVAWRSMLLQASFNYERM
QASGWLYGLLPALKKIHTNKRDLARAMKGHMGFFNTHPFLVTFVIGIILAMERSKQDVNS
IQSTKIAVGAPLGGIGDAMFWLTLLPICGGIGASLALQGSILGAVVFIVLFNVVHLGLRF
GLAHYAYRMGVAAIPLIKANTKKVGHAASIVGMTVIGALVATYVRLSTTLEITAGDAVVK
LQADVIDKLMPAFLPLVYTLTMFWLVRRGWSPLRLIAITVVLGIVGKFCHFL
>LFGLNPFC_04809 N-acetylgalactosamine permease IIC component 1
MEISLLQAFALGIIAFIAGLDMFNGLTHMHRPVVLGPLVGLVLGDLHTGILTGGTLELVW
MGLAPLAGAQPPNVIIGTIVGTAFAITTGVKPDVAVGVAVPFAVAVQMGITFLFSVMSGV
MSRCDRMAENADTRGIERVNYLALLALGTFYFLCAFLPIYFGAEHAKTIIDVLPQRLIDG
LGVAGGIMPAIGFAVLLKIMMKNVYIPYFILGFVAAAWLKLPVLAIAAAALAMALIDLLR
KSPEPTQPAAQKEEFEDGI
>LFGLNPFC_04810 PTS system sorbose-specific EIIB component
MPNIVLSRIDERLIHGQVGVQWVGFAGANLVLVANDEVAEDLVQQNLMEMVLAEGIAVRF
WTLQKVIDNIHRAADRQKILLVCKTPADFLTLVKGGVPVNRINVGNMHYANGKQQIAKTV
SVDAGDIAAFNDLKAAGVECFVQGVPTEPAVDLFKLL
>LFGLNPFC_04811 D-tagatose-1,6-bisphosphate aldolase subunit KbaZ
MKHLTEMVRQHKAGKTNGIYAVCSAHPLVLEAAIRYASANQTPLLIEATSNQVDQFGGYT
GMTPADFRGFVCQLADSLNFPQDALILGGDHLGPNRWQNLPAAQAMANADDLIKSYVAAG
FKKIHLDCSMSCQDDPIPLTDDIVAERAARLAKVAEETCREHFGEADLEYVIGTEVPVPG
GAHETLSELAVTTPDAARATLEAHRHAFEKQGLNAIWPRIIALVVQPGVEFDHTNVIDYQ
PAKAAALSQMVENYETLIFEAHSTDYQTPQSLRQLVIDHFAILKVGPALTFALREALFSL
AAIEEELVPAKACSGLRQVLENVMLDRPEYWQSHYHGDGNARRLARGYSYSDRVRYYWPD
SQIDDAFAHLVRNLADSPIPLPLISQYLPLQYVKVRSGELQPTPRELIINHIQDILAQYH
TACEGQ
>LFGLNPFC_04812 Glucitol operon repressor
MSNTDASGEKRVTGTSERREQIIQRLRQQGSVQVNDLSALYGVSTVTIRNDLAFLEKQGI
AVRAYGGALICDSTTPSVEPSVEDKSALNTAMKRSVAKAAVELIQPGHRVILDSGTTTFE
IARLMRKHTDVIAMTNGMNVANALLEAEGVELLMTGGHLRRQSQSFYGDQAEQSLQNYHF
DMLFLGVDAIDLERGVSTHNEDEARLNRRMCEVAERIIVVTDSSKFNRSSLHKIIDTQRI
DMIIVDEGIPSDSLEGLRKAGVEVILVGDHASSL
>LFGLNPFC_04813 Galactarate dehydratase (L-threo-forming)
MANIEIRQETPTAFYIKVHDTDNVAIIVNDNGLKAGTRFPDGLELIEHIPQGHKVALLDI
PANGEIIRYGEVIGYAVRAIPRGSWIDESMVVLPEAPPLHTLPLATKVPEPLPPLEGYTF
EGYRNADGSVGTKNLLGITTSVHCVAGVVDYVVKIIERDLLPKYPNVDGVVGLNHLYGCG
VAINAPAAVVPIRTIHNISLNPNFGGEVMVIGLGCEKLQPERLLTGTDDVQAIPVESASI
VSLQDEKHVGFQSMVEDILQVAECHLQKLNQRQRETCPASELVVGMQCGGSDAFSGVTAN
PAVGYASDLLVRCGATVMFSEVTEVRDAIHLLTPRAVNEEVGKRLLEEMEWYDNYLNMGK
TDRSANPSPGNKKGGLANVVEKALGSIAKSGKSAIVEVLSPGQRPTKRGLIYAATPASDF
VCGTQQVASGITVQVFTTGRGTPYGLMAVPVIKMATRTELANRWFDLMDINAGTIATGEE
TIEEVGWKLFHFILDVASGKKKTFSDQWGLHNQLAVFNPAPVT

>LFGLNPFC_04814 putative galactarate transporter
MMLDTVDEKKKGVHTRYLILLIIFIVTAVNYADRATLSIAGTEVAKELQLSAVSMGYIFS
AFGWAYLLMQIPGGWLLDKFGSKKVYTYSLFFWSLFTFLQGFVDMFPLAWAGISMFFMRF
MLGFSEAPSFPANARIVAAWFPTKERGTASAIFNSAQYFSLALFSPLLGWLTFAWGWEHV
FTVMGVIGFVLTALWIKLIHNPTDHPRMSAEELKFISENGAVVDMDHKKPGSAAASGPKL
HYIKQLLSNRMMLGVFFGQYFINTITWFFLTWFPIYLVQEKGMSILKVGLVASIPALCGF
AGGVLGGVFSDYLIKRGLSLTLARKLPIVLGMLLASTIILCNYTNNTTLVVMLMALAFFG
KGFGALGWPVISDTAPKEIVGLCGGVFNVFGNVASIVTPLVIGYLVSELHSFNAALVFVG
CSALMAMVCYLFVVGDIKRMELQK
>LFGLNPFC_04815 5-keto-4-deoxy-D-glucarate aldolase
MNNDVFPNKFKAALAAKQVQIGCWSALSNPISTEVLGLAGFDWLVLDGEHAPNDISTFIP
QLMALKGSASAPVVRVPTNEPVIIKRLLDIGFYNFLIPFVETKEEAEQAVASTRYPPEGI
RGVSVSHRANMFGTVADYFAQSNKNITILVQIESQQGVDNVDAIAATEGVDGIFVGPSDL
AAALGHLGNASHPDVQKAIQHIFNRASAHGKPSGILAPVEADARRYLEWGATFVAVGSDL
GVFRSATQKLADTFKK
>LFGLNPFC_04816 2-hydroxy-3-oxopropionate reductase
MTMKVGFIGLGIMGKPMSKNLLKAGYSLVVADRNPEAIADVIAAGAETASTAKAIAEQCD
VIITMLPNSPHVKEVALGENGIIEGAKPGTVLIDMSSIAPLASREISEALKAKGIDMLDA
PVSGGEPKAIDGTLSVMVGGDKAIFDKYYDLMKAMAGSVVHTGEIGAGNVTKLANQVIVA
LNIAAMSEALTLATKAGVNPDLVYQAIRGGLAGSTVLDAKAPMVMDRNFKPGFRIDLHIK
DLANALDTSHGVGAQLPLTAAVMEMMQALRADGLGMADHSALACYYEKLAKVEVTR
>LFGLNPFC_04817 Glycerate 2-kinase
MKIVIAPDSYKESLSASEVAQAIEKGFREIFPDAQYVSVPVADGGEGTVEAMIAATQGAE
RHAWVTGPLGEKVNASWGISGDGKTAFIEMAAASGLELVPAEKRDPLVTTSRGTGELILQ
ALESGATNIIIGIGGSATNDGGAGMVQALGAKLCDANGNEIGFGGGSLNTLNDIDISGLD
PRLKDCVIRVACDVTNPLVGDSGASRIFGPQKGASEAMIVELDNNLSHYADVIKKALHVD
VKDVPGAGAAGGMGAALMAFLGAELKSGIEIVTTALNLEEHIHDCTLVITGEGRIDSQSI
HGKVPIGVANVAKKYHKPVIGIAGSLTNDVGVVHQHGIDAVFSVLTSIGTLDEAFRGAYD
NICRASRNIAATLAIGMRNAG
>LFGLNPFC_04818 HTH-type transcriptional regulator TsaR
MSTILLPKTQHLVVFQEVIRSGSIGSAAKELGLTQPAVSKIINDIEDYFGVELVVRKNTG
VTLTPAGQLLLSRSESITREMKNMVNEISGMSSEAVVEVSFGFPSLIGFTFMSGMINKFK
EVFPKAQVSMYEAQLSSFLPAIRDGRLDFAIGTLSAEMKLQDLHVEPLFESEFVLVASKS
RTCTGTTTLESLKNEQWVLPQTNMGYYSELLTTLQRNGISIENIVKTDSVVTIYNLVLNA
DFLTVIPCDMTSPFGSNQFITIPVEETLPVAQYAAVWSKNYRIKKAASVLVELAKEYSSY
NGCRRRQLIEVD
>LFGLNPFC_04819 L-threonine dehydratase catabolic TdcB
MHITYDLPVAIDDIIEAKQRLAGRIYKTGMPRSNYFSERCKGEIFLKFENMQRTGSFKIR
GAFNKLSSLTDAEKRKGVVACSAGNHAQGVSLSCAMLGIDGKVVMPKGAPKSKVAATCDY
SAEVVLHGDNFNDTIAKVSEIVEMEGRIFIPPYDDPKVIAGQGTIGLEIMEDLYDVDNVI
VPIGGGGLIAGIAVAIKSINPTIRVIGVQSENVHGMAASFHSGEITTHRTTGTLADGCDV
SRPGNLTYEIVRELVDDIVLVSEDEIRNSMIALIQRNKVVTEGAGALACAALLSGKLDQY
IQNRKTVSIISGGNIDLSRVSQITGFVDA
>LFGLNPFC_04820 Threonine/serine transporter TdcC
MSTSDSIVSSQTKQSSWRKSDTTWTLGLFGTAIGAGVLFFPIRAGFGGLIPILLMLVLAY
PIAFYCHRALARLCLSGSNPSGNITETVEEHFGKTGGVVITFLYFFAICPLLWIYGVTIT
NTFMTFWENQLGFAPLNRGFVALFLLLLMAFVIWFGKDLMVKVMSYLVWPFIASLVLISL
SLIPYWNSAVIDQVDLGSLSLTGHDGILITVWLGISIMVFSFNFSPIVSSFVVSKREEYE
KDFGRDFTERKCSQIISRASMLMVAVVMFFAFSCLFTLSPANMAEAKAQNIPVLSYLANH
FASMTGTKTTFAITLEYAASIIALVAIFKSFFGHYLGTLEGLNGLILKFGYKGDKTKVSL
GKLNTISMIFIMGSTWVVAYANPNILDLIEAMGAPIIASLLCLLPMYAIRKAPSLAKYRG
RLDNVFVTVIGLLTILNIVYKLF
>LFGLNPFC_04821 Propionate kinase
MNEFPVVLVINCGSSSIKFSVLNASDCEVLMSGIADGINSENAFLSVNGGEPAPLAHHSY
EGALKAIAFELEKRNLNDNVALIGHRIAHGGSIFTESAIITDEVIDNIRRVSPLAPLHNY
ANLSGIESAQQLFPGVTQVAVFDTSFHQTMAPEAYLYGLPWKYYEELGVRRYGFHGTSHR
YVSQRAHSLLNLAEDDSGLVVAHLGNGASICAVRNGQSVDTSMGMTPLEGLMMGTRSGDV
DFGAMSWVASQTNQSLGDLERVVNKESGLLGISGLSSDLRVLEKAWHEGHERAQLAIKTF
VHRIARHIAGHAASLHRLDGIIFTGGIGENSSLIRRLVMEHLAVLGVEIDTEMNNRSNSF
GERIVSSENARVICAVIPTNEEKMIALDAIHLGKVNAPAEFA
>LFGLNPFC_04822 PFL-like enzyme TdcE
MKVDIDTSDKLYADAWLGFKGTDWKNEINVRDFIQHNYTPYEGDESFLAEATPATTELWE
KVMEGIRIENATHAPLDFDTNIATTITAHDAGYIDQPLEKIVGLQTDAPLKRALHPFGGI
NMIRSSFHAYGREMDSEFEYIFTDLRKTHNQGVFDVYSPDMLRCRKSGVLTGLPDGYGRG

RIIGDYRRVALYGISYLVRERELQFADLQSRLEKGEDLEATIRLREELAEHRRALLQIQE
MAAKYGFDISRPAQNAQEAVQWLYFAYLAAVKSQNGGAMSLGRTASFLDIYIERDFKAGV
LNEQQAQELIDHFIMKIRMVRFLRTPEFDSLFSGDPIWATEVIGGMGLDGRTLVTKNSFR
YLHTLHTMGPAPEPNLTILWSEALPIAFKKYAAQVSIVTSSLQYENDDLMRTDFNSDDYA
IACCVSPMVIGKQMQFFGARANLAKTLLYAINGGMDEKLKIQVGPKTAPLMDDVLDYDKV
MDSLDHFMDWLAVQYISALNIIHYMHDKYSYEASLMALHDRDVYRTMACGIAGLSVATDS
LSAIKYARVKPIRDENGLAVDFEIDGEYPQYGNNDERVDSIACDLVERFMKKIKALPTYR
NAVPTQSILTITSNVVYGQKTGNTPDGRRAGTPFAPGANPMHGRDRKGAVASLTSVAKLP
FTYAKDGISYTFSIVPAALGKEDPIRKTNLVGLLDGYFHHEADVEGGQHLNVNVMNREML
LDAIEHPEKYPNLTIRVSGYAVRFNALTREQQQDVISRTFTQAL
>LFGLNPFC_04823 Putative reactive intermediate deaminase TdcF
MKKIIETQRAPGAIGPYVQGVDLGSMVFTSGQIPVCPQTGEIPADVQDQARLSLENVKAI
VVAAGLSVGDIIKMTVFITDLNDFATINEVYKQFFDEHQATYPTRSCVQVARLPKDVKLE
IEAIAVRSA
>LFGLNPFC_04824 L-serine dehydratase TdcG
MISAFDIFKIGIGPSSSHTVGPMNAGKSFIDRLESSGLLTATSHIVVDLYGSLSLTGKGH
ATDVAIIMGLAGNSPQDVVIDEIPAFIELVTRSGRLPVASGAHIVDFPVAKNIIFHPEML
PRHENGMRITAWKGQEELLSKTYYSVGGGFIVEEEHFGLSHDVETPVPYDFHSAGELLKM
CDYNGLSISGLMMHNELALRSKAEIDAGFARIWQVMHDGIERGMNTEGVLPGPLNVPRRA
VALRRQLVSSDNISNDPMNVIDWINMYALAVSEENAAGGRVVTAPTNGACGIIPAVLAYY
DKFRRPVNERSIARYFLAAGAIGALYKMNASISGAEVGCQGEIGVACSMAAAGLTELLGG
SPAQVCNAAEIAMEHNLGLTCDPVAGQVQIPCIERNAINAVKAVNAARMAMRRTSAPRVS
LDKVIETMYETGKDMNDKYRETSRGGLAIKVVCG
>LFGLNPFC_04825 putative serine transporter
MEIASNKGVIADASTPAGRAGMSESEWREAIKFDSTDTGWVIMSIGMAIGAGIVFLPVQV
GLMGLWVFLLSSVIGYPAMYLFQRLFINTLAESPECKDYPSVISGYLGKNWGILLGALYF
VMLVIWMFVYSTAITNDSASYLHTFGVTEGLLSDSPFYGLVLICILVAISSRGEKLLFKI
STGMVLTKLLVVAALGVSMVGMWHLYNVGSLPPLGLLVKNAIITLPFTLTSILFIQTLSP
MVISYRSREKSIEVARHKALRAMNIAFGILFVTVFFYAVSFTLAMGHDEAVKAYEQNISA
LAIAAQFISGDGAAWVKVVSVILNIFAVMTAFFGVYLGFREATQGIVMNILRRKIPAEKI
NENLVQRGIMIFAILLAWSAIVLNAPVLSFTSICSPIFGMVGCLIPAWLVYKVPALHKYK
GMSLYLIIVTGLLLCVSPFLAFS
>LFGLNPFC_04826 hypothetical protein
MFDSTLNPLWQRYILAVQEEVKPALGCTEPISLALAAAVAAAELEGPVERVEAWVSPNLM
KNGLGVTVPGTGMVGLPIAAALGALGGNANAGLEVLKDATAQAISDAKALLAAGKVSVKI
QEPCDEILFSRAKVWNGEKWACVTIVGGHTNIVHIETHNGVVFTQQACVTEGEQESPLTV
LSRTTLAEILKFVNEVPFAAIRFILDSAKLNCALSQEGLSGNWGLHIGATLEKQCARGLL
AKDLSSSIVIRTSAASDARMGGATLPAMSNSGSGNQGITATMPVVVVAEHFGADDERLAR
ALMLSHLSAIYIHNQLPRLSALCAATTAAMGAAAGMAWLVDGRYETISMAISSMIGDVSG
MICDGASNSCAMKVSTSASAAWKAVLMALDDTAVTGNEGIVAHDVEQSIANLCALASHSM
QQTDRQIIEIMASKAR
>LFGLNPFC_04827 hypothetical protein
MSKKSAKKRQPVKPVVAKEPARTAKNFGYEEMLSELEAIVADAETRLAEDEATA
>LFGLNPFC_04828 Pirin-like protein YhaK
MITTRTARQCGQADYGWLQARYTFSFGHYFDPKLLGYASLRVLNQEVLAPGAAFQPRTYP
KVDILNVILDGEAEYRDSEGNHVQASAGEALLLSTQPGVSYSEHNLSKDKSLTRMQLWLD
ACPQRENPLIQKLALNMDKQQLIASPEGGMGSLQLRQQVWLHHIVLDKGESANFQLHGPR
AYLQSIHGQFHALTHHEEKAALTCGDGAFIRDEANITLVADSPLRALLIDLPV
>LFGLNPFC_04829 HTH-type transcriptional regulator YhaJ
MAKERALTLEALRVMDAIDRRGSFAAAADELGRVPSALSYTMQKLEEELDVVLFDRSGHR
TKFTNVGRMLLERGRVLLEAADKLTTDAEALARGWETHLTIVTEALVPTPAFFPLIDKLA
AKANTQLAIITEVLAGAWERLEQGRADIVIAPDMHFRSSSEINSRKLYTLMNVYVAAPDH
PIHQEPEPLSEVTRVKYRGIAVADTARERPVLTVQLLDKQPRLTVSTIEDKRQALLAGLG
VATMPYPMVEKDIAEGRLRVVSPESTSEIDIIMAWRRDSMGEAKSWCLREIPKLFSGK
>LFGLNPFC_04830 Inner membrane protein YhaH
MDWYLKVLKNYVGFRGRARRKEYWMFILVNIIFTFVLGLLDKMLGWQRAGGEGILTTIYG
ILVFLPWWAVQFRRLHDTDRSAWWALLFLIPFIGWLIIIVFNCQAGTPGENRFGPDPKLE
QE
>LFGLNPFC_04831 Glutathionyl-hydroquinone reductase YqjG
MGQLIDGVWHDTWYDTKSTGGKFQRSASAFRNWLTADGAPGPTGKGGFAAEKDRYHLYVS
LACPWAHRTLIMRKLKGLEPFISVSVVNPLMLENGWTFDDSFPGATGDTLYQHEFLYQLY
LHADPHYSGRVTVPVLWDKKNHTIVSNESAEIIRMFNTAFDALGAKAGDYYPPALQPKID
ELNGWIYDTVNNGVYKAGFATSQQAYDEAVAKVFESLARLEQILGQHRYLTGNQLTEADI
RLWTTLVRFDPVYVTHFKCDKHRISDYLNLYGFLRDIYQMPGIAETVNFDHIRNHYFRSH

KTINPTGIISIGPWQDLDEPHGRDVRFG
>LFGLNPFC_04832 Inner membrane protein YqjF
MILSIDSNDANSAPLHKKTISSLSGAVESMMKKLEDVGVLVARILMPILFITAGWGKITG
YAGTQQYMEAMGVPGFMLPLVILLEFGGGLAILFGFLTRTTALFTAGFTLLTAFLFHSNF
AEGMNSLMFMKNLTISGGFLLLAITGPGAFSIDRLLNKKW
>LFGLNPFC_04833 hypothetical protein
MSSKVERERRKAQLLSQIQQQRLDLSASRREWLEATGAYDRRWNMLLSLRSWALVGSSVM
AIWTIRHPNMLVRWARRGFGVWSAWRLVKTTLKQQQLRG
>LFGLNPFC_04834 Inner membrane protein YqjE
MADTHHAQGPGKSVLGIGQRIVSIMVEMVETRLRLAVVELEEEKANLFQLLLMLGLTMLF
AAFGLMSLMVLVIWAVDPQYRLNAMIATTVVLLLLALIGGIWTLRKSRKSTLLRHTRHEL
ANDRQLLEEESREQ
>LFGLNPFC_04835 putative protein YqjD
MSKEHTTEHLRAELKSLSDTLEEVLSSSGEKSKEELSKIRSKAEQALKQSRYRLGETGDA
IAKQTRVAAARADEYVRENPWTGVGIGAAIGVVLGVLLSRR
>LFGLNPFC_04836 Protein YqjC
MKYRIALAVSLFALSAGSYATTLCQEKEQNILKEISYAEKHQNQNRIDGLNKALSEVRAN
CSDSQLRADHQKKIAKQKDEVAERQQDLAEAKQKGDADKIAKRERKLAEAQEELKKLKAR
DY
>LFGLNPFC_04837 Modulator protein MzrA
MQIPRMSLRQLAWSGAVLLLVSTLLLAWSAVRQQESTLAIRAVHQGTTMPDGFSIWHHLD
AHGIPFKSITPKNDTLLITFDSSDQSAAAKAVLDRTLPHGYIIAQQDNNSQAMQWLTRLR
DNSHRFG
>LFGLNPFC_04838 Inner membrane protein YqjA
MELLTQLLQALWAQDFETLANPSMIGMLYFVLFVILFLENGLLPAAFLPGDSLLVLVGVL
IAKGAMGYPQTILLLTVAASLGCWVSYIQGRWLGNTRTVQNWLSHLPAHYHQRAHHLFHK
HGLSALLIGRFIAFVRTLLPTIAGLSGLNNARFQFFNWMSGLLWVLILTTLGYMLGKTPV
FLKYEDQLMSCLMLLPVVLLVFGLAGSLVVLWKKKYGNRG
>LFGLNPFC_04839 Putative L-lactate dehydrogenase operon regulatory protein
MEITEPRRLYQQLAADLKERIEQGVYLVGDKLPAERFIADEKNVSRTVVREAIIMLEVEG
YVEVRKGSGIHVVSNQPRHQQAADNNMEFANYGPFELLQARQLIESNIAEFAATQVTKQD
IMKLMAIQEQARGEQCFRDSEWDLQFHIQVALATQNSALAAIVEKMWTQRSHNPYWKKLH
EHIDARTVDNWCDDHDQILKALIRKDPHAAKLAMWQHLENTKIMLFNETSDDFEFNADRY
LFAENPVVHLDTATSGSK
>LFGLNPFC_04840 Hexuronate transporter
MRKIKGLRWYMIALVTLGTVLGYLTRNTVAAAAPTLMEELNISTQQYSYIIAAYSAAYTV
MQPVAGYVLDVLGTKIGYAMFAVLWAVFCGATALAGSWGGLAVARGAVGAAEAAMIPAGL
KASSEWFPAKERSIAVGYFNVGSSIGAMIAPPLVVWAIVMHSWQMAFIISGALSFIWAMA
WLIFYKHPRDQKHLTDEERDYIINGQEAQHQVDTAKKMSVGQILRNRQFWGIALPRFLAE
PAWGTFNAWIPLFMFKVYGFNLKEIAMFAWMPMLFADLGCILGGYLPPLFQRWFGVNLIV
SRKMVVTLGAVLMIGPGMIGLFTNPYVAIMLLCIGGFAHQALSGALITLSSDVFGRNEVA
TANGLTGMSAWLASTLFALVVGALADTIGFSPLFAVLAVFDLLGALVIWTVLQNKPAIEV
AQESHNDPAPQH
>LFGLNPFC_04841 Uronate isomerase
MTPFMTEDFLLDTEFARRLYHDYAKDQPIFDYHCHLPPQQIAEDYRFKNLYDIWLKGDHY
KWRAMRTNGVAERLCTGDASDREKFDAWAATVPHTIGNPLYHWTHLELRRPFGITGKLLS
PSTADEIWNECNELLAQDNFSARGIMQQMNVKMVGTTDDPIDSLEHHAEIAKDGSFTIKV
LPSWRPDKAFNIEQATFNDYMAKLGEVSDTDIRRFADLQTALTKRLDHFAAHGCKVSDHA
LDVVMFAEANEAELDSILARRLAGETLSEHEVAQFKTAVLVFLGAEYARRGWVQQYHIGA
LRNNNLRQFKLLGPDVGFDSINDRPMAEELSKLLSKQNEENLLPKTILYCLNPRDNEVLG
TMIGNFQGEGMPGKMQFGSGWWFNDQKDGMERQMTQLAQLGLLSRFVGMLTDSRSFLSYT
RHEYFRRILCQMIGRWVEAGEAPADINLLGEMVKNICFNNARDYFAIELN
>LFGLNPFC_04842 Altronate dehydratase
MQYIKIHALDNVAVALADLAEGTEVSVDNQTVTLRQDVARGHKFALTDIAKGANVIKYGL
PIGYALADIAAGEHVHAHNTRTNLSDLDQYRYQPDFQDLPAQAADREVQIYRRANGDVGV
RNELWILPTVGCVNGIARQIQNRFLKETNNAEGTDGVFLFSHTYGCSQLGDDHINTRTML
QNMVRHPNAGAVLVIGLGCENNQVAAFRETLGDIDPERVHFMICQQQDDEIEAGIAHLHQ
LYSVMRNDKREPGKLSELKFGLECGGSDGLSGITANPMLGRFSDYVIANGGTTVLTEVPE
MFGAEQLLMDHCRDEATFEKLVTMVNDFKQYFIAHDQPIYENPSPGNKAGGITTLEDKSL
GCTQKAGTSVVVDVLRYGERLKTPGLNLLSAPGNDAVATSALAGAGCHMVLFSTGRGTPY
GGFVPTVKIATNSELAAKKKHWIDFDAGQLIHGKAMPQLLEEFIDTIVEFANGKQTCNER
NDFRELAIFKSGVTL
>LFGLNPFC_04843 Inner membrane protein YgjV
MTAYWLAQGVGVIAFLIGITTFFNRDERRFKKQLSVYSAVIGVHFFLLGTYPAGASAILN

AIRTLITLRTRSLWVMAIFIVLTGGIGLAKFHHPVELLPVIGTIVSTWALFRCKGLTMRC
VMWFSTCCWVIHNFWAGSIGGTMIEGSFLLMNGLNIIRFWRMQRRGIDPFKVEKTPSAVD
ERG
>LFGLNPFC_04844 Serine/threonine transporter SstT
MTTQHSPGLFRRLAHGSLVKQILAGLILGILLAWISKPAAEAVGLLGTLFVGALKAVAPI
LVLMLVMASIANHQHGQKTNIRPILFLYLLGTFSAALAAVIFSFAFPSTLHLSSSAGDIS
PPSGIVEVMRGLVMSMVSNPIDALLKGNYIGILVWAIGLGFALRHGNETTKNLVNDMSNA
VTFMVKLVIHFAPIGIFGLVSSTLATTGFSTLWGYAQLLVVLVGCMLLVALVVNPLLVWW
KIRRNPFPLVLLCLRESGVYAFFTRSSAANIPVNMALCEKLNLDRDTYSVSIPLGATINM
AGAAITITVLTLAAVNTLGIPVDLPTALLLSVVASLCACGASGVAGGSLLLIPLACNMFG
ISNDIAMQVVAVGFIIGVLQDSCETALNSSTDVLFTAAACQAEDDRLANSALRN
>LFGLNPFC_04845 Putative membrane-bound redox modulator Alx
MNTVGTPLLWGGFAVVVTIMLAIDLLLQGRRGAHAMTMKQAAAWSLVWVTLSLLFNAAFW
WYLVQTEGRAVADPQALAFLTGYLIEKSLAVDNVFVWLMLFSYFSVPAALQRRVLVYGVL
GAIVLRTIMIFTGSWLISQFDWILYIFGAFLLFTGVKMALAHEDESGIGDKPLVRWLRGH
LRMTDTIDNEHFFVRKNGLLYATPLMLVLILVELSDVIFAVDSIPAIFAVTTDPFIVLTS
NLFAILGLRAMYFLLAGVAERFSMLKYGLAVILVFIGIKMLIVDFYHIPIAVSLGVVFGI
LVMTFIINAWVNYRHDKQRVE
>LFGLNPFC_04846 scyllo-inositol 2-dehydrogenase (NADP(+)) IolU
MIRFAVIGTNWITRQFVEAAHESGKYKLTAVYSRSLEQAQHFANDFSVEHLFTSLEAMAE
SDAIDAVYIASPNSLHFSQTQLFLSHKIHVICEKPLASNLAEVDAAIACARENQVVLFEA
FKTAYLPNFHLLRQALPKVGKLRKVFFNYCQYSSRYQRYLDGENPNTFNPTFSNGSIMDI
GFYCLASAVALFGEPKSVQATASLLASGVDAHGVVVMDYGDFSVTLQHSKVSDSVLASEI
QGEAGSLVIEKLSECQKVCFVPRGSQMQDLTQPQHINTMLYEAELFATLVDEHLVNHPGL
AVSRITAKLLTEIRRQTGVIFPADNVKL
>LFGLNPFC_04847 hypothetical protein
MLRAFARLLLRICFSRRTLKLGCLLLLIAGATIFIADRVMVNASKQLTWSDVNAVPARNV
GLLLGARPGNRYFTRRIDTAAALYHAGKVKWLLVSGDNGRKNYDEASGMQQALIAKGVPA
KVIFCDYAGFSTLDSVVRANKVFSENHITIISQEFHNQRAIWLAKQYGIDAIGFNAPDLN
MKHGLYTQLREKLARVSAVIDAKILHRQPKYLGPLVMIGPFSEHGCPAKE
>LFGLNPFC_04848 UTP pyrophosphatase
MSNLTYLQGYPEQLLSQVRTLINEQRLGDVLAKRYPGTHDYATDKALWQYTQDLKNQFLR
NAPPINKVMYDNKIHVLKNALGLHTAVSRVQGGKLKAKAEIRVATVFRNAPEPFLRMIVV
HELAHLKEKEHNKAFYQLCCHMEPQYHQLEFDTRLWLTQLSLGQDKI
>LFGLNPFC_04849 Ribosomal RNA large subunit methyltransferase G
MSHLDNGFRSLTLQRFPATDDVNPLQAWEAADEYLLQQLDDTEIRGPVLILNDAFGALSC
ALAEHKPYSIGDSYISELATRENLRLNGIDESSVKFLDSTADYPQQPGVVLIKVPKTLAL
LEQQLRALRKVVTPDTRIIAGAKARDIHTSTLELFEKVLGPTTTTLAWKKARLINCTFNE
PPLADAPQTVSWKLEGTDWTIHNHANVFSRTGLDIGARFFMQHLPENLEGEIVDLGCGNG
VIGLTLLDKNPQAKVVFVDESPMAVASSRLNVETNMPEALDRCEFMINNALSGVEPFRFN
AVLCNPPFHQQHALTDNVAWEMFHHARRCLKINGELYIVANRHLDYFHKLKKIFGNCTTI
ATNNKFVVLKAVKLGRRR
>LFGLNPFC_04850 2,4-dienoyl-CoA reductase
MSYPSLFAPLDLGFTTLKNRVLMGSMHTGLEEYPDGAERLAAFYAERARHGVALIVSGGI
APDLTGVGMEGGAMLNDANQIPHHRTITEAVHQEGGKIALQILHTGRYSYQPHLVAPSAL
QAPINRFVPHELTHEEILQLIDDFAHCAQLAREAGYDGVEVMGSEGYLINEFLTLRTNQR
SDQWGGDYRNRMRFAVEVVGAVRERVGNDFIIIYRLSMLDLVEGGGTFAETVELAQAIEA
AGATIINTGIGWHEARIPTIATPVPRGAFSWVTRKLKGHVSLPLVTTNRINDPQVADDIL
SRGDADMVSMARPFLADAELLSKAQSGRADEINTCIGCNQACLDQIFVGKVTSCLVNPRA
CHETKMPILPAVQKKNLAVVGAGPAGLAFAINAAARGHQVTLFDAHSEIGGQFNIAKQIP
GKEEFYETLRYYRRMIEVTGVTLKLNHTVTADQLKAFDETILASGIVPRIPPIDGIDHPK
VLSYLDVLRDKAQVGNKVAIIGCGGIGFDTAMYLSQPGESTSQNIAGFCNEWGIDSSLQQ
AGGLSPQGMQIPRSPRQIVMLQRKASKPGQGLGKTTGWIHRTTLLSRGVKMIPGVSYQKI
DDDGLHVVINGETQVLAVDNVVICAGQEPNRALAQPLIDSGKTVHLIGGCDVAMELDARR
AIAQGTRLALEI
>LFGLNPFC_04851 Glucosidase YgjK
MKIKTILTPVACALLMSFSAHAANADNYKNVINRTGAPQYMKDYDYDDHQRFNPFFDLGA
WHGHLLPDGPNTMGGFPGVALLTEEYINFMASNFDRLTVWQDGKKVDFTLEAYSIPGALV
QKLISKDVQVEMILRFATPRTSLLETKITSDKPLDLVWDGELLEKLEAKEGKPLSDKTIA
GEYPDYQRKISATRDGLKVTFGKVRATWDLLTSGESEYQVHKSLPVQTEINGNRFTSKAH
INGSTTLYTTYSHLLTAQEVSKEQMQIRDILARPAFYLTASQQRWEEYLKKGLTNPDATP
EQTRVAVKAIETLNGNWRSPGGAVKYNTVTPSVTGRWFSGNQTWPWDTWKQAFAMAHFNP
DIAKENIRAVFSWQIQPGDSVRPQDVGFVPDLIAWNLSPERGGDGGNWNERNTKPSLAAW
SVMEVYNVTQDKTWLAEMYPKLVAYHDWWLRNRDHNGNGVPEYGATRDKAHNTESGEMLF

TVKKGDKEETRSGLNNYARVVEKGQYDSLEIPAQVAASWESGRDDAAVFGFIDKEQLDKY
VANGGKRSDWTVKFAENRSQDGTLLGYSLLQESVDQASYMYSDNHYLAEMATILGKPEEA
KRYRQLAQQLADYINTCMFDPTTQFYYDVRIEDKPLANGCAGKPIVERGKGPEGWSPLFN
GAATQANADAVVKVMLDPKEFNTFVPLGTAALTNPAFGADIYWRGRVWVDQFWFGLKGME
RYGYRDDALKLADTFFQHAKGLTADGPIQENYNPLTGAQQGAPNFSWSAAHLYMLYNDFF
RKQ
>LFGLNPFC_04852 hypothetical protein
MKLITAPCRALLALPFCYAFSVTADEARPAEHDDTKTPAMTSTSSPSFRFYGELGVGGYM
DLEGENKHKYSDGTYIEGGLEMKYGSWFGLIYGEGWTVQADHDGNAWVPDHSWGGFEGGI
NRFYGGYRTNDGTEIMLSLRQDSSLDDLQWWGDFTPDLGYVIPNTRDIMTALKVQNLSGN
FRYSVTATPAGHHDESKAWLHFGKYDRYDDKYTYPAMMNGYIQYDLAEGITWMNGLEITD
GTGQLYLTGLLTPNFAARAWHHTGRADGLDVSGSESGMMVSAMYEALKGVYLSTAYTYAK
HRPDHADDETTSFMQFGIWYEYGGGRFATAFDSRFYMKNASHDPSDQLFLMQYFYW
>LFGLNPFC_04853 Inner membrane transporter YgjI
MSDTKRNTIGKFGLLSLTFAAVYSFNNVINNNIELGLASAPMFFLATIFYFIPFCLIIAE
FVSLNKNSEAGVYAWVKSSLGGRWAFITAYTYWFVNLFFFTSLLPRVIAYASYAFLGYEY
IMTPVATTIISMVLFAFSTWVSTNGAKMLGPITSVTSTLMLLLTLSYILLAGTALVGGVQ
PADPITVDAMIPNFNWAFLGVTTWIFMAAGGAESVAVYVNDVKGGSKSFVKVIILAGIFI
GVLYSVSSVLINVFVSSKELKFTGGSVQVFHGMAAYFGLPEALMNRFVGLVSFTAMFGSL
LMWTATPVKIFFSEIPEGIFGKKTVELNENGVPARAAWIQFLIVIPLMIIPMLGSNTVQD
LMNTIINMTAAASMLPPLFIMLAYLNLRAKLDHLPRDFRMGSRRTGIIVVSMLIAIFAVG
FVASTFPTGANILTIIFYNVGGIVIFLGFAWWKYSKYIKGLTAEERHIEATPARNVD
>LFGLNPFC_04854 Evolved beta-galactosidase subunit beta
MRIIDNLEQFRQIYASGKKWQRCVEAIENIDNIQPGVAHSIGDSLTYRVETDSATDALFT
GHRRYFEVHYYLQGQQKIEYAPKETLQVVEYYRDETDREYLKGCGETVEVHEGQIVICDI
HEAYRFICNNAVKKVVLKVTIEDGYFHNK
>LFGLNPFC_04855 Evolved beta-galactosidase subunit alpha
MNRWENIQLTHENRLAPRAYFFSYDSVAQARTFARETSSLFLLLSGQWNFHFFDHPLQVP
EAFTSELMADWGHITVPAMWQMEGHGKLQYTDEGFPFPIDVPFVPSDNPTGAYQRIFTLS
DGWQGKQTLIKFDGVETYFEVYVNGQYVGFSKGSRLTAEFDISAMVKTGDNLLCVRVMQW
ADSTYVEDQDMWWSAGIFRDVYLIGKQLTHINDFTVRTDFDEAYCDATLSCEVVLENLAA
SPVVTTLEYTLFDGEHVVHSSAIDHLAIEKLTSASFAFTVEQPQQWSAESPYLYHLVMTL
KDADGNVLEVVPQRVGFRDIKVRDGLFWINNRYVMLHGVNRHDNDHRKGRAVGMDRVEKD
LQLMKQHNINSVRTAHYPNDPRFYELCDIYGLFVMAETDVESHGFANVGDISRITDDPQW
EKVYVERIVRHIHAQKNHPSIIIWSLGNESGYGCNIRAMYHAAKALDDTRLVHYEEDRDA
EVVDIISTMYTRVPLMNEFGEYPHPKPRIICEYAHAMGNGPGGLTEYQNVFYKHDCIQGH
YVWEWCDHGIQAQDDNGNVWYKFGGDYGDYPNNYNFCLDGLIYSDQTPGPGLKEYKQVIA
PVKIHALDLTRGELKVENKLWFTTLDDYTLHAEVRAEGETLATQQIKLRDVAPNSEAPLQ
ITLPQLDAREAFLNITVTKDSRTRYSEAGHSIATYQFPLKENTAQPVPFAPNNARPLTLE
DDRLSCTVRGYNFAITFSKTSGKPTSWQVNGESLLTREPKINFFKPMIDNHKQEYEGLWQ
PNHLQIMQEHLRDFVVEQSDDEVLLVSRTVIAPPVFDFGMRCTYIWRIAADGQVNVALSG
ERYGDYPHIIPCIGFTMGINGEYDQVAYYGRGPGENYADSQQANIIDIWRSTVDAMFENY
PFPQNNGNRQHVRWTALTNRHGNGLLVVPQRPINFSAWHYTQENIHAAQHCNELQRSDDI
TLNLDHQLLGLGSNSWGSEVLDSWRVWFRDFSYGFTLLPVSGGEATAQSLASYEFGAGFF
STNLHSENKQ
>LFGLNPFC_04856 HTH-type transcriptional repressor MelR
MATLKDIAIEAGVSLATVSRVLNDDPTLNVKEETKHRILEIAEKLEYKTSSARKLQTGAV
NQHHILAIYSYQQELEINDPYYLAIRHGIETQCEKLAIELTNCYEHNGLPDIKNVTGILI
VGKPTSALRAAACALTDNICFIDFHEPGSGYDAVDIDLARISKEIIDFYINQGVNRIGFI
GGEDLPGKADIREVAFAEYGRLKHVVREEDIWRGGFSSSSGYELAKQMLAREDYPKALFV
ASDSIAIGVLRAIHERGLNIPQDISLISVNDIPTARFTFPPLSTVRIHSEMMGSQGVNLV
YEKARDGRALPLLVFVPSKLKLRGTTR
>LFGLNPFC_04857 tRNA-binding protein YgjH
METVAYADFARLEMRVGKIVEVKRHENADKLYIVQVDVGEKTLQTVTSLVPYYSEEELMG
KTVVVLCNLQKAKMRGETSECMLLCAETDDGSESVLLTPERMMPAGVRIV
>LFGLNPFC_04858 Putrescine aminotransferase
MFVFIPIFAIAAGVAQSLQYLNRYHVIREPPEHILNRLPSSASALACSAHALNLIEKRTL
DHEEMKALNREVIEYFKEHVNPGFLEYRKSVTAGGDYGAVEWQAGGLNTLVDTQGQEFID
CLGGFGIFNVGHRNPVVVSAVQNQLAKQPLHSQELLDPLRAMLAKTLAALTPGKLKYSFF
CNSGTESVEAALKLAKAYQSPRGKFTFIATSGAFHGKSLGALSATAKSTFRKPFMPLLPG
FRHVPFGNIEAMRTALNECKKTGDDVAAVILEPIQGEGGVILPPPGYLTAVRKLCDEFGA
LMILDEVQTGMGRTGKMFACEHENVQPDILCLAKALGGGVMPIGATIATEEVFSVLFDNP
FLHTTTFGGNPLACAAALATINVLLEQNLPAQAEQKGDMLLDGFRQLAREYPDLVQEARG
KGMLMAIEFVDNEIGYNFASEMFRQRVLVAGTLNNAKTIRIEPPLTLTIEQCELVIKAAR

KALAAMRVSVEEA
>LFGLNPFC_04859 Aerotaxis receptor
MSSHPYVTQQNTPLADDTTLMSTTDLQSYITHANDTFVQVSGYTLQELQGQPHNMVRHPD
MPKAAFADMWFTLKKGEPWSGIVKNRRKNGDHYWVRANAVPMVREGKISGYMSIRTRATD
EEIAAVEPLYKALNAGRTSKRIHKGLVVRKGWLGKLPSLPLRWRARGVMTLMFILLAVML
WFVAAPVVTYILCALVVLLASACFEWQIVRPIENVARQALKVATGERNSVEHLNRSDELG
LTLRAVGQLGLMCRWLINDVSSQVSSVRNGSETLAKGTDELNEHTQQTVDNVQQTVATMN
QMAASVKQNSATASAADKLSITASNAAVQGGEAMTTVIKTMDDIADSTQRIGTITSLIND
IAFQTNILALNAAVEAARAGEQGKGFAVVAGEVRHLASRSANAANDIRKLIDASADKVQS
GSQQVHAAGRTMEDIVAQVKNVTQLIAQISHSTLEQADGLSSLTRAVDELNLITQKNAEL
VEESAQVSAMVKHRASRLEDAVTVLH
>LFGLNPFC_04860 Transcriptional regulator YqjI
MSYHHEGCCKHEGQPRHEGCCKGEKSEHEHCGHGHQHEHGQCCGGRHGRGGGRRQRFFGH
GELRLVILDILSRDDSHGYELIKAIENLTQGNYTPSPGVIYPTLDFLQEQSLITIREEEG
GKKQIALTEQGAQWLEENREQVEMIEERIKARCVGAALRQNPQMKRALDNFKAVLDLRVN
QSDITDAQIKKIIAVIDRAAFDITQLD
>LFGLNPFC_04861 NADPH-dependent ferric-chelate reductase
MNNSPRYPQRVRNDLRFRELTVLRAERISAGFQRIVLGGEALDGFTSRGFDDHSKLFFPQ
SDAHFVPPTVTEEGIVWPEGPRPPSRDYTPLYDELRHELAIDFFIHDGGVASGWAMQAQP
GDKLTVAGPRGSLVVPEDYAYQLYVCDESGMPALRRRLETLSKLAVKPQVSALVSVRDNA
CQDYLAHLDGFNIEWLAHDEQAVDERLAQMQIPADDYFIWITGEGKVVKNLSRRFEAEQY
DPQRVRAAAYWHAK
>LFGLNPFC_04863 G/U mismatch-specific DNA glycosylase
MVEDILAPGLRVVFCGINPGLSSAGTGFPFAHPANRFWKVIYQAGFTDRQLKPQEAQHLL
DYRCGVTKLVDRPTVQANEVSKQELHAGGRKLIEKIEDYQPQALAILGKQAYEQGFSQRG
AQWGKQTLTIGSTQIWVLPNPSGLSRVSLEKLVEAYRELDQALVVRGR
>LFGLNPFC_04864 RNA polymerase sigma factor RpoD
MEQNPQSQLKLLVTRGKEQGYLTYAEVNDHLPEDIVDSDQIEDIIQMINDMGIQVMEEAP
DADDLMLAENTADEDAAEAAAQVLSSVESEIGRTTDPVRMYMREMGTVELLTREGEIDIA
KRIEDGINQVQCSVAEYPEAITYLLEQYDRVEAEEARLSDLITGFVDPNAEEDLAPTATH
VGSELSQEDLDDDEDEDEEDGDDDTADDDNSIDPELAREKFAELRAQYIVTRDTIKAKGR
SHAAAQEEILKLSEVFKQFRLVPKQFDYLVNSMRVMMDRVRTQERLIMKLCVEQCKMPKK
NFITLFTGNETSDTWFNAAIAMNKPWSEKLHDVSEEVHRALQKLQQIEEETGLTIEQVKD
INRRMSIGEAKARRAKKEMVEANLRLVISIAKKYTNRGLQFLDLIQEGNIGLMKAVDKFE
YRRGYKFSTYATWWIRQAITRSIADQARTIRIPVHMIETINKLNRISRQMLQEMGREPTP
EELAERMLMPEDKIRKVLKIAKEPISMETPIGDDEDSHLGDFIEDTTLELPLDSATTESL
RAATHDVLAGLTAREAKVLRMRFGIDMNTDHTLEEVGKQFDVTRERIRQIEAKALRKLRH
PSRSEVLRSFLDD
>LFGLNPFC_04865 DNA primase
MAGRIPRVFINDLLARTDIVDLIDARVKLKKQGKNFHACCPFHNEKTPSFTVNGEKQFYH
CFGCGAHGNAIDFLMNYDKLEFVETVEELAAMHNLEVPFEAGSGPSQIERHQRQTLYQLM
DGLNTFYQQSLQQPVATSARQYLEKRGLSHEVIARFAIGFAPPGWDNVLKRFGGNPENRQ
SLVDAGMLVTNDQGRSYDRFRERVMFPIRDKRGRVIGFGGRVLGNDTPKYLNSPETDIFH
KGRQLYGLYEAQQDNAEPNRLLVVEGYMDVVALAQYGINYAVASLGTSTTADHIQLLFRA
TNNVICCYDGDRAGRDAAWRALETALPYMTDGRQLRFMFLPDGEDPDTLVRKEGKEAFEA
RMEQAMPLSAFLFNSLMPQVDLSTPDGRARLSTLALPLISQVPGETLRIYLRQELGNKLG
ILDDSQLERLMPKAAESGVSRPVPQLKRTTMRILIGLLVQNPELATLVPPLENLDENKLP
GLGLFRELVNTCLSQPGLTTGQLLEHYRGTNNAATLEKLSMWDDIADKNIAEQTFTDSLN
HMFDSLLELRQEELIARERTHGLSNEERLELWTLNQELAKK
>LFGLNPFC_04866 30S ribosomal protein S21
MPVIKVRENEPFDVALRRFKRSCEKAGVLAEVRRREFYEKPTTERKRAKASAVKRHAKKL
ARENARRTRLY
>LFGLNPFC_04867 tRNA N6-adenosine threonylcarbamoyltransferase
MRVLGIETSCDETGIAIYDDEKGLLANQLYSQVKLHADYGGVVPELASRDHVRKTVPLIQ
EALKESGLTAKDIDAVAYTAGPGLVGALLVGATVGRSLAFAWDVPAIPVHHMEGHLLAPM
LEDNPPEFPFVALLVSGGHTQLISVTGIGQYELLGESIDDAAGEAFDKTAKLLGLDYPGG
PLLSKMAAQGTAGRFVFPRPMTDRPGLDFSFSGLKTFAANTIRDNGTDDQTRADIARAFE
DAVVDTLMIKCKRALDQTGFKRLVMAGGVSANRTLRAKLAEMMKKRRGEVFYARPEFCTD
NGAMIAYAGMVRFKAGATADLGVSVRPRWPLAELPAA
>LFGLNPFC_04868 L-tartrate/succinate antiporter
MKPSTEWWRYLAPLAVIAIIALLPLPAGLESHTWLYFAVFTGVIVGLILEPVPGAVVAMV
GISIIAILSPWLLFSPEQLAQPGFKFTAKSLSWAVSGFSNSVIWLIFAAFMFGTGYEKTG
LGRRIALILVKKMGHRTLFLGYAVMFSELILAPVTPSNSARGAGIIYPIIRNLPPLYQSQ
PNDSSSRSIGSYIMWMGIVADCVTSAIFLTAMAPNLLLIGLMKSASNATLSWGDWFLGML

PLSILLVLLVPWLAYVLYPPILKSGDQVPRWAETELQAMGPLCSREKRMLGLMVGALVLW
IFGGDYIDAAMVGYSVVALMLLLRIICWDDIVSNKAAWNVFFWLASLITLATGLNNTGFI
SWFGKLLAGSLSGYSPTIVMVALIVVFYLLRYFFASATAYTSALAPMMIAAALAMPEIPL
PVFCLMVGAAIGLGSILTPYATGPSPIYYGSGYLPTVDYWRLGAIFGLIFLVLLVITGLL
WMPMVLL
>LFGLNPFC_04869 L(+)-tartrate dehydratase subunit beta
MKKILTTPIKAEDLQDIRVGDVIYLTGTLVTCRDVCHRRLIELKRPIPYDLNGKAIFHAG
PIVRKNGDKWEMVSVGPTTSMRMESFEREFIEQTGVKLVVGKGGMGPLTEEGCQKFKALH
VIFPAGCAVLAATQVEEIEEVHWTELGMPESLWVCRVKEFGPLIVSIDTHGNNLIAENKK
LFAERRDPIVEEICEHVHYIK
>LFGLNPFC_04870 L(+)-tartrate dehydratase subunit alpha
MMSESNKQQAVNKLTEIVANFTAMISTRMPDDVVDKLKQLKDAETSSMGKIIYHTMFDNM
QKAIDLNRPACQDTGEIMFFVKVGSRFPLLGELQSILKQAVEEATVKAPLRHNAVEIFDE
VNTGKNTGSGVPWVTWDIVPDGDDAEIEVYMAGGGCTLPGRSKVLMPSEGYEGVVKFVFE
NISTLAVNACPPVLVGVGIATSVETAAVLSRKAILRPIGSRHPNPKAAELELRLEEGLNR
LGIGPQGLTGNSSVMGVHIESAARHPSTIGVAVSTGCWAHRRGTLRVHADLTFENLSHTR
SAL
>LFGLNPFC_04871 HTH-type transcriptional regulator DmlR
MLNSWPLAKDLQVLVEIVHSGSFSAAAATLGQTPAFVTKRIQILENTLATTLLNRSARGV
ALTESGQRCYEHALEILTQYQRLVDDVTQIKTRPEGMIRIGCSFGFGRSHIAPAITELMR
NYPELQVHFELFDRQIDLVQDNIDLDIRINDAIPDYYIAHLLTKNKRILCAAPEYLQKYP
QPQSLQELSRHDCLVTKERDMTHGIWELGNGQEKKSVKVSGHLSSNSGEIVLQWALEGKG
IMLRSEWDVLPFLESGKLVRVLPEYAQSANIWAVYREPLYRSMKLRVCVEFLAAWCQQRL
GKPDEGYQVM
>LFGLNPFC_04872 putative glycerol-3-phosphate acyltransferase
MSAIAPGMILIAYLCGSISSAILVCRLCGLPDPRTSGSGNPGATNVLRIGGKGAAVAVLI
FDVLKGMLPVWGAYELGVSPFWLGLIAIAACLGHIWPVFFGFKGGKGVATAFGAIAPIGW
DLTGVMAGTWLLTVLLSGYSSLGAIVSALIAPFYVWWFKPQFTFPVSMLSCLILLRHHDN
IQRLWRRQETKIWTKFKRKREKDPE
>LFGLNPFC_04873 Dihydroneopterin aldolase
MDIVFIEQLSVITTIGVYDWEQTIEQKLVFDIEMAWDNRKAAKSDDVADCLSYADIAETV
VSHVEGARFALVERVAEEVAELLLARFNSPWVRIKLSKPGAVARAANVGVIIERGNNLKE
NN
>LFGLNPFC_04874 Undecaprenyl-diphosphatase
MSDMHSLLIAAILGVVEGLTEFLPVSSTGHMIIVGHLLGFEGDTAKTFEVVIQLGSILAV
VVMFWRRLFGLIGIHFGRPLQHEGESKGRLTLIHILLGMIPAVVLGLLFHDTIKSLFNPI
NVMYALVVGGLLLIAAECLKPKEPRAPGLDDMTYRQAFMIGCFQCLALWPGFSRSGATIS
GGMLMGVSRYAASEFSFLLAVPMMMGATALDLYKSWGFLTTGDIPMFAVGFITAFVVALI
AIKTFLQLIKRISFIPFAIYRFIVAAAVYVVFF
>LFGLNPFC_04875 Multifunctional CCA protein
MKIYLVGGAVRDALLGLPVKDRDWVVVGSTPQEMLDAGYQQVGRDFPVFLHPQTHEEYAL
ARTERKSGSGYTGFTCYAAPDVTLEDDLKRRDLTINALAQDDNGEIIDPYNGLGDLQNRL
LRHVSPAFGEDPLRVLRVARFAARYAHLCFRIADETLALMREMTHAGELEHLTPERVWKE
TENALTTRNPQVFFQVLRDCGALRVLFPEIDALFGVPAPARWHPEIDTGIHTLMTLSMAA
MLSPQVDVRFATLCHDLGKGLTPPELWPRHHGHGPAGVKLVEQLCQRLRVPNEIRDLARL
VAEFHDLIHTFPMLNPKTIVKLFDSIDAWRKPQRVEQLALTSEADVRGRTGFESADYPQG
RWLREAWEVAQSVPTKAVVEAGFKGVEIREELTRRRIAAVAGWKEQRCPKPE
>LFGLNPFC_04876 hypothetical protein
MPKLRLIGLTLLALSATAVSHAEETRYVSDELNTWVRSGPGDHYRLVGTVNAGEEVTLLQ
TDANTNYAQVKDSSGRTAWIPLKQLSTEPSLRSRVPDLENQVKTLTDKLTNIDNTWNQRT
AEMQQKVAQSDSVINGLKEENQKLKNELIVAQKKVDAASVQLDDKQRTIIMQWFMYGGGV
LGLGLLLGLVLPHLIPSRKRKDRWMN
>LFGLNPFC_04877 Inorganic triphosphatase
MAQEIELKFIVNHSAVEALRDHLNTLDGEHHDPVQLLNIYYETPDNWLRGHDMGLRIRGE
NGRYEMTMKVAGRVTGGLHQRPEYNVALSAPTLDLAQLPTEVWPNGELPADLASRVQPLF
STDFYREKWLVEVDGSQIEIALDQGEVKAGEFAEPICELELELLSGDMRAVLKLANQLVS
QTGLRQGSLSKAARGYHLAQGNPAREIKPTTILHVAAKADVEQGLEAALELALAQWQYHE
ELWVRGNDAAKEQVLAAIGLVRHTLMLFGGIVPRKASTHLRDLLTQCEATIASAVSAVTA
VYSTETAMAKLALTEWLVSKAWQPFLDAKAQGKMSDSFKRFADIHLSRHAAELKSVFCQP
LGDRYRDQLPRLTRDIDSILLLAGYYDPVVAQDWLENWQGLRHAIATGQRIEIEHFRNEA
NNQEPFWLHSGKR
>LFGLNPFC_04878 Bifunctional glutamine synthetase adenylyltransferase/adenylyl-removing enzyme
MKPLSSPLQQYWQTVVERLPEPLAEESLSAQAKSVLTFSDFVQDSVIAHPEWLTELESQP

PQADEWQHYAAWLQEALSNVSDEAGLMRELRLFRRRIMVRIAWAQTLALVTEESILQQLS
HLAETLIVAARDWLYDACCREWGTPCNAQGEAQPLLILGMGKLGGGELNFSSDIDLIFAW
PEHGCTQGGRRELDNAQFFTRMGQRLIKVLDQPTQDGFVYRVDMRLRPFGESGPLVLSFA
ALEDYYQEQGRDWERYAMVKARIMGDSDGVYANELRAMLRPFVFRRYIDFSVIQSLRNMK
GMIAREVRRRGLTDNIKLGAGGIREIEFIVQVFQLIRGGREPSLQSRALLPTLSAIAALH
LLSENDAEQLRVAYLFLRRLENLLQSINDEQTQTLPSDELTRARLAWAMDFADWPQLTGV
LTAHMANVRRVFNELIGDDESETQEESLSEQWRELWQDALQEDDTTPVLAHLSEDERKQV
LMLIADFRKELDKRTIGPRGRQVLDHLMPHLLSDVCAREDAAVTLSRITALLVGIVTRTT
YLELLSEFPAALKHLISLCAASPMIASQLARYPLLLDELLDPNTLYQPTATDAYRDELRQ
YLLRVPEDDEEQQLEALRQFKQAQLLRIAAADIAGTLPVMKVSDHLTWLAEAMIDAVVQQ
AWVQMVARYGKPNHLNEREGRGFAVVGYGKLGGWELGYSSDLDLIFLHDCPMDAMTDGER
EIDGRQFYLRLAQRIMHLFSTRTSSGILYEVDARLRPSGAAGMLVTSAEAFADYQKNEAW
TWEHQALVRARVVYGDPQLTAHFDAVRREIMTLPREGKTLQTEVREMREKMRAHLGNKHR
DRFDIKADEGGITDIEFITQYLVLRYAHEKPKLTRWSDNVRILELLAQNDIMEEQEAMAL
TRAYTTLRDELHHLALQELPGHVSEDCFTAERELVRASWQKWLVEE
>LFGLNPFC_04879 Bifunctional protein HldE
MKVTLPEFERAGVMVVGDVMLDRYWYGPTSRISPEAPVPVVKVNTIEERPGGAANVAMNI
ASLGANARLVGLTGIDDAARALSKSLADVNVKCDFVSVPTHPTITKLRVLSRNQQLIRLD
FEEGFEGVDPQPLHERINQALSSIGALVLSDYAKGALASVQQMIQLARKAGVPVLIDPKG
TDFERYRGATLLTPNLSEFEAVVGKCKTEEEIVERGMKLIADYELSALLVTRSEQGMSLL
QPGKAPLHMPTQAQEVYDVTGAGDTVIGVLAATLAAGNSLEEACFFANAAAGVVVGKLGT
STVSPIELENAVRGRADTGFGVMTEEELKLAVAAARKRGEKVVMTNGVFDILHAGHVSYL
ANARKLGDRLIVAVNSDASTKRLKGDSRPVNPLEQRMIVLGALEAVDWVVSFEEDTPQRL
IAGILPDLLVKGGDYKPEEIAGSKEVWANGGEVLVLNFEDGCSTTNIIKKIQLDKKG
>LFGLNPFC_04880 Inner membrane protein YqiK
MDDIVNSVPSWMFTAIIAVCILFIIGIIFARLYRRASAEQAFVRTGLSGQKVVMSGGAIV
MPIFHEIIPINMNTLKLEVSRSTIDSLITKDRMRVDVVVAFFVRVKPSVEGIATAAQTLG
QRTLSPEDLRMLVEDKFVDALRATAAQMTMHELQDTRENFVQGVQNTVAEDLSKNGLELE
SVSLTNFNQTSKEHFNPNNAFDAEGLTKLTQETERRRRERNEVEQDVEVAVREKNRDALS
RKLEIEQQEAFMTLEQEQQVKTRTAEQNAKIAAFEAERRREAEQTRILAERQIQETEIDR
EQAVRSRKVEAEREVRIKEIEQQQVTEIANQTKSIAIAAKSEQQSQAEARANLALAEAVS
AQQNVETTRQTAEADRAKQVALIAAAQDAETKAVELTVRAKAEKEAAEMQAAAIVELAEA
TRKKGLAEAEAQRALNDAINVLSDEQTSLKFKLALLQALPAVIEKSVEPMKAIDGIKIIQ
VDGLNRGGATGDANTGNVGGGNLAEQALSAALSYRTQAPLIDSLLNEIGVSGGSLAALTS
SLSSTTPVEEKAE
>LFGLNPFC_04881 Inner membrane protein YqiJ
MILFADYNTPYLFAISFVLLIGLLEIFALICGHMLSGALDAHLDHYDSITTGHISQALHY
LNIGRLPALVVLCLLAGFFGLIGILLQHACVTLWQSPLPNLFVVPVSLLFTIIAVHYTGK
VVAPWIPRDHSSAITEEEYVGSMALITGHQATSGNPCEGKLTDQFGQIHYLLLEPEEGKT
FTKGDKVLIICRLSATRYLAENNPWPQIL
>LFGLNPFC_04882 Surface composition regulator
MDHSLNSLNNFDFLARSFARMHAEGRPVDILAVTGNMDEEHRTWFCARYAWYCQQMMQTR
ELELEH
>LFGLNPFC_04883 Ubiquinone biosynthesis accessory factor UbiK
MIDPKKIEQIARQVHESMPKGIREFGEDVEKKIRQTLQAQLTRLDLVSREEFDVQTQVLL
RTREKLALLEQRISELEARNNPIAELQSPPAIPPVDKAE
>LFGLNPFC_04884 3,4-dihydroxy-2-butanone 4-phosphate synthase
MNQTLLSSFGTPFERVENALAALREGRGVMVLDDEDRENEGDMIFPAETMTVEQMALTIR
HGSGIVCLCITDDRRKQLDLPMMVENNTSAYGTGFTVTIEAAEGVTTGVSAADRITTVRA
AIADGAKPSDLNRPGHVFPLRAQAGGVLTRGGHTEATIDLMTLAGFKPAGVLCELTNDDG
TMARAPECIEFANKHNMALVTIEDLVAYRQAHERKAS
>LFGLNPFC_04885 hypothetical protein
MITLFRLLAILCLFFNVSAFAVDCYQDGYRGTTLINGDLPAFKIPENAQPGQKIWESGDI
NITVYCDNAPGWTTDNPSENIYAWIKLPQINSADMLNNPYLTFGVTYNGVDYEGINERID
THACLDKYEQYYNGYYYDPVCNGSTLQKNVTFNAHFRVYVKFKSRPAGDQTVNFGIVNVL
QFDGEGGANMSSNAKNLRYVITGLDNISFLDCSVDVRIFPESQIVNFGQIAANSIATYRP
KAAFSVSTIKDVAADCTEQFDVATSFYTTDTLHDDTHLEMGNGLLMRITDQKTQEDIKFN
QYKRFTTYIPGQTGAMVTRDYQAELSQKPGETLVYGPFKKDLIVKINYN
>LFGLNPFC_04886 putative fimbrial chaperone YfcS
MKSPLITTGMICLLGICNFAQATVSPDRTRIIFNASNKSATVRLTNQSKIDPYLAQSWIE
DASGKKTRDYISTLPPMERIEPDEQIQIRLMALASLNDLPQDRETLFYYNVREIPPRAKE
QNVMQIAMQSRLKLFWRPKAIELKEGEMIPLQKVTITRTAAGLTLNNPTPYHITVGYIGT
NGKTLMPGADSIMVVPFTSATQHLSSLPSTFQLGFVADYGGLEMFKVECNSIQSLCQSSP
AKKGKI

>LFGLNPFC_04887 Outer membrane usher protein PapC
MNNKNTFSRDKLSHAIKNALSGVVCSLLFVLPVHAVEFNVDMIDAEDRENIDISRFEKKG
YIPPGRYLVRVQINKNMLPQTLILEWVKADNESGSLLCLTKENLTNFGLNTEFIESLQNI
AGSECLDLSQRQELTTRLDKATMILSLSVPQAWLKYQATNWTPPEFWDTGIAGFILDYNV
YASQYAPHHGDSTQNVSSYGTLGFNLGAWRLRSDYQYNQNFADGRSVNRDSEFARTYLFR
PIPSWSSKFTMGQYDLSSNLYDTFHFTGASLESDESMLPPDLQGYAPQITGIAQTNAKVT
VAQNGRVLYQTTVAPGPFTISDLGQSFQGLLDVTVEEEDGRTSTFQVGSASIPYLTRKGQ
VRYKTSLGKPTSVGHNDINNPFFWTAEASWGWLNNVSLYGGGMFTADDYQAITTGIGFNL
NQFGSLSFDVTGADASLQQQNSGNLRGYSYRFNYAKHFESTGSQITFAGYRFSDKDYVSM
SEYLSSRNGDESIDNEKESYVISLNQYFETLELNSYLNVTRNTYWDSASNTNYSVSVSKN
FDIGDFKGISASLAVSRIRWDDDEENQYYFSFSLPLQQNRNISYSMQRTGSSNTSQMISW
YDSSDRNNIWNISASATDDNIRDGEPTLRGSYQHYSPWGRLNINGSVQPNQYNSVTAGWY
GSLTATRHGIALHDYSYGDNARMMVDTDGISGIEINSNRTVTNGLGIAVIPSLSNYTTSM
LRVNNNDLPEGVDVENSVIRTTLTQGAIGYAKLNATTGYQIVGVIRQENGRFPPLGVNVT
DKATGKDVGLVAEDGFVYLSGIQENSTLHLTWGDNTCEVTPPNQSNISESAIILPCKTVK
>LFGLNPFC_04888 putative fimbrial-like protein YbgD
MSIAELILNSNLYFPHPFIKDYSHVKKTLLSMFATALLSGVAFNALADDANQGSGKITFK
GEVIDAPCSIAPGDEDQTINLGEVADTVLKSGQKSLPVDVTIHLQDCILSDGTNTVDKVK
ITFSSASVDATDSNLLKNTLEGNIGGATDVGVRLVKSDNTNVTLGTPITINFPTTNSYQE
LNFKARMESLGRTATPGNVQAQANYVLDYK
>LFGLNPFC_04889 Protein YqiD
MFIAWYWIVLIVLLAGGYFLHLKRYCKAFRHDRDALLAARSKLDKRSDKEVFEKHQ
>LFGLNPFC_04890 Zinc transporter ZupT
MSVPLILTILAGAATFIGAFLGVLGQKPSNRLLAFSLGFAAGIMLLISLMEMLPAALAAE
GMSPVLGYGMFIFGLLGYFGLDRMLPHAHPQDLMQKSVQPLPKSIKRTAILLTLGISLHN
FPEGIATFVTASSNLELGFGIALAVALHNIPEGLAVAGPVYAATGSKRTAILWAGISGLA
EILGGVLAWLILGSMISPVVMAAIMAAVAGIMVALSVDELMPLAKEIDPNNNPSYGVLCG
MSVMGFSLVLLQTAGIG
>LFGLNPFC_04891 4,5-DOPA dioxygenase extradiol
MTPLVKDIIMSSTRMPALFLGHGSPMNVLEDNLYTRSWQTLGMTLPRPKAIVVVSAHWFT
RGTGVTAMETPPTIHDFGGFPQALYDTHYPAPGSPALAQRLVELLAPVPVALDKEAWGFD
HGSWGVLIKMYPDADIPMVQLSIDSSKPAAWHFEMGRKLAALRDEGIMLVASGNVVHNLR
TVKWHGDSSPYPWATSFNEYVKANLTWQGPVEQHPLVNYLDHEGGALSNPTPEHYLPLLY
VLGAWDGQEPITIPVDGIEMGSLSMLSVQIG
>LFGLNPFC_04892 Protein-disulfide oxidoreductase DsbI
MGIKGMWKDLRTSPVDTLVRWQEQRLLWLLMAVAMGALIIILAHSFFQIYLYMAPCEQCVY
IRYAMFVMVIGGLVAAINPKNIILKLIGCVMAFYGSILGLKFSLKLNDIHHAVHNPDPDS
LFGVQGCSTDPTFPFNLPLAQWAPNWFKPTGDCGYDAPIVPDGVTLSSTQQWFVEMYQQS
EGWYLLPPWHFMNMAQACMLAFGMCLVLLVIMSGAWALKIIRG
>LFGLNPFC_04893 Thiol:disulfide interchange protein DsbL
MSKLGISSLFKTILLTAALAVSFTASAFTEGTDYMVLEKPIPNADKTLIKVFSYACPFCY
KYDKAVTGPVSEKVKDIVAFTPFHLETKGEYGKQASEVFAVLINKDKAAGISLFDANSQF
KKAKFAYYAAYHDKKERWSDGKDPAAFIKTGLDAAGMSQADFEAALKEPAVQETLKKWKA
SYDVAKIQGVPAYVVNGKYLIYTKSIKSIDAMADLIRELASK
>LFGLNPFC_04894 Arylsulfate sulfotransferase AssT
MFDKYRKTLVAGTVAITLGLSASGVMAAGFKPAPPAGQLGAVIVDPYGNAPLTALVDLDS
HVISDVKVTVHGKGEKGVEISYPVGQESLKTYDGVPIFGLYQKFANKVTVEWKENGKVMK
DDYVVHTSAIVNNYMDNRSISDLQQTKVIKVAPGFEDRLYLVNTHTFTAQGSDLHWHGEK
DKNAGILDAGPATGALPFDIAPFTFIVDTEGEYRWWLDQDTFYDGRDRDINKRGYLMGIR
ETPRGTFTAVQGQHWYEFDMMGQVLEDHKLPRGFADATHESIETPNGTVLLRVGKSNYRR
DDGVHVTTIRDHILEVDKSGRVVDVWDLTKILDPKRDALLGALDAGAVCVNVDLAHAGQQ
AKLEPDTPFGDALGVGPGRNWAHVNSIAYDAKDDSIILSSRHQGVVKIGRDKQVKWILAP
SKGWEKPLASKLLKPVDANGKPITCNENGLCENSDFDFTYTQHTAWISSKGTLTIFDNGD
GRHLEQPALPTMKYSRFVEYKIDEKKGTVQQVWEYGKERGYDFYSPITSIIEYQADRNTM
FGFGGSIHLFDVGQPTVGKLNEIDYKTKEVKVEIDVLSDKPNQTHYRALLVRPQQMFK
>LFGLNPFC_04895 Putative acid--amine ligase YgiC
MERVSITERPDWREKAHEYGFNFHTMYGEPYWCEDAYYKLTLAQVEKLEEVTAELHQMCL
KVVEKVIASDELMTKFRIPKHTWSFVRQSWLTHQPSLYSRLDLAWDGTGEPKLLENNADT
PTSLYEAAFFQWIWLEDQLNAGNLPEGSDQFNSLQEKLIDRFVELREQYGFQLLHLTCCR
DTVEDRGTIQYLQDCATEAEIATEFLYIDDIGLGEKGQFTDLQDQVISNLFKLYPWEFML
REMFSTKLEDAGVRWLEPAWKSIISNKALLPLLWEMFPNHPNLLPAYFAEDDHPQMEKYV
VKPIFSREGANVSIIENGKTIAAAEGPYGEEGMIVQQFHPLPKFGDSYMLIGSWLVNDQP
AGIGIREDRALITQDMSRFYPHIFVE
>LFGLNPFC_04896 hypothetical protein

MKRTKSIRHASFRKNWSARHLTPVALAVATVFMLAGCEKSDETVSLYQNADDCSAANPGK
SAECTTAYNNALKEAERTAPKYATREDCVAEFGEGQCQQAPAQAGMAPENQAQAQQSSGS
FWMPLMAGYMMGRLMGGGAGFAQQPLFSSKNPASPAYGKYTDATGKNYGAAQPGRTMTVP
KTAMAPKPATTTTVTRGGFGESVAKQSTMQRSATGTSSRSMGG
>LFGLNPFC_04897 Outer membrane protein TolC
MKKLLPILIGLSLSGFSSLSQAENLMQVYQQARLSNPELRKSAADRDAAFEKINEARSPL
LPQLGLGADYTYSNGYRDANGINSNATSASLQLTQSIFDMSKWRALTLQEKAAGIQDVTY
QTDQQTLILNTATAYFNVLNAIDVLSYTQAQKEAIYRQLDQTTQRFNVGLVAITDVQNAR
AQYDTVLANEVTARNNLDNAVEQLRQITGNYYPELAALNVENFKTDKPQPVNTLLKEAEK
RNLSLLQARLSQDLAREQIRQAQDGHLPTLDLTASTGISDTSYSGSKTRGAAGTQYDDSN
MGQNKVGLSFSLPIYQGGMVNSQVKQAQYNFVGASEQLESAHRSVVQTVRSSFNNINASI
SSINAYKQAVVSAQSSLDAMEAGYSVGTRTIVDVLDATTTLYNAKQELANARYNYLINQL
NIKSALGTLNEQDLLALNNALSKPVSTNPENVAPQTPEQNAIADGYAPDSPAPVVQQTSA
RTTTSNGHNPFRN
>LFGLNPFC_04898 ADP-ribose pyrophosphatase
MLKPDNLPVTFGKNDVEIIARETLYRGFFSLDLYRFRHRLFNGQMSHEVRREIFERGHAA
VLLPFDPVRDEVVLIEQIRIAAYDTSETPWLLEMVAGMIEEGESVEDVARREAIEEAGLI
VKRTKPVLSFLASPGGTSERSSIMVGEVDATTASGIHGLADENEDIRVHVVSREQAYQWV
EEGKIDNAASVIALQWLQLHHQALKNEWA
>LFGLNPFC_04899 hypothetical protein
MKRYTPDFPEMMRLCEMNFSQLRRLLPRNDAPGETVSYQVANAQYRLTIVESTRYTTLVT
IEQTAPAISYWSLPSMTVRLYHDAMVAEVCSSQQIFRFKARYDYPNKKLHQRDEKHQINQ
FLADWLRYCLAHGAMAIPVY
>LFGLNPFC_04900 3’,5’-cyclic adenosine monophosphate phosphodiesterase CpdA
MESLLTLPLAGEARVRILQITDTHLFAQKHEALLGVNTWESYQAVLEAIRPHQHEFDLIV
ATGDLAQDQSSAAYQHFAEGIASFRAPCVWLPGNHDFQPAMYSALQDAGISPAKRVFIGE
QWQILLLDSQVFGVPHGELSEFQLEWLERKLADAPERHTLLLLHHHPLPAGCSWLDQHSL
RNAGELDTVLAKFPHVKYLLCGHIHQELDLDWNGRRLLATPSTCVQFKPHCSNFTLDTIA
PGWRTLELHADGTLTTEVHRLADARFQPDTASEGY
>LFGLNPFC_04901 hypothetical protein
MSTLLYLHGFNSSPRSAKASLLKNWLAEHHPDVEMIIPQLPPYPSDAAELLESIVLEHGG
DSLGIVGSSLGGYYATWLSQCFMLPAVVVNPAVRPFELLTDYLGQNENPYTGQQYVLESR
HIYDLKVMQIDPLEAPDLIWLLQQTGDEVLDYRQAVAYYASCRQTVIEGGNHAFTGFEDY
FNPIVDFLGLHHL
>LFGLNPFC_04902 DNA topoisomerase 4 subunit B
MTQTYNADAIEVLTGLEPVRRRPGMYTDTTRPNHLGQEVIDNSVDEALAGHAKRVDVILH
ADQSLEVIDDGRGMPVDIHPEEGVPAVELILCRLHAGGKFSNKNYQFSGGLHGVGISVVN
ALSKRVEVNVRRDGQIYNIAFENGEKVQDLQVVGTCGKRNTGTSVHFWPDETFFDSPRFS
VSRLTHVLKAKAVLCPGVEITFKDEINNTEQRWCYQDGLNDYLAEAVNGLPTLPEKPFIG
NFAGDTEAVDWALLWLPEGGELLTESYVNLIPTMQGGTHVNGLRQGLLDAMREFCEYRNI
LPRGVKLSAEDIWDRCAYVLSVKMQDPQFAGQTKERLSSRQCAAFVSGVVKDAFILWLNQ
NVQAAELLAEMAISSAQRRMRAAKKVVRKKLTSGPALPGKLADCTAQDLNRTELFLVEGD
SAGGSAKQARDREYQAIMPLKGKILNTWEVSSDEVLASQEVHDISVAIGIDPDSDDLSQL
RYGKICILADADSDGLHIATLLCALFVKHFRALVKHGHVYVALPPLYRIDLGKEVYYALT
EEEKEGVLEQLKRKKGKPNVQRFKGLGEMNPMQLRETTLDPNTRRLVQLTIDDEDDQRTD
AMMDMLLAKKRSEDRRNWLQEKGDMAEIEV
>LFGLNPFC_04903 Metal-pseudopaline receptor CntO
MAMFTPSFSGLKGRALFSLLFAAPMIHATDSVTTKDGETITVTADANTATEATDGYQPLS
TSTATLTDMPMLDIPQVVNTVSDQVLENQNATTLDEALYNVSNVVQTNTLGGTQDAFVRR
GFGANRDGSIMTNGLRTVLPRSFNAATERVEVLKGPASTLYGILDPGGLINVVTKRPEKT
FHGSVSATSSSFGGGTGQLDITGPIEGTQLAYRLTGEVQDEDYWRNFGKERSTFIAPSLT
WFGDNATVTMLYSHRDYKTPFDRGTIFDLTTKQPVNVDRKIRFDEPFNITDGQSDLAQLN
AEYHLNSQWTARFDYSYSQDKYSDNQARVTAYDATTGTLTRRVDATQGSTQRMHATRADL
QGNVDIAGFYNEILGGVSYEYYDLLRTDMIRCKKAKDFNIYNPVYGNTSKCTTVSASDSD
QTIKQENYSAYAQDALYLTDNWIAVAGIRYQYYTQYAGKGRPFNVNTDSRDEQWTPKLGL
VYKLTPSVSLFANYSQTFMPQSSIASYIGDLPPESSNAYEVGAKFELFDGITADIALFDI
HKRNVLYTESIGDETIAKTAGRVRSRGVEVDLAGALTENINIIASYGYTDAKVLEDPDYA
GKPLPNVPRHTGSLFLTYDIHNMPGNNTLTFGGGGHGVSRRSATNGADYYLPGYFVADAF
AAYKMKLQYPVTLQLNVKNLFDKTYYTSSIATNNLGNQIGDPREVQFTVKMEF
>LFGLNPFC_04904 putative siderophore transport system ATP-binding protein YusV
MAHNAKGQGLILDNVSAGYHKKIIVDGVSFSVPTEKMTVLVGANGCGKSTLLSTIARILQ
PMGGSILLDGKAIHEQPTKALSRRLGILPQSPLLPEGLTVYELVSRGRFPWQNFIRQWSD
ADELAVEEALKLTGTQEFAHLPVEKLSGGQRQRCWIAMVLAQKTPYILLDEPTTWLDLRY
QVEILELLHDLTRHHGRTVVVVLHDLNFAVNYGDTLIFLRQGKVVRVLNEGEHCTPELVK

AVFDVDVHASINPLTGKPYFMPFRGVEKV
>LFGLNPFC_04905 putative siderophore transport system permease protein YfiZ
MMRTGLVTVIFLALLLVGCVVYPGIGARFIAPQTVLQAFLHFDPQNFDHNVIVRLRLPRL
AAALLTGASLGVAGALLQAVIRNPLGEPHILGLNAGAALAVVAASALGLAFPVGRPLLAS
TGGALLFLLILLLSSAGRSGLTPMKVTLCGVALSAFVSSITAAILILDEQTLLAMRTWLA
GDLAGQDWATLGTSAWFSLGGFVLALYLAPSLNMLALGDRMAQGLGVSVLRTRTFTLLAI
ALLCGAAVSIAGPIGFVGLLVPQIVRRLVSADLRVLLPLSACVGALLLLLADIIARTLFT
PHELATGVMTALVGAPVFVIMATRMFK
>LFGLNPFC_04906 Iron-uptake system permease protein FeuC
MSRIMNVGLRPLRVGKFSTLVRPKNLLLLGGLFLLAVGILIFGLMQGSFSVPASEVGRAL
FAPENVSTDARYIVQDIRLPRVIMALLCGAMLGMAGAAMQSIARNGLADPGLIGVKEGCS
VAVLWLIFQFPMLGMFWRPVAGLAGGLLVALIVIFCARDISRPRFVLIGIGVSWFFAAGI
GVFMTTADVRDVQTALMWLSGSLHAANWMLVGISACWMLPAALLLLFTARTADIALLGHQ
VATGLGVNSSRLALLRVAAPIILTAVCVSCVGNIGFVGLIAPHISRFILRGGQTTLLLGS
AVSGALLVILADSIGRLAFLPLQLPAGIIISLIGGPFFLLLLWQRRNSF
>LFGLNPFC_04907 hypothetical protein
MRLFFSLLILLSFFSRATEPVQVFTDDLGRKVTVPAHPKRIVSLHDLDITIPLIELGVPP
VASHGRTRPDGSHFIRSGALLTGVDFDNSSIAFIGTADIDIEAIVAAKPDLIITEPTRNT
PIEQLEKIAPTVSIDHLKGGAPEIYRKLAELTGTQSQLAILERRYQAQINALKATLDSQK
ITVSVIQANQGKINVMHSYHSLGRVLRDAGFRFPPLIESIPEGGRMDVSAERLPELDADF
VFATWRGDTGGKPQDELAAMEKVMPGWCQFLTACRSGRYVLISREEAISNSFASLGLMAA
QIQSQIAGRPLPEAK
>LFGLNPFC_04908 HTH-type transcriptional regulator MurR
MIQKDKARVDIFGERFRTRASQLTPGLRAVASYINEHREVVLEQTAMEIAATLNTSDATV
IRAIQALGFAGLRDLKRTLEQWLGPALSSSEKMSTTVSNLTSDVNTAIDFVLEGHLYTCN
VLSEPENRHALAQAVALLVQARQVAIFGIGASGILADYTARLFNRIGLPATALNRTGIGL
AEQLIALQRGDVLIMMAQKSAHREGLTTLREARRLGIPVILLTNALDSRFSKDASIVIHV
PRGDEKGKTPLHGTVLLCLEMIVWSVASAVPQRAVKTIKRINDFHRGLKTGRKNG
>LFGLNPFC_04909 putative quinol monooxygenase YgiN
MITVIAEIRTRPGQHHRQAVLDQFAKIVPTVLKEEGCHGYAPMVDCAAGVSFQSMAPDSI
VMIEQWESIAHLEAHLQTPHMKAYSEAVKGDVLEMNIRILQPGL
>LFGLNPFC_04910 NADPH:quinone oxidoreductase MdaB
MSNILIINGAKKFAHSNGQLNDTLTEVADGTLRDLGHDVRIVRADSDYDVKAEVQNFLWA
DVVIWQMPGWWMGAPWTVKKYIDDVFTEGHGTLYASDGRTRKDPSKKYGSGGLVQGKKYM
LSLTWNAPMEAFTEKDQFFHGVGVDGVYLPFHKANQFLGMEPLPTFIANDVIKMPDVPRY
TEEYRKHLVEIFG
>LFGLNPFC_04911 Major exported protein
MSNMIYLSIKGKIQGLISEGCGSYASIGNKYQINHVDEIFVLQFDHSLSREYNVVHHPIK
FYKPIDKSSPLLNAALSENEELSAVFNFYRINSGGCIERFYTIELQGAHLSNILTCYPHS
ITHAGNQPEDVIVLNYKNITWKHHIASTESYSCWEERIF
>LFGLNPFC_04912 hypothetical protein
MKSDLTIKNRYCTIPQAKFRKWDEMDVLLWKLGKNDSRRRSGVYYLNAYKDAYVQYNRDK
IIKHAYAAGIRPELLGGVAWIESGGMPENYKFQIYETKRMIGLLDMPENKTSFGSMGIQI
RTAAITLGLDPSELTTRNQLELATCLMEDDFTFQIAATYLRDLVLFDYPSSATLYMTNEQ
YIMAGIRYNRGVERDLGFFICLINNLPARDTDDYKFISYGMRLLEIREHIKKLINE
>LFGLNPFC_04913 Inner membrane protein YgiZ
MLKKQLLSIFEALLYIMLTYWLIDSFFAFNKYDWMLESGDNICSIPSVSGEDRILQAMIA
AFFLLTPLIILILRKLFMREMFEFWLYVFSLVICLGCGWWLFWGRFIFCY
>LFGLNPFC_04914 Sensor protein QseC
MKFTQRLSLRVRLTLIFLILASVTWLLSSFVAWKQTTDNVDELFDTQLMLFAKRLSTLDL
NEINAADRMAQTPNKLKHGHVDDDALTFAIFTHDGRMVLNDGDNGEDIPYSYQREGFADG
QLVGEDDPWRFVWMTSPDGKYRIVVGQEWEYREDMALAIVAGQLIPWLVALPIMLIIMMV
LLGRELAPLNKLALALRMRDPDSEKPLNATGVPSEVRPLVESLNQLFARTHAMMVRERRF
TSDAAHELRSPLTALKVQTEVAQLSDDDPQARKKALLQLHSGIDRATRLVDQLLTLSRLD
SLDNLQDVAEIPLEDLLQSSVMDIYHTAQQANIDVRLTLNANGIKRTGQPLLLSLLVRNL
LDNAVRYSPQGSVVDVTLNADNFIVRDNGPGVTPEALARIGERFYRPPGQTATGSGLGLS
IVQRIAKLHDMNVEFGNAEQGGFEAKVSW
>LFGLNPFC_04915 Transcriptional regulatory protein QseB
MRILLIEDDMLIGDGIKTGLSKMGFSVDWFTQGRQGKEALYSAPYDAVILDLTLPGMDGR
DILREWREKGQREPVLILTARDALAERVEGLRLGADDYLCKPFALIEVAARLEALMRRTN
GQASNELRHGNVMLDPGKRIATLAGEPLTLKPKEFALLELLMRNAGRVLPRKLIEEKLYT
WDEEVTSNAVEVHVHHLRRKLGSDFIRTVHGIGYTLGEK
>LFGLNPFC_04916 Protein YgiW
MKKFAAVIAVMALCSAPVMAAEQGGFSGPSATQSQAGGFQGPNGSVTTVESAKSLRDDTW

VTLRGNIVERISDDLYVFKDASGTINVDIDHKRWNGVTVTPKDTVEIQGEVDKDWNSVEI
DVKQIRKVNP
>LFGLNPFC_04917 DNA gyrase inhibitor
MTNLTLDVNIIDFPSIPVAMLPHRCSPELLNYSVAKFIMWRKETGLSPVNQSQTFGVAWD
DPATTAPEAFRFDICGSVSEPIPDNRYGVSNGELTGGRYAVARHVGELDDISHTIWGIIR
HWLPASGEKMRKAPILFHYTNLAEGMTERRLETDIYVPLA
>LFGLNPFC_04918 putative deoxycholate-binding periplasmic protein YgiS
MYTRNLLWLVSLVSAAPLYAADVPANTPLAPQQVFRYNNHSDPGTLDPQKVEENTAAQIV
LDLFEGLVWMDGEGQVQPAQAERWEILDDGKRYIFHLRSGLQWSDGQPLTAEDFVLGWQR
AVDPKTASPFAGYLAQAHINNAAAIVAGKADVTSLGVKATDDRTLEVTLEQPVPWFTTML
AWPTLFPVPHHVIAKHGDSWSKPENMVYNGAFVLDKWVVNEKITARKNPKYRDAQHTVLQ
QVEYLALDNSVTGYNRYRAGEVDLTWVPAQQIPAIEKSQPGELRIIPRLNSEYYNFNLEK
PPFNDVRVRRALYLTVDRQLIAQKVLGLRTPATTLTPPEVKGFSATTFDELQKPMSERVA
MAKVLLKQAGYDASHPLRFELFYNKYDLHEKTAIALSSEWKKWLGAQVTLRTMEWKTYLD
ARRAGDFMLSRQSWDATYNDASSFLNTLKSDSEENVGHWKNAQYDALLNQATQITDATKR
NALYQQAEVIINQQAPLIPIYYQPLIKLLKPYVGGFPLHNPQDYVYSKELYIKAH
>LFGLNPFC_04919 DNA topoisomerase 4 subunit A
MSDMAERLALHEFTENAYLNYSMYVIMDRALPFIGDGLKPVQRRIVYAMSELGLNASAKF
KKSARTVGDVLGKYHPHGDSACYEAMVLMAQPFSYRYPLVDGQGNWGAPDDPKSFAAMRY
TESRLSKYSELLLSELGQGTADWVPNFDGTLQEPKMLPARLPNILLNGTTGIAVGMATDI
PPHNLREVAQAAIALIDQPKTTLDQLLDIVQGPDYPTEAEIITSRAEIRKIYENGRGSVR
MRAVWKKEDGAVVISALPHQVSGARVLEQIAAQMRNKKLPMVDDLRDESDHENPTRLVIV
PRSNRVDMDQVMNHLFATTDLEKSYRINLNMIGLDGRPAVKNLLEILSEWLVFRRDTVRR
RLNYRLEKVLKRLHILEGLLVAFLNIDEVIEIIRNEDEPKPALMSRFGLTETQAEAILEL
KLRHLAKLEEMKIRGEQSELEKERDQLQGILASERKMNNLLKKELQADAQAYGDERRSPL
QEREEAKAMSEHDMLPSEPVTIVLSQMGWVRSAKGHDIDAPGLNYKAGDSFKAAVKGKSN
QPVVFVDSTGRSYAIDPITLPSARGQGEPLTGKLTLPPGATVDHMLMESDDQKLLMASDA
GYGFVCTFNDLVARNRAGKALITLPENAHVMPPVVIEDASDMLLAITQAGRMLMFPVSDL
PQLSKGKGNKIINIPSAEAARGEDGLAQLYVLPPQSTLTIHVGKRKIKLRPEELQKVAGE
RGRRGTLMRGLQRIDRVEIDSPRRASSGDSEE
>LFGLNPFC_04920 1-acyl-sn-glycerol-3-phosphate acyltransferase
MLYIFRLIITVIYSILVCVFGSIYCLFSPRNPKHVATFGHMFGRLAPLFGLKVECRKPAD
AESYGNAIYIANHQNNYDMVTASNIVQPPTVTVGKKSLLWIPFFGQLYWLTGNLLIDRNN
RTKAHGTIAEVVNHFKKRRISIWMFPEGTRSRGRGLLPFKTGAFHAAIAAGVPIIPVCVS
TTSNKINLNRLHNGLVIVEMLPPIDVSQYGKDQVRELAAHCRSIMEQKIAELDKEVAERE
AAGKV
>LFGLNPFC_04921 Cell division protein FtsP
MSLSRRQFIQASGIALCAGAVPLKASAAGQQQPLPVPPLLESRRGQPLFMTVQRAHWSFT
PGTRASVWGINGRYLGPTIRVWKGDDVKLIYSNRLTENVSMTVAGLQVPGPLMGGPARMM
SPNADWAPVLPIRQNAATLWYHANTPNRTAQQVYNGLAGMWLVEDEVSKSLPIPNHYGVD
DFPVIIQDKRLDNFGTPEYNEPGSGGFVGDTLLVNGVQSPYVEVSRGWVRLRLLNASNSR
RYQLQMSDGRPLHVISGDQGFLPAPVSVKQLSLAPGERREILVDMSNGDEVSITCGEAAS
IVDRIRGFFEPSSILVSTLVLTLRPTGLLPLVTDSLPMRLLPTEIMAGSPIRSRDISLGD
DPGINGQLWDVNRIDVTAQQGTWERWTVRADEPQAFHIEGVMFQIRNVNGAMPFPEDRGW
KDTVWVDGQVELLVYFGQPSWAHFPFYFNSQTLEMADRGSIGQLLVNPVP
>LFGLNPFC_04922 C4-dicarboxylate TRAP transporter large permease protein DctM
MEFEYIYPVLILFGSFAVMLAIGVPITFAIGLSSLLSIITALPPDAAISVISQKMTVGLD
GFTLLAIPFFVLAGNIMNTGGIARRLVNLAQALVGRLPGSLAHCNILANTLFGAISGSAV
ASAAAVGGIMSPLQEKEGYDPAFSAAVNIASAPIGLMIPPSNVLIVYSLASGGTSVAALF
LAGYLPGILTAVALMFVAALYARRHHYPVAERINYRQFLQVFRESIPSLMLIFIIIGGII
AGVFTPTEASAIAVIYSLALAMIYREITLKKLNDILLDSVVTSSIVLLLVGCSMGMSWAM
TNADVPELINELITRVSDNKWVILFIINIILLIVGTFMDITPAILIFTPIFLPIAQHLGI
DPIHFGIIMVFNLTIGLCTPPVGTILFVGCSIGKVSIDRAIKPLLPMFLALFVVMAIICY
FPQLSLMLPGLFST
>LFGLNPFC_04923 hypothetical protein
MRTLTHILNKILAGCCCIILAIMVFCVTWQVIARFIFNAPSTVLDEFTQILFMWMILLGG
VYTAGLKKHLAIDLLAQKLPAASVLTLDSFIQIIITVFAVIFMIYGGNIVVEKAQHVGQI
SPVLKWPMDKVYWVMPVSGLILVWYSVMNIIDNYRKRNSH
>LFGLNPFC_04924 Solute-binding protein
MKALRPLTASLMLLTSCLLISNTTLAKTTLKLSHNQDKSHAVHKALSYLADKTKEYSNGE
LVIRIYPNATLGNERESLELMNSGALQMVKVNAASLESFAPDYSLFSLPFLFRDRDHYYR
VLQSDLGKKILNSSERKGFVGITYYDGGARSFYSNKPITKPEDLAGMKIRVQQSPSAIAM
MKALGGVATPMAQGELYTALQQGVVDGGENNTVVYADMRHAEVAKVYSRDEHTMVPDVLI
ISTDVLNKLGDKERTALLKAADESMMQMKDVIWPAAEKEAYDKMKGMNATVVDVDKSAFK

ERVKPLYDEFKAKDAQSAKNLELVESM
>LFGLNPFC_04925 putative oxidoreductase YjmC
MTTVYVSEENLKSLVHHKLHTAGLDTDTIQQVTDVLVHADITGVHSHGVMRVEHYCTRLA
AGGLNKAPQFSIEQISPSVAILDSDDGMGHSALISATEHAIKLAQQEGLGFVSVKNTSHC
GALSYFAEMITHKGLVAIVMTQTDTCVAPHGGAERFLGTNPIAFGFPVENSHPMIVDMAT
SATAFGKILHAKETGKHIGEGLAIDKNGYGTTDPYKIENLLPFGQHKGSGIALAIDALTG
VLMNANFGNHIVRMYGDYDKMRKLASLVIAIDPKKLGNPVFAKTMAKMVTELHAVKPAPG
VEKVLAPNDPQMHYKEKCQQEGIPVPAGIFHYLAEN
>LFGLNPFC_04926 L-galactonate-5-dehydrogenase
MKTLICQQPGVMEYVEKDIPTPADNEVLLKIKAVGICGTDIHAFAGRQPFFSYPRVLGHE
ICAEAVSRGSQCQTAQSGQRYSVIPCIPCGECAACREEKTNCCERVSLYGVHQDGGFSEY
LAVREDNLVPLPDEVSDSAGALVECFAIGAHAVRRAEIKAEQNVLVIGAGPIGLATAAIA
RAKGAHVVVADIDCQRRQHVVDHLAINVFDPTQEGFIAALSEVFGGELACVVLDATGNKA
SMSHDVNLIRHGGKIVFIGLYIGELVIDDPTFHKKETTLLSSRNATREDFALVIELMRSN
KIHENLMKNQAFNFFSVGEDYQRNVVENKNMVKGVITF
>LFGLNPFC_04927 Mannitol 2-dehydrogenase
MSGNESIAAAHYDQVTTWPRDGLQADIVHIGFGAFHRGHQAVYTDLANQLSDTRWGIFEI
NLFGDAQLIENLNAQNGLFSVVETSASQSTSRLVRSVAGGIHTPRDGIAAAIHKLAEPQV
KIVSLTITAKGYCLDPQTRSLDLTNGLINHDLQNPDAPQSAIGVIVCALQQRKAAGLAAF
SVLSCDNLPDNGHLTRNAVLGFARQLDQPLAQWIEENVSFPGTMVDRIVPAMTESQFALL
ETKTGYADPCGIVCESFRQWVIEDNFVRGRPEWDKAGAMFVSNVQPYEEMKLRMLNGSHS
FLAYNGSLAGYEFIWQCMEDANFRSITHQLMINEQARTLNPDLNINIQEYADLLIERFSN
RNVAHRTGQIAMDGSQKLPQRALTPWLKLHQQKQNNAVLSLLVAGWLHYVIDAVEKSQSV
ADPMNDQFQALIKEQQDAWQQALALLHLSAIFGDLSNHQPFINEIKIAFANIKNKGIKAT
ISQLLSDEQK
>LFGLNPFC_04928 Pyruvate dehydrogenase complex repressor
MEQVITKRRYYDIGLQIEELLYSGVFKAGERLPSERELSERFNTSRTTIREAIIMLELKG
VLNVKQGSGIFFVDSTDKLNQKSLMPYSEIGPFELLQARQVIESNITGFAASQISFNELQ
ELKKIISLQENAIAAESDRFEDLDHRFHSIIAEATQNRVLIKQAAELWRAVRTENPRWKK
LNYKYLHEKHLRLQWLEDHRAIFLALQQKDSELAREASWRHLENSKNELIKIFKQDASIS
DFDDFFFAR
>LFGLNPFC_04929 hypothetical protein
MSSISLIQPDRDLFSWPQYWAACFGPAPFLPMSREEMDQLGWDSCDIILVTGDAYVDHPS
FGMAICGRMLEAQGFRVGIIAQPDWSSKDDFMRLGKPNLFFGVTAGNMDSMINRYTADRR
LRHDDAYTPDNVAGKRPDRATLVYTQRCKEAWKDVPVILGGIEASLRRTAHYDYWSDTVR
RSVLVDSKADMLMFGNGERPLVEVAHRLAMGEPISEIRDVRNTAIIVKEALPGWSGVDST
RLDTPGKIDPIPHPYGEDLPCADNKPVAPKKQEAKAVTVQPPRPKPWEKTYVLLPSFEKV
KGDKVLYAHASRILHHETNPGCARALMQKHGDRYVWINPPAIPLSTEEMDSVFALPYKRV
PHPAYGNARIPAYEMIRFSVNIMRGCFGGCSFCSITEHEGRIIQSRSEDSIINEIEAIRD
TVPGFTGVISDLGGPTANMYMLRCKSPRAEQTCRRLSCVYPDICPHMDTNHEPTINLYRR
ARDLKGIKKILIASGVRYDIAVEDPRYIKELATHHVGGYLKIAPEHTEEGPLSKMMKPGM
GSYDRFKELFDTYSKQAGKEQYLIPYFISAHPGTRDEDMVNLALWLKKHRFRLDQVQNFY
PSPLANSTTMYYTGKNPLAKIGYKSEDVFVPKGDKQRRLHKALLRYHDPANWPLIRQALE
AMGKKHLIGSRRDCLVPAPTIEEMREARRQNRNTRPALTKHTPMATQRQTPATAKKASST
QSRLQNAGAKKRPKAAVGR
>LFGLNPFC_04930 Major outer membrane lipoprotein Lpp 1
MKTIFTVGAVVLATCLLSGCVNEQKINQLASNVQTLNAKIARLEQDMKALRPQIYAAKSE
ANRANTRLDAQDYFDCLRCLRMYAE
>LFGLNPFC_04931 hypothetical protein
MKIILLFLAALASFTVHAQPPSQTVEQTVRHIYQNYKSDATAPYFGETGERAITSARIQQ
ALTLNDNLTLPGNIGWLDYDPVCDCQDFGDLVLESVAITQTDADHADAVVRFRIFKDDKE
KTSQTLKMVAENGRWVIGDIVSNHGSVLQAVNSENEKTLAAIASLQKEQPEAFVAELFEH
IADYSWPWTWVVSDSYRQAVNAFYKTTFKTANNPDEDMQIERQFIYDNPICFGEESLFSR
VDEIRVLEKTTDSARIHVRFTLTNGNNEEQELILQRREGKWEIADFIRPNSGSLLKQIEA
KTAARLKQ
>LFGLNPFC_04932 2,5-diketo-D-gluconic acid reductase A
MANPTVIKLQDGNVMPQLGLGVWQASNEEVITAIQKALEVGYRSIDTAAAYKNEEGVGKA
LKNASVNREELFITTKLWNDDHKRPREALLDSLKKLQLDYIDLYLMHWPVPAIDHYVEAW
KGMIELQKEGLIKSIGVCNFQIHHLQRLIDETGVTPVINQIELHPLMQQRQLHAWNATHK
IQTESWSPLAQGGKGVFDQKVIRDLADKYGKTPAQIVIRWHLDSGLVVIPKSVTPSRIVE
NFDVWDFRLDKDELGEIAKLDQGKRLGPDPDQFGG
>LFGLNPFC_04933 Alcohol dehydrogenase YqhD
MNNFNLHTPTRILFGKGAIAGLREQIPHDARVLITYGGGSVKKTGVLDQVLDALKGMDVL
EFGGIEPNPAYETLMNAVKLVREQKVTFLLAVGGGSVLDGTKFIAAAANYPENIDPWHIL

QTGGKEIKSAIPMGCVLTLPATGSESNAGAVISRKTTGDKQAFHSAHVQPVFAVLDPVYT
YTLPSRQVANGVVDAFVHTVEQYVTKPVDAKIQDRFAEGILLTLIEDGPKALKEPENYDV
RANVMWAATQALNGLIGAGVPQDWATHMLGHELTAMHGLDHAQTLAIVLPALWNEKRDTK
RAKLLQYAERVWNITEGSDDERIDAAIAATRNFFEQLGVPTHLSDYGLDGSSIPALLKKL
EEHGMTQLGENHDITLDVSRRIYEAAR
>LFGLNPFC_04934 HTH-type transcriptional activator RhaS
MLQNCAQSNCRIIPKKLRDMKREEICRLLADKVNKLKNKENSLSELLPDVRLLYGETPFA
RTPVMYEPGIIILFSGHKIGYINERVFRYDANEYLLLTVPLPFECETYATSEVPLAGLRL
NVDILQLQELLMDIGEDEHFQPSMAASGINSATLSEEILCAAERLLDVMERPLDARILGK
QIIREILYYVLTGPCGGALLALVSRQTHFSLISRVLKRIENKYTENLSVEQLAAEANMSV
SAFHHNFKSVTSTSPLQYLKNYRLHKARMMIIHDGMKASAAAMRVGYESASQFSREFKRY
FGVTPGEDAARMRAMQGN
>LFGLNPFC_04935 Inner membrane protein YghB
MAVIQDIIAALWQHDFAALADPHIVSVVYFVMFATLFLENGLLPASFLPGDSLLILAGAL
IAQGVMDFLPTIAILTAAASLGCWLSYIQGRWLGNTKTVKGWLAQLPAKYHQRATCMFDR
HGLLALLAGRFLAFVRTLLPTMAGISGLPNRRFQFFNWLSGLLWVSVVTSFGYALSMIPF
VKRHEDQVMTFLMILPIALLTAGLLGTLFVVIKKKYCNA
>LFGLNPFC_04936 Cystathionine beta-lyase MetC
MADKKLDTQLVNAGRSKKYTLGAVNSVIQRASSLVFDSVEAKKHATRNRANGELFYGRRG
TLTHFSLQQAMCELEGGAGCVLFPCGAAAVANSILAFVEQGDHVLMTNTAYEPSQDFCSK
ILSKLGVTTSWFDPLIGADIVNHLQPNTKIVFLESPGSITMEVHDVPAIVAAVRSVVPDA
IIMIDNTWAAGVLFKALDFGIDVSIQAATKYLVGHSDAMIGTAVCNARCWEQLRENAYLM
GQMVDADTAYITSRGLRTLGVRLRQHHESSLKVAEWLAEHPQVARVNHPALPGSKGYEFW
KRDFTGSSGLFSFVLKKKLNDEELANYLDNFSLFSMAYSWGGYESLILANQPEHIAAIRP
QGKIDFSGTLIRLHIGLEDVDDLIADLDAGFARIV
>LFGLNPFC_04937 Biopolymer transport protein ExbB
MGNNLMQTDLSVWGMYQHADIVVKCVMIGLILASVVTWAIFFSKSVEFFNQKRRLKREQQ
LLAEARSLNQANDIAADFGSKSLSLHLLNEAQNELELSEGSDDNEGIKERTSFRLERRVA
AVGRQMGRGNGYLATIGAISPFVGLFGTVWGIMNSFIGIAQTQTTNLAVVAPGIAEALLA
TAIGLVAAIPAVVIYNVFARQIGGFKAMLGDVAAQVLLLQSRDLDLEASAAAHPVRVAQK
LRAG
>LFGLNPFC_04938 Biopolymer transport protein ExbD
MAMHLNENLDDNGEMHDINVTPFIDVMLVLLIIFMVAAPLATVDVKVNLPASTSTPQPRP
EKPVYLSVKADNSMFIGNDPVTDETMITALNALTEGKKDTTIFFRADKTVDYETLMKVMD
TLHQAGYLKIGLVGEETAKAK
>LFGLNPFC_04939 putative oxidoreductase YghA
MSHLKDPTTQYYTGEYPKQKQPTPGIQAKMTPVPDCGEKTYVGSGRLKDRKALVTGGDSG
IGRAAAIAYAREGADVAISYLPVEEEDAQDVKKIIEECGRKAVLLPGDLSDEKFARSLVH
EAHKALGGLDIMALVAGKQVAIPDIADLTSEQFQKTFAINVFALFWLTQEAIPLLPKGAS
IITTSSIQAYQPSPHLLDYAATKAAILNYSRGLAKQVAEKGIRVNIVAPGPIWTALQISG
GQTQDKIPQFGQKTPMKRAGQPAELAPVYVYLASQESSYVTAEVHGVCGGEHLG
>LFGLNPFC_04940 hypothetical protein
MERFLENAMYASRWLLAPVYFGLSLALVALALKFFQEIIHVLPNIFSMAESDLILVLLSL
VDMTLVGGLLVMVMFSGYENFVSQLDISENKEKLNWLGKMDATSLKNKVAASIVAISSIH
LLRVFMDAKNVPDNKLMWYVIIHLTFVLSAFVMGYLDRLTRHNH
>LFGLNPFC_04941 L-glyceraldehyde 3-phosphate reductase
MVWLANPERYGRMQYRYCGKSGLRLPALSLGLWHNFGHVNALESQRAILRKAFDLGITHF
DLANNYGPPPGSAEENFGRLLREDFAAYRDELIISTKAGYDMWPGPYGSGGSRKYLLASL
DQSLKRMGLEYVDIFYSHRVDENTPMEETASALAHSVQSGKALYVGISSYSPERTQKMVE
LLREWKIPLLIHQPSYNLLNRWVDKSGLLDTLQNNGVGCIAFTPLAQGLLTGKYLNGIPQ
DSRMHREGNKVRGLTPKMLTEANLNSLRLLHEMAQQRGQSMAQMALSWLLKDERVTSVLI
GASRAEQLEENVQALNNLTFSTEELAQIDQHIADGELNLWQASSDK
>LFGLNPFC_04942 hypothetical protein
MPRLTAKDFPQELLDYYDYYAHGKISKREFLNLAAKYAVGGMTALALFDLLKPNYALATQ
VEFTDPEIVAEYITYPSPNGHGEVRGYLVKPAKMSGKTPAVVVVHENRGLNPYIEDVTRR
VAKAGYIALAPDGLSSVGGYPGNDDKGRELQQQVDPTKLMNDFFAAIEFMQRYPQATGKV
GISGFCYGGGVSNAAAVAYPELACAVPFYGRQAPTADVAKIEAPLLLHYAELDSRINEGW
PAYEAALKANNKVYEAYIYPEVNHGFHNDSTPRYDKSAADLAWQRTLKWFDKYLS
>LFGLNPFC_04943 hypothetical protein
MNNHFGKGLMAGLKATHADSAVNVTKFCADYKRGFVLGYSHRMYEKTGDRQLSAWEAGIL
TRRYGLDKEMVMDFFRENNSCSTLRFFMAGYRLEN
>LFGLNPFC_04944 Hydrogenase-2 small chain
MTGDNTLIHSHGINRRDFMKLCAALAATMGLSSKAAAEMAESVTNPQRPPVIWIGAQECT
GCTESLLRATHPTVENLVLETISLEYHEVLSAAFGHQVEENKHNALEKYKGQYVLVVDGS

IPLKDNGIYCMVAGEPIVDHIRKAAEGAAAIIAIGSCSAWGGVAAAGVNPTGAVSLQEVL
PGKTVINIPGCPPNPHNFLATVAHIITYGKPPKLDDKNRPTFAYGRLIHEHCERRPHFDA
GRFAKEFGDEGHREGWCLYHLGCKGPETYGNCSTLQFCDVGGVWPVAIGHPCYGCNEEGI
GFHKGIHQLANVENQTPRSQKPDVNAKEGGNVSAGAIGLLGGVVGLVAGVSVMAVRELGR
QQKKDNADSRGE
>LFGLNPFC_04945 Formate dehydrogenase, nitrate-inducible, iron-sulfur subunit
MNRRNFIKAASCGALLTGALPSVSHAAAENRPPIPGSLGMLYDSTLCVGCQACVTKCQDI
NFPERNPQGEQTWSNNDKLSPYTNNIIQVWTSGTGVNKDQEENGYAYIKKQCMHCVDPNC
VSVCPVSALKKDPKTGIVHYDKDVCTGCRYCMVACPYNVPKYDYNNPFGALHKCELCNQK
GVERLDKGGLPGCVEVCPAGAVIFGTREELMAEAKKRLALKPGSEYHYPRQTLKSGDTYL
HTVPKYYPHLYGEKEGGGTQVLVLTGVPYENLDLPKLDDLSTGARSEHVQHTLYKGMMLP
LAVLAGLTVLVRRNTKNDHHDGGDDHES
>LFGLNPFC_04946 putative Ni/Fe-hydrogenase 2 b-type cytochrome subunit
MSHDPQPLGGKIISKPVMIFGPLIVICMLLIVKRLVFGLGSVSDLNGGFPWGVWIAFDLL
IGTGFACGGWALAWAVYVFNRGQYHPLVRPALLASLFGYSLGGLSITIDVGRYWNLPYFY
IPGHFNVNSVLFETAVCMTIYIGVMALEFAPALFERLGWKLSLKRLNKVMFFIIALGALL
PTMHQSSMGSLMISAGYKVHPLWQSYEMLPLFSLLTAFIMGFSIVIFEGSLVQAGLRGNG
PDEKSLFVKLTNTISVLLAIFIVLRFGELIYRDKLSLAFAGDFYSVMFWIEVLLMLFPLV
VLRVAKLRNDSRMLFLSALSALLGCATWRLTYSLVAFNPGGGYAYFPTWEELLISIGFVA
IEICAYIVLIRLLPILPPLKQNDHNRHEASKA
>LFGLNPFC_04947 Hydrogenase-2 large chain
MSQRITIDPVTRIEGHLRIDCEIENGVVSKAWASGTMWRGMEEIVKNRDPRDAWMIVQRI
CGVCTTTHALSSVRAAESALNIDVPVNAQYIRNIILAAHTTHDHIVHFYQLSALDWVDIT
SALQADPTKASEMLKGVSTWHLNSPEEFTKVQNKIKDLVASGQLGIFANGYWGHPAMKLP
PEVNLIAVAHYLQALECQRDANRVVALLGGKTPHIQNLAVGGVANPINLDGLGVLNLERL
MYIKSFIDKLSDFVEQVYKVDTAVIAAFYPEWLTRGKGAVNYLSVPEFPTDSKNGSFLFP
GGYIENADLSSYRPITSHSDEYLIKGIQESAKHSWYKDEAPQAPWEGTTIPAYDGWSDDG
KYSWVKSPTFYGKTVEVGPLANMLVKLAAGRESTQNKLNEIVAIYQKLTGNTLEVAQLHS
TLGRIIGRTVHCCELQDILQNQYSALITNIGKGDHTTFVKPNIPATGEFKGVGFLEAPRG
MLSHWMVIKDGIISNYQAVVPSTWNSGPRNFNDDVGPYEQSLVGTPVADPNKPLEVVRTI
HSFDPCMACAVHVVDADGNEVVSVKVL
>LFGLNPFC_04948 Hydrogenase 2 maturation protease
MRILVLGVGNILLTDEAIGVRIVEALEQRYILPDYVEILDGGTAGMELLGDMANRDHLII
ADAIVSKKNAPGTMMILRDEEVPALFTNKISPHQLGLADVLSALRFTGEFPKKLTLVGVI
PESLEPHIGLTPTVEAMIEPALEQVLAALRESGVEAIPREATHD
>LFGLNPFC_04949 Hydrogenase-2 operon protein HybE
MTEEIAGFQTSPKVQVQAAFEEIARRSMHDLSFLHPSMPVYVSDFTLFEGQWTGCVITPW
MLSAVIFPGPDQLWPLRKVSEKIGLQLPYGTMTFTVGELDGVSQYLSCSLMSPLSHSMSI
EEGQRLTDDCARMILSLPVTNPDVPHAGRRALLFGRRSGENA
>LFGLNPFC_04950 Hydrogenase maturation factor HybF
MHELSLCQSAVEIIQRQAEQHDVKRVTAVWLEIGALSCVEESAVRFSFEIVCHGTVAQGC
DLHIVYKPAQAWCWDCSQVVEIHQHDAQCPICHGERLRVDTGDSLIVKSIEVE
>LFGLNPFC_04951 Hydrogenase maturation factor HybG
MCIGVPGQVLAVGEDIHQLAQVEVCGIKRDVNIALICEGNPADLLGQWVLVHVGFAMSII
DEDEAKATLDALRQMDYDITSA
>LFGLNPFC_04952 Disulfide-bond oxidoreductase YghU
MTDNTYQPAKVWTWDKSAGGAFANINRPVSGPTHEKTLPVGKHPLQLYSLGTPNGQKVTI
MLEELLALGVTGAEYDAWLIRIGDGDQFSSGFVEVNPNSKIPALRDHTHNPPIRVFESGS
ILLYLAEKFGYFLPQDLAKRTETMSWLFWLQGAAPFLGGGFGHFYHYAPVKIEYAINRFT
MEAKRLLDVLDKQLAQHKFVAGDEYTIADMAIWPWFGNVVLGGVYDAAEFLDAGSYKHVQ
RWAKEVGERPAVKRGRIVNRINGPLNEQLHERHDASDFETNTEDKRQG
>LFGLNPFC_04953 Bifunctional glutathionylspermidine synthetase/amidase
MSKGTTSQDAPFGTLLGYAPGGVAIYSSDYSSLDPQEYEDDAVFRSYIDDEYMGHKWQCV
EFARRFLFLNYGVVFTDVGMAWEIFSLRFLREVVNDNILPLQAFPNGSPRAPVAGALLIW
DKGGEFKDTGHVAIITQLHGNKVRIAEQNVIHTPLPQGQQWTRELEMVVENGGYTLKDTF
DDTTILGWMIQTEDTEYSLPQPEIAGELLKISGARLENKGQFDGKWLDEKDPLQNAYVQA
NGQVINQDPYHYYTITESAEQELIKATNELHLMYLHATDKVLKDDNLLALFDIPKILWPR
LRLSWQRRRHHMITGRMDFCMDERGLKVYEYNADSASCHTEAGLILERWAEQGYKGNGFN
PAEGLINELAGAWKHSRARPFVHIMQDKDIEENYHAQFMEQALQQAGFETRILRGLEELR
WDAAGQLIDGEGRQVNCVWKTWAWETAFDQIREVSDREFAAVPIRTGHPQNEVRLIDVLL
RPEVLVFEPLWTVIPGNKAILPILWSLFPHHRYLLDTDFTVNDELVKTGYAVKPIAGRCG
SNIDLVSHHEEVLDKTSGKFAEQKNIYQQLWCLPKVDGKYIQVCTFTVGGNYGGTCLRGD
ESLVIKKESDIEPLIVVKE
>LFGLNPFC_04954 Low-affinity inorganic phosphate transporter 1

MLNLFVGLDIYTGLLLLLALAFVLFYEAINGFHDTANAVATVIYTRAMQPQLAVVMAAFF
NFFGVLLGGLSVAYAIVHMLPTDLLLNMGSTHGLAMVFSMLLAAIIWNLGTWFFGLPASS
SHTLIGAIIGIGLTNALLTGSSVMDALNLREVTKIFSSLIVSPIVGLVIAGGLIFLLRRF
WSGTKKRDRIHRIPEDRKKKKGKRKPPFWTRIALIVSAAGVAFSHGANDGQKGIGLVMLV
LVGIAPAGFVVNMNASGYEITRTRDAVTNFEHYLQQHPELPQKLITMEPPLPAASTDGAQ
VTEFHCHPANTFDAIARVKTMLPGNMESYEPLSVSQRSQLRRIMLCISDTSAKLAKLPGV
SKEDQNLLKKLRSDMLSTIEYAPVWIIMAVALALGIGTMIGWRRVAMTIGEKIGKRGMTY
AQGMAAQMTAAVSIGLASYIGMPVSTTHVLSSAVAGTMVVDGGGLQRKTVTSILMAWVFT
LPAAIFLSGGLYWIALQLI
>LFGLNPFC_04955 Thymidylate kinase
MQSIIPPLIAVIGSDGSGKSTVCEHLITVVEKYGAAERVHLGKQAGNVGRAVTKLPLMGK
SLHKTIERNQVKTAKKLPGPVPALVITAFVARRLLRFRHMLACRRRGLIVLTDRYPQDQI
PGAYDGTVFPPNVEGGRFVSWLASQERKAFHWMASHKPDLVIKLNVDLEVACARKPDHKR
ESLARKIAITPQLTFGGAQLVDIDANQPLEKVLVDAEKAITDFMTARGYH
>LFGLNPFC_04956 hypothetical protein
MSIINSTPVRVIAIVGCDGSGKSTLTASLVNELASRMPTEHIYLGQSSGRIGEWISQLPV
IGAPFGRYLRSKAAHVHEKPSTPPGNITALVIYLLSCWRAYKFRKMLCKNQQGYLLITDR
YPQVEVPGFRFDGPQLAKTTGGNGWIKMLRQRELKLYQWMASYLPVLLIRLGIDEQTAFA
RKPDHQLAALQEKIAVTPQLTFNGARILELDGRQPADEILQASLRAIHAALS
>LFGLNPFC_04957 Thymidylate kinase
MIKRMDALQTQTVKSTTAPQPNYIPGLIAVVGCDGTGKSTLTNDLVKSLQQHWQTERRYL
GLLSGEDGDKIKRLPLVGVWLERRLAAKSSKTQSMKTKSPALWAAVIMYCFSLRRMANLR
KVQRLAQSGVLVVSDRFPQAEISGFYYDGPGIGVERATGKISRFLAQRERRLYQKMAQYR
PELIIRLGIDIDTAISRKPDHDYAELQDKIGVMSTIGYNGTKILEIDSRAPYSEVLEQAQ
KAVSLVAIVSDRRSLT
>LFGLNPFC_04958 hypothetical protein
MAVFNIKHWFADGAFRTIIRNSAWLGSSNVVSALLGLLALSCAGKGMTPAMFGVLVIVQS
YAKSISDFIKFQTWQLVVQYGTPALTNNDPQQFRNVVSFSFSLDIVSGAVAIVGGIALLP
FLSHSLGLDDQSFWLAALYCTLIPSMASSTPTGILRAVDRFDLIAVQQATKPFLRAAGSV
VAWYFDFGFAGFVIAWYVSNLVGGTMYWWFAARELRRRNIHNAFKLNLFESARHIKGAWS
FVWSTNIAHSIWSARNSCSTVLVGIVLGPAAAGLFKIAMTFFDAAGTPAGLLGKSFYPEV
MRLDPRTTRPWLLGVKSGLLAGGIGILVALAVLIVGKPLISLVFGVKYLEAYDLIQVMLG
AIVISMLGFPQESLLLMAGKQRAFLVAQTIASIGYIVLLFMFCHLFGVLGAAFAYFGGQC
LDVVLSLIPTLKAFFQRHSLLYNSTGEKS
>LFGLNPFC_04959 hypothetical protein
MMRKYFPLEASERLFVAIEEDDVVDAQVSLPPTIALSCTTEIIHDNYALCLKFWLDGVNR
QELLRLIRKQAKGDELTTDERKQFKYMRARYKHLRFAQRLYLKKHQAGFLFGKTTVFLGH
FQDGFRNGKKNIVSFYGNLLRVYLSSPVWWLVNYSLRHGQLETVNGFIAYRQKQMYILKE
IIAKPQLTGREFHDVRKIISQQVSYYDTLRSLDPENKEALLISRFLAAINGLMGDKHDDM
VADDMDNRRSYDAPVALDSDIRQRLELLISRFPLYPEQ
>LFGLNPFC_04960 putative protein YtfJ
MPSRWIVALIIALLAPGAAAHNFVTGKTVTPVYIQEGGELLLNSDDEIHYQKWNSTQLTG
KVRIIQYIAGRKSAKKKNSLLIKAVEAANFSQDRFQPTTIVNTDDAIFGTGYFVVGKIEK
NKRRYPWAQFVIDGNGLGRVAWQLPEQSSTILVLNKAGRIQWAKDGSLTPEEVDHVIALA
QKLINE
>LFGLNPFC_04961 hypothetical protein
MKLVEHYIMRGTRRLVLIIVGFLIFIFASYSAQRYLTEAANGTLALDVVLDIVFYKVLIA
LEMLLPVGLYASVGVTLGQMYTDSEITAISAAGGSPGRLYKAVLYLAIPLSIFVTLLSMY
GRPWAYAQIYQLEQQSQSELDVRQLRAKKFNTNDNGRMILSQTVDQDNNRLTDALIYTST
ANRTRIFRARSVDVVDPSPEKPTVMLHNGTAYLLDHQGRDDNEQIYRNLQLHLNPLDQSP
NVKRKAKSVTELARSVFPADHAELQWRQSRGLTALLMALLAISLSRVKPRQGRFSTLLPL
TLLFVAIFYGGDVCRTLVANGAIPLIPGLWLVPGLMLMGLLMLVARDFSLLQKFPR
>LFGLNPFC_04962 Lipopolysaccharide export system permease protein LptG
MNVFSRYLIRHLFLGFAAAAGLLLPLFTTFNLINELDDVSPGGYRWTQAVLVVLMTLPRT
LVELSPFIALLGGIVGLGQLSKNSELTAIRSTGFSIFRIALVALVAGILWTVSLGAIDEW
VASPLQQQALQIKSTATALGEDNDIIGNMLWARRGNEFVTVKSLNEQGQPVGVEIFHYRD
DLSLESYIYARSATIEDDKTWILHGVNYKKWLNGKETLETLDNLAWQSAFTSMNLEELSM
PGNTFSVRQLNHYIHYLQDTGQPSSEYHLALWEKLGQPILTLAMILLAVPFTFSVPRSPG
MGSRLAVGVIVGLLTWISYQIMVNLGLLFALSAPVTALGLPVAFVLVALSLVYWYDRQH
>LFGLNPFC_04963 8-amino-7-oxononanoate synthase
MGLYDKYARLAGERLQFSDNGLTPFGTCIDEVYSATEGRIGNKKVILAGTNNYLGLTFNH
DAIREGQAALAAQGTGTTGSRMANGSYAPHLALEKEIAEFFNRPTAIVFSTGYTANLGVI
STLADHNAVVLLDADSHASIYDACSLGGAEIIRFRHNDVNDLERRMVRLGERAKEAIIIV
EGIYSMLGDVAPLAEIVDIKRRLGGYLIVDEAHSFGVLGATGRGLAEAVGVEDDVDIIVG

TFSKSLASIGGFAVGSEAMEVLRYGSRPYIFTASPSPSCIATVRSSLRTIASQPELRQKL
MDNANHLYDGLQKLGYELSSHISPVVPVIIGSKEEGLRIWRKLISLGVYVNLILPPAAPA
GITLLRCSVNAAHSREQIDAIIQAFATQKQ
>LFGLNPFC_04964 Acyl carrier protein
MVNREIVMDYILSCLQDLVENGVEIKPDSDLVNDLGLESIKVMDLLMMLEDRFDISIPIN
ILLDVKTPAQLMETLLPWLENN
>LFGLNPFC_04965 2-alkyl-3-oxoalkanoate reductase
MNQTVAVTGATGFIGKYIIDNLLARGFHVRALTRTARAHVNDNLTWVRGSLEDTHSLSEL
VAGASAVVHCAGQVRGHKEEIFTRCNVDGSLRLMQAAKESGFCQRFLFISSLAARHPELS
WYANSKHVAEQRLTAMADEITLGVFRPTAVYGPGDKELKPLFDWMLRGLLPRLGTPETQL
SFLHVTDFAQAVSQWLSAETVQTQTYELCDGVAGGYDWQRVQQLVADVRCGSVRMVGIPQ
PLLTCLADISTALNRLAGKEPMLTRSKIRELTHADWSASNNRISEDINWFPGISLEHALR
NGLF
>LFGLNPFC_04966 Long-chain-fatty-acid--AMP ligase FadD26
MRYADFPTLVDALDYAALSSAGMNFYDRRCQLEDQLEYQTLKARAEAGAKRLLSLNLKKG
DRVALIAETSSGFVEAFFACQYAGLVAVPLAIPMGVGQRDSWSAKLQGLLASCQPAAIIT
GDEWLPLVNAATHNNNPELHVLSHAWFKALPEADVVLQRPVPNDIAYLQYTSGSTRFPRG
VIITHHEVMANLRAISHDGIKLRPGDRCVSWLPFYHDMGLVGFLLTPVATQLSVDYLRTQ
DFAMRPLQWLKLISKNRGTVSVAPPFGYELCQRRVNEKDLAELDLSCWRVAGIGAEPISA
EQLHQFAECFRQVNFDDKTFMPCYGLAENALAVSFSDEACGVVVNEVDRDILEYQGKAVA
PGAETRAVSTFVNCGKALPEHGIEIRNEADIPVAERVVGHICISGPSLMSGYFGDQVSQD
EIAATGWLDTGDLGYLLDGYLYVTGRIKDLIIIRGRNIWPQDIEYIAEQEPEIHSGDAIA
FVTAQEKIILQIQCRISDEERRGQLIHALAARIQSEFGVTADIDLLPPHSIPRTSSGKPA
RAEAKKRYQKAYAASLHVQESLA
>LFGLNPFC_04967 Protein YghO
MECDLLMIKIEKVINKKDLKAFIAFPSSLYPDDPNWIPPLFIERSEHLSAKNPGTDHIIW
QAWVAKKAGQIVGRITAQIDTLHRERYGEDTGHFGMIDSIDDPQVFAALFGTAEAWLKSQ
GASKISGPFSLNINQESGLLIEGFDTPPCAMMPHGKPWYAAHIEQLGYHKGIDLLAWWMQ
RTDLTFSPALKKLMDQVRKKVTIRCINRQRFAEDMQILREIFNSGWQHNWGFVPFTEHEF
ATMGDQLKYLVPDDMIYIAEIDSAPCAFIVGLPNINEAIVDLNGSLFPFGWAKLLWRLKV
SGVRTARVPLMGVRDEYQFSRIGPVIALLLIEALHDPFARRKIDALEMSWILETNTGMNN
MLERIGAEPYKRYRLYEKQI
>LFGLNPFC_04968 Glc operon transcriptional activator
MKDERRPICEVVAESIERLIIDGVLKVGQPLPSERRLCEKLGFSRSALREGLTVLRGRGI
IETAQGRDSRVARLNRVQDASPLIHLFSTQPRTLYDLLDVRALLEGESARLAATLGTQAD
FVVITRCYEKMLAASENNKEISLIEHAQLDHAFHLAICQASHNQVLVFTLQSLTDLMFNS
VFASVNNLYHRPQQKKQIDRQHARIYNAVLQRLPHVAQRAARDHVRTVKKNLHDIELEGH
HLIRSAVPLEMKQG
>LFGLNPFC_04969 putative FAD-linked oxidoreductase
MSILYEERLDGALPEVDRTSVLMALREHVPGLEILHTDEEIIPYECDGLSAYRTRPLLVV
LPKQMEQVTAILAVCHRLRVPVVTRGAGTGLSGGALPLEKGVLLVMARFKEILDINPIGR
RARVQPGVRNLAISQAVAPHNLYYAPDPSSQIACSIGGNVAENAGGVHCLKYGLTVHNLL
KIEVQMLDGEALTLGSDALDSPGFDLLALFTGSEGMLGVTTEVTVKLLPKPPVARVLLAS
FDSVEKAGLAVGDIIANGIIPGGLEMMDNLSIRAAEDFIHAGYPVDAEAILLCELDGVES
DVQEDCERVNDILLNAGATDVRLAQDEAERVRFWAGRKNAFPAVGRISPDYYCMDGTIPR
RALPGVLEGIARLSQQYDLRVANVFHAGDGNMHPLILFDANEPGEFARAEELGGKILELC
VEVGGSISGEHGIGREKINQMCAQFNSDEITTFHAVKAAFDPDGLLNPGKNIPTLHRCAE
FGAMHVHHGHLPFPELERF
>LFGLNPFC_04970 hypothetical protein
MLRECDYSQALLEQVNQAISDKTPLVIQGSNSKAFLGRPVTGQTLDVRCHRGIVNYDPTE
LVITARAGTPLVAIEAALESAGQMLPCEPPHYGEEATWGGMVACGLAGPRRPWSGSVRDF
VLGTRIITGTGKHLRFGGEVMKNVAGYDLSRLMAGSYGCLGVLTEISMKVLPRPRASLSL
RREISLQEAMNEIAQWQLQPLPISGLCYFDNALWIRLEGGEGSVKAARELLGGEEVAGQF
WQQLREQQLPFFSLPGTLWRISLPSDAPMMDLPGEQLIDWGGALRWLKSTADDNQIHRIA
RNAGGHATRFSAGDGGFAPLPAPLFRYHQQLKQQLDPCGVFNPGRMYAEL
>LFGLNPFC_04971 Lactate utilization protein A
MQTRLTEEMRQNARALEADSILRACVHCGFCTATCPTYQLLGDELDGPRGRIYLIKQVLE
GNEVTLKTQEHLDRCLTCRNCETTCPSGVRYHNLLDIGRDIVEQKVKRPLPERMLREGLR
QVVPRPAVFRALTQVGLVLRPFLPEQVRAKLPAETVKAKPRPPLRHKRRVLMLEGCAQPT
LSPNTNAATARVLDRLGISVMPANEAGCCGAVDYHLNAQEKGLARARNNIDAWWPAIEAG
AEAILQTASGCGAFVKEYGQMLKNDALYADKARQVSELAVDLVELLREEPLEKLAIRGDK
KLAFHCPCTLQHAQKLNGEVEKVLLRLGFTLTDVPDSHLCCGSAGTYALTHPDLARQLRD
NKMNALESGKPEMIVTANIGCQTHLASAGRTSVRHWIEIVEQALEKE
>LFGLNPFC_04972 hypothetical protein

MKTKVILSQQMASAIIAAGQEEAQKNNWSVSIAVADDGGHLLALSRMDDCAPIAAYISQE
KARTAALGRRETKGYEEMVNNGRTAFVTAPLLTSLEGGVPVVVDGQIIGAVGVSGLTGAQ
DAQVAKAAAAVLAK
>LFGLNPFC_04973 Malate synthase G
MSQTITQGRLRIDANFKRFVDEEVLPGVELDAAAFWHNVDEIVHDLAPENRQLLAERDRI
QAALDEWHRSNPGPVKDKAAYKSFLRELGYLVPQPDHVTVETTGIDSEITSQAGPQLVVP
AMNARYALNAANARWGSLYDALYGSDIIPQEGAMVSGYDPQRGEQVIAWVRRFLDESLPL
ENGSYQDVVAFKVVDKQLRIQLKNGKETTLRTPAQFVGYRGDTAAPTCILLKNNGLHIEL
QIDANGRIGKDDPAHINDVIVEAAISTILDCEDSVAAVDAEDKILLYRNLLGLMQGTLQE
KMEKNGRQIVRKLNDDRQYTAADGSEISLHGRSLLFIRNVGHLMTIPVIWDSEGNEIPEG
ILDGVMTGAIALYDLKVQKNSRTGSVYIVKPKMHGPQEVAFANKLFSRVETMLGMAPNTL
KMGIMDEERRTSLNLRSCIAQARNRVAFINTGFLDRTGDEMHSVMEAGPMLRKNQMKSTP
WIKAYERNNVLSGLFCGLRGKAQIGKGMWAMPDLMADMYSQKGDQLRAGANTAWVPSPTA
ATLHALHYHQTNVQSVQANIAQTEFNAEFEPLLDDLLTIPVAENANWSAQEIQQELDNNV
QGILGYVVRWVEQGIGCSKVPDIHNVALMEDRATLRISSQHIANWLRHGILTKDQVQASL
ENMAKVVDQQNAGDPAYRPMVENFANSCAFKAACDLIFLGVKQPNGYTEPLLHAWRLREK
ENH
